# Comparative Analysis of Kabuli Chickpea Transcriptome with Desi and Wild Chickpea Provides a Rich Resource for Development of Functional Markers

**DOI:** 10.1371/journal.pone.0052443

**Published:** 2012-12-27

**Authors:** Gaurav Agarwal, Shalu Jhanwar, Pushp Priya, Vikash K. Singh, Maneesha S. Saxena, Swarup K. Parida, Rohini Garg, Akhilesh K. Tyagi, Mukesh Jain

**Affiliations:** Functional and Applied Genomics Laboratory, National Institute of Plant Genome Research (NIPGR), New Delhi, India; Tulane University, United States of America

## Abstract

Chickpea (*Cicer arietinum* L.) is an important crop legume plant with high nutritional value. The transcriptomes of desi and wild chickpea have already been sequenced. In this study, we sequenced the transcriptome of kabuli chickpea, *C. arietinum* (genotype ICCV2), having higher commercial value, using GS-FLX Roche 454 and Illumina technologies. The assemblies of both Roche 454 and Illumina datasets were optimized using various assembly programs and parameters. The final optimized hybrid assembly generated 43,389 transcripts with an average length of 1065 bp and N50 length of 1653 bp representing 46.2 Mb of kabuli chickpea transcriptome. We identified a total of 5409 simple sequence repeats (SSRs) in these transcript sequences. Among these, at least 130 and 493 SSRs were polymorphic with desi (ICC4958) and wild (PI489777) chickpea, respectively. In addition, a total of 1986 and 37,954 single nucleotide polymorphisms (SNPs) were predicted in kabuli/desi and kabuli/wild genotypes, respectively. The SNP frequency was 0.043 SNP per kb for kabuli/desi and 0.821 SNP per kb for kabuli/wild, reflecting very low genetic diversity in chickpea. Further, SSRs and SNPs present in tissue-specific and transcription factor encoding transcripts have been identified. The experimental validation of a selected set of polymorphic SSRs and SNPs exhibited high intra-specific polymorphism potential between desi and kabuli chickpea, suggesting their utility in large-scale genotyping applications. The kabuli chickpea gene index assembled, and SSRs and SNPs identified in this study will serve as useful genomic resource for genetic improvement of chickpea.

## Introduction

Chickpea is a cool season legume crop grown in arid and semi-arid regions of the world. It is a self-pollinated and diploid plant with an estimated genome size of ∼740 Mb. Chickpea is important not only for its nutritive value, but also for its ability to maintain soil fertility by fixing atmospheric nitrogen. Despite several efforts over past decades, chickpea production has been very low worldwide. The narrow genetic diversity in cultivated chickpea germplasm, incompatibility with wild *Cicer* accessions and limited availability of genomic resources are important factors responsible for limited progress in the improvement of chickpea yield [1]–[3].

Recently, chickpea has attracted the attention of researchers worldwide and several genomic resources have been generated in the past few years. The complete transcriptome of cultivated and wild chickpeas has been sequenced using high-throughput sequencing technologies [4]–[6]. A public web resource for mining the transcriptome data is also available [5]. The identification and functional characterization of a few stress-related genes has been undertaken [7]–[12]. A considerable progress has been made in the development of genetic linkage maps of chickpea as well [13]–[18]. Further, the genetic and physical maps of chickpea genome have been integrated to assign the genetic linkage groups to particular chromosomes [19]. A significant progress in the chickpea improvement is expected to be made in coming years based on these genomic resources. However, there is still need for availability of genomic resources from diverse genotypes of chickpea for construction of dense genetic linkage map, understanding evolution and discovery of more genetic variations related to important agronomic traits.

There are two major types of chickpea distinguished by seed-size, shape and color. One, desi-type is characterized by relatively smaller seeds of angular shape with dark seed coat, whereas other kabuli-type is characterized by large owl/ram-head-shaped seeds with beige-colored seed coat. Kabuli-type chickpea is considered more economically important as it receives higher market price than desi-type. However, most of the genomic resources have been generated for desi-type chickpea so far. In this study, we sequenced the transcriptome of kabuli chickpea using GS-FLX Roche 454 and Illumina next generation sequencing (NGS) technologies and characterized in detail. Several genomic variations, polymorphic simple sequence repeats (SSRs) and single nucleotide polymorphisms (SNPs), between kabuli/desi and kabuli/wild were identified in comparative transcriptome analyses. A set of polymorphic SSRs and SNPs were evaluated for their amplification efficiency and polymorphism potential in cultivated chickpea. This study will serve as an important genomic resource for further studies in chickpea.

## Results

### Deep Sequencing of Kabuli Chickpea Transcriptome

The transcriptome of kabuli chickpea, *C. arietinum* (genotype ICCV2), was sequenced using GS-FLX Titanium and Illumina GA IIx sequencers. A total of about 1.8 million reads were generated on Roche 454 platform and about 121 million reads (44 million single-end reads of 101 bp length and 77 million paired-end reads of 100 bp length) were generated on Illumina platform. After stringent quality filtering, a total of 1,643,836 high-quality Roche 454 reads with an average length of 365 bp and 108,429,800 high-quality Illumina reads were obtained. The sequence data have been deposited in the Sequence Read Archive (SRA) database at NCBI under the accession number SRA052099. The summary of sequencing data generated and quality filtering results is given in Table S1. The length and average quality score distribution showed that about 78% of the Roche 454 reads were larger than 300 bp in length and 87% reads were of at least 30 average Phred quality score (Figure S1). For Illumina reads, the average Phred quality score was more than 30 at each base position for paired-end reads and more than 20 for single-end reads (Figure S2).

### Optimization of Transcriptome Assembly

To generate the assembly of kabuli chickpea transcriptome, first the assemblies of Roche 454 and Illumina data were optimized individually using different approaches, softwares and parameters, followed by hybrid assembly of the two datasets. For the assembly of Roche 454 data, three approaches, including *de novo*, reference-based and combined approach, were undertaken. The *de novo* assembly was performed using Newbler, CLC and TGICL softwares. The reference-based assembly was performed using Newbler and CLC softwares using the transcriptome of desi chickpea [5] as reference. In the combined approach, assembly of contigs generated in the reference assembly was combined with unassembled reads and merged assembly was performed using TGICL. Various criteria, including assembly statistics, total number of mapped reads, number of uniquely mapped reads, and alignment and coverage of reference proteome were considered to assess the assembly output from all approaches (Table S2). Based on these criteria, merged assembly of CLC reference assembly and unassembled reads (merged CLC) was considered to be the best, which generated 36,408 contigs with an average length of 1037 bp, N50 length of 1501 bp using more than 91% of the reads, of which about 86% were uniquely mapped. This assembly represented a total of 35% soybean proteins, of which 17% showed at least 80% coverage (Table 1).

**Table 1:** Assembly optimization/validation of Illumina and Roche 454 data of kabuli chickpea.

	Illumina				Roche 454	
	Assembly using total reads		Assembly using non-redundant reads		Merged assembly	
	Oases K39	ABySS K79	Oases K41	ABySS K87	Newbler	CLC
Total contigs (≥100 bp)	42,371	43,886	37,219	54,087	39,517	36,408
Large contigs (≥1000 bp)	17,168	19,042	16,877	11,408	10,315	13,492
Total size (Mb)	48.04	48.31	46.30	35.45	31.34	37.76
Maximum contig length (bp)	16,914	15,651	16,835	15,549	15,587	15,601
Average contig length (bp)	1133.9	1100.9	1244.1	655.6	793.1	1037
N50 contig length (bp)	2075	1758	2108	1297	1273	1501
Total number of reads mapped (%)	78.7	93.9	80.0	85.8	81.3	91.2
Number of uniquely mapped reads (%)	78.6	63.7	79.9	85.1	81.0	85.6
Contigs with significant hits (%)^1^	57.4	83.5	62.7	74.6	69.4	75.3
Unique soybean proteins with significant hits (%)^2^	34.2	34.3	33.4	36.3	35.0	35.0
Number of unique soybean protein with ≥80%coverage (%)^3^	18.9	22.9	19.1	19.2	15.6	17.1

1 Contigs showing significant hit (*E*-value ≤1e−5) with soybean proteins.

2 Unique soybean proteins to which contigs show significant hit (*E*-value ≤1e−5).

3 Unique soybean proteins to which contigs show ≥80% coverage.

For the assembly of Illumina reads, two datasets were used, first, comprised of all the high-quality reads (108,429,800 reads) and second, non-redundant dataset (32,646,926 reads) containing only non-clonal reads (after removing duplicate reads generated by PCR amplification). *De novo* assemblies were performed for both datasets using Velvet, Oases, ABySS and SOAPdenovo softwares using different *k*-mer lengths (Table S3). Based on the statistics, the assembly output of the best *k*-mer length was selected to evaluate the assembly outputs from different softwares for both datasets at various criteria as described above (Table 1, Table S4). Two best assemblies for each dataset (Oases K39 and ABySS K79 for total dataset, and Oases K41 and ABySS K87 for non-redundant dataset), which generated comparable output, were selected.

Finally, hybrid assembly of the selected best assemblies of Illumina dataset was performed with best Roche 454 data assembly (merged CLC) using TGICL (Table 2). Based upon the assessment criteria mentioned above, the hybrid assembly of ABySS K87 from non-redundant Illumina data with merged CLC from Roche 454 data was considered to be the best and used for downstream analyses. Overall, a total of 43,389 contigs with an average length of 1065 bp and N50 length of 1653 bp were generated. This assembly included about 92% and 96% of the Roche 454 and Illumina reads, respectively, and represented about 37% of the reference soybean proteins (Table 2).

**Table 2:** Statistics/validation of hybrid assembly of Ilumina and Roche 454 data of kabuli chickpea.

	Assembly using Illumina total reads		Assembly using Illumina non-redundant reads	
	Oases K39	ABySS K79	Oases K41	ABySS K87
Total contigs (≥100 bp)	59,644	43,315	54,408	43,389
Total size (Mb)	71.88	56.9	69.72	46.21
Large contigs (≥1000 bp)	26,386	22,128	25,915	16,967
Maximum contig length (bp)	16,935	15,654	16,856	15,605
Average contig length (bp)	1205.3	1313.7	1281.5	1065.1
N50 contig length (bp)	2001	1885	2020	1653
Total number of Roche 454 reads mapped (%)	93.2	93.2	93.2	92.3
Total number of uniquely mapped Roche 454 reads (%)	64.3	69.2	64.3	81.5
Total number of Illumina reads mapped (%)	96.8	95.0	96.8	95.5
Total number of uniquely mapped Illumina reads (%)	47.9	56.3	47.9	75.1
Contigs with significant hits (%)^1^	64.7	79.3	68.8	74.5
Unique soybean proteins with significant hits (%)^2^	39.5	36.5	38.9	36.9
Number of unique soybean protein with ≥80% coverage (%)^3^	24.2	23.2	24.2	21.4

1 Contigs showing significant hit (*E*-value ≤1e−5) with soybean proteins.

2 Unique soybean proteins to which contigs show significant hit (*E*-value ≤1e−5).

3 Unique soybean proteins to which contigs show ≥80% coverage.

### Characterization of the Kabuli Chickpea Transcriptome

**Figure 1:**
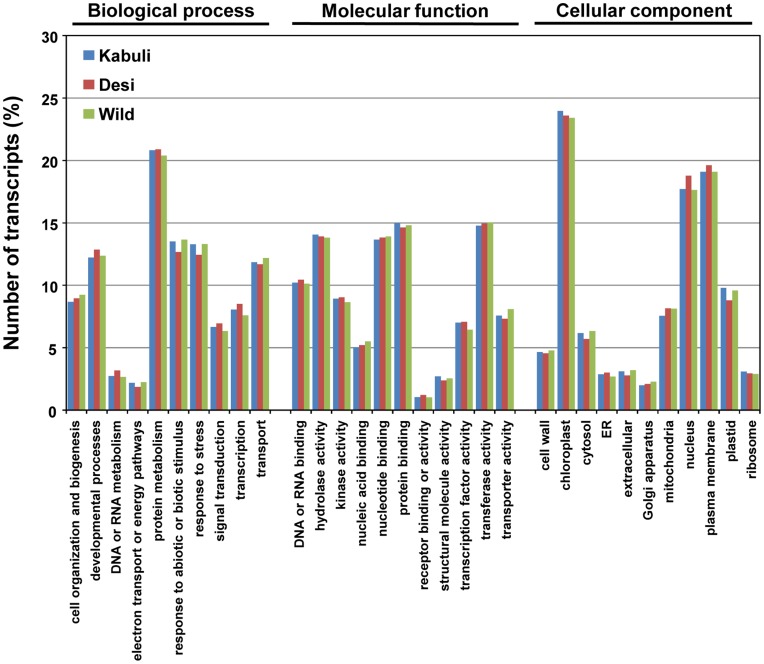
GOSlim term assignment to the transcripts of three chickpea types in different categories of biological process, molecular function and cellular component. Data for desi and wild chickpeas are from our previous studies [5], [6].

The contigs generated were designated as *C. arietinum* kabuli tentative consensus (CakTC) transcripts and assigned unique identifier numbers from CakTC00001 to CakTC43389. The complete transcriptome sequence can be downloaded from the Chickpea Transcriptome Database (CTDB; http://www.nipgr.res.in/ctdb.html). The average transcript length was 1065 bp, representing about 6.2% (46.2 Mb) of the chickpea genome sequence. About three-fourth of the transcripts were longer than 500 bp and 39% transcripts were longer than 1000 bp (Figure S3). The average GC content of the kabuli transcripts was 38% with overall distribution similar to that of desi and wild chickpea transcriptomes (Figure S4). To characterize and provide an overview of the biological functions encoded by the kabuli chickpea transcripts, their functional annotation and gene ontology (GO) analysis were performed. Out of 43,389 transcripts, 26,450 (∼61%) showed homology to at least one Arabidopsis protein and other 4287 (∼10%) transcripts showed homology with at least one protein/domain in UniRef, NR, PFAM, SMART, KEGG or COG databases. Overall, a total of 71% transcripts could be assigned with a putative function. Further, about 68% transcripts could be assigned at least one GOSlim category. Among the transcripts, to which biological process GOSlim was assigned, protein metabolism (20.8%) represented the most abundant category followed by response to biotic/abiotic stimulus (13.5%), response to stress (13.3%) and developmental processes (12.2%) (Fig. 1). Among the transcripts annotated in molecular function category, largest number were represented by protein binding (15%) followed by transferase activity (14.8%) and hydrolase activity (14%). The transcripts associated with chloroplast (24%), plasma membrane (19.1%) and nucleus (17.7%) were most abundant in cellular component GOSlim categories. Overall, different GOSlim categories represented in kabuli, desi and wild chickpea transcripts showed very similar distribution (Fig. 1). About 5% of the transcripts were predicted to encode for transcription factors belonging to 87 different families (Figure S5). Among these, MYB family transcription factors were most abundant followed by tetratricopeptide repeat (TPR), basic helix-loop-helix (bHLH) and Apetala2 (AP2)-domain containing proteins.

### Frequency and Distribution of SSRs in Kabuli Chickpea Transcriptome

**Figure 2:**
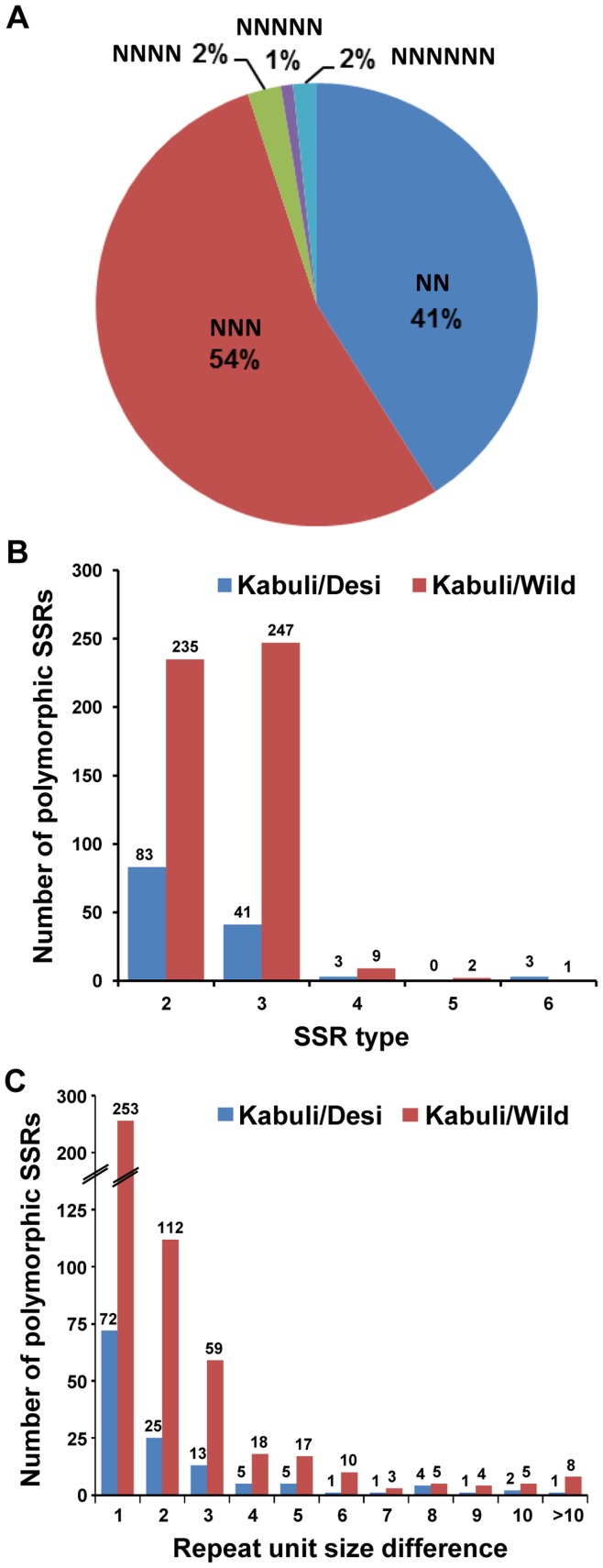
SSR distribution in kabuli, desi and wild chickpea types. (A) Distribution of total number of SSRs identified in kabuli chickpea in different classes. (B) Distribution of polymorphic SSRs identified in kabuli/desi and kabuli/wild chickpea. (C) Frequency difference of different classes of polymorphic SSRs identified in kabuli/desi and kabuli/wild chickpea.

We identified 5409 SSRs in the kabuli chickpea transcriptome, including di-nucleotide (minimum 12 nucleotides in length) and tri- to hexa-nucleotide (minimum 15 nucleotides in length) repeats representing a total of ∼1 Mb sequence (Table S5). In total, about 10% (4398) of the transcripts harbored at least one SSR and 743 transcripts contained two or more SSRs. A total of 478 SSRs were categorized as compound repeats. The number of repeat units varied from five to 63 and repeat length from 12 to 189 nucleotides. The density of SSRs was estimated to be one SSR per 8.54 kb of transcriptome sequence, which is similar to desi and wild chickpea transcriptomes predicted earlier [5], [6]. Tri-nucleotide (54%) SSRs were most abundant followed by di-nucleotide (41%) SSRs (Fig. 2A). Other types of SSRs (tetra-, penta- and hexa-nucleotide) were much less in number representing only 5% of total microsatellites. Among di-nucleotide SSRs, AG/CT was most abundant (70%). The AT-rich tri-nucleotide SSRs (AAC/GTT, AAG/CTT, AAT/ATT, ACT/ATG and AGT/ATC; 81%) were more abundant than GC-rich tri-nucleotide SSRs (ACC/GGT, ACG/CTG, AGC/CGT, AGG/CCT and CCG/CGG; 19%). The frequency of different SSR types is given in Table S6. At least 1476 (27.3%) SSRs were ≥20 nucleotides in length. Further, we designed forward and reverse primers from the flanking sequences of the identified SSR motifs, which had at least 100 bp flanking sequence on both sides. Overall, primers could be designed for a total of 2645 SSRs. The details of all SSRs along with primer sequences designed are available in the Table S7.

### Frequency and Distribution of Polymorphic SSRs

**Figure 3:**
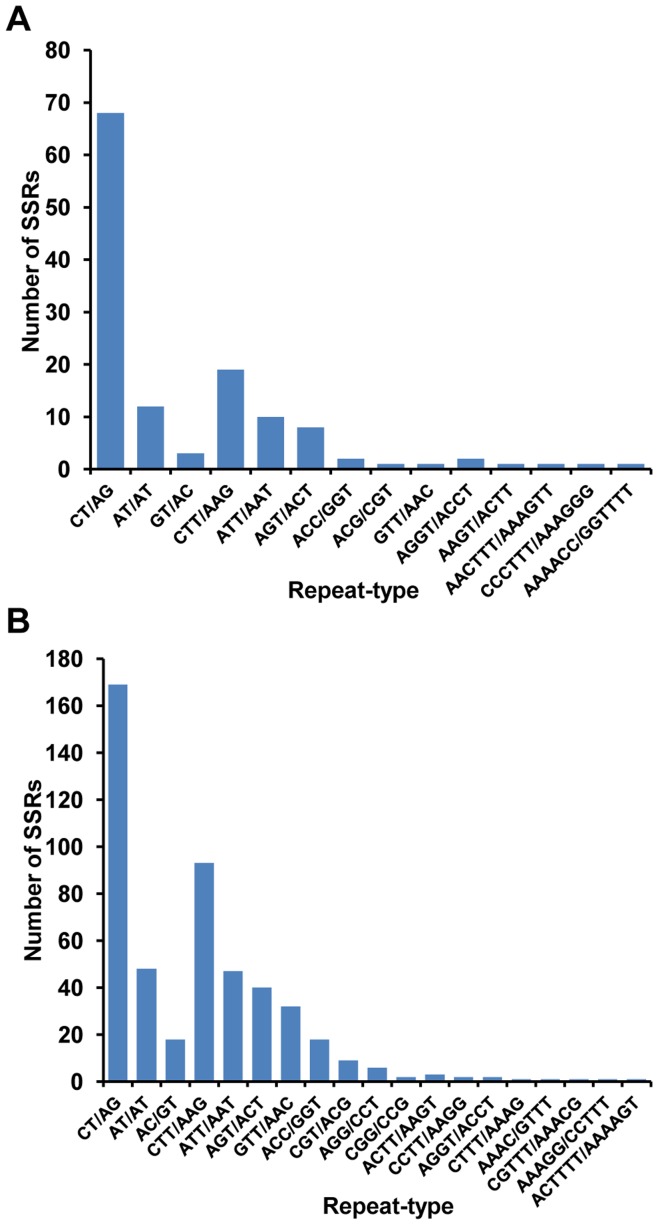
Frequency of different polymorphic SSR types. Number of polymorphic SSRs in kabuli/desi (A) and kabuli/wild (B) chickpea representing different repeat types are shown.

We found a larger average repeat length and average repeat unit size for all types of SSRs in kabuli as compared to desi and wild chickpea (Table 3). The average repeat length and average repeat unit size was least for wild chickpea as compared to cultivated (kabuli and desi) chickpeas. We determined polymorphism between SSRs identified in kabuli chickpea transcriptome in this study with SSRs reported in desi and wild chickpea transcriptomes [5], [6]. A total of 130 and 493 candidate polymorphic SSRs were identified for kabuli/desi and kabuli/wild chickpea, respectively. Di-nucleotide polymorphic SSRs were most abundant in kabuli/desi, whereas tri-nucleotide polymorphic SSRs were most abundant in kabuli/wild (Fig. 2B). Only a few polymorphic SSRs were of tetra-, penta- and hexa-nucleotide types (six in kabuli/desi, and 12 in kabuli/wild). The difference in number of repeat units varied from one to 11 for kabuli/desi and one to 40 for kabuli/wild. More than 50% of the polymorphic SSRs showed a difference of only one repeat unit (Fig. 2C). At least 33 and 129 polymorphic SSRs in kabuli/desi and kabuli/wild, respectively, were having repeat unit difference of at least three (minimum length difference of 6 nucleotides). Three and 13 polymorphic SSRs in kabuli/desi and kabuli/wild, respectively, exhibited difference in number of repeat units of at least 10 (minimum length difference of 20 nucleotides). The di-nucleotide SSR, CT/AG (52.3% in kabuli/desi and 34.3% in kabuli/wild), was most abundant followed by tri-nucleotide repeat, CTT/AAG (14.6% in kabuli/desi and 18.9% in kabuli/wild). The frequency of different repeat types among polymorphic SSRs in kabuli/desi and kabuli/wild chickpea is shown in Figure 3. We also analyzed the physical distribution of polymorphic SSRs on kabuli transcripts. In general, the polymorphic SSRs were distributed over the entire length of transcripts with some bias towards 5’ end (Figure S6).

**Table 3:** Average repeat length and unit size of different SSR-types in three chickpea genotypes.

SSR-type	Average repeat length			Average unit size		
	Kabuli	Desi	Wild	Kabuli	Desi	Wild
Di-nucleotide	17.33	17.21	16.66	8.69	8.66	8.45
Tri-nucleotide	18.04	17.63	17.68	6.05	5.88	5.94
Tetra-nucleotide	22.74	23.12	22.03	5.69	5.78	5.49
Penta-nucleotide	29.40	27.58	27.06	5.79	5.47	5.35
Hexa-nucleotide	35.42	33.29	32.23	5.78	5.51	5.35

We identified orthologous transcripts in kabuli chickpea, which were predicted to be expressed in tissue-specific (shoot, root, mature leaf, flower bud and young pod) manner in desi chickpea earlier [5] and mined the presence of polymorphic SSRs in them. At least 9 and 31 polymorphic SSRs in kabuli/desi and kabuli/wild, respectively, were present in tissue-specific transcripts. In addition, 16 and 68 polymorphic SSRs in kabuli/desi and kabuli/wild, respectively, were identified in the transcription factor encoding transcripts. The complete list of polymorphic SSRs in kabuli/desi and kabuli/wild chickpeas, including tissue specificity and transcription factors family, are given in the Tables S8 and S9, respectively.

### SNP Identification

**Figure 4:**
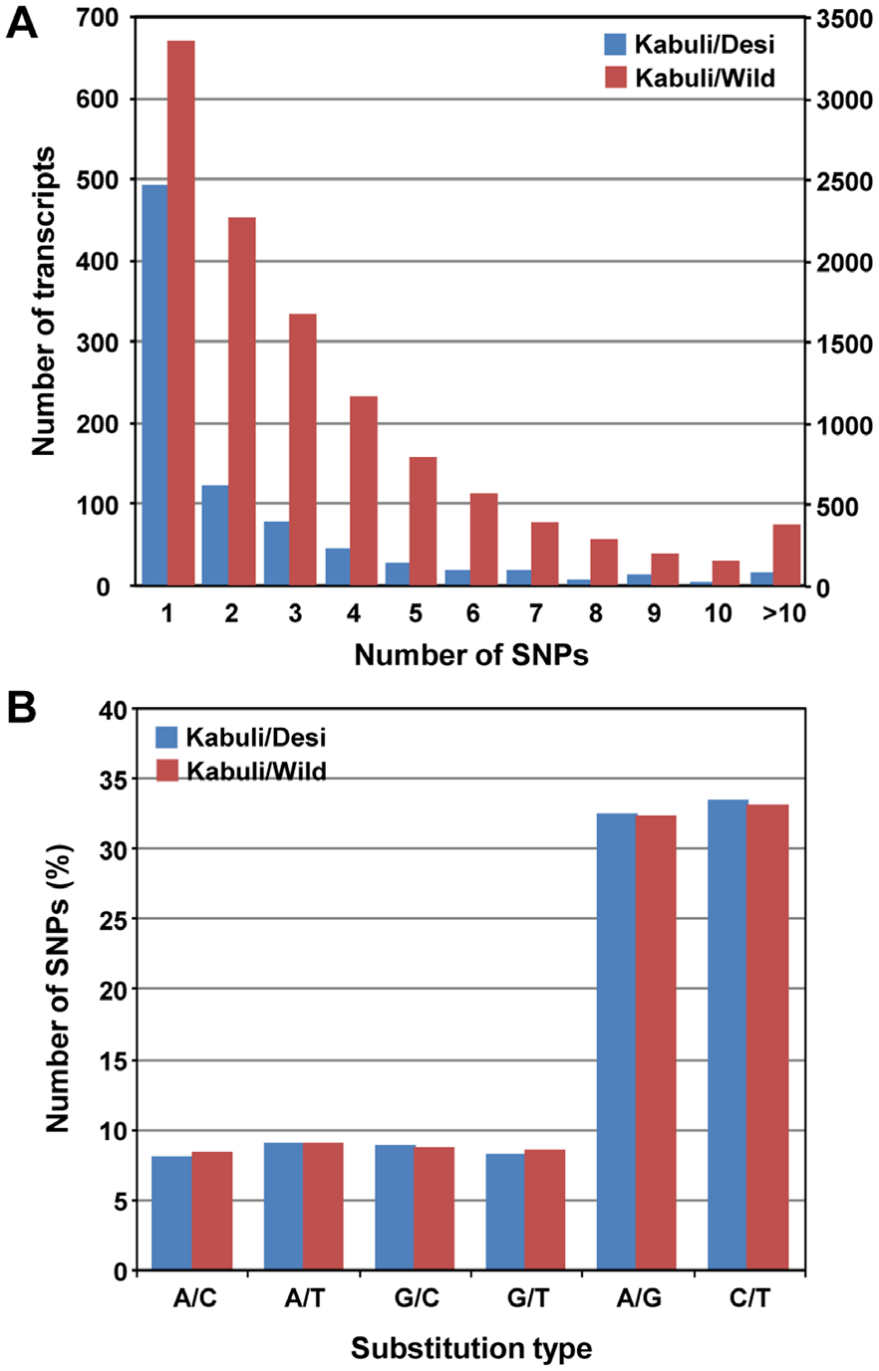
Frequency and substitution types of the identified SNPs. (A) The total number of transcripts (primary axis) and SNPs (secondary axis) having different SNP frequency is given. (B) The frequency of different substitution types is shown.

SNPs between kabuli/desi and kabuli/wild chickpea were identified using GigaBayes algorithm. To minimize the false detection, several stringent criteria, including the probability score cut-off of 0.95, SNP base Phred quality score cut-off of 30 and read-depth of at least three in the two chickpea types under analysis, consensus base ratio of 1 (number of reads supporting the SNP base to the total number of reads mapped), and removing SNPs located closely (three or more SNPs in any 10 bp window) and close to potential indels (within flanking region of 3 bp), were applied for SNP detection. This resulted in the identification of a total of 1986 and 37,954 SNPs in kabuli/desi and kabuli/wild chickpea, respectively, with a probability score of more than 0.99. The Phred quality score for more than 76% of SNP base was at least 35 (Figure S7). More than 70% of identified SNPs were supported by at least five reads in both chickpea genotypes being compared (Figure S8). These analyses indicated the high-quality of SNPs identified. The SNP density for kabuli/desi was one SNP per 23.27 kb and one SNP per 1.22 kb for kabuli/wild chickpea (Table 4). The number of SNPs detected ranged from one to 19 for kabuli/desi and one to 30 for kabuli/wild chickpea per transcript. At least 353 and 7795 transcripts harbored more than one SNP for kabuli/desi and kabuli/wild chickpeas (Fig. 4A). More than 10 SNPs were detected in 17 and 364 transcripts for kabuli/desi and kabuli/wild chickpea, respectively. The physical distribution analysis showed that SNPs were present throughout the length of transcripts (Figure S9). Further, we analyzed the type of base substitution at SNP position. The frequency of transitions (66%) was more (∼1.9 times) than the transversions (34%) for both kabuli/desi and kabuli/wild chickpeas (Fig. 4B).

**Table 4:** SNP frequency among different chickpea genotypes.

	Number of SNPs	Number of transcripts^1^	SNP density^2^	SNP frequency^3^
Kabuli/Desi	1986	846	23.27	0.043
Kabuli/Wild	37,954	11,125	1.22	0.821

1 Number of transcripts containing at least one SNP.

2 Length (in kb) of transcriptome containing one SNP.

3 Number of SNPs present per kb.

**Figure 5:**
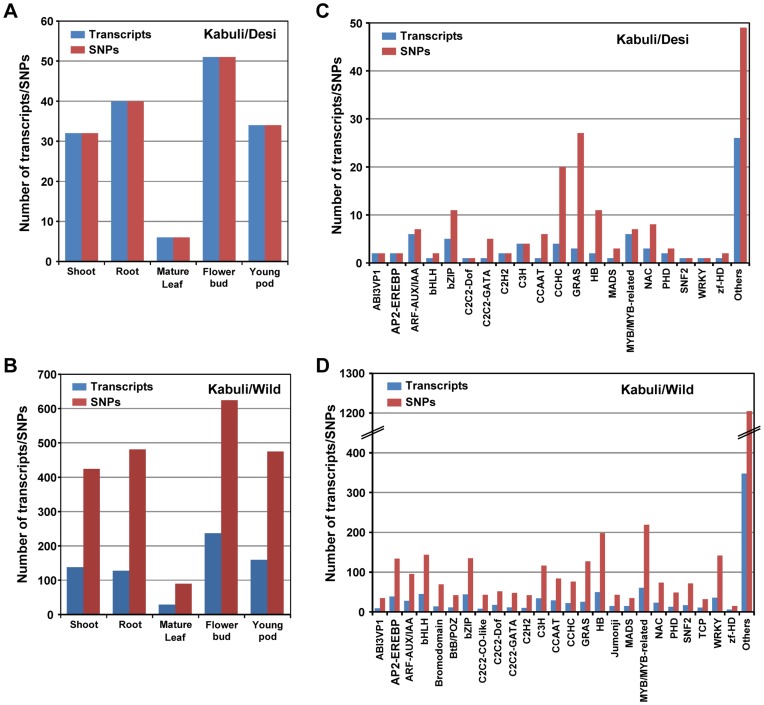
Distribution of SNPs in tissue-specific and transcription factor encoding transcripts. The number of tissue-specific (A,B) and transcription factor encoding (C,D) transcripts containing at least one SNP and total number of SNPs present in them are represented for kabuli/desi (A,C) and kabuli/wild (B,D).

We analyzed the distribution of SNPs in the tissue-specific transcripts and the transcripts encoding transcription factors. At least 163 (present in 163 transcripts) and 2094 (present in 691 transcripts) SNPs in kabuli/desi and kabuli/wild, respectively, were present in tissue-specific transcripts (Fig. 5A,B). Largest number of SNPs was detected in the transcripts expressed in flower bud followed by root and pod. A total of 174 (present in 75 transcripts) and 3333 (present in 945 transcripts) SNPs in kabuli/desi and kabuli/wild, respectively, were present in transcription factor encoding transcripts (Fig. 5C,D). Highest number of SNPs for kabuli/desi was detected in the transcripts encoding for GRAS and CCHC family transcription factors, whereas number of SNPs detected for kabuli/wild was highest in MYB/MYB-related and homeobox families. The complete information about the SNPs identified in kabuli/desi and kabuli/wild chickpeas are given in Tables S10 and S11, respectively.

### Large Scale Validation and Polymorphic Potential of SSRs

**Figure 6:**
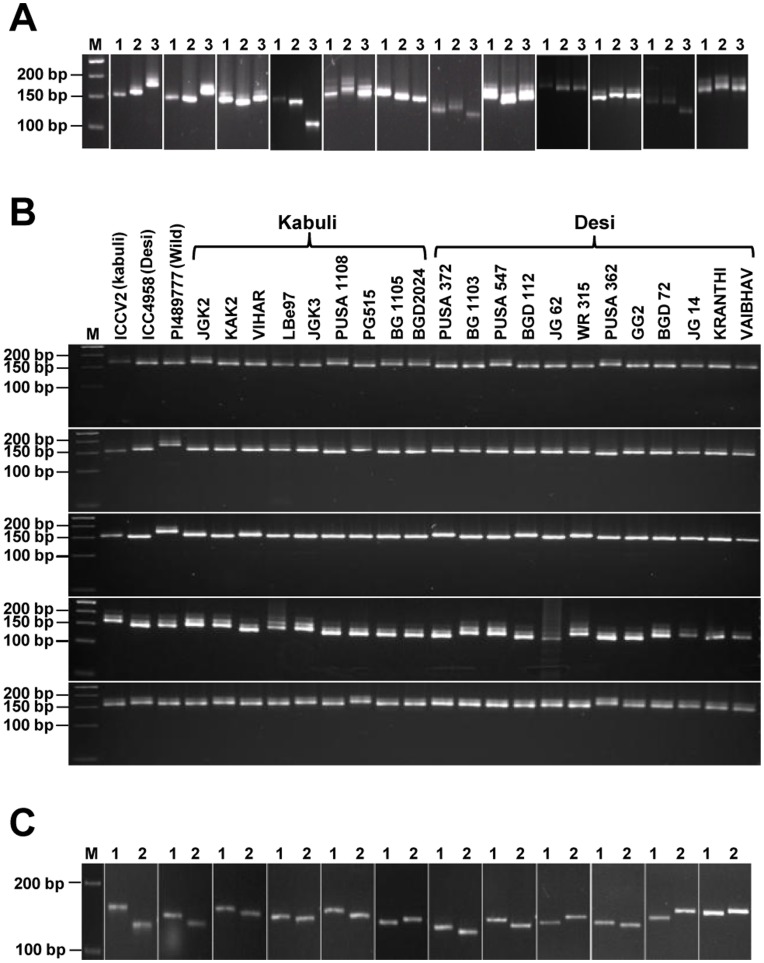
Experimental validation of *in silico* identified polymorphic SSRs and SNPs. (A) Experimental validation of polymorphic SSRs. Representative gels showing PCR amplification of polymorphic SSRs validating the length polymorphism between three chickpea genotypes, kabuli (1), desi (2) and wild (3) as expected. The PCR amplicons were resolved in 3.5% MetaPhor agarose gel. (B) Large-scale validation and polymorphism potential of selected SSRs in kabuli and desi genotypes. Representative gels showing PCR amplification of selected SSRs validating the length polymorphism in various chickpea genotypes (as indicated on the top of each lane). The PCR amplicons were resolved in 3.5% MetaPhor agarose gel. (C) Experimental validation of polymorphic SNPs by allele-specific PCR genotyping assay. Representative gels showing allele-specific fragment length variations of SNPs validating the polymorphism between kabuli (1) and desi (2) as expected. The PCR amplicons were resolved in 2.5% MetaPhor agarose gel. M, 50 bp DNA ladder as size standard.

Primers for polymorphic SSRs in kabuli/desi were designed and 40 primer pairs were selected randomly to assess their potential to amplify the target sequences and detect polymorphism among the three chickpea types (Table S12). All the primer pairs analyzed gave amplification in all the three chickpea genotypes. Among these, 25 primer pairs exhibited polymorphism in kabuli/desi and 29 pairs showed polymorphism in kabuli/wild even in low resolution agarose gel, as expected from the *in silico* analysis above. A representative gel showing the PCR amplification and polymorphism of selected SSRs is given in Figure 6A. Among the SSRs which gave clear polymorphism between kabuli and desi in metaphor agarose gel, 14 SSRs were selected for large-scale validation and genotyping in 21 additional desi and kabuli genotypes. Remarkably, all the 14 SSRs showed polymorphism and potential to differentiate at least two genotypes of desi and kabuli even in low resolution agarose gel (Table S12, Fig. 6B). The number of alleles amplified by each SSR varied from two to three and a total of 29 alleles were amplified by 14 SSRs among 21 desi and kabuli genotypes with an average of 2.1 alleles per SSR locus.

### Validation of SNPs using Allele-specific PCR Genotyping Assay

Twenty-two SNPs between kabuli and desi identified in transcription factor encoding transcripts (Table S13) were validated through allele-specific PCR genotyping assay using three primer combinations, in which allele-specific primer was used to discriminate the single nucleotide substitution. Seventeen (77.3%) of the 22 SNP loci discovered *in silico* could be validated successfully based on fragment length polymorphism (Table S13, Fig. 6C) and confirmed the presence of target SNP between kabuli and desi transcripts.

### Divergence of Kabuli Chickpea with Desi and Wild Chickpea

**Figure 7:**
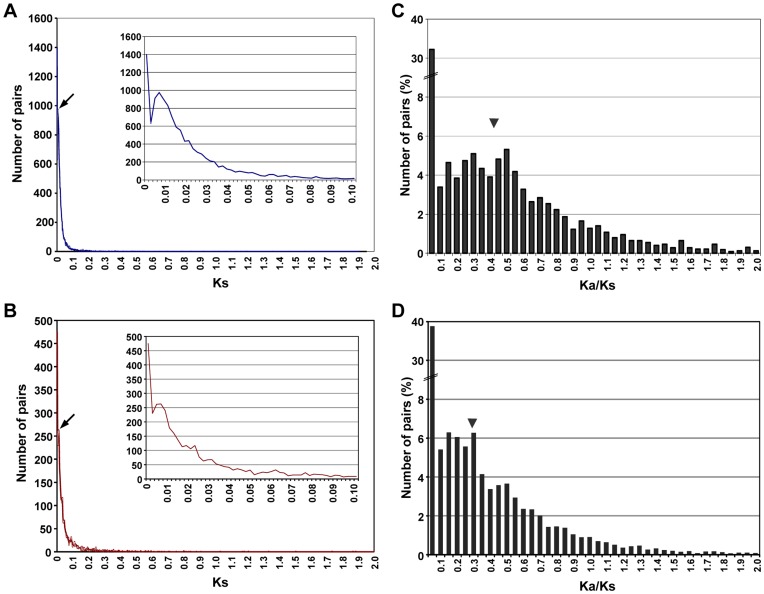
Distribution of synonymous substitution rate (Ks) value and Ka/Ks ratio among the orthologous transcript pairs between different chickpea types. (A,B) Distribution of Ks value among the orthologous transcript pairs between kabuli and desi (A), and kabuli and wild (B). The secondary peak (marked by arrow) in Ks distribution of orthologs indicates the speciation event. Inset represents enlarged version of the graph showing secondary peak. (C,D) Frequency distribution of Ka/Ks ratio in orthologous pairs between kabuli and desi (C) and kabuli and wild (D). The average value of Ka/Ks ratio is shown by arrow head.

To estimate the divergence time of kabuli chickpea with desi and wild chickpea, orthologous transcript pairs were identified. A total of 18,825 and 18,752 orthologous transcript pairs could be identified for kabuli/desi and kabuli/wild chickpea, respectively. The distribution analysis of synonymous (Ks) substitution rate of orthologous pairs showed a peak value at 0.008 for kabuli/wild chickpea (Fig. 7A), which corresponds to divergence time of about 0.53 million years ago between them. However, the peak was little diffused between Ks value of 0.006–0.008 for kabuli/desi chickpea (Fig. 7B), corresponding to divergence time of about 0.20–0.26 million years ago. A large fraction of orthologous transcript pairs between both cultivated (kabuli and desi) chickpea showed Ks value of zero, indicating non-detectable divergence.

We further investigated the selection pressure on the orthologous transcript pairs by calculating their ratio of non-synonymous (Ka) and Ks substitution rates. For this analysis, only the transcript pairs (12,254 for kabuli/wild and 3765 for kabuli/desi) having Ks value greater than zero and ≤2 were analyzed. The average Ka/Ks value for orthologous pairs was 0.41 and 0.28 for kabuli/desi and kabuli/wild chickpea, respectively (Fig. 7C,D), indicating that majority of them are under purifying selection. Only a small fraction (550 for kabuli/wild and 321 for kabuli/desi) of these transcript pairs were predicted to be under diversifying selection (Ka/Ks >1) (Fig. 7C,D). The GO analysis revealed that the terms ATP binding and protein binding were most abundant in the transcripts under diversifying selection for both kabuli/desi and kabuli/wild chickpea.

## Discussion

Kabuli chickpea characterized by large beige colored seeds is highly nutritious and has high commercial value. Depending on seed size, the market price of kabuli chickpea is upto three times higher than that of desi chickpea. Kabuli chickpea is mainly grown in South Asia, whereas desi type is grown in Mediterranean region. The nutritional assessment of both types of chickpea for ruminants showed higher nutritive value of kabuli-type than desi-type [20]. Based on chemical analysis and nutrient digestibility, kabuli chickpea were found to be better source of protein and energy for use in swine rations than desi chickpea [21]. The kabuli chickpea ICCV2 genotype is a widely cultivated, small-seeded cultivar with 100-seed weight of 18-19g. This genotype has several desirable traits, such as high yield, resistance to *Fusarium* wilt and extra-early (∼85 days) maturity [22], [23]. In addition, this genotype has been used in crosses with several other genotypes to generate mapping population for construction of genetic maps and development of improved varieties. These characteristics make the genotype ICCV2, used in this study, an appropriate choice for the generation of genomic resources for kabuli chickpea.

Here, we sequenced the transcriptome of kabuli chickpea at high depth using high-throughput Roche 454 and Illumina sequencing technologies. The assembly optimization of both datasets followed by their hybrid assembly resulted in 43,389 transcripts in total. Our results clearly showed that using both long-read and short-read sequencing technologies and a combination of different assembly strategies (*de novo*, reference-based and combined) give better assembly results. The average transcript length and total transcriptome size of kabuli chickpea transcripts was more than that of desi and wild chickpea transcripts reported earlier [5], [6], which may be due to more sequencing data used for the assembly. More than 6% of the chickpea genome sequence was represented in the transcriptome generated in this study. The functional annotation indicated that a wide range of functional diversity (molecular function, biological process and cellular component categories) was represented in the kabuli chickpea transcripts. The genes/gene families involved in various pathways and those encoding transcription factors were well represented in the transcriptome.

The divergence time between kabuli and wild estimated in this study (about 0.53 million years) was same as that for desi and wild reported earlier [6]. The divergence time between kabuli and desi was predicted to be about 0.20–0.26 million years ago. Considering the higher similarity of transcripts in kabuli/desi as compared to kabuli/wild and desi/wild, very less genetic diversity in kabuli/desi as compared to kabuli/wild and desi/wild and estimated divergence time, it appears that the cultivated chickpea diverged from wild chickpea and the two cultivated chickpea types (kabuli and desi) diverged from each other soon after that. However, the possibility of divergence of both kabuli and desi chickpea types directly from the wild chickpea can also not be ruled out. The exact picture will be clear once the genome/transcriptome sequence data from several accessions of cultivated and wild chickpea will become available. The average Ka/Ks value for the orthologous pairs in kabuli/desi and kabuli/wild was comparable to other such studies [6], [24], [25]. Most of the transcript pairs were found to be under purifying selection. A large number of transcripts putatively predicted to be involved in signaling pathways and regulation of transcription were under purifying selection as they are supposed to be conserved [26].

Until recently, the limited availability of molecular markers and their low polymorphism in different accessions has been a serious constraint for the construction of dense genetic linkage maps and tagging of important traits in chickpea. The availability of inexpensive high-throughput sequencing technologies has provided a rapid method for gene discovery and detection of molecular markers at whole genome/transcriptome level in non-model organisms [27]. The potential of such technologies in marker discovery has already been well demonstrated in several studies [6], [28]–[30]. We identified 5409 SSRs in the kabuli chickpea transcriptome, of which primers could be designed for 2645 SSRs. A comparative analysis of SSR content in three chickpea types suggested the presence of highest repetitive content in kabuli chickpea and least in wild chickpea. These results might provide a clue towards genome complexity of these chickpea types. Based on the karyotype studies, the nuclear DNA content of *C. reticulatum* (wild) was found to be lower than *C. arietinum* (cultivated), but no significant difference in DNA content was found between desi and kabuli chickpea despite clear phenotypic differences in them [3], [31]. We found the highest frequency of tri-nucleotide SSRs in chickpea transcripts consistent with other studies, which might be due to the selection against frameshift mutations to limit the expansion of other SSR types [6], [32]–[34]. *In silico* analysis identified lesser number of polymorphic SSRs in kabuli/desi (130) as compared to kabuli/wild (493), which is expected because both kabuli and desi represent same chickpea species (*C. arietinum*), whereas wild represents a different chickpea species (*C. reticulatum*). SSRs have been found to be abundant in regulatory proteins in other organisms and referred as evolutionary knobs, which fine-tune the transcription factor function [35], [36]. We also found the presence of many of SSRs in regulatory protein encoding transcripts in chickpea. In addition, a few SSRs were present in tissue-specific transcripts also. These SSR markers associated with agronomic traits would facilitate marker assisted breeding for chickpea improvement.

The experimental validation of a set of polymorphic SSRs showed very high amplification success rate and polymorphism in kabuli, desi and wild chickpea. A selected set of validated SSRs exhibited 100% polymorphic potential among 21 desi and kabuli genotypes even in low resolution agarose gel. This estimation of intra-specific polymorphic potential between desi and kabuli chickpea genotypes is much higher than that documented earlier using the genic (25–30%) and genomic (40–65%) microsatellite markers [13], [18], [37]–[39]. It thus suggests the utility of kabuli transcript sequences for developing polymorphic genic microsatellite markers and their large-scale genotyping applications specifically in desi and kabuli chickpea. The genic microsatellite markers being derived from the conserved expressed component of the genome usually revealed low intra-specific polymorphism among desi and kabuli genotypes, which limited their use in genetic association mapping and construction of high-resolution intra-specific genetic linkage (transcript) map in chickpea. However, the genic microsatellite markers developed in the present study from the kabuli chickpea transcript sequences with relatively higher intra-specific polymorphic potential would be of immense use for many applications in chickpea genetics, genomics and breeding and thus would expedite genetic/association mapping for identification of useful genes/QTLs controlling traits of agricultural importance in chickpea.

The knowledge of genetic diversity is critical for any crop improvement programs. SNP discovery among different varieties of chickpea is very important to provide tools for breeding programs and genetic mapping of quantitative trait loci for its improvement. Similar to polymorphic SSRs, very less number of SNPs was identified in kabuli/desi (1986) as compared to kabuli/wild (37,954). All of these identified SNPs were monomorphic within, but polymorphic between the two chickpea types. The SNP frequency was very less in kabuli/desi (0.043 SNP per kb) as compared to kabuli/wild (0.821 SNP per kb). The SNP frequency in kabuli/desi was much less than that of desi/wild (1.034 SNP per kb) also, as predicted earlier [6]. The SNP frequency detected in chickpea is much less than SNP frequency reported in cereal crops (4.2 SNPs per kb in rice to 16.5 SNPs per kb in wheat) [40], but similar to other legume soybean (∼1 SNP per kb) [41]. Our results confirmed the earlier reports of narrow genetic diversity in chickpea [1], [2]. The greater number of transitions as compared to transversions is consistent with previous reports [6], [42], [43]. Hyper-mutability of CpG islands and more synonymous mutations resulting due to transitions are the primary reasons attributed to higher proportion of transitions than transversions [44]–[46]. A significant number of these SNPs were detected in tissue-specific and transcription factor encoding transcripts. The SNPs detected in tissue-specific transcripts may be associated with the phenotypic variability among the different chickpea types and the SNPs detected in transcription factor encoding transcripts might play some regulatory role. The presence of SNPs in transcription factor encoding genes has also been associated with traits of agronomic importance such as stress tolerance, flowering time and seed development [47]–[49]. The identification of such SNPs is advantageous to prioritize them for efficient genotyping for important agronomic traits such as root, flower and pod development especially for a crop plant like chickpea, where SNP diversity is very less. The validation of 77.3% SNP loci discovered between kabuli and desi using allele-specific PCR genotyping assay reflected the reliability of developed SNP genetic marker resource in this study for their large-scale validation and genotyping in desi and kabuli chickpea. The efficacy of allele-specific PCR genotyping assay for large-scale validation and genotyping of genic SNP loci have been demonstrated successfully earlier in many crop species [50], [51]. Overall, the SNPs identified here represent an important resource to evaluate polymorphisms within and between wild and cultivated chickpea germplasm. With the discovery of large number of genetic markers, it will now be possible to conduct extensive diversity analysis in chickpea to identify the diverse germplasm with good agronomic traits for use in crop improvement. Further, the SNPs identified in this study, once validated and genotyped using a larger set of germplasm, could be utilized for large-scale trait association mapping and construction of high-density intra-specific transcript map to accelerate marker-assisted breeding in chickpea.

In this study, we reported the transcriptome of kabuli chickpea, which has high nutritional and market value. We presented an assembly strategy with a combination of reference-based, *de novo* and hybrid approaches for the transcriptome characterization, which can be used for other plant species too. Additionally, we demonstrated that comparison of transcriptomes from multiple genotypes/species provides an efficient method for detection of functional markers, which can be used in crop improvement programmes. A very high intra-specific polymorphic potential of SSRs identified in this study would be very useful in many applications and is expected to expedite genetic/association mapping for identification of useful genes/QTLs in chickpea. We anticipate that data and results presented here will further strengthen the molecular and genetic analysis research in chickpea.

## Materials and Methods

### Plant Material and RNA Isolation

Kabuli chickpea (*C. arietinum* L. genotype ICCV2) seeds were grown as described [52]. Root and shoot tissue samples were collected from 15-day-old seedlings. The plants were grown in field for collection of mature leaves and flower buds. At least three biological replicates of each tissue sample were harvested. Total RNA was extracted using TRI Reagent (Sigma Life Science, USA). The quality and quantity of RNA samples was assessed using NanoDrop spectrophotometer (NanoDrop Technologies) and Agilent 2100 Bioanalyzer (Agilent Technologies, Singapore) as described [52].

### Sequencing and Quality Control

Total RNA from the three biological replicates of root, shoot, mature leaf and flower bud were pooled in equal quantities for sequencing. For Roche 454 sequencing, mRNA purification, removal of rRNA contamination and double-stranded cDNA synthesis was performed as described previously [5]. The cDNA library preparation and sequencing was performed using Roche GS FLX Titanium series reagents essentially following the manufacturer’s instructions (Roche Diagnostics GmbH, Mannheim, Germany) as described previously [5]. The library preparation and sequencing (paired-end and single-end) on Illumina GA IIx platform was performed using Illumina protocols from pooled total RNA by commercial sequencing service provider (Genotypic Technologies Pvt. Ltd., Bangalore, India).

The fasta format files of Roche 454 data were generated from Standard Flowgram Format (SFF) files with quality score of Q20. For Illumina data, Fastq format files were obtained. High-quality Roche 454 reads were obtained after filtering low-quality reads, trimming of reads containing primer/adaptor sequences, trimming of reads containing homopolymers of more than seven bases and removal of reads with length of <100 bp using NGS QC tool kit [53]. Likewise, high-quality Illumina reads were obtained after filtering low-quality reads and reads containing primer/adaptor sequences using NGS QC tool kit with default parameters.

### Transcriptome Assembly and its Assessment

*De novo* assembly of Roche 454 data was performed using TGICL v2.0, Newbler v2.3 and CLC Genomics Workbench v4.7.2 softwares with parameters as described previously [5]. For reference assembly of Roche 454 data, Newbler and CLC Genomics Workbench were used with default parameters. The non-redundant (non-clonal) Illumina reads were filtered using CD-HIT program (http://weizhong-lab.ucsd.edu/cd-hit/). *De novo* assembly of Illumina data was performed using Velvet v1.1.05, Oases v0.2.01, ABySS v1.1.2, SOAPdenovo v1.04 and CLC Genomics Workbench softwares at different k-mer lengths. The assembly statistics was generated using N50 perl script of NGS QC toolkit. The mapping of reads was performed using CLC Genomics Workbench software using the criterion of minimum of 90% coverage of the total length for Roche 454 reads and allowing two mismatches for Illumina reads. The soybean proteome sequence downloaded from Phytozome database was used as reference for assembly assessment. The percentage of total length of soybean proteins aligned with the transcripts in BLASTX search (*E* value ≤1e−5) was calculated to determine the coverage of reference proteins.

### Functional Annotation

The assembled transcript sequences were used for BLAST searches against various databases (TAIR10, UniRef90, UniRef100, NR, PFAM, SMART, KEGG and COG) to assign putative functions to them using AutoFact pipeline. GOSlim terms associated with the Arabidopsis protein were assigned to the corresponding chickpea transcript showing the best hit in BLASTX search. The transcripts encoding for various transcription factors families were identified using hidden Markov model profile of the conserved domains as described earlier [5].

### Identification of SSRs and SNPs

SSR motifs were identified using MISA (MIcroSAtellite; http://pgrc.ipk-gatersleben.de/misa/) tool as described [6]. The polymorphic SSRs in kabuli/desi and kabuli/wild chickpea were detected using custom perl script as described [6]. SNP detection was performed using GigaBayes (http://bioinformatics.bc.edu/marthlab/Software_Release) with default parameters as described previously [6]. Various optimized parameters, including probability score assigned by GigaBayes, average quality score of the base representing the position of SNP, read depth (number of reads corresponding to the SNP) and consensus base ratio, were used to identify the final set of SNPs. In addition, three or more SNPs located in any 10 bp window and the SNPs within the 3 bp flanking region around a potential indel were also discarded.

### Primer Design and Validation of Polymorphic SSRs

Primers were designed from the flanking transcript sequences of all the SSR motifs using BatchPrimer3 software (http://probes.pw.usda.gov/batchprimer3/) [54]. Only the SSRs having a minimum of 100 bp flanking sequence on both sides were considered for primer designing. To validate the polymorphic SSRs, primers pairs were synthesized for the selected 40 SSRs (Sigma-Aldrich, Bangalore, India). Genomic DNA was isolated from leaf tissue of various chickpea genotypes using CTAB method. PCR amplification of the 40 SSRs was carried out from genomic DNA of the three genotypes, kabuli (ICCV2), desi (ICC4958) and wild (PI489777), and 14 selected informative SSRs in 21 additional genotypes (nine kabuli and 12 desi genotypes) using touch down thermal cycling conditions as described previously [6]. The fragment length polymorphism between the chickpea genotypes was analyzed by running the PCR products on 3.5% MetaPhor agarose gel.

### Validation of SNPs

For experimental validation, a set of 22 SNPs between ICCV2 and ICC4958 present in the transcription factor encoding transcripts were selected and genotyped using the allele-specific PCR assay. The transcript sequences from either side of the flanking SNP loci were curated and three PCR primers comprised of two allele flanking and one allele-specific primer(s) targeting each SNP loci were designed. These primers were used for PCR amplification in ICCV2 and ICC4958 genotypes using the standard PCR constituents and optimized touchdown thermal cycling parameters. The amplified allele-specific PCR products were resolved in 2.5% agarose gel and their band-sizing was determined against 100 bp DNA ladder as size standard. The fragment length polymorphism was inferred based on allele-specific PCR genotyping assay.

### Estimation of Divergence Time

Putative orthologous transcripts between kabuli and desi, and kabuli and wild chickpea were identified using reciprocal blast searches with a similarity of *E*-value ≤1e−20 and alignment length of ≥300 bp as described [6], [55]. The sequences of orthologous pairs were aligned as described previously and Ks and Ka values for each pair were calculated using CODEML program of the PAML 4.4e package [56]. For the calculation of divergence time, the synonymous substitution for dicots (1.5×10^−8^ substitution/synonymous site/year) was considered as described [57].

## Supporting Information

Figure S1 **Length (A) and average quality score (B) distribution of total number of unfiltered and filtered high-quality Roche 454 reads generated for kabuli chickpea.**
(PDF)

Figure S2 **Average quality score at each base position of total number of unfiltered and filtered high-quality Illumina reads generated for kabuli chickpea.**
(PDF)

Figure S3 **Length distribution of transcripts generated from optimized assembly for kabuli chickpea.**
(PDF)

Figure S4 **GC content distribution in the transcripts from different chickpea genotypes.** The average GC content of each transcript was calculated and percentage of transcripts with GC content within a range are represented.
(PDF)

Figure S5 **Percentage distribution of transcription factor encoding transcripts representing different families.**
(PDF)

Figure S6 **Distribution of polymorphic SSRs between kabuli/desi and kabuli/wild chickpea along the transcript length.**
(PDF)

Figure S7 **Phred quality score distribution of the SNP base in the SNPs identified in kabuli/desi and kabuli/wild chickpea.**
(PDF)

Figure S8 **Read depth distribution of the SNP base in the SNPs identified in kabuli/desi and kabuli/wild chickpea.**
(PDF)

Figure S9 **Distribution of SNPs in kabuli/desi and kabuli/wild chickpea along the transcript length.**
(PDF)

Table S1 **Quality filtering of sequencing data generated for transcriptome of kabuli-type chickpea.**
(PDF)

Table S2 **Assembly statistics/validation of Roche 454 data using different assembly programs.**
(PDF)

Table S3 **Assembly statistics of short-read Illumina data using different assembly programs at different** ***K*** **-mer values.**
(PDF)

Table S4 **Assembly validation of short-read Illumina data from different assembly programs.**
(PDF)

Table S5 **Statistics of SSRs identified in kabuli chickpea transcripts.**
(PDF)

Table S6 **Frequency of SSRs identified in kabuli chickpea transcripts.**
(PDF)

Table S7 **Primers designed for SSRs identified in the kabuli chickpea transcriptome.**
(PDF)

Table S8 **List of polymorphic SSRs identified between kabuli and desi chickpea.**
(PDF)

Table S9 **List of polymorphic SSRs identified between kabuli and wild chickpea.**
(PDF)

Table S10 **List of SNPs identified between kabuli and desi chickpea.**
(PDF)

Table S11 **List of SNPs identified between kabuli and wild chickpea.**
(PDF)

Table S12 **List of selected SSRs validated for polymorphism in chickpea genotypes.**
(PDF)

Table S13 **List of 22 selected SNPs between kabuli and desi validated using the allele-specific PCR.**
(PDF)
