# Supplementary material for: Comparative Analysis of Kabuli Chickpea Transcriptome with Desi and Wild Chickpea Provides a Rich Resource for Development of Functional Markers
Source: PLoS One. 2012 Dec 27;7(12):e52443. doi: 10.1371/journal.pone.0052443 (PMC3531472; doi:10.1371/journal.pone.0052443)

Figure S3. Length distribution of transcripts generated from optimized assembly for kabuli chickpea.

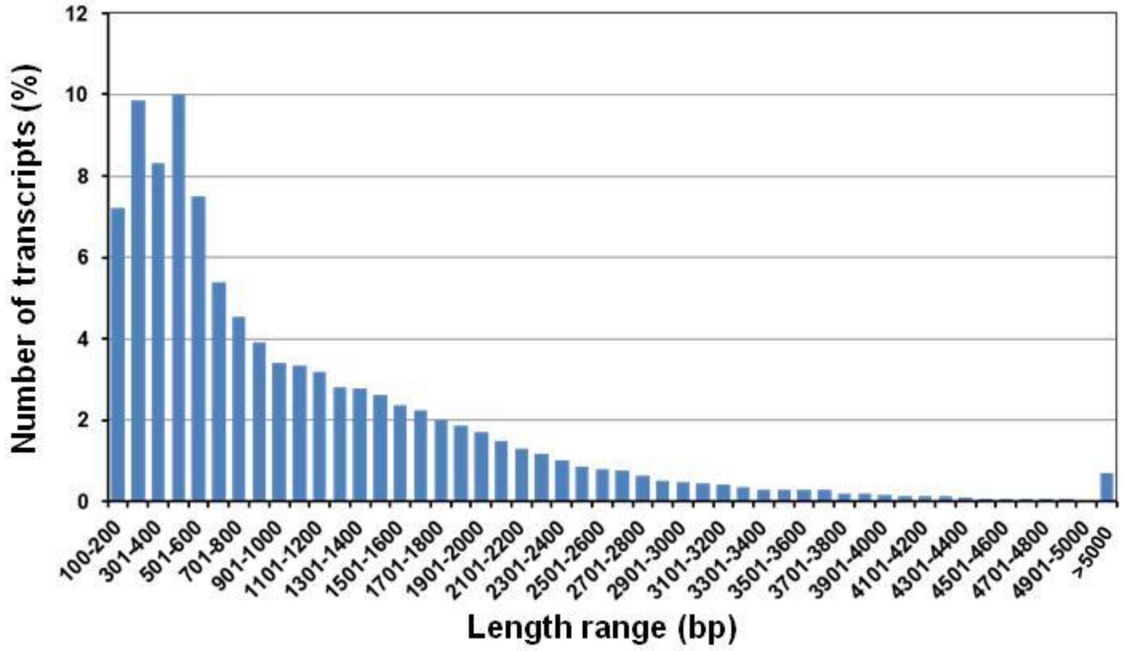

Supplement: Figure S3 — Length distribution of transcripts generated from optimized assembly for kabuli chickpea. (PDF) [file pone.0052443.s003.pdf]
