# Supplementary material for: Comparative Analysis of Kabuli Chickpea Transcriptome with Desi and Wild Chickpea Provides a Rich Resource for Development of Functional Markers
Source: PLoS One. 2012 Dec 27;7(12):e52443. doi: 10.1371/journal.pone.0052443 (PMC3531472; doi:10.1371/journal.pone.0052443)

Figure S4. GC content distribution in the transcripts from different chickpea genotypes. The average GC content of each transcript was calculated and percentage of transcripts with GC content within a range are represented.

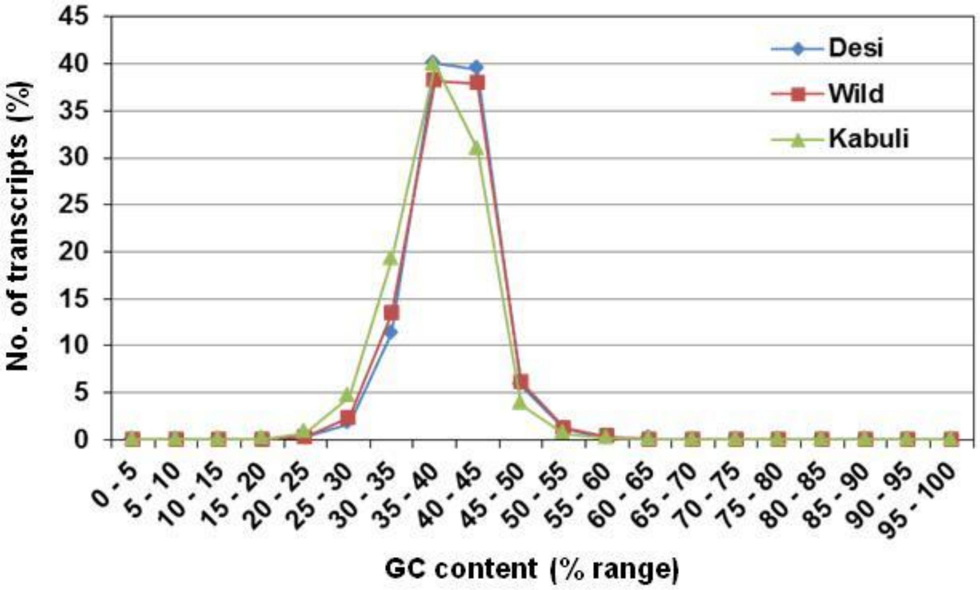

Supplement: Figure S4 — GC content distribution in the transcripts from different chickpea genotypes. The average GC content of each transcript was calculated and percentage of transcripts with GC content within a range are represented. (PDF) [file pone.0052443.s004.pdf]
