# Supplementary material for: Comparative Analysis of Kabuli Chickpea Transcriptome with Desi and Wild Chickpea Provides a Rich Resource for Development of Functional Markers
Source: PLoS One. 2012 Dec 27;7(12):e52443. doi: 10.1371/journal.pone.0052443 (PMC3531472; doi:10.1371/journal.pone.0052443)

Figure S7. Phred quality score distribution of the SNP base in the SNPs identified in kabuli/desi and kabuli/wild chickpea.

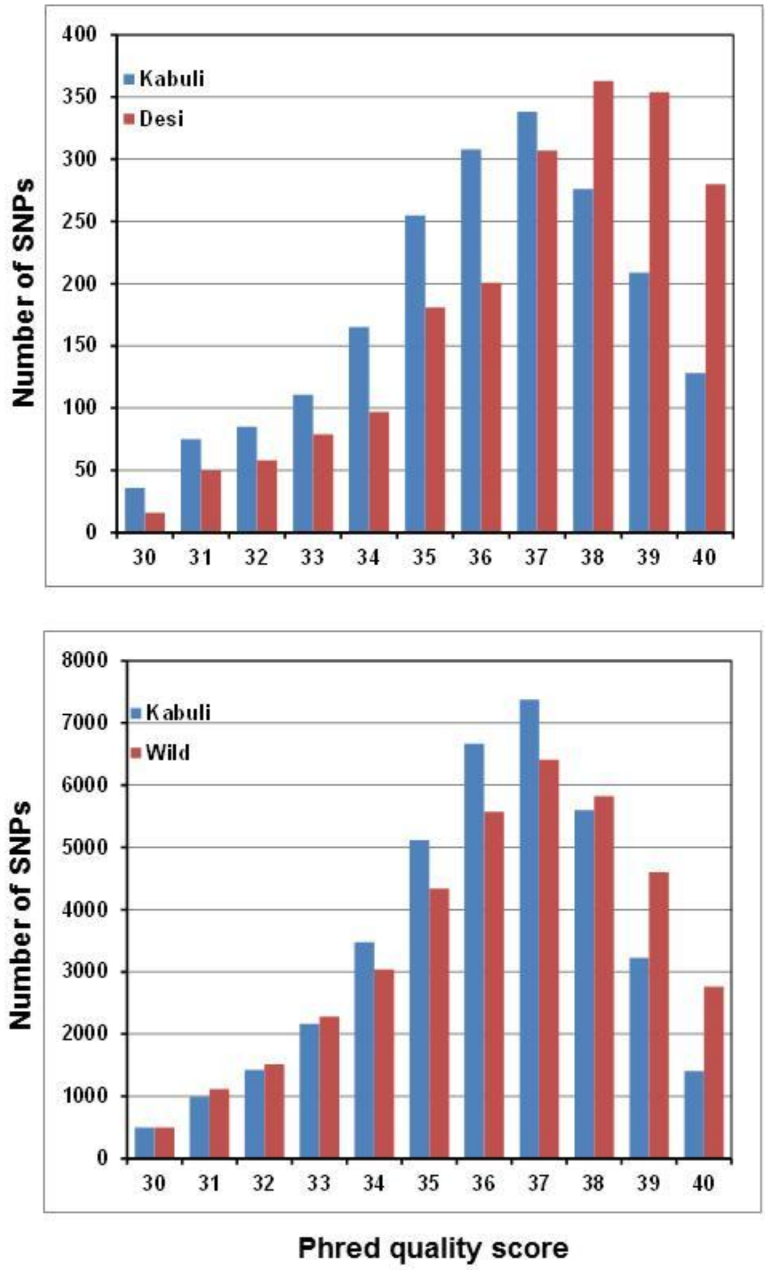

Supplement: Figure S7 — Phred quality score distribution of the SNP base in the SNPs identified in kabuli/desi and kabuli/wild chickpea. (PDF) [file pone.0052443.s007.pdf]
