# Supplementary material for: Comparative Analysis of Kabuli Chickpea Transcriptome with Desi and Wild Chickpea Provides a Rich Resource for Development of Functional Markers
Source: PLoS One. 2012 Dec 27;7(12):e52443. doi: 10.1371/journal.pone.0052443 (PMC3531472; doi:10.1371/journal.pone.0052443)

Figure S9. Distribution of SNPs in kabuli/desi and kabuli/wild chickpea along the transcript length.

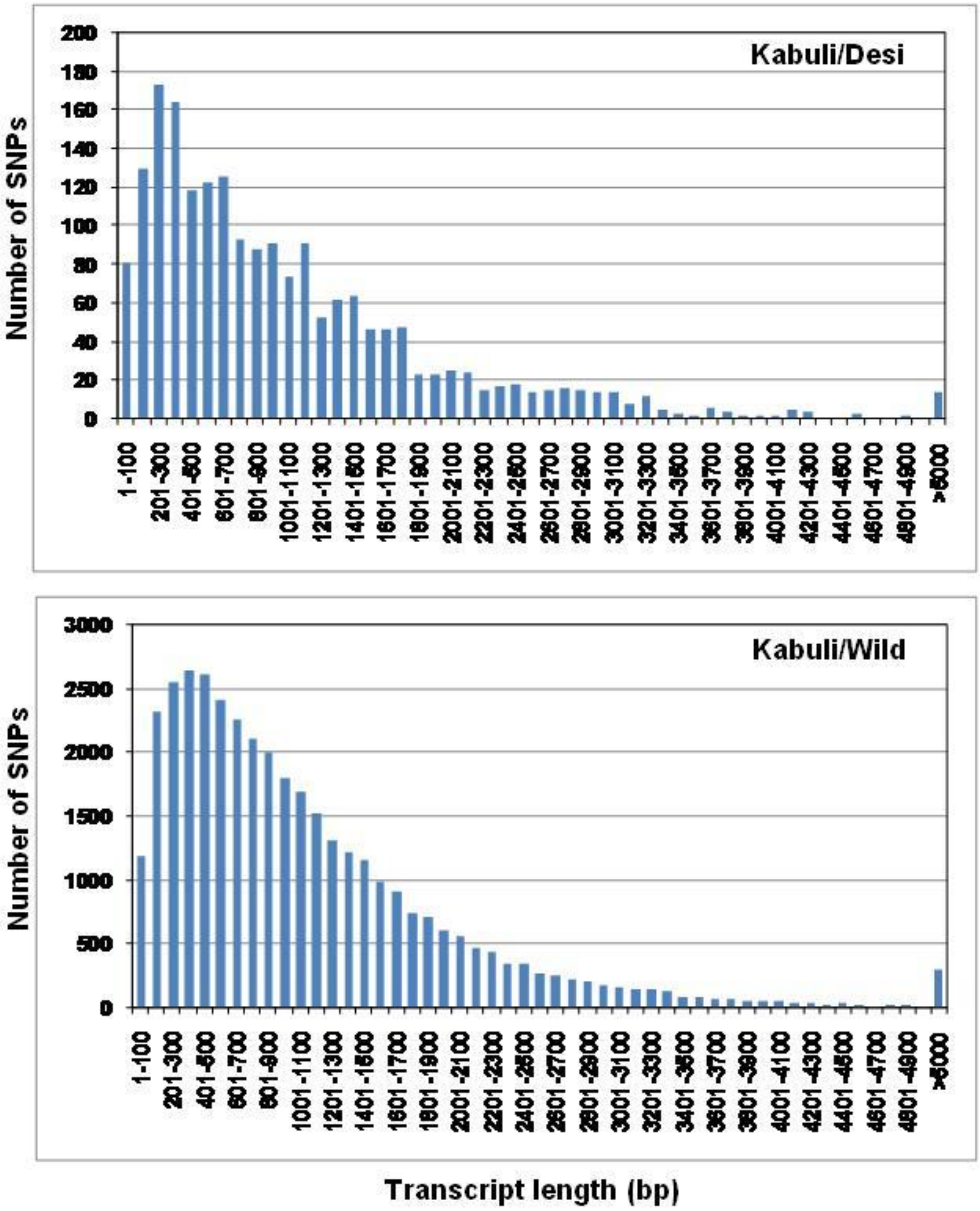

Supplement: Figure S9 — Distribution of SNPs in kabuli/desi and kabuli/wild chickpea along the transcript length. (PDF) [file pone.0052443.s009.pdf]
