# Supplementary material for: Comparative Analysis of Kabuli Chickpea Transcriptome with Desi and Wild Chickpea Provides a Rich Resource for Development of Functional Markers
Source: PLoS One. 2012 Dec 27;7(12):e52443. doi: 10.1371/journal.pone.0052443 (PMC3531472; doi:10.1371/journal.pone.0052443)
Supplement: Table S1 — Quality filtering of sequencing data generated for transcriptome of kabuli-type chickpea. (PDF) [file pone.0052443.s011.pdf]

Table S1. Quality filtering of sequencing data generated for transcriptome of kabuli-type chickpea.

Illumina data

Library/Tissue type	Total no. of reads	Fastq file size (GB)	No. of reads after filtering low quality reads[1]	No. of reads after removing primer/adapter containing reads[2]
Mixed (101 bp single-end)	44107639	11.68	37584774	37502174
Mixed (100 bp paired-end)	76970476	20.38	70927890	70927626
Total	121078115	32.06	108512664	108429800

[1]Number of read after removing low-quality reads with Phred quality score <20 in more than 30% of bases
[2]Number of reads after removing reads containing primer/adapter sequence

Roche 454 data

Library/Tissue type	Total reads	Low quality reads[1]	Trimmed reads[2]	Trashed reads[3]	High quality mRNA reads[4]	Average length[5]
Mixed	1775115	25975	56229	105304	1643836	365.3

[1]Phred quality score <20 in more than 30% of bases
[2]No. of trimmed reads containing primer/adapter sequence and more than 7-bp-long homopolymer starting from the first bp of the homopolymer
[3]No. of short reads less than 100 bp removed
[4]No. of high-quality reads corresponding to m-RNA sequences used for further analysis
[5]Average length of high-quality mRNA reads in bp
