# Supplementary material for: Comparative Analysis of Kabuli Chickpea Transcriptome with Desi and Wild Chickpea Provides a Rich Resource for Development of Functional Markers
Source: PLoS One. 2012 Dec 27;7(12):e52443. doi: 10.1371/journal.pone.0052443 (PMC3531472; doi:10.1371/journal.pone.0052443)
Supplement: Table S2 — Assembly statistics/validation of Roche 454 data using different assembly programs. (PDF) [file pone.0052443.s012.pdf]

Table S2. Assembly statistics/validation of Roche 454 data using different assembly programs for kabuli chickpea.

	De novo assembly			Reference assembly		Merged assembly	
	**Newbler**	**CLC**	**TGICL**	**Newbler**	**CLC**	**Newbler + unmapped reads**	**CLC + unmapped reads**
Total contigs (≥100 bp)	32088	38318	49300	32728	26353	39517	36408
Large contigs (≥1000 bp)	14706	12020	13318	10073	12410	10315	13492
Total size (Mb)	37.47	35.43	41.77	27.94	30.72	31.34	37.76
Maximum contig length (bp)	15488	13246	9091	15587	15601	15587	15601
Average contig length (bp)	1167.84	924.67	847.43	853.82	1165.77	793.05	1037
N50 contig length (bp)	1587	1237	1020	1399	1682	1273	1501
Total number of reads mapped (%)	1377154 (83.8)	1428531 (86.9)	1536791 (93.5)	1304966 (79.4)	1384000 (84.2)	1336398 (81.3)	1499259 (91.2)
Total number of uniquely mapped reads (%)	1044081 (63.5)	1415804 (86.1)	1439283 (87.6)	1299566 (79.1)	1275808 (77.6)	1331833 (81.0)	1406584 (85.6)
Contigs with significant hits (%)[1]	26795 (83.5)	28116 (73.4)	39403 (79.9)	25683 (78.5)	22505 (85.4)	27408 (69.4)	27406 (75.3)
Number of contigs with ≥80% coverage of soybean proteome (%)[2]	10534 (32.8)	7980 (20.8)	8404 (17.1)	9056 (27.7)	10188 (38.6)	9206 (23.3)	10354 (28.4)
Unique soybean proteins with significant hits (%)[3]	16468 (29.5)	19857 (35.6)	22316 (40.0)	18525 (33.2)	17142 (30.7)	19511 (35.0)	19544 (35.0)
Number of unique soybean proteins with ≥80% coverage (%)[4]	8487 (15.2)	7631 (13.7)	7736 (13.9)	8569 (15.4)	9275 (16.6)	8706 (15.6)	9545 (17.1)

[1] Contigs showing significant hit (*E*-value ≤1e-5) with soybean proteins
[2] Contigs showing ≥80% coverage of soybean proteins
[3] Unique soybean proteins to which contigs show significant hit (*E*-value ≤1e-5)
[4] Unique soybean proteins to which contigs show ≥80% coverage
