# Supplementary material for: Comparative Analysis of Kabuli Chickpea Transcriptome with Desi and Wild Chickpea Provides a Rich Resource for Development of Functional Markers
Source: PLoS One. 2012 Dec 27;7(12):e52443. doi: 10.1371/journal.pone.0052443 (PMC3531472; doi:10.1371/journal.pone.0052443)
Supplement: Table S3 — Assembly statistics of short-read Illumina data using different assembly programs at different K -mer values. (PDF) [file pone.0052443.s013.pdf]

Table S3. Assembly statistics of short-read Illumina data using different assembly programs at different K-mer values for kabuli chickpea.

(A) Assembly statistics using all the high-quality Illumina data (total input reads 108,429,800)

Assembly statistics using Velvet with different K-mer values

	K35	K45	K55	K65	K75	K77	K83	K85	K87	K89	K91	K93	K95
Total contigs (≥100 bp)	298342	256365	203948	123037	75552	68133	49436	44262	40040	36138	32666	28820	21535
Assembly size (Mb)	65.82	66.13	63.76	53.45	43.91	41.96	35.86	33.61	31.14	28.29	24.70	2013	13.35
Contigs (>1000 bp)	1931	3600	7685	10117	11177	11203	10845	10523	9857	8995	7542	5582	3036
Min. contig length (bp)	100	100	109	129	149	153	165	169	173	177	181	185	189
Max. contig length (bp)	4196	5714	9822	10849	8406	8056	11837	13691	13653	12156	8799	7497	5542
Average contig length (bp)	220.63	257.98	312.67	434.45	581.28	615.93	725.58	759.41	777.89	783.07	756.25	698.7	620.33
N50 contig length	237	289	404	585	833	893	1072	1115	1123	1106	1024	883	713

Assembly statistics using Oases with different K-mer values

	K31	K35	K37	K39	K41	K43	K45	K47	K55	K65
Total contigs (≥100 bp)	42970	42832	42680	42371	42317	42108	42151	42063	41247	39192
Assembly size (Mb)	47.76	48.04	48.16	48.04	47.95	47.75	47.65	47.40	45.78	42.82
Contigs (>1000 bp)	16949	17096	17170	17168	17137	17096	17055	17023	16481	15568
Min. contig length (bp)	100	100	101	100	100	100	100	100	100	100
Max. contig length (bp)	16914	17399	17400	16914	16914	16835	16914	16835	27972	15691
Average contig length (bp)	1111.49	1123.99	1128.49	1133.86	1138.02	1134.16	1130.55	1127.05	1109.97	1092.68
N50 contig length	2114	2102	2090	2075	2065	2050	2031	2006	1968	1891

Assembly statistics using SOAPdenovo with different K-mer values

	K69	K73	K75	K79	K83	K85	K87	K89	K91	K93	K95
Total contigs (≥100 bp)	134916	107009	94494	72210	52594	43963	36385	29776	24219	19338	13355
Assembly size (Mb)	57.84	52.55	49.88	44.39	38.68	35.69	32.56	29.08	24.85	19.69	12.17
Contigs (>1000 bp)	14758	14685	14538	13971	13145	12604	11843	10918	9493	7522	4437
Min. contig length (bp)	100	100	100	100	100	100	100	100	100	100	100
Max. contig length (bp)	15945	16206	16824	15262	15615	15274	13847	14106	16564	11730	8761
Average contig length (bp)	428.71	491.1	527.87	614.87	735.58	812.01	895.13	976.76	1026.1	1018.41	911.88
N50 contig length	920	1076	1157	1313	1462	1520	1596	1617	1603	1489	1275

Assembly statistics using ABySS with different K-mer values

	K69	K73	K75	K77	K79	K83	K85	K87	K93
Total contigs (≥100 bp)	54510	49698	47836	45915	43886	42449	39315	37055	31797
Assembly size (Mb)	56.29	53.11	51.62	50.15	48.31	45.60	42.39	39.37	28.12
Contigs (>1000 bp)	21771	20770	20248	19728	19042	17918	16688	15465	10544
Min. contig length (bp)	100	100	100	100	100	100	100	100	100
Max. contig length (bp)	15737	15737	15651	15651	15651	15651	15651	15580	15580
Average contig length (bp)	1032.68	1068.69	1079.12	1092.33	1100.85	1074.33	1078.32	1062.5	884.67
N50 contig length	1743	1752	1751	1759	1758	1729	1731	1715	1537

(B) Assembly statistics using non-redundant high-quality Illumina data (total input reads 32,646,926)

Assembly statistics using Velvet with different K-mer values

	K35	K45	K55	K65	K75	K77	K83	K85	K87	K89	K91	K93	K95
Total contigs (≥100 bp)	296657	255159	201425	121873	75167	68000	49352	44163	39890	36085	32687	28727	21365
Assembly size (Mb)	65.55	65.93	63.45	53.27	43.85	41.94	35.85	33.58	31.11	28.29	24.7	20.09	13.22
Contigs (>1000 bp)	1943	3558	7716	10155	11188	11199	10835	10528	9873	9010	7541	5553	2999
Min. contig length (bp)	100	100	109	129	149	153	165	169	173	177	181	185	189
Max. contig length (bp)	4718	8003	9822	10849	8098	8381	11837	13688	13673	12162	8799	7433	5542
Average contig length (bp)	220.98	258.39	315.01	437.13	583.39	616.83	726.52	760.43	780.14	784.24	755.94	699.52	618.79
N50 contig length	238	291	407	588	834	893	1071	1117	1124	1106	1023	883	708

Assembly statistics using Oases with different K-mer values

	K31	K35	K37	K39	K41	K43	K45	K47	K55	K65
Total contigs (≥100 bp)	37456	37693	37576	37326	37219	37235	37305	37431	36296	34835
Assembly size (Mb)	45.98	46.41	46.39	46.36	46.30	46.13	46.05	45.92	44.37	41.63
Contigs (>1000 bp)	16632	16854	16861	16886	16877	16891	16885	16901	16492	15588
Min. contig length (bp)	100	100	100	100	100	101	100	102	100	100
Max. contig length (bp)	16914	17584	17587	17587	16835	16835	16835	16835	15691	16403
Average contig length (bp)	1127.79	1231.53	1234.65	1242.17	1244.05	1238.98	1234.65	1226.88	1222.58	1195.12
N50 contig length	2153	2139	2127	2120	2108	2083	2058	2029	2006	1930

Assembly statistics using SOAPdenovo with different K-mer values

	K69	K73	K75	K79	K83	K85	K87	K89	K91	K93
Total contigs (≥100 bp)	149493	121607	108564	84583	63884	54810	47102	40317	34681	28822
Assembly size (Mb)	55.70	50.83	43.84	43.20	37.80	34.91	31.83	28.37	24.09	18.71
Contigs (>1000 bp)	11123	11334	11412	11309	10829	10425	9689	8721	6990	4747
Min. contig length (bp)	100	100	100	100	100	100	100	100	100	100
Max. contig length (bp)	8224	8077	8051	9534	11836	13687	13661	8799	8799	7370
Average contig length (bp)	372.59	418.03	445.32	510.81	591.76	637.06	675.94	703.84	694.87	649.21
N50 contig length	599	692	750	881	1004	1053	1068	1049	955	798

Assembly statistics using ABySS with different K-mer values

	K69	K73	K75	K77	K79	K83	K85	K87	K89	K93
Total contigs (≥100 bp)	93093	93028	92208	84667	77257	66082	61360	54087	93056	74558
Assembly size (Mb)	46.43	46.73	46.28	44.64	42.89	39.68	37.94	35.45	39.17	30.94
Contigs (>1000 bp)	12781	12739	12601	12554	12395	12087	11774	11408	10398	8226
Min. contig length (bp)	100	100	100	100	100	100	100	100	100	100
Max. contig length (bp)	15643	15643	15643	15643	15643	15643	15643	15549	13687	15582
Average contig length (bp)	498.82	502.35	501.96	527.35	555.24	600.51	618.43	655.58	421.01	415.02
N50 contig length	1022	1011	1010	1078	1135	1216	1242	1297	960	908
