# Supplementary material for: Comparative Analysis of Kabuli Chickpea Transcriptome with Desi and Wild Chickpea Provides a Rich Resource for Development of Functional Markers
Source: PLoS One. 2012 Dec 27;7(12):e52443. doi: 10.1371/journal.pone.0052443 (PMC3531472; doi:10.1371/journal.pone.0052443)
Supplement: Table S4 — Assembly validation of short-read Illumina data from different assembly programs. (PDF) [file pone.0052443.s014.pdf]

Table S4. Assembly validation of short-read Illumina data from different assembly programs for kabuli chickpea.

(A) Validation of assembly generated using all the high-quality Illumina data (total input reads 108,429,800)

	**Velvet K89**	**Oases K39**	**Abyss K79**	**SOAPdenovo K91**	**CLC**
Total contigs (≥100 bp)	36138	42371	43886	24219	86472
Large contigs (≥1000 bp)	8995	17168	19042	9493	19489
Total size (Mb)	28.29	48.04	48.31	24.85	64.64
Maximum contig length (bp)	12156	16914	15651	16564	15776
Average contig length (bp)	783.07	1133.86	1100.85	1026.1	747.58
N50 contig length (bp)	1106	2075	1758	1603	1293
Total number of reads mapped (%)	90574343 (83.5)	85359468 (78.7)	101800502 (93.9)	90953370 (83.9)	97198031 (89.6)
Total number of uniquely mapped reads (%)	90355336 (83.3)	85212654 (78.6)	69076400 (63.7)	90757537 (83.7)	96615321 (89.1)
Contigs with significant hits (%)[1]	30676 (84.9)	24304 (57.4)	36652 (83.5)	21550 (89.0)	33801 (39.1)
Number of contigs with ≥80% coverage of soybean proteome (%)[2]	8201 (22.7)	11187 (26.4)	15521 (35.4)	7563 (31.2)	15080 (17.4)
Unique soybean proteins with significant hits (%)[3]	19227 (34.5)	19054 (34.2)	19122 (34.3)	15816 (28.4)	22004 (39.4)
Number of unique soybean proteins with ≥80% coverage (%)[4]	7821 (14.0)	10561 (18.9)	12755 (22.9)	7225 (13.0)	13941 (25.0)

[1] Contigs showing significant hit (*E*-value ≤1e-5) with soybean proteins
[2] Contigs showing ≥80% coverage of soybean proteins
[3] Unique soybean proteins to which contigs show significant hit (*E*-value ≤1e-5)
[4] Unique soybean proteins to which contigs show ≥80% coverage

(B) Validation of assembly generated using non-redundant high-quality Illumina data (total input reads 32,646,926)

	Velvet K87	Oases K41	Abyss K87	SOAPdenovo K87	CLC
Total contigs (≥100 bp)	39890	37219	54087	47102	92139
Large contigs (≥1000 bp)	9873	16877	11408	9689	18341
Total size (Mb)	31.11	46.30	35.45	31.83	64.00
Maximum contig length (bp)	13673	16835	15549	13661	15776
Average contig length (bp)	780.14	1244.05	655.58	675.94	694.7
N50 contig length (bp)	1124	2108	1297	1068	1087
Total number of reads mapped (%)	25698591 (78.7)	26101627 (80.0)	28025738 (85.8)	26463504 (81.1)	28452150 (87.2)
Total number of uniquely mapped reads (%)	25602922 (78.4)	26078193 (79.9)	27775145 (85.1)	26406693 (80.9)	28366186 (86.9)
Contigs with significant hits (%)[1]	32843 (82.3)	23342 (62.7)	40325 (74.6)	38417 (81.6)	36966 (40.0)
Number of contigs with ≥80% coverage of soybean proteome (%)[2]	8800 (22.1)	11307 (30.4)	11283 (20.9)	8722 (18.5)	12942 (14.1)
Unique soybean proteins with significant hits (%)[3]	20105 (36.0)	18619 (33.4)	20269 (36.3)	20553 (36.8)	23081 (41.4)
Number of unique soybean proteins with ≥80% coverage (%)[4]	8403 (15.1)	10672 (19.1)	10691 (19.2)	8321 (14.9)	12051 (21.6)

[1] Contigs showing significant hit (*E*-value ≤1e-5) with soybean proteins
[2] Contigs showing ≥80% coverage of soybean proteins
[3] Unique soybean proteins to which contigs show significant hit (*E*-value ≤1e-5)
[4] Unique soybean proteins to which contigs show ≥80% coverage
