# Supplementary material for: Comparative Analysis of Kabuli Chickpea Transcriptome with Desi and Wild Chickpea Provides a Rich Resource for Development of Functional Markers
Source: PLoS One. 2012 Dec 27;7(12):e52443. doi: 10.1371/journal.pone.0052443 (PMC3531472; doi:10.1371/journal.pone.0052443)
Supplement: Table S5 — Statistics of SSRs identified in kabuli chickpea transcripts. (PDF) [file pone.0052443.s015.pdf]

Table S5. Statistics of SSRs identified in kabuli chickpea transcripts.

**SSR mining**	
Total number of sequences examined	43,389
Total size of examined sequences (bp)	46,212,215
Total number of identified SSRs	5,409
Number of SSR containing sequences	4,398 (10.14%)
Number of sequences containing more than one SSR	743
Number of SSRs present in compound formation	478
Frequency of SSRs	One per 8.54 kb
**Distribution of SSRs in different repeat types**	
Di-nucleotide	2,216 (40.97%)
Tri-nucleotide	2,920 (53.98%)
Tetra-nucleotide	132 (2.44%)
Penta-nucleotide	48 (0.89%)
Hexa-nucleotide	93 (1.72%)
