# Supplementary material for: Comparative Analysis of Kabuli Chickpea Transcriptome with Desi and Wild Chickpea Provides a Rich Resource for Development of Functional Markers
Source: PLoS One. 2012 Dec 27;7(12):e52443. doi: 10.1371/journal.pone.0052443 (PMC3531472; doi:10.1371/journal.pone.0052443)
Supplement: Table S6 — Frequency of SSRs identified in kabuli chickpea transcripts. (PDF) [file pone.0052443.s016.pdf]

Table S6. Frequency of SSRs identified in kabuli chickpea transcripts.

Repeats	5	6	7	8	9	10	11	12	13	14	15	16	17	18	19	20	21	22	23	24	25	26	27	28	29	30	31	32	33	34	35	36	37	38	39	40	41	42	43	44	45	46	47	48	49	50	51	52	53	54	55	56	57	58	59	60	61	62	63	total
AC/GT	-	111	54	34	19	13	5	7	5	1				1																																														250
AG/CT	-	417	287	220	137	127	87	61	40	31	27	20	14	20	16	7	8	3	1	2	5	3	1	3	1				1	1	1					1								1																1543
AT/AT	-	165	75	59	46	30	12	5	8	3	6	6	1	1	1	1	1			1	1																																							422
CG/CG	-	1																																																										1
AAC/GTT	278	126	56	18	3		1	1	1																											1																								485
AAG/CTT	396	243	94	55	31	11	9	6	4	2	3	3	3		1	2		1	1																																									865
AAT/ATT	232	124	60	32	16	7	10	5	2	2	2	3	3	2	2			1					1		1				1		1		1																											508
ACC/GGT	156	66	21	11	2	3																																																						259
ACG/CTG	43	12	2		1																																																							58
ACT/ATG	143	69	26	8	4	3	4				1													1																																			1	260
AGC/CGT	57	12	7																																																									76
AGG/CCT	93	33	9	3	4	1																																																						143
AGT/ATC	147	52	18	13	6		1																																																					237
CCG/CGG	23	3		3																																																								29
AAAC/GTTT	6	3																																																										9
AAAG/CTTT	23	6	1	3				1																																																				34
AAAT/ATTT	27	5	3	2		1				1																																																		39
AACT/ATTG	5	1																																																										6
AAGG/CCTT			1	2																																																								3
AAGT/ATTC	2	1																																																										3
AATC/AGTT		1																																																										1
AATG/ACTT	6																																																											6
AATT/AATT	5	3		1																																																								9
ACAT/ATGT	2																																																											2
ACCT/ATGG	1			1																																																								2
ACGT/ATGC	2																																																											2
ACTC/AGTG	5	3			2																																																							10
AGAT/ATCT	3		1																																																									4
AGGG/CCCT	1																																																											1
AGTC/AGTC	1																																																											1
AAAAC/GTTTT	1																																																											1
AAAAG/CTTTT	2	1	1																																																									4
AAAAT/ATTTT	2		2	1														1																																										6
AAACG/CTTTG	1																																																											1
AAACT/ATTTG	1																																																											1
AAAGG/CCTTT	4																																																											4
AAATG/ACTTT	1																																																											1
AAATT/AATTT	1	2	1																																																									4
AACAC/GTGTT	2																																																											2
AACTC/AGTTG	4																																																											4
AAGAC/CTGTT	1	1																																																										2
AAGAG/CTCTT	4	2																																																										6
AAGCT/ATTCG		1																																																										1
AAGTT/AATTC	1		1																																																									2
AATAG/ATCTT	1																																																											1
AATCC/AGGTT	1																																																											1
AATGG/ACCTT	1																																																											1
ACACC/GGTGT	1	1																																																										2
ACCCT/ATGGG	1																																																											1
AGCGT/ATCGC	2																																																											2
AGTAT/ATATC	1																																																											1
AAAAAC/GTTTTT	1																																																											1
AAAAAT/ATTTTT	1																																																											1
AAAACC/GGTTTT	2																																																											2
AAAACT/ATTTTG	2	3																																																										5

AAAAGG/CCTTTT	1																																																											1
AAAAGT/ATTTTC		2																																																										2
AAAATC/AGTTTT	2																																																											2
AAAATT/AATTTT		1																																																										1
AAACAC/GTGTTT			1																																																									1
AAACCT/ATTTGG	1																																																										1
AAAGAG/CTCTTT	3	2																																																									5
AAAGTG/ACTTTC	1																																																											1
AAAGTT/AATTTC	2	1	1	1																																																								5
AAATGC/ACGTTT			1																																																									1
AAATTC/AAGTTT	1																																																											1
AAATTG/AACTTT	2																																																											2
AACAAT/ATTGTT	1																																																											1
AACACT/ATTGTG	1																																																											1
AACAGT/ATTGTC	1																																																											1
AACATG/ACTTGT	2	1																																																										3
AACCCT/ATTGGG		1																																																										1
AACCTC/AGTTGG	1																																																											1
AACGCC/CGGTTG		1																																																										1
AACTAC/ATGTTG	1																																																											1
AACTGC/ACGTTG	1																																																											1
AAGACC/CTGGTT				1						1																																																		2
AAGAGC/CGTTCT	1																																																											1
AAGAGG/CCTTCT	1		1																																																									2
AAGAGT/ATTCTC	2																																																											2
AAGATG/ACTTCT	1																																																											1
AAGCAG/CGTCTT	2																																																											2
AAGCTC/AGTTCG	1																																																											1
AAGGAG/CCTCTT		1			1																																																							2
AAGGGT/ATTCCC	1																																																											1
AAGGTT/AATTCC	1																																																											1
AAGTAG/ATCTTC	1																																																											1
AAGTAT/ATATTC	1		1																																																									2
AAGTCC/AGGTTC	2																																																											2
AAGTGG/ACCTTC	1																																																											1
AAGTGT/ACATTC	2		2																																																									4
AATAGT/ATCATT		1																																																										1
AATCCC/AGGGTT		1																																																										1
AATCGC/AGCGTT	1																																																											1
AATCGT/AGCATT	1																																																											1
AATCTC/AGAGTT	1																																																											1
AATCTG/ACTTAG		1																																																										1
AATCTT/AATTAG	1																																																											1
AATGAT/ACTATT	1	1																																																										2
AATGTG/ACACTT	1																																																											1
ACACTG/ACTGTG	1				1																																																							2
ACCACT/ATGGTG	2																																																											2
ACCGCC/CGGTGG	1																																																											1
ACTCGG/AGCCTG	1																																																											1
ACTCTC/AGAGTG								2																																																				2
AGAGAT/ATCTCT	2																																																											2
AGAGGG/CCCTCT	2																																																											2
AGCAGG/CCTCGT	1																																																											1
AGCATC/AGTCGT	1																																																											1
AGGGGT/ATCCCC						1																																																						1
Total	1751	1482	727	468	273	197	129	88	60	41	39	32	21	24	20	10	9	6	2	3	6	3	2	4	2	0	0	0	2	1	2	0	1	0	0	2	0	0	0	0	0	0	0	1	0	0	0	0	0	0	0	0	0	0	0	0	0	0	1	5409
