# Supplementary material for: Comparative Analysis of Kabuli Chickpea Transcriptome with Desi and Wild Chickpea Provides a Rich Resource for Development of Functional Markers
Source: PLoS One. 2012 Dec 27;7(12):e52443. doi: 10.1371/journal.pone.0052443 (PMC3531472; doi:10.1371/journal.pone.0052443)
Supplement: Table S7 — Primers designed for SSRs identified in the kabuli chickpea transcriptome. (PDF) [file pone.0052443.s017.pdf]

Table S7. Primers designed for SSRs identified in the kabuli chickpea transcriptome.

SSR_ID	Transcript ID	SSR	Start	End	Tissue specificity	TF family	Forward primer (5'-3')	Reverse primer (5'-3')	Annealing temperature (°C)	Product size (bp)
CakTSSR00001	CakTC00018	(CTT)9	59	85	--	--				
CakTSSR00002	CakTC00026	(TC)8	15	30	Flower bud	--				
CakTSSR00003	CakTC00028	(GA)6	1141	1152	--	--	CTGCAGTAACACAGTTGATGA	CGGATTATAGTTTTGGGAAAT	54.85,55	150
CakTSSR00004	CakTC00046	(TGA)5	87	101	--	--				
CakTSSR00005	CakTC00088	(GGT)6	2	19	--	--				
CakTSSR00006	CakTC00093	(TTTA)5	1	20	--	--				
CakTSSR00007	CakTC00095	(TC)7	8	21	--	PLATZ				
CakTSSR00008	CakTC00096	(GAA)5	346	360	--	--	TCTGCAAGTCTAAAAGCATGT	ATCGGGGATTCTAAATGTATC	55.38,54.74	149
CakTSSR00009	CakTC00152	(GA)11	204	225	--	--				
CakTSSR00010	CakTC00195	(ATA)7	1175	1195	--	WRKY	CACCATTTCCTACTGTCACAC	TATAAAGTGCTTGTGCAATGA	55.51,54.66	150
CakTSSR00011	CakTC00196	(TA)8	81	96	--	--				
CakTSSR00012	CakTC00220	(ATA)5	274	288	--	--				
CakTSSR00013	CakTC00244	(AG)8	95	110	--	--				
CakTSSR00014	CakTC00336	(CT)8	799	814	--	--				
CakTSSR00015	CakTC00352	(AG)8	67	82	--	C3H				
CakTSSR00016	CakTC00372	(CT)13	93	118	--	--				
CakTSSR00017	CakTC00378	(GGT)5	188	202	--	--				
CakTSSR00018	CakTC00402	(GA)10	914	933	--	C2C2-GATA				
CakTSSR00019	CakTC00492	(ATA)6	137	154	--	--				
CakTSSR00020	CakTC00504	(CTC)5	637	651	--	--	TCTTCCTTTTCTTGTGCCTAT	TTAAGAGGGAGGCTAGTGATT	55.78,54.92	141
CakTSSR00021	CakTC00509	(CT)15	17	46	--	--				
CakTSSR00022	CakTC00530	(CT)7	690	703	--	MADS				
CakTSSR00023	CakTC00577	(GA)6	170	181	--	--	AGACCCTTTTGTCTCCTCTC	AGCTTTGATGTTCCTCTTCTT	54.98,54.94	148
CakTSSR00024	CakTC00579	(TTG)5	369	383	--	--	CCACCTTTAATTTTTCACCTT	TGAATGATGAAGAAATGTTCC	55.1,55.11	148
CakTSSR00025	CakTC00589	(TTC)6	416	433	--	--				
CakTSSR00026	CakTC00646	(GA)7	1984	1997	--	--				
CakTSSR00027	CakTC00669	(GT)7	119	132	--	--				
CakTSSR00028	CakTC00672	(AG)8	1966	1981	--	--				
CakTSSR00029	CakTC00689	(CAC)5	34	48	--	--				
CakTSSR00030	CakTC00695	(TTC)6	143	160	--	--	AAAAGCTCTGTTTTTGTTGTG	CACTATCAAATCACCTCAACC	54.87,54.64	163
CakTSSR00031	CakTC00713	(AGA)5	100	114	--	--				
CakTSSR00032	CakTC00745	(TC)16	96	127	--	--				
CakTSSR00033	CakTC00748	(AAT)5	353	367	--	PHD	CCTCTCGATCATCTCTACTCC	GAATCAATTTCTTCACCGTTA	55.55,54.49	149

CakTSSR00034	CakTC00771	(TCT)6	38	55	--	--				
CakTSSR00035	CakTC00775	(TTCT)5	180	199	--	--				
CakTSSR00036	CakTC00823	(GGT)6	144	161	--	--	AGAGGTCTTGAAGAGGATCTG	CAACATCAATTCATTCCAAGT	55.17,55.06	150
CakTSSR00037	CakTC00894	(AGA)5	1201	1215	--	BSD				
CakTSSR00038	CakTC00917	(TGA)5	1077	1091	--	--	TTTACAATGGTTTCAGCTTGT	ACACACTTCAAAACAACCAAA	55.07,55.72	150
CakTSSR00039	CakTC00962	(AGG)7	247	267	--	--	CACATGGAAACATCAATGAA	TTGAAGACAAAAAGCACAGAT	55.21,55.18	153
CakTSSR00040	CakTC00977	(AC)7	58	71	--	--				
CakTSSR00041	CakTC00979	(AG)16	274	305	--	--	AGGAACAGAGAGTTTGAGACC	GTGTGAAGTTTTTGTTCTTTTG	55.07,54.31	138
CakTSSR00042	CakTC00982	(GTG)8	2833	2856	--	--	CTTCTTTTAATTGAAGGGTCA	CCCTCTTTTAAACCAAAACAT	53.86,55.1	158
CakTSSR00043	CakTC00990	(CT)7	388	401	--	--				
CakTSSR00044	CakTC01004	(GA)7	130	143	--	--	CGGTTTTACAATGCTACTGAC	ACAACAGTACCCAACATCATC	55.11,54.81	145
CakTSSR00045	CakTC01014	(CTT)5	2982	2996	--	--	TCCCATGTCAGTTTCTGTTAC	CAAAGTTAAGTGCTGCTGAGT	55.13,55.04	152
CakTSSR00046	CakTC01039	(AG)11	97	118	--	--				
CakTSSR00047	CakTC01042	(AT)7	1	14	--	--				
CakTSSR00048	CakTC01044	(AG)21	1809	1850	--	--				
CakTSSR00049	CakTC01081	(CT)14	103	130	--	--	CAATCAAATCATTAACCATCG	CTTCTCTGTTTTTGGGGTACT	55.61,55.15	152
CakTSSR00050	CakTC01101	(ATT)6	367	384	--	Trihelix	CCTCTTTGCATTTCTCTTGTA	TTGGCATTGATAAACCTAAAG	54.88,54.72	159
CakTSSR00051	CakTC01101	(TCA)6	1079	1096	--	Trihelix	AACACTTGAACATTTCAAACC	ATGGTGATGTTGTTACCAAAG	54.28,55.02	153
CakTSSR00052	CakTC01189	(TTC)6	228	245	--	--				
CakTSSR00053	CakTC01191	(CT)6	26	37	--	--				
CakTSSR00054	CakTC01198	(AAC)6	321	338	--	--	AGAGATTTTGAAACAACGACA	AATGATATCCGGATTATGTCC	55.02,55.4	126
CakTSSR00055	CakTC01203	(GAA)5	302	316	--	--	GAGCCTTAAAGGTAGTGAAGG	TATGGCGGATACTACTTTGTC	55,54.56	145
CakTSSR00056	CakTC01211	(TTG)7	352	372	--	--	ACTGATGATTGGTACTGATGG	TCGAAAAACCTATCCTTTCAT	54.91,55.55	153
CakTSSR00057	CakTC01218	(CAC)5	930	944	--	--	CTCATGATCAGAATCCATGTT	TTGTGATCTCTCTTTGTTGCT	54.96,55.17	153
CakTSSR00058	CakTC01226	(GAT)5	885	899	--	--	AAAGAAGTTGTTGCTGTTGAC	ACCGAGCAAGTTTAGCATTAT	54.67,55.83	147
CakTSSR00059	CakTC01268	(GT)7	18	31	Mature Leaf	--				
CakTSSR00060	CakTC01281	(CT)7	110	123	--	--	CGGTTTTCTCCTTCTTCTAAT	ATGCATTAGAAGGACTCATTG	54.52,54.49	150
CakTSSR00061	CakTC01285	(GAA)5	310	324	--	--	AGAGCCACTGTCCTCTCTG	GTGTGTGGTGATTCAATTTCT	55.42,54.95	155
CakTSSR00062	CakTC01304	(TGA)5	218	232	--	--				
CakTSSR00063	CakTC01357	(TC)7	79	92	--	--				
CakTSSR00064	CakTC01402	(AG)11	11	32	--	--				
CakTSSR00065	CakTC01414	(CT)7	1	14	--	--				
CakTSSR00066	CakTC01458	(GAA)6	1672	1689	--	--				
CakTSSR00067	CakTC01462	(AT)6	2	13	--	--				
CakTSSR00068	CakTC01487	(CT)7	16	29	--	--				
CakTSSR00069	CakTC01488	(TGA)8	430	453	--	--				
CakTSSR00070	CakTC01502	(GA)6	1764	1775	--	--				

CakTSSR00071	CakTC01567	(AG)7	1565	1578	--	--				
CakTSSR00072	CakTC01590	(AC)9	805	822	--	--				
CakTSSR00073	CakTC01619	(TTG)8	275	298	--	C2C2-Dof	ATTTGGTGTTTGTTGATTCAC	TTGGAAACAGAACTTATTGGA	54.97,54.97	146
CakTSSR00074	CakTC01688	(CTC)5	143	157	--	--	TCCAATTCATTTCGGATACTA	CTTCATTTTCTCCATTTCCTT	54.86,55.13	162
CakTSSR00075	CakTC01693	(CAG)5	755	769	--	AP2-EREBP	CACACTTTTCCACATCTTCAT	CAGTTGCTAACAAATGTGGTG	55.13,56.8	151
CakTSSR00076	CakTC01782	(TCCT)8	162	193	--	--				
CakTSSR00077	CakTC01785	(AAT)5	73	87	--	--				
CakTSSR00078	CakTC01798	(TC)7	93	106	--	--				
CakTSSR00079	CakTC01869	(CTT)5	376	390	--	--	TTGAAAAGAGGATGGTAAAGA	AGCAATATCAATGCAAGAAGA	54.22,55.23	147
CakTSSR00080	CakTC01915	(TC)13	43	68	--	--				
CakTSSR00081	CakTC01946	(TAA)5	127	141	--	--	CATCCCTAAACAACAACAAAA	TGTAAAGACTGATGGGTTGAC	55.33,55.13	154
CakTSSR00082	CakTC01970	(CTAA)5	143	162	--	--	ATTCTTGCTTTAACCGTTTTC	TCGTAGAATTTTGGGTGTATG	55.39,55.29	149
CakTSSR00083	CakTC01996	(TC)9	128	145	--	--	CAAGAGTAGTGATTGGTTTGG	CATTTTCTTTTTCATTCTCTGC	54.9,55.9	150
CakTSSR00084	CakTC02011	(AT)7	138	151	--	--				
CakTSSR00085	CakTC02064	(TC)8	2	17	--	--				
CakTSSR00086	CakTC02070	(TG)10	617	636	--	--				
CakTSSR00087	CakTC02088	(TAT)5	16	30	--	MYB-related				
CakTSSR00088	CakTC02101	(TTAAT)5	63	87	--	--				
CakTSSR00089	CakTC02112	(TC)19	49	86	--	--				
CakTSSR00090	CakTC02127	(ATT)5	3060	3074	--	--				
CakTSSR00091	CakTC02163	(AG)18	2264	2299	--	--				
CakTSSR00092	CakTC02215	(AAGAGC)5	62	91	--	--				
CakTSSR00093	CakTC02229	(TA)6	117	128	--	--				
CakTSSR00094	CakTC02242	(AG)8	25	40	--	--				
CakTSSR00095	CakTC02282	(CCA)5	228	242	--	--				
CakTSSR00096	CakTC02333	(TA)6	8	19	--	--				
CakTSSR00097	CakTC02351	(AG)6	143	154	--	--				
CakTSSR00098	CakTC02374	(CA)7	1	14	--	--				
CakTSSR00099	CakTC02409	(TC)6	3047	3058	--	--	CACCATTTGAAAGATACCAAC	TATCACAGATTTGGGAGAAGA	54.67,54.86	153
CakTSSR00100	CakTC02521	(AGA)5	63	77	--	--				
CakTSSR00101	CakTC02543	(GGT)5	848	862	--	--	AGAGTGCAAGGTTAACATCAG	GTGAACCAAACTGTGAAACAT	54.63,55.04	156
CakTSSR00102	CakTC02543	(CAA)5	967	981	--	--	ATGTTTCACAGTTTGGTTCAC	TCCCATATCTGAAATTTGTTG	55.04,55.17	143
CakTSSR00103	CakTC02562	(GAT)6	4373	4390	--	--	AGATTGAAGAGGGAATTTCAA	AACCCCACTCTCTCTTCTCTT	55.52,56.18	140
CakTSSR00104	CakTC02570	(AG)6	32	43	--	--				
CakTSSR00105	CakTC02617	(TG)6	258	269	--	--				
CakTSSR00106	CakTC02618	(CAC)5	426	440	Mature Leaf	--	CAACTCCTCCACCATGTC	GATAGGAACATCTCAATGTCG	54.45,54.72	149
CakTSSR00107	CakTC02626	(AC)11	12	33	--	--				

CakTSSR00108	CakTC02695	(TTG)7	1116	1136	--	bHLH	GAGGCATGTAATCAACAATTC	ATGCCTTCAAGTTTCAACAC	54.73,55.28	148
CakTSSR00109	CakTC02731	(AG)9	244	261	--	--	CTTCGCTTTCTTCATTTTCTT	CTTGAAGAAATTCCAGACCTT	55.66,55.2	142
CakTSSR00110	CakTC02773	(TC)13	10	35	--	--				
CakTSSR00111	CakTC02781	(AG)10	580	599	--	--				
CakTSSR00112	CakTC02788	(GT)10	110	129	--	--				
CakTSSR00113	CakTC02827	(TCTGGT)14	609	692	--	--	GTAATCCTGGAAGTAGGAGGA	GGTCACTCAATTCAACATCAG	54.99,55.56	153
CakTSSR00114	CakTC02861	(TG)6	222	233	--	--	GATTGATTTGGGGAAGAGAC	CCTCTCACATCATCTTCTCAC	56.04,54.71	152
CakTSSR00115	CakTC02883	(GAA)5	48	62	--	--				
CakTSSR00116	CakTC02937	(CT)14	1	28	--	--				
CakTSSR00117	CakTC02955	(TTA)5	109	123	--	--	CACTTATGAGGACAGTCCAGT	GTTATCCAATTCATCAATCCA	54.25,54.92	152
CakTSSR00118	CakTC03007	(AGA)7	132	152	--	--	ACGATTTCCGATTCATCTTA	GTTCACCACAGAATTCATCAT	54.81,54.86	143
CakTSSR00119	CakTC03132	(TC)14	111	138	--	--				
CakTSSR00120	CakTC03133	(TC)10	79	98	--	--				
CakTSSR00121	CakTC03187	(CT)6	1	12	--	--				
CakTSSR00122	CakTC03230	(TC)13	18	43	--	--				
CakTSSR00123	CakTC03252	(TGA)11	506	538	--	--	AATCGTACATCACAACTCGAT	CAGCATTTACTTTCACCAATC	54.66,54.99	151
CakTSSR00124	CakTC03307	(AG)6	155	166	--	--				
CakTSSR00125	CakTC03355	(CT)19	188	225	--	--				
CakTSSR00126	CakTC03394	(TTC)5	235	249	--	--	AGCTCGAAGCTGTTTAAGATT	AAGAAAGAGAATGCGAAGAAT	55.3,55.03	147
CakTSSR00127	CakTC03420	(AG)6	21	32	Shoot	--				
CakTSSR00128	CakTC03432	(TG)8	89	104	--	--				
CakTSSR00129	CakTC03436	(CAC)6	204	221	--	--	CCCAACTTTCTAACCCTTTAC	GGAAGGAATCAAGAGAGGTAG	54.72,54.71	164
CakTSSR00130	CakTC03445	(AC)7	10	23	--	--				
CakTSSR00131	CakTC03451	(AAC)6	323	340	--	--	GGCTATCCATCAACAACATAA	AAGTGAACAACAGCAATGTTT	55.19,54.96	150
CakTSSR00132	CakTC03458	(CAA)5	90	104	--	--				
CakTSSR00133	CakTC03496	(CT)6	44	55	--	--				
CakTSSR00134	CakTC03498	(AG)12	33	56	--	--				
CakTSSR00135	CakTC03502	(AAT)6	214	231	--	bHLH	TAAATTCCGACCTTATCAATG	TCCATCAAAACAAGAGAAAGA	54.49,55.08	158
CakTSSR00136	CakTC03585	(CT)10	106	125	--	--	TTCTCTTACCTCAAGTGCAAC	AATATGGGAAGAGAAGACCAG	54.76,54.99	157
CakTSSR00137	CakTC03608	(AG)6	4294	4305	--	--	TTTGGTTTGAAGAGAATTGTG	GAACAGCTTCATATTGGTTGT	55.45,54.4	164
CakTSSR00138	CakTC03613	(TC)9	85	102	--	--				
CakTSSR00139	CakTC03654	(GAA)5	413	427	--	--	TGCTAAGTCTTCGAATTTCAG	TTACTCTTCCGTTCCTTATCC	55.04,55.16	180
CakTSSR00140	CakTC03676	(GA)12	99	122	--	--				
CakTSSR00141	CakTC03725	(TG)7	101	114	--	--	TTTTCTCTGCTTTCATGTTTC	AAAGTGAGTCTGCAAAAACAG	54.83,54.85	146
CakTSSR00142	CakTC03762	(GAA)8	1451	1474	--	--	ATTAGTGTGCATTGTCGTCTC	AAACATGAGATTCCAGATCCT	55.32,55.22	154
CakTSSR00143	CakTC03764	(CT)7	67	80	--	--				
CakTSSR00144	CakTC03766	(TA)7	68	81	--	--				

CakTSSR00145	CakTC03771	(CCA)5	34	48	--	--				
CakTSSR00146	CakTC03801	(GCA)5	84	98	--	--				
CakTSSR00147	CakTC03814	(TC)7	3	16	--	--				
CakTSSR00148	CakTC03814	(CAA)6	351	368	--	--	AAGAGAAAGAGATGAAGCACA	TCAATTCCTCTTCTTTGTTGA	54.37,55.08	150
CakTSSR00149	CakTC03814	(CTA)6	799	816	--	--	CCACCTGTTACTATTCCTTCA	AAGGAGAATCAGAACCTTCAC	54.43,55.01	151
CakTSSR00150	CakTC03814	(GAA)6	1116	1133	--	--	AGGGTAGCATTACAAGTTGGT	TAGAGGAAAATGCCAATGATA	55.45,55.02	140
CakTSSR00151	CakTC03835	(GAA)8	134	157	--	--				
CakTSSR00152	CakTC03848	(GA)6	87	98	--	--				
CakTSSR00153	CakTC03850	(AG)6	29	40	--	--				
CakTSSR00154	CakTC03853	(TA)7	57	70	--	--				
CakTSSR00155	CakTC03902	(TCC)7	90	110	--	--				
CakTSSR00156	CakTC03941	(TA)6	204	215	--	--				
CakTSSR00157	CakTC03971	(GTT)7	1185	1205	--	bHLH	GTTTTGGAACTGAAATCAACA	TGAACAACGTTTCTTCTCTTC	55.28,54.72	148
CakTSSR00158	CakTC03971	(AG)7	1659	1672	--	bHLH				
CakTSSR00159	CakTC04020	(GCT)5	726	740	--	--	CTTTTGTAGTTCTGTCGCATT	TCTTGTTTCCTGATCTTTCAA	54.82,55.08	152
CakTSSR00160	CakTC04029	(AG)7	1755	1768	--	--				
CakTSSR00161	CakTC04033	(AG)6	126	137	--	--				
CakTSSR00162	CakTC04070	(CTT)7	1415	1435	--	--				
CakTSSR00163	CakTC04076	(AGA)6	82	99	--	--				
CakTSSR00164	CakTC04093	(CAT)5	125	139	--	--	TCTTGATCTTGAAAACTCCAA	CCTTCTCCATCCATTATTCTT	55.08,54.93	175
CakTSSR00165	CakTC04114	(AT)7	2090	2103	--	--				
CakTSSR00166	CakTC04143	(CT)6	1	12	--	--				
CakTSSR00167	CakTC04146	(CAG)5	28	42	--	--				
CakTSSR00168	CakTC04159	(TTG)5	95	109	--	--				
CakTSSR00169	CakTC04162	(TGT)7	182	202	--	AP2-EREBP				
CakTSSR00170	CakTC04196	(GA)6	162	173	--	--				
CakTSSR00171	CakTC04226	(CT)9	149	166	--	--				
CakTSSR00172	CakTC04294	(CCT)5	65	79	--	--				
CakTSSR00173	CakTC04323	(TCT)7	47	67	--	--				
CakTSSR00174	CakTC04368	(ATCT)5	140	159	--	--				
CakTSSR00175	CakTC04376	(GAT)5	128	142	--	--	CAGGTCATGCTTCTCAGAAAT	ACACTATCTCCGTCTCCATCT	57,55.32	148
CakTSSR00176	CakTC04389	(CT)17	115	148	--	--				
CakTSSR00177	CakTC04394	(AGA)5	28	42	--	--				
CakTSSR00178	CakTC04397	(ACA)5	307	321	--	--	TTCTGATTACTCAGGTGAAGC	CATTCTCATCCAAAACAGTTC	54.67,54.77	149
CakTSSR00179	CakTC04461	(CAC)5	296	310	--	--	CAAAATCAAACACTTCTCCAC	GCTCCAAAATCTAACACATTG	54.85,54.99	154
CakTSSR00180	CakTC04464	(CA)6	31	42	--	--				
CakTSSR00181	CakTC04473	(CAA)5	92	106	--	--				

CakTSSR00182	CakTC04474	(GA)8	2020	2035	--	--				
CakTSSR00183	CakTC04525	(AGA)6	1	18	--	--				
CakTSSR00184	CakTC04586	(TGA)6	376	393	--	--	CGATGATCAACTCAATGTCTC	CCTCCAGGAGTCGTAATAGTT	55.65,55.11	144
CakTSSR00185	CakTC04606	(CA)7	129	142	--	--	TAAGCGCTGATAAAGTGTTTT	AAGTCATGATAATCCAGAGCA	54.7,54.88	166
CakTSSR00186	CakTC04641	(ATT)5	51	65	--	--				
CakTSSR00187	CakTC04664	(GGT)5	600	614	--	zf-HD	GGAGAAGAGGGTACCATAGAA	GAGTGGTGATGGTGGTAGTTA	54.99,55	137
CakTSSR00188	CakTC04664	(CTG)5	739	753	--	zf-HD	TAACTACCACCATCACCACTC	CACATGTGGAGGTACAACTCT	55,55.07	169
CakTSSR00189	CakTC04691	(CA)8	69	84	--	--				
CakTSSR00190	CakTC04694	(AGA)5	262	276	--	--				
CakTSSR00191	CakTC04726	(CTC)5	2091	2105	--	--	TGAGGTACTCTTGAAGTGCTC	GATGTTGAATGGAAAAGGACT	54.73,55.71	150
CakTSSR00192	CakTC04741	(CTT)8	92	115	--	--				
CakTSSR00193	CakTC04745	(CT)7	20	33	--	--				
CakTSSR00194	CakTC04750	(TTTC)6	134	157	--	--				
CakTSSR00195	CakTC04770	(TTC)6	365	382	--	--	GAATTTGCACAAAGAAGAAAC	TGAATCTGGTTGGTGTGTAAT	54.27,55.43	248
CakTSSR00196	CakTC04868	(AC)7	100	113	--	--				
CakTSSR00197	CakTC04907	(TG)8	981	996	--	C2C2-YABBY				
CakTSSR00198	CakTC04931	(AAC)7	545	565	--	--	AAATTTTCAGGATTGGGTATC	TAAGCTGATTGTTCTCTTCCA	54.79,55.28	175
CakTSSR00199	CakTC04957	(ATTTTG)5	277	306	--	--	CATCATCAATCTCAATTAGACCT	CATGTCCTTGTCCCAATATAA	55.03,55.05	157
CakTSSR00200	CakTC04987	(AT)10	14	33	--	--				
CakTSSR00201	CakTC04990	(GT)7	141	154	--	--				
CakTSSR00202	CakTC05018	(GA)6	587	598	--	--				
CakTSSR00203	CakTC05022	(TGT)6	66	83	--	--				
CakTSSR00204	CakTC05106	(TC)9	199	216	--	--	CATCTGAGAAAAAGCTGAAAA	GTTTTCAAGGTCAAACTCCAT	55,55.78	148
CakTSSR00205	CakTC05111	(TTA)5	140	154	--	--	CGATTCTTTTCCTTTCTCTTT	TGTGTGTGAATGGTTACAGAA	54.64,54.95	157
CakTSSR00206	CakTC05115	(TTC)7	150	170	--	--				
CakTSSR00207	CakTC05148	(GT)6	5	16	--	--				
CakTSSR00208	CakTC05152	(TG)7	222	235	--	--				
CakTSSR00209	CakTC05187	(GAA)5	155	169	--	--	ACATTGCAGTTTCTTTCTCAA	TCTGAACATCAGGGAATAGAA	55.18,54.86	167
CakTSSR00210	CakTC05202	(TC)6	569	580	--	MYB	AAGTTTCACTTTTGTGCCTTA	TTTGGATATATGAGGATGGTG	54.41,54.96	177
CakTSSR00211	CakTC05216	(TTA)6	98	115	--	--				
CakTSSR00212	CakTC05245	(GGAAA)5	223	247	--	--				
CakTSSR00213	CakTC05268	(AAG)5	424	438	--	--				
CakTSSR00214	CakTC05276	(GA)6	7	18	--	--				
CakTSSR00215	CakTC05277	(GA)16	2043	2074	--	--				
CakTSSR00216	CakTC05287	(GAA)5	159	173	--	--	CTCTACTCCAACAATGTCACC	TCCTTCTTCTCCTTCTTCTTC	54.69,54.55	151
CakTSSR00217	CakTC05316	(AG)6	87	98	--	--				
CakTSSR00218	CakTC05322	(TCA)5	322	336	--	--	CCACTTTTACGTTTTCTTTTTC	ATTCCCTCAGATATGGAAGAG	55.09,54.91	152

CakTSSR00219	CakTC05322	(CAT)5	1130	1144	--	--	CATTATCAGCAGATACGGACT	GATTCTGCTGGAAGTGAAGAT	54.53,56.04	148
CakTSSR00220	CakTC05332	(AAC)5	98	112	--	--				
CakTSSR00221	CakTC05412	(TC)6	24	35	--	--				
CakTSSR00222	CakTC05507	(TCC)5	15	29	--	--				
CakTSSR00223	CakTC05512	(GAA)5	62	76	--	--				
CakTSSR00224	CakTC05543	(TCT)5	33	47	--	--				
CakTSSR00225	CakTC05573	(GTT)5	917	931	Young_pod	--	ATGAAGTAGGTTGTGGTGTTG	ATTTGGAGCTTCTTCCACTT	55.08,55.61	161
CakTSSR00226	CakTC05601	(TA)7	1675	1688	--	--				
CakTSSR00227	CakTC05636	(GT)9	35	52	--	--				
CakTSSR00228	CakTC05636	(GAT)6	1190	1207	--	--	TCTCATCTAGCAAAAGGAAAA	TGAGCTTCATTTGGAGTAGAA	54.54,55.28	154
CakTSSR00229	CakTC05652	(GAA)5	113	127	--	--				
CakTSSR00230	CakTC05655	(GAA)8	98	121	--	--				
CakTSSR00231	CakTC05656	(AGA)6	2964	2981	--	--				
CakTSSR00232	CakTC05697	(GA)7	2820	2833	--	--				
CakTSSR00233	CakTC05698	(CT)18	51	86	--	--				
CakTSSR00234	CakTC05710	(CAT)9	89	115	--	--				
CakTSSR00235	CakTC05726	(CA)6	1	12	--	--				
CakTSSR00236	CakTC05731	(ACA)6	210	227	--	--				
CakTSSR00237	CakTC05775	(CT)8	102	117	--	--	CAAACACCTCTCTTTCCTTCT	CACACACATAAGATGGAACCT	55.26,55	140
CakTSSR00238	CakTC05873	(AGA)5	103	117	--	--	GTCTCTCATGTCTTGCATCTC	CTTTCGATGAAGAGTTCTTTG	54.87,54.43	156
CakTSSR00239	CakTC05951	(TTG)5	147	161	Flower bud	--				
CakTSSR00240	CakTC05959	(TCT)7	60	80	--	--				
CakTSSR00241	CakTC06068	(CAA)5	38	52	--	--				
CakTSSR00242	CakTC06087	(AAC)5	71	85	--	--				
CakTSSR00243	CakTC06104	(AAG)6	512	529	--	--	CGATCTGGTGAAGAAGAATAA	TCACAACCGTCCATTAGTATC	54.63,55.1	148
CakTSSR00244	CakTC06127	(CT)18	4	39	--	WRKY				
CakTSSR00245	CakTC06143	(AG)7	351	364	--	--	TCGATAAAGGAACAATGAAGA	AGTCACACCTTCAACACGATA	55.05,55.68	163
CakTSSR00246	CakTC06143	(GA)9	481	498	--	--	ATATCGTGTTGAAGGTGTGAC	CGAGATCCTCTCTTCTTCTTC	54.99,54.97	150
CakTSSR00247	CakTC06154	(TC)11	11	32	--	HMG				
CakTSSR00248	CakTC06186	(TA)6	129	140	--	--	AGAATTACAGTTTCCCCACTC	ATTTTCTTTCCCTAAAAGCTG	54.9,54.7	167
CakTSSR00249	CakTC06190	(TTA)7	12	32	--	--				
CakTSSR00250	CakTC06196	(GA)6	155	166	--	--				
CakTSSR00251	CakTC06222	(ACA)6	167	184	--	--	ATATCCTCTCTCCAACCAGAA	TTGGAGTAAGGAAAGGAGACT	55.39,54.79	175
CakTSSR00252	CakTC06248	(GA)6	404	415	--	--				
CakTSSR00253	CakTC06279	(ATC)5	448	462	--	--	TCTACTTCTTCACACCCTTCA	TTCCATCTTCCGTGTATAAAA	55.01,54.94	149
CakTSSR00254	CakTC06417	(TG)7	37	50	--	--				
CakTSSR00255	CakTC06423	(AAT)17	9	59	--	TPR				

CakTSSR00256	CakTC06441	(TGT)5	159	173	--	--				
CakTSSR00257	CakTC06444	(TCT)5	4	18	--	--				
CakTSSR00258	CakTC06505	(AT)6	2097	2108	--	--	CCTTCCATCCTCATCAATAGT	GCTGAACTTTGTGGAAAATTA	55.68,54.73	157
CakTSSR00259	CakTC06505	(AG)8	2345	2360	--	--	TTATGGTGTGATTCAAAAACC	CCTTTCTTTTCCACAATCTCT	55.08,55.2	136
CakTSSR00260	CakTC06592	(TCA)5	1609	1623	--	--	CACACTTCTTTCTCACACACA	GCCTATTTCACCAAATTCTTT	54.69,55.17	150
CakTSSR00261	CakTC06657	(TTG)5	144	158	--	--				
CakTSSR00262	CakTC06683	(TTG)6	111	128	--	--	ATTGATGCAACCAACACTTAT	TGAAATTCCTCAAGAAAGAGA	54.69,54.3	142
CakTSSR00263	CakTC06687	(AT)9	338	355	--	--				
CakTSSR00264	CakTC06702	(AG)11	1603	1624	--	--				
CakTSSR00265	CakTC06711	(AG)8	94	109	--	--				
CakTSSR00266	CakTC06715	(TTA)8	1	24	--	--				
CakTSSR00267	CakTC06720	(GTT)5	134	148	--	--	AGTTTTTCAGAAATGTGAGGA	TTGGAATGAACCATTTACTACA	54.08,54.88	157
CakTSSR00268	CakTC06721	(CT)6	26	37	--	--				
CakTSSR00269	CakTC06728	(CT)6	373	384	--	--				
CakTSSR00270	CakTC06739	(GAT)5	60	74	--	--				
CakTSSR00271	CakTC06744	(TCA)6	531	548	--	--	GATCTCAAAACTTTTCCCAAT	GGCATGAAAATGTACTCAAGA	54.97,55.39	137
CakTSSR00272	CakTC06802	(TC)7	76	89	--	--				
CakTSSR00273	CakTC06806	(ATA)6	1062	1079	--	--	AAGGGACAAGGAGACACTTT	GATCAACCTAGTGATTTGCAG	55.36,54.97	150
CakTSSR00274	CakTC06996	(ATC)5	121	135	--	--	ATTCCTTCTCCTCTGCTCTTA	TGCTGTTATTTTTCCTTTCTG	55.03,54.9	149
CakTSSR00275	CakTC07040	(AGA)6	145	162	--	--				
CakTSSR00276	CakTC07048	(ATA)6	76	93	--	--				
CakTSSR00277	CakTC07078	(TA)6	229	240	--	--				
CakTSSR00278	CakTC07091	(TGT)7	526	546	--	--	GAGAAATCTATGGAGGCAGAG	TTCAATGGCAATAGCAATAAC	55.71,55.52	155
CakTSSR00279	CakTC07109	(CT)7	3	16	--	--				
CakTSSR00280	CakTC07135	(AT)6	138	149	--	--				
CakTSSR00281	CakTC07300	(ATC)5	219	233	--	--	ATCATTCATCAAATCAGCATC	AGATCGATGGATAGGAAAAAG	55.05,55.09	180
CakTSSR00282	CakTC07315	(TCA)5	783	797	--	--	AAGTTTCAACCCATCTTCATT	GATTGCCTAGTGTTGAGAAAA	55.31,54.7	155
CakTSSR00283	CakTC07323	(TTC)7	1354	1374	--	--	GTTCTTGTTTTTCGAATGATG	CAACCTAAAGATGAAGTCGAA	54.96,54.72	149
CakTSSR00284	CakTC07342	(GA)9	1058	1075	--	--				
CakTSSR00285	CakTC07365	(TC)6	86	97	--	--				
CakTSSR00286	CakTC07377	(CT)8	33	48	--	bHLH				
CakTSSR00287	CakTC07377	(ATT)5	662	676	--	bHLH	GAGGAACCATTTTCTTTCTTC	AAAAACCCTTCTCATTGTGTT	54.69,55.38	167
CakTSSR00288	CakTC07403	(AG)9	2388	2405	--	EIL				
CakTSSR00289	CakTC07489	(AGA)9	122	148	--	--				
CakTSSR00290	CakTC07556	(GAA)7	2559	2579	--	--	ACCAAAAGGGTCAATTTCTAT	AGAGAAGCTGGTTTTTCAAGT	54.48,55.02	144
CakTSSR00291	CakTC07557	(GAA)8	30	53	--	--				
CakTSSR00292	CakTC07561	(TC)8	20	35	--	--				

CakTSSR00293	CakTC07582	(TA)6	733	744	--	--				
CakTSSR00294	CakTC07598	(TTC)5	1575	1589	--	--	TTTCAACGATTCTTCTTCAAC	GTTTTAGAACAACCACAACCA	54.67,55.23	150
CakTSSR00295	CakTC07598	(TGT)5	1982	1996	--	--	GGGAAGAATGAGGTAGTAGGA	GTCTCGATCCAAACAACAAC	54.99,55.54	160
CakTSSR00296	CakTC07648	(ATT)5	2827	2841	--	C3H	TGCATACTTTCAAAAGCAGTT	TTAACCTGTGGTTCAGCTTAC	55.39,54.66	149
CakTSSR00297	CakTC07649	(GCA)6	2647	2664	--	--	AGGGTTTACAATGAATGAATG	ACACAAGATTCAACCTTTTCA	54.2,54.85	155
CakTSSR00298	CakTC07653	(TTA)5	245	259	--	--	GTTAGCCAAATAAACCACATTT	TGGTTGACTAATAACTTGTGGA	55.15,54.94	139
CakTSSR00299	CakTC07661	(TC)8	37	52	--	ARR-B				
CakTSSR00300	CakTC07724	(AG)10	1	20	--	--				
CakTSSR00301	CakTC07724	(TC)8	259	274	--	--	TTCTTTTTCCAGTCCCTAATC	TTGTTGTTGTTGGTGTCTGTA	55.13,55.04	136
CakTSSR00302	CakTC07775	(CTT)6	592	609	--	--	TGAAATGTTGTTAGCTTCCAT	AACCTGGAGTTCTTGTGAAGT	54.99,55.45	157
CakTSSR00303	CakTC07782	(TTG)6	628	645	--	--	CAGGTTCTAATCTACCCTGCT	GTGTAAAGCCATGGAAGAGAT	55.26,55.9	152
CakTSSR00304	CakTC07784	(GA)13	344	369	--	--	CCTTCAAGATACGTTTCGAC	ACCATTTCTTTCTGATTTTCC	54.96,54.97	160
CakTSSR00305	CakTC07813	(TTGT)5	2571	2590	--	--				
CakTSSR00306	CakTC07838	(GAA)7	225	245	--	bHLH	AGAATAGAAAACGGCAATGTT	AGTGATTCTCTGTGTCTCTGC	55.65,54.51	155
CakTSSR00307	CakTC07885	(TGT)6	310	327	--	SBP	GTGTCTGTTGTTGTTGTGTTG	AAGCTTCTTCCTTAAACCAAA	54.92,55.14	153
CakTSSR00308	CakTC07892	(TGT)5	2920	2934	--	--	AATATCGTAGCTTCGAAAACC	CCAGATAGCTTTCATTTACTCC	55.35,54.95	162
CakTSSR00309	CakTC07909	(AG)11	3655	3676	--	--				
CakTSSR00310	CakTC07970	(TTA)8	738	761	--	C3H	TGATGATTATTTCGTTTCCTC	GCAACTAGAAGACGCTGATAA	54.4,55.06	152
CakTSSR00311	CakTC07975	(AAC)5	477	491	--	--	ACTCAAAAATCACACCTTTCA	ACACCAACAACATTAACAACC	54.85,54.93	145
CakTSSR00312	CakTC07988	(GAA)5	2332	2346	--	--				
CakTSSR00313	CakTC07994	(GGT)5	3050	3064	--	--	TCTTGCTTTAGAATGTTCGAT	AGAAATCGCGTTATTGGTAAT	54.4,55.61	148
CakTSSR00314	CakTC08000	(TCC)8	1735	1758	--	--				
CakTSSR00315	CakTC08008	(GA)6	6351	6362	--	SET				
CakTSSR00316	CakTC08058	(ATT)5	763	777	--	--	TTCTCAATGACTGTTGGTTTT	GTGATTCACCTCAAACTCAAA	54.85,55.26	157
CakTSSR00317	CakTC08067	(TA)7	6	19	--	--				
CakTSSR00318	CakTC08071	(ATT)6	167	184	--	--	ATTAAGGGTATTTGGGGTCTT	GAACGTGTGAAAGAGAAACAG	55.76,55.08	148
CakTSSR00319	CakTC08075	(TCA)6	1670	1687	--	--	ATCAACCTCATTAACATGTCG	GCTACAGAGACTTCCTTCCTC	55.1,54.92	154
CakTSSR00320	CakTC08132	(TTC)5	257	271	--	--	ACAAATCTTGTGTGTTTTCCA	CTGAGGAAGATGAAGAGGACT	55.64,55.17	154
CakTSSR00321	CakTC08174	(AT)6	44	55	--	--				
CakTSSR00322	CakTC08175	(GAT)6	1644	1661	Flower bud	--	ATGAAGATACAGAGGCCAAGT	TCACATTTGCATACTTTGGAT	55.49,55.68	150
CakTSSR00323	CakTC08181	(TGT)5	28	42	--	NAC				
CakTSSR00324	CakTC08199	(AGG)5	304	318	--	--	GGTGAAAGTGTAAATGCAAAG	ATTAACGCCCAATTCTTTACT	55.07,54.84	147
CakTSSR00325	CakTC08202	(GA)6	3776	3787	--	--				
CakTSSR00326	CakTC08203	(CT)9	81	98	--	--				
CakTSSR00327	CakTC08214	(TGA)28	133	216	--	--	GATGATGATGGTGATGATGA	GAGCAACAAATTGAGAGAGAA	54.8,54.8	150
CakTSSR00328	CakTC08216	(CA)6	277	288	--	bZIP	CCAAATCAAGAATCATGTAGC	TGTGTCAAAAAGTTGAGTGTG	54.91,54.73	126
CakTSSR00329	CakTC08232	(TGC)5	684	698	--	--	TAGTGAGTTGGTTGAAGCAGT	TGCGTCTAAATGAAGAGAAAG	55.12,55.04	170

CakTSSR00330	CakTC08240	(TGA)5	2490	2504	--	--	GACAGTTATGCAGCTTTCACT	CAGAAGATGTTCAATCCATGT	54.78,55.05	150
CakTSSR00331	CakTC08246	(ATG)6	1834	1851	--	--	TGAGTCCGATCTGAGAATAGA	CAAATCTTAATGCCAACAATG	55.01,55.84	147
CakTSSR00332	CakTC08250	(ACA)6	228	245	--	Trihelix	TCTAAAGTTTCTTCCTTTTTCG	GTGAGGATTTTGTTGAATTTG	54.91,54.79	158
CakTSSR00333	CakTC08250	(AAC)6	416	433	--	Trihelix	ACTGACAACAACACACATCAA	TGGCTCATCTTCATCACTAAG	54.82,55.56	144
CakTSSR00334	CakTC08270	(TG)10	308	327	--	--	CGACTTCTTGAATTTATGTGG	CTAGGAAAGGTACAAAAGCAA	55,54.14	160
CakTSSR00335	CakTC08279	(TG)7	302	315	--	--				
CakTSSR00336	CakTC08287	(ATG)6	2572	2589	--	--				
CakTSSR00337	CakTC08318	(GA)8	3613	3628	--	--				
CakTSSR00338	CakTC08324	(GA)7	4	17	--	--				
CakTSSR00339	CakTC08339	(TC)10	3762	3781	--	--	ATGCCTTGCTCAGAGATTC	ATCACTACACACACCGTCTCT	55.43,54.6	157
CakTSSR00340	CakTC08400	(ACT)6	947	964	Flower bud	--	AAAGCTCTCACCTCGTACTCT	AGTTGTTGTTGAACATTACCG	55.03,55.26	168
CakTSSR00341	CakTC08416	(TGT)6	1430	1447	--	--				
CakTSSR00342	CakTC08458	(TGA)5	73	87	--	TRAF				
CakTSSR00343	CakTC08458	(TTA)6	1525	1542	--	TRAF				
CakTSSR00344	CakTC08487	(CAG)6	1221	1238	--	NAC	CCAATCCAATCTCAACTATCA	TCCACTTCCACCTATTTTATG	55.16,54.38	149
CakTSSR00345	CakTC08487	(AAC)5	1551	1565	--	NAC	CTATACACTTCGGTGCTTCTC	GAGAGTCTTAGGTCGTTCTCC	54.32,54.75	151
CakTSSR00346	CakTC08489	(ATT)6	730	747	--	TCP	GTCCCCTCTGTGAAAGATACT	CCGGTGCTACTATTGATACTG	54.88,55.07	162
CakTSSR00347	CakTC08489	(TTC)5	1465	1479	--	TCP	ATCCAGACTCGGACACTTTAT	ACTTTCATTTTTCCACTTTGG	55.33,55.94	147
CakTSSR00348	CakTC08490	(AG)8	2839	2854	--	--				
CakTSSR00349	CakTC08495	(TG)6	345	356	--	--	TAGAGCTGGAAGGAGTATGTG	ACCCTCTTACTTCAATTGGTC	54.72,54.9	127
CakTSSR00350	CakTC08510	(AG)6	184	195	--	HB	AATCACACATCCTTCTCACAA	CAAAAACAAACGAAGAAGAAG	55.56,54.55	149
CakTSSR00351	CakTC08529	(TGT)5	1623	1637	--	--	CCAGATGGAATTAGATCCTTT	TCAAAGAGAGAGAAAAAGCAA	54.93,54.72	151
CakTSSR00352	CakTC08531	(ATT)7	52	72	--	--				
CakTSSR00353	CakTC08548	(TC)7	66	79	--	--				
CakTSSR00354	CakTC08548	(CAA)6	421	438	--	--	CATAAAACCAAACTCAACCAC	CTCTCCCCACTGTATGAGAC	54.75,54.56	167
CakTSSR00355	CakTC08583	(GA)6	1	12	--	--				
CakTSSR00356	CakTC08597	(TA)8	22	37	--	--				
CakTSSR00357	CakTC08611	(TTC)6	111	128	--	--	AATCAAAACAACACTTCCTTG	GAATCTTGATCAGAAAGCAAA	54.46,54.74	162
CakTSSR00358	CakTC08639	(GA)14	3512	3539	--	CAMTA	AGTCAATCCGTAAGAAGAAGC	ATAGCGAAAACAACTGAAACA	55.37,55.23	160
CakTSSR00359	CakTC08658	(GA)18	432	467	--	--	GCATTTTAAAACTTCACCTTG	CTCACTCAGTCAGTTTTACCC	54.36,53.99	158
CakTSSR00360	CakTC08661	(TC)6	570	581	--	--	TGCATCTCTTCTGAATTTGAT	ATGGTTTTGGAGATGGATAAT	55.02,55.04	140
CakTSSR00361	CakTC08663	(TCT)5	344	358	--	--	AACATATGCATTCCCTACCTC	GGTTCAATCCACATTCATAGA	55.7,54.98	150
CakTSSR00362	CakTC08673	(AG)7	1790	1803	--	--				
CakTSSR00363	CakTC08719	(TC)9	4199	4216	--	--				
CakTSSR00364	CakTC08729	(CGA)5	408	422	--	--	GTCACAGTTTTCATCATCCAC	AAATCCGATGACGAAGAGTAG	55.38,56.06	146
CakTSSR00365	CakTC08732	(AAG)5	3121	3135	--	--				
CakTSSR00366	CakTC08743	(CCA)5	719	733	--	--	TGAGGAAAGGTTTTCATACAA	CAGACTTGGAGAAGCTTATGA	54.97,54.85	160

CakTSSR00367	CakTC08743	(GA)7	2011	2024	--	--				
CakTSSR00368	CakTC08782	(TC)21	17	58	--	--				
CakTSSR00369	CakTC08807	(ATC)5	1365	1379	--	--	GATGAAGACGAAGACACAAGA	AAGTGGGTTATGATGATTGTG	55.42,54.93	139
CakTSSR00370	CakTC08807	(ATAA)5	1959	1978	--	--	CAAACAAAACCCTAGTTTTCA	TTTTCAGGGTAGGATAAAACC	54.67,55.03	149
CakTSSR00371	CakTC08807	(GAA)8	2217	2240	--	--				
CakTSSR00372	CakTC08812	(GGT)5	438	452	--	--				
CakTSSR00373	CakTC08817	(GAA)5	3599	3613	--	--	CAAAAAGATGGTCAGTGAAAC	TCTTTCTCTGTTTTGAATGGA	54.85,55.08	158
CakTSSR00374	CakTC08832	(CTT)8	294	317	--	--	GGCTCTGGAAATTTAAACAA	AATTCCAAGACAAAGTTCCAT	54.64,55.31	209
CakTSSR00375	CakTC08847	(TTA)6	359	376	--	--	TGTTGGTTTTACCAAAATGAG	ATCTTCTTCTGTTTTGCATGA	55.33,55.1	146
CakTSSR00376	CakTC08855	(GAA)6	2749	2766	--	--	CTGTAGGTGAGGAGATGAAGA	GAAATGTATCACCCTTTGTGA	54.48,55.06	150
CakTSSR00377	CakTC08877	(ACA)5	1	15	--	--				
CakTSSR00378	CakTC08885	(ATC)6	284	301	--	--	ATTCCCTTGAATCACTTTCTC	GAGGTGAAGGAAAGTGGTAAT	54.95,54.9	159
CakTSSR00379	CakTC08892	(AAT)5	481	495	--	--	GGGGTACCCTTTATAGAGACA	TAAACCAATCAAACAAAGGTG	54.89,55.33	147
CakTSSR00380	CakTC08897	(AT)7	29	42	Mature Leaf	--				
CakTSSR00381	CakTC08924	(TCCTCT)9	2364	2417	--	--	CATTGTTGTCATCTTCATCCT	TCAAAGTGATCTGATGGATTC	55.05,55.09	152
CakTSSR00382	CakTC08958	(TTG)5	389	403	--	--				
CakTSSR00383	CakTC09005	(GA)10	1920	1939	--	--				
CakTSSR00384	CakTC09007	(TC)6	2859	2870	Flower bud	--	GTTCAGTCAACTTTCGTGTTC	AAGGGAGAGACAAGTGAAGAG	54.9,55.25	152
CakTSSR00385	CakTC09026	(TGT)5	515	529	--	--	GCACACTATTCCAAGAACAAG	GTACCAACGGTTTCATCTTC	55.05,54.61	150
CakTSSR00386	CakTC09031	(AC)6	6	17	--	--				
CakTSSR00387	CakTC09046	(AT)6	283	294	--	NOZZLE	AGCTGGACTTAAAGTTTGACC	CAACCTTTGTACAGCTTCATT	55.3,54.66	150
CakTSSR00388	CakTC09051	(AG)7	2012	2025	--	--				
CakTSSR00389	CakTC09062	(GAT)6	540	557	--	--	AGAAAAAGGCATCCAATTAAG	TGCTGAGAATCAACTTCAAAT	55.33,55.1	168
CakTSSR00390	CakTC09068	(GAA)6	510	527	--	--	AAGGGATGATTTGAATAAAGG	TAACTCAGCTCCCACATTTAC	54.95,54.58	160
CakTSSR00391	CakTC09109	(GAA)7	323	343	--	--	TATCCTCTAGGGAAACACCTC	ATTTGAAGCTGTTCTTGTGAA	54.99,55.18	152
CakTSSR00392	CakTC09122	(TC)11	67	88	--	--				
CakTSSR00393	CakTC09132	(GT)6	583	594	--	--	AATAAACATAGCCCTTTGAGG	GTCAGCCTGTTGTACATGAAT	55.21,55.16	141
CakTSSR00394	CakTC09170	(AG)6	76	87	--	--				
CakTSSR00395	CakTC09181	(TCT)5	116	130	--	--	CTTCGTGACTGTGACTTTCTT	GGAATTAAAGCATTGACTGAG	54.65,54.24	149
CakTSSR00396	CakTC09190	(TC)6	1	12	--	--				
CakTSSR00397	CakTC09191	(TA)10	1413	1432	--	--				
CakTSSR00398	CakTC09209	(TA)6	1287	1298	--	--				
CakTSSR00399	CakTC09211	(AAG)5	85	99	--	--				
CakTSSR00400	CakTC09265	(TC)10	739	758	Flower bud	--	CCATTTCTTTGGCATATTGTA	CTTGCATACACAACGTGAATA	55.37,54.85	147
CakTSSR00401	CakTC09285	(AAAG)5	77	96	Young_pod	--				
CakTSSR00402	CakTC09296	(GA)12	1069	1092	--	--				
CakTSSR00403	CakTC09333	(CA)6	1181	1192	--	--	AATTAGCATAGTTCAGCACCA	TGTAGTTCATTTTCTGTCTGGA	55.18,55.04	143

CakTSSR00404	CakTC09341	(AATGTG)5	647	676	--	--	GGGTTATGTTACCAACTGTGA	TCACTCTTTCCAATTCTCAAA	55.02,55.08	176
CakTSSR00405	CakTC09352	(CT)9	1	18	--	--				
CakTSSR00406	CakTC09355	(CAC)7	371	391	--	--	TTAATAGCCAATTCCCTTCTC	GGAAGAGAAAATTCAACATGA	55.25,54.42	159
CakTSSR00407	CakTC09374	(CT)9	63	80	--	--				
CakTSSR00408	CakTC09375	(TC)8	110	125	--	--	TATCTTTCCACACCAACAAAT	TGATTGAAATAAGCACCCATA	54.67,55.77	150
CakTSSR00409	CakTC09397	(TTC)6	220	237	--	--	GTGTCTCTGACCTTAAAACGA	ATGATGGAAATGAAAGGAAGT	54.59,55.23	150
CakTSSR00410	CakTC09425	(ATG)6	1521	1538	--	--				
CakTSSR00411	CakTC09477	(CCA)6	1175	1192	--	bZIP	GAACATTGTCAGAAAACATGG	AGTGACTCTATTGCCTTCCTC	55.56,55.21	136
CakTSSR00412	CakTC09505	(CAC)5	546	560	--	--	CAACTCCTCCACCATGTC	GATAGGAACATCTCAATGTCG	54.45,54.72	149
CakTSSR00413	CakTC09550	(TC)7	22	35	--	--				
CakTSSR00414	CakTC09552	(TC)7	8	21	--	--				
CakTSSR00415	CakTC09555	(CTT)7	170	190	--	--	AGAGAAGCTGGTTTTTCAAGT	ACCAAAAGGGTCAATTTCTAT	55.02,54.48	144
CakTSSR00416	CakTC09562	(AG)11	1205	1226	--	--	GGGAAGTAGTTGAGAACCTGT	GAGAATCGAAGTGATGCTCT	54.96,54.52	134
CakTSSR00417	CakTC09575	(GAT)5	1126	1140	--	--	TCAGATTGATTTAGTTGCACA	TCCAAAACAAACAGTAAGCAT	54.42,55.07	148
CakTSSR00418	CakTC09584	(CT)17	114	147	--	--	CAAGTGTTCTCATTTCTTTCG	AGCTGAGTAAAATTCATCGTG	55.19,54.74	160
CakTSSR00419	CakTC09585	(ATA)8	1556	1579	--	--				
CakTSSR00420	CakTC09601	(CTT)5	2713	2727	--	--	TCCCATGTCAGTTTCTGTTAC	CAAAGTTAAGTGCTGCTGAGT	55.13,55.04	152
CakTSSR00421	CakTC09611	(AG)6	598	609	--	--				
CakTSSR00422	CakTC09612	(AT)8	3129	3144	--	--				
CakTSSR00423	CakTC09637	(AT)7	1638	1651	--	--				
CakTSSR00424	CakTC09645	(CT)18	1	36	--	--				
CakTSSR00425	CakTC09653	(GATAGA)5	240	269	--	--	ATAGGGTTGAAGATGATGGTT	CCTATCCCTATCCCTTTCTCT	55.11,55.58	182
CakTSSR00426	CakTC09669	(TGT)5	245	259	--	--	TGAAGTGGTTTCTGAGTTGTT	ATTCAACCTCACAGTTTCAGA	54.92,54.83	151
CakTSSR00427	CakTC09676	(TC)8	26	41	--	--				
CakTSSR00428	CakTC09678	(TCAC)9	1204	1239	--	--	TGTAGACGAAGTTTTCACGAT	GAGACGGATGAGAGTGAGAG	55.05,54.85	153
CakTSSR00429	CakTC09692	(GA)6	806	817	--	--	AAGGGAGAGACAAGTGAAGAG	GTTCAGTCAACTTTCGTGTTC	55.25,54.9	152
CakTSSR00430	CakTC09700	(CA)6	1308	1319	--	--				
CakTSSR00431	CakTC09706	(TC)6	5	16	--	--				
CakTSSR00432	CakTC09712	(GCT)5	1588	1602	--	--	GGAGTGCTGCTTGTAGTAATG	CTTCAACATTTTCCTCTCCAT	55.25,55.88	144
CakTSSR00433	CakTC09715	(GA)7	1939	1952	--	--				
CakTSSR00434	CakTC09739	(TGC)6	209	226	--	--	ACACAAGATTCAACCTTTTCA	AGGGTTTACAATGAATGAATG	54.85,54.2	155
CakTSSR00435	CakTC09746	(AAG)5	60	74	--	--				
CakTSSR00436	CakTC09763	(TC)7	10	23	--	--				
CakTSSR00437	CakTC09779	(CTT)6	1340	1357	--	--	AAATCACCTAGGTTTTGGTTC	TTCTGAAGTTGAAAAAGTGGA	54.92,55.16	144
CakTSSR00438	CakTC09793	(CT)10	1424	1443	--	--	GAGCGAGTTTGAGAAATGATA	TTTGGGGATTAATTGATGAT	54.78,54.43	155
CakTSSR00439	CakTC09794	(CT)11	1435	1456	--	--	GAGCGAGTTTGAGAAATGATA	AGCTATGAGGAGAAACGAAAT	54.78,54.91	148
CakTSSR00440	CakTC09795	(TCT)6	49	66	--	C2H2				

CakTSSR00441	CakTC09810	(AACA)5	71	90	--	--				
CakTSSR00442	CakTC09821	(TC)11	1559	1580	--	--				
CakTSSR00443	CakTC09822	(AGA)6	2868	2885	--	--				
CakTSSR00444	CakTC09824	(TTC)7	536	556	--	--	TTGATTCATATTTCCATCCAC	CAGAGAAGAATGAGGATGATG	54.92,54.84	144
CakTSSR00445	CakTC09827	(ATG)5	2318	2332	--	--	TCAACCATTTCTCAGACTTGT	AGATTGAATCATCATCACCAC	54.83,54.77	147
CakTSSR00446	CakTC09844	(GGA)5	1856	1870	--	--	AGATGTTCCAATTCCTTCTGT	TTGTTCCACAGTTTTCCTGTA	55.3,55.81	153
CakTSSR00447	CakTC09856	(GAAGA)6	7	36	--	--				
CakTSSR00448	CakTC09857	(GAA)6	2695	2712	--	--	TGTAGGTGAGGAGATGAAGAG	GAAATGTATCACCCTTTGTGA	54.48,55.06	150
CakTSSR00449	CakTC09870	(TAT)7	1647	1667	--	--				
CakTSSR00450	CakTC09873	(GAA)5	1187	1201	--	--	GGTTCAATCCACATTCATAGA	AACATATGCATTCCCTACCTC	54.98,55.7	150
CakTSSR00451	CakTC09874	(GAT)7	1041	1061	--	--	AAGGAGAGAAACCCTAAGTTG	GTTTCCCTTTTCATTCATTCT	54.42,54.97	149
CakTSSR00452	CakTC09874	(GA)8	1242	1257	--	--	GGAAACAACAGAATACAGCAC	GTAGTAGTGCTCCACAAATGC	54.88,55.08	155
CakTSSR00453	CakTC09877	(CT)9	1062	1079	--	--	CTTGAAGAAATTCCAGACCTT	GGTTGTGACTTGTGAGTTGTT	55.2,55.1	148
CakTSSR00454	CakTC09878	(TCT)5	104	118	--	--	CTTCGTGACTGTGACTTTCTT	GGAATTAAAGCATTGACTGAG	54.65,54.24	149
CakTSSR00455	CakTC09896	(CAA)5	223	237	--	--	GTAGCTTCTGAATCAAACACG	ACCTTTGAGTAACCCTTGAAC	55.22,54.98	169
CakTSSR00456	CakTC09899	(TA)8	119	134	--	--	CATTCCCTTCACCTCTAATTT	CTAGGTTAAAAGCCAGGAATC	55.01,54.94	152
CakTSSR00457	CakTC09918	(TTA)5	914	928	--	--				
CakTSSR00458	CakTC09920	(TC)10	3377	3396	--	--				
CakTSSR00459	CakTC09923	(AAG)5	42	56	--	--				
CakTSSR00460	CakTC09927	(CTG)5	468	482	--	--	TGAAAACAAACATTCTTCTCC	ACTGAGAGACTTGGATTTTCC	54.5,55.01	150
CakTSSR00461	CakTC09934	(ATT)6	440	457	--	Trihelix	CCTCTTTGCATTTCTCTTGTA	TTGGCATTGATAAACCTAAAG	54.88,54.72	159
CakTSSR00462	CakTC09934	(TCA)6	1152	1169	--	Trihelix	AACACTTGAACATTTCAAACC	ATGGTGATGTTGTTACCAAAG	54.28,55.02	153
CakTSSR00463	CakTC09936	(CAG)6	1200	1217	--	NAC	TTCCAAATCCAATCTCAACTA	CTTCCACTTTCCACCTATTTT	54.89,55.09	155
CakTSSR00464	CakTC09936	(AAAGAG)5	1364	1393	--	NAC	TGCACATCATCTTCTTCTGAT	TCATCATGACACAGAAAACAA	55.82,55	165
CakTSSR00465	CakTC09936	(AAC)5	1682	1696	--	NAC	CTATACACTTCGGTGCTTCTC	GAGAGTCTTAGGTCGTTCTCC	54.32,54.75	150
CakTSSR00466	CakTC09942	(AG)12	1	24	--	--				
CakTSSR00467	CakTC09965	(AAG)6	291	308	--	--	CACAGAACAAAGAACAACACA	CAGACTGAAAAAGTTCGTGTC	54.73,55.08	153
CakTSSR00468	CakTC09966	(CAA)5	661	675	--	--				
CakTSSR00469	CakTC09971	(TC)7	9	22	--	--				
CakTSSR00470	CakTC09974	(TTCACA)7	58	99	Young_pod	--				
CakTSSR00471	CakTC09982	(AG)8	2138	2153	--	ARR-B	GAAGTTCTGCCACTGAGTTC	TTAGGAACAGCCTTGTCAAT	54.95,54.96	153
CakTSSR00472	CakTC09997	(AG)6	80	91	--	--				
CakTSSR00473	CakTC09997	(AGA)5	341	355	--	--	GATGAAACTCAACAGAGTTGG	AGTAACAAAACCAGCAACAAA	54.83,55.15	153
CakTSSR00474	CakTC10012	(AG)8	2073	2088	--	--				
CakTSSR00475	CakTC10014	(TCA)6	580	597	--	--	GATCTCAAAACTTTTCCCAAT	GGCATGAAAATGTACTCAAGA	54.97,55.39	137
CakTSSR00476	CakTC10023	(GGT)5	61	75	--	--				
CakTSSR00477	CakTC10026	(AGT)5	3490	3504	--	--	AGAGAGAAGGTGAAGGTGAAG	TCGTCATCTCTCTTTTCAAAC	55.25,54.64	138

CakTSSR00478	CakTC10027	(TTC)8	92	115	--	--				
CakTSSR00479	CakTC10035	(TA)6	2116	2127	--	--				
CakTSSR00480	CakTC10047	(GGAAA)5	209	233	--	--	CATTTCAAGTCGGGAATCTA	TGCGAAGAATAAACCATTTAG	55.31,54.88	151
CakTSSR00481	CakTC10059	(TTC)6	128	145	--	--	CTCATTCTTCATTCTGCAAGT	AGTACATGATCGGTGAGTGAA	54.67,55.6	152
CakTSSR00482	CakTC10059	(TTA)5	3816	3830	--	--	TTCAGTAGAGAAAGCCAAGAA	ATATAACTTGCAATGCCCTTA	54.59,54.3	146
CakTSSR00483	CakTC10062	(ATG)5	3047	3061	Young_pod	--	TTCCATCTTCCGTGTATAAAA	TCTACTTCTTCACACCCTTCA	54.94,55.01	149
CakTSSR00484	CakTC10066	(ATT)5	2869	2883	--	--				
CakTSSR00485	CakTC10078	(ATT)5	297	311	--	bHLH	GAGGAACCATTTTCTTTCTTC	AAAAACCCTTCTCATTGTGTT	54.69,55.38	167
CakTSSR00486	CakTC10088	(TTA)6	394	411	--	--	TAAACCCTTTCTTCCTCATTC	ATTGTTTTGACCACGAAACTT	55.13,56.29	173
CakTSSR00487	CakTC10092	(TC)9	171	188	--	--	CATCTGAGAAAAAGCTGAAAA	GTTTTCAAGGTCAAACTCCAT	55,55.78	148
CakTSSR00488	CakTC10107	(ACC)5	274	288	--	--	AGAAATCGCGTTATTGGTAAT	TCTTGCTTTAGAATGTTCGAT	55.61,54.4	148
CakTSSR00489	CakTC10108	(GAA)5	1753	1767	--	--				
CakTSSR00490	CakTC10114	(CAA)8	225	248	--	C2C2-Dof	TTGGAAACAGAACTTATTGGA	ATTTGGTGTTTGTTGATTCAC	54.97,54.97	146
CakTSSR00491	CakTC10117	(CA)7	206	219	--	--	CCAAAGTTGTTTGAACCATAG	CGGAAAGATTTGTGAACTTTA	54.92,54.74	147
CakTSSR00492	CakTC10119	(AAG)5	1062	1076	--	TRAF	CATCCAAGACAAAATTCCTTA	GGCTCTGGAAATTTAAACAA	54.5,54.64	157
CakTSSR00493	CakTC10120	(AAC)5	1148	1162	--	--	TGAATGATGAAGAAATGTTCC	CCACCTTTAATTTTTCACCTT	55.11,55.1	148
CakTSSR00494	CakTC10135	(AAT)5	2548	2562	--	--	TTGAAGGGCACAAATTAGATA	GATAAGATTTGGTGCTTTCCT	55.1,55.15	214
CakTSSR00495	CakTC10136	(ATG)6	1382	1399	--	--	TGAGTCCGATCTGAGAATAGA	CAAATCTTAATGCCAACAATG	55.01,55.84	147
CakTSSR00496	CakTC10142	(GCA)5	363	377	--	zf-HD	CACATGTGGAGGTACAACTCT	TAACTACCACCATCACCACTC	55.07,55	169
CakTSSR00497	CakTC10142	(ACC)5	503	517	--	zf-HD	GAGTGGTGATGGTGGTAGTTA	GGAGAAGAGGGTACCATAGAA	55,54.99	137
CakTSSR00498	CakTC10142	(CTTTG)5	1095	1119	--	zf-HD				
CakTSSR00499	CakTC10148	(AG)7	1510	1523	--	--				
CakTSSR00500	CakTC10153	(GA)8	1519	1534	--	--				
CakTSSR00501	CakTC10170	(TCT)5	1	15	--	--				
CakTSSR00502	CakTC10174	(TAT)6	134	151	--	--	GATCAACCTAGTGATTTGCAG	AAGGGACAAGGAGACACTTT	54.97,55.36	150
CakTSSR00503	CakTC10180	(AG)9	4882	4899	--	--				
CakTSSR00504	CakTC10183	(TCT)8	321	344	--	--	AAACATGAGATTCCAGATCCT	ATTAGTGTGCATTGTCGTCTC	55.22,55.32	154
CakTSSR00505	CakTC10190	(AGG)7	238	258	--	--	CACATGGAAACATCAATGAA	TTGAAGACAAAAAGCACAGAT	55.21,55.18	153
CakTSSR00506	CakTC10198	(AGA)6	753	770	--	--	AAGTCTAGGGTTTGTGCATTA	CACTTCACTCATTTCAATTCC	54.2,54.77	153
CakTSSR00507	CakTC10199	(AGA)5	241	255	--	TCP	ACTTTCATTTTTCCACTTTGG	ATCCAGACTCGGACACTTTAT	55.94,55.33	147
CakTSSR00508	CakTC10199	(TAA)6	973	990	--	TCP	CCGGTGCTACTATTGATACTG	GTCCCCTCTGTGAAAGATACT	55.07,54.88	162
CakTSSR00509	CakTC10211	(TAT)5	831	845	--	--	ATGTGTATGCTATGTCCATCC	ATCCACAGCTAATCTGAAACA	54.87,54.97	140
CakTSSR00510	CakTC10222	(ATT)6	845	862	--	bHLH	TCCATCAAAACAAGAGAAAGA	TAAATTCCGACCTTATCAATG	55.08,54.49	158
CakTSSR00511	CakTC10232	(TCT)6	226	243	--	--	CGAAACGACCATCTCTACTAA	AATACTACCGGGAGAGTAACG	54.75,55.15	152
CakTSSR00512	CakTC10232	(CAA)5	454	468	--	--	AAGAACAAATCAGATCCCAAT	TTGAAGTGGTTACCAGGACTA	55.23,54.9	150
CakTSSR00513	CakTC10233	(AT)8	727	742	--	--	CACAGTTCAAAGGTTCAAAAG	AGATGGCACAATTGAAACTAA	55.11,54.99	148
CakTSSR00514	CakTC10248	(CCG)6	35	52	--	--				

CakTSSR00515	CakTC10251	(AAT)6	48	65	--	TRAF				
CakTSSR00516	CakTC10251	(ATC)5	1467	1481	--	TRAF				
CakTSSR00517	CakTC10256	(TA)6	78	89	--	--				
CakTSSR00518	CakTC10258	(ATG)6	1316	1333	--	--				
CakTSSR00519	CakTC10262	(AAG)6	409	426	--	--	GTCTGTCAAATGTTTCCTACG	CCCCTTTACAAACGTTAAGAC	54.89,55.58	148
CakTSSR00520	CakTC10263	(GA)7	2649	2662	--	--				
CakTSSR00521	CakTC10270	(ACA)6	45	62	--	--				
CakTSSR00522	CakTC10272	(GTT)5	1218	1232	--	--	AGTTTTTCAGAAATGTGAGGA	TTGGAATGAACCATTTACTACA	54.08,54.88	157
CakTSSR00523	CakTC10275	(TCC)5	120	134	--	CCHC	CAAACCCTAACACTAACACCA	AAAATAGCGAAGAACGTAACC	55.27,55.42	167
CakTSSR00524	CakTC10288	(CA)10	87	106	--	--				
CakTSSR00525	CakTC10288	(CAA)5	481	495	--	--	GTCTCGATCCAAACAACAAC	GGGAAGAATGAGGTAGTAGGA	55.54,54.99	160
CakTSSR00526	CakTC10288	(AGA)5	889	903	--	--	GTTTTAGAACAACCACAACCA	TTTCAACGATTCTTCTTCAAC	55.23,54.67	150
CakTSSR00527	CakTC10306	(ATA)5	332	346	--	--	TGTTCATCATTCAAGAGCTTC	ACAATTCACTGTCAGCATCTT	55.52,54.85	150
CakTSSR00528	CakTC10310	(AAT)5	466	480	--	--	GGGGTACCCTTTATAGAGACA	AACCAATGATACAAAGGTGTG	54.89,55.02	144
CakTSSR00529	CakTC10312	(AT)15	1676	1705	--	--				
CakTSSR00530	CakTC10321	(TTC)6	10	27	--	--				
CakTSSR00531	CakTC10321	(GAA)6	154	171	--	--	TGATGATAACGTTTTTGGTAGA	CTCTTTTAACATTCCCAGACC	55.03,55.47	154
CakTSSR00532	CakTC10321	(GAAAGA)5	331	360	--	--	CATAACATCGGGTTAAGTGTC	AAAGAGAACGAACTGAGGTCT	54.7,54.84	156
CakTSSR00533	CakTC10332	(TGA)11	468	500	--	--	AATCGTACATCACAACTCGAT	CAGCATTTACTTTCACCAATC	54.66,54.99	151
CakTSSR00534	CakTC10340	(ACC)8	160	183	--	--	CAACATTTCATCAAACACCTT	GTGGTTTTGGTTTCAACATT	55.15,54.96	162
CakTSSR00535	CakTC10348	(CTC)5	179	193	--	--	ACCACATCTTAATTCCTCTCC	GGAGGAGGGAGTATGAGAAAC	54.82,56.78	155
CakTSSR00536	CakTC10354	(GA)9	1021	1038	--	--				
CakTSSR00537	CakTC10366	(TGA)5	992	1006	--	--				
CakTSSR00538	CakTC10368	(AAC)7	275	295	--	MYB-related	CAGTATCATGATTTTCGGTGT	GTGGTTGTTCGTATTGAGAGA	55.1,55.3	119
CakTSSR00539	CakTC10377	(GA)9	1	18	--	--				
CakTSSR00540	CakTC10379	(TG)6	1	12	--	--				
CakTSSR00541	CakTC10390	(CAG)7	2225	2245	--	C3H	TTGGTTAAGGATGTTCCTTCT	CTACCATCTGCTCTTGCTCTA	55.47,55.05	154
CakTSSR00542	CakTC10403	(TCCTCT)6	2289	2324	--	--	TCATCATTGTTGTCATCTTCA	TCAAAGTGATCTGATGGATTC	54.91,55.09	138
CakTSSR00543	CakTC10409	(TA)8	3	18	--	--				
CakTSSR00544	CakTC10417	(TCA)5	98	112	--	Pseudo_ARR-B				
CakTSSR00545	CakTC10427	(ATT)5	3267	3281	--	PHD	GAATCAATTTCTTCACCGTTA	CCTCTCGATCATCTCTACTCC	54.49,55.55	148
CakTSSR00546	CakTC10445	(AGA)5	1064	1078	--	BSD	GATCTTGAGTGGGATGAGATT	CATCATCTTCAATATCCCAAC	55.63,54.49	151
CakTSSR00547	CakTC10450	(ACA)6	1338	1355	--	SBP	AAGCTTCTTCCTTAAACCAAA	GTGTCTGTTGTTGTTGTGTTG	55.14,54.92	153
CakTSSR00548	CakTC10459	(AG)11	3566	3587	--	--				
CakTSSR00549	CakTC10461	(AAT)5	453	467	--	--	GTGATTCACCTCAAACTCAAA	TTCTCAATGACTGTTGGTTTT	55.26,54.85	157
CakTSSR00550	CakTC10469	(GGT)5	709	723	--	C2H2	AGAGTGCAAGGTTAACATCAG	GTGAACCAAACTGTGAAACAT	54.63,55.04	156
CakTSSR00551	CakTC10469	(CAA)5	828	842	--	C2H2	ATGTTTCACAGTTTGGTTCAC	TCCCATATCTGAAATTTGTTG	55.04,55.17	143

CakTSSR00552	CakTC10470	(GGT)6	553	570	--	--	CGGTGAAGAATTTAAGGAACT	AAACCCTAATGGACGATACTC	55.24,54.88	156
CakTSSR00553	CakTC10473	(GAA)6	9	26	--	--				
CakTSSR00554	CakTC10493	(GAT)5	4	18	Flower bud	bHLH				
CakTSSR00555	CakTC10506	(TTAT)5	1238	1257	--	--				
CakTSSR00556	CakTC10519	(TGG)5	1485	1499	--	--	CTGAAATTGCTGGAATAATGA	CGTTCTCAGTTTCTTCTCCTT	55.49,55.42	150
CakTSSR00557	CakTC10534	(ACA)6	43	60	--	--				
CakTSSR00558	CakTC10541	(AC)6	109	120	--	--	TGAGCTCTATGAAAGAGGTTG	GGATTTCAAAAGAGCTTGAAT	54.85,55.27	143
CakTSSR00559	CakTC10590	(GAT)5	392	406	--	--	CTAGTGCCTTTGAAAGTGTTG	CATTTCCACAATCAGGTAAAG	55.31,54.84	147
CakTSSR00560	CakTC10591	(CAA)7	167	187	--	bHLH	ATGCCTTCAAGTTTCAACAC	GAGGCATGTAATCAACAATTC	55.28,54.73	149
CakTSSR00561	CakTC10600	(GAA)5	447	461	--	--				
CakTSSR00562	CakTC10607	(GGA)5	1364	1378	--	TCP	TCCAAACGAATAACCTAACAA	GTCTTCTTTTTCTTCCCATGT	55.02,55.03	153
CakTSSR00563	CakTC10607	(CTT)8	2010	2033	--	TCP	TCGACATGATAACACAAGTCA	TAAGATCAAACAAACCTCACG	55.04,55.48	160
CakTSSR00564	CakTC10649	(TCA)5	486	500	--	MYB	CTGAAGAAATTGCAAACAGAT	TGAAAGTGCTTCACTAAGAGC	54.7,55.09	156
CakTSSR00565	CakTC10651	(CTG)6	90	107	--	--				
CakTSSR00566	CakTC10655	(GAA)5	2263	2277	--	--				
CakTSSR00567	CakTC10676	(TGT)7	471	491	--	HB	ATATTTTTGTTGTTGGTGTCG	GAAGCCAATGGTAGAAGAAAT	55.2,55.15	155
CakTSSR00568	CakTC10676	(CCT)6	863	880	--	HB	TTGATTAAAAGCTCTCTGCTG	GCACAGGTGAAGCATAGTAGT	55.2,54.65	150
CakTSSR00569	CakTC10676	(GAA)8	2231	2254	--	HB	TCTTGGGACATAGAATTTGAA	GATGATTTCAACATGGGAATA	54.89,54.92	149
CakTSSR00570	CakTC10678	(AATTTC)5	96	125	--	--				
CakTSSR00571	CakTC10682	(GTT)6	1460	1477	--	--	ACACCGTTTCACACACACT	GTTTCCAAGGAAATCAAAGAA	54.6,55.99	147
CakTSSR00572	CakTC10682	(GA)7	1820	1833	--	--				
CakTSSR00573	CakTC10685	(TC)17	272	305	--	--	GTGTGAAGTTTTTGTTCTTTTG	AGGAACAGAGAGTTTGAGACC	54.31,55.07	140
CakTSSR00574	CakTC10699	(CTT)7	1309	1329	--	--				
CakTSSR00575	CakTC10700	(TG)10	263	282	--	--	CGACTTCTTGAATTTATGTGG	CTAGGAAAGGTACAAAAGCAA	55,54.14	160
CakTSSR00576	CakTC10727	(TCA)5	244	258	--	--	TTTGTCAACTTTTTGATCCTC	ATTGGTGATGTTAGTGATGCT	54.5,54.66	159
CakTSSR00577	CakTC10727	(CAT)5	1051	1065	--	--	CATTATCAGCAGATACGGACT	GATTCTGCTGGAAGTGAAGAT	54.53,56.04	148
CakTSSR00578	CakTC10741	(AG)9	1986	2003	--	--	TGGACCAAGAAAATAACTTGA	TTACCTTTGCAATGTTTGTCT	54.97,55.07	143
CakTSSR00579	CakTC10743	(AAC)5	452	466	--	--	CAAATCGCAGACCTTTATTTA	GCGAGTTTTTAGGTGTTGATA	54.88,54.77	152
CakTSSR00580	CakTC10743	(TAA)5	1435	1449	--	--	TGTTGTTGTTGATGTTTCTGA	AATCCCTCTTTCTCAAGGTAA	55.09,54.74	150
CakTSSR00581	CakTC10751	(TA)6	47	58	--	--				
CakTSSR00582	CakTC10752	(TC)6	233	244	--	--				
CakTSSR00583	CakTC10754	(AAT)5	265	279	--	--	AGGAGGAGGTTGGTGATAGTA	CGGAGTCTAGAAAGAAAGAGG	55.34,55.12	152
CakTSSR00584	CakTC10754	(GA)48	1942	2037	--	--				
CakTSSR00585	CakTC10761	(AG)7	680	693	--	--	GGAGCTTAGAAATAGGTGGAA	TTTGCCAACTGAATCGTAGT	55.32,55.91	150
CakTSSR00586	CakTC10765	(AG)9	1786	1803	--	--				
CakTSSR00587	CakTC10782	(TG)6	8	19	--	--				
CakTSSR00588	CakTC10792	(AAG)6	94	111	Flower bud	--				

CakTSSR00589	CakTC10795	(TTG)5	611	625	--	--	TTCTCCAAGAGTTTAGGGTTT	TGATAAACCTCTCACGAAATC	54.82,54.45	159
CakTSSR00590	CakTC10797	(GGA)6	352	369	--	G2-like	GTCGAATGTGGTAGGTGTTTA	CGTAGGAGGAATGAGAGAAAT	55.18,55.15	133
CakTSSR00591	CakTC10799	(ATT)6	27	44	--	--				
CakTSSR00592	CakTC10802	(TC)10	34	53	--	--				
CakTSSR00593	CakTC10817	(GT)6	1596	1607	--	--				
CakTSSR00594	CakTC10824	(GAA)5	62	76	--	--				
CakTSSR00595	CakTC10832	(ATC)6	119	136	--	C2C2-YABBY	CATATATCCAAGGTGTGGTTG	GTGGTCCAGTGATAATGTTGT	55.4,54.81	150
CakTSSR00596	CakTC10890	(GAG)5	309	323	--	--				
CakTSSR00597	CakTC10898	(ACA)6	105	122	Flower bud	--	GAGAGAGGACCCAGATACAAT	CAAACCAACTTGAAACTTGAC	54.79,54.94	153
CakTSSR00598	CakTC10914	(GAA)5	89	103	--	--				
CakTSSR00599	CakTC10923	(TC)6	136	147	--	--	ATACCCACTCTCACAACACAC	CTTGGGACATGTCTTTGACT	54.88,55.1	159
CakTSSR00600	CakTC10938	(AG)12	936	959	--	--	AGAGGTGGAGGTGAGGTATAG	ATTCTTTGCATTCTCAACAAG	54.93,54.7	159
CakTSSR00601	CakTC10946	(TGA)5	733	747	--	FHA	TTGGAAGAAACACTGAATGAT	CTTTTTCTTCGTACGGTCATA	54.77,54.78	150
CakTSSR00602	CakTC10949	(AGAAGC)5	842	871	--	--	ATGATGCAGATTTGGTTAGAA	GTGGTAGTGTAGGGGAAGTTT	54.91,54.86	153
CakTSSR00603	CakTC10959	(AG)8	52	67	--	--				
CakTSSR00604	CakTC10966	(TTG)6	673	690	--	--				
CakTSSR00605	CakTC10967	(AG)6	1378	1389	--	MYB	TTTGGATATATGAGGATGGTG	AGTTTCACTTTTTGTGCCTTA	54.96,54.41	177
CakTSSR00606	CakTC10973	(GA)6	667	678	--	--	GGCTACTGAAGAACAAATTCA	TATAAGCATTCTCCGGTTATG	54.7,54.68	157
CakTSSR00607	CakTC10974	(TGA)6	357	374	--	--	CGATGATCAACTCAATGTCTC	CCTCCAGGAGTCGTAATAGTT	55.65,55.11	144
CakTSSR00608	CakTC10982	(AAG)8	117	140	--	--	GAGGTGAATTCGATTGATGTA	AGAAGAAATGGCAATTTTAGG	55.15,55.33	138
CakTSSR00609	CakTC10995	(AG)28	988	1043	--	--				
CakTSSR00610	CakTC11001	(CAC)6	196	213	--	--	CCCAACTTTCTAACCCTTTAC	GGAAGGAATCAAGAGAGGTAG	54.72,54.71	164
CakTSSR00611	CakTC11014	(TAA)5	50	64	--	Tify				
CakTSSR00612	CakTC11019	(GAA)5	436	450	--	--				
CakTSSR00613	CakTC11024	(ATGC)5	265	284	--	--	ATGACTGGGTAATGGAAGATG	CCACTCTATCCATGAGTTGAA	56.46,55.23	175
CakTSSR00614	CakTC11032	(AAT)6	173	190	--	AUX/IAA	TCATTTCTTGTCCCTAATGAA	AGGTCGAGGCTAGTATAGGAA	54.89,54.96	152
CakTSSR00615	CakTC11037	(CA)8	58	73	Young_pod	--				
CakTSSR00616	CakTC11047	(CAA)7	858	878	--	--	TCGAAAAACCTATCCTTTCAT	ACTGATGATTGGTACTGATGG	55.55,54.91	153
CakTSSR00617	CakTC11048	(TG)8	542	557	--	C2C2-YABBY				
CakTSSR00618	CakTC11054	(TCTGGT)8	620	667	--	--	GTAATCCTGGAAGTAGGAGGA	GAGGTTCAACCATAACCTCA	54.99,55.03	147
CakTSSR00619	CakTC11066	(AG)27	2928	2981	--	--				
CakTSSR00620	CakTC11070	(GAA)5	457	471	Shoot	--				
CakTSSR00621	CakTC11084	(CT)15	13	42	--	--				
CakTSSR00622	CakTC11087	(TG)6	1	12	--	--				
CakTSSR00623	CakTC11087	(AC)7	946	959	--	--	GTGTGCATACATTACATGTGC	AGCCAAGAAAAGGAGATTAAA	54.93,55.06	156
CakTSSR00624	CakTC11091	(GAA)6	183	200	--	--	GGGCTTTGACTTTCAAATAA	GGATTGGAAAGAATTCAAGTT	54.64,54.97	145
CakTSSR00625	CakTC11097	(CAC)5	115	129	--	--	TCTAGAGCCACTACCAGTGAA	ATTGAAGAGGGATTGAGAGAG	55.22,55.1	149

CakTSSR00626	CakTC11099	(TCA)5	617	631	--	--	CAGAAGATGTTCAATCCATGT	GACAGTTATGCAGCTTTCACT	55.05,54.78	150
CakTSSR00627	CakTC11102	(GAA)5	526	540	--	--	CTGAGGAAGATGAAGAGGACT	ACAAATCTTGTGTGTTTTCCA	55.17,55.64	154
CakTSSR00628	CakTC11116	(ATC)6	298	315	--	--	CCTTTTCTGATTTCCTTGAAT	GCCGAAAATTCAACTGAGTA	55.13,55.54	159
CakTSSR00629	CakTC11140	(CT)6	189	200	--	--	ACCAAACAAGGAACAATCTCT	GTTTTGAAGGGTGATGATATG	55.38,54.58	150
CakTSSR00630	CakTC11163	(TC)6	92	103	Young_pod	--				
CakTSSR00631	CakTC11174	(GA)14	1027	1054	--	--				
CakTSSR00632	CakTC11206	(GA)10	801	820	--	--	CAACCTCTTAAAATCCCTCAT	CCCACAAACACTTATTCATTT	55.01,54.29	150
CakTSSR00633	CakTC11208	(ATG)8	21	44	Shoot	--				
CakTSSR00634	CakTC11216	(AAC)5	442	456	--	--				
CakTSSR00635	CakTC11236	(CCT)5	253	267	Flower bud	--	ACGACTTCTCGTATACCTTCC	TAGAAGTATCTGAGCGATGGA	55.1,55.23	158
CakTSSR00636	CakTC11237	(CTT)6	431	448	--	--				
CakTSSR00637	CakTC11243	(GAT)5	528	542	--	--				
CakTSSR00638	CakTC11271	(TTA)5	114	128	--	--	AAGATCAGTGAAATGCCTAAT	TCAATCTTTCAAATGTTAGTGC	53.19,54.74	146
CakTSSR00639	CakTC11336	(AAC)6	34	51	Shoot	--				
CakTSSR00640	CakTC11354	(TGT)6	518	535	--	--				
CakTSSR00641	CakTC11367	(CT)10	116	135	--	--	TTCTCTTACCTCAAGTGCAAC	AATATGGGAAGAGAAGACCAG	54.76,54.99	157
CakTSSR00642	CakTC11369	(AAT)5	1333	1347	--	--				
CakTSSR00643	CakTC11370	(TAA)10	1	30	--	--				
CakTSSR00644	CakTC11371	(GAA)7	280	300	--	--	CAACCTAAAGATGAAGTCGAA	GTTCTTGTTTTTCGAATGATG	54.72,54.96	149
CakTSSR00645	CakTC11374	(TG)7	1187	1200	--	--				
CakTSSR00646	CakTC11384	(GA)14	1246	1273	--	--	GAGGCATCTTCCTTTACTTGT	TTTCCACTGATAGCATAGCAT	55.22,55.1	147
CakTSSR00647	CakTC11388	(TTC)8	72	95	--	--				
CakTSSR00648	CakTC11390	(AAG)5	166	180	--	--	ATGAGATGGCGAAGGTATTAT	CACGAAGACTGCTCTGATAAC	55.38,55.2	163
CakTSSR00649	CakTC11396	(TTG)5	1236	1250	--	--	GTTTTGAATTTCTTGTCAGCA	TCTCAATGGTTACAAGTCAGG	55.6,55.31	150
CakTSSR00650	CakTC11403	(CGA)5	554	568	--	--	GTCACAGTTTTCATCATCCAC	AAATCCGATGACGAAGAGTAG	55.38,56.06	146
CakTSSR00651	CakTC11411	(TTG)5	1731	1745	--	--	TCTTCTTGTTCTTGAGTTCCA	TCTCACTCTCACAGACACACA	55.14,55.13	149
CakTSSR00652	CakTC11411	(TTC)6	1985	2002	--	--	GTGTCTCTGACCTTAAAACGA	ATGATGGAAATGAAAGGAAGT	54.59,55.23	150
CakTSSR00653	CakTC11415	(TCA)7	103	123	--	--	GAGAAGCATTTAGTTCACACG	ATTTACACAGCAACAGAAGGA	55.22,55.05	150
CakTSSR00654	CakTC11421	(TCT)6	102	119	--	--	GATTGGGTTCACTTACCTTTT	ATGGAGAGAAAGGAGAAGAAA	54.92,54.83	150
CakTSSR00655	CakTC11450	(TC)18	1	36	--	--				
CakTSSR00656	CakTC11466	(TTC)6	1678	1695	--	--				
CakTSSR00657	CakTC11473	(TTC)5	1195	1209	--	--	TTACTCTTCCGTTCCTTATCC	TGCTAAGTCTTCGAATTTCAG	55.16,55.04	180
CakTSSR00658	CakTC11475	(CT)7	1842	1855	--	--				
CakTSSR00659	CakTC11482	(TG)6	1830	1841	--	--				
CakTSSR00660	CakTC11484	(GAA)6	1656	1673	--	--				
CakTSSR00661	CakTC11486	(CT)10	1731	1750	--	--				
CakTSSR00662	CakTC11529	(CCA)5	96	110	Young_pod	--				

CakTSSR00663	CakTC11531	(CTT)5	1	15	--	--				
CakTSSR00664	CakTC11532	(TC)8	187	202	--	--	ATTTCTTTCTCCAATCTCCAC	AATCTTGAGGTGATTTCCATT	54.95,55.23	163
CakTSSR00665	CakTC11532	(CAA)6	498	515	--	--	ACCAACAACAAAACCTAATCA	TTGAGACTGTTCCTTCTTTCA	54.75,55.14	153
CakTSSR00666	CakTC11532	(ACA)6	689	706	--	--	GGAACAGTCTCAAGAACAGTG	CTAAGAGCAAGTGATGGAAAA	54.89,54.88	154
CakTSSR00667	CakTC11549	(AGA)7	40	60	--	--				
CakTSSR00668	CakTC11551	(CT)7	57	70	--	--				
CakTSSR00669	CakTC11555	(TTC)5	2057	2071	--	--	TCCTTCTTCTCCTTCTTCTTC	CTCTACTCCAACAATGTCACC	54.55,54.69	151
CakTSSR00670	CakTC11582	(AGA)5	69	83	--	--				
CakTSSR00671	CakTC11588	(TG)6	27	38	--	--				
CakTSSR00672	CakTC11589	(TA)7	948	961	--	--	ATTATACAATAGGCACGCAGA	TTCACACACACAAGGCTCTAT	55.15,55.85	157
CakTSSR00673	CakTC11595	(TGG)5	782	796	--	--				
CakTSSR00674	CakTC11620	(CAC)5	763	777	--	--				
CakTSSR00675	CakTC11621	(CAA)5	397	411	--	--	ATTTGGAGCTTCTTCCACTT	ATGAAGTAGGTTGTGGTGTTG	55.61,55.08	161
CakTSSR00676	CakTC11623	(TGA)5	141	155	--	--	TAGTGGTAGTGGGCATAGTGT	AGAGACACTTGAGTTGGGTTT	54.8,55.45	179
CakTSSR00677	CakTC11633	(GAA)5	287	301	--	WRKY	GCGAAGGATAAAGATGAAGAT	TGTCTTGAACGAAACTCTTGT	55.23,55.08	147
CakTSSR00678	CakTC11648	(AT)6	483	494	--	--				
CakTSSR00679	CakTC11680	(TTC)7	1590	1610	--	--	GATTGGGGAATATTCTGATTT	ACAACACTCACACTCCACACT	54.71,55.49	151
CakTSSR00680	CakTC11682	(TTC)5	164	178	--	--	CTTTAGCATTGTCTGAGCTGT	AAAGGGTTGTTTACATCTTCC	54.96,54.92	151
CakTSSR00681	CakTC11689	(TTTAT)7	85	119	--	--				
CakTSSR00682	CakTC11726	(TGG)5	154	168	--	PBF-2-like	ATAGCACTGCACTGTCTCTTC	ACTCCTTCAACTTCAAACACA	54.76,54.92	148
CakTSSR00683	CakTC11727	(TC)9	1462	1479	--	--	GCCATTTTTGAGAGTTTACAA	GTAATTGGAAGAGGATGGAGT	54.73,54.82	141
CakTSSR00684	CakTC11745	(AG)6	512	523	--	--	TCTCTTGAGTCAAAATATGCAA	AAAATTAGGATTGAGGGTTTG	55.3,55.03	149
CakTSSR00685	CakTC11747	(ATA)12	2286	2321	--	--				
CakTSSR00686	CakTC11766	(GAA)5	53	67	--	--				
CakTSSR00687	CakTC11766	(ATAC)5	823	842	--	--				
CakTSSR00688	CakTC11780	(CT)7	19	32	--	--				
CakTSSR00689	CakTC11792	(TG)8	1494	1509	--	--	GAACAGAGATAGGGAACCATT	GTAGTCGGTTACCATCCTACC	54.82,55.22	158
CakTSSR00690	CakTC11794	(GCT)5	1391	1405	--	--	AGTGCTGCTTGTAGTAATGGA	ATCAAAATAATGGATCTGCTG	55.25,54.44	161
CakTSSR00691	CakTC11806	(GA)11	438	459	--	--				
CakTSSR00692	CakTC11821	(TGT)5	708	722	--	--	CTAGTCCTCCAACAAAAACCT	TGCATAAACTAGAAGCAGGAC	55.15,54.9	138
CakTSSR00693	CakTC11841	(ACC)10	638	667	Root	--	ATGAATGAATTCCCCTAAATC	GGTCATTAACGTTATCACGAG	54.71,54.86	152
CakTSSR00694	CakTC11874	(TGG)5	590	604	--	--	CAGACTTGGAGAAGCTTATGA	TGAGGAAAGGTTTTCATACAA	54.85,54.97	160
CakTSSR00695	CakTC11875	(CT)19	1	38	--	--				
CakTSSR00696	CakTC11911	(CAT)5	304	318	--	--	TAAAAAGCGAGTGTACAGGAA	GCATTTGTATACTGGAAAACG	55.4,55.04	170
CakTSSR00697	CakTC11924	(AAG)6	132	149	--	--	TGATGAGATTGAAATTGAGGA	AATGGCATTATGTTCGTGTAG	55.71,55.31	142
CakTSSR00698	CakTC11932	(TTC)5	12	26	--	--				
CakTSSR00699	CakTC11935	(AG)8	2	17	--	--				

CakTSSR00700	CakTC11942	(TCA)5	197	211	--	--	GTCATTGTTTTCTTCCTCCTC	TATGTCAATCGGAAACAACTT	55.42,54.83	151
CakTSSR00701	CakTC11960	(CAT)5	79	93	Young_pod	--				
CakTSSR00702	CakTC11997	(TTA)5	270	284	Young_pod	--	GCCATAGAATCATCACTTTCA	TAGTGAATCTGGATGGACAAT	55.31,54.55	156
CakTSSR00703	CakTC12013	(TGT)5	138	152	--	--	AGTGGTTTCTGAGTTGTTTCA	ATTCAACCTCACAGTTTCAGA	54.92,54.83	149
CakTSSR00704	CakTC12034	(ATT)9	230	256	--	--				
CakTSSR00705	CakTC12085	(TTA)7	2060	2080	--	--	ATGGAACCTTCTCTTTTATCG	TGTGATCCATTAGAAATCTAGG	55.16,53.65	166
CakTSSR00706	CakTC12092	(TC)6	3	14	--	--				
CakTSSR00707	CakTC12097	(CT)8	123	138	--	--	CTCTTCTCCGACTCACTCAG	GCATTTTAAAACTTCACCTTG	55.14,54.36	152
CakTSSR00708	CakTC12122	(TA)6	791	802	--	--	AAATGACAGCATCGAAGATT	GCACAAAAAGAGGTATTAGCA	54.86,54.92	161
CakTSSR00709	CakTC12180	(AAT)5	331	345	--	--				
CakTSSR00710	CakTC12184	(TC)6	12	23	--	--				
CakTSSR00711	CakTC12201	(CCA)7	1837	1857	--	--	TCCTTCTCACGTTTCTCTACA	GGAGAAAGTGATTGGAGAATG	55.18,56.3	161
CakTSSR00712	CakTC12223	(AAC)7	461	481	--	bHLH	TGAACAACGTTTCTTCTCTTC	GTTTTGGAACTGAAATCAACA	54.72,55.28	148
CakTSSR00713	CakTC12242	(TTA)5	1181	1195	--	G2-like	GCTTAGGTTGTGATGTAGTCG	GATGATGATTCTCTTCAACCA	55.09,55.09	152
CakTSSR00714	CakTC12282	(TCT)5	201	215	--	--	ATCCCAATTCCCACTTCTAT	TCAGGTTCAACACAGATGAAT	55.01,55.56	150
CakTSSR00715	CakTC12304	(GAC)5	3406	3420	--	--	ATCTCCTGTTGAGATTGATGA	CCCAATTTCACATCATGTACT	54.65,54.93	153
CakTSSR00716	CakTC12307	(AG)12	4321	4344	--	--				
CakTSSR00717	CakTC12336	(CT)6	539	550	--	--	GCTCCCTCTTAAGTTTACGTC	CCAAACGTAGAAAAGATGATG	54.99,55	150
CakTSSR00718	CakTC12339	(TGA)7	928	948	--	TCP	GGCTCTTGATACCCTTACAAT	CAACCAAAAAGACAAGAATGA	55.03,55.45	160
CakTSSR00719	CakTC12339	(TG)7	1202	1215	--	TCP				
CakTSSR00720	CakTC12409	(TC)6	245	256	--	--	AGAAAGGCTATGACTTTCACA	CCCGTCTTCCAAATTAAAATA	54.28,55.97	151
CakTSSR00721	CakTC12420	(GCG)8	125	148	--	--	GAGGTCAATCTGCTACAAGAA	TATTGGAAAAAGAAGCATCAG	54.67,54.82	151
CakTSSR00722	CakTC12420	(TC)8	337	352	--	--				
CakTSSR00723	CakTC12438	(CT)8	106	121	--	--	TGGTATCTCGGATTCTATCTTC	TGTAGGGCTCATATCAAGTGT	55.1,54.84	139
CakTSSR00724	CakTC12446	(CAT)5	135	149	--	--	ACACACTTCAGAGCAAAGAGA	GCTTTTGGACATGTATTTGAG	55.24,54.99	150
CakTSSR00725	CakTC12447	(CAT)5	112	126	--	--	CACACTTCAGAGCAAAGAGAC	TTTACAATGGTTTCAGCTTGT	55.24,55.07	164
CakTSSR00726	CakTC12452	(TTC)5	18	32	Shoot	--				
CakTSSR00727	CakTC12459	(CT)14	80	107	--	--				
CakTSSR00728	CakTC12462	(AGG)5	1057	1071	--	--	TAATCAGAAGTGAAACGGTGA	GATTACGAACCTGGTGAACTT	55.9,55.82	152
CakTSSR00729	CakTC12463	(TA)14	49	76	--	--				
CakTSSR00730	CakTC12491	(TAT)8	62	85	--	--				
CakTSSR00731	CakTC12504	(CCA)5	312	326	--	--	AAGAAGCCGTACAAATACCAT	AGGAGGAGATTTGTAAACTGG	55.53,55.07	150
CakTSSR00732	CakTC12506	(GA)7	1922	1935	Young_pod	--				
CakTSSR00733	CakTC12507	(AG)8	1576	1591	--	--	TTGAAGAGGTAGTGAATGGTG	CTTTTCCTTTTCCTTTCACAT	55.31,55.21	146
CakTSSR00734	CakTC12551	(CCA)5	1450	1464	--	--				
CakTSSR00735	CakTC12564	(ATG)5	1339	1353	--	Pseudo_ARR-B	TATGTCAATCGGAAACAACTT	CATTGTTTTCTTCCTCCTCTT	54.83,55.2	148
CakTSSR00736	CakTC12583	(GAA)5	1315	1329	--	--				

CakTSSR00737	CakTC12585	(AG)7	646	659	--	--	CATGGGAGAAACAAAAGTAGA	CACATCACAGAAGAGGAGAAC	54.55,54.8	149
CakTSSR00738	CakTC12738	(GAA)6	434	451	--	--	TTCAATGTGTCATGTTACTGG	TGAAAGAGTGGCTAAGCTAAA	54.44,54.81	148
CakTSSR00739	CakTC12740	(CTT)5	197	211	--	--				
CakTSSR00740	CakTC12778	(TCA)5	46	60	--	--				
CakTSSR00741	CakTC12823	(GA)11	2	23	--	--				
CakTSSR00742	CakTC12863	(CT)6	7	18	--	--				
CakTSSR00743	CakTC12875	(GGA)5	7	21	--	--				
CakTSSR00744	CakTC12877	(CT)7	159	172	--	--				
CakTSSR00745	CakTC12883	(TA)6	1111	1122	--	--				
CakTSSR00746	CakTC12927	(TGG)5	19	33	--	--				
CakTSSR00747	CakTC12937	(GCTCAA)5	112	141	--	--	AAACAAAGGAAGGAACAACAT	TGTCCAATAATTAGGAGCTTG	55.38,54.7	153
CakTSSR00748	CakTC12979	(GA)9	84	101	Flower bud	--				
CakTSSR00749	CakTC13023	(ACA)6	1	18	--	--				
CakTSSR00750	CakTC13080	(AG)21	1458	1499	--	--				
CakTSSR00751	CakTC13081	(GGT)5	493	507	--	--	CGGGTAGAAATTCTTTTGTTT	TCTCTGCACATACTGTTGTTG	55.25,54.85	151
CakTSSR00752	CakTC13120	(AGA)6	855	872	--	--	AATAAAGGAGGATTTTGATGG	CTTGTTCGTCTACTTCCATTG	54.95,55.07	159
CakTSSR00753	CakTC13147	(TCT)8	303	326	--	--	CTCTGCCAAAACTTCTATCAA	AGAAGAAGAAGAAGCGAAAAC	54.88,54.84	148
CakTSSR00754	CakTC13159	(TCT)8	312	335	--	--				
CakTSSR00755	CakTC13161	(GAA)6	41	58	--	--				
CakTSSR00756	CakTC13174	(TA)6	389	400	--	--				
CakTSSR00757	CakTC13187	(TGA)8	595	618	--	--	CTGAATTCGATTCGTTTTGTA	TTGCTTGATATGCTTCAAACT	55.57,55.31	150
CakTSSR00758	CakTC13269	(GT)8	1256	1271	--	--	CTAAAATAGTGGGAAAATATGC	GAGTTGTTGAGATGGAAGAAA	52.79,54.47	151
CakTSSR00759	CakTC13273	(TGA)5	594	608	--	--				
CakTSSR00760	CakTC13317	(TAA)14	1055	1096	Flower bud	--				
CakTSSR00761	CakTC13318	(TA)11	101	122	--	--	TGCGACGAGAGTAGATATTTT	GAATTTTGCTTTCTTTCCTTC	54.43,55.02	147
CakTSSR00762	CakTC13366	(TTG)5	2	16	--	--				
CakTSSR00763	CakTC13377	(GTT)8	575	598	--	--	TGCAACAGGGTTTAACTCTAA	ACATAAGCACTCCACATGAAC	55.24,55.16	149
CakTSSR00764	CakTC13377	(GT)9	938	955	--	--				
CakTSSR00765	CakTC13388	(TCA)8	646	669	--	--				
CakTSSR00766	CakTC13440	(AAT)6	493	510	--	--				
CakTSSR00767	CakTC13449	(AATAA)22	1	110	--	--				
CakTSSR00768	CakTC13457	(TC)7	293	306	--	--				
CakTSSR00769	CakTC13502	(AT)6	604	615	--	--	CACCTTATCAAGTGGTTATGC	ACTTTGCTTGGTGATTCTGT	54.86,54.84	135
CakTSSR00770	CakTC13523	(TTTA)7	316	343	--	--	TGCATATAATCCTAACCCACA	TGGTGTGAACACGTACACTTA	55.65,55.08	148
CakTSSR00771	CakTC13531	(TA)6	184	195	--	--	TAATGACAGGGATCTGTTTTC	ACTTTACCTCCTCAGTTGCAG	54.29,56.22	150
CakTSSR00772	CakTC13532	(AT)12	180	203	--	--	GGAATTTGGTTGTAAAGTAATCT	GTCCTGAAATGATGGGATTAT	53.6,55.43	145
CakTSSR00773	CakTC13535	(ATT)7	183	203	--	--	CTCTTCCACTCAAACAACAAC	GACAAGAGAGTGTCGAGATTG	54.92,54.98	152

CakTSSR00774	CakTC13535	(CAA)5	322	336	--	--	CATCAAATGCATTCTCTCATT	TCAGTAGAAAACATGGGGATA	55.31,54.76	151
CakTSSR00775	CakTC13553	(ATT)7	319	339	--	WRKY	TATAAAGTGCTTGTGCAATGA	CACCATTTCCTACTGTCACAC	54.66,55.51	150
CakTSSR00776	CakTC13564	(TTAAA)6	267	296	--	--	AACGAATTGGAAGTACTAGTGG	TCCATAAAATACCCAATTAAGG	55.21,55.04	145
CakTSSR00777	CakTC13570	(CTT)14	245	286	--	--	ATGCATCTTGGTACATGGTAA	TAAGAGCCTATGACGATTCAA	55.55,55.24	152
CakTSSR00778	CakTC13615	(ACA)6	339	356	--	--				
CakTSSR00779	CakTC13642	(TGT)5	83	97	--	--				
CakTSSR00780	CakTC13656	(ATG)5	437	451	--	--	GTTTCATTGATCTTGTGGAAG	TGTAGCATTAACTTGTGCTGA	54.77,54.72	149
CakTSSR00781	CakTC13669	(AT)7	1112	1125	--	--	TAACAATGGATATGACGGAAA	TCCATTCCCTATTTTTATTCC	55.61,54.9	144
CakTSSR00782	CakTC13669	(AT)6	1569	1580	--	--	GCAAAAATCCATTAAAGTTC	CTTTCCCTGTCGTTGTTAAAT	51.79,55.97	297
CakTSSR00783	CakTC13670	(TTC)5	34	48	--	--				
CakTSSR00784	CakTC13699	(CCA)8	194	217	--	--	CAATACCAAAACAATCTCTGC	AGTTAGGTTGAAGTTGGTGGT	54.99,55.33	144
CakTSSR00785	CakTC13699	(CAC)5	378	392	--	--	AAGATTAAAGCTCATTCGTGA	GATGGTAAATTGGATTGGAAG	54.4,56.07	152
CakTSSR00786	CakTC13704	(AT)6	278	289	--	--	CAAATGAGTATGATGGGAGAA	GCGTTGGTGTATTCATATGTT	55.16,55.14	153
CakTSSR00787	CakTC13712	(CAC)5	206	220	--	--	GATATCATTTCCCCATGAAA	TCTCTTTCGTCTTTTGTTGTT	54.82,54.34	150
CakTSSR00788	CakTC13764	(TTC)6	290	307	--	--	GGTAAACTCATCAAAGCACAG	GTATGCACTTGTCACTGCTCT	55.05,55.57	164
CakTSSR00789	CakTC13853	(TTG)6	45	62	--	--				
CakTSSR00790	CakTC13886	(CTT)8	263	286	--	LOB	TCAAGAACTTTTCACCTTCAA	GGTTGTTGTTTCTGTGATGAT	55.16,54.95	165
CakTSSR00791	CakTC13893	(TA)6	312	323	--	--	CCGAATTGTAATATTTTATACCG	CCAGATAGTAATTTTCTCAATCA	54.9,53.11	294
CakTSSR00792	CakTC13907	(AT)7	657	670	--	--	TGTAGGTCTAGCAATTTGGAC	AATCTAACCATATCCCCATTC	54.58,54.59	141
CakTSSR00793	CakTC13908	(GA)15	938	967	--	--				
CakTSSR00794	CakTC13915	(AT)6	230	241	--	--	GAGACTCCCAAAGACTGAGAG	AGGGATTCGATTGTATAGGAG	55.67,54.97	153
CakTSSR00795	CakTC13920	(GT)7	13	26	--	--				
CakTSSR00796	CakTC13986	(AT)6	231	242	--	--	AAGTATGGGGACAAACTTCTT	AAAACGAAGATGGGTAAGTGT	54.53,55.42	150
CakTSSR00797	CakTC13986	(AG)6	812	823	--	--				
CakTSSR00798	CakTC14001	(CT)7	113	126	--	--				
CakTSSR00799	CakTC14036	(TCA)5	5	19	--	--				
CakTSSR00800	CakTC14037	(AG)8	1570	1585	--	--				
CakTSSR00801	CakTC14051	(TTC)23	455	523	--	--				
CakTSSR00802	CakTC14070	(AT)6	88	99	--	--				
CakTSSR00803	CakTC14072	(GAG)5	269	283	--	--	TAGAACCGGAACCATACATC	CTCCTTGAGGTTGTTCTTCTT	55,55.26	144
CakTSSR00804	CakTC14084	(TCA)5	800	814	--	--	AAGTTTCAACCCATCTTCATT	GATTGCCTAGTGTTGAGAAAA	55.31,54.7	155
CakTSSR00805	CakTC14087	(TC)7	115	128	--	--				
CakTSSR00806	CakTC14109	(CAT)5	1160	1174	--	--	TCCTCCACTAGTTCAAAAACA	CGGAAGAAATAGTGAATGTTG	55.03,55	151
CakTSSR00807	CakTC14109	(CAC)5	1583	1597	--	--	TTCAATTTCTCCATCATCAAC	AAAGTTCCAATTCGATCAGAC	55.11,55.87	152
CakTSSR00808	CakTC14109	(TAT)5	1844	1858	--	--				
CakTSSR00809	CakTC14154	(AAT)5	83	97	--	PLATZ				
CakTSSR00810	CakTC14166	(AG)7	768	781	--	--				

CakTSSR00811	CakTC14201	(TC)6	183	194	--	--	ATAGCAGCATCACTGAGTCAC	AAATTTGAAACCTTTCTCGTC	55.49,55.2	148
CakTSSR00812	CakTC14201	(GAT)5	383	397	--	--	CGAATCAGAAGAAAACAGAGA	ACTTCCTCCATTAACACTTCC	54.82,54.9	152
CakTSSR00813	CakTC14219	(AAC)5	205	219	--	--	GGCATAGCCTTCATCTAAAAT	CAGCAACAAAATAAAAACCTC	55.27,54.36	149
CakTSSR00814	CakTC14285	(TA)6	101	112	--	--	CATTGGATTGGTTTATTGGTA	GCATATTATGTGCAGTTTGAA	55.06,54.01	143
CakTSSR00815	CakTC14339	(TA)13	139	164	--	--	GGGAATTGTAGAGAGGAGAAA	TGCCTATGAGTATCTTTGCAT	55.12,55.1	173
CakTSSR00816	CakTC14367	(AAG)5	672	686	--	--				
CakTSSR00817	CakTC14371	(CTC)5	673	687	--	--	TCTTCCTTTTCTTGTGCCTAT	TTAAGAGGGAGGCTAGTGATT	55.78,54.92	141
CakTSSR00818	CakTC14372	(CAG)5	549	563	--	--	ATGCGTGTAATAGGCTTCTCT	CAACATCGTTCTTCTGTCACT	55.83,55.37	139
CakTSSR00819	CakTC14377	(GAAA)5	215	234	--	--	AGGAAGATTCCTAACTCAAGC	TAAGCTCTTTTCGGGTATTTT	54.48,55.11	154
CakTSSR00820	CakTC14407	(AC)6	447	458	--	--	TCGAAACATATACCGCTTTAC	TATTCAATTCGTCATTCTGGT	54.74,54.74	147
CakTSSR00821	CakTC14412	(AG)6	255	266	--	--				
CakTSSR00822	CakTC14414	(AG)8	856	871	--	--				
CakTSSR00823	CakTC14440	(TC)8	403	418	--	--	AGTCTTAACAAGGTTCCATGC	CACATGTGTTTCATCTCTCAA	55.97,54.54	138
CakTSSR00824	CakTC14444	(TGA)5	1533	1547	--	--				
CakTSSR00825	CakTC14469	(AG)6	19	30	--	--				
CakTSSR00826	CakTC14469	(TAT)11	307	339	--	--	TGATTTGAACTTCGACGTAAT	ATTGTTTGAAATGACTTGCAC	54.99,55.3	154
CakTSSR00827	CakTC14515	(TAC)7	119	139	--	--	GAAGTTTTCTTCGATAGGAATG	GGTCTAAGGTTTTCTCATGCT	54.92,55.22	147
CakTSSR00828	CakTC14534	(TCC)5	557	571	--	--	TGGAGAGAGAGATTGTGACTG	GTGAACAATTACTGCGTCTTT	55.43,54.65	154
CakTSSR00829	CakTC14562	(ATC)5	441	455	--	--	ACTTCATCACACATCTCCAAT	GTGGCATCATCATCAAGTAAT	54.41,55	156
CakTSSR00830	CakTC14565	(ATA)9	1455	1481	--	--	TCTTCTTCTGTTTTGCATGAT	TGTTGGTTTTACCAAAATGAG	55.1,55.33	154
CakTSSR00831	CakTC14573	(CTC)6	413	430	--	--	TTCTGGTATGGATTCATGTTT	AGCAACTATATCTGGATGCAA	54.58,55.1	157
CakTSSR00832	CakTC14585	(TA)6	143	154	--	--	AATTTTTGATGATGATGGAC	CTCAATTTTATTCCTAACAACCA	52.29,55.29	145
CakTSSR00833	CakTC14588	(CA)7	242	255	--	--	CTTGATTGAATGCTTGAGACT	TGTCAAGGATTGGATAGAAAA	54.67,54.89	156
CakTSSR00834	CakTC14599	(TTTA)14	200	255	--	--				
CakTSSR00835	CakTC14636	(AAC)5	15	29	--	--				
CakTSSR00836	CakTC14649	(AAG)5	154	168	--	--	CCATCTCTCAAAACTCACAAG	CTGAAATCTTGTGTTCCAAAG	55.01,55.03	164
CakTSSR00837	CakTC14655	(TAT)7	118	138	--	--	AGATGTTGATTACTGCTTGGA	AAGCAAAGAAATTGATTGGA	54.97,55.02	149
CakTSSR00838	CakTC14662	(AT)6	61	72	--	--				
CakTSSR00839	CakTC14688	(TTTTC)5	605	629	--	--				
CakTSSR00840	CakTC14712	(ACA)6	377	394	--	--	TTCCATCATAGACGACAGAAC	TAGCCCTTTTAAATCATCTCC	55.22,55.25	173
CakTSSR00841	CakTC14739	(TTTTCC)5	343	372	--	--	CACCAGTAGCAAACATTCTTC	GATTCCAACAAGTTCAATCAA	55.05,55.19	154
CakTSSR00842	CakTC14747	(CAC)5	35	49	--	--				
CakTSSR00843	CakTC14761	(TC)7	93	106	--	--				
CakTSSR00844	CakTC14800	(CTTT)5	5627	5646	--	--	TCTGCTTTTGTTCCTGAGTAG	TGAATGAATGACAGACACTGA	54.94,54.98	173
CakTSSR00845	CakTC14823	(ATT)6	133	150	--	--	TGGTTTGGTTTTACTTTTGTG	TGATTCGTACCGTAGAAGAAG	55.41,54.75	179
CakTSSR00846	CakTC14837	(TA)6	113	124	--	--	GGCTTGTAAATCTTACTTCCAC	CACTCCAGATGGTTTGAATAA	54.87,55.24	160
CakTSSR00847	CakTC14860	(TC)7	930	943	--	--	TAAATCATGATGGGATTTCAG	TGACCATAAGAATGGAATTTG	55.09,55.17	133

CakTSSR00848	CakTC14908	(TGG)5	187	201	--	--	GAGAGTAGGGAGGTGTTCATC	CTTTTCTTCGAATATCATCCA	55.29,54.58	127
CakTSSR00849	CakTC14932	(CAA)7	417	437	--	bHLH	TCAACTGTTCAACAACATCCT	GAGGAGGAGCACAAGAACTAT	55.64,55.21	163
CakTSSR00850	CakTC14936	(AT)6	26	37	--	--				
CakTSSR00851	CakTC14998	(AT)7	437	450	--	--	AACCTTGCATACATAGGATGA	TGTCTCCACAAGAGTCTGTTT	54.78,54.89	195
CakTSSR00852	CakTC15015	(GAT)63	407	595	--	--				
CakTSSR00853	CakTC15022	(TC)10	214	233	--	--	CGAAAACGAAATAACAATCTG	TATGAAGGGGTTGGAGTTAAT	55.18,55.28	176
CakTSSR00854	CakTC15034	(AAG)5	485	499	--	--	TTTCTAGCAAGTTCATTGGAG	GGTGTGAAATGTGAATACGAG	54.88,55.6	150
CakTSSR00855	CakTC15061	(TAA)5	214	228	--	--	CTCCTTTGCTAACTGAAAACA	GCCAAATCATATTCTTTGATG	54.96,54.85	176
CakTSSR00856	CakTC15138	(AG)28	630	685	--	--				
CakTSSR00857	CakTC15139	(AG)29	85	142	--	--				
CakTSSR00858	CakTC15150	(TAC)6	2349	2366	Shoot	--	TTTGAAGCTACGAAGAAGATG	TTCTTCACAAAGCAACAATG	55.04,54.91	148
CakTSSR00859	CakTC15156	(ATC)6	411	428	--	--	CCATTAACTTAGCCACAACAC	GAAGATCGGTTTGTAGTGAGA	54.95,54.5	153
CakTSSR00860	CakTC15158	(AGA)9	817	843	--	--	GGCTTTGTCAAGAGAGAGAGT	ATTCCTCTCCTAATTTGCATT	55.4,54.7	147
CakTSSR00861	CakTC15169	(CT)8	447	462	--	--				
CakTSSR00862	CakTC15192	(GAT)5	167	181	--	--	GACAACAATCCAAATTATCCA	AACAGCTAGAAGTGATGATGG	55,54.54	149
CakTSSR00863	CakTC15201	(GT)7	115	128	--	--	GGGAGAGATTGCTTGAACTAT	AAACAAACCATGAGCCTTAAT	55.14,55.5	156
CakTSSR00864	CakTC15214	(GA)6	495	506	--	--				
CakTSSR00865	CakTC15250	(TTC)8	406	429	--	--	AACCAATACAATGAATGATGC	ACACATTCCACAGTATTACGC	55.02,55.21	149
CakTSSR00866	CakTC15250	(ATA)5	560	574	--	--	GCGTAATACTGTGGAATGTGT	AGGAGGTGGAAACTCAAAATA	55.21,55.47	153
CakTSSR00867	CakTC15258	(GA)14	3523	3550	--	CAMTA	AGTCAATCCGTAAGAAGAAGC	ATAGCGAAAACAACTGAAACA	55.37,55.23	160
CakTSSR00868	CakTC15277	(ATTA)8	56	87	--	--				
CakTSSR00869	CakTC15283	(AAT)5	77	91	--	--				
CakTSSR00870	CakTC15296	(AAT)6	390	407	--	--				
CakTSSR00871	CakTC15340	(TA)8	143	158	--	--	CTTTGCAATTTGTCCTTACTT	AACAATTACCGTTGATTGAAG	53.96,54.45	152
CakTSSR00872	CakTC15360	(AAT)7	405	425	--	--	TGCCAGAAAGTATGATGAGTT	CTAAAGACAAGCAAGCAACTG	54.97,55.63	151
CakTSSR00873	CakTC15361	(ATT)7	179	199	--	--	CTAAAGACAAGCAAGCAACTG	TGCCAGAAAGTATGATGAGTT	55.63,54.97	151
CakTSSR00874	CakTC15365	(TC)6	190	201	--	--	GCAAGCTTAATTAACTCACACA	CCTTCAAGGAAGAGCTTTTAT	55.01,54.68	157
CakTSSR00875	CakTC15389	(GGT)5	182	196	--	--	GGAATAGTTCGAGTTTTTGGT	TCAACGACCTGAATAAAAGAA	55.08,55.13	157
CakTSSR00876	CakTC15393	(ATC)6	1430	1447	--	--	TGAGCTTCATTTGGAGTAGAA	TCTCATCTAGCAAAAGGAAAA	55.28,54.54	154
CakTSSR00877	CakTC15393	(AC)9	2562	2579	--	--				
CakTSSR00878	CakTC15408	(TA)24	1	48	--	--				
CakTSSR00879	CakTC15412	(ATA)7	885	905	--	--				
CakTSSR00880	CakTC15447	(AT)7	463	476	--	--	ATCTCCAGAGGCTTATAGACG	TTGATGGACACATTCTTCAAT	55.34,55.48	164
CakTSSR00881	CakTC15453	(CAC)6	232	249	Young_pod	--	TTTATTCCCTCCACTCTATCC	AACTCGGCCAATAGAGTTAGT	54.93,54.87	152
CakTSSR00882	CakTC15461	(TC)6	23	34	--	--				
CakTSSR00883	CakTC15466	(AG)7	518	531	--	--				
CakTSSR00884	CakTC15477	(AG)6	84	95	--	--				

CakTSSR00885	CakTC15508	(AGA)7	222	242	--	--				
CakTSSR00886	CakTC15524	(TC)7	5	18	--	--				
CakTSSR00887	CakTC15544	(AAC)5	225	239	--	--	ACAACAACAACAGAGAACGAC	TTTTCCTCTTTGACGTTTACA	55.28,55.21	140
CakTSSR00888	CakTC15586	(AGA)7	204	224	--	--	CGAGAAGAACAAAATTCATCA	ACCGGAATCACTGTTACCTAT	55.53,55.22	156
CakTSSR00889	CakTC15637	(TAA)8	14	37	--	--				
CakTSSR00890	CakTC15639	(TAT)29	319	405	--	--				
CakTSSR00891	CakTC15645	(AT)7	635	648	--	--	CATTCACATCTTTCATTTGTG	AAGAAAGGGATCCTTCTATCC	54.07,55.54	180
CakTSSR00892	CakTC15662	(TA)6	249	260	--	--				
CakTSSR00893	CakTC15683	(AT)7	1	14	Shoot	--				
CakTSSR00894	CakTC15730	(TTA)27	174	254	--	--				
CakTSSR00895	CakTC15736	(CCT)5	195	209	--	--	AGGTGTTAAAGGCTCTACTCG	ACATTGTTTGACCCTTCTACA	55.49,54.72	156
CakTSSR00896	CakTC15766	(CAA)6	1	18	--	C2C2-Dof				
CakTSSR00897	CakTC15774	(TA)21	127	168	--	--				
CakTSSR00898	CakTC15781	(TG)6	208	219	--	--	GGAAAATAGGGAGGATAAAAA	TTAGCAAGATCGAAACAACAT	54.28,55.15	154
CakTSSR00899	CakTC15812	(TAA)5	268	282	--	--				
CakTSSR00900	CakTC15813	(AT)7	291	304	--	--				
CakTSSR00901	CakTC15824	(TTC)5	264	278	--	SBP	CACAAATCTCACAATACTTTGG	AGTGACTCCTTTATTGTTGGTT	54.94,54.62	150
CakTSSR00902	CakTC15834	(CTT)5	292	306	--	SRS	TGTAACATGCTTCATCTCAAA	CCGAAACAGTGTTATGAATTT	54.42,54.45	140
CakTSSR00903	CakTC15848	(ATT)14	1	42	--	--				
CakTSSR00904	CakTC15850	(TTCCT)5	65	89	--	--				
CakTSSR00905	CakTC15850	(CA)6	426	437	--	--	TATGACTTTGGACGCATAACT	TGAACCCTTATGTGTTTGATT	55.02,54.67	217
CakTSSR00906	CakTC15850	(AG)7	898	911	--	--				
CakTSSR00907	CakTC15860	(CTT)5	43	57	--	--				
CakTSSR00908	CakTC15904	(TGAT)6	267	290	--	--	TGTCTGTGTCAGTGCTTCATA	GTGAGGCATTACCTTCTTCTT	55.29,55.22	152
CakTSSR00909	CakTC15909	(TA)9	455	472	--	--	TCTATAATGCAGGTGAAGGAA	AAACAAAGAAACTGACAATCCT	55.08,54.74	147
CakTSSR00910	CakTC15922	(TGG)5	239	253	--	--	ATAACACAACAAAGGTCATGG	GTCGGTTTCTAATGAACTCCT	55.02,55.06	155
CakTSSR00911	CakTC15933	(TCC)5	114	128	--	--	TCTAAAAGTAAATGGAGCAATG	ACAACTTGGAATTGGGTAAAG	54.11,55.82	151
CakTSSR00912	CakTC15934	(AAG)5	357	371	--	--				
CakTSSR00913	CakTC15942	(TGT)5	229	243	--	--	GGTTATATGGCTTTTCCAAAT	TTTTATCCAAACAGTCCCATA	54.99,54.78	146
CakTSSR00914	CakTC15944	(TC)10	67	86	--	Jumonji				
CakTSSR00915	CakTC15968	(TC)7	30	43	--	--				
CakTSSR00916	CakTC15978	(CA)7	151	164	--	--	AGTTCGTCCCAGTAGTAGTCC	TACAAGTTCAGTTCGATGAGC	55,55.63	147
CakTSSR00917	CakTC15993	(AC)6	382	393	--	--	TCTAGTCCTGAGACACCAGAA	TGTTGATTTTTCACCGATACT	54.99,54.83	151
CakTSSR00918	CakTC16009	(ATC)5	1198	1212	--	--	GATGAAGACGAAGACACAAGA	AAGTGGGTTATGATGATTGTG	55.42,54.93	139
CakTSSR00919	CakTC16009	(ATAA)5	1792	1811	--	--	CAAACAAAACCCTAGTTTTCA	TTTTCAGGGTAGGATAAAACC	54.67,55.03	149
CakTSSR00920	CakTC16009	(GAA)8	2050	2073	--	--				
CakTSSR00921	CakTC16012	(CAG)5	1925	1939	--	--	ACATCAAGCGTATTACCACAT	GTCGCAATTGAATATAATGCT	54.73,54.65	148

CakTSSR00922	CakTC16067	(ATT)10	91	120	--	--				
CakTSSR00923	CakTC16067	(ACT)5	984	998	--	--				
CakTSSR00924	CakTC16078	(TA)9	59	76	--	--				
CakTSSR00925	CakTC16078	(AT)6	375	386	--	--	CCTTCATAAAACTAGTGGGTGT	AACTGAATGGATTTTCAACTG	55.06,54.38	150
CakTSSR00926	CakTC16085	(GA)6	423	434	--	--				
CakTSSR00927	CakTC16122	(TGT)5	108	122	--	NAC	CAAGCTGAGGAAGCTGTATAA	GATCTAATCTCAAGGCAACCT	55.07,55.14	145
CakTSSR00928	CakTC16142	(GT)6	266	277	--	AUX/IAA	AATAAAAGGTGTTTGGGGTAG	GCATTCTGATTTTCTGTTTTG	54.99,55.12	143
CakTSSR00929	CakTC16142	(TCA)5	982	996	--	AUX/IAA	TCCACCAAGTAGTGAAGATTG	CCTTCATAGGTTGATTTGATG	55.31,54.76	152
CakTSSR00930	CakTC16173	(ACTA)5	392	411	--	--	TCCATTTTAATCTAACGGTTG	TGGGTTTTCTATTTAGCATGT	54.57,54.16	200
CakTSSR00931	CakTC16179	(GAAA)5	423	442	--	--	GTGGAACTTCAATTTGTTTCA	CACAACATGTCTCAGTTAGGG	55.28,55.69	153
CakTSSR00932	CakTC16195	(TCT)7	81	101	--	--				
CakTSSR00933	CakTC16207	(TTG)5	653	667	--	--				
CakTSSR00934	CakTC16210	(TA)10	259	278	--	--				
CakTSSR00935	CakTC16229	(TTA)5	78	92	--	--				
CakTSSR00936	CakTC16239	(GT)6	359	370	--	--	GGTCAACACCAATCTGTAAAC	TCTTAACCTGTTTGGGCTTAT	54.54,55.66	151
CakTSSR00937	CakTC16327	(AC)6	73	84	--	C3H				
CakTSSR00938	CakTC16349	(TGT)6	21	38	--	--				
CakTSSR00939	CakTC16349	(ATT)11	213	245	--	--	ATAATTTGGTTGTGTGGAACA	AATTTATTCCCAACCATCACT	55.44,55.12	165
CakTSSR00940	CakTC16349	(GAT)6	445	462	--	--	GGAATTTTGGTGATGATTTTC	CAACATATGAAGGAACACACA	56.03,54.44	149
CakTSSR00941	CakTC16359	(GA)6	353	364	--	--	ATGTTTGTCAACTACGAAAGC	CAATATTGTTGCTCTCCTCTG	54.65,55.15	149
CakTSSR00942	CakTC16388	(AC)6	537	548	--	--				
CakTSSR00943	CakTC16399	(AG)8	139	154	--	--	TTGTTGTTGTTGGTGTCTGTA	AGCATCATCCTATCTCATTCA	55.04,54.8	176
CakTSSR00944	CakTC16425	(AAAT)8	72	103	--	--				
CakTSSR00945	CakTC16433	(GA)8	509	524	--	--	CTACAGCATCCAGAGAGAAAA	CTTGTGGGTCACACTTCTATC	54.85,54.69	142
CakTSSR00946	CakTC16449	(CTT)6	113	130	--	MYB-related	ACAAGAGCAAAAATCTCCTTT	AGAATCGAAATGAAAGAGGAA	54.96,55.67	152
CakTSSR00947	CakTC16455	(GAA)5	218	232	--	--	AGAGCCACTGTCCTCTCTG	GAATGATTCAATTTCTGCTCA	55.42,55.44	150
CakTSSR00948	CakTC16460	(TG)6	419	430	--	--	GATATTGGATGGAAGGGTAAC	TTCTTAAATTGCGTTTGCTAC	55.04,55.09	148
CakTSSR00949	CakTC16475	(GGT)7	99	119	--	--				
CakTSSR00950	CakTC16480	(GA)6	5949	5960	Root	--				
CakTSSR00951	CakTC16487	(CTT)6	3154	3171	--	CCAAT	TCTTTATCTTCTGCAAAACCA	CAAGAGCAAGAACAAGAAGAG	55.29,54.64	149
CakTSSR00952	CakTC16497	(AG)8	152	167	--	--	TCTACATGTTGCAGCTAGAGTC	TGGAAAATTGAGAACCAAGT	54.97,54.76	154
CakTSSR00953	CakTC16513	(CCA)5	518	532	--	--	AGAAGCCGTACAAATACCATT	AGGAGGAGATTTGTAAACTGG	55.53,55.07	150
CakTSSR00954	CakTC16514	(GAA)7	9	29	--	--				
CakTSSR00955	CakTC16549	(TTC)6	216	233	--	--	TTGCGTTATTACTCGACATTT	CTCGGATGCGTAACTACAAC	55.2,55.95	152
CakTSSR00956	CakTC16578	(AG)7	167	180	--	--	TCCCTTTTTATAAGACCCACT	CCCGTTAATTTATGATTGACA	54.64,55.22	166
CakTSSR00957	CakTC16627	(GTTGGA)5	240	269	--	--	ATGTTGTTGGAGTTGGATGT	CGAACAAAACTCAAAGGACTA	55.23,54.8	152
CakTSSR00958	CakTC16639	(CT)6	1138	1149	--	--	CATTATTTTCTCCTCCTTGCT	GAGATATGACTGCTGTTAGGC	55.32,54.22	144

CakTSSR00959	CakTC16672	(CTT)5	150	164	--	--	CTCCTCAAACCTTTTTCAACT	GCAGAAAAATAACAAGCAGAA	55.27,55.04	151
CakTSSR00960	CakTC16681	(CAC)5	494	508	--	--	ATTGATGGATTCACTTCAAGA	GAGCTACATACCAAGGTGTTG	54.68,54.93	141
CakTSSR00961	CakTC16759	(CAG)5	52	66	--	--				
CakTSSR00962	CakTC16766	(CT)10	37	56	--	--				
CakTSSR00963	CakTC16784	(AT)10	199	218	--	--	TGATCTCACAGGCATTTCTAC	CACCCAGTGATATTCTTATGC	55.38,54.78	157
CakTSSR00964	CakTC16813	(TCT)5	246	260	--	--	ATGATTGTGGAAATAGCAGTG	CACATAGTCCAAACTCCAATC	55.26,54.64	151
CakTSSR00965	CakTC16824	(AT)7	283	296	--	--				
CakTSSR00966	CakTC16838	(AT)9	286	303	--	--	TTCTTTAGCATCTCATTGTCC	TTCTACCGTTCTATGTTTTCG	54.62,54.78	132
CakTSSR00967	CakTC16841	(AT)10	93	112	--	--				
CakTSSR00968	CakTC16846	(CTT)5	187	201	--	--	AAATGGTTCACCTTCTTTTTC	TCCATCTTTATGCTTCATGTT	55.04,54.91	152
CakTSSR00969	CakTC16847	(TCT)8	450	473	--	AP2-EREBP				
CakTSSR00970	CakTC16863	(ATA)7	430	450	--	--	TGTTCTTGTTCTTCTTTCACC	TCCCAATAGTATCCATGACTG	54.56,55.03	147
CakTSSR00971	CakTC16867	(AT)8	49	64	--	--				
CakTSSR00972	CakTC16867	(AAC)5	291	305	--	--				
CakTSSR00973	CakTC16869	(AAT)6	313	330	--	CCAAT	TTCAGAATGCTTCTTTTCATC	TAAAAGCACCACCAGAATTAG	54.74,54.78	149
CakTSSR00974	CakTC16873	(AT)11	172	193	--	--	AAACCACACTAGCAGTCTTGA	CCAACACTTAGTCTGGAATCA	55.12,55.31	145
CakTSSR00975	CakTC16897	(TC)10	200	219	--	--				
CakTSSR00976	CakTC16937	(TA)6	41	52	--	--				
CakTSSR00977	CakTC16957	(AG)6	541	552	--	C2C2-Dof	CAAATAGCTTAATCCCTTGGT	AAGCGCTTCTTTAATATTTCC	55.21,54.82	142
CakTSSR00978	CakTC16981	(GGT)6	609	626	--	--	GATGGAGGAGGTGGTAGATAG	GCCTCTCCAACACTCATAACT	55.26,55.98	156
CakTSSR00979	CakTC16981	(GA)13	732	757	--	--	GTGTTAAGGTGACGGAGATG	ATCGTTACCTCACACAGACAC	55.58,55.05	152
CakTSSR00980	CakTC16985	(AT)7	402	415	--	--				
CakTSSR00981	CakTC16989	(GT)6	7	18	--	--				
CakTSSR00982	CakTC17026	(AG)8	15	30	--	--				
CakTSSR00983	CakTC17026	(GCC)8	381	404	--	--				
CakTSSR00984	CakTC17095	(TGC)5	286	300	--	--	AGGTGTACTTTTTGGAGGTGT	ATAACTATAATGCGGCACAAC	55.33,54.61	155
CakTSSR00985	CakTC17136	(AG)6	1216	1227	--	--	CTCCATTGTTGGAACTACAAG	CGATGTAGACGAAGTTTTCAC	54.9,55.05	144
CakTSSR00986	CakTC17152	(AG)6	143	154	--	--	ATAGAGGTTGACCCCGATAAT	TCACAAGTGGCAGTTAGTTCT	56.57,55.12	136
CakTSSR00987	CakTC17182	(TAG)5	404	418	--	--				
CakTSSR00988	CakTC17256	(TC)7	19	32	--	--				
CakTSSR00989	CakTC17280	(AGA)6	15	32	--	--				
CakTSSR00990	CakTC17308	(TA)9	204	221	--	--				
CakTSSR00991	CakTC17347	(AAC)6	288	305	--	--	TTAACTGATCCACCCATACAC	GGGGTAGAAAGAAGGTTAAAG	54.93,54.02	146
CakTSSR00992	CakTC17374	(CAA)5	323	337	--	--				
CakTSSR00993	CakTC17379	(TC)7	242	255	--	bHLH	GAAAGAAAACAAAAGTGAATGC	GTTGTTCATTCTGGACATCAT	55.82,54.86	152
CakTSSR00994	CakTC17397	(TG)7	3	16	--	--				
CakTSSR00995	CakTC17400	(CTC)5	523	537	--	--	CGTAAATTCTACAAGGACGTG	TTAGAAATGCAATAGGGACAA	55.12,55.1	151

CakTSSR00996	CakTC17420	(AG)40	165	244	--	--				
CakTSSR00997	CakTC17443	(TAT)6	367	384	--	--	CGGTCACTGCCTATACAAATA	AGAATGAACCGAACCTGTTAT	55.48,55.34	158
CakTSSR00998	CakTC17453	(GT)10	80	99	--	--				
CakTSSR00999	CakTC17471	(CAT)5	9	23	--	--				
CakTSSR01000	CakTC17501	(AAG)5	32	46	--	--				
CakTSSR01001	CakTC17534	(TTA)9	157	183	Flower bud	HB	GCTTCCTTTGTTCGTAGTTTC	GAAGAATGAGAATGCAAAATG	55.84,55.03	131
CakTSSR01002	CakTC17534	(AG)6	368	379	Flower bud	HB	AATCACACATCCTTCTCACAA	CAAAAACAAACGAAGAAGAAG	55.56,54.55	149
CakTSSR01003	CakTC17552	(TA)6	1357	1368	--	--				
CakTSSR01004	CakTC17568	(AGA)6	1954	1971	--	--				
CakTSSR01005	CakTC17576	(TA)15	94	123	--	--				
CakTSSR01006	CakTC17583	(AAT)5	320	334	--	--				
CakTSSR01007	CakTC17587	(AGA)5	278	292	--	--	TTTCTCTTTGAATGTGGTGTT	CCTCACTCTCAACAATGTTTC	54.85,54.83	148
CakTSSR01008	CakTC17588	(TTTA)5	59	78	--	--				
CakTSSR01009	CakTC17613	(ATC)5	3558	3572	--	--	TAAGGATGCTTCGAGTAACTG	CTCCCAATTATCCCTCTCTTA	54.91,55.1	161
CakTSSR01010	CakTC17617	(TC)7	13	26	--	--				
CakTSSR01011	CakTC17641	(AT)13	41	66	--	--				
CakTSSR01012	CakTC17677	(TG)6	420	431	--	--	CTTAACATTTCCCCTCAAGTT	TTGGGTAGATCTTTCATCCTT	55.09,55.4	152
CakTSSR01013	CakTC17684	(TA)7	115	128	--	--	TGATTCTGAGCACAAGTGTTA	TGATTCCATTTGAGATTGAGA	54.48,55.71	143
CakTSSR01014	CakTC17701	(GTT)6	452	469	--	--	GGATAGGAATAAGGATTGGAG	ACCAACCCTAACAACAGAATC	54.48,55.66	150
CakTSSR01015	CakTC17778	(AAT)12	195	230	--	--	TGCTAAAATCCTATTTTGAGGT	GTTTTCAGGCTCAAAGGTTAT	54.97,55.23	239
CakTSSR01016	CakTC17812	(GGA)5	720	734	--	--				
CakTSSR01017	CakTC17850	(TCT)9	191	217	--	--	ATATCAATGGCCTTAATCTCC	TTCAAAGCATGATTCTGAGAC	54.9,55.52	152
CakTSSR01018	CakTC17855	(TA)6	87	98	--	--				
CakTSSR01019	CakTC17859	(GAA)5	471	485	--	--				
CakTSSR01020	CakTC17881	(TAA)5	391	405	--	--	GCATGACATAACATATGGACA	CCACACGTTTATTTTAGTTGG	54.31,54.98	162
CakTSSR01021	CakTC17884	(TA)6	130	141	--	--	TATGAGCAATTTACACGAATG	CACGACATGTATTGTGAGAAA	54.03,54.61	149
CakTSSR01022	CakTC17969	(AT)16	288	319	--	--				
CakTSSR01023	CakTC17979	(TTC)6	386	403	--	--				
CakTSSR01024	CakTC17990	(TTAT)5	51	70	--	--				
CakTSSR01025	CakTC18018	(TC)7	26	39	--	--				
CakTSSR01026	CakTC18050	(TCA)6	134	151	--	--	TTCTTCTTCATCAGAGTTGGA	CTTACTTCAAAGGGAGATGGT	55.06,55.07	134
CakTSSR01027	CakTC18073	(AT)11	393	414	--	--				
CakTSSR01028	CakTC18076	(TTA)18	1031	1084	--	--				
CakTSSR01029	CakTC18136	(ATG)5	585	599	--	--	CTCCCAATTATCCCTCTCTTA	TAAGGATGCTTCGAGTAACTG	55.1,54.91	161
CakTSSR01030	CakTC18176	(AT)8	397	412	--	--				
CakTSSR01031	CakTC18178	(TA)6	464	475	--	--				
CakTSSR01032	CakTC18212	(CA)6	244	255	--	--	ACCCTCTTACTTCAATTGGTC	TAGAGCTGGAAGGAGTATGTG	54.9,54.72	127

CakTSSR01033	CakTC18231	(CT)18	102	137	--	--				
CakTSSR01034	CakTC18239	(AG)7	1713	1726	--	--				
CakTSSR01035	CakTC18268	(TC)10	446	465	--	--				
CakTSSR01036	CakTC18272	(AAT)5	239	253	--	--	GTTATCCAATTCATCAATCCA	TGTGTTAGCTGACATTTGAAG	54.92,54.07	154
CakTSSR01037	CakTC18277	(CTT)15	142	186	--	--				
CakTSSR01038	CakTC18308	(TAA)5	2	16	--	--				
CakTSSR01039	CakTC18313	(GCT)6	87	104	--	--				
CakTSSR01040	CakTC18354	(GA)8	188	203	--	--	GCTTTCAACTTTTATTGTCCA	GCTACTTGGCTTGGAGTAAGT	54.73,55.48	142
CakTSSR01041	CakTC18364	(TGT)5	485	499	--	C3H				
CakTSSR01042	CakTC18371	(TC)12	138	161	--	--				
CakTSSR01043	CakTC18396	(TA)8	149	164	--	--	ATCATTTAATCTCAGCCCTTT	TTTGCTAATGCAGAACAGATT	54.7,55.31	150
CakTSSR01044	CakTC18399	(CT)6	121	132	--	--				
CakTSSR01045	CakTC18420	(AC)9	282	299	--	--	ATACACAAAGAGGGAAAATGC	TTTCTTGATGTGTGACGTGTA	55.89,55.13	140
CakTSSR01046	CakTC18489	(TGT)6	191	208	--	--	GACATCTTTTCGGTTTGTTC	TGTTTCGGTAAGAAATTCAAG	54.74,54.74	148
CakTSSR01047	CakTC18494	(TA)17	118	151	--	--				
CakTSSR01048	CakTC18551	(AGAGA)5	333	357	--	--	GTGGTAACTGGTAACTGATGC	GGAGTTAGAGAGGAGGTGACT	54.75,54.16	154
CakTSSR01049	CakTC18552	(AG)8	19	34	--	--				
CakTSSR01050	CakTC18558	(AG)6	27	38	--	--				
CakTSSR01051	CakTC18643	(TTA)5	30	44	--	--				
CakTSSR01052	CakTC18662	(CAG)5	1623	1637	--	--	TCTTGTTTCCTGATCTTTCAA	CTTTTGTAGTTCTGTCGCATT	55.08,54.82	152
CakTSSR01053	CakTC18664	(AT)13	229	254	--	--				
CakTSSR01054	CakTC18688	(GA)17	1131	1164	--	--	AGATAGCTCTTTTCAGCATCC	TTGTATTGTGTTTGTGTTGTGA	55.45,55.07	150
CakTSSR01055	CakTC18688	(CAT)5	1289	1303	--	--	TCTTGATCTTGAAAACTCCAA	CCTTCTCCATCCATTATTCTT	55.08,54.93	168
CakTSSR01056	CakTC18707	(ATA)5	56	70	--	--				
CakTSSR01057	CakTC18720	(TA)6	98	109	--	--				
CakTSSR01058	CakTC18778	(TAC)5	69	83	--	--				
CakTSSR01059	CakTC18786	(TA)15	70	99	--	--				
CakTSSR01060	CakTC18808	(TGTTGA)5	256	285	--	--	ATCACTGGAAGGATGTTTTTC	TGTGAAATGTTTGTCTGAATG	55.71,54.57	159
CakTSSR01061	CakTC18837	(AG)23	136	181	--	--				
CakTSSR01062	CakTC18862	(TC)11	390	411	--	--				
CakTSSR01063	CakTC18871	(AT)15	2	31	--	--				
CakTSSR01064	CakTC18881	(TA)11	11	32	--	--				
CakTSSR01065	CakTC18885	(TC)9	96	113	--	--				
CakTSSR01066	CakTC18931	(TTAGGG)6	451	486	Flower bud	--	CTTGATTAAGCGTGGAGATTA	ACACGTTTTTCTCCTCAAAA	54.85,55.01	158
CakTSSR01067	CakTC18944	(GTG)5	37	51	--	--				
CakTSSR01068	CakTC18978	(CT)6	530	541	--	LUG				
CakTSSR01069	CakTC18994	(TA)9	139	156	--	--	AGGGTTTTAGAGGGACTGACT	AGGTGAGTTGGGACTTGTTAT	56.04,55.25	164

CakTSSR01070	CakTC19019	(TCT)9	103	129	--	--				
CakTSSR01071	CakTC19110	(TTG)6	369	386	--	--	GGTGATCTAGGGTTCCTTCTA	GCATACTCAGAATCAGGACAC	54.99,54.76	158
CakTSSR01072	CakTC19190	(TA)6	177	188	--	--	CGTGGCGACGGTATATTA	TTGTAACAACACGATAGAATCA	55.57,53.5	198
CakTSSR01073	CakTC19248	(TG)7	329	342	--	--	GCCTGATTTACCTCTCAAAGT	TGAAACTGTTTGTTCATACCC	55.22,55.15	154
CakTSSR01074	CakTC19278	(TAA)6	74	91	--	--				
CakTSSR01075	CakTC19330	(GAA)5	309	323	--	--				
CakTSSR01076	CakTC19404	(TTA)5	398	412	--	--				
CakTSSR01077	CakTC19413	(TC)21	3	44	--	--				
CakTSSR01078	CakTC19435	(TTA)5	446	460	--	--				
CakTSSR01079	CakTC19467	(AAC)5	25	39	--	--				
CakTSSR01080	CakTC19470	(TA)14	378	405	--	--				
CakTSSR01081	CakTC19510	(TG)6	148	159	--	--	TTGATGGTATTCTGTGTTAAGG	TCGAGTAGCATCATATGACAA	54.47,54.37	145
CakTSSR01082	CakTC19539	(GA)9	94	111	--	--				
CakTSSR01083	CakTC19605	(TAA)6	211	228	--	--	ATATCTTGGGGTTGAGTTAGG	AGGATCCAGGATTTTGTAAAC	54.89,54.84	150
CakTSSR01084	CakTC19626	(GA)7	435	448	--	--				
CakTSSR01085	CakTC19657	(TC)9	382	399	--	--				
CakTSSR01086	CakTC19669	(TCT)6	481	498	--	CCAAT				
CakTSSR01087	CakTC19704	(AGA)6	8	25	--	--				
CakTSSR01088	CakTC19723	(TTC)6	32	49	--	--				
CakTSSR01089	CakTC19843	(AT)16	97	128	--	--				
CakTSSR01090	CakTC20109	(AT)7	407	420	--	--				
CakTSSR01091	CakTC20149	(GAA)6	2221	2238	Flower bud	--				
CakTSSR01092	CakTC20157	(AG)8	1923	1938	--	--	TGGTCATAGATTCCAGAAGAA	ACTATGGCAACATCCAAATC	54.86,54.99	166
CakTSSR01093	CakTC20157	(CCT)6	3038	3055	--	--	GCCATCAGAACCTATTGATAA	GCATTTCTTTCCTCTATGGAT	54.44,55.08	159
CakTSSR01094	CakTC20161	(ATT)5	2757	2771	--	C3H	TGCATACTTTCAAAAGCAGTT	TTAACCTGTGGTTCAGCTTAC	55.39,54.66	148
CakTSSR01095	CakTC20165	(GAA)6	1264	1281	Young_pod	--	AGGTTCTCTTTCGGTTTTAAG	CTATTTCATCTCCACCTCACA	54.6,55.23	152
CakTSSR01096	CakTC20168	(GAA)6	1582	1599	--	--	ACATGCAATCTAACTCTGGAA	TCCAACCCTCTCTCTCTATTC	54.97,55.1	157
CakTSSR01097	CakTC20168	(AGA)6	1743	1760	--	--				
CakTSSR01098	CakTC20171	(TC)7	77	90	--	--				
CakTSSR01099	CakTC20211	(TC)7	515	528	--	--				
CakTSSR01100	CakTC20264	(AG)6	872	883	--	--	TCCTTGCATCTTAGCTGTCTA	GGAACAAGTCAAGTGTTTCTG	55.47,54.92	129
CakTSSR01101	CakTC20273	(AT)8	455	470	--	--				
CakTSSR01102	CakTC20305	(TG)6	556	567	--	--	AAATCCTTCGGTGTTTCTTAG	TCTTCCTTCCCTGACTTACTC	55.24,55.18	144
CakTSSR01103	CakTC20315	(TA)6	202	213	--	--				
CakTSSR01104	CakTC20356	(AAAT)5	301	320	--	--				
CakTSSR01105	CakTC20393	(ATA)6	66	83	--	--				
CakTSSR01106	CakTC20428	(AT)6	4	15	--	--				

CakTSSR01107	CakTC20433	(TC)11	21	42	--	--				
CakTSSR01108	CakTC20467	(GTT)6	457	474	--	--				
CakTSSR01109	CakTC20581	(AT)9	91	108	--	--				
CakTSSR01110	CakTC20721	(CAT)6	181	198	--	--	TCACATCATAAAGTTCCACAA	AGGGAATTTCAATAAGGAAGA	54.07,54.68	150
CakTSSR01111	CakTC20726	(TC)6	12	23	--	--				
CakTSSR01112	CakTC20727	(GA)9	118	135	--	--	AGGGACATGAGACAATAGAGAG	TCCTCACTAAACCATAGGACA	55.07,54.82	159
CakTSSR01113	CakTC20743	(GA)6	30	41	--	--				
CakTSSR01114	CakTC20743	(TTA)6	247	264	--	--	TCTTCAATTACATCACTGTCGT	GAAGTTGGAGGTGGTTTTAAT	54.9,54.92	145
CakTSSR01115	CakTC20830	(CT)7	25	38	--	--				
CakTSSR01116	CakTC20831	(CGG)5	563	577	--	--				
CakTSSR01117	CakTC20872	(AC)6	181	192	--	--	CCAGAGGTCTTTATTTCGATT	TGGACACAGACGAGACTAGAG	55.16,55.55	165
CakTSSR01118	CakTC20975	(GAA)8	229	252	--	--				
CakTSSR01119	CakTC21008	(TC)8	92	107	--	--				
CakTSSR01120	CakTC21047	(GGA)5	432	446	--	--	CAAATTATTCGATCTGGACTG	ATCCATTTCCTGAGGAGTTT	54.92,55.21	153
CakTSSR01121	CakTC21065	(ATT)6	1	18	--	--				
CakTSSR01122	CakTC21083	(CAC)5	340	354	--	--	TAAATGAAAAACCCATGTGAG	ATTATTATCTGAAAGGGTTCG	55.25,52.86	184
CakTSSR01123	CakTC21085	(CA)18	201	236	--	--				
CakTSSR01124	CakTC21114	(AG)19	383	420	--	--				
CakTSSR01125	CakTC21141	(AT)15	338	367	--	--				
CakTSSR01126	CakTC21175	(AT)9	158	175	--	--	TGACTTGTTGATGTCTCACTG	ACCATCGACAACAGTATGAAC	54.6,54.99	148
CakTSSR01127	CakTC21185	(TCA)5	272	286	--	--				
CakTSSR01128	CakTC21186	(TTA)6	117	134	--	--	AACCCATTTTCAAAGACAACT	ACTTGCAGAAAGAAAGGATCT	55.38,54.94	148
CakTSSR01129	CakTC21236	(TG)6	126	137	--	--	TCTGTTCTAGACAAAACACTGC	GTCATGAAAACCTGATTCAAA	54.86,55.19	154
CakTSSR01130	CakTC21258	(AGA)5	49	63	--	--				
CakTSSR01131	CakTC21263	(TG)7	143	156	--	--	CATTTCTCTCTGGGGTTTTAT	GACCCAGAAAACTTTATTTGTAA	55.01,54.54	161
CakTSSR01132	CakTC21280	(AT)10	20	39	--	--				
CakTSSR01133	CakTC21281	(TA)8	365	380	--	--				
CakTSSR01134	CakTC21312	(CTT)5	223	237	--	--	GGTTGCCAACTCTTATTTTCT	CCTTTTCCTTCTTCTCTTCAC	55.23,54.92	150
CakTSSR01135	CakTC21344	(TA)6	295	306	--	--	TTCATTTTATTCGGATTGCT	GTCAACGAATCCCCGTAT	55,55.69	154
CakTSSR01136	CakTC21402	(CT)11	366	387	--	--				
CakTSSR01137	CakTC21426	(AT)6	31	42	--	--				
CakTSSR01138	CakTC21459	(AT)6	2161	2172	--	--	CCTTCCATCCTCATCAATAGT	GCTGAACTTTGTGGAAAATTA	55.68,54.73	157
CakTSSR01139	CakTC21459	(AG)21	2559	2600	--	--				
CakTSSR01140	CakTC21486	(AC)9	318	335	--	--				
CakTSSR01141	CakTC21487	(CT)8	381	396	--	--				
CakTSSR01142	CakTC21534	(GTTA)6	263	286	--	--	ATGAAAGAGAGAGAGGGAAAA	CTCACTTCATTCATTCAACATC	54.83,54.78	144
CakTSSR01143	CakTC21540	(CA)10	666	685	--	G2-like				

CakTSSR01144	CakTC21543	(TCT)5	441	455	--	AUX/IAA	GGCTTCTCAATGTAAAACTCA	GAGTGAGAGTTTTTCGGATTT	54.7,55.18	151
CakTSSR01145	CakTC21544	(AG)19	437	474	--	--				
CakTSSR01146	CakTC21556	(GA)11	1055	1076	--	--	GTTTTAGAGGAAGAGCTGGTT	ACTCTCTCTACCCTCCCTCTC	54.56,55.69	146
CakTSSR01147	CakTC21561	(GAA)5	238	252	--	--	TCCCAAATTCGAAATCTCTAT	ATTCTGTTTCTTCTTCCGACT	55.48,54.78	163
CakTSSR01148	CakTC21568	(TCA)6	372	389	--	--				
CakTSSR01149	CakTC21576	(CTT)7	2	22	--	--				
CakTSSR01150	CakTC21628	(TTG)5	103	117	--	--	GTGTTTTCGATGACTGTTGTT	GAAGCTCATCAAAAGAACGTA	55.2,54.86	114
CakTSSR01151	CakTC21640	(ATA)7	127	147	--	--	CGGATTTTAAGAAAGTGTTCC	ATGACACAAGAAAGTTGGAAG	55.63,54.43	151
CakTSSR01152	CakTC21644	(GAA)6	267	284	--	--				
CakTSSR01153	CakTC21661	(GC)6	95	106	--	--				
CakTSSR01154	CakTC21693	(TA)8	225	240	--	--				
CakTSSR01155	CakTC21716	(TTA)22	399	464	--	--				
CakTSSR01156	CakTC21719	(CTT)6	3	20	--	--				
CakTSSR01157	CakTC21737	(TAT)5	189	203	--	--	GGGTGTGTGCTCATACTATTT	ACTCCAATTGATTGTCCTTCT	54.27,55.3	154
CakTSSR01158	CakTC21748	(AT)9	81	98	--	--				
CakTSSR01159	CakTC21748	(AGA)9	324	350	--	--	TATCTCCAGCATCCAAAAATA	TCCTTGTGTCTTCTTCTGCTA	55.02,55.35	151
CakTSSR01160	CakTC21815	(GAC)6	319	336	--	--	GAACCAACAGTAATCCACGTA	CTTCAGCTGACTTTGACAATC	55.18,55.17	178
CakTSSR01161	CakTC21848	(CT)8	107	122	--	--	CTTTCTCTGATCCTTTTCGAT	GATGCCATGATATTGAATCTG	55.27,55.52	154
CakTSSR01162	CakTC21873	(TA)7	320	333	--	--	ATGTCATCCGACAAGTGATTA	CTAATTAAATGGGGAACAGGT	55.52,54.91	167
CakTSSR01163	CakTC21950	(AG)14	204	231	--	--				
CakTSSR01164	CakTC21971	(ATT)6	35	52	--	--				
CakTSSR01165	CakTC22035	(CT)6	344	355	--	--	TAACTTCTGGCCATAGTTCAA	CCAGAAAACCAGTGTCATCTA	55.16,55.31	162
CakTSSR01166	CakTC22037	(TAG)5	172	186	--	--	CACTTTTTATAAGGCACTCGT	AGTTTGACAAGTTTGCTCTTG	54,54.85	188
CakTSSR01167	CakTC22092	(AT)18	310	345	--	AP2-EREBP				
CakTSSR01168	CakTC22158	(TAT)5	364	378	--	--				
CakTSSR01169	CakTC22163	(TG)9	14	31	--	--				
CakTSSR01170	CakTC22178	(ATT)5	21	35	--	--				
CakTSSR01171	CakTC22186	(TC)7	230	243	--	--	AGTTGAACTGGAAGTGATCTG	TGTTTTCGGGAAAGTGTAATA	54.39,55.02	155
CakTSSR01172	CakTC22198	(AT)6	158	169	--	--	GCTTTGAAGTTCCATCACATA	GCAGAAATTGTTCATTTCAAG	55.39,55.12	159
CakTSSR01173	CakTC22202	(TC)11	349	370	--	--				
CakTSSR01174	CakTC22227	(TA)7	149	162	--	--	TCAAGACCATGAAGATGATTC	GGGAGTACTGGTAGGGATTTA	55.09,54.89	150
CakTSSR01175	CakTC22239	(CCA)8	1552	1575	Flower bud	--	ATTAAGGGAAAGGAAGAACAC	GACTACCTTTTGGAGGAGTTG	54.2,55.55	159
CakTSSR01176	CakTC22244	(AGA)7	352	372	--	--				
CakTSSR01177	CakTC22247	(ATA)6	136	153	--	--	CAAACACATCAACGACAAGTA	CCCTTGATCTGATTTTTCATT	54.7,55.8	150
CakTSSR01178	CakTC22251	(ATT)5	2077	2091	--	--				
CakTSSR01179	CakTC22256	(TTA)6	2279	2296	--	--	CCCTTGATCTGATTTTTCATT	CAAACACATCAACGACAAGTA	55.8,54.7	150
CakTSSR01180	CakTC22276	(GGA)5	1129	1143	--	--	GTGTATGGGGAATAGGAGATG	TCCACCACCATACTACTACAAA	55.96,54.81	154

CakTSSR01181	CakTC22297	(TTC)6	366	383	--	--	TCTAGCATTTGTTGGGATCTA	TTTCGTCATGTGTCTCTTTTT	55.08,55.02	155
CakTSSR01182	CakTC22297	(GT)6	2022	2033	--	--	TTATTGTGTTTGGTGGAAGAG	GCAACAACTCTCACTCATCTC	55.32,54.96	150
CakTSSR01183	CakTC22302	(TCA)8	2538	2561	--	--	ATTCTCTGTGCAAAAACTGAC	TCAACCGTTTCTATTTCTCAA	54.58,55.13	150
CakTSSR01184	CakTC22312	(GGA)5	1220	1234	--	--	ATGGAGGGTTTGATATCACTT	TATTACAAGTCTCCACCACCA	55.11,55.61	145
CakTSSR01185	CakTC22317	(CTC)5	567	581	--	--	ATCACCACCACCTCCATAC	GGGTGGTGACTTGTAGACATA	55.43,55	136
CakTSSR01186	CakTC22350	(ATA)9	265	291	--	--	CCACCTTCAAGGAAGAGATTA	AGCAAGGGAGGAATTATTAGA	56.04,54.87	152
CakTSSR01187	CakTC22355	(ATC)5	756	770	--	--	AACAGCTAGAAGTGATGATGG	GACAACAATCCAAATTATCCA	54.54,55	149
CakTSSR01188	CakTC22357	(CAT)6	2714	2731	--	--	ATCCTTTCTTTCCCTTTGTTA	TGAGGATTGTGATGGTAGAAG	54.76,55.23	152
CakTSSR01189	CakTC22358	(GGA)5	762	776	Root	--	GGGTGGTGACTTGTAGACATA	ATCACCACCACCTCCATAC	55,55.43	136
CakTSSR01190	CakTC22362	(TGG)5	1239	1253	--	--	TGGTGGAGAGTGATAATGGTA	CTAAGGTGTACCCTCCAGTTT	55.53,55.03	143
CakTSSR01191	CakTC22364	(CAT)5	811	825	--	--	AGCACAATAGGTTTTAACTCG	TCAAAGTTCAAGCTGAATGAT	54,55.1	149
CakTSSR01192	CakTC22393	(CAC)5	323	337	--	--				
CakTSSR01193	CakTC22397	(AT)7	4088	4101	Young_pod	CCHC	CTTGCTGTTTTGCTGTTTTAT	AGCATAGGAAATGAAAGCATA	55,54.39	155
CakTSSR01194	CakTC22417	(CT)6	44	55	--	--				
CakTSSR01195	CakTC22448	(CAA)7	249	269	--	--	CTCTGACCAAATTCGAAATAA	AATTCGTTGAAGACTCGTTCT	54.66,55.69	150
CakTSSR01196	CakTC22449	(AT)11	6568	6589	Mature Leaf	--	ATGTCTTGGTGTTTTGTTGTT	TGTGCAGTGTATCAGATCAAA	54.63,55.21	160
CakTSSR01197	CakTC22470	(CA)8	1214	1229	--	--	GAGTTGTTGAGATGGAAGAAA	TATGCATATGATGAAGAGTCA	54.47,51.64	131
CakTSSR01198	CakTC22484	(CTT)7	1706	1726	Young_pod	--	GAATTAGGGTTAGGGATTGAA	GTTTTCCAGCTTCTTTAGCTT	54.95,54.89	163
CakTSSR01199	CakTC22484	(CTT)6	1896	1913	Young_pod	--	TGATCATCATCAAACGACATA	TCTCACCCATTCTAGAAATCA	54.88,54.86	145
CakTSSR01200	CakTC22489	(GT)7	911	924	--	--	TACTGTGTGACTGTTCAACCA	GAGTTGTTGAGATGGAAGAAA	55.02,54.47	162
CakTSSR01201	CakTC22519	(AG)6	69	80	--	--				
CakTSSR01202	CakTC22519	(AGA)5	330	344	--	--				
CakTSSR01203	CakTC22521	(TC)6	139	150	--	WI/SNF-BAF60	CTCTCAAATTCCTCACTCTCC	GTCTTGAAAGATGGAAGCAC	55.59,54.89	151
CakTSSR01204	CakTC22521	(CA)6	1081	1092	--	WI/SNF-BAF60	TTTCCTTCTGTTTATTCATCG	GGTGTTTGAGGAGACATTTTT	54.66,55.78	154
CakTSSR01205	CakTC22555	(AG)8	957	972	--	--	TGGGAGAGGAGAGGATATTAG	TTCTAACGGTGCCTTTAACTT	55.08,55.88	145
CakTSSR01206	CakTC22560	(AG)6	5339	5350	--	--				
CakTSSR01207	CakTC22585	(TC)6	94	105	--	--				
CakTSSR01208	CakTC22596	(TA)8	224	239	--	--	GCTCGATAAACTTTTAAAGCA	ATGGCAATTACAACAATCACT	54.39,54.69	155
CakTSSR01209	CakTC22607	(TAGTCG)5	169	198	--	--	CGTAGTCGTAGTCGTAGTCGT	GGATGTGGGTAAAACAACAC	54.79,55.24	184
CakTSSR01210	CakTC22621	(GGT)6	466	483	--	--	TGGGCGTAGCTATGAGTATAA	TAATATAGCTGCTGCAAATCC	55.32,54.97	145
CakTSSR01211	CakTC22631	(TTA)5	1619	1633	--	--	TTATCCTGAAAGTGAATCACAA	TCTCTAGATGCTTCTGGGTTA	54.98,54.38	165
CakTSSR01212	CakTC22633	(CAA)6	187	204	--	SET	AACCTTCGAGAGTTTCCCTA	AAACTGGTGATGGTGAAATTA	55.62,54.67	152
CakTSSR01213	CakTC22634	(CAC)7	36	56	--	--				
CakTSSR01214	CakTC22634	(TA)11	1274	1295	--	--	TAAGTGGGAATAGTGCTTTGA	ACAGAGAACAAGTTCTTCAACA	55.16,54.23	165
CakTSSR01215	CakTC22636	(GTG)6	1191	1208	--	--	ATTGGTTGTCTCTCTGGGTAT	CAAGACTAAGCATAGGAACGA	55.17,54.91	151
CakTSSR01216	CakTC22636	(TTA)5	2564	2578	--	--				
CakTSSR01217	CakTC22643	(TAT)7	602	622	--	--	CATTAGGAACATGGAATGAAA	AACCCTTAATCAATTCACCTC	55.17,54.84	159

CakTSSR01218	CakTC22646	(CAT)6	64	81	--	--				
CakTSSR01219	CakTC22648	(AAT)6	348	365	--	--	ACCGAAATACAACACTTGAAA	CAAGTTGTAATCAGAGCCATT	54.91,54.58	157
CakTSSR01220	CakTC22670	(TA)8	1889	1904	--	--				
CakTSSR01221	CakTC22672	(TA)8	61	76	--	--				
CakTSSR01222	CakTC22738	(GGA)5	775	789	--	--	GAGGTGGAGGTTATAATCGTG	CTCCACCACTACCGTATCC	56.19,55.35	177
CakTSSR01223	CakTC22739	(CT)9	28	45	--	--				
CakTSSR01224	CakTC22739	(GGT)5	651	665	--	--				
CakTSSR01225	CakTC22742	(GGT)5	803	817	--	--	TACAATAGAAGCAGTGGTGGT	ACACTAATCTCTCCAGTTTCC	54.93,52.56	177
CakTSSR01226	CakTC22745	(TCC)5	106	120	--	--	CTCCACCACTACCCGTATC	GAGGTGGAGGTTATAATCGTG	55.35,56.19	177
CakTSSR01227	CakTC22746	(GA)12	341	364	--	--	GCTGCTGTTCTTATTGTTGTT	CGATATTTTCCTTCTCTCTCC	54.81,54.81	149
CakTSSR01228	CakTC22762	(AG)6	93	104	--	--				
CakTSSR01229	CakTC22768	(GTGA)9	1580	1615	--	--				
CakTSSR01230	CakTC22769	(TTC)7	453	473	--	--	CCACCTCTATCGTTTCTCTTT	AGAGGGTTTTGAGTATTTGAGA	55.23,54.83	138
CakTSSR01231	CakTC22784	(TCT)8	226	249	--	--	CTCTGCCAAAACTTCTATCAA	AGAAGAAGAAGAAGCGAAAAC	54.88,54.84	148
CakTSSR01232	CakTC22785	(AAG)5	447	461	--	--	TAGAGAGACATGGCACAAAAT	ACACTTTCTCAACTGAAGCAG	54.97,54.82	166
CakTSSR01233	CakTC22787	(TCT)8	552	575	--	--	CTCTGCCAAAACTTCTATCAA	GAAGAAGAAGAAGCGAAAAAC	54.88,55.23	149
CakTSSR01234	CakTC22795	(GAA)6	1035	1052	--	NAC				
CakTSSR01235	CakTC22805	(CA)6	776	787	--	--	TCTAAGATTCCTGATTGTGGA	AAGGACGTTACACTACACTGC	54.86,54.57	158
CakTSSR01236	CakTC22822	(TTG)5	567	581	--	--	TTCTCCAAGAGTTTAGGGTTT	TGATAAACCTCTCACGAAATC	54.82,54.45	161
CakTSSR01237	CakTC22852	(GTG)5	370	384	--	--	CCTACCTGAGATGGTTCTTCT	GGTTGTTTATGAGGATGATGA	55.06,54.98	150
CakTSSR01238	CakTC22852	(GAA)5	779	793	--	--				
CakTSSR01239	CakTC22877	(AAT)7	53	73	--	--				
CakTSSR01240	CakTC22897	(AAT)6	3477	3494	--	--	AGAATGAACCGAACCTGTTAT	CGGTCACTGCCTATACAAATA	55.34,55.48	158
CakTSSR01241	CakTC22907	(TA)10	1003	1022	--	--	GGTGCTCAACTTGATTGATTA	AAAGATGCAATTGTGCTCTAC	55.39,54.73	154
CakTSSR01242	CakTC22916	(TC)10	1424	1443	Flower bud	--				
CakTSSR01243	CakTC22933	(AATA)6	108	131	--	--	ACTGTCCAAATTCTGCAATAA	CGGGTGTTACTGTTAAGGATA	54.99,54.5	150
CakTSSR01244	CakTC22934	(CT)6	1305	1316	--	--	TGGCTAATAAATCTTCACCAA	CCTCAATAGTTTTGAAGAACG	55.1,54.34	152
CakTSSR01245	CakTC22935	(GA)6	1866	1877	--	--	GAGATATGACTGCTGTTAGGC	CATTATTTTCTCCTCCTTGCT	54.22,55.32	143
CakTSSR01246	CakTC22937	(GA)6	2432	2443	--	--	CCTCAATAGTTTTGAAGAACG	TGGCTAATAAATCTTCACCAA	54.34,55.1	152
CakTSSR01247	CakTC22939	(CTT)5	1455	1469	--	--	AGATAACCAATCCACACACAC	AGAGTTTGAGTTTGGAGGAAC	54.81,55.09	145
CakTSSR01248	CakTC22942	(CT)20	89	128	--	--				
CakTSSR01249	CakTC22958	(GA)7	499	512	--	--				
CakTSSR01250	CakTC22966	(GGAAA)5	117	141	--	--	CATTTCAAGTCGGGAATCTA	TGCGAAGAATAAACCATTTAG	55.31,54.88	151
CakTSSR01251	CakTC22971	(CAACTG)5	3365	3394	--	--				
CakTSSR01252	CakTC23014	(TC)7	144	157	--	--	CGATCAGATCTCTGAGTTCAA	AAACAAAACACCGATCTACAA	55.52,54.91	144
CakTSSR01253	CakTC23028	(TA)7	1059	1072	--	--				
CakTSSR01254	CakTC23040	(AT)6	817	828	--	--	TGAGATTCTGGGAACAAATTA	CTGACTCGTGCTTCTCATACT	54.89,54.77	159

CakTSSR01255	CakTC23047	(TA)7	121	134	--	--	AAAGACATTTCCAAAGTCACA	CCCTCTTAATAATTGGGCTAC	54.85,54.77	146
CakTSSR01256	CakTC23058	(TA)6	53	64	--	--				
CakTSSR01257	CakTC23070	(TAA)5	2447	2461	--	HB	CTAATTTCATTGCTGGTTTTG	CTCATTTGGCATAAAACTTTG	55.18,55.18	152
CakTSSR01258	CakTC23072	(GAA)6	417	434	--	--	TGAATCTGGTTGGTGTGTAAT	GAATTTGCACAAAGAAGAAAC	55.43,54.27	248
CakTSSR01259	CakTC23077	(TCAAC)5	506	530	--	--	GGGGAGTTGTTAGAGAATCAG	CCATGGTGAATGAAGAGAGT	55.47,55.01	156
CakTSSR01260	CakTC23088	(TTA)5	1548	1562	--	--	CATGAACTCCTCCAATATGAA	CTTAAATAATCGTCAGCTTCG	55.16,54.64	147
CakTSSR01261	CakTC23090	(TC)8	25	40	--	--				
CakTSSR01262	CakTC23096	(TGA)5	1780	1794	--	--				
CakTSSR01263	CakTC23098	(AT)6	147	158	--	--	AAGGTTAATGCTTGCTACAGA	TACCATTGCTTCTGGAAGTT	54.52,54.96	152
CakTSSR01264	CakTC23098	(AG)8	1727	1742	--	--	ATCATTTTAGGAAGAGGGCTA	ACAACAATTTTCTCTCCCTTT	54.87,54.65	153
CakTSSR01265	CakTC23100	(CTT)13	337	375	--	--	CAGCTTTCTTATCCACATCAG	AGAAAGAAAGGGTAATTGTGG	55.15,55.09	150
CakTSSR01266	CakTC23100	(GA)6	1583	1594	--	--	AGAACTATGGCGGTTTTGTAT	CTTCTCCATAACTCCCTCATT	55.53,54.99	154
CakTSSR01267	CakTC23114	(TATT)7	1359	1386	Young_pod	--	TTTTCATTGTAGCTGATTGCT	TGGTGAAATCAAATGATGAGT	55.31,55.48	155
CakTSSR01268	CakTC23121	(TA)6	3653	3664	--	--				
CakTSSR01269	CakTC23122	(TA)6	217	228	Shoot	--	TTCCCATGTACTTTACCTCCT	TAATGACAGGGATCTGTTTTC	55.35,54.29	156
CakTSSR01270	CakTC23122	(TA)6	5020	5031	Shoot	--	CAGGAGCTCTGATACCATCTT	TTAGGATCAATCCCATAATGA	56.08,54.62	134
CakTSSR01271	CakTC23141	(CA)8	1210	1225	--	--	TCCTGGTGTTTTCTTTGAATA	CAAAAGTATTTTGGGCTATGA	54.97,54.72	172
CakTSSR01272	CakTC23145	(CAT)5	336	350	--	--	AGGTTCTACTCAAATGCATCA	TGATCCTAACTTGCACTTTGT	54.97,55.05	166
CakTSSR01273	CakTC23146	(CAT)5	261	275	--	--	ATCCAACACAAGCTCTACTCA	ACTTGCACTTTGTTGAATCTC	55.04,54.58	154
CakTSSR01274	CakTC23156	(GGAT)5	1242	1261	--	--	CCACTAGGAAGGATTGGTTAT	TCCAATACTTTGAGGTTGAGA	54.89,54.95	158
CakTSSR01275	CakTC23159	(TA)16	1242	1273	--	--	GTGGTCATCTTGACTCTCAAT	CATCAACATCGCTTATGTACC	54.1,55.71	147
CakTSSR01276	CakTC23161	(ATT)6	1926	1943	--	--	TAGAGATTGTTCGGACAGAGA	GGTGAACTGAGTGTTTGGAT	55.1,54.91	160
CakTSSR01277	CakTC23168	(TA)7	426	439	--	--	ATATGCCTTTTCTTTTCATCC	GTTATAGGCAAACCAAGACAA	55.09,54.61	138
CakTSSR01278	CakTC23169	(GAT)7	1029	1049	--	--				
CakTSSR01279	CakTC23175	(CACAAA)7	1634	1675	--	--				
CakTSSR01280	CakTC23222	(AGA)9	1284	1310	Flower bud	--	TTCAAAGCATGATTCTGAGAC	ATATCAATGGCCTTAATCTCC	55.52,54.9	152
CakTSSR01281	CakTC23259	(TTC)6	579	596	Flower bud	--				
CakTSSR01282	CakTC23281	(CA)7	1621	1634	--	--	GTAGTCGGTTACCATCCTACC	AACATGACAATCAAGATTTGG	55.22,55.06	139
CakTSSR01283	CakTC23285	(TG)7	1350	1363	Flower bud	--	GAACAGAGATAGGGAACCATT	GTAGTCGGTTACCATCCTACC	54.82,55.22	156
CakTSSR01284	CakTC23296	(AATA)5	784	803	--	--				
CakTSSR01285	CakTC23297	(TATT)5	647	666	--	--	TCATTCAAATGCAGTTCTTCT	CTGAACAAACCAATCATCTGT	55.1,55.13	145
CakTSSR01286	CakTC23317	(TTC)5	1795	1809	--	--	GATAAGCCTGCTAAAGAGAGG	GAACAGAATCGTCAATTTCAA	55.23,55.36	151
CakTSSR01287	CakTC23317	(CT)8	2493	2508	--	--				
CakTSSR01288	CakTC23318	(GAA)5	584	598	--	--	GAACAGAATCGTCAATTTCAA	GATAAGCCTGCTAAAGAGAGG	55.36,55.23	151
CakTSSR01289	CakTC23319	(GAA)5	596	610	--	--	GAACAGAATCGTCAATTTCAA	GATAAGCCTGCTAAAGAGAGG	55.36,55.23	151
CakTSSR01290	CakTC23328	(ACA)6	92	109	--	--				
CakTSSR01291	CakTC23329	(TGT)6	1515	1532	--	--	TTGAAGACAAGTCTTTCAGGA	AATGAAACACTCACCACTCTG	55.14,55.2	151

CakTSSR01292	CakTC23330	(ACA)6	36	53	--	--				
CakTSSR01293	CakTC23332	(TA)9	1016	1033	--	--	TCAAGCAAGGATTGATTATGT	ATTAAGTCCAGAAGCTCCAAG	54.91,55.38	133
CakTSSR01294	CakTC23344	(TTG)5	1300	1314	--	--	GTTTTGAATTTCTTGTCAGCA	TCTCAATGGTTACAAGTCAGG	55.6,55.31	150
CakTSSR01295	CakTC23345	(TTG)7	881	901	--	--	TAGCATCCTTATTCTTGTTGG	CAATATCAATTTCAAGGTTGG	54.7,54.78	152
CakTSSR01296	CakTC23347	(CT)12	983	1006	--	--	CTTGAAGAAATTCCAGACCTT	AGAAGCATTTGTCGGAGTC	55.2,55.34	158
CakTSSR01297	CakTC23348	(CT)9	896	913	--	--	CTTGAAGAAATTCCAGACCTT	CTTCGCTTTCTTCATTTTCTT	55.2,55.66	142
CakTSSR01298	CakTC23350	(GA)8	2378	2393	--	--				
CakTSSR01299	CakTC23351	(TC)11	1539	1560	--	--	TCCCTAACTCCTGCATAAACT	CGGTGTAGTGAGTGAGAGAAA	55.66,55.56	153
CakTSSR01300	CakTC23353	(TGAT)5	815	834	--	--	GTATTGGCTTCCTGTTTAGGT	AAACCCTAATTTTTCAACAGG	55.11,55.1	136
CakTSSR01301	CakTC23354	(TA)6	1057	1068	--	--	GGTCGCTTATATTTTGTGAAA	GCGACTTCTAAAAGAGTGACA	54.71,54.93	172
CakTSSR01302	CakTC23356	(TGAT)5	1249	1268	--	--	GTATTGGCTTCCTGTTTAGGT	AAACCCTAATTTTTCAACAGG	55.11,55.1	136
CakTSSR01303	CakTC23356	(GA)10	1423	1442	--	--				
CakTSSR01304	CakTC23367	(CTT)5	208	222	--	NAC	CCAATCTCTGCTTCCTTTAAT	GATGGACCACAGTCAATAGAA	55.32,55.05	142
CakTSSR01305	CakTC23382	(AAAAT)8	536	575	Root	bHLH	TGGTGTGAACACGTACACTTA	GCATATAATCCTAACCCACAA	55.08,54.35	154
CakTSSR01306	CakTC23382	(TTG)7	1649	1669	Root	bHLH	GAGGAGGAGCACAAGAACTAT	TCAACTGTTCAACAACATCCT	55.21,55.64	163
CakTSSR01307	CakTC23383	(AT)6	160	171	--	bHLH	ATTTTTCTCACCACTCCTTTT	CTTTGAAATTTGGTGAAGCTA	54.65,54.9	144
CakTSSR01308	CakTC23402	(TA)7	12	25	--	--				
CakTSSR01309	CakTC23410	(TC)7	1918	1931	Young_pod	--				
CakTSSR01310	CakTC23411	(CT)9	2114	2131	--	--				
CakTSSR01311	CakTC23412	(GA)8	2584	2599	--	--				
CakTSSR01312	CakTC23413	(GA)13	799	824	--	--				
CakTSSR01313	CakTC23420	(ATG)5	1174	1188	--	--	GGGACTATGGAGATTAATTTGA	CATGGTACTGTCCTTGTGATT	54.96,55	171
CakTSSR01314	CakTC23435	(AG)7	2359	2372	--	--	CATGGGAGAAACAAAAGTAGA	CACATCACAGAAGAGGAGAAC	54.55,54.8	149
CakTSSR01315	CakTC23439	(TC)16	2031	2062	--	--	GTGAACTCAAGTGTTGTCTCC	GAAGCAGAAGGAGAGAGAATC	54.7,54.96	154
CakTSSR01316	CakTC23448	(AC)10	1956	1975	Flower bud	--	TGACCCAGAAACAGATTCATA	CTTGCTTTTCCCTAATTGACT	55.66,55.39	157
CakTSSR01317	CakTC23448	(TTAT)5	2384	2403	Flower bud	--				
CakTSSR01318	CakTC23450	(TA)6	1198	1209	--	--	CCTTCATCTTATGGAGGATTT	ATTATTACCTCGTCGACCAAA	54.93,56.32	153
CakTSSR01319	CakTC23462	(AAT)5	437	451	--	--	TCTTCCGTTGTCTGTTTGTAT	CAAGTGGAACCCACTAATTTT	54.89,55.82	150
CakTSSR01320	CakTC23466	(AG)8	1125	1140	--	--	CTTCAATTACGCATCCTTTAG	TACGAGTTTGTTGGCATAGAT	54.48,55.02	164
CakTSSR01321	CakTC23472	(ATG)6	4048	4065	--	--	CAGAAGATGATCCTAGCAATG	ATCATCGTAATCAACGTCATC	55.06,55	143
CakTSSR01322	CakTC23478	(AG)15	2237	2266	--	--				
CakTSSR01323	CakTC23479	(TC)6	1278	1289	--	--	TGGTTCAATTCGTCTTCATAG	TCCTTCATGCAGATTTATGAT	55.4,54.82	137
CakTSSR01324	CakTC23480	(GA)13	2684	2709	--	--				
CakTSSR01325	CakTC23481	(TTAAA)6	239	268	--	--	AACGAATTGGAAGTACTAGTGG	TCCATAAAATACCCAATTAAGG	55.21,55.04	145
CakTSSR01326	CakTC23493	(AG)11	916	937	--	--	GGTGGTGGAGAAGATATAGGA	AAGATTTGATTAGGAGCGATG	55.68,56.13	152
CakTSSR01327	CakTC23495	(AG)11	181	202	--	--	AAGATTTGATTAGGAGCGATG	AGATGAAGATGAAGCCATGTA	56.13,54.88	153
CakTSSR01328	CakTC23506	(AGA)9	479	505	--	--	GGTCATTGTTGAGTATTTTGG	CCTTCTTCTACCACTTGCTCT	54.67,55.46	160

CakTSSR01329	CakTC23511	(GAA)9	657	683	--	--	GTCTGATGCAGAGAAAACTTC	AAAACTAACCACGTTACACGA	54.19,55.03	143
CakTSSR01330	CakTC23511	(CT)7	839	852	--	--	TCGTGTAACGTGGTTAGTTTT	AGCCATCTTCAGAGAGAGAAG	55.03,55.5	149
CakTSSR01331	CakTC23514	(TC)6	3191	3202	--	--	CAACAATTACAGCCACTCAAT	GTCATGTGATTTGGAGTTCAT	55.34,54.86	144
CakTSSR01332	CakTC23516	(AG)12	2078	2101	--	--				
CakTSSR01333	CakTC23528	(CAT)5	69	83	--	--				
CakTSSR01334	CakTC23530	(GA)7	1374	1387	--	--				
CakTSSR01335	CakTC23532	(TC)7	12	25	--	--				
CakTSSR01336	CakTC23541	(AC)6	3510	3521	--	Pseudo_ARR-B	TTAACTTCTGCAAACAAAACC	TTACTCTCCATGTTAATTGTGG	54.81,54.47	151
CakTSSR01337	CakTC23542	(AC)6	1620	1631	--	--	TTAACTTCTGCAAACAAAACC	TTACTCTCCATGTTAATTGTGG	54.81,54.47	151
CakTSSR01338	CakTC23551	(TTC)6	40	57	--	--				
CakTSSR01339	CakTC23554	(GAT)5	361	375	--	AP2-EREBP	GTTTTTGTTGGACGATGATT	AAAGACTTGGTGTTGTTTGAA	55.04,54.94	150
CakTSSR01340	CakTC23555	(TAA)5	409	423	--	--	CGCAACTAGATTCGATAGAGA	TGCTTTAGATTGCTCATTGAT	54.99,55.23	150
CakTSSR01341	CakTC23567	(AT)8	583	598	--	--	TATGGTGTTTTTCTGTCCAAT	ACCTCTGTCTCCCTTAGAAGA	54.67,54.77	160
CakTSSR01342	CakTC23586	(AG)7	173	186	--	--				
CakTSSR01343	CakTC23593	(TTC)7	282	302	--	--	AAAATGTCAAAGACCCTTTTC	ACAGTTTGTTTTGCTTCTGAG	55.04,54.85	149
CakTSSR01344	CakTC23593	(TGA)7	429	449	--	--	CTCAGAAGCAAAACAAACTGT	TGAAACAAGACAATCCATCTC	54.85,55.18	154
CakTSSR01345	CakTC23594	(TTC)7	234	254	--	--	AAAATGTCAAAGACCCTTTTC	ACAGTTTGTTTTGCTTCTGAG	55.04,54.85	149
CakTSSR01346	CakTC23594	(TGA)7	381	401	--	--	CTCAGAAGCAAAACAAACTGT	TGAAACAAGACAATCCATCTC	54.85,55.18	154
CakTSSR01347	CakTC23605	(GA)15	1371	1400	--	PLATZ				
CakTSSR01348	CakTC23606	(CT)15	77	106	--	--				
CakTSSR01349	CakTC23613	(GA)12	816	839	--	--	TGATATGCAGAAGGGAGAATA	CCAATTCTAGGGTTTTCATTT	55,55.03	159
CakTSSR01350	CakTC23613	(GA)7	1012	1025	--	--	AAATGAAAACCCTAGAATTGG	CATCCCATATTCCTTTTACCT	55.03,54.83	159
CakTSSR01351	CakTC23613	(AG)7	1129	1142	--	--	TATTGGAGTGAGAAGGTGAAA	TCATCATCAACAACAACCATA	54.95,54.79	163
CakTSSR01352	CakTC23616	(CT)9	1068	1085	--	--	ACGAAAATACAGAGGGATAGG	TGATATGCAGAAGGGAGAATA	55.04,55	149
CakTSSR01353	CakTC23628	(AT)10	518	537	Flower bud	--				
CakTSSR01354	CakTC23633	(ATT)6	172	189	--	--	CCTACCTAACCCACAATTCTC	AATACCCAAAATACCCTTCAC	55.35,54.75	149
CakTSSR01355	CakTC23636	(ATC)5	1530	1544	--	--	CCAACCACCATCTTCATAGTA	CGAAGGATTGGTTTATTATTG	55.11,54.13	156
CakTSSR01356	CakTC23645	(AG)7	11243	11256	--	--	TCCTTTTCAACTTCATTTAGG	ATCAATCTTTCAATTCCCTTC	53.86,54.89	150
CakTSSR01357	CakTC23648	(GA)6	10702	10713	--	--	CTGCAGTAACACAGTTGATGA	CGGATTATAGTTTTGGGAAAT	54.85,55	150
CakTSSR01358	CakTC23652	(TA)7	408	421	--	--	ACCTTTAGGTTGTCTTTGTCA	CATTCCCACTGTAAGTTGTTT	54.04,54.34	136
CakTSSR01359	CakTC23673	(AG)8	1091	1106	--	--				
CakTSSR01360	CakTC23678	(AC)6	52	63	--	--				
CakTSSR01361	CakTC23680	(TA)6	190	201	--	AP2-EREBP	CCTCCACCATTAATTTTCATT	TTCTAGCTTCCACCAAAAATA	55.67,54.45	152
CakTSSR01362	CakTC23680	(TTC)6	1398	1415	--	AP2-EREBP	GCAGCTATTCATTCATCTTCT	AAAAACTCTATTTGCTGACCA	53.88,54.32	158
CakTSSR01363	CakTC23681	(AT)6	1	12	--	AP2-EREBP				
CakTSSR01364	CakTC23685	(TC)7	82	95	--	--				
CakTSSR01365	CakTC23688	(ATG)5	946	960	--	--				

CakTSSR01366	CakTC23696	(AG)7	1811	1824	--	--				
CakTSSR01367	CakTC23700	(GAGAGT)12	1073	1144	--	--				
CakTSSR01368	CakTC23703	(CTCTCA)12	103	174	--	--	CTCTCACTCTCACCCTCACT	CGTGGAAGGTATGTATCAAAA	54.29,55.29	174
CakTSSR01369	CakTC23704	(GA)6	914	925	--	--				
CakTSSR01370	CakTC23705	(AG)22	1966	2009	--	--	GAGCTCTCTCTTTGAATCTGA	TTCTGTTACACCACCGTTTAT	53.97,54.79	149
CakTSSR01371	CakTC23706	(CT)6	106	117	--	--	TCGTTTAACGGTTACTGTTTT	CAGAGAGCTCAGAATCCAATA	54.33,54.77	158
CakTSSR01372	CakTC23707	(AG)18	1420	1455	--	--	GAATCCAATAGATCGCAGAG	ACCGTTTATTATTCCGTTTTC	54.95,55.07	147
CakTSSR01373	CakTC23713	(AT)7	1499	1512	--	--	CACCGGTTTTCATAATACTTG	TCTTTCTGCTGTTTTTCAAAG	54.9,55.08	147
CakTSSR01374	CakTC23721	(AGT)5	1117	1131	--	--				
CakTSSR01375	CakTC23723	(AGT)5	1969	1983	--	--				
CakTSSR01376	CakTC23725	(ATT)5	1392	1406	--	--				
CakTSSR01377	CakTC23727	(AG)8	2681	2696	--	--	CGCAGAGATCCATAACATAAG	ATATTAACCCCTTCCATACCA	55.12,55.1	153
CakTSSR01378	CakTC23728	(CT)7	1	14	--	--				
CakTSSR01379	CakTC23735	(AG)6	1625	1636	--	--				
CakTSSR01380	CakTC23736	(GA)12	1372	1395	--	--				
CakTSSR01381	CakTC23748	(CA)9	39	56	--	--				
CakTSSR01382	CakTC23760	(CT)8	4	19	--	--				
CakTSSR01383	CakTC23773	(CAA)5	297	311	--	C3H	CAACAACACCTTCTTCAACTC	TCAGACATAGTAGCCCAAGAA	54.92,55.15	157
CakTSSR01384	CakTC23774	(AG)6	282	293	--	C3H	TGTGAGTGAATGAAAGAAACA	TTTTGTTGTGAGATGAGTGTG	54.26,54.63	150
CakTSSR01385	CakTC23774	(CAA)5	691	705	--	C3H	CAACAACACCTTCTTCAACTC	TCAGACATAGTAGCCCAAGAA	54.92,55.15	157
CakTSSR01386	CakTC23775	(GTT)5	1769	1783	--	C3H	TCAGACATAGTAGCCCAAGAA	CAACAACACCTTCTTCAACTC	55.15,54.92	157
CakTSSR01387	CakTC23775	(TC)6	2182	2193	--	C3H	TTTTGTTGTGAGATGAGTGTG	TGTGAGTGAATGAAAGAAACA	54.63,54.26	151
CakTSSR01388	CakTC23785	(GAT)5	523	537	--	--	GATTCATCATTCCCAAAAACT	ATCCAAACGATATTTGGTCTC	55.63,55.67	153
CakTSSR01389	CakTC23786	(ATC)5	563	577	--	--	ATCCAAACGATATTTGGTCTC	GATTCATCATTCCCAAAAACT	55.67,55.63	153
CakTSSR01390	CakTC23787	(ATC)6	364	381	--	--	TCACATTTGCATACTTTGGAT	ATGAAGATACAGAGGCCAAGT	55.68,55.49	150
CakTSSR01391	CakTC23790	(ATA)7	237	257	--	--	CATAAGATGAACTCCACAACC	ACTTTGATGAACAACCTTGAA	54.64,54.85	164
CakTSSR01392	CakTC23798	(GA)6	1930	1941	--	--	CGGTTTTACAATGCTACTGAC	GTGGCTTGACGTATCTGATT	55.11,55.23	150
CakTSSR01393	CakTC23799	(GA)6	1837	1848	--	--	CGGTTTTACAATGCTACTGAC	ATCCCTCCTCTTGAAGAACTC	55.11,56.51	148
CakTSSR01394	CakTC23803	(TGA)15	3735	3779	--	--	GTTGGATTGAAGATTTCGAG	TGCAACAACACTATTGAAGAA	54.84,54.51	158
CakTSSR01395	CakTC23812	(AT)7	2124	2137	--	--				
CakTSSR01396	CakTC23815	(AAT)5	81	95	--	--				
CakTSSR01397	CakTC23817	(GAT)5	1861	1875	--	--				
CakTSSR01398	CakTC23819	(TCA)5	1310	1324	--	--	TACTTAAGACTGCCGCTACAT	GACCATCCAGTTCTTCACC	54.55,55.25	151
CakTSSR01399	CakTC23833	(TCA)5	89	103	--	--				
CakTSSR01400	CakTC23833	(AG)9	256	273	--	--	TATTCATACAAGCCAAAGGAA	GAACTCCTAAAAGTGCTGTCA	55.1,54.76	152
CakTSSR01401	CakTC23835	(TC)9	455	472	--	--				
CakTSSR01402	CakTC23839	(TGA)5	1230	1244	--	--	GTGGAGTTGTCTGTTGTTGAT	AAATAGCCTCATCTCCATTTC	55.02,55.08	140

CakTSSR01403	CakTC23843	(TCT)5	1427	1441	--	DBP	TCGTGAATATTGCTTCCTTTA	TTTCAACCAGTTTTCGTTAGA	55.25,55.21	149
CakTSSR01404	CakTC23845	(AT)8	1366	1381	Flower bud	--	ATTAACAAACGACGGATTTCT	TCTTTTATTTGGAGTTGCTTG	55.5,54.9	154
CakTSSR01405	CakTC23845	(GAA)5	1615	1629	Flower bud	--	GAGAAGGAGATGAACGAAGAT	TCTCTCTCAATTGATCGCTAC	55.09,54.75	156
CakTSSR01406	CakTC23846	(CTT)5	327	341	--	--	TCTCTCTCAATTGATCGCTAC	GAGAAGGAGATGAACGAAGAT	54.75,55.09	154
CakTSSR01407	CakTC23847	(TCC)6	698	715	--	--	TCGTTTGTGATCCCTTATATC	AGTCACAAATCTGAGGTGAAA	54.28,54.83	150
CakTSSR01408	CakTC23866	(TC)8	316	331	--	--	AAGAGAGAAGGTTCGCTTAAA	AGAGTGGTTGGTTAGGTTGAT	55.27,55.25	150
CakTSSR01409	CakTC23866	(TTA)5	1711	1725	--	--	CAGTCAAGACAATCTGTGTCA	GTAGGAAGGAGGTTGGTAGTG	54.6,55.42	158
CakTSSR01410	CakTC23872	(TG)12	2358	2381	--	--				
CakTSSR01411	CakTC23885	(AAG)6	399	416	--	--	GTCTGTCAAATGTTTCCTACG	CCCCTTTACAAACGTTAAGAC	54.89,55.58	148
CakTSSR01412	CakTC23905	(TA)8	2572	2587	--	--	ATACACAACCATATGCCTCTT	TTGTTGCATCTACTCTTATGAAA	53.81,54.55	150
CakTSSR01413	CakTC23914	(AT)10	245	264	--	--	TTCAGAGTGCATACAACAAGA	CCAATTGATTCTTCGAGTTTA	54.48,54.66	142
CakTSSR01414	CakTC23915	(GA)11	3149	3170	--	--	ATGAGAGAATTTGATGTGGTG	TCTTCAACCCAGATCTCACTA	55.05,54.92	162
CakTSSR01415	CakTC23916	(ACC)5	93	107	--	--				
CakTSSR01416	CakTC23917	(AT)6	924	935	--	--	CATGGTGGTCATAAACTTCTT	ATACGAGCTCCAATTCACATA	54.25,54.94	156
CakTSSR01417	CakTC23960	(AT)9	14	31	--	--				
CakTSSR01418	CakTC23960	(TAC)5	1647	1661	--	--	GGCTTCGTAGAATAATGATGA	GTGGGTTGAAGGTATTGAAA	54.6,55.05	145
CakTSSR01419	CakTC23965	(CTG)6	395	412	--	--	TCAGTATCAGTCCACCAACTC	TTAGCAAAATCACCACCTAGA	55.11,55.16	159
CakTSSR01420	CakTC23970	(AT)7	561	574	--	--	TTGCCACAGCATTATTAGTCT	GACCATTTCTTATCATCTGACC	55.18,55.24	142
CakTSSR01421	CakTC23971	(AAT)8	4242	4265	--	--	AATCTATTTCAATGGCCTCTC	AAAATTCCGGTATGATACGAT	55.08,55.24	152
CakTSSR01422	CakTC23979	(AAT)5	3336	3350	--	--				
CakTSSR01423	CakTC23984	(CAA)8	138	161	--	--	TCTTCTCTCTTCTCGAATCAA	TTTAGAAGGTGGATGATGATG	54.43,55.16	150
CakTSSR01424	CakTC24026	(AG)6	2511	2522	--	--				
CakTSSR01425	CakTC24027	(CT)6	62	73	Shoot	--				
CakTSSR01426	CakTC24028	(CT)6	27	38	--	--				
CakTSSR01427	CakTC24033	(AAG)5	127	141	--	--	TAGAATTGGAGACACACCAAG	GAATCTAAATCCCATCGTTTT	55.31,54.94	156
CakTSSR01428	CakTC24037	(ATA)8	1867	1890	--	--				
CakTSSR01429	CakTC24039	(ACC)5	791	805	--	--	ATCAAAGTTCCTTTTTGGTGT	TGCCTTCAGATAAATGGAATA	55.38,55.02	140
CakTSSR01430	CakTC24062	(TCT)5	599	613	--	--	CACGAAGACTGCTCTGATAAC	ATGAGATGGCGAAGGTATTAT	55.2,55.38	163
CakTSSR01431	CakTC24064	(TC)14	3	30	--	AP2-EREBP				
CakTSSR01432	CakTC24070	(GA)6	1184	1195	--	--				
CakTSSR01433	CakTC24071	(TC)6	20	31	--	--				
CakTSSR01434	CakTC24122	(AGA)5	71	85	Root	--				
CakTSSR01435	CakTC24134	(GT)8	2679	2694	--	--	ACCCTTTCTGTCAATTTAACC	GGTGTTGATTAGGCAAGTAAA	54.92,54.61	150
CakTSSR01436	CakTC24143	(TCTT)8	81	112	Shoot	--				
CakTSSR01437	CakTC24149	(AG)11	44	65	--	--				
CakTSSR01438	CakTC24159	(CT)6	170	181	Shoot	--	TTCAGTATTTCCAACGGTTTA	CGAATCGAGATCAAGTAAAAA	55.02,54.82	157
CakTSSR01439	CakTC24160	(GA)9	1	18	--	--				

CakTSSR01440	CakTC24176	(GAA)6	693	710	--	--	AGGTTCTCTTTCGGTTTTAAG	CTATTTCATCTCCACCTCACA	54.6,55.23	152
CakTSSR01441	CakTC24183	(CT)6	132	143	--	--	TCTAAAGATGTTCCTGCTGAA	GGATAAAAAGCGGATACTGAT	55.28,55.12	158
CakTSSR01442	CakTC24186	(TC)7	1119	1132	--	--	TGTTTTCGTTTTCTTCGTATC	ATTAGGGTTTGAAATGAAAGG	54.73,55.03	170
CakTSSR01443	CakTC24190	(TC)6	25	36	--	--				
CakTSSR01444	CakTC24234	(TTG)5	73	87	Mature Leaf	--				
CakTSSR01445	CakTC24242	(CAT)6	330	347	--	--	TAATGGAGCAGAGCAATTTTA	TGAGCTTTGAAACATTCCTTA	55.41,55.29	159
CakTSSR01446	CakTC24244	(GGA)5	1409	1423	--	--	GGCTGTTCTCCTAGTTTAGGT	CCACCCTTTAGATATGAAACC	54.44,55.28	171
CakTSSR01447	CakTC24254	(CT)7	62	75	--	--				
CakTSSR01448	CakTC24261	(CT)7	3	16	--	--				
CakTSSR01449	CakTC24261	(TGA)5	602	616	--	--	AATCTGTTGACGACATTTCAC	AGTCAAAATCATCCATTATGC	55.12,54.26	153
CakTSSR01450	CakTC24261	(AAT)6	1427	1444	--	--	GCAAGTGCGTAACTCATTATT	ACACAGTTGAAAGGTCAAAGA	54.79,54.92	147
CakTSSR01451	CakTC24262	(TGA)5	625	639	--	--	AATCTGTTGACGACATTTCAC	AGTCAAAATCATCCATTATGC	55.12,54.26	153
CakTSSR01452	CakTC24270	(GAA)6	126	143	--	--	CACAATGAAAGAAACCGTAAT	TCAGATTCCAGCAAATAAAAC	54.45,54.65	152
CakTSSR01453	CakTC24285	(ACA)6	19	36	--	--				
CakTSSR01454	CakTC24299	(TTA)5	1308	1322	--	--	TTTTTGATCTTCAGAAACCTG	GCTTGTACCGTTAAAAAGTCA	54.68,54.85	156
CakTSSR01455	CakTC24299	(AGA)5	1475	1489	--	--	TGACTTTTTAACGGTACAAGC	ATCCTCTCAAAAACACACAAA	54.85,54.85	153
CakTSSR01456	CakTC24309	(TCT)6	244	261	--	--	CAATTTACCTACTGGCTACGA	GATGATGACGATGATGAAGAT	54.81,54.85	150
CakTSSR01457	CakTC24316	(TC)7	126	139	--	--	TTCTTTCTCTACACGTCTCCA	AAAATGGAAAGAGTGAAAGATG	55.18,55.22	150
CakTSSR01458	CakTC24318	(TGG)5	1458	1472	Root	--	GGGTTTGTGATTTCACATTAG	CATTGTCTCAAAACAAAGGAC	54.67,54.85	174
CakTSSR01459	CakTC24330	(TAT)7	202	222	--	--	CACAAACACAAACACAAACAC	TGCAGAATTAGGTTAAAAGCA	54.94,55.48	152
CakTSSR01460	CakTC24337	(GAA)5	1722	1736	--	--	AGAACGAGATGTCACAAGAAA	TCTGTTTGAAATACCCTTGAA	55,54.97	151
CakTSSR01461	CakTC24343	(GAA)8	3097	3120	--	--	AGAGATGAAATTGAGCCTTTT	CCCATCTTTTTCTTCTTCAAT	54.88,55.13	175
CakTSSR01462	CakTC24344	(GA)10	277	296	--	--	ACACTCCAATGGAATTCATAA	AATATCAGAATGGAGCCCTAC	54.58,54.95	158
CakTSSR01463	CakTC24346	(TC)6	6	17	--	--				
CakTSSR01464	CakTC24351	(TC)15	93	122	Young_pod	--				
CakTSSR01465	CakTC24358	(CA)8	53	68	--	--				
CakTSSR01466	CakTC24360	(TC)9	807	824	--	--	TGTCTGGTCAATTTTCCTATG	TGATAATCATCAGCAATTTCC	55.24,55.24	144
CakTSSR01467	CakTC24366	(AG)7	255	268	--	--	TTCTGGAGAAAATACATGCAC	CAGGTTTCTGTCAGGTACAAG	55.39,54.97	158
CakTSSR01468	CakTC24367	(AGA)9	1316	1342	--	--	ACGAGAGCGTATGTTAGTGAG	AAAGTTTGGGAGAAAGAGAAG	54.83,54.54	145
CakTSSR01469	CakTC24380	(TCT)5	1833	1847	Flower bud	--				
CakTSSR01470	CakTC24395	(GTG)5	428	442	--	--	AGAGGAAGGGCTTTTGATAC	AAACTCAATTCAGATGCCATA	55.13,54.91	152
CakTSSR01471	CakTC24408	(TC)7	93	106	--	--				
CakTSSR01472	CakTC24415	(AG)6	2051	2062	--	--				
CakTSSR01473	CakTC24420	(TAA)10	251	280	--	--	ATTAGGATTTCGCTTGCTATT	CCTTGTGAGTTTGTGACTAGG	55.06,54.97	151
CakTSSR01474	CakTC24436	(ATT)5	1277	1291	--	--	TATTATTTCTCAACCGTCCAA	ATCCGTTCTTCCTAATGAAAC	54.94,55	208
CakTSSR01475	CakTC24439	(AT)9	2967	2984	--	--				
CakTSSR01476	CakTC24450	(CT)6	60	71	--	--				

CakTSSR01477	CakTC24460	(ATCATT)6	22	57	--	--				
CakTSSR01478	CakTC24470	(TTC)22	1120	1185	--	--				
CakTSSR01479	CakTC24491	(TCT)5	39	53	--	--				
CakTSSR01480	CakTC24491	(CTT)5	558	572	--	--	GTTTTGGTAGTTTCCCTTGAG	TTTCGTCAGAGACAGAAGAAG	55.55,54.88	141
CakTSSR01481	CakTC24492	(TTTCAA)6	134	169	--	--	TTGAACACCCAAAAGTTACAC	TTCATTTGTTCCTCTGTATGC	55.23,55.39	160
CakTSSR01482	CakTC24495	(TGT)6	1898	1915	--	--	GAATGGTTCGATTAAGGTTTT	CATTTAATCCTTTTGGTGACA	55.02,55.25	147
CakTSSR01483	CakTC24509	(CT)11	96	117	--	--				
CakTSSR01484	CakTC24510	(CT)11	97	118	--	--				
CakTSSR01485	CakTC24535	(AT)6	98	109	--	--				
CakTSSR01486	CakTC24535	(TGGA)8	862	893	--	--	TACAGCCCCTACTATTGAATG	AGAACAAACCACCTCAGTCTA	54.57,53.99	149
CakTSSR01487	CakTC24542	(TGG)5	1374	1388	--	--	GCCATCCCTTTGTAGATTACT	AGGAGAATAATTGTGGTGGAG	55.03,55.75	147
CakTSSR01488	CakTC24545	(AG)9	2849	2866	--	--	CGAAAGAGAAAGAGAGTGTGA	GTAAATGAAATTCGGGTTACG	54.88,56.3	152
CakTSSR01489	CakTC24549	(AT)6	799	810	--	--	CTTATTTAACTCGAGGGTTGC	GGGTCCACTCTCAACATTAGT	55.81,55.66	151
CakTSSR01490	CakTC24566	(AT)6	3234	3245	--	--				
CakTSSR01491	CakTC24572	(AAAAC)5	364	388	--	HB	CGTTGCTTAAGATACCAAAAC	AGAGCTTCCAAGTCTCTTCAT	54.4,54.92	179
CakTSSR01492	CakTC24575	(TGG)5	387	401	--	--	AGACGGATGCTTCATATTCTT	TGTAGAGGAGGCAGATATTGA	55.58,55.06	154
CakTSSR01493	CakTC24577	(TGG)5	368	382	Root	--	AGACGGATGCTTCATATTCTT	TGTAGAGGAGGCAGATATTGA	55.58,55.06	154
CakTSSR01494	CakTC24583	(AG)6	763	774	--	NAC	GAGATCTCTCCTTCCTCAAAC	CCATCTCAACAACAGATAAGG	54.63,54.82	158
CakTSSR01495	CakTC24583	(AG)9	942	959	--	NAC				
CakTSSR01496	CakTC24586	(AAG)6	1495	1512	--	--				
CakTSSR01497	CakTC24608	(AGC)5	1655	1669	--	--	TTAAAAGGGTAGCAGGAAAAT	ATGCTTTCTAAAGGGAACAAT	54.96,54.78	151
CakTSSR01498	CakTC24614	(TTTGA)5	2829	2853	--	--	GGATCAAAGAGAGGGAGTTT	TTCATACATCCCAATAACCAA	54.91,55.46	146
CakTSSR01499	CakTC24616	(ACA)6	229	246	--	--	CTACCTTCACAACACATCCAT	CATGCAACTTAGGTTGAAGAC	55,55.05	140
CakTSSR01500	CakTC24617	(ATG)6	855	872	Flower bud	--	GATTTTGCTGATGAAAAGATG	GATGTTGCTGCAATGAGTTAT	55.03,55.42	150
CakTSSR01501	CakTC24636	(CAA)40	1	120	--	--				
CakTSSR01502	CakTC24639	(TA)7	2595	2608	--	--				
CakTSSR01503	CakTC24676	(TA)8	679	694	--	Bromodomain	GCTTCTCCCCTAAAATTCATA	TCTAAAGAGTCTCCCTCAGAAA	55.25,55.1	141
CakTSSR01504	CakTC24676	(CCA)6	1407	1424	--	Bromodomain	AATCACTTCCTGAAGAACTCC	TCCTGTTGAGATTGAGAAAGA	55.01,55.06	149
CakTSSR01505	CakTC24677	(TA)8	650	665	--	--	GCTTCTCCCCTAAAATTCATA	TCTAAAGAGTCTCCCTCAGAAA	55.25,55.1	141
CakTSSR01506	CakTC24680	(AT)7	539	552	Young_pod	--	GTCCCCAAATCCAATATTATC	ACTGTTTGCTTTTTCACTTCA	54.98,55.26	156
CakTSSR01507	CakTC24688	(CAC)5	354	368	--	--	ACACTTTCCATCTCCTCTCTC	TGAAGTAGTTGGGTCTTTTTG	54.99,54.64	151
CakTSSR01508	CakTC24689	(CAT)6	102	119	--	--	AACATTTTTCCTTTCCTTCC	TCCATTGATTACAGGAGATTG	54.96,55.16	145
CakTSSR01509	CakTC24689	(TTA)5	3334	3348	--	--	GACTAGCATTTGTCCTGATTG	AAGGAAAGTAGGAAAGCATTG	54.97,55.39	160
CakTSSR01510	CakTC24692	(AGA)8	2358	2381	--	HB				
CakTSSR01511	CakTC24704	(AT)8	646	661	--	--	CCTTACACTCTGGTTGCAATA	ATTGAATAAATGGCCACAAA	55.52,55.59	158
CakTSSR01512	CakTC24713	(CA)6	98	109	Shoot	--				
CakTSSR01513	CakTC24719	(AG)8	1062	1077	--	bZIP	ATCTATTGGGTTTGGTTGAAT	TCATTTATAGAAGCAGCACCT	55.12,54.43	148

CakTSSR01514	CakTC24736	(TTG)6	1157	1174	--	--	TTTCCATTATCCAAGAGTTGA	CAAAGACAATTCAAGCAAGTT	54.89,54.79	147
CakTSSR01515	CakTC24745	(GAC)5	3467	3481	--	--	ATCTCCTGTTGAGATTGATGA	CCCAATTTCACATCATGTACT	54.65,54.93	153
CakTSSR01516	CakTC24767	(TTA)5	52	66	--	--				
CakTSSR01517	CakTC24768	(CTT)6	1055	1072	--	--	CATTCCCATTGAAATACAGAG	TGGGTAAAGTGAAGTGAGAAG	54.76,54.61	143
CakTSSR01518	CakTC24768	(CCA)7	1234	1254	--	--	CATCCCTCTTTTCATTCTCTT	TTTTTCAACGAGTTCTGAGAG	55.12,54.9	151
CakTSSR01519	CakTC24772	(TGA)5	1515	1529	--	--	TGATGATGATGAGAGTGATGA	GTCATTTTCATCGAGATCGT	54.78,55.1	157
CakTSSR01520	CakTC24775	(TA)9	1004	1021	Shoot	--	CTCAATTCTAAAACCCAGCAT	CGTACCTCATGAATAACCAAA	56.05,55.29	150
CakTSSR01521	CakTC24775	(CTC)5	7743	7757	Shoot	--	CCAAATAAGCATTGAATATGG	GAATAGGCGAACTCTTCTGAT	54.92,55.3	145
CakTSSR01522	CakTC24781	(AG)7	2767	2780	--	--	CCGTTACGAAGATCCTTACTT	GAAAAACCAAACCAAATCAAC	55.27,56.17	172
CakTSSR01523	CakTC24785	(TA)6	318	329	--	--	CCGAATTGTAATATTTTATACCG	CCAGATAGTAATTTTCTCAATCA	54.9,53.11	296
CakTSSR01524	CakTC24788	(TAA)5	76	90	--	--				
CakTSSR01525	CakTC24792	(CT)7	62	75	--	--				
CakTSSR01526	CakTC24804	(AG)12	119	142	--	--	TAGTTCATTCAAAGCCTCTCA	GATGCCATGTTACAACAACTT	55.28,55.17	148
CakTSSR01527	CakTC24815	(TAT)6	1018	1035	--	AUX/IAA	AGGTCGAGGCTAGTATAGGAA	TCATTTCTTGTCCCTAATGAA	54.96,54.89	153
CakTSSR01528	CakTC24819	(TA)8	432	447	Flower bud	--	GAAGAAGGGGAAAGAGACATA	CTGTCCTACACTGCACTTACC	55.12,54.99	142
CakTSSR01529	CakTC24824	(TA)8	3424	3439	--	MYB	GGCTTCAGCTGTTACAATTAC	TAAATAAGAGGGTGCATTTGA	54.35,55.1	149
CakTSSR01530	CakTC24834	(TC)6	219	230	--	--	TCATTCCAATCCAACTGAATA	GTGAGTTGAGAAGGGAAGAGT	55.58,55.07	146
CakTSSR01531	CakTC24835	(TAG)5	1037	1051	Flower bud	--	TAAATAGTGGACCAAAATTGC	ACTCCTGGCTATTTGTTTACC	54.56,55.11	161
CakTSSR01532	CakTC24837	(CCA)5	245	259	--	--	CCTTCTTACACTAGTGCTCCA	TCTAGACATTGGAGGAGGTG	54.81,55.2	165
CakTSSR01533	CakTC24838	(TAT)6	119	136	--	--	GAAAAGGAAAACACACATTCA	TTGTGTTTCTGTTTTGTGTGA	55.28,55.18	140
CakTSSR01534	CakTC24850	(TC)13	188	213	--	--	CCACGCTTTCTTTCTTTATTT	TGAAGATTTGAAGGAGCATAA	55.54,55.21	151
CakTSSR01535	CakTC24855	(AAC)5	685	699	--	--	TTGAATTAAACACTCTCTTTGC	TAATCTTACAGGAAGGCAACA	54.1,55.16	138
CakTSSR01536	CakTC24858	(TTC)5	1702	1716	--	TUB	ATGTGATTCGAGATGAAGAGA	TCAGCTGTGATAGTGTGAAAA	54.82,54.48	164
CakTSSR01537	CakTC24858	(ATA)5	3614	3628	--	TUB				
CakTSSR01538	CakTC24863	(AG)7	108	121	--	--	GAATTAAAAGAGTGGCTCACA	AAAAGAAGGGTGAGTGGTAAT	54.7,54.53	155
CakTSSR01539	CakTC24870	(TC)7	1329	1342	--	--	CAATAGGTTGGATCTTCAATG	GGAAGATGAATGTGTGTTGAC	54.76,55.38	153
CakTSSR01540	CakTC24875	(TC)13	1	26	--	--				
CakTSSR01541	CakTC24885	(TAA)5	239	253	--	--	AAAGTACAGTACACCGACACAA	CTTTTCAACTTGTTTTGGAAG	54.94,54.39	161
CakTSSR01542	CakTC24891	(AAT)11	122	154	--	--	CCTTGCTCTTCTTTCACTTCT	AGCTAAAAAGGTGAATTCGTT	55.58,55.01	165
CakTSSR01543	CakTC24908	(GAA)6	1431	1448	--	--				
CakTSSR01544	CakTC24910	(TCA)8	122	145	--	--	ACCACTAAGCAATGTCTTCCT	ATTGATCTTTAGCTGGGTAGG	55.57,55.2	153
CakTSSR01545	CakTC24928	(TA)6	1110	1121	--	--	CAAATAATCGGATTCAGACAG	CAGGGCCAGTATACTAATGAA	54.92,54.57	150
CakTSSR01546	CakTC24929	(CAC)5	264	278	--	--	CAAAATCAAACACTTCTCCAC	GCTCCAAAATCTAACACATTG	54.85,54.99	154
CakTSSR01547	CakTC24934	(TA)10	236	255	--	--	TCTCTTATCACAAGGATGCAC	GTTTCAAAATTGAACACCGTA	55.38,55.32	151
CakTSSR01548	CakTC24937	(AG)11	2358	2379	--	--				
CakTSSR01549	CakTC24942	(CTT)5	388	402	--	TRAF	CTCTGGAAATTTAAACAAGCA	CATCCAAGACAAAATTCCTTA	54.9,54.5	158
CakTSSR01550	CakTC24960	(AG)13	858	883	--	--				

CakTSSR01551	CakTC24961	(AC)9	123	140	--	--	CTTGTTCTCTCTTTCCAATCA	TTTTGTTCTGTTCAACAAGGT	54.65,54.94	136
CakTSSR01552	CakTC24968	(AG)9	783	800	--	--	GAACCTTGAATTCTGTTTCTTC	TCTCAACAATCCTTCTTTCAA	54.78,55.08	151
CakTSSR01553	CakTC24977	(ATTTCA)7	209	250	Flower bud	--	TGAAAACTGAAGCAGAACAAT	CCATTATCCATCATCACAAAT	55.18,54.79	148
CakTSSR01554	CakTC24985	(ATG)5	997	1011	Flower bud	--	TATGTCAATCGGAAACAACTT	CATTGTTTTCTTCCTCCTCTT	54.83,55.2	148
CakTSSR01555	CakTC25007	(CAA)5	906	920	--	--	AACCTCAGAGTGCTTCTCATT	TTGCTAGAATAGGCAATAACG	55.7,55.06	151
CakTSSR01556	CakTC25017	(AT)6	1102	1113	--	--	GCCACGAAATTACTAGATTGA	CGTCCCAAAATATAAGCAAA	54.68,55.51	169
CakTSSR01557	CakTC25022	(TTC)6	218	235	--	--	AATGCTTTGTACTTCAACTCG	TGTTTGGAAGAGAAGAGAATG	54.82,54.65	149
CakTSSR01558	CakTC25026	(ATA)6	2550	2567	--	--				
CakTSSR01559	CakTC25027	(TGA)5	936	950	--	C2H2	TTAGGGTTTTGTGAGAATTGA	CAACAACAAAACCAAAGTTTC	54.97,54.96	140
CakTSSR01560	CakTC25027	(AAG)5	2085	2099	--	C2H2	TTTAAGCTTGCTCTTCACAGT	TGTTCCTTCCTTCATTAAAAG	54.7,53.86	157
CakTSSR01561	CakTC25036	(AGT)5	729	743	--	WRKY	GTGGTGCAACGATAGTAACTC	CTGTGAAAAATAACCCCTTTC	54.91,55.48	149
CakTSSR01562	CakTC25037	(AGT)5	730	744	--	WRKY	GTGGTGCAACGATAGTAACTC	CAAAAAGCTGTGAAAAATAGC	54.91,54.67	156
CakTSSR01563	CakTC25042	(AG)14	1	28	--	--				
CakTSSR01564	CakTC25043	(TTAT)5	1975	1994	--	--				
CakTSSR01565	CakTC25044	(TA)6	453	464	--	--	AAAGAGAATGGCTTGATTACA	GCATTGTGGTTTTCAACTTTA	54.24,55.48	155
CakTSSR01566	CakTC25052	(TC)8	15	30	--	LUG				
CakTSSR01567	CakTC25057	(GAT)5	1190	1204	--	--	AGTCGAAGATGATGAAGATGA	CATTCCCTCTCAAGGTAGATT	54.82,54.99	153
CakTSSR01568	CakTC25059	(TCTCTA)5	25	54	--	--				
CakTSSR01569	CakTC25063	(CT)7	44	57	--	--				
CakTSSR01570	CakTC25081	(TC)7	6	19	--	--				
CakTSSR01571	CakTC25085	(TC)9	237	254	Young_pod	--	CGAGATCCTCTCTTCTTCTTC	ATATCGTGTTGAAGGTGTGAC	54.97,54.99	150
CakTSSR01572	CakTC25085	(CT)7	371	384	Young_pod	--	AGTCACACCTTCAACACGATA	TCGATAAAGGAACAATGAAGA	55.68,55.05	163
CakTSSR01573	CakTC25092	(CTT)5	16	30	--	--				
CakTSSR01574	CakTC25098	(TCT)5	2058	2072	--	FHA	ATCACTTCAAACCCTTTTCTC	GCATGTGAAAGTGAAAGCTAC	55.03,55.21	150
CakTSSR01575	CakTC25099	(AG)10	2304	2323	--	--				
CakTSSR01576	CakTC25109	(CT)9	318	335	--	--	TCTCCCATTTCTTCTTCTCTC	AAAAATGGGTCAGATCTCAAT	55.23,55.23	147
CakTSSR01577	CakTC25127	(ATT)7	216	236	--	--	GGAAAGAAACAACAAGAGGAT	TTTTCCAGAAACAACTCACAT	55.03,54.85	170
CakTSSR01578	CakTC25136	(ATT)5	1010	1024	--	--	CAGTGCATTCCTTATTCAATC	GTTGATTCAATTTACCGACAA	54.91,55.24	155
CakTSSR01579	CakTC25141	(TCA)5	362	376	--	--	TCTCCATATTTCTTACGTCCA	GAAACCTAGCTATGGAAGGAG	54.92,54.92	153
CakTSSR01580	CakTC25144	(TTC)5	82	96	--	--				
CakTSSR01581	CakTC25147	(AG)9	300	317	--	--				
CakTSSR01582	CakTC25161	(TC)7	9	22	--	--				
CakTSSR01583	CakTC25167	(AT)7	12	25	--	--				
CakTSSR01584	CakTC25189	(TA)10	121	140	--	MYB	ATTCATAGTAGGCGTCACCTT	TCTAATTTCCATGAAGCAGAG	55.53,54.79	138
CakTSSR01585	CakTC25190	(TC)6	497	508	--	--	CTATTGCATGCACTCTCTTTT	GTCCTTCAAAATCTTTATTGGT	54.9,54.25	159
CakTSSR01586	CakTC25190	(GA)13	1326	1351	--	--	TGTCCCACGTTATTTTTGTAT	CAAAAATTAGATAGGGTTTGTCA	54.73,55.29	155
CakTSSR01587	CakTC25192	(GA)11	1654	1675	--	--				

CakTSSR01588	CakTC25199	(CTT)6	3827	3844	--	CCAAT	TCTTTATCTTCTGCAAAACCA	CAAGAGCAAGAACAAGAAGAG	55.29,54.64	149
CakTSSR01589	CakTC25208	(AT)6	453	464	Mature Leaf	--	GGTTGAGTGAACATCAGGATA	AATTACGTATTGCAAGGTCAA	55.05,55.04	161
CakTSSR01590	CakTC25214	(AAT)5	3074	3088	--	--	TTGAAGGGCACAAATTAGATA	GATAAGATTTGGTGCTTTCCT	55.1,55.15	214
CakTSSR01591	CakTC25218	(AT)6	480	491	--	--	TTGTTCTGTTTTTGGTTGAGT	AGCCAAGATGTTGTCATTAAA	54.94,54.99	136
CakTSSR01592	CakTC25225	(TCTTC)5	1382	1406	Young_pod	--	CCCATCTTTCTGATTCTACCT	TCTAGGACGAAGAAGATGTGA	54.99,55.1	154
CakTSSR01593	CakTC25225	(GA)7	2334	2347	Young_pod	--	CTCTCTTTCCTTTTGGTTTGT	AAGTTGCATGCATCAGTAAAT	55.27,55.01	152
CakTSSR01594	CakTC25228	(TA)20	6	45	--	--				
CakTSSR01595	CakTC25235	(AAT)11	315	347	Root	bZIP	GGTTCTTCATCATCAACTTCA	ATCTAGCTTGGCTTTGAGAAG	55.18,55.69	141
CakTSSR01596	CakTC25236	(AAT)11	295	327	--	bZIP	GGTTCTTCATCATCAACTTCA	ATCTAGCTTGGCTTTGAGAAG	55.18,55.69	141
CakTSSR01597	CakTC25250	(TAT)6	1409	1426	--	--				
CakTSSR01598	CakTC25251	(GAA)6	2725	2742	--	--	AGTCTTGTTTTAGGGCAATTT	TCGTCAAGACATTGTTTCTTT	54.86,55.02	166
CakTSSR01599	CakTC25253	(GAA)6	2817	2834	Flower bud	--	AGTCTTGTTTTAGGGCAATTT	TCGTCAAGACATTGTTTCTTT	54.86,55.02	166
CakTSSR01600	CakTC25276	(AT)6	134	145	--	--	CCAAATATGACCTGCAAATTA	TCGAAGAACTATAATCAACACAA	55.37,54.4	158
CakTSSR01601	CakTC25280	(GGT)7	883	903	--	--	GGAGAAAGTGATTGGAGAATG	TCCTTCTCACGTTTCTCTACA	56.3,55.18	161
CakTSSR01602	CakTC25290	(AG)11	230	251	--	--				
CakTSSR01603	CakTC25296	(TA)6	878	889	--	--	TCATCAACTTCCATTTTGTTC	AATGAAGACATTGCCAAAAG	55.19,55.38	159
CakTSSR01604	CakTC25314	(CT)6	144	155	Root	--	AGCTGTGTCCGTTATGAATC	GTCAGTTTGGTGTGTGTCTTT	55.23,55.1	134
CakTSSR01605	CakTC25329	(TC)7	741	754	--	TCP	TCACCCTCATTTATCATCATC	GAAACTTACATGCTTGTGCTT	54.89,54.81	142
CakTSSR01606	CakTC25330	(CT)6	209	220	--	--	ACCAAACAAGGAACAATCTCT	GTTTTGAAGGGTGATGATATG	55.38,54.58	149
CakTSSR01607	CakTC25330	(TC)7	363	376	--	--				
CakTSSR01608	CakTC25331	(GAA)11	2379	2411	--	EIL	CAAGTGAATGAATTCTCAAGC	CTCTCTTTCCTCTCATCTTCC	55.1,54.81	152
CakTSSR01609	CakTC25334	(CCA)5	1851	1865	--	--	ACTCCTTCAACTTCAAACACA	ATAGCACTGCACTGTCTCTTC	54.92,54.76	148
CakTSSR01610	CakTC25336	(GCT)6	1647	1664	--	--				
CakTSSR01611	CakTC25339	(ACA)5	112	126	--	--	CTTCTTCACAAGCCTCAGATA	CCATTGTTCCTCTCATATTCA	54.85,55.16	154
CakTSSR01612	CakTC25339	(AAC)5	2162	2176	--	--	TTCGTCTCTTTCTGCTTTATG	TTTGTCCCTTTCTCCTAAGAC	55.04,55.2	150
CakTSSR01613	CakTC25343	(CT)9	10	27	--	--				
CakTSSR01614	CakTC25344	(AT)11	669	690	Young_pod	--	GGCATCACATTCCTACTATCA	GAGTTAGACCAAACCCAAAAT	55.18,54.92	156
CakTSSR01615	CakTC25345	(AG)7	3193	3206	--	--				
CakTSSR01616	CakTC25356	(AG)10	3597	3616	--	--				
CakTSSR01617	CakTC25381	(AG)7	898	911	--	--	AAAGGGAAAGTTGAGAAAGG	TGCTTCATTCCTCTAGAAACA	55.18,55.28	155
CakTSSR01618	CakTC25384	(AG)6	2365	2376	--	--	AGGAAGACTGTGTTTGAGTGA	TGTTGTTCCTTTTTCAACTTC	54.89,54.59	150
CakTSSR01619	CakTC25385	(AG)6	2349	2360	--	--	AGGAAGACTGTGTTTGAGTGA	TGTTGTTCCTTTTTCAACTTC	54.89,54.59	150
CakTSSR01620	CakTC25391	(AT)7	1923	1936	--	--	GGTCACTGATGCAGTTTTAAG	TTATGGAAGAAGCCAAAATAG	55.05,54.01	157
CakTSSR01621	CakTC25414	(TCT)9	20	46	--	--				
CakTSSR01622	CakTC25438	(CA)6	102	113	--	--	ACAACAACATCGAAAACAGAG	CCATGCTTAGCGTTTTTATT	55.38,54.89	153
CakTSSR01623	CakTC25440	(CTT)6	2020	2037	--	--				
CakTSSR01624	CakTC25442	(AGC)6	791	808	--	--	AAACTTTTCATGCAACAACAC	GATGAGTTTTCTGATGATGGA	55.38,55.09	192

CakTSSR01625	CakTC25449	(TTG)5	299	313	--	ARF	TTCACAAAGACAAAAAGGTGT	GCAATCTTTTCCCATTGTACT	54.94,55.89	148
CakTSSR01626	CakTC25450	(AG)12	1775	1798	--	--	GGGGTTACAAAGTTAAGGAAA	AGCACCATTCAATTCAATTAC	55.1,54.35	146
CakTSSR01627	CakTC25451	(AT)6	2547	2558	--	--	TGAAAATGATTCCTATCAAGC	CTGGCAATTGACTACTACGAC	54.56,55.09	137
CakTSSR01628	CakTC25451	(ATTA)6	3364	3387	--	--	GATTAATCTCATAGCCGGAAC	CATTATCAGGTGTGGCATTAT	55.5,55.07	143
CakTSSR01629	CakTC25458	(TTG)7	325	345	--	--	TTGTGGGACAATCATAACTTT	TTTTATGTGCAGAATCAACCT	54.67,54.99	150
CakTSSR01630	CakTC25462	(AG)9	1020	1037	--	--				
CakTSSR01631	CakTC25464	(GAA)5	294	308	--	--	TATCATACATCGAAAGCTGGT	GATGAACCCTTACTCTTTCGT	54.94,55.06	176
CakTSSR01632	CakTC25465	(GAA)5	565	579	--	--	TATCATACATCGAAAGCTGGT	GATGAACCCTTACTCTTTCGT	54.94,55.06	176
CakTSSR01633	CakTC25470	(CTT)6	1269	1286	--	--	ATACGGATCCCCATCTTATAG	CCCACACAAACATAAACTCAA	54.79,56.1	141
CakTSSR01634	CakTC25472	(CTT)6	1139	1156	--	--	ATACGGATCCCCATCTTATAG	CCCACACAAACATAAACTCAA	54.79,56.1	141
CakTSSR01635	CakTC25474	(GA)6	2552	2563	--	--	TTGATGAAGAACAAGACCCTA	TAATTTCCATTTCAGTGGTTC	54.95,54.33	146
CakTSSR01636	CakTC25475	(TTA)5	264	278	--	--	CATTTTCTTCGTTTGACTCTG	CAAACCAGACATTCCATAATC	55.19,54.58	136
CakTSSR01637	CakTC25481	(AAG)5	59	73	--	--				
CakTSSR01638	CakTC25493	(TC)8	221	236	--	--	TTACTCTGTAGGCCATGTCAC	TTCCTTCTTCTTCAGCTACG	55.34,55.01	150
CakTSSR01639	CakTC25495	(TC)8	214	229	--	SET	TTACTCTGTAGGCCATGTCAC	GTTCCTTCTTCTTCAGCTACG	55.34,56.01	150
CakTSSR01640	CakTC25498	(ACC)5	331	345	--	--	GACATCTCATTCCGATACAAC	AGCTACTTAGGGAGGAATTGA	54.53,54.92	122
CakTSSR01641	CakTC25509	(AAT)5	1491	1505	--	--				
CakTSSR01642	CakTC25515	(GAT)5	1394	1408	--	--				
CakTSSR01643	CakTC25516	(GAA)5	1232	1246	--	--	ATCTTCCTTTGGCACATTTAG	GGTTTTAGGGTTTGAAGACAT	56.05,54.92	145
CakTSSR01644	CakTC25519	(AAAT)6	1471	1494	--	--	TGTTGTGAGGGGAATAGAGTA	CCATTGGCTTCTGAAGTAGTA	54.82,54.75	162
CakTSSR01645	CakTC25520	(GGT)7	1324	1344	--	--	TAGAGTTATGATTTCCGGGTA	TCTTACTCTGTTCCCTCTTCC	54.09,55.18	162
CakTSSR01646	CakTC25526	(AG)10	1435	1454	--	--				
CakTSSR01647	CakTC25528	(TAA)8	66	89	--	ARF				
CakTSSR01648	CakTC25531	(AG)6	1195	1206	--	--	ACACTTGCTTCTGCATCTAAA	CTCACCCCTTTTCATTTTATC	55.38,55.4	151
CakTSSR01649	CakTC25531	(TA)6	1662	1673	--	--	ACCAAAACCCATTATTCAGAT	CTCCAAGGGAGTGATACTTTC	55.12,55.47	154
CakTSSR01650	CakTC25531	(GA)12	1976	1999	--	--				
CakTSSR01651	CakTC25538	(TA)6	165	176	Flower bud	--	CCCCTTTTCTTGTACTTTTGT	AAGAGGACCAATATGGAGTGT	55.17,55.17	153
CakTSSR01652	CakTC25542	(ATTC)5	278	297	--	--	AAAATCATCTTTTGGACACCT	ACCCATTTCTTCCTCTCTCTA	55.31,54.71	150
CakTSSR01653	CakTC25543	(TTA)8	612	635	--	HB	AAAATGTCACTTCTTGCTGAA	AAATGGATTGAAGGAATCAGT	55.18,55.23	153
CakTSSR01654	CakTC25543	(AT)8	1320	1335	--	HB	TCGAACCATTTGTAAAAACAC	TCATCCATTCATTCAGCTTAT	55.32,54.82	135
CakTSSR01655	CakTC25551	(GT)6	114	125	--	--	AAAAACCACACTCCATTACCT	AAAGAGAATAGGGGACGTATG	55.26,55.04	159
CakTSSR01656	CakTC25554	(CT)14	79	106	--	--				
CakTSSR01657	CakTC25577	(AG)15	1403	1432	Young_pod	--				
CakTSSR01658	CakTC25580	(CA)6	2260	2271	--	--	ATTGATTCTTATGCTGGTCAA	CTATTTTGCGACAGTGAAGTT	54.91,54.82	151
CakTSSR01659	CakTC25584	(TC)13	1180	1205	--	TRAF	TTTCTTCCACAATTCTCGTTA	TACAAAAGAATCAAACCCAAG	55.13,54.59	165
CakTSSR01660	CakTC25584	(AG)6	1427	1438	--	TRAF	TTGTTAATCAAGTTGGTTGCT	CCATCCCATACCAATAACATA	55.07,54.86	132
CakTSSR01661	CakTC25585	(TC)13	1994	2019	--	TRAF				

CakTSSR01662	CakTC25586	(CT)10	513	532	--	--				
CakTSSR01663	CakTC25591	(TC)8	2250	2265	--	--	GTACTTTCACTTTGCTCACCA	CCATCTCAATGTAAAAGTTGC	55.55,54.99	148
CakTSSR01664	CakTC25598	(TGT)6	1897	1914	--	--	TAGCTAATCCCCTTAATCGTT	TCTCCTTCAGAGTTCAAATCA	54.92,55.06	143
CakTSSR01665	CakTC25598	(TAA)7	3787	3807	--	--	TGAAGTTTTGTACGGAATGAT	ATTCTTGACTTGCCATCACTA	54.83,54.97	147
CakTSSR01666	CakTC25598	(CAG)5	4941	4955	--	--	CAACACATTGCTACATTGCT	GTGGACGGTACAACACTAAGA	54.8,55.25	142
CakTSSR01667	CakTC25598	(GCT)5	5369	5383	--	--	CCATCTTCTACATCCAATTCA	TGGAAAAGGGTAAGGATTATT	55.16,54.59	150
CakTSSR01668	CakTC25598	(GCA)6	5794	5811	--	--	TGTCACTAAAAATCCACCATC	GACTGTCAGCAGTAGATGGAC	55.06,54.83	155
CakTSSR01669	CakTC25604	(AG)10	2902	2921	--	--				
CakTSSR01670	CakTC25613	(GTG)5	3917	3931	--	--	GATGGTAAATTGGATTGGAAG	AAGATTAAAGCTCATTCGTGA	56.07,54.4	152
CakTSSR01671	CakTC25613	(GTG)8	4091	4114	--	--				
CakTSSR01672	CakTC25620	(TGC)7	584	604	Flower bud	TCP	GAGACAGAGAATTGGACTGAA	GGTGAAAATCAGAATCATCAG	54.43,54.68	157
CakTSSR01673	CakTC25620	(TGT)5	917	931	Flower bud	TCP	AGTGAATGAAGCTGTTTGAGA	GTCAAGGTGTGATATTCGTGT	55.17,54.99	151
CakTSSR01674	CakTC25620	(TG)13	2291	2316	Flower bud	TCP				
CakTSSR01675	CakTC25621	(AC)13	304	329	--	TCP	CTCACTCTTTCTCTCTCACCA	TAGCTTTTCCCTGTCTTCTTT	54.68,55.12	155
CakTSSR01676	CakTC25621	(CAA)5	1365	1379	--	TCP	GTCAAGGTGTGATATTCGTGT	AGTGAATGAAGCTGTTTGAGA	54.99,55.17	151
CakTSSR01677	CakTC25621	(GCA)7	1694	1714	--	TCP	GGTGAAAATCAGAATCATCAG	GAGACAGAGAATTGGACTGAA	54.68,54.43	157
CakTSSR01678	CakTC25629	(AGAA)5	1	20	--	--				
CakTSSR01679	CakTC25634	(CAG)5	2705	2719	--	--	CTACAACAACAGCAACAACAA	GAACATAGGTTTGACCCATTC	54.96,55.99	199
CakTSSR01680	CakTC25654	(AG)12	78	101	--	--				
CakTSSR01681	CakTC25655	(TTTC)5	492	511	--	--	CCTCCTTTTAGCAATTTGAAC	ATTGGAGGTGATGGTCTTTAT	55.78,55.11	156
CakTSSR01682	CakTC25656	(CT)9	64	81	--	--				
CakTSSR01683	CakTC25657	(AG)7	869	882	--	--				
CakTSSR01684	CakTC25669	(TA)7	264	277	--	--	ACAAACCACCACTAACACTTG	TTCGAGCTTAAGATCCTTTTT	55.17,55.21	150
CakTSSR01685	CakTC25669	(GCA)5	2009	2023	--	--	TAATATGGGAGCAAAAACAGA	TTCTTTGGTTCAATTATCTCG	55.1,54.66	158
CakTSSR01686	CakTC25676	(TC)6	7	18	--	--				
CakTSSR01687	CakTC25676	(TA)9	1738	1755	--	--	ATGTTTGTGTGCTTCTGCTAT	TACAACTAACCGGCTTAATGA	55.08,55.21	157
CakTSSR01688	CakTC25678	(TC)6	80	91	--	--				
CakTSSR01689	CakTC25679	(GT)6	822	833	--	--				
CakTSSR01690	CakTC25694	(AG)6	31	42	--	--				
CakTSSR01691	CakTC25694	(ATT)6	237	254	--	--	TGAATTTGAGGTTGTGAAATC	AACCCTTCTTACAAATCCAAC	55.19,54.92	148
CakTSSR01692	CakTC25695	(AAT)6	5747	5764	--	--	AACCCTTCTTACAAATCCAAC	TGAATTTGAGGTTGTGAAATC	54.92,55.19	148
CakTSSR01693	CakTC25695	(TC)6	5956	5967	--	--				
CakTSSR01694	CakTC25699	(CT)16	799	830	--	Pseudo_ARR-B	TTTCTGCTTCACTTGATCTTC	AGGTTGGTTGTCTTAAGTTGA	54.8,54.04	149
CakTSSR01695	CakTC25708	(GAT)5	4024	4038	--	--	AAAGAGAAACGAACTCAAACC	TTCCAGTAGAAACGAAATTCA	55.26,55.13	157
CakTSSR01696	CakTC25709	(TGA)5	522	536	--	--	AATGACCCATCTTCTGTTTCT	AAAACAGAATCACCCAAAAGT	55.3,55.38	140
CakTSSR01697	CakTC25721	(TCA)7	1571	1591	--	--	GGATCACCATTCTGAGTAACA	TGCTTGATGAAGAGATAGAGC	55.05,54.92	151
CakTSSR01698	CakTC25722	(AT)8	4624	4639	--	--				

CakTSSR01699	CakTC25727	(TG)7	868	881	--	--	AAAGGGAGACATTAAAAACCA	ACACCAATTCTAATCGCTTTA	55.48,54.31	147
CakTSSR01700	CakTC25732	(ATTTT)5	244	268	--	--	CTTGCATAACACTATGTCCTGT	TGGTTCTGAAATAAAACTTGC	54.55,54.73	230
CakTSSR01701	CakTC25742	(CA)6	220	231	Shoot	--	TCCCTACAGGTTCATGACTTA	CTCTTTGCTATGGAATGATGA	54.82,55.48	150
CakTSSR01702	CakTC25757	(ATC)5	191	205	Shoot	--	TCACAATTCCAAAACCAATAC	GGTTGTGAGATAAGACAACGA	55.08,55.3	136
CakTSSR01703	CakTC25764	(GA)10	128	147	--	--	CCACCTATATTTGTGAGTGGA	CGTTTTCACATTCAATCACTT	55.11,55.31	145
CakTSSR01704	CakTC25772	(TC)13	36	61	--	HB				
CakTSSR01705	CakTC25809	(CCA)6	18	35	--	--				
CakTSSR01706	CakTC25813	(AAG)5	1820	1834	--	--	GAGACGGAGAAGCATTACATA	GCAGCAGATCTCATTTATCAT	54.65,54.55	149
CakTSSR01707	CakTC25815	(GA)6	2431	2442	--	--				
CakTSSR01708	CakTC25818	(GCT)5	782	796	--	--	TGAAAGATTCTTGGAAGTCAA	ATCATGACCAAGAACAAAAAG	55.08,54.38	161
CakTSSR01709	CakTC25825	(CCG)5	467	481	--	WRKY	TCATCTACACTCCGATCTCAC	CTGAGGATTTTGAAATTGAAC	55.2,54.04	145
CakTSSR01710	CakTC25831	(GAA)5	1924	1938	--	--	CTACATTTCTCCCACCAGTCT	GAATTCGACTTCAACGAATC	55.83,54.82	141
CakTSSR01711	CakTC25831	(TTG)5	2089	2103	--	--	ATTGGGGTTCATAATAGGTGT	CCGAAGATACACAACAAATTC	55,54.83	164
CakTSSR01712	CakTC25838	(AAAT)5	254	273	--	--	TCGTGAGATAATCAAAACTTGT	CGTCTCAATCATTTTCTTTCA	54.17,55.53	230
CakTSSR01713	CakTC25840	(ACC)5	113	127	--	C2C2-CO-like	ACAAATGTTGGAGCAAGAAC	ACATAAGTAAGCCAAGTCTGC	55.28,53.93	154
CakTSSR01714	CakTC25840	(GTG)5	717	731	--	C2C2-CO-like	TTGTTTAATGGTGAAGTTGATG	AAAATTCTGTACTTGCTGCTG	55.34,54.98	147
CakTSSR01715	CakTC25851	(CCG)5	1764	1778	--	--	AACGCTTCTAAACCTTTCAAT	GACGGAAGAAAGAAGACTGAT	55.01,55.17	161
CakTSSR01716	CakTC25852	(AC)8	1697	1712	--	--				
CakTSSR01717	CakTC25853	(AC)8	1576	1591	--	--				
CakTSSR01718	CakTC25854	(CA)6	1170	1181	--	Tify	AAAATTTAGGATGACACATGC	CCTATCCTTTCTCTTCTCCAG	54.35,54.88	154
CakTSSR01719	CakTC25854	(ATT)5	1960	1974	--	Tify	ATCTGTTTTTGCCAGAACTTT	AAGCCAAAGTTACAATGACAA	55.69,55.07	170
CakTSSR01720	CakTC25855	(TAA)11	46	78	--	--				
CakTSSR01721	CakTC25859	(AG)6	1128	1139	--	--	TTGTTGTGTGTAGTTTGGTCA	CATCACTCCAACCTCTAGTCA	55.04,55.3	150
CakTSSR01722	CakTC25871	(TTC)5	1736	1750	--	--	TGAAACATTGTTGCTTGAGTA	CTGGCTTCAACACTTTTACAC	54.51,55.14	149
CakTSSR01723	CakTC25876	(GGT)5	2749	2763	--	--	TGATAGGACCTTGTGTTTGAG	TCACACAGGAAGACCATTTAT	55.31,54.64	150
CakTSSR01724	CakTC25887	(AG)7	1024	1037	--	--	ATAATGAATCTGTTGCGTCAC	TCTCTAAAATCTCCATGTTGC	55.25,54.62	156
CakTSSR01725	CakTC25887	(TC)10	1263	1282	--	--				
CakTSSR01726	CakTC25888	(TC)7	30	43	--	--				
CakTSSR01727	CakTC25898	(TCT)5	1332	1346	--	--	ATCGACGAGGAAGATAAAAAT	CACAACAATAACAAACCGTCT	54.7,55.26	149
CakTSSR01728	CakTC25904	(AAT)6	3265	3282	--	--	TTGATCTCTTCAAGGCTGATA	ACCTTTTCACTAACCACATGA	55.19,54.72	150
CakTSSR01729	CakTC25913	(GAG)5	235	249	--	--	TAGAACCGGAACCATACATC	CTCCTTGAGGTTGTTCTTCTT	55,55.26	144
CakTSSR01730	CakTC25913	(TC)7	2509	2522	--	--	TGTTTGTAGCCTCAGTTTGAT	TGAATGAGTGAAACTTGATGA	55.05,54.16	155
CakTSSR01731	CakTC25914	(TTC)6	181	198	--	--	TTGCGTTATTACTCGACATTT	CTCGGATGCGTAACTACAAC	55.2,55.95	152
CakTSSR01732	CakTC25919	(TC)12	1	24	--	--				
CakTSSR01733	CakTC25922	(ATG)5	1201	1215	Flower bud	--	AGGTAAGGATGGTGAAGAAAA	CCTCCACATCTTTATCCTTCT	55.47,54.99	152
CakTSSR01734	CakTC25926	(CAT)5	489	503	--	--	AACATGCTTTTCTTCAATGTG	GTTTTCCATTTGAAACAACAC	55.47,54.7	150
CakTSSR01735	CakTC25926	(GAA)5	935	949	--	--	GAGCCTTAAAGGTAGTGAAGG	TATGGCGGATACTACTTTGTC	55,54.56	145

CakTSSR01736	CakTC25927	(TC)9	75	92	--	--				
CakTSSR01737	CakTC25951	(ATA)5	241	255	--	--	TAGAACATTTGCCTTGGATAA	GCCATTCAGAGCTAGTATCAG	55.1,54.4	151
CakTSSR01738	CakTC25960	(AT)7	1192	1205	--	--	GCGTATCACCAAAGTAAAACA	CACCTAGTAGGAAAAGGCTTC	55.51,55	143
CakTSSR01739	CakTC25964	(ACC)5	516	530	--	--	ATTCCACCTGAACTCGATAAT	CAGCAACAGGTTTTGGTAGT	55.26,55.41	148
CakTSSR01740	CakTC25964	(GA)6	1660	1671	--	--				
CakTSSR01741	CakTC25970	(TTA)5	813	827	--	--	GCTTCTGAATTTTCATTGTTG	TATGCGACGATAGAAGAGAGA	55.12,55.39	163
CakTSSR01742	CakTC25970	(GTT)5	1444	1458	--	--	TTAGCAAGGTTAAGACATTCG	AATCTTGACAAATTCCTCGAT	54.93,55.39	146
CakTSSR01743	CakTC25972	(TTA)6	59	76	--	--				
CakTSSR01744	CakTC25975	(TG)6	893	904	--	--				
CakTSSR01745	CakTC25981	(AAG)5	182	196	--	--	CAAAAACAAAGCAGAAGAGAA	ACCAACTGGTTATTGTTGTTG	55.08,55.1	147
CakTSSR01746	CakTC26013	(CT)10	1	20	--	--				
CakTSSR01747	CakTC26014	(GA)9	1613	1630	Young_pod	--				
CakTSSR01748	CakTC26015	(AG)6	1149	1160	--	IWS1	TCAAAATAGTTCTTCCTTTGG	CAATCTCAGACCTTAGTGTGC	53.86,55.04	155
CakTSSR01749	CakTC26028	(TCT)5	392	406	--	C2H2	TTATTCGGAAGGAAATGACTT	ACGCAAAGATTTAGAGCAATA	55.55,54.62	150
CakTSSR01750	CakTC26029	(AAT)5	214	228	--	--	AGGAGGAGGTTGGTGATAGTA	CGGAGTCTAGAAAGAAAGAGG	55.34,55.12	152
CakTSSR01751	CakTC26030	(GAA)6	1406	1423	--	--	TGCTAGCCAAGATTCAAGTAG	AAGGAAAAAGAATCACACGAT	55.07,55.46	157
CakTSSR01752	CakTC26031	(TTC)6	129	146	--	--	AAACGAAAGAAACATAGCAGA	ATTACCACCTTATGCGAAGTA	54.48,54.19	152
CakTSSR01753	CakTC26035	(TC)8	2437	2452	--	--				
CakTSSR01754	CakTC26043	(TTG)5	211	225	--	--	CTTCCAAACCCTAAACCTTAC	AAACGGAGGTCACAAAACTAT	54.72,55.42	148
CakTSSR01755	CakTC26045	(CT)6	3481	3492	--	--				
CakTSSR01756	CakTC26049	(GA)9	4331	4348	--	--				
CakTSSR01757	CakTC26050	(AG)10	3407	3426	--	--				
CakTSSR01758	CakTC26051	(AG)8	1089	1104	--	--	CATTATTATTGGTTGGATGGA	GTTTTTCTTCGCTATTTCTCC	54.98,55.05	151
CakTSSR01759	CakTC26052	(CTT)7	88	108	--	--				
CakTSSR01760	CakTC26053	(TGA)6	2692	2709	--	--	GCACAATTGGAGAAGTTAGAA	CCACACTCTCTAACATGAAGC	54.7,55.04	149
CakTSSR01761	CakTC26063	(GCC)5	917	931	--	--	CTTGACTTTGTTGATCAGAGG	ATCTCCAACATTCTCTTCCTT	55.01,54.53	149
CakTSSR01762	CakTC26065	(GA)8	384	399	--	--	TGTCTTTGTCTCTCACTCTCC	ACTTTGTTGAGCCATATGAAA	54.48,54.99	146
CakTSSR01763	CakTC26074	(CA)10	125	144	--	--	CCCACAATAAGCTTACACAAA	GATCCAACCACAGATGAATTA	55.52,54.98	185
CakTSSR01764	CakTC26082	(TTC)5	2295	2309	--	--	AACCCTCAACCTCTCTTACAC	TGTTAGGGTTCTTTCTGATGA	54.96,54.95	149
CakTSSR01765	CakTC26091	(AG)6	4858	4869	--	--				
CakTSSR01766	CakTC26096	(AT)6	50	61	--	--				
CakTSSR01767	CakTC26097	(CAT)9	74	100	--	--				
CakTSSR01768	CakTC26103	(AT)7	3585	3598	--	--	AAAGTGAATTATTTGGTGTGG	CTTTAATTTCTCTGCAACTCG	54.29,54.66	145
CakTSSR01769	CakTC26147	(TTG)5	2160	2174	--	--				
CakTSSR01770	CakTC26148	(GA)11	1080	1101	--	--	GTCAATGGTGTTCAATCTGTT	ACCTTAAGCCTAATCTTCCAA	54.95,54.94	156
CakTSSR01771	CakTC26148	(TTG)5	2208	2222	--	--				
CakTSSR01772	CakTC26149	(CAC)5	3344	3358	--	--	CAGTGACATTGCTAACATCAA	CGCTCTTCTATGGAGAACTTT	54.79,55.54	140

CakTSSR01773	CakTC26149	(TTC)5	4166	4180	--	--	TAGGAAACTTTTTCACCAACA	CAAGAAACAACAAAATGGAAG	55.05,55.05	151
CakTSSR01774	CakTC26153	(CAC)5	254	268	Young_pod	--	GGTTAAACTCCTCAAATGGAT	GGAGAAGTGGTGGATCTACTG	54.84,56.27	129
CakTSSR01775	CakTC26153	(GGT)5	617	631	Young_pod	--	GGAGAAGTGGTGGATCTACTG	GGTTAAACTCCTCAAATGGAT	56.27,54.84	129
CakTSSR01776	CakTC26154	(GGT)6	462	479	--	--	CATCTACTGGAAGACGGAGTA	CTCATTTTTCCGGTTAAACTC	54.56,55.63	131
CakTSSR01777	CakTC26159	(TC)7	31	44	--	TPR				
CakTSSR01778	CakTC26197	(CTTTT)5	1375	1399	--	--				
CakTSSR01779	CakTC26205	(TTG)5	1591	1605	Young_pod	HB	GAATACATATTCGTCGTCGTC	AAGACCAAGTCAAGGACTTTC	54.77,55.09	144
CakTSSR01780	CakTC26207	(TA)13	559	584	--	--	CCATCGTCTCTCATTCTTTTA	ACTGGTTTTGGTTACATCCTT	54.63,55.26	151
CakTSSR01781	CakTC26221	(AT)12	1	24	--	--				
CakTSSR01782	CakTC26221	(TCA)5	824	838	--	--	ATTAAGAAAATCAGCGCAAG	TCCTTTAAGAAAATGGATGTG	54.99,54.5	162
CakTSSR01783	CakTC26227	(AG)8	1532	1547	--	--				
CakTSSR01784	CakTC26228	(GA)6	1447	1458	--	--				
CakTSSR01785	CakTC26229	(CT)10	49	68	--	ABI3VP1				
CakTSSR01786	CakTC26234	(TCT)8	3344	3367	--	--	CAACAAGATTTGTTCCTTGAG	TCATATTTCCGTTACTTCGAC	55.03,54.52	152
CakTSSR01787	CakTC26236	(GGT)10	1168	1197	--	--	GGTCATTAACGTTATCACGAG	ATGAATGAATTCCCCTAAATC	54.86,54.71	152
CakTSSR01788	CakTC26250	(AAT)19	797	853	--	AUX/IAA	ACATTATAAAATGCCACATCG	GAAGGAGATAGGATGCTTGTT	55.24,55.14	203
CakTSSR01789	CakTC26251	(GT)6	123	134	--	AUX/IAA	AATAAAAGGTGTTTGGGGTAG	GCATTCTGATTTTCTGTTTTG	54.99,55.12	143
CakTSSR01790	CakTC26251	(TCA)5	838	852	--	AUX/IAA	TCCACCAAGTAGTGAAGATTG	CCTTCATAGGTTGATTTGATG	55.31,54.76	152
CakTSSR01791	CakTC26263	(TGG)8	276	299	--	--	AAGGTTCTGCTGGTAAGAAAT	TGTGGTCACGTCCTCTAATAC	54.84,55.17	159
CakTSSR01792	CakTC26264	(CT)9	53	70	--	--				
CakTSSR01793	CakTC26269	(GA)7	2059	2072	--	--	AGAAGTTGCAAAACCTAATCC	CTACTCTCTCCTCTCCCTCAA	55.23,55.35	166
CakTSSR01794	CakTC26293	(CAA)5	207	221	--	--	ACGGAATCTCTTTCTCTTCTC	TGTCCATTTTCAGTCACTTTT	54.41,54.85	153
CakTSSR01795	CakTC26297	(CT)9	88	105	--	--				
CakTSSR01796	CakTC26298	(GA)6	29	40	--	--				
CakTSSR01797	CakTC26299	(TGG)5	183	197	--	MADS	GTTGACAGAAGGAAACAAGTG	GAGTAATTACATGGCAGGATG	54.92,54.78	181
CakTSSR01798	CakTC26301	(CT)10	216	235	--	--	AGAAACTGTCTCTTCCTTCGT	CGGCTGTAGATATAGGCAAG	54.84,55.3	150
CakTSSR01799	CakTC26301	(TAC)5	1872	1886	--	--	TTCAGTTGAGTGAGGACACTT	CCATAACAAAAAGAAATGCAC	54.89,55.01	145
CakTSSR01800	CakTC26303	(GAA)6	1541	1558	--	--	ACATGCAATCTAACTCTGGAA	TCCAACCCTCTCTCTCTATTC	54.97,55.1	157
CakTSSR01801	CakTC26303	(AGA)6	1702	1719	--	--				
CakTSSR01802	CakTC26308	(AG)19	1446	1483	--	--				
CakTSSR01803	CakTC26337	(TG)6	1727	1738	--	--				
CakTSSR01804	CakTC26341	(AG)6	6622	6633	--	--	GCCTCCTTTAACAACTTCTCT	AATAAAAAGAAACCACCCAAC	54.56,54.94	148
CakTSSR01805	CakTC26349	(CT)8	122	137	--	--	TAGGACCGCTCCTTCTATACT	GATCGAACACTCACTCACAAT	54.96,55.1	150
CakTSSR01806	CakTC26354	(AT)9	36	53	--	--				
CakTSSR01807	CakTC26361	(GA)11	1518	1539	--	--				
CakTSSR01808	CakTC26377	(AG)7	1318	1331	--	--	CCGACATTTTTCTTCTTCTTT	ACCCTAGAATTCAATTTCCAC	55.36,54.84	141
CakTSSR01809	CakTC26378	(TC)7	57	70	--	--				

CakTSSR01810	CakTC26397	(TC)8	46	61	--	--				
CakTSSR01811	CakTC26400	(TTTC)5	3	22	--	--				
CakTSSR01812	CakTC26417	(TC)8	1	16	--	--				
CakTSSR01813	CakTC26421	(CT)6	153	164	--	AP2-EREBP	CTTCACTCTTCACTCTCCTCA	TGTTTAGCTGCATGAAGTTGT	54.68,56.16	148
CakTSSR01814	CakTC26431	(TC)8	14	29	--	--				
CakTSSR01815	CakTC26433	(AG)6	3117	3128	--	--				
CakTSSR01816	CakTC26435	(ACCAC)5	21	45	--	--				
CakTSSR01817	CakTC26442	(AAG)7	2074	2094	--	--	GGAGTTGTTTAAGGGTAAAGC	CATCTTCCGGTGATTCAG	54.86,54.79	152
CakTSSR01818	CakTC26444	(CA)7	12	25	--	AP2-EREBP				
CakTSSR01819	CakTC26446	(CT)7	2648	2661	--	--				
CakTSSR01820	CakTC26448	(GAT)8	3428	3451	--	--				
CakTSSR01821	CakTC26451	(GA)6	1614	1625	--	--				
CakTSSR01822	CakTC26455	(CT)14	52	79	--	--				
CakTSSR01823	CakTC26456	(TC)6	75	86	--	--				
CakTSSR01824	CakTC26460	(GA)10	74	93	--	--				
CakTSSR01825	CakTC26460	(AGC)5	199	213	--	--	GGTCATTCTTTGAACCCTAAT	CTGTTGTTGAAGAAGAGCTTG	54.84,55.43	163
CakTSSR01826	CakTC26461	(TGT)5	857	871	--	--	AACCATATTGCTGATTCTGTG	GAAGAAATGGTGATGTGAATG	55.26,55.48	138
CakTSSR01827	CakTC26463	(TC)10	443	462	--	--	CTTTGCTTCCACTACAATTTC	CATTTTTGCTGTTTAGAAGGA	54.32,54.9	151
CakTSSR01828	CakTC26463	(ACA)7	590	610	--	--	TCCTTCTAAACAGCAAAAATG	GATCATAACCCCTGTTTCTCT	54.9,54.82	155
CakTSSR01829	CakTC26463	(CAA)5	834	848	--	--	CCATTCACTTTTCAATCTGAG	GTTGTTGTTGAAATTGTAGCC	54.95,54.9	151
CakTSSR01830	CakTC26464	(GTT)7	81	101	--	--				
CakTSSR01831	CakTC26464	(GA)10	319	338	--	--	TATTGAGCTTGAACAGCCATA	TCCTCCTTTAATTCCTTCATC	56.16,55.05	150
CakTSSR01832	CakTC26465	(TC)6	131	142	--	--	CTAACTCAACACCCCTTTTCT	TCCATAACACAAACAGTGTGA	55.15,54.95	142
CakTSSR01833	CakTC26492	(GA)9	1433	1450	--	--	GTAATTGGAAGAGGATGGAGT	GCCATTTTTGAGAGTTTACAA	54.82,54.73	141
CakTSSR01834	CakTC26511	(TA)7	644	657	--	--	TGGAAGAATTAAAGCAGCTAA	ATACACTTGTTCGGGATCTCT	54.76,55.33	155
CakTSSR01835	CakTC26513	(CTT)9	186	212	--	G2-like	AGAGCAAAACACTGCAAGTAG	TTGTATTGGAAATGGTAAAGG	55.04,54.41	150
CakTSSR01836	CakTC26513	(AT)6	1878	1889	--	G2-like				
CakTSSR01837	CakTC26514	(AG)9	423	440	--	--	AAGACAAGCAAGAAAAGGACT	ACCAAGCAAAAGTGACTCATA	55.02,55.05	152
CakTSSR01838	CakTC26515	(TC)10	14	33	Flower bud	--				
CakTSSR01839	CakTC26519	(AT)14	1932	1959	--	--	GCTGCTGCAAATAGCTTATAC	TTAATGGTTGGTAAGAATGGA	54.8,54.78	146
CakTSSR01840	CakTC26521	(TC)8	27	42	--	--				
CakTSSR01841	CakTC26529	(GA)25	2090	2139	--	--				
CakTSSR01842	CakTC26535	(CTT)8	1	24	--	--				
CakTSSR01843	CakTC26536	(AG)9	5	22	--	--				
CakTSSR01844	CakTC26537	(AG)8	15	30	--	--				
CakTSSR01845	CakTC26538	(GAA)6	636	653	--	--	ATGGAGAGAAAGGAGAAGAAA	GATTGGGTTCACTTACCTTTT	54.83,54.92	150
CakTSSR01846	CakTC26546	(AAG)7	1840	1860	--	bZIP	TCCATGTGATTGTTGAGTGTA	GTTTCAGATTAACGGTTGTTG	54.86,54.91	151

CakTSSR01847	CakTC26559	(TA)10	2130	2149	Young_pod	--	TTTGGCAGCTTACTCAACTAT	TCAGCCACATCTCAAAAATAC	54.52,55.39	150
CakTSSR01848	CakTC26562	(CT)6	1855	1866	--	--	ATCTTTGTTTTCGGCTTCTT	TTCCAGAATATAAGCACAAGC	55.76,54.84	152
CakTSSR01849	CakTC26580	(ATA)7	1575	1595	Flower bud	--				
CakTSSR01850	CakTC26583	(AAG)7	2064	2084	--	--				
CakTSSR01851	CakTC26595	(GAA)9	2021	2047	--	--	CCAGTCTGAATCTGAACCATA	TTTCAACAATCACCACTTCTT	55.23,54.85	148
CakTSSR01852	CakTC26596	(GAA)9	1074	1100	--	--	CCAGTCTGAATCTGAACCATA	TTTCAACAATCACCACTTCTT	55.23,54.85	148
CakTSSR01853	CakTC26608	(GAA)5	56	70	--	--				
CakTSSR01854	CakTC26622	(TA)6	54	65	--	--				
CakTSSR01855	CakTC26632	(GA)6	215	226	--	--	CCAAAGTTTGTTTTGATATGC	CCTTTGTTTGTTGAGTTTGTT	55.01,54.55	151
CakTSSR01856	CakTC26645	(GAA)7	1116	1136	--	--	TGGTTGTAAGGAGTAATCGTG	ATCCATCTAATGTCCTCCTTC	55.36,54.74	159
CakTSSR01857	CakTC26648	(AT)6	121	132	--	--	ATATAAGGATGGCAAAAATCC	GTAGTTTTTGGGGGACTAAGA	54.92,55.09	149
CakTSSR01858	CakTC26649	(CT)14	96	123	--	--				
CakTSSR01859	CakTC26679	(AG)10	2545	2564	--	--				
CakTSSR01860	CakTC26680	(GAG)5	164	178	--	--	AGGAAGAAGCTACACTCCATC	TCTTCAGGTCTACAGTCATGG	55.21,55.3	162
CakTSSR01861	CakTC26681	(GAG)5	164	178	--	--	AGGAAGAAGCTACACTCCATC	TCTTCAGGTCTACAGTCATGG	55.21,55.3	162
CakTSSR01862	CakTC26690	(AG)17	1763	1796	--	--	TTGAGATTGGAGAGGTTGTTA	GCATAAATCACCTTCTTCTCC	54.95,55.54	146
CakTSSR01863	CakTC26693	(GGA)5	1534	1548	--	--	GTGGTGGTGATTGTTGTAGTC	CCCGTTACGTTACGTTTAACT	55.33,56.09	151
CakTSSR01864	CakTC26701	(ATA)5	198	212	--	--	TAAGATGGAAGAAAACAACCA	CAGCTTTTGGAACACTTGTAG	54.97,55.31	131
CakTSSR01865	CakTC26710	(CCT)6	2096	2113	--	--	TAGGAGACATTCCTGTCTGAA	AGCACCTGATATGAAGAAACA	54.92,54.97	149
CakTSSR01866	CakTC26719	(TC)9	210	227	--	--	TTACCTTTGCAATGTTTGTCT	TGGACCAAGAAAATAACTTGA	55.07,54.97	143
CakTSSR01867	CakTC26722	(TC)6	125	136	--	--	GTTCAGCATTTACATGCACTT	GCAGAGTTATCAGAGGGAGAT	55.49,55.12	147
CakTSSR01868	CakTC26759	(ATC)6	229	246	--	--	TTCTCTTCTTCAAAATCCAGA	ATGAATCAGAGACGAGCATT	54.3,54.83	175
CakTSSR01869	CakTC26765	(AG)8	1086	1101	--	--				
CakTSSR01870	CakTC26775	(CT)9	48	65	--	C3H				
CakTSSR01871	CakTC26779	(CTT)5	157	171	--	SBP	TTACTCGCACACTTCATAAGC	AAAGTGTACACCGAAGCAAT	55.82,54.9	150
CakTSSR01872	CakTC26793	(AG)9	650	667	--	--	CATCACCTGAAACGAAATTAG	CAAGAACTTCCCTTTCTTCTT	55,54.54	145
CakTSSR01873	CakTC26807	(ATA)6	445	462	--	--	CGAAACAGATTCTGGTCTAAG	GGGCTACCATTTGACTAACTT	54.3,55.11	156
CakTSSR01874	CakTC26816	(ATT)5	235	249	--	--	TATGCTATCATCTGAGCCATT	CACTGATTCCAAATTCTAAGG	55.02,54.09	149
CakTSSR01875	CakTC26831	(AG)9	1106	1123	--	--				
CakTSSR01876	CakTC26834	(TC)6	50	61	--	--				
CakTSSR01877	CakTC26850	(ACACTG)9	224	277	--	--	ACGCATTTCAAATCTAGAACA	CAAAATTGAAGAGAGACGAGA	55.15,54.82	148
CakTSSR01878	CakTC26856	(AT)6	47	58	Flower bud	WRKY				
CakTSSR01879	CakTC26857	(TTC)6	3118	3135	--	--	GGATTGGAAAGAATTCAAGTT	GGGCTTTGACTTTCAAATAA	54.97,54.64	145
CakTSSR01880	CakTC26869	(AGC)5	294	308	--	--	TCTTGTCATCCACACTCTCTT	TGTTGACTGAGGGATAAACAC	54.8,55.13	150
CakTSSR01881	CakTC26870	(TGC)5	319	333	--	--	TGCTTTTGATGTGAAGAAGAT	CAGCAGAGAAAAATATCCTTG	55.1,54.41	151
CakTSSR01882	CakTC26872	(ACA)6	179	196	--	--	TTCCCTCTCCATCATTCTTAT	CTGCTATTGAAGTTGCTTTTT	55.32,54.28	148
CakTSSR01883	CakTC26877	(AGG)5	485	499	--	--	GAATGAGGTTCACTTTGGTG	GATTACCCAAGGTGTTAAAGG	55.55,55.36	144

CakTSSR01884	CakTC26895	(GAATG)5	355	379	--	--	TGCTGCTTCATATACTTAGGG	GGAATGAGTTCCATCCTACTT	54.89,54.82	142
CakTSSR01885	CakTC26896	(AT)7	144	157	--	--	AACAAGTTCCCCAAAGTCTAC	TGTGCTTATTGGCTCATAACT	54.98,55.18	136
CakTSSR01886	CakTC26905	(GA)7	782	795	--	--	GCAGCAGCAGTAGTTGTAGTT	CACCATCTCTCTCGTGACC	55.07,56.53	154
CakTSSR01887	CakTC26908	(TC)18	1	36	--	--				
CakTSSR01888	CakTC26912	(TTA)5	130	144	Flower bud	--	AAACTGACACAATTTCCCTTT	AGCTAGTTTCCCTCAAAAATC	55.38,54.51	150
CakTSSR01889	CakTC26921	(TCA)6	355	372	--	--	TTCTTCTTCATCAGAGTTGGA	AGATAACCCTCTCTTCAAAGG	55.06,54.33	142
CakTSSR01890	CakTC26934	(AT)10	3	22	--	--				
CakTSSR01891	CakTC26937	(TAT)6	658	675	--	--	ATTAACCGTACAACCACAAAA	TTTCTCCACCCTTTATGATTT	54.81,55.4	151
CakTSSR01892	CakTC26942	(AAG)6	3080	3097	--	MYB-related				
CakTSSR01893	CakTC26962	(TCC)5	3515	3529	--	--	AAAGGAAAAGGAAAGTGGTAA	AAAAGATCAAAATCAGTGACG	54.84,54.54	153
CakTSSR01894	CakTC26966	(AAG)5	967	981	--	--	TGTTGTCATATGGAGACCAAT	GTACCCCAATCCTCAATGTAT	55.35,55.39	152
CakTSSR01895	CakTC26974	(AAT)5	4016	4030	--	--	GAGCTTAATGGTGAAGGAAAT	GTCTAGGTCCATCTTCAGCAT	55.15,55.91	154
CakTSSR01896	CakTC26975	(AAT)5	4630	4644	--	--	GAGCTTAATGGTGAAGGAAAT	GTCTAGGTCCATCTTCAGCAT	55.15,55.91	154
CakTSSR01897	CakTC26980	(CT)9	1436	1453	Flower bud	--	CTAACGTTGGAAATCAAGAAG	CATTGCAGAAGCTCAAATAAG	54.34,55.48	141
CakTSSR01898	CakTC26981	(CCA)5	2754	2768	--	HB	AATGCTTATTACCATCCTTGC	CAAAATGCGTAGTGAGTCTTC	55.99,55.22	149
CakTSSR01899	CakTC26990	(TTTA)10	2	41	--	--				
CakTSSR01900	CakTC26991	(AG)8	2253	2268	--	--	TGTAGCGAAGAAGAAAATGAG	GAAACATCCTTGACAGTCCTT	55.04,55.79	171
CakTSSR01901	CakTC26999	(TC)6	74	85	--	--				
CakTSSR01902	CakTC27001	(GT)6	1615	1626	Young_pod	--				
CakTSSR01903	CakTC27003	(TC)6	60	71	--	--				
CakTSSR01904	CakTC27017	(TC)8	72	87	--	TRAF				
CakTSSR01905	CakTC27019	(TGA)5	1667	1681	--	--	GATCCATGTTTCGAATTGTT	GAAAACGGTTGTTCAATTTAC	54.95,54.04	146
CakTSSR01906	CakTC27021	(ATT)6	95	112	--	--				
CakTSSR01907	CakTC27022	(AAT)7	511	531	--	--	TTTTAACCGCATTAACACAAC	TTTTTAGCTTCCTTAGCCATA	55.51,53.98	153
CakTSSR01908	CakTC27023	(ATT)5	1292	1306	--	--				
CakTSSR01909	CakTC27027	(TC)7	251	264	--	HSF	CGTAACTAACGCAGTACCTTC	GACGAGTTTTGTTGTGAAGAG	54.48,55.08	143
CakTSSR01910	CakTC27029	(TTC)5	2207	2221	--	SET	TTCCTTAACCAGACTTTCCTC	AGGTCGTAGAGGAGAAAGAAA	55.2,54.95	161
CakTSSR01911	CakTC27030	(GT)8	6	21	--	LIM				
CakTSSR01912	CakTC27036	(TAA)5	48	62	--	--				
CakTSSR01913	CakTC27040	(AT)8	86	101	--	--				
CakTSSR01914	CakTC27041	(ACA)6	269	286	--	--	GCTAACGATTTTTCTTCCAAT	CAGATGTCGAATCATCAGAAT	55.32,55.13	148
CakTSSR01915	CakTC27062	(CT)10	1802	1821	--	--	CCGCTTTTGTTTAAATTGAT	ACTTTGGCACTAGTTACATGC	54.7,54.68	151
CakTSSR01916	CakTC27085	(CA)8	101	116	--	--	TCGTACAAATTTCTCTCCAAA	CAACCAGTGATGAAAAAGAAG	55.13,55.03	142
CakTSSR01917	CakTC27088	(AG)8	1556	1571	Flower bud	--				
CakTSSR01918	CakTC27095	(CAC)6	543	560	--	--	GCGCATAAAAACACTTTCTAA	CCAACAAGTGAAGAATCAAAG	55.09,55.03	159
CakTSSR01919	CakTC27102	(AT)6	172	183	--	--	AACAGAACTGGCATGAAACTA	AATTGAATCTTGATGGAGTGTT	55.05,55.32	141
CakTSSR01920	CakTC27108	(AG)7	217	230	--	--	GGTGTTTGGAATTGTGTAGAA	TAGCATCAACTTCATCCATTT	55.15,54.91	149

CakTSSR01921	CakTC27122	(TC)10	97	116	--	--				
CakTSSR01922	CakTC27130	(AACATG)6	313	348	--	--	TTTTGCTAAGGATGTTATGGA	AATTCTTTTGAACCTTTGACC	55.1,55.04	155
CakTSSR01923	CakTC27138	(GA)16	2531	2562	--	--	TAACTTTGTTGTGATGGGAAG	TACCATTCATCATCTCTCACC	55.32,54.96	143
CakTSSR01924	CakTC27147	(AG)7	767	780	--	C2C2-GATA				
CakTSSR01925	CakTC27160	(AAG)7	913	933	--	--	AGAGGTATGATTTGGAGGAAG	TTTGTTTTCCTTCATCACTCT	54.99,54.08	151
CakTSSR01926	CakTC27164	(GAA)5	1716	1730	--	--	TGCAAACATCACATATCTCAA	GAGATTCCACTTGTGTCAATG	55.14,55.56	141
CakTSSR01927	CakTC27173	(ATT)6	230	247	--	--	CCTACACCGTTGACAAAATTA	AGCTTGCACTCTCTCTAGTCA	55.36,54.64	149
CakTSSR01928	CakTC27222	(CT)13	1776	1801	--	--	GGTTCATGGCTAAAAATCTTC	TTTGCATTACAAAAGCTCCTA	55.54,55.48	149
CakTSSR01929	CakTC27229	(TTC)6	465	482	Root	WRKY	GAAGGAACCAACAACATCTTT	CTTGTTGTTGTGATGTTGAAA	55.78,54.67	152
CakTSSR01930	CakTC27229	(AGG)5	840	854	Root	WRKY	ATGTTTCACCCAACATCAATA	AGGAATACAAACCACCTTTTT	55.36,54.56	149
CakTSSR01931	CakTC27233	(CCT)5	609	623	--	--	GTCTGGACCTGCTGTTAGAC	TGAAAATTGTTGGAAAGGTC	54.81,55.18	145
CakTSSR01932	CakTC27236	(CT)12	94	117	--	--				
CakTSSR01933	CakTC27236	(TA)6	3411	3422	--	--	TTTCCTATCTGCACCTTGATA	TTACATCAAGAAGGGTTTCCT	55.08,55.47	151
CakTSSR01934	CakTC27237	(CT)12	7	30	--	--				
CakTSSR01935	CakTC27241	(TC)14	45	72	--	--				
CakTSSR01936	CakTC27241	(TGA)6	6738	6755	--	--	CATGAGGATGACATTGAAAAT	CGTCTTCTTCCAAACCATTAT	54.98,55.9	163
CakTSSR01937	CakTC27243	(CTC)5	152	166	--	--	CACCTCTTTCTCTTTCTCTCC	ACGAAAGTGAAGTTGAAGGTT	54.89,55.61	150
CakTSSR01938	CakTC27254	(CCT)5	2019	2033	--	--	CTGAGGATGAATTTATTGTGC	GATGGGATAACATGATAGCAA	54.91,55.11	163
CakTSSR01939	CakTC27260	(AT)6	411	422	--	--	AAGGCAGTGGAGAAGAATAAT	TTCAGGCTTGTATGGTACATT	54.76,54.86	147
CakTSSR01940	CakTC27272	(GA)14	1245	1272	--	--				
CakTSSR01941	CakTC27274	(GGT)5	1549	1563	--	--	ACCAGAAGAGACTTGGAATTT	GGAAAAAGTCCTTCAACAAGT	54.62,55.11	159
CakTSSR01942	CakTC27276	(ATG)6	1716	1733	--	--	GCTATTCTTTTTGCTCTTCCT	CTATTGTTCAGGTGTTCTGGA	54.81,55.31	151
CakTSSR01943	CakTC27276	(AG)9	2438	2455	--	--	GCAAGTTTTTAGCTTTTGGAT	TTTCTCCATTTTCAACTTGTC	55.53,54.5	153
CakTSSR01944	CakTC27276	(AG)7	2584	2597	--	--				
CakTSSR01945	CakTC27284	(ATT)9	105	131	Shoot	--	ATCATGTTCTCTTCCCTCTTC	ATTAGAGGTGGATGATGGTTT	54.92,55.11	126
CakTSSR01946	CakTC27285	(TC)22	1	44	--	--				
CakTSSR01947	CakTC27286	(TC)22	215	258	--	--	GAACCTTCCTCTGTTTATTCC	TTGTTAGAAATCAGCAAAAGC	54.57,55.04	152
CakTSSR01948	CakTC27289	(ATG)5	3037	3051	--	--	TCAACCATTTCTCAGACTTGT	AGATTGAATCATCATCACCAC	54.83,54.77	147
CakTSSR01949	CakTC27313	(TC)7	1	14	--	--				
CakTSSR01950	CakTC27322	(GA)7	752	765	--	--				
CakTSSR01951	CakTC27328	(GT)8	1695	1710	--	FHA	AGATGCTAGTGGTTATGCTTG	TGTGATCTTCTGTCATCTTCA	54.77,54.11	137
CakTSSR01952	CakTC27329	(AG)6	158	169	--	--	GAAAAGAAAACAGCAACAGAA	AAGATATGCAACACCTTATGC	54.91,54.55	158
CakTSSR01953	CakTC27334	(CAG)7	2209	2229	--	C3H	TTGGTTAAGGATGTTCCTTCT	CTACCATCTGCTCTTGCTCTA	55.47,55.05	154
CakTSSR01954	CakTC27335	(TC)12	81	104	--	bZIP				
CakTSSR01955	CakTC27335	(CAG)5	1372	1386	--	bZIP	TCCAAATTCATCCTCAGAAG	CTCTCATTTTCAAATCACCTG	55.26,54.95	162
CakTSSR01956	CakTC27341	(TG)6	1906	1917	--	C2C2-CO-like	TCTATAAGCAAAAAGGGGTTT	CATTCATACACCTTCCTTTTC	54.96,53.91	166
CakTSSR01957	CakTC27342	(TGA)5	509	523	--	--	AACTTTCTCTTTCATCGAAGC	TCAATCGTCGTACCAATAAGT	55.5,54.86	162

CakTSSR01958	CakTC27343	(GA)21	1442	1483	--	BES1	CGTTGTGTTCTGGTTTATGA	AACTCATCAAAGCCTTCTCTT	54.6,54.94	145
CakTSSR01959	CakTC27346	(AG)6	2332	2343	--	--	AGACAACCTCTCTTTGTCTCA	GTTTGCATGCACTCTCTTAAT	54.07,54.73	151
CakTSSR01960	CakTC27347	(GAT)5	4670	4684	--	--	GGCAAACTCATCACAGAATTA	TCTTTCTTTCTCTCCCAAAAT	55.39,54.86	146
CakTSSR01961	CakTC27355	(AG)12	30	53	--	--				
CakTSSR01962	CakTC27356	(TA)6	39	50	--	--				
CakTSSR01963	CakTC27367	(AAAT)5	159	178	--	--	GTGTTGTTCTCTCCCTCTTCT	GCGATGAATCTCTCTGATTTA	55.07,54.7	150
CakTSSR01964	CakTC27371	(GTT)7	1218	1238	--	G2-like	GTTGTTGAGTTGTGGTGATTT	CAAGCTCACACTGAACTCTCT	55.04,54.79	146
CakTSSR01965	CakTC27407	(CT)6	1	12	--	bZIP				
CakTSSR01966	CakTC27426	(AAC)7	321	341	--	MYB-related	CAGTATCATGATTTTCGGTGT	GTGGTTGTTCGTATTGAGAGA	55.1,55.3	119
CakTSSR01967	CakTC27434	(TA)10	75	94	--	--				
CakTSSR01968	CakTC27443	(AGA)5	685	699	--	--	TTATTTTGGTTCTTGAGCTTG	GAACATACACTACTCGGTGGA	54.9,55.17	145
CakTSSR01969	CakTC27467	(TAT)5	236	250	--	--	AGTGCTTCTACACTCCTCCTC	TGGTTACTGTTGTTACCGTTT	55.28,54.87	141
CakTSSR01970	CakTC27469	(AGG)5	2903	2917	--	--	TGATCCAAAGCTGAGTTAAAA	GATTACGAACCTGGTGAACTT	55.29,55.82	146
CakTSSR01971	CakTC27472	(AAT)6	294	311	--	G2-like	AAGAACAAGGTGAGGACACTT	ATTTTTCTCATCATCACATGC	55.45,55.13	155
CakTSSR01972	CakTC27477	(GAA)5	162	176	--	--	TCTCTCCAAACATTCCAATTA	GTCTTGCTCTTGCTCTTGTT	54.89,54.88	151
CakTSSR01973	CakTC27478	(AG)6	2156	2167	--	--				
CakTSSR01974	CakTC27481	(AAT)5	1950	1964	--	--	GTGTTGACCATAGGAATCAAA	AAAACCACCTAAGAAAGTTCC	55.06,54.29	150
CakTSSR01975	CakTC27491	(TA)6	120	131	--	--	ATATGATTTTTGGTTGAGTGG	TGAACCTTAGTTTCTTTTGGA	54.2,54.3	150
CakTSSR01976	CakTC27511	(AAG)5	392	406	--	--	ATATTGGTGGCTTAAACAATG	ATCTTCGTCCACAACTAACAA	54.44,54.89	150
CakTSSR01977	CakTC27515	(AAG)7	1366	1386	--	--				
CakTSSR01978	CakTC27525	(GA)12	1729	1752	--	--				
CakTSSR01979	CakTC27526	(CA)6	2771	2782	Young_pod	PHD	TTCTTAAATTGCGTTTGCTAC	AGATTGGATGAGAAGGGTAAC	55.09,54.82	148
CakTSSR01980	CakTC27529	(CAC)5	1670	1684	--	AP2-EREBP	GGATTTCGATTTTTCTCAAAC	TTCCTCTGTAGAGGTTTTTCC	55.51,55.2	140
CakTSSR01981	CakTC27530	(TC)12	1219	1242	--	--				
CakTSSR01982	CakTC27547	(ACA)6	57	74	--	--				
CakTSSR01983	CakTC27549	(AG)6	2410	2421	--	WI/SNF-BAF60	GGGAGTTAATTGTGGATTAGG	CCACAATCTCTTTCTCTTTCA	55.28,54.65	148
CakTSSR01984	CakTC27550	(GA)11	4686	4707	--	--	AGTGTTCGATCAAAACAAAAG	ACCGTTTCTCTTCTCATTTTT	54.63,54.8	152
CakTSSR01985	CakTC27556	(AGA)7	74	94	--	C2H2				
CakTSSR01986	CakTC27556	(GAT)5	486	500	--	C2H2	GTTTGTGATTGTGATGAGGTT	TCTTTAAGCATCCATGAAAAC	54.95,54.65	137
CakTSSR01987	CakTC27556	(AG)7	1556	1569	--	C2H2	CTCCAGCAGATAAAACAAAAA	TTTCAAACAAGTAGTGGAAACA	54.9,55.14	122
CakTSSR01988	CakTC27557	(GTT)5	1	15	--	--				
CakTSSR01989	CakTC27557	(GAT)5	149	163	--	--	GTTTGTGATTGTGATGAGGTT	TCTTTAAGCATCCATGAAAAC	54.95,54.65	137
CakTSSR01990	CakTC27582	(TC)14	990	1017	--	Alfin-like				
CakTSSR01991	CakTC27590	(AGA)7	1482	1502	--	--				
CakTSSR01992	CakTC27591	(TCT)7	45	65	--	--				
CakTSSR01993	CakTC27601	(AGA)10	1201	1230	--	--	TAAAGTGAAGACCAAGAGTGC	AATTGCAATGAATGTTAGCC	54.76,55.33	154
CakTSSR01994	CakTC27609	(AT)6	84	95	--	WRKY				

CakTSSR01995	CakTC27609	(TCT)5	792	806	--	WRKY	TGGATCCAACCATTAATCATA	AAATTAAGATCCGTGGTCAAT	55.38,55.27	154
CakTSSR01996	CakTC27620	(CT)17	157	190	--	--	CCTCACAACAACAAATTCTTC	TAGTCTCTAGCTTCTGGCTGA	54.85,54.76	150
CakTSSR01997	CakTC27622	(AG)6	1465	1476	--	--				
CakTSSR01998	CakTC27634	(GAA)6	2866	2883	--	--				
CakTSSR01999	CakTC27645	(AT)6	676	687	--	--	GGTGGCATATTGATTACAAAA	TAGCCCTCATTTATTCACAAG	55.21,54.7	169
CakTSSR02000	CakTC27653	(TTC)5	353	367	--	--	TTTCTCTCCACCTTTTCTTCT	TACAAAATCGTGGTGAGAGTT	54.92,54.89	143
CakTSSR02001	CakTC27655	(TTC)5	37	51	--	ABI3VP1				
CakTSSR02002	CakTC27678	(TTC)5	2443	2457	--	--	ATAAACAAACCCAGAAGGAAG	GGTGAAATTTGGATATTTGGT	55.09,55.51	150
CakTSSR02003	CakTC27681	(CA)6	1619	1630	--	--	CCCTCTGAGTTTCCATCTAAG	GAAGAACAAACAGAACCAGAA	55.64,54.56	153
CakTSSR02004	CakTC27682	(CTT)6	382	399	--	--	CCTTCTGTGTTGTTCTGGTAG	CAGCTCGTTTTAAAATATCCA	54.97,54.88	151
CakTSSR02005	CakTC27684	(AG)7	92	105	--	--				
CakTSSR02006	CakTC27685	(AG)7	2584	2597	--	--	AATTCCGTTAATCAAGGAGAG	GTTTTGAGGTCCATCATTTCT	55.16,55.71	155
CakTSSR02007	CakTC27686	(GAA)6	1157	1174	--	--				
CakTSSR02008	CakTC27687	(CTT)6	54	71	Flower bud	--				
CakTSSR02009	CakTC27692	(TC)17	1015	1048	--	--				
CakTSSR02010	CakTC27697	(TAA)6	231	248	--	--	TGGTTTGTGAAGAAGAATCTC	CTCCTACCCATATTCTCGTTT	54.47,55.04	153
CakTSSR02011	CakTC27715	(GAA)7	1114	1134	Young_pod	--	CTGTGTCTGTGAATGTGACTG	GCAGGAATCGAATGATAATAA	55,54.55	167
CakTSSR02012	CakTC27716	(GT)7	281	294	--	--	AGTGTTTGTTTTGTTGAGCAT	GTTGGAATCAAACTCAGAATC	54.96,53.8	139
CakTSSR02013	CakTC27717	(TG)7	1016	1029	--	--				
CakTSSR02014	CakTC27727	(AG)10	348	367	--	--	ATTTGTGTAACTGGGGTTGTA	CTTTAGGCATGATTGAATGAA	54.63,55.49	149
CakTSSR02015	CakTC27742	(GGAGCA)5	325	354	--	--	GTGAAGGATATGCAAAAACC	CCCACCTATTAATCTTCAACC	54.72,55.28	155
CakTSSR02016	CakTC27749	(AATGA)5	980	1004	--	--				
CakTSSR02017	CakTC27750	(ACA)8	140	163	--	--	ACTGAACCCTCACAATCTCA	TTAGGGTTAGGGTTAGGGTTT	55.55,55.97	140
CakTSSR02018	CakTC27751	(CT)8	196	211	Flower bud	--	GAACTTTCCTTTCCTGTCTTC	CAAAGCAAAAACAGTGATGAT	54.74,55.47	152
CakTSSR02019	CakTC27761	(CTT)5	445	459	--	--	CTTTCTCAACCCTTTTACACC	TTGGCTCATACCAATTTTATC	55.55,54.47	148
CakTSSR02020	CakTC27764	(AGTG)5	110	129	--	--	CCAAGTTAACTGAAAAATGGA	AGTATTACCCTTCATGGGTTG	54.59,55.63	149
CakTSSR02021	CakTC27771	(ATA)8	638	661	--	TPR	AGCAATGTAATGTTGTTTGGA	GTTCGTTGAAATTGATGGTAG	55.76,54.83	145
CakTSSR02022	CakTC27778	(TGA)5	352	366	Young_pod	--	CTGATTACTCTGGTGAAGCTG	GAACCGGATCAATCTCTATCT	55.22,54.9	150
CakTSSR02023	CakTC27823	(AAC)6	26	43	--	--				
CakTSSR02024	CakTC27824	(CTT)6	358	375	--	--	CATCACTTCCTTCAACAACAT	CTTTGAACCCTTACTCACCTT	55.13,55.15	143
CakTSSR02025	CakTC27836	(TCA)6	2953	2970	--	--	AAGTGTTCATAAAGAGCCATCT	AAGAAGGAGAATGAAAAGAGG	54.85,54.46	153
CakTSSR02026	CakTC27840	(TCT)5	1531	1545	--	--				
CakTSSR02027	CakTC27841	(AGA)5	79	93	--	--				
CakTSSR02028	CakTC27849	(TAA)5	192	206	--	--	ACTTTTTCTCACCTTCAGCTT	GCCACCATTATGTGTAGACTC	55.02,54.66	150
CakTSSR02029	CakTC27850	(TC)6	51	62	--	--				
CakTSSR02030	CakTC27871	(ACC)6	79	96	Root	--				
CakTSSR02031	CakTC27879	(TC)6	6	17	--	--				

CakTSSR02032	CakTC27891	(CT)6	25	36	--	--				
CakTSSR02033	CakTC27891	(TCC)7	2000	2020	--	--	GCATTATAGGTTTCAAAAGCA	AATAAGAAGCAGATCCACCTT	54.86,54.76	176
CakTSSR02034	CakTC27893	(TAT)5	686	700	--	--	CTATTAGGTGTCCCCTCTGAT	GAGTACAAAGACACGTTGGAG	54.87,54.95	162
CakTSSR02035	CakTC27898	(GCA)5	843	857	Shoot	--	AGCAATCACAGAATCAACAAC	AACAGATTGCTGAGATGATTG	55.29,55.38	140
CakTSSR02036	CakTC27898	(CAG)5	2522	2536	Shoot	--	TTCAAACAACAATATCAGCAG	CTTGTCGCATTTTCATATCAT	54.03,55.35	169
CakTSSR02037	CakTC27899	(CT)14	38	65	--	--				
CakTSSR02038	CakTC27899	(TC)6	3383	3394	--	--	TTAGTGTAGCAATCTCTTCCAA	CACCGTTAACTCTAATCCAAA	54.51,54.62	180
CakTSSR02039	CakTC27904	(TTA)5	192	206	--	--	TCCAAAATTACAACTTCTCCA	CTTATATGAAGAGCCATGGTG	54.97,54.96	151
CakTSSR02040	CakTC27905	(TC)6	24	35	--	--				
CakTSSR02041	CakTC27905	(TC)6	171	182	--	--	ATTGAAATCACCCTCTTCTTC	TAGTGTGAAGCAACCAAAATC	54.95,55.47	151
CakTSSR02042	CakTC27908	(TTC)6	165	182	--	--	ATGTACTCTTCTCCCATTCGT	TGACTTTGAGAAAGACGAAGA	55.33,55.31	146
CakTSSR02043	CakTC27909	(GAT)5	431	445	--	--	TGAAGAAATATGGTCTGCACT	TTTTGCTCCTATCTGTTTCAG	54.97,54.88	146
CakTSSR02044	CakTC27910	(TC)6	589	600	--	--				
CakTSSR02045	CakTC27916	(AC)9	283	300	--	--	GTCCTAAAAAGGTTACCGAAA	GCTGATAGGCTTTTCTTTCTT	55.25,54.81	139
CakTSSR02046	CakTC27919	(ATA)5	342	356	--	--	CAACTCCAACACAATCTTCTC	AACGGTGCTAAATACAGAGGT	54.83,55.6	158
CakTSSR02047	CakTC27942	(TTG)5	1637	1651	--	WRKY	CCATTGGTGCTGACTATAGAA	CATGGATTCAGATCCAATAGA	55.44,55.07	148
CakTSSR02048	CakTC27947	(CTT)9	786	812	--	--				
CakTSSR02049	CakTC27953	(AG)7	358	371	--	--	GTTCTCTCAAACAGACTCACAA	TTGTTTTCACACATCCTTGTT	54.62,55.64	157
CakTSSR02050	CakTC27956	(AGG)5	687	701	--	--	GGTGAAAGTGTAAATGCAAAG	ATTAACGCCCAATTCTTTACT	55.07,54.84	147
CakTSSR02051	CakTC27980	(TGA)5	750	764	--	--	GGATGAGGAGGATAACATACC	ATTCTGTGAAAACTTTGCTGA	55.02,55.18	148
CakTSSR02052	CakTC27980	(GAC)5	1060	1074	--	--	TCCAACTTCATCAGTTATTGC	TTCTTCATGGAAGATGGTTTA	55.39,54.89	147
CakTSSR02053	CakTC27983	(AG)19	2844	2881	--	--				
CakTSSR02054	CakTC27984	(AT)7	2771	2784	--	--	GTTCTGAACCAAAACTTGATG	AGACCATTATCCTCTCTCTGC	54.85,55.12	150
CakTSSR02055	CakTC27985	(AT)6	2922	2933	--	--	TGGCCTTATGAATGATGTATT	TCATCAACCAGTTCTTTCATC	54.72,55.18	163
CakTSSR02056	CakTC27989	(AGAAGC)5	1113	1142	--	--				
CakTSSR02057	CakTC27998	(GTCATT)5	455	484	--	--	GCTTCCCAAGTTGATATGATT	GGCACTTGTATCAGAGAAAAA	55.82,54.7	151
CakTSSR02058	CakTC28015	(AT)6	3	14	--	--				
CakTSSR02059	CakTC28020	(TTC)6	177	194	--	ARF	GAGTATTGGGTCAGGTTCTTT	GGTGTTGTTTGAGTAGTGGAG	54.9,54.78	146
CakTSSR02060	CakTC28049	(TGA)5	1427	1441	--	GRAS				
CakTSSR02061	CakTC28050	(AG)8	2151	2166	--	--				
CakTSSR02062	CakTC28062	(ATA)7	948	968	Shoot	MYB	ATGAAAATGGGTTTGGTTTA	AACTGGTTGCTGTTATCTGAA	54.6,55.05	151
CakTSSR02063	CakTC28067	(AG)16	1304	1335	--	--				
CakTSSR02064	CakTC28076	(AT)6	2930	2941	--	--				
CakTSSR02065	CakTC28082	(ATTA)5	68	87	--	--				
CakTSSR02066	CakTC28083	(ATTA)5	939	958	Flower bud	--				
CakTSSR02067	CakTC28084	(ATA)5	253	267	--	--	GAATGAAAGGAAATCATTGTG	CTAGTGGAAGCTTCAGAGACA	54.71,54.92	149
CakTSSR02068	CakTC28092	(TC)16	1762	1793	--	--	GTGGGTTATCTGGACTTGAA	ATGTGGGAGTTATGAGTGTTG	55.03,55	173

CakTSSR02069	CakTC28092	(ACC)6	1899	1916	--	--	GCCTCTCCAACACTCATAACT	GATGGAGGAGGTGGTAGATAG	55.98,55.26	156
CakTSSR02070	CakTC28094	(TC)6	145	156	--	--	AATGTTCGCTCACATTTCTAC	CACATGAAGAGGCTAAAGAAA	54.56,54.88	158
CakTSSR02071	CakTC28100	(CTG)5	1111	1125	--	--	TTTAGTGACTGGATGTCTTGC	ATCACTGGCACTCACACTTAC	55.47,55.23	156
CakTSSR02072	CakTC28103	(AG)7	5677	5690	--	--				
CakTSSR02073	CakTC28110	(AG)7	5619	5632	--	PHD				
CakTSSR02074	CakTC28116	(GTG)8	1614	1637	--	--				
CakTSSR02075	CakTC28127	(AAC)5	133	147	--	--	CACTTTGCAGTGTCAATTTTA	TGAAATTAGGTTTTCCGTGTA	54.12,55.02	167
CakTSSR02076	CakTC28136	(AT)9	2348	2365	--	--				
CakTSSR02077	CakTC28139	(AT)6	112	123	--	Alfin-like	TAGTCAATGGTCCTTGAAAAG	ATCCTTCATAGGTTCATACCC	54.55,54.64	138
CakTSSR02078	CakTC28150	(CT)14	18	45	--	--				
CakTSSR02079	CakTC28157	(TC)6	11	22	--	ABI3VP1				
CakTSSR02080	CakTC28157	(AAT)5	1434	1448	--	ABI3VP1	CCTCCCGATCAATTCTACTAT	AACCGTTTTCCTCCTGTATT	54.97,55.33	140
CakTSSR02081	CakTC28157	(AAGA)6	1721	1744	--	ABI3VP1	ACTTCAGTGCTGTTTGATTTG	CCAACTAACAAACTTGGAAAC	55.55,54.09	155
CakTSSR02082	CakTC28164	(GA)6	1999	2010	--	--	AAGTCGATAATCCCTGTTGAT	TACGAAAACTTCAATCTCACC	55.26,54.54	143
CakTSSR02083	CakTC28171	(AT)7	56	69	--	WRKY				
CakTSSR02084	CakTC28172	(TCC)5	7	21	--	--				
CakTSSR02085	CakTC28184	(TC)6	107	118	--	--	CAAGTCAATGACAATTTTTCC	CAGTGAGAAGAGAGATCAGGA	54.79,54.58	143
CakTSSR02086	CakTC28197	(TA)6	145	156	--	--	AAATGGCACAACATAATGGTA	TTTTTACTTTGTGCAATCGTC	55.56,55.63	143
CakTSSR02087	CakTC28200	(GAA)5	1322	1336	--	--	GAAGAGGAAGAGGAAGATGAG	TTCGAAGGTCAATAACATCAG	54.81,55.4	136
CakTSSR02088	CakTC28222	(TC)6	34	45	--	--				
CakTSSR02089	CakTC28242	(TCCT)8	2595	2626	--	--	AAACTTTACGCAATGAATCAG	AAGTTGCAGTGAGTGAGTGAC	54.77,55.44	142
CakTSSR02090	CakTC28242	(AAT)5	2829	2843	--	--				
CakTSSR02091	CakTC28244	(GAG)6	737	754	--	--	TGGGACTTCTCTTCATTTGTA	TTCTCTTTCTCTTTCCCTCTC	54.95,54.55	156
CakTSSR02092	CakTC28261	(TCT)5	2685	2699	--	Jumonji	CAATTGCACATCAATATCCTT	CATCGTCAGAACAAAGAGAAG	55.19,55.18	150
CakTSSR02093	CakTC28270	(AGA)7	334	354	--	WRKY	AACGATTCCAAAAGTTACGAT	GGGTTTCTGAATTAGGTTCAT	55.5,54.84	145
CakTSSR02094	CakTC28272	(ATG)5	1425	1439	--	--	ATCCTTCTTTGTTTGATCCAT	ATACAGAAAGCAGACCCTGTT	55.23,55.57	165
CakTSSR02095	CakTC28276	(GTT)5	1889	1903	--	GRAS	CCTCTTCCACTTCTTCTTGTT	GATGAGAGTTCCTCCTGTTTC	55.26,55.42	200
CakTSSR02096	CakTC28277	(ATA)5	49	63	--	--				
CakTSSR02097	CakTC28279	(AGC)5	445	459	Flower bud	--	ATCATGTGATTGGAATCTTGA	GAAGGAAAAGGTGATGAATTT	55.4,54.97	153
CakTSSR02098	CakTC28287	(TGT)7	1868	1888	--	--	TTTTGATCAGAAGTGGTGTTT	TCAAACCATCAACAATCTCTC	54.85,55.18	151
CakTSSR02099	CakTC28295	(CAG)5	1753	1767	--	AP2-EREBP	CACACTTTTCCACATCTTCAT	CAGTTGCTAACAAATGTGGTG	55.13,56.8	151
CakTSSR02100	CakTC28303	(TTC)5	1065	1079	--	--				
CakTSSR02101	CakTC28304	(TTC)5	389	403	--	--				
CakTSSR02102	CakTC28305	(GT)6	134	145	--	--	GGAAAAATCAGGAGCATAAAT	TTGCTCAATATGTTGGCTAAT	55.09,55.12	154
CakTSSR02103	CakTC28333	(AC)6	99	110	--	SBP				
CakTSSR02104	CakTC28333	(TC)7	348	361	--	SBP	TCTTTCTTCATTCTTTTCACG	AGAAACCAAATTCAACTTTCC	54.84,55.04	140
CakTSSR02105	CakTC28333	(TG)6	466	477	--	SBP	AGGAAAGTTGAATTTGGTTTC	CAACTTTGCATACCCTCATAA	55.04,55.44	132

CakTSSR02106	CakTC28333	(TGA)5	864	878	--	SBP	TCATAATCAAGGTTTTGAGGA	GCAGCCATAACAACATCACTA	54.89,56.37	141
CakTSSR02107	CakTC28336	(TCC)6	42	59	--	--				
CakTSSR02108	CakTC28348	(AT)9	946	963	Shoot	--				
CakTSSR02109	CakTC28354	(AG)10	3710	3729	--	--	TTGTTCTTGTGTTGTTTGAGA	CTCCTCTCGTAATTTTTCACA	54.35,54.72	137
CakTSSR02110	CakTC28366	(CT)8	67	82	--	--				
CakTSSR02111	CakTC28376	(CTT)7	5	25	--	--				
CakTSSR02112	CakTC28383	(GAA)5	44	58	--	--				
CakTSSR02113	CakTC28383	(AAT)6	1372	1389	--	--	AGGTTCTGAAAGTCCTTCTTC	ACTATCCTGAGAAGACCCTTG	54.33,55.06	148
CakTSSR02114	CakTC28383	(TTC)5	1695	1709	--	--	TGGTGTGAAAAGTAATGTGGT	GATCCACACCATCTAAAACAA	55.51,55.06	152
CakTSSR02115	CakTC28384	(ATGA)5	2072	2091	--	--				
CakTSSR02116	CakTC28388	(CAC)8	1222	1245	--	C3H	CAGAACCCAGTTCTCATGTAA	ATCCGGTAAATCAACATGAAT	55.31,55.94	157
CakTSSR02117	CakTC28388	(TC)10	1591	1610	--	C3H				
CakTSSR02118	CakTC28393	(TTC)5	135	149	--	--	GTTCCTCTCTTACATGCACAC	ATGGTGTGAGGTTGTTGAATA	54.85,55.43	170
CakTSSR02119	CakTC28394	(ATA)11	143	175	--	bHLH	TTGAGCACAAAGTTTCCTCT	TCCCTTCAATAGGTAAATTCC	55.16,54.95	151
CakTSSR02120	CakTC28400	(ATT)6	123	140	--	--	AACCCCAACCCATATAATTT	TCTGGGAAGTAGATCTCACAA	54.93,54.92	164
CakTSSR02121	CakTC28401	(ATG)6	1110	1127	--	--	TACAGTGATCCTGAGGTTTTG	AACCCCTTTCAGATAGAATTG	55.31,55.01	163
CakTSSR02122	CakTC28402	(TTAT)6	186	209	--	--	TCTATGGTGCAAGAAGTTTGT	TTGCAATAGCAATCGAATATC	55.05,55.59	144
CakTSSR02123	CakTC28407	(AAC)7	654	674	Root	--	TTATGGAGAATGGTTACGATG	CCTTCATAACCTGAAACAAGT	55.21,53.58	153
CakTSSR02124	CakTC28411	(TAC)6	293	310	Flower bud	--	TTGACAAGAGCAAACAACATA	TGAAGCAATGTTGGATTTAGT	54.51,54.99	150
CakTSSR02125	CakTC28414	(CT)7	15	28	--	--				
CakTSSR02126	CakTC28423	(ATT)5	111	125	--	--	ATAAATGGAGGCGAGAGTTAT	TGCTTCAGAGTTAAAGGTTGA	54.74,55.36	132
CakTSSR02127	CakTC28423	(GAA)5	3290	3304	--	--	GAATTCCTGAGATGGTGTAAA	CTATCATGTCACACTTCTCTCG	54.29,55.06	147
CakTSSR02128	CakTC28424	(ATA)5	242	256	--	--	AAATAAGCCACACACAAACTT	TGGTAGTTGTTTGAAGCTAGG	54.11,54.84	132
CakTSSR02129	CakTC28424	(CT)9	553	570	--	--	CATTATTTGTGCTGAACAAGC	AGGGAGAGAGAAAATGGTATG	56.07,54.99	151
CakTSSR02130	CakTC28430	(AG)11	2416	2437	Young_pod	bHLH				
CakTSSR02131	CakTC28435	(TC)14	2067	2094	--	--	TTGCATAGTCATAAATTCCTCA	GCAAGACACTGTAGCTGAAAT	55.11,54.78	138
CakTSSR02132	CakTC28436	(AG)14	276	303	--	--	GCAAGACACTGTAGCTGAAAT	TTGCATAGTCATAAATTCCTCA	54.78,55.11	138
CakTSSR02133	CakTC28441	(ATA)5	565	579	--	--	GAAGGAACATGGTAGCATACA	TGCCTACTCCTTATTATCCAG	55.26,53.84	162
CakTSSR02134	CakTC28444	(TA)12	101	124	--	--				
CakTSSR02135	CakTC28445	(TA)11	100	121	--	--				
CakTSSR02136	CakTC28458	(GAA)6	3867	3884	--	--	CCGATCTCGTCTTTATCCTAT	CGGTTTGAAGAAATTGAGTC	55.12,54.92	158
CakTSSR02137	CakTC28459	(GAA)11	1152	1184	--	--	GGTGTAGGAACCGAATAACTC	TTGGGATTCTATTGATGTACG	55.34,55.21	142
CakTSSR02138	CakTC28462	(ATT)5	202	216	--	--	AAAGAATCCATTTCTTGCTCT	TCCTAAGAAGACAGAGGGAAT	54.88,54.71	153
CakTSSR02139	CakTC28462	(TCC)5	412	426	--	--	TCCATTTGCTTCTAATTTGC	AAGAGGTGCAATATTGTTGTG	55.62,55.34	151
CakTSSR02140	CakTC28468	(CA)6	266	277	Shoot	--	TCTAAACCATAACCATTTTGC	CATTTGTTAACTTTGGATTGG	54.56,54.87	133
CakTSSR02141	CakTC28475	(CT)6	33	44	--	--				
CakTSSR02142	CakTC28476	(TA)6	319	330	--	--	CTCTCCTTCTTCTCCTCATTC	ATTGGTTCTTTCTTTCTCCAC	54.81,55.03	153

CakTSSR02143	CakTC28476	(GAA)6	1470	1487	--	--	TAATGAAAATTGTGGGAGAAG	TGACCCTCTTGTCATACTCAT	54.5,54.6	153
CakTSSR02144	CakTC28495	(GT)6	3500	3511	--	--				
CakTSSR02145	CakTC28509	(TA)6	1471	1482	--	--	TATTGTTTGTGTTGGGAAGAC	ACAAATTAGAAGCTGAAAGCA	55.15,54.64	163
CakTSSR02146	CakTC28520	(AG)8	1510	1525	--	Trihelix				
CakTSSR02147	CakTC28527	(AG)7	1181	1194	--	--				
CakTSSR02148	CakTC28528	(AAT)5	275	289	--	--	AACATAAGCTCAAATCAACCA	GATAAACAAATTGCAATGTCAC	54.99,54.85	125
CakTSSR02149	CakTC28528	(AG)16	760	791	--	--	TAGTGAGATCTTTTGGGAACA	ATCTTCTCATTTCTCCTTTGC	54.95,55.26	150
CakTSSR02150	CakTC28548	(AT)9	127	144	--	--	CAATGAGTTAAGTTCCTTCACA	TGCCTTCATATGCAGAGTAGT	54.65,55.17	141
CakTSSR02151	CakTC28557	(TC)6	86	97	--	--				
CakTSSR02152	CakTC28560	(TGT)5	8	22	--	LOB				
CakTSSR02153	CakTC28561	(CAA)5	48	62	--	--				
CakTSSR02154	CakTC28584	(TAT)6	168	185	--	--	AAAAGAAGGGGTTTACATCAC	TGGAGCTTATTGAATCTGGTA	54.92,55.08	152
CakTSSR02155	CakTC28591	(CTA)6	333	350	--	--	TCAATCATGGCTAGTGAGACT	CATCATGTTTGTCTCCATCTT	54.95,55.05	151
CakTSSR02156	CakTC28599	(CAG)5	143	157	--	--	GTTATTGCTGCTTCCTCCTAT	TTTAAGTGGAGGGTTGGTTAT	55.34,55.36	150
CakTSSR02157	CakTC28601	(AGA)8	2034	2057	--	--				
CakTSSR02158	CakTC28609	(AAG)7	2389	2409	--	--	AACCCTAATTTGAAACAGAAGA	AAACAACTCTTCCTTCCTCAC	54.85,55.09	149
CakTSSR02159	CakTC28613	(CAT)5	80	94	--	--				
CakTSSR02160	CakTC28613	(GA)6	985	996	--	--	TAGTCCTGATTCGAAGGAAGT	ACCTTCACCAAAATCTCTACC	55.63,54.9	154
CakTSSR02161	CakTC28619	(CTTC)7	56	83	--	HSF				
CakTSSR02162	CakTC28652	(GAA)7	493	513	--	AP2-EREBP	CTCCAATCCAATTTCCATAAT	AATGGAAGTGGAAGTAGTGGT	55.6,55.25	139
CakTSSR02163	CakTC28682	(AG)6	2032	2043	--	--				
CakTSSR02164	CakTC28687	(CTA)6	521	538	--	WRKY	GAAATTCAATCTGAACATCCA	TTGTCAACATTCACAGAAGAA	55.11,54.26	146
CakTSSR02165	CakTC28691	(TAA)6	395	412	--	--	TCGTTTAATAGTTCCGTACCA	CTTAAAAACCTTAATCCCAACA	55.05,55.28	156
CakTSSR02166	CakTC28701	(CAT)5	342	356	--	--	TCAATCCTTCTACCAGAATCA	TTGGATAGTCATAGGCTTGAG	54.86,54.67	155
CakTSSR02167	CakTC28702	(CT)6	165	176	--	--	AGTATATGCACCAACAGTGCT	ATTTGATATTCAGGTGCTGTG	54.95,55.26	142
CakTSSR02168	CakTC28712	(GTT)6	648	665	--	--	GTTGGATGAGATTCCTATTCC	GGTCTCTTCAAAACTGTTCTTT	55.15,54.46	152
CakTSSR02169	CakTC28712	(AG)8	886	901	--	--				
CakTSSR02170	CakTC28713	(ACA)5	254	268	--	bHLH	TTAGGGGTTTATTTCCTGAAG	GTTCCTGGATTAATGTTGTTG	55.19,54.67	158
CakTSSR02171	CakTC28715	(AG)8	65	80	--	--				
CakTSSR02172	CakTC28723	(AG)10	2121	2140	Flower bud	--				
CakTSSR02173	CakTC28727	(TGC)5	363	377	--	--	CCAACAGGTAACAAACCATAG	GTCTGGTAGTGATCCCAAGAT	54.8,55.58	167
CakTSSR02174	CakTC28734	(AAT)5	1876	1890	Flower bud	--	AAAAATACCCCTAAGTCGTTG	TCGTGTTGGTAACTTTATTTTG	55.14,54.74	156
CakTSSR02175	CakTC28746	(TA)16	842	873	--	--				
CakTSSR02176	CakTC28747	(GA)11	1653	1674	--	--				
CakTSSR02177	CakTC28752	(CAA)5	65	79	--	--				
CakTSSR02178	CakTC28759	(TTG)7	733	753	--	--	GCCAATCTTCATCTGAGTAAA	AAGAAGAAGAACCCTTCACAT	54.62,54.62	152
CakTSSR02179	CakTC28765	(GAT)5	1527	1541	--	--	AGCAGTGTTGCCTCATATAAA	AATTGATCAACAGATTCATGG	55.18,54.98	167

CakTSSR02180	CakTC28775	(TTC)5	362	376	--	--	TTCCTTTACCTGTTTCCTCTC	GAACTCTTTGAATTTTCAGCA	55.2,54.83	151
CakTSSR02181	CakTC28792	(CTT)5	340	354	--	--	CACCCTCTCTATCCCTAGATG	CAATGCCAAACAAAGTAACTC	55.44,55.07	161
CakTSSR02182	CakTC28793	(CTT)5	339	353	--	--	CACCCTCTCTATCCCTAGATG	CAATGCCAAACAAAGTAACTC	55.44,55.07	161
CakTSSR02183	CakTC28797	(TC)6	1275	1286	--	--				
CakTSSR02184	CakTC28798	(CCA)7	370	390	--	C3H	ATGATGGCTGTTAAATCATTG	CATGGTTACCAGGTTGTCTAC	55.19,54.59	172
CakTSSR02185	CakTC28798	(CTT)5	623	637	--	C3H	ACAATTGTTGTGTTTGGAGAG	TCATCTAACCTTCTGCTGCTA	55.22,55.47	150
CakTSSR02186	CakTC28800	(TTC)7	604	624	Flower bud	--	TTGATTCATATTTCCATCCAC	CAGAGAAGAATGAGGATGATG	54.92,54.84	144
CakTSSR02187	CakTC28811	(GT)6	1300	1311	--	--	GAGCATGTGCTACAACCTATC	CCTAAAACAATCATCAGTCCA	54.99,55.24	155
CakTSSR02188	CakTC28814	(AGA)11	2055	2087	--	--	TTTTAAATCAAGGAAGGTGTG	TAACACGATGCTTCTTTCTTC	54.59,54.86	155
CakTSSR02189	CakTC28815	(TC)10	9	28	--	--				
CakTSSR02190	CakTC28821	(TTC)5	685	699	--	--	GCATGTGCAATTTCAACTAAT	ATAAAGCTGCAAGAGGGTATT	55.42,54.97	157
CakTSSR02191	CakTC28822	(TGT)7	885	905	--	--	GGTTGTTGAAGAAAGTGAAGA	GAAAGAGTTCAAAACCCATCT	54.56,55.03	156
CakTSSR02192	CakTC28829	(GGA)5	579	593	--	--	GAGTATCGAAGGGAGAAGAAG	TCCCTGATCCCTATCTCTATC	54.87,54.83	150
CakTSSR02193	CakTC28831	(AAG)6	99	116	--	--				
CakTSSR02194	CakTC28834	(AAG)6	523	540	--	--	AGAGCCTCATCAAGAAGAAGA	ATGTGTTTCAATGCTGTTTCT	55.92,54.88	147
CakTSSR02195	CakTC28846	(CCT)5	494	508	--	Trihelix	CATCATCTGTTGCCTCTTAGT	ATGAAGATGATGATTTCGATG	54.54,55.06	167
CakTSSR02196	CakTC28846	(TGT)5	1256	1270	--	Trihelix	TTCAATACCGTCTTCATCTTC	AATTCGCACCTTACAATATCA	54.45,54.96	151
CakTSSR02197	CakTC28851	(GTT)6	789	806	--	--	TGTGAAGATGGAAGAGAAAAG	AATCTCACCCTCATATTCCTC	54.65,54.74	153
CakTSSR02198	CakTC28881	(ATT)5	2092	2106	Shoot	--				
CakTSSR02199	CakTC28885	(GTT)8	927	950	--	--	TGCAACAGGGTTTAACTCTAA	ACATAAGCACTCCACATGAAC	55.24,55.16	149
CakTSSR02200	CakTC28885	(GT)9	1290	1307	--	--				
CakTSSR02201	CakTC28886	(AT)7	306	319	--	--	GGCCCTCCTAATTATTACAAA	GAAGCTCCTGGTATCAACTTT	55.15,55.22	161
CakTSSR02202	CakTC28886	(TTG)6	1400	1417	--	--	AAAGGAATCTTGGTGATTGTT	TAAGCCATAGCAAACTGAGTC	55.31,54.9	154
CakTSSR02203	CakTC28899	(TC)10	1118	1137	--	--				
CakTSSR02204	CakTC28910	(TAT)5	1109	1123	--	--				
CakTSSR02205	CakTC28918	(AG)6	2	13	--	--				
CakTSSR02206	CakTC28920	(TAT)6	1482	1499	--	--	GGAATCAAAAACCCTAACACT	ATGTGGTTTTCTCCCTTAGAA	54.92,55.47	165
CakTSSR02207	CakTC28922	(TCA)5	1758	1772	--	FHA	CTTTTTCTTCGTACGGTCATA	TTGGAAGAAACACTGAATGAT	54.78,54.77	150
CakTSSR02208	CakTC28922	(GGT)5	2676	2690	--	FHA				
CakTSSR02209	CakTC28923	(CCA)5	351	365	--	--				
CakTSSR02210	CakTC28925	(CAA)6	699	716	--	--	TTAATGGCTGATCACAGTTCT	TGGTCAGTTCCAAAAAGAGTA	54.97,55.03	143
CakTSSR02211	CakTC28934	(GAA)7	1449	1469	--	--				
CakTSSR02212	CakTC28936	(TC)8	44	59	--	--				
CakTSSR02213	CakTC28936	(CAC)5	538	552	--	--	TGTCCAAATTAAGGATAACCA	TGAGCTAATAAAACTGCCTGA	54.78,55.48	156
CakTSSR02214	CakTC28946	(TG)6	40	51	--	--				
CakTSSR02215	CakTC28946	(GAA)6	488	505	--	--	TACATTGGATATCGGTGTGTT	CCACTGCAGCTACTAATATGG	55.39,55.23	151
CakTSSR02216	CakTC28972	(CT)9	77	94	--	--				

CakTSSR02217	CakTC28972	(AAG)5	318	332	--	--	ACAGAAAATCTGTCGGTTTCT	TCATCTTCTTCAACATGATCC	55.54,55.09	149
CakTSSR02218	CakTC28976	(TTG)6	319	336	--	WRKY	TCATCTTTAGGTTCATCCTTG	CCTTTTAACCCTGAGATGATT	54.47,55.01	155
CakTSSR02219	CakTC28978	(GGT)6	1644	1661	--	--	AAGCAAACCACTCTTGATTTT	AGAACTCTCCATTACCAAAGG	55.69,55.07	151
CakTSSR02220	CakTC28997	(TCC)5	454	468	--	--	GTTGTCAAACAAGAAAGTTGG	GTAGAGCTGCAGAAAAGTTCA	54.94,55.09	159
CakTSSR02221	CakTC28998	(GAT)5	1470	1484	--	--	GGAGTCTGTACCACTGAATGA	TATGTCCACAGTCTCTTCCTC	55.11,54.29	151
CakTSSR02222	CakTC29000	(TTC)10	171	200	--	--	CACCAATTGTAACGAATCAAC	AGAGGTTGAAAAATGGAAGAG	55.6,55.2	149
CakTSSR02223	CakTC29009	(AGA)5	19	33	Flower bud	GNAT				
CakTSSR02224	CakTC29010	(TC)7	39	52	--	--				
CakTSSR02225	CakTC29020	(CTT)5	710	724	--	AP2-EREBP	AGTTCTTCCACAAATTCAACC	CCAAGAACAGAACAAGAAGAA	55.78,54.74	152
CakTSSR02226	CakTC29020	(TAT)5	1884	1898	--	AP2-EREBP				
CakTSSR02227	CakTC29031	(TCA)5	524	538	--	--	CTTTAACCGATCAACAATGAA	TTAGAGGCACAAAGGTTATGA	55.41,55.16	143
CakTSSR02228	CakTC29035	(TCT)6	988	1005	--	--	GTTTCAACTTCTTCGTTTTCA	AGTCGTTAGGTGACTCAGTTG	54.75,54.52	145
CakTSSR02229	CakTC29036	(CT)9	1	18	--	--				
CakTSSR02230	CakTC29039	(CAA)5	2132	2146	--	--	GTACACCTCAAGTTTCTGACG	GCGATCTCTTTTCTCTCTCTT	54.95,54.73	169
CakTSSR02231	CakTC29047	(GAT)5	145	159	--	--	CTAGTGCCTTTGAAAGTGTTG	CATTTCCACAATCAGGTAAAG	55.31,54.84	147
CakTSSR02232	CakTC29049	(GGT)7	1521	1541	--	TCP	TACATTTTATGCCGAGGTTTA	CAAATTTGAATCAGAAACTGC	55.15,55.12	149
CakTSSR02233	CakTC29049	(TCTCAT)5	1776	1805	--	TCP	GGTGCTACTGATTTCAATGTT	TTCACTTTCAGTAGGTTGAGG	54.4,54.61	150
CakTSSR02234	CakTC29049	(AG)10	2115	2134	--	TCP				
CakTSSR02235	CakTC29070	(GAA)14	1221	1262	--	--	GGTTCTTGTACCTGGCTTAAT	CTGAACTTGAGTTTCAAAAGG	55.11,54.35	154
CakTSSR02236	CakTC29077	(CAG)5	531	545	--	--	CTCAAACCTCAGAGAGAACCT	AGGACATACCTCCAAATCAAT	55.25,55.11	145
CakTSSR02237	CakTC29096	(CTT)7	1172	1192	--	--	TGAAACCAAACTCTGAGTCAT	ATGATGACGATCAAGATCAAG	54.83,55.13	129
CakTSSR02238	CakTC29099	(CAT)11	537	569	--	WRKY	GGAAGAGAACATGAAGTGTGA	TACATGTGATGATGTTGATGG	55.25,55.09	157
CakTSSR02239	CakTC29099	(AAC)6	1301	1318	--	WRKY	ATATTTCCACCATTCACCTTT	GACAAGCTGAAAATGTTTGAC	55.12,55.01	158
CakTSSR02240	CakTC29117	(TCC)5	548	562	--	--	AACACCTTGGCTACAAACATA	AGAAACCAGTAGCACCAAAA	54.95,55.04	146
CakTSSR02241	CakTC29130	(CT)6	12	23	Flower bud	--				
CakTSSR02242	CakTC29130	(TA)7	795	808	Flower bud	--				
CakTSSR02243	CakTC29131	(TA)6	165	176	--	--	TTTCTACCTTCTGCTTCTTCA	TCAATGATCCATAAGTGTTCC	54.59,54.98	149
CakTSSR02244	CakTC29135	(TG)9	2541	2558	--	--				
CakTSSR02245	CakTC29136	(TTTC)5	171	190	--	--	CCATATACAGCTGATTTCGTT	AACGAGTCAACTCAACTGAAC	54.56,54.46	156
CakTSSR02246	CakTC29146	(TAGA)5	2424	2443	--	--				
CakTSSR02247	CakTC29149	(GA)8	1148	1163	--	--				
CakTSSR02248	CakTC29155	(TGA)9	237	263	--	--	AACCTTCAAAACCTCAACTTC	TAAGGATTTAGAGCAGCAATG	55.11,55.01	152
CakTSSR02249	CakTC29156	(TGA)9	269	295	--	--	AACCTTCAAAACCTCAACTTC	TAAGGATTTAGAGCAGCAATG	55.11,55.01	152
CakTSSR02250	CakTC29164	(TA)8	313	328	--	--	TTTCTTCTCTTCTTCCCAAGT	CATCCTCTTCCATCATATTCA	54.92,55.07	150
CakTSSR02251	CakTC29169	(ACA)8	190	213	--	--	GTCGTTTCACAAGAACAACAT	TGATGAAATGCTTCTGAGAGT	55.2,55.08	143
CakTSSR02252	CakTC29172	(ATC)6	839	856	--	--				
CakTSSR02253	CakTC29175	(CT)6	121	132	--	--	ACGGAAGGATCTCTAGAATTG	TGGTGATTGAATTTTCTCAAG	55.15,55.37	158

CakTSSR02254	CakTC29183	(GAA)6	48	65	--	--				
CakTSSR02255	CakTC29184	(CAT)5	1685	1699	--	--				
CakTSSR02256	CakTC29197	(CTT)7	108	128	--	mTERF	CCCTTCTCCTTCTCGTTAATA	GAAAAGGAGATAAGGTTGAGG	55.33,54.74	149
CakTSSR02257	CakTC29224	(CT)8	261	276	--	--	TTCTCTCTCCTTGATTTGTCA	GATTCGGAAATTTGAAATAGG	55.06,55.48	179
CakTSSR02258	CakTC29228	(AG)19	1248	1285	--	--	GTACTGCTCCATCAAAAACCT	TTCTTCTTCTTCCTCTTCGAT	55.97,54.99	152
CakTSSR02259	CakTC29248	(GA)7	1976	1989	--	--				
CakTSSR02260	CakTC29252	(ATT)5	533	547	--	--	TCAAATTAGGACCTCTCACAA	GCCTTCAAGTTAGAATGGTTT	54.95,55.23	153
CakTSSR02261	CakTC29252	(TAT)7	859	879	--	--	GCTGACCTTAAATCAATAGCA	AATGGTTAGTCCAATGAATGA	54.84,54.58	150
CakTSSR02262	CakTC29255	(CT)12	239	262	--	--	AGACAACCAAACCCTAAAATC	TAATACTCCAGCCATCAAGG	54.92,55.35	150
CakTSSR02263	CakTC29263	(TC)14	1428	1455	--	C2C2-Dof	ACCACAAGATGAGTCTTCAAA	TAGAAGTGGGTGTTGTGTTTT	54.83,54.81	142
CakTSSR02264	CakTC29266	(CAT)5	923	937	--	--	TCTGAAAATCCAACCAACTTA	TGATAAGGATAGAGTGGAGAGG	54.97,55.17	152
CakTSSR02265	CakTC29278	(CAG)5	970	984	--	--	ATGTTGGTTCTGAAAGGGTTA	GCTTCTGATTAACACCAACAC	56.23,54.88	150
CakTSSR02266	CakTC29300	(AAC)5	928	942	--	--	AGATTATCGTTCCACACTTCA	ATCCTTCACTCAGTCCAATTT	54.8,55.3	152
CakTSSR02267	CakTC29304	(GA)6	2065	2076	--	--				
CakTSSR02268	CakTC29310	(CA)6	65	76	--	--				
CakTSSR02269	CakTC29320	(GTG)5	1439	1453	--	bHLH	TCTCTTTCGTCTTTTGTTGTT	GATATCATTTCCCCATGAAA	54.34,54.82	150
CakTSSR02270	CakTC29328	(AG)6	14	25	--	--				
CakTSSR02271	CakTC29330	(TC)6	125	136	--	--	CCCTTTCTTTATTTCACCATT	CTAACCTTGATGTTGATTGGA	55.03,55.24	153
CakTSSR02272	CakTC29352	(AG)10	2362	2381	--	--				
CakTSSR02273	CakTC29355	(AGG)5	1400	1414	--	--				
CakTSSR02274	CakTC29357	(CGC)5	213	227	--	--	GACCTCTGTCAACAGCTCAT	GAGTCTAGGGACTCAGACGAC	55.25,55.53	179
CakTSSR02275	CakTC29377	(TCA)5	505	519	--	MYB	CTGAAGAAATTGCAAACAGAT	TGAAAGTGCTTCACTAAGAGC	54.7,55.09	156
CakTSSR02276	CakTC29378	(CAAAAC)5	109	138	--	--	ATGAGGACCAAAATAAAAACC	AGAACTAGGGAGGAGATGTTG	54.87,55.06	164
CakTSSR02277	CakTC29387	(CCA)5	1	15	--	--				
CakTSSR02278	CakTC29405	(TCT)5	275	289	--	--	TGCCATAAAGTCTCTTCAAAA	TCTCAAAGATTCCTCTGATTG	55.29,54.56	154
CakTSSR02279	CakTC29415	(ATC)5	619	633	Shoot	--	TTTTTCCTTTTGTGTGGTTAG	GAATCGGTAAAATCAAAACCT	54.67,55.02	149
CakTSSR02280	CakTC29415	(AAC)8	921	944	Shoot	--	AACACAAACGAAGATGAAGAA	GTTATAACGATGGGTGGTTTT	55.02,55.62	146
CakTSSR02281	CakTC29415	(GCA)5	1512	1526	Shoot	--	GAAGATGAACAAGGTGATGAA	ACCTGATGTTGTAAATGTTGC	55.18,55.17	149
CakTSSR02282	CakTC29415	(CCT)5	1674	1688	Shoot	--				
CakTSSR02283	CakTC29416	(TC)13	31	56	--	--				
CakTSSR02284	CakTC29417	(TTC)5	311	325	--	--	CAATGCCAAATACCATAAAAG	GGTTGACATGTGATCTGAAAT	54.99,54.86	132
CakTSSR02285	CakTC29417	(GA)7	1400	1413	--	--				
CakTSSR02286	CakTC29420	(GA)7	101	114	--	--	TGACTTAAACCTTCCCTTCTT	CAAAAACACACTTGTTTCACA	54.82,54.76	143
CakTSSR02287	CakTC29427	(TTCACA)7	67	108	--	--				
CakTSSR02288	CakTC29436	(CT)9	1210	1227	--	--				
CakTSSR02289	CakTC29439	(AG)6	273	284	--	--	TATTTGATGACCCATGTTGAT	AATCTCCACGAACTCTTCTTC	55.28,55.17	155
CakTSSR02290	CakTC29446	(AAC)5	53	67	--	--				

CakTSSR02291	CakTC29460	(CT)6	2	13	--	--				
CakTSSR02292	CakTC29464	(ACA)5	230	244	--	--	AGAATCTCTTCATCAGCAACA	GTGGAGTTTGGAGATCTCTTT	55.08,55.01	176
CakTSSR02293	CakTC29465	(TA)6	263	274	Shoot	--	ATTTTTCCCTTTAGACAAACC	TTGAGGTTGAACACAACAAGT	54.24,55.72	147
CakTSSR02294	CakTC29468	(AAG)8	39	62	--	--				
CakTSSR02295	CakTC29469	(TC)6	40	51	--	--				
CakTSSR02296	CakTC29475	(TC)14	1509	1536	--	--	TGTGAGATTTGGTTTCGTAAT	AAGAACCAGCACTAGTGACAA	54.83,55.12	147
CakTSSR02297	CakTC29481	(CCT)5	155	169	--	--	AATTCGTTTCCCTCTTTATTG	TCAGAAGACATTCGACCTTTA	55.18,55.11	153
CakTSSR02298	CakTC29482	(GTT)6	36	53	--	--				
CakTSSR02299	CakTC29484	(TGG)5	261	275	--	--	ATGGGAGACCCATCTAGTAAA	ACAACAACAACCTTTCATGTC	55.27,55.04	157
CakTSSR02300	CakTC29487	(ACT)5	622	636	--	mTERF	GTTCCCTACAAAATCAAGGAT	AGAGATGTTGCAGGTTAGTGA	54.84,55.04	150
CakTSSR02301	CakTC29492	(ATT)6	2569	2586	--	--	ATAGATAGCTGGAGAATGCAA	AAAGATCAGGACTCCCTTTC	54.35,54.91	154
CakTSSR02302	CakTC29497	(ACA)6	1012	1029	--	MYB	ACATTGATGAATGGGTAGAAA	TTAGATGAATTGGATGGTTCT	54.58,53.82	151
CakTSSR02303	CakTC29498	(GATTTG)5	275	304	--	NAC	GCATCAGCAAAGTAGACAAAT	TCTCCAAAATTCAGCAGTTAG	54.73,54.88	152
CakTSSR02304	CakTC29498	(TCC)6	496	513	--	NAC	GGGAGAGGGAGTAACAAAGTA	TGACAACAGTTCTTGGTCTTT	55.07,54.92	148
CakTSSR02305	CakTC29498	(ATC)6	1248	1265	--	NAC	TTCTTCTTCAGTTGGATGAAA	ATGAGTCAAAGCCATAGTCAC	55.08,54.36	153
CakTSSR02306	CakTC29499	(TA)7	72	85	--	--				
CakTSSR02307	CakTC29500	(TCT)5	255	269	--	--	GTCTCATCTTTGGTCCTTCTT	TTGTTGTTGTTGAGAATGTTG	55.01,54.67	158
CakTSSR02308	CakTC29514	(TC)11	68	89	--	--				
CakTSSR02309	CakTC29528	(AC)6	75	86	--	--				
CakTSSR02310	CakTC29534	(GCC)5	1377	1391	--	--				
CakTSSR02311	CakTC29541	(AT)6	3067	3078	--	--	TGAAATGATATCCTTCCGAAT	CAGTCTCTACTGGGGGTTATT	56.15,54.95	140
CakTSSR02312	CakTC29549	(TTA)5	1262	1276	--	Trihelix				
CakTSSR02313	CakTC29553	(AG)16	1359	1390	--	--				
CakTSSR02314	CakTC29554	(GAA)5	2339	2353	--	--	AAATCAACAGAGACATTGGAA	AAAGTCGAAGTTGCTCTCTCT	54.77,55.16	146
CakTSSR02315	CakTC29557	(ATT)9	169	195	--	--	TGCTACCTGTTGTTTCTCCTA	ATGTATGGCAAGGAAAGTATG	55.23,54.4	165
CakTSSR02316	CakTC29572	(AG)9	876	893	--	--				
CakTSSR02317	CakTC29573	(AG)6	57	68	--	--				
CakTSSR02318	CakTC29574	(TGT)5	1125	1139	--	--	TCAGTAGAAAACATGGGGATA	CATCAAATGCATTCTCTCATT	54.76,55.31	151
CakTSSR02319	CakTC29574	(AAT)7	1260	1280	--	--				
CakTSSR02320	CakTC29576	(TC)13	1	26	Shoot	--				
CakTSSR02321	CakTC29580	(CT)18	1	36	--	--				
CakTSSR02322	CakTC29586	(CAA)5	63	77	--	--				
CakTSSR02323	CakTC29591	(AT)8	947	962	--	--	TCACTCTTCGGGATCTCTAGT	CAATTACTCCATCCAAATTCA	55.63,55.17	173
CakTSSR02324	CakTC29598	(AGA)5	1329	1343	--	--				
CakTSSR02325	CakTC29600	(TCT)6	123	140	--	--	TCTTCCCTTTCCTCATCTTAC	GGTGTGATTGGGATCTAAAA	55.12,54.97	152
CakTSSR02326	CakTC29601	(AGA)5	1460	1474	--	--				
CakTSSR02327	CakTC29608	(AAC)5	180	194	--	--	ACATCAGTGAGATGTCAACCT	GCTCAAAATTATCGTTAGCAG	54.47,54.62	148

CakTSSR02328	CakTC29615	(AG)10	2269	2288	--	--	TCTTGCATTGTTCGAGTATTT	TCTGTGGAACACTCTTTTCAT	55.15,54.83	165
CakTSSR02329	CakTC29620	(TC)8	7	22	--	--				
CakTSSR02330	CakTC29654	(GA)7	64	77	--	--				
CakTSSR02331	CakTC29655	(GTT)7	582	602	--	--	TGTTCAGGAGGATCATGTTT	ATCAAAACCAACAACAATCTC	55.46,54.19	150
CakTSSR02332	CakTC29662	(TTG)5	812	826	--	--	ATTTCCCACTTTTGTCTAACC	TATTAAAGGTGTTGCAAGTGG	54.92,55.52	154
CakTSSR02333	CakTC29667	(TTC)5	404	418	--	--	ACCAATAAGGATCAGGCTTAG	AAGATGGTTGTTGATTTGTTG	55.2,55.15	148
CakTSSR02334	CakTC29684	(CT)6	105	116	--	--	GTAAGTGACACTGCTTCAACC	CGAAACTTAGACTTTGGAGGT	54.94,55.31	144
CakTSSR02335	CakTC29689	(AG)12	540	563	--	--				
CakTSSR02336	CakTC29692	(GTG)9	493	519	--	--	AGAACATGGCATAGGATACG	ATCTCCTCCTGCACCACTA	55.2,55.66	148
CakTSSR02337	CakTC29708	(TTC)6	240	257	--	--	GTAGTTGTTGGGGTATGTCCT	GGCTTTCTGTTTATGGAACTT	55.54,55.23	152
CakTSSR02338	CakTC29714	(AT)10	826	845	--	--	AAATGGAAGTTTAGGGTGATG	TCAAATTGGAGACCTCATAGA	55.75,54.86	180
CakTSSR02339	CakTC29725	(TAC)6	1021	1038	--	--	GGAAAGGAAGGTTGAGAAGTA	CAGCACAATTCATCATTCTCT	55.2,55.38	165
CakTSSR02340	CakTC29738	(TCA)5	121	135	--	--	GGTCTCTAAGACATTGGGTTT	AGCTAAGGAGAAATCACGAGT	54.9,54.97	155
CakTSSR02341	CakTC29740	(CT)10	78	97	--	SWI/SNF-SWI3				
CakTSSR02342	CakTC29776	(ATA)5	1071	1085	--	ARF	TCATGTTTGTTAACCATAGGAA	TCTTGAGATTTGTTGCTCAGT	54.88,55.17	150
CakTSSR02343	CakTC29780	(AG)7	920	933	--	--				
CakTSSR02344	CakTC29781	(CT)10	1	20	--	--				
CakTSSR02345	CakTC29782	(CT)6	73	84	--	--				
CakTSSR02346	CakTC29801	(AGA)6	1898	1915	--	--				
CakTSSR02347	CakTC29805	(AT)9	61	78	Shoot	zf-HD				
CakTSSR02348	CakTC29808	(TA)6	1890	1901	--	--				
CakTSSR02349	CakTC29811	(AGA)5	63	77	--	--				
CakTSSR02350	CakTC29831	(AG)8	105	120	--	--	GCTGTCACAAGTAAAATCCAA	GTTTGCTTCATCATCGTTTAC	55.47,54.98	153
CakTSSR02351	CakTC29831	(TCT)6	1939	1956	--	--	TGTCTAACTCCACTGCAACTT	CATTGGCATCAACTACAAAAT	55.12,55.27	151
CakTSSR02352	CakTC29834	(CTT)5	230	244	--	--	TGGCACTCAGCTCACTACTAC	AAATAGCCTCATTTCCAAGTC	55.75,55.15	156
CakTSSR02353	CakTC29843	(GCA)7	1638	1658	--	Sigma70-like	ATGAGGTTGCTTTAAAGTGTG	CTTCAAACCAAAACAAGACAC	54.66,54.94	169
CakTSSR02354	CakTC29846	(AG)6	5172	5183	--	--				
CakTSSR02355	CakTC29849	(GA)7	2659	2672	--	--				
CakTSSR02356	CakTC29856	(TCA)5	2384	2398	--	--	TTACTTCTGCAAAGAGGACAC	GGCTATGATGTTGATGTTGAT	54.76,55	154
CakTSSR02357	CakTC29877	(GAA)7	1698	1718	--	--	AGTGGTACAAACATGAACAGG	CATTGGATGAAAGGACAGTTA	55.08,55.24	159
CakTSSR02358	CakTC29882	(CTT)5	419	433	--	--	AATGCTTTCTTTATTGCCTCT	CCTTGGAATTGTTGAAGAGTA	55.08,54.55	145
CakTSSR02359	CakTC29884	(GAA)5	47	61	--	--				
CakTSSR02360	CakTC29888	(TA)10	153	172	--	--	AAATCAAAGGAGGATTTCAAG	GAATTGTTCATTCCAGACCTT	55.13,55.71	142
CakTSSR02361	CakTC29890	(AGA)7	3046	3066	--	--	CTAGAAAAACCCACCTTGATT	ATTCTAATTCTGCATCAACCA	55.09,54.91	166
CakTSSR02362	CakTC29891	(ATA)6	335	352	Flower bud	--	CTTCACATCTGTTTTTCAAGG	AATTTCAGCCAGTTTCTCACT	55.03,55.69	149
CakTSSR02363	CakTC29895	(TCT)7	113	133	--	--	GCTACGCTTTTTAATCCTTTT	CCAAAGTAATCGAAGATTGAA	54.89,54.66	132
CakTSSR02364	CakTC29896	(TC)8	122	137	--	--	GCTTTTGATAGCTACCTTCCT	AAAATGTGTATCGGTTTCTGA	54.7,54.83	153

CakTSSR02365	CakTC29899	(AGA)5	4026	4040	--	--				
CakTSSR02366	CakTC29900	(TGAATC)6	2845	2880	Flower bud	SNF2	TGCGTTTTCTTTTAGTGCTTA	TGCAACTAGAGAAAGCTATGG	55.7,55.07	156
CakTSSR02367	CakTC29906	(TA)7	3996	4009	--	--				
CakTSSR02368	CakTC29912	(AAGA)6	53	76	--	--				
CakTSSR02369	CakTC29917	(AAAT)5	3803	3822	--	--				
CakTSSR02370	CakTC29919	(TAT)5	161	175	--	--	TGCGCACTATCATTTTTAATC	AGATTTGCACGTACAGCTATC	55.67,54.76	152
CakTSSR02371	CakTC29931	(CCA)6	2085	2102	--	--	GCTGTTTTACAATTTGGACAT	CCAATTTCCATCTACAACTGA	54.43,55.24	147
CakTSSR02372	CakTC29931	(GA)10	2707	2726	--	--	CGTTAATTTATGATGGATTGC	CACCGATAAACACTCTCAAAA	54.9,55.48	152
CakTSSR02373	CakTC29932	(AG)7	75	88	--	--				
CakTSSR02374	CakTC29942	(TTG)6	2131	2148	--	--	TAATGGACTTGGACTGAAAAA	TTGAAACCTCACAAAGAGAAA	54.97,55.16	158
CakTSSR02375	CakTC29945	(GCG)5	168	182	--	--	ACTTCGGGTAACGTGATTACT	TTCTCGTGTTGTTTTCAAAGT	55.45,55.1	160
CakTSSR02376	CakTC29959	(CT)8	137	152	--	--	TCATATCCACTCTCACTCACC	AGAAAGGGGAAGAGTTATGTG	55.03,55.07	146
CakTSSR02377	CakTC29960	(GAAA)5	29	48	--	--				
CakTSSR02378	CakTC29961	(AAT)8	347	370	--	C3H	GCAACTAGAAGACGCTGATAA	TGATGATTATTTCGTTTCCTC	55.06,54.4	152
CakTSSR02379	CakTC29962	(TC)15	45	74	--	--				
CakTSSR02380	CakTC29964	(AT)10	3386	3405	--	--	CTAGTTTGTCGACACATCCTC	TTGATTGTATAGGTGCTGTGA	54.87,54.29	152
CakTSSR02381	CakTC29965	(TC)20	22	61	--	HB				
CakTSSR02382	CakTC29965	(GAA)6	1441	1458	--	HB	AGTAATGATGCAATGAAGACG	GCATCAATTAAGTTTTGTTCG	55.43,55.16	149
CakTSSR02383	CakTC29969	(TTC)8	220	243	--	--	ACACCTCTACGATTCTCATCA	ACAGAGCTATCTTCGATGTCA	54.78,55.12	135
CakTSSR02384	CakTC29974	(GA)8	5039	5054	--	--				
CakTSSR02385	CakTC29979	(AG)8	18	33	--	--				
CakTSSR02386	CakTC29981	(CT)6	374	385	--	--	AGAAGCCAATTCCTCTTCTAA	AGAAAGAGGTTCTTGCAAAGT	55.04,55.02	158
CakTSSR02387	CakTC29981	(AGA)8	2245	2268	--	--	CCCATAAAGAGAAAAGTCCTC	GCTTTACCCATTTCTCTTACC	54.74,54.78	147
CakTSSR02388	CakTC29983	(TGA)5	293	307	--	--	ACAGTGGTAGCTTTTCAGATG	TCGTTGCTTCTAATGTCTTTT	54.63,54.48	156
CakTSSR02389	CakTC29989	(GAA)6	974	991	--	GRAS	TGAAGGGTAGAAAGAATCACA	TTCCACATTAAGCAACACTTT	54.95,55.07	149
CakTSSR02390	CakTC29998	(CA)6	48	59	--	--				
CakTSSR02391	CakTC29998	(TTA)5	2759	2773	--	--				
CakTSSR02392	CakTC30006	(AG)8	3196	3211	--	--				
CakTSSR02393	CakTC30018	(TA)6	2019	2030	--	--	TGCGATCCATAAATATTCTTC	TACCACCGTCTATTACCTACG	54.55,54.53	156
CakTSSR02394	CakTC30021	(TCT)5	1341	1355	--	--	TGGCTACATTTTCATGTTCTT	GCCATTGTTGTAGGAGACTTA	54.99,54.58	151
CakTSSR02395	CakTC30021	(TTG)6	1507	1524	--	--	CCAGCTAAGTCTCCTACAACA	AATTTCACCAACACTTCTTCA	54.81,54.85	147
CakTSSR02396	CakTC30029	(TA)10	55	74	Root	--				
CakTSSR02397	CakTC30030	(AAT)5	17	31	--	AUX/IAA				
CakTSSR02398	CakTC30031	(GA)7	290	303	Flower bud	--	ACTCAGAAGCTGATTGATTGA	GAACAGAGAATAAAGCCACAG	55.08,54.28	140
CakTSSR02399	CakTC30033	(AG)10	1096	1115	--	--	CTGTTTCTCCATTTTTCTTCA	AAAAGCAACTTCCCTTTTACT	54.68,54.24	146
CakTSSR02400	CakTC30038	(AG)10	118	137	--	--	CCCTTCTTTTATTTTCTCCAA	TGGTAGTGATTTGGTTTTTGT	55.3,54.75	153
CakTSSR02401	CakTC30039	(TTC)7	892	912	--	bHLH	AGTGATTCTCTGTGTCTCTGC	AGAATAGAAAACGGCAATGTT	54.51,55.65	155

CakTSSR02402	CakTC30043	(AAG)5	1833	1847	--	--				
CakTSSR02403	CakTC30060	(TCT)6	1260	1277	Root	--				
CakTSSR02404	CakTC30063	(TC)7	97	110	--	--				
CakTSSR02405	CakTC30069	(ATA)5	60	74	--	bHLH				
CakTSSR02406	CakTC30071	(AAG)5	70	84	--	--				
CakTSSR02407	CakTC30074	(TG)6	1025	1036	--	--	TTTTGTTAGGGTTTGTTGATG	TCACTTTCATCTCAAGTACCG	55.33,55.48	160
CakTSSR02408	CakTC30076	(TTG)6	358	375	--	--	GTGGATAAAGTGAGTGTTGGA	TCATCAAGAACAAGTCCAAGT	55.13,54.83	157
CakTSSR02409	CakTC30080	(CT)12	124	147	--	--	TCACTAGCCCCATAAATCTCT	GCTGAATCATCTTCTACACTGA	55.59,54.68	153
CakTSSR02410	CakTC30083	(AG)6	168	179	--	--	AGTTACCCCAACTAGACCAGA	CATCACTGCCCATTATTTATT	55.42,54.35	134
CakTSSR02411	CakTC30093	(CT)6	2146	2157	Root	--				
CakTSSR02412	CakTC30119	(ATC)5	182	196	Young_pod	--	AAGGAAGGGTATGAATGAATC	CTTGGAAGGAAACAAATTAGG	54.76,55.64	155
CakTSSR02413	CakTC30124	(TGG)6	1007	1024	--	C3H	GGTTGTCCTTATGGAGAAAGT	CTACCGCTACCAGTACTACCA	54.9,54.75	152
CakTSSR02414	CakTC30125	(CAC)7	323	343	--	--	GAAGGTTCATCCCAATTATGT	GGAGGTGAGGGATTATTCTT	55.51,54.73	143
CakTSSR02415	CakTC30126	(TTC)6	139	156	--	--	CCCTTCACTCTTCTCACTCTT	AGTTGATTAGGGTTGTTGCTT	55.25,55.57	149
CakTSSR02416	CakTC30128	(TAA)5	170	184	--	--	CACTACAATAGCAAGCAACCT	GTGGTTGTTGTGAAGTTGAAT	54.86,55.04	170
CakTSSR02417	CakTC30138	(TC)8	2389	2404	--	--				
CakTSSR02418	CakTC30147	(CT)12	215	238	--	HSF	CTCATTTCATTCATTCATTCG	GTGGAATGATCTTCAACCATA	55.74,54.98	150
CakTSSR02419	CakTC30152	(AAT)7	33	53	--	--				
CakTSSR02420	CakTC30152	(ATC)5	1056	1070	--	--	TGTCATTGTCATCAATATCCA	ACTTCTGGTGGATGAGAGTTC	54.7,55.8	149
CakTSSR02421	CakTC30160	(TAT)7	1398	1418	--	--	CGAGATTTTCGAGGAAGAG	CAACCTTCATTACATGGAGAC	55.15,54.64	161
CakTSSR02422	CakTC30161	(TCA)5	1141	1155	--	bHLH	TCAGGTTGTTGAAGGAATATG	AACATAACCCACTTTCTCCAT	55.24,55.18	150
CakTSSR02423	CakTC30165	(TA)6	1164	1175	--	--				
CakTSSR02424	CakTC30167	(TC)9	3523	3540	Flower bud	--	GTGTATGAAGCGTTGAGAAAG	AAAACTAGAGGGAGGGACAA	55.22,55.46	141
CakTSSR02425	CakTC30170	(GAC)6	1131	1148	--	--	CATCTCAGGAAATTCAATCAG	TGGCTTCTTTCAAATATTCAG	54.86,54.82	147
CakTSSR02426	CakTC30185	(ACCCTA)6	410	445	--	--	CCGTTACTTCAAATCCTTCTT	TGAGTAAGAGTAAACCGTTGC	55.24,54.82	152
CakTSSR02427	CakTC30187	(TA)9	1613	1630	Root	WRKY	TGATATGAATATCGGATTTGG	ACACTGCATTGTGTAGAGGAT	55.06,54.72	133
CakTSSR02428	CakTC30200	(AT)6	420	431	--	--	CACCAAGAGGTACAATATGTCA	GAGTTTTGTGCCATAGAAATG	55.21,54.99	146
CakTSSR02429	CakTC30207	(TCT)5	54	68	--	--				
CakTSSR02430	CakTC30211	(TTG)6	808	825	--	--				
CakTSSR02431	CakTC30212	(GA)6	622	633	--	--	TTGTTCTCTCAATTGTTGCTT	AAAGACAAGGCCAAATAGGTA	55.18,55.66	138
CakTSSR02432	CakTC30218	(AG)9	2931	2948	--	--				
CakTSSR02433	CakTC30227	(GTT)7	520	540	--	C2C2-Dof	CACTACCAAATTGAAACAACC	ATCAAATGGTATTGGTGAACA	54.75,55.36	152
CakTSSR02434	CakTC30239	(CATCCC)10	306	365	--	--	GTAATCACCAAAATCACCATC	GTAGAAGGGGACGTAGAAGG	54.4,55.49	172
CakTSSR02435	CakTC30244	(AAC)5	102	116	--	--	AAAAACACAACAACCTACGAA	TCCTGAATAAGGGTACAATGA	54.99,54.76	144
CakTSSR02436	CakTC30246	(TAA)19	1	57	--	C3H				
CakTSSR02437	CakTC30274	(GAG)5	1469	1483	--	--	AAGGGTTGATAGCGTTTTTAC	TTGCTCCTCTTCATTATCTTG	55.27,54.79	143
CakTSSR02438	CakTC30280	(AT)12	762	785	--	--	GGTTGCTCATGTTAGAGAAGA	CACCAATTAACTGAAGGATCA	54.67,55.24	158

CakTSSR02439	CakTC30282	(TC)10	199	218	--	--	TCTTTCTTCTACCCAACAACA	TGAAACATGTAGGGCATTTTA	55.03,55.84	159
CakTSSR02440	CakTC30282	(ATC)5	500	514	--	--	CCAAAGCTTGACTTAAAACTG	GTTGGTTGTTTCATCTTCTTG	54.58,54.85	153
CakTSSR02441	CakTC30282	(AAC)5	750	764	--	--	TGCTTTCTGAAGATGATCCTA	TAGAAGCAGAGAATGCAGAAC	55.19,55.01	155
CakTSSR02442	CakTC30286	(CTT)6	275	292	--	HSF	GAGTTTTTAACCGCTCTTCTC	CCTTCAACTTTTCTTTTTCCT	55.11,54.57	157
CakTSSR02443	CakTC30286	(GGT)5	1061	1075	--	HSF	GAGTTGGTGGGTTTTCTAAAG	ACCAAACAGTTTCAAACAATC	55.55,54.28	156
CakTSSR02444	CakTC30291	(AGT)6	157	174	--	--	CCAATTTTGAACTGTTTTGTC	CGATGATAACTTCGATTTTGA	54.88,55.49	158
CakTSSR02445	CakTC30292	(ACA)6	236	253	--	--	CACGACCTACTCCTCTAAGAA	GGTGTGGTGTGAATTACCTTA	53.88,55.02	148
CakTSSR02446	CakTC30292	(CAT)5	355	369	--	--	GTGACTTCATGAACCTCGTT	CTTTGTTGCACTTGTTTCTTT	55.09,54.87	160
CakTSSR02447	CakTC30303	(TTG)5	961	975	--	--				
CakTSSR02448	CakTC30314	(CATTCC)5	298	327	--	--	TTCTTAGTAGAATCGGGCTCT	GTAGAAGGGTAGGTGGAGAGA	55.08,55.06	157
CakTSSR02449	CakTC30318	(TAA)8	13	36	--	--				
CakTSSR02450	CakTC30327	(GTT)7	1574	1594	--	--	AAACATCAAGTGAAAGCAAAC	GGGTTCTCAACAAAAACATAA	54.61,54.41	161
CakTSSR02451	CakTC30327	(AAG)8	1849	1872	--	--	TCCTCGTCATTTTTCTAATCA	CCAAAGCTCAAGAAACAGTAA	55.05,54.96	167
CakTSSR02452	CakTC30331	(TCAC)6	38	61	--	--				
CakTSSR02453	CakTC30338	(CT)8	151	166	--	--	CCTCTCATCATCCTATCAAAA	GGTGGAAAGTCGTAATTGAA	54.38,55.22	155
CakTSSR02454	CakTC30350	(TGA)6	208	225	Flower bud	TCP	TGAAATTTGTTGCTTCCTTAG	TGAAACACCAAGAGATGATTT	54.9,54.77	140
CakTSSR02455	CakTC30357	(GAT)5	683	697	--	--	GAGCTCGTCAGACAATACATC	TCAGAATTGCTGTCAATATCA	54.94,54.33	148
CakTSSR02456	CakTC30359	(AGAA)8	1640	1671	--	TCP				
CakTSSR02457	CakTC30360	(AAT)6	5	22	--	--				
CakTSSR02458	CakTC30363	(CA)6	80	91	--	--				
CakTSSR02459	CakTC30368	(AG)11	1246	1267	--	--				
CakTSSR02460	CakTC30386	(CAA)8	326	349	Young_pod	--	CAACCGAACAATATTTACCAT	CAGCTTCATACCTTCTCAAAG	54.28,54.46	150
CakTSSR02461	CakTC30396	(TAA)11	385	417	--	--	TTGTTGTTGCTTATGGTCTTT	AATAGTTGAAGAGGGGAAAAA	55.07,54.76	150
CakTSSR02462	CakTC30400	(ATC)5	1051	1065	--	--	AAGGAGCAGGTCATAGGTAAC	TAAGAAGTCTGATCCTGATGG	55.1,54.43	143
CakTSSR02463	CakTC30407	(TA)6	1527	1538	--	--	GCTTAAATCTTAGGAGGCTGT	ATTTGTTCCTTCTGTTTCCTC	54.7,55.03	149
CakTSSR02464	CakTC30411	(CTA)5	206	220	Shoot	--	CGCTTCACAATAGAGAATATCA	ATGTCCTTTTCCTTCATTGTT	54.86,55.31	152
CakTSSR02465	CakTC30420	(CAA)6	85	102	Young_pod	--				
CakTSSR02466	CakTC30426	(TA)8	1776	1791	Root	--				
CakTSSR02467	CakTC30437	(CAA)13	81	119	--	--				
CakTSSR02468	CakTC30441	(GTG)5	1258	1272	--	WRKY	GATGATGTTGAAGGTTGGTAA	TCTAACGGTTATGGAAGAAGA	55.06,54.25	150
CakTSSR02469	CakTC30441	(GA)10	1386	1405	--	WRKY				
CakTSSR02470	CakTC30442	(TTC)10	186	215	--	--	TCACTTTCACCTTCTTTTTCA	GCATCTCCTTCTCATACTCCT	55.16,55.12	161
CakTSSR02471	CakTC30446	(AAC)5	390	404	--	--	TACTACTTCGATCTCCGATCA	ACTTCTTCATCATCGAAACCT	55.06,55.46	152
CakTSSR02472	CakTC30454	(GA)12	2303	2326	--	--	ATTGCTACCTTAACCTTACCG	GGAATTTCGAGATAGGAGAGA	55.31,55.2	171
CakTSSR02473	CakTC30455	(ACA)5	136	150	Shoot	--	TTACTTCACCAACCAAGAAAC	ACTTCTGGTCTTGCATTATGA	54.46,54.97	144
CakTSSR02474	CakTC30460	(GA)6	2439	2450	--	--				
CakTSSR02475	CakTC30466	(GGCGGT)5	1270	1299	--	--	ACGTTGAATTTCTCATTCTGA	GCTATTCTCCAATTCTTCCAC	54.93,55.54	162

CakTSSR02476	CakTC30483	(TCA)5	1848	1862	--	--				
CakTSSR02477	CakTC30484	(AAC)5	389	403	Shoot	--	GTAGGTAAGGCTTGGAGGTTA	ATCAAACCACAAATGAGTGAC	55.27,54.95	146
CakTSSR02478	CakTC30491	(TTC)13	559	597	--	--	ATTTACACACCGCTTAGCAT	ACACATCCATCCATAAAATCA	55.04,55.28	158
CakTSSR02479	CakTC30499	(TC)15	2641	2670	--	--				
CakTSSR02480	CakTC30525	(TTC)5	1486	1500	--	--	ATTTCCAGTAGTTCCAGTTCC	CTATCGGAATCAGAATCGTT	54.9,54.39	152
CakTSSR02481	CakTC30527	(CT)6	77	88	--	--				
CakTSSR02482	CakTC30542	(AAT)8	722	745	--	--	ATCACCTCTTTCTCAAATTCC	GTACCAATTATGTTCGCATTC	54.95,54.79	152
CakTSSR02483	CakTC30568	(GCC)5	1258	1272	--	--	CTTGAGAAGGAGGTAGTGGTT	GAAGACCCTTCTCAGTTGATT	55.14,55.01	148
CakTSSR02484	CakTC30570	(AAG)6	45	62	--	bZIP				
CakTSSR02485	CakTC30575	(CAT)6	126	143	Young_pod	ARR-B	CTTCTTCCTGAAAACCTAACC	TGAGAGATCTAGAAACGGAAG	54.82,53.91	138
CakTSSR02486	CakTC30581	(GTA)6	816	833	--	--				
CakTSSR02487	CakTC30587	(AT)16	271	302	--	--	CTCCCAAGCTTATTTTTCTTC	AAACTTCATACAGAGCCATCA	55.06,54.97	157
CakTSSR02488	CakTC30592	(TA)7	157	170	--	--	GGTTAAGGTTTCTGTGAGGAT	CGATTTGAATGTGAAATGAA	54.9,54.59	150
CakTSSR02489	CakTC30623	(TC)7	122	135	--	--	TATTTCATTTCCTTCCCTCTC	ATGTTGGATCACAAGTACTGC	55.05,55.16	151
CakTSSR02490	CakTC30636	(TGA)6	151	168	--	--	AACCATTATTTCCAATCCACT	TGCTCCCAATCAGATGTAATA	55.12,55.77	152
CakTSSR02491	CakTC30638	(CT)8	821	836	--	--	GTCAAATGCACATTTAAAACC	TCATTAAATGGATGGTAGGAA	54.84,54.7	154
CakTSSR02492	CakTC30643	(AG)9	1903	1920	--	--	TCATTCAGAAATGAGGTCAAG	GCAAATCCTCTTTTCTCTCTC	55.36,54.98	166
CakTSSR02493	CakTC30648	(GA)9	1924	1941	--	--				
CakTSSR02494	CakTC30664	(TGA)7	1005	1025	--	--	ACAAGAGGAAGATGACAATGA	ACGTTTAGACTTGGACGACTT	54.74,55.65	148
CakTSSR02495	CakTC30666	(TGT)5	274	288	--	--	GTTGTTGTTGGAACATCATTT	AGGCTATGGACATGAAGAAA	54.97,54.87	161
CakTSSR02496	CakTC30668	(ATC)7	486	506	--	--	GTTGAAATGATCGAGATGAAG	TAAGATCCTGAACCCGATACT	54.85,55.43	151
CakTSSR02497	CakTC30675	(AT)7	183	196	--	--	CAACCTTCATCACAAGTTTTC	AGGGATCCAGGTTAGAACTTA	54.85,54.61	158
CakTSSR02498	CakTC30694	(AAC)5	267	281	--	--	GTGAAAGAAGAAAGCAAAATG	CTGTCGAAATTGGTGTTACTC	54.45,54.89	149
CakTSSR02499	CakTC30698	(ATG)7	391	411	--	--	AAAAAGCAACAAGAAACACAG	CAACCATAGCAAAATTGAGAG	54.87,55.16	157
CakTSSR02500	CakTC30701	(TA)13	109	134	--	--	ACTTCCAACTTCCAAAAATCT	AATTTGCCACATCTTCAACTA	54.65,54.99	155
CakTSSR02501	CakTC30706	(AG)6	1531	1542	--	--				
CakTSSR02502	CakTC30707	(CTC)6	1503	1520	--	MYB	CCATGGACCTTTCTTTAAACT	TTGAATGAAGTTGTTGAAGGT	55.09,54.85	149
CakTSSR02503	CakTC30713	(AAAAAC)5	179	208	--	SRS	CTCCTCCTCCTATCTTCACAT	ATGATGGAGTTTGAGCTATGA	54.97,54.88	170
CakTSSR02504	CakTC30713	(ATA)5	794	808	--	SRS	TGGTGGTTCTAATGGAAGTAA	CAGCATCTTGATGACAAGAAC	54.84,55.9	155
CakTSSR02505	CakTC30718	(CAT)6	1044	1061	--	--	CTTCAAGTGGAAATACCAAAA	TGAAAATGATGACACTGATGA	54.59,54.91	155
CakTSSR02506	CakTC30718	(GAT)6	1469	1486	--	--	GGGTTGAAGAGTTAGCTAGAGA	ACCTCTCAATCCCAATTACTG	54.74,55.75	171
CakTSSR02507	CakTC30740	(AAT)6	1744	1761	--	--	TAATTGAGTGGAGGTTCATGT	CCTTTAAGCAGCAACAATAAA	54.64,55.1	141
CakTSSR02508	CakTC30741	(GAA)5	1684	1698	--	--	CTTTCTCTCCCTTTGAAAGAT	AGAATCCAACTGAAAAAGGTT	54.46,54.65	170
CakTSSR02509	CakTC30747	(TCATA)5	223	247	--	--	GTTGACTCCAACAAAAACAAA	GATTCATGATCCCTCTTTTTC	55.36,55.27	147
CakTSSR02510	CakTC30750	(ATT)6	1096	1113	--	RWP-RK	GTTATTCCCTCCAAAGGTAAA	ACAATCCAAACTAAATCACCA	55.03,54.67	143
CakTSSR02511	CakTC30757	(TGT)5	1178	1192	--	--	GGAGAGAAGAGACAGTGGAAT	ACCTCGTACAAACAATTTCCT	54.99,55.42	135
CakTSSR02512	CakTC30757	(GGA)6	1307	1324	--	--				

CakTSSR02513	CakTC30765	(TGG)5	2520	2534	--	--	TTCATCCTTATCTGTTGTTGG	TTCATGGTGCCACTAATACTT	55.24,54.86	141
CakTSSR02514	CakTC30779	(GA)10	131	150	--	VARL	TGTCTTCTGAAGCAGTTGGT	AGGGTCAGAGAAAGTTAGGAA	55.98,54.79	150
CakTSSR02515	CakTC30781	(CAA)5	184	198	--	--	CACTCAGTACGGAAAAACTTG	GGAGGATTTATCAACAACCTT	55.15,54.84	146
CakTSSR02516	CakTC30784	(AATAG)5	3922	3946	--	--				
CakTSSR02517	CakTC30793	(GGC)5	134	148	--	--	ATGCGACTAGGAAAAAGAAAT	TAAAACGGTTGTGTTTAAGGA	54.93,55.1	147
CakTSSR02518	CakTC30793	(AG)6	1428	1439	--	--	GTTGATAAGGACAATGTGTGG	AACAACCTTAACTTCCGTCTC	55.43,55.14	162
CakTSSR02519	CakTC30814	(AG)6	1437	1448	--	--				
CakTSSR02520	CakTC30820	(AGT)5	118	132	--	AP2-EREBP	TACAATTCACCCTATGCAACT	GCCTAAGATTATTGGAGCTTT	54.86,54.65	152
CakTSSR02521	CakTC30832	(GAA)5	189	203	--	ABI3VP1	TCTTCATGAGGTACCTTGAAA	TGCAAAAAGACTAAGGTTGTT	54.95,54.41	144
CakTSSR02522	CakTC30849	(TA)6	227	238	Root	HB	ACTCAATCGAAAGGGTTACTT	TATGTATGACCCCTGAAATTG	54.68,55.05	147
CakTSSR02523	CakTC30859	(GTT)5	2055	2069	--	--	TTGCTACGATGAGTTCGTAAT	CTTAAACTCACCAAACCCAAT	55.18,55.82	165
CakTSSR02524	CakTC30862	(GT)7	507	520	--	--				
CakTSSR02525	CakTC30869	(TCA)5	456	470	--	--	TCTGATTATTTGATCCTTCCA	TGATTCAGATTGGTCTATTGG	54.8,55.16	146
CakTSSR02526	CakTC30869	(TAT)5	1095	1109	--	--	ACCAATTGATAAAAGCCAAA	TTCTTCAAGTTGCTTAATTGG	54.92,54.9	157
CakTSSR02527	CakTC30869	(TC)8	1676	1691	--	--				
CakTSSR02528	CakTC30874	(ACTC)6	134	157	--	--	AAGAGATCCAATCACCTTTTC	TAGTGTTAAGGTGGTGAAGGA	54.95,54.9	144
CakTSSR02529	CakTC30875	(TTC)5	2358	2372	Root	--	AACAGAGGGAAGTTTAGCTGT	TTATCCAAAGCTAAAGCAAGA	54.9,54.76	150
CakTSSR02530	CakTC30882	(GCA)5	613	627	--	Trihelix	TCTTCTTCGTGTTCATGTTCT	ATAATGGTTACTTTCGGAGGA	55,55.44	151
CakTSSR02531	CakTC30883	(TC)9	227	244	--	--	GTGCATTTTTCAGTTCCATTA	ACGATAATGCTGAGAAGATGA	55.4,55.05	146
CakTSSR02532	CakTC30885	(ATA)7	822	842	--	--	ACTGGTTTACTGAGGGTTTTC	GAAACCAGGCAATTGTAGTAA	54.98,54.61	175
CakTSSR02533	CakTC30904	(TA)9	1499	1516	Shoot	--	TCACACTCTCTCGTTCTCATT	GGGAAATGTATATACGGAAAAA	54.98,55.03	152
CakTSSR02534	CakTC30906	(CAA)6	151	168	--	GRAS	CATTAATGGCTACTTGTGCTT	GAAGCATGTTGTTGTGTTGTA	54.8,54.69	150
CakTSSR02535	CakTC30906	(AAC)8	1235	1258	--	GRAS	ACACACTTCAAGAAACTTCCA	CTAAATCAGCAGCAGTAGGTG	54.92,55.42	150
CakTSSR02536	CakTC30909	(ATG)5	814	828	--	--	GTTTCATTGATCTTGTGGAAG	TGTAGCATTAACTTGTGCTGA	54.77,54.72	149
CakTSSR02537	CakTC30914	(GTA)7	687	707	--	HB	CCATGATAAGAAGACAACCTG	CACAAGGGTTTATGATAGTGC	54.82,54.86	150
CakTSSR02538	CakTC30929	(TTG)6	94	111	--	G2-like				
CakTSSR02539	CakTC30929	(GAA)8	227	250	--	G2-like	AACATCATCATCAAGTTGGAG	TGAAGAAGAAGCACTTAATGG	55.05,54.88	154
CakTSSR02540	CakTC30929	(CTT)5	582	596	--	G2-like	CATCAAAAGGCTTCTCCTAAT	CTCTCATGCTACGTTTTGAAG	55.32,55.39	157
CakTSSR02541	CakTC30943	(CAA)5	69	83	--	--				
CakTSSR02542	CakTC30945	(TG)9	514	531	--	--	TACGGAATTTCAGGCATAATA	GGGTTCGGGATATTTTATTTA	55.07,54.95	138
CakTSSR02543	CakTC30946	(TAT)8	119	142	--	--	CCACCATTTTCATTTTCATT	GGGAAGTTACGGTTAGATTGT	54.99,54.96	164
CakTSSR02544	CakTC30953	(AGA)6	669	686	--	C2C2-Dof	ATTGGGAGCTATTTCAATGTT	GTTCCCACAAAGACATAACAA	55.43,55.15	151
CakTSSR02545	CakTC30957	(AT)7	703	716	--	NAC	GGCTGAATATGGCTTTACTCT	TGTAAATCCAAAGTTTTGCTG	55.34,55.64	167
CakTSSR02546	CakTC30966	(CAA)7	577	597	--	--	CAACAAAATAGATGTTGATGGA	TGGTAGTTCCTTTTCCTCTTC	55.26,55.2	150
CakTSSR02547	CakTC30970	(CAT)5	129	143	--	--	ACCTGTAACAAAGCAATCTCA	CAAGTGCTAGGACAGTTCATT	55.05,54.63	153
CakTSSR02548	CakTC30970	(AG)6	2555	2566	--	--				
CakTSSR02549	CakTC30972	(TCA)5	317	331	--	--	ATAACTGGTTGGGTTTTTCTC	GTGTAGGAACAGTGTTGGAAG	54.92,54.78	150

CakTSSR02550	CakTC30981	(TC)9	27	44	--	--				
CakTSSR02551	CakTC30998	(TA)8	1084	1099	--	MYB-related				
CakTSSR02552	CakTC31004	(ATTCAT)5	1545	1574	--	--	CATATTCTGCATTGGATAAGG	AGCTCAAATGAATTTGTCTCA	54.9,55.1	142
CakTSSR02553	CakTC31007	(ATAA)5	466	485	--	--	CTCTATGAAGCCAATGAGATG	CATTTTCTCTCCACACTCAAC	55.06,54.83	143
CakTSSR02554	CakTC31014	(AAC)5	698	712	--	--	GGTTGTTTCGACATGTTTTTA	CTTGAACAACAAGATCACCAT	55.32,55.13	150
CakTSSR02555	CakTC31022	(GTT)5	1626	1640	--	Trihelix	GTTTCCTCTGAAGATGTTGAA	CATCAATTAACCTCACAAACC	54.47,54.67	154
CakTSSR02556	CakTC31022	(TC)7	2380	2393	--	Trihelix	TTTCATCATCTCCTGAATTTG	AACAAGGGAGAGAAAGAGAGA	55.29,54.89	190
CakTSSR02557	CakTC31029	(TC)6	127	138	--	--	CAATTCAATCATGCTCACAC	AAATTACAACGAAGATGATGG	54.92,54.36	143
CakTSSR02558	CakTC31034	(CT)6	43	54	--	--				
CakTSSR02559	CakTC31034	(CAC)6	333	350	--	--	AAGAAGAATTCTCCATCAACC	GTATTCGTGGAAGAAAGGAAT	54.95,55	124
CakTSSR02560	CakTC31034	(AAC)7	626	646	--	--	GTAACGTTAGGAATGTTGTCG	AGCAGTTGAATTTGAATCTTG	54.95,54.7	153
CakTSSR02561	CakTC31047	(TCC)7	741	761	Shoot	--	GAAAAGGCTTCATCATCTACA	AAGAGAATTCTTGATCCCAAC	54.62,54.95	146
CakTSSR02562	CakTC31048	(TC)19	1957	1994	--	--	AGCTGCTACCTTCTCCATAGT	TCTCACTCACACTCACACTCA	55.01,55.13	150
CakTSSR02563	CakTC31053	(TA)8	194	209	Shoot	--	AACAATATATGCAAGGGAACA	TTGGAGTCCAGAAGATAAAGA	54.81,54.17	150
CakTSSR02564	CakTC31053	(GA)6	1475	1486	Shoot	--				
CakTSSR02565	CakTC31066	(CTA)6	249	266	--	--	AACAGCACAAGATACTCCAAA	GTGTTGGTTTTCCCATATTTA	55.05,54.24	149
CakTSSR02566	CakTC31067	(ATA)5	13	27	--	--				
CakTSSR02567	CakTC31075	(TAC)5	518	532	--	--	TAGTTAAACCATTGAGCAGGA	ATATCAAGGAAGGGAAATTCA	55.16,55.33	150
CakTSSR02568	CakTC31081	(GTA)5	1302	1316	--	GRF	GGCGGAATAATTGAAGTAGTAG	GTTGTCGATCAAAAGTATTGC	55.01,54.98	158
CakTSSR02569	CakTC31081	(ATC)5	1967	1981	--	GRF	CTAAGCATTTGTTGTTGGTGT	GTTTCAAACTTGCAAAAACTC	55.42,54.36	154
CakTSSR02570	CakTC31081	(GA)8	2309	2324	--	GRF				
CakTSSR02571	CakTC31093	(TTC)5	136	150	--	--	GGACGAAACATTCTCTAACAA	ATAACCGATGAGGAGAAGAAG	54.54,55.15	158
CakTSSR02572	CakTC31101	(GTA)6	378	395	Flower bud	--	CAGAACCACATATGGAACACT	ATGATCCAACAATGAATACCA	55,55.28	117
CakTSSR02573	CakTC31103	(ACA)6	201	218	--	Trihelix	TCTAAAGTTTCTTCCTTTTTCG	GTGAGGATTTTGTTGAATTTG	54.91,54.79	158
CakTSSR02574	CakTC31103	(AAC)6	389	406	--	Trihelix	CTGACAACAACACACATCAAC	CCTTACCATTGTATCTGTCCA	54.82,55.11	150
CakTSSR02575	CakTC31107	(AAT)8	1369	1392	--	--	AAGGAAAAAGGGGTAACAGTA	GTTGGGTTTGAGAGTCTGAGT	54.72,55.87	163
CakTSSR02576	CakTC31109	(GT)12	2140	2163	--	--	GGGTGTTTTGTTTTGTTGTT	TCTTTCTCTCCTCATTCATCA	55.04,54.97	166
CakTSSR02577	CakTC31122	(AG)8	98	113	--	--				
CakTSSR02578	CakTC31123	(CT)13	12	37	--	--				
CakTSSR02579	CakTC31126	(CT)6	4	15	Flower bud	--				
CakTSSR02580	CakTC31135	(AAC)5	658	672	--	MYB	CAATTGCAAGTCAAGAAAAAG	GGATTCAATCTCGTTGACTAA	55.36,54.45	155
CakTSSR02581	CakTC31138	(TCA)5	16	30	--	CCAAT				
CakTSSR02582	CakTC31139	(CAA)6	425	442	--	SRS	AACAGAAGACAACAACAAGGA	GTGGATTAAATTCGTTGGACT	54.92,55.74	138
CakTSSR02583	CakTC31139	(TGG)6	1174	1191	--	SRS	ATCCTTCACACGATGTTTATG	TACCTTGTTGAACTTGGAAAA	55.1,55.05	153
CakTSSR02584	CakTC31142	(CAT)6	146	163	--	--	AGGACAAAGTGTCACACAAGT	ATGAATTTGCTAACCCTCTTT	54.66,54.78	149
CakTSSR02585	CakTC31145	(CT)6	4	15	--	HSF				
CakTSSR02586	CakTC31145	(TTCT)5	1203	1222	--	HSF				

CakTSSR02587	CakTC31146	(TCA)5	286	300	--	--	CTGGTACTCAGAAAAGGAAAA	TGTATTGATGATGTCATGGTG	53.89,55.09	155
CakTSSR02588	CakTC31147	(TGA)7	367	387	--	--	CCTCCTTTGTAATGGTCTTCT	TGGCTGATCAACATCAAGTA	55.07,55.11	151
CakTSSR02589	CakTC31154	(TG)6	331	342	--	--	CCTCTGTTCTCTGTTTTGTTG	GTCAAGATAGGGGATGAAAAT	55.1,54.76	145
CakTSSR02590	CakTC31156	(CAC)6	106	123	--	mTERF	CCACAACATCATCTTCAATTC	ACGAAAGAGTTTGGTTCAGAT	55.48,55.54	150
CakTSSR02591	CakTC31158	(CT)8	1	16	--	--				
CakTSSR02592	CakTC31159	(AT)6	408	419	--	--	TTTTAACCCAGTTGCTTACAC	TAAATGTAAAACGCACCTGTC	54.69,55.51	143
CakTSSR02593	CakTC31164	(TC)7	2249	2262	--	--				
CakTSSR02594	CakTC31167	(TA)10	430	449	--	--	TACATGAAGCAACATGCTAAA	TGAATGTGGTTAAGGTATTGG	54.66,55.13	159
CakTSSR02595	CakTC31179	(TGG)6	1257	1274	--	--	AAGTGGCAAATAAACTGGAG	ACCAGTGTACTCTCCACCAC	54.57,54.84	151
CakTSSR02596	CakTC31180	(AAATTG)5	70	99	--	--				
CakTSSR02597	CakTC31204	(ATT)5	2348	2362	--	--	TCACCAGAAGAATCAAAAGAA	GTGGTAAAGGGTTCAGAGATT	55.08,54.9	167
CakTSSR02598	CakTC31216	(GAA)7	2425	2445	--	HB				
CakTSSR02599	CakTC31222	(AT)11	1183	1204	--	--				
CakTSSR02600	CakTC31233	(CAG)5	1018	1032	--	--				
CakTSSR02601	CakTC31238	(CTC)6	361	378	--	--	TTCCTCCAATTCACTCACTAA	TCTTCAACGTCCTTAACTGAA	54.95,55.19	141
CakTSSR02602	CakTC31260	(TC)9	3681	3698	--	--				
CakTSSR02603	CakTC31281	(TCACCT)5	220	249	--	--	TGATTGTTAAGTCTCCCACAT	CAACAAAGAGCTTGTTAGAGG	54.64,54.55	150
CakTSSR02604	CakTC31290	(ATT)5	774	788	--	--	AGAGAAGAGAGTGCATGATGA	TAATAATCCCCTTTCCATGTT	55.06,55.22	147
CakTSSR02605	CakTC31292	(TAAT)5	348	367	--	--	AAGCGCTGTATTGTATGAATC	TGAGGAACCAATTAGATCAAG	54.7,54.47	151
CakTSSR02606	CakTC31295	(GA)8	15	30	--	--				
CakTSSR02607	CakTC31296	(CCA)5	378	392	--	--	TACCAATCCCGTATAACAGAA	TCTTTGGGAGCTTTTAAACTT	54.82,55.14	162
CakTSSR02608	CakTC31301	(GAC)5	859	873	--	--	CAGTACATCCCAAAGACCATA	TTGGTTGTGCTAGAAGGATTA	55.11,55.16	165
CakTSSR02609	CakTC31305	(TCT)7	64	84	--	--				
CakTSSR02610	CakTC31313	(CCA)5	290	304	--	--	CAATTCATGTACCTCGTCATC	TAGTTGAGCTATGTGGTCAGG	55.52,55.51	145
CakTSSR02611	CakTC31333	(ATC)5	85	99	Young_pod	--				
CakTSSR02612	CakTC31338	(AT)12	97	120	--	LOB				
CakTSSR02613	CakTC31353	(GAG)7	893	913	--	--				
CakTSSR02614	CakTC31370	(TCT)10	3521	3550	--	--				
CakTSSR02615	CakTC31375	(GA)15	95	124	--	--				
CakTSSR02616	CakTC31378	(GAA)5	1331	1345	--	--	CCTAATACCCCAAGAAACAGT	ATGTCCCACTTTATCAAACAA	54.97,54.67	159
CakTSSR02617	CakTC31385	(TC)8	252	267	--	--	GCTGTGATTGTAACGAAAAAC	CAAAGTTGATCCATTTCCTCT	55.06,55.88	159
CakTSSR02618	CakTC31394	(GA)10	1305	1324	--	--				
CakTSSR02619	CakTC31396	(TTAT)8	2828	2859	--	--	ATTGGTGAATAGTGCCAATAG	CATTACCTGTGCAGATTTCTC	54.4,54.97	159
CakTSSR02620	CakTC31401	(ACA)6	193	210	--	--	GTATGCGTCCAGAAATATTCA	AGAGTAACACCAGCCATTGTA	55.35,54.93	148
CakTSSR02621	CakTC31416	(GAG)5	620	634	--	--	GTTGAATAACGAGAAGGGTTC	CCTCTTCCACTACTCCTTCAT	55.46,55.06	151
CakTSSR02622	CakTC31433	(GT)12	3927	3950	--	--				
CakTSSR02623	CakTC31438	(AG)7	18	31	--	--				

CakTSSR02624	CakTC31443	(AAC)5	209	223	Root	--	AGCCTTCATCTAAAATCAACC	GTTGTCAGAACAACCAAACAG	55.15,55.72	164
CakTSSR02625	CakTC31447	(TAA)5	681	695	Root	--				
CakTSSR02626	CakTC31449	(AAC)5	137	151	--	--	AAACTTCAATCACAATCTTGC	TTTTGCTTCTTTGGAGAGTAA	54.53,54.62	140
CakTSSR02627	CakTC31455	(TTG)5	1230	1244	Flower bud	--	ATGCTTGAATGAGTCTACGAA	CAGAGAAAACATCGAAAAATG	55.13,55.13	152
CakTSSR02628	CakTC31457	(TTC)5	909	923	--	Coactivatorp15	ACAACTTACTGTCGTTGGAAA	ATTTCAAATCCTTCGTGAAAC	54.97,55.86	147
CakTSSR02629	CakTC31465	(ATG)5	738	752	--	--				
CakTSSR02630	CakTC31466	(AC)8	1	16	Young_pod	--				
CakTSSR02631	CakTC31467	(GAC)5	824	838	Young_pod	--	CACCAAGTGAGTCACCTAAAG	GTTTCAATCATCATCCTCTGA	54.97,55.09	149
CakTSSR02632	CakTC31481	(GAG)6	717	734	--	C3H	GGAAGCAGAAACAGTAGAGGT	TGTTCTTCCTCCTCTTTAACC	55.29,55.2	165
CakTSSR02633	CakTC31486	(AT)7	2904	2917	--	TPR				
CakTSSR02634	CakTC31487	(AG)10	1687	1706	--	CCAAT				
CakTSSR02635	CakTC31488	(TA)13	152	177	--	--	TCCCCACCTAGCTTATACTTT	CGGATATAACTTTCGGGTAAT	54.83,55.03	161
CakTSSR02636	CakTC31492	(AG)6	1439	1450	--	--	CTAGCAGAAGATGAGCATGAG	TTCAAATTCAGAGACACTGCT	55.47,55.17	151
CakTSSR02637	CakTC31492	(GATA)7	1607	1634	--	--	AGTTAGTGGTGTGCTGAAGAA	ACATTGTAGGGGTAGGTTAGC	55.12,54.99	142
CakTSSR02638	CakTC31497	(ATT)6	1154	1171	--	MYB	GCGTTTCCTATTTTAATTGTG	TTCTCGTACAATATTCCCAAA	54.35,54.94	147
CakTSSR02639	CakTC31505	(GTT)5	1185	1199	--	--	CCAATTTGATATCATCAAGGA	TAACGATAACTTCACCCTCAA	55.09,55	140
CakTSSR02640	CakTC31512	(GGC)5	637	651	--	--	CAATTAGCGAGAGTGCAATAC	CACTGAACCAAGATCAACATC	55.34,55.56	150
CakTSSR02641	CakTC31537	(TGG)5	742	756	--	--	GATATGGTAGTGGTGGAGGAG	CATTCTAAGCTCAGGGGTAAT	56.05,55.2	177
CakTSSR02642	CakTC31541	(CAA)5	463	477	--	--	GAACCTTCAACAAACAAAAGA	TTTCATTGAGGTAAAGGGATT	54.59,55.4	148
CakTSSR02643	CakTC31550	(TTG)6	700	717	--	HB	GAAATGCAACAGTCAAAAGAG	TGAATGTGGTTCTAAATGGAG	55.18,55.24	150
CakTSSR02644	CakTC31550	(GAA)9	3311	3337	--	HB				
CakTSSR02645	CakTC31551	(GAG)5	1822	1836	Flower bud	--				
CakTSSR02646	CakTC31553	(GTT)5	1136	1150	--	--	ATTAGCTGCATAAAATGCTGT	TGGTCTTCCACATTTAGGATA	54.49,54.76	150
CakTSSR02647	CakTC31554	(TCA)6	269	286	--	--	CATGCCATACTTCTTTGTTTC	CATCAACAACAAGACAAGACA	54.99,54.63	156
CakTSSR02648	CakTC31555	(CAC)5	425	439	--	--	ACCCCTAATGCTACTAACACC	TTGGAAGAAGATGATTTGTGT	54.99,54.77	152
CakTSSR02649	CakTC31555	(CCA)7	1831	1851	--	--	ACATGGTGTTGTTTCTCAAAG	GAGAAGGTACTTCCCATTGTT	55.22,54.9	161
CakTSSR02650	CakTC31555	(CTA)5	2816	2830	--	--	GATGGGAAAACAACTCTTCTT	ACTACTTGAAGGCCAAGTCTC	55.03,55.29	164
CakTSSR02651	CakTC31557	(CA)6	1342	1353	--	--				
CakTSSR02652	CakTC31567	(ATA)5	57	71	--	--				
CakTSSR02653	CakTC31574	(AGA)5	3103	3117	--	--	CAAATTTGGGATCACACTAAG	GGAGACATGAAAACCAACTCT	54.84,55.79	147
CakTSSR02654	CakTC31575	(AGA)6	12	29	--	--				
CakTSSR02655	CakTC31576	(AAG)6	1455	1472	--	--	TTGTCAGAAGTTGATGGTTCT	GAATCAAAATCTAGCAGCTCA	54.83,54.94	149
CakTSSR02656	CakTC31594	(CT)33	14	79	--	--				
CakTSSR02657	CakTC31596	(TTC)5	4098	4112	--	--				
CakTSSR02658	CakTC31612	(GA)20	2035	2074	--	--				
CakTSSR02659	CakTC31615	(TC)8	24	39	Young_pod	--				
CakTSSR02660	CakTC31620	(CAT)7	636	656	--	--	TCAACACTCAAATTGATTTCC	GATACACTTTCCCTGACCTTC	55.19,55.3	147

CakTSSR02661	CakTC31637	(AAAT)5	756	775	Flower bud	--	AATTGCATAAGACTCCACAGA	TTAAACTTCTTTAGGCCGATT	54.97,55.11	155
CakTSSR02662	CakTC31638	(TAT)5	612	626	--	--	TACACCTCATCAATCATAGGC	GAACTATACTACGCAGCAAGC	55.18,54.7	149
CakTSSR02663	CakTC31643	(AG)8	269	284	--	--	CTTTGTGTTGGAAATCACTGT	TCAACTCCTTCACTTCAAAAA	55.22,55.16	158
CakTSSR02664	CakTC31643	(TCT)6	481	498	--	--	CTATCAATGATTTTGGTGTGG	GCAATGAAAAAGACACAAAAG	55.53,55.2	164
CakTSSR02665	CakTC31653	(GA)6	1296	1307	--	--				
CakTSSR02666	CakTC31655	(TC)6	12	23	--	--				
CakTSSR02667	CakTC31656	(CTT)5	64	78	--	--				
CakTSSR02668	CakTC31668	(TTA)5	2457	2471	--	--	GGGATTATCAGTTAAGCCTTT	AAAACTTACAGGACTCGGAAT	54.35,54.68	139
CakTSSR02669	CakTC31679	(TA)7	1267	1280	--	--	CTTCACAATTTTAACCAACGA	GCCAAGAAGAAAAGAAAAAGA	55.49,55.63	151
CakTSSR02670	CakTC31682	(TTA)6	165	182	--	ARR-B	CTTTTCCCCATTATTTTCCT	GAGTGTGTTTGTTTGAGAAGC	54.94,55.07	143
CakTSSR02671	CakTC31683	(CT)8	2168	2183	Flower bud	--	CAACATTGCAGAATTAAGGTT	AAACAGATAGCCATCACCATA	54.61,54.78	148
CakTSSR02672	CakTC31686	(AT)6	7	18	--	--				
CakTSSR02673	CakTC31693	(AC)13	1328	1353	--	--	TGTTACGTTTCAAGGAATGTT	GCCACCCATAAATTGTTATCT	54.91,55.7	145
CakTSSR02674	CakTC31696	(ACC)5	377	391	--	--	ACACAATTCATTGAAGCAGTT	TGGAAGTGAATGAGTATAGGC	54.88,54.49	140
CakTSSR02675	CakTC31696	(TA)7	1296	1309	--	--				
CakTSSR02676	CakTC31707	(AT)6	2631	2642	--	bHLH	TAATGGATGCTCCTTTCTTTT	CTATGGAATTTCTCATGGTTG	55.7,54.76	173
CakTSSR02677	CakTC31709	(TTC)8	1143	1166	--	--				
CakTSSR02678	CakTC31718	(CCAACG)6	111	146	--	--	TCTTCATCTCCATTCAAACAC	GTTGAGTAAGAGGAGCCAATC	55.18,55.62	153
CakTSSR02679	CakTC31719	(AAC)5	317	331	--	--	TCATCCTCAAATTATTCCTCA	GTAACCGGGTCTGATTTTTAG	54.8,55.51	151
CakTSSR02680	CakTC31719	(TC)13	967	992	--	--				
CakTSSR02681	CakTC31728	(GGT)5	856	870	--	--	TAAGGGAGGTTATGGTCTAGG	ACCCTCTCTCTCACAGTCTTC	55.06,55.06	147
CakTSSR02682	CakTC31736	(CCA)5	490	504	--	--	CAAATATTTTACCGACTGCAC	AGCCACATAAACATGGAGATA	55.04,54.78	150
CakTSSR02683	CakTC31739	(AGA)5	148	162	--	MYB	TTGCATCTCCTGGATTATTAG	GAGCAGGGTCAAGGTACTATT	54.61,55.1	154
CakTSSR02684	CakTC31741	(TTC)7	506	526	--	--	ATTTGAAGCTGTTCTTGTGAA	TATCCTCTAGGGAAACACCTC	55.18,54.99	152
CakTSSR02685	CakTC31742	(GAA)6	108	125	Flower bud	--	TCGGTAGTGGAAATCATTTAG	CATTTCTCTGTGGTTGATGAT	54.54,55.05	147
CakTSSR02686	CakTC31751	(TC)8	35	50	--	--				
CakTSSR02687	CakTC31756	(AAT)5	305	319	Flower bud	bZIP	ATTGCTCAAGTTGTCAATGAT	TGTATTGCAGCTTCCTAACTC	54.79,54.9	148
CakTSSR02688	CakTC31758	(TGT)6	509	526	--	AP2-EREBP	CCAATTCATCTGAGTCTTGAA	AAAATTGAAGTACCAATGCTG	55.36,54.61	148
CakTSSR02689	CakTC31774	(AT)6	1	12	Root	--				
CakTSSR02690	CakTC31789	(TGA)6	842	859	--	--	GACTTTTCACGTATGATGGAG	AATCAAAGAAGTTGCCTTTCT	54.8,54.96	181
CakTSSR02691	CakTC31793	(ATC)5	91	105	--	--				
CakTSSR02692	CakTC31795	(AG)6	97	108	--	--				
CakTSSR02693	CakTC31797	(GA)8	72	87	--	--				
CakTSSR02694	CakTC31798	(ATA)6	2161	2178	--	--	GGGAGGGAGATTTAATAATCA	ATTAATCTTGCTTGCTGCAT	54.87,55.24	207
CakTSSR02695	CakTC31803	(TCC)5	148	162	--	Trihelix	AACCTAGCTTCTCCAACATTC	TTGATAGGCTGTGATGAGATT	55.22,54.88	150
CakTSSR02696	CakTC31803	(TGA)6	666	683	--	Trihelix	ATAGGGGAAAGCGTAGTTTTA	ATAATTCTCTCCGCAAAATCT	54.99,54.85	157
CakTSSR02697	CakTC31810	(GAA)6	1654	1671	--	--				

CakTSSR02698	CakTC31812	(CAA)5	29	43	--	--				
CakTSSR02699	CakTC31828	(CT)6	14	25	--	--				
CakTSSR02700	CakTC31844	(AAG)5	152	166	--	--	TCTCTAATTGACGCAGAGAAG	CGATTTCAATTTCTGTTTGC	55.02,55.95	147
CakTSSR02701	CakTC31846	(ACA)5	648	662	--	--	CAACAAGATTGTCGAGAAAAC	GACAAATTGTTTTTGTGTTCC	55.02,54.7	146
CakTSSR02702	CakTC31846	(TGG)5	1095	1109	--	--	TATCAACAATCCTATGCCAAT	TTAGCATACCTCAATGACAGC	54.72,55.59	144
CakTSSR02703	CakTC31852	(AT)11	190	211	--	--	GCTAGGGATGCATCTAAAAGT	ACCTTTCATGGGAAAAGTTAG	55.34,55.09	132
CakTSSR02704	CakTC31860	(CTG)5	531	545	Shoot	--	TATGATAGTGATGGTGCTGGT	AGCATTCATAAACAGTTCCAA	55.55,54.99	154
CakTSSR02705	CakTC31864	(TCA)5	460	474	--	--	TGCATTGAGTCTATTTTTGGT	TTTTGTTGTGAGGAACTTGAT	54.99,54.85	143
CakTSSR02706	CakTC31866	(AG)18	2317	2352	--	--	TTGTGGATCAGAGAGAAAAAG	TTCATTCTGTGTTGATGATGA	54.65,54.91	169
CakTSSR02707	CakTC31868	(CT)6	108	119	Shoot	MYB	AAAGAAAAGGGAGAAAGAACA	AGAAGGAAGAAAGGGATAAGG	54.94,55.77	138
CakTSSR02708	CakTC31874	(TTA)5	1212	1226	--	--	AGAGTGTGGGAATTGAGATTT	AGCCTTCTTCACATAAACACA	55.3,55.05	153
CakTSSR02709	CakTC31878	(GA)11	1	22	--	--				
CakTSSR02710	CakTC31910	(TGA)6	4805	4822	--	SAP				
CakTSSR02711	CakTC31931	(TA)6	202	213	--	--	TGACTACTTTTAACATGGGACA	TTAACAGCCACAACCTCTTAC	54.94,54.66	158
CakTSSR02712	CakTC31936	(TTA)5	1183	1197	--	TCP	TAGTATCCGCAATGTCAGTGT	CGAAGAATACCACAACAATTT	55.8,54.45	151
CakTSSR02713	CakTC31936	(TGG)5	1764	1778	--	TCP	GTTGTTTCTCCCATTTTTCTT	ATATAGAACCAGAGGCAGAGC	55.04,55.33	157
CakTSSR02714	CakTC31949	(AAC)5	48	62	--	--				
CakTSSR02715	CakTC31979	(CA)7	232	245	--	--	TTAAGCTCCCAAACCAATTAT	GCTATGAAGGCCTTATCAAGT	55.59,55.34	158
CakTSSR02716	CakTC32003	(TTC)10	54	83	--	HB				
CakTSSR02717	CakTC32005	(TA)6	48	59	--	--				
CakTSSR02718	CakTC32006	(AAT)5	585	599	--	--				
CakTSSR02719	CakTC32010	(CAG)5	1224	1238	--	--	ATCAACAGCATTCTCAACAAC	CACATGTCCAATACCACCTAT	55.29,54.8	155
CakTSSR02720	CakTC32010	(AT)6	3551	3562	--	--	GTCGGGTAGTCATAGGTCTTT	TGTGCTTAATGGATAAGGTTG	54.94,55.44	193
CakTSSR02721	CakTC32014	(TGA)5	385	399	--	--	TGTCAATGATGATGTTGAAGA	TCTAACCAATGGGTGTCTAAA	54.91,54.84	160
CakTSSR02722	CakTC32021	(TC)12	15	38	--	--				
CakTSSR02723	CakTC32035	(AAT)5	250	264	Shoot	--	CATCCTCTTCTTCATCTACACC	GACCGAATAAGGAGGAGAAT	55.48,54.89	159
CakTSSR02724	CakTC32035	(GTG)5	512	526	Shoot	--	AAAGTTCCAATTCGATCAGAC	TTCAATTTCTCCATCATCAAC	55.87,55.11	152
CakTSSR02725	CakTC32035	(ATG)5	935	949	Shoot	--	CGGAAGAAATAGTGAATGTTG	TCCTCCACTAGTTCAAAAACA	55,55.03	151
CakTSSR02726	CakTC32051	(TTC)6	1627	1644	--	--				
CakTSSR02727	CakTC32066	(ATC)5	231	245	--	--	AAGACCAGGAAGAGAGAGAAA	GAAAAAGTGAGACCCAGAAAT	54.89,55.03	149
CakTSSR02728	CakTC32074	(AT)6	2232	2243	--	--	GCTGACAAAAATATTTCAACG	ATGCTTTTACCAAACCACTTA	55.16,54.24	134
CakTSSR02729	CakTC32077	(TCT)6	136	153	--	--	CGAAACGACCATCTCTACTAA	AAAATCTACCGGGAGAGTAAC	54.75,54.24	152
CakTSSR02730	CakTC32077	(CAA)5	465	479	--	--	AAGAACAAATCAGATCCCAAT	TTGAAGTGGTTACCAGGACTA	55.23,54.9	150
CakTSSR02731	CakTC32089	(GTA)5	821	835	--	--	GTTATGTGACGGAATCGTCTA	ACGAATTCTTTTTACCCACTT	55.26,54.71	150
CakTSSR02732	CakTC32095	(ATC)8	1	24	--	--				
CakTSSR02733	CakTC32101	(TA)6	1141	1152	--	--	TTAGTTTGGGTCTGTGAGAAA	GTGTTGTGAGTGAGAGAAAGG	55.03,54.89	156
CakTSSR02734	CakTC32111	(CT)6	2	13	--	--				

CakTSSR02735	CakTC32111	(AG)7	323	336	--	--	TTGGTTTTTCAATCACTCCTA	TTTGAGATAAGCACCCAAGTA	54.97,55.16	152
CakTSSR02736	CakTC32111	(AT)9	1390	1407	--	--	CTACTTCTTGCCTAACAACGA	TTTAGTCATGTATGGCCTTGT	54.99,54.86	144
CakTSSR02737	CakTC32122	(ATA)5	7	21	--	WRKY				
CakTSSR02738	CakTC32132	(AG)6	401	412	Root	--	ATTACCACTCCTTCTGGTTTT	AGGCAACCTTATTGATTGAA	54.53,54.9	162
CakTSSR02739	CakTC32132	(TCT)5	688	702	Root	--	ATGAATTACCACCTCCAACTC	GTCCTGTTATGGTTATTGCTG	55.58,54.86	156
CakTSSR02740	CakTC32139	(TCT)5	67	81	--	--				
CakTSSR02741	CakTC32139	(CCA)5	718	732	--	--	TCTCATTTTCAGCATTTTGTC	TTGAAGAATGCTGTGAGTTTT	55.52,55.18	157
CakTSSR02742	CakTC32140	(AAT)6	195	212	--	--	ATCCTAATGCATCATGTTCAG	GAAGTTGGAGAAGAAGATGGT	55.18,55.01	180
CakTSSR02743	CakTC32143	(TTA)5	21	35	Root	--				
CakTSSR02744	CakTC32145	(TTG)5	4809	4823	--	--	TGTTTGTGTTTGTGTTGTTGT	TTTTGGTTACCCTTTTTCTTC	54.94,55.22	150
CakTSSR02745	CakTC32147	(TC)11	656	677	--	--				
CakTSSR02746	CakTC32148	(AGT)5	375	389	--	MYB	GAAGATTCAATCCATCATCAA	CACCACCTTCAATATTGTCAT	55.02,54.93	150
CakTSSR02747	CakTC32148	(TAT)5	614	628	--	MYB	GGCAAATATGGTTGTTGAGTA	CAACAACAAGCACGTAATAGA	55.27,54.16	149
CakTSSR02748	CakTC32157	(TGT)5	1677	1691	--	TCP	TTGGCATACGTATTCTTCTTC	AAACAACTAACGCCGTAACTA	54.68,54.51	153
CakTSSR02749	CakTC32166	(TC)6	1573	1584	--	--	CTAGATTTTGATTGTGCCAAG	GTAACAAGCCCCAAGTTTTAG	55.16,55.73	164
CakTSSR02750	CakTC32167	(AG)6	933	944	--	--	TACCAAAGAATGATGGAAGAA	TTTGCTTCATAATCCATAACC	54.89,54.47	149
CakTSSR02751	CakTC32172	(TTC)5	314	328	--	MYB-related	GAAAATCTTCGGTGTTAGGTT	CAAGGTGAACCAGGATTAAG	55.08,54.81	144
CakTSSR02752	CakTC32176	(TAT)5	1840	1854	--	--				
CakTSSR02753	CakTC32177	(TA)6	150	161	--	--	TTTGTAAGAAGTTTCCGTTTG	TGGGTTAGGTCCACTTACTTT	54.82,55.43	231
CakTSSR02754	CakTC32181	(TCT)5	1150	1164	--	--	TTGAGTGTGTGTGAGATGTGT	TCATCACCTACCAAATCTCTG	54.79,55.23	159
CakTSSR02755	CakTC32189	(AT)6	130	141	--	--	CAGTGCTTGTTGCTTCTTACT	TGTTTGTGACAATGGAGTACA	55.04,54.95	144
CakTSSR02756	CakTC32192	(GT)6	127	138	--	--	AAACCTTTGCTTGTGATAGTG	CCGAAAGAGAAAGATGAAGAT	54.66,55.27	159
CakTSSR02757	CakTC32200	(TGG)5	931	945	--	Trihelix	GTGGACTGATAAGATGGTGAG	CAGCCATAACCTTTGATACAG	54.6,55.04	155
CakTSSR02758	CakTC32202	(TAG)7	1023	1043	--	--	AATCGTTTTTGGACTATGGAC	CACAATCCTACGGTTACAGAT	55.74,54.28	152
CakTSSR02759	CakTC32218	(CT)8	261	276	--	--	AGTTATGTGCAACTCACCAAC	GGAGGAGGAGAAAAGATAACA	55.24,55.12	153
CakTSSR02760	CakTC32219	(ATT)5	446	460	--	HB	AGAGCAACTGTTGTTGTTGTT	TTATCAACGGTGGAACTTAAA	55.03,55.02	149
CakTSSR02761	CakTC32219	(ATT)5	744	758	--	HB	CACGGAAATTTTTGTTCTAAG	GAAGAAACAACAAAGCAAAAG	54.38,54.53	165
CakTSSR02762	CakTC32223	(TTC)6	1493	1510	--	--	CTTCTGAGGTGAGACGTATTG	CACAAAACAACTTCCCTTGTA	55.05,55.4	144
CakTSSR02763	CakTC32225	(GAA)5	258	272	Flower bud	--	CAGGAGGAGTTTTTCAAGAAT	TACCTCTTCCCTCAATCTCTC	55.2,55.1	150
CakTSSR02764	CakTC32226	(GTG)6	846	863	--	--	GCAGGTTAATCAGTTGTCAAG	TTCTTCTACCACTCTCCATCA	55.05,54.92	149
CakTSSR02765	CakTC32238	(CTT)6	835	852	Root	--	CTCCACCTCTAATCGGTTATT	GTTGTTTTGTGCTTCTAAAGG	55.04,54.41	143
CakTSSR02766	CakTC32245	(CT)9	17	34	--	--				
CakTSSR02767	CakTC32248	(TG)6	873	884	--	--				
CakTSSR02768	CakTC32250	(AG)9	196	213	--	--	GTCTTGTTTTGTGGTTCAAAG	ATGTCAACCCTTGAAAGAAAT	54.94,55.31	169
CakTSSR02769	CakTC32261	(CT)20	113	152	--	--	TTCATGTAAAAACCACCAATC	AAAGTGGGGTGTTGTATTGTA	55.08,54.63	155
CakTSSR02770	CakTC32264	(TGT)5	1163	1177	--	--	TAGGGTTTTGTTGAAGATTGA	GATGCAAGAACAACAACAAAG	54.97,55.97	164
CakTSSR02771	CakTC32264	(AG)12	1363	1386	--	--	CGAATCAAGAGAGAGGAAAAG	GAGTGATGACAGGAACACAAT	55.92,54.93	169

CakTSSR02772	CakTC32265	(AG)6	115	126	--	--	GTAACAAGCCCCAAGTTTTTA	TTTCAAGGGAAACACATCTC	56.11,55.17	151
CakTSSR02773	CakTC32270	(TAAT)6	1650	1673	--	--				
CakTSSR02774	CakTC32272	(AAC)6	145	162	--	WRKY	GCGTTTGATAGTTCTGTGAAT	TCCAAAGTACAGACCTTGATG	54.56,55.31	148
CakTSSR02775	CakTC32273	(AGAA)5	6	25	--	--				
CakTSSR02776	CakTC32273	(AAC)5	223	237	--	--	AAACGTTCAGAAAACCTTCTT	CAAAAACAGAGGAAAACAGAA	54.88,54.77	147
CakTSSR02777	CakTC32273	(TCT)7	3151	3171	--	--				
CakTSSR02778	CakTC32280	(TTC)6	186	203	--	--	TGGCTGTTCTTCTTCTTCTTA	TGTCACAATCTTTGAGGTTCT	54.59,54.83	152
CakTSSR02779	CakTC32283	(AACA)6	381	404	Flower bud	--	GCCTACAATTTGGAAACCTAC	AAGCTCAAGGGATCTCATTAC	55.5,55.14	146
CakTSSR02780	CakTC32284	(AAC)5	127	141	--	--	CTCTCAAAACGCTAAAATCAA	CGAAATGATGATAAAGCAAAG	55.05,55.25	145
CakTSSR02781	CakTC32294	(AG)11	1144	1165	--	C3H	GATCCAAAGAATGGAATAACC	TCTCTCATCCTCTTTCTTTCC	55.16,55.23	148
CakTSSR02782	CakTC32301	(TC)6	851	862	--	--	CCTGTACAGAGTTTCTTCCAA	CACGATTTGCTAAGGAGAATA	54.61,54.85	150
CakTSSR02783	CakTC32304	(GAA)6	520	537	--	--	TCTAGACTCATCCAAAGCAGA	TTGATGTTGTTCAAGAGGATT	55.26,54.77	140
CakTSSR02784	CakTC32307	(TTA)6	408	425	--	--	GTAGCAGCATCAGTTCATCAT	TAGAGAGAGAAAGTGGTCCAA	55.41,54.22	154
CakTSSR02785	CakTC32314	(TCT)10	46	75	--	--				
CakTSSR02786	CakTC32315	(TAA)5	1529	1543	--	--	ATTGAGGGATTTCATATTGCT	GTTCAGATAACTTGCCAAAAT	55.35,53.7	147
CakTSSR02787	CakTC32316	(GAT)6	683	700	--	--	TCTGTTTCTTGTGATTTAGGG	CCCAATCCTAATCTCTTCAAT	54.55,54.93	149
CakTSSR02788	CakTC32317	(TC)6	136	147	--	--	CAAAGTAACACTCACAGCACA	GAGAGAGAAAGAGGGAGTGAG	54.94,54.87	135
CakTSSR02789	CakTC32320	(AAG)5	816	830	--	--	AATTCTCCACCTTCATATTCC	TGGAGAAGTATCTTGAGGTGA	54.76,54.92	154
CakTSSR02790	CakTC32323	(AT)9	407	424	--	--	TATTTTAGCAGCTTCGTTGAC	ACTAACGAAAACAAGAAATGC	55.08,53.95	194
CakTSSR02791	CakTC32323	(CTT)5	1676	1690	--	--	GTTAAGGCATAACCAACAACA	ATTTGAATCAGGGGTATTTTC	55.35,54.79	146
CakTSSR02792	CakTC32330	(AAG)5	4004	4018	--	--	TTCTTCACGATTATTGTGCTT	CACTCCATAGTTCATTTCGAG	55.15,54.98	147
CakTSSR02793	CakTC32338	(AAC)5	270	284	--	--	AACTTGATCATGCATTTCACT	GTTTGCAACATTTGATATTGG	54.79,55.68	157
CakTSSR02794	CakTC32340	(CCG)6	222	239	Young_pod	--	CGTTACAAAACAACAACAACA	ATAGTCTCTCGGCATTGTTTA	54.82,54.27	151
CakTSSR02795	CakTC32348	(CCA)6	3717	3734	--	--	CACAATGTTTGAGAAGAATCC	GTACGACATGGATCAGAGGTA	54.77,55.08	150
CakTSSR02796	CakTC32354	(AT)10	2275	2294	--	--				
CakTSSR02797	CakTC32357	(AC)8	583	598	--	--				
CakTSSR02798	CakTC32366	(GT)12	4192	4215	--	--				
CakTSSR02799	CakTC32376	(TTTTA)5	26	50	--	--				
CakTSSR02800	CakTC32388	(AG)6	24	35	--	--				
CakTSSR02801	CakTC32389	(CTT)8	51	74	--	--				
CakTSSR02802	CakTC32400	(TC)11	30	51	--	--				
CakTSSR02803	CakTC32412	(GT)6	2219	2230	--	--				
CakTSSR02804	CakTC32416	(AAC)5	163	177	--	--	GTTCAGCTAAAACATCGAACA	CAAAACAACCTTAATTTGGTG	55.63,54.95	150
CakTSSR02805	CakTC32429	(CTT)6	202	219	--	--	TTTCTGCTTCACTTGATCTTC	ATTGTTGTCCACATTTCATTC	54.8,54.88	148
CakTSSR02806	CakTC32453	(TC)6	604	615	--	--				
CakTSSR02807	CakTC32456	(TGA)7	1945	1965	--	--	CAATACTGCATTCAGGAAAAC	ATCTTTGGTTCTGAGAAGGAC	54.99,55.01	148
CakTSSR02808	CakTC32460	(TTC)5	104	118	Mature Leaf	--	CCTTCCACATGCTAAATATGA	TTGATAGGAGTTTGGGGTAAT	55.36,55.28	151

CakTSSR02809	CakTC32461	(TTC)9	1313	1339	--	MYB	TGCTATTGTGATTCAGTTCCT	AGCATGACAAGTTTAAAAGCA	54.97,55.39	147
CakTSSR02810	CakTC32464	(GGT)5	14	28	Mature Leaf	--				
CakTSSR02811	CakTC32466	(GCC)5	246	260	--	--	CAAAGCCACCACCCTTAC	ATTGGAGGTGGTATAGGAAAA	56.49,55.28	154
CakTSSR02812	CakTC32488	(GGA)5	1016	1030	--	TCP	TCCAAACGAATAACCTAACAA	GTCTTCTTTTTCTTCCCATGT	55.02,55.03	153
CakTSSR02813	CakTC32488	(CTT)8	1662	1685	--	TCP	TCGACATGATAACACAAGTCA	TAAGATCAAACAAACCTCACG	55.04,55.48	160
CakTSSR02814	CakTC32494	(GA)6	13	24	--	C3H				
CakTSSR02815	CakTC32495	(CT)10	8	27	--	--				
CakTSSR02816	CakTC32496	(TTC)6	603	620	--	LOB	TCATCAATGCCTAATTCAACT	GAAGCAGAAGATGAAGACAAA	54.91,54.8	131
CakTSSR02817	CakTC32502	(AAT)5	14	28	--	--				
CakTSSR02818	CakTC32522	(TCA)5	281	295	--	--	CAGAATGCTGAAGTCAAAGTC	CAGATACCCATAACAAATCCA	55.17,55.05	176
CakTSSR02819	CakTC32533	(AC)8	187	202	--	--	TTCTTGAGTTGAAAAATGAGC	ATTAAATCAACCCCAAGATGT	54.83,55.12	167
CakTSSR02820	CakTC32535	(GAATTA)5	1793	1822	--	--	AGTAAAAACCTTTGCGAGATT	CAATCAACCACCTTATTCATC	55.01,54.58	149
CakTSSR02821	CakTC32567	(TTG)5	10	24	--	HB				
CakTSSR02822	CakTC32573	(TTC)6	140	157	--	--	GAGAGAGATTTGGGGTGATAC	CTGATCATGGATCCTACCATA	55.22,54.94	149
CakTSSR02823	CakTC32573	(ATT)7	503	523	--	--				
CakTSSR02824	CakTC32576	(ATT)16	2243	2290	--	--				
CakTSSR02825	CakTC32588	(TAT)5	575	589	--	--				
CakTSSR02826	CakTC32595	(CAC)6	1400	1417	--	--	TCCACCTTCATAAGCATACAC	ATGGAAGTTTTAATCGTAGCC	55.26,55.2	149
CakTSSR02827	CakTC32595	(ATT)15	1821	1865	--	--	TAGGGTGGATAATAGGGTTGT	TATTCTCCATACCCTCCTCAT	55.16,55.2	173
CakTSSR02828	CakTC32614	(CT)11	1	22	--	--				
CakTSSR02829	CakTC32626	(GAT)7	3684	3704	--	--	TCTCCTGTTGAAATTGATGAG	CCCAATTTCACATCATGTACT	55.36,54.93	152
CakTSSR02830	CakTC32627	(AAC)5	221	235	--	--	TACCCTTTTCGTCTTTCTTCT	AAAAGGGTGTAACGAGAATTT	54.97,54.71	152
CakTSSR02831	CakTC32630	(GA)9	541	558	--	--	TCAAAAGTGCGAATAACTAGC	TCCATCTTGTCTCTCTTTCAA	55.08,55.06	150
CakTSSR02832	CakTC32631	(AGA)6	2068	2085	--	--	CGTGTGAATGGATAGATGAGA	CCACTCACTTACTCACTCCAG	56.13,54.94	158
CakTSSR02833	CakTC32641	(GAT)5	407	421	--	--	GATATTGATGAAAGCGATGAC	ATGATCTTCCTCATCATCCTC	54.81,55.55	186
CakTSSR02834	CakTC32642	(TCA)5	1892	1906	--	--	TTATTTTCACTACCACCCAAA	GTTGTGGAATTTTTATGCAAG	54.87,55.01	145
CakTSSR02835	CakTC32666	(TGA)7	238	258	Young_pod	--	CTCGTTTAGAGGAACAAACAA	ATCTTTCTCAAGCTGTTCCTT	54.8,54.94	147
CakTSSR02836	CakTC32670	(CAT)6	797	814	--	--	ATCAGCTTCAACATTCAAATC	TTCTAGCAGTACTTCCAAACG	54.44,54.99	148
CakTSSR02837	CakTC32671	(TTC)8	2057	2080	Root	--	ACGTCCTTCTTTTACACATCA	TGATGAAGGAAGGTAGATTTG	54.89,54.47	152
CakTSSR02838	CakTC32671	(AT)13	2214	2239	Root	--	ATCAGATCCTCGACTACCTTC	GATCTGAACACCTTCCCTATT	54.96,54.82	143
CakTSSR02839	CakTC32675	(TC)12	616	639	--	--				
CakTSSR02840	CakTC32681	(TAC)6	2362	2379	Shoot	--	TTTGAAGCTACGAAGAAGATG	TTCTTCACAAAGCAACAATG	55.04,54.91	148
CakTSSR02841	CakTC32682	(CT)6	1	12	--	TRAF				
CakTSSR02842	CakTC32682	(CAT)5	303	317	--	TRAF	CAACCATCTTTTGAGTTCATC	TGGTTGAGGAAGTTATGTTGT	54.77,54.72	150
CakTSSR02843	CakTC32694	(CT)7	1391	1404	--	--				
CakTSSR02844	CakTC32695	(AGA)5	846	860	--	HB	CATTTGTTGATGGTATTGCTT	AATAGTGTTGCTGATGCTGAT	55.27,54.99	145
CakTSSR02845	CakTC32701	(TGATTT)6	1862	1897	--	--	TGATTCTGATTGTGATTGTGA	AAAGGGTCAGAGGTAGAACAC	54.91,54.96	152

CakTSSR02846	CakTC32718	(ATT)6	350	367	--	--	CCATAAACATCGTAAGCTTTG	CTAGCTGTTCAAGTTGGATTG	55.21,55.23	159
CakTSSR02847	CakTC32729	(AAT)9	2045	2071	--	--	AATAAGCAGTGTCAGATCCAA	ACTGTCAACTTCACTTTCCAA	54.97,54.92	143
CakTSSR02848	CakTC32749	(TC)7	2	15	--	--				
CakTSSR02849	CakTC32749	(AAC)5	613	627	--	--	ACAATAACCAGAACAACATGC	TTTTGTCCTTGTTGATGATTC	55.17,55.19	142
CakTSSR02850	CakTC32749	(GGA)7	1522	1542	--	--	CAGGTATGATGAACAATGGAT	TCATCATTGACATGTTGTTTC	54.85,54.28	171
CakTSSR02851	CakTC32759	(GAT)5	531	545	--	--				
CakTSSR02852	CakTC32763	(TTC)5	17	31	--	--				
CakTSSR02853	CakTC32764	(TC)11	19	40	--	--				
CakTSSR02854	CakTC32771	(GAA)5	94	108	--	--				
CakTSSR02855	CakTC32776	(AGA)6	590	607	--	--				
CakTSSR02856	CakTC32781	(GA)12	514	537	--	--	TGGTGAAGAGAGAGAAAGTGA	ATTCGAGCAAGATTCTTCATT	55.13,55.7	149
CakTSSR02857	CakTC32793	(ATC)5	112	126	--	--	TCAATATCTTTCCTTCCAACA	GGAAATTCTGATGCATCTTC	54.89,54.73	147
CakTSSR02858	CakTC32797	(CAA)5	58	72	--	TPR				
CakTSSR02859	CakTC32802	(CT)6	1589	1600	--	--	TATAAGCATTCTCCGGTTATG	GGCTACTGAAGAACAAATTCA	54.68,54.7	157
CakTSSR02860	CakTC32803	(AGC)5	1385	1399	--	--	AAGCAAGATGAAGGAACTCTT	TTATCCAGATCAGAATCAACG	54.94,55.32	158
CakTSSR02861	CakTC32816	(TTC)5	71	85	--	--				
CakTSSR02862	CakTC32818	(TTG)6	281	298	--	--	AAGTACGACGATGTTGTTGTC	ACTTCAATTTTGTTGTTGTGG	55.24,55.23	160
CakTSSR02863	CakTC32818	(AG)8	472	487	--	--				
CakTSSR02864	CakTC32830	(AAG)6	47	64	--	--				
CakTSSR02865	CakTC32830	(AAG)5	1141	1155	--	--	CACACAGACCAAGTGTCAATA	ATCATTTGGGACTACGACTCT	54.49,55.33	155
CakTSSR02866	CakTC32840	(TC)10	358	377	--	--	GGTTTGTCCTTGATTCTTTCT	TAAACTTGCATCATTTTGCTC	55.03,55.71	158
CakTSSR02867	CakTC32868	(CAA)5	1326	1340	--	--	TTGGATTTTATCCAGCTCAG	ATTGTTGGTTACTGTCTGTGG	55.47,55.08	149
CakTSSR02868	CakTC32878	(TTCTG)6	565	594	--	--				
CakTSSR02869	CakTC32879	(TA)10	411	430	--	--				
CakTSSR02870	CakTC32884	(TC)7	219	232	--	TPR	TTCTCTTCAAACATGCTTTTC	ATCGTATTGTGAAAGGGTTTT	54.83,55.35	153
CakTSSR02871	CakTC32901	(AG)9	2861	2878	--	FHA				
CakTSSR02872	CakTC32905	(TGA)6	2795	2812	--	FAR1	CTTTATCATGTTCACCAGAGG	TAATCTCTTCTTCCACCCTTT	54.82,54.74	157
CakTSSR02873	CakTC32914	(CTT)6	3906	3923	--	WI/SNF-BAF60	TCTTTATCTTCTGCAAAACCA	CAAGAGCAAGAACAAGAAGAG	55.29,54.64	149
CakTSSR02874	CakTC32937	(TC)10	223	242	--	--	TACTGTTTCTGCGATCACTTT	GAGTTTGTGCACTTTCCATAG	55.22,55.05	144
CakTSSR02875	CakTC32939	(AG)10	56	75	--	--				
CakTSSR02876	CakTC32947	(TC)8	19	34	--	--				
CakTSSR02877	CakTC32962	(TGC)5	2618	2632	--	Alfin-like	ATTTTCTTCGGTTTTGAGTCT	TCATAATTCCGAAAACGATAG	54.8,54.64	154
CakTSSR02878	CakTC32962	(GA)9	2855	2872	--	Alfin-like	GAGAGAGAGTAGGGTTTTGGA	CGTCACATTTTCTGAGCTAGT	55.18,54.8	150
CakTSSR02879	CakTC32963	(CAG)5	2901	2915	--	--	ATGTCCCAAGGACTTCTCTC	AGATCTCTGGAACTGTGGATT	55.7,55.29	150
CakTSSR02880	CakTC32991	(CTA)5	153	167	--	--	ACCCTTTTGTTTCAAGAAAGT	AATTGTTGGCTCAATAGTTGT	54.73,54.02	147
CakTSSR02881	CakTC32992	(TA)6	4113	4124	--	--	TCCACTTGAGAAGTTGGTAAA	CTACAACTTTTCCCCTGAACT	55.03,55.15	146
CakTSSR02882	CakTC33002	(CTT)8	237	260	--	HB	GATGATTTCAACATGGGAATA	TCTTGGGACATAGAATTTGAA	54.92,54.89	149

CakTSSR02883	CakTC33002	(AGG)6	1612	1629	--	HB	GCACAGGTGAAGCATAGTAGT	TTGATTAAAAGCTCTCTGCTG	54.65,55.2	150
CakTSSR02884	CakTC33002	(AAC)7	2000	2020	--	HB	GAAGCCAATGGTAGAAGAAAT	ATATTTTTGTTGTTGGTGTCG	55.15,55.2	155
CakTSSR02885	CakTC33007	(AAC)5	353	367	--	--	CACTCTGAACCTAGCCCTAAT	CTTCGAATCTCGATTCATTT	55.26,54.52	148
CakTSSR02886	CakTC33013	(AG)13	2881	2906	--	--				
CakTSSR02887	CakTC33018	(CAA)6	2711	2728	--	--	CAATCCTGTCCAACTTCATTA	CTCAAAAACCATCAAAACTTG	55.24,55.05	145
CakTSSR02888	CakTC33027	(TC)7	1	14	--	--				
CakTSSR02889	CakTC33027	(GGT)5	623	637	--	--	TAGAGGAAGCTAAAGCAAAGG	GCACTTTGAAGAAAGGGTTAT	55.85,55.23	160
CakTSSR02890	CakTC33039	(ATG)6	107	124	--	--	AAGCTTATGTTTCTGCTTCTG	GAGTGAAAGGGTCGTTGTAGT	54.24,55.9	131
CakTSSR02891	CakTC33039	(TCT)11	266	298	--	--	TGCTTCAATTCATCACTACAA	GTTTCAAATCTGATGAAGGAA	54.42,54.42	147
CakTSSR02892	CakTC33048	(GA)6	10	21	--	--				
CakTSSR02893	CakTC33050	(AGA)5	2778	2792	--	--	TGAGAACCATTGAGAGTACAGA	GTCCTTACCGAAAACTAGAGG	55.02,54.85	144
CakTSSR02894	CakTC33055	(AT)10	601	620	--	--				
CakTSSR02895	CakTC33060	(TCA)5	219	233	--	--	CTAATTCCTACCCAAACCAAT	GAGTGATCCAAACGATGATAG	54.91,54.72	172
CakTSSR02896	CakTC33083	(TTC)5	262	276	--	--	AGAAAGAACAGTTTGGAGCTT	GATAAATCGGAAATTGAAAGG	55.02,55.48	157
CakTSSR02897	CakTC33100	(TAA)5	357	371	Shoot	--	TGGAAAAGGAAGTTATTAAGGA	TGAATACCATCATCCACACTT	54.95,55.35	178
CakTSSR02898	CakTC33105	(TA)8	2388	2403	--	--				
CakTSSR02899	CakTC33108	(AGA)7	1773	1793	--	--	GCTCCTTTCCTTTGTGTATCT	TTCAGAGTCAAAGAGGTTCAA	55.22,55.14	155
CakTSSR02900	CakTC33120	(AG)25	1	50	--	--				
CakTSSR02901	CakTC33123	(ATG)5	401	415	--	--	TAATATTGGCTGGGTCAACTA	GGCCTAATTTTAAGCATGAAT	54.98,55.28	159
CakTSSR02902	CakTC33129	(GT)7	2330	2343	Mature Leaf	--				
CakTSSR02903	CakTC33135	(TTCT)5	904	923	--	--	TCACCTATAATCAATGCAACC	ATTAATGGGTTTGCATATTCC	55.19,55.62	147
CakTSSR02904	CakTC33144	(TCT)5	629	643	--	WRKY	TGTCTTGAACGAAACTCTTGT	GCGAAGGATAAAGATGAAGAT	55.08,55.23	147
CakTSSR02905	CakTC33153	(TTC)6	17	34	--	--				
CakTSSR02906	CakTC33153	(GCA)5	1717	1731	--	--	AGAGGATACCGTAGAGTCAGC	TTCTTTGGTTCAATTATCTCG	55.24,54.66	156
CakTSSR02907	CakTC33163	(GAA)5	6	20	--	--				
CakTSSR02908	CakTC33165	(ACT)6	436	453	--	--				
CakTSSR02909	CakTC33175	(TCC)5	1143	1157	--	--	ATGGCAGTTCATTAGAGAGTG	GGAAGTGTTGGATGATGATAA	54.54,54.98	139
CakTSSR02910	CakTC33191	(TA)6	407	418	--	--				
CakTSSR02911	CakTC33197	(ATC)5	255	269	--	--	CACTGATCCAAGTGTTTGACT	ATAGTGTAAGTGAGCCTGACG	55.2,54.67	149
CakTSSR02912	CakTC33197	(AGA)5	6203	6217	--	--				
CakTSSR02913	CakTC33198	(CT)6	719	730	--	--	GCTCCCTCTTAAGTTTACGTC	CCAAACGTAGAAAAGATGATG	54.99,55	150
CakTSSR02914	CakTC33205	(CAT)6	336	353	--	--	AGTCAGTTGGGAGCAATAGAT	TATGAAGTACGTCCGAGCTTA	55.49,55.35	157
CakTSSR02915	CakTC33207	(CCA)5	157	171	--	--	TGATTAATTGATTGGAGCAGT	GGAGAATTTAGGGATTTGGTA	54.91,54.95	133
CakTSSR02916	CakTC33213	(TCA)6	1277	1294	--	--	CATCATAGCCATCATCTTGTT	TGATGAGAGTGATGGTGATTT	55.18,55.48	148
CakTSSR02917	CakTC33214	(GCT)5	31	45	--	--				
CakTSSR02918	CakTC33220	(TCT)5	342	356	--	--	CCCTTTCTTTGTTCTTCTGAT	GACGAAGTAACAGGGAAAAAT	55.2,55.08	152
CakTSSR02919	CakTC33236	(AG)8	656	671	--	--				

CakTSSR02920	CakTC33240	(AG)10	1956	1975	--	--	GACAAAATCGGAAGAGAAAAA	AAAGAAAAGAGAATCCGTTTG	56.14,55.36	156
CakTSSR02921	CakTC33242	(TTC)5	164	178	--	--	TTACGATGATCAAGTGTGTGA	TATTTTGGGCTTTGACATTAG	55.04,54.72	144
CakTSSR02922	CakTC33246	(AGA)5	1530	1544	--	--	TGTAAAGTTGTTTGAGTTGAGC	GACTTAATAGGAGGCAAAAGG	54.88,54.94	153
CakTSSR02923	CakTC33251	(TCAAT)7	69	103	--	--				
CakTSSR02924	CakTC33253	(ACC)5	114	128	--	--	GGCTTCCAACAAATTCTCTAT	GAGTAGGACAAGGCTTAGGTG	55.15,55.74	140
CakTSSR02925	CakTC33256	(GCA)5	220	234	--	--	TCTTAGCGAGTTTAAGCAAGA	CATACTTCACTATCACCGCTTA	54.97,54.82	143
CakTSSR02926	CakTC33266	(GTG)6	2186	2203	--	--				
CakTSSR02927	CakTC33267	(AAG)5	804	818	--	--	GATGAGGACAAGGAGAAAGAT	TTCTTCCCAGTCATTACTCAA	54.92,54.95	168
CakTSSR02928	CakTC33270	(TTA)8	65	88	--	--				
CakTSSR02929	CakTC33289	(CTT)6	98	115	--	--				
CakTSSR02930	CakTC33291	(AG)13	4884	4909	--	--				
CakTSSR02931	CakTC33294	(GTGACT)5	879	908	--	--	AAGGACGTGACTGTAAATGTG	TACACAACCAATTAAGCGTTC	55.25,55.51	176
CakTSSR02932	CakTC33298	(TC)7	4	17	--	--				
CakTSSR02933	CakTC33303	(TCT)5	63	77	--	--				
CakTSSR02934	CakTC33310	(AT)7	2236	2249	--	--	AGTGTTCTCAGTCCATTGTTG	CGAAAGACCTCTGTAATCTCA	55.2,54.69	148
CakTSSR02935	CakTC33331	(TC)6	37	48	--	--				
CakTSSR02936	CakTC33339	(CTT)5	1933	1947	--	--	GCCAACTCTTATTTTCTCTCC	GTGACGTAACTTCCTTGAGC	54.88,55.01	166
CakTSSR02937	CakTC33342	(TGA)5	313	327	--	--	GGAGGATCAACTGAAGACTTT	CAATGGATATCAAAGAAGTCG	55.01,54.92	163
CakTSSR02938	CakTC33348	(CAA)5	1005	1019	--	--				
CakTSSR02939	CakTC33354	(AAT)5	656	670	--	--	TGATGGATCTGAAGGTAAGAA	TCAAGTTAGAACCATTTACACG	54.86,54.71	146
CakTSSR02940	CakTC33357	(TCA)5	322	336	--	--	CCTTGATGGGTTAAAGAAGTT	GCTCTGCAGCTATGTAAAGTG	55.09,55.57	149
CakTSSR02941	CakTC33359	(AC)6	226	237	--	--	TAGGATACACCAAATCCACAC	GTTATTGTGGTGGAAATTGAA	54.93,55.08	153
CakTSSR02942	CakTC33364	(TTC)7	2209	2229	--	--				
CakTSSR02943	CakTC33382	(ATG)5	269	283	--	--	AATTGTAAAAGGGACGAAGTT	AGCAATATTAGTGCATTGTCC	54.71,54.55	140
CakTSSR02944	CakTC33393	(AT)6	57	68	--	--				
CakTSSR02945	CakTC33404	(AAC)6	1154	1171	--	--	AACCTCTTCAACATTCCTTTC	CTACTTGTTCCTGATCCACTG	55.03,54.88	149
CakTSSR02946	CakTC33404	(TGG)5	1564	1578	--	--	GTAATTTGCAGGATTTGATGA	TCTCACTATCTGACCACCAAC	55.32,55.11	149
CakTSSR02947	CakTC33408	(AAC)5	891	905	--	--	AAATGATGTTCTGCTTCCTCT	ATCTTCATTCAACTTCCGTTT	55.62,55.46	151
CakTSSR02948	CakTC33419	(TCA)5	90	104	--	bZIP				
CakTSSR02949	CakTC33419	(TGG)6	480	497	--	bZIP	AAGGGTATCATCAACCACACT	GAACATTGTCAGAAAACATGG	55.95,55.56	150
CakTSSR02950	CakTC33420	(AG)7	154	167	--	--	AACAAAATCACCCTTTTCTTC	ACGTCGAATTAGCCTTAAAAC	55.04,55.42	141
CakTSSR02951	CakTC33422	(TTC)7	526	546	--	--	ATCCATTCCTAAGTCTCTTGC	TGATGATGAACAAGAACAACA	55.14,55	151
CakTSSR02952	CakTC33440	(AG)7	1890	1903	--	--				
CakTSSR02953	CakTC33459	(AC)6	295	306	--	--	AGTTCAAATTCTAGCCGAAAT	ACTGCTAAGCTTGTTTCATTG	54.93,54.98	144
CakTSSR02954	CakTC33477	(CT)11	97	118	--	--				
CakTSSR02955	CakTC33501	(TA)9	585	602	Root	--	CAAGGCATAGCTCCATAATAA	AAGGATCCCATAAAGCAAATA	54.83,55.52	159
CakTSSR02956	CakTC33503	(AGA)6	1880	1897	Young_pod	--				

CakTSSR02957	CakTC33510	(GGC)6	639	656	--	--	GTCACAAAAGGTGAAAAATTG	TGACACAATATCCATCATCAA	54.88,54.7	161
CakTSSR02958	CakTC33515	(ATA)7	157	177	--	--	CTAACAGATTCCCTTTCTGCT	GTTGATGGAAGAAAACACAGA	55.38,55.26	128
CakTSSR02959	CakTC33538	(AG)7	778	791	--	--				
CakTSSR02960	CakTC33544	(CAA)6	7554	7571	--	--	GGCTTTTCTCTAGCACTCAAT	AAAATGGTGTAATTCCGTACA	55.53,54.73	148
CakTSSR02961	CakTC33550	(CAT)7	471	491	Shoot	--	TTTCACATCATGGAACAAGA	CCTCTCTATTTCCTTTCGATT	54.89,54.44	151
CakTSSR02962	CakTC33559	(GAT)5	328	342	--	--	GTGTTGAAAAACCAGATCAAG	CCCGAACTGTTTATAGTGTTG	54.85,54.96	157
CakTSSR02963	CakTC33570	(CT)25	1	50	--	--				
CakTSSR02964	CakTC33572	(AG)6	666	677	--	--				
CakTSSR02965	CakTC33602	(GGT)5	96	110	Flower bud	--				
CakTSSR02966	CakTC33609	(AT)8	290	305	--	--	CTCCTAAAAGAAGCACCAAGT	CATATGTTTCCATCACACACA	55.46,55.18	139
CakTSSR02967	CakTC33616	(ATC)6	761	778	--	C2C2-Dof	CCAGTTCAAGATTTTAAGCAA	ATCCTCAAAAGGAAACAAAAC	54.9,55.04	144
CakTSSR02968	CakTC33624	(AG)7	32	45	Flower bud	--				
CakTSSR02969	CakTC33631	(CTC)5	156	170	--	--	GGTCTTCCGTTCCTAAAATAC	GGAGTTTTTGAAGGATTGTTT	54.63,55.04	166
CakTSSR02970	CakTC33640	(TA)10	2154	2173	--	TPR	CAATTCTTGGTTTTGACTGAA	CTTTCTTTCTCACTCCCTCTC	55.45,54.89	150
CakTSSR02971	CakTC33643	(CAA)5	394	408	--	SET	GGAGGTGAATATTCTTCCAAT	TTAAACCATTCTCAGTCTTCG	54.76,54.72	149
CakTSSR02972	CakTC33645	(TC)6	97	108	--	--				
CakTSSR02973	CakTC33648	(GGA)5	1109	1123	--	--	GGCTGTTCTCCTAGTTTAGGT	CCACCCTTTAGATATGAAACC	54.44,55.28	171
CakTSSR02974	CakTC33656	(CT)7	108	121	--	--	GTCTGTGAGATCATCCCAAT	CGAGCGTGGTTAGTGTTATTA	54.73,55.7	149
CakTSSR02975	CakTC33680	(CA)8	70	85	--	--				
CakTSSR02976	CakTC33687	(ACA)6	330	347	--	--	TCCACTTTCTCTTCCTCTTCT	TGCATAGTTGTTGAAATTGTG	54.89,54.81	153
CakTSSR02977	CakTC33688	(CT)10	2744	2763	Flower bud	ARF				
CakTSSR02978	CakTC33692	(AT)8	244	259	--	--	CATGATCTTCCTTGCTATCTG	CATACATGGATGGTTGAGATT	55.06,54.85	157
CakTSSR02979	CakTC33703	(TCT)5	541	555	--	SBP	CAACATGGTAACACTGATCCT	GGGTGAGGAAAAGAAAATCTA	55,55.13	165
CakTSSR02980	CakTC33708	(TA)6	232	243	Shoot	--	GCGGTGCAAATATATGAAAT	ATCATCAAGCCAAGTATGTGT	55.3,54.66	167
CakTSSR02981	CakTC33715	(TGT)7	258	278	--	--	GAATCGGGGTATTGAATAAGT	CTTAACTAACCGCTTCCTTTC	54.82,55.17	171
CakTSSR02982	CakTC33720	(GA)10	6926	6945	--	--				
CakTSSR02983	CakTC33727	(CT)8	2185	2200	--	C3H	AACGTGTGAATGAAGGAACTA	GAAGGGAGAGAAGAGAAGAGA	54.89,54.51	151
CakTSSR02984	CakTC33731	(TC)9	61	78	--	--				
CakTSSR02985	CakTC33733	(GGT)5	583	597	--	--	CGGGTAGAAATTCTTTTGTTT	TCTCTGCACATACTGTTGTTG	55.25,54.85	151
CakTSSR02986	CakTC33740	(CT)7	31	44	Flower bud	--				
CakTSSR02987	CakTC33747	(CT)8	652	667	--	--				
CakTSSR02988	CakTC33757	(AG)6	713	724	--	WI/SNF-BAF60b				
CakTSSR02989	CakTC33773	(GAA)5	863	877	--	--	CTTGACGATTTAGTGAATTGG	TGGACTGTCTTTTCTTCTTCA	55,55.14	145
CakTSSR02990	CakTC33777	(CAC)5	301	315	--	--	TATCGTCGAAAGAATCGAATA	GAGTCGATTTGGAGAACTCTT	55.17,55.17	156
CakTSSR02991	CakTC33792	(GAA)6	364	381	--	--	AACCTGGAGTTCTTGTGAAGT	TGAAATGTTGTTAGCTTCCAT	55.45,54.99	157
CakTSSR02992	CakTC33793	(AG)25	2395	2444	--	--				
CakTSSR02993	CakTC33799	(TTC)6	190	207	--	--	TCACCCTACCAACAATTACAC	CTACTCAGCGAACGAATTTAC	55.02,54.52	150

CakTSSR02994	CakTC33803	(AGA)6	348	365	--	--	CCTCCTATTCTCCACCTCTAC	TCTTTGTTCACATCAGTGTCA	54.58,55.07	159
CakTSSR02995	CakTC33806	(TCA)5	443	457	--	--	ATCTTTTTCTTGCACTTTTCC	CCTCCTTCTTTCAATCTTGTT	55.35,55.2	158
CakTSSR02996	CakTC33810	(AATTT)7	356	390	--	--	GAATGGATTGGTGATTCATTA	CCCTGATCAGAAGAGCTAAAT	54.92,55.31	152
CakTSSR02997	CakTC33820	(AT)25	2	51	--	--				
CakTSSR02998	CakTC33840	(ATT)7	68	88	--	Pseudo_ARR-B				
CakTSSR02999	CakTC33845	(TC)7	1	14	--	--				
CakTSSR03000	CakTC33850	(TCA)6	395	412	--	--	ACTTCATCAATTCACAAAAGC	AATCACCACCTTACCTCTGTT	54.53,55.25	148
CakTSSR03001	CakTC33858	(CT)10	2318	2337	--	--				
CakTSSR03002	CakTC33861	(GA)6	2176	2187	--	--	CGAGAGACGAAAACTATAGGA	CATCGAATTCATTCTCTTTGA	54,55.45	148
CakTSSR03003	CakTC33861	(TC)6	2447	2458	--	--				
CakTSSR03004	CakTC33870	(ATT)6	918	935	--	--				
CakTSSR03005	CakTC33896	(TTC)6	414	431	--	--	GTCACCGTCTTCATTTCAAC	AGTTTTCTGAATCCGAGGTT	55.54,55.45	154
CakTSSR03006	CakTC33903	(AC)7	46	59	--	--				
CakTSSR03007	CakTC33907	(TCC)6	232	249	--	--	GGTTCCATACATGAACAAAGA	CAATGACAAGAGGTGAGAAAT	55.06,54.34	152
CakTSSR03008	CakTC33922	(GA)7	126	139	--	--	CTTTCATCCATTCATCCAATA	CTCTGGTGTGAGAGAGAACAG	55.09,55.06	148
CakTSSR03009	CakTC33930	(TC)6	61	72	--	--				
CakTSSR03010	CakTC33947	(GA)8	459	474	--	--	GCAATTGTGACAGTGTTTTCT	TGATTCATAGGAGTGTGGAAG	55.37,55.23	150
CakTSSR03011	CakTC33947	(ATT)8	605	628	--	--				
CakTSSR03012	CakTC33954	(CAA)5	599	613	--	--	CGACGAGTATGATACATTGGT	CAAAGTTCAATGGACAAAGAC	55.13,54.85	148
CakTSSR03013	CakTC33964	(AGT)5	812	826	--	--				
CakTSSR03014	CakTC33967	(TTG)6	475	492	Flower bud	--	GCTTCTTTGTATTCCGATCTT	ACATGATGACAAAACAAAACC	55.31,54.97	157
CakTSSR03015	CakTC33971	(CT)6	43	54	--	--				
CakTSSR03016	CakTC33978	(GTG)5	2001	2015	--	--	ATTTTACGGATCGGAAGTTT	TCCAACTACAAATCAAACACTC	55.41,54.47	148
CakTSSR03017	CakTC33985	(AT)8	99	114	--	--				
CakTSSR03018	CakTC34002	(CTG)6	607	624	--	LOB	AGTTTCGAACATCAACAACAG	CAAAGTCAAAGAAGGAGACAA	55.38,54.74	156
CakTSSR03019	CakTC34004	(ATC)8	480	503	--	--	ATTGTCTTTCCCTTTTCAATC	GATGCTGATGATTTTGTGAGT	54.97,55.2	149
CakTSSR03020	CakTC34005	(AG)9	6	23	--	--				
CakTSSR03021	CakTC34010	(GA)7	2045	2058	--	--				
CakTSSR03022	CakTC34012	(TA)9	165	182	--	--	TAAGGGAATAACATCCATTTG	GCAGTTCATGTAATGCTTTATT	54.33,53.96	152
CakTSSR03023	CakTC34031	(CT)7	37	50	--	--				
CakTSSR03024	CakTC34032	(TGA)6	460	477	--	--	GATCTTAACCCTCATATTTGGA	TTGAACAATCATTAGCTTCAGT	54.96,54.34	154
CakTSSR03025	CakTC34041	(AGA)5	1965	1979	--	GNAT	AGATAAGGGTTTTGAGGAATG	GTAAATCCCTAATGGCTCACT	55.01,55.03	149
CakTSSR03026	CakTC34044	(TA)8	531	546	--	--				
CakTSSR03027	CakTC34061	(TC)8	37	52	--	--				
CakTSSR03028	CakTC34086	(CAA)6	295	312	--	--	AAGAGAAAGAGATGAAGCACA	TCAATTCCTCTTCTTTGTTGA	54.37,55.08	150
CakTSSR03029	CakTC34086	(CTA)6	743	760	--	--	CCACCTGTTACTATTCCTTCA	AAGGAGAATCAGAACCTTCAC	54.43,55.01	151
CakTSSR03030	CakTC34086	(GAA)6	1060	1077	--	--	AGGGTAGCATTACAAGTTGGT	TAGAGGAAAATGCCAATGATA	55.45,55.02	141

CakTSSR03031	CakTC34086	(AGA)8	1435	1458	--	--	CGTTTTTGAAATTTGCTAGG	GTCAGTGAGTTTATCGAATGC	55.32,54.96	147
CakTSSR03032	CakTC34099	(TG)8	135	150	--	--				
CakTSSR03033	CakTC34103	(ACC)6	33	50	--	--				
CakTSSR03034	CakTC34105	(GA)12	2138	2161	--	--				
CakTSSR03035	CakTC34113	(TAAA)5	2139	2158	--	bZIP	AAAGCATGCACTAATCACAGT	CAAGTCTTTGTCATCTTTGCT	55.08,54.76	139
CakTSSR03036	CakTC34118	(CAA)5	448	462	--	--	GTTGATGGAGATCACGAGTAG	TTCTCATGTGACCACCTAAAG	54.78,55.31	146
CakTSSR03037	CakTC34134	(AAG)6	77	94	--	--				
CakTSSR03038	CakTC34145	(AGG)5	569	583	Young_pod	--	CCATCTACACAACCAAACAAT	TTTTGCTATAGTGCTGGAGAG	55.02,55.07	145
CakTSSR03039	CakTC34158	(ATG)6	230	247	--	--	ATGATATTGAAGGTGGTGATG	ATTAGGACACCTTTGAAATCC	54.85,54.84	146
CakTSSR03040	CakTC34190	(TGG)6	2320	2337	Root	--	GAGTGCTACTTTGGATTCGTA	TATCACACGTCTGAACCCTAC	54.74,55.17	146
CakTSSR03041	CakTC34192	(TGT)6	7403	7420	--	--				
CakTSSR03042	CakTC34217	(AT)9	307	324	Root	--				
CakTSSR03043	CakTC34221	(CTT)6	1688	1705	--	--	TGCTTACCAAGTTCTTCTTTG	AAAAAGAACAGAAGTCGGAGT	54.96,54.86	171
CakTSSR03044	CakTC34231	(CAA)5	230	244	Root	--	TAGGGTTTAACTACGCCTTTT	CTGGAACAAATTAGTCTGGTG	55.07,54.9	139
CakTSSR03045	CakTC34231	(CAG)7	368	388	Root	--	CACCAGACTAATTTGTTCCAG	GCAGGAGTTCTATCGTTAGTG	54.9,54.32	143
CakTSSR03046	CakTC34252	(TCT)5	41	55	--	--				
CakTSSR03047	CakTC34258	(ATA)7	63	83	--	TPR				
CakTSSR03048	CakTC34258	(GCA)5	479	493	--	TPR	GACTGACATGTCTCTTCAACC	TTTTAGATGAAGAAGGTGCTG	54.61,54.88	145
CakTSSR03049	CakTC34281	(TCA)6	1384	1401	--	--	ATCAACCTCATTAACATGTCG	GCTACAGAGACTTCCTTCCTC	55.1,54.92	154
CakTSSR03050	CakTC34281	(AGA)10	1951	1980	--	--				
CakTSSR03051	CakTC34287	(AAC)6	446	463	--	C2C2-Dof	TGGGTTTGAGTTCTAAACAAG	CATTTCACAGCTTCTGATTGT	54.64,55.47	151
CakTSSR03052	CakTC34289	(GATTC)6	942	971	--	--	AGTCGTCTCCTCAGTCACTCT	AGAAATTGGGTTGTGAGAAAC	55.61,55.78	155
CakTSSR03053	CakTC34291	(GAC)5	312	326	Flower bud	--	CCATCCTATAAGGTCTCTTCC	ACTAGCCTCCATTTAACCCTA	54.53,54.83	152
CakTSSR03054	CakTC34304	(GAT)5	833	847	--	--	AAGGGAGATTGAGGTTATGAT	TTTCTTCAACTCCAAATTCAG	54.36,54.68	149
CakTSSR03055	CakTC34304	(AGA)5	1216	1230	--	--	TGAACAACAACAAGAACATGA	AGCTTGTGTTTCTCTTTCACA	55.09,55.25	148
CakTSSR03056	CakTC34306	(AC)6	21	32	--	--				
CakTSSR03057	CakTC34326	(CTT)5	196	210	--	--	TCACCTCCTCCCATTTATTAT	TCCATCTAGAACCCTATGAAA	55.21,54	147
CakTSSR03058	CakTC34330	(TAG)5	455	469	Flower bud	--	CTTGCTTGGTTAGAAGACGTA	GTGATCCATGATGAGACAAAC	54.99,55.29	149
CakTSSR03059	CakTC34350	(CCT)5	2682	2696	--	--	AATAGCTCAACCAGACACAGA	CATTACCGTTATCTTCCCTTT	55.04,55.06	164
CakTSSR03060	CakTC34354	(TC)9	489	506	--	PHD	CTTCTTCTTCTCCACCACTCT	TGTTGTTGTTGATGTTTGTGT	55.25,54.85	155
CakTSSR03061	CakTC34354	(TC)7	636	649	--	PHD	CTTCTTCTTCTCCACCACTCT	TGTTGTTGTTGATGTTTGTGT	55.25,54.85	155
CakTSSR03062	CakTC34358	(TA)6	1823	1834	--	--	CATAGTCGGAGGTGATAACTG	CGATGTAGCCAAATGTAAAAG	54.85,55.21	143
CakTSSR03063	CakTC34364	(CT)11	2202	2223	--	--				
CakTSSR03064	CakTC34371	(CAA)5	146	160	--	--	GAAAATTTCGCAGACTCATAC	TGTTGAAGTTACGAGTCCAAT	54.22,54.89	183
CakTSSR03065	CakTC34377	(TTCATT)6	1713	1748	--	--	AGCGATATAATCTCACGTTTG	AATCAACACTCTCTCCTCACC	54.72,55.8	152
CakTSSR03066	CakTC34380	(AG)6	308	319	--	--	TCGCTGCTTTATAACTTCAAC	ACCAATTCACTTTCACACACT	55.08,54.6	160
CakTSSR03067	CakTC34383	(AAT)7	1862	1882	--	--	GTCGCTTAAAAGCATGAGATA	GAAATAGAAATCACGATCACG	55,54.91	155

CakTSSR03068	CakTC34389	(GAA)5	445	459	--	--	TTAAATTGAAGGAGGAAGAGG	CTTCACAACACCAACATTCTC	55.3,55.64	137
CakTSSR03069	CakTC34394	(CTT)7	618	638	Flower bud	--				
CakTSSR03070	CakTC34407	(GAT)6	1930	1947	--	--	GGAATTGAGGTCAAAGAAGAT	TCCTTTTCTCTCTTTCTCACA	54.95,54.35	177
CakTSSR03071	CakTC34412	(ATG)6	2187	2204	--	--				
CakTSSR03072	CakTC34427	(AAC)7	448	468	--	GRAS	CCCAAAACATCAACAATCTTA	CCTGTTGGAAATTTCTAGGTT	55.25,55.09	132
CakTSSR03073	CakTC34433	(CTT)5	268	282	--	--	CTTAAGTGCTTTCAGCTTTTG	TGAGCACAAACACAAAACATA	54.89,55.31	152
CakTSSR03074	CakTC34449	(AAC)5	344	358	--	--	TCATCATCATCACAACAACAC	CGAAGCTCTTGTTCTCTTCTT	55.11,55.74	147
CakTSSR03075	CakTC34462	(TTTA)5	4	23	--	--				
CakTSSR03076	CakTC34470	(GA)16	2088	2119	--	--				
CakTSSR03077	CakTC34473	(AAT)9	113	139	--	--	TGAGAATATGTACGTCTGCAA	TGAGTGTTGACTCTCAGGTCT	54.46,54.87	146
CakTSSR03078	CakTC34490	(TA)6	58	69	--	--				
CakTSSR03079	CakTC34498	(CTG)5	609	623	--	--	GGAATGATGTTTCGTCTACTG	CTCTTTCCTTCTCAAGGCTAT	54.8,54.65	138
CakTSSR03080	CakTC34507	(AC)8	119	134	--	--	CAGGGTCACAGGTTACATTT	TGCGTATGCAACTTACTACAA	54.99,54.78	165
CakTSSR03081	CakTC34513	(GAA)8	45	68	--	--				
CakTSSR03082	CakTC34516	(TGG)7	1024	1044	--	--	TGTCACGTGAGAAAATGAGTA	CTCCACTTTCCCAATAAAAAT	54.3,55.03	161
CakTSSR03083	CakTC34522	(GGT)6	135	152	--	--	AATAAAGGGAGGATTGATGAG	ATCCATTTTAGATCCACCACT	54.93,55.11	146
CakTSSR03084	CakTC34530	(ATT)5	115	129	--	--	TGATCTTCAAGACAAAACCAT	CATCGGTTTATTTCAATTGTC	54.77,54.77	153
CakTSSR03085	CakTC34533	(TCT)5	187	201	--	SET	TACGCTTTTATCTCCGATCTA	TCGAGATTAGAAAACCCTAGC	54.56,55.47	149
CakTSSR03086	CakTC34542	(TC)6	11	22	--	--				
CakTSSR03087	CakTC34546	(CTT)5	161	175	--	--	TTTCATCTAATGGCTTCTTCA	TGAAACGAAGAAGAATCTCTG	55.21,54.82	148
CakTSSR03088	CakTC34548	(CTT)5	23	37	--	--				
CakTSSR03089	CakTC34556	(CT)10	5	24	--	bHLH				
CakTSSR03090	CakTC34556	(ATTCAT)7	200	241	--	bHLH	AACTCACCCTTTTTCTTCAAA	AGGATAAAGGTGAAGCAAATC	55.67,55.15	148
CakTSSR03091	CakTC34562	(ACA)5	351	365	--	--	AAAAACAGAATCTCATGCTCA	GTTGTTGTTTGGGTTTTGTT	55.1,55.04	160
CakTSSR03092	CakTC34568	(CAC)5	2255	2269	--	--				
CakTSSR03093	CakTC34569	(ATG)6	1904	1921	--	--	CTGAAGATGACAATTCAAAGG	CAGTATTGCCTTCACTTGTTT	54.95,54.66	150
CakTSSR03094	CakTC34582	(GA)10	48	67	--	--				
CakTSSR03095	CakTC34586	(CT)9	143	160	--	--	AAGTTCCACCTCTTTCTTCC	GTTAAGCTAAAGCAACCAACA	55,55	151
CakTSSR03096	CakTC34589	(AG)6	2060	2071	--	--				
CakTSSR03097	CakTC34590	(ATA)8	15	38	--	--				
CakTSSR03098	CakTC34601	(TGTT)6	399	422	--	--	ACTACACCCCCTTTGTGATAC	ACACAACCAAAGATACACACC	55.54,54.9	149
CakTSSR03099	CakTC34624	(AG)10	579	598	--	--				
CakTSSR03100	CakTC34630	(GAA)5	366	380	--	--	GATGAACACGGTTTTGTATGT	CCTCTCTTCTAATTGGGACTC	55.01,54.71	154
CakTSSR03101	CakTC34637	(TA)8	141	156	--	WRKY	GACGAGTGAGAAATGCATAAG	TTCAAGACATTGTCCCTTCTA	55.13,54.95	157
CakTSSR03102	CakTC34640	(AT)6	227	238	--	--				
CakTSSR03103	CakTC34645	(AAC)5	90	104	--	--				
CakTSSR03104	CakTC34649	(GAC)6	329	346	--	--	TATTTCCGACATCATTCTCTC	GAAGGGCTAAGTAGCAAAGAC	54.36,55.14	155

CakTSSR03105	CakTC34659	(AAT)8	6657	6680	--	--	GGATCTTGACAAACATAATCG	CGGATAGCATTATAGAGAGTTG	54.74,53.66	150
CakTSSR03106	CakTC34684	(GT)6	3703	3714	--	--				
CakTSSR03107	CakTC34693	(TTC)7	15	35	--	--				
CakTSSR03108	CakTC34696	(TCT)6	173	190	--	--	CTTTCAACTCAACTTCACCAC	TTTCTTGAAATGAACTCACCT	54.92,54.08	149
CakTSSR03109	CakTC34702	(TC)6	573	584	--	--	TGAGTCTACCATTTCGTCTTG	TTTCAGCTCAGGATAACAAAG	55.48,54.88	131
CakTSSR03110	CakTC34702	(AG)8	3833	3848	--	--	ATGGAGGAAGAGTAAACCCTA	AATAGTCCAAATCCAAACCAC	54.61,55.59	142
CakTSSR03111	CakTC34704	(ATG)10	404	433	Flower bud	--	TGGTTGAGGACAAAGAATATG	CTTCCACTTTCAACTCTTCCT	55.24,55.26	153
CakTSSR03112	CakTC34705	(TC)7	46	59	--	--				
CakTSSR03113	CakTC34717	(TGC)5	2749	2763	Flower bud	--	AGATGATAGAGTTGCCATCAA	ATATGAATCGAAGCGAAGAA	54.88,55.14	150
CakTSSR03114	CakTC34718	(AAC)5	793	807	--	--	GAGCTTTGGAATTATGTTTCA	TTGTTCCAATTCAACTTTCAC	54.65,55.28	194
CakTSSR03115	CakTC34727	(GTG)9	1	27	--	--				
CakTSSR03116	CakTC34729	(CTT)10	127	156	--	--	TGTGTGTCTTTCTTTCCATCT	AGTAAGAAAAATTGGCAGAGG	54.83,55.39	148
CakTSSR03117	CakTC34751	(AT)10	192	211	--	--	TCACTTTCTCCTATACCAATCC	ACCAATGAAAGCTAGGCTAAT	55.02,54.97	136
CakTSSR03118	CakTC34754	(AG)12	3280	3303	--	--				
CakTSSR03119	CakTC34758	(GGT)5	318	332	--	--				
CakTSSR03120	CakTC34765	(TTC)20	182	241	--	Bromodomain	GGTGAAATTGTAAGAGTCCAA	GCGAGTGGATAAACAAATAAG	54.38,54.31	160
CakTSSR03121	CakTC34799	(TC)13	97	122	--	--				
CakTSSR03122	CakTC34811	(AC)6	133	144	--	--	AGAAACTTGGAACTTCTCCAC	TTACCATGTACACGAGGTTTT	55.09,54.79	153
CakTSSR03123	CakTC34816	(GAT)5	406	420	Shoot	--	CTCATTCCCCACATATTATCA	GAGTTGTGGCAGAGTAATCAT	54.96,54.36	135
CakTSSR03124	CakTC34826	(GAG)5	315	329	--	--	TCTTCTTCTTCAGTGGCTATG	GTTTCTTCAATCTCCGATCAT	54.85,55.79	157
CakTSSR03125	CakTC34832	(ACATGA)5	72	101	--	--				
CakTSSR03126	CakTC34833	(AT)6	176	187	--	SBP	GGCTTACTATCTTGCCAATG	GGAAGAGTGAAAGTCTTGGTT	55.09,55.09	150
CakTSSR03127	CakTC34838	(TC)18	301	336	--	--				
CakTSSR03128	CakTC34839	(GT)6	3527	3538	--	--				
CakTSSR03129	CakTC34844	(AGTG)5	3699	3718	--	--	TTGTGGAATCTACGAGTGAGT	TGGGGTAGAGTAGAATGAGAA	54.87,54.04	153
CakTSSR03130	CakTC34847	(AG)8	1436	1451	--	HB	AAAAGGAAGCGATAGAGAAAG	TGAAGAGTTCTCAAACATCGT	54.83,55	150
CakTSSR03131	CakTC34847	(ACC)5	1870	1884	--	HB	CCACAATTGAAAACCAAAAT	GGGAAATTGAACATCTCTGAT	55.07,55.63	163
CakTSSR03132	CakTC34847	(TCT)6	2615	2632	--	HB	GGTGGATTAGGAACGATTAAC	CCAACACTACAAAACTCTTCC	55.27,54.04	149
CakTSSR03133	CakTC34847	(ATA)6	3450	3467	--	HB	GCAGAAGGATCAGATACAGAA	TTGGATCTCTTTGATGTTGTT	54.58,54.77	152
CakTSSR03134	CakTC34847	(TAT)5	3975	3989	--	HB	GACTTTGGAGGCATTTAATTT	CTTCTTCCTTTCCTTCCATAA	55.17,55.3	259
CakTSSR03135	CakTC34864	(ATT)6	27	44	--	--				
CakTSSR03136	CakTC34866	(TC)10	99	118	--	--				
CakTSSR03137	CakTC34873	(AG)6	886	897	--	--	TGCAAGAGGATTTATTGTGTT	ACAGGATTGAAACTCAAGGAC	54.99,55.79	160
CakTSSR03138	CakTC34874	(CAAATG)7	34	75	--	--				
CakTSSR03139	CakTC34876	(TGT)5	382	396	--	--	CTAGTCCTCCAACAAAAACCT	TGCATAAACTAGAAGCAGGAC	55.15,54.9	138
CakTSSR03140	CakTC34887	(AT)6	999	1010	--	--	CGAATGCTAACCCTAGAAAAT	TGTTTGTAGTGGAGATCTTGC	55.36,55.47	134
CakTSSR03141	CakTC34888	(CT)7	128	141	--	CAMTA	GGAAAAAGGAAAAACGAATAC	AGCTTCAAATTGTAATTGCTG	54.77,54.92	153

CakTSSR03142	CakTC34891	(AG)6	1874	1885	--	--	CTTGGGACATGTCTTTGACT	ATACCCACTCTCACAACACAC	55.1,54.88	159
CakTSSR03143	CakTC34899	(AG)6	432	443	--	--				
CakTSSR03144	CakTC34903	(CAT)5	113	127	--	--	AAAATCTCCACCTACAAGTCC	TGGTGAAAATGGTAGAGATTG	54.9,55.24	152
CakTSSR03145	CakTC34915	(GTGATG)5	3080	3109	--	--	GCAGATAAATTTTGAGAAGCA	CACAGAGTAAGCGAGAACTCA	54.96,55.89	166
CakTSSR03146	CakTC34920	(TGA)8	698	721	--	--	CTGAATTCGATTCGTTTTGTA	TTGCTTGATATGCTTCAAACT	55.57,55.31	150
CakTSSR03147	CakTC34941	(TTG)5	182	196	Flower bud	--	AGCAAGAGAATGTGATGAAGA	GGAACTTCACACCAATTATGA	55.08,55.06	157
CakTSSR03148	CakTC34971	(TGC)5	2275	2289	--	--	AAGCCTCAATACATAGCACAA	CTTTTAATCCTTTTGGCTTTC	55.18,55.08	157
CakTSSR03149	CakTC34986	(TTA)6	428	445	Mature Leaf	--	TTTGAAATGAGTGTCAAATCC	CCTCTCCCCTTTCTGATAATA	55.19,55.1	146
CakTSSR03150	CakTC34987	(CAT)5	362	376	--	--	CTATGGGAGCAACCTACATAA	AAGTGATGATGACGAAGATGA	54.57,55.65	150
CakTSSR03151	CakTC35004	(TTC)6	599	616	Root	--				
CakTSSR03152	CakTC35005	(TCT)5	68	82	--	--				
CakTSSR03153	CakTC35010	(AG)13	2793	2818	--	--				
CakTSSR03154	CakTC35024	(AT)6	197	208	--	--	ACTAACCCCTCCCTGTATATG	ATTTCTTTTGTTGGAAGATGG	54.77,55.87	152
CakTSSR03155	CakTC35041	(CA)8	4	19	--	--				
CakTSSR03156	CakTC35052	(TC)8	9	24	--	--				
CakTSSR03157	CakTC35057	(CAC)6	79	96	--	--				
CakTSSR03158	CakTC35065	(TTG)6	2271	2288	--	--	GAATTTGCGATTGGAGTAATA	CTTCTCCCACTTTTCACTTTC	54.63,55.67	158
CakTSSR03159	CakTC35086	(TA)6	7	18	--	--				
CakTSSR03160	CakTC35090	(TC)8	104	119	--	--	CTTCACCCATTCTCTTCTCTT	ATCCTACTTAGGCTGTCGAAT	55.18,54.79	157
CakTSSR03161	CakTC35095	(GAA)6	55	72	--	--				
CakTSSR03162	CakTC35097	(ATG)8	1316	1339	--	--	AACTTGTTGATCTCAATGGTG	AGGTATCTCATCTCCCAAAAC	55.13,54.82	143
CakTSSR03163	CakTC35111	(TAA)6	3892	3909	--	--				
CakTSSR03164	CakTC35113	(CT)7	1	14	--	--				
CakTSSR03165	CakTC35119	(GTG)5	968	982	--	--	ATGAAGATACAGCATCGTTTC	TTCTCATTCACACTCCCTCTA	54.47,54.92	149
CakTSSR03166	CakTC35119	(ATG)5	2715	2729	--	--	ATCATGTCACTGTGTTCAGGT	AAATAAGTCCGAATTTCATCC	55.32,54.94	164
CakTSSR03167	CakTC35124	(TA)6	117	128	--	G2-like	CCAGAAACATTCTATGATTGC	CAGCAACAACAGCTAGAAAAG	54.91,55.63	126
CakTSSR03168	CakTC35131	(TCT)5	168	182	--	--	CTTCTTCACCATTCTTCTTCA	TGTTGTTGTTGTGATGTTGTT	54.65,54.85	150
CakTSSR03169	CakTC35131	(TGT)5	723	737	--	--	TGCCATCTTTACCTAGCAATA	TAAATTTGACCTCTGAAGCTG	55.28,54.88	169
CakTSSR03170	CakTC35134	(TA)6	3004	3015	--	--				
CakTSSR03171	CakTC35154	(TC)8	4019	4034	--	--				
CakTSSR03172	CakTC35156	(AT)8	148	163	--	--	AATTAACTTAACTGCCATAAACG	CTGTTTCTGTTACGTCTCTGC	54.77,55.29	136
CakTSSR03173	CakTC35156	(GA)9	434	451	--	--				
CakTSSR03174	CakTC35159	(AG)8	942	957	--	WRKY				
CakTSSR03175	CakTC35160	(GAGT)5	3869	3888	--	--				
CakTSSR03176	CakTC35163	(ATA)7	142	162	--	--	TTGCTTTTTATTCTCTGCAAC	GTGAGATCCTTGAGATCTGTG	55.04,54.71	150
CakTSSR03177	CakTC35189	(TC)7	82	95	--	--				
CakTSSR03178	CakTC35190	(TTTA)5	116	135	--	--	AACTATTGCGTAGGGTAATGA	GAAGAAAGAGTGAGGAAGCAT	54.19,55.33	180

CakTSSR03179	CakTC35199	(AG)8	2813	2828	--	--				
CakTSSR03180	CakTC35202	(CT)8	21	36	--	--				
CakTSSR03181	CakTC35202	(GAA)5	1362	1376	--	--	GTCCCCTCTGTACATTCTGA	ATTTTGTGCAGTGTCATCTCT	55.01,54.85	153
CakTSSR03182	CakTC35209	(TA)8	1	16	Shoot	--				
CakTSSR03183	CakTC35209	(TTG)5	491	505	Shoot	--	TAGTGGGGAAGAAGGTAATCT	ATTCAGCAATCTCATGTCAAG	54.61,55.38	138
CakTSSR03184	CakTC35220	(TCT)6	985	1002	--	--	TCACTATCCTCACTCATGCTT	GAATATCTCAAGAAACGCAAG	54.95,54.4	155
CakTSSR03185	CakTC35220	(TCA)5	1249	1263	--	--	TGTCAAAGTCTGCCTCATAAT	TCATCTCCAGAATCATTTGAC	54.97,55.09	151
CakTSSR03186	CakTC35225	(GA)6	96	107	--	--				
CakTSSR03187	CakTC35240	(TAT)6	3239	3256	--	--	AACACTCAATCAAGTTGGGTA	GCTCTTCAATCTTTTATGTCC	54.72,53.32	169
CakTSSR03188	CakTC35249	(CTT)7	49	69	--	--				
CakTSSR03189	CakTC35250	(TAA)6	210	227	--	--	TGCTTCAATACAAAAATGGTC	CTCACTTCCTCCTCAAAACTT	55.4,55.26	135
CakTSSR03190	CakTC35289	(AG)8	53	68	--	--				
CakTSSR03191	CakTC35302	(TC)7	143	156	--	--	TTCGTTCTTCACTACGCTATT	AGAGAAGAGAGCGATGAGAAT	54.52,55	173
CakTSSR03192	CakTC35304	(GA)12	3379	3402	--	--				
CakTSSR03193	CakTC35309	(AGA)9	658	684	--	Bromodomain	TGAAGAAGAGGAAGAAGAAGG	GAATCAACTTTTGTGCCATTA	55.48,55.4	153
CakTSSR03194	CakTC35319	(AAT)16	690	737	--	--				
CakTSSR03195	CakTC35328	(AC)6	368	379	--	--	CAGCAAGACAACAGAAAGAAT	TTTTGTGTACTACTGCTGCTG	54.76,54.38	150
CakTSSR03196	CakTC35334	(AG)7	47	60	--	--				
CakTSSR03197	CakTC35341	(ATC)6	694	711	--	--	CCTCTCTTTAAAACCTTTTGG	AGCTTCGGGAGTTTATGTAGT	55,54.87	159
CakTSSR03198	CakTC35344	(TGT)5	2630	2644	--	--	AATATCGTAGCTTCGAAAACC	CCAGATAGCTTTCATTTACTCC	55.35,54.95	162
CakTSSR03199	CakTC35344	(AT)10	3048	3067	--	--	CTGCTTCGTCTGTTAAACATT	CCACATAACATCAAAAGAAAATC	54.82,55.5	152
CakTSSR03200	CakTC35344	(AG)6	3306	3317	--	--	GTTTTTGCTTTTTGGGTTTAG	AAGCTACAACCTCATCCTTTT	55.84,54.84	141
CakTSSR03201	CakTC35349	(CT)8	256	271	--	CAMTA	CTTTCTCTGATCCTTTTCGAT	GATGCCATGATATTGAATCTG	55.27,55.52	154
CakTSSR03202	CakTC35350	(AC)6	658	669	--	--	TCCAGAGCAACAAATAAACAT	CGCTCTGATTGTAAGATGAGT	54.99,54.71	150
CakTSSR03203	CakTC35400	(TCT)7	139	159	--	--	TCTTGCAACATTCTTCAATCT	TTTTTGGTCAAGTATGAGCTG	55.1,55.64	146
CakTSSR03204	CakTC35405	(TC)8	92	107	--	--				
CakTSSR03205	CakTC35406	(TC)7	108	121	--	--	CCTTCAGACTCTCTCACACAC	TTGAAGGTTTCGTGTTTTCTA	54.87,55.21	152
CakTSSR03206	CakTC35410	(GTT)6	2468	2485	--	--	TAGAAGGAAGAAGAAGGAGGA	ATCTTCAATCGTCTACGAACA	55,54.97	159
CakTSSR03207	CakTC35410	(TCG)5	2724	2738	--	--	GTGGTGAACCGTTGTAGTAAC	AACCAGACCATGAATTAGACC	54.67,55.58	138
CakTSSR03208	CakTC35417	(CAC)6	1777	1794	--	--	TATTCTATGCCTCTTCCACCT	CATTAGTCACTCTGTGGGAAG	55.59,54.88	145
CakTSSR03209	CakTC35426	(CT)7	43	56	--	--				
CakTSSR03210	CakTC35426	(AGC)5	2731	2745	--	--	ATCAAAATAATGGATCTGCTG	AGTGCTGCTTGTAGTAATGGA	54.44,55.25	161
CakTSSR03211	CakTC35431	(AG)10	3588	3607	--	MYB-related				
CakTSSR03212	CakTC35435	(ATT)5	3364	3378	--	--	TGGGCTTCTAAAATACTCACA	ATCCCGTCTAATTTTAAGTGG	55.16,55.06	150
CakTSSR03213	CakTC35436	(TTC)13	211	249	--	--	TCTTTAATTCCATTTCCCTCT	ATTTCTTTCTTCGTCTTCGAC	54.68,55.34	152
CakTSSR03214	CakTC35451	(TA)9	233	250	--	--	CACCTATGAGAAAACCCAATA	GCTTTTAGGAACTTTCCAATC	54.38,54.9	177
CakTSSR03215	CakTC35452	(TC)7	74	87	--	--				

CakTSSR03216	CakTC35454	(GTT)8	273	296	--	--	ATATTGGGTCAACGCTATGTA	CCAGAACATGAAGACAAAAAC	54.84,54.85	135
CakTSSR03217	CakTC35460	(AG)8	4080	4095	--	--				
CakTSSR03218	CakTC35480	(TAA)5	376	390	--	--	CAAAGCTCTCATGTCCACTAC	GAATCAACCGTAACAAACAAA	55.04,55.32	139
CakTSSR03219	CakTC35481	(GAT)5	795	809	--	--	CTGAGAGGATGTTGTTGAGTT	AGACTGCAGCTAAGAGGTTTT	54.39,55.21	146
CakTSSR03220	CakTC35487	(AG)6	164	175	--	ARF	AACACTGAGAACACAAGCAAC	TTGTTAGTGAGAGAGCGAAAC	55.45,54.93	156
CakTSSR03221	CakTC35489	(TA)6	18	29	Flower bud	--				
CakTSSR03222	CakTC35493	(GTT)5	1712	1726	--	--	ACTTCTTCATCATCGAAACCT	TACTACTTCGATCTCCGATCA	55.46,55.06	152
CakTSSR03223	CakTC35498	(TAG)6	297	314	Young_pod	--	TTATGCTCTTTCTCCTCCTCT	CAACTATGCCAAAAACAAGAC	55.03,55.07	119
CakTSSR03224	CakTC35501	(AAT)5	241	255	--	--	ATATAACTTGCAATGCCCTTA	TTCAGTAGAGAAAGCCAAGAA	54.3,54.59	146
CakTSSR03225	CakTC35527	(TC)6	107	118	--	TPR	ACAGGAGTGGTTCTTTTGAAG	TGGAATGAAAGATTGGTGTAT	56.03,54.58	144
CakTSSR03226	CakTC35537	(TAT)8	157	180	--	--	AAACCTTGTGACATGTTTCTC	TTGTGGTATGGAAGTCAAATC	54.24,55.06	149
CakTSSR03227	CakTC35540	(CCT)5	2341	2355	--	--	CATCTGCTACATCTGTTCCTC	GAAGTATCCTTCTTTGCTTCC	54.95,54.88	184
CakTSSR03228	CakTC35555	(AC)7	498	511	--	--				
CakTSSR03229	CakTC35558	(CA)6	57	68	--	--				
CakTSSR03230	CakTC35564	(TTC)6	4076	4093	--	--	ACTCTGTTACTTTTCGTTTCG	CTTTCTTTCTCCATCTCCATT	54,55.12	147
CakTSSR03231	CakTC35566	(CAG)5	2733	2747	--	--	CAATTCAAACAACAATATCAGC	CATCATTCATTTGGTGAAGTT	55.02,55.06	145
CakTSSR03232	CakTC35586	(CTCAA)5	24	48	--	--				
CakTSSR03233	CakTC35594	(AC)6	124	135	--	--	GTCTGGTCTTAACCTGGAATC	ATAGAACAAATAGCCCCAATC	55.3,54.97	154
CakTSSR03234	CakTC35600	(GA)9	3317	3334	--	TPR				
CakTSSR03235	CakTC35618	(AAC)6	495	512	--	Jumonji	TGGAAAGAGAAGTTGAAAAGA	CCATTTGGTAATTCTTTCCTC	54.39,55.4	157
CakTSSR03236	CakTC35624	(AAG)7	1092	1112	--	--				
CakTSSR03237	CakTC35628	(CTC)6	278	295	--	--	CCTAACTAGGGCTCAACTTTC	TCTTGAAGACGGTTCACTAAA	55,55.19	143
CakTSSR03238	CakTC35635	(AT)9	239	256	--	--	TCACTACCAAAAGAGAGAGGA	ATAATTTTGGCTGACATGTTG	54.22,55.27	145
CakTSSR03239	CakTC35640	(ATG)6	3332	3349	--	--	TTCACTTCCAAACCTAATCCT	ATTCTTCGCAATCACTCTTTC	55.47,56.18	152
CakTSSR03240	CakTC35642	(GTG)7	267	287	--	--	GTTTTCCCCTTCAAGAATTT	GTAAAACCTACCAACCCAAAC	54.96,55.27	150
CakTSSR03241	CakTC35649	(TC)6	152	163	--	--	TTAAAAACAGAGAGAGCCAAA	GTAGGATTTCAGACAGAGGTG	54.62,53.9	155
CakTSSR03242	CakTC35649	(TAG)5	3106	3120	--	--	CTGCAATTATTTTGGAAGATG	GTATTGAACAAAATGGTCTCG	55.1,54.83	150
CakTSSR03243	CakTC35651	(GA)6	1671	1682	--	--	TGTCAATCGAGAGTTGGTTAC	CCTTTTAGCACCCTTCTTATT	55.3,54.59	147
CakTSSR03244	CakTC35657	(CTT)5	271	285	--	--	CTGAAATCTGCAATAATCGAC	TATACTTGGGAACAAGGAACA	55.07,54.84	151
CakTSSR03245	CakTC35665	(TAA)7	231	251	--	--	TTATCCATCTTACCCCTTAAT	AGCCAAAAAGTTCTGATTCTT	52.29,54.96	146
CakTSSR03246	CakTC35676	(TCT)6	341	358	--	--	CTAAGTTTCCTGTTGCAGCTA	TCCTTATTGTCACAACACCTC	55.15,55.13	156
CakTSSR03247	CakTC35678	(TCTT)6	204	227	--	TPR	ATTCCAACAACACTCACATTC	CGAAGAAGACATGAAGAAGAA	54.95,54.82	145
CakTSSR03248	CakTC35684	(CAC)8	435	458	--	SBP	CTAAAGAATGGAATTGGGATT	CTCGTTTGTTTGCTCTATTGT	54.95,54.82	153
CakTSSR03249	CakTC35690	(GCC)5	3182	3196	--	--	TTCTGTGCTTGTTGTTGTTTA	CAGAGGTAGTTCCTCATCCTC	54.6,55.46	150
CakTSSR03250	CakTC35694	(AG)9	691	708	--	--				
CakTSSR03251	CakTC35705	(ATG)5	3274	3288	--	--	CAACAAATGAGGGAACTTGTA	GTTGTGGAGGAAACTGATTCT	55.32,55.79	166
CakTSSR03252	CakTC35707	(GAC)5	2119	2133	--	Bromodomain	GGATACAACTGATGGAATGAA	TTCACGTACCTTTGCTAAATC	54.98,54.77	145

CakTSSR03253	CakTC35722	(AGA)6	4169	4186	Mature Leaf	--	AGTACATGATCGGTGAGTGAA	CTCATTCTTCATTCTGCAAGT	55.6,54.67	152
CakTSSR03254	CakTC35737	(ACT)6	226	243	--	--	TTCAATTAGTTCCAACAGCTC	TATTAGAAAACGAAAGCGACA	54.7,55.48	146
CakTSSR03255	CakTC35745	(AAGAGA)5	2725	2754	--	Alfin-like	CTGCTTTAGTGAATTTGGTTG	ATTTAATTTTGGGAGCAACA	55.24,54.92	144
CakTSSR03256	CakTC35748	(CTT)5	350	364	--	--	TGTTTCTTCTCAACCGTAAGA	TGGTGGTTAAAAAGTATGTGG	55.19,55.21	151
CakTSSR03257	CakTC35749	(TCA)7	3068	3088	--	--	CTTCACTTCCCTCTTCTTCAT	TGATCCTGAGATTTATCCTGA	55.18,54.77	152
CakTSSR03258	CakTC35752	(ATG)6	144	161	--	--	GGTTCCTTTTTCTCTTATTCG	CATCACCATCACCATTATCTT	54.91,54.85	155
CakTSSR03259	CakTC35755	(GA)7	1282	1295	--	--	TGACCATAAGAATGGAATTTG	TAAATCATGATGGGATTTCAG	55.17,55.09	133
CakTSSR03260	CakTC35758	(CAG)5	2300	2314	--	ARF	TTACAAACGCCGCTAAAC	CTGTTGTTGTTGTTGTTGATG	55.05,55.04	168
CakTSSR03261	CakTC35758	(CAA)5	2417	2431	--	ARF	CCCAACAGACTCAACAGAAT	TACTGGTTGCTGTACACACTG	55.1,54.8	146
CakTSSR03262	CakTC35770	(TG)7	3852	3865	--	--				
CakTSSR03263	CakTC35785	(AG)6	3467	3478	--	--				
CakTSSR03264	CakTC35787	(AAT)5	77	91	--	--				
CakTSSR03265	CakTC35792	(AT)6	298	309	--	--				
CakTSSR03266	CakTC35803	(ACC)10	260	289	--	--	ATGAATGAATTCCCCTAAATC	GGTCATTAACGTTATCACGAG	54.71,54.86	152
CakTSSR03267	CakTC35806	(TC)8	261	276	--	--	ACCCTCACAATTCACAAACTA	GGCTAACCTGTGTGTGTAAAA	54.72,55.42	151
CakTSSR03268	CakTC35808	(ACC)5	212	226	--	--	GCTGTTCTTCTTTTCATTTCC	GACTAGAGAAAGTGGGAGGAG	55.74,54.77	149
CakTSSR03269	CakTC35816	(GCA)6	61	78	Flower bud	MYB-related				
CakTSSR03270	CakTC35820	(GGT)5	87	101	--	--				
CakTSSR03271	CakTC35824	(TGT)5	330	344	Young_pod	--	GTTGAAGAAGAAGCAAGTTGA	CAGTTTGAGAGCCAAAGAATA	54.89,54.88	150
CakTSSR03272	CakTC35837	(TAT)7	125	145	--	--	AAGAAACATACCAAATTCCAAC	ATATCATGTATTTGGGGTGTG	54.95,54.74	157
CakTSSR03273	CakTC35859	(CAT)5	287	301	--	--	TTTGAGGAGAAGACACACAAT	TTTGTGGTGATTAAGGTTGTT	54.83,54.75	149
CakTSSR03274	CakTC35871	(TC)8	41	56	--	--				
CakTSSR03275	CakTC35883	(CT)14	3341	3368	--	--				
CakTSSR03276	CakTC35891	(TCT)5	382	396	--	--	ATGATTGTGGAAATAGCAGTG	CACATAGTCCAAACTCCAATC	55.26,54.64	151
CakTSSR03277	CakTC35898	(CAT)5	2315	2329	--	--	AGTTTCAATTTTGTCTTGACG	CCAAAGATGTGTATGAGGAAG	54.63,54.82	161
CakTSSR03278	CakTC35898	(TCT)5	5222	5236	--	--	GATATTATTGTCCTCCAGCAA	GTTGATGACATTGAGGAAGAA	54.44,55.18	147
CakTSSR03279	CakTC35900	(TA)6	9	20	--	--				
CakTSSR03280	CakTC35922	(TTC)5	418	432	--	--	ATTTACATCTGTCCCCATTCT	GCTTCACTTATTCCTTTGTGA	55.11,54.7	155
CakTSSR03281	CakTC35926	(AACTC)5	22	46	--	--				
CakTSSR03282	CakTC35935	(GAA)5	100	114	--	--				
CakTSSR03283	CakTC35950	(AGAA)6	402	425	--	--	GAAGAGTATTGGATCCCACTT	CAGACCAAAAACAATAACCAT	54.82,54.29	150
CakTSSR03284	CakTC35953	(AG)6	32	43	--	--				
CakTSSR03285	CakTC35958	(TAA)7	369	389	--	--	ATTCCACTCTCTTCTCCATTC	GAAACATAACCCCATAAAGAAA	54.92,55.06	159
CakTSSR03286	CakTC35960	(ATTGTT)5	1373	1402	--	bHLH	CAAAACTCAGAATCTCACCAG	TCACAGAAGCAGAATCAAAGT	55.01,55.17	148
CakTSSR03287	CakTC35960	(GTT)6	1575	1592	--	bHLH	CTTGGAATTGAAATTGAAGTG	GCAGTTAGGGTTAGGTTCTTC	54.97,54.84	156
CakTSSR03288	CakTC35960	(AG)6	2552	2563	--	bHLH	AAAACAAGATGACAACCTCAC	TCTCAAATTCAACCTAAATGC	54.24,54.65	146
CakTSSR03289	CakTC35964	(AAC)6	434	451	Shoot	WRKY	CCAAATTAGCTCAAACACAAG	TGAAGAGAGATTTTCAACTGG	55.24,54.65	152

CakTSSR03290	CakTC35975	(GA)6	4549	4560	--	--				
CakTSSR03291	CakTC35977	(GGA)9	1	27	Flower bud	--				
CakTSSR03292	CakTC35990	(ATG)5	352	366	Mature Leaf	--	CTTCTTAAGGTCGGAAAAGAC	TCACCACAATTCTCATTTTTC	54.97,55.19	159
CakTSSR03293	CakTC36003	(GA)7	60	73	--	--				
CakTSSR03294	CakTC36006	(GAA)10	3113	3142	--	--				
CakTSSR03295	CakTC36008	(AAC)6	196	213	--	--	AAACAAAGACATGAGTGATGG	AATTAGAACCCCATGACATTT	55.13,55.12	149
CakTSSR03296	CakTC36011	(TC)19	21	58	--	--				
CakTSSR03297	CakTC36011	(TTA)5	279	293	--	--	CTTTCCCCAACACACTTTTAT	ACCGAAGTTGAAATAACCAA	55.82,54.82	158
CakTSSR03298	CakTC36016	(TCT)7	2903	2923	--	--				
CakTSSR03299	CakTC36019	(CCCT)5	281	300	--	SBP	TCCTTCTCTTTTCTCTTCCAC	CTTCAGAAGAAGAGTTGCTTG	55.31,54.64	151
CakTSSR03300	CakTC36020	(AT)8	130	145	--	--	CACTAAATCTCACCGTTGACT	TTGGAAGTTAAGAATGGACCT	54.46,55.47	175
CakTSSR03301	CakTC36021	(TAA)5	475	489	--	--	ACCGCTTACTCTCACTTTCTT	CTGTTAAGGGCAAAATTGTAA	55.05,54.8	139
CakTSSR03302	CakTC36022	(TGC)5	2060	2074	--	--	CATCATATGGACGCTTTACAC	GCAACAACAAATCTTTCAGTC	55.71,55.01	152
CakTSSR03303	CakTC36030	(AAG)5	111	125	--	--	TGGCACTTTGGAGTACTTATC	TTTTACCCCACTTAGTGAAGA	54.58,54.18	160
CakTSSR03304	CakTC36039	(TCA)5	2072	2086	--	--	TCGATATCTTCTTTGTGGAAG	GCTTCAATCAAAACTAAGCAA	54.63,55.04	148
CakTSSR03305	CakTC36060	(GAA)11	141	173	Flower bud	--	AAGAGAAAAAGCATGGAGAGT	CTGTTGACAACTGAGAAGAGC	54.94,55.24	145
CakTSSR03306	CakTC36072	(TG)8	5420	5435	--	--				
CakTSSR03307	CakTC36073	(TTG)5	60	74	--	--				
CakTSSR03308	CakTC36073	(GTT)6	408	425	--	--				
CakTSSR03309	CakTC36082	(TTG)5	2578	2592	--	--				
CakTSSR03310	CakTC36085	(GA)8	175	190	--	--	AAGTGTTTGTAGGCAATGTGT	TAGACATCTTTAGGCCATCAG	54.83,54.67	156
CakTSSR03311	CakTC36111	(ATG)7	75	95	--	--				
CakTSSR03312	CakTC36126	(AG)9	16	33	--	ARF				
CakTSSR03313	CakTC36134	(AAC)5	127	141	--	--	ACCAGATTAAGGGACTTTCAA	CCCCAGTAAACATCAATATCA	55.47,55.05	163
CakTSSR03314	CakTC36140	(ATC)5	570	584	--	--	ATTGAACTCCCCTCTTTTTC	CAGCTCTAAAAGATGACCAAA	54.93,54.88	162
CakTSSR03315	CakTC36142	(AG)6	38	49	--	--				
CakTSSR03316	CakTC36170	(ATTCAC)5	88	117	--	--				
CakTSSR03317	CakTC36179	(GA)19	2716	2753	--	--	GGTGATTTGAGATTTGGAGAT	ATATGATCTATCCTCGCCAGT	55.63,55.38	155
CakTSSR03318	CakTC36192	(TGT)5	467	481	--	--	GATTGGTATGTGACAGTGGTT	ATTCAATTCCTCTCAACAACA	54.81,54.77	157
CakTSSR03319	CakTC36197	(GA)7	2888	2901	--	--	AGGGTTGTTTTAAAAGACTGG	CTATCCCCAATTTGTTGATTT	55.17,55.67	156
CakTSSR03320	CakTC36200	(TGA)5	3613	3627	--	--	GGAGGATGTCAGAGGAGATAC	CAGATTGAAGTTCACAAGAGG	55.2,55.01	164
CakTSSR03321	CakTC36201	(TC)8	134	149	--	--	TTAGGGTTTGGTTTAATGTTG	GATCAAAACGACAGCGTATAG	54.5,55.18	142
CakTSSR03322	CakTC36201	(GTG)5	656	670	--	--	GGTGATGAATCTAGGGTTTCT	TCTCTGCTCCTCAATTTCAT	54.82,54.98	159
CakTSSR03323	CakTC36201	(GGA)5	961	975	--	--	TTAGTAGCGTTGGTACTGGAG	ACCTCACCTCCTTAACTTGTC	54.87,54.96	162
CakTSSR03324	CakTC36222	(GGT)5	3095	3109	--	--	TTGATGATGTGAGGGTAAATC	CCACAAACAATGTTAACTCCT	54.98,54.34	158
CakTSSR03325	CakTC36227	(AG)9	63	80	--	--				
CakTSSR03326	CakTC36238	(TC)9	75	92	--	C3H				

CakTSSR03327	CakTC36253	(AGC)5	367	381	--	--	TTCATTGCAAGAAAGTCATCT	TTAAATCGAAACACGAAAGTC	55.1,54.73	145
CakTSSR03328	CakTC36266	(CAA)6	258	275	--	--	TCTTTCCACAGATCATCAGAC	GAAGTTCTGCATATGATGCTT	55.16,54.64	149
CakTSSR03329	CakTC36267	(TC)6	3048	3059	--	--				
CakTSSR03330	CakTC36272	(AAC)12	503	538	--	--	GAGAGATATGCTCAAGAGTGG	ATCCCAAACGTCTTAAAGATT	54.15,54.63	149
CakTSSR03331	CakTC36281	(AGAAAG)6	177	212	--	--	CGTATTTTGGTCTTTTCCTTT	AAAGAGAACGAACTGAGGTCT	55.25,54.84	163
CakTSSR03332	CakTC36282	(TCA)6	251	268	--	--	TAGAAACTTGTGGTGGAGATG	ACCCAACTTTGTACCTACCAT	55.31,55.14	155
CakTSSR03333	CakTC36286	(GAT)6	785	802	--	--	AGAAAAAGGCATCCAATTAAG	TGCTGAGAATCAACTTCAAAT	55.33,55.1	167
CakTSSR03334	CakTC36288	(TG)10	4675	4694	--	--				
CakTSSR03335	CakTC36289	(TCT)6	74	91	--	C3H				
CakTSSR03336	CakTC36304	(TC)11	1	22	--	--				
CakTSSR03337	CakTC36306	(TTG)5	318	332	--	--	GCACACAAACAAACTTGAAAT	GAGAACATTGATAATGGTGGA	55.38,54.98	160
CakTSSR03338	CakTC36306	(TC)9	3047	3064	--	--				
CakTSSR03339	CakTC36315	(CCA)6	577	594	--	--	GAGGTGATGAAAAGGAGATTT	GTAGAAACCGTTCAAGAAAGC	54.95,55.84	149
CakTSSR03340	CakTC36329	(TC)6	15	26	--	--				
CakTSSR03341	CakTC36338	(TC)10	109	128	--	--	ACCTCCAATCAAATCACTTTT	AATATCCATCAACCAGCTACA	55.31,54.78	150
CakTSSR03342	CakTC36341	(TA)7	65	78	--	--				
CakTSSR03343	CakTC36369	(CT)7	78	91	--	--				
CakTSSR03344	CakTC36401	(TC)7	2883	2896	--	HB	GTGAGTGAGAATCGAAGATGA	TTAAGTGAAAACTGAGGCAAC	55.34,54.79	145
CakTSSR03345	CakTC36401	(AG)6	3140	3151	--	HB				
CakTSSR03346	CakTC36416	(TAT)6	10	27	--	--				
CakTSSR03347	CakTC36417	(CT)6	112	123	--	--	GAAGAAACCCATTAAGTAGCTG	GAAGGGTACAGAGACGGTTAC	55.03,55.41	150
CakTSSR03348	CakTC36432	(TA)6	66	77	--	--				
CakTSSR03349	CakTC36434	(AG)6	3111	3122	--	--				
CakTSSR03350	CakTC36436	(AG)10	3192	3211	--	--				
CakTSSR03351	CakTC36452	(AG)6	473	484	--	--				
CakTSSR03352	CakTC36464	(CAT)5	2056	2070	--	--	TTGTTGTCTAACACCACTTCC	GGGGGAGTTTTAAGAGTAATG	55.22,54.64	149
CakTSSR03353	CakTC36464	(AAC)7	2705	2725	--	--	TGTCTAGGGTGCATACTGTTT	TTGGGTGTAGTTTTTGTGTTT	54.93,54.84	157
CakTSSR03354	CakTC36467	(ATG)7	599	619	--	--	TGAGAAACTGAAGAAATTTGG	AAACCCACCTTTTGTATTAGG	54.68,54.99	149
CakTSSR03355	CakTC36473	(CA)9	67	84	--	--				
CakTSSR03356	CakTC36486	(TTTTC)7	26	60	Young_pod	--				
CakTSSR03357	CakTC36504	(CTT)17	283	333	Young_pod	--	CATCTTCATCCATACACCCTA	GATAAACCCATAATCATGCTG	55.03,54.72	165
CakTSSR03358	CakTC36504	(GAA)6	1020	1037	Young_pod	--	AATCAGGGGTGTTACTGGTAT	TGAAAGAGTGGCTAAGCTAAA	55.06,54.81	145
CakTSSR03359	CakTC36512	(TA)7	435	448	--	--				
CakTSSR03360	CakTC36519	(CAT)9	358	384	Young_pod	--	AGAAGATGTCGTCGATCAATA	CTTGAATCTCACACGCTACTC	54.88,55.2	158
CakTSSR03361	CakTC36534	(TC)13	127	152	--	--	GTGGATGTTTCATTACTCCAA	TCGAAGTAAATTGGTGAGTGT	55.06,54.89	173
CakTSSR03362	CakTC36541	(TC)19	161	198	--	--	AATAATCTTCGTCAAACCACA	GGGAAGTAGTTGAGAACCTGT	54.83,54.96	142
CakTSSR03363	CakTC36549	(AT)8	420	435	--	--	ACAATTTAAACACACCCACAC	GGTTTAAAATCTGTTGTCGTG	54.93,54.91	137

CakTSSR03364	CakTC36559	(TTA)12	2369	2404	--	--	GAAAACGAAGGAGACCTACTC	CTCTACGATGGTGGTTTTGTA	54.78,55.36	148
CakTSSR03365	CakTC36561	(AG)6	2768	2779	--	--				
CakTSSR03366	CakTC36562	(CCA)6	518	535	--	--				
CakTSSR03367	CakTC36563	(AG)6	6701	6712	--	--				
CakTSSR03368	CakTC36564	(GGT)5	2915	2929	--	--	TGATGATGTCCAAAAGAGTTC	TGTGCTTTTATGAGGAAATGT	55.18,54.99	162
CakTSSR03369	CakTC36592	(TTC)5	76	90	--	--				
CakTSSR03370	CakTC36596	(TC)8	7	22	--	--				
CakTSSR03371	CakTC36601	(TGA)5	2958	2972	--	--	GAGGAAGAAGCTGAAGATGAT	CTGTTCAGCCAACTTTCTAAG	55.25,54.55	150
CakTSSR03372	CakTC36621	(AAG)5	68	82	--	--				
CakTSSR03373	CakTC36629	(TG)6	1053	1064	--	--				
CakTSSR03374	CakTC36662	(TC)6	65	76	--	--				
CakTSSR03375	CakTC36662	(GAT)5	265	279	--	--	CGAATCAGAAGAAAACAGAGA	ACTTCCTCCATTAACACTTCC	54.82,54.9	153
CakTSSR03376	CakTC36668	(GAA)5	502	516	Shoot	--	TATGTCAAAGTTTGGCTCAAG	CAAGTGTTTGTGAATTCCTTT	55.64,54.46	159
CakTSSR03377	CakTC36676	(TC)7	418	431	--	--	TCTCTAAATCCACCACTTTCA	CTAACCAGTGTCGAAACAAAC	54.95,54.97	161
CakTSSR03378	CakTC36683	(GAC)5	2804	2818	--	--	AATTGCCAGTAGAGGCTTTAG	AGTTAGAAAGCCGTCCATATC	55.57,55.19	152
CakTSSR03379	CakTC36721	(GA)11	1	22	Young_pod	--				
CakTSSR03380	CakTC36731	(GA)8	533	548	--	--	CCATAAGAAGAAAAGGGTAGG	GTGTTTGTTCATTGTTCCTTT	54.81,54.28	154
CakTSSR03381	CakTC36739	(GTC)6	2645	2662	--	--	GTATGGATCTTCCTGAGCTTC	CACTCTTGTTTGAAAAATGGA	55.54,55.45	169
CakTSSR03382	CakTC36746	(GCA)5	2263	2277	--	--	TACTCGTCCCATTTGTACATC	GCTGTTGGTGACCTAATTTTT	55.1,55.96	162
CakTSSR03383	CakTC36754	(CGG)5	109	123	--	--	TGAGAGGAGAAGTAGTGACGA	GCTAAGCCATTTGATTTAACA	55.17,54.86	148
CakTSSR03384	CakTC36758	(AGA)5	1129	1143	--	--	TATACACTTGGCAAGAGAAGG	CCCAGTACTTCTCAACAACAG	54.75,54.97	138
CakTSSR03385	CakTC36765	(AAT)5	1044	1058	--	bHLH	CAGTATTAGCCACGTTTCCTA	TATCTCAGACTCTTCCCACAA	54.81,54.92	172
CakTSSR03386	CakTC36770	(AAC)5	18	32	--	--				
CakTSSR03387	CakTC36774	(ACA)9	139	165	--	--	ATCAAGTTTCTTCCATTTTCC	CTCTTAGAGGAGGAGAAGGTG	54.97,54.94	146
CakTSSR03388	CakTC36774	(GAA)7	342	362	--	--				
CakTSSR03389	CakTC36776	(TA)9	2656	2673	--	--				
CakTSSR03390	CakTC36792	(CA)7	44	57	--	--				
CakTSSR03391	CakTC36796	(TTA)7	17	37	--	--				
CakTSSR03392	CakTC36803	(GAA)5	156	170	--	--	TAGAAGAAGCAATGTTGGAAG	CAATACAAGAATGGCAAAGAA	54.88,55.57	151
CakTSSR03393	CakTC36825	(GA)6	2649	2660	--	Alfin-like				
CakTSSR03394	CakTC36828	(TC)6	17	28	--	--				
CakTSSR03395	CakTC36839	(GA)7	2728	2741	--	--				
CakTSSR03396	CakTC36848	(TC)7	69	82	--	--				
CakTSSR03397	CakTC36851	(TC)7	489	502	--	--				
CakTSSR03398	CakTC36863	(TC)7	2641	2654	--	--				
CakTSSR03399	CakTC36887	(GTT)5	296	310	--	--	GAAGAATGATTTGGCTTAACA	TTGCTATCTCAAAAATGGAAG	54.65,54.82	148
CakTSSR03400	CakTC36890	(ATC)7	2381	2401	--	--	CAGAAATCGGAATGAAAGACT	TCAAACTCACTTTCAGAGGAA	56.03,55.14	147

CakTSSR03401	CakTC36903	(ATG)5	117	131	--	HB	AATTCAGGTCCTACAAAATCC	GAGCAGTGTGCCTATGATATT	54.84,54.51	151
CakTSSR03402	CakTC36905	(AG)6	99	110	--	C3H				
CakTSSR03403	CakTC36905	(CAT)5	2505	2519	--	C3H	AGAGAAGAATCAGAGGAAGTCA	CGTGAGTTTTGGTAAATGAGT	54.9,54.5	156
CakTSSR03404	CakTC36911	(ATG)6	620	637	--	--	ACATGCTTGTTGATTCTTTGT	TCTGATGATAGCATACCCAGT	54.88,54.75	154
CakTSSR03405	CakTC36913	(AG)21	2658	2699	--	--				
CakTSSR03406	CakTC36924	(AAG)5	502	516	--	--				
CakTSSR03407	CakTC36929	(TC)9	99	116	--	--				
CakTSSR03408	CakTC36936	(AT)7	340	353	--	--				
CakTSSR03409	CakTC36942	(TTA)6	167	184	--	--	TTTGTGAGTGAGAGAAAGAGC	CCATTAATTCGAGAGTGAAGA	54.87,54.63	142
CakTSSR03410	CakTC36944	(GA)10	4187	4206	--	--				
CakTSSR03411	CakTC36948	(AAG)5	246	260	--	--	GATTTTTGGGAAAGATCATGT	TTGTGGGAGTTCTCACTCTTA	55.63,55.01	151
CakTSSR03412	CakTC36953	(CAA)7	585	605	--	--	TATTCTACCTTGGAGGGAATC	AAGACGCTGTTACCTTCATTT	54.93,55.72	148
CakTSSR03413	CakTC36953	(GAA)5	1006	1020	--	--	CAAAAGTGGAGAAAATACCG	GCACTGACAAATTGTTTCTTC	54.99,55.01	156
CakTSSR03414	CakTC36953	(AAT)7	1669	1689	--	--				
CakTSSR03415	CakTC36992	(AAG)5	91	105	--	--				
CakTSSR03416	CakTC37006	(TA)13	1	26	--	--				
CakTSSR03417	CakTC37009	(CGG)5	315	329	--	--	GATTCTCTCCATTTCCTCATC	AGAGAATTCTTGACCAGAAGG	55.26,55.18	154
CakTSSR03418	CakTC37025	(TTG)5	489	503	--	--				
CakTSSR03419	CakTC37041	(CCA)5	1747	1761	--	--	GGCGATCTAAAATTTTCTCTT	TCATTCTGCTTTTGTTAGGAC	54.61,54.7	141
CakTSSR03420	CakTC37042	(GTT)5	2573	2587	--	--	AGCACATTACAGATAGCGAGA	ATCACGATCTTCAACATCAAC	55.33,55.03	160
CakTSSR03421	CakTC37046	(TTA)12	308	343	--	--				
CakTSSR03422	CakTC37047	(TG)10	2692	2711	--	--				
CakTSSR03423	CakTC37048	(TC)11	116	137	--	--	TAAGAGCACATACGGTTTTGT	GAATCATTGTCAACCATTTTG	55.11,55.48	151
CakTSSR03424	CakTC37054	(TG)6	15	26	--	--				
CakTSSR03425	CakTC37064	(TTG)5	2132	2146	--	--				
CakTSSR03426	CakTC37068	(TCA)5	664	678	--	--	AATCCTCTGCATTCTTTCTCT	GAAGAAAGAGGTGTGTTGAAA	54.86,54.56	153
CakTSSR03427	CakTC37068	(AGA)5	2031	2045	--	--	AGCTTTTGATGGTTTTTGACT	CAACAAGTAGTTCACATGCAA	55.69,54.88	158
CakTSSR03428	CakTC37070	(GAA)5	239	253	--	--	CCTCAATTCATTCTCAAGACA	CAAACATGTTGGTTTCATAGG	55.36,55.61	150
CakTSSR03429	CakTC37094	(AAC)6	208	225	--	--	GGCTATCCATCAACAACATAA	AAGTGAACAACAGCAATGTTT	55.19,54.96	150
CakTSSR03430	CakTC37100	(AT)7	5	18	Shoot	--				
CakTSSR03431	CakTC37102	(AT)6	155	166	--	--	TCCATCATGGTTCTACTTATTCT	GCCGAAACTTCGTCTTTATTA	54.66,56.33	164
CakTSSR03432	CakTC37103	(TC)6	181	192	--	--	AGATTCAATCCACAAGTGAAG	CAAGGAATATCCAGAAAAGGT	54.34,55.01	147
CakTSSR03433	CakTC37120	(AAG)10	1480	1509	--	--	GGCTAGGCTTTTATCTTCAAC	TATATGAACAATGCTCGTCCT	55.08,54.94	140
CakTSSR03434	CakTC37123	(CT)6	14	25	--	--				
CakTSSR03435	CakTC37128	(TTA)5	1471	1485	--	--	AAACCATGCCTCTTCAATACT	AAGAATTGGATTGAGGAGAAG	55.5,55.12	154
CakTSSR03436	CakTC37133	(TCC)5	201	215	--	--	ATCCGCTCTCCATTTATCTAC	GATTTCAATGAATCTGAATCG	55.11,54.79	142
CakTSSR03437	CakTC37140	(CTT)5	143	157	--	--	GAGAAAAATGACCTCAACCTT	TGGAAACTTGTTCAGACATTT	55.03,54.85	151

CakTSSR03438	CakTC37144	(TGTT)5	1185	1204	--	--				
CakTSSR03439	CakTC37147	(GAA)9	996	1022	--	--	GCTCTACCACCATATTGAATG	CTCATTCACCATAATCCAAAA	54.78,55.17	150
CakTSSR03440	CakTC37151	(AGA)5	1600	1614	--	--				
CakTSSR03441	CakTC37154	(TTG)6	1189	1206	--	--				
CakTSSR03442	CakTC37158	(TCT)10	415	444	--	--	CTTCTGAAGATAACGTTGGAC	TCAACAAGTAGAGGAAGAGGA	54.12,54.22	152
CakTSSR03443	CakTC37160	(CAC)7	1084	1104	--	--	CAATCTGCTGAAAATCAAATC	GTATGTTGTTGTCGGAGGAG	55.03,55.58	147
CakTSSR03444	CakTC37165	(TGC)5	1066	1080	--	--	TGATTAGATTATTCGCCATTC	TGAGATCTCTGCTTTCTCTTG	54.55,54.96	156
CakTSSR03445	CakTC37167	(CAC)6	148	165	--	--	ACACACACTCTCCACTCTCAC	TACGACGACTTTGTTTTGTTT	55.07,55.15	144
CakTSSR03446	CakTC37168	(TTC)9	82	108	--	--				
CakTSSR03447	CakTC37190	(AT)7	261	274	--	bZIP	GGACTAAGGACAATGATACCC	CCATCAAAGTAAATGATTTGG	55.11,54.78	152
CakTSSR03448	CakTC37200	(TA)6	1639	1650	--	--				
CakTSSR03449	CakTC37204	(AG)7	10	23	--	--				
CakTSSR03450	CakTC37208	(TTC)5	1118	1132	--	--	TGTTCAATCCCTCTACAACAC	CCTTTATCTTACGGTGGTCTT	55.13,55.12	150
CakTSSR03451	CakTC37211	(AG)7	1165	1178	--	--				
CakTSSR03452	CakTC37212	(GAA)8	81	104	--	--				
CakTSSR03453	CakTC37214	(GGC)5	7087	7101	--	PHD	GAGAATAGAGCGAGTGTCTTG	AATCCCTAACTCGTTCTCAAC	54.41,55.06	147
CakTSSR03454	CakTC37215	(AAG)5	257	271	--	--	GAAAACTTCCTCTCCTACTGC	CATATCCTCAAGATCTGCATC	54.94,54.8	151
CakTSSR03455	CakTC37224	(AT)6	24	35	--	--				
CakTSSR03456	CakTC37227	(TAT)6	242	259	Flower bud	MADS	GGTTTGTTCCCTTTTACTTGT	CTCTTCAACTGAACCCTACCT	55,55.14	143
CakTSSR03457	CakTC37227	(AT)6	1041	1052	Flower bud	MADS				
CakTSSR03458	CakTC37228	(TGG)5	840	854	--	--	TACTGATTGGTTTCTCGGATT	GTTCTGACATCTTTTCCCTTT	56.31,55.03	147
CakTSSR03459	CakTC37236	(TTC)6	439	456	--	--	CATCTTTGTTTTCTTCATCGT	TGCAGAAGTAACACTTTTGGT	54.54,55.14	143
CakTSSR03460	CakTC37238	(TC)7	23	36	--	--				
CakTSSR03461	CakTC37247	(CAT)7	915	935	--	--	ATCTACTTTTGCTTGTGATGC	CAGATACAGTTCCATTTCCAG	54.73,54.82	161
CakTSSR03462	CakTC37250	(AAT)7	221	241	Root	bHLH	ACCTTATCAATGATTGCTTCA	CAAGACAAAGAGGAAATTGTG	54.91,55.03	154
CakTSSR03463	CakTC37251	(AAT)6	1791	1808	--	MYB	AATCAAGAGATCCACTTCACC	TGTTCATGTGCATGATTATTG	55.71,55.45	150
CakTSSR03464	CakTC37263	(TTG)6	469	486	--	--	CTAAGAGCAAGTGATGGAAAA	GGAACAGTCTCAAGAACAGTG	54.88,54.89	154
CakTSSR03465	CakTC37263	(GTT)6	660	677	--	--	TTGAGACTGTTCCTTCTTTCA	ACCAACAACAAAACCTAATCA	55.14,54.75	153
CakTSSR03466	CakTC37263	(GA)8	974	989	--	--	AATCTTGAGGTGATTTCCATT	ATTTCTTTCTCCAATCTCCAC	55.23,54.95	165
CakTSSR03467	CakTC37266	(AG)9	1153	1170	--	bHLH	GGAAGAATGGCATAAAAATAGA	CTCTCGAGGTAGGTGTGTTTA	55.27,54.64	155
CakTSSR03468	CakTC37291	(AAT)6	4	21	--	--				
CakTSSR03469	CakTC37295	(CT)6	178	189	--	zf-HD	TCAACCCTACAACACTTCATC	TTTAGCATCTGCATCTATGGT	55.13,55.1	133
CakTSSR03470	CakTC37297	(CT)7	1142	1155	--	--				
CakTSSR03471	CakTC37313	(TTC)9	16	42	--	--				
CakTSSR03472	CakTC37323	(AG)6	1702	1713	--	--				
CakTSSR03473	CakTC37329	(TGA)5	1120	1134	--	--	AGGAGATGTGTGGTTAGGATT	TTGTTTTTCAACAGCAAGTCT	55.17,55.26	151
CakTSSR03474	CakTC37333	(GAAT)5	1009	1028	--	--	GGAATTTAGGTTTCCTCTTCA	CACATTCATTCATATCCAACC	55.13,55.28	156

CakTSSR03475	CakTC37336	(TG)6	952	963	--	--	TCATTCCTAAACCTGATCTGA	GCAAGCACTGGTAGAGATATG	54.86,55.17	173
CakTSSR03476	CakTC37346	(AAG)6	292	309	--	--	CACAGAACAAAGAACAACACA	CAGACTGAAAAAGTTCGTGTC	54.73,55.08	153
CakTSSR03477	CakTC37350	(GATGGT)5	933	962	--	--	TAAATGGAGTGAGCAGAAGAA	TACGAGGAAGAATTAGGGTTT	55.28,54.79	161
CakTSSR03478	CakTC37355	(TC)9	1370	1387	--	--				
CakTSSR03479	CakTC37362	(AGA)5	661	675	--	--	AGAGGAGACAACGACCTACTT	GTCGAAGATCCTGAGAGAGAT	54.71,55.07	143
CakTSSR03480	CakTC37363	(ATC)8	526	549	--	--	ATCCAGAGGGTAAAAACAAAG	CTTCTGATACGTCTTTTGGAA	55.09,54.72	151
CakTSSR03481	CakTC37366	(ATGA)5	1154	1173	--	--				
CakTSSR03482	CakTC37375	(GAT)5	379	393	--	--	GTTTGGTGATATGAGTCCTGA	TAACTTCAGTGACAGCTCCTT	55.05,54.33	161
CakTSSR03483	CakTC37398	(CT)6	173	184	--	--	TTATTACACAACACCCTCACC	GTCATTGATCTGGTTGTGTTT	55.02,54.95	152
CakTSSR03484	CakTC37398	(AT)8	350	365	--	--	AAACACAACCAGATCAATGAC	TAAGAGGAGGAGTTGTTCCTT	54.95,54.79	156
CakTSSR03485	CakTC37408	(AAG)13	1112	1150	--	--				
CakTSSR03486	CakTC37409	(CT)17	4	37	Young_pod	--				
CakTSSR03487	CakTC37409	(CT)8	142	157	Young_pod	--	AATCACAGCAACTCAAAAGAA	TGGAAAAATAGAATGCGTATG	55.18,55.53	125
CakTSSR03488	CakTC37415	(TA)7	1562	1575	--	--				
CakTSSR03489	CakTC37420	(TC)12	103	126	--	--	GTGGGTATGTAGACTGCAGAA	CACGATGAGAGAATTGAAAAC	55.34,54.93	141
CakTSSR03490	CakTC37420	(TTG)5	283	297	--	--	GTTTTCAATTCTCTCATCGTG	TCCATTCTTCTTCTTGTTCAG	54.93,54.65	156
CakTSSR03491	CakTC37423	(ACA)5	115	129	--	--	CTTCAACCACAATTTTCACAT	GTTGTTGTAGTCGTTGTCCAT	55.15,55.07	156
CakTSSR03492	CakTC37425	(AAG)5	127	141	--	--	TTTGTCATCTCTAACGGTCTT	TTACAAGTTCATGTCATTCACC	54.12,55.16	158
CakTSSR03493	CakTC37427	(TTC)5	41	55	--	--				
CakTSSR03494	CakTC37434	(GAA)5	287	301	--	--	AACACACAATGGTTTCTATGC	ATTAAGTGATCCCTGAACCAT	55.17,55.11	148
CakTSSR03495	CakTC37448	(TCT)6	215	232	--	--	TACGCAAACTTAGAGCATTTT	CAGACCTTTCACCATTGATT	54.7,55.03	150
CakTSSR03496	CakTC37458	(CTT)5	962	976	--	--	AAGCAAGTGCTTCTTCTTCTT	CAGTTTCTGATGGGATTGATA	55.33,55.16	150
CakTSSR03497	CakTC37462	(AAGA)5	1171	1190	--	--				
CakTSSR03498	CakTC37466	(TCGCAA)5	19	48	--	--				
CakTSSR03499	CakTC37467	(TTG)6	84	101	--	--				
CakTSSR03500	CakTC37469	(GA)8	10	25	--	bZIP				
CakTSSR03501	CakTC37470	(TC)19	971	1008	--	bZIP	CAGATATGTTCCTGCATTGAT	ACCATTAACAGTCAACCATTG	55.18,55.02	153
CakTSSR03502	CakTC37472	(CT)6	3731	3742	--	--	CTTCTCTGAACAACCATTCAC	TTGAAAACAACAACAAGAAGC	54.83,55.67	156
CakTSSR03503	CakTC37477	(CT)6	37	48	--	--				
CakTSSR03504	CakTC37478	(AG)12	100	123	--	--				
CakTSSR03505	CakTC37481	(TTC)6	60	77	--	--				
CakTSSR03506	CakTC37487	(TTC)11	71	103	--	--				
CakTSSR03507	CakTC37488	(TC)8	1	16	--	--				
CakTSSR03508	CakTC37490	(GA)7	1179	1192	--	--				
CakTSSR03509	CakTC37491	(TTC)7	390	410	--	--	ACAACAAACACCCAAAATAGA	AAAATATCTTTCCATCGCTTG	54.75,56.12	138
CakTSSR03510	CakTC37496	(CAA)5	209	223	--	bHLH	AGCAAGTGTTCTACACCAAGA	AACAGGGGAAAGATCTTGTAA	55.12,55.47	139
CakTSSR03511	CakTC37498	(GAT)9	555	581	--	--	AGAAGAAGGAGGAGTAGCTGA	AAGGTACTCTGTTCCCAAATC	55.09,54.9	169

CakTSSR03512	CakTC37499	(AG)14	1721	1748	--	--				
CakTSSR03513	CakTC37500	(AT)8	1813	1828	--	--	TTGAGGAACTGGTGGTATATG	AGATGAAATGCCAGGAGTAAT	55.11,55.42	149
CakTSSR03514	CakTC37513	(TC)7	1202	1215	--	Alfin-like				
CakTSSR03515	CakTC37515	(CT)11	26	47	--	--				
CakTSSR03516	CakTC37520	(TAA)6	59	76	--	--				
CakTSSR03517	CakTC37525	(TC)6	191	202	--	--	GAATATCTCAACCGTTTCTCC	GATAAGTCTATTGGCGTCGTT	55.39,56.07	144
CakTSSR03518	CakTC37527	(GAA)8	260	283	--	--	AAGAAGAAGAAGAGGATGAGG	CAAAGTGTTTATCTCCTGGTG	54.42,54.9	149
CakTSSR03519	CakTC37539	(AT)9	1150	1167	--	--				
CakTSSR03520	CakTC37541	(TAA)5	961	975	--	NAC	GCTTAGACCCTTTATCATGTG	CAGAACTTCCTTCTCCTAAGC	54.11,55.11	150
CakTSSR03521	CakTC37545	(GA)17	1609	1642	--	--				
CakTSSR03522	CakTC37552	(TAA)7	188	208	--	--	ACACTCACAATTATTCCTCCA	GTTACCACCCTTAGAAGCAAT	54.64,55.11	152
CakTSSR03523	CakTC37559	(AT)6	1578	1589	Flower bud	Jumonji				
CakTSSR03524	CakTC37561	(GA)9	1227	1244	--	--				
CakTSSR03525	CakTC37563	(TAA)5	1585	1599	--	--				
CakTSSR03526	CakTC37565	(TTG)7	824	844	--	--	TTTGTTGGGTAGATCTTTGAA	CAAACAAAACCTCAAACTCAC	54.97,54.94	150
CakTSSR03527	CakTC37578	(GA)8	1214	1229	--	--				
CakTSSR03528	CakTC37579	(TCG)5	103	117	--	--	AGCGATGGGATCTATTCAC	TACTTTGTGAGTGTTGGTGGT	55.04,55.59	146
CakTSSR03529	CakTC37590	(ATA)6	179	196	--	WRKY	GACAAGTCATGAATGAAGGTG	GATTCTGAAGTGGAAGGATTC	55.56,55.34	148
CakTSSR03530	CakTC37590	(TCC)6	795	812	--	WRKY	CCTTAGCTTTGACAATTCCTT	CAGAGAGGTAATCATTGTTGG	55.39,54.82	155
CakTSSR03531	CakTC37598	(TTG)7	927	947	--	--	AGACCTTCTTCTTCAATGACA	GTTATCAGTCCAATGGGTTCT	54.03,55.58	146
CakTSSR03532	CakTC37598	(TTG)6	1177	1194	--	--	TAACCTCTGAGTCTGTTTTGG	ATTACTTGTTGGGTCCTCTTT	54.61,54.53	149
CakTSSR03533	CakTC37605	(TTC)7	67	87	--	--				
CakTSSR03534	CakTC37616	(GA)7	1245	1258	--	HSF				
CakTSSR03535	CakTC37620	(AG)8	1213	1228	--	--				
CakTSSR03536	CakTC37621	(CTT)5	72	86	--	--				
CakTSSR03537	CakTC37633	(TGT)7	1608	1628	--	GRAS	TGATAGTTTTGTTTGGAGGAA	GATGTGGGTCTCAATCTCATA	54.97,54.96	157
CakTSSR03538	CakTC37639	(TAG)5	176	190	--	--	AAGAGTCCAAAGATGTTGTTG	TGAGACAATTGATGTTGTTGA	54.43,55	139
CakTSSR03539	CakTC37665	(ACTC)5	1612	1631	--	--				
CakTSSR03540	CakTC37667	(AT)15	1222	1251	--	--	TGGAGAATATTATGTGACCTGA	AGCCTTACTTCAAAAATTGGT	54.77,54.86	147
CakTSSR03541	CakTC37672	(CT)6	63	74	--	--				
CakTSSR03542	CakTC37674	(ATA)5	9	23	Young_pod	--				
CakTSSR03543	CakTC37675	(CTC)5	178	192	--	--	AGTGGCCCTAGAAGCTAGTTA	ATTTAGTTGTTGGTGGTTGG	55.19,55.01	175
CakTSSR03544	CakTC37675	(TCC)6	497	514	--	--	GACTTCGAGTTCCGTCTTC	GGTGGTTGTTTTAGGAGAAGT	54.85,54.98	147
CakTSSR03545	CakTC37675	(TTC)5	805	819	--	--	TCTTCGTCGTTTGATAACACT	CTGAATCGAGAGAGAGTCTAGG	55.05,55.11	148
CakTSSR03546	CakTC37680	(GAG)9	1415	1441	--	--	TAATTAAGCCTATTTCGGATG	CAACTTACCCCCTTCTAAATC	54.01,54.64	170
CakTSSR03547	CakTC37681	(AAT)7	86	106	--	--				
CakTSSR03548	CakTC37684	(TCT)5	349	363	--	--	CCTTTCTCTTCCTCAACATCT	GAATTAGGGTTAGGGTTCTGA	55.18,55.01	149

CakTSSR03549	CakTC37685	(AG)11	2029	2050	--	--				
CakTSSR03550	CakTC37706	(ACA)7	306	326	--	C2C2-GATA	TTTCAGTGTTTGTGGAAGACT	TGACTCATTTTGGTTTTGTCT	54.92,54.85	146
CakTSSR03551	CakTC37708	(ATA)5	237	251	--	--	GATGAGCTCGTCAGTTGTATT	CAACTGCTTTTCCTTGAATTA	54.53,54.9	174
CakTSSR03552	CakTC37721	(CT)18	1	36	--	--				
CakTSSR03553	CakTC37726	(TC)6	1029	1040	--	--				
CakTSSR03554	CakTC37728	(TC)6	73	84	--	--				
CakTSSR03555	CakTC37738	(TAT)18	103	156	--	--	AAGTACAACTGACACCACCAC	TGATTCAACTACTTTCAAGGAA	54.97,54.31	149
CakTSSR03556	CakTC37742	(GA)10	169	188	Flower bud	--	TTTTGTTAGCAAGAGGTGAGA	GCTTCTTATCCTTTCGCAGTA	55.36,56.5	154
CakTSSR03557	CakTC37743	(ATT)5	1179	1193	--	Tify				
CakTSSR03558	CakTC37747	(TAT)6	863	880	--	--	TCAGGAAGATTGGTAGAATCA	AGATCCTAACCTTCACGAAAA	54.86,55.63	138
CakTSSR03559	CakTC37748	(TC)7	111	124	--	--	CTTTTGCCTCCTCATACAAAT	TGGTTTCAGGTAAAGGAAAAT	56.05,55.48	162
CakTSSR03560	CakTC37765	(TTC)6	42	59	--	--				
CakTSSR03561	CakTC37766	(AGT)5	29	43	Flower bud	zf-HD				
CakTSSR03562	CakTC37771	(AAC)5	266	280	Shoot	--	AACATGTTCAATTGCAGCTAA	AAAGAACCATCAGAAACATCA	56.07,54.77	144
CakTSSR03563	CakTC37771	(ATT)5	1561	1575	Shoot	--	GGACACAAAATTAACCACAAA	CTAGAGCACATCATTCTGCTT	55.16,54.88	163
CakTSSR03564	CakTC37775	(TTG)5	675	689	--	--	TTCCTGATTCTGTTCTTGAAA	ACAGTCAATGGATGAAAACAG	55.08,55.13	146
CakTSSR03565	CakTC37776	(CTT)7	60	80	--	--				
CakTSSR03566	CakTC37777	(TC)6	102	113	--	bHLH	ATTCCATTCTACATTCCACAA	TGAGAATGATTTTGAAGAGGA	54.58,54.99	154
CakTSSR03567	CakTC37778	(ATC)5	975	989	--	--	GTATGAAAGCGTTGAAGAAAA	AAACTCGCCACTGAACTCT	54.89,54.98	148
CakTSSR03568	CakTC37779	(GGT)6	677	694	--	--	TGATAGCAGCAAGAAGATGAT	AGAGGTTGTTTCAAGGAGAAC	55.22,55.09	164
CakTSSR03569	CakTC37782	(TGG)5	793	807	--	--	CACATGTTACAAAGTGAAGCA	CAATAAGATTCCTGTGCAAAC	54.88,54.99	149
CakTSSR03570	CakTC37782	(GTG)5	925	939	--	--	GTTTGCACAGGAATCTTATTG	AGATAGCTAGTTCCCATCGTC	54.99,55.17	145
CakTSSR03571	CakTC37793	(GTT)5	266	280	--	--	TGGTACTTTCATATCCGAGAA	CAAATCTCTCATCAAATCTGG	54.92,54.86	155
CakTSSR03572	CakTC37810	(AGA)5	1071	1085	--	--	GGCTTAAGATGGTTGTTACCT	TAACCCCTCAACTTCTCTCTC	55.11,55.18	144
CakTSSR03573	CakTC37812	(TGT)5	1624	1638	--	--	GTCGTGGAAAGTCTAGGTTCT	AACACCGTGAATGATAGTGAG	55.13,55.17	150
CakTSSR03574	CakTC37821	(TC)8	23	38	--	--				
CakTSSR03575	CakTC37830	(GTG)5	515	529	--	HB	TCTTTTCAGATGGATCTGTGT	ACCACCACAGTTATCATCATC	54.74,54.72	153
CakTSSR03576	CakTC37831	(TTC)6	296	313	--	--	GCGTTTAATCATTTCTCTCAA	CATGTCATGAACTTGAGGTCT	54.81,55.11	150
CakTSSR03577	CakTC37832	(CTCCTT)7	1091	1132	Root	--	GCAAATGAACTCATTCACAAA	GTGTTTCAGTGTTGTGGAGAT	56.31,55.02	149
CakTSSR03578	CakTC37834	(TCC)5	182	196	--	--	AAAGTCACACAGAAGCTTTCC	AAGTTAATCGAGGGGATAATG	56.18,54.98	146
CakTSSR03579	CakTC37844	(AAT)7	69	89	--	--				
CakTSSR03580	CakTC37847	(TCT)5	845	859	--	--	TTTGATGAATATCCACATGGT	GATGAAGATATGGCTGAGATG	55.28,54.8	154
CakTSSR03581	CakTC37849	(CAG)6	553	570	--	--	CTATATGCAACATCCTCAAGC	TATTAGGTCTAAGCCCCATTT	55.1,54.77	167
CakTSSR03582	CakTC37859	(TC)6	675	686	--	--	AAATTCGGTGTATAAACGTCA	CAGATGGACAAAAACAGAGAG	54.89,55.01	147
CakTSSR03583	CakTC37861	(GAA)5	475	489	--	--	CCCCGAGCTAGTATCATTATT	ACCAACTTCCGAAAGAAATTA	55.17,55.63	148
CakTSSR03584	CakTC37868	(GCA)5	738	752	--	--	CCTATTTTTACTGGTGATGGA	TTCTTCCATTGAAACTCTCAG	54.38,54.65	150
CakTSSR03585	CakTC37872	(TGA)5	809	823	--	--	AATGTGGAAATTGGAGAAAGT	TTGGAAGTTCTTATTTGGTTG	55.31,54.59	143

CakTSSR03586	CakTC37885	(CT)6	81	92	--	--				
CakTSSR03587	CakTC37887	(GAT)7	259	279	--	--	ACTTCAATAATGCAAGAACCA	ATCTAAAGCATCTTCCCAAGT	54.99,54.76	152
CakTSSR03588	CakTC37889	(GA)10	1668	1687	--	--				
CakTSSR03589	CakTC37894	(TA)8	57	72	--	--				
CakTSSR03590	CakTC37894	(CCT)6	910	927	--	--	GTTGGTGGAGAAGAGGATTAG	ATTGCATAAAAGGAGAAGACC	55.47,55.15	155
CakTSSR03591	CakTC37895	(AG)6	1177	1188	--	bHLH				
CakTSSR03592	CakTC37896	(AAG)6	899	916	--	--	ACGAAAAGTTGGTTAGGAAAA	CTTCCTCCATCAATGACTTTA	55.7,54.47	152
CakTSSR03593	CakTC37899	(CT)7	90	103	--	--				
CakTSSR03594	CakTC37915	(TCT)5	362	376	--	--	AGTAGCAACTCTCCATCATCA	TGATGGTGACTCTATTTGGTC	54.95,55.05	153
CakTSSR03595	CakTC37925	(TC)7	952	965	--	--				
CakTSSR03596	CakTC37929	(AGC)5	412	426	--	C2C2-GATA	AGTGCGGATACAAAGATGATA	CAGAACAAACCCTAACAGTTG	54.94,54.99	139
CakTSSR03597	CakTC37938	(ATG)6	654	671	--	HMG	CATGAAGGCCTATAACAAGAA	GAGCTTCTACTCGTCATCATC	54.7,54.13	145
CakTSSR03598	CakTC37941	(TTC)6	430	447	--	--	CCAACTCAAGAAAAGAAAACA	TACTTGATGGTTGTGTACGTG	54.77,54.57	148
CakTSSR03599	CakTC37942	(AT)9	48	65	--	FAR1				
CakTSSR03600	CakTC37946	(ACC)5	1084	1098	--	--	AGGAGGCATAAGACCATAAAC	TGGTGGTGCTAAATTTAAAGA	55.03,55.18	136
CakTSSR03601	CakTC37964	(CAA)5	186	200	--	--	CACACACTTTTCAATTAAGCA	TGAAGTACTGGTCTTCCATTG	54.12,55.31	153
CakTSSR03602	CakTC37970	(AC)8	1	16	--	--				
CakTSSR03603	CakTC37970	(GGA)9	944	970	--	--	CACAAGAAACTCCAAGAATTG	ACAAAAGCTTAGCATCATCAG	55.03,54.9	144
CakTSSR03604	CakTC37979	(CAC)5	79	93	--	--				
CakTSSR03605	CakTC37980	(TTA)5	956	970	--	--	TGGTTTTCTCTTCTTTTTCCT	CCAGGCATTGTTTACAATTAG	54.94,55.05	134
CakTSSR03606	CakTC37983	(AG)6	170	181	--	SRS	TCTACAGACACAAAAGGGGTA	CACTTATCTGTCGCACTCTTT	54.9,54.8	150
CakTSSR03607	CakTC37983	(TAG)5	573	587	--	SRS	GGGTTAAATTTGATTGGTTGT	TCCTTCGTTATTGTTGTTGTT	55.59,54.91	150
CakTSSR03608	CakTC37983	(ACA)5	1044	1058	--	SRS	AAGAAGGATTGTCCTCATGTT	TTATGATGAGTGTGTTGGTGA	55.3,54.86	146
CakTSSR03609	CakTC37988	(CT)11	13	34	--	--				
CakTSSR03610	CakTC37994	(GAA)6	267	284	--	--	CCTCTCTTCTTCTCTCCACAT	CCTCTTCTGCTTCTTTTTCTT	55.17,54.85	200
CakTSSR03611	CakTC37995	(ACA)5	1583	1597	--	--	ACAACAAGGACACTTTTGAGA	AATGATTATTGTTGGTGTGGA	54.92,55.36	150
CakTSSR03612	CakTC38001	(AG)6	489	500	--	--				
CakTSSR03613	CakTC38007	(AG)10	1191	1210	--	--				
CakTSSR03614	CakTC38018	(TATT)5	155	174	--	--	CTTCAGCACTAATGACTTTGG	CACTTCTCGTTTTTGATTTTG	55.23,55.21	151
CakTSSR03615	CakTC38022	(AT)19	1087	1124	--	--	TCTCCACGGATCTCATTATTA	TGACCCTAATTGCAAAGATAA	54.84,55.1	140
CakTSSR03616	CakTC38031	(TTC)6	1704	1721	--	--				
CakTSSR03617	CakTC38041	(TG)6	1700	1711	--	--				
CakTSSR03618	CakTC38044	(GA)6	1	12	--	--				
CakTSSR03619	CakTC38044	(AG)7	1610	1623	--	--	CGAACCTTCCATATTTAACAA	CAAGGTTTTCTAACCTGAACA	54.57,54.64	155
CakTSSR03620	CakTC38071	(AT)6	22	33	--	--				
CakTSSR03621	CakTC38074	(AAG)5	80	94	--	--				
CakTSSR03622	CakTC38076	(AAT)6	130	147	--	HB	TATGTTATGGCTTTTGGAGAA	GGAAGAGAGAGTTTGTATTTGC	55.1,54.97	169

CakTSSR03623	CakTC38080	(AG)8	1689	1704	--	--				
CakTSSR03624	CakTC38081	(TC)8	1105	1120	--	Alfin-like				
CakTSSR03625	CakTC38096	(AT)16	2228	2259	--	AP2-EREBP	TTGTCACCATTATCTCCACTC	CCAATATCTATTCATCTTCACCT	55.05,54.23	143
CakTSSR03626	CakTC38102	(GAAT)5	1079	1098	--	--				
CakTSSR03627	CakTC38115	(AGG)5	1186	1200	--	--	TGAGTGTTGAAACAGACAGTG	ATCTTCCATGCCTCTAAAAAT	54.69,54.7	145
CakTSSR03628	CakTC38119	(TTG)7	923	943	--	--	AGAAGGTGAAGAGGAAGAAGA	CTCAATACCGTTATGGAAACA	54.89,55.29	150
CakTSSR03629	CakTC38119	(GA)12	1053	1076	--	--				
CakTSSR03630	CakTC38121	(TC)7	23	36	--	--				
CakTSSR03631	CakTC38129	(TC)8	1	16	--	--				
CakTSSR03632	CakTC38134	(CAC)5	1397	1411	--	--	TGGAGCAGCATATGATTTATT	TAGAAGCTGATCTTGCTGAAG	55.04,55.18	140
CakTSSR03633	CakTC38135	(TA)9	995	1012	--	--				
CakTSSR03634	CakTC38136	(TC)14	110	137	--	C3H	TTACATCACATGTTCGTTTCA	TAATTGGGAGTTGATTGAATG	55.06,55.17	153
CakTSSR03635	CakTC38136	(ATG)5	479	493	--	C3H	TTTTGAAGTTAGGGATGAACA	CATCAACCTTCTCACTCACAT	54.97,55.11	149
CakTSSR03636	CakTC38136	(AT)6	1740	1751	--	C3H				
CakTSSR03637	CakTC38143	(TTC)12	1028	1063	--	--				
CakTSSR03638	CakTC38179	(ACA)5	277	291	--	--	GAACAACGTGTTCTCTCCAT	CAAAGAGATCTGAGAGTTGGA	55.09,54.62	175
CakTSSR03639	CakTC38187	(GA)14	1760	1787	--	--				
CakTSSR03640	CakTC38205	(TTC)7	1465	1485	--	--	TGGTGAAGAAGACAAGTCCTA	CATTTCGAATAAAGCTACTGC	55.01,54.62	152
CakTSSR03641	CakTC38241	(TTG)5	569	583	--	C3H	CAGCAATTGTTGTTATTGTGA	CTGAAGCTTGTAAAGATGGAA	54.81,54.88	154
CakTSSR03642	CakTC38244	(CTA)6	91	108	--	C2H2				
CakTSSR03643	CakTC38247	(CT)6	12	23	--	HB				
CakTSSR03644	CakTC38250	(TAA)9	529	555	--	Trihelix	TTCCAACACAACATGTTACAA	TTAGGAGCTAGTGGTTGATGA	54.97,55.15	154
CakTSSR03645	CakTC38252	(AG)10	1536	1555	--	--				
CakTSSR03646	CakTC38262	(CAA)5	97	111	--	--				
CakTSSR03647	CakTC38275	(AGA)8	1016	1039	Flower bud	--				
CakTSSR03648	CakTC38277	(CAT)5	268	282	--	--	TTTACCCATGACCATCACTAC	TTCGTGGTAATGATTTGATTC	54.93,55.16	176
CakTSSR03649	CakTC38288	(TTC)6	217	234	--	--	TCTAACAAGATTTTGGCAATC	AGTGTTTCACTGCAGATAGGA	54.65,55.04	150
CakTSSR03650	CakTC38300	(CT)18	11	46	--	--				
CakTSSR03651	CakTC38303	(TCT)5	137	151	--	--	ACAAAAGCACGATAGAAGTCA	CTTCAATGTCGTTTTGAGAAG	55.22,55.19	134
CakTSSR03652	CakTC38307	(ATT)5	1623	1637	--	--	TGAGTTCCAGGATAAAGTTGA	TCCAAAAGAACAACAACAAGT	54.95,54.94	178
CakTSSR03653	CakTC38307	(CGG)5	1796	1810	--	--				
CakTSSR03654	CakTC38311	(TTG)11	1105	1137	--	--	AAAGGGTAGAGAAAAGGGTTT	CCTATTTCTCCATTCCCTAAC	55.31,54.56	134
CakTSSR03655	CakTC38324	(CA)6	2341	2352	--	--				
CakTSSR03656	CakTC38326	(GT)6	1594	1605	--	--	ATTAGAAAGCAACATGTCCAA	AAAGTTACTCAGTGCGACAAG	54.99,54.88	151
CakTSSR03657	CakTC38339	(CCA)6	928	945	--	--	AAAAGGACAAGGATTATGGAG	TAAGGCCTCACATTTTGAATA	55.01,55.1	152
CakTSSR03658	CakTC38340	(AG)7	64	77	--	TPR				
CakTSSR03659	CakTC38341	(ATC)5	3781	3795	--	--	TAAGGATGCTTCGAGTAACTG	CTCCCAATTATCCCTCTCTTA	54.91,55.1	161

CakTSSR03660	CakTC38347	(TGT)5	1174	1188	--	--	GCGAGTTTTTAGGTGTTGATA	CAAATCGCAGACCTTTATTTA	54.77,54.88	152
CakTSSR03661	CakTC38352	(ACA)6	258	275	--	--	CATCAACATCTTTCACTCCAC	ATCTTGATCACCAAAGGTTTT	55.56,55.31	149
CakTSSR03662	CakTC38353	(CT)15	18	47	--	--				
CakTSSR03663	CakTC38383	(AGT)5	991	1005	--	--	GGTGATGATTGAGCTAAAGTG	AATAACACCAACACCAATTCA	54.97,55.44	152
CakTSSR03664	CakTC38388	(ATG)7	205	225	--	--	AAGAACACACCACCTCAATAA	CATAGTCCATCGAGGTTCAT	54.72,54.98	151
CakTSSR03665	CakTC38393	(GA)9	1828	1845	Young_pod	--				
CakTSSR03666	CakTC38412	(GGC)5	1409	1423	--	--	AGGAATGTAGTGATGAGGTGA	GGACCAACAGAATCAACAAC	54.6,55.36	157
CakTSSR03667	CakTC38412	(GA)15	1686	1715	--	--				
CakTSSR03668	CakTC38413	(CCA)5	325	339	--	--	CCATTTATATCACTTCCACCA	AATCGATGCTTTTGAGTCTTT	55.05,55.77	156
CakTSSR03669	CakTC38439	(TC)11	67	88	--	--				
CakTSSR03670	CakTC38442	(AAC)5	150	164	--	--	CAAAATCTCTCAACACCAAAC	AAATGAGAAGGCAAGAGATTC	54.85,55.26	149
CakTSSR03671	CakTC38442	(ATC)5	543	557	--	--	TCTCTCTACCACCAAATCAAA	TTCTGTATGATGAGCAAGGAT	54.95,54.88	134
CakTSSR03672	CakTC38449	(TC)6	1685	1696	--	--				
CakTSSR03673	CakTC38450	(TTC)7	1138	1158	--	--				
CakTSSR03674	CakTC38454	(TTA)5	1089	1103	--	bHLH	AAGGAGGGGTTTTCTCTTTAT	AATCCTATTCTCCCCTTCTCT	55.24,55.15	135
CakTSSR03675	CakTC38460	(CCA)5	784	798	--	--	GCTGATCATTGAATTGGTTAC	AACAATATTACCCCAGGAGAG	54.73,54.89	156
CakTSSR03676	CakTC38469	(CA)6	1311	1322	--	--				
CakTSSR03677	CakTC38497	(TC)9	37	54	--	--				
CakTSSR03678	CakTC38520	(CT)7	1637	1650	--	--				
CakTSSR03679	CakTC38524	(TTC)6	83	100	--	--				
CakTSSR03680	CakTC38533	(AG)7	1185	1198	--	--				
CakTSSR03681	CakTC38535	(CT)9	1091	1108	--	--				
CakTSSR03682	CakTC38536	(ACA)6	1297	1314	--	LUG	TCTCCTTCAGAGTTCAAATCA	TAGCTAATCCCCTTAATCGTT	55.06,54.92	143
CakTSSR03683	CakTC38539	(TGT)6	1340	1357	--	--	ATTGTTCTTGCTGATGATGTC	GTTTTCTCTTTTCTGGGTCAT	55.2,55.03	173
CakTSSR03684	CakTC38539	(TC)7	1631	1644	--	--				
CakTSSR03685	CakTC38545	(ACA)5	197	211	--	--	CTACCTTGAGAACGAAGAACA	CCGAGAGATTAAACTTCCATT	54.77,55.16	151
CakTSSR03686	CakTC38552	(ATA)5	1598	1612	--	--	TATGGTTTCTTAATGGCAGAA	GGTCTATTTTCCATCTTCTTCA	55.1,55.14	152
CakTSSR03687	CakTC38554	(AGC)5	1108	1122	--	Alfin-like				
CakTSSR03688	CakTC38560	(ACG)5	159	173	--	TRAF	CGAAAGATAACCAACACAATC	GAAAGACACCTCATGGTGATA	54.83,55.05	136
CakTSSR03689	CakTC38563	(ACA)6	68	85	--	--				
CakTSSR03690	CakTC38573	(TC)15	49	78	--	--				
CakTSSR03691	CakTC38582	(CTT)5	35	49	--	--				
CakTSSR03692	CakTC38590	(TCT)6	198	215	--	--	CTTCAATTCTCTTCCAATTCC	AAGAGAGGTTAAGGTCAGCAT	55.52,54.81	148
CakTSSR03693	CakTC38602	(TC)6	1199	1210	--	--				
CakTSSR03694	CakTC38604	(TGA)5	348	362	--	--	ACCAGTTGTTAGCAGGTCTTA	CTTCATGATTCTGCACCTTAC	54.25,54.97	152
CakTSSR03695	CakTC38604	(GAT)6	1339	1356	--	--	GCCTTATGGTGGAAACTTTAT	CAATGATTGAATTATGATGTCC	55.05,54.54	149
CakTSSR03696	CakTC38609	(CCT)6	296	313	--	--	GCACCCTATTCAATTATTCAC	CCGCTAGTCCTAAGAAGAGTT	53.89,54.76	141

CakTSSR03697	CakTC38616	(ATA)5	30	44	--	--				
CakTSSR03698	CakTC38620	(TC)12	2797	2820	--	--				
CakTSSR03699	CakTC38632	(AAAC)5	219	238	--	--	TTGAACAACACAAAAATACCC	GATGCTGAGAAAGCTAAGGTC	55.16,55.93	150
CakTSSR03700	CakTC38637	(TC)18	1	36	--	--				
CakTSSR03701	CakTC38647	(GAT)5	473	487	--	--	GGTGATGAAGAAGGAGGTACT	GCTCTCATCATCCTTATCCTT	54.88,55.06	153
CakTSSR03702	CakTC38648	(AG)6	969	980	--	ULT	TGCTTCAAAGAGAAGATGTTG	GCTATACGCCAGCTAATTACA	55.77,55.08	150
CakTSSR03703	CakTC38652	(AAT)5	16	30	--	--				
CakTSSR03704	CakTC38656	(ATT)10	100	129	--	--				
CakTSSR03705	CakTC38658	(TC)7	1023	1036	--	--	TTGAGGAATATATGGTCGAAA	ACATACATGCAACAATCTCAA	54.86,54.1	152
CakTSSR03706	CakTC38666	(TTA)5	383	397	--	--	CTCTGCCATGTTATGTTTTTC	ATTTGGTGGAGAAAGTTCCTA	54.99,55.47	158
CakTSSR03707	CakTC38670	(ATG)5	689	703	--	--	TAAACGTGTTCGATTTGATTC	GTGACTTTGATGGCTGATTTA	55.4,55.39	143
CakTSSR03708	CakTC38671	(AG)6	1725	1736	--	--				
CakTSSR03709	CakTC38673	(CT)9	1	18	--	--				
CakTSSR03710	CakTC38679	(TCT)5	242	256	--	--	ATCCCAATTCCCACTTCTAT	TCAGGTTCAACACAGATGAAT	55.01,55.56	150
CakTSSR03711	CakTC38690	(TA)9	163	180	--	--	GCATAAACCTGACAAAGAAAA	GCTCTTCCAATACAGAATCAA	54.73,54.62	136
CakTSSR03712	CakTC38690	(AAG)5	1438	1452	--	--	ATTAGTCCATTAGACCCTTCG	ACATGTAAACGAGGAACCTTT	55.04,55.42	146
CakTSSR03713	CakTC38694	(CT)8	1391	1406	--	--				
CakTSSR03714	CakTC38696	(TTC)8	148	171	--	--	TGATTAGAGGAGAGATGAGCA	ATCTTTGGATTTTGAGGAGAG	55.18,55.12	146
CakTSSR03715	CakTC38698	(AGA)5	1400	1414	--	--				
CakTSSR03716	CakTC38701	(AGA)5	1236	1250	--	--	GGAAGGTCAACAAAACCTAAT	AGTGTTTTTCTCAACACATGG	54.92,55.22	148
CakTSSR03717	CakTC38704	(CAA)6	1150	1167	--	--	AACCATTGTTCTTCCTTCTTC	CCCATCTAATTGGACTCTTTT	55.03,55.01	149
CakTSSR03718	CakTC38707	(GAA)7	601	621	--	--	GACAAAGAATGCAGAAGAAGA	TACTCCTGAAGGACATCTTGA	54.8,54.92	147
CakTSSR03719	CakTC38715	(GAA)5	863	877	--	--	GGTGGAGATCTTGTATCCATT	CAAGAAAGCTCAGTGAACAAC	55.51,55.25	167
CakTSSR03720	CakTC38720	(TG)6	1179	1190	--	--	CCCAATTTTAGTCCCAAATAC	CAGCAATTTAATCAGAAAGGA	55.29,54.82	157
CakTSSR03721	CakTC38734	(GA)15	1476	1505	--	--				
CakTSSR03722	CakTC38739	(TTA)12	1712	1747	--	--	GAATCTGATTTCGGAATATTTG	AGTGTGATTTCAACATCTGCT	55.5,54.85	158
CakTSSR03723	CakTC38740	(GAA)5	1486	1500	--	--				
CakTSSR03724	CakTC38744	(CAA)7	375	395	--	--	CACCACAAGTTTTGTCTTCAT	ACAAGCAAGTGAAGTAAACCA	55.22,55.14	157
CakTSSR03725	CakTC38749	(AC)8	169	184	--	--	TGCTCCATCTATAACTCCTCA	ATGAAAGTCAACACTGCATCT	55.06,54.85	137
CakTSSR03726	CakTC38753	(AG)11	391	412	--	HB	TGAGAAAAATTAGCACACAAAG	ACATATTCATGCTGTTGTTCC	54.99,55.09	143
CakTSSR03727	CakTC38756	(GAA)5	500	514	--	--	AAGACTGTGATTTGGCATTAG	ACCAGAAGCAGAAAAAGAAAC	54.58,55.42	176
CakTSSR03728	CakTC38763	(TGA)5	971	985	--	--	GTGGTCCAACATAGAACCATA	TCTTTCTTGTTTGTTGTCGTT	54.93,55.1	155
CakTSSR03729	CakTC38763	(GTG)6	1150	1167	--	--	GTGATGATTTTGTGATGGTTT	AAAGATTGCAGTGTTTGTTTC	54.88,54.61	140
CakTSSR03730	CakTC38771	(TC)6	6	17	--	--				
CakTSSR03731	CakTC38780	(TGA)5	1314	1328	--	C3H	AAATTTATCATCCCAAGGACT	AAAGAACCATTTCCAAGAGAC	54.39,55.03	152
CakTSSR03732	CakTC38786	(AAT)6	30	47	--	--				
CakTSSR03733	CakTC38786	(TTC)6	1405	1422	--	--	CAAGTTTGTATGATGGTGGAT	AAAAAGGGAATGTGAAGCTAT	54.93,54.78	147

CakTSSR03734	CakTC38789	(GAA)5	82	96	--	--				
CakTSSR03735	CakTC38796	(ATCGC)5	1282	1306	--	--	AAAAATATCCAAAGGGTATCG	GAAATGAAAAGAGATGGTCAA	55,54.42	152
CakTSSR03736	CakTC38799	(TC)8	355	370	--	--	TTCACGAAAATACATCACACA	GGAGGACTATTAGCCATTGAA	55.06,55.98	172
CakTSSR03737	CakTC38803	(ATC)5	738	752	--	MYB	GCAAGTCATGCACAAAAGTAT	TATGTTGGCCTGTTATTGGTA	55.49,55.72	148
CakTSSR03738	CakTC38805	(AG)8	3	18	--	--				
CakTSSR03739	CakTC38806	(AAC)6	1370	1387	--	--	GTTCAGATTGAGGTGAAACAG	AATAAAGGCTGCAATAACACA	54.83,55.2	149
CakTSSR03740	CakTC38817	(TGT)5	1136	1150	--	bHLH	AGATTCTTCATTGTTGGCATA	TGGATTCAACAAGTATTGGAC	54.91,55.06	140
CakTSSR03741	CakTC38830	(AAT)6	829	846	--	MYB	CCTTCTATGGTTTCATCATCA	GTTGGTGGAACATTCTCATTA	55.16,55.06	155
CakTSSR03742	CakTC38832	(CT)10	6	25	--	--				
CakTSSR03743	CakTC38833	(TCT)5	353	367	--	bHLH	TAGCAGCTGATAGTTGCTCTT	GCTACAATTGGCATATGAAAG	54.81,55.12	151
CakTSSR03744	CakTC38834	(AG)16	2980	3011	--	--	AGCTGAGTAAAATTCATCGTG	CAAGTGTTCTCATTTCTTTCG	54.74,55.19	158
CakTSSR03745	CakTC38850	(TAT)6	99	116	--	--				
CakTSSR03746	CakTC38857	(CT)6	53	64	--	--				
CakTSSR03747	CakTC38866	(TTA)6	1249	1266	--	G2-like	GCTTAAGCTTTGTTGTTGTTG	CATCATCACTTCCAAAACATT	55.46,55.06	150
CakTSSR03748	CakTC38866	(ATG)5	1394	1408	--	G2-like	TGGAAGAAGTGTTGTTGAAAT	CAAGAAGTGAAGATGAAATGG	54.85,54.95	153
CakTSSR03749	CakTC38870	(ATG)5	362	376	--	--	CAACAACAAGGAAACTGTGAT	TTGGTATCCTTAACAACAACC	55.22,54.29	138
CakTSSR03750	CakTC38872	(ACC)6	321	338	--	--	AAATTCTTCAACAACCTCCTC	GCCAATGATTTAGGTTAGGAT	55.03,54.97	157
CakTSSR03751	CakTC38879	(AG)6	3	14	--	--				
CakTSSR03752	CakTC38880	(AAGA)5	1214	1233	--	AP2-EREBP	TTCTTCATTGAATGGGTCTAA	GAATTTTGGATTGAAACAGGT	54.89,55.71	140
CakTSSR03753	CakTC38896	(TAT)5	1248	1262	--	--				
CakTSSR03754	CakTC38901	(TA)7	1418	1431	--	--	GGGGAATGTTTCTGTTTTTAT	CAGTGATGAGACCACCAGATA	54.87,56.06	152
CakTSSR03755	CakTC38904	(AAT)6	271	288	--	--	CTTCAAGAGTTATTGCTCCAA	GCTTGATTAGCCACAAGATTA	54.88,54.84	148
CakTSSR03756	CakTC38907	(TA)10	1265	1284	--	--				
CakTSSR03757	CakTC38915	(TC)9	1289	1306	--	--				
CakTSSR03758	CakTC38916	(TC)10	102	121	--	--	GTCGACTTCTGTGTCTCGTAG	TGACAACTCCATTTTAACACC	55.1,55.15	151
CakTSSR03759	CakTC38916	(AG)8	1502	1517	--	--				
CakTSSR03760	CakTC38917	(AAG)5	1292	1306	--	--	ACGAGAATTGTTTAAGGGAGT	CATTATCATCAGCAGCTTTCT	54.68,54.82	170
CakTSSR03761	CakTC38925	(TC)9	31	48	--	--				
CakTSSR03762	CakTC38925	(AAT)6	162	179	--	--	CAACAACAGTCTCACAGTCAC	CCATTGATGAGTGGGTATTTA	54.01,55.05	149
CakTSSR03763	CakTC38934	(CGC)5	654	668	--	G2-like	CCATGTTTTGTTGTAGGTGAT	GTGTTATTGTTGGACCTCAAG	55.02,54.72	150
CakTSSR03764	CakTC38934	(TC)7	1322	1335	--	G2-like				
CakTSSR03765	CakTC38936	(AT)8	1464	1479	--	--	CACAGTTCAAAGGTTCAAAAG	AGATGGCACAATTGAAACTAA	55.11,54.99	148
CakTSSR03766	CakTC38937	(AAG)5	1424	1438	--	--	GGGTTTCCAGTAACAACAATA	ATTAGTACCAATCCCACCTTC	54.29,54.72	147
CakTSSR03767	CakTC38944	(TC)7	56	69	--	--				
CakTSSR03768	CakTC38945	(TA)6	52	63	--	--				
CakTSSR03769	CakTC38960	(GA)7	1176	1189	--	--				
CakTSSR03770	CakTC38966	(GCC)5	699	713	--	--	GTGGTGATGAGGACTACGAT	CATAAAGCTGAAGCTGAACAT	54.86,54.9	150

CakTSSR03771	CakTC38979	(TGG)6	473	490	--	--	AAGGATTTGGCTTTGTTACTT	GTGTTTCCATAAGATCCATCA	54.86,54.98	169
CakTSSR03772	CakTC38983	(CT)6	593	604	--	--	TCTGTGCAACCCTAGCTATT	TAAATGTAAGCGGAATTTGAG	55.16,54.88	148
CakTSSR03773	CakTC38984	(CAC)5	420	434	--	Tify	CCAGCAAGTTATTTCTCTCAA	CACGAAAACCAGTATCTTCAG	54.88,55.07	164
CakTSSR03774	CakTC38987	(ACA)5	249	263	--	bHLH	TCAACCAACTTTGTCTATGAAG	GTTGGTGTTGGTTGTAACATT	54.65,54.93	143
CakTSSR03775	CakTC38994	(AAC)5	142	156	--	--	AAACACTCTTTGCCTCTTCTT	TGACATCCTTAACAACATTGG	55.02,56.03	149
CakTSSR03776	CakTC38997	(TC)10	1435	1454	--	WI/SNF-BAF60b				
CakTSSR03777	CakTC38998	(GA)11	1189	1210	--	--	TTGTAAGTATGGAAGGGGTTT	ATTGACAGCTTTTATCCATCA	55.36,54.91	149
CakTSSR03778	CakTC39003	(TAT)8	133	156	--	--	CAAGGAAATATCACAAAGGTG	TACAAAGGATCATGAGTTTCA	54.84,53.28	188
CakTSSR03779	CakTC39003	(TCA)8	562	585	--	--	TGAATCCATGTTCAAGAGTTT	AGACCAATTAGAAGAGCCAAT	54.77,54.76	158
CakTSSR03780	CakTC39005	(GGT)7	887	907	--	--	TGGGTTTGATGAGAAGAGTAG	TAAAACTGAATCACCACCATC	54.52,55.06	151
CakTSSR03781	CakTC39011	(TGA)5	274	288	--	--	AACAAGAGTTCAAGCACTCAA	CTCGTCTCTCTTCAAGATTCA	55.25,54.79	146
CakTSSR03782	CakTC39020	(TTC)7	949	969	--	--	CTTTGTTACGATTTCTTCGTC	GTAAGGAAAATAAAGGCAAGG	54.32,54.96	149
CakTSSR03783	CakTC39026	(GAA)7	1114	1134	--	--	GTTATTGTCTTTGGTTTCTGG	CTACTCCCATTTCTGCTTCTT	54,55.38	151
CakTSSR03784	CakTC39028	(CACT)5	1405	1424	--	--				
CakTSSR03785	CakTC39031	(TC)8	169	184	--	--	CTCTCAAATCCCTCACTTTTT	CTGACACCTAAAATGGATCAG	55.2,54.82	150
CakTSSR03786	CakTC39038	(TTC)20	35	94	--	--				
CakTSSR03787	CakTC39040	(TC)8	22	37	--	--				
CakTSSR03788	CakTC39042	(GT)7	1341	1354	--	--				
CakTSSR03789	CakTC39044	(CT)12	1446	1469	--	--				
CakTSSR03790	CakTC39055	(TA)6	1334	1345	--	--	AGCTAGTTTTGTTGAGCTTGA	GTGTTCGAACTTCCATTTACA	54.7,55.31	144
CakTSSR03791	CakTC39069	(TAA)13	438	476	--	--	CCATCAAAGACTTCTCCTTTAG	CCGTGAGAAAGATATTGACTG	54.99,54.98	157
CakTSSR03792	CakTC39082	(AAT)6	15	32	--	bHLH				
CakTSSR03793	CakTC39104	(AG)6	23	34	--	--				
CakTSSR03794	CakTC39106	(TAA)5	1499	1513	Flower bud	--				
CakTSSR03795	CakTC39108	(AAC)5	181	195	--	--	TCGTAATAATCAATGGAATCG	AAATATCTTTCCAGGACTAGCA	55.3,54.95	153
CakTSSR03796	CakTC39108	(TGG)5	854	868	--	--	GTATGGAGATGCTTATGAAGG	CATATCCAGCAAATTCACTTC	54.02,54.91	134
CakTSSR03797	CakTC39115	(TCT)6	257	274	--	--	TAATTCCAACAACAATGTTCC	TTAATGATTTTCCTCCCTCTC	55.08,55.05	147
CakTSSR03798	CakTC39122	(TTA)7	488	508	--	--	AAAGAAGATGGATCTGGAATC	ATGAAAGACTTCCACTTGATG	54.86,54.34	166
CakTSSR03799	CakTC39122	(TCT)6	1028	1045	--	--	ACCTTCTCTTGGTTCATCTTC	GTAGCAATTCTGGCTAATCCT	55.01,55.34	159
CakTSSR03800	CakTC39123	(CAA)5	90	104	--	--				
CakTSSR03801	CakTC39128	(TAA)7	705	725	--	--	AGGCCACAATAATTACAACAA	CATTCAAGTCGAAGTCAAAAG	54.89,55.19	146
CakTSSR03802	CakTC39146	(GTA)5	1243	1257	--	--	GTGAGTGATTTGGAATCTGAA	CAAGGTAGCCAAACTTAGAAA	55.18,54.14	145
CakTSSR03803	CakTC39148	(AGA)8	1476	1499	--	--				
CakTSSR03804	CakTC39156	(TTTA)5	1579	1598	--	--				
CakTSSR03805	CakTC39158	(AATT)6	325	348	--	SBP	GCTCCTCCATTAACTCATTTC	CAACTTCAAGCTCAATGTTTT	55.54,54.79	168
CakTSSR03806	CakTC39158	(TC)9	1574	1591	--	SBP				
CakTSSR03807	CakTC39161	(ACC)6	1399	1416	--	--	TTGAATACAACTCAACCCATT	CTTTTCAACTCAATTTCATGC	54.67,55.12	152

CakTSSR03808	CakTC39178	(ACC)5	241	255	--	--	AAGGTTGCAAACAAACAGTTA	TATGAGAATGATTTTGGATGG	55.15,55.09	142
CakTSSR03809	CakTC39179	(TCTT)5	82	101	--	--				
CakTSSR03810	CakTC39183	(ACT)5	1371	1385	--	--	AGGTGAAGTTTCCATCTTTTC	TCATTGTTCCTACCAAAGAGA	55.03,54.95	149
CakTSSR03811	CakTC39190	(GAA)5	1099	1113	--	--	CAATTGTCCATGAACATCAC	AGTCTTCGATTCGCTAATTCT	54.55,55.07	148
CakTSSR03812	CakTC39194	(AGA)8	331	354	--	--	CATCTTTGATTTTCTTGATGC	CCATCATCTTCATCATTCATC	55.03,55.31	153
CakTSSR03813	CakTC39200	(AG)10	1532	1551	--	--				
CakTSSR03814	CakTC39201	(TA)6	2534	2545	Young_pod	--	TTGTTGAATGCCAGTAAAGAC	ATGAACTTCCTTTTGGTGAAT	55.47,55.31	154
CakTSSR03815	CakTC39206	(CT)7	115	128	--	AUX/IAA	GCCCTTGAGTTGAGTTAAGAC	AAACTATGGAAATGGAAGGTT	55.69,54.48	147
CakTSSR03816	CakTC39212	(CTT)6	1358	1375	--	--	CTCCTTTATCCATCATTCCTT	TCATCCCTATTTTGTTTCTCA	54.93,54.89	142
CakTSSR03817	CakTC39222	(TTTA)5	1393	1412	--	--	TGTTGCTGATTTACTCTGATG	CAGACCACTTGTGTTTGATCT	53.98,55.2	152
CakTSSR03818	CakTC39223	(TCT)6	60	77	--	C2H2				
CakTSSR03819	CakTC39223	(CAA)5	1250	1264	--	C2H2	TTGGGTCAACAAATATGAGTC	TTGTTCTTGAACCCTTAACAA	55.06,55.05	149
CakTSSR03820	CakTC39223	(ATA)6	1535	1552	--	C2H2	AACCATGCAACTAAACAAATC	TTCACCACCTTCAGTTGTTAT	54.43,54.72	150
CakTSSR03821	CakTC39225	(TC)11	149	170	--	--	CCGTCCTCTACCCTTATTCTA	CCATTTCGAATGAATGTAATC	55.21,54.69	148
CakTSSR03822	CakTC39230	(TC)26	1	52	--	--				
CakTSSR03823	CakTC39235	(AAG)5	100	114	--	--				
CakTSSR03824	CakTC39237	(TCT)12	174	209	--	--	ACAGAGCGTTCAAAGTTTTAT	AATTGGAGAAGAAGAGTTGCT	53.54,54.94	154
CakTSSR03825	CakTC39245	(TGA)5	107	121	--	--	ATACGCAAATGCAATAGAGAG	CTGATGATGTAGCAACCAAGT	54.87,55.34	145
CakTSSR03826	CakTC39247	(AG)12	1566	1589	--	--	AGCTGAATTGAATCTTCCTCT	ATCATTCTCACTTCCCTTCAT	54.86,55.22	141
CakTSSR03827	CakTC39255	(ACT)6	5839	5856	--	ARID	CTAGGTTTACCCTTTCCCATA	TTGACTCTTCTTCTTCAAACG	55.07,54.9	161
CakTSSR03828	CakTC39256	(ATT)5	1531	1545	--	--	CTAACTGGTGGTGAAGAATTG	CATAGTTGCAAAACAAAATCC	54.9,55.01	156
CakTSSR03829	CakTC39262	(AG)20	1520	1559	--	--				
CakTSSR03830	CakTC39264	(TTC)7	1433	1453	--	--				
CakTSSR03831	CakTC39266	(CAG)5	1035	1049	--	--	ATCTTCCCAGTTGCTTTCTAA	AGCTCATGAATAGTGGACTCA	55.78,54.95	150
CakTSSR03832	CakTC39280	(CAA)5	87	101	--	--				
CakTSSR03833	CakTC39286	(AAG)5	1379	1393	--	--	CGCCTTTAATCTTCTCTTTTA	AAACGACACTTGAATATTTGC	53.56,54.59	152
CakTSSR03834	CakTC39287	(TGA)5	171	185	--	--	TTCCTTTTGAACAAAGTATGC	AGAGGGGTTTTTGTTGTGTAT	54.73,55.26	159
CakTSSR03835	CakTC39302	(AG)6	1531	1542	Root	--				
CakTSSR03836	CakTC39303	(CAA)5	88	102	Flower bud	--				
CakTSSR03837	CakTC39307	(ATG)5	1215	1229	--	HB	GAAATGATGATGAGTTGGAAA	TTCTTCTTTATCCATGGCTTT	55.11,55.7	145
CakTSSR03838	CakTC39307	(GA)11	1480	1501	--	HB				
CakTSSR03839	CakTC39312	(GGT)5	428	442	--	--	GATTCAGGAACATTGCATAAG	CCATCAACAATCTGTATGGAG	54.91,55.53	166
CakTSSR03840	CakTC39318	(GA)12	1563	1586	--	--				
CakTSSR03841	CakTC39325	(CAA)5	1007	1021	--	G2-like	CAAGAGATCATTCAAAATGGA	TTTCACTTCATCACTACACCA	55.29,54.12	153
CakTSSR03842	CakTC39336	(GTT)5	110	124	--	--	AGAGAGAGAACCAACCAAAGT	TCAATCACTTGAACTTGTCCT	54.67,54.83	148
CakTSSR03843	CakTC39345	(GTG)6	293	310	--	--	TCTTTGGATCCACATTCATAC	TGGCATCAAGGTTACTAATTC	54.98,54.52	152
CakTSSR03844	CakTC39353	(GA)8	1489	1504	--	--				

CakTSSR03845	CakTC39365	(AG)8	22	37	--	--				
CakTSSR03846	CakTC39372	(TC)8	100	115	Flower bud	--				
CakTSSR03847	CakTC39382	(CT)7	1	14	--	--				
CakTSSR03848	CakTC39387	(GT)8	1420	1435	--	--				
CakTSSR03849	CakTC39388	(TC)6	1211	1222	--	--	TACGAAGTCGTTATGAGGGTA	CAACTCAAACACAAATCAACA	55.04,54.67	157
CakTSSR03850	CakTC39393	(GAA)9	1346	1372	--	--	AGAGAAGCCATTTGAGAGAAT	TTTTCTCCCTTCTTCATTACC	54.86,55.13	148
CakTSSR03851	CakTC39408	(CT)7	343	356	--	--	CATCCTCATCATATTCAGCAG	GGAAGAGGAGAGTGTGGTTAC	55.78,55.37	150
CakTSSR03852	CakTC39410	(GAG)5	1429	1443	--	GeBP	TTCTACTTCGTCTTCTGGTTG	TCAAACCCTAACACCAATTCT	54.77,56.23	149
CakTSSR03853	CakTC39411	(AG)7	15	28	--	--				
CakTSSR03854	CakTC39421	(GA)8	1365	1380	--	--	GTTGGGATACCTCATAGGACT	TGCCAGAAACAGTGAGATATT	54.69,54.97	138
CakTSSR03855	CakTC39424	(TC)7	80	93	--	--				
CakTSSR03856	CakTC39430	(CTT)5	1409	1423	--	--				
CakTSSR03857	CakTC39438	(AC)6	130	141	--	--	ATAGAGACAAAATCGGAAAGC	GAAAATGGATGATGTGAGAGA	55.31,55.09	142
CakTSSR03858	CakTC39439	(CTTCTC)5	786	815	--	--	TATGATCAGTGGGTTCTTCAA	GATGATGCTGAGAAGAAGAAA	55.66,54.72	151
CakTSSR03859	CakTC39439	(TTC)5	1000	1014	--	--	CACTATCCTCTCCTTTTCCTT	AAGAATGGGTTTGATAGGAAG	54.33,55.01	151
CakTSSR03860	CakTC39446	(TTG)5	1644	1658	--	--				
CakTSSR03861	CakTC39457	(TCT)6	45	62	--	--				
CakTSSR03862	CakTC39468	(ATA)6	93	110	--	--				
CakTSSR03863	CakTC39470	(GAA)5	940	954	--	--	TTACTTCCGTGTGATAGTGCT	TACCGATCCTGTTTTTAATTG	55.09,54.57	151
CakTSSR03864	CakTC39476	(AG)6	1219	1230	--	--	ACCCAGGAGTGAAGATAAGAT	AGTTGGGTGTGTGTCTGTTAC	54.41,54.97	144
CakTSSR03865	CakTC39479	(GAT)7	1277	1297	--	--	AAGGAGAGAAACCCTAAGTTG	GTTTCCCTTTTCATTCATTCT	54.42,54.97	149
CakTSSR03866	CakTC39487	(TC)14	1	28	--	--				
CakTSSR03867	CakTC39488	(CT)16	77	108	--	--				
CakTSSR03868	CakTC39489	(TTC)6	1230	1247	--	--				
CakTSSR03869	CakTC39490	(GT)6	4	15	--	--				
CakTSSR03870	CakTC39496	(TTA)5	410	424	--	--	CACATACAACAACACCCTTTT	GCAATGACGATAAGAGAATGA	55.1,55.47	148
CakTSSR03871	CakTC39503	(ATC)5	47	61	--	--				
CakTSSR03872	CakTC39503	(TCA)5	238	252	--	--	GCTAATTGGAGAAAACAACAA	CCAATTGTAGCACAAAACTTC	54.73,55.07	152
CakTSSR03873	CakTC39514	(AG)11	84	105	--	--				
CakTSSR03874	CakTC39517	(GAG)5	1076	1090	--	FHA	CGGATTGAACTTTCTTCAAC	AAGAGAGAAGCAAAGACCTTC	54.92,54.65	134
CakTSSR03875	CakTC39527	(TTA)6	161	178	--	--	TTTTCTGTTTTGGGTTTGTC	CAATTATGGGCACTTTACAAT	55.26,54.44	166
CakTSSR03876	CakTC39534	(TAT)8	188	211	--	--	AAACCTTGTGACATGTTTCTC	TTGTGGTATGGAAGTCAAATC	54.24,55.06	149
CakTSSR03877	CakTC39539	(CAA)7	210	230	--	--	TGTGGTTCCTCTAAACGTATC	AGGGAGTGAAGGTTTATCTTG	54.41,55.07	171
CakTSSR03878	CakTC39563	(AAT)6	1360	1377	--	--				
CakTSSR03879	CakTC39571	(AG)8	1333	1348	--	--				
CakTSSR03880	CakTC39602	(CT)6	1113	1124	--	--	TACTTTTGGTCAGATGGAAAA	ATACCAGGGTGATGGTATGAT	54.97,55.67	153
CakTSSR03881	CakTC39607	(AGA)8	75	98	--	--				

CakTSSR03882	CakTC39608	(ACC)6	92	109	--	--				
CakTSSR03883	CakTC39613	(GAA)5	46	60	--	--				
CakTSSR03884	CakTC39615	(ACA)5	24	38	--	--				
CakTSSR03885	CakTC39626	(GA)11	1227	1248	--	--	CAATGAATAGGATCTTGGAGA	CCTGAGCAAACATCATTAGTC	54.38,54.97	154
CakTSSR03886	CakTC39638	(GAT)5	328	342	--	--	AAAGAGTTGTCCGATAGACCT	TTTTTATCCTCCTTTTCCTTG	54.65,55.3	149
CakTSSR03887	CakTC39645	(TGT)5	1091	1105	--	--	AAGAGGGTCAGTGAGTGATCT	CTTTACAAACAACATCAGCAA	55.36,54.12	153
CakTSSR03888	CakTC39653	(TTA)5	1001	1015	--	--	TGTTTCTTCAATGTTCTGCTT	GTCTCTCAAACAAACCAAAAA	55.18,54.59	151
CakTSSR03889	CakTC39653	(TTC)5	1311	1325	--	--				
CakTSSR03890	CakTC39655	(TTC)7	1795	1815	--	--	GATTGGGGAATATTCTGATTT	ACAACACTCACACTCCACACT	54.71,55.49	151
CakTSSR03891	CakTC39656	(TTC)17	45	95	--	--				
CakTSSR03892	CakTC39659	(AGA)5	80	94	--	--				
CakTSSR03893	CakTC39660	(CCA)7	241	261	--	--	GTGCAGAAAAGAACATAGCAT	ATGAAGAAGAGAGAGCTTCGT	54.73,55.08	159
CakTSSR03894	CakTC39675	(TTC)5	3288	3302	--	--	TCCTTCTTCTCCTTCTTCTTC	CTCTACTCCAACAATGTCACC	54.55,54.69	151
CakTSSR03895	CakTC39700	(TAT)5	81	95	--	--				
CakTSSR03896	CakTC39700	(CTT)5	251	265	--	--	GTTGAATTTTCAGATCTCGTG	AAAAACGAGAAGAATGAGGAT	54.93,54.72	153
CakTSSR03897	CakTC39704	(AAAATC)5	168	197	--	--	CCTCTAAGTCAATTTGGGTCT	TGAGAGTCGTCAGAGAGAGAG	55.07,54.83	155
CakTSSR03898	CakTC39706	(AG)8	193	208	--	--	ACTCCCATACAACACAGTACG	CAGAGTTTAATCTCAGCGAAA	55.12,55.04	156
CakTSSR03899	CakTC39710	(ATA)10	1334	1363	--	--				
CakTSSR03900	CakTC39719	(TGTAAG)5	1137	1166	--	--	TGAGATTGAGATTGAGATTGC	ATTGAGGGAATATGATGAGGT	55.43,55.02	171
CakTSSR03901	CakTC39723	(AG)6	75	86	--	--				
CakTSSR03902	CakTC39725	(CT)6	157	168	--	--	GCAAGTAAATGAAATGAAAACTC	CATCTTCAACAAGAAATCCAG	55.02,54.95	141
CakTSSR03903	CakTC39734	(CAC)5	754	768	--	--	ATAAGGAAAAAGGAAGCTTTG	AGGCAGGAACTACTTTTGAAG	54.7,55.46	151
CakTSSR03904	CakTC39738	(AG)9	1254	1271	--	--				
CakTSSR03905	CakTC39741	(CT)8	101	116	--	SBP	TATTCTTTCTACAGGCAACGA	ATTGAGGTTATCCACCAAGAT	55.32,55.11	144
CakTSSR03906	CakTC39748	(TCT)5	2623	2637	--	zf-HD				
CakTSSR03907	CakTC39752	(CTT)15	123	167	--	--	AATTCACCAATGGAAGTTTCT	CCTAACAGCCTTATCGATCTC	55.31,55.74	150
CakTSSR03908	CakTC39754	(TGC)5	789	803	--	C2C2-Dof	CTTCTCATTTGTCAGCTATGG	TTAGCAAACCCTGTTGAATTA	55.15,55.18	157
CakTSSR03909	CakTC39758	(GAA)6	17	34	--	--				
CakTSSR03910	CakTC39760	(TGTT)5	215	234	Mature Leaf	--	AGACACTTAACTCTGCAGCAA	CGAAGAAAGGTAAAAGGAAGA	55.45,55.52	150
CakTSSR03911	CakTC39768	(GGA)7	180	200	--	--	ATTCCTTCTATTGCTTGGTCT	TCTCTTTATCTCCTCCTCCTC	54.76,54.33	164
CakTSSR03912	CakTC39768	(CCA)8	453	476	--	--	GACGATATTGCTCATCACATT	GAAGGATGAAGAAAAGAATCC	55.17,54.6	149
CakTSSR03913	CakTC39775	(CCA)5	1171	1185	--	--	CGGTACGACTATAGATTCCTG	CACATCTTCCCTGTTCATATC	54.16,54.55	153
CakTSSR03914	CakTC39776	(GA)6	1430	1441	--	--	CTATATCAGCCAACTCAATCG	CTTTTCTTTCTCCCTCATCTC	55.12,54.83	162
CakTSSR03915	CakTC39780	(TTC)5	38	52	--	--				
CakTSSR03916	CakTC39783	(CAT)8	889	912	--	--	CCTTTTTCCTACTTGACACAG	GATGATCACGTCAAAAATGAT	54.22,54.97	145
CakTSSR03917	CakTC39784	(GAA)5	1452	1466	--	--	GAGGGATAGATGGAGAGAAGA	AGACTCAGATCTTTCGAATCC	55.02,55.09	138
CakTSSR03918	CakTC39785	(AG)6	1262	1273	--	Alfin-like				

CakTSSR03919	CakTC39788	(ATA)5	111	125	--	--	CACTTCAAGAATGGGAATTTT	ATAGTTCTTACGCGCACATAG	55.87,54.99	136
CakTSSR03920	CakTC39791	(AG)11	123	144	--	--	CGGTCTCTGCAAAAGTATAAG	GAAGTTGAGGATTCCATTTCT	54.53,54.95	144
CakTSSR03921	CakTC39795	(TTTCAT)6	106	141	--	--	CCTTTTCATTTCACCCTAAAT	TCGAAAGAAAGAAAGGCTAAT	55.03,55.21	145
CakTSSR03922	CakTC39796	(AATCC)5	271	295	Young_pod	--	CAGATTACATTGTTTGGATGC	TTCTTCCCAAGTAGTAACTCG	55.68,53.93	151
CakTSSR03923	CakTC39799	(GAA)19	1300	1356	--	bZIP	AACTTGTCGAAGAAAGACTCC	ATTCAATCACTCATCACCATC	55.25,54.77	158
CakTSSR03924	CakTC39802	(CTT)5	460	474	--	--	CTGACTCCGAACCTGACTC	CTTCACCTTCTCGCTTTCTAT	55.71,55.54	159
CakTSSR03925	CakTC39809	(CCA)7	275	295	--	--	CTTCATCTCTCTCTCACAAGC	CAGAGTCTGGAATCCTCTTG	54.33,54.89	158
CakTSSR03926	CakTC39811	(TC)6	232	243	--	--	ATCATCACATTCCTCAACAAC	ATATTCCCACACACATGAAAG	54.86,54.93	138
CakTSSR03927	CakTC39846	(CTT)6	130	147	--	--	TCTTGATAGTTGAAGCAAAGC	TTTCTACTTCCTCCCAAATTC	55.02,55.13	153
CakTSSR03928	CakTC39850	(GA)11	1264	1285	--	TUB				
CakTSSR03929	CakTC39858	(TTG)6	545	562	--	--	ATATGTTAGCACGTTCCATGT	TCCACCACCATAAGTACAAGT	54.73,54.59	163
CakTSSR03930	CakTC39861	(AAT)6	122	139	--	--	CAATCATTTTGAAGTTGATCC	ATGAGAGAGGTTGAAGTTTCC	54.71,55.01	144
CakTSSR03931	CakTC39864	(CAC)5	350	364	Root	--	GTTGTTGTCCATTTTGAACTC	GAAGAGAATGGTGAAGGTGAT	54.67,55.71	166
CakTSSR03932	CakTC39864	(GTT)7	939	959	Root	--	GAGGAGTTTGTGTTTGTTTGA	TCTCCATGAATAACGAAAGTG	55.35,55.4	133
CakTSSR03933	CakTC39864	(AT)9	1111	1128	Root	--	CCATAAACTGAACCGATAAAA	CTTCCCATCACTCACTACCTA	54.57,54.39	166
CakTSSR03934	CakTC39867	(TCT)5	1351	1365	Flower bud	bHLH	ACTCGAATATCTTCTGGCATT	AAGATGACATGGACGAAGAC	55.58,55	172
CakTSSR03935	CakTC39869	(TGG)5	581	595	--	--	CAGGAGCACCAACATAATAAG	AATTCAGCATGTTTACCCATA	55.04,54.81	140
CakTSSR03936	CakTC39879	(TGC)5	508	522	--	FHA	AGATCGGTGATAAGGAGTTTT	ACAGGCAGATGTCCATAACTA	54.6,54.84	157
CakTSSR03937	CakTC39897	(ATT)6	688	705	--	C2C2-Dof	CTGAGAAAGTTGTTTCCAAAA	TACCAGAAGCTAAGGTCATCA	54.77,55.15	151
CakTSSR03938	CakTC39899	(ACA)5	136	150	--	G2-like	ACTGTCACTTGCCGAGTATTA	TACTACTTCACCTGGCTTTTG	55.09,54.84	176
CakTSSR03939	CakTC39899	(AG)7	1501	1514	--	G2-like	AGGGTAGAAAACATGGAAAAG	ATTTTGATTCTCCTCAGATCC	55.09,54.86	145
CakTSSR03940	CakTC39902	(AGA)5	1104	1118	--	--	AGATTGAAAGAGGAGGTGAAT	GAAACAAATAGGGTGATCCAT	54.53,55.51	147
CakTSSR03941	CakTC39906	(AG)6	74	85	--	--				
CakTSSR03942	CakTC39929	(TTC)5	92	106	--	--				
CakTSSR03943	CakTC39933	(CAA)5	1	15	--	--				
CakTSSR03944	CakTC39951	(GA)9	1547	1564	--	--				
CakTSSR03945	CakTC39967	(ATA)5	300	314	--	--	GAACAATTACCACCAACAGAA	GGAAATGAGAAGAGGGAGAT	55.15,54.83	138
CakTSSR03946	CakTC39969	(TGA)5	1255	1269	--	--	CAAGAGTTTGAGAGAGAAACG	TCTCTCTCTTCCTCCTTTCAT	54.48,54.81	154
CakTSSR03947	CakTC39969	(AGA)5	1437	1451	--	--	TGAAGGAAGGTATCAAAGAGA	TTTTCCTAAAGCTTCCATCTT	54.17,55.06	147
CakTSSR03948	CakTC39970	(AAC)5	98	112	--	--				
CakTSSR03949	CakTC39975	(AG)7	27	40	--	--				
CakTSSR03950	CakTC39992	(CTT)7	124	144	--	--	ACTACCAAAACCATTTTCCTT	TCGGATATAATGTCGCTTTAC	54.56,54.66	149
CakTSSR03951	CakTC40000	(GA)13	1162	1187	--	LOB	ATGGAGGCGAATATATAGGAG	TTTTAAGAAACAACTGCGTTC	55.09,54.97	151
CakTSSR03952	CakTC40003	(CT)13	17	42	--	--				
CakTSSR03953	CakTC40021	(TTC)5	219	233	--	--	TGGATAAGATGAAGCAAAAAG	ATTTCCCAGTCTTGTCTTAGC	54.82,55.22	151
CakTSSR03954	CakTC40022	(GA)8	34	49	--	--				
CakTSSR03955	CakTC40024	(TGG)5	1581	1595	--	--	TGAAGAAGGGAAGAGAAAGTT	CCCAAAGAGAAAGGAATAAGA	54.92,55.3	151

CakTSSR03956	CakTC40031	(TC)12	1	24	--	--				
CakTSSR03957	CakTC40034	(GAG)6	79	96	--	--				
CakTSSR03958	CakTC40039	(GCT)5	668	682	--	--	CCTCCTCAACGTCTTCTATTT	ATTTGAATAACTGAAGCACCA	55.23,54.99	158
CakTSSR03959	CakTC40043	(GAA)5	208	222	--	--	AAGCTTGCTTGGGTTAGTAAT	GGTGGTGATTCTCCTTATTCT	55.04,54.82	161
CakTSSR03960	CakTC40044	(CTT)6	706	723	--	--	TCTTTCGTAACGATTCACATT	GGTGAGACCGAATAGTTCAC	54.99,54.58	149
CakTSSR03961	CakTC40061	(TCTTC)5	1050	1074	--	--	GTTTGATCTCATGTCTCTCCA	TGTTGTATTAGAAGCGGTGAT	55.16,55.02	146
CakTSSR03962	CakTC40076	(TC)6	41	52	--	--				
CakTSSR03963	CakTC40077	(AAAAAT)5	1115	1144	--	--	CGAGTTAATCTTCTTCGTGAG	TTCGATTCAAAGAACCATCTA	54.46,55.05	153
CakTSSR03964	CakTC40083	(TC)15	1342	1371	--	--				
CakTSSR03965	CakTC40084	(CT)9	19	36	--	--				
CakTSSR03966	CakTC40091	(TA)9	44	61	--	--				
CakTSSR03967	CakTC40115	(GA)7	1575	1588	--	--				
CakTSSR03968	CakTC40116	(CAA)5	383	397	--	zf-HD	TAGATGTTAGAGCCATGGACA	GATTTTTGGTGTTGTTTGTGT	55.86,55.05	152
CakTSSR03969	CakTC40137	(TA)7	61	74	--	--				
CakTSSR03970	CakTC40146	(TGA)9	1298	1324	--	--	GAGTTGTGGTTGGTAGTTTGA	CTCACTTCTCATCACCATCAT	55.22,55.03	157
CakTSSR03971	CakTC40153	(AT)6	218	229	--	--	GAGTGAAAGAATGCACCATTA	TCTTAGTGACGATGTTGATGA	55.39,54.2	150
CakTSSR03972	CakTC40157	(TGA)6	1201	1218	--	--	TCAAGTGTAGCTGAGGACAAT	GCTGTAAAAATGTGGACTCTG	55.04,55.05	145
CakTSSR03973	CakTC40164	(TC)7	35	48	--	--				
CakTSSR03974	CakTC40170	(CCT)5	445	459	--	--	TAGACGACTTCTCCTTCTCCT	AGAGGGAGGTTAGTGATTCAG	54.93,55.06	167
CakTSSR03975	CakTC40177	(GAA)5	605	619	--	--	ATTGAGGTTGAGGTTGAAGTT	AACACCCCTCTCTAAATTGTT	55.38,54.53	150
CakTSSR03976	CakTC40191	(AAG)5	986	1000	--	--	CTGTGAATGAAAAGGAAGAGA	ACCTTTGCTTCTTCAACTTCT	54.65,55.02	148
CakTSSR03977	CakTC40191	(CATG)5	1232	1251	--	--	AAAATAAGGTTCTTGCTTTGG	TTCTGAAGAGACACTATGTTGC	55.4,54.77	154
CakTSSR03978	CakTC40192	(TC)6	39	50	--	--				
CakTSSR03979	CakTC40200	(GA)6	1148	1159	--	--	GAGTAGGGAATCTGGGAGAG	AAAGGCTACGAATTTTCAGTT	55.37,55.01	159
CakTSSR03980	CakTC40201	(GAA)12	1695	1730	--	G2-like				
CakTSSR03981	CakTC40206	(CTC)5	758	772	--	--	TCTTAATCTCAAATCGTCGTC	TAGGTGAAGTTTCGCTACAAG	54.62,54.99	152
CakTSSR03982	CakTC40209	(GAA)6	1121	1138	--	--	GGGAATGTGAGTTTGTTTGTA	ATTCCACAGTTCCTCTGAGAC	55.15,55.8	169
CakTSSR03983	CakTC40213	(GCC)5	68	82	--	C3H				
CakTSSR03984	CakTC40214	(TTG)5	870	884	--	--	TGTTCTGATTTTCCATGTTCT	TACCCAAATGAAATCAGTGAC	54.77,55.06	145
CakTSSR03985	CakTC40214	(GT)6	1314	1325	--	--	TTGAAAGGGTTGTGAAGATTA	AGGAATGGTTCTTGATCTTGT	54.97,55.3	140
CakTSSR03986	CakTC40222	(AGC)5	36	50	--	--				
CakTSSR03987	CakTC40222	(TCT)5	187	201	--	--	AAATTCAAATCATCGGAGACT	GTGAAAGTGAACTTTTGTTGG	55.39,54.94	134
CakTSSR03988	CakTC40225	(TAA)6	776	793	--	TCP	CAATCATCATCTGCTCATTCT	GATGTAAAACTCATTCCACCA	55.3,55.06	149
CakTSSR03989	CakTC40235	(CCA)5	106	120	--	--	CCTGAACATTCAGCAATTAAC	GGTGGCTAAGTTTTTGTTTAG	54.99,53.62	149
CakTSSR03990	CakTC40238	(GGT)5	719	733	--	--	AGGAAATTAATGGGTGAAGAT	TTCTCTTCCAATGTTTTCTCA	54.39,55.08	146
CakTSSR03991	CakTC40245	(TGT)5	410	424	--	OFP	TCCTGGTGAATTAACAGAGTT	ACCCAACAATAAAGAACACAA	53.95,54.75	157
CakTSSR03992	CakTC40245	(GAA)6	731	748	--	OFP	TGTTCTGAATTCTGTTTCAGG	ACTTCCTCTGTCTATGCAGGT	55.44,55.57	150

CakTSSR03993	CakTC40248	(TCA)5	77	91	Shoot	--				
CakTSSR03994	CakTC40254	(GAA)5	67	81	--	--				
CakTSSR03995	CakTC40265	(TTA)5	7	21	--	--				
CakTSSR03996	CakTC40275	(TCA)5	115	129	--	--	CCATCATTTCACGAAGAATAA	GAAAAGTGGAAAAGGAAGAAG	55.33,54.94	150
CakTSSR03997	CakTC40285	(GA)6	748	759	--	--				
CakTSSR03998	CakTC40287	(TC)15	33	62	--	--				
CakTSSR03999	CakTC40312	(AAT)9	135	161	--	PLATZ	AAAGGCTCCTGTGATATTTTT	TCAATATTCACCTTTGGATCA	54.78,55.58	139
CakTSSR04000	CakTC40312	(AG)10	993	1012	--	PLATZ				
CakTSSR04001	CakTC40313	(TA)8	256	271	--	--	CCGAATGTGTTATCGATTTAT	TGAAGAAGACGGAATAGTGAG	54.35,54.69	136
CakTSSR04002	CakTC40315	(GGA)6	853	870	--	--	GCATTTCTTTCCTCTATGGAT	GCCATCAGAACCTATTGATAA	55.08,54.44	159
CakTSSR04003	CakTC40315	(TC)9	1968	1985	--	--				
CakTSSR04004	CakTC40316	(CTT)6	178	195	Flower bud	bHLH	CCCTCTTTTCATTTCTCTCAT	TCTCTTTGAGGAAGAACGTG	55.12,55.59	156
CakTSSR04005	CakTC40321	(TGG)5	598	612	--	--	GTGTTTTGTTATTGGGTCAAA	CTAAATCCAGAACACCCATTA	55.16,54.38	158
CakTSSR04006	CakTC40329	(TCG)5	1880	1894	--	--	GCTTGAATTTCTGTTCTCTGA	GAAGAACAAAACGACGACTAC	54.8,54.19	145
CakTSSR04007	CakTC40337	(GA)9	197	214	--	--	GGTGAAGTCTCAACTCAAGTG	CGTCGATGAAGTACTCAAAGT	54.89,54.63	154
CakTSSR04008	CakTC40338	(TA)6	2148	2159	--	--				
CakTSSR04009	CakTC40342	(GAAAGT)5	341	370	--	--	CGATAAAAATGAGAACGAGAA	CTGCTAGAACCAGATGATGAC	54.82,54.95	153
CakTSSR04010	CakTC40343	(AGA)6	289	306	--	CCHC	CCGTATTGAATGTACCAACAG	TCCTTTTCTTCTTCTTCAACC	55.64,55.32	136
CakTSSR04011	CakTC40344	(TC)6	1	12	--	--				
CakTSSR04012	CakTC40350	(GTG)6	636	653	--	--	GCATGTGCTTGATTACTTTTC	AACACCAAATCTCAAATTTCC	55.14,55.71	168
CakTSSR04013	CakTC40352	(AG)6	739	750	--	--				
CakTSSR04014	CakTC40358	(CAA)5	384	398	--	GRAS	ACTTTAGCAGAATTCCAGACC	AGATAAAGGGGAATTGTTTTG	55.22,55.03	147
CakTSSR04015	CakTC40361	(TAT)5	336	350	--	--	ATGAAAACATGCAAACTGACT	AGGCAAGAAAGTGGATAAAGT	54.88,54.84	158
CakTSSR04016	CakTC40363	(TCA)5	468	482	--	--	AATTGTTTATGCTCTCCAACA	CCTTGTTAGCATCACCATTAG	54.99,55.04	151
CakTSSR04017	CakTC40366	(CTC)5	540	554	--	--	CTCATTTCTTTATTGCACACC	AGATGGAAAAGGATGAAAAAG	54.99,55.13	150
CakTSSR04018	CakTC40380	(GTT)7	671	691	Flower bud	--	CACAACAAGAGCTTTAGATTCA	TCTCTGTCTTTCAACACCAAT	54.97,54.83	151
CakTSSR04019	CakTC40386	(TGT)5	1835	1849	--	WI/SNF-BAF60	TCGAACGCTTGGTAGTAGTAG	ACAACAACCATCACATTTTTC	55.03,54.97	155
CakTSSR04020	CakTC40390	(GA)11	1	22	--	--				
CakTSSR04021	CakTC40402	(TC)6	65	76	--	--				
CakTSSR04022	CakTC40402	(ATC)5	624	638	--	--	TGCAGAAAGTTCAAATCAAAC	ATCTCCAACTTGAGAAGTGCT	55.6,55.7	150
CakTSSR04023	CakTC40408	(TTC)5	96	110	--	--				
CakTSSR04024	CakTC40411	(GAA)5	2183	2197	--	--				
CakTSSR04025	CakTC40414	(ACA)6	196	213	Flower bud	--	AAACCCCTCTTCTAACTTCAA	TGAAGCCTAAGAGATTCAAAA	54.82,54.54	150
CakTSSR04026	CakTC40422	(CAC)5	327	341	--	--	CCCCTCAACTACAACTAATTC	TAAGAAAGTGTTCCAGTCACG	53.14,55.56	139
CakTSSR04027	CakTC40423	(AAC)6	210	227	--	--	AGGATGAAGCTTCTAGACGAG	CTCTTCCTTCCTCTTCCTTTA	55.54,54.63	161
CakTSSR04028	CakTC40424	(AAG)6	107	124	--	--	CGTTCAACATTTGTTTAAGATG	CAACTTTGTATCCCATGAAAC	55.11,54.67	148
CakTSSR04029	CakTC40427	(TC)10	56	75	--	--				

CakTSSR04030	CakTC40434	(AATA)7	731	758	--	--				
CakTSSR04031	CakTC40450	(TTC)6	1973	1990	--	--				
CakTSSR04032	CakTC40464	(TC)7	60	73	--	--				
CakTSSR04033	CakTC40469	(GA)9	923	940	--	--				
CakTSSR04034	CakTC40480	(GAA)5	91	105	--	Bromodomain				
CakTSSR04035	CakTC40480	(GAC)5	776	790	--	Bromodomain	ACGTGTTCTTGGTATGCTCTA	GTTGTGGTGGTTCACTATCAT	55.09,54.81	159
CakTSSR04036	CakTC40497	(TGT)5	745	759	Shoot	--	GGAGTTTGTGCAGAGACATAG	CAAAAACATGATTACCCTCAA	55.04,55.25	145
CakTSSR04037	CakTC40512	(CA)7	195	208	--	--	CATCACTCTTCTCCACCACTA	CAATTGAAAAAGGAAGTGGAT	55.3,55.87	147
CakTSSR04038	CakTC40517	(TAT)5	395	409	--	--	GTAGTCATGTGGGGATTGTAA	AAAAGAGCCAGAGAAGAAAAA	54.93,55.24	141
CakTSSR04039	CakTC40520	(AG)10	705	724	--	--				
CakTSSR04040	CakTC40534	(TCT)7	132	152	--	--	TGTGGTAGATGATGAAGAAGG	AGAATTGCATTACGGTACTTG	55.23,54.64	147
CakTSSR04041	CakTC40544	(AG)15	2398	2427	--	--				
CakTSSR04042	CakTC40547	(GTT)5	231	245	--	--	TGGGAGGGTTTTTGTAGTAGT	ACCCTTATAATCCAACACCAT	55.43,55	138
CakTSSR04043	CakTC40547	(TG)8	627	642	--	--	AAGATGGGAAACCAAAAATAG	GTGACAAAACACATGTAACCA	55.03,54.34	163
CakTSSR04044	CakTC40555	(TC)7	13	26	--	--				
CakTSSR04045	CakTC40557	(TC)12	641	664	--	--				
CakTSSR04046	CakTC40560	(ATA)5	406	420	--	--	TCTTTCACTATGCAACCAAGT	TCCTATTCCCTTTCTTACCAC	55.05,55.01	150
CakTSSR04047	CakTC40562	(TAT)6	171	188	--	--	ATAAACCAGATCTGACATCCA	TGCAGTTACTCGGATACTTGT	54.55,55.09	211
CakTSSR04048	CakTC40566	(TC)8	45	60	--	--				
CakTSSR04049	CakTC40577	(ATC)6	1875	1892	--	--	AAGAAGTTGCAGAGACAGTTG	TAATTAACACGCAAAAACTCC	54.82,54.79	160
CakTSSR04050	CakTC40583	(CCT)6	87	104	--	--				
CakTSSR04051	CakTC40585	(TC)6	49	60	--	--				
CakTSSR04052	CakTC40587	(ATTC)6	86	109	--	--				
CakTSSR04053	CakTC40598	(ATA)6	410	427	--	--	TCTTAATCCCTCTCTCTCCAC	TCAACATATCTGGATCCATTC	55.1,54.89	144
CakTSSR04054	CakTC40603	(CAT)5	758	772	--	--	ACATTAGGCCCTTCAAGTTAC	TCCATGTGATTCTTCTCTTTG	55.11,55.36	169
CakTSSR04055	CakTC40604	(TTC)6	698	715	--	--				
CakTSSR04056	CakTC40612	(AGA)5	385	399	--	--	GCTGAAGATTCATTTTGCTTA	GGCATGAAACTTTCTGTTAAA	54.96,54.73	152
CakTSSR04057	CakTC40628	(GAC)6	533	550	--	--	TAGAAAGTTCCAACAATCTGC	AACTCCAGAACCTCTAGCAAC	54.7,55.29	154
CakTSSR04058	CakTC40637	(GA)20	2026	2065	--	WRKY				
CakTSSR04059	CakTC40642	(CT)10	1	20	--	--				
CakTSSR04060	CakTC40642	(CAA)5	782	796	--	--	CTCCGTTAGAGAAATGTGAAG	GCTCTTTTTCATGGCTTTACT	54.3,55.53	146
CakTSSR04061	CakTC40647	(AAT)5	957	971	--	--	GAAATTAGGGAGGGAATTGTA	ATTCTTCAATCCCAAAAACTC	54.95,54.97	149
CakTSSR04062	CakTC40666	(TGG)6	776	793	--	OFP	GTCGTCGGAGAAATAGTCTTC	CTCAAATATCTCTCCCAACCT	55.62,54.99	153
CakTSSR04063	CakTC40669	(AAT)17	6	56	--	--				
CakTSSR04064	CakTC40671	(TAA)6	7	24	Mature Leaf	--				
CakTSSR04065	CakTC40672	(TA)6	550	561	--	G2-like	AACATAGTTGTTTGGCAACAT	GTAAATTACCGTGTTTGTTCG	54.77,54.97	144
CakTSSR04066	CakTC40672	(GCT)5	973	987	--	G2-like	GAAGCATTAGCTGATTCCATA	GACACTTTGAGAATCCCTTTT	54.76,55.03	142

CakTSSR04067	CakTC40672	(AG)10	1930	1949	--	G2-like				
CakTSSR04068	CakTC40682	(TTG)5	1545	1559	--	--	ACCTTTGAGTAACCCTTGAAC	GTAGCTTCTGAATCAAACACG	54.98,55.22	169
CakTSSR04069	CakTC40685	(TCT)5	245	259	--	Trihelix	AACTGTGTCCACTGATGAAAG	TGAAGTTGAAGCAAAAGAAGA	55.2,55.48	151
CakTSSR04070	CakTC40688	(TTC)7	104	124	--	--	AAGAACTAACGCAATGTTTTG	GAGTGAAGAAGGTTTTTGGTT	54.85,55.11	151
CakTSSR04071	CakTC40693	(CT)6	18	29	--	--				
CakTSSR04072	CakTC40695	(TA)9	56	73	--	--				
CakTSSR04073	CakTC40700	(ATA)5	176	190	--	--	CAGATTTATCCTTTTCGTGTG	CCGTTTATGATGATGATTGTT	55,55.03	159
CakTSSR04074	CakTC40702	(AT)10	653	672	--	--				
CakTSSR04075	CakTC40704	(ATT)7	194	214	--	--	TTTCCGGATTTATTTTCAGA	GATAACGACGTGTTGATGATT	54.97,55.09	152
CakTSSR04076	CakTC40711	(CCA)5	950	964	--	--	AATATGCCTATGAGCATCTGA	TTGAAGGTGTTAAATTGGTTG	55.02,55.33	148
CakTSSR04077	CakTC40717	(CAT)5	101	115	Flower bud	--	TTTGGGAATAGGAAATAAAGG	GTGATGGAATGATTGATTTTG	55.13,55.4	146
CakTSSR04078	CakTC40720	(TGT)5	233	247	--	--	AGCTCTTCCTTGTAGTTGGAG	TCAAATATTTCAAGAGCAAGC	55.46,54.96	154
CakTSSR04079	CakTC40747	(CAA)7	301	321	--	--	CGGAAACTTCTTTCTCTTACC	GGTCTGAAGGGTTGATAGAGT	54.97,54.88	171
CakTSSR04080	CakTC40771	(CT)6	1	12	--	--				
CakTSSR04081	CakTC40774	(CAG)5	761	775	--	LIM	CTGAACCAATTGAAGTTGAAG	AAACCTAGCTCCAAAACAAAG	55.03,55.47	151
CakTSSR04082	CakTC40790	(TAA)5	186	200	--	--	CAGTTGAAGGAAGACAAAATG	TAACTCCAACGAATGGTGAT	55.03,55.5	150
CakTSSR04083	CakTC40793	(TCA)8	534	557	--	--	CAAGTCCCTACCTTTGAGAGT	AGGAACACAAGGGGTATAAAA	55.14,55.36	177
CakTSSR04084	CakTC40794	(CTT)6	115	132	--	--	TTCTTCTGTTTCCTCCTTCTT	GGTTCACTATGAAATCAGCAC	54.92,54.79	154
CakTSSR04085	CakTC40797	(GAT)5	1778	1792	--	--	ACCTGTGATGAAGTGAATGAG	GACACAACACAATACAGAGCA	55.11,54.66	149
CakTSSR04086	CakTC40824	(AAC)5	229	243	--	--	ACACCAAGAAGTTTGTTGAAA	AATGGAAGCCCTATTATTGTT	54.94,54.61	168
CakTSSR04087	CakTC40836	(GAA)5	1894	1908	--	--				
CakTSSR04088	CakTC40842	(TC)6	41	52	--	--				
CakTSSR04089	CakTC40846	(GAA)5	566	580	--	--	CAAAAAGTGGTGAGAAAGATG	GTGCAAGACAATGGCTATATT	55.03,54.55	158
CakTSSR04090	CakTC40857	(TC)6	49	60	--	--				
CakTSSR04091	CakTC40858	(CT)7	37	50	--	--				
CakTSSR04092	CakTC40873	(CAT)7	1627	1647	--	--	CTGCTGAAACTTCTCAAAAAT	GTCCATTTGGATCAACAATTA	54.04,55	159
CakTSSR04093	CakTC40873	(AG)6	1998	2009	--	--				
CakTSSR04094	CakTC40875	(AG)6	2016	2027	--	--	CGTAAATTCACAAACAAGAGG	AAACAAACAATCCCTTTCTTC	55.08,55.04	148
CakTSSR04095	CakTC40877	(GAA)5	645	659	--	--	AATAGGGACTTTGGTGTTGTT	AAACTCTTCGTTGAGTTGTGA	55.26,55.08	144
CakTSSR04096	CakTC40880	(CA)9	55	72	--	--				
CakTSSR04097	CakTC40889	(TCT)7	86	106	--	--				
CakTSSR04098	CakTC40890	(CAA)5	1626	1640	--	TPR	GGATGTCTGCTATGTTGTTGT	AACACTAACATGTCCATTTGC	55.16,55.17	158
CakTSSR04099	CakTC40891	(CT)9	15	32	--	--				
CakTSSR04100	CakTC40893	(AGA)6	218	235	--	--	TAAGGAAGGAAGAAAAGAGGA	GTAAATTTGAAAACCCTTTCC	55.02,54.63	144
CakTSSR04101	CakTC40906	(AT)7	775	788	--	--				
CakTSSR04102	CakTC40907	(CCA)5	698	712	--	MYB	TTCCTTTCTTCCTCTCGTTAT	GGTTGAGGTACCGAGTTATTC	54.89,55.34	134
CakTSSR04103	CakTC40908	(ATA)9	253	279	--	--	GGCAACTTCTCAAAAGAAAA	GGGGTCTTTATGGCTATGTAT	54.82,54.85	147

CakTSSR04104	CakTC40910	(CCA)6	1311	1328	--	--	AGCAGGAATAGGTTTCTTACC	GATAATTGGGCTTCGGTTA	54.39,55.25	156
CakTSSR04105	CakTC40917	(TC)11	9	30	--	--				
CakTSSR04106	CakTC40919	(AG)8	2190	2205	--	--				
CakTSSR04107	CakTC40922	(AAG)5	1929	1943	--	--	TCGCTATCATCTTAATTGGTT	CCCAGAGAGAGAAAGAGAGAG	54.23,55.05	144
CakTSSR04108	CakTC40925	(TTC)9	809	835	--	--				
CakTSSR04109	CakTC40940	(AGA)6	1718	1735	--	--	TTCGTCGTTTAGACAACTCAT	TCCTCTCATTACTGTCACCAC	55.05,55.11	147
CakTSSR04110	CakTC40940	(GA)9	2027	2044	--	--				
CakTSSR04111	CakTC40943	(CTT)7	74	94	--	--				
CakTSSR04112	CakTC40946	(ATT)5	1920	1934	--	--	TCATCTTCCAATTCTTGAAAC	CTTGTTATCTCAAAAGCAAGC	54.42,54.64	135
CakTSSR04113	CakTC40962	(AAAAT)7	586	620	--	--	AATCTCTGACATGTTTGCCTA	GGGAAAACAAAAATAACACAAG	54.97,55.56	159
CakTSSR04114	CakTC40967	(AG)7	2135	2148	--	--				
CakTSSR04115	CakTC40987	(GAC)5	1978	1992	--	--				
CakTSSR04116	CakTC40991	(TCT)5	1728	1742	--	--	GGATGTGATCGGTATTATTGA	GGATCTTCTTCCATTCACAAT	54.95,55.63	157
CakTSSR04117	CakTC40994	(TTC)9	1791	1817	--	--	CCAAAAGAGGAAGTGAGAGAT	TCTACTCCAACTCCAACTCAA	55.18,55.01	159
CakTSSR04118	CakTC41004	(GA)7	560	573	--	--				
CakTSSR04119	CakTC41015	(AG)13	576	601	--	--				
CakTSSR04120	CakTC41019	(TAT)5	1893	1907	--	--				
CakTSSR04121	CakTC41037	(ATT)6	1970	1987	--	--				
CakTSSR04122	CakTC41048	(CAT)7	1733	1753	--	--	CTCTTCTTCTTCTTCGAGCTT	GCTAGTGAGGACTCCTTGTCT	54.98,55.28	155
CakTSSR04123	CakTC41054	(GAA)5	516	530	--	--	AAATGGAAGTCGACAGTTACA	CAGGCAAAGAAGAGTTTCAC	54.89,55.16	144
CakTSSR04124	CakTC41077	(AAG)5	654	668	--	--				
CakTSSR04125	CakTC41099	(AG)7	674	687	--	--				
CakTSSR04126	CakTC41104	(CAA)6	273	290	--	--	AGGAGAAACACCTTCAGTTCT	TATAAGTGATCGTTCCTCCAA	54.67,54.92	156
CakTSSR04127	CakTC41105	(TC)7	6	19	--	--				
CakTSSR04128	CakTC41106	(GTG)6	2544	2561	--	--	ATGAAGAAATGTTGTTGTTGG	TACATGGTGATGAGATTGAGG	55.15,55.96	147
CakTSSR04129	CakTC41109	(AGAA)5	74	93	--	--				
CakTSSR04130	CakTC41131	(TC)8	27	42	--	--				
CakTSSR04131	CakTC41132	(TTC)6	1209	1226	--	--	GAAGAGGAGTTTTTCATCCAC	CTGGTGAAGAACAGAATGAAG	55.42,55.01	155
CakTSSR04132	CakTC41144	(AT)6	2203	2214	--	--				
CakTSSR04133	CakTC41147	(ATG)5	624	638	--	--	AATGCAGTCAAATGTAAGGAA	TAGCATCCGAATCATATTCAT	54.99,54.8	146
CakTSSR04134	CakTC41162	(TTA)7	656	676	--	--	GCGAATGCAGGTTAAGTATAA	ATTCATTCATTCACTCGCTTA	54.9,55.07	156
CakTSSR04135	CakTC41164	(AG)26	650	701	--	--				
CakTSSR04136	CakTC41169	(AAG)8	2065	2088	--	--	AAAAGATCGAGGTTAAGGTTG	ACGAAGAACAAAAAGAGATCC	55.24,55.18	141
CakTSSR04137	CakTC41185	(CT)10	2403	2422	--	--				
CakTSSR04138	CakTC41189	(AT)6	527	538	--	--	CGTACATATGAAGCAAGGAAC	GGATGAATATGTCATTTGGAA	55.02,54.92	165
CakTSSR04139	CakTC41194	(CT)10	134	153	--	--	TTCTATGTTCACCTTTTGACG	CATGTTACTCGCATACACAGA	55.48,54.82	150
CakTSSR04140	CakTC41194	(CTG)6	1232	1249	--	--	ATCCTGAATCTGAAGTTCCTC	AATACCAACTTCTTCACTCCAC	54.92,55	166

CakTSSR04141	CakTC41199	(TC)12	178	201	--	--	TCAAGCAAACTCTTCTTTCAG	CAGTAACTGCTCCAATCAAAG	55.07,55.23	147
CakTSSR04142	CakTC41206	(GCA)5	461	475	--	--	ACGGTCCTCCTTTTATACCTC	CTGTTGCTGGAGAAGAATATG	56.37,55.15	152
CakTSSR04143	CakTC41208	(CAC)5	1530	1544	--	--	CACCTCGAATAGTGTCACCT	GAGTAGATGACGGTGGAGATA	55.14,54.28	159
CakTSSR04144	CakTC41212	(TTC)5	123	137	--	--	TTTTGTGTCCTCCTGAAAATA	GAGACCAGAATATGTGAATGC	54.97,54.7	149
CakTSSR04145	CakTC41214	(TCT)7	2328	2348	--	--				
CakTSSR04146	CakTC41218	(TCA)6	91	108	--	--				
CakTSSR04147	CakTC41219	(GA)13	2470	2495	--	--				
CakTSSR04148	CakTC41228	(GAA)5	858	872	--	--	AATACAACCCAACTACAACGA	GTCATCACAGAGTCTTTTTCG	54.79,55	159
CakTSSR04149	CakTC41230	(CT)6	2423	2434	--	--				
CakTSSR04150	CakTC41232	(AG)8	324	339	--	HB	AAAAAGGAGCGATAGAGAAAG	TGAAGAGTTCTCAAACATCGT	54.83,55	150
CakTSSR04151	CakTC41232	(ACC)5	758	772	--	HB	CCACAATTGAAAACCAAAAT	GGGAAATTGAACATCTCTGAT	55.07,55.63	163
CakTSSR04152	CakTC41232	(TCT)6	1503	1520	--	HB	GGTGGATTAGGAACGATTAAC	CCAACACTACAAAACTCTTCC	55.27,54.04	149
CakTSSR04153	CakTC41232	(ATA)6	2338	2355	--	HB	GCAGAAGGATCAGATACAGAA	TTGGATCTCTTTGATGTTGTT	54.58,54.77	152
CakTSSR04154	CakTC41232	(TAT)5	2864	2878	--	HB				
CakTSSR04155	CakTC41242	(AGG)6	65	82	--	--				
CakTSSR04156	CakTC41243	(AG)6	2501	2512	--	--				
CakTSSR04157	CakTC41245	(TG)6	609	620	--	--				
CakTSSR04158	CakTC41246	(TG)7	368	381	--	--	AGGGATGATTAAGGAATTTTG	TATCAGTCTCACCCATACTCC	54.95,54.11	148
CakTSSR04159	CakTC41248	(CTT)6	2365	2382	--	--	TAAACGAACTTCTTCAACAGG	AACCTTCACCTAAACAAATCC	54.8,54.92	155
CakTSSR04160	CakTC41249	(GAA)6	206	223	--	--	TCACATTACCCTGTTTATGCT	GGATATTGAAGACCAGAGCTT	54.86,55.14	163
CakTSSR04161	CakTC41253	(ACT)6	8	25	--	ARR-B				
CakTSSR04162	CakTC41254	(AAG)5	2368	2382	--	--	TTGGGATGAAAGTGTTAGAAA	AGTTGATCGATCTTCAATCCT	54.97,55.38	142
CakTSSR04163	CakTC41262	(TC)7	653	666	--	--				
CakTSSR04164	CakTC41263	(TC)9	21	38	--	--				
CakTSSR04165	CakTC41281	(ACT)5	546	560	--	bHLH	CAGTGCTCAAGCAGCTATAAT	TACAAACCAAACCCTACACAG	55.09,55.27	152
CakTSSR04166	CakTC41288	(TGC)5	246	260	--	WRKY	ATTGATGATGAACCATTATGC	ACAACAACAACATCAACAACA	54.94,54.85	144
CakTSSR04167	CakTC41294	(TC)7	1391	1404	--	--				
CakTSSR04168	CakTC41302	(TGA)5	2510	2524	--	--				
CakTSSR04169	CakTC41304	(AGAA)5	126	145	--	GRAS	GTGATCTGTCATCGTTTCAAT	AGGAAGCTTTGAGAAAGAGAA	55.03,55.23	140
CakTSSR04170	CakTC41304	(ACT)7	490	510	--	GRAS	TGGATAACCCTTTTCTTCTTC	AGCATTTGGTTTATGTAACGA	55.13,55.04	145
CakTSSR04171	CakTC41304	(TAG)7	899	919	--	GRAS	AAGCCTTCTATATTGCAAACC	CAAAATCCATCCCTAGAACTT	55.35,55.01	151
CakTSSR04172	CakTC41313	(CAT)5	330	344	--	--	TAAAAAGCGAGTGTACAGGAA	GCATTTGTATACTGGAAAACG	55.4,55.04	170
CakTSSR04173	CakTC41314	(TC)6	332	343	--	--	ATTTCTCTCTCTCGCTTTTGT	GGCGACTGATCTGAAATAATC	55.1,56.39	149
CakTSSR04174	CakTC41315	(TA)7	58	71	--	--				
CakTSSR04175	CakTC41335	(TAA)5	715	729	--	--				
CakTSSR04176	CakTC41338	(TTC)9	418	444	--	--	CTCGTTTCAACAAAAAGTGAT	GAATCCTCAAAAATCATCAGA	54.63,54.33	150
CakTSSR04177	CakTC41343	(TGT)5	574	588	--	MYB	GGAAACTCCTTGAGACTCTTC	TCAACAACAACACCAGAAAAT	54.72,55.64	147

CakTSSR04178	CakTC41351	(TGT)5	995	1009	--	--				
CakTSSR04179	CakTC41363	(TTG)5	92	106	--	--				
CakTSSR04180	CakTC41377	(AG)6	682	693	--	--				
CakTSSR04181	CakTC41380	(AAC)5	246	260	--	ARF	ATACCATGAAACTTGCCTAGC	AAGCTCAGACACAACTGAGAC	56.07,54.6	143
CakTSSR04182	CakTC41397	(CT)6	15	26	--	--				
CakTSSR04183	CakTC41404	(GA)10	615	634	--	AP2-EREBP				
CakTSSR04184	CakTC41413	(CT)9	4	21	--	--				
CakTSSR04185	CakTC41423	(TC)8	20	35	--	--				
CakTSSR04186	CakTC41425	(TA)6	643	654	--	--				
CakTSSR04187	CakTC41433	(TAA)5	179	193	--	--	CGTGCAAAAGCAATTAGTAGT	GAGCTATAAGAGGGAAAATCG	55.03,55.02	146
CakTSSR04188	CakTC41439	(TC)6	121	132	--	--	ACAAGAAGGAAGAGAAGCAGT	TGGATTATGTGTTGTTGATGA	55,54.79	150
CakTSSR04189	CakTC41441	(AG)18	2	37	--	--				
CakTSSR04190	CakTC41443	(TTC)6	345	362	--	--	ACTAGTATTCCCCAACCAAAA	AATTCTGGAGCTTCTTTGAAT	55.36,54.88	134
CakTSSR04191	CakTC41446	(GA)6	703	714	--	--				
CakTSSR04192	CakTC41452	(TTA)5	27	41	--	--				
CakTSSR04193	CakTC41452	(CCAATT)5	280	309	--	--	CATCGGGTAACATAGTCTTCA	GAGAAAAGATGATTGTCGTTG	55.28,54.93	124
CakTSSR04194	CakTC41474	(TGC)6	1771	1788	--	--	CTTTTCAACCTCATCAACAAC	ACTATGTGCAGAGACAAATGG	54.85,55.34	131
CakTSSR04195	CakTC41474	(GA)6	2168	2179	--	--	ACGAAACAAAACAACGAAGTA	TAAAACACGCTCATACACACA	55.15,55.35	152
CakTSSR04196	CakTC41482	(AGA)8	267	290	--	--	TTGATTCAACACTTGTGTCAG	GACAAGCTTTTTCTCTTAGGC	54.63,55.26	154
CakTSSR04197	CakTC41482	(TGATTG)5	586	615	--	--	ACAAAATCGTTTGAGAATGAG	CATGTTGTGGTTCTGAAATCT	54.54,55.13	147
CakTSSR04198	CakTC41501	(CCA)5	185	199	--	--	AACCCAATTTCTACTTTCCAC	GAGAAAATGGATGAGATGGA	54.92,54.99	186
CakTSSR04199	CakTC41501	(CCA)6	335	352	--	--	TCCATCTCATCCATTTTCTC	GATGATCACAATGACGATGA	54.99,55.09	172
CakTSSR04200	CakTC41502	(AG)7	2290	2303	--	--				
CakTSSR04201	CakTC41505	(TA)7	267	280	--	--	CCAAAGCATCACATTCATATT	CCCAAAGTGTTGAGTGTTAAG	55.19,54.99	145
CakTSSR04202	CakTC41507	(TTC)8	138	161	--	--	TCTTCACTTCTCTCTTCAACG	AAATGGAGGACTAGTTGGTTC	54.88,54.9	159
CakTSSR04203	CakTC41516	(TGT)5	294	308	Flower bud	GRF	TGAAGTGGTTTCTGAGTTGTT	ATTCAACCTCACAGTTTCAGA	54.92,54.83	151
CakTSSR04204	CakTC41516	(AAT)5	1328	1342	Flower bud	GRF				
CakTSSR04205	CakTC41518	(TCT)9	306	332	--	--	TATCATCGCCATCTTCTACTT	TCTTCTTTAATTTTGGAGTCG	54.19,54.01	163
CakTSSR04206	CakTC41521	(AGA)6	1775	1792	--	GRAS	AGCACTGAGGTCATATTCAGA	AAATCTCATCGATCCTTCTTC	54.95,55.02	145
CakTSSR04207	CakTC41522	(TA)6	1954	1965	--	--	ATTTTCTTTCCCTAAAAGCTG	AGAATTACAGTTTCCCCACTC	54.7,54.9	167
CakTSSR04208	CakTC41535	(TGT)5	278	292	--	bHLH	AATCTTTCTTTCTTTCCCAAG	GTTGTTCCTTCATGTTTTCAG	54.49,54.85	151
CakTSSR04209	CakTC41542	(AAG)6	894	911	Young_pod	--	GTTCATTCCTTGAATTTCCTT	TCATCACCAATAACATTCCTC	54.97,54.98	156
CakTSSR04210	CakTC41565	(GAT)6	676	693	--	--				
CakTSSR04211	CakTC41577	(CAA)5	159	173	--	SNF2	TATGATGGAACCAGAAGAGTG	TTGCAGAATGAGAATGAGAAT	55.23,55.02	149
CakTSSR04212	CakTC41591	(CCG)5	713	727	--	GNAT				
CakTSSR04213	CakTC41598	(ACC)5	512	526	--	Trihelix	ACTATTATGCACCCACTGAAG	GCAGAGACGAAGAAGATGATA	54.45,54.75	138
CakTSSR04214	CakTC41608	(GAA)5	600	614	--	--	AAAGAGACAATGAAAGGGGTA	TAAAAGATGCTGCAAGAAGTG	55.47,55.96	141

CakTSSR04215	CakTC41615	(TA)6	2199	2210	--	--	GCTAAGAGAAAAGCTGGAAGT	GTTTCTGCACAGTTTTCTGAC	54.87,55.07	149
CakTSSR04216	CakTC41622	(ATA)5	2121	2135	--	--	CCTTTTGTTTCTTGTTCGTAA	CACCATTCTTCATTTCTTCAC	54.82,54.77	152
CakTSSR04217	CakTC41626	(ACA)8	31	54	--	--				
CakTSSR04218	CakTC41628	(CT)6	238	249	--	--	AGAGACATAGCATTCCTTTCC	ACGGGTGCTAATAAGAACTTT	55.14,54.89	131
CakTSSR04219	CakTC41630	(TTC)7	259	279	--	--	CACCAACAAAATATGTGGACT	GGGTGCCTTGAATTAATTATC	55.02,55.36	167
CakTSSR04220	CakTC41634	(AC)11	115	136	--	--	GCAATTTCTGTTTTGCAACTA	TTCAATATGTTTGATCCACCT	55.78,54.58	149
CakTSSR04221	CakTC41639	(TC)7	21	34	--	--				
CakTSSR04222	CakTC41660	(TC)6	715	726	--	--				
CakTSSR04223	CakTC41664	(TA)6	69	80	--	--				
CakTSSR04224	CakTC41672	(CTT)5	491	505	--	--	CGTAACATCCTCATCTCTCAC	TGTTGTTCTTGTTCTTCTGGT	54.78,54.92	152
CakTSSR04225	CakTC41679	(ATG)5	1905	1919	--	--	TGACTTGGATAGAGCTGAAAG	GAGAGTTAAGCCAACTGATCC	54.85,55.62	149
CakTSSR04226	CakTC41692	(CAA)6	563	580	--	--	CTTTCACAAAACACAACAACA	TCATTGTTGTTTTCTCCATTC	54.76,55.19	170
CakTSSR04227	CakTC41718	(AT)7	75	88	--	--				
CakTSSR04228	CakTC41718	(GA)6	2241	2252	--	--				
CakTSSR04229	CakTC41722	(AAC)5	110	124	--	bZIP	AAGAGCCACAGAGAGTAAACC	TCTTCATCGCTATCTTCAAAC	55.29,54.78	162
CakTSSR04230	CakTC41724	(TC)6	101	112	--	--	TATCGAAAACTCGAAAACTTC	ATCTTAGAAAAGCGAGGAGAG	54,54.8	168
CakTSSR04231	CakTC41741	(GAA)5	19	33	--	--				
CakTSSR04232	CakTC41752	(TCT)5	30	44	--	--				
CakTSSR04233	CakTC41758	(TTC)5	2315	2329	--	--	CATCATAGCTGCTTTTTCACT	CCAACAGTAACTTGTGCTTTC	54.9,55.14	150
CakTSSR04234	CakTC41759	(GA)6	797	808	--	--	AGGGGAAACAGAATGAATATG	AGATTCTGGTTCTGGATTGTT	55.67,55.3	148
CakTSSR04235	CakTC41765	(ATC)5	529	543	--	--	AACAACTCATCAAACAATGCT	GGAGGGTGTCCTTTATCACTA	54.88,55.75	152
CakTSSR04236	CakTC41773	(CT)8	11	26	--	bHLH				
CakTSSR04237	CakTC41780	(TTC)6	136	153	--	--	GTTTTCTTCAATTCCAATTCC	TTACAAGTGAGACCGTTGTTT	55.36,54.97	150
CakTSSR04238	CakTC41785	(AG)6	27	38	--	--				
CakTSSR04239	CakTC41786	(AT)8	704	719	--	--				
CakTSSR04240	CakTC41792	(GA)12	2265	2288	--	--	CAGATGAAGAAGCAAGGTTAC	CAACCCAAACTAAACCTCTCT	54.28,55.15	137
CakTSSR04241	CakTC41793	(GA)6	588	599	--	--	AAAGGCCTTATTGATAGGAAA	CACACCAGAGATAGTCTCGAA	54.89,55.47	150
CakTSSR04242	CakTC41797	(TC)10	2294	2313	--	--				
CakTSSR04243	CakTC41808	(AG)12	46	69	--	GRAS				
CakTSSR04244	CakTC41818	(TAT)5	2013	2027	--	--	AATTATGGGTTGGTTATTTCC	CACAGGCAATAAAATGAAAGA	54.7,55.57	163
CakTSSR04245	CakTC41821	(CAA)5	149	163	--	--	GAACACACCTATGTCAAAAGC	GTAAATGGTGTGTGTTTGGAT	54.88,54.84	129
CakTSSR04246	CakTC41831	(ATA)6	155	172	Flower bud	--	TTATGCATCTAGCAGAAGGAA	TCTGCTAGGACAGTACCAGTT	55.4,54.2	139
CakTSSR04247	CakTC41840	(GCT)5	1544	1558	--	--	TCCAACATTGTACTTCAGGTC	GGTTGTTCTACTTCTTGCTGA	55.13,54.76	153
CakTSSR04248	CakTC41844	(TTC)5	41	55	--	--				
CakTSSR04249	CakTC41848	(AT)11	569	590	--	--	CACATCCACAAACACAACATA	GTTTGGGTAGCGGATTTAATA	55.26,55.99	174
CakTSSR04250	CakTC41849	(GAT)6	800	817	--	--	CCAAATCCCCTTACTTATTTC	ACACTATTCTTCCTCCTCGTC	54.59,55.04	144
CakTSSR04251	CakTC41861	(TAG)6	186	203	--	--	ATAATGAAGCAACTGTTCCAA	CAACCTCTTTTTGTTCATGTT	54.99,54.46	150

CakTSSR04252	CakTC41883	(TA)6	73	84	--	--				
CakTSSR04253	CakTC41888	(GAAATT)5	1788	1817	--	--				
CakTSSR04254	CakTC41899	(TC)25	1	50	--	--				
CakTSSR04255	CakTC41904	(TTC)8	42	65	--	--				
CakTSSR04256	CakTC41904	(CCA)6	218	235	--	--	AACACTATCCTCAACCTCTCC	ATTCGTGGAAGAATTGTTGA	54.88,55.64	148
CakTSSR04257	CakTC41906	(CT)7	59	72	Root	--				
CakTSSR04258	CakTC41915	(CTT)5	220	234	--	--	CGCTTCATTAACTTCAACAAC	AAGAAGACATAGCTCGAGGAC	55.23,55.37	154
CakTSSR04259	CakTC41915	(TCT)5	336	350	--	--	CAAATTCTTCACTTTCTGCAC	GGTGTTTTTGATGGTTTCTTC	55.18,56.19	163
CakTSSR04260	CakTC41928	(TC)6	5	16	--	--				
CakTSSR04261	CakTC41928	(CCA)6	290	307	--	--	CACTTGTAAATTCCCATCAAT	TCTTGAATGGAGAGACTAACG	54.2,54.69	147
CakTSSR04262	CakTC41929	(AAC)5	148	162	--	--	ATCACCAATTACCGATCAAG	ACTGCTTCTGATGTTGATGTT	55,54.85	153
CakTSSR04263	CakTC41933	(TA)6	1121	1132	--	--				
CakTSSR04264	CakTC41944	(ATC)7	476	496	--	C2C2-Dof	ACCAACCCTACAAGTCTCTTC	GTTATGCATCTCATGGTGAAC	54.96,55.51	142
CakTSSR04265	CakTC41944	(TAA)17	627	677	--	C2C2-Dof	CAAACTCACTTTCAGCTCTTG	GGGAATCCATTAGGATAAAGA	55.43,54.87	163
CakTSSR04266	CakTC41945	(AG)6	1224	1235	--	C2C2-Dof				
CakTSSR04267	CakTC41946	(TCT)5	122	136	--	--	AAAGAGGTCACAACACAACAC	GAGTGAACAAAGCATACGAAG	55.1,55.22	144
CakTSSR04268	CakTC41955	(CT)16	9	40	--	--				
CakTSSR04269	CakTC41959	(TTC)5	151	165	--	--	AGTGAGAAAATGCAAGCTCTA	GGAAGAAAAGGTTGTGAGTTT	54.61,55.11	140
CakTSSR04270	CakTC41973	(AAG)11	1054	1086	--	--				
CakTSSR04271	CakTC41982	(TTC)5	1574	1588	--	--	GTGTACGAAGGTAGGTTGAAG	GTGTAACATTCACCGTTTAGC	54.08,54.93	149
CakTSSR04272	CakTC41987	(AATGAT)6	755	790	--	NAC	TGGAGGTTCAAGTAGCACTAA	CCCAACAAGATATTGGGTTA	55.23,55.04	150
CakTSSR04273	CakTC41998	(AG)6	187	198	--	MADS	ATGGTTTTGCTCACATTCTTA	TCTCAATTCTCTTCAATTCCA	54.99,54.99	150
CakTSSR04274	CakTC42019	(CT)8	1828	1843	--	TUB	TGCTAACATCTTCAGTTCCTC	TGCAGAGAAAGAGTGAGAAAG	54.67,55.05	150
CakTSSR04275	CakTC42020	(GTG)6	783	800	--	--	GTTAGAGGTTGTTGGTGTTTG	AAAGAACACCACAAAAATGG	54.81,55.14	147
CakTSSR04276	CakTC42021	(TA)7	275	288	--	--	CCAAACCTCTTCATTAACACA	CTTTATTCAACGCCATTTCTA	55.32,54.88	146
CakTSSR04277	CakTC42026	(CT)15	850	879	--	--	ACAACACCTTCAGTAGTTGGA	GTTGGGTAATTTGAGAGAGTG	54.78,53.95	147
CakTSSR04278	CakTC42030	(AAG)5	514	528	--	--	GGGAAAAGGTTGAGGTTAATA	TCCTCTTCTTCTTCTCGTTCT	55.03,55.06	154
CakTSSR04279	CakTC42038	(CT)9	933	950	--	--				
CakTSSR04280	CakTC42042	(TTTA)5	1943	1962	--	--				
CakTSSR04281	CakTC42043	(AC)7	53	66	Young_pod	--				
CakTSSR04282	CakTC42052	(GA)7	1080	1093	--	--				
CakTSSR04283	CakTC42061	(TCT)5	311	325	--	--	GTTCTATACGCCAACTCAAGA	CTGGGTGATCTACGATCAATA	54.74,55.19	136
CakTSSR04284	CakTC42067	(CAT)6	335	352	--	--	TTCATTTTCTCAAAACCTCAC	AATGCATTGACTAATGATGGT	54.5,54.6	147
CakTSSR04285	CakTC42068	(TA)8	207	222	--	--	CTAAAAAGCCTCCAAAATACA	CCCTCTTTCATATGTGAGCTT	54.1,56.06	150
CakTSSR04286	CakTC42090	(TTG)5	846	860	--	--	TGGTGATATCTTCATTGGTTC	AAAAAGGAAAGAGGTTCAAGA	54.98,54.94	144
CakTSSR04287	CakTC42100	(CTT)6	1319	1336	--	--	ACCTAAGACATCCCATCTTTC	GACAACCATGATGAAGAAGAC	54.82,54.55	150
CakTSSR04288	CakTC42101	(GAA)6	101	118	--	--	AGAGAGAGATTTGGTGGAAAC	GAATCAGAATCAAACATTTCG	55.01,54.87	159

CakTSSR04289	CakTC42106	(TA)6	1074	1085	Shoot	--				
CakTSSR04290	CakTC42107	(TTA)8	893	916	--	--				
CakTSSR04291	CakTC42112	(TGT)6	843	860	--	--	GAGTGTGAGTGTTCCTTTTTG	CATCTGCTTCTTCCATAATTG	54.92,55.08	157
CakTSSR04292	CakTC42118	(AG)13	966	991	--	--				
CakTSSR04293	CakTC42122	(GA)24	2680	2727	--	--	CAGGATTTTGTGATAATTGGA	CAGGGTACTGTTTGTTTGTGT	55.17,55.17	151
CakTSSR04294	CakTC42129	(AT)9	24	41	--	--				
CakTSSR04295	CakTC42131	(TTGA)5	59	78	--	--				
CakTSSR04296	CakTC42146	(AAG)6	402	419	--	--	CAAAATCGAAGTTTCAGAGAA	CTTTTCCTCTTCTTCTTCGTC	54.84,55.07	144
CakTSSR04297	CakTC42153	(CGA)5	830	844	--	--	ATCAACCTCCAACACTCATAA	ATGAGTTTCCAAGGAAAAGTT	54.64,54.65	144
CakTSSR04298	CakTC42155	(TC)10	121	140	--	--	TATCACTCCTCCTTACAAACG	TTTGACCAGAAGAAGAACTCA	54.59,55.14	156
CakTSSR04299	CakTC42159	(GA)12	1035	1058	--	--	TCAATACTCTCTCCAAATTCCT	CATTGAATTTGATAGGCAAAG	54.75,55.1	152
CakTSSR04300	CakTC42170	(CT)10	1808	1827	--	--				
CakTSSR04301	CakTC42177	(CAT)7	384	404	--	--	TTTGTGATCTGGGTATATGCT	CCTTGTTTGTTGAGGTGATAA	54.78,55.32	153
CakTSSR04302	CakTC42185	(CAA)5	107	121	--	--	AACTCCAAAACTGAACAAACA	TGAGAACAAGAGGTCGTAGAG	54.94,54.75	159
CakTSSR04303	CakTC42190	(TTCA)5	1768	1787	--	--				
CakTSSR04304	CakTC42191	(TTC)6	762	779	--	--	AGAAGGAGTTGGTATCCAAAA	ACATTGGATTGCCTAACACTA	55.47,54.86	149
CakTSSR04305	CakTC42197	(TC)7	172	185	--	--	TCAATTTCCATGTGACTAACC	GAGGGAAGTATATGTCAAAAGC	55.06,54.79	152
CakTSSR04306	CakTC42200	(GA)13	1574	1599	--	bHLH	TGCTTTGTAGTGAGTGTGTGT	GTAACCCCAGTTGAGAAGTTT	54.29,54.98	149
CakTSSR04307	CakTC42202	(CT)7	874	887	--	--				
CakTSSR04308	CakTC42207	(GTC)5	125	139	--	--	ATCGAATAACGGATAATTGGT	CTTTGCTACTCTCAGCCATT	55.24,54.87	153
CakTSSR04309	CakTC42207	(ATA)5	252	266	--	--	GAACACCCCTTTCTTCATTAG	TTAACCTTCCCTAAAAGTTGG	55.47,55.26	142
CakTSSR04310	CakTC42207	(TGA)11	604	636	--	--	ACGGTAGGGAAATAGAATCAG	GCGTCATCTCTACCAGAACTA	55.04,54.71	151
CakTSSR04311	CakTC42222	(TC)7	147	160	--	--	ACTTTCATCACACCACTGTTC	CGCTTTGTGGTTGTATTATGT	55.02,55.39	164
CakTSSR04312	CakTC42223	(AG)15	1835	1864	--	--				
CakTSSR04313	CakTC42233	(GA)10	5	24	--	--				
CakTSSR04314	CakTC42237	(AG)8	1693	1708	--	--				
CakTSSR04315	CakTC42242	(ATA)7	114	134	--	--	TCAAGTTGAAAATGGGAAAT	ACTTCAGTGGTCTTAGGGAAG	54.7,55.14	155
CakTSSR04316	CakTC42242	(TCA)5	931	945	--	--	AGCTTTTAACACACCAAGTGA	AATGGAAAAGTGATGATGATG	55.14,54.98	149
CakTSSR04317	CakTC42254	(CT)6	63	74	--	--				
CakTSSR04318	CakTC42257	(TCA)6	135	152	--	--	GCCATAAACTAAAATCCTGCT	AGGAGGTAAAAGTGAGAAACG	55.35,55.31	151
CakTSSR04319	CakTC42262	(GA)7	2423	2436	--	--				
CakTSSR04320	CakTC42269	(AAAG)5	1255	1274	--	--				
CakTSSR04321	CakTC42272	(AT)6	804	815	--	--				
CakTSSR04322	CakTC42276	(CAA)5	740	754	Shoot	AP2-EREBP	CAACAAAGTGGTTGTGTTTCT	CTACTAGCTTGGTGGATGATG	55.3,55.02	159
CakTSSR04323	CakTC42278	(AG)8	32	47	--	--				
CakTSSR04324	CakTC42296	(TTC)6	1594	1611	--	--	AATGGCATTATGTTCGTGTAG	TGATGAGATTGAAATTGAGGA	55.31,55.71	142
CakTSSR04325	CakTC42300	(GAT)6	1833	1850	--	--	AGTCAACGAGTTGATTATGGA	CAAATCAAATTTCGTCTCAAC	54.8,54.96	149

CakTSSR04326	CakTC42306	(AAG)12	208	243	--	--	GTCTTTAGAAACAAGGGCAAT	CCACTTCTTGATTGAAGTTGA	55.23,55.44	156
CakTSSR04327	CakTC42316	(GT)9	43	60	--	--				
CakTSSR04328	CakTC42320	(AG)7	30	43	--	--				
CakTSSR04329	CakTC42321	(AAG)7	846	866	Root	--				
CakTSSR04330	CakTC42322	(AAC)5	432	446	--	--	ACTCAAAAATCACACCTTTCA	ACACCAACAACATTAACAACC	54.85,54.93	145
CakTSSR04331	CakTC42322	(TGG)7	804	824	--	--	GGAAAAATGGTTACTGTTCCT	CTCTTGCAGGTGACATAGAAC	54.92,55.04	157
CakTSSR04332	CakTC42332	(AG)6	2093	2104	--	--				
CakTSSR04333	CakTC42336	(GA)11	995	1016	--	--				
CakTSSR04334	CakTC42338	(CGA)9	454	480	--	--	TGATGATGAAGAGGAAGAAGA	AAGGTACTCTGTCCCCAAAT	54.97,55.16	145
CakTSSR04335	CakTC42346	(TAA)7	455	475	--	bHLH	TGAGATAATGGAGCTTGAATC	AGCTTGTTGAAGTGACACAGT	54.53,55.01	152
CakTSSR04336	CakTC42350	(GTG)5	288	302	--	--	TATTATGCTCACCTTTGATGG	TGGTTGCTATTTGTTTCATTC	55.36,55.4	135
CakTSSR04337	CakTC42356	(GAT)5	142	156	--	--	ATCATGATGAGCCTGTAGTTG	ATTGCCTTACGACTCTTCTTT	55.25,54.99	157
CakTSSR04338	CakTC42362	(TCATCT)5	316	345	--	--	GAGCAAGATTTCCTCAACATT	GGTGGTTGAAGTTGAACTATG	56.02,54.72	150
CakTSSR04339	CakTC42362	(CT)8	1682	1697	--	--	ACAGGATATGCATTGTTTTTG	CCGTGAAACTTTTCCATTACT	55.27,55.97	142
CakTSSR04340	CakTC42363	(AG)10	80	99	--	bZIP				
CakTSSR04341	CakTC42366	(GTT)6	1545	1562	--	--	GAGAGAGTTTGGTGATGGTTA	ACCAACCCTAACAACAGAATC	54.34,55.66	134
CakTSSR04342	CakTC42388	(TC)10	95	114	--	--				
CakTSSR04343	CakTC42390	(TCC)6	774	791	--	--	AGGGAAACAGCTTGTAAGTTT	AGATTTAGGTTAGGGGTTTGA	54.92,54.64	139
CakTSSR04344	CakTC42401	(GAA)5	85	99	--	--				
CakTSSR04345	CakTC42409	(ACTCGG)5	1803	1832	--	--				
CakTSSR04346	CakTC42424	(GAT)5	743	757	--	--	TCCAATCTTGCTTTCTTATCA	TCTTTTTCTAGCTCCTCAACA	55.21,54.59	148
CakTSSR04347	CakTC42430	(ATA)5	95	109	--	--				
CakTSSR04348	CakTC42437	(GA)8	1	16	--	--				
CakTSSR04349	CakTC42447	(AT)10	434	453	--	--				
CakTSSR04350	CakTC42461	(CAA)6	437	454	--	--	ACAAACAACAACAACAAATCC	TTGTTTCTGCTTTCTCTTCTG	55.05,55.07	176
CakTSSR04351	CakTC42462	(AAAC)6	76	99	--	--				
CakTSSR04352	CakTC42466	(CT)8	21	36	--	--				
CakTSSR04353	CakTC42467	(CTT)6	5	22	Flower bud	G2-like				
CakTSSR04354	CakTC42467	(ATA)5	453	467	Flower bud	G2-like	GATGATGATTCTCTTCAACCA	GTCGACACAGAATTAGGAAGA	55.09,54.5	155
CakTSSR04355	CakTC42468	(AG)6	108	119	--	--	CGTTTTACTTTTCGAGTTCAA	AGAGAAGAAAGCGAAGAGTTG	54.98,55.74	153
CakTSSR04356	CakTC42471	(TTTTC)6	1041	1070	Flower bud	NAC				
CakTSSR04357	CakTC42474	(TTC)6	293	310	--	--	TTAGGTCCTCCTCTCTATTGG	CACAATCTCCAAATGAAATGT	55.16,55.06	163
CakTSSR04358	CakTC42483	(TATT)5	199	218	--	--	AATCGAACAGAAACATACACG	CATCACAAAGGAATCATGGTA	55.34,55.95	152
CakTSSR04359	CakTC42484	(TC)12	4	27	--	--				
CakTSSR04360	CakTC42489	(GA)15	1	30	Shoot	--				
CakTSSR04361	CakTC42495	(AAC)6	48	65	--	--				
CakTSSR04362	CakTC42503	(ATA)5	64	78	--	--				

CakTSSR04363	CakTC42510	(GA)11	1	22	--	--				
CakTSSR04364	CakTC42513	(ACT)5	770	784	--	--	GGTCAACTGATATCACCAAGA	CGTAAGTCGAGAGTTTATCCA	55.05,54.75	160
CakTSSR04365	CakTC42513	(CAA)5	1131	1145	--	--	AAAACAAGCCAACAGTATCAA	GTGCGTCAATTAGAGTTTCTG	55.07,55.22	147
CakTSSR04366	CakTC42517	(TTC)6	823	840	--	--				
CakTSSR04367	CakTC42518	(AG)10	2016	2035	Mature Leaf	--				
CakTSSR04368	CakTC42521	(TC)6	33	44	--	MYB				
CakTSSR04369	CakTC42521	(ATC)6	695	712	--	MYB	CAATTCCTTCATCAACAACAC	GGACATCTTTCTGAAACTGAA	55.56,54.47	154
CakTSSR04370	CakTC42546	(AG)6	35	46	--	--				
CakTSSR04371	CakTC42558	(TTG)5	513	527	--	C2H2	ATGATGGTGTTGATCATTCTC	AAAAAGCTGCTCAAATAGGTT	54.77,55.16	146
CakTSSR04372	CakTC42558	(GTT)5	846	860	--	C2H2	AAAAGGTGAAAGTGATGATGA	AACTTTCCAAGACCTTCAATC	54.77,55.03	151
CakTSSR04373	CakTC42567	(ATTCA)5	67	91	--	--				
CakTSSR04374	CakTC42573	(TC)11	37	58	--	--				
CakTSSR04375	CakTC42575	(AG)8	1969	1984	--	--				
CakTSSR04376	CakTC42589	(GAA)6	1634	1651	--	--				
CakTSSR04377	CakTC42594	(CAA)5	1382	1396	--	--	GTTCTATAATGGATGGGAAGG	GACCGATGAATGATACTGATG	55.21,55.43	148
CakTSSR04378	CakTC42597	(CTC)6	151	168	--	--	CTACAACTTTTGTCCACAACC	ACGGCCACTACTGGATTAC	54.81,55.08	148
CakTSSR04379	CakTC42599	(AC)7	167	180	--	--	GCTCCAAAATTATGAAGGTGT	TTCCATATTTTAAGGTCAGCA	55.89,55.1	144
CakTSSR04380	CakTC42602	(CT)6	97	108	--	--				
CakTSSR04381	CakTC42608	(AG)7	866	879	--	NAC				
CakTSSR04382	CakTC42611	(ATT)6	488	505	--	--	TTCATAATCCACATTGTAGCA	ATGTTGCAATTATTCCCTTCT	54.24,55.43	149
CakTSSR04383	CakTC42613	(CAG)7	127	147	--	--	AATCAAAATCGGTGTTACTCA	CTTTCTTGTTCAGCTCTTTCA	54.83,55.07	148
CakTSSR04384	CakTC42616	(ATA)6	1424	1441	--	--				
CakTSSR04385	CakTC42619	(AGC)5	414	428	--	--	CTTCGACAAATTCAATGGATA	CTCTTTTGCTGTTGTTATTGC	55.33,55.39	139
CakTSSR04386	CakTC42630	(TA)11	80	101	--	--				
CakTSSR04387	CakTC42636	(AT)7	80	93	--	--				
CakTSSR04388	CakTC42644	(AG)6	205	216	--	--	ACCAATTGGAGTAGAAATTGT	GGTAAATAGCTGGTTGGATTC	52.95,55.43	139
CakTSSR04389	CakTC42648	(AG)6	113	124	--	--	TCAATCAGCTTTTACTCTCTCA	AAACACTTCCTCCATTTCTTC	54.6,55.03	154
CakTSSR04390	CakTC42655	(CT)15	2	31	--	Sigma70-like				
CakTSSR04391	CakTC42656	(TGA)6	794	811	--	--	GCATCTCTAAACCCAATTCTT	TGTGCAAGAAGTTAACGAGAT	55.15,55.22	150
CakTSSR04392	CakTC42660	(GA)8	120	135	--	--	TTATTCTCCTGAGACACATGC	TACCAAGAAGGCTTTGTATGA	55.38,55.16	150
CakTSSR04393	CakTC42667	(CAC)6	178	195	--	Trihelix	AATCCTCCGTGACGATAAC	GATTTCCGTAATCATCGGTAT	54.96,55.62	143
CakTSSR04394	CakTC42668	(AT)6	5	16	--	--				
CakTSSR04395	CakTC42668	(TTG)5	1748	1762	--	--	TCTTCTTGTTCTTGAGTTCCA	TCTCACTCTCACAGACACACA	55.14,55.13	148
CakTSSR04396	CakTC42674	(TC)7	182	195	--	--	CCACCCACTTGTAGAAAGAA	CCACAACCTCCATATTATCAG	55.3,54.63	168
CakTSSR04397	CakTC42675	(AAG)9	52	78	--	--				
CakTSSR04398	CakTC42680	(TAT)9	347	373	Flower bud	AP2-EREBP	AGAAGATTCTCCACCAAAATC	GGGAATTCAAATTGAGAAAAC	54.95,55.36	146
CakTSSR04399	CakTC42684	(AGTG)6	280	303	--	--	AAAATTAAAACCATGGAGTCG	TGAATATGTGGATGAAGAACC	55.89,54.98	163

CakTSSR04400	CakTC42688	(AAG)6	232	249	--	--	TCACCATTGGATCTTAAACAC	TTGGGGTTGAGTTGATTACTA	55.06,54.84	148
CakTSSR04401	CakTC42689	(TTG)7	691	711	--	--	ATATTCAACCAGTAGGCCTGT	TGATTAGTGTAGTGACAATTCCA	55.37,54.95	147
CakTSSR04402	CakTC42693	(GAA)5	1725	1739	--	--	CAGTAACCTCTTGGGTGTAAA	ATATATTTGAATGGCACAACC	54.51,54.18	154
CakTSSR04403	CakTC42693	(TTC)5	1888	1902	--	--				
CakTSSR04404	CakTC42701	(TCT)7	856	876	--	--				
CakTSSR04405	CakTC42710	(CT)11	6	27	--	--				
CakTSSR04406	CakTC42718	(GAT)6	197	214	--	--	TCCAACAAAAGCTGAATCTTA	CAGGATGAAGAGGGTTAATCT	55.29,54.99	147
CakTSSR04407	CakTC42722	(AG)6	14	25	--	--				
CakTSSR04408	CakTC42729	(AAG)5	225	239	--	--	TAGGCCATCAAAGATATCAAA	AGTGAGTTCCGATGTATTCAA	55.02,54.8	149
CakTSSR04409	CakTC42743	(TA)6	933	944	--	--				
CakTSSR04410	CakTC42745	(TAT)5	140	154	--	--	ATAAACAATGAAACTGGCTTG	TCACATTAAGTTGATGCTCCT	54.61,54.97	147
CakTSSR04411	CakTC42746	(ACA)5	421	435	--	--	GGAGCTCATAAACAGTTCCTC	TCTTGGCTATTGATCGTAAAA	55.62,55.25	150
CakTSSR04412	CakTC42754	(TC)7	876	889	--	--				
CakTSSR04413	CakTC42755	(TTG)9	924	950	--	C2H2	TGTCATGTTCTTCATCAATCA	AAGGAGATGGAGAAAGAGAAG	54.91,54.42	144
CakTSSR04414	CakTC42763	(TCA)5	118	132	Shoot	--	TATTTTTCCACAAACAACACC	TCTCTTCTTTGACTTGGTGAA	55.16,55.14	157
CakTSSR04415	CakTC42765	(TAA)5	1829	1843	--	--				
CakTSSR04416	CakTC42766	(AG)8	798	813	--	--	CTAGGTACCAGTGTTTGGTTG	TCTCTGTTACTCATTTCCAACA	54.86,55.04	156
CakTSSR04417	CakTC42772	(AT)6	1627	1638	--	bZIP	CGAGATCAGACCATCATTACT	AGTGACATTGTTTGTAAAGCAG	54.28,54.76	157
CakTSSR04418	CakTC42784	(CTA)5	779	793	--	C2C2-GATA	AAGAAGAAGCCTCTTGTGAAT	CCTTCAAAATCTGAATTTTCC	54.94,55.52	161
CakTSSR04419	CakTC42788	(TGA)5	793	807	--	--	ACAGTGAGGTAGCAAGCATAA	CGCAACAATACGATAAACATC	55.25,55.87	149
CakTSSR04420	CakTC42795	(ATC)8	139	162	--	--	TTCATCACCATAACACTCACA	GTGAAGACAGGTGTCGAAGTA	54.86,55.37	142
CakTSSR04421	CakTC42806	(AG)16	1917	1948	--	--				
CakTSSR04422	CakTC42809	(AT)8	2401	2416	--	--				
CakTSSR04423	CakTC42811	(AG)6	1970	1981	--	--				
CakTSSR04424	CakTC42819	(TTAT)5	250	269	--	--	AAATTGATTCGAATGCCTAGT	GATTTGGAGGATTTGAGTTTT	55.58,54.97	218
CakTSSR04425	CakTC42823	(ATA)6	132	149	--	--	CTTAAGCCAACAACTCCACTA	TTGGAAGGAAGGTTTTAGAAT	54.84,54.76	150
CakTSSR04426	CakTC42829	(AT)9	69	86	--	--				
CakTSSR04427	CakTC42832	(GAA)5	740	754	--	--	AGATGCAGAAGAAGATCAACA	GTGGTGAGTGCAACTTCTTAG	55.08,55.12	143
CakTSSR04428	CakTC42840	(GTG)5	498	512	--	--	CCAATCATAGCTAATCTTCCA	CTGTAAACCCAGTTACACCAG	54.61,54.86	151
CakTSSR04429	CakTC42840	(ACC)5	1051	1065	--	--	CTGTAAACCCAGTTACACCAG	CCAATCATAGCTAATCTTCCA	54.86,54.61	151
CakTSSR04430	CakTC42844	(TAA)7	574	594	Shoot	--	AAATTCCAGAATTACGAGGAG	CTGTGAGAGTACCTTTTGGTG	55.16,54.97	167
CakTSSR04431	CakTC42848	(AG)6	1921	1932	--	--				
CakTSSR04432	CakTC42853	(TTTC)7	1913	1940	--	--				
CakTSSR04433	CakTC42854	(CT)6	27	38	--	CCAAT				
CakTSSR04434	CakTC42855	(GTG)5	506	520	--	--	GAGAAACATTCGTGTGTCCTA	AAATTGAAAGATCGCATCAC	55.3,55.29	146
CakTSSR04435	CakTC42865	(ATG)6	637	654	--	--	CAGGTCCAAGTGTAGCAGTAG	TCCACTCCTTTGTGCTAGTAA	55.17,55.23	148
CakTSSR04436	CakTC42868	(GTG)7	12	32	--	--				

CakTSSR04437	CakTC42882	(CTC)6	71	88	--	--				
CakTSSR04438	CakTC42894	(TAT)5	269	283	--	--	ATAAAAAGCCACCTCACTCTC	CGATCTCAAAAATAACACCAT	55.22,54.36	161
CakTSSR04439	CakTC42906	(TCC)5	125	139	--	--	AACTCGCCTCTCTCATTCTT	GCGGATAGGTCTTGTTTTAG	55.77,54.26	150
CakTSSR04440	CakTC42914	(TCA)5	36	50	--	--				
CakTSSR04441	CakTC42922	(AAC)5	159	173	Flower bud	--	TCATGAGGAGAGAGAAAAACA	TTCATGAGAGGAATGAACAAC	55.06,55.18	160
CakTSSR04442	CakTC42925	(GA)8	869	884	--	--				
CakTSSR04443	CakTC42941	(TAA)7	187	207	--	--	AGCTCCAAAACACATAATCAA	TTCTTTGGCCTTTTTCTAAGT	54.99,55.14	151
CakTSSR04444	CakTC42942	(CT)7	25	38	--	--				
CakTSSR04445	CakTC42950	(TAA)5	1596	1610	--	C2H2	TCAAAGCAATAGTGTTGGAGT	CCTCCTCCTCCATAGAAATTA	55.05,55.1	153
CakTSSR04446	CakTC42971	(CTT)6	117	134	--	AP2-EREBP	TCCAATACTCCACAACACTAAA	TGTTTCCCTAAACTTCTTCCT	54.94,54.82	147
CakTSSR04447	CakTC42979	(AG)9	46	63	--	--				
CakTSSR04448	CakTC42980	(TCT)5	692	706	--	--	CAGTTCTGGTAAGACCTCAAA	AAAACAACGGAAGTGACATAA	54.61,54.91	163
CakTSSR04449	CakTC42985	(TGA)11	1878	1910	--	--				
CakTSSR04450	CakTC42998	(TCT)6	176	193	--	--	CCTTACGGAGAAGAGATTGAC	AGGATGGAGAGAAGAGAGAGA	55.63,54.78	157
CakTSSR04451	CakTC42998	(TGC)5	815	829	--	--				
CakTSSR04452	CakTC42999	(ACCCT)5	1473	1497	--	TUB	TTCCTAAACGACATCTTCAAC	CAGTTTCTTCCACAAAAGCTA	54.54,54.96	153
CakTSSR04453	CakTC42999	(ATG)7	1716	1736	--	TUB	GAAACTGAGCACTAGGAGTTG	CTTCTCTCTGCTTCATCTTCA	54.33,54.96	169
CakTSSR04454	CakTC43001	(AG)10	833	852	--	--				
CakTSSR04455	CakTC43003	(AGA)5	32	46	--	--				
CakTSSR04456	CakTC43006	(CTT)5	263	277	--	--	ACCTGAGATCACAATCAATCA	TCATCGGATTGAGTAGAGAAA	55.48,55.03	159
CakTSSR04457	CakTC43019	(AG)9	15	32	Young_pod	--				
CakTSSR04458	CakTC43023	(TC)6	50	61	--	--				
CakTSSR04459	CakTC43038	(TC)17	155	188	--	--	TAGTCCTCACAAGGGAACATA	CCTTGAGAGGTTTACAAAACA	54.82,54.64	149
CakTSSR04460	CakTC43040	(GA)7	1	14	--	--				
CakTSSR04461	CakTC43042	(TG)7	3343	3356	Young_pod	--				
CakTSSR04462	CakTC43043	(GAA)5	1781	1795	--	--	ATAAAAGACTTTCCTCCATCG	AAAATCCTAATGTGAGGGAAC	55.16,54.84	154
CakTSSR04463	CakTC43045	(ACA)6	729	746	Shoot	--	AAATTCAATCCAACGTCAAC	AGAGAGAGAAGAAGGGTGGAT	55.04,56.1	146
CakTSSR04464	CakTC43053	(TC)8	1908	1923	--	--				
CakTSSR04465	CakTC43054	(TGC)5	343	357	Mature Leaf	LIM	ACCTGTCTCTGTGTCTGATGT	AGCCTCACTTTTCTCAGTTGT	54.53,55.77	152
CakTSSR04466	CakTC43067	(AAC)5	195	209	--	--	TTACTTCATCGGTTCTCGTTA	TCGAGTGGTTCTTTAAGTTTG	55.16,54.8	153
CakTSSR04467	CakTC43073	(AG)11	1016	1037	--	--				
CakTSSR04468	CakTC43076	(ATG)5	972	986	--	--	GCCATAAGGCAATAAAAAGTA	TATCTTCCAAACAAAATGCAC	54.08,55.4	150
CakTSSR04469	CakTC43078	(GAT)5	293	307	--	--	AGGTTTCCATTTAACACTTCC	AGTACCTGCTTTGAGTCCTTT	54.92,54.9	142
CakTSSR04470	CakTC43078	(TGG)8	720	743	--	--	GTTCACTGTGATGGTGATGAT	ATTTGGTAGGGTTAAGCATGT	55.69,55.38	161
CakTSSR04471	CakTC43094	(AT)6	13	24	Young_pod	--				
CakTSSR04472	CakTC43096	(CAA)5	184	198	--	--	TGTAAAACAAAGTGGAAGGAA	CGGTTGAAGAAGAAGTACAGA	55.05,54.77	152
CakTSSR04473	CakTC43107	(ATG)5	1371	1385	--	HB	TTGAAAACATTGACTTGATCC	AGGCCAAATTTCTTGTACTCT	55.19,54.84	147

CakTSSR04474	CakTC43107	(TA)6	1716	1727	--	HB	AATCATGGAAAGGTCAAGAAC	TTCAAGTGTACCTCTTCAACC	55.71,54.43	152
CakTSSR04475	CakTC43111	(CA)7	8	21	--	TCP				
CakTSSR04476	CakTC43111	(TCA)7	276	296	--	TCP	CAACCAAAAAGACAAGAATGA	GGCTCTTGATACCCTTACAAT	55.45,55.03	161
CakTSSR04477	CakTC43112	(CT)24	1	48	Mature Leaf	bHLH				
CakTSSR04478	CakTC43112	(CAA)7	566	586	Mature Leaf	bHLH	TCTTACACCAAAACACAAACA	GAAGTGTTGTTGTTTCCCATA	54.26,55.15	154
CakTSSR04479	CakTC43112	(CAC)6	727	744	Mature Leaf	bHLH	TATGGGAAACAACAACACTTC	ATCGAAAAAGAAGCGTTAAA	55.15,54.58	153
CakTSSR04480	CakTC43117	(ATA)5	550	564	--	LOB	ACAAGAATCACCACAACAATC	GTAGTTGTCCTTCATGTCCAA	54.95,55.13	156
CakTSSR04481	CakTC43118	(TCA)5	694	708	--	--	CAAGTTAATTGCAGGAAGAAA	AATCCTCCTCTTTTTGTTTCA	54.9,55.6	148
CakTSSR04482	CakTC43119	(AT)7	1867	1880	--	--				
CakTSSR04483	CakTC43127	(TTTA)5	960	979	--	--				
CakTSSR04484	CakTC43129	(CAG)5	3077	3091	--	--	ACATCAAGCGTATTACCACAT	GTCGCAATTGAATATAATGCT	54.73,54.65	148
CakTSSR04485	CakTC43131	(AAG)6	45	62	--	--				
CakTSSR04486	CakTC43135	(AG)7	30	43	--	--				
CakTSSR04487	CakTC43137	(CTC)5	388	402	--	Trihelix	CTAACCACACCCTCCTAAAAT	GGCGTAGGATAGTAAAGAAGG	54.97,54.98	125
CakTSSR04488	CakTC43146	(TCT)6	128	145	--	--	CACTTCACTCATTTCAATTCC	AAGTCTAGGGTTTGTGCATTA	54.77,54.2	153
CakTSSR04489	CakTC43160	(AG)6	943	954	--	--				
CakTSSR04490	CakTC43178	(TGA)5	285	299	--	--	TTGTTGGGAATGAAGATACTG	CCTGTTTTTCCTAAGAAGCTC	55.24,55.12	143
CakTSSR04491	CakTC43179	(AG)10	1000	1019	--	--				
CakTSSR04492	CakTC43190	(GGA)6	987	1004	--	--	AGATTCACAGCCAATAGCATA	GTCAACAGAAATCAAACGAGT	55.1,54.41	148
CakTSSR04493	CakTC43192	(AT)8	176	191	--	--	CACATGCACCTTTTTATTTTC	ACCAATTACAGGGTTGTTTTT	55.01,55.27	166
CakTSSR04494	CakTC43193	(GAA)5	475	489	--	--	CACCATAGCTACACCTGAAAA	ACACCAACAACAACATCAGTT	55.52,55.41	150
CakTSSR04495	CakTC43198	(ATA)8	742	765	Shoot	MYB	TTCTAGAAGGTTGGATGAGAA	TGAAACTAAATCTCTTCCATGA	54.17,54.23	149
CakTSSR04496	CakTC43198	(CTT)5	940	954	Shoot	MYB	AACTTGTGATTCTATGCCTGA	ACCACTTCTCAAGAAATGACA	54.97,54.83	149
CakTSSR04497	CakTC43199	(TAT)5	117	131	--	--	TTTTGATTCCATACATTGCTC	AACGGTTGAAATTAGGAGAAG	55.32,55.24	147
CakTSSR04498	CakTC43210	(ACT)5	122	136	--	--	TCGTCATCTCTCTTTTCAAAC	AGAGAGAAGGTGAAGGTGAAG	54.64,55.25	138
CakTSSR04499	CakTC43213	(CT)6	911	922	--	--				
CakTSSR04500	CakTC43233	(TA)7	1919	1932	--	--				
CakTSSR04501	CakTC43238	(AG)7	968	981	--	--				
CakTSSR04502	CakTC43250	(TC)15	799	828	--	--	AGCTCCATCTATGTAACCACA	AGAAAAGTACCCAAACTCGAC	54.84,55.14	143
CakTSSR04503	CakTC43257	(TAA)5	85	99	--	--				
CakTSSR04504	CakTC43268	(AG)10	42	61	--	--				
CakTSSR04505	CakTC43278	(CAC)5	379	393	--	--	ATATCATCACCACCACCAATA	CAATACACTGAGGGTGATTTT	55.15,54.25	152
CakTSSR04506	CakTC43281	(TC)14	141	168	--	--	CGTCTCATTGAAGCACTTATT	CAGAGAGTGAAGAAGCAAAGA	54.74,55.05	147
CakTSSR04507	CakTC43282	(TCA)7	302	322	--	--	AAAATAGGTGCAGAGAAAGGT	GTGGCACTTTTGCTAATTTT	54.84,54.74	148
CakTSSR04508	CakTC43285	(CA)8	161	176	--	--	TTCTTTGTTTCTCTTTCGTCA	GAACAGTTGGAGAGAGATTCC	55.32,55.42	150
CakTSSR04509	CakTC43290	(GA)8	789	804	--	--	GGGGTTTTTATAGGTGAGAAA	TGAGTCCTTATGCCATCATTA	55.03,55.77	148
CakTSSR04510	CakTC43305	(AG)7	872	885	--	--				

CakTSSR04511	CakTC43306	(CGCAT)5	23	47	--	--				
CakTSSR04512	CakTC43325	(AAC)5	43	57	--	--				
CakTSSR04513	CakTC43337	(TG)7	452	465	--	--	AGGAACGAAGAAATTTGAGAC	AACCCTCCAGAACTTTTTAAC	55.18,54.29	153
CakTSSR04514	CakTC43345	(TAT)5	793	807	--	--	ATGAGAAGACATGGTTGATGA	ATTTCCTTCCTTTTCCTCTCT	55.48,55.35	150
CakTSSR04515	CakTC43360	(GGT)6	865	882	--	--				
CakTSSR04516	CakTC43367	(GGA)5	379	393	--	--	TGAGGACAGGACGGATATT	GAAATCAAGTGTGGATCATGT	55.4,54.86	152
CakTSSR04517	CakTC43372	(TCTCT)6	66	95	--	--				
CakTSSR04518	CakTC43379	(AC)6	18	29	--	--				
CakTcSSR04519	CakTC00095	[illegible] actccaa(AC)6aaatggcaattgaaaacca agagaccactgtcagagaaatcaagccaaag aacagaagaatcatgggtgctggaggtcc(T GA)5	126	291	--	PLATZ				
CakTcSSR04520	CakTC00742	(AG)6a(AG)6	153	177	--	--				
CakTcSSR04521	CakTC00962	(CTT)5ccatcaatattacaacttcagcacca g(CAC)6	516	575	--	--				
CakTcSSR04522	CakTC01101	(ATT)5ggaagaagaagaaggaacttgttgt aatgg(TAC)5	535	594	--	Trihelix				
CakTcSSR04523	CakTC01341	(CT)7(AT)8(GT)8	1	46	--	--				
CakTcSSR04524	CakTC01845	(AG)8c(GA)8	1133	1165	--	--				
CakTcSSR04525	CakTC01938	(AC)6agtaagcatatactcgagaagatgatc caacacaagccctactcaaatg(CAT)5	107	182	--	--				
CakTcSSR04526	CakTC02186	(GT)6(GA)9ac(GTT)5	630	676	--	--				
CakTcSSR04527	CakTC02409	(GA)6agagtagaatgaatggtttttagtaaag ttgttcacggatggatcaggtgttttgttgtgtca caaagtcccaatagagagaa(AG)12	3354	3472	--	--				
CakTcSSR04528	CakTC02601	(CAT)6caacccgatccttccctcaactccac caccaccttaacaacatcaagcagctctagatc ctcaacacaaacaccaccaacaacaacgtatc attattat(CAA)6	46	180	--	GRAS				
CakTcSSR04529	CakTC03065	(AG)6ttggtggagagagaaagtgatggatgt gtttgaaaatacaaaagctgttgaagacaaattt tttgcatcagcataatagt(AACA)5	155	266	--	--				
CakTcSSR04530	CakTC03605	(CT)10(AT)10	448	487	--	--				
CakTcSSR04531	CakTC03724	(TC)6ta(CT)12	762	799	--	--				
CakTcSSR04532	CakTC03862	(TCA)8attatgggttccgagttaaatgttcgt gatttatcatcttctatttcgaagaatgatcgtg(CTT)5	214	313	--	--				
CakTcSSR04533	CakTC03896	(AG)6aacc(CAACA)5	2248	2288	--	--				

CakTcSSR04534	CakTC03955	(TCT)11tggttgaaggaattgtagttgttcttccagttgtataagaagaccttgttgttgttttcattgttaa(TAT)8	3491	3614	--	--					
CakTcSSR04535	CakTC04190	(GGA)9tcccgaaacgcctcgtat(GGA)5	390	449	Root	--					
CakTcSSR04536	CakTC04948	(AAG)8aaacca(CAAAAT)5	41	100	--	--					
CakTcSSR04537	CakTC05291	(TA)6ttacccttagcatttatctgct(ATC)6	74	125	--	MADS					
CakTcSSR04538	CakTC05550	(TG)9(AG)9	120	155	--	--					
CakTcSSR04539	CakTC05622	(GA)10ttaacattgattgggaaatcaagacgagacgagaagaa(AG)17	6	97	--	--					
CakTcSSR04540	CakTC05841	(CT)9catccccactc(ACA)5	9	52	--	--					
CakTcSSR04541	CakTC06135	(AGT)5aatattgttagac(TTA)5	170	212	--	--					
CakTcSSR04542	CakTC07079	(TGA)5(GA)12	333	369	--	--					
CakTcSSR04543	CakTC07444	(AAC)5aataacaattacaataacaataacaataacaatgacgacaacaacagcaatgacgacaataacaacaacaataacaatggaggaaagaaagaagag(AAC)5	108	233	--	--					
CakTcSSR04544	CakTC07484	(CCG)5ctccacgaattaactgttttcgtctcgtctttcctcattctcacagacactaagtcatctgaaac(GTT)5	4	98	--	--					
CakTcSSR04545	CakTC07484	(GA)6aagaggaaaaaccccaatcgatcgtagaaataaaaactcacata(GT)11	325	402	--	--					
CakTcSSR04546	CakTC07556	(TGT)5tggaagtctattgagagcaacattgaaacaaagttcaagagctctacattgaagaatatgt(GAA)6	2849	2942	--	--					
CakTcSSR04547	CakTC07724	(TC)11(AC)8	558	595	--	--					
CakTcSSR04548	CakTC07755	(AC)10agagaaagagagatagtgacatagga(AG)11	157	224	--	--					
CakTcSSR04549	CakTC07869	(AG)7attaaacgtgaagctgaagagaggtggtagttggaaaagaaattgaaatgaagaatacaaaggtctagaaatatagagaaaatggtt(GA)11	2236	2358	--	--					
CakTcSSR04550	CakTC07952	(AAT)5aaaaaaaggaacaaagaaatactcacggtgcgtggaaaatggaaattggaaaggagagatatagtggaacagataagtgcaggggaaaaagct(GA)6	426	545	--	--					
CakTcSSR04551	CakTC08009	(CCA)6(CCT)6	741	776	--	G2-like					
CakTcSSR04552	CakTC08170	(GAA)5gacaaagtgcaaacacagacacttttgtcacagccctcgttgcaagatacattatttg(TCT)6	1255	1345	--	ARF					

CakTcSSR04553	CakTC08248	(CT)18cctaactctaattcttttgattgtgatctactagaccagtgtgtggatctatattctctttagtgaaac(AGA)5	76	195	--	--					
CakTcSSR04554	CakTC08491	(CT)13(CA)12	149	198	--	--					
CakTcSSR04555	CakTC08760	(TCT)5tgcaa(TTC)7	546	586	--	--					
CakTcSSR04556	CakTC08787	(GAA)5gacaataagttgaaaaagcccaaaatgtcttcatcatcaagcacaaatactgaaaccctta(CTT)15	31	151	--	--					
CakTcSSR04557	CakTC08807	(GAT)5aataatgatgatgatgata(ATG)6	1729	1780	--	--					
CakTcSSR04558	CakTC08871	(AG)10aaagagaggagtgcaataaaacagagtgtgtcaatgcagagagagagaagggactgttgagtggggagtgt(AG)6	5843	5945	--	Jumonji					
CakTcSSR04559	CakTC08883	(TTG)7atgatgatgatgttgttgctgaa(GTT)8	122	189	--	--					
CakTcSSR04560	CakTC08930	(CT)7cgttctgtctagttctctctctcggtctctcgttctgtctcattcgctctctctca(CT)6	107	188	Shoot	--					
CakTcSSR04561	CakTC09147	(TC)6tgaaactcactttctctttctaacacattt(TC)7	39	94	--	--					
CakTcSSR04562	CakTC09652	(TTC)5tgtttcactaaagagaatatagatccacacactggtctagtagatcacaatcaaaagaattagagttag(GA)14a(AG)6	2206	2330	--	--					
CakTcSSR04563	CakTC09663	(TTC)11ttggttgaaggaattgtagttgttcttccagttgtataagaagaccttgttgttgttttcattgttaa(TAT)8	3339	3463	--	--					
CakTcSSR04564	CakTC09756	(TCT)6cttcatctttcattccaac(ACA)5	783	834	--	--					
CakTcSSR04565	CakTC09761	[illegible]ggcaagtgcaggtcagctcaacaaccaaatcggaagataaatcaattattaatttgacattaatatttctaaga(TAT)6	1644	1775	--	--					
CakTcSSR04566	CakTC09934	(ATT)5ggaagaagaagaaggaacttgttgtaatgg(TAC)5	608	667	--	Trihelix					
CakTcSSR04567	CakTC10032	(TA)9aattatatattataatt(TA)8	1835	1885	--	--					
CakTcSSR04568	CakTC10077	(ACA)5acgcttcaggacacacctgaggatattttatatattgtaataatcattatt(TTA)5	116	196	--	--					
CakTcSSR04569	CakTC10079	(AG)7aagacaaaaagaaacagttgct(GA)11	1309	1366	--	LUG					
CakTcSSR04570	CakTC10172	(GA)6agagtagaatgaatggttttagtaaagttgttcacggatggatcaggtgtttgttgtgtcacaaagtcccaatagagagaa(AG)12	2991	3107	--	--					
CakTcSSR04571	CakTC10190	(CTT)5ccatcaatattacaacttcagcaccag(CAC)6	507	566	--	--					

CakTcSSR04572	CakTC10212	(CAG)6(CAT)5	1294	1326	--	--				
CakTcSSR04573	CakTC10232	(AC)6(TC)10	1	32	--	--				
CakTcSSR04574	CakTC10238	(AG)8tattgaatttgtat(GA)13	1061	1116	--	BBR/BPC				
CakTcSSR04575	CakTC10593	(CAT)6caacccgatccttccctcaactccaccaccaccttaacaacatcaagcagctctagatcctcaacacaaacaccaccaacaacaacgtatcattattat(CAA)6	46	180	--	GRAS				
CakTcSSR04576	CakTC10676	(TGT)5tggttgttggtatggttaccataccaattgttgttgttttctgaattacctgtaattgttgacgtttgtg(GTT)6	1796	1898	--	HB				
CakTcSSR04577	CakTC11026	(ATT)5ata(ATT)5	1008	1040	--	--				
CakTcSSR04578	CakTC11338	(GAA)6gttcataattcatatttcatatatggaacagaaagagggagtatagtac(GT)7	247	327	--	--				
CakTcSSR04579	CakTC11419	(AG)8c(GA)8	1102	1134	--	--				
CakTcSSR04580	CakTC11461	(AG)6aacc(CAACA)5	2248	2288	--	--				
CakTcSSR04581	CakTC11583	(ATG)6atagcagataaatgctaagggta(AT)6	647	699	--	MADS				
CakTcSSR04582	CakTC11700	(CT)9(AT)9	20	55	--	--				
CakTcSSR04583	CakTC11722	(TC)6agctttttcccctgcacttatctgttccactatatctctcctttccaatttccattttccacgcaccgtgagtatttctttgttcctttttt(TTA)5	23	140	--	--				
CakTcSSR04584	CakTC11813	(CT)26(CTA)5*	846	910	--	--				
CakTcSSR04585	CakTC12013	(TTTGAT)6tttgagaggtta(TTG)7	457	525	--	--				
CakTcSSR04586	CakTC12211	(TGT)5tggaagtctattgagagcaacattgaaacaaagttcaagagctctacattgaagaatatgt(GAA)6	519	612	--	--				
CakTcSSR04587	CakTC12327	(GAA)5gcacgatcattcttcgaaatagaagatgataaatcacgaacatttaactcggaacccataat(TGA)8	881	981	--	--				
CakTcSSR04588	CakTC12531	(GAA)5attgaag(AAC)5aagaggaagaggaggaggaggaagtagaagaagttgaagaagaagaagaggaggag(GAA)5gagagaaga(AG)6(GAG)5*	191	331	--	--				
CakTcSSR04589	CakTC12709	(GAA)5gacaaagtgacaaacacagacacttttgtcacagccctcgttgcaagatacattatttg(TCT)6	162	253	--	--				
CakTcSSR04590	CakTC12813	[illegible]ggtgatgcggagagtgaagttagaagattatggttggttgaggcgcaaatctcaaagaga(CT)6t(TC)6	1	127	Young_pod	--				
CakTcSSR04591	CakTC13528	(GAGAGG)5(GA)8	390	435	--	LOB				
CakTcSSR04592	CakTC13530	(TC)8(CTCTCC)5	161	201	--	--				

CakTcSSR04593	CakTC13563	(CCT)5actactacta(CTC)5	648	687	Root	--					
CakTcSSR04594	CakTC13567	(ATC)5aaagcatttgattgt(TAA)8	924	977	--	--					
CakTcSSR04595	CakTC13582	(TTG)5tgttggaatgaaagatgaag(AGA)6	135	187	--	--					
CakTcSSR04596	CakTC13755	(TAA)6ccaagttgttgaagaaattgttaataaa(AAT)7	920	986	--	--					
CakTcSSR04597	CakTC13763	(GAA)6naganananananananagag(AGA)17	168	257	--	--					
CakTcSSR04598	CakTC14546	(TA)7tttttaatcttttaaaaattgtgtacatattttatttaattac(AAT)5	137	208	--	--					
CakTcSSR04599	CakTC14584	(ATC)9aagatcttgaatc(TCA)5	421	475	--	--					
CakTcSSR04600	CakTC14778	(TTTA)5(TTA)15	55	116	--	--					
CakTcSSR04601	CakTC15120	(CA)13ctcttactctcgctctctttctct(CA)14	298	375	--	--					
CakTcSSR04602	CakTC15287	(ATGA)5tatcaattggtccattattcaataagtatatatcttttctctctctaggtctttctctctctttca(CT)6	272	369	--	--					
CakTcSSR04603	CakTC15506	(CAA)5cgt(TC)9(AC)6	114	161	--	--					
CakTcSSR04604	CakTC15587	(AG)10aaccccatgtcgatatcctcatagtcatcgtcggccatggtgaatgaagagagtt(GTTGA)5	772	871	--	--					
CakTcSSR04605	CakTC15600	(CT)8gtagagtgcaatttctctctctcttattatacttctattttcat(TC)6	1273	1344	--	--					
CakTcSSR04606	CakTC15984	(TAA)5tag(TAA)5tag(TAA)37(ATA)11	118	297	--	--					
CakTcSSR04607	CakTC16009	(GAT)5aataatgatgatgatgata(ATG)6	1562	1613	--	--					
CakTcSSR04608	CakTC16126	(AGA)5(AG)6*	2244	2267	--	--					
CakTcSSR04609	CakTC16518	(TAAA)6(AAT)5*atac(ATA)33	366	505	--	--					
CakTcSSR04610	CakTC16558	(AAG)7(TTG)5	1088	1123	--	--					
CakTcSSR04611	CakTC16567	(TC)8ttccattccttctttcaacaacaatatcatcacatcaacccaaatttctctcagagag(ATC)6	219	310	--	NAC					
CakTcSSR04612	CakTC16635	(TC)7gtccattcattca(TC)11	68	116	--	--					
CakTcSSR04613	CakTC16681	(ACA)6catacaccactttcaacaacaacatcagtcat(CAA)8	948	1021	--	--					
CakTcSSR04614	CakTC17154	(TC)13(CTA)7*	159	204	--	--					
CakTcSSR04615	CakTC17156	(CTTCT)5tctcactccaacgttatggaatcatcacacacatcaacc(TCT)7	162	246	--	--					
CakTcSSR04616	CakTC17546	(CT)7ttctctcttcaattcaacactccttctttattattaatcctaatcccatttacttataaatcctctattttcatcgcatcttatcgattcattcatt(TC)6	103	225	--	--					

CakTcSSR04617	CakTC17594	(AT)8(AC)11	2	39	--	--				
CakTcSSR04618	CakTC17633	(TC)16ttcctattctccacaagttctgttcccaaaagatcttgtggagaatagga(AG)13	156	263	--	--				
CakTcSSR04619	CakTC17706	(TA)9tc(TA)7	382	415	--	CCAAT				
CakTcSSR04620	CakTC17953	(ATT)10tcgccaccctcaccaagacacatat(AG)10	133	207	--	--				
CakTcSSR04621	CakTC18100	(TAG)5cttt(TGAA)5	1	39	--	--				
CakTcSSR04622	CakTC18118	(CT)7(TCT)5*tcctcctcctcttcttcttcttcaacttcttctacttcctcctcctcctcttcctc(TTG)5ttcttcaat(TTC)5	1207	1327	--	--				
CakTcSSR04623	CakTC18370	(CT)7atctatctct(TC)8	69	108	--	--				
CakTcSSR04624	CakTC18391	(GA)10aagaaagaggaccaaagaaaaggaaaagaaaggaaatagaaaaaagaatacagattcatataacgatca(AG)6tgtgtgcgaatagc(ATT)9	34	175	--	--				
CakTcSSR04625	CakTC19052	(AG)18gaggaggagagaggagggagg(GA)19	506	600	--	CCAAT				
CakTcSSR04626	CakTC19054	(TC)9tgtctct(TC)9	1369	1411	--	--				
CakTcSSR04627	CakTC19396	(TCA)8tgtttcaatgctactacctaattcctctggaggagctgtttcaaatattgctactacacccctacct(CCA)7	231	342	--	LOB				
CakTcSSR04628	CakTC19555	(CT)6(CA)6ct(CA)7	268	307	--	--				
CakTcSSR04629	CakTC19611	(TC)8ta(TC)7	467	498	Flower bud	--				
CakTcSSR04630	CakTC20220	(TCT)7ttccctctt(CAA)5	23	67	--	--				
CakTcSSR04631	CakTC20489	(ATT)13tattattattata(ATT)16	362	461	--	--				
CakTcSSR04632	CakTC20781	(AT)6gtgtg(TA)6(GA)6	490	530	--	--				
CakTcSSR04633	CakTC20846	(TC)15t(TC)10	1	51	--	--				
CakTcSSR04634	CakTC20854	(TC)8(TA)9	16	49	--	--				
CakTcSSR04635	CakTC21016	(CT)9(AT)8	125	158	--	--				
CakTcSSR04636	CakTC22203	(AT)6gtaa(AT)8	294	325	--	--				
CakTcSSR04637	CakTC22350	(ACA)6ccaggcttctcctactcctagtgctagtatgactattccgccctccatttctaaggatcaacatgcaaaatgatccatccatccttttctatttatat(TA)8	1122	1253	--	--				
CakTcSSR04638	CakTC22411	(TTG)5tgttggaatgaaagatgaag(AGA)6	715	767	--	--				
CakTcSSR04639	CakTC22725	(TC)8ta(TC)20	176	233	--	TPR				
CakTcSSR04640	CakTC22770	(CCT)5actactacta(CTC)5	48	87	--	--				
CakTcSSR04641	CakTC23325	(AG)6a(AG)6	1492	1516	--	--				
CakTcSSR04642	CakTC23326	(AG)10cga(AG)6	2813	2847	--	--				

CakTcSSR04643	CakTC23352	(ATT)5ata(ATT)5	1020	1052	--	--					
CakTcSSR04644	CakTC23353	(GA)10tattggtgttatttctggtaaggcaatatttggacctccacttgatgactattggaaaaagaagcatcaggaagaa(GCC)8	989	1108	--	--					
CakTcSSR04645	CakTC23387	(CT)7cgttctgtctagttctctctcggtctctcgttctgtctcattcgctctctctca(CT)6	74	155	--	--					
CakTcSSR04646	CakTC23597	(AG)7aaagagga(AG)6	58	91	--	--					
CakTcSSR04647	CakTC23599	(AG)7aaagagga(AG)6	20	53	--	--					
CakTcSSR04648	CakTC23977	(TCT)5tccgtgaaacgctggtaacctatttcctcttcaatattctcctcataagccattcttaattctcgata(CT)10	1333	1435	--	HB					
CakTcSSR04649	CakTC23995	(TTA)6tcagaagtttcccaattatgatgatgatgattatccatggtcatgtgttaatt(TTG)5	2210	2295	--	WRKY					
CakTcSSR04650	CakTC24020	(TCA)5tcgaaatccatttgaagtgaatgacactgatcatcactactagaactttgacaagataatacc(TCA)5	902	994	--	--					
CakTcSSR04651	CakTC24084	(ATC)5aggacctccagcacccatgattcttctgttctttggcttgatttctctgacagtggtctcttggttttcaattgccatt(TG)6tttggagtgaatggttatttgtattaagttaagt(GA)11	1037	1198	--	PLATZ					
CakTcSSR04652	CakTC24108	(CT)6atctctctaacttaaccttccttcggaggaccctaacattattaattcatttcatgtatctacatcatattgtatcatccttggaggggttattcctttta(TAT)6	2746	2875	--	--					
CakTcSSR04653	CakTC24109	(CT)6t(TC)12	134	170	--	--					
CakTcSSR04654	CakTC24202	(AG)10aaga(AG)15	2093	2146	Shoot	--					
CakTcSSR04655	CakTC24203	(AG)9aaga(AG)8	465	502	--	--					
CakTcSSR04656	CakTC24439	(TC)11(AC)8	1624	1661	--	--					
CakTcSSR04657	CakTC24499	(CT)15cg(CT)8	2961	3008	--	--					
CakTcSSR04658	CakTC24546	(TC)11ttcacactctctt(TC)10	663	717	--	--					
CakTcSSR04659	CakTC24699	(AAG)5cttcttcagcaatcttgcactctcatttcaacttctacatactctcatagttt(TTC)8	1	92	--	--					
CakTcSSR04660	CakTC24761	(GAAAGA)6gagagaggttgcagaggttggttttggtggctggaaggggaaaagcataaaccgaaacggaagaaccctaacctaactaac(CT)6	1	129	--	--					
CakTcSSR04661	CakTC24987	(TC)10ttcccatctcc(CT)6	168	210	--	--					
CakTcSSR04662	CakTC25036	(GT)6(GA)7	1359	1384	--	WRKY					

CakTcSSR04663	CakTC25053	(CAG)5caacagcaacaa(CAG)5caacaacaacagcagcagtcacagcatcagcagcagcagcaa(CAG)5	658	756	--	LUG				
CakTcSSR04664	CakTC25073	(GGA)6(GGT)6	532	567	--	--				
CakTcSSR04665	CakTC25216	(AAG)7acgatgatggtggttgtgttacagcagcagaaggaggacgctagat(TTA)5	1474	1555	--	--				
CakTcSSR04666	CakTC25329	(AAC)5cttaattctttgtctaaatctgcttgtagcagcacttctgagacaagtaaaggttctgagagagcaaaggagaaagataatgagtctagttttgc(ACA)6	1260	1387	--	TCP				
CakTcSSR04667	CakTC25415	(AGA)9(GGA)5	1563	1604	--	--				
CakTcSSR04668	CakTC25569	(GA)7tagagaaactactaccttgcttcccttaacactctttctctttctctctctatctctctctctatatatattctctctctca(TC)11	148	265	--	--				
CakTcSSR04669	CakTC25616	(AG)12tagagtt(TG)7agagtagaaatgaagacgcgtaagaaagaaggagatacaatacaaag(GA)8	4133	4240	--	--				
CakTcSSR04670	CakTC25620	(TGA)6atctgatgattattctccattgtaggagtagcatctgaaggtgaaggtgaagcagtagtagcagaagaagca(CCT)5	296	400	Flower bud	TCP				
CakTcSSR04671	CakTC25620	(TAA)6tgtttggtttatttattagacttgtgttgtgct(AG)6	1691	1753	Flower bud	TCP				
CakTcSSR04672	CakTC25621	(TC)6tagcacaacacaagtctaataaataaaccaaac(ATT)6	544	606	--	TCP				
CakTcSSR04673	CakTC25621	(GGA)5ggtgcttcttctgctactactgcttcaccttcaccttcagatgctactcctacaatggagaataatcatcagat(TCA)6	1896	2002	--	TCP				
CakTcSSR04674	CakTC25663	(ATG)5(ATGATA)5*atgatgattcagatgatgaagattgggggaagaatgctgtattggaggatgttgg(TGA)5	762	873	--	--				
CakTcSSR04675	CakTC25786	(GA)8a(AG)7aa(AG)6	1269	1313	--	--				
CakTcSSR04676	CakTC25940	(TG)12(AG)11	1098	1143	--	HB				
CakTcSSR04677	CakTC26102	(AG)7atgat(GA)8	1833	1867	--	--				
CakTcSSR04678	CakTC26179	(TC)7(TG)9	243	274	--	--				
CakTcSSR04679	CakTC26185	(CCT)5ccacaaatgacaatacctcctacaaagtatggaatagcatcaat(TCC)10	3043	3131	--	--				
CakTcSSR04680	CakTC26205	(TTG)5(ATG)6	1825	1857	Young_pod	HB				
CakTcSSR04681	CakTC26246	(GA)9agagagaga(AG)17	990	1050	--	--				
CakTcSSR04682	CakTC26531	(TGT)5gagtggggat(GA)9	2071	2113	Young_pod	--				

CakTcSSR04683	CakTC26534	(TTC)9tactcgtagtcaaagggatgtccgctacaggt(TC)13	155	239	--	--				
CakTcSSR04684	CakTC26539	(AG)7aa(AG)6	17	44	--	--				
CakTcSSR04685	CakTC26859	(ATT)6gttattatcgggtgagtggtcaattgaagaaagatcaaaatcaatgaaatcgcaagagatgtcttcgcaagcatcgagccagtaagaggggagga(AGCATT)5	6248	6391	--	--				
CakTcSSR04686	CakTC26984	(TAT)8tgttattgttgttgttg(TTA)5	208	263	--	--				
CakTcSSR04687	CakTC27201	(TCT)6(TC)8	1300	1330	--	ARF				
CakTcSSR04688	CakTC27324	(AAG)6aatcgaaattggaattgtggaagagaataaatgattagggttgaggaattggtgagggagggaggagaaagggaaaatggtat(TTTC)12ttgttagtttgggatagttcactccctcaggct(CA)6	1881	2074	--	--				
CakTcSSR04689	CakTC27396	(AG)7tcgatgttacgaagaacgcacgacacggcaagtgcaggtcagctcaacaaccaaatcggaagataaatcaattattaatttgacattaatatttctaaga(TAT)6	1839	1970	--	--				
CakTcSSR04690	CakTC27413	(TTC)6tcaaacattttcccgcgttttaagggattttcatgc(ATA)5	179	247	Flower bud	--				
CakTcSSR04691	CakTC27526	(TCT)6tcctcttcttcttcgtcctcttcttcatcttgttcttcgtattgttcttcatcgtcgtcgtcgtcc(TCT)5(TCA)5	2276	2389	Young_pod	PHD				
CakTcSSR04692	CakTC27526	(AC)6aaatcccaatttcaaaggtacaaagaaagtgcaacaaccaaacacacaaacacaaacacatgaatcataattaaaataaaaataaaaataaaaa(AC)7tctgacactacc(CTGTT)5	2904	3060	Young_pod	PHD				
CakTcSSR04693	CakTC27556	(TTC)6acacatactcaaaccaaaattaacatttgaggttacca(TGT)5	281	351	--	C2H2				
CakTcSSR04694	CakTC27621	(GA)11tacagtgttagtgatgggt(AG)17	954	1028	--	--				
CakTcSSR04695	CakTC27641	(GT)12(GA)16	1146	1201	--	--				
CakTcSSR04696	CakTC27769	(TG)9(AG)9ataagaagaaacaggttatggggaggacaagtctgcagagctggca(AG)19	3441	3561	--	--				
CakTcSSR04697	CakTC27777	(CT)7ttctctcttcaattcaacactccttctttattattaatcctaatcccatttacttataaatcctctattttcatcgcatcttatcgattcattcatt(TC)6	136	258	Shoot	--				
CakTcSSR04698	CakTC27957	(TCAG)5tca(CT)7ccgatgcgttagtgagagagagtgtgtgtatggttggtgtgtgtgtgggttt(GA)13	1167	1281	--	--				

CakTcSSR04699	CakTC27988	(AG)10atagggttagggttttaaaaaagaagaggtg(GAA)5	3368	3433	--	--				
CakTcSSR04700	CakTC28008	(TC)8tatctctctctgcgagtg(TA)7	2549	2598	--	--				
CakTcSSR04701	CakTC28030	(ACT)10(CT)34*	1	96	--	HB				
CakTcSSR04702	CakTC28157	(GCA)6ggaagaagatgtgggaaatgagcagcagcaagaggaagaggaggaggaggtagatatgatggagttgagactgggagggagcaagagcaagtgcag(GGA)5	605	733	--	ABI3VP1				
CakTcSSR04703	CakTC28171	(AG)6atgtagaaaa(AG)8	1918	1955	--	WRKY				
CakTcSSR04704	CakTC28226	(GA)10ttagcgcaaacatctcactgattttggggtaatttttct(TTC)16	1	107	--	--				
CakTcSSR04705	CakTC28367	(CT)9tttccattgtttcattcacttacttctcagcttttgtattttggtccttttcaatttcac(CT)8	1513	1606	--	--				
CakTcSSR04706	CakTC28402	(TC)18ta(TG)7	8	59	--	--				
CakTcSSR04707	CakTC28402	(TCA)7(TTA)7	1031	1072	--	--				
CakTcSSR04708	CakTC28432	(TTAA)5tgtccattttctgaccctaccccgctcttttgaatatggttgccccaagttcaa(GTTCAG)5	43	146	--	--				
CakTcSSR04709	CakTC28433	(TTAA)5tgtccattttctgaccctaccccgctcttttgaatatggttgccccaagttcaa(GTTCAG)5	243	346	--	--				
CakTcSSR04710	CakTC28501	(AG)11gttttgatttgagaaatgagacagtgatctgagctgaaacagtgggaataaagtgagggga(AG)10(GA)6	358	471	--	--				
CakTcSSR04711	CakTC28520	[illegible]tattcatgctagtattgaaatcttcaagtactactccacgatgacctaacatcatcatctcgtac(TGT)5	1172	1302	--	Trihelix				
CakTcSSR04712	CakTC28629	(AG)12(TG)13	1113	1162	--	--				
CakTcSSR04713	CakTC28643	(TCT)7tcgcgct(TC)8	164	207	--	--				
CakTcSSR04714	CakTC28711	(TTC)5ttgttcttttggcttcaacg(AC)7	4128	4176	--	FHA				
CakTcSSR04715	CakTC28712	(TCT)5tcatcc(TCT)5	298	333	--	--				
CakTcSSR04716	CakTC28798	(TAA)5tag(TAA)6	792	827	--	C3H				
CakTcSSR04717	CakTC28832	(AG)11atggttttttt(AG)6atgggtttttt(AG)9	507	580	--	--				
CakTcSSR04718	CakTC28892	(AG)11atgagagagatgaggtagttattatttatagggagaagaaaa(AG)13	2021	2110	--	--				
CakTcSSR04719	CakTC29049	(TC)9ttatctttgttctttgtgtgtgtgtctgtgcctttctg(TC)8	3	74	--	TCP				

CakTcSSR04720	CakTC29049	(CAA)5gaatcatcttcttctccttcaa(ATTCTC)5atcaacaacaccttgtggtccccttttgatggaagaccacccccctttcattatgggctccatctccatccaacaaccttcttcaaac(TCT)5	339	506	--	TCP				
CakTcSSR04721	CakTC29191	(CT)11tcctgtaatattttgtttgtgtgtgtgttctttcttt(AG)7	8	79	--	--				
CakTcSSR04722	CakTC29198	(TGT)5(TTC)5	1569	1598	--	--				
CakTcSSR04723	CakTC29368	(TTC)5tttctctcgagtaacacgcaagtcaata(TTC)5	76	133	--	--				
CakTcSSR04724	CakTC29433	(AAT)9atatgtttaccaatgctacatcatggagtaatattgaagc(AAG)6	918	1002	--	C2H2				
CakTcSSR04725	CakTC29478	(CTT)5ccttctttcgtcgctctctcaa(TCT)6	30	84	--	--				
CakTcSSR04726	CakTC29478	(CAT)5ccaatttggaatattcatac(CCT)8	535	593	--	--				
CakTcSSR04727	CakTC29579	(TCT)6(TCTCAA)5*	427	471	--	GRF				
CakTcSSR04728	CakTC29683	(TTG)6gaacatcat(TTG)7	254	301	Flower bud	--				
CakTcSSR04729	CakTC29805	(AG)11aaa(AG)9	504	546	Shoot	zf-HD				
CakTcSSR04730	CakTC29903	(CT)8ccactttctctctctacaaattcctttacaacaactgaagtaaagaac(CT)9	8	89	--	--				
CakTcSSR04731	CakTC29919	(TTCT)5(TTC)6	3	36	--	--				
CakTcSSR04732	CakTC30069	(TTG)7agggataagaaatggattatcatggttgaaagtt(TGA)5	919	988	--	bHLH				
CakTcSSR04733	CakTC30217	(CAA)7cctcaaa(AAC)5	962	1004	--	--				
CakTcSSR04734	CakTC30271	(TAA)6ttatg(TA)8	1521	1559	--	--				
CakTcSSR04735	CakTC30341	(TA)6(TG)10	1385	1416	--	--				
CakTcSSR04736	CakTC30431	(ATT)8aacaatcaaatgctt(TGA)5	2161	2214	Young_pod	--				
CakTcSSR04737	CakTC30473	(TAA)6ccaagttgttgaagaaattgttaataaa(AAT)7	2059	2125	--	--				
CakTcSSR04738	CakTC30523	(AG)6aaagagatggaattgtgtggtttgattaaaaggaagggttttagggggaatattagagggagagccactggt(AG)6atagtggaagaggcagaggttggttttgtcgggtaatgaaacagtgtcggaagggagtc(AG)6aaagagaattgtgttttgttttgt(AG)8	3664	3870	--	--				
CakTcSSR04739	CakTC30591	(TC)8tactactacttcatggcttctctcaaaaaccttttctctctctacattcccatgttca(TCT)7	34	128	--	--				
CakTcSSR04740	CakTC30664	(TCC)5acctcctccaggactaataccaagccccaacggtggtggtcctaggcctcct(CCA)5	1557	1638	--	--				

CakTcSSR04741	CakTC30694	(CT)11cactcatagaatagttcttgcactatactactaccaatgttattgtgttctgtttga(AAG)6	17	113	--	--				
CakTcSSR04742	CakTC30698	(TAT)5atatccatt(CATA)5cacacctcttaac(CT)6	175	243	--	--				
CakTcSSR04743	CakTC30702	(GAA)5gan(AGA)5(AG)28*	1749	1834	--	--				
CakTcSSR04744	CakTC30707	(TGT)7ctatgttggagtggtgaagagagaggagaataatgatggtgttgtgttggagataaagggtggtga(TGT)6	914	1018	--	MYB				
CakTcSSR04745	CakTC30748	(TC)12acgctcaccccgtgtttgtgtgtaataatcttcttcttcctattttattc(CT)6	46	131	--	AP2-EREBP				
CakTcSSR04746	CakTC30797	(TTTC)6tcag(CT)9	17	62	--	--				
CakTcSSR04747	CakTC30832	(ATG)6agaaacaggaacagaatcaataatcatttgtgaatgagaagcattaactccaccaactac(TTG)7	353	451	--	ABI3VP1				
CakTcSSR04748	CakTC30832	(TTC)7aaattcttcttcttttgatggtgttgtgatgataa(CTT)6	1392	1465	--	ABI3VP1				
CakTcSSR04749	CakTC30906	(CAA)5ctactttagctgcaggtac(TAA)7	426	480	--	GRAS				
CakTcSSR04750	CakTC30911	(AG)14acattgaacaaaatatgcaatttttgttaaatgggtttgtaactttgtcgttaaaaatgcagaagcacggtgagaaacagagaattcaagagagag(GAA)6	2400	2541	--	--				
CakTcSSR04751	CakTC30925	(ATA)6atgatg(ATA)9	2177	2227	--	--				
CakTcSSR04752	CakTC30932	(AT)7aacaacttg(TTC)5	43	80	--	--				
CakTcSSR04753	CakTC30961	(CT)7ttcttcttcatcaatatattcttattccttcttcttatttcagtaactaatggaaatggatgaca(ATC)6	160	256	--	Trihelix				
CakTcSSR04754	CakTC30961	(TGA)7aaacgatcatactcatagcactgagggactgaaagagaatgaatgttccatggagaagaagaaaaagaggacggtgagtg(GAA)5	933	1048	--	Trihelix				
CakTcSSR04755	CakTC31110	(CAA)5ctaccctctccttcaatagaaactag(TAA)5tagtaataataacaacg(ATA)5	407	494	Shoot	AP2-EREBP				
CakTcSSR04756	CakTC31179	(GGT)5gatgcatatgagattccaacaacaggtggcaatggcccttcatatacaggagta(GGT)6gaattgtattgaat(TGG)5tgaatagactggtgctggcggtggtgatgaatatactggtggtggtgatggagatggtggtgggtgataaggtgtttggt(GTG)5	652	862	--	--				

CakTcSSR04757	CakTC31182	(TAT)5tggtaattactataagtgttgattttgggttcgcgata(ATG)5	2799	2866	--	--					
CakTcSSR04758	CakTC31228	(GAA)5gttgttgaaccctataaactacactcgattagatttactaacttcatcatttctttttgctacatattt(GA)35	3106	3259	--	CPP					
CakTcSSR04759	CakTC31402	(GA)10ggagtaggagagaaaacgcagatgctcaccagatagagaagtgagttgttggcacc(GA)7	2292	2381	--	--					
CakTcSSR04760	CakTC31481	[illegible]ggaggaggaggaagtagaagaagttgaagaagaagaagaggaggag(GAA)9gaggaggtg(GAA)5gaggtggtg(GAA)5gaggtg	252	464	--	C3H					
CakTcSSR04761	CakTC31534	(GA)9gtgattcccactgcacttgacttgttcagatcga(TTC)9	2824	2902	--	--					
CakTcSSR04762	CakTC31555	(AG)8aaaaacaaaagag(AC)6	105	145	--	--					
CakTcSSR04763	CakTC31669	(AT)10tcatctcat(TC)7	138	180	--	--					
CakTcSSR04764	CakTC31990	(TC)8t(TC)6	5	33	--	--					
CakTcSSR04765	CakTC32025	(TCT)5tcgtgttcttctatttgttcagatgaagaagctgcttgtgatgatgttgaac(CTT)6	1648	1732	--	GeBP					
CakTcSSR04766	CakTC32072	(GAA)6aacgacgaa(GAT)6	392	436	--	--					
CakTcSSR04767	CakTC32074	(TTC)12ctcattccaattctcaattcttctctattccttca(ATCT)5	1140	1230	--	--					
CakTcSSR04768	CakTC32122	(CT)6aaaaa(TC)6	2072	2100	--	WRKY					
CakTcSSR04769	CakTC32123	(TTA)5ttttcttcaaactcttgaaaactagatgaaccagaattaatattatcagaacactcccaaaaatgat(TAC)5	1104	1200	Root	bHLH					
CakTcSSR04770	CakTC32157	(AC)6atcata(AT)7	990	1021	--	TCP					
CakTcSSR04771	CakTC32204	(AG)6cac(AG)7	1528	1556	--	--					
CakTcSSR04772	CakTC32348	(CT)10gtctctctg(TC)8	49	93	--	--					
CakTcSSR04773	CakTC32409	(TGT)5(TGG)5	350	379	--	--					
CakTcSSR04774	CakTC32412	(ATT)9agtaacattt(TTA)7	952	1009	--	--					
CakTcSSR04775	CakTC32521	(TTC)5(ATC)5atattcttcttctctttgttgctggttggtttcttcagaagcatcgtcatattcgggtgcagtgaactctaagtcgaagaaaggacct(TCG)5	598	730	--	--					
CakTcSSR04776	CakTC32692	(AAC)5aattaaccacaacttcagtgactagactacttcatttatttcatctcac(GTT)5	166	244	--	--					
CakTcSSR04777	CakTC32792	(GTT)7gacgaatcagtattttgatgggggtaaagggtttccgagatgaaccatcttcagcaacccacaacatgaaaaggttgacacataaagaagaagaggg(AGA)5	298	429	Flower bud	--					

CakTcSSR04778	CakTC32806	(GAA)7gaggggtgtggtggcgtggc(GA)6agagtaattcctctctttgcggccatccgataaagtcggaagatactaaataatacaaaataaaagacccaattatacaatacaatattaaattac(ATT)8	1	173	--	--				
CakTcSSR04779	CakTC32951	(AG)16atttgttggggtttggatagatg(GAA)6agcgatggatgggccttttgatttgagggagtt(GA)7	1	120	--	--				
CakTcSSR04780	CakTC32975	(GAG)8gaagag(GAA)8	498	551	--	--				
CakTcSSR04781	CakTC33002	(AG)6aaagaga(GT)6	44	74	--	HB				
CakTcSSR04782	CakTC33002	(CAA)6ccacaaacgtcaacaattacaggtaattcagaaaacaacaacaattggtatggtaaccataccaacaac(CAA)5	593	694	--	HB				
CakTcSSR04783	CakTC33080	(GA)7gggggg(GA)6	2890	2921	--	--				
CakTcSSR04784	CakTC33139	(CCA)5ccccttccagatccgctttctcttcttcttcacatcctatcttcccaaaaagcaacaccaaactaacat(TC)6ccattttcaacaaaccaaaactcattagttctcgtcccttcaaaatctcatcctccctctcaaaatcccccaccgcaccatcctccat(AAC)6	88	289	--	--				
CakTcSSR04785	CakTC33193	(GGT)5ggaggctgatatgaattttccggcggaggaggaggtggtggtggagggtatggatagtaattcgaaggtggt(GGA)5	1845	1946	--	--				
CakTcSSR04786	CakTC33220	(TTA)5tg(ATT)5	1978	2009	--	--				
CakTcSSR04787	CakTC33270	(AAC)5tctttctcctttcatatcacctccaaatcaaacccttctctttcatggcgtttacttcatgaaaaatcaaacaatgtgaaaaaacg(AGA)6ac(AAG)5	431	566	--	--				
CakTcSSR04788	CakTC33404	(TA)9tttataatacactcatctagttgaagaagaaggttttagttatatatattttatatataagagttgagctgc(TA)9	104	211	--	--				
CakTcSSR04789	CakTC33404	(ATC)8aacatgcacctgctgcttttagtaacatgccttctatggaaaacaatgctgctgcaccatcgtctttt(GGA)5	1695	1801	--	--				
CakTcSSR04790	CakTC33456	(TAA)6caac(AAT)7tacggtaagagttatcggtttgttactgaacttgaagagcttt(ATA)10	627	742	--	Trihelix				
CakTcSSR04791	CakTC33599	(TTC)16(TAC)5	97	159	--	--				
CakTcSSR04792	CakTC33616	(CT)9acttatttcaactgcatccttttatcatca(TCT)5	7	69	--	C2C2-Dof				
CakTcSSR04793	CakTC33642	(ATA)7acgatacattacaacaaacaacaa(CT)15	173	247	--	--				

CakTcSSR04794	CakTC33755	(AG)7attaaacgtgaagctgaagagaggtggtagttggaaaagaaattgaaatgaagaatacaaaggtctagaaatatagagaaaatggtt(GA)10	2273	2393	--	--				
CakTcSSR04795	CakTC33803	(CAT)5(AAT)6	510	542	--	--				
CakTcSSR04796	CakTC33825	(TAA)35ataataaataac(AAT)5	1	132	--	--				
CakTcSSR04797	CakTC33883	(TTA)7tcggatggtaaagggaagatggtgtcttgttgattttcatggagaatgagaattgagggatcaaaatcatggataataagaggtcttct(TTC)5	1751	1875	--	--				
CakTcSSR04798	CakTC33929	(TC)19tgtaattctcttcccagcaacaaaccttctctctctctaaatgtaaatatcattttctactcttttcaaaatatctctct(TC)7	1	133	--	--				
CakTcSSR04799	CakTC34098	(AGA)5gaggaca(AAG)7	2383	2425	--	--				
CakTcSSR04800	CakTC34364	(AG)6(AGA)6*	1967	1994	--	--				
CakTcSSR04801	CakTC34378	(AAT)5(ATT)5	6684	6713	--	--				
CakTcSSR04802	CakTC34449	(CTT)8ctcactttgatacatcatcttctttctaccctcttcgtccatcaccacaaaacntg(ATC)5	559	653	--	--				
CakTcSSR04803	CakTC34534	(AG)6aagaagaaaaagaagagagagcaagtttcaacaacaaaaacaaaaacaaaaacaagagaaagaa(AG)6	33	120	--	--				
CakTcSSR04804	CakTC34716	(AATA)6ttaataaaaaaatggaacaggttgtgttcgacggtggaagaatggattatggaatggtttgtgttttagggatagagaagagaggaat(GA)7aagagatcgggattgtgatttttgcggtggtggaacggagaa(AG)12	3530	3721	--	--				
CakTcSSR04805	CakTC34751	(AG)6aagagagaga(AG)7	3487	3522	--	--				
CakTcSSR04806	CakTC34772	(CT)9ttctctctt(TC)7	28	68	--	--				
CakTcSSR04807	CakTC34839	(GA)7a(AG)9	3	35	--	--				
CakTcSSR04808	CakTC34908	(AAG)5aacgaaacgcatttttttgagtatgtatggaagaaaaatgagtgaataatgcaactatactccaa(AAC)5	3586	3681	--	--				
CakTcSSR04809	CakTC34926	(AC)6tctg(AC)6	26	53	--	--				
CakTcSSR04810	CakTC34963	(TC)6ttcaacttctcagcttcaacactcgaacttcattcgcgtgtttccgacgaagaaaaaccaagaacgtaa(TC)8	78	174	--	--				
CakTcSSR04811	CakTC34982	(GAG)6gatttggaaaaggaggtggt(GGA)5	370	422	--	--				
CakTcSSR04812	CakTC35028	(GTG)5aaaac(CAA)6	341	378	--	zf-HD				
CakTcSSR04813	CakTC35085	(AAT)5acaaac(AAT)5	2540	2575	Flower bud	--				

CakTcSSR04814	CakTC35135	(AG)7aaaagaagaagcagaaga(AG)7aagaagaagcagaaga(AG)6	28	101	--	--				
CakTcSSR04815	CakTC35166	(GAT)6ggtgactggaata(ATG)5	3171	3216	--	--				
CakTcSSR04816	CakTC35203	(TAC)5tagcagctttcgaaaacgcacatagtaatagccaactaggcaaccacattcagttgcctaaaataaatcaaacaacaagaagatcttct(TTC)6	126	247	--	--				
CakTcSSR04817	CakTC35205	(GAA)6(GAT)7	2767	2805	--	--				
CakTcSSR04818	CakTC35382	(TTC)7acatttttctacaaatacttctctttgctttgttgttcttgtgatt(TC)10	22	108	--	--				
CakTcSSR04819	CakTC35566	(CT)9(AT)9	20	55	--	--				
CakTcSSR04820	CakTC35566	(CAG)6caacagcaacaccaacaacagcagcagcaacagcaacaa(CAG)5	978	1049	--	--				
CakTcSSR04821	CakTC35622	(AC)8gcac(AT)6	25	56	--	--				
CakTcSSR04822	CakTC35713	(AG)7at(AG)18	167	218	--	--				
CakTcSSR04823	CakTC35890	(CT)6cattcatttataattatctcactctcacaaactctttctctttctatctctta(CT)8	134	214	--	--				
CakTcSSR04824	CakTC35946	(CT)8ccaaaa(TC)8	49	86	--	ABI3VP1				
CakTcSSR04825	CakTC36098	(TC)7atcttctgactctcttctcttctatctatcttataccctctccaatttgcaaaccctacc(CT)7	4	91	--	--				
CakTcSSR04826	CakTC36098	(CAG)5acagaacgacttcagcagcagacagaacgactt(CAG)5	2808	2870	--	--				
CakTcSSR04827	CakTC36187	(TTC)5tagtc(ATTTTA)6	28	83	--	--				
CakTcSSR04828	CakTC36231	(TTA)5tg(ATT)6	484	518	--	--				
CakTcSSR04829	CakTC36239	(CCT)5ccacctt(CAC)5	76	112	--	--				
CakTcSSR04830	CakTC36265	(TGA)5tggtggtgatagtgatgg(TGA)6	487	537	--	--				
CakTcSSR04831	CakTC36298	(AC)11tatgtgagtttttatttctacgatcgattggggttttcctctt(TC)6	53	129	--	--				
CakTcSSR04832	CakTC36298	(AAC)5gtttcagatgacttagtgtctgtgagaatgaggaaagacgagacgaaaacagttaattcgtgga(GCG)5	338	431	--	--				
CakTcSSR04833	CakTC36467	(TTCAAT)8ttcagtttt(AATTTC)5	76	162	--	--				
CakTcSSR04834	CakTC36610	(TGG)5agaagttgccggtgacggtgttttcgatggt(GGA)6gaaagagaaggggaaggaggaggagttacaggtactgtttgtggaggagga(GAT)10gaaggaggagga(GAT)7	2070	2247	--	--				
CakTcSSR04835	CakTC36652	(CAG)7(CAA)5	2552	2587	--	ARF				

CakTcSSR04836	CakTC36776	(TC)6tagaacagagcataaagtatctctcactct(AGA)5	135	191	--	--					
CakTcSSR04837	CakTC36961	(TC)16ta(TC)12	1	58	--	--					
CakTcSSR04838	CakTC37049	(TCT)7tt(TTC)5	166	203	--	--					
CakTcSSR04839	CakTC37058	(TC)7tttcttccttcttcactaattaaacaa(TAT)6	14	72	--	--					
CakTcSSR04840	CakTC37126	(CT)8actttgaaaaaacaacagcactt(TC)10	1066	1124	--	--					
CakTcSSR04841	CakTC37567	(AG)6aatgttttt(GA)7	1151	1185	--	--					
CakTcSSR04842	CakTC37593	(CAG)6(CAT)5	1803	1835	--	--					
CakTcSSR04843	CakTC37599	(CA)6ctt(TC)7	1790	1818	--	--					
CakTcSSR04844	CakTC37613	(AGAA)8(AG)6	1181	1224	--	--					
CakTcSSR04845	CakTC37753	(CT)7ttctccaccttctctctctcttaatcttttttctctctttccatggctaatgag(AAT)7	1	89	--	WRKY					
CakTcSSR04846	CakTC37782	(GA)8caaaggggtatcatatcatactaaaataataatactaagtgattgatataaatagtttattagtggttgagattcaactaagaaggggatat(GCA)5	189	311	--	--					
CakTcSSR04847	CakTC37802	(GA)7ttgtttagttttgagga(AAG)5	1728	1773	--	--					
CakTcSSR04848	CakTC37924	(TC)7cctcctctgccttcttcttcatca(TC)7	140	191	--	--					
CakTcSSR04849	CakTC38053	(TGT)5gaggttga(ATTTTG)5	1438	1490	--	--					
CakTcSSR04850	CakTC38061	(CT)9t(TC)7	1057	1089	--	--					
CakTcSSR04851	CakTC38076	(ACA)5ctcctggtttaggttactattttatggacaat(AAC)5	284	345	--	HB					
CakTcSSR04852	CakTC38133	(CT)11ttcaaacttatactttccttttcccctccccaacacaacacaacact(TC)6	1	81	--	--					
CakTcSSR04853	CakTC38162	(GAT)7(GATGAA)5	284	331	--	--					
CakTcSSR04854	CakTC38180	(TGT)5tcctgaagataaggttgttcgaaggagagagacatagagactgtacaaccattcttcttcttgttaactctttctcact(TC)7	905	1012	--	--					
CakTcSSR04855	CakTC38220	(GAA)5(GAT)5	414	443	--	--					
CakTcSSR04856	CakTC38533	(GAA)5tttgggtggtggttataaaacctagcagcacttgagtttgtgttgttgttccatgttggataagaagccattgatgatgatgata(ATG)7tgaatctatagtttatgaatggatgttgttgt(TGA)5	478	645	--	--					
CakTcSSR04857	CakTC38541	(ACA)5acgcttcaggacacacctgaggatattttatatattgtaataatcattatt(TTA)5	227	307	--	--					

CakTcSSR04858	CakTC38713	(CCA)7cctcagagtcagacccatcacccct tctactattccaatcccaattgctacaccaattat gtcaatcgacttcaataccatccttactatccca at(CAA)7	268	405	--	--				
CakTcSSR04859	CakTC38718	(TGA)5(TGG)5	1299	1328	--	--				
CakTcSSR04860	CakTC38743	(CAA)8atgaatttacctcttcaacaagtaggt gttggaatgagcatgggaagtgggagcagc(AAT)11	1095	1208	--	HB				
CakTcSSR04861	CakTC38761	(AG)9aaaga(AG)8	1387	1425	--	bHLH				
CakTcSSR04862	CakTC38866	(GT)7gaatggagtctcaagcattcaatcaag ttgatggagtagttgcagaggagagatttaaga ttgagaagagagatttc(AG)10	1790	1900	--	G2-like				
CakTcSSR04863	CakTC38983	(GAA)5agagtcgcaaagagtttttctagcttt cagttttatttgat(TC)8tgtgactct(TTC)5 at(CAA)8	185	306	--	--				
CakTcSSR04864	CakTC38998	(ACC)5gccaccacaacctcaaaatcagaat caaaatcaaatt(CAAAAC)5ac(TAA) 8	1384	1491	--	--				
CakTcSSR04865	CakTC39058	(CT)6(CA)8	7	34	--	--				
CakTcSSR04866	CakTC39073	(AGA)6tgatgatgatgaagatggtaatgaa gaggaagaggagaaaaaagaaagaccaaag cgttccaatagtgt(GAA)6	407	511	--	SWI/SNF-BAF60b				
CakTcSSR04867	CakTC39121	(GAA)5gagaaagagaaagagacgttgttg ctgcatcgcag(TGA)8	1390	1463	--	--				
CakTcSSR04868	CakTC39483	(TCA)9(TCT)5	152	193	--	--				
CakTcSSR04869	CakTC39564	(AG)8t(GA)7	1256	1286	--	--				
CakTcSSR04870	CakTC39603	(AG)7aagacaaaaaagaaacagttgct(G A)11	1632	1690	Shoot	LUG				
CakTcSSR04871	CakTC39665	(TC)6ta(CT)12	770	807	--	--				
CakTcSSR04872	CakTC39703	(AGAA)5aaagggattattggagaagtgtgt ctttgagttatttggattttgaagaaaa(AG)12	1114	1209	--	--				
CakTcSSR04873	CakTC39740	(CCA)5ccccttccagatccgctttctcttcttc ttcacatcctatcttcccaaaaagcaacaccaa actaacat(TC)6ccattttcaacaaaccaaaa ctcattagttctcgtcccttcaaaatctcatcctcc ctctcaaaatcccccaccgcaccatcctccat(AAC)6	1328	1529	--	--				
CakTcSSR04874	CakTC39743	(AG)11gtttatgaagatagagagaagagca tggctgaatttaag(GA)10	1234	1314	--	--				
CakTcSSR04875	CakTC39754	(TAC)5(TAA)7	621	656	--	C2C2-Dof				

CakTcSSR04876	CakTC39754	(TTA)5tggtatttagtaagcactagttttgattgaaaaaatgagttttagaattttgattgaaaggaatgagatgaagtgagtgagatataaaaatgc(ATT)5	1300	1422	--	C2C2-Dof				
CakTcSSR04877	CakTC39789	(TAC)5aaccgaagaac(ATA)6	56	99	--	--				
CakTcSSR04878	CakTC39789	(TGT)5(TGC)5	433	462	--	--				
CakTcSSR04879	CakTC39789	(ACC)6catcatcatttttgaaatccattattgttcatagcttgaacattatt(ACC)5acttattggtacagaaataccctttttacccccactgttctgattattattgttattgttattt(CCA)6ctgtttttacccctctggtatgccaccgttatttttgttacctccaccatgattctgattatt(ACC)6	582	824	--	--				
CakTcSSR04880	CakTC39897	(GGT)6ggaagtgacggtggttgtgatggtggttgtggaagatggagcttgagaattagaaggagattgtgtgagtggttgaagtaaaaca(CCT)6	1390	1510	--	C2C2-Dof				
CakTcSSR04881	CakTC40096	(TC)7acacta(CT)6	11	42	--	--				
CakTcSSR04882	CakTC40184	(TCT)7ttccctctt(CAA)5	19	63	--	--				
CakTcSSR04883	CakTC40197	(GA)7gttcgtgggttgccttgcctcgctttacttcgctgtcgtttcgccggccaattattgccgttttactgtcttcttcagaaattcctttctctccctcc(CT)17	30	175	--	--				
CakTcSSR04884	CakTC40203	(CT)8tgtttatcttaaagcctaagccgtaaccgt(TTC)5(TC)8*	261	335	--	--				
CakTcSSR04885	CakTC40260	(TCT)6taagtctcatatggcatgtttaactc(ACA)8	180	247	--	--				
CakTcSSR04886	CakTC40287	(TC)6(TA)6	833	856	--	--				
CakTcSSR04887	CakTC40337	(AG)7aaagagagagatt(AG)11	40	88	--	--				
CakTcSSR04888	CakTC40339	(AG)6ttagagtt(AG)7aagttatgggttgttgccaatcctcgttgtcaaggttgagaggaggagttagagatggagataatactaataataatat(TAA)8	98	234	--	--				
CakTcSSR04889	CakTC40460	(TCT)6tcctcttcttcttcgtcctcttcttcatcttgttcttcgtattgttcttcatcgtcgtcgtcgtcc(TCT)5(TCA)5	2299	2412	--	PHD				
CakTcSSR04890	CakTC40509	(CT)10(CA)7	6	39	--	--				
CakTcSSR04891	CakTC40574	(AG)8aaacacatatagataatcagagagatattttcacaattgtgtttgttgtgtat(CCA)6	35	121	--	--				
CakTcSSR04892	CakTC40574	(CT)7(AT)9	364	395	--	--				
CakTcSSR04893	CakTC40607	(TC)21ttattatacttctattttcat(TC)6	3	77	--	--				
CakTcSSR04894	CakTC40623	(AAT)6(AAG)6	804	839	--	--				

CakTcSSR04895	CakTC40662	(ATG)7gaaaatgtaagggaaatgtgagtgtgaatgtctttgattgtag(CAT)5	783	861	--	--				
CakTcSSR04896	CakTC40671	(TCA)6tta(TGT)5	294	329	Mature Leaf	--				
CakTcSSR04897	CakTC40671	(AG)9atgtgtagtggggtggttattgaagagaggaagcgggaagaaaggaatgaaagggtgggttttatg(GA)7	634	731	Mature Leaf	--				
CakTcSSR04898	CakTC40856	(TC)7ttattctgtttctctctctaaataattagacaacgcggataatttgaataggggaagttaaaggagcagtgatgaagctgttgttggtggtggctatgc(CT)9	1872	2002	--	--				
CakTcSSR04899	CakTC40875	(AGA)6ggcgcgtgggtttgtgtatggttgtgttggtaatggattgtttaaaaacgaagagaaatttgattcggcgcgtgaatcatactcttcattgttg(TTA)5	1470	1596	--	--				
CakTcSSR04900	CakTC40893	(GAA)16aggaaccctagaatgaagagaa(AAG)12	1	106	--	--				
CakTcSSR04901	CakTC41009	(GAA)6aataagaagaggaggaagataatagagctgaccccagcatcaaaataatcatctttttatatatatagtatgttctttggaggtttgtga(AG)11	1875	2005	--	--				
CakTcSSR04902	CakTC41032	(AG)8aatacacaaacactcacacacacattctacttcacttcatttcgattcaaac(CTTT)5	1	88	--	--				
CakTcSSR04903	CakTC41098	(TC)8agagacgccatgtatcgccacatctacaccgcccttcttcttttcattctctccatttccctctct(TCA)9	93	201	--	--				
CakTcSSR04904	CakTC41172	(GTT)5(GGT)6	1982	2014	--	--				
CakTcSSR04905	CakTC41230	(TCT)5ggtttttttcttctacttctggttcttctggatcttcttcttcctcttctggttcttcttctgga(TCT)5ggttc(TTG)6	1761	1879	--	--				
CakTcSSR04906	CakTC41345	(CT)7atctatctct(TC)8	5	44	Mature Leaf	--				
CakTcSSR04907	CakTC41394	(TTC)6tctgttattggagtgatggtgaattaatgattcttagcttgaacttct(TGAACA)5	2387	2482	--	--				
CakTcSSR04908	CakTC41516	(TTTGAT)6tttgagaggtta(TTG)7	772	840	Flower bud	GRF				
CakTcSSR04909	CakTC41712	(ATA)5aaaataataatgtttgttttattagga(AG)6	652	705	--	--				
CakTcSSR04910	CakTC41760	(TC)10(AC)7	76	109	--	--				
CakTcSSR04911	CakTC41858	(AAG)5ttaattcattccttgaaaatagaattagcgaattagatctcagaa(AG)6	105	176	--	--				
CakTcSSR04912	CakTC41965	(GTT)6(GCT)7gttgttgttgtagaagacgttgttgatgataaagctgttgttgttgaaattgagattgggttgtttgtggttgctgaaaag(GTT)8	686	829	--	--				

CakTcSSR04913	CakTC42004	(TC)16tttctgcatattcttagcatatcttaaactccaattccatttccgttgctagat(AGAA)5	21	126	--	--				
CakTcSSR04914	CakTC42011	(GAA)5ttagaagag(GAA)6	765	806	--	--				
CakTcSSR04915	CakTC42022	(TC)7tttcaatgccgcccatgcctatcagaatgaactagagagagagtgagacatttcagagacgaactaaga(AT)8acacacat(AG)8	42	164	--	--				
CakTcSSR04916	CakTC42042	(TC)8tatagcattgtagtgtggtgtggacctctttgctttgctgtcttttgagttccccattgttgggctttaatggggtttgtctctttgagcttatt(ATA)5	4	129	--	--				
CakTcSSR04917	CakTC42044	(AAG)5atgataaaaatgat(GAA)5	70	113	--	--				
CakTcSSR04918	CakTC42080	(AG)6aatgacgacaagaggtaatcaacaacaaccaccgcaaccaaacggtgaacagaggctgagg(CAA)6	40	130	--	--				
CakTcSSR04919	CakTC42137	(GA)13tg(GAA)5	6	48	--	--				
CakTcSSR04920	CakTC42169	(TC)8tatctttgatttcgattttatgattgtgaatccaagtatgagagtttgag(AGA)8	772	861	--	--				
CakTcSSR04921	CakTC42230	(GT)6(GTGTG)6*	1708	1745	--	--				
CakTcSSR04922	CakTC42413	(TC)6taaacc(CT)6	1740	1769	--	--				
CakTcSSR04923	CakTC42460	(TCAAAT)5gttaggaataagaaagttggcgcttttggtgt(TGG)5	692	768	--	--				
CakTcSSR04924	CakTC42506	(CTT)6cgtcttctccgccaatcgaggatactcctccggtgaattctcaa(CCT)5	601	677	--	--				
CakTcSSR04925	CakTC42582	(AG)7aaagagagac(AG)9	985	1026	--	--				
CakTcSSR04926	CakTC42755	(TGT)5tgctgctgg(TGT)6	175	216	--	C2H2				
CakTcSSR04927	CakTC42832	(CAA)6aaggtgctaattatccacttggccattatcaagagaataacatcat(CAA)5	1470	1548	--	--				
CakTcSSR04928	CakTC43142	(TC)6(GAT)5	1838	1864	--	--				
CakTcSSR04929	CakTC43256	(GAA)6atagtagtagt(ACA)5	433	476	--	--				
CakTcSSR04930	CakTC43356	(GAA)5aaaaacccaaataacaattttaaatttttcttttttcccatcttggg(TC)6	887	961	--	zf-HD				
CakTcSSR04931	CakTC43377	(CT)6ttaacaaaaaaaaaaataaaaaaacaaa(AG)9	108	165	--	AP2-EREBP				
