# Supplementary material for: Comparative Analysis of Kabuli Chickpea Transcriptome with Desi and Wild Chickpea Provides a Rich Resource for Development of Functional Markers
Source: PLoS One. 2012 Dec 27;7(12):e52443. doi: 10.1371/journal.pone.0052443 (PMC3531472; doi:10.1371/journal.pone.0052443)
Supplement: Table S8 — List of polymorphic SSRs identified between kabuli and desi chickpea. (PDF) [file pone.0052443.s018.pdf]

## Table S8. List of polymorphic SSRs identified between kabuli and desi chickpea.

| Kabuli SSR ID | Kabuli TC ID | SSR | Start | End | Desi SSR ID | Desi TC ID | SSR | Start | End | Repeat unit size difference | Tissue specificity | TF family |
|---|---|---|---|---|---|---|---|---|---|---|---|---|
| CakTpSSR00026 | CakTC00646 | (GA)7 | 1984 | 1997 | Ca(ICC4958)TpSSR01618 | TC09857 | (GA)10 | 2061 | 2080 | 3 | -- | -- |
| CakTpSSR00219 | CakTC05322 | (CAT)5 | 1130 | 1144 | Ca(ICC4958)TpSSR01328 | TC08180 | (CAT)7 | 1053 | 1073 | 2 | -- | -- |
| CakTpSSR00368 | CakTC08782 | (TC)21 | 17 | 58 | Ca(ICC4958)TpSSR02480 | TC15958 | (TC)13 | 56 | 81 | 8 | -- | -- |
| CakTpSSR00381 | CakTC08924 | (TCCTCT)9 | 2364 | 2417 | Ca(ICC4958)TpSSR00873 | TC05387 | (TCCTCT)6 | 2292 | 2327 | 3 | -- | -- |
| CakTpSSR00428 | CakTC09678 | (TCAC)9 | 1204 | 1239 | Ca(ICC4958)TpSSR00117 | TC00871 | (TCAC)7 | 1222 | 1249 | 2 | -- | -- |
| CakTpSSR00452 | CakTC09874 | (GA)8 | 1242 | 1257 | Ca(ICC4958)TpSSR00281 | TC01966 | (GA)10 | 1262 | 1281 | 2 | -- | -- |
| CakTpSSR00630 | CakTC11163 | (TC)6 | 92 | 103 | Ca(ICC4958)TpSSR02109 | TC13124 | (TC)17 | 204 | 237 | 11 | Young_pod | -- |
| CakTpSSR00643 | CakTC11370 | (TAA)10 | 1 | 30 | Ca(ICC4958)TpSSR02227 | TC14545 | (TAA)12 | 9 | 44 | 2 | -- | -- |
| CakTpSSR00729 | CakTC12463 | (TA)14 | 49 | 76 | Ca(ICC4958)TpSSR03164 | TC22801 | (TA)13 | 49 | 74 | 1 | -- | -- |
| CakTpSSR00739 | CakTC12740 | (CTT)5 | 197 | 211 | Ca(ICC4958)TpSSR03282 | TC26236 | (CTT)6 | 197 | 214 | 1 | -- | -- |
| CakTpSSR00744 | CakTC12877 | (CT)7 | 159 | 172 | Ca(ICC4958)TpSSR03335 | TC28683 | (CT)12 | 175 | 198 | 5 | -- | -- |
| CakTpSSR00748 | CakTC12979 | (GA)9 | 84 | 101 | Ca(ICC4958)TpSSR03401 | TC31429 | (GA)8 | 84 | 99 | 1 | Flower bud | -- |
| CakTpSSR00752 | CakTC13120 | (AGA)6 | 855 | 872 | Ca(ICC4958)TpSSR02662 | TC16903 | (AGA)8 | 45 | 68 | 2 | -- | -- |
| CakTpSSR00830 | CakTC14565 | (ATA)9 | 1455 | 1481 | Ca(ICC4958)TpSSR02007 | TC12302 | (ATA)10 | 1251 | 1280 | 1 | -- | -- |
| CakTpSSR00867 | CakTC15258 | (GA)14 | 3523 | 3550 | Ca(ICC4958)TpSSR01851 | TC11344 | (GA)16 | 3528 | 3559 | 2 | -- | CAMTA |
| CakTpSSR01146 | CakTC21556 | (GA)11 | 1055 | 1076 | Ca(ICC4958)TpSSR03214 | TC23672 | (GA)10 | 1062 | 1081 | 1 | -- | -- |
| CakTpSSR01208 | CakTC22596 | (TA)8 | 224 | 239 | Ca(ICC4958)TpSSR00814 | TC05059 | (TA)6 | 365 | 376 | 2 | -- | -- |
| CakTpSSR01214 | CakTC22634 | (TA)11 | 1274 | 1295 | Ca(ICC4958)TpSSR01194 | TC07400 | (TA)12 | 1277 | 1300 | 1 | -- | -- |
| CakTpSSR01279 | CakTC23175 | (CACAAA)7 | 1634 | 1675 | Ca(ICC4958)TpSSR00033 | TC00294 | (CACAAA)8 | 1636 | 1683 | 1 | -- | -- |
| CakTpSSR01283 | CakTC23285 | (TG)7 | 1350 | 1363 | Ca(ICC4958)TpSSR03144 | TC22550 | (TG)8 | 1493 | 1508 | 1 | Flower bud | -- |
| CakTpSSR01308 | CakTC23402 | (TA)7 | 12 | 25 | Ca(ICC4958)TpSSR02913 | TC18998 | (TA)8 | 9 | 24 | 1 | -- | -- |
| CakTpSSR01352 | CakTC23616 | (CT)9 | 1068 | 1085 | Ca(ICC4958)TpSSR00856 | TC05299 | (CT)8 | 1144 | 1159 | 1 | -- | -- |
| CakTpSSR01394 | CakTC23803 | (TGA)15 | 3735 | 3779 | Ca(ICC4958)TpSSR03439 | TC34228 | (TGA)14 | 3099 | 3140 | 1 | -- | -- |
| CakTpSSR01400 | CakTC23833 | (AG)9 | 256 | 273 | Ca(ICC4958)TpSSR00905 | TC05500 | (AG)10 | 251 | 270 | 1 | -- | -- |
| CakTpSSR01486 | CakTC24535 | (TGGA)8 | 862 | 893 | Ca(ICC4958)TpSSR02031 | TC12444 | (TGGA)7 | 924 | 951 | 1 | -- | -- |
| CakTpSSR01488 | CakTC24545 | (AG)9 | 2849 | 2866 | Ca(ICC4958)TpSSR00159 | TC01142 | (AG)6 | 2993 | 3004 | 3 | -- | -- |

| | | | | | | | | | | | | |
|---|---|---|---|---|---|---|---|---|---|---|---|---|
| CakTpSSR01500 | CakTC24617 | (ATG)6 | 855 | 872 | Ca(ICC4958)TpSSR03128 | TC22362 | (ATG)5 | 873 | 887 | 1 | Flower bud | -- |
| CakTpSSR01506 | CakTC24680 | (AT)7 | 539 | 552 | Ca(ICC4958)TpSSR01661 | TC10126 | (AT)8 | 287 | 302 | 1 | Young_pod | -- |
| CakTpSSR01526 | CakTC24804 | (AG)12 | 119 | 142 | Ca(ICC4958)TpSSR01042 | TC06457 | (AG)18 | 46 | 81 | 6 | -- | -- |
| CakTpSSR01542 | CakTC24891 | (AAT)11 | 122 | 154 | Ca(ICC4958)TpSSR00635 | TC04041 | (AAT)13 | 100 | 138 | 2 | -- | -- |
| CakTpSSR01621 | CakTC25414 | (TCT)9 | 20 | 46 | Ca(ICC4958)TpSSR02974 | TC19743 | (TCT)11 | 72 | 104 | 2 | -- | -- |
| CakTpSSR01660 | CakTC25584 | (AG)6 | 1427 | 1438 | Ca(ICC4958)TpSSR00217 | TC01569 | (AG)13 | 206 | 231 | 7 | -- | TRAF |
| CakTpSSR01696 | CakTC25709 | (TGA)5 | 522 | 536 | Ca(ICC4958)TpSSR03351 | TC29442 | (TGA)6 | 4 | 21 | 1 | -- | -- |
| CakTpSSR01720 | CakTC25855 | (TAA)11 | 46 | 78 | Ca(ICC4958)TpSSR01235 | TC07671 | (TAA)9 | 49 | 75 | 2 | -- | -- |
| CakTpSSR01802 | CakTC26308 | (AG)19 | 1446 | 1483 | Ca(ICC4958)TpSSR01142 | TC07093 | (AG)16 | 1456 | 1487 | 3 | -- | -- |
| CakTpSSR01814 | CakTC26431 | (TC)8 | 14 | 29 | Ca(ICC4958)TpSSR01356 | TC08294 | (TC)7 | 6 | 19 | 1 | -- | -- |
| CakTpSSR01841 | CakTC26529 | (GA)25 | 2090 | 2139 | Ca(ICC4958)TpSSR00962 | TC05907 | (GA)28 | 1775 | 1830 | 3 | -- | -- |
| CakTpSSR01928 | CakTC27222 | (CT)13 | 1776 | 1801 | Ca(ICC4958)TpSSR02638 | TC16687 | (CT)12 | 1782 | 1805 | 1 | -- | -- |
| CakTpSSR01932 | CakTC27236 | (CT)12 | 94 | 117 | Ca(ICC4958)TpSSR00781 | TC04854 | (CT)9 | 9 | 26 | 3 | -- | -- |
| CakTpSSR01949 | CakTC27313 | (TC)7 | 1 | 14 | Ca(ICC4958)TpSSR01566 | TC09548 | (TC)8 | 18 | 33 | 1 | -- | -- |
| CakTpSSR01958 | CakTC27343 | (GA)21 | 1442 | 1483 | Ca(ICC4958)TpSSR02238 | TC14592 | (GA)20 | 1448 | 1487 | 1 | -- | BES1 |
| CakTpSSR01981 | CakTC27530 | (TC)12 | 1219 | 1242 | Ca(ICC4958)TpSSR01152 | TC07156 | (TC)11 | 1198 | 1219 | 1 | -- | -- |
| CakTpSSR02042 | CakTC27908 | (TTC)6 | 165 | 182 | Ca(ICC4958)TpSSR02064 | TC12644 | (TTC)7 | 165 | 185 | 1 | -- | -- |
| CakTpSSR02074 | CakTC28116 | (GTG)8 | 1614 | 1637 | Ca(ICC4958)TpSSR01498 | TC09117 | (GTG)6 | 1614 | 1631 | 2 | -- | -- |
| CakTpSSR02142 | CakTC28476 | (TA)6 | 319 | 330 | Ca(ICC4958)TpSSR02228 | TC14548 | (TA)7 | 159 | 172 | 1 | -- | -- |
| CakTpSSR02149 | CakTC28528 | (AG)16 | 760 | 791 | Ca(ICC4958)TpSSR00841 | TC05210 | (AG)17 | 665 | 698 | 1 | -- | -- |
| CakTpSSR02169 | CakTC28712 | (AG)8 | 886 | 901 | Ca(ICC4958)TpSSR02887 | TC18797 | (AG)11 | 870 | 891 | 3 | -- | -- |
| CakTpSSR02188 | CakTC28814 | (AGA)11 | 2055 | 2087 | Ca(ICC4958)TpSSR02415 | TC15640 | (AGA)12 | 1996 | 2031 | 1 | -- | -- |
| CakTpSSR02222 | CakTC29000 | (TTC)10 | 171 | 200 | Ca(ICC4958)TpSSR00509 | TC03306 | (TTC)5 | 27 | 41 | 5 | -- | -- |
| CakTpSSR02459 | CakTC30368 | (AG)11 | 1246 | 1267 | Ca(ICC4958)TpSSR02824 | TC18242 | (AG)10 | 952 | 971 | 1 | -- | -- |
| CakTpSSR02497 | CakTC30675 | (AT)7 | 183 | 196 | Ca(ICC4958)TpSSR02664 | TC16913 | (AT)10 | 174 | 193 | 3 | -- | -- |
| CakTpSSR02499 | CakTC30698 | (ATG)7 | 391 | 411 | Ca(ICC4958)TpSSR02814 | TC18156 | (ATG)5 | 226 | 240 | 2 | -- | -- |
| CakTpSSR02543 | CakTC30946 | (TAT)8 | 119 | 142 | Ca(ICC4958)TpSSR02726 | TC17430 | (TAT)6 | 119 | 136 | 2 | -- | -- |
| CakTpSSR02622 | CakTC31433 | (GT)12 | 3927 | 3950 | Ca(ICC4958)TpSSR00683 | TC04310 | (GT)13 | 3925 | 3950 | 1 | -- | -- |
| CakTpSSR02637 | CakTC31492 | (GATA)7 | 1607 | 1634 | Ca(ICC4958)TpSSR01477 | TC08987 | (GATA)8 | 648 | 679 | 1 | -- | -- |
| CakTpSSR02667 | CakTC31656 | (CTT)5 | 64 | 78 | Ca(ICC4958)TpSSR01057 | TC06534 | (CTT)6 | 46 | 63 | 1 | -- | -- |

| | | | | | | | | | | | | |
|---|---|---|---|---|---|---|---|---|---|---|---|---|
| CakTpSSR02706 | CakTC31866 | (AG)18 | 2317 | 2352 | Ca(ICC4958)TpSSR00257 | TC01827 | (AG)8 | 2452 | 2467 | 10 | -- | -- |
| CakTpSSR02719 | CakTC32010 | (CAG)5 | 1224 | 1238 | Ca(ICC4958)TpSSR00219 | TC01590 | (CAG)6 | 1071 | 1088 | 1 | -- | -- |
| CakTpSSR02802 | CakTC32400 | (TC)11 | 30 | 51 | Ca(ICC4958)TpSSR01599 | TC09738 | (TC)12 | 2 | 25 | 1 | -- | -- |
| CakTpSSR02806 | CakTC32453 | (TC)6 | 604 | 615 | Ca(ICC4958)TpSSR02737 | TC17540 | (TC)8 | 604 | 619 | 2 | -- | -- |
| CakTpSSR02813 | CakTC32488 | (CTT)8 | 1662 | 1685 | Ca(ICC4958)TpSSR01141 | TC07091 | (CTT)9 | 2008 | 2034 | 1 | -- | TCP |
| CakTpSSR02824 | CakTC32576 | (ATT)16 | 2243 | 2290 | Ca(ICC4958)TpSSR01370 | TC08358 | (ATT)15 | 2241 | 2285 | 1 | -- | -- |
| CakTpSSR02886 | CakTC33013 | (AG)13 | 2881 | 2906 | Ca(ICC4958)TpSSR01648 | TC10033 | (AG)14 | 2862 | 2889 | 1 | -- | -- |
| CakTpSSR02894 | CakTC33055 | (AT)10 | 601 | 620 | Ca(ICC4958)TpSSR01649 | TC10046 | (AT)8 | 521 | 536 | 2 | -- | -- |
| CakTpSSR02898 | CakTC33105 | (TA)8 | 2388 | 2403 | Ca(ICC4958)TpSSR01488 | TC09053 | (TA)9 | 65 | 82 | 1 | -- | -- |
| CakTpSSR02900 | CakTC33120 | (AG)25 | 1 | 50 | Ca(ICC4958)TpSSR01159 | TC07211 | (AG)23 | 56 | 101 | 2 | -- | -- |
| CakTpSSR02930 | CakTC33291 | (AG)13 | 4884 | 4909 | Ca(ICC4958)TpSSR00158 | TC01136 | (AG)12 | 4900 | 4923 | 1 | -- | -- |
| CakTpSSR02952 | CakTC33440 | (AG)7 | 1890 | 1903 | Ca(ICC4958)TpSSR00916 | TC05616 | (AG)6 | 1887 | 1898 | 1 | -- | -- |
| CakTpSSR02970 | CakTC33640 | (TA)10 | 2154 | 2173 | Ca(ICC4958)TpSSR01462 | TC08921 | (TA)9 | 1985 | 2002 | 1 | -- | TPR |
| CakTpSSR02987 | CakTC33747 | (CT)8 | 652 | 667 | Ca(ICC4958)TpSSR02709 | TC17310 | (CT)12 | 652 | 675 | 4 | -- | -- |
| CakTpSSR02992 | CakTC33793 | (AG)25 | 2395 | 2444 | Ca(ICC4958)TpSSR02459 | TC15892 | (AG)23 | 2395 | 2440 | 2 | -- | -- |
| CakTpSSR03000 | CakTC33850 | (TCA)6 | 395 | 412 | Ca(ICC4958)TpSSR02735 | TC17527 | (TCA)7 | 413 | 433 | 1 | -- | -- |
| CakTpSSR03003 | CakTC33861 | (TC)6 | 2447 | 2458 | Ca(ICC4958)TpSSR00864 | TC05340 | (TC)7 | 2453 | 2466 | 1 | -- | -- |
| CakTpSSR03090 | CakTC34556 | (ATTCAT)7 | 200 | 241 | Ca(ICC4958)TpSSR01265 | TC07827 | (ATTCAT)6 | 206 | 241 | 1 | -- | bHLH |
| CakTpSSR03117 | CakTC34751 | (AT)10 | 192 | 211 | Ca(ICC4958)TpSSR00718 | TC04509 | (AT)12 | 3479 | 3502 | 2 | -- | -- |
| CakTpSSR03118 | CakTC34754 | (AG)12 | 3280 | 3303 | Ca(ICC4958)TpSSR02469 | TC15922 | (AG)10 | 3314 | 3333 | 2 | -- | -- |
| CakTpSSR03171 | CakTC35154 | (TC)8 | 4019 | 4034 | Ca(ICC4958)TpSSR01627 | TC09901 | (TC)7 | 4018 | 4031 | 1 | -- | -- |
| CakTpSSR03193 | CakTC35309 | (AGA)9 | 658 | 684 | Ca(ICC4958)TpSSR01323 | TC08159 | (AGA)8 | 661 | 684 | 1 | -- | Bromodomain |
| CakTpSSR03312 | CakTC36126 | (AG)9 | 16 | 33 | Ca(ICC4958)TpSSR00762 | TC04777 | (AG)12 | 8 | 31 | 3 | -- | ARF |
| CakTpSSR03317 | CakTC36179 | (GA)19 | 2716 | 2753 | Ca(ICC4958)TpSSR01327 | TC08178 | (GA)24 | 2679 | 2726 | 5 | -- | -- |
| CakTpSSR03345 | CakTC36401 | (AG)6 | 3140 | 3151 | Ca(ICC4958)TpSSR00951 | TC05805 | (AG)7 | 281 | 294 | 1 | -- | HB |
| CakTpSSR03422 | CakTC37047 | (TG)10 | 2692 | 2711 | Ca(ICC4958)TpSSR01664 | TC10149 | (TG)9 | 2649 | 2666 | 1 | -- | -- |
| CakTpSSR03480 | CakTC37363 | (ATC)8 | 526 | 549 | Ca(ICC4958)TpSSR01799 | TC11053 | (ATC)10 | 423 | 452 | 2 | -- | -- |
| CakTpSSR03485 | CakTC37408 | (AAG)13 | 1112 | 1150 | Ca(ICC4958)TpSSR02832 | TC18327 | (AAG)5 | 1112 | 1126 | 8 | -- | -- |
| CakTpSSR03486 | CakTC37409 | (CT)17 | 4 | 37 | Ca(ICC4958)TpSSR03358 | TC29761 | (CT)12 | 31 | 54 | 5 | Young_pod | -- |
| CakTpSSR03521 | CakTC37545 | (GA)17 | 1609 | 1642 | Ca(ICC4958)TpSSR02586 | TC16423 | (GA)22 | 1608 | 1651 | 5 | -- | -- |

| | | | | | | | | | | | | |
|---|---|---|---|---|---|---|---|---|---|---|---|---|
| CakTpSSR03532 | CakTC37598 | (TTG)6 | 1177 | 1194 | Ca(ICC4958)TpSSR01794 | TC11038 | (TTG)5 | 1131 | 1145 | 1 | -- | -- |
| CakTpSSR03556 | CakTC37742 | (GA)10 | 169 | 188 | Ca(ICC4958)TpSSR00458 | TC03027 | (GA)8 | 2428 | 2443 | 2 | Flower bud | -- |
| CakTpSSR03565 | CakTC37776 | (CTT)7 | 60 | 80 | Ca(ICC4958)TpSSR02783 | TC17959 | (CTT)8 | 100 | 123 | 1 | -- | -- |
| CakTpSSR03588 | CakTC37889 | (GA)10 | 1668 | 1687 | Ca(ICC4958)TpSSR01776 | TC10907 | (GA)11 | 1648 | 1669 | 1 | -- | -- |
| CakTpSSR03637 | CakTC38143 | (TTC)12 | 1028 | 1063 | Ca(ICC4958)TpSSR02320 | TC15067 | (TTC)8 | 1079 | 1102 | 4 | -- | -- |
| CakTpSSR03639 | CakTC38187 | (GA)14 | 1760 | 1787 | Ca(ICC4958)TpSSR02595 | TC16474 | (GA)11 | 1689 | 1710 | 3 | -- | -- |
| CakTpSSR03662 | CakTC38353 | (CT)15 | 18 | 47 | Ca(ICC4958)TpSSR01269 | TC07866 | (CT)14 | 1 | 28 | 1 | -- | -- |
| CakTpSSR03667 | CakTC38412 | (GA)15 | 1686 | 1715 | Ca(ICC4958)TpSSR01421 | TC08653 | (GA)16 | 1770 | 1801 | 1 | -- | -- |
| CakTpSSR03684 | CakTC38539 | (TC)7 | 1631 | 1644 | Ca(ICC4958)TpSSR02536 | TC16204 | (TC)8 | 1614 | 1629 | 1 | -- | -- |
| CakTpSSR03721 | CakTC38734 | (GA)15 | 1476 | 1505 | Ca(ICC4958)TpSSR02226 | TC14544 | (GA)6 | 1408 | 1419 | 9 | -- | -- |
| CakTpSSR03756 | CakTC38907 | (TA)10 | 1265 | 1284 | Ca(ICC4958)TpSSR02343 | TC15225 | (TA)6 | 1267 | 1278 | 4 | -- | -- |
| CakTpSSR03776 | CakTC38997 | (TC)10 | 1435 | 1454 | Ca(ICC4958)TpSSR01838 | TC11273 | (TC)9 | 1445 | 1462 | 1 | -- | SWI/SNF |
| CakTpSSR03786 | CakTC39038 | (TTC)20 | 35 | 94 | Ca(ICC4958)TpSSR02216 | TC14490 | (TTC)18 | 26 | 79 | 2 | -- | -- |
| CakTpSSR03806 | CakTC39158 | (TC)9 | 1574 | 1591 | Ca(ICC4958)TpSSR02014 | TC12350 | (TC)7 | 2082 | 2095 | 2 | -- | SBP |
| CakTpSSR03815 | CakTC39206 | (CT)7 | 115 | 128 | Ca(ICC4958)TpSSR01626 | TC09898 | (CT)6 | 132 | 143 | 1 | -- | AUX/IAA |
| CakTpSSR03820 | CakTC39223 | (ATA)6 | 1535 | 1552 | Ca(ICC4958)TpSSR00204 | TC01434 | (ATA)7 | 1536 | 1556 | 1 | -- | C2H2 |
| CakTpSSR03821 | CakTC39225 | (TC)11 | 149 | 170 | Ca(ICC4958)TpSSR01817 | TC11158 | (TC)10 | 88 | 107 | 1 | -- | -- |
| CakTpSSR03822 | CakTC39230 | (TC)26 | 1 | 52 | Ca(ICC4958)TpSSR02329 | TC15147 | (TC)36 | 1 | 72 | 10 | -- | -- |
| CakTpSSR03826 | CakTC39247 | (AG)12 | 1566 | 1589 | Ca(ICC4958)TpSSR00969 | TC05928 | (AG)11 | 1571 | 1592 | 1 | -- | -- |
| CakTpSSR03829 | CakTC39262 | (AG)20 | 1520 | 1559 | Ca(ICC4958)TpSSR02047 | TC12532 | (AG)12 | 1492 | 1515 | 8 | -- | -- |
| CakTpSSR03853 | CakTC39411 | (AG)7 | 15 | 28 | Ca(ICC4958)TpSSR00845 | TC05231 | (AG)8 | 13 | 28 | 1 | -- | -- |
| CakTpSSR03891 | CakTC39656 | (TTC)17 | 45 | 95 | Ca(ICC4958)TpSSR02917 | TC19015 | (TTC)21 | 64 | 126 | 4 | -- | -- |
| CakTpSSR03899 | CakTC39710 | (ATA)10 | 1334 | 1363 | Ca(ICC4958)TpSSR02322 | TC15070 | (ATA)7 | 1291 | 1311 | 3 | -- | -- |
| CakTpSSR03923 | CakTC39799 | (GAA)19 | 1300 | 1356 | Ca(ICC4958)TpSSR01692 | TC10313 | (GAA)18 | 1097 | 1150 | 1 | -- | bZIP |
| CakTpSSR03960 | CakTC40044 | (CTT)6 | 706 | 723 | Ca(ICC4958)TpSSR01049 | TC06514 | (CTT)5 | 706 | 720 | 1 | -- | -- |
| CakTpSSR03970 | CakTC40146 | (TGA)9 | 1298 | 1324 | Ca(ICC4958)TpSSR01825 | TC11208 | (TGA)12 | 1283 | 1318 | 3 | -- | -- |
| CakTpSSR04045 | CakTC40557 | (TC)12 | 641 | 664 | Ca(ICC4958)TpSSR02685 | TC17104 | (TC)11 | 647 | 668 | 1 | -- | -- |
| CakTpSSR04058 | CakTC40637 | (GA)20 | 2026 | 2065 | Ca(ICC4958)TpSSR02422 | TC15677 | (GA)18 | 2026 | 2061 | 2 | -- | WRKY |
| CakTpSSR04076 | CakTC40711 | (CCA)5 | 950 | 964 | Ca(ICC4958)TpSSR00090 | TC00618 | (CCA)6 | 946 | 963 | 1 | -- | -- |
| CakTpSSR04141 | CakTC41199 | (TC)12 | 178 | 201 | Ca(ICC4958)TpSSR02445 | TC15793 | (TC)14 | 177 | 204 | 2 | -- | -- |

| | | | | | | | | | | | | |
|---|---|---|---|---|---|---|---|---|---|---|---|---|
| CakTpSSR04176 | CakTC41338 | (TTC)9 | 418 | 444 | Ca(ICC4958)TpSSR00663 | TC04203 | (TTC)5 | 418 | 432 | 4 | -- | -- |
| CakTpSSR04185 | CakTC41423 | (TC)8 | 20 | 35 | Ca(ICC4958)TpSSR00577 | TC03730 | (TC)7 | 55 | 68 | 1 | -- | -- |
| CakTpSSR04189 | CakTC41441 | (AG)18 | 2 | 37 | Ca(ICC4958)TpSSR02454 | TC15839 | (AG)10 | 14 | 33 | 8 | -- | -- |
| CakTpSSR04240 | CakTC41792 | (GA)12 | 2265 | 2288 | Ca(ICC4958)TpSSR01646 | TC10031 | (GA)13 | 2357 | 2382 | 1 | -- | -- |
| CakTpSSR04265 | CakTC41944 | (TAA)17 | 627 | 677 | Ca(ICC4958)TpSSR01075 | TC06664 | (TAA)14 | 594 | 635 | 3 | -- | C2C2-Dof |
| CakTpSSR04290 | CakTC42107 | (TTA)8 | 893 | 916 | Ca(ICC4958)TpSSR02959 | TC19502 | (TTA)7 | 894 | 914 | 1 | -- | -- |
| CakTpSSR04307 | CakTC42202 | (CT)7 | 874 | 887 | Ca(ICC4958)TpSSR01443 | TC08808 | (CT)8 | 1033 | 1048 | 1 | -- | -- |
| CakTpSSR04360 | CakTC42489 | (GA)15 | 1 | 30 | Ca(ICC4958)TpSSR00334 | TC02328 | (GA)14 | 4 | 31 | 1 | Shoot | -- |
| CakTpSSR04389 | CakTC42648 | (AG)6 | 113 | 124 | Ca(ICC4958)TpSSR02552 | TC16250 | (AG)7 | 113 | 126 | 1 | -- | -- |
| CakTpSSR04404 | CakTC42701 | (TCT)7 | 856 | 876 | Ca(ICC4958)TpSSR02791 | TC18026 | (TCT)8 | 924 | 947 | 1 | -- | -- |
| CakTpSSR04421 | CakTC42806 | (AG)16 | 1917 | 1948 | Ca(ICC4958)TpSSR01802 | TC11083 | (AG)17 | 1894 | 1927 | 1 | -- | -- |
| CakTpSSR04457 | CakTC43019 | (AG)9 | 15 | 32 | Ca(ICC4958)TpSSR00305 | TC02135 | (AG)8 | 15 | 30 | 1 | Young_pod | -- |
| CakTpSSR04464 | CakTC43053 | (TC)8 | 1908 | 1923 | Ca(ICC4958)TpSSR00955 | TC05850 | (TC)7 | 1917 | 1930 | 1 | -- | -- |
| CakTpSSR04488 | CakTC43146 | (TCT)6 | 128 | 145 | Ca(ICC4958)TpSSR03203 | TC23463 | (TCT)5 | 154 | 168 | 1 | -- | -- |
