# Supplementary material for: Comparative Analysis of Kabuli Chickpea Transcriptome with Desi and Wild Chickpea Provides a Rich Resource for Development of Functional Markers
Source: PLoS One. 2012 Dec 27;7(12):e52443. doi: 10.1371/journal.pone.0052443 (PMC3531472; doi:10.1371/journal.pone.0052443)
Supplement: Table S9 — List of polymorphic SSRs identified between kabuli and wild chickpea. (PDF) [file pone.0052443.s019.pdf]

Table S9. List of polymorphic SSRs identified between kabuli and wild chickpea.

Kabuli SSR ID	Kabuli TC ID	SSR	Start	End	Wild SSR ID	Wild TC ID	SSR	Start	End	Repeat unit size difference	Tissue specificity	TF family
CakTpSSR00050	CakTC01101	(ATT)6	367	384	CrTpSSR01148	CrTC11127	(ATT)5	368	382	1	--	Trihelix
CakTpSSR00051	CakTC01101	(TCA)6	1079	1096	CrTpSSR01150	CrTC11127	(TCA)8	1078	1101	2	--	Trihelix
CakTpSSR00063	CakTC01357	(TC)7	79	92	CrTpSSR02001	CrTC21086	(TC)6	74	85	1	--	--
CakTpSSR00076	CakTC01782	(TCCT)8	162	193	CrTpSSR00144	CrTC01670	(TCCT)9	205	240	1	--	--
CakTpSSR00086	CakTC02070	(TG)10	617	636	CrTpSSR01375	CrTC13599	(TG)7	1748	1761	3	--	--
CakTpSSR00111	CakTC02781	(AG)10	580	599	CrTpSSR00455	CrTC04402	(AG)8	3	18	2	--	--
CakTpSSR00118	CakTC03007	(AGA)7	132	152	CrTpSSR00404	CrTC03926	(AGA)8	132	155	1	--	--
CakTpSSR00125	CakTC03355	(CT)19	188	225	CrTpSSR01386	CrTC13673	(CT)11	197	218	8	--	--
CakTpSSR00131	CakTC03451	(AAC)6	323	340	CrTpSSR01478	CrTC14630	(AAC)5	350	364	1	--	--
CakTpSSR00149	CakTC03814	(CTA)6	799	816	CrTpSSR01450	CrTC14353	(CTA)5	737	751	1	--	--
CakTpSSR00154	CakTC03853	(TA)7	57	70	CrTpSSR03362	CrTC35806	(TA)8	890	905	1	--	--
CakTpSSR00157	CakTC03971	(GTT)7	1185	1205	CrTpSSR01823	CrTC18519	(GTT)5	1130	1144	2	--	bHLH
CakTpSSR00171	CakTC04226	(CT)9	149	166	CrTpSSR01271	CrTC12446	(CT)8	250	265	1	--	--
CakTpSSR00178	CakTC04397	(ACA)5	307	321	CrTpSSR02421	CrTC27371	(ACA)6	108	125	1	--	--
CakTpSSR00184	CakTC04586	(TGA)6	376	393	CrTpSSR02288	CrTC25118	(TGA)5	252	266	1	--	--
CakTpSSR00192	CakTC04741	(CTT)8	92	115	CrTpSSR01479	CrTC14649	(CTT)7	86	106	1	--	--
CakTpSSR00219	CakTC05322	(CAT)5	1130	1144	CrTpSSR01309	CrTC12894	(CAT)7	1178	1198	2	--	--
CakTpSSR00255	CakTC06423	(AAT)17	9	59	CrTpSSR01443	CrTC14298	(AAT)11	39	71	6	--	TPR
CakTpSSR00278	CakTC07091	(TGT)7	526	546	CrTpSSR01351	CrTC13301	(TGT)6	431	448	1	--	--
CakTpSSR00290	CakTC07556	(GAA)7	2559	2579	CrTpSSR00728	CrTC07188	(GAA)6	1466	1483	1	--	--
CakTpSSR00291	CakTC07557	(GAA)8	30	53	CrTpSSR00734	CrTC07211	(GAA)5	440	454	3	--	--
CakTpSSR00339	CakTC08339	(TC)10	3762	3781	CrTpSSR02247	CrTC24437	(TC)9	245	262	1	--	--
CakTpSSR00340	CakTC08400	(ACT)6	947	964	CrTpSSR01297	CrTC12754	(ACT)7	480	500	1	Flower bud	--
CakTpSSR00344	CakTC08487	(CAG)6	1221	1238	CrTpSSR02427	CrTC27424	(CAG)5	643	657	1	--	NAC
CakTpSSR00356	CakTC08597	(TA)8	22	37	CrTpSSR02608	CrTC29383	(TA)7	3	16	1	--	--
CakTpSSR00375	CakTC08847	(TTA)6	359	376	CrTpSSR00406	CrTC03962	(TTA)5	434	448	1	--	--
CakTpSSR00380	CakTC08897	(AT)7	29	42	CrTpSSR00894	CrTC08657	(AT)9	1577	1594	2	Mature Leaf	--
CakTpSSR00388	CakTC09051	(AG)7	2012	2025	CrTpSSR01463	CrTC14473	(AG)8	2033	2048	1	--	--
CakTpSSR00392	CakTC09122	(TC)11	67	88	CrTpSSR00934	CrTC09162	(TC)8	20	35	3	--	--
CakTpSSR00393	CakTC09132	(GT)6	583	594	CrTpSSR01501	CrTC14909	(GT)7	257	270	1	--	--
CakTpSSR00398	CakTC09209	(TA)6	1287	1298	CrTpSSR00912	CrTC08943	(TA)7	5	18	1	--	--
CakTpSSR00428	CakTC09678	(TCAC)9	1204	1239	CrTpSSR00281	CrTC02617	(TCAC)6	59	82	3	--	--
CakTpSSR00430	CakTC09700	(CA)6	1308	1319	CrTpSSR01610	CrTC16213	(CA)7	1401	1414	1	--	--
CakTpSSR00447	CakTC09856	(GAAGA)6	7	36	CrTpSSR02404	CrTC27128	(GAAGA)5	7	31	1	--	--
CakTpSSR00449	CakTC09870	(TAT)7	1647	1667	CrTpSSR02407	CrTC27186	(TAT)6	1557	1574	1	--	--

CakTpSSR00453	CakTC09877	(CT)9	1062	1079	CrTpSSR00342	CrTC03192	(CT)8	1064	1079	1	--	--
CakTpSSR00456	CakTC09899	(TA)8	119	134	CrTpSSR02418	CrTC27305	(TA)7	230	243	1	--	--
CakTpSSR00508	CakTC10199	(TAA)6	973	990	CrTpSSR02501	CrTC28417	(TAA)7	928	948	1	--	TCP
CakTpSSR00511	CakTC10232	(TCT)6	226	243	CrTpSSR00507	CrTC04893	(TCT)8	77	100	2	--	--
CakTpSSR00548	CakTC10459	(AG)11	3566	3587	CrTpSSR02624	CrTC29612	(AG)10	1114	1133	1	--	--
CakTpSSR00565	CakTC10651	(CTG)6	90	107	CrTpSSR01277	CrTC12581	(CTG)7	1215	1235	1	--	--
CakTpSSR00573	CakTC10685	(TC)17	272	305	CrTpSSR02749	CrTC30699	(TC)12	274	297	5	--	--
CakTpSSR00575	CakTC10700	(TG)10	263	282	CrTpSSR02758	CrTC30775	(TG)7	203	216	3	--	--
CakTpSSR00583	CakTC10754	(AAT)5	265	279	CrTpSSR00218	CrTC02283	(AAT)6	166	183	1	--	--
CakTpSSR00586	CakTC10765	(AG)9	1786	1803	CrTpSSR01389	CrTC13702	(AG)49	1768	1865	40	--	--
CakTpSSR00595	CakTC10832	(ATC)6	119	136	CrTpSSR02872	CrTC31611	(ATC)5	116	130	1	--	C2C2-YABBY
CakTpSSR00597	CakTC10898	(ACA)6	105	122	CrTpSSR02301	CrTC25451	(ACA)5	165	179	1	Flower bud	--
CakTpSSR00600	CakTC10938	(AG)12	936	959	CrTpSSR02925	CrTC32160	(AG)10	940	959	2	--	--
CakTpSSR00608	CakTC10982	(AAG)8	117	140	CrTpSSR01477	CrTC14629	(AAG)5	130	144	3	--	--
CakTpSSR00612	CakTC11019	(GAA)5	436	450	CrTpSSR02980	CrTC32653	(GAA)6	362	379	1	--	--
CakTpSSR00619	CakTC11066	(AG)27	2928	2981	CrTpSSR03025	CrTC33015	(AG)15	2900	2929	12	--	--
CakTpSSR00621	CakTC11084	(CT)15	13	42	CrTpSSR00630	CrTC06171	(CT)10	67	86	5	--	--
CakTpSSR00630	CakTC11163	(TC)6	92	103	CrTpSSR03065	CrTC33449	(TC)7	198	211	1	Young_pod	--
CakTpSSR00632	CakTC11206	(GA)10	801	820	CrTpSSR00291	CrTC02728	(GA)7	797	810	3	--	--
CakTpSSR00641	CakTC11367	(CT)10	116	135	CrTpSSR02245	CrTC24418	(CT)7	113	126	3	--	--
CakTpSSR00654	CakTC11421	(TCT)6	102	119	CrTpSSR03178	CrTC34299	(TCT)7	104	124	1	--	--
CakTpSSR00676	CakTC11623	(TGA)5	141	155	CrTpSSR03397	CrTC36149	(TGA)7	201	221	2	--	--
CakTpSSR00701	CakTC11960	(CAT)5	79	93	CrTpSSR03430	CrTC36624	(CAT)6	168	185	1	Young_pod	--
CakTpSSR00732	CakTC12506	(GA)7	1922	1935	CrTpSSR00330	CrTC03075	(GA)10	1352	1371	3	Young_pod	--
CakTpSSR00739	CakTC12740	(CTT)5	197	211	CrTpSSR02000	CrTC21081	(CTT)6	110	127	1	--	--
CakTpSSR00763	CakTC13377	(GTT)8	575	598	CrTpSSR00515	CrTC04996	(GTT)7	802	822	1	--	--
CakTpSSR00781	CakTC13669	(AT)7	1112	1125	CrTpSSR02038	CrTC21453	(AT)6	223	234	1	--	--
CakTpSSR00794	CakTC13915	(AT)6	230	241	CrTpSSR00002	CrTC00024	(AT)8	270	285	2	--	--
CakTpSSR00827	CakTC14515	(TAC)7	119	139	CrTpSSR02959	CrTC32513	(TAC)5	110	124	2	--	--
CakTpSSR00848	CakTC14908	(TGG)5	187	201	CrTpSSR00811	CrTC07915	(TGG)6	743	760	1	--	--
CakTpSSR00867	CakTC15258	(GA)14	3523	3550	CrTpSSR02975	CrTC32618	(GA)16	3520	3551	2	--	CAMTA
CakTpSSR00970	CakTC16863	(ATA)7	430	450	CrTpSSR01011	CrTC09887	(ATA)10	736	765	3	--	--
CakTpSSR00997	CakTC17443	(TAT)6	367	384	CrTpSSR00162	CrTC01914	(TAT)5	149	163	1	--	--
CakTpSSR01019	CakTC17859	(GAA)5	471	485	CrTpSSR01139	CrTC11042	(GAA)6	413	430	1	--	--
CakTpSSR01087	CakTC19704	(AGA)6	8	25	CrTpSSR02162	CrTC23178	(AGA)5	31	45	1	--	--
CakTpSSR01096	CakTC20168	(GAA)6	1582	1599	CrTpSSR00473	CrTC04572	(GAA)5	1466	1480	1	--	--
CakTpSSR01126	CakTC21175	(AT)9	158	175	CrTpSSR02018	CrTC21190	(AT)8	203	218	1	--	--
CakTpSSR01146	CakTC21556	(GA)11	1055	1076	CrTpSSR03449	CrTC36970	(GA)10	1037	1056	1	--	--
CakTpSSR01178	CakTC22251	(ATT)5	2077	2091	CrTpSSR00565	CrTC05532	(ATT)8	1997	2020	3	--	--
CakTpSSR01214	CakTC22634	(TA)11	1274	1295	CrTpSSR02731	CrTC30514	(TA)8	1265	1280	3	--	--

CakTpSSR01218	CakTC22646	(CAT)6	64	81	CrTpSSR02526	CrTC28626	(CAT)5	52	66	1	--	--
CakTpSSR01255	CakTC23047	(TA)7	121	134	CrTpSSR00202	CrTC02222	(TA)8	113	128	1	--	--
CakTpSSR01299	CakTC23351	(TC)11	1539	1560	CrTpSSR00530	CrTC05075	(TC)9	1537	1554	2	--	--
CakTpSSR01324	CakTC23480	(GA)13	2684	2709	CrTpSSR00924	CrTC09059	(GA)19	2593	2630	6	--	--
CakTpSSR01326	CakTC23493	(AG)11	916	937	CrTpSSR00260	CrTC02497	(AG)9	932	949	2	--	--
CakTpSSR01352	CakTC23616	(CT)9	1068	1085	CrTpSSR00028	CrTC00391	(CT)6	1037	1048	3	--	--
CakTpSSR01373	CakTC23713	(AT)7	1499	1512	CrTpSSR02355	CrTC26291	(AT)6	67	78	1	--	--
CakTpSSR01380	CakTC23736	(GA)12	1372	1395	CrTpSSR02737	CrTC30573	(GA)10	822	841	2	--	--
CakTpSSR01394	CakTC23803	(TGA)15	3735	3779	CrTpSSR00549	CrTC05319	(TGA)6	759	776	9	--	--
CakTpSSR01416	CakTC23917	(AT)6	924	935	CrTpSSR03092	CrTC33778	(AT)8	822	837	2	--	--
CakTpSSR01420	CakTC23970	(AT)7	561	574	CrTpSSR03314	CrTC35241	(AT)8	725	740	1	--	--
CakTpSSR01438	CakTC24159	(CT)6	170	181	CrTpSSR00795	CrTC07777	(CT)7	184	197	1	Shoot	--
CakTpSSR01442	CakTC24186	(TC)7	1119	1132	CrTpSSR01095	CrTC10632	(TC)9	1097	1114	2	--	--
CakTpSSR01445	CakTC24242	(CAT)6	330	347	CrTpSSR01786	CrTC18076	(CAT)5	301	315	1	--	--
CakTpSSR01459	CakTC24330	(TAT)7	202	222	CrTpSSR01673	CrTC16817	(TAT)5	338	352	2	--	--
CakTpSSR01464	CakTC24351	(TC)15	93	122	CrTpSSR01382	CrTC13636	(TC)7	83	96	8	Young_pod	--
CakTpSSR01466	CakTC24360	(TC)9	807	824	CrTpSSR01562	CrTC15709	(TC)12	158	181	3	--	--
CakTpSSR01476	CakTC24450	(CT)6	60	71	CrTpSSR00888	CrTC08610	(CT)7	14	27	1	--	--
CakTpSSR01484	CakTC24510	(CT)11	97	118	CrTpSSR00332	CrTC03110	(CT)14	93	120	3	--	--
CakTpSSR01486	CakTC24535	(TGGA)8	862	893	CrTpSSR03038	CrTC33176	(TGGA)6	739	762	2	--	--
CakTpSSR01513	CakTC24719	(AG)8	1062	1077	CrTpSSR00230	CrTC02353	(AG)7	1056	1069	1	--	bZIP
CakTpSSR01517	CakTC24768	(CTT)6	1055	1072	CrTpSSR00285	CrTC02666	(CTT)5	366	380	1	--	--
CakTpSSR01528	CakTC24819	(TA)8	432	447	CrTpSSR02242	CrTC24354	(TA)6	431	442	2	Flower bud	--
CakTpSSR01529	CakTC24824	(TA)8	3424	3439	CrTpSSR02569	CrTC29014	(TA)6	3293	3304	2	--	MYB
CakTpSSR01543	CakTC24908	(GAA)6	1431	1448	CrTpSSR02527	CrTC28629	(GAA)7	457	477	1	--	--
CakTpSSR01558	CakTC25026	(ATA)6	2550	2567	CrTpSSR00300	CrTC02769	(ATA)5	719	733	1	--	--
CakTpSSR01566	CakTC25052	(TC)8	15	30	CrTpSSR02587	CrTC29215	(TC)9	1	18	1	--	LUG
CakTpSSR01584	CakTC25189	(TA)10	121	140	CrTpSSR00607	CrTC06025	(TA)6	43	54	4	--	MYB
CakTpSSR01585	CakTC25190	(TC)6	497	508	CrTpSSR00707	CrTC06924	(TC)8	517	532	2	--	--
CakTpSSR01596	CakTC25236	(AAT)11	295	327	CrTpSSR01098	CrTC10664	(AAT)5	166	180	6	--	bZIP
CakTpSSR01599	CakTC25253	(GAA)6	2817	2834	CrTpSSR00053	CrTC00648	(GAA)5	710	724	1	Flower bud	--
CakTpSSR01610	CakTC25336	(GCT)6	1647	1664	CrTpSSR02539	CrTC28750	(GCT)7	1600	1620	1	--	--
CakTpSSR01617	CakTC25381	(AG)7	898	911	CrTpSSR01715	CrTC17326	(AG)9	1048	1065	2	--	--
CakTpSSR01621	CakTC25414	(TCT)9	20	46	CrTpSSR03428	CrTC36527	(TCT)6	10	27	3	--	--
CakTpSSR01623	CakTC25440	(CTT)6	2020	2037	CrTpSSR02927	CrTC32187	(CTT)5	1972	1986	1	--	--
CakTpSSR01634	CakTC25472	(CTT)6	1139	1156	CrTpSSR00541	CrTC05156	(CTT)5	1216	1230	1	--	--
CakTpSSR01638	CakTC25493	(TC)8	221	236	CrTpSSR00819	CrTC07974	(TC)12	152	175	4	--	--
CakTpSSR01645	CakTC25520	(GGT)7	1324	1344	CrTpSSR00026	CrTC00377	(GGT)5	1276	1290	2	--	--
CakTpSSR01646	CakTC25526	(AG)10	1435	1454	CrTpSSR02692	CrTC30220	(AG)7	1082	1095	3	--	--
CakTpSSR01652	CakTC25542	(ATTC)5	278	297	CrTpSSR02796	CrTC31047	(ATTC)6	98	121	1	--	--

CakTpSSR01653	CakTC25543	(TTA)8	612	635	CrTpSSR00773	CrTC07601	(TTA)11	609	641	3	--	HB
CakTpSSR01658	CakTC25580	(CA)6	2260	2271	CrTpSSR00973	CrTC09486	(CA)7	2065	2078	1	--	--
CakTpSSR01687	CakTC25676	(TA)9	1738	1755	CrTpSSR02483	CrTC28122	(TA)6	62	73	3	--	--
CakTpSSR01697	CakTC25721	(TCA)7	1571	1591	CrTpSSR01655	CrTC16621	(TCA)6	1727	1744	1	--	--
CakTpSSR01720	CakTC25855	(TAA)11	46	78	CrTpSSR00725	CrTC07153	(TAA)8	481	504	3	--	--
CakTpSSR01724	CakTC25887	(AG)7	1024	1037	CrTpSSR03384	CrTC36025	(AG)6	1005	1016	1	--	--
CakTpSSR01731	CakTC25914	(TTC)6	181	198	CrTpSSR03180	CrTC34343	(TTC)7	144	164	1	--	--
CakTpSSR01735	CakTC25926	(GAA)5	935	949	CrTpSSR01751	CrTC17790	(GAA)8	857	880	3	--	--
CakTpSSR01741	CakTC25970	(TTA)5	813	827	CrTpSSR00130	CrTC01443	(TTA)6	2243	2260	1	--	--
CakTpSSR01760	CakTC26053	(TGA)6	2692	2709	CrTpSSR00920	CrTC09014	(TGA)7	1153	1173	1	--	--
CakTpSSR01763	CakTC26074	(CA)10	125	144	CrTpSSR00124	CrTC01403	(CA)7	65	78	3	--	--
CakTpSSR01768	CakTC26103	(AT)7	3585	3598	CrTpSSR03230	CrTC34681	(AT)8	3583	3598	1	--	--
CakTpSSR01791	CakTC26263	(TGG)8	276	299	CrTpSSR03335	CrTC35472	(TGG)7	272	292	1	--	--
CakTpSSR01796	CakTC26298	(GA)6	29	40	CrTpSSR00568	CrTC05565	(GA)9	2822	2839	3	--	--
CakTpSSR01806	CakTC26354	(AT)9	36	53	CrTpSSR01625	CrTC16336	(AT)6	96	107	3	--	--
CakTpSSR01807	CakTC26361	(GA)11	1518	1539	CrTpSSR00212	CrTC02249	(GA)9	1473	1490	2	--	--
CakTpSSR01810	CakTC26397	(TC)8	46	61	CrTpSSR03011	CrTC32869	(TC)7	12	25	1	--	--
CakTpSSR01815	CakTC26433	(AG)6	3117	3128	CrTpSSR01099	CrTC10666	(AG)8	472	487	2	--	--
CakTpSSR01820	CakTC26448	(GAT)8	3428	3451	CrTpSSR01135	CrTC10945	(GAT)6	3459	3476	2	--	--
CakTpSSR01835	CakTC26513	(CTT)9	186	212	CrTpSSR02940	CrTC32348	(CTT)8	79	102	1	--	G2-like
CakTpSSR01839	CakTC26519	(AT)14	1932	1959	CrTpSSR02802	CrTC31091	(AT)13	1852	1877	1	--	--
CakTpSSR01850	CakTC26583	(AAG)7	2064	2084	CrTpSSR02564	CrTC28994	(AAG)8	2064	2087	1	--	--
CakTpSSR01851	CakTC26595	(GAA)9	2021	2047	CrTpSSR02884	CrTC31756	(GAA)6	556	573	3	--	--
CakTpSSR01852	CakTC26596	(GAA)9	1074	1100	CrTpSSR03313	CrTC35239	(GAA)6	1075	1092	3	--	--
CakTpSSR01866	CakTC26719	(TC)9	210	227	CrTpSSR01287	CrTC12711	(TC)6	191	202	3	--	--
CakTpSSR01878	CakTC26856	(AT)6	47	58	CrTpSSR01327	CrTC13124	(AT)7	830	843	1	Flower bud	WRKY
CakTpSSR01891	CakTC26937	(TAT)6	658	675	CrTpSSR02588	CrTC29217	(TAT)8	176	199	2	--	--
CakTpSSR01892	CakTC26942	(AAG)6	3080	3097	CrTpSSR00267	CrTC02580	(AAG)5	316	330	1	--	MYB-related
CakTpSSR01919	CakTC27102	(AT)6	172	183	CrTpSSR03005	CrTC32850	(AT)7	95	108	1	--	--
CakTpSSR01928	CakTC27222	(CT)13	1776	1801	CrTpSSR03304	CrTC35185	(CT)12	1730	1753	1	--	--
CakTpSSR01939	CakTC27260	(AT)6	411	422	CrTpSSR02913	CrTC32093	(AT)8	2	17	2	--	--
CakTpSSR01947	CakTC27286	(TC)22	215	258	CrTpSSR02131	CrTC22805	(TC)6	120	131	16	--	--
CakTpSSR01953	CakTC27334	(CAG)7	2209	2229	CrTpSSR00125	CrTC01406	(CAG)6	2385	2402	1	--	C3H
CakTpSSR01954	CakTC27335	(TC)12	81	104	CrTpSSR00747	CrTC07366	(TC)7	46	59	5	--	bZIP
CakTpSSR01955	CakTC27335	(CAG)5	1372	1386	CrTpSSR00748	CrTC07366	(CAG)6	1324	1341	1	--	bZIP
CakTpSSR01961	CakTC27355	(AG)12	30	53	CrTpSSR02497	CrTC28341	(AG)9	2	19	3	--	--
CakTpSSR01996	CakTC27620	(CT)17	157	190	CrTpSSR02847	CrTC31430	(CT)8	164	179	9	--	--
CakTpSSR02007	CakTC27686	(GAA)6	1157	1174	CrTpSSR00591	CrTC05790	(GAA)5	1136	1150	1	--	--
CakTpSSR02010	CakTC27697	(TAA)6	231	248	CrTpSSR01572	CrTC15813	(TAA)8	22	45	2	--	--
CakTpSSR02013	CakTC27717	(TG)7	1016	1029	CrTpSSR03198	CrTC34482	(TG)6	1085	1096	1	--	--

CakTpSSR02017	CakTC27750	(ACA)8	140	163	CrTpSSR02511	CrTC28514	(ACA)5	98	112	3	--	--
CakTpSSR02032	CakTC27891	(CT)6	25	36	CrTpSSR01637	CrTC16501	(CT)7	25	38	1	--	--
CakTpSSR02033	CakTC27891	(TCC)7	2000	2020	CrTpSSR01638	CrTC16501	(TCC)5	1949	1963	2	--	--
CakTpSSR02055	CakTC27985	(AT)6	2922	2933	CrTpSSR02766	CrTC30838	(AT)7	83	96	1	--	--
CakTpSSR02063	CakTC28067	(AG)16	1304	1335	CrTpSSR02761	CrTC30819	(AG)7	1227	1240	9	--	--
CakTpSSR02074	CakTC28116	(GTG)8	1614	1637	CrTpSSR02852	CrTC31458	(GTG)5	1614	1628	3	--	--
CakTpSSR02089	CakTC28242	(TCCT)8	2595	2626	CrTpSSR00175	CrTC02011	(TCCT)9	1416	1451	1	--	--
CakTpSSR02093	CakTC28270	(AGA)7	334	354	CrTpSSR02495	CrTC28282	(AGA)8	252	275	1	--	WRKY
CakTpSSR02109	CakTC28354	(AG)10	3710	3729	CrTpSSR02308	CrTC25571	(AG)6	419	430	4	--	--
CakTpSSR02110	CakTC28366	(CT)8	67	82	CrTpSSR01061	CrTC10376	(CT)10	39	58	2	--	--
CakTpSSR02137	CakTC28459	(GAA)11	1152	1184	CrTpSSR02644	CrTC29725	(GAA)7	1077	1097	4	--	--
CakTpSSR02143	CakTC28476	(GAA)6	1470	1487	CrTpSSR01110	CrTC10786	(GAA)8	1189	1212	2	--	--
CakTpSSR02149	CakTC28528	(AG)16	760	791	CrTpSSR02590	CrTC29239	(AG)15	565	594	1	--	--
CakTpSSR02154	CakTC28584	(TAT)6	168	185	CrTpSSR02309	CrTC25581	(TAT)7	71	91	1	--	--
CakTpSSR02184	CakTC28798	(CCA)7	370	390	CrTpSSR02837	CrTC31369	(CCA)6	254	271	1	--	C3H
CakTpSSR02186	CakTC28800	(TTC)7	604	624	CrTpSSR00534	CrTC05084	(TTC)5	722	736	2	Flower bud	--
CakTpSSR02191	CakTC28822	(TGT)7	885	905	CrTpSSR03354	CrTC35736	(TGT)5	897	911	2	--	--
CakTpSSR02194	CakTC28834	(AAG)6	523	540	CrTpSSR00574	CrTC05629	(AAG)7	137	157	1	--	--
CakTpSSR02205	CakTC28918	(AG)6	2	13	CrTpSSR01062	CrTC10383	(AG)7	275	288	1	--	--
CakTpSSR02212	CakTC28936	(TC)8	44	59	CrTpSSR03298	CrTC35145	(TC)7	69	82	1	--	--
CakTpSSR02222	CakTC29000	(TTC)10	171	200	CrTpSSR02479	CrTC28082	(TTC)5	53	67	5	--	--
CakTpSSR02229	CakTC29036	(CT)9	1	18	CrTpSSR03211	CrTC34563	(CT)8	1	16	1	--	--
CakTpSSR02234	CakTC29049	(AG)10	2115	2134	CrTpSSR00788	CrTC07718	(AG)9	2012	2029	1	--	TCP
CakTpSSR02247	CakTC29149	(GA)8	1148	1163	CrTpSSR02048	CrTC21586	(GA)6	1214	1225	2	--	--
CakTpSSR02254	CakTC29183	(GAA)6	48	65	CrTpSSR01618	CrTC16298	(GAA)7	2	22	1	--	--
CakTpSSR02270	CakTC29328	(AG)6	14	25	CrTpSSR00939	CrTC09178	(AG)11	194	215	5	--	--
CakTpSSR02281	CakTC29415	(GCA)5	1512	1526	CrTpSSR02919	CrTC32120	(GCA)6	533	550	1	Shoot	--
CakTpSSR02328	CakTC29615	(AG)10	2269	2288	CrTpSSR02068	CrTC21914	(AG)8	76	91	2	--	--
CakTpSSR02350	CakTC29831	(AG)8	105	120	CrTpSSR02642	CrTC29717	(AG)9	61	78	1	--	--
CakTpSSR02356	CakTC29856	(TCA)5	2384	2398	CrTpSSR00673	CrTC06623	(TCA)6	2381	2398	1	--	--
CakTpSSR02363	CakTC29895	(TCT)7	113	133	CrTpSSR03234	CrTC34697	(TCT)5	106	120	2	--	--
CakTpSSR02381	CakTC29965	(TC)20	22	61	CrTpSSR00976	CrTC09549	(TC)10	41	60	10	--	HB
CakTpSSR02387	CakTC29981	(AGA)8	2245	2268	CrTpSSR02795	CrTC31035	(AGA)7	2235	2255	1	--	--
CakTpSSR02393	CakTC30018	(TA)6	2019	2030	CrTpSSR03300	CrTC35166	(TA)9	1799	1816	3	--	--
CakTpSSR02396	CakTC30029	(TA)10	55	74	CrTpSSR00691	CrTC06808	(TA)6	41	52	4	Root	--
CakTpSSR02399	CakTC30033	(AG)10	1096	1115	CrTpSSR02893	CrTC31867	(AG)6	554	565	4	--	--
CakTpSSR02406	CakTC30071	(AAG)5	70	84	CrTpSSR01047	CrTC10269	(AAG)6	71	88	1	--	--
CakTpSSR02409	CakTC30080	(CT)12	124	147	CrTpSSR00741	CrTC07300	(CT)6	129	140	6	--	--
CakTpSSR02438	CakTC30280	(AT)12	762	785	CrTpSSR02572	CrTC29031	(AT)9	658	675	3	--	--
CakTpSSR02450	CakTC30327	(GTT)7	1574	1594	CrTpSSR01674	CrTC16840	(GTT)8	1590	1613	1	--	--

CakTpSSR02457	CakTC30360	(AAT)6	5	22	CrTpSSR00985	CrTC09662	(AAT)7	22	42	1	--	--
CakTpSSR02460	CakTC30386	(CAA)8	326	349	CrTpSSR00392	CrTC03684	(CAA)10	352	381	2	Young_pod	--
CakTpSSR02481	CakTC30527	(CT)6	77	88	CrTpSSR02502	CrTC28419	(CT)9	78	95	3	--	--
CakTpSSR02484	CakTC30570	(AAG)6	45	62	CrTpSSR02964	CrTC32557	(AAG)7	26	46	1	--	bZIP
CakTpSSR02487	CakTC30587	(AT)16	271	302	CrTpSSR02228	CrTC24094	(AT)15	266	295	1	--	--
CakTpSSR02497	CakTC30675	(AT)7	183	196	CrTpSSR00127	CrTC01426	(AT)6	556	567	1	--	--
CakTpSSR02500	CakTC30701	(TA)13	109	134	CrTpSSR01743	CrTC17664	(TA)15	230	259	2	--	--
CakTpSSR02504	CakTC30713	(ATA)5	794	808	CrTpSSR02215	CrTC23939	(ATA)6	802	819	1	--	SRS
CakTpSSR02507	CakTC30740	(AAT)6	1744	1761	CrTpSSR00655	CrTC06481	(AAT)5	1060	1074	1	--	--
CakTpSSR02508	CakTC30741	(GAA)5	1684	1698	CrTpSSR01849	CrTC18900	(GAA)6	1522	1539	1	--	--
CakTpSSR02515	CakTC30781	(CAA)5	184	198	CrTpSSR00698	CrTC06853	(CAA)6	222	239	1	--	--
CakTpSSR02517	CakTC30793	(GGC)5	134	148	CrTpSSR02524	CrTC28607	(GGC)7	90	110	2	--	--
CakTpSSR02531	CakTC30883	(TC)9	227	244	CrTpSSR01634	CrTC16487	(TC)10	220	239	1	--	--
CakTpSSR02535	CakTC30906	(AAC)8	1235	1258	CrTpSSR00799	CrTC07810	(AAC)7	1041	1061	1	--	GRAS
CakTpSSR02539	CakTC30929	(GAA)8	227	250	CrTpSSR03090	CrTC33767	(GAA)7	193	213	1	--	G2-like
CakTpSSR02542	CakTC30945	(TG)9	514	531	CrTpSSR01475	CrTC14581	(TG)8	513	528	1	--	--
CakTpSSR02551	CakTC30998	(TA)8	1084	1099	CrTpSSR00458	CrTC04417	(TA)9	1095	1112	1	--	MYB-related
CakTpSSR02560	CakTC31034	(AAC)7	626	646	CrTpSSR02374	CrTC26729	(AAC)6	552	569	1	--	--
CakTpSSR02576	CakTC31109	(GT)12	2140	2163	CrTpSSR01940	CrTC20330	(GT)6	29	40	6	--	--
CakTpSSR02598	CakTC31216	(GAA)7	2425	2445	CrTpSSR02866	CrTC31572	(GAA)5	2439	2453	2	--	HB
CakTpSSR02602	CakTC31260	(TC)9	3681	3698	CrTpSSR02361	CrTC26419	(TC)8	687	702	1	--	--
CakTpSSR02606	CakTC31295	(GA)8	15	30	CrTpSSR01919	CrTC19924	(GA)6	6	17	2	--	--
CakTpSSR02609	CakTC31305	(TCT)7	64	84	CrTpSSR01303	CrTC12815	(TCT)10	202	231	3	--	--
CakTpSSR02622	CakTC31433	(GT)12	3927	3950	CrTpSSR02085	CrTC22238	(GT)9	2032	2049	3	--	--
CakTpSSR02637	CakTC31492	(GATA)7	1607	1634	CrTpSSR02841	CrTC31392	(GATA)8	649	680	1	--	--
CakTpSSR02655	CakTC31576	(AAG)6	1455	1472	CrTpSSR01911	CrTC19808	(AAG)7	572	592	1	--	--
CakTpSSR02658	CakTC31612	(GA)20	2035	2074	CrTpSSR01482	CrTC14686	(GA)29	2065	2122	9	--	--
CakTpSSR02667	CakTC31656	(CTT)5	64	78	CrTpSSR00050	CrTC00615	(CTT)6	457	474	1	--	--
CakTpSSR02670	CakTC31682	(TTA)6	165	182	CrTpSSR01557	CrTC15635	(TTA)8	60	83	2	--	ARR-B
CakTpSSR02673	CakTC31693	(AC)13	1328	1353	CrTpSSR00617	CrTC06095	(AC)12	1371	1394	1	--	--
CakTpSSR02687	CakTC31756	(AAT)5	305	319	CrTpSSR00688	CrTC06781	(AAT)6	41	58	1	Flower bud	bZIP
CakTpSSR02696	CakTC31803	(TGA)6	666	683	CrTpSSR02281	CrTC25030	(TGA)5	767	781	1	--	Trihelix
CakTpSSR02706	CakTC31866	(AG)18	2317	2352	CrTpSSR00303	CrTC02808	(AG)8	2456	2471	10	--	--
CakTpSSR02717	CakTC32005	(TA)6	48	59	CrTpSSR01889	CrTC19446	(TA)11	1189	1210	5	--	--
CakTpSSR02719	CakTC32010	(CAG)5	1224	1238	CrTpSSR02388	CrTC26958	(CAG)6	1023	1040	1	--	--
CakTpSSR02722	CakTC32021	(TC)12	15	38	CrTpSSR01932	CrTC20146	(TC)10	56	75	2	--	--
CakTpSSR02728	CakTC32074	(AT)6	2232	2243	CrTpSSR02120	CrTC22719	(AT)8	1122	1137	2	--	--
CakTpSSR02735	CakTC32111	(AG)7	323	336	CrTpSSR01170	CrTC11275	(AG)6	323	334	1	--	--
CakTpSSR02738	CakTC32132	(AG)6	401	412	CrTpSSR00412	CrTC03983	(AG)7	400	413	1	Root	--
CakTpSSR02747	CakTC32148	(TAT)5	614	628	CrTpSSR02021	CrTC21218	(TAT)12	99	134	7	--	MYB

CakTpSSR02755	CakTC32189	(AT)6	130	141	CrTpSSR02416	CrTC27250	(AT)7	132	145	1	--	--
CakTpSSR02759	CakTC32218	(CT)8	261	276	CrTpSSR01282	CrTC12620	(CT)6	282	293	2	--	--
CakTpSSR02780	CakTC32284	(AAC)5	127	141	CrTpSSR03184	CrTC34367	(AAC)7	103	123	2	--	--
CakTpSSR02785	CakTC32314	(TCT)10	46	75	CrTpSSR03111	CrTC33865	(TCT)9	46	72	1	--	--
CakTpSSR02795	CakTC32348	(CCA)6	3717	3734	CrTpSSR01999	CrTC21060	(CCA)7	1224	1244	1	--	--
CakTpSSR02798	CakTC32366	(GT)12	4192	4215	CrTpSSR00852	CrTC08282	(GT)8	4153	4168	4	--	--
CakTpSSR02801	CakTC32389	(CTT)8	51	74	CrTpSSR03301	CrTC35168	(CTT)7	52	72	1	--	--
CakTpSSR02807	CakTC32456	(TGA)7	1945	1965	CrTpSSR00061	CrTC00729	(TGA)8	509	532	1	--	--
CakTpSSR02813	CakTC32488	(CTT)8	1662	1685	CrTpSSR00532	CrTC05076	(CTT)7	1618	1638	1	--	TCP
CakTpSSR02816	CakTC32496	(TTC)6	603	620	CrTpSSR00827	CrTC08019	(TTC)7	622	642	1	--	LOB
CakTpSSR02827	CakTC32595	(ATT)15	1821	1865	CrTpSSR02790	CrTC31018	(ATT)9	1809	1835	6	--	--
CakTpSSR02831	CakTC32630	(GA)9	541	558	CrTpSSR03341	CrTC35571	(GA)11	456	477	2	--	--
CakTpSSR02847	CakTC32729	(AAT)9	2045	2071	CrTpSSR03212	CrTC34574	(AAT)6	2105	2122	3	--	--
CakTpSSR02863	CakTC32818	(AG)8	472	487	CrTpSSR03097	CrTC33785	(AG)10	454	473	2	--	--
CakTpSSR02868	CakTC32878	(TTCTG)6	565	594	CrTpSSR02311	CrTC25684	(TTCTG)5	517	541	1	--	--
CakTpSSR02869	CakTC32879	(TA)10	411	430	CrTpSSR01597	CrTC16066	(TA)6	20	31	4	--	--
CakTpSSR02880	CakTC32991	(CTA)5	153	167	CrTpSSR03085	CrTC33698	(CTA)8	221	244	3	--	--
CakTpSSR02891	CakTC33039	(TCT)11	266	298	CrTpSSR00969	CrTC09454	(TCT)9	262	288	2	--	--
CakTpSSR02902	CakTC33129	(GT)7	2330	2343	CrTpSSR00007	CrTC00075	(GT)8	796	811	1	Mature Leaf	--
CakTpSSR02905	CakTC33153	(TTC)6	17	34	CrTpSSR01635	CrTC16489	(TTC)5	53	67	1	--	--
CakTpSSR02920	CakTC33240	(AG)10	1956	1975	CrTpSSR03210	CrTC34561	(AG)8	1958	1973	2	--	--
CakTpSSR02926	CakTC33266	(GTG)6	2186	2203	CrTpSSR02928	CrTC32192	(GTG)5	2188	2202	1	--	--
CakTpSSR02934	CakTC33310	(AT)7	2236	2249	CrTpSSR00604	CrTC05969	(AT)6	2247	2258	1	--	--
CakTpSSR02936	CakTC33339	(CTT)5	1933	1947	CrTpSSR02286	CrTC25081	(CTT)6	221	238	1	--	--
CakTpSSR02939	CakTC33354	(AAT)5	656	670	CrTpSSR02278	CrTC24980	(AAT)8	645	668	3	--	--
CakTpSSR02953	CakTC33459	(AC)6	295	306	CrTpSSR03026	CrTC33054	(AC)7	294	307	1	--	--
CakTpSSR02957	CakTC33510	(GGC)6	639	656	CrTpSSR00718	CrTC07045	(GGC)5	92	106	1	--	--
CakTpSSR02974	CakTC33656	(CT)7	108	121	CrTpSSR00381	CrTC03602	(CT)6	106	117	1	--	--
CakTpSSR02982	CakTC33720	(GA)10	6926	6945	CrTpSSR02149	CrTC23039	(GA)8	1320	1335	2	--	--
CakTpSSR02984	CakTC33731	(TC)9	61	78	CrTpSSR03252	CrTC34816	(TC)6	1	12	3	--	--
CakTpSSR02987	CakTC33747	(CT)8	652	667	CrTpSSR00872	CrTC08423	(CT)6	820	831	2	--	--
CakTpSSR02992	CakTC33793	(AG)25	2395	2444	CrTpSSR03224	CrTC34657	(AG)18	2341	2376	7	--	--
CakTpSSR02993	CakTC33799	(TTC)6	190	207	CrTpSSR00962	CrTC09412	(TTC)11	187	219	5	--	--
CakTpSSR03003	CakTC33861	(TC)6	2447	2458	CrTpSSR00422	CrTC04070	(TC)7	2434	2447	1	--	--
CakTpSSR03019	CakTC34004	(ATC)8	480	503	CrTpSSR02743	CrTC30664	(ATC)7	471	491	1	--	--
CakTpSSR03039	CakTC34158	(ATG)6	230	247	CrTpSSR02934	CrTC32281	(ATG)5	346	360	1	--	--
CakTpSSR03048	CakTC34258	(GCA)5	479	493	CrTpSSR01064	CrTC10400	(GCA)6	534	551	1	--	TPR
CakTpSSR03063	CakTC34364	(CT)11	2202	2223	CrTpSSR01102	CrTC10702	(CT)6	1940	1951	5	--	--
CakTpSSR03065	CakTC34377	(TTCATT)6	1713	1748	CrTpSSR01067	CrTC10423	(TTCATT)7	1388	1429	1	--	--
CakTpSSR03069	CakTC34394	(CTT)7	618	638	CrTpSSR01949	CrTC20409	(CTT)5	184	198	2	Flower bud	--

CakTpSSR03093	CakTC34569	(ATG)6	1904	1921	CrTpSSR02135	CrTC22877	(ATG)8	1915	1938	2	--	--
CakTpSSR03094	CakTC34582	(GA)10	48	67	CrTpSSR01516	CrTC15089	(GA)9	35	52	1	--	--
CakTpSSR03099	CakTC34624	(AG)10	579	598	CrTpSSR01840	CrTC18757	(AG)11	572	593	1	--	--
CakTpSSR03105	CakTC34659	(AAT)8	6657	6680	CrTpSSR02560	CrTC28939	(AAT)9	330	356	1	--	--
CakTpSSR03116	CakTC34729	(CTT)10	127	156	CrTpSSR01966	CrTC20578	(CTT)6	168	185	4	--	--
CakTpSSR03117	CakTC34751	(AT)10	192	211	CrTpSSR02549	CrTC28823	(AT)7	3437	3450	3	--	--
CakTpSSR03118	CakTC34754	(AG)12	3280	3303	CrTpSSR03229	CrTC34679	(AG)11	3305	3326	1	--	--
CakTpSSR03136	CakTC34866	(TC)10	99	118	CrTpSSR01410	CrTC13919	(TC)11	95	116	1	--	--
CakTpSSR03162	CakTC35097	(ATG)8	1316	1339	CrTpSSR01160	CrTC11205	(ATG)10	1316	1345	2	--	--
CakTpSSR03180	CakTC35202	(CT)8	21	36	CrTpSSR02153	CrTC23066	(CT)7	177	190	1	--	--
CakTpSSR03187	CakTC35240	(TAT)6	3239	3256	CrTpSSR01602	CrTC16098	(TAT)5	3200	3214	1	--	--
CakTpSSR03196	CakTC35334	(AG)7	47	60	CrTpSSR00803	CrTC07826	(AG)8	26	41	1	--	--
CakTpSSR03203	CakTC35400	(TCT)7	139	159	CrTpSSR03453	CrTC37082	(TCT)8	188	211	1	--	--
CakTpSSR03213	CakTC35436	(TTC)13	211	249	CrTpSSR02489	CrTC28225	(TTC)5	211	225	8	--	--
CakTpSSR03217	CakTC35460	(AG)8	4080	4095	CrTpSSR03241	CrTC34743	(AG)13	3982	4007	5	--	--
CakTpSSR03238	CakTC35635	(AT)9	239	256	CrTpSSR00881	CrTC08543	(AT)8	2933	2948	1	--	--
CakTpSSR03246	CakTC35676	(TCT)6	341	358	CrTpSSR01688	CrTC17059	(TCT)9	330	356	3	--	--
CakTpSSR03248	CakTC35684	(CAC)8	435	458	CrTpSSR02782	CrTC30968	(CAC)6	307	324	2	--	SBP
CakTpSSR03253	CakTC35722	(AGA)6	4169	4186	CrTpSSR00498	CrTC04839	(AGA)5	2188	2202	1	Mature Leaf	--
CakTpSSR03257	CakTC35749	(TCA)7	3068	3088	CrTpSSR00875	CrTC08452	(TCA)5	3068	3082	2	--	--
CakTpSSR03258	CakTC35752	(ATG)6	144	161	CrTpSSR02369	CrTC26688	(ATG)5	108	122	1	--	--
CakTpSSR03266	CakTC35803	(ACC)10	260	289	CrTpSSR00705	CrTC06908	(ACC)9	223	249	1	--	--
CakTpSSR03276	CakTC35891	(TCT)5	382	396	CrTpSSR00917	CrTC08978	(TCT)6	437	454	1	--	--
CakTpSSR03285	CakTC35958	(TAA)7	369	389	CrTpSSR01837	CrTC18712	(TAA)5	321	335	2	--	--
CakTpSSR03294	CakTC36006	(GAA)10	3113	3142	CrTpSSR03236	CrTC34708	(GAA)5	3075	3089	5	--	--
CakTpSSR03298	CakTC36016	(TCT)7	2903	2923	CrTpSSR01855	CrTC19010	(TCT)8	2840	2863	1	--	--
CakTpSSR03305	CakTC36060	(GAA)11	141	173	CrTpSSR03032	CrTC33095	(GAA)9	41	67	2	Flower bud	--
CakTpSSR03308	CakTC36073	(GTT)6	408	425	CrTpSSR01558	CrTC15647	(GTT)5	446	460	1	--	--
CakTpSSR03321	CakTC36201	(TC)8	134	149	CrTpSSR01869	CrTC19166	(TC)9	124	141	1	--	--
CakTpSSR03332	CakTC36282	(TCA)6	251	268	CrTpSSR01251	CrTC12227	(TCA)9	222	248	3	--	--
CakTpSSR03334	CakTC36288	(TG)10	4675	4694	CrTpSSR02270	CrTC24793	(TG)7	482	495	3	--	--
CakTpSSR03335	CakTC36289	(TCT)6	74	91	CrTpSSR03247	CrTC34769	(TCT)5	48	62	1	--	C3H
CakTpSSR03338	CakTC36306	(TC)9	3047	3064	CrTpSSR03049	CrTC33238	(TC)7	2944	2957	2	--	--
CakTpSSR03340	CakTC36329	(TC)6	15	26	CrTpSSR03239	CrTC34739	(TC)7	1	14	1	--	--
CakTpSSR03355	CakTC36473	(CA)9	67	84	CrTpSSR00664	CrTC06571	(CA)8	150	165	1	--	--
CakTpSSR03359	CakTC36512	(TA)7	435	448	CrTpSSR01084	CrTC10556	(TA)21	534	575	14	--	--
CakTpSSR03360	CakTC36519	(CAT)9	358	384	CrTpSSR02394	CrTC26991	(CAT)6	348	365	3	Young_pod	--
CakTpSSR03365	CakTC36561	(AG)6	2768	2779	CrTpSSR03256	CrTC34827	(AG)8	720	735	2	--	--
CakTpSSR03375	CakTC36662	(GAT)5	265	279	CrTpSSR01656	CrTC16627	(GAT)6	242	259	1	--	--
CakTpSSR03377	CakTC36676	(TC)7	418	431	CrTpSSR00790	CrTC07731	(TC)8	125	140	1	--	--

CakTpSSR03379	CakTC36721	(GA)11	1	22	CrTpSSR00597	CrTC05862	(GA)9	44	61	2	Young_pod	--
CakTpSSR03394	CakTC36828	(TC)6	17	28	CrTpSSR03235	CrTC34700	(TC)7	1	14	1	--	--
CakTpSSR03395	CakTC36839	(GA)7	2728	2741	CrTpSSR01963	CrTC20557	(GA)9	2894	2911	2	--	--
CakTpSSR03396	CakTC36848	(TC)7	69	82	CrTpSSR03019	CrTC32952	(TC)8	31	46	1	--	--
CakTpSSR03397	CakTC36851	(TC)7	489	502	CrTpSSR00615	CrTC06078	(TC)6	996	1007	1	--	--
CakTpSSR03405	CakTC36913	(AG)21	2658	2699	CrTpSSR02060	CrTC21778	(AG)16	2470	2501	5	--	--
CakTpSSR03412	CakTC36953	(CAA)7	585	605	CrTpSSR02982	CrTC32679	(CAA)5	580	594	2	--	--
CakTpSSR03414	CakTC36953	(AAT)7	1669	1689	CrTpSSR02984	CrTC32679	(AAT)9	1655	1681	2	--	--
CakTpSSR03430	CakTC37100	(AT)7	5	18	CrTpSSR03068	CrTC33469	(AT)6	418	429	1	Shoot	--
CakTpSSR03432	CakTC37103	(TC)6	181	192	CrTpSSR00301	CrTC02791	(TC)9	218	235	3	--	--
CakTpSSR03433	CakTC37120	(AAG)10	1480	1509	CrTpSSR02484	CrTC28142	(AAG)5	1477	1491	5	--	--
CakTpSSR03442	CakTC37158	(TCT)10	415	444	CrTpSSR03164	CrTC34220	(TCT)8	257	280	2	--	--
CakTpSSR03443	CakTC37160	(CAC)7	1084	1104	CrTpSSR02284	CrTC25069	(CAC)9	1142	1168	2	--	--
CakTpSSR03460	CakTC37238	(TC)7	23	36	CrTpSSR03189	CrTC34404	(TC)6	22	33	1	--	--
CakTpSSR03463	CakTC37251	(AAT)6	1791	1808	CrTpSSR02121	CrTC22721	(AAT)7	1348	1368	1	--	MYB
CakTpSSR03467	CakTC37266	(AG)9	1153	1170	CrTpSSR03007	CrTC32857	(AG)12	1119	1142	3	--	bHLH
CakTpSSR03471	CakTC37313	(TTC)9	16	42	CrTpSSR02690	CrTC30183	(TTC)6	8	25	3	--	--
CakTpSSR03485	CakTC37408	(AAG)13	1112	1150	CrTpSSR03383	CrTC36024	(AAG)5	993	1007	8	--	--
CakTpSSR03489	CakTC37420	(TC)12	103	126	CrTpSSR01591	CrTC15997	(TC)10	123	142	2	--	--
CakTpSSR03500	CakTC37469	(GA)8	10	25	CrTpSSR02720	CrTC30449	(GA)6	15	26	2	--	bZIP
CakTpSSR03505	CakTC37481	(TTC)6	60	77	CrTpSSR02453	CrTC27761	(TTC)5	68	82	1	--	--
CakTpSSR03508	CakTC37490	(GA)7	1179	1192	CrTpSSR02979	CrTC32649	(GA)10	1076	1095	3	--	--
CakTpSSR03510	CakTC37496	(CAA)5	209	223	CrTpSSR02272	CrTC24823	(CAA)7	228	248	2	--	bHLH
CakTpSSR03511	CakTC37498	(GAT)9	555	581	CrTpSSR02596	CrTC29283	(GAT)11	509	541	2	--	--
CakTpSSR03512	CakTC37499	(AG)14	1721	1748	CrTpSSR02476	CrTC28018	(AG)13	825	850	1	--	--
CakTpSSR03519	CakTC37539	(AT)9	1150	1167	CrTpSSR01881	CrTC19366	(AT)15	3	32	6	--	--
CakTpSSR03521	CakTC37545	(GA)17	1609	1642	CrTpSSR01333	CrTC13180	(GA)10	1605	1624	7	--	--
CakTpSSR03527	CakTC37578	(GA)8	1214	1229	CrTpSSR03188	CrTC34391	(GA)9	1121	1138	1	--	--
CakTpSSR03530	CakTC37590	(TCC)6	795	812	CrTpSSR00755	CrTC07393	(TCC)5	804	818	1	--	WRKY
CakTpSSR03532	CakTC37598	(TTG)6	1177	1194	CrTpSSR01368	CrTC13498	(TTG)7	871	891	1	--	--
CakTpSSR03533	CakTC37605	(TTC)7	67	87	CrTpSSR02685	CrTC30129	(TTC)8	82	105	1	--	--
CakTpSSR03546	CakTC37680	(GAG)9	1415	1441	CrTpSSR01079	CrTC10550	(GAG)8	1471	1494	1	--	--
CakTpSSR03551	CakTC37708	(ATA)5	237	251	CrTpSSR02653	CrTC29818	(ATA)11	222	254	6	--	--
CakTpSSR03556	CakTC37742	(GA)10	169	188	CrTpSSR00414	CrTC03984	(GA)25	1	50	15	Flower bud	--
CakTpSSR03560	CakTC37765	(TTC)6	42	59	CrTpSSR03366	CrTC35879	(TTC)7	22	42	1	--	--
CakTpSSR03562	CakTC37771	(AAC)5	266	280	CrTpSSR03071	CrTC33518	(AAC)6	257	274	1	Shoot	--
CakTpSSR03568	CakTC37779	(GGT)6	677	694	CrTpSSR02605	CrTC29367	(GGT)5	637	651	1	--	--
CakTpSSR03590	CakTC37894	(CCT)6	910	927	CrTpSSR01379	CrTC13628	(CCT)5	905	919	1	--	--
CakTpSSR03593	CakTC37899	(CT)7	90	103	CrTpSSR03194	CrTC34465	(CT)9	96	113	2	--	--
CakTpSSR03595	CakTC37925	(TC)7	952	965	CrTpSSR03197	CrTC34475	(TC)8	936	951	1	--	--

CakTpSSR03599	CakTC37942	(AT)9	48	65	CrTpSSR02997	CrTC32801	(AT)8	49	64	1	--	FAR1
CakTpSSR03602	CakTC37970	(AC)8	1	16	CrTpSSR01438	CrTC14251	(AC)7	112	125	1	--	--
CakTpSSR03603	CakTC37970	(GGA)9	944	970	CrTpSSR01439	CrTC14251	(GGA)13	1055	1093	4	--	--
CakTpSSR03605	CakTC37980	(TTA)5	956	970	CrTpSSR03375	CrTC35944	(TTA)7	956	976	2	--	--
CakTpSSR03606	CakTC37983	(AG)6	170	181	CrTpSSR02534	CrTC28690	(AG)7	24	37	1	--	SRS
CakTpSSR03609	CakTC37988	(CT)11	13	34	CrTpSSR03193	CrTC34447	(CT)7	4	17	4	--	--
CakTpSSR03615	CakTC38022	(AT)19	1087	1124	CrTpSSR01632	CrTC16434	(AT)24	1077	1124	5	--	--
CakTpSSR03627	CakTC38115	(AGG)5	1186	1200	CrTpSSR00674	CrTC06633	(AGG)6	1192	1209	1	--	--
CakTpSSR03629	CakTC38119	(GA)12	1053	1076	CrTpSSR01862	CrTC19103	(GA)10	1074	1093	2	--	--
CakTpSSR03630	CakTC38121	(TC)7	23	36	CrTpSSR01066	CrTC10422	(TC)6	46	57	1	--	--
CakTpSSR03633	CakTC38135	(TA)9	995	1012	CrTpSSR02169	CrTC23282	(TA)7	1157	1170	2	--	--
CakTpSSR03637	CakTC38143	(TTC)12	1028	1063	CrTpSSR03155	CrTC34147	(TTC)9	906	932	3	--	--
CakTpSSR03639	CakTC38187	(GA)14	1760	1787	CrTpSSR03288	CrTC35030	(GA)10	1682	1701	4	--	--
CakTpSSR03642	CakTC38244	(CTA)6	91	108	CrTpSSR01454	CrTC14393	(CTA)7	97	117	1	--	C2H2
CakTpSSR03650	CakTC38300	(CT)18	11	46	CrTpSSR00885	CrTC08573	(CT)8	96	111	10	--	--
CakTpSSR03658	CakTC38340	(AG)7	64	77	CrTpSSR03047	CrTC33235	(AG)8	56	71	1	--	TPR
CakTpSSR03679	CakTC38524	(TTC)6	83	100	CrTpSSR02142	CrTC22906	(TTC)5	75	89	1	--	--
CakTpSSR03685	CakTC38545	(ACA)5	197	211	CrTpSSR03403	CrTC36259	(ACA)6	48	65	1	--	--
CakTpSSR03697	CakTC38616	(ATA)5	30	44	CrTpSSR02357	CrTC26339	(ATA)6	21	38	1	--	--
CakTpSSR03705	CakTC38658	(TC)7	1023	1036	CrTpSSR00378	CrTC03577	(TC)8	1007	1022	1	--	--
CakTpSSR03709	CakTC38673	(CT)9	1	18	CrTpSSR00944	CrTC09205	(CT)7	711	724	2	--	--
CakTpSSR03714	CakTC38696	(TTC)8	148	171	CrTpSSR03104	CrTC33843	(TTC)11	119	151	3	--	--
CakTpSSR03721	CakTC38734	(GA)15	1476	1505	CrTpSSR01049	CrTC10302	(GA)11	1253	1274	4	--	--
CakTpSSR03726	CakTC38753	(AG)11	391	412	CrTpSSR02878	CrTC31687	(AG)10	385	404	1	--	HB
CakTpSSR03729	CakTC38763	(GTG)6	1150	1167	CrTpSSR02056	CrTC21718	(GTG)8	1419	1442	2	--	--
CakTpSSR03751	CakTC38879	(AG)6	3	14	CrTpSSR01589	CrTC15955	(AG)7	2	15	1	--	--
CakTpSSR03755	CakTC38904	(AAT)6	271	288	CrTpSSR00225	CrTC02320	(AAT)5	291	305	1	--	--
CakTpSSR03757	CakTC38915	(TC)9	1289	1306	CrTpSSR01016	CrTC09950	(TC)6	1357	1368	3	--	--
CakTpSSR03765	CakTC38936	(AT)8	1464	1479	CrTpSSR00645	CrTC06397	(AT)6	1405	1416	2	--	--
CakTpSSR03798	CakTC39122	(TTA)7	488	508	CrTpSSR01825	CrTC18537	(TTA)5	460	474	2	--	--
CakTpSSR03802	CakTC39146	(GTA)5	1243	1257	CrTpSSR01354	CrTC13337	(GTA)6	1310	1327	1	--	--
CakTpSSR03813	CakTC39200	(AG)10	1532	1551	CrTpSSR01799	CrTC18165	(AG)8	1521	1536	2	--	--
CakTpSSR03815	CakTC39206	(CT)7	115	128	CrTpSSR01727	CrTC17408	(CT)6	204	215	1	--	AUX/IAA
CakTpSSR03821	CakTC39225	(TC)11	149	170	CrTpSSR01497	CrTC14855	(TC)9	148	165	2	--	--
CakTpSSR03822	CakTC39230	(TC)26	1	52	CrTpSSR03162	CrTC34194	(TC)18	1	36	8	--	--
CakTpSSR03824	CakTC39237	(TCT)12	174	209	CrTpSSR01197	CrTC11584	(TCT)9	64	90	3	--	--
CakTpSSR03840	CakTC39318	(GA)12	1563	1586	CrTpSSR01429	CrTC14108	(GA)10	1637	1656	2	--	--
CakTpSSR03853	CakTC39411	(AG)7	15	28	CrTpSSR02593	CrTC29252	(AG)8	10	25	1	--	--
CakTpSSR03854	CakTC39421	(GA)8	1365	1380	CrTpSSR03153	CrTC34135	(GA)9	1367	1384	1	--	--
CakTpSSR03860	CakTC39446	(TTG)5	1644	1658	CrTpSSR02253	CrTC24498	(TTG)6	567	584	1	--	--

CakTpSSR03864	CakTC39476	(AG)6	1219	1230	CrTpSSR02581	CrTC29174	(AG)8	1105	1120	2	--	--
CakTpSSR03868	CakTC39489	(TTC)6	1230	1247	CrTpSSR00745	CrTC07360	(TTC)5	1234	1248	1	--	--
CakTpSSR03876	CakTC39534	(TAT)8	188	211	CrTpSSR00423	CrTC04088	(TAT)7	153	173	1	--	--
CakTpSSR03890	CakTC39655	(TTC)7	1795	1815	CrTpSSR00642	CrTC06319	(TTC)6	1814	1831	1	--	--
CakTpSSR03891	CakTC39656	(TTC)17	45	95	CrTpSSR00545	CrTC05258	(TTC)7	25	45	10	--	--
CakTpSSR03893	CakTC39660	(CCA)7	241	261	CrTpSSR00540	CrTC05136	(CCA)6	987	1004	1	--	--
CakTpSSR03904	CakTC39738	(AG)9	1254	1271	CrTpSSR00234	CrTC02361	(AG)7	1276	1289	2	--	--
CakTpSSR03907	CakTC39752	(CTT)15	123	167	CrTpSSR00677	CrTC06651	(CTT)5	159	173	10	--	--
CakTpSSR03912	CakTC39768	(CCA)8	453	476	CrTpSSR02676	CrTC30045	(CCA)11	455	487	3	--	--
CakTpSSR03923	CakTC39799	(GAA)19	1300	1356	CrTpSSR02910	CrTC32063	(GAA)15	1080	1124	4	--	bZIP
CakTpSSR03927	CakTC39846	(CTT)6	130	147	CrTpSSR02867	CrTC31577	(CTT)7	117	137	1	--	--
CakTpSSR03929	CakTC39858	(TTG)6	545	562	CrTpSSR00877	CrTC08470	(TTG)5	885	899	1	--	--
CakTpSSR03937	CakTC39897	(ATT)6	688	705	CrTpSSR02509	CrTC28495	(ATT)10	420	449	4	--	C2C2-Dof
CakTpSSR03940	CakTC39902	(AGA)5	1104	1118	CrTpSSR02807	CrTC31139	(AGA)6	1067	1084	1	--	--
CakTpSSR03950	CakTC39992	(CTT)7	124	144	CrTpSSR03307	CrTC35204	(CTT)6	110	127	1	--	--
CakTpSSR03951	CakTC40000	(GA)13	1162	1187	CrTpSSR01536	CrTC15428	(GA)9	1144	1161	4	--	LOB
CakTpSSR03956	CakTC40031	(TC)12	1	24	CrTpSSR00598	CrTC05863	(TC)9	359	376	3	--	--
CakTpSSR03975	CakTC40177	(GAA)5	605	619	CrTpSSR02632	CrTC29663	(GAA)6	585	602	1	--	--
CakTpSSR04018	CakTC40380	(GTT)7	671	691	CrTpSSR00678	CrTC06666	(GTT)6	673	690	1	Flower bud	--
CakTpSSR04023	CakTC40408	(TTC)5	96	110	CrTpSSR03416	CrTC36377	(TTC)6	103	120	1	--	--
CakTpSSR04031	CakTC40450	(TTC)6	1973	1990	CrTpSSR02836	CrTC31360	(TTC)5	308	322	1	--	--
CakTpSSR04040	CakTC40534	(TCT)7	132	152	CrTpSSR02932	CrTC32270	(TCT)6	98	115	1	--	--
CakTpSSR04045	CakTC40557	(TC)12	641	664	CrTpSSR03332	CrTC35424	(TC)7	646	659	5	--	--
CakTpSSR04047	CakTC40562	(TAT)6	171	188	CrTpSSR03338	CrTC35519	(TAT)5	132	146	1	--	--
CakTpSSR04052	CakTC40587	(ATTC)6	86	109	CrTpSSR03333	CrTC35446	(ATTC)5	50	69	1	--	--
CakTpSSR04058	CakTC40637	(GA)20	2026	2065	CrTpSSR03204	CrTC34515	(GA)14	434	461	6	--	WRKY
CakTpSSR04062	CakTC40666	(TGG)6	776	793	CrTpSSR03014	CrTC32894	(TGG)5	399	413	1	--	OFP
CakTpSSR04072	CakTC40695	(TA)9	56	73	CrTpSSR02553	CrTC28869	(TA)6	2022	2033	3	--	--
CakTpSSR04075	CakTC40704	(ATT)7	194	214	CrTpSSR03018	CrTC32941	(ATT)9	167	193	2	--	--
CakTpSSR04076	CakTC40711	(CCA)5	950	964	CrTpSSR02356	CrTC26301	(CCA)6	908	925	1	--	--
CakTpSSR04080	CakTC40771	(CT)6	1	12	CrTpSSR01455	CrTC14396	(CT)9	69	86	3	--	--
CakTpSSR04090	CakTC40857	(TC)6	49	60	CrTpSSR01207	CrTC11698	(TC)11	45	66	5	--	--
CakTpSSR04098	CakTC40890	(CAA)5	1626	1640	CrTpSSR01097	CrTC10658	(CAA)7	2106	2126	2	--	TPR
CakTpSSR04119	CakTC41015	(AG)13	576	601	CrTpSSR02883	CrTC31749	(AG)15	566	595	2	--	--
CakTpSSR04134	CakTC41162	(TTA)7	656	676	CrTpSSR02306	CrTC25563	(TTA)5	650	664	2	--	--
CakTpSSR04135	CakTC41164	(AG)26	650	701	CrTpSSR00243	CrTC02392	(AG)10	638	657	16	--	--
CakTpSSR04136	CakTC41169	(AAG)8	2065	2088	CrTpSSR01960	CrTC20528	(AAG)10	1996	2025	2	--	--
CakTpSSR04141	CakTC41199	(TC)12	178	201	CrTpSSR03216	CrTC34598	(TC)14	19	46	2	--	--
CakTpSSR04171	CakTC41304	(TAG)7	899	919	CrTpSSR01307	CrTC12864	(TAG)6	177	194	1	--	GRAS
CakTpSSR04172	CakTC41313	(CAT)5	330	344	CrTpSSR00327	CrTC03053	(CAT)7	298	318	2	--	--

CakTpSSR04189	CakTC41441	(AG)18	2	37	CrTpSSR03221	CrTC34629	(AG)7	1	14	11	--	--
CakTpSSR04191	CakTC41446	(GA)6	703	714	CrTpSSR03320	CrTC35271	(GA)7	686	699	1	--	--
CakTpSSR04205	CakTC41518	(TCT)9	306	332	CrTpSSR00833	CrTC08057	(TCT)10	297	326	1	--	--
CakTpSSR04210	CakTC41565	(GAT)6	676	693	CrTpSSR03411	CrTC36320	(GAT)5	673	687	1	--	--
CakTpSSR04211	CakTC41577	(CAA)5	159	173	CrTpSSR02491	CrTC28238	(CAA)7	137	157	2	--	SNF2
CakTpSSR04214	CakTC41608	(GAA)5	600	614	CrTpSSR01813	CrTC18384	(GAA)7	404	424	2	--	--
CakTpSSR04217	CakTC41626	(ACA)8	31	54	CrTpSSR02088	CrTC22292	(ACA)6	118	135	2	--	--
CakTpSSR04220	CakTC41634	(AC)11	115	136	CrTpSSR03329	CrTC35362	(AC)9	80	97	2	--	--
CakTpSSR04236	CakTC41773	(CT)8	11	26	CrTpSSR01943	CrTC20367	(CT)13	26	51	5	--	bHLH
CakTpSSR04238	CakTC41785	(AG)6	27	38	CrTpSSR02619	CrTC29531	(AG)8	20	35	2	--	--
CakTpSSR04242	CakTC41797	(TC)10	2294	2313	CrTpSSR01645	CrTC16549	(TC)12	2275	2298	2	--	--
CakTpSSR04243	CakTC41808	(AG)12	46	69	CrTpSSR03219	CrTC34615	(AG)13	43	68	1	--	GRAS
CakTpSSR04255	CakTC41904	(TTC)8	42	65	CrTpSSR02808	CrTC31140	(TTC)9	48	74	1	--	--
CakTpSSR04262	CakTC41929	(AAC)5	148	162	CrTpSSR00794	CrTC07760	(AAC)7	276	296	2	--	--
CakTpSSR04273	CakTC41998	(AG)6	187	198	CrTpSSR00245	CrTC02403	(AG)8	187	202	2	--	MADS
CakTpSSR04276	CakTC42021	(TA)7	275	288	CrTpSSR02820	CrTC31223	(TA)8	214	229	1	--	--
CakTpSSR04286	CakTC42090	(TTG)5	846	860	CrTpSSR00954	CrTC09292	(TTG)6	1307	1324	1	--	--
CakTpSSR04288	CakTC42101	(GAA)6	101	118	CrTpSSR02684	CrTC30128	(GAA)8	44	67	2	--	--
CakTpSSR04290	CakTC42107	(TTA)8	893	916	CrTpSSR03425	CrTC36486	(TTA)7	404	424	1	--	--
CakTpSSR04292	CakTC42118	(AG)13	966	991	CrTpSSR01415	CrTC13973	(AG)26	914	965	13	--	--
CakTpSSR04294	CakTC42129	(AT)9	24	41	CrTpSSR01693	CrTC17131	(AT)12	12	35	3	--	--
CakTpSSR04298	CakTC42155	(TC)10	121	140	CrTpSSR03419	CrTC36384	(TC)9	179	196	1	--	--
CakTpSSR04300	CakTC42170	(CT)10	1808	1827	CrTpSSR01213	CrTC11768	(CT)11	1775	1796	1	--	--
CakTpSSR04329	CakTC42321	(AAG)7	846	866	CrTpSSR03352	CrTC35721	(AAG)5	801	815	2	Root	--
CakTpSSR04331	CakTC42322	(TGG)7	804	824	CrTpSSR02471	CrTC27991	(TGG)6	73	90	1	--	--
CakTpSSR04335	CakTC42346	(TAA)7	455	475	CrTpSSR00983	CrTC09647	(TAA)8	449	472	1	--	bHLH
CakTpSSR04339	CakTC42362	(CT)8	1682	1697	CrTpSSR02512	CrTC28518	(CT)10	1620	1639	2	--	--
CakTpSSR04340	CakTC42363	(AG)10	80	99	CrTpSSR03272	CrTC34919	(AG)8	44	59	2	--	bZIP
CakTpSSR04346	CakTC42424	(GAT)5	743	757	CrTpSSR02482	CrTC28117	(GAT)6	593	610	1	--	--
CakTpSSR04350	CakTC42461	(CAA)6	437	454	CrTpSSR01850	CrTC18923	(CAA)5	443	457	1	--	--
CakTpSSR04351	CakTC42462	(AAAC)6	76	99	CrTpSSR03196	CrTC34469	(AAAC)7	42	69	1	--	--
CakTpSSR04359	CakTC42484	(TC)12	4	27	CrTpSSR02155	CrTC23117	(TC)8	18	33	4	--	--
CakTpSSR04368	CakTC42521	(TC)6	33	44	CrTpSSR00812	CrTC07928	(TC)7	20	33	1	--	MYB
CakTpSSR04383	CakTC42613	(CAG)7	127	147	CrTpSSR02658	CrTC29854	(CAG)6	115	132	1	--	--
CakTpSSR04389	CakTC42648	(AG)6	113	124	CrTpSSR03267	CrTC34891	(AG)8	88	103	2	--	--
CakTpSSR04391	CakTC42656	(TGA)6	794	811	CrTpSSR02674	CrTC30034	(TGA)7	173	193	1	--	--
CakTpSSR04398	CakTC42680	(TAT)9	347	373	CrTpSSR01633	CrTC16471	(TAT)6	98	115	3	Flower bud	AP2-EREBP
CakTpSSR04404	CakTC42701	(TCT)7	856	876	CrTpSSR03364	CrTC35844	(TCT)8	878	901	1	--	--
CakTpSSR04405	CakTC42710	(CT)11	6	27	CrTpSSR00001	CrTC00010	(CT)13	138	163	2	--	--
CakTpSSR04406	CakTC42718	(GAT)6	197	214	CrTpSSR03415	CrTC36365	(GAT)7	156	176	1	--	--

CakTpSSR04407	CakTC42722	(AG)6	14	25	CrTpSSR03063	CrTC33426	(AG)8	1	16	2	--	--
CakTpSSR04412	CakTC42754	(TC)7	876	889	CrTpSSR03355	CrTC35742	(TC)6	761	772	1	--	--
CakTpSSR04416	CakTC42766	(AG)8	798	813	CrTpSSR01697	CrTC17183	(AG)9	658	675	1	--	--
CakTpSSR04432	CakTC42853	(TTTC)7	1913	1940	CrTpSSR00636	CrTC06210	(TTTC)5	1994	2013	2	--	--
CakTpSSR04435	CakTC42865	(ATG)6	637	654	CrTpSSR03389	CrTC36085	(ATG)5	615	629	1	--	--
CakTpSSR04441	CakTC42922	(AAC)5	159	173	CrTpSSR01004	CrTC09816	(AAC)6	141	158	1	Flower bud	--
CakTpSSR04443	CakTC42941	(TAA)7	187	207	CrTpSSR03016	CrTC32909	(TAA)12	199	234	5	--	--
CakTpSSR04445	CakTC42950	(TAA)5	1596	1610	CrTpSSR00242	CrTC02386	(TAA)8	1361	1384	3	--	C2H2
CakTpSSR04449	CakTC42985	(TGA)11	1878	1910	CrTpSSR01811	CrTC18369	(TGA)5	1877	1891	6	--	--
CakTpSSR04453	CakTC42999	(ATG)7	1716	1736	CrTpSSR02257	CrTC24552	(ATG)6	1910	1927	1	--	TUB
CakTpSSR04454	CakTC43001	(AG)10	833	852	CrTpSSR03414	CrTC36356	(AG)12	813	836	2	--	--
CakTpSSR04464	CakTC43053	(TC)8	1908	1923	CrTpSSR02627	CrTC29628	(TC)7	1906	1919	1	--	--
CakTpSSR04470	CakTC43078	(TGG)8	720	743	CrTpSSR00112	CrTC01308	(TGG)6	902	919	2	--	--
CakTpSSR04478	CakTC43112	(CAA)7	566	586	CrTpSSR01734	CrTC17518	(CAA)8	509	532	1	Mature Leaf	bHLH
CakTpSSR04488	CakTC43146	(TCT)6	128	145	CrTpSSR03448	CrTC36959	(TCT)5	85	99	1	--	--
CakTpSSR04491	CakTC43179	(AG)10	1000	1019	CrTpSSR01969	CrTC20608	(AG)11	894	915	1	--	--
CakTpSSR04502	CakTC43250	(TC)15	799	828	CrTpSSR03417	CrTC36379	(TC)11	787	808	4	--	--
CakTpSSR04510	CakTC43305	(AG)7	872	885	CrTpSSR02849	CrTC31446	(AG)9	778	795	2	--	--
