# Supplementary material for: Comparative Analysis of Kabuli Chickpea Transcriptome with Desi and Wild Chickpea Provides a Rich Resource for Development of Functional Markers
Source: PLoS One. 2012 Dec 27;7(12):e52443. doi: 10.1371/journal.pone.0052443 (PMC3531472; doi:10.1371/journal.pone.0052443)
Supplement: Table S10 — List of SNPs identified between kabuli and desi chickpea. (PDF) [file pone.0052443.s020.pdf]

Table S10. List of SNPs identified between kabuli and desi chickpea.

Kabuli transcript ID	SNP_ID	SNP position (kabuli)	Kabuli allele	Desi allele	Read depth kabuli	Read depth desi	Tissue specificity	TF family
CakTC28377	Ca(ICCV2/ICC4958)SNP_00001	272	C	T	3	3	--	--
	Ca(ICCV2/ICC4958)SNP_00002	370	C	T	3	5	--	--
	Ca(ICCV2/ICC4958)SNP_00003	948	G	A	3	3	--	--
	Ca(ICCV2/ICC4958)SNP_00004	971	C	A	3	5	--	--
	Ca(ICCV2/ICC4958)SNP_00005	973	A	T	3	5	--	--
	Ca(ICCV2/ICC4958)SNP_00006	1223	C	A	3	3	--	--
	Ca(ICCV2/ICC4958)SNP_00007	1289	G	A	3	3	--	--
CakTC34874	Ca(ICCV2/ICC4958)SNP_00008	611	C	G	8	4	--	--
CakTC38944	Ca(ICCV2/ICC4958)SNP_00009	715	T	G	7	5	--	--
	Ca(ICCV2/ICC4958)SNP_00010	754	T	G	10	6	--	--
	Ca(ICCV2/ICC4958)SNP_00011	1117	T	A	19	12	--	--
	Ca(ICCV2/ICC4958)SNP_00012	1144	C	T	18	11	--	--
	Ca(ICCV2/ICC4958)SNP_00013	1264	G	A	15	13	--	--
	Ca(ICCV2/ICC4958)SNP_00014	1466	A	G	9	4	--	--
	Ca(ICCV2/ICC4958)SNP_00015	1489	A	G	9	4	--	--
	Ca(ICCV2/ICC4958)SNP_00016	1542	G	T	5	4	--	--
CakTC09809	Ca(ICCV2/ICC4958)SNP_00017	1445	T	G	3	3	--	--
CakTC13136	Ca(ICCV2/ICC4958)SNP_00018	291	T	C	4	4	--	--
	Ca(ICCV2/ICC4958)SNP_00019	299	G	C	4	4	--	--
	Ca(ICCV2/ICC4958)SNP_00020	334	G	A	5	7	--	--
CakTC25292	Ca(ICCV2/ICC4958)SNP_00021	610	C	T	11	10	--	--
	Ca(ICCV2/ICC4958)SNP_00022	1226	G	T	12	10	--	--
CakTC34422	Ca(ICCV2/ICC4958)SNP_00023	55	C	A	7	5	--	GRAS
	Ca(ICCV2/ICC4958)SNP_00024	622	T	G	21	10	--	GRAS
CakTC40414	Ca(ICCV2/ICC4958)SNP_00025	1913	A	T	6	3	Flower bud	--
CakTC31444	Ca(ICCV2/ICC4958)SNP_00026	185	C	T	5	3	--	--
	Ca(ICCV2/ICC4958)SNP_00027	272	C	T	7	9	--	--
	Ca(ICCV2/ICC4958)SNP_00028	302	C	A	8	11	--	--
	Ca(ICCV2/ICC4958)SNP_00029	395	C	T	8	10	--	--
	Ca(ICCV2/ICC4958)SNP_00030	398	C	T	5	9	--	--
	Ca(ICCV2/ICC4958)SNP_00031	482	T	C	5	8	--	--
	Ca(ICCV2/ICC4958)SNP_00032	515	T	C	5	5	--	--
	Ca(ICCV2/ICC4958)SNP_00033	548	T	C	4	5	--	--
	Ca(ICCV2/ICC4958)SNP_00034	578	C	A	5	5	--	--
	Ca(ICCV2/ICC4958)SNP_00035	728	T	C	6	4	--	--
	Ca(ICCV2/ICC4958)SNP_00036	801	A	G	6	4	--	--
	Ca(ICCV2/ICC4958)SNP_00037	803	T	C	6	5	--	--
	Ca(ICCV2/ICC4958)SNP_00038	833	C	T	6	5	--	--
	Ca(ICCV2/ICC4958)SNP_00039	1359	G	A	8	10	--	--
	Ca(ICCV2/ICC4958)SNP_00040	1400	C	T	8	10	--	--
	Ca(ICCV2/ICC4958)SNP_00041	1437	C	A	9	11	--	--
	Ca(ICCV2/ICC4958)SNP_00042	1490	A	T	7	11	--	--
	Ca(ICCV2/ICC4958)SNP_00043	1607	G	A	8	8	--	--
CakTC10834	Ca(ICCV2/ICC4958)SNP_00044	578	C	A	11	9	--	AUX/IAA
CakTC12266	Ca(ICCV2/ICC4958)SNP_00045	237	G	A	7	9	--	--
CakTC13722	Ca(ICCV2/ICC4958)SNP_00046	1752	G	A	35	9	--	--
CakTC11127	Ca(ICCV2/ICC4958)SNP_00047	258	C	G	13	13	--	CCHC
	Ca(ICCV2/ICC4958)SNP_00048	269	G	A	11	13	--	CCHC
	Ca(ICCV2/ICC4958)SNP_00049	318	T	C	16	12	--	CCHC
	Ca(ICCV2/ICC4958)SNP_00050	1254	G	A	7	13	--	CCHC
	Ca(ICCV2/ICC4958)SNP_00051	1606	A	G	10	13	--	CCHC
	Ca(ICCV2/ICC4958)SNP_00052	2091	T	G	9	12	--	CCHC
	Ca(ICCV2/ICC4958)SNP_00053	2280	A	G	8	11	--	CCHC
	Ca(ICCV2/ICC4958)SNP_00054	2331	G	A	11	11	--	CCHC
	Ca(ICCV2/ICC4958)SNP_00055	2349	C	T	14	11	--	CCHC
	Ca(ICCV2/ICC4958)SNP_00056	3065	T	C	10	32	--	CCHC
	Ca(ICCV2/ICC4958)SNP_00057	3111	A	C	14	35	--	CCHC
CakTC14353	Ca(ICCV2/ICC4958)SNP_00058	189	G	A	3	5	Young_pod	--
CakTC39323	Ca(ICCV2/ICC4958)SNP_00059	1319	A	G	3	8	--	--
CakTC30832	Ca(ICCV2/ICC4958)SNP_00060	1610	G	T	7	4	--	ABI3VP1
CakTC34113	Ca(ICCV2/ICC4958)SNP_00061	569	G	A	28	10	--	bZIP
	Ca(ICCV2/ICC4958)SNP_00062	867	A	G	30	13	--	bZIP
	Ca(ICCV2/ICC4958)SNP_00063	1127	T	C	27	13	--	bZIP
	Ca(ICCV2/ICC4958)SNP_00064	1352	C	T	10	14	--	bZIP
CakTC26979	Ca(ICCV2/ICC4958)SNP_00065	1118	G	C	13	11	--	bZIP
	Ca(ICCV2/ICC4958)SNP_00066	1127	A	G	12	11	--	bZIP
	Ca(ICCV2/ICC4958)SNP_00067	1133	A	G	13	11	--	bZIP
CakTC32079	Ca(ICCV2/ICC4958)SNP_00068	788	T	C	3	3	--	--

CakTC23460	Ca(ICCV2/ICC4958)SNP_00069	223	G	A	6	3	--	--
CakTC27374	Ca(ICCV2/ICC4958)SNP_00070	748	G	T	6	7	--	--
	Ca(ICCV2/ICC4958)SNP_00071	1301	C	T	4	8	--	--
CakTC09698	Ca(ICCV2/ICC4958)SNP_00072	2963	A	T	3	10	--	--
CakTC23234	Ca(ICCV2/ICC4958)SNP_00073	173	A	G	4	5	--	--
CakTC32696	Ca(ICCV2/ICC4958)SNP_00074	1621	G	A	5	15	--	CCHC
CakTC31023	Ca(ICCV2/ICC4958)SNP_00075	1748	C	T	4	4	--	--
	Ca(ICCV2/ICC4958)SNP_00076	1876	G	C	4	6	--	--
	Ca(ICCV2/ICC4958)SNP_00077	1879	A	G	4	6	--	--
	Ca(ICCV2/ICC4958)SNP_00078	2092	C	T	3	3	--	--
CakTC41609	Ca(ICCV2/ICC4958)SNP_00079	625	A	G	5	7	--	--
CakTC23206	Ca(ICCV2/ICC4958)SNP_00080	785	G	A	14	3	--	--
	Ca(ICCV2/ICC4958)SNP_00081	914	C	A	9	3	--	--
	Ca(ICCV2/ICC4958)SNP_00082	1002	T	G	9	3	--	--
CakTC37297	Ca(ICCV2/ICC4958)SNP_00083	182	T	C	5	11	--	--
	Ca(ICCV2/ICC4958)SNP_00084	313	G	A	8	14	--	--
CakTC28758	Ca(ICCV2/ICC4958)SNP_00085	88	G	A	13	10	--	--
	Ca(ICCV2/ICC4958)SNP_00086	171	T	A	19	18	--	--
	Ca(ICCV2/ICC4958)SNP_00087	432	A	T	61	54	--	--
	Ca(ICCV2/ICC4958)SNP_00088	623	G	A	79	57	--	--
	Ca(ICCV2/ICC4958)SNP_00089	1122	A	G	39	26	--	--
CakTC11944	Ca(ICCV2/ICC4958)SNP_00090	75	G	A	4	7	--	--
	Ca(ICCV2/ICC4958)SNP_00091	390	C	A	6	12	--	--
	Ca(ICCV2/ICC4958)SNP_00092	654	A	G	3	9	--	--
	Ca(ICCV2/ICC4958)SNP_00093	816	C	G	4	4	--	--
	Ca(ICCV2/ICC4958)SNP_00094	856	T	C	4	4	--	--
	Ca(ICCV2/ICC4958)SNP_00095	950	A	T	4	3	--	--
CakTC41163	Ca(ICCV2/ICC4958)SNP_00096	549	T	G	5	3	--	--
CakTC42187	Ca(ICCV2/ICC4958)SNP_00097	546	C	G	38	43	--	--
	Ca(ICCV2/ICC4958)SNP_00098	702	C	T	26	26	--	--
CakTC16891	Ca(ICCV2/ICC4958)SNP_00099	1154	A	G	3	3	--	--
	Ca(ICCV2/ICC4958)SNP_00100	1161	A	G	3	4	--	--
CakTC22734	Ca(ICCV2/ICC4958)SNP_00101	1005	G	A	22	23	--	--
CakTC27562	Ca(ICCV2/ICC4958)SNP_00102	1005	A	G	28	12	--	bZIP
CakTC12298	Ca(ICCV2/ICC4958)SNP_00103	33	G	A	3	3	--	--
CakTC41053	Ca(ICCV2/ICC4958)SNP_00104	2374	G	T	5	13	Shoot	--
	Ca(ICCV2/ICC4958)SNP_00105	2377	A	G	5	14	Shoot	--
CakTC30491	Ca(ICCV2/ICC4958)SNP_00106	914	C	T	14	7	--	--
CakTC38380	Ca(ICCV2/ICC4958)SNP_00107	1009	C	T	4	14	--	C2C2-Dof
CakTC39677	Ca(ICCV2/ICC4958)SNP_00108	782	C	G	9	9	--	--
	Ca(ICCV2/ICC4958)SNP_00109	956	G	T	7	13	--	--
	Ca(ICCV2/ICC4958)SNP_00110	1107	A	G	5	6	--	--
CakTC40798	Ca(ICCV2/ICC4958)SNP_00111	1030	T	C	6	4	--	--
CakTC14765	Ca(ICCV2/ICC4958)SNP_00112	666	G	T	4	3	--	--
	Ca(ICCV2/ICC4958)SNP_00113	667	C	T	4	3	--	--
	Ca(ICCV2/ICC4958)SNP_00114	818	A	G	3	3	--	--
CakTC23091	Ca(ICCV2/ICC4958)SNP_00115	955	T	G	3	4	--	--
	Ca(ICCV2/ICC4958)SNP_00116	1117	T	G	3	3	--	--
CakTC26525	Ca(ICCV2/ICC4958)SNP_00117	234	G	C	51	29	--	--
	Ca(ICCV2/ICC4958)SNP_00118	541	T	A	66	52	--	--
	Ca(ICCV2/ICC4958)SNP_00119	1267	T	C	35	25	--	--
	Ca(ICCV2/ICC4958)SNP_00120	1497	A	T	4	10	--	--
CakTC40385	Ca(ICCV2/ICC4958)SNP_00121	1480	A	G	15	5	--	--
CakTC22389	Ca(ICCV2/ICC4958)SNP_00122	240	C	A	3	3	--	--
	Ca(ICCV2/ICC4958)SNP_00123	448	G	T	4	4	--	--
	Ca(ICCV2/ICC4958)SNP_00124	497	C	A	4	5	--	--
	Ca(ICCV2/ICC4958)SNP_00125	559	C	T	9	6	--	--
	Ca(ICCV2/ICC4958)SNP_00126	681	A	G	14	7	--	--
	Ca(ICCV2/ICC4958)SNP_00127	785	A	T	14	5	--	--
	Ca(ICCV2/ICC4958)SNP_00128	914	A	T	26	13	--	--
	Ca(ICCV2/ICC4958)SNP_00129	1193	C	T	60	16	--	--
	Ca(ICCV2/ICC4958)SNP_00130	1292	T	C	58	17	--	--
	Ca(ICCV2/ICC4958)SNP_00131	1341	T	C	69	25	--	--
	Ca(ICCV2/ICC4958)SNP_00132	1355	C	T	70	24	--	--
	Ca(ICCV2/ICC4958)SNP_00133	1358	T	C	69	23	--	--
	Ca(ICCV2/ICC4958)SNP_00134	1373	G	T	67	23	--	--
	Ca(ICCV2/ICC4958)SNP_00135	1454	C	T	62	25	--	--
	Ca(ICCV2/ICC4958)SNP_00136	1466	T	C	49	24	--	--
	Ca(ICCV2/ICC4958)SNP_00137	1493	T	G	48	24	--	--
	Ca(ICCV2/ICC4958)SNP_00138	1673	G	T	51	16	--	--
	Ca(ICCV2/ICC4958)SNP_00139	1760	G	A	51	18	--	--
	Ca(ICCV2/ICC4958)SNP_00140	1778	C	T	51	23	--	--
	Ca(ICCV2/ICC4958)SNP_00141	1995	A	G	19	15	--	--
CakTC40015	Ca(ICCV2/ICC4958)SNP_00142	312	T	C	8	15	--	--

CakTC25051	Ca(ICCV2/ICC4958)SNP_00143	152	T	G	6	7	--	--
CakTC17917	Ca(ICCV2/ICC4958)SNP_00144	1199	A	T	6	3	--	mTERF
	Ca(ICCV2/ICC4958)SNP_00145	1222	G	A	4	4	--	mTERF
CakTC10294	Ca(ICCV2/ICC4958)SNP_00146	416	T	C	3	3	--	--
CakTC35898	Ca(ICCV2/ICC4958)SNP_00147	3702	C	T	13	18	--	--
CakTC36730	Ca(ICCV2/ICC4958)SNP_00148	2257	C	T	8	8	--	--
CakTC08793	Ca(ICCV2/ICC4958)SNP_00149	448	T	C	4	3	--	--
	Ca(ICCV2/ICC4958)SNP_00150	967	C	T	15	8	--	--
	Ca(ICCV2/ICC4958)SNP_00151	1726	C	G	5	3	--	--
	Ca(ICCV2/ICC4958)SNP_00152	1774	A	T	5	3	--	--
	Ca(ICCV2/ICC4958)SNP_00153	2707	C	T	3	4	--	--
	Ca(ICCV2/ICC4958)SNP_00154	2959	C	T	7	6	--	--
CakTC20005	Ca(ICCV2/ICC4958)SNP_00155	180	C	T	3	3	--	--
	Ca(ICCV2/ICC4958)SNP_00156	309	G	A	3	3	--	--
CakTC29229	Ca(ICCV2/ICC4958)SNP_00157	894	A	C	64	60	--	--
CakTC26561	Ca(ICCV2/ICC4958)SNP_00158	260	C	T	3	4	--	--
CakTC41423	Ca(ICCV2/ICC4958)SNP_00159	902	C	T	11	10	--	--
	Ca(ICCV2/ICC4958)SNP_00160	1564	G	A	6	6	--	--
	Ca(ICCV2/ICC4958)SNP_00161	1571	T	C	7	5	--	--
CakTC38895	Ca(ICCV2/ICC4958)SNP_00162	545	C	T	38	15	--	--
	Ca(ICCV2/ICC4958)SNP_00163	584	C	A	41	17	--	--
	Ca(ICCV2/ICC4958)SNP_00164	590	T	C	43	16	--	--
CakTC37634	Ca(ICCV2/ICC4958)SNP_00165	68	G	A	4	4	--	--
	Ca(ICCV2/ICC4958)SNP_00166	383	C	A	11	11	--	--
	Ca(ICCV2/ICC4958)SNP_00167	595	C	T	8	13	--	--
	Ca(ICCV2/ICC4958)SNP_00168	611	A	G	6	11	--	--
	Ca(ICCV2/ICC4958)SNP_00169	647	A	G	13	12	--	--
CakTC31897	Ca(ICCV2/ICC4958)SNP_00170	3625	G	A	9	12	--	--
CakTC37319	Ca(ICCV2/ICC4958)SNP_00171	47	C	G	4	9	--	--
	Ca(ICCV2/ICC4958)SNP_00172	242	G	A	10	20	--	--
	Ca(ICCV2/ICC4958)SNP_00173	360	T	C	10	25	--	--
	Ca(ICCV2/ICC4958)SNP_00174	1108	G	A	19	26	--	--
CakTC32023	Ca(ICCV2/ICC4958)SNP_00175	415	C	G	4	3	--	--
	Ca(ICCV2/ICC4958)SNP_00176	533	T	C	3	3	--	--
CakTC39596	Ca(ICCV2/ICC4958)SNP_00177	180	T	A	20	16	--	--
	Ca(ICCV2/ICC4958)SNP_00178	276	C	T	19	18	--	--
	Ca(ICCV2/ICC4958)SNP_00179	806	G	A	9	14	--	--
	Ca(ICCV2/ICC4958)SNP_00180	952	C	G	16	16	--	--
CakTC23119	Ca(ICCV2/ICC4958)SNP_00181	1022	T	G	18	9	--	--
	Ca(ICCV2/ICC4958)SNP_00182	1029	A	T	18	9	--	--
	Ca(ICCV2/ICC4958)SNP_00183	1041	C	G	16	9	--	--
CakTC23892	Ca(ICCV2/ICC4958)SNP_00184	200	T	A	296	298	--	--
	Ca(ICCV2/ICC4958)SNP_00185	266	C	T	377	339	--	--
	Ca(ICCV2/ICC4958)SNP_00186	398	C	T	368	360	--	--
	Ca(ICCV2/ICC4958)SNP_00187	413	G	C	373	368	--	--
	Ca(ICCV2/ICC4958)SNP_00188	678	T	C	461	357	--	--
	Ca(ICCV2/ICC4958)SNP_00189	777	A	G	528	424	--	--
	Ca(ICCV2/ICC4958)SNP_00190	978	G	T	579	452	--	--
	Ca(ICCV2/ICC4958)SNP_00191	1344	G	T	531	465	--	--
	Ca(ICCV2/ICC4958)SNP_00192	1699	A	T	314	375	--	--
CakTC41505	Ca(ICCV2/ICC4958)SNP_00193	628	A	T	44	27	--	--
	Ca(ICCV2/ICC4958)SNP_00194	691	A	G	27	14	--	--
CakTC28528	Ca(ICCV2/ICC4958)SNP_00195	301	G	A	22	23	--	--
CakTC24442	Ca(ICCV2/ICC4958)SNP_00196	1578	C	G	28	31	--	--
CakTC16500	Ca(ICCV2/ICC4958)SNP_00197	2064	G	A	3	3	--	--
CakTC29895	Ca(ICCV2/ICC4958)SNP_00198	1069	T	C	16	13	--	--
CakTC38663	Ca(ICCV2/ICC4958)SNP_00199	1395	G	A	5	9	--	--
CakTC27233	Ca(ICCV2/ICC4958)SNP_00200	2698	C	A	36	23	--	--
CakTC35429	Ca(ICCV2/ICC4958)SNP_00201	1379	C	T	15	19	--	--
CakTC29297	Ca(ICCV2/ICC4958)SNP_00202	198	A	T	3	4	--	HRT
	Ca(ICCV2/ICC4958)SNP_00203	200	G	C	3	4	--	HRT
CakTC31950	Ca(ICCV2/ICC4958)SNP_00204	358	G	A	3	14	Root	--
	Ca(ICCV2/ICC4958)SNP_00205	506	C	A	3	6	Root	--
CakTC08592	Ca(ICCV2/ICC4958)SNP_00206	374	G	A	8	11	--	--
	Ca(ICCV2/ICC4958)SNP_00207	427	T	A	11	12	--	--
	Ca(ICCV2/ICC4958)SNP_00208	451	T	A	11	12	--	--
	Ca(ICCV2/ICC4958)SNP_00209	479	A	T	11	12	--	--
	Ca(ICCV2/ICC4958)SNP_00210	489	C	A	10	11	--	--
	Ca(ICCV2/ICC4958)SNP_00211	550	G	C	10	9	--	--
CakTC22605	Ca(ICCV2/ICC4958)SNP_00212	2848	A	G	4	11	--	--
CakTC24361	Ca(ICCV2/ICC4958)SNP_00213	1060	C	T	3	6	Young_pod	--
	Ca(ICCV2/ICC4958)SNP_00214	1096	C	T	3	9	Young_pod	--
	Ca(ICCV2/ICC4958)SNP_00215	1178	C	T	3	8	Young_pod	--
CakTC39411	Ca(ICCV2/ICC4958)SNP_00216	1109	A	C	4	11	--	--

CakTC24695	Ca(ICCV2/ICC4958)SNP_00217	1012	G	A	15	7	Mature Leaf	--
CakTC29219	Ca(ICCV2/ICC4958)SNP_00218	32	A	C	13	10	--	--
	Ca(ICCV2/ICC4958)SNP_00219	498	A	G	10	13	--	--
	Ca(ICCV2/ICC4958)SNP_00220	594	C	T	11	10	--	--
	Ca(ICCV2/ICC4958)SNP_00221	657	T	C	9	9	--	--
	Ca(ICCV2/ICC4958)SNP_00222	721	T	G	8	9	--	--
CakTC41707	Ca(ICCV2/ICC4958)SNP_00223	2586	G	A	6	9	--	--
CakTC43139	Ca(ICCV2/ICC4958)SNP_00224	366	C	T	27	30	--	--
	Ca(ICCV2/ICC4958)SNP_00225	427	T	A	19	26	--	--
CakTC20209	Ca(ICCV2/ICC4958)SNP_00226	137	G	A	3	7	Young_pod	--
CakTC34774	Ca(ICCV2/ICC4958)SNP_00227	672	C	T	7	9	--	--
CakTC17161	Ca(ICCV2/ICC4958)SNP_00228	159	C	A	3	4	--	--
	Ca(ICCV2/ICC4958)SNP_00229	163	G	A	3	4	--	--
CakTC30407	Ca(ICCV2/ICC4958)SNP_00230	1579	A	G	3	6	--	--
CakTC29741	Ca(ICCV2/ICC4958)SNP_00231	1415	C	T	6	10	--	--
	Ca(ICCV2/ICC4958)SNP_00232	1588	G	C	4	8	--	--
CakTC33866	Ca(ICCV2/ICC4958)SNP_00233	986	A	T	6	5	--	--
CakTC37968	Ca(ICCV2/ICC4958)SNP_00234	194	C	T	5	7	--	--
CakTC16394	Ca(ICCV2/ICC4958)SNP_00235	70	G	C	3	3	--	--
	Ca(ICCV2/ICC4958)SNP_00236	91	C	T	3	3	--	--
CakTC38349	Ca(ICCV2/ICC4958)SNP_00237	555	C	T	5	33	--	--
	Ca(ICCV2/ICC4958)SNP_00238	765	A	G	16	62	--	--
	Ca(ICCV2/ICC4958)SNP_00239	1147	G	A	4	3	--	--
	Ca(ICCV2/ICC4958)SNP_00240	1175	C	T	4	4	--	--
CakTC13613	Ca(ICCV2/ICC4958)SNP_00241	333	C	T	8	3	--	--
	Ca(ICCV2/ICC4958)SNP_00242	339	C	A	8	3	--	--
CakTC32730	Ca(ICCV2/ICC4958)SNP_00243	642	T	C	7	3	--	--
	Ca(ICCV2/ICC4958)SNP_00244	955	G	C	5	5	--	--
	Ca(ICCV2/ICC4958)SNP_00245	1399	A	G	10	3	--	--
	Ca(ICCV2/ICC4958)SNP_00246	2103	A	G	9	5	--	--
CakTC40607	Ca(ICCV2/ICC4958)SNP_00247	845	A	G	8	19	--	--
CakTC41933	Ca(ICCV2/ICC4958)SNP_00248	713	T	C	14	5	--	--
CakTC42064	Ca(ICCV2/ICC4958)SNP_00249	445	C	G	27	7	--	--
CakTC35408	Ca(ICCV2/ICC4958)SNP_00250	1415	G	C	8	6	--	--
CakTC42116	Ca(ICCV2/ICC4958)SNP_00251	396	T	C	4	4	Flower bud	--
	Ca(ICCV2/ICC4958)SNP_00252	429	C	T	4	4	Flower bud	--
	Ca(ICCV2/ICC4958)SNP_00253	733	A	G	4	17	Flower bud	--
CakTC25884	Ca(ICCV2/ICC4958)SNP_00254	528	C	T	13	7	Young_pod	--
	Ca(ICCV2/ICC4958)SNP_00255	636	A	G	15	7	Young_pod	--
	Ca(ICCV2/ICC4958)SNP_00256	734	C	T	9	6	Young_pod	--
CakTC10574	Ca(ICCV2/ICC4958)SNP_00257	86	C	A	5	7	--	--
	Ca(ICCV2/ICC4958)SNP_00258	150	C	G	5	7	--	--
CakTC26020	Ca(ICCV2/ICC4958)SNP_00259	350	T	C	50	142	--	--
CakTC38231	Ca(ICCV2/ICC4958)SNP_00260	1118	A	G	4	4	--	--
CakTC40053	Ca(ICCV2/ICC4958)SNP_00261	978	A	G	52	29	--	--
CakTC25919	Ca(ICCV2/ICC4958)SNP_00262	571	T	C	5	15	--	--
CakTC11774	Ca(ICCV2/ICC4958)SNP_00263	660	A	G	8	4	--	--
	Ca(ICCV2/ICC4958)SNP_00264	696	G	A	11	4	--	--
	Ca(ICCV2/ICC4958)SNP_00265	1311	A	C	7	11	--	--
CakTC34840	Ca(ICCV2/ICC4958)SNP_00266	809	A	G	7	8	--	--
	Ca(ICCV2/ICC4958)SNP_00267	824	A	G	12	11	--	--
	Ca(ICCV2/ICC4958)SNP_00268	875	C	T	10	12	--	--
	Ca(ICCV2/ICC4958)SNP_00269	1107	G	A	12	11	--	--
	Ca(ICCV2/ICC4958)SNP_00270	1247	C	T	13	17	--	--
	Ca(ICCV2/ICC4958)SNP_00271	1599	A	T	4	13	--	--
	Ca(ICCV2/ICC4958)SNP_00272	1730	A	G	9	6	--	--
	Ca(ICCV2/ICC4958)SNP_00273	2203	C	T	9	5	--	--
	Ca(ICCV2/ICC4958)SNP_00274	2488	C	T	4	11	--	--
	Ca(ICCV2/ICC4958)SNP_00275	2494	A	G	3	11	--	--
	Ca(ICCV2/ICC4958)SNP_00276	2595	A	G	3	9	--	--
	Ca(ICCV2/ICC4958)SNP_00277	2633	G	A	6	8	--	--
	Ca(ICCV2/ICC4958)SNP_00278	2766	A	G	7	11	--	--
	Ca(ICCV2/ICC4958)SNP_00279	2852	T	C	10	11	--	--
	Ca(ICCV2/ICC4958)SNP_00280	2870	C	T	11	9	--	--
	Ca(ICCV2/ICC4958)SNP_00281	3082	G	A	10	17	--	--
	Ca(ICCV2/ICC4958)SNP_00282	3169	G	C	12	18	--	--
	Ca(ICCV2/ICC4958)SNP_00283	3535	T	C	10	22	--	--
	Ca(ICCV2/ICC4958)SNP_00284	3645	A	G	7	12	--	--
	Ca(ICCV2/ICC4958)SNP_00285	3863	G	T	4	5	--	--
CakTC33548	Ca(ICCV2/ICC4958)SNP_00286	418	G	A	8	12	--	--
CakTC22807	Ca(ICCV2/ICC4958)SNP_00287	4584	G	A	4	7	--	--
CakTC36305	Ca(ICCV2/ICC4958)SNP_00288	971	G	T	13	6	--	--
CakTC13792	Ca(ICCV2/ICC4958)SNP_00289	282	C	T	10	3	--	--
CakTC30590	Ca(ICCV2/ICC4958)SNP_00290	1368	C	G	3	3	Flower bud	--

CakTC42575	Ca(ICCV2/ICC4958)SNP_00291	854	G	A	5	8	--	--
	Ca(ICCV2/ICC4958)SNP_00292	974	C	T	6	6	--	--
CakTC27209	Ca(ICCV2/ICC4958)SNP_00293	145	C	T	10	11	--	--
	Ca(ICCV2/ICC4958)SNP_00294	181	A	G	13	14	--	--
	Ca(ICCV2/ICC4958)SNP_00295	385	G	C	17	54	--	--
	Ca(ICCV2/ICC4958)SNP_00296	642	C	T	47	77	--	--
	Ca(ICCV2/ICC4958)SNP_00297	986	G	A	59	47	--	--
CakTC29587	Ca(ICCV2/ICC4958)SNP_00298	1157	A	G	4	3	Shoot	--
CakTC39399	Ca(ICCV2/ICC4958)SNP_00299	992	A	C	11	17	--	--
CakTC38341	Ca(ICCV2/ICC4958)SNP_00300	2930	C	T	3	5	--	--
CakTC26917	Ca(ICCV2/ICC4958)SNP_00301	403	A	T	7	13	--	--
	Ca(ICCV2/ICC4958)SNP_00302	588	C	T	4	17	--	--
	Ca(ICCV2/ICC4958)SNP_00303	762	T	C	5	9	--	--
CakTC18498	Ca(ICCV2/ICC4958)SNP_00304	900	C	T	3	5	--	C2H2
CakTC18903	Ca(ICCV2/ICC4958)SNP_00305	3497	C	G	5	3	--	--
CakTC29853	Ca(ICCV2/ICC4958)SNP_00306	578	A	G	4	3	--	--
	Ca(ICCV2/ICC4958)SNP_00307	613	A	C	4	3	--	--
	Ca(ICCV2/ICC4958)SNP_00308	622	T	C	3	3	--	--
	Ca(ICCV2/ICC4958)SNP_00309	723	C	G	4	3	--	--
	Ca(ICCV2/ICC4958)SNP_00310	743	G	A	4	3	--	--
	Ca(ICCV2/ICC4958)SNP_00311	746	A	T	4	3	--	--
CakTC23055	Ca(ICCV2/ICC4958)SNP_00312	94	C	T	3	7	--	--
	Ca(ICCV2/ICC4958)SNP_00313	97	T	C	3	7	--	--
	Ca(ICCV2/ICC4958)SNP_00314	126	T	C	3	7	--	--
CakTC30888	Ca(ICCV2/ICC4958)SNP_00315	517	G	A	5	3	--	bHLH
	Ca(ICCV2/ICC4958)SNP_00316	1470	T	C	11	13	--	bHLH
CakTC26987	Ca(ICCV2/ICC4958)SNP_00317	622	T	C	16	7	--	--
	Ca(ICCV2/ICC4958)SNP_00318	640	G	A	16	6	--	--
	Ca(ICCV2/ICC4958)SNP_00319	1189	A	G	13	3	--	--
	Ca(ICCV2/ICC4958)SNP_00320	1892	T	G	5	7	--	--
CakTC38056	Ca(ICCV2/ICC4958)SNP_00321	962	A	G	3	8	--	--
CakTC42472	Ca(ICCV2/ICC4958)SNP_00322	817	C	T	5	8	--	--
	Ca(ICCV2/ICC4958)SNP_00323	885	G	A	4	7	--	--
CakTC31791	Ca(ICCV2/ICC4958)SNP_00324	1207	T	C	10	10	--	--
CakTC09751	Ca(ICCV2/ICC4958)SNP_00325	3972	G	T	4	5	--	--
CakTC31351	Ca(ICCV2/ICC4958)SNP_00326	889	A	G	5	4	Flower bud	MYB
CakTC42877	Ca(ICCV2/ICC4958)SNP_00327	52	G	A	10	3	--	--
	Ca(ICCV2/ICC4958)SNP_00328	173	A	T	21	7	--	--
	Ca(ICCV2/ICC4958)SNP_00329	585	A	G	44	46	--	--
CakTC38412	Ca(ICCV2/ICC4958)SNP_00330	430	T	C	14	27	--	--
CakTC25453	Ca(ICCV2/ICC4958)SNP_00331	191	C	T	3	9	--	--
CakTC38074	Ca(ICCV2/ICC4958)SNP_00332	353	G	T	5	5	--	--
	Ca(ICCV2/ICC4958)SNP_00333	908	C	A	4	8	--	--
CakTC29185	Ca(ICCV2/ICC4958)SNP_00334	518	C	G	8	3	--	--
	Ca(ICCV2/ICC4958)SNP_00335	529	G	A	8	5	--	--
	Ca(ICCV2/ICC4958)SNP_00336	620	C	G	3	4	--	--
CakTC33952	Ca(ICCV2/ICC4958)SNP_00337	771	T	A	14	5	Shoot	MYB
CakTC39762	Ca(ICCV2/ICC4958)SNP_00338	36	C	A	6	8	--	--
CakTC33125	Ca(ICCV2/ICC4958)SNP_00339	124	G	A	18	21	--	--
	Ca(ICCV2/ICC4958)SNP_00340	239	G	A	47	30	--	--
	Ca(ICCV2/ICC4958)SNP_00341	437	A	T	50	41	--	--
	Ca(ICCV2/ICC4958)SNP_00342	1028	A	C	55	42	--	--
	Ca(ICCV2/ICC4958)SNP_00343	1078	A	G	52	47	--	--
	Ca(ICCV2/ICC4958)SNP_00344	1637	A	G	34	33	--	--
	Ca(ICCV2/ICC4958)SNP_00345	1672	G	C	37	36	--	--
	Ca(ICCV2/ICC4958)SNP_00346	1673	G	A	36	36	--	--
CakTC35076	Ca(ICCV2/ICC4958)SNP_00347	1121	C	A	14	9	--	--
CakTC28555	Ca(ICCV2/ICC4958)SNP_00348	1382	G	C	17	8	--	--
CakTC27339	Ca(ICCV2/ICC4958)SNP_00349	1546	G	A	19	24	--	--
CakTC39061	Ca(ICCV2/ICC4958)SNP_00350	285	C	A	11	5	--	--
	Ca(ICCV2/ICC4958)SNP_00351	350	T	C	11	10	--	--
	Ca(ICCV2/ICC4958)SNP_00352	627	A	G	8	11	--	--
	Ca(ICCV2/ICC4958)SNP_00353	794	C	T	10	11	--	--
	Ca(ICCV2/ICC4958)SNP_00354	1177	C	T	20	7	--	--
	Ca(ICCV2/ICC4958)SNP_00355	1198	G	A	19	8	--	--
	Ca(ICCV2/ICC4958)SNP_00356	1338	A	T	20	10	--	--
	Ca(ICCV2/ICC4958)SNP_00357	1430	T	C	22	11	--	--
	Ca(ICCV2/ICC4958)SNP_00358	1441	C	G	21	11	--	--
	Ca(ICCV2/ICC4958)SNP_00359	1446	T	C	21	11	--	--
	Ca(ICCV2/ICC4958)SNP_00360	1480	G	A	21	9	--	--
	Ca(ICCV2/ICC4958)SNP_00361	1487	C	T	19	9	--	--
CakTC40639	Ca(ICCV2/ICC4958)SNP_00362	153	G	C	9	11	--	--
CakTC23851	Ca(ICCV2/ICC4958)SNP_00363	659	T	A	7	9	--	--
	Ca(ICCV2/ICC4958)SNP_00364	1511	G	A	8	11	--	--

	Ca(ICCV2/ICC4958)SNP_00365	2056	A	G	4	6	--	--
	Ca(ICCV2/ICC4958)SNP_00366	2123	C	A	3	4	--	--
CakTC30394	Ca(ICCV2/ICC4958)SNP_00367	1495	A	G	26	29	--	--
CakTC35229	Ca(ICCV2/ICC4958)SNP_00368	225	T	C	5	3	--	--
	Ca(ICCV2/ICC4958)SNP_00369	1956	A	G	8	5	--	--
	Ca(ICCV2/ICC4958)SNP_00370	2731	T	C	3	3	--	--
CakTC23861	Ca(ICCV2/ICC4958)SNP_00371	376	C	A	4	8	--	AUX/IAA
CakTC28536	Ca(ICCV2/ICC4958)SNP_00372	928	T	G	22	12	--	NAC
CakTC29356	Ca(ICCV2/ICC4958)SNP_00373	247	C	T	3	4	--	NAC
	Ca(ICCV2/ICC4958)SNP_00374	287	C	T	14	4	--	NAC
	Ca(ICCV2/ICC4958)SNP_00375	330	A	G	15	4	--	NAC
	Ca(ICCV2/ICC4958)SNP_00376	389	A	G	13	4	--	NAC
	Ca(ICCV2/ICC4958)SNP_00377	392	A	G	12	4	--	NAC
CakTC26949	Ca(ICCV2/ICC4958)SNP_00378	1636	G	A	4	9	--	--
	Ca(ICCV2/ICC4958)SNP_00379	2500	A	G	29	14	--	--
CakTC33153	Ca(ICCV2/ICC4958)SNP_00380	3078	T	A	22	26	--	--
CakTC34278	Ca(ICCV2/ICC4958)SNP_00381	16	T	A	3	4	--	--
CakTC37569	Ca(ICCV2/ICC4958)SNP_00382	142	A	G	9	10	--	--
	Ca(ICCV2/ICC4958)SNP_00383	172	T	C	9	10	--	--
	Ca(ICCV2/ICC4958)SNP_00384	804	A	G	5	13	--	--
CakTC07323	Ca(ICCV2/ICC4958)SNP_00385	1189	G	A	4	3	--	--
CakTC30647	Ca(ICCV2/ICC4958)SNP_00386	600	A	C	9	5	--	--
CakTC32345	Ca(ICCV2/ICC4958)SNP_00387	1244	T	C	10	5	--	--
CakTC10541	Ca(ICCV2/ICC4958)SNP_00388	188	G	A	3	3	--	--
CakTC36198	Ca(ICCV2/ICC4958)SNP_00389	875	G	A	15	6	--	--
	Ca(ICCV2/ICC4958)SNP_00390	1596	G	A	6	7	--	--
CakTC30780	Ca(ICCV2/ICC4958)SNP_00391	1696	A	C	3	5	--	--
CakTC41150	Ca(ICCV2/ICC4958)SNP_00392	907	G	T	4	6	--	--
CakTC42271	Ca(ICCV2/ICC4958)SNP_00393	928	C	T	6	4	--	--
CakTC22913	Ca(ICCV2/ICC4958)SNP_00394	1038	G	A	3	3	--	--
CakTC30128	Ca(ICCV2/ICC4958)SNP_00395	461	G	C	4	3	--	--
CakTC39434	Ca(ICCV2/ICC4958)SNP_00396	474	A	G	16	23	--	--
CakTC42035	Ca(ICCV2/ICC4958)SNP_00397	29	G	C	7	6	--	--
	Ca(ICCV2/ICC4958)SNP_00398	73	T	C	8	7	--	--
	Ca(ICCV2/ICC4958)SNP_00399	203	C	T	9	9	--	--
CakTC12283	Ca(ICCV2/ICC4958)SNP_00400	131	A	G	3	4	--	--
CakTC28092	Ca(ICCV2/ICC4958)SNP_00401	311	C	T	8	9	--	--
CakTC29337	Ca(ICCV2/ICC4958)SNP_00402	231	G	C	3	4	--	--
	Ca(ICCV2/ICC4958)SNP_00403	303	G	A	3	5	--	--
CakTC18325	Ca(ICCV2/ICC4958)SNP_00404	1122	T	G	8	5	--	--
CakTC31095	Ca(ICCV2/ICC4958)SNP_00405	169	A	T	3	4	--	--
	Ca(ICCV2/ICC4958)SNP_00406	211	A	T	6	5	--	--
	Ca(ICCV2/ICC4958)SNP_00407	304	C	T	6	6	--	--
	Ca(ICCV2/ICC4958)SNP_00408	460	G	A	7	5	--	--
	Ca(ICCV2/ICC4958)SNP_00409	898	C	A	3	4	--	--
	Ca(ICCV2/ICC4958)SNP_00410	974	G	A	3	4	--	--
	Ca(ICCV2/ICC4958)SNP_00411	985	G	T	3	4	--	--
	Ca(ICCV2/ICC4958)SNP_00412	1052	C	A	3	4	--	--
CakTC35131	Ca(ICCV2/ICC4958)SNP_00413	378	C	T	8	8	--	--
	Ca(ICCV2/ICC4958)SNP_00414	1973	G	C	6	4	--	--
	Ca(ICCV2/ICC4958)SNP_00415	1997	T	G	5	5	--	--
CakTC10326	Ca(ICCV2/ICC4958)SNP_00416	388	C	T	13	11	--	--
	Ca(ICCV2/ICC4958)SNP_00417	611	T	C	9	7	--	--
	Ca(ICCV2/ICC4958)SNP_00418	1278	T	G	18	17	--	--
CakTC29071	Ca(ICCV2/ICC4958)SNP_00419	941	T	A	5	4	--	--
CakTC37106	Ca(ICCV2/ICC4958)SNP_00420	1751	T	C	3	9	--	--
CakTC41190	Ca(ICCV2/ICC4958)SNP_00421	280	G	C	13	27	--	--
CakTC41753	Ca(ICCV2/ICC4958)SNP_00422	660	C	T	14	20	--	--
CakTC27106	Ca(ICCV2/ICC4958)SNP_00423	1317	T	C	9	10	--	--
CakTC23673	Ca(ICCV2/ICC4958)SNP_00424	729	A	C	3	3	--	--
	Ca(ICCV2/ICC4958)SNP_00425	735	T	C	4	4	--	--
CakTC31227	Ca(ICCV2/ICC4958)SNP_00426	765	T	C	6	7	--	--
CakTC33643	Ca(ICCV2/ICC4958)SNP_00427	2995	T	C	5	7	--	SET
	Ca(ICCV2/ICC4958)SNP_00428	2996	G	T	5	7	--	SET
CakTC38945	Ca(ICCV2/ICC4958)SNP_00429	575	T	C	8	3	--	--
	Ca(ICCV2/ICC4958)SNP_00430	1117	C	T	10	10	--	--
	Ca(ICCV2/ICC4958)SNP_00431	1256	T	A	8	10	--	--
CakTC26213	Ca(ICCV2/ICC4958)SNP_00432	250	C	T	23	21	--	--
CakTC33899	Ca(ICCV2/ICC4958)SNP_00433	568	T	C	6	12	--	--
	Ca(ICCV2/ICC4958)SNP_00434	694	A	G	7	12	--	--
	Ca(ICCV2/ICC4958)SNP_00435	913	C	A	8	11	--	--
	Ca(ICCV2/ICC4958)SNP_00436	1309	G	A	8	11	--	--
	Ca(ICCV2/ICC4958)SNP_00437	1384	A	G	11	11	--	--
	Ca(ICCV2/ICC4958)SNP_00438	1590	G	A	12	12	--	--

CakTC27626	Ca(ICCV2/ICC4958)SNP_00439	839	G	A	4	9	--	--
CakTC31122	Ca(ICCV2/ICC4958)SNP_00440	1402	A	C	4	3	--	--
	Ca(ICCV2/ICC4958)SNP_00441	2580	C	G	6	6	--	--
CakTC37772	Ca(ICCV2/ICC4958)SNP_00442	879	T	C	5	6	--	--
	Ca(ICCV2/ICC4958)SNP_00443	1278	T	C	3	3	--	--
CakTC30378	Ca(ICCV2/ICC4958)SNP_00444	269	T	A	5	7	Shoot	--
	Ca(ICCV2/ICC4958)SNP_00445	270	T	C	5	6	Shoot	--
	Ca(ICCV2/ICC4958)SNP_00446	305	G	A	9	7	Shoot	--
	Ca(ICCV2/ICC4958)SNP_00447	355	T	A	12	7	Shoot	--
	Ca(ICCV2/ICC4958)SNP_00448	359	T	C	12	5	Shoot	--
CakTC39649	Ca(ICCV2/ICC4958)SNP_00449	1138	A	G	3	11	--	--
CakTC40942	Ca(ICCV2/ICC4958)SNP_00450	732	G	A	5	8	--	--
CakTC39423	Ca(ICCV2/ICC4958)SNP_00451	548	A	G	4	7	--	--
CakTC41722	Ca(ICCV2/ICC4958)SNP_00452	514	T	G	3	7	--	bZIP
CakTC35438	Ca(ICCV2/ICC4958)SNP_00453	3097	C	T	5	10	--	C3H
CakTC09306	Ca(ICCV2/ICC4958)SNP_00454	564	T	G	23	15	--	CCHC
	Ca(ICCV2/ICC4958)SNP_00455	610	A	G	25	10	--	CCHC
	Ca(ICCV2/ICC4958)SNP_00456	1339	G	A	12	12	--	CCHC
	Ca(ICCV2/ICC4958)SNP_00457	1357	C	T	12	8	--	CCHC
	Ca(ICCV2/ICC4958)SNP_00458	2082	T	C	16	8	--	CCHC
	Ca(ICCV2/ICC4958)SNP_00459	3370	A	G	6	8	--	CCHC
	Ca(ICCV2/ICC4958)SNP_00460	3419	C	T	6	6	--	CCHC
CakTC34318	Ca(ICCV2/ICC4958)SNP_00461	1691	C	T	12	25	--	--
	Ca(ICCV2/ICC4958)SNP_00462	1983	C	T	8	7	--	--
	Ca(ICCV2/ICC4958)SNP_00463	2010	G	C	8	6	--	--
CakTC09312	Ca(ICCV2/ICC4958)SNP_00464	474	G	T	3	3	--	--
CakTC32617	Ca(ICCV2/ICC4958)SNP_00465	529	A	T	6	15	--	--
CakTC34395	Ca(ICCV2/ICC4958)SNP_00466	61	T	C	9	12	--	--
CakTC30093	Ca(ICCV2/ICC4958)SNP_00467	1150	C	T	8	9	Root	--
	Ca(ICCV2/ICC4958)SNP_00468	1174	G	A	9	9	Root	--
	Ca(ICCV2/ICC4958)SNP_00469	1264	G	A	10	12	Root	--
	Ca(ICCV2/ICC4958)SNP_00470	1405	T	C	14	14	Root	--
	Ca(ICCV2/ICC4958)SNP_00471	1641	C	T	10	6	Root	--
CakTC08642	Ca(ICCV2/ICC4958)SNP_00472	135	G	A	4	3	--	--
	Ca(ICCV2/ICC4958)SNP_00473	141	T	G	4	3	--	--
	Ca(ICCV2/ICC4958)SNP_00474	181	T	A	5	5	--	--
	Ca(ICCV2/ICC4958)SNP_00475	930	T	C	3	11	--	--
	Ca(ICCV2/ICC4958)SNP_00476	1019	C	T	3	9	--	--
	Ca(ICCV2/ICC4958)SNP_00477	2063	G	A	8	12	--	--
	Ca(ICCV2/ICC4958)SNP_00478	2067	T	C	8	12	--	--
CakTC09968	Ca(ICCV2/ICC4958)SNP_00479	1765	G	A	10	5	--	--
CakTC31771	Ca(ICCV2/ICC4958)SNP_00480	360	A	G	6	8	--	--
CakTC28369	Ca(ICCV2/ICC4958)SNP_00481	1749	G	A	5	8	--	--
	Ca(ICCV2/ICC4958)SNP_00482	1755	G	A	5	8	--	--
CakTC31885	Ca(ICCV2/ICC4958)SNP_00483	5461	A	G	12	34	--	--
	Ca(ICCV2/ICC4958)SNP_00484	8362	T	C	19	20	--	--
CakTC11371	Ca(ICCV2/ICC4958)SNP_00485	199	G	T	3	4	--	--
	Ca(ICCV2/ICC4958)SNP_00486	1260	T	C	5	4	--	--
CakTC33923	Ca(ICCV2/ICC4958)SNP_00487	77	T	C	11	10	--	--
CakTC38639	Ca(ICCV2/ICC4958)SNP_00488	1308	A	G	14	35	--	--
CakTC12500	Ca(ICCV2/ICC4958)SNP_00489	355	T	C	3	256	--	--
	Ca(ICCV2/ICC4958)SNP_00490	370	T	C	3	243	--	--
	Ca(ICCV2/ICC4958)SNP_00491	374	C	A	3	193	--	--
	Ca(ICCV2/ICC4958)SNP_00492	507	T	C	4	72	--	--
	Ca(ICCV2/ICC4958)SNP_00493	519	C	T	4	67	--	--
	Ca(ICCV2/ICC4958)SNP_00494	646	A	G	4	9	--	--
CakTC24620	Ca(ICCV2/ICC4958)SNP_00495	1830	T	C	7	13	--	--
	Ca(ICCV2/ICC4958)SNP_00496	2520	C	T	9	13	--	--
	Ca(ICCV2/ICC4958)SNP_00497	3205	T	A	4	6	--	--
CakTC29046	Ca(ICCV2/ICC4958)SNP_00498	451	A	T	4	3	Root	--
	Ca(ICCV2/ICC4958)SNP_00499	454	C	T	4	3	Root	--
	Ca(ICCV2/ICC4958)SNP_00500	463	C	G	4	3	Root	--
	Ca(ICCV2/ICC4958)SNP_00501	470	G	A	4	3	Root	--
	Ca(ICCV2/ICC4958)SNP_00502	482	G	A	4	4	Root	--
	Ca(ICCV2/ICC4958)SNP_00503	506	C	T	4	4	Root	--
CakTC27177	Ca(ICCV2/ICC4958)SNP_00504	828	G	A	7	11	Young_pod	--
	Ca(ICCV2/ICC4958)SNP_00505	1830	A	G	7	5	Young_pod	--
	Ca(ICCV2/ICC4958)SNP_00506	3014	T	C	4	3	Young_pod	--
	Ca(ICCV2/ICC4958)SNP_00507	3122	A	G	4	3	Young_pod	--
	Ca(ICCV2/ICC4958)SNP_00508	3295	A	G	3	3	Young_pod	--
	Ca(ICCV2/ICC4958)SNP_00509	3323	G	T	3	3	Young_pod	--
CakTC37669	Ca(ICCV2/ICC4958)SNP_00510	380	G	A	5	3	--	--
CakTC28361	Ca(ICCV2/ICC4958)SNP_00511	2658	G	A	5	9	Flower bud	--
CakTC10633	Ca(ICCV2/ICC4958)SNP_00512	171	T	C	3	3	--	--

	Ca(ICCV2/ICC4958)SNP_00513	217	T	G	3	3	--	--
	Ca(ICCV2/ICC4958)SNP_00514	711	G	A	5	3	--	--
CakTC35496	Ca(ICCV2/ICC4958)SNP_00515	3713	G	C	8	24	--	--
CakTC31819	Ca(ICCV2/ICC4958)SNP_00516	645	C	T	3	3	--	--
	Ca(ICCV2/ICC4958)SNP_00517	1069	A	G	3	3	--	--
CakTC32644	Ca(ICCV2/ICC4958)SNP_00518	521	T	C	3	3	--	--
CakTC29808	Ca(ICCV2/ICC4958)SNP_00519	1649	G	T	4	3	--	--
	Ca(ICCV2/ICC4958)SNP_00520	1658	G	A	5	3	--	--
	Ca(ICCV2/ICC4958)SNP_00521	1704	C	G	5	3	--	--
	Ca(ICCV2/ICC4958)SNP_00522	1745	C	T	4	3	--	--
	Ca(ICCV2/ICC4958)SNP_00523	1752	C	T	3	3	--	--
	Ca(ICCV2/ICC4958)SNP_00524	1785	T	C	3	3	--	--
CakTC32821	Ca(ICCV2/ICC4958)SNP_00525	2332	C	T	5	8	--	--
CakTC20145	Ca(ICCV2/ICC4958)SNP_00526	390	A	G	3	3	--	--
CakTC29435	Ca(ICCV2/ICC4958)SNP_00527	163	T	C	18	36	--	--
	Ca(ICCV2/ICC4958)SNP_00528	223	A	G	34	54	--	--
	Ca(ICCV2/ICC4958)SNP_00529	307	A	G	129	156	--	--
	Ca(ICCV2/ICC4958)SNP_00530	400	T	G	188	219	--	--
	Ca(ICCV2/ICC4958)SNP_00531	471	C	T	225	289	--	--
	Ca(ICCV2/ICC4958)SNP_00532	631	C	G	217	369	--	--
CakTC33469	Ca(ICCV2/ICC4958)SNP_00533	200	G	A	12	8	--	--
	Ca(ICCV2/ICC4958)SNP_00534	338	C	T	15	12	--	--
	Ca(ICCV2/ICC4958)SNP_00535	464	C	T	15	6	--	--
CakTC11238	Ca(ICCV2/ICC4958)SNP_00536	105	A	T	14	11	--	--
CakTC31451	Ca(ICCV2/ICC4958)SNP_00537	1053	G	C	12	4	--	--
CakTC24891	Ca(ICCV2/ICC4958)SNP_00538	242	A	G	11	7	--	--
CakTC32017	Ca(ICCV2/ICC4958)SNP_00539	97	A	G	5	7	--	--
	Ca(ICCV2/ICC4958)SNP_00540	951	G	A	6	4	--	--
	Ca(ICCV2/ICC4958)SNP_00541	991	T	C	6	3	--	--
	Ca(ICCV2/ICC4958)SNP_00542	1501	G	A	8	6	--	--
	Ca(ICCV2/ICC4958)SNP_00543	2192	T	A	6	10	--	--
	Ca(ICCV2/ICC4958)SNP_00544	2320	C	T	3	3	--	--
	Ca(ICCV2/ICC4958)SNP_00545	2334	A	G	4	3	--	--
CakTC40447	Ca(ICCV2/ICC4958)SNP_00546	374	G	A	25	8	--	--
	Ca(ICCV2/ICC4958)SNP_00547	407	C	T	26	9	--	--
	Ca(ICCV2/ICC4958)SNP_00548	422	G	A	29	8	--	--
	Ca(ICCV2/ICC4958)SNP_00549	437	A	G	24	8	--	--
	Ca(ICCV2/ICC4958)SNP_00550	576	G	A	31	8	--	--
	Ca(ICCV2/ICC4958)SNP_00551	584	A	G	33	8	--	--
	Ca(ICCV2/ICC4958)SNP_00552	681	T	C	29	6	--	--
	Ca(ICCV2/ICC4958)SNP_00553	689	C	A	26	4	--	--
	Ca(ICCV2/ICC4958)SNP_00554	719	T	G	22	5	--	--
	Ca(ICCV2/ICC4958)SNP_00555	751	A	G	20	4	--	--
	Ca(ICCV2/ICC4958)SNP_00556	875	C	T	13	3	--	--
	Ca(ICCV2/ICC4958)SNP_00557	897	C	A	14	3	--	--
CakTC28337	Ca(ICCV2/ICC4958)SNP_00558	409	G	A	3	3	Root	--
	Ca(ICCV2/ICC4958)SNP_00559	580	T	C	3	4	Root	--
	Ca(ICCV2/ICC4958)SNP_00560	2028	G	C	14	7	Root	--
	Ca(ICCV2/ICC4958)SNP_00561	2085	C	T	17	9	Root	--
	Ca(ICCV2/ICC4958)SNP_00562	2398	A	C	14	3	Root	--
	Ca(ICCV2/ICC4958)SNP_00563	2439	A	C	14	4	Root	--
	Ca(ICCV2/ICC4958)SNP_00564	2693	T	C	16	5	Root	--
	Ca(ICCV2/ICC4958)SNP_00565	2706	A	T	9	5	Root	--
	Ca(ICCV2/ICC4958)SNP_00566	2724	T	C	16	6	Root	--
	Ca(ICCV2/ICC4958)SNP_00567	3140	G	A	4	4	Root	--
CakTC00750	Ca(ICCV2/ICC4958)SNP_00568	297	T	A	3	4	--	--
CakTC09869	Ca(ICCV2/ICC4958)SNP_00569	2219	C	T	4	6	--	--
CakTC43213	Ca(ICCV2/ICC4958)SNP_00570	163	G	A	4	5	--	--
CakTC38024	Ca(ICCV2/ICC4958)SNP_00571	38	G	C	23	31	--	--
	Ca(ICCV2/ICC4958)SNP_00572	487	C	T	41	39	--	--
	Ca(ICCV2/ICC4958)SNP_00573	676	T	A	46	34	--	--
	Ca(ICCV2/ICC4958)SNP_00574	1123	C	T	34	63	--	--
	Ca(ICCV2/ICC4958)SNP_00575	1432	T	A	19	25	--	--
CakTC11410	Ca(ICCV2/ICC4958)SNP_00576	241	G	C	7	17	--	--
	Ca(ICCV2/ICC4958)SNP_00577	576	G	A	13	10	--	--
CakTC10476	Ca(ICCV2/ICC4958)SNP_00578	380	G	A	3	3	--	--
	Ca(ICCV2/ICC4958)SNP_00579	404	A	G	3	3	--	--
CakTC42946	Ca(ICCV2/ICC4958)SNP_00580	580	A	G	3	5	--	--
	Ca(ICCV2/ICC4958)SNP_00581	786	T	C	3	7	--	--
	Ca(ICCV2/ICC4958)SNP_00582	880	A	G	5	11	--	--
CakTC40437	Ca(ICCV2/ICC4958)SNP_00583	1963	C	T	4	3	--	--
CakTC35659	Ca(ICCV2/ICC4958)SNP_00584	80	C	G	12	10	--	--
CakTC34994	Ca(ICCV2/ICC4958)SNP_00585	4215	G	A	4	4	--	--
CakTC28206	Ca(ICCV2/ICC4958)SNP_00586	460	A	T	16	19	--	--

	Ca(ICCV2/ICC4958)SNP_00587	1216	C	T	28	12	--	--
	Ca(ICCV2/ICC4958)SNP_00588	1252	T	C	20	9	--	--
	Ca(ICCV2/ICC4958)SNP_00589	1694	T	C	12	16	--	--
CakTC25149	Ca(ICCV2/ICC4958)SNP_00590	781	C	T	54	43	--	--
CakTC40643	Ca(ICCV2/ICC4958)SNP_00591	633	G	A	9	19	--	--
CakTC08979	Ca(ICCV2/ICC4958)SNP_00592	1712	T	A	4	3	--	--
	Ca(ICCV2/ICC4958)SNP_00593	1984	T	C	3	5	--	--
	Ca(ICCV2/ICC4958)SNP_00594	1988	C	T	3	4	--	--
CakTC23920	Ca(ICCV2/ICC4958)SNP_00595	2775	G	A	4	11	--	--
CakTC25449	Ca(ICCV2/ICC4958)SNP_00596	735	T	C	18	8	--	ARF
CakTC32689	Ca(ICCV2/ICC4958)SNP_00597	578	A	G	5	12	--	--
CakTC33458	Ca(ICCV2/ICC4958)SNP_00598	34	T	C	4	6	--	--
	Ca(ICCV2/ICC4958)SNP_00599	539	C	T	25	25	--	--
	Ca(ICCV2/ICC4958)SNP_00600	588	G	C	24	32	--	--
	Ca(ICCV2/ICC4958)SNP_00601	651	T	C	27	33	--	--
CakTC32025	Ca(ICCV2/ICC4958)SNP_00602	409	C	T	3	5	--	GeBP
CakTC09183	Ca(ICCV2/ICC4958)SNP_00603	2826	A	G	3	4	--	--
CakTC10569	Ca(ICCV2/ICC4958)SNP_00604	281	A	G	4	5	Root	--
CakTC38093	Ca(ICCV2/ICC4958)SNP_00605	365	G	T	7	8	--	--
CakTC32334	Ca(ICCV2/ICC4958)SNP_00606	1344	T	G	7	13	--	--
CakTC12042	Ca(ICCV2/ICC4958)SNP_00607	153	T	C	3	7	--	--
CakTC39733	Ca(ICCV2/ICC4958)SNP_00608	53	A	G	6	3	--	--
	Ca(ICCV2/ICC4958)SNP_00609	341	C	T	5	6	--	--
	Ca(ICCV2/ICC4958)SNP_00610	998	A	T	6	3	--	--
CakTC24619	Ca(ICCV2/ICC4958)SNP_00611	1187	A	T	6	4	--	--
	Ca(ICCV2/ICC4958)SNP_00612	1872	G	A	7	6	--	--
	Ca(ICCV2/ICC4958)SNP_00613	2328	C	G	8	8	--	--
	Ca(ICCV2/ICC4958)SNP_00614	2562	A	G	13	12	--	--
	Ca(ICCV2/ICC4958)SNP_00615	3012	T	C	8	11	--	--
	Ca(ICCV2/ICC4958)SNP_00616	4024	C	T	8	4	--	--
	Ca(ICCV2/ICC4958)SNP_00617	4089	A	G	11	7	--	--
CakTC14889	Ca(ICCV2/ICC4958)SNP_00618	1931	A	G	3	3	--	--
CakTC02884	Ca(ICCV2/ICC4958)SNP_00619	483	C	T	5	3	--	--
	Ca(ICCV2/ICC4958)SNP_00620	538	T	C	4	3	--	--
	Ca(ICCV2/ICC4958)SNP_00621	565	C	T	3	3	--	--
	Ca(ICCV2/ICC4958)SNP_00622	1898	T	C	3	4	--	--
CakTC39127	Ca(ICCV2/ICC4958)SNP_00623	274	G	A	21	8	--	bZIP
	Ca(ICCV2/ICC4958)SNP_00624	1171	T	G	24	41	--	bZIP
CakTC09083	Ca(ICCV2/ICC4958)SNP_00625	1411	T	G	8	3	--	--
CakTC25879	Ca(ICCV2/ICC4958)SNP_00626	107	C	T	15	37	--	--
	Ca(ICCV2/ICC4958)SNP_00627	140	C	A	15	37	--	--
	Ca(ICCV2/ICC4958)SNP_00628	182	T	C	14	40	--	--
	Ca(ICCV2/ICC4958)SNP_00629	224	C	A	13	40	--	--
	Ca(ICCV2/ICC4958)SNP_00630	308	A	C	10	28	--	--
CakTC38766	Ca(ICCV2/ICC4958)SNP_00631	56	G	C	14	14	--	--
	Ca(ICCV2/ICC4958)SNP_00632	1105	A	G	18	27	--	--
	Ca(ICCV2/ICC4958)SNP_00633	1200	G	A	10	13	--	--
CakTC43107	Ca(ICCV2/ICC4958)SNP_00634	837	C	T	12	11	--	HB
	Ca(ICCV2/ICC4958)SNP_00635	965	C	T	11	8	--	HB
	Ca(ICCV2/ICC4958)SNP_00636	1265	G	A	9	10	--	HB
	Ca(ICCV2/ICC4958)SNP_00637	1386	G	A	13	10	--	HB
	Ca(ICCV2/ICC4958)SNP_00638	1745	C	G	4	6	--	HB
	Ca(ICCV2/ICC4958)SNP_00639	1755	A	G	4	6	--	HB
	Ca(ICCV2/ICC4958)SNP_00640	1757	A	G	3	4	--	HB
CakTC11680	Ca(ICCV2/ICC4958)SNP_00641	1710	T	C	3	5	--	--
	Ca(ICCV2/ICC4958)SNP_00642	1818	G	A	3	3	--	--
CakTC28715	Ca(ICCV2/ICC4958)SNP_00643	390	T	C	11	7	--	--
CakTC38910	Ca(ICCV2/ICC4958)SNP_00644	933	C	T	6	3	Shoot	--
	Ca(ICCV2/ICC4958)SNP_00645	1225	T	A	4	8	Shoot	--
	Ca(ICCV2/ICC4958)SNP_00646	1289	A	G	3	7	Shoot	--
CakTC10926	Ca(ICCV2/ICC4958)SNP_00647	325	A	G	16	8	--	GRAS
	Ca(ICCV2/ICC4958)SNP_00648	502	G	A	10	5	--	GRAS
	Ca(ICCV2/ICC4958)SNP_00649	883	A	G	9	6	--	GRAS
	Ca(ICCV2/ICC4958)SNP_00650	907	T	C	7	5	--	GRAS
	Ca(ICCV2/ICC4958)SNP_00651	1161	C	T	5	3	--	GRAS
	Ca(ICCV2/ICC4958)SNP_00652	1260	A	G	7	4	--	GRAS
	Ca(ICCV2/ICC4958)SNP_00653	1315	C	T	5	3	--	GRAS
	Ca(ICCV2/ICC4958)SNP_00654	1426	A	G	3	3	--	GRAS
	Ca(ICCV2/ICC4958)SNP_00655	1473	C	T	5	3	--	GRAS
	Ca(ICCV2/ICC4958)SNP_00656	1694	A	C	10	3	--	GRAS
	Ca(ICCV2/ICC4958)SNP_00657	1758	G	A	10	3	--	GRAS
	Ca(ICCV2/ICC4958)SNP_00658	2471	T	A	10	6	--	GRAS
CakTC32144	Ca(ICCV2/ICC4958)SNP_00659	428	G	T	8	7	--	--
	Ca(ICCV2/ICC4958)SNP_00660	696	A	G	8	8	--	--

CakTC39397	Ca(ICCV2/ICC4958)SNP_00661	864	G	A	35	20	--	--
	Ca(ICCV2/ICC4958)SNP_00662	938	G	A	34	20	--	--
	Ca(ICCV2/ICC4958)SNP_00663	1025	T	A	28	17	--	--
	Ca(ICCV2/ICC4958)SNP_00664	1033	T	C	27	21	--	--
	Ca(ICCV2/ICC4958)SNP_00665	1232	C	T	9	17	--	--
	Ca(ICCV2/ICC4958)SNP_00666	1265	A	G	8	11	--	--
	Ca(ICCV2/ICC4958)SNP_00667	1280	G	C	8	11	--	--
	Ca(ICCV2/ICC4958)SNP_00668	1433	T	G	5	9	--	--
	Ca(ICCV2/ICC4958)SNP_00669	1467	C	T	5	9	--	--
CakTC38702	Ca(ICCV2/ICC4958)SNP_00670	8	G	A	3	4	--	--
CakTC34664	Ca(ICCV2/ICC4958)SNP_00671	738	C	A	4	6	--	--
CakTC08061	Ca(ICCV2/ICC4958)SNP_00672	2687	G	A	7	7	--	--
	Ca(ICCV2/ICC4958)SNP_00673	2696	C	T	7	6	--	--
CakTC41687	Ca(ICCV2/ICC4958)SNP_00674	1593	G	T	3	5	--	--
CakTC09432	Ca(ICCV2/ICC4958)SNP_00675	1538	G	A	3	7	--	--
CakTC29743	Ca(ICCV2/ICC4958)SNP_00676	275	A	C	5	8	--	--
	Ca(ICCV2/ICC4958)SNP_00677	292	A	G	6	9	--	--
	Ca(ICCV2/ICC4958)SNP_00678	418	G	A	7	8	--	--
	Ca(ICCV2/ICC4958)SNP_00679	493	G	A	6	8	--	--
CakTC08324	Ca(ICCV2/ICC4958)SNP_00680	239	C	T	36	68	--	--
	Ca(ICCV2/ICC4958)SNP_00681	347	A	G	39	83	--	--
CakTC22251	Ca(ICCV2/ICC4958)SNP_00682	340	G	A	3	3	--	--
CakTC30913	Ca(ICCV2/ICC4958)SNP_00683	503	T	G	7	3	--	--
CakTC14942	Ca(ICCV2/ICC4958)SNP_00684	205	T	C	4	4	--	--
CakTC26361	Ca(ICCV2/ICC4958)SNP_00685	181	T	C	3	3	--	--
CakTC39381	Ca(ICCV2/ICC4958)SNP_00686	466	C	T	23	28	--	--
	Ca(ICCV2/ICC4958)SNP_00687	684	T	C	29	18	--	--
	Ca(ICCV2/ICC4958)SNP_00688	865	A	G	25	19	--	--
	Ca(ICCV2/ICC4958)SNP_00689	874	C	T	28	20	--	--
CakTC25721	Ca(ICCV2/ICC4958)SNP_00690	2926	T	C	17	25	--	--
CakTC26607	Ca(ICCV2/ICC4958)SNP_00691	3006	T	C	3	4	--	--
CakTC41157	Ca(ICCV2/ICC4958)SNP_00692	327	A	G	3	6	Root	--
CakTC33787	Ca(ICCV2/ICC4958)SNP_00693	377	A	C	4	10	--	--
CakTC35633	Ca(ICCV2/ICC4958)SNP_00694	1195	T	C	3	7	--	--
CakTC05322	Ca(ICCV2/ICC4958)SNP_00695	334	T	G	6	7	--	--
	Ca(ICCV2/ICC4958)SNP_00696	1958	A	T	3	7	--	--
CakTC36992	Ca(ICCV2/ICC4958)SNP_00697	1428	G	T	9	9	--	--
	Ca(ICCV2/ICC4958)SNP_00698	1691	G	A	4	4	--	--
	Ca(ICCV2/ICC4958)SNP_00699	1736	T	C	5	5	--	--
CakTC37983	Ca(ICCV2/ICC4958)SNP_00700	283	C	T	10	7	--	SRS
CakTC12085	Ca(ICCV2/ICC4958)SNP_00701	330	T	C	17	37	--	--
CakTC33129	Ca(ICCV2/ICC4958)SNP_00702	1929	C	G	11	5	Mature Leaf	--
	Ca(ICCV2/ICC4958)SNP_00703	2193	G	A	15	7	Mature Leaf	--
	Ca(ICCV2/ICC4958)SNP_00704	2233	G	C	13	4	Mature Leaf	--
CakTC40198	Ca(ICCV2/ICC4958)SNP_00705	122	T	C	7	11	--	--
	Ca(ICCV2/ICC4958)SNP_00706	132	C	T	9	12	--	--
CakTC42092	Ca(ICCV2/ICC4958)SNP_00707	540	T	C	30	25	Flower bud	MADS
	Ca(ICCV2/ICC4958)SNP_00708	615	G	A	29	26	Flower bud	MADS
	Ca(ICCV2/ICC4958)SNP_00709	684	C	G	28	26	Flower bud	MADS
CakTC40367	Ca(ICCV2/ICC4958)SNP_00710	639	G	A	26	7	--	--
CakTC28059	Ca(ICCV2/ICC4958)SNP_00711	1751	G	A	13	6	--	--
CakTC30893	Ca(ICCV2/ICC4958)SNP_00712	465	C	T	3	3	Flower bud	--
	Ca(ICCV2/ICC4958)SNP_00713	501	T	C	3	3	Flower bud	--
	Ca(ICCV2/ICC4958)SNP_00714	521	C	T	3	3	Flower bud	--
CakTC39180	Ca(ICCV2/ICC4958)SNP_00715	533	G	A	55	28	--	Tify
CakTC39941	Ca(ICCV2/ICC4958)SNP_00716	931	G	A	3	5	--	--
CakTC41294	Ca(ICCV2/ICC4958)SNP_00717	794	C	A	4	3	--	--
CakTC37356	Ca(ICCV2/ICC4958)SNP_00718	228	T	C	5	6	--	--
CakTC29365	Ca(ICCV2/ICC4958)SNP_00719	1115	T	C	3	3	--	--
	Ca(ICCV2/ICC4958)SNP_00720	1150	C	T	3	3	--	--
	Ca(ICCV2/ICC4958)SNP_00721	1408	T	C	5	3	--	--
	Ca(ICCV2/ICC4958)SNP_00722	1417	C	T	5	3	--	--
	Ca(ICCV2/ICC4958)SNP_00723	1512	C	A	4	3	--	--
	Ca(ICCV2/ICC4958)SNP_00724	1540	A	C	4	3	--	--
CakTC23372	Ca(ICCV2/ICC4958)SNP_00725	93	T	C	3	3	--	--
CakTC24621	Ca(ICCV2/ICC4958)SNP_00726	449	C	T	8	3	--	--
	Ca(ICCV2/ICC4958)SNP_00727	561	C	T	4	3	--	--
CakTC10720	Ca(ICCV2/ICC4958)SNP_00728	2552	G	A	8	3	--	--
CakTC27796	Ca(ICCV2/ICC4958)SNP_00729	373	T	C	15	20	--	--
	Ca(ICCV2/ICC4958)SNP_00730	2790	G	T	10	5	--	--
CakTC18104	Ca(ICCV2/ICC4958)SNP_00731	1466	G	T	3	8	--	--
CakTC13914	Ca(ICCV2/ICC4958)SNP_00732	792	A	G	3	5	--	--
CakTC41340	Ca(ICCV2/ICC4958)SNP_00733	647	G	A	10	3	--	--
	Ca(ICCV2/ICC4958)SNP_00734	699	A	G	10	6	--	--

	Ca(ICCV2/ICC4958)SNP_00735	873	G	A	3	6	--	--
CakTC18835	Ca(ICCV2/ICC4958)SNP_00736	465	A	T	3	9	Young_pod	--
CakTC36968	Ca(ICCV2/ICC4958)SNP_00737	32	G	A	4	22	--	--
	Ca(ICCV2/ICC4958)SNP_00738	101	C	A	4	44	--	--
	Ca(ICCV2/ICC4958)SNP_00739	342	G	A	3	49	--	--
CakTC11336	Ca(ICCV2/ICC4958)SNP_00740	240	T	C	3	9	Shoot	--
	Ca(ICCV2/ICC4958)SNP_00741	363	C	T	4	5	Shoot	--
	Ca(ICCV2/ICC4958)SNP_00742	422	T	A	3	4	Shoot	--
CakTC09754	Ca(ICCV2/ICC4958)SNP_00743	795	A	G	8	12	--	--
	Ca(ICCV2/ICC4958)SNP_00744	824	A	G	9	10	--	--
	Ca(ICCV2/ICC4958)SNP_00745	827	G	A	9	10	--	--
CakTC39135	Ca(ICCV2/ICC4958)SNP_00746	934	A	G	23	11	--	--
CakTC12538	Ca(ICCV2/ICC4958)SNP_00747	461	A	C	8	3	--	--
	Ca(ICCV2/ICC4958)SNP_00748	756	G	A	16	8	--	--
	Ca(ICCV2/ICC4958)SNP_00749	923	T	C	14	10	--	--
	Ca(ICCV2/ICC4958)SNP_00750	1200	A	G	3	6	--	--
CakTC27317	Ca(ICCV2/ICC4958)SNP_00751	2662	A	G	5	3	--	--
CakTC31925	Ca(ICCV2/ICC4958)SNP_00752	862	C	T	4	7	--	--
CakTC27755	Ca(ICCV2/ICC4958)SNP_00753	611	T	G	4	3	--	--
	Ca(ICCV2/ICC4958)SNP_00754	624	G	C	4	3	--	--
	Ca(ICCV2/ICC4958)SNP_00755	670	G	C	8	3	--	--
	Ca(ICCV2/ICC4958)SNP_00756	1502	C	T	6	7	--	--
CakTC24364	Ca(ICCV2/ICC4958)SNP_00757	1607	T	G	10	15	--	--
	Ca(ICCV2/ICC4958)SNP_00758	1660	G	C	7	14	--	--
	Ca(ICCV2/ICC4958)SNP_00759	1710	C	T	8	10	--	--
CakTC27950	Ca(ICCV2/ICC4958)SNP_00760	449	T	C	8	21	--	--
CakTC32891	Ca(ICCV2/ICC4958)SNP_00761	280	A	G	5	7	--	--
CakTC32108	Ca(ICCV2/ICC4958)SNP_00762	59	A	G	9	15	--	--
	Ca(ICCV2/ICC4958)SNP_00763	64	G	C	9	15	--	--
	Ca(ICCV2/ICC4958)SNP_00764	253	A	T	14	18	--	--
	Ca(ICCV2/ICC4958)SNP_00765	286	C	A	13	15	--	--
	Ca(ICCV2/ICC4958)SNP_00766	484	C	G	7	11	--	--
	Ca(ICCV2/ICC4958)SNP_00767	1861	A	T	4	15	--	--
	Ca(ICCV2/ICC4958)SNP_00768	2031	A	G	5	16	--	--
	Ca(ICCV2/ICC4958)SNP_00769	2118	A	G	6	11	--	--
	Ca(ICCV2/ICC4958)SNP_00770	2292	G	A	6	6	--	--
CakTC34810	Ca(ICCV2/ICC4958)SNP_00771	940	T	C	10	12	--	--
CakTC33518	Ca(ICCV2/ICC4958)SNP_00772	312	C	G	17	4	--	--
CakTC25687	Ca(ICCV2/ICC4958)SNP_00773	473	C	A	4	3	Flower bud	--
	Ca(ICCV2/ICC4958)SNP_00774	521	C	A	4	3	Flower bud	--
	Ca(ICCV2/ICC4958)SNP_00775	530	A	C	4	3	Flower bud	--
	Ca(ICCV2/ICC4958)SNP_00776	540	A	C	4	3	Flower bud	--
	Ca(ICCV2/ICC4958)SNP_00777	543	T	G	4	3	Flower bud	--
CakTC14670	Ca(ICCV2/ICC4958)SNP_00778	732	T	C	9	4	--	--
	Ca(ICCV2/ICC4958)SNP_00779	1251	C	T	9	13	--	--
	Ca(ICCV2/ICC4958)SNP_00780	1836	C	T	7	10	--	--
	Ca(ICCV2/ICC4958)SNP_00781	2010	C	G	13	9	--	--
	Ca(ICCV2/ICC4958)SNP_00782	2109	G	T	13	7	--	--
	Ca(ICCV2/ICC4958)SNP_00783	2190	G	A	15	19	--	--
	Ca(ICCV2/ICC4958)SNP_00784	2988	C	T	7	8	--	--
	Ca(ICCV2/ICC4958)SNP_00785	3240	C	T	10	6	--	--
CakTC12116	Ca(ICCV2/ICC4958)SNP_00786	374	T	A	4	3	--	--
	Ca(ICCV2/ICC4958)SNP_00787	437	A	G	4	3	--	--
CakTC36457	Ca(ICCV2/ICC4958)SNP_00788	2635	A	G	30	59	--	--
CakTC37419	Ca(ICCV2/ICC4958)SNP_00789	414	C	T	4	4	--	--
	Ca(ICCV2/ICC4958)SNP_00790	703	T	A	4	8	--	--
CakTC35897	Ca(ICCV2/ICC4958)SNP_00791	417	G	A	5	13	--	--
CakTC09773	Ca(ICCV2/ICC4958)SNP_00792	677	C	A	4	3	--	--
CakTC00293	Ca(ICCV2/ICC4958)SNP_00793	1041	C	T	7	5	--	--
CakTC35224	Ca(ICCV2/ICC4958)SNP_00794	906	C	T	13	9	--	SET
CakTC40768	Ca(ICCV2/ICC4958)SNP_00795	960	T	G	7	9	--	--
CakTC22469	Ca(ICCV2/ICC4958)SNP_00796	686	T	G	3	3	--	--
CakTC42236	Ca(ICCV2/ICC4958)SNP_00797	1471	T	C	13	14	--	--
CakTC25003	Ca(ICCV2/ICC4958)SNP_00798	548	C	T	6	4	--	--
CakTC28500	Ca(ICCV2/ICC4958)SNP_00799	788	A	G	18	13	--	--
CakTC37966	Ca(ICCV2/ICC4958)SNP_00800	394	G	A	10	10	--	--
CakTC39662	Ca(ICCV2/ICC4958)SNP_00801	220	A	T	6	7	--	--
	Ca(ICCV2/ICC4958)SNP_00802	316	A	G	6	10	--	--
	Ca(ICCV2/ICC4958)SNP_00803	755	T	C	4	12	--	--
	Ca(ICCV2/ICC4958)SNP_00804	773	T	C	3	12	--	--
	Ca(ICCV2/ICC4958)SNP_00805	968	A	C	7	13	--	--
	Ca(ICCV2/ICC4958)SNP_00806	1181	T	G	8	15	--	--
	Ca(ICCV2/ICC4958)SNP_00807	1250	A	G	6	17	--	--
	Ca(ICCV2/ICC4958)SNP_00808	1472	A	G	4	15	--	--

	Ca(ICCV2/ICC4958)SNP_00809	1478	A	G	4	16	--	--
CakTC40388	Ca(ICCV2/ICC4958)SNP_00810	757	C	T	3	6	--	--
CakTC33086	Ca(ICCV2/ICC4958)SNP_00811	1532	A	C	14	13	--	--
CakTC41022	Ca(ICCV2/ICC4958)SNP_00812	156	G	T	4	4	--	--
CakTC14068	Ca(ICCV2/ICC4958)SNP_00813	363	G	A	6	3	--	--
CakTC32264	Ca(ICCV2/ICC4958)SNP_00814	1388	C	G	5	4	--	--
CakTC27189	Ca(ICCV2/ICC4958)SNP_00815	473	A	G	4	3	Root	--
CakTC10820	Ca(ICCV2/ICC4958)SNP_00816	1331	T	C	3	5	--	--
CakTC12698	Ca(ICCV2/ICC4958)SNP_00817	612	T	C	11	9	--	--
	Ca(ICCV2/ICC4958)SNP_00818	2339	T	C	5	4	--	--
CakTC31898	Ca(ICCV2/ICC4958)SNP_00819	1218	C	T	7	3	--	--
	Ca(ICCV2/ICC4958)SNP_00820	1261	C	T	4	3	--	--
	Ca(ICCV2/ICC4958)SNP_00821	1263	C	G	4	3	--	--
	Ca(ICCV2/ICC4958)SNP_00822	1283	C	T	4	3	--	--
CakTC38830	Ca(ICCV2/ICC4958)SNP_00823	1005	C	T	13	27	--	MYB
	Ca(ICCV2/ICC4958)SNP_00824	1362	C	T	5	5	--	MYB
CakTC33767	Ca(ICCV2/ICC4958)SNP_00825	145	A	G	5	15	--	--
CakTC10369	Ca(ICCV2/ICC4958)SNP_00826	946	T	C	12	11	--	--
CakTC31181	Ca(ICCV2/ICC4958)SNP_00827	814	C	T	3	4	--	--
	Ca(ICCV2/ICC4958)SNP_00828	901	A	G	4	4	--	--
CakTC28772	Ca(ICCV2/ICC4958)SNP_00829	1057	C	T	6	4	--	--
CakTC36652	Ca(ICCV2/ICC4958)SNP_00830	2548	G	A	50	49	--	ARF
CakTC32172	Ca(ICCV2/ICC4958)SNP_00831	1173	T	A	7	10	--	MYB-related
CakTC33315	Ca(ICCV2/ICC4958)SNP_00832	286	A	G	5	10	--	--
	Ca(ICCV2/ICC4958)SNP_00833	293	A	C	6	13	--	--
	Ca(ICCV2/ICC4958)SNP_00834	386	C	G	10	17	--	--
	Ca(ICCV2/ICC4958)SNP_00835	396	C	T	10	18	--	--
	Ca(ICCV2/ICC4958)SNP_00836	488	C	G	9	17	--	--
	Ca(ICCV2/ICC4958)SNP_00837	501	A	G	9	17	--	--
	Ca(ICCV2/ICC4958)SNP_00838	624	C	T	4	17	--	--
CakTC41436	Ca(ICCV2/ICC4958)SNP_00839	1409	A	G	8	7	--	--
CakTC25653	Ca(ICCV2/ICC4958)SNP_00840	1739	C	A	117	34	--	--
CakTC36960	Ca(ICCV2/ICC4958)SNP_00841	396	T	A	5	5	--	--
CakTC32088	Ca(ICCV2/ICC4958)SNP_00842	1455	C	T	4	9	--	--
	Ca(ICCV2/ICC4958)SNP_00843	1683	C	T	4	13	--	--
CakTC42296	Ca(ICCV2/ICC4958)SNP_00844	1134	T	C	12	9	--	--
CakTC14534	Ca(ICCV2/ICC4958)SNP_00845	914	A	G	3	5	--	--
CakTC43032	Ca(ICCV2/ICC4958)SNP_00846	277	A	G	4	7	--	--
	Ca(ICCV2/ICC4958)SNP_00847	1515	G	T	12	18	--	--
CakTC35723	Ca(ICCV2/ICC4958)SNP_00848	454	T	C	7	8	--	--
CakTC25254	Ca(ICCV2/ICC4958)SNP_00849	1061	A	G	3	4	--	--
	Ca(ICCV2/ICC4958)SNP_00850	1076	T	C	3	5	--	--
	Ca(ICCV2/ICC4958)SNP_00851	1371	G	A	5	3	--	--
CakTC28858	Ca(ICCV2/ICC4958)SNP_00852	451	T	C	4	3	--	--
	Ca(ICCV2/ICC4958)SNP_00853	454	T	C	4	3	--	--
	Ca(ICCV2/ICC4958)SNP_00854	466	T	C	4	3	--	--
	Ca(ICCV2/ICC4958)SNP_00855	494	T	C	4	3	--	--
	Ca(ICCV2/ICC4958)SNP_00856	523	A	G	3	4	--	--
CakTC29897	Ca(ICCV2/ICC4958)SNP_00857	648	A	G	6	12	--	--
CakTC35762	Ca(ICCV2/ICC4958)SNP_00858	2970	A	C	10	12	--	--
	Ca(ICCV2/ICC4958)SNP_00859	3003	T	C	10	6	--	--
	Ca(ICCV2/ICC4958)SNP_00860	3045	C	T	8	7	--	--
CakTC27107	Ca(ICCV2/ICC4958)SNP_00861	2034	T	C	7	7	--	--
CakTC23000	Ca(ICCV2/ICC4958)SNP_00862	285	G	C	6	3	--	--
CakTC40093	Ca(ICCV2/ICC4958)SNP_00863	596	G	A	14	9	--	BBR/BPC
CakTC42070	Ca(ICCV2/ICC4958)SNP_00864	182	C	A	7	4	--	--
	Ca(ICCV2/ICC4958)SNP_00865	260	C	T	6	4	--	--
	Ca(ICCV2/ICC4958)SNP_00866	340	C	G	11	3	--	--
	Ca(ICCV2/ICC4958)SNP_00867	559	G	T	14	4	--	--
	Ca(ICCV2/ICC4958)SNP_00868	652	C	T	11	5	--	--
	Ca(ICCV2/ICC4958)SNP_00869	700	G	T	11	6	--	--
	Ca(ICCV2/ICC4958)SNP_00870	738	G	C	11	6	--	--
	Ca(ICCV2/ICC4958)SNP_00871	838	G	C	7	7	--	--
	Ca(ICCV2/ICC4958)SNP_00872	1530	G	T	7	3	--	--
	Ca(ICCV2/ICC4958)SNP_00873	1540	A	C	8	3	--	--
CakTC35321	Ca(ICCV2/ICC4958)SNP_00874	282	A	G	4	20	--	--
	Ca(ICCV2/ICC4958)SNP_00875	1172	G	T	8	16	--	--
	Ca(ICCV2/ICC4958)SNP_00876	2661	A	G	3	8	--	--
	Ca(ICCV2/ICC4958)SNP_00877	2686	A	G	3	5	--	--
	Ca(ICCV2/ICC4958)SNP_00878	2961	A	G	4	10	--	--
CakTC23148	Ca(ICCV2/ICC4958)SNP_00879	25	A	T	3	4	--	--
	Ca(ICCV2/ICC4958)SNP_00880	38	T	C	3	4	--	--
	Ca(ICCV2/ICC4958)SNP_00881	73	A	C	4	4	--	--
CakTC22587	Ca(ICCV2/ICC4958)SNP_00882	1126	G	A	3	3	--	--

	Ca(ICCV2/ICC4958)SNP_00883	1128	T	A	3	5	--	--
	Ca(ICCV2/ICC4958)SNP_00884	1144	A	G	3	4	--	--
	Ca(ICCV2/ICC4958)SNP_00885	1145	A	G	3	4	--	--
CakTC12400	Ca(ICCV2/ICC4958)SNP_00886	384	A	C	3	3	--	--
	Ca(ICCV2/ICC4958)SNP_00887	393	C	T	4	3	--	--
CakTC37311	Ca(ICCV2/ICC4958)SNP_00888	1659	C	T	5	4	--	--
CakTC39931	Ca(ICCV2/ICC4958)SNP_00889	815	C	T	3	3	--	--
	Ca(ICCV2/ICC4958)SNP_00890	843	G	A	3	3	--	--
	Ca(ICCV2/ICC4958)SNP_00891	879	T	C	3	6	--	--
CakTC34289	Ca(ICCV2/ICC4958)SNP_00892	321	G	C	14	7	--	--
	Ca(ICCV2/ICC4958)SNP_00893	1007	T	G	8	23	--	--
	Ca(ICCV2/ICC4958)SNP_00894	1571	C	G	8	15	--	--
	Ca(ICCV2/ICC4958)SNP_00895	1740	A	G	6	10	--	--
CakTC10646	Ca(ICCV2/ICC4958)SNP_00896	82	A	T	3	3	--	--
	Ca(ICCV2/ICC4958)SNP_00897	131	A	T	3	5	--	--
	Ca(ICCV2/ICC4958)SNP_00898	277	G	A	6	6	--	--
CakTC25878	Ca(ICCV2/ICC4958)SNP_00899	138	C	T	188	286	--	--
	Ca(ICCV2/ICC4958)SNP_00900	171	C	A	189	266	--	--
	Ca(ICCV2/ICC4958)SNP_00901	213	T	C	187	305	--	--
	Ca(ICCV2/ICC4958)SNP_00902	255	C	A	195	309	--	--
	Ca(ICCV2/ICC4958)SNP_00903	339	A	C	210	308	--	--
	Ca(ICCV2/ICC4958)SNP_00904	423	T	A	155	236	--	--
	Ca(ICCV2/ICC4958)SNP_00905	435	C	T	115	219	--	--
	Ca(ICCV2/ICC4958)SNP_00906	473	G	A	22	101	--	--
	Ca(ICCV2/ICC4958)SNP_00907	563	G	A	12	53	--	--
CakTC29842	Ca(ICCV2/ICC4958)SNP_00908	1660	G	A	4	3	--	--
	Ca(ICCV2/ICC4958)SNP_00909	2427	G	T	4	3	--	--
CakTC12180	Ca(ICCV2/ICC4958)SNP_00910	87	A	C	3	3	--	--
	Ca(ICCV2/ICC4958)SNP_00911	122	C	T	3	3	--	--
	Ca(ICCV2/ICC4958)SNP_00912	132	A	C	3	3	--	--
	Ca(ICCV2/ICC4958)SNP_00913	149	C	T	3	3	--	--
	Ca(ICCV2/ICC4958)SNP_00914	174	T	C	3	3	--	--
	Ca(ICCV2/ICC4958)SNP_00915	226	A	G	3	3	--	--
	Ca(ICCV2/ICC4958)SNP_00916	240	A	T	3	3	--	--
	Ca(ICCV2/ICC4958)SNP_00917	346	T	A	3	3	--	--
	Ca(ICCV2/ICC4958)SNP_00918	347	T	A	3	3	--	--
CakTC11091	Ca(ICCV2/ICC4958)SNP_00919	346	C	T	6	3	--	--
CakTC40725	Ca(ICCV2/ICC4958)SNP_00920	435	G	A	12	13	--	--
	Ca(ICCV2/ICC4958)SNP_00921	495	G	A	8	9	--	--
CakTC10545	Ca(ICCV2/ICC4958)SNP_00922	497	T	G	3	6	--	--
CakTC37918	Ca(ICCV2/ICC4958)SNP_00923	747	G	A	12	9	--	HSF
CakTC38636	Ca(ICCV2/ICC4958)SNP_00924	497	C	G	9	10	--	--
CakTC22357	Ca(ICCV2/ICC4958)SNP_00925	3204	G	T	5	5	--	--
	Ca(ICCV2/ICC4958)SNP_00926	3219	C	A	5	5	--	--
	Ca(ICCV2/ICC4958)SNP_00927	3224	C	A	4	5	--	--
	Ca(ICCV2/ICC4958)SNP_00928	3243	C	T	5	5	--	--
	Ca(ICCV2/ICC4958)SNP_00929	3278	C	A	5	5	--	--
	Ca(ICCV2/ICC4958)SNP_00930	3290	A	T	4	5	--	--
	Ca(ICCV2/ICC4958)SNP_00931	3312	G	T	4	5	--	--
	Ca(ICCV2/ICC4958)SNP_00932	3338	G	A	5	5	--	--
	Ca(ICCV2/ICC4958)SNP_00933	3343	G	A	3	5	--	--
CakTC42044	Ca(ICCV2/ICC4958)SNP_00934	1494	G	A	12	5	--	--
	Ca(ICCV2/ICC4958)SNP_00935	1884	A	G	6	7	--	--
CakTC10919	Ca(ICCV2/ICC4958)SNP_00936	149	G	C	7	13	--	AP2-EREBP
CakTC29382	Ca(ICCV2/ICC4958)SNP_00937	899	C	G	3	3	--	--
CakTC09975	Ca(ICCV2/ICC4958)SNP_00938	414	G	T	4	7	--	--
	Ca(ICCV2/ICC4958)SNP_00939	420	G	A	6	6	--	--
	Ca(ICCV2/ICC4958)SNP_00940	480	T	C	11	3	--	--
	Ca(ICCV2/ICC4958)SNP_00941	608	T	C	11	4	--	--
	Ca(ICCV2/ICC4958)SNP_00942	612	T	C	12	6	--	--
	Ca(ICCV2/ICC4958)SNP_00943	1713	G	A	6	12	--	--
	Ca(ICCV2/ICC4958)SNP_00944	1968	A	G	6	9	--	--
CakTC28564	Ca(ICCV2/ICC4958)SNP_00945	821	A	G	6	7	--	--
CakTC30819	Ca(ICCV2/ICC4958)SNP_00946	160	A	T	9	3	--	HB
	Ca(ICCV2/ICC4958)SNP_00947	178	T	G	8	3	--	HB
	Ca(ICCV2/ICC4958)SNP_00948	184	C	T	7	3	--	HB
	Ca(ICCV2/ICC4958)SNP_00949	343	T	C	8	3	--	HB
CakTC09763	Ca(ICCV2/ICC4958)SNP_00950	4476	A	C	4	4	--	--
CakTC27705	Ca(ICCV2/ICC4958)SNP_00951	391	A	T	10	3	Flower bud	--
	Ca(ICCV2/ICC4958)SNP_00952	1000	G	A	9	6	Flower bud	--
	Ca(ICCV2/ICC4958)SNP_00953	2817	T	G	9	4	Flower bud	--
CakTC12086	Ca(ICCV2/ICC4958)SNP_00954	646	G	A	3	6	--	--
	Ca(ICCV2/ICC4958)SNP_00955	667	C	T	3	4	--	--
	Ca(ICCV2/ICC4958)SNP_00956	718	T	C	3	3	--	--

	Ca(ICCV2/ICC4958)SNP_00957	756	C	A	4	4	--	--
	Ca(ICCV2/ICC4958)SNP_00958	774	A	G	4	4	--	--
	Ca(ICCV2/ICC4958)SNP_00959	790	A	G	4	3	--	--
	Ca(ICCV2/ICC4958)SNP_00960	827	A	G	4	3	--	--
	Ca(ICCV2/ICC4958)SNP_00961	876	G	T	3	3	--	--
CakTC41855	Ca(ICCV2/ICC4958)SNP_00962	290	G	C	6	8	--	--
CakTC42635	Ca(ICCV2/ICC4958)SNP_00963	197	C	T	4	12	--	--
	Ca(ICCV2/ICC4958)SNP_00964	231	T	C	3	15	--	--
	Ca(ICCV2/ICC4958)SNP_00965	887	A	G	5	18	--	--
	Ca(ICCV2/ICC4958)SNP_00966	899	G	A	4	18	--	--
	Ca(ICCV2/ICC4958)SNP_00967	973	G	C	6	21	--	--
	Ca(ICCV2/ICC4958)SNP_00968	1184	A	G	17	18	--	--
	Ca(ICCV2/ICC4958)SNP_00969	1679	A	G	6	11	--	--
	Ca(ICCV2/ICC4958)SNP_00970	1694	C	T	6	11	--	--
	Ca(ICCV2/ICC4958)SNP_00971	1701	A	G	6	11	--	--
CakTC41649	Ca(ICCV2/ICC4958)SNP_00972	471	T	C	3	10	Shoot	--
	Ca(ICCV2/ICC4958)SNP_00973	595	T	G	8	12	Shoot	--
	Ca(ICCV2/ICC4958)SNP_00974	1039	T	C	9	19	Shoot	--
	Ca(ICCV2/ICC4958)SNP_00975	1497	G	A	10	24	Shoot	--
	Ca(ICCV2/ICC4958)SNP_00976	1502	A	G	13	26	Shoot	--
	Ca(ICCV2/ICC4958)SNP_00977	1962	T	A	14	29	Shoot	--
	Ca(ICCV2/ICC4958)SNP_00978	2187	G	A	8	5	Shoot	--
CakTC23770	Ca(ICCV2/ICC4958)SNP_00979	3063	G	T	22	16	--	--
CakTC40548	Ca(ICCV2/ICC4958)SNP_00980	204	T	A	8	9	--	--
CakTC34146	Ca(ICCV2/ICC4958)SNP_00981	1143	C	A	7	12	--	C3H
CakTC34446	Ca(ICCV2/ICC4958)SNP_00982	1345	A	G	11	13	--	--
CakTC24545	Ca(ICCV2/ICC4958)SNP_00983	47	C	T	11	4	--	--
	Ca(ICCV2/ICC4958)SNP_00984	258	T	C	32	14	--	--
	Ca(ICCV2/ICC4958)SNP_00985	420	C	T	33	19	--	--
	Ca(ICCV2/ICC4958)SNP_00986	2862	G	A	11	39	--	--
	Ca(ICCV2/ICC4958)SNP_00987	3151	A	T	38	46	--	--
CakTC27015	Ca(ICCV2/ICC4958)SNP_00988	2549	G	A	54	157	--	--
	Ca(ICCV2/ICC4958)SNP_00989	2735	A	G	61	135	--	--
CakTC29740	Ca(ICCV2/ICC4958)SNP_00990	715	T	G	6	7	--	SWI/SNF-SWI3
	Ca(ICCV2/ICC4958)SNP_00991	925	C	T	7	6	--	SWI/SNF-SWI3
	Ca(ICCV2/ICC4958)SNP_00992	978	A	T	9	7	--	SWI/SNF-SWI3
	Ca(ICCV2/ICC4958)SNP_00993	990	T	C	9	7	--	SWI/SNF-SWI3
	Ca(ICCV2/ICC4958)SNP_00994	1120	C	T	6	7	--	SWI/SNF-SWI3
	Ca(ICCV2/ICC4958)SNP_00995	1154	G	A	11	8	--	SWI/SNF-SWI3
	Ca(ICCV2/ICC4958)SNP_00996	2572	T	A	3	6	--	SWI/SNF-SWI3
	Ca(ICCV2/ICC4958)SNP_00997	3622	T	C	9	5	--	SWI/SNF-SWI3
	Ca(ICCV2/ICC4958)SNP_00998	4676	C	A	6	9	--	SWI/SNF-SWI3
	Ca(ICCV2/ICC4958)SNP_00999	4856	C	T	7	7	--	SWI/SNF-SWI3
	Ca(ICCV2/ICC4958)SNP_01000	5558	G	A	9	13	--	SWI/SNF-SWI3
	Ca(ICCV2/ICC4958)SNP_01001	5648	T	A	10	9	--	SWI/SNF-SWI3
	Ca(ICCV2/ICC4958)SNP_01002	6145	T	C	13	5	--	SWI/SNF-SWI3
	Ca(ICCV2/ICC4958)SNP_01003	6397	C	T	8	6	--	SWI/SNF-SWI3
CakTC25045	Ca(ICCV2/ICC4958)SNP_01004	510	A	G	5	8	--	--
CakTC27295	Ca(ICCV2/ICC4958)SNP_01005	440	T	C	3	3	--	--
CakTC37097	Ca(ICCV2/ICC4958)SNP_01006	2480	G	C	17	47	--	--
CakTC43389	Ca(ICCV2/ICC4958)SNP_01007	116	T	A	10	7	--	--
	Ca(ICCV2/ICC4958)SNP_01008	637	G	A	21	5	--	--
	Ca(ICCV2/ICC4958)SNP_01009	779	G	C	21	5	--	--
	Ca(ICCV2/ICC4958)SNP_01010	881	G	A	15	5	--	--
CakTC40847	Ca(ICCV2/ICC4958)SNP_01011	223	A	G	66	175	--	--
CakTC22404	Ca(ICCV2/ICC4958)SNP_01012	7233	G	A	3	4	--	--
CakTC27398	Ca(ICCV2/ICC4958)SNP_01013	6253	C	T	7	4	Flower bud	--
CakTC40707	Ca(ICCV2/ICC4958)SNP_01014	437	A	G	6	9	--	--
CakTC38863	Ca(ICCV2/ICC4958)SNP_01015	190	A	G	8	14	Flower bud	--
	Ca(ICCV2/ICC4958)SNP_01016	414	T	C	31	24	Flower bud	--
	Ca(ICCV2/ICC4958)SNP_01017	576	C	T	61	46	Flower bud	--
	Ca(ICCV2/ICC4958)SNP_01018	1356	C	A	37	19	Flower bud	--
CakTC32215	Ca(ICCV2/ICC4958)SNP_01019	282	C	T	4	5	--	--
	Ca(ICCV2/ICC4958)SNP_01020	321	A	G	4	5	--	--
CakTC32117	Ca(ICCV2/ICC4958)SNP_01021	1702	A	T	5	4	Shoot	SET
CakTC41046	Ca(ICCV2/ICC4958)SNP_01022	308	T	G	12	20	--	--
	Ca(ICCV2/ICC4958)SNP_01023	310	A	T	12	19	--	--
	Ca(ICCV2/ICC4958)SNP_01024	472	A	T	22	20	--	--
CakTC29410	Ca(ICCV2/ICC4958)SNP_01025	403	A	G	5	6	Mature Leaf	--
	Ca(ICCV2/ICC4958)SNP_01026	766	C	T	4	3	Mature Leaf	--
CakTC11585	Ca(ICCV2/ICC4958)SNP_01027	167	G	A	6	8	--	--
	Ca(ICCV2/ICC4958)SNP_01028	613	C	T	8	8	--	--
	Ca(ICCV2/ICC4958)SNP_01029	787	T	C	11	11	--	--
	Ca(ICCV2/ICC4958)SNP_01030	913	C	T	6	7	--	--

CakTC11747	Ca(ICCV2/ICC4958)SNP_01031	2312	A	T	5	5	--	--
	Ca(ICCV2/ICC4958)SNP_01032	2324	T	A	4	5	--	--
CakTC29401	Ca(ICCV2/ICC4958)SNP_01033	154	G	A	33	11	--	--
CakTC40335	Ca(ICCV2/ICC4958)SNP_01034	641	G	C	12	18	--	TPR
	Ca(ICCV2/ICC4958)SNP_01035	672	C	G	12	17	--	TPR
	Ca(ICCV2/ICC4958)SNP_01036	682	C	G	12	16	--	TPR
CakTC09582	Ca(ICCV2/ICC4958)SNP_01037	865	C	T	7	4	--	--
CakTC30335	Ca(ICCV2/ICC4958)SNP_01038	563	C	A	7	5	Shoot	--
CakTC36486	Ca(ICCV2/ICC4958)SNP_01039	336	T	C	4	6	Young_pod	--
	Ca(ICCV2/ICC4958)SNP_01040	341	A	C	4	6	Young_pod	--
	Ca(ICCV2/ICC4958)SNP_01041	348	C	T	4	5	Young_pod	--
CakTC24397	Ca(ICCV2/ICC4958)SNP_01042	768	G	A	16	18	--	G2-like
CakTC08782	Ca(ICCV2/ICC4958)SNP_01043	360	C	T	6	5	--	--
	Ca(ICCV2/ICC4958)SNP_01044	633	C	T	3	6	--	--
	Ca(ICCV2/ICC4958)SNP_01045	945	T	C	7	4	--	--
CakTC29824	Ca(ICCV2/ICC4958)SNP_01046	251	A	T	82	214	--	--
	Ca(ICCV2/ICC4958)SNP_01047	721	A	T	239	381	--	--
CakTC14579	Ca(ICCV2/ICC4958)SNP_01048	522	A	G	3	3	--	--
	Ca(ICCV2/ICC4958)SNP_01049	536	C	G	3	3	--	--
CakTC28122	Ca(ICCV2/ICC4958)SNP_01050	637	T	C	3	5	--	--
	Ca(ICCV2/ICC4958)SNP_01051	939	A	C	4	4	--	--
CakTC37176	Ca(ICCV2/ICC4958)SNP_01052	1556	C	T	6	5	--	--
CakTC04539	Ca(ICCV2/ICC4958)SNP_01053	943	G	C	6	6	--	--
	Ca(ICCV2/ICC4958)SNP_01054	1029	A	G	4	6	--	--
	Ca(ICCV2/ICC4958)SNP_01055	1094	C	A	3	5	--	--
CakTC29030	Ca(ICCV2/ICC4958)SNP_01056	34	A	G	5	6	--	--
CakTC35907	Ca(ICCV2/ICC4958)SNP_01057	230	C	T	3	8	--	--
CakTC11512	Ca(ICCV2/ICC4958)SNP_01058	150	C	T	29	57	--	--
CakTC26598	Ca(ICCV2/ICC4958)SNP_01059	372	C	T	13	13	--	--
	Ca(ICCV2/ICC4958)SNP_01060	622	C	T	5	5	--	--
	Ca(ICCV2/ICC4958)SNP_01061	1219	A	G	10	11	--	--
CakTC17425	Ca(ICCV2/ICC4958)SNP_01062	298	G	C	3	6	--	--
CakTC26859	Ca(ICCV2/ICC4958)SNP_01063	2738	C	T	6	6	--	--
CakTC11450	Ca(ICCV2/ICC4958)SNP_01064	338	C	T	5	7	--	--
CakTC09728	Ca(ICCV2/ICC4958)SNP_01065	935	A	G	24	20	--	TPR
CakTC08925	Ca(ICCV2/ICC4958)SNP_01066	411	A	G	3	4	--	--
	Ca(ICCV2/ICC4958)SNP_01067	523	T	C	3	6	--	--
CakTC10668	Ca(ICCV2/ICC4958)SNP_01068	209	T	C	27	23	--	--
	Ca(ICCV2/ICC4958)SNP_01069	228	A	C	25	24	--	--
CakTC37679	Ca(ICCV2/ICC4958)SNP_01070	1375	G	A	4	9	--	--
CakTC40886	Ca(ICCV2/ICC4958)SNP_01071	367	T	A	24	27	--	--
	Ca(ICCV2/ICC4958)SNP_01072	687	T	C	22	31	--	--
CakTC13258	Ca(ICCV2/ICC4958)SNP_01073	220	C	T	8	3	--	--
	Ca(ICCV2/ICC4958)SNP_01074	286	C	G	7	3	--	--
CakTC42665	Ca(ICCV2/ICC4958)SNP_01075	282	C	T	22	7	--	--
CakTC41834	Ca(ICCV2/ICC4958)SNP_01076	283	G	T	6	11	--	--
CakTC39043	Ca(ICCV2/ICC4958)SNP_01077	231	A	G	4	5	--	--
	Ca(ICCV2/ICC4958)SNP_01078	415	C	G	3	4	--	--
CakTC27433	Ca(ICCV2/ICC4958)SNP_01079	127	C	T	10	7	--	--
CakTC31788	Ca(ICCV2/ICC4958)SNP_01080	1442	C	T	4	6	--	--
CakTC40918	Ca(ICCV2/ICC4958)SNP_01081	55	C	T	22	38	--	--
	Ca(ICCV2/ICC4958)SNP_01082	221	A	T	31	56	--	--
	Ca(ICCV2/ICC4958)SNP_01083	617	C	T	6	20	--	--
CakTC24866	Ca(ICCV2/ICC4958)SNP_01084	3196	C	A	20	3	--	ARF
CakTC29878	Ca(ICCV2/ICC4958)SNP_01085	1775	A	C	17	5	Shoot	--
	Ca(ICCV2/ICC4958)SNP_01086	2128	G	A	14	6	Shoot	--
CakTC09260	Ca(ICCV2/ICC4958)SNP_01087	1816	G	A	3	3	--	--
CakTC38529	Ca(ICCV2/ICC4958)SNP_01088	618	C	T	8	11	--	--
CakTC31757	Ca(ICCV2/ICC4958)SNP_01089	90	G	T	3	3	--	--
CakTC33350	Ca(ICCV2/ICC4958)SNP_01090	169	C	A	6	5	--	--
CakTC34923	Ca(ICCV2/ICC4958)SNP_01091	255	A	G	3	3	--	--
CakTC24647	Ca(ICCV2/ICC4958)SNP_01092	242	A	G	6	3	--	--
CakTC24653	Ca(ICCV2/ICC4958)SNP_01093	48	C	A	4	6	Young_pod	--
	Ca(ICCV2/ICC4958)SNP_01094	753	C	T	11	7	Young_pod	--
	Ca(ICCV2/ICC4958)SNP_01095	950	G	T	12	10	Young_pod	--
	Ca(ICCV2/ICC4958)SNP_01096	1583	C	T	4	3	Young_pod	--
CakTC27565	Ca(ICCV2/ICC4958)SNP_01097	484	C	A	7	9	--	--
CakTC18438	Ca(ICCV2/ICC4958)SNP_01098	209	A	G	3	3	--	--
CakTC20149	Ca(ICCV2/ICC4958)SNP_01099	2171	C	T	7	5	Flower bud	--
CakTC11836	Ca(ICCV2/ICC4958)SNP_01100	111	A	T	6	4	Young_pod	--
CakTC29986	Ca(ICCV2/ICC4958)SNP_01101	3769	G	C	4	17	--	--
CakTC31319	Ca(ICCV2/ICC4958)SNP_01102	91	G	A	9	10	--	--
	Ca(ICCV2/ICC4958)SNP_01103	93	A	G	9	11	--	--
CakTC35416	Ca(ICCV2/ICC4958)SNP_01104	483	G	A	15	9	--	--

	Ca(ICCV2/ICC4958)SNP_01105	670	T	C	3	6	--	--
	Ca(ICCV2/ICC4958)SNP_01106	779	A	C	13	17	--	--
	Ca(ICCV2/ICC4958)SNP_01107	1250	T	G	16	10	--	--
	Ca(ICCV2/ICC4958)SNP_01108	1351	T	A	11	9	--	--
	Ca(ICCV2/ICC4958)SNP_01109	1952	T	A	14	9	--	--
	Ca(ICCV2/ICC4958)SNP_01110	2912	A	G	8	9	--	--
CakTC42465	Ca(ICCV2/ICC4958)SNP_01111	437	G	A	3	6	--	--
	Ca(ICCV2/ICC4958)SNP_01112	438	C	G	3	6	--	--
CakTC41859	Ca(ICCV2/ICC4958)SNP_01113	33	C	A	4	4	--	--
	Ca(ICCV2/ICC4958)SNP_01114	153	A	T	12	9	--	--
	Ca(ICCV2/ICC4958)SNP_01115	225	A	G	16	11	--	--
	Ca(ICCV2/ICC4958)SNP_01116	256	G	A	56	11	--	--
CakTC35508	Ca(ICCV2/ICC4958)SNP_01117	308	T	C	12	10	--	--
CakTC26081	Ca(ICCV2/ICC4958)SNP_01118	2226	G	A	45	47	--	--
CakTC25170	Ca(ICCV2/ICC4958)SNP_01119	275	G	A	10	13	--	--
	Ca(ICCV2/ICC4958)SNP_01120	1182	G	T	10	9	--	--
	Ca(ICCV2/ICC4958)SNP_01121	1192	C	A	11	9	--	--
	Ca(ICCV2/ICC4958)SNP_01122	1236	C	A	10	8	--	--
CakTC25457	Ca(ICCV2/ICC4958)SNP_01123	484	T	C	5	3	--	--
CakTC32283	Ca(ICCV2/ICC4958)SNP_01124	1711	C	T	16	6	Flower bud	--
CakTC33135	Ca(ICCV2/ICC4958)SNP_01125	2061	G	A	27	10	--	--
CakTC29816	Ca(ICCV2/ICC4958)SNP_01126	1068	T	C	3	11	--	--
	Ca(ICCV2/ICC4958)SNP_01127	1320	A	G	3	6	--	--
	Ca(ICCV2/ICC4958)SNP_01128	1428	C	T	3	6	--	--
CakTC38046	Ca(ICCV2/ICC4958)SNP_01129	808	T	C	7	12	--	--
	Ca(ICCV2/ICC4958)SNP_01130	813	G	A	7	12	--	--
	Ca(ICCV2/ICC4958)SNP_01131	832	G	A	7	12	--	--
	Ca(ICCV2/ICC4958)SNP_01132	1110	C	G	3	6	--	--
	Ca(ICCV2/ICC4958)SNP_01133	1115	T	C	3	8	--	--
CakTC28006	Ca(ICCV2/ICC4958)SNP_01134	945	G	A	11	14	--	--
CakTC38940	Ca(ICCV2/ICC4958)SNP_01135	137	A	T	19	5	--	--
CakTC31134	Ca(ICCV2/ICC4958)SNP_01136	375	A	T	11	3	Shoot	--
CakTC38268	Ca(ICCV2/ICC4958)SNP_01137	751	A	G	10	25	--	--
CakTC40441	Ca(ICCV2/ICC4958)SNP_01138	880	C	G	3	4	--	--
CakTC13362	Ca(ICCV2/ICC4958)SNP_01139	1176	T	C	3	3	--	--
CakTC40836	Ca(ICCV2/ICC4958)SNP_01140	212	G	A	5	4	--	--
	Ca(ICCV2/ICC4958)SNP_01141	216	T	G	6	4	--	--
	Ca(ICCV2/ICC4958)SNP_01142	291	C	G	6	3	--	--
	Ca(ICCV2/ICC4958)SNP_01143	299	C	T	6	4	--	--
	Ca(ICCV2/ICC4958)SNP_01144	513	T	C	5	7	--	--
	Ca(ICCV2/ICC4958)SNP_01145	561	G	T	6	7	--	--
	Ca(ICCV2/ICC4958)SNP_01146	873	C	T	9	6	--	--
	Ca(ICCV2/ICC4958)SNP_01147	945	G	A	9	4	--	--
	Ca(ICCV2/ICC4958)SNP_01148	1047	C	A	6	3	--	--
	Ca(ICCV2/ICC4958)SNP_01149	1749	T	A	7	16	--	--
	Ca(ICCV2/ICC4958)SNP_01150	1821	A	G	7	15	--	--
	Ca(ICCV2/ICC4958)SNP_01151	1935	G	T	6	12	--	--
CakTC40211	Ca(ICCV2/ICC4958)SNP_01152	1234	C	T	4	6	--	--
CakTC40515	Ca(ICCV2/ICC4958)SNP_01153	38	T	C	4	3	--	--
CakTC10689	Ca(ICCV2/ICC4958)SNP_01154	738	T	A	10	3	--	--
CakTC41792	Ca(ICCV2/ICC4958)SNP_01155	2101	A	G	24	35	--	--
CakTC32641	Ca(ICCV2/ICC4958)SNP_01156	1099	C	T	14	14	--	--
CakTC22474	Ca(ICCV2/ICC4958)SNP_01157	244	G	T	4	3	--	--
CakTC37404	Ca(ICCV2/ICC4958)SNP_01158	653	C	G	3	7	Young_pod	--
CakTC28478	Ca(ICCV2/ICC4958)SNP_01159	908	C	T	4	3	--	--
CakTC36414	Ca(ICCV2/ICC4958)SNP_01160	302	A	G	3	3	--	--
CakTC09680	Ca(ICCV2/ICC4958)SNP_01161	1081	G	A	8	4	--	--
CakTC30076	Ca(ICCV2/ICC4958)SNP_01162	547	G	T	7	3	--	--
	Ca(ICCV2/ICC4958)SNP_01163	556	G	A	8	3	--	--
CakTC24546	Ca(ICCV2/ICC4958)SNP_01164	379	T	A	64	27	--	--
	Ca(ICCV2/ICC4958)SNP_01165	668	C	T	84	4	--	--
CakTC24713	Ca(ICCV2/ICC4958)SNP_01166	1760	T	C	6	6	Shoot	--
CakTC22501	Ca(ICCV2/ICC4958)SNP_01167	873	T	A	5	4	Flower bud	--
CakTC25152	Ca(ICCV2/ICC4958)SNP_01168	4136	A	G	3	3	--	--
	Ca(ICCV2/ICC4958)SNP_01169	4195	C	T	3	4	--	--
	Ca(ICCV2/ICC4958)SNP_01170	4214	A	G	3	6	--	--
	Ca(ICCV2/ICC4958)SNP_01171	4242	G	A	3	8	--	--
	Ca(ICCV2/ICC4958)SNP_01172	4255	C	A	3	8	--	--
	Ca(ICCV2/ICC4958)SNP_01173	4314	G	A	3	10	--	--
	Ca(ICCV2/ICC4958)SNP_01174	4552	T	G	3	5	--	--
	Ca(ICCV2/ICC4958)SNP_01175	4860	T	C	3	5	--	--
	Ca(ICCV2/ICC4958)SNP_01176	5080	G	T	3	3	--	--
CakTC14736	Ca(ICCV2/ICC4958)SNP_01177	1872	G	A	14	7	--	--
CakTC10709	Ca(ICCV2/ICC4958)SNP_01178	853	T	C	3	3	--	--

	Ca(ICCV2/ICC4958)SNP_01179	905	A	G	3	3	--	--
	Ca(ICCV2/ICC4958)SNP_01180	913	T	C	3	5	--	--
	Ca(ICCV2/ICC4958)SNP_01181	926	T	C	3	4	--	--
	Ca(ICCV2/ICC4958)SNP_01182	965	T	C	3	6	--	--
CakTC42165	Ca(ICCV2/ICC4958)SNP_01183	6	G	C	3	4	--	--
	Ca(ICCV2/ICC4958)SNP_01184	107	C	A	11	22	--	--
	Ca(ICCV2/ICC4958)SNP_01185	1169	T	C	18	16	--	--
	Ca(ICCV2/ICC4958)SNP_01186	1503	C	G	7	19	--	--
	Ca(ICCV2/ICC4958)SNP_01187	1525	G	T	6	16	--	--
CakTC33640	Ca(ICCV2/ICC4958)SNP_01188	1158	G	A	12	6	--	TPR
CakTC29618	Ca(ICCV2/ICC4958)SNP_01189	350	A	C	15	15	--	--
CakTC33541	Ca(ICCV2/ICC4958)SNP_01190	623	A	G	4	8	--	--
CakTC04485	Ca(ICCV2/ICC4958)SNP_01191	90	G	A	4	9	--	--
CakTC24299	Ca(ICCV2/ICC4958)SNP_01192	948	C	G	13	12	--	--
CakTC41663	Ca(ICCV2/ICC4958)SNP_01193	417	A	G	14	22	--	--
CakTC40209	Ca(ICCV2/ICC4958)SNP_01194	793	T	C	56	93	--	--
CakTC29105	Ca(ICCV2/ICC4958)SNP_01195	985	G	T	5	4	--	--
CakTC36042	Ca(ICCV2/ICC4958)SNP_01196	93	A	G	9	7	--	--
	Ca(ICCV2/ICC4958)SNP_01197	347	A	G	5	12	--	--
	Ca(ICCV2/ICC4958)SNP_01198	494	T	A	7	13	--	--
	Ca(ICCV2/ICC4958)SNP_01199	1118	G	A	12	22	--	--
	Ca(ICCV2/ICC4958)SNP_01200	1754	G	A	10	13	--	--
	Ca(ICCV2/ICC4958)SNP_01201	2105	T	A	4	9	--	--
	Ca(ICCV2/ICC4958)SNP_01202	2310	G	A	9	14	--	--
CakTC36103	Ca(ICCV2/ICC4958)SNP_01203	2092	A	T	7	4	Root	--
CakTC40523	Ca(ICCV2/ICC4958)SNP_01204	482	C	T	25	11	--	--
	Ca(ICCV2/ICC4958)SNP_01205	535	C	T	23	11	--	--
	Ca(ICCV2/ICC4958)SNP_01206	552	T	G	20	10	--	--
	Ca(ICCV2/ICC4958)SNP_01207	558	A	G	20	7	--	--
CakTC31258	Ca(ICCV2/ICC4958)SNP_01208	783	A	G	9	24	--	--
	Ca(ICCV2/ICC4958)SNP_01209	819	G	A	9	25	--	--
	Ca(ICCV2/ICC4958)SNP_01210	852	C	T	12	23	--	--
	Ca(ICCV2/ICC4958)SNP_01211	1566	G	A	16	25	--	--
	Ca(ICCV2/ICC4958)SNP_01212	1671	A	G	15	22	--	--
CakTC27485	Ca(ICCV2/ICC4958)SNP_01213	2939	G	C	10	5	--	--
CakTC27566	Ca(ICCV2/ICC4958)SNP_01214	900	C	A	6	3	Shoot	--
CakTC30512	Ca(ICCV2/ICC4958)SNP_01215	1370	G	A	3	15	--	--
CakTC11234	Ca(ICCV2/ICC4958)SNP_01216	1010	G	A	13	13	--	--
	Ca(ICCV2/ICC4958)SNP_01217	2020	C	T	8	15	--	--
CakTC31291	Ca(ICCV2/ICC4958)SNP_01218	2175	A	C	13	28	--	--
CakTC43053	Ca(ICCV2/ICC4958)SNP_01219	268	G	T	8	5	--	--
	Ca(ICCV2/ICC4958)SNP_01220	634	T	G	5	3	--	--
	Ca(ICCV2/ICC4958)SNP_01221	895	T	C	8	6	--	--
	Ca(ICCV2/ICC4958)SNP_01222	1021	C	T	10	9	--	--
	Ca(ICCV2/ICC4958)SNP_01223	1051	A	C	8	10	--	--
	Ca(ICCV2/ICC4958)SNP_01224	1288	A	G	6	9	--	--
	Ca(ICCV2/ICC4958)SNP_01225	1462	G	A	5	4	--	--
CakTC32667	Ca(ICCV2/ICC4958)SNP_01226	504	C	T	3	3	--	--
CakTC37717	Ca(ICCV2/ICC4958)SNP_01227	331	G	T	13	8	--	--
CakTC39014	Ca(ICCV2/ICC4958)SNP_01228	364	G	A	3	5	--	--
CakTC31748	Ca(ICCV2/ICC4958)SNP_01229	279	G	A	4	6	Root	--
	Ca(ICCV2/ICC4958)SNP_01230	289	A	G	4	6	Root	--
	Ca(ICCV2/ICC4958)SNP_01231	716	C	T	4	5	Root	--
CakTC30393	Ca(ICCV2/ICC4958)SNP_01232	291	C	T	58	27	--	--
	Ca(ICCV2/ICC4958)SNP_01233	1767	G	C	18	7	--	--
CakTC41935	Ca(ICCV2/ICC4958)SNP_01234	563	A	G	4	3	--	--
CakTC09568	Ca(ICCV2/ICC4958)SNP_01235	1008	C	T	3	3	--	--
	Ca(ICCV2/ICC4958)SNP_01236	1018	T	C	3	3	--	--
	Ca(ICCV2/ICC4958)SNP_01237	1028	A	C	3	3	--	--
	Ca(ICCV2/ICC4958)SNP_01238	1047	A	G	3	3	--	--
	Ca(ICCV2/ICC4958)SNP_01239	1071	T	C	3	3	--	--
	Ca(ICCV2/ICC4958)SNP_01240	1091	A	G	3	3	--	--
CakTC29809	Ca(ICCV2/ICC4958)SNP_01241	1132	T	C	19	8	--	--
	Ca(ICCV2/ICC4958)SNP_01242	1196	T	C	17	7	--	--
	Ca(ICCV2/ICC4958)SNP_01243	1210	C	T	18	7	--	--
	Ca(ICCV2/ICC4958)SNP_01244	1244	T	G	17	7	--	--
CakTC28031	Ca(ICCV2/ICC4958)SNP_01245	534	T	C	12	10	--	--
CakTC43285	Ca(ICCV2/ICC4958)SNP_01246	1253	C	T	5	4	--	--
CakTC39655	Ca(ICCV2/ICC4958)SNP_01247	406	T	A	4	9	--	--
CakTC28839	Ca(ICCV2/ICC4958)SNP_01248	609	C	T	5	16	--	--
CakTC28696	Ca(ICCV2/ICC4958)SNP_01249	1495	G	A	3	3	Flower bud	--
CakTC29249	Ca(ICCV2/ICC4958)SNP_01250	2416	G	C	3	5	--	--
CakTC04081	Ca(ICCV2/ICC4958)SNP_01251	257	T	C	78	106	--	--
CakTC32947	Ca(ICCV2/ICC4958)SNP_01252	1677	T	C	7	7	--	--

CakTC33824	Ca(ICCV2/ICC4958)SNP_01253	3189	A	C	3	6	--	--
CakTC30712	Ca(ICCV2/ICC4958)SNP_01254	644	G	A	6	3	--	--
	Ca(ICCV2/ICC4958)SNP_01255	653	C	T	6	3	--	--
	Ca(ICCV2/ICC4958)SNP_01256	683	C	A	5	3	--	--
	Ca(ICCV2/ICC4958)SNP_01257	701	G	A	6	3	--	--
CakTC23401	Ca(ICCV2/ICC4958)SNP_01258	160	G	A	24	5	--	--
	Ca(ICCV2/ICC4958)SNP_01259	172	A	G	25	5	--	--
CakTC35642	Ca(ICCV2/ICC4958)SNP_01260	1779	T	C	12	6	--	--
	Ca(ICCV2/ICC4958)SNP_01261	2473	G	C	7	17	--	--
CakTC26597	Ca(ICCV2/ICC4958)SNP_01262	273	G	T	7	11	--	--
	Ca(ICCV2/ICC4958)SNP_01263	459	C	G	6	12	--	--
	Ca(ICCV2/ICC4958)SNP_01264	952	A	T	21	8	--	--
	Ca(ICCV2/ICC4958)SNP_01265	2280	C	T	4	4	--	--
CakTC24637	Ca(ICCV2/ICC4958)SNP_01266	1071	T	C	28	18	--	--
CakTC31300	Ca(ICCV2/ICC4958)SNP_01267	1571	C	A	3	19	Young_pod	--
CakTC24759	Ca(ICCV2/ICC4958)SNP_01268	742	C	T	3	3	--	--
CakTC30438	Ca(ICCV2/ICC4958)SNP_01269	207	A	T	5	3	--	--
	Ca(ICCV2/ICC4958)SNP_01270	244	A	T	4	3	--	--
CakTC10128	Ca(ICCV2/ICC4958)SNP_01271	1883	A	G	4	6	--	--
CakTC11704	Ca(ICCV2/ICC4958)SNP_01272	123	G	A	4	5	--	--
	Ca(ICCV2/ICC4958)SNP_01273	161	A	T	4	5	--	--
	Ca(ICCV2/ICC4958)SNP_01274	163	C	A	4	5	--	--
	Ca(ICCV2/ICC4958)SNP_01275	178	T	G	4	5	--	--
	Ca(ICCV2/ICC4958)SNP_01276	207	G	A	4	5	--	--
	Ca(ICCV2/ICC4958)SNP_01277	208	C	T	4	5	--	--
	Ca(ICCV2/ICC4958)SNP_01278	249	A	G	4	4	--	--
	Ca(ICCV2/ICC4958)SNP_01279	255	C	G	3	4	--	--
	Ca(ICCV2/ICC4958)SNP_01280	275	A	G	4	4	--	--
	Ca(ICCV2/ICC4958)SNP_01281	276	G	A	4	3	--	--
	Ca(ICCV2/ICC4958)SNP_01282	304	C	T	4	4	--	--
CakTC24444	Ca(ICCV2/ICC4958)SNP_01283	994	C	T	4	3	--	--
CakTC31697	Ca(ICCV2/ICC4958)SNP_01284	303	A	T	6	25	Young_pod	--
	Ca(ICCV2/ICC4958)SNP_01285	305	G	A	6	25	Young_pod	--
	Ca(ICCV2/ICC4958)SNP_01286	328	T	A	6	26	Young_pod	--
	Ca(ICCV2/ICC4958)SNP_01287	332	G	A	5	26	Young_pod	--
CakTC29322	Ca(ICCV2/ICC4958)SNP_01288	388	C	T	4	3	Young_pod	--
	Ca(ICCV2/ICC4958)SNP_01289	568	A	G	4	3	Young_pod	--
CakTC40460	Ca(ICCV2/ICC4958)SNP_01290	140	C	G	7	5	--	PHD
	Ca(ICCV2/ICC4958)SNP_01291	2458	T	G	17	11	--	PHD
CakTC17397	Ca(ICCV2/ICC4958)SNP_01292	170	C	T	3	3	--	--
CakTC32060	Ca(ICCV2/ICC4958)SNP_01293	625	A	C	4	7	--	--
	Ca(ICCV2/ICC4958)SNP_01294	646	A	G	5	7	--	--
	Ca(ICCV2/ICC4958)SNP_01295	822	C	T	9	4	--	--
	Ca(ICCV2/ICC4958)SNP_01296	916	C	G	13	6	--	--
	Ca(ICCV2/ICC4958)SNP_01297	1053	G	A	11	4	--	--
	Ca(ICCV2/ICC4958)SNP_01298	1130	A	G	12	4	--	--
CakTC38992	Ca(ICCV2/ICC4958)SNP_01299	37	C	T	22	12	--	--
	Ca(ICCV2/ICC4958)SNP_01300	83	T	C	33	13	--	--
	Ca(ICCV2/ICC4958)SNP_01301	91	G	A	34	15	--	--
	Ca(ICCV2/ICC4958)SNP_01302	868	G	A	41	27	--	--
CakTC39104	Ca(ICCV2/ICC4958)SNP_01303	461	A	G	19	17	--	--
	Ca(ICCV2/ICC4958)SNP_01304	560	C	G	17	10	--	--
	Ca(ICCV2/ICC4958)SNP_01305	875	T	C	14	12	--	--
	Ca(ICCV2/ICC4958)SNP_01306	1031	T	C	13	16	--	--
	Ca(ICCV2/ICC4958)SNP_01307	1037	T	G	13	16	--	--
CakTC29978	Ca(ICCV2/ICC4958)SNP_01308	4518	A	C	3	5	--	--
CakTC23813	Ca(ICCV2/ICC4958)SNP_01309	633	T	A	12	3	--	--
	Ca(ICCV2/ICC4958)SNP_01310	866	A	G	7	3	--	--
	Ca(ICCV2/ICC4958)SNP_01311	1117	T	A	5	5	--	--
	Ca(ICCV2/ICC4958)SNP_01312	1126	C	T	5	5	--	--
CakTC42593	Ca(ICCV2/ICC4958)SNP_01313	1486	A	G	19	17	--	--
CakTC24197	Ca(ICCV2/ICC4958)SNP_01314	622	C	T	32	14	--	--
	Ca(ICCV2/ICC4958)SNP_01315	830	T	C	63	18	--	--
	Ca(ICCV2/ICC4958)SNP_01316	867	A	G	55	22	--	--
CakTC39862	Ca(ICCV2/ICC4958)SNP_01317	885	T	C	7	15	--	--
CakTC31469	Ca(ICCV2/ICC4958)SNP_01318	336	G	A	5	3	--	--
	Ca(ICCV2/ICC4958)SNP_01319	405	A	G	5	6	--	--
CakTC41155	Ca(ICCV2/ICC4958)SNP_01320	2398	A	T	7	8	--	--
CakTC30048	Ca(ICCV2/ICC4958)SNP_01321	519	T	C	9	7	--	--
	Ca(ICCV2/ICC4958)SNP_01322	993	A	G	19	10	--	--
	Ca(ICCV2/ICC4958)SNP_01323	1647	A	T	12	11	--	--
CakTC30279	Ca(ICCV2/ICC4958)SNP_01324	213	C	T	5	6	--	--
	Ca(ICCV2/ICC4958)SNP_01325	327	T	G	6	12	--	--
	Ca(ICCV2/ICC4958)SNP_01326	1253	A	G	9	10	--	--

CakTC12043	Ca(ICCV2/ICC4958)SNP_01327	50	C	G	3	8	Flower bud	--
	Ca(ICCV2/ICC4958)SNP_01328	56	A	G	3	8	Flower bud	--
	Ca(ICCV2/ICC4958)SNP_01329	86	G	T	3	8	Flower bud	--
	Ca(ICCV2/ICC4958)SNP_01330	96	A	T	3	8	Flower bud	--
	Ca(ICCV2/ICC4958)SNP_01331	174	C	T	4	8	Flower bud	--
	Ca(ICCV2/ICC4958)SNP_01332	192	C	T	4	8	Flower bud	--
	Ca(ICCV2/ICC4958)SNP_01333	193	T	C	3	8	Flower bud	--
	Ca(ICCV2/ICC4958)SNP_01334	229	A	G	4	7	Flower bud	--
	Ca(ICCV2/ICC4958)SNP_01335	235	G	A	4	8	Flower bud	--
	Ca(ICCV2/ICC4958)SNP_01336	264	T	G	5	7	Flower bud	--
	Ca(ICCV2/ICC4958)SNP_01337	265	G	A	5	6	Flower bud	--
	Ca(ICCV2/ICC4958)SNP_01338	278	T	G	5	8	Flower bud	--
	Ca(ICCV2/ICC4958)SNP_01339	346	C	T	3	5	Flower bud	--
CakTC39821	Ca(ICCV2/ICC4958)SNP_01340	1167	G	A	4	11	--	--
CakTC38978	Ca(ICCV2/ICC4958)SNP_01341	937	A	G	34	55	--	--
CakTC29545	Ca(ICCV2/ICC4958)SNP_01342	2163	T	A	3	4	--	--
CakTC26854	Ca(ICCV2/ICC4958)SNP_01343	312	A	G	17	14	--	--
	Ca(ICCV2/ICC4958)SNP_01344	372	A	G	25	16	--	--
	Ca(ICCV2/ICC4958)SNP_01345	399	A	G	23	16	--	--
	Ca(ICCV2/ICC4958)SNP_01346	429	T	C	24	14	--	--
	Ca(ICCV2/ICC4958)SNP_01347	612	G	A	15	15	--	--
	Ca(ICCV2/ICC4958)SNP_01348	717	A	G	18	7	--	--
	Ca(ICCV2/ICC4958)SNP_01349	872	C	G	15	6	--	--
	Ca(ICCV2/ICC4958)SNP_01350	953	C	T	24	7	--	--
	Ca(ICCV2/ICC4958)SNP_01351	1065	C	T	25	11	--	--
	Ca(ICCV2/ICC4958)SNP_01352	1113	G	A	27	13	--	--
	Ca(ICCV2/ICC4958)SNP_01353	1272	T	A	19	9	--	--
	Ca(ICCV2/ICC4958)SNP_01354	1283	C	T	22	10	--	--
	Ca(ICCV2/ICC4958)SNP_01355	1532	A	C	19	8	--	--
	Ca(ICCV2/ICC4958)SNP_01356	1650	G	C	17	11	--	--
	Ca(ICCV2/ICC4958)SNP_01357	2136	T	C	11	16	--	--
	Ca(ICCV2/ICC4958)SNP_01358	2742	G	A	20	8	--	--
	Ca(ICCV2/ICC4958)SNP_01359	2797	T	G	18	6	--	--
	Ca(ICCV2/ICC4958)SNP_01360	2816	A	T	19	6	--	--
CakTC24618	Ca(ICCV2/ICC4958)SNP_01361	1725	G	A	16	6	--	--
	Ca(ICCV2/ICC4958)SNP_01362	2413	A	G	7	9	--	--
	Ca(ICCV2/ICC4958)SNP_01363	3874	C	T	5	8	--	--
	Ca(ICCV2/ICC4958)SNP_01364	3939	A	G	6	9	--	--
CakTC28203	Ca(ICCV2/ICC4958)SNP_01365	380	G	A	4	3	--	--
	Ca(ICCV2/ICC4958)SNP_01366	616	A	G	31	3	--	--
	Ca(ICCV2/ICC4958)SNP_01367	659	T	G	29	3	--	--
	Ca(ICCV2/ICC4958)SNP_01368	897	A	G	29	3	--	--
	Ca(ICCV2/ICC4958)SNP_01369	989	A	C	3	4	--	--
	Ca(ICCV2/ICC4958)SNP_01370	1181	C	T	5	7	--	--
	Ca(ICCV2/ICC4958)SNP_01371	1450	T	C	7	12	--	--
	Ca(ICCV2/ICC4958)SNP_01372	1506	A	G	6	12	--	--
	Ca(ICCV2/ICC4958)SNP_01373	1515	A	G	7	11	--	--
	Ca(ICCV2/ICC4958)SNP_01374	1520	G	C	8	11	--	--
	Ca(ICCV2/ICC4958)SNP_01375	1525	T	C	7	11	--	--
CakTC27528	Ca(ICCV2/ICC4958)SNP_01376	1343	C	T	13	5	--	TUB
CakTC29484	Ca(ICCV2/ICC4958)SNP_01377	677	G	T	8	4	--	--
CakTC37642	Ca(ICCV2/ICC4958)SNP_01378	394	C	A	6	4	--	--
CakTC40874	Ca(ICCV2/ICC4958)SNP_01379	447	T	C	7	5	--	WRKY
CakTC13752	Ca(ICCV2/ICC4958)SNP_01380	3298	G	A	6	3	--	--
CakTC24946	Ca(ICCV2/ICC4958)SNP_01381	682	T	C	13	11	--	--
CakTC31945	Ca(ICCV2/ICC4958)SNP_01382	1596	C	T	13	12	--	C3H
CakTC25228	Ca(ICCV2/ICC4958)SNP_01383	212	C	T	58	23	--	--
	Ca(ICCV2/ICC4958)SNP_01384	241	A	T	82	69	--	--
	Ca(ICCV2/ICC4958)SNP_01385	357	T	C	124	100	--	--
	Ca(ICCV2/ICC4958)SNP_01386	520	C	T	151	112	--	--
	Ca(ICCV2/ICC4958)SNP_01387	574	C	A	166	121	--	--
	Ca(ICCV2/ICC4958)SNP_01388	580	C	T	161	122	--	--
	Ca(ICCV2/ICC4958)SNP_01389	655	A	C	122	77	--	--
	Ca(ICCV2/ICC4958)SNP_01390	676	C	G	105	80	--	--
	Ca(ICCV2/ICC4958)SNP_01391	730	A	C	98	70	--	--
CakTC12141	Ca(ICCV2/ICC4958)SNP_01392	312	C	T	3	3	--	--
CakTC23831	Ca(ICCV2/ICC4958)SNP_01393	1387	T	C	3	6	--	--
	Ca(ICCV2/ICC4958)SNP_01394	1390	T	C	3	6	--	--
CakTC09957	Ca(ICCV2/ICC4958)SNP_01395	1045	C	G	3	6	--	--
	Ca(ICCV2/ICC4958)SNP_01396	1259	C	T	4	4	--	--
CakTC27308	Ca(ICCV2/ICC4958)SNP_01397	736	A	T	3	7	--	--
CakTC30300	Ca(ICCV2/ICC4958)SNP_01398	374	T	C	12	10	--	--
CakTC36220	Ca(ICCV2/ICC4958)SNP_01399	1196	T	C	20	3	--	--
CakTC26491	Ca(ICCV2/ICC4958)SNP_01400	227	T	C	7	11	--	--

CakTC26175	Ca(ICCV2/ICC4958)SNP_01401	689	G	T	6	8	--	--
	Ca(ICCV2/ICC4958)SNP_01402	701	C	T	6	7	--	--
	Ca(ICCV2/ICC4958)SNP_01403	734	A	G	6	6	--	--
	Ca(ICCV2/ICC4958)SNP_01404	782	T	C	6	10	--	--
	Ca(ICCV2/ICC4958)SNP_01405	1170	A	G	4	6	--	--
	Ca(ICCV2/ICC4958)SNP_01406	1172	G	C	4	6	--	--
CakTC41501	Ca(ICCV2/ICC4958)SNP_01407	155	A	G	4	5	--	--
	Ca(ICCV2/ICC4958)SNP_01408	491	C	G	7	4	--	--
CakTC10038	Ca(ICCV2/ICC4958)SNP_01409	915	T	C	3	6	--	--
	Ca(ICCV2/ICC4958)SNP_01410	1004	C	T	4	7	--	--
	Ca(ICCV2/ICC4958)SNP_01411	1285	T	G	4	3	--	--
CakTC36304	Ca(ICCV2/ICC4958)SNP_01412	8	C	T	3	4	--	--
	Ca(ICCV2/ICC4958)SNP_01413	1577	C	T	12	23	--	--
	Ca(ICCV2/ICC4958)SNP_01414	1715	G	A	9	27	--	--
	Ca(ICCV2/ICC4958)SNP_01415	2165	T	C	13	13	--	--
	Ca(ICCV2/ICC4958)SNP_01416	2273	G	A	10	14	--	--
CakTC22348	Ca(ICCV2/ICC4958)SNP_01417	2503	G	C	11	7	--	FHA
CakTC36433	Ca(ICCV2/ICC4958)SNP_01418	1052	T	C	3	14	--	--
	Ca(ICCV2/ICC4958)SNP_01419	2347	A	G	10	7	--	--
	Ca(ICCV2/ICC4958)SNP_01420	2440	T	C	11	8	--	--
	Ca(ICCV2/ICC4958)SNP_01421	2512	A	G	11	6	--	--
	Ca(ICCV2/ICC4958)SNP_01422	2617	C	T	9	7	--	--
	Ca(ICCV2/ICC4958)SNP_01423	2621	C	A	8	7	--	--
	Ca(ICCV2/ICC4958)SNP_01424	2824	C	T	7	7	--	--
CakTC39912	Ca(ICCV2/ICC4958)SNP_01425	762	C	T	6	9	--	--
CakTC41296	Ca(ICCV2/ICC4958)SNP_01426	905	C	T	4	7	--	--
CakTC31552	Ca(ICCV2/ICC4958)SNP_01427	754	T	C	4	5	--	Alfin-like
CakTC25885	Ca(ICCV2/ICC4958)SNP_01428	212	A	G	3	6	--	--
CakTC39832	Ca(ICCV2/ICC4958)SNP_01429	288	T	C	5	22	--	--
	Ca(ICCV2/ICC4958)SNP_01430	657	T	C	5	7	--	--
	Ca(ICCV2/ICC4958)SNP_01431	1302	C	T	9	15	--	--
CakTC10459	Ca(ICCV2/ICC4958)SNP_01432	454	C	T	4	4	--	--
CakTC34249	Ca(ICCV2/ICC4958)SNP_01433	74	T	C	4	7	--	--
CakTC36164	Ca(ICCV2/ICC4958)SNP_01434	197	A	G	3	48	--	--
CakTC42021	Ca(ICCV2/ICC4958)SNP_01435	339	A	T	14	8	--	--
CakTC09156	Ca(ICCV2/ICC4958)SNP_01436	550	A	T	3	5	--	--
	Ca(ICCV2/ICC4958)SNP_01437	553	C	T	3	5	--	--
	Ca(ICCV2/ICC4958)SNP_01438	562	A	G	3	5	--	--
	Ca(ICCV2/ICC4958)SNP_01439	616	A	T	4	5	--	--
	Ca(ICCV2/ICC4958)SNP_01440	625	A	C	4	5	--	--
	Ca(ICCV2/ICC4958)SNP_01441	664	A	G	4	5	--	--
	Ca(ICCV2/ICC4958)SNP_01442	667	G	A	3	5	--	--
	Ca(ICCV2/ICC4958)SNP_01443	675	G	T	4	5	--	--
	Ca(ICCV2/ICC4958)SNP_01444	679	A	T	4	5	--	--
	Ca(ICCV2/ICC4958)SNP_01445	690	G	C	4	5	--	--
	Ca(ICCV2/ICC4958)SNP_01446	696	G	A	4	5	--	--
CakTC30708	Ca(ICCV2/ICC4958)SNP_01447	1635	G	A	4	12	--	--
CakTC22597	Ca(ICCV2/ICC4958)SNP_01448	1941	T	G	7	3	--	--
CakTC28703	Ca(ICCV2/ICC4958)SNP_01449	1014	A	T	5	6	Shoot	--
	Ca(ICCV2/ICC4958)SNP_01450	1026	G	A	6	6	Shoot	--
CakTC07671	Ca(ICCV2/ICC4958)SNP_01451	336	A	G	5	3	--	--
CakTC31893	Ca(ICCV2/ICC4958)SNP_01452	281	C	T	24	49	--	--
	Ca(ICCV2/ICC4958)SNP_01453	289	C	T	24	49	--	--
	Ca(ICCV2/ICC4958)SNP_01454	418	C	A	25	45	--	--
	Ca(ICCV2/ICC4958)SNP_01455	470	T	G	26	47	--	--
	Ca(ICCV2/ICC4958)SNP_01456	522	G	T	27	46	--	--
	Ca(ICCV2/ICC4958)SNP_01457	1113	T	C	15	27	--	--
	Ca(ICCV2/ICC4958)SNP_01458	1167	C	T	14	20	--	--
	Ca(ICCV2/ICC4958)SNP_01459	1367	A	T	9	6	--	--
	Ca(ICCV2/ICC4958)SNP_01460	1372	C	T	9	6	--	--
CakTC37883	Ca(ICCV2/ICC4958)SNP_01461	245	A	C	6	4	--	--
CakTC42690	Ca(ICCV2/ICC4958)SNP_01462	761	C	T	29	22	--	--
	Ca(ICCV2/ICC4958)SNP_01463	822	C	T	10	10	--	--
	Ca(ICCV2/ICC4958)SNP_01464	854	G	C	6	3	--	--
CakTC23525	Ca(ICCV2/ICC4958)SNP_01465	535	T	A	16	4	--	--
CakTC39376	Ca(ICCV2/ICC4958)SNP_01466	541	T	G	3	13	--	--
CakTC33087	Ca(ICCV2/ICC4958)SNP_01467	171	T	C	9	8	--	--
	Ca(ICCV2/ICC4958)SNP_01468	288	G	A	10	8	--	--
	Ca(ICCV2/ICC4958)SNP_01469	306	C	T	10	8	--	--
	Ca(ICCV2/ICC4958)SNP_01470	445	C	G	8	13	--	--
	Ca(ICCV2/ICC4958)SNP_01471	1033	T	C	6	20	--	--
	Ca(ICCV2/ICC4958)SNP_01472	1163	C	A	13	22	--	--
	Ca(ICCV2/ICC4958)SNP_01473	2214	A	G	13	17	--	--
CakTC37769	Ca(ICCV2/ICC4958)SNP_01474	45	C	G	7	7	--	--

	Ca(ICCV2/ICC4958)SNP_01475	100	C	T	25	36	--	--
CakTC40851	Ca(ICCV2/ICC4958)SNP_01476	678	T	A	20	38	--	--
	Ca(ICCV2/ICC4958)SNP_01477	772	G	C	7	24	--	--
CakTC32010	Ca(ICCV2/ICC4958)SNP_01478	1019	A	T	27	21	--	--
	Ca(ICCV2/ICC4958)SNP_01479	2360	C	T	22	21	--	--
	Ca(ICCV2/ICC4958)SNP_01480	2405	T	C	22	25	--	--
	Ca(ICCV2/ICC4958)SNP_01481	2483	T	A	30	29	--	--
	Ca(ICCV2/ICC4958)SNP_01482	3266	T	A	10	34	--	--
CakTC26058	Ca(ICCV2/ICC4958)SNP_01483	32	A	G	11	5	--	--
	Ca(ICCV2/ICC4958)SNP_01484	323	T	C	18	9	--	--
	Ca(ICCV2/ICC4958)SNP_01485	351	A	G	19	11	--	--
	Ca(ICCV2/ICC4958)SNP_01486	354	C	T	20	11	--	--
CakTC25927	Ca(ICCV2/ICC4958)SNP_01487	744	C	T	16	17	--	--
	Ca(ICCV2/ICC4958)SNP_01488	747	C	T	15	17	--	--
	Ca(ICCV2/ICC4958)SNP_01489	780	C	T	19	18	--	--
	Ca(ICCV2/ICC4958)SNP_01490	939	C	T	27	13	--	--
	Ca(ICCV2/ICC4958)SNP_01491	1457	T	C	41	23	--	--
CakTC32784	Ca(ICCV2/ICC4958)SNP_01492	1629	G	C	7	16	--	ARF
	Ca(ICCV2/ICC4958)SNP_01493	1710	G	A	8	20	--	ARF
CakTC13631	Ca(ICCV2/ICC4958)SNP_01494	712	A	G	5	7	--	--
	Ca(ICCV2/ICC4958)SNP_01495	1424	G	C	10	21	--	--
CakTC26836	Ca(ICCV2/ICC4958)SNP_01496	1486	G	A	29	16	--	--
	Ca(ICCV2/ICC4958)SNP_01497	1747	C	T	7	3	--	--
CakTC09908	Ca(ICCV2/ICC4958)SNP_01498	85	T	C	3	3	--	--
	Ca(ICCV2/ICC4958)SNP_01499	171	C	G	6	6	--	--
CakTC38843	Ca(ICCV2/ICC4958)SNP_01500	285	C	T	8	5	--	C3H
CakTC42270	Ca(ICCV2/ICC4958)SNP_01501	24	A	C	5	4	--	--
	Ca(ICCV2/ICC4958)SNP_01502	50	G	T	7	9	--	--
	Ca(ICCV2/ICC4958)SNP_01503	71	C	T	8	15	--	--
	Ca(ICCV2/ICC4958)SNP_01504	111	T	G	8	17	--	--
CakTC10681	Ca(ICCV2/ICC4958)SNP_01505	880	C	T	10	8	--	BBR/BPC
CakTC27526	Ca(ICCV2/ICC4958)SNP_01506	2435	T	G	13	13	Young_pod	PHD
CakTC34844	Ca(ICCV2/ICC4958)SNP_01507	2765	T	C	8	6	--	--
	Ca(ICCV2/ICC4958)SNP_01508	3096	A	G	14	11	--	--
CakTC24550	Ca(ICCV2/ICC4958)SNP_01509	318	C	T	9	4	--	--
	Ca(ICCV2/ICC4958)SNP_01510	1877	A	G	13	3	--	--
CakTC32002	Ca(ICCV2/ICC4958)SNP_01511	193	T	G	3	5	--	SNF2
CakTC30255	Ca(ICCV2/ICC4958)SNP_01512	1402	G	A	11	4	--	--
CakTC02614	Ca(ICCV2/ICC4958)SNP_01513	2252	A	G	3	5	--	--
CakTC36366	Ca(ICCV2/ICC4958)SNP_01514	209	C	A	6	4	--	--
	Ca(ICCV2/ICC4958)SNP_01515	251	G	A	7	4	--	--
	Ca(ICCV2/ICC4958)SNP_01516	521	T	G	3	3	--	--
CakTC31459	Ca(ICCV2/ICC4958)SNP_01517	105	A	G	5	6	Root	--
	Ca(ICCV2/ICC4958)SNP_01518	174	C	G	7	5	Root	--
	Ca(ICCV2/ICC4958)SNP_01519	203	C	T	7	5	Root	--
	Ca(ICCV2/ICC4958)SNP_01520	270	G	A	6	5	Root	--
	Ca(ICCV2/ICC4958)SNP_01521	278	G	A	6	5	Root	--
	Ca(ICCV2/ICC4958)SNP_01522	289	A	G	7	5	Root	--
	Ca(ICCV2/ICC4958)SNP_01523	311	G	A	7	5	Root	--
	Ca(ICCV2/ICC4958)SNP_01524	315	A	G	7	5	Root	--
CakTC25334	Ca(ICCV2/ICC4958)SNP_01525	509	T	C	69	105	--	--
CakTC34659	Ca(ICCV2/ICC4958)SNP_01526	2716	A	G	12	10	--	--
	Ca(ICCV2/ICC4958)SNP_01527	3097	T	C	10	11	--	--
	Ca(ICCV2/ICC4958)SNP_01528	3469	C	T	14	9	--	--
	Ca(ICCV2/ICC4958)SNP_01529	6116	A	C	6	14	--	--
CakTC26143	Ca(ICCV2/ICC4958)SNP_01530	2645	A	G	33	34	--	--
	Ca(ICCV2/ICC4958)SNP_01531	3072	C	T	39	17	--	--
	Ca(ICCV2/ICC4958)SNP_01532	6096	A	G	5	3	--	--
	Ca(ICCV2/ICC4958)SNP_01533	6181	G	A	3	3	--	--
CakTC38869	Ca(ICCV2/ICC4958)SNP_01534	95	A	G	12	7	--	Trihelix
CakTC40174	Ca(ICCV2/ICC4958)SNP_01535	45	T	A	5	3	--	--
	Ca(ICCV2/ICC4958)SNP_01536	66	T	G	5	4	--	--
	Ca(ICCV2/ICC4958)SNP_01537	71	G	C	6	5	--	--
	Ca(ICCV2/ICC4958)SNP_01538	101	C	A	6	5	--	--
	Ca(ICCV2/ICC4958)SNP_01539	111	A	G	6	5	--	--
	Ca(ICCV2/ICC4958)SNP_01540	114	G	C	5	5	--	--
	Ca(ICCV2/ICC4958)SNP_01541	311	C	T	7	9	--	--
	Ca(ICCV2/ICC4958)SNP_01542	983	C	T	12	12	--	--
CakTC38413	Ca(ICCV2/ICC4958)SNP_01543	1161	T	A	4	4	--	--
CakTC40923	Ca(ICCV2/ICC4958)SNP_01544	513	C	A	6	7	--	--
CakTC30152	Ca(ICCV2/ICC4958)SNP_01545	443	A	G	11	13	--	--
CakTC07013	Ca(ICCV2/ICC4958)SNP_01546	185	C	G	4	6	--	--
CakTC36706	Ca(ICCV2/ICC4958)SNP_01547	231	A	T	3	3	--	--
CakTC34493	Ca(ICCV2/ICC4958)SNP_01548	1020	G	A	5	3	--	--

	Ca(ICCV2/ICC4958)SNP_01549	1022	C	T	5	3	--	--
CakTC10405	Ca(ICCV2/ICC4958)SNP_01550	3654	T	G	10	3	--	--
CakTC23915	Ca(ICCV2/ICC4958)SNP_01551	4781	C	T	3	4	--	--
	Ca(ICCV2/ICC4958)SNP_01552	7702	C	G	19	11	--	--
CakTC08608	Ca(ICCV2/ICC4958)SNP_01553	649	A	G	3	6	--	--
CakTC10739	Ca(ICCV2/ICC4958)SNP_01554	822	C	G	3	4	--	--
CakTC35465	Ca(ICCV2/ICC4958)SNP_01555	288	A	C	18	13	--	--
	Ca(ICCV2/ICC4958)SNP_01556	454	G	C	28	21	--	--
	Ca(ICCV2/ICC4958)SNP_01557	705	C	T	20	31	--	--
	Ca(ICCV2/ICC4958)SNP_01558	1016	A	G	16	24	--	--
	Ca(ICCV2/ICC4958)SNP_01559	1845	G	T	39	24	--	--
	Ca(ICCV2/ICC4958)SNP_01560	2388	A	C	17	24	--	--
	Ca(ICCV2/ICC4958)SNP_01561	2565	C	T	22	24	--	--
	Ca(ICCV2/ICC4958)SNP_01562	2703	A	G	21	27	--	--
	Ca(ICCV2/ICC4958)SNP_01563	2736	C	T	23	24	--	--
	Ca(ICCV2/ICC4958)SNP_01564	4116	G	A	19	10	--	--
	Ca(ICCV2/ICC4958)SNP_01565	4182	A	G	17	8	--	--
CakTC24654	Ca(ICCV2/ICC4958)SNP_01566	899	G	A	3	5	Flower bud	--
CakTC30460	Ca(ICCV2/ICC4958)SNP_01567	71	G	A	3	5	--	--
	Ca(ICCV2/ICC4958)SNP_01568	389	A	G	219	568	--	--
	Ca(ICCV2/ICC4958)SNP_01569	662	A	G	253	489	--	--
	Ca(ICCV2/ICC4958)SNP_01570	866	C	T	208	269	--	--
	Ca(ICCV2/ICC4958)SNP_01571	1178	C	T	215	442	--	--
	Ca(ICCV2/ICC4958)SNP_01572	1535	T	G	212	409	--	--
	Ca(ICCV2/ICC4958)SNP_01573	1730	G	A	248	455	--	--
	Ca(ICCV2/ICC4958)SNP_01574	1733	T	C	252	447	--	--
	Ca(ICCV2/ICC4958)SNP_01575	1838	C	T	214	416	--	--
	Ca(ICCV2/ICC4958)SNP_01576	1889	T	C	188	341	--	--
	Ca(ICCV2/ICC4958)SNP_01577	1910	A	G	176	374	--	--
CakTC13591	Ca(ICCV2/ICC4958)SNP_01578	184	T	A	3	3	--	--
CakTC08179	Ca(ICCV2/ICC4958)SNP_01579	645	C	T	3	6	--	--
CakTC25367	Ca(ICCV2/ICC4958)SNP_01580	563	T	C	38	39	--	--
	Ca(ICCV2/ICC4958)SNP_01581	680	C	T	29	26	--	--
	Ca(ICCV2/ICC4958)SNP_01582	917	C	T	39	38	--	--
	Ca(ICCV2/ICC4958)SNP_01583	1151	A	G	46	31	--	--
	Ca(ICCV2/ICC4958)SNP_01584	1304	C	T	51	39	--	--
	Ca(ICCV2/ICC4958)SNP_01585	1604	T	C	30	34	--	--
	Ca(ICCV2/ICC4958)SNP_01586	1775	C	T	50	35	--	--
	Ca(ICCV2/ICC4958)SNP_01587	2228	C	T	52	38	--	--
	Ca(ICCV2/ICC4958)SNP_01588	2804	T	C	62	74	--	--
	Ca(ICCV2/ICC4958)SNP_01589	3590	G	A	83	59	--	--
CakTC07160	Ca(ICCV2/ICC4958)SNP_01590	572	T	C	4	3	--	--
CakTC37700	Ca(ICCV2/ICC4958)SNP_01591	217	T	G	16	9	--	--
	Ca(ICCV2/ICC4958)SNP_01592	385	T	C	15	6	--	--
	Ca(ICCV2/ICC4958)SNP_01593	517	A	G	7	5	--	--
CakTC38174	Ca(ICCV2/ICC4958)SNP_01594	375	C	G	8	13	--	--
	Ca(ICCV2/ICC4958)SNP_01595	445	G	T	10	15	--	--
	Ca(ICCV2/ICC4958)SNP_01596	645	A	G	12	13	--	--
	Ca(ICCV2/ICC4958)SNP_01597	670	C	T	14	13	--	--
	Ca(ICCV2/ICC4958)SNP_01598	786	A	T	17	18	--	--
	Ca(ICCV2/ICC4958)SNP_01599	787	T	G	17	17	--	--
	Ca(ICCV2/ICC4958)SNP_01600	1448	C	G	10	15	--	--
	Ca(ICCV2/ICC4958)SNP_01601	1505	T	C	7	10	--	--
CakTC13412	Ca(ICCV2/ICC4958)SNP_01602	844	T	C	11	4	--	--
CakTC34716	Ca(ICCV2/ICC4958)SNP_01603	335	G	A	13	24	--	--
	Ca(ICCV2/ICC4958)SNP_01604	1651	A	C	22	13	--	--
	Ca(ICCV2/ICC4958)SNP_01605	1961	G	T	20	14	--	--
	Ca(ICCV2/ICC4958)SNP_01606	1967	C	T	20	12	--	--
	Ca(ICCV2/ICC4958)SNP_01607	2972	G	A	14	10	--	--
	Ca(ICCV2/ICC4958)SNP_01608	3290	T	A	8	15	--	--
CakTC39940	Ca(ICCV2/ICC4958)SNP_01609	90	G	C	10	11	--	--
	Ca(ICCV2/ICC4958)SNP_01610	130	T	C	10	8	--	--
	Ca(ICCV2/ICC4958)SNP_01611	440	T	A	23	8	--	--
CakTC42973	Ca(ICCV2/ICC4958)SNP_01612	1381	C	G	5	4	--	--
CakTC26334	Ca(ICCV2/ICC4958)SNP_01613	221	G	A	3	6	--	--
	Ca(ICCV2/ICC4958)SNP_01614	308	G	A	3	6	--	--
	Ca(ICCV2/ICC4958)SNP_01615	355	C	T	7	8	--	--
	Ca(ICCV2/ICC4958)SNP_01616	760	G	A	14	15	--	--
CakTC25536	Ca(ICCV2/ICC4958)SNP_01617	1277	A	G	3	3	--	C2H2
CakTC02493	Ca(ICCV2/ICC4958)SNP_01618	388	G	T	7	10	--	--
	Ca(ICCV2/ICC4958)SNP_01619	394	G	A	13	10	--	--
	Ca(ICCV2/ICC4958)SNP_01620	442	C	T	17	6	--	--
	Ca(ICCV2/ICC4958)SNP_01621	454	T	C	22	7	--	--
	Ca(ICCV2/ICC4958)SNP_01622	582	T	C	20	5	--	--

	Ca(ICCV2/ICC4958)SNP_01623	586	T	C	19	7	--	--
	Ca(ICCV2/ICC4958)SNP_01624	954	A	G	11	5	--	--
	Ca(ICCV2/ICC4958)SNP_01625	1687	G	A	11	3	--	--
	Ca(ICCV2/ICC4958)SNP_01626	1942	A	G	11	5	--	--
CakTC04962	Ca(ICCV2/ICC4958)SNP_01627	130	C	T	3	3	--	--
	Ca(ICCV2/ICC4958)SNP_01628	224	A	G	3	3	--	--
CakTC13472	Ca(ICCV2/ICC4958)SNP_01629	167	G	C	3	3	--	--
CakTC34378	Ca(ICCV2/ICC4958)SNP_01630	400	C	T	9	7	--	--
CakTC14939	Ca(ICCV2/ICC4958)SNP_01631	263	T	C	3	3	--	--
	Ca(ICCV2/ICC4958)SNP_01632	268	A	T	3	3	--	--
	Ca(ICCV2/ICC4958)SNP_01633	293	T	C	3	3	--	--
CakTC12359	Ca(ICCV2/ICC4958)SNP_01634	164	G	A	3	5	--	--
CakTC40299	Ca(ICCV2/ICC4958)SNP_01635	221	G	A	4	5	--	--
CakTC23204	Ca(ICCV2/ICC4958)SNP_01636	200	A	G	242	64	--	--
	Ca(ICCV2/ICC4958)SNP_01637	641	C	T	425	63	--	--
	Ca(ICCV2/ICC4958)SNP_01638	692	T	C	432	75	--	--
CakTC34460	Ca(ICCV2/ICC4958)SNP_01639	917	A	G	4	4	Young_pod	--
CakTC26962	Ca(ICCV2/ICC4958)SNP_01640	253	T	G	16	11	--	--
CakTC39977	Ca(ICCV2/ICC4958)SNP_01641	563	A	G	13	7	--	--
	Ca(ICCV2/ICC4958)SNP_01642	587	G	T	12	8	--	--
	Ca(ICCV2/ICC4958)SNP_01643	964	C	A	9	6	--	--
	Ca(ICCV2/ICC4958)SNP_01644	1319	A	G	8	13	--	--
	Ca(ICCV2/ICC4958)SNP_01645	1466	A	T	6	9	--	--
	Ca(ICCV2/ICC4958)SNP_01646	1477	A	G	6	10	--	--
	Ca(ICCV2/ICC4958)SNP_01647	1482	G	A	7	9	--	--
CakTC31990	Ca(ICCV2/ICC4958)SNP_01648	260	G	C	6	5	--	--
	Ca(ICCV2/ICC4958)SNP_01649	1343	A	C	3	6	--	--
	Ca(ICCV2/ICC4958)SNP_01650	1373	C	T	3	6	--	--
CakTC40412	Ca(ICCV2/ICC4958)SNP_01651	69	T	C	4	8	--	--
CakTC27723	Ca(ICCV2/ICC4958)SNP_01652	1065	A	C	19	4	--	--
CakTC26349	Ca(ICCV2/ICC4958)SNP_01653	372	C	G	25	39	--	--
CakTC38794	Ca(ICCV2/ICC4958)SNP_01654	175	T	C	6	9	--	--
CakTC39793	Ca(ICCV2/ICC4958)SNP_01655	275	G	A	5	5	--	--
	Ca(ICCV2/ICC4958)SNP_01656	284	T	C	5	5	--	--
	Ca(ICCV2/ICC4958)SNP_01657	340	T	C	6	5	--	--
	Ca(ICCV2/ICC4958)SNP_01658	515	A	G	5	5	--	--
	Ca(ICCV2/ICC4958)SNP_01659	563	C	G	3	5	--	--
CakTC36542	Ca(ICCV2/ICC4958)SNP_01660	1544	T	C	52	39	--	--
CakTC40013	Ca(ICCV2/ICC4958)SNP_01661	212	G	C	7	7	--	--
CakTC11911	Ca(ICCV2/ICC4958)SNP_01662	1379	C	T	4	5	--	--
CakTC27901	Ca(ICCV2/ICC4958)SNP_01663	201	G	C	4	5	Young_pod	--
CakTC09859	Ca(ICCV2/ICC4958)SNP_01664	4172	G	A	7	3	--	SET
CakTC38314	Ca(ICCV2/ICC4958)SNP_01665	648	A	G	9	5	--	--
CakTC14073	Ca(ICCV2/ICC4958)SNP_01666	155	T	C	3	3	--	--
	Ca(ICCV2/ICC4958)SNP_01667	173	G	A	3	3	--	--
	Ca(ICCV2/ICC4958)SNP_01668	203	T	A	3	3	--	--
	Ca(ICCV2/ICC4958)SNP_01669	212	A	T	3	3	--	--
CakTC36522	Ca(ICCV2/ICC4958)SNP_01670	296	T	C	16	20	--	--
CakTC29414	Ca(ICCV2/ICC4958)SNP_01671	1040	A	G	7	3	--	--
	Ca(ICCV2/ICC4958)SNP_01672	1363	G	A	5	3	--	--
	Ca(ICCV2/ICC4958)SNP_01673	2818	C	T	3	3	--	--
CakTC25427	Ca(ICCV2/ICC4958)SNP_01674	42	T	C	15	28	Root	--
CakTC39161	Ca(ICCV2/ICC4958)SNP_01675	392	G	T	16	10	--	--
CakTC38007	Ca(ICCV2/ICC4958)SNP_01676	380	G	A	5	6	--	--
	Ca(ICCV2/ICC4958)SNP_01677	569	C	T	12	10	--	--
	Ca(ICCV2/ICC4958)SNP_01678	571	C	A	12	10	--	--
	Ca(ICCV2/ICC4958)SNP_01679	581	G	A	13	9	--	--
CakTC38014	Ca(ICCV2/ICC4958)SNP_01680	4	G	A	4	4	--	--
CakTC11743	Ca(ICCV2/ICC4958)SNP_01681	823	A	T	3	4	--	--
CakTC20931	Ca(ICCV2/ICC4958)SNP_01682	187	G	A	5	3	--	--
CakTC09772	Ca(ICCV2/ICC4958)SNP_01683	326	G	A	4	4	--	--
	Ca(ICCV2/ICC4958)SNP_01684	378	C	T	5	5	--	--
CakTC26866	Ca(ICCV2/ICC4958)SNP_01685	275	T	A	24	43	--	--
CakTC24824	Ca(ICCV2/ICC4958)SNP_01686	1098	G	A	6	11	--	MYB
CakTC39497	Ca(ICCV2/ICC4958)SNP_01687	292	C	G	5	4	Shoot	--
CakTC30759	Ca(ICCV2/ICC4958)SNP_01688	868	T	C	11	9	--	--
	Ca(ICCV2/ICC4958)SNP_01689	2484	A	G	4	4	--	--
CakTC29294	Ca(ICCV2/ICC4958)SNP_01690	1611	A	G	4	3	--	NAC
	Ca(ICCV2/ICC4958)SNP_01691	1637	A	T	4	3	--	NAC
CakTC37604	Ca(ICCV2/ICC4958)SNP_01692	490	A	G	39	40	--	--
	Ca(ICCV2/ICC4958)SNP_01693	1152	A	G	23	13	--	--
CakTC41279	Ca(ICCV2/ICC4958)SNP_01694	1467	G	A	6	15	--	--
CakTC25707	Ca(ICCV2/ICC4958)SNP_01695	1489	G	A	209	99	--	--
CakTC10457	Ca(ICCV2/ICC4958)SNP_01696	168	G	A	6	5	--	--

	Ca(ICCV2/ICC4958)SNP_01697	340	G	A	6	6	--	--
CakTC21542	Ca(ICCV2/ICC4958)SNP_01698	448	A	G	19	8	--	--
CakTC37570	Ca(ICCV2/ICC4958)SNP_01699	987	G	C	35	32	--	--
CakTC25154	Ca(ICCV2/ICC4958)SNP_01700	116	G	C	4	5	--	--
	Ca(ICCV2/ICC4958)SNP_01701	1427	C	T	15	7	--	--
CakTC06975	Ca(ICCV2/ICC4958)SNP_01702	327	C	T	4	3	--	--
CakTC41659	Ca(ICCV2/ICC4958)SNP_01703	121	A	C	3	11	--	--
CakTC24345	Ca(ICCV2/ICC4958)SNP_01704	1263	T	A	3	6	--	--
	Ca(ICCV2/ICC4958)SNP_01705	1448	G	A	4	7	--	--
CakTC10443	Ca(ICCV2/ICC4958)SNP_01706	843	A	G	11	4	--	--
CakTC27400	Ca(ICCV2/ICC4958)SNP_01707	1028	C	T	6	9	--	--
CakTC25937	Ca(ICCV2/ICC4958)SNP_01708	1390	C	T	19	19	--	--
CakTC25176	Ca(ICCV2/ICC4958)SNP_01709	2435	T	C	13	11	--	--
CakTC31139	Ca(ICCV2/ICC4958)SNP_01710	335	A	G	5	5	--	SRS
CakTC43202	Ca(ICCV2/ICC4958)SNP_01711	74	T	G	14	13	--	--
CakTC35140	Ca(ICCV2/ICC4958)SNP_01712	2058	A	T	9	21	--	--
CakTC23314	Ca(ICCV2/ICC4958)SNP_01713	61	C	T	4	3	--	CCHC
CakTC08541	Ca(ICCV2/ICC4958)SNP_01714	431	T	C	3	5	--	--
CakTC32575	Ca(ICCV2/ICC4958)SNP_01715	916	C	G	16	17	--	--
CakTC22560	Ca(ICCV2/ICC4958)SNP_01716	666	A	T	6	8	--	--
CakTC25060	Ca(ICCV2/ICC4958)SNP_01717	1299	T	A	3	4	--	--
	Ca(ICCV2/ICC4958)SNP_01718	1602	T	G	3	3	--	--
CakTC26651	Ca(ICCV2/ICC4958)SNP_01719	651	C	A	5	21	--	--
CakTC36371	Ca(ICCV2/ICC4958)SNP_01720	546	A	C	5	3	--	--
CakTC37460	Ca(ICCV2/ICC4958)SNP_01721	145	T	C	18	16	--	--
CakTC29482	Ca(ICCV2/ICC4958)SNP_01722	1599	G	T	8	7	--	--
CakTC09778	Ca(ICCV2/ICC4958)SNP_01723	196	T	G	4	11	--	--
	Ca(ICCV2/ICC4958)SNP_01724	266	G	A	7	10	--	--
CakTC23051	Ca(ICCV2/ICC4958)SNP_01725	93	T	C	12	3	--	--
CakTC23814	Ca(ICCV2/ICC4958)SNP_01726	641	C	G	8	5	--	--
CakTC33900	Ca(ICCV2/ICC4958)SNP_01727	333	C	T	4	21	--	--
CakTC37581	Ca(ICCV2/ICC4958)SNP_01728	520	C	T	13	137	--	--
CakTC39741	Ca(ICCV2/ICC4958)SNP_01729	457	G	A	8	3	--	SBP
CakTC26909	Ca(ICCV2/ICC4958)SNP_01730	950	C	T	20	19	--	--
	Ca(ICCV2/ICC4958)SNP_01731	1166	C	T	21	19	--	--
CakTC08847	Ca(ICCV2/ICC4958)SNP_01732	1177	T	A	16	5	--	--
	Ca(ICCV2/ICC4958)SNP_01733	1183	C	T	14	5	--	--
	Ca(ICCV2/ICC4958)SNP_01734	1371	G	A	7	10	--	--
CakTC34598	Ca(ICCV2/ICC4958)SNP_01735	1453	C	T	23	17	--	--
CakTC33325	Ca(ICCV2/ICC4958)SNP_01736	394	T	C	13	9	--	--
	Ca(ICCV2/ICC4958)SNP_01737	1567	G	A	18	15	--	--
	Ca(ICCV2/ICC4958)SNP_01738	1605	A	G	22	12	--	--
	Ca(ICCV2/ICC4958)SNP_01739	1755	T	A	11	8	--	--
	Ca(ICCV2/ICC4958)SNP_01740	1855	A	T	12	3	--	--
	Ca(ICCV2/ICC4958)SNP_01741	2060	A	T	11	5	--	--
	Ca(ICCV2/ICC4958)SNP_01742	2071	T	G	12	5	--	--
CakTC29746	Ca(ICCV2/ICC4958)SNP_01743	860	G	C	5	6	--	--
CakTC21124	Ca(ICCV2/ICC4958)SNP_01744	143	T	C	4	8	--	--
	Ca(ICCV2/ICC4958)SNP_01745	240	G	T	3	8	--	--
	Ca(ICCV2/ICC4958)SNP_01746	241	A	C	4	8	--	--
CakTC34127	Ca(ICCV2/ICC4958)SNP_01747	260	G	A	8	8	--	--
	Ca(ICCV2/ICC4958)SNP_01748	346	T	C	10	9	--	--
CakTC10785	Ca(ICCV2/ICC4958)SNP_01749	124	A	G	3	10	--	--
	Ca(ICCV2/ICC4958)SNP_01750	241	T	C	6	11	--	--
	Ca(ICCV2/ICC4958)SNP_01751	415	T	G	14	11	--	--
	Ca(ICCV2/ICC4958)SNP_01752	421	T	C	14	12	--	--
	Ca(ICCV2/ICC4958)SNP_01753	442	C	T	15	11	--	--
	Ca(ICCV2/ICC4958)SNP_01754	709	T	A	13	12	--	--
	Ca(ICCV2/ICC4958)SNP_01755	815	A	G	15	18	--	--
	Ca(ICCV2/ICC4958)SNP_01756	1030	A	G	9	14	--	--
	Ca(ICCV2/ICC4958)SNP_01757	1381	A	G	10	6	--	--
	Ca(ICCV2/ICC4958)SNP_01758	1539	T	C	4	4	--	--
CakTC32008	Ca(ICCV2/ICC4958)SNP_01759	216	C	A	3	4	--	--
CakTC40602	Ca(ICCV2/ICC4958)SNP_01760	659	A	G	44	119	--	--
CakTC42182	Ca(ICCV2/ICC4958)SNP_01761	618	C	T	3	5	--	--
CakTC10990	Ca(ICCV2/ICC4958)SNP_01762	377	A	G	4	4	--	--
CakTC37181	Ca(ICCV2/ICC4958)SNP_01763	690	C	G	5	8	--	--
CakTC23040	Ca(ICCV2/ICC4958)SNP_01764	614	G	T	29	6	--	--
CakTC38563	Ca(ICCV2/ICC4958)SNP_01765	769	T	G	20	10	--	--
CakTC29573	Ca(ICCV2/ICC4958)SNP_01766	301	C	T	9	3	--	--
CakTC38182	Ca(ICCV2/ICC4958)SNP_01767	103	C	G	5	8	--	--
	Ca(ICCV2/ICC4958)SNP_01768	112	C	G	5	8	--	--
	Ca(ICCV2/ICC4958)SNP_01769	303	C	G	6	5	--	--
	Ca(ICCV2/ICC4958)SNP_01770	346	G	A	5	6	--	--

CakTC30706	Ca(ICCV2/ICC4958)SNP_01771	687	G	C	7	5	--	--
	Ca(ICCV2/ICC4958)SNP_01772	771	A	G	7	8	--	--
CakTC41841	Ca(ICCV2/ICC4958)SNP_01773	49	T	G	29	18	--	CCAAT
	Ca(ICCV2/ICC4958)SNP_01774	324	A	G	38	22	--	CCAAT
	Ca(ICCV2/ICC4958)SNP_01775	360	C	T	47	23	--	CCAAT
	Ca(ICCV2/ICC4958)SNP_01776	570	G	C	25	22	--	CCAAT
	Ca(ICCV2/ICC4958)SNP_01777	588	T	C	24	22	--	CCAAT
	Ca(ICCV2/ICC4958)SNP_01778	724	C	G	15	19	--	CCAAT
CakTC39027	Ca(ICCV2/ICC4958)SNP_01779	551	A	G	4	11	--	--
	Ca(ICCV2/ICC4958)SNP_01780	749	C	T	4	6	--	--
CakTC24425	Ca(ICCV2/ICC4958)SNP_01781	103	C	A	5	3	--	--
CakTC40475	Ca(ICCV2/ICC4958)SNP_01782	59	G	A	7	11	--	--
	Ca(ICCV2/ICC4958)SNP_01783	142	A	T	7	18	--	--
	Ca(ICCV2/ICC4958)SNP_01784	304	C	T	7	22	--	--
	Ca(ICCV2/ICC4958)SNP_01785	2102	G	A	11	29	--	--
CakTC33491	Ca(ICCV2/ICC4958)SNP_01786	1234	T	C	16	10	--	--
	Ca(ICCV2/ICC4958)SNP_01787	1396	C	A	14	11	--	--
CakTC34201	Ca(ICCV2/ICC4958)SNP_01788	3724	C	A	7	11	--	--
CakTC36196	Ca(ICCV2/ICC4958)SNP_01789	649	T	C	3	8	--	--
CakTC29183	Ca(ICCV2/ICC4958)SNP_01790	896	G	A	16	11	--	--
CakTC42484	Ca(ICCV2/ICC4958)SNP_01791	193	C	T	8	8	--	--
CakTC36203	Ca(ICCV2/ICC4958)SNP_01792	2240	T	C	15	12	--	--
CakTC38725	Ca(ICCV2/ICC4958)SNP_01793	2829	A	G	5	5	--	--
CakTC26978	Ca(ICCV2/ICC4958)SNP_01794	169	G	C	4	4	--	--
	Ca(ICCV2/ICC4958)SNP_01795	178	A	G	4	4	--	--
	Ca(ICCV2/ICC4958)SNP_01796	184	A	G	4	4	--	--
CakTC41706	Ca(ICCV2/ICC4958)SNP_01797	28	T	C	3	3	--	--
CakTC39980	Ca(ICCV2/ICC4958)SNP_01798	1487	G	A	3	3	--	--
CakTC27037	Ca(ICCV2/ICC4958)SNP_01799	1407	C	G	12	8	--	--
CakTC27502	Ca(ICCV2/ICC4958)SNP_01800	1177	T	G	5	3	--	--
CakTC24781	Ca(ICCV2/ICC4958)SNP_01801	574	T	C	28	26	--	--
	Ca(ICCV2/ICC4958)SNP_01802	706	A	G	33	25	--	--
	Ca(ICCV2/ICC4958)SNP_01803	1682	G	A	13	7	--	--
	Ca(ICCV2/ICC4958)SNP_01804	1843	A	G	14	14	--	--
	Ca(ICCV2/ICC4958)SNP_01805	2172	G	T	8	12	--	--
	Ca(ICCV2/ICC4958)SNP_01806	2173	A	T	8	12	--	--
	Ca(ICCV2/ICC4958)SNP_01807	2575	G	T	13	15	--	--
CakTC26560	Ca(ICCV2/ICC4958)SNP_01808	1504	A	G	8	3	--	--
CakTC28037	Ca(ICCV2/ICC4958)SNP_01809	673	G	A	8	8	--	--
	Ca(ICCV2/ICC4958)SNP_01810	1153	A	C	4	3	--	--
	Ca(ICCV2/ICC4958)SNP_01811	1175	G	C	4	3	--	--
CakTC11642	Ca(ICCV2/ICC4958)SNP_01812	287	T	C	5	7	--	--
	Ca(ICCV2/ICC4958)SNP_01813	396	G	A	4	6	--	--
CakTC06109	Ca(ICCV2/ICC4958)SNP_01814	186	A	C	7	7	--	--
	Ca(ICCV2/ICC4958)SNP_01815	853	A	G	14	4	--	--
	Ca(ICCV2/ICC4958)SNP_01816	1077	G	A	18	6	--	--
CakTC12115	Ca(ICCV2/ICC4958)SNP_01817	95	C	G	11	3	--	--
	Ca(ICCV2/ICC4958)SNP_01818	135	C	T	11	4	--	--
CakTC23247	Ca(ICCV2/ICC4958)SNP_01819	275	G	A	44	55	--	--
CakTC38102	Ca(ICCV2/ICC4958)SNP_01820	398	T	C	5	4	--	--
CakTC39355	Ca(ICCV2/ICC4958)SNP_01821	1334	A	G	8	7	--	--
CakTC02396	Ca(ICCV2/ICC4958)SNP_01822	197	A	G	5	3	--	--
	Ca(ICCV2/ICC4958)SNP_01823	355	T	C	9	5	--	--
	Ca(ICCV2/ICC4958)SNP_01824	706	T	C	8	18	--	--
	Ca(ICCV2/ICC4958)SNP_01825	921	T	C	16	23	--	--
	Ca(ICCV2/ICC4958)SNP_01826	1294	G	A	12	11	--	--
	Ca(ICCV2/ICC4958)SNP_01827	1321	A	C	13	9	--	--
	Ca(ICCV2/ICC4958)SNP_01828	1432	A	G	8	6	--	--
	Ca(ICCV2/ICC4958)SNP_01829	1495	A	G	7	7	--	--
	Ca(ICCV2/ICC4958)SNP_01830	1612	T	C	6	4	--	--
CakTC39748	Ca(ICCV2/ICC4958)SNP_01831	1113	T	C	35	93	--	zf-HD
	Ca(ICCV2/ICC4958)SNP_01832	1221	G	A	31	71	--	zf-HD
CakTC39377	Ca(ICCV2/ICC4958)SNP_01833	1120	T	C	4	11	--	--
CakTC10454	Ca(ICCV2/ICC4958)SNP_01834	1636	T	A	3	5	--	--
	Ca(ICCV2/ICC4958)SNP_01835	2116	T	C	6	3	--	--
CakTC05656	Ca(ICCV2/ICC4958)SNP_01836	1611	T	C	3	3	--	--
CakTC42926	Ca(ICCV2/ICC4958)SNP_01837	852	A	G	8	27	--	MBF1
CakTC30521	Ca(ICCV2/ICC4958)SNP_01838	213	T	A	5	7	--	--
	Ca(ICCV2/ICC4958)SNP_01839	1011	T	C	4	4	--	--
	Ca(ICCV2/ICC4958)SNP_01840	1308	A	G	4	4	--	--
	Ca(ICCV2/ICC4958)SNP_01841	1323	C	A	4	7	--	--
CakTC35851	Ca(ICCV2/ICC4958)SNP_01842	2300	T	C	12	7	--	--
CakTC37004	Ca(ICCV2/ICC4958)SNP_01843	208	T	C	18	14	--	--
	Ca(ICCV2/ICC4958)SNP_01844	237	C	A	17	15	--	--

	Ca(ICCV2/ICC4958)SNP_01845	305	T	C	20	21	--	--
	Ca(ICCV2/ICC4958)SNP_01846	1091	G	A	16	11	--	--
	Ca(ICCV2/ICC4958)SNP_01847	1115	T	C	14	11	--	--
	Ca(ICCV2/ICC4958)SNP_01848	1202	C	G	13	13	--	--
	Ca(ICCV2/ICC4958)SNP_01849	1727	A	G	18	18	--	--
CakTC10429	Ca(ICCV2/ICC4958)SNP_01850	446	T	C	3	3	--	--
CakTC35601	Ca(ICCV2/ICC4958)SNP_01851	364	A	T	4	3	--	--
	Ca(ICCV2/ICC4958)SNP_01852	398	A	T	4	3	--	--
CakTC26761	Ca(ICCV2/ICC4958)SNP_01853	142	T	C	5	3	--	--
CakTC33992	Ca(ICCV2/ICC4958)SNP_01854	5158	A	G	10	10	--	--
CakTC24853	Ca(ICCV2/ICC4958)SNP_01855	978	C	A	18	10	--	--
	Ca(ICCV2/ICC4958)SNP_01856	1110	C	G	8	7	--	--
CakTC39880	Ca(ICCV2/ICC4958)SNP_01857	212	T	C	11	48	--	--
CakTC30674	Ca(ICCV2/ICC4958)SNP_01858	1359	T	G	3	55	--	--
	Ca(ICCV2/ICC4958)SNP_01859	1365	A	G	3	54	--	--
	Ca(ICCV2/ICC4958)SNP_01860	1416	G	A	3	46	--	--
CakTC39260	Ca(ICCV2/ICC4958)SNP_01861	1010	T	A	7	3	--	MYB-related
CakTC29906	Ca(ICCV2/ICC4958)SNP_01862	2574	G	T	25	19	--	--
	Ca(ICCV2/ICC4958)SNP_01863	3693	G	A	26	17	--	--
CakTC32982	Ca(ICCV2/ICC4958)SNP_01864	505	A	T	15	3	--	--
	Ca(ICCV2/ICC4958)SNP_01865	2643	C	G	5	6	--	--
CakTC26144	Ca(ICCV2/ICC4958)SNP_01866	248	T	C	23	19	--	--
	Ca(ICCV2/ICC4958)SNP_01867	267	A	C	23	20	--	--
CakTC17123	Ca(ICCV2/ICC4958)SNP_01868	538	C	G	4	3	--	--
CakTC32675	Ca(ICCV2/ICC4958)SNP_01869	248	T	C	30	70	--	--
	Ca(ICCV2/ICC4958)SNP_01870	257	A	G	34	71	--	--
	Ca(ICCV2/ICC4958)SNP_01871	311	A	G	34	64	--	--
	Ca(ICCV2/ICC4958)SNP_01872	518	C	T	46	101	--	--
	Ca(ICCV2/ICC4958)SNP_01873	578	G	T	47	74	--	--
CakTC34197	Ca(ICCV2/ICC4958)SNP_01874	217	G	C	18	59	--	TPR
	Ca(ICCV2/ICC4958)SNP_01875	232	T	C	17	58	--	TPR
	Ca(ICCV2/ICC4958)SNP_01876	863	G	A	26	64	--	TPR
	Ca(ICCV2/ICC4958)SNP_01877	1520	G	T	42	63	--	TPR
	Ca(ICCV2/ICC4958)SNP_01878	1787	G	A	31	49	--	TPR
	Ca(ICCV2/ICC4958)SNP_01879	2052	C	T	9	12	--	TPR
CakTC39160	Ca(ICCV2/ICC4958)SNP_01880	159	T	C	4	9	--	--
CakTC29299	Ca(ICCV2/ICC4958)SNP_01881	1657	A	G	4	3	--	--
	Ca(ICCV2/ICC4958)SNP_01882	2071	C	A	3	3	--	--
	Ca(ICCV2/ICC4958)SNP_01883	2272	T	A	4	3	--	--
CakTC10242	Ca(ICCV2/ICC4958)SNP_01884	1379	C	T	3	4	--	--
CakTC25708	Ca(ICCV2/ICC4958)SNP_01885	754	T	C	3	4	--	--
	Ca(ICCV2/ICC4958)SNP_01886	1461	C	T	4	12	--	--
	Ca(ICCV2/ICC4958)SNP_01887	1912	A	G	6	4	--	--
	Ca(ICCV2/ICC4958)SNP_01888	2158	G	A	7	11	--	--
	Ca(ICCV2/ICC4958)SNP_01889	3012	C	A	5	3	--	--
CakTC25702	Ca(ICCV2/ICC4958)SNP_01890	1876	G	A	7	10	--	--
	Ca(ICCV2/ICC4958)SNP_01891	1897	C	T	7	9	--	--
	Ca(ICCV2/ICC4958)SNP_01892	2016	C	A	5	5	--	--
	Ca(ICCV2/ICC4958)SNP_01893	2030	G	A	3	5	--	--
	Ca(ICCV2/ICC4958)SNP_01894	2033	G	A	3	5	--	--
CakTC42785	Ca(ICCV2/ICC4958)SNP_01895	217	A	G	11	3	--	--
	Ca(ICCV2/ICC4958)SNP_01896	239	T	A	11	3	--	--
	Ca(ICCV2/ICC4958)SNP_01897	418	T	C	3	3	--	--
	Ca(ICCV2/ICC4958)SNP_01898	537	C	A	3	3	--	--
CakTC37403	Ca(ICCV2/ICC4958)SNP_01899	198	T	C	38	49	--	--
CakTC10282	Ca(ICCV2/ICC4958)SNP_01900	980	T	C	6	3	--	ABI3VP1
CakTC34533	Ca(ICCV2/ICC4958)SNP_01901	3622	C	G	8	10	--	SET
CakTC35711	Ca(ICCV2/ICC4958)SNP_01902	3151	G	A	39	69	--	--
CakTC19644	Ca(ICCV2/ICC4958)SNP_01903	751	T	C	4	3	--	--
CakTC39777	Ca(ICCV2/ICC4958)SNP_01904	746	G	C	10	21	--	--
CakTC28475	Ca(ICCV2/ICC4958)SNP_01905	260	T	A	5	4	--	--
	Ca(ICCV2/ICC4958)SNP_01906	411	C	T	5	3	--	--
	Ca(ICCV2/ICC4958)SNP_01907	2729	G	A	8	16	--	--
CakTC11757	Ca(ICCV2/ICC4958)SNP_01908	693	A	G	4	3	Root	--
CakTC10219	Ca(ICCV2/ICC4958)SNP_01909	471	T	G	5	5	--	--
CakTC21942	Ca(ICCV2/ICC4958)SNP_01910	320	A	G	8	4	--	--
CakTC37315	Ca(ICCV2/ICC4958)SNP_01911	833	A	G	46	27	--	--
CakTC32764	Ca(ICCV2/ICC4958)SNP_01912	577	G	A	5	4	--	--
	Ca(ICCV2/ICC4958)SNP_01913	2068	A	T	7	3	--	--
	Ca(ICCV2/ICC4958)SNP_01914	2179	G	A	5	4	--	--
CakTC03897	Ca(ICCV2/ICC4958)SNP_01915	427	A	G	8	4	--	GRAS
	Ca(ICCV2/ICC4958)SNP_01916	604	G	A	7	7	--	GRAS
	Ca(ICCV2/ICC4958)SNP_01917	985	A	G	11	8	--	GRAS
	Ca(ICCV2/ICC4958)SNP_01918	1009	T	C	9	7	--	GRAS

	Ca(ICCV2/ICC4958)SNP_01919	1263	C	T	10	4	--	GRAS
	Ca(ICCV2/ICC4958)SNP_01920	1362	A	G	10	6	--	GRAS
	Ca(ICCV2/ICC4958)SNP_01921	1417	C	T	7	5	--	GRAS
	Ca(ICCV2/ICC4958)SNP_01922	1528	A	G	7	3	--	GRAS
	Ca(ICCV2/ICC4958)SNP_01923	1592	C	G	9	4	--	GRAS
	Ca(ICCV2/ICC4958)SNP_01924	1624	T	C	9	4	--	GRAS
	Ca(ICCV2/ICC4958)SNP_01925	1625	G	A	9	4	--	GRAS
	Ca(ICCV2/ICC4958)SNP_01926	1796	A	C	8	4	--	GRAS
	Ca(ICCV2/ICC4958)SNP_01927	2623	T	A	3	3	--	GRAS
CakTC10854	Ca(ICCV2/ICC4958)SNP_01928	297	T	C	3	3	--	--
CakTC36380	Ca(ICCV2/ICC4958)SNP_01929	110	A	G	18	3	--	--
CakTC26857	Ca(ICCV2/ICC4958)SNP_01930	1577	G	A	13	12	--	--
	Ca(ICCV2/ICC4958)SNP_01931	2882	T	C	8	8	--	--
	Ca(ICCV2/ICC4958)SNP_01932	2972	G	A	9	8	--	--
CakTC38606	Ca(ICCV2/ICC4958)SNP_01933	267	A	G	4	7	--	C2C2-GATA
	Ca(ICCV2/ICC4958)SNP_01934	279	T	A	5	7	--	C2C2-GATA
	Ca(ICCV2/ICC4958)SNP_01935	490	G	C	11	4	--	C2C2-GATA
	Ca(ICCV2/ICC4958)SNP_01936	661	C	T	15	5	--	C2C2-GATA
	Ca(ICCV2/ICC4958)SNP_01937	820	G	A	17	7	--	C2C2-GATA
CakTC39282	Ca(ICCV2/ICC4958)SNP_01938	849	T	C	5	8	--	--
CakTC40336	Ca(ICCV2/ICC4958)SNP_01939	121	C	A	6	11	--	--
CakTC04658	Ca(ICCV2/ICC4958)SNP_01940	600	T	C	6	11	--	--
CakTC42568	Ca(ICCV2/ICC4958)SNP_01941	1061	A	C	7	11	--	--
CakTC27441	Ca(ICCV2/ICC4958)SNP_01942	26	C	T	15	14	--	--
	Ca(ICCV2/ICC4958)SNP_01943	68	G	C	18	17	--	--
	Ca(ICCV2/ICC4958)SNP_01944	355	T	A	43	24	--	--
	Ca(ICCV2/ICC4958)SNP_01945	1001	A	G	47	31	--	--
	Ca(ICCV2/ICC4958)SNP_01946	1030	C	T	45	32	--	--
	Ca(ICCV2/ICC4958)SNP_01947	1099	G	A	31	26	--	--
	Ca(ICCV2/ICC4958)SNP_01948	1101	T	C	34	26	--	--
CakTC35770	Ca(ICCV2/ICC4958)SNP_01949	2881	T	C	6	3	--	--
CakTC27681	Ca(ICCV2/ICC4958)SNP_01950	975	G	A	4	3	--	--
CakTC23916	Ca(ICCV2/ICC4958)SNP_01951	1073	T	G	7	5	--	--
	Ca(ICCV2/ICC4958)SNP_01952	1684	G	A	5	7	--	--
	Ca(ICCV2/ICC4958)SNP_01953	1760	T	C	4	4	--	--
	Ca(ICCV2/ICC4958)SNP_01954	2861	T	C	4	5	--	--
	Ca(ICCV2/ICC4958)SNP_01955	2960	C	G	4	4	--	--
CakTC42135	Ca(ICCV2/ICC4958)SNP_01956	325	T	C	15	15	--	AP2-EREBP
CakTC22939	Ca(ICCV2/ICC4958)SNP_01957	2896	C	T	228	229	--	--
CakTC31747	Ca(ICCV2/ICC4958)SNP_01958	2159	C	G	4	3	--	--
CakTC26955	Ca(ICCV2/ICC4958)SNP_01959	2414	A	G	4	5	--	--
CakTC40688	Ca(ICCV2/ICC4958)SNP_01960	202	G	T	5	9	--	--
CakTC39393	Ca(ICCV2/ICC4958)SNP_01961	603	C	A	31	39	--	--
	Ca(ICCV2/ICC4958)SNP_01962	714	G	C	45	43	--	--
	Ca(ICCV2/ICC4958)SNP_01963	1026	T	A	26	52	--	--
	Ca(ICCV2/ICC4958)SNP_01964	1331	G	T	20	41	--	--
CakTC42868	Ca(ICCV2/ICC4958)SNP_01965	568	A	G	6	3	--	--
CakTC13274	Ca(ICCV2/ICC4958)SNP_01966	234	A	T	5	3	Flower bud	--
	Ca(ICCV2/ICC4958)SNP_01967	235	T	G	4	3	Flower bud	--
	Ca(ICCV2/ICC4958)SNP_01968	248	T	A	4	3	Flower bud	--
	Ca(ICCV2/ICC4958)SNP_01969	251	A	G	3	3	Flower bud	--
	Ca(ICCV2/ICC4958)SNP_01970	273	A	C	4	3	Flower bud	--
	Ca(ICCV2/ICC4958)SNP_01971	343	A	G	8	3	Flower bud	--
CakTC33936	Ca(ICCV2/ICC4958)SNP_01972	353	T	C	5	8	--	--
CakTC40247	Ca(ICCV2/ICC4958)SNP_01973	787	T	C	4	10	--	--
	Ca(ICCV2/ICC4958)SNP_01974	986	A	G	5	8	--	--
CakTC38762	Ca(ICCV2/ICC4958)SNP_01975	1142	C	G	11	13	--	--
CakTC13926	Ca(ICCV2/ICC4958)SNP_01976	1289	A	G	4	3	--	--
CakTC33875	Ca(ICCV2/ICC4958)SNP_01977	1986	C	T	7	13	--	--
	Ca(ICCV2/ICC4958)SNP_01978	2160	G	A	4	11	--	--
CakTC09935	Ca(ICCV2/ICC4958)SNP_01979	225	T	C	6	6	Flower bud	--
CakTC29977	Ca(ICCV2/ICC4958)SNP_01980	531	C	T	33	38	--	--
CakTC32000	Ca(ICCV2/ICC4958)SNP_01981	1602	T	G	6	10	--	--
CakTC10543	Ca(ICCV2/ICC4958)SNP_01982	167	T	G	4	3	--	--
	Ca(ICCV2/ICC4958)SNP_01983	171	T	G	4	3	--	--
	Ca(ICCV2/ICC4958)SNP_01984	186	C	A	5	4	--	--
	Ca(ICCV2/ICC4958)SNP_01985	206	T	A	5	4	--	--
CakTC05357	Ca(ICCV2/ICC4958)SNP_01986	290	G	A	16	11	--	--
