# Supplementary material for: Comparative Analysis of Kabuli Chickpea Transcriptome with Desi and Wild Chickpea Provides a Rich Resource for Development of Functional Markers
Source: PLoS One. 2012 Dec 27;7(12):e52443. doi: 10.1371/journal.pone.0052443 (PMC3531472; doi:10.1371/journal.pone.0052443)
Supplement: Table S11 — List of SNPs identified between kabuli and wild chickpea. (PDF) [file pone.0052443.s021.pdf]

Table S11. List of SNPs identified between kabuli and wild chickpea.

Kabuli transcript ID	SNP_IDS	SNP position (kabuli)	Kabuli allele	Wild allele	Read depth kabuli	Read depth wild	Tissue specificity	TF family
CakTC31493	Ca(ICCV2/PI489777)SNP_00001	977	G	A	8	3	--	--
CakTC42908	Ca(ICCV2/PI489777)SNP_00002	296	C	T	7	14	--	--
	Ca(ICCV2/PI489777)SNP_00003	997	A	G	36	10	--	--
	Ca(ICCV2/PI489777)SNP_00004	1673	A	C	12	3	--	--
	Ca(ICCV2/PI489777)SNP_00005	1768	G	C	10	5	--	--
	Ca(ICCV2/PI489777)SNP_00006	1847	C	G	9	7	--	--
CakTC38477	Ca(ICCV2/PI489777)SNP_00007	69	G	T	23	11	--	--
	Ca(ICCV2/PI489777)SNP_00008	83	T	C	27	13	--	--
	Ca(ICCV2/PI489777)SNP_00009	252	T	C	34	14	--	--
CakTC22840	Ca(ICCV2/PI489777)SNP_00010	1444	A	T	6	8	--	--
	Ca(ICCV2/PI489777)SNP_00011	1471	C	A	6	6	--	--
	Ca(ICCV2/PI489777)SNP_00012	1478	A	C	6	7	--	--
	Ca(ICCV2/PI489777)SNP_00013	1510	T	A	7	8	--	--
	Ca(ICCV2/PI489777)SNP_00014	1512	C	T	7	8	--	--
	Ca(ICCV2/PI489777)SNP_00015	1579	G	A	5	4	--	--
	Ca(ICCV2/PI489777)SNP_00016	1602	A	G	8	8	--	--
	Ca(ICCV2/PI489777)SNP_00017	1604	A	C	8	8	--	--
	Ca(ICCV2/PI489777)SNP_00018	1613	T	C	6	7	--	--
	Ca(ICCV2/PI489777)SNP_00019	1642	C	T	6	8	--	--
	Ca(ICCV2/PI489777)SNP_00020	1643	T	C	7	7	--	--
	Ca(ICCV2/PI489777)SNP_00021	1678	G	T	8	7	--	--
	Ca(ICCV2/PI489777)SNP_00022	1736	A	G	9	7	--	--
	Ca(ICCV2/PI489777)SNP_00023	1743	C	A	9	7	--	--
CakTC28571	Ca(ICCV2/PI489777)SNP_00024	1688	C	T	24	18	--	CCHC
	Ca(ICCV2/PI489777)SNP_00025	1961	A	G	33	11	--	CCHC
	Ca(ICCV2/PI489777)SNP_00026	1964	G	T	32	11	--	CCHC
	Ca(ICCV2/PI489777)SNP_00027	2192	A	T	36	7	--	CCHC
CakTC40343	Ca(ICCV2/PI489777)SNP_00028	353	G	A	13	9	--	CCHC
	Ca(ICCV2/PI489777)SNP_00029	623	A	G	5	5	--	CCHC
	Ca(ICCV2/PI489777)SNP_00030	632	T	C	5	5	--	CCHC
	Ca(ICCV2/PI489777)SNP_00031	654	G	A	6	6	--	CCHC
	Ca(ICCV2/PI489777)SNP_00032	791	A	G	12	9	--	CCHC
	Ca(ICCV2/PI489777)SNP_00033	1115	T	C	8	8	--	CCHC
	Ca(ICCV2/PI489777)SNP_00034	1715	C	T	16	4	--	CCHC
	Ca(ICCV2/PI489777)SNP_00035	1782	A	G	14	5	--	CCHC
	Ca(ICCV2/PI489777)SNP_00036	1926	G	A	5	3	--	CCHC
CakTC22593	Ca(ICCV2/PI489777)SNP_00037	579	A	T	13	3	Young_pod	--
	Ca(ICCV2/PI489777)SNP_00038	1620	C	T	5	6	Young_pod	--
	Ca(ICCV2/PI489777)SNP_00039	1861	T	G	7	8	Young_pod	--
CakTC23721	Ca(ICCV2/PI489777)SNP_00040	488	C	G	9	9	--	--
CakTC38575	Ca(ICCV2/PI489777)SNP_00041	167	G	A	13	8	--	--
	Ca(ICCV2/PI489777)SNP_00042	533	T	C	14	5	--	--
	Ca(ICCV2/PI489777)SNP_00043	735	A	G	9	7	--	--
CakTC35170	Ca(ICCV2/PI489777)SNP_00044	704	C	T	4	5	Shoot	MYB
	Ca(ICCV2/PI489777)SNP_00045	717	C	G	5	6	Shoot	MYB
	Ca(ICCV2/PI489777)SNP_00046	2667	T	C	8	4	Shoot	MYB
	Ca(ICCV2/PI489777)SNP_00047	3033	C	T	5	5	Shoot	MYB
CakTC43109	Ca(ICCV2/PI489777)SNP_00048	503	T	C	7	8	--	--
	Ca(ICCV2/PI489777)SNP_00049	602	A	G	6	4	--	--
CakTC42297	Ca(ICCV2/PI489777)SNP_00050	70	G	C	8	6	--	bZIP
	Ca(ICCV2/PI489777)SNP_00051	111	G	C	9	8	--	bZIP
	Ca(ICCV2/PI489777)SNP_00052	208	C	G	10	8	--	bZIP
	Ca(ICCV2/PI489777)SNP_00053	422	T	A	12	10	--	bZIP
CakTC35177	Ca(ICCV2/PI489777)SNP_00054	204	C	T	6	5	--	--
	Ca(ICCV2/PI489777)SNP_00055	322	A	T	3	5	--	--
	Ca(ICCV2/PI489777)SNP_00056	1117	T	A	7	10	--	--
	Ca(ICCV2/PI489777)SNP_00057	1276	C	T	7	5	--	--
	Ca(ICCV2/PI489777)SNP_00058	1351	T	C	5	4	--	--
	Ca(ICCV2/PI489777)SNP_00059	1910	T	C	5	5	--	--
	Ca(ICCV2/PI489777)SNP_00060	2112	G	A	8	3	--	--
CakTC26261	Ca(ICCV2/PI489777)SNP_00061	304	A	G	13	17	--	--
	Ca(ICCV2/PI489777)SNP_00062	385	G	C	10	10	--	--
	Ca(ICCV2/PI489777)SNP_00063	744	T	A	9	10	--	--
	Ca(ICCV2/PI489777)SNP_00064	757	C	T	8	10	--	--
	Ca(ICCV2/PI489777)SNP_00065	911	T	C	5	9	--	--
CakTC23948	Ca(ICCV2/PI489777)SNP_00066	1235	C	G	173	81	--	--
CakTC39219	Ca(ICCV2/PI489777)SNP_00067	104	C	A	13	7	--	--
	Ca(ICCV2/PI489777)SNP_00068	591	A	T	13	13	--	--
	Ca(ICCV2/PI489777)SNP_00069	954	C	T	14	12	--	--

	Ca(ICCV2/PI489777)SNP_00070	979	A	G	13	17	--	--
	Ca(ICCV2/PI489777)SNP_00071	1056	G	A	10	17	--	--
	Ca(ICCV2/PI489777)SNP_00072	1331	C	T	18	11	--	--
	Ca(ICCV2/PI489777)SNP_00073	1385	T	C	10	9	--	--
	Ca(ICCV2/PI489777)SNP_00074	1390	G	A	13	9	--	--
	Ca(ICCV2/PI489777)SNP_00075	1472	T	G	10	8	--	--
CakTC06071	Ca(ICCV2/PI489777)SNP_00076	4262	C	T	9	5	--	--
	Ca(ICCV2/PI489777)SNP_00077	4285	G	A	9	6	--	--
CakTC22879	Ca(ICCV2/PI489777)SNP_00078	594	C	T	13	12	--	--
	Ca(ICCV2/PI489777)SNP_00079	975	G	A	16	10	--	--
	Ca(ICCV2/PI489777)SNP_00080	1180	C	A	8	7	--	--
CakTC32038	Ca(ICCV2/PI489777)SNP_00081	75	G	C	10	7	--	--
	Ca(ICCV2/PI489777)SNP_00082	108	T	C	9	7	--	--
	Ca(ICCV2/PI489777)SNP_00083	617	A	G	9	5	--	--
	Ca(ICCV2/PI489777)SNP_00084	1022	T	C	6	4	--	--
	Ca(ICCV2/PI489777)SNP_00085	1688	C	A	10	8	--	--
	Ca(ICCV2/PI489777)SNP_00086	1710	G	A	8	8	--	--
	Ca(ICCV2/PI489777)SNP_00087	1834	A	G	10	5	--	--
	Ca(ICCV2/PI489777)SNP_00088	2835	G	A	3	3	--	--
	Ca(ICCV2/PI489777)SNP_00089	2836	C	G	3	3	--	--
	Ca(ICCV2/PI489777)SNP_00090	3531	G	A	7	4	--	--
	Ca(ICCV2/PI489777)SNP_00091	4119	A	C	15	3	--	--
	Ca(ICCV2/PI489777)SNP_00092	4383	A	G	19	4	--	--
	Ca(ICCV2/PI489777)SNP_00093	4464	G	C	15	9	--	--
	Ca(ICCV2/PI489777)SNP_00094	4793	G	A	16	10	--	--
CakTC31333	Ca(ICCV2/PI489777)SNP_00095	1162	G	C	7	5	Young_pod	--
CakTC43001	Ca(ICCV2/PI489777)SNP_00096	172	G	A	32	24	--	--
CakTC29187	Ca(ICCV2/PI489777)SNP_00097	1229	A	G	29	5	--	CCHC
	Ca(ICCV2/PI489777)SNP_00098	1238	A	G	30	5	--	CCHC
	Ca(ICCV2/PI489777)SNP_00099	1280	A	G	31	5	--	CCHC
	Ca(ICCV2/PI489777)SNP_00100	1340	G	T	27	6	--	CCHC
	Ca(ICCV2/PI489777)SNP_00101	1350	A	C	26	5	--	CCHC
	Ca(ICCV2/PI489777)SNP_00102	1412	G	C	23	6	--	CCHC
	Ca(ICCV2/PI489777)SNP_00103	1421	C	T	22	6	--	CCHC
	Ca(ICCV2/PI489777)SNP_00104	1442	G	T	18	5	--	CCHC
CakTC39975	Ca(ICCV2/PI489777)SNP_00105	982	A	C	17	11	--	--
CakTC39048	Ca(ICCV2/PI489777)SNP_00106	933	G	T	6	8	--	--
	Ca(ICCV2/PI489777)SNP_00107	1029	G	A	6	14	--	--
CakTC10736	Ca(ICCV2/PI489777)SNP_00108	313	C	T	13	17	--	--
	Ca(ICCV2/PI489777)SNP_00109	662	G	C	13	9	--	--
	Ca(ICCV2/PI489777)SNP_00110	1265	A	C	5	5	--	--
CakTC39125	Ca(ICCV2/PI489777)SNP_00111	1341	A	C	8	9	--	bZIP
CakTC24040	Ca(ICCV2/PI489777)SNP_00112	599	A	G	14	8	--	--
	Ca(ICCV2/PI489777)SNP_00113	866	G	A	17	22	--	--
	Ca(ICCV2/PI489777)SNP_00114	1891	T	C	23	6	--	--
CakTC38380	Ca(ICCV2/PI489777)SNP_00115	429	A	G	4	5	--	C2C2-Dof
	Ca(ICCV2/PI489777)SNP_00116	498	T	C	5	4	--	C2C2-Dof
	Ca(ICCV2/PI489777)SNP_00117	980	T	C	4	5	--	C2C2-Dof
	Ca(ICCV2/PI489777)SNP_00118	1027	T	C	4	5	--	C2C2-Dof
CakTC24318	Ca(ICCV2/PI489777)SNP_00119	1007	T	C	3	3	Root	--
CakTC23605	Ca(ICCV2/PI489777)SNP_00120	70	C	T	11	5	--	PLATZ
	Ca(ICCV2/PI489777)SNP_00121	374	T	C	77	28	--	PLATZ
	Ca(ICCV2/PI489777)SNP_00122	901	A	G	126	53	--	PLATZ
	Ca(ICCV2/PI489777)SNP_00123	1000	G	A	163	53	--	PLATZ
CakTC42392	Ca(ICCV2/PI489777)SNP_00124	256	C	G	13	3	--	--
CakTC39988	Ca(ICCV2/PI489777)SNP_00125	395	T	C	14	9	--	--
	Ca(ICCV2/PI489777)SNP_00126	425	A	G	10	8	--	--
	Ca(ICCV2/PI489777)SNP_00127	961	G	C	20	9	--	--
	Ca(ICCV2/PI489777)SNP_00128	971	T	C	18	9	--	--
	Ca(ICCV2/PI489777)SNP_00129	973	G	C	17	8	--	--
	Ca(ICCV2/PI489777)SNP_00130	1008	T	C	17	9	--	--
	Ca(ICCV2/PI489777)SNP_00131	1087	A	T	16	7	--	--
CakTC07561	Ca(ICCV2/PI489777)SNP_00132	539	A	C	7	3	--	--
	Ca(ICCV2/PI489777)SNP_00133	623	G	C	7	5	--	--
CakTC03573	Ca(ICCV2/PI489777)SNP_00134	770	A	T	4	3	--	--
CakTC41142	Ca(ICCV2/PI489777)SNP_00135	532	A	T	14	11	--	--
	Ca(ICCV2/PI489777)SNP_00136	540	T	A	9	12	--	--
CakTC43065	Ca(ICCV2/PI489777)SNP_00137	874	T	A	3	4	--	--
CakTC32837	Ca(ICCV2/PI489777)SNP_00138	171	C	A	14	7	--	--
	Ca(ICCV2/PI489777)SNP_00139	368	G	T	18	14	--	--
	Ca(ICCV2/PI489777)SNP_00140	1924	T	C	9	14	--	--
	Ca(ICCV2/PI489777)SNP_00141	2771	G	A	9	7	--	--
CakTC39358	Ca(ICCV2/PI489777)SNP_00142	389	A	G	9	4	--	--
	Ca(ICCV2/PI489777)SNP_00143	744	A	G	13	6	--	--

	Ca(ICCV2/PI489777)SNP_00144	1122	A	G	13	4	--	--
CakTC38756	Ca(ICCV2/PI489777)SNP_00145	464	G	A	59	16	--	--
	Ca(ICCV2/PI489777)SNP_00146	1069	C	T	38	13	--	--
	Ca(ICCV2/PI489777)SNP_00147	1262	C	T	25	11	--	--
CakTC38895	Ca(ICCV2/PI489777)SNP_00148	512	A	C	34	11	--	--
	Ca(ICCV2/PI489777)SNP_00149	590	T	C	43	31	--	--
	Ca(ICCV2/PI489777)SNP_00150	818	T	G	64	47	--	--
CakTC29974	Ca(ICCV2/PI489777)SNP_00151	1028	A	G	8	8	--	--
	Ca(ICCV2/PI489777)SNP_00152	1512	A	G	14	12	--	--
	Ca(ICCV2/PI489777)SNP_00153	1779	T	C	20	16	--	--
	Ca(ICCV2/PI489777)SNP_00154	1859	C	T	16	10	--	--
	Ca(ICCV2/PI489777)SNP_00155	2115	C	T	20	19	--	--
	Ca(ICCV2/PI489777)SNP_00156	3843	C	G	17	13	--	--
	Ca(ICCV2/PI489777)SNP_00157	4012	A	G	25	9	--	--
	Ca(ICCV2/PI489777)SNP_00158	4442	C	T	21	13	--	--
	Ca(ICCV2/PI489777)SNP_00159	4815	A	G	10	9	--	--
	Ca(ICCV2/PI489777)SNP_00160	4936	T	G	3	8	--	--
	Ca(ICCV2/PI489777)SNP_00161	5008	A	T	4	4	--	--
CakTC42521	Ca(ICCV2/PI489777)SNP_00162	703	C	A	14	9	--	MYB
	Ca(ICCV2/PI489777)SNP_00163	715	A	C	12	9	--	MYB
CakTC37634	Ca(ICCV2/PI489777)SNP_00164	464	G	T	8	3	--	--
	Ca(ICCV2/PI489777)SNP_00165	815	G	A	13	11	--	--
CakTC09982	Ca(ICCV2/PI489777)SNP_00166	294	T	C	7	10	--	ARR-B
	Ca(ICCV2/PI489777)SNP_00167	1004	T	C	18	8	--	ARR-B
CakTC26868	Ca(ICCV2/PI489777)SNP_00168	81	G	T	5	3	--	--
	Ca(ICCV2/PI489777)SNP_00169	649	G	C	3	3	--	--
	Ca(ICCV2/PI489777)SNP_00170	682	C	T	4	5	--	--
	Ca(ICCV2/PI489777)SNP_00171	934	A	G	6	4	--	--
CakTC05101	Ca(ICCV2/PI489777)SNP_00172	1431	T	C	4	8	--	--
CakTC29462	Ca(ICCV2/PI489777)SNP_00173	190	T	G	23	12	--	--
	Ca(ICCV2/PI489777)SNP_00174	742	A	G	18	7	--	--
	Ca(ICCV2/PI489777)SNP_00175	760	G	A	16	7	--	--
	Ca(ICCV2/PI489777)SNP_00176	799	A	T	24	7	--	--
	Ca(ICCV2/PI489777)SNP_00177	1060	A	G	13	5	--	--
	Ca(ICCV2/PI489777)SNP_00178	1068	G	T	13	5	--	--
CakTC31984	Ca(ICCV2/PI489777)SNP_00179	1863	T	C	16	4	--	SBP
CakTC28147	Ca(ICCV2/PI489777)SNP_00180	431	A	T	6	3	--	--
	Ca(ICCV2/PI489777)SNP_00181	1325	C	T	19	5	--	--
	Ca(ICCV2/PI489777)SNP_00182	1354	A	T	19	5	--	--
	Ca(ICCV2/PI489777)SNP_00183	1508	G	A	7	5	--	--
	Ca(ICCV2/PI489777)SNP_00184	2208	A	G	10	6	--	--
	Ca(ICCV2/PI489777)SNP_00185	2328	A	G	8	4	--	--
CakTC28495	Ca(ICCV2/PI489777)SNP_00186	180	C	A	16	7	--	--
	Ca(ICCV2/PI489777)SNP_00187	1819	G	C	32	9	--	--
	Ca(ICCV2/PI489777)SNP_00188	2828	C	T	25	6	--	--
CakTC39166	Ca(ICCV2/PI489777)SNP_00189	1377	A	G	10	3	--	--
	Ca(ICCV2/PI489777)SNP_00190	1505	T	C	8	3	--	--
CakTC36184	Ca(ICCV2/PI489777)SNP_00191	3668	A	T	11	6	--	--
	Ca(ICCV2/PI489777)SNP_00192	4364	G	T	9	4	--	--
	Ca(ICCV2/PI489777)SNP_00193	4554	A	G	8	14	--	--
	Ca(ICCV2/PI489777)SNP_00194	4698	A	T	17	13	--	--
CakTC43237	Ca(ICCV2/PI489777)SNP_00195	179	C	T	20	3	--	--
	Ca(ICCV2/PI489777)SNP_00196	182	T	G	19	4	--	--
CakTC29251	Ca(ICCV2/PI489777)SNP_00197	452	T	G	12	11	--	--
CakTC40473	Ca(ICCV2/PI489777)SNP_00198	67	C	A	3	3	--	--
	Ca(ICCV2/PI489777)SNP_00199	193	T	A	6	3	--	--
	Ca(ICCV2/PI489777)SNP_00200	201	G	C	7	3	--	--
	Ca(ICCV2/PI489777)SNP_00201	319	C	A	9	5	--	--
	Ca(ICCV2/PI489777)SNP_00202	1471	A	G	7	6	--	--
	Ca(ICCV2/PI489777)SNP_00203	1759	C	T	8	9	--	--
	Ca(ICCV2/PI489777)SNP_00204	1930	T	C	9	12	--	--
CakTC22816	Ca(ICCV2/PI489777)SNP_00205	51	T	A	3	8	--	--
CakTC23848	Ca(ICCV2/PI489777)SNP_00206	441	G	T	4	3	--	--
CakTC42134	Ca(ICCV2/PI489777)SNP_00207	334	A	C	7	9	--	--
	Ca(ICCV2/PI489777)SNP_00208	642	T	C	9	7	--	--
	Ca(ICCV2/PI489777)SNP_00209	674	C	T	9	6	--	--
	Ca(ICCV2/PI489777)SNP_00210	1475	G	A	5	9	--	--
CakTC27274	Ca(ICCV2/PI489777)SNP_00211	1371	C	T	5	3	--	--
CakTC09500	Ca(ICCV2/PI489777)SNP_00212	84	C	A	22	14	--	--
	Ca(ICCV2/PI489777)SNP_00213	990	A	T	20	22	--	--
CakTC25597	Ca(ICCV2/PI489777)SNP_00214	549	T	C	9	4	--	--
	Ca(ICCV2/PI489777)SNP_00215	614	G	C	7	3	--	--
	Ca(ICCV2/PI489777)SNP_00216	618	T	A	7	3	--	--
CakTC01341	Ca(ICCV2/PI489777)SNP_00217	189	T	A	3	3	--	--

CakTC25988	Ca(ICCV2/PI489777)SNP_00218	215	T	C	5	4	--	--
	Ca(ICCV2/PI489777)SNP_00219	396	G	C	5	5	--	--
	Ca(ICCV2/PI489777)SNP_00220	428	A	G	4	5	--	--
	Ca(ICCV2/PI489777)SNP_00221	470	C	T	6	5	--	--
	Ca(ICCV2/PI489777)SNP_00222	603	G	A	7	4	--	--
CakTC26229	Ca(ICCV2/PI489777)SNP_00223	799	A	G	3	4	--	ABI3VP1
	Ca(ICCV2/PI489777)SNP_00224	2171	C	T	15	3	--	ABI3VP1
	Ca(ICCV2/PI489777)SNP_00225	2578	G	C	8	3	--	ABI3VP1
CakTC37050	Ca(ICCV2/PI489777)SNP_00226	263	A	G	5	4	--	--
	Ca(ICCV2/PI489777)SNP_00227	659	C	T	7	3	--	--
	Ca(ICCV2/PI489777)SNP_00228	1034	G	A	7	4	--	--
CakTC40607	Ca(ICCV2/PI489777)SNP_00229	182	A	C	9	7	--	--
	Ca(ICCV2/PI489777)SNP_00230	205	C	A	9	5	--	--
	Ca(ICCV2/PI489777)SNP_00231	281	G	T	12	7	--	--
	Ca(ICCV2/PI489777)SNP_00232	303	A	T	11	6	--	--
	Ca(ICCV2/PI489777)SNP_00233	798	A	G	7	4	--	--
	Ca(ICCV2/PI489777)SNP_00234	1215	T	C	12	15	--	--
	Ca(ICCV2/PI489777)SNP_00235	1245	G	A	10	13	--	--
	Ca(ICCV2/PI489777)SNP_00236	1380	A	G	7	13	--	--
	Ca(ICCV2/PI489777)SNP_00237	1473	T	C	7	14	--	--
CakTC29020	Ca(ICCV2/PI489777)SNP_00238	142	C	G	95	61	--	AP2-EREBP
	Ca(ICCV2/PI489777)SNP_00239	386	G	A	109	49	--	AP2-EREBP
	Ca(ICCV2/PI489777)SNP_00240	672	C	T	147	45	--	AP2-EREBP
	Ca(ICCV2/PI489777)SNP_00241	726	A	T	166	64	--	AP2-EREBP
	Ca(ICCV2/PI489777)SNP_00242	1170	G	C	253	117	--	AP2-EREBP
	Ca(ICCV2/PI489777)SNP_00243	1389	G	C	88	56	--	AP2-EREBP
	Ca(ICCV2/PI489777)SNP_00244	1520	T	A	51	49	--	AP2-EREBP
	Ca(ICCV2/PI489777)SNP_00245	1557	C	T	42	39	--	AP2-EREBP
	Ca(ICCV2/PI489777)SNP_00246	1671	T	G	23	23	--	AP2-EREBP
CakTC41239	Ca(ICCV2/PI489777)SNP_00247	134	G	A	8	4	--	--
	Ca(ICCV2/PI489777)SNP_00248	265	C	T	8	7	--	--
	Ca(ICCV2/PI489777)SNP_00249	298	A	T	9	8	--	--
	Ca(ICCV2/PI489777)SNP_00250	364	C	T	10	8	--	--
	Ca(ICCV2/PI489777)SNP_00251	712	G	C	10	12	--	--
	Ca(ICCV2/PI489777)SNP_00252	799	G	A	10	14	--	--
	Ca(ICCV2/PI489777)SNP_00253	898	T	C	13	14	--	--
	Ca(ICCV2/PI489777)SNP_00254	1216	G	A	19	20	--	--
	Ca(ICCV2/PI489777)SNP_00255	1843	C	T	6	4	--	--
	Ca(ICCV2/PI489777)SNP_00256	1945	T	C	4	7	--	--
	Ca(ICCV2/PI489777)SNP_00257	2012	A	C	7	6	--	--
	Ca(ICCV2/PI489777)SNP_00258	2377	T	C	13	8	--	--
	Ca(ICCV2/PI489777)SNP_00259	2428	T	A	8	5	--	--
	Ca(ICCV2/PI489777)SNP_00260	2472	G	A	9	3	--	--
CakTC08612	Ca(ICCV2/PI489777)SNP_00261	2843	T	C	4	5	--	--
CakTC38885	Ca(ICCV2/PI489777)SNP_00262	227	T	C	3	4	--	--
	Ca(ICCV2/PI489777)SNP_00263	800	T	C	6	9	--	--
	Ca(ICCV2/PI489777)SNP_00264	804	G	C	6	9	--	--
CakTC35774	Ca(ICCV2/PI489777)SNP_00265	388	C	T	4	19	--	--
	Ca(ICCV2/PI489777)SNP_00266	794	A	T	4	10	--	--
CakTC39521	Ca(ICCV2/PI489777)SNP_00267	35	A	T	4	5	--	--
CakTC39885	Ca(ICCV2/PI489777)SNP_00268	252	G	T	49	32	--	--
	Ca(ICCV2/PI489777)SNP_00269	316	A	G	57	48	--	--
CakTC40943	Ca(ICCV2/PI489777)SNP_00270	851	G	T	9	4	--	--
CakTC38215	Ca(ICCV2/PI489777)SNP_00271	44	A	C	3	5	--	--
	Ca(ICCV2/PI489777)SNP_00272	245	G	T	6	7	--	--
	Ca(ICCV2/PI489777)SNP_00273	479	G	A	7	3	--	--
	Ca(ICCV2/PI489777)SNP_00274	596	C	T	6	5	--	--
CakTC33094	Ca(ICCV2/PI489777)SNP_00275	691	C	A	13	14	--	--
CakTC36872	Ca(ICCV2/PI489777)SNP_00276	157	C	G	25	6	--	--
	Ca(ICCV2/PI489777)SNP_00277	159	T	A	20	6	--	--
CakTC33548	Ca(ICCV2/PI489777)SNP_00278	418	G	A	8	12	--	--
	Ca(ICCV2/PI489777)SNP_00279	1091	A	C	10	7	--	--
CakTC41073	Ca(ICCV2/PI489777)SNP_00280	61	T	G	3	4	--	--
	Ca(ICCV2/PI489777)SNP_00281	470	A	C	12	9	--	--
	Ca(ICCV2/PI489777)SNP_00282	2060	G	A	27	14	--	--
	Ca(ICCV2/PI489777)SNP_00283	2299	G	A	15	7	--	--
CakTC42228	Ca(ICCV2/PI489777)SNP_00284	257	T	C	21	19	--	LIM
	Ca(ICCV2/PI489777)SNP_00285	295	C	A	27	22	--	LIM
CakTC41813	Ca(ICCV2/PI489777)SNP_00286	509	C	A	9	4	--	bHLH
CakTC38274	Ca(ICCV2/PI489777)SNP_00287	1918	T	C	3	4	--	--
	Ca(ICCV2/PI489777)SNP_00288	3964	A	G	6	5	--	--
CakTC22327	Ca(ICCV2/PI489777)SNP_00289	333	G	A	296	107	--	--
CakTC26901	Ca(ICCV2/PI489777)SNP_00290	292	G	T	23	4	--	--
	Ca(ICCV2/PI489777)SNP_00291	373	G	C	32	6	--	--

	Ca(ICCV2/PI489777)SNP_00292	850	C	T	34	5	--	--
	Ca(ICCV2/PI489777)SNP_00293	1096	G	T	23	5	--	--
CakTC37697	Ca(ICCV2/PI489777)SNP_00294	315	C	G	10	5	--	--
CakTC35927	Ca(ICCV2/PI489777)SNP_00295	403	G	T	5	5	--	--
CakTC34611	Ca(ICCV2/PI489777)SNP_00296	1365	T	A	3	7	--	--
CakTC40777	Ca(ICCV2/PI489777)SNP_00297	274	C	A	4	3	--	--
	Ca(ICCV2/PI489777)SNP_00298	624	C	A	3	3	--	--
CakTC25952	Ca(ICCV2/PI489777)SNP_00299	661	C	G	4	3	--	--
	Ca(ICCV2/PI489777)SNP_00300	1150	A	C	4	7	--	--
	Ca(ICCV2/PI489777)SNP_00301	1362	C	T	3	4	--	--
CakTC43249	Ca(ICCV2/PI489777)SNP_00302	267	C	T	9	6	--	--
	Ca(ICCV2/PI489777)SNP_00303	497	G	A	12	9	--	--
CakTC36244	Ca(ICCV2/PI489777)SNP_00304	2907	C	A	19	11	--	--
CakTC42986	Ca(ICCV2/PI489777)SNP_00305	144	T	C	3	9	--	--
CakTC42727	Ca(ICCV2/PI489777)SNP_00306	252	C	T	11	6	--	--
	Ca(ICCV2/PI489777)SNP_00307	332	A	G	14	9	--	--
	Ca(ICCV2/PI489777)SNP_00308	334	T	C	14	9	--	--
	Ca(ICCV2/PI489777)SNP_00309	976	C	G	10	9	--	--
	Ca(ICCV2/PI489777)SNP_00310	1181	A	G	5	6	--	--
	Ca(ICCV2/PI489777)SNP_00311	1372	G	T	8	12	--	--
	Ca(ICCV2/PI489777)SNP_00312	1564	G	A	6	11	--	--
	Ca(ICCV2/PI489777)SNP_00313	1574	C	T	6	11	--	--
CakTC33354	Ca(ICCV2/PI489777)SNP_00314	238	G	A	38	15	--	--
	Ca(ICCV2/PI489777)SNP_00315	673	C	T	56	20	--	--
	Ca(ICCV2/PI489777)SNP_00316	873	C	T	37	11	--	--
	Ca(ICCV2/PI489777)SNP_00317	1648	G	A	73	23	--	--
	Ca(ICCV2/PI489777)SNP_00318	1867	C	T	70	28	--	--
	Ca(ICCV2/PI489777)SNP_00319	1870	T	G	61	29	--	--
CakTC27620	Ca(ICCV2/PI489777)SNP_00320	390	G	T	10	3	--	--
	Ca(ICCV2/PI489777)SNP_00321	402	G	C	11	4	--	--
	Ca(ICCV2/PI489777)SNP_00322	576	C	A	15	5	--	--
	Ca(ICCV2/PI489777)SNP_00323	644	G	A	11	4	--	--
	Ca(ICCV2/PI489777)SNP_00324	747	A	G	19	3	--	--
	Ca(ICCV2/PI489777)SNP_00325	912	A	G	21	3	--	--
	Ca(ICCV2/PI489777)SNP_00326	1179	C	T	26	5	--	--
	Ca(ICCV2/PI489777)SNP_00327	2164	G	A	8	6	--	--
CakTC40501	Ca(ICCV2/PI489777)SNP_00328	361	C	G	18	12	--	--
	Ca(ICCV2/PI489777)SNP_00329	534	T	C	13	10	--	--
	Ca(ICCV2/PI489777)SNP_00330	547	C	A	13	11	--	--
	Ca(ICCV2/PI489777)SNP_00331	591	G	A	17	17	--	--
	Ca(ICCV2/PI489777)SNP_00332	747	G	A	12	10	--	--
	Ca(ICCV2/PI489777)SNP_00333	816	C	A	13	7	--	--
	Ca(ICCV2/PI489777)SNP_00334	1218	A	G	11	6	--	--
	Ca(ICCV2/PI489777)SNP_00335	1810	C	T	9	7	--	--
CakTC38684	Ca(ICCV2/PI489777)SNP_00336	238	T	C	17	13	--	--
CakTC26792	Ca(ICCV2/PI489777)SNP_00337	537	T	C	129	57	--	--
	Ca(ICCV2/PI489777)SNP_00338	741	G	C	95	56	--	--
	Ca(ICCV2/PI489777)SNP_00339	804	A	G	94	44	--	--
CakTC30350	Ca(ICCV2/PI489777)SNP_00340	95	A	T	4	5	Flower	bud
	Ca(ICCV2/PI489777)SNP_00341	107	T	C	5	5	Flower	bud
CakTC39287	Ca(ICCV2/PI489777)SNP_00342	389	C	T	11	4	--	--
	Ca(ICCV2/PI489777)SNP_00343	876	T	A	26	8	--	--
CakTC30035	Ca(ICCV2/PI489777)SNP_00344	324	C	T	11	8	--	--
	Ca(ICCV2/PI489777)SNP_00345	918	C	A	10	3	--	--
	Ca(ICCV2/PI489777)SNP_00346	1151	G	T	9	11	--	--
CakTC27443	Ca(ICCV2/PI489777)SNP_00347	330	T	C	9	4	--	--
CakTC35067	Ca(ICCV2/PI489777)SNP_00348	120	G	A	3	3	--	--
CakTC27942	Ca(ICCV2/PI489777)SNP_00349	1087	A	G	8	4	--	WRKY
CakTC11673	Ca(ICCV2/PI489777)SNP_00350	2499	C	T	4	4	--	--
	Ca(ICCV2/PI489777)SNP_00351	2885	G	A	5	5	--	--
CakTC33125	Ca(ICCV2/PI489777)SNP_00352	105	A	T	15	10	--	--
	Ca(ICCV2/PI489777)SNP_00353	124	G	A	18	10	--	--
	Ca(ICCV2/PI489777)SNP_00354	437	A	T	50	18	--	--
	Ca(ICCV2/PI489777)SNP_00355	914	C	T	42	14	--	--
	Ca(ICCV2/PI489777)SNP_00356	1028	A	C	55	12	--	--
	Ca(ICCV2/PI489777)SNP_00357	1154	C	T	40	12	--	--
	Ca(ICCV2/PI489777)SNP_00358	1544	T	G	39	23	--	--
	Ca(ICCV2/PI489777)SNP_00359	1672	G	C	37	22	--	--
	Ca(ICCV2/PI489777)SNP_00360	1899	T	A	9	6	--	--
	Ca(ICCV2/PI489777)SNP_00361	1949	T	C	5	4	--	--
CakTC30077	Ca(ICCV2/PI489777)SNP_00362	369	G	A	11	5	--	--
	Ca(ICCV2/PI489777)SNP_00363	501	A	G	11	5	--	--
	Ca(ICCV2/PI489777)SNP_00364	2614	A	G	5	3	--	--
	Ca(ICCV2/PI489777)SNP_00365	2620	A	G	5	3	--	--

	Ca(ICCV2/PI489777)SNP_00366	3074	T	G	6	5	--	--
	Ca(ICCV2/PI489777)SNP_00367	3152	C	G	5	4	--	--
	Ca(ICCV2/PI489777)SNP_00368	3162	A	C	3	4	--	--
CakTC04000	Ca(ICCV2/PI489777)SNP_00369	510	C	T	100	73	--	--
CakTC26626	Ca(ICCV2/PI489777)SNP_00370	517	A	G	11	3	--	--
CakTC33435	Ca(ICCV2/PI489777)SNP_00371	304	C	T	33	4	--	--
	Ca(ICCV2/PI489777)SNP_00372	322	A	G	35	5	--	--
CakTC35120	Ca(ICCV2/PI489777)SNP_00373	351	A	T	3	3	Flower	bud
CakTC10206	Ca(ICCV2/PI489777)SNP_00374	282	T	A	7	3	--	--
CakTC37718	Ca(ICCV2/PI489777)SNP_00375	198	T	C	5	6	--	--
	Ca(ICCV2/PI489777)SNP_00376	318	G	A	4	6	--	--
	Ca(ICCV2/PI489777)SNP_00377	350	C	T	6	6	--	--
	Ca(ICCV2/PI489777)SNP_00378	374	C	T	6	8	--	--
	Ca(ICCV2/PI489777)SNP_00379	434	C	T	5	8	--	--
	Ca(ICCV2/PI489777)SNP_00380	506	A	G	6	5	--	--
	Ca(ICCV2/PI489777)SNP_00381	521	T	C	6	5	--	--
	Ca(ICCV2/PI489777)SNP_00382	1313	T	G	5	5	--	--
CakTC26404	Ca(ICCV2/PI489777)SNP_00383	1230	G	T	4	3	--	--
CakTC10811	Ca(ICCV2/PI489777)SNP_00384	480	T	C	19	16	--	--
	Ca(ICCV2/PI489777)SNP_00385	513	A	G	17	15	--	--
CakTC40265	Ca(ICCV2/PI489777)SNP_00386	1250	G	A	5	3	--	--
	Ca(ICCV2/PI489777)SNP_00387	1329	T	C	6	3	--	--
	Ca(ICCV2/PI489777)SNP_00388	1694	A	G	7	3	--	--
	Ca(ICCV2/PI489777)SNP_00389	1879	T	G	10	3	--	--
	Ca(ICCV2/PI489777)SNP_00390	1949	A	G	11	3	--	--
	Ca(ICCV2/PI489777)SNP_00391	1953	T	C	11	3	--	--
CakTC34762	Ca(ICCV2/PI489777)SNP_00392	354	A	G	7	6	--	--
	Ca(ICCV2/PI489777)SNP_00393	532	G	A	6	7	--	--
	Ca(ICCV2/PI489777)SNP_00394	2726	T	C	11	7	--	--
	Ca(ICCV2/PI489777)SNP_00395	3468	G	A	4	4	--	--
	Ca(ICCV2/PI489777)SNP_00396	3492	T	C	4	4	--	--
CakTC41912	Ca(ICCV2/PI489777)SNP_00397	240	A	T	10	5	--	--
	Ca(ICCV2/PI489777)SNP_00398	241	A	T	10	5	--	--
	Ca(ICCV2/PI489777)SNP_00399	263	T	G	12	8	--	--
	Ca(ICCV2/PI489777)SNP_00400	615	A	G	47	41	--	--
	Ca(ICCV2/PI489777)SNP_00401	678	A	C	52	44	--	--
	Ca(ICCV2/PI489777)SNP_00402	708	G	A	59	52	--	--
	Ca(ICCV2/PI489777)SNP_00403	1494	C	T	54	37	--	--
	Ca(ICCV2/PI489777)SNP_00404	1797	G	A	18	11	--	--
CakTC43305	Ca(ICCV2/PI489777)SNP_00405	628	A	G	11	4	--	--
CakTC39982	Ca(ICCV2/PI489777)SNP_00406	766	A	G	5	3	--	--
CakTC37199	Ca(ICCV2/PI489777)SNP_00407	195	A	T	4	4	--	--
	Ca(ICCV2/PI489777)SNP_00408	380	C	T	12	7	--	--
	Ca(ICCV2/PI489777)SNP_00409	384	G	T	14	7	--	--
	Ca(ICCV2/PI489777)SNP_00410	759	C	T	32	18	--	--
	Ca(ICCV2/PI489777)SNP_00411	828	A	T	30	16	--	--
	Ca(ICCV2/PI489777)SNP_00412	933	T	C	23	15	--	--
CakTC29903	Ca(ICCV2/PI489777)SNP_00413	340	T	C	5	5	--	--
	Ca(ICCV2/PI489777)SNP_00414	701	A	T	17	6	--	--
	Ca(ICCV2/PI489777)SNP_00415	709	C	G	19	6	--	--
CakTC35877	Ca(ICCV2/PI489777)SNP_00416	1280	C	T	12	6	--	--
CakTC35083	Ca(ICCV2/PI489777)SNP_00417	1097	C	G	30	17	--	TPR
	Ca(ICCV2/PI489777)SNP_00418	1124	C	A	31	20	--	TPR
	Ca(ICCV2/PI489777)SNP_00419	2817	C	A	19	6	--	TPR
CakTC32345	Ca(ICCV2/PI489777)SNP_00420	1244	T	C	10	12	--	--
CakTC38929	Ca(ICCV2/PI489777)SNP_00421	315	A	G	4	12	--	--
	Ca(ICCV2/PI489777)SNP_00422	464	C	G	6	13	--	--
	Ca(ICCV2/PI489777)SNP_00423	489	T	C	6	12	--	--
	Ca(ICCV2/PI489777)SNP_00424	666	T	A	9	22	--	--
	Ca(ICCV2/PI489777)SNP_00425	1001	A	C	5	8	--	--
CakTC35701	Ca(ICCV2/PI489777)SNP_00426	103	G	T	8	8	--	--
	Ca(ICCV2/PI489777)SNP_00427	153	T	C	8	6	--	--
	Ca(ICCV2/PI489777)SNP_00428	1323	C	A	10	9	--	--
	Ca(ICCV2/PI489777)SNP_00429	1726	C	A	11	7	--	--
	Ca(ICCV2/PI489777)SNP_00430	2820	T	C	16	12	--	--
	Ca(ICCV2/PI489777)SNP_00431	3096	G	A	25	14	--	--
	Ca(ICCV2/PI489777)SNP_00432	3221	G	C	22	12	--	--
	Ca(ICCV2/PI489777)SNP_00433	3247	T	A	22	14	--	--
	Ca(ICCV2/PI489777)SNP_00434	3429	C	A	8	10	--	--
	Ca(ICCV2/PI489777)SNP_00435	3471	A	T	8	8	--	--
	Ca(ICCV2/PI489777)SNP_00436	3476	G	A	7	9	--	--
	Ca(ICCV2/PI489777)SNP_00437	3517	A	T	6	6	--	--
	Ca(ICCV2/PI489777)SNP_00438	3683	C	T	4	11	--	--
	Ca(ICCV2/PI489777)SNP_00439	3684	T	A	4	11	--	--

CakTC26269	Ca(ICCV2/PI489777)SNP_00440	1806	A	G	7	11	--	--
CakTC24500	Ca(ICCV2/PI489777)SNP_00441	1264	T	A	3	4	--	--
CakTC35131	Ca(ICCV2/PI489777)SNP_00442	378	C	T	8	4	--	--
	Ca(ICCV2/PI489777)SNP_00443	1299	A	G	3	6	--	--
	Ca(ICCV2/PI489777)SNP_00444	1688	T	C	5	4	--	--
	Ca(ICCV2/PI489777)SNP_00445	1784	T	C	5	7	--	--
	Ca(ICCV2/PI489777)SNP_00446	1973	G	C	6	7	--	--
	Ca(ICCV2/PI489777)SNP_00447	1997	T	G	5	7	--	--
	Ca(ICCV2/PI489777)SNP_00448	2491	G	A	8	5	--	--
	Ca(ICCV2/PI489777)SNP_00449	3104	G	A	10	16	--	--
CakTC02106	Ca(ICCV2/PI489777)SNP_00450	363	T	C	4	3	--	--
	Ca(ICCV2/PI489777)SNP_00451	930	T	C	3	3	--	--
CakTC43294	Ca(ICCV2/PI489777)SNP_00452	683	G	A	3	3	--	--
	Ca(ICCV2/PI489777)SNP_00453	1448	T	C	8	3	--	--
	Ca(ICCV2/PI489777)SNP_00454	1450	G	A	8	3	--	--
CakTC11417	Ca(ICCV2/PI489777)SNP_00455	1033	A	T	30	23	--	--
CakTC37491	Ca(ICCV2/PI489777)SNP_00456	801	T	G	14	6	--	--
	Ca(ICCV2/PI489777)SNP_00457	852	C	T	17	7	--	--
	Ca(ICCV2/PI489777)SNP_00458	867	C	T	16	7	--	--
	Ca(ICCV2/PI489777)SNP_00459	1000	A	G	15	6	--	--
CakTC43267	Ca(ICCV2/PI489777)SNP_00460	775	G	A	15	11	--	--
	Ca(ICCV2/PI489777)SNP_00461	860	T	A	11	3	--	--
	Ca(ICCV2/PI489777)SNP_00462	887	A	T	14	3	--	--
CakTC39952	Ca(ICCV2/PI489777)SNP_00463	172	C	T	77	32	--	--
	Ca(ICCV2/PI489777)SNP_00464	186	T	C	92	34	--	--
	Ca(ICCV2/PI489777)SNP_00465	252	G	T	104	35	--	--
	Ca(ICCV2/PI489777)SNP_00466	256	A	G	103	34	--	--
	Ca(ICCV2/PI489777)SNP_00467	408	G	A	108	26	--	--
	Ca(ICCV2/PI489777)SNP_00468	686	T	C	85	37	--	--
	Ca(ICCV2/PI489777)SNP_00469	1078	A	G	144	52	--	--
	Ca(ICCV2/PI489777)SNP_00470	1210	T	C	81	28	--	--
CakTC42704	Ca(ICCV2/PI489777)SNP_00471	489	C	T	23	8	--	--
	Ca(ICCV2/PI489777)SNP_00472	663	A	G	27	7	--	--
	Ca(ICCV2/PI489777)SNP_00473	1305	A	C	16	5	--	--
	Ca(ICCV2/PI489777)SNP_00474	1416	T	A	15	9	--	--
	Ca(ICCV2/PI489777)SNP_00475	1525	A	G	13	8	--	--
	Ca(ICCV2/PI489777)SNP_00476	1539	C	T	10	5	--	--
CakTC27054	Ca(ICCV2/PI489777)SNP_00477	617	T	C	37	18	--	--
CakTC23799	Ca(ICCV2/PI489777)SNP_00478	898	C	T	12	13	--	--
CakTC37682	Ca(ICCV2/PI489777)SNP_00479	270	C	A	4	6	--	--
	Ca(ICCV2/PI489777)SNP_00480	365	T	C	3	8	--	--
	Ca(ICCV2/PI489777)SNP_00481	592	T	G	9	10	--	--
	Ca(ICCV2/PI489777)SNP_00482	893	A	G	14	7	--	--
	Ca(ICCV2/PI489777)SNP_00483	905	G	A	11	7	--	--
	Ca(ICCV2/PI489777)SNP_00484	1155	T	C	10	15	--	--
	Ca(ICCV2/PI489777)SNP_00485	1337	T	G	13	21	--	--
	Ca(ICCV2/PI489777)SNP_00486	1366	C	T	14	25	--	--
	Ca(ICCV2/PI489777)SNP_00487	1394	T	A	8	23	--	--
	Ca(ICCV2/PI489777)SNP_00488	1507	T	C	11	27	--	--
CakTC38208	Ca(ICCV2/PI489777)SNP_00489	20	T	C	10	4	--	--
	Ca(ICCV2/PI489777)SNP_00490	31	A	T	22	7	--	--
	Ca(ICCV2/PI489777)SNP_00491	867	A	C	10	21	--	--
	Ca(ICCV2/PI489777)SNP_00492	944	G	T	7	15	--	--
CakTC33775	Ca(ICCV2/PI489777)SNP_00493	1095	G	A	7	7	--	--
CakTC31729	Ca(ICCV2/PI489777)SNP_00494	1268	C	T	9	6	--	--
	Ca(ICCV2/PI489777)SNP_00495	1424	A	C	9	4	--	--
	Ca(ICCV2/PI489777)SNP_00496	1770	G	A	7	9	--	--
CakTC33818	Ca(ICCV2/PI489777)SNP_00497	63	A	G	12	4	Shoot	--
	Ca(ICCV2/PI489777)SNP_00498	81	G	T	12	8	Shoot	--
CakTC25970	Ca(ICCV2/PI489777)SNP_00499	149	A	G	17	17	--	--
	Ca(ICCV2/PI489777)SNP_00500	209	A	G	16	20	--	--
	Ca(ICCV2/PI489777)SNP_00501	248	C	T	25	27	--	--
	Ca(ICCV2/PI489777)SNP_00502	278	G	A	26	33	--	--
	Ca(ICCV2/PI489777)SNP_00503	791	A	G	94	35	--	--
	Ca(ICCV2/PI489777)SNP_00504	929	A	T	86	63	--	--
	Ca(ICCV2/PI489777)SNP_00505	953	T	A	88	61	--	--
	Ca(ICCV2/PI489777)SNP_00506	959	G	A	88	62	--	--
	Ca(ICCV2/PI489777)SNP_00507	981	T	C	78	59	--	--
	Ca(ICCV2/PI489777)SNP_00508	1028	G	C	70	53	--	--
	Ca(ICCV2/PI489777)SNP_00509	1040	G	A	80	53	--	--
	Ca(ICCV2/PI489777)SNP_00510	1774	T	G	9	4	--	--
CakTC40043	Ca(ICCV2/PI489777)SNP_00511	112	A	G	15	5	--	--
	Ca(ICCV2/PI489777)SNP_00512	513	A	G	16	13	--	--
	Ca(ICCV2/PI489777)SNP_00513	761	T	G	9	5	--	--

	Ca(ICCV2/PI489777)SNP_00514	1018	C	G	7	3	--	--
CakTC41708	Ca(ICCV2/PI489777)SNP_00515	466	T	A	101	168	--	--
	Ca(ICCV2/PI489777)SNP_00516	693	G	A	7	15	--	--
CakTC31296	Ca(ICCV2/PI489777)SNP_00517	559	A	G	17	9	--	--
	Ca(ICCV2/PI489777)SNP_00518	1525	G	A	43	10	--	--
CakTC34005	Ca(ICCV2/PI489777)SNP_00519	2225	A	G	4	4	--	--
	Ca(ICCV2/PI489777)SNP_00520	8516	T	C	6	5	--	--
	Ca(ICCV2/PI489777)SNP_00521	8792	C	T	6	4	--	--
	Ca(ICCV2/PI489777)SNP_00522	10603	A	G	7	7	--	--
	Ca(ICCV2/PI489777)SNP_00523	11522	G	A	10	9	--	--
	Ca(ICCV2/PI489777)SNP_00524	11665	C	T	7	6	--	--
CakTC27203	Ca(ICCV2/PI489777)SNP_00525	1740	C	A	3	3	--	--
CakTC31698	Ca(ICCV2/PI489777)SNP_00526	55	G	A	3	3	Flower	bud
	Ca(ICCV2/PI489777)SNP_00527	103	C	G	4	4	Flower	bud
CakTC09306	Ca(ICCV2/PI489777)SNP_00528	539	G	A	21	7	--	CCHC
	Ca(ICCV2/PI489777)SNP_00529	564	T	G	24	4	--	CCHC
	Ca(ICCV2/PI489777)SNP_00530	2867	A	C	9	6	--	CCHC
	Ca(ICCV2/PI489777)SNP_00531	2872	A	G	10	6	--	CCHC
	Ca(ICCV2/PI489777)SNP_00532	3370	A	G	6	3	--	CCHC
	Ca(ICCV2/PI489777)SNP_00533	3419	C	T	6	3	--	CCHC
CakTC29135	Ca(ICCV2/PI489777)SNP_00534	618	C	A	8	3	--	--
	Ca(ICCV2/PI489777)SNP_00535	1437	C	T	7	4	--	--
	Ca(ICCV2/PI489777)SNP_00536	1776	C	T	3	8	--	--
	Ca(ICCV2/PI489777)SNP_00537	2275	A	G	3	4	--	--
CakTC38335	Ca(ICCV2/PI489777)SNP_00538	444	T	C	5	15	--	--
	Ca(ICCV2/PI489777)SNP_00539	605	G	A	7	7	--	--
	Ca(ICCV2/PI489777)SNP_00540	619	G	A	9	6	--	--
	Ca(ICCV2/PI489777)SNP_00541	759	G	A	12	5	--	--
	Ca(ICCV2/PI489777)SNP_00542	1104	T	G	19	5	--	--
	Ca(ICCV2/PI489777)SNP_00543	1179	A	G	15	5	--	--
	Ca(ICCV2/PI489777)SNP_00544	1285	G	T	17	5	--	--
	Ca(ICCV2/PI489777)SNP_00545	1323	A	G	10	4	--	--
	Ca(ICCV2/PI489777)SNP_00546	1326	A	C	10	4	--	--
	Ca(ICCV2/PI489777)SNP_00547	1369	A	T	10	3	--	--
	Ca(ICCV2/PI489777)SNP_00548	1445	G	T	9	4	--	--
	Ca(ICCV2/PI489777)SNP_00549	1628	G	A	7	3	--	--
	Ca(ICCV2/PI489777)SNP_00550	1642	G	T	3	3	--	--
	Ca(ICCV2/PI489777)SNP_00551	1643	T	G	3	3	--	--
CakTC25577	Ca(ICCV2/PI489777)SNP_00552	956	A	C	25	26	Young_pod	--
	Ca(ICCV2/PI489777)SNP_00553	976	G	T	22	26	Young_pod	--
	Ca(ICCV2/PI489777)SNP_00554	1004	T	C	22	37	Young_pod	--
CakTC43302	Ca(ICCV2/PI489777)SNP_00555	1558	T	A	22	9	--	--
	Ca(ICCV2/PI489777)SNP_00556	1600	T	G	24	15	--	--
	Ca(ICCV2/PI489777)SNP_00557	1645	A	G	23	13	--	--
	Ca(ICCV2/PI489777)SNP_00558	1720	T	G	23	10	--	--
	Ca(ICCV2/PI489777)SNP_00559	1721	T	G	23	13	--	--
CakTC23592	Ca(ICCV2/PI489777)SNP_00560	181	T	A	16	4	Root	--
	Ca(ICCV2/PI489777)SNP_00561	259	C	T	16	6	Root	--
	Ca(ICCV2/PI489777)SNP_00562	265	C	T	16	6	Root	--
	Ca(ICCV2/PI489777)SNP_00563	294	C	T	17	7	Root	--
	Ca(ICCV2/PI489777)SNP_00564	297	G	A	17	6	Root	--
	Ca(ICCV2/PI489777)SNP_00565	328	C	T	13	4	Root	--
CakTC28488	Ca(ICCV2/PI489777)SNP_00566	1164	G	T	7	3	--	--
	Ca(ICCV2/PI489777)SNP_00567	1365	T	A	4	4	--	--
	Ca(ICCV2/PI489777)SNP_00568	1416	T	G	7	4	--	--
CakTC38934	Ca(ICCV2/PI489777)SNP_00569	730	G	T	21	9	--	G2-like
CakTC11093	Ca(ICCV2/PI489777)SNP_00570	1163	G	A	14	6	--	--
	Ca(ICCV2/PI489777)SNP_00571	1322	C	T	9	7	--	--
	Ca(ICCV2/PI489777)SNP_00572	1408	C	G	5	10	--	--
	Ca(ICCV2/PI489777)SNP_00573	1682	T	G	8	11	--	--
	Ca(ICCV2/PI489777)SNP_00574	1803	G	T	14	8	--	--
	Ca(ICCV2/PI489777)SNP_00575	1806	T	A	15	8	--	--
	Ca(ICCV2/PI489777)SNP_00576	1820	G	T	15	7	--	--
CakTC42620	Ca(ICCV2/PI489777)SNP_00577	491	T	C	7	4	--	--
	Ca(ICCV2/PI489777)SNP_00578	570	T	C	6	3	--	--
	Ca(ICCV2/PI489777)SNP_00579	711	C	G	4	3	--	--
	Ca(ICCV2/PI489777)SNP_00580	886	A	G	4	3	--	--
	Ca(ICCV2/PI489777)SNP_00581	897	C	A	3	3	--	--
CakTC38639	Ca(ICCV2/PI489777)SNP_00582	822	T	G	10	21	--	--
	Ca(ICCV2/PI489777)SNP_00583	861	A	T	12	20	--	--
	Ca(ICCV2/PI489777)SNP_00584	1308	A	G	14	14	--	--
CakTC42966	Ca(ICCV2/PI489777)SNP_00585	167	G	T	14	5	--	--
	Ca(ICCV2/PI489777)SNP_00586	176	T	G	14	8	--	--
	Ca(ICCV2/PI489777)SNP_00587	437	C	T	13	4	--	--

	Ca(ICCV2/PI489777)SNP_00588	585	G	T	6	5	--	--
	Ca(ICCV2/PI489777)SNP_00589	599	A	G	5	6	--	--
CakTC28021	Ca(ICCV2/PI489777)SNP_00590	625	G	A	11	7	--	--
	Ca(ICCV2/PI489777)SNP_00591	799	T	C	15	6	--	--
	Ca(ICCV2/PI489777)SNP_00592	817	T	C	15	6	--	--
	Ca(ICCV2/PI489777)SNP_00593	907	A	G	13	5	--	--
	Ca(ICCV2/PI489777)SNP_00594	970	T	C	10	5	--	--
	Ca(ICCV2/PI489777)SNP_00595	976	C	T	11	5	--	--
	Ca(ICCV2/PI489777)SNP_00596	1027	C	T	8	5	--	--
CakTC10157	Ca(ICCV2/PI489777)SNP_00597	762	T	C	3	3	--	Coactivatorp15
CakTC11773	Ca(ICCV2/PI489777)SNP_00598	1247	C	G	5	3	--	--
CakTC40245	Ca(ICCV2/PI489777)SNP_00599	932	T	G	31	7	--	OFP
CakTC37693	Ca(ICCV2/PI489777)SNP_00600	1050	T	C	5	6	--	--
CakTC31819	Ca(ICCV2/PI489777)SNP_00601	645	C	T	3	8	--	--
CakTC35751	Ca(ICCV2/PI489777)SNP_00602	408	C	G	7	5	--	--
	Ca(ICCV2/PI489777)SNP_00603	465	T	A	7	8	--	--
	Ca(ICCV2/PI489777)SNP_00604	917	T	C	4	13	--	--
	Ca(ICCV2/PI489777)SNP_00605	1019	A	G	6	11	--	--
	Ca(ICCV2/PI489777)SNP_00606	1255	G	T	9	13	--	--
	Ca(ICCV2/PI489777)SNP_00607	2266	T	C	12	3	--	--
	Ca(ICCV2/PI489777)SNP_00608	2566	G	A	5	3	--	--
	Ca(ICCV2/PI489777)SNP_00609	2622	C	T	6	3	--	--
CakTC39395	Ca(ICCV2/PI489777)SNP_00610	49	A	C	8	5	--	--
	Ca(ICCV2/PI489777)SNP_00611	595	A	T	18	9	--	--
	Ca(ICCV2/PI489777)SNP_00612	742	T	C	9	13	--	--
	Ca(ICCV2/PI489777)SNP_00613	985	C	T	8	12	--	--
	Ca(ICCV2/PI489777)SNP_00614	989	T	C	7	13	--	--
CakTC22745	Ca(ICCV2/PI489777)SNP_00615	540	T	C	27	39	--	--
CakTC39188	Ca(ICCV2/PI489777)SNP_00616	285	C	T	4	6	--	NAC
CakTC35920	Ca(ICCV2/PI489777)SNP_00617	775	A	G	26	15	--	--
	Ca(ICCV2/PI489777)SNP_00618	778	T	G	25	15	--	--
	Ca(ICCV2/PI489777)SNP_00619	1836	A	G	24	15	--	--
	Ca(ICCV2/PI489777)SNP_00620	2355	A	T	52	23	--	--
	Ca(ICCV2/PI489777)SNP_00621	2571	C	T	54	16	--	--
CakTC28036	Ca(ICCV2/PI489777)SNP_00622	13	A	C	3	3	--	--
	Ca(ICCV2/PI489777)SNP_00623	717	A	G	28	14	--	--
CakTC26089	Ca(ICCV2/PI489777)SNP_00624	525	T	G	12	5	--	--
	Ca(ICCV2/PI489777)SNP_00625	685	G	A	14	12	--	--
CakTC37770	Ca(ICCV2/PI489777)SNP_00626	1642	T	G	3	4	Young_pod	--
	Ca(ICCV2/PI489777)SNP_00627	1971	T	C	4	4	Young_pod	--
CakTC26527	Ca(ICCV2/PI489777)SNP_00628	1643	T	C	5	4	--	SAP
CakTC38653	Ca(ICCV2/PI489777)SNP_00629	820	T	G	9	3	--	GNAT
	Ca(ICCV2/PI489777)SNP_00630	942	A	T	6	4	--	GNAT
	Ca(ICCV2/PI489777)SNP_00631	977	A	C	8	5	--	GNAT
	Ca(ICCV2/PI489777)SNP_00632	987	T	C	7	5	--	GNAT
CakTC24676	Ca(ICCV2/PI489777)SNP_00633	616	A	G	20	10	--	Bromodomain
	Ca(ICCV2/PI489777)SNP_00634	652	A	G	22	9	--	Bromodomain
	Ca(ICCV2/PI489777)SNP_00635	724	T	A	25	6	--	Bromodomain
	Ca(ICCV2/PI489777)SNP_00636	1880	G	T	66	38	--	Bromodomain
	Ca(ICCV2/PI489777)SNP_00637	2000	A	T	71	54	--	Bromodomain
	Ca(ICCV2/PI489777)SNP_00638	2060	G	A	79	53	--	Bromodomain
	Ca(ICCV2/PI489777)SNP_00639	2678	G	A	51	32	--	Bromodomain
CakTC43042	Ca(ICCV2/PI489777)SNP_00640	2019	A	G	6	7	Young_pod	--
	Ca(ICCV2/PI489777)SNP_00641	2115	T	C	7	7	Young_pod	--
	Ca(ICCV2/PI489777)SNP_00642	2309	A	G	4	8	Young_pod	--
	Ca(ICCV2/PI489777)SNP_00643	2475	G	A	6	4	Young_pod	--
CakTC38543	Ca(ICCV2/PI489777)SNP_00644	107	C	G	9	5	--	--
	Ca(ICCV2/PI489777)SNP_00645	143	A	C	9	5	--	--
	Ca(ICCV2/PI489777)SNP_00646	153	G	C	9	5	--	--
	Ca(ICCV2/PI489777)SNP_00647	887	G	C	7	3	--	--
	Ca(ICCV2/PI489777)SNP_00648	888	G	A	7	3	--	--
CakTC39535	Ca(ICCV2/PI489777)SNP_00649	166	T	C	8	10	--	--
	Ca(ICCV2/PI489777)SNP_00650	1299	T	C	13	7	--	--
CakTC40092	Ca(ICCV2/PI489777)SNP_00651	567	A	G	46	36	--	--
	Ca(ICCV2/PI489777)SNP_00652	964	T	G	34	23	--	--
CakTC09845	Ca(ICCV2/PI489777)SNP_00653	261	A	T	7	3	--	--
CakTC37452	Ca(ICCV2/PI489777)SNP_00654	113	C	T	28	5	--	--
	Ca(ICCV2/PI489777)SNP_00655	279	G	C	33	10	--	--
	Ca(ICCV2/PI489777)SNP_00656	317	A	T	29	10	--	--
	Ca(ICCV2/PI489777)SNP_00657	351	A	T	34	11	--	--
	Ca(ICCV2/PI489777)SNP_00658	671	T	C	18	6	--	--
	Ca(ICCV2/PI489777)SNP_00659	760	G	A	20	3	--	--
CakTC28309	Ca(ICCV2/PI489777)SNP_00660	1310	T	A	4	3	Shoot	--
	Ca(ICCV2/PI489777)SNP_00661	1481	C	T	4	3	Shoot	--

	Ca(ICCV2/PI489777)SNP_00662	1547	A	T	4	3	Shoot	--
CakTC41784	Ca(ICCV2/PI489777)SNP_00663	1437	G	A	3	3	--	--
	Ca(ICCV2/PI489777)SNP_00664	2271	T	C	4	5	--	--
CakTC40437	Ca(ICCV2/PI489777)SNP_00665	486	T	C	16	8	--	--
	Ca(ICCV2/PI489777)SNP_00666	1051	A	G	25	7	--	--
	Ca(ICCV2/PI489777)SNP_00667	1794	G	A	9	4	--	--
	Ca(ICCV2/PI489777)SNP_00668	1806	A	G	10	4	--	--
CakTC37980	Ca(ICCV2/PI489777)SNP_00669	377	T	C	22	21	--	--
	Ca(ICCV2/PI489777)SNP_00670	660	A	T	5	9	--	--
	Ca(ICCV2/PI489777)SNP_00671	930	T	C	3	5	--	--
CakTC32872	Ca(ICCV2/PI489777)SNP_00672	683	A	G	10	3	--	--
	Ca(ICCV2/PI489777)SNP_00673	2759	G	A	5	3	--	--
	Ca(ICCV2/PI489777)SNP_00674	2906	C	G	6	3	--	--
CakTC43219	Ca(ICCV2/PI489777)SNP_00675	538	A	G	8	8	--	--
	Ca(ICCV2/PI489777)SNP_00676	811	T	C	13	7	--	--
	Ca(ICCV2/PI489777)SNP_00677	859	T	G	11	8	--	--
CakTC27429	Ca(ICCV2/PI489777)SNP_00678	217	A	C	4	6	--	--
	Ca(ICCV2/PI489777)SNP_00679	281	A	G	4	6	--	--
	Ca(ICCV2/PI489777)SNP_00680	442	A	C	5	5	--	--
CakTC10809	Ca(ICCV2/PI489777)SNP_00681	547	G	T	30	10	--	--
	Ca(ICCV2/PI489777)SNP_00682	574	T	C	24	7	--	--
	Ca(ICCV2/PI489777)SNP_00683	678	A	T	15	5	--	--
CakTC40787	Ca(ICCV2/PI489777)SNP_00684	701	C	T	4	6	--	--
CakTC32689	Ca(ICCV2/PI489777)SNP_00685	1805	C	T	6	6	--	--
	Ca(ICCV2/PI489777)SNP_00686	1973	G	A	4	5	--	--
CakTC37743	Ca(ICCV2/PI489777)SNP_00687	71	T	A	5	3	--	Tify
	Ca(ICCV2/PI489777)SNP_00688	95	C	G	9	3	--	Tify
	Ca(ICCV2/PI489777)SNP_00689	620	A	G	27	26	--	Tify
	Ca(ICCV2/PI489777)SNP_00690	632	C	T	27	25	--	Tify
CakTC30803	Ca(ICCV2/PI489777)SNP_00691	827	C	G	7	3	Root	--
CakTC39110	Ca(ICCV2/PI489777)SNP_00692	324	G	A	48	45	--	TPR
	Ca(ICCV2/PI489777)SNP_00693	623	A	T	46	18	--	TPR
	Ca(ICCV2/PI489777)SNP_00694	944	A	G	54	21	--	TPR
	Ca(ICCV2/PI489777)SNP_00695	1199	A	C	39	37	--	TPR
CakTC38307	Ca(ICCV2/PI489777)SNP_00696	658	T	C	4	4	--	--
	Ca(ICCV2/PI489777)SNP_00697	1000	T	A	4	4	--	--
CakTC38093	Ca(ICCV2/PI489777)SNP_00698	365	G	T	7	9	--	--
	Ca(ICCV2/PI489777)SNP_00699	471	C	G	4	6	--	--
CakTC13170	Ca(ICCV2/PI489777)SNP_00700	500	T	C	8	3	--	--
	Ca(ICCV2/PI489777)SNP_00701	727	A	T	20	5	--	--
	Ca(ICCV2/PI489777)SNP_00702	795	C	T	22	5	--	--
	Ca(ICCV2/PI489777)SNP_00703	811	G	C	19	5	--	--
	Ca(ICCV2/PI489777)SNP_00704	824	G	T	22	5	--	--
	Ca(ICCV2/PI489777)SNP_00705	865	T	A	21	5	--	--
	Ca(ICCV2/PI489777)SNP_00706	972	C	T	19	3	--	--
CakTC39209	Ca(ICCV2/PI489777)SNP_00707	762	T	C	9	4	--	--
CakTC26568	Ca(ICCV2/PI489777)SNP_00708	239	G	A	65	68	--	--
CakTC40632	Ca(ICCV2/PI489777)SNP_00709	343	C	T	5	11	--	--
	Ca(ICCV2/PI489777)SNP_00710	1945	G	A	5	3	--	--
CakTC29562	Ca(ICCV2/PI489777)SNP_00711	842	T	C	3	4	--	--
CakTC24061	Ca(ICCV2/PI489777)SNP_00712	3204	G	T	4	3	--	--
CakTC30522	Ca(ICCV2/PI489777)SNP_00713	1474	C	T	5	3	--	--
CakTC23769	Ca(ICCV2/PI489777)SNP_00714	2039	G	A	8	7	--	--
	Ca(ICCV2/PI489777)SNP_00715	2907	G	A	3	4	--	--
	Ca(ICCV2/PI489777)SNP_00716	2937	G	A	4	4	--	--
	Ca(ICCV2/PI489777)SNP_00717	2952	G	A	4	4	--	--
	Ca(ICCV2/PI489777)SNP_00718	2956	G	A	4	4	--	--
	Ca(ICCV2/PI489777)SNP_00719	2962	G	C	4	4	--	--
	Ca(ICCV2/PI489777)SNP_00720	2996	C	T	4	4	--	--
	Ca(ICCV2/PI489777)SNP_00721	3013	A	G	5	4	--	--
CakTC29152	Ca(ICCV2/PI489777)SNP_00722	339	G	A	4	5	Young_pod	--
	Ca(ICCV2/PI489777)SNP_00723	478	T	C	6	4	Young_pod	--
CakTC31419	Ca(ICCV2/PI489777)SNP_00724	979	T	C	6	3	--	--
	Ca(ICCV2/PI489777)SNP_00725	2543	A	G	6	6	--	--
	Ca(ICCV2/PI489777)SNP_00726	2576	C	T	6	8	--	--
	Ca(ICCV2/PI489777)SNP_00727	2789	T	A	5	9	--	--
CakTC11605	Ca(ICCV2/PI489777)SNP_00728	32	G	T	23	12	--	--
	Ca(ICCV2/PI489777)SNP_00729	51	T	G	28	21	--	--
	Ca(ICCV2/PI489777)SNP_00730	223	T	A	48	30	--	--
	Ca(ICCV2/PI489777)SNP_00731	244	A	G	50	31	--	--
	Ca(ICCV2/PI489777)SNP_00732	394	G	C	48	26	--	--
	Ca(ICCV2/PI489777)SNP_00733	433	A	T	46	36	--	--
CakTC09596	Ca(ICCV2/PI489777)SNP_00734	50	C	T	61	42	--	--
	Ca(ICCV2/PI489777)SNP_00735	59	C	T	61	44	--	--

CakTC40651	Ca(ICCV2/PI489777)SNP_00736	175	T	C	22	27	--	--
	Ca(ICCV2/PI489777)SNP_00737	627	T	C	96	84	--	--
	Ca(ICCV2/PI489777)SNP_00738	1089	A	C	50	30	--	--
	Ca(ICCV2/PI489777)SNP_00739	1458	A	G	35	26	--	--
CakTC39319	Ca(ICCV2/PI489777)SNP_00740	335	C	G	6	4	--	--
	Ca(ICCV2/PI489777)SNP_00741	357	G	T	7	4	--	--
	Ca(ICCV2/PI489777)SNP_00742	501	G	A	5	3	--	--
	Ca(ICCV2/PI489777)SNP_00743	1136	G	T	5	9	--	--
	Ca(ICCV2/PI489777)SNP_00744	1227	C	T	4	9	--	--
	Ca(ICCV2/PI489777)SNP_00745	1395	C	T	3	5	--	--
CakTC30002	Ca(ICCV2/PI489777)SNP_00746	2218	G	C	5	5	--	--
	Ca(ICCV2/PI489777)SNP_00747	2338	C	A	4	4	--	--
	Ca(ICCV2/PI489777)SNP_00748	2560	T	C	4	20	--	--
	Ca(ICCV2/PI489777)SNP_00749	2722	T	C	4	22	--	--
CakTC18097	Ca(ICCV2/PI489777)SNP_00750	2306	G	T	12	16	--	--
	Ca(ICCV2/PI489777)SNP_00751	2349	G	A	10	15	--	--
CakTC01693	Ca(ICCV2/PI489777)SNP_00752	296	A	G	7	4	--	AP2-EREBP
	Ca(ICCV2/PI489777)SNP_00753	603	C	A	8	6	--	AP2-EREBP
	Ca(ICCV2/PI489777)SNP_00754	636	A	C	9	7	--	AP2-EREBP
	Ca(ICCV2/PI489777)SNP_00755	737	G	C	8	4	--	AP2-EREBP
CakTC29180	Ca(ICCV2/PI489777)SNP_00756	664	G	A	7	3	--	--
CakTC41487	Ca(ICCV2/PI489777)SNP_00757	1046	T	A	7	4	--	--
	Ca(ICCV2/PI489777)SNP_00758	2338	G	A	4	5	--	--
CakTC39684	Ca(ICCV2/PI489777)SNP_00759	39	A	T	5	3	--	--
	Ca(ICCV2/PI489777)SNP_00760	42	T	C	5	3	--	--
	Ca(ICCV2/PI489777)SNP_00761	58	G	A	9	6	--	--
	Ca(ICCV2/PI489777)SNP_00762	1048	G	A	8	3	--	--
CakTC27977	Ca(ICCV2/PI489777)SNP_00763	2625	A	G	6	4	--	--
	Ca(ICCV2/PI489777)SNP_00764	2799	T	C	8	4	--	--
	Ca(ICCV2/PI489777)SNP_00765	2886	T	G	9	3	--	--
CakTC34401	Ca(ICCV2/PI489777)SNP_00766	346	T	A	9	6	--	--
	Ca(ICCV2/PI489777)SNP_00767	404	C	T	10	6	--	--
	Ca(ICCV2/PI489777)SNP_00768	493	A	G	10	8	--	--
	Ca(ICCV2/PI489777)SNP_00769	707	G	T	7	7	--	--
	Ca(ICCV2/PI489777)SNP_00770	2053	G	A	5	10	--	--
CakTC34664	Ca(ICCV2/PI489777)SNP_00771	738	C	A	4	4	--	--
	Ca(ICCV2/PI489777)SNP_00772	748	A	G	4	4	--	--
CakTC39433	Ca(ICCV2/PI489777)SNP_00773	927	T	C	12	14	--	--
CakTC31316	Ca(ICCV2/PI489777)SNP_00774	917	G	A	17	3	--	--
	Ca(ICCV2/PI489777)SNP_00775	1036	A	G	14	6	--	--
	Ca(ICCV2/PI489777)SNP_00776	1278	T	C	14	7	--	--
	Ca(ICCV2/PI489777)SNP_00777	1454	G	A	30	7	--	--
	Ca(ICCV2/PI489777)SNP_00778	1628	T	C	12	7	--	--
	Ca(ICCV2/PI489777)SNP_00779	1709	A	T	11	5	--	--
	Ca(ICCV2/PI489777)SNP_00780	1992	G	A	9	8	--	--
	Ca(ICCV2/PI489777)SNP_00781	2006	A	C	7	9	--	--
CakTC25406	Ca(ICCV2/PI489777)SNP_00782	3816	C	T	5	3	--	--
	Ca(ICCV2/PI489777)SNP_00783	3817	A	G	5	3	--	--
CakTC42686	Ca(ICCV2/PI489777)SNP_00784	33	C	T	36	4	--	--
	Ca(ICCV2/PI489777)SNP_00785	529	T	C	13	7	--	--
	Ca(ICCV2/PI489777)SNP_00786	556	C	T	13	6	--	--
	Ca(ICCV2/PI489777)SNP_00787	627	A	G	7	3	--	--
	Ca(ICCV2/PI489777)SNP_00788	637	G	A	7	3	--	--
	Ca(ICCV2/PI489777)SNP_00789	639	C	T	7	3	--	--
CakTC39192	Ca(ICCV2/PI489777)SNP_00790	807	C	T	11	6	--	--
	Ca(ICCV2/PI489777)SNP_00791	942	G	C	18	8	--	--
	Ca(ICCV2/PI489777)SNP_00792	954	C	T	16	9	--	--
	Ca(ICCV2/PI489777)SNP_00793	1128	C	T	17	12	--	--
	Ca(ICCV2/PI489777)SNP_00794	1391	T	C	7	8	--	--
CakTC30913	Ca(ICCV2/PI489777)SNP_00795	503	T	G	7	7	--	--
	Ca(ICCV2/PI489777)SNP_00796	1531	C	G	3	3	--	--
CakTC12693	Ca(ICCV2/PI489777)SNP_00797	228	C	A	59	18	--	--
	Ca(ICCV2/PI489777)SNP_00798	361	G	T	68	17	--	--
	Ca(ICCV2/PI489777)SNP_00799	436	G	A	59	16	--	--
CakTC07705	Ca(ICCV2/PI489777)SNP_00800	92	C	T	17	6	Flower	bud
	Ca(ICCV2/PI489777)SNP_00801	110	A	G	17	4	Flower	bud
	Ca(ICCV2/PI489777)SNP_00802	278	C	A	25	6	Flower	bud
CakTC33946	Ca(ICCV2/PI489777)SNP_00803	301	G	A	53	33	--	--
	Ca(ICCV2/PI489777)SNP_00804	750	G	C	58	54	--	--
	Ca(ICCV2/PI489777)SNP_00805	1664	A	G	32	28	--	--
CakTC26974	Ca(ICCV2/PI489777)SNP_00806	1836	C	G	14	6	--	--
	Ca(ICCV2/PI489777)SNP_00807	2384	G	A	8	7	--	--
CakTC36339	Ca(ICCV2/PI489777)SNP_00808	45	C	G	34	3	--	--
	Ca(ICCV2/PI489777)SNP_00809	82	A	T	49	4	--	--

	Ca(ICCV2/PI489777)SNP_00810	94	C	T	54	7	--	--
	Ca(ICCV2/PI489777)SNP_00811	193	G	A	129	9	--	--
	Ca(ICCV2/PI489777)SNP_00812	436	T	G	80	14	--	--
	Ca(ICCV2/PI489777)SNP_00813	444	A	G	108	15	--	--
CakTC34634	Ca(ICCV2/PI489777)SNP_00814	225	G	A	4	4	--	--
	Ca(ICCV2/PI489777)SNP_00815	419	C	T	5	4	--	--
	Ca(ICCV2/PI489777)SNP_00816	496	C	T	5	4	--	--
CakTC39258	Ca(ICCV2/PI489777)SNP_00817	517	A	G	4	3	--	--
	Ca(ICCV2/PI489777)SNP_00818	1506	T	G	4	7	--	--
CakTC33896	Ca(ICCV2/PI489777)SNP_00819	372	A	G	17	4	--	--
CakTC36599	Ca(ICCV2/PI489777)SNP_00820	680	T	C	15	9	--	--
	Ca(ICCV2/PI489777)SNP_00821	707	G	A	17	9	--	--
	Ca(ICCV2/PI489777)SNP_00822	890	T	C	13	8	--	--
	Ca(ICCV2/PI489777)SNP_00823	1073	A	T	8	10	--	--
	Ca(ICCV2/PI489777)SNP_00824	1259	C	G	13	7	--	--
	Ca(ICCV2/PI489777)SNP_00825	1340	A	G	13	4	--	--
	Ca(ICCV2/PI489777)SNP_00826	1526	C	T	13	4	--	--
	Ca(ICCV2/PI489777)SNP_00827	1574	A	G	10	4	--	--
	Ca(ICCV2/PI489777)SNP_00828	1694	C	T	9	5	--	--
	Ca(ICCV2/PI489777)SNP_00829	1718	A	G	10	4	--	--
	Ca(ICCV2/PI489777)SNP_00830	1829	G	A	14	7	--	--
	Ca(ICCV2/PI489777)SNP_00831	2651	G	A	8	3	--	--
CakTC35749	Ca(ICCV2/PI489777)SNP_00832	316	A	G	8	20	--	--
	Ca(ICCV2/PI489777)SNP_00833	1044	C	T	15	14	--	--
	Ca(ICCV2/PI489777)SNP_00834	1144	C	T	12	15	--	--
	Ca(ICCV2/PI489777)SNP_00835	2626	A	G	11	4	--	--
	Ca(ICCV2/PI489777)SNP_00836	3271	A	G	3	6	--	--
CakTC35309	Ca(ICCV2/PI489777)SNP_00837	231	T	G	7	5	--	Bromodomain
	Ca(ICCV2/PI489777)SNP_00838	427	G	A	17	6	--	Bromodomain
	Ca(ICCV2/PI489777)SNP_00839	610	T	C	23	9	--	Bromodomain
	Ca(ICCV2/PI489777)SNP_00840	1030	T	G	14	8	--	Bromodomain
	Ca(ICCV2/PI489777)SNP_00841	1197	C	T	11	7	--	Bromodomain
	Ca(ICCV2/PI489777)SNP_00842	1198	G	A	11	7	--	Bromodomain
	Ca(ICCV2/PI489777)SNP_00843	1706	C	A	11	8	--	Bromodomain
	Ca(ICCV2/PI489777)SNP_00844	2098	T	C	10	9	--	Bromodomain
	Ca(ICCV2/PI489777)SNP_00845	2110	G	A	10	9	--	Bromodomain
	Ca(ICCV2/PI489777)SNP_00846	2284	T	A	17	10	--	Bromodomain
	Ca(ICCV2/PI489777)SNP_00847	2345	C	T	21	9	--	Bromodomain
	Ca(ICCV2/PI489777)SNP_00848	3058	A	G	6	9	--	Bromodomain
	Ca(ICCV2/PI489777)SNP_00849	3296	G	A	3	3	--	Bromodomain
CakTC26388	Ca(ICCV2/PI489777)SNP_00850	192	A	T	11	6	--	LIM
	Ca(ICCV2/PI489777)SNP_00851	858	T	C	17	12	--	LIM
CakTC09265	Ca(ICCV2/PI489777)SNP_00852	1299	G	A	9	4	Flower	bud
CakTC29525	Ca(ICCV2/PI489777)SNP_00853	1514	C	T	3	3	Flower	bud
	Ca(ICCV2/PI489777)SNP_00854	1701	A	G	5	3	Flower	bud
	Ca(ICCV2/PI489777)SNP_00855	1948	T	C	4	5	Flower	bud
	Ca(ICCV2/PI489777)SNP_00856	1959	G	T	7	3	Flower	bud
	Ca(ICCV2/PI489777)SNP_00857	1984	C	G	6	5	Flower	bud
	Ca(ICCV2/PI489777)SNP_00858	2555	T	C	3	8	Flower	bud
CakTC01807	Ca(ICCV2/PI489777)SNP_00859	163	T	C	4	4	--	--
CakTC43335	Ca(ICCV2/PI489777)SNP_00860	441	A	G	9	3	--	HSF
	Ca(ICCV2/PI489777)SNP_00861	1213	A	G	5	3	--	HSF
	Ca(ICCV2/PI489777)SNP_00862	1244	T	C	5	4	--	HSF
	Ca(ICCV2/PI489777)SNP_00863	1257	C	T	4	4	--	HSF
	Ca(ICCV2/PI489777)SNP_00864	1259	A	G	4	4	--	HSF
	Ca(ICCV2/PI489777)SNP_00865	1467	C	G	3	3	--	HSF
	Ca(ICCV2/PI489777)SNP_00866	1530	T	C	5	3	--	HSF
CakTC11120	Ca(ICCV2/PI489777)SNP_00867	204	G	A	8	4	--	--
	Ca(ICCV2/PI489777)SNP_00868	209	T	C	9	4	--	--
	Ca(ICCV2/PI489777)SNP_00869	345	G	A	9	6	--	--
	Ca(ICCV2/PI489777)SNP_00870	802	T	G	11	9	--	--
	Ca(ICCV2/PI489777)SNP_00871	803	T	A	11	9	--	--
	Ca(ICCV2/PI489777)SNP_00872	813	A	G	13	7	--	--
CakTC32929	Ca(ICCV2/PI489777)SNP_00873	384	G	A	6	3	--	--
	Ca(ICCV2/PI489777)SNP_00874	639	G	A	6	4	--	--
CakTC37747	Ca(ICCV2/PI489777)SNP_00875	591	A	G	3	4	--	--
	Ca(ICCV2/PI489777)SNP_00876	894	G	A	4	3	--	--
	Ca(ICCV2/PI489777)SNP_00877	915	T	A	3	3	--	--
CakTC37525	Ca(ICCV2/PI489777)SNP_00878	47	A	T	16	8	--	--
	Ca(ICCV2/PI489777)SNP_00879	53	C	G	16	10	--	--
	Ca(ICCV2/PI489777)SNP_00880	770	A	G	6	3	--	--
	Ca(ICCV2/PI489777)SNP_00881	878	C	T	9	7	--	--
	Ca(ICCV2/PI489777)SNP_00882	912	G	A	10	7	--	--
	Ca(ICCV2/PI489777)SNP_00883	1052	C	A	12	7	--	--

	Ca(ICCV2/PI489777)SNP_00884	1172	G	A	12	8	--	--
	Ca(ICCV2/PI489777)SNP_00885	1184	G	A	12	9	--	--
	Ca(ICCV2/PI489777)SNP_00886	1295	A	G	4	7	--	--
CakTC24423	Ca(ICCV2/PI489777)SNP_00887	342	A	G	7	3	--	--
	Ca(ICCV2/PI489777)SNP_00888	795	A	G	4	14	--	--
	Ca(ICCV2/PI489777)SNP_00889	864	G	A	10	12	--	--
	Ca(ICCV2/PI489777)SNP_00890	1178	T	A	15	6	--	--
	Ca(ICCV2/PI489777)SNP_00891	1397	C	T	16	6	--	--
CakTC40428	Ca(ICCV2/PI489777)SNP_00892	158	C	T	8	11	--	--
	Ca(ICCV2/PI489777)SNP_00893	161	A	T	8	12	--	--
	Ca(ICCV2/PI489777)SNP_00894	191	G	C	10	12	--	--
	Ca(ICCV2/PI489777)SNP_00895	458	A	G	14	7	--	--
	Ca(ICCV2/PI489777)SNP_00896	482	G	A	13	4	--	--
	Ca(ICCV2/PI489777)SNP_00897	1430	G	C	9	5	--	--
CakTC38557	Ca(ICCV2/PI489777)SNP_00898	353	T	C	15	24	--	--
	Ca(ICCV2/PI489777)SNP_00899	419	A	C	15	25	--	--
	Ca(ICCV2/PI489777)SNP_00900	481	T	C	19	35	--	--
	Ca(ICCV2/PI489777)SNP_00901	590	A	G	26	43	--	--
	Ca(ICCV2/PI489777)SNP_00902	614	C	T	26	48	--	--
	Ca(ICCV2/PI489777)SNP_00903	671	T	C	22	47	--	--
	Ca(ICCV2/PI489777)SNP_00904	683	G	A	22	41	--	--
	Ca(ICCV2/PI489777)SNP_00905	714	G	T	19	49	--	--
	Ca(ICCV2/PI489777)SNP_00906	727	C	G	21	46	--	--
	Ca(ICCV2/PI489777)SNP_00907	803	A	G	15	41	--	--
	Ca(ICCV2/PI489777)SNP_00908	852	G	A	19	39	--	--
	Ca(ICCV2/PI489777)SNP_00909	948	G	A	3	18	--	--
CakTC37200	Ca(ICCV2/PI489777)SNP_00910	86	T	C	6	3	--	--
	Ca(ICCV2/PI489777)SNP_00911	109	C	G	9	4	--	--
	Ca(ICCV2/PI489777)SNP_00912	295	A	T	23	6	--	--
	Ca(ICCV2/PI489777)SNP_00913	733	G	A	11	12	--	--
	Ca(ICCV2/PI489777)SNP_00914	1258	G	A	5	4	--	--
CakTC35984	Ca(ICCV2/PI489777)SNP_00915	746	G	A	6	3	--	--
CakTC25140	Ca(ICCV2/PI489777)SNP_00916	522	C	T	8	8	--	--
CakTC33479	Ca(ICCV2/PI489777)SNP_00917	255	G	A	7	3	--	--
	Ca(ICCV2/PI489777)SNP_00918	263	T	A	7	3	--	--
	Ca(ICCV2/PI489777)SNP_00919	279	A	G	6	3	--	--
	Ca(ICCV2/PI489777)SNP_00920	371	T	A	5	3	--	--
	Ca(ICCV2/PI489777)SNP_00921	372	A	C	5	3	--	--
	Ca(ICCV2/PI489777)SNP_00922	392	T	C	3	3	--	--
CakTC34810	Ca(ICCV2/PI489777)SNP_00923	733	G	A	7	7	--	--
	Ca(ICCV2/PI489777)SNP_00924	1531	T	G	10	9	--	--
	Ca(ICCV2/PI489777)SNP_00925	1939	T	C	3	6	--	--
	Ca(ICCV2/PI489777)SNP_00926	3028	C	T	10	6	--	--
CakTC10524	Ca(ICCV2/PI489777)SNP_00927	384	T	A	10	3	--	--
CakTC43195	Ca(ICCV2/PI489777)SNP_00928	1033	A	G	5	6	--	--
	Ca(ICCV2/PI489777)SNP_00929	1126	A	T	6	4	--	--
	Ca(ICCV2/PI489777)SNP_00930	1167	C	T	6	4	--	--
	Ca(ICCV2/PI489777)SNP_00931	1183	A	G	6	3	--	--
	Ca(ICCV2/PI489777)SNP_00932	1300	T	C	6	4	--	--
	Ca(ICCV2/PI489777)SNP_00933	1637	G	A	15	3	--	--
	Ca(ICCV2/PI489777)SNP_00934	1648	A	T	15	3	--	--
	Ca(ICCV2/PI489777)SNP_00935	1667	G	A	14	3	--	--
CakTC09942	Ca(ICCV2/PI489777)SNP_00936	678	T	C	8	3	--	--
	Ca(ICCV2/PI489777)SNP_00937	885	T	A	14	5	--	--
	Ca(ICCV2/PI489777)SNP_00938	1363	T	G	6	3	--	--
CakTC30887	Ca(ICCV2/PI489777)SNP_00939	115	G	A	10	12	--	--
	Ca(ICCV2/PI489777)SNP_00940	226	A	G	12	13	--	--
	Ca(ICCV2/PI489777)SNP_00941	312	G	T	13	14	--	--
	Ca(ICCV2/PI489777)SNP_00942	757	G	A	13	5	--	--
	Ca(ICCV2/PI489777)SNP_00943	1036	C	T	18	6	--	--
	Ca(ICCV2/PI489777)SNP_00944	1582	G	A	10	7	--	--
	Ca(ICCV2/PI489777)SNP_00945	1632	T	G	13	6	--	--
	Ca(ICCV2/PI489777)SNP_00946	1678	T	C	16	6	--	--
	Ca(ICCV2/PI489777)SNP_00947	1891	G	A	23	7	--	--
	Ca(ICCV2/PI489777)SNP_00948	2038	T	C	11	5	--	--
	Ca(ICCV2/PI489777)SNP_00949	2044	A	T	12	3	--	--
	Ca(ICCV2/PI489777)SNP_00950	2449	G	A	8	4	--	--
	Ca(ICCV2/PI489777)SNP_00951	2458	G	A	6	4	--	--
CakTC33083	Ca(ICCV2/PI489777)SNP_00952	168	C	G	9	4	--	--
	Ca(ICCV2/PI489777)SNP_00953	390	C	G	16	4	--	--
	Ca(ICCV2/PI489777)SNP_00954	488	A	T	16	3	--	--
CakTC26481	Ca(ICCV2/PI489777)SNP_00955	110	C	T	12	3	--	--
	Ca(ICCV2/PI489777)SNP_00956	271	A	G	19	6	--	--
	Ca(ICCV2/PI489777)SNP_00957	1562	T	G	16	8	--	--

	Ca(ICCV2/PI489777)SNP_00958	1598	T	A	16	8	--	--
	Ca(ICCV2/PI489777)SNP_00959	1975	G	C	7	12	--	--
	Ca(ICCV2/PI489777)SNP_00960	2057	C	T	4	14	--	--
	Ca(ICCV2/PI489777)SNP_00961	2192	T	C	9	16	--	--
	Ca(ICCV2/PI489777)SNP_00962	2269	T	C	12	5	--	--
CakTC41009	Ca(ICCV2/PI489777)SNP_00963	1048	G	A	168	71	--	--
	Ca(ICCV2/PI489777)SNP_00964	1231	C	T	176	89	--	--
	Ca(ICCV2/PI489777)SNP_00965	1850	G	A	48	46	--	--
	Ca(ICCV2/PI489777)SNP_00966	1878	G	C	40	24	--	--
	Ca(ICCV2/PI489777)SNP_00967	1895	T	G	37	25	--	--
CakTC10985	Ca(ICCV2/PI489777)SNP_00968	66	T	C	3	4	--	--
	Ca(ICCV2/PI489777)SNP_00969	532	A	G	25	32	--	--
CakTC28500	Ca(ICCV2/PI489777)SNP_00970	168	T	A	15	9	--	--
	Ca(ICCV2/PI489777)SNP_00971	247	A	G	19	8	--	--
	Ca(ICCV2/PI489777)SNP_00972	788	A	G	18	13	--	--
	Ca(ICCV2/PI489777)SNP_00973	849	T	C	16	14	--	--
	Ca(ICCV2/PI489777)SNP_00974	1407	C	G	10	5	--	--
CakTC31722	Ca(ICCV2/PI489777)SNP_00975	741	T	C	7	11	--	--
CakTC43288	Ca(ICCV2/PI489777)SNP_00976	209	A	G	18	3	--	--
	Ca(ICCV2/PI489777)SNP_00977	215	C	T	18	4	--	--
	Ca(ICCV2/PI489777)SNP_00978	373	T	C	18	8	--	--
	Ca(ICCV2/PI489777)SNP_00979	374	C	G	18	8	--	--
CakTC40019	Ca(ICCV2/PI489777)SNP_00980	378	C	T	6	7	--	--
	Ca(ICCV2/PI489777)SNP_00981	426	G	A	6	6	--	--
	Ca(ICCV2/PI489777)SNP_00982	534	G	A	5	6	--	--
	Ca(ICCV2/PI489777)SNP_00983	627	A	G	7	8	--	--
CakTC40081	Ca(ICCV2/PI489777)SNP_00984	58	T	C	6	7	--	--
	Ca(ICCV2/PI489777)SNP_00985	256	A	T	29	32	--	--
	Ca(ICCV2/PI489777)SNP_00986	415	C	T	32	42	--	--
	Ca(ICCV2/PI489777)SNP_00987	532	C	T	26	44	--	--
	Ca(ICCV2/PI489777)SNP_00988	564	A	G	28	44	--	--
	Ca(ICCV2/PI489777)SNP_00989	886	G	A	22	37	--	--
	Ca(ICCV2/PI489777)SNP_00990	943	T	A	22	28	--	--
	Ca(ICCV2/PI489777)SNP_00991	1148	G	A	8	13	--	--
	Ca(ICCV2/PI489777)SNP_00992	1181	G	T	6	9	--	--
	Ca(ICCV2/PI489777)SNP_00993	1236	T	G	5	7	--	--
	Ca(ICCV2/PI489777)SNP_00994	1311	T	A	4	3	--	--
CakTC29867	Ca(ICCV2/PI489777)SNP_00995	917	C	T	6	4	--	--
	Ca(ICCV2/PI489777)SNP_00996	1043	T	C	7	3	--	--
	Ca(ICCV2/PI489777)SNP_00997	1213	T	C	13	4	--	--
	Ca(ICCV2/PI489777)SNP_00998	1421	T	C	8	4	--	--
CakTC34335	Ca(ICCV2/PI489777)SNP_00999	319	C	T	3	7	--	--
	Ca(ICCV2/PI489777)SNP_01000	344	T	C	3	7	--	--
CakTC38355	Ca(ICCV2/PI489777)SNP_01001	57	T	G	14	7	--	--
	Ca(ICCV2/PI489777)SNP_01002	431	C	T	19	14	--	--
	Ca(ICCV2/PI489777)SNP_01003	626	C	T	24	9	--	--
CakTC40278	Ca(ICCV2/PI489777)SNP_01004	632	G	C	13	8	--	--
CakTC33551	Ca(ICCV2/PI489777)SNP_01005	4707	A	C	8	4	--	--
	Ca(ICCV2/PI489777)SNP_01006	4858	A	G	6	7	--	--
	Ca(ICCV2/PI489777)SNP_01007	4948	T	C	7	6	--	--
	Ca(ICCV2/PI489777)SNP_01008	5118	T	A	7	7	--	--
	Ca(ICCV2/PI489777)SNP_01009	5341	T	C	9	6	--	--
	Ca(ICCV2/PI489777)SNP_01010	5656	G	A	4	5	--	--
	Ca(ICCV2/PI489777)SNP_01011	6647	C	T	3	4	--	--
	Ca(ICCV2/PI489777)SNP_01012	6663	A	T	4	4	--	--
	Ca(ICCV2/PI489777)SNP_01013	7002	C	T	9	4	--	--
	Ca(ICCV2/PI489777)SNP_01014	7476	A	G	7	3	--	--
	Ca(ICCV2/PI489777)SNP_01015	7989	G	A	5	5	--	--
CakTC26102	Ca(ICCV2/PI489777)SNP_01016	860	A	T	22	10	--	--
CakTC29069	Ca(ICCV2/PI489777)SNP_01017	939	T	C	5	8	--	--
CakTC41637	Ca(ICCV2/PI489777)SNP_01018	1308	G	T	13	3	--	--
	Ca(ICCV2/PI489777)SNP_01019	1385	T	C	10	3	--	--
	Ca(ICCV2/PI489777)SNP_01020	1559	G	A	16	9	--	--
CakTC16164	Ca(ICCV2/PI489777)SNP_01021	405	G	T	3	3	--	--
CakTC27560	Ca(ICCV2/PI489777)SNP_01022	298	A	G	8	5	--	--
	Ca(ICCV2/PI489777)SNP_01023	1153	A	T	11	4	--	--
	Ca(ICCV2/PI489777)SNP_01024	1159	G	A	12	3	--	--
CakTC39338	Ca(ICCV2/PI489777)SNP_01025	616	T	C	10	10	--	--
	Ca(ICCV2/PI489777)SNP_01026	1479	G	A	6	5	--	--
	Ca(ICCV2/PI489777)SNP_01027	1508	G	A	6	4	--	--
CakTC31277	Ca(ICCV2/PI489777)SNP_01028	3755	G	A	11	3	--	--
CakTC40983	Ca(ICCV2/PI489777)SNP_01029	318	T	A	5	5	--	--
CakTC02060	Ca(ICCV2/PI489777)SNP_01030	229	G	A	72	51	--	--
	Ca(ICCV2/PI489777)SNP_01031	230	T	C	73	51	--	--

	Ca(ICCV2/PI489777)SNP_01032	326	G	A	101	57	--	--
CakTC42605	Ca(ICCV2/PI489777)SNP_01033	493	C	T	80	33	--	--
	Ca(ICCV2/PI489777)SNP_01034	725	G	A	57	16	--	--
	Ca(ICCV2/PI489777)SNP_01035	734	T	C	57	16	--	--
	Ca(ICCV2/PI489777)SNP_01036	864	A	G	18	3	--	--
	Ca(ICCV2/PI489777)SNP_01037	910	C	T	8	4	--	--
CakTC34196	Ca(ICCV2/PI489777)SNP_01038	345	C	T	26	10	--	--
	Ca(ICCV2/PI489777)SNP_01039	367	G	T	19	9	--	--
CakTC43032	Ca(ICCV2/PI489777)SNP_01040	277	A	G	4	5	--	--
	Ca(ICCV2/PI489777)SNP_01041	1515	G	T	12	17	--	--
CakTC34095	Ca(ICCV2/PI489777)SNP_01042	35	C	T	4	10	--	--
	Ca(ICCV2/PI489777)SNP_01043	43	C	T	4	11	--	--
	Ca(ICCV2/PI489777)SNP_01044	61	C	T	6	14	--	--
	Ca(ICCV2/PI489777)SNP_01045	140	G	A	6	14	--	--
	Ca(ICCV2/PI489777)SNP_01046	142	G	A	7	16	--	--
	Ca(ICCV2/PI489777)SNP_01047	177	G	A	8	17	--	--
	Ca(ICCV2/PI489777)SNP_01048	182	C	T	9	14	--	--
	Ca(ICCV2/PI489777)SNP_01049	260	T	C	9	14	--	--
	Ca(ICCV2/PI489777)SNP_01050	1916	T	C	4	3	--	--
CakTC28858	Ca(ICCV2/PI489777)SNP_01051	1120	G	T	3	5	--	--
	Ca(ICCV2/PI489777)SNP_01052	1126	G	A	3	6	--	--
CakTC31544	Ca(ICCV2/PI489777)SNP_01053	366	G	C	3	3	--	--
CakTC42697	Ca(ICCV2/PI489777)SNP_01054	82	G	A	5	9	--	--
CakTC10496	Ca(ICCV2/PI489777)SNP_01055	573	C	T	6	12	--	--
	Ca(ICCV2/PI489777)SNP_01056	582	A	G	7	12	--	--
	Ca(ICCV2/PI489777)SNP_01057	654	A	G	5	13	--	--
	Ca(ICCV2/PI489777)SNP_01058	875	G	A	3	8	--	--
	Ca(ICCV2/PI489777)SNP_01059	1179	C	T	9	3	--	--
CakTC43095	Ca(ICCV2/PI489777)SNP_01060	283	A	G	30	18	--	--
	Ca(ICCV2/PI489777)SNP_01061	367	G	A	33	28	--	--
	Ca(ICCV2/PI489777)SNP_01062	982	T	C	53	31	--	--
	Ca(ICCV2/PI489777)SNP_01063	1666	A	G	11	13	--	--
	Ca(ICCV2/PI489777)SNP_01064	1723	G	C	8	6	--	--
CakTC07919	Ca(ICCV2/PI489777)SNP_01065	585	T	C	3	5	--	--
CakTC25439	Ca(ICCV2/PI489777)SNP_01066	1881	A	T	3	3	--	Jumonji
CakTC15179	Ca(ICCV2/PI489777)SNP_01067	543	C	T	48	17	--	--
	Ca(ICCV2/PI489777)SNP_01068	845	A	G	18	3	--	--
CakTC39962	Ca(ICCV2/PI489777)SNP_01069	918	T	C	4	3	--	--
CakTC28019	Ca(ICCV2/PI489777)SNP_01070	720	G	C	5	3	--	C2C2-Dof
CakTC29754	Ca(ICCV2/PI489777)SNP_01071	955	G	A	9	9	--	--
	Ca(ICCV2/PI489777)SNP_01072	1483	G	A	5	5	--	--
	Ca(ICCV2/PI489777)SNP_01073	1611	G	T	3	3	--	--
	Ca(ICCV2/PI489777)SNP_01074	1636	T	G	3	4	--	--
	Ca(ICCV2/PI489777)SNP_01075	1676	A	G	4	4	--	--
CakTC39224	Ca(ICCV2/PI489777)SNP_01076	170	T	C	5	4	--	--
	Ca(ICCV2/PI489777)SNP_01077	285	C	G	7	3	--	--
	Ca(ICCV2/PI489777)SNP_01078	539	G	A	22	14	--	--
	Ca(ICCV2/PI489777)SNP_01079	548	C	G	16	13	--	--
	Ca(ICCV2/PI489777)SNP_01080	1064	A	G	27	9	--	--
	Ca(ICCV2/PI489777)SNP_01081	1148	C	T	26	12	--	--
	Ca(ICCV2/PI489777)SNP_01082	1157	A	C	27	14	--	--
	Ca(ICCV2/PI489777)SNP_01083	1177	A	T	27	13	--	--
	Ca(ICCV2/PI489777)SNP_01084	1250	T	C	24	10	--	--
	Ca(ICCV2/PI489777)SNP_01085	1358	T	G	25	11	--	--
CakTC35569	Ca(ICCV2/PI489777)SNP_01086	858	G	A	39	12	--	--
	Ca(ICCV2/PI489777)SNP_01087	959	A	G	25	10	--	--
	Ca(ICCV2/PI489777)SNP_01088	971	C	G	23	12	--	--
	Ca(ICCV2/PI489777)SNP_01089	3193	C	G	9	7	--	--
CakTC32952	Ca(ICCV2/PI489777)SNP_01090	486	C	T	4	7	--	ARF
	Ca(ICCV2/PI489777)SNP_01091	753	C	T	6	4	--	ARF
	Ca(ICCV2/PI489777)SNP_01092	856	C	T	4	6	--	ARF
	Ca(ICCV2/PI489777)SNP_01093	896	A	G	4	6	--	ARF
	Ca(ICCV2/PI489777)SNP_01094	1325	C	T	5	5	--	ARF
	Ca(ICCV2/PI489777)SNP_01095	1535	A	G	3	3	--	ARF
CakTC38064	Ca(ICCV2/PI489777)SNP_01096	839	T	C	4	7	--	--
CakTC29062	Ca(ICCV2/PI489777)SNP_01097	82	C	A	7	4	--	--
CakTC39421	Ca(ICCV2/PI489777)SNP_01098	286	T	C	10	8	--	--
	Ca(ICCV2/PI489777)SNP_01099	1118	T	C	4	5	--	--
CakTC37879	Ca(ICCV2/PI489777)SNP_01100	623	C	A	19	17	--	--
	Ca(ICCV2/PI489777)SNP_01101	642	A	C	19	16	--	--
	Ca(ICCV2/PI489777)SNP_01102	799	A	G	9	13	--	--
CakTC24103	Ca(ICCV2/PI489777)SNP_01103	255	C	T	21	4	--	--
	Ca(ICCV2/PI489777)SNP_01104	380	G	T	19	6	--	--
	Ca(ICCV2/PI489777)SNP_01105	497	A	C	21	6	--	--

	Ca(ICCV2/PI489777)SNP_01106	632	G	A	19	5	--	--
	Ca(ICCV2/PI489777)SNP_01107	917	T	C	36	9	--	--
	Ca(ICCV2/PI489777)SNP_01108	1024	G	A	36	9	--	--
	Ca(ICCV2/PI489777)SNP_01109	1154	G	A	37	10	--	--
	Ca(ICCV2/PI489777)SNP_01110	1427	T	C	64	13	--	--
	Ca(ICCV2/PI489777)SNP_01111	1466	C	T	70	9	--	--
	Ca(ICCV2/PI489777)SNP_01112	1752	G	A	73	20	--	--
	Ca(ICCV2/PI489777)SNP_01113	2069	G	A	18	11	--	--
	Ca(ICCV2/PI489777)SNP_01114	2179	A	G	31	8	--	--
CakTC39894	Ca(ICCV2/PI489777)SNP_01115	1076	G	A	8	5	--	--
	Ca(ICCV2/PI489777)SNP_01116	1080	T	C	7	5	--	--
CakTC31026	Ca(ICCV2/PI489777)SNP_01117	875	T	G	4	5	--	ABI3VP1
	Ca(ICCV2/PI489777)SNP_01118	954	C	G	4	6	--	ABI3VP1
	Ca(ICCV2/PI489777)SNP_01119	1114	T	A	3	4	--	ABI3VP1
	Ca(ICCV2/PI489777)SNP_01120	1191	C	T	3	4	--	ABI3VP1
CakTC34888	Ca(ICCV2/PI489777)SNP_01121	267	A	G	9	3	--	CAMTA
	Ca(ICCV2/PI489777)SNP_01122	1180	C	T	11	4	--	CAMTA
	Ca(ICCV2/PI489777)SNP_01123	1458	A	G	12	4	--	CAMTA
	Ca(ICCV2/PI489777)SNP_01124	2497	G	T	25	11	--	CAMTA
	Ca(ICCV2/PI489777)SNP_01125	2621	A	G	31	14	--	CAMTA
CakTC12315	Ca(ICCV2/PI489777)SNP_01126	368	G	C	4	4	--	--
CakTC30614	Ca(ICCV2/PI489777)SNP_01127	552	C	T	12	5	--	--
CakTC42321	Ca(ICCV2/PI489777)SNP_01128	93	G	T	5	3	Root	--
	Ca(ICCV2/PI489777)SNP_01129	391	C	G	6	19	Root	--
	Ca(ICCV2/PI489777)SNP_01130	443	G	A	11	18	Root	--
	Ca(ICCV2/PI489777)SNP_01131	561	T	C	4	17	Root	--
CakTC26989	Ca(ICCV2/PI489777)SNP_01132	285	G	C	11	10	--	--
	Ca(ICCV2/PI489777)SNP_01133	530	G	T	19	13	--	--
	Ca(ICCV2/PI489777)SNP_01134	593	A	G	19	15	--	--
	Ca(ICCV2/PI489777)SNP_01135	647	T	C	18	14	--	--
	Ca(ICCV2/PI489777)SNP_01136	1331	G	A	26	19	--	--
	Ca(ICCV2/PI489777)SNP_01137	1413	C	A	22	15	--	--
	Ca(ICCV2/PI489777)SNP_01138	1428	A	T	18	16	--	--
	Ca(ICCV2/PI489777)SNP_01139	2711	C	T	27	9	--	--
	Ca(ICCV2/PI489777)SNP_01140	2780	A	C	27	9	--	--
	Ca(ICCV2/PI489777)SNP_01141	2789	A	C	29	10	--	--
CakTC32300	Ca(ICCV2/PI489777)SNP_01142	326	G	A	3	3	--	--
CakTC42751	Ca(ICCV2/PI489777)SNP_01143	852	A	C	6	5	--	--
CakTC32122	Ca(ICCV2/PI489777)SNP_01144	471	A	G	5	4	--	WRKY
	Ca(ICCV2/PI489777)SNP_01145	552	A	T	4	4	--	WRKY
	Ca(ICCV2/PI489777)SNP_01146	1204	G	A	5	4	--	WRKY
CakTC23770	Ca(ICCV2/PI489777)SNP_01147	271	G	A	11	6	--	--
	Ca(ICCV2/PI489777)SNP_01148	461	G	C	17	8	--	--
	Ca(ICCV2/PI489777)SNP_01149	2403	C	A	13	3	--	--
	Ca(ICCV2/PI489777)SNP_01150	2445	C	T	15	5	--	--
	Ca(ICCV2/PI489777)SNP_01151	2453	A	G	14	5	--	--
CakTC02306	Ca(ICCV2/PI489777)SNP_01152	1198	T	G	3	5	--	--
CakTC03418	Ca(ICCV2/PI489777)SNP_01153	372	T	C	11	4	--	--
CakTC09734	Ca(ICCV2/PI489777)SNP_01154	2160	A	G	3	4	--	--
CakTC09507	Ca(ICCV2/PI489777)SNP_01155	3641	G	T	6	4	Young_pod	--
	Ca(ICCV2/PI489777)SNP_01156	3654	T	C	6	4	Young_pod	--
CakTC40516	Ca(ICCV2/PI489777)SNP_01157	390	A	T	8	6	--	--
	Ca(ICCV2/PI489777)SNP_01158	401	G	C	7	5	--	--
	Ca(ICCV2/PI489777)SNP_01159	604	A	G	5	4	--	--
	Ca(ICCV2/PI489777)SNP_01160	608	G	A	5	4	--	--
	Ca(ICCV2/PI489777)SNP_01161	633	C	T	3	3	--	--
CakTC35510	Ca(ICCV2/PI489777)SNP_01162	2275	A	G	14	14	--	--
CakTC22858	Ca(ICCV2/PI489777)SNP_01163	171	A	G	3	3	--	--
CakTC37312	Ca(ICCV2/PI489777)SNP_01164	28	G	A	4	3	--	--
	Ca(ICCV2/PI489777)SNP_01165	217	A	G	8	4	--	--
	Ca(ICCV2/PI489777)SNP_01166	361	T	A	12	8	--	--
	Ca(ICCV2/PI489777)SNP_01167	971	A	G	33	15	--	--
	Ca(ICCV2/PI489777)SNP_01168	1414	T	G	15	17	--	--
	Ca(ICCV2/PI489777)SNP_01169	1525	A	G	11	17	--	--
	Ca(ICCV2/PI489777)SNP_01170	1729	C	T	5	4	--	--
	Ca(ICCV2/PI489777)SNP_01171	1779	T	A	3	4	--	--
CakTC31560	Ca(ICCV2/PI489777)SNP_01172	911	G	C	15	3	--	MYB
CakTC34304	Ca(ICCV2/PI489777)SNP_01173	631	C	T	7	7	--	--
	Ca(ICCV2/PI489777)SNP_01174	1429	C	A	13	9	--	--
	Ca(ICCV2/PI489777)SNP_01175	1430	G	T	13	9	--	--
	Ca(ICCV2/PI489777)SNP_01176	1516	C	T	14	12	--	--
	Ca(ICCV2/PI489777)SNP_01177	2003	G	C	6	5	--	--
CakTC33075	Ca(ICCV2/PI489777)SNP_01178	421	A	C	6	8	--	--
CakTC38185	Ca(ICCV2/PI489777)SNP_01179	159	T	A	20	6	--	--

	Ca(ICCV2/PI489777)SNP_01180	280	T	A	33	8	--	--
	Ca(ICCV2/PI489777)SNP_01181	416	G	A	45	7	--	--
	Ca(ICCV2/PI489777)SNP_01182	770	A	G	27	8	--	--
	Ca(ICCV2/PI489777)SNP_01183	1187	G	C	45	6	--	--
	Ca(ICCV2/PI489777)SNP_01184	1274	T	C	41	5	--	--
CakTC27864	Ca(ICCV2/PI489777)SNP_01185	177	T	C	3	3	--	--
CakTC02200	Ca(ICCV2/PI489777)SNP_01186	405	C	T	3	5	--	--
CakTC02389	Ca(ICCV2/PI489777)SNP_01187	118	G	A	7	4	--	--
CakTC40581	Ca(ICCV2/PI489777)SNP_01188	360	G	A	4	4	--	--
	Ca(ICCV2/PI489777)SNP_01189	398	G	A	4	4	--	--
CakTC41970	Ca(ICCV2/PI489777)SNP_01190	453	T	C	5	12	--	--
	Ca(ICCV2/PI489777)SNP_01191	635	T	G	7	5	--	--
CakTC28322	Ca(ICCV2/PI489777)SNP_01192	269	A	G	29	9	--	--
	Ca(ICCV2/PI489777)SNP_01193	347	G	A	33	17	--	--
	Ca(ICCV2/PI489777)SNP_01194	1154	A	T	56	44	--	--
	Ca(ICCV2/PI489777)SNP_01195	1532	C	G	13	4	--	--
CakTC26214	Ca(ICCV2/PI489777)SNP_01196	321	G	T	95	48	--	--
	Ca(ICCV2/PI489777)SNP_01197	439	A	T	112	49	--	--
	Ca(ICCV2/PI489777)SNP_01198	442	A	T	113	48	--	--
	Ca(ICCV2/PI489777)SNP_01199	588	T	A	88	33	--	--
CakTC25365	Ca(ICCV2/PI489777)SNP_01200	807	G	A	15	4	--	--
CakTC31642	Ca(ICCV2/PI489777)SNP_01201	385	C	A	13	3	--	--
	Ca(ICCV2/PI489777)SNP_01202	527	A	G	13	4	--	--
	Ca(ICCV2/PI489777)SNP_01203	584	A	G	13	3	--	--
	Ca(ICCV2/PI489777)SNP_01204	601	T	C	16	3	--	--
CakTC37906	Ca(ICCV2/PI489777)SNP_01205	282	A	G	15	13	--	--
	Ca(ICCV2/PI489777)SNP_01206	437	T	C	16	12	--	--
	Ca(ICCV2/PI489777)SNP_01207	936	G	A	14	19	--	--
	Ca(ICCV2/PI489777)SNP_01208	951	C	T	15	18	--	--
	Ca(ICCV2/PI489777)SNP_01209	978	A	T	16	17	--	--
	Ca(ICCV2/PI489777)SNP_01210	1077	G	A	8	18	--	--
	Ca(ICCV2/PI489777)SNP_01211	1080	A	G	9	19	--	--
CakTC38600	Ca(ICCV2/PI489777)SNP_01212	464	T	C	46	34	--	bHLH
CakTC10066	Ca(ICCV2/PI489777)SNP_01213	2356	T	C	12	3	--	--
	Ca(ICCV2/PI489777)SNP_01214	2700	G	T	5	5	--	--
CakTC10179	Ca(ICCV2/PI489777)SNP_01215	202	G	C	9	5	--	--
CakTC41206	Ca(ICCV2/PI489777)SNP_01216	99	T	C	4	7	--	--
	Ca(ICCV2/PI489777)SNP_01217	312	G	A	7	8	--	--
	Ca(ICCV2/PI489777)SNP_01218	606	G	T	7	5	--	--
	Ca(ICCV2/PI489777)SNP_01219	839	A	G	7	6	--	--
	Ca(ICCV2/PI489777)SNP_01220	893	T	C	10	8	--	--
	Ca(ICCV2/PI489777)SNP_01221	1662	T	G	9	17	--	--
	Ca(ICCV2/PI489777)SNP_01222	2087	A	C	9	9	--	--
CakTC35882	Ca(ICCV2/PI489777)SNP_01223	549	G	A	18	40	--	--
	Ca(ICCV2/PI489777)SNP_01224	1711	T	C	15	19	--	--
	Ca(ICCV2/PI489777)SNP_01225	2205	G	A	16	22	--	--
	Ca(ICCV2/PI489777)SNP_01226	2352	A	G	13	11	--	--
	Ca(ICCV2/PI489777)SNP_01227	2515	T	G	10	13	--	--
	Ca(ICCV2/PI489777)SNP_01228	2635	A	T	14	16	--	--
	Ca(ICCV2/PI489777)SNP_01229	2741	C	T	14	17	--	--
	Ca(ICCV2/PI489777)SNP_01230	3171	G	T	10	17	--	--
CakTC10139	Ca(ICCV2/PI489777)SNP_01231	343	G	A	20	17	--	--
	Ca(ICCV2/PI489777)SNP_01232	1365	C	T	8	3	--	--
	Ca(ICCV2/PI489777)SNP_01233	2157	T	C	28	3	--	--
CakTC25281	Ca(ICCV2/PI489777)SNP_01234	731	C	T	3	5	--	--
	Ca(ICCV2/PI489777)SNP_01235	822	C	A	3	6	--	--
	Ca(ICCV2/PI489777)SNP_01236	1691	T	A	50	19	--	--
CakTC39310	Ca(ICCV2/PI489777)SNP_01237	363	C	G	27	20	--	--
	Ca(ICCV2/PI489777)SNP_01238	388	C	G	26	20	--	--
CakTC13052	Ca(ICCV2/PI489777)SNP_01239	34	C	G	40	25	--	--
	Ca(ICCV2/PI489777)SNP_01240	338	T	C	65	58	--	--
	Ca(ICCV2/PI489777)SNP_01241	497	C	T	52	55	--	--
	Ca(ICCV2/PI489777)SNP_01242	769	T	C	82	44	--	--
	Ca(ICCV2/PI489777)SNP_01243	782	C	T	74	42	--	--
CakTC24533	Ca(ICCV2/PI489777)SNP_01244	1187	C	T	4	5	--	--
	Ca(ICCV2/PI489777)SNP_01245	1933	C	T	9	5	--	--
CakTC25373	Ca(ICCV2/PI489777)SNP_01246	622	G	A	4	3	--	--
	Ca(ICCV2/PI489777)SNP_01247	625	C	T	4	3	--	--
CakTC40918	Ca(ICCV2/PI489777)SNP_01248	55	C	T	22	17	--	--
	Ca(ICCV2/PI489777)SNP_01249	617	C	T	6	13	--	--
CakTC39751	Ca(ICCV2/PI489777)SNP_01250	1305	A	G	11	5	--	--
CakTC13113	Ca(ICCV2/PI489777)SNP_01251	809	C	G	15	14	--	--
	Ca(ICCV2/PI489777)SNP_01252	1335	A	C	52	32	--	--
	Ca(ICCV2/PI489777)SNP_01253	1370	T	C	44	43	--	--

	Ca(ICCV2/PI489777)SNP_01254	1470	T	C	33	26	--	--
	Ca(ICCV2/PI489777)SNP_01255	1658	G	A	4	3	--	--
CakTC09238	Ca(ICCV2/PI489777)SNP_01256	390	A	T	4	9	--	--
CakTC38840	Ca(ICCV2/PI489777)SNP_01257	1260	G	T	6	3	--	--
	Ca(ICCV2/PI489777)SNP_01258	1648	G	A	3	3	--	--
CakTC34151	Ca(ICCV2/PI489777)SNP_01259	119	A	G	3	5	--	--
	Ca(ICCV2/PI489777)SNP_01260	131	T	C	3	8	--	--
	Ca(ICCV2/PI489777)SNP_01261	241	C	A	5	7	--	--
	Ca(ICCV2/PI489777)SNP_01262	457	G	C	5	5	--	--
	Ca(ICCV2/PI489777)SNP_01263	467	A	G	5	5	--	--
	Ca(ICCV2/PI489777)SNP_01264	500	G	A	5	4	--	--
CakTC31810	Ca(ICCV2/PI489777)SNP_01265	432	G	A	7	6	--	--
CakTC01044	Ca(ICCV2/PI489777)SNP_01266	159	G	A	4	4	--	--
CakTC22950	Ca(ICCV2/PI489777)SNP_01267	27	A	C	39	13	--	--
CakTC22257	Ca(ICCV2/PI489777)SNP_01268	1639	T	A	5	3	--	--
CakTC10193	Ca(ICCV2/PI489777)SNP_01269	575	G	A	14	6	--	--
CakTC17238	Ca(ICCV2/PI489777)SNP_01270	1761	A	G	7	4	--	--
CakTC28413	Ca(ICCV2/PI489777)SNP_01271	193	C	T	8	3	--	--
	Ca(ICCV2/PI489777)SNP_01272	378	T	C	19	7	--	--
CakTC29354	Ca(ICCV2/PI489777)SNP_01273	235	C	T	15	10	--	--
	Ca(ICCV2/PI489777)SNP_01274	442	T	G	17	24	--	--
	Ca(ICCV2/PI489777)SNP_01275	444	A	G	17	24	--	--
	Ca(ICCV2/PI489777)SNP_01276	1644	G	C	4	4	--	--
CakTC27110	Ca(ICCV2/PI489777)SNP_01277	1041	C	A	5	5	--	--
	Ca(ICCV2/PI489777)SNP_01278	3890	C	T	4	6	--	--
	Ca(ICCV2/PI489777)SNP_01279	3984	A	G	4	6	--	--
CakTC40962	Ca(ICCV2/PI489777)SNP_01280	73	C	T	40	5	--	--
	Ca(ICCV2/PI489777)SNP_01281	234	A	G	52	11	--	--
	Ca(ICCV2/PI489777)SNP_01282	388	T	C	41	15	--	--
	Ca(ICCV2/PI489777)SNP_01283	406	T	A	14	14	--	--
	Ca(ICCV2/PI489777)SNP_01284	451	C	T	8	13	--	--
	Ca(ICCV2/PI489777)SNP_01285	523	C	T	7	11	--	--
CakTC41534	Ca(ICCV2/PI489777)SNP_01286	649	G	A	3	3	--	--
CakTC26247	Ca(ICCV2/PI489777)SNP_01287	487	G	C	4	3	Root	--
CakTC39625	Ca(ICCV2/PI489777)SNP_01288	336	G	C	17	5	--	--
	Ca(ICCV2/PI489777)SNP_01289	410	T	C	21	5	--	--
	Ca(ICCV2/PI489777)SNP_01290	588	C	T	24	5	--	--
	Ca(ICCV2/PI489777)SNP_01291	656	G	C	29	5	--	--
CakTC39195	Ca(ICCV2/PI489777)SNP_01292	497	A	G	5	4	--	--
CakTC28724	Ca(ICCV2/PI489777)SNP_01293	76	T	C	8	3	--	--
	Ca(ICCV2/PI489777)SNP_01294	310	C	T	14	10	--	--
CakTC34330	Ca(ICCV2/PI489777)SNP_01295	115	T	A	5	6	Flower	bud
	Ca(ICCV2/PI489777)SNP_01296	127	T	C	7	6	Flower	bud
CakTC11406	Ca(ICCV2/PI489777)SNP_01297	853	A	G	10	7	--	--
	Ca(ICCV2/PI489777)SNP_01298	975	G	A	10	7	--	--
CakTC10222	Ca(ICCV2/PI489777)SNP_01299	809	A	G	10	5	--	bHLH
	Ca(ICCV2/PI489777)SNP_01300	938	G	A	10	6	--	bHLH
CakTC27984	Ca(ICCV2/PI489777)SNP_01301	320	G	T	8	3	--	--
	Ca(ICCV2/PI489777)SNP_01302	1950	G	A	28	9	--	--
	Ca(ICCV2/PI489777)SNP_01303	1989	T	C	19	4	--	--
	Ca(ICCV2/PI489777)SNP_01304	2172	T	C	29	4	--	--
	Ca(ICCV2/PI489777)SNP_01305	2203	G	A	25	4	--	--
	Ca(ICCV2/PI489777)SNP_01306	2241	C	T	29	7	--	--
	Ca(ICCV2/PI489777)SNP_01307	2248	C	T	32	8	--	--
	Ca(ICCV2/PI489777)SNP_01308	2275	G	T	30	6	--	--
	Ca(ICCV2/PI489777)SNP_01309	2292	G	A	25	4	--	--
	Ca(ICCV2/PI489777)SNP_01310	2975	A	G	5	3	--	--
CakTC34023	Ca(ICCV2/PI489777)SNP_01311	616	T	C	5	3	--	--
CakTC11446	Ca(ICCV2/PI489777)SNP_01312	392	T	C	9	7	--	--
CakTC30185	Ca(ICCV2/PI489777)SNP_01313	854	G	C	11	3	--	--
	Ca(ICCV2/PI489777)SNP_01314	1098	A	G	14	8	--	--
	Ca(ICCV2/PI489777)SNP_01315	1132	A	G	14	8	--	--
	Ca(ICCV2/PI489777)SNP_01316	1156	A	G	13	6	--	--
	Ca(ICCV2/PI489777)SNP_01317	1296	G	A	6	5	--	--
	Ca(ICCV2/PI489777)SNP_01318	1309	C	A	6	5	--	--
CakTC20170	Ca(ICCV2/PI489777)SNP_01319	325	C	T	4	3	--	--
	Ca(ICCV2/PI489777)SNP_01320	340	T	C	4	3	--	--
CakTC25460	Ca(ICCV2/PI489777)SNP_01321	1980	T	C	8	3	--	--
CakTC23209	Ca(ICCV2/PI489777)SNP_01322	1159	T	C	5	5	--	--
CakTC36550	Ca(ICCV2/PI489777)SNP_01323	1431	G	A	3	4	--	--
	Ca(ICCV2/PI489777)SNP_01324	1697	G	T	4	3	--	--
	Ca(ICCV2/PI489777)SNP_01325	2352	T	A	3	5	--	--
	Ca(ICCV2/PI489777)SNP_01326	2445	T	A	3	7	--	--
CakTC32536	Ca(ICCV2/PI489777)SNP_01327	187	T	G	9	4	--	--

	Ca(ICCV2/PI489777)SNP_01328	214	T	C	9	4	--	--
	Ca(ICCV2/PI489777)SNP_01329	264	C	G	7	4	--	--
CakTC38998	Ca(ICCV2/PI489777)SNP_01330	1396	A	G	19	5	--	--
	Ca(ICCV2/PI489777)SNP_01331	1460	C	A	13	4	--	--
	Ca(ICCV2/PI489777)SNP_01332	2002	G	A	19	10	--	--
	Ca(ICCV2/PI489777)SNP_01333	2014	T	A	19	11	--	--
	Ca(ICCV2/PI489777)SNP_01334	2044	G	A	18	10	--	--
	Ca(ICCV2/PI489777)SNP_01335	2048	C	A	18	12	--	--
	Ca(ICCV2/PI489777)SNP_01336	2059	A	G	18	12	--	--
	Ca(ICCV2/PI489777)SNP_01337	2098	T	G	16	9	--	--
	Ca(ICCV2/PI489777)SNP_01338	2230	T	A	3	11	--	--
	Ca(ICCV2/PI489777)SNP_01339	2271	A	C	4	9	--	--
	Ca(ICCV2/PI489777)SNP_01340	2374	T	G	4	9	--	--
	Ca(ICCV2/PI489777)SNP_01341	2398	C	G	3	9	--	--
CakTC29687	Ca(ICCV2/PI489777)SNP_01342	1373	T	G	6	6	--	--
CakTC26820	Ca(ICCV2/PI489777)SNP_01343	177	C	T	4	4	--	--
	Ca(ICCV2/PI489777)SNP_01344	363	C	G	10	8	--	--
	Ca(ICCV2/PI489777)SNP_01345	420	A	G	11	7	--	--
CakTC37946	Ca(ICCV2/PI489777)SNP_01346	808	A	G	17	8	--	--
CakTC26749	Ca(ICCV2/PI489777)SNP_01347	740	C	G	11	5	--	--
	Ca(ICCV2/PI489777)SNP_01348	781	C	A	10	3	--	--
	Ca(ICCV2/PI489777)SNP_01349	929	T	C	8	3	--	--
CakTC14664	Ca(ICCV2/PI489777)SNP_01350	670	G	A	6	3	--	--
CakTC28538	Ca(ICCV2/PI489777)SNP_01351	2401	C	G	8	5	Shoot	--
CakTC42379	Ca(ICCV2/PI489777)SNP_01352	261	T	C	6	4	--	--
CakTC25152	Ca(ICCV2/PI489777)SNP_01353	2142	C	T	22	13	--	--
CakTC41285	Ca(ICCV2/PI489777)SNP_01354	153	C	T	4	5	--	--
CakTC37711	Ca(ICCV2/PI489777)SNP_01355	146	A	G	8	9	--	--
	Ca(ICCV2/PI489777)SNP_01356	234	T	C	14	12	--	--
	Ca(ICCV2/PI489777)SNP_01357	248	T	C	10	13	--	--
	Ca(ICCV2/PI489777)SNP_01358	300	G	A	13	13	--	--
	Ca(ICCV2/PI489777)SNP_01359	435	C	T	14	13	--	--
	Ca(ICCV2/PI489777)SNP_01360	525	A	C	14	10	--	--
	Ca(ICCV2/PI489777)SNP_01361	681	A	C	17	3	--	--
	Ca(ICCV2/PI489777)SNP_01362	855	A	T	14	5	--	--
	Ca(ICCV2/PI489777)SNP_01363	866	C	T	17	6	--	--
	Ca(ICCV2/PI489777)SNP_01364	1209	C	T	16	10	--	--
	Ca(ICCV2/PI489777)SNP_01365	1425	T	C	13	7	--	--
	Ca(ICCV2/PI489777)SNP_01366	1544	C	G	13	3	--	--
CakTC32238	Ca(ICCV2/PI489777)SNP_01367	932	T	C	4	3	Root	--
	Ca(ICCV2/PI489777)SNP_01368	934	G	C	6	4	Root	--
	Ca(ICCV2/PI489777)SNP_01369	1135	C	T	7	5	Root	--
CakTC36895	Ca(ICCV2/PI489777)SNP_01370	628	G	A	12	5	--	--
	Ca(ICCV2/PI489777)SNP_01371	1264	G	A	3	8	--	--
	Ca(ICCV2/PI489777)SNP_01372	1292	A	G	4	8	--	--
	Ca(ICCV2/PI489777)SNP_01373	1411	G	A	4	3	--	--
CakTC23347	Ca(ICCV2/PI489777)SNP_01374	999	C	G	5	13	--	--
	Ca(ICCV2/PI489777)SNP_01375	1002	T	G	3	13	--	--
CakTC36676	Ca(ICCV2/PI489777)SNP_01376	308	A	G	3	5	--	--
CakTC11615	Ca(ICCV2/PI489777)SNP_01377	98	C	T	3	4	--	--
	Ca(ICCV2/PI489777)SNP_01378	102	G	A	3	4	--	--
	Ca(ICCV2/PI489777)SNP_01379	109	A	T	3	5	--	--
	Ca(ICCV2/PI489777)SNP_01380	208	G	A	9	6	--	--
	Ca(ICCV2/PI489777)SNP_01381	326	A	C	9	6	--	--
	Ca(ICCV2/PI489777)SNP_01382	460	T	C	12	6	--	--
	Ca(ICCV2/PI489777)SNP_01383	609	C	G	12	10	--	--
	Ca(ICCV2/PI489777)SNP_01384	659	T	C	14	7	--	--
CakTC24822	Ca(ICCV2/PI489777)SNP_01385	67	G	A	4	4	--	--
CakTC32202	Ca(ICCV2/PI489777)SNP_01386	231	T	C	7	3	--	--
	Ca(ICCV2/PI489777)SNP_01387	379	A	G	9	7	--	--
	Ca(ICCV2/PI489777)SNP_01388	488	G	C	12	8	--	--
	Ca(ICCV2/PI489777)SNP_01389	679	T	A	14	6	--	--
	Ca(ICCV2/PI489777)SNP_01390	682	T	G	13	6	--	--
	Ca(ICCV2/PI489777)SNP_01391	1131	G	C	19	5	--	--
CakTC28456	Ca(ICCV2/PI489777)SNP_01392	471	G	T	3	3	--	AP2-EREBP
CakTC10127	Ca(ICCV2/PI489777)SNP_01393	327	A	G	9	6	--	--
	Ca(ICCV2/PI489777)SNP_01394	690	A	G	3	3	--	--
CakTC41663	Ca(ICCV2/PI489777)SNP_01395	417	A	G	14	13	--	--
	Ca(ICCV2/PI489777)SNP_01396	2270	C	G	8	5	--	--
CakTC24531	Ca(ICCV2/PI489777)SNP_01397	693	A	G	32	10	Shoot	--
CakTC29858	Ca(ICCV2/PI489777)SNP_01398	707	G	T	10	5	--	--
	Ca(ICCV2/PI489777)SNP_01399	778	T	A	7	4	--	--
	Ca(ICCV2/PI489777)SNP_01400	927	G	A	9	6	--	--
	Ca(ICCV2/PI489777)SNP_01401	949	T	C	10	6	--	--

	Ca(ICCV2/PI489777)SNP_01402	966	G	A	9	5	--	--
	Ca(ICCV2/PI489777)SNP_01403	2189	T	C	17	19	--	--
	Ca(ICCV2/PI489777)SNP_01404	2515	T	A	13	9	--	--
	Ca(ICCV2/PI489777)SNP_01405	2851	G	T	6	8	--	--
CakTC24186	Ca(ICCV2/PI489777)SNP_01406	374	A	T	63	35	--	--
	Ca(ICCV2/PI489777)SNP_01407	1390	A	C	10	8	--	--
	Ca(ICCV2/PI489777)SNP_01408	1804	G	C	16	10	--	--
	Ca(ICCV2/PI489777)SNP_01409	1853	C	T	16	13	--	--
	Ca(ICCV2/PI489777)SNP_01410	2075	A	G	17	19	--	--
	Ca(ICCV2/PI489777)SNP_01411	2143	A	G	16	19	--	--
	Ca(ICCV2/PI489777)SNP_01412	2977	G	C	26	11	--	--
	Ca(ICCV2/PI489777)SNP_01413	3148	G	A	34	11	--	--
	Ca(ICCV2/PI489777)SNP_01414	3701	T	A	26	21	--	--
	Ca(ICCV2/PI489777)SNP_01415	4127	A	G	31	7	--	--
	Ca(ICCV2/PI489777)SNP_01416	4339	C	T	24	12	--	--
	Ca(ICCV2/PI489777)SNP_01417	4720	A	G	36	24	--	--
	Ca(ICCV2/PI489777)SNP_01418	6241	T	C	43	30	--	--
	Ca(ICCV2/PI489777)SNP_01419	6660	G	A	44	16	--	--
	Ca(ICCV2/PI489777)SNP_01420	7222	G	A	55	18	--	--
	Ca(ICCV2/PI489777)SNP_01421	8179	G	A	40	12	--	--
CakTC43153	Ca(ICCV2/PI489777)SNP_01422	1374	G	A	34	15	--	--
	Ca(ICCV2/PI489777)SNP_01423	1482	T	A	34	11	--	--
	Ca(ICCV2/PI489777)SNP_01424	1718	G	C	25	5	--	--
	Ca(ICCV2/PI489777)SNP_01425	1741	A	T	21	5	--	--
	Ca(ICCV2/PI489777)SNP_01426	1757	T	C	20	5	--	--
CakTC40164	Ca(ICCV2/PI489777)SNP_01427	172	A	G	74	138	--	--
	Ca(ICCV2/PI489777)SNP_01428	187	A	T	74	140	--	--
CakTC27334	Ca(ICCV2/PI489777)SNP_01429	963	T	A	100	39	--	C3H
	Ca(ICCV2/PI489777)SNP_01430	975	A	T	146	41	--	C3H
	Ca(ICCV2/PI489777)SNP_01431	1116	C	A	157	57	--	C3H
	Ca(ICCV2/PI489777)SNP_01432	1122	T	C	170	55	--	C3H
	Ca(ICCV2/PI489777)SNP_01433	1629	C	T	99	35	--	C3H
	Ca(ICCV2/PI489777)SNP_01434	1743	G	A	112	21	--	C3H
CakTC36513	Ca(ICCV2/PI489777)SNP_01435	5	G	C	3	14	--	--
	Ca(ICCV2/PI489777)SNP_01436	154	T	G	6	16	--	--
	Ca(ICCV2/PI489777)SNP_01437	319	T	G	5	15	--	--
	Ca(ICCV2/PI489777)SNP_01438	1242	T	G	7	10	--	--
CakTC34908	Ca(ICCV2/PI489777)SNP_01439	621	G	T	13	24	--	--
	Ca(ICCV2/PI489777)SNP_01440	649	T	A	13	30	--	--
	Ca(ICCV2/PI489777)SNP_01441	691	T	C	16	29	--	--
	Ca(ICCV2/PI489777)SNP_01442	1606	C	T	26	6	--	--
	Ca(ICCV2/PI489777)SNP_01443	2230	T	C	11	11	--	--
	Ca(ICCV2/PI489777)SNP_01444	3298	G	A	6	8	--	--
	Ca(ICCV2/PI489777)SNP_01445	3370	A	G	6	7	--	--
	Ca(ICCV2/PI489777)SNP_01446	3386	T	C	6	6	--	--
	Ca(ICCV2/PI489777)SNP_01447	3397	G	A	6	6	--	--
	Ca(ICCV2/PI489777)SNP_01448	3514	G	A	7	3	--	--
CakTC13632	Ca(ICCV2/PI489777)SNP_01449	640	A	C	29	12	--	--
CakTC40661	Ca(ICCV2/PI489777)SNP_01450	125	C	T	87	41	--	--
CakTC36263	Ca(ICCV2/PI489777)SNP_01451	1089	C	T	36	9	--	--
	Ca(ICCV2/PI489777)SNP_01452	1116	T	C	34	8	--	--
CakTC32959	Ca(ICCV2/PI489777)SNP_01453	1291	T	A	5	14	--	--
CakTC34089	Ca(ICCV2/PI489777)SNP_01454	291	A	G	7	4	--	--
	Ca(ICCV2/PI489777)SNP_01455	426	A	G	9	6	--	--
	Ca(ICCV2/PI489777)SNP_01456	732	C	T	9	9	--	--
	Ca(ICCV2/PI489777)SNP_01457	769	T	C	7	7	--	--
	Ca(ICCV2/PI489777)SNP_01458	777	A	G	9	8	--	--
	Ca(ICCV2/PI489777)SNP_01459	816	G	A	7	11	--	--
	Ca(ICCV2/PI489777)SNP_01460	828	G	T	8	12	--	--
	Ca(ICCV2/PI489777)SNP_01461	1527	A	G	7	7	--	--
	Ca(ICCV2/PI489777)SNP_01462	1562	T	G	5	8	--	--
CakTC33746	Ca(ICCV2/PI489777)SNP_01463	940	A	T	26	5	--	--
	Ca(ICCV2/PI489777)SNP_01464	960	G	A	26	4	--	--
	Ca(ICCV2/PI489777)SNP_01465	1415	G	A	16	3	--	--
	Ca(ICCV2/PI489777)SNP_01466	1808	C	T	17	10	--	--
CakTC38130	Ca(ICCV2/PI489777)SNP_01467	1024	C	G	5	3	--	--
CakTC42896	Ca(ICCV2/PI489777)SNP_01468	214	C	T	3	3	--	--
	Ca(ICCV2/PI489777)SNP_01469	400	A	C	8	6	--	--
	Ca(ICCV2/PI489777)SNP_01470	827	C	T	7	6	--	--
	Ca(ICCV2/PI489777)SNP_01471	845	G	A	6	6	--	--
	Ca(ICCV2/PI489777)SNP_01472	861	A	G	6	5	--	--
CakTC33527	Ca(ICCV2/PI489777)SNP_01473	118	T	C	3	5	--	--
	Ca(ICCV2/PI489777)SNP_01474	167	G	A	3	5	--	--
	Ca(ICCV2/PI489777)SNP_01475	1228	A	G	5	3	--	--

	Ca(ICCV2/PI489777)SNP_01476	1695	A	G	4	6	--	--
	Ca(ICCV2/PI489777)SNP_01477	1719	G	A	4	6	--	--
	Ca(ICCV2/PI489777)SNP_01478	1905	A	G	3	4	--	--
CakTC38084	Ca(ICCV2/PI489777)SNP_01479	260	T	C	24	65	Young_pod	--
	Ca(ICCV2/PI489777)SNP_01480	290	C	T	60	66	Young_pod	--
	Ca(ICCV2/PI489777)SNP_01481	408	A	G	64	70	Young_pod	--
	Ca(ICCV2/PI489777)SNP_01482	587	A	G	49	34	Young_pod	--
	Ca(ICCV2/PI489777)SNP_01483	590	C	A	44	33	Young_pod	--
	Ca(ICCV2/PI489777)SNP_01484	662	A	G	64	27	Young_pod	--
	Ca(ICCV2/PI489777)SNP_01485	689	A	G	70	23	Young_pod	--
	Ca(ICCV2/PI489777)SNP_01486	725	T	C	70	21	Young_pod	--
	Ca(ICCV2/PI489777)SNP_01487	1014	T	G	35	20	Young_pod	--
CakTC28268	Ca(ICCV2/PI489777)SNP_01488	1484	G	C	15	12	--	--
	Ca(ICCV2/PI489777)SNP_01489	1638	G	C	14	4	--	--
CakTC41446	Ca(ICCV2/PI489777)SNP_01490	145	A	G	4	4	--	--
	Ca(ICCV2/PI489777)SNP_01491	214	G	A	14	9	--	--
	Ca(ICCV2/PI489777)SNP_01492	274	C	T	12	16	--	--
	Ca(ICCV2/PI489777)SNP_01493	609	A	T	12	14	--	--
	Ca(ICCV2/PI489777)SNP_01494	638	T	G	10	13	--	--
CakTC26737	Ca(ICCV2/PI489777)SNP_01495	679	A	G	7	7	--	--
	Ca(ICCV2/PI489777)SNP_01496	1072	A	C	9	11	--	--
	Ca(ICCV2/PI489777)SNP_01497	1223	T	A	9	4	--	--
CakTC37859	Ca(ICCV2/PI489777)SNP_01498	954	A	G	12	4	--	--
	Ca(ICCV2/PI489777)SNP_01499	1065	A	G	17	4	--	--
CakTC40303	Ca(ICCV2/PI489777)SNP_01500	262	C	T	15	11	--	LIM
	Ca(ICCV2/PI489777)SNP_01501	335	A	G	13	11	--	LIM
	Ca(ICCV2/PI489777)SNP_01502	780	T	C	21	6	--	LIM
	Ca(ICCV2/PI489777)SNP_01503	864	C	T	22	3	--	LIM
	Ca(ICCV2/PI489777)SNP_01504	1536	A	G	23	6	--	LIM
CakTC33242	Ca(ICCV2/PI489777)SNP_01505	317	T	C	8	4	--	--
CakTC34750	Ca(ICCV2/PI489777)SNP_01506	1037	T	G	11	11	--	--
	Ca(ICCV2/PI489777)SNP_01507	2457	T	C	40	12	--	--
	Ca(ICCV2/PI489777)SNP_01508	2933	T	C	23	14	--	--
CakTC29592	Ca(ICCV2/PI489777)SNP_01509	1755	G	A	5	4	Shoot	--
	Ca(ICCV2/PI489777)SNP_01510	1964	A	G	4	3	Shoot	--
CakTC08960	Ca(ICCV2/PI489777)SNP_01511	313	T	A	3	5	--	--
CakTC30747	Ca(ICCV2/PI489777)SNP_01512	462	T	C	6	6	--	--
	Ca(ICCV2/PI489777)SNP_01513	501	C	G	5	6	--	--
	Ca(ICCV2/PI489777)SNP_01514	1234	C	T	13	14	--	--
	Ca(ICCV2/PI489777)SNP_01515	1333	A	C	14	13	--	--
	Ca(ICCV2/PI489777)SNP_01516	1398	T	G	18	11	--	--
	Ca(ICCV2/PI489777)SNP_01517	1686	G	A	14	12	--	--
	Ca(ICCV2/PI489777)SNP_01518	1800	A	G	9	15	--	--
CakTC07472	Ca(ICCV2/PI489777)SNP_01519	285	T	C	3	14	Flower	bud
	Ca(ICCV2/PI489777)SNP_01520	294	T	C	3	14	Flower	bud
	Ca(ICCV2/PI489777)SNP_01521	454	T	A	3	11	Flower	bud
	Ca(ICCV2/PI489777)SNP_01522	489	T	C	3	11	Flower	bud
	Ca(ICCV2/PI489777)SNP_01523	513	C	A	3	8	Flower	bud
	Ca(ICCV2/PI489777)SNP_01524	540	C	A	3	9	Flower	bud
CakTC25288	Ca(ICCV2/PI489777)SNP_01525	205	A	C	5	4	--	--
CakTC27348	Ca(ICCV2/PI489777)SNP_01526	319	C	T	3	4	Flower	bud
	Ca(ICCV2/PI489777)SNP_01527	754	T	C	8	3	Flower	bud
CakTC41729	Ca(ICCV2/PI489777)SNP_01528	1611	G	T	24	20	--	--
CakTC41252	Ca(ICCV2/PI489777)SNP_01529	79	T	G	5	4	--	--
CakTC37820	Ca(ICCV2/PI489777)SNP_01530	677	T	C	4	21	--	--
	Ca(ICCV2/PI489777)SNP_01531	740	A	T	8	17	--	--
CakTC28905	Ca(ICCV2/PI489777)SNP_01532	486	C	G	6	12	--	--
	Ca(ICCV2/PI489777)SNP_01533	1174	T	C	16	21	--	--
	Ca(ICCV2/PI489777)SNP_01534	1607	T	C	24	18	--	--
	Ca(ICCV2/PI489777)SNP_01535	2009	C	T	18	16	--	--
	Ca(ICCV2/PI489777)SNP_01536	2405	C	T	15	32	--	--
	Ca(ICCV2/PI489777)SNP_01537	2590	A	G	16	40	--	--
	Ca(ICCV2/PI489777)SNP_01538	2717	T	A	15	37	--	--
	Ca(ICCV2/PI489777)SNP_01539	2917	G	T	10	14	--	--
	Ca(ICCV2/PI489777)SNP_01540	2977	T	C	7	7	--	--
CakTC43078	Ca(ICCV2/PI489777)SNP_01541	666	G	T	6	5	--	--
	Ca(ICCV2/PI489777)SNP_01542	746	C	T	8	5	--	--
	Ca(ICCV2/PI489777)SNP_01543	791	A	G	13	9	--	--
	Ca(ICCV2/PI489777)SNP_01544	1028	G	A	9	11	--	--
	Ca(ICCV2/PI489777)SNP_01545	1360	A	T	3	8	--	--
	Ca(ICCV2/PI489777)SNP_01546	1439	G	A	6	9	--	--
CakTC32145	Ca(ICCV2/PI489777)SNP_01547	105	G	A	4	10	--	--
	Ca(ICCV2/PI489777)SNP_01548	167	C	G	5	9	--	--
	Ca(ICCV2/PI489777)SNP_01549	242	T	C	9	13	--	--

	Ca(ICCV2/PI489777)SNP_01550	1088	T	C	24	11	--	--
	Ca(ICCV2/PI489777)SNP_01551	1335	A	G	14	30	--	--
	Ca(ICCV2/PI489777)SNP_01552	1979	T	C	18	17	--	--
	Ca(ICCV2/PI489777)SNP_01553	2902	T	C	27	11	--	--
	Ca(ICCV2/PI489777)SNP_01554	4830	A	G	15	10	--	--
CakTC39419	Ca(ICCV2/PI489777)SNP_01555	602	A	G	18	8	--	--
	Ca(ICCV2/PI489777)SNP_01556	821	C	T	14	13	--	--
	Ca(ICCV2/PI489777)SNP_01557	1317	T	A	22	11	--	--
CakTC33045	Ca(ICCV2/PI489777)SNP_01558	103	G	T	10	3	--	--
CakTC23394	Ca(ICCV2/PI489777)SNP_01559	747	C	A	7	15	--	--
	Ca(ICCV2/PI489777)SNP_01560	1633	G	T	13	14	--	--
	Ca(ICCV2/PI489777)SNP_01561	1767	C	A	8	13	--	--
CakTC40048	Ca(ICCV2/PI489777)SNP_01562	422	T	C	7	8	--	--
	Ca(ICCV2/PI489777)SNP_01563	440	G	A	10	8	--	--
	Ca(ICCV2/PI489777)SNP_01564	591	C	T	9	4	--	--
	Ca(ICCV2/PI489777)SNP_01565	881	A	G	14	8	--	--
	Ca(ICCV2/PI489777)SNP_01566	974	T	A	9	7	--	--
	Ca(ICCV2/PI489777)SNP_01567	1476	A	G	3	3	--	--
CakTC26516	Ca(ICCV2/PI489777)SNP_01568	1772	C	T	10	9	--	--
	Ca(ICCV2/PI489777)SNP_01569	1832	C	T	13	12	--	--
	Ca(ICCV2/PI489777)SNP_01570	1835	A	G	12	12	--	--
	Ca(ICCV2/PI489777)SNP_01571	1896	G	A	8	10	--	--
	Ca(ICCV2/PI489777)SNP_01572	1898	C	A	10	11	--	--
	Ca(ICCV2/PI489777)SNP_01573	2061	G	A	6	7	--	--
CakTC41167	Ca(ICCV2/PI489777)SNP_01574	541	C	T	18	7	--	--
CakTC12700	Ca(ICCV2/PI489777)SNP_01575	1269	A	C	31	23	--	--
	Ca(ICCV2/PI489777)SNP_01576	1326	A	G	29	42	--	--
	Ca(ICCV2/PI489777)SNP_01577	1432	C	T	30	35	--	--
	Ca(ICCV2/PI489777)SNP_01578	1496	T	C	19	21	--	--
	Ca(ICCV2/PI489777)SNP_01579	1553	C	T	17	18	--	--
CakTC25137	Ca(ICCV2/PI489777)SNP_01580	605	T	C	3	4	--	--
CakTC43197	Ca(ICCV2/PI489777)SNP_01581	174	T	C	19	10	--	--
	Ca(ICCV2/PI489777)SNP_01582	425	C	T	22	33	--	--
	Ca(ICCV2/PI489777)SNP_01583	741	G	T	5	6	--	--
	Ca(ICCV2/PI489777)SNP_01584	826	G	A	4	3	--	--
CakTC38660	Ca(ICCV2/PI489777)SNP_01585	351	C	T	4	4	--	--
	Ca(ICCV2/PI489777)SNP_01586	840	G	A	5	4	--	--
CakTC39104	Ca(ICCV2/PI489777)SNP_01587	461	A	G	19	11	--	--
	Ca(ICCV2/PI489777)SNP_01588	560	C	G	17	10	--	--
	Ca(ICCV2/PI489777)SNP_01589	875	T	C	14	11	--	--
	Ca(ICCV2/PI489777)SNP_01590	1031	T	C	13	15	--	--
	Ca(ICCV2/PI489777)SNP_01591	1037	T	G	13	15	--	--
	Ca(ICCV2/PI489777)SNP_01592	1061	C	T	14	12	--	--
CakTC38992	Ca(ICCV2/PI489777)SNP_01593	37	C	T	22	21	--	--
	Ca(ICCV2/PI489777)SNP_01594	51	G	A	23	22	--	--
	Ca(ICCV2/PI489777)SNP_01595	83	T	C	33	25	--	--
	Ca(ICCV2/PI489777)SNP_01596	256	T	C	52	41	--	--
CakTC32682	Ca(ICCV2/PI489777)SNP_01597	376	T	A	41	4	--	TRAF
	Ca(ICCV2/PI489777)SNP_01598	407	A	G	41	7	--	TRAF
	Ca(ICCV2/PI489777)SNP_01599	544	A	G	8	7	--	TRAF
	Ca(ICCV2/PI489777)SNP_01600	688	C	T	7	5	--	TRAF
	Ca(ICCV2/PI489777)SNP_01601	1303	G	A	3	8	--	TRAF
	Ca(ICCV2/PI489777)SNP_01602	1318	G	A	3	7	--	TRAF
	Ca(ICCV2/PI489777)SNP_01603	1909	A	G	8	8	--	TRAF
	Ca(ICCV2/PI489777)SNP_01604	1930	C	T	8	7	--	TRAF
	Ca(ICCV2/PI489777)SNP_01605	2062	A	G	8	5	--	TRAF
CakTC32060	Ca(ICCV2/PI489777)SNP_01606	625	A	C	4	3	--	--
	Ca(ICCV2/PI489777)SNP_01607	646	A	G	5	3	--	--
	Ca(ICCV2/PI489777)SNP_01608	822	C	T	9	8	--	--
	Ca(ICCV2/PI489777)SNP_01609	916	C	G	13	11	--	--
	Ca(ICCV2/PI489777)SNP_01610	1053	G	A	11	8	--	--
	Ca(ICCV2/PI489777)SNP_01611	1130	A	G	12	6	--	--
	Ca(ICCV2/PI489777)SNP_01612	1320	C	A	8	3	--	--
CakTC42048	Ca(ICCV2/PI489777)SNP_01613	486	C	A	12	8	--	--
CakTC40220	Ca(ICCV2/PI489777)SNP_01614	413	G	A	10	6	--	--
	Ca(ICCV2/PI489777)SNP_01615	419	C	T	13	6	--	--
	Ca(ICCV2/PI489777)SNP_01616	428	C	G	14	7	--	--
	Ca(ICCV2/PI489777)SNP_01617	460	G	C	12	10	--	--
	Ca(ICCV2/PI489777)SNP_01618	484	G	C	15	10	--	--
	Ca(ICCV2/PI489777)SNP_01619	530	T	C	16	10	--	--
	Ca(ICCV2/PI489777)SNP_01620	674	T	C	15	10	--	--
	Ca(ICCV2/PI489777)SNP_01621	1322	A	G	15	9	--	--
CakTC11594	Ca(ICCV2/PI489777)SNP_01622	758	T	C	7	7	--	--
	Ca(ICCV2/PI489777)SNP_01623	799	G	A	5	6	--	--

CakTC40147	Ca(ICCV2/PI489777)SNP_01624	973	A	G	5	3	--	--
	Ca(ICCV2/PI489777)SNP_01625	1126	C	G	5	4	--	--
	Ca(ICCV2/PI489777)SNP_01626	1147	C	A	4	4	--	--
	Ca(ICCV2/PI489777)SNP_01627	1156	G	T	3	4	--	--
CakTC37063	Ca(ICCV2/PI489777)SNP_01628	403	C	T	8	7	--	--
	Ca(ICCV2/PI489777)SNP_01629	523	A	T	8	6	--	--
CakTC42853	Ca(ICCV2/PI489777)SNP_01630	1893	G	A	8	3	--	--
CakTC23286	Ca(ICCV2/PI489777)SNP_01631	137	C	A	4	5	--	--
	Ca(ICCV2/PI489777)SNP_01632	162	C	T	4	6	--	--
	Ca(ICCV2/PI489777)SNP_01633	345	A	G	12	12	--	--
	Ca(ICCV2/PI489777)SNP_01634	990	T	C	6	9	--	--
	Ca(ICCV2/PI489777)SNP_01635	2220	T	G	5	7	--	--
	Ca(ICCV2/PI489777)SNP_01636	2268	A	G	5	9	--	--
CakTC33522	Ca(ICCV2/PI489777)SNP_01637	115	T	C	27	7	--	--
	Ca(ICCV2/PI489777)SNP_01638	157	C	T	44	3	--	--
CakTC25725	Ca(ICCV2/PI489777)SNP_01639	611	A	G	34	26	--	--
	Ca(ICCV2/PI489777)SNP_01640	1019	T	G	19	16	--	--
CakTC27515	Ca(ICCV2/PI489777)SNP_01641	262	T	G	9	7	--	--
CakTC29919	Ca(ICCV2/PI489777)SNP_01642	1445	G	A	8	3	--	--
	Ca(ICCV2/PI489777)SNP_01643	2065	C	T	7	3	--	--
CakTC42273	Ca(ICCV2/PI489777)SNP_01644	1604	C	A	3	4	--	--
	Ca(ICCV2/PI489777)SNP_01645	1726	T	C	3	5	--	--
	Ca(ICCV2/PI489777)SNP_01646	1804	T	C	5	10	--	--
	Ca(ICCV2/PI489777)SNP_01647	1855	T	C	3	15	--	--
	Ca(ICCV2/PI489777)SNP_01648	1876	C	T	6	8	--	--
	Ca(ICCV2/PI489777)SNP_01649	2170	T	C	4	20	--	--
	Ca(ICCV2/PI489777)SNP_01650	2582	C	T	3	3	--	--
CakTC38296	Ca(ICCV2/PI489777)SNP_01651	992	T	C	18	8	--	--
CakTC40483	Ca(ICCV2/PI489777)SNP_01652	370	G	C	6	12	--	--
	Ca(ICCV2/PI489777)SNP_01653	744	T	C	5	4	--	--
CakTC37616	Ca(ICCV2/PI489777)SNP_01654	439	C	T	17	5	--	HSF
	Ca(ICCV2/PI489777)SNP_01655	485	A	C	20	4	--	HSF
CakTC25429	Ca(ICCV2/PI489777)SNP_01656	524	A	G	13	9	--	--
	Ca(ICCV2/PI489777)SNP_01657	1205	T	C	10	10	--	--
CakTC38326	Ca(ICCV2/PI489777)SNP_01658	725	A	G	5	10	--	--
	Ca(ICCV2/PI489777)SNP_01659	1149	C	T	5	4	--	--
	Ca(ICCV2/PI489777)SNP_01660	1379	A	G	6	3	--	--
CakTC34736	Ca(ICCV2/PI489777)SNP_01661	922	C	T	25	7	--	ARF
	Ca(ICCV2/PI489777)SNP_01662	1483	G	T	20	19	--	ARF
	Ca(ICCV2/PI489777)SNP_01663	2104	A	G	45	17	--	ARF
	Ca(ICCV2/PI489777)SNP_01664	2842	C	T	36	6	--	ARF
	Ca(ICCV2/PI489777)SNP_01665	3447	A	G	25	8	--	ARF
	Ca(ICCV2/PI489777)SNP_01666	3476	C	T	25	7	--	ARF
CakTC40878	Ca(ICCV2/PI489777)SNP_01667	2896	A	C	3	4	--	--
CakTC31527	Ca(ICCV2/PI489777)SNP_01668	1473	T	G	3	5	--	--
CakTC23979	Ca(ICCV2/PI489777)SNP_01669	27	G	A	8	4	--	--
	Ca(ICCV2/PI489777)SNP_01670	52	A	C	11	7	--	--
	Ca(ICCV2/PI489777)SNP_01671	185	A	C	33	14	--	--
	Ca(ICCV2/PI489777)SNP_01672	361	C	T	39	15	--	--
	Ca(ICCV2/PI489777)SNP_01673	2210	A	G	25	11	--	--
	Ca(ICCV2/PI489777)SNP_01674	3297	A	G	11	8	--	--
CakTC42348	Ca(ICCV2/PI489777)SNP_01675	449	G	A	69	43	--	--
	Ca(ICCV2/PI489777)SNP_01676	623	T	C	85	41	--	--
	Ca(ICCV2/PI489777)SNP_01677	688	T	C	72	42	--	--
CakTC39972	Ca(ICCV2/PI489777)SNP_01678	17	G	T	37	14	--	--
CakTC37672	Ca(ICCV2/PI489777)SNP_01679	90	C	T	4	9	--	--
	Ca(ICCV2/PI489777)SNP_01680	553	C	T	7	10	--	--
	Ca(ICCV2/PI489777)SNP_01681	622	T	C	8	12	--	--
CakTC26234	Ca(ICCV2/PI489777)SNP_01682	383	A	G	11	11	--	--
	Ca(ICCV2/PI489777)SNP_01683	551	T	C	7	9	--	--
	Ca(ICCV2/PI489777)SNP_01684	711	C	T	6	7	--	--
	Ca(ICCV2/PI489777)SNP_01685	1439	C	T	4	4	--	--
	Ca(ICCV2/PI489777)SNP_01686	3064	T	C	27	6	--	--
	Ca(ICCV2/PI489777)SNP_01687	3243	C	T	57	25	--	--
	Ca(ICCV2/PI489777)SNP_01688	3303	A	G	54	25	--	--
	Ca(ICCV2/PI489777)SNP_01689	3407	A	C	40	24	--	--
CakTC33059	Ca(ICCV2/PI489777)SNP_01690	213	A	C	5	7	--	--
CakTC28549	Ca(ICCV2/PI489777)SNP_01691	528	T	G	7	5	--	--
	Ca(ICCV2/PI489777)SNP_01692	597	C	G	8	4	--	--
	Ca(ICCV2/PI489777)SNP_01693	703	T	C	9	4	--	--
	Ca(ICCV2/PI489777)SNP_01694	711	C	T	9	5	--	--
	Ca(ICCV2/PI489777)SNP_01695	843	A	G	4	3	--	--
CakTC33054	Ca(ICCV2/PI489777)SNP_01696	324	T	C	4	6	--	--
	Ca(ICCV2/PI489777)SNP_01697	474	T	C	3	7	--	--

CakTC28083	Ca(ICCV2/PI489777)SNP_01698	197	A	G	7	3	Flower	bud
	Ca(ICCV2/PI489777)SNP_01699	334	T	C	7	3	Flower	bud
CakTC27898	Ca(ICCV2/PI489777)SNP_01700	1147	T	C	4	5	Shoot	--
CakTC32855	Ca(ICCV2/PI489777)SNP_01701	1169	T	C	14	6	--	--
	Ca(ICCV2/PI489777)SNP_01702	1643	T	C	14	4	--	--
	Ca(ICCV2/PI489777)SNP_01703	1689	A	G	15	3	--	--
	Ca(ICCV2/PI489777)SNP_01704	1697	C	T	15	3	--	--
	Ca(ICCV2/PI489777)SNP_01705	1988	T	C	9	3	--	--
	Ca(ICCV2/PI489777)SNP_01706	2167	G	C	8	5	--	--
	Ca(ICCV2/PI489777)SNP_01707	2173	G	T	8	5	--	--
CakTC41049	Ca(ICCV2/PI489777)SNP_01708	448	C	T	45	58	--	--
CakTC32824	Ca(ICCV2/PI489777)SNP_01709	363	C	A	8	3	--	--
	Ca(ICCV2/PI489777)SNP_01710	414	G	A	9	4	--	--
	Ca(ICCV2/PI489777)SNP_01711	1141	T	C	5	5	--	--
	Ca(ICCV2/PI489777)SNP_01712	1465	A	C	3	4	--	--
	Ca(ICCV2/PI489777)SNP_01713	2341	C	T	6	4	--	--
	Ca(ICCV2/PI489777)SNP_01714	2393	C	G	7	4	--	--
	Ca(ICCV2/PI489777)SNP_01715	2440	T	C	9	4	--	--
	Ca(ICCV2/PI489777)SNP_01716	2443	C	T	9	4	--	--
	Ca(ICCV2/PI489777)SNP_01717	2525	G	A	10	4	--	--
	Ca(ICCV2/PI489777)SNP_01718	2573	C	G	9	4	--	--
CakTC39917	Ca(ICCV2/PI489777)SNP_01719	555	C	G	9	6	--	--
	Ca(ICCV2/PI489777)SNP_01720	1284	G	A	12	10	--	--
	Ca(ICCV2/PI489777)SNP_01721	1346	A	C	10	5	--	--
CakTC22886	Ca(ICCV2/PI489777)SNP_01722	651	A	G	662	120	--	--
	Ca(ICCV2/PI489777)SNP_01723	672	A	G	629	120	--	--
	Ca(ICCV2/PI489777)SNP_01724	894	G	A	325	67	--	--
	Ca(ICCV2/PI489777)SNP_01725	1656	A	G	242	35	--	--
CakTC27122	Ca(ICCV2/PI489777)SNP_01726	375	T	C	17	7	--	--
CakTC23508	Ca(ICCV2/PI489777)SNP_01727	276	A	C	7	3	--	--
	Ca(ICCV2/PI489777)SNP_01728	288	G	T	7	3	--	--
CakTC28844	Ca(ICCV2/PI489777)SNP_01729	772	G	A	20	4	--	--
	Ca(ICCV2/PI489777)SNP_01730	1240	C	T	16	9	--	--
CakTC40755	Ca(ICCV2/PI489777)SNP_01731	1231	T	C	10	3	--	--
	Ca(ICCV2/PI489777)SNP_01732	1461	T	C	13	4	--	--
	Ca(ICCV2/PI489777)SNP_01733	1638	C	T	13	6	--	--
	Ca(ICCV2/PI489777)SNP_01734	1644	A	C	14	6	--	--
	Ca(ICCV2/PI489777)SNP_01735	1734	A	G	13	4	--	--
	Ca(ICCV2/PI489777)SNP_01736	1740	C	T	13	5	--	--
CakTC38811	Ca(ICCV2/PI489777)SNP_01737	881	T	C	10	3	--	--
	Ca(ICCV2/PI489777)SNP_01738	976	A	G	12	4	--	--
	Ca(ICCV2/PI489777)SNP_01739	1220	A	G	10	7	--	--
	Ca(ICCV2/PI489777)SNP_01740	1298	A	G	11	7	--	--
CakTC40530	Ca(ICCV2/PI489777)SNP_01741	1337	A	G	13	8	--	--
	Ca(ICCV2/PI489777)SNP_01742	1397	A	G	14	10	--	--
	Ca(ICCV2/PI489777)SNP_01743	1559	A	T	15	7	--	--
	Ca(ICCV2/PI489777)SNP_01744	2164	G	C	11	7	--	--
	Ca(ICCV2/PI489777)SNP_01745	2221	T	A	10	7	--	--
	Ca(ICCV2/PI489777)SNP_01746	2234	A	G	10	6	--	--
	Ca(ICCV2/PI489777)SNP_01747	2335	T	C	9	3	--	--
	Ca(ICCV2/PI489777)SNP_01748	2350	T	C	8	4	--	--
	Ca(ICCV2/PI489777)SNP_01749	2363	A	G	8	3	--	--
	Ca(ICCV2/PI489777)SNP_01750	2372	G	A	7	4	--	--
CakTC35403	Ca(ICCV2/PI489777)SNP_01751	2614	T	C	12	10	--	--
	Ca(ICCV2/PI489777)SNP_01752	3367	G	A	3	6	--	--
CakTC38988	Ca(ICCV2/PI489777)SNP_01753	185	C	G	6	7	--	--
	Ca(ICCV2/PI489777)SNP_01754	262	A	G	8	7	--	--
	Ca(ICCV2/PI489777)SNP_01755	1253	C	A	19	25	--	--
CakTC25420	Ca(ICCV2/PI489777)SNP_01756	228	G	A	38	8	--	--
	Ca(ICCV2/PI489777)SNP_01757	270	C	T	36	8	--	--
	Ca(ICCV2/PI489777)SNP_01758	839	T	C	29	7	--	--
	Ca(ICCV2/PI489777)SNP_01759	887	C	T	27	6	--	--
	Ca(ICCV2/PI489777)SNP_01760	1026	C	T	37	8	--	--
	Ca(ICCV2/PI489777)SNP_01761	1037	T	C	35	10	--	--
	Ca(ICCV2/PI489777)SNP_01762	1232	G	A	47	10	--	--
	Ca(ICCV2/PI489777)SNP_01763	2052	G	T	35	10	--	--
	Ca(ICCV2/PI489777)SNP_01764	2347	G	A	3	3	--	--
CakTC27791	Ca(ICCV2/PI489777)SNP_01765	149	G	A	48	31	--	--
	Ca(ICCV2/PI489777)SNP_01766	154	T	C	58	31	--	--
	Ca(ICCV2/PI489777)SNP_01767	170	A	G	38	32	--	--
	Ca(ICCV2/PI489777)SNP_01768	477	A	G	45	29	--	--
CakTC41352	Ca(ICCV2/PI489777)SNP_01769	762	G	C	6	3	--	--
	Ca(ICCV2/PI489777)SNP_01770	763	G	A	6	3	--	--
	Ca(ICCV2/PI489777)SNP_01771	928	G	A	5	4	--	--

	Ca(ICCV2/PI489777)SNP_01772	938	G	C	5	3	--	--
	Ca(ICCV2/PI489777)SNP_01773	1566	A	C	3	3	--	--
CakTC38298	Ca(ICCV2/PI489777)SNP_01774	418	G	T	6	3	--	--
CakTC24681	Ca(ICCV2/PI489777)SNP_01775	1842	C	G	4	4	--	--
	Ca(ICCV2/PI489777)SNP_01776	1892	A	G	4	4	--	--
CakTC28209	Ca(ICCV2/PI489777)SNP_01777	390	T	C	7	7	--	--
	Ca(ICCV2/PI489777)SNP_01778	453	A	G	7	6	--	--
	Ca(ICCV2/PI489777)SNP_01779	1467	A	T	19	9	--	--
CakTC43222	Ca(ICCV2/PI489777)SNP_01780	41	A	T	5	3	Young_pod	--
	Ca(ICCV2/PI489777)SNP_01781	81	G	C	7	4	Young_pod	--
	Ca(ICCV2/PI489777)SNP_01782	116	T	C	7	4	Young_pod	--
	Ca(ICCV2/PI489777)SNP_01783	133	T	C	7	5	Young_pod	--
	Ca(ICCV2/PI489777)SNP_01784	420	C	A	3	10	Young_pod	--
	Ca(ICCV2/PI489777)SNP_01785	792	T	C	4	4	Young_pod	--
CakTC31774	Ca(ICCV2/PI489777)SNP_01786	559	T	C	3	5	Root	--
CakTC25545	Ca(ICCV2/PI489777)SNP_01787	1414	G	A	116	89	Young_pod	--
	Ca(ICCV2/PI489777)SNP_01788	1769	A	G	100	64	Young_pod	--
CakTC37274	Ca(ICCV2/PI489777)SNP_01789	245	C	T	8	3	Flower	bud
CakTC24066	Ca(ICCV2/PI489777)SNP_01790	738	A	G	8	3	Flower	bud
	Ca(ICCV2/PI489777)SNP_01791	1347	G	A	29	22	Flower	bud
	Ca(ICCV2/PI489777)SNP_01792	1534	C	T	28	14	Flower	bud
	Ca(ICCV2/PI489777)SNP_01793	1703	C	T	26	15	Flower	bud
CakTC29453	Ca(ICCV2/PI489777)SNP_01794	313	A	G	5	3	--	--
	Ca(ICCV2/PI489777)SNP_01795	925	G	A	7	7	--	--
	Ca(ICCV2/PI489777)SNP_01796	1063	G	A	6	10	--	--
	Ca(ICCV2/PI489777)SNP_01797	1150	T	C	5	15	--	--
	Ca(ICCV2/PI489777)SNP_01798	1255	A	G	4	14	--	--
CakTC37354	Ca(ICCV2/PI489777)SNP_01799	32	C	G	13	6	--	--
	Ca(ICCV2/PI489777)SNP_01800	242	G	A	18	10	--	--
	Ca(ICCV2/PI489777)SNP_01801	434	T	C	14	8	--	--
CakTC23615	Ca(ICCV2/PI489777)SNP_01802	478	G	A	31	5	--	--
	Ca(ICCV2/PI489777)SNP_01803	605	A	G	21	8	--	--
	Ca(ICCV2/PI489777)SNP_01804	1024	A	G	20	7	--	--
	Ca(ICCV2/PI489777)SNP_01805	1436	T	C	26	6	--	--
	Ca(ICCV2/PI489777)SNP_01806	1678	T	C	21	7	--	--
	Ca(ICCV2/PI489777)SNP_01807	2443	T	A	17	8	--	--
	Ca(ICCV2/PI489777)SNP_01808	2641	G	A	18	11	--	--
	Ca(ICCV2/PI489777)SNP_01809	2707	C	A	22	10	--	--
	Ca(ICCV2/PI489777)SNP_01810	3375	G	A	21	11	--	--
	Ca(ICCV2/PI489777)SNP_01811	3384	A	T	21	10	--	--
	Ca(ICCV2/PI489777)SNP_01812	3619	C	T	14	7	--	--
	Ca(ICCV2/PI489777)SNP_01813	3877	A	G	16	4	--	--
	Ca(ICCV2/PI489777)SNP_01814	4393	T	G	22	11	--	--
	Ca(ICCV2/PI489777)SNP_01815	4549	A	G	25	8	--	--
	Ca(ICCV2/PI489777)SNP_01816	4812	A	T	16	5	--	--
	Ca(ICCV2/PI489777)SNP_01817	4947	C	T	13	5	--	--
	Ca(ICCV2/PI489777)SNP_01818	5022	T	C	10	4	--	--
	Ca(ICCV2/PI489777)SNP_01819	5136	T	G	7	5	--	--
	Ca(ICCV2/PI489777)SNP_01820	5482	C	A	8	5	--	--
	Ca(ICCV2/PI489777)SNP_01821	5896	C	T	26	26	--	--
	Ca(ICCV2/PI489777)SNP_01822	6580	T	A	9	29	--	--
CakTC30482	Ca(ICCV2/PI489777)SNP_01823	112	A	G	5	6	Root	--
	Ca(ICCV2/PI489777)SNP_01824	207	G	A	5	9	Root	--
CakTC38047	Ca(ICCV2/PI489777)SNP_01825	1370	C	T	5	4	--	--
	Ca(ICCV2/PI489777)SNP_01826	1521	T	C	5	3	--	--
	Ca(ICCV2/PI489777)SNP_01827	1542	T	G	5	3	--	--
CakTC09454	Ca(ICCV2/PI489777)SNP_01828	1702	A	T	3	3	--	--
CakTC39474	Ca(ICCV2/PI489777)SNP_01829	928	G	T	20	9	--	--
	Ca(ICCV2/PI489777)SNP_01830	1110	T	G	16	8	--	--
	Ca(ICCV2/PI489777)SNP_01831	1123	T	A	16	5	--	--
	Ca(ICCV2/PI489777)SNP_01832	1249	C	A	4	3	--	--
	Ca(ICCV2/PI489777)SNP_01833	1250	T	A	4	3	--	--
CakTC27526	Ca(ICCV2/PI489777)SNP_01834	2521	A	C	15	3	Young_pod	PHD
	Ca(ICCV2/PI489777)SNP_01835	2954	C	G	9	7	Young_pod	PHD
	Ca(ICCV2/PI489777)SNP_01836	2960	C	T	9	7	Young_pod	PHD
	Ca(ICCV2/PI489777)SNP_01837	3280	T	A	21	7	Young_pod	PHD
	Ca(ICCV2/PI489777)SNP_01838	4096	T	G	6	10	Young_pod	PHD
	Ca(ICCV2/PI489777)SNP_01839	4326	T	C	4	8	Young_pod	PHD
CakTC28598	Ca(ICCV2/PI489777)SNP_01840	114	A	C	43	32	--	--
	Ca(ICCV2/PI489777)SNP_01841	478	G	A	126	36	--	--
	Ca(ICCV2/PI489777)SNP_01842	508	C	G	126	44	--	--
	Ca(ICCV2/PI489777)SNP_01843	544	C	T	101	43	--	--
	Ca(ICCV2/PI489777)SNP_01844	856	G	T	152	70	--	--
	Ca(ICCV2/PI489777)SNP_01845	1096	A	G	53	27	--	--

CakTC43051	Ca(ICCV2/PI489777)SNP_01846	86	T	A	5	5	--	--
	Ca(ICCV2/PI489777)SNP_01847	107	T	G	7	4	--	--
	Ca(ICCV2/PI489777)SNP_01848	657	G	A	27	23	--	--
CakTC38040	Ca(ICCV2/PI489777)SNP_01849	334	T	A	79	59	--	--
CakTC23972	Ca(ICCV2/PI489777)SNP_01850	679	C	G	19	22	--	--
CakTC26763	Ca(ICCV2/PI489777)SNP_01851	192	C	G	7	8	--	RWP-RK
	Ca(ICCV2/PI489777)SNP_01852	542	A	T	5	4	--	RWP-RK
	Ca(ICCV2/PI489777)SNP_01853	2842	G	C	5	4	--	RWP-RK
	Ca(ICCV2/PI489777)SNP_01854	2915	T	A	5	4	--	RWP-RK
CakTC42067	Ca(ICCV2/PI489777)SNP_01855	574	G	A	3	9	--	--
	Ca(ICCV2/PI489777)SNP_01856	591	T	C	4	10	--	--
	Ca(ICCV2/PI489777)SNP_01857	761	A	G	6	11	--	--
CakTC42401	Ca(ICCV2/PI489777)SNP_01858	598	C	G	9	10	--	--
CakTC43058	Ca(ICCV2/PI489777)SNP_01859	440	G	A	11	7	--	--
	Ca(ICCV2/PI489777)SNP_01860	662	G	A	8	10	--	--
	Ca(ICCV2/PI489777)SNP_01861	671	T	C	8	10	--	--
CakTC22241	Ca(ICCV2/PI489777)SNP_01862	384	A	G	12	23	--	--
CakTC26489	Ca(ICCV2/PI489777)SNP_01863	498	C	T	17	3	--	--
	Ca(ICCV2/PI489777)SNP_01864	1897	G	A	45	15	--	--
	Ca(ICCV2/PI489777)SNP_01865	1967	A	G	30	11	--	--
	Ca(ICCV2/PI489777)SNP_01866	2034	C	G	25	4	--	--
CakTC32407	Ca(ICCV2/PI489777)SNP_01867	3276	G	A	11	11	--	--
	Ca(ICCV2/PI489777)SNP_01868	3951	T	C	3	6	--	--
CakTC26577	Ca(ICCV2/PI489777)SNP_01869	497	T	C	28	10	Mature	Leaf
	Ca(ICCV2/PI489777)SNP_01870	1310	T	A	35	12	Mature	Leaf
	Ca(ICCV2/PI489777)SNP_01871	1405	A	G	24	7	Mature	Leaf
CakTC39557	Ca(ICCV2/PI489777)SNP_01872	31	G	A	3	3	--	--
CakTC33270	Ca(ICCV2/PI489777)SNP_01873	466	C	T	13	7	--	--
	Ca(ICCV2/PI489777)SNP_01874	643	C	G	8	3	--	--
	Ca(ICCV2/PI489777)SNP_01875	1342	C	G	15	14	--	--
CakTC28164	Ca(ICCV2/PI489777)SNP_01876	476	T	C	40	16	--	--
CakTC24057	Ca(ICCV2/PI489777)SNP_01877	280	A	G	4	4	Shoot	--
	Ca(ICCV2/PI489777)SNP_01878	445	G	C	4	3	Shoot	--
	Ca(ICCV2/PI489777)SNP_01879	3354	C	T	3	4	Shoot	--
CakTC34380	Ca(ICCV2/PI489777)SNP_01880	246	C	G	10	5	--	--
	Ca(ICCV2/PI489777)SNP_01881	1812	T	C	15	13	--	--
	Ca(ICCV2/PI489777)SNP_01882	1869	G	A	29	15	--	--
	Ca(ICCV2/PI489777)SNP_01883	1995	T	A	13	10	--	--
CakTC42418	Ca(ICCV2/PI489777)SNP_01884	563	T	C	5	6	--	--
CakTC38233	Ca(ICCV2/PI489777)SNP_01885	642	T	C	7	3	--	--
	Ca(ICCV2/PI489777)SNP_01886	690	G	T	6	3	--	--
CakTC37628	Ca(ICCV2/PI489777)SNP_01887	471	T	C	6	4	--	--
CakTC31173	Ca(ICCV2/PI489777)SNP_01888	747	T	C	3	3	Root	--
CakTC33162	Ca(ICCV2/PI489777)SNP_01889	924	A	C	4	8	--	--
	Ca(ICCV2/PI489777)SNP_01890	978	A	T	4	8	--	--
CakTC30507	Ca(ICCV2/PI489777)SNP_01891	274	T	C	5	4	--	--
	Ca(ICCV2/PI489777)SNP_01892	375	G	A	5	6	--	--
	Ca(ICCV2/PI489777)SNP_01893	388	T	C	5	6	--	--
	Ca(ICCV2/PI489777)SNP_01894	390	A	G	5	6	--	--
	Ca(ICCV2/PI489777)SNP_01895	608	A	C	6	7	--	--
	Ca(ICCV2/PI489777)SNP_01896	1167	G	A	3	3	--	--
	Ca(ICCV2/PI489777)SNP_01897	1245	A	G	8	6	--	--
CakTC23376	Ca(ICCV2/PI489777)SNP_01898	351	T	C	4	4	Flower	bud
	Ca(ICCV2/PI489777)SNP_01899	501	T	C	3	4	Flower	bud
	Ca(ICCV2/PI489777)SNP_01900	625	G	A	4	4	Flower	bud
CakTC42285	Ca(ICCV2/PI489777)SNP_01901	480	A	C	6	6	--	--
CakTC26091	Ca(ICCV2/PI489777)SNP_01902	274	C	A	27	7	--	--
	Ca(ICCV2/PI489777)SNP_01903	1363	A	G	17	10	--	--
	Ca(ICCV2/PI489777)SNP_01904	1867	T	C	11	8	--	--
	Ca(ICCV2/PI489777)SNP_01905	2555	C	G	25	8	--	--
	Ca(ICCV2/PI489777)SNP_01906	2675	G	A	25	15	--	--
	Ca(ICCV2/PI489777)SNP_01907	2794	G	A	24	12	--	--
CakTC37223	Ca(ICCV2/PI489777)SNP_01908	276	T	C	11	8	--	--
CakTC41348	Ca(ICCV2/PI489777)SNP_01909	497	T	C	12	6	--	--
	Ca(ICCV2/PI489777)SNP_01910	1796	T	C	22	7	--	--
CakTC10880	Ca(ICCV2/PI489777)SNP_01911	530	T	C	3	6	--	--
CakTC27604	Ca(ICCV2/PI489777)SNP_01912	516	A	G	65	42	--	--
	Ca(ICCV2/PI489777)SNP_01913	1224	C	T	85	41	--	--
	Ca(ICCV2/PI489777)SNP_01914	2151	A	G	80	19	--	--
	Ca(ICCV2/PI489777)SNP_01915	2160	A	G	77	23	--	--
CakTC29246	Ca(ICCV2/PI489777)SNP_01916	32	T	C	7	5	Flower	bud
	Ca(ICCV2/PI489777)SNP_01917	64	T	A	17	12	Flower	bud
	Ca(ICCV2/PI489777)SNP_01918	75	T	G	19	13	Flower	bud
	Ca(ICCV2/PI489777)SNP_01919	124	T	G	20	15	Flower	bud

	Ca(ICCV2/PI489777)SNP_01920	610	T	C	37	14	Flower	bud
	Ca(ICCV2/PI489777)SNP_01921	652	C	T	46	14	Flower	bud
	Ca(ICCV2/PI489777)SNP_01922	845	C	A	56	13	Flower	bud
CakTC39940	Ca(ICCV2/PI489777)SNP_01923	90	G	C	10	10	--	--
	Ca(ICCV2/PI489777)SNP_01924	130	T	C	10	10	--	--
	Ca(ICCV2/PI489777)SNP_01925	898	G	A	7	7	--	--
CakTC28261	Ca(ICCV2/PI489777)SNP_01926	517	A	G	4	3	--	Jumonji
	Ca(ICCV2/PI489777)SNP_01927	655	T	C	5	3	--	Jumonji
	Ca(ICCV2/PI489777)SNP_01928	1813	C	T	3	6	--	Jumonji
	Ca(ICCV2/PI489777)SNP_01929	2047	C	T	3	3	--	Jumonji
CakTC25536	Ca(ICCV2/PI489777)SNP_01930	120	G	A	13	5	--	C2H2
	Ca(ICCV2/PI489777)SNP_01931	248	T	C	19	5	--	C2H2
	Ca(ICCV2/PI489777)SNP_01932	366	T	G	19	6	--	C2H2
	Ca(ICCV2/PI489777)SNP_01933	418	A	G	21	3	--	C2H2
	Ca(ICCV2/PI489777)SNP_01934	497	A	G	18	3	--	C2H2
	Ca(ICCV2/PI489777)SNP_01935	523	T	C	17	5	--	C2H2
	Ca(ICCV2/PI489777)SNP_01936	616	A	G	12	5	--	C2H2
	Ca(ICCV2/PI489777)SNP_01937	763	T	C	25	8	--	C2H2
	Ca(ICCV2/PI489777)SNP_01938	838	C	T	23	7	--	C2H2
	Ca(ICCV2/PI489777)SNP_01939	1006	C	A	23	4	--	C2H2
CakTC00909	Ca(ICCV2/PI489777)SNP_01940	461	G	C	12	5	--	--
CakTC28526	Ca(ICCV2/PI489777)SNP_01941	455	A	C	10	8	--	--
CakTC29912	Ca(ICCV2/PI489777)SNP_01942	1437	C	T	17	7	--	--
	Ca(ICCV2/PI489777)SNP_01943	1689	G	A	7	8	--	--
	Ca(ICCV2/PI489777)SNP_01944	2262	T	C	6	6	--	--
	Ca(ICCV2/PI489777)SNP_01945	2425	C	T	5	11	--	--
CakTC34946	Ca(ICCV2/PI489777)SNP_01946	94	C	T	4	7	--	--
	Ca(ICCV2/PI489777)SNP_01947	96	C	T	5	7	--	--
	Ca(ICCV2/PI489777)SNP_01948	323	T	C	7	11	--	--
	Ca(ICCV2/PI489777)SNP_01949	722	G	A	3	5	--	--
	Ca(ICCV2/PI489777)SNP_01950	974	C	G	6	9	--	--
	Ca(ICCV2/PI489777)SNP_01951	1640	G	A	10	3	--	--
	Ca(ICCV2/PI489777)SNP_01952	2753	C	A	12	6	--	--
	Ca(ICCV2/PI489777)SNP_01953	3342	C	G	12	6	--	--
CakTC37924	Ca(ICCV2/PI489777)SNP_01954	1323	T	C	12	6	--	--
	Ca(ICCV2/PI489777)SNP_01955	1584	C	T	8	3	--	--
CakTC40466	Ca(ICCV2/PI489777)SNP_01956	233	C	G	20	10	--	--
	Ca(ICCV2/PI489777)SNP_01957	455	A	G	18	13	--	--
	Ca(ICCV2/PI489777)SNP_01958	484	G	A	17	14	--	--
	Ca(ICCV2/PI489777)SNP_01959	907	A	G	31	20	--	--
	Ca(ICCV2/PI489777)SNP_01960	946	T	C	28	17	--	--
	Ca(ICCV2/PI489777)SNP_01961	1030	C	T	33	20	--	--
	Ca(ICCV2/PI489777)SNP_01962	1890	T	A	13	5	--	--
CakTC26349	Ca(ICCV2/PI489777)SNP_01963	366	G	T	25	12	--	--
	Ca(ICCV2/PI489777)SNP_01964	411	C	T	24	15	--	--
CakTC42060	Ca(ICCV2/PI489777)SNP_01965	56	T	C	18	16	--	--
	Ca(ICCV2/PI489777)SNP_01966	62	T	C	20	16	--	--
	Ca(ICCV2/PI489777)SNP_01967	190	A	G	26	27	--	--
	Ca(ICCV2/PI489777)SNP_01968	598	T	G	23	21	--	--
CakTC09686	Ca(ICCV2/PI489777)SNP_01969	1637	C	T	7	4	--	--
CakTC28200	Ca(ICCV2/PI489777)SNP_01970	304	G	T	19	13	--	--
CakTC31850	Ca(ICCV2/PI489777)SNP_01971	105	A	T	9	12	--	--
	Ca(ICCV2/PI489777)SNP_01972	188	G	C	10	12	--	--
	Ca(ICCV2/PI489777)SNP_01973	294	G	A	9	9	--	--
	Ca(ICCV2/PI489777)SNP_01974	398	T	G	10	11	--	--
	Ca(ICCV2/PI489777)SNP_01975	413	G	A	8	11	--	--
	Ca(ICCV2/PI489777)SNP_01976	937	T	C	17	17	--	--
	Ca(ICCV2/PI489777)SNP_01977	967	T	C	18	19	--	--
	Ca(ICCV2/PI489777)SNP_01978	1063	G	T	17	23	--	--
	Ca(ICCV2/PI489777)SNP_01979	1116	A	G	16	26	--	--
	Ca(ICCV2/PI489777)SNP_01980	1156	T	A	17	24	--	--
	Ca(ICCV2/PI489777)SNP_01981	1234	C	T	15	23	--	--
	Ca(ICCV2/PI489777)SNP_01982	1311	C	T	6	20	--	--
	Ca(ICCV2/PI489777)SNP_01983	1369	C	A	6	13	--	--
	Ca(ICCV2/PI489777)SNP_01984	1551	G	T	4	3	--	--
CakTC37382	Ca(ICCV2/PI489777)SNP_01985	517	C	T	12	3	--	--
	Ca(ICCV2/PI489777)SNP_01986	808	C	T	8	10	--	--
	Ca(ICCV2/PI489777)SNP_01987	844	A	G	8	10	--	--
CakTC36020	Ca(ICCV2/PI489777)SNP_01988	731	G	T	24	3	--	--
	Ca(ICCV2/PI489777)SNP_01989	780	G	C	21	4	--	--
CakTC29517	Ca(ICCV2/PI489777)SNP_01990	1234	C	T	5	8	--	--
CakTC33102	Ca(ICCV2/PI489777)SNP_01991	168	G	C	24	16	--	--
	Ca(ICCV2/PI489777)SNP_01992	169	C	G	24	16	--	--
CakTC25622	Ca(ICCV2/PI489777)SNP_01993	261	T	C	10	5	--	--

	Ca(ICCV2/PI489777)SNP_01994	338	G	A	10	4	--	--
	Ca(ICCV2/PI489777)SNP_01995	416	G	A	13	3	--	--
	Ca(ICCV2/PI489777)SNP_01996	790	T	C	14	6	--	--
	Ca(ICCV2/PI489777)SNP_01997	1097	C	A	9	12	--	--
	Ca(ICCV2/PI489777)SNP_01998	1135	C	A	4	9	--	--
CakTC34293	Ca(ICCV2/PI489777)SNP_01999	1178	A	G	7	4	--	--
	Ca(ICCV2/PI489777)SNP_02000	1286	G	C	5	6	--	--
CakTC39367	Ca(ICCV2/PI489777)SNP_02001	660	C	A	15	17	--	--
	Ca(ICCV2/PI489777)SNP_02002	1296	C	T	10	10	--	--
	Ca(ICCV2/PI489777)SNP_02003	1365	G	A	3	4	--	--
CakTC41205	Ca(ICCV2/PI489777)SNP_02004	157	A	C	8	3	--	--
	Ca(ICCV2/PI489777)SNP_02005	1426	A	G	9	6	--	--
	Ca(ICCV2/PI489777)SNP_02006	1427	G	T	8	6	--	--
	Ca(ICCV2/PI489777)SNP_02007	1533	G	A	9	10	--	--
	Ca(ICCV2/PI489777)SNP_02008	1650	G	T	12	15	--	--
	Ca(ICCV2/PI489777)SNP_02009	2264	G	A	5	9	--	--
CakTC42526	Ca(ICCV2/PI489777)SNP_02010	569	G	A	83	35	--	--
	Ca(ICCV2/PI489777)SNP_02011	686	A	G	90	23	--	--
	Ca(ICCV2/PI489777)SNP_02012	941	G	A	53	13	--	--
CakTC29530	Ca(ICCV2/PI489777)SNP_02013	96	T	C	11	13	--	--
	Ca(ICCV2/PI489777)SNP_02014	101	A	G	18	20	--	--
	Ca(ICCV2/PI489777)SNP_02015	528	A	G	57	32	--	--
CakTC38470	Ca(ICCV2/PI489777)SNP_02016	141	T	C	21	7	Flower	bud
	Ca(ICCV2/PI489777)SNP_02017	674	A	G	17	7	Flower	bud
	Ca(ICCV2/PI489777)SNP_02018	740	T	C	19	6	Flower	bud
	Ca(ICCV2/PI489777)SNP_02019	965	G	A	10	4	Flower	bud
	Ca(ICCV2/PI489777)SNP_02020	1047	T	G	9	4	Flower	bud
	Ca(ICCV2/PI489777)SNP_02021	1076	G	C	9	4	Flower	bud
CakTC42399	Ca(ICCV2/PI489777)SNP_02022	415	G	A	4	4	--	--
	Ca(ICCV2/PI489777)SNP_02023	436	C	T	4	3	--	--
	Ca(ICCV2/PI489777)SNP_02024	640	C	A	10	5	--	--
	Ca(ICCV2/PI489777)SNP_02025	964	C	T	6	3	--	--
	Ca(ICCV2/PI489777)SNP_02026	1118	C	T	6	4	--	--
	Ca(ICCV2/PI489777)SNP_02027	1127	T	G	6	4	--	--
	Ca(ICCV2/PI489777)SNP_02028	1160	G	C	5	3	--	--
	Ca(ICCV2/PI489777)SNP_02029	1576	T	A	6	5	--	--
CakTC27891	Ca(ICCV2/PI489777)SNP_02030	1073	A	G	9	7	--	--
	Ca(ICCV2/PI489777)SNP_02031	1586	T	C	18	16	--	--
	Ca(ICCV2/PI489777)SNP_02032	1635	G	A	17	14	--	--
	Ca(ICCV2/PI489777)SNP_02033	1637	T	G	17	14	--	--
	Ca(ICCV2/PI489777)SNP_02034	1721	T	C	14	11	--	--
CakTC42351	Ca(ICCV2/PI489777)SNP_02035	151	C	A	13	3	Shoot	--
	Ca(ICCV2/PI489777)SNP_02036	391	C	T	19	4	Shoot	--
	Ca(ICCV2/PI489777)SNP_02037	427	A	G	17	5	Shoot	--
CakTC10457	Ca(ICCV2/PI489777)SNP_02038	168	G	A	6	3	--	--
CakTC28158	Ca(ICCV2/PI489777)SNP_02039	424	T	C	20	14	--	--
	Ca(ICCV2/PI489777)SNP_02040	436	C	T	19	13	--	--
	Ca(ICCV2/PI489777)SNP_02041	592	G	T	25	7	--	--
	Ca(ICCV2/PI489777)SNP_02042	700	C	G	18	12	--	--
	Ca(ICCV2/PI489777)SNP_02043	947	A	G	30	17	--	--
	Ca(ICCV2/PI489777)SNP_02044	1057	C	A	27	11	--	--
	Ca(ICCV2/PI489777)SNP_02045	1781	C	T	21	9	--	--
	Ca(ICCV2/PI489777)SNP_02046	1808	G	A	20	9	--	--
	Ca(ICCV2/PI489777)SNP_02047	1916	G	A	23	9	--	--
	Ca(ICCV2/PI489777)SNP_02048	2056	C	A	36	10	--	--
	Ca(ICCV2/PI489777)SNP_02049	2258	C	T	18	7	--	--
	Ca(ICCV2/PI489777)SNP_02050	2347	G	A	15	7	--	--
CakTC30308	Ca(ICCV2/PI489777)SNP_02051	24	G	C	4	3	--	--
	Ca(ICCV2/PI489777)SNP_02052	45	G	A	4	3	--	--
	Ca(ICCV2/PI489777)SNP_02053	94	A	G	4	3	--	--
	Ca(ICCV2/PI489777)SNP_02054	103	G	C	4	3	--	--
	Ca(ICCV2/PI489777)SNP_02055	108	G	A	4	3	--	--
	Ca(ICCV2/PI489777)SNP_02056	133	T	C	4	3	--	--
	Ca(ICCV2/PI489777)SNP_02057	190	A	C	4	4	--	--
	Ca(ICCV2/PI489777)SNP_02058	312	A	G	5	4	--	--
CakTC38398	Ca(ICCV2/PI489777)SNP_02059	477	G	A	5	6	--	--
	Ca(ICCV2/PI489777)SNP_02060	511	G	A	5	5	--	--
	Ca(ICCV2/PI489777)SNP_02061	741	T	C	3	4	--	--
CakTC37844	Ca(ICCV2/PI489777)SNP_02062	212	C	T	12	3	--	--
	Ca(ICCV2/PI489777)SNP_02063	418	C	G	24	20	--	--
	Ca(ICCV2/PI489777)SNP_02064	878	C	T	16	15	--	--
CakTC31172	Ca(ICCV2/PI489777)SNP_02065	1117	G	T	5	7	--	--
CakTC38880	Ca(ICCV2/PI489777)SNP_02066	245	A	G	3	3	--	AP2-EREBP
	Ca(ICCV2/PI489777)SNP_02067	343	G	A	15	5	--	AP2-EREBP

	Ca(ICCV2/PI489777)SNP_02068	361	G	T	16	5	--	AP2-EREBP
	Ca(ICCV2/PI489777)SNP_02069	364	C	T	15	5	--	AP2-EREBP
	Ca(ICCV2/PI489777)SNP_02070	379	C	A	16	5	--	AP2-EREBP
	Ca(ICCV2/PI489777)SNP_02071	826	G	C	20	4	--	AP2-EREBP
	Ca(ICCV2/PI489777)SNP_02072	1153	A	G	11	8	--	AP2-EREBP
	Ca(ICCV2/PI489777)SNP_02073	1385	G	C	18	3	--	AP2-EREBP
	Ca(ICCV2/PI489777)SNP_02074	1403	G	A	18	3	--	AP2-EREBP
CakTC40202	Ca(ICCV2/PI489777)SNP_02075	114	A	C	5	4	--	--
	Ca(ICCV2/PI489777)SNP_02076	242	T	G	5	4	--	--
CakTC41461	Ca(ICCV2/PI489777)SNP_02077	73	T	C	3	3	--	--
	Ca(ICCV2/PI489777)SNP_02078	363	A	T	10	7	--	--
	Ca(ICCV2/PI489777)SNP_02079	425	T	C	13	6	--	--
	Ca(ICCV2/PI489777)SNP_02080	646	T	C	15	4	--	--
	Ca(ICCV2/PI489777)SNP_02081	896	A	G	12	4	--	--
	Ca(ICCV2/PI489777)SNP_02082	905	A	G	12	4	--	--
	Ca(ICCV2/PI489777)SNP_02083	983	A	G	14	4	--	--
	Ca(ICCV2/PI489777)SNP_02084	1225	C	T	11	3	--	--
	Ca(ICCV2/PI489777)SNP_02085	1914	C	G	7	4	--	--
	Ca(ICCV2/PI489777)SNP_02086	2039	C	T	10	4	--	--
	Ca(ICCV2/PI489777)SNP_02087	2089	C	A	9	4	--	--
CakTC39799	Ca(ICCV2/PI489777)SNP_02088	1206	T	C	26	10	--	bZIP
	Ca(ICCV2/PI489777)SNP_02089	1262	C	T	26	9	--	bZIP
CakTC31257	Ca(ICCV2/PI489777)SNP_02090	2485	T	A	14	5	--	--
CakTC31139	Ca(ICCV2/PI489777)SNP_02091	691	A	G	5	3	--	SRS
	Ca(ICCV2/PI489777)SNP_02092	781	T	C	5	5	--	SRS
	Ca(ICCV2/PI489777)SNP_02093	949	A	T	9	6	--	SRS
	Ca(ICCV2/PI489777)SNP_02094	985	A	T	10	6	--	SRS
CakTC33266	Ca(ICCV2/PI489777)SNP_02095	200	A	C	7	5	--	--
	Ca(ICCV2/PI489777)SNP_02096	640	G	A	17	9	--	--
	Ca(ICCV2/PI489777)SNP_02097	2161	G	T	6	11	--	--
CakTC28769	Ca(ICCV2/PI489777)SNP_02098	130	C	T	6	3	--	--
	Ca(ICCV2/PI489777)SNP_02099	669	A	T	4	5	--	--
	Ca(ICCV2/PI489777)SNP_02100	1908	T	C	7	5	--	--
CakTC11489	Ca(ICCV2/PI489777)SNP_02101	49	C	T	3	9	--	--
	Ca(ICCV2/PI489777)SNP_02102	121	A	G	3	11	--	--
	Ca(ICCV2/PI489777)SNP_02103	200	A	C	3	12	--	--
	Ca(ICCV2/PI489777)SNP_02104	223	A	G	5	12	--	--
	Ca(ICCV2/PI489777)SNP_02105	241	G	A	5	12	--	--
	Ca(ICCV2/PI489777)SNP_02106	460	T	C	7	6	--	--
	Ca(ICCV2/PI489777)SNP_02107	1228	C	G	10	5	--	--
	Ca(ICCV2/PI489777)SNP_02108	1294	C	T	6	6	--	--
CakTC28585	Ca(ICCV2/PI489777)SNP_02109	794	A	T	34	24	--	--
	Ca(ICCV2/PI489777)SNP_02110	838	G	A	32	22	--	--
	Ca(ICCV2/PI489777)SNP_02111	863	C	T	26	24	--	--
CakTC43142	Ca(ICCV2/PI489777)SNP_02112	412	C	T	58	30	--	--
	Ca(ICCV2/PI489777)SNP_02113	619	C	T	39	38	--	--
	Ca(ICCV2/PI489777)SNP_02114	910	A	G	43	10	--	--
	Ca(ICCV2/PI489777)SNP_02115	974	G	A	39	10	--	--
	Ca(ICCV2/PI489777)SNP_02116	1031	T	C	46	7	--	--
	Ca(ICCV2/PI489777)SNP_02117	1032	G	A	51	8	--	--
	Ca(ICCV2/PI489777)SNP_02118	1169	T	C	30	11	--	--
	Ca(ICCV2/PI489777)SNP_02119	1448	A	G	21	20	--	--
	Ca(ICCV2/PI489777)SNP_02120	1699	G	A	19	11	--	--
	Ca(ICCV2/PI489777)SNP_02121	1763	A	G	15	9	--	--
	Ca(ICCV2/PI489777)SNP_02122	3551	C	T	4	6	--	--
CakTC33758	Ca(ICCV2/PI489777)SNP_02123	570	T	C	5	10	--	--
CakTC42312	Ca(ICCV2/PI489777)SNP_02124	641	A	G	15	3	Flower	bud
CakTC39077	Ca(ICCV2/PI489777)SNP_02125	384	G	A	6	36	--	--
	Ca(ICCV2/PI489777)SNP_02126	436	C	G	7	31	--	--
	Ca(ICCV2/PI489777)SNP_02127	546	G	C	10	25	--	--
	Ca(ICCV2/PI489777)SNP_02128	915	G	A	6	9	--	--
	Ca(ICCV2/PI489777)SNP_02129	1011	A	T	7	10	--	--
	Ca(ICCV2/PI489777)SNP_02130	1022	C	T	7	11	--	--
	Ca(ICCV2/PI489777)SNP_02131	1170	A	C	4	11	--	--
	Ca(ICCV2/PI489777)SNP_02132	1227	A	G	3	11	--	--
CakTC31396	Ca(ICCV2/PI489777)SNP_02133	402	C	T	20	7	--	--
	Ca(ICCV2/PI489777)SNP_02134	413	A	C	19	7	--	--
	Ca(ICCV2/PI489777)SNP_02135	725	C	T	19	6	--	--
	Ca(ICCV2/PI489777)SNP_02136	965	A	G	23	7	--	--
	Ca(ICCV2/PI489777)SNP_02137	1118	C	G	21	5	--	--
	Ca(ICCV2/PI489777)SNP_02138	1119	G	T	14	5	--	--
	Ca(ICCV2/PI489777)SNP_02139	1298	A	G	21	7	--	--
	Ca(ICCV2/PI489777)SNP_02140	1911	C	T	15	3	--	--
	Ca(ICCV2/PI489777)SNP_02141	2207	A	G	9	5	--	--

CakTC33325	Ca(ICCV2/PI489777)SNP_02142	394	T	C	13	9	--	--
	Ca(ICCV2/PI489777)SNP_02143	427	G	C	11	11	--	--
	Ca(ICCV2/PI489777)SNP_02144	1228	T	G	20	11	--	--
	Ca(ICCV2/PI489777)SNP_02145	1567	G	A	18	8	--	--
	Ca(ICCV2/PI489777)SNP_02146	1605	A	G	22	7	--	--
	Ca(ICCV2/PI489777)SNP_02147	1755	T	A	11	4	--	--
	Ca(ICCV2/PI489777)SNP_02148	1855	A	T	12	6	--	--
	Ca(ICCV2/PI489777)SNP_02149	2060	A	T	11	5	--	--
	Ca(ICCV2/PI489777)SNP_02150	2071	T	G	12	5	--	--
CakTC33494	Ca(ICCV2/PI489777)SNP_02151	364	G	C	10	5	Mature	Leaf
	Ca(ICCV2/PI489777)SNP_02152	578	C	T	6	3	Mature	Leaf
	Ca(ICCV2/PI489777)SNP_02153	5147	T	G	16	9	Mature	Leaf
	Ca(ICCV2/PI489777)SNP_02154	6413	G	A	20	7	Mature	Leaf
CakTC28614	Ca(ICCV2/PI489777)SNP_02155	492	T	A	5	5	--	--
	Ca(ICCV2/PI489777)SNP_02156	504	T	C	4	4	--	--
	Ca(ICCV2/PI489777)SNP_02157	1281	G	A	4	4	--	--
	Ca(ICCV2/PI489777)SNP_02158	1290	C	T	4	4	--	--
	Ca(ICCV2/PI489777)SNP_02159	2269	C	A	11	4	--	--
	Ca(ICCV2/PI489777)SNP_02160	2351	G	C	6	3	--	--
CakTC29746	Ca(ICCV2/PI489777)SNP_02161	860	G	C	5	3	--	--
CakTC34127	Ca(ICCV2/PI489777)SNP_02162	260	G	A	7	4	--	--
	Ca(ICCV2/PI489777)SNP_02163	346	T	C	10	4	--	--
	Ca(ICCV2/PI489777)SNP_02164	736	C	T	11	4	--	--
	Ca(ICCV2/PI489777)SNP_02165	940	T	A	9	6	--	--
	Ca(ICCV2/PI489777)SNP_02166	1166	A	G	5	4	--	--
	Ca(ICCV2/PI489777)SNP_02167	1175	C	T	5	3	--	--
	Ca(ICCV2/PI489777)SNP_02168	1191	A	G	5	4	--	--
CakTC10683	Ca(ICCV2/PI489777)SNP_02169	1055	A	G	7	4	--	--
CakTC39401	Ca(ICCV2/PI489777)SNP_02170	185	G	T	3	10	--	--
	Ca(ICCV2/PI489777)SNP_02171	257	G	C	4	11	--	--
	Ca(ICCV2/PI489777)SNP_02172	524	G	A	16	16	--	--
	Ca(ICCV2/PI489777)SNP_02173	573	G	C	16	17	--	--
	Ca(ICCV2/PI489777)SNP_02174	725	C	T	21	14	--	--
	Ca(ICCV2/PI489777)SNP_02175	1175	C	T	15	4	--	--
CakTC10785	Ca(ICCV2/PI489777)SNP_02176	241	T	C	6	5	--	--
	Ca(ICCV2/PI489777)SNP_02177	304	T	C	6	6	--	--
	Ca(ICCV2/PI489777)SNP_02178	421	T	C	10	5	--	--
	Ca(ICCV2/PI489777)SNP_02179	475	G	A	13	9	--	--
	Ca(ICCV2/PI489777)SNP_02180	730	T	C	14	7	--	--
	Ca(ICCV2/PI489777)SNP_02181	1381	A	G	5	10	--	--
CakTC11480	Ca(ICCV2/PI489777)SNP_02182	544	A	T	9	7	--	--
	Ca(ICCV2/PI489777)SNP_02183	688	A	T	10	12	--	--
	Ca(ICCV2/PI489777)SNP_02184	1744	T	G	12	4	--	--
CakTC30656	Ca(ICCV2/PI489777)SNP_02185	387	T	C	10	3	--	MYB
	Ca(ICCV2/PI489777)SNP_02186	1083	T	C	3	5	--	MYB
	Ca(ICCV2/PI489777)SNP_02187	1816	T	C	3	5	--	MYB
CakTC25342	Ca(ICCV2/PI489777)SNP_02188	85	T	C	15	3	--	--
CakTC29849	Ca(ICCV2/PI489777)SNP_02189	403	A	G	9	4	--	--
CakTC31016	Ca(ICCV2/PI489777)SNP_02190	579	G	A	39	13	Flower	bud
	Ca(ICCV2/PI489777)SNP_02191	694	T	C	38	15	Flower	bud
CakTC42182	Ca(ICCV2/PI489777)SNP_02192	519	A	C	4	3	--	--
	Ca(ICCV2/PI489777)SNP_02193	618	C	T	3	3	--	--
	Ca(ICCV2/PI489777)SNP_02194	857	A	G	4	3	--	--
CakTC10990	Ca(ICCV2/PI489777)SNP_02195	377	A	G	4	3	--	--
CakTC40175	Ca(ICCV2/PI489777)SNP_02196	1540	A	G	26	14	--	--
CakTC24323	Ca(ICCV2/PI489777)SNP_02197	168	T	C	6	3	--	--
	Ca(ICCV2/PI489777)SNP_02198	185	T	G	6	3	--	--
	Ca(ICCV2/PI489777)SNP_02199	207	G	A	6	3	--	--
	Ca(ICCV2/PI489777)SNP_02200	232	G	C	6	3	--	--
CakTC28558	Ca(ICCV2/PI489777)SNP_02201	326	A	G	24	15	--	--
	Ca(ICCV2/PI489777)SNP_02202	564	C	G	21	17	--	--
	Ca(ICCV2/PI489777)SNP_02203	1467	T	C	18	8	--	--
	Ca(ICCV2/PI489777)SNP_02204	3172	C	A	25	24	--	--
	Ca(ICCV2/PI489777)SNP_02205	3177	A	T	26	23	--	--
CakTC38563	Ca(ICCV2/PI489777)SNP_02206	769	T	G	20	16	--	--
CakTC37159	Ca(ICCV2/PI489777)SNP_02207	749	G	A	9	13	--	--
	Ca(ICCV2/PI489777)SNP_02208	766	G	A	9	11	--	--
	Ca(ICCV2/PI489777)SNP_02209	846	T	A	7	6	--	--
	Ca(ICCV2/PI489777)SNP_02210	1160	C	T	4	5	--	--
CakTC34377	Ca(ICCV2/PI489777)SNP_02211	1427	C	T	6	5	--	--
	Ca(ICCV2/PI489777)SNP_02212	1470	A	G	7	5	--	--
	Ca(ICCV2/PI489777)SNP_02213	1938	A	T	14	3	--	--
CakTC42484	Ca(ICCV2/PI489777)SNP_02214	193	C	T	8	5	--	--
CakTC43345	Ca(ICCV2/PI489777)SNP_02215	240	G	A	35	15	--	--

	Ca(ICCV2/PI489777)SNP_02216	243	T	C	34	13	--	--
	Ca(ICCV2/PI489777)SNP_02217	432	G	A	48	21	--	--
CakTC09707	Ca(ICCV2/PI489777)SNP_02218	685	T	C	30	9	--	--
CakTC22849	Ca(ICCV2/PI489777)SNP_02219	140	C	A	4	3	--	--
CakTC37384	Ca(ICCV2/PI489777)SNP_02220	948	A	G	3	16	Root	--
	Ca(ICCV2/PI489777)SNP_02221	959	C	T	3	15	Root	--
CakTC38481	Ca(ICCV2/PI489777)SNP_02222	1130	T	C	31	21	--	--
	Ca(ICCV2/PI489777)SNP_02223	1546	T	C	14	28	--	--
	Ca(ICCV2/PI489777)SNP_02224	1624	G	T	8	4	--	--
CakTC37619	Ca(ICCV2/PI489777)SNP_02225	59	C	T	33	7	--	--
	Ca(ICCV2/PI489777)SNP_02226	975	C	T	41	15	--	--
	Ca(ICCV2/PI489777)SNP_02227	1420	T	G	11	13	--	--
CakTC25587	Ca(ICCV2/PI489777)SNP_02228	1059	C	A	20	10	--	--
	Ca(ICCV2/PI489777)SNP_02229	1211	T	C	15	6	--	--
	Ca(ICCV2/PI489777)SNP_02230	1336	T	G	7	3	--	--
CakTC24716	Ca(ICCV2/PI489777)SNP_02231	1193	G	A	22	3	--	--
	Ca(ICCV2/PI489777)SNP_02232	1219	C	T	22	5	--	--
	Ca(ICCV2/PI489777)SNP_02233	1528	T	A	9	4	--	--
CakTC39138	Ca(ICCV2/PI489777)SNP_02234	1570	A	G	5	4	--	--
CakTC29328	Ca(ICCV2/PI489777)SNP_02235	75	T	C	3	6	--	--
	Ca(ICCV2/PI489777)SNP_02236	234	C	G	3	5	--	--
	Ca(ICCV2/PI489777)SNP_02237	470	T	C	6	5	--	--
	Ca(ICCV2/PI489777)SNP_02238	620	T	C	7	5	--	--
	Ca(ICCV2/PI489777)SNP_02239	842	T	C	9	4	--	--
	Ca(ICCV2/PI489777)SNP_02240	910	A	C	10	5	--	--
	Ca(ICCV2/PI489777)SNP_02241	1008	A	C	10	5	--	--
CakTC30850	Ca(ICCV2/PI489777)SNP_02242	2271	G	C	3	7	--	--
CakTC32274	Ca(ICCV2/PI489777)SNP_02243	319	T	C	4	3	--	--
	Ca(ICCV2/PI489777)SNP_02244	818	T	C	4	4	--	--
CakTC40296	Ca(ICCV2/PI489777)SNP_02245	125	T	C	4	5	--	--
CakTC35138	Ca(ICCV2/PI489777)SNP_02246	704	C	T	8	6	--	--
	Ca(ICCV2/PI489777)SNP_02247	943	T	G	12	11	--	--
	Ca(ICCV2/PI489777)SNP_02248	945	A	T	13	11	--	--
	Ca(ICCV2/PI489777)SNP_02249	1144	T	G	14	12	--	--
	Ca(ICCV2/PI489777)SNP_02250	1923	C	T	13	9	--	--
	Ca(ICCV2/PI489777)SNP_02251	2476	G	A	6	3	--	--
	Ca(ICCV2/PI489777)SNP_02252	2828	A	G	16	6	--	--
	Ca(ICCV2/PI489777)SNP_02253	3393	G	A	9	5	--	--
	Ca(ICCV2/PI489777)SNP_02254	3397	T	C	8	5	--	--
	Ca(ICCV2/PI489777)SNP_02255	3435	G	T	8	5	--	--
CakTC29196	Ca(ICCV2/PI489777)SNP_02256	230	T	C	54	19	--	--
	Ca(ICCV2/PI489777)SNP_02257	254	C	T	60	18	--	--
	Ca(ICCV2/PI489777)SNP_02258	258	A	G	58	19	--	--
	Ca(ICCV2/PI489777)SNP_02259	731	A	C	47	31	--	--
	Ca(ICCV2/PI489777)SNP_02260	866	G	A	54	23	--	--
CakTC38102	Ca(ICCV2/PI489777)SNP_02261	242	A	G	4	4	--	--
	Ca(ICCV2/PI489777)SNP_02262	613	A	G	4	4	--	--
	Ca(ICCV2/PI489777)SNP_02263	626	G	C	4	5	--	--
CakTC39565	Ca(ICCV2/PI489777)SNP_02264	413	C	T	43	21	--	--
	Ca(ICCV2/PI489777)SNP_02265	488	T	C	40	26	--	--
	Ca(ICCV2/PI489777)SNP_02266	560	G	T	25	22	--	--
CakTC07910	Ca(ICCV2/PI489777)SNP_02267	1394	A	T	6	7	--	--
	Ca(ICCV2/PI489777)SNP_02268	2006	T	C	13	5	--	--
CakTC22934	Ca(ICCV2/PI489777)SNP_02269	1158	G	A	26	3	--	--
	Ca(ICCV2/PI489777)SNP_02270	1398	G	A	32	18	--	--
CakTC25259	Ca(ICCV2/PI489777)SNP_02271	171	G	C	8	3	--	--
	Ca(ICCV2/PI489777)SNP_02272	399	C	T	12	8	--	--
	Ca(ICCV2/PI489777)SNP_02273	2436	A	G	9	3	--	--
CakTC28097	Ca(ICCV2/PI489777)SNP_02274	1172	T	G	6	3	--	--
CakTC25886	Ca(ICCV2/PI489777)SNP_02275	1021	A	C	11	11	--	--
CakTC31673	Ca(ICCV2/PI489777)SNP_02276	472	G	C	11	9	--	--
	Ca(ICCV2/PI489777)SNP_02277	528	A	T	12	7	--	--
	Ca(ICCV2/PI489777)SNP_02278	739	A	G	12	6	--	--
CakTC43126	Ca(ICCV2/PI489777)SNP_02279	640	T	C	25	10	--	--
	Ca(ICCV2/PI489777)SNP_02280	775	C	T	32	20	--	--
CakTC38401	Ca(ICCV2/PI489777)SNP_02281	999	T	G	6	20	--	--
CakTC25785	Ca(ICCV2/PI489777)SNP_02282	1221	T	C	15	5	--	--
	Ca(ICCV2/PI489777)SNP_02283	1580	T	A	6	5	--	--
	Ca(ICCV2/PI489777)SNP_02284	1863	G	C	15	11	--	--
	Ca(ICCV2/PI489777)SNP_02285	1866	G	A	15	10	--	--
	Ca(ICCV2/PI489777)SNP_02286	1979	G	C	14	8	--	--
CakTC26971	Ca(ICCV2/PI489777)SNP_02287	119	A	G	4	5	--	--
	Ca(ICCV2/PI489777)SNP_02288	582	C	T	5	3	--	--
CakTC29299	Ca(ICCV2/PI489777)SNP_02289	374	T	G	7	6	--	--

	Ca(ICCV2/PI489777)SNP_02290	1657	A	G	4	4	--	--
CakTC29074	Ca(ICCV2/PI489777)SNP_02291	900	G	C	16	19	Flower	bud
CakTC42306	Ca(ICCV2/PI489777)SNP_02292	984	C	T	9	8	--	--
	Ca(ICCV2/PI489777)SNP_02293	1287	C	T	8	6	--	--
CakTC22667	Ca(ICCV2/PI489777)SNP_02294	2963	C	T	10	3	--	--
	Ca(ICCV2/PI489777)SNP_02295	2989	A	G	9	3	--	--
	Ca(ICCV2/PI489777)SNP_02296	3141	C	T	15	3	--	--
	Ca(ICCV2/PI489777)SNP_02297	3192	T	C	15	3	--	--
CakTC29370	Ca(ICCV2/PI489777)SNP_02298	860	G	A	63	40	--	--
	Ca(ICCV2/PI489777)SNP_02299	1880	G	A	45	13	--	--
	Ca(ICCV2/PI489777)SNP_02300	1933	C	T	67	50	--	--
	Ca(ICCV2/PI489777)SNP_02301	1936	T	C	63	47	--	--
	Ca(ICCV2/PI489777)SNP_02302	1990	T	C	81	62	--	--
CakTC34802	Ca(ICCV2/PI489777)SNP_02303	301	C	T	25	3	--	--
CakTC39657	Ca(ICCV2/PI489777)SNP_02304	200	C	A	5	4	--	--
	Ca(ICCV2/PI489777)SNP_02305	243	A	C	6	4	--	--
	Ca(ICCV2/PI489777)SNP_02306	936	C	T	6	4	--	--
CakTC38188	Ca(ICCV2/PI489777)SNP_02307	1659	A	G	4	9	--	--
CakTC38704	Ca(ICCV2/PI489777)SNP_02308	301	A	C	6	6	--	--
	Ca(ICCV2/PI489777)SNP_02309	364	T	C	6	6	--	--
	Ca(ICCV2/PI489777)SNP_02310	403	A	T	4	5	--	--
	Ca(ICCV2/PI489777)SNP_02311	414	C	A	4	5	--	--
CakTC24710	Ca(ICCV2/PI489777)SNP_02312	1297	T	C	117	20	--	bZIP
	Ca(ICCV2/PI489777)SNP_02313	1356	G	A	116	19	--	bZIP
	Ca(ICCV2/PI489777)SNP_02314	1357	C	T	115	19	--	bZIP
	Ca(ICCV2/PI489777)SNP_02315	1427	A	G	88	18	--	bZIP
	Ca(ICCV2/PI489777)SNP_02316	1675	C	G	110	14	--	bZIP
	Ca(ICCV2/PI489777)SNP_02317	2020	T	C	73	12	--	bZIP
CakTC41101	Ca(ICCV2/PI489777)SNP_02318	1214	C	T	31	15	--	--
	Ca(ICCV2/PI489777)SNP_02319	1443	C	T	23	21	--	--
CakTC36409	Ca(ICCV2/PI489777)SNP_02320	235	A	T	15	18	--	--
	Ca(ICCV2/PI489777)SNP_02321	760	C	G	12	13	--	--
	Ca(ICCV2/PI489777)SNP_02322	1348	A	T	17	5	--	--
	Ca(ICCV2/PI489777)SNP_02323	1366	G	A	17	3	--	--
	Ca(ICCV2/PI489777)SNP_02324	1432	A	G	16	3	--	--
	Ca(ICCV2/PI489777)SNP_02325	1465	T	C	17	6	--	--
	Ca(ICCV2/PI489777)SNP_02326	1794	T	A	21	4	--	--
CakTC08909	Ca(ICCV2/PI489777)SNP_02327	1718	T	C	9	3	--	--
	Ca(ICCV2/PI489777)SNP_02328	1722	C	T	9	3	--	--
	Ca(ICCV2/PI489777)SNP_02329	1926	G	A	9	5	--	--
	Ca(ICCV2/PI489777)SNP_02330	2039	A	G	8	3	--	--
CakTC28229	Ca(ICCV2/PI489777)SNP_02331	816	A	G	15	3	--	--
	Ca(ICCV2/PI489777)SNP_02332	848	C	G	15	5	--	--
	Ca(ICCV2/PI489777)SNP_02333	912	C	T	10	5	--	--
	Ca(ICCV2/PI489777)SNP_02334	2030	C	T	8	10	--	--
	Ca(ICCV2/PI489777)SNP_02335	2436	C	T	7	6	--	--
CakTC40210	Ca(ICCV2/PI489777)SNP_02336	766	A	T	12	3	--	HSF
	Ca(ICCV2/PI489777)SNP_02337	831	A	G	12	5	--	HSF
	Ca(ICCV2/PI489777)SNP_02338	1147	C	T	14	8	--	HSF
	Ca(ICCV2/PI489777)SNP_02339	1369	A	C	16	5	--	HSF
CakTC41160	Ca(ICCV2/PI489777)SNP_02340	1640	T	A	5	3	--	--
CakTC24052	Ca(ICCV2/PI489777)SNP_02341	474	G	T	15	5	Flower	bud
	Ca(ICCV2/PI489777)SNP_02342	622	G	T	28	5	Flower	bud
	Ca(ICCV2/PI489777)SNP_02343	744	A	C	20	3	Flower	bud
CakTC28439	Ca(ICCV2/PI489777)SNP_02344	703	C	A	61	47	--	LOB
	Ca(ICCV2/PI489777)SNP_02345	754	A	T	49	52	--	LOB
	Ca(ICCV2/PI489777)SNP_02346	1029	T	A	42	30	--	LOB
	Ca(ICCV2/PI489777)SNP_02347	1072	C	G	29	23	--	LOB
CakTC10751	Ca(ICCV2/PI489777)SNP_02348	1289	A	C	5	15	--	--
CakTC30924	Ca(ICCV2/PI489777)SNP_02349	704	A	G	11	3	--	--
	Ca(ICCV2/PI489777)SNP_02350	1113	G	A	8	5	--	--
	Ca(ICCV2/PI489777)SNP_02351	1172	A	G	4	5	--	--
	Ca(ICCV2/PI489777)SNP_02352	1183	C	T	5	6	--	--
	Ca(ICCV2/PI489777)SNP_02353	1316	G	A	4	6	--	--
	Ca(ICCV2/PI489777)SNP_02354	1816	T	C	3	3	--	--
	Ca(ICCV2/PI489777)SNP_02355	1858	C	A	3	4	--	--
CakTC26857	Ca(ICCV2/PI489777)SNP_02356	545	C	G	17	5	--	--
	Ca(ICCV2/PI489777)SNP_02357	2043	C	G	10	3	--	--
	Ca(ICCV2/PI489777)SNP_02358	2133	C	T	12	4	--	--
CakTC35850	Ca(ICCV2/PI489777)SNP_02359	164	T	G	5	7	--	MYB
	Ca(ICCV2/PI489777)SNP_02360	379	T	C	6	13	--	MYB
	Ca(ICCV2/PI489777)SNP_02361	2768	G	T	12	14	--	MYB
	Ca(ICCV2/PI489777)SNP_02362	3178	G	C	20	4	--	MYB
CakTC39794	Ca(ICCV2/PI489777)SNP_02363	676	A	G	11	10	--	--

	Ca(ICCV2/PI489777)SNP_02364	853	G	A	7	14	--	--
	Ca(ICCV2/PI489777)SNP_02365	1107	A	G	10	10	--	--
CakTC22753	Ca(ICCV2/PI489777)SNP_02366	187	T	A	112	61	--	--
	Ca(ICCV2/PI489777)SNP_02367	214	A	G	197	90	--	--
	Ca(ICCV2/PI489777)SNP_02368	358	G	T	212	94	--	--
CakTC35261	Ca(ICCV2/PI489777)SNP_02369	24	A	G	6	3	--	--
	Ca(ICCV2/PI489777)SNP_02370	1451	C	T	11	5	--	--
	Ca(ICCV2/PI489777)SNP_02371	1973	C	T	7	4	--	--
CakTC28507	Ca(ICCV2/PI489777)SNP_02372	83	T	G	8	4	--	--
	Ca(ICCV2/PI489777)SNP_02373	1713	A	G	28	22	--	--
CakTC40563	Ca(ICCV2/PI489777)SNP_02374	419	T	A	4	8	--	--
CakTC27001	Ca(ICCV2/PI489777)SNP_02375	638	T	G	28	11	Young_pod	--
	Ca(ICCV2/PI489777)SNP_02376	656	G	A	26	12	Young_pod	--
CakTC37470	Ca(ICCV2/PI489777)SNP_02377	78	G	T	14	9	--	bZIP
	Ca(ICCV2/PI489777)SNP_02378	354	G	C	27	38	--	bZIP
	Ca(ICCV2/PI489777)SNP_02379	378	C	A	25	29	--	bZIP
	Ca(ICCV2/PI489777)SNP_02380	459	T	C	30	35	--	bZIP
	Ca(ICCV2/PI489777)SNP_02381	968	C	A	17	18	--	bZIP
CakTC36534	Ca(ICCV2/PI489777)SNP_02382	581	T	C	22	10	--	--
	Ca(ICCV2/PI489777)SNP_02383	2406	A	G	34	23	--	--
	Ca(ICCV2/PI489777)SNP_02384	2488	A	C	33	20	--	--
CakTC41512	Ca(ICCV2/PI489777)SNP_02385	276	A	G	12	4	--	--
	Ca(ICCV2/PI489777)SNP_02386	749	G	A	18	4	--	--
	Ca(ICCV2/PI489777)SNP_02387	1883	A	G	20	21	--	--
CakTC30068	Ca(ICCV2/PI489777)SNP_02388	1268	A	G	8	6	--	--
CakTC36130	Ca(ICCV2/PI489777)SNP_02389	260	C	G	17	9	--	--
	Ca(ICCV2/PI489777)SNP_02390	1537	A	G	48	18	--	--
	Ca(ICCV2/PI489777)SNP_02391	1675	A	C	46	19	--	--
	Ca(ICCV2/PI489777)SNP_02392	1927	C	T	20	24	--	--
	Ca(ICCV2/PI489777)SNP_02393	3045	A	C	16	23	--	--
	Ca(ICCV2/PI489777)SNP_02394	3568	A	G	26	20	--	--
	Ca(ICCV2/PI489777)SNP_02395	4792	G	A	8	8	--	--
	Ca(ICCV2/PI489777)SNP_02396	5017	A	G	12	14	--	--
	Ca(ICCV2/PI489777)SNP_02397	5401	C	A	7	5	--	--
CakTC38868	Ca(ICCV2/PI489777)SNP_02398	230	C	T	8	7	--	bHLH
	Ca(ICCV2/PI489777)SNP_02399	685	T	A	9	3	--	bHLH
CakTC11393	Ca(ICCV2/PI489777)SNP_02400	1507	C	A	7	7	--	--
	Ca(ICCV2/PI489777)SNP_02401	1562	T	C	7	8	--	--
CakTC26074	Ca(ICCV2/PI489777)SNP_02402	243	G	C	63	44	--	--
	Ca(ICCV2/PI489777)SNP_02403	570	G	C	61	53	--	--
CakTC40003	Ca(ICCV2/PI489777)SNP_02404	627	T	C	18	10	--	--
	Ca(ICCV2/PI489777)SNP_02405	765	A	G	15	7	--	--
CakTC41097	Ca(ICCV2/PI489777)SNP_02406	2312	G	A	11	5	--	--
CakTC05829	Ca(ICCV2/PI489777)SNP_02407	891	G	T	3	6	--	TAZ
	Ca(ICCV2/PI489777)SNP_02408	909	T	G	3	6	--	TAZ
CakTC39353	Ca(ICCV2/PI489777)SNP_02409	516	T	C	7	5	--	--
CakTC34422	Ca(ICCV2/PI489777)SNP_02410	55	C	A	7	7	--	GRAS
	Ca(ICCV2/PI489777)SNP_02411	622	T	G	21	12	--	GRAS
	Ca(ICCV2/PI489777)SNP_02412	756	A	G	24	17	--	GRAS
	Ca(ICCV2/PI489777)SNP_02413	1632	G	A	23	23	--	GRAS
	Ca(ICCV2/PI489777)SNP_02414	1659	A	G	22	18	--	GRAS
CakTC01025	Ca(ICCV2/PI489777)SNP_02415	585	C	A	3	3	Root	--
	Ca(ICCV2/PI489777)SNP_02416	801	G	A	4	3	Root	--
	Ca(ICCV2/PI489777)SNP_02417	1086	A	G	8	9	Root	--
	Ca(ICCV2/PI489777)SNP_02418	1284	T	C	4	6	Root	--
	Ca(ICCV2/PI489777)SNP_02419	1458	A	T	12	6	Root	--
	Ca(ICCV2/PI489777)SNP_02420	1526	C	T	11	4	Root	--
	Ca(ICCV2/PI489777)SNP_02421	1704	G	A	9	4	Root	--
	Ca(ICCV2/PI489777)SNP_02422	2015	C	T	10	3	Root	--
	Ca(ICCV2/PI489777)SNP_02423	2466	C	T	8	3	Root	--
	Ca(ICCV2/PI489777)SNP_02424	2520	C	T	8	4	Root	--
	Ca(ICCV2/PI489777)SNP_02425	2547	C	A	8	3	Root	--
	Ca(ICCV2/PI489777)SNP_02426	3844	G	A	4	3	Root	--
	Ca(ICCV2/PI489777)SNP_02427	3942	A	G	6	3	Root	--
	Ca(ICCV2/PI489777)SNP_02428	3981	C	T	6	3	Root	--
	Ca(ICCV2/PI489777)SNP_02429	4029	C	T	6	4	Root	--
	Ca(ICCV2/PI489777)SNP_02430	4729	C	T	3	4	Root	--
CakTC11468	Ca(ICCV2/PI489777)SNP_02431	982	T	C	5	3	--	--
	Ca(ICCV2/PI489777)SNP_02432	1019	A	G	6	3	--	--
CakTC11568	Ca(ICCV2/PI489777)SNP_02433	106	A	T	31	5	--	--
CakTC37258	Ca(ICCV2/PI489777)SNP_02434	315	C	T	4	10	--	--
	Ca(ICCV2/PI489777)SNP_02435	317	C	T	4	10	--	--
	Ca(ICCV2/PI489777)SNP_02436	496	A	G	14	13	--	--
	Ca(ICCV2/PI489777)SNP_02437	538	T	C	18	14	--	--

	Ca(ICCV2/PI489777)SNP_02438	691	C	G	17	16	--	--
CakTC34359	Ca(ICCV2/PI489777)SNP_02439	1831	C	A	24	4	--	--
	Ca(ICCV2/PI489777)SNP_02440	1881	G	A	34	11	--	--
CakTC29164	Ca(ICCV2/PI489777)SNP_02441	934	G	A	8	7	--	--
	Ca(ICCV2/PI489777)SNP_02442	1190	C	G	16	3	--	--
CakTC24732	Ca(ICCV2/PI489777)SNP_02443	1259	T	C	41	10	--	--
	Ca(ICCV2/PI489777)SNP_02444	1325	C	T	41	8	--	--
CakTC24141	Ca(ICCV2/PI489777)SNP_02445	154	G	A	28	24	--	--
CakTC22282	Ca(ICCV2/PI489777)SNP_02446	3796	G	A	5	3	--	--
CakTC27408	Ca(ICCV2/PI489777)SNP_02447	372	T	G	5	6	--	--
	Ca(ICCV2/PI489777)SNP_02448	1549	A	G	12	4	--	--
	Ca(ICCV2/PI489777)SNP_02449	2004	A	G	32	10	--	--
	Ca(ICCV2/PI489777)SNP_02450	2142	T	G	28	10	--	--
CakTC31738	Ca(ICCV2/PI489777)SNP_02451	1253	C	T	4	8	--	--
	Ca(ICCV2/PI489777)SNP_02452	1331	G	A	9	8	--	--
	Ca(ICCV2/PI489777)SNP_02453	1394	G	C	8	8	--	--
	Ca(ICCV2/PI489777)SNP_02454	1513	G	A	9	5	--	--
	Ca(ICCV2/PI489777)SNP_02455	1608	A	C	5	5	--	--
	Ca(ICCV2/PI489777)SNP_02456	2420	T	C	10	3	--	--
	Ca(ICCV2/PI489777)SNP_02457	2625	A	G	6	5	--	--
	Ca(ICCV2/PI489777)SNP_02458	2714	C	G	4	6	--	--
	Ca(ICCV2/PI489777)SNP_02459	2771	T	A	3	5	--	--
CakTC28851	Ca(ICCV2/PI489777)SNP_02460	445	A	T	4	3	--	--
	Ca(ICCV2/PI489777)SNP_02461	893	G	C	3	4	--	--
CakTC24741	Ca(ICCV2/PI489777)SNP_02462	614	A	G	30	17	--	--
	Ca(ICCV2/PI489777)SNP_02463	759	T	C	39	21	--	--
	Ca(ICCV2/PI489777)SNP_02464	901	A	G	52	19	--	--
	Ca(ICCV2/PI489777)SNP_02465	914	G	A	43	24	--	--
	Ca(ICCV2/PI489777)SNP_02466	1136	G	T	31	27	--	--
	Ca(ICCV2/PI489777)SNP_02467	1175	G	C	30	26	--	--
CakTC37967	Ca(ICCV2/PI489777)SNP_02468	36	T	C	6	12	--	--
	Ca(ICCV2/PI489777)SNP_02469	697	G	A	12	25	--	--
	Ca(ICCV2/PI489777)SNP_02470	808	A	T	13	16	--	--
	Ca(ICCV2/PI489777)SNP_02471	952	T	C	36	34	--	--
	Ca(ICCV2/PI489777)SNP_02472	1052	G	A	37	31	--	--
	Ca(ICCV2/PI489777)SNP_02473	1297	G	T	36	23	--	--
	Ca(ICCV2/PI489777)SNP_02474	1462	C	G	7	3	--	--
CakTC29823	Ca(ICCV2/PI489777)SNP_02475	1535	C	T	4	3	--	--
CakTC41383	Ca(ICCV2/PI489777)SNP_02476	463	T	C	16	3	--	--
	Ca(ICCV2/PI489777)SNP_02477	1689	C	T	3	3	--	--
CakTC33716	Ca(ICCV2/PI489777)SNP_02478	160	G	T	6	4	--	--
CakTC38805	Ca(ICCV2/PI489777)SNP_02479	1108	T	A	12	7	--	--
CakTC40807	Ca(ICCV2/PI489777)SNP_02480	82	A	G	5	5	--	--
	Ca(ICCV2/PI489777)SNP_02481	83	T	A	5	5	--	--
	Ca(ICCV2/PI489777)SNP_02482	1900	T	A	5	4	--	--
CakTC32223	Ca(ICCV2/PI489777)SNP_02483	508	A	G	3	5	--	--
	Ca(ICCV2/PI489777)SNP_02484	784	T	A	7	5	--	--
CakTC29110	Ca(ICCV2/PI489777)SNP_02485	281	C	T	27	32	--	--
	Ca(ICCV2/PI489777)SNP_02486	560	C	T	22	21	--	--
	Ca(ICCV2/PI489777)SNP_02487	651	A	G	24	12	--	--
	Ca(ICCV2/PI489777)SNP_02488	704	C	A	13	9	--	--
	Ca(ICCV2/PI489777)SNP_02489	713	C	A	14	7	--	--
CakTC27782	Ca(ICCV2/PI489777)SNP_02490	202	A	G	3	4	--	--
	Ca(ICCV2/PI489777)SNP_02491	996	A	G	5	8	--	--
	Ca(ICCV2/PI489777)SNP_02492	2482	T	C	5	6	--	--
	Ca(ICCV2/PI489777)SNP_02493	2593	G	A	14	4	--	--
	Ca(ICCV2/PI489777)SNP_02494	2658	C	T	11	6	--	--
	Ca(ICCV2/PI489777)SNP_02495	2854	G	A	11	5	--	--
CakTC37540	Ca(ICCV2/PI489777)SNP_02496	45	A	G	3	5	--	--
	Ca(ICCV2/PI489777)SNP_02497	207	T	C	10	8	--	--
	Ca(ICCV2/PI489777)SNP_02498	625	A	T	12	8	--	--
	Ca(ICCV2/PI489777)SNP_02499	639	C	T	13	8	--	--
	Ca(ICCV2/PI489777)SNP_02500	642	C	A	13	8	--	--
	Ca(ICCV2/PI489777)SNP_02501	668	G	C	13	7	--	--
	Ca(ICCV2/PI489777)SNP_02502	708	G	A	14	7	--	--
CakTC29934	Ca(ICCV2/PI489777)SNP_02503	651	A	T	32	6	--	--
	Ca(ICCV2/PI489777)SNP_02504	720	A	T	35	5	--	--
	Ca(ICCV2/PI489777)SNP_02505	942	A	G	25	5	--	--
	Ca(ICCV2/PI489777)SNP_02506	1128	A	T	28	8	--	--
	Ca(ICCV2/PI489777)SNP_02507	1557	G	A	12	4	--	--
CakTC26344	Ca(ICCV2/PI489777)SNP_02508	328	T	C	35	20	--	--
	Ca(ICCV2/PI489777)SNP_02509	479	T	C	46	31	--	--
	Ca(ICCV2/PI489777)SNP_02510	1221	A	G	19	16	--	--
CakTC42967	Ca(ICCV2/PI489777)SNP_02511	1070	G	A	8	7	--	--

CakTC24123	Ca(ICCV2/PI489777)SNP_02512	635	C	T	6	5	--	--
	Ca(ICCV2/PI489777)SNP_02513	676	A	G	6	5	--	--
	Ca(ICCV2/PI489777)SNP_02514	749	A	G	6	7	--	--
CakTC33696	Ca(ICCV2/PI489777)SNP_02515	493	G	T	31	19	--	--
	Ca(ICCV2/PI489777)SNP_02516	690	T	C	27	24	--	--
	Ca(ICCV2/PI489777)SNP_02517	735	G	A	19	18	--	--
	Ca(ICCV2/PI489777)SNP_02518	852	T	C	24	15	--	--
	Ca(ICCV2/PI489777)SNP_02519	864	T	C	26	14	--	--
	Ca(ICCV2/PI489777)SNP_02520	1992	G	T	7	17	--	--
CakTC40846	Ca(ICCV2/PI489777)SNP_02521	315	T	A	6	5	--	--
CakTC26309	Ca(ICCV2/PI489777)SNP_02522	1027	G	A	22	8	--	--
	Ca(ICCV2/PI489777)SNP_02523	1300	T	C	19	6	--	--
CakTC09674	Ca(ICCV2/PI489777)SNP_02524	585	T	C	7	3	--	--
	Ca(ICCV2/PI489777)SNP_02525	1784	C	T	10	4	--	--
	Ca(ICCV2/PI489777)SNP_02526	2181	G	A	33	15	--	--
	Ca(ICCV2/PI489777)SNP_02527	2283	A	G	24	12	--	--
	Ca(ICCV2/PI489777)SNP_02528	2446	T	C	17	5	--	--
CakTC39284	Ca(ICCV2/PI489777)SNP_02529	1000	C	T	17	12	--	--
	Ca(ICCV2/PI489777)SNP_02530	1198	C	A	23	15	--	--
	Ca(ICCV2/PI489777)SNP_02531	1330	T	A	17	11	--	--
	Ca(ICCV2/PI489777)SNP_02532	1345	A	G	18	11	--	--
	Ca(ICCV2/PI489777)SNP_02533	1598	T	G	28	6	--	--
	Ca(ICCV2/PI489777)SNP_02534	1606	A	T	28	6	--	--
CakTC41422	Ca(ICCV2/PI489777)SNP_02535	1389	C	T	10	14	--	--
	Ca(ICCV2/PI489777)SNP_02536	1390	C	G	11	14	--	--
	Ca(ICCV2/PI489777)SNP_02537	1701	G	C	15	13	--	--
CakTC33720	Ca(ICCV2/PI489777)SNP_02538	1223	T	C	11	7	--	--
	Ca(ICCV2/PI489777)SNP_02539	5214	A	G	16	6	--	--
	Ca(ICCV2/PI489777)SNP_02540	5406	A	G	9	5	--	--
	Ca(ICCV2/PI489777)SNP_02541	5456	G	A	9	5	--	--
	Ca(ICCV2/PI489777)SNP_02542	6395	G	A	5	4	--	--
	Ca(ICCV2/PI489777)SNP_02543	6524	C	G	5	3	--	--
CakTC25271	Ca(ICCV2/PI489777)SNP_02544	412	T	A	8	5	--	BSD
CakTC23072	Ca(ICCV2/PI489777)SNP_02545	1349	T	G	117	111	--	--
	Ca(ICCV2/PI489777)SNP_02546	1940	G	T	5	5	--	--
	Ca(ICCV2/PI489777)SNP_02547	2729	C	T	3	9	--	--
	Ca(ICCV2/PI489777)SNP_02548	2837	T	A	4	10	--	--
	Ca(ICCV2/PI489777)SNP_02549	2960	G	A	6	7	--	--
	Ca(ICCV2/PI489777)SNP_02550	2972	C	G	6	6	--	--
	Ca(ICCV2/PI489777)SNP_02551	2983	G	T	6	6	--	--
	Ca(ICCV2/PI489777)SNP_02552	3203	A	T	3	4	--	--
CakTC37940	Ca(ICCV2/PI489777)SNP_02553	994	A	T	3	3	--	--
CakTC35826	Ca(ICCV2/PI489777)SNP_02554	298	T	G	3	8	--	--
CakTC24888	Ca(ICCV2/PI489777)SNP_02555	49	A	G	9	3	Young_pod	--
CakTC22381	Ca(ICCV2/PI489777)SNP_02556	286	T	G	3	14	--	--
CakTC11455	Ca(ICCV2/PI489777)SNP_02557	747	C	T	15	7	--	--
	Ca(ICCV2/PI489777)SNP_02558	906	C	T	18	10	--	--
	Ca(ICCV2/PI489777)SNP_02559	1014	A	G	17	12	--	--
	Ca(ICCV2/PI489777)SNP_02560	1116	G	A	19	12	--	--
	Ca(ICCV2/PI489777)SNP_02561	1404	G	C	17	13	--	--
CakTC23823	Ca(ICCV2/PI489777)SNP_02562	735	C	A	9	7	--	--
	Ca(ICCV2/PI489777)SNP_02563	2604	T	C	19	6	--	--
	Ca(ICCV2/PI489777)SNP_02564	2762	A	C	14	3	--	--
CakTC22637	Ca(ICCV2/PI489777)SNP_02565	334	T	A	4	3	--	--
CakTC00553	Ca(ICCV2/PI489777)SNP_02566	451	A	G	21	5	--	--
	Ca(ICCV2/PI489777)SNP_02567	565	G	A	24	7	--	--
	Ca(ICCV2/PI489777)SNP_02568	673	A	G	26	8	--	--
CakTC42702	Ca(ICCV2/PI489777)SNP_02569	155	G	C	3	15	--	--
	Ca(ICCV2/PI489777)SNP_02570	407	C	T	7	21	--	--
	Ca(ICCV2/PI489777)SNP_02571	1667	G	C	9	11	--	--
CakTC31897	Ca(ICCV2/PI489777)SNP_02572	2471	A	T	11	4	--	--
	Ca(ICCV2/PI489777)SNP_02573	3727	G	A	8	7	--	--
	Ca(ICCV2/PI489777)SNP_02574	3755	C	G	8	7	--	--
	Ca(ICCV2/PI489777)SNP_02575	4154	T	C	6	6	--	--
	Ca(ICCV2/PI489777)SNP_02576	5061	G	A	12	8	--	--
	Ca(ICCV2/PI489777)SNP_02577	5088	G	A	14	8	--	--
	Ca(ICCV2/PI489777)SNP_02578	5226	G	A	17	8	--	--
	Ca(ICCV2/PI489777)SNP_02579	6646	C	T	7	5	--	--
	Ca(ICCV2/PI489777)SNP_02580	6906	T	A	15	8	--	--
	Ca(ICCV2/PI489777)SNP_02581	6966	A	G	17	11	--	--
	Ca(ICCV2/PI489777)SNP_02582	7077	C	A	18	10	--	--
	Ca(ICCV2/PI489777)SNP_02583	7086	C	T	18	12	--	--
	Ca(ICCV2/PI489777)SNP_02584	7134	A	G	15	11	--	--
	Ca(ICCV2/PI489777)SNP_02585	7143	T	C	15	10	--	--

CakTC10130	Ca(ICCV2/PI489777)SNP_02586	26	T	C	6	10	--	--
	Ca(ICCV2/PI489777)SNP_02587	212	G	A	10	18	--	--
CakTC32261	Ca(ICCV2/PI489777)SNP_02588	1100	C	T	6	4	--	--
	Ca(ICCV2/PI489777)SNP_02589	1169	G	A	6	5	--	--
	Ca(ICCV2/PI489777)SNP_02590	1289	C	T	6	5	--	--
CakTC23080	Ca(ICCV2/PI489777)SNP_02591	105	C	G	10	4	--	--
	Ca(ICCV2/PI489777)SNP_02592	107	G	T	9	4	--	--
CakTC39064	Ca(ICCV2/PI489777)SNP_02593	1082	C	T	9	9	--	--
	Ca(ICCV2/PI489777)SNP_02594	1121	T	C	10	7	--	--
	Ca(ICCV2/PI489777)SNP_02595	1301	T	A	4	3	--	--
CakTC38255	Ca(ICCV2/PI489777)SNP_02596	360	G	C	13	10	--	--
	Ca(ICCV2/PI489777)SNP_02597	535	T	C	8	11	--	--
CakTC27396	Ca(ICCV2/PI489777)SNP_02598	1113	G	A	8	5	--	--
CakTC23467	Ca(ICCV2/PI489777)SNP_02599	1205	C	T	14	11	--	--
	Ca(ICCV2/PI489777)SNP_02600	1601	G	A	34	11	--	--
CakTC24784	Ca(ICCV2/PI489777)SNP_02601	282	G	A	4	4	Flower	bud
	Ca(ICCV2/PI489777)SNP_02602	391	A	G	5	4	Flower	bud
	Ca(ICCV2/PI489777)SNP_02603	468	C	T	4	3	Flower	bud
	Ca(ICCV2/PI489777)SNP_02604	896	G	A	10	3	Flower	bud
	Ca(ICCV2/PI489777)SNP_02605	908	T	G	10	3	Flower	bud
	Ca(ICCV2/PI489777)SNP_02606	916	C	T	11	3	Flower	bud
CakTC09768	Ca(ICCV2/PI489777)SNP_02607	104	A	C	12	14	--	--
	Ca(ICCV2/PI489777)SNP_02608	305	A	T	24	14	--	--
CakTC33291	Ca(ICCV2/PI489777)SNP_02609	363	T	A	11	4	--	--
	Ca(ICCV2/PI489777)SNP_02610	1478	T	C	6	9	--	--
	Ca(ICCV2/PI489777)SNP_02611	1722	T	G	6	4	--	--
	Ca(ICCV2/PI489777)SNP_02612	1732	G	A	7	4	--	--
	Ca(ICCV2/PI489777)SNP_02613	1737	G	C	7	4	--	--
	Ca(ICCV2/PI489777)SNP_02614	2580	T	C	8	3	--	--
	Ca(ICCV2/PI489777)SNP_02615	2594	A	G	7	3	--	--
	Ca(ICCV2/PI489777)SNP_02616	2741	A	G	4	3	--	--
	Ca(ICCV2/PI489777)SNP_02617	4620	T	C	3	3	--	--
CakTC36016	Ca(ICCV2/PI489777)SNP_02618	139	A	T	30	18	--	--
	Ca(ICCV2/PI489777)SNP_02619	923	A	G	51	43	--	--
	Ca(ICCV2/PI489777)SNP_02620	941	G	A	49	41	--	--
	Ca(ICCV2/PI489777)SNP_02621	1175	C	T	88	45	--	--
	Ca(ICCV2/PI489777)SNP_02622	1400	G	A	76	34	--	--
	Ca(ICCV2/PI489777)SNP_02623	1907	T	C	55	34	--	--
CakTC42310	Ca(ICCV2/PI489777)SNP_02624	280	C	T	6	6	--	--
	Ca(ICCV2/PI489777)SNP_02625	1265	T	C	14	5	--	--
CakTC22590	Ca(ICCV2/PI489777)SNP_02626	5150	T	C	20	12	--	--
	Ca(ICCV2/PI489777)SNP_02627	5286	A	G	13	9	--	--
CakTC42293	Ca(ICCV2/PI489777)SNP_02628	544	G	A	12	4	--	--
	Ca(ICCV2/PI489777)SNP_02629	545	T	A	13	4	--	--
	Ca(ICCV2/PI489777)SNP_02630	610	A	T	15	5	--	--
	Ca(ICCV2/PI489777)SNP_02631	697	T	A	13	5	--	--
	Ca(ICCV2/PI489777)SNP_02632	714	G	C	18	5	--	--
	Ca(ICCV2/PI489777)SNP_02633	726	A	G	13	5	--	--
CakTC30085	Ca(ICCV2/PI489777)SNP_02634	529	A	G	10	3	--	--
CakTC08106	Ca(ICCV2/PI489777)SNP_02635	816	G	A	57	44	--	--
CakTC11228	Ca(ICCV2/PI489777)SNP_02636	241	A	G	20	4	--	--
	Ca(ICCV2/PI489777)SNP_02637	734	G	T	16	7	--	--
CakTC41341	Ca(ICCV2/PI489777)SNP_02638	563	C	T	5	3	--	--
	Ca(ICCV2/PI489777)SNP_02639	566	G	T	5	3	--	--
	Ca(ICCV2/PI489777)SNP_02640	575	C	T	5	3	--	--
	Ca(ICCV2/PI489777)SNP_02641	1371	A	G	10	5	--	--
CakTC31929	Ca(ICCV2/PI489777)SNP_02642	418	C	A	26	8	--	--
	Ca(ICCV2/PI489777)SNP_02643	899	A	G	17	3	--	--
	Ca(ICCV2/PI489777)SNP_02644	1024	A	G	18	4	--	--
CakTC39411	Ca(ICCV2/PI489777)SNP_02645	189	G	A	8	3	--	--
	Ca(ICCV2/PI489777)SNP_02646	255	A	G	8	3	--	--
CakTC24834	Ca(ICCV2/PI489777)SNP_02647	513	A	T	9	9	--	--
	Ca(ICCV2/PI489777)SNP_02648	524	A	G	8	7	--	--
	Ca(ICCV2/PI489777)SNP_02649	568	C	T	12	10	--	--
	Ca(ICCV2/PI489777)SNP_02650	1738	T	A	14	4	--	--
CakTC27406	Ca(ICCV2/PI489777)SNP_02651	1137	T	G	27	7	--	--
	Ca(ICCV2/PI489777)SNP_02652	1170	G	A	21	6	--	--
CakTC42880	Ca(ICCV2/PI489777)SNP_02653	503	C	T	78	38	--	--
	Ca(ICCV2/PI489777)SNP_02654	593	G	T	76	38	--	--
	Ca(ICCV2/PI489777)SNP_02655	692	A	C	95	39	--	--
	Ca(ICCV2/PI489777)SNP_02656	950	G	A	127	38	--	--
	Ca(ICCV2/PI489777)SNP_02657	1115	G	C	113	47	--	--
	Ca(ICCV2/PI489777)SNP_02658	1392	C	T	107	43	--	--
	Ca(ICCV2/PI489777)SNP_02659	1743	G	A	39	11	--	--

	Ca(ICCV2/PI489777)SNP_02660	1757	C	T	40	8	--	--
CakTC38100	Ca(ICCV2/PI489777)SNP_02661	855	A	C	9	5	--	Alfin-like
CakTC30740	Ca(ICCV2/PI489777)SNP_02662	1196	G	A	15	5	--	--
	Ca(ICCV2/PI489777)SNP_02663	1349	C	T	16	6	--	--
	Ca(ICCV2/PI489777)SNP_02664	1367	A	C	16	4	--	--
	Ca(ICCV2/PI489777)SNP_02665	1370	A	G	19	4	--	--
CakTC42482	Ca(ICCV2/PI489777)SNP_02666	584	A	G	9	4	--	--
CakTC27850	Ca(ICCV2/PI489777)SNP_02667	1079	T	C	13	7	--	--
CakTC42343	Ca(ICCV2/PI489777)SNP_02668	252	A	T	20	5	--	--
CakTC33462	Ca(ICCV2/PI489777)SNP_02669	232	A	G	8	4	--	--
	Ca(ICCV2/PI489777)SNP_02670	1674	A	G	10	14	--	--
CakTC02543	Ca(ICCV2/PI489777)SNP_02671	976	C	T	3	8	--	--
CakTC31389	Ca(ICCV2/PI489777)SNP_02672	703	A	G	3	3	--	--
CakTC24584	Ca(ICCV2/PI489777)SNP_02673	177	G	A	26	19	--	NAC
	Ca(ICCV2/PI489777)SNP_02674	208	G	A	23	17	--	NAC
	Ca(ICCV2/PI489777)SNP_02675	228	C	T	25	20	--	NAC
	Ca(ICCV2/PI489777)SNP_02676	327	C	T	26	26	--	NAC
	Ca(ICCV2/PI489777)SNP_02677	525	A	G	51	28	--	NAC
CakTC35952	Ca(ICCV2/PI489777)SNP_02678	286	C	T	13	5	--	--
	Ca(ICCV2/PI489777)SNP_02679	471	C	A	21	16	--	--
	Ca(ICCV2/PI489777)SNP_02680	636	A	G	25	15	--	--
CakTC09370	Ca(ICCV2/PI489777)SNP_02681	337	G	A	3	3	--	--
CakTC40053	Ca(ICCV2/PI489777)SNP_02682	817	A	G	68	13	--	--
	Ca(ICCV2/PI489777)SNP_02683	978	A	G	52	4	--	--
CakTC10384	Ca(ICCV2/PI489777)SNP_02684	1333	G	T	8	5	--	--
	Ca(ICCV2/PI489777)SNP_02685	1431	T	G	11	14	--	--
	Ca(ICCV2/PI489777)SNP_02686	1492	A	C	9	13	--	--
	Ca(ICCV2/PI489777)SNP_02687	1549	T	G	7	13	--	--
	Ca(ICCV2/PI489777)SNP_02688	1731	C	G	8	13	--	--
	Ca(ICCV2/PI489777)SNP_02689	1753	G	A	8	13	--	--
	Ca(ICCV2/PI489777)SNP_02690	1765	G	A	8	9	--	--
CakTC35038	Ca(ICCV2/PI489777)SNP_02691	127	A	C	21	3	Mature	Leaf
	Ca(ICCV2/PI489777)SNP_02692	151	C	G	18	3	Mature	Leaf
CakTC24312	Ca(ICCV2/PI489777)SNP_02693	94	C	T	12	3	Young_pod	--
	Ca(ICCV2/PI489777)SNP_02694	365	C	A	27	9	Young_pod	--
CakTC32671	Ca(ICCV2/PI489777)SNP_02695	1867	A	T	18	12	Root	--
	Ca(ICCV2/PI489777)SNP_02696	2011	C	T	18	9	Root	--
	Ca(ICCV2/PI489777)SNP_02697	2110	G	A	9	3	Root	--
	Ca(ICCV2/PI489777)SNP_02698	2592	T	C	20	10	Root	--
	Ca(ICCV2/PI489777)SNP_02699	2664	C	T	23	13	Root	--
CakTC37308	Ca(ICCV2/PI489777)SNP_02700	354	A	C	25	11	--	--
	Ca(ICCV2/PI489777)SNP_02701	552	C	T	27	12	--	--
	Ca(ICCV2/PI489777)SNP_02702	990	A	G	22	10	--	--
	Ca(ICCV2/PI489777)SNP_02703	1138	C	T	16	13	--	--
CakTC25047	Ca(ICCV2/PI489777)SNP_02704	742	G	T	30	16	--	--
CakTC03955	Ca(ICCV2/PI489777)SNP_02705	2167	G	A	4	4	--	--
CakTC38341	Ca(ICCV2/PI489777)SNP_02706	535	C	T	7	3	--	--
CakTC41736	Ca(ICCV2/PI489777)SNP_02707	267	A	T	12	7	--	C2C2-YABBY
	Ca(ICCV2/PI489777)SNP_02708	534	C	T	6	3	--	C2C2-YABBY
CakTC41865	Ca(ICCV2/PI489777)SNP_02709	356	T	C	16	4	--	CCAAT
	Ca(ICCV2/PI489777)SNP_02710	773	C	G	4	3	--	CCAAT
CakTC28815	Ca(ICCV2/PI489777)SNP_02711	399	T	G	18	9	--	--
CakTC27916	Ca(ICCV2/PI489777)SNP_02712	552	A	C	78	30	--	--
CakTC26907	Ca(ICCV2/PI489777)SNP_02713	438	C	T	7	7	--	--
CakTC29928	Ca(ICCV2/PI489777)SNP_02714	671	A	C	26	8	--	--
	Ca(ICCV2/PI489777)SNP_02715	849	C	T	28	9	--	--
	Ca(ICCV2/PI489777)SNP_02716	1407	A	G	10	12	--	--
CakTC33340	Ca(ICCV2/PI489777)SNP_02717	320	A	C	50	55	--	--
CakTC23141	Ca(ICCV2/PI489777)SNP_02718	1854	T	G	5	3	--	--
CakTC37796	Ca(ICCV2/PI489777)SNP_02719	306	A	T	4	4	--	G2-like
	Ca(ICCV2/PI489777)SNP_02720	567	G	C	7	4	--	G2-like
CakTC42264	Ca(ICCV2/PI489777)SNP_02721	798	C	G	15	12	--	--
	Ca(ICCV2/PI489777)SNP_02722	861	A	G	16	11	--	--
	Ca(ICCV2/PI489777)SNP_02723	939	A	G	19	12	--	--
	Ca(ICCV2/PI489777)SNP_02724	1020	C	T	22	12	--	--
	Ca(ICCV2/PI489777)SNP_02725	1042	A	G	22	11	--	--
	Ca(ICCV2/PI489777)SNP_02726	1697	A	G	6	7	--	--
CakTC41824	Ca(ICCV2/PI489777)SNP_02727	920	G	C	31	7	--	--
CakTC36771	Ca(ICCV2/PI489777)SNP_02728	592	C	A	17	3	--	--
	Ca(ICCV2/PI489777)SNP_02729	838	C	T	19	6	--	--
	Ca(ICCV2/PI489777)SNP_02730	1057	G	A	19	11	--	--
	Ca(ICCV2/PI489777)SNP_02731	1513	A	C	17	6	--	--
	Ca(ICCV2/PI489777)SNP_02732	1742	G	T	26	11	--	--
	Ca(ICCV2/PI489777)SNP_02733	1865	T	C	22	7	--	--

	Ca(ICCV2/PI489777)SNP_02734	2637	C	T	37	5	--	--
	Ca(ICCV2/PI489777)SNP_02735	2931	G	A	27	6	--	--
	Ca(ICCV2/PI489777)SNP_02736	3247	C	T	16	3	--	--
	Ca(ICCV2/PI489777)SNP_02737	3565	A	G	14	6	--	--
	Ca(ICCV2/PI489777)SNP_02738	3938	C	T	4	3	--	--
CakTC23575	Ca(ICCV2/PI489777)SNP_02739	84	G	A	7	8	--	--
	Ca(ICCV2/PI489777)SNP_02740	1033	A	G	8	7	--	--
CakTC40177	Ca(ICCV2/PI489777)SNP_02741	442	T	C	9	8	--	--
	Ca(ICCV2/PI489777)SNP_02742	757	C	T	11	5	--	--
	Ca(ICCV2/PI489777)SNP_02743	1006	A	G	18	3	--	--
CakTC38378	Ca(ICCV2/PI489777)SNP_02744	373	A	T	4	5	--	--
	Ca(ICCV2/PI489777)SNP_02745	472	T	C	5	5	--	--
	Ca(ICCV2/PI489777)SNP_02746	665	G	C	10	4	--	--
	Ca(ICCV2/PI489777)SNP_02747	846	A	G	9	4	--	--
	Ca(ICCV2/PI489777)SNP_02748	984	A	G	4	3	--	--
CakTC31430	Ca(ICCV2/PI489777)SNP_02749	564	C	T	3	3	--	--
	Ca(ICCV2/PI489777)SNP_02750	597	C	T	4	4	--	--
CakTC35076	Ca(ICCV2/PI489777)SNP_02751	465	C	T	9	7	--	--
	Ca(ICCV2/PI489777)SNP_02752	510	A	G	11	7	--	--
	Ca(ICCV2/PI489777)SNP_02753	1554	C	T	15	13	--	--
	Ca(ICCV2/PI489777)SNP_02754	1756	T	G	19	9	--	--
CakTC29565	Ca(ICCV2/PI489777)SNP_02755	751	G	C	19	11	--	--
	Ca(ICCV2/PI489777)SNP_02756	790	A	G	18	9	--	--
CakTC28331	Ca(ICCV2/PI489777)SNP_02757	180	G	C	7	5	--	--
	Ca(ICCV2/PI489777)SNP_02758	642	A	G	11	4	--	--
	Ca(ICCV2/PI489777)SNP_02759	707	G	C	9	5	--	--
	Ca(ICCV2/PI489777)SNP_02760	3123	G	A	11	4	--	--
	Ca(ICCV2/PI489777)SNP_02761	3211	C	A	6	3	--	--
CakTC24007	Ca(ICCV2/PI489777)SNP_02762	1405	G	T	52	25	--	--
	Ca(ICCV2/PI489777)SNP_02763	2265	A	T	38	17	--	--
	Ca(ICCV2/PI489777)SNP_02764	2352	T	C	26	15	--	--
CakTC08517	Ca(ICCV2/PI489777)SNP_02765	1699	A	C	3	4	--	--
	Ca(ICCV2/PI489777)SNP_02766	1848	T	G	3	5	--	--
CakTC41303	Ca(ICCV2/PI489777)SNP_02767	437	T	C	68	121	--	--
	Ca(ICCV2/PI489777)SNP_02768	525	G	C	40	88	--	--
	Ca(ICCV2/PI489777)SNP_02769	593	A	T	3	4	--	--
CakTC34215	Ca(ICCV2/PI489777)SNP_02770	578	G	A	3	8	--	--
	Ca(ICCV2/PI489777)SNP_02771	687	T	C	3	5	--	--
CakTC27339	Ca(ICCV2/PI489777)SNP_02772	1345	G	A	16	10	--	--
	Ca(ICCV2/PI489777)SNP_02773	1546	G	A	19	12	--	--
	Ca(ICCV2/PI489777)SNP_02774	2198	G	C	33	15	--	--
CakTC25167	Ca(ICCV2/PI489777)SNP_02775	195	A	G	5	3	--	--
	Ca(ICCV2/PI489777)SNP_02776	550	T	C	9	8	--	--
	Ca(ICCV2/PI489777)SNP_02777	1128	G	A	30	13	--	--
	Ca(ICCV2/PI489777)SNP_02778	1488	T	A	30	6	--	--
	Ca(ICCV2/PI489777)SNP_02779	1749	T	A	13	4	--	--
	Ca(ICCV2/PI489777)SNP_02780	2028	G	A	9	3	--	--
	Ca(ICCV2/PI489777)SNP_02781	2471	T	C	10	6	--	--
	Ca(ICCV2/PI489777)SNP_02782	4572	A	G	10	5	--	--
	Ca(ICCV2/PI489777)SNP_02783	5297	T	A	6	4	--	--
CakTC10685	Ca(ICCV2/PI489777)SNP_02784	268	T	C	5	6	--	--
	Ca(ICCV2/PI489777)SNP_02785	1535	C	G	14	4	--	--
CakTC26815	Ca(ICCV2/PI489777)SNP_02786	1365	A	C	5	5	--	--
CakTC40930	Ca(ICCV2/PI489777)SNP_02787	725	A	G	5	8	--	--
	Ca(ICCV2/PI489777)SNP_02788	881	T	C	4	10	--	--
	Ca(ICCV2/PI489777)SNP_02789	1577	A	G	11	15	--	--
	Ca(ICCV2/PI489777)SNP_02790	1607	T	C	9	15	--	--
	Ca(ICCV2/PI489777)SNP_02791	1748	C	A	5	4	--	--
CakTC27451	Ca(ICCV2/PI489777)SNP_02792	1211	G	A	41	16	--	--
CakTC27555	Ca(ICCV2/PI489777)SNP_02793	100	T	C	13	6	--	--
	Ca(ICCV2/PI489777)SNP_02794	436	C	T	14	7	--	--
	Ca(ICCV2/PI489777)SNP_02795	463	C	T	16	16	--	--
	Ca(ICCV2/PI489777)SNP_02796	687	G	A	17	6	--	--
	Ca(ICCV2/PI489777)SNP_02797	715	C	T	16	7	--	--
	Ca(ICCV2/PI489777)SNP_02798	1374	T	C	9	8	--	--
CakTC10396	Ca(ICCV2/PI489777)SNP_02799	167	G	T	4	6	--	--
	Ca(ICCV2/PI489777)SNP_02800	702	G	A	4	3	--	--
	Ca(ICCV2/PI489777)SNP_02801	775	A	G	7	5	--	--
	Ca(ICCV2/PI489777)SNP_02802	798	G	A	7	5	--	--
	Ca(ICCV2/PI489777)SNP_02803	862	T	A	7	3	--	--
	Ca(ICCV2/PI489777)SNP_02804	884	G	A	6	3	--	--
	Ca(ICCV2/PI489777)SNP_02805	974	G	C	6	3	--	--
	Ca(ICCV2/PI489777)SNP_02806	977	A	T	6	3	--	--
CakTC40828	Ca(ICCV2/PI489777)SNP_02807	507	T	C	4	6	--	--

CakTC37009	Ca(ICCV2/PI489777)SNP_02808	300	T	A	3	10	--	--
	Ca(ICCV2/PI489777)SNP_02809	330	A	C	3	10	--	--
	Ca(ICCV2/PI489777)SNP_02810	2109	C	T	15	12	--	--
	Ca(ICCV2/PI489777)SNP_02811	2594	A	G	4	6	--	--
CakTC10857	Ca(ICCV2/PI489777)SNP_02812	126	C	T	14	4	--	--
	Ca(ICCV2/PI489777)SNP_02813	139	T	C	14	4	--	--
	Ca(ICCV2/PI489777)SNP_02814	146	A	C	14	4	--	--
	Ca(ICCV2/PI489777)SNP_02815	154	G	T	14	4	--	--
	Ca(ICCV2/PI489777)SNP_02816	160	T	G	14	4	--	--
	Ca(ICCV2/PI489777)SNP_02817	273	G	A	13	6	--	--
CakTC23024	Ca(ICCV2/PI489777)SNP_02818	432	T	C	3	6	--	--
	Ca(ICCV2/PI489777)SNP_02819	674	A	G	7	6	--	--
CakTC03896	Ca(ICCV2/PI489777)SNP_02820	349	T	C	15	20	--	--
CakTC25502	Ca(ICCV2/PI489777)SNP_02821	422	G	A	10	9	--	--
	Ca(ICCV2/PI489777)SNP_02822	477	T	C	10	10	--	--
	Ca(ICCV2/PI489777)SNP_02823	560	T	C	12	11	--	--
	Ca(ICCV2/PI489777)SNP_02824	996	G	A	13	5	--	--
	Ca(ICCV2/PI489777)SNP_02825	1107	G	C	13	4	--	--
CakTC22913	Ca(ICCV2/PI489777)SNP_02826	327	T	A	3	3	--	--
CakTC18325	Ca(ICCV2/PI489777)SNP_02827	573	A	G	20	4	--	--
	Ca(ICCV2/PI489777)SNP_02828	654	A	C	19	4	--	--
	Ca(ICCV2/PI489777)SNP_02829	768	C	A	13	3	--	--
	Ca(ICCV2/PI489777)SNP_02830	783	G	C	12	3	--	--
	Ca(ICCV2/PI489777)SNP_02831	852	T	C	6	3	--	--
	Ca(ICCV2/PI489777)SNP_02832	1292	C	T	12	3	--	--
CakTC27765	Ca(ICCV2/PI489777)SNP_02833	429	A	G	21	13	--	--
	Ca(ICCV2/PI489777)SNP_02834	939	T	C	16	16	--	--
CakTC38906	Ca(ICCV2/PI489777)SNP_02835	344	T	C	13	12	--	--
CakTC37475	Ca(ICCV2/PI489777)SNP_02836	1276	A	T	68	25	--	--
	Ca(ICCV2/PI489777)SNP_02837	1438	C	T	91	16	--	--
	Ca(ICCV2/PI489777)SNP_02838	1454	A	G	80	16	--	--
	Ca(ICCV2/PI489777)SNP_02839	1488	G	C	89	18	--	--
	Ca(ICCV2/PI489777)SNP_02840	1551	A	G	90	19	--	--
CakTC31437	Ca(ICCV2/PI489777)SNP_02841	392	A	G	3	4	Shoot	--
CakTC43347	Ca(ICCV2/PI489777)SNP_02842	301	T	C	7	3	--	--
	Ca(ICCV2/PI489777)SNP_02843	309	G	T	7	4	--	--
	Ca(ICCV2/PI489777)SNP_02844	320	C	A	8	3	--	--
	Ca(ICCV2/PI489777)SNP_02845	361	G	T	7	4	--	--
	Ca(ICCV2/PI489777)SNP_02846	410	A	G	9	4	--	--
	Ca(ICCV2/PI489777)SNP_02847	471	A	G	14	6	--	--
	Ca(ICCV2/PI489777)SNP_02848	573	G	A	16	11	--	--
	Ca(ICCV2/PI489777)SNP_02849	620	C	A	19	9	--	--
	Ca(ICCV2/PI489777)SNP_02850	810	A	G	9	10	--	--
	Ca(ICCV2/PI489777)SNP_02851	876	A	T	7	10	--	--
	Ca(ICCV2/PI489777)SNP_02852	903	G	C	7	9	--	--
CakTC40126	Ca(ICCV2/PI489777)SNP_02853	242	C	T	12	10	--	--
	Ca(ICCV2/PI489777)SNP_02854	1235	G	A	11	13	--	--
	Ca(ICCV2/PI489777)SNP_02855	1476	G	A	5	4	--	--
CakTC40368	Ca(ICCV2/PI489777)SNP_02856	712	A	G	19	28	--	--
CakTC24634	Ca(ICCV2/PI489777)SNP_02857	52	G	T	8	9	--	--
	Ca(ICCV2/PI489777)SNP_02858	137	T	C	23	18	--	--
	Ca(ICCV2/PI489777)SNP_02859	291	T	C	50	45	--	--
	Ca(ICCV2/PI489777)SNP_02860	633	A	G	197	134	--	--
	Ca(ICCV2/PI489777)SNP_02861	1104	A	G	232	128	--	--
	Ca(ICCV2/PI489777)SNP_02862	1288	G	C	184	139	--	--
CakTC24018	Ca(ICCV2/PI489777)SNP_02863	1076	A	T	13	3	--	--
	Ca(ICCV2/PI489777)SNP_02864	1131	A	T	11	3	--	--
CakTC42688	Ca(ICCV2/PI489777)SNP_02865	124	A	C	5	6	--	--
	Ca(ICCV2/PI489777)SNP_02866	906	A	C	6	3	--	--
	Ca(ICCV2/PI489777)SNP_02867	947	T	C	5	4	--	--
	Ca(ICCV2/PI489777)SNP_02868	1154	T	C	7	7	--	--
	Ca(ICCV2/PI489777)SNP_02869	1467	G	T	9	4	--	--
CakTC28188	Ca(ICCV2/PI489777)SNP_02870	378	C	G	73	6	Shoot	C2C2-CO-like
	Ca(ICCV2/PI489777)SNP_02871	499	G	A	41	5	Shoot	C2C2-CO-like
	Ca(ICCV2/PI489777)SNP_02872	701	G	A	24	4	Shoot	C2C2-CO-like
	Ca(ICCV2/PI489777)SNP_02873	1254	C	T	53	6	Shoot	C2C2-CO-like
	Ca(ICCV2/PI489777)SNP_02874	1288	C	T	49	7	Shoot	C2C2-CO-like
	Ca(ICCV2/PI489777)SNP_02875	1504	A	G	18	5	Shoot	C2C2-CO-like
	Ca(ICCV2/PI489777)SNP_02876	1509	G	C	29	5	Shoot	C2C2-CO-like
	Ca(ICCV2/PI489777)SNP_02877	1594	C	T	18	5	Shoot	C2C2-CO-like
CakTC25895	Ca(ICCV2/PI489777)SNP_02878	474	T	C	22	9	--	--
	Ca(ICCV2/PI489777)SNP_02879	480	A	C	20	10	--	--
	Ca(ICCV2/PI489777)SNP_02880	799	G	C	14	5	--	--
	Ca(ICCV2/PI489777)SNP_02881	1024	G	A	7	3	--	--

	Ca(ICCV2/PI489777)SNP_02882	1215	A	G	15	8	--	--
	Ca(ICCV2/PI489777)SNP_02883	1238	C	T	22	9	--	--
	Ca(ICCV2/PI489777)SNP_02884	1789	C	T	42	12	--	--
CakTC39597	Ca(ICCV2/PI489777)SNP_02885	42	A	G	4	6	--	--
	Ca(ICCV2/PI489777)SNP_02886	524	A	T	11	10	--	--
	Ca(ICCV2/PI489777)SNP_02887	987	T	G	22	8	--	--
CakTC37530	Ca(ICCV2/PI489777)SNP_02888	81	A	G	59	14	--	--
	Ca(ICCV2/PI489777)SNP_02889	179	C	G	80	23	--	--
	Ca(ICCV2/PI489777)SNP_02890	1172	G	T	11	9	--	--
CakTC40571	Ca(ICCV2/PI489777)SNP_02891	498	G	A	5	3	--	--
CakTC32923	Ca(ICCV2/PI489777)SNP_02892	50	C	T	4	4	--	--
	Ca(ICCV2/PI489777)SNP_02893	242	C	G	12	7	--	--
	Ca(ICCV2/PI489777)SNP_02894	1119	C	A	11	15	--	--
	Ca(ICCV2/PI489777)SNP_02895	1182	T	C	13	23	--	--
	Ca(ICCV2/PI489777)SNP_02896	1677	C	T	24	18	--	--
CakTC36504	Ca(ICCV2/PI489777)SNP_02897	848	G	A	5	3	Young_pod	--
	Ca(ICCV2/PI489777)SNP_02898	4126	A	G	3	3	Young_pod	--
	Ca(ICCV2/PI489777)SNP_02899	5025	T	C	12	17	Young_pod	--
CakTC22322	Ca(ICCV2/PI489777)SNP_02900	498	G	A	9	8	--	--
CakTC26421	Ca(ICCV2/PI489777)SNP_02901	283	A	T	36	3	--	AP2-EREBP
	Ca(ICCV2/PI489777)SNP_02902	605	T	C	22	8	--	AP2-EREBP
	Ca(ICCV2/PI489777)SNP_02903	699	T	A	18	8	--	AP2-EREBP
	Ca(ICCV2/PI489777)SNP_02904	723	A	C	19	7	--	AP2-EREBP
	Ca(ICCV2/PI489777)SNP_02905	792	T	C	15	7	--	AP2-EREBP
	Ca(ICCV2/PI489777)SNP_02906	829	T	G	20	7	--	AP2-EREBP
CakTC39526	Ca(ICCV2/PI489777)SNP_02907	129	T	G	7	3	--	--
CakTC29886	Ca(ICCV2/PI489777)SNP_02908	421	T	C	11	3	--	--
CakTC37656	Ca(ICCV2/PI489777)SNP_02909	238	T	C	8	3	--	--
CakTC39756	Ca(ICCV2/PI489777)SNP_02910	888	A	G	15	4	--	--
	Ca(ICCV2/PI489777)SNP_02911	890	A	G	14	5	--	--
CakTC26493	Ca(ICCV2/PI489777)SNP_02912	3074	C	A	3	5	--	--
CakTC31951	Ca(ICCV2/PI489777)SNP_02913	1268	T	C	4	4	--	--
CakTC33335	Ca(ICCV2/PI489777)SNP_02914	1738	G	A	15	7	--	--
	Ca(ICCV2/PI489777)SNP_02915	1899	C	T	21	6	--	--
	Ca(ICCV2/PI489777)SNP_02916	1925	G	A	18	8	--	--
	Ca(ICCV2/PI489777)SNP_02917	2275	C	G	15	12	--	--
	Ca(ICCV2/PI489777)SNP_02918	4669	A	G	6	5	--	--
CakTC26437	Ca(ICCV2/PI489777)SNP_02919	116	T	A	8	5	--	--
	Ca(ICCV2/PI489777)SNP_02920	125	T	G	9	5	--	--
	Ca(ICCV2/PI489777)SNP_02921	605	C	G	8	3	--	--
CakTC31717	Ca(ICCV2/PI489777)SNP_02922	411	C	G	7	8	--	--
	Ca(ICCV2/PI489777)SNP_02923	1042	A	G	11	5	--	--
	Ca(ICCV2/PI489777)SNP_02924	1443	C	A	3	6	--	--
CakTC39244	Ca(ICCV2/PI489777)SNP_02925	457	C	G	10	11	--	--
	Ca(ICCV2/PI489777)SNP_02926	687	A	C	3	12	--	--
	Ca(ICCV2/PI489777)SNP_02927	1133	G	A	13	3	--	--
CakTC34395	Ca(ICCV2/PI489777)SNP_02928	61	T	C	9	5	--	--
CakTC28153	Ca(ICCV2/PI489777)SNP_02929	331	A	G	4	8	--	--
	Ca(ICCV2/PI489777)SNP_02930	383	C	T	8	10	--	--
	Ca(ICCV2/PI489777)SNP_02931	1159	C	T	21	10	--	--
	Ca(ICCV2/PI489777)SNP_02932	1277	T	C	22	7	--	--
	Ca(ICCV2/PI489777)SNP_02933	1430	A	G	20	10	--	--
	Ca(ICCV2/PI489777)SNP_02934	1601	C	T	9	4	--	--
	Ca(ICCV2/PI489777)SNP_02935	1610	A	C	8	4	--	--
	Ca(ICCV2/PI489777)SNP_02936	1688	C	T	4	3	--	--
	Ca(ICCV2/PI489777)SNP_02937	1752	T	C	7	3	--	--
	Ca(ICCV2/PI489777)SNP_02938	1807	G	A	9	6	--	--
	Ca(ICCV2/PI489777)SNP_02939	1826	T	C	14	6	--	--
	Ca(ICCV2/PI489777)SNP_02940	1973	T	C	20	3	--	--
	Ca(ICCV2/PI489777)SNP_02941	2465	T	C	12	11	--	--
	Ca(ICCV2/PI489777)SNP_02942	2831	A	G	7	13	--	--
	Ca(ICCV2/PI489777)SNP_02943	2939	G	A	3	11	--	--
	Ca(ICCV2/PI489777)SNP_02944	3209	A	C	20	9	--	--
	Ca(ICCV2/PI489777)SNP_02945	3253	T	C	18	11	--	--
	Ca(ICCV2/PI489777)SNP_02946	3656	G	A	16	8	--	--
	Ca(ICCV2/PI489777)SNP_02947	3879	C	T	13	4	--	--
CakTC37997	Ca(ICCV2/PI489777)SNP_02948	298	C	G	11	3	--	TPR
	Ca(ICCV2/PI489777)SNP_02949	337	T	A	11	4	--	TPR
	Ca(ICCV2/PI489777)SNP_02950	764	A	C	4	3	--	TPR
	Ca(ICCV2/PI489777)SNP_02951	1156	G	T	7	3	--	TPR
CakTC41591	Ca(ICCV2/PI489777)SNP_02952	518	T	C	8	10	--	GNAT
CakTC42332	Ca(ICCV2/PI489777)SNP_02953	1417	A	G	3	4	--	--
CakTC38002	Ca(ICCV2/PI489777)SNP_02954	1158	G	A	4	5	--	--
	Ca(ICCV2/PI489777)SNP_02955	1253	C	T	3	3	--	--

CakTC03729	Ca(ICCV2/PI489777)SNP_02956	215	C	G	9	8	--	--
	Ca(ICCV2/PI489777)SNP_02957	540	G	A	6	9	--	--
	Ca(ICCV2/PI489777)SNP_02958	1159	G	A	4	6	--	--
	Ca(ICCV2/PI489777)SNP_02959	1180	G	A	3	7	--	--
	Ca(ICCV2/PI489777)SNP_02960	1253	C	A	4	7	--	--
CakTC42249	Ca(ICCV2/PI489777)SNP_02961	172	A	C	11	10	--	--
	Ca(ICCV2/PI489777)SNP_02962	792	G	A	21	11	--	--
CakTC04358	Ca(ICCV2/PI489777)SNP_02963	1674	C	A	4	3	--	--
CakTC22990	Ca(ICCV2/PI489777)SNP_02964	1232	A	G	5	3	Young_pod	--
CakTC10706	Ca(ICCV2/PI489777)SNP_02965	540	C	T	77	19	--	MYB-related
	Ca(ICCV2/PI489777)SNP_02966	720	T	C	64	20	--	MYB-related
	Ca(ICCV2/PI489777)SNP_02967	849	C	T	93	18	--	MYB-related
	Ca(ICCV2/PI489777)SNP_02968	1002	A	C	69	17	--	MYB-related
CakTC03637	Ca(ICCV2/PI489777)SNP_02969	182	G	C	31	65	--	--
CakTC31961	Ca(ICCV2/PI489777)SNP_02970	1153	A	T	8	11	--	--
	Ca(ICCV2/PI489777)SNP_02971	1318	A	G	9	12	--	--
	Ca(ICCV2/PI489777)SNP_02972	1558	A	G	6	6	--	--
	Ca(ICCV2/PI489777)SNP_02973	2161	C	T	6	3	--	--
	Ca(ICCV2/PI489777)SNP_02974	2242	G	A	8	4	--	--
	Ca(ICCV2/PI489777)SNP_02975	2320	C	T	5	6	--	--
	Ca(ICCV2/PI489777)SNP_02976	2448	C	T	4	7	--	--
	Ca(ICCV2/PI489777)SNP_02977	2464	A	G	3	7	--	--
CakTC29464	Ca(ICCV2/PI489777)SNP_02978	166	C	A	8	3	--	--
	Ca(ICCV2/PI489777)SNP_02979	198	T	C	8	3	--	--
	Ca(ICCV2/PI489777)SNP_02980	731	C	T	8	4	--	--
	Ca(ICCV2/PI489777)SNP_02981	1482	A	G	4	5	--	--
CakTC38572	Ca(ICCV2/PI489777)SNP_02982	269	G	A	3	8	Flower	bud
	Ca(ICCV2/PI489777)SNP_02983	295	A	T	4	8	Flower	bud
	Ca(ICCV2/PI489777)SNP_02984	313	A	G	4	8	Flower	bud
	Ca(ICCV2/PI489777)SNP_02985	361	C	T	4	5	Flower	bud
CakTC29119	Ca(ICCV2/PI489777)SNP_02986	172	A	C	7	3	--	--
	Ca(ICCV2/PI489777)SNP_02987	567	G	C	18	8	--	--
	Ca(ICCV2/PI489777)SNP_02988	572	G	C	18	8	--	--
	Ca(ICCV2/PI489777)SNP_02989	714	C	A	20	10	--	--
	Ca(ICCV2/PI489777)SNP_02990	928	A	G	18	8	--	--
	Ca(ICCV2/PI489777)SNP_02991	932	C	G	18	8	--	--
CakTC07784	Ca(ICCV2/PI489777)SNP_02992	39	A	G	14	25	--	--
CakTC41954	Ca(ICCV2/PI489777)SNP_02993	438	G	A	3	5	Root	--
	Ca(ICCV2/PI489777)SNP_02994	784	C	T	6	3	Root	--
CakTC22665	Ca(ICCV2/PI489777)SNP_02995	1207	C	T	11	4	--	--
CakTC29116	Ca(ICCV2/PI489777)SNP_02996	96	C	G	4	3	--	--
CakTC35713	Ca(ICCV2/PI489777)SNP_02997	182	T	G	6	6	--	--
	Ca(ICCV2/PI489777)SNP_02998	827	C	T	10	5	--	--
	Ca(ICCV2/PI489777)SNP_02999	855	C	T	10	6	--	--
	Ca(ICCV2/PI489777)SNP_03000	996	C	T	9	7	--	--
CakTC30349	Ca(ICCV2/PI489777)SNP_03001	340	A	T	3	24	--	--
	Ca(ICCV2/PI489777)SNP_03002	352	T	C	3	23	--	--
	Ca(ICCV2/PI489777)SNP_03003	1034	A	G	7	21	--	--
	Ca(ICCV2/PI489777)SNP_03004	1259	C	T	7	24	--	--
	Ca(ICCV2/PI489777)SNP_03005	1286	G	A	7	26	--	--
	Ca(ICCV2/PI489777)SNP_03006	1364	C	G	6	24	--	--
	Ca(ICCV2/PI489777)SNP_03007	1928	T	C	5	33	--	--
	Ca(ICCV2/PI489777)SNP_03008	2018	G	A	7	20	--	--
	Ca(ICCV2/PI489777)SNP_03009	2045	T	C	6	15	--	--
	Ca(ICCV2/PI489777)SNP_03010	2089	A	C	6	15	--	--
CakTC23686	Ca(ICCV2/PI489777)SNP_03011	540	G	A	17	9	--	--
	Ca(ICCV2/PI489777)SNP_03012	588	A	G	18	10	--	--
	Ca(ICCV2/PI489777)SNP_03013	656	A	G	19	11	--	--
CakTC13103	Ca(ICCV2/PI489777)SNP_03014	1619	C	T	48	41	--	--
CakTC39472	Ca(ICCV2/PI489777)SNP_03015	574	G	T	53	33	--	--
	Ca(ICCV2/PI489777)SNP_03016	1468	G	C	27	27	--	--
CakTC09016	Ca(ICCV2/PI489777)SNP_03017	551	A	G	6	7	--	--
CakTC42086	Ca(ICCV2/PI489777)SNP_03018	306	T	C	8	5	--	--
	Ca(ICCV2/PI489777)SNP_03019	528	C	A	4	5	--	--
CakTC26452	Ca(ICCV2/PI489777)SNP_03020	10	G	A	4	4	--	--
	Ca(ICCV2/PI489777)SNP_03021	226	A	T	9	15	--	--
	Ca(ICCV2/PI489777)SNP_03022	227	G	T	9	15	--	--
	Ca(ICCV2/PI489777)SNP_03023	243	C	T	11	16	--	--
	Ca(ICCV2/PI489777)SNP_03024	546	T	C	29	30	--	--
	Ca(ICCV2/PI489777)SNP_03025	763	G	A	23	34	--	--
	Ca(ICCV2/PI489777)SNP_03026	1009	A	G	30	42	--	--
	Ca(ICCV2/PI489777)SNP_03027	1031	C	T	31	38	--	--
	Ca(ICCV2/PI489777)SNP_03028	1114	T	C	27	37	--	--
	Ca(ICCV2/PI489777)SNP_03029	1122	G	A	30	35	--	--

CakTC26205	Ca(ICCV2/PI489777)SNP_03030	547	G	A	46	12	Young_pod	HB
	Ca(ICCV2/PI489777)SNP_03031	589	C	T	46	11	Young_pod	HB
	Ca(ICCV2/PI489777)SNP_03032	943	C	T	47	3	Young_pod	HB
	Ca(ICCV2/PI489777)SNP_03033	2199	A	G	19	4	Young_pod	HB
	Ca(ICCV2/PI489777)SNP_03034	2217	A	G	21	4	Young_pod	HB
CakTC17912	Ca(ICCV2/PI489777)SNP_03035	612	C	T	3	3	--	--
	Ca(ICCV2/PI489777)SNP_03036	804	C	T	3	3	--	--
CakTC40559	Ca(ICCV2/PI489777)SNP_03037	175	C	T	5	4	--	--
CakTC33469	Ca(ICCV2/PI489777)SNP_03038	200	G	A	12	21	--	--
	Ca(ICCV2/PI489777)SNP_03039	338	C	T	15	26	--	--
	Ca(ICCV2/PI489777)SNP_03040	464	C	T	15	16	--	--
CakTC40809	Ca(ICCV2/PI489777)SNP_03041	292	C	T	3	3	--	--
CakTC39389	Ca(ICCV2/PI489777)SNP_03042	102	T	C	6	3	--	--
	Ca(ICCV2/PI489777)SNP_03043	238	C	T	11	6	--	--
	Ca(ICCV2/PI489777)SNP_03044	327	A	C	13	8	--	--
	Ca(ICCV2/PI489777)SNP_03045	1370	T	C	12	21	--	--
CakTC34148	Ca(ICCV2/PI489777)SNP_03046	1389	A	G	3	4	--	--
	Ca(ICCV2/PI489777)SNP_03047	1464	G	A	3	5	--	--
	Ca(ICCV2/PI489777)SNP_03048	3007	T	C	8	4	--	--
	Ca(ICCV2/PI489777)SNP_03049	3275	A	G	8	5	--	--
CakTC36890	Ca(ICCV2/PI489777)SNP_03050	903	T	C	32	7	--	--
	Ca(ICCV2/PI489777)SNP_03051	1245	G	A	21	13	--	--
	Ca(ICCV2/PI489777)SNP_03052	1386	T	C	22	7	--	--
	Ca(ICCV2/PI489777)SNP_03053	1518	A	C	31	5	--	--
	Ca(ICCV2/PI489777)SNP_03054	1638	T	G	36	9	--	--
	Ca(ICCV2/PI489777)SNP_03055	1734	C	A	38	9	--	--
	Ca(ICCV2/PI489777)SNP_03056	2097	C	T	14	12	--	--
	Ca(ICCV2/PI489777)SNP_03057	2343	A	G	12	7	--	--
	Ca(ICCV2/PI489777)SNP_03058	2358	A	G	11	10	--	--
	Ca(ICCV2/PI489777)SNP_03059	2386	C	T	11	8	--	--
	Ca(ICCV2/PI489777)SNP_03060	2419	C	T	8	4	--	--
	Ca(ICCV2/PI489777)SNP_03061	2445	C	T	7	3	--	--
	Ca(ICCV2/PI489777)SNP_03062	2446	C	A	7	3	--	--
CakTC00383	Ca(ICCV2/PI489777)SNP_03063	558	T	C	7	9	--	HSF
	Ca(ICCV2/PI489777)SNP_03064	589	G	A	7	9	--	HSF
CakTC41844	Ca(ICCV2/PI489777)SNP_03065	42	T	A	4	6	--	--
	Ca(ICCV2/PI489777)SNP_03066	108	T	C	6	9	--	--
	Ca(ICCV2/PI489777)SNP_03067	433	T	C	9	5	--	--
CakTC42781	Ca(ICCV2/PI489777)SNP_03068	134	C	T	8	6	--	--
	Ca(ICCV2/PI489777)SNP_03069	547	C	G	40	18	--	--
CakTC38592	Ca(ICCV2/PI489777)SNP_03070	390	T	C	4	4	--	--
	Ca(ICCV2/PI489777)SNP_03071	435	C	T	4	3	--	--
	Ca(ICCV2/PI489777)SNP_03072	690	A	T	4	7	--	--
	Ca(ICCV2/PI489777)SNP_03073	702	T	C	4	7	--	--
	Ca(ICCV2/PI489777)SNP_03074	996	C	A	5	3	--	--
	Ca(ICCV2/PI489777)SNP_03075	1224	T	C	4	4	--	--
	Ca(ICCV2/PI489777)SNP_03076	1308	T	A	3	5	--	--
	Ca(ICCV2/PI489777)SNP_03077	2093	C	T	6	5	--	--
	Ca(ICCV2/PI489777)SNP_03078	2115	A	G	6	5	--	--
	Ca(ICCV2/PI489777)SNP_03079	2234	G	T	5	3	--	--
	Ca(ICCV2/PI489777)SNP_03080	2318	G	C	4	3	--	--
CakTC27418	Ca(ICCV2/PI489777)SNP_03081	149	G	A	6	3	--	--
	Ca(ICCV2/PI489777)SNP_03082	162	A	G	6	3	--	--
	Ca(ICCV2/PI489777)SNP_03083	207	T	A	4	3	--	--
	Ca(ICCV2/PI489777)SNP_03084	263	A	G	6	5	--	--
CakTC39545	Ca(ICCV2/PI489777)SNP_03085	870	A	G	12	5	--	--
CakTC32188	Ca(ICCV2/PI489777)SNP_03086	593	C	T	6	6	--	--
	Ca(ICCV2/PI489777)SNP_03087	854	C	T	7	4	--	--
	Ca(ICCV2/PI489777)SNP_03088	905	G	A	9	4	--	--
	Ca(ICCV2/PI489777)SNP_03089	1232	G	A	7	4	--	--
	Ca(ICCV2/PI489777)SNP_03090	1273	C	A	6	5	--	--
	Ca(ICCV2/PI489777)SNP_03091	1333	T	C	4	5	--	--
CakTC39747	Ca(ICCV2/PI489777)SNP_03092	227	T	C	4	6	--	--
CakTC37150	Ca(ICCV2/PI489777)SNP_03093	261	G	T	13	16	--	--
	Ca(ICCV2/PI489777)SNP_03094	354	A	G	20	21	--	--
	Ca(ICCV2/PI489777)SNP_03095	928	G	A	61	17	--	--
	Ca(ICCV2/PI489777)SNP_03096	1312	C	T	84	69	--	--
	Ca(ICCV2/PI489777)SNP_03097	1423	A	G	68	64	--	--
	Ca(ICCV2/PI489777)SNP_03098	1548	A	G	39	29	--	--
	Ca(ICCV2/PI489777)SNP_03099	1612	T	C	4	14	--	--
	Ca(ICCV2/PI489777)SNP_03100	1622	C	A	5	13	--	--
	Ca(ICCV2/PI489777)SNP_03101	1647	C	T	5	11	--	--
CakTC29485	Ca(ICCV2/PI489777)SNP_03102	668	G	A	6	3	Mature	Leaf
CakTC25386	Ca(ICCV2/PI489777)SNP_03103	417	C	T	64	16	--	--

CakTC37169	Ca(ICCV2/PI489777)SNP_03104	1131	C	T	30	15	--	--
CakTC37343	Ca(ICCV2/PI489777)SNP_03105	846	T	G	51	15	--	--
	Ca(ICCV2/PI489777)SNP_03106	978	G	A	50	13	--	--
	Ca(ICCV2/PI489777)SNP_03107	990	A	G	48	13	--	--
	Ca(ICCV2/PI489777)SNP_03108	1047	G	A	45	12	--	--
CakTC36276	Ca(ICCV2/PI489777)SNP_03109	2547	C	T	18	12	--	--
CakTC31993	Ca(ICCV2/PI489777)SNP_03110	772	C	T	4	4	--	--
	Ca(ICCV2/PI489777)SNP_03111	1212	A	T	6	3	--	--
	Ca(ICCV2/PI489777)SNP_03112	1355	A	G	3	3	--	--
CakTC26164	Ca(ICCV2/PI489777)SNP_03113	48	A	C	5	3	--	--
CakTC32143	Ca(ICCV2/PI489777)SNP_03114	393	A	G	5	23	Root	--
	Ca(ICCV2/PI489777)SNP_03115	401	C	T	5	21	Root	--
	Ca(ICCV2/PI489777)SNP_03116	410	A	C	4	25	Root	--
	Ca(ICCV2/PI489777)SNP_03117	813	C	G	3	33	Root	--
	Ca(ICCV2/PI489777)SNP_03118	1218	G	A	4	25	Root	--
	Ca(ICCV2/PI489777)SNP_03119	1271	A	G	5	21	Root	--
	Ca(ICCV2/PI489777)SNP_03120	1529	A	T	7	38	Root	--
CakTC28294	Ca(ICCV2/PI489777)SNP_03121	558	G	T	16	21	Flower	bud
CakTC10060	Ca(ICCV2/PI489777)SNP_03122	1129	T	C	6	3	--	--
	Ca(ICCV2/PI489777)SNP_03123	1460	A	T	13	4	--	--
	Ca(ICCV2/PI489777)SNP_03124	1540	G	A	12	6	--	--
CakTC40111	Ca(ICCV2/PI489777)SNP_03125	490	T	C	29	6	--	--
	Ca(ICCV2/PI489777)SNP_03126	580	A	G	32	8	--	--
CakTC27949	Ca(ICCV2/PI489777)SNP_03127	349	C	G	22	9	--	--
	Ca(ICCV2/PI489777)SNP_03128	362	T	C	18	9	--	--
	Ca(ICCV2/PI489777)SNP_03129	394	C	A	40	20	--	--
	Ca(ICCV2/PI489777)SNP_03130	481	T	C	47	20	--	--
	Ca(ICCV2/PI489777)SNP_03131	608	A	G	73	10	--	--
	Ca(ICCV2/PI489777)SNP_03132	632	G	T	76	14	--	--
	Ca(ICCV2/PI489777)SNP_03133	646	T	G	72	15	--	--
	Ca(ICCV2/PI489777)SNP_03134	753	C	T	77	23	--	--
	Ca(ICCV2/PI489777)SNP_03135	881	G	A	96	36	--	--
	Ca(ICCV2/PI489777)SNP_03136	1105	G	C	82	21	--	--
	Ca(ICCV2/PI489777)SNP_03137	1109	G	A	75	22	--	--
	Ca(ICCV2/PI489777)SNP_03138	1181	C	T	53	16	--	--
	Ca(ICCV2/PI489777)SNP_03139	1183	A	G	53	15	--	--
CakTC42815	Ca(ICCV2/PI489777)SNP_03140	578	G	A	5	10	--	--
	Ca(ICCV2/PI489777)SNP_03141	1283	T	C	7	3	--	--
	Ca(ICCV2/PI489777)SNP_03142	1361	C	T	8	3	--	--
	Ca(ICCV2/PI489777)SNP_03143	1730	T	G	7	5	--	--
	Ca(ICCV2/PI489777)SNP_03144	1780	C	T	9	5	--	--
CakTC11084	Ca(ICCV2/PI489777)SNP_03145	172	T	C	20	14	--	--
	Ca(ICCV2/PI489777)SNP_03146	319	C	T	19	19	--	--
	Ca(ICCV2/PI489777)SNP_03147	412	C	T	19	19	--	--
	Ca(ICCV2/PI489777)SNP_03148	436	T	C	17	17	--	--
	Ca(ICCV2/PI489777)SNP_03149	586	A	G	28	26	--	--
	Ca(ICCV2/PI489777)SNP_03150	946	C	T	34	24	--	--
	Ca(ICCV2/PI489777)SNP_03151	1015	C	G	29	19	--	--
	Ca(ICCV2/PI489777)SNP_03152	1954	A	G	49	37	--	--
	Ca(ICCV2/PI489777)SNP_03153	2041	T	C	44	37	--	--
CakTC39647	Ca(ICCV2/PI489777)SNP_03154	357	A	G	13	9	--	--
	Ca(ICCV2/PI489777)SNP_03155	395	T	C	15	10	--	--
	Ca(ICCV2/PI489777)SNP_03156	945	C	T	25	13	--	--
	Ca(ICCV2/PI489777)SNP_03157	1270	T	G	15	6	--	--
CakTC40138	Ca(ICCV2/PI489777)SNP_03158	104	A	T	5	12	--	--
	Ca(ICCV2/PI489777)SNP_03159	668	A	G	12	16	--	--
	Ca(ICCV2/PI489777)SNP_03160	1115	T	G	11	9	--	--
CakTC37951	Ca(ICCV2/PI489777)SNP_03161	337	G	C	50	62	Flower	bud
	Ca(ICCV2/PI489777)SNP_03162	353	T	C	43	58	Flower	bud
	Ca(ICCV2/PI489777)SNP_03163	406	G	A	29	56	Flower	bud
	Ca(ICCV2/PI489777)SNP_03164	472	T	A	35	44	Flower	bud
	Ca(ICCV2/PI489777)SNP_03165	878	G	A	10	5	Flower	bud
CakTC34047	Ca(ICCV2/PI489777)SNP_03166	1696	C	T	6	6	--	--
	Ca(ICCV2/PI489777)SNP_03167	1765	G	A	5	6	--	--
CakTC33052	Ca(ICCV2/PI489777)SNP_03168	908	C	T	3	10	--	--
	Ca(ICCV2/PI489777)SNP_03169	1173	G	A	3	5	--	--
CakTC30711	Ca(ICCV2/PI489777)SNP_03170	354	T	G	4	10	--	--
	Ca(ICCV2/PI489777)SNP_03171	723	G	C	3	4	--	--
	Ca(ICCV2/PI489777)SNP_03172	1572	C	T	4	14	--	--
	Ca(ICCV2/PI489777)SNP_03173	1677	T	C	3	10	--	--
CakTC27820	Ca(ICCV2/PI489777)SNP_03174	90	C	G	3	3	--	--
CakTC36322	Ca(ICCV2/PI489777)SNP_03175	763	G	A	12	11	--	--
	Ca(ICCV2/PI489777)SNP_03176	1133	A	T	8	15	--	--
	Ca(ICCV2/PI489777)SNP_03177	2182	C	T	9	7	--	--

	Ca(ICCV2/PI489777)SNP_03178	3988	A	T	12	9	--	--
	Ca(ICCV2/PI489777)SNP_03179	4009	G	A	10	9	--	--
	Ca(ICCV2/PI489777)SNP_03180	4119	A	G	12	6	--	--
	Ca(ICCV2/PI489777)SNP_03181	4545	A	C	3	7	--	--
CakTC17056	Ca(ICCV2/PI489777)SNP_03182	179	G	A	24	7	--	--
	Ca(ICCV2/PI489777)SNP_03183	470	T	C	29	15	--	--
CakTC07212	Ca(ICCV2/PI489777)SNP_03184	301	C	T	12	9	--	--
	Ca(ICCV2/PI489777)SNP_03185	346	G	T	11	7	--	--
	Ca(ICCV2/PI489777)SNP_03186	472	A	T	3	4	--	--
	Ca(ICCV2/PI489777)SNP_03187	525	G	A	3	4	--	--
CakTC09761	Ca(ICCV2/PI489777)SNP_03188	1332	A	G	8	3	--	--
	Ca(ICCV2/PI489777)SNP_03189	1459	G	T	13	3	--	--
	Ca(ICCV2/PI489777)SNP_03190	1481	A	G	10	3	--	--
CakTC41563	Ca(ICCV2/PI489777)SNP_03191	622	T	G	11	3	--	--
CakTC36854	Ca(ICCV2/PI489777)SNP_03192	232	T	C	10	4	Young_pod	--
CakTC22822	Ca(ICCV2/PI489777)SNP_03193	500	T	A	13	8	--	--
	Ca(ICCV2/PI489777)SNP_03194	795	T	C	34	18	--	--
	Ca(ICCV2/PI489777)SNP_03195	975	G	A	51	24	--	--
	Ca(ICCV2/PI489777)SNP_03196	1515	C	T	62	24	--	--
	Ca(ICCV2/PI489777)SNP_03197	1573	C	T	48	24	--	--
	Ca(ICCV2/PI489777)SNP_03198	1683	A	G	36	18	--	--
CakTC36944	Ca(ICCV2/PI489777)SNP_03199	557	T	A	6	4	--	--
CakTC42742	Ca(ICCV2/PI489777)SNP_03200	345	T	C	3	3	--	--
CakTC37725	Ca(ICCV2/PI489777)SNP_03201	14	T	A	6	5	--	--
	Ca(ICCV2/PI489777)SNP_03202	564	G	A	4	3	--	--
	Ca(ICCV2/PI489777)SNP_03203	704	T	C	5	6	--	--
	Ca(ICCV2/PI489777)SNP_03204	705	G	A	5	6	--	--
CakTC41677	Ca(ICCV2/PI489777)SNP_03205	399	G	A	8	4	--	--
	Ca(ICCV2/PI489777)SNP_03206	471	C	T	10	6	--	--
	Ca(ICCV2/PI489777)SNP_03207	1591	T	C	10	5	--	--
	Ca(ICCV2/PI489777)SNP_03208	1687	A	G	11	6	--	--
	Ca(ICCV2/PI489777)SNP_03209	1696	T	A	14	6	--	--
	Ca(ICCV2/PI489777)SNP_03210	1744	A	G	12	6	--	--
	Ca(ICCV2/PI489777)SNP_03211	1792	A	T	12	5	--	--
	Ca(ICCV2/PI489777)SNP_03212	1831	T	A	13	5	--	--
	Ca(ICCV2/PI489777)SNP_03213	1958	A	C	7	6	--	--
CakTC35289	Ca(ICCV2/PI489777)SNP_03214	688	T	C	24	15	--	--
	Ca(ICCV2/PI489777)SNP_03215	1186	T	C	14	11	--	--
	Ca(ICCV2/PI489777)SNP_03216	1453	C	T	14	11	--	--
	Ca(ICCV2/PI489777)SNP_03217	1499	G	T	15	9	--	--
	Ca(ICCV2/PI489777)SNP_03218	1807	C	T	13	7	--	--
	Ca(ICCV2/PI489777)SNP_03219	2329	C	T	22	13	--	--
	Ca(ICCV2/PI489777)SNP_03220	2371	A	G	20	13	--	--
	Ca(ICCV2/PI489777)SNP_03221	3031	C	T	31	12	--	--
	Ca(ICCV2/PI489777)SNP_03222	3034	A	G	29	12	--	--
	Ca(ICCV2/PI489777)SNP_03223	3223	C	T	39	17	--	--
	Ca(ICCV2/PI489777)SNP_03224	3394	G	C	36	18	--	--
	Ca(ICCV2/PI489777)SNP_03225	3718	G	A	28	14	--	--
	Ca(ICCV2/PI489777)SNP_03226	3910	C	T	15	16	--	--
	Ca(ICCV2/PI489777)SNP_03227	3916	A	G	13	17	--	--
	Ca(ICCV2/PI489777)SNP_03228	4195	T	C	3	10	--	--
CakTC38242	Ca(ICCV2/PI489777)SNP_03229	192	T	A	13	5	--	--
	Ca(ICCV2/PI489777)SNP_03230	242	C	A	14	5	--	--
	Ca(ICCV2/PI489777)SNP_03231	321	A	T	15	5	--	--
CakTC38787	Ca(ICCV2/PI489777)SNP_03232	69	T	C	16	5	--	--
	Ca(ICCV2/PI489777)SNP_03233	525	T	C	3	3	--	--
	Ca(ICCV2/PI489777)SNP_03234	999	C	A	9	12	--	--
	Ca(ICCV2/PI489777)SNP_03235	1071	T	C	9	12	--	--
	Ca(ICCV2/PI489777)SNP_03236	1090	C	T	8	11	--	--
	Ca(ICCV2/PI489777)SNP_03237	1265	G	A	4	4	--	--
CakTC39381	Ca(ICCV2/PI489777)SNP_03238	466	C	T	23	18	--	--
	Ca(ICCV2/PI489777)SNP_03239	684	T	C	29	24	--	--
	Ca(ICCV2/PI489777)SNP_03240	865	A	G	25	32	--	--
	Ca(ICCV2/PI489777)SNP_03241	1321	C	T	14	27	--	--
	Ca(ICCV2/PI489777)SNP_03242	1419	C	T	5	11	--	--
CakTC30379	Ca(ICCV2/PI489777)SNP_03243	101	C	A	18	6	--	--
	Ca(ICCV2/PI489777)SNP_03244	162	C	T	20	6	--	--
	Ca(ICCV2/PI489777)SNP_03245	167	C	G	20	6	--	--
	Ca(ICCV2/PI489777)SNP_03246	359	G	C	16	6	--	--
CakTC37593	Ca(ICCV2/PI489777)SNP_03247	1308	C	G	4	4	--	--
CakTC26607	Ca(ICCV2/PI489777)SNP_03248	1356	A	G	4	4	--	--
	Ca(ICCV2/PI489777)SNP_03249	1465	A	G	5	7	--	--
	Ca(ICCV2/PI489777)SNP_03250	1524	C	T	4	6	--	--
	Ca(ICCV2/PI489777)SNP_03251	1705	C	A	9	8	--	--

	Ca(ICCV2/PI489777)SNP_03252	1788	C	A	10	5	--	--
	Ca(ICCV2/PI489777)SNP_03253	1927	G	A	19	3	--	--
	Ca(ICCV2/PI489777)SNP_03254	2541	A	G	6	6	--	--
	Ca(ICCV2/PI489777)SNP_03255	2694	C	A	10	9	--	--
	Ca(ICCV2/PI489777)SNP_03256	2700	C	T	9	9	--	--
	Ca(ICCV2/PI489777)SNP_03257	3367	G	T	7	4	--	--
CakTC37345	Ca(ICCV2/PI489777)SNP_03258	489	T	G	3	3	--	bHLH
CakTC22629	Ca(ICCV2/PI489777)SNP_03259	2040	G	A	13	4	--	--
	Ca(ICCV2/PI489777)SNP_03260	2406	C	T	14	5	--	--
	Ca(ICCV2/PI489777)SNP_03261	2640	A	T	10	5	--	--
	Ca(ICCV2/PI489777)SNP_03262	3320	C	T	20	11	--	--
CakTC29019	Ca(ICCV2/PI489777)SNP_03263	34	T	C	10	3	--	--
	Ca(ICCV2/PI489777)SNP_03264	293	C	T	35	17	--	--
	Ca(ICCV2/PI489777)SNP_03265	680	C	T	35	13	--	--
CakTC38623	Ca(ICCV2/PI489777)SNP_03266	757	A	G	15	16	--	--
CakTC40747	Ca(ICCV2/PI489777)SNP_03267	35	A	G	8	13	--	--
	Ca(ICCV2/PI489777)SNP_03268	198	C	G	66	24	--	--
	Ca(ICCV2/PI489777)SNP_03269	309	A	G	77	24	--	--
CakTC32428	Ca(ICCV2/PI489777)SNP_03270	77	A	G	3	4	Flower	bud
CakTC13082	Ca(ICCV2/PI489777)SNP_03271	670	C	T	4	4	--	--
CakTC34098	Ca(ICCV2/PI489777)SNP_03272	885	C	T	7	6	--	--
	Ca(ICCV2/PI489777)SNP_03273	951	C	T	8	6	--	--
	Ca(ICCV2/PI489777)SNP_03274	1077	T	G	7	9	--	--
	Ca(ICCV2/PI489777)SNP_03275	1885	G	A	3	4	--	--
	Ca(ICCV2/PI489777)SNP_03276	2038	G	T	3	5	--	--
	Ca(ICCV2/PI489777)SNP_03277	2211	T	C	3	3	--	--
	Ca(ICCV2/PI489777)SNP_03278	2212	G	A	3	3	--	--
	Ca(ICCV2/PI489777)SNP_03279	2293	G	T	4	3	--	--
CakTC40416	Ca(ICCV2/PI489777)SNP_03280	46	A	G	8	13	--	--
CakTC37175	Ca(ICCV2/PI489777)SNP_03281	16	A	T	4	8	--	--
	Ca(ICCV2/PI489777)SNP_03282	178	C	T	28	28	--	--
	Ca(ICCV2/PI489777)SNP_03283	220	T	A	28	35	--	--
	Ca(ICCV2/PI489777)SNP_03284	286	T	C	25	41	--	--
	Ca(ICCV2/PI489777)SNP_03285	295	C	T	30	41	--	--
	Ca(ICCV2/PI489777)SNP_03286	298	C	T	28	40	--	--
	Ca(ICCV2/PI489777)SNP_03287	796	G	A	36	17	--	--
	Ca(ICCV2/PI489777)SNP_03288	799	C	T	37	15	--	--
	Ca(ICCV2/PI489777)SNP_03289	1282	C	G	39	28	--	--
	Ca(ICCV2/PI489777)SNP_03290	1570	C	T	26	17	--	--
CakTC30687	Ca(ICCV2/PI489777)SNP_03291	124	T	C	4	6	--	--
	Ca(ICCV2/PI489777)SNP_03292	242	T	C	5	6	--	--
	Ca(ICCV2/PI489777)SNP_03293	1046	G	A	5	3	--	--
CakTC23451	Ca(ICCV2/PI489777)SNP_03294	889	A	G	11	4	--	--
CakTC32441	Ca(ICCV2/PI489777)SNP_03295	201	A	G	10	8	--	AP2-EREBP
CakTC27625	Ca(ICCV2/PI489777)SNP_03296	136	C	T	105	44	--	--
	Ca(ICCV2/PI489777)SNP_03297	149	A	T	104	48	--	--
	Ca(ICCV2/PI489777)SNP_03298	193	T	G	125	59	--	--
	Ca(ICCV2/PI489777)SNP_03299	1092	C	T	230	125	--	--
CakTC23903	Ca(ICCV2/PI489777)SNP_03300	608	C	A	6	3	--	--
	Ca(ICCV2/PI489777)SNP_03301	631	C	T	7	3	--	--
	Ca(ICCV2/PI489777)SNP_03302	677	C	T	5	3	--	--
	Ca(ICCV2/PI489777)SNP_03303	740	T	C	5	3	--	--
	Ca(ICCV2/PI489777)SNP_03304	1269	C	G	22	3	--	--
	Ca(ICCV2/PI489777)SNP_03305	1900	A	T	37	15	--	--
	Ca(ICCV2/PI489777)SNP_03306	1982	C	T	37	17	--	--
	Ca(ICCV2/PI489777)SNP_03307	2102	C	G	46	23	--	--
CakTC25957	Ca(ICCV2/PI489777)SNP_03308	1547	T	C	10	3	--	--
CakTC39029	Ca(ICCV2/PI489777)SNP_03309	656	A	G	3	5	--	--
CakTC28976	Ca(ICCV2/PI489777)SNP_03310	235	T	C	12	4	--	WRKY
	Ca(ICCV2/PI489777)SNP_03311	466	C	T	23	3	--	WRKY
	Ca(ICCV2/PI489777)SNP_03312	1293	A	G	20	3	--	WRKY
	Ca(ICCV2/PI489777)SNP_03313	1398	T	A	16	3	--	WRKY
CakTC39135	Ca(ICCV2/PI489777)SNP_03314	934	A	G	23	4	--	--
	Ca(ICCV2/PI489777)SNP_03315	1299	G	A	6	5	--	--
	Ca(ICCV2/PI489777)SNP_03316	1359	G	A	6	5	--	--
	Ca(ICCV2/PI489777)SNP_03317	1425	G	C	5	5	--	--
	Ca(ICCV2/PI489777)SNP_03318	1476	T	A	7	5	--	--
	Ca(ICCV2/PI489777)SNP_03319	1590	A	T	3	4	--	--
CakTC32616	Ca(ICCV2/PI489777)SNP_03320	356	G	A	8	14	--	CCAAT
	Ca(ICCV2/PI489777)SNP_03321	374	C	T	9	8	--	CCAAT
CakTC26852	Ca(ICCV2/PI489777)SNP_03322	708	A	G	30	29	--	--
	Ca(ICCV2/PI489777)SNP_03323	735	G	A	30	28	--	--
CakTC28640	Ca(ICCV2/PI489777)SNP_03324	301	T	C	3	3	--	--
	Ca(ICCV2/PI489777)SNP_03325	455	G	A	3	4	--	--

CakTC38437	Ca(ICCV2/PI489777)SNP_03326	396	T	C	5	9	--	--
	Ca(ICCV2/PI489777)SNP_03327	399	C	T	5	8	--	--
CakTC34255	Ca(ICCV2/PI489777)SNP_03328	115	G	A	12	9	--	--
	Ca(ICCV2/PI489777)SNP_03329	244	A	C	24	13	--	--
	Ca(ICCV2/PI489777)SNP_03330	836	G	A	62	21	--	--
	Ca(ICCV2/PI489777)SNP_03331	1178	T	C	14	19	--	--
	Ca(ICCV2/PI489777)SNP_03332	1433	G	A	25	21	--	--
	Ca(ICCV2/PI489777)SNP_03333	1812	A	G	22	20	--	--
CakTC32891	Ca(ICCV2/PI489777)SNP_03334	178	G	C	3	5	--	--
	Ca(ICCV2/PI489777)SNP_03335	280	A	G	5	6	--	--
	Ca(ICCV2/PI489777)SNP_03336	330	T	C	5	7	--	--
CakTC24611	Ca(ICCV2/PI489777)SNP_03337	674	G	T	16	13	--	--
	Ca(ICCV2/PI489777)SNP_03338	887	G	T	26	13	--	--
	Ca(ICCV2/PI489777)SNP_03339	3111	G	A	18	12	--	--
	Ca(ICCV2/PI489777)SNP_03340	3359	T	C	20	11	--	--
	Ca(ICCV2/PI489777)SNP_03341	3503	A	C	20	13	--	--
CakTC25809	Ca(ICCV2/PI489777)SNP_03342	2484	A	G	7	3	--	--
CakTC08458	Ca(ICCV2/PI489777)SNP_03343	1338	C	T	4	4	--	TRAF
	Ca(ICCV2/PI489777)SNP_03344	1446	A	G	5	4	--	TRAF
CakTC25199	Ca(ICCV2/PI489777)SNP_03345	3087	C	T	7	5	--	CCAAT
	Ca(ICCV2/PI489777)SNP_03346	4527	T	C	28	105	--	CCAAT
	Ca(ICCV2/PI489777)SNP_03347	4566	G	A	21	96	--	CCAAT
	Ca(ICCV2/PI489777)SNP_03348	4757	A	C	13	58	--	CCAAT
	Ca(ICCV2/PI489777)SNP_03349	4777	G	T	12	54	--	CCAAT
	Ca(ICCV2/PI489777)SNP_03350	4833	T	G	11	28	--	CCAAT
	Ca(ICCV2/PI489777)SNP_03351	4836	A	C	11	29	--	CCAAT
	Ca(ICCV2/PI489777)SNP_03352	4860	G	C	10	24	--	CCAAT
CakTC14670	Ca(ICCV2/PI489777)SNP_03353	732	T	C	5	3	--	--
	Ca(ICCV2/PI489777)SNP_03354	1429	C	T	10	9	--	--
	Ca(ICCV2/PI489777)SNP_03355	1467	A	G	7	9	--	--
	Ca(ICCV2/PI489777)SNP_03356	2988	C	T	4	5	--	--
CakTC29428	Ca(ICCV2/PI489777)SNP_03357	1116	T	C	4	3	Shoot	--
CakTC36457	Ca(ICCV2/PI489777)SNP_03358	585	T	C	38	20	--	--
	Ca(ICCV2/PI489777)SNP_03359	673	G	C	43	19	--	--
	Ca(ICCV2/PI489777)SNP_03360	2635	A	G	30	31	--	--
CakTC23715	Ca(ICCV2/PI489777)SNP_03361	791	A	G	47	25	--	--
	Ca(ICCV2/PI489777)SNP_03362	1237	A	G	111	31	--	--
CakTC07881	Ca(ICCV2/PI489777)SNP_03363	5631	T	C	3	5	--	SNF2
CakTC29889	Ca(ICCV2/PI489777)SNP_03364	191	T	A	5	3	--	--
	Ca(ICCV2/PI489777)SNP_03365	1226	A	C	12	11	--	--
	Ca(ICCV2/PI489777)SNP_03366	2686	T	A	3	3	--	--
CakTC29855	Ca(ICCV2/PI489777)SNP_03367	191	T	G	3	3	--	CCHC
	Ca(ICCV2/PI489777)SNP_03368	990	A	G	10	3	--	CCHC
CakTC32509	Ca(ICCV2/PI489777)SNP_03369	2154	C	A	6	3	--	--
CakTC01006	Ca(ICCV2/PI489777)SNP_03370	178	A	G	4	3	--	--
	Ca(ICCV2/PI489777)SNP_03371	217	C	T	5	3	--	--
	Ca(ICCV2/PI489777)SNP_03372	607	G	A	5	4	--	--
CakTC38247	Ca(ICCV2/PI489777)SNP_03373	502	A	T	8	8	--	HB
	Ca(ICCV2/PI489777)SNP_03374	599	G	A	10	6	--	HB
CakTC30658	Ca(ICCV2/PI489777)SNP_03375	267	C	T	3	3	--	--
	Ca(ICCV2/PI489777)SNP_03376	316	C	A	4	3	--	--
CakTC33027	Ca(ICCV2/PI489777)SNP_03377	2584	T	G	12	17	--	--
CakTC32697	Ca(ICCV2/PI489777)SNP_03378	383	A	G	11	18	--	--
	Ca(ICCV2/PI489777)SNP_03379	517	A	C	3	8	--	--
CakTC36228	Ca(ICCV2/PI489777)SNP_03380	1067	C	T	29	13	--	--
CakTC34582	Ca(ICCV2/PI489777)SNP_03381	304	A	C	31	17	--	--
	Ca(ICCV2/PI489777)SNP_03382	381	C	T	20	9	--	--
	Ca(ICCV2/PI489777)SNP_03383	384	A	T	19	6	--	--
	Ca(ICCV2/PI489777)SNP_03384	489	G	T	5	3	--	--
	Ca(ICCV2/PI489777)SNP_03385	492	T	A	4	3	--	--
CakTC37262	Ca(ICCV2/PI489777)SNP_03386	759	G	C	9	7	--	--
	Ca(ICCV2/PI489777)SNP_03387	852	G	A	9	4	--	--
	Ca(ICCV2/PI489777)SNP_03388	1495	A	G	8	6	--	--
CakTC23308	Ca(ICCV2/PI489777)SNP_03389	340	C	A	16	9	--	--
	Ca(ICCV2/PI489777)SNP_03390	375	A	G	14	13	--	--
CakTC42185	Ca(ICCV2/PI489777)SNP_03391	17	T	C	17	10	--	--
	Ca(ICCV2/PI489777)SNP_03392	18	C	T	17	10	--	--
CakTC30004	Ca(ICCV2/PI489777)SNP_03393	365	C	G	9	5	--	--
CakTC11886	Ca(ICCV2/PI489777)SNP_03394	281	C	G	21	14	--	--
	Ca(ICCV2/PI489777)SNP_03395	438	A	G	22	17	--	--
	Ca(ICCV2/PI489777)SNP_03396	1331	A	G	6	19	--	--
CakTC27720	Ca(ICCV2/PI489777)SNP_03397	1164	T	C	7	3	--	--
CakTC28278	Ca(ICCV2/PI489777)SNP_03398	1297	C	A	21	13	--	IWS1
	Ca(ICCV2/PI489777)SNP_03399	1662	T	C	29	24	--	IWS1

	Ca(ICCV2/PI489777)SNP_03400	1681	C	A	29	21	--	IWS1
CakTC32790	Ca(ICCV2/PI489777)SNP_03401	155	T	C	17	5	--	--
	Ca(ICCV2/PI489777)SNP_03402	2276	C	T	14	14	--	--
	Ca(ICCV2/PI489777)SNP_03403	2481	G	A	15	11	--	--
	Ca(ICCV2/PI489777)SNP_03404	2487	G	A	15	11	--	--
	Ca(ICCV2/PI489777)SNP_03405	2493	G	A	15	10	--	--
	Ca(ICCV2/PI489777)SNP_03406	2513	T	G	15	11	--	--
	Ca(ICCV2/PI489777)SNP_03407	2706	C	A	9	9	--	--
CakTC35051	Ca(ICCV2/PI489777)SNP_03408	6	T	C	5	4	--	--
	Ca(ICCV2/PI489777)SNP_03409	146	G	C	6	4	--	--
CakTC36807	Ca(ICCV2/PI489777)SNP_03410	566	G	T	22	4	--	--
	Ca(ICCV2/PI489777)SNP_03411	647	A	G	27	4	--	--
	Ca(ICCV2/PI489777)SNP_03412	744	G	A	28	5	--	--
	Ca(ICCV2/PI489777)SNP_03413	761	T	C	29	5	--	--
	Ca(ICCV2/PI489777)SNP_03414	2291	G	A	43	8	--	--
CakTC23281	Ca(ICCV2/PI489777)SNP_03415	94	A	C	11	14	--	--
	Ca(ICCV2/PI489777)SNP_03416	218	A	G	19	16	--	--
	Ca(ICCV2/PI489777)SNP_03417	283	G	A	19	17	--	--
	Ca(ICCV2/PI489777)SNP_03418	2560	G	A	12	5	--	--
	Ca(ICCV2/PI489777)SNP_03419	2908	T	C	7	4	--	--
	Ca(ICCV2/PI489777)SNP_03420	3008	G	C	4	3	--	--
CakTC41452	Ca(ICCV2/PI489777)SNP_03421	439	A	G	14	6	--	--
	Ca(ICCV2/PI489777)SNP_03422	554	A	G	15	6	--	--
	Ca(ICCV2/PI489777)SNP_03423	728	T	C	8	5	--	--
CakTC38371	Ca(ICCV2/PI489777)SNP_03424	1137	T	G	46	18	--	DBP
CakTC12845	Ca(ICCV2/PI489777)SNP_03425	30	C	T	6	3	--	--
CakTC37557	Ca(ICCV2/PI489777)SNP_03426	302	T	C	12	4	--	--
CakTC40626	Ca(ICCV2/PI489777)SNP_03427	63	G	T	6	6	--	--
CakTC23034	Ca(ICCV2/PI489777)SNP_03428	495	C	T	10	6	--	--
	Ca(ICCV2/PI489777)SNP_03429	603	A	C	12	21	--	--
	Ca(ICCV2/PI489777)SNP_03430	762	T	A	32	45	--	--
	Ca(ICCV2/PI489777)SNP_03431	921	C	T	39	43	--	--
	Ca(ICCV2/PI489777)SNP_03432	1071	T	C	55	40	--	--
	Ca(ICCV2/PI489777)SNP_03433	1257	A	G	47	29	--	--
	Ca(ICCV2/PI489777)SNP_03434	1479	C	T	61	77	--	--
CakTC37311	Ca(ICCV2/PI489777)SNP_03435	657	C	T	10	5	--	--
	Ca(ICCV2/PI489777)SNP_03436	982	T	C	8	9	--	--
	Ca(ICCV2/PI489777)SNP_03437	1247	T	C	89	14	--	--
CakTC31335	Ca(ICCV2/PI489777)SNP_03438	518	A	G	4	4	--	HB
	Ca(ICCV2/PI489777)SNP_03439	545	T	C	4	4	--	HB
CakTC31405	Ca(ICCV2/PI489777)SNP_03440	468	C	A	8	3	--	--
	Ca(ICCV2/PI489777)SNP_03441	527	A	T	8	3	--	--
	Ca(ICCV2/PI489777)SNP_03442	551	C	T	7	3	--	--
CakTC27713	Ca(ICCV2/PI489777)SNP_03443	818	G	A	7	4	Shoot	--
CakTC24198	Ca(ICCV2/PI489777)SNP_03444	1185	T	C	7	3	--	--
	Ca(ICCV2/PI489777)SNP_03445	1194	T	A	7	3	--	--
	Ca(ICCV2/PI489777)SNP_03446	1350	C	A	5	4	--	--
	Ca(ICCV2/PI489777)SNP_03447	1502	T	C	6	4	--	--
	Ca(ICCV2/PI489777)SNP_03448	1505	G	A	3	4	--	--
	Ca(ICCV2/PI489777)SNP_03449	1609	T	A	4	4	--	--
	Ca(ICCV2/PI489777)SNP_03450	1610	A	T	4	4	--	--
CakTC28652	Ca(ICCV2/PI489777)SNP_03451	1272	A	T	3	3	--	AP2-EREBP
CakTC41254	Ca(ICCV2/PI489777)SNP_03452	468	T	C	6	6	--	--
	Ca(ICCV2/PI489777)SNP_03453	1929	T	G	5	4	--	--
CakTC37856	Ca(ICCV2/PI489777)SNP_03454	840	G	A	15	16	--	--
CakTC10484	Ca(ICCV2/PI489777)SNP_03455	885	A	G	3	7	--	GRAS
	Ca(ICCV2/PI489777)SNP_03456	1192	C	T	4	4	--	GRAS
	Ca(ICCV2/PI489777)SNP_03457	1245	T	C	4	4	--	GRAS
CakTC40262	Ca(ICCV2/PI489777)SNP_03458	258	G	C	3	7	--	--
	Ca(ICCV2/PI489777)SNP_03459	507	A	G	7	9	--	--
CakTC13507	Ca(ICCV2/PI489777)SNP_03460	162	A	G	5	3	--	--
	Ca(ICCV2/PI489777)SNP_03461	211	G	T	7	4	--	--
	Ca(ICCV2/PI489777)SNP_03462	213	G	A	7	4	--	--
	Ca(ICCV2/PI489777)SNP_03463	308	T	C	8	4	--	--
CakTC24889	Ca(ICCV2/PI489777)SNP_03464	401	A	G	6	8	--	--
	Ca(ICCV2/PI489777)SNP_03465	1016	T	C	5	3	--	--
CakTC41248	Ca(ICCV2/PI489777)SNP_03466	275	G	A	11	3	--	--
	Ca(ICCV2/PI489777)SNP_03467	1031	G	A	10	3	--	--
	Ca(ICCV2/PI489777)SNP_03468	1133	A	G	9	3	--	--
	Ca(ICCV2/PI489777)SNP_03469	1219	T	G	11	3	--	--
	Ca(ICCV2/PI489777)SNP_03470	2021	C	G	10	9	--	--
	Ca(ICCV2/PI489777)SNP_03471	2099	C	T	7	6	--	--
	Ca(ICCV2/PI489777)SNP_03472	2171	G	A	7	7	--	--
CakTC39785	Ca(ICCV2/PI489777)SNP_03473	103	A	G	5	4	--	Alfin-like

	Ca(ICCV2/PI489777)SNP_03474	396	T	C	17	3	--	Alfin-like
CakTC42443	Ca(ICCV2/PI489777)SNP_03475	369	G	A	25	32	--	--
	Ca(ICCV2/PI489777)SNP_03476	1082	G	A	43	29	--	--
	Ca(ICCV2/PI489777)SNP_03477	1122	A	G	45	25	--	--
	Ca(ICCV2/PI489777)SNP_03478	1141	A	G	44	28	--	--
	Ca(ICCV2/PI489777)SNP_03479	1477	G	A	24	11	--	--
	Ca(ICCV2/PI489777)SNP_03480	1534	C	G	19	11	--	--
CakTC33835	Ca(ICCV2/PI489777)SNP_03481	1376	G	C	6	11	--	--
	Ca(ICCV2/PI489777)SNP_03482	1968	G	A	12	3	--	--
CakTC28806	Ca(ICCV2/PI489777)SNP_03483	1097	C	G	4	5	--	--
	Ca(ICCV2/PI489777)SNP_03484	1109	G	A	3	5	--	--
CakTC06734	Ca(ICCV2/PI489777)SNP_03485	178	A	G	17	5	--	--
	Ca(ICCV2/PI489777)SNP_03486	273	C	T	20	7	--	--
	Ca(ICCV2/PI489777)SNP_03487	358	A	G	8	6	--	--
CakTC40596	Ca(ICCV2/PI489777)SNP_03488	607	A	G	35	23	--	--
	Ca(ICCV2/PI489777)SNP_03489	671	G	C	29	18	--	--
CakTC29533	Ca(ICCV2/PI489777)SNP_03490	827	T	C	5	6	--	--
CakTC23160	Ca(ICCV2/PI489777)SNP_03491	314	T	C	6	3	Shoot	--
	Ca(ICCV2/PI489777)SNP_03492	358	T	C	6	3	Shoot	--
	Ca(ICCV2/PI489777)SNP_03493	389	G	A	6	3	Shoot	--
	Ca(ICCV2/PI489777)SNP_03494	392	A	G	6	3	Shoot	--
	Ca(ICCV2/PI489777)SNP_03495	431	T	A	5	3	Shoot	--
	Ca(ICCV2/PI489777)SNP_03496	452	C	A	5	3	Shoot	--
CakTC26871	Ca(ICCV2/PI489777)SNP_03497	144	T	G	30	4	--	--
CakTC41466	Ca(ICCV2/PI489777)SNP_03498	802	A	T	14	8	--	--
	Ca(ICCV2/PI489777)SNP_03499	1865	G	C	10	13	--	--
CakTC39295	Ca(ICCV2/PI489777)SNP_03500	324	A	T	24	27	--	--
	Ca(ICCV2/PI489777)SNP_03501	409	T	C	26	26	--	--
	Ca(ICCV2/PI489777)SNP_03502	763	T	C	37	11	--	--
CakTC36306	Ca(ICCV2/PI489777)SNP_03503	417	A	T	14	11	--	--
	Ca(ICCV2/PI489777)SNP_03504	444	T	A	14	11	--	--
	Ca(ICCV2/PI489777)SNP_03505	630	G	A	23	19	--	--
	Ca(ICCV2/PI489777)SNP_03506	697	G	A	20	17	--	--
	Ca(ICCV2/PI489777)SNP_03507	1338	C	T	17	10	--	--
	Ca(ICCV2/PI489777)SNP_03508	1413	C	T	14	14	--	--
	Ca(ICCV2/PI489777)SNP_03509	1530	A	G	15	17	--	--
	Ca(ICCV2/PI489777)SNP_03510	2079	C	T	15	10	--	--
	Ca(ICCV2/PI489777)SNP_03511	2214	C	T	14	5	--	--
	Ca(ICCV2/PI489777)SNP_03512	2462	A	C	6	4	--	--
	Ca(ICCV2/PI489777)SNP_03513	2472	A	C	6	4	--	--
CakTC28843	Ca(ICCV2/PI489777)SNP_03514	921	T	C	3	3	Flower	bud
CakTC25246	Ca(ICCV2/PI489777)SNP_03515	348	T	C	31	10	--	--
	Ca(ICCV2/PI489777)SNP_03516	789	T	C	32	10	--	--
	Ca(ICCV2/PI489777)SNP_03517	1071	C	G	39	6	--	--
	Ca(ICCV2/PI489777)SNP_03518	1281	A	G	31	5	--	--
	Ca(ICCV2/PI489777)SNP_03519	1294	A	G	33	6	--	--
	Ca(ICCV2/PI489777)SNP_03520	1300	A	C	33	5	--	--
	Ca(ICCV2/PI489777)SNP_03521	1515	T	C	35	7	--	--
	Ca(ICCV2/PI489777)SNP_03522	1539	T	G	33	7	--	--
	Ca(ICCV2/PI489777)SNP_03523	1722	G	A	52	4	--	--
	Ca(ICCV2/PI489777)SNP_03524	2073	A	G	26	3	--	--
CakTC22888	Ca(ICCV2/PI489777)SNP_03525	918	C	T	59	24	--	--
	Ca(ICCV2/PI489777)SNP_03526	1206	A	G	28	10	--	--
CakTC41682	Ca(ICCV2/PI489777)SNP_03527	434	A	G	20	17	--	--
	Ca(ICCV2/PI489777)SNP_03528	494	C	A	18	16	--	--
CakTC36202	Ca(ICCV2/PI489777)SNP_03529	334	T	C	34	25	--	--
	Ca(ICCV2/PI489777)SNP_03530	1006	A	G	34	26	--	--
	Ca(ICCV2/PI489777)SNP_03531	1243	A	T	46	28	--	--
	Ca(ICCV2/PI489777)SNP_03532	1558	T	C	46	36	--	--
	Ca(ICCV2/PI489777)SNP_03533	1874	G	A	50	30	--	--
	Ca(ICCV2/PI489777)SNP_03534	2327	T	A	40	43	--	--
CakTC23578	Ca(ICCV2/PI489777)SNP_03535	303	C	A	7	5	Flower	bud
CakTC23407	Ca(ICCV2/PI489777)SNP_03536	301	T	G	16	4	--	--
CakTC33561	Ca(ICCV2/PI489777)SNP_03537	203	G	A	3	3	--	--
	Ca(ICCV2/PI489777)SNP_03538	277	A	G	3	3	--	--
	Ca(ICCV2/PI489777)SNP_03539	278	C	T	3	3	--	--
	Ca(ICCV2/PI489777)SNP_03540	343	C	A	4	4	--	--
CakTC27161	Ca(ICCV2/PI489777)SNP_03541	160	G	A	4	5	--	--
	Ca(ICCV2/PI489777)SNP_03542	772	C	T	46	10	--	--
	Ca(ICCV2/PI489777)SNP_03543	1597	G	A	24	14	--	--
	Ca(ICCV2/PI489777)SNP_03544	1975	A	G	16	12	--	--
	Ca(ICCV2/PI489777)SNP_03545	2290	C	T	12	14	--	--
	Ca(ICCV2/PI489777)SNP_03546	2548	G	A	18	13	--	--
	Ca(ICCV2/PI489777)SNP_03547	2737	A	T	16	6	--	--

CakTC37416	Ca(ICCV2/PI489777)SNP_03548	208	G	A	29	12	--	--
	Ca(ICCV2/PI489777)SNP_03549	981	G	A	67	49	--	--
	Ca(ICCV2/PI489777)SNP_03550	993	G	T	63	46	--	--
	Ca(ICCV2/PI489777)SNP_03551	1038	G	A	56	40	--	--
	Ca(ICCV2/PI489777)SNP_03552	1041	T	A	58	40	--	--
CakTC33679	Ca(ICCV2/PI489777)SNP_03553	1122	T	C	9	11	--	--
	Ca(ICCV2/PI489777)SNP_03554	1837	T	C	4	14	--	--
CakTC24857	Ca(ICCV2/PI489777)SNP_03555	124	T	C	46	21	--	--
	Ca(ICCV2/PI489777)SNP_03556	195	G	A	53	27	--	--
	Ca(ICCV2/PI489777)SNP_03557	259	T	C	60	29	--	--
	Ca(ICCV2/PI489777)SNP_03558	423	C	G	57	22	--	--
	Ca(ICCV2/PI489777)SNP_03559	429	C	T	65	33	--	--
	Ca(ICCV2/PI489777)SNP_03560	582	A	G	33	15	--	--
CakTC26925	Ca(ICCV2/PI489777)SNP_03561	188	T	C	3	7	--	TPR
	Ca(ICCV2/PI489777)SNP_03562	607	A	G	4	3	--	TPR
CakTC23288	Ca(ICCV2/PI489777)SNP_03563	2374	G	A	68	6	--	--
	Ca(ICCV2/PI489777)SNP_03564	2410	A	G	67	10	--	--
	Ca(ICCV2/PI489777)SNP_03565	2437	C	A	64	13	--	--
	Ca(ICCV2/PI489777)SNP_03566	2482	A	C	56	19	--	--
CakTC24572	Ca(ICCV2/PI489777)SNP_03567	826	G	A	125	80	--	HB
	Ca(ICCV2/PI489777)SNP_03568	865	G	A	132	87	--	HB
	Ca(ICCV2/PI489777)SNP_03569	1363	C	G	46	69	--	HB
	Ca(ICCV2/PI489777)SNP_03570	1603	A	G	23	15	--	HB
CakTC41893	Ca(ICCV2/PI489777)SNP_03571	257	A	G	7	6	--	--
	Ca(ICCV2/PI489777)SNP_03572	261	C	T	8	5	--	--
	Ca(ICCV2/PI489777)SNP_03573	331	T	G	8	4	--	--
	Ca(ICCV2/PI489777)SNP_03574	421	G	T	7	6	--	--
	Ca(ICCV2/PI489777)SNP_03575	851	A	G	3	9	--	--
CakTC42013	Ca(ICCV2/PI489777)SNP_03576	418	T	C	7	4	Shoot	--
	Ca(ICCV2/PI489777)SNP_03577	614	A	G	7	3	Shoot	--
	Ca(ICCV2/PI489777)SNP_03578	617	G	A	8	3	Shoot	--
CakTC27382	Ca(ICCV2/PI489777)SNP_03579	21	C	G	8	4	--	--
	Ca(ICCV2/PI489777)SNP_03580	132	C	G	26	6	--	--
	Ca(ICCV2/PI489777)SNP_03581	1016	G	A	24	4	--	--
	Ca(ICCV2/PI489777)SNP_03582	1097	A	G	23	7	--	--
	Ca(ICCV2/PI489777)SNP_03583	1115	C	T	21	9	--	--
	Ca(ICCV2/PI489777)SNP_03584	1251	T	G	9	10	--	--
	Ca(ICCV2/PI489777)SNP_03585	1433	G	T	9	7	--	--
CakTC29234	Ca(ICCV2/PI489777)SNP_03586	998	C	A	8	5	--	--
	Ca(ICCV2/PI489777)SNP_03587	1145	T	C	5	3	--	--
CakTC38168	Ca(ICCV2/PI489777)SNP_03588	55	T	C	16	3	--	--
CakTC22542	Ca(ICCV2/PI489777)SNP_03589	66	T	A	10	5	Shoot	--
	Ca(ICCV2/PI489777)SNP_03590	145	C	G	15	7	Shoot	--
	Ca(ICCV2/PI489777)SNP_03591	169	T	C	14	8	Shoot	--
	Ca(ICCV2/PI489777)SNP_03592	1378	C	G	24	14	Shoot	--
	Ca(ICCV2/PI489777)SNP_03593	1433	C	A	24	15	Shoot	--
CakTC20166	Ca(ICCV2/PI489777)SNP_03594	461	A	G	14	10	--	--
	Ca(ICCV2/PI489777)SNP_03595	596	A	G	19	11	--	--
	Ca(ICCV2/PI489777)SNP_03596	1211	A	G	15	6	--	--
CakTC27903	Ca(ICCV2/PI489777)SNP_03597	406	A	G	6	3	Shoot	--
CakTC42913	Ca(ICCV2/PI489777)SNP_03598	231	A	G	18	16	--	--
	Ca(ICCV2/PI489777)SNP_03599	408	G	A	18	11	--	--
	Ca(ICCV2/PI489777)SNP_03600	491	C	A	19	13	--	--
	Ca(ICCV2/PI489777)SNP_03601	589	T	C	18	13	--	--
CakTC28375	Ca(ICCV2/PI489777)SNP_03602	117	T	C	6	10	--	--
	Ca(ICCV2/PI489777)SNP_03603	351	T	C	11	12	--	--
	Ca(ICCV2/PI489777)SNP_03604	374	T	G	9	12	--	--
	Ca(ICCV2/PI489777)SNP_03605	853	T	C	10	5	--	--
	Ca(ICCV2/PI489777)SNP_03606	876	A	C	12	5	--	--
	Ca(ICCV2/PI489777)SNP_03607	1039	G	A	20	9	--	--
	Ca(ICCV2/PI489777)SNP_03608	1192	C	T	30	9	--	--
	Ca(ICCV2/PI489777)SNP_03609	1273	T	C	31	6	--	--
	Ca(ICCV2/PI489777)SNP_03610	1375	T	C	26	7	--	--
	Ca(ICCV2/PI489777)SNP_03611	1432	C	A	29	6	--	--
	Ca(ICCV2/PI489777)SNP_03612	1886	A	G	29	14	--	--
	Ca(ICCV2/PI489777)SNP_03613	2001	T	C	15	9	--	--
CakTC30446	Ca(ICCV2/PI489777)SNP_03614	776	A	T	6	4	--	--
	Ca(ICCV2/PI489777)SNP_03615	793	C	T	5	4	--	--
	Ca(ICCV2/PI489777)SNP_03616	954	G	T	3	4	--	--
	Ca(ICCV2/PI489777)SNP_03617	1380	C	T	7	6	--	--
	Ca(ICCV2/PI489777)SNP_03618	2149	C	T	8	4	--	--
	Ca(ICCV2/PI489777)SNP_03619	2228	C	T	8	4	--	--
	Ca(ICCV2/PI489777)SNP_03620	2641	T	C	7	5	--	--
	Ca(ICCV2/PI489777)SNP_03621	4252	T	A	7	5	--	--

	Ca(ICCV2/PI489777)SNP_03622	4284	A	T	7	4	--	--
CakTC21290	Ca(ICCV2/PI489777)SNP_03623	430	T	G	6	3	--	SET
	Ca(ICCV2/PI489777)SNP_03624	548	C	T	3	3	--	SET
	Ca(ICCV2/PI489777)SNP_03625	553	T	C	3	3	--	SET
	Ca(ICCV2/PI489777)SNP_03626	649	G	C	8	5	--	SET
	Ca(ICCV2/PI489777)SNP_03627	769	C	T	10	6	--	SET
	Ca(ICCV2/PI489777)SNP_03628	859	C	T	9	3	--	SET
	Ca(ICCV2/PI489777)SNP_03629	883	T	A	8	3	--	SET
CakTC22684	Ca(ICCV2/PI489777)SNP_03630	241	A	G	3	13	--	--
CakTC40255	Ca(ICCV2/PI489777)SNP_03631	1129	A	G	3	3	Young_pod	--
CakTC22243	Ca(ICCV2/PI489777)SNP_03632	1022	G	A	417	155	--	--
CakTC43049	Ca(ICCV2/PI489777)SNP_03633	792	A	G	28	19	--	--
	Ca(ICCV2/PI489777)SNP_03634	879	A	G	18	12	--	--
CakTC09728	Ca(ICCV2/PI489777)SNP_03635	316	A	C	3	3	--	TPR
	Ca(ICCV2/PI489777)SNP_03636	322	T	C	3	3	--	TPR
	Ca(ICCV2/PI489777)SNP_03637	417	C	A	7	3	--	TPR
	Ca(ICCV2/PI489777)SNP_03638	435	G	A	7	3	--	TPR
	Ca(ICCV2/PI489777)SNP_03639	935	A	G	24	8	--	TPR
	Ca(ICCV2/PI489777)SNP_03640	1081	T	A	24	9	--	TPR
	Ca(ICCV2/PI489777)SNP_03641	1112	C	G	23	9	--	TPR
CakTC24528	Ca(ICCV2/PI489777)SNP_03642	657	C	T	3	3	--	--
	Ca(ICCV2/PI489777)SNP_03643	681	G	T	3	3	--	--
CakTC37518	Ca(ICCV2/PI489777)SNP_03644	466	A	G	10	4	--	--
CakTC42650	Ca(ICCV2/PI489777)SNP_03645	566	G	A	28	12	--	--
CakTC42659	Ca(ICCV2/PI489777)SNP_03646	580	T	C	9	9	--	--
	Ca(ICCV2/PI489777)SNP_03647	650	G	C	10	12	--	--
	Ca(ICCV2/PI489777)SNP_03648	1085	C	T	8	11	--	--
	Ca(ICCV2/PI489777)SNP_03649	1573	C	T	17	7	--	--
CakTC15590	Ca(ICCV2/PI489777)SNP_03650	470	T	C	3	4	--	--
CakTC27073	Ca(ICCV2/PI489777)SNP_03651	945	T	C	26	21	--	--
	Ca(ICCV2/PI489777)SNP_03652	959	C	A	26	25	--	--
	Ca(ICCV2/PI489777)SNP_03653	960	T	C	26	25	--	--
CakTC10956	Ca(ICCV2/PI489777)SNP_03654	722	T	C	3	3	--	--
CakTC42564	Ca(ICCV2/PI489777)SNP_03655	334	G	A	6	4	--	--
	Ca(ICCV2/PI489777)SNP_03656	848	A	C	7	6	--	--
CakTC31375	Ca(ICCV2/PI489777)SNP_03657	536	C	A	19	14	--	--
CakTC22429	Ca(ICCV2/PI489777)SNP_03658	1013	C	G	8	8	--	--
CakTC39996	Ca(ICCV2/PI489777)SNP_03659	541	C	T	114	72	--	--
	Ca(ICCV2/PI489777)SNP_03660	610	A	G	115	65	--	--
	Ca(ICCV2/PI489777)SNP_03661	688	A	G	107	56	--	--
	Ca(ICCV2/PI489777)SNP_03662	721	A	G	101	46	--	--
	Ca(ICCV2/PI489777)SNP_03663	1132	T	A	63	34	--	--
	Ca(ICCV2/PI489777)SNP_03664	1202	A	G	49	26	--	--
CakTC29050	Ca(ICCV2/PI489777)SNP_03665	102	G	A	4	3	--	--
CakTC36101	Ca(ICCV2/PI489777)SNP_03666	543	T	G	5	3	--	--
	Ca(ICCV2/PI489777)SNP_03667	927	C	G	4	5	--	--
	Ca(ICCV2/PI489777)SNP_03668	1009	C	G	4	3	--	--
	Ca(ICCV2/PI489777)SNP_03669	1077	T	C	4	3	--	--
	Ca(ICCV2/PI489777)SNP_03670	1421	G	A	7	8	--	--
	Ca(ICCV2/PI489777)SNP_03671	1517	C	T	8	8	--	--
	Ca(ICCV2/PI489777)SNP_03672	2349	T	G	7	4	--	--
	Ca(ICCV2/PI489777)SNP_03673	2428	G	A	4	4	--	--
CakTC27326	Ca(ICCV2/PI489777)SNP_03674	1524	C	T	3	3	--	--
CakTC29544	Ca(ICCV2/PI489777)SNP_03675	106	T	A	8	8	--	--
	Ca(ICCV2/PI489777)SNP_03676	280	C	T	8	12	--	--
	Ca(ICCV2/PI489777)SNP_03677	619	G	C	9	9	--	--
CakTC40680	Ca(ICCV2/PI489777)SNP_03678	511	T	C	3	3	--	--
CakTC28139	Ca(ICCV2/PI489777)SNP_03679	838	G	C	29	8	--	Alfin-like
	Ca(ICCV2/PI489777)SNP_03680	1151	A	T	18	10	--	Alfin-like
	Ca(ICCV2/PI489777)SNP_03681	2816	A	C	7	4	--	Alfin-like
	Ca(ICCV2/PI489777)SNP_03682	3809	C	T	8	4	--	Alfin-like
CakTC08143	Ca(ICCV2/PI489777)SNP_03683	691	G	A	4	7	--	--
CakTC09133	Ca(ICCV2/PI489777)SNP_03684	1423	T	C	3	3	--	--
	Ca(ICCV2/PI489777)SNP_03685	2096	G	C	4	3	--	--
	Ca(ICCV2/PI489777)SNP_03686	3843	C	G	4	4	--	--
CakTC12543	Ca(ICCV2/PI489777)SNP_03687	559	T	G	4	3	--	--
CakTC37809	Ca(ICCV2/PI489777)SNP_03688	422	T	C	4	4	--	--
	Ca(ICCV2/PI489777)SNP_03689	830	C	T	4	4	--	--
	Ca(ICCV2/PI489777)SNP_03690	839	A	G	4	4	--	--
CakTC26375	Ca(ICCV2/PI489777)SNP_03691	267	G	A	6	5	--	--
	Ca(ICCV2/PI489777)SNP_03692	465	A	G	5	6	--	--
	Ca(ICCV2/PI489777)SNP_03693	518	G	A	7	9	--	--
	Ca(ICCV2/PI489777)SNP_03694	626	G	A	5	6	--	--
	Ca(ICCV2/PI489777)SNP_03695	944	G	T	5	5	--	--

CakTC29960	Ca(ICCV2/PI489777)SNP_03696	253	G	A	51	17	--	--
	Ca(ICCV2/PI489777)SNP_03697	279	A	T	59	25	--	--
	Ca(ICCV2/PI489777)SNP_03698	281	C	G	62	24	--	--
	Ca(ICCV2/PI489777)SNP_03699	1371	C	T	64	27	--	--
	Ca(ICCV2/PI489777)SNP_03700	1401	G	T	59	26	--	--
	Ca(ICCV2/PI489777)SNP_03701	1488	A	G	51	25	--	--
	Ca(ICCV2/PI489777)SNP_03702	1839	G	C	40	7	--	--
CakTC26641	Ca(ICCV2/PI489777)SNP_03703	1096	T	C	9	3	--	--
	Ca(ICCV2/PI489777)SNP_03704	1339	G	A	11	3	--	--
CakTC28193	Ca(ICCV2/PI489777)SNP_03705	193	C	A	15	3	Young_pod	FHA
	Ca(ICCV2/PI489777)SNP_03706	497	A	G	7	5	Young_pod	FHA
	Ca(ICCV2/PI489777)SNP_03707	700	A	G	8	7	Young_pod	FHA
	Ca(ICCV2/PI489777)SNP_03708	1234	A	T	10	4	Young_pod	FHA
	Ca(ICCV2/PI489777)SNP_03709	1268	G	A	8	3	Young_pod	FHA
	Ca(ICCV2/PI489777)SNP_03710	1902	C	G	11	10	Young_pod	FHA
	Ca(ICCV2/PI489777)SNP_03711	1912	C	T	10	10	Young_pod	FHA
	Ca(ICCV2/PI489777)SNP_03712	1943	T	G	10	8	Young_pod	FHA
	Ca(ICCV2/PI489777)SNP_03713	1960	G	A	10	8	Young_pod	FHA
	Ca(ICCV2/PI489777)SNP_03714	1967	C	T	10	8	Young_pod	FHA
CakTC23760	Ca(ICCV2/PI489777)SNP_03715	1184	G	A	52	8	--	--
CakTC26197	Ca(ICCV2/PI489777)SNP_03716	1053	T	A	4	5	--	--
	Ca(ICCV2/PI489777)SNP_03717	1075	A	G	5	7	--	--
	Ca(ICCV2/PI489777)SNP_03718	1078	G	A	5	7	--	--
	Ca(ICCV2/PI489777)SNP_03719	1093	G	A	5	6	--	--
	Ca(ICCV2/PI489777)SNP_03720	1098	A	G	5	6	--	--
	Ca(ICCV2/PI489777)SNP_03721	1236	G	C	4	4	--	--
	Ca(ICCV2/PI489777)SNP_03722	1257	G	A	4	4	--	--
	Ca(ICCV2/PI489777)SNP_03723	1260	A	G	4	3	--	--
CakTC42156	Ca(ICCV2/PI489777)SNP_03724	435	A	G	26	12	--	--
	Ca(ICCV2/PI489777)SNP_03725	968	G	A	26	14	--	--
	Ca(ICCV2/PI489777)SNP_03726	1110	T	A	32	9	--	--
CakTC28423	Ca(ICCV2/PI489777)SNP_03727	3082	T	C	28	8	--	--
	Ca(ICCV2/PI489777)SNP_03728	3344	T	G	20	5	--	--
CakTC38739	Ca(ICCV2/PI489777)SNP_03729	521	G	A	5	3	--	--
	Ca(ICCV2/PI489777)SNP_03730	790	A	C	6	4	--	--
	Ca(ICCV2/PI489777)SNP_03731	1203	T	G	3	5	--	--
CakTC23435	Ca(ICCV2/PI489777)SNP_03732	240	T	C	12	3	--	--
	Ca(ICCV2/PI489777)SNP_03733	266	G	C	13	3	--	--
CakTC41791	Ca(ICCV2/PI489777)SNP_03734	89	T	C	8	5	--	--
	Ca(ICCV2/PI489777)SNP_03735	101	A	G	8	5	--	--
	Ca(ICCV2/PI489777)SNP_03736	114	C	T	9	5	--	--
	Ca(ICCV2/PI489777)SNP_03737	170	C	T	10	5	--	--
CakTC40061	Ca(ICCV2/PI489777)SNP_03738	295	A	T	9	4	--	--
	Ca(ICCV2/PI489777)SNP_03739	366	C	T	10	5	--	--
	Ca(ICCV2/PI489777)SNP_03740	394	A	G	10	6	--	--
	Ca(ICCV2/PI489777)SNP_03741	470	T	C	8	6	--	--
	Ca(ICCV2/PI489777)SNP_03742	740	T	C	7	18	--	--
	Ca(ICCV2/PI489777)SNP_03743	741	C	T	7	18	--	--
CakTC28358	Ca(ICCV2/PI489777)SNP_03744	1327	C	T	10	3	--	--
CakTC38268	Ca(ICCV2/PI489777)SNP_03745	385	A	T	19	16	--	--
	Ca(ICCV2/PI489777)SNP_03746	544	A	T	9	12	--	--
CakTC26055	Ca(ICCV2/PI489777)SNP_03747	238	T	C	4	6	--	--
	Ca(ICCV2/PI489777)SNP_03748	527	T	C	10	13	--	--
	Ca(ICCV2/PI489777)SNP_03749	605	C	T	10	17	--	--
CakTC27184	Ca(ICCV2/PI489777)SNP_03750	533	T	A	62	21	--	--
	Ca(ICCV2/PI489777)SNP_03751	634	G	A	60	11	--	--
CakTC39202	Ca(ICCV2/PI489777)SNP_03752	82	C	T	5	6	--	--
	Ca(ICCV2/PI489777)SNP_03753	189	A	G	11	7	--	--
	Ca(ICCV2/PI489777)SNP_03754	975	G	T	38	7	--	--
CakTC40025	Ca(ICCV2/PI489777)SNP_03755	516	A	G	81	20	--	--
	Ca(ICCV2/PI489777)SNP_03756	1213	C	A	20	7	--	--
CakTC28925	Ca(ICCV2/PI489777)SNP_03757	127	C	G	8	6	--	--
	Ca(ICCV2/PI489777)SNP_03758	140	T	G	10	7	--	--
	Ca(ICCV2/PI489777)SNP_03759	233	A	C	15	11	--	--
	Ca(ICCV2/PI489777)SNP_03760	405	C	A	11	12	--	--
	Ca(ICCV2/PI489777)SNP_03761	457	G	A	16	11	--	--
	Ca(ICCV2/PI489777)SNP_03762	489	T	C	14	9	--	--
	Ca(ICCV2/PI489777)SNP_03763	501	G	A	11	7	--	--
CakTC42503	Ca(ICCV2/PI489777)SNP_03764	1046	A	G	5	9	--	--
CakTC26013	Ca(ICCV2/PI489777)SNP_03765	212	A	G	10	5	--	--
	Ca(ICCV2/PI489777)SNP_03766	419	T	C	6	6	--	--
CakTC30483	Ca(ICCV2/PI489777)SNP_03767	1479	A	G	28	38	--	--
CakTC41273	Ca(ICCV2/PI489777)SNP_03768	126	G	C	10	17	--	--
	Ca(ICCV2/PI489777)SNP_03769	231	A	C	13	17	--	--

	Ca(ICCV2/PI489777)SNP_03770	260	A	G	15	18	--	--
CakTC08399	Ca(ICCV2/PI489777)SNP_03771	95	A	T	7	6	--	--
	Ca(ICCV2/PI489777)SNP_03772	565	C	G	13	31	--	--
	Ca(ICCV2/PI489777)SNP_03773	573	C	A	13	31	--	--
CakTC34788	Ca(ICCV2/PI489777)SNP_03774	376	T	G	4	4	--	--
	Ca(ICCV2/PI489777)SNP_03775	484	T	C	9	6	--	--
	Ca(ICCV2/PI489777)SNP_03776	2627	T	C	5	3	--	--
	Ca(ICCV2/PI489777)SNP_03777	2715	A	C	4	3	--	--
CakTC27932	Ca(ICCV2/PI489777)SNP_03778	103	A	T	3	5	--	--
	Ca(ICCV2/PI489777)SNP_03779	144	T	A	5	6	--	--
	Ca(ICCV2/PI489777)SNP_03780	502	G	A	8	3	--	--
	Ca(ICCV2/PI489777)SNP_03781	946	G	A	15	11	--	--
CakTC26273	Ca(ICCV2/PI489777)SNP_03782	371	C	T	15	12	--	--
	Ca(ICCV2/PI489777)SNP_03783	520	A	G	10	15	--	--
	Ca(ICCV2/PI489777)SNP_03784	599	T	C	13	13	--	--
	Ca(ICCV2/PI489777)SNP_03785	635	T	G	25	14	--	--
	Ca(ICCV2/PI489777)SNP_03786	653	T	C	19	14	--	--
	Ca(ICCV2/PI489777)SNP_03787	863	A	G	42	14	--	--
	Ca(ICCV2/PI489777)SNP_03788	1013	T	C	35	17	--	--
	Ca(ICCV2/PI489777)SNP_03789	1520	C	A	8	7	--	--
CakTC40685	Ca(ICCV2/PI489777)SNP_03790	430	C	T	42	28	--	Trihelix
	Ca(ICCV2/PI489777)SNP_03791	462	G	C	34	25	--	Trihelix
	Ca(ICCV2/PI489777)SNP_03792	897	G	A	53	16	--	Trihelix
	Ca(ICCV2/PI489777)SNP_03793	940	A	G	62	18	--	Trihelix
	Ca(ICCV2/PI489777)SNP_03794	1126	C	T	53	18	--	Trihelix
	Ca(ICCV2/PI489777)SNP_03795	1162	G	A	54	17	--	Trihelix
	Ca(ICCV2/PI489777)SNP_03796	1210	G	A	47	20	--	Trihelix
	Ca(ICCV2/PI489777)SNP_03797	1237	A	G	40	19	--	Trihelix
	Ca(ICCV2/PI489777)SNP_03798	1325	A	G	40	15	--	Trihelix
	Ca(ICCV2/PI489777)SNP_03799	1349	A	G	43	15	--	Trihelix
	Ca(ICCV2/PI489777)SNP_03800	1372	A	G	41	18	--	Trihelix
	Ca(ICCV2/PI489777)SNP_03801	1834	G	A	14	14	--	Trihelix
CakTC42510	Ca(ICCV2/PI489777)SNP_03802	384	T	C	9	9	--	--
	Ca(ICCV2/PI489777)SNP_03803	387	T	C	15	9	--	--
	Ca(ICCV2/PI489777)SNP_03804	556	T	A	8	8	--	--
	Ca(ICCV2/PI489777)SNP_03805	963	T	G	17	6	--	--
CakTC26146	Ca(ICCV2/PI489777)SNP_03806	174	G	C	10	11	--	--
CakTC42165	Ca(ICCV2/PI489777)SNP_03807	107	C	A	11	5	--	--
	Ca(ICCV2/PI489777)SNP_03808	230	T	G	7	7	--	--
	Ca(ICCV2/PI489777)SNP_03809	1169	T	C	18	8	--	--
	Ca(ICCV2/PI489777)SNP_03810	1525	G	T	6	8	--	--
CakTC28197	Ca(ICCV2/PI489777)SNP_03811	516	A	G	13	11	--	--
	Ca(ICCV2/PI489777)SNP_03812	540	C	A	15	11	--	--
	Ca(ICCV2/PI489777)SNP_03813	649	T	C	13	10	--	--
	Ca(ICCV2/PI489777)SNP_03814	748	T	C	18	13	--	--
	Ca(ICCV2/PI489777)SNP_03815	1039	T	G	9	11	--	--
	Ca(ICCV2/PI489777)SNP_03816	1104	T	C	7	10	--	--
	Ca(ICCV2/PI489777)SNP_03817	1833	T	A	13	10	--	--
	Ca(ICCV2/PI489777)SNP_03818	2322	C	T	5	6	--	--
	Ca(ICCV2/PI489777)SNP_03819	2353	T	C	3	6	--	--
CakTC36900	Ca(ICCV2/PI489777)SNP_03820	1170	T	C	12	6	--	--
	Ca(ICCV2/PI489777)SNP_03821	1889	C	T	12	14	--	--
CakTC22794	Ca(ICCV2/PI489777)SNP_03822	1101	G	C	16	6	--	--
	Ca(ICCV2/PI489777)SNP_03823	1157	C	T	15	6	--	--
	Ca(ICCV2/PI489777)SNP_03824	1173	G	C	15	7	--	--
	Ca(ICCV2/PI489777)SNP_03825	1192	C	T	14	5	--	--
	Ca(ICCV2/PI489777)SNP_03826	1236	C	G	12	5	--	--
	Ca(ICCV2/PI489777)SNP_03827	1242	C	T	12	5	--	--
	Ca(ICCV2/PI489777)SNP_03828	1349	C	A	13	5	--	--
	Ca(ICCV2/PI489777)SNP_03829	1417	T	A	13	4	--	--
	Ca(ICCV2/PI489777)SNP_03830	1443	T	G	10	4	--	--
	Ca(ICCV2/PI489777)SNP_03831	1523	G	A	10	4	--	--
	Ca(ICCV2/PI489777)SNP_03832	1635	C	G	10	3	--	--
	Ca(ICCV2/PI489777)SNP_03833	1640	T	C	10	3	--	--
CakTC37431	Ca(ICCV2/PI489777)SNP_03834	993	G	A	101	27	--	--
	Ca(ICCV2/PI489777)SNP_03835	1071	T	C	23	7	--	--
CakTC08369	Ca(ICCV2/PI489777)SNP_03836	1711	A	C	5	8	--	--
	Ca(ICCV2/PI489777)SNP_03837	1720	T	C	6	6	--	--
	Ca(ICCV2/PI489777)SNP_03838	1749	A	G	7	7	--	--
	Ca(ICCV2/PI489777)SNP_03839	2715	A	G	3	3	--	--
CakTC43240	Ca(ICCV2/PI489777)SNP_03840	1231	G	A	22	16	--	--
CakTC28058	Ca(ICCV2/PI489777)SNP_03841	199	T	A	16	4	--	--
	Ca(ICCV2/PI489777)SNP_03842	201	C	T	16	6	--	--
	Ca(ICCV2/PI489777)SNP_03843	511	A	G	16	9	--	--

CakTC42885	Ca(ICCV2/PI489777)SNP_03844	771	T	A	5	7	--	--
CakTC25324	Ca(ICCV2/PI489777)SNP_03845	738	C	T	7	3	--	--
	Ca(ICCV2/PI489777)SNP_03846	746	G	A	8	4	--	--
	Ca(ICCV2/PI489777)SNP_03847	908	T	C	8	3	--	--
	Ca(ICCV2/PI489777)SNP_03848	1233	T	C	3	3	--	--
CakTC30811	Ca(ICCV2/PI489777)SNP_03849	1781	G	T	4	3	--	--
CakTC24490	Ca(ICCV2/PI489777)SNP_03850	144	G	T	318	101	--	--
CakTC26997	Ca(ICCV2/PI489777)SNP_03851	560	C	G	17	16	--	--
	Ca(ICCV2/PI489777)SNP_03852	992	A	C	17	25	--	--
	Ca(ICCV2/PI489777)SNP_03853	1163	A	G	25	16	--	--
	Ca(ICCV2/PI489777)SNP_03854	1316	G	T	21	12	--	--
CakTC36103	Ca(ICCV2/PI489777)SNP_03855	2209	G	A	6	4	Root	--
CakTC27319	Ca(ICCV2/PI489777)SNP_03856	418	T	C	11	5	--	--
CakTC09452	Ca(ICCV2/PI489777)SNP_03857	1483	A	C	4	3	--	--
CakTC09874	Ca(ICCV2/PI489777)SNP_03858	592	C	T	4	3	--	--
	Ca(ICCV2/PI489777)SNP_03859	734	T	C	3	3	--	--
	Ca(ICCV2/PI489777)SNP_03860	1259	C	A	32	23	--	--
	Ca(ICCV2/PI489777)SNP_03861	1614	C	T	20	14	--	--
	Ca(ICCV2/PI489777)SNP_03862	1672	G	C	18	21	--	--
CakTC28027	Ca(ICCV2/PI489777)SNP_03863	1655	G	A	6	3	--	--
	Ca(ICCV2/PI489777)SNP_03864	1751	A	G	5	4	--	--
	Ca(ICCV2/PI489777)SNP_03865	2106	G	A	9	4	--	--
CakTC41605	Ca(ICCV2/PI489777)SNP_03866	305	T	C	17	8	--	--
	Ca(ICCV2/PI489777)SNP_03867	1833	A	G	16	14	--	--
CakTC37462	Ca(ICCV2/PI489777)SNP_03868	558	C	T	9	4	--	--
	Ca(ICCV2/PI489777)SNP_03869	559	C	A	9	4	--	--
	Ca(ICCV2/PI489777)SNP_03870	663	G	A	10	5	--	--
	Ca(ICCV2/PI489777)SNP_03871	1007	T	C	15	3	--	--
CakTC28336	Ca(ICCV2/PI489777)SNP_03872	1994	A	G	15	16	--	--
	Ca(ICCV2/PI489777)SNP_03873	2191	A	C	8	3	--	--
	Ca(ICCV2/PI489777)SNP_03874	2212	T	G	6	3	--	--
CakTC24250	Ca(ICCV2/PI489777)SNP_03875	648	C	T	8	8	--	--
	Ca(ICCV2/PI489777)SNP_03876	1683	C	T	10	5	--	--
	Ca(ICCV2/PI489777)SNP_03877	1788	C	G	7	6	--	--
	Ca(ICCV2/PI489777)SNP_03878	1884	T	C	9	11	--	--
	Ca(ICCV2/PI489777)SNP_03879	2325	G	A	8	7	--	--
	Ca(ICCV2/PI489777)SNP_03880	2477	A	T	19	9	--	--
	Ca(ICCV2/PI489777)SNP_03881	2760	G	A	18	8	--	--
	Ca(ICCV2/PI489777)SNP_03882	2881	G	A	16	6	--	--
CakTC23681	Ca(ICCV2/PI489777)SNP_03883	498	T	C	11	9	--	AP2-EREBP
	Ca(ICCV2/PI489777)SNP_03884	697	C	G	20	15	--	AP2-EREBP
	Ca(ICCV2/PI489777)SNP_03885	761	C	G	19	15	--	AP2-EREBP
CakTC30615	Ca(ICCV2/PI489777)SNP_03886	430	T	G	3	7	--	--
	Ca(ICCV2/PI489777)SNP_03887	537	T	C	3	7	--	--
CakTC33099	Ca(ICCV2/PI489777)SNP_03888	31	A	C	14	11	--	--
	Ca(ICCV2/PI489777)SNP_03889	159	T	C	14	17	--	--
	Ca(ICCV2/PI489777)SNP_03890	232	G	C	25	20	--	--
	Ca(ICCV2/PI489777)SNP_03891	295	T	C	25	19	--	--
	Ca(ICCV2/PI489777)SNP_03892	298	T	C	23	20	--	--
	Ca(ICCV2/PI489777)SNP_03893	434	C	T	15	8	--	--
	Ca(ICCV2/PI489777)SNP_03894	464	T	C	9	6	--	--
	Ca(ICCV2/PI489777)SNP_03895	500	G	A	10	8	--	--
CakTC42354	Ca(ICCV2/PI489777)SNP_03896	107	T	C	6	4	--	--
CakTC12911	Ca(ICCV2/PI489777)SNP_03897	631	G	C	3	4	--	--
CakTC39712	Ca(ICCV2/PI489777)SNP_03898	477	G	A	27	11	--	--
	Ca(ICCV2/PI489777)SNP_03899	997	G	A	24	15	--	--
	Ca(ICCV2/PI489777)SNP_03900	1137	T	C	20	8	--	--
	Ca(ICCV2/PI489777)SNP_03901	1242	T	G	4	3	--	--
CakTC43182	Ca(ICCV2/PI489777)SNP_03902	253	G	C	21	15	--	--
	Ca(ICCV2/PI489777)SNP_03903	496	A	G	7	7	--	--
	Ca(ICCV2/PI489777)SNP_03904	793	G	A	16	9	--	--
CakTC39221	Ca(ICCV2/PI489777)SNP_03905	397	T	C	8	8	--	--
	Ca(ICCV2/PI489777)SNP_03906	688	T	C	8	6	--	--
	Ca(ICCV2/PI489777)SNP_03907	955	T	C	8	3	--	--
CakTC30393	Ca(ICCV2/PI489777)SNP_03908	291	C	T	58	22	--	--
	Ca(ICCV2/PI489777)SNP_03909	1754	A	C	19	3	--	--
	Ca(ICCV2/PI489777)SNP_03910	1767	G	C	18	3	--	--
CakTC30910	Ca(ICCV2/PI489777)SNP_03911	480	C	G	13	21	--	--
	Ca(ICCV2/PI489777)SNP_03912	650	C	A	9	7	--	--
	Ca(ICCV2/PI489777)SNP_03913	812	G	A	6	4	--	--
CakTC36450	Ca(ICCV2/PI489777)SNP_03914	159	A	T	11	3	--	--
	Ca(ICCV2/PI489777)SNP_03915	183	A	T	15	3	--	--
	Ca(ICCV2/PI489777)SNP_03916	503	C	T	31	10	--	--
	Ca(ICCV2/PI489777)SNP_03917	1615	A	G	23	5	--	--

	Ca(ICCV2/PI489777)SNP_03918	2658	G	A	10	9	--	--
CakTC32488	Ca(ICCV2/PI489777)SNP_03919	382	G	A	12	5	--	TCP
	Ca(ICCV2/PI489777)SNP_03920	922	T	A	6	3	--	TCP
CakTC21235	Ca(ICCV2/PI489777)SNP_03921	931	T	C	3	15	--	--
CakTC37871	Ca(ICCV2/PI489777)SNP_03922	268	A	G	11	6	--	--
	Ca(ICCV2/PI489777)SNP_03923	292	T	C	11	6	--	--
	Ca(ICCV2/PI489777)SNP_03924	1018	A	G	3	3	--	--
CakTC33013	Ca(ICCV2/PI489777)SNP_03925	1967	A	G	41	22	--	--
	Ca(ICCV2/PI489777)SNP_03926	2144	G	A	45	20	--	--
	Ca(ICCV2/PI489777)SNP_03927	2294	A	T	24	20	--	--
	Ca(ICCV2/PI489777)SNP_03928	2597	G	A	24	10	--	--
CakTC09049	Ca(ICCV2/PI489777)SNP_03929	878	T	C	3	8	--	--
	Ca(ICCV2/PI489777)SNP_03930	887	C	T	3	8	--	--
	Ca(ICCV2/PI489777)SNP_03931	947	A	G	3	3	--	--
CakTC26282	Ca(ICCV2/PI489777)SNP_03932	246	C	G	43	24	--	--
	Ca(ICCV2/PI489777)SNP_03933	477	C	T	71	33	--	--
	Ca(ICCV2/PI489777)SNP_03934	1486	A	C	63	10	--	--
CakTC31534	Ca(ICCV2/PI489777)SNP_03935	500	A	G	10	6	--	--
	Ca(ICCV2/PI489777)SNP_03936	644	G	A	11	12	--	--
	Ca(ICCV2/PI489777)SNP_03937	1075	T	C	21	19	--	--
	Ca(ICCV2/PI489777)SNP_03938	1493	T	G	11	9	--	--
	Ca(ICCV2/PI489777)SNP_03939	1556	C	T	14	9	--	--
	Ca(ICCV2/PI489777)SNP_03940	1844	C	T	17	11	--	--
	Ca(ICCV2/PI489777)SNP_03941	1955	G	A	21	14	--	--
	Ca(ICCV2/PI489777)SNP_03942	1988	C	A	22	13	--	--
	Ca(ICCV2/PI489777)SNP_03943	2129	G	A	8	12	--	--
	Ca(ICCV2/PI489777)SNP_03944	2452	T	C	15	8	--	--
	Ca(ICCV2/PI489777)SNP_03945	2566	A	T	16	7	--	--
	Ca(ICCV2/PI489777)SNP_03946	2690	T	C	17	5	--	--
CakTC39935	Ca(ICCV2/PI489777)SNP_03947	536	A	G	6	14	--	C3H
CakTC37131	Ca(ICCV2/PI489777)SNP_03948	113	T	C	8	6	--	--
	Ca(ICCV2/PI489777)SNP_03949	593	G	A	8	7	--	--
CakTC41223	Ca(ICCV2/PI489777)SNP_03950	333	T	C	31	14	--	--
	Ca(ICCV2/PI489777)SNP_03951	336	C	T	32	14	--	--
	Ca(ICCV2/PI489777)SNP_03952	475	G	T	39	17	--	--
	Ca(ICCV2/PI489777)SNP_03953	489	G	A	39	17	--	--
	Ca(ICCV2/PI489777)SNP_03954	2420	A	G	19	14	--	--
CakTC03498	Ca(ICCV2/PI489777)SNP_03955	274	A	G	11	6	--	--
CakTC29333	Ca(ICCV2/PI489777)SNP_03956	137	A	G	14	4	--	--
	Ca(ICCV2/PI489777)SNP_03957	389	G	C	6	3	--	--
	Ca(ICCV2/PI489777)SNP_03958	405	T	C	7	3	--	--
CakTC35887	Ca(ICCV2/PI489777)SNP_03959	155	T	C	3	3	Shoot	--
CakTC24111	Ca(ICCV2/PI489777)SNP_03960	176	C	T	7	4	--	--
CakTC33931	Ca(ICCV2/PI489777)SNP_03961	247	G	C	26	19	--	--
CakTC39905	Ca(ICCV2/PI489777)SNP_03962	654	T	C	18	22	--	--
	Ca(ICCV2/PI489777)SNP_03963	735	G	A	17	25	--	--
	Ca(ICCV2/PI489777)SNP_03964	747	G	A	18	23	--	--
	Ca(ICCV2/PI489777)SNP_03965	783	G	A	15	21	--	--
	Ca(ICCV2/PI489777)SNP_03966	1308	G	A	29	21	--	--
CakTC26648	Ca(ICCV2/PI489777)SNP_03967	826	T	C	21	3	--	--
	Ca(ICCV2/PI489777)SNP_03968	1494	C	G	17	4	--	--
	Ca(ICCV2/PI489777)SNP_03969	1650	G	C	24	4	--	--
	Ca(ICCV2/PI489777)SNP_03970	2483	G	A	31	5	--	--
	Ca(ICCV2/PI489777)SNP_03971	2740	C	T	15	6	--	--
	Ca(ICCV2/PI489777)SNP_03972	2765	G	A	23	6	--	--
	Ca(ICCV2/PI489777)SNP_03973	2790	T	C	21	6	--	--
CakTC30245	Ca(ICCV2/PI489777)SNP_03974	336	G	A	213	59	--	--
CakTC31300	Ca(ICCV2/PI489777)SNP_03975	795	G	A	3	3	Young_pod	--
CakTC41835	Ca(ICCV2/PI489777)SNP_03976	1384	A	C	4	4	--	--
	Ca(ICCV2/PI489777)SNP_03977	1463	C	G	5	5	--	--
	Ca(ICCV2/PI489777)SNP_03978	1613	A	G	4	5	--	--
	Ca(ICCV2/PI489777)SNP_03979	2153	T	A	4	3	--	--
	Ca(ICCV2/PI489777)SNP_03980	2218	T	C	3	4	--	--
	Ca(ICCV2/PI489777)SNP_03981	2410	T	G	3	3	--	--
	Ca(ICCV2/PI489777)SNP_03982	2473	G	A	3	3	--	--
CakTC40180	Ca(ICCV2/PI489777)SNP_03983	458	G	A	4	3	--	--
CakTC30438	Ca(ICCV2/PI489777)SNP_03984	1390	G	A	4	5	--	--
CakTC08977	Ca(ICCV2/PI489777)SNP_03985	852	C	T	3	4	--	Jumonji
CakTC27754	Ca(ICCV2/PI489777)SNP_03986	1694	G	A	4	3	Shoot	--
	Ca(ICCV2/PI489777)SNP_03987	1779	G	A	3	4	Shoot	--
CakTC41135	Ca(ICCV2/PI489777)SNP_03988	1601	G	A	3	3	--	--
CakTC27778	Ca(ICCV2/PI489777)SNP_03989	939	A	T	3	4	Young_pod	--
CakTC35808	Ca(ICCV2/PI489777)SNP_03990	83	T	C	19	8	--	--
	Ca(ICCV2/PI489777)SNP_03991	271	C	T	42	35	--	--

	Ca(ICCV2/PI489777)SNP_03992	514	T	C	18	34	--	--
	Ca(ICCV2/PI489777)SNP_03993	556	G	T	33	38	--	--
	Ca(ICCV2/PI489777)SNP_03994	715	G	T	39	20	--	--
	Ca(ICCV2/PI489777)SNP_03995	1085	G	A	23	28	--	--
	Ca(ICCV2/PI489777)SNP_03996	1094	C	T	24	30	--	--
	Ca(ICCV2/PI489777)SNP_03997	1576	A	G	41	27	--	--
	Ca(ICCV2/PI489777)SNP_03998	1627	A	G	55	28	--	--
	Ca(ICCV2/PI489777)SNP_03999	1640	A	C	54	23	--	--
	Ca(ICCV2/PI489777)SNP_04000	1647	G	C	53	25	--	--
	Ca(ICCV2/PI489777)SNP_04001	1873	T	C	57	25	--	--
	Ca(ICCV2/PI489777)SNP_04002	2113	T	C	40	32	--	--
	Ca(ICCV2/PI489777)SNP_04003	2233	T	C	41	31	--	--
	Ca(ICCV2/PI489777)SNP_04004	2923	C	T	30	24	--	--
CakTC08312	Ca(ICCV2/PI489777)SNP_04005	1540	T	C	4	3	--	--
	Ca(ICCV2/PI489777)SNP_04006	1615	G	A	4	3	--	--
CakTC27032	Ca(ICCV2/PI489777)SNP_04007	1140	C	G	3	4	Flower	bud
CakTC35431	Ca(ICCV2/PI489777)SNP_04008	502	C	T	5	3	--	MYB-related
	Ca(ICCV2/PI489777)SNP_04009	1810	A	G	9	6	--	MYB-related
	Ca(ICCV2/PI489777)SNP_04010	2002	T	A	4	6	--	MYB-related
	Ca(ICCV2/PI489777)SNP_04011	2049	T	C	5	5	--	MYB-related
CakTC38779	Ca(ICCV2/PI489777)SNP_04012	189	A	C	8	5	--	--
	Ca(ICCV2/PI489777)SNP_04013	340	C	T	13	12	--	--
	Ca(ICCV2/PI489777)SNP_04014	427	A	G	13	13	--	--
	Ca(ICCV2/PI489777)SNP_04015	670	C	T	13	11	--	--
CakTC26077	Ca(ICCV2/PI489777)SNP_04016	1370	T	A	3	3	Flower	bud
	Ca(ICCV2/PI489777)SNP_04017	2159	T	G	5	4	Flower	bud
CakTC08278	Ca(ICCV2/PI489777)SNP_04018	215	C	T	4	4	--	--
CakTC35553	Ca(ICCV2/PI489777)SNP_04019	724	C	T	12	15	--	--
	Ca(ICCV2/PI489777)SNP_04020	1036	A	C	14	16	--	--
	Ca(ICCV2/PI489777)SNP_04021	1459	A	G	14	10	--	--
	Ca(ICCV2/PI489777)SNP_04022	1541	C	T	16	9	--	--
	Ca(ICCV2/PI489777)SNP_04023	1750	T	C	18	7	--	--
	Ca(ICCV2/PI489777)SNP_04024	1762	G	A	18	5	--	--
	Ca(ICCV2/PI489777)SNP_04025	2311	G	A	28	10	--	--
	Ca(ICCV2/PI489777)SNP_04026	2461	G	A	29	18	--	--
	Ca(ICCV2/PI489777)SNP_04027	2674	A	T	37	11	--	--
	Ca(ICCV2/PI489777)SNP_04028	4043	T	C	4	3	--	--
CakTC27272	Ca(ICCV2/PI489777)SNP_04029	176	G	A	24	8	--	--
	Ca(ICCV2/PI489777)SNP_04030	399	C	G	45	22	--	--
	Ca(ICCV2/PI489777)SNP_04031	525	A	T	43	25	--	--
	Ca(ICCV2/PI489777)SNP_04032	584	C	T	40	19	--	--
	Ca(ICCV2/PI489777)SNP_04033	615	C	T	27	17	--	--
	Ca(ICCV2/PI489777)SNP_04034	984	G	A	21	10	--	--
CakTC32718	Ca(ICCV2/PI489777)SNP_04035	325	C	A	44	19	--	--
	Ca(ICCV2/PI489777)SNP_04036	460	C	A	40	19	--	--
CakTC32473	Ca(ICCV2/PI489777)SNP_04037	330	T	G	5	15	--	--
CakTC11389	Ca(ICCV2/PI489777)SNP_04038	337	T	C	8	3	--	--
	Ca(ICCV2/PI489777)SNP_04039	1435	C	G	18	9	--	--
CakTC28635	Ca(ICCV2/PI489777)SNP_04040	1114	T	C	26	15	--	--
	Ca(ICCV2/PI489777)SNP_04041	1363	G	A	27	4	--	--
	Ca(ICCV2/PI489777)SNP_04042	1447	T	C	14	7	--	--
	Ca(ICCV2/PI489777)SNP_04043	1843	A	G	15	8	--	--
	Ca(ICCV2/PI489777)SNP_04044	2014	A	G	16	9	--	--
	Ca(ICCV2/PI489777)SNP_04045	2100	A	G	20	10	--	--
	Ca(ICCV2/PI489777)SNP_04046	2332	A	C	24	12	--	--
	Ca(ICCV2/PI489777)SNP_04047	2353	G	A	23	13	--	--
	Ca(ICCV2/PI489777)SNP_04048	2406	G	A	21	12	--	--
	Ca(ICCV2/PI489777)SNP_04049	2570	G	A	4	5	--	--
CakTC36725	Ca(ICCV2/PI489777)SNP_04050	251	T	C	3	4	--	--
	Ca(ICCV2/PI489777)SNP_04051	385	T	G	3	4	--	--
CakTC31469	Ca(ICCV2/PI489777)SNP_04052	405	A	G	5	3	--	--
	Ca(ICCV2/PI489777)SNP_04053	1334	T	G	5	8	--	--
	Ca(ICCV2/PI489777)SNP_04054	1586	C	T	3	4	--	--
CakTC38605	Ca(ICCV2/PI489777)SNP_04055	245	A	C	18	7	--	--
	Ca(ICCV2/PI489777)SNP_04056	854	G	A	8	8	--	--
	Ca(ICCV2/PI489777)SNP_04057	983	A	G	4	8	--	--
CakTC22556	Ca(ICCV2/PI489777)SNP_04058	117	G	T	5	4	Flower	bud
CakTC09855	Ca(ICCV2/PI489777)SNP_04059	248	T	C	6	8	--	--
	Ca(ICCV2/PI489777)SNP_04060	2179	A	G	5	9	--	--
	Ca(ICCV2/PI489777)SNP_04061	2935	A	G	6	3	--	--
CakTC27650	Ca(ICCV2/PI489777)SNP_04062	579	C	T	15	4	--	--
	Ca(ICCV2/PI489777)SNP_04063	694	T	A	8	3	--	--
	Ca(ICCV2/PI489777)SNP_04064	712	A	G	7	3	--	--
	Ca(ICCV2/PI489777)SNP_04065	875	A	G	4	3	--	--

	Ca(ICCV2/PI489777)SNP_04066	1733	G	A	11	20	--	--
	Ca(ICCV2/PI489777)SNP_04067	1940	T	C	10	8	--	--
CakTC02409	Ca(ICCV2/PI489777)SNP_04068	838	T	C	8	3	--	--
	Ca(ICCV2/PI489777)SNP_04069	998	G	A	6	5	--	--
	Ca(ICCV2/PI489777)SNP_04070	1076	G	A	5	4	--	--
CakTC29877	Ca(ICCV2/PI489777)SNP_04071	728	C	G	5	4	--	--
	Ca(ICCV2/PI489777)SNP_04072	827	G	A	5	6	--	--
	Ca(ICCV2/PI489777)SNP_04073	869	T	C	6	6	--	--
	Ca(ICCV2/PI489777)SNP_04074	878	C	T	7	6	--	--
	Ca(ICCV2/PI489777)SNP_04075	887	T	C	6	6	--	--
	Ca(ICCV2/PI489777)SNP_04076	1058	C	T	12	10	--	--
	Ca(ICCV2/PI489777)SNP_04077	1103	C	T	14	10	--	--
	Ca(ICCV2/PI489777)SNP_04078	1190	C	A	14	9	--	--
	Ca(ICCV2/PI489777)SNP_04079	2088	G	A	5	3	--	--
CakTC26750	Ca(ICCV2/PI489777)SNP_04080	212	G	A	4	3	--	--
	Ca(ICCV2/PI489777)SNP_04081	324	A	C	4	3	--	--
CakTC38978	Ca(ICCV2/PI489777)SNP_04082	803	G	A	25	11	--	--
	Ca(ICCV2/PI489777)SNP_04083	823	A	T	30	15	--	--
	Ca(ICCV2/PI489777)SNP_04084	854	T	C	35	15	--	--
	Ca(ICCV2/PI489777)SNP_04085	937	A	G	34	11	--	--
	Ca(ICCV2/PI489777)SNP_04086	985	G	T	28	10	--	--
	Ca(ICCV2/PI489777)SNP_04087	1092	C	T	10	4	--	--
CakTC33077	Ca(ICCV2/PI489777)SNP_04088	24	T	A	4	5	--	--
CakTC41863	Ca(ICCV2/PI489777)SNP_04089	208	T	C	4	4	--	--
	Ca(ICCV2/PI489777)SNP_04090	766	G	A	6	4	--	--
CakTC12897	Ca(ICCV2/PI489777)SNP_04091	70	C	T	15	22	--	--
	Ca(ICCV2/PI489777)SNP_04092	870	T	C	26	14	--	--
	Ca(ICCV2/PI489777)SNP_04093	958	C	G	10	5	--	--
CakTC27686	Ca(ICCV2/PI489777)SNP_04094	193	G	A	14	10	--	--
	Ca(ICCV2/PI489777)SNP_04095	269	G	A	17	16	--	--
	Ca(ICCV2/PI489777)SNP_04096	348	C	T	19	15	--	--
	Ca(ICCV2/PI489777)SNP_04097	418	G	A	20	15	--	--
CakTC37296	Ca(ICCV2/PI489777)SNP_04098	223	A	C	21	31	--	--
	Ca(ICCV2/PI489777)SNP_04099	230	C	T	21	29	--	--
CakTC40401	Ca(ICCV2/PI489777)SNP_04100	837	C	A	16	9	--	--
	Ca(ICCV2/PI489777)SNP_04101	959	A	T	16	8	--	--
	Ca(ICCV2/PI489777)SNP_04102	974	G	A	15	9	--	--
	Ca(ICCV2/PI489777)SNP_04103	1127	C	G	12	13	--	--
	Ca(ICCV2/PI489777)SNP_04104	1556	C	T	9	9	--	--
	Ca(ICCV2/PI489777)SNP_04105	1647	A	C	10	12	--	--
	Ca(ICCV2/PI489777)SNP_04106	1956	A	C	8	4	--	--
CakTC43026	Ca(ICCV2/PI489777)SNP_04107	1432	C	T	13	17	--	--
CakTC33827	Ca(ICCV2/PI489777)SNP_04108	56	G	C	3	3	--	--
	Ca(ICCV2/PI489777)SNP_04109	183	G	A	22	12	--	--
	Ca(ICCV2/PI489777)SNP_04110	205	G	C	24	13	--	--
	Ca(ICCV2/PI489777)SNP_04111	784	G	A	14	6	--	--
CakTC34070	Ca(ICCV2/PI489777)SNP_04112	292	C	T	61	40	--	--
	Ca(ICCV2/PI489777)SNP_04113	1753	C	A	70	59	--	--
CakTC37044	Ca(ICCV2/PI489777)SNP_04114	1586	T	C	18	16	--	--
CakTC41765	Ca(ICCV2/PI489777)SNP_04115	617	A	T	8	11	--	--
CakTC18105	Ca(ICCV2/PI489777)SNP_04116	244	G	A	15	4	--	--
	Ca(ICCV2/PI489777)SNP_04117	434	G	C	25	3	--	--
	Ca(ICCV2/PI489777)SNP_04118	909	G	C	22	12	--	--
	Ca(ICCV2/PI489777)SNP_04119	1085	T	G	9	4	--	--
CakTC24222	Ca(ICCV2/PI489777)SNP_04120	1327	C	T	106	20	--	--
	Ca(ICCV2/PI489777)SNP_04121	1495	A	G	136	11	--	--
CakTC26326	Ca(ICCV2/PI489777)SNP_04122	55	C	G	4	3	--	--
CakTC27308	Ca(ICCV2/PI489777)SNP_04123	199	G	C	11	11	--	--
CakTC27302	Ca(ICCV2/PI489777)SNP_04124	33	C	A	4	4	--	--
	Ca(ICCV2/PI489777)SNP_04125	79	A	C	5	7	--	--
	Ca(ICCV2/PI489777)SNP_04126	80	A	G	5	7	--	--
	Ca(ICCV2/PI489777)SNP_04127	98	C	T	5	8	--	--
CakTC36965	Ca(ICCV2/PI489777)SNP_04128	1468	T	G	11	3	--	--
CakTC33037	Ca(ICCV2/PI489777)SNP_04129	461	G	A	4	3	--	--
	Ca(ICCV2/PI489777)SNP_04130	839	G	A	3	5	--	--
CakTC10278	Ca(ICCV2/PI489777)SNP_04131	702	A	G	5	6	--	--
	Ca(ICCV2/PI489777)SNP_04132	1973	T	C	6	8	--	--
	Ca(ICCV2/PI489777)SNP_04133	1991	A	G	6	5	--	--
CakTC28546	Ca(ICCV2/PI489777)SNP_04134	170	A	G	13	8	--	Tify
	Ca(ICCV2/PI489777)SNP_04135	299	A	G	20	13	--	Tify
	Ca(ICCV2/PI489777)SNP_04136	453	A	G	28	9	--	Tify
	Ca(ICCV2/PI489777)SNP_04137	454	G	C	29	9	--	Tify
	Ca(ICCV2/PI489777)SNP_04138	585	G	T	30	11	--	Tify
	Ca(ICCV2/PI489777)SNP_04139	616	C	T	30	13	--	Tify

	Ca(ICCV2/PI489777)SNP_04140	1111	C	G	28	16	--	Tify
	Ca(ICCV2/PI489777)SNP_04141	1185	G	A	31	17	--	Tify
CakTC34574	Ca(ICCV2/PI489777)SNP_04142	1274	A	G	21	15	--	--
	Ca(ICCV2/PI489777)SNP_04143	1454	G	C	27	11	--	--
	Ca(ICCV2/PI489777)SNP_04144	1505	T	C	31	13	--	--
	Ca(ICCV2/PI489777)SNP_04145	1554	G	A	27	9	--	--
	Ca(ICCV2/PI489777)SNP_04146	1626	T	A	27	12	--	--
	Ca(ICCV2/PI489777)SNP_04147	1707	C	T	24	15	--	--
	Ca(ICCV2/PI489777)SNP_04148	1719	C	T	23	15	--	--
	Ca(ICCV2/PI489777)SNP_04149	1723	A	T	23	16	--	--
	Ca(ICCV2/PI489777)SNP_04150	1749	C	T	22	16	--	--
	Ca(ICCV2/PI489777)SNP_04151	2106	A	G	13	9	--	--
	Ca(ICCV2/PI489777)SNP_04152	2181	C	G	12	5	--	--
	Ca(ICCV2/PI489777)SNP_04153	2433	A	G	10	10	--	--
	Ca(ICCV2/PI489777)SNP_04154	2652	C	T	8	4	--	--
	Ca(ICCV2/PI489777)SNP_04155	2715	A	G	5	7	--	--
CakTC38483	Ca(ICCV2/PI489777)SNP_04156	790	A	G	6	4	--	--
CakTC37332	Ca(ICCV2/PI489777)SNP_04157	366	T	G	10	8	--	--
	Ca(ICCV2/PI489777)SNP_04158	1443	A	C	12	3	--	--
CakTC29619	Ca(ICCV2/PI489777)SNP_04159	14	G	T	6	3	--	--
	Ca(ICCV2/PI489777)SNP_04160	493	C	T	14	14	--	--
	Ca(ICCV2/PI489777)SNP_04161	703	A	G	22	7	--	--
	Ca(ICCV2/PI489777)SNP_04162	806	T	C	30	14	--	--
	Ca(ICCV2/PI489777)SNP_04163	849	A	G	28	13	--	--
	Ca(ICCV2/PI489777)SNP_04164	850	A	G	28	13	--	--
	Ca(ICCV2/PI489777)SNP_04165	875	A	G	24	14	--	--
	Ca(ICCV2/PI489777)SNP_04166	879	A	G	24	14	--	--
	Ca(ICCV2/PI489777)SNP_04167	954	G	T	15	3	--	--
CakTC37916	Ca(ICCV2/PI489777)SNP_04168	440	T	C	9	10	--	--
	Ca(ICCV2/PI489777)SNP_04169	476	G	T	8	7	--	--
CakTC25838	Ca(ICCV2/PI489777)SNP_04170	709	G	T	11	4	--	--
CakTC26293	Ca(ICCV2/PI489777)SNP_04171	884	A	G	48	21	--	--
CakTC25797	Ca(ICCV2/PI489777)SNP_04172	898	A	G	15	8	--	--
	Ca(ICCV2/PI489777)SNP_04173	1498	T	G	6	3	--	--
	Ca(ICCV2/PI489777)SNP_04174	2369	C	T	10	8	--	--
	Ca(ICCV2/PI489777)SNP_04175	2686	A	C	11	8	--	--
	Ca(ICCV2/PI489777)SNP_04176	3468	T	C	8	4	--	--
	Ca(ICCV2/PI489777)SNP_04177	5226	T	C	6	4	--	--
	Ca(ICCV2/PI489777)SNP_04178	6373	G	A	4	4	--	--
CakTC36446	Ca(ICCV2/PI489777)SNP_04179	174	C	G	3	3	--	--
	Ca(ICCV2/PI489777)SNP_04180	298	C	A	3	3	--	--
CakTC39159	Ca(ICCV2/PI489777)SNP_04181	1434	A	G	10	6	--	bZIP
CakTC39212	Ca(ICCV2/PI489777)SNP_04182	1061	A	T	20	6	--	--
CakTC40888	Ca(ICCV2/PI489777)SNP_04183	366	A	G	40	39	--	--
CakTC37396	Ca(ICCV2/PI489777)SNP_04184	87	G	C	4	3	--	TPR
	Ca(ICCV2/PI489777)SNP_04185	187	C	A	19	10	--	TPR
CakTC37504	Ca(ICCV2/PI489777)SNP_04186	303	G	A	10	3	--	--
CakTC29088	Ca(ICCV2/PI489777)SNP_04187	302	A	T	10	3	--	--
CakTC39271	Ca(ICCV2/PI489777)SNP_04188	372	A	T	10	22	--	--
	Ca(ICCV2/PI489777)SNP_04189	374	G	T	9	21	--	--
	Ca(ICCV2/PI489777)SNP_04190	536	A	G	9	14	--	--
	Ca(ICCV2/PI489777)SNP_04191	893	T	C	13	12	--	--
	Ca(ICCV2/PI489777)SNP_04192	1142	C	G	9	14	--	--
	Ca(ICCV2/PI489777)SNP_04193	1297	A	G	7	8	--	--
	Ca(ICCV2/PI489777)SNP_04194	1372	T	C	8	6	--	--
CakTC41929	Ca(ICCV2/PI489777)SNP_04195	164	G	A	9	8	--	--
CakTC42400	Ca(ICCV2/PI489777)SNP_04196	511	A	G	10	5	--	TUB
	Ca(ICCV2/PI489777)SNP_04197	1060	T	C	24	10	--	TUB
	Ca(ICCV2/PI489777)SNP_04198	1180	T	C	20	12	--	TUB
CakTC10225	Ca(ICCV2/PI489777)SNP_04199	519	C	G	9	11	--	--
	Ca(ICCV2/PI489777)SNP_04200	1011	G	T	9	5	--	--
CakTC33169	Ca(ICCV2/PI489777)SNP_04201	1253	A	G	12	5	--	--
	Ca(ICCV2/PI489777)SNP_04202	1867	T	G	4	3	--	--
CakTC23640	Ca(ICCV2/PI489777)SNP_04203	111	T	C	31	11	--	--
CakTC15471	Ca(ICCV2/PI489777)SNP_04204	354	T	A	8	11	Shoot	--
	Ca(ICCV2/PI489777)SNP_04205	487	A	G	5	4	Shoot	--
	Ca(ICCV2/PI489777)SNP_04206	524	A	G	5	6	Shoot	--
CakTC36055	Ca(ICCV2/PI489777)SNP_04207	2001	A	G	6	4	--	--
	Ca(ICCV2/PI489777)SNP_04208	2452	T	A	8	4	--	--
CakTC11612	Ca(ICCV2/PI489777)SNP_04209	73	C	T	32	13	--	--
	Ca(ICCV2/PI489777)SNP_04210	196	T	C	51	38	--	--
	Ca(ICCV2/PI489777)SNP_04211	885	G	C	27	37	--	--
CakTC32565	Ca(ICCV2/PI489777)SNP_04212	173	A	G	6	3	--	--
CakTC34287	Ca(ICCV2/PI489777)SNP_04213	681	A	C	3	4	--	C2C2-Dof

	Ca(ICCV2/PI489777)SNP_04214	694	A	T	3	3	--	C2C2-Dof
CakTC38119	Ca(ICCV2/PI489777)SNP_04215	507	T	C	19	8	--	--
	Ca(ICCV2/PI489777)SNP_04216	626	C	G	25	9	--	--
CakTC29191	Ca(ICCV2/PI489777)SNP_04217	247	A	C	23	9	--	--
	Ca(ICCV2/PI489777)SNP_04218	640	A	G	13	7	--	--
	Ca(ICCV2/PI489777)SNP_04219	913	A	G	17	8	--	--
	Ca(ICCV2/PI489777)SNP_04220	943	C	T	13	10	--	--
	Ca(ICCV2/PI489777)SNP_04221	1003	C	T	16	9	--	--
	Ca(ICCV2/PI489777)SNP_04222	1018	G	A	12	8	--	--
CakTC31872	Ca(ICCV2/PI489777)SNP_04223	1332	A	G	7	5	--	--
	Ca(ICCV2/PI489777)SNP_04224	3287	A	T	3	14	--	--
	Ca(ICCV2/PI489777)SNP_04225	3347	G	A	4	9	--	--
CakTC42407	Ca(ICCV2/PI489777)SNP_04226	83	A	T	28	29	--	--
	Ca(ICCV2/PI489777)SNP_04227	165	C	T	30	29	--	--
	Ca(ICCV2/PI489777)SNP_04228	222	T	C	37	32	--	--
	Ca(ICCV2/PI489777)SNP_04229	234	A	G	37	39	--	--
	Ca(ICCV2/PI489777)SNP_04230	307	A	G	38	37	--	--
	Ca(ICCV2/PI489777)SNP_04231	315	C	T	38	36	--	--
	Ca(ICCV2/PI489777)SNP_04232	1146	C	T	26	28	--	--
CakTC33327	Ca(ICCV2/PI489777)SNP_04233	1304	G	T	3	4	--	--
CakTC24913	Ca(ICCV2/PI489777)SNP_04234	993	A	T	7	4	--	--
	Ca(ICCV2/PI489777)SNP_04235	1117	A	T	4	5	--	--
	Ca(ICCV2/PI489777)SNP_04236	1291	C	T	15	3	--	--
	Ca(ICCV2/PI489777)SNP_04237	1576	A	G	3	4	--	--
	Ca(ICCV2/PI489777)SNP_04238	1583	T	C	3	3	--	--
CakTC30938	Ca(ICCV2/PI489777)SNP_04239	138	A	G	49	26	--	--
	Ca(ICCV2/PI489777)SNP_04240	291	T	C	51	39	--	--
	Ca(ICCV2/PI489777)SNP_04241	805	G	A	35	19	--	--
CakTC35199	Ca(ICCV2/PI489777)SNP_04242	1494	C	T	38	37	--	--
CakTC42473	Ca(ICCV2/PI489777)SNP_04243	140	C	T	7	4	--	--
	Ca(ICCV2/PI489777)SNP_04244	142	T	C	7	4	--	--
	Ca(ICCV2/PI489777)SNP_04245	397	A	G	8	4	--	--
CakTC41270	Ca(ICCV2/PI489777)SNP_04246	417	T	C	7	3	--	--
	Ca(ICCV2/PI489777)SNP_04247	588	T	C	4	4	--	--
	Ca(ICCV2/PI489777)SNP_04248	636	T	C	5	4	--	--
	Ca(ICCV2/PI489777)SNP_04249	1188	G	C	6	3	--	--
CakTC42430	Ca(ICCV2/PI489777)SNP_04250	377	C	T	16	12	--	--
	Ca(ICCV2/PI489777)SNP_04251	668	C	A	23	14	--	--
	Ca(ICCV2/PI489777)SNP_04252	734	A	G	17	10	--	--
	Ca(ICCV2/PI489777)SNP_04253	1514	A	G	15	12	--	--
CakTC10422	Ca(ICCV2/PI489777)SNP_04254	144	T	C	22	29	--	--
CakTC36570	Ca(ICCV2/PI489777)SNP_04255	374	A	G	23	22	--	CCHC
	Ca(ICCV2/PI489777)SNP_04256	420	T	A	25	24	--	CCHC
	Ca(ICCV2/PI489777)SNP_04257	473	G	T	26	24	--	CCHC
	Ca(ICCV2/PI489777)SNP_04258	1286	T	G	73	22	--	CCHC
	Ca(ICCV2/PI489777)SNP_04259	2588	C	T	14	8	--	CCHC
	Ca(ICCV2/PI489777)SNP_04260	2646	G	A	10	6	--	CCHC
CakTC24908	Ca(ICCV2/PI489777)SNP_04261	426	A	G	5	3	--	--
	Ca(ICCV2/PI489777)SNP_04262	529	A	G	6	5	--	--
	Ca(ICCV2/PI489777)SNP_04263	570	C	T	9	5	--	--
	Ca(ICCV2/PI489777)SNP_04264	585	A	C	10	5	--	--
	Ca(ICCV2/PI489777)SNP_04265	592	T	C	9	5	--	--
	Ca(ICCV2/PI489777)SNP_04266	599	G	A	10	5	--	--
	Ca(ICCV2/PI489777)SNP_04267	609	A	G	10	5	--	--
CakTC31476	Ca(ICCV2/PI489777)SNP_04268	485	A	G	4	3	--	--
	Ca(ICCV2/PI489777)SNP_04269	924	G	A	6	3	--	--
CakTC32763	Ca(ICCV2/PI489777)SNP_04270	97	C	T	36	17	--	--
	Ca(ICCV2/PI489777)SNP_04271	260	A	T	31	15	--	--
	Ca(ICCV2/PI489777)SNP_04272	287	C	G	25	14	--	--
	Ca(ICCV2/PI489777)SNP_04273	378	T	G	21	14	--	--
CakTC37246	Ca(ICCV2/PI489777)SNP_04274	131	C	T	111	106	--	--
	Ca(ICCV2/PI489777)SNP_04275	464	T	C	111	117	--	--
	Ca(ICCV2/PI489777)SNP_04276	521	C	T	107	106	--	--
	Ca(ICCV2/PI489777)SNP_04277	584	T	G	116	101	--	--
CakTC05800	Ca(ICCV2/PI489777)SNP_04278	2310	G	T	18	21	--	--
	Ca(ICCV2/PI489777)SNP_04279	2353	G	A	13	20	--	--
CakTC32123	Ca(ICCV2/PI489777)SNP_04280	613	A	T	3	14	Root	bHLH
	Ca(ICCV2/PI489777)SNP_04281	662	C	T	3	11	Root	bHLH
	Ca(ICCV2/PI489777)SNP_04282	664	A	C	3	12	Root	bHLH
CakTC38167	Ca(ICCV2/PI489777)SNP_04283	335	C	A	9	5	--	--
	Ca(ICCV2/PI489777)SNP_04284	551	A	C	8	3	--	--
	Ca(ICCV2/PI489777)SNP_04285	635	G	T	6	4	--	--
	Ca(ICCV2/PI489777)SNP_04286	801	T	C	10	5	--	--
	Ca(ICCV2/PI489777)SNP_04287	1194	G	A	8	14	--	--

	Ca(ICCV2/PI489777)SNP_04288	1229	A	C	10	12	--	--
CakTC25343	Ca(ICCV2/PI489777)SNP_04289	586	C	T	26	6	--	--
	Ca(ICCV2/PI489777)SNP_04290	1036	G	A	36	12	--	--
	Ca(ICCV2/PI489777)SNP_04291	1211	G	A	30	14	--	--
CakTC11427	Ca(ICCV2/PI489777)SNP_04292	961	A	T	26	9	--	--
	Ca(ICCV2/PI489777)SNP_04293	1018	G	A	25	8	--	--
CakTC24464	Ca(ICCV2/PI489777)SNP_04294	700	T	A	35	10	--	--
CakTC38784	Ca(ICCV2/PI489777)SNP_04295	127	C	T	9	3	--	C2C2-Dof
	Ca(ICCV2/PI489777)SNP_04296	220	G	A	11	4	--	C2C2-Dof
	Ca(ICCV2/PI489777)SNP_04297	562	T	C	4	4	--	C2C2-Dof
	Ca(ICCV2/PI489777)SNP_04298	929	A	C	18	5	--	C2C2-Dof
CakTC23299	Ca(ICCV2/PI489777)SNP_04299	340	G	A	9	10	--	--
	Ca(ICCV2/PI489777)SNP_04300	732	A	T	8	7	--	--
CakTC39264	Ca(ICCV2/PI489777)SNP_04301	880	T	G	8	4	--	--
CakTC25602	Ca(ICCV2/PI489777)SNP_04302	278	T	C	23	24	--	--
CakTC43118	Ca(ICCV2/PI489777)SNP_04303	143	A	T	5	4	--	--
	Ca(ICCV2/PI489777)SNP_04304	204	T	G	11	7	--	--
	Ca(ICCV2/PI489777)SNP_04305	622	A	G	33	21	--	--
	Ca(ICCV2/PI489777)SNP_04306	869	C	A	23	6	--	--
	Ca(ICCV2/PI489777)SNP_04307	889	G	A	18	6	--	--
	Ca(ICCV2/PI489777)SNP_04308	901	T	A	20	5	--	--
CakTC30013	Ca(ICCV2/PI489777)SNP_04309	61	G	C	4	5	--	--
	Ca(ICCV2/PI489777)SNP_04310	109	A	T	4	5	--	--
	Ca(ICCV2/PI489777)SNP_04311	422	A	G	8	4	--	--
	Ca(ICCV2/PI489777)SNP_04312	1112	C	T	6	7	--	--
	Ca(ICCV2/PI489777)SNP_04313	1490	T	C	9	4	--	--
	Ca(ICCV2/PI489777)SNP_04314	2235	G	T	7	3	--	--
	Ca(ICCV2/PI489777)SNP_04315	2535	T	A	4	5	--	--
CakTC33983	Ca(ICCV2/PI489777)SNP_04316	579	A	G	5	7	--	--
CakTC34202	Ca(ICCV2/PI489777)SNP_04317	903	G	A	11	9	Young_pod	--
	Ca(ICCV2/PI489777)SNP_04318	1401	G	T	34	10	Young_pod	--
	Ca(ICCV2/PI489777)SNP_04319	1772	T	C	18	6	Young_pod	--
	Ca(ICCV2/PI489777)SNP_04320	2168	A	G	4	3	Young_pod	--
CakTC37700	Ca(ICCV2/PI489777)SNP_04321	48	C	T	7	4	--	--
	Ca(ICCV2/PI489777)SNP_04322	841	T	A	8	6	--	--
CakTC41317	Ca(ICCV2/PI489777)SNP_04323	606	C	A	56	38	--	--
CakTC27806	Ca(ICCV2/PI489777)SNP_04324	472	G	A	12	4	--	--
	Ca(ICCV2/PI489777)SNP_04325	530	G	A	8	8	--	--
	Ca(ICCV2/PI489777)SNP_04326	1180	G	A	20	14	--	--
CakTC37810	Ca(ICCV2/PI489777)SNP_04327	1014	G	T	49	22	--	--
	Ca(ICCV2/PI489777)SNP_04328	1015	T	A	49	19	--	--
CakTC24965	Ca(ICCV2/PI489777)SNP_04329	319	A	G	65	38	--	--
	Ca(ICCV2/PI489777)SNP_04330	593	G	A	90	30	--	--
	Ca(ICCV2/PI489777)SNP_04331	979	C	T	104	38	--	--
	Ca(ICCV2/PI489777)SNP_04332	1345	C	G	17	8	--	--
	Ca(ICCV2/PI489777)SNP_04333	1907	C	T	25	10	--	--
	Ca(ICCV2/PI489777)SNP_04334	1925	A	T	24	11	--	--
	Ca(ICCV2/PI489777)SNP_04335	2433	T	A	35	16	--	--
	Ca(ICCV2/PI489777)SNP_04336	2436	A	G	37	17	--	--
	Ca(ICCV2/PI489777)SNP_04337	3025	G	A	44	18	--	--
	Ca(ICCV2/PI489777)SNP_04338	3050	G	A	43	16	--	--
	Ca(ICCV2/PI489777)SNP_04339	3114	G	T	38	13	--	--
	Ca(ICCV2/PI489777)SNP_04340	3185	A	C	28	12	--	--
	Ca(ICCV2/PI489777)SNP_04341	3926	A	G	18	6	--	--
	Ca(ICCV2/PI489777)SNP_04342	4368	G	T	38	23	--	--
CakTC33530	Ca(ICCV2/PI489777)SNP_04343	1276	G	A	17	10	--	--
	Ca(ICCV2/PI489777)SNP_04344	1334	T	A	14	11	--	--
CakTC39620	Ca(ICCV2/PI489777)SNP_04345	390	T	C	12	13	--	--
	Ca(ICCV2/PI489777)SNP_04346	730	C	T	14	34	--	--
	Ca(ICCV2/PI489777)SNP_04347	840	C	T	12	39	--	--
	Ca(ICCV2/PI489777)SNP_04348	853	G	A	12	34	--	--
CakTC10204	Ca(ICCV2/PI489777)SNP_04349	177	G	A	3	4	--	--
CakTC28064	Ca(ICCV2/PI489777)SNP_04350	384	T	C	120	74	--	--
	Ca(ICCV2/PI489777)SNP_04351	768	C	G	24	7	--	--
CakTC10762	Ca(ICCV2/PI489777)SNP_04352	613	A	G	25	6	--	BES1
	Ca(ICCV2/PI489777)SNP_04353	1274	G	C	4	4	--	BES1
CakTC35267	Ca(ICCV2/PI489777)SNP_04354	110	A	G	8	9	--	--
	Ca(ICCV2/PI489777)SNP_04355	141	A	G	8	7	--	--
CakTC22475	Ca(ICCV2/PI489777)SNP_04356	357	C	T	3	3	--	--
CakTC10943	Ca(ICCV2/PI489777)SNP_04357	1145	A	G	6	5	--	--
	Ca(ICCV2/PI489777)SNP_04358	1298	A	G	3	4	--	--
CakTC10353	Ca(ICCV2/PI489777)SNP_04359	651	A	G	17	4	--	HB
	Ca(ICCV2/PI489777)SNP_04360	885	G	A	19	13	--	HB
	Ca(ICCV2/PI489777)SNP_04361	924	T	C	19	18	--	HB

CakTC34329	Ca(ICCV2/PI489777)SNP_04362	76	A	C	3	10	--	--
CakTC36813	Ca(ICCV2/PI489777)SNP_04363	261	C	G	5	6	--	--
	Ca(ICCV2/PI489777)SNP_04364	492	G	C	16	4	--	--
	Ca(ICCV2/PI489777)SNP_04365	1534	T	G	15	16	--	--
	Ca(ICCV2/PI489777)SNP_04366	1884	T	C	21	19	--	--
	Ca(ICCV2/PI489777)SNP_04367	2073	G	C	17	8	--	--
	Ca(ICCV2/PI489777)SNP_04368	2483	T	C	6	4	--	--
CakTC23986	Ca(ICCV2/PI489777)SNP_04369	294	A	C	14	21	--	--
	Ca(ICCV2/PI489777)SNP_04370	387	G	T	23	22	--	--
	Ca(ICCV2/PI489777)SNP_04371	553	G	T	44	18	--	--
	Ca(ICCV2/PI489777)SNP_04372	591	A	G	45	18	--	--
	Ca(ICCV2/PI489777)SNP_04373	612	A	T	41	13	--	--
	Ca(ICCV2/PI489777)SNP_04374	832	A	C	16	5	--	--
	Ca(ICCV2/PI489777)SNP_04375	856	G	A	17	7	--	--
	Ca(ICCV2/PI489777)SNP_04376	973	A	G	19	8	--	--
	Ca(ICCV2/PI489777)SNP_04377	985	A	G	21	8	--	--
	Ca(ICCV2/PI489777)SNP_04378	1114	C	G	17	8	--	--
	Ca(ICCV2/PI489777)SNP_04379	1126	G	A	16	6	--	--
	Ca(ICCV2/PI489777)SNP_04380	1267	T	C	20	3	--	--
	Ca(ICCV2/PI489777)SNP_04381	1506	A	G	14	3	--	--
	Ca(ICCV2/PI489777)SNP_04382	1671	G	A	14	4	--	--
CakTC43063	Ca(ICCV2/PI489777)SNP_04383	693	T	A	23	10	--	--
	Ca(ICCV2/PI489777)SNP_04384	1228	A	C	38	45	--	--
CakTC25692	Ca(ICCV2/PI489777)SNP_04385	1751	T	C	3	3	--	--
CakTC25909	Ca(ICCV2/PI489777)SNP_04386	702	T	C	10	3	--	--
CakTC09540	Ca(ICCV2/PI489777)SNP_04387	775	A	T	3	5	--	--
CakTC25183	Ca(ICCV2/PI489777)SNP_04388	424	C	T	33	37	--	--
	Ca(ICCV2/PI489777)SNP_04389	718	C	T	46	26	--	--
	Ca(ICCV2/PI489777)SNP_04390	994	C	T	49	34	--	--
CakTC26351	Ca(ICCV2/PI489777)SNP_04391	382	T	C	12	10	--	--
	Ca(ICCV2/PI489777)SNP_04392	502	C	T	14	15	--	--
	Ca(ICCV2/PI489777)SNP_04393	565	G	C	22	15	--	--
	Ca(ICCV2/PI489777)SNP_04394	716	G	T	25	10	--	--
	Ca(ICCV2/PI489777)SNP_04395	1128	G	A	23	5	--	--
	Ca(ICCV2/PI489777)SNP_04396	1531	C	T	10	19	--	--
	Ca(ICCV2/PI489777)SNP_04397	1586	A	G	7	13	--	--
	Ca(ICCV2/PI489777)SNP_04398	1675	A	G	8	11	--	--
	Ca(ICCV2/PI489777)SNP_04399	1678	C	T	8	10	--	--
	Ca(ICCV2/PI489777)SNP_04400	1884	C	T	13	9	--	--
	Ca(ICCV2/PI489777)SNP_04401	2324	T	C	10	5	--	--
	Ca(ICCV2/PI489777)SNP_04402	2330	A	G	10	5	--	--
	Ca(ICCV2/PI489777)SNP_04403	2494	T	C	8	3	--	--
	Ca(ICCV2/PI489777)SNP_04404	2648	G	A	18	4	--	--
	Ca(ICCV2/PI489777)SNP_04405	2666	C	A	18	4	--	--
	Ca(ICCV2/PI489777)SNP_04406	3026	A	G	19	8	--	--
	Ca(ICCV2/PI489777)SNP_04407	3351	C	G	14	9	--	--
	Ca(ICCV2/PI489777)SNP_04408	4079	T	C	6	8	--	--
	Ca(ICCV2/PI489777)SNP_04409	4102	A	G	7	10	--	--
	Ca(ICCV2/PI489777)SNP_04410	4289	T	A	13	10	--	--
	Ca(ICCV2/PI489777)SNP_04411	4404	C	T	14	9	--	--
	Ca(ICCV2/PI489777)SNP_04412	4458	G	T	16	6	--	--
	Ca(ICCV2/PI489777)SNP_04413	4474	C	G	16	5	--	--
	Ca(ICCV2/PI489777)SNP_04414	4717	C	G	7	4	--	--
	Ca(ICCV2/PI489777)SNP_04415	4760	C	T	8	4	--	--
	Ca(ICCV2/PI489777)SNP_04416	4832	T	C	9	8	--	--
	Ca(ICCV2/PI489777)SNP_04417	5105	A	G	11	5	--	--
	Ca(ICCV2/PI489777)SNP_04418	5375	G	A	17	7	--	--
	Ca(ICCV2/PI489777)SNP_04419	5403	A	G	17	4	--	--
	Ca(ICCV2/PI489777)SNP_04420	5460	T	G	8	4	--	--
CakTC43008	Ca(ICCV2/PI489777)SNP_04421	536	C	T	29	36	--	--
	Ca(ICCV2/PI489777)SNP_04422	542	G	T	29	37	--	--
	Ca(ICCV2/PI489777)SNP_04423	758	T	C	32	26	--	--
	Ca(ICCV2/PI489777)SNP_04424	906	A	G	45	25	--	--
CakTC11743	Ca(ICCV2/PI489777)SNP_04425	823	A	C	3	9	--	--
CakTC26596	Ca(ICCV2/PI489777)SNP_04426	739	A	T	12	4	--	--
CakTC23839	Ca(ICCV2/PI489777)SNP_04427	1063	A	G	77	17	--	--
	Ca(ICCV2/PI489777)SNP_04428	1209	T	A	73	13	--	--
CakTC27159	Ca(ICCV2/PI489777)SNP_04429	335	T	C	21	7	--	--
	Ca(ICCV2/PI489777)SNP_04430	347	A	C	23	8	--	--
	Ca(ICCV2/PI489777)SNP_04431	350	A	G	23	8	--	--
	Ca(ICCV2/PI489777)SNP_04432	359	T	C	23	8	--	--
	Ca(ICCV2/PI489777)SNP_04433	362	C	T	23	8	--	--
	Ca(ICCV2/PI489777)SNP_04434	440	T	C	25	9	--	--
	Ca(ICCV2/PI489777)SNP_04435	470	G	A	27	10	--	--

	Ca(ICCV2/PI489777)SNP_04436	656	A	G	23	12	--	--
	Ca(ICCV2/PI489777)SNP_04437	882	C	T	8	11	--	--
	Ca(ICCV2/PI489777)SNP_04438	1576	G	C	25	15	--	--
	Ca(ICCV2/PI489777)SNP_04439	1578	T	C	22	15	--	--
	Ca(ICCV2/PI489777)SNP_04440	1759	G	T	15	10	--	--
CakTC09147	Ca(ICCV2/PI489777)SNP_04441	1723	C	T	4	6	--	--
	Ca(ICCV2/PI489777)SNP_04442	1782	A	G	4	9	--	--
CakTC39497	Ca(ICCV2/PI489777)SNP_04443	292	C	G	5	6	Shoot	--
	Ca(ICCV2/PI489777)SNP_04444	413	A	G	7	8	Shoot	--
CakTC22902	Ca(ICCV2/PI489777)SNP_04445	2157	A	T	3	3	--	--
	Ca(ICCV2/PI489777)SNP_04446	2162	T	C	3	3	--	--
	Ca(ICCV2/PI489777)SNP_04447	4239	C	T	39	5	--	--
	Ca(ICCV2/PI489777)SNP_04448	4438	T	C	49	8	--	--
CakTC27313	Ca(ICCV2/PI489777)SNP_04449	2200	A	T	25	5	--	--
CakTC24725	Ca(ICCV2/PI489777)SNP_04450	1417	G	A	45	44	--	--
	Ca(ICCV2/PI489777)SNP_04451	1418	A	G	45	44	--	--
CakTC34160	Ca(ICCV2/PI489777)SNP_04452	1085	A	G	14	12	--	--
	Ca(ICCV2/PI489777)SNP_04453	1249	A	C	14	13	--	--
	Ca(ICCV2/PI489777)SNP_04454	1394	T	C	13	13	--	--
CakTC21542	Ca(ICCV2/PI489777)SNP_04455	432	G	A	27	9	--	--
	Ca(ICCV2/PI489777)SNP_04456	585	C	T	13	13	--	--
	Ca(ICCV2/PI489777)SNP_04457	609	C	G	16	14	--	--
	Ca(ICCV2/PI489777)SNP_04458	1273	T	A	24	6	--	--
CakTC09122	Ca(ICCV2/PI489777)SNP_04459	251	G	T	13	4	--	--
	Ca(ICCV2/PI489777)SNP_04460	1268	A	C	10	6	--	--
	Ca(ICCV2/PI489777)SNP_04461	1286	T	C	11	7	--	--
	Ca(ICCV2/PI489777)SNP_04462	1296	A	G	13	6	--	--
	Ca(ICCV2/PI489777)SNP_04463	1457	C	T	7	7	--	--
	Ca(ICCV2/PI489777)SNP_04464	1500	C	T	9	6	--	--
	Ca(ICCV2/PI489777)SNP_04465	1685	G	T	9	6	--	--
	Ca(ICCV2/PI489777)SNP_04466	2256	A	G	9	7	--	--
	Ca(ICCV2/PI489777)SNP_04467	3944	A	G	19	4	--	--
	Ca(ICCV2/PI489777)SNP_04468	4072	C	T	20	3	--	--
	Ca(ICCV2/PI489777)SNP_04469	4091	G	A	19	4	--	--
	Ca(ICCV2/PI489777)SNP_04470	4459	G	A	12	3	--	--
CakTC28017	Ca(ICCV2/PI489777)SNP_04471	1081	G	C	12	9	--	--
	Ca(ICCV2/PI489777)SNP_04472	1122	G	A	9	6	--	--
CakTC32589	Ca(ICCV2/PI489777)SNP_04473	1459	T	C	7	3	--	--
	Ca(ICCV2/PI489777)SNP_04474	1460	C	G	7	3	--	--
	Ca(ICCV2/PI489777)SNP_04475	1609	C	T	5	3	--	--
CakTC33070	Ca(ICCV2/PI489777)SNP_04476	2568	G	A	12	8	--	--
	Ca(ICCV2/PI489777)SNP_04477	2698	T	C	6	7	--	--
CakTC31663	Ca(ICCV2/PI489777)SNP_04478	392	T	C	13	4	--	--
	Ca(ICCV2/PI489777)SNP_04479	395	A	G	11	4	--	--
	Ca(ICCV2/PI489777)SNP_04480	875	T	C	13	6	--	--
CakTC39891	Ca(ICCV2/PI489777)SNP_04481	397	A	G	4	9	--	--
	Ca(ICCV2/PI489777)SNP_04482	995	T	G	13	12	--	--
	Ca(ICCV2/PI489777)SNP_04483	1115	A	T	15	9	--	--
CakTC22760	Ca(ICCV2/PI489777)SNP_04484	444	A	G	4	3	--	--
CakTC36172	Ca(ICCV2/PI489777)SNP_04485	234	G	A	7	3	--	--
	Ca(ICCV2/PI489777)SNP_04486	2330	A	G	6	4	--	--
CakTC33429	Ca(ICCV2/PI489777)SNP_04487	669	A	G	61	34	--	--
	Ca(ICCV2/PI489777)SNP_04488	731	C	G	9	3	--	--
CakTC29148	Ca(ICCV2/PI489777)SNP_04489	281	T	C	5	7	--	--
	Ca(ICCV2/PI489777)SNP_04490	318	T	C	4	6	--	--
	Ca(ICCV2/PI489777)SNP_04491	451	A	T	6	5	--	--
CakTC25445	Ca(ICCV2/PI489777)SNP_04492	234	A	G	6	18	--	--
	Ca(ICCV2/PI489777)SNP_04493	321	T	C	3	19	--	--
CakTC34352	Ca(ICCV2/PI489777)SNP_04494	293	A	T	43	13	--	--
CakTC11552	Ca(ICCV2/PI489777)SNP_04495	458	G	A	26	11	--	--
	Ca(ICCV2/PI489777)SNP_04496	470	T	C	26	10	--	--
	Ca(ICCV2/PI489777)SNP_04497	531	T	A	17	9	--	--
CakTC26702	Ca(ICCV2/PI489777)SNP_04498	417	T	C	7	7	--	--
CakTC37454	Ca(ICCV2/PI489777)SNP_04499	1483	G	A	10	9	--	--
CakTC43133	Ca(ICCV2/PI489777)SNP_04500	1361	C	T	25	18	--	--
	Ca(ICCV2/PI489777)SNP_04501	1647	T	A	15	12	--	--
	Ca(ICCV2/PI489777)SNP_04502	1701	C	G	6	3	--	--
CakTC36563	Ca(ICCV2/PI489777)SNP_04503	702	G	A	4	4	--	--
	Ca(ICCV2/PI489777)SNP_04504	987	A	G	9	6	--	--
	Ca(ICCV2/PI489777)SNP_04505	1185	T	C	3	8	--	--
	Ca(ICCV2/PI489777)SNP_04506	1359	A	T	6	5	--	--
	Ca(ICCV2/PI489777)SNP_04507	1427	C	T	6	5	--	--
	Ca(ICCV2/PI489777)SNP_04508	1916	C	T	7	5	--	--
	Ca(ICCV2/PI489777)SNP_04509	2367	C	T	6	3	--	--

	Ca(ICCV2/PI489777)SNP_04510	2421	C	T	5	3	--	--
	Ca(ICCV2/PI489777)SNP_04511	3930	C	T	3	4	--	--
	Ca(ICCV2/PI489777)SNP_04512	4086	T	C	6	3	--	--
	Ca(ICCV2/PI489777)SNP_04513	4097	C	T	6	4	--	--
	Ca(ICCV2/PI489777)SNP_04514	4316	C	T	4	5	--	--
	Ca(ICCV2/PI489777)SNP_04515	4317	C	T	4	4	--	--
	Ca(ICCV2/PI489777)SNP_04516	4881	A	G	4	5	--	--
	Ca(ICCV2/PI489777)SNP_04517	5001	G	A	3	3	--	--
	Ca(ICCV2/PI489777)SNP_04518	5496	A	G	4	6	--	--
CakTC26397	Ca(ICCV2/PI489777)SNP_04519	390	T	C	30	28	--	--
	Ca(ICCV2/PI489777)SNP_04520	815	C	A	51	21	--	--
	Ca(ICCV2/PI489777)SNP_04521	1965	T	C	59	39	--	--
	Ca(ICCV2/PI489777)SNP_04522	2863	G	A	18	14	--	--
CakTC22727	Ca(ICCV2/PI489777)SNP_04523	930	G	A	24	12	--	--
	Ca(ICCV2/PI489777)SNP_04524	1121	C	T	13	8	--	--
	Ca(ICCV2/PI489777)SNP_04525	1138	C	G	12	8	--	--
	Ca(ICCV2/PI489777)SNP_04526	1239	G	A	10	6	--	--
	Ca(ICCV2/PI489777)SNP_04527	1635	C	T	16	13	--	--
	Ca(ICCV2/PI489777)SNP_04528	1691	G	C	22	17	--	--
	Ca(ICCV2/PI489777)SNP_04529	1695	G	T	21	17	--	--
	Ca(ICCV2/PI489777)SNP_04530	1739	C	G	24	18	--	--
	Ca(ICCV2/PI489777)SNP_04531	1947	A	C	21	6	--	--
	Ca(ICCV2/PI489777)SNP_04532	2095	A	G	18	9	--	--
	Ca(ICCV2/PI489777)SNP_04533	2342	A	G	19	7	--	--
	Ca(ICCV2/PI489777)SNP_04534	2843	G	A	18	14	--	--
	Ca(ICCV2/PI489777)SNP_04535	2845	A	G	19	13	--	--
CakTC33900	Ca(ICCV2/PI489777)SNP_04536	303	G	C	3	13	--	--
CakTC39267	Ca(ICCV2/PI489777)SNP_04537	681	C	T	6	36	--	--
CakTC35792	Ca(ICCV2/PI489777)SNP_04538	94	C	T	7	3	--	--
CakTC39847	Ca(ICCV2/PI489777)SNP_04539	68	G	T	3	5	--	--
	Ca(ICCV2/PI489777)SNP_04540	88	C	T	3	5	--	--
	Ca(ICCV2/PI489777)SNP_04541	212	A	T	5	10	--	--
	Ca(ICCV2/PI489777)SNP_04542	250	T	G	6	10	--	--
	Ca(ICCV2/PI489777)SNP_04543	1463	C	G	4	3	--	--
CakTC38714	Ca(ICCV2/PI489777)SNP_04544	400	G	T	6	4	--	--
	Ca(ICCV2/PI489777)SNP_04545	676	G	A	14	3	--	--
	Ca(ICCV2/PI489777)SNP_04546	730	A	G	15	4	--	--
CakTC39440	Ca(ICCV2/PI489777)SNP_04547	395	A	G	13	10	--	--
	Ca(ICCV2/PI489777)SNP_04548	451	A	C	11	13	--	--
	Ca(ICCV2/PI489777)SNP_04549	543	C	A	9	11	--	--
	Ca(ICCV2/PI489777)SNP_04550	545	G	A	10	10	--	--
	Ca(ICCV2/PI489777)SNP_04551	563	G	A	11	11	--	--
	Ca(ICCV2/PI489777)SNP_04552	611	G	A	9	9	--	--
	Ca(ICCV2/PI489777)SNP_04553	719	T	C	11	4	--	--
CakTC39930	Ca(ICCV2/PI489777)SNP_04554	1153	G	A	10	13	--	--
CakTC39036	Ca(ICCV2/PI489777)SNP_04555	175	T	A	8	6	--	--
	Ca(ICCV2/PI489777)SNP_04556	437	A	C	10	8	--	--
	Ca(ICCV2/PI489777)SNP_04557	1063	T	C	12	6	--	--
CakTC11077	Ca(ICCV2/PI489777)SNP_04558	667	C	T	12	11	--	--
	Ca(ICCV2/PI489777)SNP_04559	697	G	A	10	10	--	--
	Ca(ICCV2/PI489777)SNP_04560	714	T	G	10	8	--	--
	Ca(ICCV2/PI489777)SNP_04561	715	A	T	10	8	--	--
CakTC27222	Ca(ICCV2/PI489777)SNP_04562	175	G	T	9	3	--	--
	Ca(ICCV2/PI489777)SNP_04563	648	A	T	7	5	--	--
	Ca(ICCV2/PI489777)SNP_04564	1146	T	C	16	3	--	--
	Ca(ICCV2/PI489777)SNP_04565	1597	G	C	14	7	--	--
CakTC31452	Ca(ICCV2/PI489777)SNP_04566	592	T	C	13	8	Shoot	--
	Ca(ICCV2/PI489777)SNP_04567	641	C	A	15	8	Shoot	--
	Ca(ICCV2/PI489777)SNP_04568	851	A	G	16	4	Shoot	--
	Ca(ICCV2/PI489777)SNP_04569	1797	T	A	8	3	Shoot	--
	Ca(ICCV2/PI489777)SNP_04570	1798	G	T	7	4	Shoot	--
CakTC35532	Ca(ICCV2/PI489777)SNP_04571	388	G	C	21	7	--	--
	Ca(ICCV2/PI489777)SNP_04572	741	T	C	25	14	--	--
	Ca(ICCV2/PI489777)SNP_04573	768	C	A	30	11	--	--
	Ca(ICCV2/PI489777)SNP_04574	889	A	G	24	13	--	--
	Ca(ICCV2/PI489777)SNP_04575	1176	C	T	23	17	--	--
	Ca(ICCV2/PI489777)SNP_04576	1950	C	T	13	8	--	--
	Ca(ICCV2/PI489777)SNP_04577	2255	G	A	10	9	--	--
	Ca(ICCV2/PI489777)SNP_04578	3207	A	G	9	10	--	--
	Ca(ICCV2/PI489777)SNP_04579	3247	G	A	10	11	--	--
	Ca(ICCV2/PI489777)SNP_04580	3503	G	T	7	7	--	--
CakTC29625	Ca(ICCV2/PI489777)SNP_04581	647	C	T	63	38	--	--
CakTC34783	Ca(ICCV2/PI489777)SNP_04582	148	A	G	5	4	--	Bromodomain
	Ca(ICCV2/PI489777)SNP_04583	950	A	G	5	4	--	Bromodomain

	Ca(ICCV2/PI489777)SNP_04584	2309	T	A	6	5	--	Bromodomain
	Ca(ICCV2/PI489777)SNP_04585	2510	A	G	5	4	--	Bromodomain
	Ca(ICCV2/PI489777)SNP_04586	2609	T	C	8	6	--	Bromodomain
	Ca(ICCV2/PI489777)SNP_04587	2673	A	T	9	4	--	Bromodomain
	Ca(ICCV2/PI489777)SNP_04588	2763	A	C	8	5	--	Bromodomain
	Ca(ICCV2/PI489777)SNP_04589	3291	G	T	4	13	--	Bromodomain
	Ca(ICCV2/PI489777)SNP_04590	3734	T	C	4	6	--	Bromodomain
CakTC22771	Ca(ICCV2/PI489777)SNP_04591	462	T	C	214	119	--	--
CakTC26402	Ca(ICCV2/PI489777)SNP_04592	100	T	C	3	4	--	--
CakTC27464	Ca(ICCV2/PI489777)SNP_04593	1143	T	C	12	10	--	--
	Ca(ICCV2/PI489777)SNP_04594	1380	G	A	16	11	--	--
CakTC39017	Ca(ICCV2/PI489777)SNP_04595	340	T	C	4	7	--	--
CakTC35832	Ca(ICCV2/PI489777)SNP_04596	828	T	A	6	3	--	--
	Ca(ICCV2/PI489777)SNP_04597	1275	A	G	6	8	--	--
	Ca(ICCV2/PI489777)SNP_04598	2955	C	G	10	5	--	--
CakTC28246	Ca(ICCV2/PI489777)SNP_04599	141	G	A	42	17	--	--
CakTC33080	Ca(ICCV2/PI489777)SNP_04600	1502	C	T	4	11	--	--
	Ca(ICCV2/PI489777)SNP_04601	2141	C	T	10	3	--	--
	Ca(ICCV2/PI489777)SNP_04602	2258	A	T	15	3	--	--
CakTC26886	Ca(ICCV2/PI489777)SNP_04603	583	C	T	17	11	--	--
	Ca(ICCV2/PI489777)SNP_04604	705	G	T	11	5	--	--
CakTC29129	Ca(ICCV2/PI489777)SNP_04605	126	C	G	8	7	--	--
	Ca(ICCV2/PI489777)SNP_04606	1531	G	A	27	3	--	--
	Ca(ICCV2/PI489777)SNP_04607	1627	T	C	29	5	--	--
	Ca(ICCV2/PI489777)SNP_04608	2117	C	G	4	5	--	--
CakTC41639	Ca(ICCV2/PI489777)SNP_04609	142	A	C	14	5	--	--
	Ca(ICCV2/PI489777)SNP_04610	217	A	G	15	6	--	--
CakTC33259	Ca(ICCV2/PI489777)SNP_04611	694	A	G	17	6	--	--
CakTC07832	Ca(ICCV2/PI489777)SNP_04612	405	C	A	6	15	--	--
CakTC27037	Ca(ICCV2/PI489777)SNP_04613	327	T	C	33	15	--	--
	Ca(ICCV2/PI489777)SNP_04614	516	T	C	21	11	--	--
	Ca(ICCV2/PI489777)SNP_04615	546	G	T	20	10	--	--
	Ca(ICCV2/PI489777)SNP_04616	1407	C	G	12	10	--	--
	Ca(ICCV2/PI489777)SNP_04617	1528	T	C	5	7	--	--
CakTC27669	Ca(ICCV2/PI489777)SNP_04618	1609	T	C	12	4	--	--
	Ca(ICCV2/PI489777)SNP_04619	1699	T	C	7	5	--	--
CakTC39572	Ca(ICCV2/PI489777)SNP_04620	78	A	G	6	4	--	--
	Ca(ICCV2/PI489777)SNP_04621	1026	G	A	11	9	--	--
CakTC37146	Ca(ICCV2/PI489777)SNP_04622	863	C	T	20	3	--	--
CakTC27811	Ca(ICCV2/PI489777)SNP_04623	136	C	T	6	5	--	--
	Ca(ICCV2/PI489777)SNP_04624	454	A	G	29	23	--	--
	Ca(ICCV2/PI489777)SNP_04625	1287	G	A	24	18	--	--
CakTC40616	Ca(ICCV2/PI489777)SNP_04626	433	A	T	4	4	--	NAC
	Ca(ICCV2/PI489777)SNP_04627	696	A	G	4	4	--	NAC
	Ca(ICCV2/PI489777)SNP_04628	752	T	C	4	3	--	NAC
CakTC38027	Ca(ICCV2/PI489777)SNP_04629	1520	A	G	3	3	--	--
CakTC37753	Ca(ICCV2/PI489777)SNP_04630	641	G	A	3	4	--	WRKY
	Ca(ICCV2/PI489777)SNP_04631	686	T	C	7	5	--	WRKY
	Ca(ICCV2/PI489777)SNP_04632	692	G	T	7	5	--	WRKY
	Ca(ICCV2/PI489777)SNP_04633	725	T	C	9	5	--	WRKY
	Ca(ICCV2/PI489777)SNP_04634	851	T	C	8	4	--	WRKY
CakTC38218	Ca(ICCV2/PI489777)SNP_04635	234	C	A	15	7	--	--
	Ca(ICCV2/PI489777)SNP_04636	252	T	A	15	9	--	--
	Ca(ICCV2/PI489777)SNP_04637	609	G	C	14	18	--	--
	Ca(ICCV2/PI489777)SNP_04638	1096	T	C	10	4	--	--
CakTC37238	Ca(ICCV2/PI489777)SNP_04639	950	A	C	7	11	--	--
CakTC28399	Ca(ICCV2/PI489777)SNP_04640	374	A	C	33	30	--	--
	Ca(ICCV2/PI489777)SNP_04641	611	A	C	41	42	--	--
	Ca(ICCV2/PI489777)SNP_04642	680	T	C	42	38	--	--
	Ca(ICCV2/PI489777)SNP_04643	1112	C	G	43	14	--	--
	Ca(ICCV2/PI489777)SNP_04644	2344	G	T	11	3	--	--
	Ca(ICCV2/PI489777)SNP_04645	2471	C	G	17	8	--	--
	Ca(ICCV2/PI489777)SNP_04646	2539	G	A	17	7	--	--
	Ca(ICCV2/PI489777)SNP_04647	2561	G	A	16	7	--	--
CakTC35302	Ca(ICCV2/PI489777)SNP_04648	92	T	A	4	4	--	--
	Ca(ICCV2/PI489777)SNP_04649	949	C	T	17	19	--	--
	Ca(ICCV2/PI489777)SNP_04650	1690	C	T	23	26	--	--
	Ca(ICCV2/PI489777)SNP_04651	2977	T	C	26	18	--	--
	Ca(ICCV2/PI489777)SNP_04652	3001	C	T	22	16	--	--
	Ca(ICCV2/PI489777)SNP_04653	3628	C	G	36	18	--	--
	Ca(ICCV2/PI489777)SNP_04654	4021	G	C	17	18	--	--
CakTC30231	Ca(ICCV2/PI489777)SNP_04655	531	A	G	13	3	--	--
	Ca(ICCV2/PI489777)SNP_04656	1107	G	A	25	3	--	--
	Ca(ICCV2/PI489777)SNP_04657	1146	A	C	14	3	--	--

	Ca(ICCV2/PI489777)SNP_04658	1188	A	C	12	3	--	--
CakTC40387	Ca(ICCV2/PI489777)SNP_04659	1046	G	T	11	3	--	--
CakTC24130	Ca(ICCV2/PI489777)SNP_04660	278	G	A	13	3	--	--
	Ca(ICCV2/PI489777)SNP_04661	347	T	C	10	3	--	--
	Ca(ICCV2/PI489777)SNP_04662	577	T	C	14	4	--	--
CakTC30674	Ca(ICCV2/PI489777)SNP_04663	1359	T	G	3	6	--	--
	Ca(ICCV2/PI489777)SNP_04664	1365	A	G	3	6	--	--
CakTC24679	Ca(ICCV2/PI489777)SNP_04665	421	G	C	8	7	--	--
	Ca(ICCV2/PI489777)SNP_04666	1025	T	C	8	3	--	--
	Ca(ICCV2/PI489777)SNP_04667	2139	G	T	23	10	--	--
	Ca(ICCV2/PI489777)SNP_04668	2182	A	C	15	9	--	--
	Ca(ICCV2/PI489777)SNP_04669	2318	C	T	4	5	--	--
CakTC24901	Ca(ICCV2/PI489777)SNP_04670	578	C	T	35	38	--	--
CakTC40736	Ca(ICCV2/PI489777)SNP_04671	166	A	G	18	22	--	--
	Ca(ICCV2/PI489777)SNP_04672	231	T	C	18	21	--	--
	Ca(ICCV2/PI489777)SNP_04673	243	A	G	17	24	--	--
	Ca(ICCV2/PI489777)SNP_04674	606	C	T	30	21	--	--
	Ca(ICCV2/PI489777)SNP_04675	681	A	G	28	11	--	--
	Ca(ICCV2/PI489777)SNP_04676	1181	T	A	14	10	--	--
	Ca(ICCV2/PI489777)SNP_04677	1220	G	T	15	11	--	--
CakTC38555	Ca(ICCV2/PI489777)SNP_04678	1466	C	T	9	8	--	--
	Ca(ICCV2/PI489777)SNP_04679	1599	C	T	8	5	--	--
CakTC32181	Ca(ICCV2/PI489777)SNP_04680	1062	A	T	9	6	--	--
CakTC37589	Ca(ICCV2/PI489777)SNP_04681	177	C	T	8	7	--	--
	Ca(ICCV2/PI489777)SNP_04682	194	T	G	12	9	--	--
	Ca(ICCV2/PI489777)SNP_04683	206	A	C	12	11	--	--
	Ca(ICCV2/PI489777)SNP_04684	380	A	G	14	13	--	--
	Ca(ICCV2/PI489777)SNP_04685	443	T	A	19	23	--	--
	Ca(ICCV2/PI489777)SNP_04686	556	G	A	19	25	--	--
	Ca(ICCV2/PI489777)SNP_04687	595	C	G	15	22	--	--
CakTC30681	Ca(ICCV2/PI489777)SNP_04688	2814	A	G	7	5	--	--
CakTC34911	Ca(ICCV2/PI489777)SNP_04689	350	A	G	7	8	--	MYB
CakTC24808	Ca(ICCV2/PI489777)SNP_04690	286	T	C	115	14	--	--
	Ca(ICCV2/PI489777)SNP_04691	289	G	A	121	14	--	--
	Ca(ICCV2/PI489777)SNP_04692	407	G	T	149	17	--	--
CakTC28475	Ca(ICCV2/PI489777)SNP_04693	1924	T	C	15	3	--	--
	Ca(ICCV2/PI489777)SNP_04694	2031	A	G	11	4	--	--
	Ca(ICCV2/PI489777)SNP_04695	2350	T	G	11	7	--	--
	Ca(ICCV2/PI489777)SNP_04696	2557	A	G	7	4	--	--
	Ca(ICCV2/PI489777)SNP_04697	2568	C	T	6	4	--	--
	Ca(ICCV2/PI489777)SNP_04698	2729	G	A	8	5	--	--
	Ca(ICCV2/PI489777)SNP_04699	2873	G	A	9	5	--	--
CakTC23777	Ca(ICCV2/PI489777)SNP_04700	117	A	G	4	4	--	--
CakTC24696	Ca(ICCV2/PI489777)SNP_04701	1659	G	A	7	3	--	--
CakTC36380	Ca(ICCV2/PI489777)SNP_04702	51	G	T	12	9	--	--
	Ca(ICCV2/PI489777)SNP_04703	110	A	G	18	13	--	--
CakTC43284	Ca(ICCV2/PI489777)SNP_04704	84	A	G	13	5	--	PBF-2-like
	Ca(ICCV2/PI489777)SNP_04705	175	C	G	13	5	--	PBF-2-like
CakTC25194	Ca(ICCV2/PI489777)SNP_04706	531	G	C	5	6	--	--
CakTC04658	Ca(ICCV2/PI489777)SNP_04707	600	T	C	3	3	--	--
CakTC20159	Ca(ICCV2/PI489777)SNP_04708	877	G	A	11	5	Root	--
	Ca(ICCV2/PI489777)SNP_04709	943	G	A	9	5	Root	--
CakTC29266	Ca(ICCV2/PI489777)SNP_04710	1145	T	C	5	5	--	--
CakTC34893	Ca(ICCV2/PI489777)SNP_04711	617	A	G	3	4	--	--
CakTC31866	Ca(ICCV2/PI489777)SNP_04712	208	G	A	5	3	--	--
	Ca(ICCV2/PI489777)SNP_04713	276	G	A	12	6	--	--
	Ca(ICCV2/PI489777)SNP_04714	527	T	C	9	7	--	--
	Ca(ICCV2/PI489777)SNP_04715	1397	T	C	5	6	--	--
	Ca(ICCV2/PI489777)SNP_04716	2045	T	C	5	4	--	--
	Ca(ICCV2/PI489777)SNP_04717	2075	G	A	6	4	--	--
	Ca(ICCV2/PI489777)SNP_04718	2248	G	T	7	3	--	--
CakTC37488	Ca(ICCV2/PI489777)SNP_04719	659	A	T	5	3	--	--
CakTC25125	Ca(ICCV2/PI489777)SNP_04720	468	G	A	11	3	--	--
	Ca(ICCV2/PI489777)SNP_04721	496	G	A	15	3	--	--
	Ca(ICCV2/PI489777)SNP_04722	742	T	C	16	6	--	--
	Ca(ICCV2/PI489777)SNP_04723	908	C	T	4	5	--	--
	Ca(ICCV2/PI489777)SNP_04724	956	A	C	3	5	--	--
CakTC28676	Ca(ICCV2/PI489777)SNP_04725	244	G	A	3	7	--	--
	Ca(ICCV2/PI489777)SNP_04726	803	A	G	8	7	--	--
	Ca(ICCV2/PI489777)SNP_04727	898	C	T	12	7	--	--
	Ca(ICCV2/PI489777)SNP_04728	976	A	G	9	5	--	--
	Ca(ICCV2/PI489777)SNP_04729	1563	A	G	7	5	--	--
	Ca(ICCV2/PI489777)SNP_04730	2394	T	A	4	4	--	--
CakTC00760	Ca(ICCV2/PI489777)SNP_04731	69	G	T	12	3	--	--

	Ca(ICCV2/PI489777)SNP_04732	172	A	G	11	8	--	--
	Ca(ICCV2/PI489777)SNP_04733	176	A	T	12	8	--	--
	Ca(ICCV2/PI489777)SNP_04734	300	T	A	15	12	--	--
	Ca(ICCV2/PI489777)SNP_04735	359	C	T	12	10	--	--
CakTC29952	Ca(ICCV2/PI489777)SNP_04736	268	A	C	4	3	--	--
	Ca(ICCV2/PI489777)SNP_04737	1065	A	G	13	5	--	--
	Ca(ICCV2/PI489777)SNP_04738	1212	G	T	10	5	--	--
	Ca(ICCV2/PI489777)SNP_04739	1257	A	T	9	4	--	--
	Ca(ICCV2/PI489777)SNP_04740	1428	C	T	10	4	--	--
	Ca(ICCV2/PI489777)SNP_04741	1470	A	C	7	4	--	--
CakTC39959	Ca(ICCV2/PI489777)SNP_04742	25	C	G	6	3	--	--
	Ca(ICCV2/PI489777)SNP_04743	83	T	C	13	5	--	--
	Ca(ICCV2/PI489777)SNP_04744	254	C	T	12	6	--	--
	Ca(ICCV2/PI489777)SNP_04745	290	T	C	15	6	--	--
	Ca(ICCV2/PI489777)SNP_04746	635	A	G	15	4	--	--
	Ca(ICCV2/PI489777)SNP_04747	1150	G	A	13	6	--	--
	Ca(ICCV2/PI489777)SNP_04748	1153	T	C	14	6	--	--
	Ca(ICCV2/PI489777)SNP_04749	1184	G	T	14	5	--	--
	Ca(ICCV2/PI489777)SNP_04750	1208	G	A	14	4	--	--
	Ca(ICCV2/PI489777)SNP_04751	1226	A	G	12	6	--	--
CakTC29988	Ca(ICCV2/PI489777)SNP_04752	93	C	T	34	3	--	--
	Ca(ICCV2/PI489777)SNP_04753	1304	G	A	49	21	--	--
	Ca(ICCV2/PI489777)SNP_04754	1444	G	A	72	12	--	--
	Ca(ICCV2/PI489777)SNP_04755	2134	T	C	69	24	--	--
	Ca(ICCV2/PI489777)SNP_04756	2139	G	C	70	26	--	--
	Ca(ICCV2/PI489777)SNP_04757	2271	T	G	59	25	--	--
	Ca(ICCV2/PI489777)SNP_04758	2301	G	C	62	28	--	--
	Ca(ICCV2/PI489777)SNP_04759	2481	G	A	21	5	--	--
CakTC33571	Ca(ICCV2/PI489777)SNP_04760	589	G	A	4	9	--	--
	Ca(ICCV2/PI489777)SNP_04761	1762	G	A	4	3	--	--
	Ca(ICCV2/PI489777)SNP_04762	1833	C	T	3	3	--	--
CakTC34108	Ca(ICCV2/PI489777)SNP_04763	114	A	G	17	5	--	--
	Ca(ICCV2/PI489777)SNP_04764	132	C	T	43	20	--	--
CakTC41011	Ca(ICCV2/PI489777)SNP_04765	385	C	A	3	3	Flower	bud
CakTC25981	Ca(ICCV2/PI489777)SNP_04766	813	T	C	46	30	--	--
CakTC34408	Ca(ICCV2/PI489777)SNP_04767	226	A	T	7	3	--	--
	Ca(ICCV2/PI489777)SNP_04768	406	C	T	5	3	--	--
	Ca(ICCV2/PI489777)SNP_04769	543	C	T	7	6	--	--
	Ca(ICCV2/PI489777)SNP_04770	791	A	T	6	9	--	--
	Ca(ICCV2/PI489777)SNP_04771	933	G	T	8	8	--	--
	Ca(ICCV2/PI489777)SNP_04772	941	T	C	8	9	--	--
	Ca(ICCV2/PI489777)SNP_04773	996	G	A	8	7	--	--
	Ca(ICCV2/PI489777)SNP_04774	1000	G	A	8	7	--	--
CakTC40158	Ca(ICCV2/PI489777)SNP_04775	221	T	C	11	10	--	--
CakTC33199	Ca(ICCV2/PI489777)SNP_04776	705	T	G	23	4	--	GRAS
	Ca(ICCV2/PI489777)SNP_04777	738	C	T	24	6	--	GRAS
	Ca(ICCV2/PI489777)SNP_04778	829	C	A	37	3	--	GRAS
	Ca(ICCV2/PI489777)SNP_04779	1176	T	C	38	4	--	GRAS
	Ca(ICCV2/PI489777)SNP_04780	1389	T	C	36	7	--	GRAS
	Ca(ICCV2/PI489777)SNP_04781	1608	G	A	38	5	--	GRAS
	Ca(ICCV2/PI489777)SNP_04782	1755	G	C	20	9	--	GRAS
	Ca(ICCV2/PI489777)SNP_04783	1809	A	G	19	9	--	GRAS
	Ca(ICCV2/PI489777)SNP_04784	1921	C	G	18	9	--	GRAS
	Ca(ICCV2/PI489777)SNP_04785	2071	T	G	3	4	--	GRAS
CakTC37286	Ca(ICCV2/PI489777)SNP_04786	680	G	A	59	26	--	--
CakTC32803	Ca(ICCV2/PI489777)SNP_04787	94	C	T	11	4	--	--
	Ca(ICCV2/PI489777)SNP_04788	253	T	C	11	6	--	--
	Ca(ICCV2/PI489777)SNP_04789	442	C	T	12	6	--	--
	Ca(ICCV2/PI489777)SNP_04790	557	G	A	15	4	--	--
	Ca(ICCV2/PI489777)SNP_04791	583	A	G	16	4	--	--
	Ca(ICCV2/PI489777)SNP_04792	605	C	G	17	4	--	--
	Ca(ICCV2/PI489777)SNP_04793	827	A	C	18	3	--	--
	Ca(ICCV2/PI489777)SNP_04794	940	A	G	13	5	--	--
	Ca(ICCV2/PI489777)SNP_04795	1389	G	A	22	12	--	--
	Ca(ICCV2/PI489777)SNP_04796	1399	C	T	18	12	--	--
	Ca(ICCV2/PI489777)SNP_04797	2664	G	A	6	4	--	--
	Ca(ICCV2/PI489777)SNP_04798	2709	A	C	5	3	--	--
CakTC42772	Ca(ICCV2/PI489777)SNP_04799	271	T	C	8	7	--	bZIP
	Ca(ICCV2/PI489777)SNP_04800	870	G	A	14	12	--	bZIP
	Ca(ICCV2/PI489777)SNP_04801	1036	T	C	13	14	--	bZIP
	Ca(ICCV2/PI489777)SNP_04802	1366	C	A	26	14	--	bZIP
	Ca(ICCV2/PI489777)SNP_04803	1378	G	A	21	17	--	bZIP
	Ca(ICCV2/PI489777)SNP_04804	1438	C	A	26	20	--	bZIP
CakTC10654	Ca(ICCV2/PI489777)SNP_04805	1011	A	G	3	3	--	--

CakTC32000	Ca(ICCV2/PI489777)SNP_04806	655	C	A	4	3	--	--
	Ca(ICCV2/PI489777)SNP_04807	1261	A	G	6	6	--	--
	Ca(ICCV2/PI489777)SNP_04808	1284	T	C	5	7	--	--
	Ca(ICCV2/PI489777)SNP_04809	1541	C	G	6	5	--	--
	Ca(ICCV2/PI489777)SNP_04810	1602	T	G	6	5	--	--
	Ca(ICCV2/PI489777)SNP_04811	1612	C	T	6	3	--	--
CakTC42673	Ca(ICCV2/PI489777)SNP_04812	754	A	G	3	3	--	--
CakTC27047	Ca(ICCV2/PI489777)SNP_04813	468	C	A	21	3	--	--
CakTC41488	Ca(ICCV2/PI489777)SNP_04814	135	T	C	5	4	--	--
	Ca(ICCV2/PI489777)SNP_04815	140	A	G	5	4	--	--
CakTC42527	Ca(ICCV2/PI489777)SNP_04816	808	C	T	20	13	--	--
	Ca(ICCV2/PI489777)SNP_04817	1034	C	T	22	15	--	--
	Ca(ICCV2/PI489777)SNP_04818	1156	G	A	17	14	--	--
	Ca(ICCV2/PI489777)SNP_04819	1576	G	A	31	11	--	--
	Ca(ICCV2/PI489777)SNP_04820	1609	A	T	30	12	--	--
CakTC36204	Ca(ICCV2/PI489777)SNP_04821	1462	G	A	4	5	--	--
	Ca(ICCV2/PI489777)SNP_04822	2089	C	G	4	5	--	--
CakTC41111	Ca(ICCV2/PI489777)SNP_04823	179	A	C	8	10	--	--
CakTC27478	Ca(ICCV2/PI489777)SNP_04824	493	T	C	23	6	--	--
	Ca(ICCV2/PI489777)SNP_04825	901	G	A	7	5	--	--
	Ca(ICCV2/PI489777)SNP_04826	1010	C	T	4	4	--	--
	Ca(ICCV2/PI489777)SNP_04827	2036	T	C	55	13	--	--
CakTC39327	Ca(ICCV2/PI489777)SNP_04828	58	A	C	19	8	--	--
	Ca(ICCV2/PI489777)SNP_04829	91	C	T	26	5	--	--
CakTC30524	Ca(ICCV2/PI489777)SNP_04830	111	G	A	3	3	--	C3H
	Ca(ICCV2/PI489777)SNP_04831	133	A	G	3	3	--	C3H
	Ca(ICCV2/PI489777)SNP_04832	150	G	T	3	3	--	C3H
	Ca(ICCV2/PI489777)SNP_04833	256	T	C	5	3	--	C3H
	Ca(ICCV2/PI489777)SNP_04834	1313	G	C	9	5	--	C3H
CakTC10834	Ca(ICCV2/PI489777)SNP_04835	73	C	A	13	19	--	AUX/IAA
	Ca(ICCV2/PI489777)SNP_04836	97	A	C	11	19	--	AUX/IAA
CakTC42745	Ca(ICCV2/PI489777)SNP_04837	422	C	T	25	10	--	--
	Ca(ICCV2/PI489777)SNP_04838	747	A	G	33	19	--	--
CakTC41313	Ca(ICCV2/PI489777)SNP_04839	412	C	G	13	10	--	--
	Ca(ICCV2/PI489777)SNP_04840	508	C	T	16	10	--	--
	Ca(ICCV2/PI489777)SNP_04841	2255	G	A	10	7	--	--
	Ca(ICCV2/PI489777)SNP_04842	2306	A	G	8	6	--	--
CakTC11127	Ca(ICCV2/PI489777)SNP_04843	816	T	C	8	4	--	CCHC
	Ca(ICCV2/PI489777)SNP_04844	821	T	G	8	4	--	CCHC
	Ca(ICCV2/PI489777)SNP_04845	1245	C	T	7	5	--	CCHC
	Ca(ICCV2/PI489777)SNP_04846	1606	A	G	14	4	--	CCHC
	Ca(ICCV2/PI489777)SNP_04847	3111	A	C	13	19	--	CCHC
CakTC42957	Ca(ICCV2/PI489777)SNP_04848	1056	T	C	15	7	--	--
CakTC40992	Ca(ICCV2/PI489777)SNP_04849	302	A	C	15	18	Young_pod	--
	Ca(ICCV2/PI489777)SNP_04850	473	T	C	10	12	Young_pod	--
	Ca(ICCV2/PI489777)SNP_04851	476	T	G	10	12	Young_pod	--
	Ca(ICCV2/PI489777)SNP_04852	515	T	C	11	7	Young_pod	--
	Ca(ICCV2/PI489777)SNP_04853	528	C	G	11	7	Young_pod	--
	Ca(ICCV2/PI489777)SNP_04854	566	A	G	10	7	Young_pod	--
	Ca(ICCV2/PI489777)SNP_04855	585	G	A	4	7	Young_pod	--
CakTC28405	Ca(ICCV2/PI489777)SNP_04856	118	A	G	22	5	--	--
CakTC42087	Ca(ICCV2/PI489777)SNP_04857	543	A	T	24	28	--	--
CakTC39974	Ca(ICCV2/PI489777)SNP_04858	161	G	A	3	4	--	--
	Ca(ICCV2/PI489777)SNP_04859	916	T	C	11	3	--	--
	Ca(ICCV2/PI489777)SNP_04860	933	T	C	11	3	--	--
	Ca(ICCV2/PI489777)SNP_04861	936	T	C	11	4	--	--
	Ca(ICCV2/PI489777)SNP_04862	948	A	T	11	4	--	--
CakTC40640	Ca(ICCV2/PI489777)SNP_04863	1481	T	C	8	3	--	--
	Ca(ICCV2/PI489777)SNP_04864	1483	A	G	8	3	--	--
	Ca(ICCV2/PI489777)SNP_04865	1876	A	G	4	9	--	--
	Ca(ICCV2/PI489777)SNP_04866	2291	A	G	6	4	--	--
	Ca(ICCV2/PI489777)SNP_04867	2669	C	T	6	4	--	--
	Ca(ICCV2/PI489777)SNP_04868	2717	G	A	7	5	--	--
	Ca(ICCV2/PI489777)SNP_04869	2921	G	A	5	3	--	--
CakTC42019	Ca(ICCV2/PI489777)SNP_04870	157	A	C	13	4	--	TUB
	Ca(ICCV2/PI489777)SNP_04871	388	G	A	19	20	--	TUB
	Ca(ICCV2/PI489777)SNP_04872	421	C	T	29	23	--	TUB
CakTC41481	Ca(ICCV2/PI489777)SNP_04873	1349	G	A	7	26	--	--
	Ca(ICCV2/PI489777)SNP_04874	1822	C	T	14	6	--	--
	Ca(ICCV2/PI489777)SNP_04875	2005	A	G	10	4	--	--
CakTC31023	Ca(ICCV2/PI489777)SNP_04876	628	G	C	5	5	--	--
	Ca(ICCV2/PI489777)SNP_04877	631	C	T	5	5	--	--
	Ca(ICCV2/PI489777)SNP_04878	1708	G	C	5	5	--	--
CakTC43076	Ca(ICCV2/PI489777)SNP_04879	456	C	T	3	3	--	--

CakTC32096	Ca(ICCV2/PI489777)SNP_04880	545	C	T	6	6	--	--
	Ca(ICCV2/PI489777)SNP_04881	1278	G	C	16	5	--	--
	Ca(ICCV2/PI489777)SNP_04882	1308	T	C	16	5	--	--
	Ca(ICCV2/PI489777)SNP_04883	1347	G	A	16	5	--	--
CakTC40697	Ca(ICCV2/PI489777)SNP_04884	279	C	T	77	7	--	--
	Ca(ICCV2/PI489777)SNP_04885	302	C	A	67	7	--	--
	Ca(ICCV2/PI489777)SNP_04886	363	C	T	59	7	--	--
	Ca(ICCV2/PI489777)SNP_04887	412	G	T	21	4	--	--
CakTC39181	Ca(ICCV2/PI489777)SNP_04888	1272	A	T	9	7	--	--
CakTC41198	Ca(ICCV2/PI489777)SNP_04889	197	T	G	11	8	--	--
	Ca(ICCV2/PI489777)SNP_04890	216	A	G	13	8	--	--
CakTC37207	Ca(ICCV2/PI489777)SNP_04891	243	G	T	30	11	--	--
CakTC22678	Ca(ICCV2/PI489777)SNP_04892	209	C	T	5	6	Young_pod	--
CakTC27562	Ca(ICCV2/PI489777)SNP_04893	361	C	T	18	8	--	bZIP
	Ca(ICCV2/PI489777)SNP_04894	436	C	T	15	12	--	bZIP
	Ca(ICCV2/PI489777)SNP_04895	1005	A	G	28	18	--	bZIP
CakTC39686	Ca(ICCV2/PI489777)SNP_04896	376	A	G	15	3	--	--
	Ca(ICCV2/PI489777)SNP_04897	780	A	G	15	5	--	--
CakTC42283	Ca(ICCV2/PI489777)SNP_04898	151	G	A	18	11	--	--
	Ca(ICCV2/PI489777)SNP_04899	189	T	C	19	11	--	--
	Ca(ICCV2/PI489777)SNP_04900	795	A	T	19	14	--	--
	Ca(ICCV2/PI489777)SNP_04901	955	C	T	37	24	--	--
CakTC40754	Ca(ICCV2/PI489777)SNP_04902	1130	T	C	6	8	--	--
CakTC40385	Ca(ICCV2/PI489777)SNP_04903	497	T	A	5	3	--	--
	Ca(ICCV2/PI489777)SNP_04904	1187	C	G	16	8	--	--
	Ca(ICCV2/PI489777)SNP_04905	1348	C	G	16	7	--	--
	Ca(ICCV2/PI489777)SNP_04906	1480	A	G	15	9	--	--
	Ca(ICCV2/PI489777)SNP_04907	1523	C	A	17	7	--	--
	Ca(ICCV2/PI489777)SNP_04908	1570	A	G	15	6	--	--
	Ca(ICCV2/PI489777)SNP_04909	1666	A	C	16	5	--	--
CakTC26692	Ca(ICCV2/PI489777)SNP_04910	1039	G	A	9	4	--	--
CakTC02697	Ca(ICCV2/PI489777)SNP_04911	777	C	T	4	4	--	--
CakTC29786	Ca(ICCV2/PI489777)SNP_04912	278	C	T	12	3	--	--
	Ca(ICCV2/PI489777)SNP_04913	474	A	G	14	5	--	--
	Ca(ICCV2/PI489777)SNP_04914	708	C	T	6	3	--	--
	Ca(ICCV2/PI489777)SNP_04915	1234	C	T	4	3	--	--
CakTC11678	Ca(ICCV2/PI489777)SNP_04916	985	C	G	4	5	--	--
	Ca(ICCV2/PI489777)SNP_04917	1423	C	T	14	8	--	--
CakTC38353	Ca(ICCV2/PI489777)SNP_04918	1195	A	G	20	11	--	--
	Ca(ICCV2/PI489777)SNP_04919	1252	C	T	14	7	--	--
	Ca(ICCV2/PI489777)SNP_04920	1471	C	A	12	10	--	--
CakTC37846	Ca(ICCV2/PI489777)SNP_04921	1055	G	A	14	8	--	--
	Ca(ICCV2/PI489777)SNP_04922	1088	A	G	13	8	--	--
CakTC06048	Ca(ICCV2/PI489777)SNP_04923	1179	C	T	3	5	--	--
	Ca(ICCV2/PI489777)SNP_04924	1258	G	C	4	4	--	--
	Ca(ICCV2/PI489777)SNP_04925	1391	T	C	3	3	--	--
CakTC43337	Ca(ICCV2/PI489777)SNP_04926	85	C	G	3	4	--	--
	Ca(ICCV2/PI489777)SNP_04927	182	C	A	3	10	--	--
	Ca(ICCV2/PI489777)SNP_04928	381	A	T	13	8	--	--
	Ca(ICCV2/PI489777)SNP_04929	1599	C	T	10	8	--	--
	Ca(ICCV2/PI489777)SNP_04930	1676	G	A	7	6	--	--
CakTC41629	Ca(ICCV2/PI489777)SNP_04931	466	C	G	11	14	--	--
	Ca(ICCV2/PI489777)SNP_04932	475	T	C	10	14	--	--
	Ca(ICCV2/PI489777)SNP_04933	597	C	T	8	9	--	--
CakTC25815	Ca(ICCV2/PI489777)SNP_04934	614	C	A	3	6	--	--
CakTC39186	Ca(ICCV2/PI489777)SNP_04935	867	T	C	43	18	--	C3H
	Ca(ICCV2/PI489777)SNP_04936	876	T	A	43	19	--	C3H
	Ca(ICCV2/PI489777)SNP_04937	1729	C	T	3	6	--	C3H
	Ca(ICCV2/PI489777)SNP_04938	1960	A	G	4	5	--	C3H
CakTC40094	Ca(ICCV2/PI489777)SNP_04939	1023	G	T	4	4	--	--
CakTC38655	Ca(ICCV2/PI489777)SNP_04940	114	C	T	7	5	--	--
	Ca(ICCV2/PI489777)SNP_04941	171	G	A	9	6	--	--
	Ca(ICCV2/PI489777)SNP_04942	427	G	T	11	9	--	--
	Ca(ICCV2/PI489777)SNP_04943	448	C	T	10	15	--	--
	Ca(ICCV2/PI489777)SNP_04944	532	G	A	14	14	--	--
CakTC32512	Ca(ICCV2/PI489777)SNP_04945	421	G	A	4	6	--	--
CakTC33922	Ca(ICCV2/PI489777)SNP_04946	483	C	T	31	11	--	--
CakTC16941	Ca(ICCV2/PI489777)SNP_04947	269	T	C	3	3	--	--
	Ca(ICCV2/PI489777)SNP_04948	383	A	G	3	3	--	--
CakTC42024	Ca(ICCV2/PI489777)SNP_04949	10	C	G	6	3	--	--
	Ca(ICCV2/PI489777)SNP_04950	19	T	C	6	3	--	--
	Ca(ICCV2/PI489777)SNP_04951	107	A	G	13	5	--	--
	Ca(ICCV2/PI489777)SNP_04952	253	G	A	22	8	--	--
	Ca(ICCV2/PI489777)SNP_04953	376	T	A	19	8	--	--

	Ca(ICCV2/PI489777)SNP_04954	771	A	C	11	7	--	--
	Ca(ICCV2/PI489777)SNP_04955	839	A	G	15	7	--	--
CakTC28486	Ca(ICCV2/PI489777)SNP_04956	262	T	A	8	8	--	--
CakTC24442	Ca(ICCV2/PI489777)SNP_04957	1051	C	T	25	6	--	--
	Ca(ICCV2/PI489777)SNP_04958	1064	C	A	33	6	--	--
	Ca(ICCV2/PI489777)SNP_04959	1166	G	T	27	5	--	--
	Ca(ICCV2/PI489777)SNP_04960	1214	A	T	32	6	--	--
CakTC32330	Ca(ICCV2/PI489777)SNP_04961	159	A	G	7	10	--	--
	Ca(ICCV2/PI489777)SNP_04962	246	G	A	7	13	--	--
	Ca(ICCV2/PI489777)SNP_04963	327	A	G	4	13	--	--
	Ca(ICCV2/PI489777)SNP_04964	393	C	T	11	15	--	--
	Ca(ICCV2/PI489777)SNP_04965	569	T	A	35	19	--	--
	Ca(ICCV2/PI489777)SNP_04966	1059	A	T	47	24	--	--
	Ca(ICCV2/PI489777)SNP_04967	1193	G	T	49	33	--	--
	Ca(ICCV2/PI489777)SNP_04968	1197	A	T	48	35	--	--
	Ca(ICCV2/PI489777)SNP_04969	1212	G	C	56	35	--	--
	Ca(ICCV2/PI489777)SNP_04970	1704	A	T	30	17	--	--
	Ca(ICCV2/PI489777)SNP_04971	1785	G	A	40	22	--	--
	Ca(ICCV2/PI489777)SNP_04972	1807	T	C	40	21	--	--
	Ca(ICCV2/PI489777)SNP_04973	2898	T	C	37	16	--	--
	Ca(ICCV2/PI489777)SNP_04974	3725	G	T	24	21	--	--
CakTC25122	Ca(ICCV2/PI489777)SNP_04975	182	A	T	9	3	--	--
	Ca(ICCV2/PI489777)SNP_04976	226	C	T	8	5	--	--
	Ca(ICCV2/PI489777)SNP_04977	294	A	T	16	4	--	--
	Ca(ICCV2/PI489777)SNP_04978	474	C	G	15	5	--	--
	Ca(ICCV2/PI489777)SNP_04979	647	A	C	17	4	--	--
CakTC39041	Ca(ICCV2/PI489777)SNP_04980	178	C	T	30	13	--	--
	Ca(ICCV2/PI489777)SNP_04981	631	C	T	23	30	--	--
CakTC35005	Ca(ICCV2/PI489777)SNP_04982	2046	G	A	24	5	--	--
	Ca(ICCV2/PI489777)SNP_04983	2994	G	A	21	10	--	--
CakTC42699	Ca(ICCV2/PI489777)SNP_04984	1622	A	G	43	59	--	--
	Ca(ICCV2/PI489777)SNP_04985	1833	T	A	10	9	--	--
CakTC40326	Ca(ICCV2/PI489777)SNP_04986	606	C	T	6	4	--	--
	Ca(ICCV2/PI489777)SNP_04987	1190	A	T	17	8	--	--
	Ca(ICCV2/PI489777)SNP_04988	1197	T	C	20	8	--	--
	Ca(ICCV2/PI489777)SNP_04989	1202	T	C	20	8	--	--
CakTC40035	Ca(ICCV2/PI489777)SNP_04990	648	T	C	6	11	--	--
	Ca(ICCV2/PI489777)SNP_04991	972	G	C	7	6	--	--
CakTC37192	Ca(ICCV2/PI489777)SNP_04992	743	C	A	3	12	--	--
	Ca(ICCV2/PI489777)SNP_04993	794	G	T	6	10	--	--
	Ca(ICCV2/PI489777)SNP_04994	1108	G	A	7	5	--	--
	Ca(ICCV2/PI489777)SNP_04995	1127	C	A	7	5	--	--
	Ca(ICCV2/PI489777)SNP_04996	1128	A	T	8	5	--	--
CakTC24819	Ca(ICCV2/PI489777)SNP_04997	846	A	T	8	3	Flower	bud
	Ca(ICCV2/PI489777)SNP_04998	865	C	G	9	3	Flower	bud
CakTC42638	Ca(ICCV2/PI489777)SNP_04999	118	G	C	3	4	--	--
	Ca(ICCV2/PI489777)SNP_05000	228	T	C	5	5	--	--
	Ca(ICCV2/PI489777)SNP_05001	240	C	T	5	5	--	--
	Ca(ICCV2/PI489777)SNP_05002	381	C	T	4	5	--	--
	Ca(ICCV2/PI489777)SNP_05003	627	T	C	5	3	--	--
CakTC29965	Ca(ICCV2/PI489777)SNP_05004	376	G	C	5	6	--	HB
	Ca(ICCV2/PI489777)SNP_05005	767	T	C	10	4	--	HB
	Ca(ICCV2/PI489777)SNP_05006	1158	C	A	9	12	--	HB
	Ca(ICCV2/PI489777)SNP_05007	1505	G	A	13	7	--	HB
	Ca(ICCV2/PI489777)SNP_05008	1531	C	A	12	8	--	HB
	Ca(ICCV2/PI489777)SNP_05009	2383	A	G	11	7	--	HB
CakTC39991	Ca(ICCV2/PI489777)SNP_05010	134	T	G	13	9	--	--
	Ca(ICCV2/PI489777)SNP_05011	557	C	T	8	4	--	--
	Ca(ICCV2/PI489777)SNP_05012	572	T	C	7	3	--	--
	Ca(ICCV2/PI489777)SNP_05013	584	C	A	8	3	--	--
CakTC36197	Ca(ICCV2/PI489777)SNP_05014	256	T	A	5	4	--	--
	Ca(ICCV2/PI489777)SNP_05015	313	G	A	4	4	--	--
	Ca(ICCV2/PI489777)SNP_05016	1090	C	T	8	5	--	--
	Ca(ICCV2/PI489777)SNP_05017	1453	G	A	9	5	--	--
	Ca(ICCV2/PI489777)SNP_05018	1473	T	A	10	5	--	--
	Ca(ICCV2/PI489777)SNP_05019	1663	C	T	9	9	--	--
	Ca(ICCV2/PI489777)SNP_05020	1819	G	T	4	12	--	--
	Ca(ICCV2/PI489777)SNP_05021	1873	T	G	3	11	--	--
	Ca(ICCV2/PI489777)SNP_05022	2200	A	G	6	5	--	--
CakTC34476	Ca(ICCV2/PI489777)SNP_05023	546	C	T	7	10	--	--
	Ca(ICCV2/PI489777)SNP_05024	765	T	C	3	3	--	--
	Ca(ICCV2/PI489777)SNP_05025	1603	C	T	5	7	--	--
	Ca(ICCV2/PI489777)SNP_05026	1739	A	T	10	9	--	--
CakTC42169	Ca(ICCV2/PI489777)SNP_05027	515	A	G	21	16	--	--

	Ca(ICCV2/PI489777)SNP_05028	536	C	T	20	14	--	--
CakTC40677	Ca(ICCV2/PI489777)SNP_05029	86	A	G	3	10	--	--
	Ca(ICCV2/PI489777)SNP_05030	216	A	T	7	14	--	--
CakTC22403	Ca(ICCV2/PI489777)SNP_05031	2322	A	G	7	3	Flower	bud
CakTC09647	Ca(ICCV2/PI489777)SNP_05032	813	T	C	15	6	--	--
CakTC40504	Ca(ICCV2/PI489777)SNP_05033	521	A	G	10	8	--	--
CakTC17684	Ca(ICCV2/PI489777)SNP_05034	387	C	T	3	4	--	--
CakTC39280	Ca(ICCV2/PI489777)SNP_05035	301	C	T	6	10	--	--
	Ca(ICCV2/PI489777)SNP_05036	1229	G	A	5	7	--	--
CakTC37749	Ca(ICCV2/PI489777)SNP_05037	605	C	A	33	19	--	--
CakTC11057	Ca(ICCV2/PI489777)SNP_05038	221	A	G	9	8	--	--
	Ca(ICCV2/PI489777)SNP_05039	298	G	A	8	9	--	--
	Ca(ICCV2/PI489777)SNP_05040	709	G	A	7	7	--	--
	Ca(ICCV2/PI489777)SNP_05041	1114	C	A	5	8	--	--
CakTC28808	Ca(ICCV2/PI489777)SNP_05042	1700	A	C	6	5	--	--
CakTC36584	Ca(ICCV2/PI489777)SNP_05043	363	C	A	22	20	--	--
	Ca(ICCV2/PI489777)SNP_05044	444	T	C	22	19	--	--
	Ca(ICCV2/PI489777)SNP_05045	495	C	T	17	16	--	--
	Ca(ICCV2/PI489777)SNP_05046	618	A	G	15	18	--	--
	Ca(ICCV2/PI489777)SNP_05047	636	G	A	17	19	--	--
	Ca(ICCV2/PI489777)SNP_05048	1554	G	A	25	19	--	--
	Ca(ICCV2/PI489777)SNP_05049	2201	A	G	34	18	--	--
CakTC10725	Ca(ICCV2/PI489777)SNP_05050	694	G	C	8	9	--	--
	Ca(ICCV2/PI489777)SNP_05051	928	C	T	22	8	--	--
	Ca(ICCV2/PI489777)SNP_05052	961	A	G	20	7	--	--
	Ca(ICCV2/PI489777)SNP_05053	1429	T	C	16	12	--	--
	Ca(ICCV2/PI489777)SNP_05054	2335	A	T	31	39	--	--
CakTC23584	Ca(ICCV2/PI489777)SNP_05055	2563	C	T	8	5	Mature	Leaf
	Ca(ICCV2/PI489777)SNP_05056	2732	C	A	4	7	Mature	Leaf
	Ca(ICCV2/PI489777)SNP_05057	2763	T	C	5	6	Mature	Leaf
	Ca(ICCV2/PI489777)SNP_05058	2781	C	T	5	5	Mature	Leaf
CakTC43340	Ca(ICCV2/PI489777)SNP_05059	695	G	T	11	9	--	--
	Ca(ICCV2/PI489777)SNP_05060	755	A	G	10	7	--	--
	Ca(ICCV2/PI489777)SNP_05061	855	C	T	8	6	--	--
CakTC26020	Ca(ICCV2/PI489777)SNP_05062	350	T	C	50	44	--	--
	Ca(ICCV2/PI489777)SNP_05063	794	C	G	101	51	--	--
CakTC07722	Ca(ICCV2/PI489777)SNP_05064	238	G	T	7	3	--	--
CakTC26174	Ca(ICCV2/PI489777)SNP_05065	143	G	A	50	32	--	--
	Ca(ICCV2/PI489777)SNP_05066	739	T	C	16	9	--	--
	Ca(ICCV2/PI489777)SNP_05067	794	T	C	11	7	--	--
CakTC42233	Ca(ICCV2/PI489777)SNP_05068	531	A	G	31	34	--	--
	Ca(ICCV2/PI489777)SNP_05069	1062	C	T	29	20	--	--
CakTC42858	Ca(ICCV2/PI489777)SNP_05070	69	A	C	20	10	--	--
CakTC20986	Ca(ICCV2/PI489777)SNP_05071	200	T	G	3	5	--	--
	Ca(ICCV2/PI489777)SNP_05072	222	G	A	3	5	--	--
CakTC36928	Ca(ICCV2/PI489777)SNP_05073	1990	A	T	17	5	--	--
	Ca(ICCV2/PI489777)SNP_05074	2287	A	G	12	8	--	--
	Ca(ICCV2/PI489777)SNP_05075	2554	A	G	15	5	--	--
CakTC42051	Ca(ICCV2/PI489777)SNP_05076	556	G	C	7	5	Root	--
	Ca(ICCV2/PI489777)SNP_05077	895	C	T	6	5	Root	--
	Ca(ICCV2/PI489777)SNP_05078	929	C	G	6	5	Root	--
CakTC34562	Ca(ICCV2/PI489777)SNP_05079	602	C	T	33	9	--	--
	Ca(ICCV2/PI489777)SNP_05080	812	T	G	20	6	--	--
	Ca(ICCV2/PI489777)SNP_05081	1346	T	C	24	10	--	--
	Ca(ICCV2/PI489777)SNP_05082	1644	C	T	30	9	--	--
	Ca(ICCV2/PI489777)SNP_05083	1742	C	G	23	7	--	--
CakTC11825	Ca(ICCV2/PI489777)SNP_05084	1852	T	C	4	3	--	--
CakTC26087	Ca(ICCV2/PI489777)SNP_05085	181	T	C	8	4	Young_pod	HSF
	Ca(ICCV2/PI489777)SNP_05086	305	G	A	11	3	Young_pod	HSF
	Ca(ICCV2/PI489777)SNP_05087	651	T	A	3	3	Young_pod	HSF
	Ca(ICCV2/PI489777)SNP_05088	817	G	A	6	3	Young_pod	HSF
	Ca(ICCV2/PI489777)SNP_05089	1355	G	A	13	4	Young_pod	HSF
	Ca(ICCV2/PI489777)SNP_05090	1362	T	C	12	4	Young_pod	HSF
	Ca(ICCV2/PI489777)SNP_05091	1690	C	T	9	4	Young_pod	HSF
	Ca(ICCV2/PI489777)SNP_05092	1739	A	G	13	6	Young_pod	HSF
	Ca(ICCV2/PI489777)SNP_05093	1788	T	C	15	7	Young_pod	HSF
	Ca(ICCV2/PI489777)SNP_05094	1824	T	C	15	7	Young_pod	HSF
	Ca(ICCV2/PI489777)SNP_05095	2462	G	C	10	4	Young_pod	HSF
	Ca(ICCV2/PI489777)SNP_05096	2465	T	C	10	3	Young_pod	HSF
	Ca(ICCV2/PI489777)SNP_05097	2533	T	C	12	3	Young_pod	HSF
	Ca(ICCV2/PI489777)SNP_05098	2810	C	T	10	5	Young_pod	HSF
	Ca(ICCV2/PI489777)SNP_05099	2815	C	T	10	4	Young_pod	HSF
	Ca(ICCV2/PI489777)SNP_05100	2844	A	G	12	4	Young_pod	HSF
	Ca(ICCV2/PI489777)SNP_05101	2856	G	C	11	7	Young_pod	HSF

	Ca(ICCV2/PI489777)SNP_05102	2938	G	A	14	6	Young_pod	HSF
	Ca(ICCV2/PI489777)SNP_05103	2969	G	A	13	7	Young_pod	HSF
	Ca(ICCV2/PI489777)SNP_05104	3062	G	A	8	3	Young_pod	HSF
	Ca(ICCV2/PI489777)SNP_05105	3147	C	T	9	7	Young_pod	HSF
CakTC32567	Ca(ICCV2/PI489777)SNP_05106	614	G	A	9	4	--	HB
	Ca(ICCV2/PI489777)SNP_05107	759	T	C	9	8	--	HB
	Ca(ICCV2/PI489777)SNP_05108	1777	A	T	14	10	--	HB
	Ca(ICCV2/PI489777)SNP_05109	1877	C	T	7	6	--	HB
CakTC26107	Ca(ICCV2/PI489777)SNP_05110	512	A	T	13	10	--	--
	Ca(ICCV2/PI489777)SNP_05111	969	T	C	17	22	--	--
	Ca(ICCV2/PI489777)SNP_05112	1424	A	G	7	9	--	--
CakTC37662	Ca(ICCV2/PI489777)SNP_05113	930	G	A	17	22	--	--
	Ca(ICCV2/PI489777)SNP_05114	941	A	G	13	21	--	--
	Ca(ICCV2/PI489777)SNP_05115	974	T	A	11	20	--	--
	Ca(ICCV2/PI489777)SNP_05116	986	T	A	7	17	--	--
CakTC30619	Ca(ICCV2/PI489777)SNP_05117	322	G	A	3	7	--	--
CakTC35540	Ca(ICCV2/PI489777)SNP_05118	3275	T	C	18	5	--	--
CakTC27975	Ca(ICCV2/PI489777)SNP_05119	526	T	C	4	3	--	--
	Ca(ICCV2/PI489777)SNP_05120	794	C	G	9	6	--	--
	Ca(ICCV2/PI489777)SNP_05121	832	A	G	9	7	--	--
	Ca(ICCV2/PI489777)SNP_05122	956	G	A	7	6	--	--
	Ca(ICCV2/PI489777)SNP_05123	977	C	A	8	7	--	--
	Ca(ICCV2/PI489777)SNP_05124	1046	C	G	7	6	--	--
	Ca(ICCV2/PI489777)SNP_05125	1160	A	G	8	3	--	--
CakTC39072	Ca(ICCV2/PI489777)SNP_05126	574	G	A	27	11	--	--
	Ca(ICCV2/PI489777)SNP_05127	617	G	A	31	16	--	--
	Ca(ICCV2/PI489777)SNP_05128	1031	G	A	15	15	--	--
	Ca(ICCV2/PI489777)SNP_05129	1184	G	T	14	14	--	--
	Ca(ICCV2/PI489777)SNP_05130	1188	C	T	15	15	--	--
CakTC32984	Ca(ICCV2/PI489777)SNP_05131	161	G	A	5	4	--	--
	Ca(ICCV2/PI489777)SNP_05132	258	A	G	7	4	--	--
	Ca(ICCV2/PI489777)SNP_05133	2774	C	T	5	6	--	--
CakTC24812	Ca(ICCV2/PI489777)SNP_05134	210	G	A	11	6	--	--
	Ca(ICCV2/PI489777)SNP_05135	240	A	G	11	6	--	--
	Ca(ICCV2/PI489777)SNP_05136	351	T	G	13	8	--	--
	Ca(ICCV2/PI489777)SNP_05137	359	G	A	14	8	--	--
	Ca(ICCV2/PI489777)SNP_05138	390	T	C	15	35	--	--
	Ca(ICCV2/PI489777)SNP_05139	659	T	C	10	50	--	--
	Ca(ICCV2/PI489777)SNP_05140	731	T	C	8	48	--	--
	Ca(ICCV2/PI489777)SNP_05141	740	G	A	7	47	--	--
CakTC09993	Ca(ICCV2/PI489777)SNP_05142	936	A	G	5	4	--	--
	Ca(ICCV2/PI489777)SNP_05143	1638	G	A	3	3	--	--
	Ca(ICCV2/PI489777)SNP_05144	1937	T	C	4	9	--	--
CakTC22997	Ca(ICCV2/PI489777)SNP_05145	399	A	C	7	6	--	--
CakTC37428	Ca(ICCV2/PI489777)SNP_05146	167	A	G	5	5	Root	--
	Ca(ICCV2/PI489777)SNP_05147	361	A	C	10	16	Root	--
	Ca(ICCV2/PI489777)SNP_05148	565	C	T	9	12	Root	--
	Ca(ICCV2/PI489777)SNP_05149	976	G	A	15	21	Root	--
	Ca(ICCV2/PI489777)SNP_05150	1040	T	C	15	15	Root	--
CakTC23261	Ca(ICCV2/PI489777)SNP_05151	1082	G	A	6	8	--	--
	Ca(ICCV2/PI489777)SNP_05152	1375	G	A	6	11	--	--
	Ca(ICCV2/PI489777)SNP_05153	1987	G	C	3	3	--	--
	Ca(ICCV2/PI489777)SNP_05154	1997	A	T	3	3	--	--
CakTC37688	Ca(ICCV2/PI489777)SNP_05155	90	T	C	4	4	--	--
	Ca(ICCV2/PI489777)SNP_05156	684	G	A	15	4	--	--
	Ca(ICCV2/PI489777)SNP_05157	1081	G	A	8	3	--	--
CakTC09713	Ca(ICCV2/PI489777)SNP_05158	1693	C	T	4	4	--	--
	Ca(ICCV2/PI489777)SNP_05159	1772	A	C	6	4	--	--
	Ca(ICCV2/PI489777)SNP_05160	1871	T	C	5	4	--	--
	Ca(ICCV2/PI489777)SNP_05161	2483	C	A	10	4	--	--
	Ca(ICCV2/PI489777)SNP_05162	2682	A	G	8	3	--	--
CakTC07613	Ca(ICCV2/PI489777)SNP_05163	335	C	T	15	4	--	--
	Ca(ICCV2/PI489777)SNP_05164	398	G	C	18	4	--	--
	Ca(ICCV2/PI489777)SNP_05165	461	A	G	19	3	--	--
	Ca(ICCV2/PI489777)SNP_05166	476	T	C	18	3	--	--
	Ca(ICCV2/PI489777)SNP_05167	731	T	C	23	6	--	--
	Ca(ICCV2/PI489777)SNP_05168	1142	T	C	36	8	--	--
	Ca(ICCV2/PI489777)SNP_05169	1409	T	G	24	11	--	--
CakTC26987	Ca(ICCV2/PI489777)SNP_05170	622	T	C	12	9	--	--
	Ca(ICCV2/PI489777)SNP_05171	640	G	A	13	9	--	--
	Ca(ICCV2/PI489777)SNP_05172	1593	C	T	10	7	--	--
	Ca(ICCV2/PI489777)SNP_05173	1892	T	G	5	3	--	--
CakTC25500	Ca(ICCV2/PI489777)SNP_05174	567	A	G	30	24	--	--
	Ca(ICCV2/PI489777)SNP_05175	1013	G	A	15	11	--	--

CakTC31417	Ca(ICCV2/PI489777)SNP_05176	329	C	A	10	3	Young_pod	--
	Ca(ICCV2/PI489777)SNP_05177	1810	A	C	3	4	Young_pod	--
CakTC28882	Ca(ICCV2/PI489777)SNP_05178	292	T	C	6	6	--	--
	Ca(ICCV2/PI489777)SNP_05179	447	T	C	6	11	--	--
	Ca(ICCV2/PI489777)SNP_05180	694	G	C	10	7	--	--
	Ca(ICCV2/PI489777)SNP_05181	708	C	T	10	6	--	--
	Ca(ICCV2/PI489777)SNP_05182	765	A	G	9	4	--	--
	Ca(ICCV2/PI489777)SNP_05183	804	C	T	8	4	--	--
	Ca(ICCV2/PI489777)SNP_05184	822	T	G	9	3	--	--
	Ca(ICCV2/PI489777)SNP_05185	863	C	A	6	4	--	--
CakTC22827	Ca(ICCV2/PI489777)SNP_05186	1181	C	T	8	3	--	--
CakTC25672	Ca(ICCV2/PI489777)SNP_05187	73	C	A	25	68	--	--
	Ca(ICCV2/PI489777)SNP_05188	226	A	G	72	138	--	--
	Ca(ICCV2/PI489777)SNP_05189	301	A	T	56	142	--	--
	Ca(ICCV2/PI489777)SNP_05190	318	C	T	90	144	--	--
CakTC43149	Ca(ICCV2/PI489777)SNP_05191	160	T	G	6	8	--	--
	Ca(ICCV2/PI489777)SNP_05192	302	C	T	11	31	--	--
CakTC35462	Ca(ICCV2/PI489777)SNP_05193	817	G	A	17	7	--	--
	Ca(ICCV2/PI489777)SNP_05194	1276	T	C	8	16	--	--
	Ca(ICCV2/PI489777)SNP_05195	2384	C	G	10	6	--	--
	Ca(ICCV2/PI489777)SNP_05196	2872	C	T	17	14	--	--
	Ca(ICCV2/PI489777)SNP_05197	3484	G	A	9	7	--	--
	Ca(ICCV2/PI489777)SNP_05198	3798	T	A	5	6	--	--
CakTC29868	Ca(ICCV2/PI489777)SNP_05199	851	T	G	10	11	--	--
	Ca(ICCV2/PI489777)SNP_05200	1049	C	T	11	19	--	--
	Ca(ICCV2/PI489777)SNP_05201	1055	A	G	11	19	--	--
	Ca(ICCV2/PI489777)SNP_05202	1328	T	A	16	14	--	--
	Ca(ICCV2/PI489777)SNP_05203	1699	A	T	7	11	--	--
	Ca(ICCV2/PI489777)SNP_05204	1923	C	T	7	5	--	--
	Ca(ICCV2/PI489777)SNP_05205	1951	G	C	7	5	--	--
	Ca(ICCV2/PI489777)SNP_05206	1954	G	C	7	4	--	--
CakTC40135	Ca(ICCV2/PI489777)SNP_05207	87	T	C	8	3	--	--
CakTC40017	Ca(ICCV2/PI489777)SNP_05208	924	A	G	14	29	--	--
CakTC16902	Ca(ICCV2/PI489777)SNP_05209	686	A	C	20	4	--	--
	Ca(ICCV2/PI489777)SNP_05210	691	C	G	19	4	--	--
CakTC42424	Ca(ICCV2/PI489777)SNP_05211	166	G	A	10	3	--	--
	Ca(ICCV2/PI489777)SNP_05212	206	C	T	12	4	--	--
CakTC42214	Ca(ICCV2/PI489777)SNP_05213	642	T	C	12	18	--	--
CakTC38648	Ca(ICCV2/PI489777)SNP_05214	871	C	A	5	8	--	ULT
CakTC24513	Ca(ICCV2/PI489777)SNP_05215	894	T	C	5	5	--	bZIP
CakTC41055	Ca(ICCV2/PI489777)SNP_05216	184	T	C	12	4	--	--
	Ca(ICCV2/PI489777)SNP_05217	196	A	C	12	4	--	--
	Ca(ICCV2/PI489777)SNP_05218	444	G	A	10	6	--	--
	Ca(ICCV2/PI489777)SNP_05219	546	G	C	4	4	--	--
CakTC23990	Ca(ICCV2/PI489777)SNP_05220	351	C	T	14	3	--	--
	Ca(ICCV2/PI489777)SNP_05221	879	C	T	6	7	--	--
	Ca(ICCV2/PI489777)SNP_05222	1035	A	C	4	6	--	--
CakTC33457	Ca(ICCV2/PI489777)SNP_05223	494	T	A	15	10	--	--
CakTC34443	Ca(ICCV2/PI489777)SNP_05224	113	A	T	15	7	--	--
	Ca(ICCV2/PI489777)SNP_05225	159	C	T	19	8	--	--
	Ca(ICCV2/PI489777)SNP_05226	179	T	C	18	8	--	--
	Ca(ICCV2/PI489777)SNP_05227	197	T	C	18	7	--	--
CakTC30317	Ca(ICCV2/PI489777)SNP_05228	180	A	G	9	12	Shoot	--
	Ca(ICCV2/PI489777)SNP_05229	292	C	T	15	6	Shoot	--
	Ca(ICCV2/PI489777)SNP_05230	370	A	T	15	11	Shoot	--
	Ca(ICCV2/PI489777)SNP_05231	492	C	T	13	10	Shoot	--
	Ca(ICCV2/PI489777)SNP_05232	800	C	G	23	10	Shoot	--
CakTC25599	Ca(ICCV2/PI489777)SNP_05233	207	C	G	88	82	--	--
CakTC43034	Ca(ICCV2/PI489777)SNP_05234	139	A	G	3	4	--	--
	Ca(ICCV2/PI489777)SNP_05235	186	G	A	5	4	--	--
	Ca(ICCV2/PI489777)SNP_05236	430	A	T	9	5	--	--
CakTC37406	Ca(ICCV2/PI489777)SNP_05237	74	C	T	18	5	--	--
CakTC29527	Ca(ICCV2/PI489777)SNP_05238	1527	G	A	7	9	--	--
	Ca(ICCV2/PI489777)SNP_05239	1534	G	A	7	9	--	--
	Ca(ICCV2/PI489777)SNP_05240	1560	G	A	7	9	--	--
CakTC26258	Ca(ICCV2/PI489777)SNP_05241	17	G	T	35	10	--	--
	Ca(ICCV2/PI489777)SNP_05242	57	G	T	64	16	--	--
	Ca(ICCV2/PI489777)SNP_05243	63	A	T	61	17	--	--
	Ca(ICCV2/PI489777)SNP_05244	184	G	T	71	30	--	--
	Ca(ICCV2/PI489777)SNP_05245	196	A	G	75	31	--	--
	Ca(ICCV2/PI489777)SNP_05246	1793	A	G	89	89	--	--
CakTC04785	Ca(ICCV2/PI489777)SNP_05247	497	T	C	10	13	--	--
	Ca(ICCV2/PI489777)SNP_05248	569	T	C	15	19	--	--
	Ca(ICCV2/PI489777)SNP_05249	602	C	T	18	18	--	--

	Ca(ICCV2/PI489777)SNP_05250	899	T	C	23	17	--	--
	Ca(ICCV2/PI489777)SNP_05251	1157	C	T	29	23	--	--
CakTC39298	Ca(ICCV2/PI489777)SNP_05252	104	G	C	19	9	--	--
	Ca(ICCV2/PI489777)SNP_05253	371	G	A	26	11	--	--
CakTC30319	Ca(ICCV2/PI489777)SNP_05254	388	C	T	3	6	--	--
CakTC31505	Ca(ICCV2/PI489777)SNP_05255	366	C	T	3	4	--	--
	Ca(ICCV2/PI489777)SNP_05256	600	A	G	3	4	--	--
	Ca(ICCV2/PI489777)SNP_05257	774	G	A	3	7	--	--
	Ca(ICCV2/PI489777)SNP_05258	783	C	T	3	7	--	--
	Ca(ICCV2/PI489777)SNP_05259	787	G	A	3	7	--	--
CakTC30024	Ca(ICCV2/PI489777)SNP_05260	595	A	G	9	4	--	Alfin-like
	Ca(ICCV2/PI489777)SNP_05261	1171	C	T	13	9	--	Alfin-like
	Ca(ICCV2/PI489777)SNP_05262	2551	T	C	12	9	--	Alfin-like
	Ca(ICCV2/PI489777)SNP_05263	2774	C	T	12	6	--	Alfin-like
	Ca(ICCV2/PI489777)SNP_05264	2923	C	T	16	5	--	Alfin-like
CakTC34589	Ca(ICCV2/PI489777)SNP_05265	125	A	C	17	17	--	--
	Ca(ICCV2/PI489777)SNP_05266	219	C	A	22	26	--	--
	Ca(ICCV2/PI489777)SNP_05267	1124	G	C	35	14	--	--
	Ca(ICCV2/PI489777)SNP_05268	1589	G	A	14	35	--	--
	Ca(ICCV2/PI489777)SNP_05269	1811	G	A	18	32	--	--
	Ca(ICCV2/PI489777)SNP_05270	2093	G	T	11	7	--	--
CakTC33153	Ca(ICCV2/PI489777)SNP_05271	19	C	G	7	3	--	--
	Ca(ICCV2/PI489777)SNP_05272	60	A	G	10	5	--	--
	Ca(ICCV2/PI489777)SNP_05273	636	T	C	31	20	--	--
	Ca(ICCV2/PI489777)SNP_05274	789	T	C	34	25	--	--
	Ca(ICCV2/PI489777)SNP_05275	930	C	T	36	29	--	--
	Ca(ICCV2/PI489777)SNP_05276	1208	C	G	29	23	--	--
	Ca(ICCV2/PI489777)SNP_05277	2259	C	G	24	10	--	--
	Ca(ICCV2/PI489777)SNP_05278	3078	T	A	22	20	--	--
	Ca(ICCV2/PI489777)SNP_05279	4741	G	A	16	11	--	--
	Ca(ICCV2/PI489777)SNP_05280	4815	T	G	9	8	--	--
CakTC40005	Ca(ICCV2/PI489777)SNP_05281	190	C	T	9	4	Mature	Leaf
	Ca(ICCV2/PI489777)SNP_05282	198	C	T	8	4	Mature	Leaf
	Ca(ICCV2/PI489777)SNP_05283	218	C	T	7	4	Mature	Leaf
	Ca(ICCV2/PI489777)SNP_05284	872	T	C	10	8	Mature	Leaf
	Ca(ICCV2/PI489777)SNP_05285	948	G	C	5	5	Mature	Leaf
CakTC37569	Ca(ICCV2/PI489777)SNP_05286	331	T	C	9	9	--	--
	Ca(ICCV2/PI489777)SNP_05287	804	A	G	5	13	--	--
CakTC42953	Ca(ICCV2/PI489777)SNP_05288	154	A	T	27	9	--	--
	Ca(ICCV2/PI489777)SNP_05289	1164	G	A	24	4	--	--
	Ca(ICCV2/PI489777)SNP_05290	1369	T	C	14	19	--	--
CakTC26104	Ca(ICCV2/PI489777)SNP_05291	1577	G	A	13	10	--	--
CakTC27255	Ca(ICCV2/PI489777)SNP_05292	569	T	C	11	4	--	--
	Ca(ICCV2/PI489777)SNP_05293	693	C	T	10	3	--	--
CakTC39549	Ca(ICCV2/PI489777)SNP_05294	150	C	T	44	15	--	--
	Ca(ICCV2/PI489777)SNP_05295	268	C	T	55	17	--	--
	Ca(ICCV2/PI489777)SNP_05296	1205	T	C	9	6	--	--
CakTC42825	Ca(ICCV2/PI489777)SNP_05297	550	G	T	47	57	--	--
CakTC42035	Ca(ICCV2/PI489777)SNP_05298	12	T	C	6	4	--	--
	Ca(ICCV2/PI489777)SNP_05299	13	C	T	6	4	--	--
	Ca(ICCV2/PI489777)SNP_05300	44	T	C	7	11	--	--
	Ca(ICCV2/PI489777)SNP_05301	73	T	C	8	13	--	--
	Ca(ICCV2/PI489777)SNP_05302	203	C	T	9	17	--	--
	Ca(ICCV2/PI489777)SNP_05303	632	T	C	17	7	--	--
	Ca(ICCV2/PI489777)SNP_05304	1295	G	T	11	10	--	--
	Ca(ICCV2/PI489777)SNP_05305	1307	A	G	8	9	--	--
	Ca(ICCV2/PI489777)SNP_05306	1313	G	T	11	9	--	--
	Ca(ICCV2/PI489777)SNP_05307	1517	A	T	9	11	--	--
CakTC30253	Ca(ICCV2/PI489777)SNP_05308	700	C	T	3	3	--	--
	Ca(ICCV2/PI489777)SNP_05309	701	A	G	3	3	--	--
CakTC43163	Ca(ICCV2/PI489777)SNP_05310	698	C	T	3	3	--	--
CakTC30320	Ca(ICCV2/PI489777)SNP_05311	843	G	C	7	10	--	--
	Ca(ICCV2/PI489777)SNP_05312	846	A	G	7	10	--	--
	Ca(ICCV2/PI489777)SNP_05313	1278	C	T	6	10	--	--
	Ca(ICCV2/PI489777)SNP_05314	1331	T	C	8	8	--	--
	Ca(ICCV2/PI489777)SNP_05315	1425	C	G	8	11	--	--
	Ca(ICCV2/PI489777)SNP_05316	1458	G	A	8	12	--	--
	Ca(ICCV2/PI489777)SNP_05317	2034	A	G	4	9	--	--
	Ca(ICCV2/PI489777)SNP_05318	2101	C	G	6	10	--	--
CakTC29908	Ca(ICCV2/PI489777)SNP_05319	828	G	A	5	7	--	--
	Ca(ICCV2/PI489777)SNP_05320	2089	A	G	3	3	--	--
	Ca(ICCV2/PI489777)SNP_05321	2823	T	C	7	11	--	--
CakTC24674	Ca(ICCV2/PI489777)SNP_05322	283	T	C	10	5	--	--
	Ca(ICCV2/PI489777)SNP_05323	285	C	G	10	5	--	--

	Ca(ICCV2/PI489777)SNP_05324	1485	T	C	3	3	--	--
CakTC39660	Ca(ICCV2/PI489777)SNP_05325	312	C	A	10	5	--	--
	Ca(ICCV2/PI489777)SNP_05326	384	G	A	16	7	--	--
	Ca(ICCV2/PI489777)SNP_05327	415	G	A	15	7	--	--
	Ca(ICCV2/PI489777)SNP_05328	462	T	C	14	4	--	--
	Ca(ICCV2/PI489777)SNP_05329	567	C	T	14	3	--	--
	Ca(ICCV2/PI489777)SNP_05330	894	A	G	18	8	--	--
CakTC43014	Ca(ICCV2/PI489777)SNP_05331	1232	A	G	9	9	--	--
	Ca(ICCV2/PI489777)SNP_05332	1320	G	A	5	9	--	--
	Ca(ICCV2/PI489777)SNP_05333	1570	C	G	7	3	--	--
CakTC38049	Ca(ICCV2/PI489777)SNP_05334	303	G	T	19	13	--	--
CakTC30976	Ca(ICCV2/PI489777)SNP_05335	1155	G	A	4	3	--	--
CakTC39588	Ca(ICCV2/PI489777)SNP_05336	1510	A	G	5	4	--	--
CakTC27115	Ca(ICCV2/PI489777)SNP_05337	144	G	C	14	9	--	--
CakTC28730	Ca(ICCV2/PI489777)SNP_05338	72	C	G	41	14	--	--
CakTC41753	Ca(ICCV2/PI489777)SNP_05339	430	G	T	13	22	--	--
	Ca(ICCV2/PI489777)SNP_05340	463	C	T	13	20	--	--
	Ca(ICCV2/PI489777)SNP_05341	660	C	T	14	18	--	--
	Ca(ICCV2/PI489777)SNP_05342	747	A	G	9	11	--	--
	Ca(ICCV2/PI489777)SNP_05343	770	C	T	6	9	--	--
CakTC39197	Ca(ICCV2/PI489777)SNP_05344	729	G	A	15	3	--	--
	Ca(ICCV2/PI489777)SNP_05345	864	T	A	15	8	--	--
CakTC42603	Ca(ICCV2/PI489777)SNP_05346	242	C	T	6	5	--	--
CakTC38750	Ca(ICCV2/PI489777)SNP_05347	84	T	C	8	3	--	--
	Ca(ICCV2/PI489777)SNP_05348	321	G	A	13	8	--	--
	Ca(ICCV2/PI489777)SNP_05349	499	C	T	12	7	--	--
	Ca(ICCV2/PI489777)SNP_05350	663	G	A	6	5	--	--
	Ca(ICCV2/PI489777)SNP_05351	721	T	C	7	8	--	--
	Ca(ICCV2/PI489777)SNP_05352	1074	A	G	15	7	--	--
	Ca(ICCV2/PI489777)SNP_05353	1095	G	A	14	8	--	--
	Ca(ICCV2/PI489777)SNP_05354	1138	C	T	14	7	--	--
	Ca(ICCV2/PI489777)SNP_05355	1149	G	T	14	4	--	--
CakTC39928	Ca(ICCV2/PI489777)SNP_05356	357	T	A	17	11	--	--
	Ca(ICCV2/PI489777)SNP_05357	1002	T	C	9	3	--	--
CakTC33656	Ca(ICCV2/PI489777)SNP_05358	1839	C	T	12	6	--	--
	Ca(ICCV2/PI489777)SNP_05359	3037	A	T	8	5	--	--
	Ca(ICCV2/PI489777)SNP_05360	3721	T	C	5	14	--	--
	Ca(ICCV2/PI489777)SNP_05361	5224	C	T	12	8	--	--
	Ca(ICCV2/PI489777)SNP_05362	5647	G	A	11	17	--	--
CakTC34039	Ca(ICCV2/PI489777)SNP_05363	176	C	T	30	16	--	--
	Ca(ICCV2/PI489777)SNP_05364	272	T	C	35	19	--	--
	Ca(ICCV2/PI489777)SNP_05365	1349	T	C	9	22	--	--
	Ca(ICCV2/PI489777)SNP_05366	1586	G	A	19	24	--	--
	Ca(ICCV2/PI489777)SNP_05367	1595	A	C	19	32	--	--
	Ca(ICCV2/PI489777)SNP_05368	1604	T	G	22	29	--	--
	Ca(ICCV2/PI489777)SNP_05369	1853	C	T	17	16	--	--
CakTC14884	Ca(ICCV2/PI489777)SNP_05370	340	G	A	5	3	--	--
	Ca(ICCV2/PI489777)SNP_05371	884	C	A	12	5	--	--
	Ca(ICCV2/PI489777)SNP_05372	1019	T	A	7	4	--	--
	Ca(ICCV2/PI489777)SNP_05373	1046	T	C	5	3	--	--
CakTC29416	Ca(ICCV2/PI489777)SNP_05374	609	T	C	64	43	--	--
	Ca(ICCV2/PI489777)SNP_05375	678	C	T	63	36	--	--
	Ca(ICCV2/PI489777)SNP_05376	890	C	A	38	24	--	--
CakTC37439	Ca(ICCV2/PI489777)SNP_05377	489	A	G	46	14	--	--
	Ca(ICCV2/PI489777)SNP_05378	549	G	T	44	15	--	--
	Ca(ICCV2/PI489777)SNP_05379	672	A	C	34	17	--	--
	Ca(ICCV2/PI489777)SNP_05380	1095	A	G	23	13	--	--
	Ca(ICCV2/PI489777)SNP_05381	1149	A	G	46	15	--	--
CakTC21118	Ca(ICCV2/PI489777)SNP_05382	187	T	A	10	4	--	--
	Ca(ICCV2/PI489777)SNP_05383	647	A	G	22	11	--	--
CakTC40104	Ca(ICCV2/PI489777)SNP_05384	656	C	T	3	3	--	--
CakTC29950	Ca(ICCV2/PI489777)SNP_05385	35	C	T	16	5	--	--
	Ca(ICCV2/PI489777)SNP_05386	703	T	C	26	14	--	--
	Ca(ICCV2/PI489777)SNP_05387	1003	C	T	29	18	--	--
	Ca(ICCV2/PI489777)SNP_05388	1011	C	A	29	23	--	--
CakTC33228	Ca(ICCV2/PI489777)SNP_05389	355	G	T	7	18	--	--
	Ca(ICCV2/PI489777)SNP_05390	478	C	T	14	20	--	--
	Ca(ICCV2/PI489777)SNP_05391	1199	G	A	17	10	--	--
CakTC23772	Ca(ICCV2/PI489777)SNP_05392	650	C	A	29	6	--	--
	Ca(ICCV2/PI489777)SNP_05393	1260	T	C	15	4	--	--
	Ca(ICCV2/PI489777)SNP_05394	1268	G	A	17	4	--	--
	Ca(ICCV2/PI489777)SNP_05395	1566	T	C	11	3	--	--
	Ca(ICCV2/PI489777)SNP_05396	2775	C	G	13	5	--	--
	Ca(ICCV2/PI489777)SNP_05397	3250	C	G	19	11	--	--

	Ca(ICCV2/PI489777)SNP_05398	3440	C	T	13	8	--	--
CakTC37677	Ca(ICCV2/PI489777)SNP_05399	648	C	T	14	8	Mature	Leaf
	Ca(ICCV2/PI489777)SNP_05400	652	G	A	13	8	Mature	Leaf
	Ca(ICCV2/PI489777)SNP_05401	1170	C	T	4	3	Mature	Leaf
CakTC14908	Ca(ICCV2/PI489777)SNP_05402	186	C	G	4	3	--	--
CakTC34318	Ca(ICCV2/PI489777)SNP_05403	1133	C	T	22	8	--	--
	Ca(ICCV2/PI489777)SNP_05404	1681	G	A	12	4	--	--
	Ca(ICCV2/PI489777)SNP_05405	1691	C	T	12	4	--	--
CakTC38062	Ca(ICCV2/PI489777)SNP_05406	82	A	G	13	24	--	--
	Ca(ICCV2/PI489777)SNP_05407	444	C	T	18	53	--	--
	Ca(ICCV2/PI489777)SNP_05408	639	T	C	15	41	--	--
	Ca(ICCV2/PI489777)SNP_05409	777	T	C	11	32	--	--
	Ca(ICCV2/PI489777)SNP_05410	859	C	G	10	24	--	--
	Ca(ICCV2/PI489777)SNP_05411	994	C	T	4	11	--	--
CakTC24174	Ca(ICCV2/PI489777)SNP_05412	111	T	C	4	7	--	--
	Ca(ICCV2/PI489777)SNP_05413	272	A	T	6	6	--	--
	Ca(ICCV2/PI489777)SNP_05414	1087	T	C	13	14	--	--
CakTC11060	Ca(ICCV2/PI489777)SNP_05415	372	G	T	9	7	--	--
CakTC29979	Ca(ICCV2/PI489777)SNP_05416	23	G	A	4	3	--	--
	Ca(ICCV2/PI489777)SNP_05417	132	T	C	18	9	--	--
	Ca(ICCV2/PI489777)SNP_05418	1246	C	T	39	10	--	--
CakTC27529	Ca(ICCV2/PI489777)SNP_05419	350	G	T	33	27	--	AP2-EREBP
	Ca(ICCV2/PI489777)SNP_05420	729	T	C	29	14	--	AP2-EREBP
	Ca(ICCV2/PI489777)SNP_05421	885	C	A	31	9	--	AP2-EREBP
CakTC34079	Ca(ICCV2/PI489777)SNP_05422	1977	A	T	11	10	--	--
CakTC34537	Ca(ICCV2/PI489777)SNP_05423	546	A	T	4	7	--	--
CakTC32559	Ca(ICCV2/PI489777)SNP_05424	257	G	A	30	26	--	--
CakTC29752	Ca(ICCV2/PI489777)SNP_05425	541	A	G	3	6	--	--
CakTC34886	Ca(ICCV2/PI489777)SNP_05426	311	C	T	5	3	--	--
CakTC39092	Ca(ICCV2/PI489777)SNP_05427	417	T	A	33	17	--	--
	Ca(ICCV2/PI489777)SNP_05428	612	A	G	24	16	--	--
	Ca(ICCV2/PI489777)SNP_05429	707	G	T	24	6	--	--
	Ca(ICCV2/PI489777)SNP_05430	953	T	C	17	5	--	--
CakTC24063	Ca(ICCV2/PI489777)SNP_05431	707	A	C	5	4	--	--
CakTC40924	Ca(ICCV2/PI489777)SNP_05432	235	C	T	11	29	--	--
	Ca(ICCV2/PI489777)SNP_05433	259	A	C	12	27	--	--
	Ca(ICCV2/PI489777)SNP_05434	343	A	T	12	26	--	--
	Ca(ICCV2/PI489777)SNP_05435	502	T	A	14	32	--	--
	Ca(ICCV2/PI489777)SNP_05436	573	G	A	13	16	--	--
	Ca(ICCV2/PI489777)SNP_05437	580	T	C	11	23	--	--
	Ca(ICCV2/PI489777)SNP_05438	1910	A	G	11	13	--	--
CakTC33809	Ca(ICCV2/PI489777)SNP_05439	112	T	A	8	22	--	--
	Ca(ICCV2/PI489777)SNP_05440	190	G	A	15	38	--	--
	Ca(ICCV2/PI489777)SNP_05441	601	C	G	25	24	--	--
CakTC29664	Ca(ICCV2/PI489777)SNP_05442	93	C	T	5	5	--	--
	Ca(ICCV2/PI489777)SNP_05443	169	A	G	5	6	--	--
	Ca(ICCV2/PI489777)SNP_05444	243	T	C	5	6	--	--
	Ca(ICCV2/PI489777)SNP_05445	292	G	T	6	8	--	--
	Ca(ICCV2/PI489777)SNP_05446	429	G	A	4	5	--	--
CakTC10633	Ca(ICCV2/PI489777)SNP_05447	933	C	T	5	5	--	--
	Ca(ICCV2/PI489777)SNP_05448	955	T	A	5	5	--	--
	Ca(ICCV2/PI489777)SNP_05449	987	G	T	3	5	--	--
	Ca(ICCV2/PI489777)SNP_05450	1014	A	C	3	3	--	--
	Ca(ICCV2/PI489777)SNP_05451	1309	C	T	3	4	--	--
	Ca(ICCV2/PI489777)SNP_05452	1365	C	T	6	4	--	--
CakTC39291	Ca(ICCV2/PI489777)SNP_05453	66	G	A	4	3	--	--
	Ca(ICCV2/PI489777)SNP_05454	194	T	C	5	4	--	--
	Ca(ICCV2/PI489777)SNP_05455	987	T	G	8	4	--	--
CakTC07516	Ca(ICCV2/PI489777)SNP_05456	680	A	G	5	10	--	--
CakTC09736	Ca(ICCV2/PI489777)SNP_05457	420	C	T	5	5	--	--
CakTC23458	Ca(ICCV2/PI489777)SNP_05458	849	A	C	9	3	--	CCHC
	Ca(ICCV2/PI489777)SNP_05459	873	A	G	7	4	--	CCHC
	Ca(ICCV2/PI489777)SNP_05460	875	C	T	9	4	--	CCHC
	Ca(ICCV2/PI489777)SNP_05461	893	G	T	8	4	--	CCHC
CakTC28908	Ca(ICCV2/PI489777)SNP_05462	356	A	G	5	3	--	--
	Ca(ICCV2/PI489777)SNP_05463	650	T	C	7	8	--	--
	Ca(ICCV2/PI489777)SNP_05464	663	T	C	8	6	--	--
CakTC28081	Ca(ICCV2/PI489777)SNP_05465	221	T	G	5	4	--	--
	Ca(ICCV2/PI489777)SNP_05466	338	C	A	7	7	--	--
	Ca(ICCV2/PI489777)SNP_05467	368	C	T	6	8	--	--
CakTC10377	Ca(ICCV2/PI489777)SNP_05468	1033	T	C	6	4	--	--
CakTC10741	Ca(ICCV2/PI489777)SNP_05469	1702	A	C	3	6	--	--
CakTC26802	Ca(ICCV2/PI489777)SNP_05470	183	G	C	14	7	--	--
	Ca(ICCV2/PI489777)SNP_05471	441	A	G	17	7	--	--

CakTC34809	Ca(ICCV2/PI489777)SNP_05472	227	G	T	4	8	--	--
	Ca(ICCV2/PI489777)SNP_05473	1489	T	C	5	3	--	--
	Ca(ICCV2/PI489777)SNP_05474	1640	C	A	6	5	--	--
	Ca(ICCV2/PI489777)SNP_05475	1887	G	A	5	4	--	--
	Ca(ICCV2/PI489777)SNP_05476	2144	A	G	5	3	--	--
	Ca(ICCV2/PI489777)SNP_05477	2148	C	T	6	3	--	--
	Ca(ICCV2/PI489777)SNP_05478	2292	A	G	8	3	--	--
	Ca(ICCV2/PI489777)SNP_05479	2931	T	G	8	5	--	--
	Ca(ICCV2/PI489777)SNP_05480	3148	A	G	7	7	--	--
	Ca(ICCV2/PI489777)SNP_05481	3225	T	C	7	7	--	--
CakTC32399	Ca(ICCV2/PI489777)SNP_05482	435	G	A	5	10	--	--
	Ca(ICCV2/PI489777)SNP_05483	514	G	A	7	10	--	--
	Ca(ICCV2/PI489777)SNP_05484	551	A	G	6	8	--	--
	Ca(ICCV2/PI489777)SNP_05485	627	G	T	7	8	--	--
	Ca(ICCV2/PI489777)SNP_05486	899	G	A	5	5	--	--
	Ca(ICCV2/PI489777)SNP_05487	1735	C	T	6	4	--	--
	Ca(ICCV2/PI489777)SNP_05488	2968	T	C	6	3	--	--
	Ca(ICCV2/PI489777)SNP_05489	3247	C	T	3	3	--	--
CakTC42529	Ca(ICCV2/PI489777)SNP_05490	283	T	A	3	3	--	TPR
	Ca(ICCV2/PI489777)SNP_05491	653	C	T	5	3	--	TPR
	Ca(ICCV2/PI489777)SNP_05492	681	C	T	4	3	--	TPR
	Ca(ICCV2/PI489777)SNP_05493	1105	A	G	14	3	--	TPR
CakTC39754	Ca(ICCV2/PI489777)SNP_05494	723	T	A	7	6	--	C2C2-Dof
	Ca(ICCV2/PI489777)SNP_05495	861	C	T	7	4	--	C2C2-Dof
CakTC38699	Ca(ICCV2/PI489777)SNP_05496	120	G	C	4	9	--	--
	Ca(ICCV2/PI489777)SNP_05497	177	T	C	4	9	--	--
	Ca(ICCV2/PI489777)SNP_05498	793	T	C	10	3	--	--
CakTC39217	Ca(ICCV2/PI489777)SNP_05499	308	C	T	4	13	--	--
	Ca(ICCV2/PI489777)SNP_05500	791	T	C	3	10	--	--
	Ca(ICCV2/PI489777)SNP_05501	842	T	C	4	13	--	--
	Ca(ICCV2/PI489777)SNP_05502	893	T	G	4	15	--	--
	Ca(ICCV2/PI489777)SNP_05503	1142	A	T	7	9	--	--
	Ca(ICCV2/PI489777)SNP_05504	1518	T	G	3	3	--	--
CakTC31153	Ca(ICCV2/PI489777)SNP_05505	416	C	G	4	3	--	--
	Ca(ICCV2/PI489777)SNP_05506	445	G	T	4	3	--	--
	Ca(ICCV2/PI489777)SNP_05507	453	A	G	4	3	--	--
CakTC11410	Ca(ICCV2/PI489777)SNP_05508	1073	C	T	3	3	--	--
CakTC10861	Ca(ICCV2/PI489777)SNP_05509	569	A	G	6	3	--	--
	Ca(ICCV2/PI489777)SNP_05510	704	A	G	4	3	--	--
	Ca(ICCV2/PI489777)SNP_05511	836	A	T	6	3	--	--
	Ca(ICCV2/PI489777)SNP_05512	950	T	C	7	3	--	--
	Ca(ICCV2/PI489777)SNP_05513	1766	A	G	7	6	--	--
CakTC42609	Ca(ICCV2/PI489777)SNP_05514	138	A	G	4	8	--	--
	Ca(ICCV2/PI489777)SNP_05515	168	T	C	4	10	--	--
	Ca(ICCV2/PI489777)SNP_05516	323	T	C	7	8	--	--
	Ca(ICCV2/PI489777)SNP_05517	1274	C	T	4	3	--	--
CakTC40471	Ca(ICCV2/PI489777)SNP_05518	145	A	G	45	47	--	--
CakTC36590	Ca(ICCV2/PI489777)SNP_05519	2011	T	C	19	13	Young_pod	--
CakTC29838	Ca(ICCV2/PI489777)SNP_05520	776	C	G	3	4	--	--
	Ca(ICCV2/PI489777)SNP_05521	781	C	G	3	5	--	--
	Ca(ICCV2/PI489777)SNP_05522	1011	C	G	4	5	--	--
	Ca(ICCV2/PI489777)SNP_05523	1149	A	C	4	6	--	--
CakTC29039	Ca(ICCV2/PI489777)SNP_05524	656	G	A	20	10	--	--
	Ca(ICCV2/PI489777)SNP_05525	1301	T	C	17	14	--	--
	Ca(ICCV2/PI489777)SNP_05526	1825	T	C	18	11	--	--
CakTC32025	Ca(ICCV2/PI489777)SNP_05527	354	T	C	3	5	--	GeBP
	Ca(ICCV2/PI489777)SNP_05528	568	G	C	6	11	--	GeBP
	Ca(ICCV2/PI489777)SNP_05529	757	C	T	21	20	--	GeBP
	Ca(ICCV2/PI489777)SNP_05530	1318	A	G	23	18	--	GeBP
	Ca(ICCV2/PI489777)SNP_05531	1366	C	G	31	25	--	GeBP
	Ca(ICCV2/PI489777)SNP_05532	1392	G	T	30	19	--	GeBP
CakTC23134	Ca(ICCV2/PI489777)SNP_05533	83	G	T	5	14	--	--
	Ca(ICCV2/PI489777)SNP_05534	1431	T	A	37	8	--	--
CakTC43371	Ca(ICCV2/PI489777)SNP_05535	49	C	A	11	16	--	--
	Ca(ICCV2/PI489777)SNP_05536	50	T	C	10	16	--	--
	Ca(ICCV2/PI489777)SNP_05537	59	A	G	11	16	--	--
CakTC29948	Ca(ICCV2/PI489777)SNP_05538	3158	T	C	6	3	--	--
	Ca(ICCV2/PI489777)SNP_05539	3407	A	C	6	4	--	--
	Ca(ICCV2/PI489777)SNP_05540	4266	G	A	8	8	--	--
	Ca(ICCV2/PI489777)SNP_05541	4496	A	G	9	11	--	--
	Ca(ICCV2/PI489777)SNP_05542	5206	C	T	10	3	--	--
	Ca(ICCV2/PI489777)SNP_05543	5513	C	T	3	3	--	--
CakTC41035	Ca(ICCV2/PI489777)SNP_05544	443	G	T	87	48	--	--
CakTC41024	Ca(ICCV2/PI489777)SNP_05545	227	A	G	20	11	--	--

	Ca(ICCV2/PI489777)SNP_05546	500	G	A	18	4	--	--
CakTC32132	Ca(ICCV2/PI489777)SNP_05547	1480	A	G	7	11	Root	--
CakTC30644	Ca(ICCV2/PI489777)SNP_05548	198	C	T	27	12	--	--
	Ca(ICCV2/PI489777)SNP_05549	576	C	T	11	8	--	--
	Ca(ICCV2/PI489777)SNP_05550	588	C	T	9	7	--	--
	Ca(ICCV2/PI489777)SNP_05551	633	T	G	12	3	--	--
	Ca(ICCV2/PI489777)SNP_05552	645	C	T	11	3	--	--
	Ca(ICCV2/PI489777)SNP_05553	669	A	G	14	4	--	--
CakTC40319	Ca(ICCV2/PI489777)SNP_05554	571	T	C	17	5	--	--
	Ca(ICCV2/PI489777)SNP_05555	810	A	G	22	10	--	--
	Ca(ICCV2/PI489777)SNP_05556	1898	C	T	33	14	--	--
CakTC26652	Ca(ICCV2/PI489777)SNP_05557	127	T	C	5	4	--	--
	Ca(ICCV2/PI489777)SNP_05558	568	C	A	6	3	--	--
	Ca(ICCV2/PI489777)SNP_05559	1024	A	G	9	4	--	--
	Ca(ICCV2/PI489777)SNP_05560	1129	A	T	8	7	--	--
	Ca(ICCV2/PI489777)SNP_05561	1330	T	C	15	9	--	--
	Ca(ICCV2/PI489777)SNP_05562	1585	G	A	14	4	--	--
	Ca(ICCV2/PI489777)SNP_05563	1798	C	T	21	10	--	--
	Ca(ICCV2/PI489777)SNP_05564	2702	C	T	10	5	--	--
CakTC26570	Ca(ICCV2/PI489777)SNP_05565	530	G	C	3	6	--	--
	Ca(ICCV2/PI489777)SNP_05566	827	A	T	5	5	--	--
	Ca(ICCV2/PI489777)SNP_05567	1043	T	C	20	5	--	--
	Ca(ICCV2/PI489777)SNP_05568	1102	T	C	23	6	--	--
	Ca(ICCV2/PI489777)SNP_05569	1474	A	T	4	6	--	--
CakTC32390	Ca(ICCV2/PI489777)SNP_05570	683	G	C	112	89	Flower	bud
CakTC39200	Ca(ICCV2/PI489777)SNP_05571	119	G	C	7	11	--	--
CakTC09332	Ca(ICCV2/PI489777)SNP_05572	929	A	G	6	3	--	--
	Ca(ICCV2/PI489777)SNP_05573	2120	C	A	3	6	--	--
CakTC32522	Ca(ICCV2/PI489777)SNP_05574	199	T	C	7	3	--	--
	Ca(ICCV2/PI489777)SNP_05575	1684	C	T	6	3	--	--
CakTC43265	Ca(ICCV2/PI489777)SNP_05576	1251	C	T	16	22	--	--
	Ca(ICCV2/PI489777)SNP_05577	1290	C	T	17	20	--	--
	Ca(ICCV2/PI489777)SNP_05578	1410	G	A	15	15	--	--
	Ca(ICCV2/PI489777)SNP_05579	1494	A	T	11	11	--	--
	Ca(ICCV2/PI489777)SNP_05580	1563	A	G	10	11	--	--
	Ca(ICCV2/PI489777)SNP_05581	1649	T	C	5	3	--	--
	Ca(ICCV2/PI489777)SNP_05582	1652	T	C	3	4	--	--
	Ca(ICCV2/PI489777)SNP_05583	1699	C	T	4	4	--	--
CakTC39127	Ca(ICCV2/PI489777)SNP_05584	131	T	C	15	21	--	bZIP
	Ca(ICCV2/PI489777)SNP_05585	262	C	T	17	26	--	bZIP
	Ca(ICCV2/PI489777)SNP_05586	274	G	A	21	20	--	bZIP
	Ca(ICCV2/PI489777)SNP_05587	1171	T	G	24	54	--	bZIP
CakTC02884	Ca(ICCV2/PI489777)SNP_05588	58	G	A	12	8	--	--
	Ca(ICCV2/PI489777)SNP_05589	153	A	C	13	7	--	--
	Ca(ICCV2/PI489777)SNP_05590	943	A	G	3	7	--	--
	Ca(ICCV2/PI489777)SNP_05591	1944	T	C	6	5	--	--
	Ca(ICCV2/PI489777)SNP_05592	2081	C	T	7	4	--	--
CakTC24765	Ca(ICCV2/PI489777)SNP_05593	103	A	C	49	26	--	--
	Ca(ICCV2/PI489777)SNP_05594	224	T	A	55	38	--	--
	Ca(ICCV2/PI489777)SNP_05595	272	T	A	64	29	--	--
	Ca(ICCV2/PI489777)SNP_05596	302	C	T	73	41	--	--
	Ca(ICCV2/PI489777)SNP_05597	1211	T	G	80	33	--	--
	Ca(ICCV2/PI489777)SNP_05598	1730	C	T	25	20	--	--
	Ca(ICCV2/PI489777)SNP_05599	1753	C	G	34	10	--	--
	Ca(ICCV2/PI489777)SNP_05600	1897	C	G	24	6	--	--
	Ca(ICCV2/PI489777)SNP_05601	1965	T	C	22	4	--	--
CakTC31657	Ca(ICCV2/PI489777)SNP_05602	978	G	C	7	3	--	--
	Ca(ICCV2/PI489777)SNP_05603	1191	G	C	6	3	--	--
	Ca(ICCV2/PI489777)SNP_05604	2197	C	G	7	6	--	--
CakTC26056	Ca(ICCV2/PI489777)SNP_05605	1231	G	A	3	3	--	--
	Ca(ICCV2/PI489777)SNP_05606	1264	A	G	3	3	--	--
CakTC26036	Ca(ICCV2/PI489777)SNP_05607	487	A	G	4	5	--	G2-like
	Ca(ICCV2/PI489777)SNP_05608	535	G	A	4	4	--	G2-like
	Ca(ICCV2/PI489777)SNP_05609	583	G	C	4	5	--	G2-like
	Ca(ICCV2/PI489777)SNP_05610	1116	A	G	11	7	--	G2-like
CakTC32340	Ca(ICCV2/PI489777)SNP_05611	846	G	C	6	8	Young_pod	--
CakTC13059	Ca(ICCV2/PI489777)SNP_05612	289	G	A	4	4	--	--
	Ca(ICCV2/PI489777)SNP_05613	445	C	T	3	5	--	--
	Ca(ICCV2/PI489777)SNP_05614	544	A	G	3	5	--	--
	Ca(ICCV2/PI489777)SNP_05615	905	G	A	3	6	--	--
	Ca(ICCV2/PI489777)SNP_05616	986	A	G	3	9	--	--
	Ca(ICCV2/PI489777)SNP_05617	1217	A	T	5	7	--	--
	Ca(ICCV2/PI489777)SNP_05618	1241	G	T	5	4	--	--
	Ca(ICCV2/PI489777)SNP_05619	1374	C	G	5	4	--	--

	Ca(ICCV2/PI489777)SNP_05620	1404	G	C	6	4	--	--
CakTC23844	Ca(ICCV2/PI489777)SNP_05621	285	C	T	34	11	Shoot	--
	Ca(ICCV2/PI489777)SNP_05622	317	T	G	35	12	Shoot	--
	Ca(ICCV2/PI489777)SNP_05623	528	T	C	20	8	Shoot	--
CakTC38317	Ca(ICCV2/PI489777)SNP_05624	102	G	A	10	11	--	--
	Ca(ICCV2/PI489777)SNP_05625	440	T	C	15	21	--	--
	Ca(ICCV2/PI489777)SNP_05626	1075	C	A	10	8	--	--
CakTC25477	Ca(ICCV2/PI489777)SNP_05627	331	T	C	7	11	--	--
	Ca(ICCV2/PI489777)SNP_05628	479	A	G	7	9	--	--
CakTC38561	Ca(ICCV2/PI489777)SNP_05629	324	A	G	11	4	--	--
CakTC42136	Ca(ICCV2/PI489777)SNP_05630	671	G	A	10	6	Shoot	--
	Ca(ICCV2/PI489777)SNP_05631	824	T	A	12	13	Shoot	--
	Ca(ICCV2/PI489777)SNP_05632	881	A	G	9	11	Shoot	--
CakTC29460	Ca(ICCV2/PI489777)SNP_05633	2449	A	G	5	4	--	--
	Ca(ICCV2/PI489777)SNP_05634	2474	A	C	5	4	--	--
CakTC35396	Ca(ICCV2/PI489777)SNP_05635	829	C	T	15	11	--	--
	Ca(ICCV2/PI489777)SNP_05636	1316	A	G	13	17	--	--
	Ca(ICCV2/PI489777)SNP_05637	2116	T	C	25	21	--	--
	Ca(ICCV2/PI489777)SNP_05638	2167	G	C	20	12	--	--
	Ca(ICCV2/PI489777)SNP_05639	3079	A	G	9	7	--	--
CakTC35596	Ca(ICCV2/PI489777)SNP_05640	735	A	T	5	3	--	--
	Ca(ICCV2/PI489777)SNP_05641	1196	A	C	10	11	--	--
	Ca(ICCV2/PI489777)SNP_05642	1430	T	G	9	9	--	--
	Ca(ICCV2/PI489777)SNP_05643	1439	C	T	8	9	--	--
	Ca(ICCV2/PI489777)SNP_05644	1994	G	A	14	4	--	--
	Ca(ICCV2/PI489777)SNP_05645	3191	G	A	12	4	--	--
CakTC41797	Ca(ICCV2/PI489777)SNP_05646	83	C	G	4	3	--	--
	Ca(ICCV2/PI489777)SNP_05647	1167	A	C	42	30	--	--
	Ca(ICCV2/PI489777)SNP_05648	1168	G	A	42	30	--	--
	Ca(ICCV2/PI489777)SNP_05649	1866	C	G	21	11	--	--
CakTC41811	Ca(ICCV2/PI489777)SNP_05650	130	C	T	3	5	--	--
	Ca(ICCV2/PI489777)SNP_05651	148	T	C	3	5	--	--
	Ca(ICCV2/PI489777)SNP_05652	191	C	T	3	4	--	--
	Ca(ICCV2/PI489777)SNP_05653	223	A	C	3	4	--	--
	Ca(ICCV2/PI489777)SNP_05654	253	T	C	3	5	--	--
	Ca(ICCV2/PI489777)SNP_05655	268	T	C	3	6	--	--
	Ca(ICCV2/PI489777)SNP_05656	328	C	T	3	9	--	--
	Ca(ICCV2/PI489777)SNP_05657	352	C	A	3	8	--	--
	Ca(ICCV2/PI489777)SNP_05658	652	G	A	7	9	--	--
	Ca(ICCV2/PI489777)SNP_05659	688	G	A	10	5	--	--
	Ca(ICCV2/PI489777)SNP_05660	919	T	C	13	9	--	--
	Ca(ICCV2/PI489777)SNP_05661	934	T	C	16	10	--	--
	Ca(ICCV2/PI489777)SNP_05662	1504	G	A	19	12	--	--
	Ca(ICCV2/PI489777)SNP_05663	1561	G	T	17	10	--	--
	Ca(ICCV2/PI489777)SNP_05664	2005	T	A	9	9	--	--
	Ca(ICCV2/PI489777)SNP_05665	2263	A	G	3	9	--	--
CakTC10073	Ca(ICCV2/PI489777)SNP_05666	268	T	C	5	3	--	--
CakTC38205	Ca(ICCV2/PI489777)SNP_05667	502	A	G	4	4	--	--
	Ca(ICCV2/PI489777)SNP_05668	532	T	C	3	3	--	--
	Ca(ICCV2/PI489777)SNP_05669	628	A	G	3	8	--	--
	Ca(ICCV2/PI489777)SNP_05670	841	C	T	3	6	--	--
	Ca(ICCV2/PI489777)SNP_05671	1099	T	G	4	6	--	--
	Ca(ICCV2/PI489777)SNP_05672	1105	T	C	4	4	--	--
	Ca(ICCV2/PI489777)SNP_05673	1262	A	G	3	4	--	--
	Ca(ICCV2/PI489777)SNP_05674	1406	C	G	3	4	--	--
CakTC26602	Ca(ICCV2/PI489777)SNP_05675	780	C	T	4	14	--	--
	Ca(ICCV2/PI489777)SNP_05676	885	T	A	17	18	--	--
	Ca(ICCV2/PI489777)SNP_05677	993	T	C	29	22	--	--
	Ca(ICCV2/PI489777)SNP_05678	1212	G	A	28	21	--	--
CakTC08287	Ca(ICCV2/PI489777)SNP_05679	309	T	G	11	4	--	--
	Ca(ICCV2/PI489777)SNP_05680	541	A	G	11	9	--	--
	Ca(ICCV2/PI489777)SNP_05681	868	T	C	8	8	--	--
	Ca(ICCV2/PI489777)SNP_05682	943	A	T	8	8	--	--
	Ca(ICCV2/PI489777)SNP_05683	960	C	T	8	8	--	--
	Ca(ICCV2/PI489777)SNP_05684	1953	T	C	5	4	--	--
	Ca(ICCV2/PI489777)SNP_05685	2008	C	T	4	7	--	--
	Ca(ICCV2/PI489777)SNP_05686	2021	A	G	4	7	--	--
	Ca(ICCV2/PI489777)SNP_05687	2151	C	T	6	5	--	--
	Ca(ICCV2/PI489777)SNP_05688	2193	G	C	5	5	--	--
	Ca(ICCV2/PI489777)SNP_05689	2207	G	C	5	3	--	--
CakTC32003	Ca(ICCV2/PI489777)SNP_05690	2127	C	T	5	3	--	HB
	Ca(ICCV2/PI489777)SNP_05691	2145	C	T	6	3	--	HB
	Ca(ICCV2/PI489777)SNP_05692	2256	C	T	7	3	--	HB
CakTC36026	Ca(ICCV2/PI489777)SNP_05693	80	T	C	52	40	--	--

CakTC00458	Ca(ICCV2/PI489777)SNP_05694	203	G	A	16	13	--	--
	Ca(ICCV2/PI489777)SNP_05695	417	T	G	50	27	--	--
	Ca(ICCV2/PI489777)SNP_05696	498	A	G	54	25	--	--
	Ca(ICCV2/PI489777)SNP_05697	744	T	A	12	4	--	--
CakTC35916	Ca(ICCV2/PI489777)SNP_05698	667	T	C	4	4	Shoot	--
	Ca(ICCV2/PI489777)SNP_05699	915	T	C	6	9	Shoot	--
	Ca(ICCV2/PI489777)SNP_05700	1039	T	A	5	10	Shoot	--
	Ca(ICCV2/PI489777)SNP_05701	1048	A	G	4	10	Shoot	--
CakTC10803	Ca(ICCV2/PI489777)SNP_05702	246	G	A	29	18	--	--
	Ca(ICCV2/PI489777)SNP_05703	460	T	G	60	30	--	--
	Ca(ICCV2/PI489777)SNP_05704	541	A	G	60	29	--	--
	Ca(ICCV2/PI489777)SNP_05705	787	T	A	44	29	--	--
	Ca(ICCV2/PI489777)SNP_05706	886	C	T	55	34	--	--
	Ca(ICCV2/PI489777)SNP_05707	925	T	G	62	38	--	--
	Ca(ICCV2/PI489777)SNP_05708	928	G	A	63	38	--	--
	Ca(ICCV2/PI489777)SNP_05709	1099	A	G	51	31	--	--
CakTC29043	Ca(ICCV2/PI489777)SNP_05710	88	G	C	6	3	--	--
	Ca(ICCV2/PI489777)SNP_05711	753	T	G	11	23	--	--
	Ca(ICCV2/PI489777)SNP_05712	774	T	C	9	21	--	--
	Ca(ICCV2/PI489777)SNP_05713	927	C	T	16	17	--	--
	Ca(ICCV2/PI489777)SNP_05714	1208	G	A	12	6	--	--
CakTC27645	Ca(ICCV2/PI489777)SNP_05715	685	T	G	3	3	--	--
CakTC37832	Ca(ICCV2/PI489777)SNP_05716	61	G	A	12	3	Root	--
	Ca(ICCV2/PI489777)SNP_05717	87	T	G	14	4	Root	--
	Ca(ICCV2/PI489777)SNP_05718	1276	T	C	4	3	Root	--
CakTC25768	Ca(ICCV2/PI489777)SNP_05719	1089	T	C	3	3	--	--
CakTC31123	Ca(ICCV2/PI489777)SNP_05720	2172	C	T	15	10	--	--
	Ca(ICCV2/PI489777)SNP_05721	2374	A	T	5	5	--	--
CakTC28302	Ca(ICCV2/PI489777)SNP_05722	81	T	A	6	16	--	--
	Ca(ICCV2/PI489777)SNP_05723	876	T	C	14	16	--	--
CakTC00995	Ca(ICCV2/PI489777)SNP_05724	212	A	G	5	5	--	--
	Ca(ICCV2/PI489777)SNP_05725	656	A	G	13	12	--	--
	Ca(ICCV2/PI489777)SNP_05726	815	G	A	12	14	--	--
	Ca(ICCV2/PI489777)SNP_05727	857	G	T	8	10	--	--
CakTC38904	Ca(ICCV2/PI489777)SNP_05728	705	A	G	3	3	--	--
	Ca(ICCV2/PI489777)SNP_05729	951	T	A	6	3	--	--
	Ca(ICCV2/PI489777)SNP_05730	1140	C	T	7	8	--	--
	Ca(ICCV2/PI489777)SNP_05731	1292	A	G	4	7	--	--
CakTC25275	Ca(ICCV2/PI489777)SNP_05732	220	A	T	72	10	--	--
	Ca(ICCV2/PI489777)SNP_05733	444	C	T	87	11	--	--
	Ca(ICCV2/PI489777)SNP_05734	639	T	A	80	5	--	--
	Ca(ICCV2/PI489777)SNP_05735	1274	T	C	63	16	--	--
	Ca(ICCV2/PI489777)SNP_05736	1306	G	A	78	15	--	--
	Ca(ICCV2/PI489777)SNP_05737	1386	G	T	101	15	--	--
CakTC24719	Ca(ICCV2/PI489777)SNP_05738	453	G	A	27	23	--	bZIP
	Ca(ICCV2/PI489777)SNP_05739	571	G	A	42	37	--	bZIP
	Ca(ICCV2/PI489777)SNP_05740	739	G	T	63	64	--	bZIP
	Ca(ICCV2/PI489777)SNP_05741	1204	G	C	6	19	--	bZIP
CakTC28589	Ca(ICCV2/PI489777)SNP_05742	517	T	C	28	19	--	--
CakTC11636	Ca(ICCV2/PI489777)SNP_05743	17	A	G	4	3	--	--
	Ca(ICCV2/PI489777)SNP_05744	33	T	G	4	3	--	--
CakTC40609	Ca(ICCV2/PI489777)SNP_05745	42	G	C	53	10	Flower	bud
	Ca(ICCV2/PI489777)SNP_05746	171	G	A	65	33	Flower	bud
	Ca(ICCV2/PI489777)SNP_05747	240	A	G	81	53	Flower	bud
	Ca(ICCV2/PI489777)SNP_05748	345	T	A	141	108	Flower	bud
	Ca(ICCV2/PI489777)SNP_05749	567	A	G	125	106	Flower	bud
	Ca(ICCV2/PI489777)SNP_05750	600	G	C	118	85	Flower	bud
CakTC27796	Ca(ICCV2/PI489777)SNP_05751	295	A	G	12	9	--	--
	Ca(ICCV2/PI489777)SNP_05752	373	T	C	15	14	--	--
	Ca(ICCV2/PI489777)SNP_05753	656	A	C	9	8	--	--
	Ca(ICCV2/PI489777)SNP_05754	1257	C	T	35	4	--	--
	Ca(ICCV2/PI489777)SNP_05755	1780	A	G	10	7	--	--
CakTC33096	Ca(ICCV2/PI489777)SNP_05756	421	G	A	63	28	--	--
CakTC31105	Ca(ICCV2/PI489777)SNP_05757	804	A	G	6	10	--	--
	Ca(ICCV2/PI489777)SNP_05758	919	T	C	7	10	--	--
	Ca(ICCV2/PI489777)SNP_05759	1088	A	G	9	6	--	--
CakTC22650	Ca(ICCV2/PI489777)SNP_05760	482	T	C	3	4	--	--
	Ca(ICCV2/PI489777)SNP_05761	505	A	T	4	3	--	--
	Ca(ICCV2/PI489777)SNP_05762	569	T	A	4	3	--	--
	Ca(ICCV2/PI489777)SNP_05763	579	C	G	4	3	--	--
CakTC05382	Ca(ICCV2/PI489777)SNP_05764	229	G	C	9	6	--	--
CakTC42363	Ca(ICCV2/PI489777)SNP_05765	1031	G	A	20	13	--	bZIP
CakTC31038	Ca(ICCV2/PI489777)SNP_05766	170	G	C	4	4	--	C2C2-Dof
	Ca(ICCV2/PI489777)SNP_05767	226	T	C	3	4	--	C2C2-Dof

CakTC41776	Ca(ICCV2/PI489777)SNP_05768	482	A	G	24	43	--	--
CakTC39476	Ca(ICCV2/PI489777)SNP_05769	195	T	C	3	4	--	--
	Ca(ICCV2/PI489777)SNP_05770	262	C	A	5	3	--	--
CakTC28109	Ca(ICCV2/PI489777)SNP_05771	124	G	T	10	3	--	--
	Ca(ICCV2/PI489777)SNP_05772	1749	C	G	6	3	--	--
	Ca(ICCV2/PI489777)SNP_05773	1754	T	A	6	3	--	--
CakTC08500	Ca(ICCV2/PI489777)SNP_05774	393	T	C	3	6	--	--
	Ca(ICCV2/PI489777)SNP_05775	579	A	T	11	5	--	--
	Ca(ICCV2/PI489777)SNP_05776	723	A	T	15	9	--	--
	Ca(ICCV2/PI489777)SNP_05777	1779	T	G	8	3	--	--
CakTC37413	Ca(ICCV2/PI489777)SNP_05778	219	A	G	11	3	--	--
	Ca(ICCV2/PI489777)SNP_05779	261	T	C	11	3	--	--
	Ca(ICCV2/PI489777)SNP_05780	270	A	C	11	3	--	--
CakTC26531	Ca(ICCV2/PI489777)SNP_05781	1212	G	A	3	4	Young_pod	--
CakTC03882	Ca(ICCV2/PI489777)SNP_05782	495	T	A	6	3	--	--
	Ca(ICCV2/PI489777)SNP_05783	608	T	C	3	3	--	--
	Ca(ICCV2/PI489777)SNP_05784	1142	A	G	3	3	--	--
	Ca(ICCV2/PI489777)SNP_05785	1210	T	C	3	3	--	--
CakTC38429	Ca(ICCV2/PI489777)SNP_05786	1397	C	T	7	6	--	--
	Ca(ICCV2/PI489777)SNP_05787	1506	T	G	16	7	--	--
CakTC24868	Ca(ICCV2/PI489777)SNP_05788	522	A	G	21	4	--	--
CakTC22877	Ca(ICCV2/PI489777)SNP_05789	820	T	A	38	3	--	--
CakTC31975	Ca(ICCV2/PI489777)SNP_05790	1266	T	C	4	6	Flower	bud
CakTC42078	Ca(ICCV2/PI489777)SNP_05791	497	A	G	16	12	--	--
CakTC37219	Ca(ICCV2/PI489777)SNP_05792	1071	G	T	5	8	--	--
CakTC22603	Ca(ICCV2/PI489777)SNP_05793	1137	C	T	16	4	--	GNAT
	Ca(ICCV2/PI489777)SNP_05794	3021	C	T	8	14	--	GNAT
	Ca(ICCV2/PI489777)SNP_05795	3169	C	T	24	22	--	GNAT
	Ca(ICCV2/PI489777)SNP_05796	3269	T	C	28	21	--	GNAT
CakTC40775	Ca(ICCV2/PI489777)SNP_05797	404	T	G	16	8	--	--
	Ca(ICCV2/PI489777)SNP_05798	661	C	A	11	8	--	--
	Ca(ICCV2/PI489777)SNP_05799	711	A	G	4	7	--	--
	Ca(ICCV2/PI489777)SNP_05800	753	T	C	3	7	--	--
CakTC23590	Ca(ICCV2/PI489777)SNP_05801	191	A	G	99	26	--	--
	Ca(ICCV2/PI489777)SNP_05802	204	C	T	103	22	--	--
	Ca(ICCV2/PI489777)SNP_05803	340	A	C	122	26	--	--
CakTC24011	Ca(ICCV2/PI489777)SNP_05804	530	C	T	3	6	--	--
	Ca(ICCV2/PI489777)SNP_05805	766	T	G	20	7	--	--
	Ca(ICCV2/PI489777)SNP_05806	796	T	C	21	8	--	--
	Ca(ICCV2/PI489777)SNP_05807	1311	C	T	24	7	--	--
	Ca(ICCV2/PI489777)SNP_05808	2120	C	G	12	6	--	--
	Ca(ICCV2/PI489777)SNP_05809	2151	A	T	12	6	--	--
CakTC34772	Ca(ICCV2/PI489777)SNP_05810	1300	A	G	10	7	--	--
	Ca(ICCV2/PI489777)SNP_05811	3073	T	C	18	11	--	--
	Ca(ICCV2/PI489777)SNP_05812	3339	G	A	12	6	--	--
CakTC40768	Ca(ICCV2/PI489777)SNP_05813	897	C	T	8	8	--	--
	Ca(ICCV2/PI489777)SNP_05814	1272	A	G	12	6	--	--
	Ca(ICCV2/PI489777)SNP_05815	1685	G	C	15	3	--	--
CakTC31192	Ca(ICCV2/PI489777)SNP_05816	1052	C	T	5	3	--	--
	Ca(ICCV2/PI489777)SNP_05817	1230	T	A	4	3	--	--
CakTC25003	Ca(ICCV2/PI489777)SNP_05818	593	G	C	9	3	--	--
	Ca(ICCV2/PI489777)SNP_05819	737	A	T	9	7	--	--
CakTC03007	Ca(ICCV2/PI489777)SNP_05820	1165	G	C	6	8	--	--
	Ca(ICCV2/PI489777)SNP_05821	1632	A	G	6	5	--	--
CakTC22703	Ca(ICCV2/PI489777)SNP_05822	886	C	T	6	7	--	--
	Ca(ICCV2/PI489777)SNP_05823	903	C	G	6	7	--	--
	Ca(ICCV2/PI489777)SNP_05824	1004	G	A	9	10	--	--
CakTC43088	Ca(ICCV2/PI489777)SNP_05825	146	G	C	4	3	--	--
	Ca(ICCV2/PI489777)SNP_05826	447	A	G	10	9	--	--
	Ca(ICCV2/PI489777)SNP_05827	496	T	A	9	9	--	--
	Ca(ICCV2/PI489777)SNP_05828	502	C	T	9	18	--	--
	Ca(ICCV2/PI489777)SNP_05829	774	T	G	18	25	--	--
	Ca(ICCV2/PI489777)SNP_05830	838	A	T	17	22	--	--
	Ca(ICCV2/PI489777)SNP_05831	1450	T	A	6	7	--	--
CakTC33680	Ca(ICCV2/PI489777)SNP_05832	1288	T	C	29	23	--	--
CakTC33799	Ca(ICCV2/PI489777)SNP_05833	476	T	C	6	5	--	--
	Ca(ICCV2/PI489777)SNP_05834	2090	T	C	13	4	--	--
	Ca(ICCV2/PI489777)SNP_05835	9998	A	G	15	4	--	--
CakTC37052	Ca(ICCV2/PI489777)SNP_05836	194	C	A	4	7	--	--
	Ca(ICCV2/PI489777)SNP_05837	1399	T	A	19	10	--	--
CakTC08596	Ca(ICCV2/PI489777)SNP_05838	113	T	C	15	10	--	--
	Ca(ICCV2/PI489777)SNP_05839	171	A	T	42	16	--	--
	Ca(ICCV2/PI489777)SNP_05840	900	T	A	65	21	--	--
	Ca(ICCV2/PI489777)SNP_05841	957	T	C	61	20	--	--

	Ca(ICCV2/PI489777)SNP_05842	1150	A	T	70	22	--	--
CakTC28347	Ca(ICCV2/PI489777)SNP_05843	519	T	C	3	3	--	--
	Ca(ICCV2/PI489777)SNP_05844	1790	G	A	4	3	--	--
CakTC25979	Ca(ICCV2/PI489777)SNP_05845	444	A	G	4	4	--	--
	Ca(ICCV2/PI489777)SNP_05846	1886	C	T	3	3	--	--
	Ca(ICCV2/PI489777)SNP_05847	1955	C	T	4	3	--	--
CakTC42861	Ca(ICCV2/PI489777)SNP_05848	191	T	C	13	5	--	--
	Ca(ICCV2/PI489777)SNP_05849	416	G	A	20	6	--	--
	Ca(ICCV2/PI489777)SNP_05850	810	T	G	14	10	--	--
CakTC37762	Ca(ICCV2/PI489777)SNP_05851	562	A	G	16	12	--	--
	Ca(ICCV2/PI489777)SNP_05852	607	T	C	18	17	--	--
	Ca(ICCV2/PI489777)SNP_05853	682	G	T	19	13	--	--
CakTC34972	Ca(ICCV2/PI489777)SNP_05854	646	A	G	10	7	--	--
CakTC23320	Ca(ICCV2/PI489777)SNP_05855	1292	A	T	5	4	--	--
CakTC38213	Ca(ICCV2/PI489777)SNP_05856	158	T	C	7	6	--	--
	Ca(ICCV2/PI489777)SNP_05857	638	T	C	59	47	--	--
CakTC10708	Ca(ICCV2/PI489777)SNP_05858	1061	C	T	19	14	--	--
CakTC37614	Ca(ICCV2/PI489777)SNP_05859	95	A	G	28	9	--	--
CakTC24266	Ca(ICCV2/PI489777)SNP_05860	1682	G	A	20	6	--	--
CakTC14228	Ca(ICCV2/PI489777)SNP_05861	2562	C	T	5	6	--	--
CakTC13196	Ca(ICCV2/PI489777)SNP_05862	316	A	C	10	9	--	--
	Ca(ICCV2/PI489777)SNP_05863	390	C	G	21	12	--	--
	Ca(ICCV2/PI489777)SNP_05864	526	C	T	12	12	--	--
	Ca(ICCV2/PI489777)SNP_05865	711	A	G	14	8	--	--
CakTC26712	Ca(ICCV2/PI489777)SNP_05866	586	A	G	27	16	--	--
CakTC25620	Ca(ICCV2/PI489777)SNP_05867	565	T	A	3	3	Flower	bud
CakTC36547	Ca(ICCV2/PI489777)SNP_05868	1498	C	G	3	5	--	--
CakTC33437	Ca(ICCV2/PI489777)SNP_05869	291	C	T	7	11	--	--
	Ca(ICCV2/PI489777)SNP_05870	527	G	C	7	13	--	--
CakTC43297	Ca(ICCV2/PI489777)SNP_05871	510	A	G	50	13	--	--
	Ca(ICCV2/PI489777)SNP_05872	968	T	C	23	20	--	--
CakTC15365	Ca(ICCV2/PI489777)SNP_05873	992	T	C	6	6	--	--
	Ca(ICCV2/PI489777)SNP_05874	1094	T	C	8	5	--	--
	Ca(ICCV2/PI489777)SNP_05875	1559	T	C	9	4	--	--
	Ca(ICCV2/PI489777)SNP_05876	3260	C	T	11	5	--	--
	Ca(ICCV2/PI489777)SNP_05877	3707	T	C	3	6	--	--
CakTC23956	Ca(ICCV2/PI489777)SNP_05878	546	T	C	3	3	--	--
	Ca(ICCV2/PI489777)SNP_05879	551	A	G	3	3	--	--
	Ca(ICCV2/PI489777)SNP_05880	557	G	C	3	3	--	--
CakTC40534	Ca(ICCV2/PI489777)SNP_05881	1078	G	A	4	3	--	--
CakTC26668	Ca(ICCV2/PI489777)SNP_05882	1376	C	T	13	21	--	--
	Ca(ICCV2/PI489777)SNP_05883	1409	G	T	15	23	--	--
	Ca(ICCV2/PI489777)SNP_05884	1493	G	A	13	22	--	--
	Ca(ICCV2/PI489777)SNP_05885	1538	G	A	14	26	--	--
	Ca(ICCV2/PI489777)SNP_05886	1836	G	A	6	5	--	--
CakTC11873	Ca(ICCV2/PI489777)SNP_05887	495	T	C	5	8	--	C3H
CakTC42995	Ca(ICCV2/PI489777)SNP_05888	165	G	A	10	10	--	--
	Ca(ICCV2/PI489777)SNP_05889	1614	G	T	11	7	--	--
CakTC30899	Ca(ICCV2/PI489777)SNP_05890	1121	G	A	5	4	--	--
CakTC11091	Ca(ICCV2/PI489777)SNP_05891	346	C	T	5	6	--	--
	Ca(ICCV2/PI489777)SNP_05892	436	A	G	6	6	--	--
	Ca(ICCV2/PI489777)SNP_05893	1177	C	T	11	6	--	--
	Ca(ICCV2/PI489777)SNP_05894	1185	G	A	12	6	--	--
	Ca(ICCV2/PI489777)SNP_05895	1275	G	C	11	4	--	--
	Ca(ICCV2/PI489777)SNP_05896	2773	G	C	16	5	--	--
CakTC43253	Ca(ICCV2/PI489777)SNP_05897	840	T	A	16	6	--	--
CakTC31185	Ca(ICCV2/PI489777)SNP_05898	719	A	C	5	5	--	--
CakTC35847	Ca(ICCV2/PI489777)SNP_05899	163	G	T	12	4	--	--
	Ca(ICCV2/PI489777)SNP_05900	746	T	C	16	7	--	--
	Ca(ICCV2/PI489777)SNP_05901	1853	C	A	14	8	--	--
CakTC22357	Ca(ICCV2/PI489777)SNP_05902	741	C	T	6	4	--	--
	Ca(ICCV2/PI489777)SNP_05903	1635	G	A	6	5	--	--
	Ca(ICCV2/PI489777)SNP_05904	1815	C	T	4	6	--	--
	Ca(ICCV2/PI489777)SNP_05905	2312	T	C	9	3	--	--
CakTC36441	Ca(ICCV2/PI489777)SNP_05906	499	A	T	9	13	--	SNF2
	Ca(ICCV2/PI489777)SNP_05907	629	A	C	14	8	--	SNF2
	Ca(ICCV2/PI489777)SNP_05908	786	A	G	26	5	--	SNF2
	Ca(ICCV2/PI489777)SNP_05909	900	G	A	20	4	--	SNF2
	Ca(ICCV2/PI489777)SNP_05910	1238	T	G	13	6	--	SNF2
	Ca(ICCV2/PI489777)SNP_05911	1533	A	T	16	4	--	SNF2
	Ca(ICCV2/PI489777)SNP_05912	1845	T	A	24	7	--	SNF2
	Ca(ICCV2/PI489777)SNP_05913	2271	A	G	13	10	--	SNF2
	Ca(ICCV2/PI489777)SNP_05914	2412	G	A	9	8	--	SNF2
	Ca(ICCV2/PI489777)SNP_05915	2718	A	G	13	13	--	SNF2

	Ca(ICCV2/PI489777)SNP_05916	3644	G	A	20	5	--	SNF2
	Ca(ICCV2/PI489777)SNP_05917	3722	T	C	19	5	--	SNF2
	Ca(ICCV2/PI489777)SNP_05918	3854	G	A	18	6	--	SNF2
CakTC27513	Ca(ICCV2/PI489777)SNP_05919	604	T	G	12	8	--	--
	Ca(ICCV2/PI489777)SNP_05920	1078	C	T	13	10	--	--
	Ca(ICCV2/PI489777)SNP_05921	1105	C	A	14	11	--	--
	Ca(ICCV2/PI489777)SNP_05922	1180	A	G	9	12	--	--
CakTC26295	Ca(ICCV2/PI489777)SNP_05923	635	A	G	10	7	--	GRAS
CakTC08088	Ca(ICCV2/PI489777)SNP_05924	747	A	G	6	3	--	--
CakTC42044	Ca(ICCV2/PI489777)SNP_05925	86	T	A	10	7	--	--
	Ca(ICCV2/PI489777)SNP_05926	230	T	C	11	7	--	--
	Ca(ICCV2/PI489777)SNP_05927	932	A	G	5	3	--	--
	Ca(ICCV2/PI489777)SNP_05928	1039	T	C	5	4	--	--
	Ca(ICCV2/PI489777)SNP_05929	1064	T	C	4	4	--	--
	Ca(ICCV2/PI489777)SNP_05930	1494	G	A	8	4	--	--
	Ca(ICCV2/PI489777)SNP_05931	1970	C	T	3	3	--	--
CakTC38230	Ca(ICCV2/PI489777)SNP_05932	48	A	T	5	13	--	--
	Ca(ICCV2/PI489777)SNP_05933	327	T	C	31	55	--	--
	Ca(ICCV2/PI489777)SNP_05934	333	T	C	32	56	--	--
	Ca(ICCV2/PI489777)SNP_05935	1009	C	T	8	5	--	--
CakTC37370	Ca(ICCV2/PI489777)SNP_05936	395	C	T	4	6	--	--
	Ca(ICCV2/PI489777)SNP_05937	817	A	C	8	6	--	--
CakTC09975	Ca(ICCV2/PI489777)SNP_05938	1314	C	A	13	4	--	--
CakTC29901	Ca(ICCV2/PI489777)SNP_05939	926	T	C	27	8	--	C3H
	Ca(ICCV2/PI489777)SNP_05940	1334	T	C	27	18	--	C3H
CakTC36159	Ca(ICCV2/PI489777)SNP_05941	604	G	A	5	4	--	--
	Ca(ICCV2/PI489777)SNP_05942	919	C	T	6	3	--	--
	Ca(ICCV2/PI489777)SNP_05943	1108	G	A	6	6	--	--
	Ca(ICCV2/PI489777)SNP_05944	2306	T	C	3	3	--	--
CakTC33884	Ca(ICCV2/PI489777)SNP_05945	256	T	A	16	12	--	--
	Ca(ICCV2/PI489777)SNP_05946	719	T	C	23	17	--	--
	Ca(ICCV2/PI489777)SNP_05947	1567	A	G	10	13	--	--
	Ca(ICCV2/PI489777)SNP_05948	1723	G	A	18	17	--	--
CakTC42706	Ca(ICCV2/PI489777)SNP_05949	347	T	C	13	22	--	--
CakTC42724	Ca(ICCV2/PI489777)SNP_05950	209	T	A	70	29	--	--
	Ca(ICCV2/PI489777)SNP_05951	214	T	A	77	33	--	--
	Ca(ICCV2/PI489777)SNP_05952	224	T	C	75	29	--	--
	Ca(ICCV2/PI489777)SNP_05953	275	T	C	76	35	--	--
	Ca(ICCV2/PI489777)SNP_05954	660	C	G	14	9	--	--
	Ca(ICCV2/PI489777)SNP_05955	791	C	T	10	3	--	--
	Ca(ICCV2/PI489777)SNP_05956	861	A	T	7	3	--	--
CakTC29599	Ca(ICCV2/PI489777)SNP_05957	174	A	T	3	10	Root	--
	Ca(ICCV2/PI489777)SNP_05958	628	T	G	4	7	Root	--
	Ca(ICCV2/PI489777)SNP_05959	1344	T	C	9	19	Root	--
	Ca(ICCV2/PI489777)SNP_05960	1569	C	A	4	6	Root	--
CakTC10007	Ca(ICCV2/PI489777)SNP_05961	1523	T	C	10	3	--	--
	Ca(ICCV2/PI489777)SNP_05962	1727	A	G	12	6	--	--
CakTC26540	Ca(ICCV2/PI489777)SNP_05963	724	T	C	3	4	--	--
	Ca(ICCV2/PI489777)SNP_05964	847	G	A	5	4	--	--
	Ca(ICCV2/PI489777)SNP_05965	1021	A	G	6	8	--	--
CakTC42984	Ca(ICCV2/PI489777)SNP_05966	355	A	G	10	4	--	--
CakTC28253	Ca(ICCV2/PI489777)SNP_05967	253	T	C	7	4	--	--
	Ca(ICCV2/PI489777)SNP_05968	366	G	A	8	6	--	--
	Ca(ICCV2/PI489777)SNP_05969	963	G	C	8	4	--	--
CakTC42622	Ca(ICCV2/PI489777)SNP_05970	61	T	G	21	6	--	Tify
	Ca(ICCV2/PI489777)SNP_05971	64	T	G	21	6	--	Tify
	Ca(ICCV2/PI489777)SNP_05972	333	C	G	25	11	--	Tify
CakTC34146	Ca(ICCV2/PI489777)SNP_05973	1597	A	G	4	4	--	C3H
CakTC24566	Ca(ICCV2/PI489777)SNP_05974	671	G	A	85	17	--	--
	Ca(ICCV2/PI489777)SNP_05975	850	G	A	93	29	--	--
	Ca(ICCV2/PI489777)SNP_05976	1606	G	A	56	20	--	--
	Ca(ICCV2/PI489777)SNP_05977	3092	A	G	58	19	--	--
	Ca(ICCV2/PI489777)SNP_05978	3114	T	G	54	17	--	--
CakTC05276	Ca(ICCV2/PI489777)SNP_05979	615	T	A	81	89	--	--
CakTC23374	Ca(ICCV2/PI489777)SNP_05980	1485	A	G	3	4	--	--
	Ca(ICCV2/PI489777)SNP_05981	2281	G	A	8	6	--	--
	Ca(ICCV2/PI489777)SNP_05982	2459	T	C	8	3	--	--
CakTC39262	Ca(ICCV2/PI489777)SNP_05983	1073	A	G	21	8	--	--
CakTC10137	Ca(ICCV2/PI489777)SNP_05984	80	T	C	8	6	--	--
	Ca(ICCV2/PI489777)SNP_05985	108	C	T	8	6	--	--
	Ca(ICCV2/PI489777)SNP_05986	171	T	C	8	7	--	--
	Ca(ICCV2/PI489777)SNP_05987	175	C	T	10	7	--	--
	Ca(ICCV2/PI489777)SNP_05988	218	G	A	10	6	--	--
	Ca(ICCV2/PI489777)SNP_05989	268	T	C	10	6	--	--

	Ca(ICCV2/PI489777)SNP_05990	304	G	T	11	6	--	--
	Ca(ICCV2/PI489777)SNP_05991	308	T	C	11	6	--	--
	Ca(ICCV2/PI489777)SNP_05992	326	G	A	11	6	--	--
	Ca(ICCV2/PI489777)SNP_05993	343	C	T	9	6	--	--
	Ca(ICCV2/PI489777)SNP_05994	448	A	G	9	4	--	--
	Ca(ICCV2/PI489777)SNP_05995	504	C	T	5	3	--	--
CakTC42894	Ca(ICCV2/PI489777)SNP_05996	129	A	C	12	16	--	--
	Ca(ICCV2/PI489777)SNP_05997	250	T	C	24	21	--	--
CakTC09839	Ca(ICCV2/PI489777)SNP_05998	2096	T	C	8	3	--	--
CakTC36831	Ca(ICCV2/PI489777)SNP_05999	1562	T	C	23	9	--	--
	Ca(ICCV2/PI489777)SNP_06000	2257	G	A	11	9	--	--
CakTC39745	Ca(ICCV2/PI489777)SNP_06001	292	C	T	16	4	--	--
	Ca(ICCV2/PI489777)SNP_06002	403	C	T	13	3	--	--
CakTC28580	Ca(ICCV2/PI489777)SNP_06003	564	T	C	6	6	--	--
	Ca(ICCV2/PI489777)SNP_06004	582	G	A	7	7	--	--
	Ca(ICCV2/PI489777)SNP_06005	1168	C	A	7	12	--	--
CakTC30046	Ca(ICCV2/PI489777)SNP_06006	1310	T	C	7	6	--	--
CakTC39289	Ca(ICCV2/PI489777)SNP_06007	300	C	T	4	4	--	--
	Ca(ICCV2/PI489777)SNP_06008	398	A	G	7	4	--	--
	Ca(ICCV2/PI489777)SNP_06009	407	A	G	3	3	--	--
CakTC23076	Ca(ICCV2/PI489777)SNP_06010	104	C	T	23	3	--	--
	Ca(ICCV2/PI489777)SNP_06011	107	C	G	24	3	--	--
CakTC12146	Ca(ICCV2/PI489777)SNP_06012	210	C	T	8	5	--	--
	Ca(ICCV2/PI489777)SNP_06013	266	G	A	10	5	--	--
CakTC34618	Ca(ICCV2/PI489777)SNP_06014	54	C	A	4	7	--	--
	Ca(ICCV2/PI489777)SNP_06015	395	T	A	8	8	--	--
	Ca(ICCV2/PI489777)SNP_06016	396	T	G	8	8	--	--
	Ca(ICCV2/PI489777)SNP_06017	472	A	G	5	5	--	--
CakTC31410	Ca(ICCV2/PI489777)SNP_06018	928	T	C	9	3	--	bZIP
CakTC40492	Ca(ICCV2/PI489777)SNP_06019	476	T	G	12	13	--	--
	Ca(ICCV2/PI489777)SNP_06020	710	C	G	8	9	--	--
CakTC23506	Ca(ICCV2/PI489777)SNP_06021	263	A	G	6	4	--	--
CakTC08782	Ca(ICCV2/PI489777)SNP_06022	639	T	C	4	5	--	--
	Ca(ICCV2/PI489777)SNP_06023	885	T	C	6	3	--	--
	Ca(ICCV2/PI489777)SNP_06024	981	C	G	7	3	--	--
	Ca(ICCV2/PI489777)SNP_06025	1101	C	T	7	3	--	--
	Ca(ICCV2/PI489777)SNP_06026	1362	C	T	11	9	--	--
	Ca(ICCV2/PI489777)SNP_06027	1483	T	G	10	13	--	--
	Ca(ICCV2/PI489777)SNP_06028	1591	G	T	10	7	--	--
	Ca(ICCV2/PI489777)SNP_06029	1626	T	C	10	8	--	--
	Ca(ICCV2/PI489777)SNP_06030	1631	T	G	10	8	--	--
	Ca(ICCV2/PI489777)SNP_06031	1654	G	A	8	6	--	--
CakTC26542	Ca(ICCV2/PI489777)SNP_06032	438	G	C	49	14	--	--
CakTC39171	Ca(ICCV2/PI489777)SNP_06033	1434	G	T	8	3	--	--
CakTC09253	Ca(ICCV2/PI489777)SNP_06034	1031	C	T	3	4	--	--
CakTC29824	Ca(ICCV2/PI489777)SNP_06035	251	A	T	82	82	--	--
	Ca(ICCV2/PI489777)SNP_06036	1132	A	G	124	87	--	--
CakTC41473	Ca(ICCV2/PI489777)SNP_06037	1802	T	G	28	27	--	--
CakTC24127	Ca(ICCV2/PI489777)SNP_06038	1992	C	T	35	6	--	--
	Ca(ICCV2/PI489777)SNP_06039	2652	T	C	26	7	--	--
CakTC40069	Ca(ICCV2/PI489777)SNP_06040	236	A	G	52	13	--	--
	Ca(ICCV2/PI489777)SNP_06041	830	T	A	38	22	--	--
	Ca(ICCV2/PI489777)SNP_06042	854	A	T	46	20	--	--
CakTC27709	Ca(ICCV2/PI489777)SNP_06043	386	G	A	3	4	--	--
CakTC24277	Ca(ICCV2/PI489777)SNP_06044	128	A	G	17	15	--	--
	Ca(ICCV2/PI489777)SNP_06045	498	G	A	40	31	--	--
	Ca(ICCV2/PI489777)SNP_06046	500	G	A	39	27	--	--
CakTC22280	Ca(ICCV2/PI489777)SNP_06047	180	A	G	17	25	--	--
	Ca(ICCV2/PI489777)SNP_06048	244	G	A	31	41	--	--
	Ca(ICCV2/PI489777)SNP_06049	350	T	C	43	42	--	--
	Ca(ICCV2/PI489777)SNP_06050	407	T	G	49	22	--	--
	Ca(ICCV2/PI489777)SNP_06051	1214	A	G	31	17	--	--
CakTC31158	Ca(ICCV2/PI489777)SNP_06052	544	T	C	8	7	--	--
	Ca(ICCV2/PI489777)SNP_06053	736	G	C	15	3	--	--
	Ca(ICCV2/PI489777)SNP_06054	1046	C	T	11	3	--	--
	Ca(ICCV2/PI489777)SNP_06055	1069	C	T	13	4	--	--
CakTC25308	Ca(ICCV2/PI489777)SNP_06056	1560	G	A	20	9	Young_pod	--
CakTC42775	Ca(ICCV2/PI489777)SNP_06057	120	T	C	8	4	--	TPR
	Ca(ICCV2/PI489777)SNP_06058	337	G	A	15	15	--	TPR
	Ca(ICCV2/PI489777)SNP_06059	1390	G	T	12	11	--	TPR
CakTC28222	Ca(ICCV2/PI489777)SNP_06060	1160	C	T	9	5	--	--
	Ca(ICCV2/PI489777)SNP_06061	1175	C	T	9	4	--	--
	Ca(ICCV2/PI489777)SNP_06062	2113	T	C	32	9	--	--
CakTC28450	Ca(ICCV2/PI489777)SNP_06063	126	A	T	12	6	--	--

	Ca(ICCV2/PI489777)SNP_06064	212	G	A	20	5	--	--
	Ca(ICCV2/PI489777)SNP_06065	269	G	A	24	10	--	--
	Ca(ICCV2/PI489777)SNP_06066	305	C	T	29	11	--	--
	Ca(ICCV2/PI489777)SNP_06067	319	A	C	28	10	--	--
	Ca(ICCV2/PI489777)SNP_06068	481	A	G	27	9	--	--
	Ca(ICCV2/PI489777)SNP_06069	622	C	T	23	6	--	--
	Ca(ICCV2/PI489777)SNP_06070	769	C	T	24	5	--	--
	Ca(ICCV2/PI489777)SNP_06071	858	G	A	29	4	--	--
	Ca(ICCV2/PI489777)SNP_06072	1088	T	G	30	8	--	--
	Ca(ICCV2/PI489777)SNP_06073	1715	A	G	13	3	--	--
CakTC31788	Ca(ICCV2/PI489777)SNP_06074	1229	T	C	4	3	--	--
	Ca(ICCV2/PI489777)SNP_06075	1238	G	A	5	3	--	--
	Ca(ICCV2/PI489777)SNP_06076	1240	T	C	5	3	--	--
CakTC29622	Ca(ICCV2/PI489777)SNP_06077	158	A	G	6	4	--	--
	Ca(ICCV2/PI489777)SNP_06078	983	T	C	8	10	--	--
CakTC37626	Ca(ICCV2/PI489777)SNP_06079	500	G	C	24	13	--	--
	Ca(ICCV2/PI489777)SNP_06080	902	A	G	18	18	--	--
	Ca(ICCV2/PI489777)SNP_06081	1001	G	T	12	7	--	--
CakTC43307	Ca(ICCV2/PI489777)SNP_06082	131	G	A	3	8	--	--
	Ca(ICCV2/PI489777)SNP_06083	532	G	A	4	3	--	--
	Ca(ICCV2/PI489777)SNP_06084	541	G	A	4	3	--	--
	Ca(ICCV2/PI489777)SNP_06085	586	A	G	3	3	--	--
CakTC42684	Ca(ICCV2/PI489777)SNP_06086	558	T	A	15	12	--	--
	Ca(ICCV2/PI489777)SNP_06087	972	C	T	29	6	--	--
	Ca(ICCV2/PI489777)SNP_06088	1329	C	A	16	4	--	--
	Ca(ICCV2/PI489777)SNP_06089	1692	T	A	7	3	--	--
CakTC37754	Ca(ICCV2/PI489777)SNP_06090	273	T	A	6	8	--	--
	Ca(ICCV2/PI489777)SNP_06091	335	G	C	5	7	--	--
	Ca(ICCV2/PI489777)SNP_06092	346	T	C	5	7	--	--
	Ca(ICCV2/PI489777)SNP_06093	351	G	A	6	7	--	--
CakTC29693	Ca(ICCV2/PI489777)SNP_06094	132	G	T	36	28	--	--
	Ca(ICCV2/PI489777)SNP_06095	426	A	G	174	71	--	--
CakTC34572	Ca(ICCV2/PI489777)SNP_06096	126	A	G	5	13	--	--
CakTC27262	Ca(ICCV2/PI489777)SNP_06097	109	C	T	3	4	--	--
	Ca(ICCV2/PI489777)SNP_06098	187	T	C	4	3	--	--
	Ca(ICCV2/PI489777)SNP_06099	365	C	G	4	5	--	--
	Ca(ICCV2/PI489777)SNP_06100	411	G	A	4	6	--	--
	Ca(ICCV2/PI489777)SNP_06101	592	G	A	90	42	--	--
	Ca(ICCV2/PI489777)SNP_06102	1298	G	A	93	15	--	--
CakTC07433	Ca(ICCV2/PI489777)SNP_06103	518	T	C	20	14	--	--
	Ca(ICCV2/PI489777)SNP_06104	551	A	G	22	14	--	--
CakTC37731	Ca(ICCV2/PI489777)SNP_06105	122	G	T	4	6	--	--
CakTC37695	Ca(ICCV2/PI489777)SNP_06106	894	G	T	13	10	--	--
CakTC28371	Ca(ICCV2/PI489777)SNP_06107	735	A	G	3	3	--	--
	Ca(ICCV2/PI489777)SNP_06108	771	C	T	3	5	--	--
	Ca(ICCV2/PI489777)SNP_06109	955	A	G	5	11	--	--
	Ca(ICCV2/PI489777)SNP_06110	1035	T	C	5	11	--	--
	Ca(ICCV2/PI489777)SNP_06111	1396	G	T	3	3	--	--
CakTC39443	Ca(ICCV2/PI489777)SNP_06112	805	T	G	9	4	--	--
	Ca(ICCV2/PI489777)SNP_06113	1038	C	T	7	11	--	--
CakTC29560	Ca(ICCV2/PI489777)SNP_06114	30	A	C	8	12	--	MYB-related
	Ca(ICCV2/PI489777)SNP_06115	40	C	T	11	11	--	MYB-related
	Ca(ICCV2/PI489777)SNP_06116	87	C	G	13	10	--	MYB-related
CakTC13158	Ca(ICCV2/PI489777)SNP_06117	332	C	A	33	4	--	--
CakTC42268	Ca(ICCV2/PI489777)SNP_06118	792	G	C	29	9	--	C2C2-GATA
	Ca(ICCV2/PI489777)SNP_06119	916	A	G	21	5	--	C2C2-GATA
	Ca(ICCV2/PI489777)SNP_06120	947	A	G	17	4	--	C2C2-GATA
	Ca(ICCV2/PI489777)SNP_06121	1113	G	C	12	3	--	C2C2-GATA
	Ca(ICCV2/PI489777)SNP_06122	1170	A	C	9	3	--	C2C2-GATA
	Ca(ICCV2/PI489777)SNP_06123	1428	G	A	5	3	--	C2C2-GATA
	Ca(ICCV2/PI489777)SNP_06124	1488	G	A	3	3	--	C2C2-GATA
	Ca(ICCV2/PI489777)SNP_06125	1521	A	G	4	3	--	C2C2-GATA
CakTC31319	Ca(ICCV2/PI489777)SNP_06126	91	G	A	9	3	--	--
	Ca(ICCV2/PI489777)SNP_06127	93	A	G	9	3	--	--
	Ca(ICCV2/PI489777)SNP_06128	131	C	G	20	16	--	--
CakTC34726	Ca(ICCV2/PI489777)SNP_06129	3246	G	C	7	5	--	--
CakTC31347	Ca(ICCV2/PI489777)SNP_06130	129	A	T	4	6	Flower	bud
CakTC30382	Ca(ICCV2/PI489777)SNP_06131	1697	C	G	4	4	--	TPR
CakTC28202	Ca(ICCV2/PI489777)SNP_06132	66	C	G	17	13	--	--
	Ca(ICCV2/PI489777)SNP_06133	215	A	G	34	17	--	--
	Ca(ICCV2/PI489777)SNP_06134	495	C	A	27	5	--	--
	Ca(ICCV2/PI489777)SNP_06135	650	C	T	41	14	--	--
	Ca(ICCV2/PI489777)SNP_06136	848	T	C	55	36	--	--
	Ca(ICCV2/PI489777)SNP_06137	1448	T	A	24	21	--	--

	Ca(ICCV2/PI489777)SNP_06138	1497	A	G	16	20	--	--
	Ca(ICCV2/PI489777)SNP_06139	1533	T	C	14	16	--	--
CakTC25170	Ca(ICCV2/PI489777)SNP_06140	275	G	A	10	6	--	--
	Ca(ICCV2/PI489777)SNP_06141	1021	T	C	9	14	--	--
	Ca(ICCV2/PI489777)SNP_06142	1108	G	A	7	13	--	--
	Ca(ICCV2/PI489777)SNP_06143	1192	C	A	11	15	--	--
CakTC39317	Ca(ICCV2/PI489777)SNP_06144	228	A	G	16	3	--	--
	Ca(ICCV2/PI489777)SNP_06145	282	C	T	20	6	--	--
	Ca(ICCV2/PI489777)SNP_06146	517	C	T	17	6	--	--
	Ca(ICCV2/PI489777)SNP_06147	787	T	C	9	9	--	--
	Ca(ICCV2/PI489777)SNP_06148	805	G	A	14	9	--	--
	Ca(ICCV2/PI489777)SNP_06149	958	C	G	15	14	--	--
	Ca(ICCV2/PI489777)SNP_06150	1034	T	A	16	16	--	--
	Ca(ICCV2/PI489777)SNP_06151	1225	C	T	22	15	--	--
	Ca(ICCV2/PI489777)SNP_06152	1464	C	T	20	9	--	--
CakTC29816	Ca(ICCV2/PI489777)SNP_06153	1320	A	G	3	4	--	--
CakTC42385	Ca(ICCV2/PI489777)SNP_06154	831	G	A	8	5	--	--
CakTC22851	Ca(ICCV2/PI489777)SNP_06155	675	T	C	12	3	--	--
	Ca(ICCV2/PI489777)SNP_06156	780	A	G	15	3	--	--
CakTC39560	Ca(ICCV2/PI489777)SNP_06157	460	C	A	11	6	--	--
	Ca(ICCV2/PI489777)SNP_06158	823	A	C	9	9	--	--
	Ca(ICCV2/PI489777)SNP_06159	847	A	G	9	7	--	--
	Ca(ICCV2/PI489777)SNP_06160	871	C	T	8	6	--	--
	Ca(ICCV2/PI489777)SNP_06161	1518	C	A	15	4	--	--
	Ca(ICCV2/PI489777)SNP_06162	1531	G	C	17	4	--	--
	Ca(ICCV2/PI489777)SNP_06163	1637	G	A	9	4	--	--
CakTC32294	Ca(ICCV2/PI489777)SNP_06164	763	C	A	11	10	--	C3H
CakTC40803	Ca(ICCV2/PI489777)SNP_06165	934	C	T	107	129	--	--
CakTC40488	Ca(ICCV2/PI489777)SNP_06166	69	T	C	4	8	--	--
	Ca(ICCV2/PI489777)SNP_06167	94	T	A	6	8	--	--
	Ca(ICCV2/PI489777)SNP_06168	145	A	T	11	11	--	--
	Ca(ICCV2/PI489777)SNP_06169	208	T	A	19	30	--	--
	Ca(ICCV2/PI489777)SNP_06170	316	A	T	26	34	--	--
	Ca(ICCV2/PI489777)SNP_06171	317	T	G	26	33	--	--
	Ca(ICCV2/PI489777)SNP_06172	347	A	C	30	39	--	--
	Ca(ICCV2/PI489777)SNP_06173	662	C	G	23	27	--	--
CakTC39732	Ca(ICCV2/PI489777)SNP_06174	115	T	C	10	11	--	--
CakTC34578	Ca(ICCV2/PI489777)SNP_06175	36	C	A	4	4	--	--
	Ca(ICCV2/PI489777)SNP_06176	109	G	A	5	8	--	--
	Ca(ICCV2/PI489777)SNP_06177	139	G	T	5	7	--	--
	Ca(ICCV2/PI489777)SNP_06178	539	T	G	3	7	--	--
CakTC34291	Ca(ICCV2/PI489777)SNP_06179	338	C	T	7	6	Flower	bud
	Ca(ICCV2/PI489777)SNP_06180	464	C	T	3	5	Flower	bud
	Ca(ICCV2/PI489777)SNP_06181	527	G	C	4	7	Flower	bud
	Ca(ICCV2/PI489777)SNP_06182	1198	A	G	6	5	Flower	bud
	Ca(ICCV2/PI489777)SNP_06183	1331	C	T	8	6	Flower	bud
CakTC30280	Ca(ICCV2/PI489777)SNP_06184	161	C	T	8	3	--	--
	Ca(ICCV2/PI489777)SNP_06185	748	T	C	12	13	--	--
CakTC26825	Ca(ICCV2/PI489777)SNP_06186	83	A	G	6	5	--	--
	Ca(ICCV2/PI489777)SNP_06187	175	T	C	10	14	--	--
	Ca(ICCV2/PI489777)SNP_06188	249	C	A	20	12	--	--
	Ca(ICCV2/PI489777)SNP_06189	293	C	T	23	16	--	--
	Ca(ICCV2/PI489777)SNP_06190	2863	T	C	4	3	--	--
CakTC25584	Ca(ICCV2/PI489777)SNP_06191	453	C	T	14	5	--	TRAF
	Ca(ICCV2/PI489777)SNP_06192	501	G	A	15	5	--	TRAF
CakTC27068	Ca(ICCV2/PI489777)SNP_06193	389	C	T	5	3	--	--
	Ca(ICCV2/PI489777)SNP_06194	996	A	G	3	5	--	--
CakTC10288	Ca(ICCV2/PI489777)SNP_06195	237	C	T	5	5	--	--
	Ca(ICCV2/PI489777)SNP_06196	2268	C	T	5	4	--	--
CakTC30951	Ca(ICCV2/PI489777)SNP_06197	1462	T	C	14	19	--	--
CakTC36892	Ca(ICCV2/PI489777)SNP_06198	469	G	A	5	5	--	--
	Ca(ICCV2/PI489777)SNP_06199	712	G	A	8	9	--	--
	Ca(ICCV2/PI489777)SNP_06200	1621	C	T	15	4	--	--
	Ca(ICCV2/PI489777)SNP_06201	1792	T	C	19	5	--	--
	Ca(ICCV2/PI489777)SNP_06202	2463	T	C	9	4	--	--
	Ca(ICCV2/PI489777)SNP_06203	2472	T	G	12	4	--	--
CakTC37221	Ca(ICCV2/PI489777)SNP_06204	414	A	C	15	9	--	--
	Ca(ICCV2/PI489777)SNP_06205	807	T	C	18	9	--	--
	Ca(ICCV2/PI489777)SNP_06206	844	T	C	9	11	--	--
	Ca(ICCV2/PI489777)SNP_06207	873	T	C	11	13	--	--
CakTC40155	Ca(ICCV2/PI489777)SNP_06208	1343	C	T	20	15	--	--
CakTC36601	Ca(ICCV2/PI489777)SNP_06209	1324	A	G	23	11	--	--
CakTC28765	Ca(ICCV2/PI489777)SNP_06210	1175	A	T	24	5	--	--
	Ca(ICCV2/PI489777)SNP_06211	1289	C	A	25	9	--	--

	Ca(ICCV2/PI489777)SNP_06212	1445	G	A	117	52	--	--
	Ca(ICCV2/PI489777)SNP_06213	1473	G	T	114	42	--	--
CakTC31232	Ca(ICCV2/PI489777)SNP_06214	429	A	T	6	5	Young_pod	--
CakTC24507	Ca(ICCV2/PI489777)SNP_06215	709	C	T	16	16	--	C3H
	Ca(ICCV2/PI489777)SNP_06216	1784	G	A	56	37	--	C3H
CakTC23018	Ca(ICCV2/PI489777)SNP_06217	707	A	G	19	4	Shoot	--
CakTC42252	Ca(ICCV2/PI489777)SNP_06218	241	T	C	26	4	--	--
	Ca(ICCV2/PI489777)SNP_06219	250	A	G	25	4	--	--
	Ca(ICCV2/PI489777)SNP_06220	259	A	C	26	6	--	--
	Ca(ICCV2/PI489777)SNP_06221	409	G	A	20	8	--	--
	Ca(ICCV2/PI489777)SNP_06222	421	T	C	12	9	--	--
CakTC37986	Ca(ICCV2/PI489777)SNP_06223	250	A	G	5	6	--	HB
	Ca(ICCV2/PI489777)SNP_06224	975	G	A	12	7	--	HB
CakTC25685	Ca(ICCV2/PI489777)SNP_06225	183	G	T	65	30	--	--
CakTC23223	Ca(ICCV2/PI489777)SNP_06226	408	C	T	37	119	--	--
	Ca(ICCV2/PI489777)SNP_06227	1106	T	G	103	58	--	--
	Ca(ICCV2/PI489777)SNP_06228	1157	A	T	97	70	--	--
CakTC34252	Ca(ICCV2/PI489777)SNP_06229	88	G	C	39	22	--	--
CakTC39789	Ca(ICCV2/PI489777)SNP_06230	1197	C	T	20	27	--	--
	Ca(ICCV2/PI489777)SNP_06231	1200	A	G	20	26	--	--
	Ca(ICCV2/PI489777)SNP_06232	1387	C	G	33	25	--	--
	Ca(ICCV2/PI489777)SNP_06233	1536	A	T	38	19	--	--
	Ca(ICCV2/PI489777)SNP_06234	1715	A	C	9	9	--	--
CakTC43211	Ca(ICCV2/PI489777)SNP_06235	1168	G	T	4	56	--	--
	Ca(ICCV2/PI489777)SNP_06236	1512	G	C	4	6	--	--
	Ca(ICCV2/PI489777)SNP_06237	1610	A	T	5	6	--	--
	Ca(ICCV2/PI489777)SNP_06238	1648	C	T	5	5	--	--
	Ca(ICCV2/PI489777)SNP_06239	1730	C	T	4	5	--	--
CakTC39796	Ca(ICCV2/PI489777)SNP_06240	730	A	G	3	14	Young_pod	--
	Ca(ICCV2/PI489777)SNP_06241	850	C	T	3	11	Young_pod	--
	Ca(ICCV2/PI489777)SNP_06242	1148	T	C	4	6	Young_pod	--
	Ca(ICCV2/PI489777)SNP_06243	1160	A	G	3	6	Young_pod	--
	Ca(ICCV2/PI489777)SNP_06244	1333	C	A	3	6	Young_pod	--
CakTC33639	Ca(ICCV2/PI489777)SNP_06245	1574	T	A	5	4	--	--
	Ca(ICCV2/PI489777)SNP_06246	1757	C	T	7	8	--	--
CakTC39618	Ca(ICCV2/PI489777)SNP_06247	204	T	G	11	10	--	--
	Ca(ICCV2/PI489777)SNP_06248	309	G	C	14	14	--	--
	Ca(ICCV2/PI489777)SNP_06249	1118	T	G	8	4	--	--
CakTC30698	Ca(ICCV2/PI489777)SNP_06250	668	A	G	22	19	--	--
CakTC26559	Ca(ICCV2/PI489777)SNP_06251	1569	T	C	14	3	Young_pod	--
CakTC40645	Ca(ICCV2/PI489777)SNP_06252	1336	T	C	21	15	--	--
	Ca(ICCV2/PI489777)SNP_06253	1477	G	T	13	11	--	--
	Ca(ICCV2/PI489777)SNP_06254	1529	G	T	12	8	--	--
	Ca(ICCV2/PI489777)SNP_06255	1811	G	A	10	9	--	--
CakTC40209	Ca(ICCV2/PI489777)SNP_06256	31	G	A	3	4	--	--
	Ca(ICCV2/PI489777)SNP_06257	109	C	T	6	15	--	--
	Ca(ICCV2/PI489777)SNP_06258	782	C	G	56	40	--	--
	Ca(ICCV2/PI489777)SNP_06259	793	T	C	56	46	--	--
	Ca(ICCV2/PI489777)SNP_06260	1034	G	A	84	28	--	--
	Ca(ICCV2/PI489777)SNP_06261	1077	G	A	65	28	--	--
	Ca(ICCV2/PI489777)SNP_06262	1096	A	T	77	29	--	--
	Ca(ICCV2/PI489777)SNP_06263	1097	T	G	76	30	--	--
CakTC38882	Ca(ICCV2/PI489777)SNP_06264	60	T	A	12	7	--	--
	Ca(ICCV2/PI489777)SNP_06265	451	T	G	23	16	--	--
	Ca(ICCV2/PI489777)SNP_06266	470	G	A	23	16	--	--
	Ca(ICCV2/PI489777)SNP_06267	571	A	C	18	18	--	--
	Ca(ICCV2/PI489777)SNP_06268	765	A	G	50	34	--	--
	Ca(ICCV2/PI489777)SNP_06269	947	A	G	59	37	--	--
	Ca(ICCV2/PI489777)SNP_06270	948	A	C	57	38	--	--
	Ca(ICCV2/PI489777)SNP_06271	1002	G	C	58	38	--	--
	Ca(ICCV2/PI489777)SNP_06272	1196	A	C	37	10	--	--
	Ca(ICCV2/PI489777)SNP_06273	1207	A	G	35	12	--	--
CakTC37386	Ca(ICCV2/PI489777)SNP_06274	113	C	T	7	10	--	--
	Ca(ICCV2/PI489777)SNP_06275	224	C	T	7	13	--	--
	Ca(ICCV2/PI489777)SNP_06276	230	T	C	6	12	--	--
CakTC26678	Ca(ICCV2/PI489777)SNP_06277	1291	G	A	97	39	--	--
	Ca(ICCV2/PI489777)SNP_06278	1582	G	A	59	26	--	--
CakTC24893	Ca(ICCV2/PI489777)SNP_06279	1288	A	G	24	7	--	--
	Ca(ICCV2/PI489777)SNP_06280	1533	G	A	23	3	--	--
	Ca(ICCV2/PI489777)SNP_06281	2205	A	G	11	6	--	--
CakTC28263	Ca(ICCV2/PI489777)SNP_06282	547	C	T	3	5	--	--
	Ca(ICCV2/PI489777)SNP_06283	549	A	G	4	5	--	--
	Ca(ICCV2/PI489777)SNP_06284	3310	A	T	3	7	--	--
CakTC39252	Ca(ICCV2/PI489777)SNP_06285	755	T	C	4	3	--	--

CakTC40252	Ca(ICCV2/PI489777)SNP_06286	167	T	C	5	3	--	--
	Ca(ICCV2/PI489777)SNP_06287	229	G	T	14	6	--	--
	Ca(ICCV2/PI489777)SNP_06288	247	G	A	15	10	--	--
	Ca(ICCV2/PI489777)SNP_06289	264	T	C	15	10	--	--
	Ca(ICCV2/PI489777)SNP_06290	634	T	G	21	13	--	--
	Ca(ICCV2/PI489777)SNP_06291	640	G	A	22	13	--	--
	Ca(ICCV2/PI489777)SNP_06292	736	A	G	23	13	--	--
	Ca(ICCV2/PI489777)SNP_06293	877	A	T	21	13	--	--
	Ca(ICCV2/PI489777)SNP_06294	880	A	T	23	13	--	--
	Ca(ICCV2/PI489777)SNP_06295	1439	G	T	17	9	--	--
	Ca(ICCV2/PI489777)SNP_06296	1450	T	C	18	8	--	--
CakTC39079	Ca(ICCV2/PI489777)SNP_06297	1163	T	C	3	5	--	--
CakTC19800	Ca(ICCV2/PI489777)SNP_06298	655	A	G	26	25	--	--
	Ca(ICCV2/PI489777)SNP_06299	715	G	A	20	21	--	--
	Ca(ICCV2/PI489777)SNP_06300	722	G	T	16	22	--	--
	Ca(ICCV2/PI489777)SNP_06301	856	G	C	33	18	--	--
	Ca(ICCV2/PI489777)SNP_06302	882	C	G	37	22	--	--
	Ca(ICCV2/PI489777)SNP_06303	883	A	T	37	22	--	--
	Ca(ICCV2/PI489777)SNP_06304	1558	A	G	45	13	--	--
	Ca(ICCV2/PI489777)SNP_06305	1588	C	T	32	11	--	--
CakTC40461	Ca(ICCV2/PI489777)SNP_06306	430	G	T	10	18	--	GNAT
	Ca(ICCV2/PI489777)SNP_06307	517	A	G	4	16	--	GNAT
CakTC34427	Ca(ICCV2/PI489777)SNP_06308	726	A	G	33	14	--	GRAS
	Ca(ICCV2/PI489777)SNP_06309	1071	G	A	13	11	--	GRAS
	Ca(ICCV2/PI489777)SNP_06310	1422	G	A	18	13	--	GRAS
	Ca(ICCV2/PI489777)SNP_06311	1434	A	T	17	13	--	GRAS
	Ca(ICCV2/PI489777)SNP_06312	1933	A	T	12	13	--	GRAS
CakTC11487	Ca(ICCV2/PI489777)SNP_06313	979	T	C	6	4	--	--
	Ca(ICCV2/PI489777)SNP_06314	1540	G	A	9	6	--	--
CakTC33504	Ca(ICCV2/PI489777)SNP_06315	451	A	G	6	10	--	--
CakTC28587	Ca(ICCV2/PI489777)SNP_06316	100	T	C	3	4	--	--
	Ca(ICCV2/PI489777)SNP_06317	301	T	C	9	5	--	--
	Ca(ICCV2/PI489777)SNP_06318	363	C	T	10	6	--	--
	Ca(ICCV2/PI489777)SNP_06319	595	G	A	11	6	--	--
	Ca(ICCV2/PI489777)SNP_06320	845	C	G	9	12	--	--
	Ca(ICCV2/PI489777)SNP_06321	1219	T	G	11	13	--	--
	Ca(ICCV2/PI489777)SNP_06322	1552	G	A	19	17	--	--
	Ca(ICCV2/PI489777)SNP_06323	1762	A	G	5	7	--	--
	Ca(ICCV2/PI489777)SNP_06324	1829	G	T	5	5	--	--
	Ca(ICCV2/PI489777)SNP_06325	1834	G	C	6	6	--	--
CakTC25311	Ca(ICCV2/PI489777)SNP_06326	718	A	G	41	16	--	--
CakTC23478	Ca(ICCV2/PI489777)SNP_06327	238	A	G	17	18	--	--
	Ca(ICCV2/PI489777)SNP_06328	325	T	C	30	27	--	--
	Ca(ICCV2/PI489777)SNP_06329	1693	T	C	27	21	--	--
	Ca(ICCV2/PI489777)SNP_06330	1843	A	G	17	11	--	--
	Ca(ICCV2/PI489777)SNP_06331	1867	G	A	23	14	--	--
	Ca(ICCV2/PI489777)SNP_06332	1960	G	A	25	18	--	--
	Ca(ICCV2/PI489777)SNP_06333	2107	A	G	15	21	--	--
CakTC25181	Ca(ICCV2/PI489777)SNP_06334	103	C	A	6	8	--	--
	Ca(ICCV2/PI489777)SNP_06335	830	T	G	32	14	--	--
CakTC17813	Ca(ICCV2/PI489777)SNP_06336	752	T	C	3	4	--	--
CakTC33970	Ca(ICCV2/PI489777)SNP_06337	421	C	T	9	3	--	--
	Ca(ICCV2/PI489777)SNP_06338	422	A	C	8	3	--	--
	Ca(ICCV2/PI489777)SNP_06339	699	G	A	6	4	--	--
	Ca(ICCV2/PI489777)SNP_06340	732	C	T	5	4	--	--
	Ca(ICCV2/PI489777)SNP_06341	996	T	A	9	4	--	--
	Ca(ICCV2/PI489777)SNP_06342	1137	T	C	10	4	--	--
	Ca(ICCV2/PI489777)SNP_06343	1407	A	G	13	9	--	--
	Ca(ICCV2/PI489777)SNP_06344	1818	T	C	8	4	--	--
CakTC42829	Ca(ICCV2/PI489777)SNP_06345	713	T	C	62	19	--	--
CakTC28013	Ca(ICCV2/PI489777)SNP_06346	67	A	G	3	7	--	--
	Ca(ICCV2/PI489777)SNP_06347	93	C	T	4	6	--	--
	Ca(ICCV2/PI489777)SNP_06348	966	T	C	7	6	--	--
	Ca(ICCV2/PI489777)SNP_06349	972	A	T	7	6	--	--
CakTC28568	Ca(ICCV2/PI489777)SNP_06350	389	T	C	42	22	--	--
	Ca(ICCV2/PI489777)SNP_06351	503	A	C	39	25	--	--
	Ca(ICCV2/PI489777)SNP_06352	803	T	C	34	18	--	--
	Ca(ICCV2/PI489777)SNP_06353	1088	G	T	35	27	--	--
	Ca(ICCV2/PI489777)SNP_06354	1985	A	G	17	20	--	--
CakTC24242	Ca(ICCV2/PI489777)SNP_06355	332	T	G	14	3	--	--
	Ca(ICCV2/PI489777)SNP_06356	368	T	C	16	3	--	--
	Ca(ICCV2/PI489777)SNP_06357	1535	C	T	15	5	--	--
	Ca(ICCV2/PI489777)SNP_06358	1594	G	T	20	5	--	--
	Ca(ICCV2/PI489777)SNP_06359	2510	G	C	19	3	--	--

	Ca(ICCV2/PI489777)SNP_06360	2535	A	G	18	3	--	--
	Ca(ICCV2/PI489777)SNP_06361	2653	G	C	12	3	--	--
CakTC36530	Ca(ICCV2/PI489777)SNP_06362	785	T	C	4	4	--	--
	Ca(ICCV2/PI489777)SNP_06363	1456	G	A	15	13	--	--
	Ca(ICCV2/PI489777)SNP_06364	1511	T	C	8	12	--	--
	Ca(ICCV2/PI489777)SNP_06365	2088	G	A	6	4	--	--
	Ca(ICCV2/PI489777)SNP_06366	2455	T	A	10	3	--	--
CakTC34644	Ca(ICCV2/PI489777)SNP_06367	1899	T	C	26	12	--	--
	Ca(ICCV2/PI489777)SNP_06368	1982	G	A	24	10	--	--
CakTC11780	Ca(ICCV2/PI489777)SNP_06369	271	T	C	5	9	--	--
CakTC41237	Ca(ICCV2/PI489777)SNP_06370	832	C	T	15	3	--	--
	Ca(ICCV2/PI489777)SNP_06371	1006	A	C	22	5	--	--
	Ca(ICCV2/PI489777)SNP_06372	1209	G	T	12	3	--	--
	Ca(ICCV2/PI489777)SNP_06373	1262	C	T	12	3	--	--
	Ca(ICCV2/PI489777)SNP_06374	1381	A	C	17	4	--	--
	Ca(ICCV2/PI489777)SNP_06375	1529	T	C	12	7	--	--
	Ca(ICCV2/PI489777)SNP_06376	1754	C	T	12	10	--	--
CakTC34348	Ca(ICCV2/PI489777)SNP_06377	680	A	T	7	12	--	--
	Ca(ICCV2/PI489777)SNP_06378	757	A	G	5	12	--	--
	Ca(ICCV2/PI489777)SNP_06379	816	C	T	5	9	--	--
CakTC37338	Ca(ICCV2/PI489777)SNP_06380	1122	A	G	13	6	--	--
CakTC12579	Ca(ICCV2/PI489777)SNP_06381	284	A	G	4	4	--	--
CakTC36877	Ca(ICCV2/PI489777)SNP_06382	213	C	G	19	6	--	--
	Ca(ICCV2/PI489777)SNP_06383	227	C	T	20	6	--	--
CakTC32041	Ca(ICCV2/PI489777)SNP_06384	130	A	C	7	3	--	--
	Ca(ICCV2/PI489777)SNP_06385	221	C	T	7	3	--	--
	Ca(ICCV2/PI489777)SNP_06386	285	A	C	8	3	--	--
	Ca(ICCV2/PI489777)SNP_06387	5623	C	T	8	3	--	--
CakTC29263	Ca(ICCV2/PI489777)SNP_06388	1845	C	G	11	5	--	C2C2-Dof
	Ca(ICCV2/PI489777)SNP_06389	2034	T	C	10	6	--	C2C2-Dof
CakTC27879	Ca(ICCV2/PI489777)SNP_06390	1698	G	C	29	17	--	--
CakTC32947	Ca(ICCV2/PI489777)SNP_06391	348	A	G	8	5	--	--
	Ca(ICCV2/PI489777)SNP_06392	360	C	T	9	5	--	--
	Ca(ICCV2/PI489777)SNP_06393	537	G	A	3	5	--	--
	Ca(ICCV2/PI489777)SNP_06394	546	G	A	4	5	--	--
	Ca(ICCV2/PI489777)SNP_06395	2635	G	A	8	8	--	--
CakTC09609	Ca(ICCV2/PI489777)SNP_06396	1213	A	T	3	3	--	E2F-DP
CakTC41071	Ca(ICCV2/PI489777)SNP_06397	249	T	C	4	4	--	--
CakTC39870	Ca(ICCV2/PI489777)SNP_06398	577	G	A	51	21	--	--
	Ca(ICCV2/PI489777)SNP_06399	1159	C	G	27	7	--	--
	Ca(ICCV2/PI489777)SNP_06400	1168	G	C	25	7	--	--
	Ca(ICCV2/PI489777)SNP_06401	1187	T	C	17	6	--	--
	Ca(ICCV2/PI489777)SNP_06402	1192	A	G	19	6	--	--
	Ca(ICCV2/PI489777)SNP_06403	1213	T	A	11	6	--	--
	Ca(ICCV2/PI489777)SNP_06404	1214	G	A	16	6	--	--
CakTC26922	Ca(ICCV2/PI489777)SNP_06405	1250	A	C	4	4	--	--
	Ca(ICCV2/PI489777)SNP_06406	2369	A	C	7	6	--	--
	Ca(ICCV2/PI489777)SNP_06407	2917	C	A	10	4	--	--
	Ca(ICCV2/PI489777)SNP_06408	2992	T	G	5	4	--	--
CakTC10014	Ca(ICCV2/PI489777)SNP_06409	223	T	A	5	6	--	--
	Ca(ICCV2/PI489777)SNP_06410	282	A	T	5	8	--	--
	Ca(ICCV2/PI489777)SNP_06411	415	T	C	6	10	--	--
	Ca(ICCV2/PI489777)SNP_06412	429	T	G	4	10	--	--
	Ca(ICCV2/PI489777)SNP_06413	588	A	G	4	11	--	--
	Ca(ICCV2/PI489777)SNP_06414	726	A	C	4	4	--	--
CakTC26385	Ca(ICCV2/PI489777)SNP_06415	472	T	C	28	11	--	--
	Ca(ICCV2/PI489777)SNP_06416	713	G	A	27	6	--	--
	Ca(ICCV2/PI489777)SNP_06417	736	A	G	27	6	--	--
	Ca(ICCV2/PI489777)SNP_06418	794	T	C	25	5	--	--
	Ca(ICCV2/PI489777)SNP_06419	939	T	C	23	4	--	--
	Ca(ICCV2/PI489777)SNP_06420	985	C	T	18	3	--	--
	Ca(ICCV2/PI489777)SNP_06421	1002	T	C	21	4	--	--
	Ca(ICCV2/PI489777)SNP_06422	1006	A	C	20	3	--	--
	Ca(ICCV2/PI489777)SNP_06423	1014	C	T	17	4	--	--
CakTC28622	Ca(ICCV2/PI489777)SNP_06424	3108	G	C	5	3	--	--
CakTC38796	Ca(ICCV2/PI489777)SNP_06425	230	T	G	21	4	--	--
	Ca(ICCV2/PI489777)SNP_06426	254	G	A	21	4	--	--
	Ca(ICCV2/PI489777)SNP_06427	353	A	G	28	6	--	--
	Ca(ICCV2/PI489777)SNP_06428	1037	G	A	35	10	--	--
	Ca(ICCV2/PI489777)SNP_06429	1094	C	T	27	9	--	--
	Ca(ICCV2/PI489777)SNP_06430	1173	T	G	20	6	--	--
	Ca(ICCV2/PI489777)SNP_06431	1175	G	C	20	6	--	--
	Ca(ICCV2/PI489777)SNP_06432	1214	A	C	18	5	--	--
CakTC31658	Ca(ICCV2/PI489777)SNP_06433	288	A	T	7	4	--	--

CakTC32245	Ca(ICCV2/PI489777)SNP_06434	1304	C	T	28	37	--	--
	Ca(ICCV2/PI489777)SNP_06435	1353	G	C	13	28	--	--
CakTC26629	Ca(ICCV2/PI489777)SNP_06436	1716	C	T	4	4	--	--
	Ca(ICCV2/PI489777)SNP_06437	1729	C	T	4	3	--	--
	Ca(ICCV2/PI489777)SNP_06438	1734	G	A	3	3	--	--
CakTC39773	Ca(ICCV2/PI489777)SNP_06439	194	G	A	7	4	--	--
	Ca(ICCV2/PI489777)SNP_06440	336	T	G	9	3	--	--
	Ca(ICCV2/PI489777)SNP_06441	455	G	A	7	4	--	--
	Ca(ICCV2/PI489777)SNP_06442	914	T	A	9	5	--	--
CakTC37617	Ca(ICCV2/PI489777)SNP_06443	809	C	A	5	3	--	--
CakTC26275	Ca(ICCV2/PI489777)SNP_06444	146	C	A	17	12	--	--
	Ca(ICCV2/PI489777)SNP_06445	421	A	G	8	8	--	--
	Ca(ICCV2/PI489777)SNP_06446	422	C	A	9	8	--	--
CakTC43227	Ca(ICCV2/PI489777)SNP_06447	306	A	G	8	7	--	--
CakTC28733	Ca(ICCV2/PI489777)SNP_06448	154	C	T	13	4	--	--
	Ca(ICCV2/PI489777)SNP_06449	211	T	A	11	4	--	--
CakTC39862	Ca(ICCV2/PI489777)SNP_06450	545	A	G	5	3	--	--
	Ca(ICCV2/PI489777)SNP_06451	885	T	C	7	10	--	--
CakTC24591	Ca(ICCV2/PI489777)SNP_06452	904	A	T	47	16	Flower	bud
	Ca(ICCV2/PI489777)SNP_06453	952	C	A	50	13	Flower	bud
	Ca(ICCV2/PI489777)SNP_06454	1104	C	T	58	12	Flower	bud
	Ca(ICCV2/PI489777)SNP_06455	1869	C	G	13	7	Flower	bud
CakTC33174	Ca(ICCV2/PI489777)SNP_06456	376	T	A	19	6	--	--
CakTC38338	Ca(ICCV2/PI489777)SNP_06457	1532	T	C	4	6	Young_pod	LIM
	Ca(ICCV2/PI489777)SNP_06458	1669	C	G	7	4	Young_pod	LIM
	Ca(ICCV2/PI489777)SNP_06459	1699	T	G	6	4	Young_pod	LIM
CakTC39811	Ca(ICCV2/PI489777)SNP_06460	1109	A	C	15	13	--	--
	Ca(ICCV2/PI489777)SNP_06461	1161	G	T	13	9	--	--
CakTC38539	Ca(ICCV2/PI489777)SNP_06462	630	A	G	23	17	--	--
	Ca(ICCV2/PI489777)SNP_06463	765	G	A	19	16	--	--
	Ca(ICCV2/PI489777)SNP_06464	1530	A	G	11	6	--	--
CakTC30279	Ca(ICCV2/PI489777)SNP_06465	1253	A	G	9	3	--	--
	Ca(ICCV2/PI489777)SNP_06466	1403	T	C	14	4	--	--
	Ca(ICCV2/PI489777)SNP_06467	1748	C	T	3	7	--	--
CakTC25071	Ca(ICCV2/PI489777)SNP_06468	827	A	G	11	9	--	--
	Ca(ICCV2/PI489777)SNP_06469	989	T	A	13	7	--	--
	Ca(ICCV2/PI489777)SNP_06470	992	C	T	13	6	--	--
	Ca(ICCV2/PI489777)SNP_06471	1090	T	C	12	3	--	--
CakTC23617	Ca(ICCV2/PI489777)SNP_06472	408	C	G	9	4	--	--
	Ca(ICCV2/PI489777)SNP_06473	413	G	A	9	3	--	--
	Ca(ICCV2/PI489777)SNP_06474	526	G	C	4	4	--	--
	Ca(ICCV2/PI489777)SNP_06475	559	A	T	4	4	--	--
CakTC27872	Ca(ICCV2/PI489777)SNP_06476	432	A	T	69	48	--	--
	Ca(ICCV2/PI489777)SNP_06477	534	G	A	56	33	--	--
CakTC23709	Ca(ICCV2/PI489777)SNP_06478	133	C	A	9	8	--	--
	Ca(ICCV2/PI489777)SNP_06479	842	T	G	8	5	--	--
CakTC25340	Ca(ICCV2/PI489777)SNP_06480	1209	A	G	3	3	--	--
CakTC24946	Ca(ICCV2/PI489777)SNP_06481	481	C	G	17	6	--	--
	Ca(ICCV2/PI489777)SNP_06482	826	T	G	19	3	--	--
	Ca(ICCV2/PI489777)SNP_06483	1194	A	T	7	6	--	--
	Ca(ICCV2/PI489777)SNP_06484	1276	T	C	3	3	--	--
CakTC29372	Ca(ICCV2/PI489777)SNP_06485	13	T	A	6	12	--	--
	Ca(ICCV2/PI489777)SNP_06486	457	G	A	20	61	--	--
	Ca(ICCV2/PI489777)SNP_06487	720	T	A	20	52	--	--
	Ca(ICCV2/PI489777)SNP_06488	1033	G	T	16	54	--	--
	Ca(ICCV2/PI489777)SNP_06489	1339	A	T	4	16	--	--
CakTC25144	Ca(ICCV2/PI489777)SNP_06490	127	A	T	12	4	--	--
	Ca(ICCV2/PI489777)SNP_06491	221	G	A	14	4	--	--
	Ca(ICCV2/PI489777)SNP_06492	679	T	C	16	4	--	--
	Ca(ICCV2/PI489777)SNP_06493	718	G	A	14	4	--	--
	Ca(ICCV2/PI489777)SNP_06494	730	G	T	13	4	--	--
	Ca(ICCV2/PI489777)SNP_06495	818	A	G	10	4	--	--
CakTC33277	Ca(ICCV2/PI489777)SNP_06496	493	A	G	166	7	--	--
CakTC34630	Ca(ICCV2/PI489777)SNP_06497	485	G	A	51	9	--	--
CakTC05841	Ca(ICCV2/PI489777)SNP_06498	909	C	T	7	5	--	--
	Ca(ICCV2/PI489777)SNP_06499	1271	G	A	5	6	--	--
CakTC09819	Ca(ICCV2/PI489777)SNP_06500	2272	A	T	7	6	--	--
	Ca(ICCV2/PI489777)SNP_06501	2327	T	C	3	7	--	--
CakTC42969	Ca(ICCV2/PI489777)SNP_06502	219	A	T	3	5	--	--
	Ca(ICCV2/PI489777)SNP_06503	247	C	T	3	5	--	--
	Ca(ICCV2/PI489777)SNP_06504	283	C	A	3	5	--	--
	Ca(ICCV2/PI489777)SNP_06505	355	C	T	8	4	--	--
CakTC29953	Ca(ICCV2/PI489777)SNP_06506	3151	C	G	3	8	--	Bromodomain
	Ca(ICCV2/PI489777)SNP_06507	3224	A	G	4	5	--	Bromodomain

	Ca(ICCV2/PI489777)SNP_06508	5354	A	C	6	4	--	Bromodomain
CakTC11755	Ca(ICCV2/PI489777)SNP_06509	1186	A	T	7	6	--	--
CakTC26354	Ca(ICCV2/PI489777)SNP_06510	200	T	C	10	3	--	--
	Ca(ICCV2/PI489777)SNP_06511	245	G	A	10	3	--	--
	Ca(ICCV2/PI489777)SNP_06512	329	A	T	11	3	--	--
	Ca(ICCV2/PI489777)SNP_06513	860	G	A	13	3	--	--
	Ca(ICCV2/PI489777)SNP_06514	876	G	C	13	3	--	--
	Ca(ICCV2/PI489777)SNP_06515	882	A	T	13	3	--	--
	Ca(ICCV2/PI489777)SNP_06516	891	A	C	14	3	--	--
	Ca(ICCV2/PI489777)SNP_06517	917	C	T	10	3	--	--
CakTC22395	Ca(ICCV2/PI489777)SNP_06518	2334	T	C	3	3	--	--
CakTC37728	Ca(ICCV2/PI489777)SNP_06519	211	A	C	17	12	--	--
CakTC27056	Ca(ICCV2/PI489777)SNP_06520	1426	G	A	7	3	--	--
CakTC25222	Ca(ICCV2/PI489777)SNP_06521	618	T	C	61	33	--	--
	Ca(ICCV2/PI489777)SNP_06522	951	C	T	47	36	--	--
CakTC39807	Ca(ICCV2/PI489777)SNP_06523	253	T	C	22	5	--	--
	Ca(ICCV2/PI489777)SNP_06524	736	G	A	10	5	--	--
	Ca(ICCV2/PI489777)SNP_06525	876	A	G	22	6	--	--
	Ca(ICCV2/PI489777)SNP_06526	1107	G	A	18	9	--	--
	Ca(ICCV2/PI489777)SNP_06527	1162	T	A	17	8	--	--
CakTC30289	Ca(ICCV2/PI489777)SNP_06528	855	T	C	4	5	--	--
CakTC38740	Ca(ICCV2/PI489777)SNP_06529	762	G	T	60	28	--	--
	Ca(ICCV2/PI489777)SNP_06530	898	A	G	40	20	--	--
CakTC26475	Ca(ICCV2/PI489777)SNP_06531	1105	T	C	5	3	--	--
	Ca(ICCV2/PI489777)SNP_06532	1570	G	A	4	3	--	--
	Ca(ICCV2/PI489777)SNP_06533	2895	A	G	3	7	--	--
CakTC07064	Ca(ICCV2/PI489777)SNP_06534	984	T	C	3	4	--	--
CakTC37098	Ca(ICCV2/PI489777)SNP_06535	627	G	A	11	4	--	MYB-related
	Ca(ICCV2/PI489777)SNP_06536	992	T	C	14	9	--	MYB-related
	Ca(ICCV2/PI489777)SNP_06537	1006	G	C	13	8	--	MYB-related
	Ca(ICCV2/PI489777)SNP_06538	1163	G	A	7	6	--	MYB-related
	Ca(ICCV2/PI489777)SNP_06539	1190	T	G	6	7	--	MYB-related
	Ca(ICCV2/PI489777)SNP_06540	1760	T	A	5	10	--	MYB-related
	Ca(ICCV2/PI489777)SNP_06541	2490	G	A	8	7	--	MYB-related
CakTC33331	Ca(ICCV2/PI489777)SNP_06542	3619	T	C	10	9	--	--
CakTC26630	Ca(ICCV2/PI489777)SNP_06543	63	T	C	6	6	--	--
	Ca(ICCV2/PI489777)SNP_06544	98	A	T	11	8	--	--
	Ca(ICCV2/PI489777)SNP_06545	149	A	G	14	10	--	--
	Ca(ICCV2/PI489777)SNP_06546	1178	A	G	48	36	--	--
	Ca(ICCV2/PI489777)SNP_06547	1349	G	A	35	27	--	--
	Ca(ICCV2/PI489777)SNP_06548	1365	T	C	38	40	--	--
	Ca(ICCV2/PI489777)SNP_06549	1397	C	T	37	38	--	--
	Ca(ICCV2/PI489777)SNP_06550	1438	T	A	32	33	--	--
	Ca(ICCV2/PI489777)SNP_06551	1448	C	T	33	35	--	--
	Ca(ICCV2/PI489777)SNP_06552	1451	G	T	35	36	--	--
CakTC32626	Ca(ICCV2/PI489777)SNP_06553	2023	T	C	29	27	--	--
	Ca(ICCV2/PI489777)SNP_06554	3069	C	T	50	15	--	--
	Ca(ICCV2/PI489777)SNP_06555	3146	A	G	49	19	--	--
	Ca(ICCV2/PI489777)SNP_06556	5914	A	C	40	32	--	--
CakTC37189	Ca(ICCV2/PI489777)SNP_06557	596	G	A	6	5	--	--
CakTC33240	Ca(ICCV2/PI489777)SNP_06558	827	T	G	69	35	--	--
	Ca(ICCV2/PI489777)SNP_06559	875	G	A	60	37	--	--
	Ca(ICCV2/PI489777)SNP_06560	1406	G	C	41	32	--	--
CakTC43128	Ca(ICCV2/PI489777)SNP_06561	746	T	C	59	37	--	--
CakTC38765	Ca(ICCV2/PI489777)SNP_06562	2839	G	A	5	3	--	--
	Ca(ICCV2/PI489777)SNP_06563	2916	C	T	4	3	--	--
CakTC28964	Ca(ICCV2/PI489777)SNP_06564	1551	G	T	5	7	--	--
	Ca(ICCV2/PI489777)SNP_06565	1763	A	G	5	6	--	--
CakTC42123	Ca(ICCV2/PI489777)SNP_06566	378	A	T	5	5	--	--
	Ca(ICCV2/PI489777)SNP_06567	404	C	G	5	5	--	--
CakTC42498	Ca(ICCV2/PI489777)SNP_06568	687	T	C	5	4	--	AP2-EREBP
CakTC22710	Ca(ICCV2/PI489777)SNP_06569	598	T	C	4	10	--	--
	Ca(ICCV2/PI489777)SNP_06570	603	T	C	4	10	--	--
	Ca(ICCV2/PI489777)SNP_06571	790	A	G	12	8	--	--
CakTC37926	Ca(ICCV2/PI489777)SNP_06572	284	A	G	3	3	--	--
	Ca(ICCV2/PI489777)SNP_06573	285	C	T	3	3	--	--
	Ca(ICCV2/PI489777)SNP_06574	307	A	G	3	3	--	--
	Ca(ICCV2/PI489777)SNP_06575	330	T	C	4	3	--	--
CakTC26058	Ca(ICCV2/PI489777)SNP_06576	78	G	A	18	5	--	--
	Ca(ICCV2/PI489777)SNP_06577	141	G	A	24	6	--	--
	Ca(ICCV2/PI489777)SNP_06578	323	T	C	18	7	--	--
	Ca(ICCV2/PI489777)SNP_06579	351	A	G	19	5	--	--
	Ca(ICCV2/PI489777)SNP_06580	354	C	T	20	5	--	--
CakTC29510	Ca(ICCV2/PI489777)SNP_06581	639	A	G	4	3	--	NAC

CakTC28509	Ca(ICCV2/PI489777)SNP_06582	1142	G	A	6	7	--	--
CakTC41411	Ca(ICCV2/PI489777)SNP_06583	273	C	A	33	8	--	--
	Ca(ICCV2/PI489777)SNP_06584	397	T	C	36	14	--	--
	Ca(ICCV2/PI489777)SNP_06585	585	A	T	27	14	--	--
	Ca(ICCV2/PI489777)SNP_06586	1038	A	G	16	8	--	--
	Ca(ICCV2/PI489777)SNP_06587	1850	G	A	13	5	--	--
	Ca(ICCV2/PI489777)SNP_06588	1865	C	T	13	5	--	--
	Ca(ICCV2/PI489777)SNP_06589	2037	C	T	16	12	--	--
	Ca(ICCV2/PI489777)SNP_06590	2219	C	G	22	18	--	--
CakTC30926	Ca(ICCV2/PI489777)SNP_06591	1979	A	T	6	4	--	--
CakTC26554	Ca(ICCV2/PI489777)SNP_06592	628	A	G	3	3	--	--
CakTC26936	Ca(ICCV2/PI489777)SNP_06593	93	C	T	11	12	--	--
	Ca(ICCV2/PI489777)SNP_06594	309	C	G	29	11	--	--
	Ca(ICCV2/PI489777)SNP_06595	399	T	G	29	10	--	--
CakTC33446	Ca(ICCV2/PI489777)SNP_06596	509	C	T	4	7	--	--
	Ca(ICCV2/PI489777)SNP_06597	536	G	A	4	7	--	--
CakTC40514	Ca(ICCV2/PI489777)SNP_06598	762	T	A	48	14	--	--
	Ca(ICCV2/PI489777)SNP_06599	828	T	C	46	14	--	--
CakTC39567	Ca(ICCV2/PI489777)SNP_06600	197	A	G	5	16	--	--
	Ca(ICCV2/PI489777)SNP_06601	210	G	T	3	19	--	--
	Ca(ICCV2/PI489777)SNP_06602	734	T	A	15	3	--	--
CakTC36576	Ca(ICCV2/PI489777)SNP_06603	437	T	C	16	6	--	--
	Ca(ICCV2/PI489777)SNP_06604	1121	C	T	14	9	--	--
	Ca(ICCV2/PI489777)SNP_06605	1222	G	T	18	4	--	--
	Ca(ICCV2/PI489777)SNP_06606	1626	T	C	27	11	--	--
	Ca(ICCV2/PI489777)SNP_06607	2085	T	C	9	6	--	--
	Ca(ICCV2/PI489777)SNP_06608	2154	C	G	7	3	--	--
	Ca(ICCV2/PI489777)SNP_06609	2235	G	A	9	5	--	--
	Ca(ICCV2/PI489777)SNP_06610	2343	A	G	11	8	--	--
	Ca(ICCV2/PI489777)SNP_06611	2636	G	A	7	7	--	--
CakTC41938	Ca(ICCV2/PI489777)SNP_06612	45	C	A	10	12	--	--
	Ca(ICCV2/PI489777)SNP_06613	198	C	A	14	17	--	--
	Ca(ICCV2/PI489777)SNP_06614	538	C	T	21	13	--	--
	Ca(ICCV2/PI489777)SNP_06615	630	C	T	35	19	--	--
	Ca(ICCV2/PI489777)SNP_06616	727	C	A	36	25	--	--
	Ca(ICCV2/PI489777)SNP_06617	954	C	G	28	45	--	--
	Ca(ICCV2/PI489777)SNP_06618	1347	C	G	21	42	--	--
CakTC37457	Ca(ICCV2/PI489777)SNP_06619	186	A	C	18	7	--	WRKY
	Ca(ICCV2/PI489777)SNP_06620	189	A	C	16	7	--	WRKY
	Ca(ICCV2/PI489777)SNP_06621	216	T	C	16	7	--	WRKY
	Ca(ICCV2/PI489777)SNP_06622	465	C	T	12	6	--	WRKY
	Ca(ICCV2/PI489777)SNP_06623	558	T	C	10	7	--	WRKY
	Ca(ICCV2/PI489777)SNP_06624	777	T	C	9	9	--	WRKY
	Ca(ICCV2/PI489777)SNP_06625	846	C	A	7	9	--	WRKY
CakTC27102	Ca(ICCV2/PI489777)SNP_06626	160	G	A	75	34	--	--
	Ca(ICCV2/PI489777)SNP_06627	392	A	T	138	205	--	--
	Ca(ICCV2/PI489777)SNP_06628	433	G	A	152	242	--	--
	Ca(ICCV2/PI489777)SNP_06629	478	G	A	179	256	--	--
CakTC40929	Ca(ICCV2/PI489777)SNP_06630	1565	T	C	8	7	--	--
	Ca(ICCV2/PI489777)SNP_06631	1820	A	C	12	5	--	--
	Ca(ICCV2/PI489777)SNP_06632	1870	T	C	10	5	--	--
	Ca(ICCV2/PI489777)SNP_06633	1934	T	C	11	3	--	--
CakTC40568	Ca(ICCV2/PI489777)SNP_06634	299	G	A	13	7	--	--
	Ca(ICCV2/PI489777)SNP_06635	338	T	C	16	6	--	--
	Ca(ICCV2/PI489777)SNP_06636	348	C	T	16	6	--	--
	Ca(ICCV2/PI489777)SNP_06637	367	A	G	15	8	--	--
	Ca(ICCV2/PI489777)SNP_06638	463	G	C	17	6	--	--
	Ca(ICCV2/PI489777)SNP_06639	566	T	A	7	8	--	--
CakTC37582	Ca(ICCV2/PI489777)SNP_06640	1092	C	A	6	4	--	--
	Ca(ICCV2/PI489777)SNP_06641	1116	A	C	6	4	--	--
	Ca(ICCV2/PI489777)SNP_06642	1117	A	C	6	4	--	--
CakTC31759	Ca(ICCV2/PI489777)SNP_06643	628	T	G	9	25	--	--
	Ca(ICCV2/PI489777)SNP_06644	644	A	G	9	18	--	--
CakTC30255	Ca(ICCV2/PI489777)SNP_06645	2146	A	T	16	6	--	--
CakTC43039	Ca(ICCV2/PI489777)SNP_06646	627	G	A	6	4	--	--
	Ca(ICCV2/PI489777)SNP_06647	1222	T	C	6	3	--	--
CakTC24155	Ca(ICCV2/PI489777)SNP_06648	240	A	G	5	5	--	--
	Ca(ICCV2/PI489777)SNP_06649	242	A	C	5	5	--	--
	Ca(ICCV2/PI489777)SNP_06650	630	G	T	10	5	--	--
	Ca(ICCV2/PI489777)SNP_06651	679	T	A	10	6	--	--
	Ca(ICCV2/PI489777)SNP_06652	1150	T	A	7	4	--	--
CakTC25835	Ca(ICCV2/PI489777)SNP_06653	1520	A	G	15	8	--	--
	Ca(ICCV2/PI489777)SNP_06654	1679	G	A	13	10	--	--
	Ca(ICCV2/PI489777)SNP_06655	1721	G	T	12	11	--	--

	Ca(ICCV2/PI489777)SNP_06656	2330	T	C	45	20	--	--
	Ca(ICCV2/PI489777)SNP_06657	2717	T	G	62	29	--	--
	Ca(ICCV2/PI489777)SNP_06658	2950	G	A	70	24	--	--
	Ca(ICCV2/PI489777)SNP_06659	2957	A	G	66	22	--	--
	Ca(ICCV2/PI489777)SNP_06660	3011	A	T	67	23	--	--
	Ca(ICCV2/PI489777)SNP_06661	3050	G	A	53	25	--	--
	Ca(ICCV2/PI489777)SNP_06662	3134	A	G	48	37	--	--
	Ca(ICCV2/PI489777)SNP_06663	3146	C	T	59	35	--	--
CakTC10220	Ca(ICCV2/PI489777)SNP_06664	1540	G	A	4	3	--	--
CakTC38122	Ca(ICCV2/PI489777)SNP_06665	269	G	C	10	5	--	MYB
	Ca(ICCV2/PI489777)SNP_06666	278	C	T	11	5	--	MYB
	Ca(ICCV2/PI489777)SNP_06667	308	C	T	13	6	--	MYB
	Ca(ICCV2/PI489777)SNP_06668	389	A	C	11	6	--	MYB
	Ca(ICCV2/PI489777)SNP_06669	439	A	T	10	7	--	MYB
	Ca(ICCV2/PI489777)SNP_06670	708	A	C	7	8	--	MYB
CakTC30541	Ca(ICCV2/PI489777)SNP_06671	535	G	A	10	6	--	TPR
	Ca(ICCV2/PI489777)SNP_06672	581	A	G	9	5	--	TPR
	Ca(ICCV2/PI489777)SNP_06673	798	G	A	12	13	--	TPR
	Ca(ICCV2/PI489777)SNP_06674	883	A	C	19	28	--	TPR
	Ca(ICCV2/PI489777)SNP_06675	1344	T	C	13	4	--	TPR
	Ca(ICCV2/PI489777)SNP_06676	1988	A	G	20	6	--	TPR
	Ca(ICCV2/PI489777)SNP_06677	2480	T	A	17	11	--	TPR
	Ca(ICCV2/PI489777)SNP_06678	3049	T	A	13	11	--	TPR
	Ca(ICCV2/PI489777)SNP_06679	3103	T	C	13	13	--	TPR
	Ca(ICCV2/PI489777)SNP_06680	3194	G	A	14	11	--	TPR
	Ca(ICCV2/PI489777)SNP_06681	3214	C	G	14	13	--	TPR
	Ca(ICCV2/PI489777)SNP_06682	3313	A	T	9	9	--	TPR
CakTC28364	Ca(ICCV2/PI489777)SNP_06683	366	C	T	9	12	Flower	bud
CakTC27742	Ca(ICCV2/PI489777)SNP_06684	321	C	A	45	5	--	--
	Ca(ICCV2/PI489777)SNP_06685	452	T	C	42	9	--	--
	Ca(ICCV2/PI489777)SNP_06686	472	A	G	64	9	--	--
	Ca(ICCV2/PI489777)SNP_06687	591	T	A	58	11	--	--
	Ca(ICCV2/PI489777)SNP_06688	593	T	A	58	11	--	--
CakTC32317	Ca(ICCV2/PI489777)SNP_06689	369	C	T	4	3	--	--
CakTC23164	Ca(ICCV2/PI489777)SNP_06690	745	G	A	8	4	--	--
	Ca(ICCV2/PI489777)SNP_06691	781	G	A	10	4	--	--
	Ca(ICCV2/PI489777)SNP_06692	834	C	T	15	4	--	--
	Ca(ICCV2/PI489777)SNP_06693	862	G	T	19	4	--	--
	Ca(ICCV2/PI489777)SNP_06694	890	C	T	16	4	--	--
	Ca(ICCV2/PI489777)SNP_06695	1318	G	A	16	6	--	--
CakTC31423	Ca(ICCV2/PI489777)SNP_06696	825	A	G	7	7	--	--
	Ca(ICCV2/PI489777)SNP_06697	849	A	G	7	8	--	--
CakTC24050	Ca(ICCV2/PI489777)SNP_06698	323	G	A	16	4	--	--
	Ca(ICCV2/PI489777)SNP_06699	567	T	C	10	5	--	--
CakTC35113	Ca(ICCV2/PI489777)SNP_06700	944	T	C	37	20	--	--
CakTC33694	Ca(ICCV2/PI489777)SNP_06701	798	C	T	3	4	--	--
	Ca(ICCV2/PI489777)SNP_06702	1091	A	G	3	4	--	--
	Ca(ICCV2/PI489777)SNP_06703	1322	C	T	3	3	--	--
CakTC30834	Ca(ICCV2/PI489777)SNP_06704	897	A	G	16	5	--	--
	Ca(ICCV2/PI489777)SNP_06705	1292	G	A	12	3	--	--
CakTC41745	Ca(ICCV2/PI489777)SNP_06706	65	C	G	3	8	--	--
	Ca(ICCV2/PI489777)SNP_06707	211	T	C	11	11	--	--
CakTC36402	Ca(ICCV2/PI489777)SNP_06708	256	G	T	4	7	Young_pod	--
CakTC25367	Ca(ICCV2/PI489777)SNP_06709	680	C	T	29	17	--	--
	Ca(ICCV2/PI489777)SNP_06710	917	C	T	39	27	--	--
	Ca(ICCV2/PI489777)SNP_06711	1151	A	G	46	30	--	--
	Ca(ICCV2/PI489777)SNP_06712	1304	C	T	51	18	--	--
	Ca(ICCV2/PI489777)SNP_06713	1604	T	C	30	26	--	--
	Ca(ICCV2/PI489777)SNP_06714	1775	C	T	50	38	--	--
	Ca(ICCV2/PI489777)SNP_06715	2228	C	T	52	37	--	--
	Ca(ICCV2/PI489777)SNP_06716	2804	T	C	62	28	--	--
	Ca(ICCV2/PI489777)SNP_06717	2865	C	G	44	24	--	--
	Ca(ICCV2/PI489777)SNP_06718	3590	G	A	83	40	--	--
CakTC38174	Ca(ICCV2/PI489777)SNP_06719	196	C	T	6	10	--	--
	Ca(ICCV2/PI489777)SNP_06720	445	G	T	10	15	--	--
	Ca(ICCV2/PI489777)SNP_06721	670	C	T	14	8	--	--
	Ca(ICCV2/PI489777)SNP_06722	1448	C	G	10	5	--	--
	Ca(ICCV2/PI489777)SNP_06723	1505	T	C	7	3	--	--
CakTC38132	Ca(ICCV2/PI489777)SNP_06724	448	A	G	18	11	--	--
CakTC26787	Ca(ICCV2/PI489777)SNP_06725	1110	C	G	5	4	--	--
CakTC14223	Ca(ICCV2/PI489777)SNP_06726	152	G	A	4	3	--	--
CakTC23352	Ca(ICCV2/PI489777)SNP_06727	188	C	T	7	11	--	--
CakTC40166	Ca(ICCV2/PI489777)SNP_06728	656	A	G	33	24	--	--
CakTC01243	Ca(ICCV2/PI489777)SNP_06729	514	T	A	28	9	--	--

	Ca(ICCV2/PI489777)SNP_06730	703	C	T	15	5	--	--
CakTC31331	Ca(ICCV2/PI489777)SNP_06731	312	T	C	5	14	--	--
	Ca(ICCV2/PI489777)SNP_06732	385	G	A	8	14	--	--
	Ca(ICCV2/PI489777)SNP_06733	991	A	G	24	13	--	--
	Ca(ICCV2/PI489777)SNP_06734	1238	T	C	21	12	--	--
	Ca(ICCV2/PI489777)SNP_06735	1347	A	T	16	15	--	--
	Ca(ICCV2/PI489777)SNP_06736	1472	G	T	8	15	--	--
CakTC35899	Ca(ICCV2/PI489777)SNP_06737	262	A	G	26	8	--	--
	Ca(ICCV2/PI489777)SNP_06738	344	G	C	27	8	--	--
	Ca(ICCV2/PI489777)SNP_06739	354	C	A	27	8	--	--
	Ca(ICCV2/PI489777)SNP_06740	403	T	C	22	8	--	--
	Ca(ICCV2/PI489777)SNP_06741	420	T	A	14	7	--	--
	Ca(ICCV2/PI489777)SNP_06742	444	G	A	7	4	--	--
CakTC40532	Ca(ICCV2/PI489777)SNP_06743	2096	T	G	13	12	--	--
	Ca(ICCV2/PI489777)SNP_06744	2117	G	A	12	12	--	--
	Ca(ICCV2/PI489777)SNP_06745	2120	G	A	14	12	--	--
	Ca(ICCV2/PI489777)SNP_06746	2265	A	G	8	9	--	--
CakTC27677	Ca(ICCV2/PI489777)SNP_06747	263	T	C	3	3	--	--
CakTC22643	Ca(ICCV2/PI489777)SNP_06748	331	A	C	69	14	--	--
CakTC39947	Ca(ICCV2/PI489777)SNP_06749	118	T	C	12	5	--	--
	Ca(ICCV2/PI489777)SNP_06750	244	G	A	14	9	--	--
	Ca(ICCV2/PI489777)SNP_06751	849	C	T	17	26	--	--
CakTC23204	Ca(ICCV2/PI489777)SNP_06752	200	A	G	242	114	--	--
	Ca(ICCV2/PI489777)SNP_06753	641	C	T	425	151	--	--
	Ca(ICCV2/PI489777)SNP_06754	692	T	C	432	151	--	--
CakTC25298	Ca(ICCV2/PI489777)SNP_06755	330	C	T	13	3	--	--
	Ca(ICCV2/PI489777)SNP_06756	1644	G	A	14	3	--	--
CakTC09534	Ca(ICCV2/PI489777)SNP_06757	970	G	A	16	6	--	--
	Ca(ICCV2/PI489777)SNP_06758	1690	C	T	17	10	--	--
	Ca(ICCV2/PI489777)SNP_06759	1795	G	A	21	8	--	--
	Ca(ICCV2/PI489777)SNP_06760	2180	C	G	14	5	--	--
CakTC34692	Ca(ICCV2/PI489777)SNP_06761	201	T	G	8	3	--	TPR
	Ca(ICCV2/PI489777)SNP_06762	220	A	T	9	3	--	TPR
	Ca(ICCV2/PI489777)SNP_06763	1858	C	T	10	6	--	TPR
	Ca(ICCV2/PI489777)SNP_06764	1957	G	T	9	8	--	TPR
	Ca(ICCV2/PI489777)SNP_06765	2308	T	C	17	9	--	TPR
	Ca(ICCV2/PI489777)SNP_06766	2605	A	G	13	9	--	TPR
	Ca(ICCV2/PI489777)SNP_06767	2665	A	G	16	9	--	TPR
	Ca(ICCV2/PI489777)SNP_06768	3426	G	A	15	7	--	TPR
CakTC33061	Ca(ICCV2/PI489777)SNP_06769	142	G	A	12	6	--	--
	Ca(ICCV2/PI489777)SNP_06770	144	T	G	10	6	--	--
	Ca(ICCV2/PI489777)SNP_06771	381	A	G	7	6	--	--
	Ca(ICCV2/PI489777)SNP_06772	484	C	A	3	4	--	--
CakTC26442	Ca(ICCV2/PI489777)SNP_06773	1044	T	G	8	13	--	--
	Ca(ICCV2/PI489777)SNP_06774	1176	G	C	11	9	--	--
	Ca(ICCV2/PI489777)SNP_06775	1797	G	A	7	7	--	--
	Ca(ICCV2/PI489777)SNP_06776	1821	T	C	6	6	--	--
CakTC26216	Ca(ICCV2/PI489777)SNP_06777	1066	A	G	32	21	--	--
	Ca(ICCV2/PI489777)SNP_06778	1092	G	T	21	13	--	--
	Ca(ICCV2/PI489777)SNP_06779	1222	G	C	7	4	--	--
CakTC41444	Ca(ICCV2/PI489777)SNP_06780	512	A	T	48	7	--	--
	Ca(ICCV2/PI489777)SNP_06781	524	T	A	47	9	--	--
	Ca(ICCV2/PI489777)SNP_06782	530	C	T	46	9	--	--
	Ca(ICCV2/PI489777)SNP_06783	547	T	C	41	9	--	--
CakTC33613	Ca(ICCV2/PI489777)SNP_06784	621	G	A	21	15	--	--
	Ca(ICCV2/PI489777)SNP_06785	956	A	G	16	7	--	--
CakTC39582	Ca(ICCV2/PI489777)SNP_06786	377	T	C	12	7	--	--
	Ca(ICCV2/PI489777)SNP_06787	737	C	T	9	9	--	--
	Ca(ICCV2/PI489777)SNP_06788	902	G	A	8	8	--	--
CakTC38147	Ca(ICCV2/PI489777)SNP_06789	489	G	A	4	3	--	--
CakTC28788	Ca(ICCV2/PI489777)SNP_06790	1146	T	C	5	4	--	--
CakTC31644	Ca(ICCV2/PI489777)SNP_06791	1015	G	A	7	4	--	--
	Ca(ICCV2/PI489777)SNP_06792	1406	C	T	5	4	--	--
	Ca(ICCV2/PI489777)SNP_06793	1412	T	C	5	4	--	--
CakTC09559	Ca(ICCV2/PI489777)SNP_06794	440	G	A	3	3	Young_pod	--
CakTC42491	Ca(ICCV2/PI489777)SNP_06795	252	C	A	6	7	--	--
CakTC25543	Ca(ICCV2/PI489777)SNP_06796	137	C	G	5	5	--	HB
	Ca(ICCV2/PI489777)SNP_06797	181	C	G	8	15	--	HB
	Ca(ICCV2/PI489777)SNP_06798	217	G	A	9	11	--	HB
	Ca(ICCV2/PI489777)SNP_06799	280	C	T	17	18	--	HB
	Ca(ICCV2/PI489777)SNP_06800	845	C	G	127	57	--	HB
	Ca(ICCV2/PI489777)SNP_06801	857	G	A	118	57	--	HB
	Ca(ICCV2/PI489777)SNP_06802	884	C	T	113	49	--	HB
	Ca(ICCV2/PI489777)SNP_06803	1495	G	A	42	34	--	HB

CakTC23428	Ca(ICCV2/PI489777)SNP_06804	984	A	C	70	9	--	HMG
	Ca(ICCV2/PI489777)SNP_06805	991	T	C	64	11	--	HMG
	Ca(ICCV2/PI489777)SNP_06806	1136	C	A	81	11	--	HMG
	Ca(ICCV2/PI489777)SNP_06807	1141	G	A	79	9	--	HMG
	Ca(ICCV2/PI489777)SNP_06808	1200	G	A	72	10	--	HMG
	Ca(ICCV2/PI489777)SNP_06809	1515	C	T	16	9	--	HMG
CakTC26256	Ca(ICCV2/PI489777)SNP_06810	165	G	C	17	5	--	--
	Ca(ICCV2/PI489777)SNP_06811	501	G	A	30	10	--	--
	Ca(ICCV2/PI489777)SNP_06812	789	T	C	29	9	--	--
CakTC08454	Ca(ICCV2/PI489777)SNP_06813	112	G	C	33	6	--	--
	Ca(ICCV2/PI489777)SNP_06814	235	C	A	38	8	--	--
	Ca(ICCV2/PI489777)SNP_06815	299	A	C	31	8	--	--
	Ca(ICCV2/PI489777)SNP_06816	461	G	A	27	7	--	--
	Ca(ICCV2/PI489777)SNP_06817	569	G	T	14	6	--	--
	Ca(ICCV2/PI489777)SNP_06818	914	C	T	52	13	--	--
	Ca(ICCV2/PI489777)SNP_06819	930	T	C	54	14	--	--
	Ca(ICCV2/PI489777)SNP_06820	984	A	C	54	12	--	--
CakTC41617	Ca(ICCV2/PI489777)SNP_06821	430	G	A	19	5	--	--
	Ca(ICCV2/PI489777)SNP_06822	563	G	A	14	3	--	--
CakTC25054	Ca(ICCV2/PI489777)SNP_06823	159	T	C	29	5	--	--
CakTC26781	Ca(ICCV2/PI489777)SNP_06824	674	C	T	3	3	--	--
CakTC25427	Ca(ICCV2/PI489777)SNP_06825	42	T	C	15	29	Root	--
CakTC41019	Ca(ICCV2/PI489777)SNP_06826	1045	C	T	19	8	--	--
	Ca(ICCV2/PI489777)SNP_06827	1501	C	T	26	8	--	--
CakTC35085	Ca(ICCV2/PI489777)SNP_06828	150	G	C	10	4	Flower	bud
	Ca(ICCV2/PI489777)SNP_06829	620	A	G	9	7	Flower	bud
	Ca(ICCV2/PI489777)SNP_06830	2350	G	A	9	9	Flower	bud
CakTC27751	Ca(ICCV2/PI489777)SNP_06831	1382	G	A	9	11	Flower	bud
	Ca(ICCV2/PI489777)SNP_06832	1387	A	G	9	11	Flower	bud
	Ca(ICCV2/PI489777)SNP_06833	4221	T	C	4	10	Flower	bud
CakTC30125	Ca(ICCV2/PI489777)SNP_06834	306	A	T	13	6	--	--
CakTC24824	Ca(ICCV2/PI489777)SNP_06835	1047	C	T	6	4	--	MYB
	Ca(ICCV2/PI489777)SNP_06836	1098	G	A	6	4	--	MYB
	Ca(ICCV2/PI489777)SNP_06837	1170	T	A	9	7	--	MYB
	Ca(ICCV2/PI489777)SNP_06838	1357	G	A	18	8	--	MYB
	Ca(ICCV2/PI489777)SNP_06839	1600	G	A	17	8	--	MYB
	Ca(ICCV2/PI489777)SNP_06840	1652	T	C	16	9	--	MYB
	Ca(ICCV2/PI489777)SNP_06841	1802	T	A	9	9	--	MYB
	Ca(ICCV2/PI489777)SNP_06842	1937	G	A	12	10	--	MYB
	Ca(ICCV2/PI489777)SNP_06843	2333	T	C	5	5	--	MYB
	Ca(ICCV2/PI489777)SNP_06844	2695	T	C	5	3	--	MYB
	Ca(ICCV2/PI489777)SNP_06845	3134	A	G	7	5	--	MYB
	Ca(ICCV2/PI489777)SNP_06846	3146	A	G	7	5	--	MYB
CakTC31691	Ca(ICCV2/PI489777)SNP_06847	306	A	C	6	4	--	--
	Ca(ICCV2/PI489777)SNP_06848	342	G	A	6	5	--	--
	Ca(ICCV2/PI489777)SNP_06849	526	T	A	7	6	--	--
	Ca(ICCV2/PI489777)SNP_06850	702	G	A	7	5	--	--
	Ca(ICCV2/PI489777)SNP_06851	876	A	G	8	4	--	--
	Ca(ICCV2/PI489777)SNP_06852	894	G	A	8	5	--	--
	Ca(ICCV2/PI489777)SNP_06853	972	A	G	10	4	--	--
CakTC11408	Ca(ICCV2/PI489777)SNP_06854	353	C	G	3	3	--	--
CakTC28688	Ca(ICCV2/PI489777)SNP_06855	938	T	C	13	5	--	--
	Ca(ICCV2/PI489777)SNP_06856	1863	A	T	8	4	--	--
CakTC26795	Ca(ICCV2/PI489777)SNP_06857	426	G	T	13	3	--	--
	Ca(ICCV2/PI489777)SNP_06858	494	T	C	14	3	--	--
	Ca(ICCV2/PI489777)SNP_06859	993	C	T	20	16	--	--
	Ca(ICCV2/PI489777)SNP_06860	2422	G	A	22	9	--	--
CakTC40276	Ca(ICCV2/PI489777)SNP_06861	132	T	C	7	8	--	--
CakTC32175	Ca(ICCV2/PI489777)SNP_06862	674	G	A	11	3	--	--
	Ca(ICCV2/PI489777)SNP_06863	814	T	A	6	4	--	--
CakTC43209	Ca(ICCV2/PI489777)SNP_06864	1380	G	A	8	8	--	--
	Ca(ICCV2/PI489777)SNP_06865	1542	A	G	11	5	--	--
	Ca(ICCV2/PI489777)SNP_06866	1608	C	T	10	4	--	--
CakTC25707	Ca(ICCV2/PI489777)SNP_06867	256	A	T	106	3	--	--
	Ca(ICCV2/PI489777)SNP_06868	345	C	T	148	13	--	--
	Ca(ICCV2/PI489777)SNP_06869	369	C	T	134	15	--	--
	Ca(ICCV2/PI489777)SNP_06870	429	C	T	169	19	--	--
	Ca(ICCV2/PI489777)SNP_06871	453	A	C	181	22	--	--
	Ca(ICCV2/PI489777)SNP_06872	630	C	T	185	26	--	--
	Ca(ICCV2/PI489777)SNP_06873	636	C	T	192	29	--	--
	Ca(ICCV2/PI489777)SNP_06874	783	G	C	234	26	--	--
	Ca(ICCV2/PI489777)SNP_06875	1489	G	A	209	38	--	--
	Ca(ICCV2/PI489777)SNP_06876	1672	C	T	159	12	--	--
	Ca(ICCV2/PI489777)SNP_06877	1687	C	G	165	20	--	--

	Ca(ICCV2/PI489777)SNP_06878	1793	C	T	153	9	--	--
	Ca(ICCV2/PI489777)SNP_06879	1907	A	G	94	4	--	--
CakTC25358	Ca(ICCV2/PI489777)SNP_06880	402	C	T	4	3	--	--
CakTC38633	Ca(ICCV2/PI489777)SNP_06881	390	C	T	9	9	--	--
CakTC37535	Ca(ICCV2/PI489777)SNP_06882	195	A	T	19	10	--	--
	Ca(ICCV2/PI489777)SNP_06883	328	C	T	18	12	--	--
	Ca(ICCV2/PI489777)SNP_06884	346	T	C	18	10	--	--
	Ca(ICCV2/PI489777)SNP_06885	565	T	C	12	7	--	--
CakTC36841	Ca(ICCV2/PI489777)SNP_06886	874	G	T	20	7	--	--
	Ca(ICCV2/PI489777)SNP_06887	990	A	G	24	6	--	--
	Ca(ICCV2/PI489777)SNP_06888	993	C	T	24	6	--	--
	Ca(ICCV2/PI489777)SNP_06889	1050	A	G	20	6	--	--
	Ca(ICCV2/PI489777)SNP_06890	1972	C	A	29	11	--	--
	Ca(ICCV2/PI489777)SNP_06891	2520	C	T	25	18	--	--
	Ca(ICCV2/PI489777)SNP_06892	2523	T	C	26	18	--	--
	Ca(ICCV2/PI489777)SNP_06893	2544	C	T	25	15	--	--
	Ca(ICCV2/PI489777)SNP_06894	3306	C	T	20	22	--	--
	Ca(ICCV2/PI489777)SNP_06895	3825	G	A	24	12	--	--
	Ca(ICCV2/PI489777)SNP_06896	4080	T	G	19	19	--	--
	Ca(ICCV2/PI489777)SNP_06897	4506	T	C	21	11	--	--
	Ca(ICCV2/PI489777)SNP_06898	4740	G	C	4	6	--	--
	Ca(ICCV2/PI489777)SNP_06899	4745	C	A	3	6	--	--
CakTC05776	Ca(ICCV2/PI489777)SNP_06900	307	G	A	22	3	--	--
	Ca(ICCV2/PI489777)SNP_06901	394	A	T	14	5	--	--
	Ca(ICCV2/PI489777)SNP_06902	424	G	A	16	5	--	--
CakTC16253	Ca(ICCV2/PI489777)SNP_06903	488	G	A	8	6	--	--
CakTC09639	Ca(ICCV2/PI489777)SNP_06904	1151	T	G	5	5	--	--
	Ca(ICCV2/PI489777)SNP_06905	1190	T	A	5	5	--	--
	Ca(ICCV2/PI489777)SNP_06906	1196	T	A	5	5	--	--
	Ca(ICCV2/PI489777)SNP_06907	1214	A	G	7	5	--	--
CakTC41916	Ca(ICCV2/PI489777)SNP_06908	232	C	G	9	8	--	--
CakTC40187	Ca(ICCV2/PI489777)SNP_06909	456	G	C	7	11	--	IWS1
CakTC09862	Ca(ICCV2/PI489777)SNP_06910	822	G	T	3	3	--	--
CakTC39826	Ca(ICCV2/PI489777)SNP_06911	214	C	G	10	11	--	--
	Ca(ICCV2/PI489777)SNP_06912	1229	C	G	11	7	--	--
	Ca(ICCV2/PI489777)SNP_06913	1284	A	G	6	5	--	--
	Ca(ICCV2/PI489777)SNP_06914	1293	A	G	7	5	--	--
CakTC42795	Ca(ICCV2/PI489777)SNP_06915	183	A	G	7	3	--	--
	Ca(ICCV2/PI489777)SNP_06916	707	T	C	6	7	--	--
	Ca(ICCV2/PI489777)SNP_06917	720	A	G	6	8	--	--
	Ca(ICCV2/PI489777)SNP_06918	1283	C	T	14	6	--	--
	Ca(ICCV2/PI489777)SNP_06919	1508	G	T	15	5	--	--
CakTC36870	Ca(ICCV2/PI489777)SNP_06920	85	C	G	5	3	--	--
CakTC37350	Ca(ICCV2/PI489777)SNP_06921	438	C	A	7	10	--	--
	Ca(ICCV2/PI489777)SNP_06922	549	A	G	10	10	--	--
	Ca(ICCV2/PI489777)SNP_06923	854	A	G	13	11	--	--
	Ca(ICCV2/PI489777)SNP_06924	861	A	G	10	10	--	--
CakTC23890	Ca(ICCV2/PI489777)SNP_06925	247	A	G	140	49	--	--
	Ca(ICCV2/PI489777)SNP_06926	382	A	C	210	73	--	--
	Ca(ICCV2/PI489777)SNP_06927	655	T	A	266	66	--	--
	Ca(ICCV2/PI489777)SNP_06928	898	C	T	377	81	--	--
CakTC22748	Ca(ICCV2/PI489777)SNP_06929	176	G	A	88	30	--	--
CakTC40960	Ca(ICCV2/PI489777)SNP_06930	314	C	T	62	39	--	--
	Ca(ICCV2/PI489777)SNP_06931	475	G	T	40	27	--	--
	Ca(ICCV2/PI489777)SNP_06932	508	G	T	34	19	--	--
	Ca(ICCV2/PI489777)SNP_06933	569	A	T	18	3	--	--
CakTC34971	Ca(ICCV2/PI489777)SNP_06934	266	A	G	7	7	--	--
	Ca(ICCV2/PI489777)SNP_06935	549	T	C	5	6	--	--
	Ca(ICCV2/PI489777)SNP_06936	874	A	G	20	16	--	--
	Ca(ICCV2/PI489777)SNP_06937	1096	G	A	20	7	--	--
	Ca(ICCV2/PI489777)SNP_06938	1306	G	A	23	4	--	--
	Ca(ICCV2/PI489777)SNP_06939	1744	A	G	25	11	--	--
	Ca(ICCV2/PI489777)SNP_06940	1957	G	C	16	10	--	--
	Ca(ICCV2/PI489777)SNP_06941	2108	T	A	7	12	--	--
	Ca(ICCV2/PI489777)SNP_06942	2263	A	T	11	13	--	--
CakTC30218	Ca(ICCV2/PI489777)SNP_06943	565	A	G	13	7	--	--
	Ca(ICCV2/PI489777)SNP_06944	776	A	T	13	7	--	--
	Ca(ICCV2/PI489777)SNP_06945	871	C	T	15	7	--	--
	Ca(ICCV2/PI489777)SNP_06946	974	G	A	22	7	--	--
	Ca(ICCV2/PI489777)SNP_06947	1451	C	T	18	7	--	--
	Ca(ICCV2/PI489777)SNP_06948	2136	G	T	10	4	--	--
	Ca(ICCV2/PI489777)SNP_06949	2513	A	G	6	4	--	--
CakTC25115	Ca(ICCV2/PI489777)SNP_06950	228	A	T	17	8	--	--
	Ca(ICCV2/PI489777)SNP_06951	445	G	A	80	30	--	--

	Ca(ICCV2/PI489777)SNP_06952	640	T	C	114	34	--	--
CakTC33244	Ca(ICCV2/PI489777)SNP_06953	68	C	T	20	4	--	--
	Ca(ICCV2/PI489777)SNP_06954	263	A	G	72	27	--	--
	Ca(ICCV2/PI489777)SNP_06955	315	C	G	73	27	--	--
CakTC39489	Ca(ICCV2/PI489777)SNP_06956	326	T	C	16	40	--	--
	Ca(ICCV2/PI489777)SNP_06957	392	G	T	14	39	--	--
	Ca(ICCV2/PI489777)SNP_06958	419	A	G	10	39	--	--
	Ca(ICCV2/PI489777)SNP_06959	1064	T	A	9	23	--	--
	Ca(ICCV2/PI489777)SNP_06960	1190	C	T	5	19	--	--
CakTC40668	Ca(ICCV2/PI489777)SNP_06961	432	A	G	4	3	--	--
CakTC30246	Ca(ICCV2/PI489777)SNP_06962	1389	A	T	66	24	--	C3H
CakTC24115	Ca(ICCV2/PI489777)SNP_06963	1879	A	G	19	10	--	--
CakTC34123	Ca(ICCV2/PI489777)SNP_06964	15	C	G	7	3	--	--
CakTC25825	Ca(ICCV2/PI489777)SNP_06965	586	C	A	27	4	--	WRKY
	Ca(ICCV2/PI489777)SNP_06966	706	T	C	16	10	--	WRKY
	Ca(ICCV2/PI489777)SNP_06967	727	A	G	14	9	--	WRKY
	Ca(ICCV2/PI489777)SNP_06968	807	C	T	14	3	--	WRKY
CakTC28502	Ca(ICCV2/PI489777)SNP_06969	532	C	T	7	6	Flower	bud
	Ca(ICCV2/PI489777)SNP_06970	1300	A	T	10	4	Flower	bud
	Ca(ICCV2/PI489777)SNP_06971	1693	T	C	11	9	Flower	bud
CakTC33714	Ca(ICCV2/PI489777)SNP_06972	96	C	T	18	5	--	--
CakTC10172	Ca(ICCV2/PI489777)SNP_06973	1268	T	G	3	3	--	--
	Ca(ICCV2/PI489777)SNP_06974	1623	G	A	5	5	--	--
	Ca(ICCV2/PI489777)SNP_06975	1848	G	A	5	11	--	--
CakTC37365	Ca(ICCV2/PI489777)SNP_06976	138	G	C	18	9	--	--
	Ca(ICCV2/PI489777)SNP_06977	297	T	C	33	24	--	--
	Ca(ICCV2/PI489777)SNP_06978	324	C	A	30	24	--	--
	Ca(ICCV2/PI489777)SNP_06979	357	C	T	35	24	--	--
	Ca(ICCV2/PI489777)SNP_06980	489	T	C	22	20	--	--
	Ca(ICCV2/PI489777)SNP_06981	531	T	C	20	18	--	--
	Ca(ICCV2/PI489777)SNP_06982	552	G	A	21	17	--	--
	Ca(ICCV2/PI489777)SNP_06983	591	A	G	21	17	--	--
CakTC25656	Ca(ICCV2/PI489777)SNP_06984	262	T	C	5	12	--	--
	Ca(ICCV2/PI489777)SNP_06985	334	A	G	5	13	--	--
CakTC38125	Ca(ICCV2/PI489777)SNP_06986	962	C	T	13	5	--	--
CakTC27926	Ca(ICCV2/PI489777)SNP_06987	2626	A	G	7	6	--	--
	Ca(ICCV2/PI489777)SNP_06988	2688	A	C	6	4	--	--
	Ca(ICCV2/PI489777)SNP_06989	2707	T	C	3	4	--	--
CakTC08751	Ca(ICCV2/PI489777)SNP_06990	486	C	A	5	7	--	--
	Ca(ICCV2/PI489777)SNP_06991	3000	C	T	3	8	--	--
CakTC36630	Ca(ICCV2/PI489777)SNP_06992	193	T	A	10	4	--	--
	Ca(ICCV2/PI489777)SNP_06993	194	C	T	11	4	--	--
	Ca(ICCV2/PI489777)SNP_06994	235	A	C	19	7	--	--
	Ca(ICCV2/PI489777)SNP_06995	236	C	A	19	7	--	--
	Ca(ICCV2/PI489777)SNP_06996	1427	A	T	12	8	--	--
	Ca(ICCV2/PI489777)SNP_06997	1658	T	C	10	3	--	--
	Ca(ICCV2/PI489777)SNP_06998	1751	C	T	5	4	--	--
	Ca(ICCV2/PI489777)SNP_06999	2385	T	C	14	18	--	--
	Ca(ICCV2/PI489777)SNP_07000	2459	G	A	16	17	--	--
CakTC32680	Ca(ICCV2/PI489777)SNP_07001	642	A	T	51	22	--	--
	Ca(ICCV2/PI489777)SNP_07002	1374	G	A	26	14	--	--
	Ca(ICCV2/PI489777)SNP_07003	1458	T	G	27	15	--	--
CakTC26236	Ca(ICCV2/PI489777)SNP_07004	1415	G	T	180	56	--	--
	Ca(ICCV2/PI489777)SNP_07005	1555	G	A	157	34	--	--
	Ca(ICCV2/PI489777)SNP_07006	1576	G	A	149	46	--	--
	Ca(ICCV2/PI489777)SNP_07007	1590	G	A	147	45	--	--
	Ca(ICCV2/PI489777)SNP_07008	1673	C	T	134	43	--	--
	Ca(ICCV2/PI489777)SNP_07009	1800	A	G	45	9	--	--
CakTC09651	Ca(ICCV2/PI489777)SNP_07010	1279	A	G	3	4	Young_pod	--
CakTC26487	Ca(ICCV2/PI489777)SNP_07011	26	T	C	34	6	--	--
	Ca(ICCV2/PI489777)SNP_07012	105	G	A	48	9	--	--
	Ca(ICCV2/PI489777)SNP_07013	263	C	G	67	15	--	--
	Ca(ICCV2/PI489777)SNP_07014	371	C	T	39	17	--	--
	Ca(ICCV2/PI489777)SNP_07015	708	T	A	23	9	--	--
	Ca(ICCV2/PI489777)SNP_07016	904	G	A	12	6	--	--
CakTC40222	Ca(ICCV2/PI489777)SNP_07017	1402	G	A	17	7	--	--
CakTC42486	Ca(ICCV2/PI489777)SNP_07018	638	T	C	9	5	--	--
	Ca(ICCV2/PI489777)SNP_07019	1048	A	G	11	3	--	--
CakTC09658	Ca(ICCV2/PI489777)SNP_07020	301	G	T	108	58	--	--
	Ca(ICCV2/PI489777)SNP_07021	895	T	C	126	51	--	--
	Ca(ICCV2/PI489777)SNP_07022	1133	C	T	24	22	--	--
	Ca(ICCV2/PI489777)SNP_07023	1196	A	G	15	10	--	--
CakTC08256	Ca(ICCV2/PI489777)SNP_07024	288	T	C	10	6	--	--
CakTC36123	Ca(ICCV2/PI489777)SNP_07025	652	C	G	8	10	--	--

	Ca(ICCV2/PI489777)SNP_07026	1408	C	T	7	8	--	--
	Ca(ICCV2/PI489777)SNP_07027	1928	T	C	16	8	--	--
	Ca(ICCV2/PI489777)SNP_07028	2036	T	C	17	5	--	--
CakTC00784	Ca(ICCV2/PI489777)SNP_07029	84	C	A	3	5	--	--
	Ca(ICCV2/PI489777)SNP_07030	129	A	G	4	3	--	--
	Ca(ICCV2/PI489777)SNP_07031	173	A	C	4	4	--	--
CakTC29573	Ca(ICCV2/PI489777)SNP_07032	94	G	A	14	6	--	--
	Ca(ICCV2/PI489777)SNP_07033	181	A	C	13	8	--	--
	Ca(ICCV2/PI489777)SNP_07034	223	A	C	13	10	--	--
	Ca(ICCV2/PI489777)SNP_07035	301	C	T	9	10	--	--
	Ca(ICCV2/PI489777)SNP_07036	367	G	A	7	15	--	--
CakTC30675	Ca(ICCV2/PI489777)SNP_07037	1081	T	G	5	5	--	--
	Ca(ICCV2/PI489777)SNP_07038	1216	T	C	5	4	--	--
	Ca(ICCV2/PI489777)SNP_07039	1218	G	A	5	4	--	--
CakTC38291	Ca(ICCV2/PI489777)SNP_07040	479	T	C	9	7	--	--
	Ca(ICCV2/PI489777)SNP_07041	781	A	G	10	6	--	--
CakTC31781	Ca(ICCV2/PI489777)SNP_07042	570	T	C	4	3	--	--
	Ca(ICCV2/PI489777)SNP_07043	2051	G	A	4	3	--	--
	Ca(ICCV2/PI489777)SNP_07044	2350	T	C	3	4	--	--
	Ca(ICCV2/PI489777)SNP_07045	2482	T	C	5	4	--	--
	Ca(ICCV2/PI489777)SNP_07046	2492	A	G	4	4	--	--
	Ca(ICCV2/PI489777)SNP_07047	2500	C	T	5	4	--	--
	Ca(ICCV2/PI489777)SNP_07048	2691	A	G	4	5	--	--
	Ca(ICCV2/PI489777)SNP_07049	2795	G	A	5	4	--	--
	Ca(ICCV2/PI489777)SNP_07050	3001	C	A	7	5	--	--
	Ca(ICCV2/PI489777)SNP_07051	3104	G	A	11	4	--	--
	Ca(ICCV2/PI489777)SNP_07052	3636	T	C	6	8	--	--
	Ca(ICCV2/PI489777)SNP_07053	3654	T	C	6	8	--	--
	Ca(ICCV2/PI489777)SNP_07054	3759	C	T	4	5	--	--
	Ca(ICCV2/PI489777)SNP_07055	4380	T	C	5	5	--	--
CakTC27634	Ca(ICCV2/PI489777)SNP_07056	1409	C	T	42	19	--	--
	Ca(ICCV2/PI489777)SNP_07057	1709	A	G	32	4	--	--
	Ca(ICCV2/PI489777)SNP_07058	2595	T	C	15	6	--	--
	Ca(ICCV2/PI489777)SNP_07059	2613	A	G	12	7	--	--
	Ca(ICCV2/PI489777)SNP_07060	2625	C	A	16	7	--	--
CakTC41321	Ca(ICCV2/PI489777)SNP_07061	2260	T	C	8	3	--	--
CakTC35690	Ca(ICCV2/PI489777)SNP_07062	1448	A	G	8	3	--	--
	Ca(ICCV2/PI489777)SNP_07063	3290	A	G	3	3	--	--
CakTC36863	Ca(ICCV2/PI489777)SNP_07064	1793	T	C	13	7	--	--
	Ca(ICCV2/PI489777)SNP_07065	1979	C	T	9	5	--	--
CakTC42275	Ca(ICCV2/PI489777)SNP_07066	202	C	T	7	5	--	--
	Ca(ICCV2/PI489777)SNP_07067	1000	C	G	14	10	--	--
CakTC42403	Ca(ICCV2/PI489777)SNP_07068	462	C	T	5	3	Shoot	--
	Ca(ICCV2/PI489777)SNP_07069	490	A	T	8	3	Shoot	--
	Ca(ICCV2/PI489777)SNP_07070	514	G	T	8	3	Shoot	--
	Ca(ICCV2/PI489777)SNP_07071	634	G	T	6	3	Shoot	--
	Ca(ICCV2/PI489777)SNP_07072	660	A	G	4	3	Shoot	--
CakTC23284	Ca(ICCV2/PI489777)SNP_07073	275	G	A	17	9	--	--
	Ca(ICCV2/PI489777)SNP_07074	296	A	C	15	8	--	--
	Ca(ICCV2/PI489777)SNP_07075	1709	C	T	27	21	--	--
	Ca(ICCV2/PI489777)SNP_07076	1843	A	G	27	12	--	--
	Ca(ICCV2/PI489777)SNP_07077	1904	G	A	31	15	--	--
CakTC42429	Ca(ICCV2/PI489777)SNP_07078	233	C	T	9	8	--	--
	Ca(ICCV2/PI489777)SNP_07079	253	A	G	6	8	--	--
	Ca(ICCV2/PI489777)SNP_07080	1146	C	T	6	12	--	--
	Ca(ICCV2/PI489777)SNP_07081	1368	G	A	10	11	--	--
	Ca(ICCV2/PI489777)SNP_07082	1718	G	A	3	5	--	--
CakTC39611	Ca(ICCV2/PI489777)SNP_07083	468	G	A	12	15	--	--
	Ca(ICCV2/PI489777)SNP_07084	1027	A	C	13	8	--	--
	Ca(ICCV2/PI489777)SNP_07085	1029	G	T	11	8	--	--
CakTC42093	Ca(ICCV2/PI489777)SNP_07086	545	C	T	3	22	--	--
CakTC40375	Ca(ICCV2/PI489777)SNP_07087	151	T	C	23	7	--	--
	Ca(ICCV2/PI489777)SNP_07088	229	C	T	21	10	--	--
	Ca(ICCV2/PI489777)SNP_07089	1731	T	C	10	5	--	--
CakTC32739	Ca(ICCV2/PI489777)SNP_07090	1476	C	T	8	3	--	HB
CakTC34752	Ca(ICCV2/PI489777)SNP_07091	396	G	A	5	4	--	--
	Ca(ICCV2/PI489777)SNP_07092	405	A	G	4	3	--	--
CakTC39711	Ca(ICCV2/PI489777)SNP_07093	603	T	C	23	21	--	--
CakTC38042	Ca(ICCV2/PI489777)SNP_07094	43	A	C	22	10	--	--
	Ca(ICCV2/PI489777)SNP_07095	132	A	G	20	17	--	--
	Ca(ICCV2/PI489777)SNP_07096	306	G	A	33	22	--	--
	Ca(ICCV2/PI489777)SNP_07097	441	C	T	24	26	--	--
	Ca(ICCV2/PI489777)SNP_07098	572	T	A	16	18	--	--
	Ca(ICCV2/PI489777)SNP_07099	600	A	G	22	16	--	--

	Ca(ICCV2/PI489777)SNP_07100	1404	C	G	29	37	--	--
CakTC38544	Ca(ICCV2/PI489777)SNP_07101	600	G	T	10	5	--	--
CakTC42592	Ca(ICCV2/PI489777)SNP_07102	337	G	A	18	4	--	--
	Ca(ICCV2/PI489777)SNP_07103	539	C	T	14	7	--	--
	Ca(ICCV2/PI489777)SNP_07104	543	G	A	15	7	--	--
	Ca(ICCV2/PI489777)SNP_07105	555	T	A	13	6	--	--
	Ca(ICCV2/PI489777)SNP_07106	982	A	C	3	11	--	--
CakTC37575	Ca(ICCV2/PI489777)SNP_07107	492	C	T	58	63	--	--
	Ca(ICCV2/PI489777)SNP_07108	564	C	T	76	31	--	--
	Ca(ICCV2/PI489777)SNP_07109	612	C	A	63	28	--	--
	Ca(ICCV2/PI489777)SNP_07110	678	C	A	48	30	--	--
	Ca(ICCV2/PI489777)SNP_07111	1032	C	G	24	30	--	--
CakTC22743	Ca(ICCV2/PI489777)SNP_07112	2383	C	A	9	5	--	--
CakTC23247	Ca(ICCV2/PI489777)SNP_07113	275	G	A	44	43	--	--
	Ca(ICCV2/PI489777)SNP_07114	743	T	G	143	45	--	--
CakTC28871	Ca(ICCV2/PI489777)SNP_07115	956	T	G	5	3	--	--
	Ca(ICCV2/PI489777)SNP_07116	975	A	G	4	3	--	--
	Ca(ICCV2/PI489777)SNP_07117	978	T	G	4	3	--	--
	Ca(ICCV2/PI489777)SNP_07118	1069	T	C	4	6	--	--
	Ca(ICCV2/PI489777)SNP_07119	1087	T	C	4	5	--	--
	Ca(ICCV2/PI489777)SNP_07120	1221	C	T	6	5	--	--
	Ca(ICCV2/PI489777)SNP_07121	1243	T	C	6	4	--	--
	Ca(ICCV2/PI489777)SNP_07122	1250	T	A	6	4	--	--
	Ca(ICCV2/PI489777)SNP_07123	1300	G	A	6	5	--	--
	Ca(ICCV2/PI489777)SNP_07124	1373	C	T	6	4	--	--
	Ca(ICCV2/PI489777)SNP_07125	1494	A	G	10	3	--	--
	Ca(ICCV2/PI489777)SNP_07126	1655	C	G	9	4	--	--
	Ca(ICCV2/PI489777)SNP_07127	1660	G	A	9	4	--	--
	Ca(ICCV2/PI489777)SNP_07128	1808	C	T	7	4	--	--
	Ca(ICCV2/PI489777)SNP_07129	1865	T	C	6	3	--	--
	Ca(ICCV2/PI489777)SNP_07130	1880	C	T	6	4	--	--
	Ca(ICCV2/PI489777)SNP_07131	1952	G	T	4	3	--	--
	Ca(ICCV2/PI489777)SNP_07132	1978	G	T	4	3	--	--
	Ca(ICCV2/PI489777)SNP_07133	1987	G	A	4	3	--	--
CakTC39257	Ca(ICCV2/PI489777)SNP_07134	344	T	C	6	4	--	--
	Ca(ICCV2/PI489777)SNP_07135	568	A	T	14	5	--	--
	Ca(ICCV2/PI489777)SNP_07136	616	C	T	15	4	--	--
CakTC41522	Ca(ICCV2/PI489777)SNP_07137	551	G	C	10	3	--	--
	Ca(ICCV2/PI489777)SNP_07138	921	C	T	61	23	--	--
	Ca(ICCV2/PI489777)SNP_07139	993	G	A	71	26	--	--
CakTC42326	Ca(ICCV2/PI489777)SNP_07140	655	A	T	22	13	--	--
	Ca(ICCV2/PI489777)SNP_07141	1456	T	C	15	14	--	--
	Ca(ICCV2/PI489777)SNP_07142	1506	A	G	14	8	--	--
	Ca(ICCV2/PI489777)SNP_07143	1755	G	A	5	4	--	--
	Ca(ICCV2/PI489777)SNP_07144	1798	T	C	5	4	--	--
CakTC23359	Ca(ICCV2/PI489777)SNP_07145	13	C	A	157	114	--	--
	Ca(ICCV2/PI489777)SNP_07146	60	T	C	282	153	--	--
CakTC42800	Ca(ICCV2/PI489777)SNP_07147	146	C	T	35	20	--	--
	Ca(ICCV2/PI489777)SNP_07148	598	A	T	27	19	--	--
	Ca(ICCV2/PI489777)SNP_07149	637	C	G	21	18	--	--
CakTC40229	Ca(ICCV2/PI489777)SNP_07150	36	T	A	6	5	Root	--
	Ca(ICCV2/PI489777)SNP_07151	150	T	C	12	13	Root	--
	Ca(ICCV2/PI489777)SNP_07152	249	C	T	20	24	Root	--
	Ca(ICCV2/PI489777)SNP_07153	607	G	A	41	41	Root	--
	Ca(ICCV2/PI489777)SNP_07154	612	A	G	44	38	Root	--
	Ca(ICCV2/PI489777)SNP_07155	665	T	C	41	35	Root	--
	Ca(ICCV2/PI489777)SNP_07156	666	G	A	41	33	Root	--
	Ca(ICCV2/PI489777)SNP_07157	741	T	A	38	40	Root	--
	Ca(ICCV2/PI489777)SNP_07158	813	A	G	38	36	Root	--
	Ca(ICCV2/PI489777)SNP_07159	831	C	T	35	40	Root	--
	Ca(ICCV2/PI489777)SNP_07160	840	C	T	32	39	Root	--
	Ca(ICCV2/PI489777)SNP_07161	842	G	A	37	40	Root	--
CakTC41166	Ca(ICCV2/PI489777)SNP_07162	215	A	G	6	3	--	TPR
	Ca(ICCV2/PI489777)SNP_07163	626	T	C	5	5	--	TPR
	Ca(ICCV2/PI489777)SNP_07164	1238	A	G	18	13	--	TPR
	Ca(ICCV2/PI489777)SNP_07165	1256	C	G	20	12	--	TPR
	Ca(ICCV2/PI489777)SNP_07166	1520	C	T	20	23	--	TPR
	Ca(ICCV2/PI489777)SNP_07167	1561	G	C	20	20	--	TPR
CakTC30266	Ca(ICCV2/PI489777)SNP_07168	715	T	G	28	18	--	--
	Ca(ICCV2/PI489777)SNP_07169	846	A	G	33	18	--	--
	Ca(ICCV2/PI489777)SNP_07170	3986	T	A	5	10	--	--
CakTC42534	Ca(ICCV2/PI489777)SNP_07171	685	G	A	18	3	--	--
	Ca(ICCV2/PI489777)SNP_07172	847	G	A	24	5	--	--
	Ca(ICCV2/PI489777)SNP_07173	1122	C	A	19	7	--	--

	Ca(ICCV2/PI489777)SNP_07174	1123	C	G	19	7	--	--
	Ca(ICCV2/PI489777)SNP_07175	1480	A	G	27	10	--	--
CakTC26761	Ca(ICCV2/PI489777)SNP_07176	1030	C	A	6	5	--	--
CakTC41520	Ca(ICCV2/PI489777)SNP_07177	1074	A	G	19	12	--	--
	Ca(ICCV2/PI489777)SNP_07178	1164	A	T	21	14	--	--
	Ca(ICCV2/PI489777)SNP_07179	1188	G	A	21	15	--	--
	Ca(ICCV2/PI489777)SNP_07180	1533	G	A	30	7	--	--
	Ca(ICCV2/PI489777)SNP_07181	1703	G	C	21	6	--	--
	Ca(ICCV2/PI489777)SNP_07182	2188	A	G	10	4	--	--
CakTC38514	Ca(ICCV2/PI489777)SNP_07183	647	T	C	7	8	--	--
	Ca(ICCV2/PI489777)SNP_07184	1147	G	A	7	5	--	--
CakTC42062	Ca(ICCV2/PI489777)SNP_07185	184	A	G	20	4	--	--
CakTC03325	Ca(ICCV2/PI489777)SNP_07186	258	C	T	4	5	--	--
	Ca(ICCV2/PI489777)SNP_07187	421	C	T	3	5	--	--
CakTC35406	Ca(ICCV2/PI489777)SNP_07188	584	T	C	20	29	--	--
	Ca(ICCV2/PI489777)SNP_07189	791	C	T	19	32	--	--
	Ca(ICCV2/PI489777)SNP_07190	800	A	T	19	28	--	--
	Ca(ICCV2/PI489777)SNP_07191	1034	G	A	44	48	--	--
	Ca(ICCV2/PI489777)SNP_07192	1144	C	G	47	40	--	--
	Ca(ICCV2/PI489777)SNP_07193	2165	C	A	52	40	--	--
	Ca(ICCV2/PI489777)SNP_07194	3364	A	T	11	5	--	--
CakTC40407	Ca(ICCV2/PI489777)SNP_07195	796	C	T	6	6	--	--
	Ca(ICCV2/PI489777)SNP_07196	1409	A	G	14	7	--	--
	Ca(ICCV2/PI489777)SNP_07197	1585	C	T	13	20	--	--
CakTC37999	Ca(ICCV2/PI489777)SNP_07198	222	T	C	3	5	--	--
	Ca(ICCV2/PI489777)SNP_07199	242	C	T	3	6	--	--
CakTC32675	Ca(ICCV2/PI489777)SNP_07200	311	A	G	34	20	--	--
	Ca(ICCV2/PI489777)SNP_07201	641	C	A	17	11	--	--
CakTC33898	Ca(ICCV2/PI489777)SNP_07202	386	A	G	7	8	--	--
	Ca(ICCV2/PI489777)SNP_07203	1265	A	T	11	19	--	--
	Ca(ICCV2/PI489777)SNP_07204	1424	A	T	15	14	--	--
	Ca(ICCV2/PI489777)SNP_07205	1677	C	T	15	4	--	--
CakTC42654	Ca(ICCV2/PI489777)SNP_07206	705	A	C	15	6	--	--
	Ca(ICCV2/PI489777)SNP_07207	1274	A	G	28	18	--	--
	Ca(ICCV2/PI489777)SNP_07208	1508	T	C	24	17	--	--
	Ca(ICCV2/PI489777)SNP_07209	1526	G	A	24	15	--	--
CakTC39915	Ca(ICCV2/PI489777)SNP_07210	609	A	G	14	9	--	--
CakTC33867	Ca(ICCV2/PI489777)SNP_07211	2244	C	T	6	11	--	--
	Ca(ICCV2/PI489777)SNP_07212	2325	C	G	4	5	--	--
CakTC22884	Ca(ICCV2/PI489777)SNP_07213	1170	A	G	11	5	--	--
CakTC29577	Ca(ICCV2/PI489777)SNP_07214	180	G	A	22	15	--	--
	Ca(ICCV2/PI489777)SNP_07215	373	A	C	20	11	--	--
	Ca(ICCV2/PI489777)SNP_07216	707	A	G	9	3	--	--
CakTC07192	Ca(ICCV2/PI489777)SNP_07217	872	G	A	11	6	--	--
	Ca(ICCV2/PI489777)SNP_07218	1235	A	G	11	3	--	--
	Ca(ICCV2/PI489777)SNP_07219	1590	G	A	12	6	--	--
	Ca(ICCV2/PI489777)SNP_07220	1749	T	C	9	4	--	--
	Ca(ICCV2/PI489777)SNP_07221	1794	A	T	6	4	--	--
CakTC29570	Ca(ICCV2/PI489777)SNP_07222	1093	T	A	128	73	--	--
CakTC30000	Ca(ICCV2/PI489777)SNP_07223	1419	C	T	11	14	--	--
	Ca(ICCV2/PI489777)SNP_07224	2103	A	G	6	4	--	--
CakTC40816	Ca(ICCV2/PI489777)SNP_07225	391	G	A	4	5	--	--
	Ca(ICCV2/PI489777)SNP_07226	515	A	T	5	4	--	--
CakTC38025	Ca(ICCV2/PI489777)SNP_07227	450	C	T	8	16	--	--
	Ca(ICCV2/PI489777)SNP_07228	467	A	G	8	11	--	--
	Ca(ICCV2/PI489777)SNP_07229	690	A	C	3	9	--	--
	Ca(ICCV2/PI489777)SNP_07230	961	A	G	5	4	--	--
	Ca(ICCV2/PI489777)SNP_07231	991	A	G	4	3	--	--
	Ca(ICCV2/PI489777)SNP_07232	1016	T	A	4	3	--	--
CakTC29055	Ca(ICCV2/PI489777)SNP_07233	386	A	G	17	8	--	--
	Ca(ICCV2/PI489777)SNP_07234	751	C	T	9	13	--	--
	Ca(ICCV2/PI489777)SNP_07235	905	G	T	34	11	--	--
	Ca(ICCV2/PI489777)SNP_07236	1068	G	A	43	9	--	--
	Ca(ICCV2/PI489777)SNP_07237	1115	A	G	41	7	--	--
	Ca(ICCV2/PI489777)SNP_07238	1324	A	T	16	8	--	--
	Ca(ICCV2/PI489777)SNP_07239	1346	C	T	17	7	--	--
	Ca(ICCV2/PI489777)SNP_07240	1556	A	G	16	13	--	--
	Ca(ICCV2/PI489777)SNP_07241	1586	C	T	14	12	--	--
	Ca(ICCV2/PI489777)SNP_07242	1639	T	C	16	10	--	--
	Ca(ICCV2/PI489777)SNP_07243	1641	A	C	15	11	--	--
	Ca(ICCV2/PI489777)SNP_07244	1793	T	C	9	6	--	--
	Ca(ICCV2/PI489777)SNP_07245	1910	C	T	6	3	--	--
	Ca(ICCV2/PI489777)SNP_07246	2018	T	C	4	3	--	--
	Ca(ICCV2/PI489777)SNP_07247	2095	G	A	4	3	--	--

	Ca(ICCV2/PI489777)SNP_07248	2111	G	A	4	3	--	--
	Ca(ICCV2/PI489777)SNP_07249	2169	C	T	4	3	--	--
CakTC23889	Ca(ICCV2/PI489777)SNP_07250	530	C	G	10	4	Flower	bud
	Ca(ICCV2/PI489777)SNP_07251	537	T	C	12	3	Flower	bud
	Ca(ICCV2/PI489777)SNP_07252	579	T	G	12	4	Flower	bud
CakTC39681	Ca(ICCV2/PI489777)SNP_07253	139	G	T	8	8	--	--
	Ca(ICCV2/PI489777)SNP_07254	960	A	T	27	9	--	--
	Ca(ICCV2/PI489777)SNP_07255	972	T	C	27	9	--	--
	Ca(ICCV2/PI489777)SNP_07256	1344	C	G	6	7	--	--
CakTC41286	Ca(ICCV2/PI489777)SNP_07257	422	G	A	3	11	--	--
CakTC29851	Ca(ICCV2/PI489777)SNP_07258	1116	T	C	21	14	--	--
	Ca(ICCV2/PI489777)SNP_07259	1170	T	G	17	12	--	--
	Ca(ICCV2/PI489777)SNP_07260	2024	C	A	17	15	--	--
	Ca(ICCV2/PI489777)SNP_07261	2065	A	T	26	14	--	--
	Ca(ICCV2/PI489777)SNP_07262	2268	G	A	8	6	--	--
	Ca(ICCV2/PI489777)SNP_07263	2361	C	T	9	5	--	--
	Ca(ICCV2/PI489777)SNP_07264	2421	C	T	6	4	--	--
CakTC43365	Ca(ICCV2/PI489777)SNP_07265	1549	G	A	9	7	--	--
	Ca(ICCV2/PI489777)SNP_07266	1747	A	G	8	3	--	--
CakTC39869	Ca(ICCV2/PI489777)SNP_07267	854	C	T	39	27	--	--
	Ca(ICCV2/PI489777)SNP_07268	913	T	C	40	24	--	--
	Ca(ICCV2/PI489777)SNP_07269	973	T	C	34	18	--	--
CakTC11435	Ca(ICCV2/PI489777)SNP_07270	410	C	T	4	5	Root	--
	Ca(ICCV2/PI489777)SNP_07271	413	A	C	4	5	Root	--
	Ca(ICCV2/PI489777)SNP_07272	480	A	G	3	5	Root	--
	Ca(ICCV2/PI489777)SNP_07273	489	G	A	3	5	Root	--
	Ca(ICCV2/PI489777)SNP_07274	492	T	C	3	5	Root	--
	Ca(ICCV2/PI489777)SNP_07275	541	G	A	3	9	Root	--
	Ca(ICCV2/PI489777)SNP_07276	597	A	G	3	11	Root	--
	Ca(ICCV2/PI489777)SNP_07277	606	A	G	3	10	Root	--
	Ca(ICCV2/PI489777)SNP_07278	608	A	T	3	10	Root	--
	Ca(ICCV2/PI489777)SNP_07279	1392	T	C	3	3	Root	--
	Ca(ICCV2/PI489777)SNP_07280	1419	A	T	3	3	Root	--
CakTC27906	Ca(ICCV2/PI489777)SNP_07281	2599	G	T	9	4	Shoot	--
	Ca(ICCV2/PI489777)SNP_07282	2605	G	C	9	5	Shoot	--
CakTC43199	Ca(ICCV2/PI489777)SNP_07283	120	T	A	4	5	--	--
	Ca(ICCV2/PI489777)SNP_07284	151	G	T	6	5	--	--
	Ca(ICCV2/PI489777)SNP_07285	181	C	G	5	5	--	--
	Ca(ICCV2/PI489777)SNP_07286	1349	G	A	6	7	--	--
CakTC22842	Ca(ICCV2/PI489777)SNP_07287	546	T	A	8	11	--	--
	Ca(ICCV2/PI489777)SNP_07288	972	C	T	19	3	--	--
CakTC27601	Ca(ICCV2/PI489777)SNP_07289	1203	A	C	23	16	--	--
CakTC30692	Ca(ICCV2/PI489777)SNP_07290	2069	T	C	16	8	Flower	bud
	Ca(ICCV2/PI489777)SNP_07291	2171	G	A	12	8	Flower	bud
	Ca(ICCV2/PI489777)SNP_07292	2363	T	C	5	9	Flower	bud
	Ca(ICCV2/PI489777)SNP_07293	2749	C	A	9	4	Flower	bud
CakTC41985	Ca(ICCV2/PI489777)SNP_07294	990	T	C	11	4	--	--
	Ca(ICCV2/PI489777)SNP_07295	1056	T	G	10	4	--	--
	Ca(ICCV2/PI489777)SNP_07296	1089	C	T	10	4	--	--
	Ca(ICCV2/PI489777)SNP_07297	1204	A	C	7	3	--	--
CakTC39088	Ca(ICCV2/PI489777)SNP_07298	561	C	A	12	4	--	--
	Ca(ICCV2/PI489777)SNP_07299	625	C	T	18	11	--	--
CakTC28041	Ca(ICCV2/PI489777)SNP_07300	203	T	C	10	5	--	--
CakTC39106	Ca(ICCV2/PI489777)SNP_07301	540	A	G	16	7	Flower	bud
CakTC27832	Ca(ICCV2/PI489777)SNP_07302	184	C	A	14	4	--	--
	Ca(ICCV2/PI489777)SNP_07303	214	T	C	13	5	--	--
CakTC41880	Ca(ICCV2/PI489777)SNP_07304	499	A	G	10	16	--	--
CakTC22939	Ca(ICCV2/PI489777)SNP_07305	2236	T	G	193	67	--	--
	Ca(ICCV2/PI489777)SNP_07306	2395	G	A	189	71	--	--
	Ca(ICCV2/PI489777)SNP_07307	2404	G	A	184	72	--	--
	Ca(ICCV2/PI489777)SNP_07308	2443	C	T	221	82	--	--
CakTC37352	Ca(ICCV2/PI489777)SNP_07309	684	C	T	17	3	--	--
	Ca(ICCV2/PI489777)SNP_07310	999	A	T	11	10	--	--
CakTC40904	Ca(ICCV2/PI489777)SNP_07311	195	A	G	12	5	--	PLATZ
CakTC37652	Ca(ICCV2/PI489777)SNP_07312	410	G	A	11	13	--	--
	Ca(ICCV2/PI489777)SNP_07313	884	T	C	4	10	--	--
	Ca(ICCV2/PI489777)SNP_07314	1334	T	C	22	5	--	--
	Ca(ICCV2/PI489777)SNP_07315	1415	A	G	24	4	--	--
	Ca(ICCV2/PI489777)SNP_07316	1583	G	A	7	4	--	--
CakTC38076	Ca(ICCV2/PI489777)SNP_07317	1035	G	A	6	3	--	HB
CakTC23383	Ca(ICCV2/PI489777)SNP_07318	679	A	G	20	6	--	bHLH
	Ca(ICCV2/PI489777)SNP_07319	1048	G	A	28	10	--	bHLH
	Ca(ICCV2/PI489777)SNP_07320	1050	C	T	28	11	--	bHLH
CakTC40247	Ca(ICCV2/PI489777)SNP_07321	533	C	T	11	12	--	--

	Ca(ICCV2/PI489777)SNP_07322	986	A	G	5	5	--	--
CakTC41145	Ca(ICCV2/PI489777)SNP_07323	314	T	G	16	7	--	--
	Ca(ICCV2/PI489777)SNP_07324	329	G	C	18	7	--	--
	Ca(ICCV2/PI489777)SNP_07325	332	G	C	16	6	--	--
	Ca(ICCV2/PI489777)SNP_07326	972	T	C	29	4	--	--
	Ca(ICCV2/PI489777)SNP_07327	1372	T	A	28	9	--	--
	Ca(ICCV2/PI489777)SNP_07328	1422	A	G	25	9	--	--
	Ca(ICCV2/PI489777)SNP_07329	1477	A	G	25	8	--	--
	Ca(ICCV2/PI489777)SNP_07330	1565	G	A	28	8	--	--
	Ca(ICCV2/PI489777)SNP_07331	2136	G	A	17	5	--	--
CakTC32409	Ca(ICCV2/PI489777)SNP_07332	346	G	T	6	3	--	--
	Ca(ICCV2/PI489777)SNP_07333	349	C	T	6	3	--	--
	Ca(ICCV2/PI489777)SNP_07334	375	G	T	6	3	--	--
	Ca(ICCV2/PI489777)SNP_07335	4879	T	G	8	5	--	--
	Ca(ICCV2/PI489777)SNP_07336	5320	G	A	8	3	--	--
	Ca(ICCV2/PI489777)SNP_07337	5638	G	A	7	8	--	--
	Ca(ICCV2/PI489777)SNP_07338	5830	G	A	8	7	--	--
	Ca(ICCV2/PI489777)SNP_07339	6136	A	G	5	5	--	--
CakTC30454	Ca(ICCV2/PI489777)SNP_07340	2590	T	C	83	88	--	--
CakTC42069	Ca(ICCV2/PI489777)SNP_07341	423	A	T	5	6	--	AP2-EREBP
	Ca(ICCV2/PI489777)SNP_07342	529	C	T	7	5	--	AP2-EREBP
CakTC27730	Ca(ICCV2/PI489777)SNP_07343	411	C	T	5	3	--	--
	Ca(ICCV2/PI489777)SNP_07344	507	A	G	4	4	--	--
	Ca(ICCV2/PI489777)SNP_07345	584	A	G	7	3	--	--
CakTC38818	Ca(ICCV2/PI489777)SNP_07346	1432	T	C	4	7	--	--
CakTC26759	Ca(ICCV2/PI489777)SNP_07347	126	T	C	7	6	--	--
	Ca(ICCV2/PI489777)SNP_07348	307	A	C	13	11	--	--
	Ca(ICCV2/PI489777)SNP_07349	308	C	T	13	11	--	--
	Ca(ICCV2/PI489777)SNP_07350	444	T	C	13	10	--	--
CakTC23004	Ca(ICCV2/PI489777)SNP_07351	151	T	G	14	13	--	--
	Ca(ICCV2/PI489777)SNP_07352	786	G	C	35	13	--	--
	Ca(ICCV2/PI489777)SNP_07353	1021	C	T	32	8	--	--
	Ca(ICCV2/PI489777)SNP_07354	1120	T	A	29	6	--	--
CakTC32200	Ca(ICCV2/PI489777)SNP_07355	895	A	G	8	5	--	Trihelix
	Ca(ICCV2/PI489777)SNP_07356	1183	T	C	7	6	--	Trihelix
	Ca(ICCV2/PI489777)SNP_07357	1315	A	G	13	8	--	Trihelix
CakTC32728	Ca(ICCV2/PI489777)SNP_07358	187	T	C	9	3	--	--
	Ca(ICCV2/PI489777)SNP_07359	384	T	G	20	7	--	--
	Ca(ICCV2/PI489777)SNP_07360	405	G	C	23	11	--	--
	Ca(ICCV2/PI489777)SNP_07361	432	G	T	24	10	--	--
	Ca(ICCV2/PI489777)SNP_07362	587	A	G	22	22	--	--
	Ca(ICCV2/PI489777)SNP_07363	798	C	T	21	25	--	--
	Ca(ICCV2/PI489777)SNP_07364	1146	A	G	23	19	--	--
	Ca(ICCV2/PI489777)SNP_07365	1308	G	A	27	16	--	--
	Ca(ICCV2/PI489777)SNP_07366	1325	C	T	25	15	--	--
	Ca(ICCV2/PI489777)SNP_07367	1569	G	T	15	13	--	--
	Ca(ICCV2/PI489777)SNP_07368	1625	C	T	12	12	--	--
	Ca(ICCV2/PI489777)SNP_07369	1725	G	A	10	12	--	--
	Ca(ICCV2/PI489777)SNP_07370	1815	A	G	17	14	--	--
	Ca(ICCV2/PI489777)SNP_07371	1826	A	T	16	17	--	--
	Ca(ICCV2/PI489777)SNP_07372	1959	G	C	16	15	--	--
CakTC32997	Ca(ICCV2/PI489777)SNP_07373	1930	T	G	25	6	--	--
	Ca(ICCV2/PI489777)SNP_07374	2065	C	T	23	6	--	--
	Ca(ICCV2/PI489777)SNP_07375	2074	A	G	22	6	--	--
	Ca(ICCV2/PI489777)SNP_07376	2442	G	C	14	10	--	--
CakTC26929	Ca(ICCV2/PI489777)SNP_07377	502	A	T	6	19	Root	--
	Ca(ICCV2/PI489777)SNP_07378	541	A	C	6	16	Root	--
	Ca(ICCV2/PI489777)SNP_07379	558	G	A	6	15	Root	--
	Ca(ICCV2/PI489777)SNP_07380	898	C	T	3	28	Root	--
	Ca(ICCV2/PI489777)SNP_07381	1043	G	C	3	25	Root	--
	Ca(ICCV2/PI489777)SNP_07382	1256	C	T	3	24	Root	--
	Ca(ICCV2/PI489777)SNP_07383	1552	A	T	4	11	Root	--
	Ca(ICCV2/PI489777)SNP_07384	1571	T	G	4	8	Root	--
CakTC27040	Ca(ICCV2/PI489777)SNP_07385	312	A	G	7	12	--	--
CakTC32963	Ca(ICCV2/PI489777)SNP_07386	189	C	G	6	4	--	--
	Ca(ICCV2/PI489777)SNP_07387	1355	G	A	6	4	--	--
	Ca(ICCV2/PI489777)SNP_07388	1549	C	T	7	3	--	--
	Ca(ICCV2/PI489777)SNP_07389	3737	G	A	8	9	--	--
CakTC34272	Ca(ICCV2/PI489777)SNP_07390	1903	A	C	4	11	--	--
CakTC37904	Ca(ICCV2/PI489777)SNP_07391	132	T	C	4	3	--	--
CakTC24337	Ca(ICCV2/PI489777)SNP_07392	1142	G	A	21	10	--	--
	Ca(ICCV2/PI489777)SNP_07393	1229	C	T	10	8	--	--
CakTC29286	Ca(ICCV2/PI489777)SNP_07394	182	T	G	5	4	--	--
	Ca(ICCV2/PI489777)SNP_07395	443	A	G	13	9	--	--

	Ca(ICCV2/PI489777)SNP_07396	587	C	T	13	10	--	--
	Ca(ICCV2/PI489777)SNP_07397	593	A	G	13	10	--	--
	Ca(ICCV2/PI489777)SNP_07398	1148	C	T	16	5	--	--
	Ca(ICCV2/PI489777)SNP_07399	1624	A	G	5	3	--	--
CakTC24068	Ca(ICCV2/PI489777)SNP_07400	99	A	C	98	34	--	--
	Ca(ICCV2/PI489777)SNP_07401	748	T	C	19	4	--	--
	Ca(ICCV2/PI489777)SNP_07402	751	G	A	18	4	--	--
CakTC39540	Ca(ICCV2/PI489777)SNP_07403	901	G	A	21	5	--	--
	Ca(ICCV2/PI489777)SNP_07404	1017	G	A	9	7	--	--
CakTC31050	Ca(ICCV2/PI489777)SNP_07405	1107	G	A	4	3	Flower	bud
CakTC13722	Ca(ICCV2/PI489777)SNP_07406	1049	A	G	34	18	--	--
	Ca(ICCV2/PI489777)SNP_07407	1307	T	G	76	36	--	--
	Ca(ICCV2/PI489777)SNP_07408	1752	G	A	35	15	--	--
CakTC32355	Ca(ICCV2/PI489777)SNP_07409	921	T	C	9	12	--	--
CakTC40624	Ca(ICCV2/PI489777)SNP_07410	683	T	A	11	3	--	--
CakTC34705	Ca(ICCV2/PI489777)SNP_07411	420	C	T	30	33	--	--
	Ca(ICCV2/PI489777)SNP_07412	1830	C	T	30	18	--	--
	Ca(ICCV2/PI489777)SNP_07413	1971	A	C	24	21	--	--
	Ca(ICCV2/PI489777)SNP_07414	3610	T	A	6	7	--	--
	Ca(ICCV2/PI489777)SNP_07415	3611	C	T	6	7	--	--
	Ca(ICCV2/PI489777)SNP_07416	3661	C	T	3	5	--	--
CakTC10548	Ca(ICCV2/PI489777)SNP_07417	293	T	A	29	18	--	--
	Ca(ICCV2/PI489777)SNP_07418	421	T	A	64	48	--	--
	Ca(ICCV2/PI489777)SNP_07419	580	G	C	69	43	--	--
	Ca(ICCV2/PI489777)SNP_07420	636	T	C	52	44	--	--
	Ca(ICCV2/PI489777)SNP_07421	645	A	C	46	42	--	--
CakTC25244	Ca(ICCV2/PI489777)SNP_07422	715	A	C	6	4	--	--
	Ca(ICCV2/PI489777)SNP_07423	744	G	C	6	5	--	--
	Ca(ICCV2/PI489777)SNP_07424	1120	A	G	9	8	--	--
	Ca(ICCV2/PI489777)SNP_07425	1528	C	G	25	9	--	--
	Ca(ICCV2/PI489777)SNP_07426	1952	A	G	10	3	--	--
CakTC26199	Ca(ICCV2/PI489777)SNP_07427	850	A	G	36	17	--	--
	Ca(ICCV2/PI489777)SNP_07428	857	C	G	44	19	--	--
	Ca(ICCV2/PI489777)SNP_07429	868	A	G	44	21	--	--
	Ca(ICCV2/PI489777)SNP_07430	877	G	A	54	21	--	--
	Ca(ICCV2/PI489777)SNP_07431	914	C	T	63	19	--	--
	Ca(ICCV2/PI489777)SNP_07432	928	G	A	68	21	--	--
	Ca(ICCV2/PI489777)SNP_07433	937	G	T	70	20	--	--
	Ca(ICCV2/PI489777)SNP_07434	939	A	C	66	19	--	--
	Ca(ICCV2/PI489777)SNP_07435	977	T	C	70	27	--	--
CakTC29025	Ca(ICCV2/PI489777)SNP_07436	324	T	A	9	13	--	HB
	Ca(ICCV2/PI489777)SNP_07437	934	G	C	3	5	--	HB
CakTC31995	Ca(ICCV2/PI489777)SNP_07438	700	T	C	3	3	--	--
CakTC32696	Ca(ICCV2/PI489777)SNP_07439	405	A	T	4	4	--	CCHC
	Ca(ICCV2/PI489777)SNP_07440	1621	G	A	5	3	--	CCHC
	Ca(ICCV2/PI489777)SNP_07441	1827	C	A	9	3	--	CCHC
CakTC40549	Ca(ICCV2/PI489777)SNP_07442	312	T	C	4	7	Young_pod	--
	Ca(ICCV2/PI489777)SNP_07443	345	G	A	5	8	Young_pod	--
CakTC27304	Ca(ICCV2/PI489777)SNP_07444	94	G	C	12	5	--	--
	Ca(ICCV2/PI489777)SNP_07445	924	C	G	26	16	--	--
	Ca(ICCV2/PI489777)SNP_07446	1057	C	T	19	13	--	--
	Ca(ICCV2/PI489777)SNP_07447	1237	T	C	12	5	--	--
	Ca(ICCV2/PI489777)SNP_07448	1474	A	T	6	4	--	--
CakTC30176	Ca(ICCV2/PI489777)SNP_07449	92	C	T	6	4	--	--
	Ca(ICCV2/PI489777)SNP_07450	156	C	G	9	5	--	--
	Ca(ICCV2/PI489777)SNP_07451	225	G	T	9	6	--	--
	Ca(ICCV2/PI489777)SNP_07452	595	T	C	7	5	--	--
	Ca(ICCV2/PI489777)SNP_07453	603	G	T	7	5	--	--
CakTC33050	Ca(ICCV2/PI489777)SNP_07454	518	A	C	106	39	--	--
	Ca(ICCV2/PI489777)SNP_07455	1677	C	T	105	44	--	--
	Ca(ICCV2/PI489777)SNP_07456	1964	T	G	145	39	--	--
	Ca(ICCV2/PI489777)SNP_07457	2820	A	C	85	25	--	--
	Ca(ICCV2/PI489777)SNP_07458	2901	G	T	29	8	--	--
CakTC34812	Ca(ICCV2/PI489777)SNP_07459	277	A	G	7	11	--	--
	Ca(ICCV2/PI489777)SNP_07460	322	T	C	9	10	--	--
	Ca(ICCV2/PI489777)SNP_07461	1174	C	T	24	9	--	--
	Ca(ICCV2/PI489777)SNP_07462	1231	T	C	29	17	--	--
	Ca(ICCV2/PI489777)SNP_07463	1873	T	C	13	12	--	--
	Ca(ICCV2/PI489777)SNP_07464	2686	C	T	26	13	--	--
	Ca(ICCV2/PI489777)SNP_07465	2767	A	G	23	10	--	--
	Ca(ICCV2/PI489777)SNP_07466	3247	C	T	12	17	--	--
	Ca(ICCV2/PI489777)SNP_07467	3273	T	C	18	20	--	--
CakTC32841	Ca(ICCV2/PI489777)SNP_07468	520	T	C	19	7	--	--
	Ca(ICCV2/PI489777)SNP_07469	570	G	A	21	4	--	--

	Ca(ICCV2/PI489777)SNP_07470	619	G	T	27	5	--	--
	Ca(ICCV2/PI489777)SNP_07471	882	G	A	18	14	--	--
	Ca(ICCV2/PI489777)SNP_07472	1486	A	G	9	9	--	--
	Ca(ICCV2/PI489777)SNP_07473	1587	T	A	5	10	--	--
	Ca(ICCV2/PI489777)SNP_07474	1694	C	T	9	5	--	--
	Ca(ICCV2/PI489777)SNP_07475	1983	A	C	10	9	--	--
	Ca(ICCV2/PI489777)SNP_07476	2062	C	T	8	8	--	--
	Ca(ICCV2/PI489777)SNP_07477	2070	G	A	8	8	--	--
	Ca(ICCV2/PI489777)SNP_07478	2090	G	T	8	8	--	--
	Ca(ICCV2/PI489777)SNP_07479	2299	A	G	10	7	--	--
	Ca(ICCV2/PI489777)SNP_07480	2412	G	A	14	8	--	--
	Ca(ICCV2/PI489777)SNP_07481	2625	G	A	14	8	--	--
CakTC10062	Ca(ICCV2/PI489777)SNP_07482	2946	T	C	17	3	Young_pod	--
	Ca(ICCV2/PI489777)SNP_07483	3091	A	G	18	7	Young_pod	--
CakTC37723	Ca(ICCV2/PI489777)SNP_07484	540	A	G	31	10	Flower	bud
	Ca(ICCV2/PI489777)SNP_07485	804	T	C	22	4	Flower	bud
	Ca(ICCV2/PI489777)SNP_07486	1276	T	C	9	9	Flower	bud
	Ca(ICCV2/PI489777)SNP_07487	1416	C	T	9	12	Flower	bud
	Ca(ICCV2/PI489777)SNP_07488	1497	C	T	9	7	Flower	bud
CakTC38625	Ca(ICCV2/PI489777)SNP_07489	169	G	T	6	3	--	--
	Ca(ICCV2/PI489777)SNP_07490	405	T	C	11	3	--	--
	Ca(ICCV2/PI489777)SNP_07491	858	C	T	12	3	--	--
CakTC38181	Ca(ICCV2/PI489777)SNP_07492	849	A	G	7	5	--	--
	Ca(ICCV2/PI489777)SNP_07493	1055	G	A	7	8	--	--
	Ca(ICCV2/PI489777)SNP_07494	1072	G	A	7	9	--	--
	Ca(ICCV2/PI489777)SNP_07495	1798	G	A	11	3	--	--
CakTC11437	Ca(ICCV2/PI489777)SNP_07496	286	T	C	8	5	--	--
CakTC28329	Ca(ICCV2/PI489777)SNP_07497	1636	G	A	12	3	--	--
CakTC37490	Ca(ICCV2/PI489777)SNP_07498	1003	G	A	20	15	--	--
CakTC39968	Ca(ICCV2/PI489777)SNP_07499	199	A	G	21	11	--	MED7
	Ca(ICCV2/PI489777)SNP_07500	308	C	G	25	11	--	MED7
	Ca(ICCV2/PI489777)SNP_07501	319	G	C	26	13	--	MED7
	Ca(ICCV2/PI489777)SNP_07502	435	G	A	30	7	--	MED7
	Ca(ICCV2/PI489777)SNP_07503	517	G	A	21	10	--	MED7
	Ca(ICCV2/PI489777)SNP_07504	574	C	T	21	16	--	MED7
	Ca(ICCV2/PI489777)SNP_07505	601	A	G	18	14	--	MED7
	Ca(ICCV2/PI489777)SNP_07506	682	G	A	18	14	--	MED7
	Ca(ICCV2/PI489777)SNP_07507	793	G	A	17	8	--	MED7
	Ca(ICCV2/PI489777)SNP_07508	817	C	T	14	4	--	MED7
	Ca(ICCV2/PI489777)SNP_07509	931	A	G	11	3	--	MED7
CakTC38534	Ca(ICCV2/PI489777)SNP_07510	122	T	C	12	11	--	--
	Ca(ICCV2/PI489777)SNP_07511	237	G	A	13	11	--	--
	Ca(ICCV2/PI489777)SNP_07512	597	C	T	11	4	--	--
	Ca(ICCV2/PI489777)SNP_07513	663	T	G	15	8	--	--
	Ca(ICCV2/PI489777)SNP_07514	756	T	G	13	8	--	--
	Ca(ICCV2/PI489777)SNP_07515	801	A	G	11	10	--	--
	Ca(ICCV2/PI489777)SNP_07516	865	C	G	9	10	--	--
CakTC24708	Ca(ICCV2/PI489777)SNP_07517	1466	C	T	43	9	--	--
	Ca(ICCV2/PI489777)SNP_07518	2724	C	T	42	5	--	--
CakTC42783	Ca(ICCV2/PI489777)SNP_07519	463	G	A	3	3	--	--
CakTC30548	Ca(ICCV2/PI489777)SNP_07520	879	C	T	8	4	Young_pod	--
	Ca(ICCV2/PI489777)SNP_07521	1149	C	T	17	6	Young_pod	--
	Ca(ICCV2/PI489777)SNP_07522	1768	G	A	16	8	Young_pod	--
	Ca(ICCV2/PI489777)SNP_07523	2554	G	A	9	4	Young_pod	--
	Ca(ICCV2/PI489777)SNP_07524	2931	C	T	6	4	Young_pod	--
	Ca(ICCV2/PI489777)SNP_07525	3282	T	G	8	5	Young_pod	--
	Ca(ICCV2/PI489777)SNP_07526	3630	T	C	6	5	Young_pod	--
	Ca(ICCV2/PI489777)SNP_07527	3762	A	T	5	3	Young_pod	--
	Ca(ICCV2/PI489777)SNP_07528	3816	C	T	3	5	Young_pod	--
CakTC41405	Ca(ICCV2/PI489777)SNP_07529	2215	C	T	19	19	--	--
CakTC23398	Ca(ICCV2/PI489777)SNP_07530	567	A	G	33	12	--	--
	Ca(ICCV2/PI489777)SNP_07531	576	T	C	40	12	--	--
	Ca(ICCV2/PI489777)SNP_07532	873	C	T	62	17	--	--
	Ca(ICCV2/PI489777)SNP_07533	990	G	A	65	25	--	--
	Ca(ICCV2/PI489777)SNP_07534	1155	G	T	65	19	--	--
	Ca(ICCV2/PI489777)SNP_07535	1158	G	A	65	25	--	--
	Ca(ICCV2/PI489777)SNP_07536	1383	G	A	48	20	--	--
CakTC35104	Ca(ICCV2/PI489777)SNP_07537	57	A	C	3	7	--	--
CakTC38191	Ca(ICCV2/PI489777)SNP_07538	554	T	C	5	7	--	--
CakTC28181	Ca(ICCV2/PI489777)SNP_07539	349	G	C	9	10	--	--
CakTC42084	Ca(ICCV2/PI489777)SNP_07540	1475	G	T	4	4	--	--
CakTC41262	Ca(ICCV2/PI489777)SNP_07541	191	T	G	26	41	--	--
	Ca(ICCV2/PI489777)SNP_07542	564	C	G	42	43	--	--
CakTC26439	Ca(ICCV2/PI489777)SNP_07543	141	A	C	9	4	--	--

	Ca(ICCV2/PI489777)SNP_07544	433	C	A	38	4	--	--
	Ca(ICCV2/PI489777)SNP_07545	443	A	G	37	3	--	--
CakTC35607	Ca(ICCV2/PI489777)SNP_07546	647	A	G	7	5	--	--
	Ca(ICCV2/PI489777)SNP_07547	728	A	G	9	7	--	--
	Ca(ICCV2/PI489777)SNP_07548	1114	A	T	10	7	--	--
	Ca(ICCV2/PI489777)SNP_07549	1192	T	C	12	9	--	--
	Ca(ICCV2/PI489777)SNP_07550	2801	G	A	10	12	--	--
	Ca(ICCV2/PI489777)SNP_07551	2915	C	T	10	13	--	--
CakTC40145	Ca(ICCV2/PI489777)SNP_07552	480	A	C	7	3	--	--
	Ca(ICCV2/PI489777)SNP_07553	589	C	T	8	5	--	--
	Ca(ICCV2/PI489777)SNP_07554	1194	T	C	4	3	--	--
CakTC40556	Ca(ICCV2/PI489777)SNP_07555	1006	G	A	15	7	--	--
	Ca(ICCV2/PI489777)SNP_07556	1218	G	T	15	6	--	--
	Ca(ICCV2/PI489777)SNP_07557	1357	C	T	12	4	--	--
	Ca(ICCV2/PI489777)SNP_07558	1636	T	C	14	3	--	--
	Ca(ICCV2/PI489777)SNP_07559	1641	A	G	14	4	--	--
	Ca(ICCV2/PI489777)SNP_07560	1943	G	C	12	10	--	--
	Ca(ICCV2/PI489777)SNP_07561	2005	C	A	9	8	--	--
CakTC30146	Ca(ICCV2/PI489777)SNP_07562	761	C	G	3	4	--	--
CakTC36272	Ca(ICCV2/PI489777)SNP_07563	778	C	T	13	3	--	--
	Ca(ICCV2/PI489777)SNP_07564	811	G	A	13	4	--	--
	Ca(ICCV2/PI489777)SNP_07565	1341	A	G	14	13	--	--
	Ca(ICCV2/PI489777)SNP_07566	1594	A	G	17	6	--	--
CakTC42813	Ca(ICCV2/PI489777)SNP_07567	343	A	C	39	10	--	--
	Ca(ICCV2/PI489777)SNP_07568	551	G	A	48	15	--	--
	Ca(ICCV2/PI489777)SNP_07569	1634	G	A	28	23	--	--
	Ca(ICCV2/PI489777)SNP_07570	1688	A	G	26	16	--	--
CakTC41561	Ca(ICCV2/PI489777)SNP_07571	328	T	C	11	4	--	--
CakTC42744	Ca(ICCV2/PI489777)SNP_07572	295	C	T	4	3	--	--
	Ca(ICCV2/PI489777)SNP_07573	364	C	G	6	3	--	--
	Ca(ICCV2/PI489777)SNP_07574	418	A	G	9	4	--	--
	Ca(ICCV2/PI489777)SNP_07575	529	G	A	11	3	--	--
	Ca(ICCV2/PI489777)SNP_07576	544	C	T	11	5	--	--
	Ca(ICCV2/PI489777)SNP_07577	547	G	A	11	5	--	--
	Ca(ICCV2/PI489777)SNP_07578	618	C	T	13	5	--	--
	Ca(ICCV2/PI489777)SNP_07579	630	T	C	11	5	--	--
	Ca(ICCV2/PI489777)SNP_07580	652	A	C	11	4	--	--
	Ca(ICCV2/PI489777)SNP_07581	673	G	A	9	6	--	--
	Ca(ICCV2/PI489777)SNP_07582	787	G	A	5	5	--	--
	Ca(ICCV2/PI489777)SNP_07583	795	A	G	5	5	--	--
CakTC41571	Ca(ICCV2/PI489777)SNP_07584	592	G	T	95	99	--	--
	Ca(ICCV2/PI489777)SNP_07585	694	A	T	91	83	--	--
CakTC09438	Ca(ICCV2/PI489777)SNP_07586	874	A	G	3	4	--	--
CakTC10281	Ca(ICCV2/PI489777)SNP_07587	318	C	T	4	5	--	--
CakTC38225	Ca(ICCV2/PI489777)SNP_07588	73	C	G	10	3	--	--
	Ca(ICCV2/PI489777)SNP_07589	647	G	C	39	24	--	--
	Ca(ICCV2/PI489777)SNP_07590	876	G	A	29	31	--	--
	Ca(ICCV2/PI489777)SNP_07591	897	G	A	31	28	--	--
	Ca(ICCV2/PI489777)SNP_07592	972	T	C	12	11	--	--
CakTC34169	Ca(ICCV2/PI489777)SNP_07593	49	A	C	6	3	--	--
	Ca(ICCV2/PI489777)SNP_07594	64	T	C	7	4	--	--
	Ca(ICCV2/PI489777)SNP_07595	778	A	G	4	11	--	--
	Ca(ICCV2/PI489777)SNP_07596	785	T	C	3	10	--	--
CakTC42892	Ca(ICCV2/PI489777)SNP_07597	183	G	A	16	12	--	--
	Ca(ICCV2/PI489777)SNP_07598	307	G	C	14	12	--	--
CakTC23653	Ca(ICCV2/PI489777)SNP_07599	337	A	T	25	18	--	--
	Ca(ICCV2/PI489777)SNP_07600	1211	A	C	68	15	--	--
	Ca(ICCV2/PI489777)SNP_07601	1256	G	T	64	20	--	--
	Ca(ICCV2/PI489777)SNP_07602	1840	A	T	71	24	--	--
CakTC32210	Ca(ICCV2/PI489777)SNP_07603	341	A	G	14	11	--	--
	Ca(ICCV2/PI489777)SNP_07604	452	C	A	7	12	--	--
	Ca(ICCV2/PI489777)SNP_07605	740	T	C	23	3	--	--
	Ca(ICCV2/PI489777)SNP_07606	1114	T	C	13	12	--	--
CakTC41497	Ca(ICCV2/PI489777)SNP_07607	254	T	C	7	5	Flower	bud
	Ca(ICCV2/PI489777)SNP_07608	469	C	G	10	13	Flower	bud
	Ca(ICCV2/PI489777)SNP_07609	563	C	A	7	10	Flower	bud
CakTC35126	Ca(ICCV2/PI489777)SNP_07610	676	T	A	10	10	--	--
	Ca(ICCV2/PI489777)SNP_07611	1098	C	T	15	15	--	--
	Ca(ICCV2/PI489777)SNP_07612	2209	T	C	26	12	--	--
	Ca(ICCV2/PI489777)SNP_07613	2286	C	A	32	9	--	--
CakTC36285	Ca(ICCV2/PI489777)SNP_07614	255	T	G	19	5	Young_pod	--
	Ca(ICCV2/PI489777)SNP_07615	279	G	A	16	7	Young_pod	--
	Ca(ICCV2/PI489777)SNP_07616	378	A	G	20	8	Young_pod	--
CakTC14260	Ca(ICCV2/PI489777)SNP_07617	198	C	G	6	3	--	--

	Ca(ICCV2/PI489777)SNP_07618	326	C	A	10	5	--	--
	Ca(ICCV2/PI489777)SNP_07619	364	T	A	12	8	--	--
	Ca(ICCV2/PI489777)SNP_07620	751	A	T	17	9	--	--
CakTC32486	Ca(ICCV2/PI489777)SNP_07621	1167	G	A	11	10	--	--
CakTC37591	Ca(ICCV2/PI489777)SNP_07622	542	T	C	8	11	--	--
CakTC25664	Ca(ICCV2/PI489777)SNP_07623	452	A	C	22	10	--	WRKY
	Ca(ICCV2/PI489777)SNP_07624	491	C	T	17	11	--	WRKY
	Ca(ICCV2/PI489777)SNP_07625	620	C	T	54	8	--	WRKY
	Ca(ICCV2/PI489777)SNP_07626	776	A	G	52	12	--	WRKY
CakTC38114	Ca(ICCV2/PI489777)SNP_07627	341	A	G	8	4	--	--
	Ca(ICCV2/PI489777)SNP_07628	1448	G	A	4	4	--	--
CakTC40298	Ca(ICCV2/PI489777)SNP_07629	48	C	A	4	5	--	--
	Ca(ICCV2/PI489777)SNP_07630	49	T	C	4	5	--	--
	Ca(ICCV2/PI489777)SNP_07631	222	A	G	18	11	--	--
	Ca(ICCV2/PI489777)SNP_07632	482	C	T	19	10	--	--
	Ca(ICCV2/PI489777)SNP_07633	714	A	G	22	5	--	--
	Ca(ICCV2/PI489777)SNP_07634	1807	A	G	12	8	--	--
	Ca(ICCV2/PI489777)SNP_07635	1917	G	A	8	5	--	--
	Ca(ICCV2/PI489777)SNP_07636	2026	A	G	3	5	--	--
CakTC25133	Ca(ICCV2/PI489777)SNP_07637	191	C	A	3	3	--	--
	Ca(ICCV2/PI489777)SNP_07638	244	A	G	3	5	--	--
CakTC09089	Ca(ICCV2/PI489777)SNP_07639	70	G	A	4	4	Shoot	--
CakTC25356	Ca(ICCV2/PI489777)SNP_07640	353	G	A	46	17	--	--
	Ca(ICCV2/PI489777)SNP_07641	560	T	C	53	22	--	--
	Ca(ICCV2/PI489777)SNP_07642	659	G	A	52	11	--	--
	Ca(ICCV2/PI489777)SNP_07643	995	G	A	29	14	--	--
	Ca(ICCV2/PI489777)SNP_07644	1266	T	G	30	14	--	--
	Ca(ICCV2/PI489777)SNP_07645	1407	G	A	18	8	--	--
	Ca(ICCV2/PI489777)SNP_07646	1423	A	G	31	10	--	--
	Ca(ICCV2/PI489777)SNP_07647	1526	C	T	32	4	--	--
	Ca(ICCV2/PI489777)SNP_07648	1530	A	C	34	4	--	--
	Ca(ICCV2/PI489777)SNP_07649	1658	T	C	29	4	--	--
	Ca(ICCV2/PI489777)SNP_07650	1983	A	G	29	7	--	--
	Ca(ICCV2/PI489777)SNP_07651	2010	G	A	26	8	--	--
	Ca(ICCV2/PI489777)SNP_07652	2297	A	G	31	6	--	--
	Ca(ICCV2/PI489777)SNP_07653	2453	C	T	23	14	--	--
	Ca(ICCV2/PI489777)SNP_07654	2469	T	C	25	14	--	--
	Ca(ICCV2/PI489777)SNP_07655	2694	C	T	28	13	--	--
	Ca(ICCV2/PI489777)SNP_07656	2736	C	T	28	8	--	--
	Ca(ICCV2/PI489777)SNP_07657	3113	C	T	8	3	--	--
CakTC25032	Ca(ICCV2/PI489777)SNP_07658	610	A	G	57	27	--	--
	Ca(ICCV2/PI489777)SNP_07659	861	C	T	70	25	--	--
	Ca(ICCV2/PI489777)SNP_07660	916	G	T	73	34	--	--
	Ca(ICCV2/PI489777)SNP_07661	1888	G	T	41	20	--	--
	Ca(ICCV2/PI489777)SNP_07662	1960	G	A	50	24	--	--
	Ca(ICCV2/PI489777)SNP_07663	2053	G	C	49	25	--	--
	Ca(ICCV2/PI489777)SNP_07664	2687	C	G	17	9	--	--
	Ca(ICCV2/PI489777)SNP_07665	2822	G	T	11	5	--	--
CakTC24656	Ca(ICCV2/PI489777)SNP_07666	237	G	A	3	5	--	--
CakTC27623	Ca(ICCV2/PI489777)SNP_07667	180	A	C	21	21	--	--
	Ca(ICCV2/PI489777)SNP_07668	578	C	T	18	9	--	--
CakTC28425	Ca(ICCV2/PI489777)SNP_07669	177	T	A	16	9	--	--
	Ca(ICCV2/PI489777)SNP_07670	272	T	C	13	11	--	--
	Ca(ICCV2/PI489777)SNP_07671	553	C	A	11	4	--	--
CakTC39495	Ca(ICCV2/PI489777)SNP_07672	136	T	C	3	17	--	--
	Ca(ICCV2/PI489777)SNP_07673	722	T	C	17	29	--	--
	Ca(ICCV2/PI489777)SNP_07674	739	A	T	13	25	--	--
	Ca(ICCV2/PI489777)SNP_07675	867	T	C	19	29	--	--
CakTC30019	Ca(ICCV2/PI489777)SNP_07676	19	C	T	9	4	--	--
	Ca(ICCV2/PI489777)SNP_07677	899	G	T	24	19	--	--
	Ca(ICCV2/PI489777)SNP_07678	1825	T	C	32	12	--	--
	Ca(ICCV2/PI489777)SNP_07679	1873	C	A	33	13	--	--
	Ca(ICCV2/PI489777)SNP_07680	1996	C	T	42	11	--	--
	Ca(ICCV2/PI489777)SNP_07681	2476	C	G	33	16	--	--
CakTC26007	Ca(ICCV2/PI489777)SNP_07682	695	T	A	8	10	--	--
	Ca(ICCV2/PI489777)SNP_07683	1598	A	T	24	8	--	--
CakTC42717	Ca(ICCV2/PI489777)SNP_07684	274	G	T	7	3	--	--
CakTC38789	Ca(ICCV2/PI489777)SNP_07685	171	G	A	37	12	--	--
CakTC41464	Ca(ICCV2/PI489777)SNP_07686	361	G	C	13	5	--	--
	Ca(ICCV2/PI489777)SNP_07687	600	C	T	11	5	--	--
CakTC30367	Ca(ICCV2/PI489777)SNP_07688	296	C	T	4	3	--	--
	Ca(ICCV2/PI489777)SNP_07689	622	G	T	3	4	--	--
	Ca(ICCV2/PI489777)SNP_07690	716	G	T	4	4	--	--
CakTC32789	Ca(ICCV2/PI489777)SNP_07691	431	A	G	10	5	--	--

	Ca(ICCV2/PI489777)SNP_07692	565	T	C	15	3	--	--
	Ca(ICCV2/PI489777)SNP_07693	843	T	G	17	12	--	--
	Ca(ICCV2/PI489777)SNP_07694	2912	T	C	11	5	--	--
CakTC30426	Ca(ICCV2/PI489777)SNP_07695	1036	C	T	4	15	Root	--
	Ca(ICCV2/PI489777)SNP_07696	1163	C	A	5	9	Root	--
	Ca(ICCV2/PI489777)SNP_07697	1237	T	C	5	8	Root	--
	Ca(ICCV2/PI489777)SNP_07698	1250	T	A	5	7	Root	--
	Ca(ICCV2/PI489777)SNP_07699	1252	T	C	4	6	Root	--
CakTC08125	Ca(ICCV2/PI489777)SNP_07700	706	T	C	3	4	--	--
	Ca(ICCV2/PI489777)SNP_07701	782	G	C	4	5	--	--
	Ca(ICCV2/PI489777)SNP_07702	957	T	C	3	10	--	--
CakTC34189	Ca(ICCV2/PI489777)SNP_07703	1696	G	A	16	6	--	--
	Ca(ICCV2/PI489777)SNP_07704	1777	A	C	13	6	--	--
CakTC16382	Ca(ICCV2/PI489777)SNP_07705	466	G	A	3	15	--	--
CakTC40818	Ca(ICCV2/PI489777)SNP_07706	316	G	C	19	7	--	--
	Ca(ICCV2/PI489777)SNP_07707	589	T	A	4	8	--	--
	Ca(ICCV2/PI489777)SNP_07708	874	C	T	8	9	--	--
	Ca(ICCV2/PI489777)SNP_07709	907	T	C	13	10	--	--
	Ca(ICCV2/PI489777)SNP_07710	977	T	C	14	10	--	--
CakTC41504	Ca(ICCV2/PI489777)SNP_07711	1325	G	A	4	5	--	--
CakTC33516	Ca(ICCV2/PI489777)SNP_07712	120	C	A	11	12	--	--
	Ca(ICCV2/PI489777)SNP_07713	140	G	T	12	13	--	--
	Ca(ICCV2/PI489777)SNP_07714	277	A	G	17	19	--	--
	Ca(ICCV2/PI489777)SNP_07715	464	G	C	23	18	--	--
CakTC32423	Ca(ICCV2/PI489777)SNP_07716	138	A	C	5	3	--	--
CakTC33424	Ca(ICCV2/PI489777)SNP_07717	1084	A	G	9	4	--	--
CakTC23178	Ca(ICCV2/PI489777)SNP_07718	483	A	G	3	6	--	--
CakTC41195	Ca(ICCV2/PI489777)SNP_07719	235	T	C	30	18	--	--
CakTC22890	Ca(ICCV2/PI489777)SNP_07720	992	C	T	9	6	--	--
	Ca(ICCV2/PI489777)SNP_07721	1106	C	T	7	4	--	--
	Ca(ICCV2/PI489777)SNP_07722	1181	T	C	7	4	--	--
	Ca(ICCV2/PI489777)SNP_07723	2197	A	G	8	16	--	--
	Ca(ICCV2/PI489777)SNP_07724	2708	T	C	11	13	--	--
	Ca(ICCV2/PI489777)SNP_07725	2903	A	C	19	10	--	--
	Ca(ICCV2/PI489777)SNP_07726	3014	A	C	21	10	--	--
	Ca(ICCV2/PI489777)SNP_07727	3146	T	A	23	9	--	--
	Ca(ICCV2/PI489777)SNP_07728	3147	G	T	23	9	--	--
	Ca(ICCV2/PI489777)SNP_07729	3206	C	T	15	8	--	--
	Ca(ICCV2/PI489777)SNP_07730	3269	G	T	11	5	--	--
	Ca(ICCV2/PI489777)SNP_07731	3332	G	T	6	5	--	--
	Ca(ICCV2/PI489777)SNP_07732	3343	T	A	6	5	--	--
CakTC33952	Ca(ICCV2/PI489777)SNP_07733	771	T	A	14	7	Shoot	MYB
CakTC01326	Ca(ICCV2/PI489777)SNP_07734	591	C	T	6	3	--	--
	Ca(ICCV2/PI489777)SNP_07735	621	G	A	6	3	--	--
	Ca(ICCV2/PI489777)SNP_07736	798	T	G	5	3	--	--
CakTC09532	Ca(ICCV2/PI489777)SNP_07737	931	G	A	4	5	--	--
CakTC37481	Ca(ICCV2/PI489777)SNP_07738	1058	G	A	3	3	--	--
CakTC40719	Ca(ICCV2/PI489777)SNP_07739	156	G	A	9	10	--	--
	Ca(ICCV2/PI489777)SNP_07740	654	C	T	6	10	--	--
	Ca(ICCV2/PI489777)SNP_07741	675	A	C	5	10	--	--
	Ca(ICCV2/PI489777)SNP_07742	687	T	G	5	10	--	--
CakTC34517	Ca(ICCV2/PI489777)SNP_07743	127	A	G	15	16	--	--
	Ca(ICCV2/PI489777)SNP_07744	571	A	T	22	7	--	--
CakTC37958	Ca(ICCV2/PI489777)SNP_07745	345	G	A	39	3	--	--
	Ca(ICCV2/PI489777)SNP_07746	361	T	C	42	3	--	--
	Ca(ICCV2/PI489777)SNP_07747	390	T	A	37	3	--	--
	Ca(ICCV2/PI489777)SNP_07748	479	T	C	20	3	--	--
	Ca(ICCV2/PI489777)SNP_07749	492	A	G	19	3	--	--
CakTC28555	Ca(ICCV2/PI489777)SNP_07750	42	A	G	13	7	--	--
	Ca(ICCV2/PI489777)SNP_07751	203	A	T	20	9	--	--
	Ca(ICCV2/PI489777)SNP_07752	1017	A	C	18	3	--	--
	Ca(ICCV2/PI489777)SNP_07753	1152	T	C	19	8	--	--
	Ca(ICCV2/PI489777)SNP_07754	1161	T	C	18	11	--	--
	Ca(ICCV2/PI489777)SNP_07755	1326	T	A	18	18	--	--
CakTC41530	Ca(ICCV2/PI489777)SNP_07756	151	T	G	17	31	--	--
	Ca(ICCV2/PI489777)SNP_07757	356	G	A	16	25	--	--
	Ca(ICCV2/PI489777)SNP_07758	376	T	C	14	31	--	--
	Ca(ICCV2/PI489777)SNP_07759	498	A	G	6	19	--	--
CakTC28373	Ca(ICCV2/PI489777)SNP_07760	378	G	T	30	17	--	--
	Ca(ICCV2/PI489777)SNP_07761	790	T	C	22	9	--	--
	Ca(ICCV2/PI489777)SNP_07762	898	C	G	28	11	--	--
	Ca(ICCV2/PI489777)SNP_07763	1016	T	C	35	14	--	--
	Ca(ICCV2/PI489777)SNP_07764	1139	G	T	37	13	--	--
	Ca(ICCV2/PI489777)SNP_07765	1355	A	G	27	8	--	--

	Ca(ICCV2/PI489777)SNP_07766	1613	A	G	24	7	--	--
CakTC39061	Ca(ICCV2/PI489777)SNP_07767	285	C	A	7	4	--	--
	Ca(ICCV2/PI489777)SNP_07768	794	C	T	9	3	--	--
	Ca(ICCV2/PI489777)SNP_07769	1430	T	C	22	3	--	--
	Ca(ICCV2/PI489777)SNP_07770	1441	C	G	21	3	--	--
	Ca(ICCV2/PI489777)SNP_07771	1446	T	C	21	3	--	--
	Ca(ICCV2/PI489777)SNP_07772	1476	A	G	18	3	--	--
	Ca(ICCV2/PI489777)SNP_07773	1480	G	A	21	3	--	--
	Ca(ICCV2/PI489777)SNP_07774	1487	C	T	19	3	--	--
	Ca(ICCV2/PI489777)SNP_07775	1579	A	G	13	3	--	--
	Ca(ICCV2/PI489777)SNP_07776	1583	C	G	11	3	--	--
CakTC08416	Ca(ICCV2/PI489777)SNP_07777	324	T	G	4	3	--	--
	Ca(ICCV2/PI489777)SNP_07778	576	C	T	3	5	--	--
	Ca(ICCV2/PI489777)SNP_07779	732	A	G	3	8	--	--
CakTC35474	Ca(ICCV2/PI489777)SNP_07780	888	A	T	17	4	--	--
CakTC29293	Ca(ICCV2/PI489777)SNP_07781	485	A	G	34	8	--	--
CakTC35643	Ca(ICCV2/PI489777)SNP_07782	188	A	T	4	8	--	--
	Ca(ICCV2/PI489777)SNP_07783	383	G	A	6	5	--	--
	Ca(ICCV2/PI489777)SNP_07784	911	T	A	4	5	--	--
	Ca(ICCV2/PI489777)SNP_07785	2347	C	T	8	11	--	--
	Ca(ICCV2/PI489777)SNP_07786	2363	G	A	7	13	--	--
	Ca(ICCV2/PI489777)SNP_07787	2527	C	A	8	5	--	--
	Ca(ICCV2/PI489777)SNP_07788	2535	A	G	8	11	--	--
	Ca(ICCV2/PI489777)SNP_07789	2676	T	A	12	12	--	--
	Ca(ICCV2/PI489777)SNP_07790	3194	C	T	9	23	--	--
	Ca(ICCV2/PI489777)SNP_07791	3497	T	C	12	21	--	--
	Ca(ICCV2/PI489777)SNP_07792	3787	C	A	11	6	--	--
	Ca(ICCV2/PI489777)SNP_07793	4088	G	A	6	12	--	--
CakTC39156	Ca(ICCV2/PI489777)SNP_07794	343	C	T	9	5	--	--
	Ca(ICCV2/PI489777)SNP_07795	406	T	C	8	8	--	--
	Ca(ICCV2/PI489777)SNP_07796	439	C	T	10	8	--	--
	Ca(ICCV2/PI489777)SNP_07797	493	A	C	9	8	--	--
	Ca(ICCV2/PI489777)SNP_07798	780	G	A	5	7	--	--
	Ca(ICCV2/PI489777)SNP_07799	811	A	C	7	5	--	--
	Ca(ICCV2/PI489777)SNP_07800	1075	T	C	3	4	--	--
CakTC10605	Ca(ICCV2/PI489777)SNP_07801	553	A	G	208	136	--	--
CakTC42925	Ca(ICCV2/PI489777)SNP_07802	435	T	C	15	15	--	--
	Ca(ICCV2/PI489777)SNP_07803	474	A	G	10	13	--	--
	Ca(ICCV2/PI489777)SNP_07804	695	A	G	4	3	--	--
CakTC23861	Ca(ICCV2/PI489777)SNP_07805	450	A	T	6	3	--	AUX/IAA
CakTC39632	Ca(ICCV2/PI489777)SNP_07806	144	G	C	3	4	--	--
CakTC08215	Ca(ICCV2/PI489777)SNP_07807	672	T	A	8	17	--	--
	Ca(ICCV2/PI489777)SNP_07808	1578	G	T	20	5	--	--
CakTC26984	Ca(ICCV2/PI489777)SNP_07809	2283	T	G	10	3	--	--
CakTC36616	Ca(ICCV2/PI489777)SNP_07810	266	G	A	38	11	--	--
	Ca(ICCV2/PI489777)SNP_07811	278	T	C	33	11	--	--
	Ca(ICCV2/PI489777)SNP_07812	368	A	G	33	11	--	--
	Ca(ICCV2/PI489777)SNP_07813	383	G	A	33	11	--	--
CakTC23758	Ca(ICCV2/PI489777)SNP_07814	1391	A	C	30	6	Root	--
	Ca(ICCV2/PI489777)SNP_07815	1844	C	T	44	4	Root	--
CakTC09044	Ca(ICCV2/PI489777)SNP_07816	195	G	T	4	4	--	AP2-EREBP
CakTC40587	Ca(ICCV2/PI489777)SNP_07817	395	C	T	22	37	--	--
	Ca(ICCV2/PI489777)SNP_07818	437	C	T	22	39	--	--
	Ca(ICCV2/PI489777)SNP_07819	590	A	G	18	20	--	--
CakTC40594	Ca(ICCV2/PI489777)SNP_07820	538	C	T	68	59	--	--
CakTC23516	Ca(ICCV2/PI489777)SNP_07821	583	T	G	9	4	--	--
	Ca(ICCV2/PI489777)SNP_07822	667	A	G	9	6	--	--
	Ca(ICCV2/PI489777)SNP_07823	727	A	T	8	6	--	--
CakTC29319	Ca(ICCV2/PI489777)SNP_07824	910	C	T	3	5	Flower	bud
CakTC38795	Ca(ICCV2/PI489777)SNP_07825	443	C	G	6	4	--	--
CakTC41065	Ca(ICCV2/PI489777)SNP_07826	574	G	A	14	10	--	--
CakTC42470	Ca(ICCV2/PI489777)SNP_07827	136	G	C	22	12	--	--
	Ca(ICCV2/PI489777)SNP_07828	320	T	G	58	13	--	--
CakTC36805	Ca(ICCV2/PI489777)SNP_07829	2705	T	C	20	18	--	--
CakTC27715	Ca(ICCV2/PI489777)SNP_07830	40	T	A	7	4	Young_pod	--
	Ca(ICCV2/PI489777)SNP_07831	41	T	C	8	4	Young_pod	--
	Ca(ICCV2/PI489777)SNP_07832	151	G	C	8	7	Young_pod	--
	Ca(ICCV2/PI489777)SNP_07833	158	A	C	6	7	Young_pod	--
	Ca(ICCV2/PI489777)SNP_07834	210	A	C	3	6	Young_pod	--
	Ca(ICCV2/PI489777)SNP_07835	1195	G	C	19	11	Young_pod	--
	Ca(ICCV2/PI489777)SNP_07836	1210	C	A	17	11	Young_pod	--
CakTC08134	Ca(ICCV2/PI489777)SNP_07837	1367	C	T	3	3	Young_pod	--
	Ca(ICCV2/PI489777)SNP_07838	1369	T	G	3	3	Young_pod	--
	Ca(ICCV2/PI489777)SNP_07839	1490	T	A	4	3	Young_pod	--

	Ca(ICCV2/PI489777)SNP_07840	1513	G	A	4	3	Young_pod	--
	Ca(ICCV2/PI489777)SNP_07841	1564	C	T	4	3	Young_pod	--
	Ca(ICCV2/PI489777)SNP_07842	1688	C	T	3	4	Young_pod	--
	Ca(ICCV2/PI489777)SNP_07843	2192	G	A	3	3	Young_pod	--
	Ca(ICCV2/PI489777)SNP_07844	2273	G	T	3	3	Young_pod	--
CakTC25849	Ca(ICCV2/PI489777)SNP_07845	263	A	G	217	52	--	--
	Ca(ICCV2/PI489777)SNP_07846	1688	G	A	16	27	--	--
	Ca(ICCV2/PI489777)SNP_07847	1700	A	G	13	27	--	--
	Ca(ICCV2/PI489777)SNP_07848	1770	A	T	19	26	--	--
	Ca(ICCV2/PI489777)SNP_07849	1979	T	C	15	22	--	--
	Ca(ICCV2/PI489777)SNP_07850	2053	C	A	15	20	--	--
	Ca(ICCV2/PI489777)SNP_07851	2123	T	C	11	14	--	--
	Ca(ICCV2/PI489777)SNP_07852	2213	A	G	10	14	--	--
	Ca(ICCV2/PI489777)SNP_07853	2255	G	A	6	10	--	--
	Ca(ICCV2/PI489777)SNP_07854	2309	G	A	6	8	--	--
	Ca(ICCV2/PI489777)SNP_07855	2465	T	C	9	3	--	--
	Ca(ICCV2/PI489777)SNP_07856	2520	G	T	7	4	--	--
	Ca(ICCV2/PI489777)SNP_07857	2633	T	C	9	4	--	--
CakTC39631	Ca(ICCV2/PI489777)SNP_07858	498	A	G	29	20	--	--
	Ca(ICCV2/PI489777)SNP_07859	795	A	G	23	17	--	--
	Ca(ICCV2/PI489777)SNP_07860	870	A	G	21	17	--	--
	Ca(ICCV2/PI489777)SNP_07861	1062	A	C	13	12	--	--
	Ca(ICCV2/PI489777)SNP_07862	1371	G	A	35	12	--	--
	Ca(ICCV2/PI489777)SNP_07863	1559	C	G	16	7	--	--
	Ca(ICCV2/PI489777)SNP_07864	1584	A	G	13	3	--	--
CakTC27394	Ca(ICCV2/PI489777)SNP_07865	3034	A	G	46	45	--	--
CakTC38887	Ca(ICCV2/PI489777)SNP_07866	521	C	T	6	7	--	--
	Ca(ICCV2/PI489777)SNP_07867	776	G	A	4	3	--	--
	Ca(ICCV2/PI489777)SNP_07868	1064	G	A	3	4	--	--
CakTC33643	Ca(ICCV2/PI489777)SNP_07869	1386	C	T	11	4	--	SET
	Ca(ICCV2/PI489777)SNP_07870	1466	T	C	6	5	--	SET
	Ca(ICCV2/PI489777)SNP_07871	1942	A	G	3	6	--	SET
	Ca(ICCV2/PI489777)SNP_07872	2733	C	A	6	11	--	SET
	Ca(ICCV2/PI489777)SNP_07873	2801	C	T	6	12	--	SET
	Ca(ICCV2/PI489777)SNP_07874	3173	T	C	3	8	--	SET
	Ca(ICCV2/PI489777)SNP_07875	3567	G	A	6	5	--	SET
	Ca(ICCV2/PI489777)SNP_07876	4614	G	A	4	6	--	SET
	Ca(ICCV2/PI489777)SNP_07877	5217	A	T	5	4	--	SET
	Ca(ICCV2/PI489777)SNP_07878	5248	C	T	3	3	--	SET
	Ca(ICCV2/PI489777)SNP_07879	5320	C	G	4	3	--	SET
	Ca(ICCV2/PI489777)SNP_07880	6018	G	A	7	7	--	SET
	Ca(ICCV2/PI489777)SNP_07881	6078	G	A	11	8	--	SET
CakTC38570	Ca(ICCV2/PI489777)SNP_07882	563	T	C	58	44	--	--
	Ca(ICCV2/PI489777)SNP_07883	776	G	A	41	27	--	--
	Ca(ICCV2/PI489777)SNP_07884	868	C	T	26	13	--	--
	Ca(ICCV2/PI489777)SNP_07885	919	G	C	19	11	--	--
CakTC27380	Ca(ICCV2/PI489777)SNP_07886	465	T	G	13	7	--	--
CakTC28312	Ca(ICCV2/PI489777)SNP_07887	410	C	T	28	16	--	--
	Ca(ICCV2/PI489777)SNP_07888	3593	A	C	6	8	--	--
CakTC07647	Ca(ICCV2/PI489777)SNP_07889	492	T	C	8	4	--	--
CakTC28627	Ca(ICCV2/PI489777)SNP_07890	989	G	A	4	6	Young_pod	--
CakTC24350	Ca(ICCV2/PI489777)SNP_07891	1566	G	A	5	4	Young_pod	--
CakTC42158	Ca(ICCV2/PI489777)SNP_07892	453	A	G	9	3	--	--
CakTC00828	Ca(ICCV2/PI489777)SNP_07893	873	G	A	7	5	--	--
CakTC28287	Ca(ICCV2/PI489777)SNP_07894	1635	A	G	3	3	--	--
CakTC26923	Ca(ICCV2/PI489777)SNP_07895	677	A	C	17	9	--	--
	Ca(ICCV2/PI489777)SNP_07896	1186	C	T	46	13	--	--
	Ca(ICCV2/PI489777)SNP_07897	1201	T	C	48	12	--	--
	Ca(ICCV2/PI489777)SNP_07898	1249	A	T	43	12	--	--
	Ca(ICCV2/PI489777)SNP_07899	1384	T	C	28	12	--	--
	Ca(ICCV2/PI489777)SNP_07900	1477	G	A	32	15	--	--
	Ca(ICCV2/PI489777)SNP_07901	1594	T	C	20	13	--	--
	Ca(ICCV2/PI489777)SNP_07902	1718	C	G	22	10	--	--
	Ca(ICCV2/PI489777)SNP_07903	2337	G	C	25	8	--	--
	Ca(ICCV2/PI489777)SNP_07904	3106	C	T	15	6	--	--
	Ca(ICCV2/PI489777)SNP_07905	3582	A	G	33	27	--	--
	Ca(ICCV2/PI489777)SNP_07906	3636	G	A	19	6	--	--
	Ca(ICCV2/PI489777)SNP_07907	3640	C	T	17	6	--	--
	Ca(ICCV2/PI489777)SNP_07908	4120	G	A	24	9	--	--
	Ca(ICCV2/PI489777)SNP_07909	4889	C	G	34	8	--	--
	Ca(ICCV2/PI489777)SNP_07910	5508	G	C	34	10	--	--
	Ca(ICCV2/PI489777)SNP_07911	5632	A	G	34	11	--	--
	Ca(ICCV2/PI489777)SNP_07912	5749	C	T	37	16	--	--
	Ca(ICCV2/PI489777)SNP_07913	5842	A	G	28	16	--	--

	Ca(ICCV2/PI489777)SNP_07914	5977	T	A	40	16	--	--
	Ca(ICCV2/PI489777)SNP_07915	6025	A	G	42	17	--	--
	Ca(ICCV2/PI489777)SNP_07916	6040	G	A	38	17	--	--
	Ca(ICCV2/PI489777)SNP_07917	6549	T	G	21	10	--	--
CakTC37542	Ca(ICCV2/PI489777)SNP_07918	980	G	C	13	16	--	--
	Ca(ICCV2/PI489777)SNP_07919	1012	G	C	15	16	--	--
CakTC06591	Ca(ICCV2/PI489777)SNP_07920	920	T	C	10	15	--	--
	Ca(ICCV2/PI489777)SNP_07921	1056	A	G	16	8	--	--
CakTC10833	Ca(ICCV2/PI489777)SNP_07922	681	C	T	26	22	--	--
CakTC09098	Ca(ICCV2/PI489777)SNP_07923	2819	T	C	3	8	--	--
	Ca(ICCV2/PI489777)SNP_07924	2847	T	C	3	7	--	--
	Ca(ICCV2/PI489777)SNP_07925	2852	T	C	3	7	--	--
CakTC23197	Ca(ICCV2/PI489777)SNP_07926	2274	T	C	7	4	Mature	Leaf
CakTC34470	Ca(ICCV2/PI489777)SNP_07927	1871	G	A	3	4	--	--
	Ca(ICCV2/PI489777)SNP_07928	1944	T	C	3	3	--	--
CakTC42441	Ca(ICCV2/PI489777)SNP_07929	184	G	T	14	3	--	--
	Ca(ICCV2/PI489777)SNP_07930	694	C	A	3	3	--	--
CakTC10516	Ca(ICCV2/PI489777)SNP_07931	48	A	G	5	4	--	--
	Ca(ICCV2/PI489777)SNP_07932	133	A	C	8	5	--	--
CakTC31064	Ca(ICCV2/PI489777)SNP_07933	1899	G	A	9	7	--	--
	Ca(ICCV2/PI489777)SNP_07934	1907	G	A	8	7	--	--
CakTC42545	Ca(ICCV2/PI489777)SNP_07935	219	A	C	15	5	Flower	bud
	Ca(ICCV2/PI489777)SNP_07936	561	A	T	21	5	Flower	bud
	Ca(ICCV2/PI489777)SNP_07937	618	A	G	23	4	Flower	bud
	Ca(ICCV2/PI489777)SNP_07938	675	A	T	24	4	Flower	bud
	Ca(ICCV2/PI489777)SNP_07939	1311	G	A	14	6	Flower	bud
	Ca(ICCV2/PI489777)SNP_07940	1704	T	G	4	3	Flower	bud
CakTC10020	Ca(ICCV2/PI489777)SNP_07941	3238	A	T	18	4	--	TPR
CakTC10084	Ca(ICCV2/PI489777)SNP_07942	1515	C	A	4	4	--	--
CakTC41977	Ca(ICCV2/PI489777)SNP_07943	142	T	C	12	8	--	--
	Ca(ICCV2/PI489777)SNP_07944	233	G	A	30	15	--	--
CakTC42015	Ca(ICCV2/PI489777)SNP_07945	662	A	G	6	5	--	--
	Ca(ICCV2/PI489777)SNP_07946	763	A	T	5	5	--	--
	Ca(ICCV2/PI489777)SNP_07947	797	G	A	5	4	--	--
CakTC09512	Ca(ICCV2/PI489777)SNP_07948	186	T	G	8	3	--	--
	Ca(ICCV2/PI489777)SNP_07949	192	C	G	6	3	--	--
	Ca(ICCV2/PI489777)SNP_07950	418	C	T	12	6	--	--
	Ca(ICCV2/PI489777)SNP_07951	502	T	C	17	6	--	--
CakTC11129	Ca(ICCV2/PI489777)SNP_07952	727	A	T	5	3	--	--
	Ca(ICCV2/PI489777)SNP_07953	771	A	G	6	3	--	--
	Ca(ICCV2/PI489777)SNP_07954	904	G	A	5	8	--	--
	Ca(ICCV2/PI489777)SNP_07955	1003	A	T	9	8	--	--
	Ca(ICCV2/PI489777)SNP_07956	1164	C	T	8	9	--	--
	Ca(ICCV2/PI489777)SNP_07957	1197	A	G	9	9	--	--
	Ca(ICCV2/PI489777)SNP_07958	1459	A	G	7	3	--	--
	Ca(ICCV2/PI489777)SNP_07959	2108	A	G	5	5	--	--
	Ca(ICCV2/PI489777)SNP_07960	2248	G	C	7	4	--	--
	Ca(ICCV2/PI489777)SNP_07961	2262	C	G	7	4	--	--
	Ca(ICCV2/PI489777)SNP_07962	2295	G	T	5	4	--	--
	Ca(ICCV2/PI489777)SNP_07963	2406	T	C	8	5	--	--
	Ca(ICCV2/PI489777)SNP_07964	2642	G	A	7	3	--	--
CakTC42091	Ca(ICCV2/PI489777)SNP_07965	853	T	C	5	7	--	GNAT
CakTC40610	Ca(ICCV2/PI489777)SNP_07966	191	T	C	8	7	--	--
	Ca(ICCV2/PI489777)SNP_07967	309	A	C	10	7	--	--
CakTC27091	Ca(ICCV2/PI489777)SNP_07968	471	G	A	15	10	--	--
CakTC38003	Ca(ICCV2/PI489777)SNP_07969	446	T	C	9	3	--	--
CakTC41490	Ca(ICCV2/PI489777)SNP_07970	718	A	G	7	8	--	--
	Ca(ICCV2/PI489777)SNP_07971	1056	C	T	14	8	--	--
	Ca(ICCV2/PI489777)SNP_07972	1371	C	G	17	10	--	--
CakTC32821	Ca(ICCV2/PI489777)SNP_07973	582	A	G	5	4	--	--
	Ca(ICCV2/PI489777)SNP_07974	666	C	T	8	6	--	--
	Ca(ICCV2/PI489777)SNP_07975	1910	G	T	5	3	--	--
CakTC38711	Ca(ICCV2/PI489777)SNP_07976	464	T	G	9	8	--	--
	Ca(ICCV2/PI489777)SNP_07977	570	A	G	13	4	--	--
	Ca(ICCV2/PI489777)SNP_07978	717	C	T	14	6	--	--
	Ca(ICCV2/PI489777)SNP_07979	844	C	G	17	3	--	--
	Ca(ICCV2/PI489777)SNP_07980	1110	G	C	14	11	--	--
CakTC33300	Ca(ICCV2/PI489777)SNP_07981	419	C	A	12	9	--	--
	Ca(ICCV2/PI489777)SNP_07982	1238	A	G	10	9	--	--
CakTC41898	Ca(ICCV2/PI489777)SNP_07983	353	A	T	19	5	--	--
	Ca(ICCV2/PI489777)SNP_07984	363	G	A	15	5	--	--
CakTC11238	Ca(ICCV2/PI489777)SNP_07985	790	C	T	8	4	--	--
CakTC27632	Ca(ICCV2/PI489777)SNP_07986	59	C	G	43	25	--	--
	Ca(ICCV2/PI489777)SNP_07987	322	G	T	64	36	--	--

CakTC34617	Ca(ICCV2/PI489777)SNP_07988	209	C	T	41	27	--	--
	Ca(ICCV2/PI489777)SNP_07989	230	A	G	41	29	--	--
	Ca(ICCV2/PI489777)SNP_07990	524	T	C	43	26	--	--
CakTC36011	Ca(ICCV2/PI489777)SNP_07991	1280	A	G	16	9	--	--
	Ca(ICCV2/PI489777)SNP_07992	1856	A	T	28	9	--	--
	Ca(ICCV2/PI489777)SNP_07993	2225	T	C	27	7	--	--
	Ca(ICCV2/PI489777)SNP_07994	2381	T	C	34	9	--	--
	Ca(ICCV2/PI489777)SNP_07995	2395	A	G	34	9	--	--
CakTC24574	Ca(ICCV2/PI489777)SNP_07996	757	C	G	91	31	--	--
	Ca(ICCV2/PI489777)SNP_07997	777	C	A	74	19	--	--
CakTC07053	Ca(ICCV2/PI489777)SNP_07998	813	G	T	6	6	--	--
	Ca(ICCV2/PI489777)SNP_07999	861	T	A	6	4	--	--
	Ca(ICCV2/PI489777)SNP_08000	1077	G	A	12	6	--	--
	Ca(ICCV2/PI489777)SNP_08001	1230	T	C	12	3	--	--
	Ca(ICCV2/PI489777)SNP_08002	1446	A	T	4	4	--	--
CakTC24743	Ca(ICCV2/PI489777)SNP_08003	120	C	A	16	7	--	--
CakTC39652	Ca(ICCV2/PI489777)SNP_08004	573	T	C	8	3	--	CCAAT
	Ca(ICCV2/PI489777)SNP_08005	703	C	T	15	6	--	CCAAT
	Ca(ICCV2/PI489777)SNP_08006	960	C	A	27	15	--	CCAAT
	Ca(ICCV2/PI489777)SNP_08007	1683	G	C	13	3	--	CCAAT
CakTC24201	Ca(ICCV2/PI489777)SNP_08008	470	A	G	20	14	--	--
	Ca(ICCV2/PI489777)SNP_08009	686	G	A	31	11	--	--
	Ca(ICCV2/PI489777)SNP_08010	1268	C	T	28	14	--	--
	Ca(ICCV2/PI489777)SNP_08011	2075	T	C	16	3	--	--
CakTC30497	Ca(ICCV2/PI489777)SNP_08012	904	G	A	5	3	--	--
CakTC23325	Ca(ICCV2/PI489777)SNP_08013	489	G	A	365	185	--	--
	Ca(ICCV2/PI489777)SNP_08014	579	G	A	354	198	--	--
	Ca(ICCV2/PI489777)SNP_08015	1059	G	A	105	36	--	--
	Ca(ICCV2/PI489777)SNP_08016	2305	T	C	111	62	--	--
CakTC24526	Ca(ICCV2/PI489777)SNP_08017	860	A	G	6	3	--	--
CakTC28052	Ca(ICCV2/PI489777)SNP_08018	157	A	G	7	5	--	--
	Ca(ICCV2/PI489777)SNP_08019	568	C	T	43	18	--	--
	Ca(ICCV2/PI489777)SNP_08020	665	A	G	45	27	--	--
	Ca(ICCV2/PI489777)SNP_08021	918	C	T	32	29	--	--
CakTC31743	Ca(ICCV2/PI489777)SNP_08022	476	C	T	4	5	--	--
	Ca(ICCV2/PI489777)SNP_08023	512	T	G	6	5	--	--
	Ca(ICCV2/PI489777)SNP_08024	583	G	A	6	5	--	--
	Ca(ICCV2/PI489777)SNP_08025	619	T	C	7	8	--	--
	Ca(ICCV2/PI489777)SNP_08026	677	T	G	7	9	--	--
	Ca(ICCV2/PI489777)SNP_08027	866	G	A	10	12	--	--
	Ca(ICCV2/PI489777)SNP_08028	893	C	T	8	14	--	--
	Ca(ICCV2/PI489777)SNP_08029	1144	T	C	3	7	--	--
	Ca(ICCV2/PI489777)SNP_08030	1145	G	A	3	6	--	--
CakTC24247	Ca(ICCV2/PI489777)SNP_08031	1387	T	C	7	5	--	--
CakTC33434	Ca(ICCV2/PI489777)SNP_08032	470	T	C	24	11	--	--
	Ca(ICCV2/PI489777)SNP_08033	647	T	C	32	17	--	--
	Ca(ICCV2/PI489777)SNP_08034	949	T	C	23	15	--	--
	Ca(ICCV2/PI489777)SNP_08035	1899	C	G	11	9	--	--
CakTC39550	Ca(ICCV2/PI489777)SNP_08036	467	C	T	6	3	--	--
	Ca(ICCV2/PI489777)SNP_08037	752	C	T	12	6	--	--
CakTC27366	Ca(ICCV2/PI489777)SNP_08038	430	A	T	36	13	--	--
	Ca(ICCV2/PI489777)SNP_08039	535	T	C	35	19	--	--
CakTC37794	Ca(ICCV2/PI489777)SNP_08040	588	C	T	9	8	--	--
CakTC40063	Ca(ICCV2/PI489777)SNP_08041	428	C	G	13	3	--	TPR
CakTC39696	Ca(ICCV2/PI489777)SNP_08042	156	A	T	3	4	--	--
CakTC37134	Ca(ICCV2/PI489777)SNP_08043	692	T	A	13	5	--	--
	Ca(ICCV2/PI489777)SNP_08044	758	A	G	16	6	--	--
	Ca(ICCV2/PI489777)SNP_08045	957	A	C	9	9	--	--
	Ca(ICCV2/PI489777)SNP_08046	1022	G	T	11	7	--	--
	Ca(ICCV2/PI489777)SNP_08047	1064	G	C	15	6	--	--
	Ca(ICCV2/PI489777)SNP_08048	1173	T	G	12	4	--	--
	Ca(ICCV2/PI489777)SNP_08049	1271	G	A	16	4	--	--
CakTC31587	Ca(ICCV2/PI489777)SNP_08050	3365	A	G	15	3	--	--
CakTC42341	Ca(ICCV2/PI489777)SNP_08051	808	G	A	31	19	--	--
CakTC31779	Ca(ICCV2/PI489777)SNP_08052	200	G	A	62	87	--	--
CakTC24871	Ca(ICCV2/PI489777)SNP_08053	127	C	G	9	11	--	--
	Ca(ICCV2/PI489777)SNP_08054	141	T	C	9	12	--	--
	Ca(ICCV2/PI489777)SNP_08055	1561	G	A	16	13	--	--
	Ca(ICCV2/PI489777)SNP_08056	1943	A	T	10	4	--	--
	Ca(ICCV2/PI489777)SNP_08057	1944	A	C	10	3	--	--
	Ca(ICCV2/PI489777)SNP_08058	2429	G	A	8	7	--	--
	Ca(ICCV2/PI489777)SNP_08059	2467	T	A	7	9	--	--
	Ca(ICCV2/PI489777)SNP_08060	2837	C	A	11	8	--	--
	Ca(ICCV2/PI489777)SNP_08061	2978	T	C	8	8	--	--

	Ca(ICCV2/PI489777)SNP_08062	3090	A	G	5	5	--	--
CakTC39250	Ca(ICCV2/PI489777)SNP_08063	563	C	T	16	6	--	--
	Ca(ICCV2/PI489777)SNP_08064	1217	C	T	3	18	--	--
CakTC22316	Ca(ICCV2/PI489777)SNP_08065	403	T	G	21	12	--	--
	Ca(ICCV2/PI489777)SNP_08066	1354	C	T	14	13	--	--
	Ca(ICCV2/PI489777)SNP_08067	2083	G	A	4	5	--	--
	Ca(ICCV2/PI489777)SNP_08068	2509	C	A	8	14	--	--
	Ca(ICCV2/PI489777)SNP_08069	2625	G	C	3	10	--	--
CakTC23610	Ca(ICCV2/PI489777)SNP_08070	364	T	C	7	10	--	--
	Ca(ICCV2/PI489777)SNP_08071	1412	C	A	10	6	--	--
	Ca(ICCV2/PI489777)SNP_08072	1537	A	T	12	8	--	--
	Ca(ICCV2/PI489777)SNP_08073	1572	C	T	14	9	--	--
	Ca(ICCV2/PI489777)SNP_08074	1576	G	A	13	11	--	--
	Ca(ICCV2/PI489777)SNP_08075	1746	C	A	9	12	--	--
	Ca(ICCV2/PI489777)SNP_08076	1928	G	A	9	7	--	--
	Ca(ICCV2/PI489777)SNP_08077	2005	G	A	8	8	--	--
	Ca(ICCV2/PI489777)SNP_08078	2071	C	T	15	9	--	--
	Ca(ICCV2/PI489777)SNP_08079	2152	G	A	19	9	--	--
	Ca(ICCV2/PI489777)SNP_08080	2250	C	G	22	8	--	--
	Ca(ICCV2/PI489777)SNP_08081	2290	A	T	23	8	--	--
	Ca(ICCV2/PI489777)SNP_08082	2296	T	A	24	8	--	--
	Ca(ICCV2/PI489777)SNP_08083	2323	A	G	24	6	--	--
CakTC26121	Ca(ICCV2/PI489777)SNP_08084	1472	T	C	6	5	--	--
CakTC38920	Ca(ICCV2/PI489777)SNP_08085	90	C	A	23	16	--	--
	Ca(ICCV2/PI489777)SNP_08086	163	C	A	38	37	--	--
	Ca(ICCV2/PI489777)SNP_08087	286	C	A	45	34	--	--
	Ca(ICCV2/PI489777)SNP_08088	371	T	C	46	38	--	--
	Ca(ICCV2/PI489777)SNP_08089	1053	C	G	34	18	--	--
CakTC25164	Ca(ICCV2/PI489777)SNP_08090	618	T	C	3	4	--	--
	Ca(ICCV2/PI489777)SNP_08091	638	C	T	3	4	--	--
CakTC40074	Ca(ICCV2/PI489777)SNP_08092	1008	C	T	34	5	--	--
	Ca(ICCV2/PI489777)SNP_08093	1043	G	A	33	5	--	--
	Ca(ICCV2/PI489777)SNP_08094	1098	G	C	32	5	--	--
CakTC31387	Ca(ICCV2/PI489777)SNP_08095	606	T	C	4	4	--	FAR1
	Ca(ICCV2/PI489777)SNP_08096	1078	G	A	9	3	--	FAR1
CakTC32263	Ca(ICCV2/PI489777)SNP_08097	264	C	A	4	6	--	--
CakTC39351	Ca(ICCV2/PI489777)SNP_08098	1299	T	C	3	4	--	--
CakTC28950	Ca(ICCV2/PI489777)SNP_08099	593	T	C	6	8	--	--
	Ca(ICCV2/PI489777)SNP_08100	799	C	T	6	3	--	--
CakTC34806	Ca(ICCV2/PI489777)SNP_08101	382	G	T	4	6	--	--
	Ca(ICCV2/PI489777)SNP_08102	403	G	A	4	6	--	--
	Ca(ICCV2/PI489777)SNP_08103	535	G	A	4	6	--	--
	Ca(ICCV2/PI489777)SNP_08104	537	C	T	4	6	--	--
CakTC38253	Ca(ICCV2/PI489777)SNP_08105	103	A	T	4	6	--	--
	Ca(ICCV2/PI489777)SNP_08106	393	T	G	26	18	--	--
	Ca(ICCV2/PI489777)SNP_08107	516	A	G	27	18	--	--
	Ca(ICCV2/PI489777)SNP_08108	714	C	A	19	24	--	--
	Ca(ICCV2/PI489777)SNP_08109	846	C	G	24	18	--	--
	Ca(ICCV2/PI489777)SNP_08110	1056	T	A	28	35	--	--
CakTC27081	Ca(ICCV2/PI489777)SNP_08111	875	C	T	95	51	--	--
CakTC22937	Ca(ICCV2/PI489777)SNP_08112	355	A	T	24	34	--	--
	Ca(ICCV2/PI489777)SNP_08113	486	C	G	29	25	--	--
	Ca(ICCV2/PI489777)SNP_08114	508	T	C	31	29	--	--
	Ca(ICCV2/PI489777)SNP_08115	634	C	T	19	16	--	--
	Ca(ICCV2/PI489777)SNP_08116	2591	C	T	17	6	--	--
	Ca(ICCV2/PI489777)SNP_08117	3479	A	G	5	3	--	--
CakTC27448	Ca(ICCV2/PI489777)SNP_08118	1001	A	T	6	13	--	--
CakTC29936	Ca(ICCV2/PI489777)SNP_08119	792	T	C	9	3	Young_pod	--
	Ca(ICCV2/PI489777)SNP_08120	1052	C	A	11	3	Young_pod	--
	Ca(ICCV2/PI489777)SNP_08121	1071	A	C	9	4	Young_pod	--
	Ca(ICCV2/PI489777)SNP_08122	1109	A	T	9	5	Young_pod	--
	Ca(ICCV2/PI489777)SNP_08123	1173	T	G	9	5	Young_pod	--
	Ca(ICCV2/PI489777)SNP_08124	1201	A	T	8	5	Young_pod	--
	Ca(ICCV2/PI489777)SNP_08125	1242	T	C	9	4	Young_pod	--
CakTC41687	Ca(ICCV2/PI489777)SNP_08126	934	T	C	6	3	--	--
	Ca(ICCV2/PI489777)SNP_08127	1593	G	T	3	7	--	--
	Ca(ICCV2/PI489777)SNP_08128	1805	T	C	5	7	--	--
CakTC26837	Ca(ICCV2/PI489777)SNP_08129	834	C	T	4	3	--	--
CakTC27404	Ca(ICCV2/PI489777)SNP_08130	413	A	C	8	12	--	--
	Ca(ICCV2/PI489777)SNP_08131	479	A	C	11	12	--	--
	Ca(ICCV2/PI489777)SNP_08132	2049	C	T	13	11	--	--
	Ca(ICCV2/PI489777)SNP_08133	3096	C	A	11	4	--	--
CakTC26395	Ca(ICCV2/PI489777)SNP_08134	194	C	T	5	3	--	--
CakTC34208	Ca(ICCV2/PI489777)SNP_08135	587	C	T	7	8	--	--

	Ca(ICCV2/PI489777)SNP_08136	1280	G	T	4	4	--	--
	Ca(ICCV2/PI489777)SNP_08137	1286	A	C	5	4	--	--
	Ca(ICCV2/PI489777)SNP_08138	1938	G	A	4	4	--	--
CakTC23413	Ca(ICCV2/PI489777)SNP_08139	338	T	C	15	4	--	--
CakTC27343	Ca(ICCV2/PI489777)SNP_08140	729	G	A	51	16	--	BES1
	Ca(ICCV2/PI489777)SNP_08141	1074	T	C	72	29	--	BES1
CakTC23032	Ca(ICCV2/PI489777)SNP_08142	263	G	C	6	3	--	--
CakTC38855	Ca(ICCV2/PI489777)SNP_08143	701	G	A	5	3	--	--
	Ca(ICCV2/PI489777)SNP_08144	788	C	T	3	4	--	--
CakTC38957	Ca(ICCV2/PI489777)SNP_08145	287	A	G	9	3	--	--
CakTC08131	Ca(ICCV2/PI489777)SNP_08146	2131	C	T	4	4	--	--
	Ca(ICCV2/PI489777)SNP_08147	2152	T	A	4	5	--	--
	Ca(ICCV2/PI489777)SNP_08148	2234	T	C	4	5	--	--
	Ca(ICCV2/PI489777)SNP_08149	2366	G	A	3	4	--	--
	Ca(ICCV2/PI489777)SNP_08150	2375	T	C	3	3	--	--
CakTC36937	Ca(ICCV2/PI489777)SNP_08151	1170	G	A	21	14	--	--
	Ca(ICCV2/PI489777)SNP_08152	1190	A	C	16	15	--	--
	Ca(ICCV2/PI489777)SNP_08153	1432	G	A	31	15	--	--
	Ca(ICCV2/PI489777)SNP_08154	1443	T	C	31	14	--	--
	Ca(ICCV2/PI489777)SNP_08155	1694	A	T	46	16	--	--
	Ca(ICCV2/PI489777)SNP_08156	2207	C	T	21	16	--	--
CakTC25907	Ca(ICCV2/PI489777)SNP_08157	330	G	A	4	3	--	--
CakTC42678	Ca(ICCV2/PI489777)SNP_08158	821	C	T	5	6	--	--
CakTC43324	Ca(ICCV2/PI489777)SNP_08159	235	C	G	87	65	--	--
	Ca(ICCV2/PI489777)SNP_08160	747	C	A	45	41	--	--
	Ca(ICCV2/PI489777)SNP_08161	755	T	G	29	37	--	--
CakTC38721	Ca(ICCV2/PI489777)SNP_08162	1274	T	G	18	22	--	--
CakTC42588	Ca(ICCV2/PI489777)SNP_08163	908	G	T	83	72	--	--
CakTC29779	Ca(ICCV2/PI489777)SNP_08164	377	T	C	3	9	--	--
	Ca(ICCV2/PI489777)SNP_08165	485	G	A	4	7	--	--
	Ca(ICCV2/PI489777)SNP_08166	1352	T	C	12	4	--	--
	Ca(ICCV2/PI489777)SNP_08167	1422	A	T	10	4	--	--
CakTC28462	Ca(ICCV2/PI489777)SNP_08168	610	G	T	77	32	--	--
	Ca(ICCV2/PI489777)SNP_08169	792	T	G	77	28	--	--
	Ca(ICCV2/PI489777)SNP_08170	891	A	T	83	35	--	--
	Ca(ICCV2/PI489777)SNP_08171	1041	C	T	97	47	--	--
	Ca(ICCV2/PI489777)SNP_08172	1045	A	G	90	53	--	--
	Ca(ICCV2/PI489777)SNP_08173	1715	T	C	4	3	--	--
	Ca(ICCV2/PI489777)SNP_08174	1748	T	C	3	3	--	--
CakTC29821	Ca(ICCV2/PI489777)SNP_08175	473	C	T	3	3	Shoot	--
	Ca(ICCV2/PI489777)SNP_08176	1052	A	T	3	3	Shoot	--
CakTC09754	Ca(ICCV2/PI489777)SNP_08177	795	A	G	8	4	--	--
CakTC26888	Ca(ICCV2/PI489777)SNP_08178	1102	C	G	3	4	--	--
	Ca(ICCV2/PI489777)SNP_08179	1117	T	G	3	4	--	--
CakTC26789	Ca(ICCV2/PI489777)SNP_08180	275	T	A	13	11	--	--
	Ca(ICCV2/PI489777)SNP_08181	509	T	G	13	6	--	--
	Ca(ICCV2/PI489777)SNP_08182	1583	T	C	14	4	--	--
CakTC29652	Ca(ICCV2/PI489777)SNP_08183	1829	T	A	4	5	--	--
	Ca(ICCV2/PI489777)SNP_08184	1832	A	T	4	5	--	--
	Ca(ICCV2/PI489777)SNP_08185	1949	C	A	3	4	--	--
CakTC39509	Ca(ICCV2/PI489777)SNP_08186	145	T	A	4	14	--	--
	Ca(ICCV2/PI489777)SNP_08187	431	T	C	17	11	--	--
	Ca(ICCV2/PI489777)SNP_08188	632	A	G	18	19	--	--
CakTC31925	Ca(ICCV2/PI489777)SNP_08189	862	C	T	4	3	--	--
CakTC39998	Ca(ICCV2/PI489777)SNP_08190	376	G	C	8	3	--	--
	Ca(ICCV2/PI489777)SNP_08191	653	C	T	8	4	--	--
	Ca(ICCV2/PI489777)SNP_08192	713	G	A	13	5	--	--
	Ca(ICCV2/PI489777)SNP_08193	950	G	C	16	5	--	--
	Ca(ICCV2/PI489777)SNP_08194	988	C	A	10	5	--	--
	Ca(ICCV2/PI489777)SNP_08195	1102	G	A	7	3	--	--
CakTC28873	Ca(ICCV2/PI489777)SNP_08196	526	C	G	6	7	--	--
	Ca(ICCV2/PI489777)SNP_08197	672	A	G	3	3	--	--
	Ca(ICCV2/PI489777)SNP_08198	1840	C	T	3	7	--	--
CakTC42477	Ca(ICCV2/PI489777)SNP_08199	940	T	C	22	8	--	--
	Ca(ICCV2/PI489777)SNP_08200	1156	A	G	16	8	--	--
	Ca(ICCV2/PI489777)SNP_08201	1261	A	C	20	9	--	--
	Ca(ICCV2/PI489777)SNP_08202	1340	G	C	15	9	--	--
	Ca(ICCV2/PI489777)SNP_08203	1341	C	A	14	9	--	--
	Ca(ICCV2/PI489777)SNP_08204	1358	A	G	14	9	--	--
	Ca(ICCV2/PI489777)SNP_08205	1443	G	T	10	8	--	--
CakTC23186	Ca(ICCV2/PI489777)SNP_08206	487	G	C	8	24	--	--
	Ca(ICCV2/PI489777)SNP_08207	1365	T	C	13	13	--	--
CakTC09539	Ca(ICCV2/PI489777)SNP_08208	988	G	A	12	6	--	--
CakTC35669	Ca(ICCV2/PI489777)SNP_08209	565	G	T	75	62	--	--

CakTC02214	Ca(ICCV2/PI489777)SNP_08210	34	C	T	18	18	--	CCAAT
	Ca(ICCV2/PI489777)SNP_08211	124	T	A	35	25	--	CCAAT
	Ca(ICCV2/PI489777)SNP_08212	157	C	T	40	32	--	CCAAT
CakTC25955	Ca(ICCV2/PI489777)SNP_08213	335	T	G	69	133	--	--
	Ca(ICCV2/PI489777)SNP_08214	407	G	T	124	157	--	--
	Ca(ICCV2/PI489777)SNP_08215	838	C	T	103	112	--	--
CakTC22320	Ca(ICCV2/PI489777)SNP_08216	180	A	G	3	3	--	--
	Ca(ICCV2/PI489777)SNP_08217	767	A	G	15	7	--	--
	Ca(ICCV2/PI489777)SNP_08218	1075	G	A	5	6	--	--
CakTC31466	Ca(ICCV2/PI489777)SNP_08219	398	G	A	5	3	Young_pod	--
	Ca(ICCV2/PI489777)SNP_08220	400	C	T	5	3	Young_pod	--
	Ca(ICCV2/PI489777)SNP_08221	736	T	C	7	8	Young_pod	--
CakTC28971	Ca(ICCV2/PI489777)SNP_08222	825	C	T	66	34	--	--
CakTC22697	Ca(ICCV2/PI489777)SNP_08223	1028	A	T	8	9	--	--
CakTC33305	Ca(ICCV2/PI489777)SNP_08224	131	A	C	4	11	--	--
CakTC37291	Ca(ICCV2/PI489777)SNP_08225	655	T	C	3	6	--	--
	Ca(ICCV2/PI489777)SNP_08226	910	C	T	10	5	--	--
	Ca(ICCV2/PI489777)SNP_08227	1075	A	G	9	5	--	--
CakTC07782	Ca(ICCV2/PI489777)SNP_08228	456	T	C	4	3	--	--
CakTC13861	Ca(ICCV2/PI489777)SNP_08229	183	A	G	3	3	--	--
CakTC26153	Ca(ICCV2/PI489777)SNP_08230	138	G	A	4	3	Young_pod	--
	Ca(ICCV2/PI489777)SNP_08231	284	A	C	3	3	Young_pod	--
	Ca(ICCV2/PI489777)SNP_08232	951	C	T	4	3	Young_pod	--
CakTC27224	Ca(ICCV2/PI489777)SNP_08233	334	G	A	6	9	--	--
	Ca(ICCV2/PI489777)SNP_08234	337	T	C	6	9	--	--
	Ca(ICCV2/PI489777)SNP_08235	430	T	A	7	7	--	--
	Ca(ICCV2/PI489777)SNP_08236	444	C	T	7	7	--	--
	Ca(ICCV2/PI489777)SNP_08237	880	C	T	5	3	--	--
	Ca(ICCV2/PI489777)SNP_08238	1714	C	G	6	3	--	--
CakTC36377	Ca(ICCV2/PI489777)SNP_08239	1163	A	G	6	4	--	--
CakTC10489	Ca(ICCV2/PI489777)SNP_08240	248	G	T	21	10	--	FHA
	Ca(ICCV2/PI489777)SNP_08241	327	C	A	17	9	--	FHA
	Ca(ICCV2/PI489777)SNP_08242	335	G	C	21	12	--	FHA
	Ca(ICCV2/PI489777)SNP_08243	355	A	G	22	12	--	FHA
	Ca(ICCV2/PI489777)SNP_08244	473	A	G	21	13	--	FHA
	Ca(ICCV2/PI489777)SNP_08245	649	C	T	14	10	--	FHA
	Ca(ICCV2/PI489777)SNP_08246	778	C	T	10	4	--	FHA
CakTC29402	Ca(ICCV2/PI489777)SNP_08247	1101	C	T	6	8	--	--
	Ca(ICCV2/PI489777)SNP_08248	1115	T	C	6	7	--	--
	Ca(ICCV2/PI489777)SNP_08249	1152	A	T	5	8	--	--
	Ca(ICCV2/PI489777)SNP_08250	1178	T	C	6	7	--	--
CakTC35501	Ca(ICCV2/PI489777)SNP_08251	565	T	A	12	8	--	--
	Ca(ICCV2/PI489777)SNP_08252	782	G	A	16	11	--	--
	Ca(ICCV2/PI489777)SNP_08253	1172	G	C	4	4	--	--
	Ca(ICCV2/PI489777)SNP_08254	1882	G	T	11	6	--	--
	Ca(ICCV2/PI489777)SNP_08255	2168	G	A	7	9	--	--
	Ca(ICCV2/PI489777)SNP_08256	3221	G	A	22	6	--	--
	Ca(ICCV2/PI489777)SNP_08257	3807	T	G	8	4	--	--
CakTC23934	Ca(ICCV2/PI489777)SNP_08258	405	C	T	83	18	--	--
	Ca(ICCV2/PI489777)SNP_08259	2062	C	A	99	24	--	--
	Ca(ICCV2/PI489777)SNP_08260	2106	T	G	105	23	--	--
	Ca(ICCV2/PI489777)SNP_08261	3115	T	A	72	25	--	--
	Ca(ICCV2/PI489777)SNP_08262	3144	T	G	64	22	--	--
	Ca(ICCV2/PI489777)SNP_08263	3710	C	G	57	13	--	--
	Ca(ICCV2/PI489777)SNP_08264	3925	A	G	6	3	--	--
CakTC31597	Ca(ICCV2/PI489777)SNP_08265	87	G	A	7	12	--	--
	Ca(ICCV2/PI489777)SNP_08266	194	T	C	121	177	--	--
CakTC24613	Ca(ICCV2/PI489777)SNP_08267	583	G	T	20	11	--	--
	Ca(ICCV2/PI489777)SNP_08268	796	G	T	30	14	--	--
	Ca(ICCV2/PI489777)SNP_08269	3020	G	A	23	14	--	--
	Ca(ICCV2/PI489777)SNP_08270	3268	T	C	18	16	--	--
CakTC30221	Ca(ICCV2/PI489777)SNP_08271	256	T	C	8	3	Flower	bud
	Ca(ICCV2/PI489777)SNP_08272	277	A	G	9	3	Flower	bud
CakTC40324	Ca(ICCV2/PI489777)SNP_08273	248	T	C	5	5	--	--
	Ca(ICCV2/PI489777)SNP_08274	1406	G	A	11	11	--	--
CakTC43220	Ca(ICCV2/PI489777)SNP_08275	177	A	G	28	8	--	--
	Ca(ICCV2/PI489777)SNP_08276	737	G	A	18	13	--	--
	Ca(ICCV2/PI489777)SNP_08277	773	G	A	15	13	--	--
CakTC26430	Ca(ICCV2/PI489777)SNP_08278	507	T	G	26	3	--	--
	Ca(ICCV2/PI489777)SNP_08279	1407	C	A	10	6	--	--
CakTC38383	Ca(ICCV2/PI489777)SNP_08280	43	A	G	7	3	--	--
	Ca(ICCV2/PI489777)SNP_08281	167	C	T	10	8	--	--
	Ca(ICCV2/PI489777)SNP_08282	296	A	G	15	11	--	--
	Ca(ICCV2/PI489777)SNP_08283	399	T	A	12	8	--	--

	Ca(ICCV2/PI489777)SNP_08284	495	A	G	8	5	--	--
CakTC28071	Ca(ICCV2/PI489777)SNP_08285	127	C	T	55	45	--	--
CakTC23735	Ca(ICCV2/PI489777)SNP_08286	1370	C	T	41	26	--	--
	Ca(ICCV2/PI489777)SNP_08287	1519	G	A	36	23	--	--
CakTC24509	Ca(ICCV2/PI489777)SNP_08288	337	A	G	16	14	--	--
	Ca(ICCV2/PI489777)SNP_08289	343	T	C	21	15	--	--
	Ca(ICCV2/PI489777)SNP_08290	1053	A	G	12	11	--	--
	Ca(ICCV2/PI489777)SNP_08291	1337	A	C	6	7	--	--
	Ca(ICCV2/PI489777)SNP_08292	1381	A	T	3	7	--	--
CakTC35723	Ca(ICCV2/PI489777)SNP_08293	454	T	C	7	9	--	--
CakTC29994	Ca(ICCV2/PI489777)SNP_08294	145	A	G	13	18	--	--
	Ca(ICCV2/PI489777)SNP_08295	399	T	C	11	20	--	--
	Ca(ICCV2/PI489777)SNP_08296	1309	G	A	16	23	--	--
	Ca(ICCV2/PI489777)SNP_08297	1755	A	C	17	20	--	--
	Ca(ICCV2/PI489777)SNP_08298	1842	C	T	9	19	--	--
	Ca(ICCV2/PI489777)SNP_08299	2352	A	T	18	29	--	--
	Ca(ICCV2/PI489777)SNP_08300	2829	T	C	16	20	--	--
	Ca(ICCV2/PI489777)SNP_08301	3348	T	C	12	35	--	--
	Ca(ICCV2/PI489777)SNP_08302	3352	T	C	14	37	--	--
	Ca(ICCV2/PI489777)SNP_08303	3887	G	A	8	8	--	--
CakTC31491	Ca(ICCV2/PI489777)SNP_08304	1085	A	G	6	3	--	--
CakTC22388	Ca(ICCV2/PI489777)SNP_08305	862	G	T	21	6	--	--
CakTC09812	Ca(ICCV2/PI489777)SNP_08306	812	C	T	4	4	--	--
	Ca(ICCV2/PI489777)SNP_08307	814	C	G	4	4	--	--
CakTC36187	Ca(ICCV2/PI489777)SNP_08308	1125	T	C	3	4	--	--
	Ca(ICCV2/PI489777)SNP_08309	3710	C	T	11	7	--	--
	Ca(ICCV2/PI489777)SNP_08310	3722	G	A	11	6	--	--
	Ca(ICCV2/PI489777)SNP_08311	3828	A	G	10	11	--	--
	Ca(ICCV2/PI489777)SNP_08312	4584	C	T	17	6	--	--
	Ca(ICCV2/PI489777)SNP_08313	5278	A	G	9	7	--	--
CakTC28265	Ca(ICCV2/PI489777)SNP_08314	182	A	C	39	6	--	--
	Ca(ICCV2/PI489777)SNP_08315	222	G	A	46	4	--	--
	Ca(ICCV2/PI489777)SNP_08316	275	G	A	46	4	--	--
	Ca(ICCV2/PI489777)SNP_08317	2354	G	A	28	6	--	--
	Ca(ICCV2/PI489777)SNP_08318	2491	A	T	22	5	--	--
	Ca(ICCV2/PI489777)SNP_08319	2684	T	G	26	4	--	--
	Ca(ICCV2/PI489777)SNP_08320	2982	G	C	24	4	--	--
	Ca(ICCV2/PI489777)SNP_08321	3861	G	A	7	7	--	--
	Ca(ICCV2/PI489777)SNP_08322	5565	C	T	14	3	--	--
	Ca(ICCV2/PI489777)SNP_08323	5675	T	C	13	3	--	--
	Ca(ICCV2/PI489777)SNP_08324	8907	T	C	5	4	--	--
CakTC27829	Ca(ICCV2/PI489777)SNP_08325	1755	G	T	14	3	--	--
	Ca(ICCV2/PI489777)SNP_08326	1795	C	A	15	3	--	--
	Ca(ICCV2/PI489777)SNP_08327	1805	C	G	14	3	--	--
	Ca(ICCV2/PI489777)SNP_08328	1843	G	T	14	3	--	--
	Ca(ICCV2/PI489777)SNP_08329	1844	T	A	14	3	--	--
	Ca(ICCV2/PI489777)SNP_08330	1853	A	T	14	3	--	--
	Ca(ICCV2/PI489777)SNP_08331	1864	A	T	14	3	--	--
	Ca(ICCV2/PI489777)SNP_08332	1937	A	G	15	3	--	--
CakTC08873	Ca(ICCV2/PI489777)SNP_08333	360	C	T	3	10	--	--
CakTC41867	Ca(ICCV2/PI489777)SNP_08334	865	G	A	9	8	--	--
CakTC02071	Ca(ICCV2/PI489777)SNP_08335	298	A	G	8	6	--	--
CakTC43172	Ca(ICCV2/PI489777)SNP_08336	142	C	T	23	7	--	--
CakTC25790	Ca(ICCV2/PI489777)SNP_08337	44	A	C	3	5	--	--
	Ca(ICCV2/PI489777)SNP_08338	374	T	A	3	9	--	--
CakTC27285	Ca(ICCV2/PI489777)SNP_08339	1578	C	A	23	8	--	--
CakTC32595	Ca(ICCV2/PI489777)SNP_08340	164	C	T	4	4	--	--
	Ca(ICCV2/PI489777)SNP_08341	912	G	T	44	16	--	--
	Ca(ICCV2/PI489777)SNP_08342	969	T	C	44	12	--	--
	Ca(ICCV2/PI489777)SNP_08343	1078	A	G	49	17	--	--
	Ca(ICCV2/PI489777)SNP_08344	1297	A	G	33	14	--	--
	Ca(ICCV2/PI489777)SNP_08345	1344	A	G	32	13	--	--
	Ca(ICCV2/PI489777)SNP_08346	1353	C	G	30	13	--	--
	Ca(ICCV2/PI489777)SNP_08347	1495	T	C	16	9	--	--
	Ca(ICCV2/PI489777)SNP_08348	1604	C	G	19	11	--	--
	Ca(ICCV2/PI489777)SNP_08349	1687	A	T	19	7	--	--
CakTC24421	Ca(ICCV2/PI489777)SNP_08350	433	A	G	11	21	--	--
	Ca(ICCV2/PI489777)SNP_08351	463	A	G	13	20	--	--
	Ca(ICCV2/PI489777)SNP_08352	818	G	A	5	6	--	--
	Ca(ICCV2/PI489777)SNP_08353	859	A	G	7	6	--	--
	Ca(ICCV2/PI489777)SNP_08354	895	G	A	6	6	--	--
CakTC38440	Ca(ICCV2/PI489777)SNP_08355	1159	C	T	10	15	--	--
	Ca(ICCV2/PI489777)SNP_08356	1182	A	C	7	15	--	--
CakTC36296	Ca(ICCV2/PI489777)SNP_08357	1869	T	G	22	14	--	--

CakTC37864	Ca(ICCV2/PI489777)SNP_08358	83	C	G	5	5	--	--
	Ca(ICCV2/PI489777)SNP_08359	976	A	C	9	5	--	--
	Ca(ICCV2/PI489777)SNP_08360	1531	T	C	14	7	--	--
	Ca(ICCV2/PI489777)SNP_08361	1563	A	G	14	7	--	--
	Ca(ICCV2/PI489777)SNP_08362	1577	C	T	12	7	--	--
CakTC29201	Ca(ICCV2/PI489777)SNP_08363	642	C	T	4	3	Shoot	--
	Ca(ICCV2/PI489777)SNP_08364	801	G	A	4	3	Shoot	--
CakTC33279	Ca(ICCV2/PI489777)SNP_08365	165	G	T	3	3	--	--
CakTC23801	Ca(ICCV2/PI489777)SNP_08366	493	T	C	73	83	--	--
	Ca(ICCV2/PI489777)SNP_08367	1736	T	C	366	153	--	--
	Ca(ICCV2/PI489777)SNP_08368	2162	A	C	9	4	--	--
CakTC25488	Ca(ICCV2/PI489777)SNP_08369	145	G	C	12	4	--	--
	Ca(ICCV2/PI489777)SNP_08370	478	G	T	5	3	--	--
CakTC12336	Ca(ICCV2/PI489777)SNP_08371	1578	A	T	3	3	--	--
	Ca(ICCV2/PI489777)SNP_08372	2725	G	A	13	15	--	--
CakTC28384	Ca(ICCV2/PI489777)SNP_08373	422	A	G	6	6	--	--
	Ca(ICCV2/PI489777)SNP_08374	444	C	T	6	6	--	--
	Ca(ICCV2/PI489777)SNP_08375	511	C	T	6	7	--	--
	Ca(ICCV2/PI489777)SNP_08376	524	G	C	6	6	--	--
	Ca(ICCV2/PI489777)SNP_08377	986	G	T	8	3	--	--
CakTC41432	Ca(ICCV2/PI489777)SNP_08378	999	T	C	13	7	--	--
CakTC08499	Ca(ICCV2/PI489777)SNP_08379	782	A	G	3	3	--	--
	Ca(ICCV2/PI489777)SNP_08380	1121	A	G	3	4	--	--
	Ca(ICCV2/PI489777)SNP_08381	1214	C	T	4	6	--	--
	Ca(ICCV2/PI489777)SNP_08382	1265	A	G	4	5	--	--
CakTC28491	Ca(ICCV2/PI489777)SNP_08383	115	C	A	13	12	--	--
CakTC27914	Ca(ICCV2/PI489777)SNP_08384	1676	A	G	11	6	--	--
	Ca(ICCV2/PI489777)SNP_08385	1694	T	G	14	5	--	--
CakTC38599	Ca(ICCV2/PI489777)SNP_08386	493	T	C	30	12	--	--
CakTC31433	Ca(ICCV2/PI489777)SNP_08387	3762	A	G	5	3	--	--
CakTC25045	Ca(ICCV2/PI489777)SNP_08388	442	A	G	7	7	--	--
	Ca(ICCV2/PI489777)SNP_08389	510	A	G	6	4	--	--
CakTC42509	Ca(ICCV2/PI489777)SNP_08390	458	G	A	114	65	--	--
	Ca(ICCV2/PI489777)SNP_08391	1106	A	G	91	55	--	--
CakTC43389	Ca(ICCV2/PI489777)SNP_08392	377	G	A	15	5	--	--
	Ca(ICCV2/PI489777)SNP_08393	539	A	G	12	4	--	--
	Ca(ICCV2/PI489777)SNP_08394	728	A	G	23	3	--	--
	Ca(ICCV2/PI489777)SNP_08395	758	A	G	21	3	--	--
CakTC27398	Ca(ICCV2/PI489777)SNP_08396	971	T	C	3	4	Flower	bud
	Ca(ICCV2/PI489777)SNP_08397	4708	A	G	3	7	Flower	bud
	Ca(ICCV2/PI489777)SNP_08398	6253	C	T	10	5	Flower	bud
	Ca(ICCV2/PI489777)SNP_08399	6394	A	G	6	5	Flower	bud
	Ca(ICCV2/PI489777)SNP_08400	6440	A	G	5	5	Flower	bud
CakTC39128	Ca(ICCV2/PI489777)SNP_08401	943	G	A	9	4	--	--
	Ca(ICCV2/PI489777)SNP_08402	1098	A	G	7	8	--	--
CakTC40366	Ca(ICCV2/PI489777)SNP_08403	190	G	C	25	11	--	--
	Ca(ICCV2/PI489777)SNP_08404	723	A	G	13	3	--	--
	Ca(ICCV2/PI489777)SNP_08405	855	T	C	12	5	--	--
	Ca(ICCV2/PI489777)SNP_08406	1165	A	G	32	10	--	--
	Ca(ICCV2/PI489777)SNP_08407	1227	C	A	24	12	--	--
	Ca(ICCV2/PI489777)SNP_08408	1658	T	G	18	12	--	--
CakTC34625	Ca(ICCV2/PI489777)SNP_08409	23	T	C	5	3	--	--
	Ca(ICCV2/PI489777)SNP_08410	449	A	G	11	9	--	--
	Ca(ICCV2/PI489777)SNP_08411	545	C	T	11	8	--	--
	Ca(ICCV2/PI489777)SNP_08412	716	A	G	14	7	--	--
	Ca(ICCV2/PI489777)SNP_08413	1022	G	A	8	6	--	--
	Ca(ICCV2/PI489777)SNP_08414	1049	G	A	8	5	--	--
	Ca(ICCV2/PI489777)SNP_08415	1091	A	G	13	7	--	--
CakTC37532	Ca(ICCV2/PI489777)SNP_08416	675	C	T	11	3	--	bHLH
CakTC40573	Ca(ICCV2/PI489777)SNP_08417	52	G	T	3	3	--	--
	Ca(ICCV2/PI489777)SNP_08418	109	A	G	6	5	--	--
	Ca(ICCV2/PI489777)SNP_08419	136	T	G	7	7	--	--
	Ca(ICCV2/PI489777)SNP_08420	205	T	A	6	7	--	--
CakTC31439	Ca(ICCV2/PI489777)SNP_08421	40	T	C	6	3	--	--
	Ca(ICCV2/PI489777)SNP_08422	89	A	T	7	6	--	--
	Ca(ICCV2/PI489777)SNP_08423	103	C	T	9	6	--	--
	Ca(ICCV2/PI489777)SNP_08424	121	A	C	9	6	--	--
	Ca(ICCV2/PI489777)SNP_08425	136	T	A	8	5	--	--
	Ca(ICCV2/PI489777)SNP_08426	182	C	T	11	6	--	--
	Ca(ICCV2/PI489777)SNP_08427	343	T	C	18	7	--	--
	Ca(ICCV2/PI489777)SNP_08428	388	T	C	19	8	--	--
	Ca(ICCV2/PI489777)SNP_08429	462	C	A	20	10	--	--
	Ca(ICCV2/PI489777)SNP_08430	514	G	A	19	4	--	--
	Ca(ICCV2/PI489777)SNP_08431	520	C	T	17	7	--	--

	Ca(ICCV2/PI489777)SNP_08432	526	T	C	18	7	--	--
	Ca(ICCV2/PI489777)SNP_08433	746	G	A	12	7	--	--
	Ca(ICCV2/PI489777)SNP_08434	754	T	G	8	7	--	--
CakTC33733	Ca(ICCV2/PI489777)SNP_08435	145	A	G	10	4	--	--
	Ca(ICCV2/PI489777)SNP_08436	2341	A	G	14	11	--	--
CakTC37341	Ca(ICCV2/PI489777)SNP_08437	247	G	C	9	10	--	--
	Ca(ICCV2/PI489777)SNP_08438	374	C	T	13	12	--	--
CakTC24735	Ca(ICCV2/PI489777)SNP_08439	717	G	A	5	9	--	--
	Ca(ICCV2/PI489777)SNP_08440	3069	C	T	3	4	--	--
CakTC25732	Ca(ICCV2/PI489777)SNP_08441	324	G	A	30	9	--	--
	Ca(ICCV2/PI489777)SNP_08442	386	T	C	36	13	--	--
CakTC12244	Ca(ICCV2/PI489777)SNP_08443	535	C	G	25	9	--	--
	Ca(ICCV2/PI489777)SNP_08444	809	G	T	34	18	--	--
	Ca(ICCV2/PI489777)SNP_08445	812	C	T	35	17	--	--
	Ca(ICCV2/PI489777)SNP_08446	860	C	T	35	17	--	--
	Ca(ICCV2/PI489777)SNP_08447	914	A	C	33	15	--	--
	Ca(ICCV2/PI489777)SNP_08448	1397	T	C	66	44	--	--
CakTC08882	Ca(ICCV2/PI489777)SNP_08449	71	C	T	3	3	--	--
CakTC33392	Ca(ICCV2/PI489777)SNP_08450	359	T	G	11	5	--	--
	Ca(ICCV2/PI489777)SNP_08451	659	C	G	12	5	--	--
	Ca(ICCV2/PI489777)SNP_08452	1012	C	G	7	5	--	--
	Ca(ICCV2/PI489777)SNP_08453	1694	T	C	6	4	--	--
	Ca(ICCV2/PI489777)SNP_08454	2042	G	A	4	10	--	--
CakTC28563	Ca(ICCV2/PI489777)SNP_08455	628	T	C	30	9	--	--
	Ca(ICCV2/PI489777)SNP_08456	664	G	A	30	7	--	--
	Ca(ICCV2/PI489777)SNP_08457	1411	A	C	7	3	--	--
CakTC23694	Ca(ICCV2/PI489777)SNP_08458	124	G	A	3	4	--	--
	Ca(ICCV2/PI489777)SNP_08459	168	C	T	17	4	--	--
	Ca(ICCV2/PI489777)SNP_08460	597	G	T	38	33	--	--
CakTC27550	Ca(ICCV2/PI489777)SNP_08461	713	T	A	19	6	--	--
	Ca(ICCV2/PI489777)SNP_08462	1197	G	A	26	16	--	--
	Ca(ICCV2/PI489777)SNP_08463	1288	G	A	21	15	--	--
	Ca(ICCV2/PI489777)SNP_08464	1321	G	A	21	17	--	--
	Ca(ICCV2/PI489777)SNP_08465	1900	A	G	32	11	--	--
	Ca(ICCV2/PI489777)SNP_08466	3210	A	G	16	14	--	--
CakTC24937	Ca(ICCV2/PI489777)SNP_08467	517	G	A	9	9	--	--
CakTC34454	Ca(ICCV2/PI489777)SNP_08468	483	A	C	20	4	--	--
	Ca(ICCV2/PI489777)SNP_08469	624	G	C	15	3	--	--
	Ca(ICCV2/PI489777)SNP_08470	702	A	C	14	3	--	--
	Ca(ICCV2/PI489777)SNP_08471	780	A	G	9	3	--	--
	Ca(ICCV2/PI489777)SNP_08472	933	C	T	13	10	--	--
	Ca(ICCV2/PI489777)SNP_08473	963	C	T	10	9	--	--
	Ca(ICCV2/PI489777)SNP_08474	1016	A	G	8	10	--	--
	Ca(ICCV2/PI489777)SNP_08475	1194	C	T	12	16	--	--
	Ca(ICCV2/PI489777)SNP_08476	1314	G	A	11	28	--	--
	Ca(ICCV2/PI489777)SNP_08477	1435	A	G	14	32	--	--
	Ca(ICCV2/PI489777)SNP_08478	1835	T	C	6	7	--	--
	Ca(ICCV2/PI489777)SNP_08479	1867	A	T	6	6	--	--
CakTC27895	Ca(ICCV2/PI489777)SNP_08480	1931	T	G	5	3	Root	--
	Ca(ICCV2/PI489777)SNP_08481	2438	T	A	6	3	Root	--
CakTC41924	Ca(ICCV2/PI489777)SNP_08482	509	G	A	4	3	--	--
CakTC36567	Ca(ICCV2/PI489777)SNP_08483	2106	A	C	10	10	--	--
CakTC11512	Ca(ICCV2/PI489777)SNP_08484	23	C	G	9	10	--	--
CakTC26598	Ca(ICCV2/PI489777)SNP_08485	372	C	T	13	4	--	--
	Ca(ICCV2/PI489777)SNP_08486	485	A	G	7	7	--	--
	Ca(ICCV2/PI489777)SNP_08487	1147	G	A	10	4	--	--
	Ca(ICCV2/PI489777)SNP_08488	1531	G	T	20	5	--	--
	Ca(ICCV2/PI489777)SNP_08489	1555	G	A	17	5	--	--
CakTC22930	Ca(ICCV2/PI489777)SNP_08490	497	G	A	4	15	--	--
	Ca(ICCV2/PI489777)SNP_08491	868	A	G	4	31	--	--
	Ca(ICCV2/PI489777)SNP_08492	1053	G	A	7	21	--	--
CakTC31564	Ca(ICCV2/PI489777)SNP_08493	2563	C	G	3	4	--	--
CakTC33204	Ca(ICCV2/PI489777)SNP_08494	196	C	T	13	9	--	--
	Ca(ICCV2/PI489777)SNP_08495	676	T	C	18	6	--	--
	Ca(ICCV2/PI489777)SNP_08496	1200	G	C	22	7	--	--
	Ca(ICCV2/PI489777)SNP_08497	1486	T	A	15	18	--	--
	Ca(ICCV2/PI489777)SNP_08498	1812	A	C	20	20	--	--
CakTC18440	Ca(ICCV2/PI489777)SNP_08499	1058	G	A	11	12	--	CCAAT
	Ca(ICCV2/PI489777)SNP_08500	1099	G	T	9	11	--	CCAAT
	Ca(ICCV2/PI489777)SNP_08501	1120	A	C	7	10	--	CCAAT
	Ca(ICCV2/PI489777)SNP_08502	1157	T	A	7	8	--	CCAAT
CakTC27646	Ca(ICCV2/PI489777)SNP_08503	547	G	C	5	6	--	--
CakTC43181	Ca(ICCV2/PI489777)SNP_08504	101	T	G	12	13	--	--
	Ca(ICCV2/PI489777)SNP_08505	126	C	T	12	13	--	--

	Ca(ICCV2/PI489777)SNP_08506	196	T	C	12	13	--	--
CakTC30293	Ca(ICCV2/PI489777)SNP_08507	364	A	G	4	3	--	--
	Ca(ICCV2/PI489777)SNP_08508	391	A	G	4	4	--	--
CakTC34231	Ca(ICCV2/PI489777)SNP_08509	223	G	A	4	7	Root	--
	Ca(ICCV2/PI489777)SNP_08510	316	A	G	5	5	Root	--
	Ca(ICCV2/PI489777)SNP_08511	619	C	T	4	8	Root	--
	Ca(ICCV2/PI489777)SNP_08512	645	C	G	4	5	Root	--
	Ca(ICCV2/PI489777)SNP_08513	772	C	T	8	7	Root	--
	Ca(ICCV2/PI489777)SNP_08514	791	A	G	8	5	Root	--
	Ca(ICCV2/PI489777)SNP_08515	794	A	G	8	5	Root	--
	Ca(ICCV2/PI489777)SNP_08516	1124	C	A	4	13	Root	--
	Ca(ICCV2/PI489777)SNP_08517	1252	T	A	7	10	Root	--
	Ca(ICCV2/PI489777)SNP_08518	1300	G	A	8	9	Root	--
	Ca(ICCV2/PI489777)SNP_08519	1360	G	A	6	10	Root	--
	Ca(ICCV2/PI489777)SNP_08520	1457	A	G	5	4	Root	--
	Ca(ICCV2/PI489777)SNP_08521	1487	A	G	5	4	Root	--
	Ca(ICCV2/PI489777)SNP_08522	1517	C	T	5	3	Root	--
	Ca(ICCV2/PI489777)SNP_08523	1559	G	A	4	4	Root	--
CakTC24596	Ca(ICCV2/PI489777)SNP_08524	118	C	A	17	3	--	--
	Ca(ICCV2/PI489777)SNP_08525	174	G	T	35	5	--	--
	Ca(ICCV2/PI489777)SNP_08526	220	T	C	37	6	--	--
	Ca(ICCV2/PI489777)SNP_08527	221	G	T	37	6	--	--
	Ca(ICCV2/PI489777)SNP_08528	255	A	C	38	7	--	--
	Ca(ICCV2/PI489777)SNP_08529	351	A	T	20	3	--	--
	Ca(ICCV2/PI489777)SNP_08530	496	T	C	41	7	--	--
	Ca(ICCV2/PI489777)SNP_08531	502	G	A	40	8	--	--
	Ca(ICCV2/PI489777)SNP_08532	646	A	G	40	13	--	--
	Ca(ICCV2/PI489777)SNP_08533	760	C	T	34	15	--	--
	Ca(ICCV2/PI489777)SNP_08534	1453	T	C	36	18	--	--
	Ca(ICCV2/PI489777)SNP_08535	1495	A	C	37	16	--	--
CakTC11376	Ca(ICCV2/PI489777)SNP_08536	114	G	T	11	4	--	--
	Ca(ICCV2/PI489777)SNP_08537	146	A	G	12	5	--	--
	Ca(ICCV2/PI489777)SNP_08538	504	C	T	22	9	--	--
	Ca(ICCV2/PI489777)SNP_08539	1053	A	T	22	8	--	--
	Ca(ICCV2/PI489777)SNP_08540	1094	C	T	26	8	--	--
CakTC30786	Ca(ICCV2/PI489777)SNP_08541	280	C	T	6	4	--	--
	Ca(ICCV2/PI489777)SNP_08542	288	G	A	6	4	--	--
	Ca(ICCV2/PI489777)SNP_08543	292	C	T	6	4	--	--
	Ca(ICCV2/PI489777)SNP_08544	306	A	G	7	4	--	--
	Ca(ICCV2/PI489777)SNP_08545	317	A	G	6	4	--	--
	Ca(ICCV2/PI489777)SNP_08546	402	T	C	6	4	--	--
	Ca(ICCV2/PI489777)SNP_08547	624	G	A	16	4	--	--
	Ca(ICCV2/PI489777)SNP_08548	641	T	C	15	4	--	--
	Ca(ICCV2/PI489777)SNP_08549	702	A	T	15	3	--	--
	Ca(ICCV2/PI489777)SNP_08550	735	A	C	15	4	--	--
	Ca(ICCV2/PI489777)SNP_08551	786	G	A	17	3	--	--
CakTC29899	Ca(ICCV2/PI489777)SNP_08552	471	A	T	25	15	--	--
	Ca(ICCV2/PI489777)SNP_08553	2235	T	G	11	11	--	--
	Ca(ICCV2/PI489777)SNP_08554	3507	C	T	7	10	--	--
	Ca(ICCV2/PI489777)SNP_08555	3550	G	A	5	9	--	--
CakTC33873	Ca(ICCV2/PI489777)SNP_08556	115	T	G	24	4	--	--
	Ca(ICCV2/PI489777)SNP_08557	151	T	C	30	7	--	--
	Ca(ICCV2/PI489777)SNP_08558	154	C	T	30	7	--	--
	Ca(ICCV2/PI489777)SNP_08559	241	A	G	41	22	--	--
	Ca(ICCV2/PI489777)SNP_08560	505	A	G	39	22	--	--
	Ca(ICCV2/PI489777)SNP_08561	538	A	G	39	25	--	--
	Ca(ICCV2/PI489777)SNP_08562	1010	T	G	33	21	--	--
	Ca(ICCV2/PI489777)SNP_08563	1426	C	A	35	20	--	--
	Ca(ICCV2/PI489777)SNP_08564	1441	C	A	32	17	--	--
	Ca(ICCV2/PI489777)SNP_08565	1453	G	A	35	16	--	--
	Ca(ICCV2/PI489777)SNP_08566	1471	G	A	42	15	--	--
	Ca(ICCV2/PI489777)SNP_08567	1525	A	G	41	10	--	--
	Ca(ICCV2/PI489777)SNP_08568	1792	A	G	28	11	--	--
	Ca(ICCV2/PI489777)SNP_08569	1809	G	A	27	12	--	--
	Ca(ICCV2/PI489777)SNP_08570	2027	G	C	37	10	--	--
	Ca(ICCV2/PI489777)SNP_08571	2137	A	G	31	8	--	--
	Ca(ICCV2/PI489777)SNP_08572	2163	A	C	32	8	--	--
	Ca(ICCV2/PI489777)SNP_08573	2195	G	T	31	6	--	--
CakTC40122	Ca(ICCV2/PI489777)SNP_08574	779	T	A	26	3	--	--
CakTC34162	Ca(ICCV2/PI489777)SNP_08575	118	G	A	24	18	--	--
	Ca(ICCV2/PI489777)SNP_08576	265	T	C	21	13	--	--
	Ca(ICCV2/PI489777)SNP_08577	315	T	G	18	12	--	--
CakTC23842	Ca(ICCV2/PI489777)SNP_08578	460	A	G	4	4	--	--
	Ca(ICCV2/PI489777)SNP_08579	1222	T	C	9	3	--	--

CakTC42178	Ca(ICCV2/PI489777)SNP_08580	861	A	T	7	4	--	--
CakTC27006	Ca(ICCV2/PI489777)SNP_08581	1210	C	T	10	8	Shoot	--
	Ca(ICCV2/PI489777)SNP_08582	1373	G	A	6	5	Shoot	--
	Ca(ICCV2/PI489777)SNP_08583	1514	C	T	5	4	Shoot	--
CakTC40237	Ca(ICCV2/PI489777)SNP_08584	720	A	T	3	3	--	--
	Ca(ICCV2/PI489777)SNP_08585	763	T	C	3	3	--	--
CakTC41315	Ca(ICCV2/PI489777)SNP_08586	115	A	C	17	3	--	--
	Ca(ICCV2/PI489777)SNP_08587	301	T	C	28	18	--	--
	Ca(ICCV2/PI489777)SNP_08588	474	G	C	28	12	--	--
CakTC28498	Ca(ICCV2/PI489777)SNP_08589	66	A	T	25	9	--	--
	Ca(ICCV2/PI489777)SNP_08590	546	G	A	60	22	--	--
	Ca(ICCV2/PI489777)SNP_08591	627	A	G	62	23	--	--
CakTC37563	Ca(ICCV2/PI489777)SNP_08592	161	T	C	10	6	--	--
	Ca(ICCV2/PI489777)SNP_08593	177	G	A	14	9	--	--
	Ca(ICCV2/PI489777)SNP_08594	632	G	T	28	17	--	--
	Ca(ICCV2/PI489777)SNP_08595	1270	G	A	25	16	--	--
	Ca(ICCV2/PI489777)SNP_08596	1396	G	A	25	11	--	--
CakTC29986	Ca(ICCV2/PI489777)SNP_08597	3769	G	C	4	13	--	--
	Ca(ICCV2/PI489777)SNP_08598	3932	C	T	11	9	--	--
CakTC31956	Ca(ICCV2/PI489777)SNP_08599	1286	A	G	4	3	--	TPR
	Ca(ICCV2/PI489777)SNP_08600	1297	G	T	4	3	--	TPR
CakTC42033	Ca(ICCV2/PI489777)SNP_08601	467	A	C	3	9	--	--
	Ca(ICCV2/PI489777)SNP_08602	2320	A	G	5	3	--	--
	Ca(ICCV2/PI489777)SNP_08603	2421	G	A	4	3	--	--
CakTC39888	Ca(ICCV2/PI489777)SNP_08604	378	A	T	3	4	--	--
	Ca(ICCV2/PI489777)SNP_08605	597	G	A	15	5	--	--
CakTC38091	Ca(ICCV2/PI489777)SNP_08606	142	G	T	17	17	--	--
	Ca(ICCV2/PI489777)SNP_08607	158	C	G	16	18	--	--
	Ca(ICCV2/PI489777)SNP_08608	734	C	A	11	15	--	--
CakTC04388	Ca(ICCV2/PI489777)SNP_08609	146	T	C	8	6	--	--
	Ca(ICCV2/PI489777)SNP_08610	148	C	T	8	6	--	--
	Ca(ICCV2/PI489777)SNP_08611	174	A	T	8	6	--	--
	Ca(ICCV2/PI489777)SNP_08612	265	T	C	4	5	--	--
CakTC42489	Ca(ICCV2/PI489777)SNP_08613	247	T	G	5	7	Shoot	--
CakTC35508	Ca(ICCV2/PI489777)SNP_08614	391	T	G	12	3	--	--
CakTC11420	Ca(ICCV2/PI489777)SNP_08615	129	C	T	4	7	--	--
	Ca(ICCV2/PI489777)SNP_08616	163	C	T	4	7	--	--
	Ca(ICCV2/PI489777)SNP_08617	199	C	T	5	9	--	--
	Ca(ICCV2/PI489777)SNP_08618	261	G	T	5	9	--	--
	Ca(ICCV2/PI489777)SNP_08619	293	A	G	5	8	--	--
CakTC30668	Ca(ICCV2/PI489777)SNP_08620	423	A	G	5	3	--	--
	Ca(ICCV2/PI489777)SNP_08621	762	C	T	7	10	--	--
	Ca(ICCV2/PI489777)SNP_08622	1128	A	C	4	4	--	--
	Ca(ICCV2/PI489777)SNP_08623	1179	A	G	4	4	--	--
	Ca(ICCV2/PI489777)SNP_08624	1714	C	A	9	7	--	--
	Ca(ICCV2/PI489777)SNP_08625	1778	T	C	13	7	--	--
	Ca(ICCV2/PI489777)SNP_08626	2107	C	T	7	3	--	--
	Ca(ICCV2/PI489777)SNP_08627	2225	G	A	9	6	--	--
	Ca(ICCV2/PI489777)SNP_08628	2350	G	A	9	3	--	--
	Ca(ICCV2/PI489777)SNP_08629	2401	T	A	8	6	--	--
	Ca(ICCV2/PI489777)SNP_08630	2472	C	G	7	6	--	--
	Ca(ICCV2/PI489777)SNP_08631	2482	T	C	9	6	--	--
	Ca(ICCV2/PI489777)SNP_08632	2485	T	C	8	6	--	--
	Ca(ICCV2/PI489777)SNP_08633	2494	T	G	9	8	--	--
	Ca(ICCV2/PI489777)SNP_08634	2611	G	A	5	7	--	--
	Ca(ICCV2/PI489777)SNP_08635	2620	C	A	5	8	--	--
	Ca(ICCV2/PI489777)SNP_08636	2652	C	A	4	8	--	--
CakTC43112	Ca(ICCV2/PI489777)SNP_08637	236	T	C	6	4	Mature	Leaf
	Ca(ICCV2/PI489777)SNP_08638	560	T	C	14	5	Mature	Leaf
	Ca(ICCV2/PI489777)SNP_08639	983	T	C	27	3	Mature	Leaf
	Ca(ICCV2/PI489777)SNP_08640	1004	A	T	25	5	Mature	Leaf
	Ca(ICCV2/PI489777)SNP_08641	1061	A	G	32	5	Mature	Leaf
	Ca(ICCV2/PI489777)SNP_08642	1457	T	C	10	8	Mature	Leaf
CakTC07583	Ca(ICCV2/PI489777)SNP_08643	614	C	T	6	3	--	--
	Ca(ICCV2/PI489777)SNP_08644	617	G	A	6	3	--	--
CakTC39806	Ca(ICCV2/PI489777)SNP_08645	73	T	C	10	4	--	--
	Ca(ICCV2/PI489777)SNP_08646	218	G	A	19	7	--	--
	Ca(ICCV2/PI489777)SNP_08647	513	G	A	27	16	--	--
	Ca(ICCV2/PI489777)SNP_08648	668	G	A	18	13	--	--
	Ca(ICCV2/PI489777)SNP_08649	1172	A	C	20	8	--	--
	Ca(ICCV2/PI489777)SNP_08650	1226	A	G	19	9	--	--
	Ca(ICCV2/PI489777)SNP_08651	1242	C	A	19	6	--	--
	Ca(ICCV2/PI489777)SNP_08652	1249	G	A	20	6	--	--
CakTC32374	Ca(ICCV2/PI489777)SNP_08653	452	A	G	13	4	--	--

	Ca(ICCV2/PI489777)SNP_08654	755	A	G	7	4	--	--
	Ca(ICCV2/PI489777)SNP_08655	923	A	G	9	6	--	--
	Ca(ICCV2/PI489777)SNP_08656	995	A	C	6	11	--	--
CakTC28006	Ca(ICCV2/PI489777)SNP_08657	1685	T	A	19	9	--	--
	Ca(ICCV2/PI489777)SNP_08658	2516	A	T	13	13	--	--
	Ca(ICCV2/PI489777)SNP_08659	2645	C	A	15	19	--	--
	Ca(ICCV2/PI489777)SNP_08660	2678	G	A	15	20	--	--
	Ca(ICCV2/PI489777)SNP_08661	2910	T	G	15	13	--	--
CakTC40738	Ca(ICCV2/PI489777)SNP_08662	340	G	C	26	11	--	GRAS
	Ca(ICCV2/PI489777)SNP_08663	452	T	C	26	12	--	GRAS
	Ca(ICCV2/PI489777)SNP_08664	506	C	G	29	12	--	GRAS
	Ca(ICCV2/PI489777)SNP_08665	872	A	G	46	7	--	GRAS
	Ca(ICCV2/PI489777)SNP_08666	1085	A	G	43	9	--	GRAS
	Ca(ICCV2/PI489777)SNP_08667	1432	G	T	37	3	--	GRAS
	Ca(ICCV2/PI489777)SNP_08668	1523	G	A	34	3	--	GRAS
	Ca(ICCV2/PI489777)SNP_08669	1556	A	C	27	3	--	GRAS
	Ca(ICCV2/PI489777)SNP_08670	2406	G	A	3	3	--	GRAS
CakTC16249	Ca(ICCV2/PI489777)SNP_08671	626	C	G	3	3	--	--
	Ca(ICCV2/PI489777)SNP_08672	652	T	C	3	4	--	--
CakTC22599	Ca(ICCV2/PI489777)SNP_08673	555	A	T	6	3	Flower	bud
CakTC30011	Ca(ICCV2/PI489777)SNP_08674	4447	C	T	21	16	--	DBP
	Ca(ICCV2/PI489777)SNP_08675	4735	A	G	6	13	--	DBP
	Ca(ICCV2/PI489777)SNP_08676	4870	T	A	6	10	--	DBP
	Ca(ICCV2/PI489777)SNP_08677	4872	A	G	6	9	--	DBP
CakTC39881	Ca(ICCV2/PI489777)SNP_08678	550	A	T	4	5	--	--
	Ca(ICCV2/PI489777)SNP_08679	619	T	C	8	6	--	--
	Ca(ICCV2/PI489777)SNP_08680	683	T	C	8	4	--	--
	Ca(ICCV2/PI489777)SNP_08681	1181	A	G	7	4	--	--
CakTC39137	Ca(ICCV2/PI489777)SNP_08682	113	C	T	4	3	Shoot	--
	Ca(ICCV2/PI489777)SNP_08683	407	A	G	5	6	Shoot	--
	Ca(ICCV2/PI489777)SNP_08684	447	T	C	4	6	Shoot	--
	Ca(ICCV2/PI489777)SNP_08685	1219	A	G	4	4	Shoot	--
CakTC27193	Ca(ICCV2/PI489777)SNP_08686	341	C	T	10	7	--	--
	Ca(ICCV2/PI489777)SNP_08687	716	G	A	11	7	--	--
	Ca(ICCV2/PI489777)SNP_08688	737	T	C	12	8	--	--
	Ca(ICCV2/PI489777)SNP_08689	942	C	T	12	4	--	--
CakTC38070	Ca(ICCV2/PI489777)SNP_08690	1202	A	G	3	5	--	--
CakTC36037	Ca(ICCV2/PI489777)SNP_08691	363	G	A	3	3	--	--
CakTC31450	Ca(ICCV2/PI489777)SNP_08692	769	T	C	3	5	--	--
CakTC42905	Ca(ICCV2/PI489777)SNP_08693	381	T	G	25	14	--	--
	Ca(ICCV2/PI489777)SNP_08694	420	G	A	19	13	--	--
CakTC14736	Ca(ICCV2/PI489777)SNP_08695	1371	A	T	17	16	--	--
	Ca(ICCV2/PI489777)SNP_08696	1752	A	C	19	28	--	--
	Ca(ICCV2/PI489777)SNP_08697	2738	C	T	92	58	--	--
	Ca(ICCV2/PI489777)SNP_08698	3136	T	C	9	8	--	--
	Ca(ICCV2/PI489777)SNP_08699	3213	C	A	10	6	--	--
CakTC27311	Ca(ICCV2/PI489777)SNP_08700	734	G	A	4	39	--	--
	Ca(ICCV2/PI489777)SNP_08701	1455	A	G	12	3	--	--
	Ca(ICCV2/PI489777)SNP_08702	2958	A	C	5	6	--	--
	Ca(ICCV2/PI489777)SNP_08703	3403	A	G	13	3	--	--
	Ca(ICCV2/PI489777)SNP_08704	4191	T	C	7	5	--	--
CakTC34257	Ca(ICCV2/PI489777)SNP_08705	194	G	A	5	7	--	--
CakTC42339	Ca(ICCV2/PI489777)SNP_08706	733	G	A	34	10	--	--
	Ca(ICCV2/PI489777)SNP_08707	1040	C	A	6	5	--	--
CakTC34007	Ca(ICCV2/PI489777)SNP_08708	81	A	G	10	19	--	--
CakTC25212	Ca(ICCV2/PI489777)SNP_08709	596	T	C	10	8	--	--
	Ca(ICCV2/PI489777)SNP_08710	1649	C	T	22	8	--	--
	Ca(ICCV2/PI489777)SNP_08711	1817	C	T	24	8	--	--
	Ca(ICCV2/PI489777)SNP_08712	2504	A	C	21	8	--	--
CakTC23008	Ca(ICCV2/PI489777)SNP_08713	347	A	G	59	54	--	--
CakTC39622	Ca(ICCV2/PI489777)SNP_08714	710	A	G	11	18	--	bZIP
CakTC34053	Ca(ICCV2/PI489777)SNP_08715	357	A	G	16	4	--	Sigma70-like
	Ca(ICCV2/PI489777)SNP_08716	1254	T	C	15	4	--	Sigma70-like
	Ca(ICCV2/PI489777)SNP_08717	1377	C	T	22	4	--	Sigma70-like
	Ca(ICCV2/PI489777)SNP_08718	1897	T	A	18	6	--	Sigma70-like
CakTC25690	Ca(ICCV2/PI489777)SNP_08719	604	T	C	35	48	--	--
CakTC37144	Ca(ICCV2/PI489777)SNP_08720	378	C	A	37	26	--	--
	Ca(ICCV2/PI489777)SNP_08721	991	C	T	104	42	--	--
CakTC27051	Ca(ICCV2/PI489777)SNP_08722	212	C	A	21	18	--	TPR
	Ca(ICCV2/PI489777)SNP_08723	262	C	T	25	21	--	TPR
	Ca(ICCV2/PI489777)SNP_08724	1416	T	C	21	9	--	TPR
	Ca(ICCV2/PI489777)SNP_08725	1863	G	A	23	22	--	TPR
	Ca(ICCV2/PI489777)SNP_08726	2412	C	T	27	24	--	TPR
	Ca(ICCV2/PI489777)SNP_08727	2667	C	T	42	17	--	TPR

	Ca(ICCV2/PI489777)SNP_08728	2790	C	T	35	19	--	TPR
	Ca(ICCV2/PI489777)SNP_08729	3024	T	G	28	18	--	TPR
CakTC05727	Ca(ICCV2/PI489777)SNP_08730	235	C	T	4	4	--	--
CakTC16639	Ca(ICCV2/PI489777)SNP_08731	777	A	G	27	9	--	--
	Ca(ICCV2/PI489777)SNP_08732	2753	A	T	15	5	--	--
CakTC12506	Ca(ICCV2/PI489777)SNP_08733	1070	T	C	5	8	Young_pod	--
CakTC28215	Ca(ICCV2/PI489777)SNP_08734	1281	G	A	12	3	--	--
	Ca(ICCV2/PI489777)SNP_08735	1714	T	C	6	4	--	--
CakTC09279	Ca(ICCV2/PI489777)SNP_08736	991	T	A	6	4	--	--
CakTC30533	Ca(ICCV2/PI489777)SNP_08737	99	G	T	3	5	--	--
	Ca(ICCV2/PI489777)SNP_08738	271	A	G	4	5	--	--
	Ca(ICCV2/PI489777)SNP_08739	1291	C	T	5	6	--	--
	Ca(ICCV2/PI489777)SNP_08740	1408	T	C	5	5	--	--
CakTC38270	Ca(ICCV2/PI489777)SNP_08741	400	C	A	9	19	Root	--
	Ca(ICCV2/PI489777)SNP_08742	463	G	A	11	21	Root	--
	Ca(ICCV2/PI489777)SNP_08743	701	G	A	19	14	Root	--
CakTC26424	Ca(ICCV2/PI489777)SNP_08744	98	G	A	19	14	--	--
	Ca(ICCV2/PI489777)SNP_08745	119	C	T	20	14	--	--
	Ca(ICCV2/PI489777)SNP_08746	137	T	A	20	14	--	--
	Ca(ICCV2/PI489777)SNP_08747	1225	A	G	7	15	--	--
CakTC40621	Ca(ICCV2/PI489777)SNP_08748	143	A	T	81	8	--	--
	Ca(ICCV2/PI489777)SNP_08749	476	G	A	169	45	--	--
	Ca(ICCV2/PI489777)SNP_08750	532	C	T	181	58	--	--
	Ca(ICCV2/PI489777)SNP_08751	1657	C	T	148	52	--	--
	Ca(ICCV2/PI489777)SNP_08752	1691	G	T	146	43	--	--
	Ca(ICCV2/PI489777)SNP_08753	1697	C	A	141	39	--	--
	Ca(ICCV2/PI489777)SNP_08754	1787	A	G	118	37	--	--
CakTC40841	Ca(ICCV2/PI489777)SNP_08755	67	G	A	6	4	--	--
	Ca(ICCV2/PI489777)SNP_08756	71	C	G	6	4	--	--
	Ca(ICCV2/PI489777)SNP_08757	702	A	G	17	6	--	--
	Ca(ICCV2/PI489777)SNP_08758	1284	T	C	17	12	--	--
	Ca(ICCV2/PI489777)SNP_08759	1321	C	G	20	12	--	--
	Ca(ICCV2/PI489777)SNP_08760	1338	C	T	18	15	--	--
	Ca(ICCV2/PI489777)SNP_08761	1542	T	C	19	12	--	--
	Ca(ICCV2/PI489777)SNP_08762	1693	G	C	11	6	--	--
CakTC25391	Ca(ICCV2/PI489777)SNP_08763	323	A	G	16	17	--	--
	Ca(ICCV2/PI489777)SNP_08764	452	T	C	28	19	--	--
	Ca(ICCV2/PI489777)SNP_08765	821	T	C	26	14	--	--
CakTC36565	Ca(ICCV2/PI489777)SNP_08766	838	A	G	10	8	--	--
	Ca(ICCV2/PI489777)SNP_08767	1429	A	G	5	3	--	--
	Ca(ICCV2/PI489777)SNP_08768	1523	C	T	3	4	--	--
	Ca(ICCV2/PI489777)SNP_08769	1879	G	A	8	4	--	--
	Ca(ICCV2/PI489777)SNP_08770	2330	A	C	11	10	--	--
	Ca(ICCV2/PI489777)SNP_08771	2418	C	T	10	9	--	--
CakTC07464	Ca(ICCV2/PI489777)SNP_08772	450	T	C	4	12	--	--
	Ca(ICCV2/PI489777)SNP_08773	460	C	T	4	12	--	--
	Ca(ICCV2/PI489777)SNP_08774	479	T	C	5	11	--	--
	Ca(ICCV2/PI489777)SNP_08775	842	G	A	7	3	--	--
	Ca(ICCV2/PI489777)SNP_08776	1169	A	G	7	8	--	--
	Ca(ICCV2/PI489777)SNP_08777	1724	T	G	3	7	--	--
	Ca(ICCV2/PI489777)SNP_08778	1739	G	A	3	7	--	--
	Ca(ICCV2/PI489777)SNP_08779	2027	T	C	4	3	--	--
CakTC34354	Ca(ICCV2/PI489777)SNP_08780	2232	T	C	7	4	--	PHD
	Ca(ICCV2/PI489777)SNP_08781	5357	T	C	13	6	--	PHD
	Ca(ICCV2/PI489777)SNP_08782	5504	C	T	11	4	--	PHD
	Ca(ICCV2/PI489777)SNP_08783	7865	G	A	3	3	--	PHD
CakTC30228	Ca(ICCV2/PI489777)SNP_08784	228	A	G	6	7	--	--
	Ca(ICCV2/PI489777)SNP_08785	713	T	G	4	8	--	--
CakTC35635	Ca(ICCV2/PI489777)SNP_08786	1364	A	G	13	14	--	--
	Ca(ICCV2/PI489777)SNP_08787	2041	C	T	8	4	--	--
	Ca(ICCV2/PI489777)SNP_08788	2902	T	C	13	7	--	--
	Ca(ICCV2/PI489777)SNP_08789	3010	A	G	11	4	--	--
CakTC41243	Ca(ICCV2/PI489777)SNP_08790	2184	G	T	35	34	--	--
	Ca(ICCV2/PI489777)SNP_08791	2185	A	C	33	34	--	--
CakTC28314	Ca(ICCV2/PI489777)SNP_08792	2415	A	G	12	3	--	--
	Ca(ICCV2/PI489777)SNP_08793	2460	A	G	17	3	--	--
	Ca(ICCV2/PI489777)SNP_08794	2750	G	A	20	5	--	--
	Ca(ICCV2/PI489777)SNP_08795	3094	G	A	18	3	--	--
CakTC34546	Ca(ICCV2/PI489777)SNP_08796	142	A	G	7	7	--	--
	Ca(ICCV2/PI489777)SNP_08797	1389	A	G	8	4	--	--
	Ca(ICCV2/PI489777)SNP_08798	1404	C	A	6	3	--	--
	Ca(ICCV2/PI489777)SNP_08799	1455	G	A	6	5	--	--
	Ca(ICCV2/PI489777)SNP_08800	1920	C	A	10	6	--	--
	Ca(ICCV2/PI489777)SNP_08801	1962	A	C	9	5	--	--

	Ca(ICCV2/PI489777)SNP_08802	1995	A	C	7	4	--	--
	Ca(ICCV2/PI489777)SNP_08803	2062	C	A	5	4	--	--
	Ca(ICCV2/PI489777)SNP_08804	2066	G	A	5	4	--	--
CakTC22844	Ca(ICCV2/PI489777)SNP_08805	911	T	A	13	9	--	--
CakTC42660	Ca(ICCV2/PI489777)SNP_08806	1390	C	T	5	4	--	--
CakTC07690	Ca(ICCV2/PI489777)SNP_08807	1307	T	C	6	3	--	--
CakTC28031	Ca(ICCV2/PI489777)SNP_08808	518	C	T	9	3	--	--
	Ca(ICCV2/PI489777)SNP_08809	520	A	G	11	3	--	--
	Ca(ICCV2/PI489777)SNP_08810	534	T	C	12	3	--	--
	Ca(ICCV2/PI489777)SNP_08811	1013	A	G	19	6	--	--
	Ca(ICCV2/PI489777)SNP_08812	1346	G	C	13	6	--	--
CakTC38941	Ca(ICCV2/PI489777)SNP_08813	42	C	A	4	4	--	--
	Ca(ICCV2/PI489777)SNP_08814	158	A	G	6	5	--	--
	Ca(ICCV2/PI489777)SNP_08815	350	C	A	9	7	--	--
	Ca(ICCV2/PI489777)SNP_08816	614	A	G	15	12	--	--
	Ca(ICCV2/PI489777)SNP_08817	794	C	T	17	12	--	--
	Ca(ICCV2/PI489777)SNP_08818	839	A	G	22	10	--	--
	Ca(ICCV2/PI489777)SNP_08819	905	G	A	14	14	--	--
	Ca(ICCV2/PI489777)SNP_08820	1352	A	G	16	14	--	--
	Ca(ICCV2/PI489777)SNP_08821	1421	T	C	17	11	--	--
	Ca(ICCV2/PI489777)SNP_08822	1464	A	G	13	7	--	--
CakTC40749	Ca(ICCV2/PI489777)SNP_08823	423	T	C	12	4	--	--
	Ca(ICCV2/PI489777)SNP_08824	615	T	A	9	6	--	--
	Ca(ICCV2/PI489777)SNP_08825	1314	C	T	25	9	--	--
	Ca(ICCV2/PI489777)SNP_08826	1738	G	A	12	5	--	--
	Ca(ICCV2/PI489777)SNP_08827	1820	C	T	12	3	--	--
CakTC28678	Ca(ICCV2/PI489777)SNP_08828	145	A	T	11	3	--	--
	Ca(ICCV2/PI489777)SNP_08829	305	G	A	12	8	--	--
	Ca(ICCV2/PI489777)SNP_08830	315	C	T	9	7	--	--
	Ca(ICCV2/PI489777)SNP_08831	575	G	T	9	12	--	--
	Ca(ICCV2/PI489777)SNP_08832	728	A	G	9	16	--	--
	Ca(ICCV2/PI489777)SNP_08833	761	A	G	8	15	--	--
CakTC38611	Ca(ICCV2/PI489777)SNP_08834	260	A	T	16	3	--	--
	Ca(ICCV2/PI489777)SNP_08835	266	G	A	13	3	--	--
	Ca(ICCV2/PI489777)SNP_08836	272	C	G	16	3	--	--
	Ca(ICCV2/PI489777)SNP_08837	284	C	T	16	3	--	--
	Ca(ICCV2/PI489777)SNP_08838	292	A	G	15	3	--	--
	Ca(ICCV2/PI489777)SNP_08839	662	T	C	17	5	--	--
CakTC41927	Ca(ICCV2/PI489777)SNP_08840	44	A	T	4	7	--	--
	Ca(ICCV2/PI489777)SNP_08841	344	C	T	8	10	--	--
	Ca(ICCV2/PI489777)SNP_08842	401	C	T	9	9	--	--
	Ca(ICCV2/PI489777)SNP_08843	502	A	G	6	4	--	--
CakTC36906	Ca(ICCV2/PI489777)SNP_08844	378	C	T	7	6	--	--
	Ca(ICCV2/PI489777)SNP_08845	379	A	G	7	6	--	--
	Ca(ICCV2/PI489777)SNP_08846	428	C	T	12	10	--	--
	Ca(ICCV2/PI489777)SNP_08847	476	A	G	13	12	--	--
	Ca(ICCV2/PI489777)SNP_08848	783	G	A	24	10	--	--
	Ca(ICCV2/PI489777)SNP_08849	1188	A	G	23	12	--	--
	Ca(ICCV2/PI489777)SNP_08850	1503	A	G	26	8	--	--
	Ca(ICCV2/PI489777)SNP_08851	1686	A	G	23	17	--	--
	Ca(ICCV2/PI489777)SNP_08852	1829	G	T	39	16	--	--
CakTC24168	Ca(ICCV2/PI489777)SNP_08853	572	A	G	3	4	--	--
CakTC38773	Ca(ICCV2/PI489777)SNP_08854	212	A	G	4	3	--	--
	Ca(ICCV2/PI489777)SNP_08855	502	A	G	7	4	--	--
	Ca(ICCV2/PI489777)SNP_08856	766	C	A	7	5	--	--
	Ca(ICCV2/PI489777)SNP_08857	801	C	T	6	7	--	--
	Ca(ICCV2/PI489777)SNP_08858	805	C	T	6	7	--	--
	Ca(ICCV2/PI489777)SNP_08859	821	T	C	6	6	--	--
	Ca(ICCV2/PI489777)SNP_08860	986	T	C	4	4	--	--
	Ca(ICCV2/PI489777)SNP_08861	1012	A	G	3	6	--	--
	Ca(ICCV2/PI489777)SNP_08862	1053	A	C	4	4	--	--
CakTC26613	Ca(ICCV2/PI489777)SNP_08863	549	G	T	14	20	--	--
	Ca(ICCV2/PI489777)SNP_08864	552	C	T	12	20	--	--
	Ca(ICCV2/PI489777)SNP_08865	745	A	T	23	27	--	--
	Ca(ICCV2/PI489777)SNP_08866	814	A	C	24	27	--	--
	Ca(ICCV2/PI489777)SNP_08867	846	G	A	26	31	--	--
	Ca(ICCV2/PI489777)SNP_08868	1125	G	A	36	30	--	--
	Ca(ICCV2/PI489777)SNP_08869	1307	T	A	35	28	--	--
	Ca(ICCV2/PI489777)SNP_08870	1472	C	T	22	21	--	--
	Ca(ICCV2/PI489777)SNP_08871	1565	C	T	18	18	--	--
	Ca(ICCV2/PI489777)SNP_08872	1586	T	A	22	13	--	--
	Ca(ICCV2/PI489777)SNP_08873	1628	C	T	17	17	--	--
	Ca(ICCV2/PI489777)SNP_08874	1643	C	T	26	18	--	--
	Ca(ICCV2/PI489777)SNP_08875	2155	A	G	24	13	--	--

	Ca(ICCV2/PI489777)SNP_08876	2195	T	A	6	6	--	--
	Ca(ICCV2/PI489777)SNP_08877	2205	A	T	9	6	--	--
CakTC34416	Ca(ICCV2/PI489777)SNP_08878	992	A	G	3	6	--	--
	Ca(ICCV2/PI489777)SNP_08879	1153	A	C	5	6	--	--
	Ca(ICCV2/PI489777)SNP_08880	1745	G	A	7	6	--	--
	Ca(ICCV2/PI489777)SNP_08881	1748	A	G	7	6	--	--
CakTC41068	Ca(ICCV2/PI489777)SNP_08882	189	C	T	7	6	--	--
	Ca(ICCV2/PI489777)SNP_08883	192	G	C	7	7	--	--
CakTC42456	Ca(ICCV2/PI489777)SNP_08884	606	G	C	63	7	--	--
	Ca(ICCV2/PI489777)SNP_08885	632	C	T	66	5	--	--
	Ca(ICCV2/PI489777)SNP_08886	866	A	G	61	7	--	--
	Ca(ICCV2/PI489777)SNP_08887	1250	T	C	37	5	--	--
	Ca(ICCV2/PI489777)SNP_08888	1289	C	T	42	7	--	--
CakTC28366	Ca(ICCV2/PI489777)SNP_08889	1038	C	T	6	5	--	--
CakTC10386	Ca(ICCV2/PI489777)SNP_08890	742	G	A	7	3	--	--
CakTC40418	Ca(ICCV2/PI489777)SNP_08891	338	C	T	4	6	--	--
	Ca(ICCV2/PI489777)SNP_08892	411	A	G	6	7	--	--
	Ca(ICCV2/PI489777)SNP_08893	768	C	T	6	3	--	--
CakTC29680	Ca(ICCV2/PI489777)SNP_08894	291	G	A	34	34	--	--
	Ca(ICCV2/PI489777)SNP_08895	1200	G	A	32	28	--	--
	Ca(ICCV2/PI489777)SNP_08896	1461	G	T	26	34	--	--
	Ca(ICCV2/PI489777)SNP_08897	1581	G	A	24	27	--	--
	Ca(ICCV2/PI489777)SNP_08898	1722	G	T	17	19	--	--
CakTC21047	Ca(ICCV2/PI489777)SNP_08899	1560	T	C	8	6	--	--
	Ca(ICCV2/PI489777)SNP_08900	1561	T	A	8	6	--	--
	Ca(ICCV2/PI489777)SNP_08901	1592	C	G	12	6	--	--
	Ca(ICCV2/PI489777)SNP_08902	1708	G	A	13	5	--	--
CakTC09187	Ca(ICCV2/PI489777)SNP_08903	90	T	C	3	9	--	--
CakTC38761	Ca(ICCV2/PI489777)SNP_08904	207	G	A	3	19	--	bHLH
	Ca(ICCV2/PI489777)SNP_08905	224	G	A	5	19	--	bHLH
CakTC35798	Ca(ICCV2/PI489777)SNP_08906	77	A	T	5	4	--	--
CakTC41185	Ca(ICCV2/PI489777)SNP_08907	1358	C	G	31	16	--	--
	Ca(ICCV2/PI489777)SNP_08908	1571	C	T	27	11	--	--
	Ca(ICCV2/PI489777)SNP_08909	1640	T	C	21	11	--	--
	Ca(ICCV2/PI489777)SNP_08910	1805	C	T	33	15	--	--
	Ca(ICCV2/PI489777)SNP_08911	1857	A	G	40	18	--	--
	Ca(ICCV2/PI489777)SNP_08912	1890	G	C	34	17	--	--
	Ca(ICCV2/PI489777)SNP_08913	1903	C	T	34	18	--	--
	Ca(ICCV2/PI489777)SNP_08914	1913	A	G	33	16	--	--
	Ca(ICCV2/PI489777)SNP_08915	2139	G	A	10	11	--	--
CakTC22909	Ca(ICCV2/PI489777)SNP_08916	689	A	G	27	10	--	--
	Ca(ICCV2/PI489777)SNP_08917	1055	A	G	12	4	--	--
	Ca(ICCV2/PI489777)SNP_08918	1841	A	C	28	20	--	--
	Ca(ICCV2/PI489777)SNP_08919	2668	G	C	13	7	--	--
CakTC11548	Ca(ICCV2/PI489777)SNP_08920	371	A	G	9	3	--	--
CakTC12493	Ca(ICCV2/PI489777)SNP_08921	34	C	A	6	3	--	TPR
	Ca(ICCV2/PI489777)SNP_08922	35	T	C	6	3	--	TPR
	Ca(ICCV2/PI489777)SNP_08923	80	C	A	8	4	--	TPR
	Ca(ICCV2/PI489777)SNP_08924	544	C	T	10	5	--	TPR
	Ca(ICCV2/PI489777)SNP_08925	756	G	T	5	4	--	TPR
CakTC42833	Ca(ICCV2/PI489777)SNP_08926	1789	T	C	4	4	--	C2H2
	Ca(ICCV2/PI489777)SNP_08927	1800	C	T	4	4	--	C2H2
CakTC10059	Ca(ICCV2/PI489777)SNP_08928	1905	C	T	19	5	--	--
	Ca(ICCV2/PI489777)SNP_08929	2191	C	A	18	3	--	--
	Ca(ICCV2/PI489777)SNP_08930	3291	C	T	10	17	--	--
	Ca(ICCV2/PI489777)SNP_08931	3508	A	T	10	11	--	--
CakTC40839	Ca(ICCV2/PI489777)SNP_08932	819	T	C	20	4	--	--
	Ca(ICCV2/PI489777)SNP_08933	906	T	C	18	8	--	--
CakTC10678	Ca(ICCV2/PI489777)SNP_08934	162	T	C	4	3	--	--
CakTC38821	Ca(ICCV2/PI489777)SNP_08935	573	G	A	9	8	--	--
	Ca(ICCV2/PI489777)SNP_08936	664	C	G	8	10	--	--
	Ca(ICCV2/PI489777)SNP_08937	1002	A	G	22	4	--	--
	Ca(ICCV2/PI489777)SNP_08938	1174	A	G	21	4	--	--
	Ca(ICCV2/PI489777)SNP_08939	1194	G	C	21	4	--	--
CakTC28120	Ca(ICCV2/PI489777)SNP_08940	286	T	C	10	3	--	--
	Ca(ICCV2/PI489777)SNP_08941	1149	T	C	12	6	--	--
	Ca(ICCV2/PI489777)SNP_08942	1772	G	A	3	3	--	--
	Ca(ICCV2/PI489777)SNP_08943	3143	T	C	34	5	--	--
CakTC38915	Ca(ICCV2/PI489777)SNP_08944	235	G	A	29	18	--	--
CakTC43135	Ca(ICCV2/PI489777)SNP_08945	100	G	T	28	32	--	--
	Ca(ICCV2/PI489777)SNP_08946	354	C	A	31	36	--	--
	Ca(ICCV2/PI489777)SNP_08947	564	A	G	15	16	--	--
	Ca(ICCV2/PI489777)SNP_08948	878	A	G	5	4	--	--
CakTC08183	Ca(ICCV2/PI489777)SNP_08949	298	G	A	5	10	--	--

	Ca(ICCV2/PI489777)SNP_08950	415	G	A	6	10	--	--
	Ca(ICCV2/PI489777)SNP_08951	504	T	A	5	7	--	--
	Ca(ICCV2/PI489777)SNP_08952	558	T	G	6	5	--	--
CakTC37636	Ca(ICCV2/PI489777)SNP_08953	790	C	T	12	12	--	--
	Ca(ICCV2/PI489777)SNP_08954	1274	C	G	15	8	--	--
CakTC41345	Ca(ICCV2/PI489777)SNP_08955	714	T	C	8	3	Mature	Leaf
	Ca(ICCV2/PI489777)SNP_08956	891	T	C	10	8	Mature	Leaf
	Ca(ICCV2/PI489777)SNP_08957	1074	A	C	14	10	Mature	Leaf
	Ca(ICCV2/PI489777)SNP_08958	1176	C	T	15	9	Mature	Leaf
CakTC37839	Ca(ICCV2/PI489777)SNP_08959	230	G	A	14	9	--	--
	Ca(ICCV2/PI489777)SNP_08960	295	T	G	18	15	--	--
	Ca(ICCV2/PI489777)SNP_08961	415	C	T	23	28	--	--
	Ca(ICCV2/PI489777)SNP_08962	424	T	A	16	29	--	--
	Ca(ICCV2/PI489777)SNP_08963	487	G	A	24	35	--	--
	Ca(ICCV2/PI489777)SNP_08964	1099	T	C	34	22	--	--
	Ca(ICCV2/PI489777)SNP_08965	1480	T	A	33	16	--	--
	Ca(ICCV2/PI489777)SNP_08966	1590	T	C	27	5	--	--
	Ca(ICCV2/PI489777)SNP_08967	1628	G	C	10	3	--	--
CakTC24904	Ca(ICCV2/PI489777)SNP_08968	1227	C	T	49	14	--	AP2-EREBP
CakTC26757	Ca(ICCV2/PI489777)SNP_08969	723	G	A	8	8	--	--
	Ca(ICCV2/PI489777)SNP_08970	906	A	G	9	3	--	--
	Ca(ICCV2/PI489777)SNP_08971	969	T	C	8	3	--	--
	Ca(ICCV2/PI489777)SNP_08972	1156	C	T	11	5	--	--
CakTC35906	Ca(ICCV2/PI489777)SNP_08973	2784	C	T	13	9	--	--
CakTC28203	Ca(ICCV2/PI489777)SNP_08974	616	A	G	31	4	--	--
	Ca(ICCV2/PI489777)SNP_08975	659	T	G	29	5	--	--
	Ca(ICCV2/PI489777)SNP_08976	724	A	G	32	6	--	--
	Ca(ICCV2/PI489777)SNP_08977	897	A	G	29	10	--	--
	Ca(ICCV2/PI489777)SNP_08978	989	A	C	3	11	--	--
	Ca(ICCV2/PI489777)SNP_08979	1181	C	T	5	14	--	--
	Ca(ICCV2/PI489777)SNP_08980	1506	A	G	6	10	--	--
	Ca(ICCV2/PI489777)SNP_08981	1515	A	G	7	9	--	--
	Ca(ICCV2/PI489777)SNP_08982	1520	G	C	8	9	--	--
	Ca(ICCV2/PI489777)SNP_08983	1525	T	C	7	10	--	--
CakTC11382	Ca(ICCV2/PI489777)SNP_08984	118	C	T	10	8	--	--
	Ca(ICCV2/PI489777)SNP_08985	147	G	C	11	11	--	--
	Ca(ICCV2/PI489777)SNP_08986	199	C	T	11	11	--	--
	Ca(ICCV2/PI489777)SNP_08987	213	T	A	11	10	--	--
	Ca(ICCV2/PI489777)SNP_08988	262	A	G	11	12	--	--
CakTC24606	Ca(ICCV2/PI489777)SNP_08989	166	G	C	10	10	--	--
	Ca(ICCV2/PI489777)SNP_08990	520	A	G	27	8	--	--
	Ca(ICCV2/PI489777)SNP_08991	699	A	G	34	4	--	--
	Ca(ICCV2/PI489777)SNP_08992	743	G	A	40	3	--	--
CakTC31307	Ca(ICCV2/PI489777)SNP_08993	1282	C	G	5	3	--	--
CakTC23297	Ca(ICCV2/PI489777)SNP_08994	1352	T	C	3	4	--	--
	Ca(ICCV2/PI489777)SNP_08995	1542	A	G	3	4	--	--
CakTC11105	Ca(ICCV2/PI489777)SNP_08996	1167	T	C	6	8	--	--
	Ca(ICCV2/PI489777)SNP_08997	1494	C	T	8	5	--	--
	Ca(ICCV2/PI489777)SNP_08998	1857	A	G	4	7	--	--
	Ca(ICCV2/PI489777)SNP_08999	1886	A	G	4	7	--	--
CakTC31750	Ca(ICCV2/PI489777)SNP_09000	210	C	G	7	4	--	--
	Ca(ICCV2/PI489777)SNP_09001	213	C	G	7	4	--	--
	Ca(ICCV2/PI489777)SNP_09002	280	A	G	4	3	--	--
CakTC30765	Ca(ICCV2/PI489777)SNP_09003	1650	A	T	8	11	--	--
	Ca(ICCV2/PI489777)SNP_09004	1656	T	G	8	14	--	--
	Ca(ICCV2/PI489777)SNP_09005	1704	T	C	7	17	--	--
	Ca(ICCV2/PI489777)SNP_09006	2754	T	G	11	4	--	--
	Ca(ICCV2/PI489777)SNP_09007	3068	G	A	5	6	--	--
	Ca(ICCV2/PI489777)SNP_09008	3132	T	C	5	8	--	--
	Ca(ICCV2/PI489777)SNP_09009	3886	A	T	8	3	--	--
	Ca(ICCV2/PI489777)SNP_09010	4257	T	A	11	6	--	--
CakTC11508	Ca(ICCV2/PI489777)SNP_09011	171	A	T	24	15	--	--
CakTC36206	Ca(ICCV2/PI489777)SNP_09012	1906	T	C	13	10	--	--
CakTC38803	Ca(ICCV2/PI489777)SNP_09013	729	T	C	12	4	--	MYB
	Ca(ICCV2/PI489777)SNP_09014	1050	T	C	12	3	--	MYB
	Ca(ICCV2/PI489777)SNP_09015	1135	G	T	13	5	--	MYB
	Ca(ICCV2/PI489777)SNP_09016	1163	C	T	12	5	--	MYB
	Ca(ICCV2/PI489777)SNP_09017	1175	T	C	13	5	--	MYB
CakTC15441	Ca(ICCV2/PI489777)SNP_09018	355	G	T	3	4	--	--
CakTC37390	Ca(ICCV2/PI489777)SNP_09019	96	C	T	5	4	--	--
	Ca(ICCV2/PI489777)SNP_09020	182	T	C	5	20	--	--
	Ca(ICCV2/PI489777)SNP_09021	209	T	C	4	22	--	--
	Ca(ICCV2/PI489777)SNP_09022	211	G	A	3	21	--	--
	Ca(ICCV2/PI489777)SNP_09023	896	T	G	23	41	--	--

	Ca(ICCV2/PI489777)SNP_09024	986	C	T	24	41	--	--
	Ca(ICCV2/PI489777)SNP_09025	1072	G	A	27	36	--	--
CakTC27466	Ca(ICCV2/PI489777)SNP_09026	567	C	T	81	29	--	--
	Ca(ICCV2/PI489777)SNP_09027	783	C	T	60	28	--	--
	Ca(ICCV2/PI489777)SNP_09028	882	C	T	62	23	--	--
	Ca(ICCV2/PI489777)SNP_09029	960	C	T	44	12	--	--
CakTC24428	Ca(ICCV2/PI489777)SNP_09030	191	T	C	9	4	--	--
	Ca(ICCV2/PI489777)SNP_09031	250	C	T	10	7	--	--
	Ca(ICCV2/PI489777)SNP_09032	388	A	G	12	8	--	--
	Ca(ICCV2/PI489777)SNP_09033	572	G	A	16	6	--	--
	Ca(ICCV2/PI489777)SNP_09034	679	G	A	16	5	--	--
	Ca(ICCV2/PI489777)SNP_09035	694	G	T	16	6	--	--
CakTC28292	Ca(ICCV2/PI489777)SNP_09036	787	G	T	3	4	--	--
CakTC40770	Ca(ICCV2/PI489777)SNP_09037	307	A	G	26	31	--	--
	Ca(ICCV2/PI489777)SNP_09038	400	C	A	25	24	--	--
	Ca(ICCV2/PI489777)SNP_09039	735	T	A	27	17	--	--
	Ca(ICCV2/PI489777)SNP_09040	774	C	G	27	16	--	--
	Ca(ICCV2/PI489777)SNP_09041	815	C	T	23	6	--	--
CakTC42021	Ca(ICCV2/PI489777)SNP_09042	332	A	G	13	6	--	--
	Ca(ICCV2/PI489777)SNP_09043	339	A	T	14	9	--	--
	Ca(ICCV2/PI489777)SNP_09044	822	C	T	8	15	--	--
CakTC37812	Ca(ICCV2/PI489777)SNP_09045	494	T	C	5	22	--	--
	Ca(ICCV2/PI489777)SNP_09046	536	A	G	6	21	--	--
	Ca(ICCV2/PI489777)SNP_09047	734	C	G	7	15	--	--
	Ca(ICCV2/PI489777)SNP_09048	797	A	C	7	18	--	--
	Ca(ICCV2/PI489777)SNP_09049	893	C	A	7	11	--	--
	Ca(ICCV2/PI489777)SNP_09050	1106	G	A	6	7	--	--
	Ca(ICCV2/PI489777)SNP_09051	1731	C	T	4	4	--	--
CakTC40509	Ca(ICCV2/PI489777)SNP_09052	135	T	C	27	12	--	--
	Ca(ICCV2/PI489777)SNP_09053	420	C	A	52	15	--	--
CakTC23779	Ca(ICCV2/PI489777)SNP_09054	1877	A	G	3	3	--	--
CakTC23278	Ca(ICCV2/PI489777)SNP_09055	30	C	T	8	5	--	--
	Ca(ICCV2/PI489777)SNP_09056	144	C	A	16	10	--	--
	Ca(ICCV2/PI489777)SNP_09057	182	A	G	21	16	--	--
	Ca(ICCV2/PI489777)SNP_09058	206	G	A	20	15	--	--
CakTC36316	Ca(ICCV2/PI489777)SNP_09059	1617	G	A	51	33	--	--
	Ca(ICCV2/PI489777)SNP_09060	2421	T	A	32	32	--	--
CakTC35414	Ca(ICCV2/PI489777)SNP_09061	645	C	T	4	7	--	--
CakTC27653	Ca(ICCV2/PI489777)SNP_09062	691	A	G	10	10	--	--
	Ca(ICCV2/PI489777)SNP_09063	943	T	C	5	12	--	--
CakTC42690	Ca(ICCV2/PI489777)SNP_09064	761	C	T	29	7	--	--
CakTC35378	Ca(ICCV2/PI489777)SNP_09065	865	T	C	8	8	--	--
	Ca(ICCV2/PI489777)SNP_09066	1284	A	G	16	6	--	--
	Ca(ICCV2/PI489777)SNP_09067	1562	G	A	12	9	--	--
	Ca(ICCV2/PI489777)SNP_09068	1602	C	T	15	13	--	--
	Ca(ICCV2/PI489777)SNP_09069	1707	G	A	15	11	--	--
	Ca(ICCV2/PI489777)SNP_09070	2094	A	C	15	7	--	--
	Ca(ICCV2/PI489777)SNP_09071	2142	C	T	15	7	--	--
	Ca(ICCV2/PI489777)SNP_09072	2260	C	G	7	7	--	--
	Ca(ICCV2/PI489777)SNP_09073	3067	C	A	12	3	--	--
	Ca(ICCV2/PI489777)SNP_09074	3258	C	T	12	3	--	--
	Ca(ICCV2/PI489777)SNP_09075	3403	T	G	12	7	--	--
	Ca(ICCV2/PI489777)SNP_09076	4031	C	T	7	6	--	--
CakTC30577	Ca(ICCV2/PI489777)SNP_09077	645	G	A	23	6	--	C2C2-Dof
	Ca(ICCV2/PI489777)SNP_09078	722	C	T	25	6	--	C2C2-Dof
	Ca(ICCV2/PI489777)SNP_09079	789	T	A	18	5	--	C2C2-Dof
	Ca(ICCV2/PI489777)SNP_09080	825	T	C	14	5	--	C2C2-Dof
CakTC39376	Ca(ICCV2/PI489777)SNP_09081	226	T	G	9	3	--	--
	Ca(ICCV2/PI489777)SNP_09082	384	A	G	8	4	--	--
	Ca(ICCV2/PI489777)SNP_09083	390	A	G	8	5	--	--
	Ca(ICCV2/PI489777)SNP_09084	541	T	G	3	5	--	--
CakTC38852	Ca(ICCV2/PI489777)SNP_09085	350	G	T	3	3	--	--
	Ca(ICCV2/PI489777)SNP_09086	449	C	T	3	13	--	--
	Ca(ICCV2/PI489777)SNP_09087	630	C	A	8	12	--	--
	Ca(ICCV2/PI489777)SNP_09088	967	A	G	7	12	--	--
	Ca(ICCV2/PI489777)SNP_09089	1022	G	A	10	10	--	--
	Ca(ICCV2/PI489777)SNP_09090	1079	C	A	10	9	--	--
CakTC30337	Ca(ICCV2/PI489777)SNP_09091	41	G	A	4	3	--	--
	Ca(ICCV2/PI489777)SNP_09092	279	T	G	8	8	--	--
	Ca(ICCV2/PI489777)SNP_09093	310	C	T	8	8	--	--
	Ca(ICCV2/PI489777)SNP_09094	541	A	G	3	10	--	--
	Ca(ICCV2/PI489777)SNP_09095	675	A	T	3	5	--	--
CakTC40207	Ca(ICCV2/PI489777)SNP_09096	1289	C	G	8	4	--	--
CakTC27995	Ca(ICCV2/PI489777)SNP_09097	304	T	C	5	6	--	--

	Ca(ICCV2/PI489777)SNP_09098	430	C	T	9	5	--	--
	Ca(ICCV2/PI489777)SNP_09099	487	A	T	9	5	--	--
	Ca(ICCV2/PI489777)SNP_09100	736	C	T	8	5	--	--
CakTC30138	Ca(ICCV2/PI489777)SNP_09101	265	A	G	6	3	--	--
CakTC39937	Ca(ICCV2/PI489777)SNP_09102	55	G	C	8	4	--	--
	Ca(ICCV2/PI489777)SNP_09103	157	A	C	26	11	--	--
	Ca(ICCV2/PI489777)SNP_09104	332	A	G	37	21	--	--
	Ca(ICCV2/PI489777)SNP_09105	694	T	A	30	11	--	--
	Ca(ICCV2/PI489777)SNP_09106	1086	G	T	16	8	--	--
	Ca(ICCV2/PI489777)SNP_09107	1114	C	T	14	7	--	--
CakTC01036	Ca(ICCV2/PI489777)SNP_09108	3360	G	T	4	5	--	--
CakTC30941	Ca(ICCV2/PI489777)SNP_09109	201	G	T	7	5	Root	--
CakTC37546	Ca(ICCV2/PI489777)SNP_09110	415	C	T	10	4	--	--
CakTC42667	Ca(ICCV2/PI489777)SNP_09111	382	G	A	24	7	--	Trihelix
	Ca(ICCV2/PI489777)SNP_09112	1653	G	A	3	5	--	Trihelix
CakTC13101	Ca(ICCV2/PI489777)SNP_09113	264	A	G	12	11	--	--
	Ca(ICCV2/PI489777)SNP_09114	497	G	C	18	5	--	--
CakTC02895	Ca(ICCV2/PI489777)SNP_09115	81	A	G	3	3	--	--
CakTC39325	Ca(ICCV2/PI489777)SNP_09116	1361	G	C	8	4	--	G2-like
CakTC14943	Ca(ICCV2/PI489777)SNP_09117	89	C	A	14	20	--	--
	Ca(ICCV2/PI489777)SNP_09118	724	T	G	72	88	--	--
CakTC41188	Ca(ICCV2/PI489777)SNP_09119	59	C	G	3	3	--	--
	Ca(ICCV2/PI489777)SNP_09120	103	G	A	4	3	--	--
	Ca(ICCV2/PI489777)SNP_09121	160	G	T	4	3	--	--
	Ca(ICCV2/PI489777)SNP_09122	219	C	G	6	3	--	--
	Ca(ICCV2/PI489777)SNP_09123	319	A	G	6	3	--	--
	Ca(ICCV2/PI489777)SNP_09124	352	C	G	7	3	--	--
	Ca(ICCV2/PI489777)SNP_09125	381	C	T	7	9	--	--
	Ca(ICCV2/PI489777)SNP_09126	431	G	T	7	6	--	--
	Ca(ICCV2/PI489777)SNP_09127	976	A	G	4	4	--	--
	Ca(ICCV2/PI489777)SNP_09128	1436	A	G	10	6	--	--
	Ca(ICCV2/PI489777)SNP_09129	2207	A	T	8	7	--	--
CakTC27309	Ca(ICCV2/PI489777)SNP_09130	452	C	T	6	7	--	--
	Ca(ICCV2/PI489777)SNP_09131	1316	G	A	13	10	--	--
CakTC38413	Ca(ICCV2/PI489777)SNP_09132	170	G	A	38	18	--	--
	Ca(ICCV2/PI489777)SNP_09133	256	C	T	45	19	--	--
	Ca(ICCV2/PI489777)SNP_09134	639	A	G	34	8	--	--
	Ca(ICCV2/PI489777)SNP_09135	673	G	A	29	8	--	--
	Ca(ICCV2/PI489777)SNP_09136	925	A	G	31	12	--	--
	Ca(ICCV2/PI489777)SNP_09137	954	A	G	29	11	--	--
	Ca(ICCV2/PI489777)SNP_09138	1112	C	T	8	6	--	--
CakTC37398	Ca(ICCV2/PI489777)SNP_09139	801	C	T	53	22	--	--
	Ca(ICCV2/PI489777)SNP_09140	858	C	A	48	21	--	--
	Ca(ICCV2/PI489777)SNP_09141	864	T	C	50	16	--	--
	Ca(ICCV2/PI489777)SNP_09142	894	C	T	49	22	--	--
	Ca(ICCV2/PI489777)SNP_09143	930	C	T	41	32	--	--
	Ca(ICCV2/PI489777)SNP_09144	1287	T	C	24	21	--	--
CakTC38457	Ca(ICCV2/PI489777)SNP_09145	468	T	G	6	3	--	--
CakTC29812	Ca(ICCV2/PI489777)SNP_09146	239	C	T	3	3	--	TRAF
CakTC42309	Ca(ICCV2/PI489777)SNP_09147	560	T	C	51	18	--	--
	Ca(ICCV2/PI489777)SNP_09148	581	T	G	48	19	--	--
	Ca(ICCV2/PI489777)SNP_09149	744	C	T	20	6	--	--
CakTC35477	Ca(ICCV2/PI489777)SNP_09150	941	G	A	8	4	--	--
	Ca(ICCV2/PI489777)SNP_09151	1320	A	G	11	3	--	--
	Ca(ICCV2/PI489777)SNP_09152	2250	T	C	7	4	--	--
	Ca(ICCV2/PI489777)SNP_09153	2607	T	C	5	3	--	--
	Ca(ICCV2/PI489777)SNP_09154	2628	G	A	8	4	--	--
	Ca(ICCV2/PI489777)SNP_09155	2937	C	A	10	5	--	--
	Ca(ICCV2/PI489777)SNP_09156	3456	A	C	10	3	--	--
	Ca(ICCV2/PI489777)SNP_09157	3513	T	C	11	3	--	--
	Ca(ICCV2/PI489777)SNP_09158	3702	T	C	5	7	--	--
	Ca(ICCV2/PI489777)SNP_09159	3741	T	G	5	7	--	--
	Ca(ICCV2/PI489777)SNP_09160	3807	A	G	5	5	--	--
	Ca(ICCV2/PI489777)SNP_09161	3878	G	T	3	5	--	--
CakTC28756	Ca(ICCV2/PI489777)SNP_09162	409	G	C	5	7	Young_pod	--
	Ca(ICCV2/PI489777)SNP_09163	727	A	G	4	6	Young_pod	--
CakTC08721	Ca(ICCV2/PI489777)SNP_09164	545	C	T	3	3	--	--
CakTC36018	Ca(ICCV2/PI489777)SNP_09165	1303	C	T	13	8	--	--
CakTC28544	Ca(ICCV2/PI489777)SNP_09166	84	A	G	3	16	Root	--
	Ca(ICCV2/PI489777)SNP_09167	140	G	C	25	20	Root	--
	Ca(ICCV2/PI489777)SNP_09168	156	A	C	26	19	Root	--
	Ca(ICCV2/PI489777)SNP_09169	168	G	A	31	24	Root	--
	Ca(ICCV2/PI489777)SNP_09170	286	A	G	52	19	Root	--
	Ca(ICCV2/PI489777)SNP_09171	801	A	G	135	144	Root	--

	Ca(ICCV2/PI489777)SNP_09172	807	C	T	129	139	Root	--
	Ca(ICCV2/PI489777)SNP_09173	879	A	G	142	162	Root	--
	Ca(ICCV2/PI489777)SNP_09174	978	A	G	143	147	Root	--
	Ca(ICCV2/PI489777)SNP_09175	1275	G	A	64	55	Root	--
	Ca(ICCV2/PI489777)SNP_09176	1386	G	A	53	50	Root	--
	Ca(ICCV2/PI489777)SNP_09177	1443	C	A	48	45	Root	--
	Ca(ICCV2/PI489777)SNP_09178	1446	A	G	49	47	Root	--
CakTC35611	Ca(ICCV2/PI489777)SNP_09179	2839	G	A	9	4	--	--
CakTC26061	Ca(ICCV2/PI489777)SNP_09180	1346	A	T	7	4	Young_pod	--
	Ca(ICCV2/PI489777)SNP_09181	1431	C	T	8	4	Young_pod	--
CakTC31221	Ca(ICCV2/PI489777)SNP_09182	36	A	G	6	4	--	--
	Ca(ICCV2/PI489777)SNP_09183	72	T	A	7	4	--	--
	Ca(ICCV2/PI489777)SNP_09184	99	G	A	8	5	--	--
	Ca(ICCV2/PI489777)SNP_09185	921	T	G	4	4	--	--
CakTC37580	Ca(ICCV2/PI489777)SNP_09186	403	G	T	3	5	Root	--
	Ca(ICCV2/PI489777)SNP_09187	708	C	T	9	7	Root	--
	Ca(ICCV2/PI489777)SNP_09188	1008	C	T	7	9	Root	--
CakTC39651	Ca(ICCV2/PI489777)SNP_09189	341	A	G	23	13	--	--
	Ca(ICCV2/PI489777)SNP_09190	344	T	C	22	12	--	--
	Ca(ICCV2/PI489777)SNP_09191	494	A	G	22	16	--	--
	Ca(ICCV2/PI489777)SNP_09192	1303	G	C	5	4	--	--
CakTC23995	Ca(ICCV2/PI489777)SNP_09193	537	C	T	20	3	--	WRKY
CakTC30773	Ca(ICCV2/PI489777)SNP_09194	2085	A	C	3	4	Flower	bud
CakTC33942	Ca(ICCV2/PI489777)SNP_09195	89	C	T	6	5	Shoot	--
	Ca(ICCV2/PI489777)SNP_09196	563	C	T	4	4	Shoot	--
	Ca(ICCV2/PI489777)SNP_09197	565	A	G	4	4	Shoot	--
	Ca(ICCV2/PI489777)SNP_09198	671	A	G	3	5	Shoot	--
	Ca(ICCV2/PI489777)SNP_09199	1573	A	G	5	3	Shoot	--
	Ca(ICCV2/PI489777)SNP_09200	1728	T	G	4	3	Shoot	--
CakTC41903	Ca(ICCV2/PI489777)SNP_09201	591	T	C	3	5	--	--
	Ca(ICCV2/PI489777)SNP_09202	1026	G	T	10	6	--	--
	Ca(ICCV2/PI489777)SNP_09203	1295	C	T	7	5	--	--
	Ca(ICCV2/PI489777)SNP_09204	1392	G	A	6	9	--	--
	Ca(ICCV2/PI489777)SNP_09205	1419	A	T	6	11	--	--
	Ca(ICCV2/PI489777)SNP_09206	1526	A	C	3	11	--	--
	Ca(ICCV2/PI489777)SNP_09207	1535	C	T	3	11	--	--
	Ca(ICCV2/PI489777)SNP_09208	1622	G	A	5	8	--	--
	Ca(ICCV2/PI489777)SNP_09209	1662	G	T	5	8	--	--
	Ca(ICCV2/PI489777)SNP_09210	1665	G	A	4	8	--	--
	Ca(ICCV2/PI489777)SNP_09211	1725	T	G	5	7	--	--
	Ca(ICCV2/PI489777)SNP_09212	1792	C	G	5	6	--	--
CakTC27934	Ca(ICCV2/PI489777)SNP_09213	358	C	T	3	3	--	--
	Ca(ICCV2/PI489777)SNP_09214	422	G	A	3	3	--	--
	Ca(ICCV2/PI489777)SNP_09215	1331	T	C	5	4	--	--
	Ca(ICCV2/PI489777)SNP_09216	1386	A	G	6	4	--	--
CakTC32658	Ca(ICCV2/PI489777)SNP_09217	1048	G	C	16	9	--	--
	Ca(ICCV2/PI489777)SNP_09218	1795	A	C	22	27	--	--
	Ca(ICCV2/PI489777)SNP_09219	1992	T	G	23	23	--	--
	Ca(ICCV2/PI489777)SNP_09220	2149	T	C	23	18	--	--
CakTC32830	Ca(ICCV2/PI489777)SNP_09221	119	C	G	7	14	--	--
	Ca(ICCV2/PI489777)SNP_09222	463	G	T	10	9	--	--
	Ca(ICCV2/PI489777)SNP_09223	684	C	A	16	3	--	--
	Ca(ICCV2/PI489777)SNP_09224	804	C	T	16	3	--	--
	Ca(ICCV2/PI489777)SNP_09225	1380	G	A	19	9	--	--
	Ca(ICCV2/PI489777)SNP_09226	1440	A	T	20	7	--	--
	Ca(ICCV2/PI489777)SNP_09227	1441	A	C	20	7	--	--
	Ca(ICCV2/PI489777)SNP_09228	1646	A	C	22	7	--	--
	Ca(ICCV2/PI489777)SNP_09229	1716	T	C	20	5	--	--
	Ca(ICCV2/PI489777)SNP_09230	1835	A	G	13	5	--	--
	Ca(ICCV2/PI489777)SNP_09231	1845	A	G	12	5	--	--
	Ca(ICCV2/PI489777)SNP_09232	2429	T	C	17	6	--	--
CakTC27164	Ca(ICCV2/PI489777)SNP_09233	722	C	T	18	12	--	--
CakTC28885	Ca(ICCV2/PI489777)SNP_09234	345	G	A	17	8	--	--
CakTC39977	Ca(ICCV2/PI489777)SNP_09235	563	A	G	13	5	--	--
	Ca(ICCV2/PI489777)SNP_09236	1425	G	C	6	4	--	--
CakTC26224	Ca(ICCV2/PI489777)SNP_09237	358	T	C	10	8	--	--
	Ca(ICCV2/PI489777)SNP_09238	705	T	C	13	6	--	--
	Ca(ICCV2/PI489777)SNP_09239	1008	A	C	10	5	--	--
	Ca(ICCV2/PI489777)SNP_09240	1276	C	G	11	4	--	--
	Ca(ICCV2/PI489777)SNP_09241	1282	C	G	11	4	--	--
CakTC39960	Ca(ICCV2/PI489777)SNP_09242	667	G	A	6	6	--	--
CakTC36216	Ca(ICCV2/PI489777)SNP_09243	671	A	G	12	7	--	--
CakTC28199	Ca(ICCV2/PI489777)SNP_09244	487	T	C	33	5	--	NAC
	Ca(ICCV2/PI489777)SNP_09245	907	G	A	17	4	--	NAC

CakTC42005	Ca(ICCV2/PI489777)SNP_09246	125	A	G	32	18	--	--
	Ca(ICCV2/PI489777)SNP_09247	495	T	C	60	29	--	--
	Ca(ICCV2/PI489777)SNP_09248	853	C	T	48	26	--	--
CakTC29962	Ca(ICCV2/PI489777)SNP_09249	288	G	C	25	26	--	--
	Ca(ICCV2/PI489777)SNP_09250	816	C	T	23	18	--	--
	Ca(ICCV2/PI489777)SNP_09251	1593	T	A	18	20	--	--
	Ca(ICCV2/PI489777)SNP_09252	2028	C	T	27	27	--	--
	Ca(ICCV2/PI489777)SNP_09253	2172	T	A	24	23	--	--
	Ca(ICCV2/PI489777)SNP_09254	4344	T	C	21	13	--	--
	Ca(ICCV2/PI489777)SNP_09255	5025	A	C	16	17	--	--
	Ca(ICCV2/PI489777)SNP_09256	5644	G	C	3	6	--	--
CakTC11911	Ca(ICCV2/PI489777)SNP_09257	386	C	G	29	20	--	--
	Ca(ICCV2/PI489777)SNP_09258	482	C	T	27	21	--	--
CakTC11486	Ca(ICCV2/PI489777)SNP_09259	162	A	C	11	3	--	--
	Ca(ICCV2/PI489777)SNP_09260	287	A	G	15	6	--	--
	Ca(ICCV2/PI489777)SNP_09261	439	G	T	20	10	--	--
	Ca(ICCV2/PI489777)SNP_09262	777	C	G	17	13	--	--
	Ca(ICCV2/PI489777)SNP_09263	1194	A	T	31	13	--	--
CakTC01502	Ca(ICCV2/PI489777)SNP_09264	635	C	T	3	4	--	--
CakTC37965	Ca(ICCV2/PI489777)SNP_09265	120	A	G	25	14	--	--
	Ca(ICCV2/PI489777)SNP_09266	187	C	T	30	23	--	--
	Ca(ICCV2/PI489777)SNP_09267	330	C	A	42	39	--	--
	Ca(ICCV2/PI489777)SNP_09268	648	G	C	55	26	--	--
	Ca(ICCV2/PI489777)SNP_09269	651	T	G	56	28	--	--
	Ca(ICCV2/PI489777)SNP_09270	723	G	A	58	24	--	--
	Ca(ICCV2/PI489777)SNP_09271	732	A	T	60	26	--	--
	Ca(ICCV2/PI489777)SNP_09272	786	C	T	48	24	--	--
	Ca(ICCV2/PI489777)SNP_09273	1020	G	A	15	13	--	--
CakTC07047	Ca(ICCV2/PI489777)SNP_09274	462	C	T	3	8	--	--
CakTC29457	Ca(ICCV2/PI489777)SNP_09275	656	G	A	3	3	--	--
	Ca(ICCV2/PI489777)SNP_09276	857	T	C	7	7	--	--
	Ca(ICCV2/PI489777)SNP_09277	1124	G	C	19	10	--	--
	Ca(ICCV2/PI489777)SNP_09278	1238	C	T	16	9	--	--
	Ca(ICCV2/PI489777)SNP_09279	1268	C	T	16	4	--	--
CakTC16480	Ca(ICCV2/PI489777)SNP_09280	2239	C	T	21	11	Root	--
	Ca(ICCV2/PI489777)SNP_09281	2806	G	A	20	14	Root	--
	Ca(ICCV2/PI489777)SNP_09282	3201	A	G	13	9	Root	--
	Ca(ICCV2/PI489777)SNP_09283	4175	C	T	7	11	Root	--
	Ca(ICCV2/PI489777)SNP_09284	4217	C	T	8	11	Root	--
	Ca(ICCV2/PI489777)SNP_09285	4440	C	A	12	7	Root	--
CakTC16487	Ca(ICCV2/PI489777)SNP_09286	3651	T	C	77	60	--	CCAAT
	Ca(ICCV2/PI489777)SNP_09287	3750	A	G	75	67	--	CCAAT
	Ca(ICCV2/PI489777)SNP_09288	3762	A	C	79	68	--	CCAAT
	Ca(ICCV2/PI489777)SNP_09289	3846	G	T	74	53	--	CCAAT
	Ca(ICCV2/PI489777)SNP_09290	4020	G	A	15	11	--	CCAAT
	Ca(ICCV2/PI489777)SNP_09291	4084	A	G	7	13	--	CCAAT
CakTC09258	Ca(ICCV2/PI489777)SNP_09292	564	G	A	45	34	--	--
CakTC26866	Ca(ICCV2/PI489777)SNP_09293	275	T	A	24	19	--	--
CakTC13830	Ca(ICCV2/PI489777)SNP_09294	693	G	A	3	8	--	--
	Ca(ICCV2/PI489777)SNP_09295	1232	T	C	4	8	--	--
CakTC38011	Ca(ICCV2/PI489777)SNP_09296	400	C	G	6	6	--	--
	Ca(ICCV2/PI489777)SNP_09297	532	A	G	10	13	--	--
CakTC42330	Ca(ICCV2/PI489777)SNP_09298	270	C	T	12	4	--	--
	Ca(ICCV2/PI489777)SNP_09299	560	C	G	21	7	--	--
	Ca(ICCV2/PI489777)SNP_09300	618	T	A	19	6	--	--
	Ca(ICCV2/PI489777)SNP_09301	1002	C	T	14	8	--	--
	Ca(ICCV2/PI489777)SNP_09302	1006	T	C	13	8	--	--
CakTC37240	Ca(ICCV2/PI489777)SNP_09303	1561	G	A	42	33	--	--
CakTC31355	Ca(ICCV2/PI489777)SNP_09304	527	A	C	6	4	--	--
CakTC32932	Ca(ICCV2/PI489777)SNP_09305	618	T	C	5	4	--	--
	Ca(ICCV2/PI489777)SNP_09306	654	A	C	6	3	--	--
	Ca(ICCV2/PI489777)SNP_09307	1015	C	A	7	5	--	--
	Ca(ICCV2/PI489777)SNP_09308	1254	C	T	7	4	--	--
CakTC42566	Ca(ICCV2/PI489777)SNP_09309	656	T	C	24	27	--	--
CakTC26019	Ca(ICCV2/PI489777)SNP_09310	295	A	G	26	13	--	HSF
	Ca(ICCV2/PI489777)SNP_09311	401	T	C	32	16	--	HSF
	Ca(ICCV2/PI489777)SNP_09312	466	A	T	30	16	--	HSF
	Ca(ICCV2/PI489777)SNP_09313	545	A	G	32	17	--	HSF
	Ca(ICCV2/PI489777)SNP_09314	1135	C	G	26	13	--	HSF
	Ca(ICCV2/PI489777)SNP_09315	1344	C	T	24	3	--	HSF
	Ca(ICCV2/PI489777)SNP_09316	1568	T	C	19	15	--	HSF
	Ca(ICCV2/PI489777)SNP_09317	1571	T	A	20	14	--	HSF
	Ca(ICCV2/PI489777)SNP_09318	1662	T	C	17	13	--	HSF
CakTC28195	Ca(ICCV2/PI489777)SNP_09319	176	C	T	41	20	--	--

	Ca(ICCV2/PI489777)SNP_09320	1638	C	T	56	20	--	--
CakTC25120	Ca(ICCV2/PI489777)SNP_09321	619	C	A	27	35	--	--
CakTC37467	Ca(ICCV2/PI489777)SNP_09322	1272	C	T	23	27	--	--
CakTC38277	Ca(ICCV2/PI489777)SNP_09323	165	T	A	25	11	--	--
	Ca(ICCV2/PI489777)SNP_09324	684	A	G	59	26	--	--
	Ca(ICCV2/PI489777)SNP_09325	923	A	G	36	20	--	--
	Ca(ICCV2/PI489777)SNP_09326	933	G	C	35	18	--	--
CakTC30079	Ca(ICCV2/PI489777)SNP_09327	123	G	A	4	5	--	--
	Ca(ICCV2/PI489777)SNP_09328	250	G	A	9	5	--	--
CakTC24845	Ca(ICCV2/PI489777)SNP_09329	405	G	A	20	4	--	--
	Ca(ICCV2/PI489777)SNP_09330	579	A	G	18	8	--	--
	Ca(ICCV2/PI489777)SNP_09331	1080	T	C	26	5	--	--
	Ca(ICCV2/PI489777)SNP_09332	1135	T	A	28	7	--	--
	Ca(ICCV2/PI489777)SNP_09333	1155	C	T	27	6	--	--
CakTC28795	Ca(ICCV2/PI489777)SNP_09334	292	C	T	16	25	--	--
	Ca(ICCV2/PI489777)SNP_09335	356	C	T	24	27	--	--
	Ca(ICCV2/PI489777)SNP_09336	1447	C	T	19	28	--	--
CakTC39714	Ca(ICCV2/PI489777)SNP_09337	1160	A	G	13	5	--	--
CakTC31033	Ca(ICCV2/PI489777)SNP_09338	782	T	C	3	8	--	--
	Ca(ICCV2/PI489777)SNP_09339	790	A	C	3	8	--	--
CakTC06493	Ca(ICCV2/PI489777)SNP_09340	131	T	G	6	5	--	--
CakTC26804	Ca(ICCV2/PI489777)SNP_09341	226	C	A	3	4	--	--
	Ca(ICCV2/PI489777)SNP_09342	258	T	G	3	3	--	--
	Ca(ICCV2/PI489777)SNP_09343	930	A	G	6	7	--	--
	Ca(ICCV2/PI489777)SNP_09344	2517	C	T	7	3	--	--
	Ca(ICCV2/PI489777)SNP_09345	2538	G	A	7	3	--	--
CakTC29870	Ca(ICCV2/PI489777)SNP_09346	1189	A	G	13	7	Shoot	Alfin-like
	Ca(ICCV2/PI489777)SNP_09347	1265	T	G	10	7	Shoot	Alfin-like
	Ca(ICCV2/PI489777)SNP_09348	1540	G	T	21	4	Shoot	Alfin-like
	Ca(ICCV2/PI489777)SNP_09349	1592	T	C	20	4	Shoot	Alfin-like
	Ca(ICCV2/PI489777)SNP_09350	1629	G	A	16	3	Shoot	Alfin-like
CakTC42148	Ca(ICCV2/PI489777)SNP_09351	202	C	T	4	4	--	--
	Ca(ICCV2/PI489777)SNP_09352	889	C	T	6	8	--	--
CakTC41901	Ca(ICCV2/PI489777)SNP_09353	658	A	T	10	3	--	--
CakTC37460	Ca(ICCV2/PI489777)SNP_09354	145	T	C	18	5	--	--
CakTC37210	Ca(ICCV2/PI489777)SNP_09355	54	A	C	9	4	--	--
	Ca(ICCV2/PI489777)SNP_09356	251	C	T	9	3	--	--
CakTC22315	Ca(ICCV2/PI489777)SNP_09357	140	G	T	3	6	--	--
	Ca(ICCV2/PI489777)SNP_09358	566	C	T	5	3	--	--
	Ca(ICCV2/PI489777)SNP_09359	2703	A	C	21	19	--	--
	Ca(ICCV2/PI489777)SNP_09360	3096	A	T	4	4	--	--
CakTC33803	Ca(ICCV2/PI489777)SNP_09361	237	A	C	51	11	--	--
	Ca(ICCV2/PI489777)SNP_09362	503	G	A	37	3	--	--
	Ca(ICCV2/PI489777)SNP_09363	642	A	G	13	11	--	--
CakTC37884	Ca(ICCV2/PI489777)SNP_09364	1261	T	A	10	7	--	--
	Ca(ICCV2/PI489777)SNP_09365	1297	A	G	8	7	--	--
CakTC39058	Ca(ICCV2/PI489777)SNP_09366	679	G	A	18	7	--	--
	Ca(ICCV2/PI489777)SNP_09367	1490	T	C	4	3	--	--
	Ca(ICCV2/PI489777)SNP_09368	1492	C	T	4	3	--	--
CakTC40617	Ca(ICCV2/PI489777)SNP_09369	271	A	G	6	3	--	--
	Ca(ICCV2/PI489777)SNP_09370	1263	T	A	5	6	--	--
	Ca(ICCV2/PI489777)SNP_09371	1722	C	T	3	3	--	--
CakTC08718	Ca(ICCV2/PI489777)SNP_09372	176	T	A	10	4	--	--
	Ca(ICCV2/PI489777)SNP_09373	210	A	G	7	4	--	--
	Ca(ICCV2/PI489777)SNP_09374	370	C	G	14	7	--	--
	Ca(ICCV2/PI489777)SNP_09375	587	T	C	17	4	--	--
CakTC10409	Ca(ICCV2/PI489777)SNP_09376	215	G	A	3	3	--	--
	Ca(ICCV2/PI489777)SNP_09377	295	C	T	4	3	--	--
	Ca(ICCV2/PI489777)SNP_09378	301	T	A	4	3	--	--
CakTC33007	Ca(ICCV2/PI489777)SNP_09379	298	T	C	41	13	--	--
	Ca(ICCV2/PI489777)SNP_09380	385	G	C	43	15	--	--
	Ca(ICCV2/PI489777)SNP_09381	407	G	C	41	14	--	--
	Ca(ICCV2/PI489777)SNP_09382	1279	T	C	18	7	--	--
	Ca(ICCV2/PI489777)SNP_09383	1564	T	C	27	19	--	--
	Ca(ICCV2/PI489777)SNP_09384	1570	C	T	26	20	--	--
	Ca(ICCV2/PI489777)SNP_09385	1780	T	C	34	16	--	--
	Ca(ICCV2/PI489777)SNP_09386	2032	C	T	37	16	--	--
	Ca(ICCV2/PI489777)SNP_09387	2440	T	C	44	21	--	--
CakTC26909	Ca(ICCV2/PI489777)SNP_09388	1166	C	T	21	7	--	--
	Ca(ICCV2/PI489777)SNP_09389	1184	G	A	17	6	--	--
	Ca(ICCV2/PI489777)SNP_09390	1226	G	A	13	6	--	--
	Ca(ICCV2/PI489777)SNP_09391	1276	A	T	24	6	--	--
	Ca(ICCV2/PI489777)SNP_09392	1333	A	C	31	7	--	--
	Ca(ICCV2/PI489777)SNP_09393	1469	C	T	23	7	--	--

	Ca(ICCV2/PI489777)SNP_09394	1487	C	T	23	8	--	--
	Ca(ICCV2/PI489777)SNP_09395	1489	C	T	23	8	--	--
	Ca(ICCV2/PI489777)SNP_09396	1697	T	A	12	11	--	--
	Ca(ICCV2/PI489777)SNP_09397	1706	A	G	12	11	--	--
	Ca(ICCV2/PI489777)SNP_09398	1805	G	C	11	12	--	--
	Ca(ICCV2/PI489777)SNP_09399	1857	C	G	8	13	--	--
	Ca(ICCV2/PI489777)SNP_09400	2355	G	A	13	9	--	--
	Ca(ICCV2/PI489777)SNP_09401	2384	G	A	16	7	--	--
	Ca(ICCV2/PI489777)SNP_09402	2677	G	C	21	6	--	--
	Ca(ICCV2/PI489777)SNP_09403	3131	C	A	5	5	--	--
	Ca(ICCV2/PI489777)SNP_09404	3320	G	A	20	14	--	--
	Ca(ICCV2/PI489777)SNP_09405	3527	C	T	16	18	--	--
	Ca(ICCV2/PI489777)SNP_09406	4991	A	G	8	5	--	--
	Ca(ICCV2/PI489777)SNP_09407	5186	G	A	8	5	--	--
	Ca(ICCV2/PI489777)SNP_09408	5336	G	T	13	3	--	--
CakTC37142	Ca(ICCV2/PI489777)SNP_09409	71	C	T	4	5	--	--
CakTC40763	Ca(ICCV2/PI489777)SNP_09410	1036	C	A	7	8	--	--
CakTC38033	Ca(ICCV2/PI489777)SNP_09411	517	C	A	64	49	--	--
	Ca(ICCV2/PI489777)SNP_09412	1683	A	G	7	12	--	--
	Ca(ICCV2/PI489777)SNP_09413	1695	C	T	9	12	--	--
CakTC08337	Ca(ICCV2/PI489777)SNP_09414	1504	G	A	7	8	--	--
	Ca(ICCV2/PI489777)SNP_09415	1524	G	A	5	8	--	--
	Ca(ICCV2/PI489777)SNP_09416	1568	G	C	4	7	--	--
CakTC37433	Ca(ICCV2/PI489777)SNP_09417	168	C	A	11	9	--	--
	Ca(ICCV2/PI489777)SNP_09418	260	A	G	11	10	--	--
	Ca(ICCV2/PI489777)SNP_09419	276	G	A	13	10	--	--
	Ca(ICCV2/PI489777)SNP_09420	317	C	T	13	9	--	--
	Ca(ICCV2/PI489777)SNP_09421	367	A	G	12	13	--	--
	Ca(ICCV2/PI489777)SNP_09422	871	T	C	20	11	--	--
CakTC08559	Ca(ICCV2/PI489777)SNP_09423	21	G	C	3	3	--	PHD
	Ca(ICCV2/PI489777)SNP_09424	31	A	T	4	4	--	PHD
	Ca(ICCV2/PI489777)SNP_09425	85	A	C	6	6	--	PHD
CakTC26605	Ca(ICCV2/PI489777)SNP_09426	116	C	A	7	3	--	--
	Ca(ICCV2/PI489777)SNP_09427	925	G	A	9	14	--	--
CakTC42463	Ca(ICCV2/PI489777)SNP_09428	839	A	G	5	9	--	--
CakTC13108	Ca(ICCV2/PI489777)SNP_09429	1003	C	T	8	5	--	EIL
CakTC27761	Ca(ICCV2/PI489777)SNP_09430	286	A	G	5	9	--	--
	Ca(ICCV2/PI489777)SNP_09431	359	C	T	6	9	--	--
CakTC23469	Ca(ICCV2/PI489777)SNP_09432	269	C	T	4	7	--	mTERF
	Ca(ICCV2/PI489777)SNP_09433	1042	C	G	9	7	--	mTERF
	Ca(ICCV2/PI489777)SNP_09434	1848	A	G	10	10	--	mTERF
CakTC41841	Ca(ICCV2/PI489777)SNP_09435	49	T	G	29	8	--	CCAAT
CakTC41212	Ca(ICCV2/PI489777)SNP_09436	581	G	A	5	6	--	--
	Ca(ICCV2/PI489777)SNP_09437	611	T	G	5	6	--	--
	Ca(ICCV2/PI489777)SNP_09438	649	C	T	4	6	--	--
CakTC36079	Ca(ICCV2/PI489777)SNP_09439	330	C	G	7	5	--	--
	Ca(ICCV2/PI489777)SNP_09440	476	C	G	13	11	--	--
	Ca(ICCV2/PI489777)SNP_09441	594	A	G	18	10	--	--
	Ca(ICCV2/PI489777)SNP_09442	2057	T	C	3	10	--	--
CakTC29331	Ca(ICCV2/PI489777)SNP_09443	1110	G	A	28	7	--	--
	Ca(ICCV2/PI489777)SNP_09444	1761	G	C	9	3	--	--
	Ca(ICCV2/PI489777)SNP_09445	1827	C	G	7	3	--	--
CakTC34091	Ca(ICCV2/PI489777)SNP_09446	257	C	G	16	6	--	--
	Ca(ICCV2/PI489777)SNP_09447	492	C	A	7	3	--	--
CakTC39025	Ca(ICCV2/PI489777)SNP_09448	1168	A	C	24	8	--	--
CakTC37949	Ca(ICCV2/PI489777)SNP_09449	212	T	C	27	20	--	--
CakTC29609	Ca(ICCV2/PI489777)SNP_09450	1350	A	G	3	3	Root	--
CakTC38834	Ca(ICCV2/PI489777)SNP_09451	1156	A	T	9	5	--	--
	Ca(ICCV2/PI489777)SNP_09452	1207	T	C	9	8	--	--
CakTC02320	Ca(ICCV2/PI489777)SNP_09453	390	A	C	7	3	--	--
CakTC29774	Ca(ICCV2/PI489777)SNP_09454	1124	G	C	4	3	--	--
	Ca(ICCV2/PI489777)SNP_09455	1131	A	T	4	3	--	--
	Ca(ICCV2/PI489777)SNP_09456	1230	C	T	4	5	--	--
	Ca(ICCV2/PI489777)SNP_09457	1876	G	C	4	4	--	--
CakTC31078	Ca(ICCV2/PI489777)SNP_09458	1739	G	T	7	10	--	--
	Ca(ICCV2/PI489777)SNP_09459	2617	C	G	18	13	--	--
	Ca(ICCV2/PI489777)SNP_09460	2752	G	A	19	9	--	--
	Ca(ICCV2/PI489777)SNP_09461	2822	G	A	15	8	--	--
	Ca(ICCV2/PI489777)SNP_09462	2977	G	T	14	13	--	--
	Ca(ICCV2/PI489777)SNP_09463	3298	G	A	26	10	--	--
	Ca(ICCV2/PI489777)SNP_09464	3496	T	C	10	12	--	--
CakTC31666	Ca(ICCV2/PI489777)SNP_09465	1817	G	A	3	3	--	--
	Ca(ICCV2/PI489777)SNP_09466	1898	A	G	3	3	--	--
CakTC33215	Ca(ICCV2/PI489777)SNP_09467	555	T	C	3	4	--	--

	Ca(ICCV2/PI489777)SNP_09468	5529	A	G	4	3	--	--
	Ca(ICCV2/PI489777)SNP_09469	5586	T	C	4	3	--	--
	Ca(ICCV2/PI489777)SNP_09470	7332	A	T	4	4	--	--
	Ca(ICCV2/PI489777)SNP_09471	13368	G	T	4	7	--	--
	Ca(ICCV2/PI489777)SNP_09472	13579	G	A	5	5	--	--
CakTC23902	Ca(ICCV2/PI489777)SNP_09473	491	A	T	28	4	--	--
	Ca(ICCV2/PI489777)SNP_09474	590	G	A	19	4	--	--
	Ca(ICCV2/PI489777)SNP_09475	602	A	T	24	4	--	--
	Ca(ICCV2/PI489777)SNP_09476	670	T	C	30	6	--	--
	Ca(ICCV2/PI489777)SNP_09477	671	T	C	30	6	--	--
	Ca(ICCV2/PI489777)SNP_09478	680	C	T	30	6	--	--
	Ca(ICCV2/PI489777)SNP_09479	700	C	T	29	5	--	--
	Ca(ICCV2/PI489777)SNP_09480	716	G	T	29	6	--	--
	Ca(ICCV2/PI489777)SNP_09481	1385	G	A	10	3	--	--
	Ca(ICCV2/PI489777)SNP_09482	1388	A	T	9	3	--	--
	Ca(ICCV2/PI489777)SNP_09483	1757	G	T	11	3	--	--
	Ca(ICCV2/PI489777)SNP_09484	1952	A	G	9	4	--	--
	Ca(ICCV2/PI489777)SNP_09485	2024	T	C	9	6	--	--
	Ca(ICCV2/PI489777)SNP_09486	2033	G	A	10	6	--	--
	Ca(ICCV2/PI489777)SNP_09487	2186	A	G	10	5	--	--
	Ca(ICCV2/PI489777)SNP_09488	2226	C	T	8	5	--	--
	Ca(ICCV2/PI489777)SNP_09489	2258	T	G	9	5	--	--
	Ca(ICCV2/PI489777)SNP_09490	2345	G	A	7	4	--	--
CakTC41726	Ca(ICCV2/PI489777)SNP_09491	179	A	G	5	4	Young_pod	--
	Ca(ICCV2/PI489777)SNP_09492	394	G	C	7	7	Young_pod	--
	Ca(ICCV2/PI489777)SNP_09493	437	T	C	10	4	Young_pod	--
	Ca(ICCV2/PI489777)SNP_09494	841	G	A	17	8	Young_pod	--
	Ca(ICCV2/PI489777)SNP_09495	878	G	A	18	7	Young_pod	--
	Ca(ICCV2/PI489777)SNP_09496	911	C	T	19	9	Young_pod	--
	Ca(ICCV2/PI489777)SNP_09497	923	G	A	22	9	Young_pod	--
	Ca(ICCV2/PI489777)SNP_09498	935	C	T	21	10	Young_pod	--
CakTC12121	Ca(ICCV2/PI489777)SNP_09499	427	A	T	17	15	--	--
	Ca(ICCV2/PI489777)SNP_09500	538	A	G	23	9	--	--
CakTC35745	Ca(ICCV2/PI489777)SNP_09501	1076	A	T	12	3	--	Alfin-like
	Ca(ICCV2/PI489777)SNP_09502	1170	G	A	12	3	--	Alfin-like
	Ca(ICCV2/PI489777)SNP_09503	1742	A	G	4	4	--	Alfin-like
	Ca(ICCV2/PI489777)SNP_09504	2834	G	T	11	5	--	Alfin-like
	Ca(ICCV2/PI489777)SNP_09505	3017	T	A	11	9	--	Alfin-like
CakTC42729	Ca(ICCV2/PI489777)SNP_09506	1406	C	A	11	5	--	--
CakTC33019	Ca(ICCV2/PI489777)SNP_09507	715	T	A	7	4	--	--
	Ca(ICCV2/PI489777)SNP_09508	1882	G	A	15	9	--	--
	Ca(ICCV2/PI489777)SNP_09509	2107	C	T	17	6	--	--
	Ca(ICCV2/PI489777)SNP_09510	2488	A	C	7	5	--	--
CakTC28233	Ca(ICCV2/PI489777)SNP_09511	86	C	G	9	5	--	--
	Ca(ICCV2/PI489777)SNP_09512	135	T	A	12	7	--	--
	Ca(ICCV2/PI489777)SNP_09513	196	G	T	13	8	--	--
	Ca(ICCV2/PI489777)SNP_09514	360	G	A	16	12	--	--
	Ca(ICCV2/PI489777)SNP_09515	723	G	A	27	7	--	--
	Ca(ICCV2/PI489777)SNP_09516	747	T	C	26	6	--	--
	Ca(ICCV2/PI489777)SNP_09517	948	T	C	27	4	--	--
CakTC38994	Ca(ICCV2/PI489777)SNP_09518	304	A	C	9	5	--	--
	Ca(ICCV2/PI489777)SNP_09519	409	C	T	6	4	--	--
	Ca(ICCV2/PI489777)SNP_09520	823	G	A	6	4	--	--
	Ca(ICCV2/PI489777)SNP_09521	982	G	A	6	4	--	--
	Ca(ICCV2/PI489777)SNP_09522	1060	A	G	5	3	--	--
	Ca(ICCV2/PI489777)SNP_09523	1120	C	T	6	4	--	--
CakTC30384	Ca(ICCV2/PI489777)SNP_09524	473	A	G	6	7	--	--
CakTC28392	Ca(ICCV2/PI489777)SNP_09525	504	T	G	39	14	--	--
	Ca(ICCV2/PI489777)SNP_09526	1116	C	T	41	21	--	--
	Ca(ICCV2/PI489777)SNP_09527	1125	T	C	39	21	--	--
	Ca(ICCV2/PI489777)SNP_09528	1283	T	G	39	15	--	--
	Ca(ICCV2/PI489777)SNP_09529	1378	A	T	32	9	--	--
CakTC42870	Ca(ICCV2/PI489777)SNP_09530	461	C	T	22	9	Flower	bud
	Ca(ICCV2/PI489777)SNP_09531	513	A	C	21	12	Flower	bud
	Ca(ICCV2/PI489777)SNP_09532	717	G	A	15	7	Flower	bud
	Ca(ICCV2/PI489777)SNP_09533	786	T	A	13	5	Flower	bud
CakTC24005	Ca(ICCV2/PI489777)SNP_09534	1396	G	A	11	9	--	--
	Ca(ICCV2/PI489777)SNP_09535	1660	T	C	8	5	--	--
CakTC11174	Ca(ICCV2/PI489777)SNP_09536	217	C	T	14	9	--	--
	Ca(ICCV2/PI489777)SNP_09537	460	G	A	18	12	--	--
	Ca(ICCV2/PI489777)SNP_09538	625	A	G	11	10	--	--
	Ca(ICCV2/PI489777)SNP_09539	655	A	G	15	10	--	--
CakTC26144	Ca(ICCV2/PI489777)SNP_09540	248	T	C	23	5	--	--
	Ca(ICCV2/PI489777)SNP_09541	267	A	C	23	5	--	--

	Ca(ICCV2/PI489777)SNP_09542	1194	T	C	22	3	--	--
	Ca(ICCV2/PI489777)SNP_09543	1592	C	T	17	9	--	--
	Ca(ICCV2/PI489777)SNP_09544	1779	C	T	12	10	--	--
	Ca(ICCV2/PI489777)SNP_09545	1911	A	G	8	7	--	--
CakTC25336	Ca(ICCV2/PI489777)SNP_09546	228	G	T	55	47	--	--
	Ca(ICCV2/PI489777)SNP_09547	470	T	G	154	111	--	--
	Ca(ICCV2/PI489777)SNP_09548	704	T	A	106	77	--	--
	Ca(ICCV2/PI489777)SNP_09549	1646	A	T	91	58	--	--
CakTC40781	Ca(ICCV2/PI489777)SNP_09550	348	C	A	10	6	--	--
CakTC31701	Ca(ICCV2/PI489777)SNP_09551	967	A	T	6	3	Shoot	--
CakTC27533	Ca(ICCV2/PI489777)SNP_09552	2015	G	T	8	3	--	--
	Ca(ICCV2/PI489777)SNP_09553	2094	G	T	6	4	--	--
CakTC39456	Ca(ICCV2/PI489777)SNP_09554	1172	G	A	7	13	--	TPR
	Ca(ICCV2/PI489777)SNP_09555	1173	C	T	7	12	--	TPR
	Ca(ICCV2/PI489777)SNP_09556	1265	G	T	4	11	--	TPR
CakTC26068	Ca(ICCV2/PI489777)SNP_09557	329	G	T	56	52	--	--
CakTC29086	Ca(ICCV2/PI489777)SNP_09558	33	T	C	6	6	--	--
	Ca(ICCV2/PI489777)SNP_09559	261	C	T	30	12	--	--
	Ca(ICCV2/PI489777)SNP_09560	864	C	A	22	10	--	--
CakTC35711	Ca(ICCV2/PI489777)SNP_09561	715	A	G	50	32	--	--
	Ca(ICCV2/PI489777)SNP_09562	1999	C	T	61	58	--	--
	Ca(ICCV2/PI489777)SNP_09563	2593	T	C	60	48	--	--
	Ca(ICCV2/PI489777)SNP_09564	3662	A	G	7	16	--	--
	Ca(ICCV2/PI489777)SNP_09565	3684	T	C	5	11	--	--
CakTC26324	Ca(ICCV2/PI489777)SNP_09566	851	A	G	21	5	--	--
	Ca(ICCV2/PI489777)SNP_09567	908	G	A	17	5	--	--
	Ca(ICCV2/PI489777)SNP_09568	1152	T	C	12	6	--	--
	Ca(ICCV2/PI489777)SNP_09569	1371	T	C	13	8	--	--
CakTC42098	Ca(ICCV2/PI489777)SNP_09570	761	T	C	11	3	--	--
	Ca(ICCV2/PI489777)SNP_09571	1289	T	C	15	4	--	--
CakTC27690	Ca(ICCV2/PI489777)SNP_09572	430	T	G	35	16	--	--
	Ca(ICCV2/PI489777)SNP_09573	879	G	A	13	13	--	--
	Ca(ICCV2/PI489777)SNP_09574	1007	A	G	12	11	--	--
	Ca(ICCV2/PI489777)SNP_09575	1543	G	A	8	6	--	--
	Ca(ICCV2/PI489777)SNP_09576	1618	C	T	9	5	--	--
CakTC26944	Ca(ICCV2/PI489777)SNP_09577	1166	T	A	15	3	--	TCP
	Ca(ICCV2/PI489777)SNP_09578	1289	G	A	13	6	--	TCP
	Ca(ICCV2/PI489777)SNP_09579	1511	G	A	4	3	--	TCP
	Ca(ICCV2/PI489777)SNP_09580	1513	C	T	4	3	--	TCP
CakTC25384	Ca(ICCV2/PI489777)SNP_09581	228	T	G	15	3	--	--
CakTC25751	Ca(ICCV2/PI489777)SNP_09582	1291	T	C	64	20	--	--
	Ca(ICCV2/PI489777)SNP_09583	1330	G	A	60	18	--	--
	Ca(ICCV2/PI489777)SNP_09584	1548	A	T	19	3	--	--
	Ca(ICCV2/PI489777)SNP_09585	1549	T	C	19	3	--	--
	Ca(ICCV2/PI489777)SNP_09586	1625	G	A	16	3	--	--
CakTC37511	Ca(ICCV2/PI489777)SNP_09587	546	C	A	8	3	--	--
	Ca(ICCV2/PI489777)SNP_09588	804	C	T	11	4	--	--
CakTC28817	Ca(ICCV2/PI489777)SNP_09589	233	A	G	3	10	--	--
CakTC42071	Ca(ICCV2/PI489777)SNP_09590	151	A	C	4	4	--	--
	Ca(ICCV2/PI489777)SNP_09591	656	G	A	11	4	--	--
	Ca(ICCV2/PI489777)SNP_09592	792	G	C	6	3	--	--
	Ca(ICCV2/PI489777)SNP_09593	888	C	T	5	3	--	--
	Ca(ICCV2/PI489777)SNP_09594	928	C	T	4	3	--	--
CakTC26533	Ca(ICCV2/PI489777)SNP_09595	616	C	T	7	8	--	--
	Ca(ICCV2/PI489777)SNP_09596	661	C	T	7	6	--	--
CakTC32471	Ca(ICCV2/PI489777)SNP_09597	447	T	C	5	4	--	--
	Ca(ICCV2/PI489777)SNP_09598	469	C	A	4	4	--	--
CakTC20154	Ca(ICCV2/PI489777)SNP_09599	126	C	G	8	7	--	--
	Ca(ICCV2/PI489777)SNP_09600	134	G	A	11	11	--	--
CakTC42027	Ca(ICCV2/PI489777)SNP_09601	91	A	T	17	9	--	--
	Ca(ICCV2/PI489777)SNP_09602	1615	C	T	21	4	--	--
CakTC28738	Ca(ICCV2/PI489777)SNP_09603	409	C	G	28	15	--	--
	Ca(ICCV2/PI489777)SNP_09604	439	G	A	27	14	--	--
	Ca(ICCV2/PI489777)SNP_09605	490	G	A	36	12	--	--
	Ca(ICCV2/PI489777)SNP_09606	920	A	G	7	11	--	--
CakTC39408	Ca(ICCV2/PI489777)SNP_09607	334	C	T	32	12	--	--
	Ca(ICCV2/PI489777)SNP_09608	342	A	T	50	20	--	--
	Ca(ICCV2/PI489777)SNP_09609	386	A	C	94	29	--	--
	Ca(ICCV2/PI489777)SNP_09610	414	C	T	108	37	--	--
	Ca(ICCV2/PI489777)SNP_09611	1392	A	G	90	74	--	--
	Ca(ICCV2/PI489777)SNP_09612	1490	T	C	74	60	--	--
	Ca(ICCV2/PI489777)SNP_09613	1499	G	C	74	60	--	--
	Ca(ICCV2/PI489777)SNP_09614	1554	G	T	77	54	--	--
CakTC41156	Ca(ICCV2/PI489777)SNP_09615	2008	C	T	6	4	--	--

CakTC33537	Ca(ICCV2/PI489777)SNP_09616	807	A	G	4	4	--	--
	Ca(ICCV2/PI489777)SNP_09617	810	T	C	4	4	--	--
	Ca(ICCV2/PI489777)SNP_09618	840	T	A	5	4	--	--
CakTC30251	Ca(ICCV2/PI489777)SNP_09619	911	A	T	4	6	--	--
CakTC13553	Ca(ICCV2/PI489777)SNP_09620	196	G	A	4	7	--	WRKY
	Ca(ICCV2/PI489777)SNP_09621	256	G	C	6	8	--	WRKY
	Ca(ICCV2/PI489777)SNP_09622	340	A	G	5	7	--	WRKY
	Ca(ICCV2/PI489777)SNP_09623	382	T	C	9	7	--	WRKY
	Ca(ICCV2/PI489777)SNP_09624	514	T	C	9	3	--	WRKY
	Ca(ICCV2/PI489777)SNP_09625	1060	G	A	18	3	--	WRKY
CakTC03738	Ca(ICCV2/PI489777)SNP_09626	194	A	G	4	10	--	--
CakTC39273	Ca(ICCV2/PI489777)SNP_09627	196	A	G	4	5	--	--
CakTC38362	Ca(ICCV2/PI489777)SNP_09628	353	A	G	10	8	--	--
	Ca(ICCV2/PI489777)SNP_09629	715	T	C	11	9	--	--
	Ca(ICCV2/PI489777)SNP_09630	867	A	G	10	7	--	--
	Ca(ICCV2/PI489777)SNP_09631	998	G	T	8	7	--	--
	Ca(ICCV2/PI489777)SNP_09632	1031	G	A	6	7	--	--
CakTC22716	Ca(ICCV2/PI489777)SNP_09633	1024	G	A	3	3	--	--
CakTC08292	Ca(ICCV2/PI489777)SNP_09634	213	G	A	3	4	Young_pod	--
CakTC23688	Ca(ICCV2/PI489777)SNP_09635	475	A	T	19	14	--	--
	Ca(ICCV2/PI489777)SNP_09636	730	C	G	6	4	--	--
CakTC41483	Ca(ICCV2/PI489777)SNP_09637	979	A	G	13	4	--	--
	Ca(ICCV2/PI489777)SNP_09638	1024	T	G	13	4	--	--
	Ca(ICCV2/PI489777)SNP_09639	1789	A	T	20	8	--	--
	Ca(ICCV2/PI489777)SNP_09640	2063	T	C	24	5	--	--
CakTC25739	Ca(ICCV2/PI489777)SNP_09641	344	C	T	62	39	--	--
	Ca(ICCV2/PI489777)SNP_09642	404	C	T	66	40	--	--
CakTC32524	Ca(ICCV2/PI489777)SNP_09643	334	T	A	3	6	--	--
CakTC26707	Ca(ICCV2/PI489777)SNP_09644	105	A	C	5	4	--	--
	Ca(ICCV2/PI489777)SNP_09645	509	A	G	12	5	--	--
	Ca(ICCV2/PI489777)SNP_09646	1090	A	G	12	4	--	--
CakTC33961	Ca(ICCV2/PI489777)SNP_09647	577	T	C	9	3	--	--
	Ca(ICCV2/PI489777)SNP_09648	629	G	C	9	4	--	--
CakTC36970	Ca(ICCV2/PI489777)SNP_09649	385	A	G	44	17	--	--
	Ca(ICCV2/PI489777)SNP_09650	640	G	A	39	15	--	--
	Ca(ICCV2/PI489777)SNP_09651	797	C	T	39	14	--	--
	Ca(ICCV2/PI489777)SNP_09652	814	A	G	32	12	--	--
	Ca(ICCV2/PI489777)SNP_09653	1615	C	A	51	17	--	--
	Ca(ICCV2/PI489777)SNP_09654	1813	G	A	46	17	--	--
	Ca(ICCV2/PI489777)SNP_09655	1840	T	C	47	18	--	--
	Ca(ICCV2/PI489777)SNP_09656	2341	A	G	63	19	--	--
	Ca(ICCV2/PI489777)SNP_09657	2454	G	A	40	18	--	--
CakTC22458	Ca(ICCV2/PI489777)SNP_09658	183	C	G	8	3	--	--
	Ca(ICCV2/PI489777)SNP_09659	250	C	T	5	4	--	--
	Ca(ICCV2/PI489777)SNP_09660	340	A	T	11	5	--	--
	Ca(ICCV2/PI489777)SNP_09661	441	A	T	12	4	--	--
	Ca(ICCV2/PI489777)SNP_09662	613	C	A	17	3	--	--
	Ca(ICCV2/PI489777)SNP_09663	923	A	G	8	13	--	--
	Ca(ICCV2/PI489777)SNP_09664	1171	G	A	4	12	--	--
CakTC07480	Ca(ICCV2/PI489777)SNP_09665	624	C	G	7	3	--	--
	Ca(ICCV2/PI489777)SNP_09666	1011	T	C	7	5	--	--
CakTC25836	Ca(ICCV2/PI489777)SNP_09667	1141	T	A	65	10	--	Sigma70-like
	Ca(ICCV2/PI489777)SNP_09668	1297	G	C	48	10	--	Sigma70-like
CakTC09738	Ca(ICCV2/PI489777)SNP_09669	15	C	T	6	17	Flower	bud
	Ca(ICCV2/PI489777)SNP_09670	188	T	C	10	47	Flower	bud
CakTC42193	Ca(ICCV2/PI489777)SNP_09671	53	G	A	17	3	--	--
	Ca(ICCV2/PI489777)SNP_09672	231	C	A	78	45	--	--
	Ca(ICCV2/PI489777)SNP_09673	285	C	T	84	51	--	--
	Ca(ICCV2/PI489777)SNP_09674	315	G	A	76	49	--	--
	Ca(ICCV2/PI489777)SNP_09675	558	C	T	50	37	--	--
CakTC29977	Ca(ICCV2/PI489777)SNP_09676	334	T	C	32	20	--	--
	Ca(ICCV2/PI489777)SNP_09677	531	C	T	33	27	--	--
	Ca(ICCV2/PI489777)SNP_09678	696	T	C	53	25	--	--
	Ca(ICCV2/PI489777)SNP_09679	871	A	G	44	24	--	--
	Ca(ICCV2/PI489777)SNP_09680	1501	G	C	54	18	--	--
	Ca(ICCV2/PI489777)SNP_09681	1989	C	G	41	11	--	--
	Ca(ICCV2/PI489777)SNP_09682	2044	T	G	41	12	--	--
CakTC30444	Ca(ICCV2/PI489777)SNP_09683	797	G	A	3	7	Shoot	--
	Ca(ICCV2/PI489777)SNP_09684	1700	C	G	6	3	Shoot	--
CakTC38513	Ca(ICCV2/PI489777)SNP_09685	632	T	C	5	11	--	--
CakTC02278	Ca(ICCV2/PI489777)SNP_09686	72	C	T	15	4	--	--
CakTC01014	Ca(ICCV2/PI489777)SNP_09687	860	T	C	8	5	--	--
	Ca(ICCV2/PI489777)SNP_09688	877	C	G	8	4	--	--
	Ca(ICCV2/PI489777)SNP_09689	1039	C	T	10	6	--	--

	Ca(ICCV2/PI489777)SNP_09690	1181	C	T	12	8	--	--
	Ca(ICCV2/PI489777)SNP_09691	1189	A	G	13	11	--	--
	Ca(ICCV2/PI489777)SNP_09692	1390	C	A	10	6	--	--
	Ca(ICCV2/PI489777)SNP_09693	1840	C	T	8	3	--	--
	Ca(ICCV2/PI489777)SNP_09694	2207	T	C	3	6	--	--
CakTC28458	Ca(ICCV2/PI489777)SNP_09695	3503	T	C	28	12	--	--
	Ca(ICCV2/PI489777)SNP_09696	3558	A	G	28	10	--	--
	Ca(ICCV2/PI489777)SNP_09697	4247	A	G	4	5	--	--
CakTC16037	Ca(ICCV2/PI489777)SNP_09698	125	T	G	4	5	--	--
CakTC38459	Ca(ICCV2/PI489777)SNP_09699	793	T	C	6	9	--	--
CakTC27126	Ca(ICCV2/PI489777)SNP_09700	174	C	A	3	4	--	--
	Ca(ICCV2/PI489777)SNP_09701	186	T	A	3	4	--	--
CakTC41725	Ca(ICCV2/PI489777)SNP_09702	269	G	A	4	5	Flower	bud
	Ca(ICCV2/PI489777)SNP_09703	363	A	C	7	7	Flower	bud
	Ca(ICCV2/PI489777)SNP_09704	1293	G	A	7	4	Flower	bud
CakTC40764	Ca(ICCV2/PI489777)SNP_09705	322	C	T	17	3	--	--
	Ca(ICCV2/PI489777)SNP_09706	511	C	T	19	5	--	--
CakTC23244	Ca(ICCV2/PI489777)SNP_09707	23	C	T	5	4	--	--
CakTC38142	Ca(ICCV2/PI489777)SNP_09708	1473	A	T	13	4	--	--
CakTC25754	Ca(ICCV2/PI489777)SNP_09709	61	T	A	13	4	--	--
CakTC41037	Ca(ICCV2/PI489777)SNP_09710	770	G	A	34	19	--	--
	Ca(ICCV2/PI489777)SNP_09711	1914	A	C	10	5	--	--
CakTC08281	Ca(ICCV2/PI489777)SNP_09712	478	T	C	11	7	--	--
CakTC35965	Ca(ICCV2/PI489777)SNP_09713	409	C	T	3	10	--	--
CakTC32256	Ca(ICCV2/PI489777)SNP_09714	457	C	T	51	9	--	--
	Ca(ICCV2/PI489777)SNP_09715	482	T	C	58	10	--	--
	Ca(ICCV2/PI489777)SNP_09716	512	A	G	67	10	--	--
	Ca(ICCV2/PI489777)SNP_09717	685	A	G	83	13	--	--
	Ca(ICCV2/PI489777)SNP_09718	922	C	T	65	28	--	--
	Ca(ICCV2/PI489777)SNP_09719	925	G	C	64	28	--	--
	Ca(ICCV2/PI489777)SNP_09720	1136	T	G	60	31	--	--
	Ca(ICCV2/PI489777)SNP_09721	1531	G	A	17	7	--	--
CakTC29350	Ca(ICCV2/PI489777)SNP_09722	1179	T	C	9	3	--	--
CakTC39009	Ca(ICCV2/PI489777)SNP_09723	1303	T	C	5	4	--	--
CakTC29691	Ca(ICCV2/PI489777)SNP_09724	663	T	C	3	4	--	--
	Ca(ICCV2/PI489777)SNP_09725	848	T	C	3	5	--	--
CakTC36777	Ca(ICCV2/PI489777)SNP_09726	577	G	A	19	12	--	--
	Ca(ICCV2/PI489777)SNP_09727	1150	G	A	9	10	--	--
	Ca(ICCV2/PI489777)SNP_09728	1345	T	C	14	11	--	--
	Ca(ICCV2/PI489777)SNP_09729	1951	A	G	15	19	--	--
	Ca(ICCV2/PI489777)SNP_09730	2383	A	G	24	10	--	--
CakTC36846	Ca(ICCV2/PI489777)SNP_09731	718	C	T	14	7	--	--
	Ca(ICCV2/PI489777)SNP_09732	750	T	C	14	7	--	--
	Ca(ICCV2/PI489777)SNP_09733	758	C	G	14	8	--	--
	Ca(ICCV2/PI489777)SNP_09734	786	G	A	7	8	--	--
	Ca(ICCV2/PI489777)SNP_09735	2604	C	T	5	4	--	--
CakTC41557	Ca(ICCV2/PI489777)SNP_09736	658	C	T	43	30	--	--
CakTC34542	Ca(ICCV2/PI489777)SNP_09737	381	T	C	22	7	--	--
	Ca(ICCV2/PI489777)SNP_09738	528	C	T	10	7	--	--
	Ca(ICCV2/PI489777)SNP_09739	798	C	T	21	3	--	--
	Ca(ICCV2/PI489777)SNP_09740	807	T	C	20	4	--	--
	Ca(ICCV2/PI489777)SNP_09741	927	G	A	19	6	--	--
	Ca(ICCV2/PI489777)SNP_09742	1066	T	C	25	9	--	--
	Ca(ICCV2/PI489777)SNP_09743	1080	C	A	23	7	--	--
	Ca(ICCV2/PI489777)SNP_09744	1131	T	A	22	8	--	--
	Ca(ICCV2/PI489777)SNP_09745	1557	A	G	23	14	--	--
CakTC33806	Ca(ICCV2/PI489777)SNP_09746	403	T	A	6	6	--	--
CakTC40893	Ca(ICCV2/PI489777)SNP_09747	248	T	A	9	3	--	--
	Ca(ICCV2/PI489777)SNP_09748	498	A	G	11	3	--	--
	Ca(ICCV2/PI489777)SNP_09749	1434	C	T	26	11	--	--
CakTC23206	Ca(ICCV2/PI489777)SNP_09750	785	G	A	14	5	--	--
	Ca(ICCV2/PI489777)SNP_09751	914	C	A	9	4	--	--
	Ca(ICCV2/PI489777)SNP_09752	1002	T	G	9	5	--	--
CakTC23259	Ca(ICCV2/PI489777)SNP_09753	539	T	G	7	5	Flower	bud
CakTC37272	Ca(ICCV2/PI489777)SNP_09754	56	G	T	27	21	--	--
	Ca(ICCV2/PI489777)SNP_09755	80	A	G	40	30	--	--
	Ca(ICCV2/PI489777)SNP_09756	86	A	G	59	37	--	--
	Ca(ICCV2/PI489777)SNP_09757	242	T	C	84	42	--	--
	Ca(ICCV2/PI489777)SNP_09758	549	C	G	46	41	--	--
	Ca(ICCV2/PI489777)SNP_09759	628	A	C	48	26	--	--
	Ca(ICCV2/PI489777)SNP_09760	638	C	T	45	25	--	--
	Ca(ICCV2/PI489777)SNP_09761	1024	T	G	30	20	--	--
	Ca(ICCV2/PI489777)SNP_09762	1034	G	T	27	14	--	--
CakTC28779	Ca(ICCV2/PI489777)SNP_09763	372	G	A	20	8	--	--

CakTC38833	Ca(ICCV2/PI489777)SNP_09764	1097	G	C	8	4	--	bHLH
CakTC28610	Ca(ICCV2/PI489777)SNP_09765	141	T	A	36	39	--	--
	Ca(ICCV2/PI489777)SNP_09766	686	T	G	14	7	--	--
CakTC26232	Ca(ICCV2/PI489777)SNP_09767	344	A	G	15	4	--	--
	Ca(ICCV2/PI489777)SNP_09768	427	T	A	17	4	--	--
	Ca(ICCV2/PI489777)SNP_09769	431	C	T	18	4	--	--
	Ca(ICCV2/PI489777)SNP_09770	457	C	T	16	4	--	--
	Ca(ICCV2/PI489777)SNP_09771	485	T	C	14	4	--	--
	Ca(ICCV2/PI489777)SNP_09772	1146	T	G	7	5	--	--
CakTC42187	Ca(ICCV2/PI489777)SNP_09773	78	C	T	31	3	--	--
	Ca(ICCV2/PI489777)SNP_09774	304	T	G	37	3	--	--
	Ca(ICCV2/PI489777)SNP_09775	546	C	G	38	8	--	--
CakTC33473	Ca(ICCV2/PI489777)SNP_09776	1009	A	G	22	13	--	--
	Ca(ICCV2/PI489777)SNP_09777	1330	C	T	19	9	--	--
	Ca(ICCV2/PI489777)SNP_09778	1333	T	C	18	10	--	--
	Ca(ICCV2/PI489777)SNP_09779	1435	G	A	12	8	--	--
CakTC32149	Ca(ICCV2/PI489777)SNP_09780	418	G	A	3	5	--	AP2-EREBP
	Ca(ICCV2/PI489777)SNP_09781	442	A	G	3	5	--	AP2-EREBP
CakTC36990	Ca(ICCV2/PI489777)SNP_09782	462	C	T	5	7	Root	--
	Ca(ICCV2/PI489777)SNP_09783	555	G	A	7	9	Root	--
	Ca(ICCV2/PI489777)SNP_09784	562	T	A	6	9	Root	--
	Ca(ICCV2/PI489777)SNP_09785	567	T	A	8	11	Root	--
	Ca(ICCV2/PI489777)SNP_09786	652	T	C	15	10	Root	--
	Ca(ICCV2/PI489777)SNP_09787	849	C	A	19	16	Root	--
	Ca(ICCV2/PI489777)SNP_09788	963	G	T	16	17	Root	--
	Ca(ICCV2/PI489777)SNP_09789	2539	G	A	4	5	Root	--
	Ca(ICCV2/PI489777)SNP_09790	2556	A	C	3	5	Root	--
CakTC42924	Ca(ICCV2/PI489777)SNP_09791	24	T	A	16	3	--	--
	Ca(ICCV2/PI489777)SNP_09792	29	T	C	17	4	--	--
	Ca(ICCV2/PI489777)SNP_09793	35	A	G	17	4	--	--
	Ca(ICCV2/PI489777)SNP_09794	50	A	G	11	4	--	--
	Ca(ICCV2/PI489777)SNP_09795	57	G	T	18	4	--	--
	Ca(ICCV2/PI489777)SNP_09796	62	G	A	19	4	--	--
	Ca(ICCV2/PI489777)SNP_09797	71	A	G	18	5	--	--
	Ca(ICCV2/PI489777)SNP_09798	76	T	G	19	5	--	--
	Ca(ICCV2/PI489777)SNP_09799	130	G	A	19	7	--	--
	Ca(ICCV2/PI489777)SNP_09800	224	C	T	17	7	--	--
	Ca(ICCV2/PI489777)SNP_09801	245	A	T	19	6	--	--
	Ca(ICCV2/PI489777)SNP_09802	563	T	G	18	8	--	--
	Ca(ICCV2/PI489777)SNP_09803	606	C	G	17	9	--	--
CakTC38411	Ca(ICCV2/PI489777)SNP_09804	222	C	T	40	15	--	--
	Ca(ICCV2/PI489777)SNP_09805	384	T	C	49	25	--	--
	Ca(ICCV2/PI489777)SNP_09806	955	T	C	32	22	--	--
	Ca(ICCV2/PI489777)SNP_09807	1029	C	G	33	16	--	--
CakTC36770	Ca(ICCV2/PI489777)SNP_09808	922	T	C	7	9	--	--
	Ca(ICCV2/PI489777)SNP_09809	943	A	T	8	8	--	--
	Ca(ICCV2/PI489777)SNP_09810	1356	G	C	11	6	--	--
	Ca(ICCV2/PI489777)SNP_09811	1909	C	T	7	9	--	--
	Ca(ICCV2/PI489777)SNP_09812	2276	G	T	9	12	--	--
	Ca(ICCV2/PI489777)SNP_09813	2306	A	C	13	11	--	--
	Ca(ICCV2/PI489777)SNP_09814	2357	G	A	12	10	--	--
	Ca(ICCV2/PI489777)SNP_09815	2391	C	T	11	10	--	--
	Ca(ICCV2/PI489777)SNP_09816	2444	G	T	14	10	--	--
	Ca(ICCV2/PI489777)SNP_09817	2462	C	T	12	9	--	--
CakTC25415	Ca(ICCV2/PI489777)SNP_09818	865	C	G	18	12	--	--
CakTC26463	Ca(ICCV2/PI489777)SNP_09819	344	G	C	14	8	--	--
	Ca(ICCV2/PI489777)SNP_09820	695	G	T	25	6	--	--
	Ca(ICCV2/PI489777)SNP_09821	698	C	G	25	7	--	--
	Ca(ICCV2/PI489777)SNP_09822	818	G	T	27	5	--	--
	Ca(ICCV2/PI489777)SNP_09823	827	T	C	27	7	--	--
	Ca(ICCV2/PI489777)SNP_09824	926	T	C	22	11	--	--
	Ca(ICCV2/PI489777)SNP_09825	929	C	A	17	11	--	--
	Ca(ICCV2/PI489777)SNP_09826	1144	A	G	27	10	--	--
	Ca(ICCV2/PI489777)SNP_09827	1239	G	T	20	7	--	--
CakTC39436	Ca(ICCV2/PI489777)SNP_09828	570	G	A	41	53	--	--
	Ca(ICCV2/PI489777)SNP_09829	683	C	T	45	55	--	--
	Ca(ICCV2/PI489777)SNP_09830	702	C	T	48	48	--	--
	Ca(ICCV2/PI489777)SNP_09831	1264	A	G	28	18	--	--
CakTC28970	Ca(ICCV2/PI489777)SNP_09832	894	C	A	10	3	--	--
CakTC06432	Ca(ICCV2/PI489777)SNP_09833	1355	C	G	9	3	--	--
	Ca(ICCV2/PI489777)SNP_09834	1895	G	A	8	3	--	--
	Ca(ICCV2/PI489777)SNP_09835	2955	T	C	4	12	--	--
CakTC32992	Ca(ICCV2/PI489777)SNP_09836	1071	T	C	5	3	--	--
	Ca(ICCV2/PI489777)SNP_09837	1458	T	C	10	3	--	--

	Ca(ICCV2/PI489777)SNP_09838	1896	C	T	5	4	--	--
	Ca(ICCV2/PI489777)SNP_09839	2314	G	T	8	4	--	--
	Ca(ICCV2/PI489777)SNP_09840	2449	G	A	12	4	--	--
	Ca(ICCV2/PI489777)SNP_09841	2750	G	A	10	7	--	--
	Ca(ICCV2/PI489777)SNP_09842	2786	T	A	6	7	--	--
	Ca(ICCV2/PI489777)SNP_09843	2844	G	T	7	6	--	--
	Ca(ICCV2/PI489777)SNP_09844	3015	T	C	15	3	--	--
	Ca(ICCV2/PI489777)SNP_09845	3278	A	C	19	5	--	--
	Ca(ICCV2/PI489777)SNP_09846	3295	G	T	23	5	--	--
	Ca(ICCV2/PI489777)SNP_09847	3372	T	C	16	6	--	--
	Ca(ICCV2/PI489777)SNP_09848	3661	G	A	14	11	--	--
	Ca(ICCV2/PI489777)SNP_09849	3690	A	G	16	11	--	--
	Ca(ICCV2/PI489777)SNP_09850	3855	G	A	8	13	--	--
	Ca(ICCV2/PI489777)SNP_09851	3871	C	G	8	10	--	--
	Ca(ICCV2/PI489777)SNP_09852	4134	G	A	5	7	--	--
CakTC37180	Ca(ICCV2/PI489777)SNP_09853	120	T	C	13	10	--	--
	Ca(ICCV2/PI489777)SNP_09854	252	T	C	12	8	--	--
CakTC31693	Ca(ICCV2/PI489777)SNP_09855	78	T	C	5	3	--	--
	Ca(ICCV2/PI489777)SNP_09856	110	C	T	7	4	--	--
	Ca(ICCV2/PI489777)SNP_09857	361	C	A	14	3	--	--
	Ca(ICCV2/PI489777)SNP_09858	1154	G	A	4	3	--	--
CakTC29551	Ca(ICCV2/PI489777)SNP_09859	789	G	A	15	5	--	--
	Ca(ICCV2/PI489777)SNP_09860	1108	G	C	11	10	--	--
	Ca(ICCV2/PI489777)SNP_09861	1394	C	T	11	9	--	--
	Ca(ICCV2/PI489777)SNP_09862	1448	T	C	8	8	--	--
	Ca(ICCV2/PI489777)SNP_09863	1474	T	C	8	8	--	--
CakTC42899	Ca(ICCV2/PI489777)SNP_09864	1371	C	G	4	3	--	Bromodomain
CakTC38737	Ca(ICCV2/PI489777)SNP_09865	523	T	C	5	4	--	--
	Ca(ICCV2/PI489777)SNP_09866	568	C	G	7	4	--	--
CakTC28929	Ca(ICCV2/PI489777)SNP_09867	581	T	G	7	13	Young_pod	TCP
	Ca(ICCV2/PI489777)SNP_09868	719	A	G	9	11	Young_pod	TCP
	Ca(ICCV2/PI489777)SNP_09869	731	G	A	10	12	Young_pod	TCP
CakTC39283	Ca(ICCV2/PI489777)SNP_09870	508	G	A	11	7	--	SAP
	Ca(ICCV2/PI489777)SNP_09871	672	C	A	9	4	--	SAP
	Ca(ICCV2/PI489777)SNP_09872	1051	G	A	6	4	--	SAP
	Ca(ICCV2/PI489777)SNP_09873	1204	C	T	8	7	--	SAP
	Ca(ICCV2/PI489777)SNP_09874	1442	A	G	4	6	--	SAP
	Ca(ICCV2/PI489777)SNP_09875	1507	G	C	3	3	--	SAP
CakTC31103	Ca(ICCV2/PI489777)SNP_09876	1045	A	G	3	5	--	Trihelix
CakTC33042	Ca(ICCV2/PI489777)SNP_09877	192	T	C	12	13	--	--
	Ca(ICCV2/PI489777)SNP_09878	749	A	C	38	20	--	--
	Ca(ICCV2/PI489777)SNP_09879	854	T	C	43	24	--	--
	Ca(ICCV2/PI489777)SNP_09880	863	A	G	36	25	--	--
	Ca(ICCV2/PI489777)SNP_09881	2363	C	T	77	62	--	--
	Ca(ICCV2/PI489777)SNP_09882	2522	C	T	42	37	--	--
	Ca(ICCV2/PI489777)SNP_09883	2567	A	C	40	47	--	--
CakTC32389	Ca(ICCV2/PI489777)SNP_09884	1276	T	C	6	11	--	--
CakTC35712	Ca(ICCV2/PI489777)SNP_09885	626	A	G	24	17	--	--
	Ca(ICCV2/PI489777)SNP_09886	1148	T	C	12	13	--	--
	Ca(ICCV2/PI489777)SNP_09887	1636	T	C	14	11	--	--
	Ca(ICCV2/PI489777)SNP_09888	2143	A	T	19	12	--	--
	Ca(ICCV2/PI489777)SNP_09889	2839	C	T	12	7	--	--
	Ca(ICCV2/PI489777)SNP_09890	2867	C	T	11	4	--	--
	Ca(ICCV2/PI489777)SNP_09891	3099	C	T	10	6	--	--
	Ca(ICCV2/PI489777)SNP_09892	3155	G	C	7	6	--	--
CakTC39648	Ca(ICCV2/PI489777)SNP_09893	90	G	C	11	4	--	--
	Ca(ICCV2/PI489777)SNP_09894	153	T	C	9	5	--	--
	Ca(ICCV2/PI489777)SNP_09895	162	C	T	6	5	--	--
	Ca(ICCV2/PI489777)SNP_09896	477	C	T	16	9	--	--
CakTC37358	Ca(ICCV2/PI489777)SNP_09897	832	G	A	54	18	--	--
CakTC37319	Ca(ICCV2/PI489777)SNP_09898	162	G	C	8	7	--	--
	Ca(ICCV2/PI489777)SNP_09899	242	G	A	10	11	--	--
CakTC26485	Ca(ICCV2/PI489777)SNP_09900	268	G	A	12	14	--	--
	Ca(ICCV2/PI489777)SNP_09901	368	A	G	18	18	--	--
	Ca(ICCV2/PI489777)SNP_09902	573	G	A	44	13	--	--
	Ca(ICCV2/PI489777)SNP_09903	933	T	C	42	17	--	--
	Ca(ICCV2/PI489777)SNP_09904	1686	C	T	16	4	--	--
CakTC28906	Ca(ICCV2/PI489777)SNP_09905	175	T	G	3	6	--	--
CakTC35347	Ca(ICCV2/PI489777)SNP_09906	1029	C	T	4	5	--	--
	Ca(ICCV2/PI489777)SNP_09907	1343	A	T	9	6	--	--
CakTC03796	Ca(ICCV2/PI489777)SNP_09908	213	A	G	3	11	--	--
CakTC42493	Ca(ICCV2/PI489777)SNP_09909	1048	T	C	4	13	--	--
CakTC38315	Ca(ICCV2/PI489777)SNP_09910	246	G	T	39	25	Flower	bud
	Ca(ICCV2/PI489777)SNP_09911	1359	T	G	20	11	Flower	bud

	Ca(ICCV2/PI489777)SNP_09912	1442	A	G	13	7	Flower	bud
CakTC40095	Ca(ICCV2/PI489777)SNP_09913	403	T	C	17	4	--	--
CakTC11412	Ca(ICCV2/PI489777)SNP_09914	47	A	T	9	7	--	--
	Ca(ICCV2/PI489777)SNP_09915	175	T	C	14	13	--	--
	Ca(ICCV2/PI489777)SNP_09916	575	A	G	9	4	--	--
	Ca(ICCV2/PI489777)SNP_09917	594	T	C	8	3	--	--
	Ca(ICCV2/PI489777)SNP_09918	657	C	T	4	3	--	--
CakTC40226	Ca(ICCV2/PI489777)SNP_09919	924	C	T	15	14	--	--
	Ca(ICCV2/PI489777)SNP_09920	1371	C	T	9	5	--	--
CakTC25355	Ca(ICCV2/PI489777)SNP_09921	751	A	G	34	7	--	--
	Ca(ICCV2/PI489777)SNP_09922	1022	C	T	36	8	--	--
	Ca(ICCV2/PI489777)SNP_09923	1364	T	C	8	3	--	--
	Ca(ICCV2/PI489777)SNP_09924	2395	A	G	12	3	--	--
CakTC39871	Ca(ICCV2/PI489777)SNP_09925	631	T	C	77	35	--	bZIP
	Ca(ICCV2/PI489777)SNP_09926	792	C	G	92	52	--	bZIP
	Ca(ICCV2/PI489777)SNP_09927	1348	G	T	30	20	--	bZIP
CakTC38017	Ca(ICCV2/PI489777)SNP_09928	120	G	C	3	7	--	--
	Ca(ICCV2/PI489777)SNP_09929	207	G	T	8	7	--	--
CakTC23679	Ca(ICCV2/PI489777)SNP_09930	908	T	C	33	7	Flower	bud
CakTC42658	Ca(ICCV2/PI489777)SNP_09931	163	T	C	5	10	--	--
CakTC24361	Ca(ICCV2/PI489777)SNP_09932	239	G	A	6	3	Young_pod	--
	Ca(ICCV2/PI489777)SNP_09933	252	T	A	4	4	Young_pod	--
	Ca(ICCV2/PI489777)SNP_09934	291	A	C	3	4	Young_pod	--
	Ca(ICCV2/PI489777)SNP_09935	324	T	C	3	4	Young_pod	--
	Ca(ICCV2/PI489777)SNP_09936	369	T	C	3	4	Young_pod	--
	Ca(ICCV2/PI489777)SNP_09937	416	T	C	3	4	Young_pod	--
	Ca(ICCV2/PI489777)SNP_09938	417	T	C	3	4	Young_pod	--
	Ca(ICCV2/PI489777)SNP_09939	449	C	T	4	3	Young_pod	--
	Ca(ICCV2/PI489777)SNP_09940	451	A	G	4	4	Young_pod	--
	Ca(ICCV2/PI489777)SNP_09941	980	T	G	4	3	Young_pod	--
	Ca(ICCV2/PI489777)SNP_09942	981	G	A	4	3	Young_pod	--
	Ca(ICCV2/PI489777)SNP_09943	1060	C	T	3	3	Young_pod	--
	Ca(ICCV2/PI489777)SNP_09944	1085	A	C	3	3	Young_pod	--
	Ca(ICCV2/PI489777)SNP_09945	1096	C	T	3	3	Young_pod	--
	Ca(ICCV2/PI489777)SNP_09946	1178	C	T	3	3	Young_pod	--
CakTC40168	Ca(ICCV2/PI489777)SNP_09947	783	A	G	4	4	--	--
CakTC24642	Ca(ICCV2/PI489777)SNP_09948	183	C	A	17	4	--	--
	Ca(ICCV2/PI489777)SNP_09949	189	T	C	17	4	--	--
	Ca(ICCV2/PI489777)SNP_09950	449	G	C	38	10	--	--
	Ca(ICCV2/PI489777)SNP_09951	464	A	G	37	10	--	--
	Ca(ICCV2/PI489777)SNP_09952	650	G	A	38	20	--	--
	Ca(ICCV2/PI489777)SNP_09953	1202	A	C	35	10	--	--
CakTC11906	Ca(ICCV2/PI489777)SNP_09954	1406	A	C	24	4	Shoot	--
CakTC27989	Ca(ICCV2/PI489777)SNP_09955	1086	G	A	16	9	--	--
CakTC37474	Ca(ICCV2/PI489777)SNP_09956	153	T	A	31	21	--	--
	Ca(ICCV2/PI489777)SNP_09957	411	G	T	43	26	--	--
	Ca(ICCV2/PI489777)SNP_09958	775	T	C	23	8	--	--
	Ca(ICCV2/PI489777)SNP_09959	798	C	T	23	6	--	--
CakTC34697	Ca(ICCV2/PI489777)SNP_09960	312	C	A	12	6	--	--
	Ca(ICCV2/PI489777)SNP_09961	315	A	C	11	6	--	--
	Ca(ICCV2/PI489777)SNP_09962	2781	C	G	16	3	--	--
CakTC38350	Ca(ICCV2/PI489777)SNP_09963	697	A	C	8	3	Mature	Leaf
	Ca(ICCV2/PI489777)SNP_09964	749	C	T	11	3	Mature	Leaf
	Ca(ICCV2/PI489777)SNP_09965	844	T	A	14	4	Mature	Leaf
	Ca(ICCV2/PI489777)SNP_09966	858	T	C	13	4	Mature	Leaf
	Ca(ICCV2/PI489777)SNP_09967	888	A	C	13	4	Mature	Leaf
	Ca(ICCV2/PI489777)SNP_09968	948	A	G	11	4	Mature	Leaf
CakTC41550	Ca(ICCV2/PI489777)SNP_09969	471	C	T	38	81	--	--
CakTC38864	Ca(ICCV2/PI489777)SNP_09970	461	T	C	19	7	--	--
	Ca(ICCV2/PI489777)SNP_09971	806	C	T	17	11	--	--
CakTC14821	Ca(ICCV2/PI489777)SNP_09972	28	C	A	3	5	--	--
CakTC11372	Ca(ICCV2/PI489777)SNP_09973	197	C	T	6	3	--	--
CakTC40315	Ca(ICCV2/PI489777)SNP_09974	2029	G	A	3	5	--	--
CakTC32382	Ca(ICCV2/PI489777)SNP_09975	310	A	G	3	4	--	--
	Ca(ICCV2/PI489777)SNP_09976	611	A	T	3	7	--	--
CakTC26856	Ca(ICCV2/PI489777)SNP_09977	400	G	A	15	3	Flower	bud
	Ca(ICCV2/PI489777)SNP_09978	470	C	G	13	5	Flower	bud
	Ca(ICCV2/PI489777)SNP_09979	488	A	T	12	6	Flower	bud
CakTC27961	Ca(ICCV2/PI489777)SNP_09980	178	G	A	31	12	--	--
	Ca(ICCV2/PI489777)SNP_09981	311	T	C	32	15	--	--
	Ca(ICCV2/PI489777)SNP_09982	650	C	T	20	9	--	--
CakTC39600	Ca(ICCV2/PI489777)SNP_09983	413	T	C	14	12	--	--
	Ca(ICCV2/PI489777)SNP_09984	659	C	T	7	3	--	--
	Ca(ICCV2/PI489777)SNP_09985	917	G	T	13	6	--	--

	Ca(ICCV2/PI489777)SNP_09986	946	A	G	11	6	--	--
CakTC32814	Ca(ICCV2/PI489777)SNP_09987	429	T	G	8	10	--	--
CakTC37893	Ca(ICCV2/PI489777)SNP_09988	414	T	C	32	18	--	--
	Ca(ICCV2/PI489777)SNP_09989	492	G	A	27	17	--	--
	Ca(ICCV2/PI489777)SNP_09990	569	T	C	19	20	--	--
	Ca(ICCV2/PI489777)SNP_09991	1011	G	A	46	20	--	--
	Ca(ICCV2/PI489777)SNP_09992	1065	C	T	55	28	--	--
	Ca(ICCV2/PI489777)SNP_09993	1198	T	C	48	29	--	--
	Ca(ICCV2/PI489777)SNP_09994	1606	T	C	3	4	--	--
CakTC42536	Ca(ICCV2/PI489777)SNP_09995	1699	G	C	3	4	--	--
CakTC38369	Ca(ICCV2/PI489777)SNP_09996	1000	G	A	7	4	--	--
	Ca(ICCV2/PI489777)SNP_09997	1074	A	C	8	4	--	--
	Ca(ICCV2/PI489777)SNP_09998	1075	G	A	7	4	--	--
	Ca(ICCV2/PI489777)SNP_09999	1117	A	T	7	3	--	--
	Ca(ICCV2/PI489777)SNP_10000	1143	A	T	6	3	--	--
CakTC27629	Ca(ICCV2/PI489777)SNP_10001	1009	A	G	16	11	--	--
	Ca(ICCV2/PI489777)SNP_10002	2045	T	C	7	10	--	--
CakTC34309	Ca(ICCV2/PI489777)SNP_10003	861	A	G	27	30	--	GRAS
	Ca(ICCV2/PI489777)SNP_10004	1659	A	G	44	28	--	GRAS
	Ca(ICCV2/PI489777)SNP_10005	1755	T	G	72	45	--	GRAS
	Ca(ICCV2/PI489777)SNP_10006	1911	G	T	70	40	--	GRAS
CakTC34296	Ca(ICCV2/PI489777)SNP_10007	731	C	T	7	7	--	--
	Ca(ICCV2/PI489777)SNP_10008	1100	C	T	4	3	--	--
	Ca(ICCV2/PI489777)SNP_10009	1350	G	A	6	7	--	--
CakTC24136	Ca(ICCV2/PI489777)SNP_10010	994	A	G	8	7	--	--
	Ca(ICCV2/PI489777)SNP_10011	1645	T	C	9	10	--	--
	Ca(ICCV2/PI489777)SNP_10012	1690	A	T	9	11	--	--
	Ca(ICCV2/PI489777)SNP_10013	2269	T	C	5	5	--	--
CakTC18976	Ca(ICCV2/PI489777)SNP_10014	1944	A	G	7	4	--	--
	Ca(ICCV2/PI489777)SNP_10015	1985	A	G	7	4	--	--
CakTC23792	Ca(ICCV2/PI489777)SNP_10016	532	G	A	19	11	--	--
	Ca(ICCV2/PI489777)SNP_10017	533	C	A	19	11	--	--
	Ca(ICCV2/PI489777)SNP_10018	1524	T	C	11	7	--	--
	Ca(ICCV2/PI489777)SNP_10019	2011	T	C	18	5	--	--
	Ca(ICCV2/PI489777)SNP_10020	2028	C	T	17	3	--	--
	Ca(ICCV2/PI489777)SNP_10021	2086	T	C	16	6	--	--
	Ca(ICCV2/PI489777)SNP_10022	3371	A	G	6	3	--	--
	Ca(ICCV2/PI489777)SNP_10023	3584	A	G	3	3	--	--
CakTC23611	Ca(ICCV2/PI489777)SNP_10024	568	A	G	6	5	Young_pod	--
	Ca(ICCV2/PI489777)SNP_10025	577	C	G	6	5	Young_pod	--
CakTC29273	Ca(ICCV2/PI489777)SNP_10026	1048	C	T	9	5	--	--
CakTC18498	Ca(ICCV2/PI489777)SNP_10027	730	A	G	3	7	--	C2H2
	Ca(ICCV2/PI489777)SNP_10028	802	C	A	3	7	--	C2H2
	Ca(ICCV2/PI489777)SNP_10029	900	C	T	4	5	--	C2H2
	Ca(ICCV2/PI489777)SNP_10030	951	C	G	3	3	--	C2H2
	Ca(ICCV2/PI489777)SNP_10031	1014	A	T	3	3	--	C2H2
	Ca(ICCV2/PI489777)SNP_10032	1036	A	G	3	3	--	C2H2
	Ca(ICCV2/PI489777)SNP_10033	1045	C	T	3	3	--	C2H2
	Ca(ICCV2/PI489777)SNP_10034	1081	C	T	3	3	--	C2H2
CakTC42551	Ca(ICCV2/PI489777)SNP_10035	353	T	C	3	8	--	--
	Ca(ICCV2/PI489777)SNP_10036	742	C	T	7	12	--	--
CakTC27240	Ca(ICCV2/PI489777)SNP_10037	2163	G	T	24	14	--	--
	Ca(ICCV2/PI489777)SNP_10038	2208	G	A	16	12	--	--
	Ca(ICCV2/PI489777)SNP_10039	2244	T	G	27	10	--	--
	Ca(ICCV2/PI489777)SNP_10040	2256	A	G	27	10	--	--
	Ca(ICCV2/PI489777)SNP_10041	2319	G	A	22	6	--	--
	Ca(ICCV2/PI489777)SNP_10042	2352	C	T	17	3	--	--
CakTC29092	Ca(ICCV2/PI489777)SNP_10043	784	T	C	8	4	--	--
	Ca(ICCV2/PI489777)SNP_10044	960	T	C	6	4	--	--
	Ca(ICCV2/PI489777)SNP_10045	1220	A	T	3	3	--	--
	Ca(ICCV2/PI489777)SNP_10046	1688	A	G	5	6	--	--
	Ca(ICCV2/PI489777)SNP_10047	1777	A	C	6	5	--	--
	Ca(ICCV2/PI489777)SNP_10048	2628	G	C	7	4	--	--
	Ca(ICCV2/PI489777)SNP_10049	2634	G	C	7	5	--	--
CakTC40453	Ca(ICCV2/PI489777)SNP_10050	45	T	C	5	5	--	--
	Ca(ICCV2/PI489777)SNP_10051	398	G	A	25	14	--	--
	Ca(ICCV2/PI489777)SNP_10052	497	A	G	15	15	--	--
	Ca(ICCV2/PI489777)SNP_10053	836	A	G	8	26	--	--
	Ca(ICCV2/PI489777)SNP_10054	1376	G	A	18	8	--	--
CakTC35065	Ca(ICCV2/PI489777)SNP_10055	1610	A	G	13	7	--	--
CakTC33865	Ca(ICCV2/PI489777)SNP_10056	333	A	T	143	72	--	--
	Ca(ICCV2/PI489777)SNP_10057	504	G	A	41	29	--	--
CakTC29218	Ca(ICCV2/PI489777)SNP_10058	862	C	T	4	3	--	--
CakTC13737	Ca(ICCV2/PI489777)SNP_10059	2259	C	T	3	3	--	--

CakTC33246	Ca(ICCV2/PI489777)SNP_10060	1367	G	T	8	7	--	--
	Ca(ICCV2/PI489777)SNP_10061	1419	A	G	7	5	--	--
	Ca(ICCV2/PI489777)SNP_10062	1660	C	G	4	3	--	--
	Ca(ICCV2/PI489777)SNP_10063	1934	T	A	5	4	--	--
CakTC31575	Ca(ICCV2/PI489777)SNP_10064	331	T	C	17	5	--	--
	Ca(ICCV2/PI489777)SNP_10065	565	T	G	7	6	--	--
	Ca(ICCV2/PI489777)SNP_10066	1179	A	G	8	10	--	--
CakTC10135	Ca(ICCV2/PI489777)SNP_10067	1721	A	G	26	14	--	--
	Ca(ICCV2/PI489777)SNP_10068	1898	A	T	26	18	--	--
CakTC22788	Ca(ICCV2/PI489777)SNP_10069	368	C	T	157	38	--	--
	Ca(ICCV2/PI489777)SNP_10070	461	T	G	109	28	--	--
	Ca(ICCV2/PI489777)SNP_10071	515	C	T	53	17	--	--
CakTC26913	Ca(ICCV2/PI489777)SNP_10072	513	C	A	12	3	--	--
	Ca(ICCV2/PI489777)SNP_10073	2278	A	C	28	13	--	--
	Ca(ICCV2/PI489777)SNP_10074	2388	C	T	30	16	--	--
	Ca(ICCV2/PI489777)SNP_10075	2473	A	G	33	11	--	--
	Ca(ICCV2/PI489777)SNP_10076	2742	C	T	26	12	--	--
	Ca(ICCV2/PI489777)SNP_10077	2970	A	G	22	9	--	--
CakTC27951	Ca(ICCV2/PI489777)SNP_10078	746	G	A	26	11	--	--
CakTC41969	Ca(ICCV2/PI489777)SNP_10079	441	G	T	11	15	--	--
CakTC38537	Ca(ICCV2/PI489777)SNP_10080	167	G	A	27	4	--	--
	Ca(ICCV2/PI489777)SNP_10081	488	G	A	47	17	--	--
CakTC31239	Ca(ICCV2/PI489777)SNP_10082	308	G	A	5	3	--	--
CakTC42292	Ca(ICCV2/PI489777)SNP_10083	286	T	A	15	15	--	--
	Ca(ICCV2/PI489777)SNP_10084	500	T	C	3	7	--	--
	Ca(ICCV2/PI489777)SNP_10085	505	C	A	3	6	--	--
	Ca(ICCV2/PI489777)SNP_10086	739	T	C	4	4	--	--
CakTC39364	Ca(ICCV2/PI489777)SNP_10087	97	A	G	6	4	--	--
	Ca(ICCV2/PI489777)SNP_10088	287	G	A	70	70	--	--
	Ca(ICCV2/PI489777)SNP_10089	292	T	C	73	68	--	--
	Ca(ICCV2/PI489777)SNP_10090	559	G	A	65	52	--	--
	Ca(ICCV2/PI489777)SNP_10091	790	A	C	70	41	--	--
CakTC23315	Ca(ICCV2/PI489777)SNP_10092	1108	T	C	4	4	--	--
	Ca(ICCV2/PI489777)SNP_10093	1150	C	T	3	4	--	--
	Ca(ICCV2/PI489777)SNP_10094	1158	C	A	3	4	--	--
CakTC27362	Ca(ICCV2/PI489777)SNP_10095	433	T	C	10	5	--	bHLH
	Ca(ICCV2/PI489777)SNP_10096	724	C	T	14	4	--	bHLH
	Ca(ICCV2/PI489777)SNP_10097	892	C	G	17	4	--	bHLH
CakTC30672	Ca(ICCV2/PI489777)SNP_10098	397	A	G	9	3	--	--
	Ca(ICCV2/PI489777)SNP_10099	609	T	C	6	3	--	--
CakTC28581	Ca(ICCV2/PI489777)SNP_10100	167	T	C	9	3	--	--
	Ca(ICCV2/PI489777)SNP_10101	188	T	A	13	3	--	--
	Ca(ICCV2/PI489777)SNP_10102	209	A	C	14	3	--	--
	Ca(ICCV2/PI489777)SNP_10103	1007	A	G	24	17	--	--
	Ca(ICCV2/PI489777)SNP_10104	1565	C	T	21	21	--	--
	Ca(ICCV2/PI489777)SNP_10105	1625	T	G	14	15	--	--
	Ca(ICCV2/PI489777)SNP_10106	1925	T	C	30	12	--	--
	Ca(ICCV2/PI489777)SNP_10107	1961	G	C	32	13	--	--
	Ca(ICCV2/PI489777)SNP_10108	2154	T	G	34	14	--	--
	Ca(ICCV2/PI489777)SNP_10109	2217	A	C	53	16	--	--
	Ca(ICCV2/PI489777)SNP_10110	2243	G	A	55	16	--	--
	Ca(ICCV2/PI489777)SNP_10111	3297	T	C	3	4	--	--
CakTC26243	Ca(ICCV2/PI489777)SNP_10112	1300	G	C	9	9	--	--
CakTC30450	Ca(ICCV2/PI489777)SNP_10113	381	T	C	18	4	--	--
	Ca(ICCV2/PI489777)SNP_10114	432	T	G	18	5	--	--
	Ca(ICCV2/PI489777)SNP_10115	510	C	T	16	4	--	--
	Ca(ICCV2/PI489777)SNP_10116	769	C	G	20	4	--	--
	Ca(ICCV2/PI489777)SNP_10117	1693	A	C	6	4	--	--
CakTC00369	Ca(ICCV2/PI489777)SNP_10118	517	G	A	5	3	--	--
CakTC38984	Ca(ICCV2/PI489777)SNP_10119	141	G	T	9	3	--	Tify
	Ca(ICCV2/PI489777)SNP_10120	599	C	T	9	4	--	Tify
	Ca(ICCV2/PI489777)SNP_10121	631	G	T	9	3	--	Tify
	Ca(ICCV2/PI489777)SNP_10122	718	T	C	4	3	--	Tify
CakTC28466	Ca(ICCV2/PI489777)SNP_10123	346	G	A	4	4	--	--
CakTC38996	Ca(ICCV2/PI489777)SNP_10124	252	A	G	6	9	--	--
	Ca(ICCV2/PI489777)SNP_10125	809	G	T	9	9	--	--
	Ca(ICCV2/PI489777)SNP_10126	1259	G	C	7	9	--	--
	Ca(ICCV2/PI489777)SNP_10127	1315	G	A	5	7	--	--
CakTC26308	Ca(ICCV2/PI489777)SNP_10128	1063	G	A	18	16	--	--
	Ca(ICCV2/PI489777)SNP_10129	1336	T	C	11	9	--	--
CakTC42994	Ca(ICCV2/PI489777)SNP_10130	695	G	C	3	21	--	--
	Ca(ICCV2/PI489777)SNP_10131	1322	G	A	3	15	--	--
	Ca(ICCV2/PI489777)SNP_10132	1505	T	A	5	12	--	--
	Ca(ICCV2/PI489777)SNP_10133	1901	G	A	3	12	--	--

CakTC41442	Ca(ICCV2/PI489777)SNP_10134	629	T	A	18	23	--	--
	Ca(ICCV2/PI489777)SNP_10135	2242	G	C	10	5	--	--
CakTC28016	Ca(ICCV2/PI489777)SNP_10136	58	A	G	5	10	--	--
	Ca(ICCV2/PI489777)SNP_10137	391	A	T	13	12	--	--
	Ca(ICCV2/PI489777)SNP_10138	415	A	C	13	13	--	--
	Ca(ICCV2/PI489777)SNP_10139	1227	T	C	13	9	--	--
	Ca(ICCV2/PI489777)SNP_10140	1707	A	T	46	7	--	--
	Ca(ICCV2/PI489777)SNP_10141	1767	G	C	57	8	--	--
	Ca(ICCV2/PI489777)SNP_10142	1828	A	C	56	6	--	--
	Ca(ICCV2/PI489777)SNP_10143	2139	G	A	13	8	--	--
	Ca(ICCV2/PI489777)SNP_10144	2404	A	G	14	8	--	--
	Ca(ICCV2/PI489777)SNP_10145	2667	G	C	12	6	--	--
	Ca(ICCV2/PI489777)SNP_10146	2692	G	A	13	5	--	--
CakTC34278	Ca(ICCV2/PI489777)SNP_10147	21	C	A	3	3	--	--
	Ca(ICCV2/PI489777)SNP_10148	128	C	T	25	19	--	--
CakTC23041	Ca(ICCV2/PI489777)SNP_10149	117	G	A	167	138	--	--
	Ca(ICCV2/PI489777)SNP_10150	168	T	C	243	169	--	--
CakTC38939	Ca(ICCV2/PI489777)SNP_10151	866	G	A	24	14	--	--
CakTC30946	Ca(ICCV2/PI489777)SNP_10152	291	C	T	18	5	--	--
	Ca(ICCV2/PI489777)SNP_10153	696	C	T	8	24	--	--
	Ca(ICCV2/PI489777)SNP_10154	807	A	C	11	21	--	--
	Ca(ICCV2/PI489777)SNP_10155	1094	T	C	9	11	--	--
CakTC40279	Ca(ICCV2/PI489777)SNP_10156	753	C	G	22	20	--	--
CakTC39254	Ca(ICCV2/PI489777)SNP_10157	67	T	C	5	3	--	--
CakTC09652	Ca(ICCV2/PI489777)SNP_10158	891	G	T	15	3	--	--
	Ca(ICCV2/PI489777)SNP_10159	930	A	G	15	3	--	--
	Ca(ICCV2/PI489777)SNP_10160	2067	T	G	12	5	--	--
	Ca(ICCV2/PI489777)SNP_10161	2163	A	G	8	5	--	--
	Ca(ICCV2/PI489777)SNP_10162	5192	A	G	8	3	--	--
CakTC23875	Ca(ICCV2/PI489777)SNP_10163	452	C	G	322	85	--	--
	Ca(ICCV2/PI489777)SNP_10164	836	G	A	288	19	--	--
	Ca(ICCV2/PI489777)SNP_10165	934	G	A	22	13	--	--
CakTC34623	Ca(ICCV2/PI489777)SNP_10166	108	G	C	8	3	--	--
	Ca(ICCV2/PI489777)SNP_10167	164	C	T	8	3	--	--
	Ca(ICCV2/PI489777)SNP_10168	331	T	C	7	5	--	--
	Ca(ICCV2/PI489777)SNP_10169	341	G	A	7	5	--	--
	Ca(ICCV2/PI489777)SNP_10170	439	T	A	4	5	--	--
CakTC29972	Ca(ICCV2/PI489777)SNP_10171	202	T	C	10	7	--	TPR
	Ca(ICCV2/PI489777)SNP_10172	238	A	G	10	9	--	TPR
	Ca(ICCV2/PI489777)SNP_10173	362	G	A	12	9	--	TPR
	Ca(ICCV2/PI489777)SNP_10174	363	C	G	12	10	--	TPR
	Ca(ICCV2/PI489777)SNP_10175	1201	C	A	6	3	--	TPR
	Ca(ICCV2/PI489777)SNP_10176	1302	T	C	9	3	--	TPR
	Ca(ICCV2/PI489777)SNP_10177	1844	T	C	10	7	--	TPR
	Ca(ICCV2/PI489777)SNP_10178	2306	C	A	6	6	--	TPR
	Ca(ICCV2/PI489777)SNP_10179	2527	A	G	7	7	--	TPR
	Ca(ICCV2/PI489777)SNP_10180	2646	A	G	8	6	--	TPR
	Ca(ICCV2/PI489777)SNP_10181	3019	C	A	17	6	--	TPR
CakTC28092	Ca(ICCV2/PI489777)SNP_10182	687	G	T	12	12	--	--
	Ca(ICCV2/PI489777)SNP_10183	719	T	C	13	14	--	--
	Ca(ICCV2/PI489777)SNP_10184	891	G	A	18	11	--	--
	Ca(ICCV2/PI489777)SNP_10185	1341	G	A	12	4	--	--
	Ca(ICCV2/PI489777)SNP_10186	1871	A	C	60	17	--	--
	Ca(ICCV2/PI489777)SNP_10187	1946	T	C	57	14	--	--
	Ca(ICCV2/PI489777)SNP_10188	2360	G	C	25	9	--	--
CakTC14618	Ca(ICCV2/PI489777)SNP_10189	619	C	T	4	3	--	--
CakTC32547	Ca(ICCV2/PI489777)SNP_10190	127	C	G	43	19	--	--
	Ca(ICCV2/PI489777)SNP_10191	403	A	G	63	29	--	--
	Ca(ICCV2/PI489777)SNP_10192	1626	T	C	74	31	--	--
	Ca(ICCV2/PI489777)SNP_10193	1800	T	A	41	21	--	--
	Ca(ICCV2/PI489777)SNP_10194	1857	G	T	37	20	--	--
CakTC25704	Ca(ICCV2/PI489777)SNP_10195	1062	C	T	3	4	--	--
CakTC10326	Ca(ICCV2/PI489777)SNP_10196	665	A	C	14	6	--	--
	Ca(ICCV2/PI489777)SNP_10197	683	T	C	11	7	--	--
	Ca(ICCV2/PI489777)SNP_10198	1278	T	G	14	9	--	--
CakTC24753	Ca(ICCV2/PI489777)SNP_10199	266	C	T	8	6	--	--
	Ca(ICCV2/PI489777)SNP_10200	1197	G	A	15	7	--	--
CakTC39314	Ca(ICCV2/PI489777)SNP_10201	759	C	A	5	6	--	--
CakTC24279	Ca(ICCV2/PI489777)SNP_10202	856	A	G	40	81	Root	--
	Ca(ICCV2/PI489777)SNP_10203	928	T	A	39	88	Root	--
	Ca(ICCV2/PI489777)SNP_10204	1042	G	A	38	117	Root	--
	Ca(ICCV2/PI489777)SNP_10205	1145	A	G	32	102	Root	--
	Ca(ICCV2/PI489777)SNP_10206	1288	C	T	40	100	Root	--
	Ca(ICCV2/PI489777)SNP_10207	1674	T	C	8	15	Root	--

CakTC42625	Ca(ICCV2/PI489777)SNP_10208	165	C	A	141	90	--	--
	Ca(ICCV2/PI489777)SNP_10209	176	C	T	143	90	--	--
	Ca(ICCV2/PI489777)SNP_10210	740	T	C	8	25	--	--
	Ca(ICCV2/PI489777)SNP_10211	746	T	A	8	16	--	--
CakTC34229	Ca(ICCV2/PI489777)SNP_10212	1091	G	A	6	9	--	HB
	Ca(ICCV2/PI489777)SNP_10213	1913	C	T	16	3	--	HB
	Ca(ICCV2/PI489777)SNP_10214	3170	T	G	7	13	--	HB
	Ca(ICCV2/PI489777)SNP_10215	3394	G	A	8	19	--	HB
	Ca(ICCV2/PI489777)SNP_10216	3403	G	T	7	19	--	HB
	Ca(ICCV2/PI489777)SNP_10217	4031	A	G	16	4	--	HB
	Ca(ICCV2/PI489777)SNP_10218	4520	T	C	16	7	--	HB
	Ca(ICCV2/PI489777)SNP_10219	5144	T	C	18	7	--	HB
	Ca(ICCV2/PI489777)SNP_10220	5186	C	T	20	6	--	HB
	Ca(ICCV2/PI489777)SNP_10221	5478	A	C	19	9	--	HB
	Ca(ICCV2/PI489777)SNP_10222	5537	G	T	19	3	--	HB
	Ca(ICCV2/PI489777)SNP_10223	5632	T	C	20	4	--	HB
	Ca(ICCV2/PI489777)SNP_10224	6136	T	A	5	4	--	HB
CakTC38292	Ca(ICCV2/PI489777)SNP_10225	228	A	G	9	4	--	--
	Ca(ICCV2/PI489777)SNP_10226	524	G	C	5	3	--	--
CakTC37212	Ca(ICCV2/PI489777)SNP_10227	153	C	G	15	9	--	--
	Ca(ICCV2/PI489777)SNP_10228	462	C	T	34	11	--	--
	Ca(ICCV2/PI489777)SNP_10229	672	A	G	17	10	--	--
	Ca(ICCV2/PI489777)SNP_10230	1074	T	A	22	5	--	--
CakTC33593	Ca(ICCV2/PI489777)SNP_10231	165	C	T	4	9	--	--
	Ca(ICCV2/PI489777)SNP_10232	188	T	A	4	9	--	--
	Ca(ICCV2/PI489777)SNP_10233	239	G	C	4	10	--	--
	Ca(ICCV2/PI489777)SNP_10234	310	G	C	4	10	--	--
	Ca(ICCV2/PI489777)SNP_10235	651	C	G	23	8	--	--
	Ca(ICCV2/PI489777)SNP_10236	1830	A	G	38	9	--	--
	Ca(ICCV2/PI489777)SNP_10237	1989	A	T	36	12	--	--
CakTC29302	Ca(ICCV2/PI489777)SNP_10238	341	G	A	10	11	--	--
	Ca(ICCV2/PI489777)SNP_10239	689	A	C	13	7	--	--
	Ca(ICCV2/PI489777)SNP_10240	928	G	C	9	3	--	--
	Ca(ICCV2/PI489777)SNP_10241	1243	C	G	17	9	--	--
	Ca(ICCV2/PI489777)SNP_10242	1733	A	G	15	6	--	--
	Ca(ICCV2/PI489777)SNP_10243	1835	C	T	9	9	--	--
	Ca(ICCV2/PI489777)SNP_10244	1846	C	T	8	10	--	--
CakTC08008	Ca(ICCV2/PI489777)SNP_10245	4296	G	A	6	4	--	SET
	Ca(ICCV2/PI489777)SNP_10246	4840	G	A	3	4	--	SET
	Ca(ICCV2/PI489777)SNP_10247	5402	A	G	6	4	--	SET
	Ca(ICCV2/PI489777)SNP_10248	5424	C	G	7	4	--	SET
CakTC43244	Ca(ICCV2/PI489777)SNP_10249	193	G	A	15	10	--	ABI3VP1
	Ca(ICCV2/PI489777)SNP_10250	316	G	A	22	10	--	ABI3VP1
	Ca(ICCV2/PI489777)SNP_10251	667	T	C	17	16	--	ABI3VP1
	Ca(ICCV2/PI489777)SNP_10252	1030	T	C	18	19	--	ABI3VP1
	Ca(ICCV2/PI489777)SNP_10253	1118	T	G	19	22	--	ABI3VP1
	Ca(ICCV2/PI489777)SNP_10254	1120	A	T	20	22	--	ABI3VP1
CakTC37539	Ca(ICCV2/PI489777)SNP_10255	505	A	C	34	22	--	--
	Ca(ICCV2/PI489777)SNP_10256	1147	C	T	12	4	--	--
CakTC33319	Ca(ICCV2/PI489777)SNP_10257	107	C	A	16	15	--	--
	Ca(ICCV2/PI489777)SNP_10258	208	G	A	26	15	--	--
	Ca(ICCV2/PI489777)SNP_10259	2271	G	T	16	13	--	--
CakTC39251	Ca(ICCV2/PI489777)SNP_10260	138	G	T	20	13	--	--
	Ca(ICCV2/PI489777)SNP_10261	877	A	T	9	5	--	--
	Ca(ICCV2/PI489777)SNP_10262	879	A	C	10	8	--	--
	Ca(ICCV2/PI489777)SNP_10263	951	A	T	13	10	--	--
	Ca(ICCV2/PI489777)SNP_10264	1013	T	A	13	9	--	--
	Ca(ICCV2/PI489777)SNP_10265	1057	G	A	14	9	--	--
	Ca(ICCV2/PI489777)SNP_10266	1111	T	A	15	9	--	--
	Ca(ICCV2/PI489777)SNP_10267	1234	C	T	17	7	--	--
CakTC32316	Ca(ICCV2/PI489777)SNP_10268	148	T	A	12	4	--	--
	Ca(ICCV2/PI489777)SNP_10269	149	C	G	12	4	--	--
	Ca(ICCV2/PI489777)SNP_10270	188	G	A	15	4	--	--
	Ca(ICCV2/PI489777)SNP_10271	259	T	C	15	5	--	--
	Ca(ICCV2/PI489777)SNP_10272	421	C	T	6	15	--	--
	Ca(ICCV2/PI489777)SNP_10273	427	T	C	5	16	--	--
	Ca(ICCV2/PI489777)SNP_10274	646	C	G	6	18	--	--
	Ca(ICCV2/PI489777)SNP_10275	668	G	C	7	19	--	--
	Ca(ICCV2/PI489777)SNP_10276	865	A	G	7	4	--	--
	Ca(ICCV2/PI489777)SNP_10277	1075	T	C	3	4	--	--
	Ca(ICCV2/PI489777)SNP_10278	1404	A	G	11	4	--	--
CakTC27567	Ca(ICCV2/PI489777)SNP_10279	697	T	C	7	3	--	--
CakTC23350	Ca(ICCV2/PI489777)SNP_10280	455	T	A	42	11	--	--
	Ca(ICCV2/PI489777)SNP_10281	1145	T	C	35	3	--	--

	Ca(ICCV2/PI489777)SNP_10282	1151	G	A	35	4	--	--
	Ca(ICCV2/PI489777)SNP_10283	2252	C	G	21	4	--	--
	Ca(ICCV2/PI489777)SNP_10284	2266	A	G	17	4	--	--
CakTC12242	Ca(ICCV2/PI489777)SNP_10285	588	A	T	11	4	--	G2-like
CakTC27145	Ca(ICCV2/PI489777)SNP_10286	1133	A	G	4	3	--	--
	Ca(ICCV2/PI489777)SNP_10287	1155	G	C	3	3	--	--
CakTC39692	Ca(ICCV2/PI489777)SNP_10288	399	C	T	5	10	--	--
	Ca(ICCV2/PI489777)SNP_10289	689	C	T	8	9	--	--
	Ca(ICCV2/PI489777)SNP_10290	1154	T	C	23	10	--	--
	Ca(ICCV2/PI489777)SNP_10291	1308	T	C	17	7	--	--
	Ca(ICCV2/PI489777)SNP_10292	1440	A	C	12	5	--	--
	Ca(ICCV2/PI489777)SNP_10293	1492	C	G	12	4	--	--
CakTC37897	Ca(ICCV2/PI489777)SNP_10294	51	C	A	3	8	--	--
	Ca(ICCV2/PI489777)SNP_10295	167	A	C	7	13	--	--
	Ca(ICCV2/PI489777)SNP_10296	898	G	A	41	15	--	--
CakTC07418	Ca(ICCV2/PI489777)SNP_10297	555	A	T	4	6	--	--
CakTC05061	Ca(ICCV2/PI489777)SNP_10298	453	G	A	5	3	--	--
	Ca(ICCV2/PI489777)SNP_10299	672	T	C	3	3	--	--
CakTC37601	Ca(ICCV2/PI489777)SNP_10300	289	T	C	9	5	--	--
	Ca(ICCV2/PI489777)SNP_10301	340	A	G	11	5	--	--
	Ca(ICCV2/PI489777)SNP_10302	443	T	C	11	4	--	--
	Ca(ICCV2/PI489777)SNP_10303	948	T	C	19	10	--	--
	Ca(ICCV2/PI489777)SNP_10304	949	C	T	18	10	--	--
	Ca(ICCV2/PI489777)SNP_10305	977	A	G	11	9	--	--
	Ca(ICCV2/PI489777)SNP_10306	995	C	A	10	9	--	--
	Ca(ICCV2/PI489777)SNP_10307	998	C	T	8	9	--	--
	Ca(ICCV2/PI489777)SNP_10308	1022	G	A	7	8	--	--
	Ca(ICCV2/PI489777)SNP_10309	1103	A	G	5	7	--	--
	Ca(ICCV2/PI489777)SNP_10310	1110	G	T	5	8	--	--
	Ca(ICCV2/PI489777)SNP_10311	1151	A	C	5	6	--	--
	Ca(ICCV2/PI489777)SNP_10312	1178	C	T	5	5	--	--
	Ca(ICCV2/PI489777)SNP_10313	1210	T	G	4	5	--	--
CakTC33049	Ca(ICCV2/PI489777)SNP_10314	321	C	G	5	6	--	--
CakTC42428	Ca(ICCV2/PI489777)SNP_10315	79	A	G	4	5	--	--
	Ca(ICCV2/PI489777)SNP_10316	121	G	A	3	7	--	--
	Ca(ICCV2/PI489777)SNP_10317	256	C	T	4	6	--	--
	Ca(ICCV2/PI489777)SNP_10318	265	C	T	3	7	--	--
	Ca(ICCV2/PI489777)SNP_10319	491	G	C	3	4	--	--
CakTC38746	Ca(ICCV2/PI489777)SNP_10320	678	T	C	3	5	--	--
CakTC37715	Ca(ICCV2/PI489777)SNP_10321	225	G	A	7	9	--	--
	Ca(ICCV2/PI489777)SNP_10322	229	G	A	7	8	--	--
	Ca(ICCV2/PI489777)SNP_10323	523	G	A	3	15	--	--
CakTC41758	Ca(ICCV2/PI489777)SNP_10324	248	A	C	4	9	--	--
	Ca(ICCV2/PI489777)SNP_10325	401	G	T	6	9	--	--
	Ca(ICCV2/PI489777)SNP_10326	410	C	T	6	9	--	--
	Ca(ICCV2/PI489777)SNP_10327	1550	C	T	9	11	--	--
	Ca(ICCV2/PI489777)SNP_10328	1553	A	G	9	11	--	--
	Ca(ICCV2/PI489777)SNP_10329	1709	A	G	15	6	--	--
	Ca(ICCV2/PI489777)SNP_10330	1817	G	C	13	7	--	--
	Ca(ICCV2/PI489777)SNP_10331	1946	A	G	21	7	--	--
CakTC24775	Ca(ICCV2/PI489777)SNP_10332	7944	G	A	745	250	Shoot	--
	Ca(ICCV2/PI489777)SNP_10333	7965	G	C	740	225	Shoot	--
	Ca(ICCV2/PI489777)SNP_10334	7984	A	G	621	226	Shoot	--
	Ca(ICCV2/PI489777)SNP_10335	8048	A	G	563	214	Shoot	--
CakTC38044	Ca(ICCV2/PI489777)SNP_10336	669	G	T	3	3	--	--
	Ca(ICCV2/PI489777)SNP_10337	1482	T	C	4	5	--	--
CakTC31416	Ca(ICCV2/PI489777)SNP_10338	89	G	C	5	3	--	--
CakTC42046	Ca(ICCV2/PI489777)SNP_10339	770	G	A	19	8	--	--
CakTC04450	Ca(ICCV2/PI489777)SNP_10340	974	C	T	11	8	--	--
	Ca(ICCV2/PI489777)SNP_10341	1180	T	G	10	5	--	--
	Ca(ICCV2/PI489777)SNP_10342	1182	G	A	10	5	--	--
CakTC25541	Ca(ICCV2/PI489777)SNP_10343	1844	A	C	12	8	--	--
	Ca(ICCV2/PI489777)SNP_10344	2032	G	A	23	12	--	--
CakTC09633	Ca(ICCV2/PI489777)SNP_10345	2001	A	G	15	17	--	--
CakTC09729	Ca(ICCV2/PI489777)SNP_10346	441	A	G	3	3	--	--
CakTC42278	Ca(ICCV2/PI489777)SNP_10347	323	A	G	31	15	--	--
	Ca(ICCV2/PI489777)SNP_10348	569	A	G	19	9	--	--
	Ca(ICCV2/PI489777)SNP_10349	1280	A	G	20	18	--	--
	Ca(ICCV2/PI489777)SNP_10350	1610	G	T	24	12	--	--
CakTC37902	Ca(ICCV2/PI489777)SNP_10351	140	C	G	5	4	--	--
	Ca(ICCV2/PI489777)SNP_10352	146	T	C	5	5	--	--
	Ca(ICCV2/PI489777)SNP_10353	222	C	A	4	4	--	--
	Ca(ICCV2/PI489777)SNP_10354	236	T	A	5	5	--	--
	Ca(ICCV2/PI489777)SNP_10355	238	T	A	5	5	--	--

	Ca(ICCV2/PI489777)SNP_10356	558	G	A	6	6	--	--
	Ca(ICCV2/PI489777)SNP_10357	1158	A	G	3	4	--	--
	Ca(ICCV2/PI489777)SNP_10358	1464	G	A	7	4	--	--
CakTC12632	Ca(ICCV2/PI489777)SNP_10359	113	G	T	10	12	--	--
	Ca(ICCV2/PI489777)SNP_10360	322	T	C	14	17	--	--
	Ca(ICCV2/PI489777)SNP_10361	517	T	G	8	13	--	--
	Ca(ICCV2/PI489777)SNP_10362	677	G	A	7	6	--	--
CakTC26682	Ca(ICCV2/PI489777)SNP_10363	562	C	T	3	5	--	--
	Ca(ICCV2/PI489777)SNP_10364	573	C	A	3	4	--	--
	Ca(ICCV2/PI489777)SNP_10365	1925	T	C	5	6	--	--
	Ca(ICCV2/PI489777)SNP_10366	2038	T	C	3	7	--	--
	Ca(ICCV2/PI489777)SNP_10367	2043	C	T	3	7	--	--
CakTC09702	Ca(ICCV2/PI489777)SNP_10368	68	C	T	29	11	--	--
	Ca(ICCV2/PI489777)SNP_10369	74	C	T	29	10	--	--
	Ca(ICCV2/PI489777)SNP_10370	149	T	C	32	13	--	--
	Ca(ICCV2/PI489777)SNP_10371	158	A	C	32	13	--	--
	Ca(ICCV2/PI489777)SNP_10372	164	T	A	32	13	--	--
	Ca(ICCV2/PI489777)SNP_10373	405	C	A	15	11	--	--
CakTC27444	Ca(ICCV2/PI489777)SNP_10374	523	A	G	5	5	--	--
	Ca(ICCV2/PI489777)SNP_10375	738	A	G	5	6	--	--
CakTC34753	Ca(ICCV2/PI489777)SNP_10376	206	A	C	21	10	--	--
	Ca(ICCV2/PI489777)SNP_10377	1225	A	G	29	12	--	--
	Ca(ICCV2/PI489777)SNP_10378	3244	G	A	15	11	--	--
CakTC37824	Ca(ICCV2/PI489777)SNP_10379	122	T	C	6	4	--	--
	Ca(ICCV2/PI489777)SNP_10380	189	A	G	7	4	--	--
	Ca(ICCV2/PI489777)SNP_10381	264	T	C	9	4	--	--
	Ca(ICCV2/PI489777)SNP_10382	300	C	T	9	5	--	--
CakTC34086	Ca(ICCV2/PI489777)SNP_10383	735	C	T	5	5	--	--
	Ca(ICCV2/PI489777)SNP_10384	1530	A	C	45	26	--	--
	Ca(ICCV2/PI489777)SNP_10385	1852	C	T	10	5	--	--
CakTC31957	Ca(ICCV2/PI489777)SNP_10386	516	T	C	48	18	--	--
	Ca(ICCV2/PI489777)SNP_10387	562	G	T	56	26	--	--
CakTC04069	Ca(ICCV2/PI489777)SNP_10388	770	G	A	12	8	--	--
	Ca(ICCV2/PI489777)SNP_10389	1070	G	A	14	10	--	--
CakTC23147	Ca(ICCV2/PI489777)SNP_10390	537	C	T	19	12	--	--
	Ca(ICCV2/PI489777)SNP_10391	1295	T	A	7	9	--	--
CakTC28594	Ca(ICCV2/PI489777)SNP_10392	58	T	A	12	9	--	--
CakTC23282	Ca(ICCV2/PI489777)SNP_10393	188	C	G	5	6	--	--
CakTC39798	Ca(ICCV2/PI489777)SNP_10394	215	T	C	12	13	--	--
	Ca(ICCV2/PI489777)SNP_10395	705	G	A	75	39	--	--
	Ca(ICCV2/PI489777)SNP_10396	720	C	G	76	34	--	--
	Ca(ICCV2/PI489777)SNP_10397	822	A	G	107	44	--	--
CakTC02339	Ca(ICCV2/PI489777)SNP_10398	467	C	A	4	4	--	--
	Ca(ICCV2/PI489777)SNP_10399	793	A	G	10	4	--	--
	Ca(ICCV2/PI489777)SNP_10400	907	A	C	6	7	--	--
	Ca(ICCV2/PI489777)SNP_10401	936	A	G	6	7	--	--
	Ca(ICCV2/PI489777)SNP_10402	1043	G	T	6	4	--	--
CakTC41451	Ca(ICCV2/PI489777)SNP_10403	1043	C	T	21	11	--	--
	Ca(ICCV2/PI489777)SNP_10404	1103	G	A	20	8	--	--
	Ca(ICCV2/PI489777)SNP_10405	1355	T	C	32	12	--	--
	Ca(ICCV2/PI489777)SNP_10406	1417	G	A	36	14	--	--
CakTC34599	Ca(ICCV2/PI489777)SNP_10407	587	T	C	24	18	--	--
CakTC38450	Ca(ICCV2/PI489777)SNP_10408	241	T	C	3	3	--	--
	Ca(ICCV2/PI489777)SNP_10409	345	A	G	4	3	--	--
CakTC25057	Ca(ICCV2/PI489777)SNP_10410	928	T	C	24	6	--	--
CakTC32017	Ca(ICCV2/PI489777)SNP_10411	951	G	A	6	3	--	--
	Ca(ICCV2/PI489777)SNP_10412	991	T	C	6	3	--	--
	Ca(ICCV2/PI489777)SNP_10413	1278	T	C	6	5	--	--
	Ca(ICCV2/PI489777)SNP_10414	1979	T	C	6	10	--	--
	Ca(ICCV2/PI489777)SNP_10415	2192	T	A	6	9	--	--
	Ca(ICCV2/PI489777)SNP_10416	2241	G	A	6	8	--	--
CakTC27277	Ca(ICCV2/PI489777)SNP_10417	503	G	A	14	20	--	--
	Ca(ICCV2/PI489777)SNP_10418	559	G	C	21	18	--	--
	Ca(ICCV2/PI489777)SNP_10419	808	G	T	29	10	--	--
	Ca(ICCV2/PI489777)SNP_10420	935	G	A	36	18	--	--
CakTC15503	Ca(ICCV2/PI489777)SNP_10421	1054	T	G	117	33	--	--
CakTC28554	Ca(ICCV2/PI489777)SNP_10422	229	A	G	6	4	--	--
	Ca(ICCV2/PI489777)SNP_10423	247	G	A	6	4	--	--
	Ca(ICCV2/PI489777)SNP_10424	310	C	A	6	4	--	--
	Ca(ICCV2/PI489777)SNP_10425	397	G	T	7	5	--	--
	Ca(ICCV2/PI489777)SNP_10426	406	G	A	7	4	--	--
	Ca(ICCV2/PI489777)SNP_10427	502	C	G	7	3	--	--
	Ca(ICCV2/PI489777)SNP_10428	1165	T	C	4	3	--	--
CakTC31281	Ca(ICCV2/PI489777)SNP_10429	596	C	G	7	4	--	--

	Ca(ICCV2/PI489777)SNP_10430	657	G	A	5	4	--	--
CakTC39840	Ca(ICCV2/PI489777)SNP_10431	397	C	A	4	3	--	--
	Ca(ICCV2/PI489777)SNP_10432	456	C	T	12	3	--	--
CakTC34836	Ca(ICCV2/PI489777)SNP_10433	144	T	C	10	9	--	--
	Ca(ICCV2/PI489777)SNP_10434	189	C	T	10	8	--	--
	Ca(ICCV2/PI489777)SNP_10435	263	T	C	10	10	--	--
CakTC23736	Ca(ICCV2/PI489777)SNP_10436	199	G	T	4	3	--	--
	Ca(ICCV2/PI489777)SNP_10437	394	G	A	36	27	--	--
	Ca(ICCV2/PI489777)SNP_10438	759	A	G	11	3	--	--
	Ca(ICCV2/PI489777)SNP_10439	1067	A	G	24	8	--	--
CakTC43123	Ca(ICCV2/PI489777)SNP_10440	1596	G	T	13	29	--	--
	Ca(ICCV2/PI489777)SNP_10441	1851	C	T	3	7	--	--
CakTC18041	Ca(ICCV2/PI489777)SNP_10442	1506	G	A	3	5	--	--
	Ca(ICCV2/PI489777)SNP_10443	2040	T	C	3	5	--	--
CakTC25149	Ca(ICCV2/PI489777)SNP_10444	486	A	G	14	7	--	--
	Ca(ICCV2/PI489777)SNP_10445	504	T	C	18	6	--	--
	Ca(ICCV2/PI489777)SNP_10446	546	G	C	21	10	--	--
	Ca(ICCV2/PI489777)SNP_10447	684	G	A	40	9	--	--
	Ca(ICCV2/PI489777)SNP_10448	720	T	C	48	12	--	--
	Ca(ICCV2/PI489777)SNP_10449	759	A	C	58	14	--	--
	Ca(ICCV2/PI489777)SNP_10450	812	A	C	57	15	--	--
	Ca(ICCV2/PI489777)SNP_10451	888	T	C	40	13	--	--
CakTC28206	Ca(ICCV2/PI489777)SNP_10452	460	A	T	16	12	--	--
	Ca(ICCV2/PI489777)SNP_10453	1252	T	C	11	6	--	--
CakTC40643	Ca(ICCV2/PI489777)SNP_10454	242	C	G	3	10	--	--
	Ca(ICCV2/PI489777)SNP_10455	633	G	A	9	9	--	--
CakTC31641	Ca(ICCV2/PI489777)SNP_10456	137	T	C	4	6	--	--
	Ca(ICCV2/PI489777)SNP_10457	165	C	A	4	6	--	--
	Ca(ICCV2/PI489777)SNP_10458	489	C	T	11	3	--	--
CakTC40870	Ca(ICCV2/PI489777)SNP_10459	569	G	A	3	3	--	--
	Ca(ICCV2/PI489777)SNP_10460	1310	A	T	3	5	--	--
	Ca(ICCV2/PI489777)SNP_10461	1415	A	G	3	4	--	--
CakTC39816	Ca(ICCV2/PI489777)SNP_10462	279	A	T	8	4	--	--
	Ca(ICCV2/PI489777)SNP_10463	420	G	C	10	4	--	--
	Ca(ICCV2/PI489777)SNP_10464	1116	G	A	9	4	--	--
	Ca(ICCV2/PI489777)SNP_10465	1124	C	T	11	4	--	--
	Ca(ICCV2/PI489777)SNP_10466	1158	A	C	4	5	--	--
	Ca(ICCV2/PI489777)SNP_10467	1449	T	C	7	3	--	--
	Ca(ICCV2/PI489777)SNP_10468	1467	T	A	7	3	--	--
CakTC08111	Ca(ICCV2/PI489777)SNP_10469	454	T	C	3	3	--	--
CakTC35963	Ca(ICCV2/PI489777)SNP_10470	248	A	T	4	4	--	--
	Ca(ICCV2/PI489777)SNP_10471	823	T	C	9	3	--	--
CakTC11591	Ca(ICCV2/PI489777)SNP_10472	705	A	C	14	14	--	--
	Ca(ICCV2/PI489777)SNP_10473	880	C	A	7	3	--	--
CakTC32334	Ca(ICCV2/PI489777)SNP_10474	1344	T	G	7	13	--	--
CakTC36495	Ca(ICCV2/PI489777)SNP_10475	315	G	A	8	4	--	--
	Ca(ICCV2/PI489777)SNP_10476	361	T	G	7	5	--	--
	Ca(ICCV2/PI489777)SNP_10477	813	C	T	4	7	--	--
	Ca(ICCV2/PI489777)SNP_10478	1360	T	C	6	4	--	--
CakTC23539	Ca(ICCV2/PI489777)SNP_10479	133	G	C	7	10	--	--
	Ca(ICCV2/PI489777)SNP_10480	144	C	T	7	10	--	--
	Ca(ICCV2/PI489777)SNP_10481	328	A	C	11	11	--	--
	Ca(ICCV2/PI489777)SNP_10482	416	T	C	8	7	--	--
CakTC23618	Ca(ICCV2/PI489777)SNP_10483	1067	G	A	29	17	--	--
	Ca(ICCV2/PI489777)SNP_10484	1706	G	A	50	16	--	--
	Ca(ICCV2/PI489777)SNP_10485	2132	G	A	33	25	--	--
	Ca(ICCV2/PI489777)SNP_10486	2490	G	A	7	6	--	--
CakTC38942	Ca(ICCV2/PI489777)SNP_10487	386	T	C	11	6	--	--
	Ca(ICCV2/PI489777)SNP_10488	416	T	G	12	6	--	--
	Ca(ICCV2/PI489777)SNP_10489	470	C	T	12	5	--	--
	Ca(ICCV2/PI489777)SNP_10490	479	G	A	11	8	--	--
	Ca(ICCV2/PI489777)SNP_10491	545	C	G	10	7	--	--
	Ca(ICCV2/PI489777)SNP_10492	596	T	C	12	7	--	--
	Ca(ICCV2/PI489777)SNP_10493	1049	T	C	8	6	--	--
	Ca(ICCV2/PI489777)SNP_10494	1298	A	G	10	6	--	--
	Ca(ICCV2/PI489777)SNP_10495	1457	G	C	5	7	--	--
	Ca(ICCV2/PI489777)SNP_10496	1517	C	T	3	6	--	--
CakTC38404	Ca(ICCV2/PI489777)SNP_10497	997	C	G	54	13	--	--
CakTC08662	Ca(ICCV2/PI489777)SNP_10498	1094	G	A	4	10	--	--
CakTC30271	Ca(ICCV2/PI489777)SNP_10499	488	C	T	19	9	--	--
	Ca(ICCV2/PI489777)SNP_10500	584	C	G	17	6	--	--
	Ca(ICCV2/PI489777)SNP_10501	630	T	C	10	4	--	--
CakTC35589	Ca(ICCV2/PI489777)SNP_10502	114	C	T	11	9	--	--
	Ca(ICCV2/PI489777)SNP_10503	513	G	T	23	9	--	--

	Ca(ICCV2/PI489777)SNP_10504	588	C	T	19	10	--	--
	Ca(ICCV2/PI489777)SNP_10505	609	A	G	21	12	--	--
	Ca(ICCV2/PI489777)SNP_10506	1863	C	T	46	42	--	--
	Ca(ICCV2/PI489777)SNP_10507	2395	A	C	48	42	--	--
CakTC33879	Ca(ICCV2/PI489777)SNP_10508	326	C	T	9	8	--	--
	Ca(ICCV2/PI489777)SNP_10509	480	T	C	11	7	--	--
	Ca(ICCV2/PI489777)SNP_10510	481	G	A	11	7	--	--
	Ca(ICCV2/PI489777)SNP_10511	495	A	G	11	6	--	--
	Ca(ICCV2/PI489777)SNP_10512	906	G	T	7	3	--	--
	Ca(ICCV2/PI489777)SNP_10513	954	C	T	8	5	--	--
	Ca(ICCV2/PI489777)SNP_10514	999	G	A	9	5	--	--
CakTC27877	Ca(ICCV2/PI489777)SNP_10515	523	A	G	13	4	Root	--
	Ca(ICCV2/PI489777)SNP_10516	1054	G	A	14	3	Root	--
	Ca(ICCV2/PI489777)SNP_10517	1125	A	T	14	4	Root	--
CakTC34857	Ca(ICCV2/PI489777)SNP_10518	292	C	T	38	17	--	--
	Ca(ICCV2/PI489777)SNP_10519	425	T	C	47	26	--	--
	Ca(ICCV2/PI489777)SNP_10520	440	A	C	46	29	--	--
	Ca(ICCV2/PI489777)SNP_10521	454	G	C	47	25	--	--
	Ca(ICCV2/PI489777)SNP_10522	484	G	A	48	28	--	--
	Ca(ICCV2/PI489777)SNP_10523	1483	G	A	42	21	--	--
CakTC25781	Ca(ICCV2/PI489777)SNP_10524	1389	C	T	9	3	--	--
	Ca(ICCV2/PI489777)SNP_10525	1502	C	G	6	11	--	--
	Ca(ICCV2/PI489777)SNP_10526	1607	T	C	6	13	--	--
	Ca(ICCV2/PI489777)SNP_10527	1632	G	A	10	14	--	--
	Ca(ICCV2/PI489777)SNP_10528	1665	G	A	12	11	--	--
	Ca(ICCV2/PI489777)SNP_10529	1741	A	C	14	9	--	--
	Ca(ICCV2/PI489777)SNP_10530	1766	G	C	11	5	--	--
	Ca(ICCV2/PI489777)SNP_10531	1781	C	G	14	6	--	--
	Ca(ICCV2/PI489777)SNP_10532	1869	C	T	11	4	--	--
	Ca(ICCV2/PI489777)SNP_10533	1904	A	T	11	3	--	--
	Ca(ICCV2/PI489777)SNP_10534	1915	G	A	10	3	--	--
	Ca(ICCV2/PI489777)SNP_10535	1948	T	C	10	3	--	--
CakTC16142	Ca(ICCV2/PI489777)SNP_10536	1482	A	T	20	17	--	AUX/IAA
CakTC41598	Ca(ICCV2/PI489777)SNP_10537	692	C	T	34	16	--	Trihelix
	Ca(ICCV2/PI489777)SNP_10538	701	T	C	29	17	--	Trihelix
	Ca(ICCV2/PI489777)SNP_10539	710	A	G	27	17	--	Trihelix
	Ca(ICCV2/PI489777)SNP_10540	1632	T	G	29	8	--	Trihelix
	Ca(ICCV2/PI489777)SNP_10541	1805	T	C	28	8	--	Trihelix
	Ca(ICCV2/PI489777)SNP_10542	2381	A	G	11	9	--	Trihelix
CakTC39510	Ca(ICCV2/PI489777)SNP_10543	714	G	A	35	48	--	--
	Ca(ICCV2/PI489777)SNP_10544	725	T	C	31	47	--	--
	Ca(ICCV2/PI489777)SNP_10545	741	G	A	36	35	--	--
CakTC34568	Ca(ICCV2/PI489777)SNP_10546	563	C	T	9	7	--	--
	Ca(ICCV2/PI489777)SNP_10547	722	G	A	6	15	--	--
	Ca(ICCV2/PI489777)SNP_10548	959	G	A	6	14	--	--
	Ca(ICCV2/PI489777)SNP_10549	1565	G	C	6	7	--	--
	Ca(ICCV2/PI489777)SNP_10550	1974	T	C	5	3	--	--
CakTC28522	Ca(ICCV2/PI489777)SNP_10551	547	T	G	39	11	--	MYB
	Ca(ICCV2/PI489777)SNP_10552	645	C	G	35	10	--	MYB
	Ca(ICCV2/PI489777)SNP_10553	648	C	G	33	12	--	MYB
	Ca(ICCV2/PI489777)SNP_10554	797	G	A	16	10	--	MYB
	Ca(ICCV2/PI489777)SNP_10555	893	A	G	14	7	--	MYB
CakTC22757	Ca(ICCV2/PI489777)SNP_10556	345	T	C	12	3	--	--
	Ca(ICCV2/PI489777)SNP_10557	355	A	T	10	3	--	--
	Ca(ICCV2/PI489777)SNP_10558	379	G	A	17	3	--	--
CakTC33274	Ca(ICCV2/PI489777)SNP_10559	1489	A	G	9	10	--	--
	Ca(ICCV2/PI489777)SNP_10560	1849	C	T	9	5	--	--
	Ca(ICCV2/PI489777)SNP_10561	1918	A	C	9	10	--	--
	Ca(ICCV2/PI489777)SNP_10562	2002	C	T	7	10	--	--
CakTC37756	Ca(ICCV2/PI489777)SNP_10563	901	G	A	28	14	--	--
CakTC42102	Ca(ICCV2/PI489777)SNP_10564	104	A	G	4	3	--	--
	Ca(ICCV2/PI489777)SNP_10565	312	C	T	9	6	--	--
	Ca(ICCV2/PI489777)SNP_10566	349	G	A	10	4	--	--
	Ca(ICCV2/PI489777)SNP_10567	378	A	G	12	4	--	--
	Ca(ICCV2/PI489777)SNP_10568	555	C	A	17	6	--	--
	Ca(ICCV2/PI489777)SNP_10569	828	C	T	13	7	--	--
	Ca(ICCV2/PI489777)SNP_10570	1104	T	C	12	5	--	--
	Ca(ICCV2/PI489777)SNP_10571	1672	G	A	9	6	--	--
	Ca(ICCV2/PI489777)SNP_10572	1685	C	T	8	6	--	--
CakTC15679	Ca(ICCV2/PI489777)SNP_10573	81	T	C	11	3	--	--
	Ca(ICCV2/PI489777)SNP_10574	100	G	A	17	4	--	--
	Ca(ICCV2/PI489777)SNP_10575	285	C	T	17	7	--	--
	Ca(ICCV2/PI489777)SNP_10576	300	C	G	17	7	--	--
	Ca(ICCV2/PI489777)SNP_10577	636	A	G	20	9	--	--

	Ca(ICCV2/PI489777)SNP_10578	813	C	G	18	6	--	--
	Ca(ICCV2/PI489777)SNP_10579	864	C	A	15	5	--	--
	Ca(ICCV2/PI489777)SNP_10580	918	T	G	14	4	--	--
	Ca(ICCV2/PI489777)SNP_10581	957	T	C	11	4	--	--
CakTC35644	Ca(ICCV2/PI489777)SNP_10582	602	A	G	18	4	--	--
	Ca(ICCV2/PI489777)SNP_10583	832	T	C	15	6	--	--
	Ca(ICCV2/PI489777)SNP_10584	1045	G	T	17	13	--	--
	Ca(ICCV2/PI489777)SNP_10585	1240	T	C	31	11	--	--
	Ca(ICCV2/PI489777)SNP_10586	2168	C	G	15	8	--	--
	Ca(ICCV2/PI489777)SNP_10587	2235	T	C	11	11	--	--
	Ca(ICCV2/PI489777)SNP_10588	2251	T	C	12	10	--	--
	Ca(ICCV2/PI489777)SNP_10589	2320	A	G	11	13	--	--
	Ca(ICCV2/PI489777)SNP_10590	2602	C	G	18	20	--	--
	Ca(ICCV2/PI489777)SNP_10591	2620	C	T	20	20	--	--
	Ca(ICCV2/PI489777)SNP_10592	2702	A	G	17	17	--	--
CakTC26811	Ca(ICCV2/PI489777)SNP_10593	744	G	A	16	5	--	--
	Ca(ICCV2/PI489777)SNP_10594	1347	T	C	7	13	--	--
	Ca(ICCV2/PI489777)SNP_10595	1450	A	G	12	13	--	--
	Ca(ICCV2/PI489777)SNP_10596	1806	T	G	15	5	--	--
	Ca(ICCV2/PI489777)SNP_10597	2052	A	T	13	11	--	--
CakTC38892	Ca(ICCV2/PI489777)SNP_10598	198	C	A	4	3	--	--
CakTC10926	Ca(ICCV2/PI489777)SNP_10599	325	A	G	15	4	--	GRAS
	Ca(ICCV2/PI489777)SNP_10600	688	C	T	6	4	--	GRAS
	Ca(ICCV2/PI489777)SNP_10601	1359	C	A	5	5	--	GRAS
	Ca(ICCV2/PI489777)SNP_10602	1473	C	T	5	5	--	GRAS
	Ca(ICCV2/PI489777)SNP_10603	1522	T	C	8	4	--	GRAS
	Ca(ICCV2/PI489777)SNP_10604	1546	G	A	8	4	--	GRAS
CakTC25928	Ca(ICCV2/PI489777)SNP_10605	218	A	G	3	5	--	--
	Ca(ICCV2/PI489777)SNP_10606	271	C	T	4	5	--	--
	Ca(ICCV2/PI489777)SNP_10607	783	T	C	10	5	--	--
	Ca(ICCV2/PI489777)SNP_10608	892	C	A	10	7	--	--
CakTC32137	Ca(ICCV2/PI489777)SNP_10609	1225	A	G	13	6	--	--
CakTC27590	Ca(ICCV2/PI489777)SNP_10610	527	C	T	10	10	--	--
	Ca(ICCV2/PI489777)SNP_10611	682	C	T	5	6	--	--
	Ca(ICCV2/PI489777)SNP_10612	694	T	G	5	6	--	--
	Ca(ICCV2/PI489777)SNP_10613	705	G	A	5	6	--	--
	Ca(ICCV2/PI489777)SNP_10614	748	G	A	3	6	--	--
	Ca(ICCV2/PI489777)SNP_10615	816	T	C	3	5	--	--
	Ca(ICCV2/PI489777)SNP_10616	1009	A	T	4	5	--	--
CakTC31313	Ca(ICCV2/PI489777)SNP_10617	267	G	A	6	3	--	--
	Ca(ICCV2/PI489777)SNP_10618	603	C	T	4	3	--	--
CakTC25124	Ca(ICCV2/PI489777)SNP_10619	408	A	G	13	5	--	--
	Ca(ICCV2/PI489777)SNP_10620	613	T	G	10	3	--	--
CakTC42356	Ca(ICCV2/PI489777)SNP_10621	12	C	T	11	5	--	--
	Ca(ICCV2/PI489777)SNP_10622	55	G	A	112	67	--	--
	Ca(ICCV2/PI489777)SNP_10623	162	C	T	169	105	--	--
	Ca(ICCV2/PI489777)SNP_10624	192	C	T	166	109	--	--
	Ca(ICCV2/PI489777)SNP_10625	567	T	C	86	73	--	--
CakTC10185	Ca(ICCV2/PI489777)SNP_10626	518	A	G	10	10	--	--
	Ca(ICCV2/PI489777)SNP_10627	584	T	C	11	10	--	--
CakTC39116	Ca(ICCV2/PI489777)SNP_10628	94	T	C	8	6	--	--
	Ca(ICCV2/PI489777)SNP_10629	100	G	T	9	4	--	--
	Ca(ICCV2/PI489777)SNP_10630	116	C	T	11	6	--	--
	Ca(ICCV2/PI489777)SNP_10631	168	T	C	11	5	--	--
	Ca(ICCV2/PI489777)SNP_10632	250	G	A	11	4	--	--
	Ca(ICCV2/PI489777)SNP_10633	624	A	G	9	4	--	--
	Ca(ICCV2/PI489777)SNP_10634	734	T	A	11	3	--	--
CakTC33506	Ca(ICCV2/PI489777)SNP_10635	357	G	T	26	15	--	--
	Ca(ICCV2/PI489777)SNP_10636	480	A	C	25	13	--	--
	Ca(ICCV2/PI489777)SNP_10637	510	G	A	22	13	--	--
CakTC28471	Ca(ICCV2/PI489777)SNP_10638	86	A	C	8	8	--	--
CakTC41997	Ca(ICCV2/PI489777)SNP_10639	502	G	T	20	4	--	--
CakTC30248	Ca(ICCV2/PI489777)SNP_10640	69	G	T	4	3	--	--
	Ca(ICCV2/PI489777)SNP_10641	85	T	C	4	4	--	--
	Ca(ICCV2/PI489777)SNP_10642	378	C	T	10	10	--	--
	Ca(ICCV2/PI489777)SNP_10643	843	T	C	19	6	--	--
	Ca(ICCV2/PI489777)SNP_10644	994	T	A	16	12	--	--
	Ca(ICCV2/PI489777)SNP_10645	1056	T	G	14	9	--	--
	Ca(ICCV2/PI489777)SNP_10646	1274	T	C	24	10	--	--
	Ca(ICCV2/PI489777)SNP_10647	1611	G	A	26	9	--	--
	Ca(ICCV2/PI489777)SNP_10648	1671	C	T	25	13	--	--
	Ca(ICCV2/PI489777)SNP_10649	1695	C	T	20	14	--	--
	Ca(ICCV2/PI489777)SNP_10650	2047	G	A	18	10	--	--
	Ca(ICCV2/PI489777)SNP_10651	2232	A	G	17	9	--	--

	Ca(ICCV2/PI489777)SNP_10652	2325	A	T	13	11	--	--
	Ca(ICCV2/PI489777)SNP_10653	2395	T	C	11	9	--	--
	Ca(ICCV2/PI489777)SNP_10654	2714	T	A	7	8	--	--
	Ca(ICCV2/PI489777)SNP_10655	2822	G	T	6	7	--	--
	Ca(ICCV2/PI489777)SNP_10656	3306	G	A	7	4	--	--
	Ca(ICCV2/PI489777)SNP_10657	3670	T	C	4	3	--	--
	Ca(ICCV2/PI489777)SNP_10658	3689	A	T	4	3	--	--
	Ca(ICCV2/PI489777)SNP_10659	3695	C	T	5	6	--	--
CakTC43342	Ca(ICCV2/PI489777)SNP_10660	178	T	G	12	3	--	--
CakTC35974	Ca(ICCV2/PI489777)SNP_10661	3319	A	T	27	7	--	TPR
CakTC05847	Ca(ICCV2/PI489777)SNP_10662	496	A	G	34	28	--	--
CakTC27618	Ca(ICCV2/PI489777)SNP_10663	272	C	T	28	13	--	LOB
	Ca(ICCV2/PI489777)SNP_10664	371	A	G	29	18	--	LOB
CakTC42708	Ca(ICCV2/PI489777)SNP_10665	76	G	A	3	3	--	--
	Ca(ICCV2/PI489777)SNP_10666	420	G	A	3	3	--	--
CakTC35860	Ca(ICCV2/PI489777)SNP_10667	1205	A	G	9	4	--	--
CakTC08461	Ca(ICCV2/PI489777)SNP_10668	937	G	T	3	5	--	--
	Ca(ICCV2/PI489777)SNP_10669	1804	T	C	3	3	--	--
	Ca(ICCV2/PI489777)SNP_10670	1831	A	G	4	3	--	--
	Ca(ICCV2/PI489777)SNP_10671	3250	C	T	4	4	--	--
CakTC29610	Ca(ICCV2/PI489777)SNP_10672	522	A	G	3	7	--	--
	Ca(ICCV2/PI489777)SNP_10673	1299	T	C	3	4	--	--
CakTC42692	Ca(ICCV2/PI489777)SNP_10674	686	G	T	3	3	--	--
CakTC10212	Ca(ICCV2/PI489777)SNP_10675	799	C	G	3	4	--	--
CakTC39378	Ca(ICCV2/PI489777)SNP_10676	626	T	G	20	5	--	--
	Ca(ICCV2/PI489777)SNP_10677	669	C	G	22	5	--	--
	Ca(ICCV2/PI489777)SNP_10678	674	G	T	21	5	--	--
	Ca(ICCV2/PI489777)SNP_10679	961	A	T	23	9	--	--
CakTC38743	Ca(ICCV2/PI489777)SNP_10680	479	T	C	8	3	--	HB
	Ca(ICCV2/PI489777)SNP_10681	1083	T	C	4	3	--	HB
CakTC36747	Ca(ICCV2/PI489777)SNP_10682	157	T	C	14	6	--	--
	Ca(ICCV2/PI489777)SNP_10683	172	C	T	14	6	--	--
	Ca(ICCV2/PI489777)SNP_10684	451	A	G	12	4	--	--
	Ca(ICCV2/PI489777)SNP_10685	1621	C	A	13	10	--	--
	Ca(ICCV2/PI489777)SNP_10686	1987	G	A	5	6	--	--
	Ca(ICCV2/PI489777)SNP_10687	1994	G	T	5	6	--	--
	Ca(ICCV2/PI489777)SNP_10688	2096	A	G	8	8	--	--
CakTC24407	Ca(ICCV2/PI489777)SNP_10689	697	A	T	46	19	Mature	Leaf
	Ca(ICCV2/PI489777)SNP_10690	1033	T	C	22	11	Mature	Leaf
CakTC25898	Ca(ICCV2/PI489777)SNP_10691	277	T	C	4	10	--	--
	Ca(ICCV2/PI489777)SNP_10692	587	A	G	7	7	--	--
	Ca(ICCV2/PI489777)SNP_10693	629	G	A	7	7	--	--
	Ca(ICCV2/PI489777)SNP_10694	1120	T	G	8	14	--	--
	Ca(ICCV2/PI489777)SNP_10695	1364	C	G	3	5	--	--
CakTC26497	Ca(ICCV2/PI489777)SNP_10696	539	C	T	22	7	--	--
	Ca(ICCV2/PI489777)SNP_10697	683	A	T	73	11	--	--
	Ca(ICCV2/PI489777)SNP_10698	982	G	A	81	10	--	--
	Ca(ICCV2/PI489777)SNP_10699	989	G	A	75	10	--	--
	Ca(ICCV2/PI489777)SNP_10700	993	G	C	77	10	--	--
	Ca(ICCV2/PI489777)SNP_10701	1015	A	T	76	10	--	--
	Ca(ICCV2/PI489777)SNP_10702	1058	G	C	70	10	--	--
	Ca(ICCV2/PI489777)SNP_10703	1100	T	C	72	10	--	--
CakTC27373	Ca(ICCV2/PI489777)SNP_10704	125	G	A	12	3	--	--
	Ca(ICCV2/PI489777)SNP_10705	2176	G	C	17	16	--	--
	Ca(ICCV2/PI489777)SNP_10706	2233	A	T	17	15	--	--
CakTC23976	Ca(ICCV2/PI489777)SNP_10707	334	G	A	27	5	--	--
	Ca(ICCV2/PI489777)SNP_10708	341	A	G	23	5	--	--
	Ca(ICCV2/PI489777)SNP_10709	1022	C	T	10	3	--	--
	Ca(ICCV2/PI489777)SNP_10710	1053	A	T	12	3	--	--
CakTC37449	Ca(ICCV2/PI489777)SNP_10711	547	T	C	5	4	--	--
CakTC39612	Ca(ICCV2/PI489777)SNP_10712	298	T	C	24	14	--	--
	Ca(ICCV2/PI489777)SNP_10713	332	C	T	19	17	--	--
	Ca(ICCV2/PI489777)SNP_10714	385	A	G	26	17	--	--
	Ca(ICCV2/PI489777)SNP_10715	856	C	T	17	18	--	--
	Ca(ICCV2/PI489777)SNP_10716	979	T	C	8	18	--	--
CakTC28728	Ca(ICCV2/PI489777)SNP_10717	450	A	T	18	9	--	--
	Ca(ICCV2/PI489777)SNP_10718	512	A	T	16	7	--	--
	Ca(ICCV2/PI489777)SNP_10719	636	G	A	16	15	--	--
	Ca(ICCV2/PI489777)SNP_10720	669	T	C	10	13	--	--
	Ca(ICCV2/PI489777)SNP_10721	1116	C	A	9	17	--	--
CakTC31732	Ca(ICCV2/PI489777)SNP_10722	495	T	G	60	45	--	--
	Ca(ICCV2/PI489777)SNP_10723	873	A	G	43	30	--	--
	Ca(ICCV2/PI489777)SNP_10724	2250	C	T	30	18	--	--
CakTC26991	Ca(ICCV2/PI489777)SNP_10725	154	G	A	3	5	--	--

	Ca(ICCV2/PI489777)SNP_10726	473	T	C	11	6	--	--
	Ca(ICCV2/PI489777)SNP_10727	1341	T	C	5	4	--	--
CakTC38689	Ca(ICCV2/PI489777)SNP_10728	113	A	G	31	12	--	HB
CakTC29914	Ca(ICCV2/PI489777)SNP_10729	1095	C	T	19	12	--	--
	Ca(ICCV2/PI489777)SNP_10730	1461	G	A	21	10	--	--
	Ca(ICCV2/PI489777)SNP_10731	1515	A	T	19	11	--	--
	Ca(ICCV2/PI489777)SNP_10732	1623	T	C	7	10	--	--
	Ca(ICCV2/PI489777)SNP_10733	1993	C	T	11	6	--	--
	Ca(ICCV2/PI489777)SNP_10734	2231	G	A	11	8	--	--
CakTC31096	Ca(ICCV2/PI489777)SNP_10735	1189	T	C	3	3	--	--
	Ca(ICCV2/PI489777)SNP_10736	1201	C	T	4	3	--	--
CakTC31878	Ca(ICCV2/PI489777)SNP_10737	1080	C	T	7	12	--	--
CakTC38630	Ca(ICCV2/PI489777)SNP_10738	914	A	C	4	6	--	--
	Ca(ICCV2/PI489777)SNP_10739	952	C	T	5	5	--	--
	Ca(ICCV2/PI489777)SNP_10740	1075	A	G	3	4	--	--
CakTC26740	Ca(ICCV2/PI489777)SNP_10741	340	G	C	14	5	--	--
	Ca(ICCV2/PI489777)SNP_10742	433	T	C	5	5	--	--
	Ca(ICCV2/PI489777)SNP_10743	665	T	C	21	22	--	--
	Ca(ICCV2/PI489777)SNP_10744	698	C	T	18	23	--	--
	Ca(ICCV2/PI489777)SNP_10745	727	G	A	20	25	--	--
	Ca(ICCV2/PI489777)SNP_10746	943	T	G	26	21	--	--
	Ca(ICCV2/PI489777)SNP_10747	955	C	T	24	22	--	--
CakTC34904	Ca(ICCV2/PI489777)SNP_10748	64	A	C	3	3	--	--
	Ca(ICCV2/PI489777)SNP_10749	83	T	C	3	3	--	--
CakTC28116	Ca(ICCV2/PI489777)SNP_10750	300	G	C	20	9	--	--
	Ca(ICCV2/PI489777)SNP_10751	1561	G	T	17	4	--	--
CakTC36632	Ca(ICCV2/PI489777)SNP_10752	396	A	G	33	14	--	--
	Ca(ICCV2/PI489777)SNP_10753	447	C	T	34	12	--	--
	Ca(ICCV2/PI489777)SNP_10754	894	A	C	34	14	--	--
	Ca(ICCV2/PI489777)SNP_10755	957	T	C	32	17	--	--
	Ca(ICCV2/PI489777)SNP_10756	1572	G	T	25	14	--	--
	Ca(ICCV2/PI489777)SNP_10757	1573	C	T	26	14	--	--
	Ca(ICCV2/PI489777)SNP_10758	2016	G	A	26	16	--	--
	Ca(ICCV2/PI489777)SNP_10759	2046	G	T	27	19	--	--
	Ca(ICCV2/PI489777)SNP_10760	2085	T	A	24	13	--	--
	Ca(ICCV2/PI489777)SNP_10761	2425	A	G	20	8	--	--
	Ca(ICCV2/PI489777)SNP_10762	2496	A	T	14	3	--	--
	Ca(ICCV2/PI489777)SNP_10763	2528	G	A	11	3	--	--
	Ca(ICCV2/PI489777)SNP_10764	2641	G	A	3	3	--	--
CakTC35224	Ca(ICCV2/PI489777)SNP_10765	1350	C	T	8	5	--	SET
	Ca(ICCV2/PI489777)SNP_10766	1356	C	T	8	5	--	SET
	Ca(ICCV2/PI489777)SNP_10767	1362	A	T	8	5	--	SET
	Ca(ICCV2/PI489777)SNP_10768	1437	C	T	6	8	--	SET
CakTC35202	Ca(ICCV2/PI489777)SNP_10769	312	A	G	8	5	--	--
	Ca(ICCV2/PI489777)SNP_10770	536	A	G	7	3	--	--
	Ca(ICCV2/PI489777)SNP_10771	2165	G	A	13	6	--	--
	Ca(ICCV2/PI489777)SNP_10772	2290	C	A	9	4	--	--
	Ca(ICCV2/PI489777)SNP_10773	2507	T	C	8	12	--	--
CakTC40106	Ca(ICCV2/PI489777)SNP_10774	323	T	C	10	9	--	--
	Ca(ICCV2/PI489777)SNP_10775	587	G	A	9	8	--	--
	Ca(ICCV2/PI489777)SNP_10776	716	T	C	11	6	--	--
	Ca(ICCV2/PI489777)SNP_10777	728	C	T	12	6	--	--
	Ca(ICCV2/PI489777)SNP_10778	1428	A	G	4	9	--	--
	Ca(ICCV2/PI489777)SNP_10779	1467	T	A	3	9	--	--
	Ca(ICCV2/PI489777)SNP_10780	1638	T	C	7	7	--	--
CakTC33767	Ca(ICCV2/PI489777)SNP_10781	145	A	G	5	12	--	--
	Ca(ICCV2/PI489777)SNP_10782	548	G	T	11	18	--	--
CakTC29845	Ca(ICCV2/PI489777)SNP_10783	619	G	T	7	3	--	--
	Ca(ICCV2/PI489777)SNP_10784	1194	C	T	20	6	--	--
	Ca(ICCV2/PI489777)SNP_10785	1421	A	T	14	12	--	--
	Ca(ICCV2/PI489777)SNP_10786	1717	A	C	7	8	--	--
	Ca(ICCV2/PI489777)SNP_10787	1816	G	A	8	7	--	--
	Ca(ICCV2/PI489777)SNP_10788	2632	A	G	7	3	--	--
	Ca(ICCV2/PI489777)SNP_10789	2704	C	A	7	9	--	--
	Ca(ICCV2/PI489777)SNP_10790	2835	T	C	5	5	--	--
CakTC37423	Ca(ICCV2/PI489777)SNP_10791	170	T	A	34	10	--	--
	Ca(ICCV2/PI489777)SNP_10792	353	C	T	62	30	--	--
	Ca(ICCV2/PI489777)SNP_10793	888	A	G	33	15	--	--
	Ca(ICCV2/PI489777)SNP_10794	998	C	G	25	12	--	--
CakTC37442	Ca(ICCV2/PI489777)SNP_10795	461	G	A	8	4	--	--
CakTC25440	Ca(ICCV2/PI489777)SNP_10796	389	T	C	24	13	--	--
	Ca(ICCV2/PI489777)SNP_10797	399	T	C	28	12	--	--
	Ca(ICCV2/PI489777)SNP_10798	1342	C	T	28	4	--	--
	Ca(ICCV2/PI489777)SNP_10799	1543	T	C	35	16	--	--

CakTC29486	Ca(ICCV2/PI489777)SNP_10800	117	G	C	4	3	--	--
	Ca(ICCV2/PI489777)SNP_10801	616	G	A	14	16	--	--
	Ca(ICCV2/PI489777)SNP_10802	677	T	G	13	14	--	--
	Ca(ICCV2/PI489777)SNP_10803	678	C	G	13	14	--	--
	Ca(ICCV2/PI489777)SNP_10804	712	T	A	8	11	--	--
	Ca(ICCV2/PI489777)SNP_10805	724	G	C	7	7	--	--
CakTC07869	Ca(ICCV2/PI489777)SNP_10806	837	T	G	41	46	--	--
	Ca(ICCV2/PI489777)SNP_10807	1915	C	G	13	27	--	--
CakTC25653	Ca(ICCV2/PI489777)SNP_10808	1827	T	C	133	13	--	--
	Ca(ICCV2/PI489777)SNP_10809	1983	T	C	112	9	--	--
	Ca(ICCV2/PI489777)SNP_10810	2823	A	G	187	39	--	--
	Ca(ICCV2/PI489777)SNP_10811	2869	G	A	192	32	--	--
	Ca(ICCV2/PI489777)SNP_10812	2996	A	T	133	15	--	--
	Ca(ICCV2/PI489777)SNP_10813	3003	C	A	132	20	--	--
	Ca(ICCV2/PI489777)SNP_10814	3059	A	G	66	4	--	--
CakTC26123	Ca(ICCV2/PI489777)SNP_10815	382	G	A	7	5	--	--
	Ca(ICCV2/PI489777)SNP_10816	393	A	G	6	3	--	--
	Ca(ICCV2/PI489777)SNP_10817	428	T	A	9	6	--	--
	Ca(ICCV2/PI489777)SNP_10818	477	T	C	9	5	--	--
	Ca(ICCV2/PI489777)SNP_10819	496	C	T	7	5	--	--
	Ca(ICCV2/PI489777)SNP_10820	582	A	T	10	6	--	--
	Ca(ICCV2/PI489777)SNP_10821	630	A	T	10	7	--	--
	Ca(ICCV2/PI489777)SNP_10822	840	G	A	5	11	--	--
CakTC25303	Ca(ICCV2/PI489777)SNP_10823	1130	A	C	142	175	--	--
	Ca(ICCV2/PI489777)SNP_10824	1526	A	T	27	36	--	--
	Ca(ICCV2/PI489777)SNP_10825	1550	G	C	26	21	--	--
CakTC25985	Ca(ICCV2/PI489777)SNP_10826	347	A	G	63	58	--	--
	Ca(ICCV2/PI489777)SNP_10827	1455	A	G	89	64	--	--
	Ca(ICCV2/PI489777)SNP_10828	1811	A	T	127	93	--	--
	Ca(ICCV2/PI489777)SNP_10829	1973	A	G	122	89	--	--
	Ca(ICCV2/PI489777)SNP_10830	2030	C	T	119	83	--	--
	Ca(ICCV2/PI489777)SNP_10831	2759	T	C	56	43	--	--
	Ca(ICCV2/PI489777)SNP_10832	2786	C	A	47	38	--	--
CakTC32905	Ca(ICCV2/PI489777)SNP_10833	2312	T	C	3	4	--	FAR1
CakTC02731	Ca(ICCV2/PI489777)SNP_10834	75	C	A	3	4	--	--
	Ca(ICCV2/PI489777)SNP_10835	243	T	G	9	4	--	--
CakTC41950	Ca(ICCV2/PI489777)SNP_10836	31	A	T	12	11	--	--
	Ca(ICCV2/PI489777)SNP_10837	742	C	T	68	80	--	--
CakTC41084	Ca(ICCV2/PI489777)SNP_10838	258	C	A	18	5	--	--
	Ca(ICCV2/PI489777)SNP_10839	1959	A	G	10	6	--	--
	Ca(ICCV2/PI489777)SNP_10840	2050	G	A	15	6	--	--
CakTC39177	Ca(ICCV2/PI489777)SNP_10841	518	C	T	25	14	--	--
	Ca(ICCV2/PI489777)SNP_10842	1228	G	C	8	8	--	--
CakTC33329	Ca(ICCV2/PI489777)SNP_10843	442	C	T	48	5	--	--
CakTC37116	Ca(ICCV2/PI489777)SNP_10844	171	A	T	23	10	--	--
	Ca(ICCV2/PI489777)SNP_10845	392	T	G	33	12	--	--
	Ca(ICCV2/PI489777)SNP_10846	772	G	T	24	15	--	--
	Ca(ICCV2/PI489777)SNP_10847	1316	T	C	59	21	--	--
	Ca(ICCV2/PI489777)SNP_10848	1355	C	T	59	16	--	--
	Ca(ICCV2/PI489777)SNP_10849	1873	A	C	97	48	--	--
	Ca(ICCV2/PI489777)SNP_10850	2123	A	G	90	55	--	--
CakTC31392	Ca(ICCV2/PI489777)SNP_10851	213	A	T	51	13	--	--
	Ca(ICCV2/PI489777)SNP_10852	344	A	G	68	19	--	--
	Ca(ICCV2/PI489777)SNP_10853	887	C	T	62	29	--	--
	Ca(ICCV2/PI489777)SNP_10854	893	G	T	63	28	--	--
	Ca(ICCV2/PI489777)SNP_10855	1043	G	C	62	34	--	--
	Ca(ICCV2/PI489777)SNP_10856	1250	A	G	69	42	--	--
	Ca(ICCV2/PI489777)SNP_10857	1442	A	T	83	45	--	--
	Ca(ICCV2/PI489777)SNP_10858	1505	G	T	90	42	--	--
CakTC28632	Ca(ICCV2/PI489777)SNP_10859	342	T	C	18	15	--	--
	Ca(ICCV2/PI489777)SNP_10860	559	T	G	29	14	--	--
CakTC31520	Ca(ICCV2/PI489777)SNP_10861	502	T	C	5	4	--	--
CakTC32464	Ca(ICCV2/PI489777)SNP_10862	155	G	A	9	7	Mature	Leaf
	Ca(ICCV2/PI489777)SNP_10863	330	T	C	8	5	Mature	Leaf
CakTC16057	Ca(ICCV2/PI489777)SNP_10864	267	A	G	3	3	--	--
CakTC27107	Ca(ICCV2/PI489777)SNP_10865	120	C	T	14	4	--	--
	Ca(ICCV2/PI489777)SNP_10866	258	T	A	13	4	--	--
	Ca(ICCV2/PI489777)SNP_10867	1401	C	T	11	14	--	--
	Ca(ICCV2/PI489777)SNP_10868	2034	T	C	4	7	--	--
	Ca(ICCV2/PI489777)SNP_10869	2373	C	A	11	9	--	--
	Ca(ICCV2/PI489777)SNP_10870	3240	G	A	6	11	--	--
CakTC23749	Ca(ICCV2/PI489777)SNP_10871	139	A	G	38	8	--	--
	Ca(ICCV2/PI489777)SNP_10872	265	A	C	52	11	--	--
CakTC39569	Ca(ICCV2/PI489777)SNP_10873	187	A	G	18	12	--	--

	Ca(ICCV2/PI489777)SNP_10874	659	A	G	14	8	--	--
	Ca(ICCV2/PI489777)SNP_10875	1132	A	G	28	9	--	--
	Ca(ICCV2/PI489777)SNP_10876	1139	T	G	27	9	--	--
CakTC41886	Ca(ICCV2/PI489777)SNP_10877	459	A	C	12	11	--	--
CakTC42241	Ca(ICCV2/PI489777)SNP_10878	126	C	A	16	3	--	--
	Ca(ICCV2/PI489777)SNP_10879	278	T	C	15	4	--	--
CakTC29282	Ca(ICCV2/PI489777)SNP_10880	578	A	G	3	5	--	--
CakTC42747	Ca(ICCV2/PI489777)SNP_10881	988	T	C	6	3	Young_pod	--
	Ca(ICCV2/PI489777)SNP_10882	1264	A	G	7	3	Young_pod	--
	Ca(ICCV2/PI489777)SNP_10883	1398	T	G	10	3	Young_pod	--
	Ca(ICCV2/PI489777)SNP_10884	1480	T	C	9	3	Young_pod	--
CakTC35834	Ca(ICCV2/PI489777)SNP_10885	2239	G	A	12	7	--	--
	Ca(ICCV2/PI489777)SNP_10886	3263	C	T	5	6	--	--
CakTC34384	Ca(ICCV2/PI489777)SNP_10887	814	G	A	25	7	--	--
	Ca(ICCV2/PI489777)SNP_10888	964	A	G	25	11	--	--
	Ca(ICCV2/PI489777)SNP_10889	1030	C	A	24	11	--	--
CakTC06367	Ca(ICCV2/PI489777)SNP_10890	229	T	A	4	5	--	--
CakTC08968	Ca(ICCV2/PI489777)SNP_10891	677	C	T	3	4	Young_pod	--
	Ca(ICCV2/PI489777)SNP_10892	807	C	T	3	4	Young_pod	--
	Ca(ICCV2/PI489777)SNP_10893	855	T	C	3	4	Young_pod	--
CakTC26345	Ca(ICCV2/PI489777)SNP_10894	490	C	T	12	8	--	--
	Ca(ICCV2/PI489777)SNP_10895	535	T	C	17	6	--	--
	Ca(ICCV2/PI489777)SNP_10896	1084	T	G	15	11	--	--
	Ca(ICCV2/PI489777)SNP_10897	1420	C	G	23	13	--	--
	Ca(ICCV2/PI489777)SNP_10898	1610	T	A	15	8	--	--
CakTC22857	Ca(ICCV2/PI489777)SNP_10899	399	T	C	9	4	--	--
	Ca(ICCV2/PI489777)SNP_10900	499	C	G	13	5	--	--
	Ca(ICCV2/PI489777)SNP_10901	533	A	T	14	3	--	--
CakTC25103	Ca(ICCV2/PI489777)SNP_10902	191	G	C	18	9	--	--
	Ca(ICCV2/PI489777)SNP_10903	716	G	A	24	12	--	--
	Ca(ICCV2/PI489777)SNP_10904	1387	C	T	18	9	--	--
	Ca(ICCV2/PI489777)SNP_10905	1388	G	A	18	9	--	--
	Ca(ICCV2/PI489777)SNP_10906	1424	C	T	16	5	--	--
CakTC24552	Ca(ICCV2/PI489777)SNP_10907	53	A	T	5	3	--	--
	Ca(ICCV2/PI489777)SNP_10908	166	A	T	6	5	--	--
	Ca(ICCV2/PI489777)SNP_10909	313	C	T	8	3	--	--
CakTC40300	Ca(ICCV2/PI489777)SNP_10910	221	C	T	5	5	--	--
	Ca(ICCV2/PI489777)SNP_10911	287	G	A	6	5	--	--
	Ca(ICCV2/PI489777)SNP_10912	1502	C	T	13	18	--	--
CakTC38755	Ca(ICCV2/PI489777)SNP_10913	1325	A	G	5	6	--	--
	Ca(ICCV2/PI489777)SNP_10914	1340	G	A	5	6	--	--
CakTC28008	Ca(ICCV2/PI489777)SNP_10915	1146	G	A	86	50	--	--
	Ca(ICCV2/PI489777)SNP_10916	1148	C	G	86	50	--	--
CakTC08469	Ca(ICCV2/PI489777)SNP_10917	401	G	T	3	8	--	--
CakTC37554	Ca(ICCV2/PI489777)SNP_10918	216	C	T	9	6	--	--
	Ca(ICCV2/PI489777)SNP_10919	410	G	T	12	10	--	--
	Ca(ICCV2/PI489777)SNP_10920	587	A	G	12	10	--	--
	Ca(ICCV2/PI489777)SNP_10921	761	G	C	19	9	--	--
	Ca(ICCV2/PI489777)SNP_10922	1007	C	A	10	3	--	--
CakTC31404	Ca(ICCV2/PI489777)SNP_10923	162	G	A	11	12	--	--
	Ca(ICCV2/PI489777)SNP_10924	1008	C	T	3	3	--	--
	Ca(ICCV2/PI489777)SNP_10925	1373	C	T	6	8	--	--
	Ca(ICCV2/PI489777)SNP_10926	1530	C	A	5	6	--	--
CakTC26609	Ca(ICCV2/PI489777)SNP_10927	563	T	C	9	5	--	--
	Ca(ICCV2/PI489777)SNP_10928	661	C	G	12	3	--	--
	Ca(ICCV2/PI489777)SNP_10929	664	G	A	15	3	--	--
	Ca(ICCV2/PI489777)SNP_10930	913	G	C	19	7	--	--
	Ca(ICCV2/PI489777)SNP_10931	940	G	T	16	6	--	--
CakTC37549	Ca(ICCV2/PI489777)SNP_10932	1154	T	C	18	11	--	--
CakTC23645	Ca(ICCV2/PI489777)SNP_10933	6675	A	G	6	8	--	--
	Ca(ICCV2/PI489777)SNP_10934	7206	T	C	12	8	--	--
	Ca(ICCV2/PI489777)SNP_10935	9520	G	C	5	4	--	--
	Ca(ICCV2/PI489777)SNP_10936	11255	A	T	4	3	--	--
CakTC26168	Ca(ICCV2/PI489777)SNP_10937	724	C	T	10	13	--	--
CakTC27672	Ca(ICCV2/PI489777)SNP_10938	65	T	C	25	5	--	--
	Ca(ICCV2/PI489777)SNP_10939	115	T	C	31	5	--	--
CakTC35758	Ca(ICCV2/PI489777)SNP_10940	808	C	T	8	9	--	ARF
	Ca(ICCV2/PI489777)SNP_10941	2197	C	T	15	4	--	ARF
	Ca(ICCV2/PI489777)SNP_10942	2389	G	A	11	9	--	ARF
	Ca(ICCV2/PI489777)SNP_10943	2449	G	A	21	10	--	ARF
	Ca(ICCV2/PI489777)SNP_10944	3067	G	A	14	5	--	ARF
	Ca(ICCV2/PI489777)SNP_10945	3250	T	G	7	6	--	ARF
	Ca(ICCV2/PI489777)SNP_10946	3376	G	T	9	8	--	ARF
	Ca(ICCV2/PI489777)SNP_10947	4269	T	C	5	7	--	ARF

CakTC33681	Ca(ICCV2/PI489777)SNP_10948	253	G	A	103	30	--	--
CakTC32227	Ca(ICCV2/PI489777)SNP_10949	986	C	T	3	3	--	--
	Ca(ICCV2/PI489777)SNP_10950	1100	G	A	6	3	--	--
CakTC35217	Ca(ICCV2/PI489777)SNP_10951	235	T	C	34	22	--	--
CakTC03999	Ca(ICCV2/PI489777)SNP_10952	749	A	G	4	3	--	--
	Ca(ICCV2/PI489777)SNP_10953	869	A	G	6	4	--	--
CakTC22735	Ca(ICCV2/PI489777)SNP_10954	42	C	G	28	28	--	--
	Ca(ICCV2/PI489777)SNP_10955	819	A	T	122	82	--	--
CakTC38647	Ca(ICCV2/PI489777)SNP_10956	406	T	C	7	12	--	--
CakTC25607	Ca(ICCV2/PI489777)SNP_10957	555	A	T	6	4	Root	--
	Ca(ICCV2/PI489777)SNP_10958	615	A	G	6	4	Root	--
	Ca(ICCV2/PI489777)SNP_10959	626	C	T	5	4	Root	--
CakTC28694	Ca(ICCV2/PI489777)SNP_10960	1567	G	A	3	4	--	--
CakTC28119	Ca(ICCV2/PI489777)SNP_10961	787	A	G	42	13	--	--
	Ca(ICCV2/PI489777)SNP_10962	1117	A	G	23	16	--	--
	Ca(ICCV2/PI489777)SNP_10963	1144	G	C	25	16	--	--
	Ca(ICCV2/PI489777)SNP_10964	1201	A	G	23	16	--	--
	Ca(ICCV2/PI489777)SNP_10965	1330	G	T	23	10	--	--
	Ca(ICCV2/PI489777)SNP_10966	1680	C	T	9	11	--	--
	Ca(ICCV2/PI489777)SNP_10967	1740	C	T	13	10	--	--
	Ca(ICCV2/PI489777)SNP_10968	2247	G	A	18	3	--	--
	Ca(ICCV2/PI489777)SNP_10969	2428	A	C	23	5	--	--
	Ca(ICCV2/PI489777)SNP_10970	2580	A	G	20	5	--	--
	Ca(ICCV2/PI489777)SNP_10971	2604	A	G	18	5	--	--
	Ca(ICCV2/PI489777)SNP_10972	3216	T	C	26	10	--	--
	Ca(ICCV2/PI489777)SNP_10973	3384	A	G	29	10	--	--
	Ca(ICCV2/PI489777)SNP_10974	3525	G	A	22	11	--	--
	Ca(ICCV2/PI489777)SNP_10975	3579	C	T	19	12	--	--
	Ca(ICCV2/PI489777)SNP_10976	3772	A	G	16	12	--	--
	Ca(ICCV2/PI489777)SNP_10977	4155	C	T	32	8	--	--
	Ca(ICCV2/PI489777)SNP_10978	4533	G	C	14	8	--	--
CakTC30335	Ca(ICCV2/PI489777)SNP_10979	563	C	A	7	8	Shoot	--
	Ca(ICCV2/PI489777)SNP_10980	635	A	G	6	5	Shoot	--
	Ca(ICCV2/PI489777)SNP_10981	1176	G	C	4	5	Shoot	--
CakTC29324	Ca(ICCV2/PI489777)SNP_10982	25	C	T	33	44	--	CCAAT
CakTC32278	Ca(ICCV2/PI489777)SNP_10983	414	C	G	3	4	--	--
	Ca(ICCV2/PI489777)SNP_10984	559	A	G	6	6	--	--
	Ca(ICCV2/PI489777)SNP_10985	1378	T	G	7	7	--	--
	Ca(ICCV2/PI489777)SNP_10986	1412	A	T	8	4	--	--
	Ca(ICCV2/PI489777)SNP_10987	1504	C	A	8	7	--	--
	Ca(ICCV2/PI489777)SNP_10988	1518	T	C	8	6	--	--
	Ca(ICCV2/PI489777)SNP_10989	1739	C	A	10	9	--	--
	Ca(ICCV2/PI489777)SNP_10990	1760	T	A	9	11	--	--
CakTC39562	Ca(ICCV2/PI489777)SNP_10991	1067	G	A	7	9	--	--
	Ca(ICCV2/PI489777)SNP_10992	1082	A	C	7	9	--	--
	Ca(ICCV2/PI489777)SNP_10993	1693	T	C	3	5	--	--
CakTC29374	Ca(ICCV2/PI489777)SNP_10994	65	G	C	8	8	--	NAC
	Ca(ICCV2/PI489777)SNP_10995	621	A	G	19	17	--	NAC
	Ca(ICCV2/PI489777)SNP_10996	838	T	A	8	14	--	NAC
	Ca(ICCV2/PI489777)SNP_10997	920	G	T	3	8	--	NAC
	Ca(ICCV2/PI489777)SNP_10998	1092	A	G	9	5	--	NAC
	Ca(ICCV2/PI489777)SNP_10999	1094	T	C	10	5	--	NAC
	Ca(ICCV2/PI489777)SNP_11000	1102	A	G	10	5	--	NAC
CakTC25285	Ca(ICCV2/PI489777)SNP_11001	98	C	A	13	4	Flower	bud
CakTC34006	Ca(ICCV2/PI489777)SNP_11002	333	T	C	32	5	--	--
	Ca(ICCV2/PI489777)SNP_11003	369	T	C	41	5	--	--
	Ca(ICCV2/PI489777)SNP_11004	2142	G	A	15	11	--	--
	Ca(ICCV2/PI489777)SNP_11005	2550	G	A	16	11	--	--
	Ca(ICCV2/PI489777)SNP_11006	3269	A	G	18	15	--	--
	Ca(ICCV2/PI489777)SNP_11007	5687	C	G	16	7	--	--
	Ca(ICCV2/PI489777)SNP_11008	6699	A	T	18	14	--	--
	Ca(ICCV2/PI489777)SNP_11009	7270	A	G	9	9	--	--
	Ca(ICCV2/PI489777)SNP_11010	7311	A	G	8	8	--	--
CakTC25548	Ca(ICCV2/PI489777)SNP_11011	93	C	T	29	7	--	--
	Ca(ICCV2/PI489777)SNP_11012	415	T	C	159	104	--	--
	Ca(ICCV2/PI489777)SNP_11013	527	A	G	190	154	--	--
CakTC42518	Ca(ICCV2/PI489777)SNP_11014	525	T	C	6	3	Mature	Leaf
CakTC36111	Ca(ICCV2/PI489777)SNP_11015	482	A	G	12	7	--	--
CakTC30354	Ca(ICCV2/PI489777)SNP_11016	250	A	C	3	3	--	--
CakTC28225	Ca(ICCV2/PI489777)SNP_11017	441	C	T	46	11	--	--
CakTC32496	Ca(ICCV2/PI489777)SNP_11018	118	A	C	3	3	--	LOB
CakTC26311	Ca(ICCV2/PI489777)SNP_11019	574	G	A	7	8	--	bZIP
	Ca(ICCV2/PI489777)SNP_11020	656	T	C	5	10	--	bZIP
CakTC43083	Ca(ICCV2/PI489777)SNP_11021	540	G	A	10	7	--	--

	Ca(ICCV2/PI489777)SNP_11022	859	C	G	3	6	--	--
	Ca(ICCV2/PI489777)SNP_11023	888	C	G	3	6	--	--
CakTC39752	Ca(ICCV2/PI489777)SNP_11024	1524	G	T	9	5	--	--
	Ca(ICCV2/PI489777)SNP_11025	1606	A	G	6	5	--	--
	Ca(ICCV2/PI489777)SNP_11026	1627	G	A	6	5	--	--
	Ca(ICCV2/PI489777)SNP_11027	1667	G	T	5	5	--	--
	Ca(ICCV2/PI489777)SNP_11028	1675	T	G	4	5	--	--
	Ca(ICCV2/PI489777)SNP_11029	1709	C	A	4	5	--	--
CakTC18943	Ca(ICCV2/PI489777)SNP_11030	3078	C	T	7	5	--	--
CakTC23946	Ca(ICCV2/PI489777)SNP_11031	157	C	T	19	4	--	MYB
	Ca(ICCV2/PI489777)SNP_11032	521	T	A	26	8	--	MYB
	Ca(ICCV2/PI489777)SNP_11033	863	T	G	29	6	--	MYB
CakTC06400	Ca(ICCV2/PI489777)SNP_11034	587	C	T	4	4	--	--
CakTC35493	Ca(ICCV2/PI489777)SNP_11035	780	C	T	6	3	--	--
	Ca(ICCV2/PI489777)SNP_11036	869	A	T	5	4	--	--
	Ca(ICCV2/PI489777)SNP_11037	1821	G	A	3	6	--	--
	Ca(ICCV2/PI489777)SNP_11038	2031	G	C	4	6	--	--
	Ca(ICCV2/PI489777)SNP_11039	2052	A	G	4	6	--	--
CakTC42681	Ca(ICCV2/PI489777)SNP_11040	248	G	A	5	3	--	--
	Ca(ICCV2/PI489777)SNP_11041	954	G	A	11	9	--	--
	Ca(ICCV2/PI489777)SNP_11042	1576	G	A	5	4	--	--
CakTC37630	Ca(ICCV2/PI489777)SNP_11043	163	G	T	61	17	--	--
	Ca(ICCV2/PI489777)SNP_11044	181	A	C	92	20	--	--
	Ca(ICCV2/PI489777)SNP_11045	431	C	T	157	36	--	--
	Ca(ICCV2/PI489777)SNP_11046	1214	C	T	58	5	--	--
	Ca(ICCV2/PI489777)SNP_11047	1215	T	C	48	5	--	--
CakTC24288	Ca(ICCV2/PI489777)SNP_11048	148	G	A	4	5	--	--
	Ca(ICCV2/PI489777)SNP_11049	153	C	T	4	5	--	--
	Ca(ICCV2/PI489777)SNP_11050	265	G	A	5	5	--	--
	Ca(ICCV2/PI489777)SNP_11051	354	T	A	4	6	--	--
	Ca(ICCV2/PI489777)SNP_11052	408	T	G	4	6	--	--
CakTC37220	Ca(ICCV2/PI489777)SNP_11053	735	T	C	10	5	--	--
	Ca(ICCV2/PI489777)SNP_11054	825	T	C	3	4	--	--
CakTC15949	Ca(ICCV2/PI489777)SNP_11055	285	C	T	4	4	--	--
CakTC38642	Ca(ICCV2/PI489777)SNP_11056	368	C	T	15	7	--	--
CakTC31500	Ca(ICCV2/PI489777)SNP_11057	2590	G	A	6	3	--	--
	Ca(ICCV2/PI489777)SNP_11058	3236	T	C	3	8	--	--
CakTC24866	Ca(ICCV2/PI489777)SNP_11059	203	C	T	16	9	--	ARF
	Ca(ICCV2/PI489777)SNP_11060	571	T	C	17	12	--	ARF
	Ca(ICCV2/PI489777)SNP_11061	2527	G	T	18	15	--	ARF
	Ca(ICCV2/PI489777)SNP_11062	2649	G	C	17	13	--	ARF
CakTC29444	Ca(ICCV2/PI489777)SNP_11063	1819	T	C	19	7	--	--
CakTC42125	Ca(ICCV2/PI489777)SNP_11064	286	G	A	3	22	--	--
	Ca(ICCV2/PI489777)SNP_11065	360	G	A	3	21	--	--
CakTC11098	Ca(ICCV2/PI489777)SNP_11066	45	T	C	5	4	--	--
	Ca(ICCV2/PI489777)SNP_11067	269	G	A	19	11	--	--
	Ca(ICCV2/PI489777)SNP_11068	860	G	A	15	5	--	--
CakTC39121	Ca(ICCV2/PI489777)SNP_11069	721	G	A	9	6	--	--
CakTC33476	Ca(ICCV2/PI489777)SNP_11070	162	C	G	14	12	--	--
	Ca(ICCV2/PI489777)SNP_11071	607	T	G	9	7	--	--
	Ca(ICCV2/PI489777)SNP_11072	639	G	C	3	6	--	--
	Ca(ICCV2/PI489777)SNP_11073	651	A	C	3	6	--	--
CakTC33330	Ca(ICCV2/PI489777)SNP_11074	262	G	A	10	7	--	--
	Ca(ICCV2/PI489777)SNP_11075	479	T	G	9	6	--	--
	Ca(ICCV2/PI489777)SNP_11076	493	G	A	9	5	--	--
	Ca(ICCV2/PI489777)SNP_11077	595	C	T	5	4	--	--
CakTC38917	Ca(ICCV2/PI489777)SNP_11078	215	T	C	4	5	--	--
	Ca(ICCV2/PI489777)SNP_11079	496	C	A	24	26	--	--
	Ca(ICCV2/PI489777)SNP_11080	742	T	C	16	20	--	--
	Ca(ICCV2/PI489777)SNP_11081	808	C	T	15	17	--	--
	Ca(ICCV2/PI489777)SNP_11082	814	C	T	15	16	--	--
	Ca(ICCV2/PI489777)SNP_11083	1324	G	A	6	19	--	--
CakTC29651	Ca(ICCV2/PI489777)SNP_11084	752	C	T	44	17	--	--
	Ca(ICCV2/PI489777)SNP_11085	836	T	C	52	20	--	--
	Ca(ICCV2/PI489777)SNP_11086	1070	G	A	23	19	--	--
CakTC08095	Ca(ICCV2/PI489777)SNP_11087	1056	T	C	84	18	--	--
CakTC43068	Ca(ICCV2/PI489777)SNP_11088	459	C	G	12	16	--	--
CakTC31489	Ca(ICCV2/PI489777)SNP_11089	659	T	C	4	5	--	--
	Ca(ICCV2/PI489777)SNP_11090	1076	T	G	3	5	--	--
CakTC42607	Ca(ICCV2/PI489777)SNP_11091	744	G	T	38	23	Mature	Leaf
	Ca(ICCV2/PI489777)SNP_11092	993	T	C	18	30	Mature	Leaf
CakTC30968	Ca(ICCV2/PI489777)SNP_11093	338	G	C	7	5	--	--
CakTC41408	Ca(ICCV2/PI489777)SNP_11094	371	C	A	12	10	--	--
CakTC24124	Ca(ICCV2/PI489777)SNP_11095	1105	T	C	35	14	--	--

	Ca(ICCV2/PI489777)SNP_11096	1308	A	T	26	5	--	--
CakTC24268	Ca(ICCV2/PI489777)SNP_11097	182	C	T	4	3	Shoot	--
	Ca(ICCV2/PI489777)SNP_11098	201	G	C	3	3	Shoot	--
CakTC24998	Ca(ICCV2/PI489777)SNP_11099	1209	C	T	38	32	--	--
CakTC31883	Ca(ICCV2/PI489777)SNP_11100	1521	C	T	6	3	--	--
CakTC31694	Ca(ICCV2/PI489777)SNP_11101	413	T	C	6	4	--	--
CakTC41172	Ca(ICCV2/PI489777)SNP_11102	537	T	C	12	13	--	--
	Ca(ICCV2/PI489777)SNP_11103	726	T	C	22	8	--	--
	Ca(ICCV2/PI489777)SNP_11104	801	T	C	23	11	--	--
	Ca(ICCV2/PI489777)SNP_11105	1140	A	G	20	3	--	--
	Ca(ICCV2/PI489777)SNP_11106	1998	G	T	17	14	--	--
	Ca(ICCV2/PI489777)SNP_11107	2020	A	G	16	5	--	--
	Ca(ICCV2/PI489777)SNP_11108	2023	C	G	15	5	--	--
	Ca(ICCV2/PI489777)SNP_11109	2076	A	G	13	9	--	--
CakTC10743	Ca(ICCV2/PI489777)SNP_11110	372	C	T	11	7	--	--
	Ca(ICCV2/PI489777)SNP_11111	378	T	A	9	6	--	--
	Ca(ICCV2/PI489777)SNP_11112	527	C	A	9	9	--	--
	Ca(ICCV2/PI489777)SNP_11113	1480	A	G	4	3	--	--
CakTC03575	Ca(ICCV2/PI489777)SNP_11114	95	T	C	4	3	--	--
	Ca(ICCV2/PI489777)SNP_11115	153	G	A	7	3	--	--
CakTC32779	Ca(ICCV2/PI489777)SNP_11116	1086	C	T	19	7	--	--
	Ca(ICCV2/PI489777)SNP_11117	1525	G	A	25	11	--	--
	Ca(ICCV2/PI489777)SNP_11118	1606	G	C	30	11	--	--
	Ca(ICCV2/PI489777)SNP_11119	2857	G	C	8	3	--	--
CakTC38282	Ca(ICCV2/PI489777)SNP_11120	35	C	A	21	18	--	--
	Ca(ICCV2/PI489777)SNP_11121	730	T	G	60	33	--	--
	Ca(ICCV2/PI489777)SNP_11122	733	A	T	57	33	--	--
CakTC33159	Ca(ICCV2/PI489777)SNP_11123	652	C	T	6	15	--	--
	Ca(ICCV2/PI489777)SNP_11124	766	G	A	8	9	--	--
	Ca(ICCV2/PI489777)SNP_11125	1886	C	T	3	7	--	--
CakTC26512	Ca(ICCV2/PI489777)SNP_11126	199	A	C	11	3	--	E2F-DP
	Ca(ICCV2/PI489777)SNP_11127	1183	T	A	14	5	--	E2F-DP
CakTC37806	Ca(ICCV2/PI489777)SNP_11128	990	T	C	20	23	--	--
	Ca(ICCV2/PI489777)SNP_11129	1009	T	C	16	22	--	--
CakTC11460	Ca(ICCV2/PI489777)SNP_11130	983	A	G	5	6	--	--
	Ca(ICCV2/PI489777)SNP_11131	1011	T	A	4	8	--	--
	Ca(ICCV2/PI489777)SNP_11132	1397	T	C	4	6	--	--
	Ca(ICCV2/PI489777)SNP_11133	1511	A	G	4	6	--	--
	Ca(ICCV2/PI489777)SNP_11134	1586	C	T	4	6	--	--
CakTC38204	Ca(ICCV2/PI489777)SNP_11135	477	A	G	6	7	--	--
	Ca(ICCV2/PI489777)SNP_11136	1993	G	T	6	4	--	--
CakTC40341	Ca(ICCV2/PI489777)SNP_11137	471	A	G	3	3	--	--
CakTC37686	Ca(ICCV2/PI489777)SNP_11138	855	T	C	40	20	--	HB
	Ca(ICCV2/PI489777)SNP_11139	1019	A	G	29	15	--	HB
	Ca(ICCV2/PI489777)SNP_11140	1157	A	T	16	8	--	HB
CakTC41780	Ca(ICCV2/PI489777)SNP_11141	104	A	C	28	20	--	--
	Ca(ICCV2/PI489777)SNP_11142	223	T	C	39	25	--	--
	Ca(ICCV2/PI489777)SNP_11143	304	G	A	37	26	--	--
CakTC31724	Ca(ICCV2/PI489777)SNP_11144	480	T	A	4	16	--	--
	Ca(ICCV2/PI489777)SNP_11145	630	T	C	3	15	--	--
CakTC26919	Ca(ICCV2/PI489777)SNP_11146	845	G	A	4	4	Young_pod	--
CakTC37229	Ca(ICCV2/PI489777)SNP_11147	474	C	T	6	4	--	--
	Ca(ICCV2/PI489777)SNP_11148	540	A	G	5	4	--	--
	Ca(ICCV2/PI489777)SNP_11149	573	T	A	4	4	--	--
	Ca(ICCV2/PI489777)SNP_11150	575	A	G	4	4	--	--
	Ca(ICCV2/PI489777)SNP_11151	896	A	C	3	3	--	--
CakTC12248	Ca(ICCV2/PI489777)SNP_11152	208	T	G	31	6	--	--
	Ca(ICCV2/PI489777)SNP_11153	265	C	T	28	16	--	--
CakTC15738	Ca(ICCV2/PI489777)SNP_11154	1841	C	T	4	4	--	Jumonji
	Ca(ICCV2/PI489777)SNP_11155	1936	A	C	7	6	--	Jumonji
	Ca(ICCV2/PI489777)SNP_11156	1983	G	C	7	5	--	Jumonji
CakTC36758	Ca(ICCV2/PI489777)SNP_11157	746	C	T	14	13	--	--
	Ca(ICCV2/PI489777)SNP_11158	896	T	G	22	16	--	--
	Ca(ICCV2/PI489777)SNP_11159	1123	A	C	22	30	--	--
	Ca(ICCV2/PI489777)SNP_11160	1124	T	G	22	30	--	--
	Ca(ICCV2/PI489777)SNP_11161	1676	T	C	27	22	--	--
	Ca(ICCV2/PI489777)SNP_11162	2254	A	G	18	39	--	--
	Ca(ICCV2/PI489777)SNP_11163	2657	C	G	5	10	--	--
CakTC01763	Ca(ICCV2/PI489777)SNP_11164	81	C	T	45	11	--	--
	Ca(ICCV2/PI489777)SNP_11165	204	C	T	53	10	--	--
	Ca(ICCV2/PI489777)SNP_11166	363	T	C	43	11	--	--
CakTC33913	Ca(ICCV2/PI489777)SNP_11167	774	A	T	3	7	--	SBP
CakTC43150	Ca(ICCV2/PI489777)SNP_11168	536	A	G	6	6	--	--
	Ca(ICCV2/PI489777)SNP_11169	697	T	C	9	3	--	--

CakTC26370	Ca(ICCV2/PI489777)SNP_11170	363	C	G	13	24	--	--
	Ca(ICCV2/PI489777)SNP_11171	375	T	A	13	22	--	--
	Ca(ICCV2/PI489777)SNP_11172	528	C	T	15	12	--	--
	Ca(ICCV2/PI489777)SNP_11173	961	A	G	7	9	--	--
	Ca(ICCV2/PI489777)SNP_11174	1860	C	T	6	6	--	--
CakTC40674	Ca(ICCV2/PI489777)SNP_11175	248	G	A	3	3	--	--
	Ca(ICCV2/PI489777)SNP_11176	350	T	C	4	4	--	--
	Ca(ICCV2/PI489777)SNP_11177	421	C	A	4	4	--	--
	Ca(ICCV2/PI489777)SNP_11178	436	C	T	4	4	--	--
CakTC42331	Ca(ICCV2/PI489777)SNP_11179	352	A	G	24	23	--	--
	Ca(ICCV2/PI489777)SNP_11180	357	A	T	28	24	--	--
	Ca(ICCV2/PI489777)SNP_11181	369	A	G	34	25	--	--
	Ca(ICCV2/PI489777)SNP_11182	380	T	C	33	28	--	--
	Ca(ICCV2/PI489777)SNP_11183	406	C	G	38	28	--	--
	Ca(ICCV2/PI489777)SNP_11184	419	A	G	43	25	--	--
	Ca(ICCV2/PI489777)SNP_11185	692	T	A	21	22	--	--
	Ca(ICCV2/PI489777)SNP_11186	750	T	C	5	7	--	--
CakTC25593	Ca(ICCV2/PI489777)SNP_11187	292	G	T	77	22	--	--
	Ca(ICCV2/PI489777)SNP_11188	814	T	C	75	43	--	--
CakTC38331	Ca(ICCV2/PI489777)SNP_11189	545	T	C	15	5	Young_pod	--
	Ca(ICCV2/PI489777)SNP_11190	659	G	A	16	5	Young_pod	--
	Ca(ICCV2/PI489777)SNP_11191	830	A	C	24	5	Young_pod	--
	Ca(ICCV2/PI489777)SNP_11192	917	C	G	30	5	Young_pod	--
	Ca(ICCV2/PI489777)SNP_11193	918	T	C	27	5	Young_pod	--
	Ca(ICCV2/PI489777)SNP_11194	1089	T	A	22	5	Young_pod	--
CakTC34300	Ca(ICCV2/PI489777)SNP_11195	321	G	C	13	12	Shoot	--
CakTC23802	Ca(ICCV2/PI489777)SNP_11196	181	G	C	29	3	--	--
CakTC26225	Ca(ICCV2/PI489777)SNP_11197	756	C	G	99	33	--	--
CakTC24299	Ca(ICCV2/PI489777)SNP_11198	603	G	A	18	10	--	--
	Ca(ICCV2/PI489777)SNP_11199	813	G	A	16	9	--	--
	Ca(ICCV2/PI489777)SNP_11200	948	C	T	12	12	--	--
	Ca(ICCV2/PI489777)SNP_11201	1095	C	T	6	13	--	--
	Ca(ICCV2/PI489777)SNP_11202	1500	T	A	5	4	--	--
CakTC41816	Ca(ICCV2/PI489777)SNP_11203	291	C	G	4	3	--	S1Fa-like
	Ca(ICCV2/PI489777)SNP_11204	822	G	A	7	3	--	S1Fa-like
	Ca(ICCV2/PI489777)SNP_11205	888	G	A	7	4	--	S1Fa-like
	Ca(ICCV2/PI489777)SNP_11206	999	G	A	11	7	--	S1Fa-like
CakTC38525	Ca(ICCV2/PI489777)SNP_11207	823	T	C	11	6	--	--
	Ca(ICCV2/PI489777)SNP_11208	1216	A	G	9	3	--	--
CakTC26299	Ca(ICCV2/PI489777)SNP_11209	351	T	C	13	10	--	MADS
	Ca(ICCV2/PI489777)SNP_11210	393	T	C	11	11	--	MADS
CakTC42827	Ca(ICCV2/PI489777)SNP_11211	527	C	T	5	9	--	--
	Ca(ICCV2/PI489777)SNP_11212	699	A	G	4	10	--	--
	Ca(ICCV2/PI489777)SNP_11213	1732	G	A	3	5	--	--
CakTC35087	Ca(ICCV2/PI489777)SNP_11214	664	G	C	12	5	--	--
	Ca(ICCV2/PI489777)SNP_11215	805	A	G	11	5	--	--
	Ca(ICCV2/PI489777)SNP_11216	974	A	G	16	4	--	--
	Ca(ICCV2/PI489777)SNP_11217	1096	G	A	12	4	--	--
	Ca(ICCV2/PI489777)SNP_11218	1306	C	T	25	9	--	--
	Ca(ICCV2/PI489777)SNP_11219	2393	G	C	5	4	--	--
	Ca(ICCV2/PI489777)SNP_11220	2520	G	T	5	6	--	--
CakTC40062	Ca(ICCV2/PI489777)SNP_11221	982	A	G	19	10	--	--
CakTC25761	Ca(ICCV2/PI489777)SNP_11222	342	C	T	9	8	Young_pod	--
	Ca(ICCV2/PI489777)SNP_11223	724	C	T	14	3	Young_pod	--
	Ca(ICCV2/PI489777)SNP_11224	1723	A	G	9	5	Young_pod	--
	Ca(ICCV2/PI489777)SNP_11225	1843	T	C	8	3	Young_pod	--
	Ca(ICCV2/PI489777)SNP_11226	2033	A	T	11	7	Young_pod	--
CakTC24251	Ca(ICCV2/PI489777)SNP_11227	564	C	T	7	4	--	--
	Ca(ICCV2/PI489777)SNP_11228	1599	C	T	15	6	--	--
	Ca(ICCV2/PI489777)SNP_11229	1704	C	G	14	4	--	--
	Ca(ICCV2/PI489777)SNP_11230	1800	T	C	8	4	--	--
	Ca(ICCV2/PI489777)SNP_11231	3433	A	C	8	4	--	--
CakTC40523	Ca(ICCV2/PI489777)SNP_11232	482	C	T	25	6	--	--
	Ca(ICCV2/PI489777)SNP_11233	535	C	T	23	6	--	--
	Ca(ICCV2/PI489777)SNP_11234	552	T	G	20	5	--	--
	Ca(ICCV2/PI489777)SNP_11235	558	A	G	20	5	--	--
CakTC29034	Ca(ICCV2/PI489777)SNP_11236	558	C	A	23	3	--	--
	Ca(ICCV2/PI489777)SNP_11237	660	C	A	21	7	--	--
	Ca(ICCV2/PI489777)SNP_11238	663	T	A	21	7	--	--
	Ca(ICCV2/PI489777)SNP_11239	742	G	C	21	7	--	--
	Ca(ICCV2/PI489777)SNP_11240	927	T	C	18	7	--	--
	Ca(ICCV2/PI489777)SNP_11241	1158	G	C	33	8	--	--
CakTC38687	Ca(ICCV2/PI489777)SNP_11242	159	T	C	8	3	--	--
	Ca(ICCV2/PI489777)SNP_11243	168	C	T	7	3	--	--

	Ca(ICCV2/PI489777)SNP_11244	329	T	G	13	4	--	--
	Ca(ICCV2/PI489777)SNP_11245	382	A	G	13	3	--	--
	Ca(ICCV2/PI489777)SNP_11246	390	A	C	13	4	--	--
	Ca(ICCV2/PI489777)SNP_11247	418	T	A	15	3	--	--
	Ca(ICCV2/PI489777)SNP_11248	648	G	T	13	3	--	--
	Ca(ICCV2/PI489777)SNP_11249	687	G	A	11	4	--	--
	Ca(ICCV2/PI489777)SNP_11250	831	A	G	11	4	--	--
	Ca(ICCV2/PI489777)SNP_11251	843	G	A	11	4	--	--
	Ca(ICCV2/PI489777)SNP_11252	894	A	G	11	4	--	--
	Ca(ICCV2/PI489777)SNP_11253	966	G	A	12	5	--	--
	Ca(ICCV2/PI489777)SNP_11254	1242	T	C	9	5	--	--
	Ca(ICCV2/PI489777)SNP_11255	1245	G	A	11	5	--	--
	Ca(ICCV2/PI489777)SNP_11256	1280	C	T	15	5	--	--
	Ca(ICCV2/PI489777)SNP_11257	1371	C	T	13	8	--	--
	Ca(ICCV2/PI489777)SNP_11258	1564	G	T	9	11	--	--
	Ca(ICCV2/PI489777)SNP_11259	1572	G	A	10	11	--	--
CakTC43280	Ca(ICCV2/PI489777)SNP_11260	1259	G	A	18	10	--	--
CakTC37866	Ca(ICCV2/PI489777)SNP_11261	211	A	T	25	25	--	--
	Ca(ICCV2/PI489777)SNP_11262	1554	T	C	13	8	--	--
	Ca(ICCV2/PI489777)SNP_11263	1563	C	T	12	8	--	--
	Ca(ICCV2/PI489777)SNP_11264	1604	C	G	3	6	--	--
CakTC31291	Ca(ICCV2/PI489777)SNP_11265	2175	A	C	13	7	--	--
CakTC39694	Ca(ICCV2/PI489777)SNP_11266	806	A	G	7	4	--	--
CakTC14057	Ca(ICCV2/PI489777)SNP_11267	284	G	A	7	6	--	--
CakTC28481	Ca(ICCV2/PI489777)SNP_11268	862	G	A	5	3	--	--
	Ca(ICCV2/PI489777)SNP_11269	1275	C	T	8	3	--	--
	Ca(ICCV2/PI489777)SNP_11270	2986	C	T	5	5	--	--
CakTC28039	Ca(ICCV2/PI489777)SNP_11271	989	A	G	15	5	--	--
	Ca(ICCV2/PI489777)SNP_11272	1028	T	A	10	5	--	--
	Ca(ICCV2/PI489777)SNP_11273	1090	A	G	11	5	--	--
	Ca(ICCV2/PI489777)SNP_11274	1589	T	A	6	7	--	--
	Ca(ICCV2/PI489777)SNP_11275	1590	T	C	6	7	--	--
	Ca(ICCV2/PI489777)SNP_11276	1722	T	A	9	4	--	--
	Ca(ICCV2/PI489777)SNP_11277	1843	G	A	15	12	--	--
	Ca(ICCV2/PI489777)SNP_11278	1936	A	G	17	12	--	--
	Ca(ICCV2/PI489777)SNP_11279	2149	C	T	19	12	--	--
	Ca(ICCV2/PI489777)SNP_11280	2189	C	G	19	9	--	--
	Ca(ICCV2/PI489777)SNP_11281	2227	A	G	23	11	--	--
	Ca(ICCV2/PI489777)SNP_11282	2246	G	C	22	10	--	--
	Ca(ICCV2/PI489777)SNP_11283	2258	A	G	18	5	--	--
	Ca(ICCV2/PI489777)SNP_11284	2505	A	G	10	6	--	--
	Ca(ICCV2/PI489777)SNP_11285	2746	C	A	7	6	--	--
CakTC32877	Ca(ICCV2/PI489777)SNP_11286	1070	T	C	19	12	--	--
CakTC31141	Ca(ICCV2/PI489777)SNP_11287	2057	T	G	14	12	--	--
	Ca(ICCV2/PI489777)SNP_11288	2390	T	C	3	3	--	--
CakTC14072	Ca(ICCV2/PI489777)SNP_11289	1807	A	G	8	8	--	--
	Ca(ICCV2/PI489777)SNP_11290	1843	A	G	5	8	--	--
CakTC02406	Ca(ICCV2/PI489777)SNP_11291	981	T	C	5	7	--	--
CakTC01069	Ca(ICCV2/PI489777)SNP_11292	953	C	T	8	19	--	--
	Ca(ICCV2/PI489777)SNP_11293	1066	A	C	4	15	--	--
	Ca(ICCV2/PI489777)SNP_11294	1072	C	A	3	13	--	--
	Ca(ICCV2/PI489777)SNP_11295	1080	T	G	4	12	--	--
	Ca(ICCV2/PI489777)SNP_11296	1087	A	G	4	9	--	--
	Ca(ICCV2/PI489777)SNP_11297	1100	G	A	3	7	--	--
CakTC25564	Ca(ICCV2/PI489777)SNP_11298	1415	T	A	5	4	--	GRAS
CakTC26035	Ca(ICCV2/PI489777)SNP_11299	779	A	G	22	15	--	--
	Ca(ICCV2/PI489777)SNP_11300	2114	A	G	23	6	--	--
	Ca(ICCV2/PI489777)SNP_11301	2197	C	G	43	7	--	--
CakTC42726	Ca(ICCV2/PI489777)SNP_11302	53	A	G	7	3	--	--
	Ca(ICCV2/PI489777)SNP_11303	319	T	G	11	3	--	--
	Ca(ICCV2/PI489777)SNP_11304	377	G	A	10	5	--	--
	Ca(ICCV2/PI489777)SNP_11305	534	C	G	6	3	--	--
CakTC25206	Ca(ICCV2/PI489777)SNP_11306	37	T	G	12	9	--	--
	Ca(ICCV2/PI489777)SNP_11307	222	G	A	23	18	--	--
	Ca(ICCV2/PI489777)SNP_11308	331	G	T	26	16	--	--
	Ca(ICCV2/PI489777)SNP_11309	422	G	T	24	18	--	--
	Ca(ICCV2/PI489777)SNP_11310	484	C	G	17	15	--	--
	Ca(ICCV2/PI489777)SNP_11311	585	C	T	22	12	--	--
CakTC33545	Ca(ICCV2/PI489777)SNP_11312	474	A	T	4	7	--	bHLH
	Ca(ICCV2/PI489777)SNP_11313	957	C	G	9	5	--	bHLH
	Ca(ICCV2/PI489777)SNP_11314	1773	A	C	4	4	--	bHLH
CakTC39162	Ca(ICCV2/PI489777)SNP_11315	1309	G	A	16	17	--	--
	Ca(ICCV2/PI489777)SNP_11316	1394	C	A	10	9	--	--
CakTC31034	Ca(ICCV2/PI489777)SNP_11317	853	G	A	6	4	--	--

	Ca(ICCV2/PI489777)SNP_11318	862	T	C	6	5	--	--
	Ca(ICCV2/PI489777)SNP_11319	985	A	T	15	4	--	--
	Ca(ICCV2/PI489777)SNP_11320	1075	G	A	14	7	--	--
	Ca(ICCV2/PI489777)SNP_11321	1271	G	A	10	9	--	--
	Ca(ICCV2/PI489777)SNP_11322	1406	C	T	9	7	--	--
	Ca(ICCV2/PI489777)SNP_11323	1500	C	T	9	5	--	--
	Ca(ICCV2/PI489777)SNP_11324	2473	T	C	6	8	--	--
CakTC42223	Ca(ICCV2/PI489777)SNP_11325	151	T	C	8	4	--	--
	Ca(ICCV2/PI489777)SNP_11326	335	T	C	23	7	--	--
	Ca(ICCV2/PI489777)SNP_11327	730	G	A	20	4	--	--
	Ca(ICCV2/PI489777)SNP_11328	881	A	G	14	7	--	--
	Ca(ICCV2/PI489777)SNP_11329	1741	C	G	3	3	--	--
CakTC35612	Ca(ICCV2/PI489777)SNP_11330	196	A	C	11	9	--	--
	Ca(ICCV2/PI489777)SNP_11331	381	G	T	12	11	--	--
	Ca(ICCV2/PI489777)SNP_11332	702	T	C	14	6	--	--
	Ca(ICCV2/PI489777)SNP_11333	868	G	A	15	10	--	--
	Ca(ICCV2/PI489777)SNP_11334	2163	G	T	25	26	--	--
	Ca(ICCV2/PI489777)SNP_11335	3030	C	T	20	17	--	--
	Ca(ICCV2/PI489777)SNP_11336	3426	G	A	25	30	--	--
	Ca(ICCV2/PI489777)SNP_11337	3642	T	A	12	4	--	--
CakTC33960	Ca(ICCV2/PI489777)SNP_11338	371	C	G	8	9	--	bHLH
	Ca(ICCV2/PI489777)SNP_11339	376	C	A	11	8	--	bHLH
	Ca(ICCV2/PI489777)SNP_11340	494	C	G	11	12	--	bHLH
	Ca(ICCV2/PI489777)SNP_11341	578	G	T	11	8	--	bHLH
	Ca(ICCV2/PI489777)SNP_11342	737	T	C	15	12	--	bHLH
CakTC25631	Ca(ICCV2/PI489777)SNP_11343	307	G	A	16	22	--	--
	Ca(ICCV2/PI489777)SNP_11344	329	G	A	17	25	--	--
	Ca(ICCV2/PI489777)SNP_11345	334	C	T	21	26	--	--
	Ca(ICCV2/PI489777)SNP_11346	455	G	A	37	52	--	--
	Ca(ICCV2/PI489777)SNP_11347	534	C	T	50	58	--	--
	Ca(ICCV2/PI489777)SNP_11348	735	C	T	63	68	--	--
	Ca(ICCV2/PI489777)SNP_11349	1597	G	A	44	56	--	--
	Ca(ICCV2/PI489777)SNP_11350	2036	G	A	37	56	--	--
	Ca(ICCV2/PI489777)SNP_11351	2451	G	A	47	34	--	--
	Ca(ICCV2/PI489777)SNP_11352	2611	G	A	43	35	--	--
	Ca(ICCV2/PI489777)SNP_11353	2740	A	T	31	33	--	--
	Ca(ICCV2/PI489777)SNP_11354	2833	T	C	33	36	--	--
	Ca(ICCV2/PI489777)SNP_11355	2989	T	C	41	31	--	--
	Ca(ICCV2/PI489777)SNP_11356	3658	C	G	36	37	--	--
	Ca(ICCV2/PI489777)SNP_11357	3778	A	C	42	39	--	--
	Ca(ICCV2/PI489777)SNP_11358	3944	C	T	30	53	--	--
	Ca(ICCV2/PI489777)SNP_11359	4234	T	C	21	38	--	--
	Ca(ICCV2/PI489777)SNP_11360	4485	C	G	11	15	--	--
CakTC39956	Ca(ICCV2/PI489777)SNP_11361	326	G	A	10	7	--	--
	Ca(ICCV2/PI489777)SNP_11362	488	C	T	3	7	--	--
	Ca(ICCV2/PI489777)SNP_11363	812	A	G	16	6	--	--
	Ca(ICCV2/PI489777)SNP_11364	1286	T	C	3	6	--	--
CakTC30606	Ca(ICCV2/PI489777)SNP_11365	515	C	A	3	5	--	--
	Ca(ICCV2/PI489777)SNP_11366	569	C	T	5	4	--	--
	Ca(ICCV2/PI489777)SNP_11367	1046	C	A	3	4	--	--
	Ca(ICCV2/PI489777)SNP_11368	1060	C	G	3	4	--	--
CakTC24939	Ca(ICCV2/PI489777)SNP_11369	1097	C	T	3	3	--	Tify
CakTC26544	Ca(ICCV2/PI489777)SNP_11370	914	G	A	5	3	--	--
	Ca(ICCV2/PI489777)SNP_11371	1512	G	A	10	5	--	--
	Ca(ICCV2/PI489777)SNP_11372	2615	A	T	3	4	--	--
	Ca(ICCV2/PI489777)SNP_11373	2723	T	C	3	3	--	--
	Ca(ICCV2/PI489777)SNP_11374	2926	T	C	3	4	--	--
	Ca(ICCV2/PI489777)SNP_11375	3697	C	A	7	3	--	--
CakTC33843	Ca(ICCV2/PI489777)SNP_11376	545	A	C	4	4	--	--
CakTC41634	Ca(ICCV2/PI489777)SNP_11377	440	G	A	28	23	--	--
CakTC35271	Ca(ICCV2/PI489777)SNP_11378	141	T	G	4	4	--	--
	Ca(ICCV2/PI489777)SNP_11379	1951	A	G	9	8	--	--
	Ca(ICCV2/PI489777)SNP_11380	2446	C	T	18	9	--	--
	Ca(ICCV2/PI489777)SNP_11381	3061	T	G	15	6	--	--
	Ca(ICCV2/PI489777)SNP_11382	3085	T	G	11	8	--	--
	Ca(ICCV2/PI489777)SNP_11383	3218	T	G	13	8	--	--
CakTC28040	Ca(ICCV2/PI489777)SNP_11384	342	A	G	3	3	--	--
CakTC23856	Ca(ICCV2/PI489777)SNP_11385	374	G	A	21	4	--	--
	Ca(ICCV2/PI489777)SNP_11386	1394	C	T	18	3	--	--
	Ca(ICCV2/PI489777)SNP_11387	1871	G	A	6	11	--	--
	Ca(ICCV2/PI489777)SNP_11388	1901	C	T	5	12	--	--
	Ca(ICCV2/PI489777)SNP_11389	2818	G	A	16	5	--	--
CakTC24652	Ca(ICCV2/PI489777)SNP_11390	1400	T	C	6	10	--	--
	Ca(ICCV2/PI489777)SNP_11391	1402	G	A	6	11	--	--

	Ca(ICCV2/PI489777)SNP_11392	1646	A	T	6	8	--	--
	Ca(ICCV2/PI489777)SNP_11393	1731	G	A	3	6	--	--
CakTC30048	Ca(ICCV2/PI489777)SNP_11394	519	T	C	9	13	--	--
CakTC39102	Ca(ICCV2/PI489777)SNP_11395	1340	T	C	13	6	--	--
CakTC37204	Ca(ICCV2/PI489777)SNP_11396	1167	G	T	13	13	--	--
CakTC39065	Ca(ICCV2/PI489777)SNP_11397	635	T	C	4	3	--	--
	Ca(ICCV2/PI489777)SNP_11398	1238	G	A	4	4	--	--
	Ca(ICCV2/PI489777)SNP_11399	1274	A	C	5	4	--	--
CakTC25774	Ca(ICCV2/PI489777)SNP_11400	3026	G	A	4	3	--	--
	Ca(ICCV2/PI489777)SNP_11401	3929	C	T	4	3	--	--
CakTC36665	Ca(ICCV2/PI489777)SNP_11402	1460	A	G	27	14	--	--
	Ca(ICCV2/PI489777)SNP_11403	1595	G	A	34	13	--	--
	Ca(ICCV2/PI489777)SNP_11404	2622	A	G	47	23	--	--
	Ca(ICCV2/PI489777)SNP_11405	3066	T	C	18	21	--	--
	Ca(ICCV2/PI489777)SNP_11406	3589	G	T	10	9	--	--
	Ca(ICCV2/PI489777)SNP_11407	3624	T	C	11	7	--	--
	Ca(ICCV2/PI489777)SNP_11408	3625	G	T	11	7	--	--
CakTC32693	Ca(ICCV2/PI489777)SNP_11409	150	T	G	4	7	--	--
	Ca(ICCV2/PI489777)SNP_11410	599	T	A	13	5	--	--
	Ca(ICCV2/PI489777)SNP_11411	1060	G	A	5	7	--	--
	Ca(ICCV2/PI489777)SNP_11412	1338	T	C	6	6	--	--
CakTC40346	Ca(ICCV2/PI489777)SNP_11413	276	C	T	13	7	--	--
	Ca(ICCV2/PI489777)SNP_11414	293	A	T	13	7	--	--
	Ca(ICCV2/PI489777)SNP_11415	709	T	C	16	4	--	--
	Ca(ICCV2/PI489777)SNP_11416	855	G	A	20	5	--	--
	Ca(ICCV2/PI489777)SNP_11417	1500	C	T	21	4	--	--
	Ca(ICCV2/PI489777)SNP_11418	1563	T	A	17	4	--	--
	Ca(ICCV2/PI489777)SNP_11419	1647	A	G	14	3	--	--
	Ca(ICCV2/PI489777)SNP_11420	1671	A	C	12	3	--	--
CakTC31900	Ca(ICCV2/PI489777)SNP_11421	1079	C	T	9	3	--	--
	Ca(ICCV2/PI489777)SNP_11422	1143	G	A	9	3	--	--
	Ca(ICCV2/PI489777)SNP_11423	2066	A	C	4	4	--	--
	Ca(ICCV2/PI489777)SNP_11424	2177	T	C	5	4	--	--
CakTC40874	Ca(ICCV2/PI489777)SNP_11425	236	A	G	6	4	--	WRKY
	Ca(ICCV2/PI489777)SNP_11426	447	T	C	7	7	--	WRKY
	Ca(ICCV2/PI489777)SNP_11427	1013	T	C	21	7	--	WRKY
	Ca(ICCV2/PI489777)SNP_11428	1130	T	C	21	5	--	WRKY
	Ca(ICCV2/PI489777)SNP_11429	1481	G	A	22	8	--	WRKY
	Ca(ICCV2/PI489777)SNP_11430	1532	A	G	21	9	--	WRKY
CakTC05863	Ca(ICCV2/PI489777)SNP_11431	344	A	G	5	4	--	--
	Ca(ICCV2/PI489777)SNP_11432	851	G	A	16	11	--	--
	Ca(ICCV2/PI489777)SNP_11433	1248	C	A	4	6	--	--
	Ca(ICCV2/PI489777)SNP_11434	1751	A	T	21	10	--	--
	Ca(ICCV2/PI489777)SNP_11435	2406	A	T	15	9	--	--
	Ca(ICCV2/PI489777)SNP_11436	2441	G	C	15	10	--	--
CakTC10233	Ca(ICCV2/PI489777)SNP_11437	489	G	T	8	3	--	--
	Ca(ICCV2/PI489777)SNP_11438	506	T	A	7	3	--	--
	Ca(ICCV2/PI489777)SNP_11439	508	T	G	7	3	--	--
	Ca(ICCV2/PI489777)SNP_11440	518	T	C	7	3	--	--
CakTC37394	Ca(ICCV2/PI489777)SNP_11441	132	G	A	5	4	--	--
	Ca(ICCV2/PI489777)SNP_11442	214	G	T	8	5	--	--
	Ca(ICCV2/PI489777)SNP_11443	382	C	A	10	8	--	--
	Ca(ICCV2/PI489777)SNP_11444	1178	T	C	11	6	--	--
	Ca(ICCV2/PI489777)SNP_11445	1322	T	C	9	3	--	--
CakTC27291	Ca(ICCV2/PI489777)SNP_11446	1186	T	C	10	12	Root	--
	Ca(ICCV2/PI489777)SNP_11447	1228	C	T	4	12	Root	--
	Ca(ICCV2/PI489777)SNP_11448	1337	C	T	7	11	Root	--
	Ca(ICCV2/PI489777)SNP_11449	1429	A	C	6	7	Root	--
CakTC29136	Ca(ICCV2/PI489777)SNP_11450	464	T	C	3	4	--	--
	Ca(ICCV2/PI489777)SNP_11451	1067	A	G	6	8	--	--
CakTC42162	Ca(ICCV2/PI489777)SNP_11452	527	C	T	7	3	--	--
CakTC39989	Ca(ICCV2/PI489777)SNP_11453	86	T	C	4	6	--	--
	Ca(ICCV2/PI489777)SNP_11454	125	A	G	6	7	--	--
	Ca(ICCV2/PI489777)SNP_11455	374	A	C	7	10	--	--
	Ca(ICCV2/PI489777)SNP_11456	855	C	T	5	8	--	--
CakTC10121	Ca(ICCV2/PI489777)SNP_11457	328	T	A	3	3	Flower	bud
	Ca(ICCV2/PI489777)SNP_11458	548	C	A	11	7	Flower	bud
	Ca(ICCV2/PI489777)SNP_11459	559	G	C	11	9	Flower	bud
	Ca(ICCV2/PI489777)SNP_11460	571	C	T	11	9	Flower	bud
	Ca(ICCV2/PI489777)SNP_11461	694	A	T	7	6	Flower	bud
	Ca(ICCV2/PI489777)SNP_11462	802	G	A	4	5	Flower	bud
CakTC40140	Ca(ICCV2/PI489777)SNP_11463	830	A	G	9	5	--	--
	Ca(ICCV2/PI489777)SNP_11464	1028	A	C	11	5	--	--
	Ca(ICCV2/PI489777)SNP_11465	1234	A	G	6	5	--	--

	Ca(ICCV2/PI489777)SNP_11466	1252	G	C	9	4	--	--
CakTC39933	Ca(ICCV2/PI489777)SNP_11467	56	T	A	9	16	--	--
	Ca(ICCV2/PI489777)SNP_11468	59	A	T	9	15	--	--
	Ca(ICCV2/PI489777)SNP_11469	234	C	G	11	17	--	--
	Ca(ICCV2/PI489777)SNP_11470	254	A	C	11	18	--	--
	Ca(ICCV2/PI489777)SNP_11471	478	T	C	5	13	--	--
	Ca(ICCV2/PI489777)SNP_11472	542	T	C	3	7	--	--
	Ca(ICCV2/PI489777)SNP_11473	554	C	G	5	7	--	--
	Ca(ICCV2/PI489777)SNP_11474	968	T	A	7	11	--	--
	Ca(ICCV2/PI489777)SNP_11475	980	A	G	4	11	--	--
CakTC33136	Ca(ICCV2/PI489777)SNP_11476	491	T	G	20	13	--	--
	Ca(ICCV2/PI489777)SNP_11477	629	C	A	28	19	--	--
	Ca(ICCV2/PI489777)SNP_11478	923	T	C	20	18	--	--
CakTC30696	Ca(ICCV2/PI489777)SNP_11479	1452	T	C	6	4	Shoot	--
	Ca(ICCV2/PI489777)SNP_11480	1471	A	G	6	4	Shoot	--
CakTC07768	Ca(ICCV2/PI489777)SNP_11481	136	T	C	3	6	--	--
	Ca(ICCV2/PI489777)SNP_11482	536	A	G	8	4	--	--
	Ca(ICCV2/PI489777)SNP_11483	555	T	C	7	3	--	--
	Ca(ICCV2/PI489777)SNP_11484	618	C	T	9	4	--	--
CakTC41296	Ca(ICCV2/PI489777)SNP_11485	343	T	A	11	5	--	--
	Ca(ICCV2/PI489777)SNP_11486	1371	T	C	11	3	--	--
	Ca(ICCV2/PI489777)SNP_11487	2062	C	T	5	3	--	--
	Ca(ICCV2/PI489777)SNP_11488	2367	C	T	7	4	--	--
CakTC41230	Ca(ICCV2/PI489777)SNP_11489	468	C	T	10	12	--	--
	Ca(ICCV2/PI489777)SNP_11490	752	A	C	10	8	--	--
	Ca(ICCV2/PI489777)SNP_11491	809	T	G	16	6	--	--
	Ca(ICCV2/PI489777)SNP_11492	854	T	G	17	4	--	--
	Ca(ICCV2/PI489777)SNP_11493	947	G	A	19	3	--	--
	Ca(ICCV2/PI489777)SNP_11494	1864	G	C	10	7	--	--
	Ca(ICCV2/PI489777)SNP_11495	1934	C	G	8	9	--	--
	Ca(ICCV2/PI489777)SNP_11496	2158	A	G	9	9	--	--
	Ca(ICCV2/PI489777)SNP_11497	2247	G	A	8	9	--	--
	Ca(ICCV2/PI489777)SNP_11498	2393	T	G	6	6	--	--
CakTC29796	Ca(ICCV2/PI489777)SNP_11499	20	T	C	3	4	--	--
	Ca(ICCV2/PI489777)SNP_11500	72	T	C	9	8	--	--
	Ca(ICCV2/PI489777)SNP_11501	176	A	T	13	10	--	--
CakTC32472	Ca(ICCV2/PI489777)SNP_11502	437	G	A	6	3	--	--
CakTC42798	Ca(ICCV2/PI489777)SNP_11503	92	A	G	4	3	--	--
	Ca(ICCV2/PI489777)SNP_11504	487	T	C	7	7	--	--
	Ca(ICCV2/PI489777)SNP_11505	599	G	A	10	5	--	--
	Ca(ICCV2/PI489777)SNP_11506	612	C	G	10	5	--	--
CakTC36082	Ca(ICCV2/PI489777)SNP_11507	516	C	T	45	12	--	--
	Ca(ICCV2/PI489777)SNP_11508	690	C	T	68	10	--	--
	Ca(ICCV2/PI489777)SNP_11509	1071	T	C	49	18	--	--
	Ca(ICCV2/PI489777)SNP_11510	1254	G	A	69	16	--	--
	Ca(ICCV2/PI489777)SNP_11511	2070	A	C	50	10	--	--
	Ca(ICCV2/PI489777)SNP_11512	2076	C	T	50	11	--	--
	Ca(ICCV2/PI489777)SNP_11513	2124	G	A	30	12	--	--
	Ca(ICCV2/PI489777)SNP_11514	2486	T	G	43	24	--	--
	Ca(ICCV2/PI489777)SNP_11515	2490	C	T	43	24	--	--
	Ca(ICCV2/PI489777)SNP_11516	2504	T	A	42	22	--	--
	Ca(ICCV2/PI489777)SNP_11517	2505	A	T	40	19	--	--
CakTC26549	Ca(ICCV2/PI489777)SNP_11518	237	C	G	11	6	--	--
	Ca(ICCV2/PI489777)SNP_11519	834	C	G	14	3	--	--
	Ca(ICCV2/PI489777)SNP_11520	998	A	G	13	3	--	--
	Ca(ICCV2/PI489777)SNP_11521	1033	A	T	12	3	--	--
CakTC41104	Ca(ICCV2/PI489777)SNP_11522	351	G	T	5	4	--	--
	Ca(ICCV2/PI489777)SNP_11523	1881	A	G	5	5	--	--
CakTC39013	Ca(ICCV2/PI489777)SNP_11524	62	T	A	11	3	--	--
	Ca(ICCV2/PI489777)SNP_11525	308	T	C	23	11	--	--
CakTC38172	Ca(ICCV2/PI489777)SNP_11526	486	T	C	4	6	--	CCAAT
CakTC35558	Ca(ICCV2/PI489777)SNP_11527	535	T	C	7	3	--	--
	Ca(ICCV2/PI489777)SNP_11528	886	C	T	7	5	--	--
CakTC22621	Ca(ICCV2/PI489777)SNP_11529	800	C	T	3	6	--	--
CakTC37521	Ca(ICCV2/PI489777)SNP_11530	460	A	G	32	17	--	--
	Ca(ICCV2/PI489777)SNP_11531	1111	G	A	42	8	--	--
CakTC31128	Ca(ICCV2/PI489777)SNP_11532	1268	G	C	29	38	--	--
	Ca(ICCV2/PI489777)SNP_11533	1349	T	G	19	34	--	--
CakTC42756	Ca(ICCV2/PI489777)SNP_11534	206	G	C	12	12	--	--
	Ca(ICCV2/PI489777)SNP_11535	271	A	C	17	14	--	--
	Ca(ICCV2/PI489777)SNP_11536	278	A	C	17	14	--	--
	Ca(ICCV2/PI489777)SNP_11537	1144	T	C	16	37	--	--
CakTC40494	Ca(ICCV2/PI489777)SNP_11538	357	A	C	4	10	--	bHLH
	Ca(ICCV2/PI489777)SNP_11539	560	A	T	4	5	--	bHLH

	Ca(ICCV2/PI489777)SNP_11540	744	C	T	11	5	--	bHLH
CakTC31455	Ca(ICCV2/PI489777)SNP_11541	416	C	T	3	3	Flower	bud
	Ca(ICCV2/PI489777)SNP_11542	880	C	A	6	4	Flower	bud
	Ca(ICCV2/PI489777)SNP_11543	962	A	G	6	10	Flower	bud
CakTC41376	Ca(ICCV2/PI489777)SNP_11544	36	C	T	3	3	--	--
	Ca(ICCV2/PI489777)SNP_11545	95	T	C	4	4	--	--
	Ca(ICCV2/PI489777)SNP_11546	581	G	A	4	3	--	--
	Ca(ICCV2/PI489777)SNP_11547	641	A	G	3	3	--	--
	Ca(ICCV2/PI489777)SNP_11548	2170	T	G	8	4	--	--
	Ca(ICCV2/PI489777)SNP_11549	2201	C	T	9	3	--	--
CakTC31328	Ca(ICCV2/PI489777)SNP_11550	90	C	T	5	5	--	--
	Ca(ICCV2/PI489777)SNP_11551	169	G	A	5	4	--	--
	Ca(ICCV2/PI489777)SNP_11552	280	C	T	5	4	--	--
CakTC35683	Ca(ICCV2/PI489777)SNP_11553	222	C	T	3	5	--	GNAT
CakTC32160	Ca(ICCV2/PI489777)SNP_11554	295	G	A	21	7	--	--
	Ca(ICCV2/PI489777)SNP_11555	907	A	T	30	26	--	--
CakTC29100	Ca(ICCV2/PI489777)SNP_11556	1362	T	G	3	3	--	--
	Ca(ICCV2/PI489777)SNP_11557	1380	C	T	4	3	--	--
	Ca(ICCV2/PI489777)SNP_11558	1520	C	T	4	4	--	--
	Ca(ICCV2/PI489777)SNP_11559	1551	G	A	3	3	--	--
	Ca(ICCV2/PI489777)SNP_11560	1554	C	A	4	3	--	--
	Ca(ICCV2/PI489777)SNP_11561	1624	T	C	7	6	--	--
	Ca(ICCV2/PI489777)SNP_11562	1803	T	C	5	6	--	--
	Ca(ICCV2/PI489777)SNP_11563	1968	C	T	5	3	--	--
	Ca(ICCV2/PI489777)SNP_11564	2100	C	T	4	5	--	--
	Ca(ICCV2/PI489777)SNP_11565	2329	T	A	3	3	--	--
CakTC24837	Ca(ICCV2/PI489777)SNP_11566	1865	G	A	12	9	--	--
	Ca(ICCV2/PI489777)SNP_11567	1867	G	A	12	9	--	--
	Ca(ICCV2/PI489777)SNP_11568	2115	T	G	11	7	--	--
	Ca(ICCV2/PI489777)SNP_11569	2116	T	G	11	7	--	--
CakTC38183	Ca(ICCV2/PI489777)SNP_11570	138	G	A	14	6	--	--
	Ca(ICCV2/PI489777)SNP_11571	263	A	G	17	7	--	--
	Ca(ICCV2/PI489777)SNP_11572	297	C	A	13	8	--	--
	Ca(ICCV2/PI489777)SNP_11573	435	C	T	11	4	--	--
	Ca(ICCV2/PI489777)SNP_11574	923	T	G	4	12	--	--
CakTC05502	Ca(ICCV2/PI489777)SNP_11575	153	G	C	19	10	--	--
CakTC41288	Ca(ICCV2/PI489777)SNP_11576	989	A	G	17	11	--	WRKY
	Ca(ICCV2/PI489777)SNP_11577	1019	T	C	21	10	--	WRKY
	Ca(ICCV2/PI489777)SNP_11578	1355	T	C	30	10	--	WRKY
	Ca(ICCV2/PI489777)SNP_11579	1448	G	A	19	9	--	WRKY
	Ca(ICCV2/PI489777)SNP_11580	1541	G	A	11	8	--	WRKY
	Ca(ICCV2/PI489777)SNP_11581	1595	G	A	11	5	--	WRKY
	Ca(ICCV2/PI489777)SNP_11582	1696	C	T	7	9	--	WRKY
	Ca(ICCV2/PI489777)SNP_11583	1851	C	T	9	7	--	WRKY
	Ca(ICCV2/PI489777)SNP_11584	1856	A	G	9	7	--	WRKY
CakTC27020	Ca(ICCV2/PI489777)SNP_11585	2124	T	C	3	6	--	--
CakTC08318	Ca(ICCV2/PI489777)SNP_11586	283	C	T	5	3	--	--
	Ca(ICCV2/PI489777)SNP_11587	1179	T	A	4	3	--	--
CakTC39179	Ca(ICCV2/PI489777)SNP_11588	575	A	G	26	10	--	--
CakTC24265	Ca(ICCV2/PI489777)SNP_11589	342	C	T	13	5	--	--
CakTC38023	Ca(ICCV2/PI489777)SNP_11590	167	C	T	19	8	--	--
	Ca(ICCV2/PI489777)SNP_11591	559	A	T	17	8	--	--
	Ca(ICCV2/PI489777)SNP_11592	571	A	G	18	11	--	--
CakTC39479	Ca(ICCV2/PI489777)SNP_11593	826	C	T	8	7	--	--
	Ca(ICCV2/PI489777)SNP_11594	969	T	C	8	5	--	--
CakTC41044	Ca(ICCV2/PI489777)SNP_11595	234	T	A	36	16	--	--
CakTC34413	Ca(ICCV2/PI489777)SNP_11596	1750	G	A	7	5	Mature	Leaf
	Ca(ICCV2/PI489777)SNP_11597	1800	G	A	5	5	Mature	Leaf
CakTC04188	Ca(ICCV2/PI489777)SNP_11598	465	T	A	4	4	--	--
CakTC37577	Ca(ICCV2/PI489777)SNP_11599	1037	C	T	6	6	--	--
CakTC30152	Ca(ICCV2/PI489777)SNP_11600	1041	A	G	12	5	--	--
CakTC42455	Ca(ICCV2/PI489777)SNP_11601	416	A	G	33	12	--	--
CakTC39430	Ca(ICCV2/PI489777)SNP_11602	229	C	A	7	6	--	--
	Ca(ICCV2/PI489777)SNP_11603	831	G	A	24	16	--	--
	Ca(ICCV2/PI489777)SNP_11604	1119	A	C	33	20	--	--
	Ca(ICCV2/PI489777)SNP_11605	1136	T	C	21	16	--	--
	Ca(ICCV2/PI489777)SNP_11606	1347	A	T	28	18	--	--
	Ca(ICCV2/PI489777)SNP_11607	1364	C	A	28	18	--	--
	Ca(ICCV2/PI489777)SNP_11608	1446	T	A	25	13	--	--
CakTC37875	Ca(ICCV2/PI489777)SNP_11609	378	T	C	7	6	--	--
	Ca(ICCV2/PI489777)SNP_11610	741	C	T	13	13	--	--
	Ca(ICCV2/PI489777)SNP_11611	963	T	C	3	4	--	--
CakTC29418	Ca(ICCV2/PI489777)SNP_11612	129	A	C	19	8	--	--
	Ca(ICCV2/PI489777)SNP_11613	636	A	T	35	7	--	--

	Ca(ICCV2/PI489777)SNP_11614	888	T	C	37	6	--	--
CakTC10981	Ca(ICCV2/PI489777)SNP_11615	472	G	A	6	6	--	--
CakTC38497	Ca(ICCV2/PI489777)SNP_11616	228	A	C	64	23	--	--
CakTC35978	Ca(ICCV2/PI489777)SNP_11617	656	C	T	3	3	--	--
	Ca(ICCV2/PI489777)SNP_11618	2753	G	A	14	4	--	--
	Ca(ICCV2/PI489777)SNP_11619	2859	G	A	9	5	--	--
	Ca(ICCV2/PI489777)SNP_11620	3084	T	C	12	4	--	--
	Ca(ICCV2/PI489777)SNP_11621	3695	T	C	8	7	--	--
	Ca(ICCV2/PI489777)SNP_11622	3715	G	A	11	7	--	--
	Ca(ICCV2/PI489777)SNP_11623	3763	G	A	14	10	--	--
	Ca(ICCV2/PI489777)SNP_11624	3849	A	C	14	10	--	--
	Ca(ICCV2/PI489777)SNP_11625	4616	G	T	12	13	--	--
CakTC26775	Ca(ICCV2/PI489777)SNP_11626	854	A	G	7	11	--	C3H
	Ca(ICCV2/PI489777)SNP_11627	1386	T	A	10	3	--	C3H
	Ca(ICCV2/PI489777)SNP_11628	5153	G	A	17	9	--	C3H
CakTC38060	Ca(ICCV2/PI489777)SNP_11629	511	A	C	10	4	--	--
	Ca(ICCV2/PI489777)SNP_11630	758	C	G	18	3	--	--
	Ca(ICCV2/PI489777)SNP_11631	854	T	C	14	4	--	--
CakTC32554	Ca(ICCV2/PI489777)SNP_11632	127	A	G	4	3	--	--
	Ca(ICCV2/PI489777)SNP_11633	151	G	C	3	3	--	--
	Ca(ICCV2/PI489777)SNP_11634	897	T	C	9	3	--	--
CakTC05636	Ca(ICCV2/PI489777)SNP_11635	1049	C	T	4	3	--	--
CakTC05326	Ca(ICCV2/PI489777)SNP_11636	1263	T	A	4	7	--	--
	Ca(ICCV2/PI489777)SNP_11637	1359	A	G	4	5	--	--
CakTC32570	Ca(ICCV2/PI489777)SNP_11638	215	C	G	25	7	--	--
	Ca(ICCV2/PI489777)SNP_11639	373	T	C	32	19	--	--
	Ca(ICCV2/PI489777)SNP_11640	427	G	A	36	18	--	--
	Ca(ICCV2/PI489777)SNP_11641	1816	T	C	15	16	--	--
	Ca(ICCV2/PI489777)SNP_11642	1882	C	G	12	10	--	--
CakTC10508	Ca(ICCV2/PI489777)SNP_11643	83	T	G	3	3	--	--
CakTC11814	Ca(ICCV2/PI489777)SNP_11644	1685	G	A	27	19	--	TPR
	Ca(ICCV2/PI489777)SNP_11645	1874	T	A	34	18	--	TPR
	Ca(ICCV2/PI489777)SNP_11646	2234	C	T	20	15	--	TPR
	Ca(ICCV2/PI489777)SNP_11647	2414	A	T	33	18	--	TPR
	Ca(ICCV2/PI489777)SNP_11648	2489	C	T	32	18	--	TPR
	Ca(ICCV2/PI489777)SNP_11649	2612	C	T	34	17	--	TPR
	Ca(ICCV2/PI489777)SNP_11650	2846	T	G	34	19	--	TPR
	Ca(ICCV2/PI489777)SNP_11651	3258	G	A	7	7	--	TPR
CakTC38302	Ca(ICCV2/PI489777)SNP_11652	971	G	A	6	3	--	--
CakTC28032	Ca(ICCV2/PI489777)SNP_11653	42	T	A	15	4	--	--
	Ca(ICCV2/PI489777)SNP_11654	750	C	T	8	7	--	--
	Ca(ICCV2/PI489777)SNP_11655	774	C	T	11	8	--	--
CakTC43281	Ca(ICCV2/PI489777)SNP_11656	1281	C	T	7	3	--	--
CakTC28599	Ca(ICCV2/PI489777)SNP_11657	435	A	G	48	28	--	--
CakTC24436	Ca(ICCV2/PI489777)SNP_11658	403	C	T	27	19	--	--
	Ca(ICCV2/PI489777)SNP_11659	405	C	A	28	19	--	--
	Ca(ICCV2/PI489777)SNP_11660	596	A	C	59	21	--	--
	Ca(ICCV2/PI489777)SNP_11661	864	G	A	24	11	--	--
CakTC34926	Ca(ICCV2/PI489777)SNP_11662	699	G	A	12	5	--	--
CakTC31242	Ca(ICCV2/PI489777)SNP_11663	998	T	C	4	4	--	--
	Ca(ICCV2/PI489777)SNP_11664	1004	T	C	5	4	--	--
	Ca(ICCV2/PI489777)SNP_11665	1024	C	T	5	4	--	--
CakTC10433	Ca(ICCV2/PI489777)SNP_11666	2276	G	A	6	5	--	--
	Ca(ICCV2/PI489777)SNP_11667	2439	G	A	7	8	--	--
	Ca(ICCV2/PI489777)SNP_11668	2595	C	T	4	6	--	--
CakTC30660	Ca(ICCV2/PI489777)SNP_11669	443	G	T	3	4	--	WRKY
CakTC28047	Ca(ICCV2/PI489777)SNP_11670	41	A	G	10	4	--	--
	Ca(ICCV2/PI489777)SNP_11671	512	A	G	41	25	--	--
	Ca(ICCV2/PI489777)SNP_11672	566	G	A	29	19	--	--
	Ca(ICCV2/PI489777)SNP_11673	1445	C	T	55	17	--	--
CakTC39913	Ca(ICCV2/PI489777)SNP_11674	737	A	G	126	54	--	--
	Ca(ICCV2/PI489777)SNP_11675	907	T	C	78	55	--	--
CakTC24356	Ca(ICCV2/PI489777)SNP_11676	197	C	T	18	7	--	--
	Ca(ICCV2/PI489777)SNP_11677	352	A	G	15	7	--	--
	Ca(ICCV2/PI489777)SNP_11678	1100	T	C	7	4	--	--
CakTC31001	Ca(ICCV2/PI489777)SNP_11679	1159	C	G	10	7	--	--
	Ca(ICCV2/PI489777)SNP_11680	1520	G	C	6	6	--	--
	Ca(ICCV2/PI489777)SNP_11681	1540	A	G	6	6	--	--
	Ca(ICCV2/PI489777)SNP_11682	1583	A	G	6	6	--	--
	Ca(ICCV2/PI489777)SNP_11683	1744	C	A	9	6	--	--
	Ca(ICCV2/PI489777)SNP_11684	1812	C	T	8	8	--	--
	Ca(ICCV2/PI489777)SNP_11685	1826	A	C	9	8	--	--
	Ca(ICCV2/PI489777)SNP_11686	1839	A	G	8	8	--	--
	Ca(ICCV2/PI489777)SNP_11687	1853	G	A	9	8	--	--

CakTC41113	Ca(ICCV2/PI489777)SNP_11688	34	C	T	6	4	--	--
	Ca(ICCV2/PI489777)SNP_11689	40	T	A	6	4	--	--
	Ca(ICCV2/PI489777)SNP_11690	405	G	A	14	8	--	--
	Ca(ICCV2/PI489777)SNP_11691	424	T	C	14	8	--	--
	Ca(ICCV2/PI489777)SNP_11692	553	A	T	5	4	--	--
CakTC27758	Ca(ICCV2/PI489777)SNP_11693	1574	C	G	4	3	--	--
	Ca(ICCV2/PI489777)SNP_11694	1610	A	G	4	3	--	--
	Ca(ICCV2/PI489777)SNP_11695	1962	T	G	4	4	--	--
	Ca(ICCV2/PI489777)SNP_11696	1983	A	G	3	5	--	--
CakTC33283	Ca(ICCV2/PI489777)SNP_11697	826	T	G	10	5	--	--
	Ca(ICCV2/PI489777)SNP_11698	1039	T	C	23	11	--	--
	Ca(ICCV2/PI489777)SNP_11699	1114	C	T	24	11	--	--
	Ca(ICCV2/PI489777)SNP_11700	1798	C	T	9	3	--	--
	Ca(ICCV2/PI489777)SNP_11701	1960	C	T	6	3	--	--
CakTC39547	Ca(ICCV2/PI489777)SNP_11702	358	T	C	4	3	--	--
CakTC40748	Ca(ICCV2/PI489777)SNP_11703	853	A	G	26	11	--	--
	Ca(ICCV2/PI489777)SNP_11704	1060	C	G	26	7	--	--
CakTC30073	Ca(ICCV2/PI489777)SNP_11705	277	T	C	3	5	--	--
CakTC00434	Ca(ICCV2/PI489777)SNP_11706	202	A	G	7	13	--	CCAAT
CakTC39198	Ca(ICCV2/PI489777)SNP_11707	164	G	T	15	14	--	--
	Ca(ICCV2/PI489777)SNP_11708	778	C	G	14	13	--	--
	Ca(ICCV2/PI489777)SNP_11709	1146	C	T	10	9	--	--
	Ca(ICCV2/PI489777)SNP_11710	1182	G	T	12	10	--	--
	Ca(ICCV2/PI489777)SNP_11711	1282	A	T	11	11	--	--
	Ca(ICCV2/PI489777)SNP_11712	1295	G	T	9	12	--	--
	Ca(ICCV2/PI489777)SNP_11713	1378	C	A	12	8	--	--
CakTC38466	Ca(ICCV2/PI489777)SNP_11714	65	T	A	3	4	--	--
	Ca(ICCV2/PI489777)SNP_11715	124	C	T	8	6	--	--
	Ca(ICCV2/PI489777)SNP_11716	130	G	A	8	6	--	--
	Ca(ICCV2/PI489777)SNP_11717	150	C	G	10	7	--	--
	Ca(ICCV2/PI489777)SNP_11718	253	T	C	8	7	--	--
	Ca(ICCV2/PI489777)SNP_11719	256	C	T	9	7	--	--
	Ca(ICCV2/PI489777)SNP_11720	266	T	C	9	7	--	--
	Ca(ICCV2/PI489777)SNP_11721	301	G	A	11	6	--	--
	Ca(ICCV2/PI489777)SNP_11722	308	T	G	10	8	--	--
	Ca(ICCV2/PI489777)SNP_11723	501	T	C	11	16	--	--
	Ca(ICCV2/PI489777)SNP_11724	527	A	C	9	16	--	--
CakTC39734	Ca(ICCV2/PI489777)SNP_11725	97	C	T	8	4	--	--
	Ca(ICCV2/PI489777)SNP_11726	153	T	A	13	13	--	--
CakTC42215	Ca(ICCV2/PI489777)SNP_11727	234	C	A	3	3	--	--
	Ca(ICCV2/PI489777)SNP_11728	235	A	C	3	3	--	--
	Ca(ICCV2/PI489777)SNP_11729	467	G	A	10	4	--	--
CakTC41994	Ca(ICCV2/PI489777)SNP_11730	412	C	T	3	11	--	--
	Ca(ICCV2/PI489777)SNP_11731	799	A	G	3	5	--	--
	Ca(ICCV2/PI489777)SNP_11732	871	T	C	3	4	--	--
CakTC11531	Ca(ICCV2/PI489777)SNP_11733	786	C	T	6	3	--	--
CakTC30041	Ca(ICCV2/PI489777)SNP_11734	1360	T	C	8	8	--	--
	Ca(ICCV2/PI489777)SNP_11735	2594	A	G	9	11	--	--
CakTC42550	Ca(ICCV2/PI489777)SNP_11736	316	C	T	14	5	--	--
	Ca(ICCV2/PI489777)SNP_11737	871	G	C	5	3	--	--
CakTC39341	Ca(ICCV2/PI489777)SNP_11738	662	G	A	5	7	--	--
	Ca(ICCV2/PI489777)SNP_11739	877	G	A	8	7	--	--
CakTC25023	Ca(ICCV2/PI489777)SNP_11740	569	G	A	15	7	Young_pod	--
	Ca(ICCV2/PI489777)SNP_11741	587	G	A	16	6	Young_pod	--
	Ca(ICCV2/PI489777)SNP_11742	752	T	C	16	4	Young_pod	--
	Ca(ICCV2/PI489777)SNP_11743	806	A	T	19	4	Young_pod	--
	Ca(ICCV2/PI489777)SNP_11744	1019	T	C	15	4	Young_pod	--
	Ca(ICCV2/PI489777)SNP_11745	1033	C	T	17	4	Young_pod	--
	Ca(ICCV2/PI489777)SNP_11746	1079	C	T	16	4	Young_pod	--
	Ca(ICCV2/PI489777)SNP_11747	1128	A	G	17	3	Young_pod	--
	Ca(ICCV2/PI489777)SNP_11748	1142	A	G	19	3	Young_pod	--
	Ca(ICCV2/PI489777)SNP_11749	1155	C	A	18	3	Young_pod	--
	Ca(ICCV2/PI489777)SNP_11750	1202	A	C	9	3	Young_pod	--
CakTC40033	Ca(ICCV2/PI489777)SNP_11751	175	G	C	24	12	--	--
	Ca(ICCV2/PI489777)SNP_11752	214	A	C	24	13	--	--
	Ca(ICCV2/PI489777)SNP_11753	492	C	T	35	18	--	--
	Ca(ICCV2/PI489777)SNP_11754	588	C	T	26	11	--	--
	Ca(ICCV2/PI489777)SNP_11755	660	T	C	23	9	--	--
	Ca(ICCV2/PI489777)SNP_11756	1424	G	A	12	3	--	--
CakTC39857	Ca(ICCV2/PI489777)SNP_11757	525	G	T	9	4	--	--
	Ca(ICCV2/PI489777)SNP_11758	1250	T	C	11	3	--	--
CakTC03119	Ca(ICCV2/PI489777)SNP_11759	560	C	G	6	7	--	--
CakTC30286	Ca(ICCV2/PI489777)SNP_11760	281	C	A	4	3	--	HSF
	Ca(ICCV2/PI489777)SNP_11761	592	C	T	22	13	--	HSF

CakTC29294	Ca(ICCV2/PI489777)SNP_11762	797	G	A	3	3	--	NAC
CakTC09504	Ca(ICCV2/PI489777)SNP_11763	1387	T	C	8	15	--	--
	Ca(ICCV2/PI489777)SNP_11764	1408	C	A	7	16	--	--
	Ca(ICCV2/PI489777)SNP_11765	1546	T	G	9	14	--	--
CakTC23362	Ca(ICCV2/PI489777)SNP_11766	1554	T	C	6	3	--	--
	Ca(ICCV2/PI489777)SNP_11767	1633	C	A	6	3	--	--
CakTC40044	Ca(ICCV2/PI489777)SNP_11768	830	A	T	6	4	--	--
	Ca(ICCV2/PI489777)SNP_11769	848	A	G	6	4	--	--
	Ca(ICCV2/PI489777)SNP_11770	872	T	G	6	4	--	--
	Ca(ICCV2/PI489777)SNP_11771	1014	A	C	4	3	--	--
CakTC37570	Ca(ICCV2/PI489777)SNP_11772	226	C	T	48	17	--	--
CakTC39391	Ca(ICCV2/PI489777)SNP_11773	115	C	A	25	10	--	--
	Ca(ICCV2/PI489777)SNP_11774	290	T	C	28	14	--	--
	Ca(ICCV2/PI489777)SNP_11775	302	A	G	28	15	--	--
	Ca(ICCV2/PI489777)SNP_11776	443	C	T	20	15	--	--
	Ca(ICCV2/PI489777)SNP_11777	575	G	A	19	18	--	--
	Ca(ICCV2/PI489777)SNP_11778	1195	A	T	13	13	--	--
CakTC32939	Ca(ICCV2/PI489777)SNP_11779	998	A	G	12	7	--	--
	Ca(ICCV2/PI489777)SNP_11780	1033	C	A	13	6	--	--
	Ca(ICCV2/PI489777)SNP_11781	1096	T	A	10	6	--	--
	Ca(ICCV2/PI489777)SNP_11782	1615	T	C	12	4	--	--
	Ca(ICCV2/PI489777)SNP_11783	1618	C	T	11	4	--	--
	Ca(ICCV2/PI489777)SNP_11784	1625	A	G	13	4	--	--
	Ca(ICCV2/PI489777)SNP_11785	1708	A	G	13	5	--	--
	Ca(ICCV2/PI489777)SNP_11786	1879	T	C	15	5	--	--
	Ca(ICCV2/PI489777)SNP_11787	2110	A	G	17	10	--	--
	Ca(ICCV2/PI489777)SNP_11788	2281	T	A	16	9	--	--
	Ca(ICCV2/PI489777)SNP_11789	2317	T	G	15	9	--	--
	Ca(ICCV2/PI489777)SNP_11790	2397	G	T	21	6	--	--
	Ca(ICCV2/PI489777)SNP_11791	2414	G	A	20	6	--	--
	Ca(ICCV2/PI489777)SNP_11792	2488	G	A	12	5	--	--
	Ca(ICCV2/PI489777)SNP_11793	2698	G	A	8	3	--	--
	Ca(ICCV2/PI489777)SNP_11794	2714	A	G	10	3	--	--
CakTC24415	Ca(ICCV2/PI489777)SNP_11795	1087	C	A	5	6	--	--
	Ca(ICCV2/PI489777)SNP_11796	1223	C	T	6	5	--	--
	Ca(ICCV2/PI489777)SNP_11797	1229	A	G	5	5	--	--
	Ca(ICCV2/PI489777)SNP_11798	1396	T	A	4	4	--	--
CakTC24160	Ca(ICCV2/PI489777)SNP_11799	1216	C	T	25	7	--	--
	Ca(ICCV2/PI489777)SNP_11800	1618	A	G	23	10	--	--
	Ca(ICCV2/PI489777)SNP_11801	2170	G	A	15	7	--	--
	Ca(ICCV2/PI489777)SNP_11802	2593	C	T	17	5	--	--
CakTC09902	Ca(ICCV2/PI489777)SNP_11803	953	A	C	4	3	--	--
CakTC33001	Ca(ICCV2/PI489777)SNP_11804	123	T	C	468	28	--	--
CakTC11660	Ca(ICCV2/PI489777)SNP_11805	904	G	C	7	9	--	--
	Ca(ICCV2/PI489777)SNP_11806	1266	A	G	3	5	--	--
CakTC24813	Ca(ICCV2/PI489777)SNP_11807	1795	G	A	3	3	--	--
CakTC26026	Ca(ICCV2/PI489777)SNP_11808	343	T	C	4	4	--	--
CakTC05122	Ca(ICCV2/PI489777)SNP_11809	919	G	T	3	5	--	--
CakTC41193	Ca(ICCV2/PI489777)SNP_11810	1387	T	C	9	5	--	--
	Ca(ICCV2/PI489777)SNP_11811	1464	G	C	10	6	--	--
CakTC26673	Ca(ICCV2/PI489777)SNP_11812	678	T	C	15	16	--	--
	Ca(ICCV2/PI489777)SNP_11813	1236	A	G	10	8	--	--
	Ca(ICCV2/PI489777)SNP_11814	2076	C	G	12	7	--	--
CakTC38460	Ca(ICCV2/PI489777)SNP_11815	1842	T	A	37	26	--	--
CakTC36922	Ca(ICCV2/PI489777)SNP_11816	472	A	T	6	13	--	--
	Ca(ICCV2/PI489777)SNP_11817	1254	T	C	20	14	--	--
	Ca(ICCV2/PI489777)SNP_11818	1427	C	A	25	8	--	--
	Ca(ICCV2/PI489777)SNP_11819	1773	C	G	28	10	--	--
	Ca(ICCV2/PI489777)SNP_11820	2219	G	A	17	11	--	--
	Ca(ICCV2/PI489777)SNP_11821	2363	T	C	10	13	--	--
CakTC34097	Ca(ICCV2/PI489777)SNP_11822	47	A	G	5	3	--	--
CakTC38223	Ca(ICCV2/PI489777)SNP_11823	892	G	C	7	5	--	SAP
	Ca(ICCV2/PI489777)SNP_11824	1223	T	C	3	5	--	SAP
CakTC12483	Ca(ICCV2/PI489777)SNP_11825	387	A	G	7	4	--	CCHC
	Ca(ICCV2/PI489777)SNP_11826	528	G	A	3	6	--	CCHC
CakTC41311	Ca(ICCV2/PI489777)SNP_11827	1208	C	G	5	7	--	--
	Ca(ICCV2/PI489777)SNP_11828	1541	C	T	5	9	--	--
CakTC41128	Ca(ICCV2/PI489777)SNP_11829	689	C	A	21	33	--	--
	Ca(ICCV2/PI489777)SNP_11830	767	T	C	14	18	--	--
	Ca(ICCV2/PI489777)SNP_11831	1238	T	C	31	27	--	--
	Ca(ICCV2/PI489777)SNP_11832	1739	C	G	26	18	--	--
	Ca(ICCV2/PI489777)SNP_11833	2428	A	C	10	9	--	--
CakTC37283	Ca(ICCV2/PI489777)SNP_11834	979	C	T	12	7	--	--
	Ca(ICCV2/PI489777)SNP_11835	1099	G	A	10	9	--	--

	Ca(ICCV2/PI489777)SNP_11836	1182	G	C	8	5	--	--
CakTC22672	Ca(ICCV2/PI489777)SNP_11837	802	A	G	94	57	--	--
	Ca(ICCV2/PI489777)SNP_11838	931	A	G	103	73	--	--
	Ca(ICCV2/PI489777)SNP_11839	1179	A	T	95	69	--	--
	Ca(ICCV2/PI489777)SNP_11840	1245	A	C	77	39	--	--
	Ca(ICCV2/PI489777)SNP_11841	1308	C	T	73	37	--	--
CakTC38094	Ca(ICCV2/PI489777)SNP_11842	727	C	T	3	3	--	--
	Ca(ICCV2/PI489777)SNP_11843	893	G	T	3	5	--	--
	Ca(ICCV2/PI489777)SNP_11844	973	A	G	3	3	--	--
CakTC37417	Ca(ICCV2/PI489777)SNP_11845	299	G	A	11	7	--	--
	Ca(ICCV2/PI489777)SNP_11846	545	G	A	15	10	--	--
	Ca(ICCV2/PI489777)SNP_11847	680	C	T	11	12	--	--
	Ca(ICCV2/PI489777)SNP_11848	704	C	T	11	15	--	--
	Ca(ICCV2/PI489777)SNP_11849	1097	T	C	11	6	--	--
	Ca(ICCV2/PI489777)SNP_11850	1100	T	A	11	5	--	--
CakTC36367	Ca(ICCV2/PI489777)SNP_11851	1021	T	G	19	13	--	--
	Ca(ICCV2/PI489777)SNP_11852	1446	C	T	15	12	--	--
	Ca(ICCV2/PI489777)SNP_11853	1807	G	A	16	18	--	--
	Ca(ICCV2/PI489777)SNP_11854	2218	T	C	11	6	--	--
	Ca(ICCV2/PI489777)SNP_11855	2993	G	A	15	8	--	--
CakTC22733	Ca(ICCV2/PI489777)SNP_11856	2214	A	G	450	205	--	--
	Ca(ICCV2/PI489777)SNP_11857	2676	G	C	395	191	--	--
	Ca(ICCV2/PI489777)SNP_11858	2785	G	A	235	111	--	--
CakTC37943	Ca(ICCV2/PI489777)SNP_11859	259	C	G	4	5	--	--
	Ca(ICCV2/PI489777)SNP_11860	610	A	G	5	4	--	--
CakTC39044	Ca(ICCV2/PI489777)SNP_11861	1226	A	G	5	4	--	--
CakTC29203	Ca(ICCV2/PI489777)SNP_11862	88	C	T	8	3	--	HB
	Ca(ICCV2/PI489777)SNP_11863	175	C	T	12	3	--	HB
	Ca(ICCV2/PI489777)SNP_11864	199	T	A	14	3	--	HB
	Ca(ICCV2/PI489777)SNP_11865	212	C	G	14	3	--	HB
	Ca(ICCV2/PI489777)SNP_11866	292	C	T	20	3	--	HB
	Ca(ICCV2/PI489777)SNP_11867	523	G	A	17	4	--	HB
	Ca(ICCV2/PI489777)SNP_11868	652	G	T	11	5	--	HB
	Ca(ICCV2/PI489777)SNP_11869	912	G	C	29	3	--	HB
	Ca(ICCV2/PI489777)SNP_11870	1302	G	A	24	7	--	HB
	Ca(ICCV2/PI489777)SNP_11871	1319	T	A	23	6	--	HB
	Ca(ICCV2/PI489777)SNP_11872	1521	C	G	7	4	--	HB
	Ca(ICCV2/PI489777)SNP_11873	1528	T	A	5	3	--	HB
CakTC10817	Ca(ICCV2/PI489777)SNP_11874	574	C	T	16	10	--	--
	Ca(ICCV2/PI489777)SNP_11875	853	C	T	18	9	--	--
	Ca(ICCV2/PI489777)SNP_11876	939	T	G	12	6	--	--
CakTC23562	Ca(ICCV2/PI489777)SNP_11877	195	C	G	28	12	--	--
	Ca(ICCV2/PI489777)SNP_11878	762	C	T	19	11	--	--
	Ca(ICCV2/PI489777)SNP_11879	1831	C	T	20	15	--	--
	Ca(ICCV2/PI489777)SNP_11880	2092	C	T	23	16	--	--
CakTC43036	Ca(ICCV2/PI489777)SNP_11881	148	C	T	7	10	--	--
	Ca(ICCV2/PI489777)SNP_11882	450	C	G	10	6	--	--
	Ca(ICCV2/PI489777)SNP_11883	881	G	A	18	5	--	--
	Ca(ICCV2/PI489777)SNP_11884	899	T	C	20	3	--	--
CakTC23690	Ca(ICCV2/PI489777)SNP_11885	429	A	C	8	3	--	--
	Ca(ICCV2/PI489777)SNP_11886	448	C	G	8	3	--	--
	Ca(ICCV2/PI489777)SNP_11887	485	C	T	8	3	--	--
	Ca(ICCV2/PI489777)SNP_11888	548	C	G	5	3	--	--
	Ca(ICCV2/PI489777)SNP_11889	624	A	C	3	3	--	--
	Ca(ICCV2/PI489777)SNP_11890	668	C	T	4	3	--	--
	Ca(ICCV2/PI489777)SNP_11891	695	A	G	4	3	--	--
	Ca(ICCV2/PI489777)SNP_11892	906	A	G	6	3	--	--
	Ca(ICCV2/PI489777)SNP_11893	984	T	C	6	3	--	--
	Ca(ICCV2/PI489777)SNP_11894	1180	C	T	18	4	--	--
CakTC39326	Ca(ICCV2/PI489777)SNP_11895	1263	G	T	7	4	--	--
CakTC37676	Ca(ICCV2/PI489777)SNP_11896	129	T	A	17	11	--	--
	Ca(ICCV2/PI489777)SNP_11897	1042	C	T	15	15	--	--
CakTC40030	Ca(ICCV2/PI489777)SNP_11898	449	C	T	3	5	--	--
	Ca(ICCV2/PI489777)SNP_11899	459	G	A	3	5	--	--
	Ca(ICCV2/PI489777)SNP_11900	543	T	C	9	8	--	--
	Ca(ICCV2/PI489777)SNP_11901	985	C	T	13	6	--	--
	Ca(ICCV2/PI489777)SNP_11902	1408	C	T	9	3	--	--
CakTC40072	Ca(ICCV2/PI489777)SNP_11903	34	C	T	4	10	--	--
	Ca(ICCV2/PI489777)SNP_11904	111	A	T	37	53	--	--
	Ca(ICCV2/PI489777)SNP_11905	126	C	A	39	45	--	--
	Ca(ICCV2/PI489777)SNP_11906	500	G	T	81	115	--	--
	Ca(ICCV2/PI489777)SNP_11907	611	T	A	55	91	--	--
CakTC34549	Ca(ICCV2/PI489777)SNP_11908	2010	A	C	3	14	--	--
CakTC29183	Ca(ICCV2/PI489777)SNP_11909	452	C	G	25	9	--	--

	Ca(ICCV2/PI489777)SNP_11910	926	A	G	12	5	--	--
	Ca(ICCV2/PI489777)SNP_11911	1336	T	C	13	5	--	--
	Ca(ICCV2/PI489777)SNP_11912	1476	G	A	9	8	--	--
CakTC34068	Ca(ICCV2/PI489777)SNP_11913	445	C	T	14	9	--	--
	Ca(ICCV2/PI489777)SNP_11914	454	A	T	14	10	--	--
CakTC26726	Ca(ICCV2/PI489777)SNP_11915	2636	A	T	7	3	Root	--
CakTC40133	Ca(ICCV2/PI489777)SNP_11916	469	A	G	30	7	--	--
	Ca(ICCV2/PI489777)SNP_11917	941	C	G	21	4	--	--
	Ca(ICCV2/PI489777)SNP_11918	1179	T	A	18	5	--	--
CakTC32603	Ca(ICCV2/PI489777)SNP_11919	1686	A	G	27	18	--	--
	Ca(ICCV2/PI489777)SNP_11920	1692	A	G	28	18	--	--
	Ca(ICCV2/PI489777)SNP_11921	1794	G	A	27	21	--	--
CakTC40085	Ca(ICCV2/PI489777)SNP_11922	862	G	T	8	3	--	--
CakTC24108	Ca(ICCV2/PI489777)SNP_11923	467	A	G	9	10	--	--
	Ca(ICCV2/PI489777)SNP_11924	794	T	C	12	6	--	--
	Ca(ICCV2/PI489777)SNP_11925	869	A	T	11	9	--	--
	Ca(ICCV2/PI489777)SNP_11926	886	C	T	11	8	--	--
	Ca(ICCV2/PI489777)SNP_11927	1425	A	G	10	6	--	--
	Ca(ICCV2/PI489777)SNP_11928	1448	T	A	10	5	--	--
	Ca(ICCV2/PI489777)SNP_11929	2344	T	A	126	91	--	--
	Ca(ICCV2/PI489777)SNP_11930	2776	T	A	77	112	--	--
	Ca(ICCV2/PI489777)SNP_11931	2933	G	C	26	7	--	--
	Ca(ICCV2/PI489777)SNP_11932	2940	T	C	28	7	--	--
	Ca(ICCV2/PI489777)SNP_11933	2946	C	A	26	7	--	--
	Ca(ICCV2/PI489777)SNP_11934	2989	C	T	27	7	--	--
CakTC38050	Ca(ICCV2/PI489777)SNP_11935	66	C	T	27	12	--	--
	Ca(ICCV2/PI489777)SNP_11936	86	C	T	42	16	--	--
	Ca(ICCV2/PI489777)SNP_11937	187	T	A	58	23	--	--
CakTC28567	Ca(ICCV2/PI489777)SNP_11938	131	T	C	4	4	--	--
	Ca(ICCV2/PI489777)SNP_11939	593	A	G	7	3	--	--
	Ca(ICCV2/PI489777)SNP_11940	666	T	C	7	5	--	--
	Ca(ICCV2/PI489777)SNP_11941	1071	G	A	4	8	--	--
CakTC39074	Ca(ICCV2/PI489777)SNP_11942	1320	C	G	9	7	--	--
CakTC39355	Ca(ICCV2/PI489777)SNP_11943	1334	A	G	8	7	--	--
	Ca(ICCV2/PI489777)SNP_11944	1336	A	C	8	7	--	--
CakTC39448	Ca(ICCV2/PI489777)SNP_11945	405	G	C	11	9	--	--
CakTC35041	Ca(ICCV2/PI489777)SNP_11946	835	A	G	8	7	--	--
CakTC05656	Ca(ICCV2/PI489777)SNP_11947	2244	A	C	3	4	--	--
	Ca(ICCV2/PI489777)SNP_11948	2515	C	T	5	4	--	--
	Ca(ICCV2/PI489777)SNP_11949	2546	C	T	4	4	--	--
CakTC31006	Ca(ICCV2/PI489777)SNP_11950	1408	G	C	7	5	--	--
	Ca(ICCV2/PI489777)SNP_11951	1999	T	C	6	4	--	--
	Ca(ICCV2/PI489777)SNP_11952	2177	T	C	6	4	--	--
CakTC26503	Ca(ICCV2/PI489777)SNP_11953	63	A	G	48	3	--	--
	Ca(ICCV2/PI489777)SNP_11954	571	A	G	22	10	--	--
CakTC39860	Ca(ICCV2/PI489777)SNP_11955	283	A	T	4	5	--	--
CakTC38336	Ca(ICCV2/PI489777)SNP_11956	687	T	C	34	18	--	--
	Ca(ICCV2/PI489777)SNP_11957	754	T	A	26	16	--	--
	Ca(ICCV2/PI489777)SNP_11958	756	T	C	26	17	--	--
	Ca(ICCV2/PI489777)SNP_11959	787	A	T	25	8	--	--
	Ca(ICCV2/PI489777)SNP_11960	847	A	T	31	16	--	--
	Ca(ICCV2/PI489777)SNP_11961	850	T	C	30	17	--	--
	Ca(ICCV2/PI489777)SNP_11962	875	A	G	25	13	--	--
	Ca(ICCV2/PI489777)SNP_11963	1020	G	A	12	8	--	--
CakTC12064	Ca(ICCV2/PI489777)SNP_11964	2526	T	C	6	6	--	--
	Ca(ICCV2/PI489777)SNP_11965	2708	G	A	13	4	--	--
CakTC28030	Ca(ICCV2/PI489777)SNP_11966	497	T	C	24	28	--	HB
	Ca(ICCV2/PI489777)SNP_11967	523	G	A	28	29	--	HB
	Ca(ICCV2/PI489777)SNP_11968	532	C	G	29	26	--	HB
	Ca(ICCV2/PI489777)SNP_11969	859	T	A	53	24	--	HB
CakTC29906	Ca(ICCV2/PI489777)SNP_11970	378	T	C	16	8	--	--
	Ca(ICCV2/PI489777)SNP_11971	1066	T	G	14	6	--	--
	Ca(ICCV2/PI489777)SNP_11972	2574	G	T	25	11	--	--
	Ca(ICCV2/PI489777)SNP_11973	2610	A	C	33	10	--	--
	Ca(ICCV2/PI489777)SNP_11974	2907	A	G	30	16	--	--
	Ca(ICCV2/PI489777)SNP_11975	3573	G	T	23	10	--	--
	Ca(ICCV2/PI489777)SNP_11976	3576	A	G	23	10	--	--
CakTC40007	Ca(ICCV2/PI489777)SNP_11977	80	T	C	11	10	--	WRKY
	Ca(ICCV2/PI489777)SNP_11978	91	C	T	18	10	--	WRKY
	Ca(ICCV2/PI489777)SNP_11979	321	C	T	30	19	--	WRKY
	Ca(ICCV2/PI489777)SNP_11980	330	T	C	32	19	--	WRKY
	Ca(ICCV2/PI489777)SNP_11981	448	A	G	34	18	--	WRKY
	Ca(ICCV2/PI489777)SNP_11982	498	T	C	29	16	--	WRKY
	Ca(ICCV2/PI489777)SNP_11983	597	A	G	19	21	--	WRKY

CakTC26023	Ca(ICCV2/PI489777)SNP_11984	392	G	T	8	10	--	--
CakTC31330	Ca(ICCV2/PI489777)SNP_11985	2607	C	T	21	3	--	--
CakTC13673	Ca(ICCV2/PI489777)SNP_11986	329	G	A	5	6	--	--
	Ca(ICCV2/PI489777)SNP_11987	368	G	A	5	4	--	--
CakTC39897	Ca(ICCV2/PI489777)SNP_11988	850	A	T	9	4	--	C2C2-Dof
	Ca(ICCV2/PI489777)SNP_11989	958	G	C	8	3	--	C2C2-Dof
CakTC37078	Ca(ICCV2/PI489777)SNP_11990	77	G	A	5	5	--	--
	Ca(ICCV2/PI489777)SNP_11991	152	G	A	13	7	--	--
	Ca(ICCV2/PI489777)SNP_11992	173	C	G	11	8	--	--
	Ca(ICCV2/PI489777)SNP_11993	443	C	T	21	14	--	--
	Ca(ICCV2/PI489777)SNP_11994	728	T	C	31	20	--	--
	Ca(ICCV2/PI489777)SNP_11995	1031	C	T	31	34	--	--
	Ca(ICCV2/PI489777)SNP_11996	1100	G	T	25	41	--	--
	Ca(ICCV2/PI489777)SNP_11997	1901	G	A	27	34	--	--
	Ca(ICCV2/PI489777)SNP_11998	1910	A	G	35	35	--	--
	Ca(ICCV2/PI489777)SNP_11999	1988	G	C	27	28	--	--
	Ca(ICCV2/PI489777)SNP_12000	2258	A	G	14	14	--	--
CakTC33120	Ca(ICCV2/PI489777)SNP_12001	1082	T	C	24	12	--	--
	Ca(ICCV2/PI489777)SNP_12002	1089	C	T	21	12	--	--
	Ca(ICCV2/PI489777)SNP_12003	1699	G	C	11	6	--	--
	Ca(ICCV2/PI489777)SNP_12004	2059	C	T	32	20	--	--
	Ca(ICCV2/PI489777)SNP_12005	2392	G	A	25	21	--	--
	Ca(ICCV2/PI489777)SNP_12006	2507	C	G	21	24	--	--
	Ca(ICCV2/PI489777)SNP_12007	2509	G	C	24	23	--	--
	Ca(ICCV2/PI489777)SNP_12008	2566	C	T	17	12	--	--
	Ca(ICCV2/PI489777)SNP_12009	2714	G	A	21	12	--	--
	Ca(ICCV2/PI489777)SNP_12010	2791	A	G	18	15	--	--
	Ca(ICCV2/PI489777)SNP_12011	3120	G	C	24	15	--	--
	Ca(ICCV2/PI489777)SNP_12012	3168	G	A	26	15	--	--
	Ca(ICCV2/PI489777)SNP_12013	4010	A	G	33	25	--	--
	Ca(ICCV2/PI489777)SNP_12014	4020	A	T	38	23	--	--
	Ca(ICCV2/PI489777)SNP_12015	4158	T	A	31	9	--	--
	Ca(ICCV2/PI489777)SNP_12016	4192	G	A	32	11	--	--
	Ca(ICCV2/PI489777)SNP_12017	4426	A	G	36	9	--	--
	Ca(ICCV2/PI489777)SNP_12018	4450	G	T	38	9	--	--
	Ca(ICCV2/PI489777)SNP_12019	5302	G	A	58	21	--	--
CakTC24686	Ca(ICCV2/PI489777)SNP_12020	1813	A	G	27	16	--	--
	Ca(ICCV2/PI489777)SNP_12021	1834	G	A	27	17	--	--
CakTC40274	Ca(ICCV2/PI489777)SNP_12022	102	G	A	13	13	--	--
	Ca(ICCV2/PI489777)SNP_12023	157	C	A	18	16	--	--
CakTC25665	Ca(ICCV2/PI489777)SNP_12024	1183	G	A	7	3	--	--
	Ca(ICCV2/PI489777)SNP_12025	1465	C	T	26	4	--	--
CakTC40505	Ca(ICCV2/PI489777)SNP_12026	689	C	A	7	10	--	--
	Ca(ICCV2/PI489777)SNP_12027	737	C	A	8	12	--	--
	Ca(ICCV2/PI489777)SNP_12028	995	T	C	6	11	--	--
CakTC38068	Ca(ICCV2/PI489777)SNP_12029	113	C	A	4	3	--	--
	Ca(ICCV2/PI489777)SNP_12030	254	G	A	7	3	--	--
	Ca(ICCV2/PI489777)SNP_12031	353	T	A	9	3	--	--
	Ca(ICCV2/PI489777)SNP_12032	401	G	A	6	3	--	--
	Ca(ICCV2/PI489777)SNP_12033	739	C	T	8	3	--	--
CakTC41647	Ca(ICCV2/PI489777)SNP_12034	267	C	T	13	6	--	--
	Ca(ICCV2/PI489777)SNP_12035	520	G	A	6	6	--	--
CakTC31358	Ca(ICCV2/PI489777)SNP_12036	1005	C	G	33	18	--	--
CakTC40336	Ca(ICCV2/PI489777)SNP_12037	405	G	A	5	5	--	--
	Ca(ICCV2/PI489777)SNP_12038	722	A	G	8	5	--	--
CakTC22889	Ca(ICCV2/PI489777)SNP_12039	184	T	A	12	3	--	--
	Ca(ICCV2/PI489777)SNP_12040	541	G	A	40	8	--	--
CakTC41711	Ca(ICCV2/PI489777)SNP_12041	1424	T	G	38	24	--	--
CakTC39701	Ca(ICCV2/PI489777)SNP_12042	756	T	G	17	8	--	--
CakTC28701	Ca(ICCV2/PI489777)SNP_12043	406	A	G	57	40	--	--
	Ca(ICCV2/PI489777)SNP_12044	481	A	T	58	39	--	--
	Ca(ICCV2/PI489777)SNP_12045	484	G	A	54	40	--	--
	Ca(ICCV2/PI489777)SNP_12046	759	C	T	12	12	--	--
	Ca(ICCV2/PI489777)SNP_12047	1372	T	C	31	28	--	--
CakTC09309	Ca(ICCV2/PI489777)SNP_12048	2354	A	G	3	3	--	--
	Ca(ICCV2/PI489777)SNP_12049	2377	G	T	3	3	--	--
CakTC35770	Ca(ICCV2/PI489777)SNP_12050	3212	A	G	6	4	--	--
CakTC29588	Ca(ICCV2/PI489777)SNP_12051	1173	T	A	6	3	--	--
	Ca(ICCV2/PI489777)SNP_12052	1952	C	T	15	6	--	--
	Ca(ICCV2/PI489777)SNP_12053	1967	C	T	16	6	--	--
	Ca(ICCV2/PI489777)SNP_12054	2242	T	G	20	3	--	--
	Ca(ICCV2/PI489777)SNP_12055	2371	A	G	16	5	--	--
CakTC42508	Ca(ICCV2/PI489777)SNP_12056	865	T	C	4	12	--	--
	Ca(ICCV2/PI489777)SNP_12057	970	C	T	7	13	--	--

	Ca(ICCV2/PI489777)SNP_12058	979	G	A	7	13	--	--
CakTC27414	Ca(ICCV2/PI489777)SNP_12059	99	T	G	6	4	--	--
	Ca(ICCV2/PI489777)SNP_12060	1082	C	T	7	8	--	--
	Ca(ICCV2/PI489777)SNP_12061	1755	G	A	7	5	--	--
CakTC39068	Ca(ICCV2/PI489777)SNP_12062	606	T	C	7	7	--	--
	Ca(ICCV2/PI489777)SNP_12063	826	A	G	9	4	--	--
	Ca(ICCV2/PI489777)SNP_12064	1005	T	A	6	6	--	--
	Ca(ICCV2/PI489777)SNP_12065	1010	T	A	6	6	--	--
	Ca(ICCV2/PI489777)SNP_12066	1455	T	A	5	3	--	--
CakTC38627	Ca(ICCV2/PI489777)SNP_12067	1047	A	G	34	35	--	--
CakTC08411	Ca(ICCV2/PI489777)SNP_12068	1328	A	G	4	9	--	--
	Ca(ICCV2/PI489777)SNP_12069	1376	T	A	4	8	--	--
	Ca(ICCV2/PI489777)SNP_12070	1442	A	T	4	3	--	--
CakTC28100	Ca(ICCV2/PI489777)SNP_12071	2271	T	A	5	4	--	--
	Ca(ICCV2/PI489777)SNP_12072	2301	C	T	5	5	--	--
	Ca(ICCV2/PI489777)SNP_12073	2319	T	C	4	6	--	--
CakTC31180	Ca(ICCV2/PI489777)SNP_12074	558	G	C	4	7	--	--
CakTC08632	Ca(ICCV2/PI489777)SNP_12075	777	A	C	31	17	--	--
	Ca(ICCV2/PI489777)SNP_12076	936	G	T	18	12	--	--
CakTC26650	Ca(ICCV2/PI489777)SNP_12077	161	T	C	28	11	--	--
	Ca(ICCV2/PI489777)SNP_12078	1116	G	T	6	6	--	--
CakTC40513	Ca(ICCV2/PI489777)SNP_12079	88	C	T	16	4	--	--
	Ca(ICCV2/PI489777)SNP_12080	622	G	T	193	109	--	--
	Ca(ICCV2/PI489777)SNP_12081	632	C	G	181	95	--	--
	Ca(ICCV2/PI489777)SNP_12082	635	G	A	191	109	--	--
	Ca(ICCV2/PI489777)SNP_12083	673	T	C	154	75	--	--
CakTC06018	Ca(ICCV2/PI489777)SNP_12084	66	A	G	4	4	--	--
CakTC25611	Ca(ICCV2/PI489777)SNP_12085	1228	T	G	8	4	--	--
	Ca(ICCV2/PI489777)SNP_12086	1307	G	A	10	5	--	--
	Ca(ICCV2/PI489777)SNP_12087	1472	T	A	8	6	--	--
	Ca(ICCV2/PI489777)SNP_12088	1639	C	A	6	4	--	--
CakTC38149	Ca(ICCV2/PI489777)SNP_12089	206	C	T	12	5	--	--
	Ca(ICCV2/PI489777)SNP_12090	264	G	A	13	6	--	--
	Ca(ICCV2/PI489777)SNP_12091	711	A	C	4	4	--	--
CakTC22491	Ca(ICCV2/PI489777)SNP_12092	206	T	C	9	7	--	--
CakTC26265	Ca(ICCV2/PI489777)SNP_12093	789	A	G	4	4	Flower	bud
	Ca(ICCV2/PI489777)SNP_12094	861	T	A	3	4	Flower	bud
	Ca(ICCV2/PI489777)SNP_12095	967	C	G	4	5	Flower	bud
	Ca(ICCV2/PI489777)SNP_12096	4188	A	T	4	3	Flower	bud
	Ca(ICCV2/PI489777)SNP_12097	4202	A	G	3	3	Flower	bud
	Ca(ICCV2/PI489777)SNP_12098	4209	C	T	4	3	Flower	bud
CakTC31619	Ca(ICCV2/PI489777)SNP_12099	702	A	G	15	25	--	--
	Ca(ICCV2/PI489777)SNP_12100	876	T	C	28	28	--	--
CakTC30287	Ca(ICCV2/PI489777)SNP_12101	683	T	C	3	5	--	--
CakTC41789	Ca(ICCV2/PI489777)SNP_12102	166	G	C	7	3	--	--
	Ca(ICCV2/PI489777)SNP_12103	292	C	G	8	3	--	--
	Ca(ICCV2/PI489777)SNP_12104	501	G	A	4	3	--	--
CakTC41537	Ca(ICCV2/PI489777)SNP_12105	380	T	C	8	6	--	--
CakTC39357	Ca(ICCV2/PI489777)SNP_12106	951	A	G	6	4	--	--
	Ca(ICCV2/PI489777)SNP_12107	997	G	A	5	7	--	--
CakTC40039	Ca(ICCV2/PI489777)SNP_12108	76	A	G	12	14	--	--
	Ca(ICCV2/PI489777)SNP_12109	123	A	T	12	21	--	--
	Ca(ICCV2/PI489777)SNP_12110	913	C	T	29	17	--	--
CakTC33337	Ca(ICCV2/PI489777)SNP_12111	160	G	A	11	7	--	--
	Ca(ICCV2/PI489777)SNP_12112	2071	T	C	5	4	--	--
CakTC41922	Ca(ICCV2/PI489777)SNP_12113	490	C	G	18	12	--	--
	Ca(ICCV2/PI489777)SNP_12114	550	T	C	14	14	--	--
	Ca(ICCV2/PI489777)SNP_12115	653	G	T	15	15	--	--
	Ca(ICCV2/PI489777)SNP_12116	1068	A	G	17	8	--	--
	Ca(ICCV2/PI489777)SNP_12117	1416	C	T	13	8	--	--
	Ca(ICCV2/PI489777)SNP_12118	1441	G	C	13	8	--	--
CakTC29272	Ca(ICCV2/PI489777)SNP_12119	2147	T	C	7	6	Flower	bud
	Ca(ICCV2/PI489777)SNP_12120	2242	T	C	7	5	Flower	bud
	Ca(ICCV2/PI489777)SNP_12121	2407	T	C	7	4	Flower	bud
CakTC29167	Ca(ICCV2/PI489777)SNP_12122	263	T	C	7	13	--	--
	Ca(ICCV2/PI489777)SNP_12123	403	A	G	5	17	--	--
	Ca(ICCV2/PI489777)SNP_12124	683	C	T	6	8	--	--
CakTC35195	Ca(ICCV2/PI489777)SNP_12125	380	C	T	15	4	--	--
	Ca(ICCV2/PI489777)SNP_12126	2245	C	T	16	12	--	--
	Ca(ICCV2/PI489777)SNP_12127	2681	T	C	34	4	--	--
	Ca(ICCV2/PI489777)SNP_12128	3012	T	A	16	15	--	--
	Ca(ICCV2/PI489777)SNP_12129	3122	G	C	15	21	--	--
CakTC23900	Ca(ICCV2/PI489777)SNP_12130	377	C	T	11	5	--	--
	Ca(ICCV2/PI489777)SNP_12131	633	T	C	12	12	--	--

	Ca(ICCV2/PI489777)SNP_12132	1006	G	A	21	23	--	--
	Ca(ICCV2/PI489777)SNP_12133	1043	G	A	24	22	--	--
	Ca(ICCV2/PI489777)SNP_12134	1155	C	A	26	12	--	--
	Ca(ICCV2/PI489777)SNP_12135	1163	G	C	25	12	--	--
	Ca(ICCV2/PI489777)SNP_12136	1377	A	G	22	9	--	--
	Ca(ICCV2/PI489777)SNP_12137	1481	A	G	22	7	--	--
	Ca(ICCV2/PI489777)SNP_12138	1704	T	C	17	13	--	--
	Ca(ICCV2/PI489777)SNP_12139	1839	T	C	9	8	--	--
CakTC25292	Ca(ICCV2/PI489777)SNP_12140	334	T	A	14	7	--	--
	Ca(ICCV2/PI489777)SNP_12141	610	C	T	11	4	--	--
CakTC31444	Ca(ICCV2/PI489777)SNP_12142	272	C	T	7	7	--	--
	Ca(ICCV2/PI489777)SNP_12143	395	C	T	8	7	--	--
	Ca(ICCV2/PI489777)SNP_12144	398	C	T	5	8	--	--
	Ca(ICCV2/PI489777)SNP_12145	515	T	C	5	9	--	--
	Ca(ICCV2/PI489777)SNP_12146	539	A	G	4	4	--	--
	Ca(ICCV2/PI489777)SNP_12147	548	T	C	4	6	--	--
	Ca(ICCV2/PI489777)SNP_12148	549	A	G	4	6	--	--
	Ca(ICCV2/PI489777)SNP_12149	578	C	A	5	6	--	--
	Ca(ICCV2/PI489777)SNP_12150	1359	G	A	8	10	--	--
	Ca(ICCV2/PI489777)SNP_12151	1400	C	T	8	12	--	--
	Ca(ICCV2/PI489777)SNP_12152	1437	C	A	9	10	--	--
	Ca(ICCV2/PI489777)SNP_12153	1490	A	T	7	12	--	--
CakTC10305	Ca(ICCV2/PI489777)SNP_12154	111	G	A	5	6	--	--
CakTC39491	Ca(ICCV2/PI489777)SNP_12155	54	A	T	5	3	--	--
	Ca(ICCV2/PI489777)SNP_12156	77	G	A	8	4	--	--
	Ca(ICCV2/PI489777)SNP_12157	115	T	A	10	4	--	--
	Ca(ICCV2/PI489777)SNP_12158	780	A	G	58	45	--	--
	Ca(ICCV2/PI489777)SNP_12159	1116	A	G	67	28	--	--
	Ca(ICCV2/PI489777)SNP_12160	1211	T	G	57	21	--	--
CakTC29983	Ca(ICCV2/PI489777)SNP_12161	674	T	A	6	8	--	--
	Ca(ICCV2/PI489777)SNP_12162	1269	G	A	29	3	--	--
	Ca(ICCV2/PI489777)SNP_12163	1505	T	G	21	6	--	--
CakTC34714	Ca(ICCV2/PI489777)SNP_12164	2500	C	T	24	9	--	--
CakTC33933	Ca(ICCV2/PI489777)SNP_12165	416	C	T	47	23	--	--
	Ca(ICCV2/PI489777)SNP_12166	591	A	G	18	4	--	--
CakTC40405	Ca(ICCV2/PI489777)SNP_12167	879	A	C	25	5	--	--
	Ca(ICCV2/PI489777)SNP_12168	1062	C	T	17	5	--	--
	Ca(ICCV2/PI489777)SNP_12169	1617	A	G	11	5	--	--
CakTC10338	Ca(ICCV2/PI489777)SNP_12170	96	C	G	14	3	--	--
	Ca(ICCV2/PI489777)SNP_12171	2589	G	A	105	22	--	--
CakTC38113	Ca(ICCV2/PI489777)SNP_12172	284	C	T	7	5	--	--
	Ca(ICCV2/PI489777)SNP_12173	419	A	G	6	3	--	--
	Ca(ICCV2/PI489777)SNP_12174	604	C	T	6	8	--	--
	Ca(ICCV2/PI489777)SNP_12175	715	G	A	8	7	--	--
	Ca(ICCV2/PI489777)SNP_12176	796	C	A	8	14	--	--
CakTC36106	Ca(ICCV2/PI489777)SNP_12177	153	C	G	33	22	--	--
	Ca(ICCV2/PI489777)SNP_12178	500	A	G	82	53	--	--
	Ca(ICCV2/PI489777)SNP_12179	782	A	G	73	52	--	--
	Ca(ICCV2/PI489777)SNP_12180	1541	A	T	90	50	--	--
	Ca(ICCV2/PI489777)SNP_12181	1610	C	T	78	53	--	--
	Ca(ICCV2/PI489777)SNP_12182	1832	A	G	78	51	--	--
CakTC10510	Ca(ICCV2/PI489777)SNP_12183	756	T	C	4	3	--	--
CakTC32432	Ca(ICCV2/PI489777)SNP_12184	196	C	T	82	29	--	--
	Ca(ICCV2/PI489777)SNP_12185	406	T	C	58	24	--	--
	Ca(ICCV2/PI489777)SNP_12186	471	C	T	7	10	--	--
	Ca(ICCV2/PI489777)SNP_12187	545	G	A	6	4	--	--
CakTC32439	Ca(ICCV2/PI489777)SNP_12188	8	A	G	4	3	--	--
CakTC09878	Ca(ICCV2/PI489777)SNP_12189	482	A	G	7	7	--	--
	Ca(ICCV2/PI489777)SNP_12190	505	T	C	4	7	--	--
	Ca(ICCV2/PI489777)SNP_12191	563	G	A	5	6	--	--
	Ca(ICCV2/PI489777)SNP_12192	606	C	T	5	5	--	--
	Ca(ICCV2/PI489777)SNP_12193	722	T	C	3	6	--	--
CakTC43136	Ca(ICCV2/PI489777)SNP_12194	1050	A	G	22	7	--	--
CakTC22463	Ca(ICCV2/PI489777)SNP_12195	73	C	G	7	4	--	--
	Ca(ICCV2/PI489777)SNP_12196	212	T	C	5	6	--	--
	Ca(ICCV2/PI489777)SNP_12197	218	C	T	10	6	--	--
	Ca(ICCV2/PI489777)SNP_12198	314	G	T	6	10	--	--
	Ca(ICCV2/PI489777)SNP_12199	471	G	A	10	13	--	--
	Ca(ICCV2/PI489777)SNP_12200	482	C	T	10	13	--	--
	Ca(ICCV2/PI489777)SNP_12201	551	G	A	10	11	--	--
CakTC43054	Ca(ICCV2/PI489777)SNP_12202	733	T	C	7	12	Mature	Leaf
	Ca(ICCV2/PI489777)SNP_12203	742	C	T	10	12	Mature	Leaf
	Ca(ICCV2/PI489777)SNP_12204	817	C	T	11	11	Mature	Leaf
	Ca(ICCV2/PI489777)SNP_12205	829	G	A	12	10	Mature	Leaf

CakTC30633	Ca(ICCV2/PI489777)SNP_12206	1616	C	T	3	6	--	--
CakTC37517	Ca(ICCV2/PI489777)SNP_12207	481	G	C	12	6	--	--
	Ca(ICCV2/PI489777)SNP_12208	1225	G	T	7	4	--	--
CakTC36519	Ca(ICCV2/PI489777)SNP_12209	1015	G	A	3	3	Young_pod	--
	Ca(ICCV2/PI489777)SNP_12210	2517	T	C	9	5	Young_pod	--
CakTC34959	Ca(ICCV2/PI489777)SNP_12211	551	T	C	22	14	--	--
	Ca(ICCV2/PI489777)SNP_12212	1682	T	C	19	18	--	--
	Ca(ICCV2/PI489777)SNP_12213	1850	C	T	19	27	--	--
	Ca(ICCV2/PI489777)SNP_12214	2249	C	T	32	18	--	--
	Ca(ICCV2/PI489777)SNP_12215	3071	G	A	21	27	--	--
	Ca(ICCV2/PI489777)SNP_12216	3191	T	G	22	37	--	--
	Ca(ICCV2/PI489777)SNP_12217	3329	C	T	25	30	--	--
	Ca(ICCV2/PI489777)SNP_12218	3446	A	G	14	7	--	--
CakTC33235	Ca(ICCV2/PI489777)SNP_12219	1430	C	A	3	5	--	--
	Ca(ICCV2/PI489777)SNP_12220	1659	G	A	4	3	--	--
	Ca(ICCV2/PI489777)SNP_12221	1660	A	T	4	3	--	--
	Ca(ICCV2/PI489777)SNP_12222	1799	A	C	5	6	--	--
CakTC30209	Ca(ICCV2/PI489777)SNP_12223	496	A	G	7	3	--	--
	Ca(ICCV2/PI489777)SNP_12224	696	T	C	4	3	--	--
	Ca(ICCV2/PI489777)SNP_12225	820	C	T	3	3	--	--
	Ca(ICCV2/PI489777)SNP_12226	1372	G	A	4	4	--	--
	Ca(ICCV2/PI489777)SNP_12227	1385	C	T	5	4	--	--
	Ca(ICCV2/PI489777)SNP_12228	1429	T	C	4	4	--	--
	Ca(ICCV2/PI489777)SNP_12229	1688	T	C	4	5	--	--
	Ca(ICCV2/PI489777)SNP_12230	1916	G	T	6	6	--	--
	Ca(ICCV2/PI489777)SNP_12231	2007	T	C	7	5	--	--
	Ca(ICCV2/PI489777)SNP_12232	2113	G	A	6	3	--	--
CakTC26063	Ca(ICCV2/PI489777)SNP_12233	835	T	C	15	9	--	--
CakTC39884	Ca(ICCV2/PI489777)SNP_12234	1221	G	A	7	4	--	--
CakTC22722	Ca(ICCV2/PI489777)SNP_12235	1534	A	G	8	22	--	--
CakTC32481	Ca(ICCV2/PI489777)SNP_12236	251	T	G	48	26	--	--
CakTC27692	Ca(ICCV2/PI489777)SNP_12237	166	T	C	5	6	--	--
	Ca(ICCV2/PI489777)SNP_12238	188	G	A	6	6	--	--
	Ca(ICCV2/PI489777)SNP_12239	325	T	C	16	4	--	--
	Ca(ICCV2/PI489777)SNP_12240	406	C	T	8	6	--	--
	Ca(ICCV2/PI489777)SNP_12241	439	C	T	13	6	--	--
	Ca(ICCV2/PI489777)SNP_12242	456	T	C	13	5	--	--
	Ca(ICCV2/PI489777)SNP_12243	457	A	G	13	5	--	--
	Ca(ICCV2/PI489777)SNP_12244	475	A	G	12	5	--	--
	Ca(ICCV2/PI489777)SNP_12245	502	G	A	13	4	--	--
	Ca(ICCV2/PI489777)SNP_12246	653	G	A	8	4	--	--
CakTC06744	Ca(ICCV2/PI489777)SNP_12247	233	A	T	3	5	--	--
CakTC29581	Ca(ICCV2/PI489777)SNP_12248	2016	A	G	4	3	--	--
CakTC27188	Ca(ICCV2/PI489777)SNP_12249	2702	G	T	3	3	--	--
CakTC30955	Ca(ICCV2/PI489777)SNP_12250	1372	T	C	7	4	--	--
	Ca(ICCV2/PI489777)SNP_12251	1859	A	G	6	3	--	--
	Ca(ICCV2/PI489777)SNP_12252	1942	G	A	6	4	--	--
CakTC33891	Ca(ICCV2/PI489777)SNP_12253	1104	G	A	32	35	--	--
	Ca(ICCV2/PI489777)SNP_12254	1755	C	A	21	32	--	--
	Ca(ICCV2/PI489777)SNP_12255	1788	G	A	24	29	--	--
	Ca(ICCV2/PI489777)SNP_12256	1806	G	A	24	26	--	--
	Ca(ICCV2/PI489777)SNP_12257	1827	A	C	17	26	--	--
	Ca(ICCV2/PI489777)SNP_12258	1923	T	C	17	19	--	--
CakTC38838	Ca(ICCV2/PI489777)SNP_12259	438	C	T	10	10	--	--
	Ca(ICCV2/PI489777)SNP_12260	474	C	T	10	7	--	--
	Ca(ICCV2/PI489777)SNP_12261	501	C	T	11	8	--	--
	Ca(ICCV2/PI489777)SNP_12262	610	T	C	4	6	--	--
	Ca(ICCV2/PI489777)SNP_12263	645	C	T	7	8	--	--
	Ca(ICCV2/PI489777)SNP_12264	744	A	G	3	6	--	--
	Ca(ICCV2/PI489777)SNP_12265	768	T	C	4	5	--	--
	Ca(ICCV2/PI489777)SNP_12266	789	T	C	3	6	--	--
CakTC23707	Ca(ICCV2/PI489777)SNP_12267	472	G	A	16	7	--	--
	Ca(ICCV2/PI489777)SNP_12268	1330	G	A	6	5	--	--
CakTC40281	Ca(ICCV2/PI489777)SNP_12269	297	T	C	6	8	--	--
CakTC27330	Ca(ICCV2/PI489777)SNP_12270	69	T	C	11	6	--	--
	Ca(ICCV2/PI489777)SNP_12271	1482	A	G	27	39	--	--
	Ca(ICCV2/PI489777)SNP_12272	1554	A	G	23	9	--	--
	Ca(ICCV2/PI489777)SNP_12273	1557	C	T	23	9	--	--
CakTC25263	Ca(ICCV2/PI489777)SNP_12274	273	A	G	18	30	--	--
CakTC27936	Ca(ICCV2/PI489777)SNP_12275	584	A	G	26	19	--	TPR
	Ca(ICCV2/PI489777)SNP_12276	746	C	A	22	17	--	TPR
	Ca(ICCV2/PI489777)SNP_12277	962	T	C	32	10	--	TPR
	Ca(ICCV2/PI489777)SNP_12278	1265	T	G	23	11	--	TPR
	Ca(ICCV2/PI489777)SNP_12279	1398	G	A	17	10	--	TPR

CakTC26881	Ca(ICCV2/PI489777)SNP_12280	114	T	C	11	3	--	--
	Ca(ICCV2/PI489777)SNP_12281	358	T	C	9	3	--	--
	Ca(ICCV2/PI489777)SNP_12282	399	T	C	8	3	--	--
CakTC42873	Ca(ICCV2/PI489777)SNP_12283	516	G	C	18	8	--	--
	Ca(ICCV2/PI489777)SNP_12284	594	G	A	11	7	--	--
CakTC23883	Ca(ICCV2/PI489777)SNP_12285	449	G	A	53	12	--	--
	Ca(ICCV2/PI489777)SNP_12286	1389	A	G	78	19	--	--
	Ca(ICCV2/PI489777)SNP_12287	2307	G	T	95	29	--	--
	Ca(ICCV2/PI489777)SNP_12288	3898	G	A	94	29	--	--
	Ca(ICCV2/PI489777)SNP_12289	4287	C	T	91	27	--	--
	Ca(ICCV2/PI489777)SNP_12290	4309	G	T	100	27	--	--
CakTC40306	Ca(ICCV2/PI489777)SNP_12291	39	G	C	3	3	--	--
CakTC39879	Ca(ICCV2/PI489777)SNP_12292	745	C	T	34	8	--	FHA
CakTC27162	Ca(ICCV2/PI489777)SNP_12293	180	T	C	6	3	--	--
	Ca(ICCV2/PI489777)SNP_12294	373	A	C	6	5	--	--
	Ca(ICCV2/PI489777)SNP_12295	882	A	G	6	5	--	--
	Ca(ICCV2/PI489777)SNP_12296	1187	T	G	11	9	--	--
CakTC42115	Ca(ICCV2/PI489777)SNP_12297	495	T	G	6	15	--	--
	Ca(ICCV2/PI489777)SNP_12298	642	G	A	10	17	--	--
	Ca(ICCV2/PI489777)SNP_12299	796	G	C	9	14	--	--
	Ca(ICCV2/PI489777)SNP_12300	958	G	A	3	5	--	--
CakTC26094	Ca(ICCV2/PI489777)SNP_12301	237	G	T	11	12	--	--
	Ca(ICCV2/PI489777)SNP_12302	955	C	T	22	17	--	--
	Ca(ICCV2/PI489777)SNP_12303	1385	T	C	45	22	--	--
	Ca(ICCV2/PI489777)SNP_12304	2188	T	C	36	15	--	--
	Ca(ICCV2/PI489777)SNP_12305	2558	A	G	49	18	--	--
	Ca(ICCV2/PI489777)SNP_12306	2870	G	A	56	27	--	--
	Ca(ICCV2/PI489777)SNP_12307	3412	G	A	25	17	--	--
CakTC22688	Ca(ICCV2/PI489777)SNP_12308	2880	G	A	4	5	--	--
CakTC11051	Ca(ICCV2/PI489777)SNP_12309	383	A	T	9	4	--	--
	Ca(ICCV2/PI489777)SNP_12310	1454	T	C	35	20	--	--
	Ca(ICCV2/PI489777)SNP_12311	1781	C	A	21	16	--	--
	Ca(ICCV2/PI489777)SNP_12312	1888	A	G	23	14	--	--
	Ca(ICCV2/PI489777)SNP_12313	1897	A	G	19	10	--	--
	Ca(ICCV2/PI489777)SNP_12314	1927	G	A	12	11	--	--
	Ca(ICCV2/PI489777)SNP_12315	2032	A	T	16	10	--	--
CakTC32119	Ca(ICCV2/PI489777)SNP_12316	93	A	G	3	4	--	--
	Ca(ICCV2/PI489777)SNP_12317	103	G	C	3	4	--	--
	Ca(ICCV2/PI489777)SNP_12318	163	G	T	6	5	--	--
	Ca(ICCV2/PI489777)SNP_12319	259	T	G	9	7	--	--
	Ca(ICCV2/PI489777)SNP_12320	360	G	A	9	7	--	--
	Ca(ICCV2/PI489777)SNP_12321	744	A	G	6	3	--	--
	Ca(ICCV2/PI489777)SNP_12322	928	C	T	4	6	--	--
CakTC23344	Ca(ICCV2/PI489777)SNP_12323	1475	T	C	21	11	--	--
	Ca(ICCV2/PI489777)SNP_12324	1482	A	G	22	11	--	--
	Ca(ICCV2/PI489777)SNP_12325	1583	C	A	20	8	--	--
CakTC37348	Ca(ICCV2/PI489777)SNP_12326	125	C	G	10	3	--	--
	Ca(ICCV2/PI489777)SNP_12327	127	C	T	10	3	--	--
	Ca(ICCV2/PI489777)SNP_12328	161	A	C	11	12	--	--
	Ca(ICCV2/PI489777)SNP_12329	204	C	T	11	11	--	--
	Ca(ICCV2/PI489777)SNP_12330	243	T	G	10	10	--	--
CakTC09925	Ca(ICCV2/PI489777)SNP_12331	414	C	T	3	4	--	--
	Ca(ICCV2/PI489777)SNP_12332	2293	G	T	4	4	--	--
	Ca(ICCV2/PI489777)SNP_12333	2770	C	G	5	3	--	--
CakTC22731	Ca(ICCV2/PI489777)SNP_12334	1416	T	C	5	5	--	--
CakTC37392	Ca(ICCV2/PI489777)SNP_12335	714	C	T	53	13	--	--
	Ca(ICCV2/PI489777)SNP_12336	837	A	G	56	24	--	--
	Ca(ICCV2/PI489777)SNP_12337	936	A	G	65	25	--	--
CakTC11033	Ca(ICCV2/PI489777)SNP_12338	601	A	T	7	3	--	--
CakTC23003	Ca(ICCV2/PI489777)SNP_12339	404	T	G	11	3	--	--
	Ca(ICCV2/PI489777)SNP_12340	719	T	C	10	7	--	--
	Ca(ICCV2/PI489777)SNP_12341	1079	A	G	22	7	--	--
	Ca(ICCV2/PI489777)SNP_12342	1316	A	G	19	11	--	--
	Ca(ICCV2/PI489777)SNP_12343	1607	T	C	15	8	--	--
	Ca(ICCV2/PI489777)SNP_12344	1888	G	A	35	17	--	--
	Ca(ICCV2/PI489777)SNP_12345	2005	C	T	37	18	--	--
	Ca(ICCV2/PI489777)SNP_12346	2040	T	G	34	19	--	--
	Ca(ICCV2/PI489777)SNP_12347	2125	T	G	28	17	--	--
	Ca(ICCV2/PI489777)SNP_12348	2143	A	G	25	16	--	--
CakTC22720	Ca(ICCV2/PI489777)SNP_12349	872	A	G	12	11	--	--
	Ca(ICCV2/PI489777)SNP_12350	1629	G	A	18	18	--	--
	Ca(ICCV2/PI489777)SNP_12351	3098	T	C	3	4	--	--
CakTC38696	Ca(ICCV2/PI489777)SNP_12352	412	G	T	22	10	--	--
	Ca(ICCV2/PI489777)SNP_12353	1004	T	G	16	4	--	--

CakTC13053	Ca(ICCV2/PI489777)SNP_12354	175	G	A	15	3	--	--
CakTC33772	Ca(ICCV2/PI489777)SNP_12355	402	C	A	57	15	--	--
	Ca(ICCV2/PI489777)SNP_12356	712	G	T	45	14	--	--
CakTC27183	Ca(ICCV2/PI489777)SNP_12357	44	A	T	4	3	Young_pod	--
CakTC31668	Ca(ICCV2/PI489777)SNP_12358	431	C	G	4	6	--	--
	Ca(ICCV2/PI489777)SNP_12359	920	C	T	4	3	--	--
	Ca(ICCV2/PI489777)SNP_12360	943	A	C	5	3	--	--
	Ca(ICCV2/PI489777)SNP_12361	1371	T	G	11	3	--	--
	Ca(ICCV2/PI489777)SNP_12362	1707	C	T	12	6	--	--
CakTC31441	Ca(ICCV2/PI489777)SNP_12363	320	A	G	3	5	--	--
	Ca(ICCV2/PI489777)SNP_12364	605	A	T	3	6	--	--
CakTC30511	Ca(ICCV2/PI489777)SNP_12365	638	C	T	17	4	--	--
	Ca(ICCV2/PI489777)SNP_12366	674	G	A	21	5	--	--
	Ca(ICCV2/PI489777)SNP_12367	1013	C	G	16	3	--	--
	Ca(ICCV2/PI489777)SNP_12368	1042	G	A	19	4	--	--
CakTC38287	Ca(ICCV2/PI489777)SNP_12369	1070	A	T	32	20	--	--
	Ca(ICCV2/PI489777)SNP_12370	1169	C	T	20	12	--	--
CakTC27732	Ca(ICCV2/PI489777)SNP_12371	752	A	G	40	41	Shoot	--
	Ca(ICCV2/PI489777)SNP_12372	839	C	T	44	40	Shoot	--
CakTC30071	Ca(ICCV2/PI489777)SNP_12373	1557	A	C	8	3	--	--
CakTC28578	Ca(ICCV2/PI489777)SNP_12374	426	G	A	3	7	--	MYB
CakTC09526	Ca(ICCV2/PI489777)SNP_12375	549	G	A	10	5	Shoot	--
	Ca(ICCV2/PI489777)SNP_12376	1382	A	T	15	7	Shoot	--
	Ca(ICCV2/PI489777)SNP_12377	1911	A	C	10	4	Shoot	--
	Ca(ICCV2/PI489777)SNP_12378	1953	T	C	12	4	Shoot	--
CakTC18239	Ca(ICCV2/PI489777)SNP_12379	818	G	A	4	4	--	--
	Ca(ICCV2/PI489777)SNP_12380	820	A	G	4	4	--	--
CakTC27003	Ca(ICCV2/PI489777)SNP_12381	1479	G	A	16	12	--	--
CakTC11072	Ca(ICCV2/PI489777)SNP_12382	906	T	C	8	3	--	E2F-DP
CakTC39606	Ca(ICCV2/PI489777)SNP_12383	181	A	C	22	6	--	--
	Ca(ICCV2/PI489777)SNP_12384	708	C	T	15	7	--	--
CakTC41112	Ca(ICCV2/PI489777)SNP_12385	368	G	A	6	4	--	--
	Ca(ICCV2/PI489777)SNP_12386	625	A	C	10	5	--	--
	Ca(ICCV2/PI489777)SNP_12387	647	T	C	10	6	--	--
	Ca(ICCV2/PI489777)SNP_12388	651	A	G	9	6	--	--
	Ca(ICCV2/PI489777)SNP_12389	1040	G	A	4	5	--	--
	Ca(ICCV2/PI489777)SNP_12390	1106	T	C	6	9	--	--
	Ca(ICCV2/PI489777)SNP_12391	1121	A	T	5	7	--	--
	Ca(ICCV2/PI489777)SNP_12392	1482	T	C	13	5	--	--
CakTC36933	Ca(ICCV2/PI489777)SNP_12393	360	T	C	49	33	--	--
	Ca(ICCV2/PI489777)SNP_12394	597	A	G	36	22	--	--
	Ca(ICCV2/PI489777)SNP_12395	822	A	G	31	23	--	--
	Ca(ICCV2/PI489777)SNP_12396	1413	A	T	43	27	--	--
	Ca(ICCV2/PI489777)SNP_12397	1557	T	G	44	23	--	--
	Ca(ICCV2/PI489777)SNP_12398	2448	G	C	7	5	--	--
	Ca(ICCV2/PI489777)SNP_12399	2451	G	C	7	5	--	--
CakTC40058	Ca(ICCV2/PI489777)SNP_12400	179	G	C	7	4	--	--
	Ca(ICCV2/PI489777)SNP_12401	181	T	A	6	4	--	--
	Ca(ICCV2/PI489777)SNP_12402	439	C	T	44	12	--	--
CakTC26917	Ca(ICCV2/PI489777)SNP_12403	588	C	T	5	11	--	--
CakTC38680	Ca(ICCV2/PI489777)SNP_12404	754	T	C	10	6	--	--
	Ca(ICCV2/PI489777)SNP_12405	762	G	A	10	5	--	--
	Ca(ICCV2/PI489777)SNP_12406	846	T	G	11	5	--	--
CakTC35900	Ca(ICCV2/PI489777)SNP_12407	291	G	C	4	13	--	--
CakTC37658	Ca(ICCV2/PI489777)SNP_12408	1222	G	A	3	8	--	--
CakTC28082	Ca(ICCV2/PI489777)SNP_12409	981	T	C	6	4	--	--
	Ca(ICCV2/PI489777)SNP_12410	1179	C	T	8	4	--	--
	Ca(ICCV2/PI489777)SNP_12411	1238	C	T	6	5	--	--
CakTC26931	Ca(ICCV2/PI489777)SNP_12412	127	G	C	4	4	--	--
	Ca(ICCV2/PI489777)SNP_12413	521	G	A	6	4	--	--
	Ca(ICCV2/PI489777)SNP_12414	581	A	G	4	3	--	--
	Ca(ICCV2/PI489777)SNP_12415	1871	T	A	6	3	--	--
CakTC40023	Ca(ICCV2/PI489777)SNP_12416	136	A	G	23	15	--	--
	Ca(ICCV2/PI489777)SNP_12417	147	G	A	20	17	--	--
	Ca(ICCV2/PI489777)SNP_12418	179	A	G	23	17	--	--
CakTC35436	Ca(ICCV2/PI489777)SNP_12419	775	C	T	20	13	--	--
	Ca(ICCV2/PI489777)SNP_12420	1217	G	A	20	17	--	--
	Ca(ICCV2/PI489777)SNP_12421	1736	G	A	11	8	--	--
	Ca(ICCV2/PI489777)SNP_12422	1808	G	T	19	9	--	--
	Ca(ICCV2/PI489777)SNP_12423	1847	C	T	20	9	--	--
	Ca(ICCV2/PI489777)SNP_12424	1976	G	A	17	8	--	--
	Ca(ICCV2/PI489777)SNP_12425	2030	G	A	23	14	--	--
	Ca(ICCV2/PI489777)SNP_12426	2179	A	C	21	14	--	--
	Ca(ICCV2/PI489777)SNP_12427	2266	T	A	18	13	--	--

	Ca(ICCV2/PI489777)SNP_12428	2570	T	C	14	6	--	--
	Ca(ICCV2/PI489777)SNP_12429	2798	T	C	12	13	--	--
	Ca(ICCV2/PI489777)SNP_12430	3481	A	C	16	14	--	--
	Ca(ICCV2/PI489777)SNP_12431	3707	C	T	31	9	--	--
	Ca(ICCV2/PI489777)SNP_12432	3756	T	C	31	11	--	--
CakTC33902	Ca(ICCV2/PI489777)SNP_12433	29	A	C	7	9	--	--
CakTC26753	Ca(ICCV2/PI489777)SNP_12434	780	C	T	31	11	--	--
	Ca(ICCV2/PI489777)SNP_12435	906	C	T	44	14	--	--
	Ca(ICCV2/PI489777)SNP_12436	921	C	T	39	13	--	--
	Ca(ICCV2/PI489777)SNP_12437	1056	A	G	39	21	--	--
	Ca(ICCV2/PI489777)SNP_12438	1197	G	A	32	20	--	--
	Ca(ICCV2/PI489777)SNP_12439	1410	C	G	21	12	--	--
CakTC39688	Ca(ICCV2/PI489777)SNP_12440	130	G	A	20	10	--	--
	Ca(ICCV2/PI489777)SNP_12441	379	T	C	25	14	--	--
	Ca(ICCV2/PI489777)SNP_12442	1205	T	C	9	4	--	--
	Ca(ICCV2/PI489777)SNP_12443	1270	C	T	4	3	--	--
CakTC36059	Ca(ICCV2/PI489777)SNP_12444	2241	T	C	4	4	--	--
	Ca(ICCV2/PI489777)SNP_12445	3081	G	A	6	6	--	--
	Ca(ICCV2/PI489777)SNP_12446	3291	T	G	5	5	--	--
CakTC30752	Ca(ICCV2/PI489777)SNP_12447	894	T	C	4	4	--	--
CakTC25453	Ca(ICCV2/PI489777)SNP_12448	62	T	C	3	3	--	--
	Ca(ICCV2/PI489777)SNP_12449	77	T	G	3	3	--	--
	Ca(ICCV2/PI489777)SNP_12450	191	C	T	3	5	--	--
CakTC27907	Ca(ICCV2/PI489777)SNP_12451	124	C	T	4	3	--	--
	Ca(ICCV2/PI489777)SNP_12452	544	C	T	19	20	--	--
	Ca(ICCV2/PI489777)SNP_12453	562	T	C	23	24	--	--
	Ca(ICCV2/PI489777)SNP_12454	1186	T	A	13	18	--	--
	Ca(ICCV2/PI489777)SNP_12455	1804	G	A	8	9	--	--
CakTC39039	Ca(ICCV2/PI489777)SNP_12456	101	C	T	12	5	--	--
	Ca(ICCV2/PI489777)SNP_12457	256	T	A	22	12	--	--
	Ca(ICCV2/PI489777)SNP_12458	337	C	T	30	18	--	--
	Ca(ICCV2/PI489777)SNP_12459	370	T	C	33	21	--	--
	Ca(ICCV2/PI489777)SNP_12460	847	A	G	22	11	--	--
	Ca(ICCV2/PI489777)SNP_12461	913	G	A	24	12	--	--
	Ca(ICCV2/PI489777)SNP_12462	970	C	A	28	22	--	--
	Ca(ICCV2/PI489777)SNP_12463	1040	C	T	30	21	--	--
	Ca(ICCV2/PI489777)SNP_12464	1205	G	C	17	7	--	--
	Ca(ICCV2/PI489777)SNP_12465	1330	A	G	11	7	--	--
CakTC27886	Ca(ICCV2/PI489777)SNP_12466	637	G	C	33	13	--	--
	Ca(ICCV2/PI489777)SNP_12467	1160	T	C	39	18	--	--
CakTC11398	Ca(ICCV2/PI489777)SNP_12468	192	C	G	8	7	--	--
	Ca(ICCV2/PI489777)SNP_12469	517	G	A	4	4	--	--
	Ca(ICCV2/PI489777)SNP_12470	1134	G	A	6	4	--	--
	Ca(ICCV2/PI489777)SNP_12471	1228	C	A	3	3	--	--
CakTC41831	Ca(ICCV2/PI489777)SNP_12472	193	G	C	3	5	Flower	bud
CakTC39033	Ca(ICCV2/PI489777)SNP_12473	547	T	C	5	4	--	--
	Ca(ICCV2/PI489777)SNP_12474	1022	T	C	7	3	--	--
	Ca(ICCV2/PI489777)SNP_12475	1424	A	G	4	3	--	--
	Ca(ICCV2/PI489777)SNP_12476	1443	T	C	4	3	--	--
CakTC09533	Ca(ICCV2/PI489777)SNP_12477	2587	G	T	5	11	--	--
CakTC39824	Ca(ICCV2/PI489777)SNP_12478	1392	G	A	3	3	--	--
CakTC27557	Ca(ICCV2/PI489777)SNP_12479	208	T	C	5	14	--	--
CakTC02273	Ca(ICCV2/PI489777)SNP_12480	639	T	C	3	5	--	--
CakTC10764	Ca(ICCV2/PI489777)SNP_12481	240	T	G	4	5	--	--
CakTC29493	Ca(ICCV2/PI489777)SNP_12482	399	G	T	51	84	--	--
CakTC41300	Ca(ICCV2/PI489777)SNP_12483	1656	A	C	12	12	--	--
	Ca(ICCV2/PI489777)SNP_12484	1788	G	A	17	16	--	--
	Ca(ICCV2/PI489777)SNP_12485	2305	G	C	19	10	--	--
CakTC40331	Ca(ICCV2/PI489777)SNP_12486	1621	T	C	6	5	--	--
	Ca(ICCV2/PI489777)SNP_12487	1951	T	C	8	3	--	--
CakTC41772	Ca(ICCV2/PI489777)SNP_12488	126	T	A	7	4	--	--
CakTC25832	Ca(ICCV2/PI489777)SNP_12489	283	A	G	9	6	--	--
	Ca(ICCV2/PI489777)SNP_12490	348	A	G	10	6	--	--
	Ca(ICCV2/PI489777)SNP_12491	708	A	C	20	4	--	--
	Ca(ICCV2/PI489777)SNP_12492	718	T	G	14	4	--	--
CakTC30457	Ca(ICCV2/PI489777)SNP_12493	1102	T	C	6	3	--	--
CakTC23860	Ca(ICCV2/PI489777)SNP_12494	799	C	T	15	7	--	AUX/IAA
	Ca(ICCV2/PI489777)SNP_12495	1121	A	G	15	6	--	AUX/IAA
CakTC26050	Ca(ICCV2/PI489777)SNP_12496	115	A	T	5	7	--	--
	Ca(ICCV2/PI489777)SNP_12497	154	C	G	5	8	--	--
CakTC37886	Ca(ICCV2/PI489777)SNP_12498	605	G	T	14	12	--	mTERF
	Ca(ICCV2/PI489777)SNP_12499	870	A	C	16	10	--	mTERF
	Ca(ICCV2/PI489777)SNP_12500	1098	A	C	16	7	--	mTERF
CakTC09629	Ca(ICCV2/PI489777)SNP_12501	1495	T	C	7	3	--	--

	Ca(ICCV2/PI489777)SNP_12502	1517	G	A	6	3	--	--
CakTC42560	Ca(ICCV2/PI489777)SNP_12503	107	C	A	23	7	--	--
	Ca(ICCV2/PI489777)SNP_12504	853	G	A	43	14	--	--
CakTC31603	Ca(ICCV2/PI489777)SNP_12505	713	C	T	19	6	--	--
	Ca(ICCV2/PI489777)SNP_12506	1164	C	G	20	3	--	--
	Ca(ICCV2/PI489777)SNP_12507	1366	A	G	16	5	--	--
	Ca(ICCV2/PI489777)SNP_12508	1546	G	T	13	3	--	--
CakTC40987	Ca(ICCV2/PI489777)SNP_12509	115	T	A	3	5	--	--
	Ca(ICCV2/PI489777)SNP_12510	326	G	A	20	21	--	--
	Ca(ICCV2/PI489777)SNP_12511	336	A	C	24	19	--	--
	Ca(ICCV2/PI489777)SNP_12512	366	T	C	23	19	--	--
	Ca(ICCV2/PI489777)SNP_12513	417	G	A	23	21	--	--
	Ca(ICCV2/PI489777)SNP_12514	480	T	C	25	21	--	--
	Ca(ICCV2/PI489777)SNP_12515	484	T	G	22	21	--	--
	Ca(ICCV2/PI489777)SNP_12516	1024	G	A	19	15	--	--
	Ca(ICCV2/PI489777)SNP_12517	1341	A	G	11	14	--	--
	Ca(ICCV2/PI489777)SNP_12518	1543	G	A	18	10	--	--
	Ca(ICCV2/PI489777)SNP_12519	1633	G	A	18	26	--	--
CakTC41150	Ca(ICCV2/PI489777)SNP_12520	472	C	T	4	5	--	--
	Ca(ICCV2/PI489777)SNP_12521	487	G	A	4	4	--	--
	Ca(ICCV2/PI489777)SNP_12522	553	A	G	5	3	--	--
	Ca(ICCV2/PI489777)SNP_12523	737	A	C	7	4	--	--
	Ca(ICCV2/PI489777)SNP_12524	738	G	C	8	4	--	--
	Ca(ICCV2/PI489777)SNP_12525	811	T	C	6	3	--	--
	Ca(ICCV2/PI489777)SNP_12526	814	A	C	7	3	--	--
	Ca(ICCV2/PI489777)SNP_12527	823	C	T	5	3	--	--
	Ca(ICCV2/PI489777)SNP_12528	826	G	T	4	3	--	--
	Ca(ICCV2/PI489777)SNP_12529	838	G	A	7	3	--	--
	Ca(ICCV2/PI489777)SNP_12530	961	T	A	4	3	--	--
	Ca(ICCV2/PI489777)SNP_12531	1015	G	A	4	3	--	--
	Ca(ICCV2/PI489777)SNP_12532	1245	C	A	12	3	--	--
	Ca(ICCV2/PI489777)SNP_12533	1942	G	A	7	13	--	--
	Ca(ICCV2/PI489777)SNP_12534	2106	T	C	6	3	--	--
CakTC23763	Ca(ICCV2/PI489777)SNP_12535	149	C	T	54	22	--	--
CakTC26807	Ca(ICCV2/PI489777)SNP_12536	1105	C	T	20	7	--	--
	Ca(ICCV2/PI489777)SNP_12537	1165	T	G	40	8	--	--
	Ca(ICCV2/PI489777)SNP_12538	2183	A	C	49	21	--	--
	Ca(ICCV2/PI489777)SNP_12539	2599	A	T	51	25	--	--
	Ca(ICCV2/PI489777)SNP_12540	2633	C	T	51	24	--	--
	Ca(ICCV2/PI489777)SNP_12541	2686	A	G	39	19	--	--
	Ca(ICCV2/PI489777)SNP_12542	2833	C	T	32	18	--	--
CakTC24193	Ca(ICCV2/PI489777)SNP_12543	2187	A	G	29	6	--	--
CakTC24968	Ca(ICCV2/PI489777)SNP_12544	478	T	C	66	20	--	--
	Ca(ICCV2/PI489777)SNP_12545	622	A	G	79	16	--	--
CakTC24499	Ca(ICCV2/PI489777)SNP_12546	423	T	C	7	5	--	--
	Ca(ICCV2/PI489777)SNP_12547	424	T	G	7	5	--	--
	Ca(ICCV2/PI489777)SNP_12548	455	A	G	7	6	--	--
	Ca(ICCV2/PI489777)SNP_12549	2294	G	A	15	8	--	--
	Ca(ICCV2/PI489777)SNP_12550	2351	T	C	15	8	--	--
CakTC32653	Ca(ICCV2/PI489777)SNP_12551	81	G	A	61	35	--	--
	Ca(ICCV2/PI489777)SNP_12552	140	G	T	61	38	--	--
	Ca(ICCV2/PI489777)SNP_12553	192	C	T	64	37	--	--
	Ca(ICCV2/PI489777)SNP_12554	221	A	G	63	40	--	--
	Ca(ICCV2/PI489777)SNP_12555	237	A	T	64	40	--	--
	Ca(ICCV2/PI489777)SNP_12556	328	T	G	20	9	--	--
CakTC38974	Ca(ICCV2/PI489777)SNP_12557	662	A	G	5	5	--	--
	Ca(ICCV2/PI489777)SNP_12558	831	G	A	10	4	--	--
	Ca(ICCV2/PI489777)SNP_12559	922	C	T	10	3	--	--
CakTC30844	Ca(ICCV2/PI489777)SNP_12560	863	G	A	9	14	--	--
	Ca(ICCV2/PI489777)SNP_12561	868	A	G	10	13	--	--
	Ca(ICCV2/PI489777)SNP_12562	1298	G	A	15	7	--	--
	Ca(ICCV2/PI489777)SNP_12563	1562	G	T	19	10	--	--
CakTC30588	Ca(ICCV2/PI489777)SNP_12564	399	A	G	3	4	--	--
CakTC43022	Ca(ICCV2/PI489777)SNP_12565	950	G	A	10	3	--	--
CakTC27724	Ca(ICCV2/PI489777)SNP_12566	395	C	T	6	4	Flower	bud
	Ca(ICCV2/PI489777)SNP_12567	413	C	T	4	4	Flower	bud
CakTC40234	Ca(ICCV2/PI489777)SNP_12568	465	T	G	6	4	--	--
CakTC42584	Ca(ICCV2/PI489777)SNP_12569	1262	G	A	29	19	--	--
	Ca(ICCV2/PI489777)SNP_12570	1355	C	T	35	17	--	--
	Ca(ICCV2/PI489777)SNP_12571	1388	C	T	32	15	--	--
CakTC24720	Ca(ICCV2/PI489777)SNP_12572	29	G	A	6	3	--	--
	Ca(ICCV2/PI489777)SNP_12573	514	C	T	21	6	--	--
CakTC25299	Ca(ICCV2/PI489777)SNP_12574	317	T	C	8	3	--	--
	Ca(ICCV2/PI489777)SNP_12575	337	T	C	12	3	--	--

CakTC34390	Ca(ICCV2/PI489777)SNP_12576	294	G	A	29	6	--	--
	Ca(ICCV2/PI489777)SNP_12577	404	A	T	29	6	--	--
	Ca(ICCV2/PI489777)SNP_12578	406	G	A	28	5	--	--
	Ca(ICCV2/PI489777)SNP_12579	946	C	T	28	6	--	--
	Ca(ICCV2/PI489777)SNP_12580	996	C	T	34	5	--	--
	Ca(ICCV2/PI489777)SNP_12581	1253	G	A	33	7	--	--
	Ca(ICCV2/PI489777)SNP_12582	1832	C	T	14	8	--	--
CakTC32036	Ca(ICCV2/PI489777)SNP_12583	609	C	T	5	3	--	--
	Ca(ICCV2/PI489777)SNP_12584	996	C	T	9	4	--	--
	Ca(ICCV2/PI489777)SNP_12585	1448	A	G	13	7	--	--
	Ca(ICCV2/PI489777)SNP_12586	2201	C	T	8	8	--	--
	Ca(ICCV2/PI489777)SNP_12587	2356	G	T	7	6	--	--
	Ca(ICCV2/PI489777)SNP_12588	3792	G	A	6	12	--	--
CakTC27610	Ca(ICCV2/PI489777)SNP_12589	420	A	T	22	16	--	--
	Ca(ICCV2/PI489777)SNP_12590	690	A	G	6	6	--	--
CakTC28527	Ca(ICCV2/PI489777)SNP_12591	626	A	G	5	7	--	--
	Ca(ICCV2/PI489777)SNP_12592	1139	C	T	16	7	--	--
CakTC27215	Ca(ICCV2/PI489777)SNP_12593	634	G	A	48	37	--	--
CakTC42431	Ca(ICCV2/PI489777)SNP_12594	454	C	T	5	4	--	C2C2-Dof
	Ca(ICCV2/PI489777)SNP_12595	509	T	C	5	5	--	C2C2-Dof
	Ca(ICCV2/PI489777)SNP_12596	601	T	A	5	5	--	C2C2-Dof
CakTC40178	Ca(ICCV2/PI489777)SNP_12597	211	G	C	16	9	--	MYB
	Ca(ICCV2/PI489777)SNP_12598	379	T	C	15	9	--	MYB
	Ca(ICCV2/PI489777)SNP_12599	740	T	C	33	30	--	MYB
	Ca(ICCV2/PI489777)SNP_12600	821	G	A	29	23	--	MYB
	Ca(ICCV2/PI489777)SNP_12601	872	C	A	27	29	--	MYB
	Ca(ICCV2/PI489777)SNP_12602	899	T	C	28	28	--	MYB
	Ca(ICCV2/PI489777)SNP_12603	989	A	G	45	33	--	MYB
	Ca(ICCV2/PI489777)SNP_12604	1193	C	G	36	21	--	MYB
	Ca(ICCV2/PI489777)SNP_12605	1290	C	G	39	21	--	MYB
CakTC42733	Ca(ICCV2/PI489777)SNP_12606	572	A	G	12	16	--	--
	Ca(ICCV2/PI489777)SNP_12607	686	C	T	4	10	--	--
CakTC35291	Ca(ICCV2/PI489777)SNP_12608	651	A	G	4	11	--	--
CakTC39820	Ca(ICCV2/PI489777)SNP_12609	146	A	G	9	6	--	--
CakTC40161	Ca(ICCV2/PI489777)SNP_12610	1499	G	A	90	107	--	--
CakTC32051	Ca(ICCV2/PI489777)SNP_12611	741	A	T	24	6	--	--
CakTC41158	Ca(ICCV2/PI489777)SNP_12612	386	T	C	7	4	--	--
CakTC32828	Ca(ICCV2/PI489777)SNP_12613	1617	G	A	3	3	--	--
	Ca(ICCV2/PI489777)SNP_12614	1624	T	G	3	3	--	--
CakTC38759	Ca(ICCV2/PI489777)SNP_12615	206	G	T	11	5	--	--
	Ca(ICCV2/PI489777)SNP_12616	1343	G	A	4	4	--	--
CakTC32388	Ca(ICCV2/PI489777)SNP_12617	401	C	A	8	10	--	--
	Ca(ICCV2/PI489777)SNP_12618	750	T	A	3	6	--	--
	Ca(ICCV2/PI489777)SNP_12619	6167	T	C	6	3	--	--
CakTC38360	Ca(ICCV2/PI489777)SNP_12620	364	T	C	4	4	--	--
CakTC33858	Ca(ICCV2/PI489777)SNP_12621	286	T	A	14	5	--	--
	Ca(ICCV2/PI489777)SNP_12622	499	C	T	10	14	--	--
	Ca(ICCV2/PI489777)SNP_12623	712	A	G	9	12	--	--
	Ca(ICCV2/PI489777)SNP_12624	964	G	A	9	5	--	--
	Ca(ICCV2/PI489777)SNP_12625	1126	G	A	16	5	--	--
	Ca(ICCV2/PI489777)SNP_12626	1312	C	T	9	7	--	--
	Ca(ICCV2/PI489777)SNP_12627	1549	T	C	14	9	--	--
	Ca(ICCV2/PI489777)SNP_12628	1672	A	G	14	9	--	--
CakTC29997	Ca(ICCV2/PI489777)SNP_12629	1810	G	T	3	4	--	--
	Ca(ICCV2/PI489777)SNP_12630	1918	G	A	3	4	--	--
	Ca(ICCV2/PI489777)SNP_12631	2016	T	C	3	4	--	--
	Ca(ICCV2/PI489777)SNP_12632	2021	T	C	3	3	--	--
	Ca(ICCV2/PI489777)SNP_12633	2110	C	A	3	5	--	--
CakTC24239	Ca(ICCV2/PI489777)SNP_12634	827	G	A	10	9	--	CCHC
	Ca(ICCV2/PI489777)SNP_12635	973	C	A	4	5	--	CCHC
CakTC35873	Ca(ICCV2/PI489777)SNP_12636	726	C	G	7	6	--	--
	Ca(ICCV2/PI489777)SNP_12637	2432	C	T	4	3	--	--
	Ca(ICCV2/PI489777)SNP_12638	3401	G	A	4	5	--	--
CakTC10613	Ca(ICCV2/PI489777)SNP_12639	1141	T	C	4	4	--	--
CakTC37887	Ca(ICCV2/PI489777)SNP_12640	523	T	C	16	7	--	--
CakTC40951	Ca(ICCV2/PI489777)SNP_12641	149	C	T	113	54	--	--
	Ca(ICCV2/PI489777)SNP_12642	224	C	A	137	56	--	--
	Ca(ICCV2/PI489777)SNP_12643	357	G	T	131	68	--	--
CakTC29675	Ca(ICCV2/PI489777)SNP_12644	377	C	T	5	5	--	--
	Ca(ICCV2/PI489777)SNP_12645	382	C	A	5	5	--	--
	Ca(ICCV2/PI489777)SNP_12646	1420	T	C	6	3	--	--
CakTC31332	Ca(ICCV2/PI489777)SNP_12647	256	C	A	22	9	--	--
	Ca(ICCV2/PI489777)SNP_12648	2274	C	T	16	14	--	--
CakTC32819	Ca(ICCV2/PI489777)SNP_12649	111	C	T	3	3	--	--

CakTC27098	Ca(ICCV2/PI489777)SNP_12650	734	C	A	4	5	--	--
CakTC39801	Ca(ICCV2/PI489777)SNP_12651	159	T	C	5	7	--	--
	Ca(ICCV2/PI489777)SNP_12652	473	C	T	5	8	--	--
CakTC29943	Ca(ICCV2/PI489777)SNP_12653	643	C	G	3	5	--	--
	Ca(ICCV2/PI489777)SNP_12654	676	A	T	3	5	--	--
	Ca(ICCV2/PI489777)SNP_12655	724	T	C	4	6	--	--
	Ca(ICCV2/PI489777)SNP_12656	964	T	C	7	7	--	--
	Ca(ICCV2/PI489777)SNP_12657	1034	C	A	8	7	--	--
	Ca(ICCV2/PI489777)SNP_12658	1459	A	G	7	4	--	--
	Ca(ICCV2/PI489777)SNP_12659	3958	A	G	8	6	--	--
	Ca(ICCV2/PI489777)SNP_12660	4361	T	A	11	7	--	--
	Ca(ICCV2/PI489777)SNP_12661	5263	C	T	18	11	--	--
CakTC37972	Ca(ICCV2/PI489777)SNP_12662	668	C	T	9	6	--	--
CakTC29890	Ca(ICCV2/PI489777)SNP_12663	282	C	T	5	9	--	--
	Ca(ICCV2/PI489777)SNP_12664	1208	T	A	5	3	--	--
	Ca(ICCV2/PI489777)SNP_12665	1823	A	G	7	4	--	--
CakTC38594	Ca(ICCV2/PI489777)SNP_12666	857	A	G	3	4	--	--
	Ca(ICCV2/PI489777)SNP_12667	865	C	T	3	4	--	--
CakTC40446	Ca(ICCV2/PI489777)SNP_12668	63	A	G	7	7	--	--
	Ca(ICCV2/PI489777)SNP_12669	221	G	C	7	10	--	--
CakTC38426	Ca(ICCV2/PI489777)SNP_12670	221	C	T	6	7	--	--
	Ca(ICCV2/PI489777)SNP_12671	399	C	T	12	11	--	--
	Ca(ICCV2/PI489777)SNP_12672	494	T	C	23	14	--	--
	Ca(ICCV2/PI489777)SNP_12673	588	A	G	26	11	--	--
	Ca(ICCV2/PI489777)SNP_12674	650	C	T	34	13	--	--
	Ca(ICCV2/PI489777)SNP_12675	714	C	T	33	8	--	--
	Ca(ICCV2/PI489777)SNP_12676	779	C	T	37	9	--	--
	Ca(ICCV2/PI489777)SNP_12677	809	G	A	29	9	--	--
CakTC35856	Ca(ICCV2/PI489777)SNP_12678	91	G	A	3	3	--	--
	Ca(ICCV2/PI489777)SNP_12679	92	C	T	3	3	--	--
	Ca(ICCV2/PI489777)SNP_12680	120	C	T	3	3	--	--
CakTC10262	Ca(ICCV2/PI489777)SNP_12681	615	A	T	5	3	--	--
	Ca(ICCV2/PI489777)SNP_12682	1315	C	T	9	9	--	--
CakTC27973	Ca(ICCV2/PI489777)SNP_12683	2255	T	A	9	3	--	--
	Ca(ICCV2/PI489777)SNP_12684	2329	C	T	7	3	--	--
	Ca(ICCV2/PI489777)SNP_12685	2546	C	T	9	9	--	--
	Ca(ICCV2/PI489777)SNP_12686	2570	C	T	8	8	--	--
	Ca(ICCV2/PI489777)SNP_12687	2571	A	G	6	8	--	--
	Ca(ICCV2/PI489777)SNP_12688	2892	A	T	5	7	--	--
CakTC06170	Ca(ICCV2/PI489777)SNP_12689	443	A	G	6	4	--	--
CakTC39081	Ca(ICCV2/PI489777)SNP_12690	386	C	T	15	9	--	C2C2-GATA
	Ca(ICCV2/PI489777)SNP_12691	464	T	C	14	3	--	C2C2-GATA
	Ca(ICCV2/PI489777)SNP_12692	585	A	T	17	7	--	C2C2-GATA
	Ca(ICCV2/PI489777)SNP_12693	734	T	C	14	6	--	C2C2-GATA
	Ca(ICCV2/PI489777)SNP_12694	848	C	T	16	8	--	C2C2-GATA
	Ca(ICCV2/PI489777)SNP_12695	849	A	G	16	8	--	C2C2-GATA
	Ca(ICCV2/PI489777)SNP_12696	960	G	A	16	7	--	C2C2-GATA
CakTC32974	Ca(ICCV2/PI489777)SNP_12697	217	G	C	21	4	--	--
	Ca(ICCV2/PI489777)SNP_12698	310	C	T	21	4	--	--
CakTC24599	Ca(ICCV2/PI489777)SNP_12699	901	C	T	77	34	--	--
CakTC25030	Ca(ICCV2/PI489777)SNP_12700	485	G	C	37	14	--	--
	Ca(ICCV2/PI489777)SNP_12701	1052	T	C	66	28	--	--
CakTC23412	Ca(ICCV2/PI489777)SNP_12702	2218	A	G	7	3	--	--
CakTC11026	Ca(ICCV2/PI489777)SNP_12703	176	C	T	11	10	--	--
CakTC24353	Ca(ICCV2/PI489777)SNP_12704	321	A	T	11	14	--	--
CakTC38551	Ca(ICCV2/PI489777)SNP_12705	64	G	A	5	4	--	--
	Ca(ICCV2/PI489777)SNP_12706	154	A	C	5	4	--	--
	Ca(ICCV2/PI489777)SNP_12707	222	C	T	7	6	--	--
	Ca(ICCV2/PI489777)SNP_12708	262	T	C	8	6	--	--
	Ca(ICCV2/PI489777)SNP_12709	273	C	G	8	6	--	--
	Ca(ICCV2/PI489777)SNP_12710	291	A	G	8	6	--	--
CakTC37165	Ca(ICCV2/PI489777)SNP_12711	840	A	G	17	5	--	--
	Ca(ICCV2/PI489777)SNP_12712	859	T	G	17	5	--	--
	Ca(ICCV2/PI489777)SNP_12713	1120	A	T	11	8	--	--
CakTC41828	Ca(ICCV2/PI489777)SNP_12714	53	C	T	5	6	--	--
	Ca(ICCV2/PI489777)SNP_12715	120	C	T	15	9	--	--
	Ca(ICCV2/PI489777)SNP_12716	689	A	T	6	5	--	--
	Ca(ICCV2/PI489777)SNP_12717	779	G	A	4	4	--	--
	Ca(ICCV2/PI489777)SNP_12718	815	A	G	4	4	--	--
	Ca(ICCV2/PI489777)SNP_12719	870	G	C	3	4	--	--
CakTC27460	Ca(ICCV2/PI489777)SNP_12720	318	A	C	5	3	--	--
	Ca(ICCV2/PI489777)SNP_12721	326	C	T	6	3	--	--
	Ca(ICCV2/PI489777)SNP_12722	374	C	T	8	3	--	--
	Ca(ICCV2/PI489777)SNP_12723	396	A	G	8	3	--	--

	Ca(ICCV2/PI489777)SNP_12724	399	A	T	7	3	--	--
	Ca(ICCV2/PI489777)SNP_12725	407	A	G	6	3	--	--
	Ca(ICCV2/PI489777)SNP_12726	410	A	G	7	3	--	--
	Ca(ICCV2/PI489777)SNP_12727	434	C	T	8	3	--	--
	Ca(ICCV2/PI489777)SNP_12728	438	G	T	7	3	--	--
CakTC26942	Ca(ICCV2/PI489777)SNP_12729	309	C	T	77	36	--	MYB-related
	Ca(ICCV2/PI489777)SNP_12730	372	A	G	90	59	--	MYB-related
	Ca(ICCV2/PI489777)SNP_12731	885	A	G	134	52	--	MYB-related
	Ca(ICCV2/PI489777)SNP_12732	999	C	T	89	42	--	MYB-related
	Ca(ICCV2/PI489777)SNP_12733	1028	T	G	77	40	--	MYB-related
	Ca(ICCV2/PI489777)SNP_12734	1044	T	A	63	38	--	MYB-related
	Ca(ICCV2/PI489777)SNP_12735	1279	T	G	83	31	--	MYB-related
	Ca(ICCV2/PI489777)SNP_12736	1380	G	A	69	32	--	MYB-related
	Ca(ICCV2/PI489777)SNP_12737	1550	C	T	86	30	--	MYB-related
	Ca(ICCV2/PI489777)SNP_12738	2129	A	C	69	32	--	MYB-related
	Ca(ICCV2/PI489777)SNP_12739	2795	A	G	18	22	--	MYB-related
CakTC40521	Ca(ICCV2/PI489777)SNP_12740	258	A	G	8	6	--	--
CakTC38430	Ca(ICCV2/PI489777)SNP_12741	904	A	G	6	14	--	--
CakTC30795	Ca(ICCV2/PI489777)SNP_12742	320	C	T	4	3	--	--
CakTC34237	Ca(ICCV2/PI489777)SNP_12743	297	T	C	56	21	--	--
	Ca(ICCV2/PI489777)SNP_12744	308	G	A	63	21	--	--
	Ca(ICCV2/PI489777)SNP_12745	468	A	G	65	18	--	--
	Ca(ICCV2/PI489777)SNP_12746	554	A	G	23	8	--	--
CakTC34994	Ca(ICCV2/PI489777)SNP_12747	556	C	T	4	3	--	--
CakTC39722	Ca(ICCV2/PI489777)SNP_12748	814	T	C	20	20	--	--
	Ca(ICCV2/PI489777)SNP_12749	956	T	C	20	15	--	--
	Ca(ICCV2/PI489777)SNP_12750	1078	G	A	12	10	--	--
	Ca(ICCV2/PI489777)SNP_12751	1206	G	A	5	4	--	--
CakTC40863	Ca(ICCV2/PI489777)SNP_12752	885	T	C	15	19	--	--
	Ca(ICCV2/PI489777)SNP_12753	1004	C	T	17	21	--	--
	Ca(ICCV2/PI489777)SNP_12754	1026	C	T	17	20	--	--
	Ca(ICCV2/PI489777)SNP_12755	1245	C	A	19	18	--	--
	Ca(ICCV2/PI489777)SNP_12756	1441	C	T	34	11	--	--
	Ca(ICCV2/PI489777)SNP_12757	1582	G	A	38	16	--	--
	Ca(ICCV2/PI489777)SNP_12758	2078	G	A	8	18	--	--
	Ca(ICCV2/PI489777)SNP_12759	2138	G	T	7	4	--	--
CakTC25449	Ca(ICCV2/PI489777)SNP_12760	735	T	C	18	9	--	ARF
CakTC32767	Ca(ICCV2/PI489777)SNP_12761	1536	A	G	7	7	--	--
CakTC31537	Ca(ICCV2/PI489777)SNP_12762	904	T	C	30	5	--	--
	Ca(ICCV2/PI489777)SNP_12763	1098	A	T	10	3	--	--
CakTC27849	Ca(ICCV2/PI489777)SNP_12764	444	T	C	16	12	--	--
	Ca(ICCV2/PI489777)SNP_12765	1554	C	A	15	10	--	--
	Ca(ICCV2/PI489777)SNP_12766	1800	G	C	14	5	--	--
CakTC38280	Ca(ICCV2/PI489777)SNP_12767	198	A	G	5	5	--	--
	Ca(ICCV2/PI489777)SNP_12768	295	G	A	6	7	--	--
CakTC29396	Ca(ICCV2/PI489777)SNP_12769	2442	G	A	3	3	--	--
CakTC25029	Ca(ICCV2/PI489777)SNP_12770	157	G	A	11	5	--	--
	Ca(ICCV2/PI489777)SNP_12771	1271	A	T	17	9	--	--
CakTC25434	Ca(ICCV2/PI489777)SNP_12772	1462	A	C	9	12	--	--
	Ca(ICCV2/PI489777)SNP_12773	1596	G	A	8	11	--	--
CakTC25606	Ca(ICCV2/PI489777)SNP_12774	1473	T	C	4	4	Young_pod	--
	Ca(ICCV2/PI489777)SNP_12775	1526	G	A	4	3	Young_pod	--
	Ca(ICCV2/PI489777)SNP_12776	1530	T	G	4	3	Young_pod	--
CakTC01660	Ca(ICCV2/PI489777)SNP_12777	440	C	T	9	10	--	--
	Ca(ICCV2/PI489777)SNP_12778	660	C	T	10	5	--	--
	Ca(ICCV2/PI489777)SNP_12779	713	C	G	7	4	--	--
	Ca(ICCV2/PI489777)SNP_12780	1191	G	A	16	4	--	--
	Ca(ICCV2/PI489777)SNP_12781	1997	A	T	5	3	--	--
CakTC36839	Ca(ICCV2/PI489777)SNP_12782	828	T	C	19	16	--	--
	Ca(ICCV2/PI489777)SNP_12783	2139	C	T	11	7	--	--
	Ca(ICCV2/PI489777)SNP_12784	2225	C	T	10	3	--	--
CakTC38799	Ca(ICCV2/PI489777)SNP_12785	581	C	T	10	6	--	--
	Ca(ICCV2/PI489777)SNP_12786	992	C	T	20	6	--	--
	Ca(ICCV2/PI489777)SNP_12787	1061	T	C	14	11	--	--
CakTC37704	Ca(ICCV2/PI489777)SNP_12788	755	G	A	21	3	--	--
CakTC36310	Ca(ICCV2/PI489777)SNP_12789	107	G	A	3	7	--	--
	Ca(ICCV2/PI489777)SNP_12790	123	C	T	3	9	--	--
	Ca(ICCV2/PI489777)SNP_12791	296	G	A	27	34	--	--
	Ca(ICCV2/PI489777)SNP_12792	541	G	A	16	35	--	--
CakTC37456	Ca(ICCV2/PI489777)SNP_12793	742	G	A	14	6	--	--
	Ca(ICCV2/PI489777)SNP_12794	1054	A	G	6	5	--	--
	Ca(ICCV2/PI489777)SNP_12795	1103	T	C	5	5	--	--
	Ca(ICCV2/PI489777)SNP_12796	1123	A	G	5	4	--	--
CakTC27375	Ca(ICCV2/PI489777)SNP_12797	1502	G	C	6	4	Root	--

	Ca(ICCV2/PI489777)SNP_12798	1586	A	G	8	5	Root	--
CakTC38039	Ca(ICCV2/PI489777)SNP_12799	119	A	T	5	3	--	--
CakTC24692	Ca(ICCV2/PI489777)SNP_12800	402	C	T	13	3	--	HB
	Ca(ICCV2/PI489777)SNP_12801	403	A	G	12	3	--	HB
	Ca(ICCV2/PI489777)SNP_12802	563	A	G	29	4	--	HB
	Ca(ICCV2/PI489777)SNP_12803	1333	C	T	92	11	--	HB
	Ca(ICCV2/PI489777)SNP_12804	1354	C	T	101	11	--	HB
	Ca(ICCV2/PI489777)SNP_12805	1390	T	C	106	12	--	HB
	Ca(ICCV2/PI489777)SNP_12806	1429	C	G	106	11	--	HB
	Ca(ICCV2/PI489777)SNP_12807	2174	T	G	118	20	--	HB
CakTC35837	Ca(ICCV2/PI489777)SNP_12808	2401	G	A	4	3	--	--
	Ca(ICCV2/PI489777)SNP_12809	2950	C	T	4	5	--	--
CakTC28543	Ca(ICCV2/PI489777)SNP_12810	1061	G	A	5	4	--	--
	Ca(ICCV2/PI489777)SNP_12811	1332	C	A	16	4	--	--
CakTC40689	Ca(ICCV2/PI489777)SNP_12812	155	G	A	5	7	--	--
	Ca(ICCV2/PI489777)SNP_12813	436	G	A	5	8	--	--
	Ca(ICCV2/PI489777)SNP_12814	584	T	A	3	6	--	--
	Ca(ICCV2/PI489777)SNP_12815	592	C	T	3	7	--	--
CakTC38614	Ca(ICCV2/PI489777)SNP_12816	65	C	G	8	3	--	--
	Ca(ICCV2/PI489777)SNP_12817	85	C	G	9	3	--	--
	Ca(ICCV2/PI489777)SNP_12818	131	A	G	7	8	--	--
	Ca(ICCV2/PI489777)SNP_12819	899	C	T	51	33	--	--
	Ca(ICCV2/PI489777)SNP_12820	902	C	T	51	34	--	--
CakTC34917	Ca(ICCV2/PI489777)SNP_12821	504	A	G	42	17	--	--
CakTC31981	Ca(ICCV2/PI489777)SNP_12822	414	C	T	7	5	--	--
CakTC40507	Ca(ICCV2/PI489777)SNP_12823	57	G	A	13	8	--	HMG
	Ca(ICCV2/PI489777)SNP_12824	481	T	C	4	7	--	HMG
	Ca(ICCV2/PI489777)SNP_12825	535	T	A	3	5	--	HMG
	Ca(ICCV2/PI489777)SNP_12826	536	G	A	3	5	--	HMG
CakTC26614	Ca(ICCV2/PI489777)SNP_12827	437	C	A	37	6	--	--
	Ca(ICCV2/PI489777)SNP_12828	884	C	T	19	4	--	--
	Ca(ICCV2/PI489777)SNP_12829	2290	T	G	12	3	--	--
	Ca(ICCV2/PI489777)SNP_12830	2477	A	C	13	7	--	--
CakTC17204	Ca(ICCV2/PI489777)SNP_12831	455	T	C	3	5	--	--
CakTC40890	Ca(ICCV2/PI489777)SNP_12832	917	C	T	13	25	--	TPR
	Ca(ICCV2/PI489777)SNP_12833	980	T	C	11	26	--	TPR
	Ca(ICCV2/PI489777)SNP_12834	1055	T	C	12	23	--	TPR
CakTC37444	Ca(ICCV2/PI489777)SNP_12835	757	C	A	23	17	--	--
CakTC43386	Ca(ICCV2/PI489777)SNP_12836	799	A	G	8	4	--	--
CakTC35089	Ca(ICCV2/PI489777)SNP_12837	201	C	T	15	9	--	--
	Ca(ICCV2/PI489777)SNP_12838	1400	A	G	11	7	--	--
	Ca(ICCV2/PI489777)SNP_12839	1664	T	C	25	8	--	--
	Ca(ICCV2/PI489777)SNP_12840	1742	G	A	27	8	--	--
CakTC22251	Ca(ICCV2/PI489777)SNP_12841	189	G	A	3	3	--	--
	Ca(ICCV2/PI489777)SNP_12842	190	T	G	3	3	--	--
	Ca(ICCV2/PI489777)SNP_12843	340	G	A	3	4	--	--
CakTC37234	Ca(ICCV2/PI489777)SNP_12844	510	T	C	21	13	--	--
	Ca(ICCV2/PI489777)SNP_12845	537	C	T	19	13	--	--
	Ca(ICCV2/PI489777)SNP_12846	560	C	G	17	10	--	--
	Ca(ICCV2/PI489777)SNP_12847	561	C	T	17	12	--	--
	Ca(ICCV2/PI489777)SNP_12848	588	A	T	15	9	--	--
CakTC09300	Ca(ICCV2/PI489777)SNP_12849	515	T	G	3	3	--	--
CakTC08512	Ca(ICCV2/PI489777)SNP_12850	1039	A	G	4	4	--	--
CakTC14371	Ca(ICCV2/PI489777)SNP_12851	390	T	G	10	10	--	--
	Ca(ICCV2/PI489777)SNP_12852	1446	T	A	11	7	--	--
	Ca(ICCV2/PI489777)SNP_12853	1451	A	T	11	7	--	--
CakTC34206	Ca(ICCV2/PI489777)SNP_12854	230	C	G	123	16	--	--
	Ca(ICCV2/PI489777)SNP_12855	666	T	C	156	36	--	--
CakTC38212	Ca(ICCV2/PI489777)SNP_12856	25	A	T	8	10	--	--
	Ca(ICCV2/PI489777)SNP_12857	622	C	G	41	64	--	--
CakTC43013	Ca(ICCV2/PI489777)SNP_12858	812	C	T	10	3	--	--
	Ca(ICCV2/PI489777)SNP_12859	833	C	T	11	4	--	--
	Ca(ICCV2/PI489777)SNP_12860	923	A	G	11	4	--	--
CakTC41241	Ca(ICCV2/PI489777)SNP_12861	464	C	T	8	6	--	--
	Ca(ICCV2/PI489777)SNP_12862	602	A	T	4	4	--	--
CakTC24815	Ca(ICCV2/PI489777)SNP_12863	441	G	A	43	38	--	AUX/IAA
	Ca(ICCV2/PI489777)SNP_12864	596	T	C	14	10	--	AUX/IAA
	Ca(ICCV2/PI489777)SNP_12865	644	T	C	10	4	--	AUX/IAA
CakTC37373	Ca(ICCV2/PI489777)SNP_12866	778	G	A	5	3	--	--
	Ca(ICCV2/PI489777)SNP_12867	1327	C	G	9	7	--	--
	Ca(ICCV2/PI489777)SNP_12868	1383	G	T	5	7	--	--
CakTC38163	Ca(ICCV2/PI489777)SNP_12869	106	G	A	5	3	--	--
	Ca(ICCV2/PI489777)SNP_12870	433	G	A	30	19	--	--
	Ca(ICCV2/PI489777)SNP_12871	645	A	G	23	18	--	--

	Ca(ICCV2/PI489777)SNP_12872	910	G	A	17	15	--	--
CakTC24902	Ca(ICCV2/PI489777)SNP_12873	85	C	T	36	6	--	AP2-EREBP
	Ca(ICCV2/PI489777)SNP_12874	87	C	G	37	6	--	AP2-EREBP
	Ca(ICCV2/PI489777)SNP_12875	341	C	T	50	10	--	AP2-EREBP
CakTC02589	Ca(ICCV2/PI489777)SNP_12876	223	G	C	6	3	--	--
	Ca(ICCV2/PI489777)SNP_12877	225	A	C	6	4	--	--
	Ca(ICCV2/PI489777)SNP_12878	300	A	T	4	5	--	--
	Ca(ICCV2/PI489777)SNP_12879	690	G	A	5	6	--	--
	Ca(ICCV2/PI489777)SNP_12880	702	G	C	7	6	--	--
CakTC39772	Ca(ICCV2/PI489777)SNP_12881	448	C	T	4	4	--	--
	Ca(ICCV2/PI489777)SNP_12882	476	T	C	5	4	--	--
	Ca(ICCV2/PI489777)SNP_12883	562	T	C	3	3	--	--
	Ca(ICCV2/PI489777)SNP_12884	601	G	T	4	3	--	--
	Ca(ICCV2/PI489777)SNP_12885	634	T	C	3	5	--	--
CakTC25191	Ca(ICCV2/PI489777)SNP_12886	183	A	C	5	40	--	--
	Ca(ICCV2/PI489777)SNP_12887	538	T	A	12	55	--	--
	Ca(ICCV2/PI489777)SNP_12888	542	C	G	12	57	--	--
	Ca(ICCV2/PI489777)SNP_12889	584	C	A	13	48	--	--
	Ca(ICCV2/PI489777)SNP_12890	633	C	G	12	41	--	--
	Ca(ICCV2/PI489777)SNP_12891	642	G	T	13	41	--	--
	Ca(ICCV2/PI489777)SNP_12892	656	A	G	10	42	--	--
	Ca(ICCV2/PI489777)SNP_12893	815	C	A	22	35	--	--
	Ca(ICCV2/PI489777)SNP_12894	854	G	C	22	38	--	--
	Ca(ICCV2/PI489777)SNP_12895	1468	A	G	15	50	--	--
	Ca(ICCV2/PI489777)SNP_12896	1486	T	C	13	56	--	--
	Ca(ICCV2/PI489777)SNP_12897	1726	A	C	20	60	--	--
	Ca(ICCV2/PI489777)SNP_12898	1758	G	A	21	59	--	--
	Ca(ICCV2/PI489777)SNP_12899	1894	C	T	17	53	--	--
	Ca(ICCV2/PI489777)SNP_12900	2499	T	C	13	29	--	--
	Ca(ICCV2/PI489777)SNP_12901	2500	A	T	13	29	--	--
CakTC08729	Ca(ICCV2/PI489777)SNP_12902	462	C	T	19	5	--	--
CakTC42519	Ca(ICCV2/PI489777)SNP_12903	125	C	G	5	6	--	--
	Ca(ICCV2/PI489777)SNP_12904	519	C	T	8	27	--	--
	Ca(ICCV2/PI489777)SNP_12905	767	C	T	7	12	--	--
CakTC08831	Ca(ICCV2/PI489777)SNP_12906	611	T	C	12	5	--	--
	Ca(ICCV2/PI489777)SNP_12907	629	A	G	13	6	--	--
	Ca(ICCV2/PI489777)SNP_12908	1682	G	A	4	3	--	--
	Ca(ICCV2/PI489777)SNP_12909	2043	C	T	6	4	--	--
	Ca(ICCV2/PI489777)SNP_12910	2352	A	C	3	4	--	--
CakTC10275	Ca(ICCV2/PI489777)SNP_12911	556	C	G	4	4	--	CCHC
	Ca(ICCV2/PI489777)SNP_12912	598	A	G	4	4	--	CCHC
	Ca(ICCV2/PI489777)SNP_12913	630	C	T	4	4	--	CCHC
	Ca(ICCV2/PI489777)SNP_12914	684	G	A	4	3	--	CCHC
CakTC12538	Ca(ICCV2/PI489777)SNP_12915	756	G	A	17	4	--	--
CakTC19054	Ca(ICCV2/PI489777)SNP_12916	732	G	A	6	4	--	--
	Ca(ICCV2/PI489777)SNP_12917	760	G	A	4	3	--	--
CakTC25213	Ca(ICCV2/PI489777)SNP_12918	201	A	G	7	3	Root	--
	Ca(ICCV2/PI489777)SNP_12919	211	G	A	9	3	Root	--
CakTC42461	Ca(ICCV2/PI489777)SNP_12920	389	A	T	5	5	--	--
	Ca(ICCV2/PI489777)SNP_12921	461	G	C	7	8	--	--
CakTC42387	Ca(ICCV2/PI489777)SNP_12922	28	T	C	3	6	--	--
	Ca(ICCV2/PI489777)SNP_12923	232	C	T	15	9	--	--
	Ca(ICCV2/PI489777)SNP_12924	1213	C	T	13	16	--	--
CakTC38586	Ca(ICCV2/PI489777)SNP_12925	737	T	G	14	12	--	--
	Ca(ICCV2/PI489777)SNP_12926	745	C	T	15	11	--	--
	Ca(ICCV2/PI489777)SNP_12927	750	G	T	16	10	--	--
	Ca(ICCV2/PI489777)SNP_12928	1104	A	G	8	6	--	--
CakTC26451	Ca(ICCV2/PI489777)SNP_12929	411	T	C	3	4	--	--
CakTC28999	Ca(ICCV2/PI489777)SNP_12930	770	T	C	4	4	--	--
CakTC23541	Ca(ICCV2/PI489777)SNP_12931	80	C	T	9	4	--	Pseudo_ARR-B
	Ca(ICCV2/PI489777)SNP_12932	1368	A	G	11	5	--	Pseudo_ARR-B
	Ca(ICCV2/PI489777)SNP_12933	1405	A	G	11	4	--	Pseudo_ARR-B
	Ca(ICCV2/PI489777)SNP_12934	1962	T	C	42	17	--	Pseudo_ARR-B
	Ca(ICCV2/PI489777)SNP_12935	2174	A	G	28	13	--	Pseudo_ARR-B
	Ca(ICCV2/PI489777)SNP_12936	2223	C	G	36	16	--	Pseudo_ARR-B
	Ca(ICCV2/PI489777)SNP_12937	2290	A	G	29	15	--	Pseudo_ARR-B
	Ca(ICCV2/PI489777)SNP_12938	2698	C	G	20	10	--	Pseudo_ARR-B
	Ca(ICCV2/PI489777)SNP_12939	3707	T	C	11	5	--	Pseudo_ARR-B
	Ca(ICCV2/PI489777)SNP_12940	4041	T	C	8	5	--	Pseudo_ARR-B
	Ca(ICCV2/PI489777)SNP_12941	4239	G	C	7	3	--	Pseudo_ARR-B
	Ca(ICCV2/PI489777)SNP_12942	4409	T	C	6	5	--	Pseudo_ARR-B
	Ca(ICCV2/PI489777)SNP_12943	4473	C	A	8	4	--	Pseudo_ARR-B
CakTC26422	Ca(ICCV2/PI489777)SNP_12944	910	G	A	3	26	--	--
	Ca(ICCV2/PI489777)SNP_12945	1453	C	T	12	21	--	--

	Ca(ICCV2/PI489777)SNP_12946	1620	C	G	5	15	--	--
CakTC25354	Ca(ICCV2/PI489777)SNP_12947	310	G	A	24	18	--	CCAAT
	Ca(ICCV2/PI489777)SNP_12948	550	G	A	33	9	--	CCAAT
CakTC35240	Ca(ICCV2/PI489777)SNP_12949	76	C	A	5	6	--	--
	Ca(ICCV2/PI489777)SNP_12950	221	G	A	15	7	--	--
	Ca(ICCV2/PI489777)SNP_12951	668	G	A	15	17	--	--
	Ca(ICCV2/PI489777)SNP_12952	726	A	C	13	17	--	--
	Ca(ICCV2/PI489777)SNP_12953	805	A	T	19	18	--	--
	Ca(ICCV2/PI489777)SNP_12954	809	G	A	19	19	--	--
	Ca(ICCV2/PI489777)SNP_12955	1010	A	G	26	19	--	--
	Ca(ICCV2/PI489777)SNP_12956	1114	G	A	23	19	--	--
	Ca(ICCV2/PI489777)SNP_12957	1424	A	G	26	25	--	--
	Ca(ICCV2/PI489777)SNP_12958	1479	G	A	20	28	--	--
	Ca(ICCV2/PI489777)SNP_12959	1540	G	A	17	27	--	--
	Ca(ICCV2/PI489777)SNP_12960	1604	T	C	33	26	--	--
	Ca(ICCV2/PI489777)SNP_12961	1768	C	T	35	26	--	--
	Ca(ICCV2/PI489777)SNP_12962	2392	T	C	9	30	--	--
	Ca(ICCV2/PI489777)SNP_12963	2407	T	C	10	30	--	--
	Ca(ICCV2/PI489777)SNP_12964	2818	C	T	15	16	--	--
CakTC31639	Ca(ICCV2/PI489777)SNP_12965	840	T	C	29	10	--	--
CakTC33885	Ca(ICCV2/PI489777)SNP_12966	778	G	A	16	8	--	C2C2-GATA
CakTC23625	Ca(ICCV2/PI489777)SNP_12967	660	A	T	36	3	--	--
CakTC40704	Ca(ICCV2/PI489777)SNP_12968	751	A	T	6	4	--	--
	Ca(ICCV2/PI489777)SNP_12969	766	G	A	5	4	--	--
	Ca(ICCV2/PI489777)SNP_12970	824	G	T	4	4	--	--
CakTC22746	Ca(ICCV2/PI489777)SNP_12971	366	G	A	10	11	--	--
CakTC32064	Ca(ICCV2/PI489777)SNP_12972	406	A	G	36	34	--	--
CakTC31512	Ca(ICCV2/PI489777)SNP_12973	583	A	G	7	11	--	--
CakTC43217	Ca(ICCV2/PI489777)SNP_12974	655	T	C	16	6	--	--
	Ca(ICCV2/PI489777)SNP_12975	694	T	C	19	6	--	--
CakTC39403	Ca(ICCV2/PI489777)SNP_12976	631	T	C	3	4	--	--
	Ca(ICCV2/PI489777)SNP_12977	709	T	C	3	4	--	--
	Ca(ICCV2/PI489777)SNP_12978	737	A	G	4	6	--	--
	Ca(ICCV2/PI489777)SNP_12979	742	G	A	5	6	--	--
	Ca(ICCV2/PI489777)SNP_12980	1099	A	G	5	7	--	--
CakTC39214	Ca(ICCV2/PI489777)SNP_12981	154	A	T	10	3	--	--
	Ca(ICCV2/PI489777)SNP_12982	519	G	T	8	13	--	--
CakTC32056	Ca(ICCV2/PI489777)SNP_12983	216	C	A	15	5	--	--
	Ca(ICCV2/PI489777)SNP_12984	313	C	T	23	8	--	--
	Ca(ICCV2/PI489777)SNP_12985	316	C	T	23	8	--	--
CakTC10502	Ca(ICCV2/PI489777)SNP_12986	117	G	T	4	3	--	--
	Ca(ICCV2/PI489777)SNP_12987	402	T	C	5	8	--	--
	Ca(ICCV2/PI489777)SNP_12988	930	G	A	8	4	--	--
CakTC41022	Ca(ICCV2/PI489777)SNP_12989	156	G	T	4	4	--	--
CakTC42831	Ca(ICCV2/PI489777)SNP_12990	702	G	T	4	6	--	--
	Ca(ICCV2/PI489777)SNP_12991	798	G	A	5	9	--	--
	Ca(ICCV2/PI489777)SNP_12992	816	G	A	6	7	--	--
	Ca(ICCV2/PI489777)SNP_12993	1074	T	C	7	7	--	--
	Ca(ICCV2/PI489777)SNP_12994	1150	A	G	7	6	--	--
	Ca(ICCV2/PI489777)SNP_12995	1162	A	C	7	6	--	--
	Ca(ICCV2/PI489777)SNP_12996	1166	C	T	7	6	--	--
CakTC23118	Ca(ICCV2/PI489777)SNP_12997	556	C	T	18	3	--	--
CakTC26905	Ca(ICCV2/PI489777)SNP_12998	302	G	A	10	3	--	--
	Ca(ICCV2/PI489777)SNP_12999	427	C	A	8	5	--	--
	Ca(ICCV2/PI489777)SNP_13000	439	A	C	9	7	--	--
	Ca(ICCV2/PI489777)SNP_13001	616	G	A	4	10	--	--
	Ca(ICCV2/PI489777)SNP_13002	698	C	T	4	7	--	--
	Ca(ICCV2/PI489777)SNP_13003	826	C	G	4	8	--	--
	Ca(ICCV2/PI489777)SNP_13004	841	G	T	4	9	--	--
CakTC09794	Ca(ICCV2/PI489777)SNP_13005	1217	T	C	9	7	--	--
	Ca(ICCV2/PI489777)SNP_13006	2331	G	T	4	3	--	--
CakTC14091	Ca(ICCV2/PI489777)SNP_13007	829	A	G	3	3	--	--
	Ca(ICCV2/PI489777)SNP_13008	846	A	G	3	3	--	--
	Ca(ICCV2/PI489777)SNP_13009	864	C	T	3	3	--	--
	Ca(ICCV2/PI489777)SNP_13010	876	T	A	3	3	--	--
	Ca(ICCV2/PI489777)SNP_13011	891	G	T	3	3	--	--
	Ca(ICCV2/PI489777)SNP_13012	928	G	C	3	3	--	--
CakTC36336	Ca(ICCV2/PI489777)SNP_13013	323	A	G	8	5	--	--
CakTC09842	Ca(ICCV2/PI489777)SNP_13014	509	A	G	8	18	--	--
	Ca(ICCV2/PI489777)SNP_13015	1477	G	T	10	4	--	--
CakTC27114	Ca(ICCV2/PI489777)SNP_13016	651	G	A	35	18	--	--
	Ca(ICCV2/PI489777)SNP_13017	741	C	T	20	17	--	--
	Ca(ICCV2/PI489777)SNP_13018	801	T	C	16	12	--	--
	Ca(ICCV2/PI489777)SNP_13019	906	T	C	13	5	--	--

	Ca(ICCV2/PI489777)SNP_13020	909	G	A	13	5	--	--
CakTC38057	Ca(ICCV2/PI489777)SNP_13021	810	T	C	5	4	--	--
CakTC33315	Ca(ICCV2/PI489777)SNP_13022	198	A	G	7	3	--	--
	Ca(ICCV2/PI489777)SNP_13023	220	T	C	6	6	--	--
	Ca(ICCV2/PI489777)SNP_13024	229	T	A	6	7	--	--
	Ca(ICCV2/PI489777)SNP_13025	249	C	A	7	7	--	--
	Ca(ICCV2/PI489777)SNP_13026	338	C	T	7	9	--	--
	Ca(ICCV2/PI489777)SNP_13027	488	C	G	9	9	--	--
	Ca(ICCV2/PI489777)SNP_13028	551	G	C	5	8	--	--
	Ca(ICCV2/PI489777)SNP_13029	600	T	G	6	10	--	--
CakTC30200	Ca(ICCV2/PI489777)SNP_13030	386	T	C	7	12	--	--
	Ca(ICCV2/PI489777)SNP_13031	551	G	A	6	13	--	--
	Ca(ICCV2/PI489777)SNP_13032	991	C	T	5	8	--	--
	Ca(ICCV2/PI489777)SNP_13033	2202	G	A	11	6	--	--
CakTC12246	Ca(ICCV2/PI489777)SNP_13034	249	A	G	5	4	Young_pod	--
	Ca(ICCV2/PI489777)SNP_13035	361	A	G	5	3	Young_pod	--
CakTC33971	Ca(ICCV2/PI489777)SNP_13036	882	A	G	20	9	--	--
	Ca(ICCV2/PI489777)SNP_13037	966	G	A	25	12	--	--
	Ca(ICCV2/PI489777)SNP_13038	1674	G	A	31	11	--	--
	Ca(ICCV2/PI489777)SNP_13039	1686	T	C	30	11	--	--
	Ca(ICCV2/PI489777)SNP_13040	1784	C	A	26	11	--	--
CakTC39142	Ca(ICCV2/PI489777)SNP_13041	314	C	T	9	5	--	--
	Ca(ICCV2/PI489777)SNP_13042	800	T	C	10	5	--	--
	Ca(ICCV2/PI489777)SNP_13043	1313	A	G	9	5	--	--
	Ca(ICCV2/PI489777)SNP_13044	1319	C	T	8	5	--	--
CakTC38528	Ca(ICCV2/PI489777)SNP_13045	416	G	A	13	9	--	--
CakTC33978	Ca(ICCV2/PI489777)SNP_13046	650	G	A	39	14	--	--
	Ca(ICCV2/PI489777)SNP_13047	669	G	A	40	13	--	--
	Ca(ICCV2/PI489777)SNP_13048	812	T	A	39	16	--	--
	Ca(ICCV2/PI489777)SNP_13049	1598	A	G	30	25	--	--
	Ca(ICCV2/PI489777)SNP_13050	1937	A	G	31	13	--	--
	Ca(ICCV2/PI489777)SNP_13051	2031	A	G	20	8	--	--
	Ca(ICCV2/PI489777)SNP_13052	2085	G	T	12	4	--	--
CakTC28089	Ca(ICCV2/PI489777)SNP_13053	1311	T	A	3	3	--	--
CakTC23456	Ca(ICCV2/PI489777)SNP_13054	352	T	A	43	50	--	--
	Ca(ICCV2/PI489777)SNP_13055	379	T	C	51	52	--	--
CakTC35762	Ca(ICCV2/PI489777)SNP_13056	401	A	C	3	4	--	--
	Ca(ICCV2/PI489777)SNP_13057	508	A	G	8	4	--	--
	Ca(ICCV2/PI489777)SNP_13058	511	G	A	8	4	--	--
	Ca(ICCV2/PI489777)SNP_13059	953	A	G	13	3	--	--
	Ca(ICCV2/PI489777)SNP_13060	1611	A	G	20	15	--	--
	Ca(ICCV2/PI489777)SNP_13061	1749	C	T	25	13	--	--
	Ca(ICCV2/PI489777)SNP_13062	1776	C	T	25	12	--	--
	Ca(ICCV2/PI489777)SNP_13063	1824	C	T	22	11	--	--
	Ca(ICCV2/PI489777)SNP_13064	2103	G	A	19	6	--	--
	Ca(ICCV2/PI489777)SNP_13065	2298	G	A	26	12	--	--
	Ca(ICCV2/PI489777)SNP_13066	2493	T	C	26	12	--	--
	Ca(ICCV2/PI489777)SNP_13067	2760	C	T	10	6	--	--
	Ca(ICCV2/PI489777)SNP_13068	3003	T	C	10	9	--	--
	Ca(ICCV2/PI489777)SNP_13069	3045	C	T	8	9	--	--
	Ca(ICCV2/PI489777)SNP_13070	3162	G	A	5	4	--	--
CakTC29897	Ca(ICCV2/PI489777)SNP_13071	541	T	C	6	4	--	--
	Ca(ICCV2/PI489777)SNP_13072	597	T	C	5	4	--	--
	Ca(ICCV2/PI489777)SNP_13073	648	A	G	6	4	--	--
	Ca(ICCV2/PI489777)SNP_13074	815	C	G	7	5	--	--
	Ca(ICCV2/PI489777)SNP_13075	962	G	A	9	4	--	--
	Ca(ICCV2/PI489777)SNP_13076	1155	G	A	22	9	--	--
	Ca(ICCV2/PI489777)SNP_13077	1467	A	G	16	11	--	--
	Ca(ICCV2/PI489777)SNP_13078	1496	A	G	15	8	--	--
CakTC29242	Ca(ICCV2/PI489777)SNP_13079	561	C	T	4	5	--	--
	Ca(ICCV2/PI489777)SNP_13080	951	C	T	7	4	--	--
	Ca(ICCV2/PI489777)SNP_13081	1248	G	A	28	5	--	--
	Ca(ICCV2/PI489777)SNP_13082	1293	C	T	30	5	--	--
	Ca(ICCV2/PI489777)SNP_13083	1380	T	C	30	10	--	--
	Ca(ICCV2/PI489777)SNP_13084	1419	T	C	28	13	--	--
CakTC10356	Ca(ICCV2/PI489777)SNP_13085	422	T	G	22	15	--	TPR
	Ca(ICCV2/PI489777)SNP_13086	434	C	T	22	17	--	TPR
	Ca(ICCV2/PI489777)SNP_13087	488	T	C	21	17	--	TPR
	Ca(ICCV2/PI489777)SNP_13088	683	T	C	12	7	--	TPR
CakTC33481	Ca(ICCV2/PI489777)SNP_13089	36	T	A	21	29	--	--
	Ca(ICCV2/PI489777)SNP_13090	144	A	G	26	46	--	--
CakTC10676	Ca(ICCV2/PI489777)SNP_13091	334	G	A	12	5	--	HB
	Ca(ICCV2/PI489777)SNP_13092	363	T	C	12	5	--	HB
CakTC25313	Ca(ICCV2/PI489777)SNP_13093	344	T	C	10	9	--	--

CakTC38601	Ca(ICCV2/PI489777)SNP_13094	1223	A	G	36	22	--	--
	Ca(ICCV2/PI489777)SNP_13095	1565	A	C	3	3	--	--
CakTC38418	Ca(ICCV2/PI489777)SNP_13096	468	C	A	38	22	--	--
CakTC24087	Ca(ICCV2/PI489777)SNP_13097	106	A	T	7	3	Flower	bud
	Ca(ICCV2/PI489777)SNP_13098	135	G	T	7	4	Flower	bud
CakTC39676	Ca(ICCV2/PI489777)SNP_13099	880	G	C	8	4	Root	--
	Ca(ICCV2/PI489777)SNP_13100	1395	A	G	3	6	Root	--
	Ca(ICCV2/PI489777)SNP_13101	1474	T	G	4	4	Root	--
CakTC39931	Ca(ICCV2/PI489777)SNP_13102	815	C	T	3	5	--	--
	Ca(ICCV2/PI489777)SNP_13103	843	G	A	3	5	--	--
	Ca(ICCV2/PI489777)SNP_13104	879	T	C	3	3	--	--
	Ca(ICCV2/PI489777)SNP_13105	1005	G	A	5	3	--	--
CakTC22261	Ca(ICCV2/PI489777)SNP_13106	668	G	A	5	4	--	--
	Ca(ICCV2/PI489777)SNP_13107	1805	A	G	5	5	--	--
	Ca(ICCV2/PI489777)SNP_13108	2045	T	G	8	6	--	--
	Ca(ICCV2/PI489777)SNP_13109	2084	A	G	8	5	--	--
	Ca(ICCV2/PI489777)SNP_13110	3005	C	T	4	3	--	--
CakTC40214	Ca(ICCV2/PI489777)SNP_13111	204	A	G	22	7	--	--
	Ca(ICCV2/PI489777)SNP_13112	216	C	A	28	6	--	--
	Ca(ICCV2/PI489777)SNP_13113	246	A	G	29	13	--	--
	Ca(ICCV2/PI489777)SNP_13114	250	C	A	30	12	--	--
	Ca(ICCV2/PI489777)SNP_13115	294	C	T	28	12	--	--
	Ca(ICCV2/PI489777)SNP_13116	309	T	A	33	14	--	--
	Ca(ICCV2/PI489777)SNP_13117	740	G	A	78	36	--	--
	Ca(ICCV2/PI489777)SNP_13118	782	C	G	89	37	--	--
	Ca(ICCV2/PI489777)SNP_13119	1865	G	T	8	6	--	--
	Ca(ICCV2/PI489777)SNP_13120	1913	T	A	7	4	--	--
	Ca(ICCV2/PI489777)SNP_13121	2129	G	A	12	6	--	--
	Ca(ICCV2/PI489777)SNP_13122	2282	T	C	9	3	--	--
CakTC33538	Ca(ICCV2/PI489777)SNP_13123	324	A	G	4	3	--	--
CakTC13185	Ca(ICCV2/PI489777)SNP_13124	241	A	T	43	6	--	--
CakTC20793	Ca(ICCV2/PI489777)SNP_13125	270	G	C	10	7	--	--
	Ca(ICCV2/PI489777)SNP_13126	2281	A	G	11	4	--	--
CakTC23982	Ca(ICCV2/PI489777)SNP_13127	1273	G	A	25	20	--	--
CakTC34404	Ca(ICCV2/PI489777)SNP_13128	132	C	T	9	4	--	--
	Ca(ICCV2/PI489777)SNP_13129	212	G	C	17	13	--	--
	Ca(ICCV2/PI489777)SNP_13130	510	C	T	17	13	--	--
CakTC30010	Ca(ICCV2/PI489777)SNP_13131	717	C	T	7	3	--	--
	Ca(ICCV2/PI489777)SNP_13132	1017	C	T	5	4	--	--
	Ca(ICCV2/PI489777)SNP_13133	1095	G	A	4	4	--	--
	Ca(ICCV2/PI489777)SNP_13134	1215	A	G	6	4	--	--
CakTC42172	Ca(ICCV2/PI489777)SNP_13135	257	C	G	12	3	--	--
	Ca(ICCV2/PI489777)SNP_13136	598	A	T	12	7	--	--
	Ca(ICCV2/PI489777)SNP_13137	655	T	A	12	7	--	--
	Ca(ICCV2/PI489777)SNP_13138	667	T	C	14	6	--	--
	Ca(ICCV2/PI489777)SNP_13139	727	A	T	12	6	--	--
	Ca(ICCV2/PI489777)SNP_13140	809	G	A	16	4	--	--
	Ca(ICCV2/PI489777)SNP_13141	856	C	A	10	3	--	--
	Ca(ICCV2/PI489777)SNP_13142	1339	C	T	22	6	--	--
	Ca(ICCV2/PI489777)SNP_13143	1379	C	G	22	5	--	--
	Ca(ICCV2/PI489777)SNP_13144	1459	A	G	19	3	--	--
	Ca(ICCV2/PI489777)SNP_13145	1705	A	G	14	3	--	--
CakTC39396	Ca(ICCV2/PI489777)SNP_13146	1193	C	A	7	5	--	--
	Ca(ICCV2/PI489777)SNP_13147	1273	A	C	7	5	--	--
CakTC26369	Ca(ICCV2/PI489777)SNP_13148	168	T	A	3	3	--	--
	Ca(ICCV2/PI489777)SNP_13149	1185	C	T	4	11	--	--
CakTC33150	Ca(ICCV2/PI489777)SNP_13150	1267	C	T	13	4	--	--
CakTC24683	Ca(ICCV2/PI489777)SNP_13151	249	A	G	3	4	--	--
CakTC27836	Ca(ICCV2/PI489777)SNP_13152	2184	G	A	12	6	--	--
	Ca(ICCV2/PI489777)SNP_13153	2481	G	A	9	9	--	--
CakTC39501	Ca(ICCV2/PI489777)SNP_13154	196	A	G	26	27	--	--
CakTC43186	Ca(ICCV2/PI489777)SNP_13155	223	C	T	34	14	--	--
	Ca(ICCV2/PI489777)SNP_13156	296	C	T	37	12	--	--
CakTC29659	Ca(ICCV2/PI489777)SNP_13157	388	G	T	3	3	--	--
	Ca(ICCV2/PI489777)SNP_13158	523	A	G	3	3	--	--
	Ca(ICCV2/PI489777)SNP_13159	528	G	A	3	3	--	--
CakTC25369	Ca(ICCV2/PI489777)SNP_13160	666	T	C	5	3	--	--
	Ca(ICCV2/PI489777)SNP_13161	1307	G	T	14	20	--	--
CakTC38473	Ca(ICCV2/PI489777)SNP_13162	105	G	C	8	5	--	--
	Ca(ICCV2/PI489777)SNP_13163	279	T	C	18	19	--	--
	Ca(ICCV2/PI489777)SNP_13164	592	T	C	32	22	--	--
	Ca(ICCV2/PI489777)SNP_13165	616	T	C	38	21	--	--
	Ca(ICCV2/PI489777)SNP_13166	646	C	T	39	25	--	--
	Ca(ICCV2/PI489777)SNP_13167	1198	G	A	46	49	--	--

	Ca(ICCV2/PI489777)SNP_13168	1303	G	A	47	44	--	--
	Ca(ICCV2/PI489777)SNP_13169	1409	T	A	33	40	--	--
	Ca(ICCV2/PI489777)SNP_13170	1489	A	G	33	31	--	--
	Ca(ICCV2/PI489777)SNP_13171	1531	C	T	30	26	--	--
	Ca(ICCV2/PI489777)SNP_13172	1585	G	C	22	22	--	--
CakTC33206	Ca(ICCV2/PI489777)SNP_13173	561	T	G	37	25	--	--
	Ca(ICCV2/PI489777)SNP_13174	1507	G	A	14	17	--	--
CakTC32979	Ca(ICCV2/PI489777)SNP_13175	65	T	A	4	3	--	--
	Ca(ICCV2/PI489777)SNP_13176	516	T	G	9	7	--	--
CakTC41106	Ca(ICCV2/PI489777)SNP_13177	1069	C	G	10	7	--	--
	Ca(ICCV2/PI489777)SNP_13178	2070	T	C	9	5	--	--
CakTC28054	Ca(ICCV2/PI489777)SNP_13179	1236	A	G	25	9	--	--
	Ca(ICCV2/PI489777)SNP_13180	1566	T	C	17	8	--	--
CakTC23053	Ca(ICCV2/PI489777)SNP_13181	374	T	C	208	141	--	--
CakTC39765	Ca(ICCV2/PI489777)SNP_13182	258	C	A	31	29	--	--
	Ca(ICCV2/PI489777)SNP_13183	480	T	C	39	35	--	--
	Ca(ICCV2/PI489777)SNP_13184	1317	T	C	46	20	--	--
CakTC20155	Ca(ICCV2/PI489777)SNP_13185	108	C	G	11	3	--	--
	Ca(ICCV2/PI489777)SNP_13186	262	G	A	32	14	--	--
	Ca(ICCV2/PI489777)SNP_13187	800	C	A	30	5	--	--
CakTC41649	Ca(ICCV2/PI489777)SNP_13188	471	T	C	3	5	Shoot	--
	Ca(ICCV2/PI489777)SNP_13189	595	T	G	8	8	Shoot	--
	Ca(ICCV2/PI489777)SNP_13190	625	G	T	8	8	Shoot	--
	Ca(ICCV2/PI489777)SNP_13191	967	C	T	9	8	Shoot	--
	Ca(ICCV2/PI489777)SNP_13192	970	C	T	8	8	Shoot	--
	Ca(ICCV2/PI489777)SNP_13193	1039	T	C	9	11	Shoot	--
	Ca(ICCV2/PI489777)SNP_13194	1201	C	A	15	9	Shoot	--
	Ca(ICCV2/PI489777)SNP_13195	1427	T	C	8	3	Shoot	--
	Ca(ICCV2/PI489777)SNP_13196	1497	G	A	10	3	Shoot	--
	Ca(ICCV2/PI489777)SNP_13197	1502	A	G	13	3	Shoot	--
	Ca(ICCV2/PI489777)SNP_13198	1775	A	G	15	14	Shoot	--
	Ca(ICCV2/PI489777)SNP_13199	1851	G	A	14	9	Shoot	--
	Ca(ICCV2/PI489777)SNP_13200	1947	A	G	14	10	Shoot	--
	Ca(ICCV2/PI489777)SNP_13201	1962	T	A	14	8	Shoot	--
	Ca(ICCV2/PI489777)SNP_13202	2102	A	T	8	6	Shoot	--
CakTC27318	Ca(ICCV2/PI489777)SNP_13203	159	A	G	9	6	Shoot	--
	Ca(ICCV2/PI489777)SNP_13204	520	G	T	10	3	Shoot	--
	Ca(ICCV2/PI489777)SNP_13205	750	T	C	6	4	Shoot	--
	Ca(ICCV2/PI489777)SNP_13206	804	G	T	5	4	Shoot	--
	Ca(ICCV2/PI489777)SNP_13207	837	C	T	5	4	Shoot	--
CakTC30745	Ca(ICCV2/PI489777)SNP_13208	829	G	T	36	13	--	--
CakTC43310	Ca(ICCV2/PI489777)SNP_13209	326	T	G	9	28	--	--
	Ca(ICCV2/PI489777)SNP_13210	509	G	T	8	31	--	--
CakTC28103	Ca(ICCV2/PI489777)SNP_13211	106	T	C	4	6	--	--
	Ca(ICCV2/PI489777)SNP_13212	171	G	A	6	6	--	--
	Ca(ICCV2/PI489777)SNP_13213	1377	T	C	61	47	--	--
	Ca(ICCV2/PI489777)SNP_13214	1437	G	A	63	42	--	--
	Ca(ICCV2/PI489777)SNP_13215	2709	C	A	39	23	--	--
	Ca(ICCV2/PI489777)SNP_13216	3426	C	G	53	24	--	--
	Ca(ICCV2/PI489777)SNP_13217	3636	A	G	57	23	--	--
	Ca(ICCV2/PI489777)SNP_13218	3711	C	T	47	18	--	--
	Ca(ICCV2/PI489777)SNP_13219	3795	A	T	60	21	--	--
	Ca(ICCV2/PI489777)SNP_13220	3828	C	T	43	18	--	--
	Ca(ICCV2/PI489777)SNP_13221	4158	A	G	20	15	--	--
	Ca(ICCV2/PI489777)SNP_13222	5297	G	T	26	19	--	--
CakTC38480	Ca(ICCV2/PI489777)SNP_13223	305	C	T	36	16	--	--
	Ca(ICCV2/PI489777)SNP_13224	1021	T	A	12	13	--	--
CakTC30116	Ca(ICCV2/PI489777)SNP_13225	273	A	G	10	6	--	--
	Ca(ICCV2/PI489777)SNP_13226	710	G	A	9	3	--	--
	Ca(ICCV2/PI489777)SNP_13227	1584	T	A	15	4	--	--
	Ca(ICCV2/PI489777)SNP_13228	1784	C	T	7	4	--	--
	Ca(ICCV2/PI489777)SNP_13229	1880	G	A	4	3	--	--
CakTC40154	Ca(ICCV2/PI489777)SNP_13230	529	G	C	15	7	--	--
	Ca(ICCV2/PI489777)SNP_13231	639	C	T	14	9	--	--
	Ca(ICCV2/PI489777)SNP_13232	726	G	A	15	10	--	--
	Ca(ICCV2/PI489777)SNP_13233	834	C	T	12	10	--	--
	Ca(ICCV2/PI489777)SNP_13234	843	A	G	15	10	--	--
	Ca(ICCV2/PI489777)SNP_13235	940	C	A	15	13	--	--
	Ca(ICCV2/PI489777)SNP_13236	963	C	T	13	13	--	--
CakTC26049	Ca(ICCV2/PI489777)SNP_13237	244	C	G	8	4	--	--
	Ca(ICCV2/PI489777)SNP_13238	539	C	T	21	7	--	--
	Ca(ICCV2/PI489777)SNP_13239	3042	C	T	14	6	--	--
	Ca(ICCV2/PI489777)SNP_13240	3199	A	G	11	7	--	--
	Ca(ICCV2/PI489777)SNP_13241	3277	T	C	16	6	--	--

CakTC37242	Ca(ICCV2/PI489777)SNP_13242	39	A	C	11	7	--	--
	Ca(ICCV2/PI489777)SNP_13243	170	A	T	13	13	--	--
	Ca(ICCV2/PI489777)SNP_13244	196	G	C	14	15	--	--
	Ca(ICCV2/PI489777)SNP_13245	236	T	A	16	19	--	--
CakTC09924	Ca(ICCV2/PI489777)SNP_13246	1346	C	T	3	4	--	TPR
CakTC41046	Ca(ICCV2/PI489777)SNP_13247	276	G	T	13	7	--	--
	Ca(ICCV2/PI489777)SNP_13248	308	T	G	12	8	--	--
	Ca(ICCV2/PI489777)SNP_13249	310	A	T	12	7	--	--
	Ca(ICCV2/PI489777)SNP_13250	561	A	C	21	5	--	--
CakTC35631	Ca(ICCV2/PI489777)SNP_13251	1066	C	A	5	3	--	--
CakTC42776	Ca(ICCV2/PI489777)SNP_13252	323	A	G	42	14	--	--
	Ca(ICCV2/PI489777)SNP_13253	395	G	A	44	15	--	--
	Ca(ICCV2/PI489777)SNP_13254	604	C	T	33	13	--	--
CakTC37567	Ca(ICCV2/PI489777)SNP_13255	56	T	A	7	9	--	--
	Ca(ICCV2/PI489777)SNP_13256	87	A	T	6	8	--	--
	Ca(ICCV2/PI489777)SNP_13257	97	C	T	7	7	--	--
	Ca(ICCV2/PI489777)SNP_13258	582	A	G	16	26	--	--
	Ca(ICCV2/PI489777)SNP_13259	1124	C	T	20	26	--	--
CakTC30554	Ca(ICCV2/PI489777)SNP_13260	609	A	C	4	3	--	--
CakTC39817	Ca(ICCV2/PI489777)SNP_13261	42	T	A	3	5	--	--
	Ca(ICCV2/PI489777)SNP_13262	59	G	A	8	6	--	--
	Ca(ICCV2/PI489777)SNP_13263	87	T	G	16	6	--	--
	Ca(ICCV2/PI489777)SNP_13264	418	A	G	35	16	--	--
	Ca(ICCV2/PI489777)SNP_13265	532	G	A	29	17	--	--
	Ca(ICCV2/PI489777)SNP_13266	616	C	A	30	10	--	--
	Ca(ICCV2/PI489777)SNP_13267	763	C	T	17	6	--	--
CakTC41378	Ca(ICCV2/PI489777)SNP_13268	2555	A	G	3	3	--	--
CakTC31468	Ca(ICCV2/PI489777)SNP_13269	401	T	C	30	7	--	--
	Ca(ICCV2/PI489777)SNP_13270	827	T	C	10	7	--	--
	Ca(ICCV2/PI489777)SNP_13271	1091	C	T	9	11	--	--
	Ca(ICCV2/PI489777)SNP_13272	1133	G	A	12	13	--	--
	Ca(ICCV2/PI489777)SNP_13273	1466	A	G	27	12	--	--
	Ca(ICCV2/PI489777)SNP_13274	1535	T	A	31	10	--	--
	Ca(ICCV2/PI489777)SNP_13275	2195	T	C	5	4	--	--
CakTC40812	Ca(ICCV2/PI489777)SNP_13276	625	T	C	3	16	--	--
CakTC30935	Ca(ICCV2/PI489777)SNP_13277	4475	T	C	7	3	--	Alfin-like
CakTC27242	Ca(ICCV2/PI489777)SNP_13278	874	A	G	27	4	--	--
	Ca(ICCV2/PI489777)SNP_13279	1603	A	G	3	7	--	--
	Ca(ICCV2/PI489777)SNP_13280	1637	C	T	4	9	--	--
	Ca(ICCV2/PI489777)SNP_13281	1696	A	G	4	9	--	--
	Ca(ICCV2/PI489777)SNP_13282	1698	G	C	4	9	--	--
CakTC27310	Ca(ICCV2/PI489777)SNP_13283	56	G	A	6	3	--	--
	Ca(ICCV2/PI489777)SNP_13284	269	C	T	26	13	--	--
	Ca(ICCV2/PI489777)SNP_13285	1217	G	A	10	12	--	--
	Ca(ICCV2/PI489777)SNP_13286	1457	G	A	4	5	--	--
	Ca(ICCV2/PI489777)SNP_13287	1463	A	G	4	5	--	--
CakTC24804	Ca(ICCV2/PI489777)SNP_13288	737	C	G	70	3	--	--
CakTC36193	Ca(ICCV2/PI489777)SNP_13289	156	A	G	3	5	--	--
	Ca(ICCV2/PI489777)SNP_13290	449	G	A	4	5	--	--
CakTC28122	Ca(ICCV2/PI489777)SNP_13291	637	T	C	3	4	--	--
	Ca(ICCV2/PI489777)SNP_13292	976	C	T	6	4	--	--
CakTC30267	Ca(ICCV2/PI489777)SNP_13293	1082	A	G	4	4	Flower	bud
CakTC24982	Ca(ICCV2/PI489777)SNP_13294	1893	C	T	34	9	--	--
	Ca(ICCV2/PI489777)SNP_13295	1929	C	T	41	10	--	--
	Ca(ICCV2/PI489777)SNP_13296	1992	A	G	32	12	--	--
	Ca(ICCV2/PI489777)SNP_13297	2362	T	C	26	9	--	--
CakTC31745	Ca(ICCV2/PI489777)SNP_13298	1054	C	T	7	6	--	FAR1
CakTC39238	Ca(ICCV2/PI489777)SNP_13299	1509	C	T	4	10	--	--
CakTC37963	Ca(ICCV2/PI489777)SNP_13300	232	A	G	6	3	--	--
CakTC28915	Ca(ICCV2/PI489777)SNP_13301	599	A	G	54	26	--	--
CakTC22893	Ca(ICCV2/PI489777)SNP_13302	223	G	A	15	10	--	--
	Ca(ICCV2/PI489777)SNP_13303	709	T	A	10	9	--	--
	Ca(ICCV2/PI489777)SNP_13304	778	A	T	6	8	--	--
	Ca(ICCV2/PI489777)SNP_13305	1054	T	C	7	12	--	--
	Ca(ICCV2/PI489777)SNP_13306	1184	T	C	5	6	--	--
CakTC36961	Ca(ICCV2/PI489777)SNP_13307	1588	C	T	7	9	--	--
CakTC42460	Ca(ICCV2/PI489777)SNP_13308	988	G	T	13	4	--	--
	Ca(ICCV2/PI489777)SNP_13309	1108	T	C	15	4	--	--
CakTC41393	Ca(ICCV2/PI489777)SNP_13310	101	T	C	7	7	--	--
	Ca(ICCV2/PI489777)SNP_13311	126	C	T	7	7	--	--
	Ca(ICCV2/PI489777)SNP_13312	154	T	C	6	7	--	--
	Ca(ICCV2/PI489777)SNP_13313	1337	A	T	4	8	--	--
	Ca(ICCV2/PI489777)SNP_13314	1903	G	A	11	7	--	--
	Ca(ICCV2/PI489777)SNP_13315	1992	T	C	10	4	--	--

	Ca(ICCV2/PI489777)SNP_13316	2188	C	A	6	4	--	--
CakTC32120	Ca(ICCV2/PI489777)SNP_13317	325	A	C	8	3	--	--
	Ca(ICCV2/PI489777)SNP_13318	835	C	T	14	7	--	--
	Ca(ICCV2/PI489777)SNP_13319	1438	T	A	7	3	--	--
CakTC28446	Ca(ICCV2/PI489777)SNP_13320	300	G	A	8	7	--	--
	Ca(ICCV2/PI489777)SNP_13321	357	T	A	15	7	--	--
	Ca(ICCV2/PI489777)SNP_13322	831	A	G	25	8	--	--
	Ca(ICCV2/PI489777)SNP_13323	1023	T	A	22	3	--	--
	Ca(ICCV2/PI489777)SNP_13324	1150	A	G	14	8	--	--
	Ca(ICCV2/PI489777)SNP_13325	1569	G	A	5	4	--	--
CakTC31223	Ca(ICCV2/PI489777)SNP_13326	76	A	G	12	33	--	--
	Ca(ICCV2/PI489777)SNP_13327	93	A	T	10	34	--	--
CakTC33727	Ca(ICCV2/PI489777)SNP_13328	1553	C	T	11	3	--	C3H
	Ca(ICCV2/PI489777)SNP_13329	2491	T	C	18	3	--	C3H
CakTC37869	Ca(ICCV2/PI489777)SNP_13330	172	T	C	3	5	Shoot	--
	Ca(ICCV2/PI489777)SNP_13331	446	C	A	8	3	Shoot	--
CakTC11458	Ca(ICCV2/PI489777)SNP_13332	1380	T	C	8	3	--	--
CakTC38662	Ca(ICCV2/PI489777)SNP_13333	228	T	C	11	10	--	FHA
	Ca(ICCV2/PI489777)SNP_13334	477	G	T	9	7	--	FHA
	Ca(ICCV2/PI489777)SNP_13335	1351	A	G	20	11	--	FHA
CakTC37195	Ca(ICCV2/PI489777)SNP_13336	184	C	T	11	33	--	--
	Ca(ICCV2/PI489777)SNP_13337	195	G	T	11	34	--	--
	Ca(ICCV2/PI489777)SNP_13338	928	C	G	11	6	--	--
	Ca(ICCV2/PI489777)SNP_13339	963	T	G	11	6	--	--
CakTC34862	Ca(ICCV2/PI489777)SNP_13340	612	A	G	3	3	--	--
CakTC29924	Ca(ICCV2/PI489777)SNP_13341	1881	A	G	11	3	Root	--
	Ca(ICCV2/PI489777)SNP_13342	2030	C	T	10	3	Root	--
CakTC29802	Ca(ICCV2/PI489777)SNP_13343	593	G	A	13	16	--	--
	Ca(ICCV2/PI489777)SNP_13344	878	C	T	10	5	--	--
CakTC41947	Ca(ICCV2/PI489777)SNP_13345	239	T	C	7	5	--	--
	Ca(ICCV2/PI489777)SNP_13346	345	G	T	9	6	--	--
	Ca(ICCV2/PI489777)SNP_13347	352	A	G	8	7	--	--
CakTC25249	Ca(ICCV2/PI489777)SNP_13348	1196	A	C	28	4	--	--
	Ca(ICCV2/PI489777)SNP_13349	1250	A	T	25	4	--	--
CakTC35725	Ca(ICCV2/PI489777)SNP_13350	42	G	T	17	9	--	--
	Ca(ICCV2/PI489777)SNP_13351	132	G	T	22	15	--	--
	Ca(ICCV2/PI489777)SNP_13352	557	T	C	39	19	--	--
	Ca(ICCV2/PI489777)SNP_13353	1034	G	T	39	19	--	--
	Ca(ICCV2/PI489777)SNP_13354	1166	T	C	38	19	--	--
	Ca(ICCV2/PI489777)SNP_13355	1304	T	G	31	24	--	--
	Ca(ICCV2/PI489777)SNP_13356	1887	C	T	49	13	--	--
	Ca(ICCV2/PI489777)SNP_13357	2219	T	C	37	16	--	--
	Ca(ICCV2/PI489777)SNP_13358	3506	C	A	22	13	--	--
CakTC37768	Ca(ICCV2/PI489777)SNP_13359	120	G	A	3	4	Young_pod	--
	Ca(ICCV2/PI489777)SNP_13360	782	C	A	4	7	Young_pod	--
	Ca(ICCV2/PI489777)SNP_13361	817	A	G	3	8	Young_pod	--
CakTC38046	Ca(ICCV2/PI489777)SNP_13362	832	G	A	7	3	--	--
	Ca(ICCV2/PI489777)SNP_13363	1110	C	G	3	6	--	--
	Ca(ICCV2/PI489777)SNP_13364	1115	T	C	3	6	--	--
CakTC41343	Ca(ICCV2/PI489777)SNP_13365	226	C	T	7	5	--	MYB
	Ca(ICCV2/PI489777)SNP_13366	1696	G	A	13	7	--	MYB
	Ca(ICCV2/PI489777)SNP_13367	2354	T	C	20	8	--	MYB
CakTC28668	Ca(ICCV2/PI489777)SNP_13368	553	G	A	4	4	--	--
CakTC33055	Ca(ICCV2/PI489777)SNP_13369	334	G	A	14	7	--	--
	Ca(ICCV2/PI489777)SNP_13370	433	C	T	12	5	--	--
CakTC40515	Ca(ICCV2/PI489777)SNP_13371	656	T	C	40	36	--	--
CakTC32114	Ca(ICCV2/PI489777)SNP_13372	1259	A	G	5	6	--	--
CakTC12287	Ca(ICCV2/PI489777)SNP_13373	614	C	T	5	4	--	--
CakTC38824	Ca(ICCV2/PI489777)SNP_13374	930	T	C	31	8	--	--
	Ca(ICCV2/PI489777)SNP_13375	1141	C	T	27	6	--	--
CakTC41794	Ca(ICCV2/PI489777)SNP_13376	194	T	C	10	7	Root	--
	Ca(ICCV2/PI489777)SNP_13377	718	A	C	8	16	Root	--
	Ca(ICCV2/PI489777)SNP_13378	1770	C	T	6	6	Root	--
CakTC35717	Ca(ICCV2/PI489777)SNP_13379	625	C	T	13	3	--	--
CakTC24738	Ca(ICCV2/PI489777)SNP_13380	1255	G	C	9	3	--	--
	Ca(ICCV2/PI489777)SNP_13381	1317	C	T	11	3	--	--
CakTC38342	Ca(ICCV2/PI489777)SNP_13382	552	G	A	6	8	--	G2-like
	Ca(ICCV2/PI489777)SNP_13383	968	A	G	12	6	--	G2-like
CakTC13459	Ca(ICCV2/PI489777)SNP_13384	528	G	A	4	8	--	--
	Ca(ICCV2/PI489777)SNP_13385	573	A	T	8	8	--	--
CakTC27399	Ca(ICCV2/PI489777)SNP_13386	802	T	C	23	14	--	--
	Ca(ICCV2/PI489777)SNP_13387	1555	A	C	25	9	--	--
	Ca(ICCV2/PI489777)SNP_13388	2241	T	C	20	13	--	--
	Ca(ICCV2/PI489777)SNP_13389	2279	A	G	18	13	--	--

	Ca(ICCV2/PI489777)SNP_13390	2348	G	A	15	14	--	--
	Ca(ICCV2/PI489777)SNP_13391	2507	C	A	9	7	--	--
CakTC32825	Ca(ICCV2/PI489777)SNP_13392	682	T	A	6	4	--	--
	Ca(ICCV2/PI489777)SNP_13393	2122	C	T	7	6	--	--
	Ca(ICCV2/PI489777)SNP_13394	2688	T	C	9	3	--	--
CakTC10709	Ca(ICCV2/PI489777)SNP_13395	794	G	A	3	6	--	--
	Ca(ICCV2/PI489777)SNP_13396	807	T	G	3	6	--	--
	Ca(ICCV2/PI489777)SNP_13397	860	T	C	3	7	--	--
	Ca(ICCV2/PI489777)SNP_13398	869	T	G	3	8	--	--
	Ca(ICCV2/PI489777)SNP_13399	871	G	C	3	8	--	--
	Ca(ICCV2/PI489777)SNP_13400	895	A	G	3	10	--	--
	Ca(ICCV2/PI489777)SNP_13401	896	C	T	3	11	--	--
	Ca(ICCV2/PI489777)SNP_13402	926	T	C	3	13	--	--
	Ca(ICCV2/PI489777)SNP_13403	940	A	T	3	8	--	--
	Ca(ICCV2/PI489777)SNP_13404	950	T	C	3	11	--	--
	Ca(ICCV2/PI489777)SNP_13405	955	T	C	3	13	--	--
	Ca(ICCV2/PI489777)SNP_13406	965	T	C	3	13	--	--
	Ca(ICCV2/PI489777)SNP_13407	1960	A	G	3	9	--	--
CakTC09756	Ca(ICCV2/PI489777)SNP_13408	183	A	G	6	5	--	--
	Ca(ICCV2/PI489777)SNP_13409	221	G	A	7	10	--	--
	Ca(ICCV2/PI489777)SNP_13410	416	A	T	8	9	--	--
	Ca(ICCV2/PI489777)SNP_13411	425	G	A	8	9	--	--
	Ca(ICCV2/PI489777)SNP_13412	1185	G	C	139	66	--	--
CakTC09490	Ca(ICCV2/PI489777)SNP_13413	165	A	T	3	4	--	--
	Ca(ICCV2/PI489777)SNP_13414	179	A	T	3	4	--	--
	Ca(ICCV2/PI489777)SNP_13415	208	A	G	3	4	--	--
	Ca(ICCV2/PI489777)SNP_13416	220	T	A	3	4	--	--
	Ca(ICCV2/PI489777)SNP_13417	288	T	C	5	4	--	--
	Ca(ICCV2/PI489777)SNP_13418	394	C	T	6	5	--	--
	Ca(ICCV2/PI489777)SNP_13419	400	A	T	6	6	--	--
	Ca(ICCV2/PI489777)SNP_13420	412	A	G	6	6	--	--
	Ca(ICCV2/PI489777)SNP_13421	451	G	A	5	5	--	--
	Ca(ICCV2/PI489777)SNP_13422	553	G	A	5	4	--	--
	Ca(ICCV2/PI489777)SNP_13423	571	A	C	6	4	--	--
	Ca(ICCV2/PI489777)SNP_13424	691	G	A	8	3	--	--
CakTC33848	Ca(ICCV2/PI489777)SNP_13425	400	C	G	3	7	Flower	bud
	Ca(ICCV2/PI489777)SNP_13426	428	T	C	4	7	Flower	bud
CakTC29083	Ca(ICCV2/PI489777)SNP_13427	288	C	A	10	3	--	--
CakTC24608	Ca(ICCV2/PI489777)SNP_13428	1628	A	G	7	5	--	--
	Ca(ICCV2/PI489777)SNP_13429	2073	A	G	7	4	--	--
CakTC27455	Ca(ICCV2/PI489777)SNP_13430	243	G	T	5	3	--	--
	Ca(ICCV2/PI489777)SNP_13431	1489	G	A	9	4	--	--
	Ca(ICCV2/PI489777)SNP_13432	1646	C	G	9	3	--	--
	Ca(ICCV2/PI489777)SNP_13433	2233	T	C	8	3	--	--
	Ca(ICCV2/PI489777)SNP_13434	3465	A	C	12	6	--	--
	Ca(ICCV2/PI489777)SNP_13435	3466	C	A	12	6	--	--
	Ca(ICCV2/PI489777)SNP_13436	3645	T	C	9	7	--	--
	Ca(ICCV2/PI489777)SNP_13437	3860	G	A	9	3	--	--
	Ca(ICCV2/PI489777)SNP_13438	3934	C	T	8	4	--	--
	Ca(ICCV2/PI489777)SNP_13439	4197	G	A	3	3	--	--
CakTC36012	Ca(ICCV2/PI489777)SNP_13440	132	T	A	9	6	--	--
	Ca(ICCV2/PI489777)SNP_13441	348	T	A	8	5	--	--
CakTC24588	Ca(ICCV2/PI489777)SNP_13442	352	C	G	30	5	--	--
	Ca(ICCV2/PI489777)SNP_13443	379	T	C	32	4	--	--
	Ca(ICCV2/PI489777)SNP_13444	770	A	T	252	25	--	--
CakTC42393	Ca(ICCV2/PI489777)SNP_13445	520	C	T	50	61	--	--
CakTC42139	Ca(ICCV2/PI489777)SNP_13446	711	C	T	39	5	--	--
CakTC09711	Ca(ICCV2/PI489777)SNP_13447	350	A	G	7	6	--	--
	Ca(ICCV2/PI489777)SNP_13448	599	T	C	6	3	--	--
CakTC38196	Ca(ICCV2/PI489777)SNP_13449	107	C	T	12	5	--	--
	Ca(ICCV2/PI489777)SNP_13450	126	G	A	13	6	--	--
	Ca(ICCV2/PI489777)SNP_13451	218	C	G	14	11	--	--
	Ca(ICCV2/PI489777)SNP_13452	379	A	G	24	25	--	--
	Ca(ICCV2/PI489777)SNP_13453	892	A	C	14	16	--	--
	Ca(ICCV2/PI489777)SNP_13454	1111	A	G	26	12	--	--
	Ca(ICCV2/PI489777)SNP_13455	1288	A	G	27	23	--	--
	Ca(ICCV2/PI489777)SNP_13456	1312	C	T	26	23	--	--
CakTC24745	Ca(ICCV2/PI489777)SNP_13457	493	G	A	5	5	--	--
	Ca(ICCV2/PI489777)SNP_13458	721	T	G	10	6	--	--
	Ca(ICCV2/PI489777)SNP_13459	1072	C	G	12	3	--	--
	Ca(ICCV2/PI489777)SNP_13460	1102	T	C	8	3	--	--
	Ca(ICCV2/PI489777)SNP_13461	1183	A	G	9	5	--	--
	Ca(ICCV2/PI489777)SNP_13462	1438	C	T	8	10	--	--
	Ca(ICCV2/PI489777)SNP_13463	4742	C	T	9	6	--	--

	Ca(ICCV2/PI489777)SNP_13464	5039	G	T	5	19	--	--
CakTC36042	Ca(ICCV2/PI489777)SNP_13465	494	T	A	7	5	--	--
	Ca(ICCV2/PI489777)SNP_13466	1754	G	A	10	9	--	--
	Ca(ICCV2/PI489777)SNP_13467	2105	T	A	4	4	--	--
CakTC37792	Ca(ICCV2/PI489777)SNP_13468	1025	G	T	8	10	--	--
	Ca(ICCV2/PI489777)SNP_13469	1304	G	A	15	9	--	--
	Ca(ICCV2/PI489777)SNP_13470	1718	A	G	17	8	--	--
CakTC25841	Ca(ICCV2/PI489777)SNP_13471	1035	G	C	13	7	--	--
CakTC26623	Ca(ICCV2/PI489777)SNP_13472	1647	G	T	8	4	Mature	Leaf
CakTC25376	Ca(ICCV2/PI489777)SNP_13473	139	A	C	3	3	--	--
	Ca(ICCV2/PI489777)SNP_13474	463	C	T	12	11	--	--
	Ca(ICCV2/PI489777)SNP_13475	466	G	A	12	11	--	--
	Ca(ICCV2/PI489777)SNP_13476	1078	C	A	11	3	--	--
	Ca(ICCV2/PI489777)SNP_13477	1087	T	C	9	3	--	--
	Ca(ICCV2/PI489777)SNP_13478	1630	G	A	11	4	--	--
	Ca(ICCV2/PI489777)SNP_13479	1656	G	A	13	5	--	--
	Ca(ICCV2/PI489777)SNP_13480	1700	G	A	14	4	--	--
	Ca(ICCV2/PI489777)SNP_13481	1705	G	T	13	4	--	--
	Ca(ICCV2/PI489777)SNP_13482	1911	T	C	16	4	--	--
CakTC40458	Ca(ICCV2/PI489777)SNP_13483	312	A	G	6	5	--	--
	Ca(ICCV2/PI489777)SNP_13484	362	G	C	7	5	--	--
	Ca(ICCV2/PI489777)SNP_13485	425	C	A	6	4	--	--
CakTC32800	Ca(ICCV2/PI489777)SNP_13486	1435	A	G	5	7	--	--
	Ca(ICCV2/PI489777)SNP_13487	1917	A	G	8	3	--	--
	Ca(ICCV2/PI489777)SNP_13488	2411	G	A	9	3	--	--
CakTC06271	Ca(ICCV2/PI489777)SNP_13489	119	A	C	4	4	--	--
CakTC23070	Ca(ICCV2/PI489777)SNP_13490	838	G	A	62	9	--	HB
	Ca(ICCV2/PI489777)SNP_13491	853	T	C	60	7	--	HB
	Ca(ICCV2/PI489777)SNP_13492	856	G	A	55	7	--	HB
	Ca(ICCV2/PI489777)SNP_13493	2153	T	C	145	23	--	HB
CakTC41390	Ca(ICCV2/PI489777)SNP_13494	679	G	A	12	7	--	--
	Ca(ICCV2/PI489777)SNP_13495	1933	C	T	6	13	--	--
	Ca(ICCV2/PI489777)SNP_13496	1973	T	C	9	13	--	--
	Ca(ICCV2/PI489777)SNP_13497	2268	A	G	6	12	--	--
	Ca(ICCV2/PI489777)SNP_13498	2415	A	G	3	8	--	--
CakTC10843	Ca(ICCV2/PI489777)SNP_13499	57	G	A	24	13	--	--
CakTC31216	Ca(ICCV2/PI489777)SNP_13500	548	G	A	33	8	--	HB
	Ca(ICCV2/PI489777)SNP_13501	563	G	A	37	8	--	HB
	Ca(ICCV2/PI489777)SNP_13502	575	C	T	35	6	--	HB
	Ca(ICCV2/PI489777)SNP_13503	581	A	G	35	6	--	HB
	Ca(ICCV2/PI489777)SNP_13504	629	G	A	35	5	--	HB
	Ca(ICCV2/PI489777)SNP_13505	1514	T	C	17	4	--	HB
CakTC38468	Ca(ICCV2/PI489777)SNP_13506	287	C	T	11	6	--	--
CakTC28755	Ca(ICCV2/PI489777)SNP_13507	1796	C	G	3	3	--	--
CakTC10407	Ca(ICCV2/PI489777)SNP_13508	135	C	T	4	3	--	--
CakTC38902	Ca(ICCV2/PI489777)SNP_13509	53	T	G	26	11	--	--
	Ca(ICCV2/PI489777)SNP_13510	54	T	C	26	11	--	--
	Ca(ICCV2/PI489777)SNP_13511	85	A	C	22	14	--	--
	Ca(ICCV2/PI489777)SNP_13512	1016	G	A	19	7	--	--
CakTC34689	Ca(ICCV2/PI489777)SNP_13513	512	G	A	4	4	--	SNF2
	Ca(ICCV2/PI489777)SNP_13514	2227	C	T	6	3	--	SNF2
	Ca(ICCV2/PI489777)SNP_13515	3064	C	T	5	5	--	SNF2
	Ca(ICCV2/PI489777)SNP_13516	3172	C	T	8	6	--	SNF2
	Ca(ICCV2/PI489777)SNP_13517	3298	T	G	7	5	--	SNF2
CakTC26788	Ca(ICCV2/PI489777)SNP_13518	390	T	A	6	3	--	--
	Ca(ICCV2/PI489777)SNP_13519	406	G	C	7	3	--	--
	Ca(ICCV2/PI489777)SNP_13520	624	T	G	10	3	--	--
	Ca(ICCV2/PI489777)SNP_13521	1131	A	T	13	4	--	--
	Ca(ICCV2/PI489777)SNP_13522	1386	T	C	12	5	--	--
	Ca(ICCV2/PI489777)SNP_13523	1698	T	C	11	8	--	--
CakTC23738	Ca(ICCV2/PI489777)SNP_13524	1315	C	A	4	4	--	--
	Ca(ICCV2/PI489777)SNP_13525	1345	G	C	5	4	--	--
	Ca(ICCV2/PI489777)SNP_13526	1447	C	G	6	3	--	--
	Ca(ICCV2/PI489777)SNP_13527	1477	G	T	6	3	--	--
CakTC39236	Ca(ICCV2/PI489777)SNP_13528	152	A	C	18	12	--	--
	Ca(ICCV2/PI489777)SNP_13529	170	G	A	18	12	--	--
	Ca(ICCV2/PI489777)SNP_13530	1260	G	A	27	14	--	--
CakTC23305	Ca(ICCV2/PI489777)SNP_13531	317	C	T	7	8	--	--
	Ca(ICCV2/PI489777)SNP_13532	422	T	C	9	11	--	--
CakTC37956	Ca(ICCV2/PI489777)SNP_13533	87	C	T	12	8	--	--
	Ca(ICCV2/PI489777)SNP_13534	118	T	A	10	9	--	--
	Ca(ICCV2/PI489777)SNP_13535	251	T	G	16	11	--	--
	Ca(ICCV2/PI489777)SNP_13536	322	C	T	18	12	--	--
	Ca(ICCV2/PI489777)SNP_13537	940	C	A	32	13	--	--

	Ca(ICCV2/PI489777)SNP_13538	978	A	G	38	15	--	--
	Ca(ICCV2/PI489777)SNP_13539	1012	C	T	42	21	--	--
	Ca(ICCV2/PI489777)SNP_13540	1261	T	C	29	18	--	--
CakTC36280	Ca(ICCV2/PI489777)SNP_13541	31	C	T	35	5	--	--
CakTC13205	Ca(ICCV2/PI489777)SNP_13542	261	A	G	39	7	--	--
CakTC25037	Ca(ICCV2/PI489777)SNP_13543	331	C	T	19	6	--	WRKY
	Ca(ICCV2/PI489777)SNP_13544	509	C	G	18	6	--	WRKY
CakTC42961	Ca(ICCV2/PI489777)SNP_13545	188	C	T	3	3	--	--
	Ca(ICCV2/PI489777)SNP_13546	194	T	C	3	3	--	--
	Ca(ICCV2/PI489777)SNP_13547	293	G	A	8	3	--	--
CakTC41229	Ca(ICCV2/PI489777)SNP_13548	918	T	A	23	15	--	--
	Ca(ICCV2/PI489777)SNP_13549	2017	C	T	14	10	--	--
	Ca(ICCV2/PI489777)SNP_13550	2465	A	G	8	6	--	--
	Ca(ICCV2/PI489777)SNP_13551	2494	G	A	7	4	--	--
CakTC30411	Ca(ICCV2/PI489777)SNP_13552	1259	G	A	16	3	Shoot	--
	Ca(ICCV2/PI489777)SNP_13553	1535	G	A	9	5	Shoot	--
	Ca(ICCV2/PI489777)SNP_13554	3161	T	C	3	6	Shoot	--
	Ca(ICCV2/PI489777)SNP_13555	3188	A	G	4	8	Shoot	--
CakTC23145	Ca(ICCV2/PI489777)SNP_13556	74	A	G	3	4	--	--
	Ca(ICCV2/PI489777)SNP_13557	879	T	C	8	3	--	--
	Ca(ICCV2/PI489777)SNP_13558	908	C	T	14	9	--	--
CakTC30081	Ca(ICCV2/PI489777)SNP_13559	240	T	G	4	3	--	--
CakTC26472	Ca(ICCV2/PI489777)SNP_13560	518	A	G	35	26	--	bZIP
	Ca(ICCV2/PI489777)SNP_13561	1481	C	G	18	14	--	bZIP
CakTC28150	Ca(ICCV2/PI489777)SNP_13562	1530	G	A	10	4	--	--
	Ca(ICCV2/PI489777)SNP_13563	1586	C	T	11	4	--	--
	Ca(ICCV2/PI489777)SNP_13564	1622	A	G	6	3	--	--
	Ca(ICCV2/PI489777)SNP_13565	1811	T	G	11	3	--	--
	Ca(ICCV2/PI489777)SNP_13566	1933	A	G	13	3	--	--
	Ca(ICCV2/PI489777)SNP_13567	2005	C	G	13	3	--	--
	Ca(ICCV2/PI489777)SNP_13568	2075	G	A	12	3	--	--
	Ca(ICCV2/PI489777)SNP_13569	2106	T	G	11	3	--	--
	Ca(ICCV2/PI489777)SNP_13570	2132	G	T	10	3	--	--
CakTC42202	Ca(ICCV2/PI489777)SNP_13571	742	G	A	5	10	--	--
	Ca(ICCV2/PI489777)SNP_13572	774	G	C	5	10	--	--
	Ca(ICCV2/PI489777)SNP_13573	826	T	A	3	10	--	--
CakTC27769	Ca(ICCV2/PI489777)SNP_13574	433	C	A	9	15	--	--
	Ca(ICCV2/PI489777)SNP_13575	1264	A	G	19	16	--	--
	Ca(ICCV2/PI489777)SNP_13576	1891	C	T	23	14	--	--
	Ca(ICCV2/PI489777)SNP_13577	2062	T	A	15	12	--	--
CakTC32184	Ca(ICCV2/PI489777)SNP_13578	1368	G	A	109	58	--	--
	Ca(ICCV2/PI489777)SNP_13579	1452	T	C	103	55	--	--
	Ca(ICCV2/PI489777)SNP_13580	1617	G	T	82	46	--	--
	Ca(ICCV2/PI489777)SNP_13581	1674	G	A	88	55	--	--
	Ca(ICCV2/PI489777)SNP_13582	1827	T	A	130	66	--	--
	Ca(ICCV2/PI489777)SNP_13583	1907	T	C	111	61	--	--
	Ca(ICCV2/PI489777)SNP_13584	1947	G	A	128	56	--	--
	Ca(ICCV2/PI489777)SNP_13585	2069	A	G	118	56	--	--
	Ca(ICCV2/PI489777)SNP_13586	2161	G	A	50	18	--	--
CakTC25583	Ca(ICCV2/PI489777)SNP_13587	1415	C	A	5	3	--	--
	Ca(ICCV2/PI489777)SNP_13588	1662	G	A	9	4	--	--
CakTC33824	Ca(ICCV2/PI489777)SNP_13589	650	T	A	8	5	--	--
	Ca(ICCV2/PI489777)SNP_13590	911	C	A	3	4	--	--
	Ca(ICCV2/PI489777)SNP_13591	1998	T	G	6	12	--	--
	Ca(ICCV2/PI489777)SNP_13592	3189	A	C	3	3	--	--
	Ca(ICCV2/PI489777)SNP_13593	4399	C	T	3	3	--	--
	Ca(ICCV2/PI489777)SNP_13594	5346	A	C	6	7	--	--
	Ca(ICCV2/PI489777)SNP_13595	6388	C	T	3	7	--	--
	Ca(ICCV2/PI489777)SNP_13596	7032	A	G	7	9	--	--
	Ca(ICCV2/PI489777)SNP_13597	7514	A	G	9	5	--	--
	Ca(ICCV2/PI489777)SNP_13598	8294	T	C	9	9	--	--
	Ca(ICCV2/PI489777)SNP_13599	8540	T	C	10	9	--	--
CakTC23579	Ca(ICCV2/PI489777)SNP_13600	244	A	G	26	6	--	--
	Ca(ICCV2/PI489777)SNP_13601	574	A	G	7	10	--	--
	Ca(ICCV2/PI489777)SNP_13602	727	A	G	5	8	--	--
	Ca(ICCV2/PI489777)SNP_13603	1168	T	G	5	5	--	--
	Ca(ICCV2/PI489777)SNP_13604	1186	G	T	5	5	--	--
CakTC30784	Ca(ICCV2/PI489777)SNP_13605	1226	C	A	8	9	--	--
	Ca(ICCV2/PI489777)SNP_13606	1409	A	G	16	8	--	--
	Ca(ICCV2/PI489777)SNP_13607	3083	A	G	6	23	--	--
	Ca(ICCV2/PI489777)SNP_13608	3287	A	G	4	18	--	--
CakTC29023	Ca(ICCV2/PI489777)SNP_13609	1500	T	C	4	5	Flower	bud
CakTC35642	Ca(ICCV2/PI489777)SNP_13610	1779	T	C	12	3	--	--
	Ca(ICCV2/PI489777)SNP_13611	2473	G	C	7	9	--	--

CakTC41627	Ca(ICCV2/PI489777)SNP_13612	177	C	T	17	9	--	--
	Ca(ICCV2/PI489777)SNP_13613	396	A	T	8	4	--	--
CakTC40248	Ca(ICCV2/PI489777)SNP_13614	159	C	A	32	8	Shoot	--
	Ca(ICCV2/PI489777)SNP_13615	162	G	A	32	8	Shoot	--
	Ca(ICCV2/PI489777)SNP_13616	196	G	T	36	11	Shoot	--
	Ca(ICCV2/PI489777)SNP_13617	295	T	C	39	14	Shoot	--
CakTC29731	Ca(ICCV2/PI489777)SNP_13618	450	A	C	7	5	--	--
	Ca(ICCV2/PI489777)SNP_13619	560	A	G	5	4	--	--
CakTC23039	Ca(ICCV2/PI489777)SNP_13620	1691	A	T	8	4	--	--
	Ca(ICCV2/PI489777)SNP_13621	1945	A	G	5	7	--	--
	Ca(ICCV2/PI489777)SNP_13622	1975	G	C	5	7	--	--
	Ca(ICCV2/PI489777)SNP_13623	2053	A	T	9	7	--	--
	Ca(ICCV2/PI489777)SNP_13624	2057	A	G	8	6	--	--
	Ca(ICCV2/PI489777)SNP_13625	2069	G	A	12	8	--	--
	Ca(ICCV2/PI489777)SNP_13626	2095	T	C	12	7	--	--
	Ca(ICCV2/PI489777)SNP_13627	2203	C	T	17	11	--	--
	Ca(ICCV2/PI489777)SNP_13628	2217	A	G	17	10	--	--
	Ca(ICCV2/PI489777)SNP_13629	2319	T	C	16	11	--	--
	Ca(ICCV2/PI489777)SNP_13630	2635	T	A	16	9	--	--
	Ca(ICCV2/PI489777)SNP_13631	2731	T	C	12	8	--	--
CakTC43388	Ca(ICCV2/PI489777)SNP_13632	230	G	C	17	4	--	--
	Ca(ICCV2/PI489777)SNP_13633	763	C	T	5	4	--	--
CakTC41685	Ca(ICCV2/PI489777)SNP_13634	623	G	C	10	5	--	--
	Ca(ICCV2/PI489777)SNP_13635	679	T	C	7	4	--	--
CakTC34398	Ca(ICCV2/PI489777)SNP_13636	198	A	G	5	3	--	FAR1
CakTC33633	Ca(ICCV2/PI489777)SNP_13637	232	C	A	3	3	--	--
	Ca(ICCV2/PI489777)SNP_13638	393	G	A	4	5	--	--
CakTC23181	Ca(ICCV2/PI489777)SNP_13639	890	T	C	20	25	--	--
CakTC06609	Ca(ICCV2/PI489777)SNP_13640	82	A	G	3	3	Young_pod	--
	Ca(ICCV2/PI489777)SNP_13641	92	T	C	3	4	Young_pod	--
	Ca(ICCV2/PI489777)SNP_13642	475	T	C	5	7	Young_pod	--
	Ca(ICCV2/PI489777)SNP_13643	505	C	T	3	9	Young_pod	--
	Ca(ICCV2/PI489777)SNP_13644	521	G	A	5	9	Young_pod	--
	Ca(ICCV2/PI489777)SNP_13645	625	G	A	4	4	Young_pod	--
	Ca(ICCV2/PI489777)SNP_13646	898	C	T	4	6	Young_pod	--
	Ca(ICCV2/PI489777)SNP_13647	1012	T	G	3	7	Young_pod	--
	Ca(ICCV2/PI489777)SNP_13648	1432	T	C	7	7	Young_pod	--
	Ca(ICCV2/PI489777)SNP_13649	1651	A	C	4	8	Young_pod	--
CakTC39929	Ca(ICCV2/PI489777)SNP_13650	70	T	A	5	10	--	--
	Ca(ICCV2/PI489777)SNP_13651	393	C	T	9	8	--	--
	Ca(ICCV2/PI489777)SNP_13652	729	C	T	13	4	--	--
	Ca(ICCV2/PI489777)SNP_13653	861	A	T	9	7	--	--
	Ca(ICCV2/PI489777)SNP_13654	1048	G	C	7	8	--	--
	Ca(ICCV2/PI489777)SNP_13655	1114	G	T	5	6	--	--
	Ca(ICCV2/PI489777)SNP_13656	1699	C	T	9	4	--	--
CakTC34456	Ca(ICCV2/PI489777)SNP_13657	374	T	C	4	4	--	--
	Ca(ICCV2/PI489777)SNP_13658	426	A	T	4	6	--	--
CakTC40322	Ca(ICCV2/PI489777)SNP_13659	18	C	A	8	3	--	--
	Ca(ICCV2/PI489777)SNP_13660	19	A	T	7	3	--	--
	Ca(ICCV2/PI489777)SNP_13661	169	A	T	100	36	--	--
	Ca(ICCV2/PI489777)SNP_13662	349	A	C	103	64	--	--
CakTC42972	Ca(ICCV2/PI489777)SNP_13663	65	A	G	15	5	--	--
	Ca(ICCV2/PI489777)SNP_13664	612	T	C	32	13	--	--
CakTC03736	Ca(ICCV2/PI489777)SNP_13665	107	A	C	7	3	--	--
CakTC28451	Ca(ICCV2/PI489777)SNP_13666	1327	T	C	3	3	--	--
CakTC11451	Ca(ICCV2/PI489777)SNP_13667	621	A	G	6	11	--	--
	Ca(ICCV2/PI489777)SNP_13668	1681	C	T	4	5	--	--
	Ca(ICCV2/PI489777)SNP_13669	2415	G	C	3	7	--	--
CakTC39922	Ca(ICCV2/PI489777)SNP_13670	391	G	A	15	7	--	--
	Ca(ICCV2/PI489777)SNP_13671	523	A	G	17	7	--	--
	Ca(ICCV2/PI489777)SNP_13672	865	C	T	8	7	--	--
	Ca(ICCV2/PI489777)SNP_13673	880	C	T	7	9	--	--
	Ca(ICCV2/PI489777)SNP_13674	940	A	G	8	12	--	--
	Ca(ICCV2/PI489777)SNP_13675	970	G	T	9	11	--	--
	Ca(ICCV2/PI489777)SNP_13676	1031	T	G	12	14	--	--
CakTC39834	Ca(ICCV2/PI489777)SNP_13677	135	A	G	23	7	--	--
	Ca(ICCV2/PI489777)SNP_13678	627	A	G	17	14	--	--
	Ca(ICCV2/PI489777)SNP_13679	756	C	T	14	10	--	--
	Ca(ICCV2/PI489777)SNP_13680	876	A	T	17	9	--	--
	Ca(ICCV2/PI489777)SNP_13681	952	G	A	14	5	--	--
	Ca(ICCV2/PI489777)SNP_13682	1031	C	T	13	3	--	--
	Ca(ICCV2/PI489777)SNP_13683	1060	A	T	12	4	--	--
CakTC31586	Ca(ICCV2/PI489777)SNP_13684	478	T	C	3	6	--	--
	Ca(ICCV2/PI489777)SNP_13685	532	G	A	3	6	--	--

	Ca(ICCV2/PI489777)SNP_13686	988	C	T	15	9	--	--
	Ca(ICCV2/PI489777)SNP_13687	1426	G	A	10	9	--	--
	Ca(ICCV2/PI489777)SNP_13688	1789	T	C	9	5	--	--
CakTC16999	Ca(ICCV2/PI489777)SNP_13689	30	T	C	5	6	Young_pod	--
	Ca(ICCV2/PI489777)SNP_13690	72	A	G	4	7	Young_pod	--
	Ca(ICCV2/PI489777)SNP_13691	128	T	G	7	8	Young_pod	--
CakTC43086	Ca(ICCV2/PI489777)SNP_13692	234	A	C	4	6	--	--
	Ca(ICCV2/PI489777)SNP_13693	535	G	A	9	6	--	--
	Ca(ICCV2/PI489777)SNP_13694	543	C	T	8	5	--	--
	Ca(ICCV2/PI489777)SNP_13695	553	C	T	9	6	--	--
	Ca(ICCV2/PI489777)SNP_13696	559	T	G	9	5	--	--
	Ca(ICCV2/PI489777)SNP_13697	645	T	C	9	6	--	--
	Ca(ICCV2/PI489777)SNP_13698	666	G	C	11	5	--	--
	Ca(ICCV2/PI489777)SNP_13699	686	A	C	12	5	--	--
	Ca(ICCV2/PI489777)SNP_13700	739	A	T	6	5	--	--
	Ca(ICCV2/PI489777)SNP_13701	1085	G	A	14	4	--	--
	Ca(ICCV2/PI489777)SNP_13702	1148	T	C	14	6	--	--
	Ca(ICCV2/PI489777)SNP_13703	1613	G	A	15	11	--	--
	Ca(ICCV2/PI489777)SNP_13704	1709	A	G	17	11	--	--
	Ca(ICCV2/PI489777)SNP_13705	1880	T	A	12	10	--	--
CakTC38385	Ca(ICCV2/PI489777)SNP_13706	42	G	C	10	17	--	--
	Ca(ICCV2/PI489777)SNP_13707	546	C	A	6	24	--	--
	Ca(ICCV2/PI489777)SNP_13708	736	A	C	10	17	--	--
	Ca(ICCV2/PI489777)SNP_13709	819	C	T	10	17	--	--
CakTC31706	Ca(ICCV2/PI489777)SNP_13710	1927	C	G	6	13	--	--
CakTC15944	Ca(ICCV2/PI489777)SNP_13711	658	A	T	3	3	--	Jumonji
CakTC41385	Ca(ICCV2/PI489777)SNP_13712	541	A	C	12	44	Root	--
	Ca(ICCV2/PI489777)SNP_13713	598	A	G	7	19	Root	--
	Ca(ICCV2/PI489777)SNP_13714	604	T	A	7	19	Root	--
	Ca(ICCV2/PI489777)SNP_13715	641	G	A	5	6	Root	--
	Ca(ICCV2/PI489777)SNP_13716	660	G	A	6	7	Root	--
CakTC30965	Ca(ICCV2/PI489777)SNP_13717	422	G	T	3	4	--	--
CakTC40128	Ca(ICCV2/PI489777)SNP_13718	200	G	C	15	6	--	--
	Ca(ICCV2/PI489777)SNP_13719	1009	A	G	30	20	--	--
CakTC28184	Ca(ICCV2/PI489777)SNP_13720	161	T	C	28	28	--	--
CakTC38734	Ca(ICCV2/PI489777)SNP_13721	1153	T	C	5	11	--	--
	Ca(ICCV2/PI489777)SNP_13722	1195	C	G	5	9	--	--
CakTC24630	Ca(ICCV2/PI489777)SNP_13723	379	G	C	3	3	--	--
CakTC37823	Ca(ICCV2/PI489777)SNP_13724	469	G	A	6	7	--	--
	Ca(ICCV2/PI489777)SNP_13725	707	C	T	26	20	--	--
	Ca(ICCV2/PI489777)SNP_13726	804	G	A	32	19	--	--
	Ca(ICCV2/PI489777)SNP_13727	1414	T	A	29	5	--	--
CakTC36433	Ca(ICCV2/PI489777)SNP_13728	1052	T	C	3	9	--	--
	Ca(ICCV2/PI489777)SNP_13729	1972	A	G	3	8	--	--
	Ca(ICCV2/PI489777)SNP_13730	2479	T	C	12	3	--	--
	Ca(ICCV2/PI489777)SNP_13731	2824	C	T	7	7	--	--
CakTC22348	Ca(ICCV2/PI489777)SNP_13732	2049	T	C	6	5	--	FHA
	Ca(ICCV2/PI489777)SNP_13733	2146	G	C	7	6	--	FHA
	Ca(ICCV2/PI489777)SNP_13734	2503	G	C	11	16	--	FHA
	Ca(ICCV2/PI489777)SNP_13735	2591	T	C	7	12	--	FHA
CakTC41658	Ca(ICCV2/PI489777)SNP_13736	51	C	T	9	4	--	--
	Ca(ICCV2/PI489777)SNP_13737	60	T	A	8	4	--	--
	Ca(ICCV2/PI489777)SNP_13738	409	A	T	10	5	--	--
CakTC37649	Ca(ICCV2/PI489777)SNP_13739	173	T	C	7	3	--	--
	Ca(ICCV2/PI489777)SNP_13740	207	G	C	7	4	--	--
	Ca(ICCV2/PI489777)SNP_13741	366	T	C	19	8	--	--
	Ca(ICCV2/PI489777)SNP_13742	720	C	T	16	11	--	--
	Ca(ICCV2/PI489777)SNP_13743	735	G	T	15	12	--	--
	Ca(ICCV2/PI489777)SNP_13744	921	A	G	6	9	--	--
	Ca(ICCV2/PI489777)SNP_13745	1017	T	A	8	15	--	--
	Ca(ICCV2/PI489777)SNP_13746	1124	T	G	10	13	--	--
	Ca(ICCV2/PI489777)SNP_13747	1270	G	A	6	7	--	--
CakTC40591	Ca(ICCV2/PI489777)SNP_13748	542	C	A	4	8	--	--
	Ca(ICCV2/PI489777)SNP_13749	659	G	A	3	6	--	--
	Ca(ICCV2/PI489777)SNP_13750	825	T	C	3	3	--	--
	Ca(ICCV2/PI489777)SNP_13751	1092	T	G	3	4	--	--
	Ca(ICCV2/PI489777)SNP_13752	1142	T	C	3	4	--	--
	Ca(ICCV2/PI489777)SNP_13753	1752	T	C	5	3	--	--
CakTC24145	Ca(ICCV2/PI489777)SNP_13754	377	T	C	6	3	--	--
CakTC37132	Ca(ICCV2/PI489777)SNP_13755	985	T	A	72	55	--	--
	Ca(ICCV2/PI489777)SNP_13756	1150	C	A	45	38	--	--
CakTC41417	Ca(ICCV2/PI489777)SNP_13757	283	A	G	5	10	--	--
	Ca(ICCV2/PI489777)SNP_13758	313	C	T	5	10	--	--
	Ca(ICCV2/PI489777)SNP_13759	401	G	A	8	11	--	--

	Ca(ICCV2/PI489777)SNP_13760	430	C	T	8	11	--	--
	Ca(ICCV2/PI489777)SNP_13761	2078	C	G	27	15	--	--
	Ca(ICCV2/PI489777)SNP_13762	2092	G	A	21	15	--	--
CakTC10286	Ca(ICCV2/PI489777)SNP_13763	979	T	C	5	8	--	--
	Ca(ICCV2/PI489777)SNP_13764	1052	A	G	6	7	--	--
	Ca(ICCV2/PI489777)SNP_13765	1247	G	A	3	6	--	--
	Ca(ICCV2/PI489777)SNP_13766	1344	T	C	4	6	--	--
CakTC27335	Ca(ICCV2/PI489777)SNP_13767	682	T	C	16	12	--	bZIP
	Ca(ICCV2/PI489777)SNP_13768	1089	C	T	23	11	--	bZIP
	Ca(ICCV2/PI489777)SNP_13769	1368	G	T	32	17	--	bZIP
	Ca(ICCV2/PI489777)SNP_13770	1371	T	G	32	18	--	bZIP
	Ca(ICCV2/PI489777)SNP_13771	1527	C	T	16	10	--	bZIP
CakTC36858	Ca(ICCV2/PI489777)SNP_13772	258	T	C	6	3	--	--
CakTC23750	Ca(ICCV2/PI489777)SNP_13773	4425	T	C	9	17	--	CCHC
	Ca(ICCV2/PI489777)SNP_13774	4428	C	T	8	16	--	CCHC
	Ca(ICCV2/PI489777)SNP_13775	4569	T	C	11	19	--	CCHC
CakTC32590	Ca(ICCV2/PI489777)SNP_13776	1781	G	T	15	3	--	--
	Ca(ICCV2/PI489777)SNP_13777	2092	T	C	12	7	--	--
CakTC41651	Ca(ICCV2/PI489777)SNP_13778	260	G	C	3	3	--	--
	Ca(ICCV2/PI489777)SNP_13779	335	T	C	4	3	--	--
CakTC37163	Ca(ICCV2/PI489777)SNP_13780	529	A	T	4	4	--	--
	Ca(ICCV2/PI489777)SNP_13781	727	G	A	7	6	--	--
CakTC41896	Ca(ICCV2/PI489777)SNP_13782	1706	C	G	8	4	--	--
	Ca(ICCV2/PI489777)SNP_13783	1707	A	G	8	4	--	--
CakTC28719	Ca(ICCV2/PI489777)SNP_13784	463	C	G	40	20	--	--
	Ca(ICCV2/PI489777)SNP_13785	1833	G	A	43	15	--	--
CakTC40851	Ca(ICCV2/PI489777)SNP_13786	582	A	G	29	20	--	--
	Ca(ICCV2/PI489777)SNP_13787	672	G	A	20	18	--	--
	Ca(ICCV2/PI489777)SNP_13788	692	A	G	18	16	--	--
	Ca(ICCV2/PI489777)SNP_13789	772	G	C	7	10	--	--
CakTC42978	Ca(ICCV2/PI489777)SNP_13790	957	C	A	6	3	--	--
	Ca(ICCV2/PI489777)SNP_13791	987	G	A	3	4	--	--
	Ca(ICCV2/PI489777)SNP_13792	1307	C	T	4	5	--	--
	Ca(ICCV2/PI489777)SNP_13793	1316	G	A	7	5	--	--
	Ca(ICCV2/PI489777)SNP_13794	1325	A	G	7	6	--	--
CakTC28394	Ca(ICCV2/PI489777)SNP_13795	312	C	T	15	5	--	bHLH
CakTC42362	Ca(ICCV2/PI489777)SNP_13796	1467	T	C	15	10	--	--
	Ca(ICCV2/PI489777)SNP_13797	1522	C	T	11	9	--	--
CakTC13600	Ca(ICCV2/PI489777)SNP_13798	168	T	C	5	4	--	--
CakTC39243	Ca(ICCV2/PI489777)SNP_13799	40	A	C	6	8	--	--
	Ca(ICCV2/PI489777)SNP_13800	238	A	T	12	14	--	--
	Ca(ICCV2/PI489777)SNP_13801	1398	A	G	5	19	--	--
CakTC22936	Ca(ICCV2/PI489777)SNP_13802	362	T	C	15	4	--	--
CakTC26432	Ca(ICCV2/PI489777)SNP_13803	1445	A	C	213	202	--	--
CakTC10471	Ca(ICCV2/PI489777)SNP_13804	646	G	A	4	4	--	--
CakTC23893	Ca(ICCV2/PI489777)SNP_13805	217	G	A	6	3	--	bHLH
	Ca(ICCV2/PI489777)SNP_13806	702	T	G	17	16	--	bHLH
	Ca(ICCV2/PI489777)SNP_13807	718	T	G	16	20	--	bHLH
	Ca(ICCV2/PI489777)SNP_13808	769	C	T	15	24	--	bHLH
	Ca(ICCV2/PI489777)SNP_13809	1012	T	G	26	12	--	bHLH
	Ca(ICCV2/PI489777)SNP_13810	1183	A	G	16	9	--	bHLH
	Ca(ICCV2/PI489777)SNP_13811	1461	T	C	24	19	--	bHLH
	Ca(ICCV2/PI489777)SNP_13812	1468	A	G	25	19	--	bHLH
	Ca(ICCV2/PI489777)SNP_13813	1483	T	A	25	19	--	bHLH
	Ca(ICCV2/PI489777)SNP_13814	1546	G	A	24	19	--	bHLH
	Ca(ICCV2/PI489777)SNP_13815	1582	A	G	22	17	--	bHLH
	Ca(ICCV2/PI489777)SNP_13816	1635	A	G	26	16	--	bHLH
CakTC25525	Ca(ICCV2/PI489777)SNP_13817	205	C	T	17	3	--	--
	Ca(ICCV2/PI489777)SNP_13818	886	G	C	16	3	--	--
	Ca(ICCV2/PI489777)SNP_13819	969	G	A	13	3	--	--
CakTC28811	Ca(ICCV2/PI489777)SNP_13820	275	A	T	42	40	--	--
	Ca(ICCV2/PI489777)SNP_13821	510	T	A	55	47	--	--
	Ca(ICCV2/PI489777)SNP_13822	1451	T	A	7	9	--	--
CakTC38843	Ca(ICCV2/PI489777)SNP_13823	651	G	A	13	10	--	C3H
CakTC35785	Ca(ICCV2/PI489777)SNP_13824	582	T	A	54	29	--	--
	Ca(ICCV2/PI489777)SNP_13825	749	G	A	41	31	--	--
	Ca(ICCV2/PI489777)SNP_13826	1022	G	A	35	18	--	--
	Ca(ICCV2/PI489777)SNP_13827	1044	G	C	31	13	--	--
	Ca(ICCV2/PI489777)SNP_13828	2063	C	A	23	7	--	--
	Ca(ICCV2/PI489777)SNP_13829	2780	G	A	19	11	--	--
	Ca(ICCV2/PI489777)SNP_13830	3173	G	A	6	18	--	--
	Ca(ICCV2/PI489777)SNP_13831	3252	A	G	9	16	--	--
CakTC29882	Ca(ICCV2/PI489777)SNP_13832	476	C	A	19	5	--	--
	Ca(ICCV2/PI489777)SNP_13833	557	A	G	24	4	--	--

	Ca(ICCV2/PI489777)SNP_13834	722	C	T	32	5	--	--
	Ca(ICCV2/PI489777)SNP_13835	1226	T	C	13	7	--	--
	Ca(ICCV2/PI489777)SNP_13836	1463	C	T	17	7	--	--
	Ca(ICCV2/PI489777)SNP_13837	1630	A	C	12	5	--	--
	Ca(ICCV2/PI489777)SNP_13838	1807	T	C	15	5	--	--
	Ca(ICCV2/PI489777)SNP_13839	1855	A	G	12	5	--	--
CakTC42073	Ca(ICCV2/PI489777)SNP_13840	63	T	G	13	6	--	--
	Ca(ICCV2/PI489777)SNP_13841	82	C	T	15	6	--	--
	Ca(ICCV2/PI489777)SNP_13842	696	G	A	20	8	--	--
	Ca(ICCV2/PI489777)SNP_13843	800	G	A	18	5	--	--
CakTC35312	Ca(ICCV2/PI489777)SNP_13844	1688	A	G	19	10	--	--
	Ca(ICCV2/PI489777)SNP_13845	2741	A	C	10	5	--	--
CakTC31880	Ca(ICCV2/PI489777)SNP_13846	1154	A	G	5	5	--	--
	Ca(ICCV2/PI489777)SNP_13847	1188	G	A	5	6	--	--
CakTC33466	Ca(ICCV2/PI489777)SNP_13848	235	T	C	6	6	Flower	bud
	Ca(ICCV2/PI489777)SNP_13849	303	A	T	9	6	Flower	bud
	Ca(ICCV2/PI489777)SNP_13850	417	C	T	10	6	Flower	bud
	Ca(ICCV2/PI489777)SNP_13851	418	T	A	11	6	Flower	bud
	Ca(ICCV2/PI489777)SNP_13852	467	C	T	10	3	Flower	bud
CakTC27226	Ca(ICCV2/PI489777)SNP_13853	1210	T	C	4	14	Flower	bud
	Ca(ICCV2/PI489777)SNP_13854	1444	C	A	3	11	Flower	bud
	Ca(ICCV2/PI489777)SNP_13855	1446	G	C	3	12	Flower	bud
CakTC37784	Ca(ICCV2/PI489777)SNP_13856	193	T	A	3	7	--	--
	Ca(ICCV2/PI489777)SNP_13857	223	T	C	4	5	--	--
CakTC38719	Ca(ICCV2/PI489777)SNP_13858	516	A	C	29	22	--	--
	Ca(ICCV2/PI489777)SNP_13859	621	C	T	23	12	--	--
CakTC29975	Ca(ICCV2/PI489777)SNP_13860	417	A	G	14	5	--	--
	Ca(ICCV2/PI489777)SNP_13861	576	C	A	10	5	--	--
CakTC08090	Ca(ICCV2/PI489777)SNP_13862	1538	C	T	6	6	--	TPR
CakTC22750	Ca(ICCV2/PI489777)SNP_13863	437	G	A	34	10	--	--
	Ca(ICCV2/PI489777)SNP_13864	2572	G	A	7	6	--	--
	Ca(ICCV2/PI489777)SNP_13865	2764	A	G	16	4	--	--
CakTC38869	Ca(ICCV2/PI489777)SNP_13866	95	A	G	12	3	--	Trihelix
	Ca(ICCV2/PI489777)SNP_13867	288	G	A	21	6	--	Trihelix
	Ca(ICCV2/PI489777)SNP_13868	548	T	C	21	4	--	Trihelix
	Ca(ICCV2/PI489777)SNP_13869	1028	G	A	3	4	--	Trihelix
CakTC36667	Ca(ICCV2/PI489777)SNP_13870	283	T	C	7	9	--	--
CakTC30258	Ca(ICCV2/PI489777)SNP_13871	568	T	C	11	3	--	mTERF
	Ca(ICCV2/PI489777)SNP_13872	847	T	G	4	3	--	mTERF
CakTC36851	Ca(ICCV2/PI489777)SNP_13873	238	G	A	3	10	--	--
	Ca(ICCV2/PI489777)SNP_13874	266	G	A	3	12	--	--
CakTC33980	Ca(ICCV2/PI489777)SNP_13875	81	G	T	11	10	--	--
	Ca(ICCV2/PI489777)SNP_13876	1999	A	C	7	7	--	--
CakTC28818	Ca(ICCV2/PI489777)SNP_13877	1603	T	C	15	3	--	--
	Ca(ICCV2/PI489777)SNP_13878	1641	G	A	13	4	--	--
	Ca(ICCV2/PI489777)SNP_13879	1785	A	G	8	4	--	--
CakTC23830	Ca(ICCV2/PI489777)SNP_13880	883	T	G	15	4	--	--
	Ca(ICCV2/PI489777)SNP_13881	975	C	T	21	6	--	--
	Ca(ICCV2/PI489777)SNP_13882	1456	A	C	19	6	--	--
	Ca(ICCV2/PI489777)SNP_13883	1461	G	A	19	6	--	--
	Ca(ICCV2/PI489777)SNP_13884	1623	T	C	10	6	--	--
	Ca(ICCV2/PI489777)SNP_13885	2257	G	A	25	8	--	--
	Ca(ICCV2/PI489777)SNP_13886	2465	A	G	18	9	--	--
	Ca(ICCV2/PI489777)SNP_13887	2574	C	T	23	8	--	--
CakTC40173	Ca(ICCV2/PI489777)SNP_13888	845	C	T	10	3	--	--
	Ca(ICCV2/PI489777)SNP_13889	1153	C	G	7	6	--	--
CakTC39966	Ca(ICCV2/PI489777)SNP_13890	216	G	A	27	8	--	--
	Ca(ICCV2/PI489777)SNP_13891	1247	G	A	6	3	--	--
	Ca(ICCV2/PI489777)SNP_13892	1253	T	A	7	3	--	--
CakTC34846	Ca(ICCV2/PI489777)SNP_13893	500	T	C	3	4	Flower	bud
CakTC42322	Ca(ICCV2/PI489777)SNP_13894	1034	T	C	5	4	--	--
	Ca(ICCV2/PI489777)SNP_13895	1644	T	C	4	5	--	--
	Ca(ICCV2/PI489777)SNP_13896	1812	G	A	3	4	--	--
CakTC35609	Ca(ICCV2/PI489777)SNP_13897	196	A	G	5	5	--	--
	Ca(ICCV2/PI489777)SNP_13898	418	C	A	9	4	--	--
	Ca(ICCV2/PI489777)SNP_13899	654	C	G	11	3	--	--
	Ca(ICCV2/PI489777)SNP_13900	762	G	A	10	3	--	--
	Ca(ICCV2/PI489777)SNP_13901	767	C	G	11	3	--	--
	Ca(ICCV2/PI489777)SNP_13902	2460	A	T	12	5	--	--
	Ca(ICCV2/PI489777)SNP_13903	2917	G	C	10	9	--	--
	Ca(ICCV2/PI489777)SNP_13904	2941	G	T	10	7	--	--
	Ca(ICCV2/PI489777)SNP_13905	2957	C	T	10	8	--	--
CakTC33006	Ca(ICCV2/PI489777)SNP_13906	588	G	C	11	20	--	--
	Ca(ICCV2/PI489777)SNP_13907	657	G	A	11	15	--	--

	Ca(ICCV2/PI489777)SNP_13908	742	G	C	11	20	--	--
	Ca(ICCV2/PI489777)SNP_13909	743	C	T	11	20	--	--
	Ca(ICCV2/PI489777)SNP_13910	848	T	C	10	17	--	--
CakTC43091	Ca(ICCV2/PI489777)SNP_13911	638	T	C	40	59	--	--
	Ca(ICCV2/PI489777)SNP_13912	719	A	G	41	45	--	--
	Ca(ICCV2/PI489777)SNP_13913	925	T	C	42	41	--	--
	Ca(ICCV2/PI489777)SNP_13914	937	C	A	36	41	--	--
	Ca(ICCV2/PI489777)SNP_13915	1013	T	G	28	33	--	--
	Ca(ICCV2/PI489777)SNP_13916	1039	A	T	27	31	--	--
	Ca(ICCV2/PI489777)SNP_13917	1084	C	T	34	35	--	--
	Ca(ICCV2/PI489777)SNP_13918	1214	T	C	30	42	--	--
	Ca(ICCV2/PI489777)SNP_13919	1367	A	G	36	36	--	--
	Ca(ICCV2/PI489777)SNP_13920	1499	A	T	44	33	--	--
	Ca(ICCV2/PI489777)SNP_13921	1553	G	A	48	33	--	--
CakTC25086	Ca(ICCV2/PI489777)SNP_13922	103	G	T	7	8	--	--
	Ca(ICCV2/PI489777)SNP_13923	1140	A	G	8	4	--	--
	Ca(ICCV2/PI489777)SNP_13924	1152	G	A	7	3	--	--
	Ca(ICCV2/PI489777)SNP_13925	1262	G	A	9	4	--	--
CakTC37588	Ca(ICCV2/PI489777)SNP_13926	1018	G	T	8	14	--	--
CakTC41430	Ca(ICCV2/PI489777)SNP_13927	539	G	A	25	14	--	--
CakTC40125	Ca(ICCV2/PI489777)SNP_13928	618	A	T	11	14	--	--
	Ca(ICCV2/PI489777)SNP_13929	891	G	A	14	17	--	--
	Ca(ICCV2/PI489777)SNP_13930	1146	A	C	7	14	--	--
	Ca(ICCV2/PI489777)SNP_13931	1218	T	C	5	9	--	--
CakTC26334	Ca(ICCV2/PI489777)SNP_13932	613	G	T	15	7	--	--
	Ca(ICCV2/PI489777)SNP_13933	1042	A	T	6	6	--	--
CakTC41940	Ca(ICCV2/PI489777)SNP_13934	1420	C	T	7	6	--	--
CakTC25854	Ca(ICCV2/PI489777)SNP_13935	433	C	G	6	4	--	Tify
CakTC10724	Ca(ICCV2/PI489777)SNP_13936	2444	A	G	6	5	--	--
CakTC28379	Ca(ICCV2/PI489777)SNP_13937	3269	A	T	31	12	--	--
CakTC33390	Ca(ICCV2/PI489777)SNP_13938	20	A	C	3	16	--	CCAAT
	Ca(ICCV2/PI489777)SNP_13939	520	C	G	3	3	--	CCAAT
CakTC35262	Ca(ICCV2/PI489777)SNP_13940	678	G	C	9	14	--	--
	Ca(ICCV2/PI489777)SNP_13941	694	C	G	5	13	--	--
	Ca(ICCV2/PI489777)SNP_13942	734	A	G	7	10	--	--
	Ca(ICCV2/PI489777)SNP_13943	792	C	T	10	7	--	--
	Ca(ICCV2/PI489777)SNP_13944	810	A	G	10	9	--	--
	Ca(ICCV2/PI489777)SNP_13945	843	A	G	10	8	--	--
	Ca(ICCV2/PI489777)SNP_13946	3191	T	C	4	7	--	--
	Ca(ICCV2/PI489777)SNP_13947	3280	G	A	3	6	--	--
CakTC29327	Ca(ICCV2/PI489777)SNP_13948	370	C	T	9	3	--	--
	Ca(ICCV2/PI489777)SNP_13949	433	T	C	7	3	--	--
	Ca(ICCV2/PI489777)SNP_13950	750	G	A	13	5	--	--
	Ca(ICCV2/PI489777)SNP_13951	929	A	G	11	4	--	--
	Ca(ICCV2/PI489777)SNP_13952	1190	C	G	8	9	--	--
	Ca(ICCV2/PI489777)SNP_13953	1429	G	A	6	4	--	--
CakTC39643	Ca(ICCV2/PI489777)SNP_13954	38	G	A	6	6	--	--
	Ca(ICCV2/PI489777)SNP_13955	49	A	G	10	9	--	--
	Ca(ICCV2/PI489777)SNP_13956	53	T	A	10	9	--	--
	Ca(ICCV2/PI489777)SNP_13957	89	T	C	8	10	--	--
	Ca(ICCV2/PI489777)SNP_13958	132	G	A	14	12	--	--
	Ca(ICCV2/PI489777)SNP_13959	179	T	A	15	15	--	--
	Ca(ICCV2/PI489777)SNP_13960	216	T	A	20	18	--	--
	Ca(ICCV2/PI489777)SNP_13961	500	T	C	15	15	--	--
	Ca(ICCV2/PI489777)SNP_13962	763	A	C	22	15	--	--
	Ca(ICCV2/PI489777)SNP_13963	785	T	C	24	15	--	--
	Ca(ICCV2/PI489777)SNP_13964	874	C	A	26	15	--	--
CakTC42057	Ca(ICCV2/PI489777)SNP_13965	231	A	G	11	3	--	--
	Ca(ICCV2/PI489777)SNP_13966	646	T	C	32	19	--	--
	Ca(ICCV2/PI489777)SNP_13967	1158	A	C	45	25	--	--
	Ca(ICCV2/PI489777)SNP_13968	1376	A	G	38	35	--	--
	Ca(ICCV2/PI489777)SNP_13969	1397	T	C	31	21	--	--
	Ca(ICCV2/PI489777)SNP_13970	1819	C	T	14	9	--	--
CakTC42412	Ca(ICCV2/PI489777)SNP_13971	171	A	G	19	7	--	--
	Ca(ICCV2/PI489777)SNP_13972	702	T	C	46	19	--	--
CakTC07476	Ca(ICCV2/PI489777)SNP_13973	976	C	G	3	7	--	C3H
	Ca(ICCV2/PI489777)SNP_13974	989	A	G	4	9	--	C3H
	Ca(ICCV2/PI489777)SNP_13975	1453	T	C	15	9	--	C3H
	Ca(ICCV2/PI489777)SNP_13976	1488	A	G	14	9	--	C3H
CakTC36427	Ca(ICCV2/PI489777)SNP_13977	2487	A	G	20	6	--	--
CakTC27288	Ca(ICCV2/PI489777)SNP_13978	1865	T	G	8	5	--	--
	Ca(ICCV2/PI489777)SNP_13979	2065	G	A	9	5	--	--
	Ca(ICCV2/PI489777)SNP_13980	2071	G	A	8	4	--	--
CakTC30001	Ca(ICCV2/PI489777)SNP_13981	389	C	G	7	5	--	--

	Ca(ICCV2/PI489777)SNP_13982	3500	C	A	12	8	--	--
CakTC39781	Ca(ICCV2/PI489777)SNP_13983	127	C	T	6	4	--	--
	Ca(ICCV2/PI489777)SNP_13984	269	G	A	6	4	--	--
	Ca(ICCV2/PI489777)SNP_13985	325	G	T	7	5	--	--
	Ca(ICCV2/PI489777)SNP_13986	552	A	C	8	4	--	--
CakTC37880	Ca(ICCV2/PI489777)SNP_13987	370	G	A	5	7	--	--
CakTC26848	Ca(ICCV2/PI489777)SNP_13988	28	C	T	3	7	--	--
	Ca(ICCV2/PI489777)SNP_13989	143	A	G	7	10	--	--
	Ca(ICCV2/PI489777)SNP_13990	150	T	C	7	10	--	--
	Ca(ICCV2/PI489777)SNP_13991	198	C	A	11	12	--	--
	Ca(ICCV2/PI489777)SNP_13992	281	G	A	18	20	--	--
	Ca(ICCV2/PI489777)SNP_13993	786	T	G	29	22	--	--
	Ca(ICCV2/PI489777)SNP_13994	950	A	T	3	10	--	--
CakTC31594	Ca(ICCV2/PI489777)SNP_13995	926	C	T	11	10	--	--
	Ca(ICCV2/PI489777)SNP_13996	1173	C	A	9	10	--	--
	Ca(ICCV2/PI489777)SNP_13997	1325	C	T	9	14	--	--
	Ca(ICCV2/PI489777)SNP_13998	1370	C	T	10	16	--	--
	Ca(ICCV2/PI489777)SNP_13999	1494	T	A	14	21	--	--
	Ca(ICCV2/PI489777)SNP_14000	1505	C	A	13	20	--	--
	Ca(ICCV2/PI489777)SNP_14001	1609	G	A	11	18	--	--
	Ca(ICCV2/PI489777)SNP_14002	2146	G	T	12	12	--	--
	Ca(ICCV2/PI489777)SNP_14003	2220	C	A	9	11	--	--
	Ca(ICCV2/PI489777)SNP_14004	2264	T	C	13	14	--	--
	Ca(ICCV2/PI489777)SNP_14005	2507	A	G	14	8	--	--
CakTC39658	Ca(ICCV2/PI489777)SNP_14006	481	T	C	5	3	--	--
	Ca(ICCV2/PI489777)SNP_14007	661	G	A	4	4	--	--
CakTC29369	Ca(ICCV2/PI489777)SNP_14008	112	T	G	17	18	--	--
	Ca(ICCV2/PI489777)SNP_14009	660	C	T	72	51	--	--
	Ca(ICCV2/PI489777)SNP_14010	663	C	G	75	40	--	--
	Ca(ICCV2/PI489777)SNP_14011	675	C	T	74	57	--	--
	Ca(ICCV2/PI489777)SNP_14012	741	G	A	82	61	--	--
	Ca(ICCV2/PI489777)SNP_14013	946	T	G	86	55	--	--
	Ca(ICCV2/PI489777)SNP_14014	1115	G	C	46	34	--	--
	Ca(ICCV2/PI489777)SNP_14015	1427	G	A	30	22	--	--
	Ca(ICCV2/PI489777)SNP_14016	1720	G	C	26	23	--	--
	Ca(ICCV2/PI489777)SNP_14017	1762	C	G	21	17	--	--
	Ca(ICCV2/PI489777)SNP_14018	1767	A	G	17	17	--	--
CakTC22669	Ca(ICCV2/PI489777)SNP_14019	490	T	C	5	5	--	--
	Ca(ICCV2/PI489777)SNP_14020	495	C	A	5	4	--	--
	Ca(ICCV2/PI489777)SNP_14021	500	C	T	5	4	--	--
	Ca(ICCV2/PI489777)SNP_14022	609	G	A	11	7	--	--
	Ca(ICCV2/PI489777)SNP_14023	677	G	T	12	5	--	--
CakTC30728	Ca(ICCV2/PI489777)SNP_14024	1023	C	T	5	4	--	--
CakTC25154	Ca(ICCV2/PI489777)SNP_14025	116	G	C	4	4	--	--
	Ca(ICCV2/PI489777)SNP_14026	411	T	C	44	30	--	--
	Ca(ICCV2/PI489777)SNP_14027	1829	A	G	5	3	--	--
CakTC40574	Ca(ICCV2/PI489777)SNP_14028	341	G	A	3	7	--	--
	Ca(ICCV2/PI489777)SNP_14029	1461	A	C	12	13	--	--
CakTC39515	Ca(ICCV2/PI489777)SNP_14030	90	C	G	7	7	--	--
	Ca(ICCV2/PI489777)SNP_14031	119	C	A	8	7	--	--
	Ca(ICCV2/PI489777)SNP_14032	701	G	A	13	6	--	--
	Ca(ICCV2/PI489777)SNP_14033	912	G	C	10	6	--	--
	Ca(ICCV2/PI489777)SNP_14034	947	C	G	11	6	--	--
	Ca(ICCV2/PI489777)SNP_14035	951	A	G	11	5	--	--
CakTC29780	Ca(ICCV2/PI489777)SNP_14036	443	G	A	19	7	--	--
	Ca(ICCV2/PI489777)SNP_14037	457	G	T	12	7	--	--
	Ca(ICCV2/PI489777)SNP_14038	509	G	C	15	6	--	--
	Ca(ICCV2/PI489777)SNP_14039	659	A	G	7	7	--	--
	Ca(ICCV2/PI489777)SNP_14040	896	A	C	3	4	--	--
	Ca(ICCV2/PI489777)SNP_14041	904	G	A	3	4	--	--
CakTC33404	Ca(ICCV2/PI489777)SNP_14042	751	T	C	11	9	--	--
	Ca(ICCV2/PI489777)SNP_14043	964	C	T	8	10	--	--
	Ca(ICCV2/PI489777)SNP_14044	1015	T	C	9	10	--	--
	Ca(ICCV2/PI489777)SNP_14045	1021	C	T	9	10	--	--
	Ca(ICCV2/PI489777)SNP_14046	1201	T	C	6	9	--	--
CakTC26142	Ca(ICCV2/PI489777)SNP_14047	426	T	C	28	17	--	--
	Ca(ICCV2/PI489777)SNP_14048	2296	T	C	14	16	--	--
	Ca(ICCV2/PI489777)SNP_14049	2632	C	T	25	22	--	--
	Ca(ICCV2/PI489777)SNP_14050	2634	A	G	26	22	--	--
CakTC23926	Ca(ICCV2/PI489777)SNP_14051	979	G	A	13	4	--	--
	Ca(ICCV2/PI489777)SNP_14052	1017	G	A	15	4	--	--
	Ca(ICCV2/PI489777)SNP_14053	1272	G	T	19	5	--	--
	Ca(ICCV2/PI489777)SNP_14054	1512	C	T	11	5	--	--
CakTC25645	Ca(ICCV2/PI489777)SNP_14055	552	G	C	62	31	--	--

	Ca(ICCV2/PI489777)SNP_14056	770	T	C	47	25	--	--
	Ca(ICCV2/PI489777)SNP_14057	804	T	C	51	27	--	--
	Ca(ICCV2/PI489777)SNP_14058	886	T	C	41	28	--	--
CakTC42577	Ca(ICCV2/PI489777)SNP_14059	316	C	T	8	5	--	--
	Ca(ICCV2/PI489777)SNP_14060	318	C	T	9	7	--	--
	Ca(ICCV2/PI489777)SNP_14061	699	G	A	19	16	--	--
CakTC30375	Ca(ICCV2/PI489777)SNP_14062	2890	T	C	6	6	--	TPR
CakTC14811	Ca(ICCV2/PI489777)SNP_14063	418	G	A	3	9	--	--
	Ca(ICCV2/PI489777)SNP_14064	561	C	T	3	8	--	--
	Ca(ICCV2/PI489777)SNP_14065	579	T	A	3	6	--	--
	Ca(ICCV2/PI489777)SNP_14066	621	A	G	3	7	--	--
CakTC32729	Ca(ICCV2/PI489777)SNP_14067	915	A	G	17	20	--	--
	Ca(ICCV2/PI489777)SNP_14068	1029	C	A	16	21	--	--
	Ca(ICCV2/PI489777)SNP_14069	1848	A	G	10	12	--	--
CakTC30996	Ca(ICCV2/PI489777)SNP_14070	698	A	G	8	4	--	--
CakTC41672	Ca(ICCV2/PI489777)SNP_14071	501	T	C	15	3	--	--
	Ca(ICCV2/PI489777)SNP_14072	645	T	A	11	8	--	--
	Ca(ICCV2/PI489777)SNP_14073	660	A	G	11	8	--	--
	Ca(ICCV2/PI489777)SNP_14074	765	T	A	10	7	--	--
	Ca(ICCV2/PI489777)SNP_14075	1407	G	A	15	9	--	--
	Ca(ICCV2/PI489777)SNP_14076	1464	G	T	9	7	--	--
	Ca(ICCV2/PI489777)SNP_14077	1809	A	G	6	4	--	--
	Ca(ICCV2/PI489777)SNP_14078	1872	G	A	6	7	--	--
	Ca(ICCV2/PI489777)SNP_14079	1902	T	A	6	7	--	--
	Ca(ICCV2/PI489777)SNP_14080	1905	C	T	8	7	--	--
	Ca(ICCV2/PI489777)SNP_14081	2010	T	C	7	8	--	--
	Ca(ICCV2/PI489777)SNP_14082	2189	T	C	9	8	--	--
CakTC11237	Ca(ICCV2/PI489777)SNP_14083	214	C	T	3	3	--	--
	Ca(ICCV2/PI489777)SNP_14084	257	C	T	3	4	--	--
	Ca(ICCV2/PI489777)SNP_14085	293	T	G	3	4	--	--
	Ca(ICCV2/PI489777)SNP_14086	327	A	G	3	4	--	--
CakTC42567	Ca(ICCV2/PI489777)SNP_14087	850	T	C	7	4	--	--
	Ca(ICCV2/PI489777)SNP_14088	1387	C	T	7	7	--	--
CakTC24316	Ca(ICCV2/PI489777)SNP_14089	1805	A	G	5	3	--	--
	Ca(ICCV2/PI489777)SNP_14090	1812	G	C	5	3	--	--
CakTC40717	Ca(ICCV2/PI489777)SNP_14091	257	G	A	5	3	Flower	bud
CakTC27657	Ca(ICCV2/PI489777)SNP_14092	841	A	G	45	15	--	--
CakTC24956	Ca(ICCV2/PI489777)SNP_14093	954	T	C	13	3	--	--
	Ca(ICCV2/PI489777)SNP_14094	1024	A	G	19	5	--	--
	Ca(ICCV2/PI489777)SNP_14095	1135	G	C	16	6	--	--
	Ca(ICCV2/PI489777)SNP_14096	1268	G	A	9	4	--	--
	Ca(ICCV2/PI489777)SNP_14097	1272	A	T	10	3	--	--
	Ca(ICCV2/PI489777)SNP_14098	1358	A	G	5	3	--	--
	Ca(ICCV2/PI489777)SNP_14099	1509	G	T	5	3	--	--
CakTC01457	Ca(ICCV2/PI489777)SNP_14100	728	A	T	4	8	--	--
	Ca(ICCV2/PI489777)SNP_14101	800	A	T	4	8	--	--
	Ca(ICCV2/PI489777)SNP_14102	2888	C	T	3	3	--	--
CakTC28383	Ca(ICCV2/PI489777)SNP_14103	927	T	C	9	8	--	--
	Ca(ICCV2/PI489777)SNP_14104	1100	G	C	3	8	--	--
	Ca(ICCV2/PI489777)SNP_14105	1134	A	G	3	9	--	--
	Ca(ICCV2/PI489777)SNP_14106	1137	G	A	3	10	--	--
	Ca(ICCV2/PI489777)SNP_14107	1182	A	T	3	10	--	--
	Ca(ICCV2/PI489777)SNP_14108	2151	A	G	9	13	--	--
	Ca(ICCV2/PI489777)SNP_14109	2196	G	T	10	13	--	--
	Ca(ICCV2/PI489777)SNP_14110	2206	C	T	10	12	--	--
	Ca(ICCV2/PI489777)SNP_14111	2222	G	T	12	13	--	--
	Ca(ICCV2/PI489777)SNP_14112	2224	A	T	12	13	--	--
	Ca(ICCV2/PI489777)SNP_14113	2244	C	G	13	13	--	--
	Ca(ICCV2/PI489777)SNP_14114	2259	G	A	12	10	--	--
	Ca(ICCV2/PI489777)SNP_14115	3084	C	T	7	10	--	--
	Ca(ICCV2/PI489777)SNP_14116	3173	T	C	11	14	--	--
	Ca(ICCV2/PI489777)SNP_14117	3237	G	A	13	18	--	--
	Ca(ICCV2/PI489777)SNP_14118	3331	G	A	15	19	--	--
	Ca(ICCV2/PI489777)SNP_14119	3982	G	C	10	11	--	--
	Ca(ICCV2/PI489777)SNP_14120	4067	C	T	10	13	--	--
CakTC42865	Ca(ICCV2/PI489777)SNP_14121	359	C	A	69	59	--	--
CakTC22484	Ca(ICCV2/PI489777)SNP_14122	1222	G	C	19	13	Young_pod	--
	Ca(ICCV2/PI489777)SNP_14123	1609	A	T	25	26	Young_pod	--
	Ca(ICCV2/PI489777)SNP_14124	1999	G	A	3	3	Young_pod	--
CakTC29885	Ca(ICCV2/PI489777)SNP_14125	230	C	T	21	14	--	--
	Ca(ICCV2/PI489777)SNP_14126	242	A	G	19	13	--	--
	Ca(ICCV2/PI489777)SNP_14127	2543	G	A	37	28	--	--
	Ca(ICCV2/PI489777)SNP_14128	3386	A	G	30	27	--	--
CakTC09805	Ca(ICCV2/PI489777)SNP_14129	2218	T	C	5	3	--	--

	Ca(ICCV2/PI489777)SNP_14130	2261	G	T	5	3	--	--
CakTC42571	Ca(ICCV2/PI489777)SNP_14131	466	G	T	47	36	--	--
	Ca(ICCV2/PI489777)SNP_14132	604	G	A	79	35	--	--
CakTC28275	Ca(ICCV2/PI489777)SNP_14133	284	T	C	3	8	--	Bromodomain
	Ca(ICCV2/PI489777)SNP_14134	3004	T	A	7	5	--	Bromodomain
	Ca(ICCV2/PI489777)SNP_14135	4161	A	G	5	10	--	Bromodomain
	Ca(ICCV2/PI489777)SNP_14136	4227	A	C	5	8	--	Bromodomain
	Ca(ICCV2/PI489777)SNP_14137	4416	C	T	6	6	--	Bromodomain
	Ca(ICCV2/PI489777)SNP_14138	4425	T	C	6	5	--	Bromodomain
	Ca(ICCV2/PI489777)SNP_14139	5592	G	A	15	8	--	Bromodomain
CakTC23613	Ca(ICCV2/PI489777)SNP_14140	547	C	T	9	6	--	--
	Ca(ICCV2/PI489777)SNP_14141	1214	G	T	5	3	--	--
CakTC43100	Ca(ICCV2/PI489777)SNP_14142	877	A	T	35	11	--	--
	Ca(ICCV2/PI489777)SNP_14143	1265	A	G	4	4	--	--
CakTC38089	Ca(ICCV2/PI489777)SNP_14144	462	T	G	7	5	--	--
CakTC23511	Ca(ICCV2/PI489777)SNP_14145	835	G	T	229	60	--	--
CakTC29862	Ca(ICCV2/PI489777)SNP_14146	291	G	A	6	7	--	--
	Ca(ICCV2/PI489777)SNP_14147	1056	G	A	6	8	--	--
	Ca(ICCV2/PI489777)SNP_14148	2053	T	A	28	6	--	--
CakTC30706	Ca(ICCV2/PI489777)SNP_14149	771	A	G	7	3	--	--
CakTC41769	Ca(ICCV2/PI489777)SNP_14150	422	A	G	4	12	--	AP2-EREBP
	Ca(ICCV2/PI489777)SNP_14151	437	G	A	4	11	--	AP2-EREBP
CakTC42100	Ca(ICCV2/PI489777)SNP_14152	983	T	G	9	9	--	--
	Ca(ICCV2/PI489777)SNP_14153	1335	T	A	21	4	--	--
	Ca(ICCV2/PI489777)SNP_14154	1480	C	T	15	4	--	--
CakTC10259	Ca(ICCV2/PI489777)SNP_14155	3038	C	G	8	7	--	--
	Ca(ICCV2/PI489777)SNP_14156	3049	G	A	8	7	--	--
CakTC38802	Ca(ICCV2/PI489777)SNP_14157	699	G	A	14	14	Flower	bud
	Ca(ICCV2/PI489777)SNP_14158	840	T	C	8	12	Flower	bud
CakTC23014	Ca(ICCV2/PI489777)SNP_14159	1389	G	T	7	4	--	--
	Ca(ICCV2/PI489777)SNP_14160	1466	T	C	9	5	--	--
	Ca(ICCV2/PI489777)SNP_14161	1534	G	A	8	4	--	--
CakTC27710	Ca(ICCV2/PI489777)SNP_14162	1496	A	G	3	3	--	--
	Ca(ICCV2/PI489777)SNP_14163	1886	C	A	4	4	--	--
CakTC30016	Ca(ICCV2/PI489777)SNP_14164	1954	C	T	11	6	--	--
CakTC31686	Ca(ICCV2/PI489777)SNP_14165	293	C	T	15	3	--	--
	Ca(ICCV2/PI489777)SNP_14166	416	T	C	23	4	--	--
	Ca(ICCV2/PI489777)SNP_14167	443	T	A	25	4	--	--
	Ca(ICCV2/PI489777)SNP_14168	449	G	T	26	4	--	--
	Ca(ICCV2/PI489777)SNP_14169	506	C	T	25	4	--	--
	Ca(ICCV2/PI489777)SNP_14170	522	A	G	23	4	--	--
CakTC42010	Ca(ICCV2/PI489777)SNP_14171	336	G	T	13	5	--	--
	Ca(ICCV2/PI489777)SNP_14172	536	T	C	13	16	--	--
	Ca(ICCV2/PI489777)SNP_14173	1005	C	T	19	10	--	--
	Ca(ICCV2/PI489777)SNP_14174	1545	A	G	12	17	--	--
CakTC02652	Ca(ICCV2/PI489777)SNP_14175	10	T	C	4	3	--	--
	Ca(ICCV2/PI489777)SNP_14176	210	A	G	6	4	--	--
CakTC38809	Ca(ICCV2/PI489777)SNP_14177	264	T	C	9	12	--	--
	Ca(ICCV2/PI489777)SNP_14178	306	G	A	6	14	--	--
	Ca(ICCV2/PI489777)SNP_14179	897	C	T	7	4	--	--
	Ca(ICCV2/PI489777)SNP_14180	1221	A	C	8	10	--	--
	Ca(ICCV2/PI489777)SNP_14181	1301	C	T	10	9	--	--
	Ca(ICCV2/PI489777)SNP_14182	1309	T	C	9	8	--	--
	Ca(ICCV2/PI489777)SNP_14183	1351	G	C	8	7	--	--
CakTC39368	Ca(ICCV2/PI489777)SNP_14184	165	G	C	4	14	--	--
	Ca(ICCV2/PI489777)SNP_14185	265	C	G	6	15	--	--
	Ca(ICCV2/PI489777)SNP_14186	557	T	G	9	10	--	--
	Ca(ICCV2/PI489777)SNP_14187	701	T	C	13	11	--	--
	Ca(ICCV2/PI489777)SNP_14188	929	C	T	16	5	--	--
	Ca(ICCV2/PI489777)SNP_14189	1143	C	T	27	6	--	--
	Ca(ICCV2/PI489777)SNP_14190	1277	A	G	17	4	--	--
	Ca(ICCV2/PI489777)SNP_14191	1386	T	C	11	4	--	--
CakTC25730	Ca(ICCV2/PI489777)SNP_14192	380	G	T	13	5	--	--
CakTC26841	Ca(ICCV2/PI489777)SNP_14193	528	T	A	9	4	--	--
	Ca(ICCV2/PI489777)SNP_14194	632	G	A	9	4	--	--
	Ca(ICCV2/PI489777)SNP_14195	635	A	G	11	4	--	--
	Ca(ICCV2/PI489777)SNP_14196	750	T	G	8	4	--	--
CakTC34532	Ca(ICCV2/PI489777)SNP_14197	336	T	C	3	4	--	--
CakTC38105	Ca(ICCV2/PI489777)SNP_14198	359	C	T	23	11	--	--
	Ca(ICCV2/PI489777)SNP_14199	527	C	T	11	4	--	--
	Ca(ICCV2/PI489777)SNP_14200	560	T	A	4	6	--	--
	Ca(ICCV2/PI489777)SNP_14201	1000	G	C	10	7	--	--
CakTC24792	Ca(ICCV2/PI489777)SNP_14202	500	T	G	17	8	--	--
	Ca(ICCV2/PI489777)SNP_14203	698	G	A	21	10	--	--

	Ca(ICCV2/PI489777)SNP_14204	797	T	C	15	7	--	--
	Ca(ICCV2/PI489777)SNP_14205	1202	A	G	13	9	--	--
CakTC29970	Ca(ICCV2/PI489777)SNP_14206	422	G	A	20	13	--	--
	Ca(ICCV2/PI489777)SNP_14207	520	G	A	20	9	--	--
	Ca(ICCV2/PI489777)SNP_14208	759	T	C	15	3	--	--
	Ca(ICCV2/PI489777)SNP_14209	1030	C	T	9	4	--	--
	Ca(ICCV2/PI489777)SNP_14210	1930	T	C	11	7	--	--
	Ca(ICCV2/PI489777)SNP_14211	2266	C	T	12	11	--	--
	Ca(ICCV2/PI489777)SNP_14212	2437	T	C	12	7	--	--
	Ca(ICCV2/PI489777)SNP_14213	2536	A	C	10	6	--	--
	Ca(ICCV2/PI489777)SNP_14214	2797	G	C	10	4	--	--
	Ca(ICCV2/PI489777)SNP_14215	3436	A	G	8	5	--	--
	Ca(ICCV2/PI489777)SNP_14216	3439	G	A	7	5	--	--
	Ca(ICCV2/PI489777)SNP_14217	3935	G	C	14	3	--	--
	Ca(ICCV2/PI489777)SNP_14218	3948	C	G	13	3	--	--
	Ca(ICCV2/PI489777)SNP_14219	3950	G	A	12	3	--	--
CakTC41579	Ca(ICCV2/PI489777)SNP_14220	222	T	C	30	17	--	--
	Ca(ICCV2/PI489777)SNP_14221	498	G	A	43	15	--	--
	Ca(ICCV2/PI489777)SNP_14222	516	A	G	44	16	--	--
	Ca(ICCV2/PI489777)SNP_14223	531	C	T	46	14	--	--
	Ca(ICCV2/PI489777)SNP_14224	690	C	T	52	12	--	--
	Ca(ICCV2/PI489777)SNP_14225	1095	G	A	59	20	--	--
	Ca(ICCV2/PI489777)SNP_14226	2007	A	T	34	11	--	--
CakTC34515	Ca(ICCV2/PI489777)SNP_14227	677	T	C	3	8	--	--
	Ca(ICCV2/PI489777)SNP_14228	699	T	C	3	7	--	--
	Ca(ICCV2/PI489777)SNP_14229	749	T	C	3	9	--	--
	Ca(ICCV2/PI489777)SNP_14230	879	T	C	4	5	--	--
	Ca(ICCV2/PI489777)SNP_14231	880	C	T	4	5	--	--
	Ca(ICCV2/PI489777)SNP_14232	897	T	C	4	5	--	--
	Ca(ICCV2/PI489777)SNP_14233	994	A	G	3	3	--	--
	Ca(ICCV2/PI489777)SNP_14234	996	T	C	3	3	--	--
	Ca(ICCV2/PI489777)SNP_14235	1019	A	G	3	3	--	--
CakTC40192	Ca(ICCV2/PI489777)SNP_14236	1566	C	G	9	15	--	--
	Ca(ICCV2/PI489777)SNP_14237	1567	T	G	9	15	--	--
CakTC28583	Ca(ICCV2/PI489777)SNP_14238	1219	T	G	6	7	--	--
	Ca(ICCV2/PI489777)SNP_14239	1299	G	C	7	7	--	--
CakTC35356	Ca(ICCV2/PI489777)SNP_14240	254	C	G	14	10	--	--
	Ca(ICCV2/PI489777)SNP_14241	886	G	A	54	17	--	--
	Ca(ICCV2/PI489777)SNP_14242	1360	G	A	56	20	--	--
	Ca(ICCV2/PI489777)SNP_14243	3325	C	T	31	9	--	--
	Ca(ICCV2/PI489777)SNP_14244	4106	C	G	40	16	--	--
CakTC41669	Ca(ICCV2/PI489777)SNP_14245	2055	C	G	8	6	--	--
CakTC37936	Ca(ICCV2/PI489777)SNP_14246	115	C	T	5	10	--	--
	Ca(ICCV2/PI489777)SNP_14247	538	G	A	7	6	--	--
CakTC42811	Ca(ICCV2/PI489777)SNP_14248	255	T	C	4	4	--	--
	Ca(ICCV2/PI489777)SNP_14249	929	T	C	29	12	--	--
	Ca(ICCV2/PI489777)SNP_14250	1006	G	A	28	18	--	--
	Ca(ICCV2/PI489777)SNP_14251	1388	T	C	35	7	--	--
	Ca(ICCV2/PI489777)SNP_14252	1790	T	C	30	8	--	--
CakTC23509	Ca(ICCV2/PI489777)SNP_14253	253	A	G	95	136	--	--
	Ca(ICCV2/PI489777)SNP_14254	768	G	A	290	191	--	--
CakTC12385	Ca(ICCV2/PI489777)SNP_14255	7769	T	C	7	3	--	--
CakTC33341	Ca(ICCV2/PI489777)SNP_14256	209	T	C	7	7	--	GRAS
	Ca(ICCV2/PI489777)SNP_14257	755	C	G	5	6	--	GRAS
	Ca(ICCV2/PI489777)SNP_14258	778	A	G	6	7	--	GRAS
	Ca(ICCV2/PI489777)SNP_14259	882	G	A	8	4	--	GRAS
	Ca(ICCV2/PI489777)SNP_14260	1358	A	G	7	3	--	GRAS
	Ca(ICCV2/PI489777)SNP_14261	1464	G	A	4	4	--	GRAS
CakTC37140	Ca(ICCV2/PI489777)SNP_14262	246	C	T	7	14	--	--
CakTC27234	Ca(ICCV2/PI489777)SNP_14263	109	T	C	4	6	--	--
	Ca(ICCV2/PI489777)SNP_14264	767	C	T	3	3	--	--
	Ca(ICCV2/PI489777)SNP_14265	1497	C	A	6	5	--	--
CakTC09678	Ca(ICCV2/PI489777)SNP_14266	348	T	A	50	40	--	--
CakTC36289	Ca(ICCV2/PI489777)SNP_14267	90	C	G	18	4	--	C3H
	Ca(ICCV2/PI489777)SNP_14268	226	G	C	19	5	--	C3H
	Ca(ICCV2/PI489777)SNP_14269	239	A	G	15	5	--	C3H
	Ca(ICCV2/PI489777)SNP_14270	253	G	T	17	4	--	C3H
	Ca(ICCV2/PI489777)SNP_14271	575	A	G	9	9	--	C3H
	Ca(ICCV2/PI489777)SNP_14272	796	C	G	12	3	--	C3H
	Ca(ICCV2/PI489777)SNP_14273	800	T	C	13	3	--	C3H
	Ca(ICCV2/PI489777)SNP_14274	879	G	T	9	3	--	C3H
	Ca(ICCV2/PI489777)SNP_14275	923	G	A	9	7	--	C3H
	Ca(ICCV2/PI489777)SNP_14276	1439	G	A	30	13	--	C3H
	Ca(ICCV2/PI489777)SNP_14277	1485	A	G	36	12	--	C3H

	Ca(ICCV2/PI489777)SNP_14278	1667	G	C	35	15	--	C3H
	Ca(ICCV2/PI489777)SNP_14279	2119	T	C	22	11	--	C3H
	Ca(ICCV2/PI489777)SNP_14280	2292	T	G	27	18	--	C3H
CakTC03181	Ca(ICCV2/PI489777)SNP_14281	648	C	A	3	4	Root	--
CakTC32456	Ca(ICCV2/PI489777)SNP_14282	137	A	T	5	7	--	--
	Ca(ICCV2/PI489777)SNP_14283	218	C	T	7	7	--	--
	Ca(ICCV2/PI489777)SNP_14284	810	T	C	12	10	--	--
	Ca(ICCV2/PI489777)SNP_14285	1498	A	C	13	5	--	--
CakTC43201	Ca(ICCV2/PI489777)SNP_14286	554	G	A	6	3	--	--
	Ca(ICCV2/PI489777)SNP_14287	618	G	T	3	3	--	--
CakTC26456	Ca(ICCV2/PI489777)SNP_14288	31	A	G	5	5	--	--
	Ca(ICCV2/PI489777)SNP_14289	153	G	C	35	18	--	--
	Ca(ICCV2/PI489777)SNP_14290	944	G	T	21	9	--	--
CakTC22781	Ca(ICCV2/PI489777)SNP_14291	449	C	T	498	196	--	--
	Ca(ICCV2/PI489777)SNP_14292	542	T	G	311	150	--	--
	Ca(ICCV2/PI489777)SNP_14293	596	C	T	159	101	--	--
CakTC10133	Ca(ICCV2/PI489777)SNP_14294	174	T	A	18	5	--	--
	Ca(ICCV2/PI489777)SNP_14295	206	G	A	17	5	--	--
	Ca(ICCV2/PI489777)SNP_14296	443	C	T	16	7	--	--
	Ca(ICCV2/PI489777)SNP_14297	1046	A	G	12	3	--	--
	Ca(ICCV2/PI489777)SNP_14298	1081	T	G	4	3	--	--
CakTC02937	Ca(ICCV2/PI489777)SNP_14299	124	T	C	4	4	--	--
	Ca(ICCV2/PI489777)SNP_14300	520	T	C	6	3	--	--
CakTC23609	Ca(ICCV2/PI489777)SNP_14301	162	T	C	9	11	--	--
	Ca(ICCV2/PI489777)SNP_14302	236	T	C	16	20	--	--
	Ca(ICCV2/PI489777)SNP_14303	342	G	A	29	23	--	--
	Ca(ICCV2/PI489777)SNP_14304	415	T	C	19	22	--	--
	Ca(ICCV2/PI489777)SNP_14305	798	G	A	14	8	--	--
	Ca(ICCV2/PI489777)SNP_14306	1065	C	T	52	24	--	--
	Ca(ICCV2/PI489777)SNP_14307	1077	C	T	49	21	--	--
	Ca(ICCV2/PI489777)SNP_14308	1255	A	G	30	16	--	--
CakTC26417	Ca(ICCV2/PI489777)SNP_14309	435	A	C	25	15	--	--
	Ca(ICCV2/PI489777)SNP_14310	935	A	G	22	13	--	--
CakTC41250	Ca(ICCV2/PI489777)SNP_14311	325	C	T	19	5	--	--
	Ca(ICCV2/PI489777)SNP_14312	328	C	A	19	5	--	--
	Ca(ICCV2/PI489777)SNP_14313	339	C	T	17	4	--	--
CakTC28189	Ca(ICCV2/PI489777)SNP_14314	904	A	G	4	3	--	--
CakTC41751	Ca(ICCV2/PI489777)SNP_14315	1520	G	A	19	12	--	--
	Ca(ICCV2/PI489777)SNP_14316	2330	C	T	6	7	--	--
CakTC37837	Ca(ICCV2/PI489777)SNP_14317	914	G	A	20	24	--	--
	Ca(ICCV2/PI489777)SNP_14318	959	G	C	16	19	--	--
	Ca(ICCV2/PI489777)SNP_14319	1203	G	C	8	7	--	--
	Ca(ICCV2/PI489777)SNP_14320	1219	C	G	7	5	--	--
CakTC13750	Ca(ICCV2/PI489777)SNP_14321	651	A	G	5	5	--	--
CakTC27630	Ca(ICCV2/PI489777)SNP_14322	711	T	C	12	13	--	--
	Ca(ICCV2/PI489777)SNP_14323	990	A	G	13	9	--	--
CakTC43178	Ca(ICCV2/PI489777)SNP_14324	744	G	T	11	6	--	--
CakTC29536	Ca(ICCV2/PI489777)SNP_14325	1426	A	G	30	18	--	--
CakTC30970	Ca(ICCV2/PI489777)SNP_14326	1394	C	T	7	4	--	--
	Ca(ICCV2/PI489777)SNP_14327	1409	C	A	6	4	--	--
	Ca(ICCV2/PI489777)SNP_14328	2516	C	G	4	3	--	--
CakTC42302	Ca(ICCV2/PI489777)SNP_14329	279	T	G	3	3	--	--
CakTC23916	Ca(ICCV2/PI489777)SNP_14330	470	A	T	5	9	--	--
	Ca(ICCV2/PI489777)SNP_14331	1073	T	G	7	7	--	--
	Ca(ICCV2/PI489777)SNP_14332	2912	G	A	3	6	--	--
	Ca(ICCV2/PI489777)SNP_14333	3191	C	T	3	7	--	--
	Ca(ICCV2/PI489777)SNP_14334	3463	T	A	3	3	--	--
CakTC02800	Ca(ICCV2/PI489777)SNP_14335	120	T	C	6	3	--	--
	Ca(ICCV2/PI489777)SNP_14336	147	A	T	5	3	--	--
CakTC31305	Ca(ICCV2/PI489777)SNP_14337	411	T	C	3	3	--	--
	Ca(ICCV2/PI489777)SNP_14338	802	A	G	3	3	--	--
	Ca(ICCV2/PI489777)SNP_14339	818	A	G	3	3	--	--
	Ca(ICCV2/PI489777)SNP_14340	820	G	A	3	3	--	--
	Ca(ICCV2/PI489777)SNP_14341	850	A	G	4	3	--	--
	Ca(ICCV2/PI489777)SNP_14342	903	C	T	4	3	--	--
	Ca(ICCV2/PI489777)SNP_14343	915	A	G	4	3	--	--
	Ca(ICCV2/PI489777)SNP_14344	1157	A	G	3	6	--	--
CakTC37154	Ca(ICCV2/PI489777)SNP_14345	369	A	G	33	19	--	--
	Ca(ICCV2/PI489777)SNP_14346	390	C	T	70	33	--	--
	Ca(ICCV2/PI489777)SNP_14347	553	A	C	55	24	--	--
	Ca(ICCV2/PI489777)SNP_14348	1182	C	A	5	3	--	--
CakTC38008	Ca(ICCV2/PI489777)SNP_14349	926	G	C	21	8	--	--
	Ca(ICCV2/PI489777)SNP_14350	931	C	T	21	8	--	--
	Ca(ICCV2/PI489777)SNP_14351	972	G	T	19	6	--	--

CakTC41498	Ca(ICCV2/PI489777)SNP_14352	91	A	C	5	3	--	--
	Ca(ICCV2/PI489777)SNP_14353	433	A	G	40	9	--	--
CakTC39393	Ca(ICCV2/PI489777)SNP_14354	287	T	C	18	14	--	--
	Ca(ICCV2/PI489777)SNP_14355	603	C	A	31	26	--	--
	Ca(ICCV2/PI489777)SNP_14356	714	G	C	45	27	--	--
	Ca(ICCV2/PI489777)SNP_14357	1331	G	T	20	13	--	--
CakTC40825	Ca(ICCV2/PI489777)SNP_14358	359	G	A	15	4	--	--
	Ca(ICCV2/PI489777)SNP_14359	1682	A	T	22	13	--	--
CakTC25517	Ca(ICCV2/PI489777)SNP_14360	309	G	A	15	10	--	--
CakTC34703	Ca(ICCV2/PI489777)SNP_14361	393	A	T	50	37	--	--
	Ca(ICCV2/PI489777)SNP_14362	573	C	T	53	35	--	--
	Ca(ICCV2/PI489777)SNP_14363	1062	G	A	93	39	--	--
	Ca(ICCV2/PI489777)SNP_14364	2286	C	T	46	31	--	--
	Ca(ICCV2/PI489777)SNP_14365	2513	A	C	53	30	--	--
	Ca(ICCV2/PI489777)SNP_14366	3321	A	G	43	31	--	--
	Ca(ICCV2/PI489777)SNP_14367	3576	G	C	17	19	--	--
CakTC40064	Ca(ICCV2/PI489777)SNP_14368	874	A	G	31	12	--	--
	Ca(ICCV2/PI489777)SNP_14369	929	T	C	35	11	--	--
	Ca(ICCV2/PI489777)SNP_14370	1528	G	A	15	7	--	--
CakTC34155	Ca(ICCV2/PI489777)SNP_14371	181	A	C	13	11	--	--
	Ca(ICCV2/PI489777)SNP_14372	663	C	T	14	8	--	--
	Ca(ICCV2/PI489777)SNP_14373	978	T	C	30	16	--	--
	Ca(ICCV2/PI489777)SNP_14374	1401	T	C	28	14	--	--
	Ca(ICCV2/PI489777)SNP_14375	2145	T	G	21	24	--	--
	Ca(ICCV2/PI489777)SNP_14376	2265	G	A	25	25	--	--
	Ca(ICCV2/PI489777)SNP_14377	3495	T	C	28	9	--	--
	Ca(ICCV2/PI489777)SNP_14378	3573	A	G	31	10	--	--
	Ca(ICCV2/PI489777)SNP_14379	5208	A	C	38	25	--	--
	Ca(ICCV2/PI489777)SNP_14380	5991	T	A	22	31	--	--
	Ca(ICCV2/PI489777)SNP_14381	6177	A	C	27	32	--	--
	Ca(ICCV2/PI489777)SNP_14382	6267	T	C	28	23	--	--
	Ca(ICCV2/PI489777)SNP_14383	6483	A	G	28	16	--	--
	Ca(ICCV2/PI489777)SNP_14384	7392	C	T	31	16	--	--
CakTC26831	Ca(ICCV2/PI489777)SNP_14385	564	G	A	192	83	--	--
CakTC24852	Ca(ICCV2/PI489777)SNP_14386	283	C	G	6	3	Shoot	--
CakTC35519	Ca(ICCV2/PI489777)SNP_14387	1309	T	C	8	11	--	--
	Ca(ICCV2/PI489777)SNP_14388	1427	G	A	6	9	--	--
	Ca(ICCV2/PI489777)SNP_14389	1504	C	T	5	8	--	--
	Ca(ICCV2/PI489777)SNP_14390	2465	T	C	4	4	--	--
	Ca(ICCV2/PI489777)SNP_14391	2792	C	T	8	3	--	--
CakTC13699	Ca(ICCV2/PI489777)SNP_14392	1616	A	G	4	12	--	--
CakTC10780	Ca(ICCV2/PI489777)SNP_14393	421	A	G	71	28	--	--
	Ca(ICCV2/PI489777)SNP_14394	987	C	G	121	43	--	--
	Ca(ICCV2/PI489777)SNP_14395	1510	T	A	116	35	--	--
	Ca(ICCV2/PI489777)SNP_14396	2497	A	G	83	35	--	--
	Ca(ICCV2/PI489777)SNP_14397	2640	A	G	79	31	--	--
	Ca(ICCV2/PI489777)SNP_14398	2746	G	A	61	29	--	--
CakTC40837	Ca(ICCV2/PI489777)SNP_14399	1178	T	C	120	20	--	--
	Ca(ICCV2/PI489777)SNP_14400	1209	C	T	120	21	--	--
	Ca(ICCV2/PI489777)SNP_14401	1245	A	C	130	33	--	--
	Ca(ICCV2/PI489777)SNP_14402	1310	C	T	119	32	--	--
CakTC39432	Ca(ICCV2/PI489777)SNP_14403	161	G	A	12	7	--	--
	Ca(ICCV2/PI489777)SNP_14404	436	G	A	9	10	--	--
	Ca(ICCV2/PI489777)SNP_14405	972	T	C	11	13	--	--
	Ca(ICCV2/PI489777)SNP_14406	1324	A	G	6	9	--	--
	Ca(ICCV2/PI489777)SNP_14407	1326	T	C	6	9	--	--
	Ca(ICCV2/PI489777)SNP_14408	1341	T	C	6	8	--	--
CakTC12418	Ca(ICCV2/PI489777)SNP_14409	219	A	G	4	5	--	--
	Ca(ICCV2/PI489777)SNP_14410	252	C	T	4	5	--	--
	Ca(ICCV2/PI489777)SNP_14411	288	T	C	4	6	--	--
CakTC41282	Ca(ICCV2/PI489777)SNP_14412	1033	A	C	6	14	--	--
CakTC28648	Ca(ICCV2/PI489777)SNP_14413	57	G	A	5	3	--	--
	Ca(ICCV2/PI489777)SNP_14414	411	C	T	14	4	--	--
CakTC39670	Ca(ICCV2/PI489777)SNP_14415	1008	T	A	8	4	--	--
CakTC28009	Ca(ICCV2/PI489777)SNP_14416	859	A	G	6	4	--	--
	Ca(ICCV2/PI489777)SNP_14417	1031	T	C	4	6	--	--
	Ca(ICCV2/PI489777)SNP_14418	1045	A	T	4	6	--	--
CakTC12266	Ca(ICCV2/PI489777)SNP_14419	237	G	A	6	5	--	--
CakTC32560	Ca(ICCV2/PI489777)SNP_14420	51	C	T	4	5	--	--
	Ca(ICCV2/PI489777)SNP_14421	59	G	T	5	5	--	--
	Ca(ICCV2/PI489777)SNP_14422	247	C	T	5	6	--	--
CakTC39614	Ca(ICCV2/PI489777)SNP_14423	2061	C	G	16	9	--	--
CakTC11472	Ca(ICCV2/PI489777)SNP_14424	271	A	G	38	22	--	--
CakTC12526	Ca(ICCV2/PI489777)SNP_14425	717	G	T	3	3	--	--

	Ca(ICCV2/PI489777)SNP_14426	787	G	A	3	3	--	--
CakTC10593	Ca(ICCV2/PI489777)SNP_14427	893	C	T	6	4	--	GRAS
CakTC31395	Ca(ICCV2/PI489777)SNP_14428	1660	T	C	4	3	--	--
	Ca(ICCV2/PI489777)SNP_14429	1665	A	C	5	3	--	--
	Ca(ICCV2/PI489777)SNP_14430	1753	C	G	6	6	--	--
	Ca(ICCV2/PI489777)SNP_14431	2568	A	G	6	5	--	--
CakTC34371	Ca(ICCV2/PI489777)SNP_14432	64	T	G	12	9	--	--
CakTC23234	Ca(ICCV2/PI489777)SNP_14433	280	G	T	4	5	--	--
CakTC31343	Ca(ICCV2/PI489777)SNP_14434	273	C	T	4	3	--	--
	Ca(ICCV2/PI489777)SNP_14435	288	G	A	5	3	--	--
CakTC30154	Ca(ICCV2/PI489777)SNP_14436	150	C	T	4	4	--	--
	Ca(ICCV2/PI489777)SNP_14437	240	A	G	4	4	--	--
CakTC32683	Ca(ICCV2/PI489777)SNP_14438	1140	T	C	6	7	--	--
	Ca(ICCV2/PI489777)SNP_14439	1320	C	T	3	3	--	--
	Ca(ICCV2/PI489777)SNP_14440	2259	C	T	9	3	--	--
	Ca(ICCV2/PI489777)SNP_14441	2520	T	G	11	4	--	--
	Ca(ICCV2/PI489777)SNP_14442	4539	T	C	5	5	--	--
	Ca(ICCV2/PI489777)SNP_14443	5310	T	A	4	4	--	--
CakTC07889	Ca(ICCV2/PI489777)SNP_14444	184	G	A	10	3	--	--
	Ca(ICCV2/PI489777)SNP_14445	500	T	C	10	10	--	--
	Ca(ICCV2/PI489777)SNP_14446	2850	T	C	7	4	--	--
	Ca(ICCV2/PI489777)SNP_14447	2880	G	A	4	4	--	--
	Ca(ICCV2/PI489777)SNP_14448	2910	T	C	4	5	--	--
	Ca(ICCV2/PI489777)SNP_14449	2967	A	G	4	4	--	--
	Ca(ICCV2/PI489777)SNP_14450	3132	C	T	5	7	--	--
	Ca(ICCV2/PI489777)SNP_14451	3264	T	C	7	6	--	--
CakTC37156	Ca(ICCV2/PI489777)SNP_14452	405	C	T	9	8	--	--
	Ca(ICCV2/PI489777)SNP_14453	748	T	C	5	5	--	--
CakTC38922	Ca(ICCV2/PI489777)SNP_14454	771	A	T	17	9	--	--
CakTC23951	Ca(ICCV2/PI489777)SNP_14455	229	T	C	46	7	--	--
	Ca(ICCV2/PI489777)SNP_14456	331	G	A	45	6	--	--
CakTC33877	Ca(ICCV2/PI489777)SNP_14457	195	A	G	112	26	--	--
	Ca(ICCV2/PI489777)SNP_14458	219	C	T	113	28	--	--
CakTC37401	Ca(ICCV2/PI489777)SNP_14459	184	G	A	41	26	--	--
	Ca(ICCV2/PI489777)SNP_14460	378	C	T	64	39	--	--
	Ca(ICCV2/PI489777)SNP_14461	435	G	T	69	37	--	--
	Ca(ICCV2/PI489777)SNP_14462	987	T	C	45	25	--	--
CakTC37982	Ca(ICCV2/PI489777)SNP_14463	75	C	T	14	5	--	--
	Ca(ICCV2/PI489777)SNP_14464	126	T	C	15	5	--	--
	Ca(ICCV2/PI489777)SNP_14465	133	G	A	15	4	--	--
	Ca(ICCV2/PI489777)SNP_14466	150	C	T	15	5	--	--
	Ca(ICCV2/PI489777)SNP_14467	223	T	C	14	3	--	--
	Ca(ICCV2/PI489777)SNP_14468	624	C	T	9	7	--	--
	Ca(ICCV2/PI489777)SNP_14469	996	G	C	17	6	--	--
CakTC32271	Ca(ICCV2/PI489777)SNP_14470	2585	G	A	14	3	--	--
	Ca(ICCV2/PI489777)SNP_14471	3987	A	G	14	7	--	--
	Ca(ICCV2/PI489777)SNP_14472	4515	C	T	16	4	--	--
	Ca(ICCV2/PI489777)SNP_14473	4694	T	C	19	6	--	--
	Ca(ICCV2/PI489777)SNP_14474	4915	T	C	20	10	--	--
	Ca(ICCV2/PI489777)SNP_14475	5139	C	T	12	9	--	--
	Ca(ICCV2/PI489777)SNP_14476	5161	G	A	16	12	--	--
	Ca(ICCV2/PI489777)SNP_14477	5168	T	A	13	12	--	--
	Ca(ICCV2/PI489777)SNP_14478	5249	C	G	10	12	--	--
CakTC43018	Ca(ICCV2/PI489777)SNP_14479	32	A	T	30	18	--	--
CakTC10923	Ca(ICCV2/PI489777)SNP_14480	466	T	C	66	84	--	--
	Ca(ICCV2/PI489777)SNP_14481	601	C	T	60	93	--	--
	Ca(ICCV2/PI489777)SNP_14482	736	C	T	57	83	--	--
	Ca(ICCV2/PI489777)SNP_14483	979	C	T	65	100	--	--
CakTC39866	Ca(ICCV2/PI489777)SNP_14484	1140	A	C	20	15	--	--
CakTC28232	Ca(ICCV2/PI489777)SNP_14485	734	A	C	14	11	--	--
	Ca(ICCV2/PI489777)SNP_14486	1098	G	A	72	20	--	--
	Ca(ICCV2/PI489777)SNP_14487	1428	C	T	67	30	--	--
CakTC35738	Ca(ICCV2/PI489777)SNP_14488	1186	T	G	7	9	--	--
	Ca(ICCV2/PI489777)SNP_14489	3664	G	A	4	6	--	--
	Ca(ICCV2/PI489777)SNP_14490	3893	A	C	9	8	--	--
CakTC40153	Ca(ICCV2/PI489777)SNP_14491	229	T	G	7	11	--	--
	Ca(ICCV2/PI489777)SNP_14492	305	G	A	11	9	--	--
	Ca(ICCV2/PI489777)SNP_14493	608	A	G	16	15	--	--
	Ca(ICCV2/PI489777)SNP_14494	887	A	G	10	7	--	--
	Ca(ICCV2/PI489777)SNP_14495	959	G	C	10	8	--	--
	Ca(ICCV2/PI489777)SNP_14496	989	A	G	9	9	--	--
CakTC38128	Ca(ICCV2/PI489777)SNP_14497	162	A	G	8	6	--	--
CakTC39936	Ca(ICCV2/PI489777)SNP_14498	992	T	C	7	7	--	--
	Ca(ICCV2/PI489777)SNP_14499	1204	A	C	8	8	--	--

CakTC41181	Ca(ICCV2/PI489777)SNP_14500	98	A	G	12	33	--	--
CakTC02206	Ca(ICCV2/PI489777)SNP_14501	353	C	G	10	19	--	--
	Ca(ICCV2/PI489777)SNP_14502	791	G	A	4	11	--	--
CakTC39677	Ca(ICCV2/PI489777)SNP_14503	149	G	A	5	9	--	--
	Ca(ICCV2/PI489777)SNP_14504	956	G	T	7	7	--	--
CakTC41999	Ca(ICCV2/PI489777)SNP_14505	439	C	A	7	6	Root	MADS
	Ca(ICCV2/PI489777)SNP_14506	752	G	A	6	3	Root	MADS
	Ca(ICCV2/PI489777)SNP_14507	810	G	A	9	6	Root	MADS
	Ca(ICCV2/PI489777)SNP_14508	838	C	T	8	6	Root	MADS
	Ca(ICCV2/PI489777)SNP_14509	868	T	C	7	6	Root	MADS
	Ca(ICCV2/PI489777)SNP_14510	972	T	G	5	6	Root	MADS
	Ca(ICCV2/PI489777)SNP_14511	984	T	C	5	6	Root	MADS
	Ca(ICCV2/PI489777)SNP_14512	999	T	A	5	4	Root	MADS
CakTC26525	Ca(ICCV2/PI489777)SNP_14513	234	G	C	51	9	--	--
	Ca(ICCV2/PI489777)SNP_14514	541	T	A	66	9	--	--
	Ca(ICCV2/PI489777)SNP_14515	1267	T	C	35	10	--	--
CakTC25986	Ca(ICCV2/PI489777)SNP_14516	774	G	A	5	3	--	--
CakTC16981	Ca(ICCV2/PI489777)SNP_14517	165	C	G	33	6	--	--
	Ca(ICCV2/PI489777)SNP_14518	579	A	G	57	19	--	--
	Ca(ICCV2/PI489777)SNP_14519	1354	T	A	49	51	--	--
CakTC09826	Ca(ICCV2/PI489777)SNP_14520	1024	G	C	4	3	--	--
	Ca(ICCV2/PI489777)SNP_14521	1349	A	G	5	3	--	--
	Ca(ICCV2/PI489777)SNP_14522	1572	A	G	4	3	--	--
CakTC12271	Ca(ICCV2/PI489777)SNP_14523	309	T	C	7	4	Flower	bud
CakTC10967	Ca(ICCV2/PI489777)SNP_14524	1245	T	C	5	3	--	MYB
CakTC30373	Ca(ICCV2/PI489777)SNP_14525	489	G	T	5	3	--	--
	Ca(ICCV2/PI489777)SNP_14526	588	G	A	4	3	--	--
	Ca(ICCV2/PI489777)SNP_14527	594	C	T	4	3	--	--
	Ca(ICCV2/PI489777)SNP_14528	600	G	A	4	3	--	--
	Ca(ICCV2/PI489777)SNP_14529	653	T	C	6	5	--	--
	Ca(ICCV2/PI489777)SNP_14530	675	A	G	6	4	--	--
	Ca(ICCV2/PI489777)SNP_14531	693	G	A	6	5	--	--
CakTC36780	Ca(ICCV2/PI489777)SNP_14532	528	C	G	10	6	--	--
	Ca(ICCV2/PI489777)SNP_14533	1073	T	C	18	4	--	--
	Ca(ICCV2/PI489777)SNP_14534	1075	T	A	18	4	--	--
	Ca(ICCV2/PI489777)SNP_14535	1113	A	G	14	4	--	--
	Ca(ICCV2/PI489777)SNP_14536	1345	A	C	9	5	--	--
	Ca(ICCV2/PI489777)SNP_14537	1359	T	C	10	5	--	--
	Ca(ICCV2/PI489777)SNP_14538	1513	T	C	6	5	--	--
	Ca(ICCV2/PI489777)SNP_14539	1688	A	C	11	8	--	--
	Ca(ICCV2/PI489777)SNP_14540	1783	C	A	14	6	--	--
	Ca(ICCV2/PI489777)SNP_14541	1800	A	G	13	6	--	--
	Ca(ICCV2/PI489777)SNP_14542	1862	T	G	17	5	--	--
	Ca(ICCV2/PI489777)SNP_14543	2088	C	G	13	7	--	--
	Ca(ICCV2/PI489777)SNP_14544	2591	C	T	19	5	--	--
	Ca(ICCV2/PI489777)SNP_14545	3291	C	T	15	3	--	--
CakTC39343	Ca(ICCV2/PI489777)SNP_14546	539	T	C	3	10	--	--
	Ca(ICCV2/PI489777)SNP_14547	1094	T	A	4	6	--	--
	Ca(ICCV2/PI489777)SNP_14548	1106	A	G	6	4	--	--
CakTC31798	Ca(ICCV2/PI489777)SNP_14549	570	A	G	13	4	--	--
	Ca(ICCV2/PI489777)SNP_14550	753	G	A	14	7	--	--
	Ca(ICCV2/PI489777)SNP_14551	1199	G	C	14	3	--	--
CakTC26790	Ca(ICCV2/PI489777)SNP_14552	240	C	T	36	6	--	--
CakTC29916	Ca(ICCV2/PI489777)SNP_14553	906	T	C	28	8	--	--
	Ca(ICCV2/PI489777)SNP_14554	942	A	C	23	8	--	--
	Ca(ICCV2/PI489777)SNP_14555	1248	A	T	14	8	--	--
	Ca(ICCV2/PI489777)SNP_14556	1455	A	G	8	6	--	--
	Ca(ICCV2/PI489777)SNP_14557	1599	A	G	4	10	--	--
	Ca(ICCV2/PI489777)SNP_14558	2143	G	A	8	12	--	--
	Ca(ICCV2/PI489777)SNP_14559	2639	T	C	8	9	--	--
CakTC18662	Ca(ICCV2/PI489777)SNP_14560	634	A	G	3	9	--	--
	Ca(ICCV2/PI489777)SNP_14561	640	C	T	3	9	--	--
	Ca(ICCV2/PI489777)SNP_14562	652	A	G	3	9	--	--
	Ca(ICCV2/PI489777)SNP_14563	733	T	A	6	9	--	--
	Ca(ICCV2/PI489777)SNP_14564	973	A	C	13	7	--	--
	Ca(ICCV2/PI489777)SNP_14565	1000	G	T	14	7	--	--
	Ca(ICCV2/PI489777)SNP_14566	1090	G	A	13	8	--	--
	Ca(ICCV2/PI489777)SNP_14567	2102	A	G	17	6	--	--
CakTC41980	Ca(ICCV2/PI489777)SNP_14568	409	T	C	6	3	--	--
CakTC33378	Ca(ICCV2/PI489777)SNP_14569	636	C	A	23	8	--	--
	Ca(ICCV2/PI489777)SNP_14570	951	A	G	36	15	--	--
	Ca(ICCV2/PI489777)SNP_14571	1218	A	T	30	29	--	--
	Ca(ICCV2/PI489777)SNP_14572	1626	G	A	33	12	--	--
	Ca(ICCV2/PI489777)SNP_14573	2001	G	A	33	7	--	--

	Ca(ICCV2/PI489777)SNP_14574	2004	G	T	32	8	--	--
	Ca(ICCV2/PI489777)SNP_14575	2083	A	G	13	5	--	--
CakTC38645	Ca(ICCV2/PI489777)SNP_14576	825	G	A	9	6	--	--
	Ca(ICCV2/PI489777)SNP_14577	910	T	A	7	4	--	--
	Ca(ICCV2/PI489777)SNP_14578	1627	T	G	9	3	--	--
CakTC26659	Ca(ICCV2/PI489777)SNP_14579	519	T	A	34	10	--	--
	Ca(ICCV2/PI489777)SNP_14580	567	C	T	31	3	--	--
	Ca(ICCV2/PI489777)SNP_14581	663	A	G	24	3	--	--
CakTC27160	Ca(ICCV2/PI489777)SNP_14582	240	A	G	36	4	--	--
CakTC23877	Ca(ICCV2/PI489777)SNP_14583	382	C	T	267	54	--	--
	Ca(ICCV2/PI489777)SNP_14584	415	A	G	259	59	--	--
	Ca(ICCV2/PI489777)SNP_14585	1404	A	G	169	38	--	--
	Ca(ICCV2/PI489777)SNP_14586	1468	T	C	107	28	--	--
CakTC25569	Ca(ICCV2/PI489777)SNP_14587	785	C	T	143	50	--	--
	Ca(ICCV2/PI489777)SNP_14588	919	C	T	136	57	--	--
	Ca(ICCV2/PI489777)SNP_14589	1747	G	C	139	67	--	--
CakTC33198	Ca(ICCV2/PI489777)SNP_14590	363	T	C	7	3	--	--
	Ca(ICCV2/PI489777)SNP_14591	1758	A	T	4	7	--	--
	Ca(ICCV2/PI489777)SNP_14592	5146	A	G	12	5	--	--
CakTC43224	Ca(ICCV2/PI489777)SNP_14593	288	C	T	3	5	--	--
	Ca(ICCV2/PI489777)SNP_14594	321	G	C	4	5	--	--
	Ca(ICCV2/PI489777)SNP_14595	363	T	C	3	5	--	--
CakTC31018	Ca(ICCV2/PI489777)SNP_14596	178	T	A	3	6	--	--
	Ca(ICCV2/PI489777)SNP_14597	526	T	C	6	5	--	--
CakTC11409	Ca(ICCV2/PI489777)SNP_14598	22	G	C	3	4	--	--
	Ca(ICCV2/PI489777)SNP_14599	465	G	A	7	3	--	--
CakTC37060	Ca(ICCV2/PI489777)SNP_14600	304	C	T	8	7	--	--
	Ca(ICCV2/PI489777)SNP_14601	1582	T	C	17	8	--	--
	Ca(ICCV2/PI489777)SNP_14602	2167	A	G	11	11	--	--
	Ca(ICCV2/PI489777)SNP_14603	2263	G	C	13	6	--	--
	Ca(ICCV2/PI489777)SNP_14604	2845	A	C	5	12	--	--
	Ca(ICCV2/PI489777)SNP_14605	3253	C	T	15	10	--	--
	Ca(ICCV2/PI489777)SNP_14606	3694	C	T	19	12	--	--
CakTC43193	Ca(ICCV2/PI489777)SNP_14607	35	T	C	7	15	--	--
	Ca(ICCV2/PI489777)SNP_14608	313	A	C	7	26	--	--
	Ca(ICCV2/PI489777)SNP_14609	373	C	G	7	28	--	--
	Ca(ICCV2/PI489777)SNP_14610	558	C	T	10	11	--	--
	Ca(ICCV2/PI489777)SNP_14611	600	T	C	12	11	--	--
	Ca(ICCV2/PI489777)SNP_14612	807	T	C	11	6	--	--
	Ca(ICCV2/PI489777)SNP_14613	1415	G	A	13	15	--	--
CakTC24715	Ca(ICCV2/PI489777)SNP_14614	736	G	A	24	11	--	--
	Ca(ICCV2/PI489777)SNP_14615	762	C	T	28	16	--	--
	Ca(ICCV2/PI489777)SNP_14616	1071	T	A	22	11	--	--
CakTC23280	Ca(ICCV2/PI489777)SNP_14617	335	T	C	5	4	--	--
	Ca(ICCV2/PI489777)SNP_14618	429	G	T	4	5	--	--
CakTC31650	Ca(ICCV2/PI489777)SNP_14619	313	G	A	52	12	--	--
	Ca(ICCV2/PI489777)SNP_14620	537	T	C	92	15	--	--
CakTC32055	Ca(ICCV2/PI489777)SNP_14621	195	A	T	15	21	--	--
	Ca(ICCV2/PI489777)SNP_14622	399	G	C	26	51	--	--
	Ca(ICCV2/PI489777)SNP_14623	681	G	A	94	73	--	--
	Ca(ICCV2/PI489777)SNP_14624	691	A	T	99	61	--	--
	Ca(ICCV2/PI489777)SNP_14625	881	C	T	77	65	--	--
	Ca(ICCV2/PI489777)SNP_14626	927	G	A	67	51	--	--
CakTC27654	Ca(ICCV2/PI489777)SNP_14627	70	T	G	5	6	--	--
	Ca(ICCV2/PI489777)SNP_14628	154	C	A	7	9	--	--
	Ca(ICCV2/PI489777)SNP_14629	189	C	T	7	12	--	--
	Ca(ICCV2/PI489777)SNP_14630	225	A	C	7	14	--	--
	Ca(ICCV2/PI489777)SNP_14631	265	G	A	20	22	--	--
	Ca(ICCV2/PI489777)SNP_14632	277	T	C	20	23	--	--
	Ca(ICCV2/PI489777)SNP_14633	352	G	A	35	28	--	--
	Ca(ICCV2/PI489777)SNP_14634	439	G	A	38	25	--	--
CakTC26185	Ca(ICCV2/PI489777)SNP_14635	1178	G	A	12	22	--	--
CakTC29963	Ca(ICCV2/PI489777)SNP_14636	245	T	C	22	14	--	--
	Ca(ICCV2/PI489777)SNP_14637	262	C	T	23	14	--	--
	Ca(ICCV2/PI489777)SNP_14638	475	T	C	13	16	--	--
	Ca(ICCV2/PI489777)SNP_14639	747	A	G	26	11	--	--
	Ca(ICCV2/PI489777)SNP_14640	1437	T	C	33	12	--	--
	Ca(ICCV2/PI489777)SNP_14641	2297	C	G	28	10	--	--
	Ca(ICCV2/PI489777)SNP_14642	2843	A	C	23	6	--	--
	Ca(ICCV2/PI489777)SNP_14643	3767	C	T	37	17	--	--
	Ca(ICCV2/PI489777)SNP_14644	4172	G	C	14	14	--	--
	Ca(ICCV2/PI489777)SNP_14645	4872	T	C	14	9	--	--
CakTC42614	Ca(ICCV2/PI489777)SNP_14646	806	A	G	18	5	--	--
CakTC24569	Ca(ICCV2/PI489777)SNP_14647	774	C	T	4	3	--	--

CakTC32579	Ca(ICCV2/PI489777)SNP_14648	239	T	C	4	7	--	--
	Ca(ICCV2/PI489777)SNP_14649	947	A	T	3	8	--	--
	Ca(ICCV2/PI489777)SNP_14650	1409	C	T	17	14	--	--
	Ca(ICCV2/PI489777)SNP_14651	1628	T	C	13	12	--	--
CakTC39279	Ca(ICCV2/PI489777)SNP_14652	276	C	T	13	5	--	--
	Ca(ICCV2/PI489777)SNP_14653	347	T	C	12	4	--	--
	Ca(ICCV2/PI489777)SNP_14654	415	T	C	16	3	--	--
	Ca(ICCV2/PI489777)SNP_14655	721	G	C	13	8	--	--
	Ca(ICCV2/PI489777)SNP_14656	990	T	A	10	4	--	--
	Ca(ICCV2/PI489777)SNP_14657	1035	G	A	16	7	--	--
	Ca(ICCV2/PI489777)SNP_14658	1415	T	G	5	4	--	--
CakTC42694	Ca(ICCV2/PI489777)SNP_14659	1884	T	C	6	3	--	--
CakTC38768	Ca(ICCV2/PI489777)SNP_14660	977	T	A	10	3	--	--
	Ca(ICCV2/PI489777)SNP_14661	1013	C	A	17	3	--	--
	Ca(ICCV2/PI489777)SNP_14662	1268	G	C	11	3	--	--
CakTC01288	Ca(ICCV2/PI489777)SNP_14663	51	G	T	6	3	--	--
	Ca(ICCV2/PI489777)SNP_14664	88	G	T	5	4	--	--
	Ca(ICCV2/PI489777)SNP_14665	226	T	C	11	4	--	--
	Ca(ICCV2/PI489777)SNP_14666	242	C	G	11	4	--	--
CakTC38890	Ca(ICCV2/PI489777)SNP_14667	307	T	C	8	4	--	--
CakTC39874	Ca(ICCV2/PI489777)SNP_14668	1652	A	C	15	22	--	--
CakTC16394	Ca(ICCV2/PI489777)SNP_14669	70	G	C	3	3	--	--
	Ca(ICCV2/PI489777)SNP_14670	91	C	T	3	3	--	--
CakTC22378	Ca(ICCV2/PI489777)SNP_14671	489	A	G	103	117	--	--
CakTC28343	Ca(ICCV2/PI489777)SNP_14672	273	C	A	17	21	--	--
	Ca(ICCV2/PI489777)SNP_14673	2092	C	T	10	5	--	--
CakTC27179	Ca(ICCV2/PI489777)SNP_14674	829	A	G	18	20	--	--
CakTC38637	Ca(ICCV2/PI489777)SNP_14675	527	G	T	3	3	--	--
CakTC42396	Ca(ICCV2/PI489777)SNP_14676	858	A	G	9	10	--	--
	Ca(ICCV2/PI489777)SNP_14677	914	T	C	8	9	--	--
CakTC25432	Ca(ICCV2/PI489777)SNP_14678	1851	A	G	6	3	--	--
	Ca(ICCV2/PI489777)SNP_14679	1898	C	G	5	3	--	--
	Ca(ICCV2/PI489777)SNP_14680	1927	A	G	5	3	--	--
CakTC11784	Ca(ICCV2/PI489777)SNP_14681	490	C	A	11	7	--	--
CakTC42366	Ca(ICCV2/PI489777)SNP_14682	639	C	T	24	8	--	--
	Ca(ICCV2/PI489777)SNP_14683	672	T	C	26	8	--	--
	Ca(ICCV2/PI489777)SNP_14684	861	A	T	27	12	--	--
	Ca(ICCV2/PI489777)SNP_14685	1029	A	G	13	11	--	--
	Ca(ICCV2/PI489777)SNP_14686	1324	A	T	23	13	--	--
	Ca(ICCV2/PI489777)SNP_14687	1715	C	T	21	9	--	--
	Ca(ICCV2/PI489777)SNP_14688	1720	C	T	22	8	--	--
CakTC25919	Ca(ICCV2/PI489777)SNP_14689	571	T	C	5	9	--	--
CakTC27030	Ca(ICCV2/PI489777)SNP_14690	597	T	C	9	7	--	LIM
	Ca(ICCV2/PI489777)SNP_14691	864	G	A	10	8	--	LIM
CakTC37928	Ca(ICCV2/PI489777)SNP_14692	142	T	C	10	10	Shoot	--
	Ca(ICCV2/PI489777)SNP_14693	863	A	G	4	7	Shoot	--
CakTC11475	Ca(ICCV2/PI489777)SNP_14694	1284	C	T	12	11	--	--
CakTC34662	Ca(ICCV2/PI489777)SNP_14695	207	A	G	3	3	--	--
CakTC42209	Ca(ICCV2/PI489777)SNP_14696	300	T	C	10	12	--	--
	Ca(ICCV2/PI489777)SNP_14697	507	A	T	19	31	--	--
	Ca(ICCV2/PI489777)SNP_14698	603	C	T	16	23	--	--
	Ca(ICCV2/PI489777)SNP_14699	654	C	A	14	24	--	--
CakTC39709	Ca(ICCV2/PI489777)SNP_14700	881	C	T	30	17	--	--
CakTC39226	Ca(ICCV2/PI489777)SNP_14701	1126	T	G	3	16	--	--
CakTC35382	Ca(ICCV2/PI489777)SNP_14702	477	G	C	10	9	--	--
	Ca(ICCV2/PI489777)SNP_14703	676	T	G	11	9	--	--
	Ca(ICCV2/PI489777)SNP_14704	1194	A	G	17	4	--	--
	Ca(ICCV2/PI489777)SNP_14705	1305	G	A	19	4	--	--
	Ca(ICCV2/PI489777)SNP_14706	1854	G	C	24	5	--	--
	Ca(ICCV2/PI489777)SNP_14707	2307	A	G	25	10	--	--
	Ca(ICCV2/PI489777)SNP_14708	2550	C	G	19	9	--	--
	Ca(ICCV2/PI489777)SNP_14709	2823	A	G	20	5	--	--
	Ca(ICCV2/PI489777)SNP_14710	2998	A	C	24	8	--	--
	Ca(ICCV2/PI489777)SNP_14711	3042	A	G	29	8	--	--
	Ca(ICCV2/PI489777)SNP_14712	3078	A	G	25	7	--	--
	Ca(ICCV2/PI489777)SNP_14713	3155	T	C	13	5	--	--
	Ca(ICCV2/PI489777)SNP_14714	3165	T	G	13	3	--	--
	Ca(ICCV2/PI489777)SNP_14715	3220	T	C	10	3	--	--
CakTC37425	Ca(ICCV2/PI489777)SNP_14716	143	G	A	14	11	--	--
	Ca(ICCV2/PI489777)SNP_14717	254	G	C	32	23	--	--
	Ca(ICCV2/PI489777)SNP_14718	299	T	C	34	25	--	--
	Ca(ICCV2/PI489777)SNP_14719	329	C	T	36	23	--	--
	Ca(ICCV2/PI489777)SNP_14720	335	G	A	34	23	--	--
	Ca(ICCV2/PI489777)SNP_14721	378	T	C	36	27	--	--

	Ca(ICCV2/PI489777)SNP_14722	380	T	A	35	26	--	--
	Ca(ICCV2/PI489777)SNP_14723	559	G	A	32	21	--	--
	Ca(ICCV2/PI489777)SNP_14724	947	A	G	10	3	--	--
CakTC24535	Ca(ICCV2/PI489777)SNP_14725	226	A	C	13	13	--	--
	Ca(ICCV2/PI489777)SNP_14726	579	T	C	23	7	--	--
CakTC42381	Ca(ICCV2/PI489777)SNP_14727	486	A	C	3	4	--	--
	Ca(ICCV2/PI489777)SNP_14728	575	G	T	3	4	--	--
	Ca(ICCV2/PI489777)SNP_14729	651	G	A	3	3	--	--
	Ca(ICCV2/PI489777)SNP_14730	683	T	C	5	3	--	--
	Ca(ICCV2/PI489777)SNP_14731	737	T	A	4	3	--	--
CakTC11386	Ca(ICCV2/PI489777)SNP_14732	353	A	G	5	3	--	--
	Ca(ICCV2/PI489777)SNP_14733	1007	C	T	9	3	--	--
CakTC32124	Ca(ICCV2/PI489777)SNP_14734	373	C	T	10	4	--	AP2-EREBP
	Ca(ICCV2/PI489777)SNP_14735	437	C	T	8	4	--	AP2-EREBP
	Ca(ICCV2/PI489777)SNP_14736	476	C	A	13	3	--	AP2-EREBP
	Ca(ICCV2/PI489777)SNP_14737	500	A	G	13	4	--	AP2-EREBP
	Ca(ICCV2/PI489777)SNP_14738	734	A	G	10	3	--	AP2-EREBP
CakTC23317	Ca(ICCV2/PI489777)SNP_14739	1894	T	G	35	9	--	--
CakTC35600	Ca(ICCV2/PI489777)SNP_14740	1219	T	A	9	20	--	TPR
	Ca(ICCV2/PI489777)SNP_14741	1872	C	G	14	7	--	TPR
	Ca(ICCV2/PI489777)SNP_14742	2181	T	G	14	8	--	TPR
	Ca(ICCV2/PI489777)SNP_14743	2995	A	G	8	5	--	TPR
	Ca(ICCV2/PI489777)SNP_14744	3095	T	C	8	3	--	TPR
CakTC13550	Ca(ICCV2/PI489777)SNP_14745	212	A	C	16	10	--	--
	Ca(ICCV2/PI489777)SNP_14746	293	A	G	20	11	--	--
CakTC39946	Ca(ICCV2/PI489777)SNP_14747	334	C	T	6	5	--	--
CakTC30327	Ca(ICCV2/PI489777)SNP_14748	257	A	C	3	6	--	--
	Ca(ICCV2/PI489777)SNP_14749	1596	A	T	6	3	--	--
	Ca(ICCV2/PI489777)SNP_14750	1599	T	A	7	3	--	--
CakTC38359	Ca(ICCV2/PI489777)SNP_14751	65	A	C	4	5	--	--
	Ca(ICCV2/PI489777)SNP_14752	984	A	G	39	8	--	--
CakTC25609	Ca(ICCV2/PI489777)SNP_14753	1029	C	T	7	5	Young_pod	--
CakTC37329	Ca(ICCV2/PI489777)SNP_14754	501	A	G	12	3	--	--
CakTC09397	Ca(ICCV2/PI489777)SNP_14755	374	C	T	86	22	--	--
	Ca(ICCV2/PI489777)SNP_14756	445	C	T	65	18	--	--
CakTC23888	Ca(ICCV2/PI489777)SNP_14757	810	T	C	63	24	--	--
	Ca(ICCV2/PI489777)SNP_14758	3298	A	C	10	7	--	--
	Ca(ICCV2/PI489777)SNP_14759	4210	C	T	7	6	--	--
	Ca(ICCV2/PI489777)SNP_14760	4275	C	T	8	6	--	--
CakTC38745	Ca(ICCV2/PI489777)SNP_14761	1167	A	C	3	7	--	--
CakTC23824	Ca(ICCV2/PI489777)SNP_14762	5612	A	G	28	12	--	--
	Ca(ICCV2/PI489777)SNP_14763	5778	G	A	33	8	--	--
CakTC25279	Ca(ICCV2/PI489777)SNP_14764	1038	G	A	11	5	--	--
CakTC38494	Ca(ICCV2/PI489777)SNP_14765	85	T	C	19	9	--	--
	Ca(ICCV2/PI489777)SNP_14766	518	C	A	15	13	--	--
	Ca(ICCV2/PI489777)SNP_14767	741	G	A	17	11	--	--
CakTC43155	Ca(ICCV2/PI489777)SNP_14768	936	G	T	24	11	--	--
	Ca(ICCV2/PI489777)SNP_14769	945	C	T	22	12	--	--
CakTC42999	Ca(ICCV2/PI489777)SNP_14770	146	G	T	16	15	--	TUB
	Ca(ICCV2/PI489777)SNP_14771	720	T	C	29	12	--	TUB
	Ca(ICCV2/PI489777)SNP_14772	849	A	G	26	14	--	TUB
	Ca(ICCV2/PI489777)SNP_14773	960	C	T	26	13	--	TUB
CakTC30748	Ca(ICCV2/PI489777)SNP_14774	480	G	C	4	3	--	AP2-EREBP
	Ca(ICCV2/PI489777)SNP_14775	614	C	T	7	7	--	AP2-EREBP
	Ca(ICCV2/PI489777)SNP_14776	732	C	T	8	9	--	AP2-EREBP
	Ca(ICCV2/PI489777)SNP_14777	1106	G	A	16	9	--	AP2-EREBP
	Ca(ICCV2/PI489777)SNP_14778	1186	G	T	12	7	--	AP2-EREBP
	Ca(ICCV2/PI489777)SNP_14779	1204	C	G	13	6	--	AP2-EREBP
CakTC31835	Ca(ICCV2/PI489777)SNP_14780	616	G	C	17	19	--	--
CakTC27266	Ca(ICCV2/PI489777)SNP_14781	718	C	T	25	19	--	--
	Ca(ICCV2/PI489777)SNP_14782	1799	G	A	12	9	--	--
CakTC41664	Ca(ICCV2/PI489777)SNP_14783	140	C	A	14	16	--	--
CakTC35229	Ca(ICCV2/PI489777)SNP_14784	2010	C	T	9	3	--	--
CakTC11632	Ca(ICCV2/PI489777)SNP_14785	626	C	T	7	3	--	--
	Ca(ICCV2/PI489777)SNP_14786	673	G	A	7	4	--	--
CakTC04245	Ca(ICCV2/PI489777)SNP_14787	163	T	C	7	3	--	--
	Ca(ICCV2/PI489777)SNP_14788	272	C	T	8	4	--	--
	Ca(ICCV2/PI489777)SNP_14789	326	T	C	8	4	--	--
CakTC28786	Ca(ICCV2/PI489777)SNP_14790	529	A	G	8	5	--	ARID
	Ca(ICCV2/PI489777)SNP_14791	564	G	T	9	5	--	ARID
	Ca(ICCV2/PI489777)SNP_14792	709	A	G	10	8	--	ARID
	Ca(ICCV2/PI489777)SNP_14793	764	G	A	14	4	--	ARID
	Ca(ICCV2/PI489777)SNP_14794	962	A	G	21	7	--	ARID
	Ca(ICCV2/PI489777)SNP_14795	1134	C	G	14	5	--	ARID

CakTC33599	Ca(ICCV2/PI489777)SNP_14796	143	T	A	10	6	--	--
	Ca(ICCV2/PI489777)SNP_14797	436	C	T	34	15	--	--
	Ca(ICCV2/PI489777)SNP_14798	1420	T	A	30	18	--	--
CakTC35154	Ca(ICCV2/PI489777)SNP_14799	992	A	G	7	12	--	--
	Ca(ICCV2/PI489777)SNP_14800	2129	T	C	8	3	--	--
CakTC42505	Ca(ICCV2/PI489777)SNP_14801	193	G	A	19	18	--	--
	Ca(ICCV2/PI489777)SNP_14802	211	T	C	22	17	--	--
	Ca(ICCV2/PI489777)SNP_14803	373	T	A	18	22	--	--
	Ca(ICCV2/PI489777)SNP_14804	442	C	T	8	26	--	--
	Ca(ICCV2/PI489777)SNP_14805	523	A	G	7	28	--	--
CakTC31522	Ca(ICCV2/PI489777)SNP_14806	381	C	T	6	3	--	--
	Ca(ICCV2/PI489777)SNP_14807	393	T	C	5	3	--	--
	Ca(ICCV2/PI489777)SNP_14808	408	T	A	5	5	--	--
	Ca(ICCV2/PI489777)SNP_14809	658	A	G	4	5	--	--
	Ca(ICCV2/PI489777)SNP_14810	717	A	G	4	5	--	--
CakTC39349	Ca(ICCV2/PI489777)SNP_14811	154	A	G	7	3	--	--
	Ca(ICCV2/PI489777)SNP_14812	432	T	C	3	3	--	--
CakTC09093	Ca(ICCV2/PI489777)SNP_14813	1465	A	C	19	8	--	--
	Ca(ICCV2/PI489777)SNP_14814	2102	G	A	8	6	--	--
CakTC22531	Ca(ICCV2/PI489777)SNP_14815	200	G	A	4	3	Young_pod	--
CakTC35577	Ca(ICCV2/PI489777)SNP_14816	263	G	A	22	4	--	--
	Ca(ICCV2/PI489777)SNP_14817	432	T	A	21	8	--	--
	Ca(ICCV2/PI489777)SNP_14818	443	C	T	22	10	--	--
	Ca(ICCV2/PI489777)SNP_14819	798	C	A	11	9	--	--
	Ca(ICCV2/PI489777)SNP_14820	2308	T	C	15	5	--	--
	Ca(ICCV2/PI489777)SNP_14821	4010	C	T	37	6	--	--
CakTC29248	Ca(ICCV2/PI489777)SNP_14822	181	G	A	15	4	--	--
	Ca(ICCV2/PI489777)SNP_14823	187	T	C	14	4	--	--
	Ca(ICCV2/PI489777)SNP_14824	205	A	C	16	4	--	--
	Ca(ICCV2/PI489777)SNP_14825	1564	C	A	24	4	--	--
CakTC34147	Ca(ICCV2/PI489777)SNP_14826	1854	A	C	10	14	--	--
CakTC29513	Ca(ICCV2/PI489777)SNP_14827	578	G	T	3	8	--	--
	Ca(ICCV2/PI489777)SNP_14828	1305	C	T	4	14	--	--
CakTC30905	Ca(ICCV2/PI489777)SNP_14829	455	C	T	9	3	--	--
	Ca(ICCV2/PI489777)SNP_14830	605	A	T	10	5	--	--
	Ca(ICCV2/PI489777)SNP_14831	1745	A	T	3	3	--	--
CakTC40014	Ca(ICCV2/PI489777)SNP_14832	446	T	C	4	4	--	GRAS
CakTC25820	Ca(ICCV2/PI489777)SNP_14833	1977	G	T	5	4	--	--
	Ca(ICCV2/PI489777)SNP_14834	2193	C	T	6	6	--	--
	Ca(ICCV2/PI489777)SNP_14835	2199	T	C	6	6	--	--
	Ca(ICCV2/PI489777)SNP_14836	2319	G	A	9	4	--	--
	Ca(ICCV2/PI489777)SNP_14837	2583	T	A	20	6	--	--
	Ca(ICCV2/PI489777)SNP_14838	2676	A	G	16	5	--	--
	Ca(ICCV2/PI489777)SNP_14839	2925	A	C	7	9	--	--
	Ca(ICCV2/PI489777)SNP_14840	3067	T	C	11	14	--	--
	Ca(ICCV2/PI489777)SNP_14841	3084	G	A	13	10	--	--
	Ca(ICCV2/PI489777)SNP_14842	3144	C	T	19	13	--	--
	Ca(ICCV2/PI489777)SNP_14843	3174	C	T	18	14	--	--
	Ca(ICCV2/PI489777)SNP_14844	3216	T	C	20	11	--	--
	Ca(ICCV2/PI489777)SNP_14845	3217	A	G	20	11	--	--
	Ca(ICCV2/PI489777)SNP_14846	3309	T	C	19	11	--	--
	Ca(ICCV2/PI489777)SNP_14847	3363	T	C	17	10	--	--
	Ca(ICCV2/PI489777)SNP_14848	3507	C	T	9	8	--	--
	Ca(ICCV2/PI489777)SNP_14849	4014	G	C	11	5	--	--
CakTC26946	Ca(ICCV2/PI489777)SNP_14850	202	T	C	11	3	--	--
	Ca(ICCV2/PI489777)SNP_14851	669	T	C	15	10	--	--
CakTC30551	Ca(ICCV2/PI489777)SNP_14852	565	C	A	8	3	--	--
	Ca(ICCV2/PI489777)SNP_14853	879	C	A	10	4	--	--
CakTC25226	Ca(ICCV2/PI489777)SNP_14854	936	T	C	5	4	--	--
CakTC12598	Ca(ICCV2/PI489777)SNP_14855	71	T	C	3	9	--	--
	Ca(ICCV2/PI489777)SNP_14856	569	A	G	26	52	--	--
	Ca(ICCV2/PI489777)SNP_14857	727	A	T	47	48	--	--
	Ca(ICCV2/PI489777)SNP_14858	988	A	G	28	39	--	--
	Ca(ICCV2/PI489777)SNP_14859	1197	C	G	11	19	--	--
	Ca(ICCV2/PI489777)SNP_14860	1205	C	A	8	18	--	--
CakTC26420	Ca(ICCV2/PI489777)SNP_14861	385	G	A	6	3	Shoot	--
	Ca(ICCV2/PI489777)SNP_14862	409	G	A	6	3	Shoot	--
	Ca(ICCV2/PI489777)SNP_14863	563	G	A	7	4	Shoot	--
CakTC37709	Ca(ICCV2/PI489777)SNP_14864	270	G	A	69	29	--	--
CakTC23035	Ca(ICCV2/PI489777)SNP_14865	1247	A	G	99	62	--	--
CakTC41331	Ca(ICCV2/PI489777)SNP_14866	2209	A	G	34	18	--	--
	Ca(ICCV2/PI489777)SNP_14867	2381	A	G	24	11	--	--
CakTC42038	Ca(ICCV2/PI489777)SNP_14868	172	G	A	5	5	--	--
	Ca(ICCV2/PI489777)SNP_14869	190	A	G	5	5	--	--

	Ca(ICCV2/PI489777)SNP_14870	221	G	T	5	5	--	--
	Ca(ICCV2/PI489777)SNP_14871	390	G	A	7	11	--	--
CakTC30131	Ca(ICCV2/PI489777)SNP_14872	729	G	A	186	114	--	--
	Ca(ICCV2/PI489777)SNP_14873	810	G	A	106	63	--	--
	Ca(ICCV2/PI489777)SNP_14874	820	T	G	99	58	--	--
	Ca(ICCV2/PI489777)SNP_14875	835	C	T	53	42	--	--
	Ca(ICCV2/PI489777)SNP_14876	989	T	C	10	3	--	--
CakTC09847	Ca(ICCV2/PI489777)SNP_14877	327	G	A	3	3	--	--
CakTC23530	Ca(ICCV2/PI489777)SNP_14878	456	G	A	46	15	--	--
CakTC31132	Ca(ICCV2/PI489777)SNP_14879	355	C	T	3	3	--	--
	Ca(ICCV2/PI489777)SNP_14880	406	A	G	4	3	--	--
	Ca(ICCV2/PI489777)SNP_14881	556	C	T	3	3	--	--
	Ca(ICCV2/PI489777)SNP_14882	613	T	C	3	3	--	--
	Ca(ICCV2/PI489777)SNP_14883	918	C	T	3	5	--	--
CakTC33239	Ca(ICCV2/PI489777)SNP_14884	438	C	G	3	3	--	--
CakTC43258	Ca(ICCV2/PI489777)SNP_14885	498	A	G	17	4	--	--
	Ca(ICCV2/PI489777)SNP_14886	960	A	G	14	15	--	--
	Ca(ICCV2/PI489777)SNP_14887	1016	G	A	21	19	--	--
	Ca(ICCV2/PI489777)SNP_14888	1083	T	C	20	20	--	--
	Ca(ICCV2/PI489777)SNP_14889	1201	T	C	16	22	--	--
	Ca(ICCV2/PI489777)SNP_14890	1457	C	G	22	11	--	--
	Ca(ICCV2/PI489777)SNP_14891	1563	C	T	16	9	--	--
CakTC30386	Ca(ICCV2/PI489777)SNP_14892	2306	A	G	3	4	Young_pod	--
CakTC24122	Ca(ICCV2/PI489777)SNP_14893	434	C	G	5	14	Root	--
	Ca(ICCV2/PI489777)SNP_14894	446	A	T	6	14	Root	--
	Ca(ICCV2/PI489777)SNP_14895	551	T	G	6	16	Root	--
	Ca(ICCV2/PI489777)SNP_14896	626	A	G	3	15	Root	--
CakTC21947	Ca(ICCV2/PI489777)SNP_14897	3584	A	T	3	6	--	--
	Ca(ICCV2/PI489777)SNP_14898	3746	A	C	3	3	--	--
CakTC43011	Ca(ICCV2/PI489777)SNP_14899	194	C	T	13	3	--	--
	Ca(ICCV2/PI489777)SNP_14900	586	C	T	12	4	--	--
	Ca(ICCV2/PI489777)SNP_14901	2222	G	A	4	10	--	--
	Ca(ICCV2/PI489777)SNP_14902	2657	C	T	9	6	--	--
CakTC28324	Ca(ICCV2/PI489777)SNP_14903	548	C	T	4	3	--	--
CakTC42495	Ca(ICCV2/PI489777)SNP_14904	282	A	T	15	14	--	--
CakTC25533	Ca(ICCV2/PI489777)SNP_14905	604	A	G	17	9	--	--
	Ca(ICCV2/PI489777)SNP_14906	640	A	G	20	9	--	--
	Ca(ICCV2/PI489777)SNP_14907	737	A	G	21	10	--	--
	Ca(ICCV2/PI489777)SNP_14908	974	T	C	25	15	--	--
	Ca(ICCV2/PI489777)SNP_14909	980	C	T	11	10	--	--
	Ca(ICCV2/PI489777)SNP_14910	1079	C	T	23	17	--	--
CakTC41884	Ca(ICCV2/PI489777)SNP_14911	216	C	A	10	4	--	--
	Ca(ICCV2/PI489777)SNP_14912	242	A	T	15	5	--	--
	Ca(ICCV2/PI489777)SNP_14913	381	T	C	36	13	--	--
	Ca(ICCV2/PI489777)SNP_14914	513	G	A	32	16	--	--
	Ca(ICCV2/PI489777)SNP_14915	900	C	A	24	5	--	--
	Ca(ICCV2/PI489777)SNP_14916	1347	C	A	10	14	--	--
	Ca(ICCV2/PI489777)SNP_14917	1407	G	T	15	14	--	--
	Ca(ICCV2/PI489777)SNP_14918	1510	G	A	17	18	--	--
	Ca(ICCV2/PI489777)SNP_14919	1730	A	C	9	15	--	--
CakTC27745	Ca(ICCV2/PI489777)SNP_14920	87	C	T	7	17	--	--
	Ca(ICCV2/PI489777)SNP_14921	109	G	A	8	17	--	--
	Ca(ICCV2/PI489777)SNP_14922	205	T	G	10	17	--	--
	Ca(ICCV2/PI489777)SNP_14923	216	T	C	13	17	--	--
	Ca(ICCV2/PI489777)SNP_14924	252	T	C	13	17	--	--
	Ca(ICCV2/PI489777)SNP_14925	255	T	C	14	17	--	--
	Ca(ICCV2/PI489777)SNP_14926	312	A	G	13	18	--	--
	Ca(ICCV2/PI489777)SNP_14927	376	G	C	17	19	--	--
	Ca(ICCV2/PI489777)SNP_14928	1150	C	A	9	7	--	--
	Ca(ICCV2/PI489777)SNP_14929	1645	C	A	21	10	--	--
	Ca(ICCV2/PI489777)SNP_14930	1815	T	C	24	8	--	--
CakTC27121	Ca(ICCV2/PI489777)SNP_14931	2201	T	C	6	3	--	--
CakTC38121	Ca(ICCV2/PI489777)SNP_14932	424	C	G	20	39	--	--
	Ca(ICCV2/PI489777)SNP_14933	616	T	C	22	27	--	--
	Ca(ICCV2/PI489777)SNP_14934	1500	A	G	6	6	--	--
CakTC43125	Ca(ICCV2/PI489777)SNP_14935	507	A	G	13	15	--	--
	Ca(ICCV2/PI489777)SNP_14936	749	C	T	35	11	--	--
	Ca(ICCV2/PI489777)SNP_14937	974	C	T	35	10	--	--
	Ca(ICCV2/PI489777)SNP_14938	1230	T	C	17	7	--	--
	Ca(ICCV2/PI489777)SNP_14939	1461	A	G	34	17	--	--
	Ca(ICCV2/PI489777)SNP_14940	1711	G	A	16	8	--	--
CakTC40968	Ca(ICCV2/PI489777)SNP_14941	623	T	C	6	6	--	--
	Ca(ICCV2/PI489777)SNP_14942	1977	A	T	7	9	--	--
CakTC08642	Ca(ICCV2/PI489777)SNP_14943	110	A	C	4	4	--	--

	Ca(ICCV2/PI489777)SNP_14944	135	G	A	4	4	--	--
	Ca(ICCV2/PI489777)SNP_14945	141	T	G	4	3	--	--
	Ca(ICCV2/PI489777)SNP_14946	181	T	A	4	3	--	--
	Ca(ICCV2/PI489777)SNP_14947	937	C	T	3	5	--	--
	Ca(ICCV2/PI489777)SNP_14948	949	G	A	3	3	--	--
	Ca(ICCV2/PI489777)SNP_14949	1734	T	G	4	9	--	--
	Ca(ICCV2/PI489777)SNP_14950	1846	G	A	4	9	--	--
	Ca(ICCV2/PI489777)SNP_14951	2067	T	C	4	5	--	--
CakTC38548	Ca(ICCV2/PI489777)SNP_14952	352	C	T	5	3	--	--
CakTC27592	Ca(ICCV2/PI489777)SNP_14953	2724	A	G	4	5	--	SET
CakTC31885	Ca(ICCV2/PI489777)SNP_14954	312	G	A	11	5	--	--
	Ca(ICCV2/PI489777)SNP_14955	641	T	C	15	10	--	--
	Ca(ICCV2/PI489777)SNP_14956	693	T	C	19	7	--	--
	Ca(ICCV2/PI489777)SNP_14957	987	T	C	15	6	--	--
	Ca(ICCV2/PI489777)SNP_14958	1332	A	T	13	7	--	--
	Ca(ICCV2/PI489777)SNP_14959	2130	G	A	6	4	--	--
	Ca(ICCV2/PI489777)SNP_14960	2361	C	A	8	8	--	--
	Ca(ICCV2/PI489777)SNP_14961	2450	A	G	9	9	--	--
	Ca(ICCV2/PI489777)SNP_14962	2715	C	T	5	6	--	--
	Ca(ICCV2/PI489777)SNP_14963	3336	T	C	10	7	--	--
	Ca(ICCV2/PI489777)SNP_14964	3375	C	T	10	8	--	--
	Ca(ICCV2/PI489777)SNP_14965	3381	T	G	8	8	--	--
	Ca(ICCV2/PI489777)SNP_14966	3454	G	A	6	9	--	--
	Ca(ICCV2/PI489777)SNP_14967	4634	T	C	17	6	--	--
	Ca(ICCV2/PI489777)SNP_14968	4752	A	T	15	8	--	--
	Ca(ICCV2/PI489777)SNP_14969	4797	A	G	14	9	--	--
	Ca(ICCV2/PI489777)SNP_14970	5442	C	T	14	13	--	--
	Ca(ICCV2/PI489777)SNP_14971	5461	A	G	12	13	--	--
	Ca(ICCV2/PI489777)SNP_14972	5745	A	T	13	9	--	--
	Ca(ICCV2/PI489777)SNP_14973	6087	C	T	20	11	--	--
	Ca(ICCV2/PI489777)SNP_14974	6873	A	G	12	6	--	--
	Ca(ICCV2/PI489777)SNP_14975	7491	G	A	20	12	--	--
	Ca(ICCV2/PI489777)SNP_14976	7680	C	T	20	7	--	--
	Ca(ICCV2/PI489777)SNP_14977	7935	A	G	20	15	--	--
	Ca(ICCV2/PI489777)SNP_14978	7998	A	G	21	12	--	--
	Ca(ICCV2/PI489777)SNP_14979	8362	T	C	19	23	--	--
	Ca(ICCV2/PI489777)SNP_14980	8645	G	C	11	9	--	--
CakTC26718	Ca(ICCV2/PI489777)SNP_14981	101	C	G	11	13	--	--
	Ca(ICCV2/PI489777)SNP_14982	109	G	A	14	14	--	--
CakTC29488	Ca(ICCV2/PI489777)SNP_14983	793	C	T	4	8	--	--
CakTC38753	Ca(ICCV2/PI489777)SNP_14984	1118	T	C	41	31	--	HB
CakTC38179	Ca(ICCV2/PI489777)SNP_14985	124	C	T	15	6	--	--
	Ca(ICCV2/PI489777)SNP_14986	419	C	T	18	11	--	--
	Ca(ICCV2/PI489777)SNP_14987	734	C	T	23	14	--	--
CakTC37334	Ca(ICCV2/PI489777)SNP_14988	77	T	C	24	13	--	--
	Ca(ICCV2/PI489777)SNP_14989	141	A	G	54	32	--	--
	Ca(ICCV2/PI489777)SNP_14990	351	A	G	77	36	--	--
	Ca(ICCV2/PI489777)SNP_14991	1024	T	C	20	16	--	--
	Ca(ICCV2/PI489777)SNP_14992	1043	A	G	12	9	--	--
CakTC40579	Ca(ICCV2/PI489777)SNP_14993	930	A	G	26	11	--	--
	Ca(ICCV2/PI489777)SNP_14994	960	C	T	24	11	--	--
	Ca(ICCV2/PI489777)SNP_14995	984	T	C	25	9	--	--
	Ca(ICCV2/PI489777)SNP_14996	1014	G	T	20	7	--	--
	Ca(ICCV2/PI489777)SNP_14997	1045	A	T	24	9	--	--
	Ca(ICCV2/PI489777)SNP_14998	1061	A	T	24	9	--	--
	Ca(ICCV2/PI489777)SNP_14999	1143	T	C	16	7	--	--
	Ca(ICCV2/PI489777)SNP_15000	1158	T	A	18	7	--	--
	Ca(ICCV2/PI489777)SNP_15001	1164	A	T	19	7	--	--
	Ca(ICCV2/PI489777)SNP_15002	1281	A	G	18	5	--	--
	Ca(ICCV2/PI489777)SNP_15003	1689	C	T	17	8	--	--
CakTC36828	Ca(ICCV2/PI489777)SNP_15004	187	A	G	19	9	--	--
	Ca(ICCV2/PI489777)SNP_15005	334	C	T	30	12	--	--
	Ca(ICCV2/PI489777)SNP_15006	394	G	A	24	6	--	--
	Ca(ICCV2/PI489777)SNP_15007	2211	A	G	42	41	--	--
	Ca(ICCV2/PI489777)SNP_15008	2297	T	G	35	40	--	--
	Ca(ICCV2/PI489777)SNP_15009	2355	C	T	30	24	--	--
CakTC29481	Ca(ICCV2/PI489777)SNP_15010	103	G	A	40	7	--	--
	Ca(ICCV2/PI489777)SNP_15011	369	G	A	42	12	--	--
CakTC36634	Ca(ICCV2/PI489777)SNP_15012	96	C	T	25	9	--	TRAF
	Ca(ICCV2/PI489777)SNP_15013	298	T	C	29	13	--	TRAF
	Ca(ICCV2/PI489777)SNP_15014	667	C	T	9	4	--	TRAF
CakTC28301	Ca(ICCV2/PI489777)SNP_15015	311	A	G	4	5	--	--
	Ca(ICCV2/PI489777)SNP_15016	491	C	T	14	4	--	--
	Ca(ICCV2/PI489777)SNP_15017	517	C	T	13	4	--	--

	Ca(ICCV2/PI489777)SNP_15018	524	A	G	15	4	--	--
	Ca(ICCV2/PI489777)SNP_15019	670	C	T	8	3	--	--
CakTC28390	Ca(ICCV2/PI489777)SNP_15020	664	G	A	8	4	--	--
	Ca(ICCV2/PI489777)SNP_15021	673	A	G	10	4	--	--
	Ca(ICCV2/PI489777)SNP_15022	1117	A	G	11	11	--	--
	Ca(ICCV2/PI489777)SNP_15023	1177	A	G	18	13	--	--
CakTC26180	Ca(ICCV2/PI489777)SNP_15024	479	T	C	148	128	--	--
CakTC24380	Ca(ICCV2/PI489777)SNP_15025	1486	A	G	18	4	Flower	bud
	Ca(ICCV2/PI489777)SNP_15026	1555	A	C	13	4	Flower	bud
	Ca(ICCV2/PI489777)SNP_15027	1603	C	G	15	5	Flower	bud
	Ca(ICCV2/PI489777)SNP_15028	1693	C	T	15	6	Flower	bud
	Ca(ICCV2/PI489777)SNP_15029	1796	G	A	12	6	Flower	bud
CakTC05710	Ca(ICCV2/PI489777)SNP_15030	259	T	C	3	3	--	--
CakTC25339	Ca(ICCV2/PI489777)SNP_15031	1394	T	C	14	3	--	--
	Ca(ICCV2/PI489777)SNP_15032	1898	G	A	20	6	--	--
	Ca(ICCV2/PI489777)SNP_15033	2952	A	T	7	13	--	--
	Ca(ICCV2/PI489777)SNP_15034	3296	G	A	20	22	--	--
	Ca(ICCV2/PI489777)SNP_15035	3496	G	T	13	13	--	--
	Ca(ICCV2/PI489777)SNP_15036	4334	G	A	6	10	--	--
	Ca(ICCV2/PI489777)SNP_15037	4376	A	G	8	11	--	--
	Ca(ICCV2/PI489777)SNP_15038	4685	G	A	14	9	--	--
	Ca(ICCV2/PI489777)SNP_15039	4722	A	G	11	6	--	--
CakTC31648	Ca(ICCV2/PI489777)SNP_15040	291	A	C	10	6	--	bZIP
CakTC32857	Ca(ICCV2/PI489777)SNP_15041	245	T	C	4	5	--	--
CakTC36987	Ca(ICCV2/PI489777)SNP_15042	80	A	G	3	7	--	--
CakTC37502	Ca(ICCV2/PI489777)SNP_15043	346	C	T	20	4	--	--
	Ca(ICCV2/PI489777)SNP_15044	373	G	C	16	6	--	--
	Ca(ICCV2/PI489777)SNP_15045	763	A	G	13	4	--	--
	Ca(ICCV2/PI489777)SNP_15046	978	T	C	9	3	--	--
CakTC30217	Ca(ICCV2/PI489777)SNP_15047	135	T	C	14	14	--	--
	Ca(ICCV2/PI489777)SNP_15048	280	T	C	39	26	--	--
	Ca(ICCV2/PI489777)SNP_15049	656	C	A	26	16	--	--
	Ca(ICCV2/PI489777)SNP_15050	1066	T	C	17	19	--	--
	Ca(ICCV2/PI489777)SNP_15051	1169	T	A	18	25	--	--
	Ca(ICCV2/PI489777)SNP_15052	1714	C	T	18	20	--	--
	Ca(ICCV2/PI489777)SNP_15053	1825	G	A	23	26	--	--
	Ca(ICCV2/PI489777)SNP_15054	2458	C	T	12	9	--	--
	Ca(ICCV2/PI489777)SNP_15055	2473	T	G	15	11	--	--
	Ca(ICCV2/PI489777)SNP_15056	2554	C	T	18	9	--	--
CakTC33285	Ca(ICCV2/PI489777)SNP_15057	445	G	T	17	70	--	MBF1
CakTC25422	Ca(ICCV2/PI489777)SNP_15058	110	G	A	12	11	--	--
	Ca(ICCV2/PI489777)SNP_15059	307	A	G	30	47	--	--
CakTC02740	Ca(ICCV2/PI489777)SNP_15060	516	G	T	33	14	--	--
CakTC38694	Ca(ICCV2/PI489777)SNP_15061	1152	A	G	3	6	--	--
	Ca(ICCV2/PI489777)SNP_15062	1222	C	G	5	5	--	--
CakTC40750	Ca(ICCV2/PI489777)SNP_15063	306	T	A	13	15	--	--
	Ca(ICCV2/PI489777)SNP_15064	542	A	G	19	9	--	--
	Ca(ICCV2/PI489777)SNP_15065	691	C	T	18	3	--	--
	Ca(ICCV2/PI489777)SNP_15066	1329	A	G	13	10	--	--
	Ca(ICCV2/PI489777)SNP_15067	1435	A	C	21	11	--	--
CakTC00750	Ca(ICCV2/PI489777)SNP_15068	297	T	A	3	17	--	--
	Ca(ICCV2/PI489777)SNP_15069	1316	G	A	7	10	--	--
	Ca(ICCV2/PI489777)SNP_15070	1376	A	G	11	12	--	--
	Ca(ICCV2/PI489777)SNP_15071	1400	T	G	12	11	--	--
	Ca(ICCV2/PI489777)SNP_15072	1424	A	G	11	10	--	--
CakTC37479	Ca(ICCV2/PI489777)SNP_15073	605	C	G	5	5	--	--
CakTC40920	Ca(ICCV2/PI489777)SNP_15074	69	C	G	5	4	--	--
	Ca(ICCV2/PI489777)SNP_15075	1299	T	C	6	5	--	--
	Ca(ICCV2/PI489777)SNP_15076	1302	C	G	6	5	--	--
	Ca(ICCV2/PI489777)SNP_15077	1494	T	C	5	3	--	--
CakTC09937	Ca(ICCV2/PI489777)SNP_15078	363	T	C	4	3	--	--
	Ca(ICCV2/PI489777)SNP_15079	511	T	C	4	5	--	--
CakTC34963	Ca(ICCV2/PI489777)SNP_15080	1739	G	A	10	5	--	--
	Ca(ICCV2/PI489777)SNP_15081	2145	G	A	3	3	--	--
	Ca(ICCV2/PI489777)SNP_15082	2397	G	A	9	7	--	--
	Ca(ICCV2/PI489777)SNP_15083	2537	G	A	10	7	--	--
	Ca(ICCV2/PI489777)SNP_15084	2997	G	A	10	4	--	--
CakTC41448	Ca(ICCV2/PI489777)SNP_15085	141	A	G	38	40	--	--
	Ca(ICCV2/PI489777)SNP_15086	384	A	G	47	54	--	--
	Ca(ICCV2/PI489777)SNP_15087	624	A	T	19	27	--	--
	Ca(ICCV2/PI489777)SNP_15088	651	A	G	18	21	--	--
	Ca(ICCV2/PI489777)SNP_15089	690	G	A	9	15	--	--
CakTC28256	Ca(ICCV2/PI489777)SNP_15090	816	C	T	48	14	--	--
	Ca(ICCV2/PI489777)SNP_15091	849	C	T	51	11	--	--

	Ca(ICCV2/PI489777)SNP_15092	891	A	G	48	10	--	--
	Ca(ICCV2/PI489777)SNP_15093	1134	G	T	26	11	--	--
	Ca(ICCV2/PI489777)SNP_15094	1149	A	G	28	9	--	--
	Ca(ICCV2/PI489777)SNP_15095	1182	C	T	26	10	--	--
CakTC37108	Ca(ICCV2/PI489777)SNP_15096	3031	T	G	5	9	--	--
	Ca(ICCV2/PI489777)SNP_15097	3811	T	C	11	5	--	--
CakTC25668	Ca(ICCV2/PI489777)SNP_15098	928	T	G	11	4	--	--
	Ca(ICCV2/PI489777)SNP_15099	1142	G	A	10	5	--	--
CakTC11017	Ca(ICCV2/PI489777)SNP_15100	329	C	T	21	10	--	--
	Ca(ICCV2/PI489777)SNP_15101	357	A	G	19	9	--	--
	Ca(ICCV2/PI489777)SNP_15102	563	C	T	22	8	--	--
CakTC38722	Ca(ICCV2/PI489777)SNP_15103	916	C	T	19	11	--	--
	Ca(ICCV2/PI489777)SNP_15104	1308	A	G	12	4	--	--
	Ca(ICCV2/PI489777)SNP_15105	1353	T	C	10	4	--	--
	Ca(ICCV2/PI489777)SNP_15106	1354	T	G	10	4	--	--
	Ca(ICCV2/PI489777)SNP_15107	1396	A	G	9	3	--	--
CakTC42286	Ca(ICCV2/PI489777)SNP_15108	189	A	C	3	4	--	--
	Ca(ICCV2/PI489777)SNP_15109	421	G	C	4	7	--	--
	Ca(ICCV2/PI489777)SNP_15110	493	G	A	3	5	--	--
	Ca(ICCV2/PI489777)SNP_15111	532	G	A	3	5	--	--
CakTC30830	Ca(ICCV2/PI489777)SNP_15112	1028	G	A	6	6	Flower	bud
CakTC40365	Ca(ICCV2/PI489777)SNP_15113	133	A	T	3	3	--	--
	Ca(ICCV2/PI489777)SNP_15114	420	A	G	9	21	--	--
	Ca(ICCV2/PI489777)SNP_15115	707	C	T	11	14	--	--
CakTC25418	Ca(ICCV2/PI489777)SNP_15116	1087	G	A	4	3	--	--
CakTC34029	Ca(ICCV2/PI489777)SNP_15117	673	C	T	58	38	--	--
	Ca(ICCV2/PI489777)SNP_15118	841	C	A	58	45	--	--
	Ca(ICCV2/PI489777)SNP_15119	1069	C	T	48	41	--	--
	Ca(ICCV2/PI489777)SNP_15120	1098	A	G	43	41	--	--
	Ca(ICCV2/PI489777)SNP_15121	1357	A	T	39	22	--	--
CakTC31406	Ca(ICCV2/PI489777)SNP_15122	16	C	T	11	3	--	--
	Ca(ICCV2/PI489777)SNP_15123	50	T	C	16	3	--	--
CakTC39312	Ca(ICCV2/PI489777)SNP_15124	370	T	C	28	12	--	--
	Ca(ICCV2/PI489777)SNP_15125	409	T	A	34	13	--	--
	Ca(ICCV2/PI489777)SNP_15126	784	G	T	24	11	--	--
	Ca(ICCV2/PI489777)SNP_15127	796	A	G	22	7	--	--
CakTC32420	Ca(ICCV2/PI489777)SNP_15128	65	C	T	5	3	--	--
CakTC38835	Ca(ICCV2/PI489777)SNP_15129	187	C	T	3	7	--	--
	Ca(ICCV2/PI489777)SNP_15130	849	C	T	16	12	--	--
	Ca(ICCV2/PI489777)SNP_15131	977	A	G	9	6	--	--
	Ca(ICCV2/PI489777)SNP_15132	1142	G	A	11	7	--	--
	Ca(ICCV2/PI489777)SNP_15133	1242	A	C	10	7	--	--
	Ca(ICCV2/PI489777)SNP_15134	1257	A	T	10	7	--	--
CakTC26500	Ca(ICCV2/PI489777)SNP_15135	405	G	A	13	7	--	--
	Ca(ICCV2/PI489777)SNP_15136	559	T	C	9	7	--	--
	Ca(ICCV2/PI489777)SNP_15137	560	T	A	6	7	--	--
	Ca(ICCV2/PI489777)SNP_15138	672	C	T	6	3	--	--
	Ca(ICCV2/PI489777)SNP_15139	1597	T	A	7	3	--	--
CakTC42530	Ca(ICCV2/PI489777)SNP_15140	298	T	C	12	4	--	--
CakTC25831	Ca(ICCV2/PI489777)SNP_15141	713	G	T	82	6	--	--
	Ca(ICCV2/PI489777)SNP_15142	714	A	G	83	6	--	--
	Ca(ICCV2/PI489777)SNP_15143	741	A	G	84	6	--	--
	Ca(ICCV2/PI489777)SNP_15144	751	G	A	97	7	--	--
	Ca(ICCV2/PI489777)SNP_15145	786	G	C	122	9	--	--
	Ca(ICCV2/PI489777)SNP_15146	798	C	T	123	11	--	--
	Ca(ICCV2/PI489777)SNP_15147	799	C	A	123	11	--	--
	Ca(ICCV2/PI489777)SNP_15148	823	T	G	107	14	--	--
	Ca(ICCV2/PI489777)SNP_15149	870	A	G	153	19	--	--
	Ca(ICCV2/PI489777)SNP_15150	1416	T	C	158	46	--	--
	Ca(ICCV2/PI489777)SNP_15151	1686	A	G	158	50	--	--
	Ca(ICCV2/PI489777)SNP_15152	1745	A	G	142	59	--	--
	Ca(ICCV2/PI489777)SNP_15153	1767	T	G	142	58	--	--
	Ca(ICCV2/PI489777)SNP_15154	2049	T	C	139	40	--	--
CakTC14889	Ca(ICCV2/PI489777)SNP_15155	3384	A	C	4	3	--	--
	Ca(ICCV2/PI489777)SNP_15156	3389	G	A	4	3	--	--
CakTC23208	Ca(ICCV2/PI489777)SNP_15157	121	A	G	8	4	--	--
CakTC28279	Ca(ICCV2/PI489777)SNP_15158	252	C	T	8	3	Flower	bud
	Ca(ICCV2/PI489777)SNP_15159	296	C	T	7	3	Flower	bud
	Ca(ICCV2/PI489777)SNP_15160	318	A	G	7	3	Flower	bud
CakTC24644	Ca(ICCV2/PI489777)SNP_15161	770	A	T	5	10	--	--
CakTC00660	Ca(ICCV2/PI489777)SNP_15162	135	A	T	4	4	--	--
	Ca(ICCV2/PI489777)SNP_15163	186	T	C	7	5	--	--
	Ca(ICCV2/PI489777)SNP_15164	187	C	G	7	5	--	--
CakTC40598	Ca(ICCV2/PI489777)SNP_15165	329	A	T	13	6	--	--

	Ca(ICCV2/PI489777)SNP_15166	440	C	A	17	7	--	--
CakTC25706	Ca(ICCV2/PI489777)SNP_15167	234	G	A	7	4	--	--
	Ca(ICCV2/PI489777)SNP_15168	347	T	A	15	7	--	--
	Ca(ICCV2/PI489777)SNP_15169	423	A	G	14	7	--	--
	Ca(ICCV2/PI489777)SNP_15170	609	A	G	15	5	--	--
	Ca(ICCV2/PI489777)SNP_15171	690	C	G	12	6	--	--
CakTC28223	Ca(ICCV2/PI489777)SNP_15172	1082	G	A	6	4	Flower	bud
	Ca(ICCV2/PI489777)SNP_15173	1119	T	G	5	4	Flower	bud
	Ca(ICCV2/PI489777)SNP_15174	1147	A	G	7	4	Flower	bud
	Ca(ICCV2/PI489777)SNP_15175	1150	C	T	7	4	Flower	bud
CakTC43107	Ca(ICCV2/PI489777)SNP_15176	768	T	G	11	5	--	HB
CakTC11680	Ca(ICCV2/PI489777)SNP_15177	1710	T	C	3	3	--	--
CakTC26538	Ca(ICCV2/PI489777)SNP_15178	650	A	G	16	13	--	--
CakTC32314	Ca(ICCV2/PI489777)SNP_15179	127	T	C	7	3	--	--
	Ca(ICCV2/PI489777)SNP_15180	139	A	G	7	3	--	--
	Ca(ICCV2/PI489777)SNP_15181	147	T	A	8	3	--	--
CakTC26440	Ca(ICCV2/PI489777)SNP_15182	1385	C	G	168	23	--	--
	Ca(ICCV2/PI489777)SNP_15183	1421	C	T	167	20	--	--
	Ca(ICCV2/PI489777)SNP_15184	1460	C	A	154	17	--	--
	Ca(ICCV2/PI489777)SNP_15185	1643	C	T	64	3	--	--
CakTC24378	Ca(ICCV2/PI489777)SNP_15186	139	C	A	9	6	--	--
	Ca(ICCV2/PI489777)SNP_15187	216	C	T	30	20	--	--
	Ca(ICCV2/PI489777)SNP_15188	436	G	C	33	27	--	--
	Ca(ICCV2/PI489777)SNP_15189	559	A	G	25	31	--	--
	Ca(ICCV2/PI489777)SNP_15190	685	C	A	64	37	--	--
	Ca(ICCV2/PI489777)SNP_15191	687	C	T	67	41	--	--
	Ca(ICCV2/PI489777)SNP_15192	742	A	G	49	36	--	--
	Ca(ICCV2/PI489777)SNP_15193	771	T	A	77	39	--	--
	Ca(ICCV2/PI489777)SNP_15194	793	C	T	77	41	--	--
CakTC26999	Ca(ICCV2/PI489777)SNP_15195	177	A	T	3	7	--	--
	Ca(ICCV2/PI489777)SNP_15196	2686	T	G	4	3	--	--
	Ca(ICCV2/PI489777)SNP_15197	2701	A	G	4	3	--	--
	Ca(ICCV2/PI489777)SNP_15198	2980	T	C	4	4	--	--
CakTC24300	Ca(ICCV2/PI489777)SNP_15199	258	T	C	21	3	Flower	bud
	Ca(ICCV2/PI489777)SNP_15200	579	T	G	22	6	Flower	bud
	Ca(ICCV2/PI489777)SNP_15201	759	A	G	17	5	Flower	bud
	Ca(ICCV2/PI489777)SNP_15202	909	T	C	19	6	Flower	bud
	Ca(ICCV2/PI489777)SNP_15203	1146	T	C	16	7	Flower	bud
CakTC28875	Ca(ICCV2/PI489777)SNP_15204	232	A	G	3	4	--	--
	Ca(ICCV2/PI489777)SNP_15205	1213	T	A	7	7	--	--
	Ca(ICCV2/PI489777)SNP_15206	1285	A	G	5	6	--	--
CakTC40757	Ca(ICCV2/PI489777)SNP_15207	502	C	G	38	16	--	--
CakTC25136	Ca(ICCV2/PI489777)SNP_15208	379	T	A	18	18	--	--
	Ca(ICCV2/PI489777)SNP_15209	492	G	A	18	21	--	--
	Ca(ICCV2/PI489777)SNP_15210	580	G	A	27	23	--	--
	Ca(ICCV2/PI489777)SNP_15211	615	G	C	29	22	--	--
	Ca(ICCV2/PI489777)SNP_15212	664	C	T	30	24	--	--
	Ca(ICCV2/PI489777)SNP_15213	673	T	C	31	26	--	--
	Ca(ICCV2/PI489777)SNP_15214	1180	C	T	11	12	--	--
CakTC13921	Ca(ICCV2/PI489777)SNP_15215	987	A	G	71	48	--	--
	Ca(ICCV2/PI489777)SNP_15216	1062	T	C	68	39	--	--
CakTC34559	Ca(ICCV2/PI489777)SNP_15217	109	G	A	4	3	--	--
	Ca(ICCV2/PI489777)SNP_15218	111	G	A	4	3	--	--
	Ca(ICCV2/PI489777)SNP_15219	121	C	A	4	3	--	--
	Ca(ICCV2/PI489777)SNP_15220	157	A	G	4	3	--	--
CakTC29090	Ca(ICCV2/PI489777)SNP_15221	748	A	T	3	12	--	--
CakTC34020	Ca(ICCV2/PI489777)SNP_15222	179	A	G	5	10	--	--
	Ca(ICCV2/PI489777)SNP_15223	1901	G	C	74	65	--	--
	Ca(ICCV2/PI489777)SNP_15224	1930	G	C	76	66	--	--
	Ca(ICCV2/PI489777)SNP_15225	2060	A	G	60	55	--	--
CakTC36978	Ca(ICCV2/PI489777)SNP_15226	133	T	C	3	5	--	--
CakTC38300	Ca(ICCV2/PI489777)SNP_15227	665	T	C	12	7	--	--
	Ca(ICCV2/PI489777)SNP_15228	923	T	C	24	17	--	--
CakTC30560	Ca(ICCV2/PI489777)SNP_15229	191	A	G	4	4	Flower	bud
	Ca(ICCV2/PI489777)SNP_15230	245	C	T	5	4	Flower	bud
	Ca(ICCV2/PI489777)SNP_15231	340	T	A	4	4	Flower	bud
	Ca(ICCV2/PI489777)SNP_15232	416	T	G	4	3	Flower	bud
CakTC41440	Ca(ICCV2/PI489777)SNP_15233	214	T	C	4	3	--	--
	Ca(ICCV2/PI489777)SNP_15234	751	A	G	17	8	--	--
	Ca(ICCV2/PI489777)SNP_15235	1201	G	A	10	7	--	--
	Ca(ICCV2/PI489777)SNP_15236	1287	C	T	9	3	--	--
CakTC37317	Ca(ICCV2/PI489777)SNP_15237	1014	G	T	15	5	--	--
CakTC26361	Ca(ICCV2/PI489777)SNP_15238	181	T	C	3	3	--	--
CakTC29933	Ca(ICCV2/PI489777)SNP_15239	703	T	G	3	4	--	--

	Ca(ICCV2/PI489777)SNP_15240	715	C	T	3	4	--	--
	Ca(ICCV2/PI489777)SNP_15241	1018	T	C	4	6	--	--
CakTC05202	Ca(ICCV2/PI489777)SNP_15242	115	T	C	8	5	--	MYB
	Ca(ICCV2/PI489777)SNP_15243	204	G	A	9	12	--	MYB
	Ca(ICCV2/PI489777)SNP_15244	288	G	A	12	12	--	MYB
	Ca(ICCV2/PI489777)SNP_15245	714	A	G	8	6	--	MYB
	Ca(ICCV2/PI489777)SNP_15246	1458	A	G	7	3	--	MYB
CakTC11846	Ca(ICCV2/PI489777)SNP_15247	99	T	G	3	3	--	--
	Ca(ICCV2/PI489777)SNP_15248	178	A	G	3	3	--	--
CakTC32994	Ca(ICCV2/PI489777)SNP_15249	740	G	A	10	9	--	--
	Ca(ICCV2/PI489777)SNP_15250	1862	C	T	14	3	--	--
CakTC42127	Ca(ICCV2/PI489777)SNP_15251	82	C	T	8	13	--	--
	Ca(ICCV2/PI489777)SNP_15252	218	G	C	12	22	--	--
	Ca(ICCV2/PI489777)SNP_15253	842	C	T	12	23	--	--
CakTC26227	Ca(ICCV2/PI489777)SNP_15254	759	G	A	6	6	--	--
	Ca(ICCV2/PI489777)SNP_15255	1062	G	A	7	4	--	--
CakTC28368	Ca(ICCV2/PI489777)SNP_15256	841	A	C	7	4	--	--
	Ca(ICCV2/PI489777)SNP_15257	1639	G	A	6	3	--	--
	Ca(ICCV2/PI489777)SNP_15258	1817	T	G	9	4	--	--
	Ca(ICCV2/PI489777)SNP_15259	2304	C	T	4	3	--	--
	Ca(ICCV2/PI489777)SNP_15260	2694	G	A	5	3	--	--
CakTC34540	Ca(ICCV2/PI489777)SNP_15261	480	G	A	22	7	--	--
	Ca(ICCV2/PI489777)SNP_15262	659	C	T	30	24	--	--
	Ca(ICCV2/PI489777)SNP_15263	1079	G	A	82	27	--	--
	Ca(ICCV2/PI489777)SNP_15264	1271	G	C	61	29	--	--
CakTC36596	Ca(ICCV2/PI489777)SNP_15265	823	C	A	13	5	--	--
CakTC36992	Ca(ICCV2/PI489777)SNP_15266	114	T	C	7	7	--	--
	Ca(ICCV2/PI489777)SNP_15267	1428	G	T	9	5	--	--
CakTC26171	Ca(ICCV2/PI489777)SNP_15268	595	T	C	3	4	--	--
	Ca(ICCV2/PI489777)SNP_15269	979	C	T	5	4	--	--
	Ca(ICCV2/PI489777)SNP_15270	1606	C	T	5	5	--	--
	Ca(ICCV2/PI489777)SNP_15271	1655	G	T	3	3	--	--
CakTC33846	Ca(ICCV2/PI489777)SNP_15272	19	G	A	6	4	--	--
	Ca(ICCV2/PI489777)SNP_15273	1220	T	C	18	4	--	--
	Ca(ICCV2/PI489777)SNP_15274	2082	G	T	14	5	--	--
CakTC33471	Ca(ICCV2/PI489777)SNP_15275	162	G	A	13	12	--	--
	Ca(ICCV2/PI489777)SNP_15276	334	T	C	17	11	--	--
	Ca(ICCV2/PI489777)SNP_15277	731	A	G	22	6	--	--
	Ca(ICCV2/PI489777)SNP_15278	769	G	A	12	7	--	--
	Ca(ICCV2/PI489777)SNP_15279	858	G	A	11	5	--	--
CakTC37322	Ca(ICCV2/PI489777)SNP_15280	343	G	A	17	20	--	SAP
	Ca(ICCV2/PI489777)SNP_15281	421	A	G	21	19	--	SAP
	Ca(ICCV2/PI489777)SNP_15282	565	A	C	15	10	--	SAP
CakTC37941	Ca(ICCV2/PI489777)SNP_15283	251	C	T	5	10	--	--
	Ca(ICCV2/PI489777)SNP_15284	265	A	T	6	10	--	--
	Ca(ICCV2/PI489777)SNP_15285	857	G	A	14	3	--	--
	Ca(ICCV2/PI489777)SNP_15286	1305	C	G	10	6	--	--
	Ca(ICCV2/PI489777)SNP_15287	1321	A	G	10	6	--	--
	Ca(ICCV2/PI489777)SNP_15288	1532	C	T	6	5	--	--
CakTC35349	Ca(ICCV2/PI489777)SNP_15289	330	G	A	4	4	--	CAMTA
	Ca(ICCV2/PI489777)SNP_15290	464	G	A	5	4	--	CAMTA
	Ca(ICCV2/PI489777)SNP_15291	806	G	T	7	7	--	CAMTA
	Ca(ICCV2/PI489777)SNP_15292	1150	A	T	7	5	--	CAMTA
	Ca(ICCV2/PI489777)SNP_15293	1664	G	A	9	3	--	CAMTA
	Ca(ICCV2/PI489777)SNP_15294	2525	G	T	17	4	--	CAMTA
CakTC30909	Ca(ICCV2/PI489777)SNP_15295	740	G	C	3	7	--	--
	Ca(ICCV2/PI489777)SNP_15296	770	C	T	3	9	--	--
	Ca(ICCV2/PI489777)SNP_15297	881	C	T	3	13	--	--
	Ca(ICCV2/PI489777)SNP_15298	1066	A	G	3	8	--	--
	Ca(ICCV2/PI489777)SNP_15299	1092	G	A	3	7	--	--
	Ca(ICCV2/PI489777)SNP_15300	1475	C	T	6	13	--	--
	Ca(ICCV2/PI489777)SNP_15301	1748	T	C	8	11	--	--
	Ca(ICCV2/PI489777)SNP_15302	1997	A	G	4	3	--	--
CakTC40313	Ca(ICCV2/PI489777)SNP_15303	720	T	C	33	19	--	--
	Ca(ICCV2/PI489777)SNP_15304	777	A	G	26	12	--	--
	Ca(ICCV2/PI489777)SNP_15305	840	A	G	22	14	--	--
CakTC35277	Ca(ICCV2/PI489777)SNP_15306	269	A	G	13	11	--	--
	Ca(ICCV2/PI489777)SNP_15307	718	A	G	40	24	--	--
	Ca(ICCV2/PI489777)SNP_15308	1930	G	A	21	18	--	--
	Ca(ICCV2/PI489777)SNP_15309	2983	G	A	18	15	--	--
CakTC35432	Ca(ICCV2/PI489777)SNP_15310	23	T	A	6	3	--	--
CakTC17690	Ca(ICCV2/PI489777)SNP_15311	895	C	T	47	11	--	--
	Ca(ICCV2/PI489777)SNP_15312	922	T	C	45	10	--	--
	Ca(ICCV2/PI489777)SNP_15313	1135	G	A	41	4	--	--

	Ca(ICCV2/PI489777)SNP_15314	1210	G	C	37	6	--	--
	Ca(ICCV2/PI489777)SNP_15315	1430	G	A	24	4	--	--
CakTC10196	Ca(ICCV2/PI489777)SNP_15316	105	C	T	20	5	--	--
	Ca(ICCV2/PI489777)SNP_15317	700	A	G	28	10	--	--
	Ca(ICCV2/PI489777)SNP_15318	1132	G	A	32	8	--	--
	Ca(ICCV2/PI489777)SNP_15319	1999	A	G	45	20	--	--
CakTC39485	Ca(ICCV2/PI489777)SNP_15320	551	T	A	24	17	--	--
CakTC27627	Ca(ICCV2/PI489777)SNP_15321	1977	G	A	4	4	--	--
CakTC12588	Ca(ICCV2/PI489777)SNP_15322	634	C	T	3	3	--	--
CakTC28617	Ca(ICCV2/PI489777)SNP_15323	196	A	T	8	11	--	--
	Ca(ICCV2/PI489777)SNP_15324	329	T	C	10	9	--	--
	Ca(ICCV2/PI489777)SNP_15325	331	T	C	10	9	--	--
	Ca(ICCV2/PI489777)SNP_15326	367	C	G	11	8	--	--
	Ca(ICCV2/PI489777)SNP_15327	368	G	A	10	8	--	--
CakTC27755	Ca(ICCV2/PI489777)SNP_15328	670	G	C	7	3	--	--
	Ca(ICCV2/PI489777)SNP_15329	1502	C	T	6	5	--	--
CakTC38079	Ca(ICCV2/PI489777)SNP_15330	288	G	A	36	35	--	--
	Ca(ICCV2/PI489777)SNP_15331	441	A	G	37	41	--	--
	Ca(ICCV2/PI489777)SNP_15332	771	G	A	28	33	--	--
CakTC32179	Ca(ICCV2/PI489777)SNP_15333	474	A	G	16	12	--	--
	Ca(ICCV2/PI489777)SNP_15334	655	C	G	26	15	--	--
	Ca(ICCV2/PI489777)SNP_15335	859	A	G	26	16	--	--
CakTC38883	Ca(ICCV2/PI489777)SNP_15336	86	T	A	19	3	--	--
	Ca(ICCV2/PI489777)SNP_15337	88	T	A	19	3	--	--
	Ca(ICCV2/PI489777)SNP_15338	105	T	C	21	3	--	--
	Ca(ICCV2/PI489777)SNP_15339	239	G	A	31	7	--	--
	Ca(ICCV2/PI489777)SNP_15340	247	T	C	33	7	--	--
	Ca(ICCV2/PI489777)SNP_15341	274	T	G	28	7	--	--
	Ca(ICCV2/PI489777)SNP_15342	290	A	G	39	7	--	--
	Ca(ICCV2/PI489777)SNP_15343	323	A	G	34	7	--	--
	Ca(ICCV2/PI489777)SNP_15344	374	A	G	33	9	--	--
	Ca(ICCV2/PI489777)SNP_15345	452	A	G	30	9	--	--
	Ca(ICCV2/PI489777)SNP_15346	464	G	A	38	7	--	--
CakTC23790	Ca(ICCV2/PI489777)SNP_15347	260	T	A	207	242	--	--
CakTC34294	Ca(ICCV2/PI489777)SNP_15348	1649	C	G	11	8	--	--
	Ca(ICCV2/PI489777)SNP_15349	1701	A	G	13	9	--	--
	Ca(ICCV2/PI489777)SNP_15350	2088	T	C	4	6	--	--
CakTC42255	Ca(ICCV2/PI489777)SNP_15351	513	G	T	9	7	--	--
	Ca(ICCV2/PI489777)SNP_15352	786	T	C	12	14	--	--
	Ca(ICCV2/PI489777)SNP_15353	996	G	A	13	7	--	--
CakTC27899	Ca(ICCV2/PI489777)SNP_15354	1159	T	C	16	6	--	--
	Ca(ICCV2/PI489777)SNP_15355	2257	C	T	5	4	--	--
CakTC27987	Ca(ICCV2/PI489777)SNP_15356	1716	C	T	31	31	--	--
	Ca(ICCV2/PI489777)SNP_15357	1749	A	G	34	35	--	--
	Ca(ICCV2/PI489777)SNP_15358	2175	T	C	22	21	--	--
	Ca(ICCV2/PI489777)SNP_15359	2396	G	C	15	6	--	--
	Ca(ICCV2/PI489777)SNP_15360	2450	G	A	21	7	--	--
	Ca(ICCV2/PI489777)SNP_15361	2470	A	C	20	7	--	--
CakTC23692	Ca(ICCV2/PI489777)SNP_15362	478	G	C	4	5	--	SBP
	Ca(ICCV2/PI489777)SNP_15363	1373	T	C	9	3	--	SBP
CakTC37257	Ca(ICCV2/PI489777)SNP_15364	317	G	A	5	5	--	--
	Ca(ICCV2/PI489777)SNP_15365	414	C	T	4	5	--	--
	Ca(ICCV2/PI489777)SNP_15366	960	T	C	10	4	--	--
CakTC27446	Ca(ICCV2/PI489777)SNP_15367	419	A	G	24	10	--	--
	Ca(ICCV2/PI489777)SNP_15368	657	G	C	19	5	--	--
CakTC20171	Ca(ICCV2/PI489777)SNP_15369	265	C	T	6	3	--	--
	Ca(ICCV2/PI489777)SNP_15370	271	C	T	6	3	--	--
	Ca(ICCV2/PI489777)SNP_15371	1347	C	A	5	11	--	--
CakTC26765	Ca(ICCV2/PI489777)SNP_15372	229	C	T	55	9	--	--
	Ca(ICCV2/PI489777)SNP_15373	607	C	T	85	17	--	--
	Ca(ICCV2/PI489777)SNP_15374	650	T	C	76	20	--	--
	Ca(ICCV2/PI489777)SNP_15375	961	T	G	46	16	--	--
	Ca(ICCV2/PI489777)SNP_15376	1015	G	C	28	13	--	--
CakTC09569	Ca(ICCV2/PI489777)SNP_15377	593	G	A	204	150	--	--
CakTC31960	Ca(ICCV2/PI489777)SNP_15378	670	A	G	3	6	--	--
	Ca(ICCV2/PI489777)SNP_15379	682	T	C	3	6	--	--
CakTC32549	Ca(ICCV2/PI489777)SNP_15380	402	G	C	11	7	--	--
CakTC39465	Ca(ICCV2/PI489777)SNP_15381	906	T	A	14	22	--	--
	Ca(ICCV2/PI489777)SNP_15382	1234	T	A	10	21	--	--
CakTC42851	Ca(ICCV2/PI489777)SNP_15383	494	A	T	25	19	--	--
	Ca(ICCV2/PI489777)SNP_15384	1184	C	T	35	34	--	--
	Ca(ICCV2/PI489777)SNP_15385	1301	G	A	43	16	--	--
	Ca(ICCV2/PI489777)SNP_15386	1395	G	T	28	12	--	--
	Ca(ICCV2/PI489777)SNP_15387	1556	C	T	25	10	--	--

	Ca(ICCV2/PI489777)SNP_15388	1604	A	C	13	10	--	--
CakTC39517	Ca(ICCV2/PI489777)SNP_15389	807	G	T	7	11	--	FHA
	Ca(ICCV2/PI489777)SNP_15390	837	A	G	7	11	--	FHA
	Ca(ICCV2/PI489777)SNP_15391	1005	G	C	8	4	--	FHA
CakTC07497	Ca(ICCV2/PI489777)SNP_15392	375	T	G	11	4	--	--
CakTC40388	Ca(ICCV2/PI489777)SNP_15393	276	A	G	5	3	--	--
	Ca(ICCV2/PI489777)SNP_15394	351	A	T	6	4	--	--
	Ca(ICCV2/PI489777)SNP_15395	452	A	T	7	4	--	--
	Ca(ICCV2/PI489777)SNP_15396	476	A	G	8	3	--	--
	Ca(ICCV2/PI489777)SNP_15397	631	G	C	7	3	--	--
CakTC13852	Ca(ICCV2/PI489777)SNP_15398	164	T	A	4	7	--	--
	Ca(ICCV2/PI489777)SNP_15399	245	T	C	8	7	--	--
CakTC32710	Ca(ICCV2/PI489777)SNP_15400	1331	A	G	17	11	--	ARID
	Ca(ICCV2/PI489777)SNP_15401	1427	A	G	14	10	--	ARID
CakTC35890	Ca(ICCV2/PI489777)SNP_15402	434	A	G	25	21	--	--
	Ca(ICCV2/PI489777)SNP_15403	1953	G	A	37	25	--	--
	Ca(ICCV2/PI489777)SNP_15404	2204	A	G	37	47	--	--
	Ca(ICCV2/PI489777)SNP_15405	2215	G	T	38	41	--	--
	Ca(ICCV2/PI489777)SNP_15406	2290	A	G	38	42	--	--
CakTC11959	Ca(ICCV2/PI489777)SNP_15407	155	T	C	4	8	--	--
	Ca(ICCV2/PI489777)SNP_15408	184	C	A	4	9	--	--
CakTC01568	Ca(ICCV2/PI489777)SNP_15409	187	G	T	4	3	--	--
	Ca(ICCV2/PI489777)SNP_15410	457	C	T	4	3	--	--
CakTC38748	Ca(ICCV2/PI489777)SNP_15411	688	G	A	9	4	--	FAR1
	Ca(ICCV2/PI489777)SNP_15412	925	C	T	16	6	--	FAR1
CakTC25505	Ca(ICCV2/PI489777)SNP_15413	141	A	T	6	5	--	--
	Ca(ICCV2/PI489777)SNP_15414	1695	T	C	33	29	--	--
	Ca(ICCV2/PI489777)SNP_15415	1870	G	T	21	21	--	--
CakTC36089	Ca(ICCV2/PI489777)SNP_15416	214	A	G	19	10	--	--
	Ca(ICCV2/PI489777)SNP_15417	283	C	A	24	16	--	--
	Ca(ICCV2/PI489777)SNP_15418	748	G	A	23	12	--	--
	Ca(ICCV2/PI489777)SNP_15419	890	C	T	21	13	--	--
	Ca(ICCV2/PI489777)SNP_15420	1090	T	C	27	13	--	--
	Ca(ICCV2/PI489777)SNP_15421	1392	C	T	20	14	--	--
	Ca(ICCV2/PI489777)SNP_15422	1420	C	A	17	17	--	--
	Ca(ICCV2/PI489777)SNP_15423	1531	A	G	14	9	--	--
	Ca(ICCV2/PI489777)SNP_15424	1621	T	C	20	11	--	--
	Ca(ICCV2/PI489777)SNP_15425	1831	G	A	24	12	--	--
	Ca(ICCV2/PI489777)SNP_15426	2549	T	G	4	3	--	--
	Ca(ICCV2/PI489777)SNP_15427	2561	A	G	5	3	--	--
	Ca(ICCV2/PI489777)SNP_15428	2564	A	G	5	3	--	--
CakTC42804	Ca(ICCV2/PI489777)SNP_15429	1425	C	T	32	20	--	--
CakTC31793	Ca(ICCV2/PI489777)SNP_15430	563	T	G	4	4	--	--
	Ca(ICCV2/PI489777)SNP_15431	662	G	A	4	9	--	--
CakTC31131	Ca(ICCV2/PI489777)SNP_15432	421	T	C	44	30	--	--
	Ca(ICCV2/PI489777)SNP_15433	439	C	T	37	33	--	--
CakTC32447	Ca(ICCV2/PI489777)SNP_15434	511	A	T	8	18	--	--
CakTC37608	Ca(ICCV2/PI489777)SNP_15435	166	C	T	8	6	--	--
	Ca(ICCV2/PI489777)SNP_15436	254	C	T	13	8	--	--
	Ca(ICCV2/PI489777)SNP_15437	473	A	T	27	16	--	--
	Ca(ICCV2/PI489777)SNP_15438	674	G	C	15	18	--	--
	Ca(ICCV2/PI489777)SNP_15439	854	A	T	11	15	--	--
	Ca(ICCV2/PI489777)SNP_15440	860	G	A	10	21	--	--
	Ca(ICCV2/PI489777)SNP_15441	883	T	C	10	23	--	--
	Ca(ICCV2/PI489777)SNP_15442	993	G	C	13	13	--	--
	Ca(ICCV2/PI489777)SNP_15443	1004	A	C	13	11	--	--
	Ca(ICCV2/PI489777)SNP_15444	1089	T	G	21	13	--	--
	Ca(ICCV2/PI489777)SNP_15445	1230	C	A	41	16	--	--
	Ca(ICCV2/PI489777)SNP_15446	1232	A	G	38	16	--	--
CakTC37512	Ca(ICCV2/PI489777)SNP_15447	131	C	G	8	11	--	--
	Ca(ICCV2/PI489777)SNP_15448	268	G	A	12	12	--	--
	Ca(ICCV2/PI489777)SNP_15449	572	T	C	17	6	--	--
	Ca(ICCV2/PI489777)SNP_15450	676	C	T	14	5	--	--
	Ca(ICCV2/PI489777)SNP_15451	797	A	G	11	8	--	--
	Ca(ICCV2/PI489777)SNP_15452	1132	T	C	21	13	--	--
	Ca(ICCV2/PI489777)SNP_15453	1267	G	A	13	9	--	--
CakTC29284	Ca(ICCV2/PI489777)SNP_15454	746	G	A	3	7	--	--
	Ca(ICCV2/PI489777)SNP_15455	947	C	T	7	4	--	--
	Ca(ICCV2/PI489777)SNP_15456	2063	A	T	5	3	--	--
	Ca(ICCV2/PI489777)SNP_15457	2480	G	C	6	6	--	--
CakTC34125	Ca(ICCV2/PI489777)SNP_15458	1380	G	A	9	10	--	--
	Ca(ICCV2/PI489777)SNP_15459	1553	T	C	9	8	--	--
CakTC33134	Ca(ICCV2/PI489777)SNP_15460	809	A	G	12	10	--	--
	Ca(ICCV2/PI489777)SNP_15461	1473	A	G	10	5	--	--

	Ca(ICCV2/PI489777)SNP_15462	1592	C	T	9	4	--	--
CakTC24898	Ca(ICCV2/PI489777)SNP_15463	321	T	C	29	5	--	--
	Ca(ICCV2/PI489777)SNP_15464	342	A	T	28	4	--	--
	Ca(ICCV2/PI489777)SNP_15465	343	A	G	28	4	--	--
	Ca(ICCV2/PI489777)SNP_15466	380	A	G	27	4	--	--
	Ca(ICCV2/PI489777)SNP_15467	383	A	G	25	4	--	--
CakTC30741	Ca(ICCV2/PI489777)SNP_15468	1330	C	A	3	3	--	--
CakTC33408	Ca(ICCV2/PI489777)SNP_15469	98	C	G	63	63	--	--
	Ca(ICCV2/PI489777)SNP_15470	275	T	C	99	63	--	--
	Ca(ICCV2/PI489777)SNP_15471	812	A	G	59	119	--	--
	Ca(ICCV2/PI489777)SNP_15472	819	A	C	64	112	--	--
	Ca(ICCV2/PI489777)SNP_15473	824	T	A	66	94	--	--
	Ca(ICCV2/PI489777)SNP_15474	831	G	A	66	95	--	--
	Ca(ICCV2/PI489777)SNP_15475	867	A	G	56	122	--	--
	Ca(ICCV2/PI489777)SNP_15476	970	A	G	81	136	--	--
	Ca(ICCV2/PI489777)SNP_15477	1034	A	G	92	121	--	--
	Ca(ICCV2/PI489777)SNP_15478	1055	A	G	82	116	--	--
	Ca(ICCV2/PI489777)SNP_15479	1088	A	G	78	102	--	--
	Ca(ICCV2/PI489777)SNP_15480	1580	C	T	33	63	--	--
	Ca(ICCV2/PI489777)SNP_15481	1665	A	T	32	41	--	--
	Ca(ICCV2/PI489777)SNP_15482	1708	C	A	29	43	--	--
	Ca(ICCV2/PI489777)SNP_15483	1762	T	A	19	38	--	--
CakTC27912	Ca(ICCV2/PI489777)SNP_15484	418	T	C	40	18	--	--
CakTC26241	Ca(ICCV2/PI489777)SNP_15485	287	G	T	3	7	--	--
	Ca(ICCV2/PI489777)SNP_15486	419	G	A	4	5	--	--
CakTC38937	Ca(ICCV2/PI489777)SNP_15487	919	T	C	8	3	--	--
	Ca(ICCV2/PI489777)SNP_15488	1365	C	G	14	6	--	--
CakTC03117	Ca(ICCV2/PI489777)SNP_15489	92	G	C	5	3	--	--
	Ca(ICCV2/PI489777)SNP_15490	164	C	A	4	4	--	--
	Ca(ICCV2/PI489777)SNP_15491	165	G	C	4	4	--	--
CakTC36718	Ca(ICCV2/PI489777)SNP_15492	532	T	C	7	9	--	--
	Ca(ICCV2/PI489777)SNP_15493	2299	A	G	8	13	--	--
CakTC38677	Ca(ICCV2/PI489777)SNP_15494	684	T	G	68	52	--	--
CakTC25088	Ca(ICCV2/PI489777)SNP_15495	301	G	A	57	11	--	--
	Ca(ICCV2/PI489777)SNP_15496	511	C	T	86	23	--	--
	Ca(ICCV2/PI489777)SNP_15497	794	C	G	53	10	--	--
	Ca(ICCV2/PI489777)SNP_15498	1124	C	G	59	13	--	--
	Ca(ICCV2/PI489777)SNP_15499	1317	C	T	42	10	--	--
CakTC30948	Ca(ICCV2/PI489777)SNP_15500	104	T	G	26	7	--	--
	Ca(ICCV2/PI489777)SNP_15501	555	T	G	7	9	--	--
	Ca(ICCV2/PI489777)SNP_15502	587	G	A	7	7	--	--
	Ca(ICCV2/PI489777)SNP_15503	684	G	C	5	5	--	--
CakTC36756	Ca(ICCV2/PI489777)SNP_15504	1351	G	A	7	4	--	--
	Ca(ICCV2/PI489777)SNP_15505	1369	T	C	10	4	--	--
	Ca(ICCV2/PI489777)SNP_15506	1876	A	T	23	4	--	--
CakTC39836	Ca(ICCV2/PI489777)SNP_15507	1205	G	C	44	15	--	--
CakTC37826	Ca(ICCV2/PI489777)SNP_15508	192	C	G	8	3	--	--
CakTC28002	Ca(ICCV2/PI489777)SNP_15509	85	C	T	5	4	Flower	bud
	Ca(ICCV2/PI489777)SNP_15510	93	A	G	5	4	Flower	bud
	Ca(ICCV2/PI489777)SNP_15511	1107	C	T	3	4	Flower	bud
	Ca(ICCV2/PI489777)SNP_15512	1153	C	G	4	4	Flower	bud
	Ca(ICCV2/PI489777)SNP_15513	1363	T	A	5	4	Flower	bud
CakTC42979	Ca(ICCV2/PI489777)SNP_15514	144	A	C	6	4	--	--
	Ca(ICCV2/PI489777)SNP_15515	273	T	C	5	5	--	--
	Ca(ICCV2/PI489777)SNP_15516	314	C	T	5	5	--	--
	Ca(ICCV2/PI489777)SNP_15517	415	A	G	6	4	--	--
	Ca(ICCV2/PI489777)SNP_15518	422	C	T	5	5	--	--
	Ca(ICCV2/PI489777)SNP_15519	1013	A	C	5	5	--	--
	Ca(ICCV2/PI489777)SNP_15520	1014	C	A	5	5	--	--
	Ca(ICCV2/PI489777)SNP_15521	1047	A	G	5	6	--	--
	Ca(ICCV2/PI489777)SNP_15522	1088	G	T	8	6	--	--
	Ca(ICCV2/PI489777)SNP_15523	1104	A	G	8	6	--	--
	Ca(ICCV2/PI489777)SNP_15524	1237	T	G	8	8	--	--
CakTC42141	Ca(ICCV2/PI489777)SNP_15525	117	C	T	3	5	--	--
	Ca(ICCV2/PI489777)SNP_15526	612	G	A	10	6	--	--
CakTC26113	Ca(ICCV2/PI489777)SNP_15527	178	C	T	3	4	--	--
	Ca(ICCV2/PI489777)SNP_15528	320	A	C	7	8	--	--
	Ca(ICCV2/PI489777)SNP_15529	956	T	C	7	10	--	--
	Ca(ICCV2/PI489777)SNP_15530	1119	C	T	9	5	--	--
	Ca(ICCV2/PI489777)SNP_15531	1410	T	C	14	13	--	--
	Ca(ICCV2/PI489777)SNP_15532	1476	G	T	12	15	--	--
CakTC27689	Ca(ICCV2/PI489777)SNP_15533	582	A	G	3	3	--	--
	Ca(ICCV2/PI489777)SNP_15534	608	A	G	3	3	--	--
CakTC37622	Ca(ICCV2/PI489777)SNP_15535	102	T	G	16	7	--	--

	Ca(ICCV2/PI489777)SNP_15536	133	G	C	17	12	--	--
	Ca(ICCV2/PI489777)SNP_15537	253	T	C	23	16	--	--
	Ca(ICCV2/PI489777)SNP_15538	292	A	G	26	23	--	--
	Ca(ICCV2/PI489777)SNP_15539	412	T	C	24	23	--	--
	Ca(ICCV2/PI489777)SNP_15540	421	T	G	22	28	--	--
CakTC27831	Ca(ICCV2/PI489777)SNP_15541	612	C	T	5	4	--	--
	Ca(ICCV2/PI489777)SNP_15542	816	G	A	6	3	--	--
CakTC27671	Ca(ICCV2/PI489777)SNP_15543	306	G	A	82	26	--	--
	Ca(ICCV2/PI489777)SNP_15544	482	A	G	87	33	--	--
	Ca(ICCV2/PI489777)SNP_15545	880	G	C	65	46	--	--
	Ca(ICCV2/PI489777)SNP_15546	985	G	T	28	11	--	--
CakTC34745	Ca(ICCV2/PI489777)SNP_15547	1614	G	A	5	5	Root	--
	Ca(ICCV2/PI489777)SNP_15548	2485	G	A	6	7	Root	--
	Ca(ICCV2/PI489777)SNP_15549	2515	A	C	5	7	Root	--
	Ca(ICCV2/PI489777)SNP_15550	2536	A	G	6	7	Root	--
CakTC38510	Ca(ICCV2/PI489777)SNP_15551	701	G	A	4	8	--	C2C2-GATA
	Ca(ICCV2/PI489777)SNP_15552	805	T	C	4	9	--	C2C2-GATA
CakTC37909	Ca(ICCV2/PI489777)SNP_15553	547	T	G	7	7	--	mTERF
	Ca(ICCV2/PI489777)SNP_15554	619	A	G	8	6	--	mTERF
	Ca(ICCV2/PI489777)SNP_15555	670	A	G	10	7	--	mTERF
CakTC37918	Ca(ICCV2/PI489777)SNP_15556	495	C	T	7	5	--	HSF
	Ca(ICCV2/PI489777)SNP_15557	747	G	A	12	4	--	HSF
CakTC28110	Ca(ICCV2/PI489777)SNP_15558	3290	C	T	6	3	--	PHD
	Ca(ICCV2/PI489777)SNP_15559	5035	T	C	3	3	--	PHD
CakTC33756	Ca(ICCV2/PI489777)SNP_15560	233	T	G	14	10	--	--
CakTC35352	Ca(ICCV2/PI489777)SNP_15561	69	T	A	8	5	--	--
	Ca(ICCV2/PI489777)SNP_15562	72	T	A	8	5	--	--
	Ca(ICCV2/PI489777)SNP_15563	300	T	C	9	5	--	--
	Ca(ICCV2/PI489777)SNP_15564	3472	A	C	7	8	--	--
	Ca(ICCV2/PI489777)SNP_15565	3565	A	G	8	8	--	--
	Ca(ICCV2/PI489777)SNP_15566	3751	C	T	10	7	--	--
	Ca(ICCV2/PI489777)SNP_15567	3815	C	A	8	5	--	--
CakTC27851	Ca(ICCV2/PI489777)SNP_15568	420	T	C	3	3	--	--
	Ca(ICCV2/PI489777)SNP_15569	624	T	G	3	5	--	--
	Ca(ICCV2/PI489777)SNP_15570	635	A	C	3	6	--	--
	Ca(ICCV2/PI489777)SNP_15571	678	T	G	4	5	--	--
	Ca(ICCV2/PI489777)SNP_15572	887	A	T	8	6	--	--
	Ca(ICCV2/PI489777)SNP_15573	1354	A	G	3	4	--	--
CakTC27044	Ca(ICCV2/PI489777)SNP_15574	515	A	C	12	19	--	--
CakTC10146	Ca(ICCV2/PI489777)SNP_15575	1226	T	C	5	4	--	--
CakTC09932	Ca(ICCV2/PI489777)SNP_15576	723	C	T	9	7	--	--
	Ca(ICCV2/PI489777)SNP_15577	746	G	A	9	5	--	--
	Ca(ICCV2/PI489777)SNP_15578	1730	G	A	5	5	--	--
	Ca(ICCV2/PI489777)SNP_15579	1837	T	C	4	6	--	--
	Ca(ICCV2/PI489777)SNP_15580	1896	G	C	3	5	--	--
CakTC41203	Ca(ICCV2/PI489777)SNP_15581	296	G	C	27	6	--	--
	Ca(ICCV2/PI489777)SNP_15582	524	T	C	12	6	--	--
	Ca(ICCV2/PI489777)SNP_15583	530	T	C	17	6	--	--
	Ca(ICCV2/PI489777)SNP_15584	566	G	A	13	5	--	--
	Ca(ICCV2/PI489777)SNP_15585	593	C	A	11	5	--	--
	Ca(ICCV2/PI489777)SNP_15586	1134	A	C	25	13	--	--
	Ca(ICCV2/PI489777)SNP_15587	1208	T	A	20	11	--	--
	Ca(ICCV2/PI489777)SNP_15588	1697	C	T	8	4	--	--
	Ca(ICCV2/PI489777)SNP_15589	2024	G	A	19	6	--	--
	Ca(ICCV2/PI489777)SNP_15590	2045	A	G	18	4	--	--
CakTC38294	Ca(ICCV2/PI489777)SNP_15591	660	C	G	37	29	--	--
	Ca(ICCV2/PI489777)SNP_15592	661	A	T	36	29	--	--
	Ca(ICCV2/PI489777)SNP_15593	784	T	C	27	33	--	--
	Ca(ICCV2/PI489777)SNP_15594	795	T	A	35	27	--	--
CakTC27569	Ca(ICCV2/PI489777)SNP_15595	310	T	C	216	52	--	--
	Ca(ICCV2/PI489777)SNP_15596	932	T	C	244	46	--	--
CakTC43292	Ca(ICCV2/PI489777)SNP_15597	131	G	A	9	11	--	--
	Ca(ICCV2/PI489777)SNP_15598	407	A	G	5	9	--	--
	Ca(ICCV2/PI489777)SNP_15599	433	A	G	8	9	--	--
	Ca(ICCV2/PI489777)SNP_15600	621	T	A	7	7	--	--
CakTC38982	Ca(ICCV2/PI489777)SNP_15601	878	T	C	7	8	--	--
	Ca(ICCV2/PI489777)SNP_15602	941	T	C	4	11	--	--
	Ca(ICCV2/PI489777)SNP_15603	1302	G	A	3	3	--	--
CakTC35409	Ca(ICCV2/PI489777)SNP_15604	76	C	T	4	3	--	--
	Ca(ICCV2/PI489777)SNP_15605	489	C	T	5	8	--	--
	Ca(ICCV2/PI489777)SNP_15606	1953	A	G	22	9	--	--
	Ca(ICCV2/PI489777)SNP_15607	2187	C	T	13	11	--	--
	Ca(ICCV2/PI489777)SNP_15608	2286	A	G	11	10	--	--
	Ca(ICCV2/PI489777)SNP_15609	2304	T	C	12	8	--	--

	Ca(ICCV2/PI489777)SNP_15610	2691	G	A	18	18	--	--
	Ca(ICCV2/PI489777)SNP_15611	3357	A	G	13	10	--	--
	Ca(ICCV2/PI489777)SNP_15612	3489	C	T	18	8	--	--
	Ca(ICCV2/PI489777)SNP_15613	3522	T	C	13	4	--	--
	Ca(ICCV2/PI489777)SNP_15614	4012	T	C	15	23	--	--
	Ca(ICCV2/PI489777)SNP_15615	4015	G	C	15	22	--	--
	Ca(ICCV2/PI489777)SNP_15616	4039	A	T	14	21	--	--
	Ca(ICCV2/PI489777)SNP_15617	4175	G	A	12	12	--	--
CakTC18036	Ca(ICCV2/PI489777)SNP_15618	781	A	G	30	10	--	--
CakTC24343	Ca(ICCV2/PI489777)SNP_15619	2005	G	A	8	7	--	--
CakTC40189	Ca(ICCV2/PI489777)SNP_15620	120	A	T	8	3	--	--
	Ca(ICCV2/PI489777)SNP_15621	308	T	C	26	9	--	--
CakTC29740	Ca(ICCV2/PI489777)SNP_15622	1254	C	T	8	4	--	SWI/SNF-SWI3
	Ca(ICCV2/PI489777)SNP_15623	3622	T	C	9	5	--	SWI/SNF-SWI3
	Ca(ICCV2/PI489777)SNP_15624	6040	C	T	14	3	--	SWI/SNF-SWI3
CakTC30121	Ca(ICCV2/PI489777)SNP_15625	853	T	C	8	5	--	--
	Ca(ICCV2/PI489777)SNP_15626	1001	A	T	8	3	--	--
	Ca(ICCV2/PI489777)SNP_15627	1045	A	G	9	4	--	--
	Ca(ICCV2/PI489777)SNP_15628	1178	G	A	9	7	--	--
	Ca(ICCV2/PI489777)SNP_15629	1277	A	T	6	7	--	--
	Ca(ICCV2/PI489777)SNP_15630	1733	A	G	3	3	--	--
	Ca(ICCV2/PI489777)SNP_15631	2382	A	G	5	4	--	--
	Ca(ICCV2/PI489777)SNP_15632	2536	C	G	7	3	--	--
	Ca(ICCV2/PI489777)SNP_15633	2569	G	T	6	3	--	--
	Ca(ICCV2/PI489777)SNP_15634	2680	T	C	6	5	--	--
	Ca(ICCV2/PI489777)SNP_15635	2916	G	A	11	3	--	--
CakTC38777	Ca(ICCV2/PI489777)SNP_15636	332	A	G	12	12	--	--
	Ca(ICCV2/PI489777)SNP_15637	337	T	C	12	7	--	--
	Ca(ICCV2/PI489777)SNP_15638	679	A	G	14	7	--	--
	Ca(ICCV2/PI489777)SNP_15639	771	A	G	14	4	--	--
	Ca(ICCV2/PI489777)SNP_15640	891	G	A	14	6	--	--
	Ca(ICCV2/PI489777)SNP_15641	941	A	G	16	4	--	--
	Ca(ICCV2/PI489777)SNP_15642	1218	T	C	10	12	--	--
	Ca(ICCV2/PI489777)SNP_15643	1379	G	A	17	10	--	--
CakTC39063	Ca(ICCV2/PI489777)SNP_15644	1051	A	G	4	5	--	--
	Ca(ICCV2/PI489777)SNP_15645	1491	G	A	4	6	--	--
CakTC38640	Ca(ICCV2/PI489777)SNP_15646	478	G	T	8	3	Young_pod	--
CakTC25332	Ca(ICCV2/PI489777)SNP_15647	1363	G	A	5	3	--	--
CakTC32186	Ca(ICCV2/PI489777)SNP_15648	300	G	T	18	16	--	--
	Ca(ICCV2/PI489777)SNP_15649	435	G	C	16	17	--	--
	Ca(ICCV2/PI489777)SNP_15650	647	G	C	17	20	--	--
	Ca(ICCV2/PI489777)SNP_15651	684	T	C	17	19	--	--
	Ca(ICCV2/PI489777)SNP_15652	1077	G	T	16	14	--	--
CakTC09774	Ca(ICCV2/PI489777)SNP_15653	247	A	G	10	13	--	--
CakTC41967	Ca(ICCV2/PI489777)SNP_15654	1677	G	T	29	7	--	--
CakTC41175	Ca(ICCV2/PI489777)SNP_15655	626	A	G	8	6	--	--
	Ca(ICCV2/PI489777)SNP_15656	1909	G	A	6	4	--	--
CakTC35063	Ca(ICCV2/PI489777)SNP_15657	1249	A	G	19	12	--	--
	Ca(ICCV2/PI489777)SNP_15658	1404	G	A	20	11	--	--
	Ca(ICCV2/PI489777)SNP_15659	1591	A	G	12	7	--	--
	Ca(ICCV2/PI489777)SNP_15660	1900	T	G	19	8	--	--
	Ca(ICCV2/PI489777)SNP_15661	1923	G	A	24	8	--	--
	Ca(ICCV2/PI489777)SNP_15662	2023	A	G	19	12	--	--
	Ca(ICCV2/PI489777)SNP_15663	2719	C	T	27	10	--	--
	Ca(ICCV2/PI489777)SNP_15664	3458	T	A	25	5	--	--
	Ca(ICCV2/PI489777)SNP_15665	3554	C	T	19	6	--	--
CakTC38845	Ca(ICCV2/PI489777)SNP_15666	333	G	A	3	13	--	--
	Ca(ICCV2/PI489777)SNP_15667	594	T	A	13	10	--	--
	Ca(ICCV2/PI489777)SNP_15668	642	A	T	13	9	--	--
	Ca(ICCV2/PI489777)SNP_15669	990	C	T	11	4	--	--
CakTC42623	Ca(ICCV2/PI489777)SNP_15670	420	T	C	136	118	--	--
CakTC40810	Ca(ICCV2/PI489777)SNP_15671	293	T	C	23	13	--	--
	Ca(ICCV2/PI489777)SNP_15672	1167	A	T	22	23	--	--
	Ca(ICCV2/PI489777)SNP_15673	1443	A	G	25	17	--	--
CakTC34241	Ca(ICCV2/PI489777)SNP_15674	265	T	C	5	3	--	--
CakTC43056	Ca(ICCV2/PI489777)SNP_15675	1168	C	A	24	6	--	--
CakTC24777	Ca(ICCV2/PI489777)SNP_15676	233	G	A	40	18	--	--
	Ca(ICCV2/PI489777)SNP_15677	1429	G	T	4	5	--	--
	Ca(ICCV2/PI489777)SNP_15678	1430	T	C	4	5	--	--
	Ca(ICCV2/PI489777)SNP_15679	1463	A	G	7	6	--	--
	Ca(ICCV2/PI489777)SNP_15680	1513	T	G	6	6	--	--
	Ca(ICCV2/PI489777)SNP_15681	1523	A	C	6	6	--	--
	Ca(ICCV2/PI489777)SNP_15682	1635	A	T	9	3	--	--
	Ca(ICCV2/PI489777)SNP_15683	1657	A	G	9	3	--	--

	Ca(ICCV2/PI489777)SNP_15684	1658	G	A	7	3	--	--
	Ca(ICCV2/PI489777)SNP_15685	2178	C	T	89	33	--	--
	Ca(ICCV2/PI489777)SNP_15686	2325	T	C	77	31	--	--
CakTC31109	Ca(ICCV2/PI489777)SNP_15687	897	T	C	10	7	--	--
	Ca(ICCV2/PI489777)SNP_15688	910	C	T	10	7	--	--
	Ca(ICCV2/PI489777)SNP_15689	930	A	T	10	6	--	--
	Ca(ICCV2/PI489777)SNP_15690	958	G	A	9	4	--	--
	Ca(ICCV2/PI489777)SNP_15691	985	A	T	9	4	--	--
	Ca(ICCV2/PI489777)SNP_15692	988	A	G	7	4	--	--
	Ca(ICCV2/PI489777)SNP_15693	1180	G	A	6	5	--	--
	Ca(ICCV2/PI489777)SNP_15694	1225	A	G	6	6	--	--
	Ca(ICCV2/PI489777)SNP_15695	1234	G	A	6	5	--	--
CakTC32605	Ca(ICCV2/PI489777)SNP_15696	445	G	A	13	6	Young_pod	--
	Ca(ICCV2/PI489777)SNP_15697	454	C	T	12	6	Young_pod	--
	Ca(ICCV2/PI489777)SNP_15698	457	T	C	13	6	Young_pod	--
	Ca(ICCV2/PI489777)SNP_15699	475	A	G	13	4	Young_pod	--
CakTC40682	Ca(ICCV2/PI489777)SNP_15700	429	C	T	6	9	--	--
	Ca(ICCV2/PI489777)SNP_15701	1350	G	C	5	3	--	--
CakTC25150	Ca(ICCV2/PI489777)SNP_15702	889	T	A	11	9	--	ABI3VP1
	Ca(ICCV2/PI489777)SNP_15703	964	A	G	15	17	--	ABI3VP1
	Ca(ICCV2/PI489777)SNP_15704	1118	G	T	17	16	--	ABI3VP1
	Ca(ICCV2/PI489777)SNP_15705	1175	C	T	14	11	--	ABI3VP1
	Ca(ICCV2/PI489777)SNP_15706	1244	A	T	15	10	--	ABI3VP1
	Ca(ICCV2/PI489777)SNP_15707	1272	G	C	13	8	--	ABI3VP1
CakTC39084	Ca(ICCV2/PI489777)SNP_15708	173	C	T	11	3	--	--
	Ca(ICCV2/PI489777)SNP_15709	306	C	A	10	6	--	--
	Ca(ICCV2/PI489777)SNP_15710	307	C	A	10	6	--	--
	Ca(ICCV2/PI489777)SNP_15711	784	A	T	13	4	--	--
	Ca(ICCV2/PI489777)SNP_15712	971	A	G	9	4	--	--
CakTC40002	Ca(ICCV2/PI489777)SNP_15713	977	T	A	4	8	Root	--
CakTC28463	Ca(ICCV2/PI489777)SNP_15714	455	A	T	7	3	--	--
CakTC39007	Ca(ICCV2/PI489777)SNP_15715	75	T	C	3	7	--	--
	Ca(ICCV2/PI489777)SNP_15716	91	C	T	3	9	--	--
	Ca(ICCV2/PI489777)SNP_15717	116	C	G	4	9	--	--
	Ca(ICCV2/PI489777)SNP_15718	118	A	T	4	9	--	--
	Ca(ICCV2/PI489777)SNP_15719	134	G	C	4	10	--	--
	Ca(ICCV2/PI489777)SNP_15720	927	C	T	6	6	--	--
	Ca(ICCV2/PI489777)SNP_15721	1150	T	A	5	3	--	--
	Ca(ICCV2/PI489777)SNP_15722	1154	G	A	4	3	--	--
	Ca(ICCV2/PI489777)SNP_15723	1162	G	A	5	3	--	--
CakTC26696	Ca(ICCV2/PI489777)SNP_15724	863	G	A	10	4	--	--
	Ca(ICCV2/PI489777)SNP_15725	874	C	A	10	4	--	--
	Ca(ICCV2/PI489777)SNP_15726	920	G	A	10	5	--	--
	Ca(ICCV2/PI489777)SNP_15727	995	G	A	8	6	--	--
	Ca(ICCV2/PI489777)SNP_15728	1250	G	A	19	7	--	--
	Ca(ICCV2/PI489777)SNP_15729	1382	A	G	18	10	--	--
	Ca(ICCV2/PI489777)SNP_15730	1391	A	G	19	9	--	--
	Ca(ICCV2/PI489777)SNP_15731	1569	T	C	8	6	--	--
CakTC23500	Ca(ICCV2/PI489777)SNP_15732	194	C	T	17	5	--	--
	Ca(ICCV2/PI489777)SNP_15733	427	C	T	16	5	--	--
	Ca(ICCV2/PI489777)SNP_15734	464	C	A	13	5	--	--
	Ca(ICCV2/PI489777)SNP_15735	665	T	C	4	3	--	--
	Ca(ICCV2/PI489777)SNP_15736	712	A	G	4	5	--	--
	Ca(ICCV2/PI489777)SNP_15737	843	T	C	3	6	--	--
	Ca(ICCV2/PI489777)SNP_15738	2326	T	C	31	11	--	--
	Ca(ICCV2/PI489777)SNP_15739	3376	G	T	25	14	--	--
CakTC29872	Ca(ICCV2/PI489777)SNP_15740	2694	G	A	3	3	--	--
	Ca(ICCV2/PI489777)SNP_15741	2718	A	G	3	3	--	--
CakTC33967	Ca(ICCV2/PI489777)SNP_15742	548	T	C	4	3	Flower	bud
	Ca(ICCV2/PI489777)SNP_15743	690	T	G	5	4	Flower	bud
	Ca(ICCV2/PI489777)SNP_15744	694	C	T	5	4	Flower	bud
CakTC37895	Ca(ICCV2/PI489777)SNP_15745	490	T	C	4	5	--	bHLH
CakTC26859	Ca(ICCV2/PI489777)SNP_15746	1122	T	C	3	12	--	--
	Ca(ICCV2/PI489777)SNP_15747	1673	C	G	16	4	--	--
	Ca(ICCV2/PI489777)SNP_15748	1706	C	T	14	7	--	--
	Ca(ICCV2/PI489777)SNP_15749	1997	C	T	9	13	--	--
	Ca(ICCV2/PI489777)SNP_15750	4280	C	T	7	7	--	--
	Ca(ICCV2/PI489777)SNP_15751	6281	G	A	10	7	--	--
	Ca(ICCV2/PI489777)SNP_15752	6401	G	A	9	6	--	--
CakTC40785	Ca(ICCV2/PI489777)SNP_15753	901	C	T	11	16	--	--
CakTC09181	Ca(ICCV2/PI489777)SNP_15754	473	A	G	7	6	--	--
	Ca(ICCV2/PI489777)SNP_15755	496	T	C	6	6	--	--
	Ca(ICCV2/PI489777)SNP_15756	554	G	A	10	6	--	--
	Ca(ICCV2/PI489777)SNP_15757	597	C	T	13	6	--	--

	Ca(ICCV2/PI489777)SNP_15758	713	T	C	14	5	--	--
CakTC37689	Ca(ICCV2/PI489777)SNP_15759	921	C	A	11	9	--	--
CakTC35999	Ca(ICCV2/PI489777)SNP_15760	305	A	G	32	33	--	--
	Ca(ICCV2/PI489777)SNP_15761	1273	G	A	54	39	--	--
	Ca(ICCV2/PI489777)SNP_15762	2077	A	G	21	56	--	--
	Ca(ICCV2/PI489777)SNP_15763	3083	G	C	15	28	--	--
	Ca(ICCV2/PI489777)SNP_15764	3152	T	G	10	10	--	--
CakTC43097	Ca(ICCV2/PI489777)SNP_15765	781	G	A	10	3	--	--
CakTC11046	Ca(ICCV2/PI489777)SNP_15766	488	T	C	143	146	--	--
CakTC10831	Ca(ICCV2/PI489777)SNP_15767	84	C	T	12	21	--	--
CakTC23767	Ca(ICCV2/PI489777)SNP_15768	1172	T	A	4	5	--	--
	Ca(ICCV2/PI489777)SNP_15769	1173	T	C	5	5	--	--
	Ca(ICCV2/PI489777)SNP_15770	1384	A	G	3	4	--	--
	Ca(ICCV2/PI489777)SNP_15771	2595	T	C	20	6	--	--
	Ca(ICCV2/PI489777)SNP_15772	2619	T	C	21	5	--	--
	Ca(ICCV2/PI489777)SNP_15773	2652	C	T	19	5	--	--
CakTC32632	Ca(ICCV2/PI489777)SNP_15774	1947	T	C	17	11	--	--
	Ca(ICCV2/PI489777)SNP_15775	2040	A	C	12	10	--	--
CakTC31004	Ca(ICCV2/PI489777)SNP_15776	1388	A	C	4	4	--	--
	Ca(ICCV2/PI489777)SNP_15777	1423	C	T	4	4	--	--
CakTC42336	Ca(ICCV2/PI489777)SNP_15778	747	T	C	19	19	--	--
CakTC39477	Ca(ICCV2/PI489777)SNP_15779	526	G	A	16	4	--	NAC
	Ca(ICCV2/PI489777)SNP_15780	802	C	T	25	9	--	NAC
	Ca(ICCV2/PI489777)SNP_15781	825	G	A	25	8	--	NAC
	Ca(ICCV2/PI489777)SNP_15782	931	G	A	17	7	--	NAC
CakTC22818	Ca(ICCV2/PI489777)SNP_15783	1494	T	C	9	4	--	--
CakTC24678	Ca(ICCV2/PI489777)SNP_15784	304	G	A	170	64	--	AP2-EREBP
	Ca(ICCV2/PI489777)SNP_15785	488	G	C	146	58	--	AP2-EREBP
	Ca(ICCV2/PI489777)SNP_15786	714	T	G	64	23	--	AP2-EREBP
CakTC13156	Ca(ICCV2/PI489777)SNP_15787	196	C	T	15	4	--	--
CakTC24434	Ca(ICCV2/PI489777)SNP_15788	192	A	T	8	4	--	--
	Ca(ICCV2/PI489777)SNP_15789	782	C	T	8	4	--	--
	Ca(ICCV2/PI489777)SNP_15790	812	T	C	6	3	--	--
CakTC09287	Ca(ICCV2/PI489777)SNP_15791	620	G	A	6	3	--	--
CakTC40767	Ca(ICCV2/PI489777)SNP_15792	1850	T	C	15	6	--	--
CakTC31182	Ca(ICCV2/PI489777)SNP_15793	547	G	A	24	8	--	--
	Ca(ICCV2/PI489777)SNP_15794	1199	C	G	10	10	--	--
	Ca(ICCV2/PI489777)SNP_15795	2227	G	A	16	5	--	--
	Ca(ICCV2/PI489777)SNP_15796	2429	G	A	8	3	--	--
CakTC26664	Ca(ICCV2/PI489777)SNP_15797	485	G	C	24	8	--	--
	Ca(ICCV2/PI489777)SNP_15798	1120	C	T	32	5	--	--
CakTC26495	Ca(ICCV2/PI489777)SNP_15799	575	A	C	6	5	--	--
	Ca(ICCV2/PI489777)SNP_15800	965	T	C	11	3	--	--
	Ca(ICCV2/PI489777)SNP_15801	986	G	A	10	3	--	--
	Ca(ICCV2/PI489777)SNP_15802	1070	C	A	4	3	--	--
CakTC31681	Ca(ICCV2/PI489777)SNP_15803	282	T	C	12	3	--	--
	Ca(ICCV2/PI489777)SNP_15804	362	A	G	7	4	--	--
CakTC40090	Ca(ICCV2/PI489777)SNP_15805	194	G	A	3	7	--	--
	Ca(ICCV2/PI489777)SNP_15806	1013	A	C	6	3	--	--
	Ca(ICCV2/PI489777)SNP_15807	1033	G	A	5	3	--	--
	Ca(ICCV2/PI489777)SNP_15808	1035	A	T	5	3	--	--
CakTC39512	Ca(ICCV2/PI489777)SNP_15809	742	C	T	9	4	--	--
CakTC12654	Ca(ICCV2/PI489777)SNP_15810	640	A	G	5	3	--	--
CakTC09835	Ca(ICCV2/PI489777)SNP_15811	582	C	T	9	24	Root	--
	Ca(ICCV2/PI489777)SNP_15812	765	C	T	7	9	Root	--
	Ca(ICCV2/PI489777)SNP_15813	780	A	G	7	9	Root	--
	Ca(ICCV2/PI489777)SNP_15814	852	A	T	6	10	Root	--
	Ca(ICCV2/PI489777)SNP_15815	1101	A	T	6	4	Root	--
	Ca(ICCV2/PI489777)SNP_15816	1284	A	T	10	14	Root	--
CakTC41375	Ca(ICCV2/PI489777)SNP_15817	1392	A	G	11	6	--	--
	Ca(ICCV2/PI489777)SNP_15818	1683	T	C	14	5	--	--
	Ca(ICCV2/PI489777)SNP_15819	2216	A	G	7	4	--	--
	Ca(ICCV2/PI489777)SNP_15820	2450	C	A	4	4	--	--
CakTC25819	Ca(ICCV2/PI489777)SNP_15821	1514	C	T	7	4	--	--
	Ca(ICCV2/PI489777)SNP_15822	1539	G	A	8	3	--	--
	Ca(ICCV2/PI489777)SNP_15823	1574	G	A	10	3	--	--
	Ca(ICCV2/PI489777)SNP_15824	2121	G	T	7	5	--	--
	Ca(ICCV2/PI489777)SNP_15825	2337	C	T	6	3	--	--
	Ca(ICCV2/PI489777)SNP_15826	2343	T	C	6	4	--	--
CakTC12183	Ca(ICCV2/PI489777)SNP_15827	1178	G	A	11	3	--	--
	Ca(ICCV2/PI489777)SNP_15828	1658	A	G	21	7	--	--
	Ca(ICCV2/PI489777)SNP_15829	1829	A	G	20	11	--	--
	Ca(ICCV2/PI489777)SNP_15830	1915	A	G	11	10	--	--
CakTC26011	Ca(ICCV2/PI489777)SNP_15831	1086	T	G	8	7	--	--

	Ca(ICCV2/PI489777)SNP_15832	2052	A	G	12	7	--	--
	Ca(ICCV2/PI489777)SNP_15833	2234	C	T	6	3	--	--
CakTC39294	Ca(ICCV2/PI489777)SNP_15834	988	A	T	19	12	--	--
CakTC24283	Ca(ICCV2/PI489777)SNP_15835	554	C	T	6	3	--	--
CakTC33213	Ca(ICCV2/PI489777)SNP_15836	892	G	A	43	18	--	--
	Ca(ICCV2/PI489777)SNP_15837	955	A	G	26	10	--	--
	Ca(ICCV2/PI489777)SNP_15838	1930	T	A	5	10	--	--
	Ca(ICCV2/PI489777)SNP_15839	1986	C	T	4	9	--	--
CakTC27480	Ca(ICCV2/PI489777)SNP_15840	511	C	G	37	27	--	--
	Ca(ICCV2/PI489777)SNP_15841	512	G	T	37	27	--	--
CakTC23340	Ca(ICCV2/PI489777)SNP_15842	32	T	G	12	30	--	--
	Ca(ICCV2/PI489777)SNP_15843	167	T	C	20	34	--	--
	Ca(ICCV2/PI489777)SNP_15844	261	C	G	63	87	--	--
	Ca(ICCV2/PI489777)SNP_15845	735	G	A	77	74	--	--
	Ca(ICCV2/PI489777)SNP_15846	744	T	C	82	74	--	--
	Ca(ICCV2/PI489777)SNP_15847	843	T	C	102	68	--	--
	Ca(ICCV2/PI489777)SNP_15848	924	T	A	111	71	--	--
CakTC31126	Ca(ICCV2/PI489777)SNP_15849	1078	C	T	3	4	Flower	bud
	Ca(ICCV2/PI489777)SNP_15850	1109	C	A	3	4	Flower	bud
CakTC34729	Ca(ICCV2/PI489777)SNP_15851	980	A	T	11	4	--	--
	Ca(ICCV2/PI489777)SNP_15852	1038	A	T	12	4	--	--
	Ca(ICCV2/PI489777)SNP_15853	2244	A	G	11	6	--	--
CakTC10024	Ca(ICCV2/PI489777)SNP_15854	413	C	G	5	4	--	C3H
	Ca(ICCV2/PI489777)SNP_15855	426	A	G	5	3	--	C3H
	Ca(ICCV2/PI489777)SNP_15856	1377	C	T	6	4	--	C3H
CakTC31546	Ca(ICCV2/PI489777)SNP_15857	1030	T	A	3	3	--	--
CakTC37765	Ca(ICCV2/PI489777)SNP_15858	751	T	C	16	12	--	--
	Ca(ICCV2/PI489777)SNP_15859	894	A	T	8	6	--	--
CakTC29157	Ca(ICCV2/PI489777)SNP_15860	381	A	T	50	12	--	--
CakTC34743	Ca(ICCV2/PI489777)SNP_15861	196	G	C	8	3	--	--
	Ca(ICCV2/PI489777)SNP_15862	257	T	C	5	6	--	--
	Ca(ICCV2/PI489777)SNP_15863	291	G	A	8	6	--	--
	Ca(ICCV2/PI489777)SNP_15864	317	G	C	10	6	--	--
	Ca(ICCV2/PI489777)SNP_15865	1820	T	C	19	5	--	--
	Ca(ICCV2/PI489777)SNP_15866	1966	G	A	23	5	--	--
	Ca(ICCV2/PI489777)SNP_15867	2048	T	C	20	4	--	--
	Ca(ICCV2/PI489777)SNP_15868	2049	G	A	20	4	--	--
	Ca(ICCV2/PI489777)SNP_15869	2108	T	C	21	4	--	--
	Ca(ICCV2/PI489777)SNP_15870	2206	A	G	11	5	--	--
	Ca(ICCV2/PI489777)SNP_15871	2426	C	T	7	5	--	--
	Ca(ICCV2/PI489777)SNP_15872	2543	C	T	10	8	--	--
	Ca(ICCV2/PI489777)SNP_15873	2636	T	A	9	6	--	--
	Ca(ICCV2/PI489777)SNP_15874	2676	T	A	10	4	--	--
	Ca(ICCV2/PI489777)SNP_15875	2695	T	C	11	5	--	--
	Ca(ICCV2/PI489777)SNP_15876	2897	T	C	13	4	--	--
	Ca(ICCV2/PI489777)SNP_15877	2925	G	A	11	5	--	--
	Ca(ICCV2/PI489777)SNP_15878	2952	A	G	12	5	--	--
	Ca(ICCV2/PI489777)SNP_15879	3014	G	A	12	3	--	--
	Ca(ICCV2/PI489777)SNP_15880	3020	G	A	13	3	--	--
CakTC04716	Ca(ICCV2/PI489777)SNP_15881	836	A	C	3	16	--	--
CakTC17795	Ca(ICCV2/PI489777)SNP_15882	644	G	C	8	3	--	--
	Ca(ICCV2/PI489777)SNP_15883	661	A	G	8	3	--	--
CakTC39859	Ca(ICCV2/PI489777)SNP_15884	150	T	C	10	4	--	--
	Ca(ICCV2/PI489777)SNP_15885	915	G	A	14	11	--	--
	Ca(ICCV2/PI489777)SNP_15886	978	C	T	8	10	--	--
CakTC10431	Ca(ICCV2/PI489777)SNP_15887	230	A	G	8	5	--	--
	Ca(ICCV2/PI489777)SNP_15888	636	G	A	16	4	--	--
CakTC25975	Ca(ICCV2/PI489777)SNP_15889	200	C	T	17	4	--	--
CakTC41318	Ca(ICCV2/PI489777)SNP_15890	79	A	C	4	5	--	--
CakTC34658	Ca(ICCV2/PI489777)SNP_15891	270	C	T	10	8	--	--
	Ca(ICCV2/PI489777)SNP_15892	303	C	T	11	8	--	--
	Ca(ICCV2/PI489777)SNP_15893	336	C	T	10	8	--	--
CakTC34182	Ca(ICCV2/PI489777)SNP_15894	135	C	T	3	6	--	--
	Ca(ICCV2/PI489777)SNP_15895	320	T	G	6	7	--	--
	Ca(ICCV2/PI489777)SNP_15896	347	A	C	6	3	--	--
CakTC31910	Ca(ICCV2/PI489777)SNP_15897	568	C	T	11	4	--	SAP
	Ca(ICCV2/PI489777)SNP_15898	778	G	T	12	6	--	SAP
	Ca(ICCV2/PI489777)SNP_15899	3731	G	A	9	12	--	SAP
	Ca(ICCV2/PI489777)SNP_15900	4319	G	T	9	4	--	SAP
	Ca(ICCV2/PI489777)SNP_15901	4322	A	C	9	4	--	SAP
	Ca(ICCV2/PI489777)SNP_15902	4400	T	C	9	4	--	SAP
	Ca(ICCV2/PI489777)SNP_15903	4678	G	A	12	10	--	SAP
	Ca(ICCV2/PI489777)SNP_15904	4806	G	T	8	7	--	SAP
CakTC31411	Ca(ICCV2/PI489777)SNP_15905	2463	T	C	3	3	--	C2H2

CakTC39718	Ca(ICCV2/PI489777)SNP_15906	421	A	G	22	8	--	--
	Ca(ICCV2/PI489777)SNP_15907	1216	G	A	59	11	--	--
	Ca(ICCV2/PI489777)SNP_15908	1253	A	G	36	5	--	--
	Ca(ICCV2/PI489777)SNP_15909	1256	A	G	36	5	--	--
CakTC40911	Ca(ICCV2/PI489777)SNP_15910	719	G	T	26	21	--	--
CakTC33640	Ca(ICCV2/PI489777)SNP_15911	1158	G	A	12	4	--	TPR
CakTC31601	Ca(ICCV2/PI489777)SNP_15912	1015	T	C	4	4	--	Jumonji
CakTC38210	Ca(ICCV2/PI489777)SNP_15913	320	A	G	9	24	--	--
	Ca(ICCV2/PI489777)SNP_15914	335	A	G	7	24	--	--
CakTC40797	Ca(ICCV2/PI489777)SNP_15915	1501	A	G	50	33	--	--
	Ca(ICCV2/PI489777)SNP_15916	1712	G	A	63	33	--	--
	Ca(ICCV2/PI489777)SNP_15917	1734	C	G	58	35	--	--
	Ca(ICCV2/PI489777)SNP_15918	1792	T	A	61	35	--	--
CakTC32469	Ca(ICCV2/PI489777)SNP_15919	632	A	G	5	12	--	--
	Ca(ICCV2/PI489777)SNP_15920	1185	T	A	7	4	--	--
CakTC40194	Ca(ICCV2/PI489777)SNP_15921	1227	T	G	26	33	--	--
CakTC41992	Ca(ICCV2/PI489777)SNP_15922	175	A	G	33	23	--	--
CakTC38576	Ca(ICCV2/PI489777)SNP_15923	967	A	T	46	40	--	--
	Ca(ICCV2/PI489777)SNP_15924	1053	C	T	41	26	--	--
	Ca(ICCV2/PI489777)SNP_15925	1066	C	T	40	30	--	--
CakTC26025	Ca(ICCV2/PI489777)SNP_15926	2449	T	G	4	12	--	--
	Ca(ICCV2/PI489777)SNP_15927	2466	C	T	4	12	--	--
	Ca(ICCV2/PI489777)SNP_15928	2490	C	T	3	12	--	--
CakTC39911	Ca(ICCV2/PI489777)SNP_15929	1457	A	T	10	16	--	--
CakTC42210	Ca(ICCV2/PI489777)SNP_15930	620	A	C	26	15	--	--
	Ca(ICCV2/PI489777)SNP_15931	1508	C	T	14	12	--	--
	Ca(ICCV2/PI489777)SNP_15932	1562	G	T	14	11	--	--
CakTC25236	Ca(ICCV2/PI489777)SNP_15933	369	C	T	13	4	--	bZIP
	Ca(ICCV2/PI489777)SNP_15934	403	A	T	11	5	--	bZIP
	Ca(ICCV2/PI489777)SNP_15935	444	A	G	13	6	--	bZIP
	Ca(ICCV2/PI489777)SNP_15936	445	G	T	13	6	--	bZIP
	Ca(ICCV2/PI489777)SNP_15937	482	C	T	15	4	--	bZIP
	Ca(ICCV2/PI489777)SNP_15938	652	G	A	20	6	--	bZIP
	Ca(ICCV2/PI489777)SNP_15939	798	T	A	45	28	--	bZIP
	Ca(ICCV2/PI489777)SNP_15940	813	G	A	51	29	--	bZIP
	Ca(ICCV2/PI489777)SNP_15941	859	C	G	53	28	--	bZIP
	Ca(ICCV2/PI489777)SNP_15942	1311	G	C	31	8	--	bZIP
CakTC34498	Ca(ICCV2/PI489777)SNP_15943	184	C	T	4	4	--	--
	Ca(ICCV2/PI489777)SNP_15944	187	C	T	4	4	--	--
	Ca(ICCV2/PI489777)SNP_15945	292	T	C	4	4	--	--
CakTC42002	Ca(ICCV2/PI489777)SNP_15946	34	T	A	3	3	--	--
CakTC24439	Ca(ICCV2/PI489777)SNP_15947	2159	G	A	56	25	--	--
	Ca(ICCV2/PI489777)SNP_15948	2846	G	A	13	3	--	--
CakTC30029	Ca(ICCV2/PI489777)SNP_15949	286	T	A	4	5	Root	--
	Ca(ICCV2/PI489777)SNP_15950	919	C	T	3	5	Root	--
	Ca(ICCV2/PI489777)SNP_15951	967	C	T	3	5	Root	--
CakTC09695	Ca(ICCV2/PI489777)SNP_15952	180	C	T	3	20	--	--
	Ca(ICCV2/PI489777)SNP_15953	230	T	G	3	27	--	--
	Ca(ICCV2/PI489777)SNP_15954	344	T	C	24	40	--	--
	Ca(ICCV2/PI489777)SNP_15955	596	A	G	38	71	--	--
	Ca(ICCV2/PI489777)SNP_15956	683	T	C	32	70	--	--
CakTC26748	Ca(ICCV2/PI489777)SNP_15957	627	C	A	5	3	--	--
	Ca(ICCV2/PI489777)SNP_15958	635	C	T	4	3	--	--
	Ca(ICCV2/PI489777)SNP_15959	638	T	C	4	3	--	--
CakTC29854	Ca(ICCV2/PI489777)SNP_15960	2509	G	C	15	6	--	--
CakTC35929	Ca(ICCV2/PI489777)SNP_15961	831	A	G	64	20	--	--
	Ca(ICCV2/PI489777)SNP_15962	1089	A	T	46	20	--	--
	Ca(ICCV2/PI489777)SNP_15963	2186	T	C	34	18	--	--
CakTC39003	Ca(ICCV2/PI489777)SNP_15964	538	G	A	13	7	--	--
	Ca(ICCV2/PI489777)SNP_15965	883	A	G	3	6	--	--
CakTC24413	Ca(ICCV2/PI489777)SNP_15966	1181	T	G	27	23	--	--
CakTC40693	Ca(ICCV2/PI489777)SNP_15967	35	G	C	6	4	--	--
	Ca(ICCV2/PI489777)SNP_15968	495	T	C	61	49	--	--
	Ca(ICCV2/PI489777)SNP_15969	618	A	G	9	15	--	--
CakTC40423	Ca(ICCV2/PI489777)SNP_15970	119	C	T	18	5	--	--
	Ca(ICCV2/PI489777)SNP_15971	188	G	A	21	5	--	--
	Ca(ICCV2/PI489777)SNP_15972	213	A	G	21	6	--	--
	Ca(ICCV2/PI489777)SNP_15973	1013	C	T	36	21	--	--
	Ca(ICCV2/PI489777)SNP_15974	1019	C	T	36	20	--	--
CakTC11123	Ca(ICCV2/PI489777)SNP_15975	50	G	A	6	6	--	--
	Ca(ICCV2/PI489777)SNP_15976	1068	T	C	162	51	--	--
	Ca(ICCV2/PI489777)SNP_15977	1089	A	T	154	51	--	--
	Ca(ICCV2/PI489777)SNP_15978	1090	A	G	153	48	--	--
	Ca(ICCV2/PI489777)SNP_15979	1127	A	G	149	42	--	--

	Ca(ICCV2/PI489777)SNP_15980	1130	A	G	144	42	--	--
CakTC31137	Ca(ICCV2/PI489777)SNP_15981	261	G	A	10	3	--	--
CakTC36833	Ca(ICCV2/PI489777)SNP_15982	477	T	C	20	6	--	--
	Ca(ICCV2/PI489777)SNP_15983	487	G	T	21	5	--	--
	Ca(ICCV2/PI489777)SNP_15984	497	C	A	20	6	--	--
	Ca(ICCV2/PI489777)SNP_15985	515	A	G	24	6	--	--
	Ca(ICCV2/PI489777)SNP_15986	772	A	G	32	8	--	--
	Ca(ICCV2/PI489777)SNP_15987	1170	G	A	17	10	--	--
	Ca(ICCV2/PI489777)SNP_15988	1213	A	C	17	10	--	--
	Ca(ICCV2/PI489777)SNP_15989	1604	T	C	12	10	--	--
	Ca(ICCV2/PI489777)SNP_15990	1885	T	A	14	10	--	--
	Ca(ICCV2/PI489777)SNP_15991	2662	C	T	15	11	--	--
	Ca(ICCV2/PI489777)SNP_15992	2734	T	C	18	16	--	--
	Ca(ICCV2/PI489777)SNP_15993	2845	C	T	14	13	--	--
	Ca(ICCV2/PI489777)SNP_15994	2872	T	C	18	14	--	--
	Ca(ICCV2/PI489777)SNP_15995	3244	T	C	15	9	--	--
	Ca(ICCV2/PI489777)SNP_15996	3337	T	C	12	8	--	--
	Ca(ICCV2/PI489777)SNP_15997	4381	T	C	19	13	--	--
	Ca(ICCV2/PI489777)SNP_15998	4390	C	T	19	12	--	--
	Ca(ICCV2/PI489777)SNP_15999	4411	A	C	16	9	--	--
	Ca(ICCV2/PI489777)SNP_16000	4555	A	G	14	13	--	--
	Ca(ICCV2/PI489777)SNP_16001	4638	C	T	12	10	--	--
CakTC38670	Ca(ICCV2/PI489777)SNP_16002	201	T	C	15	5	--	--
	Ca(ICCV2/PI489777)SNP_16003	205	T	C	16	5	--	--
	Ca(ICCV2/PI489777)SNP_16004	268	C	T	16	5	--	--
	Ca(ICCV2/PI489777)SNP_16005	1351	T	C	8	3	--	--
CakTC29568	Ca(ICCV2/PI489777)SNP_16006	1357	G	A	7	3	--	--
CakTC40637	Ca(ICCV2/PI489777)SNP_16007	685	A	G	23	11	--	WRKY
	Ca(ICCV2/PI489777)SNP_16008	1060	G	T	7	9	--	WRKY
	Ca(ICCV2/PI489777)SNP_16009	1387	G	T	14	4	--	WRKY
CakTC10522	Ca(ICCV2/PI489777)SNP_16010	1105	C	T	3	5	--	--
CakTC37717	Ca(ICCV2/PI489777)SNP_16011	331	G	T	13	13	--	--
	Ca(ICCV2/PI489777)SNP_16012	457	A	G	12	14	--	--
	Ca(ICCV2/PI489777)SNP_16013	490	G	A	13	12	--	--
	Ca(ICCV2/PI489777)SNP_16014	1209	A	G	9	18	--	--
CakTC40097	Ca(ICCV2/PI489777)SNP_16015	493	A	C	5	6	--	--
CakTC22960	Ca(ICCV2/PI489777)SNP_16016	447	A	G	72	64	--	--
	Ca(ICCV2/PI489777)SNP_16017	825	A	G	75	53	--	--
	Ca(ICCV2/PI489777)SNP_16018	873	G	T	65	53	--	--
	Ca(ICCV2/PI489777)SNP_16019	2067	A	G	49	48	--	--
	Ca(ICCV2/PI489777)SNP_16020	2597	T	C	17	10	--	--
	Ca(ICCV2/PI489777)SNP_16021	2605	G	A	15	5	--	--
CakTC39924	Ca(ICCV2/PI489777)SNP_16022	519	T	C	3	3	--	--
CakTC43208	Ca(ICCV2/PI489777)SNP_16023	431	T	C	5	4	--	--
	Ca(ICCV2/PI489777)SNP_16024	446	G	A	5	4	--	--
	Ca(ICCV2/PI489777)SNP_16025	467	A	G	4	4	--	--
	Ca(ICCV2/PI489777)SNP_16026	656	A	G	4	4	--	--
	Ca(ICCV2/PI489777)SNP_16027	665	A	C	4	3	--	--
	Ca(ICCV2/PI489777)SNP_16028	799	C	T	4	3	--	--
CakTC43285	Ca(ICCV2/PI489777)SNP_16029	1253	C	T	5	4	--	--
	Ca(ICCV2/PI489777)SNP_16030	1467	C	A	5	5	--	--
CakTC33816	Ca(ICCV2/PI489777)SNP_16031	242	A	G	6	8	--	--
	Ca(ICCV2/PI489777)SNP_16032	249	A	T	6	10	--	--
	Ca(ICCV2/PI489777)SNP_16033	447	T	C	7	13	--	--
	Ca(ICCV2/PI489777)SNP_16034	477	A	G	6	15	--	--
	Ca(ICCV2/PI489777)SNP_16035	729	A	G	17	20	--	--
	Ca(ICCV2/PI489777)SNP_16036	756	T	C	18	22	--	--
	Ca(ICCV2/PI489777)SNP_16037	918	T	C	16	22	--	--
CakTC10076	Ca(ICCV2/PI489777)SNP_16038	353	T	G	3	3	--	--
CakTC37565	Ca(ICCV2/PI489777)SNP_16039	1108	G	A	5	4	--	--
	Ca(ICCV2/PI489777)SNP_16040	1124	A	G	6	4	--	--
CakTC26028	Ca(ICCV2/PI489777)SNP_16041	687	C	T	13	3	--	C2H2
	Ca(ICCV2/PI489777)SNP_16042	1450	G	A	9	11	--	C2H2
	Ca(ICCV2/PI489777)SNP_16043	1489	G	T	14	11	--	C2H2
CakTC01590	Ca(ICCV2/PI489777)SNP_16044	272	C	T	3	8	--	--
	Ca(ICCV2/PI489777)SNP_16045	279	G	A	3	9	--	--
	Ca(ICCV2/PI489777)SNP_16046	323	G	A	3	10	--	--
CakTC11414	Ca(ICCV2/PI489777)SNP_16047	315	G	C	52	30	--	--
	Ca(ICCV2/PI489777)SNP_16048	316	T	C	51	30	--	--
	Ca(ICCV2/PI489777)SNP_16049	335	C	T	52	30	--	--
CakTC04081	Ca(ICCV2/PI489777)SNP_16050	1025	G	T	38	48	--	--
CakTC40079	Ca(ICCV2/PI489777)SNP_16051	112	G	A	7	6	--	--
CakTC08664	Ca(ICCV2/PI489777)SNP_16052	471	C	T	4	4	--	--
	Ca(ICCV2/PI489777)SNP_16053	758	A	T	3	3	--	--

CakTC33744	Ca(ICCV2/PI489777)SNP_16054	112	G	A	7	4	--	--
	Ca(ICCV2/PI489777)SNP_16055	142	A	G	8	4	--	--
CakTC32901	Ca(ICCV2/PI489777)SNP_16056	170	A	C	3	5	--	FHA
	Ca(ICCV2/PI489777)SNP_16057	343	T	A	15	9	--	FHA
	Ca(ICCV2/PI489777)SNP_16058	351	T	C	15	9	--	FHA
	Ca(ICCV2/PI489777)SNP_16059	416	G	T	11	10	--	FHA
	Ca(ICCV2/PI489777)SNP_16060	609	G	A	12	7	--	FHA
	Ca(ICCV2/PI489777)SNP_16061	744	A	T	11	3	--	FHA
	Ca(ICCV2/PI489777)SNP_16062	1080	A	G	12	5	--	FHA
	Ca(ICCV2/PI489777)SNP_16063	1488	C	T	22	4	--	FHA
	Ca(ICCV2/PI489777)SNP_16064	1562	G	C	18	7	--	FHA
	Ca(ICCV2/PI489777)SNP_16065	1578	G	C	20	7	--	FHA
	Ca(ICCV2/PI489777)SNP_16066	1650	C	A	6	3	--	FHA
	Ca(ICCV2/PI489777)SNP_16067	1668	T	C	6	5	--	FHA
	Ca(ICCV2/PI489777)SNP_16068	1677	A	T	6	4	--	FHA
	Ca(ICCV2/PI489777)SNP_16069	1872	T	C	8	4	--	FHA
	Ca(ICCV2/PI489777)SNP_16070	1932	A	G	6	3	--	FHA
	Ca(ICCV2/PI489777)SNP_16071	2221	G	C	5	3	--	FHA
CakTC39899	Ca(ICCV2/PI489777)SNP_16072	614	G	A	19	5	--	G2-like
	Ca(ICCV2/PI489777)SNP_16073	1201	C	T	11	5	--	G2-like
	Ca(ICCV2/PI489777)SNP_16074	1343	A	G	10	3	--	G2-like
CakTC36440	Ca(ICCV2/PI489777)SNP_16075	307	G	A	11	6	--	--
	Ca(ICCV2/PI489777)SNP_16076	339	T	C	9	6	--	--
	Ca(ICCV2/PI489777)SNP_16077	350	A	G	9	6	--	--
	Ca(ICCV2/PI489777)SNP_16078	365	T	C	9	6	--	--
	Ca(ICCV2/PI489777)SNP_16079	1290	C	G	20	4	--	--
	Ca(ICCV2/PI489777)SNP_16080	1578	T	C	24	6	--	--
	Ca(ICCV2/PI489777)SNP_16081	1611	T	C	15	4	--	--
	Ca(ICCV2/PI489777)SNP_16082	2124	C	T	18	10	--	--
	Ca(ICCV2/PI489777)SNP_16083	2292	G	T	10	9	--	--
	Ca(ICCV2/PI489777)SNP_16084	2310	G	A	12	9	--	--
	Ca(ICCV2/PI489777)SNP_16085	2547	C	T	10	10	--	--
	Ca(ICCV2/PI489777)SNP_16086	2880	G	A	11	11	--	--
CakTC26082	Ca(ICCV2/PI489777)SNP_16087	2325	T	A	16	15	--	--
CakTC31763	Ca(ICCV2/PI489777)SNP_16088	1332	G	C	3	5	Flower	bud
	Ca(ICCV2/PI489777)SNP_16089	1757	A	G	4	6	Flower	bud
	Ca(ICCV2/PI489777)SNP_16090	1854	G	A	5	4	Flower	bud
	Ca(ICCV2/PI489777)SNP_16091	2418	C	A	5	5	Flower	bud
CakTC28704	Ca(ICCV2/PI489777)SNP_16092	1090	A	G	43	11	--	--
	Ca(ICCV2/PI489777)SNP_16093	1102	C	A	45	13	--	--
	Ca(ICCV2/PI489777)SNP_16094	1396	A	G	29	15	--	--
	Ca(ICCV2/PI489777)SNP_16095	1651	G	A	34	8	--	--
	Ca(ICCV2/PI489777)SNP_16096	2047	G	A	20	7	--	--
	Ca(ICCV2/PI489777)SNP_16097	2113	G	A	27	9	--	--
	Ca(ICCV2/PI489777)SNP_16098	2176	G	T	31	12	--	--
CakTC32692	Ca(ICCV2/PI489777)SNP_16099	1376	A	G	7	5	--	--
	Ca(ICCV2/PI489777)SNP_16100	1508	A	C	8	7	--	--
	Ca(ICCV2/PI489777)SNP_16101	1511	G	T	8	7	--	--
	Ca(ICCV2/PI489777)SNP_16102	1832	T	G	4	3	--	--
CakTC27474	Ca(ICCV2/PI489777)SNP_16103	626	A	G	7	9	--	--
	Ca(ICCV2/PI489777)SNP_16104	644	T	G	7	10	--	--
	Ca(ICCV2/PI489777)SNP_16105	665	A	C	7	7	--	--
	Ca(ICCV2/PI489777)SNP_16106	1319	C	A	8	6	--	--
	Ca(ICCV2/PI489777)SNP_16107	1471	A	G	3	6	--	--
CakTC42911	Ca(ICCV2/PI489777)SNP_16108	177	G	A	8	5	--	--
	Ca(ICCV2/PI489777)SNP_16109	349	G	A	12	15	--	--
	Ca(ICCV2/PI489777)SNP_16110	353	A	T	12	14	--	--
CakTC24101	Ca(ICCV2/PI489777)SNP_16111	1250	G	A	19	3	Flower	bud
	Ca(ICCV2/PI489777)SNP_16112	1360	A	G	24	3	Flower	bud
CakTC29904	Ca(ICCV2/PI489777)SNP_16113	1239	C	A	4	3	--	--
CakTC25803	Ca(ICCV2/PI489777)SNP_16114	866	C	G	34	8	--	--
	Ca(ICCV2/PI489777)SNP_16115	911	C	T	35	9	--	--
	Ca(ICCV2/PI489777)SNP_16116	1094	A	G	26	11	--	--
	Ca(ICCV2/PI489777)SNP_16117	1135	T	G	19	11	--	--
	Ca(ICCV2/PI489777)SNP_16118	1202	C	T	21	13	--	--
CakTC40070	Ca(ICCV2/PI489777)SNP_16119	108	C	G	7	5	--	--
	Ca(ICCV2/PI489777)SNP_16120	863	C	T	11	6	--	--
	Ca(ICCV2/PI489777)SNP_16121	1344	T	C	9	5	--	--
	Ca(ICCV2/PI489777)SNP_16122	1503	G	A	5	3	--	--
CakTC09640	Ca(ICCV2/PI489777)SNP_16123	838	A	G	17	3	--	--
	Ca(ICCV2/PI489777)SNP_16124	841	C	T	16	3	--	--
	Ca(ICCV2/PI489777)SNP_16125	1164	G	A	9	4	--	--
	Ca(ICCV2/PI489777)SNP_16126	1442	T	C	8	5	--	--
	Ca(ICCV2/PI489777)SNP_16127	1532	A	C	4	4	--	--

CakTC07746	Ca(ICCV2/PI489777)SNP_16128	320	A	G	12	3	--	--
	Ca(ICCV2/PI489777)SNP_16129	366	T	C	12	3	--	--
	Ca(ICCV2/PI489777)SNP_16130	407	T	C	11	4	--	--
	Ca(ICCV2/PI489777)SNP_16131	521	C	A	10	3	--	--
CakTC37227	Ca(ICCV2/PI489777)SNP_16132	860	G	T	13	10	Flower	bud
CakTC28333	Ca(ICCV2/PI489777)SNP_16133	1397	C	T	6	3	--	SBP
	Ca(ICCV2/PI489777)SNP_16134	1587	C	T	6	5	--	SBP
CakTC40131	Ca(ICCV2/PI489777)SNP_16135	510	G	A	10	3	--	--
CakTC36622	Ca(ICCV2/PI489777)SNP_16136	149	T	C	12	4	--	--
	Ca(ICCV2/PI489777)SNP_16137	165	G	A	7	4	--	--
	Ca(ICCV2/PI489777)SNP_16138	549	T	C	8	3	--	--
	Ca(ICCV2/PI489777)SNP_16139	576	C	T	7	4	--	--
	Ca(ICCV2/PI489777)SNP_16140	745	A	G	8	8	--	--
	Ca(ICCV2/PI489777)SNP_16141	1052	G	A	17	5	--	--
CakTC37314	Ca(ICCV2/PI489777)SNP_16142	620	A	G	8	4	--	--
CakTC40955	Ca(ICCV2/PI489777)SNP_16143	45	G	T	8	4	--	--
	Ca(ICCV2/PI489777)SNP_16144	66	G	T	11	4	--	--
CakTC40528	Ca(ICCV2/PI489777)SNP_16145	81	C	A	3	7	--	--
	Ca(ICCV2/PI489777)SNP_16146	111	C	A	4	6	--	--
	Ca(ICCV2/PI489777)SNP_16147	312	T	A	5	7	--	--
CakTC30020	Ca(ICCV2/PI489777)SNP_16148	1170	G	A	6	3	--	--
	Ca(ICCV2/PI489777)SNP_16149	1238	C	T	7	3	--	--
	Ca(ICCV2/PI489777)SNP_16150	1442	G	C	7	3	--	--
	Ca(ICCV2/PI489777)SNP_16151	2063	T	C	7	3	--	--
	Ca(ICCV2/PI489777)SNP_16152	2254	C	T	4	3	--	--
	Ca(ICCV2/PI489777)SNP_16153	3023	T	A	12	4	--	--
	Ca(ICCV2/PI489777)SNP_16154	3150	A	G	8	6	--	--
	Ca(ICCV2/PI489777)SNP_16155	3216	T	C	8	4	--	--
CakTC30096	Ca(ICCV2/PI489777)SNP_16156	172	C	A	3	3	Root	--
	Ca(ICCV2/PI489777)SNP_16157	176	A	T	3	3	Root	--
	Ca(ICCV2/PI489777)SNP_16158	265	G	C	3	4	Root	--
CakTC37300	Ca(ICCV2/PI489777)SNP_16159	558	G	A	8	5	Flower	bud
CakTC39076	Ca(ICCV2/PI489777)SNP_16160	385	G	A	22	10	--	--
	Ca(ICCV2/PI489777)SNP_16161	725	G	A	10	6	--	--
CakTC23831	Ca(ICCV2/PI489777)SNP_16162	742	C	A	6	7	--	--
	Ca(ICCV2/PI489777)SNP_16163	858	C	A	10	7	--	--
	Ca(ICCV2/PI489777)SNP_16164	962	C	T	7	3	--	--
	Ca(ICCV2/PI489777)SNP_16165	1020	T	A	5	3	--	--
	Ca(ICCV2/PI489777)SNP_16166	1468	A	C	7	3	--	--
	Ca(ICCV2/PI489777)SNP_16167	3273	T	A	7	5	--	--
	Ca(ICCV2/PI489777)SNP_16168	3384	A	G	3	5	--	--
	Ca(ICCV2/PI489777)SNP_16169	4906	C	T	3	5	--	--
CakTC39843	Ca(ICCV2/PI489777)SNP_16170	581	C	T	3	3	--	--
	Ca(ICCV2/PI489777)SNP_16171	603	C	T	3	3	--	--
CakTC30064	Ca(ICCV2/PI489777)SNP_16172	1339	G	A	4	4	--	--
	Ca(ICCV2/PI489777)SNP_16173	1434	C	T	6	4	--	--
	Ca(ICCV2/PI489777)SNP_16174	1517	G	A	7	3	--	--
CakTC43361	Ca(ICCV2/PI489777)SNP_16175	368	G	T	21	34	--	--
	Ca(ICCV2/PI489777)SNP_16176	378	G	A	28	37	--	--
	Ca(ICCV2/PI489777)SNP_16177	1203	A	T	26	13	--	--
	Ca(ICCV2/PI489777)SNP_16178	1656	A	G	26	13	--	--
CakTC26015	Ca(ICCV2/PI489777)SNP_16179	1456	T	C	29	22	--	IWS1
	Ca(ICCV2/PI489777)SNP_16180	1776	C	A	27	26	--	IWS1
	Ca(ICCV2/PI489777)SNP_16181	3079	T	C	28	17	--	IWS1
	Ca(ICCV2/PI489777)SNP_16182	3864	C	T	49	27	--	IWS1
	Ca(ICCV2/PI489777)SNP_16183	4867	C	T	20	20	--	IWS1
CakTC37777	Ca(ICCV2/PI489777)SNP_16184	401	T	C	13	9	--	bHLH
	Ca(ICCV2/PI489777)SNP_16185	451	T	C	15	8	--	bHLH
	Ca(ICCV2/PI489777)SNP_16186	607	T	C	15	9	--	bHLH
	Ca(ICCV2/PI489777)SNP_16187	1376	G	A	17	5	--	bHLH
	Ca(ICCV2/PI489777)SNP_16188	1494	T	C	12	5	--	bHLH
CakTC42238	Ca(ICCV2/PI489777)SNP_16189	121	C	T	4	4	--	--
	Ca(ICCV2/PI489777)SNP_16190	1113	A	G	7	4	--	--
CakTC01695	Ca(ICCV2/PI489777)SNP_16191	647	G	A	46	28	--	--
CakTC28401	Ca(ICCV2/PI489777)SNP_16192	966	T	G	177	74	--	--
CakTC41401	Ca(ICCV2/PI489777)SNP_16193	79	T	C	6	10	--	--
	Ca(ICCV2/PI489777)SNP_16194	535	G	C	21	16	--	--
	Ca(ICCV2/PI489777)SNP_16195	573	T	G	17	13	--	--
CakTC39454	Ca(ICCV2/PI489777)SNP_16196	1358	T	C	3	4	--	--
CakTC39767	Ca(ICCV2/PI489777)SNP_16197	268	G	A	25	16	--	--
	Ca(ICCV2/PI489777)SNP_16198	881	T	C	13	22	--	--
CakTC27251	Ca(ICCV2/PI489777)SNP_16199	273	C	T	9	7	--	--
	Ca(ICCV2/PI489777)SNP_16200	312	C	T	7	7	--	--
	Ca(ICCV2/PI489777)SNP_16201	646	A	G	10	6	--	--

	Ca(ICCV2/PI489777)SNP_16202	648	G	A	10	6	--	--
	Ca(ICCV2/PI489777)SNP_16203	675	T	C	9	7	--	--
	Ca(ICCV2/PI489777)SNP_16204	753	G	A	7	6	--	--
	Ca(ICCV2/PI489777)SNP_16205	783	T	A	10	11	--	--
	Ca(ICCV2/PI489777)SNP_16206	1466	G	A	21	4	--	--
	Ca(ICCV2/PI489777)SNP_16207	1517	T	C	18	3	--	--
	Ca(ICCV2/PI489777)SNP_16208	1656	C	T	14	8	--	--
	Ca(ICCV2/PI489777)SNP_16209	2504	T	C	10	3	--	--
	Ca(ICCV2/PI489777)SNP_16210	2962	G	A	6	3	--	--
	Ca(ICCV2/PI489777)SNP_16211	3016	A	T	14	4	--	--
	Ca(ICCV2/PI489777)SNP_16212	3251	G	A	6	9	--	--
	Ca(ICCV2/PI489777)SNP_16213	3986	C	A	13	11	--	--
CakTC34256	Ca(ICCV2/PI489777)SNP_16214	759	C	A	10	7	--	--
	Ca(ICCV2/PI489777)SNP_16215	1466	A	G	9	9	--	--
	Ca(ICCV2/PI489777)SNP_16216	2608	C	A	7	5	--	--
	Ca(ICCV2/PI489777)SNP_16217	3571	A	C	12	16	--	--
	Ca(ICCV2/PI489777)SNP_16218	4133	C	G	9	6	--	--
	Ca(ICCV2/PI489777)SNP_16219	4343	T	C	21	6	--	--
	Ca(ICCV2/PI489777)SNP_16220	5080	C	A	21	7	--	--
CakTC42541	Ca(ICCV2/PI489777)SNP_16221	1389	T	C	10	7	--	--
	Ca(ICCV2/PI489777)SNP_16222	1708	G	A	11	4	--	--
CakTC37124	Ca(ICCV2/PI489777)SNP_16223	293	G	T	9	16	--	--
	Ca(ICCV2/PI489777)SNP_16224	356	G	T	13	15	--	--
	Ca(ICCV2/PI489777)SNP_16225	582	T	C	24	17	--	--
CakTC24655	Ca(ICCV2/PI489777)SNP_16226	465	G	A	16	22	--	--
	Ca(ICCV2/PI489777)SNP_16227	534	C	T	18	22	--	--
	Ca(ICCV2/PI489777)SNP_16228	793	C	G	30	16	--	--
	Ca(ICCV2/PI489777)SNP_16229	1007	A	C	21	12	--	--
	Ca(ICCV2/PI489777)SNP_16230	1648	G	A	13	11	--	--
CakTC18451	Ca(ICCV2/PI489777)SNP_16231	587	T	C	30	25	--	--
	Ca(ICCV2/PI489777)SNP_16232	2013	C	G	7	16	--	--
	Ca(ICCV2/PI489777)SNP_16233	2037	A	C	3	12	--	--
CakTC40989	Ca(ICCV2/PI489777)SNP_16234	400	T	G	5	3	--	--
	Ca(ICCV2/PI489777)SNP_16235	535	T	C	7	4	--	--
	Ca(ICCV2/PI489777)SNP_16236	548	G	A	7	5	--	--
	Ca(ICCV2/PI489777)SNP_16237	561	A	G	7	5	--	--
	Ca(ICCV2/PI489777)SNP_16238	640	G	A	11	6	--	--
CakTC40715	Ca(ICCV2/PI489777)SNP_16239	56	T	A	30	47	--	--
	Ca(ICCV2/PI489777)SNP_16240	279	G	A	70	103	--	--
	Ca(ICCV2/PI489777)SNP_16241	402	A	T	77	89	--	--
	Ca(ICCV2/PI489777)SNP_16242	480	A	G	62	75	--	--
CakTC26053	Ca(ICCV2/PI489777)SNP_16243	2047	G	A	5	16	--	--
	Ca(ICCV2/PI489777)SNP_16244	2304	G	C	4	18	--	--
	Ca(ICCV2/PI489777)SNP_16245	4836	G	A	6	4	--	--
CakTC31058	Ca(ICCV2/PI489777)SNP_16246	430	T	C	18	11	--	--
	Ca(ICCV2/PI489777)SNP_16247	433	C	T	24	11	--	--
	Ca(ICCV2/PI489777)SNP_16248	655	T	C	22	12	--	--
CakTC30708	Ca(ICCV2/PI489777)SNP_16249	814	T	C	18	5	--	--
	Ca(ICCV2/PI489777)SNP_16250	1512	T	C	5	4	--	--
	Ca(ICCV2/PI489777)SNP_16251	1720	G	A	3	3	--	--
	Ca(ICCV2/PI489777)SNP_16252	1723	C	G	3	3	--	--
	Ca(ICCV2/PI489777)SNP_16253	1746	C	A	3	3	--	--
CakTC28703	Ca(ICCV2/PI489777)SNP_16254	1014	A	T	5	7	Shoot	--
	Ca(ICCV2/PI489777)SNP_16255	1026	G	A	6	6	Shoot	--
CakTC38066	Ca(ICCV2/PI489777)SNP_16256	1419	C	A	6	5	--	--
CakTC24178	Ca(ICCV2/PI489777)SNP_16257	1139	A	G	7	5	Young_pod	--
CakTC10494	Ca(ICCV2/PI489777)SNP_16258	579	A	G	36	29	--	--
CakTC30874	Ca(ICCV2/PI489777)SNP_16259	241	C	G	51	18	--	--
	Ca(ICCV2/PI489777)SNP_16260	351	T	C	55	20	--	--
	Ca(ICCV2/PI489777)SNP_16261	358	C	G	63	22	--	--
	Ca(ICCV2/PI489777)SNP_16262	972	C	T	50	20	--	--
	Ca(ICCV2/PI489777)SNP_16263	1014	G	A	41	22	--	--
CakTC37172	Ca(ICCV2/PI489777)SNP_16264	92	T	C	27	14	--	--
CakTC38313	Ca(ICCV2/PI489777)SNP_16265	188	G	T	21	5	--	--
	Ca(ICCV2/PI489777)SNP_16266	266	A	G	21	5	--	--
	Ca(ICCV2/PI489777)SNP_16267	326	C	T	23	6	--	--
	Ca(ICCV2/PI489777)SNP_16268	327	C	T	23	6	--	--
	Ca(ICCV2/PI489777)SNP_16269	392	G	A	24	6	--	--
	Ca(ICCV2/PI489777)SNP_16270	452	A	G	22	3	--	--
	Ca(ICCV2/PI489777)SNP_16271	464	C	A	23	4	--	--
	Ca(ICCV2/PI489777)SNP_16272	487	C	T	23	4	--	--
	Ca(ICCV2/PI489777)SNP_16273	494	G	T	22	3	--	--
CakTC09663	Ca(ICCV2/PI489777)SNP_16274	1630	A	T	3	9	--	--
CakTC36612	Ca(ICCV2/PI489777)SNP_16275	590	T	G	3	3	--	--

	Ca(ICCV2/PI489777)SNP_16276	591	C	A	3	3	--	--
CakTC37660	Ca(ICCV2/PI489777)SNP_16277	628	G	A	40	16	--	--
	Ca(ICCV2/PI489777)SNP_16278	1354	G	A	49	16	--	--
	Ca(ICCV2/PI489777)SNP_16279	1446	A	G	25	14	--	--
CakTC41535	Ca(ICCV2/PI489777)SNP_16280	1162	G	A	5	3	--	bHLH
	Ca(ICCV2/PI489777)SNP_16281	1935	T	C	12	3	--	bHLH
CakTC39703	Ca(ICCV2/PI489777)SNP_16282	307	T	C	5	8	--	--
CakTC32010	Ca(ICCV2/PI489777)SNP_16283	1019	A	T	27	7	--	--
	Ca(ICCV2/PI489777)SNP_16284	1082	T	C	35	8	--	--
	Ca(ICCV2/PI489777)SNP_16285	1694	G	A	22	5	--	--
	Ca(ICCV2/PI489777)SNP_16286	2360	C	T	22	18	--	--
	Ca(ICCV2/PI489777)SNP_16287	2405	T	C	22	17	--	--
	Ca(ICCV2/PI489777)SNP_16288	2483	T	A	30	23	--	--
	Ca(ICCV2/PI489777)SNP_16289	3059	T	A	15	8	--	--
	Ca(ICCV2/PI489777)SNP_16290	3266	T	A	10	9	--	--
CakTC41291	Ca(ICCV2/PI489777)SNP_16291	496	G	A	17	23	--	--
CakTC36034	Ca(ICCV2/PI489777)SNP_16292	337	G	C	18	13	--	--
	Ca(ICCV2/PI489777)SNP_16293	1833	A	G	14	18	--	--
	Ca(ICCV2/PI489777)SNP_16294	1848	T	C	13	17	--	--
	Ca(ICCV2/PI489777)SNP_16295	1890	A	T	11	16	--	--
	Ca(ICCV2/PI489777)SNP_16296	1938	C	T	13	24	--	--
	Ca(ICCV2/PI489777)SNP_16297	1971	A	G	15	16	--	--
	Ca(ICCV2/PI489777)SNP_16298	1977	T	C	14	17	--	--
	Ca(ICCV2/PI489777)SNP_16299	2139	G	C	13	20	--	--
	Ca(ICCV2/PI489777)SNP_16300	2199	G	A	15	21	--	--
	Ca(ICCV2/PI489777)SNP_16301	3112	C	T	12	14	--	--
CakTC39328	Ca(ICCV2/PI489777)SNP_16302	827	C	T	49	17	--	--
CakTC42749	Ca(ICCV2/PI489777)SNP_16303	324	C	T	3	4	--	--
	Ca(ICCV2/PI489777)SNP_16304	375	A	T	4	4	--	--
	Ca(ICCV2/PI489777)SNP_16305	388	A	C	6	4	--	--
	Ca(ICCV2/PI489777)SNP_16306	553	G	T	8	3	--	--
	Ca(ICCV2/PI489777)SNP_16307	598	G	T	8	3	--	--
	Ca(ICCV2/PI489777)SNP_16308	828	T	G	4	7	--	--
	Ca(ICCV2/PI489777)SNP_16309	1218	G	T	7	3	--	--
	Ca(ICCV2/PI489777)SNP_16310	1242	G	T	7	5	--	--
	Ca(ICCV2/PI489777)SNP_16311	1245	A	G	7	5	--	--
CakTC24702	Ca(ICCV2/PI489777)SNP_16312	239	A	C	7	4	--	--
CakTC39446	Ca(ICCV2/PI489777)SNP_16313	426	C	T	11	4	--	--
	Ca(ICCV2/PI489777)SNP_16314	430	C	G	11	4	--	--
CakTC31244	Ca(ICCV2/PI489777)SNP_16315	564	C	T	4	3	--	--
	Ca(ICCV2/PI489777)SNP_16316	591	G	A	5	3	--	--
	Ca(ICCV2/PI489777)SNP_16317	594	T	G	5	3	--	--
	Ca(ICCV2/PI489777)SNP_16318	603	G	A	8	4	--	--
	Ca(ICCV2/PI489777)SNP_16319	836	T	C	11	5	--	--
CakTC27154	Ca(ICCV2/PI489777)SNP_16320	122	C	G	23	13	--	--
	Ca(ICCV2/PI489777)SNP_16321	548	T	C	32	37	--	--
	Ca(ICCV2/PI489777)SNP_16322	795	C	T	26	23	--	--
	Ca(ICCV2/PI489777)SNP_16323	1069	G	T	18	20	--	--
	Ca(ICCV2/PI489777)SNP_16324	1311	T	C	10	10	--	--
CakTC31852	Ca(ICCV2/PI489777)SNP_16325	1105	A	G	3	3	--	--
CakTC02614	Ca(ICCV2/PI489777)SNP_16326	462	T	A	5	9	--	--
	Ca(ICCV2/PI489777)SNP_16327	2061	C	T	3	5	--	--
CakTC34017	Ca(ICCV2/PI489777)SNP_16328	645	A	T	7	4	--	--
	Ca(ICCV2/PI489777)SNP_16329	868	A	C	8	9	--	--
	Ca(ICCV2/PI489777)SNP_16330	1050	T	C	21	13	--	--
	Ca(ICCV2/PI489777)SNP_16331	1259	G	C	21	11	--	--
	Ca(ICCV2/PI489777)SNP_16332	1277	T	C	23	11	--	--
	Ca(ICCV2/PI489777)SNP_16333	1332	C	G	18	7	--	--
CakTC24426	Ca(ICCV2/PI489777)SNP_16334	989	C	T	18	24	--	--
CakTC28512	Ca(ICCV2/PI489777)SNP_16335	416	T	C	43	17	--	--
	Ca(ICCV2/PI489777)SNP_16336	816	A	G	41	24	--	--
	Ca(ICCV2/PI489777)SNP_16337	845	C	G	39	19	--	--
	Ca(ICCV2/PI489777)SNP_16338	851	T	G	23	16	--	--
CakTC39123	Ca(ICCV2/PI489777)SNP_16339	259	C	T	15	5	--	--
	Ca(ICCV2/PI489777)SNP_16340	448	C	G	30	6	--	--
	Ca(ICCV2/PI489777)SNP_16341	661	G	C	21	3	--	--
	Ca(ICCV2/PI489777)SNP_16342	1078	C	T	12	9	--	--
	Ca(ICCV2/PI489777)SNP_16343	1084	T	C	8	11	--	--
	Ca(ICCV2/PI489777)SNP_16344	1099	C	T	11	13	--	--
	Ca(ICCV2/PI489777)SNP_16345	1234	T	A	11	11	--	--
CakTC27236	Ca(ICCV2/PI489777)SNP_16346	344	A	G	31	11	--	--
	Ca(ICCV2/PI489777)SNP_16347	1259	A	G	30	19	--	--
CakTC31696	Ca(ICCV2/PI489777)SNP_16348	151	A	G	6	3	--	--
	Ca(ICCV2/PI489777)SNP_16349	191	A	G	31	18	--	--

	Ca(ICCV2/PI489777)SNP_16350	304	T	C	44	20	--	--
	Ca(ICCV2/PI489777)SNP_16351	357	C	G	50	23	--	--
	Ca(ICCV2/PI489777)SNP_16352	397	C	T	46	24	--	--
	Ca(ICCV2/PI489777)SNP_16353	456	A	G	43	28	--	--
	Ca(ICCV2/PI489777)SNP_16354	457	A	G	43	28	--	--
	Ca(ICCV2/PI489777)SNP_16355	797	T	C	46	18	--	--
	Ca(ICCV2/PI489777)SNP_16356	817	C	T	45	16	--	--
	Ca(ICCV2/PI489777)SNP_16357	916	G	C	49	11	--	--
	Ca(ICCV2/PI489777)SNP_16358	1045	C	G	30	9	--	--
	Ca(ICCV2/PI489777)SNP_16359	1275	A	G	12	4	--	--
CakTC37232	Ca(ICCV2/PI489777)SNP_16360	160	C	T	3	3	--	--
CakTC13107	Ca(ICCV2/PI489777)SNP_16361	352	T	G	10	5	--	--
	Ca(ICCV2/PI489777)SNP_16362	511	G	A	11	5	--	--
CakTC11096	Ca(ICCV2/PI489777)SNP_16363	419	G	A	5	4	--	--
	Ca(ICCV2/PI489777)SNP_16364	1113	T	C	5	4	--	--
CakTC31768	Ca(ICCV2/PI489777)SNP_16365	1409	C	T	7	5	--	--
	Ca(ICCV2/PI489777)SNP_16366	1699	G	A	13	5	--	--
	Ca(ICCV2/PI489777)SNP_16367	1715	A	G	11	6	--	--
	Ca(ICCV2/PI489777)SNP_16368	1820	A	G	9	5	--	--
CakTC28078	Ca(ICCV2/PI489777)SNP_16369	251	C	T	3	3	--	--
	Ca(ICCV2/PI489777)SNP_16370	337	C	T	5	4	--	--
	Ca(ICCV2/PI489777)SNP_16371	928	C	T	14	4	--	--
CakTC43081	Ca(ICCV2/PI489777)SNP_16372	459	G	A	132	156	--	--
	Ca(ICCV2/PI489777)SNP_16373	471	G	C	123	126	--	--
	Ca(ICCV2/PI489777)SNP_16374	501	C	T	142	178	--	--
	Ca(ICCV2/PI489777)SNP_16375	740	T	C	117	65	--	--
	Ca(ICCV2/PI489777)SNP_16376	810	C	G	85	17	--	--
CakTC30460	Ca(ICCV2/PI489777)SNP_16377	662	A	G	253	181	--	--
	Ca(ICCV2/PI489777)SNP_16378	1535	T	G	212	189	--	--
	Ca(ICCV2/PI489777)SNP_16379	1838	C	T	214	165	--	--
	Ca(ICCV2/PI489777)SNP_16380	1910	A	G	176	154	--	--
	Ca(ICCV2/PI489777)SNP_16381	2418	T	A	87	65	--	--
CakTC43066	Ca(ICCV2/PI489777)SNP_16382	608	C	T	6	5	--	--
	Ca(ICCV2/PI489777)SNP_16383	829	T	C	8	5	--	--
CakTC30236	Ca(ICCV2/PI489777)SNP_16384	56	C	G	3	4	--	--
CakTC22616	Ca(ICCV2/PI489777)SNP_16385	118	C	A	21	4	--	--
	Ca(ICCV2/PI489777)SNP_16386	372	A	G	20	3	--	--
	Ca(ICCV2/PI489777)SNP_16387	411	T	C	20	5	--	--
	Ca(ICCV2/PI489777)SNP_16388	882	T	C	86	46	--	--
	Ca(ICCV2/PI489777)SNP_16389	1575	C	T	51	25	--	--
CakTC27554	Ca(ICCV2/PI489777)SNP_16390	366	G	T	3	5	--	--
	Ca(ICCV2/PI489777)SNP_16391	588	C	G	3	5	--	--
CakTC41075	Ca(ICCV2/PI489777)SNP_16392	1192	T	C	8	10	--	--
	Ca(ICCV2/PI489777)SNP_16393	1810	T	C	12	9	--	--
	Ca(ICCV2/PI489777)SNP_16394	1886	C	T	12	8	--	--
	Ca(ICCV2/PI489777)SNP_16395	1963	T	G	14	7	--	--
	Ca(ICCV2/PI489777)SNP_16396	2318	C	T	14	9	--	--
	Ca(ICCV2/PI489777)SNP_16397	2336	G	A	9	8	--	--
	Ca(ICCV2/PI489777)SNP_16398	2392	C	G	10	6	--	--
CakTC25699	Ca(ICCV2/PI489777)SNP_16399	343	G	A	9	9	--	Pseudo_ARR-B
	Ca(ICCV2/PI489777)SNP_16400	798	A	T	8	8	--	Pseudo_ARR-B
	Ca(ICCV2/PI489777)SNP_16401	801	C	T	8	8	--	Pseudo_ARR-B
	Ca(ICCV2/PI489777)SNP_16402	2277	T	A	40	13	--	Pseudo_ARR-B
CakTC39334	Ca(ICCV2/PI489777)SNP_16403	557	A	T	3	4	--	--
	Ca(ICCV2/PI489777)SNP_16404	560	T	C	3	4	--	--
	Ca(ICCV2/PI489777)SNP_16405	771	A	C	4	6	--	--
	Ca(ICCV2/PI489777)SNP_16406	827	T	C	3	7	--	--
	Ca(ICCV2/PI489777)SNP_16407	860	C	G	3	6	--	--
	Ca(ICCV2/PI489777)SNP_16408	872	T	G	3	6	--	--
	Ca(ICCV2/PI489777)SNP_16409	956	T	A	5	8	--	--
CakTC41215	Ca(ICCV2/PI489777)SNP_16410	1732	G	A	12	7	--	--
	Ca(ICCV2/PI489777)SNP_16411	1810	A	C	9	5	--	--
CakTC28552	Ca(ICCV2/PI489777)SNP_16412	210	G	C	24	9	--	--
	Ca(ICCV2/PI489777)SNP_16413	347	G	A	20	6	--	--
	Ca(ICCV2/PI489777)SNP_16414	434	A	G	16	8	--	--
	Ca(ICCV2/PI489777)SNP_16415	540	G	C	22	7	--	--
	Ca(ICCV2/PI489777)SNP_16416	761	C	T	26	4	--	--
	Ca(ICCV2/PI489777)SNP_16417	811	G	C	20	3	--	--
CakTC34105	Ca(ICCV2/PI489777)SNP_16418	375	A	G	31	21	--	--
	Ca(ICCV2/PI489777)SNP_16419	476	G	A	33	26	--	--
	Ca(ICCV2/PI489777)SNP_16420	1928	G	A	24	26	--	--
CakTC43269	Ca(ICCV2/PI489777)SNP_16421	530	T	G	4	3	--	PLATZ
	Ca(ICCV2/PI489777)SNP_16422	605	A	G	3	4	--	PLATZ
CakTC40412	Ca(ICCV2/PI489777)SNP_16423	96	T	C	4	3	--	--

	Ca(ICCV2/PI489777)SNP_16424	102	T	C	4	3	--	--
	Ca(ICCV2/PI489777)SNP_16425	119	A	T	4	3	--	--
	Ca(ICCV2/PI489777)SNP_16426	140	C	T	6	3	--	--
	Ca(ICCV2/PI489777)SNP_16427	195	C	T	3	3	--	--
	Ca(ICCV2/PI489777)SNP_16428	211	G	A	8	3	--	--
CakTC27267	Ca(ICCV2/PI489777)SNP_16429	600	A	G	248	58	--	--
	Ca(ICCV2/PI489777)SNP_16430	628	G	A	253	55	--	--
	Ca(ICCV2/PI489777)SNP_16431	1095	T	C	176	63	--	--
	Ca(ICCV2/PI489777)SNP_16432	1271	G	A	102	21	--	--
CakTC22946	Ca(ICCV2/PI489777)SNP_16433	349	T	C	14	19	--	--
	Ca(ICCV2/PI489777)SNP_16434	733	G	A	23	19	--	--
CakTC38082	Ca(ICCV2/PI489777)SNP_16435	943	C	T	4	3	--	--
	Ca(ICCV2/PI489777)SNP_16436	1045	A	G	5	3	--	--
	Ca(ICCV2/PI489777)SNP_16437	1064	T	C	5	5	--	--
	Ca(ICCV2/PI489777)SNP_16438	1110	G	A	3	6	--	--
	Ca(ICCV2/PI489777)SNP_16439	1118	A	T	4	6	--	--
CakTC32958	Ca(ICCV2/PI489777)SNP_16440	132	G	A	4	8	--	--
	Ca(ICCV2/PI489777)SNP_16441	279	A	T	9	10	--	--
	Ca(ICCV2/PI489777)SNP_16442	370	A	C	9	10	--	--
	Ca(ICCV2/PI489777)SNP_16443	874	T	C	12	18	--	--
	Ca(ICCV2/PI489777)SNP_16444	1484	A	T	15	12	--	--
	Ca(ICCV2/PI489777)SNP_16445	1612	A	G	15	10	--	--
CakTC18118	Ca(ICCV2/PI489777)SNP_16446	282	A	G	3	3	--	--
CakTC38919	Ca(ICCV2/PI489777)SNP_16447	618	G	A	23	18	--	--
	Ca(ICCV2/PI489777)SNP_16448	1134	C	T	44	20	--	--
	Ca(ICCV2/PI489777)SNP_16449	1283	T	G	28	21	--	--
	Ca(ICCV2/PI489777)SNP_16450	1402	A	G	8	3	--	--
CakTC41059	Ca(ICCV2/PI489777)SNP_16451	195	G	A	40	64	--	--
	Ca(ICCV2/PI489777)SNP_16452	306	T	C	53	68	--	--
CakTC40771	Ca(ICCV2/PI489777)SNP_16453	642	C	T	6	4	--	--
CakTC41170	Ca(ICCV2/PI489777)SNP_16454	224	A	G	29	9	--	--
	Ca(ICCV2/PI489777)SNP_16455	330	T	C	30	10	--	--
	Ca(ICCV2/PI489777)SNP_16456	356	T	A	35	8	--	--
	Ca(ICCV2/PI489777)SNP_16457	360	C	A	38	7	--	--
	Ca(ICCV2/PI489777)SNP_16458	391	C	A	39	13	--	--
	Ca(ICCV2/PI489777)SNP_16459	1036	G	A	14	8	--	--
	Ca(ICCV2/PI489777)SNP_16460	1234	G	C	34	12	--	--
	Ca(ICCV2/PI489777)SNP_16461	1343	C	A	60	19	--	--
	Ca(ICCV2/PI489777)SNP_16462	1875	G	A	17	12	--	--
	Ca(ICCV2/PI489777)SNP_16463	1913	T	G	6	6	--	--
	Ca(ICCV2/PI489777)SNP_16464	1946	G	T	7	4	--	--
	Ca(ICCV2/PI489777)SNP_16465	2133	C	G	3	3	--	--
CakTC26039	Ca(ICCV2/PI489777)SNP_16466	472	G	A	4	3	--	--
	Ca(ICCV2/PI489777)SNP_16467	1405	A	G	8	3	--	--
CakTC12630	Ca(ICCV2/PI489777)SNP_16468	405	T	C	22	7	--	--
CakTC32860	Ca(ICCV2/PI489777)SNP_16469	262	C	T	4	8	--	--
	Ca(ICCV2/PI489777)SNP_16470	391	T	C	4	8	--	--
CakTC33880	Ca(ICCV2/PI489777)SNP_16471	928	A	G	7	5	--	--
CakTC42164	Ca(ICCV2/PI489777)SNP_16472	783	C	A	3	10	--	--
	Ca(ICCV2/PI489777)SNP_16473	972	C	T	9	11	--	--
	Ca(ICCV2/PI489777)SNP_16474	1647	T	A	3	7	--	--
CakTC43274	Ca(ICCV2/PI489777)SNP_16475	188	C	G	26	39	--	--
	Ca(ICCV2/PI489777)SNP_16476	197	C	G	23	39	--	--
	Ca(ICCV2/PI489777)SNP_16477	395	A	C	23	58	--	--
	Ca(ICCV2/PI489777)SNP_16478	611	T	A	14	31	--	--
	Ca(ICCV2/PI489777)SNP_16479	683	T	G	16	14	--	--
	Ca(ICCV2/PI489777)SNP_16480	1188	G	A	10	22	--	--
	Ca(ICCV2/PI489777)SNP_16481	1565	A	G	5	14	--	--
CakTC38201	Ca(ICCV2/PI489777)SNP_16482	211	G	T	3	17	--	--
	Ca(ICCV2/PI489777)SNP_16483	414	T	C	10	18	--	--
	Ca(ICCV2/PI489777)SNP_16484	423	C	T	10	18	--	--
	Ca(ICCV2/PI489777)SNP_16485	438	C	T	11	17	--	--
	Ca(ICCV2/PI489777)SNP_16486	594	A	C	17	6	--	--
	Ca(ICCV2/PI489777)SNP_16487	602	G	C	17	5	--	--
	Ca(ICCV2/PI489777)SNP_16488	744	C	T	10	5	--	--
	Ca(ICCV2/PI489777)SNP_16489	753	C	T	9	5	--	--
	Ca(ICCV2/PI489777)SNP_16490	771	T	C	7	5	--	--
	Ca(ICCV2/PI489777)SNP_16491	819	T	C	8	5	--	--
	Ca(ICCV2/PI489777)SNP_16492	1101	A	G	7	4	--	--
	Ca(ICCV2/PI489777)SNP_16493	1332	A	T	8	3	--	--
CakTC38897	Ca(ICCV2/PI489777)SNP_16494	1058	T	G	37	6	--	--
CakTC24996	Ca(ICCV2/PI489777)SNP_16495	530	A	C	7	9	--	--
	Ca(ICCV2/PI489777)SNP_16496	1037	A	G	7	6	--	--
CakTC40728	Ca(ICCV2/PI489777)SNP_16497	984	C	T	21	9	--	--

	Ca(ICCV2/PI489777)SNP_16498	1164	G	C	14	6	--	--
	Ca(ICCV2/PI489777)SNP_16499	1357	T	C	15	7	--	--
	Ca(ICCV2/PI489777)SNP_16500	1449	G	A	13	4	--	--
CakTC06186	Ca(ICCV2/PI489777)SNP_16501	1101	C	T	87	26	--	--
	Ca(ICCV2/PI489777)SNP_16502	1173	G	A	79	24	--	--
CakTC23349	Ca(ICCV2/PI489777)SNP_16503	503	A	G	4	4	Root	--
	Ca(ICCV2/PI489777)SNP_16504	729	T	C	5	4	Root	--
	Ca(ICCV2/PI489777)SNP_16505	968	G	C	3	3	Root	--
	Ca(ICCV2/PI489777)SNP_16506	1303	C	T	5	4	Root	--
CakTC42388	Ca(ICCV2/PI489777)SNP_16507	400	T	C	44	23	--	--
	Ca(ICCV2/PI489777)SNP_16508	552	G	A	18	21	--	--
	Ca(ICCV2/PI489777)SNP_16509	645	C	T	5	12	--	--
CakTC27293	Ca(ICCV2/PI489777)SNP_16510	278	G	T	9	4	--	--
	Ca(ICCV2/PI489777)SNP_16511	590	T	C	10	9	--	--
	Ca(ICCV2/PI489777)SNP_16512	1055	G	C	13	7	--	--
CakTC23999	Ca(ICCV2/PI489777)SNP_16513	77	C	T	8	8	--	--
	Ca(ICCV2/PI489777)SNP_16514	690	G	A	12	4	--	--
	Ca(ICCV2/PI489777)SNP_16515	1347	T	C	13	11	--	--
	Ca(ICCV2/PI489777)SNP_16516	1435	C	G	5	3	--	--
CakTC35586	Ca(ICCV2/PI489777)SNP_16517	51	C	G	3	4	--	--
	Ca(ICCV2/PI489777)SNP_16518	449	T	C	4	5	--	--
	Ca(ICCV2/PI489777)SNP_16519	2833	G	A	12	6	--	--
	Ca(ICCV2/PI489777)SNP_16520	3098	G	C	5	4	--	--
CakTC41279	Ca(ICCV2/PI489777)SNP_16521	545	C	T	8	6	--	--
	Ca(ICCV2/PI489777)SNP_16522	818	T	C	8	3	--	--
	Ca(ICCV2/PI489777)SNP_16523	911	C	T	9	3	--	--
	Ca(ICCV2/PI489777)SNP_16524	1662	C	T	10	10	--	--
	Ca(ICCV2/PI489777)SNP_16525	2094	A	G	11	5	--	--
CakTC27386	Ca(ICCV2/PI489777)SNP_16526	127	C	G	7	3	--	--
	Ca(ICCV2/PI489777)SNP_16527	646	C	T	16	15	--	--
	Ca(ICCV2/PI489777)SNP_16528	1017	A	C	11	11	--	--
CakTC39185	Ca(ICCV2/PI489777)SNP_16529	191	A	G	7	4	--	--
	Ca(ICCV2/PI489777)SNP_16530	219	A	G	8	4	--	--
	Ca(ICCV2/PI489777)SNP_16531	567	T	A	16	8	--	--
	Ca(ICCV2/PI489777)SNP_16532	1272	C	T	17	3	--	--
	Ca(ICCV2/PI489777)SNP_16533	1464	G	T	17	3	--	--
CakTC10160	Ca(ICCV2/PI489777)SNP_16534	574	A	T	77	56	--	--
	Ca(ICCV2/PI489777)SNP_16535	698	T	A	41	23	--	--
	Ca(ICCV2/PI489777)SNP_16536	702	T	C	33	23	--	--
	Ca(ICCV2/PI489777)SNP_16537	805	C	A	21	14	--	--
	Ca(ICCV2/PI489777)SNP_16538	867	C	T	7	6	--	--
CakTC10962	Ca(ICCV2/PI489777)SNP_16539	276	A	G	3	3	--	--
	Ca(ICCV2/PI489777)SNP_16540	283	C	T	4	3	--	--
	Ca(ICCV2/PI489777)SNP_16541	488	G	A	4	3	--	--
CakTC29958	Ca(ICCV2/PI489777)SNP_16542	2178	G	T	5	3	--	SNF2
CakTC42947	Ca(ICCV2/PI489777)SNP_16543	263	T	A	13	8	--	--
	Ca(ICCV2/PI489777)SNP_16544	282	C	T	18	10	--	--
	Ca(ICCV2/PI489777)SNP_16545	725	A	G	15	22	--	--
	Ca(ICCV2/PI489777)SNP_16546	793	C	A	4	7	--	--
CakTC37632	Ca(ICCV2/PI489777)SNP_16547	114	C	T	35	64	--	--
	Ca(ICCV2/PI489777)SNP_16548	493	C	G	49	84	--	--
	Ca(ICCV2/PI489777)SNP_16549	503	G	A	46	70	--	--
	Ca(ICCV2/PI489777)SNP_16550	642	T	C	36	65	--	--
CakTC38499	Ca(ICCV2/PI489777)SNP_16551	513	C	T	36	18	--	--
	Ca(ICCV2/PI489777)SNP_16552	856	T	G	50	25	--	--
	Ca(ICCV2/PI489777)SNP_16553	925	T	G	46	19	--	--
CakTC32021	Ca(ICCV2/PI489777)SNP_16554	1434	G	A	10	11	--	--
CakTC23329	Ca(ICCV2/PI489777)SNP_16555	245	C	G	5	3	--	--
	Ca(ICCV2/PI489777)SNP_16556	250	T	G	5	3	--	--
	Ca(ICCV2/PI489777)SNP_16557	327	T	G	4	3	--	--
	Ca(ICCV2/PI489777)SNP_16558	666	C	A	14	3	--	--
	Ca(ICCV2/PI489777)SNP_16559	1030	G	C	119	35	--	--
	Ca(ICCV2/PI489777)SNP_16560	1126	A	T	171	63	--	--
	Ca(ICCV2/PI489777)SNP_16561	1234	G	A	170	65	--	--
	Ca(ICCV2/PI489777)SNP_16562	1410	T	C	112	45	--	--
	Ca(ICCV2/PI489777)SNP_16563	1498	T	G	83	26	--	--
CakTC28944	Ca(ICCV2/PI489777)SNP_16564	709	T	C	3	3	--	--
CakTC08407	Ca(ICCV2/PI489777)SNP_16565	390	C	A	3	3	--	--
CakTC43073	Ca(ICCV2/PI489777)SNP_16566	741	G	A	31	11	--	--
CakTC37637	Ca(ICCV2/PI489777)SNP_16567	193	C	T	12	11	--	--
	Ca(ICCV2/PI489777)SNP_16568	271	C	T	10	10	--	--
CakTC41727	Ca(ICCV2/PI489777)SNP_16569	3336	T	C	7	4	Flower	bud
CakTC04003	Ca(ICCV2/PI489777)SNP_16570	279	C	T	5	4	--	--
	Ca(ICCV2/PI489777)SNP_16571	596	A	G	5	3	--	--

CakTC25675	Ca(ICCV2/PI489777)SNP_16572	1356	T	A	14	7	--	--
CakTC38583	Ca(ICCV2/PI489777)SNP_16573	839	T	A	59	22	--	--
	Ca(ICCV2/PI489777)SNP_16574	926	C	G	51	16	--	--
CakTC38685	Ca(ICCV2/PI489777)SNP_16575	136	C	G	7	3	--	--
	Ca(ICCV2/PI489777)SNP_16576	310	C	T	9	3	--	--
CakTC25060	Ca(ICCV2/PI489777)SNP_16577	4324	T	A	3	7	--	--
	Ca(ICCV2/PI489777)SNP_16578	4924	C	G	5	6	--	--
CakTC09311	Ca(ICCV2/PI489777)SNP_16579	89	G	A	5	4	--	--
CakTC35173	Ca(ICCV2/PI489777)SNP_16580	84	G	T	10	6	--	--
	Ca(ICCV2/PI489777)SNP_16581	1149	T	C	57	52	--	--
	Ca(ICCV2/PI489777)SNP_16582	2376	A	G	39	28	--	--
	Ca(ICCV2/PI489777)SNP_16583	2562	T	A	36	27	--	--
	Ca(ICCV2/PI489777)SNP_16584	3229	G	A	38	31	--	--
CakTC05734	Ca(ICCV2/PI489777)SNP_16585	106	A	G	6	3	--	--
CakTC40880	Ca(ICCV2/PI489777)SNP_16586	1807	G	A	17	11	--	--
CakTC16978	Ca(ICCV2/PI489777)SNP_16587	51	C	T	3	3	--	--
	Ca(ICCV2/PI489777)SNP_16588	59	G	C	3	4	--	--
	Ca(ICCV2/PI489777)SNP_16589	301	A	C	3	5	--	--
CakTC32885	Ca(ICCV2/PI489777)SNP_16590	66	T	C	5	10	--	--
	Ca(ICCV2/PI489777)SNP_16591	68	G	A	5	10	--	--
CakTC36232	Ca(ICCV2/PI489777)SNP_16592	367	G	A	11	9	--	--
	Ca(ICCV2/PI489777)SNP_16593	540	C	T	14	4	--	--
	Ca(ICCV2/PI489777)SNP_16594	794	G	A	10	10	--	--
	Ca(ICCV2/PI489777)SNP_16595	1235	T	C	21	10	--	--
	Ca(ICCV2/PI489777)SNP_16596	1697	A	G	15	17	--	--
CakTC33358	Ca(ICCV2/PI489777)SNP_16597	247	T	A	23	8	--	--
	Ca(ICCV2/PI489777)SNP_16598	262	C	T	26	8	--	--
CakTC21124	Ca(ICCV2/PI489777)SNP_16599	240	G	T	4	3	--	--
	Ca(ICCV2/PI489777)SNP_16600	241	A	C	5	3	--	--
	Ca(ICCV2/PI489777)SNP_16601	1018	G	A	5	3	--	--
CakTC29467	Ca(ICCV2/PI489777)SNP_16602	73	G	C	3	3	Shoot	--
	Ca(ICCV2/PI489777)SNP_16603	596	T	A	3	3	Shoot	--
	Ca(ICCV2/PI489777)SNP_16604	926	C	A	5	3	Shoot	--
CakTC23123	Ca(ICCV2/PI489777)SNP_16605	2304	G	A	5	3	Flower	bud
CakTC11603	Ca(ICCV2/PI489777)SNP_16606	940	T	G	67	23	--	--
	Ca(ICCV2/PI489777)SNP_16607	976	C	G	62	20	--	--
CakTC43262	Ca(ICCV2/PI489777)SNP_16608	308	C	T	3	6	--	--
	Ca(ICCV2/PI489777)SNP_16609	782	G	A	5	4	--	--
	Ca(ICCV2/PI489777)SNP_16610	806	T	A	6	3	--	--
	Ca(ICCV2/PI489777)SNP_16611	1441	T	C	3	5	--	--
	Ca(ICCV2/PI489777)SNP_16612	1604	A	G	4	4	--	--
	Ca(ICCV2/PI489777)SNP_16613	1669	G	C	5	3	--	--
CakTC41715	Ca(ICCV2/PI489777)SNP_16614	133	A	G	5	5	--	--
	Ca(ICCV2/PI489777)SNP_16615	362	G	A	11	5	--	--
	Ca(ICCV2/PI489777)SNP_16616	1040	C	T	10	7	--	--
	Ca(ICCV2/PI489777)SNP_16617	1523	T	C	8	8	--	--
	Ca(ICCV2/PI489777)SNP_16618	1666	C	G	12	7	--	--
	Ca(ICCV2/PI489777)SNP_16619	2057	A	T	13	3	--	--
	Ca(ICCV2/PI489777)SNP_16620	2105	C	T	7	5	--	--
CakTC25204	Ca(ICCV2/PI489777)SNP_16621	2010	T	C	44	12	--	--
	Ca(ICCV2/PI489777)SNP_16622	2649	A	G	18	6	--	--
	Ca(ICCV2/PI489777)SNP_16623	2677	G	A	15	7	--	--
CakTC38139	Ca(ICCV2/PI489777)SNP_16624	351	A	G	19	18	--	--
	Ca(ICCV2/PI489777)SNP_16625	486	T	C	10	15	--	--
CakTC28487	Ca(ICCV2/PI489777)SNP_16626	1206	G	T	35	11	--	--
CakTC17916	Ca(ICCV2/PI489777)SNP_16627	590	A	G	4	3	--	--
CakTC40475	Ca(ICCV2/PI489777)SNP_16628	142	A	T	7	5	--	--
	Ca(ICCV2/PI489777)SNP_16629	304	C	T	7	6	--	--
	Ca(ICCV2/PI489777)SNP_16630	596	A	G	11	12	--	--
	Ca(ICCV2/PI489777)SNP_16631	2102	G	A	11	12	--	--
CakTC10738	Ca(ICCV2/PI489777)SNP_16632	1005	A	G	9	4	--	--
	Ca(ICCV2/PI489777)SNP_16633	1214	A	G	9	3	--	--
CakTC35010	Ca(ICCV2/PI489777)SNP_16634	145	G	A	15	9	--	--
	Ca(ICCV2/PI489777)SNP_16635	661	A	G	27	12	--	--
	Ca(ICCV2/PI489777)SNP_16636	932	A	G	20	9	--	--
	Ca(ICCV2/PI489777)SNP_16637	1606	G	A	21	9	--	--
	Ca(ICCV2/PI489777)SNP_16638	1816	C	T	22	9	--	--
CakTC37440	Ca(ICCV2/PI489777)SNP_16639	1308	C	T	6	4	--	PHD
CakTC42648	Ca(ICCV2/PI489777)SNP_16640	376	G	C	20	3	--	--
	Ca(ICCV2/PI489777)SNP_16641	612	G	A	12	3	--	--
	Ca(ICCV2/PI489777)SNP_16642	1030	A	G	20	16	--	--
	Ca(ICCV2/PI489777)SNP_16643	1083	C	T	22	16	--	--
CakTC41706	Ca(ICCV2/PI489777)SNP_16644	46	C	T	4	5	--	--
CakTC39980	Ca(ICCV2/PI489777)SNP_16645	331	T	A	12	19	--	--

	Ca(ICCV2/PI489777)SNP_16646	505	G	A	15	12	--	--
	Ca(ICCV2/PI489777)SNP_16647	1150	C	G	26	16	--	--
	Ca(ICCV2/PI489777)SNP_16648	1293	T	C	25	12	--	--
	Ca(ICCV2/PI489777)SNP_16649	1344	C	T	16	5	--	--
CakTC32395	Ca(ICCV2/PI489777)SNP_16650	148	A	C	9	4	--	--
	Ca(ICCV2/PI489777)SNP_16651	342	A	G	15	15	--	--
CakTC28037	Ca(ICCV2/PI489777)SNP_16652	436	T	C	9	3	--	--
	Ca(ICCV2/PI489777)SNP_16653	757	G	T	7	3	--	--
CakTC13058	Ca(ICCV2/PI489777)SNP_16654	882	C	T	66	61	--	--
	Ca(ICCV2/PI489777)SNP_16655	1068	C	G	27	36	--	--
	Ca(ICCV2/PI489777)SNP_16656	1164	T	C	9	12	--	--
	Ca(ICCV2/PI489777)SNP_16657	1776	C	T	4	8	--	--
CakTC11642	Ca(ICCV2/PI489777)SNP_16658	287	T	C	5	3	--	--
	Ca(ICCV2/PI489777)SNP_16659	396	G	A	4	3	--	--
CakTC06109	Ca(ICCV2/PI489777)SNP_16660	186	A	C	11	3	--	--
	Ca(ICCV2/PI489777)SNP_16661	781	A	G	9	11	--	--
	Ca(ICCV2/PI489777)SNP_16662	799	T	G	11	12	--	--
	Ca(ICCV2/PI489777)SNP_16663	853	A	G	14	8	--	--
	Ca(ICCV2/PI489777)SNP_16664	1077	G	A	15	8	--	--
CakTC25772	Ca(ICCV2/PI489777)SNP_16665	965	T	C	48	17	--	HB
	Ca(ICCV2/PI489777)SNP_16666	1124	A	C	46	16	--	HB
CakTC30114	Ca(ICCV2/PI489777)SNP_16667	659	T	C	4	4	Flower	bud
	Ca(ICCV2/PI489777)SNP_16668	710	G	C	4	5	Flower	bud
CakTC31036	Ca(ICCV2/PI489777)SNP_16669	306	C	T	3	4	--	--
CakTC02396	Ca(ICCV2/PI489777)SNP_16670	355	T	C	14	18	--	--
	Ca(ICCV2/PI489777)SNP_16671	1006	A	G	18	9	--	--
	Ca(ICCV2/PI489777)SNP_16672	1261	C	T	20	7	--	--
	Ca(ICCV2/PI489777)SNP_16673	1315	A	G	17	5	--	--
	Ca(ICCV2/PI489777)SNP_16674	1495	A	G	7	3	--	--
CakTC32911	Ca(ICCV2/PI489777)SNP_16675	224	A	G	42	6	--	--
	Ca(ICCV2/PI489777)SNP_16676	314	T	C	49	6	--	--
	Ca(ICCV2/PI489777)SNP_16677	358	T	C	45	6	--	--
	Ca(ICCV2/PI489777)SNP_16678	378	A	G	43	5	--	--
	Ca(ICCV2/PI489777)SNP_16679	402	C	G	43	5	--	--
CakTC22535	Ca(ICCV2/PI489777)SNP_16680	728	A	G	6	3	--	--
	Ca(ICCV2/PI489777)SNP_16681	1215	G	C	6	4	--	--
	Ca(ICCV2/PI489777)SNP_16682	1907	T	C	13	8	--	--
CakTC38424	Ca(ICCV2/PI489777)SNP_16683	614	C	T	8	9	--	--
	Ca(ICCV2/PI489777)SNP_16684	710	T	C	5	6	--	--
CakTC41608	Ca(ICCV2/PI489777)SNP_16685	659	T	A	14	6	--	--
	Ca(ICCV2/PI489777)SNP_16686	729	A	G	12	6	--	--
	Ca(ICCV2/PI489777)SNP_16687	738	G	A	11	6	--	--
	Ca(ICCV2/PI489777)SNP_16688	800	A	G	9	4	--	--
CakTC28085	Ca(ICCV2/PI489777)SNP_16689	1055	C	T	54	17	--	--
CakTC39373	Ca(ICCV2/PI489777)SNP_16690	77	T	C	5	4	--	--
	Ca(ICCV2/PI489777)SNP_16691	110	T	C	7	8	--	--
	Ca(ICCV2/PI489777)SNP_16692	344	A	C	13	33	--	--
CakTC41889	Ca(ICCV2/PI489777)SNP_16693	230	A	G	28	16	--	--
	Ca(ICCV2/PI489777)SNP_16694	407	C	T	30	17	--	--
	Ca(ICCV2/PI489777)SNP_16695	707	C	T	12	9	--	--
CakTC33453	Ca(ICCV2/PI489777)SNP_16696	123	G	A	3	13	--	--
CakTC31568	Ca(ICCV2/PI489777)SNP_16697	1800	A	G	11	3	Young_pod	--
	Ca(ICCV2/PI489777)SNP_16698	3162	G	T	16	6	Young_pod	--
CakTC39285	Ca(ICCV2/PI489777)SNP_16699	1254	A	T	19	15	--	--
	Ca(ICCV2/PI489777)SNP_16700	1384	C	T	16	4	--	--
CakTC33776	Ca(ICCV2/PI489777)SNP_16701	243	C	T	6	3	--	--
CakTC38284	Ca(ICCV2/PI489777)SNP_16702	110	C	G	9	6	--	--
	Ca(ICCV2/PI489777)SNP_16703	136	G	C	17	10	--	--
	Ca(ICCV2/PI489777)SNP_16704	141	G	C	20	10	--	--
CakTC09445	Ca(ICCV2/PI489777)SNP_16705	227	T	G	5	4	--	MYB
	Ca(ICCV2/PI489777)SNP_16706	393	A	T	7	4	--	MYB
CakTC40605	Ca(ICCV2/PI489777)SNP_16707	605	C	T	5	3	--	--
	Ca(ICCV2/PI489777)SNP_16708	629	A	G	5	3	--	--
	Ca(ICCV2/PI489777)SNP_16709	1652	G	A	3	7	--	--
	Ca(ICCV2/PI489777)SNP_16710	1754	A	C	5	7	--	--
	Ca(ICCV2/PI489777)SNP_16711	1915	G	T	8	6	--	--
	Ca(ICCV2/PI489777)SNP_16712	1973	C	T	8	7	--	--
	Ca(ICCV2/PI489777)SNP_16713	2035	A	T	7	3	--	--
CakTC42754	Ca(ICCV2/PI489777)SNP_16714	640	T	C	11	11	--	--
	Ca(ICCV2/PI489777)SNP_16715	741	A	G	12	6	--	--
	Ca(ICCV2/PI489777)SNP_16716	831	A	G	11	5	--	--
CakTC26915	Ca(ICCV2/PI489777)SNP_16717	386	C	G	26	6	--	--
	Ca(ICCV2/PI489777)SNP_16718	457	G	A	22	9	--	--
	Ca(ICCV2/PI489777)SNP_16719	763	C	A	14	6	--	--

CakTC39160	Ca(ICCV2/PI489777)SNP_16720	22	A	T	4	4	--	--
	Ca(ICCV2/PI489777)SNP_16721	159	T	C	4	10	--	--
	Ca(ICCV2/PI489777)SNP_16722	413	T	C	10	7	--	--
	Ca(ICCV2/PI489777)SNP_16723	515	A	G	11	3	--	--
	Ca(ICCV2/PI489777)SNP_16724	1234	A	G	7	4	--	--
	Ca(ICCV2/PI489777)SNP_16725	1431	C	G	4	3	--	--
	Ca(ICCV2/PI489777)SNP_16726	1463	G	A	4	3	--	--
	Ca(ICCV2/PI489777)SNP_16727	1490	G	A	4	3	--	--
CakTC34172	Ca(ICCV2/PI489777)SNP_16728	312	T	G	38	21	--	--
	Ca(ICCV2/PI489777)SNP_16729	386	T	A	40	22	--	--
	Ca(ICCV2/PI489777)SNP_16730	845	C	A	30	21	--	--
	Ca(ICCV2/PI489777)SNP_16731	848	G	A	31	21	--	--
	Ca(ICCV2/PI489777)SNP_16732	1676	A	G	23	28	--	--
	Ca(ICCV2/PI489777)SNP_16733	1765	A	G	35	35	--	--
	Ca(ICCV2/PI489777)SNP_16734	2066	A	C	23	13	--	--
CakTC42120	Ca(ICCV2/PI489777)SNP_16735	1008	G	C	7	6	--	--
	Ca(ICCV2/PI489777)SNP_16736	1026	T	C	8	5	--	--
CakTC22981	Ca(ICCV2/PI489777)SNP_16737	2168	T	G	70	20	--	--
	Ca(ICCV2/PI489777)SNP_16738	2267	C	G	12	17	--	--
	Ca(ICCV2/PI489777)SNP_16739	2282	A	C	9	17	--	--
	Ca(ICCV2/PI489777)SNP_16740	2308	T	G	8	14	--	--
	Ca(ICCV2/PI489777)SNP_16741	3043	G	A	6	3	--	--
	Ca(ICCV2/PI489777)SNP_16742	3046	T	A	6	3	--	--
	Ca(ICCV2/PI489777)SNP_16743	3148	T	G	5	3	--	--
	Ca(ICCV2/PI489777)SNP_16744	3183	T	C	7	5	--	--
	Ca(ICCV2/PI489777)SNP_16745	3189	T	C	7	4	--	--
	Ca(ICCV2/PI489777)SNP_16746	3227	G	A	7	4	--	--
	Ca(ICCV2/PI489777)SNP_16747	3265	T	A	7	5	--	--
	Ca(ICCV2/PI489777)SNP_16748	3313	A	G	7	5	--	--
	Ca(ICCV2/PI489777)SNP_16749	3319	A	G	7	5	--	--
	Ca(ICCV2/PI489777)SNP_16750	3328	G	C	9	5	--	--
	Ca(ICCV2/PI489777)SNP_16751	3383	A	C	6	4	--	--
	Ca(ICCV2/PI489777)SNP_16752	3405	T	C	8	4	--	--
	Ca(ICCV2/PI489777)SNP_16753	3406	C	T	8	4	--	--
CakTC38527	Ca(ICCV2/PI489777)SNP_16754	724	A	G	9	7	--	--
	Ca(ICCV2/PI489777)SNP_16755	770	A	G	9	8	--	--
	Ca(ICCV2/PI489777)SNP_16756	839	T	A	8	6	--	--
CakTC41147	Ca(ICCV2/PI489777)SNP_16757	637	T	G	7	3	--	--
	Ca(ICCV2/PI489777)SNP_16758	1417	G	A	13	11	--	--
CakTC12575	Ca(ICCV2/PI489777)SNP_16759	167	T	A	3	7	--	--
	Ca(ICCV2/PI489777)SNP_16760	249	A	T	7	7	--	--
CakTC28603	Ca(ICCV2/PI489777)SNP_16761	140	T	G	17	13	--	--
	Ca(ICCV2/PI489777)SNP_16762	150	A	G	19	13	--	--
	Ca(ICCV2/PI489777)SNP_16763	516	A	G	45	20	--	--
	Ca(ICCV2/PI489777)SNP_16764	1236	C	G	34	9	--	--
	Ca(ICCV2/PI489777)SNP_16765	1791	G	A	23	13	--	--
	Ca(ICCV2/PI489777)SNP_16766	1926	T	G	37	18	--	--
	Ca(ICCV2/PI489777)SNP_16767	2055	G	A	25	11	--	--
CakTC39777	Ca(ICCV2/PI489777)SNP_16768	112	A	C	9	4	--	--
	Ca(ICCV2/PI489777)SNP_16769	471	C	T	9	5	--	--
	Ca(ICCV2/PI489777)SNP_16770	510	C	T	10	5	--	--
	Ca(ICCV2/PI489777)SNP_16771	746	G	C	10	4	--	--
	Ca(ICCV2/PI489777)SNP_16772	839	G	C	11	3	--	--
CakTC37081	Ca(ICCV2/PI489777)SNP_16773	1625	C	T	26	12	--	--
CakTC10159	Ca(ICCV2/PI489777)SNP_16774	684	T	A	3	4	--	--
CakTC36242	Ca(ICCV2/PI489777)SNP_16775	214	C	A	6	5	--	--
	Ca(ICCV2/PI489777)SNP_16776	287	C	A	6	5	--	--
	Ca(ICCV2/PI489777)SNP_16777	299	G	A	6	5	--	--
	Ca(ICCV2/PI489777)SNP_16778	1591	C	A	3	4	--	--
	Ca(ICCV2/PI489777)SNP_16779	1617	A	C	3	5	--	--
	Ca(ICCV2/PI489777)SNP_16780	1673	T	C	3	4	--	--
	Ca(ICCV2/PI489777)SNP_16781	1766	T	G	4	5	--	--
CakTC08826	Ca(ICCV2/PI489777)SNP_16782	185	G	A	4	4	--	--
	Ca(ICCV2/PI489777)SNP_16783	316	T	A	4	5	--	--
	Ca(ICCV2/PI489777)SNP_16784	459	G	C	3	3	--	--
	Ca(ICCV2/PI489777)SNP_16785	494	G	T	3	4	--	--
CakTC30338	Ca(ICCV2/PI489777)SNP_16786	1515	G	T	3	11	--	--
	Ca(ICCV2/PI489777)SNP_16787	2280	C	G	4	3	--	--
	Ca(ICCV2/PI489777)SNP_16788	2973	A	C	7	3	--	--
	Ca(ICCV2/PI489777)SNP_16789	3072	G	A	9	4	--	--
	Ca(ICCV2/PI489777)SNP_16790	3267	T	A	5	7	--	--
	Ca(ICCV2/PI489777)SNP_16791	3903	A	T	13	4	--	--
	Ca(ICCV2/PI489777)SNP_16792	4272	C	T	8	8	--	--
	Ca(ICCV2/PI489777)SNP_16793	4330	C	T	7	10	--	--

CakTC28519	Ca(ICCV2/PI489777)SNP_16794	1408	A	C	6	3	--	--
	Ca(ICCV2/PI489777)SNP_16795	2503	A	G	4	5	--	--
	Ca(ICCV2/PI489777)SNP_16796	2677	A	C	3	4	--	--
CakTC31094	Ca(ICCV2/PI489777)SNP_16797	652	G	A	4	9	--	--
	Ca(ICCV2/PI489777)SNP_16798	1690	C	T	5	10	--	--
CakTC27025	Ca(ICCV2/PI489777)SNP_16799	88	C	A	7	8	--	--
	Ca(ICCV2/PI489777)SNP_16800	283	G	C	10	7	--	--
	Ca(ICCV2/PI489777)SNP_16801	600	A	T	14	9	--	--
	Ca(ICCV2/PI489777)SNP_16802	606	T	G	14	9	--	--
CakTC25596	Ca(ICCV2/PI489777)SNP_16803	96	G	A	16	12	--	--
	Ca(ICCV2/PI489777)SNP_16804	179	C	G	15	10	--	--
	Ca(ICCV2/PI489777)SNP_16805	272	T	C	11	10	--	--
	Ca(ICCV2/PI489777)SNP_16806	281	C	T	13	10	--	--
CakTC32817	Ca(ICCV2/PI489777)SNP_16807	1352	T	C	6	3	--	--
CakTC38509	Ca(ICCV2/PI489777)SNP_16808	240	G	A	25	22	--	bZIP
	Ca(ICCV2/PI489777)SNP_16809	263	A	G	26	20	--	bZIP
	Ca(ICCV2/PI489777)SNP_16810	272	C	T	26	22	--	bZIP
	Ca(ICCV2/PI489777)SNP_16811	1016	C	T	13	27	--	bZIP
	Ca(ICCV2/PI489777)SNP_16812	1020	T	A	13	26	--	bZIP
CakTC25580	Ca(ICCV2/PI489777)SNP_16813	379	A	G	21	11	--	--
	Ca(ICCV2/PI489777)SNP_16814	413	A	C	23	14	--	--
	Ca(ICCV2/PI489777)SNP_16815	422	A	G	24	14	--	--
	Ca(ICCV2/PI489777)SNP_16816	488	T	A	28	16	--	--
	Ca(ICCV2/PI489777)SNP_16817	568	A	T	26	17	--	--
	Ca(ICCV2/PI489777)SNP_16818	603	G	T	36	19	--	--
	Ca(ICCV2/PI489777)SNP_16819	705	T	G	34	12	--	--
	Ca(ICCV2/PI489777)SNP_16820	773	T	C	37	15	--	--
	Ca(ICCV2/PI489777)SNP_16821	780	A	G	36	10	--	--
	Ca(ICCV2/PI489777)SNP_16822	907	T	G	34	10	--	--
	Ca(ICCV2/PI489777)SNP_16823	1669	T	C	35	16	--	--
	Ca(ICCV2/PI489777)SNP_16824	1881	G	A	45	14	--	--
	Ca(ICCV2/PI489777)SNP_16825	2008	C	T	38	12	--	--
	Ca(ICCV2/PI489777)SNP_16826	2379	C	T	12	12	--	--
	Ca(ICCV2/PI489777)SNP_16827	2424	G	A	11	11	--	--
	Ca(ICCV2/PI489777)SNP_16828	2440	A	T	10	6	--	--
	Ca(ICCV2/PI489777)SNP_16829	2530	T	A	8	6	--	--
CakTC41701	Ca(ICCV2/PI489777)SNP_16830	519	C	G	8	6	--	--
CakTC37800	Ca(ICCV2/PI489777)SNP_16831	1157	G	A	7	4	--	--
CakTC27575	Ca(ICCV2/PI489777)SNP_16832	201	T	G	24	10	--	--
CakTC29594	Ca(ICCV2/PI489777)SNP_16833	169	G	A	22	7	--	--
	Ca(ICCV2/PI489777)SNP_16834	631	G	A	37	10	--	--
CakTC14543	Ca(ICCV2/PI489777)SNP_16835	147	A	G	5	4	--	--
CakTC31246	Ca(ICCV2/PI489777)SNP_16836	131	C	T	24	21	--	--
	Ca(ICCV2/PI489777)SNP_16837	1432	G	A	22	9	--	--
	Ca(ICCV2/PI489777)SNP_16838	2254	C	T	38	20	--	--
CakTC27990	Ca(ICCV2/PI489777)SNP_16839	114	G	T	9	6	--	--
CakTC35843	Ca(ICCV2/PI489777)SNP_16840	1538	T	C	13	4	--	--
	Ca(ICCV2/PI489777)SNP_16841	1610	G	A	12	6	--	--
	Ca(ICCV2/PI489777)SNP_16842	1694	A	G	11	9	--	--
	Ca(ICCV2/PI489777)SNP_16843	2063	A	T	8	3	--	--
	Ca(ICCV2/PI489777)SNP_16844	2137	T	C	9	4	--	--
	Ca(ICCV2/PI489777)SNP_16845	3026	T	C	5	4	--	--
	Ca(ICCV2/PI489777)SNP_16846	3357	A	G	12	3	--	--
	Ca(ICCV2/PI489777)SNP_16847	3711	G	A	13	7	--	--
	Ca(ICCV2/PI489777)SNP_16848	3993	A	G	15	11	--	--
CakTC26040	Ca(ICCV2/PI489777)SNP_16849	3913	A	G	21	5	--	--
CakTC43048	Ca(ICCV2/PI489777)SNP_16850	822	T	C	9	4	Root	--
	Ca(ICCV2/PI489777)SNP_16851	885	T	C	6	4	Root	--
CakTC25595	Ca(ICCV2/PI489777)SNP_16852	741	G	C	24	3	--	bHLH
CakTC04230	Ca(ICCV2/PI489777)SNP_16853	3600	T	C	4	3	--	PHD
CakTC40496	Ca(ICCV2/PI489777)SNP_16854	138	G	A	5	3	--	--
CakTC10621	Ca(ICCV2/PI489777)SNP_16855	347	G	A	3	4	Flower	bud
CakTC27426	Ca(ICCV2/PI489777)SNP_16856	130	C	T	6	9	--	MYB-related
	Ca(ICCV2/PI489777)SNP_16857	163	C	G	10	9	--	MYB-related
	Ca(ICCV2/PI489777)SNP_16858	168	A	C	10	9	--	MYB-related
	Ca(ICCV2/PI489777)SNP_16859	767	G	A	23	6	--	MYB-related
	Ca(ICCV2/PI489777)SNP_16860	844	T	C	11	9	--	MYB-related
	Ca(ICCV2/PI489777)SNP_16861	1005	C	A	10	6	--	MYB-related
CakTC13172	Ca(ICCV2/PI489777)SNP_16862	525	C	T	51	3	--	--
	Ca(ICCV2/PI489777)SNP_16863	621	A	T	46	6	--	--
CakTC39902	Ca(ICCV2/PI489777)SNP_16864	177	A	G	9	4	--	--
	Ca(ICCV2/PI489777)SNP_16865	189	A	G	12	5	--	--
	Ca(ICCV2/PI489777)SNP_16866	258	A	C	11	5	--	--
CakTC02959	Ca(ICCV2/PI489777)SNP_16867	680	A	G	3	8	--	--

	Ca(ICCV2/PI489777)SNP_16868	930	C	T	5	7	--	--
CakTC30190	Ca(ICCV2/PI489777)SNP_16869	1149	T	C	4	3	--	--
CakTC09971	Ca(ICCV2/PI489777)SNP_16870	293	A	C	5	5	--	--
CakTC37782	Ca(ICCV2/PI489777)SNP_16871	340	A	G	4	8	--	--
	Ca(ICCV2/PI489777)SNP_16872	583	G	C	4	11	--	--
CakTC27360	Ca(ICCV2/PI489777)SNP_16873	1097	A	C	24	17	--	--
	Ca(ICCV2/PI489777)SNP_16874	1260	T	C	24	24	--	--
	Ca(ICCV2/PI489777)SNP_16875	1295	A	G	24	29	--	--
	Ca(ICCV2/PI489777)SNP_16876	1385	A	G	15	20	--	--
CakTC40414	Ca(ICCV2/PI489777)SNP_16877	1199	A	G	4	7	Flower	bud
CakTC42143	Ca(ICCV2/PI489777)SNP_16878	107	C	G	5	6	--	--
	Ca(ICCV2/PI489777)SNP_16879	696	A	G	7	4	--	--
	Ca(ICCV2/PI489777)SNP_16880	819	C	T	4	8	--	--
	Ca(ICCV2/PI489777)SNP_16881	1110	C	T	10	3	--	--
	Ca(ICCV2/PI489777)SNP_16882	1200	T	C	11	6	--	--
	Ca(ICCV2/PI489777)SNP_16883	1329	C	G	6	9	--	--
CakTC06697	Ca(ICCV2/PI489777)SNP_16884	798	A	T	4	4	Flower	bud
CakTC27871	Ca(ICCV2/PI489777)SNP_16885	696	C	G	18	25	Root	--
CakTC35227	Ca(ICCV2/PI489777)SNP_16886	116	A	T	22	12	--	--
	Ca(ICCV2/PI489777)SNP_16887	149	A	G	25	12	--	--
	Ca(ICCV2/PI489777)SNP_16888	162	A	G	21	12	--	--
	Ca(ICCV2/PI489777)SNP_16889	349	G	A	34	12	--	--
	Ca(ICCV2/PI489777)SNP_16890	372	C	T	33	13	--	--
	Ca(ICCV2/PI489777)SNP_16891	493	A	C	28	11	--	--
	Ca(ICCV2/PI489777)SNP_16892	630	T	A	23	16	--	--
	Ca(ICCV2/PI489777)SNP_16893	632	A	G	24	16	--	--
	Ca(ICCV2/PI489777)SNP_16894	692	G	A	24	15	--	--
	Ca(ICCV2/PI489777)SNP_16895	1006	G	A	35	29	--	--
	Ca(ICCV2/PI489777)SNP_16896	1072	G	A	32	26	--	--
	Ca(ICCV2/PI489777)SNP_16897	1388	G	A	32	18	--	--
	Ca(ICCV2/PI489777)SNP_16898	1734	A	C	40	36	--	--
	Ca(ICCV2/PI489777)SNP_16899	2002	C	T	36	25	--	--
	Ca(ICCV2/PI489777)SNP_16900	2206	C	T	21	18	--	--
	Ca(ICCV2/PI489777)SNP_16901	2227	G	A	22	22	--	--
CakTC10659	Ca(ICCV2/PI489777)SNP_16902	40	A	G	7	4	--	--
	Ca(ICCV2/PI489777)SNP_16903	1952	C	T	52	6	--	--
CakTC41081	Ca(ICCV2/PI489777)SNP_16904	106	G	T	3	7	--	--
CakTC39304	Ca(ICCV2/PI489777)SNP_16905	731	T	C	6	3	--	TPR
	Ca(ICCV2/PI489777)SNP_16906	890	T	C	7	4	--	TPR
	Ca(ICCV2/PI489777)SNP_16907	1126	G	T	4	3	--	TPR
CakTC38037	Ca(ICCV2/PI489777)SNP_16908	165	A	G	23	59	Root	--
	Ca(ICCV2/PI489777)SNP_16909	899	G	A	19	41	Root	--
	Ca(ICCV2/PI489777)SNP_16910	1015	A	T	11	21	Root	--
	Ca(ICCV2/PI489777)SNP_16911	1067	C	T	5	14	Root	--
CakTC22618	Ca(ICCV2/PI489777)SNP_16912	456	G	A	9	4	--	--
	Ca(ICCV2/PI489777)SNP_16913	673	G	A	4	3	--	--
CakTC39749	Ca(ICCV2/PI489777)SNP_16914	227	A	G	4	5	--	--
	Ca(ICCV2/PI489777)SNP_16915	311	A	G	4	6	--	--
	Ca(ICCV2/PI489777)SNP_16916	316	A	G	4	6	--	--
	Ca(ICCV2/PI489777)SNP_16917	463	T	C	4	6	--	--
	Ca(ICCV2/PI489777)SNP_16918	790	T	G	5	5	--	--
	Ca(ICCV2/PI489777)SNP_16919	813	C	T	3	3	--	--
CakTC30958	Ca(ICCV2/PI489777)SNP_16920	549	G	A	11	4	--	--
	Ca(ICCV2/PI489777)SNP_16921	592	G	A	11	4	--	--
	Ca(ICCV2/PI489777)SNP_16922	783	G	T	3	5	--	--
	Ca(ICCV2/PI489777)SNP_16923	1191	C	T	5	11	--	--
CakTC31514	Ca(ICCV2/PI489777)SNP_16924	1397	A	G	10	4	--	--
	Ca(ICCV2/PI489777)SNP_16925	1772	T	C	8	6	--	--
CakTC39577	Ca(ICCV2/PI489777)SNP_16926	1524	G	A	14	13	--	--
CakTC28239	Ca(ICCV2/PI489777)SNP_16927	203	G	A	26	6	--	--
	Ca(ICCV2/PI489777)SNP_16928	780	A	T	24	9	--	--
	Ca(ICCV2/PI489777)SNP_16929	783	T	A	21	9	--	--
CakTC39724	Ca(ICCV2/PI489777)SNP_16930	1159	C	T	4	4	--	--
CakTC26166	Ca(ICCV2/PI489777)SNP_16931	230	G	A	4	6	--	--
	Ca(ICCV2/PI489777)SNP_16932	295	G	T	8	12	--	--
	Ca(ICCV2/PI489777)SNP_16933	316	G	A	8	13	--	--
	Ca(ICCV2/PI489777)SNP_16934	1074	G	A	6	7	--	--
	Ca(ICCV2/PI489777)SNP_16935	1146	C	T	4	7	--	--
	Ca(ICCV2/PI489777)SNP_16936	1355	T	C	7	4	--	--
	Ca(ICCV2/PI489777)SNP_16937	2145	G	A	3	3	--	--
CakTC37202	Ca(ICCV2/PI489777)SNP_16938	351	G	A	3	5	--	--
	Ca(ICCV2/PI489777)SNP_16939	381	G	C	3	4	--	--
CakTC25316	Ca(ICCV2/PI489777)SNP_16940	1751	G	A	8	4	--	--
	Ca(ICCV2/PI489777)SNP_16941	1970	G	A	10	5	--	--

CakTC41587	Ca(ICCV2/PI489777)SNP_16942	183	G	C	15	23	--	--
	Ca(ICCV2/PI489777)SNP_16943	420	C	T	19	19	--	--
CakTC38615	Ca(ICCV2/PI489777)SNP_16944	863	A	C	4	9	--	--
	Ca(ICCV2/PI489777)SNP_16945	895	C	T	4	8	--	--
	Ca(ICCV2/PI489777)SNP_16946	941	G	T	4	9	--	--
	Ca(ICCV2/PI489777)SNP_16947	943	C	T	4	9	--	--
CakTC39277	Ca(ICCV2/PI489777)SNP_16948	661	T	A	17	7	--	--
	Ca(ICCV2/PI489777)SNP_16949	949	C	T	9	9	--	--
	Ca(ICCV2/PI489777)SNP_16950	1140	T	G	13	11	--	--
CakTC11465	Ca(ICCV2/PI489777)SNP_16951	115	G	A	4	3	--	--
	Ca(ICCV2/PI489777)SNP_16952	197	C	A	4	5	--	--
	Ca(ICCV2/PI489777)SNP_16953	407	G	A	14	8	--	--
	Ca(ICCV2/PI489777)SNP_16954	467	A	G	14	12	--	--
	Ca(ICCV2/PI489777)SNP_16955	987	T	C	20	15	--	--
	Ca(ICCV2/PI489777)SNP_16956	1023	G	A	26	19	--	--
	Ca(ICCV2/PI489777)SNP_16957	1634	G	A	13	12	--	--
	Ca(ICCV2/PI489777)SNP_16958	1643	G	A	12	12	--	--
CakTC23162	Ca(ICCV2/PI489777)SNP_16959	538	G	C	203	85	--	--
	Ca(ICCV2/PI489777)SNP_16960	790	C	T	68	45	--	--
CakTC38158	Ca(ICCV2/PI489777)SNP_16961	422	C	A	19	6	--	MADS
CakTC39148	Ca(ICCV2/PI489777)SNP_16962	391	A	G	15	7	--	--
	Ca(ICCV2/PI489777)SNP_16963	507	A	C	18	13	--	--
CakTC11425	Ca(ICCV2/PI489777)SNP_16964	858	G	A	8	4	--	--
	Ca(ICCV2/PI489777)SNP_16965	888	C	T	7	4	--	--
	Ca(ICCV2/PI489777)SNP_16966	909	T	C	7	4	--	--
	Ca(ICCV2/PI489777)SNP_16967	910	G	T	7	4	--	--
	Ca(ICCV2/PI489777)SNP_16968	925	C	T	7	4	--	--
	Ca(ICCV2/PI489777)SNP_16969	926	A	G	7	4	--	--
CakTC33770	Ca(ICCV2/PI489777)SNP_16970	23	T	C	6	3	--	--
	Ca(ICCV2/PI489777)SNP_16971	97	C	T	25	12	--	--
	Ca(ICCV2/PI489777)SNP_16972	100	G	A	25	11	--	--
	Ca(ICCV2/PI489777)SNP_16973	124	C	T	28	15	--	--
	Ca(ICCV2/PI489777)SNP_16974	166	C	A	29	14	--	--
	Ca(ICCV2/PI489777)SNP_16975	280	G	A	27	13	--	--
	Ca(ICCV2/PI489777)SNP_16976	341	A	G	28	15	--	--
CakTC40051	Ca(ICCV2/PI489777)SNP_16977	794	A	C	11	8	--	--
CakTC01394	Ca(ICCV2/PI489777)SNP_16978	423	A	G	14	3	--	--
	Ca(ICCV2/PI489777)SNP_16979	687	A	G	5	6	--	--
	Ca(ICCV2/PI489777)SNP_16980	701	T	C	5	5	--	--
	Ca(ICCV2/PI489777)SNP_16981	728	G	C	7	6	--	--
	Ca(ICCV2/PI489777)SNP_16982	758	G	T	5	7	--	--
	Ca(ICCV2/PI489777)SNP_16983	767	T	C	5	6	--	--
	Ca(ICCV2/PI489777)SNP_16984	804	G	A	4	5	--	--
CakTC28396	Ca(ICCV2/PI489777)SNP_16985	69	A	G	5	5	--	--
	Ca(ICCV2/PI489777)SNP_16986	89	G	A	8	8	--	--
	Ca(ICCV2/PI489777)SNP_16987	98	C	G	7	7	--	--
	Ca(ICCV2/PI489777)SNP_16988	165	A	G	14	11	--	--
	Ca(ICCV2/PI489777)SNP_16989	206	C	A	14	13	--	--
	Ca(ICCV2/PI489777)SNP_16990	460	T	C	22	15	--	--
	Ca(ICCV2/PI489777)SNP_16991	508	A	G	22	17	--	--
CakTC11627	Ca(ICCV2/PI489777)SNP_16992	374	T	C	17	15	--	--
CakTC32663	Ca(ICCV2/PI489777)SNP_16993	1521	G	C	5	3	--	--
	Ca(ICCV2/PI489777)SNP_16994	1539	T	C	5	4	--	--
CakTC23705	Ca(ICCV2/PI489777)SNP_16995	398	A	C	40	9	--	--
	Ca(ICCV2/PI489777)SNP_16996	439	T	G	40	10	--	--
	Ca(ICCV2/PI489777)SNP_16997	592	G	A	26	18	--	--
	Ca(ICCV2/PI489777)SNP_16998	676	G	A	32	21	--	--
	Ca(ICCV2/PI489777)SNP_16999	742	T	G	31	16	--	--
	Ca(ICCV2/PI489777)SNP_17000	952	A	G	25	15	--	--
	Ca(ICCV2/PI489777)SNP_17001	1038	G	A	30	15	--	--
CakTC29911	Ca(ICCV2/PI489777)SNP_17002	559	G	A	5	4	--	--
	Ca(ICCV2/PI489777)SNP_17003	1904	C	T	5	6	--	--
CakTC25553	Ca(ICCV2/PI489777)SNP_17004	799	A	C	3	4	--	--
CakTC36920	Ca(ICCV2/PI489777)SNP_17005	1475	G	A	13	11	--	PHD
	Ca(ICCV2/PI489777)SNP_17006	1499	A	G	10	12	--	PHD
	Ca(ICCV2/PI489777)SNP_17007	1607	G	A	13	12	--	PHD
	Ca(ICCV2/PI489777)SNP_17008	2399	G	A	10	13	--	PHD
	Ca(ICCV2/PI489777)SNP_17009	2852	C	G	12	9	--	PHD
	Ca(ICCV2/PI489777)SNP_17010	3263	G	A	18	10	--	PHD
	Ca(ICCV2/PI489777)SNP_17011	4828	C	T	5	11	--	PHD
	Ca(ICCV2/PI489777)SNP_17012	4848	T	G	6	12	--	PHD
CakTC42344	Ca(ICCV2/PI489777)SNP_17013	284	A	G	22	6	--	--
	Ca(ICCV2/PI489777)SNP_17014	872	C	T	29	8	--	--
CakTC35220	Ca(ICCV2/PI489777)SNP_17015	894	C	A	16	16	--	--

	Ca(ICCV2/PI489777)SNP_17016	1002	T	C	23	14	--	--
	Ca(ICCV2/PI489777)SNP_17017	1257	A	G	15	5	--	--
CakTC29895	Ca(ICCV2/PI489777)SNP_17018	148	T	C	6	6	--	--
	Ca(ICCV2/PI489777)SNP_17019	211	G	C	9	8	--	--
	Ca(ICCV2/PI489777)SNP_17020	432	C	T	11	10	--	--
	Ca(ICCV2/PI489777)SNP_17021	1069	T	C	16	7	--	--
	Ca(ICCV2/PI489777)SNP_17022	1905	G	A	8	7	--	--
	Ca(ICCV2/PI489777)SNP_17023	2511	C	T	13	12	--	--
	Ca(ICCV2/PI489777)SNP_17024	2618	C	T	16	15	--	--
	Ca(ICCV2/PI489777)SNP_17025	2774	G	A	12	15	--	--
	Ca(ICCV2/PI489777)SNP_17026	3079	C	T	5	3	--	--
CakTC23820	Ca(ICCV2/PI489777)SNP_17027	739	C	T	38	23	--	--
	Ca(ICCV2/PI489777)SNP_17028	862	T	C	59	35	--	--
CakTC24926	Ca(ICCV2/PI489777)SNP_17029	106	T	A	16	9	--	--
	Ca(ICCV2/PI489777)SNP_17030	204	T	C	17	10	--	--
	Ca(ICCV2/PI489777)SNP_17031	245	T	C	22	10	--	--
	Ca(ICCV2/PI489777)SNP_17032	1158	C	T	17	7	--	--
	Ca(ICCV2/PI489777)SNP_17033	1444	A	G	21	9	--	--
	Ca(ICCV2/PI489777)SNP_17034	1461	T	C	20	9	--	--
	Ca(ICCV2/PI489777)SNP_17035	1483	G	A	19	9	--	--
	Ca(ICCV2/PI489777)SNP_17036	1499	C	T	18	9	--	--
CakTC34534	Ca(ICCV2/PI489777)SNP_17037	332	T	C	38	19	--	--
CakTC08592	Ca(ICCV2/PI489777)SNP_17038	203	G	A	6	3	--	--
	Ca(ICCV2/PI489777)SNP_17039	218	G	A	5	3	--	--
	Ca(ICCV2/PI489777)SNP_17040	374	G	A	8	5	--	--
	Ca(ICCV2/PI489777)SNP_17041	401	G	A	9	3	--	--
	Ca(ICCV2/PI489777)SNP_17042	451	T	A	12	4	--	--
	Ca(ICCV2/PI489777)SNP_17043	461	G	A	12	4	--	--
	Ca(ICCV2/PI489777)SNP_17044	479	A	T	12	4	--	--
	Ca(ICCV2/PI489777)SNP_17045	550	G	C	11	3	--	--
CakTC29146	Ca(ICCV2/PI489777)SNP_17046	468	C	T	11	5	--	--
	Ca(ICCV2/PI489777)SNP_17047	675	G	A	5	4	--	--
CakTC41833	Ca(ICCV2/PI489777)SNP_17048	465	A	G	8	14	--	--
	Ca(ICCV2/PI489777)SNP_17049	493	A	G	7	15	--	--
CakTC20209	Ca(ICCV2/PI489777)SNP_17050	137	G	A	3	3	Young_pod	--
CakTC24949	Ca(ICCV2/PI489777)SNP_17051	62	G	T	3	4	--	--
	Ca(ICCV2/PI489777)SNP_17052	63	C	T	3	4	--	--
	Ca(ICCV2/PI489777)SNP_17053	1588	T	A	12	10	--	--
	Ca(ICCV2/PI489777)SNP_17054	1599	G	A	13	10	--	--
	Ca(ICCV2/PI489777)SNP_17055	1628	T	A	11	10	--	--
CakTC35286	Ca(ICCV2/PI489777)SNP_17056	1427	G	A	14	10	--	--
	Ca(ICCV2/PI489777)SNP_17057	1724	G	A	7	10	--	--
	Ca(ICCV2/PI489777)SNP_17058	1736	T	G	8	10	--	--
	Ca(ICCV2/PI489777)SNP_17059	2075	G	A	9	4	--	--
	Ca(ICCV2/PI489777)SNP_17060	2249	A	G	10	7	--	--
	Ca(ICCV2/PI489777)SNP_17061	3116	G	A	11	7	--	--
CakTC25101	Ca(ICCV2/PI489777)SNP_17062	250	A	G	8	3	--	--
	Ca(ICCV2/PI489777)SNP_17063	364	A	G	14	6	--	--
	Ca(ICCV2/PI489777)SNP_17064	442	G	A	18	6	--	--
	Ca(ICCV2/PI489777)SNP_17065	1195	T	C	9	4	--	--
	Ca(ICCV2/PI489777)SNP_17066	1418	A	C	11	4	--	--
	Ca(ICCV2/PI489777)SNP_17067	1580	T	C	9	4	--	--
	Ca(ICCV2/PI489777)SNP_17068	1877	C	A	10	4	--	--
	Ca(ICCV2/PI489777)SNP_17069	1973	G	A	11	3	--	--
	Ca(ICCV2/PI489777)SNP_17070	2404	A	G	15	3	--	--
CakTC32985	Ca(ICCV2/PI489777)SNP_17071	1436	T	C	7	3	--	ARID
	Ca(ICCV2/PI489777)SNP_17072	1838	T	C	8	5	--	ARID
CakTC25693	Ca(ICCV2/PI489777)SNP_17073	749	G	C	9	3	--	--
CakTC24586	Ca(ICCV2/PI489777)SNP_17074	1464	T	C	19	24	--	--
CakTC31199	Ca(ICCV2/PI489777)SNP_17075	306	A	T	3	6	--	--
	Ca(ICCV2/PI489777)SNP_17076	344	G	A	4	6	--	--
	Ca(ICCV2/PI489777)SNP_17077	566	C	T	4	6	--	--
	Ca(ICCV2/PI489777)SNP_17078	757	A	G	5	3	--	--
	Ca(ICCV2/PI489777)SNP_17079	842	C	T	5	3	--	--
	Ca(ICCV2/PI489777)SNP_17080	941	G	A	5	3	--	--
CakTC38217	Ca(ICCV2/PI489777)SNP_17081	996	T	A	8	12	--	--
	Ca(ICCV2/PI489777)SNP_17082	1067	C	T	8	11	--	--
CakTC23360	Ca(ICCV2/PI489777)SNP_17083	1378	A	T	7	6	--	--
CakTC37691	Ca(ICCV2/PI489777)SNP_17084	680	T	C	122	61	--	--
	Ca(ICCV2/PI489777)SNP_17085	993	G	A	38	33	--	--
	Ca(ICCV2/PI489777)SNP_17086	1037	C	T	40	33	--	--
CakTC42174	Ca(ICCV2/PI489777)SNP_17087	412	C	T	16	13	--	--
CakTC23434	Ca(ICCV2/PI489777)SNP_17088	302	T	C	14	3	Young_pod	--
	Ca(ICCV2/PI489777)SNP_17089	328	G	C	12	4	Young_pod	--

CakTC23518	Ca(ICCV2/PI489777)SNP_17090	607	A	G	3	4	--	--		
CakTC28166	Ca(ICCV2/PI489777)SNP_17091	1072	G	C	3	5	--	--		
	Ca(ICCV2/PI489777)SNP_17092	2055	C	G	5	3	--	--		
CakTC42170	Ca(ICCV2/PI489777)SNP_17093	287	G	T	14	11	--	--		
	Ca(ICCV2/PI489777)SNP_17094	289	T	A	13	11	--	--		
	Ca(ICCV2/PI489777)SNP_17095	304	C	T	12	11	--	--		
	Ca(ICCV2/PI489777)SNP_17096	985	G	A	11	6	--	--		
	Ca(ICCV2/PI489777)SNP_17097	1021	C	T	10	7	--	--		
	Ca(ICCV2/PI489777)SNP_17098	1329	T	A	6	8	--	--		
	Ca(ICCV2/PI489777)SNP_17099	1540	A	G	8	10	--	--		
CakTC26898	Ca(ICCV2/PI489777)SNP_17100	328	T	C	43	31	--	--		
CakTC11774	Ca(ICCV2/PI489777)SNP_17101	660	A	G	17	4	--	--		
CakTC29441	Ca(ICCV2/PI489777)SNP_17102	1054	C	T	5	5	--	--		
CakTC42179	Ca(ICCV2/PI489777)SNP_17103	163	T	A	9	6	--	--		
	Ca(ICCV2/PI489777)SNP_17104	259	T	A	16	4	--	--		
	Ca(ICCV2/PI489777)SNP_17105	692	A	G	16	12	--	--		
CakTC43232	Ca(ICCV2/PI489777)SNP_17106	58	G	A	21	3	--	--		
	Ca(ICCV2/PI489777)SNP_17107	64	G	C	22	3	--	--		
	Ca(ICCV2/PI489777)SNP_17108	69	C	T	22	4	--	--		
	Ca(ICCV2/PI489777)SNP_17109	140	C	T	38	10	--	--		
	Ca(ICCV2/PI489777)SNP_17110	663	T	G	17	13	--	--		
	Ca(ICCV2/PI489777)SNP_17111	783	C	T	8	13	--	--		
CakTC28012	Ca(ICCV2/PI489777)SNP_17112	1101	T	C	6	5	--	--		
	Ca(ICCV2/PI489777)SNP_17113	1107	A	T	6	5	--	--		
CakTC39233	Ca(ICCV2/PI489777)SNP_17114	556	T	C	6	3	--	--		
	Ca(ICCV2/PI489777)SNP_17115	1057	T	A	9	5	--	--		
CakTC39452	Ca(ICCV2/PI489777)SNP_17116	1088	T	A	5	4	--	mTERF		
CakTC25359	Ca(ICCV2/PI489777)SNP_17117	994	G	A	38	12	--	--		
CakTC22652	Ca(ICCV2/PI489777)SNP_17118	107	T	C	5	3	--	--		
	Ca(ICCV2/PI489777)SNP_17119	163	T	C	7	3	--	--		
	Ca(ICCV2/PI489777)SNP_17120	182	C	T	8	3	--	--		
	Ca(ICCV2/PI489777)SNP_17121	237	T	A	10	4	--	--		
	Ca(ICCV2/PI489777)SNP_17122	274	C	G	9	4	--	--		
	Ca(ICCV2/PI489777)SNP_17123	1031	T	C	12	3	--	--		
	Ca(ICCV2/PI489777)SNP_17124	1040	G	A	14	3	--	--		
	Ca(ICCV2/PI489777)SNP_17125	1061	A	G	15	3	--	--		
	Ca(ICCV2/PI489777)SNP_17126	1076	A	C	11	3	--	--		
	Ca(ICCV2/PI489777)SNP_17127	1184	G	C	15	4	--	--		
	Ca(ICCV2/PI489777)SNP_17128	1305	T	C	16	4	--	--		
	Ca(ICCV2/PI489777)SNP_17129	1306	A	G	15	4	--	--		
	Ca(ICCV2/PI489777)SNP_17130	1363	T	C	13	3	--	--		
	Ca(ICCV2/PI489777)SNP_17131	1386	G	A	14	3	--	--		
	Ca(ICCV2/PI489777)SNP_17132	1404	C	G	13	3	--	--		
CakTC27857	Ca(ICCV2/PI489777)SNP_17133	282	A	T	10	4	--	--		
	Ca(ICCV2/PI489777)SNP_17134	306	C	T	10	5	--	--		
	Ca(ICCV2/PI489777)SNP_17135	504	C	G	17	13	--	--		
	Ca(ICCV2/PI489777)SNP_17136	565	G	A	25	14	--	--		
	Ca(ICCV2/PI489777)SNP_17137	786	A	G	20	14	--	--		
	Ca(ICCV2/PI489777)SNP_17138	1448	G	A	12	3	--	--		
	Ca(ICCV2/PI489777)SNP_17139	3003	C	T	14	4	--	--		
	Ca(ICCV2/PI489777)SNP_17140	3066	A	G	14	4	--	--		
	Ca(ICCV2/PI489777)SNP_17141	3249	A	G	9	4	--	--		
	Ca(ICCV2/PI489777)SNP_17142	3254	A	G	8	4	--	--		
	Ca(ICCV2/PI489777)SNP_17143	3414	G	C	7	3	--	--		
	Ca(ICCV2/PI489777)SNP_17144	3529	T	C	13	4	--	--		
	Ca(ICCV2/PI489777)SNP_17145	3542	T	G	13	4	--	--		
	Ca(ICCV2/PI489777)SNP_17146	3618	T	C	11	5	--	--		
	Ca(ICCV2/PI489777)SNP_17147	4083	A	G	9	4	--	--		
CakTC08240	Ca(ICCV2/PI489777)SNP_17148	1593	A	G	3	6	--	--		
CakTC43250	Ca(ICCV2/PI489777)SNP_17149	97	C	A	15	10	--	--		
	Ca(ICCV2/PI489777)SNP_17150	360	C	T	12	17	--	--		
	Ca(ICCV2/PI489777)SNP_17151	732	T	C	6	6	--	--		
CakTC24817	Ca(ICCV2/PI489777)SNP_17152	198	T	A	6	7	Flower	bud		
	Ca(ICCV2/PI489777)SNP_17153	270	G	A	6	7	Flower	bud		
	Ca(ICCV2/PI489777)SNP_17154	2040	G	A	4	3	Flower	bud		
CakTC23410	Ca(ICCV2/PI489777)SNP_17155	514	T	A	54	20	Young_pod	--		
CakTC30912	Ca(ICCV2/PI489777)SNP_17156	1371	A	C	13	13	--	TPR		
	Ca(ICCV2/PI489777)SNP_17157	2584	G	A	10	17	--	TPR		
CakTC29422	Ca(ICCV2/PI489777)SNP_17158	1575	A	G	18	4	--	--		
	Ca(ICCV2/PI489777)SNP_17159	1650	A	G	25	3	--	--		
	Ca(ICCV2/PI489777)SNP_17160	1971	A	G	16	3	--	--		
CakTC32673	Ca(ICCV2/PI489777)SNP_17161	263	T	A	13	3	--	--		
	Ca(ICCV2/PI489777)SNP_17162	281	T	C	13	3	--	--		
	Ca(ICCV2/PI489777)SNP_17163	1730	G	A	6	10	--	--		

	Ca(ICCV2/PI489777)SNP_17164	2139	G	C	5	6	--	--
CakTC14380	Ca(ICCV2/PI489777)SNP_17165	428	G	A	3	3	--	--
CakTC23403	Ca(ICCV2/PI489777)SNP_17166	4823	T	C	37	9	Mature	Leaf
CakTC38482	Ca(ICCV2/PI489777)SNP_17167	160	G	T	7	3	--	--
	Ca(ICCV2/PI489777)SNP_17168	861	G	A	5	10	--	--
CakTC40734	Ca(ICCV2/PI489777)SNP_17169	92	G	A	13	10	--	--
	Ca(ICCV2/PI489777)SNP_17170	489	A	G	16	12	--	--
CakTC28319	Ca(ICCV2/PI489777)SNP_17171	213	G	A	11	3	--	--
	Ca(ICCV2/PI489777)SNP_17172	287	A	C	11	15	--	--
CakTC23629	Ca(ICCV2/PI489777)SNP_17173	1049	C	A	33	46	--	--
	Ca(ICCV2/PI489777)SNP_17174	4108	A	G	15	11	--	--
CakTC10413	Ca(ICCV2/PI489777)SNP_17175	428	C	T	7	4	--	--
CakTC11611	Ca(ICCV2/PI489777)SNP_17176	53	T	G	3	3	--	--
CakTC33989	Ca(ICCV2/PI489777)SNP_17177	1651	T	G	5	7	--	HB
	Ca(ICCV2/PI489777)SNP_17178	1654	A	G	6	7	--	HB
	Ca(ICCV2/PI489777)SNP_17179	1773	T	A	3	5	--	HB
	Ca(ICCV2/PI489777)SNP_17180	1774	C	A	3	5	--	HB
	Ca(ICCV2/PI489777)SNP_17181	1834	C	T	3	14	--	HB
	Ca(ICCV2/PI489777)SNP_17182	2166	G	A	4	9	--	HB
CakTC23331	Ca(ICCV2/PI489777)SNP_17183	3777	T	C	119	41	--	--
	Ca(ICCV2/PI489777)SNP_17184	4137	T	C	115	52	--	--
	Ca(ICCV2/PI489777)SNP_17185	4965	A	G	91	40	--	--
CakTC22646	Ca(ICCV2/PI489777)SNP_17186	1251	G	A	17	18	--	--
	Ca(ICCV2/PI489777)SNP_17187	1461	A	G	24	13	--	--
	Ca(ICCV2/PI489777)SNP_17188	1986	A	T	24	22	--	--
	Ca(ICCV2/PI489777)SNP_17189	2283	T	C	27	20	--	--
CakTC35390	Ca(ICCV2/PI489777)SNP_17190	2896	C	T	3	3	--	--
CakTC39504	Ca(ICCV2/PI489777)SNP_17191	946	C	T	9	9	--	--
CakTC41031	Ca(ICCV2/PI489777)SNP_17192	811	T	C	16	6	--	--
	Ca(ICCV2/PI489777)SNP_17193	982	A	T	12	4	--	--
	Ca(ICCV2/PI489777)SNP_17194	1069	G	A	13	4	--	--
	Ca(ICCV2/PI489777)SNP_17195	1284	C	A	19	10	--	--
	Ca(ICCV2/PI489777)SNP_17196	1285	C	T	20	8	--	--
	Ca(ICCV2/PI489777)SNP_17197	1642	T	C	17	19	--	--
CakTC42243	Ca(ICCV2/PI489777)SNP_17198	1477	A	T	6	3	--	--
	Ca(ICCV2/PI489777)SNP_17199	1735	C	T	5	5	--	--
	Ca(ICCV2/PI489777)SNP_17200	2528	T	C	6	3	--	--
CakTC24690	Ca(ICCV2/PI489777)SNP_17201	711	G	A	22	8	--	--
	Ca(ICCV2/PI489777)SNP_17202	821	T	C	18	7	--	--
	Ca(ICCV2/PI489777)SNP_17203	928	A	G	3	14	--	--
CakTC00704	Ca(ICCV2/PI489777)SNP_17204	308	A	G	3	4	--	--
	Ca(ICCV2/PI489777)SNP_17205	567	G	T	4	4	--	--
	Ca(ICCV2/PI489777)SNP_17206	599	C	A	3	10	--	--
CakTC22630	Ca(ICCV2/PI489777)SNP_17207	1946	T	C	4	3	--	--
	Ca(ICCV2/PI489777)SNP_17208	2003	T	C	3	3	--	--
CakTC29476	Ca(ICCV2/PI489777)SNP_17209	355	T	C	4	15	Root	--
	Ca(ICCV2/PI489777)SNP_17210	809	C	T	5	12	Root	--
	Ca(ICCV2/PI489777)SNP_17211	1236	C	T	3	4	Root	--
CakTC36746	Ca(ICCV2/PI489777)SNP_17212	2253	C	A	22	19	--	--
	Ca(ICCV2/PI489777)SNP_17213	3090	T	C	19	10	--	--
	Ca(ICCV2/PI489777)SNP_17214	3549	C	A	10	7	--	--
CakTC15258	Ca(ICCV2/PI489777)SNP_17215	2734	A	G	11	3	--	CAMTA
CakTC37244	Ca(ICCV2/PI489777)SNP_17216	121	A	G	17	10	--	--
	Ca(ICCV2/PI489777)SNP_17217	580	T	C	22	10	--	--
CakTC42889	Ca(ICCV2/PI489777)SNP_17218	667	C	G	11	5	--	--
	Ca(ICCV2/PI489777)SNP_17219	676	A	G	11	5	--	--
	Ca(ICCV2/PI489777)SNP_17220	709	T	C	8	4	--	--
	Ca(ICCV2/PI489777)SNP_17221	831	G	A	3	5	--	--
	Ca(ICCV2/PI489777)SNP_17222	835	G	A	4	4	--	--
CakTC29185	Ca(ICCV2/PI489777)SNP_17223	529	G	A	8	3	--	--
CakTC30683	Ca(ICCV2/PI489777)SNP_17224	1243	T	A	6	5	--	--
CakTC27147	Ca(ICCV2/PI489777)SNP_17225	341	T	C	6	13	--	C2C2-GATA
	Ca(ICCV2/PI489777)SNP_17226	738	G	A	9	16	--	C2C2-GATA
	Ca(ICCV2/PI489777)SNP_17227	768	G	T	8	18	--	C2C2-GATA
CakTC41381	Ca(ICCV2/PI489777)SNP_17228	929	G	A	25	15	--	--
	Ca(ICCV2/PI489777)SNP_17229	2119	A	G	9	7	--	--
	Ca(ICCV2/PI489777)SNP_17230	2158	A	G	10	8	--	--
	Ca(ICCV2/PI489777)SNP_17231	2162	T	G	9	8	--	--
	Ca(ICCV2/PI489777)SNP_17232	2296	A	G	11	7	--	--
CakTC34580	Ca(ICCV2/PI489777)SNP_17233	1626	T	C	6	6	--	--
	Ca(ICCV2/PI489777)SNP_17234	1851	A	T	6	3	--	--
CakTC29800	Ca(ICCV2/PI489777)SNP_17235	394	T	A	68	36	--	--
	Ca(ICCV2/PI489777)SNP_17236	601	A	G	56	26	--	--
CakTC36956	Ca(ICCV2/PI489777)SNP_17237	1748	T	C	38	26	--	--

CakTC10012	Ca(ICCV2/PI489777)SNP_17238	600	T	C	10	7	--	--
CakTC39398	Ca(ICCV2/PI489777)SNP_17239	332	A	G	27	35	--	--
	Ca(ICCV2/PI489777)SNP_17240	959	G	A	15	16	--	--
	Ca(ICCV2/PI489777)SNP_17241	1214	A	G	19	32	--	--
	Ca(ICCV2/PI489777)SNP_17242	1509	A	G	7	23	--	--
	Ca(ICCV2/PI489777)SNP_17243	1584	G	A	3	5	--	--
CakTC30663	Ca(ICCV2/PI489777)SNP_17244	1121	G	A	3	3	--	--
	Ca(ICCV2/PI489777)SNP_17245	1503	G	T	3	3	--	--
	Ca(ICCV2/PI489777)SNP_17246	1507	T	C	3	3	--	--
CakTC39193	Ca(ICCV2/PI489777)SNP_17247	772	T	C	5	8	--	--
	Ca(ICCV2/PI489777)SNP_17248	787	A	C	4	8	--	--
	Ca(ICCV2/PI489777)SNP_17249	892	T	C	8	8	--	--
	Ca(ICCV2/PI489777)SNP_17250	1138	G	C	11	13	--	--
CakTC40576	Ca(ICCV2/PI489777)SNP_17251	720	G	A	4	4	--	--
	Ca(ICCV2/PI489777)SNP_17252	1407	G	A	4	3	--	--
CakTC11049	Ca(ICCV2/PI489777)SNP_17253	901	G	A	3	3	--	SRS
CakTC23454	Ca(ICCV2/PI489777)SNP_17254	307	A	G	34	8	--	--
	Ca(ICCV2/PI489777)SNP_17255	453	T	A	44	7	--	--
CakTC40393	Ca(ICCV2/PI489777)SNP_17256	1060	A	T	10	7	--	--
	Ca(ICCV2/PI489777)SNP_17257	1567	C	T	19	13	--	--
CakTC26704	Ca(ICCV2/PI489777)SNP_17258	946	G	A	8	12	--	--
CakTC23806	Ca(ICCV2/PI489777)SNP_17259	373	T	C	17	5	--	--
	Ca(ICCV2/PI489777)SNP_17260	415	C	T	19	5	--	--
CakTC42025	Ca(ICCV2/PI489777)SNP_17261	890	A	G	8	3	--	--
CakTC40338	Ca(ICCV2/PI489777)SNP_17262	769	T	C	9	5	--	--
	Ca(ICCV2/PI489777)SNP_17263	780	A	G	9	5	--	--
	Ca(ICCV2/PI489777)SNP_17264	1390	A	G	6	3	--	--
	Ca(ICCV2/PI489777)SNP_17265	1396	C	T	5	4	--	--
CakTC32416	Ca(ICCV2/PI489777)SNP_17266	123	G	A	5	5	--	--
	Ca(ICCV2/PI489777)SNP_17267	125	G	A	4	5	--	--
	Ca(ICCV2/PI489777)SNP_17268	148	G	A	9	4	--	--
	Ca(ICCV2/PI489777)SNP_17269	388	G	A	15	8	--	--
	Ca(ICCV2/PI489777)SNP_17270	399	G	A	25	9	--	--
	Ca(ICCV2/PI489777)SNP_17271	565	C	T	17	4	--	--
CakTC27892	Ca(ICCV2/PI489777)SNP_17272	1184	A	T	3	3	--	--
CakTC10055	Ca(ICCV2/PI489777)SNP_17273	599	A	G	5	3	--	--
CakTC30797	Ca(ICCV2/PI489777)SNP_17274	263	T	A	31	21	--	--
	Ca(ICCV2/PI489777)SNP_17275	437	T	C	24	30	--	--
	Ca(ICCV2/PI489777)SNP_17276	782	C	T	28	18	--	--
CakTC40469	Ca(ICCV2/PI489777)SNP_17277	426	A	G	4	6	--	--
	Ca(ICCV2/PI489777)SNP_17278	778	A	G	15	6	--	--
	Ca(ICCV2/PI489777)SNP_17279	810	G	A	17	7	--	--
CakTC14761	Ca(ICCV2/PI489777)SNP_17280	92	A	C	21	12	--	--
	Ca(ICCV2/PI489777)SNP_17281	121	G	T	38	15	--	--
	Ca(ICCV2/PI489777)SNP_17282	925	C	T	90	30	--	--
CakTC34341	Ca(ICCV2/PI489777)SNP_17283	1413	C	T	3	3	--	--
	Ca(ICCV2/PI489777)SNP_17284	1431	A	G	4	5	--	--
CakTC34170	Ca(ICCV2/PI489777)SNP_17285	274	C	T	31	22	--	--
	Ca(ICCV2/PI489777)SNP_17286	527	A	G	6	6	--	--
	Ca(ICCV2/PI489777)SNP_17287	553	G	A	6	4	--	--
CakTC40239	Ca(ICCV2/PI489777)SNP_17288	132	G	C	11	3	--	--
	Ca(ICCV2/PI489777)SNP_17289	245	C	T	11	3	--	--
	Ca(ICCV2/PI489777)SNP_17290	990	T	A	5	8	--	--
	Ca(ICCV2/PI489777)SNP_17291	999	A	G	5	8	--	--
CakTC26072	Ca(ICCV2/PI489777)SNP_17292	1461	G	A	8	10	--	--
	Ca(ICCV2/PI489777)SNP_17293	1565	G	C	4	3	--	--
	Ca(ICCV2/PI489777)SNP_17294	1583	T	G	4	3	--	--
CakTC09462	Ca(ICCV2/PI489777)SNP_17295	454	A	G	5	4	--	--
CakTC31163	Ca(ICCV2/PI489777)SNP_17296	1208	G	A	4	4	--	--
CakTC13141	Ca(ICCV2/PI489777)SNP_17297	207	C	T	4	3	--	--
	Ca(ICCV2/PI489777)SNP_17298	247	G	T	4	3	--	--
CakTC34649	Ca(ICCV2/PI489777)SNP_17299	31	A	T	10	10	--	--
	Ca(ICCV2/PI489777)SNP_17300	32	A	T	10	10	--	--
	Ca(ICCV2/PI489777)SNP_17301	185	G	A	20	15	--	--
	Ca(ICCV2/PI489777)SNP_17302	206	A	T	19	15	--	--
	Ca(ICCV2/PI489777)SNP_17303	316	A	C	25	11	--	--
	Ca(ICCV2/PI489777)SNP_17304	340	C	T	28	13	--	--
	Ca(ICCV2/PI489777)SNP_17305	490	A	T	28	12	--	--
	Ca(ICCV2/PI489777)SNP_17306	520	G	C	27	9	--	--
	Ca(ICCV2/PI489777)SNP_17307	598	C	T	23	7	--	--
	Ca(ICCV2/PI489777)SNP_17308	2053	T	C	13	21	--	--
	Ca(ICCV2/PI489777)SNP_17309	2072	T	C	9	21	--	--
CakTC37584	Ca(ICCV2/PI489777)SNP_17310	500	G	A	11	5	--	--
CakTC39053	Ca(ICCV2/PI489777)SNP_17311	468	A	C	16	8	--	--

	Ca(ICCV2/PI489777)SNP_17312	522	C	T	17	15	--	--
	Ca(ICCV2/PI489777)SNP_17313	560	A	G	22	17	--	--
	Ca(ICCV2/PI489777)SNP_17314	578	G	A	20	16	--	--
	Ca(ICCV2/PI489777)SNP_17315	590	C	T	22	16	--	--
	Ca(ICCV2/PI489777)SNP_17316	602	C	T	23	18	--	--
	Ca(ICCV2/PI489777)SNP_17317	697	C	G	17	24	--	--
	Ca(ICCV2/PI489777)SNP_17318	719	A	G	16	26	--	--
	Ca(ICCV2/PI489777)SNP_17319	947	T	C	12	23	--	--
	Ca(ICCV2/PI489777)SNP_17320	1367	A	G	8	22	--	--
CakTC35438	Ca(ICCV2/PI489777)SNP_17321	624	C	T	18	7	--	C3H
	Ca(ICCV2/PI489777)SNP_17322	1286	C	A	32	9	--	C3H
	Ca(ICCV2/PI489777)SNP_17323	1477	G	A	32	5	--	C3H
	Ca(ICCV2/PI489777)SNP_17324	2815	A	G	12	4	--	C3H
	Ca(ICCV2/PI489777)SNP_17325	3033	T	G	9	4	--	C3H
	Ca(ICCV2/PI489777)SNP_17326	3909	G	T	3	7	--	C3H
CakTC29080	Ca(ICCV2/PI489777)SNP_17327	507	T	C	154	84	--	--
CakTC30469	Ca(ICCV2/PI489777)SNP_17328	217	C	T	6	4	--	--
	Ca(ICCV2/PI489777)SNP_17329	420	G	A	10	8	--	--
	Ca(ICCV2/PI489777)SNP_17330	476	C	A	10	5	--	--
	Ca(ICCV2/PI489777)SNP_17331	489	G	A	10	5	--	--
	Ca(ICCV2/PI489777)SNP_17332	1086	A	G	3	9	--	--
	Ca(ICCV2/PI489777)SNP_17333	3150	A	T	17	10	--	--
	Ca(ICCV2/PI489777)SNP_17334	3183	A	C	18	9	--	--
	Ca(ICCV2/PI489777)SNP_17335	3198	T	C	18	10	--	--
	Ca(ICCV2/PI489777)SNP_17336	3408	G	A	6	5	--	--
	Ca(ICCV2/PI489777)SNP_17337	3550	A	T	10	3	--	--
CakTC24151	Ca(ICCV2/PI489777)SNP_17338	152	C	T	26	4	Shoot	--
	Ca(ICCV2/PI489777)SNP_17339	189	T	C	29	4	Shoot	--
	Ca(ICCV2/PI489777)SNP_17340	251	T	G	26	4	Shoot	--
	Ca(ICCV2/PI489777)SNP_17341	357	C	A	7	4	Shoot	--
CakTC23059	Ca(ICCV2/PI489777)SNP_17342	455	T	A	129	107	Young_pod	--
	Ca(ICCV2/PI489777)SNP_17343	947	C	T	107	77	Young_pod	--
CakTC13135	Ca(ICCV2/PI489777)SNP_17344	542	A	G	6	6	--	--
CakTC31167	Ca(ICCV2/PI489777)SNP_17345	1352	C	T	53	8	--	--
	Ca(ICCV2/PI489777)SNP_17346	1917	G	A	32	10	--	--
	Ca(ICCV2/PI489777)SNP_17347	2130	C	T	42	13	--	--
	Ca(ICCV2/PI489777)SNP_17348	2268	T	C	46	15	--	--
	Ca(ICCV2/PI489777)SNP_17349	2505	T	G	25	7	--	--
	Ca(ICCV2/PI489777)SNP_17350	2556	A	T	19	5	--	--
	Ca(ICCV2/PI489777)SNP_17351	2580	T	C	14	6	--	--
	Ca(ICCV2/PI489777)SNP_17352	2874	C	T	15	10	--	--
	Ca(ICCV2/PI489777)SNP_17353	2904	G	T	16	9	--	--
CakTC27602	Ca(ICCV2/PI489777)SNP_17354	150	T	A	5	3	Shoot	--
	Ca(ICCV2/PI489777)SNP_17355	222	A	T	5	4	Shoot	--
	Ca(ICCV2/PI489777)SNP_17356	387	T	C	5	8	Shoot	--
CakTC41559	Ca(ICCV2/PI489777)SNP_17357	74	C	G	11	4	--	--
CakTC11462	Ca(ICCV2/PI489777)SNP_17358	576	A	G	4	4	--	--
	Ca(ICCV2/PI489777)SNP_17359	972	C	G	6	3	--	--
	Ca(ICCV2/PI489777)SNP_17360	984	C	T	5	3	--	--
	Ca(ICCV2/PI489777)SNP_17361	1373	T	A	4	3	--	--
CakTC12500	Ca(ICCV2/PI489777)SNP_17362	34	T	C	33	5	--	--
	Ca(ICCV2/PI489777)SNP_17363	80	A	G	34	7	--	--
CakTC12163	Ca(ICCV2/PI489777)SNP_17364	235	A	G	15	4	--	--
	Ca(ICCV2/PI489777)SNP_17365	344	T	A	16	4	--	--
CakTC34350	Ca(ICCV2/PI489777)SNP_17366	281	G	A	8	9	--	--
	Ca(ICCV2/PI489777)SNP_17367	1866	G	T	6	3	--	--
	Ca(ICCV2/PI489777)SNP_17368	1898	T	C	8	3	--	--
	Ca(ICCV2/PI489777)SNP_17369	2831	C	T	13	3	--	--
	Ca(ICCV2/PI489777)SNP_17370	3284	C	T	28	8	--	--
	Ca(ICCV2/PI489777)SNP_17371	3767	T	C	14	6	--	--
	Ca(ICCV2/PI489777)SNP_17372	3768	C	A	14	6	--	--
	Ca(ICCV2/PI489777)SNP_17373	3854	C	T	16	6	--	--
	Ca(ICCV2/PI489777)SNP_17374	4050	T	C	18	3	--	--
	Ca(ICCV2/PI489777)SNP_17375	4454	G	A	16	7	--	--
	Ca(ICCV2/PI489777)SNP_17376	4565	A	G	20	5	--	--
	Ca(ICCV2/PI489777)SNP_17377	5229	A	C	19	4	--	--
	Ca(ICCV2/PI489777)SNP_17378	5342	A	T	18	4	--	--
CakTC00411	Ca(ICCV2/PI489777)SNP_17379	346	A	T	20	26	--	--
	Ca(ICCV2/PI489777)SNP_17380	352	A	G	22	26	--	--
	Ca(ICCV2/PI489777)SNP_17381	361	G	A	22	26	--	--
	Ca(ICCV2/PI489777)SNP_17382	400	A	G	24	27	--	--
	Ca(ICCV2/PI489777)SNP_17383	571	G	A	19	22	--	--
	Ca(ICCV2/PI489777)SNP_17384	676	C	T	18	17	--	--
CakTC34609	Ca(ICCV2/PI489777)SNP_17385	972	T	C	8	7	--	--

	Ca(ICCV2/PI489777)SNP_17386	1795	T	C	5	8	--	--
CakTC39503	Ca(ICCV2/PI489777)SNP_17387	1179	T	C	3	4	--	--
CakTC23984	Ca(ICCV2/PI489777)SNP_17388	989	T	C	38	19	--	--
CakTC14532	Ca(ICCV2/PI489777)SNP_17389	480	T	C	11	9	--	--
	Ca(ICCV2/PI489777)SNP_17390	552	T	C	13	9	--	--
	Ca(ICCV2/PI489777)SNP_17391	585	C	T	11	8	--	--
	Ca(ICCV2/PI489777)SNP_17392	881	T	C	16	9	--	--
	Ca(ICCV2/PI489777)SNP_17393	1139	C	T	16	19	--	--
CakTC43046	Ca(ICCV2/PI489777)SNP_17394	485	C	G	3	3	--	--
	Ca(ICCV2/PI489777)SNP_17395	534	A	G	9	3	--	--
CakTC29409	Ca(ICCV2/PI489777)SNP_17396	1803	G	A	5	4	--	--
CakTC32001	Ca(ICCV2/PI489777)SNP_17397	80	T	C	10	16	--	--
	Ca(ICCV2/PI489777)SNP_17398	923	A	G	11	18	--	--
	Ca(ICCV2/PI489777)SNP_17399	2369	T	C	14	10	--	--
	Ca(ICCV2/PI489777)SNP_17400	3563	C	A	21	18	--	--
	Ca(ICCV2/PI489777)SNP_17401	4147	C	T	17	16	--	--
	Ca(ICCV2/PI489777)SNP_17402	4199	G	A	16	22	--	--
CakTC37486	Ca(ICCV2/PI489777)SNP_17403	337	C	T	10	10	--	--
CakTC10656	Ca(ICCV2/PI489777)SNP_17404	453	T	C	4	4	--	bHLH
	Ca(ICCV2/PI489777)SNP_17405	456	C	T	4	4	--	bHLH
	Ca(ICCV2/PI489777)SNP_17406	597	C	A	3	3	--	bHLH
	Ca(ICCV2/PI489777)SNP_17407	1494	C	T	8	4	--	bHLH
	Ca(ICCV2/PI489777)SNP_17408	1683	T	G	9	5	--	bHLH
CakTC09922	Ca(ICCV2/PI489777)SNP_17409	560	T	C	5	4	--	--
CakTC27663	Ca(ICCV2/PI489777)SNP_17410	1759	G	A	9	3	--	--
CakTC29332	Ca(ICCV2/PI489777)SNP_17411	1116	T	C	3	4	--	--
	Ca(ICCV2/PI489777)SNP_17412	1719	G	A	4	3	--	--
CakTC31451	Ca(ICCV2/PI489777)SNP_17413	1053	G	C	12	4	--	--
CakTC29179	Ca(ICCV2/PI489777)SNP_17414	233	A	G	20	12	--	--
CakTC07582	Ca(ICCV2/PI489777)SNP_17415	171	T	C	4	7	--	--
CakTC38826	Ca(ICCV2/PI489777)SNP_17416	149	C	T	51	13	--	--
	Ca(ICCV2/PI489777)SNP_17417	903	G	A	91	58	--	--
	Ca(ICCV2/PI489777)SNP_17418	982	T	C	98	57	--	--
	Ca(ICCV2/PI489777)SNP_17419	1002	G	C	101	59	--	--
CakTC35082	Ca(ICCV2/PI489777)SNP_17420	2107	A	T	11	3	--	mTERF
CakTC26279	Ca(ICCV2/PI489777)SNP_17421	317	C	T	10	9	--	SET
	Ca(ICCV2/PI489777)SNP_17422	677	C	T	22	7	--	SET
	Ca(ICCV2/PI489777)SNP_17423	800	G	A	11	4	--	SET
	Ca(ICCV2/PI489777)SNP_17424	806	C	T	12	4	--	SET
	Ca(ICCV2/PI489777)SNP_17425	1346	T	A	12	5	--	SET
	Ca(ICCV2/PI489777)SNP_17426	1421	C	T	18	6	--	SET
	Ca(ICCV2/PI489777)SNP_17427	1462	G	A	18	5	--	SET
	Ca(ICCV2/PI489777)SNP_17428	1472	C	G	18	5	--	SET
	Ca(ICCV2/PI489777)SNP_17429	1588	A	G	15	3	--	SET
	Ca(ICCV2/PI489777)SNP_17430	1589	G	A	14	3	--	SET
CakTC29586	Ca(ICCV2/PI489777)SNP_17431	392	A	G	9	4	--	--
CakTC11031	Ca(ICCV2/PI489777)SNP_17432	262	T	G	6	8	--	--
CakTC40257	Ca(ICCV2/PI489777)SNP_17433	115	C	T	10	3	--	--
	Ca(ICCV2/PI489777)SNP_17434	146	G	C	8	3	--	--
	Ca(ICCV2/PI489777)SNP_17435	933	C	T	10	11	--	--
	Ca(ICCV2/PI489777)SNP_17436	1095	G	A	20	7	--	--
	Ca(ICCV2/PI489777)SNP_17437	1194	T	A	25	9	--	--
	Ca(ICCV2/PI489777)SNP_17438	1217	G	A	28	8	--	--
	Ca(ICCV2/PI489777)SNP_17439	1381	A	T	15	6	--	--
	Ca(ICCV2/PI489777)SNP_17440	1398	C	G	12	5	--	--
	Ca(ICCV2/PI489777)SNP_17441	1540	G	A	4	3	--	--
CakTC29169	Ca(ICCV2/PI489777)SNP_17442	1018	A	G	5	3	--	--
	Ca(ICCV2/PI489777)SNP_17443	1464	A	G	4	4	--	--
CakTC27181	Ca(ICCV2/PI489777)SNP_17444	99	T	A	4	3	--	--
	Ca(ICCV2/PI489777)SNP_17445	123	T	G	4	3	--	--
	Ca(ICCV2/PI489777)SNP_17446	339	A	G	6	4	--	--
CakTC15150	Ca(ICCV2/PI489777)SNP_17447	358	T	C	6	3	Shoot	--
CakTC37990	Ca(ICCV2/PI489777)SNP_17448	458	G	A	10	4	--	--
	Ca(ICCV2/PI489777)SNP_17449	973	A	T	13	8	--	--
	Ca(ICCV2/PI489777)SNP_17450	1006	A	G	15	12	--	--
CakTC09183	Ca(ICCV2/PI489777)SNP_17451	708	A	G	7	3	--	--
	Ca(ICCV2/PI489777)SNP_17452	813	C	T	5	4	--	--
	Ca(ICCV2/PI489777)SNP_17453	907	A	G	5	4	--	--
	Ca(ICCV2/PI489777)SNP_17454	1062	A	G	3	3	--	--
	Ca(ICCV2/PI489777)SNP_17455	1077	A	G	3	3	--	--
CakTC38194	Ca(ICCV2/PI489777)SNP_17456	870	A	G	7	6	--	--
CakTC02065	Ca(ICCV2/PI489777)SNP_17457	199	C	T	3	3	--	--
CakTC29628	Ca(ICCV2/PI489777)SNP_17458	468	T	A	3	9	--	--
	Ca(ICCV2/PI489777)SNP_17459	565	C	T	3	9	--	--

	Ca(ICCV2/PI489777)SNP_17460	1160	C	G	7	7	--	--
	Ca(ICCV2/PI489777)SNP_17461	1205	T	C	5	5	--	--
	Ca(ICCV2/PI489777)SNP_17462	1217	G	A	3	4	--	--
	Ca(ICCV2/PI489777)SNP_17463	1219	A	C	4	4	--	--
CakTC41478	Ca(ICCV2/PI489777)SNP_17464	1185	G	C	22	11	--	--
	Ca(ICCV2/PI489777)SNP_17465	1963	T	A	8	4	--	--
	Ca(ICCV2/PI489777)SNP_17466	2335	A	T	7	10	--	--
CakTC22897	Ca(ICCV2/PI489777)SNP_17467	1626	A	T	6	7	--	--
	Ca(ICCV2/PI489777)SNP_17468	2182	A	G	12	6	--	--
	Ca(ICCV2/PI489777)SNP_17469	2371	G	A	9	5	--	--
	Ca(ICCV2/PI489777)SNP_17470	2973	A	G	5	3	--	--
	Ca(ICCV2/PI489777)SNP_17471	3346	C	G	5	3	--	--
	Ca(ICCV2/PI489777)SNP_17472	3440	A	T	5	4	--	--
CakTC24841	Ca(ICCV2/PI489777)SNP_17473	71	A	G	70	55	--	--
	Ca(ICCV2/PI489777)SNP_17474	87	G	T	103	80	--	--
CakTC40657	Ca(ICCV2/PI489777)SNP_17475	510	G	A	6	19	--	--
CakTC26016	Ca(ICCV2/PI489777)SNP_17476	368	A	T	3	3	Shoot	--
CakTC32802	Ca(ICCV2/PI489777)SNP_17477	444	A	G	3	6	--	--
CakTC25576	Ca(ICCV2/PI489777)SNP_17478	1308	C	T	4	3	--	--
CakTC32563	Ca(ICCV2/PI489777)SNP_17479	84	A	G	6	6	--	--
	Ca(ICCV2/PI489777)SNP_17480	87	T	A	6	5	--	--
	Ca(ICCV2/PI489777)SNP_17481	182	G	T	9	6	--	--
	Ca(ICCV2/PI489777)SNP_17482	195	T	C	9	6	--	--
	Ca(ICCV2/PI489777)SNP_17483	311	C	T	9	5	--	--
CakTC27332	Ca(ICCV2/PI489777)SNP_17484	513	A	C	26	3	--	--
CakTC10332	Ca(ICCV2/PI489777)SNP_17485	1716	G	A	12	3	--	--
	Ca(ICCV2/PI489777)SNP_17486	1721	C	T	13	3	--	--
CakTC24697	Ca(ICCV2/PI489777)SNP_17487	1277	C	T	4	6	--	--
CakTC33324	Ca(ICCV2/PI489777)SNP_17488	379	T	C	3	3	--	--
CakTC42875	Ca(ICCV2/PI489777)SNP_17489	622	C	T	15	23	--	--
	Ca(ICCV2/PI489777)SNP_17490	692	C	T	14	23	--	--
	Ca(ICCV2/PI489777)SNP_17491	702	A	T	13	22	--	--
CakTC08355	Ca(ICCV2/PI489777)SNP_17492	402	A	C	9	4	--	--
	Ca(ICCV2/PI489777)SNP_17493	516	C	A	3	3	--	--
	Ca(ICCV2/PI489777)SNP_17494	531	G	C	3	3	--	--
	Ca(ICCV2/PI489777)SNP_17495	600	T	C	5	3	--	--
	Ca(ICCV2/PI489777)SNP_17496	870	T	G	4	3	--	--
	Ca(ICCV2/PI489777)SNP_17497	1040	C	T	8	4	--	--
CakTC33851	Ca(ICCV2/PI489777)SNP_17498	297	G	A	3	4	--	--
	Ca(ICCV2/PI489777)SNP_17499	393	C	T	4	6	--	--
	Ca(ICCV2/PI489777)SNP_17500	490	T	C	8	6	--	--
	Ca(ICCV2/PI489777)SNP_17501	492	C	T	9	6	--	--
	Ca(ICCV2/PI489777)SNP_17502	787	A	T	6	10	--	--
	Ca(ICCV2/PI489777)SNP_17503	1024	T	C	14	12	--	--
	Ca(ICCV2/PI489777)SNP_17504	1747	A	C	19	3	--	--
	Ca(ICCV2/PI489777)SNP_17505	2038	T	C	16	17	--	--
CakTC23472	Ca(ICCV2/PI489777)SNP_17506	177	C	G	5	9	--	--
CakTC28186	Ca(ICCV2/PI489777)SNP_17507	650	G	T	15	8	--	--
	Ca(ICCV2/PI489777)SNP_17508	752	G	A	10	5	--	--
CakTC23685	Ca(ICCV2/PI489777)SNP_17509	298	T	C	4	9	--	--
	Ca(ICCV2/PI489777)SNP_17510	1402	T	C	12	17	--	--
	Ca(ICCV2/PI489777)SNP_17511	1982	A	T	18	12	--	--
	Ca(ICCV2/PI489777)SNP_17512	2146	A	C	13	10	--	--
CakTC34590	Ca(ICCV2/PI489777)SNP_17513	347	A	C	24	14	--	--
CakTC41610	Ca(ICCV2/PI489777)SNP_17514	468	T	C	8	3	--	--
CakTC06402	Ca(ICCV2/PI489777)SNP_17515	430	A	G	4	3	--	--
CakTC15953	Ca(ICCV2/PI489777)SNP_17516	692	G	A	3	3	--	--
CakTC41471	Ca(ICCV2/PI489777)SNP_17517	562	A	G	3	8	--	--
CakTC14390	Ca(ICCV2/PI489777)SNP_17518	272	T	A	6	3	--	--
	Ca(ICCV2/PI489777)SNP_17519	376	A	T	7	3	--	--
	Ca(ICCV2/PI489777)SNP_17520	422	A	G	8	3	--	--
	Ca(ICCV2/PI489777)SNP_17521	448	C	T	8	3	--	--
	Ca(ICCV2/PI489777)SNP_17522	457	C	T	8	3	--	--
CakTC37515	Ca(ICCV2/PI489777)SNP_17523	899	A	C	11	8	--	--
	Ca(ICCV2/PI489777)SNP_17524	959	T	A	13	7	--	--
CakTC27726	Ca(ICCV2/PI489777)SNP_17525	301	G	T	3	6	--	--
	Ca(ICCV2/PI489777)SNP_17526	305	C	T	3	5	--	--
CakTC28978	Ca(ICCV2/PI489777)SNP_17527	785	C	G	12	10	--	--
	Ca(ICCV2/PI489777)SNP_17528	992	C	T	14	7	--	--
	Ca(ICCV2/PI489777)SNP_17529	1154	C	T	9	7	--	--
	Ca(ICCV2/PI489777)SNP_17530	1391	C	G	13	9	--	--
	Ca(ICCV2/PI489777)SNP_17531	1541	C	T	14	7	--	--
	Ca(ICCV2/PI489777)SNP_17532	1862	A	T	14	4	--	--
CakTC37841	Ca(ICCV2/PI489777)SNP_17533	525	C	T	13	4	--	--

	Ca(ICCV2/PI489777)SNP_17534	620	A	G	16	5	--	--
	Ca(ICCV2/PI489777)SNP_17535	640	G	A	15	6	--	--
	Ca(ICCV2/PI489777)SNP_17536	833	G	A	12	7	--	--
	Ca(ICCV2/PI489777)SNP_17537	884	G	C	13	8	--	--
	Ca(ICCV2/PI489777)SNP_17538	921	G	A	12	8	--	--
CakTC27345	Ca(ICCV2/PI489777)SNP_17539	1804	A	C	4	7	--	--
	Ca(ICCV2/PI489777)SNP_17540	1897	A	C	8	12	--	--
	Ca(ICCV2/PI489777)SNP_17541	5799	T	C	7	6	--	--
	Ca(ICCV2/PI489777)SNP_17542	6015	G	A	18	11	--	--
	Ca(ICCV2/PI489777)SNP_17543	6876	G	A	9	3	--	--
	Ca(ICCV2/PI489777)SNP_17544	7565	A	C	13	6	--	--
	Ca(ICCV2/PI489777)SNP_17545	8145	A	G	11	4	--	--
	Ca(ICCV2/PI489777)SNP_17546	9147	C	T	20	6	--	--
	Ca(ICCV2/PI489777)SNP_17547	10225	T	C	10	3	--	--
	Ca(ICCV2/PI489777)SNP_17548	10467	T	C	21	10	--	--
CakTC11699	Ca(ICCV2/PI489777)SNP_17549	520	T	C	12	20	--	--
	Ca(ICCV2/PI489777)SNP_17550	1066	C	T	23	21	--	--
CakTC13154	Ca(ICCV2/PI489777)SNP_17551	369	A	G	57	24	--	--
	Ca(ICCV2/PI489777)SNP_17552	684	A	G	9	3	--	--
	Ca(ICCV2/PI489777)SNP_17553	710	T	C	9	3	--	--
	Ca(ICCV2/PI489777)SNP_17554	711	G	A	9	3	--	--
	Ca(ICCV2/PI489777)SNP_17555	1183	T	A	169	88	--	--
CakTC10374	Ca(ICCV2/PI489777)SNP_17556	291	G	A	17	4	--	--
	Ca(ICCV2/PI489777)SNP_17557	320	A	T	16	5	--	--
	Ca(ICCV2/PI489777)SNP_17558	512	T	C	15	10	--	--
CakTC24799	Ca(ICCV2/PI489777)SNP_17559	287	T	C	3	4	--	--
CakTC34116	Ca(ICCV2/PI489777)SNP_17560	361	C	T	6	3	--	--
	Ca(ICCV2/PI489777)SNP_17561	682	C	T	4	4	--	--
	Ca(ICCV2/PI489777)SNP_17562	1644	T	C	7	5	--	--
CakTC29981	Ca(ICCV2/PI489777)SNP_17563	903	T	C	18	3	--	--
	Ca(ICCV2/PI489777)SNP_17564	1360	C	A	12	7	--	--
	Ca(ICCV2/PI489777)SNP_17565	1381	T	C	12	8	--	--
	Ca(ICCV2/PI489777)SNP_17566	1589	T	A	12	3	--	--
	Ca(ICCV2/PI489777)SNP_17567	2275	G	A	11	8	--	--
	Ca(ICCV2/PI489777)SNP_17568	2314	C	T	11	12	--	--
CakTC19409	Ca(ICCV2/PI489777)SNP_17569	358	T	C	5	12	--	--
	Ca(ICCV2/PI489777)SNP_17570	512	G	A	6	9	--	--
	Ca(ICCV2/PI489777)SNP_17571	627	C	G	3	8	--	--
CakTC39037	Ca(ICCV2/PI489777)SNP_17572	115	A	T	8	4	--	--
	Ca(ICCV2/PI489777)SNP_17573	135	G	A	10	4	--	--
	Ca(ICCV2/PI489777)SNP_17574	173	G	C	11	4	--	--
	Ca(ICCV2/PI489777)SNP_17575	178	A	G	13	4	--	--
	Ca(ICCV2/PI489777)SNP_17576	225	T	C	13	4	--	--
	Ca(ICCV2/PI489777)SNP_17577	309	C	A	16	8	--	--
	Ca(ICCV2/PI489777)SNP_17578	468	T	C	5	7	--	--
	Ca(ICCV2/PI489777)SNP_17579	480	C	T	9	8	--	--
	Ca(ICCV2/PI489777)SNP_17580	528	C	T	7	11	--	--
	Ca(ICCV2/PI489777)SNP_17581	586	C	A	3	11	--	--
	Ca(ICCV2/PI489777)SNP_17582	846	A	G	6	11	--	--
	Ca(ICCV2/PI489777)SNP_17583	876	A	G	6	12	--	--
	Ca(ICCV2/PI489777)SNP_17584	879	A	G	6	11	--	--
	Ca(ICCV2/PI489777)SNP_17585	1038	T	G	7	6	--	--
	Ca(ICCV2/PI489777)SNP_17586	1074	C	A	6	5	--	--
	Ca(ICCV2/PI489777)SNP_17587	1158	C	G	8	5	--	--
CakTC34263	Ca(ICCV2/PI489777)SNP_17588	554	A	G	25	5	Root	CCAAT
CakTC28437	Ca(ICCV2/PI489777)SNP_17589	1331	T	C	8	10	--	--
	Ca(ICCV2/PI489777)SNP_17590	1335	C	T	9	12	--	--
CakTC42663	Ca(ICCV2/PI489777)SNP_17591	477	T	G	6	4	--	--
CakTC33629	Ca(ICCV2/PI489777)SNP_17592	274	G	C	4	3	--	--
CakTC11595	Ca(ICCV2/PI489777)SNP_17593	626	T	C	75	38	--	--
	Ca(ICCV2/PI489777)SNP_17594	677	C	A	73	39	--	--
CakTC41524	Ca(ICCV2/PI489777)SNP_17595	253	A	G	9	4	--	--
	Ca(ICCV2/PI489777)SNP_17596	660	G	T	12	7	--	--
	Ca(ICCV2/PI489777)SNP_17597	741	A	G	12	7	--	--
	Ca(ICCV2/PI489777)SNP_17598	789	T	C	12	11	--	--
CakTC41667	Ca(ICCV2/PI489777)SNP_17599	253	G	C	48	15	--	--
	Ca(ICCV2/PI489777)SNP_17600	256	A	G	47	17	--	--
	Ca(ICCV2/PI489777)SNP_17601	465	A	G	36	3	--	--
	Ca(ICCV2/PI489777)SNP_17602	476	G	C	35	3	--	--
	Ca(ICCV2/PI489777)SNP_17603	481	T	G	27	5	--	--
	Ca(ICCV2/PI489777)SNP_17604	570	C	G	26	3	--	--
CakTC26320	Ca(ICCV2/PI489777)SNP_17605	1788	G	A	9	14	--	--
CakTC29874	Ca(ICCV2/PI489777)SNP_17606	693	A	G	10	17	--	--
	Ca(ICCV2/PI489777)SNP_17607	1005	C	T	34	12	--	--

	Ca(ICCV2/PI489777)SNP_17608	1122	G	A	35	11	--	--
	Ca(ICCV2/PI489777)SNP_17609	1800	C	T	28	13	--	--
CakTC40027	Ca(ICCV2/PI489777)SNP_17610	88	C	T	25	14	--	--
CakTC34357	Ca(ICCV2/PI489777)SNP_17611	373	C	T	13	5	--	--
	Ca(ICCV2/PI489777)SNP_17612	826	A	T	8	8	--	--
	Ca(ICCV2/PI489777)SNP_17613	1287	T	C	7	9	--	--
	Ca(ICCV2/PI489777)SNP_17614	1819	G	A	13	4	--	--
CakTC27784	Ca(ICCV2/PI489777)SNP_17615	198	G	A	8	3	--	TPR
	Ca(ICCV2/PI489777)SNP_17616	1483	C	G	7	5	--	TPR
	Ca(ICCV2/PI489777)SNP_17617	2482	T	G	25	7	--	TPR
CakTC35706	Ca(ICCV2/PI489777)SNP_17618	487	A	G	5	8	--	--
CakTC41365	Ca(ICCV2/PI489777)SNP_17619	161	C	T	34	27	--	--
CakTC39856	Ca(ICCV2/PI489777)SNP_17620	114	C	G	10	10	--	--
	Ca(ICCV2/PI489777)SNP_17621	128	A	G	14	11	--	--
	Ca(ICCV2/PI489777)SNP_17622	149	C	A	13	10	--	--
	Ca(ICCV2/PI489777)SNP_17623	158	A	T	10	9	--	--
	Ca(ICCV2/PI489777)SNP_17624	221	G	A	17	10	--	--
	Ca(ICCV2/PI489777)SNP_17625	289	C	G	17	9	--	--
	Ca(ICCV2/PI489777)SNP_17626	487	C	T	8	7	--	--
	Ca(ICCV2/PI489777)SNP_17627	709	A	G	5	7	--	--
	Ca(ICCV2/PI489777)SNP_17628	1051	G	A	3	3	--	--
	Ca(ICCV2/PI489777)SNP_17629	1087	T	A	3	4	--	--
CakTC43368	Ca(ICCV2/PI489777)SNP_17630	282	G	A	8	3	--	--
	Ca(ICCV2/PI489777)SNP_17631	1107	G	A	15	4	--	--
	Ca(ICCV2/PI489777)SNP_17632	1110	A	G	15	4	--	--
	Ca(ICCV2/PI489777)SNP_17633	1158	T	C	10	3	--	--
CakTC38516	Ca(ICCV2/PI489777)SNP_17634	1433	T	C	74	104	--	--
CakTC29546	Ca(ICCV2/PI489777)SNP_17635	26	G	T	3	3	--	--
CakTC23722	Ca(ICCV2/PI489777)SNP_17636	531	T	G	6	3	--	--
CakTC30078	Ca(ICCV2/PI489777)SNP_17637	836	T	C	9	5	Mature	Leaf
	Ca(ICCV2/PI489777)SNP_17638	923	T	A	6	3	Mature	Leaf
	Ca(ICCV2/PI489777)SNP_17639	1016	T	C	4	5	Mature	Leaf
CakTC42816	Ca(ICCV2/PI489777)SNP_17640	125	C	T	7	3	--	--
	Ca(ICCV2/PI489777)SNP_17641	149	T	C	9	3	--	--
	Ca(ICCV2/PI489777)SNP_17642	180	T	C	7	3	--	--
	Ca(ICCV2/PI489777)SNP_17643	245	A	G	9	3	--	--
	Ca(ICCV2/PI489777)SNP_17644	302	A	G	10	3	--	--
	Ca(ICCV2/PI489777)SNP_17645	319	C	G	11	3	--	--
	Ca(ICCV2/PI489777)SNP_17646	349	A	G	13	3	--	--
	Ca(ICCV2/PI489777)SNP_17647	1531	G	C	3	11	--	--
	Ca(ICCV2/PI489777)SNP_17648	1544	T	C	4	10	--	--
	Ca(ICCV2/PI489777)SNP_17649	1613	A	G	5	14	--	--
	Ca(ICCV2/PI489777)SNP_17650	1761	A	C	4	13	--	--
CakTC36094	Ca(ICCV2/PI489777)SNP_17651	454	C	G	13	9	--	--
	Ca(ICCV2/PI489777)SNP_17652	485	G	A	8	10	--	--
	Ca(ICCV2/PI489777)SNP_17653	645	T	C	11	8	--	--
	Ca(ICCV2/PI489777)SNP_17654	773	A	G	7	3	--	--
	Ca(ICCV2/PI489777)SNP_17655	1031	C	T	4	4	--	--
	Ca(ICCV2/PI489777)SNP_17656	1284	G	T	8	8	--	--
	Ca(ICCV2/PI489777)SNP_17657	1607	C	T	5	5	--	--
	Ca(ICCV2/PI489777)SNP_17658	1680	A	G	4	6	--	--
	Ca(ICCV2/PI489777)SNP_17659	1750	G	T	9	5	--	--
	Ca(ICCV2/PI489777)SNP_17660	1844	G	A	8	7	--	--
	Ca(ICCV2/PI489777)SNP_17661	1930	T	C	10	3	--	--
	Ca(ICCV2/PI489777)SNP_17662	2088	A	G	7	13	--	--
	Ca(ICCV2/PI489777)SNP_17663	2225	C	A	7	13	--	--
CakTC41928	Ca(ICCV2/PI489777)SNP_17664	446	T	C	3	5	--	--
CakTC40997	Ca(ICCV2/PI489777)SNP_17665	528	C	G	12	3	--	AP2-EREBP
	Ca(ICCV2/PI489777)SNP_17666	579	A	T	16	4	--	AP2-EREBP
	Ca(ICCV2/PI489777)SNP_17667	585	T	A	10	5	--	AP2-EREBP
	Ca(ICCV2/PI489777)SNP_17668	850	C	T	14	7	--	AP2-EREBP
	Ca(ICCV2/PI489777)SNP_17669	1030	G	A	6	5	--	AP2-EREBP
CakTC40359	Ca(ICCV2/PI489777)SNP_17670	262	G	T	8	16	--	--
	Ca(ICCV2/PI489777)SNP_17671	305	C	G	8	31	--	--
	Ca(ICCV2/PI489777)SNP_17672	353	A	G	33	45	--	--
	Ca(ICCV2/PI489777)SNP_17673	626	A	G	51	45	--	--
CakTC27186	Ca(ICCV2/PI489777)SNP_17674	943	A	G	5	3	--	--
	Ca(ICCV2/PI489777)SNP_17675	1301	A	G	4	5	--	--
CakTC27920	Ca(ICCV2/PI489777)SNP_17676	278	C	T	32	26	--	--
	Ca(ICCV2/PI489777)SNP_17677	279	A	T	32	26	--	--
	Ca(ICCV2/PI489777)SNP_17678	1178	G	T	25	20	--	--
	Ca(ICCV2/PI489777)SNP_17679	1600	A	C	38	23	--	--
	Ca(ICCV2/PI489777)SNP_17680	1612	G	A	35	23	--	--
	Ca(ICCV2/PI489777)SNP_17681	2110	A	G	33	18	--	--

	Ca(ICCV2/PI489777)SNP_17682	2596	G	A	36	21	--	--
CakTC24958	Ca(ICCV2/PI489777)SNP_17683	273	G	C	115	41	--	--
CakTC42513	Ca(ICCV2/PI489777)SNP_17684	56	G	C	15	4	--	--
	Ca(ICCV2/PI489777)SNP_17685	187	C	A	23	6	--	--
	Ca(ICCV2/PI489777)SNP_17686	478	C	T	25	8	--	--
	Ca(ICCV2/PI489777)SNP_17687	517	G	A	23	10	--	--
	Ca(ICCV2/PI489777)SNP_17688	748	A	G	36	11	--	--
	Ca(ICCV2/PI489777)SNP_17689	782	A	G	36	13	--	--
	Ca(ICCV2/PI489777)SNP_17690	1000	C	T	34	11	--	--
CakTC22311	Ca(ICCV2/PI489777)SNP_17691	215	A	G	7	4	--	--
	Ca(ICCV2/PI489777)SNP_17692	727	T	G	8	10	--	--
	Ca(ICCV2/PI489777)SNP_17693	753	C	G	9	10	--	--
CakTC38111	Ca(ICCV2/PI489777)SNP_17694	411	A	C	16	8	--	--
CakTC04213	Ca(ICCV2/PI489777)SNP_17695	22	T	C	5	5	--	--
CakTC25723	Ca(ICCV2/PI489777)SNP_17696	169	G	A	14	5	--	--
CakTC38325	Ca(ICCV2/PI489777)SNP_17697	718	C	A	6	9	--	--
	Ca(ICCV2/PI489777)SNP_17698	916	A	G	5	7	--	--
	Ca(ICCV2/PI489777)SNP_17699	922	C	T	5	6	--	--
	Ca(ICCV2/PI489777)SNP_17700	970	G	A	3	4	--	--
CakTC25395	Ca(ICCV2/PI489777)SNP_17701	293	A	G	10	5	Young_pod	--
	Ca(ICCV2/PI489777)SNP_17702	524	C	G	7	8	Young_pod	--
	Ca(ICCV2/PI489777)SNP_17703	764	A	G	6	8	Young_pod	--
CakTC22362	Ca(ICCV2/PI489777)SNP_17704	195	G	A	25	55	--	--
	Ca(ICCV2/PI489777)SNP_17705	410	C	T	117	135	--	--
	Ca(ICCV2/PI489777)SNP_17706	518	T	A	219	150	--	--
	Ca(ICCV2/PI489777)SNP_17707	1410	T	C	229	146	--	--
	Ca(ICCV2/PI489777)SNP_17708	1452	T	G	243	133	--	--
	Ca(ICCV2/PI489777)SNP_17709	2664	T	C	9	3	--	--
	Ca(ICCV2/PI489777)SNP_17710	2960	A	G	9	5	--	--
CakTC23174	Ca(ICCV2/PI489777)SNP_17711	1233	T	C	6	13	--	--
	Ca(ICCV2/PI489777)SNP_17712	1301	A	C	6	16	--	--
	Ca(ICCV2/PI489777)SNP_17713	3369	A	G	3	8	--	--
	Ca(ICCV2/PI489777)SNP_17714	3576	T	C	9	7	--	--
	Ca(ICCV2/PI489777)SNP_17715	3972	C	T	3	5	--	--
CakTC36068	Ca(ICCV2/PI489777)SNP_17716	390	C	T	7	4	--	--
	Ca(ICCV2/PI489777)SNP_17717	537	A	G	8	6	--	--
	Ca(ICCV2/PI489777)SNP_17718	541	T	C	8	10	--	--
	Ca(ICCV2/PI489777)SNP_17719	634	T	C	8	11	--	--
	Ca(ICCV2/PI489777)SNP_17720	1009	G	A	9	5	--	--
	Ca(ICCV2/PI489777)SNP_17721	1027	T	C	8	4	--	--
	Ca(ICCV2/PI489777)SNP_17722	1342	T	G	7	4	--	--
	Ca(ICCV2/PI489777)SNP_17723	1600	A	G	17	8	--	--
	Ca(ICCV2/PI489777)SNP_17724	2146	A	G	8	12	--	--
	Ca(ICCV2/PI489777)SNP_17725	2339	G	T	6	8	--	--
	Ca(ICCV2/PI489777)SNP_17726	2461	T	C	3	6	--	--
CakTC42360	Ca(ICCV2/PI489777)SNP_17727	103	G	C	17	17	--	--
	Ca(ICCV2/PI489777)SNP_17728	156	A	T	18	17	--	--
	Ca(ICCV2/PI489777)SNP_17729	159	A	T	18	17	--	--
	Ca(ICCV2/PI489777)SNP_17730	456	C	T	13	13	--	--
	Ca(ICCV2/PI489777)SNP_17731	543	T	C	10	10	--	--
	Ca(ICCV2/PI489777)SNP_17732	642	G	A	12	7	--	--
CakTC30846	Ca(ICCV2/PI489777)SNP_17733	86	C	T	6	3	--	--
	Ca(ICCV2/PI489777)SNP_17734	143	C	A	6	5	--	--
	Ca(ICCV2/PI489777)SNP_17735	179	T	C	5	3	--	--
	Ca(ICCV2/PI489777)SNP_17736	332	T	G	7	5	--	--
CakTC34509	Ca(ICCV2/PI489777)SNP_17737	201	G	A	42	34	--	--
CakTC12972	Ca(ICCV2/PI489777)SNP_17738	1807	T	C	4	8	--	--
CakTC39047	Ca(ICCV2/PI489777)SNP_17739	445	T	G	15	8	--	--
CakTC40028	Ca(ICCV2/PI489777)SNP_17740	387	G	T	11	7	--	--
	Ca(ICCV2/PI489777)SNP_17741	732	G	A	11	3	--	--
	Ca(ICCV2/PI489777)SNP_17742	952	C	T	11	4	--	--
	Ca(ICCV2/PI489777)SNP_17743	1063	T	G	14	7	--	--
	Ca(ICCV2/PI489777)SNP_17744	1358	A	G	8	5	--	--
CakTC38264	Ca(ICCV2/PI489777)SNP_17745	378	A	G	12	6	--	--
	Ca(ICCV2/PI489777)SNP_17746	433	A	G	14	6	--	--
	Ca(ICCV2/PI489777)SNP_17747	525	C	T	14	6	--	--
	Ca(ICCV2/PI489777)SNP_17748	567	T	C	10	5	--	--
	Ca(ICCV2/PI489777)SNP_17749	1098	T	C	4	3	--	--
	Ca(ICCV2/PI489777)SNP_17750	1123	G	A	4	4	--	--
	Ca(ICCV2/PI489777)SNP_17751	1225	T	A	5	3	--	--
	Ca(ICCV2/PI489777)SNP_17752	1275	T	C	5	3	--	--
	Ca(ICCV2/PI489777)SNP_17753	1285	C	G	5	3	--	--
CakTC40448	Ca(ICCV2/PI489777)SNP_17754	644	T	G	17	13	Root	--
CakTC38574	Ca(ICCV2/PI489777)SNP_17755	167	A	G	31	9	--	--

	Ca(ICCV2/PI489777)SNP_17756	554	G	A	18	10	--	--
	Ca(ICCV2/PI489777)SNP_17757	814	A	G	21	6	--	--
CakTC27457	Ca(ICCV2/PI489777)SNP_17758	577	G	A	8	9	--	GNAT
	Ca(ICCV2/PI489777)SNP_17759	712	T	A	10	8	--	GNAT
	Ca(ICCV2/PI489777)SNP_17760	739	T	C	10	8	--	GNAT
	Ca(ICCV2/PI489777)SNP_17761	769	T	C	10	8	--	GNAT
	Ca(ICCV2/PI489777)SNP_17762	776	T	C	9	8	--	GNAT
CakTC12698	Ca(ICCV2/PI489777)SNP_17763	612	T	C	10	7	--	--
	Ca(ICCV2/PI489777)SNP_17764	947	A	G	9	6	--	--
	Ca(ICCV2/PI489777)SNP_17765	1083	A	G	11	7	--	--
	Ca(ICCV2/PI489777)SNP_17766	1090	G	A	13	6	--	--
	Ca(ICCV2/PI489777)SNP_17767	1250	G	A	18	8	--	--
	Ca(ICCV2/PI489777)SNP_17768	1904	A	G	21	8	--	--
	Ca(ICCV2/PI489777)SNP_17769	2339	T	C	8	3	--	--
CakTC40232	Ca(ICCV2/PI489777)SNP_17770	270	T	C	17	8	--	--
	Ca(ICCV2/PI489777)SNP_17771	303	C	T	19	10	--	--
	Ca(ICCV2/PI489777)SNP_17772	318	C	A	19	11	--	--
	Ca(ICCV2/PI489777)SNP_17773	453	C	A	14	11	--	--
	Ca(ICCV2/PI489777)SNP_17774	590	G	A	9	10	--	--
	Ca(ICCV2/PI489777)SNP_17775	678	C	G	8	8	--	--
	Ca(ICCV2/PI489777)SNP_17776	1106	C	T	9	5	--	--
CakTC26187	Ca(ICCV2/PI489777)SNP_17777	255	C	A	23	16	--	bZIP
	Ca(ICCV2/PI489777)SNP_17778	1143	C	T	11	13	--	bZIP
CakTC37817	Ca(ICCV2/PI489777)SNP_17779	82	T	A	38	40	--	--
	Ca(ICCV2/PI489777)SNP_17780	302	G	T	67	58	--	--
CakTC27884	Ca(ICCV2/PI489777)SNP_17781	313	G	T	3	4	Flower	bud
	Ca(ICCV2/PI489777)SNP_17782	662	T	G	5	5	Flower	bud
	Ca(ICCV2/PI489777)SNP_17783	684	G	C	5	5	Flower	bud
	Ca(ICCV2/PI489777)SNP_17784	1226	T	C	4	3	Flower	bud
	Ca(ICCV2/PI489777)SNP_17785	4131	T	C	5	4	Flower	bud
CakTC02598	Ca(ICCV2/PI489777)SNP_17786	304	A	G	9	6	--	--
	Ca(ICCV2/PI489777)SNP_17787	1481	T	G	12	7	--	--
CakTC36652	Ca(ICCV2/PI489777)SNP_17788	343	G	A	4	6	--	ARF
	Ca(ICCV2/PI489777)SNP_17789	397	T	C	4	6	--	ARF
CakTC08772	Ca(ICCV2/PI489777)SNP_17790	2447	G	A	3	7	--	--
CakTC41436	Ca(ICCV2/PI489777)SNP_17791	1943	T	G	8	3	--	--
CakTC10887	Ca(ICCV2/PI489777)SNP_17792	293	T	C	10	8	--	--
	Ca(ICCV2/PI489777)SNP_17793	311	T	C	15	10	--	--
	Ca(ICCV2/PI489777)SNP_17794	470	A	G	15	12	--	--
	Ca(ICCV2/PI489777)SNP_17795	492	T	C	15	11	--	--
	Ca(ICCV2/PI489777)SNP_17796	509	A	G	16	12	--	--
	Ca(ICCV2/PI489777)SNP_17797	517	C	G	18	12	--	--
	Ca(ICCV2/PI489777)SNP_17798	519	G	C	18	12	--	--
	Ca(ICCV2/PI489777)SNP_17799	548	A	G	19	10	--	--
	Ca(ICCV2/PI489777)SNP_17800	560	C	T	18	11	--	--
	Ca(ICCV2/PI489777)SNP_17801	725	A	T	11	7	--	--
	Ca(ICCV2/PI489777)SNP_17802	948	A	T	10	13	--	--
	Ca(ICCV2/PI489777)SNP_17803	1010	A	G	10	13	--	--
	Ca(ICCV2/PI489777)SNP_17804	1060	C	T	7	13	--	--
	Ca(ICCV2/PI489777)SNP_17805	1181	T	A	6	5	--	--
CakTC42296	Ca(ICCV2/PI489777)SNP_17806	806	A	G	12	5	--	--
	Ca(ICCV2/PI489777)SNP_17807	1134	T	C	12	32	--	--
	Ca(ICCV2/PI489777)SNP_17808	1610	T	A	10	3	--	--
CakTC32529	Ca(ICCV2/PI489777)SNP_17809	349	G	A	11	6	--	--
	Ca(ICCV2/PI489777)SNP_17810	523	T	A	8	5	--	--
	Ca(ICCV2/PI489777)SNP_17811	637	T	C	6	3	--	--
	Ca(ICCV2/PI489777)SNP_17812	1168	G	A	8	3	--	--
CakTC42562	Ca(ICCV2/PI489777)SNP_17813	866	G	A	30	26	--	--
CakTC07357	Ca(ICCV2/PI489777)SNP_17814	1511	A	G	6	4	--	--
	Ca(ICCV2/PI489777)SNP_17815	1566	G	T	7	5	--	--
	Ca(ICCV2/PI489777)SNP_17816	1613	A	G	6	4	--	--
CakTC29335	Ca(ICCV2/PI489777)SNP_17817	2392	T	C	9	4	--	--
	Ca(ICCV2/PI489777)SNP_17818	2400	A	G	9	4	--	--
	Ca(ICCV2/PI489777)SNP_17819	2417	G	A	9	4	--	--
	Ca(ICCV2/PI489777)SNP_17820	2452	T	C	8	6	--	--
	Ca(ICCV2/PI489777)SNP_17821	2490	A	G	8	6	--	--
	Ca(ICCV2/PI489777)SNP_17822	2492	G	A	8	6	--	--
	Ca(ICCV2/PI489777)SNP_17823	2522	T	C	11	6	--	--
	Ca(ICCV2/PI489777)SNP_17824	2626	G	A	9	6	--	--
	Ca(ICCV2/PI489777)SNP_17825	2640	G	A	9	6	--	--
	Ca(ICCV2/PI489777)SNP_17826	2655	A	T	8	6	--	--
	Ca(ICCV2/PI489777)SNP_17827	2673	G	T	8	7	--	--
	Ca(ICCV2/PI489777)SNP_17828	2705	G	A	7	7	--	--
	Ca(ICCV2/PI489777)SNP_17829	2774	G	T	5	3	--	--

	Ca(ICCV2/PI489777)SNP_17830	2798	G	C	3	3	--	--
	Ca(ICCV2/PI489777)SNP_17831	2834	A	G	3	3	--	--
	Ca(ICCV2/PI489777)SNP_17832	2838	A	G	3	3	--	--
	Ca(ICCV2/PI489777)SNP_17833	2870	T	C	3	5	--	--
	Ca(ICCV2/PI489777)SNP_17834	2871	G	A	3	5	--	--
	Ca(ICCV2/PI489777)SNP_17835	4891	T	G	5	4	--	--
	Ca(ICCV2/PI489777)SNP_17836	4893	T	A	5	4	--	--
	Ca(ICCV2/PI489777)SNP_17837	4920	A	G	4	5	--	--
	Ca(ICCV2/PI489777)SNP_17838	4990	T	C	4	5	--	--
CakTC34133	Ca(ICCV2/PI489777)SNP_17839	66	A	C	3	3	--	--
	Ca(ICCV2/PI489777)SNP_17840	269	G	A	41	27	--	--
CakTC31081	Ca(ICCV2/PI489777)SNP_17841	599	T	C	8	3	--	GRF
	Ca(ICCV2/PI489777)SNP_17842	668	A	G	10	3	--	GRF
	Ca(ICCV2/PI489777)SNP_17843	689	T	A	11	3	--	GRF
	Ca(ICCV2/PI489777)SNP_17844	692	T	A	12	3	--	GRF
CakTC23135	Ca(ICCV2/PI489777)SNP_17845	409	A	C	3	3	--	--
CakTC42466	Ca(ICCV2/PI489777)SNP_17846	561	G	T	16	6	--	--
CakTC32869	Ca(ICCV2/PI489777)SNP_17847	238	T	C	37	11	--	--
	Ca(ICCV2/PI489777)SNP_17848	295	G	A	36	12	--	--
CakTC29941	Ca(ICCV2/PI489777)SNP_17849	418	G	C	7	4	--	--
	Ca(ICCV2/PI489777)SNP_17850	2253	T	C	4	6	--	--
	Ca(ICCV2/PI489777)SNP_17851	3246	C	T	6	3	--	--
CakTC41625	Ca(ICCV2/PI489777)SNP_17852	219	A	G	27	25	--	--
	Ca(ICCV2/PI489777)SNP_17853	372	T	C	28	30	--	--
	Ca(ICCV2/PI489777)SNP_17854	516	T	C	34	21	--	--
CakTC11767	Ca(ICCV2/PI489777)SNP_17855	214	G	C	4	3	--	--
CakTC41533	Ca(ICCV2/PI489777)SNP_17856	713	A	G	6	4	--	--
	Ca(ICCV2/PI489777)SNP_17857	946	C	T	4	5	--	--
	Ca(ICCV2/PI489777)SNP_17858	1241	A	G	6	4	--	--
	Ca(ICCV2/PI489777)SNP_17859	2002	G	A	16	6	--	--
	Ca(ICCV2/PI489777)SNP_17860	2035	T	C	16	5	--	--
CakTC03506	Ca(ICCV2/PI489777)SNP_17861	509	C	T	4	7	--	--
CakTC30570	Ca(ICCV2/PI489777)SNP_17862	333	C	A	9	5	--	bZIP
	Ca(ICCV2/PI489777)SNP_17863	1005	C	T	9	5	--	bZIP
	Ca(ICCV2/PI489777)SNP_17864	1062	C	T	5	4	--	bZIP
CakTC11760	Ca(ICCV2/PI489777)SNP_17865	568	A	G	41	39	--	--
	Ca(ICCV2/PI489777)SNP_17866	1153	G	A	28	20	--	--
CakTC27139	Ca(ICCV2/PI489777)SNP_17867	15	T	C	4	6	--	--
	Ca(ICCV2/PI489777)SNP_17868	29	G	C	4	6	--	--
	Ca(ICCV2/PI489777)SNP_17869	351	C	T	54	32	--	--
	Ca(ICCV2/PI489777)SNP_17870	840	G	A	24	16	--	--
CakTC25215	Ca(ICCV2/PI489777)SNP_17871	559	A	G	8	15	--	--
	Ca(ICCV2/PI489777)SNP_17872	702	A	G	18	9	--	--
	Ca(ICCV2/PI489777)SNP_17873	1815	G	C	11	3	--	--
CakTC28619	Ca(ICCV2/PI489777)SNP_17874	202	A	G	14	12	--	HSF
	Ca(ICCV2/PI489777)SNP_17875	250	C	G	15	11	--	HSF
	Ca(ICCV2/PI489777)SNP_17876	322	A	G	13	14	--	HSF
	Ca(ICCV2/PI489777)SNP_17877	698	G	A	9	14	--	HSF
	Ca(ICCV2/PI489777)SNP_17878	873	A	C	12	19	--	HSF
	Ca(ICCV2/PI489777)SNP_17879	976	C	T	10	20	--	HSF
	Ca(ICCV2/PI489777)SNP_17880	1000	T	C	11	17	--	HSF
	Ca(ICCV2/PI489777)SNP_17881	1072	A	G	7	15	--	HSF
	Ca(ICCV2/PI489777)SNP_17882	1225	C	T	4	11	--	HSF
CakTC30166	Ca(ICCV2/PI489777)SNP_17883	1109	C	A	9	5	--	--
	Ca(ICCV2/PI489777)SNP_17884	1331	G	T	18	12	--	--
	Ca(ICCV2/PI489777)SNP_17885	1369	T	G	14	13	--	--
	Ca(ICCV2/PI489777)SNP_17886	1376	A	G	16	16	--	--
	Ca(ICCV2/PI489777)SNP_17887	1526	G	A	18	16	--	--
	Ca(ICCV2/PI489777)SNP_17888	1769	G	A	8	12	--	--
CakTC42414	Ca(ICCV2/PI489777)SNP_17889	550	A	G	12	14	--	--
CakTC41227	Ca(ICCV2/PI489777)SNP_17890	619	G	A	36	5	--	--
	Ca(ICCV2/PI489777)SNP_17891	780	C	T	25	3	--	--
CakTC29171	Ca(ICCV2/PI489777)SNP_17892	157	A	C	4	4	--	--
	Ca(ICCV2/PI489777)SNP_17893	274	T	A	4	4	--	--
	Ca(ICCV2/PI489777)SNP_17894	403	C	T	5	4	--	--
	Ca(ICCV2/PI489777)SNP_17895	502	G	C	4	4	--	--
CakTC27507	Ca(ICCV2/PI489777)SNP_17896	2009	A	C	3	3	--	--
	Ca(ICCV2/PI489777)SNP_17897	2015	A	G	5	3	--	--
	Ca(ICCV2/PI489777)SNP_17898	2038	G	A	5	3	--	--
	Ca(ICCV2/PI489777)SNP_17899	2100	T	C	5	3	--	--
	Ca(ICCV2/PI489777)SNP_17900	2103	T	C	4	3	--	--
	Ca(ICCV2/PI489777)SNP_17901	2111	A	C	4	3	--	--
	Ca(ICCV2/PI489777)SNP_17902	2233	C	A	4	3	--	--
	Ca(ICCV2/PI489777)SNP_17903	2308	T	G	3	3	--	--

CakTC28537	Ca(ICCV2/PI489777)SNP_17904	135	C	A	6	4	--	--
	Ca(ICCV2/PI489777)SNP_17905	316	C	T	22	8	--	--
	Ca(ICCV2/PI489777)SNP_17906	403	T	C	33	13	--	--
	Ca(ICCV2/PI489777)SNP_17907	579	A	G	31	14	--	--
	Ca(ICCV2/PI489777)SNP_17908	733	A	G	42	15	--	--
	Ca(ICCV2/PI489777)SNP_17909	956	A	C	38	10	--	--
CakTC32655	Ca(ICCV2/PI489777)SNP_17910	156	C	T	6	3	--	--
	Ca(ICCV2/PI489777)SNP_17911	205	C	T	7	5	--	--
	Ca(ICCV2/PI489777)SNP_17912	286	G	T	11	6	--	--
	Ca(ICCV2/PI489777)SNP_17913	297	A	G	12	8	--	--
	Ca(ICCV2/PI489777)SNP_17914	298	T	A	12	8	--	--
	Ca(ICCV2/PI489777)SNP_17915	518	C	T	6	5	--	--
	Ca(ICCV2/PI489777)SNP_17916	535	T	C	5	4	--	--
	Ca(ICCV2/PI489777)SNP_17917	1267	C	T	10	8	--	--
	Ca(ICCV2/PI489777)SNP_17918	1973	A	T	5	10	--	--
	Ca(ICCV2/PI489777)SNP_17919	2069	A	G	5	7	--	--
CakTC24187	Ca(ICCV2/PI489777)SNP_17920	654	A	C	5	7	--	--
	Ca(ICCV2/PI489777)SNP_17921	750	T	G	9	8	--	--
	Ca(ICCV2/PI489777)SNP_17922	762	T	G	11	7	--	--
CakTC40490	Ca(ICCV2/PI489777)SNP_17923	784	C	T	5	4	--	--
	Ca(ICCV2/PI489777)SNP_17924	1003	G	A	3	4	--	--
CakTC27958	Ca(ICCV2/PI489777)SNP_17925	859	A	G	3	7	Root	--
	Ca(ICCV2/PI489777)SNP_17926	1349	G	A	3	4	Root	--
	Ca(ICCV2/PI489777)SNP_17927	1373	T	C	3	4	Root	--
CakTC35552	Ca(ICCV2/PI489777)SNP_17928	543	A	G	8	8	--	--
	Ca(ICCV2/PI489777)SNP_17929	925	T	C	4	5	--	--
	Ca(ICCV2/PI489777)SNP_17930	964	T	C	9	5	--	--
	Ca(ICCV2/PI489777)SNP_17931	986	C	T	9	8	--	--
	Ca(ICCV2/PI489777)SNP_17932	1051	T	C	13	7	--	--
	Ca(ICCV2/PI489777)SNP_17933	1327	T	C	23	6	--	--
	Ca(ICCV2/PI489777)SNP_17934	2624	T	C	14	11	--	--
	Ca(ICCV2/PI489777)SNP_17935	2908	C	T	14	21	--	--
	Ca(ICCV2/PI489777)SNP_17936	3274	A	C	24	13	--	--
	Ca(ICCV2/PI489777)SNP_17937	3811	C	T	14	6	--	--
CakTC23992	Ca(ICCV2/PI489777)SNP_17938	174	C	A	39	29	--	--
	Ca(ICCV2/PI489777)SNP_17939	411	A	T	37	33	--	--
	Ca(ICCV2/PI489777)SNP_17940	1164	G	C	19	16	--	--
	Ca(ICCV2/PI489777)SNP_17941	1809	C	A	24	26	--	--
	Ca(ICCV2/PI489777)SNP_17942	1916	A	G	25	25	--	--
	Ca(ICCV2/PI489777)SNP_17943	1955	G	A	20	25	--	--
	Ca(ICCV2/PI489777)SNP_17944	2060	A	T	19	25	--	--
CakTC40858	Ca(ICCV2/PI489777)SNP_17945	1614	G	C	17	43	--	--
CakTC40897	Ca(ICCV2/PI489777)SNP_17946	114	T	C	15	5	--	--
	Ca(ICCV2/PI489777)SNP_17947	271	A	G	25	10	--	--
	Ca(ICCV2/PI489777)SNP_17948	379	A	G	29	19	--	--
	Ca(ICCV2/PI489777)SNP_17949	388	C	A	29	19	--	--
	Ca(ICCV2/PI489777)SNP_17950	394	T	A	25	19	--	--
	Ca(ICCV2/PI489777)SNP_17951	478	C	G	44	19	--	--
	Ca(ICCV2/PI489777)SNP_17952	550	G	A	39	18	--	--
	Ca(ICCV2/PI489777)SNP_17953	594	A	G	37	15	--	--
	Ca(ICCV2/PI489777)SNP_17954	692	C	T	35	16	--	--
	Ca(ICCV2/PI489777)SNP_17955	736	G	A	34	15	--	--
CakTC24722	Ca(ICCV2/PI489777)SNP_17956	131	G	T	9	6	Root	--
	Ca(ICCV2/PI489777)SNP_17957	216	T	A	10	9	Root	--
	Ca(ICCV2/PI489777)SNP_17958	291	G	C	21	14	Root	--
	Ca(ICCV2/PI489777)SNP_17959	342	A	G	66	28	Root	--
	Ca(ICCV2/PI489777)SNP_17960	1110	T	A	45	13	Root	--
	Ca(ICCV2/PI489777)SNP_17961	1125	C	T	43	16	Root	--
	Ca(ICCV2/PI489777)SNP_17962	1134	T	A	41	17	Root	--
	Ca(ICCV2/PI489777)SNP_17963	1146	T	C	43	17	Root	--
	Ca(ICCV2/PI489777)SNP_17964	1245	A	C	34	14	Root	--
	Ca(ICCV2/PI489777)SNP_17965	1325	G	T	32	17	Root	--
	Ca(ICCV2/PI489777)SNP_17966	1553	G	A	16	11	Root	--
CakTC20354	Ca(ICCV2/PI489777)SNP_17967	204	G	A	3	3	--	--
CakTC33015	Ca(ICCV2/PI489777)SNP_17968	197	A	C	6	5	--	--
CakTC27840	Ca(ICCV2/PI489777)SNP_17969	674	C	T	8	4	--	--
CakTC33107	Ca(ICCV2/PI489777)SNP_17970	195	A	G	6	3	--	--
	Ca(ICCV2/PI489777)SNP_17971	345	C	T	6	3	--	--
	Ca(ICCV2/PI489777)SNP_17972	505	C	T	4	3	--	--
CakTC42130	Ca(ICCV2/PI489777)SNP_17973	430	A	G	11	7	--	--
	Ca(ICCV2/PI489777)SNP_17974	445	A	T	9	7	--	--
	Ca(ICCV2/PI489777)SNP_17975	446	A	G	9	7	--	--
	Ca(ICCV2/PI489777)SNP_17976	726	T	C	8	4	--	--
CakTC40526	Ca(ICCV2/PI489777)SNP_17977	1802	T	C	3	3	--	--

CakTC11240	Ca(ICCV2/PI489777)SNP_17978	629	T	A	33	8	--	--
	Ca(ICCV2/PI489777)SNP_17979	1763	T	G	14	8	--	--
	Ca(ICCV2/PI489777)SNP_17980	1853	C	T	12	7	--	--
	Ca(ICCV2/PI489777)SNP_17981	1883	G	A	13	7	--	--
	Ca(ICCV2/PI489777)SNP_17982	2024	G	A	13	5	--	--
	Ca(ICCV2/PI489777)SNP_17983	2284	A	G	16	7	--	--
	Ca(ICCV2/PI489777)SNP_17984	2296	G	A	17	6	--	--
	Ca(ICCV2/PI489777)SNP_17985	2331	C	T	14	8	--	--
	Ca(ICCV2/PI489777)SNP_17986	2355	G	T	15	6	--	--
	Ca(ICCV2/PI489777)SNP_17987	2359	A	G	15	6	--	--
	Ca(ICCV2/PI489777)SNP_17988	2485	G	C	15	6	--	--
CakTC33856	Ca(ICCV2/PI489777)SNP_17989	667	G	T	5	4	--	--
	Ca(ICCV2/PI489777)SNP_17990	688	C	T	5	4	--	--
	Ca(ICCV2/PI489777)SNP_17991	754	T	C	5	4	--	--
CakTC41552	Ca(ICCV2/PI489777)SNP_17992	155	G	C	4	4	--	--
	Ca(ICCV2/PI489777)SNP_17993	1165	T	C	15	4	--	--
	Ca(ICCV2/PI489777)SNP_17994	1636	A	G	15	8	--	--
	Ca(ICCV2/PI489777)SNP_17995	1798	G	A	20	12	--	--
CakTC42075	Ca(ICCV2/PI489777)SNP_17996	372	G	A	6	10	--	--
	Ca(ICCV2/PI489777)SNP_17997	485	C	T	6	7	--	--
CakTC32747	Ca(ICCV2/PI489777)SNP_17998	108	G	A	3	8	--	--
	Ca(ICCV2/PI489777)SNP_17999	331	G	A	8	15	--	--
	Ca(ICCV2/PI489777)SNP_18000	1123	A	G	6	9	--	--
CakTC28609	Ca(ICCV2/PI489777)SNP_18001	132	A	G	4	5	--	--
CakTC42433	Ca(ICCV2/PI489777)SNP_18002	1007	A	C	20	20	--	--
	Ca(ICCV2/PI489777)SNP_18003	1145	G	A	20	17	--	--
	Ca(ICCV2/PI489777)SNP_18004	1239	C	T	22	26	--	--
	Ca(ICCV2/PI489777)SNP_18005	1299	G	T	15	28	--	--
	Ca(ICCV2/PI489777)SNP_18006	1300	A	G	16	28	--	--
	Ca(ICCV2/PI489777)SNP_18007	1379	T	A	19	23	--	--
	Ca(ICCV2/PI489777)SNP_18008	1475	C	T	16	16	--	--
	Ca(ICCV2/PI489777)SNP_18009	1514	G	A	13	14	--	--
	Ca(ICCV2/PI489777)SNP_18010	1573	A	G	6	9	--	--
	Ca(ICCV2/PI489777)SNP_18011	1618	G	A	5	9	--	--
CakTC40790	Ca(ICCV2/PI489777)SNP_18012	241	C	T	5	10	--	--
CakTC25451	Ca(ICCV2/PI489777)SNP_18013	481	T	C	35	11	--	--
	Ca(ICCV2/PI489777)SNP_18014	594	A	T	37	10	--	--
	Ca(ICCV2/PI489777)SNP_18015	1044	T	G	54	22	--	--
	Ca(ICCV2/PI489777)SNP_18016	1263	G	C	50	34	--	--
	Ca(ICCV2/PI489777)SNP_18017	1698	C	T	41	28	--	--
	Ca(ICCV2/PI489777)SNP_18018	1856	T	A	32	19	--	--
CakTC38964	Ca(ICCV2/PI489777)SNP_18019	428	C	T	106	57	--	--
	Ca(ICCV2/PI489777)SNP_18020	734	T	C	65	32	--	--
CakTC27938	Ca(ICCV2/PI489777)SNP_18021	775	C	T	15	23	--	--
	Ca(ICCV2/PI489777)SNP_18022	1057	A	G	27	23	--	--
CakTC33926	Ca(ICCV2/PI489777)SNP_18023	270	G	C	6	9	--	--
	Ca(ICCV2/PI489777)SNP_18024	543	C	T	3	4	--	--
	Ca(ICCV2/PI489777)SNP_18025	636	A	G	4	5	--	--
	Ca(ICCV2/PI489777)SNP_18026	702	A	G	5	7	--	--
	Ca(ICCV2/PI489777)SNP_18027	1517	A	C	6	7	--	--
	Ca(ICCV2/PI489777)SNP_18028	1522	A	G	6	6	--	--
	Ca(ICCV2/PI489777)SNP_18029	1528	A	T	6	7	--	--
	Ca(ICCV2/PI489777)SNP_18030	1661	T	A	5	10	--	--
CakTC27321	Ca(ICCV2/PI489777)SNP_18031	153	A	T	45	36	--	--
	Ca(ICCV2/PI489777)SNP_18032	201	C	A	50	41	--	--
	Ca(ICCV2/PI489777)SNP_18033	339	T	C	54	35	--	--
	Ca(ICCV2/PI489777)SNP_18034	387	T	G	70	41	--	--
	Ca(ICCV2/PI489777)SNP_18035	429	T	C	72	51	--	--
	Ca(ICCV2/PI489777)SNP_18036	649	T	A	62	38	--	--
	Ca(ICCV2/PI489777)SNP_18037	1468	C	T	10	11	--	--
CakTC29973	Ca(ICCV2/PI489777)SNP_18038	376	T	C	4	9	--	--
	Ca(ICCV2/PI489777)SNP_18039	697	T	C	6	14	--	--
	Ca(ICCV2/PI489777)SNP_18040	979	G	A	9	8	--	--
	Ca(ICCV2/PI489777)SNP_18041	1544	G	A	8	9	--	--
	Ca(ICCV2/PI489777)SNP_18042	1691	A	G	7	11	--	--
	Ca(ICCV2/PI489777)SNP_18043	2630	T	C	4	10	--	--
	Ca(ICCV2/PI489777)SNP_18044	2674	G	A	5	9	--	--
	Ca(ICCV2/PI489777)SNP_18045	2988	A	C	5	4	--	--
CakTC35443	Ca(ICCV2/PI489777)SNP_18046	119	C	T	6	4	--	--
	Ca(ICCV2/PI489777)SNP_18047	206	C	A	7	4	--	--
	Ca(ICCV2/PI489777)SNP_18048	274	T	C	11	5	--	--
	Ca(ICCV2/PI489777)SNP_18049	408	G	A	11	4	--	--
	Ca(ICCV2/PI489777)SNP_18050	1971	T	C	8	8	--	--
	Ca(ICCV2/PI489777)SNP_18051	3965	T	A	9	5	--	--

	Ca(ICCV2/PI489777)SNP_18052	4004	C	G	6	6	--	--
CakTC41913	Ca(ICCV2/PI489777)SNP_18053	96	C	T	7	5	--	--
	Ca(ICCV2/PI489777)SNP_18054	199	G	A	13	5	--	--
	Ca(ICCV2/PI489777)SNP_18055	394	G	A	18	13	--	--
	Ca(ICCV2/PI489777)SNP_18056	430	A	G	20	14	--	--
	Ca(ICCV2/PI489777)SNP_18057	1588	C	T	34	11	--	--
	Ca(ICCV2/PI489777)SNP_18058	1645	G	T	26	11	--	--
	Ca(ICCV2/PI489777)SNP_18059	1663	G	A	26	11	--	--
CakTC33025	Ca(ICCV2/PI489777)SNP_18060	684	C	G	6	5	Flower	bud
CakTC20721	Ca(ICCV2/PI489777)SNP_18061	1298	C	T	10	8	--	--
	Ca(ICCV2/PI489777)SNP_18062	1346	C	A	9	9	--	--
	Ca(ICCV2/PI489777)SNP_18063	2339	C	T	9	5	--	--
	Ca(ICCV2/PI489777)SNP_18064	2567	C	T	10	5	--	--
	Ca(ICCV2/PI489777)SNP_18065	2656	G	A	8	4	--	--
	Ca(ICCV2/PI489777)SNP_18066	3101	A	T	17	4	--	--
	Ca(ICCV2/PI489777)SNP_18067	4365	A	C	8	8	--	--
CakTC41335	Ca(ICCV2/PI489777)SNP_18068	349	T	A	8	9	--	--
CakTC29081	Ca(ICCV2/PI489777)SNP_18069	1391	G	C	11	3	--	--
	Ca(ICCV2/PI489777)SNP_18070	1811	C	G	7	8	--	--
	Ca(ICCV2/PI489777)SNP_18071	1952	G	A	8	9	--	--
	Ca(ICCV2/PI489777)SNP_18072	2267	G	C	10	4	--	--
	Ca(ICCV2/PI489777)SNP_18073	2336	T	C	10	3	--	--
	Ca(ICCV2/PI489777)SNP_18074	2681	A	G	10	9	--	--
CakTC31309	Ca(ICCV2/PI489777)SNP_18075	539	T	C	51	45	--	--
	Ca(ICCV2/PI489777)SNP_18076	774	A	G	49	34	--	--
	Ca(ICCV2/PI489777)SNP_18077	802	C	T	32	28	--	--
CakTC09896	Ca(ICCV2/PI489777)SNP_18078	432	C	G	8	3	--	--
CakTC41656	Ca(ICCV2/PI489777)SNP_18079	592	A	G	3	3	--	--
	Ca(ICCV2/PI489777)SNP_18080	2284	A	G	3	3	--	--
	Ca(ICCV2/PI489777)SNP_18081	2298	C	T	3	3	--	--
CakTC24309	Ca(ICCV2/PI489777)SNP_18082	67	C	T	10	7	--	--
	Ca(ICCV2/PI489777)SNP_18083	676	G	A	16	16	--	--
CakTC38221	Ca(ICCV2/PI489777)SNP_18084	324	T	C	14	7	--	--
CakTC39000	Ca(ICCV2/PI489777)SNP_18085	182	A	T	3	5	--	--
	Ca(ICCV2/PI489777)SNP_18086	350	A	T	3	4	--	--
	Ca(ICCV2/PI489777)SNP_18087	425	T	C	5	4	--	--
	Ca(ICCV2/PI489777)SNP_18088	434	C	T	6	4	--	--
CakTC34940	Ca(ICCV2/PI489777)SNP_18089	276	A	T	5	7	--	--
	Ca(ICCV2/PI489777)SNP_18090	1020	T	G	11	5	--	--
	Ca(ICCV2/PI489777)SNP_18091	1290	T	C	11	8	--	--
	Ca(ICCV2/PI489777)SNP_18092	1914	C	T	16	5	--	--
	Ca(ICCV2/PI489777)SNP_18093	2456	G	C	16	7	--	--
CakTC38771	Ca(ICCV2/PI489777)SNP_18094	238	A	G	4	3	--	--
CakTC39783	Ca(ICCV2/PI489777)SNP_18095	913	A	C	12	8	--	--
	Ca(ICCV2/PI489777)SNP_18096	1144	A	C	12	4	--	--
CakTC27865	Ca(ICCV2/PI489777)SNP_18097	613	A	C	4	3	--	--
CakTC25042	Ca(ICCV2/PI489777)SNP_18098	295	T	C	40	18	--	--
	Ca(ICCV2/PI489777)SNP_18099	1905	T	C	148	70	--	--
CakTC24705	Ca(ICCV2/PI489777)SNP_18100	339	T	A	8	6	--	--
	Ca(ICCV2/PI489777)SNP_18101	408	G	A	11	7	--	--
	Ca(ICCV2/PI489777)SNP_18102	607	G	C	9	3	--	--
CakTC34965	Ca(ICCV2/PI489777)SNP_18103	20	G	C	3	8	--	--
	Ca(ICCV2/PI489777)SNP_18104	416	G	T	4	3	--	--
CakTC39641	Ca(ICCV2/PI489777)SNP_18105	71	T	A	3	5	--	--
	Ca(ICCV2/PI489777)SNP_18106	207	G	T	6	6	--	--
	Ca(ICCV2/PI489777)SNP_18107	348	C	A	8	7	--	--
	Ca(ICCV2/PI489777)SNP_18108	438	T	G	14	9	--	--
	Ca(ICCV2/PI489777)SNP_18109	473	C	T	16	8	--	--
	Ca(ICCV2/PI489777)SNP_18110	519	A	C	14	9	--	--
	Ca(ICCV2/PI489777)SNP_18111	750	C	T	9	9	--	--
	Ca(ICCV2/PI489777)SNP_18112	936	T	G	6	8	--	--
	Ca(ICCV2/PI489777)SNP_18113	1151	G	A	7	7	--	--
	Ca(ICCV2/PI489777)SNP_18114	1169	G	A	7	7	--	--
CakTC30933	Ca(ICCV2/PI489777)SNP_18115	176	A	G	17	11	--	--
	Ca(ICCV2/PI489777)SNP_18116	726	T	C	227	51	--	--
	Ca(ICCV2/PI489777)SNP_18117	1218	C	A	129	51	--	--
	Ca(ICCV2/PI489777)SNP_18118	1353	G	T	65	27	--	--
CakTC41132	Ca(ICCV2/PI489777)SNP_18119	774	T	C	13	10	--	--
	Ca(ICCV2/PI489777)SNP_18120	1332	C	T	15	9	--	--
	Ca(ICCV2/PI489777)SNP_18121	1707	A	G	20	6	--	--
	Ca(ICCV2/PI489777)SNP_18122	1716	T	C	23	3	--	--
	Ca(ICCV2/PI489777)SNP_18123	1877	T	G	16	4	--	--
	Ca(ICCV2/PI489777)SNP_18124	1887	G	A	16	4	--	--
	Ca(ICCV2/PI489777)SNP_18125	1941	G	A	18	5	--	--

	Ca(ICCV2/PI489777)SNP_18126	2004	G	C	13	5	--	--
	Ca(ICCV2/PI489777)SNP_18127	2021	T	C	8	5	--	--
	Ca(ICCV2/PI489777)SNP_18128	2175	A	T	16	5	--	--
	Ca(ICCV2/PI489777)SNP_18129	2184	T	A	15	5	--	--
CakTC31370	Ca(ICCV2/PI489777)SNP_18130	463	T	A	19	4	--	--
	Ca(ICCV2/PI489777)SNP_18131	574	T	C	17	6	--	--
	Ca(ICCV2/PI489777)SNP_18132	1369	T	A	16	11	--	--
	Ca(ICCV2/PI489777)SNP_18133	2014	G	A	5	3	--	--
CakTC35043	Ca(ICCV2/PI489777)SNP_18134	403	G	C	4	3	Shoot	--
	Ca(ICCV2/PI489777)SNP_18135	432	A	G	3	3	Shoot	--
CakTC25862	Ca(ICCV2/PI489777)SNP_18136	213	A	G	12	7	--	--
	Ca(ICCV2/PI489777)SNP_18137	501	C	T	9	7	--	--
	Ca(ICCV2/PI489777)SNP_18138	510	T	G	9	8	--	--
	Ca(ICCV2/PI489777)SNP_18139	803	T	C	5	3	--	--
	Ca(ICCV2/PI489777)SNP_18140	1001	T	C	11	3	--	--
	Ca(ICCV2/PI489777)SNP_18141	1018	G	C	11	4	--	--
	Ca(ICCV2/PI489777)SNP_18142	1108	T	C	7	4	--	--
CakTC28774	Ca(ICCV2/PI489777)SNP_18143	873	A	T	7	4	--	--
	Ca(ICCV2/PI489777)SNP_18144	891	T	C	7	3	--	--
CakTC30739	Ca(ICCV2/PI489777)SNP_18145	611	C	G	7	3	--	--
	Ca(ICCV2/PI489777)SNP_18146	636	C	G	7	4	--	--
CakTC34285	Ca(ICCV2/PI489777)SNP_18147	852	G	A	38	24	--	--
	Ca(ICCV2/PI489777)SNP_18148	2016	T	C	35	13	--	--
	Ca(ICCV2/PI489777)SNP_18149	2136	T	C	15	11	--	--
	Ca(ICCV2/PI489777)SNP_18150	2224	G	A	7	5	--	--
CakTC39964	Ca(ICCV2/PI489777)SNP_18151	149	G	A	10	10	--	--
	Ca(ICCV2/PI489777)SNP_18152	236	T	A	10	13	--	--
	Ca(ICCV2/PI489777)SNP_18153	257	T	C	10	12	--	--
	Ca(ICCV2/PI489777)SNP_18154	369	C	T	9	12	--	--
	Ca(ICCV2/PI489777)SNP_18155	377	A	G	9	12	--	--
	Ca(ICCV2/PI489777)SNP_18156	401	G	A	9	9	--	--
	Ca(ICCV2/PI489777)SNP_18157	644	G	A	3	3	--	--
	Ca(ICCV2/PI489777)SNP_18158	650	C	T	3	3	--	--
	Ca(ICCV2/PI489777)SNP_18159	1235	T	C	7	3	--	--
	Ca(ICCV2/PI489777)SNP_18160	1240	C	A	6	4	--	--
	Ca(ICCV2/PI489777)SNP_18161	1391	G	T	4	3	--	--
CakTC32977	Ca(ICCV2/PI489777)SNP_18162	387	T	C	19	10	--	--
	Ca(ICCV2/PI489777)SNP_18163	820	A	G	10	9	--	--
CakTC26332	Ca(ICCV2/PI489777)SNP_18164	204	G	C	35	12	--	--
	Ca(ICCV2/PI489777)SNP_18165	636	C	G	131	53	--	--
CakTC39638	Ca(ICCV2/PI489777)SNP_18166	313	A	T	19	18	--	--
	Ca(ICCV2/PI489777)SNP_18167	426	G	C	15	14	--	--
	Ca(ICCV2/PI489777)SNP_18168	521	A	G	17	14	--	--
CakTC43344	Ca(ICCV2/PI489777)SNP_18169	802	G	A	89	28	--	--
CakTC10609	Ca(ICCV2/PI489777)SNP_18170	981	A	G	7	4	--	--
	Ca(ICCV2/PI489777)SNP_18171	1550	T	G	4	3	--	--
CakTC39306	Ca(ICCV2/PI489777)SNP_18172	118	C	T	4	7	--	--
	Ca(ICCV2/PI489777)SNP_18173	1263	G	T	5	4	--	--
	Ca(ICCV2/PI489777)SNP_18174	1411	T	A	3	3	--	--
	Ca(ICCV2/PI489777)SNP_18175	1434	T	A	3	3	--	--
	Ca(ICCV2/PI489777)SNP_18176	1436	A	G	3	3	--	--
CakTC38239	Ca(ICCV2/PI489777)SNP_18177	78	G	C	21	7	--	--
	Ca(ICCV2/PI489777)SNP_18178	260	T	C	47	17	--	--
	Ca(ICCV2/PI489777)SNP_18179	266	T	C	50	17	--	--
	Ca(ICCV2/PI489777)SNP_18180	492	A	G	38	15	--	--
	Ca(ICCV2/PI489777)SNP_18181	573	C	T	30	14	--	--
	Ca(ICCV2/PI489777)SNP_18182	675	C	T	20	13	--	--
CakTC38700	Ca(ICCV2/PI489777)SNP_18183	310	C	T	9	4	--	--
CakTC18509	Ca(ICCV2/PI489777)SNP_18184	98	T	C	214	158	--	--
CakTC38814	Ca(ICCV2/PI489777)SNP_18185	83	A	T	19	5	--	--
	Ca(ICCV2/PI489777)SNP_18186	990	C	T	21	16	--	--
	Ca(ICCV2/PI489777)SNP_18187	1030	A	G	22	17	--	--
CakTC24878	Ca(ICCV2/PI489777)SNP_18188	566	A	G	13	20	--	--
CakTC39595	Ca(ICCV2/PI489777)SNP_18189	39	C	G	19	6	--	--
	Ca(ICCV2/PI489777)SNP_18190	874	G	A	14	5	--	--
	Ca(ICCV2/PI489777)SNP_18191	895	T	C	15	5	--	--
CakTC23140	Ca(ICCV2/PI489777)SNP_18192	326	C	T	8	8	--	--
CakTC32283	Ca(ICCV2/PI489777)SNP_18193	200	T	A	9	7	Flower	bud
	Ca(ICCV2/PI489777)SNP_18194	202	A	G	9	7	Flower	bud
	Ca(ICCV2/PI489777)SNP_18195	567	T	C	7	3	Flower	bud
	Ca(ICCV2/PI489777)SNP_18196	817	A	C	9	3	Flower	bud
	Ca(ICCV2/PI489777)SNP_18197	824	A	C	6	3	Flower	bud
	Ca(ICCV2/PI489777)SNP_18198	873	T	C	9	3	Flower	bud
	Ca(ICCV2/PI489777)SNP_18199	922	C	T	7	3	Flower	bud

	Ca(ICCV2/PI489777)SNP_18200	957	T	A	12	3	Flower	bud
	Ca(ICCV2/PI489777)SNP_18201	1004	C	T	11	3	Flower	bud
	Ca(ICCV2/PI489777)SNP_18202	1711	C	T	16	6	Flower	bud
	Ca(ICCV2/PI489777)SNP_18203	1930	T	G	13	6	Flower	bud
CakTC32945	Ca(ICCV2/PI489777)SNP_18204	777	G	A	5	3	--	--
CakTC39635	Ca(ICCV2/PI489777)SNP_18205	809	A	G	34	15	--	--
CakTC42982	Ca(ICCV2/PI489777)SNP_18206	720	C	G	9	3	--	--
CakTC24450	Ca(ICCV2/PI489777)SNP_18207	543	G	A	21	25	--	--
	Ca(ICCV2/PI489777)SNP_18208	934	G	C	22	19	--	--
	Ca(ICCV2/PI489777)SNP_18209	1196	T	C	16	18	--	--
	Ca(ICCV2/PI489777)SNP_18210	1335	C	T	37	15	--	--
	Ca(ICCV2/PI489777)SNP_18211	1502	G	C	27	25	--	--
	Ca(ICCV2/PI489777)SNP_18212	1666	G	C	21	19	--	--
CakTC10845	Ca(ICCV2/PI489777)SNP_18213	52	T	A	3	5	--	--
	Ca(ICCV2/PI489777)SNP_18214	139	C	G	11	9	--	--
	Ca(ICCV2/PI489777)SNP_18215	170	C	T	11	8	--	--
	Ca(ICCV2/PI489777)SNP_18216	181	G	C	11	10	--	--
	Ca(ICCV2/PI489777)SNP_18217	204	C	G	11	10	--	--
	Ca(ICCV2/PI489777)SNP_18218	228	A	T	17	10	--	--
CakTC41942	Ca(ICCV2/PI489777)SNP_18219	98	C	T	12	3	--	--
	Ca(ICCV2/PI489777)SNP_18220	99	T	A	12	3	--	--
	Ca(ICCV2/PI489777)SNP_18221	365	T	C	23	5	--	--
	Ca(ICCV2/PI489777)SNP_18222	581	T	C	21	7	--	--
	Ca(ICCV2/PI489777)SNP_18223	671	C	T	15	10	--	--
	Ca(ICCV2/PI489777)SNP_18224	884	G	A	33	8	--	--
	Ca(ICCV2/PI489777)SNP_18225	1148	A	G	29	14	--	--
CakTC23543	Ca(ICCV2/PI489777)SNP_18226	320	A	G	4	4	--	--
	Ca(ICCV2/PI489777)SNP_18227	359	T	G	4	4	--	--
	Ca(ICCV2/PI489777)SNP_18228	393	C	T	4	4	--	--
CakTC23924	Ca(ICCV2/PI489777)SNP_18229	982	T	A	25	7	--	--
	Ca(ICCV2/PI489777)SNP_18230	986	G	A	25	9	--	--
	Ca(ICCV2/PI489777)SNP_18231	1020	T	C	24	9	--	--
	Ca(ICCV2/PI489777)SNP_18232	1620	G	T	43	26	--	--
CakTC39779	Ca(ICCV2/PI489777)SNP_18233	217	T	C	18	10	--	--
CakTC09687	Ca(ICCV2/PI489777)SNP_18234	4473	C	T	3	4	--	--
CakTC41565	Ca(ICCV2/PI489777)SNP_18235	95	G	T	9	11	--	--
	Ca(ICCV2/PI489777)SNP_18236	142	A	T	10	12	--	--
	Ca(ICCV2/PI489777)SNP_18237	374	T	C	18	14	--	--
	Ca(ICCV2/PI489777)SNP_18238	602	C	T	11	10	--	--
	Ca(ICCV2/PI489777)SNP_18239	612	G	A	10	8	--	--
CakTC26419	Ca(ICCV2/PI489777)SNP_18240	679	T	G	14	3	--	--
CakTC23788	Ca(ICCV2/PI489777)SNP_18241	473	T	C	8	8	--	IWS1
	Ca(ICCV2/PI489777)SNP_18242	479	T	A	9	8	--	IWS1
	Ca(ICCV2/PI489777)SNP_18243	495	G	A	10	8	--	IWS1
	Ca(ICCV2/PI489777)SNP_18244	1569	C	T	26	17	--	IWS1
CakTC28832	Ca(ICCV2/PI489777)SNP_18245	146	C	T	59	18	--	--
CakTC27196	Ca(ICCV2/PI489777)SNP_18246	241	A	C	10	4	--	--
	Ca(ICCV2/PI489777)SNP_18247	353	A	G	7	4	--	--
	Ca(ICCV2/PI489777)SNP_18248	444	A	G	6	3	--	--
CakTC24084	Ca(ICCV2/PI489777)SNP_18249	2351	T	C	3	4	--	PLATZ
CakTC04172	Ca(ICCV2/PI489777)SNP_18250	191	T	A	7	7	--	--
CakTC41056	Ca(ICCV2/PI489777)SNP_18251	116	C	G	5	3	--	--
	Ca(ICCV2/PI489777)SNP_18252	175	T	C	7	7	--	--
	Ca(ICCV2/PI489777)SNP_18253	254	C	G	9	9	--	--
	Ca(ICCV2/PI489777)SNP_18254	502	G	A	14	7	--	--
	Ca(ICCV2/PI489777)SNP_18255	886	C	T	23	4	--	--
	Ca(ICCV2/PI489777)SNP_18256	919	C	A	21	5	--	--
	Ca(ICCV2/PI489777)SNP_18257	1066	G	A	22	5	--	--
	Ca(ICCV2/PI489777)SNP_18258	1348	T	A	21	11	--	--
	Ca(ICCV2/PI489777)SNP_18259	1409	T	C	15	13	--	--
	Ca(ICCV2/PI489777)SNP_18260	1435	T	C	22	12	--	--
	Ca(ICCV2/PI489777)SNP_18261	1972	A	G	55	11	--	--
	Ca(ICCV2/PI489777)SNP_18262	2135	A	C	38	10	--	--
	Ca(ICCV2/PI489777)SNP_18263	2137	G	A	35	10	--	--
	Ca(ICCV2/PI489777)SNP_18264	2261	C	A	25	7	--	--
	Ca(ICCV2/PI489777)SNP_18265	2304	C	T	19	5	--	--
	Ca(ICCV2/PI489777)SNP_18266	2327	G	A	12	5	--	--
	Ca(ICCV2/PI489777)SNP_18267	2362	A	C	3	3	--	--
	Ca(ICCV2/PI489777)SNP_18268	2368	C	A	3	3	--	--
CakTC41427	Ca(ICCV2/PI489777)SNP_18269	282	T	A	8	10	--	--
	Ca(ICCV2/PI489777)SNP_18270	459	C	T	9	8	--	--
CakTC42320	Ca(ICCV2/PI489777)SNP_18271	241	C	T	35	17	--	--
	Ca(ICCV2/PI489777)SNP_18272	514	A	G	19	17	--	--
	Ca(ICCV2/PI489777)SNP_18273	1111	C	T	50	15	--	--

CakTC32699	Ca(ICCV2/PI489777)SNP_18274	524	C	T	7	7	--	SNF2
	Ca(ICCV2/PI489777)SNP_18275	686	C	T	16	8	--	SNF2
	Ca(ICCV2/PI489777)SNP_18276	1372	C	T	21	8	--	SNF2
	Ca(ICCV2/PI489777)SNP_18277	1614	T	A	11	4	--	SNF2
	Ca(ICCV2/PI489777)SNP_18278	2034	C	G	14	7	--	SNF2
	Ca(ICCV2/PI489777)SNP_18279	2465	A	G	11	5	--	SNF2
	Ca(ICCV2/PI489777)SNP_18280	2637	G	A	9	8	--	SNF2
	Ca(ICCV2/PI489777)SNP_18281	2982	A	G	7	4	--	SNF2
	Ca(ICCV2/PI489777)SNP_18282	3933	A	G	9	9	--	SNF2
	Ca(ICCV2/PI489777)SNP_18283	4127	G	A	9	13	--	SNF2
	Ca(ICCV2/PI489777)SNP_18284	4149	T	C	7	11	--	SNF2
	Ca(ICCV2/PI489777)SNP_18285	4157	T	C	7	11	--	SNF2
CakTC43120	Ca(ICCV2/PI489777)SNP_18286	870	C	T	6	10	Young_pod	--
CakTC24546	Ca(ICCV2/PI489777)SNP_18287	121	C	T	13	6	--	--
	Ca(ICCV2/PI489777)SNP_18288	917	C	T	9	6	--	--
	Ca(ICCV2/PI489777)SNP_18289	1620	C	T	13	14	--	--
	Ca(ICCV2/PI489777)SNP_18290	2886	T	C	31	16	--	--
CakTC25485	Ca(ICCV2/PI489777)SNP_18291	70	T	C	7	8	--	--
	Ca(ICCV2/PI489777)SNP_18292	1363	G	A	24	8	--	--
	Ca(ICCV2/PI489777)SNP_18293	4161	T	C	4	7	--	--
CakTC32146	Ca(ICCV2/PI489777)SNP_18294	914	G	A	5	3	--	--
	Ca(ICCV2/PI489777)SNP_18295	987	G	A	5	4	--	--
	Ca(ICCV2/PI489777)SNP_18296	1027	G	T	4	4	--	--
	Ca(ICCV2/PI489777)SNP_18297	1731	A	G	6	4	--	--
	Ca(ICCV2/PI489777)SNP_18298	1787	G	C	5	3	--	--
CakTC26733	Ca(ICCV2/PI489777)SNP_18299	755	T	A	8	10	--	--
	Ca(ICCV2/PI489777)SNP_18300	756	C	G	8	10	--	--
	Ca(ICCV2/PI489777)SNP_18301	1096	A	C	10	17	--	--
	Ca(ICCV2/PI489777)SNP_18302	1204	A	G	16	12	--	--
	Ca(ICCV2/PI489777)SNP_18303	1222	A	C	14	12	--	--
	Ca(ICCV2/PI489777)SNP_18304	1314	C	T	15	11	--	--
	Ca(ICCV2/PI489777)SNP_18305	1357	C	T	8	3	--	--
	Ca(ICCV2/PI489777)SNP_18306	1567	C	T	4	3	--	--
	Ca(ICCV2/PI489777)SNP_18307	1594	T	A	4	3	--	--
CakTC04878	Ca(ICCV2/PI489777)SNP_18308	694	C	T	6	5	--	--
	Ca(ICCV2/PI489777)SNP_18309	697	G	A	6	5	--	--
CakTC37041	Ca(ICCV2/PI489777)SNP_18310	1114	A	G	68	38	--	--
	Ca(ICCV2/PI489777)SNP_18311	2036	C	A	49	24	--	--
	Ca(ICCV2/PI489777)SNP_18312	2044	G	C	49	24	--	--
CakTC27520	Ca(ICCV2/PI489777)SNP_18313	225	G	A	10	4	--	--
	Ca(ICCV2/PI489777)SNP_18314	609	T	C	12	7	--	--
	Ca(ICCV2/PI489777)SNP_18315	697	G	A	12	7	--	--
	Ca(ICCV2/PI489777)SNP_18316	1321	G	A	7	4	--	--
CakTC41982	Ca(ICCV2/PI489777)SNP_18317	1038	C	A	10	3	--	--
	Ca(ICCV2/PI489777)SNP_18318	1441	A	G	9	6	--	--
	Ca(ICCV2/PI489777)SNP_18319	1754	A	G	7	3	--	--
CakTC23573	Ca(ICCV2/PI489777)SNP_18320	2460	G	C	33	32	--	--
	Ca(ICCV2/PI489777)SNP_18321	2481	T	A	31	30	--	--
	Ca(ICCV2/PI489777)SNP_18322	2802	A	T	16	21	--	--
CakTC25926	Ca(ICCV2/PI489777)SNP_18323	548	C	T	13	8	--	--
	Ca(ICCV2/PI489777)SNP_18324	593	T	C	11	8	--	--
	Ca(ICCV2/PI489777)SNP_18325	680	A	G	7	7	--	--
CakTC38584	Ca(ICCV2/PI489777)SNP_18326	680	A	C	4	6	--	--
	Ca(ICCV2/PI489777)SNP_18327	867	C	T	6	8	--	--
	Ca(ICCV2/PI489777)SNP_18328	987	T	A	5	3	--	--
CakTC39383	Ca(ICCV2/PI489777)SNP_18329	1015	G	A	10	5	--	--
CakTC18569	Ca(ICCV2/PI489777)SNP_18330	485	A	G	4	4	--	--
	Ca(ICCV2/PI489777)SNP_18331	530	C	T	3	4	--	--
	Ca(ICCV2/PI489777)SNP_18332	533	G	T	3	4	--	--
	Ca(ICCV2/PI489777)SNP_18333	657	A	G	3	4	--	--
CakTC33402	Ca(ICCV2/PI489777)SNP_18334	122	T	C	9	3	--	--
	Ca(ICCV2/PI489777)SNP_18335	173	C	T	10	4	--	--
	Ca(ICCV2/PI489777)SNP_18336	450	T	C	16	8	--	--
	Ca(ICCV2/PI489777)SNP_18337	645	G	A	24	7	--	--
	Ca(ICCV2/PI489777)SNP_18338	672	G	T	24	7	--	--
	Ca(ICCV2/PI489777)SNP_18339	1008	G	A	11	3	--	--
	Ca(ICCV2/PI489777)SNP_18340	1134	A	G	12	4	--	--
	Ca(ICCV2/PI489777)SNP_18341	1716	C	T	16	21	--	--
	Ca(ICCV2/PI489777)SNP_18342	1770	C	T	13	20	--	--
CakTC36437	Ca(ICCV2/PI489777)SNP_18343	92	T	C	30	8	--	S1Fa-like
	Ca(ICCV2/PI489777)SNP_18344	239	C	G	35	10	--	S1Fa-like
CakTC40171	Ca(ICCV2/PI489777)SNP_18345	564	T	G	59	11	--	--
	Ca(ICCV2/PI489777)SNP_18346	594	C	T	53	12	--	--
	Ca(ICCV2/PI489777)SNP_18347	604	T	C	60	14	--	--

	Ca(ICCV2/PI489777)SNP_18348	1255	A	G	31	12	--	--
	Ca(ICCV2/PI489777)SNP_18349	1324	A	G	38	18	--	--
CakTC22978	Ca(ICCV2/PI489777)SNP_18350	1437	T	C	197	81	--	--
	Ca(ICCV2/PI489777)SNP_18351	2371	A	T	188	59	--	--
CakTC37039	Ca(ICCV2/PI489777)SNP_18352	490	T	C	4	3	--	--
CakTC29031	Ca(ICCV2/PI489777)SNP_18353	263	A	G	3	16	--	--
	Ca(ICCV2/PI489777)SNP_18354	264	C	T	3	16	--	--
	Ca(ICCV2/PI489777)SNP_18355	274	A	T	3	16	--	--
	Ca(ICCV2/PI489777)SNP_18356	275	A	C	3	16	--	--
	Ca(ICCV2/PI489777)SNP_18357	730	G	A	89	31	--	--
	Ca(ICCV2/PI489777)SNP_18358	756	C	G	90	26	--	--
	Ca(ICCV2/PI489777)SNP_18359	905	C	G	91	34	--	--
	Ca(ICCV2/PI489777)SNP_18360	1045	C	T	61	29	--	--
	Ca(ICCV2/PI489777)SNP_18361	1197	C	G	40	13	--	--
	Ca(ICCV2/PI489777)SNP_18362	1480	T	C	66	28	--	--
	Ca(ICCV2/PI489777)SNP_18363	1519	G	A	64	31	--	--
	Ca(ICCV2/PI489777)SNP_18364	1647	T	C	69	34	--	--
	Ca(ICCV2/PI489777)SNP_18365	1674	T	C	64	33	--	--
	Ca(ICCV2/PI489777)SNP_18366	1678	T	G	60	33	--	--
CakTC39656	Ca(ICCV2/PI489777)SNP_18367	866	C	T	9	5	--	--
CakTC13071	Ca(ICCV2/PI489777)SNP_18368	1588	T	C	7	3	--	--
CakTC31588	Ca(ICCV2/PI489777)SNP_18369	2020	A	G	13	9	--	--
	Ca(ICCV2/PI489777)SNP_18370	2179	G	T	11	12	--	--
	Ca(ICCV2/PI489777)SNP_18371	2245	A	G	8	11	--	--
	Ca(ICCV2/PI489777)SNP_18372	2713	A	G	7	7	--	--
	Ca(ICCV2/PI489777)SNP_18373	2758	C	T	9	6	--	--
CakTC26634	Ca(ICCV2/PI489777)SNP_18374	514	A	G	39	12	--	--
	Ca(ICCV2/PI489777)SNP_18375	655	T	C	36	28	--	--
	Ca(ICCV2/PI489777)SNP_18376	913	C	T	47	35	--	--
	Ca(ICCV2/PI489777)SNP_18377	922	C	G	51	36	--	--
	Ca(ICCV2/PI489777)SNP_18378	1138	C	T	42	30	--	--
	Ca(ICCV2/PI489777)SNP_18379	1150	T	C	42	31	--	--
	Ca(ICCV2/PI489777)SNP_18380	1162	T	A	33	29	--	--
	Ca(ICCV2/PI489777)SNP_18381	1177	T	C	43	28	--	--
	Ca(ICCV2/PI489777)SNP_18382	1211	A	T	42	30	--	--
	Ca(ICCV2/PI489777)SNP_18383	1273	G	A	32	28	--	--
	Ca(ICCV2/PI489777)SNP_18384	1309	A	G	32	18	--	--
	Ca(ICCV2/PI489777)SNP_18385	1318	T	C	33	16	--	--
	Ca(ICCV2/PI489777)SNP_18386	1648	C	T	26	17	--	--
	Ca(ICCV2/PI489777)SNP_18387	1700	C	T	23	17	--	--
	Ca(ICCV2/PI489777)SNP_18388	1876	C	T	13	6	--	--
	Ca(ICCV2/PI489777)SNP_18389	1898	C	T	12	6	--	--
CakTC33468	Ca(ICCV2/PI489777)SNP_18390	409	G	A	7	7	--	--
	Ca(ICCV2/PI489777)SNP_18391	455	C	T	3	5	--	--
CakTC39293	Ca(ICCV2/PI489777)SNP_18392	64	C	G	7	5	--	--
	Ca(ICCV2/PI489777)SNP_18393	287	A	G	14	8	--	--
	Ca(ICCV2/PI489777)SNP_18394	1191	A	T	15	17	--	--
CakTC24859	Ca(ICCV2/PI489777)SNP_18395	195	G	C	3	5	--	TUB
	Ca(ICCV2/PI489777)SNP_18396	265	G	A	5	6	--	TUB
	Ca(ICCV2/PI489777)SNP_18397	436	C	T	10	11	--	TUB
	Ca(ICCV2/PI489777)SNP_18398	487	A	G	9	13	--	TUB
	Ca(ICCV2/PI489777)SNP_18399	571	A	G	8	12	--	TUB
	Ca(ICCV2/PI489777)SNP_18400	742	A	C	12	11	--	TUB
	Ca(ICCV2/PI489777)SNP_18401	1240	A	G	14	13	--	TUB
	Ca(ICCV2/PI489777)SNP_18402	1372	A	C	15	4	--	TUB
	Ca(ICCV2/PI489777)SNP_18403	1429	C	A	10	5	--	TUB
CakTC23918	Ca(ICCV2/PI489777)SNP_18404	820	G	C	26	6	--	--
	Ca(ICCV2/PI489777)SNP_18405	825	C	A	25	6	--	--
	Ca(ICCV2/PI489777)SNP_18406	866	G	A	25	7	--	--
	Ca(ICCV2/PI489777)SNP_18407	873	G	C	26	7	--	--
	Ca(ICCV2/PI489777)SNP_18408	919	T	C	23	5	--	--
	Ca(ICCV2/PI489777)SNP_18409	964	G	C	27	6	--	--
	Ca(ICCV2/PI489777)SNP_18410	972	T	C	28	8	--	--
	Ca(ICCV2/PI489777)SNP_18411	1125	G	T	26	8	--	--
	Ca(ICCV2/PI489777)SNP_18412	1428	A	G	15	3	--	--
	Ca(ICCV2/PI489777)SNP_18413	1911	T	G	31	11	--	--
	Ca(ICCV2/PI489777)SNP_18414	1965	G	A	33	15	--	--
	Ca(ICCV2/PI489777)SNP_18415	2013	G	A	33	17	--	--
	Ca(ICCV2/PI489777)SNP_18416	2016	C	A	33	17	--	--
	Ca(ICCV2/PI489777)SNP_18417	2290	G	C	18	12	--	--
CakTC37989	Ca(ICCV2/PI489777)SNP_18418	98	C	T	19	18	--	--
	Ca(ICCV2/PI489777)SNP_18419	452	C	T	19	21	--	--
	Ca(ICCV2/PI489777)SNP_18420	497	C	T	24	15	--	--
	Ca(ICCV2/PI489777)SNP_18421	624	A	G	18	15	--	--

	Ca(ICCV2/PI489777)SNP_18422	754	A	C	18	15	--	--
	Ca(ICCV2/PI489777)SNP_18423	811	C	T	18	14	--	--
	Ca(ICCV2/PI489777)SNP_18424	852	A	G	15	11	--	--
	Ca(ICCV2/PI489777)SNP_18425	883	A	C	12	10	--	--
CakTC30544	Ca(ICCV2/PI489777)SNP_18426	1166	G	A	21	6	--	--
	Ca(ICCV2/PI489777)SNP_18427	1441	C	T	11	4	--	--
	Ca(ICCV2/PI489777)SNP_18428	1522	G	A	13	4	--	--
	Ca(ICCV2/PI489777)SNP_18429	1785	T	A	16	9	--	--
	Ca(ICCV2/PI489777)SNP_18430	1930	G	A	4	4	--	--
CakTC40887	Ca(ICCV2/PI489777)SNP_18431	324	C	G	6	10	--	--
	Ca(ICCV2/PI489777)SNP_18432	345	G	A	7	6	--	--
	Ca(ICCV2/PI489777)SNP_18433	470	A	C	11	22	--	--
	Ca(ICCV2/PI489777)SNP_18434	488	G	A	12	18	--	--
	Ca(ICCV2/PI489777)SNP_18435	614	A	G	7	18	--	--
	Ca(ICCV2/PI489777)SNP_18436	683	G	A	10	15	--	--
CakTC09949	Ca(ICCV2/PI489777)SNP_18437	1056	A	G	4	4	--	--
	Ca(ICCV2/PI489777)SNP_18438	1205	G	C	5	3	--	--
	Ca(ICCV2/PI489777)SNP_18439	3241	C	T	3	4	--	--
	Ca(ICCV2/PI489777)SNP_18440	3265	C	T	3	5	--	--
CakTC28586	Ca(ICCV2/PI489777)SNP_18441	415	G	T	21	9	--	--
	Ca(ICCV2/PI489777)SNP_18442	716	A	G	16	11	--	--
	Ca(ICCV2/PI489777)SNP_18443	1078	C	T	6	7	--	--
	Ca(ICCV2/PI489777)SNP_18444	1154	T	A	8	6	--	--
CakTC27712	Ca(ICCV2/PI489777)SNP_18445	935	T	C	13	10	--	--
	Ca(ICCV2/PI489777)SNP_18446	977	T	C	11	10	--	--
CakTC07642	Ca(ICCV2/PI489777)SNP_18447	500	C	G	3	3	--	--
CakTC24978	Ca(ICCV2/PI489777)SNP_18448	514	C	T	15	9	--	--
	Ca(ICCV2/PI489777)SNP_18449	711	A	G	14	6	--	--
	Ca(ICCV2/PI489777)SNP_18450	1970	T	C	6	3	--	--
	Ca(ICCV2/PI489777)SNP_18451	2294	G	T	16	5	--	--
CakTC40350	Ca(ICCV2/PI489777)SNP_18452	137	A	C	37	24	--	--
	Ca(ICCV2/PI489777)SNP_18453	653	G	A	10	6	--	--
	Ca(ICCV2/PI489777)SNP_18454	659	A	G	8	5	--	--
CakTC37118	Ca(ICCV2/PI489777)SNP_18455	768	T	C	8	20	--	--
CakTC33297	Ca(ICCV2/PI489777)SNP_18456	664	T	A	8	4	--	--
	Ca(ICCV2/PI489777)SNP_18457	1090	C	T	15	3	--	--
	Ca(ICCV2/PI489777)SNP_18458	1987	C	T	14	6	--	--
	Ca(ICCV2/PI489777)SNP_18459	2045	C	A	16	7	--	--
	Ca(ICCV2/PI489777)SNP_18460	2759	T	C	3	5	--	--
CakTC38709	Ca(ICCV2/PI489777)SNP_18461	334	T	C	34	21	--	--
	Ca(ICCV2/PI489777)SNP_18462	508	G	A	20	24	--	--
	Ca(ICCV2/PI489777)SNP_18463	631	C	T	23	21	--	--
	Ca(ICCV2/PI489777)SNP_18464	883	C	T	26	14	--	--
	Ca(ICCV2/PI489777)SNP_18465	1081	G	T	16	5	--	--
	Ca(ICCV2/PI489777)SNP_18466	1122	T	A	9	4	--	--
	Ca(ICCV2/PI489777)SNP_18467	1223	A	G	7	3	--	--
CakTC41454	Ca(ICCV2/PI489777)SNP_18468	693	A	G	25	20	--	--
CakTC26818	Ca(ICCV2/PI489777)SNP_18469	1245	A	G	15	7	--	--
CakTC25494	Ca(ICCV2/PI489777)SNP_18470	287	A	G	12	10	--	SET
CakTC38098	Ca(ICCV2/PI489777)SNP_18471	272	C	G	23	4	--	--
	Ca(ICCV2/PI489777)SNP_18472	386	C	T	23	4	--	--
	Ca(ICCV2/PI489777)SNP_18473	455	C	T	20	3	--	--
CakTC40430	Ca(ICCV2/PI489777)SNP_18474	48	T	A	13	4	--	--
	Ca(ICCV2/PI489777)SNP_18475	270	T	C	30	21	--	--
	Ca(ICCV2/PI489777)SNP_18476	289	C	A	17	22	--	--
	Ca(ICCV2/PI489777)SNP_18477	483	G	A	19	20	--	--
CakTC42712	Ca(ICCV2/PI489777)SNP_18478	1530	A	G	54	42	--	--
	Ca(ICCV2/PI489777)SNP_18479	1659	T	C	49	38	--	--
CakTC27823	Ca(ICCV2/PI489777)SNP_18480	2391	T	C	8	5	--	--
	Ca(ICCV2/PI489777)SNP_18481	2694	A	G	19	3	--	--
CakTC33761	Ca(ICCV2/PI489777)SNP_18482	238	C	A	28	6	--	--
	Ca(ICCV2/PI489777)SNP_18483	359	T	C	22	13	--	--
	Ca(ICCV2/PI489777)SNP_18484	514	A	G	31	6	--	--
CakTC34513	Ca(ICCV2/PI489777)SNP_18485	166	A	G	55	35	--	--
	Ca(ICCV2/PI489777)SNP_18486	360	G	C	38	43	--	--
CakTC30014	Ca(ICCV2/PI489777)SNP_18487	1044	A	G	6	3	--	--
CakTC38048	Ca(ICCV2/PI489777)SNP_18488	851	C	G	8	8	--	--
CakTC37075	Ca(ICCV2/PI489777)SNP_18489	1159	A	T	15	17	--	--
CakTC32443	Ca(ICCV2/PI489777)SNP_18490	61	G	A	4	3	--	--
	Ca(ICCV2/PI489777)SNP_18491	290	G	C	5	6	--	--
	Ca(ICCV2/PI489777)SNP_18492	326	A	T	5	6	--	--
	Ca(ICCV2/PI489777)SNP_18493	1055	A	T	3	5	--	--
CakTC40814	Ca(ICCV2/PI489777)SNP_18494	142	C	T	10	23	--	--
	Ca(ICCV2/PI489777)SNP_18495	425	C	T	4	21	--	--

	Ca(ICCV2/PI489777)SNP_18496	435	C	T	5	24	--	--
	Ca(ICCV2/PI489777)SNP_18497	473	T	C	5	27	--	--
CakTC24761	Ca(ICCV2/PI489777)SNP_18498	1675	C	T	24	16	--	--
CakTC32879	Ca(ICCV2/PI489777)SNP_18499	162	A	C	3	3	--	--
	Ca(ICCV2/PI489777)SNP_18500	193	C	A	3	3	--	--
CakTC38186	Ca(ICCV2/PI489777)SNP_18501	262	C	T	9	6	--	--
	Ca(ICCV2/PI489777)SNP_18502	340	T	C	7	5	--	--
	Ca(ICCV2/PI489777)SNP_18503	613	T	G	10	4	--	--
	Ca(ICCV2/PI489777)SNP_18504	685	A	G	8	5	--	--
	Ca(ICCV2/PI489777)SNP_18505	753	G	C	11	4	--	--
	Ca(ICCV2/PI489777)SNP_18506	1063	T	C	7	7	--	--
CakTC23464	Ca(ICCV2/PI489777)SNP_18507	177	T	C	9	7	--	--
	Ca(ICCV2/PI489777)SNP_18508	692	C	G	8	8	--	--
CakTC24072	Ca(ICCV2/PI489777)SNP_18509	1113	A	G	65	28	--	Bromodomain
	Ca(ICCV2/PI489777)SNP_18510	1315	G	T	62	19	--	Bromodomain
	Ca(ICCV2/PI489777)SNP_18511	2097	G	T	58	21	--	Bromodomain
	Ca(ICCV2/PI489777)SNP_18512	2377	A	C	42	16	--	Bromodomain
CakTC40100	Ca(ICCV2/PI489777)SNP_18513	1261	G	C	9	12	Flower	bud
CakTC28855	Ca(ICCV2/PI489777)SNP_18514	175	T	A	16	9	--	--
	Ca(ICCV2/PI489777)SNP_18515	223	G	T	17	11	--	--
	Ca(ICCV2/PI489777)SNP_18516	258	G	A	15	12	--	--
	Ca(ICCV2/PI489777)SNP_18517	808	C	T	5	6	--	--
CakTC24230	Ca(ICCV2/PI489777)SNP_18518	373	C	T	4	3	--	--
CakTC33430	Ca(ICCV2/PI489777)SNP_18519	55	A	C	21	12	--	--
	Ca(ICCV2/PI489777)SNP_18520	101	A	C	41	23	--	--
	Ca(ICCV2/PI489777)SNP_18521	571	T	A	24	22	--	--
	Ca(ICCV2/PI489777)SNP_18522	618	G	T	8	4	--	--
CakTC37953	Ca(ICCV2/PI489777)SNP_18523	1072	A	G	10	12	--	--
CakTC40376	Ca(ICCV2/PI489777)SNP_18524	358	A	G	13	12	--	--
	Ca(ICCV2/PI489777)SNP_18525	376	C	T	12	8	--	--
	Ca(ICCV2/PI489777)SNP_18526	463	G	C	11	12	--	--
	Ca(ICCV2/PI489777)SNP_18527	697	A	G	6	9	--	--
CakTC34411	Ca(ICCV2/PI489777)SNP_18528	286	G	A	4	6	Flower	bud
	Ca(ICCV2/PI489777)SNP_18529	628	C	A	5	5	Flower	bud
CakTC42444	Ca(ICCV2/PI489777)SNP_18530	233	T	C	32	16	--	--
	Ca(ICCV2/PI489777)SNP_18531	555	C	T	33	16	--	--
	Ca(ICCV2/PI489777)SNP_18532	831	A	G	17	7	--	--
	Ca(ICCV2/PI489777)SNP_18533	884	T	C	6	4	--	--
	Ca(ICCV2/PI489777)SNP_18534	890	T	C	6	3	--	--
	Ca(ICCV2/PI489777)SNP_18535	895	T	C	6	4	--	--
	Ca(ICCV2/PI489777)SNP_18536	901	A	G	6	4	--	--
	Ca(ICCV2/PI489777)SNP_18537	907	A	G	6	4	--	--
CakTC42630	Ca(ICCV2/PI489777)SNP_18538	651	T	G	13	8	--	--
CakTC43308	Ca(ICCV2/PI489777)SNP_18539	40	A	C	8	3	--	--
	Ca(ICCV2/PI489777)SNP_18540	111	T	C	9	6	--	--
	Ca(ICCV2/PI489777)SNP_18541	181	T	A	12	7	--	--
	Ca(ICCV2/PI489777)SNP_18542	513	G	A	5	5	--	--
	Ca(ICCV2/PI489777)SNP_18543	542	G	T	5	4	--	--
CakTC28141	Ca(ICCV2/PI489777)SNP_18544	72	T	C	9	9	--	--
	Ca(ICCV2/PI489777)SNP_18545	780	C	T	18	15	--	--
	Ca(ICCV2/PI489777)SNP_18546	1179	T	C	31	20	--	--
	Ca(ICCV2/PI489777)SNP_18547	1248	A	G	22	21	--	--
	Ca(ICCV2/PI489777)SNP_18548	1281	T	A	21	17	--	--
	Ca(ICCV2/PI489777)SNP_18549	1524	C	T	21	13	--	--
	Ca(ICCV2/PI489777)SNP_18550	1624	T	C	20	8	--	--
	Ca(ICCV2/PI489777)SNP_18551	1678	G	A	15	11	--	--
	Ca(ICCV2/PI489777)SNP_18552	1734	A	G	18	8	--	--
	Ca(ICCV2/PI489777)SNP_18553	1873	C	G	8	3	--	--
CakTC26854	Ca(ICCV2/PI489777)SNP_18554	312	A	G	17	9	--	--
	Ca(ICCV2/PI489777)SNP_18555	372	A	G	25	12	--	--
	Ca(ICCV2/PI489777)SNP_18556	399	A	G	23	15	--	--
	Ca(ICCV2/PI489777)SNP_18557	429	T	C	24	13	--	--
	Ca(ICCV2/PI489777)SNP_18558	612	G	A	15	11	--	--
	Ca(ICCV2/PI489777)SNP_18559	1042	T	A	27	5	--	--
	Ca(ICCV2/PI489777)SNP_18560	1065	C	T	25	5	--	--
	Ca(ICCV2/PI489777)SNP_18561	1113	G	A	27	4	--	--
	Ca(ICCV2/PI489777)SNP_18562	1272	T	A	19	3	--	--
	Ca(ICCV2/PI489777)SNP_18563	1283	C	T	22	3	--	--
	Ca(ICCV2/PI489777)SNP_18564	1650	G	C	17	10	--	--
	Ca(ICCV2/PI489777)SNP_18565	2808	T	A	19	10	--	--
	Ca(ICCV2/PI489777)SNP_18566	2813	C	T	19	10	--	--
	Ca(ICCV2/PI489777)SNP_18567	2830	C	G	19	9	--	--
	Ca(ICCV2/PI489777)SNP_18568	2896	G	C	10	8	--	--
	Ca(ICCV2/PI489777)SNP_18569	2997	C	G	8	4	--	--

CakTC38658	Ca(ICCV2/PI489777)SNP_18570	131	T	A	36	50	--	--
	Ca(ICCV2/PI489777)SNP_18571	191	C	T	65	93	--	--
	Ca(ICCV2/PI489777)SNP_18572	209	A	C	79	77	--	--
	Ca(ICCV2/PI489777)SNP_18573	226	C	G	64	87	--	--
	Ca(ICCV2/PI489777)SNP_18574	403	A	C	86	108	--	--
	Ca(ICCV2/PI489777)SNP_18575	433	G	A	82	112	--	--
CakTC42445	Ca(ICCV2/PI489777)SNP_18576	671	T	G	11	5	--	--
	Ca(ICCV2/PI489777)SNP_18577	805	T	A	13	5	--	--
	Ca(ICCV2/PI489777)SNP_18578	824	C	T	13	4	--	--
CakTC39030	Ca(ICCV2/PI489777)SNP_18579	119	T	G	10	5	--	--
	Ca(ICCV2/PI489777)SNP_18580	965	C	G	5	12	--	--
CakTC35189	Ca(ICCV2/PI489777)SNP_18581	411	C	T	16	8	--	--
	Ca(ICCV2/PI489777)SNP_18582	783	T	A	11	4	--	--
	Ca(ICCV2/PI489777)SNP_18583	1695	G	A	5	6	--	--
	Ca(ICCV2/PI489777)SNP_18584	2637	A	T	9	6	--	--
CakTC24577	Ca(ICCV2/PI489777)SNP_18585	1615	A	G	8	3	Root	--
	Ca(ICCV2/PI489777)SNP_18586	1942	A	C	15	5	Root	--
	Ca(ICCV2/PI489777)SNP_18587	2256	C	G	8	4	Root	--
CakTC43327	Ca(ICCV2/PI489777)SNP_18588	806	G	A	3	4	--	--
	Ca(ICCV2/PI489777)SNP_18589	914	G	A	4	4	--	--
CakTC26980	Ca(ICCV2/PI489777)SNP_18590	2703	G	A	6	8	Flower	bud
	Ca(ICCV2/PI489777)SNP_18591	3633	A	G	10	4	Flower	bud
CakTC01786	Ca(ICCV2/PI489777)SNP_18592	153	T	A	10	3	--	--
	Ca(ICCV2/PI489777)SNP_18593	229	T	G	9	3	--	--
CakTC26491	Ca(ICCV2/PI489777)SNP_18594	227	T	C	7	4	--	--
CakTC25847	Ca(ICCV2/PI489777)SNP_18595	130	C	T	10	10	--	--
	Ca(ICCV2/PI489777)SNP_18596	556	G	T	22	5	--	--
	Ca(ICCV2/PI489777)SNP_18597	658	T	C	28	16	--	--
	Ca(ICCV2/PI489777)SNP_18598	1071	A	C	61	32	--	--
	Ca(ICCV2/PI489777)SNP_18599	1363	G	A	52	26	--	--
	Ca(ICCV2/PI489777)SNP_18600	1618	C	T	31	15	--	--
	Ca(ICCV2/PI489777)SNP_18601	1805	G	A	30	6	--	--
	Ca(ICCV2/PI489777)SNP_18602	1872	G	A	27	5	--	--
	Ca(ICCV2/PI489777)SNP_18603	1924	C	T	13	4	--	--
CakTC36304	Ca(ICCV2/PI489777)SNP_18604	12	C	T	3	5	--	--
	Ca(ICCV2/PI489777)SNP_18605	31	C	A	7	7	--	--
	Ca(ICCV2/PI489777)SNP_18606	77	T	G	8	10	--	--
	Ca(ICCV2/PI489777)SNP_18607	341	A	G	6	11	--	--
	Ca(ICCV2/PI489777)SNP_18608	1133	T	A	3	11	--	--
	Ca(ICCV2/PI489777)SNP_18609	1577	C	T	12	8	--	--
	Ca(ICCV2/PI489777)SNP_18610	1715	G	A	9	6	--	--
	Ca(ICCV2/PI489777)SNP_18611	1727	C	T	11	6	--	--
CakTC41493	Ca(ICCV2/PI489777)SNP_18612	168	C	T	9	9	--	--
CakTC42786	Ca(ICCV2/PI489777)SNP_18613	373	G	T	12	10	--	--
	Ca(ICCV2/PI489777)SNP_18614	973	C	T	13	4	--	--
	Ca(ICCV2/PI489777)SNP_18615	1051	C	T	13	6	--	--
CakTC07019	Ca(ICCV2/PI489777)SNP_18616	289	A	G	29	13	--	--
	Ca(ICCV2/PI489777)SNP_18617	295	G	A	36	14	--	--
	Ca(ICCV2/PI489777)SNP_18618	412	T	C	57	22	--	--
	Ca(ICCV2/PI489777)SNP_18619	523	A	G	61	18	--	--
	Ca(ICCV2/PI489777)SNP_18620	715	G	C	43	5	--	--
CakTC23367	Ca(ICCV2/PI489777)SNP_18621	1861	T	C	11	11	--	NAC
	Ca(ICCV2/PI489777)SNP_18622	1940	C	A	11	10	--	NAC
CakTC27095	Ca(ICCV2/PI489777)SNP_18623	551	C	G	23	4	--	--
	Ca(ICCV2/PI489777)SNP_18624	700	C	T	24	3	--	--
CakTC29865	Ca(ICCV2/PI489777)SNP_18625	216	C	T	8	7	--	--
	Ca(ICCV2/PI489777)SNP_18626	996	C	T	37	9	--	--
	Ca(ICCV2/PI489777)SNP_18627	1143	A	G	30	8	--	--
CakTC20440	Ca(ICCV2/PI489777)SNP_18628	687	T	C	16	5	--	--
CakTC24567	Ca(ICCV2/PI489777)SNP_18629	1127	C	T	15	16	--	--
CakTC09824	Ca(ICCV2/PI489777)SNP_18630	461	C	G	8	6	--	--
	Ca(ICCV2/PI489777)SNP_18631	989	C	T	3	3	--	--
CakTC42548	Ca(ICCV2/PI489777)SNP_18632	53	G	T	8	5	--	--
	Ca(ICCV2/PI489777)SNP_18633	126	G	A	10	9	--	--
	Ca(ICCV2/PI489777)SNP_18634	337	T	A	13	13	--	--
	Ca(ICCV2/PI489777)SNP_18635	1192	A	G	10	5	--	--
CakTC42644	Ca(ICCV2/PI489777)SNP_18636	204	T	C	11	6	--	--
	Ca(ICCV2/PI489777)SNP_18637	351	C	G	26	7	--	--
	Ca(ICCV2/PI489777)SNP_18638	381	A	G	29	8	--	--
	Ca(ICCV2/PI489777)SNP_18639	579	A	G	15	4	--	--
	Ca(ICCV2/PI489777)SNP_18640	626	C	T	12	3	--	--
	Ca(ICCV2/PI489777)SNP_18641	823	C	T	8	4	--	--
CakTC24522	Ca(ICCV2/PI489777)SNP_18642	887	C	T	6	4	--	BSD
CakTC26203	Ca(ICCV2/PI489777)SNP_18643	748	A	G	6	4	--	BES1

	Ca(ICCV2/PI489777)SNP_18644	778	A	G	7	4	--	BES1
	Ca(ICCV2/PI489777)SNP_18645	1154	G	A	6	5	--	BES1
	Ca(ICCV2/PI489777)SNP_18646	1486	C	A	3	7	--	BES1
	Ca(ICCV2/PI489777)SNP_18647	1495	T	C	3	7	--	BES1
CakTC37388	Ca(ICCV2/PI489777)SNP_18648	358	T	G	12	12	--	--
	Ca(ICCV2/PI489777)SNP_18649	1422	A	G	8	9	--	--
	Ca(ICCV2/PI489777)SNP_18650	1548	C	G	5	8	--	--
CakTC27403	Ca(ICCV2/PI489777)SNP_18651	580	A	G	7	3	--	--
CakTC33793	Ca(ICCV2/PI489777)SNP_18652	353	A	T	19	3	--	--
	Ca(ICCV2/PI489777)SNP_18653	1074	A	G	29	10	--	--
	Ca(ICCV2/PI489777)SNP_18654	2192	T	G	9	6	--	--
	Ca(ICCV2/PI489777)SNP_18655	2207	A	G	7	6	--	--
CakTC36981	Ca(ICCV2/PI489777)SNP_18656	332	A	G	7	4	--	--
	Ca(ICCV2/PI489777)SNP_18657	337	T	C	7	3	--	--
CakTC42304	Ca(ICCV2/PI489777)SNP_18658	1501	A	G	10	8	--	--
CakTC25852	Ca(ICCV2/PI489777)SNP_18659	657	C	A	12	4	--	--
CakTC40228	Ca(ICCV2/PI489777)SNP_18660	1336	T	G	13	6	--	--
	Ca(ICCV2/PI489777)SNP_18661	1342	G	T	12	7	--	--
CakTC29310	Ca(ICCV2/PI489777)SNP_18662	182	T	A	12	18	--	--
	Ca(ICCV2/PI489777)SNP_18663	531	T	C	18	19	--	--
CakTC28801	Ca(ICCV2/PI489777)SNP_18664	142	C	G	5	3	--	--
	Ca(ICCV2/PI489777)SNP_18665	217	A	G	7	3	--	--
	Ca(ICCV2/PI489777)SNP_18666	309	C	G	7	4	--	--
	Ca(ICCV2/PI489777)SNP_18667	423	A	G	7	3	--	--
	Ca(ICCV2/PI489777)SNP_18668	933	T	C	4	16	--	--
	Ca(ICCV2/PI489777)SNP_18669	948	G	A	4	16	--	--
CakTC13631	Ca(ICCV2/PI489777)SNP_18670	712	A	G	3	3	--	--
	Ca(ICCV2/PI489777)SNP_18671	831	T	C	6	10	--	--
	Ca(ICCV2/PI489777)SNP_18672	860	C	A	7	10	--	--
	Ca(ICCV2/PI489777)SNP_18673	917	A	T	6	15	--	--
	Ca(ICCV2/PI489777)SNP_18674	986	C	T	9	13	--	--
	Ca(ICCV2/PI489777)SNP_18675	1424	G	C	8	15	--	--
CakTC30513	Ca(ICCV2/PI489777)SNP_18676	1780	G	C	41	7	--	--
	Ca(ICCV2/PI489777)SNP_18677	1962	A	C	7	3	--	--
	Ca(ICCV2/PI489777)SNP_18678	2843	A	C	14	7	--	--
	Ca(ICCV2/PI489777)SNP_18679	3057	C	A	9	3	--	--
	Ca(ICCV2/PI489777)SNP_18680	3731	C	A	4	5	--	--
CakTC31829	Ca(ICCV2/PI489777)SNP_18681	925	G	A	7	5	--	AP2-EREBP
CakTC26836	Ca(ICCV2/PI489777)SNP_18682	164	G	T	33	11	--	--
	Ca(ICCV2/PI489777)SNP_18683	706	T	C	53	30	--	--
	Ca(ICCV2/PI489777)SNP_18684	1117	T	C	69	48	--	--
	Ca(ICCV2/PI489777)SNP_18685	1255	C	A	48	32	--	--
	Ca(ICCV2/PI489777)SNP_18686	1486	G	A	29	20	--	--
	Ca(ICCV2/PI489777)SNP_18687	1800	C	T	7	4	--	--
	Ca(ICCV2/PI489777)SNP_18688	1827	C	T	6	4	--	--
CakTC41338	Ca(ICCV2/PI489777)SNP_18689	253	C	T	31	74	--	--
	Ca(ICCV2/PI489777)SNP_18690	549	T	C	11	9	--	--
CakTC38651	Ca(ICCV2/PI489777)SNP_18691	53	T	C	14	6	--	--
	Ca(ICCV2/PI489777)SNP_18692	200	T	A	38	15	--	--
	Ca(ICCV2/PI489777)SNP_18693	249	C	A	44	20	--	--
	Ca(ICCV2/PI489777)SNP_18694	412	T	C	49	21	--	--
	Ca(ICCV2/PI489777)SNP_18695	535	G	C	34	13	--	--
	Ca(ICCV2/PI489777)SNP_18696	673	C	T	47	7	--	--
	Ca(ICCV2/PI489777)SNP_18697	1336	A	G	30	16	--	--
	Ca(ICCV2/PI489777)SNP_18698	1453	A	G	38	16	--	--
CakTC39886	Ca(ICCV2/PI489777)SNP_18699	1094	T	C	11	4	--	--
CakTC40000	Ca(ICCV2/PI489777)SNP_18700	665	A	G	13	4	--	LOB
CakTC27390	Ca(ICCV2/PI489777)SNP_18701	2059	A	T	13	9	--	NAC
	Ca(ICCV2/PI489777)SNP_18702	2078	A	G	9	7	--	NAC
	Ca(ICCV2/PI489777)SNP_18703	2091	C	T	4	5	--	NAC
CakTC22864	Ca(ICCV2/PI489777)SNP_18704	954	C	T	5	3	--	GNAT
CakTC34844	Ca(ICCV2/PI489777)SNP_18705	1371	C	A	16	5	--	--
	Ca(ICCV2/PI489777)SNP_18706	2013	G	A	14	7	--	--
	Ca(ICCV2/PI489777)SNP_18707	2765	T	C	8	5	--	--
	Ca(ICCV2/PI489777)SNP_18708	2839	A	T	9	6	--	--
	Ca(ICCV2/PI489777)SNP_18709	3146	C	A	7	4	--	--
	Ca(ICCV2/PI489777)SNP_18710	3563	T	A	3	5	--	--
	Ca(ICCV2/PI489777)SNP_18711	3696	G	C	4	5	--	--
CakTC24550	Ca(ICCV2/PI489777)SNP_18712	932	A	G	23	4	--	--
	Ca(ICCV2/PI489777)SNP_18713	1272	T	C	13	4	--	--
	Ca(ICCV2/PI489777)SNP_18714	1877	A	G	13	11	--	--
CakTC26339	Ca(ICCV2/PI489777)SNP_18715	1240	G	A	14	13	--	--
CakTC04806	Ca(ICCV2/PI489777)SNP_18716	172	A	G	3	4	--	--
	Ca(ICCV2/PI489777)SNP_18717	1039	T	C	4	4	--	--

	Ca(ICCV2/PI489777)SNP_18718	1057	G	A	5	4	--	--
CakTC28389	Ca(ICCV2/PI489777)SNP_18719	227	G	C	3	6	--	C3H
CakTC41257	Ca(ICCV2/PI489777)SNP_18720	272	G	C	26	14	--	--
CakTC26434	Ca(ICCV2/PI489777)SNP_18721	367	T	C	29	15	--	--
CakTC09960	Ca(ICCV2/PI489777)SNP_18722	41	T	C	30	23	--	--
	Ca(ICCV2/PI489777)SNP_18723	67	A	G	32	23	--	--
	Ca(ICCV2/PI489777)SNP_18724	95	T	G	33	25	--	--
	Ca(ICCV2/PI489777)SNP_18725	105	G	A	33	24	--	--
	Ca(ICCV2/PI489777)SNP_18726	110	T	C	32	24	--	--
	Ca(ICCV2/PI489777)SNP_18727	119	G	A	36	22	--	--
	Ca(ICCV2/PI489777)SNP_18728	155	C	T	35	28	--	--
	Ca(ICCV2/PI489777)SNP_18729	158	A	T	36	27	--	--
	Ca(ICCV2/PI489777)SNP_18730	216	C	T	31	27	--	--
	Ca(ICCV2/PI489777)SNP_18731	251	T	G	28	21	--	--
	Ca(ICCV2/PI489777)SNP_18732	263	G	C	29	26	--	--
	Ca(ICCV2/PI489777)SNP_18733	272	G	T	27	26	--	--
	Ca(ICCV2/PI489777)SNP_18734	326	C	G	26	23	--	--
	Ca(ICCV2/PI489777)SNP_18735	470	A	T	18	17	--	--
	Ca(ICCV2/PI489777)SNP_18736	656	T	G	17	25	--	--
	Ca(ICCV2/PI489777)SNP_18737	774	A	G	33	31	--	--
	Ca(ICCV2/PI489777)SNP_18738	1022	A	T	24	10	--	--
	Ca(ICCV2/PI489777)SNP_18739	1049	C	A	15	10	--	--
	Ca(ICCV2/PI489777)SNP_18740	1069	A	G	13	9	--	--
	Ca(ICCV2/PI489777)SNP_18741	1078	A	C	12	6	--	--
	Ca(ICCV2/PI489777)SNP_18742	1088	T	A	10	4	--	--
CakTC25378	Ca(ICCV2/PI489777)SNP_18743	963	A	G	10	12	--	--
	Ca(ICCV2/PI489777)SNP_18744	1161	G	A	3	4	--	--
	Ca(ICCV2/PI489777)SNP_18745	1863	A	C	6	4	--	--
	Ca(ICCV2/PI489777)SNP_18746	1896	A	T	6	5	--	--
	Ca(ICCV2/PI489777)SNP_18747	2046	T	G	11	5	--	--
	Ca(ICCV2/PI489777)SNP_18748	2064	G	A	10	6	--	--
	Ca(ICCV2/PI489777)SNP_18749	2273	A	G	9	3	--	--
	Ca(ICCV2/PI489777)SNP_18750	2275	G	T	9	3	--	--
CakTC38953	Ca(ICCV2/PI489777)SNP_18751	455	G	A	4	3	--	--
	Ca(ICCV2/PI489777)SNP_18752	629	G	C	4	3	--	--
CakTC26470	Ca(ICCV2/PI489777)SNP_18753	473	T	A	3	3	--	--
CakTC30630	Ca(ICCV2/PI489777)SNP_18754	999	A	T	6	3	--	--
	Ca(ICCV2/PI489777)SNP_18755	1093	C	A	6	3	--	--
	Ca(ICCV2/PI489777)SNP_18756	1416	A	G	6	5	--	--
CakTC40552	Ca(ICCV2/PI489777)SNP_18757	292	T	C	9	3	--	--
CakTC38859	Ca(ICCV2/PI489777)SNP_18758	134	A	G	29	20	--	--
	Ca(ICCV2/PI489777)SNP_18759	396	G	C	33	28	--	--
	Ca(ICCV2/PI489777)SNP_18760	401	A	G	34	29	--	--
	Ca(ICCV2/PI489777)SNP_18761	522	T	C	24	22	--	--
CakTC41061	Ca(ICCV2/PI489777)SNP_18762	259	G	A	8	3	--	--
	Ca(ICCV2/PI489777)SNP_18763	413	C	T	6	3	--	--
CakTC11432	Ca(ICCV2/PI489777)SNP_18764	183	C	G	55	8	--	--
	Ca(ICCV2/PI489777)SNP_18765	375	T	C	67	11	--	--
	Ca(ICCV2/PI489777)SNP_18766	486	A	G	52	13	--	--
	Ca(ICCV2/PI489777)SNP_18767	603	C	T	39	10	--	--
	Ca(ICCV2/PI489777)SNP_18768	609	T	C	36	10	--	--
	Ca(ICCV2/PI489777)SNP_18769	1011	T	G	45	10	--	--
	Ca(ICCV2/PI489777)SNP_18770	1012	T	G	45	10	--	--
	Ca(ICCV2/PI489777)SNP_18771	1039	G	T	46	8	--	--
CakTC38909	Ca(ICCV2/PI489777)SNP_18772	64	A	T	4	13	--	--
	Ca(ICCV2/PI489777)SNP_18773	132	C	A	6	15	--	--
	Ca(ICCV2/PI489777)SNP_18774	360	C	T	10	17	--	--
	Ca(ICCV2/PI489777)SNP_18775	768	T	C	16	35	--	--
	Ca(ICCV2/PI489777)SNP_18776	1386	G	A	5	5	--	--
CakTC40146	Ca(ICCV2/PI489777)SNP_18777	1081	G	T	14	6	--	--
	Ca(ICCV2/PI489777)SNP_18778	1165	G	C	14	5	--	--
	Ca(ICCV2/PI489777)SNP_18779	1254	T	A	15	4	--	--
	Ca(ICCV2/PI489777)SNP_18780	1255	G	T	16	4	--	--
	Ca(ICCV2/PI489777)SNP_18781	1264	T	G	15	4	--	--
	Ca(ICCV2/PI489777)SNP_18782	1365	A	T	7	3	--	--
	Ca(ICCV2/PI489777)SNP_18783	1375	G	A	7	3	--	--
	Ca(ICCV2/PI489777)SNP_18784	1438	A	G	3	3	--	--
CakTC39248	Ca(ICCV2/PI489777)SNP_18785	435	T	G	12	7	--	--
	Ca(ICCV2/PI489777)SNP_18786	581	T	C	14	6	--	--
	Ca(ICCV2/PI489777)SNP_18787	1488	T	A	6	10	--	--
CakTC29742	Ca(ICCV2/PI489777)SNP_18788	732	C	T	29	22	--	--
CakTC28448	Ca(ICCV2/PI489777)SNP_18789	469	C	A	10	4	--	MYB
	Ca(ICCV2/PI489777)SNP_18790	669	G	A	16	9	--	MYB
	Ca(ICCV2/PI489777)SNP_18791	900	T	C	14	12	--	MYB

	Ca(ICCV2/PI489777)SNP_18792	1285	T	C	27	14	--	MYB
	Ca(ICCV2/PI489777)SNP_18793	1393	T	C	17	10	--	MYB
CakTC24069	Ca(ICCV2/PI489777)SNP_18794	22	A	G	90	10	--	--
CakTC38717	Ca(ICCV2/PI489777)SNP_18795	367	A	C	6	10	--	--
	Ca(ICCV2/PI489777)SNP_18796	379	G	T	6	10	--	--
CakTC40118	Ca(ICCV2/PI489777)SNP_18797	873	T	C	11	10	--	--
	Ca(ICCV2/PI489777)SNP_18798	960	T	C	16	9	--	--
	Ca(ICCV2/PI489777)SNP_18799	1020	A	G	17	8	--	--
	Ca(ICCV2/PI489777)SNP_18800	1083	G	C	14	7	--	--
CakTC30823	Ca(ICCV2/PI489777)SNP_18801	1065	A	C	14	7	--	--
	Ca(ICCV2/PI489777)SNP_18802	1599	A	G	9	8	--	--
	Ca(ICCV2/PI489777)SNP_18803	2238	G	A	15	3	--	--
	Ca(ICCV2/PI489777)SNP_18804	3752	A	T	17	8	--	--
CakTC40953	Ca(ICCV2/PI489777)SNP_18805	1511	A	G	9	6	--	--
	Ca(ICCV2/PI489777)SNP_18806	1895	C	T	3	3	--	--
CakTC31841	Ca(ICCV2/PI489777)SNP_18807	1079	G	A	3	4	--	ARF
	Ca(ICCV2/PI489777)SNP_18808	1376	A	G	4	5	--	ARF
	Ca(ICCV2/PI489777)SNP_18809	1386	A	G	5	5	--	ARF
	Ca(ICCV2/PI489777)SNP_18810	1391	C	T	5	5	--	ARF
	Ca(ICCV2/PI489777)SNP_18811	1496	G	A	5	5	--	ARF
	Ca(ICCV2/PI489777)SNP_18812	1716	C	A	14	4	--	ARF
	Ca(ICCV2/PI489777)SNP_18813	2009	T	C	14	6	--	ARF
	Ca(ICCV2/PI489777)SNP_18814	2295	A	C	17	6	--	ARF
CakTC34716	Ca(ICCV2/PI489777)SNP_18815	1562	C	T	19	14	--	--
	Ca(ICCV2/PI489777)SNP_18816	2081	A	G	22	8	--	--
	Ca(ICCV2/PI489777)SNP_18817	2972	G	A	14	4	--	--
	Ca(ICCV2/PI489777)SNP_18818	3290	T	A	8	4	--	--
CakTC42862	Ca(ICCV2/PI489777)SNP_18819	408	T	C	6	23	--	--
	Ca(ICCV2/PI489777)SNP_18820	693	G	T	8	12	--	--
	Ca(ICCV2/PI489777)SNP_18821	773	G	A	7	7	--	--
CakTC25201	Ca(ICCV2/PI489777)SNP_18822	228	G	A	44	3	--	--
	Ca(ICCV2/PI489777)SNP_18823	315	C	T	53	6	--	--
	Ca(ICCV2/PI489777)SNP_18824	326	C	A	50	6	--	--
	Ca(ICCV2/PI489777)SNP_18825	327	A	G	50	6	--	--
	Ca(ICCV2/PI489777)SNP_18826	432	T	G	50	14	--	--
	Ca(ICCV2/PI489777)SNP_18827	445	G	T	52	16	--	--
	Ca(ICCV2/PI489777)SNP_18828	536	C	T	39	16	--	--
	Ca(ICCV2/PI489777)SNP_18829	748	T	G	34	11	--	--
	Ca(ICCV2/PI489777)SNP_18830	847	C	T	27	5	--	--
	Ca(ICCV2/PI489777)SNP_18831	859	C	T	27	7	--	--
CakTC41698	Ca(ICCV2/PI489777)SNP_18832	428	G	C	232	126	--	--
	Ca(ICCV2/PI489777)SNP_18833	429	T	C	235	126	--	--
CakTC24459	Ca(ICCV2/PI489777)SNP_18834	124	T	A	33	4	--	--
	Ca(ICCV2/PI489777)SNP_18835	181	C	T	47	4	--	--
	Ca(ICCV2/PI489777)SNP_18836	729	C	G	35	3	--	--
	Ca(ICCV2/PI489777)SNP_18837	1883	A	G	30	3	--	--
CakTC22814	Ca(ICCV2/PI489777)SNP_18838	550	T	C	7	3	Shoot	--
CakTC32470	Ca(ICCV2/PI489777)SNP_18839	557	T	C	17	4	--	--
	Ca(ICCV2/PI489777)SNP_18840	603	T	C	19	4	--	--
CakTC37346	Ca(ICCV2/PI489777)SNP_18841	369	A	G	7	3	--	--
	Ca(ICCV2/PI489777)SNP_18842	434	A	T	6	3	--	--
CakTC36502	Ca(ICCV2/PI489777)SNP_18843	687	T	C	3	3	--	--
CakTC41460	Ca(ICCV2/PI489777)SNP_18844	150	C	T	49	18	--	--
	Ca(ICCV2/PI489777)SNP_18845	204	T	C	62	24	--	--
	Ca(ICCV2/PI489777)SNP_18846	236	A	G	64	26	--	--
	Ca(ICCV2/PI489777)SNP_18847	453	A	G	64	31	--	--
CakTC08446	Ca(ICCV2/PI489777)SNP_18848	3689	A	C	4	4	Young_pod	--
CakTC30621	Ca(ICCV2/PI489777)SNP_18849	898	A	C	4	4	--	--
CakTC41877	Ca(ICCV2/PI489777)SNP_18850	250	G	A	5	3	--	--
CakTC26411	Ca(ICCV2/PI489777)SNP_18851	179	G	A	32	18	--	--
	Ca(ICCV2/PI489777)SNP_18852	762	T	G	34	24	--	--
	Ca(ICCV2/PI489777)SNP_18853	1422	A	T	52	24	--	--
CakTC23964	Ca(ICCV2/PI489777)SNP_18854	109	A	G	4	3	--	--
	Ca(ICCV2/PI489777)SNP_18855	923	G	T	55	14	--	--
	Ca(ICCV2/PI489777)SNP_18856	1532	G	T	72	18	--	--
CakTC36373	Ca(ICCV2/PI489777)SNP_18857	270	A	C	5	5	--	--
CakTC15141	Ca(ICCV2/PI489777)SNP_18858	281	C	T	3	3	--	--
	Ca(ICCV2/PI489777)SNP_18859	439	C	T	4	3	--	--
CakTC22634	Ca(ICCV2/PI489777)SNP_18860	251	C	T	14	8	--	--
	Ca(ICCV2/PI489777)SNP_18861	578	C	T	8	5	--	--
	Ca(ICCV2/PI489777)SNP_18862	624	A	C	8	5	--	--
	Ca(ICCV2/PI489777)SNP_18863	806	T	C	11	9	--	--
	Ca(ICCV2/PI489777)SNP_18864	1467	C	A	4	4	--	--
CakTC37799	Ca(ICCV2/PI489777)SNP_18865	359	C	T	25	13	--	SAP

	Ca(ICCV2/PI489777)SNP_18866	809	C	T	7	5	--	SAP
CakTC29922	Ca(ICCV2/PI489777)SNP_18867	2149	C	T	7	6	--	--
CakTC28152	Ca(ICCV2/PI489777)SNP_18868	463	G	A	13	3	--	--
	Ca(ICCV2/PI489777)SNP_18869	1147	C	T	7	5	--	--
	Ca(ICCV2/PI489777)SNP_18870	1382	T	C	8	4	--	--
	Ca(ICCV2/PI489777)SNP_18871	1401	G	C	8	4	--	--
CakTC36963	Ca(ICCV2/PI489777)SNP_18872	104	T	G	3	9	--	--
	Ca(ICCV2/PI489777)SNP_18873	367	C	T	3	9	--	--
CakTC13322	Ca(ICCV2/PI489777)SNP_18874	2914	G	A	7	4	--	--
CakTC26885	Ca(ICCV2/PI489777)SNP_18875	201	C	T	9	4	--	--
	Ca(ICCV2/PI489777)SNP_18876	317	A	G	10	8	--	--
	Ca(ICCV2/PI489777)SNP_18877	424	A	G	10	8	--	--
CakTC27086	Ca(ICCV2/PI489777)SNP_18878	853	T	C	41	34	--	--
	Ca(ICCV2/PI489777)SNP_18879	1360	T	C	42	35	--	--
CakTC11066	Ca(ICCV2/PI489777)SNP_18880	2107	A	G	8	4	--	--
	Ca(ICCV2/PI489777)SNP_18881	2475	G	C	6	6	--	--
	Ca(ICCV2/PI489777)SNP_18882	2697	G	A	6	3	--	--
CakTC40853	Ca(ICCV2/PI489777)SNP_18883	1169	A	G	34	10	--	--
CakTC29929	Ca(ICCV2/PI489777)SNP_18884	78	C	T	10	9	--	--
	Ca(ICCV2/PI489777)SNP_18885	81	T	G	10	9	--	--
	Ca(ICCV2/PI489777)SNP_18886	97	A	T	11	12	--	--
	Ca(ICCV2/PI489777)SNP_18887	341	C	T	33	19	--	--
	Ca(ICCV2/PI489777)SNP_18888	875	T	C	26	11	--	--
CakTC08108	Ca(ICCV2/PI489777)SNP_18889	1444	A	G	6	13	--	--
	Ca(ICCV2/PI489777)SNP_18890	1510	A	G	6	11	--	--
	Ca(ICCV2/PI489777)SNP_18891	1516	C	T	5	11	--	--
	Ca(ICCV2/PI489777)SNP_18892	1540	A	G	7	11	--	--
	Ca(ICCV2/PI489777)SNP_18893	1618	G	T	7	9	--	--
CakTC23958	Ca(ICCV2/PI489777)SNP_18894	68	G	T	9	13	--	--
	Ca(ICCV2/PI489777)SNP_18895	344	A	G	14	13	--	--
CakTC06975	Ca(ICCV2/PI489777)SNP_18896	1492	T	C	5	4	--	--
CakTC43301	Ca(ICCV2/PI489777)SNP_18897	926	T	C	6	6	--	--
	Ca(ICCV2/PI489777)SNP_18898	1033	T	C	6	3	--	--
	Ca(ICCV2/PI489777)SNP_18899	1493	G	A	8	4	--	--
CakTC38632	Ca(ICCV2/PI489777)SNP_18900	964	A	G	8	7	--	--
CakTC40973	Ca(ICCV2/PI489777)SNP_18901	369	T	A	14	10	--	--
	Ca(ICCV2/PI489777)SNP_18902	1089	G	T	24	15	--	--
	Ca(ICCV2/PI489777)SNP_18903	1483	G	A	20	18	--	--
CakTC42132	Ca(ICCV2/PI489777)SNP_18904	323	A	G	71	14	--	--
	Ca(ICCV2/PI489777)SNP_18905	431	T	C	91	23	--	--
	Ca(ICCV2/PI489777)SNP_18906	740	G	A	94	21	--	--
	Ca(ICCV2/PI489777)SNP_18907	751	T	A	84	19	--	--
	Ca(ICCV2/PI489777)SNP_18908	759	G	T	92	18	--	--
	Ca(ICCV2/PI489777)SNP_18909	764	C	T	92	17	--	--
	Ca(ICCV2/PI489777)SNP_18910	817	T	A	58	9	--	--
CakTC30344	Ca(ICCV2/PI489777)SNP_18911	184	C	T	10	11	--	--
	Ca(ICCV2/PI489777)SNP_18912	187	C	T	10	11	--	--
	Ca(ICCV2/PI489777)SNP_18913	301	C	A	10	7	--	--
	Ca(ICCV2/PI489777)SNP_18914	553	G	T	7	18	--	--
	Ca(ICCV2/PI489777)SNP_18915	1045	C	T	6	15	--	--
	Ca(ICCV2/PI489777)SNP_18916	1147	A	T	8	20	--	--
	Ca(ICCV2/PI489777)SNP_18917	1471	T	C	8	11	--	--
CakTC39473	Ca(ICCV2/PI489777)SNP_18918	1017	C	T	3	5	--	--
CakTC25736	Ca(ICCV2/PI489777)SNP_18919	245	T	A	6	5	Young_pod	--
	Ca(ICCV2/PI489777)SNP_18920	942	T	A	3	31	Young_pod	--
	Ca(ICCV2/PI489777)SNP_18921	987	A	T	3	31	Young_pod	--
CakTC41823	Ca(ICCV2/PI489777)SNP_18922	196	C	T	11	12	--	--
	Ca(ICCV2/PI489777)SNP_18923	250	A	G	10	13	--	--
	Ca(ICCV2/PI489777)SNP_18924	262	T	C	13	13	--	--
	Ca(ICCV2/PI489777)SNP_18925	296	T	A	14	13	--	--
	Ca(ICCV2/PI489777)SNP_18926	709	G	A	25	5	--	--
CakTC31210	Ca(ICCV2/PI489777)SNP_18927	159	G	A	24	7	--	--
	Ca(ICCV2/PI489777)SNP_18928	245	A	C	34	10	--	--
	Ca(ICCV2/PI489777)SNP_18929	377	C	T	30	10	--	--
CakTC37267	Ca(ICCV2/PI489777)SNP_18930	67	C	A	5	8	--	--
	Ca(ICCV2/PI489777)SNP_18931	309	C	T	9	18	--	--
	Ca(ICCV2/PI489777)SNP_18932	522	A	G	6	36	--	--
	Ca(ICCV2/PI489777)SNP_18933	693	T	C	21	37	--	--
	Ca(ICCV2/PI489777)SNP_18934	711	T	C	14	34	--	--
CakTC42803	Ca(ICCV2/PI489777)SNP_18935	113	G	A	29	11	--	MBF1
	Ca(ICCV2/PI489777)SNP_18936	131	A	G	28	15	--	MBF1
	Ca(ICCV2/PI489777)SNP_18937	362	C	T	80	57	--	MBF1
CakTC25794	Ca(ICCV2/PI489777)SNP_18938	886	C	G	86	45	--	--
	Ca(ICCV2/PI489777)SNP_18939	1518	C	T	41	49	--	--

	Ca(ICCV2/PI489777)SNP_18940	1635	A	G	33	40	--	--
CakTC42204	Ca(ICCV2/PI489777)SNP_18941	148	C	T	36	14	--	--
	Ca(ICCV2/PI489777)SNP_18942	183	T	G	20	17	--	--
	Ca(ICCV2/PI489777)SNP_18943	442	G	A	44	20	--	--
	Ca(ICCV2/PI489777)SNP_18944	652	C	T	15	10	--	--
	Ca(ICCV2/PI489777)SNP_18945	667	G	A	15	10	--	--
	Ca(ICCV2/PI489777)SNP_18946	676	C	T	14	10	--	--
	Ca(ICCV2/PI489777)SNP_18947	691	C	A	12	9	--	--
	Ca(ICCV2/PI489777)SNP_18948	773	A	T	7	8	--	--
	Ca(ICCV2/PI489777)SNP_18949	807	G	A	6	8	--	--
CakTC25874	Ca(ICCV2/PI489777)SNP_18950	242	A	T	14	4	--	--
	Ca(ICCV2/PI489777)SNP_18951	270	C	A	14	4	--	--
	Ca(ICCV2/PI489777)SNP_18952	383	A	G	21	10	--	--
CakTC42738	Ca(ICCV2/PI489777)SNP_18953	617	A	T	24	4	--	--
	Ca(ICCV2/PI489777)SNP_18954	1460	G	T	33	18	--	--
CakTC40499	Ca(ICCV2/PI489777)SNP_18955	240	T	C	58	54	--	--
CakTC27910	Ca(ICCV2/PI489777)SNP_18956	304	C	T	20	25	--	--
	Ca(ICCV2/PI489777)SNP_18957	359	C	T	20	6	--	--
	Ca(ICCV2/PI489777)SNP_18958	362	T	C	19	14	--	--
	Ca(ICCV2/PI489777)SNP_18959	377	T	C	15	13	--	--
	Ca(ICCV2/PI489777)SNP_18960	473	A	T	16	5	--	--
	Ca(ICCV2/PI489777)SNP_18961	521	G	A	18	6	--	--
CakTC26195	Ca(ICCV2/PI489777)SNP_18962	456	C	G	30	13	--	--
CakTC28283	Ca(ICCV2/PI489777)SNP_18963	418	T	G	15	5	--	--
	Ca(ICCV2/PI489777)SNP_18964	449	T	G	15	5	--	--
	Ca(ICCV2/PI489777)SNP_18965	566	A	G	15	5	--	--
	Ca(ICCV2/PI489777)SNP_18966	1154	C	T	6	8	--	--
	Ca(ICCV2/PI489777)SNP_18967	2502	C	T	4	7	--	--
CakTC05909	Ca(ICCV2/PI489777)SNP_18968	497	T	G	10	11	--	--
CakTC37197	Ca(ICCV2/PI489777)SNP_18969	256	C	T	70	57	Young_pod	--
	Ca(ICCV2/PI489777)SNP_18970	413	G	A	56	60	Young_pod	--
	Ca(ICCV2/PI489777)SNP_18971	856	A	C	83	104	Young_pod	--
CakTC41268	Ca(ICCV2/PI489777)SNP_18972	321	T	G	45	9	--	--
CakTC43215	Ca(ICCV2/PI489777)SNP_18973	141	T	C	14	36	--	--
	Ca(ICCV2/PI489777)SNP_18974	727	A	G	32	61	--	--
	Ca(ICCV2/PI489777)SNP_18975	826	T	A	19	44	--	--
	Ca(ICCV2/PI489777)SNP_18976	829	G	A	17	45	--	--
CakTC39132	Ca(ICCV2/PI489777)SNP_18977	230	G	T	10	8	--	--
	Ca(ICCV2/PI489777)SNP_18978	873	T	C	11	7	--	--
CakTC25074	Ca(ICCV2/PI489777)SNP_18979	806	G	A	104	60	--	--
CakTC07637	Ca(ICCV2/PI489777)SNP_18980	305	C	T	3	9	--	--
	Ca(ICCV2/PI489777)SNP_18981	593	T	C	5	3	--	--
CakTC22365	Ca(ICCV2/PI489777)SNP_18982	543	A	T	4	5	--	--
	Ca(ICCV2/PI489777)SNP_18983	544	C	A	4	5	--	--
	Ca(ICCV2/PI489777)SNP_18984	1577	T	A	88	9	--	--
CakTC31458	Ca(ICCV2/PI489777)SNP_18985	829	T	C	4	3	--	--
	Ca(ICCV2/PI489777)SNP_18986	1070	A	G	12	4	--	--
CakTC36582	Ca(ICCV2/PI489777)SNP_18987	1110	C	T	5	5	--	--
	Ca(ICCV2/PI489777)SNP_18988	1204	C	G	4	4	--	--
	Ca(ICCV2/PI489777)SNP_18989	1227	T	C	4	4	--	--
CakTC13200	Ca(ICCV2/PI489777)SNP_18990	249	T	A	139	10	--	--
CakTC41597	Ca(ICCV2/PI489777)SNP_18991	8	C	A	23	41	--	--
	Ca(ICCV2/PI489777)SNP_18992	505	C	T	179	217	--	--
	Ca(ICCV2/PI489777)SNP_18993	623	C	G	18	27	--	--
CakTC43188	Ca(ICCV2/PI489777)SNP_18994	436	T	A	19	10	--	--
	Ca(ICCV2/PI489777)SNP_18995	1597	C	G	22	17	--	--
	Ca(ICCV2/PI489777)SNP_18996	1639	G	A	29	12	--	--
	Ca(ICCV2/PI489777)SNP_18997	1641	G	A	29	12	--	--
CakTC11692	Ca(ICCV2/PI489777)SNP_18998	285	C	T	4	3	Shoot	--
	Ca(ICCV2/PI489777)SNP_18999	298	T	C	4	3	Shoot	--
	Ca(ICCV2/PI489777)SNP_19000	305	A	C	5	3	Shoot	--
	Ca(ICCV2/PI489777)SNP_19001	313	G	A	5	3	Shoot	--
	Ca(ICCV2/PI489777)SNP_19002	325	A	G	5	3	Shoot	--
	Ca(ICCV2/PI489777)SNP_19003	334	T	A	5	3	Shoot	--
	Ca(ICCV2/PI489777)SNP_19004	343	G	C	5	3	Shoot	--
CakTC29566	Ca(ICCV2/PI489777)SNP_19005	282	C	T	18	5	--	--
	Ca(ICCV2/PI489777)SNP_19006	510	G	A	14	5	--	--
	Ca(ICCV2/PI489777)SNP_19007	538	A	G	15	5	--	--
CakTC22401	Ca(ICCV2/PI489777)SNP_19008	1212	C	T	5	8	--	--
CakTC28869	Ca(ICCV2/PI489777)SNP_19009	493	A	G	68	45	--	--
CakTC39019	Ca(ICCV2/PI489777)SNP_19010	940	C	T	17	6	--	zf-HD
CakTC41952	Ca(ICCV2/PI489777)SNP_19011	995	C	G	6	9	--	--
CakTC42261	Ca(ICCV2/PI489777)SNP_19012	193	C	T	7	3	--	NAC
	Ca(ICCV2/PI489777)SNP_19013	351	A	G	8	5	--	NAC

	Ca(ICCV2/PI489777)SNP_19014	1558	T	C	4	7	--	NAC
CakTC39020	Ca(ICCV2/PI489777)SNP_19015	717	G	C	9	9	--	--
CakTC39482	Ca(ICCV2/PI489777)SNP_19016	983	A	G	69	39	--	--
CakTC24497	Ca(ICCV2/PI489777)SNP_19017	555	A	G	6	9	--	--
CakTC23319	Ca(ICCV2/PI489777)SNP_19018	511	A	C	32	4	--	--
	Ca(ICCV2/PI489777)SNP_19019	2247	C	T	14	6	--	--
CakTC38487	Ca(ICCV2/PI489777)SNP_19020	890	C	T	83	81	--	--
CakTC41576	Ca(ICCV2/PI489777)SNP_19021	342	C	T	3	7	--	DDT
	Ca(ICCV2/PI489777)SNP_19022	621	C	T	11	9	--	DDT
	Ca(ICCV2/PI489777)SNP_19023	643	G	A	11	9	--	DDT
CakTC37536	Ca(ICCV2/PI489777)SNP_19024	29	C	T	45	26	--	--
CakTC39522	Ca(ICCV2/PI489777)SNP_19025	200	T	C	5	6	--	--
CakTC40182	Ca(ICCV2/PI489777)SNP_19026	312	C	A	28	9	--	--
CakTC37543	Ca(ICCV2/PI489777)SNP_19027	944	T	C	4	3	--	--
	Ca(ICCV2/PI489777)SNP_19028	1025	G	A	6	3	--	--
CakTC32490	Ca(ICCV2/PI489777)SNP_19029	2435	T	C	7	3	--	--
	Ca(ICCV2/PI489777)SNP_19030	2515	A	G	4	3	--	--
CakTC37790	Ca(ICCV2/PI489777)SNP_19031	891	T	C	46	50	--	--
CakTC27767	Ca(ICCV2/PI489777)SNP_19032	688	G	A	4	3	--	--
	Ca(ICCV2/PI489777)SNP_19033	698	A	C	5	3	--	--
CakTC29498	Ca(ICCV2/PI489777)SNP_19034	233	A	G	19	3	--	NAC
CakTC39417	Ca(ICCV2/PI489777)SNP_19035	1616	C	T	8	9	--	--
CakTC27337	Ca(ICCV2/PI489777)SNP_19036	644	A	G	7	11	--	--
	Ca(ICCV2/PI489777)SNP_19037	812	A	G	3	7	--	--
	Ca(ICCV2/PI489777)SNP_19038	886	G	A	5	5	--	--
CakTC27502	Ca(ICCV2/PI489777)SNP_19039	1133	T	C	4	6	--	--
	Ca(ICCV2/PI489777)SNP_19040	1177	T	G	5	6	--	--
	Ca(ICCV2/PI489777)SNP_19041	1901	T	C	3	4	--	--
CakTC22384	Ca(ICCV2/PI489777)SNP_19042	625	C	T	11	7	--	--
CakTC27642	Ca(ICCV2/PI489777)SNP_19043	290	C	T	5	6	--	--
CakTC37860	Ca(ICCV2/PI489777)SNP_19044	397	C	T	6	15	--	--
	Ca(ICCV2/PI489777)SNP_19045	476	A	G	7	21	--	--
	Ca(ICCV2/PI489777)SNP_19046	493	A	G	5	20	--	--
	Ca(ICCV2/PI489777)SNP_19047	858	T	C	15	13	--	--
	Ca(ICCV2/PI489777)SNP_19048	971	T	A	6	6	--	--
	Ca(ICCV2/PI489777)SNP_19049	974	C	A	6	5	--	--
CakTC09853	Ca(ICCV2/PI489777)SNP_19050	619	C	T	28	27	--	--
	Ca(ICCV2/PI489777)SNP_19051	838	T	C	12	8	--	--
CakTC06802	Ca(ICCV2/PI489777)SNP_19052	264	C	T	4	3	--	--
	Ca(ICCV2/PI489777)SNP_19053	270	C	T	5	3	--	--
CakTC31202	Ca(ICCV2/PI489777)SNP_19054	331	T	C	3	7	--	--
	Ca(ICCV2/PI489777)SNP_19055	488	C	G	5	3	--	--
	Ca(ICCV2/PI489777)SNP_19056	643	G	T	8	3	--	--
	Ca(ICCV2/PI489777)SNP_19057	785	A	C	7	5	--	--
	Ca(ICCV2/PI489777)SNP_19058	1189	G	A	5	3	--	--
	Ca(ICCV2/PI489777)SNP_19059	1273	G	A	4	4	--	--
	Ca(ICCV2/PI489777)SNP_19060	1288	A	G	3	4	--	--
CakTC32460	Ca(ICCV2/PI489777)SNP_19061	4818	A	G	13	6	Mature	Leaf
	Ca(ICCV2/PI489777)SNP_19062	5043	T	C	9	3	Mature	Leaf
CakTC40966	Ca(ICCV2/PI489777)SNP_19063	348	T	C	15	30	--	--
	Ca(ICCV2/PI489777)SNP_19064	761	A	G	24	19	--	--
CakTC42586	Ca(ICCV2/PI489777)SNP_19065	775	A	T	59	18	--	--
	Ca(ICCV2/PI489777)SNP_19066	1642	G	T	18	14	--	--
CakTC09535	Ca(ICCV2/PI489777)SNP_19067	6970	C	T	4	5	--	--
	Ca(ICCV2/PI489777)SNP_19068	7026	T	C	5	3	--	--
	Ca(ICCV2/PI489777)SNP_19069	7281	G	A	6	5	--	--
	Ca(ICCV2/PI489777)SNP_19070	11031	A	G	4	4	--	--
	Ca(ICCV2/PI489777)SNP_19071	12434	A	G	5	8	--	--
	Ca(ICCV2/PI489777)SNP_19072	12448	T	A	5	9	--	--
CakTC04638	Ca(ICCV2/PI489777)SNP_19073	50	C	T	9	10	--	--
CakTC39228	Ca(ICCV2/PI489777)SNP_19074	265	T	C	13	12	--	--
	Ca(ICCV2/PI489777)SNP_19075	315	T	C	13	12	--	--
	Ca(ICCV2/PI489777)SNP_19076	510	C	T	11	10	--	--
	Ca(ICCV2/PI489777)SNP_19077	771	A	G	7	4	--	--
	Ca(ICCV2/PI489777)SNP_19078	1259	G	C	5	12	--	--
CakTC10454	Ca(ICCV2/PI489777)SNP_19079	818	G	A	22	8	--	--
	Ca(ICCV2/PI489777)SNP_19080	1409	A	G	8	3	--	--
	Ca(ICCV2/PI489777)SNP_19081	1454	C	T	11	3	--	--
	Ca(ICCV2/PI489777)SNP_19082	1527	C	A	7	5	--	--
	Ca(ICCV2/PI489777)SNP_19083	1636	T	A	6	3	--	--
	Ca(ICCV2/PI489777)SNP_19084	1641	C	T	6	4	--	--
	Ca(ICCV2/PI489777)SNP_19085	1742	T	C	8	4	--	--
	Ca(ICCV2/PI489777)SNP_19086	2176	T	C	8	5	--	--
	Ca(ICCV2/PI489777)SNP_19087	3059	C	T	6	11	--	--

	Ca(ICCV2/PI489777)SNP_19088	3388	A	G	5	10	--	--
CakTC31298	Ca(ICCV2/PI489777)SNP_19089	511	T	C	8	3	--	--
CakTC29661	Ca(ICCV2/PI489777)SNP_19090	205	A	T	126	72	--	--
	Ca(ICCV2/PI489777)SNP_19091	240	G	C	127	57	--	--
CakTC08510	Ca(ICCV2/PI489777)SNP_19092	400	A	G	5	3	--	HB
	Ca(ICCV2/PI489777)SNP_19093	658	A	G	5	6	--	HB
	Ca(ICCV2/PI489777)SNP_19094	663	G	T	5	6	--	HB
	Ca(ICCV2/PI489777)SNP_19095	717	C	G	5	5	--	HB
	Ca(ICCV2/PI489777)SNP_19096	2454	G	T	4	5	--	HB
	Ca(ICCV2/PI489777)SNP_19097	2965	A	G	8	3	--	HB
	Ca(ICCV2/PI489777)SNP_19098	3142	A	G	5	6	--	HB
	Ca(ICCV2/PI489777)SNP_19099	4048	A	G	8	7	--	HB
	Ca(ICCV2/PI489777)SNP_19100	5168	G	A	7	6	--	HB
	Ca(ICCV2/PI489777)SNP_19101	5394	C	G	4	9	--	HB
	Ca(ICCV2/PI489777)SNP_19102	5396	G	A	4	9	--	HB
	Ca(ICCV2/PI489777)SNP_19103	5503	A	G	3	7	--	HB
CakTC28637	Ca(ICCV2/PI489777)SNP_19104	984	C	T	4	9	--	--
	Ca(ICCV2/PI489777)SNP_19105	1203	C	T	4	9	--	--
CakTC39736	Ca(ICCV2/PI489777)SNP_19106	371	A	G	57	23	--	--
	Ca(ICCV2/PI489777)SNP_19107	977	G	A	28	25	--	--
	Ca(ICCV2/PI489777)SNP_19108	1385	C	T	34	17	--	--
CakTC32876	Ca(ICCV2/PI489777)SNP_19109	75	G	A	4	3	--	--
	Ca(ICCV2/PI489777)SNP_19110	425	G	A	3	6	--	--
CakTC29639	Ca(ICCV2/PI489777)SNP_19111	606	G	A	9	7	--	--
CakTC30900	Ca(ICCV2/PI489777)SNP_19112	229	T	C	13	5	--	--
	Ca(ICCV2/PI489777)SNP_19113	310	T	G	16	6	--	--
	Ca(ICCV2/PI489777)SNP_19114	616	C	T	17	3	--	--
	Ca(ICCV2/PI489777)SNP_19115	727	C	A	20	5	--	--
	Ca(ICCV2/PI489777)SNP_19116	732	C	A	17	5	--	--
	Ca(ICCV2/PI489777)SNP_19117	781	G	A	18	4	--	--
	Ca(ICCV2/PI489777)SNP_19118	877	G	A	15	5	--	--
	Ca(ICCV2/PI489777)SNP_19119	1297	G	A	10	5	--	--
	Ca(ICCV2/PI489777)SNP_19120	1541	G	T	8	5	--	--
	Ca(ICCV2/PI489777)SNP_19121	1780	G	A	3	6	--	--
	Ca(ICCV2/PI489777)SNP_19122	1861	T	G	3	9	--	--
	Ca(ICCV2/PI489777)SNP_19123	3613	G	A	6	3	--	--
	Ca(ICCV2/PI489777)SNP_19124	3681	C	G	7	3	--	--
CakTC38666	Ca(ICCV2/PI489777)SNP_19125	242	A	C	10	6	--	--
	Ca(ICCV2/PI489777)SNP_19126	463	T	A	10	8	--	--
	Ca(ICCV2/PI489777)SNP_19127	833	C	T	9	5	--	--
	Ca(ICCV2/PI489777)SNP_19128	851	G	T	9	5	--	--
	Ca(ICCV2/PI489777)SNP_19129	876	A	G	8	5	--	--
	Ca(ICCV2/PI489777)SNP_19130	890	T	A	8	5	--	--
CakTC39260	Ca(ICCV2/PI489777)SNP_19131	83	T	C	5	3	--	MYB-related
	Ca(ICCV2/PI489777)SNP_19132	501	G	A	7	3	--	MYB-related
	Ca(ICCV2/PI489777)SNP_19133	1010	T	A	7	7	--	MYB-related
	Ca(ICCV2/PI489777)SNP_19134	1038	T	C	7	7	--	MYB-related
	Ca(ICCV2/PI489777)SNP_19135	1053	A	G	7	7	--	MYB-related
	Ca(ICCV2/PI489777)SNP_19136	1179	A	G	5	7	--	MYB-related
CakTC41302	Ca(ICCV2/PI489777)SNP_19137	2327	A	C	15	7	--	--
	Ca(ICCV2/PI489777)SNP_19138	2501	A	G	11	6	--	--
CakTC28893	Ca(ICCV2/PI489777)SNP_19139	273	C	T	3	4	--	MADS
CakTC31288	Ca(ICCV2/PI489777)SNP_19140	87	T	C	4	3	--	--
	Ca(ICCV2/PI489777)SNP_19141	1645	A	C	11	9	--	--
	Ca(ICCV2/PI489777)SNP_19142	1649	A	C	11	9	--	--
CakTC25155	Ca(ICCV2/PI489777)SNP_19143	304	C	T	16	12	--	--
CakTC30953	Ca(ICCV2/PI489777)SNP_19144	227	G	A	3	3	--	C2C2-Dof
	Ca(ICCV2/PI489777)SNP_19145	318	G	A	3	3	--	C2C2-Dof
	Ca(ICCV2/PI489777)SNP_19146	327	G	A	3	3	--	C2C2-Dof
	Ca(ICCV2/PI489777)SNP_19147	335	C	T	3	4	--	C2C2-Dof
	Ca(ICCV2/PI489777)SNP_19148	376	G	A	5	3	--	C2C2-Dof
CakTC43328	Ca(ICCV2/PI489777)SNP_19149	375	G	C	7	6	Shoot	MYB
	Ca(ICCV2/PI489777)SNP_19150	420	A	C	7	5	Shoot	MYB
	Ca(ICCV2/PI489777)SNP_19151	944	T	G	8	22	Shoot	MYB
CakTC28297	Ca(ICCV2/PI489777)SNP_19152	1871	G	T	22	8	--	MYB
	Ca(ICCV2/PI489777)SNP_19153	3668	C	T	22	11	--	MYB
CakTC40113	Ca(ICCV2/PI489777)SNP_19154	287	C	A	20	10	--	--
	Ca(ICCV2/PI489777)SNP_19155	1252	T	G	6	14	--	--
CakTC37403	Ca(ICCV2/PI489777)SNP_19156	198	T	C	38	18	--	--
	Ca(ICCV2/PI489777)SNP_19157	251	G	C	36	22	--	--
	Ca(ICCV2/PI489777)SNP_19158	293	G	A	40	24	--	--
CakTC11454	Ca(ICCV2/PI489777)SNP_19159	261	C	A	10	5	--	--
	Ca(ICCV2/PI489777)SNP_19160	467	A	G	8	5	--	--
	Ca(ICCV2/PI489777)SNP_19161	750	C	G	3	3	--	--

CakTC41949	Ca(ICCV2/PI489777)SNP_19162	661	T	C	12	22	--	--
CakTC33063	Ca(ICCV2/PI489777)SNP_19163	139	A	G	6	3	Shoot	--
	Ca(ICCV2/PI489777)SNP_19164	276	C	T	7	5	Shoot	--
CakTC34184	Ca(ICCV2/PI489777)SNP_19165	439	G	A	10	7	--	--
	Ca(ICCV2/PI489777)SNP_19166	643	C	T	4	5	--	--
	Ca(ICCV2/PI489777)SNP_19167	649	T	C	4	5	--	--
CakTC26429	Ca(ICCV2/PI489777)SNP_19168	151	A	G	5	6	--	--
	Ca(ICCV2/PI489777)SNP_19169	152	T	A	5	6	--	--
	Ca(ICCV2/PI489777)SNP_19170	636	A	T	18	8	--	--
	Ca(ICCV2/PI489777)SNP_19171	684	G	A	13	9	--	--
	Ca(ICCV2/PI489777)SNP_19172	846	G	A	17	7	--	--
	Ca(ICCV2/PI489777)SNP_19173	897	T	A	21	4	--	--
	Ca(ICCV2/PI489777)SNP_19174	927	G	T	21	6	--	--
	Ca(ICCV2/PI489777)SNP_19175	953	A	G	17	6	--	--
	Ca(ICCV2/PI489777)SNP_19176	1149	T	G	17	9	--	--
	Ca(ICCV2/PI489777)SNP_19177	1152	G	A	18	9	--	--
	Ca(ICCV2/PI489777)SNP_19178	1401	G	A	23	5	--	--
CakTC30207	Ca(ICCV2/PI489777)SNP_19179	656	A	G	9	3	--	--
	Ca(ICCV2/PI489777)SNP_19180	1209	G	A	10	3	--	--
CakTC38531	Ca(ICCV2/PI489777)SNP_19181	106	G	C	8	6	--	--
	Ca(ICCV2/PI489777)SNP_19182	136	T	C	8	5	--	--
	Ca(ICCV2/PI489777)SNP_19183	141	A	T	8	5	--	--
	Ca(ICCV2/PI489777)SNP_19184	192	T	C	9	5	--	--
	Ca(ICCV2/PI489777)SNP_19185	914	T	C	7	3	--	--
CakTC35109	Ca(ICCV2/PI489777)SNP_19186	349	C	T	3	11	--	--
	Ca(ICCV2/PI489777)SNP_19187	2392	A	G	6	14	--	--
CakTC30598	Ca(ICCV2/PI489777)SNP_19188	1179	A	G	5	3	--	--
	Ca(ICCV2/PI489777)SNP_19189	1242	C	A	5	4	--	--
CakTC37572	Ca(ICCV2/PI489777)SNP_19190	256	C	A	3	3	--	--
	Ca(ICCV2/PI489777)SNP_19191	351	C	T	4	5	--	--
CakTC30396	Ca(ICCV2/PI489777)SNP_19192	2173	C	T	3	3	--	--
	Ca(ICCV2/PI489777)SNP_19193	2215	T	C	3	3	--	--
CakTC09015	Ca(ICCV2/PI489777)SNP_19194	196	G	A	8	4	--	--
CakTC17326	Ca(ICCV2/PI489777)SNP_19195	516	C	T	472	255	--	--
	Ca(ICCV2/PI489777)SNP_19196	1203	C	T	118	25	--	--
CakTC25021	Ca(ICCV2/PI489777)SNP_19197	1234	C	G	4	3	Young_pod	--
CakTC14877	Ca(ICCV2/PI489777)SNP_19198	246	T	C	4	4	--	--
	Ca(ICCV2/PI489777)SNP_19199	421	A	T	3	4	--	--
	Ca(ICCV2/PI489777)SNP_19200	473	G	A	3	4	--	--
CakTC11367	Ca(ICCV2/PI489777)SNP_19201	355	G	A	9	12	--	--
	Ca(ICCV2/PI489777)SNP_19202	556	T	C	9	10	--	--
	Ca(ICCV2/PI489777)SNP_19203	823	G	C	21	9	--	--
	Ca(ICCV2/PI489777)SNP_19204	937	C	T	17	5	--	--
	Ca(ICCV2/PI489777)SNP_19205	1015	A	G	7	4	--	--
CakTC37167	Ca(ICCV2/PI489777)SNP_19206	235	A	C	12	3	--	--
	Ca(ICCV2/PI489777)SNP_19207	268	A	G	12	3	--	--
	Ca(ICCV2/PI489777)SNP_19208	742	T	A	7	4	--	--
CakTC38762	Ca(ICCV2/PI489777)SNP_19209	1142	C	G	11	19	--	--
CakTC27437	Ca(ICCV2/PI489777)SNP_19210	436	A	G	7	4	Root	--
CakTC42789	Ca(ICCV2/PI489777)SNP_19211	791	T	C	8	4	Root	--
	Ca(ICCV2/PI489777)SNP_19212	811	C	T	8	4	Root	--
	Ca(ICCV2/PI489777)SNP_19213	814	A	T	7	4	Root	--
	Ca(ICCV2/PI489777)SNP_19214	857	A	G	5	4	Root	--
CakTC29237	Ca(ICCV2/PI489777)SNP_19215	157	C	T	34	6	--	--
	Ca(ICCV2/PI489777)SNP_19216	839	G	A	91	10	--	--
CakTC26624	Ca(ICCV2/PI489777)SNP_19217	1113	A	G	8	3	--	C3H
	Ca(ICCV2/PI489777)SNP_19218	1356	A	C	5	4	--	C3H
	Ca(ICCV2/PI489777)SNP_19219	1441	A	G	5	4	--	C3H
CakTC30359	Ca(ICCV2/PI489777)SNP_19220	1521	G	C	3	3	--	TCP
CakTC39740	Ca(ICCV2/PI489777)SNP_19221	228	C	T	16	16	--	--
	Ca(ICCV2/PI489777)SNP_19222	364	A	T	33	22	--	--
	Ca(ICCV2/PI489777)SNP_19223	744	G	T	30	16	--	--
	Ca(ICCV2/PI489777)SNP_19224	856	C	T	42	22	--	--
	Ca(ICCV2/PI489777)SNP_19225	980	G	A	48	31	--	--
	Ca(ICCV2/PI489777)SNP_19226	989	G	C	41	30	--	--
	Ca(ICCV2/PI489777)SNP_19227	1635	C	T	9	3	--	--
	Ca(ICCV2/PI489777)SNP_19228	1647	T	C	9	3	--	--
	Ca(ICCV2/PI489777)SNP_19229	1828	A	G	7	5	--	--
	Ca(ICCV2/PI489777)SNP_19230	1947	G	A	9	3	--	--
CakTC38377	Ca(ICCV2/PI489777)SNP_19231	350	T	C	4	9	Flower	bud
	Ca(ICCV2/PI489777)SNP_19232	725	A	G	3	5	Flower	bud
	Ca(ICCV2/PI489777)SNP_19233	752	T	C	4	5	Flower	bud
	Ca(ICCV2/PI489777)SNP_19234	860	A	T	5	5	Flower	bud
	Ca(ICCV2/PI489777)SNP_19235	872	A	G	4	5	Flower	bud

CakTC40127	Ca(ICCV2/PI489777)SNP_19236	167	C	T	18	6	--	--
	Ca(ICCV2/PI489777)SNP_19237	169	C	G	18	6	--	--
	Ca(ICCV2/PI489777)SNP_19238	295	T	C	22	7	--	--
	Ca(ICCV2/PI489777)SNP_19239	590	A	G	13	7	--	--
	Ca(ICCV2/PI489777)SNP_19240	865	G	A	16	6	--	--
	Ca(ICCV2/PI489777)SNP_19241	1166	T	C	18	8	--	--
CakTC35231	Ca(ICCV2/PI489777)SNP_19242	1022	G	A	7	4	--	--
	Ca(ICCV2/PI489777)SNP_19243	1522	G	A	6	4	--	--
	Ca(ICCV2/PI489777)SNP_19244	1610	G	T	5	7	--	--
	Ca(ICCV2/PI489777)SNP_19245	1807	G	A	4	5	--	--
	Ca(ICCV2/PI489777)SNP_19246	2089	T	C	4	10	--	--
	Ca(ICCV2/PI489777)SNP_19247	4864	T	G	3	3	--	--
CakTC40057	Ca(ICCV2/PI489777)SNP_19248	288	A	G	3	5	--	--
CakTC30781	Ca(ICCV2/PI489777)SNP_19249	426	C	T	7	9	--	--
	Ca(ICCV2/PI489777)SNP_19250	465	T	C	8	8	--	--
	Ca(ICCV2/PI489777)SNP_19251	1032	C	G	5	7	--	--
	Ca(ICCV2/PI489777)SNP_19252	1149	G	A	4	8	--	--
	Ca(ICCV2/PI489777)SNP_19253	1192	C	T	3	8	--	--
CakTC25269	Ca(ICCV2/PI489777)SNP_19254	673	C	G	14	3	Root	--
	Ca(ICCV2/PI489777)SNP_19255	1605	G	A	3	3	Root	--
	Ca(ICCV2/PI489777)SNP_19256	1617	T	C	3	3	Root	--
	Ca(ICCV2/PI489777)SNP_19257	1619	G	A	3	3	Root	--
	Ca(ICCV2/PI489777)SNP_19258	1628	T	C	3	3	Root	--
CakTC42464	Ca(ICCV2/PI489777)SNP_19259	47	C	T	18	13	--	--
	Ca(ICCV2/PI489777)SNP_19260	334	G	A	26	27	--	--
	Ca(ICCV2/PI489777)SNP_19261	939	A	G	22	16	--	--
	Ca(ICCV2/PI489777)SNP_19262	1573	T	C	20	17	--	--
	Ca(ICCV2/PI489777)SNP_19263	1659	C	G	14	10	--	--
CakTC27388	Ca(ICCV2/PI489777)SNP_19264	77	T	C	18	14	--	--
CakTC26492	Ca(ICCV2/PI489777)SNP_19265	2796	T	A	11	14	--	--
CakTC26396	Ca(ICCV2/PI489777)SNP_19266	221	C	T	12	6	--	--
CakTC23570	Ca(ICCV2/PI489777)SNP_19267	151	G	A	108	9	Shoot	--
	Ca(ICCV2/PI489777)SNP_19268	421	C	A	132	9	Shoot	--
	Ca(ICCV2/PI489777)SNP_19269	644	C	A	178	9	Shoot	--
	Ca(ICCV2/PI489777)SNP_19270	1624	G	T	81	10	Shoot	--
	Ca(ICCV2/PI489777)SNP_19271	1970	C	A	10	4	Shoot	--
	Ca(ICCV2/PI489777)SNP_19272	2195	T	C	12	7	Shoot	--
CakTC30591	Ca(ICCV2/PI489777)SNP_19273	995	T	A	4	8	--	--
	Ca(ICCV2/PI489777)SNP_19274	1010	G	C	4	9	--	--
	Ca(ICCV2/PI489777)SNP_19275	1049	C	A	4	9	--	--
	Ca(ICCV2/PI489777)SNP_19276	1253	G	A	4	5	--	--
	Ca(ICCV2/PI489777)SNP_19277	1621	T	C	4	3	--	--
CakTC24233	Ca(ICCV2/PI489777)SNP_19278	287	T	C	4	6	--	--
	Ca(ICCV2/PI489777)SNP_19279	324	G	A	4	5	--	--
	Ca(ICCV2/PI489777)SNP_19280	588	C	G	11	4	--	--
CakTC36562	Ca(ICCV2/PI489777)SNP_19281	226	A	T	7	4	--	--
CakTC42318	Ca(ICCV2/PI489777)SNP_19282	219	C	T	4	3	--	--
CakTC25046	Ca(ICCV2/PI489777)SNP_19283	218	G	A	55	21	--	--
	Ca(ICCV2/PI489777)SNP_19284	1119	C	A	63	20	--	--
CakTC06310	Ca(ICCV2/PI489777)SNP_19285	602	A	T	11	6	--	--
	Ca(ICCV2/PI489777)SNP_19286	753	C	T	9	3	--	--
	Ca(ICCV2/PI489777)SNP_19287	792	T	G	9	6	--	--
	Ca(ICCV2/PI489777)SNP_19288	861	G	A	11	3	--	--
	Ca(ICCV2/PI489777)SNP_19289	951	A	G	12	11	--	--
	Ca(ICCV2/PI489777)SNP_19290	984	A	T	11	10	--	--
	Ca(ICCV2/PI489777)SNP_19291	1120	G	A	10	8	--	--
	Ca(ICCV2/PI489777)SNP_19292	1158	A	G	10	8	--	--
	Ca(ICCV2/PI489777)SNP_19293	1227	T	A	8	7	--	--
	Ca(ICCV2/PI489777)SNP_19294	1268	C	G	8	6	--	--
CakTC10403	Ca(ICCV2/PI489777)SNP_19295	513	A	C	9	4	--	--
	Ca(ICCV2/PI489777)SNP_19296	2221	T	C	3	3	--	--
	Ca(ICCV2/PI489777)SNP_19297	2587	G	A	4	5	--	--
CakTC42679	Ca(ICCV2/PI489777)SNP_19298	806	C	T	10	19	--	--
	Ca(ICCV2/PI489777)SNP_19299	808	A	G	10	17	--	--
	Ca(ICCV2/PI489777)SNP_19300	932	T	C	10	20	--	--
	Ca(ICCV2/PI489777)SNP_19301	938	T	C	11	17	--	--
	Ca(ICCV2/PI489777)SNP_19302	944	A	G	10	20	--	--
	Ca(ICCV2/PI489777)SNP_19303	1007	T	C	12	28	--	--
	Ca(ICCV2/PI489777)SNP_19304	1114	C	T	14	28	--	--
	Ca(ICCV2/PI489777)SNP_19305	1292	C	T	24	46	--	--
	Ca(ICCV2/PI489777)SNP_19306	1313	C	G	25	48	--	--
	Ca(ICCV2/PI489777)SNP_19307	1801	G	A	9	6	--	--
	Ca(ICCV2/PI489777)SNP_19308	1808	C	T	8	5	--	--
CakTC39776	Ca(ICCV2/PI489777)SNP_19309	1226	A	G	38	14	--	--

	Ca(ICCV2/PI489777)SNP_19310	1358	G	A	41	17	--	--
	Ca(ICCV2/PI489777)SNP_19311	1391	A	G	40	15	--	--
	Ca(ICCV2/PI489777)SNP_19312	1461	G	T	29	14	--	--
	Ca(ICCV2/PI489777)SNP_19313	1579	A	G	9	3	--	--
CakTC39715	Ca(ICCV2/PI489777)SNP_19314	535	A	G	12	6	--	--
	Ca(ICCV2/PI489777)SNP_19315	1483	C	T	17	13	--	--
	Ca(ICCV2/PI489777)SNP_19316	1516	G	A	14	10	--	--
CakTC23576	Ca(ICCV2/PI489777)SNP_19317	712	T	C	9	11	--	--
	Ca(ICCV2/PI489777)SNP_19318	865	T	C	8	11	--	--
	Ca(ICCV2/PI489777)SNP_19319	1136	C	A	20	6	--	--
CakTC30140	Ca(ICCV2/PI489777)SNP_19320	390	A	C	8	3	--	--
	Ca(ICCV2/PI489777)SNP_19321	399	T	G	8	3	--	--
CakTC03069	Ca(ICCV2/PI489777)SNP_19322	423	C	T	5	5	--	--
	Ca(ICCV2/PI489777)SNP_19323	570	A	G	5	4	--	--
CakTC23812	Ca(ICCV2/PI489777)SNP_19324	126	T	C	26	15	--	--
	Ca(ICCV2/PI489777)SNP_19325	1148	G	A	42	30	--	--
	Ca(ICCV2/PI489777)SNP_19326	1363	C	T	32	29	--	--
	Ca(ICCV2/PI489777)SNP_19327	1421	T	C	31	29	--	--
	Ca(ICCV2/PI489777)SNP_19328	1430	C	G	32	26	--	--
	Ca(ICCV2/PI489777)SNP_19329	1463	C	T	26	24	--	--
	Ca(ICCV2/PI489777)SNP_19330	1658	T	C	6	4	--	--
	Ca(ICCV2/PI489777)SNP_19331	1682	A	G	5	4	--	--
	Ca(ICCV2/PI489777)SNP_19332	1703	A	G	3	4	--	--
	Ca(ICCV2/PI489777)SNP_19333	1719	T	G	5	4	--	--
CakTC37933	Ca(ICCV2/PI489777)SNP_19334	1106	C	T	5	4	--	--
	Ca(ICCV2/PI489777)SNP_19335	1293	G	A	7	5	--	--
CakTC41825	Ca(ICCV2/PI489777)SNP_19336	115	G	A	16	11	--	--
	Ca(ICCV2/PI489777)SNP_19337	444	C	T	18	12	--	--
	Ca(ICCV2/PI489777)SNP_19338	972	C	A	25	14	--	--
	Ca(ICCV2/PI489777)SNP_19339	1314	C	T	31	14	--	--
CakTC27762	Ca(ICCV2/PI489777)SNP_19340	184	T	C	4	3	--	GNAT
	Ca(ICCV2/PI489777)SNP_19341	236	A	G	4	3	--	GNAT
CakTC40295	Ca(ICCV2/PI489777)SNP_19342	596	G	T	49	32	--	--
	Ca(ICCV2/PI489777)SNP_19343	629	G	C	38	31	--	--
	Ca(ICCV2/PI489777)SNP_19344	652	G	A	44	29	--	--
	Ca(ICCV2/PI489777)SNP_19345	661	A	G	44	30	--	--
CakTC26510	Ca(ICCV2/PI489777)SNP_19346	1820	G	T	12	3	--	--
	Ca(ICCV2/PI489777)SNP_19347	1821	A	T	12	3	--	--
CakTC39757	Ca(ICCV2/PI489777)SNP_19348	51	C	A	10	10	--	--
	Ca(ICCV2/PI489777)SNP_19349	318	A	G	13	11	--	--
	Ca(ICCV2/PI489777)SNP_19350	653	A	G	10	9	--	--
CakTC29995	Ca(ICCV2/PI489777)SNP_19351	304	C	T	8	4	--	--
CakTC28780	Ca(ICCV2/PI489777)SNP_19352	260	C	T	11	3	--	--
	Ca(ICCV2/PI489777)SNP_19353	1509	C	A	11	6	--	--
CakTC30491	Ca(ICCV2/PI489777)SNP_19354	1416	T	C	9	4	--	--
CakTC26507	Ca(ICCV2/PI489777)SNP_19355	856	T	A	5	7	Flower	bud
CakTC32392	Ca(ICCV2/PI489777)SNP_19356	59	A	G	23	23	--	--
CakTC39139	Ca(ICCV2/PI489777)SNP_19357	1403	A	G	17	21	--	--
CakTC22389	Ca(ICCV2/PI489777)SNP_19358	448	G	T	4	4	--	--
	Ca(ICCV2/PI489777)SNP_19359	471	C	G	4	5	--	--
	Ca(ICCV2/PI489777)SNP_19360	756	G	C	12	3	--	--
	Ca(ICCV2/PI489777)SNP_19361	1125	C	T	52	9	--	--
	Ca(ICCV2/PI489777)SNP_19362	1193	C	T	60	9	--	--
	Ca(ICCV2/PI489777)SNP_19363	1292	T	C	58	8	--	--
	Ca(ICCV2/PI489777)SNP_19364	1341	T	C	68	8	--	--
	Ca(ICCV2/PI489777)SNP_19365	1355	C	T	69	5	--	--
	Ca(ICCV2/PI489777)SNP_19366	1358	T	C	68	5	--	--
	Ca(ICCV2/PI489777)SNP_19367	1373	G	T	66	5	--	--
	Ca(ICCV2/PI489777)SNP_19368	1392	G	A	65	6	--	--
	Ca(ICCV2/PI489777)SNP_19369	1454	C	T	61	9	--	--
	Ca(ICCV2/PI489777)SNP_19370	1466	T	C	49	7	--	--
	Ca(ICCV2/PI489777)SNP_19371	1493	T	G	48	8	--	--
	Ca(ICCV2/PI489777)SNP_19372	1844	T	C	43	13	--	--
	Ca(ICCV2/PI489777)SNP_19373	1850	C	T	40	13	--	--
	Ca(ICCV2/PI489777)SNP_19374	1944	C	G	24	7	--	--
	Ca(ICCV2/PI489777)SNP_19375	1961	A	G	19	7	--	--
	Ca(ICCV2/PI489777)SNP_19376	1992	A	T	19	6	--	--
	Ca(ICCV2/PI489777)SNP_19377	1997	C	T	19	6	--	--
CakTC37269	Ca(ICCV2/PI489777)SNP_19378	1887	C	T	9	8	--	--
CakTC29920	Ca(ICCV2/PI489777)SNP_19379	836	T	C	35	37	--	--
	Ca(ICCV2/PI489777)SNP_19380	2306	T	A	31	7	--	--
CakTC30664	Ca(ICCV2/PI489777)SNP_19381	700	C	A	4	3	--	--
	Ca(ICCV2/PI489777)SNP_19382	720	C	T	4	4	--	--
	Ca(ICCV2/PI489777)SNP_19383	1092	G	A	4	5	--	--

	Ca(ICCV2/PI489777)SNP_19384	1149	T	G	3	5	--	--
	Ca(ICCV2/PI489777)SNP_19385	1323	G	T	4	4	--	--
	Ca(ICCV2/PI489777)SNP_19386	1474	G	A	3	4	--	--
CakTC08223	Ca(ICCV2/PI489777)SNP_19387	382	A	G	7	3	--	--
	Ca(ICCV2/PI489777)SNP_19388	385	G	A	8	3	--	--
	Ca(ICCV2/PI489777)SNP_19389	399	G	C	8	3	--	--
CakTC37655	Ca(ICCV2/PI489777)SNP_19390	123	A	G	10	4	--	--
	Ca(ICCV2/PI489777)SNP_19391	361	T	C	24	9	--	--
	Ca(ICCV2/PI489777)SNP_19392	1066	T	C	9	3	--	--
CakTC41423	Ca(ICCV2/PI489777)SNP_19393	1571	T	C	7	3	--	--
	Ca(ICCV2/PI489777)SNP_19394	2375	G	A	6	7	--	--
CakTC38806	Ca(ICCV2/PI489777)SNP_19395	814	C	G	16	5	--	--
CakTC09581	Ca(ICCV2/PI489777)SNP_19396	505	A	G	3	9	--	--
	Ca(ICCV2/PI489777)SNP_19397	697	A	C	4	3	--	--
CakTC37476	Ca(ICCV2/PI489777)SNP_19398	409	C	T	3	3	--	--
CakTC42591	Ca(ICCV2/PI489777)SNP_19399	102	T	C	7	5	--	--
CakTC39596	Ca(ICCV2/PI489777)SNP_19400	276	C	T	19	13	--	--
	Ca(ICCV2/PI489777)SNP_19401	370	A	G	19	8	--	--
	Ca(ICCV2/PI489777)SNP_19402	551	A	G	8	8	--	--
	Ca(ICCV2/PI489777)SNP_19403	555	A	T	7	8	--	--
	Ca(ICCV2/PI489777)SNP_19404	952	C	G	16	11	--	--
	Ca(ICCV2/PI489777)SNP_19405	1247	G	C	5	5	--	--
CakTC17136	Ca(ICCV2/PI489777)SNP_19406	2328	T	A	8	4	--	--
CakTC27790	Ca(ICCV2/PI489777)SNP_19407	750	A	G	3	3	Flower	bud
	Ca(ICCV2/PI489777)SNP_19408	834	C	T	4	3	Flower	bud
	Ca(ICCV2/PI489777)SNP_19409	858	C	T	4	3	Flower	bud
CakTC28154	Ca(ICCV2/PI489777)SNP_19410	592	G	C	57	77	--	--
CakTC31438	Ca(ICCV2/PI489777)SNP_19411	1565	T	C	3	3	--	--
	Ca(ICCV2/PI489777)SNP_19412	1640	T	C	3	3	--	--
CakTC43175	Ca(ICCV2/PI489777)SNP_19413	100	A	G	14	5	--	--
	Ca(ICCV2/PI489777)SNP_19414	104	G	C	14	6	--	--
	Ca(ICCV2/PI489777)SNP_19415	203	T	C	19	7	--	--
	Ca(ICCV2/PI489777)SNP_19416	830	C	A	52	33	--	--
	Ca(ICCV2/PI489777)SNP_19417	1742	C	T	17	17	--	--
CakTC35175	Ca(ICCV2/PI489777)SNP_19418	699	G	A	3	4	--	--
CakTC38724	Ca(ICCV2/PI489777)SNP_19419	190	C	T	14	3	--	--
	Ca(ICCV2/PI489777)SNP_19420	1086	T	C	4	4	--	--
CakTC27176	Ca(ICCV2/PI489777)SNP_19421	1034	G	A	29	3	Young_pod	--
	Ca(ICCV2/PI489777)SNP_19422	1040	G	A	27	3	Young_pod	--
CakTC31950	Ca(ICCV2/PI489777)SNP_19423	358	G	A	3	11	Root	--
CakTC39646	Ca(ICCV2/PI489777)SNP_19424	824	C	T	7	8	--	--
	Ca(ICCV2/PI489777)SNP_19425	1284	A	G	9	3	--	--
	Ca(ICCV2/PI489777)SNP_19426	1335	T	G	13	4	--	--
CakTC41881	Ca(ICCV2/PI489777)SNP_19427	653	A	G	17	12	--	--
CakTC27556	Ca(ICCV2/PI489777)SNP_19428	360	A	G	4	3	--	C2H2
CakTC26431	Ca(ICCV2/PI489777)SNP_19429	65	A	T	3	4	--	--
	Ca(ICCV2/PI489777)SNP_19430	96	A	T	8	6	--	--
	Ca(ICCV2/PI489777)SNP_19431	239	A	G	11	7	--	--
CakTC40519	Ca(ICCV2/PI489777)SNP_19432	1915	G	A	11	12	--	--
	Ca(ICCV2/PI489777)SNP_19433	1950	G	A	12	13	--	--
	Ca(ICCV2/PI489777)SNP_19434	1990	A	G	14	13	--	--
	Ca(ICCV2/PI489777)SNP_19435	2089	G	C	10	13	--	--
CakTC25969	Ca(ICCV2/PI489777)SNP_19436	758	T	C	58	35	--	--
	Ca(ICCV2/PI489777)SNP_19437	5125	T	C	29	11	--	--
CakTC26204	Ca(ICCV2/PI489777)SNP_19438	545	C	T	10	5	Shoot	BES1
CakTC37250	Ca(ICCV2/PI489777)SNP_19439	379	A	G	11	11	Root	bHLH
	Ca(ICCV2/PI489777)SNP_19440	481	C	A	10	10	Root	bHLH
CakTC25733	Ca(ICCV2/PI489777)SNP_19441	362	T	C	34	12	--	--
	Ca(ICCV2/PI489777)SNP_19442	524	C	T	47	11	--	--
	Ca(ICCV2/PI489777)SNP_19443	1271	C	T	56	7	--	--
	Ca(ICCV2/PI489777)SNP_19444	2753	T	C	90	18	--	--
CakTC28951	Ca(ICCV2/PI489777)SNP_19445	265	A	G	3	5	--	--
CakTC38349	Ca(ICCV2/PI489777)SNP_19446	765	A	G	16	19	--	--
CakTC40077	Ca(ICCV2/PI489777)SNP_19447	538	A	C	24	31	--	--
	Ca(ICCV2/PI489777)SNP_19448	1036	A	G	21	14	--	--
CakTC10056	Ca(ICCV2/PI489777)SNP_19449	1433	G	A	4	3	--	--
CakTC17327	Ca(ICCV2/PI489777)SNP_19450	1062	T	C	14	10	--	--
CakTC30343	Ca(ICCV2/PI489777)SNP_19451	950	G	A	4	3	--	--
CakTC10359	Ca(ICCV2/PI489777)SNP_19452	1399	G	A	3	3	--	--
	Ca(ICCV2/PI489777)SNP_19453	1528	G	A	3	5	--	--
	Ca(ICCV2/PI489777)SNP_19454	1597	C	T	3	4	--	--
	Ca(ICCV2/PI489777)SNP_19455	1954	T	C	6	3	--	--
	Ca(ICCV2/PI489777)SNP_19456	2005	G	A	5	4	--	--
	Ca(ICCV2/PI489777)SNP_19457	2097	T	C	5	4	--	--

	Ca(ICCV2/PI489777)SNP_19458	2248	C	T	3	5	--	--
	Ca(ICCV2/PI489777)SNP_19459	2436	T	C	3	5	--	--
	Ca(ICCV2/PI489777)SNP_19460	3153	T	A	4	6	--	--
CakTC10761	Ca(ICCV2/PI489777)SNP_19461	127	A	G	5	7	--	--
	Ca(ICCV2/PI489777)SNP_19462	304	G	A	8	9	--	--
CakTC25160	Ca(ICCV2/PI489777)SNP_19463	1419	G	T	3	3	--	--
	Ca(ICCV2/PI489777)SNP_19464	1438	C	G	4	3	--	--
	Ca(ICCV2/PI489777)SNP_19465	1617	T	C	7	4	--	--
	Ca(ICCV2/PI489777)SNP_19466	1656	C	T	7	4	--	--
	Ca(ICCV2/PI489777)SNP_19467	1665	T	C	9	4	--	--
	Ca(ICCV2/PI489777)SNP_19468	1671	T	A	9	4	--	--
CakTC40817	Ca(ICCV2/PI489777)SNP_19469	303	G	T	6	9	--	--
	Ca(ICCV2/PI489777)SNP_19470	465	G	A	6	7	--	--
	Ca(ICCV2/PI489777)SNP_19471	1015	G	A	13	3	--	--
CakTC36855	Ca(ICCV2/PI489777)SNP_19472	1688	A	G	14	9	--	--
	Ca(ICCV2/PI489777)SNP_19473	1752	A	G	18	10	--	--
CakTC41955	Ca(ICCV2/PI489777)SNP_19474	1025	G	C	17	4	--	--
	Ca(ICCV2/PI489777)SNP_19475	1538	T	C	7	6	--	--
CakTC25559	Ca(ICCV2/PI489777)SNP_19476	825	G	A	82	38	--	--
CakTC23191	Ca(ICCV2/PI489777)SNP_19477	1238	C	A	7	8	--	--
	Ca(ICCV2/PI489777)SNP_19478	1787	G	A	20	7	--	--
	Ca(ICCV2/PI489777)SNP_19479	2568	C	T	13	5	--	--
	Ca(ICCV2/PI489777)SNP_19480	3148	A	G	293	87	--	--
CakTC41304	Ca(ICCV2/PI489777)SNP_19481	1173	T	A	4	3	--	GRAS
	Ca(ICCV2/PI489777)SNP_19482	1221	C	A	6	5	--	GRAS
	Ca(ICCV2/PI489777)SNP_19483	1303	C	T	5	3	--	GRAS
	Ca(ICCV2/PI489777)SNP_19484	1896	A	T	4	3	--	GRAS
	Ca(ICCV2/PI489777)SNP_19485	2196	C	A	3	4	--	GRAS
CakTC39427	Ca(ICCV2/PI489777)SNP_19486	195	T	C	6	3	--	--
	Ca(ICCV2/PI489777)SNP_19487	255	C	T	7	3	--	--
	Ca(ICCV2/PI489777)SNP_19488	571	T	G	6	5	--	--
	Ca(ICCV2/PI489777)SNP_19489	813	A	C	5	4	--	--
	Ca(ICCV2/PI489777)SNP_19490	1039	G	A	6	5	--	--
CakTC34840	Ca(ICCV2/PI489777)SNP_19491	1599	A	T	4	6	--	--
	Ca(ICCV2/PI489777)SNP_19492	2092	C	T	10	4	--	--
	Ca(ICCV2/PI489777)SNP_19493	2204	G	A	10	3	--	--
	Ca(ICCV2/PI489777)SNP_19494	3367	T	C	15	5	--	--
	Ca(ICCV2/PI489777)SNP_19495	3535	T	C	10	11	--	--
	Ca(ICCV2/PI489777)SNP_19496	3540	G	C	12	11	--	--
	Ca(ICCV2/PI489777)SNP_19497	3645	A	G	7	8	--	--
	Ca(ICCV2/PI489777)SNP_19498	3851	C	T	5	4	--	--
CakTC39698	Ca(ICCV2/PI489777)SNP_19499	502	T	C	3	4	--	--
	Ca(ICCV2/PI489777)SNP_19500	548	G	T	3	4	--	--
CakTC38593	Ca(ICCV2/PI489777)SNP_19501	1470	A	G	4	5	Flower	bud
	Ca(ICCV2/PI489777)SNP_19502	1519	T	C	6	5	Flower	bud
	Ca(ICCV2/PI489777)SNP_19503	1632	C	T	8	5	Flower	bud
CakTC10918	Ca(ICCV2/PI489777)SNP_19504	915	A	C	9	14	--	--
CakTC10368	Ca(ICCV2/PI489777)SNP_19505	160	G	A	14	4	--	MYB-related
CakTC32354	Ca(ICCV2/PI489777)SNP_19506	724	T	C	5	3	--	--
	Ca(ICCV2/PI489777)SNP_19507	984	T	G	4	3	--	--
	Ca(ICCV2/PI489777)SNP_19508	1812	A	G	12	3	--	--
CakTC32076	Ca(ICCV2/PI489777)SNP_19509	769	C	T	5	6	--	--
CakTC24742	Ca(ICCV2/PI489777)SNP_19510	541	C	G	112	139	--	--
	Ca(ICCV2/PI489777)SNP_19511	748	C	T	22	35	--	--
CakTC37151	Ca(ICCV2/PI489777)SNP_19512	451	C	G	19	6	--	--
	Ca(ICCV2/PI489777)SNP_19513	847	G	A	11	3	--	--
	Ca(ICCV2/PI489777)SNP_19514	949	T	C	10	5	--	--
	Ca(ICCV2/PI489777)SNP_19515	1201	C	T	11	10	--	--
	Ca(ICCV2/PI489777)SNP_19516	1324	G	A	16	7	--	--
	Ca(ICCV2/PI489777)SNP_19517	1330	T	C	13	8	--	--
	Ca(ICCV2/PI489777)SNP_19518	1458	A	G	14	9	--	--
CakTC24049	Ca(ICCV2/PI489777)SNP_19519	1476	C	T	8	10	--	--
CakTC18765	Ca(ICCV2/PI489777)SNP_19520	908	C	T	5	3	--	--
CakTC37737	Ca(ICCV2/PI489777)SNP_19521	158	G	C	5	3	--	--
	Ca(ICCV2/PI489777)SNP_19522	207	G	T	7	3	--	--
	Ca(ICCV2/PI489777)SNP_19523	276	C	T	8	3	--	--
	Ca(ICCV2/PI489777)SNP_19524	302	T	A	8	3	--	--
CakTC40110	Ca(ICCV2/PI489777)SNP_19525	386	C	A	13	6	--	MYB
	Ca(ICCV2/PI489777)SNP_19526	657	T	C	23	9	--	MYB
	Ca(ICCV2/PI489777)SNP_19527	1065	G	A	7	3	--	MYB
CakTC23073	Ca(ICCV2/PI489777)SNP_19528	2146	A	G	25	12	--	HB
	Ca(ICCV2/PI489777)SNP_19529	2185	C	T	23	12	--	HB
	Ca(ICCV2/PI489777)SNP_19530	2419	T	C	13	7	--	HB
CakTC41058	Ca(ICCV2/PI489777)SNP_19531	419	T	C	4	6	--	--

	Ca(ICCV2/PI489777)SNP_19532	545	G	A	3	8	--	--
	Ca(ICCV2/PI489777)SNP_19533	552	A	G	3	9	--	--
CakTC07942	Ca(ICCV2/PI489777)SNP_19534	944	T	C	3	4	--	--
CakTC11551	Ca(ICCV2/PI489777)SNP_19535	222	C	T	16	5	--	--
	Ca(ICCV2/PI489777)SNP_19536	333	C	T	18	4	--	--
	Ca(ICCV2/PI489777)SNP_19537	792	A	G	14	3	--	--
	Ca(ICCV2/PI489777)SNP_19538	794	G	A	14	3	--	--
CakTC42472	Ca(ICCV2/PI489777)SNP_19539	192	C	T	17	15	--	--
CakTC40121	Ca(ICCV2/PI489777)SNP_19540	64	T	A	19	10	--	--
	Ca(ICCV2/PI489777)SNP_19541	1076	G	T	7	7	--	--
CakTC28180	Ca(ICCV2/PI489777)SNP_19542	317	A	T	8	4	--	--
	Ca(ICCV2/PI489777)SNP_19543	1115	A	G	9	5	--	--
	Ca(ICCV2/PI489777)SNP_19544	1436	A	G	5	5	--	--
CakTC31791	Ca(ICCV2/PI489777)SNP_19545	1207	T	C	10	8	--	--
CakTC09751	Ca(ICCV2/PI489777)SNP_19546	78	C	A	6	3	--	--
	Ca(ICCV2/PI489777)SNP_19547	122	A	T	6	3	--	--
	Ca(ICCV2/PI489777)SNP_19548	123	A	C	6	3	--	--
	Ca(ICCV2/PI489777)SNP_19549	429	G	A	6	4	--	--
	Ca(ICCV2/PI489777)SNP_19550	495	T	C	5	3	--	--
	Ca(ICCV2/PI489777)SNP_19551	1273	G	A	10	4	--	--
	Ca(ICCV2/PI489777)SNP_19552	1296	A	G	6	4	--	--
	Ca(ICCV2/PI489777)SNP_19553	1401	C	A	3	4	--	--
	Ca(ICCV2/PI489777)SNP_19554	1447	G	A	4	4	--	--
	Ca(ICCV2/PI489777)SNP_19555	1465	T	C	4	3	--	--
	Ca(ICCV2/PI489777)SNP_19556	1473	A	G	4	3	--	--
	Ca(ICCV2/PI489777)SNP_19557	1768	C	T	4	3	--	--
	Ca(ICCV2/PI489777)SNP_19558	2128	G	A	3	3	--	--
CakTC18621	Ca(ICCV2/PI489777)SNP_19559	229	A	G	9	7	--	--
	Ca(ICCV2/PI489777)SNP_19560	387	C	T	16	7	--	--
	Ca(ICCV2/PI489777)SNP_19561	450	G	C	18	5	--	--
	Ca(ICCV2/PI489777)SNP_19562	513	A	G	15	4	--	--
	Ca(ICCV2/PI489777)SNP_19563	783	T	C	23	4	--	--
	Ca(ICCV2/PI489777)SNP_19564	1194	T	C	23	14	--	--
	Ca(ICCV2/PI489777)SNP_19565	1461	T	G	35	13	--	--
CakTC34978	Ca(ICCV2/PI489777)SNP_19566	1213	C	T	11	6	--	--
	Ca(ICCV2/PI489777)SNP_19567	1763	G	A	7	7	--	--
CakTC41249	Ca(ICCV2/PI489777)SNP_19568	146	T	A	3	14	--	--
	Ca(ICCV2/PI489777)SNP_19569	307	A	C	15	13	--	--
	Ca(ICCV2/PI489777)SNP_19570	1804	G	T	17	13	--	--
	Ca(ICCV2/PI489777)SNP_19571	2022	T	C	11	13	--	--
CakTC13768	Ca(ICCV2/PI489777)SNP_19572	1793	C	T	5	4	Young_pod	--
CakTC42308	Ca(ICCV2/PI489777)SNP_19573	468	T	A	56	44	--	--
	Ca(ICCV2/PI489777)SNP_19574	772	G	A	44	37	--	--
CakTC39551	Ca(ICCV2/PI489777)SNP_19575	212	C	A	3	3	--	--
CakTC38807	Ca(ICCV2/PI489777)SNP_19576	38	T	C	8	4	--	--
	Ca(ICCV2/PI489777)SNP_19577	91	A	C	11	5	--	--
	Ca(ICCV2/PI489777)SNP_19578	236	C	A	15	15	--	--
	Ca(ICCV2/PI489777)SNP_19579	389	A	G	12	12	--	--
	Ca(ICCV2/PI489777)SNP_19580	392	T	C	8	9	--	--
	Ca(ICCV2/PI489777)SNP_19581	644	G	C	11	9	--	--
CakTC24140	Ca(ICCV2/PI489777)SNP_19582	794	C	T	8	4	--	--
	Ca(ICCV2/PI489777)SNP_19583	1215	A	G	154	70	--	--
	Ca(ICCV2/PI489777)SNP_19584	1465	G	A	15	3	--	--
	Ca(ICCV2/PI489777)SNP_19585	1518	G	T	16	3	--	--
CakTC28850	Ca(ICCV2/PI489777)SNP_19586	50	T	G	3	5	--	--
	Ca(ICCV2/PI489777)SNP_19587	125	A	G	3	14	--	--
	Ca(ICCV2/PI489777)SNP_19588	140	A	T	4	13	--	--
CakTC32517	Ca(ICCV2/PI489777)SNP_19589	712	A	G	3	4	--	--
CakTC43214	Ca(ICCV2/PI489777)SNP_19590	236	C	T	17	7	--	--
	Ca(ICCV2/PI489777)SNP_19591	345	C	T	19	14	--	--
CakTC31484	Ca(ICCV2/PI489777)SNP_19592	1130	A	C	3	3	--	--
CakTC08902	Ca(ICCV2/PI489777)SNP_19593	429	G	C	65	80	--	--
	Ca(ICCV2/PI489777)SNP_19594	1245	G	T	84	179	--	--
CakTC42372	Ca(ICCV2/PI489777)SNP_19595	1147	G	C	3	4	--	--
CakTC10846	Ca(ICCV2/PI489777)SNP_19596	217	G	A	3	4	Young_pod	--
	Ca(ICCV2/PI489777)SNP_19597	424	G	C	3	8	Young_pod	--
CakTC41873	Ca(ICCV2/PI489777)SNP_19598	135	T	C	9	11	--	--
	Ca(ICCV2/PI489777)SNP_19599	634	A	G	41	15	--	--
	Ca(ICCV2/PI489777)SNP_19600	1787	C	T	6	7	--	--
CakTC38286	Ca(ICCV2/PI489777)SNP_19601	40	T	C	5	12	Young_pod	--
	Ca(ICCV2/PI489777)SNP_19602	482	C	T	26	24	Young_pod	--
	Ca(ICCV2/PI489777)SNP_19603	615	A	G	12	17	Young_pod	--
	Ca(ICCV2/PI489777)SNP_19604	674	G	A	17	20	Young_pod	--
	Ca(ICCV2/PI489777)SNP_19605	770	C	T	24	19	Young_pod	--

	Ca(ICCV2/PI489777)SNP_19606	967	C	G	14	11	Young_pod	--
	Ca(ICCV2/PI489777)SNP_19607	968	T	C	14	11	Young_pod	--
	Ca(ICCV2/PI489777)SNP_19608	993	C	A	14	9	Young_pod	--
CakTC42131	Ca(ICCV2/PI489777)SNP_19609	354	C	T	4	6	--	--
CakTC25379	Ca(ICCV2/PI489777)SNP_19610	461	C	T	9	6	--	--
	Ca(ICCV2/PI489777)SNP_19611	465	G	C	8	6	--	--
CakTC31716	Ca(ICCV2/PI489777)SNP_19612	135	C	T	4	6	--	--
	Ca(ICCV2/PI489777)SNP_19613	141	C	T	4	9	--	--
	Ca(ICCV2/PI489777)SNP_19614	145	C	T	4	9	--	--
	Ca(ICCV2/PI489777)SNP_19615	179	T	C	3	9	--	--
	Ca(ICCV2/PI489777)SNP_19616	207	T	C	4	11	--	--
	Ca(ICCV2/PI489777)SNP_19617	217	A	G	6	9	--	--
	Ca(ICCV2/PI489777)SNP_19618	220	A	C	6	9	--	--
	Ca(ICCV2/PI489777)SNP_19619	271	G	A	9	9	--	--
CakTC08349	Ca(ICCV2/PI489777)SNP_19620	117	G	A	15	6	--	--
	Ca(ICCV2/PI489777)SNP_19621	408	T	C	22	12	--	--
CakTC39838	Ca(ICCV2/PI489777)SNP_19622	697	T	C	55	13	--	--
	Ca(ICCV2/PI489777)SNP_19623	981	G	A	36	19	--	--
	Ca(ICCV2/PI489777)SNP_19624	1370	G	A	18	7	--	--
	Ca(ICCV2/PI489777)SNP_19625	1442	T	C	14	5	--	--
	Ca(ICCV2/PI489777)SNP_19626	1474	T	C	13	5	--	--
	Ca(ICCV2/PI489777)SNP_19627	1578	T	G	8	6	--	--
CakTC37168	Ca(ICCV2/PI489777)SNP_19628	227	C	T	3	4	--	--
CakTC32772	Ca(ICCV2/PI489777)SNP_19629	627	A	G	20	3	Flower	bud
	Ca(ICCV2/PI489777)SNP_19630	643	G	A	21	3	Flower	bud
	Ca(ICCV2/PI489777)SNP_19631	652	T	C	17	3	Flower	bud
	Ca(ICCV2/PI489777)SNP_19632	653	T	G	17	3	Flower	bud
	Ca(ICCV2/PI489777)SNP_19633	668	A	G	20	3	Flower	bud
	Ca(ICCV2/PI489777)SNP_19634	776	C	T	16	5	Flower	bud
	Ca(ICCV2/PI489777)SNP_19635	803	G	A	13	5	Flower	bud
	Ca(ICCV2/PI489777)SNP_19636	1533	C	T	14	5	Flower	bud
	Ca(ICCV2/PI489777)SNP_19637	1593	G	A	13	7	Flower	bud
CakTC24026	Ca(ICCV2/PI489777)SNP_19638	1423	G	A	12	3	--	--
CakTC25270	Ca(ICCV2/PI489777)SNP_19639	1318	T	A	21	3	--	BSD
CakTC42383	Ca(ICCV2/PI489777)SNP_19640	134	C	T	13	3	--	--
CakTC26890	Ca(ICCV2/PI489777)SNP_19641	766	C	T	4	5	--	--
CakTC33638	Ca(ICCV2/PI489777)SNP_19642	373	G	A	16	9	--	--
	Ca(ICCV2/PI489777)SNP_19643	435	A	C	16	11	--	--
	Ca(ICCV2/PI489777)SNP_19644	436	T	A	16	10	--	--
CakTC41676	Ca(ICCV2/PI489777)SNP_19645	52	A	G	5	5	--	--
	Ca(ICCV2/PI489777)SNP_19646	156	T	A	8	5	--	--
	Ca(ICCV2/PI489777)SNP_19647	216	T	C	8	5	--	--
	Ca(ICCV2/PI489777)SNP_19648	357	C	G	7	6	--	--
CakTC38800	Ca(ICCV2/PI489777)SNP_19649	1406	A	G	42	23	--	--
CakTC08922	Ca(ICCV2/PI489777)SNP_19650	317	A	G	4	16	--	--
	Ca(ICCV2/PI489777)SNP_19651	327	C	T	4	15	--	--
	Ca(ICCV2/PI489777)SNP_19652	955	A	G	11	10	--	--
	Ca(ICCV2/PI489777)SNP_19653	1087	G	C	10	10	--	--
	Ca(ICCV2/PI489777)SNP_19654	1138	G	T	9	4	--	--
CakTC12615	Ca(ICCV2/PI489777)SNP_19655	1053	T	C	42	18	--	--
	Ca(ICCV2/PI489777)SNP_19656	1542	T	A	72	26	--	--
	Ca(ICCV2/PI489777)SNP_19657	1735	G	A	11	10	--	--
CakTC27063	Ca(ICCV2/PI489777)SNP_19658	50	T	C	5	3	--	--
	Ca(ICCV2/PI489777)SNP_19659	102	C	G	12	5	--	--
	Ca(ICCV2/PI489777)SNP_19660	173	T	C	13	8	--	--
	Ca(ICCV2/PI489777)SNP_19661	200	C	T	13	4	--	--
	Ca(ICCV2/PI489777)SNP_19662	229	G	C	14	7	--	--
	Ca(ICCV2/PI489777)SNP_19663	293	C	G	14	7	--	--
	Ca(ICCV2/PI489777)SNP_19664	487	G	A	18	8	--	--
	Ca(ICCV2/PI489777)SNP_19665	536	G	C	18	11	--	--
	Ca(ICCV2/PI489777)SNP_19666	564	A	C	16	9	--	--
	Ca(ICCV2/PI489777)SNP_19667	570	C	T	20	10	--	--
	Ca(ICCV2/PI489777)SNP_19668	1052	C	T	37	9	--	--
	Ca(ICCV2/PI489777)SNP_19669	1565	A	G	36	14	--	--
	Ca(ICCV2/PI489777)SNP_19670	1566	C	A	36	13	--	--
	Ca(ICCV2/PI489777)SNP_19671	1638	C	T	32	12	--	--
	Ca(ICCV2/PI489777)SNP_19672	1710	T	C	36	13	--	--
	Ca(ICCV2/PI489777)SNP_19673	2020	C	G	18	11	--	--
	Ca(ICCV2/PI489777)SNP_19674	2528	C	G	11	5	--	--
	Ca(ICCV2/PI489777)SNP_19675	2603	T	A	7	5	--	--
CakTC27861	Ca(ICCV2/PI489777)SNP_19676	795	G	A	6	4	--	--
	Ca(ICCV2/PI489777)SNP_19677	901	A	G	6	6	--	--
	Ca(ICCV2/PI489777)SNP_19678	1080	T	C	8	5	--	--
	Ca(ICCV2/PI489777)SNP_19679	1845	T	G	10	6	--	--

CakTC24575	Ca(ICCV2/PI489777)SNP_19680	429	A	G	12	5	--	--
	Ca(ICCV2/PI489777)SNP_19681	441	T	A	11	5	--	--
	Ca(ICCV2/PI489777)SNP_19682	457	A	T	9	5	--	--
CakTC33132	Ca(ICCV2/PI489777)SNP_19683	120	C	T	11	10	--	--
	Ca(ICCV2/PI489777)SNP_19684	663	T	C	22	26	--	--
	Ca(ICCV2/PI489777)SNP_19685	666	T	G	21	25	--	--
	Ca(ICCV2/PI489777)SNP_19686	753	C	T	25	31	--	--
	Ca(ICCV2/PI489777)SNP_19687	1134	T	A	31	35	--	--
	Ca(ICCV2/PI489777)SNP_19688	1620	T	C	22	34	--	--
	Ca(ICCV2/PI489777)SNP_19689	1635	C	T	21	33	--	--
	Ca(ICCV2/PI489777)SNP_19690	1890	G	A	32	26	--	--
	Ca(ICCV2/PI489777)SNP_19691	2068	C	T	25	21	--	--
	Ca(ICCV2/PI489777)SNP_19692	2092	A	T	22	20	--	--
	Ca(ICCV2/PI489777)SNP_19693	2116	A	C	20	16	--	--
	Ca(ICCV2/PI489777)SNP_19694	2135	T	C	15	12	--	--
CakTC31363	Ca(ICCV2/PI489777)SNP_19695	1595	T	C	8	5	--	--
	Ca(ICCV2/PI489777)SNP_19696	1756	A	G	7	4	--	--
	Ca(ICCV2/PI489777)SNP_19697	1781	C	T	6	4	--	--
	Ca(ICCV2/PI489777)SNP_19698	1785	G	T	6	5	--	--
	Ca(ICCV2/PI489777)SNP_19699	1830	G	A	5	5	--	--
CakTC11567	Ca(ICCV2/PI489777)SNP_19700	447	A	G	10	3	--	MYB
CakTC29766	Ca(ICCV2/PI489777)SNP_19701	372	T	C	21	5	--	--
	Ca(ICCV2/PI489777)SNP_19702	656	C	G	12	3	--	--
	Ca(ICCV2/PI489777)SNP_19703	1163	A	G	6	14	--	--
CakTC33528	Ca(ICCV2/PI489777)SNP_19704	90	C	T	63	55	--	--
CakTC33821	Ca(ICCV2/PI489777)SNP_19705	385	T	C	34	20	--	--
CakTC28514	Ca(ICCV2/PI489777)SNP_19706	550	T	C	3	7	--	LUG
CakTC28613	Ca(ICCV2/PI489777)SNP_19707	2744	A	G	5	5	--	--
CakTC29634	Ca(ICCV2/PI489777)SNP_19708	919	G	A	4	3	--	--
CakTC33723	Ca(ICCV2/PI489777)SNP_19709	676	A	G	12	25	--	--
	Ca(ICCV2/PI489777)SNP_19710	1765	G	A	18	16	--	--
CakTC00336	Ca(ICCV2/PI489777)SNP_19711	385	C	G	22	17	--	--
	Ca(ICCV2/PI489777)SNP_19712	798	G	T	67	6	--	--
CakTC27395	Ca(ICCV2/PI489777)SNP_19713	680	C	G	10	4	--	--
	Ca(ICCV2/PI489777)SNP_19714	1127	A	T	17	3	--	--
	Ca(ICCV2/PI489777)SNP_19715	1279	G	C	14	3	--	--
CakTC41342	Ca(ICCV2/PI489777)SNP_19716	1249	G	A	22	22	--	--
	Ca(ICCV2/PI489777)SNP_19717	1384	A	G	20	13	--	--
CakTC06879	Ca(ICCV2/PI489777)SNP_19718	945	A	G	6	9	--	--
	Ca(ICCV2/PI489777)SNP_19719	957	G	C	6	9	--	--
CakTC07683	Ca(ICCV2/PI489777)SNP_19720	467	G	A	20	16	--	--
	Ca(ICCV2/PI489777)SNP_19721	577	G	C	17	12	--	--
	Ca(ICCV2/PI489777)SNP_19722	824	T	G	3	4	--	--
CakTC34868	Ca(ICCV2/PI489777)SNP_19723	2255	G	A	3	5	--	--
CakTC10001	Ca(ICCV2/PI489777)SNP_19724	71	A	C	6	9	--	--
CakTC34451	Ca(ICCV2/PI489777)SNP_19725	277	C	T	4	6	--	--
CakTC32931	Ca(ICCV2/PI489777)SNP_19726	1763	T	G	17	12	--	--
	Ca(ICCV2/PI489777)SNP_19727	2486	C	T	8	4	--	--
CakTC30981	Ca(ICCV2/PI489777)SNP_19728	277	G	A	7	9	--	--
	Ca(ICCV2/PI489777)SNP_19729	411	T	C	10	9	--	--
	Ca(ICCV2/PI489777)SNP_19730	524	C	T	6	7	--	--
CakTC40429	Ca(ICCV2/PI489777)SNP_19731	653	C	A	12	6	--	--
	Ca(ICCV2/PI489777)SNP_19732	782	G	A	11	6	--	--
	Ca(ICCV2/PI489777)SNP_19733	854	G	A	9	5	--	--
CakTC40877	Ca(ICCV2/PI489777)SNP_19734	693	C	T	11	14	--	--
	Ca(ICCV2/PI489777)SNP_19735	718	T	C	9	13	--	--
	Ca(ICCV2/PI489777)SNP_19736	723	A	G	7	12	--	--
	Ca(ICCV2/PI489777)SNP_19737	749	T	A	6	10	--	--
	Ca(ICCV2/PI489777)SNP_19738	770	A	G	4	8	--	--
CakTC43369	Ca(ICCV2/PI489777)SNP_19739	932	A	G	13	3	Flower	bud
CakTC11481	Ca(ICCV2/PI489777)SNP_19740	663	T	C	3	3	--	--
CakTC33406	Ca(ICCV2/PI489777)SNP_19741	305	G	A	6	8	--	--
CakTC29515	Ca(ICCV2/PI489777)SNP_19742	319	G	T	33	4	--	--
	Ca(ICCV2/PI489777)SNP_19743	349	T	C	22	4	--	--
	Ca(ICCV2/PI489777)SNP_19744	370	G	A	37	4	--	--
	Ca(ICCV2/PI489777)SNP_19745	1288	C	T	64	8	--	--
	Ca(ICCV2/PI489777)SNP_19746	1300	A	G	63	7	--	--
	Ca(ICCV2/PI489777)SNP_19747	1450	G	T	32	9	--	--
CakTC30018	Ca(ICCV2/PI489777)SNP_19748	1404	A	G	36	20	--	--
	Ca(ICCV2/PI489777)SNP_19749	2045	G	C	28	10	--	--
CakTC30517	Ca(ICCV2/PI489777)SNP_19750	401	T	A	21	4	--	--
	Ca(ICCV2/PI489777)SNP_19751	713	A	G	24	4	--	--
	Ca(ICCV2/PI489777)SNP_19752	815	C	T	30	15	--	--
	Ca(ICCV2/PI489777)SNP_19753	920	G	C	24	15	--	--

	Ca(ICCV2/PI489777)SNP_19754	1004	T	C	22	15	--	--
CakTC42814	Ca(ICCV2/PI489777)SNP_19755	206	T	C	4	4	--	--
	Ca(ICCV2/PI489777)SNP_19756	1028	C	T	14	7	--	--
	Ca(ICCV2/PI489777)SNP_19757	1156	A	G	14	12	--	--
	Ca(ICCV2/PI489777)SNP_19758	1190	T	C	7	11	--	--
	Ca(ICCV2/PI489777)SNP_19759	1241	A	G	8	11	--	--
	Ca(ICCV2/PI489777)SNP_19760	1292	G	T	10	11	--	--
	Ca(ICCV2/PI489777)SNP_19761	1707	G	T	6	3	--	--
CakTC13081	Ca(ICCV2/PI489777)SNP_19762	870	T	C	6	4	--	--
	Ca(ICCV2/PI489777)SNP_19763	1820	C	G	11	6	--	--
CakTC42737	Ca(ICCV2/PI489777)SNP_19764	688	G	T	11	5	--	--
	Ca(ICCV2/PI489777)SNP_19765	698	C	T	12	5	--	--
CakTC42049	Ca(ICCV2/PI489777)SNP_19766	644	C	T	3	6	--	--
	Ca(ICCV2/PI489777)SNP_19767	764	G	A	3	6	--	--
CakTC23309	Ca(ICCV2/PI489777)SNP_19768	1343	T	C	5	4	--	--
	Ca(ICCV2/PI489777)SNP_19769	1378	G	T	5	4	--	--
CakTC33008	Ca(ICCV2/PI489777)SNP_19770	369	T	G	22	5	--	--
	Ca(ICCV2/PI489777)SNP_19771	1190	A	G	5	5	--	--
	Ca(ICCV2/PI489777)SNP_19772	1750	T	C	5	6	--	--
CakTC28835	Ca(ICCV2/PI489777)SNP_19773	1080	C	T	6	3	--	TPR
CakTC39146	Ca(ICCV2/PI489777)SNP_19774	33	T	A	9	7	--	--
	Ca(ICCV2/PI489777)SNP_19775	87	C	T	13	8	--	--
	Ca(ICCV2/PI489777)SNP_19776	201	T	A	17	9	--	--
	Ca(ICCV2/PI489777)SNP_19777	506	T	A	22	7	--	--
	Ca(ICCV2/PI489777)SNP_19778	618	C	T	28	7	--	--
	Ca(ICCV2/PI489777)SNP_19779	691	A	G	26	6	--	--
	Ca(ICCV2/PI489777)SNP_19780	735	T	G	22	6	--	--
	Ca(ICCV2/PI489777)SNP_19781	915	G	T	18	14	--	--
	Ca(ICCV2/PI489777)SNP_19782	923	C	A	19	16	--	--
	Ca(ICCV2/PI489777)SNP_19783	1046	A	G	11	12	--	--
	Ca(ICCV2/PI489777)SNP_19784	1178	T	C	9	9	--	--
	Ca(ICCV2/PI489777)SNP_19785	1224	G	A	12	6	--	--
CakTC09105	Ca(ICCV2/PI489777)SNP_19786	597	A	G	6	3	--	--
	Ca(ICCV2/PI489777)SNP_19787	3216	C	T	4	5	--	--
	Ca(ICCV2/PI489777)SNP_19788	4515	A	T	4	8	--	--
	Ca(ICCV2/PI489777)SNP_19789	5262	T	C	4	6	--	--
CakTC26851	Ca(ICCV2/PI489777)SNP_19790	2274	T	G	7	3	--	--
	Ca(ICCV2/PI489777)SNP_19791	2653	A	G	5	5	--	--
CakTC33419	Ca(ICCV2/PI489777)SNP_19792	260	T	C	4	5	--	bZIP
	Ca(ICCV2/PI489777)SNP_19793	636	C	T	3	3	--	bZIP
	Ca(ICCV2/PI489777)SNP_19794	756	A	G	3	3	--	bZIP
CakTC38370	Ca(ICCV2/PI489777)SNP_19795	734	A	G	21	40	--	BSD
	Ca(ICCV2/PI489777)SNP_19796	1286	C	T	19	14	--	BSD
	Ca(ICCV2/PI489777)SNP_19797	1307	A	G	16	17	--	BSD
	Ca(ICCV2/PI489777)SNP_19798	1339	A	G	16	16	--	BSD
	Ca(ICCV2/PI489777)SNP_19799	1729	C	G	10	9	--	BSD
CakTC40835	Ca(ICCV2/PI489777)SNP_19800	1645	A	G	17	3	--	--
	Ca(ICCV2/PI489777)SNP_19801	1666	T	A	15	3	--	--
	Ca(ICCV2/PI489777)SNP_19802	1667	A	G	15	3	--	--
CakTC29147	Ca(ICCV2/PI489777)SNP_19803	1667	A	T	10	9	--	--
	Ca(ICCV2/PI489777)SNP_19804	1672	T	A	8	9	--	--
	Ca(ICCV2/PI489777)SNP_19805	1715	A	G	10	6	--	--
	Ca(ICCV2/PI489777)SNP_19806	1719	A	T	10	7	--	--
	Ca(ICCV2/PI489777)SNP_19807	1770	C	T	8	8	--	--
	Ca(ICCV2/PI489777)SNP_19808	1794	A	G	9	7	--	--
	Ca(ICCV2/PI489777)SNP_19809	2044	G	T	4	4	--	--
CakTC34264	Ca(ICCV2/PI489777)SNP_19810	86	T	C	8	54	--	--
	Ca(ICCV2/PI489777)SNP_19811	386	T	C	4	44	--	--
	Ca(ICCV2/PI489777)SNP_19812	471	G	C	3	43	--	--
CakTC37158	Ca(ICCV2/PI489777)SNP_19813	729	G	A	13	10	--	--
	Ca(ICCV2/PI489777)SNP_19814	789	C	T	18	10	--	--
	Ca(ICCV2/PI489777)SNP_19815	792	C	G	18	10	--	--
	Ca(ICCV2/PI489777)SNP_19816	813	C	T	20	9	--	--
CakTC31162	Ca(ICCV2/PI489777)SNP_19817	264	G	A	42	27	--	--
	Ca(ICCV2/PI489777)SNP_19818	267	T	C	43	29	--	--
	Ca(ICCV2/PI489777)SNP_19819	1080	C	G	41	57	--	--
	Ca(ICCV2/PI489777)SNP_19820	1176	T	C	65	56	--	--
	Ca(ICCV2/PI489777)SNP_19821	1308	T	C	99	102	--	--
	Ca(ICCV2/PI489777)SNP_19822	2065	T	C	12	14	--	--
CakTC40744	Ca(ICCV2/PI489777)SNP_19823	79	G	A	10	3	--	--
	Ca(ICCV2/PI489777)SNP_19824	83	T	C	10	3	--	--
	Ca(ICCV2/PI489777)SNP_19825	103	G	A	9	4	--	--
CakTC36005	Ca(ICCV2/PI489777)SNP_19826	1312	T	A	6	6	--	--
CakTC14903	Ca(ICCV2/PI489777)SNP_19827	334	A	C	3	3	--	--

CakTC31928	Ca(ICCV2/PI489777)SNP_19828	422	T	C	3	4	--	--
	Ca(ICCV2/PI489777)SNP_19829	1125	T	G	3	3	--	--
	Ca(ICCV2/PI489777)SNP_19830	1319	T	C	4	6	--	--
	Ca(ICCV2/PI489777)SNP_19831	1547	A	G	4	5	--	--
CakTC41284	Ca(ICCV2/PI489777)SNP_19832	746	G	T	22	16	--	--
	Ca(ICCV2/PI489777)SNP_19833	1637	C	G	11	21	--	--
	Ca(ICCV2/PI489777)SNP_19834	1688	G	A	12	18	--	--
	Ca(ICCV2/PI489777)SNP_19835	1813	C	T	16	23	--	--
	Ca(ICCV2/PI489777)SNP_19836	2369	T	A	22	13	--	--
	Ca(ICCV2/PI489777)SNP_19837	2423	T	C	12	9	--	--
CakTC28259	Ca(ICCV2/PI489777)SNP_19838	109	C	T	3	4	--	--
	Ca(ICCV2/PI489777)SNP_19839	161	T	C	22	17	--	--
	Ca(ICCV2/PI489777)SNP_19840	163	C	G	21	17	--	--
	Ca(ICCV2/PI489777)SNP_19841	1504	T	C	23	21	--	--
CakTC40791	Ca(ICCV2/PI489777)SNP_19842	696	C	T	14	6	--	--
	Ca(ICCV2/PI489777)SNP_19843	702	C	T	14	5	--	--
CakTC26926	Ca(ICCV2/PI489777)SNP_19844	39	A	G	9	5	--	TPR
	Ca(ICCV2/PI489777)SNP_19845	117	C	T	18	9	--	TPR
	Ca(ICCV2/PI489777)SNP_19846	199	T	G	20	11	--	TPR
	Ca(ICCV2/PI489777)SNP_19847	404	T	C	15	11	--	TPR
CakTC22885	Ca(ICCV2/PI489777)SNP_19848	101	T	C	57	11	--	--
	Ca(ICCV2/PI489777)SNP_19849	437	A	C	153	78	--	--
CakTC37947	Ca(ICCV2/PI489777)SNP_19850	629	G	A	5	7	--	--
	Ca(ICCV2/PI489777)SNP_19851	645	G	A	4	7	--	--
	Ca(ICCV2/PI489777)SNP_19852	941	A	G	5	10	--	--
CakTC27711	Ca(ICCV2/PI489777)SNP_19853	408	G	A	21	6	--	--
	Ca(ICCV2/PI489777)SNP_19854	527	C	T	34	8	--	--
	Ca(ICCV2/PI489777)SNP_19855	738	G	A	34	7	--	--
	Ca(ICCV2/PI489777)SNP_19856	877	T	G	9	3	--	--
CakTC29927	Ca(ICCV2/PI489777)SNP_19857	2097	C	T	11	10	--	--
CakTC38248	Ca(ICCV2/PI489777)SNP_19858	114	C	T	8	9	--	--
	Ca(ICCV2/PI489777)SNP_19859	421	T	G	5	9	--	--
	Ca(ICCV2/PI489777)SNP_19860	422	T	C	4	9	--	--
	Ca(ICCV2/PI489777)SNP_19861	490	C	T	7	5	--	--
	Ca(ICCV2/PI489777)SNP_19862	908	T	G	12	5	--	--
	Ca(ICCV2/PI489777)SNP_19863	943	A	G	10	4	--	--
CakTC26670	Ca(ICCV2/PI489777)SNP_19864	97	C	G	7	3	--	--
	Ca(ICCV2/PI489777)SNP_19865	132	G	A	7	3	--	--
CakTC36711	Ca(ICCV2/PI489777)SNP_19866	82	C	T	8	8	--	--
	Ca(ICCV2/PI489777)SNP_19867	84	A	G	8	8	--	--
	Ca(ICCV2/PI489777)SNP_19868	675	A	G	6	7	--	--
	Ca(ICCV2/PI489777)SNP_19869	684	T	C	7	7	--	--
	Ca(ICCV2/PI489777)SNP_19870	771	C	T	3	6	--	--
	Ca(ICCV2/PI489777)SNP_19871	924	A	C	6	10	--	--
	Ca(ICCV2/PI489777)SNP_19872	1068	C	A	13	9	--	--
	Ca(ICCV2/PI489777)SNP_19873	1128	T	G	11	10	--	--
	Ca(ICCV2/PI489777)SNP_19874	1344	A	G	8	4	--	--
	Ca(ICCV2/PI489777)SNP_19875	1500	T	C	8	7	--	--
	Ca(ICCV2/PI489777)SNP_19876	1913	A	G	8	7	--	--
	Ca(ICCV2/PI489777)SNP_19877	1922	C	G	10	6	--	--
	Ca(ICCV2/PI489777)SNP_19878	2238	C	T	7	5	--	--
CakTC10568	Ca(ICCV2/PI489777)SNP_19879	133	A	G	6	4	--	--
	Ca(ICCV2/PI489777)SNP_19880	188	C	T	7	4	--	--
CakTC25428	Ca(ICCV2/PI489777)SNP_19881	659	A	G	17	8	--	--
	Ca(ICCV2/PI489777)SNP_19882	1069	C	T	14	4	--	--
CakTC37556	Ca(ICCV2/PI489777)SNP_19883	953	A	G	11	11	--	--
	Ca(ICCV2/PI489777)SNP_19884	1224	G	C	23	12	--	--
CakTC11121	Ca(ICCV2/PI489777)SNP_19885	302	T	C	5	5	--	--
CakTC22666	Ca(ICCV2/PI489777)SNP_19886	589	A	C	4	5	--	--
CakTC41089	Ca(ICCV2/PI489777)SNP_19887	447	T	C	18	25	--	--
	Ca(ICCV2/PI489777)SNP_19888	787	C	G	23	18	--	--
	Ca(ICCV2/PI489777)SNP_19889	909	C	T	22	22	--	--
	Ca(ICCV2/PI489777)SNP_19890	1344	C	T	33	31	--	--
	Ca(ICCV2/PI489777)SNP_19891	1638	C	T	14	22	--	--
	Ca(ICCV2/PI489777)SNP_19892	2177	T	C	20	19	--	--
	Ca(ICCV2/PI489777)SNP_19893	2328	C	T	8	6	--	--
CakTC37366	Ca(ICCV2/PI489777)SNP_19894	895	A	G	16	7	--	--
CakTC07643	Ca(ICCV2/PI489777)SNP_19895	332	A	G	5	5	--	--
	Ca(ICCV2/PI489777)SNP_19896	695	A	G	5	15	--	--
CakTC26685	Ca(ICCV2/PI489777)SNP_19897	337	A	G	9	6	--	--
	Ca(ICCV2/PI489777)SNP_19898	778	C	A	33	12	--	--
	Ca(ICCV2/PI489777)SNP_19899	853	T	C	31	12	--	--
	Ca(ICCV2/PI489777)SNP_19900	930	C	G	25	15	--	--
	Ca(ICCV2/PI489777)SNP_19901	1105	T	C	26	14	--	--

	Ca(ICCV2/PI489777)SNP_19902	1110	A	G	23	12	--	--
CakTC39311	Ca(ICCV2/PI489777)SNP_19903	541	C	G	26	8	--	--
	Ca(ICCV2/PI489777)SNP_19904	573	C	A	28	6	--	--
	Ca(ICCV2/PI489777)SNP_19905	1392	T	G	5	5	--	--
CakTC28842	Ca(ICCV2/PI489777)SNP_19906	212	G	A	3	8	--	--
	Ca(ICCV2/PI489777)SNP_19907	387	T	C	6	3	--	--
	Ca(ICCV2/PI489777)SNP_19908	443	A	G	5	4	--	--
CakTC42451	Ca(ICCV2/PI489777)SNP_19909	275	A	C	15	23	--	--
	Ca(ICCV2/PI489777)SNP_19910	289	T	A	19	17	--	--
CakTC17382	Ca(ICCV2/PI489777)SNP_19911	25	T	C	3	7	--	--
	Ca(ICCV2/PI489777)SNP_19912	67	C	T	4	7	--	--
	Ca(ICCV2/PI489777)SNP_19913	80	G	A	3	8	--	--
	Ca(ICCV2/PI489777)SNP_19914	107	A	G	4	8	--	--
	Ca(ICCV2/PI489777)SNP_19915	114	C	T	4	8	--	--
	Ca(ICCV2/PI489777)SNP_19916	224	G	C	5	8	--	--
	Ca(ICCV2/PI489777)SNP_19917	237	T	C	7	10	--	--
	Ca(ICCV2/PI489777)SNP_19918	665	T	C	15	10	--	--
CakTC39203	Ca(ICCV2/PI489777)SNP_19919	1185	A	C	11	10	Mature	Leaf
	Ca(ICCV2/PI489777)SNP_19920	1260	A	G	17	11	Mature	Leaf
	Ca(ICCV2/PI489777)SNP_19921	1265	G	A	18	9	Mature	Leaf
	Ca(ICCV2/PI489777)SNP_19922	1425	G	A	16	6	Mature	Leaf
	Ca(ICCV2/PI489777)SNP_19923	1446	A	G	14	6	Mature	Leaf
	Ca(ICCV2/PI489777)SNP_19924	1464	A	G	15	6	Mature	Leaf
CakTC27456	Ca(ICCV2/PI489777)SNP_19925	208	C	A	12	5	--	--
	Ca(ICCV2/PI489777)SNP_19926	280	G	C	33	23	--	--
	Ca(ICCV2/PI489777)SNP_19927	368	C	T	42	30	--	--
	Ca(ICCV2/PI489777)SNP_19928	410	G	C	44	27	--	--
	Ca(ICCV2/PI489777)SNP_19929	613	T	A	57	34	--	--
CakTC02963	Ca(ICCV2/PI489777)SNP_19930	1580	G	A	7	9	--	--
	Ca(ICCV2/PI489777)SNP_19931	1801	G	A	9	8	--	--
	Ca(ICCV2/PI489777)SNP_19932	1816	T	C	9	4	--	--
CakTC09202	Ca(ICCV2/PI489777)SNP_19933	1899	T	G	9	3	--	--
	Ca(ICCV2/PI489777)SNP_19934	2353	G	A	5	3	--	--
CakTC24887	Ca(ICCV2/PI489777)SNP_19935	469	G	A	6	4	--	--
	Ca(ICCV2/PI489777)SNP_19936	480	T	C	8	6	--	--
	Ca(ICCV2/PI489777)SNP_19937	819	T	C	10	3	--	--
	Ca(ICCV2/PI489777)SNP_19938	1089	A	C	13	3	--	--
	Ca(ICCV2/PI489777)SNP_19939	1254	A	G	15	8	--	--
	Ca(ICCV2/PI489777)SNP_19940	2179	A	C	13	8	--	--
CakTC14005	Ca(ICCV2/PI489777)SNP_19941	55	T	G	5	3	--	--
	Ca(ICCV2/PI489777)SNP_19942	126	C	T	5	5	--	--
	Ca(ICCV2/PI489777)SNP_19943	160	C	T	5	4	--	--
CakTC24619	Ca(ICCV2/PI489777)SNP_19944	1167	T	C	9	6	--	--
	Ca(ICCV2/PI489777)SNP_19945	1187	A	T	9	6	--	--
	Ca(ICCV2/PI489777)SNP_19946	1593	A	G	4	6	--	--
	Ca(ICCV2/PI489777)SNP_19947	1753	T	C	3	6	--	--
	Ca(ICCV2/PI489777)SNP_19948	1872	G	A	8	7	--	--
	Ca(ICCV2/PI489777)SNP_19949	2328	C	G	9	6	--	--
	Ca(ICCV2/PI489777)SNP_19950	3756	A	C	3	5	--	--
	Ca(ICCV2/PI489777)SNP_19951	4024	C	T	10	3	--	--
CakTC33557	Ca(ICCV2/PI489777)SNP_19952	423	C	T	17	46	--	--
	Ca(ICCV2/PI489777)SNP_19953	454	C	T	16	43	--	--
	Ca(ICCV2/PI489777)SNP_19954	526	C	T	11	11	--	--
CakTC30422	Ca(ICCV2/PI489777)SNP_19955	895	G	A	8	3	--	--
	Ca(ICCV2/PI489777)SNP_19956	1053	C	T	12	3	--	--
	Ca(ICCV2/PI489777)SNP_19957	1835	G	A	7	3	--	--
	Ca(ICCV2/PI489777)SNP_19958	1919	G	A	8	4	--	--
CakTC22485	Ca(ICCV2/PI489777)SNP_19959	1413	C	T	7	5	--	--
CakTC26606	Ca(ICCV2/PI489777)SNP_19960	147	T	G	8	5	--	--
	Ca(ICCV2/PI489777)SNP_19961	262	C	T	22	8	--	--
	Ca(ICCV2/PI489777)SNP_19962	302	C	G	24	8	--	--
	Ca(ICCV2/PI489777)SNP_19963	404	T	C	28	10	--	--
	Ca(ICCV2/PI489777)SNP_19964	2419	C	T	9	11	--	--
CakTC41688	Ca(ICCV2/PI489777)SNP_19965	371	A	G	5	7	Flower	bud
	Ca(ICCV2/PI489777)SNP_19966	460	A	T	7	8	Flower	bud
	Ca(ICCV2/PI489777)SNP_19967	520	A	C	6	6	Flower	bud
	Ca(ICCV2/PI489777)SNP_19968	584	G	A	4	3	Flower	bud
CakTC40261	Ca(ICCV2/PI489777)SNP_19969	351	G	A	37	23	--	--
	Ca(ICCV2/PI489777)SNP_19970	492	A	G	42	21	--	--
	Ca(ICCV2/PI489777)SNP_19971	585	G	A	36	20	--	--
	Ca(ICCV2/PI489777)SNP_19972	952	C	G	30	17	--	--
	Ca(ICCV2/PI489777)SNP_19973	985	G	A	21	10	--	--
	Ca(ICCV2/PI489777)SNP_19974	1180	C	T	16	9	--	--
	Ca(ICCV2/PI489777)SNP_19975	1399	C	T	20	14	--	--

	Ca(ICCV2/PI489777)SNP_19976	1930	C	T	17	14	--	--
	Ca(ICCV2/PI489777)SNP_19977	2098	A	G	22	10	--	--
	Ca(ICCV2/PI489777)SNP_19978	2407	T	C	29	13	--	--
CakTC26771	Ca(ICCV2/PI489777)SNP_19979	427	A	C	3	3	--	--
CakTC30657	Ca(ICCV2/PI489777)SNP_19980	1396	A	C	3	5	--	--
CakTC34518	Ca(ICCV2/PI489777)SNP_19981	522	C	T	4	7	--	--
	Ca(ICCV2/PI489777)SNP_19982	579	A	G	4	7	--	--
CakTC36201	Ca(ICCV2/PI489777)SNP_19983	220	T	A	8	7	--	--
	Ca(ICCV2/PI489777)SNP_19984	750	G	A	17	6	--	--
	Ca(ICCV2/PI489777)SNP_19985	1212	G	A	41	20	--	--
	Ca(ICCV2/PI489777)SNP_19986	1215	A	G	40	19	--	--
	Ca(ICCV2/PI489777)SNP_19987	1350	T	G	18	14	--	--
	Ca(ICCV2/PI489777)SNP_19988	1407	T	C	5	9	--	--
	Ca(ICCV2/PI489777)SNP_19989	1872	G	A	38	9	--	--
CakTC25312	Ca(ICCV2/PI489777)SNP_19990	249	C	T	16	3	--	IWS1
CakTC32144	Ca(ICCV2/PI489777)SNP_19991	696	A	G	8	5	--	--
	Ca(ICCV2/PI489777)SNP_19992	1418	C	G	3	8	--	--
CakTC39633	Ca(ICCV2/PI489777)SNP_19993	136	C	A	8	12	--	--
	Ca(ICCV2/PI489777)SNP_19994	179	T	G	11	15	--	--
CakTC39636	Ca(ICCV2/PI489777)SNP_19995	576	A	T	14	10	--	--
	Ca(ICCV2/PI489777)SNP_19996	675	C	T	14	14	--	--
	Ca(ICCV2/PI489777)SNP_19997	690	C	T	16	8	--	--
	Ca(ICCV2/PI489777)SNP_19998	768	G	A	23	23	--	--
	Ca(ICCV2/PI489777)SNP_19999	801	A	G	24	28	--	--
	Ca(ICCV2/PI489777)SNP_20000	840	C	G	25	28	--	--
CakTC24405	Ca(ICCV2/PI489777)SNP_20001	872	G	A	39	14	--	--
CakTC20165	Ca(ICCV2/PI489777)SNP_20002	1277	A	G	11	5	Young_pod	--
CakTC41853	Ca(ICCV2/PI489777)SNP_20003	62	T	C	12	11	--	--
	Ca(ICCV2/PI489777)SNP_20004	91	C	T	14	12	--	--
	Ca(ICCV2/PI489777)SNP_20005	509	T	C	6	13	--	--
	Ca(ICCV2/PI489777)SNP_20006	673	G	C	9	9	--	--
	Ca(ICCV2/PI489777)SNP_20007	742	G	T	9	8	--	--
	Ca(ICCV2/PI489777)SNP_20008	743	G	A	9	8	--	--
CakTC03343	Ca(ICCV2/PI489777)SNP_20009	291	C	T	13	11	--	--
	Ca(ICCV2/PI489777)SNP_20010	588	G	A	3	5	--	--
	Ca(ICCV2/PI489777)SNP_20011	606	C	A	3	3	--	--
CakTC23480	Ca(ICCV2/PI489777)SNP_20012	2677	T	G	6	13	--	--
CakTC35095	Ca(ICCV2/PI489777)SNP_20013	1110	C	T	4	13	--	--
	Ca(ICCV2/PI489777)SNP_20014	1284	A	G	6	14	--	--
	Ca(ICCV2/PI489777)SNP_20015	1326	G	C	7	13	--	--
CakTC38476	Ca(ICCV2/PI489777)SNP_20016	907	C	T	5	10	Root	--
CakTC29743	Ca(ICCV2/PI489777)SNP_20017	935	T	C	5	7	--	--
	Ca(ICCV2/PI489777)SNP_20018	1188	T	C	3	5	--	--
CakTC02562	Ca(ICCV2/PI489777)SNP_20019	206	T	G	8	8	--	--
	Ca(ICCV2/PI489777)SNP_20020	1293	A	G	8	8	--	--
	Ca(ICCV2/PI489777)SNP_20021	1450	T	C	9	7	--	--
	Ca(ICCV2/PI489777)SNP_20022	1470	T	A	11	9	--	--
	Ca(ICCV2/PI489777)SNP_20023	1915	C	T	10	3	--	--
	Ca(ICCV2/PI489777)SNP_20024	2004	G	A	17	3	--	--
	Ca(ICCV2/PI489777)SNP_20025	2799	C	T	11	3	--	--
	Ca(ICCV2/PI489777)SNP_20026	3225	G	T	14	8	--	--
	Ca(ICCV2/PI489777)SNP_20027	3273	G	A	15	9	--	--
CakTC42415	Ca(ICCV2/PI489777)SNP_20028	779	G	C	13	6	--	--
CakTC38851	Ca(ICCV2/PI489777)SNP_20029	1989	C	T	3	3	--	--
CakTC25168	Ca(ICCV2/PI489777)SNP_20030	95	C	T	62	33	--	--
	Ca(ICCV2/PI489777)SNP_20031	180	G	A	76	38	--	--
	Ca(ICCV2/PI489777)SNP_20032	199	C	A	54	35	--	--
	Ca(ICCV2/PI489777)SNP_20033	605	C	T	86	39	--	--
	Ca(ICCV2/PI489777)SNP_20034	646	A	G	88	37	--	--
	Ca(ICCV2/PI489777)SNP_20035	775	A	C	64	20	--	--
CakTC05322	Ca(ICCV2/PI489777)SNP_20036	334	T	G	5	5	--	--
	Ca(ICCV2/PI489777)SNP_20037	1129	C	T	5	3	--	--
	Ca(ICCV2/PI489777)SNP_20038	1958	A	T	3	6	--	--
	Ca(ICCV2/PI489777)SNP_20039	2163	C	T	3	5	--	--
CakTC32625	Ca(ICCV2/PI489777)SNP_20040	1135	A	C	16	8	--	--
	Ca(ICCV2/PI489777)SNP_20041	1281	C	T	21	11	--	--
	Ca(ICCV2/PI489777)SNP_20042	1293	T	C	22	11	--	--
	Ca(ICCV2/PI489777)SNP_20043	1567	C	T	16	4	--	--
	Ca(ICCV2/PI489777)SNP_20044	1610	G	C	15	3	--	--
	Ca(ICCV2/PI489777)SNP_20045	1612	G	A	10	3	--	--
	Ca(ICCV2/PI489777)SNP_20046	1626	C	T	10	3	--	--
	Ca(ICCV2/PI489777)SNP_20047	1629	G	C	10	3	--	--
CakTC42092	Ca(ICCV2/PI489777)SNP_20048	540	T	C	30	28	Flower	bud
	Ca(ICCV2/PI489777)SNP_20049	615	G	A	29	29	Flower	bud

	Ca(ICCV2/PI489777)SNP_20050	643	A	G	31	30	Flower	bud
	Ca(ICCV2/PI489777)SNP_20051	651	T	A	30	23	Flower	bud
CakTC26584	Ca(ICCV2/PI489777)SNP_20052	199	A	C	59	14	--	--
	Ca(ICCV2/PI489777)SNP_20053	492	G	C	93	16	--	--
	Ca(ICCV2/PI489777)SNP_20054	503	G	A	77	18	--	--
	Ca(ICCV2/PI489777)SNP_20055	1085	T	C	50	18	--	--
	Ca(ICCV2/PI489777)SNP_20056	1102	T	A	52	17	--	--
	Ca(ICCV2/PI489777)SNP_20057	1121	C	A	51	17	--	--
	Ca(ICCV2/PI489777)SNP_20058	1179	C	G	49	17	--	--
	Ca(ICCV2/PI489777)SNP_20059	1199	C	G	45	16	--	--
CakTC37186	Ca(ICCV2/PI489777)SNP_20060	568	T	C	12	7	--	--
	Ca(ICCV2/PI489777)SNP_20061	1119	A	T	11	7	--	--
	Ca(ICCV2/PI489777)SNP_20062	1269	G	C	9	9	--	--
	Ca(ICCV2/PI489777)SNP_20063	1339	T	A	8	9	--	--
CakTC27391	Ca(ICCV2/PI489777)SNP_20064	476	T	C	15	32	--	--
	Ca(ICCV2/PI489777)SNP_20065	549	A	G	18	29	--	--
	Ca(ICCV2/PI489777)SNP_20066	1761	A	G	28	15	--	--
	Ca(ICCV2/PI489777)SNP_20067	1782	G	C	28	12	--	--
CakTC28803	Ca(ICCV2/PI489777)SNP_20068	575	A	G	3	5	--	--
CakTC33374	Ca(ICCV2/PI489777)SNP_20069	130	G	A	7	3	--	--
	Ca(ICCV2/PI489777)SNP_20070	148	G	T	7	3	--	--
CakTC31506	Ca(ICCV2/PI489777)SNP_20071	227	G	A	9	3	--	--
CakTC38449	Ca(ICCV2/PI489777)SNP_20072	1155	A	T	3	4	--	--
	Ca(ICCV2/PI489777)SNP_20073	1347	T	C	7	8	--	--
	Ca(ICCV2/PI489777)SNP_20074	1397	T	C	8	7	--	--
CakTC18104	Ca(ICCV2/PI489777)SNP_20075	456	A	G	12	4	--	--
	Ca(ICCV2/PI489777)SNP_20076	1135	T	A	6	10	--	--
	Ca(ICCV2/PI489777)SNP_20077	1466	G	T	4	10	--	--
CakTC42186	Ca(ICCV2/PI489777)SNP_20078	144	C	T	3	4	--	--
	Ca(ICCV2/PI489777)SNP_20079	273	A	G	4	8	--	--
CakTC41988	Ca(ICCV2/PI489777)SNP_20080	92	G	C	11	5	--	--
	Ca(ICCV2/PI489777)SNP_20081	401	G	A	31	21	--	--
	Ca(ICCV2/PI489777)SNP_20082	617	A	C	22	17	--	--
CakTC29233	Ca(ICCV2/PI489777)SNP_20083	73	C	T	4	5	--	--
	Ca(ICCV2/PI489777)SNP_20084	154	C	T	3	4	--	--
	Ca(ICCV2/PI489777)SNP_20085	229	A	G	5	9	--	--
	Ca(ICCV2/PI489777)SNP_20086	397	G	C	3	6	--	--
	Ca(ICCV2/PI489777)SNP_20087	837	T	A	6	3	--	--
	Ca(ICCV2/PI489777)SNP_20088	855	A	G	6	3	--	--
CakTC11507	Ca(ICCV2/PI489777)SNP_20089	164	T	A	9	10	--	--
	Ca(ICCV2/PI489777)SNP_20090	215	C	T	11	13	--	--
	Ca(ICCV2/PI489777)SNP_20091	222	A	G	11	12	--	--
	Ca(ICCV2/PI489777)SNP_20092	225	T	C	10	11	--	--
	Ca(ICCV2/PI489777)SNP_20093	238	A	G	11	14	--	--
	Ca(ICCV2/PI489777)SNP_20094	251	G	C	11	14	--	--
	Ca(ICCV2/PI489777)SNP_20095	294	C	T	13	13	--	--
CakTC33671	Ca(ICCV2/PI489777)SNP_20096	392	T	C	8	6	--	--
CakTC39786	Ca(ICCV2/PI489777)SNP_20097	193	T	C	19	6	--	--
	Ca(ICCV2/PI489777)SNP_20098	583	A	T	18	3	--	--
	Ca(ICCV2/PI489777)SNP_20099	637	G	C	18	3	--	--
	Ca(ICCV2/PI489777)SNP_20100	760	C	T	23	3	--	--
	Ca(ICCV2/PI489777)SNP_20101	1119	T	C	24	24	--	--
CakTC41654	Ca(ICCV2/PI489777)SNP_20102	220	A	T	34	10	--	--
	Ca(ICCV2/PI489777)SNP_20103	222	T	G	34	10	--	--
	Ca(ICCV2/PI489777)SNP_20104	305	T	G	37	9	--	--
	Ca(ICCV2/PI489777)SNP_20105	317	C	G	35	9	--	--
	Ca(ICCV2/PI489777)SNP_20106	484	C	A	29	5	--	--
	Ca(ICCV2/PI489777)SNP_20107	504	C	A	24	5	--	--
CakTC39543	Ca(ICCV2/PI489777)SNP_20108	178	C	T	18	16	--	--
	Ca(ICCV2/PI489777)SNP_20109	280	C	T	14	16	--	--
	Ca(ICCV2/PI489777)SNP_20110	454	T	C	19	10	--	--
	Ca(ICCV2/PI489777)SNP_20111	571	T	C	25	3	--	--
	Ca(ICCV2/PI489777)SNP_20112	901	A	G	19	10	--	--
CakTC01310	Ca(ICCV2/PI489777)SNP_20113	630	G	A	5	3	--	--
CakTC10727	Ca(ICCV2/PI489777)SNP_20114	1050	C	T	4	5	--	--
	Ca(ICCV2/PI489777)SNP_20115	1134	T	C	4	3	--	--
	Ca(ICCV2/PI489777)SNP_20116	1879	A	T	5	6	--	--
	Ca(ICCV2/PI489777)SNP_20117	2381	A	G	3	3	--	--
CakTC24791	Ca(ICCV2/PI489777)SNP_20118	505	T	C	14	11	Flower	bud
	Ca(ICCV2/PI489777)SNP_20119	910	A	G	18	16	Flower	bud
	Ca(ICCV2/PI489777)SNP_20120	1009	C	T	19	16	Flower	bud
	Ca(ICCV2/PI489777)SNP_20121	1603	T	A	4	6	Flower	bud
CakTC29216	Ca(ICCV2/PI489777)SNP_20122	210	T	G	6	3	--	--
	Ca(ICCV2/PI489777)SNP_20123	250	G	A	6	3	--	--

CakTC34481	Ca(ICCV2/PI489777)SNP_20124	1290	A	T	6	8	--	--
	Ca(ICCV2/PI489777)SNP_20125	1808	C	G	12	5	--	--
CakTC37594	Ca(ICCV2/PI489777)SNP_20126	587	A	G	48	15	--	--
	Ca(ICCV2/PI489777)SNP_20127	640	C	T	51	9	--	--
CakTC10252	Ca(ICCV2/PI489777)SNP_20128	418	T	C	12	6	--	--
CakTC40386	Ca(ICCV2/PI489777)SNP_20129	922	C	T	7	6	--	WI/SNF-BAF60
	Ca(ICCV2/PI489777)SNP_20130	1099	C	A	7	7	--	WI/SNF-BAF60
CakTC26453	Ca(ICCV2/PI489777)SNP_20131	755	T	G	30	13	--	--
	Ca(ICCV2/PI489777)SNP_20132	1022	T	C	20	16	--	--
	Ca(ICCV2/PI489777)SNP_20133	1183	A	C	3	11	--	--
CakTC33067	Ca(ICCV2/PI489777)SNP_20134	369	C	A	5	6	--	--
	Ca(ICCV2/PI489777)SNP_20135	566	A	G	6	7	--	--
	Ca(ICCV2/PI489777)SNP_20136	719	T	C	8	3	--	--
	Ca(ICCV2/PI489777)SNP_20137	888	T	G	9	5	--	--
	Ca(ICCV2/PI489777)SNP_20138	1187	C	T	9	6	--	--
	Ca(ICCV2/PI489777)SNP_20139	1313	A	C	12	3	--	--
	Ca(ICCV2/PI489777)SNP_20140	1545	C	T	12	6	--	--
	Ca(ICCV2/PI489777)SNP_20141	1803	G	T	19	8	--	--
	Ca(ICCV2/PI489777)SNP_20142	2074	C	T	14	10	--	--
	Ca(ICCV2/PI489777)SNP_20143	2584	G	A	6	4	--	--
CakTC34120	Ca(ICCV2/PI489777)SNP_20144	396	A	C	3	4	--	--
	Ca(ICCV2/PI489777)SNP_20145	435	A	T	3	5	--	--
CakTC29011	Ca(ICCV2/PI489777)SNP_20146	1630	A	T	3	4	--	--
CakTC29677	Ca(ICCV2/PI489777)SNP_20147	616	G	A	28	15	--	--
	Ca(ICCV2/PI489777)SNP_20148	700	A	G	20	12	--	--
CakTC42649	Ca(ICCV2/PI489777)SNP_20149	160	A	G	7	4	--	--
	Ca(ICCV2/PI489777)SNP_20150	192	C	T	7	4	--	--
CakTC11438	Ca(ICCV2/PI489777)SNP_20151	878	C	T	6	7	--	--
	Ca(ICCV2/PI489777)SNP_20152	893	T	C	7	6	--	--
	Ca(ICCV2/PI489777)SNP_20153	1040	T	C	8	7	--	--
	Ca(ICCV2/PI489777)SNP_20154	1094	A	G	9	6	--	--
	Ca(ICCV2/PI489777)SNP_20155	1241	A	G	9	6	--	--
	Ca(ICCV2/PI489777)SNP_20156	1562	C	T	8	7	--	--
	Ca(ICCV2/PI489777)SNP_20157	1625	C	T	7	7	--	--
	Ca(ICCV2/PI489777)SNP_20158	1667	C	G	7	5	--	--
	Ca(ICCV2/PI489777)SNP_20159	1677	G	A	7	4	--	--
	Ca(ICCV2/PI489777)SNP_20160	1679	A	T	7	4	--	--
CakTC34626	Ca(ICCV2/PI489777)SNP_20161	66	G	T	7	3	--	--
	Ca(ICCV2/PI489777)SNP_20162	203	T	C	8	6	--	--
	Ca(ICCV2/PI489777)SNP_20163	242	T	C	10	7	--	--
	Ca(ICCV2/PI489777)SNP_20164	261	T	A	10	7	--	--
	Ca(ICCV2/PI489777)SNP_20165	263	C	A	10	7	--	--
	Ca(ICCV2/PI489777)SNP_20166	284	T	C	10	7	--	--
	Ca(ICCV2/PI489777)SNP_20167	1195	T	C	9	4	--	--
CakTC37966	Ca(ICCV2/PI489777)SNP_20168	394	G	A	10	8	--	--
	Ca(ICCV2/PI489777)SNP_20169	528	T	C	8	4	--	--
	Ca(ICCV2/PI489777)SNP_20170	576	A	C	6	5	--	--
	Ca(ICCV2/PI489777)SNP_20171	597	G	C	7	5	--	--
	Ca(ICCV2/PI489777)SNP_20172	1230	T	C	11	4	--	--
CakTC27983	Ca(ICCV2/PI489777)SNP_20173	277	C	T	12	19	--	--
	Ca(ICCV2/PI489777)SNP_20174	613	A	G	23	9	--	--
	Ca(ICCV2/PI489777)SNP_20175	676	A	G	21	9	--	--
	Ca(ICCV2/PI489777)SNP_20176	952	T	A	22	15	--	--
	Ca(ICCV2/PI489777)SNP_20177	2005	T	C	5	14	--	--
CakTC38813	Ca(ICCV2/PI489777)SNP_20178	168	A	T	9	7	--	--
	Ca(ICCV2/PI489777)SNP_20179	201	C	A	9	6	--	--
CakTC24200	Ca(ICCV2/PI489777)SNP_20180	1250	G	A	18	21	--	--
CakTC42479	Ca(ICCV2/PI489777)SNP_20181	1737	G	A	16	10	--	--
	Ca(ICCV2/PI489777)SNP_20182	1753	T	G	20	9	--	--
CakTC41845	Ca(ICCV2/PI489777)SNP_20183	85	G	A	22	5	--	--
CakTC14932	Ca(ICCV2/PI489777)SNP_20184	920	A	C	46	14	--	bHLH
	Ca(ICCV2/PI489777)SNP_20185	1070	C	T	56	12	--	bHLH
	Ca(ICCV2/PI489777)SNP_20186	1073	T	C	58	12	--	bHLH
CakTC32187	Ca(ICCV2/PI489777)SNP_20187	84	G	A	3	4	--	--
	Ca(ICCV2/PI489777)SNP_20188	255	C	T	4	4	--	--
CakTC34545	Ca(ICCV2/PI489777)SNP_20189	411	A	G	7	9	--	--
CakTC39471	Ca(ICCV2/PI489777)SNP_20190	683	A	G	31	6	--	--
CakTC10078	Ca(ICCV2/PI489777)SNP_20191	1354	G	A	6	5	--	bHLH
	Ca(ICCV2/PI489777)SNP_20192	1433	T	G	7	5	--	bHLH
CakTC25801	Ca(ICCV2/PI489777)SNP_20193	49	T	C	11	11	--	--
	Ca(ICCV2/PI489777)SNP_20194	62	T	A	11	11	--	--
	Ca(ICCV2/PI489777)SNP_20195	100	C	T	12	12	--	--
	Ca(ICCV2/PI489777)SNP_20196	102	G	A	13	12	--	--
	Ca(ICCV2/PI489777)SNP_20197	152	T	C	25	34	--	--

	Ca(ICCV2/PI489777)SNP_20198	158	C	A	31	39	--	--
	Ca(ICCV2/PI489777)SNP_20199	167	A	G	32	41	--	--
CakTC41720	Ca(ICCV2/PI489777)SNP_20200	1786	A	C	4	3	--	--
CakTC34150	Ca(ICCV2/PI489777)SNP_20201	446	T	C	14	21	--	--
	Ca(ICCV2/PI489777)SNP_20202	462	G	A	14	21	--	--
	Ca(ICCV2/PI489777)SNP_20203	545	T	C	13	19	--	--
CakTC34796	Ca(ICCV2/PI489777)SNP_20204	922	C	T	23	35	--	--
	Ca(ICCV2/PI489777)SNP_20205	3216	A	C	24	18	--	--
	Ca(ICCV2/PI489777)SNP_20206	3247	C	A	21	18	--	--
CakTC41367	Ca(ICCV2/PI489777)SNP_20207	755	A	G	8	4	--	--
	Ca(ICCV2/PI489777)SNP_20208	1172	C	A	6	3	--	--
	Ca(ICCV2/PI489777)SNP_20209	2243	C	T	13	6	--	--
CakTC11366	Ca(ICCV2/PI489777)SNP_20210	1121	T	C	4	4	--	--
CakTC30959	Ca(ICCV2/PI489777)SNP_20211	155	G	A	78	101	--	--
CakTC41265	Ca(ICCV2/PI489777)SNP_20212	527	T	A	12	6	--	--
	Ca(ICCV2/PI489777)SNP_20213	1418	G	T	6	7	--	--
	Ca(ICCV2/PI489777)SNP_20214	1661	T	A	10	7	--	--
	Ca(ICCV2/PI489777)SNP_20215	1811	G	C	10	4	--	--
CakTC09624	Ca(ICCV2/PI489777)SNP_20216	607	G	C	5	3	--	--
CakTC13074	Ca(ICCV2/PI489777)SNP_20217	314	C	T	6	3	--	--
CakTC33361	Ca(ICCV2/PI489777)SNP_20218	1777	C	G	10	10	--	--
CakTC38713	Ca(ICCV2/PI489777)SNP_20219	288	A	T	13	7	--	--
CakTC42197	Ca(ICCV2/PI489777)SNP_20220	195	G	C	12	7	--	--
	Ca(ICCV2/PI489777)SNP_20221	234	T	A	14	12	--	--
	Ca(ICCV2/PI489777)SNP_20222	237	A	T	8	12	--	--
	Ca(ICCV2/PI489777)SNP_20223	1049	A	G	23	15	--	--
CakTC37665	Ca(ICCV2/PI489777)SNP_20224	177	A	G	11	6	--	--
	Ca(ICCV2/PI489777)SNP_20225	559	C	A	18	13	--	--
	Ca(ICCV2/PI489777)SNP_20226	816	C	T	20	6	--	--
	Ca(ICCV2/PI489777)SNP_20227	1465	G	A	16	8	--	--
CakTC37865	Ca(ICCV2/PI489777)SNP_20228	287	C	A	23	15	--	--
	Ca(ICCV2/PI489777)SNP_20229	290	T	A	22	15	--	--
	Ca(ICCV2/PI489777)SNP_20230	927	A	G	55	10	--	--
	Ca(ICCV2/PI489777)SNP_20231	1178	T	C	21	23	--	--
CakTC42085	Ca(ICCV2/PI489777)SNP_20232	380	G	T	11	4	--	--
	Ca(ICCV2/PI489777)SNP_20233	1358	A	G	19	11	--	--
	Ca(ICCV2/PI489777)SNP_20234	1490	A	G	21	6	--	--
	Ca(ICCV2/PI489777)SNP_20235	1494	A	G	22	5	--	--
	Ca(ICCV2/PI489777)SNP_20236	1646	G	A	14	4	--	--
	Ca(ICCV2/PI489777)SNP_20237	1691	G	A	13	5	--	--
CakTC31702	Ca(ICCV2/PI489777)SNP_20238	308	C	G	4	4	--	--
	Ca(ICCV2/PI489777)SNP_20239	334	C	T	4	4	--	--
	Ca(ICCV2/PI489777)SNP_20240	344	A	G	4	4	--	--
	Ca(ICCV2/PI489777)SNP_20241	828	G	A	9	13	--	--
	Ca(ICCV2/PI489777)SNP_20242	839	C	T	9	13	--	--
	Ca(ICCV2/PI489777)SNP_20243	1037	C	T	10	9	--	--
	Ca(ICCV2/PI489777)SNP_20244	1046	A	C	3	10	--	--
	Ca(ICCV2/PI489777)SNP_20245	1206	A	G	9	3	--	--
	Ca(ICCV2/PI489777)SNP_20246	1429	A	G	3	3	--	--
CakTC29893	Ca(ICCV2/PI489777)SNP_20247	81	G	C	7	3	--	--
	Ca(ICCV2/PI489777)SNP_20248	780	G	A	52	21	--	--
CakTC09689	Ca(ICCV2/PI489777)SNP_20249	81	T	C	25	6	--	--
	Ca(ICCV2/PI489777)SNP_20250	171	C	A	121	49	--	--
CakTC29316	Ca(ICCV2/PI489777)SNP_20251	487	G	A	10	11	--	--
CakTC33872	Ca(ICCV2/PI489777)SNP_20252	757	G	A	13	9	--	--
CakTC23328	Ca(ICCV2/PI489777)SNP_20253	65	T	C	56	26	--	--
	Ca(ICCV2/PI489777)SNP_20254	126	A	C	255	81	--	--
	Ca(ICCV2/PI489777)SNP_20255	214	A	G	267	89	--	--
CakTC25878	Ca(ICCV2/PI489777)SNP_20256	423	T	A	155	63	--	--
	Ca(ICCV2/PI489777)SNP_20257	470	A	G	26	31	--	--
	Ca(ICCV2/PI489777)SNP_20258	473	G	A	22	26	--	--
	Ca(ICCV2/PI489777)SNP_20259	563	G	A	12	18	--	--
	Ca(ICCV2/PI489777)SNP_20260	587	T	C	12	17	--	--
	Ca(ICCV2/PI489777)SNP_20261	595	C	A	12	15	--	--
CakTC30725	Ca(ICCV2/PI489777)SNP_20262	854	T	C	5	10	--	--
	Ca(ICCV2/PI489777)SNP_20263	1003	T	A	4	8	--	--
CakTC39864	Ca(ICCV2/PI489777)SNP_20264	318	T	C	28	21	Root	--
	Ca(ICCV2/PI489777)SNP_20265	354	A	G	27	23	Root	--
	Ca(ICCV2/PI489777)SNP_20266	489	G	C	24	19	Root	--
	Ca(ICCV2/PI489777)SNP_20267	777	G	C	26	23	Root	--
CakTC40994	Ca(ICCV2/PI489777)SNP_20268	251	A	G	20	6	--	--
	Ca(ICCV2/PI489777)SNP_20269	308	G	A	23	7	--	--
	Ca(ICCV2/PI489777)SNP_20270	467	A	G	33	8	--	--
	Ca(ICCV2/PI489777)SNP_20271	518	C	T	27	8	--	--

	Ca(ICCV2/PI489777)SNP_20272	1648	T	G	10	4	--	--
	Ca(ICCV2/PI489777)SNP_20273	1691	G	C	7	4	--	--
	Ca(ICCV2/PI489777)SNP_20274	1757	C	G	8	4	--	--
CakTC23917	Ca(ICCV2/PI489777)SNP_20275	699	C	T	528	190	--	--
CakTC27331	Ca(ICCV2/PI489777)SNP_20276	445	A	G	16	11	--	--
	Ca(ICCV2/PI489777)SNP_20277	482	G	C	21	11	--	--
	Ca(ICCV2/PI489777)SNP_20278	483	C	T	21	11	--	--
	Ca(ICCV2/PI489777)SNP_20279	1196	A	G	13	8	--	--
	Ca(ICCV2/PI489777)SNP_20280	1197	C	T	13	8	--	--
	Ca(ICCV2/PI489777)SNP_20281	1209	T	C	13	9	--	--
	Ca(ICCV2/PI489777)SNP_20282	1395	T	A	25	5	--	--
CakTC38236	Ca(ICCV2/PI489777)SNP_20283	380	T	C	55	9	--	NAC
	Ca(ICCV2/PI489777)SNP_20284	1161	T	C	123	15	--	NAC
CakTC27736	Ca(ICCV2/PI489777)SNP_20285	443	C	T	39	33	--	--
	Ca(ICCV2/PI489777)SNP_20286	651	G	A	36	34	--	--
	Ca(ICCV2/PI489777)SNP_20287	872	C	T	27	17	--	--
	Ca(ICCV2/PI489777)SNP_20288	1115	G	C	38	11	--	--
	Ca(ICCV2/PI489777)SNP_20289	1158	A	G	45	15	--	--
	Ca(ICCV2/PI489777)SNP_20290	1673	T	C	24	6	--	--
	Ca(ICCV2/PI489777)SNP_20291	1760	T	A	24	6	--	--
	Ca(ICCV2/PI489777)SNP_20292	1821	A	G	26	9	--	--
	Ca(ICCV2/PI489777)SNP_20293	2466	G	A	14	11	--	--
	Ca(ICCV2/PI489777)SNP_20294	2523	A	G	12	9	--	--
	Ca(ICCV2/PI489777)SNP_20295	2630	A	G	12	9	--	--
	Ca(ICCV2/PI489777)SNP_20296	2769	A	G	16	10	--	--
	Ca(ICCV2/PI489777)SNP_20297	2964	C	T	18	8	--	--
	Ca(ICCV2/PI489777)SNP_20298	3132	T	C	12	4	--	--
	Ca(ICCV2/PI489777)SNP_20299	3306	T	C	9	12	--	--
	Ca(ICCV2/PI489777)SNP_20300	3324	A	T	7	14	--	--
	Ca(ICCV2/PI489777)SNP_20301	3483	A	G	14	10	--	--
	Ca(ICCV2/PI489777)SNP_20302	3915	G	T	5	13	--	--
	Ca(ICCV2/PI489777)SNP_20303	4293	C	T	11	6	--	--
	Ca(ICCV2/PI489777)SNP_20304	5110	T	C	12	3	--	--
CakTC06566	Ca(ICCV2/PI489777)SNP_20305	383	G	A	3	5	Flower	bud
	Ca(ICCV2/PI489777)SNP_20306	583	A	C	3	4	Flower	bud
CakTC38792	Ca(ICCV2/PI489777)SNP_20307	861	A	C	4	9	--	--
	Ca(ICCV2/PI489777)SNP_20308	897	C	G	6	9	--	--
	Ca(ICCV2/PI489777)SNP_20309	920	A	C	5	9	--	--
	Ca(ICCV2/PI489777)SNP_20310	970	G	C	5	11	--	--
	Ca(ICCV2/PI489777)SNP_20311	984	C	A	6	11	--	--
	Ca(ICCV2/PI489777)SNP_20312	1077	A	G	6	11	--	--
CakTC34032	Ca(ICCV2/PI489777)SNP_20313	163	G	A	6	3	--	--
CakTC40557	Ca(ICCV2/PI489777)SNP_20314	255	G	A	21	23	--	--
	Ca(ICCV2/PI489777)SNP_20315	363	C	T	19	20	--	--
	Ca(ICCV2/PI489777)SNP_20316	656	C	T	14	3	--	--
CakTC40548	Ca(ICCV2/PI489777)SNP_20317	104	G	A	6	4	--	--
	Ca(ICCV2/PI489777)SNP_20318	204	T	A	8	5	--	--
	Ca(ICCV2/PI489777)SNP_20319	387	C	A	13	3	--	--
	Ca(ICCV2/PI489777)SNP_20320	414	T	C	14	4	--	--
	Ca(ICCV2/PI489777)SNP_20321	477	C	T	16	4	--	--
	Ca(ICCV2/PI489777)SNP_20322	522	T	C	14	3	--	--
CakTC25958	Ca(ICCV2/PI489777)SNP_20323	1605	A	G	10	7	--	--
	Ca(ICCV2/PI489777)SNP_20324	1616	C	T	8	6	--	--
	Ca(ICCV2/PI489777)SNP_20325	1719	A	C	7	5	--	--
CakTC37712	Ca(ICCV2/PI489777)SNP_20326	852	A	G	5	4	--	--
CakTC40139	Ca(ICCV2/PI489777)SNP_20327	169	G	C	4	4	--	--
	Ca(ICCV2/PI489777)SNP_20328	249	T	A	4	6	--	--
	Ca(ICCV2/PI489777)SNP_20329	295	C	T	5	6	--	--
	Ca(ICCV2/PI489777)SNP_20330	350	C	T	5	7	--	--
CakTC26301	Ca(ICCV2/PI489777)SNP_20331	889	A	C	23	8	--	--
	Ca(ICCV2/PI489777)SNP_20332	1192	T	C	22	7	--	--
	Ca(ICCV2/PI489777)SNP_20333	1333	T	G	30	8	--	--
	Ca(ICCV2/PI489777)SNP_20334	1354	A	G	35	11	--	--
	Ca(ICCV2/PI489777)SNP_20335	1435	T	C	33	12	--	--
	Ca(ICCV2/PI489777)SNP_20336	1492	T	A	25	9	--	--
	Ca(ICCV2/PI489777)SNP_20337	1537	G	A	31	4	--	--
CakTC39136	Ca(ICCV2/PI489777)SNP_20338	132	G	C	11	4	--	--
	Ca(ICCV2/PI489777)SNP_20339	310	C	T	26	10	--	--
	Ca(ICCV2/PI489777)SNP_20340	356	G	A	30	11	--	--
	Ca(ICCV2/PI489777)SNP_20341	359	T	C	30	11	--	--
	Ca(ICCV2/PI489777)SNP_20342	661	T	G	30	3	--	--
	Ca(ICCV2/PI489777)SNP_20343	814	G	A	23	3	--	--
	Ca(ICCV2/PI489777)SNP_20344	825	G	T	23	4	--	--
	Ca(ICCV2/PI489777)SNP_20345	941	T	C	17	8	--	--

	Ca(ICCV2/PI489777)SNP_20346	989	G	A	23	7	--	--
	Ca(ICCV2/PI489777)SNP_20347	1088	A	G	34	11	--	--
CakTC28453	Ca(ICCV2/PI489777)SNP_20348	2181	T	C	35	5	Flower	bud
CakTC27660	Ca(ICCV2/PI489777)SNP_20349	961	T	C	7	5	--	--
CakTC38054	Ca(ICCV2/PI489777)SNP_20350	82	T	C	6	4	--	--
CakTC01882	Ca(ICCV2/PI489777)SNP_20351	97	A	G	3	3	--	--
CakTC32422	Ca(ICCV2/PI489777)SNP_20352	473	G	A	9	5	--	HMG
	Ca(ICCV2/PI489777)SNP_20353	944	G	A	20	3	--	HMG
CakTC36860	Ca(ICCV2/PI489777)SNP_20354	69	G	A	3	4	--	--
	Ca(ICCV2/PI489777)SNP_20355	111	T	C	4	4	--	--
	Ca(ICCV2/PI489777)SNP_20356	118	A	G	4	4	--	--
	Ca(ICCV2/PI489777)SNP_20357	193	C	T	3	3	--	--
	Ca(ICCV2/PI489777)SNP_20358	412	C	G	3	5	--	--
	Ca(ICCV2/PI489777)SNP_20359	413	T	C	3	5	--	--
CakTC38580	Ca(ICCV2/PI489777)SNP_20360	579	G	A	11	5	--	--
CakTC24302	Ca(ICCV2/PI489777)SNP_20361	595	G	T	6	3	--	--
	Ca(ICCV2/PI489777)SNP_20362	697	T	C	7	3	--	--
	Ca(ICCV2/PI489777)SNP_20363	709	A	C	8	3	--	--
	Ca(ICCV2/PI489777)SNP_20364	715	T	C	7	3	--	--
	Ca(ICCV2/PI489777)SNP_20365	1137	T	A	14	4	--	--
	Ca(ICCV2/PI489777)SNP_20366	1208	C	T	9	3	--	--
	Ca(ICCV2/PI489777)SNP_20367	1210	A	G	11	3	--	--
CakTC25859	Ca(ICCV2/PI489777)SNP_20368	137	G	T	4	3	--	--
	Ca(ICCV2/PI489777)SNP_20369	849	T	C	21	8	--	--
CakTC39097	Ca(ICCV2/PI489777)SNP_20370	1155	C	A	18	5	--	--
CakTC26838	Ca(ICCV2/PI489777)SNP_20371	864	T	G	25	18	--	--
	Ca(ICCV2/PI489777)SNP_20372	1095	C	T	23	14	--	--
CakTC30688	Ca(ICCV2/PI489777)SNP_20373	593	G	A	3	3	Flower	bud
	Ca(ICCV2/PI489777)SNP_20374	1259	A	C	7	3	Flower	bud
CakTC41662	Ca(ICCV2/PI489777)SNP_20375	166	G	A	13	8	--	--
	Ca(ICCV2/PI489777)SNP_20376	521	C	G	54	7	--	--
CakTC22478	Ca(ICCV2/PI489777)SNP_20377	502	A	G	10	3	--	--
CakTC28557	Ca(ICCV2/PI489777)SNP_20378	310	T	C	12	7	--	--
	Ca(ICCV2/PI489777)SNP_20379	444	C	G	15	8	--	--
	Ca(ICCV2/PI489777)SNP_20380	772	A	T	17	3	--	--
	Ca(ICCV2/PI489777)SNP_20381	1089	A	C	26	5	--	--
	Ca(ICCV2/PI489777)SNP_20382	1258	T	C	25	5	--	--
	Ca(ICCV2/PI489777)SNP_20383	1307	C	G	22	5	--	--
	Ca(ICCV2/PI489777)SNP_20384	1309	C	T	22	4	--	--
	Ca(ICCV2/PI489777)SNP_20385	1367	C	T	27	4	--	--
CakTC33116	Ca(ICCV2/PI489777)SNP_20386	157	A	G	7	13	--	--
	Ca(ICCV2/PI489777)SNP_20387	568	T	C	6	5	--	--
	Ca(ICCV2/PI489777)SNP_20388	1164	A	T	12	9	--	--
	Ca(ICCV2/PI489777)SNP_20389	1181	G	A	10	10	--	--
	Ca(ICCV2/PI489777)SNP_20390	1277	A	G	7	6	--	--
	Ca(ICCV2/PI489777)SNP_20391	1285	A	G	12	6	--	--
	Ca(ICCV2/PI489777)SNP_20392	1400	T	A	10	4	--	--
	Ca(ICCV2/PI489777)SNP_20393	1542	G	A	5	3	--	--
	Ca(ICCV2/PI489777)SNP_20394	1905	G	T	6	7	--	--
	Ca(ICCV2/PI489777)SNP_20395	1963	T	C	5	4	--	--
CakTC38256	Ca(ICCV2/PI489777)SNP_20396	835	C	T	7	4	--	PHD
	Ca(ICCV2/PI489777)SNP_20397	952	G	A	9	5	--	PHD
CakTC38932	Ca(ICCV2/PI489777)SNP_20398	195	T	C	8	3	--	--
	Ca(ICCV2/PI489777)SNP_20399	430	C	T	4	3	--	--
	Ca(ICCV2/PI489777)SNP_20400	2749	T	C	5	4	--	--
CakTC27777	Ca(ICCV2/PI489777)SNP_20401	176	T	A	5	4	Shoot	--
	Ca(ICCV2/PI489777)SNP_20402	405	A	G	4	4	Shoot	--
	Ca(ICCV2/PI489777)SNP_20403	536	C	T	14	9	Shoot	--
CakTC09579	Ca(ICCV2/PI489777)SNP_20404	687	A	G	8	9	--	--
CakTC34022	Ca(ICCV2/PI489777)SNP_20405	657	T	C	9	12	--	--
	Ca(ICCV2/PI489777)SNP_20406	1512	T	G	30	11	--	--
	Ca(ICCV2/PI489777)SNP_20407	1754	T	A	9	12	--	--
	Ca(ICCV2/PI489777)SNP_20408	1767	C	T	9	10	--	--
CakTC34321	Ca(ICCV2/PI489777)SNP_20409	675	C	A	19	12	--	--
	Ca(ICCV2/PI489777)SNP_20410	676	A	G	19	12	--	--
	Ca(ICCV2/PI489777)SNP_20411	763	A	T	14	10	--	--
	Ca(ICCV2/PI489777)SNP_20412	774	G	T	15	12	--	--
CakTC31629	Ca(ICCV2/PI489777)SNP_20413	1006	G	A	8	3	--	--
	Ca(ICCV2/PI489777)SNP_20414	2500	T	C	7	11	--	--
CakTC32892	Ca(ICCV2/PI489777)SNP_20415	137	C	T	6	6	--	--
	Ca(ICCV2/PI489777)SNP_20416	680	A	G	9	8	--	--
	Ca(ICCV2/PI489777)SNP_20417	1589	G	A	13	5	--	--
	Ca(ICCV2/PI489777)SNP_20418	1652	G	A	12	9	--	--
	Ca(ICCV2/PI489777)SNP_20419	1922	A	G	12	12	--	--

CakTC37889	Ca(ICCV2/PI489777)SNP_20420	812	G	A	33	11	--	--
	Ca(ICCV2/PI489777)SNP_20421	1572	C	G	15	5	--	--
CakTC26619	Ca(ICCV2/PI489777)SNP_20422	763	A	C	4	5	--	--
CakTC10668	Ca(ICCV2/PI489777)SNP_20423	228	A	C	25	3	--	--
	Ca(ICCV2/PI489777)SNP_20424	536	A	G	19	8	--	--
	Ca(ICCV2/PI489777)SNP_20425	1552	C	T	21	9	--	--
	Ca(ICCV2/PI489777)SNP_20426	1739	C	T	16	13	--	--
	Ca(ICCV2/PI489777)SNP_20427	1871	A	G	6	8	--	--
CakTC42265	Ca(ICCV2/PI489777)SNP_20428	247	T	G	16	16	--	--
	Ca(ICCV2/PI489777)SNP_20429	417	T	C	27	30	--	--
	Ca(ICCV2/PI489777)SNP_20430	591	C	G	33	20	--	--
CakTC26076	Ca(ICCV2/PI489777)SNP_20431	1263	C	G	59	20	--	--
CakTC35420	Ca(ICCV2/PI489777)SNP_20432	1109	G	A	7	3	--	--
	Ca(ICCV2/PI489777)SNP_20433	1110	G	T	7	3	--	--
	Ca(ICCV2/PI489777)SNP_20434	2183	A	G	3	12	--	--
	Ca(ICCV2/PI489777)SNP_20435	2481	C	T	5	9	--	--
CakTC30161	Ca(ICCV2/PI489777)SNP_20436	409	G	A	9	9	--	bHLH
	Ca(ICCV2/PI489777)SNP_20437	505	A	G	5	7	--	bHLH
	Ca(ICCV2/PI489777)SNP_20438	566	T	C	5	5	--	bHLH
	Ca(ICCV2/PI489777)SNP_20439	583	A	G	6	9	--	bHLH
	Ca(ICCV2/PI489777)SNP_20440	584	T	G	6	9	--	bHLH
	Ca(ICCV2/PI489777)SNP_20441	600	T	C	6	8	--	bHLH
	Ca(ICCV2/PI489777)SNP_20442	613	C	G	5	9	--	bHLH
	Ca(ICCV2/PI489777)SNP_20443	691	T	A	4	4	--	bHLH
CakTC08067	Ca(ICCV2/PI489777)SNP_20444	531	A	G	23	9	--	--
CakTC29429	Ca(ICCV2/PI489777)SNP_20445	418	C	T	16	4	--	--
	Ca(ICCV2/PI489777)SNP_20446	871	C	G	3	3	--	--
CakTC33795	Ca(ICCV2/PI489777)SNP_20447	271	G	A	29	6	--	--
	Ca(ICCV2/PI489777)SNP_20448	316	A	G	29	7	--	--
	Ca(ICCV2/PI489777)SNP_20449	379	G	A	34	6	--	--
	Ca(ICCV2/PI489777)SNP_20450	481	G	A	32	5	--	--
	Ca(ICCV2/PI489777)SNP_20451	568	G	A	30	3	--	--
CakTC42438	Ca(ICCV2/PI489777)SNP_20452	88	C	A	6	3	--	--
	Ca(ICCV2/PI489777)SNP_20453	97	T	C	7	4	--	--
	Ca(ICCV2/PI489777)SNP_20454	137	C	T	17	17	--	--
	Ca(ICCV2/PI489777)SNP_20455	143	A	C	15	18	--	--
	Ca(ICCV2/PI489777)SNP_20456	465	G	T	45	56	--	--
	Ca(ICCV2/PI489777)SNP_20457	534	T	C	33	52	--	--
CakTC26610	Ca(ICCV2/PI489777)SNP_20458	1084	T	C	27	50	--	--
	Ca(ICCV2/PI489777)SNP_20459	1362	G	A	14	26	--	--
CakTC41008	Ca(ICCV2/PI489777)SNP_20460	299	T	C	4	14	--	--
	Ca(ICCV2/PI489777)SNP_20461	610	G	A	54	26	--	--
	Ca(ICCV2/PI489777)SNP_20462	680	G	C	48	23	--	--
	Ca(ICCV2/PI489777)SNP_20463	1679	C	G	26	12	--	--
	Ca(ICCV2/PI489777)SNP_20464	1766	T	C	19	12	--	--
CakTC38354	Ca(ICCV2/PI489777)SNP_20465	684	A	G	13	9	--	--
	Ca(ICCV2/PI489777)SNP_20466	711	A	G	11	11	--	--
	Ca(ICCV2/PI489777)SNP_20467	846	G	A	13	13	--	--
	Ca(ICCV2/PI489777)SNP_20468	861	G	A	13	13	--	--
	Ca(ICCV2/PI489777)SNP_20469	1314	G	A	11	13	--	--
	Ca(ICCV2/PI489777)SNP_20470	1489	T	A	4	10	--	--
CakTC03344	Ca(ICCV2/PI489777)SNP_20471	637	A	G	9	5	--	--
	Ca(ICCV2/PI489777)SNP_20472	702	G	T	6	5	--	--
CakTC09051	Ca(ICCV2/PI489777)SNP_20473	1635	G	A	6	4	--	--
CakTC34111	Ca(ICCV2/PI489777)SNP_20474	545	G	A	20	7	--	--
	Ca(ICCV2/PI489777)SNP_20475	791	A	C	14	5	--	--
CakTC42365	Ca(ICCV2/PI489777)SNP_20476	245	G	A	14	5	--	WRKY
	Ca(ICCV2/PI489777)SNP_20477	283	A	G	16	4	--	WRKY
	Ca(ICCV2/PI489777)SNP_20478	315	T	G	17	5	--	WRKY
	Ca(ICCV2/PI489777)SNP_20479	376	T	G	18	5	--	WRKY
	Ca(ICCV2/PI489777)SNP_20480	378	T	G	18	5	--	WRKY
	Ca(ICCV2/PI489777)SNP_20481	1063	A	G	22	4	--	WRKY
CakTC30484	Ca(ICCV2/PI489777)SNP_20482	399	A	G	3	3	Shoot	--
	Ca(ICCV2/PI489777)SNP_20483	576	A	G	3	3	Shoot	--
	Ca(ICCV2/PI489777)SNP_20484	1837	C	T	5	3	Shoot	--
CakTC32748	Ca(ICCV2/PI489777)SNP_20485	1866	C	G	12	3	--	--
CakTC40402	Ca(ICCV2/PI489777)SNP_20486	642	T	A	10	5	--	--
CakTC33857	Ca(ICCV2/PI489777)SNP_20487	252	G	A	12	4	--	--
	Ca(ICCV2/PI489777)SNP_20488	386	C	T	13	6	--	--
	Ca(ICCV2/PI489777)SNP_20489	1637	G	A	11	4	--	--
	Ca(ICCV2/PI489777)SNP_20490	2291	T	G	5	5	--	--
	Ca(ICCV2/PI489777)SNP_20491	2325	T	C	5	5	--	--
CakTC37975	Ca(ICCV2/PI489777)SNP_20492	434	T	A	13	5	--	--
CakTC11113	Ca(ICCV2/PI489777)SNP_20493	1516	C	T	15	10	--	--

	Ca(ICCV2/PI489777)SNP_20494	1576	G	A	18	10	--	--
	Ca(ICCV2/PI489777)SNP_20495	2306	C	T	10	6	--	--
	Ca(ICCV2/PI489777)SNP_20496	2333	G	A	13	4	--	--
	Ca(ICCV2/PI489777)SNP_20497	2441	T	C	9	4	--	--
	Ca(ICCV2/PI489777)SNP_20498	2456	A	C	6	4	--	--
	Ca(ICCV2/PI489777)SNP_20499	2457	A	G	6	4	--	--
	Ca(ICCV2/PI489777)SNP_20500	2466	C	T	9	4	--	--
	Ca(ICCV2/PI489777)SNP_20501	2482	T	C	9	3	--	--
CakTC40730	Ca(ICCV2/PI489777)SNP_20502	209	A	G	7	4	--	--
	Ca(ICCV2/PI489777)SNP_20503	253	C	A	10	10	--	--
	Ca(ICCV2/PI489777)SNP_20504	323	C	G	20	21	--	--
	Ca(ICCV2/PI489777)SNP_20505	527	T	A	28	18	--	--
	Ca(ICCV2/PI489777)SNP_20506	1226	T	A	16	8	--	--
	Ca(ICCV2/PI489777)SNP_20507	1403	T	G	14	14	--	--
	Ca(ICCV2/PI489777)SNP_20508	1442	A	G	14	14	--	--
	Ca(ICCV2/PI489777)SNP_20509	1568	A	G	9	9	--	--
	Ca(ICCV2/PI489777)SNP_20510	1679	G	T	12	5	--	--
	Ca(ICCV2/PI489777)SNP_20511	1798	G	A	7	4	--	--
	Ca(ICCV2/PI489777)SNP_20512	1985	G	C	8	4	--	--
CakTC38612	Ca(ICCV2/PI489777)SNP_20513	325	G	A	4	7	--	--
CakTC30331	Ca(ICCV2/PI489777)SNP_20514	727	T	A	5	3	--	--
	Ca(ICCV2/PI489777)SNP_20515	1627	G	C	10	6	--	--
	Ca(ICCV2/PI489777)SNP_20516	1846	T	G	10	4	--	--
	Ca(ICCV2/PI489777)SNP_20517	2557	A	T	13	8	--	--
CakTC12492	Ca(ICCV2/PI489777)SNP_20518	1749	T	C	3	4	--	--
	Ca(ICCV2/PI489777)SNP_20519	2172	A	G	4	3	--	--
CakTC34071	Ca(ICCV2/PI489777)SNP_20520	152	T	C	4	11	--	--
	Ca(ICCV2/PI489777)SNP_20521	683	T	A	37	31	--	--
	Ca(ICCV2/PI489777)SNP_20522	836	C	T	41	27	--	--
	Ca(ICCV2/PI489777)SNP_20523	2021	G	A	8	8	--	--
	Ca(ICCV2/PI489777)SNP_20524	2115	C	T	5	6	--	--
	Ca(ICCV2/PI489777)SNP_20525	2118	T	C	5	7	--	--
CakTC38265	Ca(ICCV2/PI489777)SNP_20526	403	C	T	16	29	--	CSD
	Ca(ICCV2/PI489777)SNP_20527	424	G	A	24	32	--	CSD
	Ca(ICCV2/PI489777)SNP_20528	448	G	A	25	32	--	CSD
	Ca(ICCV2/PI489777)SNP_20529	671	C	T	24	32	--	CSD
CakTC34092	Ca(ICCV2/PI489777)SNP_20530	216	C	T	5	5	--	--
	Ca(ICCV2/PI489777)SNP_20531	282	C	T	6	7	--	--
	Ca(ICCV2/PI489777)SNP_20532	359	A	T	8	7	--	--
	Ca(ICCV2/PI489777)SNP_20533	374	G	T	8	7	--	--
	Ca(ICCV2/PI489777)SNP_20534	387	G	T	7	9	--	--
	Ca(ICCV2/PI489777)SNP_20535	391	A	C	5	10	--	--
	Ca(ICCV2/PI489777)SNP_20536	460	C	T	4	10	--	--
	Ca(ICCV2/PI489777)SNP_20537	525	G	T	5	10	--	--
	Ca(ICCV2/PI489777)SNP_20538	726	A	T	8	13	--	--
	Ca(ICCV2/PI489777)SNP_20539	903	T	G	13	10	--	--
	Ca(ICCV2/PI489777)SNP_20540	960	A	G	9	5	--	--
CakTC37174	Ca(ICCV2/PI489777)SNP_20541	208	G	A	5	6	--	--
CakTC32351	Ca(ICCV2/PI489777)SNP_20542	602	C	A	9	3	--	--
	Ca(ICCV2/PI489777)SNP_20543	743	T	C	11	3	--	--
	Ca(ICCV2/PI489777)SNP_20544	1947	C	T	8	3	--	--
CakTC32613	Ca(ICCV2/PI489777)SNP_20545	510	G	A	35	45	--	--
	Ca(ICCV2/PI489777)SNP_20546	514	G	A	34	45	--	--
	Ca(ICCV2/PI489777)SNP_20547	543	C	T	31	48	--	--
	Ca(ICCV2/PI489777)SNP_20548	549	T	C	30	44	--	--
	Ca(ICCV2/PI489777)SNP_20549	615	G	A	27	50	--	--
	Ca(ICCV2/PI489777)SNP_20550	627	A	G	23	44	--	--
	Ca(ICCV2/PI489777)SNP_20551	1335	C	T	111	60	--	--
	Ca(ICCV2/PI489777)SNP_20552	1362	C	T	101	70	--	--
	Ca(ICCV2/PI489777)SNP_20553	1464	G	A	85	79	--	--
	Ca(ICCV2/PI489777)SNP_20554	1560	A	G	89	69	--	--
	Ca(ICCV2/PI489777)SNP_20555	2134	T	G	8	8	--	--
	Ca(ICCV2/PI489777)SNP_20556	2165	A	T	6	8	--	--
CakTC16715	Ca(ICCV2/PI489777)SNP_20557	232	A	G	3	3	--	--
CakTC39213	Ca(ICCV2/PI489777)SNP_20558	193	T	A	26	11	--	--
	Ca(ICCV2/PI489777)SNP_20559	1205	C	T	34	14	--	--
	Ca(ICCV2/PI489777)SNP_20560	1267	C	T	28	13	--	--
	Ca(ICCV2/PI489777)SNP_20561	1412	A	G	17	11	--	--
	Ca(ICCV2/PI489777)SNP_20562	1419	C	T	17	11	--	--
CakTC27747	Ca(ICCV2/PI489777)SNP_20563	287	T	C	9	4	--	--
	Ca(ICCV2/PI489777)SNP_20564	458	C	T	5	9	--	--
	Ca(ICCV2/PI489777)SNP_20565	611	G	A	12	7	--	--
	Ca(ICCV2/PI489777)SNP_20566	637	T	C	11	8	--	--
CakTC39848	Ca(ICCV2/PI489777)SNP_20567	1107	A	T	33	36	--	--

	Ca(ICCV2/PI489777)SNP_20568	1553	C	A	9	21	--	--
CakTC22312	Ca(ICCV2/PI489777)SNP_20569	303	G	A	7	20	--	--
	Ca(ICCV2/PI489777)SNP_20570	419	A	G	5	18	--	--
	Ca(ICCV2/PI489777)SNP_20571	1418	T	C	30	35	--	--
	Ca(ICCV2/PI489777)SNP_20572	1582	T	A	13	45	--	--
CakTC40441	Ca(ICCV2/PI489777)SNP_20573	379	T	C	5	3	--	--
CakTC42175	Ca(ICCV2/PI489777)SNP_20574	49	C	T	5	4	--	--
	Ca(ICCV2/PI489777)SNP_20575	184	A	T	9	4	--	--
	Ca(ICCV2/PI489777)SNP_20576	185	C	G	9	4	--	--
	Ca(ICCV2/PI489777)SNP_20577	271	T	C	14	4	--	--
	Ca(ICCV2/PI489777)SNP_20578	574	T	C	10	6	--	--
	Ca(ICCV2/PI489777)SNP_20579	1316	C	T	5	5	--	--
CakTC33467	Ca(ICCV2/PI489777)SNP_20580	172	G	A	11	23	--	--
	Ca(ICCV2/PI489777)SNP_20581	248	G	A	13	24	--	--
	Ca(ICCV2/PI489777)SNP_20582	305	A	G	18	20	--	--
	Ca(ICCV2/PI489777)SNP_20583	552	C	G	15	13	--	--
	Ca(ICCV2/PI489777)SNP_20584	562	C	A	15	12	--	--
CakTC29771	Ca(ICCV2/PI489777)SNP_20585	246	A	G	6	3	--	--
	Ca(ICCV2/PI489777)SNP_20586	326	A	G	6	3	--	--
	Ca(ICCV2/PI489777)SNP_20587	336	A	G	3	3	--	--
	Ca(ICCV2/PI489777)SNP_20588	1544	A	G	3	3	--	--
	Ca(ICCV2/PI489777)SNP_20589	1545	T	A	3	3	--	--
CakTC29339	Ca(ICCV2/PI489777)SNP_20590	470	G	A	4	3	--	--
CakTC41792	Ca(ICCV2/PI489777)SNP_20591	40	G	A	13	16	--	--
	Ca(ICCV2/PI489777)SNP_20592	162	C	A	26	26	--	--
	Ca(ICCV2/PI489777)SNP_20593	227	G	A	37	22	--	--
	Ca(ICCV2/PI489777)SNP_20594	302	T	C	44	27	--	--
	Ca(ICCV2/PI489777)SNP_20595	950	A	T	54	38	--	--
	Ca(ICCV2/PI489777)SNP_20596	1058	C	T	45	21	--	--
	Ca(ICCV2/PI489777)SNP_20597	1061	C	T	48	26	--	--
	Ca(ICCV2/PI489777)SNP_20598	1544	A	G	27	34	--	--
	Ca(ICCV2/PI489777)SNP_20599	1928	A	G	30	38	--	--
	Ca(ICCV2/PI489777)SNP_20600	2000	G	A	25	28	--	--
	Ca(ICCV2/PI489777)SNP_20601	2101	A	G	24	23	--	--
	Ca(ICCV2/PI489777)SNP_20602	2191	G	C	18	27	--	--
CakTC36913	Ca(ICCV2/PI489777)SNP_20603	395	T	C	7	10	--	--
	Ca(ICCV2/PI489777)SNP_20604	468	C	T	8	14	--	--
	Ca(ICCV2/PI489777)SNP_20605	684	A	G	13	9	--	--
	Ca(ICCV2/PI489777)SNP_20606	934	C	T	8	6	--	--
	Ca(ICCV2/PI489777)SNP_20607	1593	A	G	9	6	--	--
	Ca(ICCV2/PI489777)SNP_20608	1731	G	A	13	5	--	--
	Ca(ICCV2/PI489777)SNP_20609	1975	A	G	11	5	--	--
CakTC24495	Ca(ICCV2/PI489777)SNP_20610	751	A	G	19	18	--	--
	Ca(ICCV2/PI489777)SNP_20611	1267	T	C	8	12	--	--
	Ca(ICCV2/PI489777)SNP_20612	1327	G	A	9	12	--	--
	Ca(ICCV2/PI489777)SNP_20613	1874	C	T	15	8	--	--
	Ca(ICCV2/PI489777)SNP_20614	1891	A	T	12	8	--	--
	Ca(ICCV2/PI489777)SNP_20615	1897	G	T	13	8	--	--
CakTC38118	Ca(ICCV2/PI489777)SNP_20616	117	C	T	3	4	--	--
CakTC28185	Ca(ICCV2/PI489777)SNP_20617	974	G	A	26	4	--	MYB-related
	Ca(ICCV2/PI489777)SNP_20618	1157	G	A	16	3	--	MYB-related
	Ca(ICCV2/PI489777)SNP_20619	3245	A	G	12	3	--	MYB-related
CakTC28859	Ca(ICCV2/PI489777)SNP_20620	305	C	G	4	3	--	--
	Ca(ICCV2/PI489777)SNP_20621	519	A	T	3	5	--	--
	Ca(ICCV2/PI489777)SNP_20622	1109	A	T	7	8	--	--
	Ca(ICCV2/PI489777)SNP_20623	1145	A	T	7	10	--	--
	Ca(ICCV2/PI489777)SNP_20624	1229	A	G	5	4	--	--
	Ca(ICCV2/PI489777)SNP_20625	1238	T	C	6	6	--	--
CakTC32670	Ca(ICCV2/PI489777)SNP_20626	256	T	C	10	4	--	--
	Ca(ICCV2/PI489777)SNP_20627	288	G	T	10	5	--	--
	Ca(ICCV2/PI489777)SNP_20628	352	T	C	11	5	--	--
	Ca(ICCV2/PI489777)SNP_20629	362	T	A	11	4	--	--
	Ca(ICCV2/PI489777)SNP_20630	684	G	A	20	4	--	--
	Ca(ICCV2/PI489777)SNP_20631	1107	G	T	13	9	--	--
	Ca(ICCV2/PI489777)SNP_20632	1842	C	T	16	11	--	--
	Ca(ICCV2/PI489777)SNP_20633	2200	G	A	13	7	--	--
	Ca(ICCV2/PI489777)SNP_20634	2226	A	G	13	4	--	--
CakTC38332	Ca(ICCV2/PI489777)SNP_20635	191	T	G	13	11	--	--
CakTC41033	Ca(ICCV2/PI489777)SNP_20636	1036	A	G	14	4	--	--
	Ca(ICCV2/PI489777)SNP_20637	1349	G	C	9	4	--	--
	Ca(ICCV2/PI489777)SNP_20638	1493	A	T	11	6	--	--
	Ca(ICCV2/PI489777)SNP_20639	1519	G	C	7	6	--	--
	Ca(ICCV2/PI489777)SNP_20640	1534	A	C	5	9	--	--
CakTC39499	Ca(ICCV2/PI489777)SNP_20641	258	T	C	7	4	--	--

	Ca(ICCV2/PI489777)SNP_20642	352	T	C	12	6	--	--
	Ca(ICCV2/PI489777)SNP_20643	370	A	G	12	6	--	--
	Ca(ICCV2/PI489777)SNP_20644	379	A	G	12	6	--	--
	Ca(ICCV2/PI489777)SNP_20645	1060	A	T	18	3	--	--
CakTC29618	Ca(ICCV2/PI489777)SNP_20646	499	T	C	18	13	--	--
	Ca(ICCV2/PI489777)SNP_20647	1434	T	C	4	3	--	--
	Ca(ICCV2/PI489777)SNP_20648	1998	A	C	8	4	--	--
	Ca(ICCV2/PI489777)SNP_20649	2058	A	C	6	4	--	--
	Ca(ICCV2/PI489777)SNP_20650	2085	C	G	5	4	--	--
	Ca(ICCV2/PI489777)SNP_20651	2112	T	C	6	4	--	--
CakTC42847	Ca(ICCV2/PI489777)SNP_20652	266	C	T	5	3	--	--
	Ca(ICCV2/PI489777)SNP_20653	436	C	T	13	10	--	--
	Ca(ICCV2/PI489777)SNP_20654	873	G	A	6	6	--	--
	Ca(ICCV2/PI489777)SNP_20655	1098	A	G	4	4	--	--
	Ca(ICCV2/PI489777)SNP_20656	1651	T	G	3	11	--	--
CakTC41802	Ca(ICCV2/PI489777)SNP_20657	646	C	G	8	12	--	--
CakTC37533	Ca(ICCV2/PI489777)SNP_20658	696	T	C	28	11	Young_pod	--
CakTC41810	Ca(ICCV2/PI489777)SNP_20659	81	A	C	10	3	--	--
	Ca(ICCV2/PI489777)SNP_20660	644	A	T	5	5	--	--
CakTC24169	Ca(ICCV2/PI489777)SNP_20661	937	A	T	3	3	--	--
CakTC31682	Ca(ICCV2/PI489777)SNP_20662	213	G	C	7	5	--	ARR-B
	Ca(ICCV2/PI489777)SNP_20663	567	A	G	12	13	--	ARR-B
	Ca(ICCV2/PI489777)SNP_20664	1383	C	T	15	10	--	ARR-B
CakTC05127	Ca(ICCV2/PI489777)SNP_20665	459	A	T	4	3	--	--
	Ca(ICCV2/PI489777)SNP_20666	666	T	C	4	3	--	--
CakTC24119	Ca(ICCV2/PI489777)SNP_20667	168	T	C	22	6	Shoot	--
CakTC39744	Ca(ICCV2/PI489777)SNP_20668	447	A	G	9	9	--	--
	Ca(ICCV2/PI489777)SNP_20669	1016	T	C	17	6	--	--
	Ca(ICCV2/PI489777)SNP_20670	1261	G	T	16	11	--	--
	Ca(ICCV2/PI489777)SNP_20671	1351	A	T	15	9	--	--
CakTC38683	Ca(ICCV2/PI489777)SNP_20672	362	C	T	17	11	--	--
	Ca(ICCV2/PI489777)SNP_20673	395	G	A	14	10	--	--
	Ca(ICCV2/PI489777)SNP_20674	406	T	C	16	11	--	--
	Ca(ICCV2/PI489777)SNP_20675	543	G	A	14	7	--	--
	Ca(ICCV2/PI489777)SNP_20676	563	C	G	13	7	--	--
CakTC06703	Ca(ICCV2/PI489777)SNP_20677	476	C	A	9	5	--	--
	Ca(ICCV2/PI489777)SNP_20678	576	A	G	6	5	--	--
	Ca(ICCV2/PI489777)SNP_20679	786	A	C	8	5	--	--
CakTC38422	Ca(ICCV2/PI489777)SNP_20680	377	C	A	54	85	--	--
	Ca(ICCV2/PI489777)SNP_20681	1045	A	G	3	12	--	--
	Ca(ICCV2/PI489777)SNP_20682	1054	T	G	3	13	--	--
CakTC00672	Ca(ICCV2/PI489777)SNP_20683	1591	G	C	4	3	--	--
CakTC39024	Ca(ICCV2/PI489777)SNP_20684	487	G	C	6	6	--	--
CakTC24344	Ca(ICCV2/PI489777)SNP_20685	420	T	A	9	4	--	--
CakTC26539	Ca(ICCV2/PI489777)SNP_20686	74	T	C	12	4	--	--
	Ca(ICCV2/PI489777)SNP_20687	86	A	T	13	4	--	--
	Ca(ICCV2/PI489777)SNP_20688	1244	T	C	8	3	--	--
CakTC29490	Ca(ICCV2/PI489777)SNP_20689	1637	T	C	3	3	--	--
CakTC28547	Ca(ICCV2/PI489777)SNP_20690	435	A	G	11	6	--	--
	Ca(ICCV2/PI489777)SNP_20691	886	T	G	6	6	--	--
CakTC27130	Ca(ICCV2/PI489777)SNP_20692	287	T	C	14	5	--	--
CakTC15604	Ca(ICCV2/PI489777)SNP_20693	537	A	G	6	3	--	--
CakTC26693	Ca(ICCV2/PI489777)SNP_20694	169	G	T	5	10	--	--
	Ca(ICCV2/PI489777)SNP_20695	417	A	C	59	26	--	--
	Ca(ICCV2/PI489777)SNP_20696	734	T	C	26	7	--	--
	Ca(ICCV2/PI489777)SNP_20697	750	C	T	24	9	--	--
	Ca(ICCV2/PI489777)SNP_20698	768	C	A	25	11	--	--
	Ca(ICCV2/PI489777)SNP_20699	819	C	T	21	14	--	--
	Ca(ICCV2/PI489777)SNP_20700	822	G	C	23	16	--	--
	Ca(ICCV2/PI489777)SNP_20701	1500	C	A	29	12	--	--
CakTC28213	Ca(ICCV2/PI489777)SNP_20702	206	C	T	18	4	--	--
	Ca(ICCV2/PI489777)SNP_20703	243	T	C	19	3	--	--
	Ca(ICCV2/PI489777)SNP_20704	428	A	G	22	7	--	--
	Ca(ICCV2/PI489777)SNP_20705	496	C	T	22	7	--	--
	Ca(ICCV2/PI489777)SNP_20706	767	A	G	10	7	--	--
	Ca(ICCV2/PI489777)SNP_20707	866	T	C	10	10	--	--
	Ca(ICCV2/PI489777)SNP_20708	1208	T	C	13	7	--	--
CakTC30385	Ca(ICCV2/PI489777)SNP_20709	53	T	A	4	3	--	--
	Ca(ICCV2/PI489777)SNP_20710	82	A	G	5	5	--	--
	Ca(ICCV2/PI489777)SNP_20711	140	C	G	5	3	--	--
	Ca(ICCV2/PI489777)SNP_20712	167	G	A	5	4	--	--
	Ca(ICCV2/PI489777)SNP_20713	678	C	T	4	4	--	--
	Ca(ICCV2/PI489777)SNP_20714	679	T	A	4	4	--	--
	Ca(ICCV2/PI489777)SNP_20715	747	C	T	7	5	--	--

	Ca(ICCV2/PI489777)SNP_20716	760	G	T	7	3	--	--
	Ca(ICCV2/PI489777)SNP_20717	762	T	A	7	5	--	--
	Ca(ICCV2/PI489777)SNP_20718	830	A	T	10	6	--	--
	Ca(ICCV2/PI489777)SNP_20719	917	G	A	10	6	--	--
	Ca(ICCV2/PI489777)SNP_20720	988	C	T	10	6	--	--
	Ca(ICCV2/PI489777)SNP_20721	1062	A	T	8	9	--	--
	Ca(ICCV2/PI489777)SNP_20722	1067	C	T	8	9	--	--
	Ca(ICCV2/PI489777)SNP_20723	1089	C	T	6	7	--	--
	Ca(ICCV2/PI489777)SNP_20724	1092	A	T	6	8	--	--
	Ca(ICCV2/PI489777)SNP_20725	1099	C	T	5	7	--	--
	Ca(ICCV2/PI489777)SNP_20726	1105	T	C	5	6	--	--
	Ca(ICCV2/PI489777)SNP_20727	1117	A	C	5	7	--	--
	Ca(ICCV2/PI489777)SNP_20728	1118	T	G	5	7	--	--
	Ca(ICCV2/PI489777)SNP_20729	1155	C	T	5	7	--	--
	Ca(ICCV2/PI489777)SNP_20730	1161	G	T	5	7	--	--
	Ca(ICCV2/PI489777)SNP_20731	1289	T	A	5	7	--	--
	Ca(ICCV2/PI489777)SNP_20732	1306	C	T	4	7	--	--
	Ca(ICCV2/PI489777)SNP_20733	1320	T	A	3	7	--	--
CakTC35358	Ca(ICCV2/PI489777)SNP_20734	678	T	C	15	5	--	--
	Ca(ICCV2/PI489777)SNP_20735	688	C	G	14	4	--	--
	Ca(ICCV2/PI489777)SNP_20736	767	T	C	9	4	--	--
	Ca(ICCV2/PI489777)SNP_20737	1130	C	T	14	5	--	--
	Ca(ICCV2/PI489777)SNP_20738	1688	T	C	6	10	--	--
	Ca(ICCV2/PI489777)SNP_20739	1825	C	T	3	9	--	--
	Ca(ICCV2/PI489777)SNP_20740	1943	G	A	5	15	--	--
	Ca(ICCV2/PI489777)SNP_20741	1957	G	A	6	15	--	--
	Ca(ICCV2/PI489777)SNP_20742	2055	G	T	5	20	--	--
	Ca(ICCV2/PI489777)SNP_20743	2079	G	A	7	20	--	--
	Ca(ICCV2/PI489777)SNP_20744	2412	C	A	8	8	--	--
	Ca(ICCV2/PI489777)SNP_20745	2858	C	T	5	6	--	--
	Ca(ICCV2/PI489777)SNP_20746	3012	G	A	12	4	--	--
	Ca(ICCV2/PI489777)SNP_20747	3093	C	T	11	4	--	--
	Ca(ICCV2/PI489777)SNP_20748	3293	T	G	8	11	--	--
	Ca(ICCV2/PI489777)SNP_20749	3375	A	G	7	10	--	--
	Ca(ICCV2/PI489777)SNP_20750	3929	G	A	12	13	--	--
	Ca(ICCV2/PI489777)SNP_20751	4282	T	C	5	13	--	--
CakTC38456	Ca(ICCV2/PI489777)SNP_20752	257	T	A	20	3	--	--
	Ca(ICCV2/PI489777)SNP_20753	561	A	G	14	3	--	--
	Ca(ICCV2/PI489777)SNP_20754	625	A	G	14	4	--	--
	Ca(ICCV2/PI489777)SNP_20755	761	C	A	9	4	--	--
	Ca(ICCV2/PI489777)SNP_20756	852	C	T	13	6	--	--
	Ca(ICCV2/PI489777)SNP_20757	1100	G	A	15	6	--	--
CakTC10483	Ca(ICCV2/PI489777)SNP_20758	866	T	A	22	14	--	C2C2-CO-like
	Ca(ICCV2/PI489777)SNP_20759	914	C	T	23	8	--	C2C2-CO-like
	Ca(ICCV2/PI489777)SNP_20760	1010	A	G	20	9	--	C2C2-CO-like
CakTC31301	Ca(ICCV2/PI489777)SNP_20761	861	C	T	5	6	--	--
CakTC41469	Ca(ICCV2/PI489777)SNP_20762	413	C	T	9	10	--	TPR
	Ca(ICCV2/PI489777)SNP_20763	516	G	A	9	11	--	TPR
	Ca(ICCV2/PI489777)SNP_20764	623	A	G	9	8	--	TPR
	Ca(ICCV2/PI489777)SNP_20765	1433	G	A	7	8	--	TPR
	Ca(ICCV2/PI489777)SNP_20766	1628	T	C	9	9	--	TPR
	Ca(ICCV2/PI489777)SNP_20767	1672	A	G	11	7	--	TPR
	Ca(ICCV2/PI489777)SNP_20768	2273	A	C	4	10	--	TPR
CakTC25887	Ca(ICCV2/PI489777)SNP_20769	164	A	G	10	16	--	--
	Ca(ICCV2/PI489777)SNP_20770	898	C	A	23	20	--	--
CakTC32039	Ca(ICCV2/PI489777)SNP_20771	636	A	G	35	38	--	--
	Ca(ICCV2/PI489777)SNP_20772	699	T	C	38	35	--	--
	Ca(ICCV2/PI489777)SNP_20773	969	T	A	45	31	--	--
CakTC33495	Ca(ICCV2/PI489777)SNP_20774	132	A	C	8	6	--	--
	Ca(ICCV2/PI489777)SNP_20775	340	G	A	11	6	--	--
	Ca(ICCV2/PI489777)SNP_20776	389	T	C	8	6	--	--
	Ca(ICCV2/PI489777)SNP_20777	790	T	C	4	3	--	--
	Ca(ICCV2/PI489777)SNP_20778	859	C	A	3	3	--	--
	Ca(ICCV2/PI489777)SNP_20779	1084	T	C	6	3	--	--
	Ca(ICCV2/PI489777)SNP_20780	1258	T	C	11	9	--	--
	Ca(ICCV2/PI489777)SNP_20781	1633	A	G	28	13	--	--
	Ca(ICCV2/PI489777)SNP_20782	1671	C	T	24	6	--	--
	Ca(ICCV2/PI489777)SNP_20783	1678	C	T	26	13	--	--
CakTC10796	Ca(ICCV2/PI489777)SNP_20784	598	A	G	3	6	--	--
	Ca(ICCV2/PI489777)SNP_20785	963	C	T	7	7	--	--
	Ca(ICCV2/PI489777)SNP_20786	988	C	A	9	7	--	--
CakTC42018	Ca(ICCV2/PI489777)SNP_20787	883	G	A	5	4	--	--
CakTC27687	Ca(ICCV2/PI489777)SNP_20788	1217	C	T	5	3	Flower	bud
	Ca(ICCV2/PI489777)SNP_20789	1287	G	A	6	3	Flower	bud

	Ca(ICCV2/PI489777)SNP_20790	1366	C	T	5	3	Flower	bud
CakTC23680	Ca(ICCV2/PI489777)SNP_20791	1101	T	C	22	5	--	AP2-EREBP
	Ca(ICCV2/PI489777)SNP_20792	1197	G	A	21	5	--	AP2-EREBP
CakTC30363	Ca(ICCV2/PI489777)SNP_20793	81	A	T	10	14	--	--
	Ca(ICCV2/PI489777)SNP_20794	453	G	A	17	20	--	--
	Ca(ICCV2/PI489777)SNP_20795	513	T	C	18	17	--	--
CakTC25616	Ca(ICCV2/PI489777)SNP_20796	2080	G	A	65	92	--	--
	Ca(ICCV2/PI489777)SNP_20797	3354	T	C	43	58	--	--
CakTC38463	Ca(ICCV2/PI489777)SNP_20798	87	G	C	8	3	--	--
CakTC37139	Ca(ICCV2/PI489777)SNP_20799	73	G	A	8	10	--	--
CakTC23436	Ca(ICCV2/PI489777)SNP_20800	526	T	C	6	7	--	--
	Ca(ICCV2/PI489777)SNP_20801	720	G	A	8	9	--	--
	Ca(ICCV2/PI489777)SNP_20802	1532	G	A	22	12	--	--
	Ca(ICCV2/PI489777)SNP_20803	1846	A	G	14	19	--	--
CakTC25385	Ca(ICCV2/PI489777)SNP_20804	1149	G	A	18	3	--	--
CakTC09948	Ca(ICCV2/PI489777)SNP_20805	196	T	C	8	15	--	--
	Ca(ICCV2/PI489777)SNP_20806	575	G	C	3	6	--	--
CakTC22371	Ca(ICCV2/PI489777)SNP_20807	2712	A	G	15	4	--	--
CakTC31615	Ca(ICCV2/PI489777)SNP_20808	668	G	A	4	4	Young_pod	--
CakTC43370	Ca(ICCV2/PI489777)SNP_20809	224	C	T	16	7	--	--
CakTC39307	Ca(ICCV2/PI489777)SNP_20810	519	T	A	17	5	--	HB
	Ca(ICCV2/PI489777)SNP_20811	969	T	C	7	4	--	HB
CakTC37245	Ca(ICCV2/PI489777)SNP_20812	309	C	A	5	4	Young_pod	--
	Ca(ICCV2/PI489777)SNP_20813	352	C	T	5	4	Young_pod	--
	Ca(ICCV2/PI489777)SNP_20814	453	C	T	6	4	Young_pod	--
	Ca(ICCV2/PI489777)SNP_20815	840	A	G	5	4	Young_pod	--
CakTC37981	Ca(ICCV2/PI489777)SNP_20816	270	G	A	15	3	--	--
	Ca(ICCV2/PI489777)SNP_20817	378	T	C	16	4	--	--
	Ca(ICCV2/PI489777)SNP_20818	733	C	T	16	3	--	--
CakTC26304	Ca(ICCV2/PI489777)SNP_20819	311	A	G	4	4	--	WRKY
	Ca(ICCV2/PI489777)SNP_20820	1139	A	T	10	4	--	WRKY
	Ca(ICCV2/PI489777)SNP_20821	1275	G	T	20	13	--	WRKY
CakTC29471	Ca(ICCV2/PI489777)SNP_20822	831	T	A	11	3	--	--
	Ca(ICCV2/PI489777)SNP_20823	1230	T	C	10	5	--	--
	Ca(ICCV2/PI489777)SNP_20824	1524	T	G	6	6	--	--
	Ca(ICCV2/PI489777)SNP_20825	2172	T	C	14	4	--	--
	Ca(ICCV2/PI489777)SNP_20826	2186	T	C	15	4	--	--
	Ca(ICCV2/PI489777)SNP_20827	2703	G	A	15	17	--	--
	Ca(ICCV2/PI489777)SNP_20828	2715	G	C	16	18	--	--
	Ca(ICCV2/PI489777)SNP_20829	2976	T	C	10	10	--	--
	Ca(ICCV2/PI489777)SNP_20830	3014	A	G	12	10	--	--
	Ca(ICCV2/PI489777)SNP_20831	3023	C	T	11	8	--	--
CakTC41878	Ca(ICCV2/PI489777)SNP_20832	427	A	C	5	4	--	--
	Ca(ICCV2/PI489777)SNP_20833	1251	C	T	11	5	--	--
CakTC24197	Ca(ICCV2/PI489777)SNP_20834	66	T	A	12	8	--	--
	Ca(ICCV2/PI489777)SNP_20835	902	G	A	65	11	--	--
	Ca(ICCV2/PI489777)SNP_20836	916	T	C	65	10	--	--
	Ca(ICCV2/PI489777)SNP_20837	1136	C	T	36	6	--	--
CakTC28820	Ca(ICCV2/PI489777)SNP_20838	15	A	T	11	3	--	--
	Ca(ICCV2/PI489777)SNP_20839	251	C	T	32	39	--	--
	Ca(ICCV2/PI489777)SNP_20840	438	C	G	42	52	--	--
	Ca(ICCV2/PI489777)SNP_20841	633	A	G	46	47	--	--
	Ca(ICCV2/PI489777)SNP_20842	650	A	T	42	43	--	--
	Ca(ICCV2/PI489777)SNP_20843	656	T	C	39	40	--	--
	Ca(ICCV2/PI489777)SNP_20844	1492	C	G	18	23	--	--
	Ca(ICCV2/PI489777)SNP_20845	1518	T	A	18	20	--	--
CakTC34596	Ca(ICCV2/PI489777)SNP_20846	387	A	G	4	3	--	--
CakTC22555	Ca(ICCV2/PI489777)SNP_20847	400	T	C	22	6	--	--
CakTC34964	Ca(ICCV2/PI489777)SNP_20848	380	T	G	5	14	--	--
	Ca(ICCV2/PI489777)SNP_20849	732	G	T	12	16	--	--
	Ca(ICCV2/PI489777)SNP_20850	734	T	A	12	16	--	--
	Ca(ICCV2/PI489777)SNP_20851	805	T	G	14	16	--	--
	Ca(ICCV2/PI489777)SNP_20852	900	C	T	8	13	--	--
	Ca(ICCV2/PI489777)SNP_20853	1160	T	C	11	14	--	--
	Ca(ICCV2/PI489777)SNP_20854	1311	C	T	18	13	--	--
	Ca(ICCV2/PI489777)SNP_20855	1417	T	G	19	11	--	--
	Ca(ICCV2/PI489777)SNP_20856	1474	T	C	23	14	--	--
	Ca(ICCV2/PI489777)SNP_20857	1604	A	G	12	13	--	--
	Ca(ICCV2/PI489777)SNP_20858	1607	G	A	18	14	--	--
	Ca(ICCV2/PI489777)SNP_20859	1632	C	T	21	15	--	--
	Ca(ICCV2/PI489777)SNP_20860	1637	C	T	23	13	--	--
	Ca(ICCV2/PI489777)SNP_20861	1715	C	A	26	14	--	--
	Ca(ICCV2/PI489777)SNP_20862	1782	T	G	23	12	--	--
	Ca(ICCV2/PI489777)SNP_20863	1928	G	A	36	21	--	--

	Ca(ICCV2/PI489777)SNP_20864	1968	A	C	24	17	--	--
	Ca(ICCV2/PI489777)SNP_20865	2349	G	T	9	21	--	--
	Ca(ICCV2/PI489777)SNP_20866	2379	T	C	9	12	--	--
	Ca(ICCV2/PI489777)SNP_20867	2598	A	G	10	20	--	--
	Ca(ICCV2/PI489777)SNP_20868	2767	A	T	6	14	--	--
CakTC24872	Ca(ICCV2/PI489777)SNP_20869	1333	T	C	88	39	--	--
CakTC26985	Ca(ICCV2/PI489777)SNP_20870	836	G	T	12	5	--	--
	Ca(ICCV2/PI489777)SNP_20871	1277	G	T	4	5	--	--
CakTC10657	Ca(ICCV2/PI489777)SNP_20872	453	G	A	9	4	--	--
CakTC29545	Ca(ICCV2/PI489777)SNP_20873	876	T	A	3	7	--	--
	Ca(ICCV2/PI489777)SNP_20874	969	T	A	3	5	--	--
	Ca(ICCV2/PI489777)SNP_20875	2163	T	A	3	5	--	--
	Ca(ICCV2/PI489777)SNP_20876	2381	T	C	3	5	--	--
CakTC31376	Ca(ICCV2/PI489777)SNP_20877	1248	T	C	3	3	Root	GNAT
CakTC23422	Ca(ICCV2/PI489777)SNP_20878	82	T	C	41	27	--	--
	Ca(ICCV2/PI489777)SNP_20879	128	T	C	87	64	--	--
	Ca(ICCV2/PI489777)SNP_20880	135	C	T	90	65	--	--
CakTC40238	Ca(ICCV2/PI489777)SNP_20881	464	G	A	7	15	--	--
	Ca(ICCV2/PI489777)SNP_20882	487	C	G	6	14	--	--
CakTC29830	Ca(ICCV2/PI489777)SNP_20883	158	G	A	13	11	--	--
	Ca(ICCV2/PI489777)SNP_20884	644	G	A	70	74	--	--
	Ca(ICCV2/PI489777)SNP_20885	794	T	C	35	39	--	--
	Ca(ICCV2/PI489777)SNP_20886	989	T	A	45	40	--	--
	Ca(ICCV2/PI489777)SNP_20887	1005	C	T	46	36	--	--
	Ca(ICCV2/PI489777)SNP_20888	1092	T	G	21	6	--	--
CakTC41219	Ca(ICCV2/PI489777)SNP_20889	118	G	A	6	12	--	--
	Ca(ICCV2/PI489777)SNP_20890	856	A	G	35	19	--	--
	Ca(ICCV2/PI489777)SNP_20891	2284	A	G	17	8	--	--
CakTC23414	Ca(ICCV2/PI489777)SNP_20892	844	G	T	3	3	--	--
CakTC22782	Ca(ICCV2/PI489777)SNP_20893	142	G	A	12	3	--	--
	Ca(ICCV2/PI489777)SNP_20894	682	C	T	52	10	--	--
	Ca(ICCV2/PI489777)SNP_20895	775	T	G	56	10	--	--
	Ca(ICCV2/PI489777)SNP_20896	829	C	T	44	10	--	--
CakTC10038	Ca(ICCV2/PI489777)SNP_20897	922	C	T	3	3	--	--
	Ca(ICCV2/PI489777)SNP_20898	1004	C	T	3	3	--	--
	Ca(ICCV2/PI489777)SNP_20899	1752	G	A	4	6	--	--
	Ca(ICCV2/PI489777)SNP_20900	1973	T	C	4	7	--	--
CakTC42133	Ca(ICCV2/PI489777)SNP_20901	442	G	A	4	13	--	--
	Ca(ICCV2/PI489777)SNP_20902	573	G	A	5	11	--	--
	Ca(ICCV2/PI489777)SNP_20903	696	G	T	5	8	--	--
CakTC39360	Ca(ICCV2/PI489777)SNP_20904	320	G	T	5	5	--	--
	Ca(ICCV2/PI489777)SNP_20905	1069	G	C	5	11	--	--
	Ca(ICCV2/PI489777)SNP_20906	1126	T	C	3	11	--	--
	Ca(ICCV2/PI489777)SNP_20907	1316	C	T	4	5	--	--
	Ca(ICCV2/PI489777)SNP_20908	1355	T	C	3	5	--	--
	Ca(ICCV2/PI489777)SNP_20909	1439	C	T	3	3	--	--
CakTC41822	Ca(ICCV2/PI489777)SNP_20910	729	G	T	29	21	--	--
CakTC39086	Ca(ICCV2/PI489777)SNP_20911	591	T	C	9	21	--	--
	Ca(ICCV2/PI489777)SNP_20912	982	C	A	14	17	--	--
	Ca(ICCV2/PI489777)SNP_20913	1114	G	A	29	19	--	--
	Ca(ICCV2/PI489777)SNP_20914	1180	C	T	30	18	--	--
	Ca(ICCV2/PI489777)SNP_20915	1294	G	A	30	23	--	--
	Ca(ICCV2/PI489777)SNP_20916	1538	C	G	5	4	--	--
CakTC32650	Ca(ICCV2/PI489777)SNP_20917	391	G	A	3	3	--	--
	Ca(ICCV2/PI489777)SNP_20918	650	T	C	9	11	--	--
	Ca(ICCV2/PI489777)SNP_20919	815	T	C	8	7	--	--
	Ca(ICCV2/PI489777)SNP_20920	880	A	C	8	6	--	--
CakTC42871	Ca(ICCV2/PI489777)SNP_20921	521	G	T	5	4	--	--
	Ca(ICCV2/PI489777)SNP_20922	741	A	G	8	10	--	--
	Ca(ICCV2/PI489777)SNP_20923	1119	A	G	11	20	--	--
	Ca(ICCV2/PI489777)SNP_20924	1430	T	G	4	20	--	--
	Ca(ICCV2/PI489777)SNP_20925	1447	G	A	4	19	--	--
	Ca(ICCV2/PI489777)SNP_20926	1453	T	C	4	18	--	--
CakTC41915	Ca(ICCV2/PI489777)SNP_20927	48	C	T	18	5	--	--
	Ca(ICCV2/PI489777)SNP_20928	323	T	C	30	5	--	--
	Ca(ICCV2/PI489777)SNP_20929	332	T	C	34	7	--	--
	Ca(ICCV2/PI489777)SNP_20930	696	G	A	26	8	--	--
	Ca(ICCV2/PI489777)SNP_20931	860	C	T	20	4	--	--
	Ca(ICCV2/PI489777)SNP_20932	899	G	A	14	4	--	--
CakTC30637	Ca(ICCV2/PI489777)SNP_20933	3264	A	G	3	3	--	--
CakTC22239	Ca(ICCV2/PI489777)SNP_20934	255	G	C	5	8	Flower	bud
	Ca(ICCV2/PI489777)SNP_20935	511	A	G	8	10	Flower	bud
	Ca(ICCV2/PI489777)SNP_20936	994	C	G	53	6	Flower	bud
	Ca(ICCV2/PI489777)SNP_20937	1001	A	T	52	7	Flower	bud

	Ca(ICCV2/PI489777)SNP_20938	1062	A	G	77	14	Flower	bud
	Ca(ICCV2/PI489777)SNP_20939	1155	T	G	100	16	Flower	bud
	Ca(ICCV2/PI489777)SNP_20940	1197	T	A	106	17	Flower	bud
CakTC29454	Ca(ICCV2/PI489777)SNP_20941	794	A	G	5	6	Flower	bud
CakTC38958	Ca(ICCV2/PI489777)SNP_20942	77	T	C	18	10	--	--
	Ca(ICCV2/PI489777)SNP_20943	196	T	C	20	12	--	--
	Ca(ICCV2/PI489777)SNP_20944	258	G	T	22	18	--	--
CakTC24925	Ca(ICCV2/PI489777)SNP_20945	68	G	A	19	6	Young_pod	--
	Ca(ICCV2/PI489777)SNP_20946	186	G	A	33	13	Young_pod	--
	Ca(ICCV2/PI489777)SNP_20947	732	T	A	44	24	Young_pod	--
CakTC36293	Ca(ICCV2/PI489777)SNP_20948	268	C	T	3	8	--	--
CakTC35294	Ca(ICCV2/PI489777)SNP_20949	191	G	C	4	5	--	--
	Ca(ICCV2/PI489777)SNP_20950	285	C	A	3	5	--	--
	Ca(ICCV2/PI489777)SNP_20951	383	G	C	4	8	--	--
	Ca(ICCV2/PI489777)SNP_20952	1579	G	A	7	3	--	--
	Ca(ICCV2/PI489777)SNP_20953	1893	G	A	7	6	--	--
	Ca(ICCV2/PI489777)SNP_20954	2474	A	T	7	9	--	--
	Ca(ICCV2/PI489777)SNP_20955	2556	T	G	4	6	--	--
	Ca(ICCV2/PI489777)SNP_20956	2688	T	C	3	3	--	--
	Ca(ICCV2/PI489777)SNP_20957	2941	A	G	6	7	--	--
CakTC38195	Ca(ICCV2/PI489777)SNP_20958	486	C	T	15	16	--	--
	Ca(ICCV2/PI489777)SNP_20959	991	G	T	3	14	--	--
CakTC00543	Ca(ICCV2/PI489777)SNP_20960	702	T	G	4	3	--	E2F-DP
CakTC05291	Ca(ICCV2/PI489777)SNP_20961	217	C	G	3	8	--	MADS
	Ca(ICCV2/PI489777)SNP_20962	440	G	C	3	3	--	MADS
CakTC26998	Ca(ICCV2/PI489777)SNP_20963	181	C	T	49	29	--	HMG
	Ca(ICCV2/PI489777)SNP_20964	566	T	C	35	31	--	HMG
	Ca(ICCV2/PI489777)SNP_20965	596	G	T	31	24	--	HMG
	Ca(ICCV2/PI489777)SNP_20966	697	G	C	20	23	--	HMG
	Ca(ICCV2/PI489777)SNP_20967	719	G	T	28	21	--	HMG
CakTC27042	Ca(ICCV2/PI489777)SNP_20968	606	G	A	5	4	--	--
	Ca(ICCV2/PI489777)SNP_20969	1206	T	C	4	3	--	--
	Ca(ICCV2/PI489777)SNP_20970	1236	A	G	6	5	--	--
CakTC40899	Ca(ICCV2/PI489777)SNP_20971	844	T	C	24	16	--	--
	Ca(ICCV2/PI489777)SNP_20972	873	G	A	22	17	--	--
	Ca(ICCV2/PI489777)SNP_20973	1218	C	T	17	26	--	--
CakTC26223	Ca(ICCV2/PI489777)SNP_20974	148	A	G	5	3	--	--
	Ca(ICCV2/PI489777)SNP_20975	2127	G	A	9	3	--	--
	Ca(ICCV2/PI489777)SNP_20976	3120	A	C	5	4	--	--
CakTC25253	Ca(ICCV2/PI489777)SNP_20977	332	A	G	5	3	Flower	bud
	Ca(ICCV2/PI489777)SNP_20978	913	C	T	21	3	Flower	bud
	Ca(ICCV2/PI489777)SNP_20979	1138	A	G	11	7	Flower	bud
	Ca(ICCV2/PI489777)SNP_20980	1246	G	A	6	7	Flower	bud
	Ca(ICCV2/PI489777)SNP_20981	2737	T	A	13	6	Flower	bud
	Ca(ICCV2/PI489777)SNP_20982	2854	A	G	13	5	Flower	bud
CakTC41606	Ca(ICCV2/PI489777)SNP_20983	854	G	A	4	6	--	--
	Ca(ICCV2/PI489777)SNP_20984	869	C	T	4	6	--	--
	Ca(ICCV2/PI489777)SNP_20985	1971	G	A	8	5	--	--
CakTC24132	Ca(ICCV2/PI489777)SNP_20986	1958	C	T	4	5	--	--
	Ca(ICCV2/PI489777)SNP_20987	2041	A	G	5	3	--	--
	Ca(ICCV2/PI489777)SNP_20988	2306	A	T	6	3	--	--
	Ca(ICCV2/PI489777)SNP_20989	2894	C	T	10	4	--	--
CakTC41525	Ca(ICCV2/PI489777)SNP_20990	830	A	C	10	11	--	--
CakTC37483	Ca(ICCV2/PI489777)SNP_20991	346	A	G	10	5	--	GNAT
	Ca(ICCV2/PI489777)SNP_20992	1027	G	A	3	8	--	GNAT
CakTC42270	Ca(ICCV2/PI489777)SNP_20993	71	C	T	8	5	--	--
CakTC39375	Ca(ICCV2/PI489777)SNP_20994	353	A	G	14	7	--	--
	Ca(ICCV2/PI489777)SNP_20995	450	T	C	8	9	--	--
	Ca(ICCV2/PI489777)SNP_20996	521	A	G	6	9	--	--
CakTC33982	Ca(ICCV2/PI489777)SNP_20997	973	A	T	10	3	--	--
	Ca(ICCV2/PI489777)SNP_20998	1009	A	G	6	5	--	--
	Ca(ICCV2/PI489777)SNP_20999	1627	A	C	12	5	--	--
	Ca(ICCV2/PI489777)SNP_21000	1840	A	G	3	4	--	--
	Ca(ICCV2/PI489777)SNP_21001	1879	C	T	5	3	--	--
CakTC25090	Ca(ICCV2/PI489777)SNP_21002	731	A	G	11	7	--	--
	Ca(ICCV2/PI489777)SNP_21003	800	C	T	25	8	--	--
	Ca(ICCV2/PI489777)SNP_21004	1064	G	A	29	13	--	--
	Ca(ICCV2/PI489777)SNP_21005	1368	C	T	20	5	--	--
	Ca(ICCV2/PI489777)SNP_21006	1651	C	G	37	14	--	--
	Ca(ICCV2/PI489777)SNP_21007	1981	C	G	45	17	--	--
	Ca(ICCV2/PI489777)SNP_21008	2174	C	T	41	15	--	--
CakTC43296	Ca(ICCV2/PI489777)SNP_21009	434	T	G	21	9	--	--
	Ca(ICCV2/PI489777)SNP_21010	559	T	C	22	14	--	--
	Ca(ICCV2/PI489777)SNP_21011	655	T	A	22	12	--	--

	Ca(ICCV2/PI489777)SNP_21012	677	C	T	22	10	--	--
	Ca(ICCV2/PI489777)SNP_21013	793	T	C	23	6	--	--
CakTC37545	Ca(ICCV2/PI489777)SNP_21014	722	G	T	57	26	--	--
	Ca(ICCV2/PI489777)SNP_21015	793	C	T	49	15	--	--
	Ca(ICCV2/PI489777)SNP_21016	1225	G	A	12	18	--	--
CakTC28651	Ca(ICCV2/PI489777)SNP_21017	1457	C	T	5	7	--	--
CakTC32487	Ca(ICCV2/PI489777)SNP_21018	534	C	T	4	3	--	--
CakTC40923	Ca(ICCV2/PI489777)SNP_21019	513	C	A	6	7	--	--
CakTC39958	Ca(ICCV2/PI489777)SNP_21020	289	A	G	7	12	--	--
	Ca(ICCV2/PI489777)SNP_21021	379	A	G	7	9	--	--
CakTC38772	Ca(ICCV2/PI489777)SNP_21022	690	T	C	35	26	--	--
CakTC39945	Ca(ICCV2/PI489777)SNP_21023	673	G	A	30	20	--	--
	Ca(ICCV2/PI489777)SNP_21024	891	T	C	30	34	--	--
CakTC41832	Ca(ICCV2/PI489777)SNP_21025	583	T	C	4	4	--	--
CakTC22468	Ca(ICCV2/PI489777)SNP_21026	781	C	A	4	9	Flower	bud
	Ca(ICCV2/PI489777)SNP_21027	802	T	C	4	5	Flower	bud
CakTC34258	Ca(ICCV2/PI489777)SNP_21028	518	C	G	49	22	--	TPR
	Ca(ICCV2/PI489777)SNP_21029	1141	C	T	51	21	--	TPR
	Ca(ICCV2/PI489777)SNP_21030	1351	A	G	62	35	--	TPR
	Ca(ICCV2/PI489777)SNP_21031	1405	A	G	68	22	--	TPR
CakTC26012	Ca(ICCV2/PI489777)SNP_21032	1028	A	G	33	27	--	--
	Ca(ICCV2/PI489777)SNP_21033	1303	G	A	39	30	--	--
	Ca(ICCV2/PI489777)SNP_21034	1310	C	T	38	29	--	--
	Ca(ICCV2/PI489777)SNP_21035	2067	G	A	34	4	--	--
	Ca(ICCV2/PI489777)SNP_21036	2367	T	A	19	6	--	--
	Ca(ICCV2/PI489777)SNP_21037	2440	A	T	19	8	--	--
	Ca(ICCV2/PI489777)SNP_21038	2459	T	C	13	6	--	--
	Ca(ICCV2/PI489777)SNP_21039	2498	C	A	21	6	--	--
	Ca(ICCV2/PI489777)SNP_21040	2662	T	A	25	9	--	--
	Ca(ICCV2/PI489777)SNP_21041	2710	T	C	25	13	--	--
	Ca(ICCV2/PI489777)SNP_21042	2755	T	C	27	14	--	--
	Ca(ICCV2/PI489777)SNP_21043	3027	C	T	17	15	--	--
	Ca(ICCV2/PI489777)SNP_21044	3211	C	T	21	6	--	--
	Ca(ICCV2/PI489777)SNP_21045	3307	C	A	22	3	--	--
CakTC27156	Ca(ICCV2/PI489777)SNP_21046	241	T	C	6	3	--	--
	Ca(ICCV2/PI489777)SNP_21047	480	T	C	7	3	--	--
CakTC09801	Ca(ICCV2/PI489777)SNP_21048	272	G	A	7	6	Flower	bud
	Ca(ICCV2/PI489777)SNP_21049	614	C	A	10	9	Flower	bud
CakTC40479	Ca(ICCV2/PI489777)SNP_21050	290	T	C	4	8	--	--
CakTC03979	Ca(ICCV2/PI489777)SNP_21051	806	C	G	18	15	--	--
	Ca(ICCV2/PI489777)SNP_21052	1645	G	A	10	10	--	--
	Ca(ICCV2/PI489777)SNP_21053	1687	G	A	9	7	--	--
	Ca(ICCV2/PI489777)SNP_21054	2661	T	C	14	15	--	--
CakTC26842	Ca(ICCV2/PI489777)SNP_21055	1364	C	T	14	3	Flower	bud
	Ca(ICCV2/PI489777)SNP_21056	1537	C	G	18	4	Flower	bud
	Ca(ICCV2/PI489777)SNP_21057	2541	A	G	18	6	Flower	bud
	Ca(ICCV2/PI489777)SNP_21058	2859	T	G	10	3	Flower	bud
CakTC40889	Ca(ICCV2/PI489777)SNP_21059	68	A	T	4	3	--	--
	Ca(ICCV2/PI489777)SNP_21060	127	A	G	4	3	--	--
	Ca(ICCV2/PI489777)SNP_21061	694	C	T	4	5	--	--
CakTC32054	Ca(ICCV2/PI489777)SNP_21062	1247	C	A	7	3	--	--
	Ca(ICCV2/PI489777)SNP_21063	1366	G	A	6	3	--	--
	Ca(ICCV2/PI489777)SNP_21064	1430	A	G	4	3	--	--
CakTC28169	Ca(ICCV2/PI489777)SNP_21065	216	C	T	12	9	--	bZIP
	Ca(ICCV2/PI489777)SNP_21066	1177	A	G	22	8	--	bZIP
	Ca(ICCV2/PI489777)SNP_21067	1296	C	T	26	11	--	bZIP
CakTC34216	Ca(ICCV2/PI489777)SNP_21068	2968	T	C	9	13	--	TAZ
	Ca(ICCV2/PI489777)SNP_21069	3673	G	A	19	10	--	TAZ
CakTC27483	Ca(ICCV2/PI489777)SNP_21070	750	T	C	3	3	--	HRT
CakTC36905	Ca(ICCV2/PI489777)SNP_21071	594	T	C	5	3	--	C3H
	Ca(ICCV2/PI489777)SNP_21072	657	G	A	4	4	--	C3H
	Ca(ICCV2/PI489777)SNP_21073	697	C	G	4	4	--	C3H
	Ca(ICCV2/PI489777)SNP_21074	705	C	T	5	4	--	C3H
	Ca(ICCV2/PI489777)SNP_21075	1191	C	T	6	3	--	C3H
	Ca(ICCV2/PI489777)SNP_21076	1206	T	C	6	3	--	C3H
	Ca(ICCV2/PI489777)SNP_21077	1272	G	A	4	4	--	C3H
	Ca(ICCV2/PI489777)SNP_21078	1299	T	C	3	4	--	C3H
	Ca(ICCV2/PI489777)SNP_21079	1329	T	C	8	4	--	C3H
	Ca(ICCV2/PI489777)SNP_21080	1374	A	G	8	5	--	C3H
	Ca(ICCV2/PI489777)SNP_21081	2109	T	C	3	6	--	C3H
	Ca(ICCV2/PI489777)SNP_21082	2135	A	G	5	5	--	C3H
	Ca(ICCV2/PI489777)SNP_21083	2137	G	A	5	5	--	C3H
CakTC34910	Ca(ICCV2/PI489777)SNP_21084	465	G	A	10	16	--	--
	Ca(ICCV2/PI489777)SNP_21085	470	G	A	11	18	--	--

	Ca(ICCV2/PI489777)SNP_21086	1833	G	A	12	14	--	--
	Ca(ICCV2/PI489777)SNP_21087	3357	T	C	17	19	--	--
	Ca(ICCV2/PI489777)SNP_21088	3460	A	G	11	9	--	--
CakTC09833	Ca(ICCV2/PI489777)SNP_21089	573	T	C	14	8	--	--
CakTC28698	Ca(ICCV2/PI489777)SNP_21090	550	C	T	11	7	--	--
	Ca(ICCV2/PI489777)SNP_21091	597	T	C	10	7	--	--
	Ca(ICCV2/PI489777)SNP_21092	1342	T	C	8	19	--	--
	Ca(ICCV2/PI489777)SNP_21093	1964	G	T	6	13	--	--
	Ca(ICCV2/PI489777)SNP_21094	1965	G	C	6	14	--	--
	Ca(ICCV2/PI489777)SNP_21095	1993	A	G	9	14	--	--
CakTC35905	Ca(ICCV2/PI489777)SNP_21096	416	G	A	13	15	--	--
	Ca(ICCV2/PI489777)SNP_21097	752	A	C	8	6	--	--
	Ca(ICCV2/PI489777)SNP_21098	887	T	G	5	4	--	--
	Ca(ICCV2/PI489777)SNP_21099	2680	T	C	4	7	--	--
CakTC42661	Ca(ICCV2/PI489777)SNP_21100	281	C	G	20	7	--	--
	Ca(ICCV2/PI489777)SNP_21101	864	A	C	12	16	--	--
CakTC31197	Ca(ICCV2/PI489777)SNP_21102	318	G	A	6	5	--	--
	Ca(ICCV2/PI489777)SNP_21103	831	G	A	7	3	--	--
	Ca(ICCV2/PI489777)SNP_21104	992	T	C	6	4	--	--
	Ca(ICCV2/PI489777)SNP_21105	1542	G	A	4	5	--	--
CakTC37797	Ca(ICCV2/PI489777)SNP_21106	853	A	C	4	11	Flower	bud
CakTC43318	Ca(ICCV2/PI489777)SNP_21107	2739	C	A	15	4	--	--
	Ca(ICCV2/PI489777)SNP_21108	2828	A	G	13	5	--	--
CakTC26969	Ca(ICCV2/PI489777)SNP_21109	385	G	A	7	4	--	--
	Ca(ICCV2/PI489777)SNP_21110	400	C	T	7	4	--	--
CakTC24317	Ca(ICCV2/PI489777)SNP_21111	408	A	C	19	6	--	--
	Ca(ICCV2/PI489777)SNP_21112	780	C	T	18	8	--	--
CakTC34833	Ca(ICCV2/PI489777)SNP_21113	1942	C	G	15	4	--	SBP
	Ca(ICCV2/PI489777)SNP_21114	2462	G	A	16	9	--	SBP
	Ca(ICCV2/PI489777)SNP_21115	2698	G	A	8	9	--	SBP
	Ca(ICCV2/PI489777)SNP_21116	2787	A	C	11	8	--	SBP
CakTC42515	Ca(ICCV2/PI489777)SNP_21117	44	T	C	5	6	--	--
	Ca(ICCV2/PI489777)SNP_21118	46	C	A	6	6	--	--
	Ca(ICCV2/PI489777)SNP_21119	69	C	A	13	13	--	--
	Ca(ICCV2/PI489777)SNP_21120	124	C	T	15	18	--	--
	Ca(ICCV2/PI489777)SNP_21121	150	G	C	14	19	--	--
	Ca(ICCV2/PI489777)SNP_21122	203	C	T	16	21	--	--
	Ca(ICCV2/PI489777)SNP_21123	210	T	C	18	21	--	--
	Ca(ICCV2/PI489777)SNP_21124	307	G	C	20	21	--	--
	Ca(ICCV2/PI489777)SNP_21125	312	A	C	21	20	--	--
	Ca(ICCV2/PI489777)SNP_21126	614	A	C	9	8	--	--
	Ca(ICCV2/PI489777)SNP_21127	621	A	G	9	12	--	--
CakTC31308	Ca(ICCV2/PI489777)SNP_21128	137	T	C	4	11	--	--
	Ca(ICCV2/PI489777)SNP_21129	600	C	T	166	153	--	--
CakTC41373	Ca(ICCV2/PI489777)SNP_21130	250	C	T	8	8	--	--
	Ca(ICCV2/PI489777)SNP_21131	754	A	G	10	13	--	--
	Ca(ICCV2/PI489777)SNP_21132	1192	G	A	13	7	--	--
	Ca(ICCV2/PI489777)SNP_21133	1227	A	T	14	8	--	--
CakTC29542	Ca(ICCV2/PI489777)SNP_21134	1947	C	T	7	3	--	--
CakTC38386	Ca(ICCV2/PI489777)SNP_21135	277	A	T	10	6	--	AUX/IAA
	Ca(ICCV2/PI489777)SNP_21136	1005	T	A	28	15	--	AUX/IAA
CakTC24662	Ca(ICCV2/PI489777)SNP_21137	321	T	C	60	16	--	--
	Ca(ICCV2/PI489777)SNP_21138	412	C	T	37	15	--	--
CakTC41360	Ca(ICCV2/PI489777)SNP_21139	183	G	T	40	25	--	--
	Ca(ICCV2/PI489777)SNP_21140	256	A	C	56	39	--	--
	Ca(ICCV2/PI489777)SNP_21141	259	G	A	56	39	--	--
	Ca(ICCV2/PI489777)SNP_21142	280	G	A	56	38	--	--
	Ca(ICCV2/PI489777)SNP_21143	394	G	A	61	55	--	--
	Ca(ICCV2/PI489777)SNP_21144	493	G	A	57	52	--	--
	Ca(ICCV2/PI489777)SNP_21145	687	A	T	38	45	--	--
CakTC27092	Ca(ICCV2/PI489777)SNP_21146	205	C	G	15	6	--	--
	Ca(ICCV2/PI489777)SNP_21147	359	G	A	14	6	--	--
	Ca(ICCV2/PI489777)SNP_21148	394	C	T	12	6	--	--
	Ca(ICCV2/PI489777)SNP_21149	406	T	C	10	6	--	--
	Ca(ICCV2/PI489777)SNP_21150	928	A	C	15	5	--	--
	Ca(ICCV2/PI489777)SNP_21151	2062	A	T	12	8	--	--
	Ca(ICCV2/PI489777)SNP_21152	3477	T	C	5	3	--	--
	Ca(ICCV2/PI489777)SNP_21153	3884	C	A	5	4	--	--
	Ca(ICCV2/PI489777)SNP_21154	4042	A	C	6	3	--	--
CakTC39400	Ca(ICCV2/PI489777)SNP_21155	1014	T	C	7	5	--	--
CakTC29265	Ca(ICCV2/PI489777)SNP_21156	990	C	A	4	6	--	--
	Ca(ICCV2/PI489777)SNP_21157	1127	C	T	5	6	--	--
CakTC23648	Ca(ICCV2/PI489777)SNP_21158	122	C	T	5	3	--	--
	Ca(ICCV2/PI489777)SNP_21159	1259	G	C	11	4	--	--

CakTC43268	Ca(ICCV2/PI489777)SNP_21160	246	A	G	25	30	--	--
	Ca(ICCV2/PI489777)SNP_21161	486	T	C	17	32	--	--
CakTC42129	Ca(ICCV2/PI489777)SNP_21162	725	G	T	26	5	--	--
CakTC34407	Ca(ICCV2/PI489777)SNP_21163	1306	A	T	15	8	--	--
	Ca(ICCV2/PI489777)SNP_21164	1390	G	A	16	11	--	--
	Ca(ICCV2/PI489777)SNP_21165	1435	G	A	18	9	--	--
	Ca(ICCV2/PI489777)SNP_21166	1492	T	G	20	7	--	--
	Ca(ICCV2/PI489777)SNP_21167	2016	G	A	19	4	--	--
CakTC35650	Ca(ICCV2/PI489777)SNP_21168	386	G	A	9	8	--	--
	Ca(ICCV2/PI489777)SNP_21169	2105	A	G	7	11	--	--
CakTC27200	Ca(ICCV2/PI489777)SNP_21170	302	C	T	13	13	--	--
	Ca(ICCV2/PI489777)SNP_21171	353	G	T	17	14	--	--
	Ca(ICCV2/PI489777)SNP_21172	1741	A	G	26	7	--	--
	Ca(ICCV2/PI489777)SNP_21173	1757	G	A	25	8	--	--
CakTC11655	Ca(ICCV2/PI489777)SNP_21174	300	A	G	26	12	--	--
	Ca(ICCV2/PI489777)SNP_21175	538	C	G	26	13	--	--
	Ca(ICCV2/PI489777)SNP_21176	864	T	C	19	15	--	--
	Ca(ICCV2/PI489777)SNP_21177	910	T	C	24	14	--	--
	Ca(ICCV2/PI489777)SNP_21178	994	G	A	32	13	--	--
	Ca(ICCV2/PI489777)SNP_21179	2527	C	T	55	28	--	--
	Ca(ICCV2/PI489777)SNP_21180	2530	C	T	55	26	--	--
CakTC37487	Ca(ICCV2/PI489777)SNP_21181	398	T	A	26	8	--	--
	Ca(ICCV2/PI489777)SNP_21182	401	G	A	25	8	--	--
	Ca(ICCV2/PI489777)SNP_21183	770	G	T	21	17	--	--
CakTC23998	Ca(ICCV2/PI489777)SNP_21184	26	C	T	14	6	--	--
	Ca(ICCV2/PI489777)SNP_21185	324	G	A	27	10	--	--
CakTC40024	Ca(ICCV2/PI489777)SNP_21186	167	G	A	3	5	--	--
	Ca(ICCV2/PI489777)SNP_21187	325	A	G	8	4	--	--
	Ca(ICCV2/PI489777)SNP_21188	556	G	C	10	8	--	--
	Ca(ICCV2/PI489777)SNP_21189	919	G	A	10	7	--	--
CakTC27607	Ca(ICCV2/PI489777)SNP_21190	1178	C	G	3	11	--	--
CakTC26045	Ca(ICCV2/PI489777)SNP_21191	855	C	T	18	8	--	--
	Ca(ICCV2/PI489777)SNP_21192	1831	G	A	23	11	--	--
	Ca(ICCV2/PI489777)SNP_21193	2296	C	T	17	9	--	--
	Ca(ICCV2/PI489777)SNP_21194	3118	C	T	9	7	--	--
	Ca(ICCV2/PI489777)SNP_21195	3494	C	G	7	4	--	--
CakTC37510	Ca(ICCV2/PI489777)SNP_21196	964	C	G	3	8	--	--
CakTC39502	Ca(ICCV2/PI489777)SNP_21197	1370	A	G	11	13	Flower	bud
	Ca(ICCV2/PI489777)SNP_21198	1381	A	G	10	13	Flower	bud
CakTC25400	Ca(ICCV2/PI489777)SNP_21199	616	G	A	6	4	--	--
	Ca(ICCV2/PI489777)SNP_21200	633	A	C	5	4	--	--
	Ca(ICCV2/PI489777)SNP_21201	737	A	C	7	3	--	--
CakTC40737	Ca(ICCV2/PI489777)SNP_21202	400	G	A	19	14	--	--
	Ca(ICCV2/PI489777)SNP_21203	614	C	G	17	8	--	--
CakTC39850	Ca(ICCV2/PI489777)SNP_21204	777	A	G	29	7	--	TUB
	Ca(ICCV2/PI489777)SNP_21205	792	C	T	22	5	--	TUB
	Ca(ICCV2/PI489777)SNP_21206	1002	A	G	24	8	--	TUB
	Ca(ICCV2/PI489777)SNP_21207	1077	A	T	23	8	--	TUB
CakTC42000	Ca(ICCV2/PI489777)SNP_21208	318	G	A	3	3	--	--
CakTC33301	Ca(ICCV2/PI489777)SNP_21209	381	C	G	7	8	--	--
	Ca(ICCV2/PI489777)SNP_21210	452	T	C	5	9	--	--
	Ca(ICCV2/PI489777)SNP_21211	614	C	T	8	9	--	--
	Ca(ICCV2/PI489777)SNP_21212	878	T	C	8	6	--	--
	Ca(ICCV2/PI489777)SNP_21213	1535	G	T	10	5	--	--
	Ca(ICCV2/PI489777)SNP_21214	1868	C	G	9	5	--	--
	Ca(ICCV2/PI489777)SNP_21215	1904	C	G	12	9	--	--
	Ca(ICCV2/PI489777)SNP_21216	2036	T	G	12	9	--	--
	Ca(ICCV2/PI489777)SNP_21217	2117	A	G	14	10	--	--
	Ca(ICCV2/PI489777)SNP_21218	2186	G	T	15	10	--	--
	Ca(ICCV2/PI489777)SNP_21219	2345	T	G	15	6	--	--
	Ca(ICCV2/PI489777)SNP_21220	2375	G	A	15	6	--	--
	Ca(ICCV2/PI489777)SNP_21221	2609	A	T	17	4	--	--
	Ca(ICCV2/PI489777)SNP_21222	2645	C	A	19	3	--	--
CakTC23419	Ca(ICCV2/PI489777)SNP_21223	146	C	G	17	3	--	--
	Ca(ICCV2/PI489777)SNP_21224	474	C	T	16	7	--	--
CakTC39573	Ca(ICCV2/PI489777)SNP_21225	52	C	G	4	5	--	--
	Ca(ICCV2/PI489777)SNP_21226	53	C	T	4	4	--	--
	Ca(ICCV2/PI489777)SNP_21227	672	A	T	62	36	--	--
	Ca(ICCV2/PI489777)SNP_21228	730	T	C	54	37	--	--
	Ca(ICCV2/PI489777)SNP_21229	840	T	C	53	41	--	--
	Ca(ICCV2/PI489777)SNP_21230	1012	T	C	32	24	--	--
	Ca(ICCV2/PI489777)SNP_21231	1127	T	G	13	12	--	--
	Ca(ICCV2/PI489777)SNP_21232	1156	A	T	17	12	--	--
	Ca(ICCV2/PI489777)SNP_21233	1246	G	A	9	4	--	--

CakTC42773	Ca(ICCV2/PI489777)SNP_21234	102	T	G	10	4	--	--
	Ca(ICCV2/PI489777)SNP_21235	407	T	G	11	10	--	--
	Ca(ICCV2/PI489777)SNP_21236	431	A	C	11	10	--	--
	Ca(ICCV2/PI489777)SNP_21237	437	A	G	10	9	--	--
	Ca(ICCV2/PI489777)SNP_21238	518	C	T	15	9	--	--
	Ca(ICCV2/PI489777)SNP_21239	536	G	A	18	8	--	--
	Ca(ICCV2/PI489777)SNP_21240	650	A	G	26	7	--	--
	Ca(ICCV2/PI489777)SNP_21241	758	C	T	35	3	--	--
	Ca(ICCV2/PI489777)SNP_21242	782	C	T	39	6	--	--
	Ca(ICCV2/PI489777)SNP_21243	794	T	G	40	8	--	--
	Ca(ICCV2/PI489777)SNP_21244	860	T	C	34	13	--	--
	Ca(ICCV2/PI489777)SNP_21245	959	C	T	32	13	--	--
	Ca(ICCV2/PI489777)SNP_21246	969	G	C	31	13	--	--
	Ca(ICCV2/PI489777)SNP_21247	1123	T	A	23	12	--	--
	Ca(ICCV2/PI489777)SNP_21248	1202	G	A	23	6	--	--
	Ca(ICCV2/PI489777)SNP_21249	1434	C	G	20	4	--	--
CakTC23720	Ca(ICCV2/PI489777)SNP_21250	1705	A	G	19	15	--	--
CakTC12509	Ca(ICCV2/PI489777)SNP_21251	13	T	A	60	14	--	--
	Ca(ICCV2/PI489777)SNP_21252	279	T	A	62	15	--	--
	Ca(ICCV2/PI489777)SNP_21253	367	G	C	73	19	--	--
	Ca(ICCV2/PI489777)SNP_21254	481	G	C	45	21	--	--
	Ca(ICCV2/PI489777)SNP_21255	678	C	T	52	19	--	--
	Ca(ICCV2/PI489777)SNP_21256	1634	G	T	32	7	--	--
	Ca(ICCV2/PI489777)SNP_21257	2282	T	C	29	6	--	--
	Ca(ICCV2/PI489777)SNP_21258	2294	T	C	29	5	--	--
CakTC33570	Ca(ICCV2/PI489777)SNP_21259	230	C	G	6	13	--	--
CakTC42118	Ca(ICCV2/PI489777)SNP_21260	902	T	C	22	7	--	--
CakTC42668	Ca(ICCV2/PI489777)SNP_21261	221	C	G	13	10	--	--
	Ca(ICCV2/PI489777)SNP_21262	1004	A	G	13	5	--	--
	Ca(ICCV2/PI489777)SNP_21263	1427	G	A	9	5	--	--
	Ca(ICCV2/PI489777)SNP_21264	1434	A	T	7	5	--	--
CakTC13189	Ca(ICCV2/PI489777)SNP_21265	760	G	A	53	57	--	--
	Ca(ICCV2/PI489777)SNP_21266	913	G	A	50	62	--	--
CakTC38620	Ca(ICCV2/PI489777)SNP_21267	1914	T	C	16	15	--	--
	Ca(ICCV2/PI489777)SNP_21268	2081	G	C	14	18	--	--
CakTC23051	Ca(ICCV2/PI489777)SNP_21269	93	T	C	13	11	--	--
CakTC23689	Ca(ICCV2/PI489777)SNP_21270	105	A	G	23	4	--	--
	Ca(ICCV2/PI489777)SNP_21271	143	A	G	48	14	--	--
CakTC38357	Ca(ICCV2/PI489777)SNP_21272	991	T	G	147	128	--	--
	Ca(ICCV2/PI489777)SNP_21273	1018	T	G	138	127	--	--
	Ca(ICCV2/PI489777)SNP_21274	1048	A	G	116	103	--	--
CakTC29005	Ca(ICCV2/PI489777)SNP_21275	911	G	A	6	3	--	--
CakTC28245	Ca(ICCV2/PI489777)SNP_21276	515	A	C	5	4	--	--
CakTC32703	Ca(ICCV2/PI489777)SNP_21277	438	C	T	12	16	--	--
	Ca(ICCV2/PI489777)SNP_21278	553	G	A	3	4	--	--
CakTC39050	Ca(ICCV2/PI489777)SNP_21279	552	C	T	21	18	--	--
CakTC37843	Ca(ICCV2/PI489777)SNP_21280	97	T	A	4	7	--	--
	Ca(ICCV2/PI489777)SNP_21281	145	G	C	6	5	--	--
	Ca(ICCV2/PI489777)SNP_21282	244	G	C	7	15	--	--
	Ca(ICCV2/PI489777)SNP_21283	673	C	A	18	20	--	--
CakTC42696	Ca(ICCV2/PI489777)SNP_21284	438	G	A	104	131	--	--
CakTC37121	Ca(ICCV2/PI489777)SNP_21285	324	A	C	11	7	--	--
	Ca(ICCV2/PI489777)SNP_21286	357	T	C	12	14	--	--
	Ca(ICCV2/PI489777)SNP_21287	363	C	T	15	15	--	--
	Ca(ICCV2/PI489777)SNP_21288	585	C	G	29	24	--	--
CakTC31891	Ca(ICCV2/PI489777)SNP_21289	968	C	T	4	3	--	--
CakTC39813	Ca(ICCV2/PI489777)SNP_21290	1000	T	C	60	41	--	--
CakTC12129	Ca(ICCV2/PI489777)SNP_21291	414	C	T	10	11	--	--
	Ca(ICCV2/PI489777)SNP_21292	459	T	C	9	9	--	--
	Ca(ICCV2/PI489777)SNP_21293	1008	T	G	6	5	--	--
CakTC34201	Ca(ICCV2/PI489777)SNP_21294	957	C	G	14	11	--	--
	Ca(ICCV2/PI489777)SNP_21295	1190	A	G	18	7	--	--
	Ca(ICCV2/PI489777)SNP_21296	1326	C	T	21	7	--	--
	Ca(ICCV2/PI489777)SNP_21297	2750	A	G	11	6	--	--
	Ca(ICCV2/PI489777)SNP_21298	2793	C	T	11	5	--	--
	Ca(ICCV2/PI489777)SNP_21299	3628	A	T	7	3	--	--
	Ca(ICCV2/PI489777)SNP_21300	3651	A	G	8	3	--	--
	Ca(ICCV2/PI489777)SNP_21301	3724	C	A	7	5	--	--
	Ca(ICCV2/PI489777)SNP_21302	3916	G	T	5	8	--	--
	Ca(ICCV2/PI489777)SNP_21303	4288	A	G	4	10	--	--
	Ca(ICCV2/PI489777)SNP_21304	4733	T	G	8	5	--	--
	Ca(ICCV2/PI489777)SNP_21305	5192	A	G	13	8	--	--
	Ca(ICCV2/PI489777)SNP_21306	5673	A	T	4	4	--	--
	Ca(ICCV2/PI489777)SNP_21307	5690	G	A	3	4	--	--

	Ca(ICCV2/PI489777)SNP_21308	5693	G	C	3	4	--	--
	Ca(ICCV2/PI489777)SNP_21309	5794	C	T	3	5	--	--
CakTC38619	Ca(ICCV2/PI489777)SNP_21310	407	G	A	4	7	Root	--
	Ca(ICCV2/PI489777)SNP_21311	475	C	T	3	7	Root	--
CakTC11384	Ca(ICCV2/PI489777)SNP_21312	400	C	G	47	18	--	--
	Ca(ICCV2/PI489777)SNP_21313	526	A	T	37	21	--	--
	Ca(ICCV2/PI489777)SNP_21314	585	C	T	33	15	--	--
	Ca(ICCV2/PI489777)SNP_21315	616	C	T	27	13	--	--
	Ca(ICCV2/PI489777)SNP_21316	985	G	A	16	12	--	--
	Ca(ICCV2/PI489777)SNP_21317	1231	G	C	9	4	--	--
	Ca(ICCV2/PI489777)SNP_21318	1319	T	C	3	3	--	--
CakTC41271	Ca(ICCV2/PI489777)SNP_21319	802	G	A	6	3	--	--
	Ca(ICCV2/PI489777)SNP_21320	1780	G	A	4	4	--	--
	Ca(ICCV2/PI489777)SNP_21321	2353	A	T	5	3	--	--
CakTC26043	Ca(ICCV2/PI489777)SNP_21322	700	G	A	10	6	--	--
CakTC36203	Ca(ICCV2/PI489777)SNP_21323	1643	T	C	8	4	--	--
	Ca(ICCV2/PI489777)SNP_21324	2574	C	T	11	5	--	--
	Ca(ICCV2/PI489777)SNP_21325	3512	G	A	8	6	--	--
CakTC41671	Ca(ICCV2/PI489777)SNP_21326	584	A	G	7	7	--	--
	Ca(ICCV2/PI489777)SNP_21327	1016	T	C	7	5	--	--
	Ca(ICCV2/PI489777)SNP_21328	1328	C	T	9	5	--	--
	Ca(ICCV2/PI489777)SNP_21329	1452	A	G	11	4	--	--
	Ca(ICCV2/PI489777)SNP_21330	1481	A	G	9	4	--	--
	Ca(ICCV2/PI489777)SNP_21331	1502	A	G	14	4	--	--
	Ca(ICCV2/PI489777)SNP_21332	1697	T	C	12	3	--	--
CakTC27050	Ca(ICCV2/PI489777)SNP_21333	284	T	G	10	7	--	--
	Ca(ICCV2/PI489777)SNP_21334	843	G	A	5	4	--	--
	Ca(ICCV2/PI489777)SNP_21335	889	T	G	8	6	--	--
	Ca(ICCV2/PI489777)SNP_21336	907	A	G	61	8	--	--
CakTC22738	Ca(ICCV2/PI489777)SNP_21337	45	A	G	23	32	--	--
	Ca(ICCV2/PI489777)SNP_21338	94	C	T	29	37	--	--
	Ca(ICCV2/PI489777)SNP_21339	276	T	A	31	48	--	--
	Ca(ICCV2/PI489777)SNP_21340	724	A	G	13	4	--	--
	Ca(ICCV2/PI489777)SNP_21341	858	A	T	39	20	--	--
	Ca(ICCV2/PI489777)SNP_21342	951	A	G	34	19	--	--
	Ca(ICCV2/PI489777)SNP_21343	1025	G	T	37	20	--	--
	Ca(ICCV2/PI489777)SNP_21344	1095	G	T	34	18	--	--
CakTC26595	Ca(ICCV2/PI489777)SNP_21345	1686	A	T	4	3	--	--
CakTC33975	Ca(ICCV2/PI489777)SNP_21346	154	G	A	11	8	--	--
	Ca(ICCV2/PI489777)SNP_21347	406	A	G	14	13	--	--
	Ca(ICCV2/PI489777)SNP_21348	466	A	G	11	6	--	--
	Ca(ICCV2/PI489777)SNP_21349	475	C	A	11	7	--	--
CakTC41778	Ca(ICCV2/PI489777)SNP_21350	88	C	G	11	13	--	--
	Ca(ICCV2/PI489777)SNP_21351	306	A	C	29	29	--	--
CakTC29363	Ca(ICCV2/PI489777)SNP_21352	189	A	T	8	12	--	--
	Ca(ICCV2/PI489777)SNP_21353	212	C	G	8	9	--	--
CakTC24639	Ca(ICCV2/PI489777)SNP_21354	77	T	C	7	8	--	--
	Ca(ICCV2/PI489777)SNP_21355	630	T	C	8	20	--	--
	Ca(ICCV2/PI489777)SNP_21356	1095	C	T	37	25	--	--
	Ca(ICCV2/PI489777)SNP_21357	1156	G	A	38	26	--	--
	Ca(ICCV2/PI489777)SNP_21358	1186	G	A	35	22	--	--
	Ca(ICCV2/PI489777)SNP_21359	1191	C	T	36	23	--	--
CakTC01733	Ca(ICCV2/PI489777)SNP_21360	81	A	G	10	3	--	--
	Ca(ICCV2/PI489777)SNP_21361	136	C	T	9	3	--	--
CakTC04070	Ca(ICCV2/PI489777)SNP_21362	659	C	T	4	13	--	--
	Ca(ICCV2/PI489777)SNP_21363	660	G	A	4	13	--	--
CakTC25601	Ca(ICCV2/PI489777)SNP_21364	636	C	T	5	5	--	--
	Ca(ICCV2/PI489777)SNP_21365	673	T	G	10	3	--	--
	Ca(ICCV2/PI489777)SNP_21366	686	C	A	9	4	--	--
	Ca(ICCV2/PI489777)SNP_21367	752	T	A	7	3	--	--
	Ca(ICCV2/PI489777)SNP_21368	763	G	A	9	5	--	--
CakTC41586	Ca(ICCV2/PI489777)SNP_21369	543	A	G	9	14	--	--
	Ca(ICCV2/PI489777)SNP_21370	980	C	G	11	5	--	--
	Ca(ICCV2/PI489777)SNP_21371	1632	T	C	13	6	--	--
	Ca(ICCV2/PI489777)SNP_21372	1755	T	C	11	7	--	--
	Ca(ICCV2/PI489777)SNP_21373	1835	G	A	14	6	--	--
	Ca(ICCV2/PI489777)SNP_21374	2208	G	A	15	12	--	--
CakTC12115	Ca(ICCV2/PI489777)SNP_21375	196	C	T	10	4	--	--
	Ca(ICCV2/PI489777)SNP_21376	588	C	T	7	4	--	--
CakTC27325	Ca(ICCV2/PI489777)SNP_21377	782	A	G	7	3	--	--
CakTC38434	Ca(ICCV2/PI489777)SNP_21378	85	T	C	18	5	--	--
	Ca(ICCV2/PI489777)SNP_21379	167	G	A	18	6	--	--
	Ca(ICCV2/PI489777)SNP_21380	1500	A	G	11	7	--	--
	Ca(ICCV2/PI489777)SNP_21381	1549	G	A	9	6	--	--

CakTC27807	Ca(ICCV2/PI489777)SNP_21382	30	G	C	28	3	--	--
CakTC09281	Ca(ICCV2/PI489777)SNP_21383	1166	G	A	3	4	--	--
CakTC23754	Ca(ICCV2/PI489777)SNP_21384	202	T	A	364	147	--	--
	Ca(ICCV2/PI489777)SNP_21385	568	C	T	48	17	--	--
	Ca(ICCV2/PI489777)SNP_21386	589	C	T	9	13	--	--
	Ca(ICCV2/PI489777)SNP_21387	623	A	G	8	11	--	--
CakTC31968	Ca(ICCV2/PI489777)SNP_21388	620	T	A	3	3	--	--
	Ca(ICCV2/PI489777)SNP_21389	830	C	T	3	3	--	--
CakTC34004	Ca(ICCV2/PI489777)SNP_21390	939	G	A	9	3	--	--
	Ca(ICCV2/PI489777)SNP_21391	1832	G	A	8	4	--	--
	Ca(ICCV2/PI489777)SNP_21392	1843	A	T	8	7	--	--
CakTC21560	Ca(ICCV2/PI489777)SNP_21393	376	A	G	6	3	--	--
	Ca(ICCV2/PI489777)SNP_21394	487	G	A	5	3	--	--
	Ca(ICCV2/PI489777)SNP_21395	1735	G	T	5	3	--	--
	Ca(ICCV2/PI489777)SNP_21396	1819	A	G	6	3	--	--
	Ca(ICCV2/PI489777)SNP_21397	2693	G	A	6	3	--	--
	Ca(ICCV2/PI489777)SNP_21398	3638	T	G	8	6	--	--
	Ca(ICCV2/PI489777)SNP_21399	4084	C	G	12	7	--	--
	Ca(ICCV2/PI489777)SNP_21400	4714	G	A	3	5	--	--
CakTC22428	Ca(ICCV2/PI489777)SNP_21401	426	A	G	45	31	--	--
	Ca(ICCV2/PI489777)SNP_21402	453	A	G	48	34	--	--
	Ca(ICCV2/PI489777)SNP_21403	945	G	A	42	32	--	--
CakTC39880	Ca(ICCV2/PI489777)SNP_21404	212	T	C	11	19	--	--
	Ca(ICCV2/PI489777)SNP_21405	1321	G	C	7	4	--	--
CakTC24593	Ca(ICCV2/PI489777)SNP_21406	572	G	A	4	3	--	--
	Ca(ICCV2/PI489777)SNP_21407	573	C	T	4	3	--	--
	Ca(ICCV2/PI489777)SNP_21408	817	G	A	5	3	--	--
	Ca(ICCV2/PI489777)SNP_21409	871	G	C	5	4	--	--
CakTC37681	Ca(ICCV2/PI489777)SNP_21410	1238	A	G	5	15	--	--
	Ca(ICCV2/PI489777)SNP_21411	1263	C	G	3	7	--	--
CakTC23776	Ca(ICCV2/PI489777)SNP_21412	1615	C	T	3	11	--	--
CakTC27597	Ca(ICCV2/PI489777)SNP_21413	595	T	C	13	3	--	--
	Ca(ICCV2/PI489777)SNP_21414	742	T	C	12	8	--	--
	Ca(ICCV2/PI489777)SNP_21415	760	C	T	10	8	--	--
	Ca(ICCV2/PI489777)SNP_21416	788	G	A	12	7	--	--
CakTC39218	Ca(ICCV2/PI489777)SNP_21417	372	A	C	7	3	--	--
CakTC38907	Ca(ICCV2/PI489777)SNP_21418	86	T	A	21	3	--	--
	Ca(ICCV2/PI489777)SNP_21419	511	C	G	22	8	--	--
CakTC41027	Ca(ICCV2/PI489777)SNP_21420	213	T	C	19	3	--	--
	Ca(ICCV2/PI489777)SNP_21421	1867	G	T	14	7	--	--
	Ca(ICCV2/PI489777)SNP_21422	1947	T	C	10	6	--	--
CakTC06132	Ca(ICCV2/PI489777)SNP_21423	88	A	G	8	9	--	--
	Ca(ICCV2/PI489777)SNP_21424	188	A	G	9	14	--	--
	Ca(ICCV2/PI489777)SNP_21425	312	G	T	19	14	--	--
	Ca(ICCV2/PI489777)SNP_21426	347	A	T	18	14	--	--
CakTC38968	Ca(ICCV2/PI489777)SNP_21427	881	T	C	23	26	--	--
	Ca(ICCV2/PI489777)SNP_21428	940	C	T	21	19	--	--
	Ca(ICCV2/PI489777)SNP_21429	982	T	A	16	15	--	--
CakTC36210	Ca(ICCV2/PI489777)SNP_21430	59	T	G	3	3	--	--
	Ca(ICCV2/PI489777)SNP_21431	186	A	G	17	10	--	--
CakTC39727	Ca(ICCV2/PI489777)SNP_21432	412	T	A	5	3	--	--
	Ca(ICCV2/PI489777)SNP_21433	649	C	T	7	3	--	--
	Ca(ICCV2/PI489777)SNP_21434	751	C	T	10	4	--	--
	Ca(ICCV2/PI489777)SNP_21435	802	A	G	9	5	--	--
	Ca(ICCV2/PI489777)SNP_21436	832	G	T	8	5	--	--
	Ca(ICCV2/PI489777)SNP_21437	1009	T	C	4	4	--	--
CakTC29940	Ca(ICCV2/PI489777)SNP_21438	83	T	C	14	5	--	--
	Ca(ICCV2/PI489777)SNP_21439	159	T	A	15	5	--	--
	Ca(ICCV2/PI489777)SNP_21440	337	G	A	19	7	--	--
	Ca(ICCV2/PI489777)SNP_21441	1959	T	C	23	3	--	--
CakTC08473	Ca(ICCV2/PI489777)SNP_21442	1287	G	C	3	3	--	--
CakTC33901	Ca(ICCV2/PI489777)SNP_21443	464	A	T	7	8	--	--
	Ca(ICCV2/PI489777)SNP_21444	477	C	T	6	9	--	--
CakTC33906	Ca(ICCV2/PI489777)SNP_21445	164	G	T	11	10	--	--
	Ca(ICCV2/PI489777)SNP_21446	457	T	G	19	12	--	--
CakTC37409	Ca(ICCV2/PI489777)SNP_21447	406	T	C	6	4	Young_pod	--
	Ca(ICCV2/PI489777)SNP_21448	465	C	A	5	3	Young_pod	--
	Ca(ICCV2/PI489777)SNP_21449	537	G	A	5	3	Young_pod	--
	Ca(ICCV2/PI489777)SNP_21450	838	G	A	7	5	Young_pod	--
	Ca(ICCV2/PI489777)SNP_21451	902	T	G	10	4	Young_pod	--
CakTC41244	Ca(ICCV2/PI489777)SNP_21452	91	C	A	28	10	--	--
	Ca(ICCV2/PI489777)SNP_21453	249	A	C	34	11	--	--
	Ca(ICCV2/PI489777)SNP_21454	436	C	T	36	10	--	--
	Ca(ICCV2/PI489777)SNP_21455	461	G	T	35	9	--	--

	Ca(ICCV2/PI489777)SNP_21456	497	C	G	18	3	--	--
CakTC35938	Ca(ICCV2/PI489777)SNP_21457	222	T	C	21	6	--	--
CakTC43132	Ca(ICCV2/PI489777)SNP_21458	1004	A	C	9	12	--	--
	Ca(ICCV2/PI489777)SNP_21459	1326	A	G	13	15	--	--
	Ca(ICCV2/PI489777)SNP_21460	1705	G	A	4	8	--	--
	Ca(ICCV2/PI489777)SNP_21461	1719	A	G	3	8	--	--
CakTC22766	Ca(ICCV2/PI489777)SNP_21462	403	C	G	15	5	--	--
CakTC39621	Ca(ICCV2/PI489777)SNP_21463	850	T	A	9	8	Flower	bud
CakTC29876	Ca(ICCV2/PI489777)SNP_21464	648	T	C	11	7	--	--
	Ca(ICCV2/PI489777)SNP_21465	1527	T	C	7	5	--	--
CakTC25009	Ca(ICCV2/PI489777)SNP_21466	498	T	G	8	5	Flower	bud
CakTC43002	Ca(ICCV2/PI489777)SNP_21467	523	C	T	10	4	--	--
CakTC38606	Ca(ICCV2/PI489777)SNP_21468	267	A	G	4	13	--	C2C2-GATA
	Ca(ICCV2/PI489777)SNP_21469	279	T	C	5	13	--	C2C2-GATA
	Ca(ICCV2/PI489777)SNP_21470	463	A	C	8	10	--	C2C2-GATA
	Ca(ICCV2/PI489777)SNP_21471	490	G	C	11	8	--	C2C2-GATA
	Ca(ICCV2/PI489777)SNP_21472	641	A	G	12	4	--	C2C2-GATA
	Ca(ICCV2/PI489777)SNP_21473	651	C	T	13	4	--	C2C2-GATA
	Ca(ICCV2/PI489777)SNP_21474	681	G	C	13	4	--	C2C2-GATA
	Ca(ICCV2/PI489777)SNP_21475	783	A	G	19	9	--	C2C2-GATA
CakTC25446	Ca(ICCV2/PI489777)SNP_21476	531	T	G	10	4	--	--
	Ca(ICCV2/PI489777)SNP_21477	669	A	G	9	5	--	--
	Ca(ICCV2/PI489777)SNP_21478	677	C	T	10	5	--	--
	Ca(ICCV2/PI489777)SNP_21479	931	G	C	6	3	--	--
	Ca(ICCV2/PI489777)SNP_21480	1463	A	G	11	4	--	--
	Ca(ICCV2/PI489777)SNP_21481	1630	T	G	7	3	--	--
	Ca(ICCV2/PI489777)SNP_21482	1912	A	C	6	5	--	--
	Ca(ICCV2/PI489777)SNP_21483	2126	G	A	5	4	--	--
	Ca(ICCV2/PI489777)SNP_21484	2438	C	T	6	3	--	--
CakTC26869	Ca(ICCV2/PI489777)SNP_21485	128	T	G	99	3	--	--
CakTC35675	Ca(ICCV2/PI489777)SNP_21486	859	A	C	14	5	--	--
	Ca(ICCV2/PI489777)SNP_21487	2513	C	T	30	7	--	--
	Ca(ICCV2/PI489777)SNP_21488	2848	G	A	34	10	--	--
	Ca(ICCV2/PI489777)SNP_21489	3197	A	G	13	12	--	--
	Ca(ICCV2/PI489777)SNP_21490	3222	G	A	13	12	--	--
	Ca(ICCV2/PI489777)SNP_21491	3272	G	C	13	11	--	--
CakTC28757	Ca(ICCV2/PI489777)SNP_21492	436	G	C	3	6	--	--
CakTC38827	Ca(ICCV2/PI489777)SNP_21493	1173	A	T	7	9	--	--
	Ca(ICCV2/PI489777)SNP_21494	1255	C	T	6	10	--	--
	Ca(ICCV2/PI489777)SNP_21495	1259	G	A	4	9	--	--
CakTC37811	Ca(ICCV2/PI489777)SNP_21496	290	G	T	4	4	--	--
	Ca(ICCV2/PI489777)SNP_21497	332	C	G	3	4	--	--
	Ca(ICCV2/PI489777)SNP_21498	1039	A	G	9	3	--	--
	Ca(ICCV2/PI489777)SNP_21499	1174	G	A	7	3	--	--
CakTC38914	Ca(ICCV2/PI489777)SNP_21500	370	T	G	19	5	--	--
	Ca(ICCV2/PI489777)SNP_21501	559	A	T	17	7	--	--
CakTC10421	Ca(ICCV2/PI489777)SNP_21502	219	T	C	107	62	--	--
	Ca(ICCV2/PI489777)SNP_21503	1143	A	C	159	80	--	--
CakTC24477	Ca(ICCV2/PI489777)SNP_21504	104	T	C	21	7	--	--
	Ca(ICCV2/PI489777)SNP_21505	105	G	A	20	6	--	--
	Ca(ICCV2/PI489777)SNP_21506	230	T	A	29	9	--	--
	Ca(ICCV2/PI489777)SNP_21507	291	A	G	32	14	--	--
	Ca(ICCV2/PI489777)SNP_21508	743	C	T	56	17	--	--
CakTC39505	Ca(ICCV2/PI489777)SNP_21509	238	G	A	12	4	--	--
	Ca(ICCV2/PI489777)SNP_21510	343	G	C	15	5	--	--
CakTC24657	Ca(ICCV2/PI489777)SNP_21511	228	T	C	20	14	--	--
	Ca(ICCV2/PI489777)SNP_21512	326	G	T	26	18	--	--
	Ca(ICCV2/PI489777)SNP_21513	1172	G	C	63	25	--	--
	Ca(ICCV2/PI489777)SNP_21514	2329	G	A	28	22	--	--
	Ca(ICCV2/PI489777)SNP_21515	3148	C	A	34	8	--	--
CakTC41710	Ca(ICCV2/PI489777)SNP_21516	48	C	T	7	5	--	--
	Ca(ICCV2/PI489777)SNP_21517	189	T	G	23	9	--	--
	Ca(ICCV2/PI489777)SNP_21518	479	C	T	34	11	--	--
	Ca(ICCV2/PI489777)SNP_21519	1070	C	T	34	5	--	--
	Ca(ICCV2/PI489777)SNP_21520	1185	T	C	31	5	--	--
	Ca(ICCV2/PI489777)SNP_21521	1514	C	T	16	13	--	--
	Ca(ICCV2/PI489777)SNP_21522	1721	A	T	22	13	--	--
	Ca(ICCV2/PI489777)SNP_21523	1739	T	G	21	12	--	--
	Ca(ICCV2/PI489777)SNP_21524	1946	A	G	24	9	--	--
	Ca(ICCV2/PI489777)SNP_21525	1969	A	T	24	10	--	--
	Ca(ICCV2/PI489777)SNP_21526	1980	G	A	21	10	--	--
	Ca(ICCV2/PI489777)SNP_21527	2285	C	T	13	3	--	--
CakTC24726	Ca(ICCV2/PI489777)SNP_21528	1324	C	G	20	9	--	--
CakTC26360	Ca(ICCV2/PI489777)SNP_21529	681	C	T	18	16	--	--

	Ca(ICCV2/PI489777)SNP_21530	1732	G	A	5	5	--	--
	Ca(ICCV2/PI489777)SNP_21531	1733	A	T	4	5	--	--
CakTC33668	Ca(ICCV2/PI489777)SNP_21532	926	G	C	3	4	--	--
	Ca(ICCV2/PI489777)SNP_21533	1143	A	T	3	3	--	--
CakTC41153	Ca(ICCV2/PI489777)SNP_21534	1490	T	C	20	4	--	--
	Ca(ICCV2/PI489777)SNP_21535	1599	G	A	16	4	--	--
	Ca(ICCV2/PI489777)SNP_21536	2068	G	A	20	5	--	--
CakTC37453	Ca(ICCV2/PI489777)SNP_21537	390	A	G	11	7	--	--
	Ca(ICCV2/PI489777)SNP_21538	820	T	C	9	9	--	--
	Ca(ICCV2/PI489777)SNP_21539	874	T	C	9	8	--	--
CakTC11631	Ca(ICCV2/PI489777)SNP_21540	85	A	T	9	6	--	--
	Ca(ICCV2/PI489777)SNP_21541	110	G	C	10	5	--	--
	Ca(ICCV2/PI489777)SNP_21542	490	G	C	16	6	--	--
	Ca(ICCV2/PI489777)SNP_21543	756	T	C	5	5	--	--
	Ca(ICCV2/PI489777)SNP_21544	796	A	T	5	5	--	--
CakTC25951	Ca(ICCV2/PI489777)SNP_21545	230	G	A	24	24	--	--
	Ca(ICCV2/PI489777)SNP_21546	689	A	G	98	54	--	--
	Ca(ICCV2/PI489777)SNP_21547	950	C	T	98	38	--	--
CakTC32161	Ca(ICCV2/PI489777)SNP_21548	355	A	C	9	6	--	--
	Ca(ICCV2/PI489777)SNP_21549	421	A	C	14	5	--	--
	Ca(ICCV2/PI489777)SNP_21550	454	A	G	13	6	--	--
	Ca(ICCV2/PI489777)SNP_21551	726	T	C	22	8	--	--
	Ca(ICCV2/PI489777)SNP_21552	1030	A	G	12	4	--	--
	Ca(ICCV2/PI489777)SNP_21553	1110	A	G	10	4	--	--
CakTC28480	Ca(ICCV2/PI489777)SNP_21554	919	A	G	12	3	--	--
	Ca(ICCV2/PI489777)SNP_21555	1000	A	G	17	3	--	--
	Ca(ICCV2/PI489777)SNP_21556	1040	G	A	15	3	--	--
CakTC40910	Ca(ICCV2/PI489777)SNP_21557	1370	C	T	16	7	--	--
	Ca(ICCV2/PI489777)SNP_21558	1475	G	A	20	10	--	--
	Ca(ICCV2/PI489777)SNP_21559	1911	C	T	14	11	--	--
CakTC26713	Ca(ICCV2/PI489777)SNP_21560	483	C	T	57	39	--	AP2-EREBP
	Ca(ICCV2/PI489777)SNP_21561	848	G	A	128	47	--	AP2-EREBP
CakTC11602	Ca(ICCV2/PI489777)SNP_21562	252	G	C	14	5	--	--
	Ca(ICCV2/PI489777)SNP_21563	767	A	G	16	13	--	--
CakTC25444	Ca(ICCV2/PI489777)SNP_21564	211	C	A	15	5	--	--
CakTC30401	Ca(ICCV2/PI489777)SNP_21565	440	T	C	10	3	--	--
	Ca(ICCV2/PI489777)SNP_21566	500	A	T	11	4	--	--
CakTC40541	Ca(ICCV2/PI489777)SNP_21567	71	T	G	25	23	--	--
	Ca(ICCV2/PI489777)SNP_21568	212	T	C	29	28	--	--
	Ca(ICCV2/PI489777)SNP_21569	647	C	T	14	11	--	--
	Ca(ICCV2/PI489777)SNP_21570	740	T	C	3	8	--	--
CakTC29987	Ca(ICCV2/PI489777)SNP_21571	1477	G	A	18	4	--	--
	Ca(ICCV2/PI489777)SNP_21572	2215	T	G	8	6	--	--
CakTC34612	Ca(ICCV2/PI489777)SNP_21573	360	C	G	3	6	--	--
CakTC36129	Ca(ICCV2/PI489777)SNP_21574	164	G	A	9	4	--	--
	Ca(ICCV2/PI489777)SNP_21575	194	G	A	8	5	--	--
	Ca(ICCV2/PI489777)SNP_21576	332	A	T	11	6	--	--
	Ca(ICCV2/PI489777)SNP_21577	401	T	C	11	5	--	--
	Ca(ICCV2/PI489777)SNP_21578	586	G	A	15	4	--	--
CakTC42166	Ca(ICCV2/PI489777)SNP_21579	399	A	G	15	6	--	--
	Ca(ICCV2/PI489777)SNP_21580	1903	C	T	5	22	--	--
	Ca(ICCV2/PI489777)SNP_21581	1976	A	G	7	19	--	--
CakTC27190	Ca(ICCV2/PI489777)SNP_21582	495	T	A	23	4	--	--
	Ca(ICCV2/PI489777)SNP_21583	750	A	G	54	15	--	--
CakTC28476	Ca(ICCV2/PI489777)SNP_21584	793	A	G	9	13	--	--
CakTC09333	Ca(ICCV2/PI489777)SNP_21585	830	G	A	4	7	--	--
	Ca(ICCV2/PI489777)SNP_21586	1063	C	T	13	3	--	--
CakTC26268	Ca(ICCV2/PI489777)SNP_21587	1107	G	A	10	5	--	--
	Ca(ICCV2/PI489777)SNP_21588	2149	A	G	5	4	--	--
CakTC42893	Ca(ICCV2/PI489777)SNP_21589	630	C	A	29	15	--	--
	Ca(ICCV2/PI489777)SNP_21590	678	G	A	28	15	--	--
	Ca(ICCV2/PI489777)SNP_21591	755	T	C	14	4	--	--
	Ca(ICCV2/PI489777)SNP_21592	802	C	G	9	4	--	--
CakTC41875	Ca(ICCV2/PI489777)SNP_21593	157	T	C	6	3	--	--
	Ca(ICCV2/PI489777)SNP_21594	537	A	T	11	3	--	--
CakTC38209	Ca(ICCV2/PI489777)SNP_21595	212	C	G	24	6	--	--
	Ca(ICCV2/PI489777)SNP_21596	385	C	A	25	5	--	--
	Ca(ICCV2/PI489777)SNP_21597	625	A	G	9	3	--	--
CakTC40778	Ca(ICCV2/PI489777)SNP_21598	1006	G	T	8	5	--	--
CakTC39227	Ca(ICCV2/PI489777)SNP_21599	172	C	G	11	3	--	--
CakTC10944	Ca(ICCV2/PI489777)SNP_21600	857	T	C	9	6	--	--
	Ca(ICCV2/PI489777)SNP_21601	1044	A	G	6	4	--	--
	Ca(ICCV2/PI489777)SNP_21602	1049	A	G	6	4	--	--
CakTC23971	Ca(ICCV2/PI489777)SNP_21603	1169	G	A	8	5	--	--

	Ca(ICCV2/PI489777)SNP_21604	3147	C	T	10	12	--	--
	Ca(ICCV2/PI489777)SNP_21605	3183	C	T	7	10	--	--
	Ca(ICCV2/PI489777)SNP_21606	3342	A	T	18	7	--	--
CakTC33742	Ca(ICCV2/PI489777)SNP_21607	512	G	T	3	12	--	CCAAT
CakTC28157	Ca(ICCV2/PI489777)SNP_21608	307	C	T	4	3	--	ABI3VP1
	Ca(ICCV2/PI489777)SNP_21609	750	A	G	13	8	--	ABI3VP1
	Ca(ICCV2/PI489777)SNP_21610	833	G	A	22	6	--	ABI3VP1
	Ca(ICCV2/PI489777)SNP_21611	1106	A	T	29	14	--	ABI3VP1
CakTC33260	Ca(ICCV2/PI489777)SNP_21612	504	A	C	12	14	--	--
	Ca(ICCV2/PI489777)SNP_21613	595	T	C	13	8	--	--
CakTC22537	Ca(ICCV2/PI489777)SNP_21614	161	C	T	6	5	--	--
	Ca(ICCV2/PI489777)SNP_21615	252	C	G	6	5	--	--
	Ca(ICCV2/PI489777)SNP_21616	380	C	A	11	8	--	--
	Ca(ICCV2/PI489777)SNP_21617	418	T	A	9	8	--	--
	Ca(ICCV2/PI489777)SNP_21618	1114	G	A	24	12	--	--
	Ca(ICCV2/PI489777)SNP_21619	1534	C	T	19	15	--	--
	Ca(ICCV2/PI489777)SNP_21620	2965	A	G	3	3	--	--
CakTC07528	Ca(ICCV2/PI489777)SNP_21621	77	A	G	18	4	--	--
	Ca(ICCV2/PI489777)SNP_21622	116	A	C	22	6	--	--
	Ca(ICCV2/PI489777)SNP_21623	170	G	T	24	6	--	--
	Ca(ICCV2/PI489777)SNP_21624	221	G	C	26	8	--	--
	Ca(ICCV2/PI489777)SNP_21625	245	G	A	26	7	--	--
	Ca(ICCV2/PI489777)SNP_21626	398	T	C	23	5	--	--
	Ca(ICCV2/PI489777)SNP_21627	734	G	C	18	7	--	--
	Ca(ICCV2/PI489777)SNP_21628	749	G	A	17	7	--	--
CakTC29902	Ca(ICCV2/PI489777)SNP_21629	1497	C	T	17	13	--	--
CakTC33139	Ca(ICCV2/PI489777)SNP_21630	174	T	C	9	6	--	--
	Ca(ICCV2/PI489777)SNP_21631	211	G	A	9	8	--	--
	Ca(ICCV2/PI489777)SNP_21632	233	A	G	11	8	--	--
	Ca(ICCV2/PI489777)SNP_21633	395	C	T	9	9	--	--
	Ca(ICCV2/PI489777)SNP_21634	407	T	C	7	5	--	--
	Ca(ICCV2/PI489777)SNP_21635	588	A	G	4	5	--	--
	Ca(ICCV2/PI489777)SNP_21636	707	G	A	8	5	--	--
	Ca(ICCV2/PI489777)SNP_21637	1895	G	T	12	9	--	--
CakTC36677	Ca(ICCV2/PI489777)SNP_21638	142	C	T	12	3	--	TPR
	Ca(ICCV2/PI489777)SNP_21639	184	G	A	13	3	--	TPR
	Ca(ICCV2/PI489777)SNP_21640	496	G	C	13	6	--	TPR
	Ca(ICCV2/PI489777)SNP_21641	1969	G	A	14	5	--	TPR
	Ca(ICCV2/PI489777)SNP_21642	1995	C	T	12	5	--	TPR
CakTC40709	Ca(ICCV2/PI489777)SNP_21643	1198	T	C	38	14	--	--
	Ca(ICCV2/PI489777)SNP_21644	1803	T	G	21	11	--	--
CakTC33844	Ca(ICCV2/PI489777)SNP_21645	443	T	C	5	6	--	--
CakTC26545	Ca(ICCV2/PI489777)SNP_21646	591	C	T	53	25	--	--
	Ca(ICCV2/PI489777)SNP_21647	631	T	C	42	23	--	--
	Ca(ICCV2/PI489777)SNP_21648	927	A	G	65	12	--	--
CakTC22931	Ca(ICCV2/PI489777)SNP_21649	476	T	C	7	35	--	--
	Ca(ICCV2/PI489777)SNP_21650	701	C	A	13	24	--	--
	Ca(ICCV2/PI489777)SNP_21651	925	A	T	7	6	--	--
	Ca(ICCV2/PI489777)SNP_21652	984	G	T	6	5	--	--
	Ca(ICCV2/PI489777)SNP_21653	2223	C	T	5	6	--	--
CakTC40779	Ca(ICCV2/PI489777)SNP_21654	1121	A	G	6	13	--	BSD
	Ca(ICCV2/PI489777)SNP_21655	1626	C	T	9	8	--	BSD
CakTC28638	Ca(ICCV2/PI489777)SNP_21656	307	C	T	93	53	--	--
CakTC43154	Ca(ICCV2/PI489777)SNP_21657	96	G	C	4	7	--	--
	Ca(ICCV2/PI489777)SNP_21658	97	C	T	5	7	--	--
	Ca(ICCV2/PI489777)SNP_21659	124	C	T	5	7	--	--
	Ca(ICCV2/PI489777)SNP_21660	190	G	A	5	6	--	--
	Ca(ICCV2/PI489777)SNP_21661	252	A	G	5	6	--	--
	Ca(ICCV2/PI489777)SNP_21662	788	A	G	3	4	--	--
CakTC01820	Ca(ICCV2/PI489777)SNP_21663	423	G	A	5	7	--	--
	Ca(ICCV2/PI489777)SNP_21664	786	C	G	3	3	--	--
CakTC31256	Ca(ICCV2/PI489777)SNP_21665	449	A	G	4	6	--	--
	Ca(ICCV2/PI489777)SNP_21666	877	T	C	9	5	--	--
	Ca(ICCV2/PI489777)SNP_21667	893	T	C	8	4	--	--
CakTC43143	Ca(ICCV2/PI489777)SNP_21668	118	C	A	7	3	--	--
CakTC42985	Ca(ICCV2/PI489777)SNP_21669	52	A	G	3	4	--	--
	Ca(ICCV2/PI489777)SNP_21670	810	C	A	48	21	--	--
	Ca(ICCV2/PI489777)SNP_21671	1215	T	C	27	9	--	--
	Ca(ICCV2/PI489777)SNP_21672	1427	C	G	35	15	--	--
	Ca(ICCV2/PI489777)SNP_21673	1449	A	G	33	17	--	--
CakTC32465	Ca(ICCV2/PI489777)SNP_21674	345	T	G	22	16	--	--
	Ca(ICCV2/PI489777)SNP_21675	494	A	G	20	30	--	--
	Ca(ICCV2/PI489777)SNP_21676	796	G	A	25	32	--	--
	Ca(ICCV2/PI489777)SNP_21677	1834	T	C	19	15	--	--

	Ca(ICCV2/PI489777)SNP_21678	1993	T	A	17	12	--	--
	Ca(ICCV2/PI489777)SNP_21679	2047	C	T	18	11	--	--
CakTC41704	Ca(ICCV2/PI489777)SNP_21680	1310	C	T	12	5	--	--
CakTC40266	Ca(ICCV2/PI489777)SNP_21681	503	G	C	5	5	--	GRAS
	Ca(ICCV2/PI489777)SNP_21682	540	C	T	9	5	--	GRAS
	Ca(ICCV2/PI489777)SNP_21683	885	C	T	12	6	--	GRAS
	Ca(ICCV2/PI489777)SNP_21684	1041	G	A	8	6	--	GRAS
	Ca(ICCV2/PI489777)SNP_21685	1104	A	G	11	5	--	GRAS
	Ca(ICCV2/PI489777)SNP_21686	1182	G	T	11	4	--	GRAS
	Ca(ICCV2/PI489777)SNP_21687	1464	G	C	15	6	--	GRAS
	Ca(ICCV2/PI489777)SNP_21688	1686	C	G	11	5	--	GRAS
CakTC08914	Ca(ICCV2/PI489777)SNP_21689	696	T	C	6	3	--	--
CakTC29945	Ca(ICCV2/PI489777)SNP_21690	1475	T	C	6	4	--	--
CakTC38820	Ca(ICCV2/PI489777)SNP_21691	290	T	C	3	3	--	--
	Ca(ICCV2/PI489777)SNP_21692	901	G	C	7	5	--	--
CakTC26550	Ca(ICCV2/PI489777)SNP_21693	332	C	T	13	5	Flower	bud
	Ca(ICCV2/PI489777)SNP_21694	1020	A	G	10	4	Flower	bud
	Ca(ICCV2/PI489777)SNP_21695	1231	T	C	8	5	Flower	bud
	Ca(ICCV2/PI489777)SNP_21696	1236	T	C	9	5	Flower	bud
	Ca(ICCV2/PI489777)SNP_21697	1762	A	G	6	3	Flower	bud
CakTC10294	Ca(ICCV2/PI489777)SNP_21698	297	C	T	5	4	--	--
CakTC07812	Ca(ICCV2/PI489777)SNP_21699	1352	T	C	4	3	--	RB
CakTC42639	Ca(ICCV2/PI489777)SNP_21700	257	C	T	30	10	--	--
	Ca(ICCV2/PI489777)SNP_21701	266	T	C	36	10	--	--
CakTC19756	Ca(ICCV2/PI489777)SNP_21702	198	A	G	3	3	Shoot	--
	Ca(ICCV2/PI489777)SNP_21703	318	A	C	3	4	Shoot	--
	Ca(ICCV2/PI489777)SNP_21704	378	C	T	5	6	Shoot	--
	Ca(ICCV2/PI489777)SNP_21705	417	C	T	4	5	Shoot	--
	Ca(ICCV2/PI489777)SNP_21706	420	T	C	4	5	Shoot	--
	Ca(ICCV2/PI489777)SNP_21707	570	A	G	5	7	Shoot	--
	Ca(ICCV2/PI489777)SNP_21708	619	T	C	6	5	Shoot	--
	Ca(ICCV2/PI489777)SNP_21709	732	C	T	5	3	Shoot	--
CakTC40102	Ca(ICCV2/PI489777)SNP_21710	1145	T	G	3	3	--	--
CakTC39124	Ca(ICCV2/PI489777)SNP_21711	784	C	T	20	3	--	--
	Ca(ICCV2/PI489777)SNP_21712	1349	G	A	15	7	--	--
	Ca(ICCV2/PI489777)SNP_21713	1400	A	G	14	11	--	--
	Ca(ICCV2/PI489777)SNP_21714	1412	A	T	14	9	--	--
	Ca(ICCV2/PI489777)SNP_21715	1421	C	T	13	8	--	--
CakTC38092	Ca(ICCV2/PI489777)SNP_21716	839	T	C	3	5	--	MADS
CakTC41141	Ca(ICCV2/PI489777)SNP_21717	130	C	G	6	7	--	--
	Ca(ICCV2/PI489777)SNP_21718	269	C	T	17	11	--	--
	Ca(ICCV2/PI489777)SNP_21719	352	A	G	28	18	--	--
	Ca(ICCV2/PI489777)SNP_21720	1774	G	A	13	29	--	--
	Ca(ICCV2/PI489777)SNP_21721	1906	A	G	24	24	--	--
	Ca(ICCV2/PI489777)SNP_21722	1918	C	G	25	27	--	--
CakTC40042	Ca(ICCV2/PI489777)SNP_21723	895	G	A	22	7	--	--
	Ca(ICCV2/PI489777)SNP_21724	1027	T	A	23	5	--	--
	Ca(ICCV2/PI489777)SNP_21725	1183	A	T	10	3	--	--
CakTC43291	Ca(ICCV2/PI489777)SNP_21726	639	A	G	13	9	--	--
CakTC42528	Ca(ICCV2/PI489777)SNP_21727	324	G	A	5	3	--	--
	Ca(ICCV2/PI489777)SNP_21728	361	G	T	6	3	--	--
	Ca(ICCV2/PI489777)SNP_21729	636	T	A	4	8	--	--
	Ca(ICCV2/PI489777)SNP_21730	708	A	G	4	6	--	--
	Ca(ICCV2/PI489777)SNP_21731	807	T	C	7	5	--	--
CakTC34673	Ca(ICCV2/PI489777)SNP_21732	325	C	A	11	10	--	--
	Ca(ICCV2/PI489777)SNP_21733	365	A	T	9	8	--	--
	Ca(ICCV2/PI489777)SNP_21734	498	A	C	9	6	--	--
	Ca(ICCV2/PI489777)SNP_21735	519	T	C	8	3	--	--
	Ca(ICCV2/PI489777)SNP_21736	690	C	T	7	5	--	--
	Ca(ICCV2/PI489777)SNP_21737	772	G	T	7	5	--	--
	Ca(ICCV2/PI489777)SNP_21738	936	G	A	10	5	--	--
	Ca(ICCV2/PI489777)SNP_21739	1059	T	G	12	7	--	--
	Ca(ICCV2/PI489777)SNP_21740	1306	C	A	19	7	--	--
	Ca(ICCV2/PI489777)SNP_21741	1478	T	C	12	5	--	--
	Ca(ICCV2/PI489777)SNP_21742	1588	G	A	11	13	--	--
	Ca(ICCV2/PI489777)SNP_21743	2040	G	C	13	20	--	--
	Ca(ICCV2/PI489777)SNP_21744	2283	A	G	16	9	--	--
	Ca(ICCV2/PI489777)SNP_21745	2520	T	C	11	16	--	--
	Ca(ICCV2/PI489777)SNP_21746	2539	G	A	6	17	--	--
	Ca(ICCV2/PI489777)SNP_21747	2613	G	A	12	19	--	--
	Ca(ICCV2/PI489777)SNP_21748	2691	T	C	13	17	--	--
	Ca(ICCV2/PI489777)SNP_21749	2715	T	C	15	18	--	--
	Ca(ICCV2/PI489777)SNP_21750	2832	T	C	16	16	--	--
	Ca(ICCV2/PI489777)SNP_21751	2841	C	G	17	16	--	--

	Ca(ICCV2/PI489777)SNP_21752	2931	C	A	15	16	--	--
	Ca(ICCV2/PI489777)SNP_21753	2958	T	C	18	17	--	--
	Ca(ICCV2/PI489777)SNP_21754	3273	T	A	11	12	--	--
	Ca(ICCV2/PI489777)SNP_21755	3330	T	A	5	12	--	--
CakTC37801	Ca(ICCV2/PI489777)SNP_21756	114	A	T	32	6	--	--
	Ca(ICCV2/PI489777)SNP_21757	151	A	G	36	8	--	--
	Ca(ICCV2/PI489777)SNP_21758	243	G	A	41	9	--	--
	Ca(ICCV2/PI489777)SNP_21759	322	A	G	39	11	--	--
	Ca(ICCV2/PI489777)SNP_21760	394	T	C	39	7	--	--
	Ca(ICCV2/PI489777)SNP_21761	594	T	G	23	6	--	--
	Ca(ICCV2/PI489777)SNP_21762	884	A	G	9	4	--	--
CakTC38921	Ca(ICCV2/PI489777)SNP_21763	1672	T	C	4	3	Flower	bud
CakTC24695	Ca(ICCV2/PI489777)SNP_21764	277	G	C	15	9	Mature	Leaf
	Ca(ICCV2/PI489777)SNP_21765	496	C	T	20	10	Mature	Leaf
	Ca(ICCV2/PI489777)SNP_21766	554	G	C	20	11	Mature	Leaf
	Ca(ICCV2/PI489777)SNP_21767	639	G	A	16	6	Mature	Leaf
CakTC29951	Ca(ICCV2/PI489777)SNP_21768	493	C	T	11	4	Shoot	HB
	Ca(ICCV2/PI489777)SNP_21769	991	T	A	12	3	Shoot	HB
	Ca(ICCV2/PI489777)SNP_21770	1600	T	C	4	3	Shoot	HB
CakTC41707	Ca(ICCV2/PI489777)SNP_21771	542	A	G	29	20	--	--
	Ca(ICCV2/PI489777)SNP_21772	866	G	A	23	25	--	--
	Ca(ICCV2/PI489777)SNP_21773	1865	G	C	15	6	--	--
	Ca(ICCV2/PI489777)SNP_21774	1910	G	C	8	4	--	--
	Ca(ICCV2/PI489777)SNP_21775	2214	C	T	4	7	--	--
	Ca(ICCV2/PI489777)SNP_21776	2265	C	A	9	8	--	--
	Ca(ICCV2/PI489777)SNP_21777	2980	G	C	10	13	--	--
	Ca(ICCV2/PI489777)SNP_21778	3063	G	A	8	12	--	--
	Ca(ICCV2/PI489777)SNP_21779	3366	C	T	5	8	--	--
	Ca(ICCV2/PI489777)SNP_21780	3786	C	T	4	6	--	--
	Ca(ICCV2/PI489777)SNP_21781	3897	T	C	5	5	--	--
	Ca(ICCV2/PI489777)SNP_21782	4016	G	A	7	5	--	--
CakTC40342	Ca(ICCV2/PI489777)SNP_21783	676	C	T	18	3	--	--
	Ca(ICCV2/PI489777)SNP_21784	1890	G	C	14	5	--	--
CakTC11672	Ca(ICCV2/PI489777)SNP_21785	924	T	G	3	6	--	--
	Ca(ICCV2/PI489777)SNP_21786	950	T	C	4	6	--	--
CakTC36699	Ca(ICCV2/PI489777)SNP_21787	1110	G	T	16	3	--	--
	Ca(ICCV2/PI489777)SNP_21788	1343	C	T	17	9	--	--
	Ca(ICCV2/PI489777)SNP_21789	1407	A	G	16	9	--	--
	Ca(ICCV2/PI489777)SNP_21790	1527	C	T	8	8	--	--
	Ca(ICCV2/PI489777)SNP_21791	1910	G	A	6	3	--	--
	Ca(ICCV2/PI489777)SNP_21792	1990	G	A	7	3	--	--
	Ca(ICCV2/PI489777)SNP_21793	2236	T	C	8	3	--	--
	Ca(ICCV2/PI489777)SNP_21794	2240	C	T	8	3	--	--
	Ca(ICCV2/PI489777)SNP_21795	2259	A	G	8	3	--	--
CakTC26152	Ca(ICCV2/PI489777)SNP_21796	331	A	C	19	5	--	--
	Ca(ICCV2/PI489777)SNP_21797	1486	A	G	15	4	--	--
	Ca(ICCV2/PI489777)SNP_21798	3412	G	A	13	5	--	--
CakTC32701	Ca(ICCV2/PI489777)SNP_21799	658	C	T	9	6	--	--
	Ca(ICCV2/PI489777)SNP_21800	885	G	A	13	6	--	--
	Ca(ICCV2/PI489777)SNP_21801	1825	T	G	4	4	--	--
	Ca(ICCV2/PI489777)SNP_21802	1831	C	G	5	4	--	--
	Ca(ICCV2/PI489777)SNP_21803	1977	T	C	6	7	--	--
CakTC41486	Ca(ICCV2/PI489777)SNP_21804	199	A	G	3	8	--	--
CakTC40524	Ca(ICCV2/PI489777)SNP_21805	94	G	A	12	4	Young_pod	--
	Ca(ICCV2/PI489777)SNP_21806	142	A	C	13	3	Young_pod	--
	Ca(ICCV2/PI489777)SNP_21807	355	A	C	17	7	Young_pod	--
	Ca(ICCV2/PI489777)SNP_21808	369	G	A	17	7	Young_pod	--
	Ca(ICCV2/PI489777)SNP_21809	551	C	A	8	6	Young_pod	--
	Ca(ICCV2/PI489777)SNP_21810	599	T	C	6	5	Young_pod	--
CakTC41010	Ca(ICCV2/PI489777)SNP_21811	380	A	G	7	6	--	--
	Ca(ICCV2/PI489777)SNP_21812	498	T	A	9	4	--	--
	Ca(ICCV2/PI489777)SNP_21813	542	T	C	8	7	--	--
	Ca(ICCV2/PI489777)SNP_21814	563	A	T	10	8	--	--
	Ca(ICCV2/PI489777)SNP_21815	726	T	C	9	3	--	--
CakTC25512	Ca(ICCV2/PI489777)SNP_21816	1331	A	G	28	11	--	--
CakTC40580	Ca(ICCV2/PI489777)SNP_21817	186	T	C	3	4	--	--
CakTC38388	Ca(ICCV2/PI489777)SNP_21818	226	G	A	19	9	--	--
	Ca(ICCV2/PI489777)SNP_21819	456	C	T	11	7	--	--
	Ca(ICCV2/PI489777)SNP_21820	491	C	T	16	6	--	--
	Ca(ICCV2/PI489777)SNP_21821	572	G	A	22	6	--	--
	Ca(ICCV2/PI489777)SNP_21822	624	T	C	24	5	--	--
	Ca(ICCV2/PI489777)SNP_21823	979	C	T	22	6	--	--
CakTC43283	Ca(ICCV2/PI489777)SNP_21824	385	A	C	54	14	--	AP2-EREBP
	Ca(ICCV2/PI489777)SNP_21825	535	T	G	43	12	--	AP2-EREBP

	Ca(ICCV2/PI489777)SNP_21826	811	G	A	34	5	--	AP2-EREBP
	Ca(ICCV2/PI489777)SNP_21827	832	C	T	22	6	--	AP2-EREBP
CakTC23710	Ca(ICCV2/PI489777)SNP_21828	1681	G	A	10	3	--	--
CakTC12635	Ca(ICCV2/PI489777)SNP_21829	223	A	G	16	7	--	--
	Ca(ICCV2/PI489777)SNP_21830	670	C	T	6	7	--	--
CakTC29007	Ca(ICCV2/PI489777)SNP_21831	1354	T	G	20	9	--	--
CakTC27202	Ca(ICCV2/PI489777)SNP_21832	446	A	G	11	4	Root	--
CakTC25910	Ca(ICCV2/PI489777)SNP_21833	615	G	A	140	43	--	--
	Ca(ICCV2/PI489777)SNP_21834	818	C	T	113	25	--	--
	Ca(ICCV2/PI489777)SNP_21835	1177	T	C	78	31	--	--
	Ca(ICCV2/PI489777)SNP_21836	1193	A	G	98	36	--	--
	Ca(ICCV2/PI489777)SNP_21837	1375	C	T	59	17	--	--
	Ca(ICCV2/PI489777)SNP_21838	1733	T	C	41	9	--	--
	Ca(ICCV2/PI489777)SNP_21839	1749	C	T	40	4	--	--
	Ca(ICCV2/PI489777)SNP_21840	1769	C	A	39	6	--	--
	Ca(ICCV2/PI489777)SNP_21841	1793	A	T	32	8	--	--
CakTC35012	Ca(ICCV2/PI489777)SNP_21842	303	A	T	56	67	--	--
	Ca(ICCV2/PI489777)SNP_21843	857	G	C	50	75	--	--
	Ca(ICCV2/PI489777)SNP_21844	864	G	A	52	77	--	--
	Ca(ICCV2/PI489777)SNP_21845	2055	G	A	49	51	--	--
CakTC41117	Ca(ICCV2/PI489777)SNP_21846	99	C	T	4	6	--	--
	Ca(ICCV2/PI489777)SNP_21847	426	T	C	10	8	--	--
CakTC10934	Ca(ICCV2/PI489777)SNP_21848	894	T	A	5	6	--	--
	Ca(ICCV2/PI489777)SNP_21849	906	T	C	5	6	--	--
	Ca(ICCV2/PI489777)SNP_21850	1262	C	T	4	4	--	--
CakTC24207	Ca(ICCV2/PI489777)SNP_21851	670	G	T	35	28	--	--
	Ca(ICCV2/PI489777)SNP_21852	736	C	G	37	29	--	--
	Ca(ICCV2/PI489777)SNP_21853	1185	C	T	79	34	--	--
	Ca(ICCV2/PI489777)SNP_21854	1426	T	C	87	29	--	--
	Ca(ICCV2/PI489777)SNP_21855	1709	T	G	93	25	--	--
	Ca(ICCV2/PI489777)SNP_21856	1970	A	G	37	26	--	--
	Ca(ICCV2/PI489777)SNP_21857	2115	C	A	84	34	--	--
	Ca(ICCV2/PI489777)SNP_21858	2184	A	G	93	37	--	--
	Ca(ICCV2/PI489777)SNP_21859	2277	C	T	101	33	--	--
	Ca(ICCV2/PI489777)SNP_21860	2418	A	G	105	22	--	--
	Ca(ICCV2/PI489777)SNP_21861	2493	G	C	90	24	--	--
	Ca(ICCV2/PI489777)SNP_21862	2547	C	G	63	16	--	--
	Ca(ICCV2/PI489777)SNP_21863	2608	A	C	62	18	--	--
CakTC02511	Ca(ICCV2/PI489777)SNP_21864	309	T	C	7	12	--	--
	Ca(ICCV2/PI489777)SNP_21865	312	T	C	7	12	--	--
	Ca(ICCV2/PI489777)SNP_21866	327	G	C	9	12	--	--
CakTC36982	Ca(ICCV2/PI489777)SNP_21867	3169	A	T	50	20	--	--
CakTC36654	Ca(ICCV2/PI489777)SNP_21868	1540	A	G	7	4	--	--
CakTC32998	Ca(ICCV2/PI489777)SNP_21869	186	T	C	8	3	--	--
CakTC36880	Ca(ICCV2/PI489777)SNP_21870	385	T	C	12	8	--	--
	Ca(ICCV2/PI489777)SNP_21871	1363	C	T	6	10	--	--
	Ca(ICCV2/PI489777)SNP_21872	2401	T	C	14	5	--	--
CakTC34303	Ca(ICCV2/PI489777)SNP_21873	1531	G	A	7	5	--	--
	Ca(ICCV2/PI489777)SNP_21874	1815	A	G	16	6	--	--
CakTC06423	Ca(ICCV2/PI489777)SNP_21875	330	G	A	10	4	--	TPR
	Ca(ICCV2/PI489777)SNP_21876	1625	T	C	6	4	--	TPR
CakTC13458	Ca(ICCV2/PI489777)SNP_21877	279	T	C	5	3	--	--
	Ca(ICCV2/PI489777)SNP_21878	281	T	A	5	3	--	--
CakTC42746	Ca(ICCV2/PI489777)SNP_21879	225	C	T	11	21	--	--
	Ca(ICCV2/PI489777)SNP_21880	486	C	T	11	8	--	--
CakTC41189	Ca(ICCV2/PI489777)SNP_21881	451	G	A	28	8	--	--
	Ca(ICCV2/PI489777)SNP_21882	505	C	A	57	8	--	--
	Ca(ICCV2/PI489777)SNP_21883	591	G	T	167	16	--	--
CakTC29186	Ca(ICCV2/PI489777)SNP_21884	438	C	T	9	3	--	CCHC
	Ca(ICCV2/PI489777)SNP_21885	806	T	G	11	9	--	CCHC
CakTC26796	Ca(ICCV2/PI489777)SNP_21886	353	T	C	33	21	--	--
	Ca(ICCV2/PI489777)SNP_21887	524	A	G	35	23	--	--
CakTC24994	Ca(ICCV2/PI489777)SNP_21888	1855	G	A	13	5	--	--
	Ca(ICCV2/PI489777)SNP_21889	1983	G	C	10	3	--	--
CakTC39762	Ca(ICCV2/PI489777)SNP_21890	537	A	T	8	8	--	--
	Ca(ICCV2/PI489777)SNP_21891	550	C	T	11	10	--	--
CakTC39201	Ca(ICCV2/PI489777)SNP_21892	2443	T	C	3	6	Young_pod	--
CakTC24909	Ca(ICCV2/PI489777)SNP_21893	838	C	T	8	5	--	--
CakTC38316	Ca(ICCV2/PI489777)SNP_21894	1527	G	A	6	3	--	--
	Ca(ICCV2/PI489777)SNP_21895	1593	C	T	7	3	--	--
CakTC25474	Ca(ICCV2/PI489777)SNP_21896	800	C	G	95	43	--	--
	Ca(ICCV2/PI489777)SNP_21897	993	T	C	88	50	--	--
	Ca(ICCV2/PI489777)SNP_21898	1006	C	T	83	43	--	--
	Ca(ICCV2/PI489777)SNP_21899	1052	C	A	93	40	--	--

	Ca(ICCV2/PI489777)SNP_21900	1057	T	G	95	47	--	--
	Ca(ICCV2/PI489777)SNP_21901	1599	C	T	82	12	--	--
	Ca(ICCV2/PI489777)SNP_21902	2239	G	T	101	29	--	--
	Ca(ICCV2/PI489777)SNP_21903	2282	C	A	102	31	--	--
CakTC41324	Ca(ICCV2/PI489777)SNP_21904	185	C	T	12	4	--	--
	Ca(ICCV2/PI489777)SNP_21905	2260	C	A	17	12	--	--
	Ca(ICCV2/PI489777)SNP_21906	2425	G	T	4	3	--	--
CakTC42282	Ca(ICCV2/PI489777)SNP_21907	88	C	T	12	4	--	--
	Ca(ICCV2/PI489777)SNP_21908	622	G	A	56	46	--	--
	Ca(ICCV2/PI489777)SNP_21909	819	T	A	11	3	--	--
CakTC40882	Ca(ICCV2/PI489777)SNP_21910	362	C	T	21	8	--	--
CakTC24986	Ca(ICCV2/PI489777)SNP_21911	277	A	G	5	10	--	--
	Ca(ICCV2/PI489777)SNP_21912	367	C	T	7	11	--	--
	Ca(ICCV2/PI489777)SNP_21913	451	G	A	6	9	--	--
	Ca(ICCV2/PI489777)SNP_21914	456	A	G	7	10	--	--
	Ca(ICCV2/PI489777)SNP_21915	622	C	T	5	9	--	--
CakTC33147	Ca(ICCV2/PI489777)SNP_21916	1219	T	C	4	4	--	--
	Ca(ICCV2/PI489777)SNP_21917	1577	C	T	3	4	--	--
	Ca(ICCV2/PI489777)SNP_21918	2375	G	C	3	3	--	--
	Ca(ICCV2/PI489777)SNP_21919	2502	G	A	3	4	--	--
CakTC09541	Ca(ICCV2/PI489777)SNP_21920	303	A	G	7	5	--	--
	Ca(ICCV2/PI489777)SNP_21921	512	C	T	14	10	--	--
	Ca(ICCV2/PI489777)SNP_21922	547	T	G	12	9	--	--
CakTC35266	Ca(ICCV2/PI489777)SNP_21923	1268	G	T	35	23	--	--
	Ca(ICCV2/PI489777)SNP_21924	2082	A	G	35	21	--	--
	Ca(ICCV2/PI489777)SNP_21925	2093	A	G	38	19	--	--
	Ca(ICCV2/PI489777)SNP_21926	2835	A	G	31	3	--	--
	Ca(ICCV2/PI489777)SNP_21927	3138	C	T	15	15	--	--
	Ca(ICCV2/PI489777)SNP_21928	3228	T	G	14	14	--	--
	Ca(ICCV2/PI489777)SNP_21929	3314	G	A	18	15	--	--
	Ca(ICCV2/PI489777)SNP_21930	3639	A	G	10	10	--	--
CakTC33810	Ca(ICCV2/PI489777)SNP_21931	231	A	T	39	9	--	--
	Ca(ICCV2/PI489777)SNP_21932	412	C	T	73	31	--	--
	Ca(ICCV2/PI489777)SNP_21933	432	G	A	80	36	--	--
	Ca(ICCV2/PI489777)SNP_21934	2199	C	T	39	43	--	--
	Ca(ICCV2/PI489777)SNP_21935	2224	G	C	34	34	--	--
CakTC27697	Ca(ICCV2/PI489777)SNP_21936	888	A	G	7	3	--	--
	Ca(ICCV2/PI489777)SNP_21937	1416	C	T	9	3	--	--
	Ca(ICCV2/PI489777)SNP_21938	1594	T	G	8	4	--	--
	Ca(ICCV2/PI489777)SNP_21939	3290	G	A	9	5	--	--
	Ca(ICCV2/PI489777)SNP_21940	3343	C	A	7	4	--	--
CakTC30917	Ca(ICCV2/PI489777)SNP_21941	894	T	G	3	4	--	--
CakTC11607	Ca(ICCV2/PI489777)SNP_21942	245	T	C	15	14	--	CCAAT
	Ca(ICCV2/PI489777)SNP_21943	246	T	C	15	15	--	CCAAT
	Ca(ICCV2/PI489777)SNP_21944	376	A	G	22	16	--	CCAAT
	Ca(ICCV2/PI489777)SNP_21945	823	C	T	42	20	--	CCAAT
	Ca(ICCV2/PI489777)SNP_21946	1108	A	G	10	5	--	CCAAT
CakTC41072	Ca(ICCV2/PI489777)SNP_21947	524	A	G	6	3	--	--
CakTC01717	Ca(ICCV2/PI489777)SNP_21948	530	T	C	17	19	--	--
	Ca(ICCV2/PI489777)SNP_21949	1656	C	T	11	10	--	--
	Ca(ICCV2/PI489777)SNP_21950	1850	A	G	8	7	--	--
CakTC10094	Ca(ICCV2/PI489777)SNP_21951	137	A	T	3	3	--	--
CakTC22246	Ca(ICCV2/PI489777)SNP_21952	382	A	G	19	18	--	--
CakTC38034	Ca(ICCV2/PI489777)SNP_21953	745	G	A	51	50	--	--
	Ca(ICCV2/PI489777)SNP_21954	1034	G	A	15	5	--	--
CakTC40789	Ca(ICCV2/PI489777)SNP_21955	480	T	C	17	8	Young_pod	--
	Ca(ICCV2/PI489777)SNP_21956	1089	A	T	9	13	Young_pod	--
	Ca(ICCV2/PI489777)SNP_21957	1443	G	A	5	8	Young_pod	--
CakTC06070	Ca(ICCV2/PI489777)SNP_21958	1179	A	G	3	4	--	--
CakTC28303	Ca(ICCV2/PI489777)SNP_21959	393	C	A	14	4	--	--
	Ca(ICCV2/PI489777)SNP_21960	637	C	G	12	7	--	--
	Ca(ICCV2/PI489777)SNP_21961	930	A	G	26	14	--	--
	Ca(ICCV2/PI489777)SNP_21962	1014	A	G	23	13	--	--
	Ca(ICCV2/PI489777)SNP_21963	1099	C	G	22	9	--	--
CakTC25537	Ca(ICCV2/PI489777)SNP_21964	210	A	G	14	38	Flower	bud
CakTC40333	Ca(ICCV2/PI489777)SNP_21965	318	G	A	4	40	--	--
	Ca(ICCV2/PI489777)SNP_21966	660	T	C	6	32	--	--
CakTC24303	Ca(ICCV2/PI489777)SNP_21967	48	A	G	4	4	--	--
	Ca(ICCV2/PI489777)SNP_21968	67	C	T	7	6	--	--
	Ca(ICCV2/PI489777)SNP_21969	90	T	C	11	8	--	--
	Ca(ICCV2/PI489777)SNP_21970	126	G	C	11	8	--	--
	Ca(ICCV2/PI489777)SNP_21971	230	C	T	11	10	--	--
	Ca(ICCV2/PI489777)SNP_21972	579	A	G	8	7	--	--
CakTC10968	Ca(ICCV2/PI489777)SNP_21973	756	T	C	3	10	--	--

CakTC23389	Ca(ICCV2/PI489777)SNP_21974	138	A	G	31	15	--	--
CakTC38399	Ca(ICCV2/PI489777)SNP_21975	531	A	G	4	3	--	--
	Ca(ICCV2/PI489777)SNP_21976	540	C	T	3	3	--	--
CakTC31826	Ca(ICCV2/PI489777)SNP_21977	467	T	G	7	3	--	--
	Ca(ICCV2/PI489777)SNP_21978	2081	C	A	8	6	--	--
CakTC09659	Ca(ICCV2/PI489777)SNP_21979	3044	C	G	4	3	--	FAR1
CakTC41722	Ca(ICCV2/PI489777)SNP_21980	364	C	T	5	12	--	bZIP
	Ca(ICCV2/PI489777)SNP_21981	514	T	G	3	6	--	bZIP
CakTC27273	Ca(ICCV2/PI489777)SNP_21982	1019	T	C	7	3	--	--
	Ca(ICCV2/PI489777)SNP_21983	2066	C	T	5	11	--	--
	Ca(ICCV2/PI489777)SNP_21984	2138	G	C	6	7	--	--
	Ca(ICCV2/PI489777)SNP_21985	2156	A	C	6	7	--	--
	Ca(ICCV2/PI489777)SNP_21986	2201	C	A	10	7	--	--
	Ca(ICCV2/PI489777)SNP_21987	2438	T	C	10	12	--	--
	Ca(ICCV2/PI489777)SNP_21988	2879	C	T	18	16	--	--
	Ca(ICCV2/PI489777)SNP_21989	2960	C	T	9	11	--	--
	Ca(ICCV2/PI489777)SNP_21990	3517	C	A	11	7	--	--
CakTC23122	Ca(ICCV2/PI489777)SNP_21991	5836	T	C	44	9	Shoot	--
	Ca(ICCV2/PI489777)SNP_21992	6446	T	C	28	11	Shoot	--
	Ca(ICCV2/PI489777)SNP_21993	6507	G	A	23	9	Shoot	--
CakTC28076	Ca(ICCV2/PI489777)SNP_21994	2843	A	G	7	5	--	--
CakTC37633	Ca(ICCV2/PI489777)SNP_21995	741	C	T	5	6	--	GRAS
	Ca(ICCV2/PI489777)SNP_21996	863	A	G	5	5	--	GRAS
	Ca(ICCV2/PI489777)SNP_21997	1641	T	G	5	4	--	GRAS
CakTC24762	Ca(ICCV2/PI489777)SNP_21998	266	A	G	26	10	--	--
	Ca(ICCV2/PI489777)SNP_21999	308	C	T	29	11	--	--
	Ca(ICCV2/PI489777)SNP_22000	568	C	T	19	9	--	--
	Ca(ICCV2/PI489777)SNP_22001	865	A	G	26	15	--	--
	Ca(ICCV2/PI489777)SNP_22002	1188	A	G	8	13	--	--
CakTC25598	Ca(ICCV2/PI489777)SNP_22003	2522	A	G	14	3	--	--
	Ca(ICCV2/PI489777)SNP_22004	3172	C	A	13	4	--	--
	Ca(ICCV2/PI489777)SNP_22005	3255	C	G	17	7	--	--
	Ca(ICCV2/PI489777)SNP_22006	4077	T	C	41	29	--	--
	Ca(ICCV2/PI489777)SNP_22007	4369	C	A	46	21	--	--
	Ca(ICCV2/PI489777)SNP_22008	4436	C	A	51	20	--	--
	Ca(ICCV2/PI489777)SNP_22009	4558	T	C	71	22	--	--
	Ca(ICCV2/PI489777)SNP_22010	4619	C	A	74	21	--	--
	Ca(ICCV2/PI489777)SNP_22011	5326	T	C	67	22	--	--
	Ca(ICCV2/PI489777)SNP_22012	6127	A	T	49	24	--	--
	Ca(ICCV2/PI489777)SNP_22013	6871	G	C	74	43	--	--
	Ca(ICCV2/PI489777)SNP_22014	7336	C	A	67	33	--	--
	Ca(ICCV2/PI489777)SNP_22015	7642	G	A	45	39	--	--
	Ca(ICCV2/PI489777)SNP_22016	8232	C	T	7	6	--	--
CakTC37280	Ca(ICCV2/PI489777)SNP_22017	1245	A	G	15	5	--	--
	Ca(ICCV2/PI489777)SNP_22018	1396	T	C	22	5	--	--
	Ca(ICCV2/PI489777)SNP_22019	1554	G	A	20	4	--	--
	Ca(ICCV2/PI489777)SNP_22020	1640	A	G	12	3	--	--
	Ca(ICCV2/PI489777)SNP_22021	1661	A	T	12	3	--	--
CakTC39520	Ca(ICCV2/PI489777)SNP_22022	176	A	G	4	3	--	--
	Ca(ICCV2/PI489777)SNP_22023	218	C	T	3	3	--	--
	Ca(ICCV2/PI489777)SNP_22024	314	C	A	5	7	--	--
	Ca(ICCV2/PI489777)SNP_22025	651	A	C	16	9	--	--
	Ca(ICCV2/PI489777)SNP_22026	769	G	A	15	8	--	--
	Ca(ICCV2/PI489777)SNP_22027	852	G	A	11	8	--	--
	Ca(ICCV2/PI489777)SNP_22028	939	G	A	13	9	--	--
	Ca(ICCV2/PI489777)SNP_22029	971	C	A	15	11	--	--
	Ca(ICCV2/PI489777)SNP_22030	1143	G	A	13	10	--	--
CakTC35148	Ca(ICCV2/PI489777)SNP_22031	1262	T	G	8	3	--	--
	Ca(ICCV2/PI489777)SNP_22032	1278	G	C	8	3	--	--
	Ca(ICCV2/PI489777)SNP_22033	1506	G	A	7	5	--	--
	Ca(ICCV2/PI489777)SNP_22034	2036	A	G	17	12	--	--
	Ca(ICCV2/PI489777)SNP_22035	2887	C	T	18	10	--	--
CakTC37094	Ca(ICCV2/PI489777)SNP_22036	1301	G	C	8	5	--	--
	Ca(ICCV2/PI489777)SNP_22037	1307	T	C	8	5	--	--
	Ca(ICCV2/PI489777)SNP_22038	1322	T	C	8	5	--	--
	Ca(ICCV2/PI489777)SNP_22039	1371	A	C	9	6	--	--
CakTC37862	Ca(ICCV2/PI489777)SNP_22040	158	A	G	7	3	--	--
CakTC41744	Ca(ICCV2/PI489777)SNP_22041	2017	A	C	26	36	--	--
	Ca(ICCV2/PI489777)SNP_22042	2028	T	C	26	34	--	--
	Ca(ICCV2/PI489777)SNP_22043	2041	T	C	28	33	--	--
	Ca(ICCV2/PI489777)SNP_22044	2086	A	G	29	27	--	--
	Ca(ICCV2/PI489777)SNP_22045	2187	G	C	24	23	--	--
	Ca(ICCV2/PI489777)SNP_22046	2199	T	C	30	22	--	--
CakTC39554	Ca(ICCV2/PI489777)SNP_22047	211	A	G	24	4	--	--

	Ca(ICCV2/PI489777)SNP_22048	237	C	T	24	4	--	--
	Ca(ICCV2/PI489777)SNP_22049	272	A	G	23	4	--	--
	Ca(ICCV2/PI489777)SNP_22050	343	A	C	22	4	--	--
	Ca(ICCV2/PI489777)SNP_22051	401	G	C	20	4	--	--
CakTC29128	Ca(ICCV2/PI489777)SNP_22052	177	G	C	25	61	--	--
	Ca(ICCV2/PI489777)SNP_22053	528	C	T	38	63	--	--
	Ca(ICCV2/PI489777)SNP_22054	610	T	C	62	66	--	--
	Ca(ICCV2/PI489777)SNP_22055	625	C	G	60	65	--	--
CakTC38489	Ca(ICCV2/PI489777)SNP_22056	1416	C	G	5	6	--	--
	Ca(ICCV2/PI489777)SNP_22057	1623	C	T	7	4	--	--
CakTC30093	Ca(ICCV2/PI489777)SNP_22058	1124	A	C	9	8	Root	--
	Ca(ICCV2/PI489777)SNP_22059	1141	G	A	9	9	Root	--
	Ca(ICCV2/PI489777)SNP_22060	1264	G	A	10	4	Root	--
CakTC41403	Ca(ICCV2/PI489777)SNP_22061	322	T	G	4	8	--	CCAAT
	Ca(ICCV2/PI489777)SNP_22062	380	G	C	5	10	--	CCAAT
	Ca(ICCV2/PI489777)SNP_22063	658	A	G	12	10	--	CCAAT
	Ca(ICCV2/PI489777)SNP_22064	697	C	A	12	8	--	CCAAT
	Ca(ICCV2/PI489777)SNP_22065	700	A	G	12	8	--	CCAAT
CakTC23321	Ca(ICCV2/PI489777)SNP_22066	3520	A	T	124	75	--	--
	Ca(ICCV2/PI489777)SNP_22067	3523	T	A	123	75	--	--
	Ca(ICCV2/PI489777)SNP_22068	4321	A	T	76	32	--	--
CakTC25127	Ca(ICCV2/PI489777)SNP_22069	898	G	A	39	3	--	--
CakTC42397	Ca(ICCV2/PI489777)SNP_22070	347	C	T	10	4	--	--
	Ca(ICCV2/PI489777)SNP_22071	1589	G	A	5	10	--	--
CakTC29857	Ca(ICCV2/PI489777)SNP_22072	2661	T	C	3	6	Flower	bud
CakTC10028	Ca(ICCV2/PI489777)SNP_22073	45	A	T	3	4	--	--
	Ca(ICCV2/PI489777)SNP_22074	172	G	C	5	4	--	--
CakTC23847	Ca(ICCV2/PI489777)SNP_22075	82	C	G	4	3	--	--
	Ca(ICCV2/PI489777)SNP_22076	571	C	T	32	9	--	--
	Ca(ICCV2/PI489777)SNP_22077	1820	C	G	27	5	--	--
	Ca(ICCV2/PI489777)SNP_22078	2016	T	C	15	9	--	--
CakTC33963	Ca(ICCV2/PI489777)SNP_22079	418	T	G	22	9	--	--
	Ca(ICCV2/PI489777)SNP_22080	483	C	A	22	9	--	--
	Ca(ICCV2/PI489777)SNP_22081	493	T	C	22	10	--	--
	Ca(ICCV2/PI489777)SNP_22082	500	G	T	22	10	--	--
	Ca(ICCV2/PI489777)SNP_22083	543	G	C	19	9	--	--
CakTC35496	Ca(ICCV2/PI489777)SNP_22084	267	T	A	8	5	--	--
	Ca(ICCV2/PI489777)SNP_22085	568	G	A	22	13	--	--
	Ca(ICCV2/PI489777)SNP_22086	1059	A	T	23	28	--	--
	Ca(ICCV2/PI489777)SNP_22087	1359	T	G	15	17	--	--
	Ca(ICCV2/PI489777)SNP_22088	1818	G	A	11	16	--	--
	Ca(ICCV2/PI489777)SNP_22089	2219	A	T	18	17	--	--
	Ca(ICCV2/PI489777)SNP_22090	2220	T	G	18	17	--	--
	Ca(ICCV2/PI489777)SNP_22091	2883	T	C	7	5	--	--
	Ca(ICCV2/PI489777)SNP_22092	2978	C	G	10	7	--	--
	Ca(ICCV2/PI489777)SNP_22093	3713	G	C	8	13	--	--
CakTC35618	Ca(ICCV2/PI489777)SNP_22094	1341	G	A	3	3	--	Jumonji
	Ca(ICCV2/PI489777)SNP_22095	1426	T	C	5	5	--	Jumonji
	Ca(ICCV2/PI489777)SNP_22096	2101	T	C	5	4	--	Jumonji
	Ca(ICCV2/PI489777)SNP_22097	3014	T	C	4	5	--	Jumonji
CakTC29788	Ca(ICCV2/PI489777)SNP_22098	241	T	C	4	3	Young_pod	--
CakTC28337	Ca(ICCV2/PI489777)SNP_22099	1644	A	G	7	4	Root	--
	Ca(ICCV2/PI489777)SNP_22100	2698	C	T	15	5	Root	--
CakTC37760	Ca(ICCV2/PI489777)SNP_22101	550	G	A	3	4	--	--
	Ca(ICCV2/PI489777)SNP_22102	561	G	A	4	4	--	--
CakTC41971	Ca(ICCV2/PI489777)SNP_22103	350	G	C	24	6	--	--
	Ca(ICCV2/PI489777)SNP_22104	447	T	C	27	8	--	--
	Ca(ICCV2/PI489777)SNP_22105	653	G	T	17	14	--	--
	Ca(ICCV2/PI489777)SNP_22106	692	C	G	13	12	--	--
	Ca(ICCV2/PI489777)SNP_22107	731	C	T	12	11	--	--
	Ca(ICCV2/PI489777)SNP_22108	754	C	T	11	10	--	--
CakTC24899	Ca(ICCV2/PI489777)SNP_22109	506	G	T	3	3	--	--
	Ca(ICCV2/PI489777)SNP_22110	731	T	C	6	4	--	--
	Ca(ICCV2/PI489777)SNP_22111	1291	C	T	51	10	--	--
CakTC33243	Ca(ICCV2/PI489777)SNP_22112	475	G	T	3	8	--	--
	Ca(ICCV2/PI489777)SNP_22113	491	A	C	4	9	--	--
	Ca(ICCV2/PI489777)SNP_22114	502	A	G	4	9	--	--
	Ca(ICCV2/PI489777)SNP_22115	505	T	C	4	9	--	--
	Ca(ICCV2/PI489777)SNP_22116	631	G	A	6	9	--	--
	Ca(ICCV2/PI489777)SNP_22117	691	C	T	7	12	--	--
CakTC35659	Ca(ICCV2/PI489777)SNP_22118	80	C	G	12	7	--	--
	Ca(ICCV2/PI489777)SNP_22119	1111	G	A	15	11	--	--
	Ca(ICCV2/PI489777)SNP_22120	1113	A	C	17	10	--	--
	Ca(ICCV2/PI489777)SNP_22121	2231	T	C	19	11	--	--

	Ca(ICCV2/PI489777)SNP_22122	2273	A	G	17	9	--	--
CakTC38569	Ca(ICCV2/PI489777)SNP_22123	319	A	G	10	7	--	--
	Ca(ICCV2/PI489777)SNP_22124	613	C	T	18	9	--	--
	Ca(ICCV2/PI489777)SNP_22125	634	T	C	18	8	--	--
CakTC30565	Ca(ICCV2/PI489777)SNP_22126	1775	T	C	15	11	--	--
	Ca(ICCV2/PI489777)SNP_22127	1880	A	T	13	6	--	--
	Ca(ICCV2/PI489777)SNP_22128	1958	C	G	4	5	--	--
CakTC09941	Ca(ICCV2/PI489777)SNP_22129	527	G	A	3	3	--	--
CakTC23920	Ca(ICCV2/PI489777)SNP_22130	2606	T	A	17	12	--	--
	Ca(ICCV2/PI489777)SNP_22131	2775	G	A	4	6	--	--
CakTC41638	Ca(ICCV2/PI489777)SNP_22132	187	T	C	3	10	--	MYB
CakTC38334	Ca(ICCV2/PI489777)SNP_22133	109	A	C	30	9	--	--
	Ca(ICCV2/PI489777)SNP_22134	763	A	G	33	4	--	--
CakTC40136	Ca(ICCV2/PI489777)SNP_22135	351	T	C	12	14	--	--
CakTC27941	Ca(ICCV2/PI489777)SNP_22136	228	T	C	15	17	--	--
	Ca(ICCV2/PI489777)SNP_22137	256	G	C	20	17	--	--
	Ca(ICCV2/PI489777)SNP_22138	764	A	G	93	23	--	--
	Ca(ICCV2/PI489777)SNP_22139	2082	T	A	16	57	--	--
	Ca(ICCV2/PI489777)SNP_22140	2163	G	C	11	5	--	--
CakTC34195	Ca(ICCV2/PI489777)SNP_22141	466	T	C	8	4	--	--
CakTC33458	Ca(ICCV2/PI489777)SNP_22142	822	G	A	37	13	--	--
CakTC38752	Ca(ICCV2/PI489777)SNP_22143	173	G	A	6	3	--	--
	Ca(ICCV2/PI489777)SNP_22144	194	T	C	6	6	--	--
	Ca(ICCV2/PI489777)SNP_22145	197	A	T	6	6	--	--
	Ca(ICCV2/PI489777)SNP_22146	231	T	G	9	6	--	--
	Ca(ICCV2/PI489777)SNP_22147	750	G	A	24	7	--	--
	Ca(ICCV2/PI489777)SNP_22148	1117	A	G	29	4	--	--
CakTC40080	Ca(ICCV2/PI489777)SNP_22149	171	T	G	9	9	--	--
CakTC28562	Ca(ICCV2/PI489777)SNP_22150	121	C	T	8	6	--	--
CakTC42996	Ca(ICCV2/PI489777)SNP_22151	218	A	G	9	11	--	--
	Ca(ICCV2/PI489777)SNP_22152	652	G	A	71	30	--	--
	Ca(ICCV2/PI489777)SNP_22153	768	C	A	36	15	--	--
CakTC26480	Ca(ICCV2/PI489777)SNP_22154	284	G	C	164	16	--	--
CakTC26742	Ca(ICCV2/PI489777)SNP_22155	1255	G	A	25	5	--	--
	Ca(ICCV2/PI489777)SNP_22156	1342	A	G	25	6	--	--
	Ca(ICCV2/PI489777)SNP_22157	1351	A	G	26	6	--	--
	Ca(ICCV2/PI489777)SNP_22158	1399	T	G	28	8	--	--
	Ca(ICCV2/PI489777)SNP_22159	1468	A	G	18	8	--	--
CakTC25438	Ca(ICCV2/PI489777)SNP_22160	3349	A	G	5	4	--	--
	Ca(ICCV2/PI489777)SNP_22161	3563	C	G	8	5	--	--
	Ca(ICCV2/PI489777)SNP_22162	4431	C	T	8	5	--	--
CakTC31723	Ca(ICCV2/PI489777)SNP_22163	426	T	G	5	3	--	--
	Ca(ICCV2/PI489777)SNP_22164	771	C	A	7	3	--	--
	Ca(ICCV2/PI489777)SNP_22165	856	G	C	6	4	--	--
	Ca(ICCV2/PI489777)SNP_22166	874	A	G	7	4	--	--
	Ca(ICCV2/PI489777)SNP_22167	887	G	A	7	4	--	--
	Ca(ICCV2/PI489777)SNP_22168	1187	G	A	11	4	--	--
	Ca(ICCV2/PI489777)SNP_22169	1246	C	T	13	4	--	--
	Ca(ICCV2/PI489777)SNP_22170	1259	T	G	12	3	--	--
CakTC23778	Ca(ICCV2/PI489777)SNP_22171	995	C	T	22	6	--	--
	Ca(ICCV2/PI489777)SNP_22172	1079	T	C	13	3	--	--
CakTC26669	Ca(ICCV2/PI489777)SNP_22173	2607	G	A	44	25	--	--
	Ca(ICCV2/PI489777)SNP_22174	2702	C	T	23	26	--	--
	Ca(ICCV2/PI489777)SNP_22175	3050	T	C	5	6	--	--
CakTC07858	Ca(ICCV2/PI489777)SNP_22176	590	A	G	45	29	--	--
	Ca(ICCV2/PI489777)SNP_22177	841	C	T	62	25	--	--
	Ca(ICCV2/PI489777)SNP_22178	896	G	T	67	30	--	--
	Ca(ICCV2/PI489777)SNP_22179	1868	G	T	31	22	--	--
	Ca(ICCV2/PI489777)SNP_22180	2033	G	C	48	23	--	--
	Ca(ICCV2/PI489777)SNP_22181	2667	C	G	21	19	--	--
	Ca(ICCV2/PI489777)SNP_22182	2685	C	T	18	18	--	--
	Ca(ICCV2/PI489777)SNP_22183	2686	G	C	17	18	--	--
	Ca(ICCV2/PI489777)SNP_22184	2802	G	T	13	13	--	--
	Ca(ICCV2/PI489777)SNP_22185	2906	G	T	12	8	--	--
CakTC23249	Ca(ICCV2/PI489777)SNP_22186	1884	T	G	433	111	--	--
	Ca(ICCV2/PI489777)SNP_22187	2467	A	C	259	112	--	--
	Ca(ICCV2/PI489777)SNP_22188	3271	A	G	365	121	--	--
CakTC29067	Ca(ICCV2/PI489777)SNP_22189	697	G	C	30	6	--	--
	Ca(ICCV2/PI489777)SNP_22190	1379	G	A	13	6	--	--
	Ca(ICCV2/PI489777)SNP_22191	1502	G	C	13	7	--	--
	Ca(ICCV2/PI489777)SNP_22192	1537	C	T	11	7	--	--
CakTC26202	Ca(ICCV2/PI489777)SNP_22193	109	C	T	9	7	--	--
	Ca(ICCV2/PI489777)SNP_22194	306	C	G	32	24	--	--
	Ca(ICCV2/PI489777)SNP_22195	465	G	A	30	17	--	--

	Ca(ICCV2/PI489777)SNP_22196	507	G	A	25	14	--	--
CakTC40638	Ca(ICCV2/PI489777)SNP_22197	676	A	C	5	10	--	--
	Ca(ICCV2/PI489777)SNP_22198	734	T	C	5	10	--	--
	Ca(ICCV2/PI489777)SNP_22199	789	T	A	7	10	--	--
	Ca(ICCV2/PI489777)SNP_22200	914	C	T	7	8	--	--
CakTC26283	Ca(ICCV2/PI489777)SNP_22201	216	G	A	41	13	--	--
	Ca(ICCV2/PI489777)SNP_22202	1196	T	C	11	4	--	--
CakTC28715	Ca(ICCV2/PI489777)SNP_22203	390	T	C	11	5	--	--
CakTC25807	Ca(ICCV2/PI489777)SNP_22204	953	C	T	9	4	--	--
CakTC27614	Ca(ICCV2/PI489777)SNP_22205	170	T	A	4	5	--	--
CakTC10138	Ca(ICCV2/PI489777)SNP_22206	252	C	T	10	5	--	--
CakTC13725	Ca(ICCV2/PI489777)SNP_22207	32	C	G	3	3	--	--
	Ca(ICCV2/PI489777)SNP_22208	51	A	G	3	4	--	--
	Ca(ICCV2/PI489777)SNP_22209	63	T	C	3	4	--	--
	Ca(ICCV2/PI489777)SNP_22210	64	T	C	3	6	--	--
	Ca(ICCV2/PI489777)SNP_22211	78	G	A	3	7	--	--
	Ca(ICCV2/PI489777)SNP_22212	97	G	A	3	6	--	--
	Ca(ICCV2/PI489777)SNP_22213	99	A	G	3	7	--	--
	Ca(ICCV2/PI489777)SNP_22214	132	G	A	3	7	--	--
	Ca(ICCV2/PI489777)SNP_22215	159	T	G	3	8	--	--
	Ca(ICCV2/PI489777)SNP_22216	188	G	A	3	8	--	--
	Ca(ICCV2/PI489777)SNP_22217	219	T	C	3	7	--	--
	Ca(ICCV2/PI489777)SNP_22218	231	G	A	3	8	--	--
	Ca(ICCV2/PI489777)SNP_22219	232	G	T	3	8	--	--
CakTC38065	Ca(ICCV2/PI489777)SNP_22220	275	G	T	8	4	--	--
	Ca(ICCV2/PI489777)SNP_22221	423	A	G	12	5	--	--
	Ca(ICCV2/PI489777)SNP_22222	474	A	C	24	9	--	--
	Ca(ICCV2/PI489777)SNP_22223	642	C	T	37	15	--	--
	Ca(ICCV2/PI489777)SNP_22224	688	C	T	33	20	--	--
	Ca(ICCV2/PI489777)SNP_22225	1173	T	G	18	14	--	--
	Ca(ICCV2/PI489777)SNP_22226	1221	A	G	16	11	--	--
	Ca(ICCV2/PI489777)SNP_22227	1257	G	C	16	14	--	--
	Ca(ICCV2/PI489777)SNP_22228	1506	T	C	12	11	--	--
	Ca(ICCV2/PI489777)SNP_22229	1537	T	G	12	9	--	--
CakTC31846	Ca(ICCV2/PI489777)SNP_22230	939	C	G	11	4	--	--
CakTC39049	Ca(ICCV2/PI489777)SNP_22231	750	G	A	6	3	--	--
CakTC33719	Ca(ICCV2/PI489777)SNP_22232	95	G	A	16	6	--	--
	Ca(ICCV2/PI489777)SNP_22233	155	G	T	17	7	--	--
	Ca(ICCV2/PI489777)SNP_22234	176	T	C	17	10	--	--
CakTC27151	Ca(ICCV2/PI489777)SNP_22235	89	A	G	31	7	--	--
CakTC26067	Ca(ICCV2/PI489777)SNP_22236	380	A	C	3	3	--	--
CakTC22736	Ca(ICCV2/PI489777)SNP_22237	399	C	T	15	10	--	--
	Ca(ICCV2/PI489777)SNP_22238	445	T	G	10	8	--	--
	Ca(ICCV2/PI489777)SNP_22239	460	A	C	10	9	--	--
	Ca(ICCV2/PI489777)SNP_22240	572	C	T	22	19	--	--
	Ca(ICCV2/PI489777)SNP_22241	797	T	C	33	5	--	--
CakTC28707	Ca(ICCV2/PI489777)SNP_22242	541	G	A	61	50	--	--
	Ca(ICCV2/PI489777)SNP_22243	550	G	C	62	49	--	--
	Ca(ICCV2/PI489777)SNP_22244	646	T	C	42	46	--	--
CakTC26405	Ca(ICCV2/PI489777)SNP_22245	841	G	C	29	24	--	--
	Ca(ICCV2/PI489777)SNP_22246	1453	T	C	27	20	--	--
CakTC39180	Ca(ICCV2/PI489777)SNP_22247	480	G	A	45	13	--	Tify
	Ca(ICCV2/PI489777)SNP_22248	533	G	A	55	18	--	Tify
	Ca(ICCV2/PI489777)SNP_22249	1071	A	G	45	18	--	Tify
	Ca(ICCV2/PI489777)SNP_22250	1116	C	T	47	18	--	Tify
	Ca(ICCV2/PI489777)SNP_22251	1388	T	C	27	8	--	Tify
CakTC43064	Ca(ICCV2/PI489777)SNP_22252	315	C	G	28	15	--	--
	Ca(ICCV2/PI489777)SNP_22253	694	G	C	33	20	--	--
	Ca(ICCV2/PI489777)SNP_22254	709	T	G	31	20	--	--
CakTC38532	Ca(ICCV2/PI489777)SNP_22255	76	T	C	3	3	--	--
	Ca(ICCV2/PI489777)SNP_22256	102	C	T	4	3	--	--
	Ca(ICCV2/PI489777)SNP_22257	169	C	T	5	3	--	--
	Ca(ICCV2/PI489777)SNP_22258	170	C	T	5	3	--	--
CakTC09742	Ca(ICCV2/PI489777)SNP_22259	1641	A	G	6	4	--	--
CakTC27727	Ca(ICCV2/PI489777)SNP_22260	272	T	A	6	9	--	--
CakTC24062	Ca(ICCV2/PI489777)SNP_22261	369	T	C	8	3	--	--
CakTC39424	Ca(ICCV2/PI489777)SNP_22262	132	T	A	4	4	--	--
	Ca(ICCV2/PI489777)SNP_22263	134	C	A	4	4	--	--
CakTC26030	Ca(ICCV2/PI489777)SNP_22264	229	G	A	5	4	--	--
	Ca(ICCV2/PI489777)SNP_22265	1149	T	C	34	9	--	--
CakTC42145	Ca(ICCV2/PI489777)SNP_22266	329	G	A	3	5	--	--
	Ca(ICCV2/PI489777)SNP_22267	470	T	C	5	5	--	--
CakTC07911	Ca(ICCV2/PI489777)SNP_22268	337	C	T	3	3	--	--
CakTC37680	Ca(ICCV2/PI489777)SNP_22269	298	T	C	11	9	--	--

	Ca(ICCV2/PI489777)SNP_22270	568	C	T	16	16	--	--
	Ca(ICCV2/PI489777)SNP_22271	923	G	A	14	12	--	--
CakTC29087	Ca(ICCV2/PI489777)SNP_22272	389	C	T	6	3	--	--
	Ca(ICCV2/PI489777)SNP_22273	469	C	G	4	3	--	--
	Ca(ICCV2/PI489777)SNP_22274	551	A	C	7	3	--	--
	Ca(ICCV2/PI489777)SNP_22275	1125	C	G	4	3	--	--
	Ca(ICCV2/PI489777)SNP_22276	1664	C	G	3	6	--	--
	Ca(ICCV2/PI489777)SNP_22277	1697	T	G	4	4	--	--
	Ca(ICCV2/PI489777)SNP_22278	1712	C	A	4	4	--	--
	Ca(ICCV2/PI489777)SNP_22279	1769	T	A	4	4	--	--
CakTC37878	Ca(ICCV2/PI489777)SNP_22280	472	C	G	5	4	--	--
CakTC35513	Ca(ICCV2/PI489777)SNP_22281	609	G	A	3	3	--	--
CakTC42759	Ca(ICCV2/PI489777)SNP_22282	54	C	A	5	3	--	--
	Ca(ICCV2/PI489777)SNP_22283	144	A	G	6	3	--	--
CakTC26567	Ca(ICCV2/PI489777)SNP_22284	395	C	T	15	7	--	--
	Ca(ICCV2/PI489777)SNP_22285	869	G	T	13	4	--	--
	Ca(ICCV2/PI489777)SNP_22286	909	A	G	9	4	--	--
	Ca(ICCV2/PI489777)SNP_22287	944	A	C	5	6	--	--
	Ca(ICCV2/PI489777)SNP_22288	1049	A	C	9	5	--	--
	Ca(ICCV2/PI489777)SNP_22289	1256	T	C	5	3	--	--
CakTC36109	Ca(ICCV2/PI489777)SNP_22290	501	A	G	5	3	--	--
	Ca(ICCV2/PI489777)SNP_22291	949	A	G	9	5	--	--
	Ca(ICCV2/PI489777)SNP_22292	2654	C	G	4	7	--	--
	Ca(ICCV2/PI489777)SNP_22293	3102	A	C	14	7	--	--
	Ca(ICCV2/PI489777)SNP_22294	5139	T	A	6	5	--	--
CakTC29715	Ca(ICCV2/PI489777)SNP_22295	362	G	A	17	8	--	--
	Ca(ICCV2/PI489777)SNP_22296	828	G	A	13	7	--	--
CakTC33445	Ca(ICCV2/PI489777)SNP_22297	418	C	T	44	42	--	--
CakTC26103	Ca(ICCV2/PI489777)SNP_22298	179	A	C	15	9	--	--
CakTC26400	Ca(ICCV2/PI489777)SNP_22299	350	T	C	13	7	--	--
CakTC43096	Ca(ICCV2/PI489777)SNP_22300	517	A	C	13	8	--	--
	Ca(ICCV2/PI489777)SNP_22301	707	C	T	6	9	--	--
	Ca(ICCV2/PI489777)SNP_22302	1031	C	T	9	5	--	--
	Ca(ICCV2/PI489777)SNP_22303	1137	A	G	8	7	--	--
CakTC40037	Ca(ICCV2/PI489777)SNP_22304	255	G	C	3	3	--	--
	Ca(ICCV2/PI489777)SNP_22305	578	C	T	7	4	--	--
	Ca(ICCV2/PI489777)SNP_22306	579	A	G	7	4	--	--
	Ca(ICCV2/PI489777)SNP_22307	1132	A	G	7	4	--	--
CakTC39170	Ca(ICCV2/PI489777)SNP_22308	1229	C	T	6	6	--	--
CakTC34031	Ca(ICCV2/PI489777)SNP_22309	1519	G	A	7	28	--	--
	Ca(ICCV2/PI489777)SNP_22310	1894	G	T	8	10	--	--
	Ca(ICCV2/PI489777)SNP_22311	2791	T	C	20	12	--	--
	Ca(ICCV2/PI489777)SNP_22312	3895	T	C	17	13	--	--
	Ca(ICCV2/PI489777)SNP_22313	4903	C	T	16	21	--	--
	Ca(ICCV2/PI489777)SNP_22314	5369	G	C	23	14	--	--
	Ca(ICCV2/PI489777)SNP_22315	6217	C	T	31	6	--	--
	Ca(ICCV2/PI489777)SNP_22316	7123	T	G	17	11	--	--
	Ca(ICCV2/PI489777)SNP_22317	7585	T	C	14	9	--	--
	Ca(ICCV2/PI489777)SNP_22318	7855	T	C	19	19	--	--
	Ca(ICCV2/PI489777)SNP_22319	10711	G	A	29	9	--	--
CakTC10390	Ca(ICCV2/PI489777)SNP_22320	991	A	T	214	48	--	C3H
	Ca(ICCV2/PI489777)SNP_22321	1132	C	A	176	58	--	C3H
	Ca(ICCV2/PI489777)SNP_22322	1138	T	C	181	56	--	C3H
	Ca(ICCV2/PI489777)SNP_22323	1645	C	T	118	37	--	C3H
	Ca(ICCV2/PI489777)SNP_22324	1744	A	G	117	34	--	C3H
CakTC30239	Ca(ICCV2/PI489777)SNP_22325	676	C	T	26	3	--	--
	Ca(ICCV2/PI489777)SNP_22326	738	C	G	31	3	--	--
CakTC37678	Ca(ICCV2/PI489777)SNP_22327	361	C	G	14	5	--	--
	Ca(ICCV2/PI489777)SNP_22328	452	A	G	38	3	--	--
	Ca(ICCV2/PI489777)SNP_22329	661	T	C	47	13	--	--
CakTC38184	Ca(ICCV2/PI489777)SNP_22330	73	A	T	7	7	--	--
	Ca(ICCV2/PI489777)SNP_22331	561	A	G	8	3	--	--
CakTC24565	Ca(ICCV2/PI489777)SNP_22332	328	T	C	5	3	--	--
	Ca(ICCV2/PI489777)SNP_22333	1164	G	T	20	3	--	--
	Ca(ICCV2/PI489777)SNP_22334	1637	T	C	14	10	--	--
	Ca(ICCV2/PI489777)SNP_22335	1643	T	C	14	10	--	--
	Ca(ICCV2/PI489777)SNP_22336	1864	A	G	14	4	--	--
	Ca(ICCV2/PI489777)SNP_22337	2545	A	G	7	8	--	--
CakTC28408	Ca(ICCV2/PI489777)SNP_22338	1762	A	T	13	11	--	--
CakTC26448	Ca(ICCV2/PI489777)SNP_22339	1207	A	T	17	14	--	--
	Ca(ICCV2/PI489777)SNP_22340	1713	C	G	12	11	--	--
	Ca(ICCV2/PI489777)SNP_22341	1756	G	A	10	13	--	--
	Ca(ICCV2/PI489777)SNP_22342	1824	T	G	13	11	--	--
	Ca(ICCV2/PI489777)SNP_22343	1914	T	C	10	9	--	--

	Ca(ICCV2/PI489777)SNP_22344	2218	G	A	8	10	--	--
	Ca(ICCV2/PI489777)SNP_22345	2742	C	G	27	6	--	--
	Ca(ICCV2/PI489777)SNP_22346	2944	C	T	17	5	--	--
CakTC09694	Ca(ICCV2/PI489777)SNP_22347	269	T	C	6	4	--	--
	Ca(ICCV2/PI489777)SNP_22348	499	T	C	4	4	--	--
	Ca(ICCV2/PI489777)SNP_22349	500	G	A	4	4	--	--
	Ca(ICCV2/PI489777)SNP_22350	521	C	A	4	4	--	--
	Ca(ICCV2/PI489777)SNP_22351	541	T	A	4	4	--	--
CakTC29159	Ca(ICCV2/PI489777)SNP_22352	194	T	G	29	7	--	--
	Ca(ICCV2/PI489777)SNP_22353	1359	A	C	28	10	--	--
	Ca(ICCV2/PI489777)SNP_22354	1692	C	G	23	12	--	--
	Ca(ICCV2/PI489777)SNP_22355	1701	G	A	22	11	--	--
	Ca(ICCV2/PI489777)SNP_22356	1757	T	C	23	12	--	--
	Ca(ICCV2/PI489777)SNP_22357	1783	C	T	27	13	--	--
	Ca(ICCV2/PI489777)SNP_22358	1793	C	G	27	13	--	--
	Ca(ICCV2/PI489777)SNP_22359	2117	G	T	4	4	--	--
CakTC42236	Ca(ICCV2/PI489777)SNP_22360	365	A	G	5	3	--	--
	Ca(ICCV2/PI489777)SNP_22361	1471	T	C	13	13	--	--
	Ca(ICCV2/PI489777)SNP_22362	1701	A	G	8	7	--	--
CakTC40420	Ca(ICCV2/PI489777)SNP_22363	309	G	T	12	92	--	--
	Ca(ICCV2/PI489777)SNP_22364	319	T	C	13	97	--	--
	Ca(ICCV2/PI489777)SNP_22365	505	A	G	10	113	--	--
	Ca(ICCV2/PI489777)SNP_22366	590	A	G	7	82	--	--
CakTC40438	Ca(ICCV2/PI489777)SNP_22367	650	A	G	7	3	--	--
CakTC38693	Ca(ICCV2/PI489777)SNP_22368	132	T	G	8	3	--	--
	Ca(ICCV2/PI489777)SNP_22369	1121	G	A	9	6	--	--
	Ca(ICCV2/PI489777)SNP_22370	1133	A	C	9	6	--	--
CakTC37363	Ca(ICCV2/PI489777)SNP_22371	186	A	T	3	5	--	--
CakTC11739	Ca(ICCV2/PI489777)SNP_22372	210	A	G	23	16	--	--
CakTC01446	Ca(ICCV2/PI489777)SNP_22373	1799	A	G	4	10	--	--
CakTC33086	Ca(ICCV2/PI489777)SNP_22374	301	A	G	8	5	--	--
	Ca(ICCV2/PI489777)SNP_22375	1235	A	G	7	6	--	--
	Ca(ICCV2/PI489777)SNP_22376	1532	A	C	14	9	--	--
CakTC43077	Ca(ICCV2/PI489777)SNP_22377	346	C	T	4	12	--	--
CakTC38652	Ca(ICCV2/PI489777)SNP_22378	89	A	C	11	4	--	--
	Ca(ICCV2/PI489777)SNP_22379	367	A	G	28	14	--	--
	Ca(ICCV2/PI489777)SNP_22380	892	C	T	11	5	--	--
CakTC39918	Ca(ICCV2/PI489777)SNP_22381	92	T	C	5	8	Shoot	--
	Ca(ICCV2/PI489777)SNP_22382	172	C	T	4	8	Shoot	--
	Ca(ICCV2/PI489777)SNP_22383	944	T	C	11	3	Shoot	--
	Ca(ICCV2/PI489777)SNP_22384	947	G	A	12	3	Shoot	--
	Ca(ICCV2/PI489777)SNP_22385	989	T	C	14	5	Shoot	--
	Ca(ICCV2/PI489777)SNP_22386	1030	C	T	16	5	Shoot	--
	Ca(ICCV2/PI489777)SNP_22387	1060	G	A	13	4	Shoot	--
CakTC06127	Ca(ICCV2/PI489777)SNP_22388	617	C	T	12	3	--	WRKY
CakTC33286	Ca(ICCV2/PI489777)SNP_22389	765	T	C	4	5	--	--
	Ca(ICCV2/PI489777)SNP_22390	871	G	C	7	3	--	--
	Ca(ICCV2/PI489777)SNP_22391	953	C	A	7	3	--	--
	Ca(ICCV2/PI489777)SNP_22392	2152	T	C	12	6	--	--
CakTC41316	Ca(ICCV2/PI489777)SNP_22393	1584	A	G	11	12	--	--
	Ca(ICCV2/PI489777)SNP_22394	1749	A	T	10	15	--	--
	Ca(ICCV2/PI489777)SNP_22395	1917	A	G	6	9	--	--
	Ca(ICCV2/PI489777)SNP_22396	2052	T	C	13	16	--	--
	Ca(ICCV2/PI489777)SNP_22397	2375	T	A	9	9	--	--
	Ca(ICCV2/PI489777)SNP_22398	2394	G	A	10	8	--	--
	Ca(ICCV2/PI489777)SNP_22399	2395	A	G	10	8	--	--
	Ca(ICCV2/PI489777)SNP_22400	2410	A	G	7	8	--	--
CakTC40691	Ca(ICCV2/PI489777)SNP_22401	332	C	T	12	3	--	--
	Ca(ICCV2/PI489777)SNP_22402	344	G	C	13	3	--	--
	Ca(ICCV2/PI489777)SNP_22403	385	T	G	17	3	--	--
	Ca(ICCV2/PI489777)SNP_22404	425	G	T	19	4	--	--
	Ca(ICCV2/PI489777)SNP_22405	989	T	C	6	7	--	--
	Ca(ICCV2/PI489777)SNP_22406	1385	T	C	14	9	--	--
	Ca(ICCV2/PI489777)SNP_22407	1484	C	T	11	6	--	--
	Ca(ICCV2/PI489777)SNP_22408	1607	G	A	16	3	--	--
CakTC42034	Ca(ICCV2/PI489777)SNP_22409	733	G	A	3	5	--	--
	Ca(ICCV2/PI489777)SNP_22410	736	A	G	3	5	--	--
CakTC12642	Ca(ICCV2/PI489777)SNP_22411	1633	A	G	9	3	--	--
CakTC11657	Ca(ICCV2/PI489777)SNP_22412	764	T	C	49	31	--	--
	Ca(ICCV2/PI489777)SNP_22413	2756	G	A	7	9	--	--
CakTC35752	Ca(ICCV2/PI489777)SNP_22414	730	A	T	5	3	--	--
	Ca(ICCV2/PI489777)SNP_22415	1491	T	C	6	3	--	--
	Ca(ICCV2/PI489777)SNP_22416	1508	T	G	6	3	--	--
	Ca(ICCV2/PI489777)SNP_22417	1709	G	A	7	3	--	--

	Ca(ICCV2/PI489777)SNP_22418	1972	G	T	10	4	--	--
	Ca(ICCV2/PI489777)SNP_22419	2322	G	A	3	3	--	--
CakTC41091	Ca(ICCV2/PI489777)SNP_22420	140	G	A	8	10	--	--
	Ca(ICCV2/PI489777)SNP_22421	453	G	A	7	6	--	--
CakTC43348	Ca(ICCV2/PI489777)SNP_22422	711	C	T	34	17	--	--
	Ca(ICCV2/PI489777)SNP_22423	747	T	C	39	16	--	--
	Ca(ICCV2/PI489777)SNP_22424	931	A	T	29	16	--	--
	Ca(ICCV2/PI489777)SNP_22425	941	G	A	47	16	--	--
	Ca(ICCV2/PI489777)SNP_22426	1467	A	T	43	29	--	--
	Ca(ICCV2/PI489777)SNP_22427	1703	A	G	30	22	--	--
	Ca(ICCV2/PI489777)SNP_22428	1771	T	C	26	21	--	--
	Ca(ICCV2/PI489777)SNP_22429	1841	T	C	21	11	--	--
CakTC41199	Ca(ICCV2/PI489777)SNP_22430	2274	A	T	3	3	--	--
CakTC38200	Ca(ICCV2/PI489777)SNP_22431	206	C	T	6	9	--	--
	Ca(ICCV2/PI489777)SNP_22432	531	A	T	14	7	--	--
CakTC42563	Ca(ICCV2/PI489777)SNP_22433	521	G	A	85	55	--	--
CakTC43160	Ca(ICCV2/PI489777)SNP_22434	152	T	C	23	3	--	--
	Ca(ICCV2/PI489777)SNP_22435	766	G	A	4	5	--	--
CakTC32331	Ca(ICCV2/PI489777)SNP_22436	115	A	G	18	6	--	--
	Ca(ICCV2/PI489777)SNP_22437	229	T	C	19	7	--	--
	Ca(ICCV2/PI489777)SNP_22438	250	C	T	19	7	--	--
	Ca(ICCV2/PI489777)SNP_22439	742	G	T	8	9	--	--
	Ca(ICCV2/PI489777)SNP_22440	994	T	C	9	10	--	--
	Ca(ICCV2/PI489777)SNP_22441	1045	C	T	7	8	--	--
CakTC28416	Ca(ICCV2/PI489777)SNP_22442	387	A	T	11	26	--	--
CakTC30723	Ca(ICCV2/PI489777)SNP_22443	828	G	C	3	3	Root	--
	Ca(ICCV2/PI489777)SNP_22444	999	T	C	4	4	Root	--
CakTC26088	Ca(ICCV2/PI489777)SNP_22445	1192	T	C	12	4	--	--
CakTC09232	Ca(ICCV2/PI489777)SNP_22446	145	T	C	3	3	--	--
	Ca(ICCV2/PI489777)SNP_22447	146	G	A	3	3	--	--
CakTC43336	Ca(ICCV2/PI489777)SNP_22448	1757	C	T	28	6	--	--
CakTC39382	Ca(ICCV2/PI489777)SNP_22449	104	G	A	9	6	--	--
	Ca(ICCV2/PI489777)SNP_22450	712	A	G	35	15	--	--
CakTC25458	Ca(ICCV2/PI489777)SNP_22451	834	T	C	30	7	--	--
	Ca(ICCV2/PI489777)SNP_22452	858	A	G	29	8	--	--
	Ca(ICCV2/PI489777)SNP_22453	930	C	G	39	10	--	--
	Ca(ICCV2/PI489777)SNP_22454	2183	C	A	32	10	--	--
	Ca(ICCV2/PI489777)SNP_22455	2224	T	C	30	10	--	--
CakTC01830	Ca(ICCV2/PI489777)SNP_22456	85	G	C	13	6	--	--
CakTC06047	Ca(ICCV2/PI489777)SNP_22457	1079	T	C	7	8	--	--
CakTC27258	Ca(ICCV2/PI489777)SNP_22458	140	C	T	4	5	--	--
	Ca(ICCV2/PI489777)SNP_22459	1015	A	G	20	27	--	--
	Ca(ICCV2/PI489777)SNP_22460	1171	G	A	22	11	--	--
CakTC37847	Ca(ICCV2/PI489777)SNP_22461	439	G	A	10	13	--	--
	Ca(ICCV2/PI489777)SNP_22462	785	G	A	24	15	--	--
CakTC38865	Ca(ICCV2/PI489777)SNP_22463	1047	A	G	5	13	--	--
	Ca(ICCV2/PI489777)SNP_22464	1142	C	T	6	14	--	--
	Ca(ICCV2/PI489777)SNP_22465	1312	A	G	5	10	--	--
CakTC34232	Ca(ICCV2/PI489777)SNP_22466	96	C	T	4	3	--	--
	Ca(ICCV2/PI489777)SNP_22467	1061	T	A	12	13	--	--
	Ca(ICCV2/PI489777)SNP_22468	1652	G	A	18	9	--	--
	Ca(ICCV2/PI489777)SNP_22469	1954	G	T	19	10	--	--
CakTC39208	Ca(ICCV2/PI489777)SNP_22470	855	G	C	5	7	--	--
	Ca(ICCV2/PI489777)SNP_22471	1068	T	C	10	16	--	--
	Ca(ICCV2/PI489777)SNP_22472	1137	G	T	10	19	--	--
CakTC37355	Ca(ICCV2/PI489777)SNP_22473	475	A	G	13	7	--	--
CakTC23841	Ca(ICCV2/PI489777)SNP_22474	419	G	A	9	5	--	--
	Ca(ICCV2/PI489777)SNP_22475	516	T	C	10	6	--	--
CakTC29478	Ca(ICCV2/PI489777)SNP_22476	183	A	C	8	5	--	--
	Ca(ICCV2/PI489777)SNP_22477	260	G	C	9	6	--	--
CakTC37564	Ca(ICCV2/PI489777)SNP_22478	356	A	C	13	23	--	--
CakTC40216	Ca(ICCV2/PI489777)SNP_22479	413	C	T	6	6	--	--
	Ca(ICCV2/PI489777)SNP_22480	584	T	C	6	5	--	--
	Ca(ICCV2/PI489777)SNP_22481	692	C	T	5	5	--	--
	Ca(ICCV2/PI489777)SNP_22482	859	G	T	7	5	--	--
CakTC40713	Ca(ICCV2/PI489777)SNP_22483	332	T	C	194	15	--	--
	Ca(ICCV2/PI489777)SNP_22484	354	C	A	196	34	--	--
	Ca(ICCV2/PI489777)SNP_22485	371	T	C	202	34	--	--
	Ca(ICCV2/PI489777)SNP_22486	464	C	T	202	34	--	--
CakTC24173	Ca(ICCV2/PI489777)SNP_22487	772	T	A	33	8	--	--
CakTC10625	Ca(ICCV2/PI489777)SNP_22488	240	G	C	29	3	--	--
CakTC23616	Ca(ICCV2/PI489777)SNP_22489	91	C	A	14	5	--	--
	Ca(ICCV2/PI489777)SNP_22490	474	C	T	91	53	--	--
	Ca(ICCV2/PI489777)SNP_22491	588	G	T	105	73	--	--

	Ca(ICCV2/PI489777)SNP_22492	660	T	A	119	83	--	--
	Ca(ICCV2/PI489777)SNP_22493	693	G	A	113	76	--	--
	Ca(ICCV2/PI489777)SNP_22494	723	C	T	114	77	--	--
	Ca(ICCV2/PI489777)SNP_22495	1120	G	T	6	22	--	--
	Ca(ICCV2/PI489777)SNP_22496	1394	G	A	15	3	--	--
	Ca(ICCV2/PI489777)SNP_22497	2455	C	A	22	24	--	--
CakTC36110	Ca(ICCV2/PI489777)SNP_22498	1035	A	G	5	5	--	--
	Ca(ICCV2/PI489777)SNP_22499	5391	A	G	7	7	--	--
CakTC36259	Ca(ICCV2/PI489777)SNP_22500	2379	T	A	17	9	--	HB
	Ca(ICCV2/PI489777)SNP_22501	2478	G	C	20	11	--	HB
CakTC41755	Ca(ICCV2/PI489777)SNP_22502	2325	T	G	3	3	--	--
CakTC24923	Ca(ICCV2/PI489777)SNP_22503	151	G	A	29	16	--	--
	Ca(ICCV2/PI489777)SNP_22504	269	G	A	37	28	--	--
	Ca(ICCV2/PI489777)SNP_22505	945	G	A	29	3	--	--
CakTC39040	Ca(ICCV2/PI489777)SNP_22506	430	G	A	10	16	--	--
	Ca(ICCV2/PI489777)SNP_22507	598	C	T	9	14	--	--
	Ca(ICCV2/PI489777)SNP_22508	628	C	T	8	12	--	--
	Ca(ICCV2/PI489777)SNP_22509	781	G	A	6	10	--	--
CakTC36726	Ca(ICCV2/PI489777)SNP_22510	766	G	A	14	8	--	--
	Ca(ICCV2/PI489777)SNP_22511	811	T	C	13	5	--	--
	Ca(ICCV2/PI489777)SNP_22512	913	A	G	15	6	--	--
	Ca(ICCV2/PI489777)SNP_22513	1228	T	G	19	5	--	--
	Ca(ICCV2/PI489777)SNP_22514	1258	G	A	17	5	--	--
	Ca(ICCV2/PI489777)SNP_22515	1357	T	C	18	4	--	--
	Ca(ICCV2/PI489777)SNP_22516	1921	C	T	10	3	--	--
	Ca(ICCV2/PI489777)SNP_22517	2110	C	T	14	6	--	--
	Ca(ICCV2/PI489777)SNP_22518	2242	A	G	22	8	--	--
CakTC35716	Ca(ICCV2/PI489777)SNP_22519	974	T	C	11	9	--	--
	Ca(ICCV2/PI489777)SNP_22520	1493	A	G	15	14	--	--
	Ca(ICCV2/PI489777)SNP_22521	1862	A	T	14	7	--	--
	Ca(ICCV2/PI489777)SNP_22522	2216	C	T	5	14	--	--
CakTC10808	Ca(ICCV2/PI489777)SNP_22523	316	A	C	13	3	--	--
CakTC25323	Ca(ICCV2/PI489777)SNP_22524	2419	T	G	7	5	--	--
	Ca(ICCV2/PI489777)SNP_22525	2830	T	C	13	7	--	--
	Ca(ICCV2/PI489777)SNP_22526	2911	G	A	7	6	--	--
	Ca(ICCV2/PI489777)SNP_22527	3184	G	A	11	5	--	--
	Ca(ICCV2/PI489777)SNP_22528	3334	T	C	9	4	--	--
	Ca(ICCV2/PI489777)SNP_22529	3396	A	G	6	4	--	--
	Ca(ICCV2/PI489777)SNP_22530	3613	A	G	3	11	--	--
	Ca(ICCV2/PI489777)SNP_22531	3880	A	C	11	12	--	--
	Ca(ICCV2/PI489777)SNP_22532	3943	A	G	28	15	--	--
CakTC25407	Ca(ICCV2/PI489777)SNP_22533	2795	G	A	6	5	Young_pod	--
CakTC32586	Ca(ICCV2/PI489777)SNP_22534	35	T	C	3	3	--	--
	Ca(ICCV2/PI489777)SNP_22535	109	T	G	3	3	--	--
	Ca(ICCV2/PI489777)SNP_22536	310	A	G	3	3	--	--
CakTC35871	Ca(ICCV2/PI489777)SNP_22537	411	A	G	4	5	--	--
CakTC01293	Ca(ICCV2/PI489777)SNP_22538	299	C	T	10	3	--	--
	Ca(ICCV2/PI489777)SNP_22539	440	T	G	8	7	--	--
	Ca(ICCV2/PI489777)SNP_22540	463	C	G	10	8	--	--
	Ca(ICCV2/PI489777)SNP_22541	681	T	A	7	4	--	--
CakTC27651	Ca(ICCV2/PI489777)SNP_22542	403	A	G	3	6	--	--
	Ca(ICCV2/PI489777)SNP_22543	526	T	C	7	7	--	--
CakTC31607	Ca(ICCV2/PI489777)SNP_22544	153	A	T	5	3	--	--
	Ca(ICCV2/PI489777)SNP_22545	168	T	C	5	3	--	--
	Ca(ICCV2/PI489777)SNP_22546	907	T	C	6	7	--	--
	Ca(ICCV2/PI489777)SNP_22547	914	T	C	3	7	--	--
	Ca(ICCV2/PI489777)SNP_22548	925	C	T	4	6	--	--
	Ca(ICCV2/PI489777)SNP_22549	1017	A	G	4	8	--	--
	Ca(ICCV2/PI489777)SNP_22550	1158	A	G	4	4	--	--
	Ca(ICCV2/PI489777)SNP_22551	2223	A	C	5	4	--	--
	Ca(ICCV2/PI489777)SNP_22552	2302	C	G	4	4	--	--
CakTC32928	Ca(ICCV2/PI489777)SNP_22553	571	T	G	8	3	--	--
	Ca(ICCV2/PI489777)SNP_22554	615	T	G	6	3	--	--
CakTC42506	Ca(ICCV2/PI489777)SNP_22555	170	C	T	5	7	--	--
	Ca(ICCV2/PI489777)SNP_22556	185	T	C	7	7	--	--
	Ca(ICCV2/PI489777)SNP_22557	425	C	T	9	11	--	--
	Ca(ICCV2/PI489777)SNP_22558	821	C	G	14	6	--	--
	Ca(ICCV2/PI489777)SNP_22559	845	G	A	14	5	--	--
	Ca(ICCV2/PI489777)SNP_22560	1028	A	G	8	7	--	--
	Ca(ICCV2/PI489777)SNP_22561	1625	G	A	10	11	--	--
CakTC35926	Ca(ICCV2/PI489777)SNP_22562	955	G	T	14	10	--	--
CakTC07794	Ca(ICCV2/PI489777)SNP_22563	83	C	T	6	9	--	--
	Ca(ICCV2/PI489777)SNP_22564	282	T	C	5	7	--	--
	Ca(ICCV2/PI489777)SNP_22565	342	C	T	6	9	--	--

CakTC28045	Ca(ICCV2/PI489777)SNP_22566	315	T	C	14	7	--	--
	Ca(ICCV2/PI489777)SNP_22567	491	G	C	10	8	--	--
	Ca(ICCV2/PI489777)SNP_22568	1275	A	C	14	10	--	--
	Ca(ICCV2/PI489777)SNP_22569	1287	G	A	15	11	--	--
	Ca(ICCV2/PI489777)SNP_22570	1642	C	T	8	7	--	--
	Ca(ICCV2/PI489777)SNP_22571	1746	A	G	8	5	--	--
	Ca(ICCV2/PI489777)SNP_22572	2121	T	C	6	3	--	--
	Ca(ICCV2/PI489777)SNP_22573	2316	G	T	17	5	--	--
	Ca(ICCV2/PI489777)SNP_22574	3189	C	T	7	7	--	--
	Ca(ICCV2/PI489777)SNP_22575	3935	C	G	8	4	--	--
CakTC34569	Ca(ICCV2/PI489777)SNP_22576	135	G	C	3	8	--	--
	Ca(ICCV2/PI489777)SNP_22577	426	A	C	3	10	--	--
	Ca(ICCV2/PI489777)SNP_22578	732	T	C	5	7	--	--
CakTC38304	Ca(ICCV2/PI489777)SNP_22579	41	A	C	6	3	--	--
	Ca(ICCV2/PI489777)SNP_22580	317	A	G	15	12	--	--
	Ca(ICCV2/PI489777)SNP_22581	381	A	G	11	12	--	--
CakTC04032	Ca(ICCV2/PI489777)SNP_22582	264	T	G	13	7	--	--
CakTC37742	Ca(ICCV2/PI489777)SNP_22583	259	C	G	35	6	Flower	bud
	Ca(ICCV2/PI489777)SNP_22584	304	C	G	46	8	Flower	bud
	Ca(ICCV2/PI489777)SNP_22585	934	T	G	38	14	Flower	bud
	Ca(ICCV2/PI489777)SNP_22586	1303	C	T	24	10	Flower	bud
	Ca(ICCV2/PI489777)SNP_22587	1627	T	C	27	17	Flower	bud
CakTC39189	Ca(ICCV2/PI489777)SNP_22588	917	G	A	3	6	--	--
	Ca(ICCV2/PI489777)SNP_22589	1016	G	A	3	5	--	--
	Ca(ICCV2/PI489777)SNP_22590	1209	T	C	3	4	--	--
CakTC39683	Ca(ICCV2/PI489777)SNP_22591	184	C	A	3	3	--	--
	Ca(ICCV2/PI489777)SNP_22592	212	G	A	3	4	--	--
	Ca(ICCV2/PI489777)SNP_22593	332	T	G	4	3	--	--
	Ca(ICCV2/PI489777)SNP_22594	792	G	A	17	13	--	--
	Ca(ICCV2/PI489777)SNP_22595	1239	G	A	9	3	--	--
	Ca(ICCV2/PI489777)SNP_22596	1309	C	A	8	3	--	--
CakTC27472	Ca(ICCV2/PI489777)SNP_22597	728	A	T	4	4	--	G2-like
	Ca(ICCV2/PI489777)SNP_22598	767	T	C	4	5	--	G2-like
CakTC27530	Ca(ICCV2/PI489777)SNP_22599	245	G	C	6	6	--	--
	Ca(ICCV2/PI489777)SNP_22600	351	A	T	9	6	--	--
	Ca(ICCV2/PI489777)SNP_22601	372	G	A	8	7	--	--
	Ca(ICCV2/PI489777)SNP_22602	374	C	T	10	7	--	--
CakTC40500	Ca(ICCV2/PI489777)SNP_22603	14	A	G	12	7	--	--
	Ca(ICCV2/PI489777)SNP_22604	141	C	T	51	54	--	--
	Ca(ICCV2/PI489777)SNP_22605	159	T	A	51	56	--	--
CakTC40116	Ca(ICCV2/PI489777)SNP_22606	562	C	A	29	21	--	zf-HD
	Ca(ICCV2/PI489777)SNP_22607	704	T	C	25	18	--	zf-HD
	Ca(ICCV2/PI489777)SNP_22608	1317	A	G	5	4	--	zf-HD
CakTC34771	Ca(ICCV2/PI489777)SNP_22609	1962	C	T	37	5	Shoot	--
	Ca(ICCV2/PI489777)SNP_22610	2129	C	A	25	9	Shoot	--
	Ca(ICCV2/PI489777)SNP_22611	2136	A	G	25	9	Shoot	--
	Ca(ICCV2/PI489777)SNP_22612	2568	T	C	34	7	Shoot	--
	Ca(ICCV2/PI489777)SNP_22613	3243	A	T	35	9	Shoot	--
	Ca(ICCV2/PI489777)SNP_22614	3701	G	C	25	5	Shoot	--
	Ca(ICCV2/PI489777)SNP_22615	3903	C	G	23	5	Shoot	--
CakTC41956	Ca(ICCV2/PI489777)SNP_22616	582	T	C	9	4	Root	--
CakTC11585	Ca(ICCV2/PI489777)SNP_22617	167	G	A	7	3	--	--
	Ca(ICCV2/PI489777)SNP_22618	195	A	C	6	3	--	--
	Ca(ICCV2/PI489777)SNP_22619	613	C	T	7	4	--	--
CakTC38273	Ca(ICCV2/PI489777)SNP_22620	1068	A	C	4	8	--	--
CakTC39429	Ca(ICCV2/PI489777)SNP_22621	452	A	G	10	4	--	--
CakTC10207	Ca(ICCV2/PI489777)SNP_22622	595	C	T	5	17	--	--
	Ca(ICCV2/PI489777)SNP_22623	1101	T	G	4	10	--	--
	Ca(ICCV2/PI489777)SNP_22624	1219	G	A	4	3	--	--
	Ca(ICCV2/PI489777)SNP_22625	1291	G	C	5	3	--	--
CakTC37226	Ca(ICCV2/PI489777)SNP_22626	369	A	G	16	20	--	--
	Ca(ICCV2/PI489777)SNP_22627	420	C	T	13	18	--	--
	Ca(ICCV2/PI489777)SNP_22628	783	A	T	19	16	--	--
CakTC11664	Ca(ICCV2/PI489777)SNP_22629	1665	G	C	33	19	--	--
CakTC31643	Ca(ICCV2/PI489777)SNP_22630	1850	T	G	22	9	--	--
	Ca(ICCV2/PI489777)SNP_22631	1949	A	G	4	5	--	--
	Ca(ICCV2/PI489777)SNP_22632	2088	C	A	3	4	--	--
CakTC36624	Ca(ICCV2/PI489777)SNP_22633	126	G	A	8	4	--	--
	Ca(ICCV2/PI489777)SNP_22634	152	A	G	9	4	--	--
	Ca(ICCV2/PI489777)SNP_22635	421	G	A	6	6	--	--
	Ca(ICCV2/PI489777)SNP_22636	2422	T	C	9	4	--	--
	Ca(ICCV2/PI489777)SNP_22637	2817	C	G	9	18	--	--
	Ca(ICCV2/PI489777)SNP_22638	3662	C	T	5	6	--	--
	Ca(ICCV2/PI489777)SNP_22639	3799	A	G	8	3	--	--

	Ca(ICCV2/PI489777)SNP_22640	3861	G	A	7	3	--	--
CakTC27029	Ca(ICCV2/PI489777)SNP_22641	53	G	T	4	4	--	SET
CakTC30466	Ca(ICCV2/PI489777)SNP_22642	544	C	T	5	4	--	--
	Ca(ICCV2/PI489777)SNP_22643	1117	T	C	9	5	--	--
	Ca(ICCV2/PI489777)SNP_22644	1128	C	A	10	3	--	--
	Ca(ICCV2/PI489777)SNP_22645	1197	G	T	10	3	--	--
CakTC42225	Ca(ICCV2/PI489777)SNP_22646	228	A	G	48	71	--	--
CakTC40684	Ca(ICCV2/PI489777)SNP_22647	1645	T	A	6	5	--	--
	Ca(ICCV2/PI489777)SNP_22648	1958	G	A	4	5	--	--
CakTC12224	Ca(ICCV2/PI489777)SNP_22649	474	A	C	11	3	--	--
	Ca(ICCV2/PI489777)SNP_22650	475	A	C	11	3	--	--
	Ca(ICCV2/PI489777)SNP_22651	723	C	T	10	3	--	--
CakTC14242	Ca(ICCV2/PI489777)SNP_22652	118	G	A	7	3	--	--
	Ca(ICCV2/PI489777)SNP_22653	285	G	A	7	5	--	--
	Ca(ICCV2/PI489777)SNP_22654	451	T	C	4	7	--	--
	Ca(ICCV2/PI489777)SNP_22655	540	G	A	4	6	--	--
CakTC25940	Ca(ICCV2/PI489777)SNP_22656	306	A	G	27	10	--	HB
	Ca(ICCV2/PI489777)SNP_22657	312	G	A	28	12	--	HB
	Ca(ICCV2/PI489777)SNP_22658	570	G	A	38	16	--	HB
CakTC25568	Ca(ICCV2/PI489777)SNP_22659	1418	C	T	4	4	--	--
CakTC41192	Ca(ICCV2/PI489777)SNP_22660	247	C	T	6	5	--	--
	Ca(ICCV2/PI489777)SNP_22661	257	G	A	6	5	--	--
	Ca(ICCV2/PI489777)SNP_22662	444	C	T	8	5	--	--
	Ca(ICCV2/PI489777)SNP_22663	532	C	T	7	3	--	--
CakTC25225	Ca(ICCV2/PI489777)SNP_22664	1857	C	T	15	8	Young_pod	--
	Ca(ICCV2/PI489777)SNP_22665	1858	A	C	15	8	Young_pod	--
	Ca(ICCV2/PI489777)SNP_22666	2054	G	C	10	8	Young_pod	--
	Ca(ICCV2/PI489777)SNP_22667	2069	C	T	11	8	Young_pod	--
	Ca(ICCV2/PI489777)SNP_22668	2100	A	G	9	7	Young_pod	--
	Ca(ICCV2/PI489777)SNP_22669	2156	C	G	7	3	Young_pod	--
CakTC22837	Ca(ICCV2/PI489777)SNP_22670	74	G	C	16	6	--	--
	Ca(ICCV2/PI489777)SNP_22671	226	A	T	35	20	--	--
	Ca(ICCV2/PI489777)SNP_22672	834	G	T	4	3	--	--
CakTC08767	Ca(ICCV2/PI489777)SNP_22673	573	C	A	4	3	--	bHLH
CakTC38515	Ca(ICCV2/PI489777)SNP_22674	416	T	C	7	8	--	--
	Ca(ICCV2/PI489777)SNP_22675	735	C	A	9	13	--	--
CakTC26366	Ca(ICCV2/PI489777)SNP_22676	1136	C	T	21	25	--	--
	Ca(ICCV2/PI489777)SNP_22677	1232	C	T	14	20	--	--
	Ca(ICCV2/PI489777)SNP_22678	1353	G	T	6	9	--	--
CakTC40009	Ca(ICCV2/PI489777)SNP_22679	265	A	G	16	4	--	--
	Ca(ICCV2/PI489777)SNP_22680	355	T	C	58	18	--	--
	Ca(ICCV2/PI489777)SNP_22681	964	A	T	36	21	--	--
	Ca(ICCV2/PI489777)SNP_22682	982	G	T	31	20	--	--
	Ca(ICCV2/PI489777)SNP_22683	1156	A	G	30	14	--	--
CakTC25894	Ca(ICCV2/PI489777)SNP_22684	110	C	A	17	9	--	--
	Ca(ICCV2/PI489777)SNP_22685	912	C	T	49	8	--	--
	Ca(ICCV2/PI489777)SNP_22686	987	A	C	47	5	--	--
	Ca(ICCV2/PI489777)SNP_22687	1114	C	G	43	9	--	--
	Ca(ICCV2/PI489777)SNP_22688	1129	C	A	38	9	--	--
	Ca(ICCV2/PI489777)SNP_22689	1241	G	C	22	9	--	--
	Ca(ICCV2/PI489777)SNP_22690	1268	G	T	17	6	--	--
CakTC27583	Ca(ICCV2/PI489777)SNP_22691	225	T	C	17	6	--	--
CakTC37507	Ca(ICCV2/PI489777)SNP_22692	151	T	C	8	4	--	--
	Ca(ICCV2/PI489777)SNP_22693	291	C	A	10	4	--	--
CakTC41777	Ca(ICCV2/PI489777)SNP_22694	132	C	A	6	7	--	--
	Ca(ICCV2/PI489777)SNP_22695	1179	T	C	8	8	--	--
	Ca(ICCV2/PI489777)SNP_22696	1200	T	G	7	7	--	--
	Ca(ICCV2/PI489777)SNP_22697	1366	C	T	15	9	--	--
CakTC31670	Ca(ICCV2/PI489777)SNP_22698	926	C	T	8	3	--	--
CakTC33755	Ca(ICCV2/PI489777)SNP_22699	451	G	A	44	52	--	--
	Ca(ICCV2/PI489777)SNP_22700	874	T	G	26	49	--	--
	Ca(ICCV2/PI489777)SNP_22701	2929	A	G	4	9	--	--
CakTC26947	Ca(ICCV2/PI489777)SNP_22702	15	C	T	30	18	--	--
	Ca(ICCV2/PI489777)SNP_22703	37	C	T	28	21	--	--
	Ca(ICCV2/PI489777)SNP_22704	81	T	C	39	31	--	--
	Ca(ICCV2/PI489777)SNP_22705	459	T	C	30	30	--	--
CakTC30693	Ca(ICCV2/PI489777)SNP_22706	207	T	C	4	5	--	--
	Ca(ICCV2/PI489777)SNP_22707	247	T	C	3	6	--	--
	Ca(ICCV2/PI489777)SNP_22708	381	T	G	3	7	--	--
CakTC35182	Ca(ICCV2/PI489777)SNP_22709	217	T	C	8	3	--	--
	Ca(ICCV2/PI489777)SNP_22710	3181	C	T	14	5	--	--
CakTC22267	Ca(ICCV2/PI489777)SNP_22711	1309	A	T	22	8	Flower	bud
	Ca(ICCV2/PI489777)SNP_22712	1435	G	T	33	9	Flower	bud
CakTC31067	Ca(ICCV2/PI489777)SNP_22713	1011	A	G	14	5	--	--

	Ca(ICCV2/PI489777)SNP_22714	1071	G	A	12	5	--	--
CakTC37741	Ca(ICCV2/PI489777)SNP_22715	397	C	A	13	8	--	--
	Ca(ICCV2/PI489777)SNP_22716	722	C	T	12	6	--	--
	Ca(ICCV2/PI489777)SNP_22717	791	C	A	6	6	--	--
CakTC41964	Ca(ICCV2/PI489777)SNP_22718	283	A	G	9	3	--	--
CakTC08257	Ca(ICCV2/PI489777)SNP_22719	1364	A	G	3	3	Flower	bud
CakTC39581	Ca(ICCV2/PI489777)SNP_22720	384	A	G	7	3	Young_pod	SNF2
CakTC37609	Ca(ICCV2/PI489777)SNP_22721	268	C	T	3	4	--	--
	Ca(ICCV2/PI489777)SNP_22722	348	A	G	3	7	--	--
	Ca(ICCV2/PI489777)SNP_22723	840	A	G	13	12	--	--
	Ca(ICCV2/PI489777)SNP_22724	927	G	A	14	8	--	--
CakTC30278	Ca(ICCV2/PI489777)SNP_22725	747	G	A	7	5	Root	--
	Ca(ICCV2/PI489777)SNP_22726	816	A	G	6	6	Root	--
	Ca(ICCV2/PI489777)SNP_22727	855	A	T	7	6	Root	--
	Ca(ICCV2/PI489777)SNP_22728	909	A	T	4	6	Root	--
	Ca(ICCV2/PI489777)SNP_22729	1124	C	G	9	8	Root	--
	Ca(ICCV2/PI489777)SNP_22730	1951	A	G	4	15	Root	--
	Ca(ICCV2/PI489777)SNP_22731	2485	C	T	5	4	Root	--
CakTC43169	Ca(ICCV2/PI489777)SNP_22732	755	C	T	5	8	--	--
CakTC41508	Ca(ICCV2/PI489777)SNP_22733	1737	G	A	5	11	--	--
	Ca(ICCV2/PI489777)SNP_22734	2233	G	A	8	26	--	--
	Ca(ICCV2/PI489777)SNP_22735	2288	A	G	6	26	--	--
	Ca(ICCV2/PI489777)SNP_22736	2314	G	A	6	17	--	--
	Ca(ICCV2/PI489777)SNP_22737	2320	T	A	3	3	--	--
CakTC41253	Ca(ICCV2/PI489777)SNP_22738	563	C	T	4	3	--	ARR-B
	Ca(ICCV2/PI489777)SNP_22739	938	T	C	9	4	--	ARR-B
	Ca(ICCV2/PI489777)SNP_22740	1059	C	G	10	4	--	ARR-B
CakTC18070	Ca(ICCV2/PI489777)SNP_22741	185	A	G	6	4	--	--
	Ca(ICCV2/PI489777)SNP_22742	317	G	C	9	4	--	--
	Ca(ICCV2/PI489777)SNP_22743	413	G	A	6	3	--	--
	Ca(ICCV2/PI489777)SNP_22744	428	C	T	4	3	--	--
	Ca(ICCV2/PI489777)SNP_22745	439	G	T	5	3	--	--
CakTC38547	Ca(ICCV2/PI489777)SNP_22746	521	A	T	7	13	--	--
	Ca(ICCV2/PI489777)SNP_22747	900	A	G	12	14	--	--
	Ca(ICCV2/PI489777)SNP_22748	963	T	G	6	10	--	--
CakTC28990	Ca(ICCV2/PI489777)SNP_22749	150	C	T	5	3	Young_pod	--
	Ca(ICCV2/PI489777)SNP_22750	190	T	G	5	3	Young_pod	--
	Ca(ICCV2/PI489777)SNP_22751	239	C	G	5	3	Young_pod	--
	Ca(ICCV2/PI489777)SNP_22752	616	A	G	4	5	Young_pod	--
	Ca(ICCV2/PI489777)SNP_22753	1039	A	G	15	3	Young_pod	--
	Ca(ICCV2/PI489777)SNP_22754	1138	T	C	11	4	Young_pod	--
	Ca(ICCV2/PI489777)SNP_22755	1363	A	G	8	4	Young_pod	--
	Ca(ICCV2/PI489777)SNP_22756	1531	C	T	3	3	Young_pod	--
	Ca(ICCV2/PI489777)SNP_22757	1970	A	G	9	3	Young_pod	--
	Ca(ICCV2/PI489777)SNP_22758	2316	A	G	8	5	Young_pod	--
	Ca(ICCV2/PI489777)SNP_22759	2516	A	G	3	5	Young_pod	--
CakTC28670	Ca(ICCV2/PI489777)SNP_22760	816	C	T	3	3	--	--
	Ca(ICCV2/PI489777)SNP_22761	972	T	C	5	3	--	--
	Ca(ICCV2/PI489777)SNP_22762	1030	C	T	4	3	--	--
	Ca(ICCV2/PI489777)SNP_22763	1975	C	T	4	3	--	--
	Ca(ICCV2/PI489777)SNP_22764	2255	G	A	3	4	--	--
CakTC38502	Ca(ICCV2/PI489777)SNP_22765	76	A	G	3	7	--	--
	Ca(ICCV2/PI489777)SNP_22766	634	A	G	26	18	--	--
	Ca(ICCV2/PI489777)SNP_22767	850	A	G	28	18	--	--
	Ca(ICCV2/PI489777)SNP_22768	996	G	T	18	13	--	--
CakTC41523	Ca(ICCV2/PI489777)SNP_22769	1460	C	T	6	3	--	--
	Ca(ICCV2/PI489777)SNP_22770	1766	C	T	15	5	--	--
	Ca(ICCV2/PI489777)SNP_22771	2260	A	G	4	4	--	--
CakTC34486	Ca(ICCV2/PI489777)SNP_22772	763	T	C	8	3	--	--
CakTC42780	Ca(ICCV2/PI489777)SNP_22773	87	A	T	4	11	--	--
	Ca(ICCV2/PI489777)SNP_22774	97	T	A	4	12	--	--
	Ca(ICCV2/PI489777)SNP_22775	188	T	A	10	24	--	--
	Ca(ICCV2/PI489777)SNP_22776	349	T	C	9	29	--	--
	Ca(ICCV2/PI489777)SNP_22777	728	A	C	10	32	--	--
	Ca(ICCV2/PI489777)SNP_22778	1142	G	A	14	31	--	--
	Ca(ICCV2/PI489777)SNP_22779	1190	A	G	15	35	--	--
	Ca(ICCV2/PI489777)SNP_22780	1598	T	A	14	13	--	--
CakTC20158	Ca(ICCV2/PI489777)SNP_22781	233	C	G	13	5	--	--
	Ca(ICCV2/PI489777)SNP_22782	578	A	G	10	6	--	--
CakTC42852	Ca(ICCV2/PI489777)SNP_22783	995	G	A	5	3	Flower	bud
CakTC27525	Ca(ICCV2/PI489777)SNP_22784	131	G	C	40	6	--	--
	Ca(ICCV2/PI489777)SNP_22785	284	G	T	71	14	--	--
	Ca(ICCV2/PI489777)SNP_22786	314	A	C	70	18	--	--
	Ca(ICCV2/PI489777)SNP_22787	390	G	C	103	29	--	--

	Ca(ICCV2/PI489777)SNP_22788	486	T	C	99	32	--	--
	Ca(ICCV2/PI489777)SNP_22789	1095	C	T	94	13	--	--
	Ca(ICCV2/PI489777)SNP_22790	1566	T	C	52	6	--	--
CakTC39673	Ca(ICCV2/PI489777)SNP_22791	85	G	C	41	6	--	C2H2
	Ca(ICCV2/PI489777)SNP_22792	208	C	A	49	7	--	C2H2
	Ca(ICCV2/PI489777)SNP_22793	272	A	C	44	7	--	C2H2
	Ca(ICCV2/PI489777)SNP_22794	434	G	A	40	8	--	C2H2
	Ca(ICCV2/PI489777)SNP_22795	887	C	T	51	11	--	C2H2
	Ca(ICCV2/PI489777)SNP_22796	903	T	C	52	11	--	C2H2
	Ca(ICCV2/PI489777)SNP_22797	957	A	C	54	9	--	C2H2
	Ca(ICCV2/PI489777)SNP_22798	3399	C	T	8	3	--	C2H2
CakTC42647	Ca(ICCV2/PI489777)SNP_22799	729	T	C	8	3	--	--
	Ca(ICCV2/PI489777)SNP_22800	777	T	A	10	3	--	--
	Ca(ICCV2/PI489777)SNP_22801	939	G	T	7	6	--	--
	Ca(ICCV2/PI489777)SNP_22802	945	C	T	7	5	--	--
	Ca(ICCV2/PI489777)SNP_22803	1020	G	T	7	3	--	--
	Ca(ICCV2/PI489777)SNP_22804	1080	A	G	11	9	--	--
	Ca(ICCV2/PI489777)SNP_22805	1089	G	A	11	9	--	--
	Ca(ICCV2/PI489777)SNP_22806	1244	T	C	9	8	--	--
	Ca(ICCV2/PI489777)SNP_22807	1662	T	G	6	8	--	--
	Ca(ICCV2/PI489777)SNP_22808	1789	G	A	6	5	--	--
CakTC29574	Ca(ICCV2/PI489777)SNP_22809	679	A	T	6	8	--	--
	Ca(ICCV2/PI489777)SNP_22810	703	T	C	6	7	--	--
CakTC26782	Ca(ICCV2/PI489777)SNP_22811	245	A	G	4	3	--	--
	Ca(ICCV2/PI489777)SNP_22812	278	A	G	4	3	--	--
	Ca(ICCV2/PI489777)SNP_22813	358	C	T	3	3	--	--
	Ca(ICCV2/PI489777)SNP_22814	427	T	C	3	4	--	--
CakTC38138	Ca(ICCV2/PI489777)SNP_22815	23	A	G	10	7	Shoot	--
	Ca(ICCV2/PI489777)SNP_22816	260	T	C	18	17	Shoot	--
	Ca(ICCV2/PI489777)SNP_22817	413	A	G	21	18	Shoot	--
	Ca(ICCV2/PI489777)SNP_22818	939	A	G	6	3	Shoot	--
CakTC27890	Ca(ICCV2/PI489777)SNP_22819	376	C	G	24	20	--	--
	Ca(ICCV2/PI489777)SNP_22820	453	T	C	32	27	--	--
	Ca(ICCV2/PI489777)SNP_22821	1105	G	A	25	14	--	--
	Ca(ICCV2/PI489777)SNP_22822	1268	T	C	32	11	--	--
	Ca(ICCV2/PI489777)SNP_22823	1336	G	T	22	9	--	--
CakTC30515	Ca(ICCV2/PI489777)SNP_22824	1284	C	T	3	3	--	--
	Ca(ICCV2/PI489777)SNP_22825	1290	C	T	3	3	--	--
	Ca(ICCV2/PI489777)SNP_22826	1365	A	C	4	3	--	--
CakTC30623	Ca(ICCV2/PI489777)SNP_22827	681	A	T	14	11	--	--
	Ca(ICCV2/PI489777)SNP_22828	927	T	G	9	5	--	--
CakTC26081	Ca(ICCV2/PI489777)SNP_22829	469	G	T	37	13	--	--
	Ca(ICCV2/PI489777)SNP_22830	2126	C	T	47	22	--	--
	Ca(ICCV2/PI489777)SNP_22831	2226	G	A	45	27	--	--
CakTC24534	Ca(ICCV2/PI489777)SNP_22832	760	C	G	5	6	Flower	bud
	Ca(ICCV2/PI489777)SNP_22833	833	A	G	5	5	Flower	bud
	Ca(ICCV2/PI489777)SNP_22834	836	C	G	5	4	Flower	bud
	Ca(ICCV2/PI489777)SNP_22835	854	G	T	5	5	Flower	bud
	Ca(ICCV2/PI489777)SNP_22836	867	G	A	5	5	Flower	bud
	Ca(ICCV2/PI489777)SNP_22837	922	A	G	4	4	Flower	bud
CakTC27123	Ca(ICCV2/PI489777)SNP_22838	167	T	C	56	24	--	--
CakTC37320	Ca(ICCV2/PI489777)SNP_22839	224	G	T	45	26	--	--
	Ca(ICCV2/PI489777)SNP_22840	574	G	A	32	35	--	--
	Ca(ICCV2/PI489777)SNP_22841	600	T	C	28	33	--	--
	Ca(ICCV2/PI489777)SNP_22842	831	C	T	47	27	--	--
	Ca(ICCV2/PI489777)SNP_22843	1020	A	C	34	11	--	--
	Ca(ICCV2/PI489777)SNP_22844	1098	G	T	22	5	--	--
CakTC25296	Ca(ICCV2/PI489777)SNP_22845	897	A	G	20	33	--	--
CakTC42349	Ca(ICCV2/PI489777)SNP_22846	437	T	C	4	3	--	bZIP
	Ca(ICCV2/PI489777)SNP_22847	860	T	C	9	7	--	bZIP
	Ca(ICCV2/PI489777)SNP_22848	1406	G	A	8	7	--	bZIP
	Ca(ICCV2/PI489777)SNP_22849	1497	G	T	6	7	--	bZIP
CakTC40149	Ca(ICCV2/PI489777)SNP_22850	377	C	G	13	6	--	--
	Ca(ICCV2/PI489777)SNP_22851	697	T	G	10	9	--	--
	Ca(ICCV2/PI489777)SNP_22852	959	T	G	8	8	--	--
CakTC34720	Ca(ICCV2/PI489777)SNP_22853	205	T	G	6	15	--	--
	Ca(ICCV2/PI489777)SNP_22854	220	C	A	9	14	--	--
	Ca(ICCV2/PI489777)SNP_22855	263	T	C	9	13	--	--
	Ca(ICCV2/PI489777)SNP_22856	600	T	C	12	11	--	--
	Ca(ICCV2/PI489777)SNP_22857	718	T	G	12	11	--	--
	Ca(ICCV2/PI489777)SNP_22858	897	T	G	18	9	--	--
	Ca(ICCV2/PI489777)SNP_22859	1077	T	C	21	11	--	--
	Ca(ICCV2/PI489777)SNP_22860	1261	G	C	31	14	--	--
	Ca(ICCV2/PI489777)SNP_22861	1869	A	G	24	6	--	--

	Ca(ICCV2/PI489777)SNP_22862	1986	T	C	25	11	--	--
	Ca(ICCV2/PI489777)SNP_22863	2253	A	C	10	6	--	--
	Ca(ICCV2/PI489777)SNP_22864	2739	T	C	24	17	--	--
	Ca(ICCV2/PI489777)SNP_22865	2907	C	T	28	20	--	--
	Ca(ICCV2/PI489777)SNP_22866	3355	A	G	14	10	--	--
	Ca(ICCV2/PI489777)SNP_22867	3426	A	T	14	8	--	--
CakTC41564	Ca(ICCV2/PI489777)SNP_22868	547	G	A	5	22	--	--
CakTC42580	Ca(ICCV2/PI489777)SNP_22869	253	G	T	3	4	--	--
	Ca(ICCV2/PI489777)SNP_22870	352	A	G	8	6	--	--
	Ca(ICCV2/PI489777)SNP_22871	387	C	T	8	6	--	--
	Ca(ICCV2/PI489777)SNP_22872	400	T	G	7	6	--	--
	Ca(ICCV2/PI489777)SNP_22873	927	C	A	12	4	--	--
	Ca(ICCV2/PI489777)SNP_22874	1031	T	C	10	3	--	--
CakTC24934	Ca(ICCV2/PI489777)SNP_22875	342	C	T	5	3	--	--
	Ca(ICCV2/PI489777)SNP_22876	359	C	T	4	3	--	--
	Ca(ICCV2/PI489777)SNP_22877	392	C	A	4	3	--	--
	Ca(ICCV2/PI489777)SNP_22878	401	A	G	5	3	--	--
	Ca(ICCV2/PI489777)SNP_22879	652	A	G	4	3	--	--
CakTC38955	Ca(ICCV2/PI489777)SNP_22880	200	A	G	11	11	--	--
	Ca(ICCV2/PI489777)SNP_22881	638	G	A	71	56	--	--
CakTC25178	Ca(ICCV2/PI489777)SNP_22882	143	C	T	15	5	--	--
	Ca(ICCV2/PI489777)SNP_22883	350	A	C	33	8	--	--
	Ca(ICCV2/PI489777)SNP_22884	378	G	A	34	15	--	--
CakTC27093	Ca(ICCV2/PI489777)SNP_22885	1049	C	T	8	4	--	--
	Ca(ICCV2/PI489777)SNP_22886	1222	A	G	10	7	--	--
	Ca(ICCV2/PI489777)SNP_22887	1458	C	T	11	10	--	--
	Ca(ICCV2/PI489777)SNP_22888	2245	A	G	13	9	--	--
	Ca(ICCV2/PI489777)SNP_22889	2306	C	T	13	10	--	--
	Ca(ICCV2/PI489777)SNP_22890	2391	A	G	10	9	--	--
	Ca(ICCV2/PI489777)SNP_22891	2404	T	C	13	9	--	--
	Ca(ICCV2/PI489777)SNP_22892	2623	A	C	11	12	--	--
	Ca(ICCV2/PI489777)SNP_22893	3313	C	T	19	4	--	--
	Ca(ICCV2/PI489777)SNP_22894	3661	G	T	14	8	--	--
	Ca(ICCV2/PI489777)SNP_22895	3662	C	A	14	8	--	--
CakTC29202	Ca(ICCV2/PI489777)SNP_22896	2557	G	T	5	4	--	--
	Ca(ICCV2/PI489777)SNP_22897	3100	G	C	3	3	--	--
CakTC37613	Ca(ICCV2/PI489777)SNP_22898	1011	C	T	25	24	--	--
	Ca(ICCV2/PI489777)SNP_22899	1109	G	A	21	19	--	--
CakTC30076	Ca(ICCV2/PI489777)SNP_22900	547	G	T	7	3	--	--
CakTC24682	Ca(ICCV2/PI489777)SNP_22901	268	G	A	3	5	--	--
	Ca(ICCV2/PI489777)SNP_22902	370	G	A	3	6	--	--
	Ca(ICCV2/PI489777)SNP_22903	385	G	A	4	6	--	--
	Ca(ICCV2/PI489777)SNP_22904	502	G	T	5	8	--	--
	Ca(ICCV2/PI489777)SNP_22905	573	T	A	3	10	--	--
	Ca(ICCV2/PI489777)SNP_22906	901	G	A	5	9	--	--
CakTC34650	Ca(ICCV2/PI489777)SNP_22907	64	A	C	11	15	--	--
	Ca(ICCV2/PI489777)SNP_22908	516	T	C	3	8	--	--
	Ca(ICCV2/PI489777)SNP_22909	542	G	T	3	3	--	--
CakTC25374	Ca(ICCV2/PI489777)SNP_22910	62	A	C	9	3	--	--
	Ca(ICCV2/PI489777)SNP_22911	77	A	C	8	4	--	--
	Ca(ICCV2/PI489777)SNP_22912	603	A	G	17	3	--	--
	Ca(ICCV2/PI489777)SNP_22913	809	C	A	16	4	--	--
	Ca(ICCV2/PI489777)SNP_22914	814	C	T	13	4	--	--
	Ca(ICCV2/PI489777)SNP_22915	858	C	T	16	3	--	--
	Ca(ICCV2/PI489777)SNP_22916	884	C	T	11	8	--	--
	Ca(ICCV2/PI489777)SNP_22917	1274	T	G	9	7	--	--
	Ca(ICCV2/PI489777)SNP_22918	1427	A	G	11	5	--	--
	Ca(ICCV2/PI489777)SNP_22919	1436	G	T	13	6	--	--
	Ca(ICCV2/PI489777)SNP_22920	2499	G	A	7	5	--	--
	Ca(ICCV2/PI489777)SNP_22921	2524	T	G	8	5	--	--
CakTC40286	Ca(ICCV2/PI489777)SNP_22922	695	T	G	3	3	--	--
	Ca(ICCV2/PI489777)SNP_22923	696	C	T	3	3	--	--
CakTC09154	Ca(ICCV2/PI489777)SNP_22924	334	T	A	3	3	--	--
CakTC42068	Ca(ICCV2/PI489777)SNP_22925	158	T	G	13	21	--	--
	Ca(ICCV2/PI489777)SNP_22926	651	T	C	22	23	--	--
CakTC32656	Ca(ICCV2/PI489777)SNP_22927	1180	G	A	12	23	--	--
	Ca(ICCV2/PI489777)SNP_22928	1275	G	C	10	22	--	--
	Ca(ICCV2/PI489777)SNP_22929	1546	T	C	20	8	--	--
	Ca(ICCV2/PI489777)SNP_22930	1888	A	T	28	11	--	--
	Ca(ICCV2/PI489777)SNP_22931	1895	T	G	26	8	--	--
CakTC41287	Ca(ICCV2/PI489777)SNP_22932	946	C	T	5	3	--	--
CakTC36985	Ca(ICCV2/PI489777)SNP_22933	1160	C	G	13	7	--	--
	Ca(ICCV2/PI489777)SNP_22934	1690	A	C	34	10	--	--
	Ca(ICCV2/PI489777)SNP_22935	2222	C	T	33	14	--	--

	Ca(ICCV2/PI489777)SNP_22936	2255	G	A	33	13	--	--
	Ca(ICCV2/PI489777)SNP_22937	2310	A	G	18	9	--	--
	Ca(ICCV2/PI489777)SNP_22938	2383	G	A	22	9	--	--
CakTC34036	Ca(ICCV2/PI489777)SNP_22939	205	T	G	4	5	--	--
CakTC40302	Ca(ICCV2/PI489777)SNP_22940	305	T	C	10	4	--	--
	Ca(ICCV2/PI489777)SNP_22941	399	A	T	10	5	--	--
	Ca(ICCV2/PI489777)SNP_22942	1616	A	G	8	5	--	--
	Ca(ICCV2/PI489777)SNP_22943	1754	C	G	8	4	--	--
CakTC38560	Ca(ICCV2/PI489777)SNP_22944	529	A	T	10	5	--	TRAF
	Ca(ICCV2/PI489777)SNP_22945	628	T	C	7	7	--	TRAF
	Ca(ICCV2/PI489777)SNP_22946	896	A	G	3	4	--	TRAF
CakTC24036	Ca(ICCV2/PI489777)SNP_22947	116	A	G	65	190	--	--
	Ca(ICCV2/PI489777)SNP_22948	160	A	G	66	207	--	--
	Ca(ICCV2/PI489777)SNP_22949	310	G	A	97	262	--	--
	Ca(ICCV2/PI489777)SNP_22950	844	C	T	42	6	--	--
CakTC14163	Ca(ICCV2/PI489777)SNP_22951	320	C	T	24	8	--	--
CakTC42370	Ca(ICCV2/PI489777)SNP_22952	136	G	A	14	3	--	--
	Ca(ICCV2/PI489777)SNP_22953	220	T	C	35	6	--	--
	Ca(ICCV2/PI489777)SNP_22954	271	A	G	39	7	--	--
	Ca(ICCV2/PI489777)SNP_22955	289	C	T	40	6	--	--
	Ca(ICCV2/PI489777)SNP_22956	313	A	G	39	13	--	--
	Ca(ICCV2/PI489777)SNP_22957	382	G	A	36	11	--	--
	Ca(ICCV2/PI489777)SNP_22958	424	T	C	35	12	--	--
	Ca(ICCV2/PI489777)SNP_22959	1606	G	A	38	19	--	--
	Ca(ICCV2/PI489777)SNP_22960	1708	G	A	30	16	--	--
	Ca(ICCV2/PI489777)SNP_22961	1721	A	T	23	11	--	--
CakTC38987	Ca(ICCV2/PI489777)SNP_22962	1118	A	C	3	5	--	bHLH
	Ca(ICCV2/PI489777)SNP_22963	1284	T	C	3	4	--	bHLH
CakTC26870	Ca(ICCV2/PI489777)SNP_22964	665	A	C	5	3	--	--
CakTC28548	Ca(ICCV2/PI489777)SNP_22965	679	A	G	7	10	--	--
CakTC41148	Ca(ICCV2/PI489777)SNP_22966	122	T	A	12	14	--	--
	Ca(ICCV2/PI489777)SNP_22967	379	A	T	98	56	--	--
	Ca(ICCV2/PI489777)SNP_22968	524	G	A	112	53	--	--
	Ca(ICCV2/PI489777)SNP_22969	614	G	A	92	46	--	--
CakTC34312	Ca(ICCV2/PI489777)SNP_22970	2805	T	C	18	18	--	--
	Ca(ICCV2/PI489777)SNP_22971	3286	T	C	10	18	--	--
	Ca(ICCV2/PI489777)SNP_22972	4639	C	T	5	6	--	--
	Ca(ICCV2/PI489777)SNP_22973	5362	A	T	10	13	--	--
	Ca(ICCV2/PI489777)SNP_22974	5455	G	A	10	7	--	--
	Ca(ICCV2/PI489777)SNP_22975	5672	T	G	15	13	--	--
CakTC42313	Ca(ICCV2/PI489777)SNP_22976	244	A	G	29	10	--	TPR
	Ca(ICCV2/PI489777)SNP_22977	273	C	G	30	10	--	TPR
	Ca(ICCV2/PI489777)SNP_22978	504	T	C	15	7	--	TPR
	Ca(ICCV2/PI489777)SNP_22979	588	A	C	16	8	--	TPR
	Ca(ICCV2/PI489777)SNP_22980	618	G	A	17	9	--	TPR
	Ca(ICCV2/PI489777)SNP_22981	924	G	A	23	17	--	TPR
	Ca(ICCV2/PI489777)SNP_22982	1468	G	A	36	13	--	TPR
CakTC40277	Ca(ICCV2/PI489777)SNP_22983	105	T	C	13	7	--	--
CakTC37188	Ca(ICCV2/PI489777)SNP_22984	108	C	A	11	5	--	--
	Ca(ICCV2/PI489777)SNP_22985	170	G	A	12	5	--	--
	Ca(ICCV2/PI489777)SNP_22986	207	A	C	13	5	--	--
	Ca(ICCV2/PI489777)SNP_22987	446	C	A	9	4	--	--
CakTC33364	Ca(ICCV2/PI489777)SNP_22988	1035	G	T	6	19	--	--
	Ca(ICCV2/PI489777)SNP_22989	1991	G	C	10	3	--	--
	Ca(ICCV2/PI489777)SNP_22990	2022	T	G	10	3	--	--
CakTC38295	Ca(ICCV2/PI489777)SNP_22991	170	C	G	9	8	--	CCAAT
	Ca(ICCV2/PI489777)SNP_22992	266	T	C	8	9	--	CCAAT
	Ca(ICCV2/PI489777)SNP_22993	1120	C	T	23	18	--	CCAAT
CakTC43196	Ca(ICCV2/PI489777)SNP_22994	365	A	G	38	78	--	--
	Ca(ICCV2/PI489777)SNP_22995	378	T	C	45	84	--	--
	Ca(ICCV2/PI489777)SNP_22996	467	G	A	40	72	--	--
	Ca(ICCV2/PI489777)SNP_22997	485	C	A	41	81	--	--
	Ca(ICCV2/PI489777)SNP_22998	569	A	T	31	67	--	--
CakTC24860	Ca(ICCV2/PI489777)SNP_22999	457	T	C	19	3	--	TUB
	Ca(ICCV2/PI489777)SNP_23000	954	T	G	12	3	--	TUB
CakTC24334	Ca(ICCV2/PI489777)SNP_23001	1505	A	G	11	3	--	--
CakTC27937	Ca(ICCV2/PI489777)SNP_23002	2150	T	G	8	5	--	--
	Ca(ICCV2/PI489777)SNP_23003	2256	T	C	6	11	--	--
CakTC35862	Ca(ICCV2/PI489777)SNP_23004	711	G	A	10	6	--	--
	Ca(ICCV2/PI489777)SNP_23005	906	A	G	8	4	--	--
	Ca(ICCV2/PI489777)SNP_23006	1053	A	G	12	5	--	--
	Ca(ICCV2/PI489777)SNP_23007	1806	C	T	14	12	--	--
	Ca(ICCV2/PI489777)SNP_23008	1959	G	A	12	9	--	--
	Ca(ICCV2/PI489777)SNP_23009	2532	T	C	7	3	--	--

CakTC08485	Ca(ICCV2/PI489777)SNP_23010	526	C	G	4	6	--	--
CakTC10979	Ca(ICCV2/PI489777)SNP_23011	335	T	C	8	3	--	--
	Ca(ICCV2/PI489777)SNP_23012	766	C	T	9	7	--	--
	Ca(ICCV2/PI489777)SNP_23013	862	G	T	6	8	--	--
	Ca(ICCV2/PI489777)SNP_23014	1159	A	G	11	7	--	--
	Ca(ICCV2/PI489777)SNP_23015	1321	T	G	25	10	--	--
	Ca(ICCV2/PI489777)SNP_23016	1648	A	T	32	12	--	--
	Ca(ICCV2/PI489777)SNP_23017	2014	C	T	10	7	--	--
	Ca(ICCV2/PI489777)SNP_23018	2092	T	C	9	8	--	--
	Ca(ICCV2/PI489777)SNP_23019	2206	T	C	11	7	--	--
CakTC34735	Ca(ICCV2/PI489777)SNP_23020	263	C	G	5	3	--	--
	Ca(ICCV2/PI489777)SNP_23021	405	G	C	4	3	--	--
	Ca(ICCV2/PI489777)SNP_23022	455	A	G	4	3	--	--
CakTC11473	Ca(ICCV2/PI489777)SNP_23023	856	T	G	15	10	--	--
	Ca(ICCV2/PI489777)SNP_23024	1271	G	T	9	7	--	--
	Ca(ICCV2/PI489777)SNP_23025	1310	C	T	6	6	--	--
CakTC29811	Ca(ICCV2/PI489777)SNP_23026	340	A	G	4	4	--	--
	Ca(ICCV2/PI489777)SNP_23027	387	T	A	3	5	--	--
CakTC26427	Ca(ICCV2/PI489777)SNP_23028	514	T	C	21	14	--	--
	Ca(ICCV2/PI489777)SNP_23029	642	G	A	25	15	--	--
	Ca(ICCV2/PI489777)SNP_23030	1807	T	C	5	7	--	--
	Ca(ICCV2/PI489777)SNP_23031	2053	A	T	12	10	--	--
	Ca(ICCV2/PI489777)SNP_23032	2752	T	C	5	4	--	--
CakTC08442	Ca(ICCV2/PI489777)SNP_23033	508	G	C	9	3	--	--
	Ca(ICCV2/PI489777)SNP_23034	684	C	G	9	3	--	--
CakTC39518	Ca(ICCV2/PI489777)SNP_23035	642	G	A	61	69	--	--
	Ca(ICCV2/PI489777)SNP_23036	795	G	A	83	108	--	--
CakTC38598	Ca(ICCV2/PI489777)SNP_23037	109	T	C	16	6	--	--
	Ca(ICCV2/PI489777)SNP_23038	424	A	G	21	13	--	--
CakTC37907	Ca(ICCV2/PI489777)SNP_23039	574	T	A	4	4	Flower	bud
CakTC05477	Ca(ICCV2/PI489777)SNP_23040	443	T	G	6	6	--	SET
	Ca(ICCV2/PI489777)SNP_23041	488	C	T	6	7	--	SET
	Ca(ICCV2/PI489777)SNP_23042	872	C	T	3	3	--	SET
CakTC10065	Ca(ICCV2/PI489777)SNP_23043	223	A	G	5	3	--	--
	Ca(ICCV2/PI489777)SNP_23044	357	T	C	7	3	--	--
	Ca(ICCV2/PI489777)SNP_23045	453	T	A	10	3	--	--
	Ca(ICCV2/PI489777)SNP_23046	807	T	C	8	10	--	--
	Ca(ICCV2/PI489777)SNP_23047	972	G	A	5	6	--	--
CakTC39316	Ca(ICCV2/PI489777)SNP_23048	470	G	A	52	59	--	--
	Ca(ICCV2/PI489777)SNP_23049	686	A	G	69	67	--	--
	Ca(ICCV2/PI489777)SNP_23050	800	A	C	55	55	--	--
	Ca(ICCV2/PI489777)SNP_23051	893	A	G	52	42	--	--
	Ca(ICCV2/PI489777)SNP_23052	908	G	A	47	47	--	--
	Ca(ICCV2/PI489777)SNP_23053	959	G	A	59	59	--	--
	Ca(ICCV2/PI489777)SNP_23054	1031	C	T	71	60	--	--
	Ca(ICCV2/PI489777)SNP_23055	1351	G	A	44	33	--	--
CakTC29891	Ca(ICCV2/PI489777)SNP_23056	1252	A	G	23	14	Flower	bud
	Ca(ICCV2/PI489777)SNP_23057	2252	T	G	21	11	Flower	bud
	Ca(ICCV2/PI489777)SNP_23058	2423	A	C	34	21	Flower	bud
	Ca(ICCV2/PI489777)SNP_23059	2521	C	G	38	23	Flower	bud
	Ca(ICCV2/PI489777)SNP_23060	3253	A	G	40	15	Flower	bud
	Ca(ICCV2/PI489777)SNP_23061	3715	C	T	20	8	Flower	bud
	Ca(ICCV2/PI489777)SNP_23062	3765	G	A	20	9	Flower	bud
	Ca(ICCV2/PI489777)SNP_23063	4167	A	C	8	3	Flower	bud
CakTC37647	Ca(ICCV2/PI489777)SNP_23064	810	A	G	11	3	--	--
	Ca(ICCV2/PI489777)SNP_23065	852	G	C	14	3	--	--
	Ca(ICCV2/PI489777)SNP_23066	866	T	C	14	4	--	--
	Ca(ICCV2/PI489777)SNP_23067	878	G	C	15	3	--	--
	Ca(ICCV2/PI489777)SNP_23068	889	T	A	15	3	--	--
	Ca(ICCV2/PI489777)SNP_23069	899	G	C	15	3	--	--
CakTC38409	Ca(ICCV2/PI489777)SNP_23070	222	T	A	16	16	--	--
	Ca(ICCV2/PI489777)SNP_23071	814	G	A	30	21	--	--
	Ca(ICCV2/PI489777)SNP_23072	841	G	C	37	31	--	--
	Ca(ICCV2/PI489777)SNP_23073	1417	A	G	23	17	--	--
CakTC35648	Ca(ICCV2/PI489777)SNP_23074	2059	G	T	9	4	--	--
CakTC28839	Ca(ICCV2/PI489777)SNP_23075	609	C	T	5	5	--	--
	Ca(ICCV2/PI489777)SNP_23076	642	T	C	4	5	--	--
	Ca(ICCV2/PI489777)SNP_23077	723	A	G	4	6	--	--
	Ca(ICCV2/PI489777)SNP_23078	876	C	T	3	7	--	--
	Ca(ICCV2/PI489777)SNP_23079	1017	T	C	3	6	--	--
	Ca(ICCV2/PI489777)SNP_23080	1458	T	A	7	3	--	--
	Ca(ICCV2/PI489777)SNP_23081	1740	A	G	8	4	--	--
	Ca(ICCV2/PI489777)SNP_23082	2034	T	G	3	7	--	--
	Ca(ICCV2/PI489777)SNP_23083	2209	G	A	6	6	--	--

CakTC38832	Ca(ICCV2/PI489777)SNP_23084	1293	A	G	8	4	--	--
CakTC37596	Ca(ICCV2/PI489777)SNP_23085	246	C	A	16	11	--	--
	Ca(ICCV2/PI489777)SNP_23086	456	C	T	33	15	--	--
	Ca(ICCV2/PI489777)SNP_23087	936	G	A	27	26	--	--
CakTC22711	Ca(ICCV2/PI489777)SNP_23088	237	T	C	7	3	--	--
	Ca(ICCV2/PI489777)SNP_23089	310	T	C	8	3	--	--
	Ca(ICCV2/PI489777)SNP_23090	375	A	G	6	3	--	--
CakTC00679	Ca(ICCV2/PI489777)SNP_23091	151	T	C	10	4	--	--
	Ca(ICCV2/PI489777)SNP_23092	331	C	T	12	5	--	--
CakTC25280	Ca(ICCV2/PI489777)SNP_23093	175	A	C	16	20	--	--
	Ca(ICCV2/PI489777)SNP_23094	219	T	C	17	22	--	--
	Ca(ICCV2/PI489777)SNP_23095	385	A	G	30	30	--	--
CakTC41447	Ca(ICCV2/PI489777)SNP_23096	100	A	C	7	3	--	--
	Ca(ICCV2/PI489777)SNP_23097	307	T	C	9	5	--	--
	Ca(ICCV2/PI489777)SNP_23098	352	A	C	8	6	--	--
	Ca(ICCV2/PI489777)SNP_23099	406	T	C	8	4	--	--
CakTC27014	Ca(ICCV2/PI489777)SNP_23100	1411	C	T	6	3	--	--
CakTC28725	Ca(ICCV2/PI489777)SNP_23101	212	T	G	12	8	--	--
	Ca(ICCV2/PI489777)SNP_23102	791	T	C	66	28	--	--
	Ca(ICCV2/PI489777)SNP_23103	902	G	A	58	40	--	--
CakTC27537	Ca(ICCV2/PI489777)SNP_23104	1402	C	A	12	12	--	ARF
	Ca(ICCV2/PI489777)SNP_23105	1766	G	A	29	6	--	ARF
	Ca(ICCV2/PI489777)SNP_23106	1873	G	A	32	5	--	ARF
	Ca(ICCV2/PI489777)SNP_23107	2356	T	C	22	8	--	ARF
	Ca(ICCV2/PI489777)SNP_23108	2492	T	C	23	10	--	ARF
	Ca(ICCV2/PI489777)SNP_23109	2886	G	A	8	7	--	ARF
	Ca(ICCV2/PI489777)SNP_23110	2904	C	A	11	5	--	ARF
	Ca(ICCV2/PI489777)SNP_23111	2966	A	G	10	5	--	ARF
	Ca(ICCV2/PI489777)SNP_23112	2976	T	C	12	7	--	ARF
	Ca(ICCV2/PI489777)SNP_23113	3030	C	T	14	8	--	ARF
	Ca(ICCV2/PI489777)SNP_23114	3171	A	C	13	8	--	ARF
	Ca(ICCV2/PI489777)SNP_23115	4016	C	T	10	5	--	ARF
	Ca(ICCV2/PI489777)SNP_23116	4151	G	A	7	3	--	ARF
CakTC36401	Ca(ICCV2/PI489777)SNP_23117	673	A	C	3	3	--	HB
	Ca(ICCV2/PI489777)SNP_23118	1624	G	A	14	5	--	HB
CakTC12571	Ca(ICCV2/PI489777)SNP_23119	823	A	G	4	3	--	--
	Ca(ICCV2/PI489777)SNP_23120	1175	A	G	17	3	--	--
	Ca(ICCV2/PI489777)SNP_23121	1389	T	C	14	5	--	--
CakTC27792	Ca(ICCV2/PI489777)SNP_23122	146	C	T	7	30	--	bHLH
	Ca(ICCV2/PI489777)SNP_23123	270	C	T	8	36	--	bHLH
	Ca(ICCV2/PI489777)SNP_23124	447	C	T	5	24	--	bHLH
	Ca(ICCV2/PI489777)SNP_23125	876	G	A	9	24	--	bHLH
	Ca(ICCV2/PI489777)SNP_23126	1072	C	T	11	18	--	bHLH
	Ca(ICCV2/PI489777)SNP_23127	1079	C	G	11	19	--	bHLH
CakTC30281	Ca(ICCV2/PI489777)SNP_23128	1064	T	C	4	3	Flower	bud
CakTC43273	Ca(ICCV2/PI489777)SNP_23129	214	A	G	4	4	--	--
CakTC38297	Ca(ICCV2/PI489777)SNP_23130	313	A	G	11	10	--	--
	Ca(ICCV2/PI489777)SNP_23131	690	C	G	34	17	--	--
	Ca(ICCV2/PI489777)SNP_23132	859	T	C	23	9	--	--
CakTC09890	Ca(ICCV2/PI489777)SNP_23133	867	G	A	3	4	--	--
CakTC09170	Ca(ICCV2/PI489777)SNP_23134	2285	T	C	5	4	--	--
	Ca(ICCV2/PI489777)SNP_23135	4853	C	T	6	6	--	--
CakTC24121	Ca(ICCV2/PI489777)SNP_23136	1450	T	C	5	3	--	TPR
	Ca(ICCV2/PI489777)SNP_23137	1484	T	C	4	3	--	TPR
	Ca(ICCV2/PI489777)SNP_23138	1648	A	C	6	4	--	TPR
CakTC42122	Ca(ICCV2/PI489777)SNP_23139	2838	A	C	3	6	--	--
	Ca(ICCV2/PI489777)SNP_23140	2862	T	C	3	6	--	--
	Ca(ICCV2/PI489777)SNP_23141	3204	T	A	29	8	--	--
	Ca(ICCV2/PI489777)SNP_23142	3465	C	T	26	8	--	--
	Ca(ICCV2/PI489777)SNP_23143	3525	G	T	19	9	--	--
	Ca(ICCV2/PI489777)SNP_23144	3537	T	C	16	8	--	--
	Ca(ICCV2/PI489777)SNP_23145	4212	A	T	14	5	--	--
	Ca(ICCV2/PI489777)SNP_23146	4260	A	T	13	5	--	--
	Ca(ICCV2/PI489777)SNP_23147	4439	G	C	4	3	--	--
CakTC26217	Ca(ICCV2/PI489777)SNP_23148	365	A	G	11	3	--	--
CakTC37851	Ca(ICCV2/PI489777)SNP_23149	41	C	T	8	3	--	--
	Ca(ICCV2/PI489777)SNP_23150	104	A	C	8	4	--	--
CakTC24821	Ca(ICCV2/PI489777)SNP_23151	331	C	T	52	27	--	--
	Ca(ICCV2/PI489777)SNP_23152	402	A	G	43	25	--	--
	Ca(ICCV2/PI489777)SNP_23153	608	G	A	65	33	--	--
	Ca(ICCV2/PI489777)SNP_23154	947	A	G	86	36	--	--
	Ca(ICCV2/PI489777)SNP_23155	1155	A	C	39	15	--	--
CakTC28323	Ca(ICCV2/PI489777)SNP_23156	1345	G	T	4	3	--	--
CakTC32815	Ca(ICCV2/PI489777)SNP_23157	14	T	C	13	4	--	--

	Ca(ICCV2/PI489777)SNP_23158	38	T	C	21	7	--	--
	Ca(ICCV2/PI489777)SNP_23159	277	T	A	22	8	--	--
	Ca(ICCV2/PI489777)SNP_23160	299	T	C	21	8	--	--
	Ca(ICCV2/PI489777)SNP_23161	319	A	G	16	8	--	--
	Ca(ICCV2/PI489777)SNP_23162	364	T	G	13	8	--	--
CakTC35791	Ca(ICCV2/PI489777)SNP_23163	1429	A	C	32	4	Mature	Leaf
	Ca(ICCV2/PI489777)SNP_23164	2016	A	G	12	10	Mature	Leaf
CakTC42593	Ca(ICCV2/PI489777)SNP_23165	1486	A	G	19	6	--	--
CakTC37976	Ca(ICCV2/PI489777)SNP_23166	849	C	T	27	13	--	--
	Ca(ICCV2/PI489777)SNP_23167	985	A	T	28	13	--	--
	Ca(ICCV2/PI489777)SNP_23168	1162	G	A	25	11	--	--
	Ca(ICCV2/PI489777)SNP_23169	1321	T	C	19	13	--	--
	Ca(ICCV2/PI489777)SNP_23170	1354	G	A	18	9	--	--
	Ca(ICCV2/PI489777)SNP_23171	1531	G	A	12	7	--	--
	Ca(ICCV2/PI489777)SNP_23172	1591	G	A	8	7	--	--
	Ca(ICCV2/PI489777)SNP_23173	1649	C	A	5	7	--	--
	Ca(ICCV2/PI489777)SNP_23174	1743	G	A	6	4	--	--
CakTC33881	Ca(ICCV2/PI489777)SNP_23175	443	G	C	6	4	--	--
	Ca(ICCV2/PI489777)SNP_23176	495	C	T	5	4	--	--
	Ca(ICCV2/PI489777)SNP_23177	516	T	C	4	4	--	--
	Ca(ICCV2/PI489777)SNP_23178	520	A	G	4	4	--	--
CakTC37496	Ca(ICCV2/PI489777)SNP_23179	502	C	T	4	5	--	bHLH
CakTC43010	Ca(ICCV2/PI489777)SNP_23180	227	A	G	3	4	--	--
	Ca(ICCV2/PI489777)SNP_23181	344	G	C	3	4	--	--
CakTC39821	Ca(ICCV2/PI489777)SNP_23182	646	T	C	7	5	--	--
CakTC26246	Ca(ICCV2/PI489777)SNP_23183	303	A	G	30	16	--	--
CakTC37606	Ca(ICCV2/PI489777)SNP_23184	501	C	T	13	8	--	--
	Ca(ICCV2/PI489777)SNP_23185	568	C	A	12	11	--	--
	Ca(ICCV2/PI489777)SNP_23186	832	C	G	11	8	--	--
	Ca(ICCV2/PI489777)SNP_23187	1235	G	C	5	3	--	--
CakTC27738	Ca(ICCV2/PI489777)SNP_23188	565	A	T	147	87	Flower	bud
	Ca(ICCV2/PI489777)SNP_23189	676	G	A	188	114	Flower	bud
	Ca(ICCV2/PI489777)SNP_23190	1249	A	C	206	149	Flower	bud
CakTC40088	Ca(ICCV2/PI489777)SNP_23191	81	A	T	11	3	--	--
	Ca(ICCV2/PI489777)SNP_23192	92	A	C	11	4	--	--
	Ca(ICCV2/PI489777)SNP_23193	93	T	C	11	4	--	--
	Ca(ICCV2/PI489777)SNP_23194	157	A	C	19	4	--	--
	Ca(ICCV2/PI489777)SNP_23195	244	T	G	18	3	--	--
	Ca(ICCV2/PI489777)SNP_23196	592	A	G	12	8	--	--
	Ca(ICCV2/PI489777)SNP_23197	610	T	A	15	9	--	--
	Ca(ICCV2/PI489777)SNP_23198	1424	C	T	10	4	--	--
	Ca(ICCV2/PI489777)SNP_23199	1497	G	A	10	5	--	--
CakTC35343	Ca(ICCV2/PI489777)SNP_23200	124	C	T	3	10	--	--
CakTC42378	Ca(ICCV2/PI489777)SNP_23201	185	C	T	4	6	--	--
	Ca(ICCV2/PI489777)SNP_23202	634	G	A	9	5	--	--
	Ca(ICCV2/PI489777)SNP_23203	746	T	C	9	4	--	--
	Ca(ICCV2/PI489777)SNP_23204	1462	T	C	8	12	--	--
	Ca(ICCV2/PI489777)SNP_23205	1584	A	C	9	9	--	--
	Ca(ICCV2/PI489777)SNP_23206	1668	G	T	9	4	--	--
	Ca(ICCV2/PI489777)SNP_23207	1694	C	G	6	4	--	--
CakTC28455	Ca(ICCV2/PI489777)SNP_23208	349	A	T	14	8	--	--
	Ca(ICCV2/PI489777)SNP_23209	1077	A	C	36	10	--	--
	Ca(ICCV2/PI489777)SNP_23210	1430	T	C	23	16	--	--
CakTC29684	Ca(ICCV2/PI489777)SNP_23211	769	T	A	7	5	--	--
CakTC39624	Ca(ICCV2/PI489777)SNP_23212	794	C	T	5	12	--	--
	Ca(ICCV2/PI489777)SNP_23213	903	T	C	6	9	--	--
	Ca(ICCV2/PI489777)SNP_23214	934	T	C	5	8	--	--
	Ca(ICCV2/PI489777)SNP_23215	1014	T	A	4	6	--	--
	Ca(ICCV2/PI489777)SNP_23216	1058	A	G	4	6	--	--
	Ca(ICCV2/PI489777)SNP_23217	1106	C	A	8	4	--	--
	Ca(ICCV2/PI489777)SNP_23218	1145	G	A	9	3	--	--
CakTC39702	Ca(ICCV2/PI489777)SNP_23219	1181	T	A	39	36	--	--
CakTC27449	Ca(ICCV2/PI489777)SNP_23220	665	T	C	93	17	--	C2C2-CO-like
	Ca(ICCV2/PI489777)SNP_23221	1025	A	G	49	19	--	C2C2-CO-like
	Ca(ICCV2/PI489777)SNP_23222	1088	A	G	48	20	--	C2C2-CO-like
CakTC23127	Ca(ICCV2/PI489777)SNP_23223	411	G	A	10	10	--	--
CakTC38764	Ca(ICCV2/PI489777)SNP_23224	201	C	T	10	13	--	--
	Ca(ICCV2/PI489777)SNP_23225	1231	T	A	6	12	--	--
CakTC10509	Ca(ICCV2/PI489777)SNP_23226	829	A	G	18	6	--	--
	Ca(ICCV2/PI489777)SNP_23227	961	G	C	16	7	--	--
CakTC31191	Ca(ICCV2/PI489777)SNP_23228	1647	T	A	8	25	--	--
	Ca(ICCV2/PI489777)SNP_23229	1971	C	T	6	15	--	--
CakTC37829	Ca(ICCV2/PI489777)SNP_23230	808	C	T	10	4	--	--
	Ca(ICCV2/PI489777)SNP_23231	940	G	A	13	6	--	--

	Ca(ICCV2/PI489777)SNP_23232	1138	A	G	21	10	--	--
	Ca(ICCV2/PI489777)SNP_23233	1285	A	G	19	10	--	--
	Ca(ICCV2/PI489777)SNP_23234	1303	G	A	20	9	--	--
	Ca(ICCV2/PI489777)SNP_23235	1335	A	G	23	8	--	--
	Ca(ICCV2/PI489777)SNP_23236	1489	T	C	20	7	--	--
	Ca(ICCV2/PI489777)SNP_23237	1529	A	G	17	5	--	--
	Ca(ICCV2/PI489777)SNP_23238	1609	G	T	11	4	--	--
CakTC38935	Ca(ICCV2/PI489777)SNP_23239	454	G	A	17	3	--	--
CakTC33747	Ca(ICCV2/PI489777)SNP_23240	607	A	C	70	22	--	--
CakTC32173	Ca(ICCV2/PI489777)SNP_23241	1170	G	A	3	3	Flower	bud
CakTC40761	Ca(ICCV2/PI489777)SNP_23242	536	T	G	72	44	--	--
	Ca(ICCV2/PI489777)SNP_23243	562	A	C	63	46	--	--
	Ca(ICCV2/PI489777)SNP_23244	815	G	T	19	12	--	--
CakTC40700	Ca(ICCV2/PI489777)SNP_23245	369	G	C	11	7	--	--
	Ca(ICCV2/PI489777)SNP_23246	1094	A	G	21	10	--	--
	Ca(ICCV2/PI489777)SNP_23247	1129	C	T	21	13	--	--
CakTC39605	Ca(ICCV2/PI489777)SNP_23248	1078	C	A	6	8	--	--
CakTC38634	Ca(ICCV2/PI489777)SNP_23249	225	A	T	3	3	--	--
	Ca(ICCV2/PI489777)SNP_23250	369	G	A	8	6	--	--
	Ca(ICCV2/PI489777)SNP_23251	469	A	T	8	7	--	--
	Ca(ICCV2/PI489777)SNP_23252	672	C	T	9	8	--	--
CakTC40408	Ca(ICCV2/PI489777)SNP_23253	665	C	T	8	5	--	--
CakTC39259	Ca(ICCV2/PI489777)SNP_23254	269	A	G	7	9	--	--
	Ca(ICCV2/PI489777)SNP_23255	982	C	T	87	24	--	--
CakTC38860	Ca(ICCV2/PI489777)SNP_23256	1129	C	T	8	8	--	--
CakTC27509	Ca(ICCV2/PI489777)SNP_23257	30	C	T	16	20	--	CCAAT
	Ca(ICCV2/PI489777)SNP_23258	330	C	T	29	34	--	CCAAT
CakTC43325	Ca(ICCV2/PI489777)SNP_23259	552	G	A	7	6	--	--
CakTC38839	Ca(ICCV2/PI489777)SNP_23260	788	A	G	9	9	--	--
	Ca(ICCV2/PI489777)SNP_23261	875	G	A	13	13	--	--
	Ca(ICCV2/PI489777)SNP_23262	1300	G	A	11	5	--	--
CakTC19569	Ca(ICCV2/PI489777)SNP_23263	940	T	C	7	8	--	--
CakTC27925	Ca(ICCV2/PI489777)SNP_23264	427	C	T	19	28	--	--
CakTC05876	Ca(ICCV2/PI489777)SNP_23265	72	A	G	3	4	--	MYB-related
CakTC32239	Ca(ICCV2/PI489777)SNP_23266	1642	G	A	3	5	--	--
	Ca(ICCV2/PI489777)SNP_23267	1645	C	T	3	5	--	--
CakTC39388	Ca(ICCV2/PI489777)SNP_23268	304	C	T	16	3	--	--
	Ca(ICCV2/PI489777)SNP_23269	430	C	T	18	4	--	--
	Ca(ICCV2/PI489777)SNP_23270	925	C	T	8	9	--	--
CakTC31426	Ca(ICCV2/PI489777)SNP_23271	1059	G	A	3	5	--	--
CakTC34126	Ca(ICCV2/PI489777)SNP_23272	85	T	C	7	5	--	--
	Ca(ICCV2/PI489777)SNP_23273	240	C	T	16	16	--	--
	Ca(ICCV2/PI489777)SNP_23274	297	C	T	18	18	--	--
	Ca(ICCV2/PI489777)SNP_23275	375	C	T	20	19	--	--
	Ca(ICCV2/PI489777)SNP_23276	552	T	C	17	17	--	--
	Ca(ICCV2/PI489777)SNP_23277	1026	T	C	33	20	--	--
	Ca(ICCV2/PI489777)SNP_23278	3639	C	A	24	32	--	--
	Ca(ICCV2/PI489777)SNP_23279	4077	G	A	39	23	--	--
	Ca(ICCV2/PI489777)SNP_23280	7028	G	C	34	22	--	--
CakTC31140	Ca(ICCV2/PI489777)SNP_23281	694	A	G	7	3	--	--
	Ca(ICCV2/PI489777)SNP_23282	711	C	T	6	3	--	--
CakTC10456	Ca(ICCV2/PI489777)SNP_23283	770	G	T	9	4	--	--
	Ca(ICCV2/PI489777)SNP_23284	784	G	A	9	5	--	--
	Ca(ICCV2/PI489777)SNP_23285	874	C	A	9	4	--	--
CakTC31780	Ca(ICCV2/PI489777)SNP_23286	439	A	T	4	5	--	--
CakTC42821	Ca(ICCV2/PI489777)SNP_23287	482	G	T	42	28	--	--
CakTC22433	Ca(ICCV2/PI489777)SNP_23288	309	T	C	14	5	--	--
	Ca(ICCV2/PI489777)SNP_23289	310	G	A	15	5	--	--
	Ca(ICCV2/PI489777)SNP_23290	475	C	A	11	4	--	--
	Ca(ICCV2/PI489777)SNP_23291	584	A	T	11	5	--	--
	Ca(ICCV2/PI489777)SNP_23292	590	A	C	9	6	--	--
	Ca(ICCV2/PI489777)SNP_23293	631	C	G	13	5	--	--
	Ca(ICCV2/PI489777)SNP_23294	650	C	T	14	7	--	--
	Ca(ICCV2/PI489777)SNP_23295	794	C	T	9	5	--	--
CakTC40374	Ca(ICCV2/PI489777)SNP_23296	209	T	G	9	9	--	--
	Ca(ICCV2/PI489777)SNP_23297	257	C	A	13	17	--	--
	Ca(ICCV2/PI489777)SNP_23298	258	G	A	13	17	--	--
	Ca(ICCV2/PI489777)SNP_23299	285	T	C	14	18	--	--
CakTC37414	Ca(ICCV2/PI489777)SNP_23300	450	C	T	4	5	--	--
	Ca(ICCV2/PI489777)SNP_23301	559	A	G	6	5	--	--
	Ca(ICCV2/PI489777)SNP_23302	598	T	C	4	3	--	--
CakTC25300	Ca(ICCV2/PI489777)SNP_23303	281	C	T	35	9	--	--
	Ca(ICCV2/PI489777)SNP_23304	332	C	T	37	11	--	--
	Ca(ICCV2/PI489777)SNP_23305	520	A	G	26	12	--	--

	Ca(ICCV2/PI489777)SNP_23306	621	G	C	18	10	--	--
	Ca(ICCV2/PI489777)SNP_23307	673	T	G	16	9	--	--
	Ca(ICCV2/PI489777)SNP_23308	701	C	T	13	8	--	--
CakTC29710	Ca(ICCV2/PI489777)SNP_23309	922	C	A	4	5	Shoot	--
	Ca(ICCV2/PI489777)SNP_23310	1038	A	G	4	8	Shoot	--
	Ca(ICCV2/PI489777)SNP_23311	1084	G	A	6	7	Shoot	--
CakTC29708	Ca(ICCV2/PI489777)SNP_23312	522	T	C	22	5	--	--
	Ca(ICCV2/PI489777)SNP_23313	696	A	G	19	5	--	--
CakTC29993	Ca(ICCV2/PI489777)SNP_23314	131	T	G	6	3	--	--
	Ca(ICCV2/PI489777)SNP_23315	1243	C	A	7	9	--	--
	Ca(ICCV2/PI489777)SNP_23316	2282	T	C	11	13	--	--
	Ca(ICCV2/PI489777)SNP_23317	2849	A	G	13	15	--	--
CakTC28365	Ca(ICCV2/PI489777)SNP_23318	239	A	G	5	5	--	--
	Ca(ICCV2/PI489777)SNP_23319	607	A	G	22	11	--	--
CakTC42531	Ca(ICCV2/PI489777)SNP_23320	447	C	T	16	12	--	--
CakTC29982	Ca(ICCV2/PI489777)SNP_23321	1109	C	A	4	6	--	--
	Ca(ICCV2/PI489777)SNP_23322	2547	C	T	6	11	--	--
	Ca(ICCV2/PI489777)SNP_23323	2649	C	G	12	9	--	--
CakTC33460	Ca(ICCV2/PI489777)SNP_23324	156	G	A	6	4	--	--
	Ca(ICCV2/PI489777)SNP_23325	250	T	A	9	7	--	--
	Ca(ICCV2/PI489777)SNP_23326	316	A	G	10	9	--	--
	Ca(ICCV2/PI489777)SNP_23327	848	A	G	9	5	--	--
	Ca(ICCV2/PI489777)SNP_23328	892	G	A	9	4	--	--
	Ca(ICCV2/PI489777)SNP_23329	967	G	A	11	4	--	--
	Ca(ICCV2/PI489777)SNP_23330	1291	A	T	21	13	--	--
	Ca(ICCV2/PI489777)SNP_23331	1690	G	A	11	9	--	--
CakTC38727	Ca(ICCV2/PI489777)SNP_23332	762	G	A	6	3	--	--
CakTC34763	Ca(ICCV2/PI489777)SNP_23333	1512	G	A	6	9	--	--
	Ca(ICCV2/PI489777)SNP_23334	1586	T	A	7	10	--	--
CakTC34659	Ca(ICCV2/PI489777)SNP_23335	502	C	T	8	4	--	--
	Ca(ICCV2/PI489777)SNP_23336	583	C	T	14	4	--	--
	Ca(ICCV2/PI489777)SNP_23337	1844	G	A	20	9	--	--
	Ca(ICCV2/PI489777)SNP_23338	2716	A	G	12	4	--	--
	Ca(ICCV2/PI489777)SNP_23339	3469	C	T	14	6	--	--
	Ca(ICCV2/PI489777)SNP_23340	3616	G	A	11	5	--	--
	Ca(ICCV2/PI489777)SNP_23341	3974	T	C	6	5	--	--
	Ca(ICCV2/PI489777)SNP_23342	4759	T	C	10	3	--	--
	Ca(ICCV2/PI489777)SNP_23343	4884	G	A	9	3	--	--
	Ca(ICCV2/PI489777)SNP_23344	6116	A	C	6	3	--	--
	Ca(ICCV2/PI489777)SNP_23345	6187	A	G	5	3	--	--
CakTC33175	Ca(ICCV2/PI489777)SNP_23346	393	G	C	15	11	--	--
	Ca(ICCV2/PI489777)SNP_23347	429	C	T	16	11	--	--
	Ca(ICCV2/PI489777)SNP_23348	537	G	C	14	7	--	--
	Ca(ICCV2/PI489777)SNP_23349	725	G	A	16	9	--	--
	Ca(ICCV2/PI489777)SNP_23350	786	C	T	16	10	--	--
	Ca(ICCV2/PI489777)SNP_23351	1050	G	A	18	11	--	--
	Ca(ICCV2/PI489777)SNP_23352	1230	G	C	11	7	--	--
	Ca(ICCV2/PI489777)SNP_23353	1404	C	T	15	14	--	--
CakTC40174	Ca(ICCV2/PI489777)SNP_23354	45	T	A	5	3	--	--
	Ca(ICCV2/PI489777)SNP_23355	66	T	G	5	4	--	--
	Ca(ICCV2/PI489777)SNP_23356	995	A	G	12	6	--	--
CakTC39708	Ca(ICCV2/PI489777)SNP_23357	839	C	T	18	40	--	--
	Ca(ICCV2/PI489777)SNP_23358	884	G	A	18	36	--	--
CakTC27002	Ca(ICCV2/PI489777)SNP_23359	36	G	A	3	4	--	--
	Ca(ICCV2/PI489777)SNP_23360	324	T	A	4	4	--	--
CakTC24947	Ca(ICCV2/PI489777)SNP_23361	1041	T	A	114	66	--	--
CakTC25503	Ca(ICCV2/PI489777)SNP_23362	873	C	A	10	6	--	--
	Ca(ICCV2/PI489777)SNP_23363	1455	A	G	10	9	--	--
CakTC41412	Ca(ICCV2/PI489777)SNP_23364	318	G	A	4	4	--	--
CakTC34142	Ca(ICCV2/PI489777)SNP_23365	545	G	A	8	5	--	--
CakTC28418	Ca(ICCV2/PI489777)SNP_23366	1379	G	A	49	13	--	--
	Ca(ICCV2/PI489777)SNP_23367	1618	T	A	24	10	--	--
CakTC08328	Ca(ICCV2/PI489777)SNP_23368	815	G	A	3	3	--	--
CakTC41266	Ca(ICCV2/PI489777)SNP_23369	228	G	A	13	5	--	--
	Ca(ICCV2/PI489777)SNP_23370	279	C	T	18	13	--	--
	Ca(ICCV2/PI489777)SNP_23371	777	G	A	12	17	--	--
	Ca(ICCV2/PI489777)SNP_23372	867	G	A	22	16	--	--
	Ca(ICCV2/PI489777)SNP_23373	880	C	T	25	14	--	--
	Ca(ICCV2/PI489777)SNP_23374	1050	A	G	26	13	--	--
	Ca(ICCV2/PI489777)SNP_23375	1200	T	A	17	24	--	--
	Ca(ICCV2/PI489777)SNP_23376	1282	C	A	16	18	--	--
	Ca(ICCV2/PI489777)SNP_23377	1296	T	A	15	18	--	--
	Ca(ICCV2/PI489777)SNP_23378	1609	G	C	25	13	--	--
	Ca(ICCV2/PI489777)SNP_23379	1716	A	G	25	15	--	--

CakTC25742	Ca(ICCV2/PI489777)SNP_23380	666	A	G	14	20	Shoot	--
	Ca(ICCV2/PI489777)SNP_23381	1053	A	G	24	14	Shoot	--
	Ca(ICCV2/PI489777)SNP_23382	1242	A	G	30	17	Shoot	--
	Ca(ICCV2/PI489777)SNP_23383	1330	C	T	27	16	Shoot	--
	Ca(ICCV2/PI489777)SNP_23384	1665	T	G	6	9	Shoot	--
	Ca(ICCV2/PI489777)SNP_23385	1667	G	T	7	8	Shoot	--
CakTC28904	Ca(ICCV2/PI489777)SNP_23386	180	C	T	6	3	--	--
	Ca(ICCV2/PI489777)SNP_23387	240	A	C	8	4	--	--
CakTC31056	Ca(ICCV2/PI489777)SNP_23388	350	G	C	5	4	--	--
	Ca(ICCV2/PI489777)SNP_23389	828	A	T	20	6	--	--
	Ca(ICCV2/PI489777)SNP_23390	829	A	T	20	6	--	--
CakTC37252	Ca(ICCV2/PI489777)SNP_23391	287	A	G	3	8	--	--
	Ca(ICCV2/PI489777)SNP_23392	488	C	G	4	7	--	--
	Ca(ICCV2/PI489777)SNP_23393	840	G	C	6	7	--	--
CakTC29371	Ca(ICCV2/PI489777)SNP_23394	489	T	C	4	7	Flower	bud
	Ca(ICCV2/PI489777)SNP_23395	555	T	C	5	6	Flower	bud
	Ca(ICCV2/PI489777)SNP_23396	567	C	T	4	5	Flower	bud
CakTC24532	Ca(ICCV2/PI489777)SNP_23397	732	G	A	14	8	--	--
	Ca(ICCV2/PI489777)SNP_23398	1303	T	G	28	11	--	--
	Ca(ICCV2/PI489777)SNP_23399	1349	G	A	22	11	--	--
CakTC29726	Ca(ICCV2/PI489777)SNP_23400	1906	A	C	10	5	--	--
	Ca(ICCV2/PI489777)SNP_23401	2082	A	G	4	4	--	--
CakTC37420	Ca(ICCV2/PI489777)SNP_23402	151	C	A	16	9	--	--
	Ca(ICCV2/PI489777)SNP_23403	349	G	A	27	16	--	--
CakTC42801	Ca(ICCV2/PI489777)SNP_23404	1801	G	C	7	3	--	--
CakTC28597	Ca(ICCV2/PI489777)SNP_23405	925	A	C	26	5	--	--
CakTC28098	Ca(ICCV2/PI489777)SNP_23406	288	A	T	3	4	--	--
	Ca(ICCV2/PI489777)SNP_23407	452	G	C	5	7	--	--
	Ca(ICCV2/PI489777)SNP_23408	564	T	C	12	6	--	--
	Ca(ICCV2/PI489777)SNP_23409	1955	G	A	8	4	--	--
CakTC27103	Ca(ICCV2/PI489777)SNP_23410	4257	G	T	7	7	--	--
	Ca(ICCV2/PI489777)SNP_23411	4546	G	A	19	6	--	--
	Ca(ICCV2/PI489777)SNP_23412	5309	C	T	17	6	--	--
CakTC24581	Ca(ICCV2/PI489777)SNP_23413	1419	C	T	9	8	--	--
	Ca(ICCV2/PI489777)SNP_23414	1894	T	C	7	3	--	--
CakTC32948	Ca(ICCV2/PI489777)SNP_23415	527	T	A	31	10	--	--
	Ca(ICCV2/PI489777)SNP_23416	591	A	G	34	13	--	--
	Ca(ICCV2/PI489777)SNP_23417	1415	G	A	6	8	--	--
	Ca(ICCV2/PI489777)SNP_23418	1957	C	T	5	8	--	--
CakTC39342	Ca(ICCV2/PI489777)SNP_23419	464	C	T	46	26	--	--
CakTC25053	Ca(ICCV2/PI489777)SNP_23420	167	C	G	12	8	--	LUG
	Ca(ICCV2/PI489777)SNP_23421	693	G	A	9	6	--	LUG
	Ca(ICCV2/PI489777)SNP_23422	732	G	T	11	8	--	LUG
	Ca(ICCV2/PI489777)SNP_23423	1296	A	G	19	13	--	LUG
	Ca(ICCV2/PI489777)SNP_23424	1815	G	A	16	15	--	LUG
	Ca(ICCV2/PI489777)SNP_23425	2148	T	C	9	8	--	LUG
	Ca(ICCV2/PI489777)SNP_23426	2181	G	A	11	7	--	LUG
	Ca(ICCV2/PI489777)SNP_23427	2400	A	G	12	10	--	LUG
	Ca(ICCV2/PI489777)SNP_23428	2841	A	C	13	12	--	LUG
	Ca(ICCV2/PI489777)SNP_23429	2984	G	A	14	7	--	LUG
	Ca(ICCV2/PI489777)SNP_23430	3054	A	T	16	6	--	LUG
CakTC29075	Ca(ICCV2/PI489777)SNP_23431	1563	T	C	4	3	--	HB
	Ca(ICCV2/PI489777)SNP_23432	1780	T	C	8	3	--	HB
CakTC37014	Ca(ICCV2/PI489777)SNP_23433	251	A	C	4	5	--	--
	Ca(ICCV2/PI489777)SNP_23434	321	G	A	6	5	--	--
	Ca(ICCV2/PI489777)SNP_23435	701	T	C	3	9	--	--
CakTC39348	Ca(ICCV2/PI489777)SNP_23436	697	A	C	111	44	--	--
	Ca(ICCV2/PI489777)SNP_23437	846	A	C	130	38	--	--
	Ca(ICCV2/PI489777)SNP_23438	907	T	C	133	50	--	--
	Ca(ICCV2/PI489777)SNP_23439	1080	T	C	96	43	--	--
CakTC29848	Ca(ICCV2/PI489777)SNP_23440	145	C	T	7	6	--	--
	Ca(ICCV2/PI489777)SNP_23441	148	G	A	7	6	--	--
	Ca(ICCV2/PI489777)SNP_23442	1688	G	A	33	16	--	--
	Ca(ICCV2/PI489777)SNP_23443	1709	G	A	30	16	--	--
	Ca(ICCV2/PI489777)SNP_23444	1779	G	C	27	15	--	--
	Ca(ICCV2/PI489777)SNP_23445	1992	A	G	35	17	--	--
	Ca(ICCV2/PI489777)SNP_23446	2257	T	G	21	9	--	--
	Ca(ICCV2/PI489777)SNP_23447	2355	C	T	22	12	--	--
	Ca(ICCV2/PI489777)SNP_23448	2373	T	G	27	11	--	--
CakTC15600	Ca(ICCV2/PI489777)SNP_23449	184	T	C	4	4	--	--
CakTC27342	Ca(ICCV2/PI489777)SNP_23450	67	G	C	22	5	--	--
	Ca(ICCV2/PI489777)SNP_23451	121	A	G	23	9	--	--
	Ca(ICCV2/PI489777)SNP_23452	138	C	T	27	27	--	--
	Ca(ICCV2/PI489777)SNP_23453	152	G	A	53	29	--	--

	Ca(ICCV2/PI489777)SNP_23454	153	T	C	54	29	--	--
	Ca(ICCV2/PI489777)SNP_23455	682	G	A	15	6	--	--
CakTC31858	Ca(ICCV2/PI489777)SNP_23456	1520	A	C	12	4	Root	--
CakTC27867	Ca(ICCV2/PI489777)SNP_23457	884	T	C	12	6	--	--
	Ca(ICCV2/PI489777)SNP_23458	959	T	C	8	7	--	--
CakTC32158	Ca(ICCV2/PI489777)SNP_23459	249	A	G	4	4	--	--
CakTC36522	Ca(ICCV2/PI489777)SNP_23460	2075	T	C	20	9	--	--
	Ca(ICCV2/PI489777)SNP_23461	3746	G	A	22	11	--	--
CakTC37671	Ca(ICCV2/PI489777)SNP_23462	163	C	A	7	8	--	--
	Ca(ICCV2/PI489777)SNP_23463	349	C	T	9	8	--	--
	Ca(ICCV2/PI489777)SNP_23464	748	T	C	21	6	--	--
	Ca(ICCV2/PI489777)SNP_23465	1000	T	C	6	4	--	--
	Ca(ICCV2/PI489777)SNP_23466	1080	A	T	4	3	--	--
	Ca(ICCV2/PI489777)SNP_23467	1114	T	C	4	3	--	--
CakTC34550	Ca(ICCV2/PI489777)SNP_23468	1177	G	A	3	8	Flower	bud
	Ca(ICCV2/PI489777)SNP_23469	1285	T	A	18	10	Flower	bud
	Ca(ICCV2/PI489777)SNP_23470	1626	A	G	3	4	Flower	bud
CakTC36072	Ca(ICCV2/PI489777)SNP_23471	532	C	T	23	14	--	--
	Ca(ICCV2/PI489777)SNP_23472	1642	G	A	13	18	--	--
	Ca(ICCV2/PI489777)SNP_23473	2238	A	C	31	13	--	--
	Ca(ICCV2/PI489777)SNP_23474	2548	A	G	25	4	--	--
	Ca(ICCV2/PI489777)SNP_23475	3714	T	C	15	10	--	--
	Ca(ICCV2/PI489777)SNP_23476	3719	C	G	15	10	--	--
	Ca(ICCV2/PI489777)SNP_23477	4702	A	G	6	12	--	--
	Ca(ICCV2/PI489777)SNP_23478	4716	G	A	6	11	--	--
	Ca(ICCV2/PI489777)SNP_23479	4918	G	A	12	17	--	--
	Ca(ICCV2/PI489777)SNP_23480	5371	A	G	5	4	--	--
CakTC38400	Ca(ICCV2/PI489777)SNP_23481	82	A	C	9	25	--	--
CakTC00195	Ca(ICCV2/PI489777)SNP_23482	424	G	C	6	6	--	WRKY
	Ca(ICCV2/PI489777)SNP_23483	456	C	T	8	10	--	WRKY
	Ca(ICCV2/PI489777)SNP_23484	570	T	A	4	9	--	WRKY
	Ca(ICCV2/PI489777)SNP_23485	697	A	C	7	4	--	WRKY
	Ca(ICCV2/PI489777)SNP_23486	707	C	G	7	4	--	WRKY
	Ca(ICCV2/PI489777)SNP_23487	708	T	G	7	4	--	WRKY
	Ca(ICCV2/PI489777)SNP_23488	849	G	A	3	8	--	WRKY
	Ca(ICCV2/PI489777)SNP_23489	1002	A	G	5	13	--	WRKY
	Ca(ICCV2/PI489777)SNP_23490	1134	A	G	4	14	--	WRKY
	Ca(ICCV2/PI489777)SNP_23491	1260	C	G	7	12	--	WRKY
	Ca(ICCV2/PI489777)SNP_23492	1320	C	T	6	12	--	WRKY
CakTC22444	Ca(ICCV2/PI489777)SNP_23493	535	T	A	3	3	--	--
	Ca(ICCV2/PI489777)SNP_23494	703	T	C	4	3	--	--
CakTC39759	Ca(ICCV2/PI489777)SNP_23495	314	C	T	20	6	--	--
	Ca(ICCV2/PI489777)SNP_23496	713	G	A	12	9	--	--
	Ca(ICCV2/PI489777)SNP_23497	1034	T	C	20	8	--	--
	Ca(ICCV2/PI489777)SNP_23498	1076	C	T	21	8	--	--
	Ca(ICCV2/PI489777)SNP_23499	1118	A	T	17	6	--	--
	Ca(ICCV2/PI489777)SNP_23500	1170	A	G	17	8	--	--
CakTC23110	Ca(ICCV2/PI489777)SNP_23501	282	A	G	9	3	Flower	bud
CakTC39589	Ca(ICCV2/PI489777)SNP_23502	472	A	C	10	4	--	--
	Ca(ICCV2/PI489777)SNP_23503	497	T	C	7	4	--	--
CakTC25318	Ca(ICCV2/PI489777)SNP_23504	74	C	T	3	7	--	--
	Ca(ICCV2/PI489777)SNP_23505	220	C	T	3	7	--	--
CakTC40756	Ca(ICCV2/PI489777)SNP_23506	1248	G	A	6	9	--	--
	Ca(ICCV2/PI489777)SNP_23507	1249	C	T	6	9	--	--
	Ca(ICCV2/PI489777)SNP_23508	1432	T	C	7	13	--	--
	Ca(ICCV2/PI489777)SNP_23509	1878	A	G	3	3	--	--
CakTC38189	Ca(ICCV2/PI489777)SNP_23510	146	C	G	6	4	--	Trihelix
CakTC26075	Ca(ICCV2/PI489777)SNP_23511	264	G	C	56	20	--	--
	Ca(ICCV2/PI489777)SNP_23512	1400	T	C	156	55	--	--
	Ca(ICCV2/PI489777)SNP_23513	1409	G	A	158	59	--	--
CakTC09978	Ca(ICCV2/PI489777)SNP_23514	607	A	C	5	3	--	--
	Ca(ICCV2/PI489777)SNP_23515	706	T	C	4	3	--	--
CakTC37326	Ca(ICCV2/PI489777)SNP_23516	387	C	T	28	29	--	--
	Ca(ICCV2/PI489777)SNP_23517	534	T	C	46	46	--	--
	Ca(ICCV2/PI489777)SNP_23518	708	A	G	39	47	--	--
CakTC27587	Ca(ICCV2/PI489777)SNP_23519	550	G	A	8	6	--	--
	Ca(ICCV2/PI489777)SNP_23520	810	C	A	22	5	--	--
	Ca(ICCV2/PI489777)SNP_23521	947	T	G	24	3	--	--
CakTC21835	Ca(ICCV2/PI489777)SNP_23522	95	C	A	6	6	Flower	bud
	Ca(ICCV2/PI489777)SNP_23523	271	T	C	6	8	Flower	bud
CakTC36781	Ca(ICCV2/PI489777)SNP_23524	1892	T	G	8	3	--	--
	Ca(ICCV2/PI489777)SNP_23525	2168	C	T	9	7	--	--
CakTC43221	Ca(ICCV2/PI489777)SNP_23526	784	T	C	13	10	--	--
	Ca(ICCV2/PI489777)SNP_23527	856	T	A	15	14	--	--

	Ca(ICCV2/PI489777)SNP_23528	1178	A	G	26	28	--	--
	Ca(ICCV2/PI489777)SNP_23529	1561	A	G	16	14	--	--
	Ca(ICCV2/PI489777)SNP_23530	1562	G	C	16	14	--	--
CakTC32349	Ca(ICCV2/PI489777)SNP_23531	388	A	C	6	3	--	--
	Ca(ICCV2/PI489777)SNP_23532	487	G	A	13	9	--	--
	Ca(ICCV2/PI489777)SNP_23533	832	T	C	8	16	--	--
	Ca(ICCV2/PI489777)SNP_23534	933	T	G	10	9	--	--
	Ca(ICCV2/PI489777)SNP_23535	1759	C	T	7	7	--	--
	Ca(ICCV2/PI489777)SNP_23536	1941	C	T	11	4	--	--
	Ca(ICCV2/PI489777)SNP_23537	1971	C	G	8	4	--	--
	Ca(ICCV2/PI489777)SNP_23538	2230	A	C	8	4	--	--
	Ca(ICCV2/PI489777)SNP_23539	2788	T	A	6	5	--	--
	Ca(ICCV2/PI489777)SNP_23540	3643	C	T	7	4	--	--
	Ca(ICCV2/PI489777)SNP_23541	4451	T	C	13	4	--	--
	Ca(ICCV2/PI489777)SNP_23542	4474	T	C	11	3	--	--
	Ca(ICCV2/PI489777)SNP_23543	5876	G	A	4	3	--	--
CakTC23392	Ca(ICCV2/PI489777)SNP_23544	803	G	A	23	3	--	--
	Ca(ICCV2/PI489777)SNP_23545	931	T	C	21	4	--	--
	Ca(ICCV2/PI489777)SNP_23546	958	C	A	22	3	--	--
CakTC25784	Ca(ICCV2/PI489777)SNP_23547	173	C	T	11	3	--	--
	Ca(ICCV2/PI489777)SNP_23548	176	C	G	12	3	--	--
	Ca(ICCV2/PI489777)SNP_23549	365	A	T	14	4	--	--
	Ca(ICCV2/PI489777)SNP_23550	406	T	C	15	4	--	--
CakTC36491	Ca(ICCV2/PI489777)SNP_23551	443	C	T	4	3	--	--
CakTC39425	Ca(ICCV2/PI489777)SNP_23552	1250	C	G	5	4	--	--
CakTC24711	Ca(ICCV2/PI489777)SNP_23553	53	G	A	3	3	Flower	bud
CakTC32575	Ca(ICCV2/PI489777)SNP_23554	377	G	A	7	7	--	--
	Ca(ICCV2/PI489777)SNP_23555	639	G	T	9	4	--	--
	Ca(ICCV2/PI489777)SNP_23556	647	A	T	7	4	--	--
	Ca(ICCV2/PI489777)SNP_23557	916	C	G	16	3	--	--
CakTC42902	Ca(ICCV2/PI489777)SNP_23558	1595	T	C	3	5	--	--
CakTC11394	Ca(ICCV2/PI489777)SNP_23559	388	G	A	7	6	--	--
CakTC26341	Ca(ICCV2/PI489777)SNP_23560	339	T	C	11	7	--	--
	Ca(ICCV2/PI489777)SNP_23561	2526	A	C	12	10	--	--
	Ca(ICCV2/PI489777)SNP_23562	2748	C	A	18	4	--	--
	Ca(ICCV2/PI489777)SNP_23563	4008	T	C	7	9	--	--
	Ca(ICCV2/PI489777)SNP_23564	4105	T	G	9	9	--	--
	Ca(ICCV2/PI489777)SNP_23565	4485	C	T	9	5	--	--
	Ca(ICCV2/PI489777)SNP_23566	4938	T	A	8	3	--	--
	Ca(ICCV2/PI489777)SNP_23567	5178	G	T	14	7	--	--
	Ca(ICCV2/PI489777)SNP_23568	5487	A	T	18	9	--	--
CakTC38478	Ca(ICCV2/PI489777)SNP_23569	35	C	T	15	6	--	--
	Ca(ICCV2/PI489777)SNP_23570	82	C	G	22	7	--	--
	Ca(ICCV2/PI489777)SNP_23571	323	T	C	21	9	--	--
	Ca(ICCV2/PI489777)SNP_23572	438	G	A	23	11	--	--
	Ca(ICCV2/PI489777)SNP_23573	468	C	T	24	13	--	--
	Ca(ICCV2/PI489777)SNP_23574	559	T	C	14	14	--	--
	Ca(ICCV2/PI489777)SNP_23575	662	T	C	18	14	--	--
	Ca(ICCV2/PI489777)SNP_23576	665	T	C	19	16	--	--
	Ca(ICCV2/PI489777)SNP_23577	989	T	A	17	18	--	--
	Ca(ICCV2/PI489777)SNP_23578	1227	C	A	11	20	--	--
CakTC23814	Ca(ICCV2/PI489777)SNP_23579	584	G	T	10	7	--	--
	Ca(ICCV2/PI489777)SNP_23580	641	C	G	8	7	--	--
	Ca(ICCV2/PI489777)SNP_23581	730	G	A	6	13	--	--
	Ca(ICCV2/PI489777)SNP_23582	1034	C	T	7	5	--	--
CakTC43346	Ca(ICCV2/PI489777)SNP_23583	128	T	C	21	30	--	--
	Ca(ICCV2/PI489777)SNP_23584	538	T	C	44	57	--	--
	Ca(ICCV2/PI489777)SNP_23585	803	G	C	18	20	--	--
CakTC23084	Ca(ICCV2/PI489777)SNP_23586	352	C	T	7	7	--	--
	Ca(ICCV2/PI489777)SNP_23587	386	T	C	7	10	--	--
	Ca(ICCV2/PI489777)SNP_23588	626	G	A	9	3	--	--
CakTC37581	Ca(ICCV2/PI489777)SNP_23589	520	C	T	13	47	--	--
	Ca(ICCV2/PI489777)SNP_23590	673	C	T	12	29	--	--
CakTC08847	Ca(ICCV2/PI489777)SNP_23591	198	C	T	6	3	--	--
	Ca(ICCV2/PI489777)SNP_23592	907	T	A	19	3	--	--
CakTC38848	Ca(ICCV2/PI489777)SNP_23593	288	C	G	5	3	Flower	bud
	Ca(ICCV2/PI489777)SNP_23594	393	C	A	5	3	Flower	bud
CakTC04834	Ca(ICCV2/PI489777)SNP_23595	433	G	T	6	11	--	bZIP
CakTC37996	Ca(ICCV2/PI489777)SNP_23596	14	A	G	3	12	--	--
	Ca(ICCV2/PI489777)SNP_23597	66	A	G	8	15	--	--
	Ca(ICCV2/PI489777)SNP_23598	93	T	A	11	9	--	--
	Ca(ICCV2/PI489777)SNP_23599	197	G	T	20	24	--	--
	Ca(ICCV2/PI489777)SNP_23600	357	A	C	24	24	--	--
	Ca(ICCV2/PI489777)SNP_23601	360	G	A	24	24	--	--

	Ca(ICCV2/PI489777)SNP_23602	456	A	G	23	16	--	--
	Ca(ICCV2/PI489777)SNP_23603	825	A	G	12	6	--	--
CakTC41691	Ca(ICCV2/PI489777)SNP_23604	182	C	G	11	14	--	--
CakTC40907	Ca(ICCV2/PI489777)SNP_23605	676	G	A	6	3	--	MYB
CakTC40808	Ca(ICCV2/PI489777)SNP_23606	509	G	C	7	13	--	--
CakTC11513	Ca(ICCV2/PI489777)SNP_23607	138	C	G	18	28	--	--
CakTC38454	Ca(ICCV2/PI489777)SNP_23608	354	C	A	33	18	--	bHLH
	Ca(ICCV2/PI489777)SNP_23609	409	A	T	41	25	--	bHLH
	Ca(ICCV2/PI489777)SNP_23610	478	G	A	48	26	--	bHLH
	Ca(ICCV2/PI489777)SNP_23611	574	G	T	40	20	--	bHLH
	Ca(ICCV2/PI489777)SNP_23612	787	T	C	30	5	--	bHLH
	Ca(ICCV2/PI489777)SNP_23613	829	A	G	21	6	--	bHLH
CakTC10770	Ca(ICCV2/PI489777)SNP_23614	1338	G	A	37	5	--	--
CakTC26679	Ca(ICCV2/PI489777)SNP_23615	876	A	G	35	9	--	--
	Ca(ICCV2/PI489777)SNP_23616	933	G	A	27	4	--	--
	Ca(ICCV2/PI489777)SNP_23617	1333	A	C	20	13	--	--
	Ca(ICCV2/PI489777)SNP_23618	2175	G	A	7	4	--	--
CakTC42061	Ca(ICCV2/PI489777)SNP_23619	378	T	C	3	4	--	--
	Ca(ICCV2/PI489777)SNP_23620	664	G	A	5	4	--	--
	Ca(ICCV2/PI489777)SNP_23621	1160	A	G	5	5	--	--
	Ca(ICCV2/PI489777)SNP_23622	1210	C	A	6	4	--	--
	Ca(ICCV2/PI489777)SNP_23623	1478	G	A	11	9	--	--
	Ca(ICCV2/PI489777)SNP_23624	1626	T	C	9	14	--	--
CakTC24506	Ca(ICCV2/PI489777)SNP_23625	771	G	A	30	14	--	--
	Ca(ICCV2/PI489777)SNP_23626	1064	A	G	32	8	--	--
	Ca(ICCV2/PI489777)SNP_23627	1705	C	G	23	12	--	--
CakTC42245	Ca(ICCV2/PI489777)SNP_23628	103	A	G	4	7	--	--
	Ca(ICCV2/PI489777)SNP_23629	454	C	T	9	13	--	--
	Ca(ICCV2/PI489777)SNP_23630	723	T	G	11	10	--	--
CakTC22843	Ca(ICCV2/PI489777)SNP_23631	201	C	A	9	3	--	--
	Ca(ICCV2/PI489777)SNP_23632	345	A	G	16	5	--	--
	Ca(ICCV2/PI489777)SNP_23633	387	T	G	33	6	--	--
	Ca(ICCV2/PI489777)SNP_23634	1444	C	T	8	4	--	--
CakTC26380	Ca(ICCV2/PI489777)SNP_23635	295	A	T	9	7	--	--
	Ca(ICCV2/PI489777)SNP_23636	302	T	C	7	7	--	--
	Ca(ICCV2/PI489777)SNP_23637	653	T	C	12	7	--	--
	Ca(ICCV2/PI489777)SNP_23638	759	G	C	10	3	--	--
	Ca(ICCV2/PI489777)SNP_23639	1294	C	T	4	4	--	--
CakTC40144	Ca(ICCV2/PI489777)SNP_23640	163	C	T	3	9	--	--
CakTC27676	Ca(ICCV2/PI489777)SNP_23641	388	C	T	16	15	--	--
	Ca(ICCV2/PI489777)SNP_23642	572	G	A	24	14	--	--
	Ca(ICCV2/PI489777)SNP_23643	625	G	A	21	15	--	--
	Ca(ICCV2/PI489777)SNP_23644	707	A	G	16	13	--	--
CakTC29355	Ca(ICCV2/PI489777)SNP_23645	1074	C	A	10	8	--	--
CakTC42546	Ca(ICCV2/PI489777)SNP_23646	1190	A	G	4	3	--	--
CakTC00046	Ca(ICCV2/PI489777)SNP_23647	161	T	G	8	9	--	--
CakTC24339	Ca(ICCV2/PI489777)SNP_23648	701	C	G	14	3	--	--
	Ca(ICCV2/PI489777)SNP_23649	757	T	C	14	5	--	--
	Ca(ICCV2/PI489777)SNP_23650	792	G	A	12	5	--	--
	Ca(ICCV2/PI489777)SNP_23651	890	C	T	9	3	--	--
CakTC30508	Ca(ICCV2/PI489777)SNP_23652	1086	A	T	5	4	--	--
CakTC42687	Ca(ICCV2/PI489777)SNP_23653	859	A	T	11	4	--	--
	Ca(ICCV2/PI489777)SNP_23654	1186	C	T	13	6	--	--
CakTC27053	Ca(ICCV2/PI489777)SNP_23655	589	C	G	7	11	--	--
	Ca(ICCV2/PI489777)SNP_23656	622	A	G	7	14	--	--
CakTC37224	Ca(ICCV2/PI489777)SNP_23657	255	G	C	43	15	--	--
	Ca(ICCV2/PI489777)SNP_23658	314	T	C	52	28	--	--
	Ca(ICCV2/PI489777)SNP_23659	371	T	C	57	25	--	--
	Ca(ICCV2/PI489777)SNP_23660	830	C	G	41	14	--	--
	Ca(ICCV2/PI489777)SNP_23661	897	G	A	38	15	--	--
	Ca(ICCV2/PI489777)SNP_23662	941	A	G	42	18	--	--
	Ca(ICCV2/PI489777)SNP_23663	1536	A	G	11	4	--	--
CakTC28686	Ca(ICCV2/PI489777)SNP_23664	644	G	A	7	9	--	--
	Ca(ICCV2/PI489777)SNP_23665	913	T	G	6	4	--	--
	Ca(ICCV2/PI489777)SNP_23666	1188	G	A	3	4	--	--
CakTC29430	Ca(ICCV2/PI489777)SNP_23667	543	T	C	14	20	--	--
	Ca(ICCV2/PI489777)SNP_23668	552	C	T	14	17	--	--
	Ca(ICCV2/PI489777)SNP_23669	648	G	A	14	12	--	--
	Ca(ICCV2/PI489777)SNP_23670	678	T	C	14	12	--	--
	Ca(ICCV2/PI489777)SNP_23671	693	A	G	13	12	--	--
	Ca(ICCV2/PI489777)SNP_23672	730	A	G	15	17	--	--
	Ca(ICCV2/PI489777)SNP_23673	993	C	T	12	13	--	--
CakTC37987	Ca(ICCV2/PI489777)SNP_23674	201	T	G	7	4	--	--
CakTC34571	Ca(ICCV2/PI489777)SNP_23675	1831	G	A	8	5	--	--

CakTC23703	Ca(ICCV2/PI489777)SNP_23676	1695	A	G	10	5	--	--
CakTC39738	Ca(ICCV2/PI489777)SNP_23677	337	C	T	45	39	--	--
	Ca(ICCV2/PI489777)SNP_23678	1018	A	G	50	71	--	--
	Ca(ICCV2/PI489777)SNP_23679	1111	G	C	36	57	--	--
CakTC38026	Ca(ICCV2/PI489777)SNP_23680	217	C	T	7	3	--	--
	Ca(ICCV2/PI489777)SNP_23681	818	A	T	8	15	--	--
CakTC24295	Ca(ICCV2/PI489777)SNP_23682	140	G	C	21	14	--	--
	Ca(ICCV2/PI489777)SNP_23683	424	G	T	39	28	--	--
	Ca(ICCV2/PI489777)SNP_23684	853	C	G	25	32	--	--
	Ca(ICCV2/PI489777)SNP_23685	3428	G	T	11	4	--	--
CakTC31093	Ca(ICCV2/PI489777)SNP_23686	1150	T	C	4	4	--	--
	Ca(ICCV2/PI489777)SNP_23687	1230	G	T	5	4	--	--
CakTC38471	Ca(ICCV2/PI489777)SNP_23688	1369	C	T	9	7	--	--
CakTC25818	Ca(ICCV2/PI489777)SNP_23689	1489	C	T	10	3	--	--
	Ca(ICCV2/PI489777)SNP_23690	1516	C	A	3	3	--	--
	Ca(ICCV2/PI489777)SNP_23691	1522	C	T	4	3	--	--
CakTC43017	Ca(ICCV2/PI489777)SNP_23692	117	T	C	3	5	--	--
	Ca(ICCV2/PI489777)SNP_23693	325	T	C	4	5	--	--
	Ca(ICCV2/PI489777)SNP_23694	477	T	G	7	4	--	--
	Ca(ICCV2/PI489777)SNP_23695	1524	A	C	6	3	--	--
CakTC40467	Ca(ICCV2/PI489777)SNP_23696	82	C	T	12	17	--	--
CakTC25999	Ca(ICCV2/PI489777)SNP_23697	374	A	G	36	31	--	--
CakTC27261	Ca(ICCV2/PI489777)SNP_23698	180	T	C	19	5	--	--
	Ca(ICCV2/PI489777)SNP_23699	936	C	T	37	13	--	--
	Ca(ICCV2/PI489777)SNP_23700	1235	G	A	48	13	--	--
CakTC29392	Ca(ICCV2/PI489777)SNP_23701	870	C	T	3	3	Flower	bud
CakTC30521	Ca(ICCV2/PI489777)SNP_23702	213	T	A	5	6	--	--
	Ca(ICCV2/PI489777)SNP_23703	1011	T	C	4	3	--	--
	Ca(ICCV2/PI489777)SNP_23704	2354	G	A	4	8	--	--
CakTC36021	Ca(ICCV2/PI489777)SNP_23705	487	T	C	5	7	--	--
	Ca(ICCV2/PI489777)SNP_23706	490	C	T	4	7	--	--
	Ca(ICCV2/PI489777)SNP_23707	1159	T	C	6	4	--	--
	Ca(ICCV2/PI489777)SNP_23708	1372	G	A	9	5	--	--
	Ca(ICCV2/PI489777)SNP_23709	1609	A	G	12	11	--	--
	Ca(ICCV2/PI489777)SNP_23710	2275	C	T	9	11	--	--
	Ca(ICCV2/PI489777)SNP_23711	2380	G	A	10	11	--	--
CakTC25621	Ca(ICCV2/PI489777)SNP_23712	1733	A	T	7	3	--	TCP
CakTC35851	Ca(ICCV2/PI489777)SNP_23713	473	A	C	11	14	--	--
	Ca(ICCV2/PI489777)SNP_23714	534	C	T	15	11	--	--
	Ca(ICCV2/PI489777)SNP_23715	908	A	G	37	17	--	--
	Ca(ICCV2/PI489777)SNP_23716	1343	T	C	21	9	--	--
	Ca(ICCV2/PI489777)SNP_23717	1997	C	T	27	8	--	--
	Ca(ICCV2/PI489777)SNP_23718	2157	C	T	17	3	--	--
	Ca(ICCV2/PI489777)SNP_23719	2164	T	C	14	3	--	--
	Ca(ICCV2/PI489777)SNP_23720	2300	T	C	10	4	--	--
	Ca(ICCV2/PI489777)SNP_23721	2635	A	G	13	7	--	--
CakTC42040	Ca(ICCV2/PI489777)SNP_23722	221	G	T	10	3	--	--
	Ca(ICCV2/PI489777)SNP_23723	555	G	A	8	5	--	--
	Ca(ICCV2/PI489777)SNP_23724	596	A	C	8	5	--	--
	Ca(ICCV2/PI489777)SNP_23725	598	A	C	7	5	--	--
	Ca(ICCV2/PI489777)SNP_23726	857	A	G	4	6	--	--
CakTC25307	Ca(ICCV2/PI489777)SNP_23727	711	G	A	30	33	--	--
CakTC33695	Ca(ICCV2/PI489777)SNP_23728	1920	T	C	5	5	--	BES1
CakTC28947	Ca(ICCV2/PI489777)SNP_23729	228	C	T	25	9	--	--
CakTC29694	Ca(ICCV2/PI489777)SNP_23730	615	C	A	4	3	--	--
CakTC37522	Ca(ICCV2/PI489777)SNP_23731	115	T	C	11	4	--	--
	Ca(ICCV2/PI489777)SNP_23732	193	A	C	19	11	--	--
	Ca(ICCV2/PI489777)SNP_23733	456	A	G	33	28	--	--
	Ca(ICCV2/PI489777)SNP_23734	552	A	C	38	30	--	--
	Ca(ICCV2/PI489777)SNP_23735	763	T	C	42	31	--	--
	Ca(ICCV2/PI489777)SNP_23736	1529	C	A	24	3	--	--
CakTC41934	Ca(ICCV2/PI489777)SNP_23737	754	A	G	19	5	--	--
CakTC42398	Ca(ICCV2/PI489777)SNP_23738	226	T	G	4	13	--	--
	Ca(ICCV2/PI489777)SNP_23739	448	G	T	4	12	--	--
CakTC41096	Ca(ICCV2/PI489777)SNP_23740	80	C	T	12	6	--	--
	Ca(ICCV2/PI489777)SNP_23741	86	T	C	12	6	--	--
	Ca(ICCV2/PI489777)SNP_23742	109	G	T	12	6	--	--
	Ca(ICCV2/PI489777)SNP_23743	110	T	C	12	6	--	--
	Ca(ICCV2/PI489777)SNP_23744	288	C	T	13	6	--	--
CakTC23507	Ca(ICCV2/PI489777)SNP_23745	291	A	G	13	5	--	--
	Ca(ICCV2/PI489777)SNP_23746	555	T	A	18	8	--	--
CakTC26585	Ca(ICCV2/PI489777)SNP_23747	1099	A	G	3	9	--	--
CakTC24894	Ca(ICCV2/PI489777)SNP_23748	146	C	A	8	3	--	--
CakTC28768	Ca(ICCV2/PI489777)SNP_23749	241	A	G	32	30	--	--

CakTC13057	Ca(ICCV2/PI489777)SNP_23750	406	A	T	21	10	--	--
	Ca(ICCV2/PI489777)SNP_23751	589	C	A	5	3	--	--
CakTC35325	Ca(ICCV2/PI489777)SNP_23752	1689	C	T	18	4	--	--
	Ca(ICCV2/PI489777)SNP_23753	1999	T	C	23	7	--	--
	Ca(ICCV2/PI489777)SNP_23754	2101	T	G	28	9	--	--
	Ca(ICCV2/PI489777)SNP_23755	2182	A	G	20	8	--	--
	Ca(ICCV2/PI489777)SNP_23756	2774	C	T	28	17	--	--
CakTC27221	Ca(ICCV2/PI489777)SNP_23757	763	T	C	12	5	--	--
CakTC40822	Ca(ICCV2/PI489777)SNP_23758	258	A	G	5	3	--	--
CakTC11011	Ca(ICCV2/PI489777)SNP_23759	395	C	T	7	5	--	--
	Ca(ICCV2/PI489777)SNP_23760	632	A	C	6	7	--	--
CakTC36527	Ca(ICCV2/PI489777)SNP_23761	370	A	T	7	5	--	HB
	Ca(ICCV2/PI489777)SNP_23762	463	A	G	7	6	--	HB
	Ca(ICCV2/PI489777)SNP_23763	1136	G	A	5	6	--	HB
	Ca(ICCV2/PI489777)SNP_23764	1526	C	T	10	4	--	HB
	Ca(ICCV2/PI489777)SNP_23765	2045	T	C	8	3	--	HB
	Ca(ICCV2/PI489777)SNP_23766	2076	G	A	8	4	--	HB
	Ca(ICCV2/PI489777)SNP_23767	2525	T	A	8	3	--	HB
CakTC23044	Ca(ICCV2/PI489777)SNP_23768	4007	G	A	9	3	--	--
	Ca(ICCV2/PI489777)SNP_23769	4175	G	A	11	3	--	--
	Ca(ICCV2/PI489777)SNP_23770	5017	G	A	8	4	--	--
CakTC40109	Ca(ICCV2/PI489777)SNP_23771	317	C	T	17	19	--	--
	Ca(ICCV2/PI489777)SNP_23772	1049	G	A	22	32	--	--
	Ca(ICCV2/PI489777)SNP_23773	1293	A	G	22	35	--	--
	Ca(ICCV2/PI489777)SNP_23774	1337	A	G	20	21	--	--
	Ca(ICCV2/PI489777)SNP_23775	1430	G	C	19	26	--	--
CakTC40608	Ca(ICCV2/PI489777)SNP_23776	338	T	C	22	4	--	--
	Ca(ICCV2/PI489777)SNP_23777	565	A	T	26	4	--	--
	Ca(ICCV2/PI489777)SNP_23778	633	C	T	25	3	--	--
	Ca(ICCV2/PI489777)SNP_23779	662	G	T	23	3	--	--
	Ca(ICCV2/PI489777)SNP_23780	703	T	A	21	4	--	--
	Ca(ICCV2/PI489777)SNP_23781	810	C	T	17	4	--	--
CakTC29291	Ca(ICCV2/PI489777)SNP_23782	1724	T	C	14	3	Shoot	--
CakTC09673	Ca(ICCV2/PI489777)SNP_23783	1086	C	G	7	3	--	--
CakTC12524	Ca(ICCV2/PI489777)SNP_23784	214	T	C	17	4	--	--
CakTC34744	Ca(ICCV2/PI489777)SNP_23785	51	T	C	4	6	--	--
	Ca(ICCV2/PI489777)SNP_23786	2194	G	A	11	19	--	--
CakTC31472	Ca(ICCV2/PI489777)SNP_23787	1477	A	G	8	6	--	--
	Ca(ICCV2/PI489777)SNP_23788	1631	T	C	11	3	--	--
CakTC10596	Ca(ICCV2/PI489777)SNP_23789	170	C	A	3	3	--	WRKY
	Ca(ICCV2/PI489777)SNP_23790	252	A	G	5	3	--	WRKY
	Ca(ICCV2/PI489777)SNP_23791	499	C	T	10	4	--	WRKY
	Ca(ICCV2/PI489777)SNP_23792	568	C	T	11	5	--	WRKY
CakTC26938	Ca(ICCV2/PI489777)SNP_23793	1396	G	T	23	7	--	--
	Ca(ICCV2/PI489777)SNP_23794	2116	C	T	13	8	--	--
	Ca(ICCV2/PI489777)SNP_23795	2588	A	G	14	7	--	--
	Ca(ICCV2/PI489777)SNP_23796	2693	G	T	12	7	--	--
	Ca(ICCV2/PI489777)SNP_23797	3566	C	A	22	7	--	--
	Ca(ICCV2/PI489777)SNP_23798	4710	A	G	21	11	--	--
	Ca(ICCV2/PI489777)SNP_23799	5524	G	A	32	20	--	--
	Ca(ICCV2/PI489777)SNP_23800	5677	G	A	29	12	--	--
	Ca(ICCV2/PI489777)SNP_23801	5822	G	A	17	7	--	--
	Ca(ICCV2/PI489777)SNP_23802	5903	C	T	17	8	--	--
CakTC16127	Ca(ICCV2/PI489777)SNP_23803	260	A	G	3	5	--	--
	Ca(ICCV2/PI489777)SNP_23804	277	G	A	3	5	--	--
	Ca(ICCV2/PI489777)SNP_23805	378	T	C	3	5	--	--
	Ca(ICCV2/PI489777)SNP_23806	409	T	C	3	5	--	--
CakTC39529	Ca(ICCV2/PI489777)SNP_23807	239	A	C	101	67	--	AP2-EREBP
	Ca(ICCV2/PI489777)SNP_23808	1056	C	T	158	68	--	AP2-EREBP
	Ca(ICCV2/PI489777)SNP_23809	1068	T	G	154	73	--	AP2-EREBP
CakTC40195	Ca(ICCV2/PI489777)SNP_23810	157	G	A	5	6	--	--
	Ca(ICCV2/PI489777)SNP_23811	304	T	C	10	8	--	--
	Ca(ICCV2/PI489777)SNP_23812	901	C	A	7	4	--	--
CakTC35166	Ca(ICCV2/PI489777)SNP_23813	2840	G	C	4	4	--	--
	Ca(ICCV2/PI489777)SNP_23814	2870	G	A	4	5	--	--
CakTC10466	Ca(ICCV2/PI489777)SNP_23815	434	C	T	35	50	--	--
	Ca(ICCV2/PI489777)SNP_23816	587	C	T	22	37	--	--
	Ca(ICCV2/PI489777)SNP_23817	593	A	T	19	22	--	--
	Ca(ICCV2/PI489777)SNP_23818	629	C	T	13	24	--	--
	Ca(ICCV2/PI489777)SNP_23819	641	T	C	11	24	--	--
CakTC40251	Ca(ICCV2/PI489777)SNP_23820	267	A	T	17	7	--	--
	Ca(ICCV2/PI489777)SNP_23821	551	A	T	8	9	--	--
CakTC33759	Ca(ICCV2/PI489777)SNP_23822	276	C	G	7	9	--	--
CakTC33503	Ca(ICCV2/PI489777)SNP_23823	1843	A	C	7	11	Young_pod	--

CakTC05173	Ca(ICCV2/PI489777)SNP_23824	1226	A	G	5	4	--	--
CakTC37021	Ca(ICCV2/PI489777)SNP_23825	78	C	T	114	17	--	--
	Ca(ICCV2/PI489777)SNP_23826	87	C	T	114	17	--	--
	Ca(ICCV2/PI489777)SNP_23827	258	C	A	114	10	--	--
	Ca(ICCV2/PI489777)SNP_23828	357	A	T	101	14	--	--
CakTC40297	Ca(ICCV2/PI489777)SNP_23829	375	G	A	6	6	--	--
	Ca(ICCV2/PI489777)SNP_23830	435	C	T	9	5	--	--
	Ca(ICCV2/PI489777)SNP_23831	444	A	G	9	5	--	--
CakTC07791	Ca(ICCV2/PI489777)SNP_23832	312	G	T	5	3	Root	--
CakTC25102	Ca(ICCV2/PI489777)SNP_23833	142	G	C	18	5	--	--
	Ca(ICCV2/PI489777)SNP_23834	667	G	A	11	6	--	--
	Ca(ICCV2/PI489777)SNP_23835	1113	T	C	13	3	--	--
	Ca(ICCV2/PI489777)SNP_23836	1427	C	T	36	9	--	--
	Ca(ICCV2/PI489777)SNP_23837	1428	G	A	36	9	--	--
	Ca(ICCV2/PI489777)SNP_23838	1464	C	T	44	9	--	--
CakTC23871	Ca(ICCV2/PI489777)SNP_23839	335	T	G	7	3	--	--
	Ca(ICCV2/PI489777)SNP_23840	1803	T	C	4	13	--	--
	Ca(ICCV2/PI489777)SNP_23841	2732	C	T	36	23	--	--
	Ca(ICCV2/PI489777)SNP_23842	2744	T	C	32	24	--	--
	Ca(ICCV2/PI489777)SNP_23843	2791	T	C	36	23	--	--
	Ca(ICCV2/PI489777)SNP_23844	2843	G	A	37	21	--	--
	Ca(ICCV2/PI489777)SNP_23845	3011	T	C	26	6	--	--
	Ca(ICCV2/PI489777)SNP_23846	3018	G	A	25	7	--	--
	Ca(ICCV2/PI489777)SNP_23847	3026	A	C	27	6	--	--
CakTC00221	Ca(ICCV2/PI489777)SNP_23848	358	A	G	3	6	--	--
CakTC38175	Ca(ICCV2/PI489777)SNP_23849	1406	C	A	10	9	--	--
CakTC26672	Ca(ICCV2/PI489777)SNP_23850	1983	G	C	7	4	--	--
CakTC41052	Ca(ICCV2/PI489777)SNP_23851	434	C	T	20	9	--	--
	Ca(ICCV2/PI489777)SNP_23852	638	T	G	17	6	--	--
	Ca(ICCV2/PI489777)SNP_23853	641	A	G	17	6	--	--
	Ca(ICCV2/PI489777)SNP_23854	653	G	A	16	6	--	--
CakTC38944	Ca(ICCV2/PI489777)SNP_23855	295	C	T	22	6	--	--
	Ca(ICCV2/PI489777)SNP_23856	391	C	T	15	6	--	--
	Ca(ICCV2/PI489777)SNP_23857	484	G	T	8	8	--	--
	Ca(ICCV2/PI489777)SNP_23858	649	G	A	10	10	--	--
	Ca(ICCV2/PI489777)SNP_23859	754	T	G	10	11	--	--
	Ca(ICCV2/PI489777)SNP_23860	1048	T	C	19	7	--	--
	Ca(ICCV2/PI489777)SNP_23861	1159	G	A	17	4	--	--
	Ca(ICCV2/PI489777)SNP_23862	1306	G	A	10	3	--	--
CakTC27344	Ca(ICCV2/PI489777)SNP_23863	818	G	A	10	5	--	--
	Ca(ICCV2/PI489777)SNP_23864	1001	G	A	18	8	--	--
	Ca(ICCV2/PI489777)SNP_23865	1103	A	G	13	32	--	--
	Ca(ICCV2/PI489777)SNP_23866	1221	G	A	18	41	--	--
	Ca(ICCV2/PI489777)SNP_23867	1229	T	C	16	43	--	--
	Ca(ICCV2/PI489777)SNP_23868	1232	T	C	14	42	--	--
	Ca(ICCV2/PI489777)SNP_23869	1344	A	G	15	44	--	--
	Ca(ICCV2/PI489777)SNP_23870	1482	A	G	11	28	--	--
	Ca(ICCV2/PI489777)SNP_23871	1661	T	C	3	6	--	--
CakTC36759	Ca(ICCV2/PI489777)SNP_23872	815	G	T	3	4	Flower	bud
CakTC42716	Ca(ICCV2/PI489777)SNP_23873	41	G	A	3	3	--	--
	Ca(ICCV2/PI489777)SNP_23874	592	G	A	20	13	--	--
CakTC23307	Ca(ICCV2/PI489777)SNP_23875	765	C	G	6	10	--	--
CakTC41196	Ca(ICCV2/PI489777)SNP_23876	99	C	T	7	13	--	--
	Ca(ICCV2/PI489777)SNP_23877	181	G	A	13	56	--	--
CakTC41652	Ca(ICCV2/PI489777)SNP_23878	608	G	T	4	3	--	--
CakTC34516	Ca(ICCV2/PI489777)SNP_23879	904	T	A	6	4	--	--
	Ca(ICCV2/PI489777)SNP_23880	1189	C	T	12	6	--	--
CakTC00503	Ca(ICCV2/PI489777)SNP_23881	1450	G	A	3	9	--	--
CakTC37722	Ca(ICCV2/PI489777)SNP_23882	358	T	A	7	8	--	--
	Ca(ICCV2/PI489777)SNP_23883	572	T	C	10	5	--	--
CakTC26829	Ca(ICCV2/PI489777)SNP_23884	161	T	G	14	3	--	--
	Ca(ICCV2/PI489777)SNP_23885	392	A	G	22	9	--	--
CakTC12384	Ca(ICCV2/PI489777)SNP_23886	1722	T	C	6	3	--	--
	Ca(ICCV2/PI489777)SNP_23887	1827	G	C	7	4	--	--
	Ca(ICCV2/PI489777)SNP_23888	1893	T	C	9	7	--	--
	Ca(ICCV2/PI489777)SNP_23889	2105	G	A	13	6	--	--
CakTC30807	Ca(ICCV2/PI489777)SNP_23890	970	T	C	7	5	--	--
CakTC39932	Ca(ICCV2/PI489777)SNP_23891	1040	A	C	8	5	--	--
	Ca(ICCV2/PI489777)SNP_23892	1265	T	C	9	6	--	--
CakTC34113	Ca(ICCV2/PI489777)SNP_23893	438	A	G	29	8	--	bZIP
	Ca(ICCV2/PI489777)SNP_23894	867	A	G	30	14	--	bZIP
	Ca(ICCV2/PI489777)SNP_23895	1062	A	G	29	9	--	bZIP
	Ca(ICCV2/PI489777)SNP_23896	1107	C	T	27	8	--	bZIP
	Ca(ICCV2/PI489777)SNP_23897	1127	T	C	27	6	--	bZIP

	Ca(ICCV2/PI489777)SNP_23898	1299	C	T	13	7	--	bZIP
	Ca(ICCV2/PI489777)SNP_23899	1349	C	T	10	9	--	bZIP
	Ca(ICCV2/PI489777)SNP_23900	2010	A	G	25	6	--	bZIP
	Ca(ICCV2/PI489777)SNP_23901	2036	A	G	21	6	--	bZIP
CakTC26979	Ca(ICCV2/PI489777)SNP_23902	337	C	T	5	8	--	bZIP
	Ca(ICCV2/PI489777)SNP_23903	511	T	C	12	14	--	bZIP
	Ca(ICCV2/PI489777)SNP_23904	1133	A	G	13	14	--	bZIP
	Ca(ICCV2/PI489777)SNP_23905	1204	T	A	11	10	--	bZIP
CakTC26517	Ca(ICCV2/PI489777)SNP_23906	1924	C	A	5	4	--	--
	Ca(ICCV2/PI489777)SNP_23907	2351	G	A	4	3	--	--
CakTC29850	Ca(ICCV2/PI489777)SNP_23908	418	G	C	5	8	Shoot	--
	Ca(ICCV2/PI489777)SNP_23909	482	C	T	6	8	Shoot	--
	Ca(ICCV2/PI489777)SNP_23910	523	G	A	6	8	Shoot	--
	Ca(ICCV2/PI489777)SNP_23911	582	G	C	6	5	Shoot	--
	Ca(ICCV2/PI489777)SNP_23912	751	G	A	6	3	Shoot	--
	Ca(ICCV2/PI489777)SNP_23913	1439	A	T	10	6	Shoot	--
	Ca(ICCV2/PI489777)SNP_23914	2192	G	T	3	7	Shoot	--
	Ca(ICCV2/PI489777)SNP_23915	2324	G	A	4	6	Shoot	--
CakTC11379	Ca(ICCV2/PI489777)SNP_23916	1270	G	A	32	8	--	--
CakTC38870	Ca(ICCV2/PI489777)SNP_23917	558	A	G	6	5	--	--
	Ca(ICCV2/PI489777)SNP_23918	559	G	T	6	5	--	--
	Ca(ICCV2/PI489777)SNP_23919	608	T	C	6	5	--	--
	Ca(ICCV2/PI489777)SNP_23920	1080	C	T	6	3	--	--
CakTC32065	Ca(ICCV2/PI489777)SNP_23921	1183	A	G	14	7	--	PLATZ
CakTC25865	Ca(ICCV2/PI489777)SNP_23922	391	A	G	26	12	--	--
	Ca(ICCV2/PI489777)SNP_23923	654	T	A	10	8	--	--
CakTC09793	Ca(ICCV2/PI489777)SNP_23924	1206	T	C	12	6	--	--
	Ca(ICCV2/PI489777)SNP_23925	2640	T	C	12	3	--	--
	Ca(ICCV2/PI489777)SNP_23926	2811	C	T	12	4	--	--
	Ca(ICCV2/PI489777)SNP_23927	3039	G	T	6	5	--	--
	Ca(ICCV2/PI489777)SNP_23928	3610	A	G	12	4	--	--
	Ca(ICCV2/PI489777)SNP_23929	4656	A	G	14	3	--	--
	Ca(ICCV2/PI489777)SNP_23930	4737	G	T	16	4	--	--
	Ca(ICCV2/PI489777)SNP_23931	5298	A	G	17	6	--	--
	Ca(ICCV2/PI489777)SNP_23932	5426	C	T	16	4	--	--
	Ca(ICCV2/PI489777)SNP_23933	5445	G	A	16	4	--	--
CakTC12403	Ca(ICCV2/PI489777)SNP_23934	1057	A	C	5	3	--	--
CakTC11944	Ca(ICCV2/PI489777)SNP_23935	75	G	A	5	5	--	--
	Ca(ICCV2/PI489777)SNP_23936	370	A	C	7	6	--	--
	Ca(ICCV2/PI489777)SNP_23937	376	A	C	3	7	--	--
	Ca(ICCV2/PI489777)SNP_23938	471	G	T	7	3	--	--
CakTC23633	Ca(ICCV2/PI489777)SNP_23939	151	C	T	9	5	--	--
	Ca(ICCV2/PI489777)SNP_23940	460	G	C	15	5	--	--
	Ca(ICCV2/PI489777)SNP_23941	686	A	G	14	22	--	--
	Ca(ICCV2/PI489777)SNP_23942	1210	C	A	10	3	--	--
	Ca(ICCV2/PI489777)SNP_23943	1217	T	C	11	3	--	--
	Ca(ICCV2/PI489777)SNP_23944	1227	C	A	11	3	--	--
CakTC41986	Ca(ICCV2/PI489777)SNP_23945	406	T	C	18	15	--	--
CakTC34388	Ca(ICCV2/PI489777)SNP_23946	631	G	A	23	9	--	--
	Ca(ICCV2/PI489777)SNP_23947	743	G	A	27	10	--	--
	Ca(ICCV2/PI489777)SNP_23948	751	T	C	27	10	--	--
	Ca(ICCV2/PI489777)SNP_23949	793	T	C	28	10	--	--
	Ca(ICCV2/PI489777)SNP_23950	1033	T	G	22	4	--	--
	Ca(ICCV2/PI489777)SNP_23951	1123	A	G	30	6	--	--
CakTC24403	Ca(ICCV2/PI489777)SNP_23952	182	A	G	7	6	--	--
	Ca(ICCV2/PI489777)SNP_23953	534	T	C	23	9	--	--
	Ca(ICCV2/PI489777)SNP_23954	729	A	T	22	6	--	--
	Ca(ICCV2/PI489777)SNP_23955	948	A	G	26	12	--	--
	Ca(ICCV2/PI489777)SNP_23956	1407	C	T	18	10	--	--
	Ca(ICCV2/PI489777)SNP_23957	1410	G	T	19	9	--	--
	Ca(ICCV2/PI489777)SNP_23958	1755	T	C	23	13	--	--
	Ca(ICCV2/PI489777)SNP_23959	2017	C	G	13	7	--	--
	Ca(ICCV2/PI489777)SNP_23960	2390	T	C	29	15	--	--
	Ca(ICCV2/PI489777)SNP_23961	2508	T	C	27	17	--	--
	Ca(ICCV2/PI489777)SNP_23962	2590	C	A	38	20	--	--
	Ca(ICCV2/PI489777)SNP_23963	2706	C	T	38	24	--	--
	Ca(ICCV2/PI489777)SNP_23964	2790	C	T	55	27	--	--
	Ca(ICCV2/PI489777)SNP_23965	3002	A	G	31	21	--	--
	Ca(ICCV2/PI489777)SNP_23966	3003	A	C	31	21	--	--
	Ca(ICCV2/PI489777)SNP_23967	3033	A	G	31	18	--	--
	Ca(ICCV2/PI489777)SNP_23968	3117	A	G	24	19	--	--
	Ca(ICCV2/PI489777)SNP_23969	3120	A	G	23	18	--	--
	Ca(ICCV2/PI489777)SNP_23970	3272	C	T	25	15	--	--
	Ca(ICCV2/PI489777)SNP_23971	3345	T	C	21	11	--	--

	Ca(ICCV2/PI489777)SNP_23972	3480	A	G	14	8	--	--
	Ca(ICCV2/PI489777)SNP_23973	3578	G	T	6	8	--	--
	Ca(ICCV2/PI489777)SNP_23974	3585	C	T	7	8	--	--
	Ca(ICCV2/PI489777)SNP_23975	3744	A	G	8	6	--	--
	Ca(ICCV2/PI489777)SNP_23976	3769	A	G	8	5	--	--
	Ca(ICCV2/PI489777)SNP_23977	3809	A	G	7	5	--	--
CakTC39157	Ca(ICCV2/PI489777)SNP_23978	392	G	C	3	4	--	Trihelix
CakTC25934	Ca(ICCV2/PI489777)SNP_23979	97	G	A	34	6	--	--
	Ca(ICCV2/PI489777)SNP_23980	585	C	T	71	36	--	--
	Ca(ICCV2/PI489777)SNP_23981	983	C	T	79	22	--	--
	Ca(ICCV2/PI489777)SNP_23982	1649	T	C	53	20	--	--
	Ca(ICCV2/PI489777)SNP_23983	1862	G	T	49	22	--	--
	Ca(ICCV2/PI489777)SNP_23984	1912	T	C	50	20	--	--
CakTC37263	Ca(ICCV2/PI489777)SNP_23985	178	C	G	7	3	--	--
	Ca(ICCV2/PI489777)SNP_23986	445	G	A	17	5	--	--
	Ca(ICCV2/PI489777)SNP_23987	565	G	A	18	4	--	--
CakTC24530	Ca(ICCV2/PI489777)SNP_23988	1770	G	T	11	3	--	--
CakTC33376	Ca(ICCV2/PI489777)SNP_23989	353	T	G	3	6	--	--
CakTC28743	Ca(ICCV2/PI489777)SNP_23990	161	G	A	3	3	--	C2C2-Dof
	Ca(ICCV2/PI489777)SNP_23991	604	A	T	3	4	--	C2C2-Dof
CakTC23170	Ca(ICCV2/PI489777)SNP_23992	1096	T	G	3	7	--	--
	Ca(ICCV2/PI489777)SNP_23993	1239	T	A	5	4	--	--
CakTC23439	Ca(ICCV2/PI489777)SNP_23994	3184	C	T	34	20	--	--
	Ca(ICCV2/PI489777)SNP_23995	4218	T	A	6	3	--	--
CakTC06605	Ca(ICCV2/PI489777)SNP_23996	194	G	C	3	3	--	--
	Ca(ICCV2/PI489777)SNP_23997	983	A	G	3	3	--	--
	Ca(ICCV2/PI489777)SNP_23998	1133	C	T	4	3	--	--
	Ca(ICCV2/PI489777)SNP_23999	1415	C	A	4	6	--	--
	Ca(ICCV2/PI489777)SNP_24000	1512	A	C	4	6	--	--
CakTC36739	Ca(ICCV2/PI489777)SNP_24001	1034	A	C	11	10	--	--
	Ca(ICCV2/PI489777)SNP_24002	2174	T	C	19	12	--	--
CakTC41503	Ca(ICCV2/PI489777)SNP_24003	252	C	T	17	5	--	HMG
	Ca(ICCV2/PI489777)SNP_24004	807	C	T	13	7	--	HMG
CakTC08793	Ca(ICCV2/PI489777)SNP_24005	665	T	C	6	3	--	--
	Ca(ICCV2/PI489777)SNP_24006	1414	C	A	5	4	--	--
CakTC38705	Ca(ICCV2/PI489777)SNP_24007	829	G	A	4	9	--	--
	Ca(ICCV2/PI489777)SNP_24008	981	G	A	11	7	--	--
	Ca(ICCV2/PI489777)SNP_24009	1020	C	A	14	4	--	--
CakTC41668	Ca(ICCV2/PI489777)SNP_24010	1857	A	G	46	18	--	--
	Ca(ICCV2/PI489777)SNP_24011	2141	A	T	20	16	--	--
CakTC36853	Ca(ICCV2/PI489777)SNP_24012	458	G	A	4	11	--	--
	Ca(ICCV2/PI489777)SNP_24013	1877	C	T	25	14	--	--
CakTC42670	Ca(ICCV2/PI489777)SNP_24014	875	G	A	8	11	--	--
	Ca(ICCV2/PI489777)SNP_24015	878	C	T	9	11	--	--
	Ca(ICCV2/PI489777)SNP_24016	1469	G	T	11	3	--	--
	Ca(ICCV2/PI489777)SNP_24017	1760	G	A	6	4	--	--
CakTC40727	Ca(ICCV2/PI489777)SNP_24018	228	C	G	8	6	--	--
	Ca(ICCV2/PI489777)SNP_24019	235	G	T	8	5	--	--
	Ca(ICCV2/PI489777)SNP_24020	544	A	G	8	7	--	--
	Ca(ICCV2/PI489777)SNP_24021	626	T	A	8	4	--	--
	Ca(ICCV2/PI489777)SNP_24022	661	C	T	9	5	--	--
	Ca(ICCV2/PI489777)SNP_24023	669	T	C	8	5	--	--
CakTC29359	Ca(ICCV2/PI489777)SNP_24024	167	G	A	12	4	--	bHLH
	Ca(ICCV2/PI489777)SNP_24025	772	T	C	7	8	--	bHLH
	Ca(ICCV2/PI489777)SNP_24026	881	A	G	8	10	--	bHLH
	Ca(ICCV2/PI489777)SNP_24027	963	T	A	9	11	--	bHLH
	Ca(ICCV2/PI489777)SNP_24028	968	A	G	8	11	--	bHLH
CakTC32623	Ca(ICCV2/PI489777)SNP_24029	424	G	A	50	7	--	--
	Ca(ICCV2/PI489777)SNP_24030	1220	G	T	31	19	--	--
CakTC18021	Ca(ICCV2/PI489777)SNP_24031	391	C	T	7	3	--	--
	Ca(ICCV2/PI489777)SNP_24032	1422	A	G	7	5	--	--
	Ca(ICCV2/PI489777)SNP_24033	1463	C	T	6	5	--	--
	Ca(ICCV2/PI489777)SNP_24034	1901	A	G	7	4	--	--
	Ca(ICCV2/PI489777)SNP_24035	1909	C	G	7	4	--	--
CakTC35410	Ca(ICCV2/PI489777)SNP_24036	665	A	C	8	6	--	--
	Ca(ICCV2/PI489777)SNP_24037	739	G	A	12	8	--	--
	Ca(ICCV2/PI489777)SNP_24038	851	A	G	14	5	--	--
	Ca(ICCV2/PI489777)SNP_24039	1265	A	G	12	5	--	--
	Ca(ICCV2/PI489777)SNP_24040	2145	G	T	17	5	--	--
	Ca(ICCV2/PI489777)SNP_24041	2764	G	C	19	10	--	--
	Ca(ICCV2/PI489777)SNP_24042	2860	C	G	17	10	--	--
CakTC37541	Ca(ICCV2/PI489777)SNP_24043	910	G	C	49	20	--	NAC
CakTC16500	Ca(ICCV2/PI489777)SNP_24044	2179	C	T	4	3	--	--
	Ca(ICCV2/PI489777)SNP_24045	2231	C	T	3	3	--	--

CakTC36485	Ca(ICCV2/PI489777)SNP_24046	1294	G	A	5	4	--	--
	Ca(ICCV2/PI489777)SNP_24047	1465	G	A	10	10	--	--
	Ca(ICCV2/PI489777)SNP_24048	2035	C	T	9	7	--	--
	Ca(ICCV2/PI489777)SNP_24049	2338	T	C	11	6	--	--
	Ca(ICCV2/PI489777)SNP_24050	2575	T	C	13	7	--	--
	Ca(ICCV2/PI489777)SNP_24051	2866	A	G	10	8	--	--
CakTC41458	Ca(ICCV2/PI489777)SNP_24052	596	G	A	15	4	--	--
	Ca(ICCV2/PI489777)SNP_24053	1486	A	G	9	4	--	--
CakTC11215	Ca(ICCV2/PI489777)SNP_24054	199	C	G	3	3	--	--
CakTC08851	Ca(ICCV2/PI489777)SNP_24055	238	T	A	17	7	--	--
	Ca(ICCV2/PI489777)SNP_24056	259	A	G	19	9	--	--
	Ca(ICCV2/PI489777)SNP_24057	409	G	C	36	18	--	--
	Ca(ICCV2/PI489777)SNP_24058	448	A	T	40	27	--	--
	Ca(ICCV2/PI489777)SNP_24059	962	G	T	9	12	--	--
CakTC26499	Ca(ICCV2/PI489777)SNP_24060	618	C	T	12	5	--	TPR
	Ca(ICCV2/PI489777)SNP_24061	1571	T	A	12	3	--	TPR
CakTC32218	Ca(ICCV2/PI489777)SNP_24062	154	C	T	9	4	--	--
	Ca(ICCV2/PI489777)SNP_24063	1225	T	G	16	11	--	--
	Ca(ICCV2/PI489777)SNP_24064	1335	C	T	8	15	--	--
	Ca(ICCV2/PI489777)SNP_24065	1566	T	C	9	25	--	--
	Ca(ICCV2/PI489777)SNP_24066	1586	T	C	10	23	--	--
	Ca(ICCV2/PI489777)SNP_24067	1830	A	G	6	6	--	--
	Ca(ICCV2/PI489777)SNP_24068	1890	C	G	6	4	--	--
CakTC41433	Ca(ICCV2/PI489777)SNP_24069	543	A	G	7	3	--	--
	Ca(ICCV2/PI489777)SNP_24070	563	C	T	7	3	--	--
	Ca(ICCV2/PI489777)SNP_24071	584	C	A	8	3	--	--
	Ca(ICCV2/PI489777)SNP_24072	1588	G	A	8	11	--	--
	Ca(ICCV2/PI489777)SNP_24073	1775	C	A	10	4	--	--
	Ca(ICCV2/PI489777)SNP_24074	1801	G	A	7	4	--	--
	Ca(ICCV2/PI489777)SNP_24075	1971	A	G	7	3	--	--
	Ca(ICCV2/PI489777)SNP_24076	2038	A	G	6	3	--	--
	Ca(ICCV2/PI489777)SNP_24077	2215	C	T	5	3	--	--
CakTC37795	Ca(ICCV2/PI489777)SNP_24078	878	A	T	18	6	--	--
	Ca(ICCV2/PI489777)SNP_24079	1484	C	A	8	6	--	--
CakTC10506	Ca(ICCV2/PI489777)SNP_24080	469	A	G	25	17	--	--
	Ca(ICCV2/PI489777)SNP_24081	937	C	T	45	36	--	--
	Ca(ICCV2/PI489777)SNP_24082	1099	A	G	26	25	--	--
CakTC42152	Ca(ICCV2/PI489777)SNP_24083	442	G	A	52	63	--	--
	Ca(ICCV2/PI489777)SNP_24084	1246	T	A	66	38	--	--
	Ca(ICCV2/PI489777)SNP_24085	1303	G	A	62	42	--	--
	Ca(ICCV2/PI489777)SNP_24086	1652	T	C	25	46	--	--
	Ca(ICCV2/PI489777)SNP_24087	1680	G	A	28	47	--	--
CakTC41976	Ca(ICCV2/PI489777)SNP_24088	559	G	C	28	39	--	--
	Ca(ICCV2/PI489777)SNP_24089	1480	A	C	8	19	--	--
	Ca(ICCV2/PI489777)SNP_24090	1729	T	C	5	16	--	--
CakTC41892	Ca(ICCV2/PI489777)SNP_24091	204	A	T	10	26	--	--
	Ca(ICCV2/PI489777)SNP_24092	860	T	C	14	20	--	--
	Ca(ICCV2/PI489777)SNP_24093	875	T	G	13	18	--	--
CakTC09511	Ca(ICCV2/PI489777)SNP_24094	1938	A	G	3	3	--	--
	Ca(ICCV2/PI489777)SNP_24095	1946	C	T	3	3	--	--
CakTC23023	Ca(ICCV2/PI489777)SNP_24096	1354	A	G	3	4	--	--
	Ca(ICCV2/PI489777)SNP_24097	1374	G	C	3	4	--	--
	Ca(ICCV2/PI489777)SNP_24098	1418	A	G	3	4	--	--
CakTC24839	Ca(ICCV2/PI489777)SNP_24099	1268	T	C	13	7	--	--
	Ca(ICCV2/PI489777)SNP_24100	1349	G	A	11	16	--	--
CakTC34849	Ca(ICCV2/PI489777)SNP_24101	991	G	A	7	4	--	--
	Ca(ICCV2/PI489777)SNP_24102	3088	T	C	6	10	--	--
CakTC31971	Ca(ICCV2/PI489777)SNP_24103	246	C	G	16	8	Shoot	--
	Ca(ICCV2/PI489777)SNP_24104	258	C	T	15	8	Shoot	--
	Ca(ICCV2/PI489777)SNP_24105	423	T	C	13	9	Shoot	--
	Ca(ICCV2/PI489777)SNP_24106	813	G	C	15	3	Shoot	--
	Ca(ICCV2/PI489777)SNP_24107	872	T	C	12	3	Shoot	--
	Ca(ICCV2/PI489777)SNP_24108	1509	C	T	10	5	Shoot	--
	Ca(ICCV2/PI489777)SNP_24109	1582	T	C	8	6	Shoot	--
	Ca(ICCV2/PI489777)SNP_24110	1863	G	A	12	3	Shoot	--
	Ca(ICCV2/PI489777)SNP_24111	2497	A	G	3	3	Shoot	--
CakTC06303	Ca(ICCV2/PI489777)SNP_24112	112	G	A	5	3	--	--
	Ca(ICCV2/PI489777)SNP_24113	113	C	T	5	3	--	--
CakTC34494	Ca(ICCV2/PI489777)SNP_24114	412	A	G	4	4	--	--
CakTC26983	Ca(ICCV2/PI489777)SNP_24115	269	A	T	32	11	--	--
	Ca(ICCV2/PI489777)SNP_24116	468	T	G	16	6	--	--
	Ca(ICCV2/PI489777)SNP_24117	475	C	T	25	6	--	--
	Ca(ICCV2/PI489777)SNP_24118	489	A	G	16	6	--	--
	Ca(ICCV2/PI489777)SNP_24119	653	T	C	15	5	--	--

CakTC28805	Ca(ICCV2/PI489777)SNP_24120	117	A	G	7	24	Root	--
	Ca(ICCV2/PI489777)SNP_24121	125	G	C	8	28	Root	--
	Ca(ICCV2/PI489777)SNP_24122	129	C	T	7	27	Root	--
	Ca(ICCV2/PI489777)SNP_24123	207	A	G	7	34	Root	--
	Ca(ICCV2/PI489777)SNP_24124	229	C	A	8	34	Root	--
	Ca(ICCV2/PI489777)SNP_24125	375	T	C	17	46	Root	--
	Ca(ICCV2/PI489777)SNP_24126	446	C	T	18	44	Root	--
	Ca(ICCV2/PI489777)SNP_24127	748	C	G	3	24	Root	--
	Ca(ICCV2/PI489777)SNP_24128	760	G	C	3	20	Root	--
	Ca(ICCV2/PI489777)SNP_24129	761	T	C	3	20	Root	--
CakTC40455	Ca(ICCV2/PI489777)SNP_24130	402	C	G	4	3	--	--
CakTC39852	Ca(ICCV2/PI489777)SNP_24131	153	G	A	21	4	--	--
	Ca(ICCV2/PI489777)SNP_24132	945	A	C	27	5	--	--
CakTC24525	Ca(ICCV2/PI489777)SNP_24133	1736	C	G	5	3	--	--
CakTC23577	Ca(ICCV2/PI489777)SNP_24134	632	T	C	9	4	--	--
CakTC27549	Ca(ICCV2/PI489777)SNP_24135	1818	G	A	6	8	--	WI/SNF-BAF60
	Ca(ICCV2/PI489777)SNP_24136	2513	A	G	8	3	--	WI/SNF-BAF60
CakTC29888	Ca(ICCV2/PI489777)SNP_24137	1011	G	A	12	5	--	--
	Ca(ICCV2/PI489777)SNP_24138	2087	T	C	5	8	--	--
	Ca(ICCV2/PI489777)SNP_24139	2291	G	T	8	6	--	--
CakTC23406	Ca(ICCV2/PI489777)SNP_24140	680	G	A	175	124	Flower	bud
	Ca(ICCV2/PI489777)SNP_24141	878	T	C	157	116	Flower	bud
	Ca(ICCV2/PI489777)SNP_24142	926	T	C	162	127	Flower	bud
	Ca(ICCV2/PI489777)SNP_24143	1079	C	T	186	110	Flower	bud
	Ca(ICCV2/PI489777)SNP_24144	1751	G	A	104	83	Flower	bud
CakTC23337	Ca(ICCV2/PI489777)SNP_24145	529	C	G	8	6	--	--
CakTC36971	Ca(ICCV2/PI489777)SNP_24146	466	C	G	13	15	--	--
	Ca(ICCV2/PI489777)SNP_24147	831	T	C	14	18	--	--
	Ca(ICCV2/PI489777)SNP_24148	1084	C	T	19	18	--	--
	Ca(ICCV2/PI489777)SNP_24149	1469	C	T	23	17	--	--
	Ca(ICCV2/PI489777)SNP_24150	1600	T	G	21	23	--	--
	Ca(ICCV2/PI489777)SNP_24151	1639	G	A	23	21	--	--
	Ca(ICCV2/PI489777)SNP_24152	1648	C	T	22	25	--	--
CakTC38009	Ca(ICCV2/PI489777)SNP_24153	184	T	A	11	4	--	--
	Ca(ICCV2/PI489777)SNP_24154	204	T	A	14	3	--	--
	Ca(ICCV2/PI489777)SNP_24155	215	T	C	14	4	--	--
	Ca(ICCV2/PI489777)SNP_24156	348	A	G	17	9	--	--
	Ca(ICCV2/PI489777)SNP_24157	542	G	A	21	11	--	--
	Ca(ICCV2/PI489777)SNP_24158	825	G	C	26	8	--	--
CakTC34340	Ca(ICCV2/PI489777)SNP_24159	304	A	C	26	6	--	--
	Ca(ICCV2/PI489777)SNP_24160	371	T	C	54	11	--	--
	Ca(ICCV2/PI489777)SNP_24161	764	C	A	32	14	--	--
	Ca(ICCV2/PI489777)SNP_24162	2108	A	G	20	14	--	--
	Ca(ICCV2/PI489777)SNP_24163	2477	A	C	26	18	--	--
	Ca(ICCV2/PI489777)SNP_24164	2513	C	A	27	19	--	--
	Ca(ICCV2/PI489777)SNP_24165	3929	C	T	16	14	--	--
	Ca(ICCV2/PI489777)SNP_24166	4394	A	G	22	9	--	--
	Ca(ICCV2/PI489777)SNP_24167	5330	G	A	9	5	--	--
CakTC28313	Ca(ICCV2/PI489777)SNP_24168	974	G	C	16	7	--	--
CakTC38888	Ca(ICCV2/PI489777)SNP_24169	156	T	C	16	9	--	--
	Ca(ICCV2/PI489777)SNP_24170	1430	A	G	15	12	--	--
	Ca(ICCV2/PI489777)SNP_24171	1502	C	T	12	9	--	--
CakTC31730	Ca(ICCV2/PI489777)SNP_24172	892	A	G	25	16	--	--
	Ca(ICCV2/PI489777)SNP_24173	1125	G	A	7	5	--	--
CakTC42159	Ca(ICCV2/PI489777)SNP_24174	453	A	G	48	52	--	--
CakTC38674	Ca(ICCV2/PI489777)SNP_24175	461	A	C	8	4	--	--
	Ca(ICCV2/PI489777)SNP_24176	605	A	G	5	5	--	--
	Ca(ICCV2/PI489777)SNP_24177	615	T	C	7	4	--	--
CakTC28295	Ca(ICCV2/PI489777)SNP_24178	295	C	G	4	3	--	AP2-EREBP
	Ca(ICCV2/PI489777)SNP_24179	331	G	C	5	3	--	AP2-EREBP
	Ca(ICCV2/PI489777)SNP_24180	1294	A	G	7	4	--	AP2-EREBP
CakTC36305	Ca(ICCV2/PI489777)SNP_24181	1161	C	T	8	3	--	--
	Ca(ICCV2/PI489777)SNP_24182	1313	G	T	8	7	--	--
	Ca(ICCV2/PI489777)SNP_24183	1659	T	C	9	3	--	--
	Ca(ICCV2/PI489777)SNP_24184	1680	C	T	9	3	--	--
	Ca(ICCV2/PI489777)SNP_24185	2124	G	A	9	5	--	--
	Ca(ICCV2/PI489777)SNP_24186	2355	T	C	15	3	--	--
	Ca(ICCV2/PI489777)SNP_24187	2481	A	G	13	4	--	--
	Ca(ICCV2/PI489777)SNP_24188	2539	T	C	12	4	--	--
	Ca(ICCV2/PI489777)SNP_24189	2603	G	C	11	3	--	--
CakTC25025	Ca(ICCV2/PI489777)SNP_24190	241	A	G	21	19	--	--
	Ca(ICCV2/PI489777)SNP_24191	562	G	T	7	23	--	--
CakTC28051	Ca(ICCV2/PI489777)SNP_24192	1896	A	G	10	8	--	--
CakTC11541	Ca(ICCV2/PI489777)SNP_24193	57	A	G	4	3	--	--

CakTC42575	Ca(ICCV2/PI489777)SNP_24194	854	G	A	5	5	--	--
CakTC27209	Ca(ICCV2/PI489777)SNP_24195	145	C	T	10	8	--	--
	Ca(ICCV2/PI489777)SNP_24196	181	A	G	13	10	--	--
	Ca(ICCV2/PI489777)SNP_24197	385	G	C	17	24	--	--
	Ca(ICCV2/PI489777)SNP_24198	642	C	T	47	34	--	--
	Ca(ICCV2/PI489777)SNP_24199	1105	T	G	52	39	--	--
	Ca(ICCV2/PI489777)SNP_24200	1270	A	G	50	41	--	--
	Ca(ICCV2/PI489777)SNP_24201	1481	G	T	25	18	--	--
CakTC23326	Ca(ICCV2/PI489777)SNP_24202	307	G	A	10	4	--	--
	Ca(ICCV2/PI489777)SNP_24203	2391	G	A	90	35	--	--
CakTC31591	Ca(ICCV2/PI489777)SNP_24204	157	A	G	18	11	--	--
	Ca(ICCV2/PI489777)SNP_24205	924	G	A	20	4	--	--
CakTC38774	Ca(ICCV2/PI489777)SNP_24206	742	A	G	7	4	--	--
	Ca(ICCV2/PI489777)SNP_24207	1350	G	C	4	5	--	--
	Ca(ICCV2/PI489777)SNP_24208	1461	T	A	3	4	--	--
CakTC39541	Ca(ICCV2/PI489777)SNP_24209	1105	T	A	23	11	--	--
CakTC39644	Ca(ICCV2/PI489777)SNP_24210	724	A	G	14	8	--	--
	Ca(ICCV2/PI489777)SNP_24211	1034	C	T	24	3	--	--
CakTC30888	Ca(ICCV2/PI489777)SNP_24212	1470	T	C	11	3	--	bHLH
CakTC37135	Ca(ICCV2/PI489777)SNP_24213	39	A	C	7	4	--	--
	Ca(ICCV2/PI489777)SNP_24214	867	T	A	25	15	--	--
	Ca(ICCV2/PI489777)SNP_24215	1026	G	C	8	3	--	--
CakTC39234	Ca(ICCV2/PI489777)SNP_24216	175	G	A	6	4	--	--
	Ca(ICCV2/PI489777)SNP_24217	193	T	A	11	6	--	--
CakTC30416	Ca(ICCV2/PI489777)SNP_24218	633	A	G	3	3	--	--
CakTC28380	Ca(ICCV2/PI489777)SNP_24219	574	C	T	4	5	Shoot	--
	Ca(ICCV2/PI489777)SNP_24220	877	G	A	4	4	Shoot	--
	Ca(ICCV2/PI489777)SNP_24221	1285	G	A	10	3	Shoot	--
	Ca(ICCV2/PI489777)SNP_24222	1463	C	G	6	3	Shoot	--
	Ca(ICCV2/PI489777)SNP_24223	1497	T	C	4	3	Shoot	--
CakTC39967	Ca(ICCV2/PI489777)SNP_24224	371	G	A	6	4	--	--
	Ca(ICCV2/PI489777)SNP_24225	388	A	T	6	5	--	--
	Ca(ICCV2/PI489777)SNP_24226	493	C	T	5	5	--	--
CakTC30957	Ca(ICCV2/PI489777)SNP_24227	561	T	A	5	4	--	NAC
	Ca(ICCV2/PI489777)SNP_24228	1256	G	A	29	5	--	NAC
	Ca(ICCV2/PI489777)SNP_24229	1278	C	T	28	8	--	NAC
	Ca(ICCV2/PI489777)SNP_24230	1630	G	T	37	11	--	NAC
	Ca(ICCV2/PI489777)SNP_24231	1640	T	A	29	10	--	NAC
	Ca(ICCV2/PI489777)SNP_24232	1955	A	C	28	10	--	NAC
	Ca(ICCV2/PI489777)SNP_24233	2072	A	C	28	13	--	NAC
	Ca(ICCV2/PI489777)SNP_24234	2255	G	A	35	13	--	NAC
	Ca(ICCV2/PI489777)SNP_24235	2598	A	C	15	6	--	NAC
CakTC23927	Ca(ICCV2/PI489777)SNP_24236	151	T	C	6	6	--	--
	Ca(ICCV2/PI489777)SNP_24237	881	C	T	18	6	--	--
	Ca(ICCV2/PI489777)SNP_24238	919	C	T	19	6	--	--
	Ca(ICCV2/PI489777)SNP_24239	1379	G	A	18	3	--	--
CakTC25759	Ca(ICCV2/PI489777)SNP_24240	1365	T	C	3	4	--	--
	Ca(ICCV2/PI489777)SNP_24241	1595	C	A	5	5	--	--
	Ca(ICCV2/PI489777)SNP_24242	1780	C	T	4	6	--	--
CakTC43137	Ca(ICCV2/PI489777)SNP_24243	592	T	C	24	15	--	Trihelix
	Ca(ICCV2/PI489777)SNP_24244	1503	C	T	15	10	--	Trihelix
CakTC31825	Ca(ICCV2/PI489777)SNP_24245	394	A	G	5	3	--	--
	Ca(ICCV2/PI489777)SNP_24246	1235	A	G	3	6	--	--
	Ca(ICCV2/PI489777)SNP_24247	1502	T	C	3	5	--	--
CakTC37547	Ca(ICCV2/PI489777)SNP_24248	832	G	A	3	8	--	--
	Ca(ICCV2/PI489777)SNP_24249	945	A	G	7	9	--	--
	Ca(ICCV2/PI489777)SNP_24250	1020	T	A	7	6	--	--
	Ca(ICCV2/PI489777)SNP_24251	1025	A	G	6	8	--	--
CakTC24067	Ca(ICCV2/PI489777)SNP_24252	1174	C	A	7	11	--	AP2-EREBP
	Ca(ICCV2/PI489777)SNP_24253	1264	A	G	8	10	--	AP2-EREBP
	Ca(ICCV2/PI489777)SNP_24254	1380	T	G	8	12	--	AP2-EREBP
	Ca(ICCV2/PI489777)SNP_24255	1505	G	A	7	11	--	AP2-EREBP
CakTC32152	Ca(ICCV2/PI489777)SNP_24256	340	G	A	11	6	--	--
	Ca(ICCV2/PI489777)SNP_24257	341	C	A	11	6	--	--
	Ca(ICCV2/PI489777)SNP_24258	458	C	G	7	4	--	--
	Ca(ICCV2/PI489777)SNP_24259	510	C	T	8	4	--	--
CakTC38829	Ca(ICCV2/PI489777)SNP_24260	48	G	A	4	4	--	--
	Ca(ICCV2/PI489777)SNP_24261	83	A	T	4	5	--	--
	Ca(ICCV2/PI489777)SNP_24262	99	A	C	4	4	--	--
	Ca(ICCV2/PI489777)SNP_24263	1139	C	A	33	13	--	--
CakTC09803	Ca(ICCV2/PI489777)SNP_24264	108	G	C	8	3	--	--
	Ca(ICCV2/PI489777)SNP_24265	239	T	C	11	4	--	--
	Ca(ICCV2/PI489777)SNP_24266	899	C	A	6	3	--	--
	Ca(ICCV2/PI489777)SNP_24267	2230	G	A	7	5	--	--

CakTC41263	Ca(ICCV2/PI489777)SNP_24268	298	A	C	15	24	--	--
	Ca(ICCV2/PI489777)SNP_24269	338	A	G	14	25	--	--
	Ca(ICCV2/PI489777)SNP_24270	467	T	C	22	32	--	--
CakTC11456	Ca(ICCV2/PI489777)SNP_24271	2208	T	G	8	3	--	--
	Ca(ICCV2/PI489777)SNP_24272	2421	T	A	3	3	--	--
	Ca(ICCV2/PI489777)SNP_24273	2466	C	A	4	3	--	--
CakTC26455	Ca(ICCV2/PI489777)SNP_24274	452	T	C	18	8	--	--
	Ca(ICCV2/PI489777)SNP_24275	482	T	C	19	7	--	--
	Ca(ICCV2/PI489777)SNP_24276	647	C	T	21	7	--	--
	Ca(ICCV2/PI489777)SNP_24277	890	G	A	12	4	--	--
CakTC36273	Ca(ICCV2/PI489777)SNP_24278	204	C	A	23	4	--	--
	Ca(ICCV2/PI489777)SNP_24279	654	G	A	16	11	--	--
	Ca(ICCV2/PI489777)SNP_24280	673	C	T	17	13	--	--
	Ca(ICCV2/PI489777)SNP_24281	1386	A	T	18	6	--	--
	Ca(ICCV2/PI489777)SNP_24282	2871	T	A	8	8	--	--
CakTC28293	Ca(ICCV2/PI489777)SNP_24283	69	T	C	55	19	--	--
	Ca(ICCV2/PI489777)SNP_24284	226	T	C	153	39	--	--
CakTC37399	Ca(ICCV2/PI489777)SNP_24285	845	A	G	45	42	--	--
CakTC08455	Ca(ICCV2/PI489777)SNP_24286	604	A	C	3	3	--	--
CakTC34396	Ca(ICCV2/PI489777)SNP_24287	223	T	G	24	31	--	--
CakTC38107	Ca(ICCV2/PI489777)SNP_24288	1706	G	A	17	12	--	--
	Ca(ICCV2/PI489777)SNP_24289	1847	T	A	3	6	--	--
CakTC10289	Ca(ICCV2/PI489777)SNP_24290	241	T	C	6	4	--	--
	Ca(ICCV2/PI489777)SNP_24291	1002	G	A	22	7	--	--
	Ca(ICCV2/PI489777)SNP_24292	1674	A	G	12	3	--	--
CakTC38180	Ca(ICCV2/PI489777)SNP_24293	965	C	T	8	4	--	--
CakTC08074	Ca(ICCV2/PI489777)SNP_24294	1966	C	T	3	4	--	--
CakTC24846	Ca(ICCV2/PI489777)SNP_24295	229	C	T	15	6	--	--
	Ca(ICCV2/PI489777)SNP_24296	245	T	C	16	7	--	--
	Ca(ICCV2/PI489777)SNP_24297	247	A	G	17	6	--	--
	Ca(ICCV2/PI489777)SNP_24298	272	T	C	18	7	--	--
	Ca(ICCV2/PI489777)SNP_24299	356	C	A	19	7	--	--
CakTC24149	Ca(ICCV2/PI489777)SNP_24300	661	G	A	21	18	--	--
	Ca(ICCV2/PI489777)SNP_24301	724	A	G	20	14	--	--
CakTC25092	Ca(ICCV2/PI489777)SNP_24302	257	A	T	35	16	--	--
	Ca(ICCV2/PI489777)SNP_24303	268	A	G	36	15	--	--
	Ca(ICCV2/PI489777)SNP_24304	280	G	A	30	13	--	--
	Ca(ICCV2/PI489777)SNP_24305	322	C	T	38	17	--	--
	Ca(ICCV2/PI489777)SNP_24306	331	G	A	33	17	--	--
	Ca(ICCV2/PI489777)SNP_24307	348	A	C	38	19	--	--
CakTC38004	Ca(ICCV2/PI489777)SNP_24308	107	C	T	10	5	--	--
CakTC42782	Ca(ICCV2/PI489777)SNP_24309	527	G	C	19	11	--	--
	Ca(ICCV2/PI489777)SNP_24310	649	C	A	16	13	--	--
	Ca(ICCV2/PI489777)SNP_24311	1200	G	A	18	7	--	--
	Ca(ICCV2/PI489777)SNP_24312	1555	C	T	9	7	--	--
CakTC38985	Ca(ICCV2/PI489777)SNP_24313	1019	G	T	3	4	--	--
	Ca(ICCV2/PI489777)SNP_24314	1085	C	T	4	4	--	--
	Ca(ICCV2/PI489777)SNP_24315	1183	A	C	3	5	--	--
CakTC28634	Ca(ICCV2/PI489777)SNP_24316	151	G	A	3	3	--	C2C2-CO-like
	Ca(ICCV2/PI489777)SNP_24317	665	G	A	10	4	--	C2C2-CO-like
	Ca(ICCV2/PI489777)SNP_24318	747	G	A	7	4	--	C2C2-CO-like
	Ca(ICCV2/PI489777)SNP_24319	858	T	C	7	12	--	C2C2-CO-like
	Ca(ICCV2/PI489777)SNP_24320	982	C	A	10	13	--	C2C2-CO-like
	Ca(ICCV2/PI489777)SNP_24321	985	G	A	10	13	--	C2C2-CO-like
	Ca(ICCV2/PI489777)SNP_24322	1030	T	C	7	16	--	C2C2-CO-like
	Ca(ICCV2/PI489777)SNP_24323	1206	C	G	14	11	--	C2C2-CO-like
	Ca(ICCV2/PI489777)SNP_24324	1232	A	G	13	10	--	C2C2-CO-like
	Ca(ICCV2/PI489777)SNP_24325	1311	C	G	13	10	--	C2C2-CO-like
	Ca(ICCV2/PI489777)SNP_24326	1364	C	G	10	8	--	C2C2-CO-like
	Ca(ICCV2/PI489777)SNP_24327	1382	G	A	10	8	--	C2C2-CO-like
CakTC33062	Ca(ICCV2/PI489777)SNP_24328	180	C	G	33	24	--	--
	Ca(ICCV2/PI489777)SNP_24329	535	G	C	3	5	--	--
CakTC41562	Ca(ICCV2/PI489777)SNP_24330	228	A	T	3	10	--	--
	Ca(ICCV2/PI489777)SNP_24331	653	G	A	4	9	--	--
CakTC09601	Ca(ICCV2/PI489777)SNP_24332	1571	C	T	4	4	--	--
	Ca(ICCV2/PI489777)SNP_24333	1645	C	T	4	3	--	--
	Ca(ICCV2/PI489777)SNP_24334	1982	T	C	3	6	--	--
CakTC25134	Ca(ICCV2/PI489777)SNP_24335	304	T	G	23	21	--	--
	Ca(ICCV2/PI489777)SNP_24336	409	C	G	20	22	--	--
	Ca(ICCV2/PI489777)SNP_24337	988	T	G	40	32	--	--
	Ca(ICCV2/PI489777)SNP_24338	1033	A	G	41	30	--	--
	Ca(ICCV2/PI489777)SNP_24339	1086	T	A	41	31	--	--
	Ca(ICCV2/PI489777)SNP_24340	1090	G	T	42	31	--	--
	Ca(ICCV2/PI489777)SNP_24341	1150	A	G	38	31	--	--

	Ca(ICCV2/PI489777)SNP_24342	1167	G	A	37	33	--	--
	Ca(ICCV2/PI489777)SNP_24343	1298	A	G	40	28	--	--
	Ca(ICCV2/PI489777)SNP_24344	1363	A	G	38	27	--	--
	Ca(ICCV2/PI489777)SNP_24345	1384	C	T	41	26	--	--
	Ca(ICCV2/PI489777)SNP_24346	1418	G	A	37	27	--	--
	Ca(ICCV2/PI489777)SNP_24347	1716	T	C	15	7	--	--
CakTC13748	Ca(ICCV2/PI489777)SNP_24348	60	A	G	3	3	--	--
	Ca(ICCV2/PI489777)SNP_24349	244	C	G	4	4	--	--
	Ca(ICCV2/PI489777)SNP_24350	273	T	C	4	4	--	--
CakTC31407	Ca(ICCV2/PI489777)SNP_24351	862	T	G	5	3	--	--
	Ca(ICCV2/PI489777)SNP_24352	963	C	T	5	4	--	--
	Ca(ICCV2/PI489777)SNP_24353	1013	T	C	5	4	--	--
CakTC29499	Ca(ICCV2/PI489777)SNP_24354	721	C	T	7	4	--	--
	Ca(ICCV2/PI489777)SNP_24355	844	T	C	4	6	--	--
	Ca(ICCV2/PI489777)SNP_24356	949	C	T	8	6	--	--
CakTC40190	Ca(ICCV2/PI489777)SNP_24357	115	G	A	3	5	--	--
	Ca(ICCV2/PI489777)SNP_24358	280	A	G	5	6	--	--
	Ca(ICCV2/PI489777)SNP_24359	937	A	G	3	3	--	--
CakTC13216	Ca(ICCV2/PI489777)SNP_24360	702	A	G	21	19	--	--
CakTC30088	Ca(ICCV2/PI489777)SNP_24361	305	T	A	6	3	--	--
	Ca(ICCV2/PI489777)SNP_24362	390	T	C	4	5	--	--
	Ca(ICCV2/PI489777)SNP_24363	1197	G	A	5	3	--	--
	Ca(ICCV2/PI489777)SNP_24364	1200	C	A	5	3	--	--
CakTC37592	Ca(ICCV2/PI489777)SNP_24365	560	C	T	3	9	--	--
	Ca(ICCV2/PI489777)SNP_24366	611	A	T	3	8	--	--
	Ca(ICCV2/PI489777)SNP_24367	701	T	G	3	10	--	--
CakTC33658	Ca(ICCV2/PI489777)SNP_24368	257	A	G	14	8	--	--
	Ca(ICCV2/PI489777)SNP_24369	734	A	G	9	8	--	--
CakTC27174	Ca(ICCV2/PI489777)SNP_24370	631	A	G	28	7	--	--
	Ca(ICCV2/PI489777)SNP_24371	735	G	A	31	10	--	--
	Ca(ICCV2/PI489777)SNP_24372	885	G	A	32	9	--	--
	Ca(ICCV2/PI489777)SNP_24373	1074	T	C	31	9	--	--
CakTC33899	Ca(ICCV2/PI489777)SNP_24374	568	T	C	6	4	--	--
	Ca(ICCV2/PI489777)SNP_24375	694	A	G	7	4	--	--
	Ca(ICCV2/PI489777)SNP_24376	913	C	A	8	3	--	--
	Ca(ICCV2/PI489777)SNP_24377	1192	T	C	12	4	--	--
CakTC07699	Ca(ICCV2/PI489777)SNP_24378	1155	G	A	3	5	--	--
	Ca(ICCV2/PI489777)SNP_24379	1380	C	T	8	5	--	--
	Ca(ICCV2/PI489777)SNP_24380	2201	A	T	9	8	--	--
CakTC41445	Ca(ICCV2/PI489777)SNP_24381	373	C	G	8	7	--	--
	Ca(ICCV2/PI489777)SNP_24382	419	T	G	8	7	--	--
	Ca(ICCV2/PI489777)SNP_24383	499	C	T	3	6	--	--
CakTC33493	Ca(ICCV2/PI489777)SNP_24384	553	G	T	23	21	Shoot	--
	Ca(ICCV2/PI489777)SNP_24385	1526	T	A	25	7	Shoot	--
CakTC03838	Ca(ICCV2/PI489777)SNP_24386	685	T	C	7	3	--	--
CakTC30378	Ca(ICCV2/PI489777)SNP_24387	305	G	A	9	3	Shoot	--
	Ca(ICCV2/PI489777)SNP_24388	355	T	A	12	3	Shoot	--
	Ca(ICCV2/PI489777)SNP_24389	642	T	C	11	3	Shoot	--
	Ca(ICCV2/PI489777)SNP_24390	724	T	G	11	4	Shoot	--
	Ca(ICCV2/PI489777)SNP_24391	792	G	A	3	3	Shoot	--
CakTC22304	Ca(ICCV2/PI489777)SNP_24392	753	C	T	8	3	--	--
	Ca(ICCV2/PI489777)SNP_24393	807	T	A	8	4	--	--
CakTC11521	Ca(ICCV2/PI489777)SNP_24394	681	A	G	17	9	--	--
	Ca(ICCV2/PI489777)SNP_24395	1644	T	C	8	3	--	--
CakTC33069	Ca(ICCV2/PI489777)SNP_24396	88	T	C	5	11	--	--
	Ca(ICCV2/PI489777)SNP_24397	155	C	G	5	12	--	--
	Ca(ICCV2/PI489777)SNP_24398	187	T	G	6	9	--	--
	Ca(ICCV2/PI489777)SNP_24399	218	G	C	6	12	--	--
	Ca(ICCV2/PI489777)SNP_24400	475	A	G	11	18	--	--
	Ca(ICCV2/PI489777)SNP_24401	493	C	T	11	16	--	--
	Ca(ICCV2/PI489777)SNP_24402	577	A	T	14	16	--	--
	Ca(ICCV2/PI489777)SNP_24403	685	A	G	20	17	--	--
	Ca(ICCV2/PI489777)SNP_24404	865	T	C	19	19	--	--
	Ca(ICCV2/PI489777)SNP_24405	907	A	G	22	16	--	--
	Ca(ICCV2/PI489777)SNP_24406	1012	T	C	21	15	--	--
	Ca(ICCV2/PI489777)SNP_24407	1099	G	T	24	16	--	--
	Ca(ICCV2/PI489777)SNP_24408	1172	C	G	29	16	--	--
	Ca(ICCV2/PI489777)SNP_24409	1186	G	A	31	17	--	--
	Ca(ICCV2/PI489777)SNP_24410	1676	G	A	24	15	--	--
	Ca(ICCV2/PI489777)SNP_24411	2374	C	T	28	29	--	--
	Ca(ICCV2/PI489777)SNP_24412	2510	G	A	23	20	--	--
	Ca(ICCV2/PI489777)SNP_24413	2713	A	G	5	7	--	--
CakTC32189	Ca(ICCV2/PI489777)SNP_24414	593	A	C	9	3	--	--
	Ca(ICCV2/PI489777)SNP_24415	788	A	G	4	5	--	--

	Ca(ICCV2/PI489777)SNP_24416	1034	C	T	7	5	--	--
	Ca(ICCV2/PI489777)SNP_24417	1065	T	A	8	3	--	--
	Ca(ICCV2/PI489777)SNP_24418	1145	T	C	8	6	--	--
CakTC43203	Ca(ICCV2/PI489777)SNP_24419	192	C	T	14	3	--	--
CakTC39062	Ca(ICCV2/PI489777)SNP_24420	787	T	C	38	31	Young_pod	--
CakTC22314	Ca(ICCV2/PI489777)SNP_24421	115	G	T	93	45	--	--
	Ca(ICCV2/PI489777)SNP_24422	301	G	A	135	66	--	--
	Ca(ICCV2/PI489777)SNP_24423	421	G	A	172	76	--	--
	Ca(ICCV2/PI489777)SNP_24424	1605	T	C	7	4	--	--
	Ca(ICCV2/PI489777)SNP_24425	1719	T	C	5	3	--	--
CakTC27779	Ca(ICCV2/PI489777)SNP_24426	1502	C	G	33	10	Root	--
	Ca(ICCV2/PI489777)SNP_24427	1710	A	G	25	18	Root	--
CakTC28409	Ca(ICCV2/PI489777)SNP_24428	139	G	T	11	13	--	AUX/IAA
	Ca(ICCV2/PI489777)SNP_24429	580	T	C	10	15	--	AUX/IAA
	Ca(ICCV2/PI489777)SNP_24430	1034	G	A	25	9	--	AUX/IAA
CakTC39018	Ca(ICCV2/PI489777)SNP_24431	52	G	A	4	8	--	--
	Ca(ICCV2/PI489777)SNP_24432	266	T	A	12	7	--	--
	Ca(ICCV2/PI489777)SNP_24433	1061	A	T	34	13	--	--
CakTC24856	Ca(ICCV2/PI489777)SNP_24434	1306	A	C	4	5	--	--
	Ca(ICCV2/PI489777)SNP_24435	1421	T	G	8	6	--	--
	Ca(ICCV2/PI489777)SNP_24436	1682	C	G	6	3	--	--
CakTC26154	Ca(ICCV2/PI489777)SNP_24437	1025	T	C	5	6	--	--
	Ca(ICCV2/PI489777)SNP_24438	1127	G	A	5	6	--	--
	Ca(ICCV2/PI489777)SNP_24439	1258	T	C	5	4	--	--
CakTC24554	Ca(ICCV2/PI489777)SNP_24440	1306	G	A	6	11	--	--
	Ca(ICCV2/PI489777)SNP_24441	1333	A	T	7	9	--	--
	Ca(ICCV2/PI489777)SNP_24442	1764	A	G	8	3	--	--
CakTC37128	Ca(ICCV2/PI489777)SNP_24443	351	A	T	18	14	--	--
	Ca(ICCV2/PI489777)SNP_24444	417	C	T	18	17	--	--
	Ca(ICCV2/PI489777)SNP_24445	516	A	G	17	12	--	--
	Ca(ICCV2/PI489777)SNP_24446	1188	G	A	6	8	--	--
	Ca(ICCV2/PI489777)SNP_24447	1268	C	A	8	13	--	--
	Ca(ICCV2/PI489777)SNP_24448	1402	A	G	7	10	--	--
CakTC39386	Ca(ICCV2/PI489777)SNP_24449	1173	A	G	30	7	--	--
	Ca(ICCV2/PI489777)SNP_24450	1415	A	G	5	3	--	--
CakTC24070	Ca(ICCV2/PI489777)SNP_24451	640	T	C	15	3	--	--
	Ca(ICCV2/PI489777)SNP_24452	665	C	T	20	3	--	--
CakTC30417	Ca(ICCV2/PI489777)SNP_24453	391	G	A	5	3	--	--
CakTC08719	Ca(ICCV2/PI489777)SNP_24454	776	T	C	13	6	--	--
	Ca(ICCV2/PI489777)SNP_24455	824	A	G	15	6	--	--
	Ca(ICCV2/PI489777)SNP_24456	834	G	T	16	6	--	--
	Ca(ICCV2/PI489777)SNP_24457	1358	A	G	11	3	--	--
	Ca(ICCV2/PI489777)SNP_24458	1795	C	T	25	10	--	--
CakTC24358	Ca(ICCV2/PI489777)SNP_24459	525	T	C	48	14	--	--
	Ca(ICCV2/PI489777)SNP_24460	857	T	C	34	9	--	--
	Ca(ICCV2/PI489777)SNP_24461	1244	T	C	31	12	--	--
	Ca(ICCV2/PI489777)SNP_24462	1563	C	T	20	11	--	--
CakTC33290	Ca(ICCV2/PI489777)SNP_24463	1797	C	T	7	4	--	--
CakTC40184	Ca(ICCV2/PI489777)SNP_24464	162	T	C	27	26	--	--
	Ca(ICCV2/PI489777)SNP_24465	195	A	G	30	26	--	--
CakTC35125	Ca(ICCV2/PI489777)SNP_24466	152	C	T	4	3	--	--
	Ca(ICCV2/PI489777)SNP_24467	200	T	C	4	5	--	--
	Ca(ICCV2/PI489777)SNP_24468	292	C	T	6	5	--	--
	Ca(ICCV2/PI489777)SNP_24469	365	A	G	5	5	--	--
CakTC36889	Ca(ICCV2/PI489777)SNP_24470	2088	G	A	14	3	--	--
	Ca(ICCV2/PI489777)SNP_24471	3277	A	G	8	8	--	--
	Ca(ICCV2/PI489777)SNP_24472	3511	G	A	15	13	--	--
	Ca(ICCV2/PI489777)SNP_24473	4206	A	G	14	11	--	--
CakTC24620	Ca(ICCV2/PI489777)SNP_24474	123	C	T	5	4	--	--
	Ca(ICCV2/PI489777)SNP_24475	368	G	A	4	4	--	--
	Ca(ICCV2/PI489777)SNP_24476	2799	T	C	9	3	--	--
	Ca(ICCV2/PI489777)SNP_24477	3205	T	A	4	3	--	--
CakTC26078	Ca(ICCV2/PI489777)SNP_24478	1552	A	T	9	6	--	Bromodomain
CakTC25663	Ca(ICCV2/PI489777)SNP_24479	3553	C	T	6	6	--	--
	Ca(ICCV2/PI489777)SNP_24480	3658	C	T	5	5	--	--
	Ca(ICCV2/PI489777)SNP_24481	3947	A	G	7	4	--	--
	Ca(ICCV2/PI489777)SNP_24482	3948	G	T	7	4	--	--
	Ca(ICCV2/PI489777)SNP_24483	4129	A	C	10	7	--	--
	Ca(ICCV2/PI489777)SNP_24484	4154	C	T	11	6	--	--
	Ca(ICCV2/PI489777)SNP_24485	4254	T	G	14	5	--	--
	Ca(ICCV2/PI489777)SNP_24486	4326	A	G	10	3	--	--
	Ca(ICCV2/PI489777)SNP_24487	4614	G	C	24	3	--	--
CakTC38190	Ca(ICCV2/PI489777)SNP_24488	165	T	C	56	21	--	--
	Ca(ICCV2/PI489777)SNP_24489	183	G	T	62	23	--	--

CakTC39507	Ca(ICCV2/PI489777)SNP_24490	400	G	A	7	12	--	--
	Ca(ICCV2/PI489777)SNP_24491	409	T	C	8	14	--	--
	Ca(ICCV2/PI489777)SNP_24492	583	T	A	12	8	--	--
	Ca(ICCV2/PI489777)SNP_24493	584	G	C	12	8	--	--
	Ca(ICCV2/PI489777)SNP_24494	767	G	C	13	3	--	--
	Ca(ICCV2/PI489777)SNP_24495	1032	G	C	13	9	--	--
	Ca(ICCV2/PI489777)SNP_24496	1064	T	C	13	10	--	--
	Ca(ICCV2/PI489777)SNP_24497	1074	C	G	12	10	--	--
	Ca(ICCV2/PI489777)SNP_24498	1212	T	C	7	8	--	--
	Ca(ICCV2/PI489777)SNP_24499	1249	A	G	8	8	--	--
CakTC25655	Ca(ICCV2/PI489777)SNP_24500	1830	T	C	11	5	--	--
	Ca(ICCV2/PI489777)SNP_24501	2125	G	T	8	3	--	--
CakTC41136	Ca(ICCV2/PI489777)SNP_24502	135	G	C	9	6	--	--
	Ca(ICCV2/PI489777)SNP_24503	391	G	T	16	8	--	--
	Ca(ICCV2/PI489777)SNP_24504	605	A	C	16	4	--	--
	Ca(ICCV2/PI489777)SNP_24505	1345	G	C	8	3	--	--
	Ca(ICCV2/PI489777)SNP_24506	1539	G	A	7	3	--	--
	Ca(ICCV2/PI489777)SNP_24507	1612	A	G	7	3	--	--
	Ca(ICCV2/PI489777)SNP_24508	1617	C	T	7	3	--	--
	Ca(ICCV2/PI489777)SNP_24509	1668	A	T	8	3	--	--
	Ca(ICCV2/PI489777)SNP_24510	1917	T	A	11	5	--	--
	Ca(ICCV2/PI489777)SNP_24511	2082	A	G	8	3	--	--
CakTC24086	Ca(ICCV2/PI489777)SNP_24512	157	C	A	3	3	--	--
CakTC00570	Ca(ICCV2/PI489777)SNP_24513	321	T	C	3	4	--	--
CakTC27622	Ca(ICCV2/PI489777)SNP_24514	1111	C	T	7	5	--	--
	Ca(ICCV2/PI489777)SNP_24515	1201	G	C	9	3	--	--
CakTC28426	Ca(ICCV2/PI489777)SNP_24516	1489	T	C	7	5	Root	--
	Ca(ICCV2/PI489777)SNP_24517	1497	A	G	8	4	Root	--
	Ca(ICCV2/PI489777)SNP_24518	1617	T	C	8	5	Root	--
	Ca(ICCV2/PI489777)SNP_24519	1776	G	A	7	3	Root	--
CakTC11683	Ca(ICCV2/PI489777)SNP_24520	1651	C	T	6	9	--	--
	Ca(ICCV2/PI489777)SNP_24521	1717	G	A	4	8	--	--
	Ca(ICCV2/PI489777)SNP_24522	1795	G	C	3	4	--	--
CakTC39525	Ca(ICCV2/PI489777)SNP_24523	768	C	T	28	6	--	--
CakTC28814	Ca(ICCV2/PI489777)SNP_24524	494	C	T	19	4	--	--
	Ca(ICCV2/PI489777)SNP_24525	581	G	A	20	5	--	--
	Ca(ICCV2/PI489777)SNP_24526	1163	A	G	27	11	--	--
CakTC39270	Ca(ICCV2/PI489777)SNP_24527	268	G	T	20	4	--	--
	Ca(ICCV2/PI489777)SNP_24528	362	C	T	17	6	--	--
	Ca(ICCV2/PI489777)SNP_24529	668	C	A	6	3	--	--
	Ca(ICCV2/PI489777)SNP_24530	1287	A	G	9	7	--	--
CakTC28872	Ca(ICCV2/PI489777)SNP_24531	835	A	G	8	3	--	--
	Ca(ICCV2/PI489777)SNP_24532	836	A	G	8	4	--	--
	Ca(ICCV2/PI489777)SNP_24533	1230	C	T	3	3	--	--
CakTC11908	Ca(ICCV2/PI489777)SNP_24534	270	A	T	6	3	Young_pod	--
	Ca(ICCV2/PI489777)SNP_24535	271	T	C	6	3	Young_pod	--
CakTC07970	Ca(ICCV2/PI489777)SNP_24536	570	C	A	3	4	--	C3H
CakTC38970	Ca(ICCV2/PI489777)SNP_24537	709	C	T	7	3	--	--
CakTC40054	Ca(ICCV2/PI489777)SNP_24538	171	A	G	13	15	--	TAZ
	Ca(ICCV2/PI489777)SNP_24539	244	A	C	17	17	--	TAZ
	Ca(ICCV2/PI489777)SNP_24540	310	T	C	18	16	--	TAZ
	Ca(ICCV2/PI489777)SNP_24541	779	T	G	21	13	--	TAZ
	Ca(ICCV2/PI489777)SNP_24542	1356	T	C	13	25	--	TAZ
	Ca(ICCV2/PI489777)SNP_24543	1380	T	C	13	21	--	TAZ
CakTC23483	Ca(ICCV2/PI489777)SNP_24544	63	T	C	16	7	--	--
	Ca(ICCV2/PI489777)SNP_24545	614	T	G	6	3	--	--
	Ca(ICCV2/PI489777)SNP_24546	848	A	G	7	4	--	--
CakTC23933	Ca(ICCV2/PI489777)SNP_24547	593	C	T	143	86	--	--
	Ca(ICCV2/PI489777)SNP_24548	682	A	G	138	97	--	--
	Ca(ICCV2/PI489777)SNP_24549	2653	G	T	150	109	--	--
CakTC28898	Ca(ICCV2/PI489777)SNP_24550	280	G	C	4	3	--	--
CakTC24015	Ca(ICCV2/PI489777)SNP_24551	72	A	T	15	22	--	--
	Ca(ICCV2/PI489777)SNP_24552	183	C	A	22	46	--	--
	Ca(ICCV2/PI489777)SNP_24553	1476	A	C	98	137	--	--
	Ca(ICCV2/PI489777)SNP_24554	1881	T	C	107	104	--	--
	Ca(ICCV2/PI489777)SNP_24555	2520	G	A	105	85	--	--
	Ca(ICCV2/PI489777)SNP_24556	2655	G	A	96	81	--	--
	Ca(ICCV2/PI489777)SNP_24557	2712	T	A	79	77	--	--
CakTC30911	Ca(ICCV2/PI489777)SNP_24558	1260	T	C	4	33	--	--
	Ca(ICCV2/PI489777)SNP_24559	1616	T	C	3	14	--	--
	Ca(ICCV2/PI489777)SNP_24560	1621	A	G	5	19	--	--
	Ca(ICCV2/PI489777)SNP_24561	1683	T	C	5	10	--	--
	Ca(ICCV2/PI489777)SNP_24562	1845	A	G	5	4	--	--
	Ca(ICCV2/PI489777)SNP_24563	1890	G	A	5	5	--	--

	Ca(ICCV2/PI489777)SNP_24564	1962	T	C	4	5	--	--
	Ca(ICCV2/PI489777)SNP_24565	2085	G	C	5	5	--	--
	Ca(ICCV2/PI489777)SNP_24566	2105	T	C	5	5	--	--
CakTC29738	Ca(ICCV2/PI489777)SNP_24567	223	A	G	4	4	--	--
	Ca(ICCV2/PI489777)SNP_24568	360	C	T	3	5	--	--
	Ca(ICCV2/PI489777)SNP_24569	1251	T	A	8	3	--	--
	Ca(ICCV2/PI489777)SNP_24570	1800	G	A	21	4	--	--
CakTC10155	Ca(ICCV2/PI489777)SNP_24571	96	A	G	4	5	--	TPR
	Ca(ICCV2/PI489777)SNP_24572	341	G	T	5	4	--	TPR
CakTC30356	Ca(ICCV2/PI489777)SNP_24573	317	T	C	12	3	--	--
	Ca(ICCV2/PI489777)SNP_24574	416	C	T	11	3	--	--
	Ca(ICCV2/PI489777)SNP_24575	559	A	G	7	7	--	--
CakTC04474	Ca(ICCV2/PI489777)SNP_24576	1178	C	T	8	3	--	--
CakTC24199	Ca(ICCV2/PI489777)SNP_24577	565	C	T	29	13	--	--
	Ca(ICCV2/PI489777)SNP_24578	839	A	G	25	7	--	--
	Ca(ICCV2/PI489777)SNP_24579	1228	T	A	14	14	--	--
	Ca(ICCV2/PI489777)SNP_24580	1231	A	G	14	14	--	--
	Ca(ICCV2/PI489777)SNP_24581	1306	C	A	8	11	--	--
CakTC24540	Ca(ICCV2/PI489777)SNP_24582	360	A	T	5	6	--	--
	Ca(ICCV2/PI489777)SNP_24583	567	T	C	12	4	--	--
	Ca(ICCV2/PI489777)SNP_24584	1315	C	T	8	5	--	--
CakTC15788	Ca(ICCV2/PI489777)SNP_24585	1584	T	C	60	23	--	--
	Ca(ICCV2/PI489777)SNP_24586	1629	G	A	57	30	--	--
CakTC41421	Ca(ICCV2/PI489777)SNP_24587	206	T	C	9	7	--	--
CakTC40884	Ca(ICCV2/PI489777)SNP_24588	192	A	G	9	11	--	--
	Ca(ICCV2/PI489777)SNP_24589	285	A	G	9	12	--	--
	Ca(ICCV2/PI489777)SNP_24590	327	G	T	20	13	--	--
CakTC39990	Ca(ICCV2/PI489777)SNP_24591	1083	G	A	13	5	--	--
CakTC38443	Ca(ICCV2/PI489777)SNP_24592	120	A	G	24	35	--	--
	Ca(ICCV2/PI489777)SNP_24593	161	T	C	36	38	--	--
	Ca(ICCV2/PI489777)SNP_24594	386	C	T	56	55	--	--
	Ca(ICCV2/PI489777)SNP_24595	413	A	C	56	50	--	--
	Ca(ICCV2/PI489777)SNP_24596	443	T	C	60	53	--	--
	Ca(ICCV2/PI489777)SNP_24597	482	T	C	51	55	--	--
	Ca(ICCV2/PI489777)SNP_24598	515	T	G	48	52	--	--
	Ca(ICCV2/PI489777)SNP_24599	815	T	C	54	29	--	--
	Ca(ICCV2/PI489777)SNP_24600	1328	G	A	32	21	--	--
CakTC43173	Ca(ICCV2/PI489777)SNP_24601	947	G	A	4	5	--	--
CakTC25520	Ca(ICCV2/PI489777)SNP_24602	304	A	G	12	14	--	--
	Ca(ICCV2/PI489777)SNP_24603	625	C	T	41	18	--	--
	Ca(ICCV2/PI489777)SNP_24604	742	T	C	36	18	--	--
	Ca(ICCV2/PI489777)SNP_24605	1003	G	A	32	19	--	--
	Ca(ICCV2/PI489777)SNP_24606	1306	C	T	24	28	--	--
	Ca(ICCV2/PI489777)SNP_24607	1384	G	A	21	28	--	--
	Ca(ICCV2/PI489777)SNP_24608	1520	A	G	16	12	--	--
	Ca(ICCV2/PI489777)SNP_24609	1537	T	A	7	8	--	--
CakTC27333	Ca(ICCV2/PI489777)SNP_24610	743	G	A	11	4	--	--
CakTC40059	Ca(ICCV2/PI489777)SNP_24611	964	G	C	9	3	--	--
	Ca(ICCV2/PI489777)SNP_24612	1052	C	T	4	3	--	--
CakTC29022	Ca(ICCV2/PI489777)SNP_24613	811	G	A	13	5	--	--
	Ca(ICCV2/PI489777)SNP_24614	1020	C	G	15	6	--	--
	Ca(ICCV2/PI489777)SNP_24615	1138	C	T	12	9	--	--
	Ca(ICCV2/PI489777)SNP_24616	1676	G	C	7	6	--	--
CakTC26604	Ca(ICCV2/PI489777)SNP_24617	563	A	G	6	6	--	--
CakTC26095	Ca(ICCV2/PI489777)SNP_24618	154	T	C	32	12	--	--
CakTC30341	Ca(ICCV2/PI489777)SNP_24619	63	T	C	3	3	--	--
	Ca(ICCV2/PI489777)SNP_24620	76	G	T	3	3	--	--
CakTC26071	Ca(ICCV2/PI489777)SNP_24621	295	A	T	3	10	--	--
CakTC26129	Ca(ICCV2/PI489777)SNP_24622	805	A	T	28	5	--	WRKY
CakTC41289	Ca(ICCV2/PI489777)SNP_24623	576	C	G	15	8	--	--
	Ca(ICCV2/PI489777)SNP_24624	591	A	G	14	9	--	--
	Ca(ICCV2/PI489777)SNP_24625	864	T	C	8	12	--	--
	Ca(ICCV2/PI489777)SNP_24626	921	G	A	7	11	--	--
	Ca(ICCV2/PI489777)SNP_24627	1167	G	A	10	3	--	--
	Ca(ICCV2/PI489777)SNP_24628	1473	G	T	8	5	--	--
CakTC01281	Ca(ICCV2/PI489777)SNP_24629	1013	C	T	4	6	--	--
CakTC26488	Ca(ICCV2/PI489777)SNP_24630	672	T	A	4	4	--	--
CakTC42450	Ca(ICCV2/PI489777)SNP_24631	192	T	A	3	3	--	--
	Ca(ICCV2/PI489777)SNP_24632	1544	C	T	12	9	--	--
CakTC38928	Ca(ICCV2/PI489777)SNP_24633	390	C	A	10	5	--	--
	Ca(ICCV2/PI489777)SNP_24634	407	A	T	11	5	--	--
CakTC38905	Ca(ICCV2/PI489777)SNP_24635	431	C	T	4	3	--	--
CakTC29118	Ca(ICCV2/PI489777)SNP_24636	369	A	G	59	68	--	--
	Ca(ICCV2/PI489777)SNP_24637	918	T	G	64	38	--	--

CakTC36252	Ca(ICCV2/PI489777)SNP_24638	283	G	C	11	6	--	--
	Ca(ICCV2/PI489777)SNP_24639	1277	C	G	22	14	--	--
	Ca(ICCV2/PI489777)SNP_24640	2603	T	C	34	15	--	--
	Ca(ICCV2/PI489777)SNP_24641	2688	G	T	55	16	--	--
	Ca(ICCV2/PI489777)SNP_24642	2887	T	C	39	10	--	--
CakTC27320	Ca(ICCV2/PI489777)SNP_24643	408	G	A	4	3	--	--
	Ca(ICCV2/PI489777)SNP_24644	862	G	A	5	5	--	--
	Ca(ICCV2/PI489777)SNP_24645	1003	A	G	7	5	--	--
	Ca(ICCV2/PI489777)SNP_24646	1024	G	A	7	5	--	--
	Ca(ICCV2/PI489777)SNP_24647	1105	A	G	4	4	--	--
CakTC41847	Ca(ICCV2/PI489777)SNP_24648	586	A	G	14	6	--	--
CakTC31530	Ca(ICCV2/PI489777)SNP_24649	212	T	C	9	3	--	--
	Ca(ICCV2/PI489777)SNP_24650	225	T	C	11	3	--	--
CakTC37340	Ca(ICCV2/PI489777)SNP_24651	649	T	G	32	15	--	--
	Ca(ICCV2/PI489777)SNP_24652	769	A	G	43	11	--	--
	Ca(ICCV2/PI489777)SNP_24653	919	G	C	33	5	--	--
CakTC25721	Ca(ICCV2/PI489777)SNP_24654	727	C	T	40	25	--	--
	Ca(ICCV2/PI489777)SNP_24655	870	A	G	35	20	--	--
	Ca(ICCV2/PI489777)SNP_24656	904	T	C	20	19	--	--
	Ca(ICCV2/PI489777)SNP_24657	944	A	T	22	18	--	--
	Ca(ICCV2/PI489777)SNP_24658	1186	T	C	28	12	--	--
	Ca(ICCV2/PI489777)SNP_24659	1505	A	G	42	23	--	--
	Ca(ICCV2/PI489777)SNP_24660	1563	C	T	48	29	--	--
	Ca(ICCV2/PI489777)SNP_24661	1721	C	T	24	31	--	--
	Ca(ICCV2/PI489777)SNP_24662	2632	A	G	22	16	--	--
	Ca(ICCV2/PI489777)SNP_24663	2727	A	G	23	14	--	--
	Ca(ICCV2/PI489777)SNP_24664	2926	T	C	17	14	--	--
CakTC37599	Ca(ICCV2/PI489777)SNP_24665	651	G	A	9	19	--	--
	Ca(ICCV2/PI489777)SNP_24666	738	C	T	11	14	--	--
	Ca(ICCV2/PI489777)SNP_24667	754	A	G	9	13	--	--
	Ca(ICCV2/PI489777)SNP_24668	807	T	C	9	10	--	--
	Ca(ICCV2/PI489777)SNP_24669	840	A	G	10	8	--	--
CakTC27090	Ca(ICCV2/PI489777)SNP_24670	170	T	A	4	6	--	--
CakTC28662	Ca(ICCV2/PI489777)SNP_24671	23	T	C	3	6	--	--
	Ca(ICCV2/PI489777)SNP_24672	27	T	C	5	7	--	--
	Ca(ICCV2/PI489777)SNP_24673	624	T	A	14	14	--	--
CakTC30433	Ca(ICCV2/PI489777)SNP_24674	492	C	T	7	10	--	--
	Ca(ICCV2/PI489777)SNP_24675	542	T	G	7	12	--	--
	Ca(ICCV2/PI489777)SNP_24676	575	C	G	5	12	--	--
	Ca(ICCV2/PI489777)SNP_24677	724	G	C	8	9	--	--
	Ca(ICCV2/PI489777)SNP_24678	1126	A	G	6	7	--	--
	Ca(ICCV2/PI489777)SNP_24679	1199	A	G	4	4	--	--
	Ca(ICCV2/PI489777)SNP_24680	1540	C	T	4	4	--	--
CakTC41231	Ca(ICCV2/PI489777)SNP_24681	590	G	A	35	35	--	--
CakTC33129	Ca(ICCV2/PI489777)SNP_24682	1740	G	A	8	5	Mature	Leaf
	Ca(ICCV2/PI489777)SNP_24683	2132	G	A	15	5	Mature	Leaf
CakTC37653	Ca(ICCV2/PI489777)SNP_24684	274	A	C	21	10	--	--
	Ca(ICCV2/PI489777)SNP_24685	647	C	T	22	13	--	--
	Ca(ICCV2/PI489777)SNP_24686	1200	T	A	23	4	--	--
CakTC31809	Ca(ICCV2/PI489777)SNP_24687	309	T	C	3	6	--	PHD
	Ca(ICCV2/PI489777)SNP_24688	1970	A	G	5	5	--	PHD
	Ca(ICCV2/PI489777)SNP_24689	2143	A	C	4	3	--	PHD
CakTC23257	Ca(ICCV2/PI489777)SNP_24690	777	T	C	13	5	--	--
	Ca(ICCV2/PI489777)SNP_24691	961	G	A	14	7	--	--
CakTC35250	Ca(ICCV2/PI489777)SNP_24692	334	T	C	7	4	--	--
	Ca(ICCV2/PI489777)SNP_24693	1232	A	G	8	6	--	--
	Ca(ICCV2/PI489777)SNP_24694	2518	T	C	11	7	--	--
CakTC38001	Ca(ICCV2/PI489777)SNP_24695	532	G	A	4	7	--	--
CakTC10720	Ca(ICCV2/PI489777)SNP_24696	1178	T	G	7	3	--	--
	Ca(ICCV2/PI489777)SNP_24697	1204	C	T	7	3	--	--
CakTC42449	Ca(ICCV2/PI489777)SNP_24698	729	G	A	4	5	--	--
	Ca(ICCV2/PI489777)SNP_24699	1136	C	T	7	3	--	--
CakTC12245	Ca(ICCV2/PI489777)SNP_24700	200	A	T	5	3	--	--
	Ca(ICCV2/PI489777)SNP_24701	247	C	A	5	3	--	--
CakTC34453	Ca(ICCV2/PI489777)SNP_24702	1576	A	G	4	3	--	--
CakTC42014	Ca(ICCV2/PI489777)SNP_24703	150	C	T	4	4	--	AP2-EREBP
	Ca(ICCV2/PI489777)SNP_24704	516	G	A	4	8	--	AP2-EREBP
CakTC41340	Ca(ICCV2/PI489777)SNP_24705	873	G	A	3	6	--	--
	Ca(ICCV2/PI489777)SNP_24706	1265	C	G	3	9	--	--
	Ca(ICCV2/PI489777)SNP_24707	1451	G	A	7	4	--	--
CakTC25798	Ca(ICCV2/PI489777)SNP_24708	497	T	C	5	5	--	Bromodomain
	Ca(ICCV2/PI489777)SNP_24709	723	A	T	6	3	--	Bromodomain
	Ca(ICCV2/PI489777)SNP_24710	2041	G	T	5	7	--	Bromodomain
	Ca(ICCV2/PI489777)SNP_24711	2620	T	C	9	4	--	Bromodomain

	Ca(ICCV2/PI489777)SNP_24712	2914	T	A	10	3	--	Bromodomain
	Ca(ICCV2/PI489777)SNP_24713	3147	T	G	4	7	--	Bromodomain
CakTC27397	Ca(ICCV2/PI489777)SNP_24714	219	A	G	3	3	--	--
CakTC16908	Ca(ICCV2/PI489777)SNP_24715	172	C	T	4	4	--	--
	Ca(ICCV2/PI489777)SNP_24716	175	A	C	4	4	--	--
CakTC27896	Ca(ICCV2/PI489777)SNP_24717	271	A	G	11	5	--	--
	Ca(ICCV2/PI489777)SNP_24718	1090	T	G	7	3	--	--
	Ca(ICCV2/PI489777)SNP_24719	1124	A	G	6	3	--	--
CakTC31079	Ca(ICCV2/PI489777)SNP_24720	876	T	C	78	41	--	--
CakTC26433	Ca(ICCV2/PI489777)SNP_24721	1650	C	G	3	4	--	--
	Ca(ICCV2/PI489777)SNP_24722	1807	A	G	8	4	--	--
	Ca(ICCV2/PI489777)SNP_24723	1880	C	A	11	3	--	--
	Ca(ICCV2/PI489777)SNP_24724	1890	G	A	12	3	--	--
	Ca(ICCV2/PI489777)SNP_24725	2322	T	C	10	6	--	--
	Ca(ICCV2/PI489777)SNP_24726	2409	G	C	9	5	--	--
	Ca(ICCV2/PI489777)SNP_24727	2919	T	G	21	3	--	--
CakTC36397	Ca(ICCV2/PI489777)SNP_24728	217	T	C	3	8	--	--
	Ca(ICCV2/PI489777)SNP_24729	225	T	G	3	9	--	--
	Ca(ICCV2/PI489777)SNP_24730	249	G	A	3	11	--	--
CakTC31610	Ca(ICCV2/PI489777)SNP_24731	1628	C	T	4	3	Flower	bud
CakTC38669	Ca(ICCV2/PI489777)SNP_24732	3392	A	C	9	3	--	--
	Ca(ICCV2/PI489777)SNP_24733	3986	A	G	6	5	--	--
	Ca(ICCV2/PI489777)SNP_24734	4084	C	A	6	5	--	--
CakTC31581	Ca(ICCV2/PI489777)SNP_24735	246	G	C	3	4	--	--
	Ca(ICCV2/PI489777)SNP_24736	333	A	G	36	32	--	--
CakTC03554	Ca(ICCV2/PI489777)SNP_24737	563	A	G	17	29	--	--
CakTC28060	Ca(ICCV2/PI489777)SNP_24738	162	C	T	54	50	--	--
	Ca(ICCV2/PI489777)SNP_24739	576	A	G	32	24	--	--
	Ca(ICCV2/PI489777)SNP_24740	690	C	T	26	21	--	--
CakTC26122	Ca(ICCV2/PI489777)SNP_24741	496	C	T	6	4	--	--
	Ca(ICCV2/PI489777)SNP_24742	582	A	T	6	3	--	--
CakTC25789	Ca(ICCV2/PI489777)SNP_24743	233	T	C	19	13	--	--
	Ca(ICCV2/PI489777)SNP_24744	2030	A	T	44	6	--	--
	Ca(ICCV2/PI489777)SNP_24745	3084	G	A	16	16	--	--
	Ca(ICCV2/PI489777)SNP_24746	3339	C	T	10	9	--	--
CakTC34616	Ca(ICCV2/PI489777)SNP_24747	120	T	A	19	27	--	--
CakTC25994	Ca(ICCV2/PI489777)SNP_24748	662	A	G	38	23	--	--
CakTC23542	Ca(ICCV2/PI489777)SNP_24749	248	C	G	11	4	--	--
	Ca(ICCV2/PI489777)SNP_24750	315	A	G	11	4	--	--
	Ca(ICCV2/PI489777)SNP_24751	807	C	G	10	4	--	--
	Ca(ICCV2/PI489777)SNP_24752	1421	G	A	9	12	--	--
	Ca(ICCV2/PI489777)SNP_24753	1455	A	C	8	12	--	--
	Ca(ICCV2/PI489777)SNP_24754	1494	T	C	7	8	--	--
CakTC22786	Ca(ICCV2/PI489777)SNP_24755	162	G	A	91	27	--	--
	Ca(ICCV2/PI489777)SNP_24756	216	A	C	138	28	--	--
CakTC38535	Ca(ICCV2/PI489777)SNP_24757	413	G	A	33	9	--	--
	Ca(ICCV2/PI489777)SNP_24758	419	T	C	32	9	--	--
	Ca(ICCV2/PI489777)SNP_24759	485	A	G	36	16	--	--
	Ca(ICCV2/PI489777)SNP_24760	569	A	G	28	22	--	--
CakTC22368	Ca(ICCV2/PI489777)SNP_24761	221	T	C	5	3	--	--
	Ca(ICCV2/PI489777)SNP_24762	256	G	C	5	3	--	--
	Ca(ICCV2/PI489777)SNP_24763	258	A	C	5	3	--	--
CakTC10426	Ca(ICCV2/PI489777)SNP_24764	853	A	G	5	5	--	--
CakTC13102	Ca(ICCV2/PI489777)SNP_24765	1087	T	C	5	3	--	--
CakTC32264	Ca(ICCV2/PI489777)SNP_24766	1388	C	G	5	3	--	--
	Ca(ICCV2/PI489777)SNP_24767	1436	A	C	4	3	--	--
CakTC31742	Ca(ICCV2/PI489777)SNP_24768	156	C	G	8	4	Flower	bud
	Ca(ICCV2/PI489777)SNP_24769	711	G	C	3	4	Flower	bud
	Ca(ICCV2/PI489777)SNP_24770	737	G	A	3	3	Flower	bud
CakTC22912	Ca(ICCV2/PI489777)SNP_24771	2629	T	C	8	4	--	--
	Ca(ICCV2/PI489777)SNP_24772	2995	T	C	26	9	--	--
CakTC31160	Ca(ICCV2/PI489777)SNP_24773	447	C	T	3	4	Shoot	--
	Ca(ICCV2/PI489777)SNP_24774	522	G	A	3	4	Shoot	--
CakTC31898	Ca(ICCV2/PI489777)SNP_24775	254	A	G	6	7	--	--
	Ca(ICCV2/PI489777)SNP_24776	302	C	T	7	8	--	--
	Ca(ICCV2/PI489777)SNP_24777	329	G	A	8	8	--	--
	Ca(ICCV2/PI489777)SNP_24778	1218	C	T	7	6	--	--
	Ca(ICCV2/PI489777)SNP_24779	1263	C	G	4	7	--	--
	Ca(ICCV2/PI489777)SNP_24780	1319	C	T	4	6	--	--
	Ca(ICCV2/PI489777)SNP_24781	1328	A	G	3	5	--	--
	Ca(ICCV2/PI489777)SNP_24782	1367	T	C	3	5	--	--
CakTC36602	Ca(ICCV2/PI489777)SNP_24783	295	T	C	16	4	--	--
	Ca(ICCV2/PI489777)SNP_24784	310	C	T	17	4	--	--
	Ca(ICCV2/PI489777)SNP_24785	319	T	C	17	4	--	--

	Ca(ICCV2/PI489777)SNP_24786	325	C	G	17	4	--	--
	Ca(ICCV2/PI489777)SNP_24787	460	C	T	17	3	--	--
	Ca(ICCV2/PI489777)SNP_24788	490	T	C	19	3	--	--
	Ca(ICCV2/PI489777)SNP_24789	1016	C	T	22	10	--	--
CakTC37558	Ca(ICCV2/PI489777)SNP_24790	575	A	T	8	4	--	--
CakTC41144	Ca(ICCV2/PI489777)SNP_24791	989	T	C	6	4	--	--
CakTC43102	Ca(ICCV2/PI489777)SNP_24792	339	T	C	67	42	--	--
CakTC36365	Ca(ICCV2/PI489777)SNP_24793	586	G	A	4	3	Young_pod	--
CakTC27230	Ca(ICCV2/PI489777)SNP_24794	103	C	T	5	7	Flower	bud
	Ca(ICCV2/PI489777)SNP_24795	202	C	T	6	7	Flower	bud
	Ca(ICCV2/PI489777)SNP_24796	1294	C	T	6	6	Flower	bud
	Ca(ICCV2/PI489777)SNP_24797	1299	A	G	8	5	Flower	bud
	Ca(ICCV2/PI489777)SNP_24798	1489	A	C	6	5	Flower	bud
CakTC28172	Ca(ICCV2/PI489777)SNP_24799	865	A	C	3	5	--	--
	Ca(ICCV2/PI489777)SNP_24800	908	T	C	5	5	--	--
	Ca(ICCV2/PI489777)SNP_24801	2002	T	C	5	4	--	--
	Ca(ICCV2/PI489777)SNP_24802	2003	T	C	5	4	--	--
CakTC08234	Ca(ICCV2/PI489777)SNP_24803	418	C	T	3	33	--	--
	Ca(ICCV2/PI489777)SNP_24804	748	T	A	4	29	--	--
	Ca(ICCV2/PI489777)SNP_24805	778	G	C	4	27	--	--
	Ca(ICCV2/PI489777)SNP_24806	785	G	T	4	25	--	--
	Ca(ICCV2/PI489777)SNP_24807	988	A	T	7	47	--	--
	Ca(ICCV2/PI489777)SNP_24808	992	C	A	7	44	--	--
	Ca(ICCV2/PI489777)SNP_24809	1004	T	C	6	46	--	--
	Ca(ICCV2/PI489777)SNP_24810	1070	G	T	7	48	--	--
	Ca(ICCV2/PI489777)SNP_24811	1197	C	T	6	44	--	--
	Ca(ICCV2/PI489777)SNP_24812	1262	C	T	4	45	--	--
	Ca(ICCV2/PI489777)SNP_24813	1366	A	G	4	26	--	--
	Ca(ICCV2/PI489777)SNP_24814	1367	T	A	4	23	--	--
CakTC37821	Ca(ICCV2/PI489777)SNP_24815	32	C	T	7	7	--	--
	Ca(ICCV2/PI489777)SNP_24816	339	A	T	15	11	--	--
	Ca(ICCV2/PI489777)SNP_24817	340	G	A	16	11	--	--
	Ca(ICCV2/PI489777)SNP_24818	421	C	G	16	12	--	--
	Ca(ICCV2/PI489777)SNP_24819	424	T	C	17	11	--	--
	Ca(ICCV2/PI489777)SNP_24820	475	C	T	15	13	--	--
	Ca(ICCV2/PI489777)SNP_24821	565	G	A	17	15	--	--
	Ca(ICCV2/PI489777)SNP_24822	571	T	G	18	11	--	--
	Ca(ICCV2/PI489777)SNP_24823	592	G	T	15	13	--	--
	Ca(ICCV2/PI489777)SNP_24824	703	T	A	17	20	--	--
	Ca(ICCV2/PI489777)SNP_24825	956	G	A	15	19	--	--
	Ca(ICCV2/PI489777)SNP_24826	1270	C	T	26	20	--	--
	Ca(ICCV2/PI489777)SNP_24827	1328	A	G	22	9	--	--
CakTC11469	Ca(ICCV2/PI489777)SNP_24828	496	A	G	8	6	--	--
CakTC27781	Ca(ICCV2/PI489777)SNP_24829	340	C	G	26	18	--	--
	Ca(ICCV2/PI489777)SNP_24830	476	C	A	32	17	--	--
	Ca(ICCV2/PI489777)SNP_24831	993	G	C	10	5	--	--
	Ca(ICCV2/PI489777)SNP_24832	1063	C	T	6	5	--	--
CakTC10705	Ca(ICCV2/PI489777)SNP_24833	716	A	G	10	5	--	--
	Ca(ICCV2/PI489777)SNP_24834	846	C	T	7	4	--	--
	Ca(ICCV2/PI489777)SNP_24835	1728	C	T	15	9	--	--
	Ca(ICCV2/PI489777)SNP_24836	1807	A	T	19	7	--	--
	Ca(ICCV2/PI489777)SNP_24837	1961	C	G	33	12	--	--
	Ca(ICCV2/PI489777)SNP_24838	1971	G	A	35	11	--	--
	Ca(ICCV2/PI489777)SNP_24839	2070	A	G	36	11	--	--
	Ca(ICCV2/PI489777)SNP_24840	2136	A	C	36	12	--	--
	Ca(ICCV2/PI489777)SNP_24841	2148	G	A	35	12	--	--
	Ca(ICCV2/PI489777)SNP_24842	2172	G	A	35	10	--	--
	Ca(ICCV2/PI489777)SNP_24843	2394	C	T	15	8	--	--
	Ca(ICCV2/PI489777)SNP_24844	2412	G	A	19	7	--	--
	Ca(ICCV2/PI489777)SNP_24845	2493	T	C	15	5	--	--
	Ca(ICCV2/PI489777)SNP_24846	2499	T	G	17	6	--	--
	Ca(ICCV2/PI489777)SNP_24847	2523	A	C	15	6	--	--
CakTC37324	Ca(ICCV2/PI489777)SNP_24848	973	C	T	6	4	--	--
CakTC41306	Ca(ICCV2/PI489777)SNP_24849	588	C	T	4	3	--	--
CakTC43278	Ca(ICCV2/PI489777)SNP_24850	238	A	T	85	34	--	--
	Ca(ICCV2/PI489777)SNP_24851	437	G	A	84	43	--	--
	Ca(ICCV2/PI489777)SNP_24852	443	A	T	77	43	--	--
	Ca(ICCV2/PI489777)SNP_24853	519	C	T	49	42	--	--
	Ca(ICCV2/PI489777)SNP_24854	587	T	C	28	23	--	--
	Ca(ICCV2/PI489777)SNP_24855	602	T	C	25	27	--	--
	Ca(ICCV2/PI489777)SNP_24856	605	G	A	26	28	--	--
	Ca(ICCV2/PI489777)SNP_24857	697	G	A	24	24	--	--
CakTC26394	Ca(ICCV2/PI489777)SNP_24858	222	C	T	8	23	--	CCHC
	Ca(ICCV2/PI489777)SNP_24859	294	C	T	14	39	--	CCHC

	Ca(ICCV2/PI489777)SNP_24860	342	T	C	27	55	--	CCHC
CakTC41827	Ca(ICCV2/PI489777)SNP_24861	481	G	A	6	7	--	--
	Ca(ICCV2/PI489777)SNP_24862	569	T	C	6	7	--	--
CakTC25562	Ca(ICCV2/PI489777)SNP_24863	389	T	C	58	33	--	--
	Ca(ICCV2/PI489777)SNP_24864	501	A	G	64	45	--	--
	Ca(ICCV2/PI489777)SNP_24865	1302	G	A	69	41	--	--
CakTC39247	Ca(ICCV2/PI489777)SNP_24866	400	G	C	21	31	--	--
	Ca(ICCV2/PI489777)SNP_24867	432	A	C	17	24	--	--
	Ca(ICCV2/PI489777)SNP_24868	475	C	G	9	23	--	--
	Ca(ICCV2/PI489777)SNP_24869	619	G	T	7	26	--	--
	Ca(ICCV2/PI489777)SNP_24870	682	G	A	6	26	--	--
	Ca(ICCV2/PI489777)SNP_24871	1246	G	A	4	11	--	--
	Ca(ICCV2/PI489777)SNP_24872	1408	G	A	3	6	--	--
	Ca(ICCV2/PI489777)SNP_24873	1442	T	G	4	7	--	--
CakTC23466	Ca(ICCV2/PI489777)SNP_24874	285	T	C	43	25	--	--
CakTC43381	Ca(ICCV2/PI489777)SNP_24875	310	C	T	14	15	--	--
	Ca(ICCV2/PI489777)SNP_24876	316	T	A	13	15	--	--
	Ca(ICCV2/PI489777)SNP_24877	630	C	T	41	24	--	--
	Ca(ICCV2/PI489777)SNP_24878	1597	C	G	38	23	--	--
CakTC09336	Ca(ICCV2/PI489777)SNP_24879	202	T	A	7	5	--	--
	Ca(ICCV2/PI489777)SNP_24880	234	G	A	9	5	--	--
	Ca(ICCV2/PI489777)SNP_24881	471	C	T	6	5	--	--
	Ca(ICCV2/PI489777)SNP_24882	1085	T	G	6	10	--	--
CakTC28359	Ca(ICCV2/PI489777)SNP_24883	186	A	G	18	7	--	--
CakTC31596	Ca(ICCV2/PI489777)SNP_24884	350	G	A	7	7	--	--
CakTC39253	Ca(ICCV2/PI489777)SNP_24885	965	G	A	26	16	--	--
	Ca(ICCV2/PI489777)SNP_24886	967	T	G	25	13	--	--
	Ca(ICCV2/PI489777)SNP_24887	1190	G	A	37	14	--	--
	Ca(ICCV2/PI489777)SNP_24888	1292	G	A	37	15	--	--
	Ca(ICCV2/PI489777)SNP_24889	1352	A	T	35	14	--	--
	Ca(ICCV2/PI489777)SNP_24890	1442	G	C	28	12	--	--
	Ca(ICCV2/PI489777)SNP_24891	1501	A	G	8	4	--	--
CakTC41733	Ca(ICCV2/PI489777)SNP_24892	551	C	T	6	3	--	--
	Ca(ICCV2/PI489777)SNP_24893	623	C	T	9	3	--	--
	Ca(ICCV2/PI489777)SNP_24894	1229	G	A	7	10	--	--
	Ca(ICCV2/PI489777)SNP_24895	2258	G	A	4	6	--	--
CakTC06743	Ca(ICCV2/PI489777)SNP_24896	531	A	G	11	9	--	--
CakTC36010	Ca(ICCV2/PI489777)SNP_24897	1478	G	C	5	7	--	CAMTA
	Ca(ICCV2/PI489777)SNP_24898	1601	G	A	15	7	--	CAMTA
	Ca(ICCV2/PI489777)SNP_24899	2522	A	G	14	10	--	CAMTA
	Ca(ICCV2/PI489777)SNP_24900	2690	A	G	13	6	--	CAMTA
	Ca(ICCV2/PI489777)SNP_24901	2975	A	G	17	10	--	CAMTA
	Ca(ICCV2/PI489777)SNP_24902	3139	C	G	13	6	--	CAMTA
CakTC24664	Ca(ICCV2/PI489777)SNP_24903	1283	G	T	16	8	--	--
CakTC39508	Ca(ICCV2/PI489777)SNP_24904	253	T	C	13	6	--	--
	Ca(ICCV2/PI489777)SNP_24905	1110	T	A	22	12	--	--
	Ca(ICCV2/PI489777)SNP_24906	1231	T	A	16	5	--	--
CakTC40415	Ca(ICCV2/PI489777)SNP_24907	1078	T	C	4	7	--	--
	Ca(ICCV2/PI489777)SNP_24908	1268	G	C	10	4	--	--
	Ca(ICCV2/PI489777)SNP_24909	1365	T	C	9	3	--	--
CakTC30510	Ca(ICCV2/PI489777)SNP_24910	237	C	A	5	6	--	--
	Ca(ICCV2/PI489777)SNP_24911	240	A	G	5	6	--	--
	Ca(ICCV2/PI489777)SNP_24912	1408	G	A	9	4	--	--
CakTC40856	Ca(ICCV2/PI489777)SNP_24913	281	G	A	9	4	--	--
	Ca(ICCV2/PI489777)SNP_24914	787	T	C	8	3	--	--
	Ca(ICCV2/PI489777)SNP_24915	895	C	T	8	6	--	--
	Ca(ICCV2/PI489777)SNP_24916	897	C	G	8	5	--	--
	Ca(ICCV2/PI489777)SNP_24917	1255	C	T	9	8	--	--
CakTC26649	Ca(ICCV2/PI489777)SNP_24918	2687	C	T	3	4	--	--
	Ca(ICCV2/PI489777)SNP_24919	3845	G	T	4	4	--	--
	Ca(ICCV2/PI489777)SNP_24920	4001	A	G	4	6	--	--
CakTC38856	Ca(ICCV2/PI489777)SNP_24921	139	C	A	4	3	--	--
	Ca(ICCV2/PI489777)SNP_24922	627	A	G	9	6	--	--
	Ca(ICCV2/PI489777)SNP_24923	696	C	G	10	5	--	--
CakTC01737	Ca(ICCV2/PI489777)SNP_24924	149	T	C	15	12	--	--
	Ca(ICCV2/PI489777)SNP_24925	2033	C	T	26	16	--	--
	Ca(ICCV2/PI489777)SNP_24926	2135	T	C	18	18	--	--
	Ca(ICCV2/PI489777)SNP_24927	2243	G	A	20	9	--	--
	Ca(ICCV2/PI489777)SNP_24928	2402	G	A	21	3	--	--
	Ca(ICCV2/PI489777)SNP_24929	2822	T	A	16	7	--	--
CakTC22591	Ca(ICCV2/PI489777)SNP_24930	3342	T	C	3	3	Young_pod	--
	Ca(ICCV2/PI489777)SNP_24931	3367	G	T	4	4	Young_pod	--
	Ca(ICCV2/PI489777)SNP_24932	3379	G	C	4	4	Young_pod	--
	Ca(ICCV2/PI489777)SNP_24933	3465	C	T	4	5	Young_pod	--

	Ca(ICCV2/PI489777)SNP_24934	3563	C	T	4	3	Young_pod	--
CakTC30519	Ca(ICCV2/PI489777)SNP_24935	2375	A	C	13	11	Flower	bud
	Ca(ICCV2/PI489777)SNP_24936	3195	A	G	15	7	Flower	bud
CakTC41855	Ca(ICCV2/PI489777)SNP_24937	1256	C	G	4	5	--	--
	Ca(ICCV2/PI489777)SNP_24938	1360	A	T	4	6	--	--
CakTC43212	Ca(ICCV2/PI489777)SNP_24939	759	C	G	3	8	--	HB
CakTC42150	Ca(ICCV2/PI489777)SNP_24940	434	C	A	6	8	--	--
	Ca(ICCV2/PI489777)SNP_24941	580	G	A	11	7	--	--
	Ca(ICCV2/PI489777)SNP_24942	700	A	C	16	6	--	--
CakTC32916	Ca(ICCV2/PI489777)SNP_24943	213	A	G	6	3	--	--
	Ca(ICCV2/PI489777)SNP_24944	218	G	C	6	3	--	--
	Ca(ICCV2/PI489777)SNP_24945	442	C	G	5	3	--	--
CakTC07094	Ca(ICCV2/PI489777)SNP_24946	1825	C	G	3	4	--	--
CakTC40847	Ca(ICCV2/PI489777)SNP_24947	223	A	G	66	123	--	--
	Ca(ICCV2/PI489777)SNP_24948	361	A	G	74	158	--	--
	Ca(ICCV2/PI489777)SNP_24949	539	C	G	59	115	--	--
CakTC26680	Ca(ICCV2/PI489777)SNP_24950	765	G	C	7	3	--	--
CakTC39695	Ca(ICCV2/PI489777)SNP_24951	430	A	G	3	3	--	--
	Ca(ICCV2/PI489777)SNP_24952	612	T	C	5	3	--	--
CakTC33517	Ca(ICCV2/PI489777)SNP_24953	185	G	T	28	19	--	--
	Ca(ICCV2/PI489777)SNP_24954	373	T	A	72	37	--	--
CakTC36586	Ca(ICCV2/PI489777)SNP_24955	1946	T	C	9	6	--	--
	Ca(ICCV2/PI489777)SNP_24956	2387	C	T	21	14	--	--
	Ca(ICCV2/PI489777)SNP_24957	2507	C	T	16	13	--	--
CakTC40068	Ca(ICCV2/PI489777)SNP_24958	293	T	A	7	8	--	--
	Ca(ICCV2/PI489777)SNP_24959	341	T	C	8	8	--	--
	Ca(ICCV2/PI489777)SNP_24960	398	G	A	8	8	--	--
	Ca(ICCV2/PI489777)SNP_24961	608	G	A	10	10	--	--
CakTC25640	Ca(ICCV2/PI489777)SNP_24962	575	T	C	31	13	--	--
	Ca(ICCV2/PI489777)SNP_24963	728	T	C	43	23	--	--
CakTC10444	Ca(ICCV2/PI489777)SNP_24964	244	C	T	8	4	--	--
	Ca(ICCV2/PI489777)SNP_24965	628	G	C	9	5	--	--
CakTC38819	Ca(ICCV2/PI489777)SNP_24966	1277	A	G	3	3	--	--
	Ca(ICCV2/PI489777)SNP_24967	1394	T	A	3	3	--	--
CakTC42457	Ca(ICCV2/PI489777)SNP_24968	287	G	T	5	11	Young_pod	--
	Ca(ICCV2/PI489777)SNP_24969	1007	T	A	5	4	Young_pod	--
	Ca(ICCV2/PI489777)SNP_24970	1097	T	C	6	3	Young_pod	--
	Ca(ICCV2/PI489777)SNP_24971	1418	T	C	6	8	Young_pod	--
	Ca(ICCV2/PI489777)SNP_24972	1454	G	A	5	10	Young_pod	--
CakTC36378	Ca(ICCV2/PI489777)SNP_24973	483	G	A	105	77	--	--
	Ca(ICCV2/PI489777)SNP_24974	786	A	G	104	53	--	--
	Ca(ICCV2/PI489777)SNP_24975	854	T	C	101	45	--	--
	Ca(ICCV2/PI489777)SNP_24976	1031	A	G	96	38	--	--
	Ca(ICCV2/PI489777)SNP_24977	1679	G	A	89	37	--	--
	Ca(ICCV2/PI489777)SNP_24978	2036	A	G	109	67	--	--
	Ca(ICCV2/PI489777)SNP_24979	2773	C	G	81	41	--	--
	Ca(ICCV2/PI489777)SNP_24980	2800	A	T	77	39	--	--
	Ca(ICCV2/PI489777)SNP_24981	2965	C	G	22	4	--	--
CakTC06077	Ca(ICCV2/PI489777)SNP_24982	516	T	G	12	14	--	--
	Ca(ICCV2/PI489777)SNP_24983	546	G	A	12	12	--	--
	Ca(ICCV2/PI489777)SNP_24984	616	G	C	10	8	--	--
CakTC34207	Ca(ICCV2/PI489777)SNP_24985	235	A	G	5	12	--	--
	Ca(ICCV2/PI489777)SNP_24986	555	T	C	20	17	--	--
	Ca(ICCV2/PI489777)SNP_24987	1477	C	A	16	10	--	--
	Ca(ICCV2/PI489777)SNP_24988	2021	A	C	9	5	--	--
CakTC25956	Ca(ICCV2/PI489777)SNP_24989	716	C	T	41	8	--	--
	Ca(ICCV2/PI489777)SNP_24990	887	C	T	39	6	--	--
	Ca(ICCV2/PI489777)SNP_24991	1457	C	A	50	24	--	--
	Ca(ICCV2/PI489777)SNP_24992	1577	A	T	31	13	--	--
	Ca(ICCV2/PI489777)SNP_24993	1683	T	C	16	4	--	--
CakTC39438	Ca(ICCV2/PI489777)SNP_24994	358	T	C	32	16	--	--
	Ca(ICCV2/PI489777)SNP_24995	524	A	C	11	19	--	--
	Ca(ICCV2/PI489777)SNP_24996	1039	A	G	25	20	--	--
	Ca(ICCV2/PI489777)SNP_24997	1418	A	G	9	4	--	--
CakTC42462	Ca(ICCV2/PI489777)SNP_24998	563	G	T	4	6	--	--
CakTC27405	Ca(ICCV2/PI489777)SNP_24999	317	A	C	10	6	--	--
	Ca(ICCV2/PI489777)SNP_25000	383	A	C	11	9	--	--
	Ca(ICCV2/PI489777)SNP_25001	3258	C	A	11	3	--	--
	Ca(ICCV2/PI489777)SNP_25002	3707	G	A	5	5	--	--
CakTC27089	Ca(ICCV2/PI489777)SNP_25003	225	T	A	17	25	--	--
	Ca(ICCV2/PI489777)SNP_25004	761	A	G	4	4	--	--
	Ca(ICCV2/PI489777)SNP_25005	870	A	T	4	4	--	--
CakTC38781	Ca(ICCV2/PI489777)SNP_25006	44	T	C	4	9	--	--
	Ca(ICCV2/PI489777)SNP_25007	98	T	C	5	11	--	--

	Ca(ICCV2/PI489777)SNP_25008	169	G	C	9	19	--	--
CakTC28346	Ca(ICCV2/PI489777)SNP_25009	144	G	A	26	8	--	--
	Ca(ICCV2/PI489777)SNP_25010	152	A	C	28	10	--	--
	Ca(ICCV2/PI489777)SNP_25011	712	A	T	38	22	--	--
CakTC40396	Ca(ICCV2/PI489777)SNP_25012	350	C	T	9	6	--	--
	Ca(ICCV2/PI489777)SNP_25013	398	T	C	12	5	--	--
	Ca(ICCV2/PI489777)SNP_25014	584	T	C	11	4	--	--
CakTC24605	Ca(ICCV2/PI489777)SNP_25015	596	G	A	34	21	--	--
	Ca(ICCV2/PI489777)SNP_25016	725	T	A	50	19	--	--
	Ca(ICCV2/PI489777)SNP_25017	854	A	G	48	11	--	--
	Ca(ICCV2/PI489777)SNP_25018	1081	C	G	27	8	--	--
	Ca(ICCV2/PI489777)SNP_25019	1090	C	G	23	7	--	--
	Ca(ICCV2/PI489777)SNP_25020	1102	C	G	16	4	--	--
CakTC04045	Ca(ICCV2/PI489777)SNP_25021	174	A	C	3	3	--	--
	Ca(ICCV2/PI489777)SNP_25022	246	C	T	3	3	--	--
	Ca(ICCV2/PI489777)SNP_25023	346	A	G	5	4	--	--
	Ca(ICCV2/PI489777)SNP_25024	901	A	G	3	3	--	--
CakTC05031	Ca(ICCV2/PI489777)SNP_25025	794	T	C	5	5	--	--
	Ca(ICCV2/PI489777)SNP_25026	831	A	G	5	4	--	--
	Ca(ICCV2/PI489777)SNP_25027	876	T	C	6	5	--	--
	Ca(ICCV2/PI489777)SNP_25028	1386	C	T	8	3	--	--
CakTC31924	Ca(ICCV2/PI489777)SNP_25029	1343	G	T	4	15	--	--
CakTC41926	Ca(ICCV2/PI489777)SNP_25030	130	C	G	23	7	--	--
	Ca(ICCV2/PI489777)SNP_25031	1126	A	C	20	7	--	--
CakTC16280	Ca(ICCV2/PI489777)SNP_25032	304	G	A	3	4	--	--
	Ca(ICCV2/PI489777)SNP_25033	313	G	A	3	4	--	--
	Ca(ICCV2/PI489777)SNP_25034	347	T	A	3	4	--	--
CakTC29149	Ca(ICCV2/PI489777)SNP_25035	277	T	C	4	15	--	--
	Ca(ICCV2/PI489777)SNP_25036	316	T	C	5	19	--	--
	Ca(ICCV2/PI489777)SNP_25037	406	T	C	6	22	--	--
	Ca(ICCV2/PI489777)SNP_25038	472	G	A	6	25	--	--
	Ca(ICCV2/PI489777)SNP_25039	541	A	G	9	27	--	--
	Ca(ICCV2/PI489777)SNP_25040	547	A	C	7	27	--	--
	Ca(ICCV2/PI489777)SNP_25041	625	A	T	8	19	--	--
	Ca(ICCV2/PI489777)SNP_25042	667	G	T	7	17	--	--
	Ca(ICCV2/PI489777)SNP_25043	673	T	A	7	19	--	--
	Ca(ICCV2/PI489777)SNP_25044	718	C	T	8	14	--	--
	Ca(ICCV2/PI489777)SNP_25045	847	A	T	13	33	--	--
	Ca(ICCV2/PI489777)SNP_25046	850	G	A	13	33	--	--
	Ca(ICCV2/PI489777)SNP_25047	961	C	A	12	31	--	--
	Ca(ICCV2/PI489777)SNP_25048	1068	T	A	13	31	--	--
	Ca(ICCV2/PI489777)SNP_25049	1138	A	G	3	10	--	--
CakTC42732	Ca(ICCV2/PI489777)SNP_25050	970	A	G	42	20	--	BSD
	Ca(ICCV2/PI489777)SNP_25051	1209	C	T	31	34	--	BSD
CakTC02412	Ca(ICCV2/PI489777)SNP_25052	386	C	T	3	5	--	--
	Ca(ICCV2/PI489777)SNP_25053	980	C	T	4	4	--	--
	Ca(ICCV2/PI489777)SNP_25054	1079	T	G	6	4	--	--
	Ca(ICCV2/PI489777)SNP_25055	1337	C	T	8	8	--	--
	Ca(ICCV2/PI489777)SNP_25056	1531	T	C	8	6	--	--
	Ca(ICCV2/PI489777)SNP_25057	1545	G	T	10	6	--	--
CakTC30336	Ca(ICCV2/PI489777)SNP_25058	245	C	T	3	5	--	--
	Ca(ICCV2/PI489777)SNP_25059	255	A	C	5	6	--	--
	Ca(ICCV2/PI489777)SNP_25060	812	A	G	5	6	--	--
	Ca(ICCV2/PI489777)SNP_25061	814	G	A	5	6	--	--
CakTC41834	Ca(ICCV2/PI489777)SNP_25062	283	G	T	6	7	--	--
CakTC42698	Ca(ICCV2/PI489777)SNP_25063	243	C	T	9	4	--	MYB-related
	Ca(ICCV2/PI489777)SNP_25064	352	T	C	13	5	--	MYB-related
	Ca(ICCV2/PI489777)SNP_25065	1327	C	T	13	4	--	MYB-related
CakTC28121	Ca(ICCV2/PI489777)SNP_25066	721	A	G	19	11	--	--
	Ca(ICCV2/PI489777)SNP_25067	1582	G	A	12	5	--	--
	Ca(ICCV2/PI489777)SNP_25068	1585	A	G	12	6	--	--
CakTC29878	Ca(ICCV2/PI489777)SNP_25069	557	C	A	11	3	Shoot	--
	Ca(ICCV2/PI489777)SNP_25070	643	T	A	16	9	Shoot	--
	Ca(ICCV2/PI489777)SNP_25071	847	A	G	19	13	Shoot	--
	Ca(ICCV2/PI489777)SNP_25072	1543	T	C	25	13	Shoot	--
	Ca(ICCV2/PI489777)SNP_25073	1954	A	G	12	14	Shoot	--
	Ca(ICCV2/PI489777)SNP_25074	2128	G	A	14	14	Shoot	--
CakTC27141	Ca(ICCV2/PI489777)SNP_25075	248	T	C	8	49	--	--
CakTC27218	Ca(ICCV2/PI489777)SNP_25076	615	T	C	9	10	--	--
	Ca(ICCV2/PI489777)SNP_25077	1147	G	A	25	5	--	--
	Ca(ICCV2/PI489777)SNP_25078	1222	A	G	24	5	--	--
	Ca(ICCV2/PI489777)SNP_25079	1259	C	T	23	7	--	--
	Ca(ICCV2/PI489777)SNP_25080	1260	C	G	23	7	--	--
	Ca(ICCV2/PI489777)SNP_25081	1294	C	T	29	9	--	--

	Ca(ICCV2/PI489777)SNP_25082	1347	A	T	36	12	--	--
	Ca(ICCV2/PI489777)SNP_25083	2881	C	A	15	11	--	--
	Ca(ICCV2/PI489777)SNP_25084	2944	G	A	14	11	--	--
	Ca(ICCV2/PI489777)SNP_25085	3163	A	G	10	8	--	--
	Ca(ICCV2/PI489777)SNP_25086	3569	A	T	9	6	--	--
CakTC39057	Ca(ICCV2/PI489777)SNP_25087	681	A	T	4	7	--	--
	Ca(ICCV2/PI489777)SNP_25088	900	T	A	5	13	--	--
CakTC25901	Ca(ICCV2/PI489777)SNP_25089	69	A	C	3	10	Flower	bud
	Ca(ICCV2/PI489777)SNP_25090	199	G	A	5	11	Flower	bud
CakTC43322	Ca(ICCV2/PI489777)SNP_25091	32	G	A	8	3	--	--
	Ca(ICCV2/PI489777)SNP_25092	625	C	T	15	6	--	--
CakTC36052	Ca(ICCV2/PI489777)SNP_25093	1842	T	C	7	3	--	--
CakTC36171	Ca(ICCV2/PI489777)SNP_25094	184	C	T	5	5	--	--
	Ca(ICCV2/PI489777)SNP_25095	247	A	G	5	6	--	--
	Ca(ICCV2/PI489777)SNP_25096	1606	G	A	8	3	--	--
CakTC11788	Ca(ICCV2/PI489777)SNP_25097	330	T	C	9	3	--	--
CakTC41346	Ca(ICCV2/PI489777)SNP_25098	612	A	G	46	15	--	--
	Ca(ICCV2/PI489777)SNP_25099	641	A	G	45	15	--	--
	Ca(ICCV2/PI489777)SNP_25100	663	G	C	43	16	--	--
	Ca(ICCV2/PI489777)SNP_25101	678	A	G	46	16	--	--
	Ca(ICCV2/PI489777)SNP_25102	752	G	C	30	13	--	--
	Ca(ICCV2/PI489777)SNP_25103	782	T	C	30	11	--	--
	Ca(ICCV2/PI489777)SNP_25104	783	G	T	28	9	--	--
CakTC38916	Ca(ICCV2/PI489777)SNP_25105	601	A	T	8	3	--	--
CakTC26867	Ca(ICCV2/PI489777)SNP_25106	387	T	C	160	64	--	--
	Ca(ICCV2/PI489777)SNP_25107	441	T	A	217	75	--	--
	Ca(ICCV2/PI489777)SNP_25108	596	A	T	232	98	--	--
CakTC43254	Ca(ICCV2/PI489777)SNP_25109	71	T	A	7	28	Root	--
	Ca(ICCV2/PI489777)SNP_25110	556	A	G	3	11	Root	--
CakTC37247	Ca(ICCV2/PI489777)SNP_25111	916	A	G	5	3	--	--
CakTC37293	Ca(ICCV2/PI489777)SNP_25112	737	C	G	22	16	--	--
	Ca(ICCV2/PI489777)SNP_25113	863	A	G	14	17	--	--
CakTC24190	Ca(ICCV2/PI489777)SNP_25114	1011	T	C	30	4	--	--
CakTC26603	Ca(ICCV2/PI489777)SNP_25115	165	C	T	15	9	--	--
	Ca(ICCV2/PI489777)SNP_25116	843	A	C	24	4	--	--
CakTC40776	Ca(ICCV2/PI489777)SNP_25117	714	G	A	13	3	--	VOZ
	Ca(ICCV2/PI489777)SNP_25118	756	C	G	13	4	--	VOZ
CakTC35416	Ca(ICCV2/PI489777)SNP_25119	670	T	C	3	14	--	--
	Ca(ICCV2/PI489777)SNP_25120	779	A	C	13	12	--	--
	Ca(ICCV2/PI489777)SNP_25121	1250	T	G	16	16	--	--
	Ca(ICCV2/PI489777)SNP_25122	1351	T	A	11	15	--	--
	Ca(ICCV2/PI489777)SNP_25123	1952	T	A	14	3	--	--
CakTC35886	Ca(ICCV2/PI489777)SNP_25124	2441	G	T	5	3	--	--
	Ca(ICCV2/PI489777)SNP_25125	2688	A	T	12	5	--	--
	Ca(ICCV2/PI489777)SNP_25126	2756	C	T	7	4	--	--
	Ca(ICCV2/PI489777)SNP_25127	2757	G	A	7	4	--	--
	Ca(ICCV2/PI489777)SNP_25128	2787	A	G	8	4	--	--
CakTC40508	Ca(ICCV2/PI489777)SNP_25129	173	T	G	9	5	--	--
	Ca(ICCV2/PI489777)SNP_25130	224	A	G	9	4	--	--
	Ca(ICCV2/PI489777)SNP_25131	343	A	T	6	8	--	--
	Ca(ICCV2/PI489777)SNP_25132	357	T	C	8	8	--	--
	Ca(ICCV2/PI489777)SNP_25133	542	A	G	6	13	--	--
	Ca(ICCV2/PI489777)SNP_25134	602	A	G	7	13	--	--
CakTC08676	Ca(ICCV2/PI489777)SNP_25135	4255	G	A	4	5	--	--
	Ca(ICCV2/PI489777)SNP_25136	4538	T	C	5	3	--	--
CakTC32412	Ca(ICCV2/PI489777)SNP_25137	92	G	C	5	5	--	--
	Ca(ICCV2/PI489777)SNP_25138	150	G	C	3	5	--	--
CakTC34915	Ca(ICCV2/PI489777)SNP_25139	344	T	C	19	13	--	--
	Ca(ICCV2/PI489777)SNP_25140	758	C	T	34	29	--	--
	Ca(ICCV2/PI489777)SNP_25141	973	C	T	26	28	--	--
	Ca(ICCV2/PI489777)SNP_25142	997	G	A	27	27	--	--
	Ca(ICCV2/PI489777)SNP_25143	1013	G	A	21	26	--	--
	Ca(ICCV2/PI489777)SNP_25144	1153	C	T	20	18	--	--
	Ca(ICCV2/PI489777)SNP_25145	1206	G	A	21	14	--	--
	Ca(ICCV2/PI489777)SNP_25146	1263	A	G	18	12	--	--
	Ca(ICCV2/PI489777)SNP_25147	1846	G	T	20	17	--	--
	Ca(ICCV2/PI489777)SNP_25148	1961	T	C	20	18	--	--
	Ca(ICCV2/PI489777)SNP_25149	2223	A	G	14	15	--	--
	Ca(ICCV2/PI489777)SNP_25150	2754	A	G	10	5	--	--
	Ca(ICCV2/PI489777)SNP_25151	2952	T	C	18	13	--	--
	Ca(ICCV2/PI489777)SNP_25152	3113	G	A	18	3	--	--
	Ca(ICCV2/PI489777)SNP_25153	3207	T	C	41	9	--	--
	Ca(ICCV2/PI489777)SNP_25154	3428	G	A	34	11	--	--
CakTC41719	Ca(ICCV2/PI489777)SNP_25155	202	C	T	13	14	--	--

	Ca(ICCV2/PI489777)SNP_25156	1508	C	T	20	7	--	--
	Ca(ICCV2/PI489777)SNP_25157	1745	C	A	11	9	--	--
CakTC29508	Ca(ICCV2/PI489777)SNP_25158	606	G	A	12	5	--	--
	Ca(ICCV2/PI489777)SNP_25159	623	A	G	12	5	--	--
	Ca(ICCV2/PI489777)SNP_25160	637	A	G	13	5	--	--
	Ca(ICCV2/PI489777)SNP_25161	646	G	T	13	5	--	--
	Ca(ICCV2/PI489777)SNP_25162	918	G	C	23	7	--	--
	Ca(ICCV2/PI489777)SNP_25163	1458	T	C	9	3	--	--
CakTC41686	Ca(ICCV2/PI489777)SNP_25164	145	C	T	3	3	--	--
CakTC23031	Ca(ICCV2/PI489777)SNP_25165	116	A	G	19	6	--	--
	Ca(ICCV2/PI489777)SNP_25166	171	T	C	20	7	--	--
	Ca(ICCV2/PI489777)SNP_25167	223	T	G	18	7	--	--
	Ca(ICCV2/PI489777)SNP_25168	306	C	G	23	7	--	--
CakTC31751	Ca(ICCV2/PI489777)SNP_25169	871	G	C	6	6	--	--
	Ca(ICCV2/PI489777)SNP_25170	1457	C	T	7	9	--	--
CakTC08398	Ca(ICCV2/PI489777)SNP_25171	1908	G	A	4	8	--	--
CakTC41222	Ca(ICCV2/PI489777)SNP_25172	228	C	A	6	5	--	--
CakTC23901	Ca(ICCV2/PI489777)SNP_25173	797	C	T	8	3	--	--
	Ca(ICCV2/PI489777)SNP_25174	842	A	G	6	3	--	--
CakTC38559	Ca(ICCV2/PI489777)SNP_25175	621	A	T	22	13	--	--
	Ca(ICCV2/PI489777)SNP_25176	704	T	C	27	15	--	--
	Ca(ICCV2/PI489777)SNP_25177	738	G	A	26	16	--	--
	Ca(ICCV2/PI489777)SNP_25178	881	G	T	18	9	--	--
CakTC28204	Ca(ICCV2/PI489777)SNP_25179	1114	A	G	20	13	--	SNF2
	Ca(ICCV2/PI489777)SNP_25180	1657	A	G	40	9	--	SNF2
CakTC22031	Ca(ICCV2/PI489777)SNP_25181	1173	C	T	7	3	--	--
	Ca(ICCV2/PI489777)SNP_25182	1669	G	C	10	5	--	--
	Ca(ICCV2/PI489777)SNP_25183	1680	C	T	12	5	--	--
	Ca(ICCV2/PI489777)SNP_25184	1692	G	A	10	5	--	--
	Ca(ICCV2/PI489777)SNP_25185	1816	A	G	9	5	--	--
CakTC39887	Ca(ICCV2/PI489777)SNP_25186	666	G	A	29	8	--	--
CakTC32419	Ca(ICCV2/PI489777)SNP_25187	370	C	T	10	4	--	--
	Ca(ICCV2/PI489777)SNP_25188	526	A	G	5	6	--	--
	Ca(ICCV2/PI489777)SNP_25189	614	T	C	3	7	--	--
	Ca(ICCV2/PI489777)SNP_25190	700	T	C	3	7	--	--
	Ca(ICCV2/PI489777)SNP_25191	859	C	A	5	8	--	--
	Ca(ICCV2/PI489777)SNP_25192	899	G	A	5	8	--	--
	Ca(ICCV2/PI489777)SNP_25193	1003	T	C	3	5	--	--
	Ca(ICCV2/PI489777)SNP_25194	1117	A	T	3	8	--	--
	Ca(ICCV2/PI489777)SNP_25195	1169	G	A	3	8	--	--
	Ca(ICCV2/PI489777)SNP_25196	1522	C	T	5	11	--	--
	Ca(ICCV2/PI489777)SNP_25197	1762	G	A	4	13	--	--
CakTC17513	Ca(ICCV2/PI489777)SNP_25198	1364	C	T	5	3	--	--
	Ca(ICCV2/PI489777)SNP_25199	1462	T	G	3	3	--	--
CakTC40011	Ca(ICCV2/PI489777)SNP_25200	362	A	C	5	4	--	--
	Ca(ICCV2/PI489777)SNP_25201	419	A	C	3	4	--	--
CakTC30799	Ca(ICCV2/PI489777)SNP_25202	1128	G	C	5	5	--	--
CakTC40615	Ca(ICCV2/PI489777)SNP_25203	518	C	A	12	15	--	--
	Ca(ICCV2/PI489777)SNP_25204	703	A	G	3	10	--	--
CakTC30012	Ca(ICCV2/PI489777)SNP_25205	1273	C	A	25	18	--	--
	Ca(ICCV2/PI489777)SNP_25206	1348	A	G	30	20	--	--
	Ca(ICCV2/PI489777)SNP_25207	1355	A	C	26	20	--	--
CakTC43000	Ca(ICCV2/PI489777)SNP_25208	390	A	G	16	7	--	--
	Ca(ICCV2/PI489777)SNP_25209	546	A	C	27	14	--	--
CakTC37415	Ca(ICCV2/PI489777)SNP_25210	925	T	C	12	5	--	--
CakTC38875	Ca(ICCV2/PI489777)SNP_25211	589	T	C	12	17	--	--
	Ca(ICCV2/PI489777)SNP_25212	1053	T	A	47	30	--	--
	Ca(ICCV2/PI489777)SNP_25213	1134	C	T	30	25	--	--
	Ca(ICCV2/PI489777)SNP_25214	1210	C	G	22	12	--	--
CakTC43110	Ca(ICCV2/PI489777)SNP_25215	841	C	T	6	9	--	--
CakTC40824	Ca(ICCV2/PI489777)SNP_25216	460	T	C	16	7	--	--
	Ca(ICCV2/PI489777)SNP_25217	1446	C	T	9	6	--	--
	Ca(ICCV2/PI489777)SNP_25218	1723	T	A	12	7	--	--
	Ca(ICCV2/PI489777)SNP_25219	1744	T	A	9	6	--	--
	Ca(ICCV2/PI489777)SNP_25220	1866	C	T	9	6	--	--
	Ca(ICCV2/PI489777)SNP_25221	1940	A	G	8	5	--	--
	Ca(ICCV2/PI489777)SNP_25222	1942	G	T	8	5	--	--
CakTC23996	Ca(ICCV2/PI489777)SNP_25223	645	A	G	8	3	Flower	bud
	Ca(ICCV2/PI489777)SNP_25224	2195	G	A	16	4	Flower	bud
CakTC31014	Ca(ICCV2/PI489777)SNP_25225	1009	A	G	3	6	--	--
CakTC30940	Ca(ICCV2/PI489777)SNP_25226	1351	T	G	8	4	--	--
	Ca(ICCV2/PI489777)SNP_25227	1399	C	A	8	4	--	--
CakTC36236	Ca(ICCV2/PI489777)SNP_25228	25	T	C	4	10	--	--
	Ca(ICCV2/PI489777)SNP_25229	447	T	A	3	20	--	--

	Ca(ICCV2/PI489777)SNP_25230	603	C	T	4	16	--	--
CakTC42338	Ca(ICCV2/PI489777)SNP_25231	706	C	T	26	18	--	--
CakTC11505	Ca(ICCV2/PI489777)SNP_25232	182	A	T	16	7	--	--
	Ca(ICCV2/PI489777)SNP_25233	639	A	G	18	8	--	--
CakTC39324	Ca(ICCV2/PI489777)SNP_25234	223	A	G	4	6	--	--
	Ca(ICCV2/PI489777)SNP_25235	306	G	A	5	6	--	--
	Ca(ICCV2/PI489777)SNP_25236	580	A	G	5	8	--	--
	Ca(ICCV2/PI489777)SNP_25237	588	G	A	4	9	--	--
	Ca(ICCV2/PI489777)SNP_25238	590	G	A	5	9	--	--
	Ca(ICCV2/PI489777)SNP_25239	598	C	A	5	8	--	--
	Ca(ICCV2/PI489777)SNP_25240	1284	C	T	6	3	--	--
CakTC29881	Ca(ICCV2/PI489777)SNP_25241	1303	A	C	21	3	--	--
CakTC09976	Ca(ICCV2/PI489777)SNP_25242	1376	A	G	8	3	--	--
CakTC24096	Ca(ICCV2/PI489777)SNP_25243	1501	G	C	7	3	--	--
CakTC23272	Ca(ICCV2/PI489777)SNP_25244	1532	T	C	4	3	--	--
CakTC27485	Ca(ICCV2/PI489777)SNP_25245	335	G	A	3	5	--	--
	Ca(ICCV2/PI489777)SNP_25246	2421	A	T	3	3	--	--
	Ca(ICCV2/PI489777)SNP_25247	2777	T	C	8	3	--	--
	Ca(ICCV2/PI489777)SNP_25248	2863	T	C	10	3	--	--
CakTC06592	Ca(ICCV2/PI489777)SNP_25249	276	A	C	4	3	--	--
	Ca(ICCV2/PI489777)SNP_25250	1012	C	T	7	5	--	--
CakTC27844	Ca(ICCV2/PI489777)SNP_25251	678	A	G	8	7	--	--
	Ca(ICCV2/PI489777)SNP_25252	899	T	C	16	7	--	--
	Ca(ICCV2/PI489777)SNP_25253	1983	C	T	7	8	--	--
CakTC30707	Ca(ICCV2/PI489777)SNP_25254	523	C	T	5	3	--	MYB
	Ca(ICCV2/PI489777)SNP_25255	550	C	T	3	3	--	MYB
CakTC36520	Ca(ICCV2/PI489777)SNP_25256	559	C	T	28	10	--	CPP
	Ca(ICCV2/PI489777)SNP_25257	575	C	T	30	10	--	CPP
	Ca(ICCV2/PI489777)SNP_25258	1972	A	T	15	10	--	CPP
	Ca(ICCV2/PI489777)SNP_25259	1976	G	T	16	10	--	CPP
	Ca(ICCV2/PI489777)SNP_25260	1988	G	A	16	9	--	CPP
	Ca(ICCV2/PI489777)SNP_25261	2130	A	G	12	12	--	CPP
	Ca(ICCV2/PI489777)SNP_25262	2197	A	G	9	9	--	CPP
	Ca(ICCV2/PI489777)SNP_25263	2239	G	C	8	9	--	CPP
	Ca(ICCV2/PI489777)SNP_25264	2552	A	C	14	10	--	CPP
	Ca(ICCV2/PI489777)SNP_25265	2556	G	C	13	10	--	CPP
CakTC33569	Ca(ICCV2/PI489777)SNP_25266	3254	A	C	9	9	--	--
CakTC41683	Ca(ICCV2/PI489777)SNP_25267	373	G	A	26	28	--	--
	Ca(ICCV2/PI489777)SNP_25268	493	T	C	23	13	--	--
	Ca(ICCV2/PI489777)SNP_25269	509	G	C	22	17	--	--
	Ca(ICCV2/PI489777)SNP_25270	546	A	G	18	13	--	--
CakTC23864	Ca(ICCV2/PI489777)SNP_25271	340	C	G	13	6	--	AUX/IAA
CakTC40998	Ca(ICCV2/PI489777)SNP_25272	1348	T	C	7	8	--	--
	Ca(ICCV2/PI489777)SNP_25273	1468	G	C	8	3	--	--
	Ca(ICCV2/PI489777)SNP_25274	1471	C	G	7	3	--	--
	Ca(ICCV2/PI489777)SNP_25275	1714	A	T	14	17	--	--
	Ca(ICCV2/PI489777)SNP_25276	1779	G	A	7	16	--	--
	Ca(ICCV2/PI489777)SNP_25277	1781	C	T	5	16	--	--
	Ca(ICCV2/PI489777)SNP_25278	1827	A	G	6	16	--	--
	Ca(ICCV2/PI489777)SNP_25279	1846	A	G	5	17	--	--
	Ca(ICCV2/PI489777)SNP_25280	1855	A	T	5	17	--	--
CakTC42079	Ca(ICCV2/PI489777)SNP_25281	352	G	T	44	37	--	--
CakTC10458	Ca(ICCV2/PI489777)SNP_25282	240	C	T	5	3	--	--
	Ca(ICCV2/PI489777)SNP_25283	650	A	G	4	3	--	--
CakTC39240	Ca(ICCV2/PI489777)SNP_25284	424	A	G	28	11	--	--
	Ca(ICCV2/PI489777)SNP_25285	535	A	G	13	10	--	--
	Ca(ICCV2/PI489777)SNP_25286	1246	T	G	6	5	--	--
	Ca(ICCV2/PI489777)SNP_25287	1347	G	A	4	3	--	--
CakTC24809	Ca(ICCV2/PI489777)SNP_25288	108	T	C	31	9	--	--
CakTC43007	Ca(ICCV2/PI489777)SNP_25289	136	T	C	3	8	--	--
	Ca(ICCV2/PI489777)SNP_25290	357	C	T	58	70	--	--
	Ca(ICCV2/PI489777)SNP_25291	572	T	C	76	96	--	--
	Ca(ICCV2/PI489777)SNP_25292	897	C	T	128	63	--	--
	Ca(ICCV2/PI489777)SNP_25293	1069	C	T	109	82	--	--
	Ca(ICCV2/PI489777)SNP_25294	1444	C	T	72	49	--	--
CakTC17523	Ca(ICCV2/PI489777)SNP_25295	240	G	C	19	3	--	--
	Ca(ICCV2/PI489777)SNP_25296	252	G	C	18	3	--	--
	Ca(ICCV2/PI489777)SNP_25297	261	T	C	21	3	--	--
CakTC38969	Ca(ICCV2/PI489777)SNP_25298	435	G	A	11	5	--	--
	Ca(ICCV2/PI489777)SNP_25299	471	A	G	9	5	--	--
	Ca(ICCV2/PI489777)SNP_25300	482	C	T	8	6	--	--
	Ca(ICCV2/PI489777)SNP_25301	1187	T	G	8	12	--	--
CakTC33313	Ca(ICCV2/PI489777)SNP_25302	67	A	T	8	3	--	--
CakTC25214	Ca(ICCV2/PI489777)SNP_25303	147	G	A	4	3	--	--

	Ca(ICCV2/PI489777)SNP_25304	163	C	G	4	3	--	--
	Ca(ICCV2/PI489777)SNP_25305	943	A	G	30	20	--	--
	Ca(ICCV2/PI489777)SNP_25306	2434	A	G	32	5	--	--
	Ca(ICCV2/PI489777)SNP_25307	3228	A	G	8	3	--	--
CakTC43119	Ca(ICCV2/PI489777)SNP_25308	566	G	A	15	5	--	--
	Ca(ICCV2/PI489777)SNP_25309	1031	T	C	12	7	--	--
	Ca(ICCV2/PI489777)SNP_25310	1215	G	A	9	5	--	--
CakTC42517	Ca(ICCV2/PI489777)SNP_25311	688	G	A	17	5	--	--
CakTC26597	Ca(ICCV2/PI489777)SNP_25312	919	T	C	19	3	--	--
	Ca(ICCV2/PI489777)SNP_25313	2057	G	A	8	3	--	--
	Ca(ICCV2/PI489777)SNP_25314	2086	C	A	10	5	--	--
	Ca(ICCV2/PI489777)SNP_25315	2350	A	C	4	3	--	--
CakTC25947	Ca(ICCV2/PI489777)SNP_25316	431	A	T	31	20	--	--
	Ca(ICCV2/PI489777)SNP_25317	785	C	T	10	3	--	--
CakTC31497	Ca(ICCV2/PI489777)SNP_25318	330	C	A	32	20	--	MYB
	Ca(ICCV2/PI489777)SNP_25319	504	C	T	20	19	--	MYB
	Ca(ICCV2/PI489777)SNP_25320	510	C	T	19	18	--	MYB
	Ca(ICCV2/PI489777)SNP_25321	606	T	C	20	17	--	MYB
CakTC12490	Ca(ICCV2/PI489777)SNP_25322	1850	A	C	17	9	--	--
CakTC08491	Ca(ICCV2/PI489777)SNP_25323	286	T	A	6	6	--	--
	Ca(ICCV2/PI489777)SNP_25324	475	T	C	5	3	--	--
CakTC30139	Ca(ICCV2/PI489777)SNP_25325	217	A	G	4	4	--	--
CakTC25680	Ca(ICCV2/PI489777)SNP_25326	156	A	G	4	12	--	--
	Ca(ICCV2/PI489777)SNP_25327	609	C	T	15	15	--	--
	Ca(ICCV2/PI489777)SNP_25328	888	C	T	15	9	--	--
	Ca(ICCV2/PI489777)SNP_25329	974	T	G	20	8	--	--
CakTC10046	Ca(ICCV2/PI489777)SNP_25330	978	G	A	9	3	--	--
CakTC34218	Ca(ICCV2/PI489777)SNP_25331	280	A	C	11	9	--	--
CakTC39805	Ca(ICCV2/PI489777)SNP_25332	722	T	C	14	21	--	--
	Ca(ICCV2/PI489777)SNP_25333	751	G	A	12	24	--	--
	Ca(ICCV2/PI489777)SNP_25334	1057	T	C	12	23	--	--
	Ca(ICCV2/PI489777)SNP_25335	1311	T	C	5	8	--	--
CakTC42347	Ca(ICCV2/PI489777)SNP_25336	105	C	A	4	3	--	--
	Ca(ICCV2/PI489777)SNP_25337	1257	A	G	20	8	--	--
	Ca(ICCV2/PI489777)SNP_25338	1334	C	T	20	9	--	--
	Ca(ICCV2/PI489777)SNP_25339	1912	A	T	7	4	--	--
CakTC36142	Ca(ICCV2/PI489777)SNP_25340	2543	T	C	5	4	--	--
CakTC26943	Ca(ICCV2/PI489777)SNP_25341	246	G	A	52	29	--	--
CakTC37333	Ca(ICCV2/PI489777)SNP_25342	61	C	T	7	3	--	--
	Ca(ICCV2/PI489777)SNP_25343	73	C	G	13	7	--	--
CakTC41003	Ca(ICCV2/PI489777)SNP_25344	480	A	C	3	3	--	--
CakTC11421	Ca(ICCV2/PI489777)SNP_25345	122	A	T	34	17	--	--
	Ca(ICCV2/PI489777)SNP_25346	1068	A	G	4	5	--	--
CakTC33255	Ca(ICCV2/PI489777)SNP_25347	191	T	C	3	7	--	--
	Ca(ICCV2/PI489777)SNP_25348	545	G	A	10	7	--	--
	Ca(ICCV2/PI489777)SNP_25349	563	G	A	12	7	--	--
	Ca(ICCV2/PI489777)SNP_25350	977	C	T	6	8	--	--
	Ca(ICCV2/PI489777)SNP_25351	1515	G	A	7	7	--	--
	Ca(ICCV2/PI489777)SNP_25352	1885	C	T	15	5	--	--
CakTC41155	Ca(ICCV2/PI489777)SNP_25353	48	A	C	5	9	--	--
	Ca(ICCV2/PI489777)SNP_25354	195	C	T	8	11	--	--
	Ca(ICCV2/PI489777)SNP_25355	745	A	G	6	7	--	--
	Ca(ICCV2/PI489777)SNP_25356	1234	A	G	14	12	--	--
	Ca(ICCV2/PI489777)SNP_25357	1984	G	A	7	17	--	--
	Ca(ICCV2/PI489777)SNP_25358	2192	T	C	10	15	--	--
	Ca(ICCV2/PI489777)SNP_25359	2309	G	A	8	11	--	--
	Ca(ICCV2/PI489777)SNP_25360	2398	A	T	7	6	--	--
CakTC27327	Ca(ICCV2/PI489777)SNP_25361	1428	T	C	5	3	Flower	bud
CakTC30203	Ca(ICCV2/PI489777)SNP_25362	116	A	G	3	3	--	--
	Ca(ICCV2/PI489777)SNP_25363	510	T	C	13	4	--	--
	Ca(ICCV2/PI489777)SNP_25364	678	A	G	5	3	--	--
	Ca(ICCV2/PI489777)SNP_25365	785	T	C	5	3	--	--
	Ca(ICCV2/PI489777)SNP_25366	1098	T	G	6	5	--	--
	Ca(ICCV2/PI489777)SNP_25367	1212	C	T	8	9	--	--
	Ca(ICCV2/PI489777)SNP_25368	1424	T	G	4	6	--	--
CakTC32532	Ca(ICCV2/PI489777)SNP_25369	250	T	G	15	13	--	--
	Ca(ICCV2/PI489777)SNP_25370	2056	C	A	19	16	--	--
CakTC37237	Ca(ICCV2/PI489777)SNP_25371	390	G	A	11	9	--	--
	Ca(ICCV2/PI489777)SNP_25372	913	G	C	4	5	--	--
CakTC23780	Ca(ICCV2/PI489777)SNP_25373	268	A	C	10	4	--	--
CakTC24905	Ca(ICCV2/PI489777)SNP_25374	479	C	T	14	7	--	--
	Ca(ICCV2/PI489777)SNP_25375	1485	A	G	15	5	--	--
	Ca(ICCV2/PI489777)SNP_25376	1545	C	T	9	4	--	--
	Ca(ICCV2/PI489777)SNP_25377	2014	C	T	18	9	--	--

CakTC25105	Ca(ICCV2/PI489777)SNP_25378	103	C	T	6	5	--	TPR
	Ca(ICCV2/PI489777)SNP_25379	757	G	T	6	12	--	TPR
CakTC11511	Ca(ICCV2/PI489777)SNP_25380	244	T	G	76	22	--	--
CakTC38622	Ca(ICCV2/PI489777)SNP_25381	623	T	A	7	5	--	--
	Ca(ICCV2/PI489777)SNP_25382	640	C	T	7	5	--	--
CakTC36566	Ca(ICCV2/PI489777)SNP_25383	203	A	G	10	10	--	--
	Ca(ICCV2/PI489777)SNP_25384	963	A	G	8	4	--	--
	Ca(ICCV2/PI489777)SNP_25385	966	T	C	8	5	--	--
	Ca(ICCV2/PI489777)SNP_25386	1102	C	A	10	5	--	--
	Ca(ICCV2/PI489777)SNP_25387	1548	T	C	12	5	--	--
	Ca(ICCV2/PI489777)SNP_25388	1905	T	G	8	5	--	--
	Ca(ICCV2/PI489777)SNP_25389	2256	C	G	7	4	--	--
CakTC37950	Ca(ICCV2/PI489777)SNP_25390	115	G	A	5	4	--	--
	Ca(ICCV2/PI489777)SNP_25391	275	C	G	16	16	--	--
	Ca(ICCV2/PI489777)SNP_25392	350	A	C	22	16	--	--
	Ca(ICCV2/PI489777)SNP_25393	359	C	G	23	18	--	--
	Ca(ICCV2/PI489777)SNP_25394	629	G	C	40	17	--	--
	Ca(ICCV2/PI489777)SNP_25395	743	A	G	62	20	--	--
	Ca(ICCV2/PI489777)SNP_25396	791	C	T	62	19	--	--
CakTC40794	Ca(ICCV2/PI489777)SNP_25397	173	C	T	3	12	--	--
	Ca(ICCV2/PI489777)SNP_25398	182	G	A	5	13	--	--
	Ca(ICCV2/PI489777)SNP_25399	385	C	G	5	18	--	--
	Ca(ICCV2/PI489777)SNP_25400	494	T	C	6	21	--	--
CakTC36038	Ca(ICCV2/PI489777)SNP_25401	1056	G	A	26	7	--	--
	Ca(ICCV2/PI489777)SNP_25402	2427	T	G	20	5	--	--
	Ca(ICCV2/PI489777)SNP_25403	2433	T	C	20	6	--	--
	Ca(ICCV2/PI489777)SNP_25404	2816	C	T	15	6	--	--
CakTC38238	Ca(ICCV2/PI489777)SNP_25405	348	A	T	27	8	--	--
CakTC42177	Ca(ICCV2/PI489777)SNP_25406	1369	C	T	38	3	--	--
CakTC36120	Ca(ICCV2/PI489777)SNP_25407	639	T	C	11	5	--	--
	Ca(ICCV2/PI489777)SNP_25408	746	T	A	14	4	--	--
	Ca(ICCV2/PI489777)SNP_25409	1496	C	T	13	4	--	--
	Ca(ICCV2/PI489777)SNP_25410	1679	T	A	5	6	--	--
	Ca(ICCV2/PI489777)SNP_25411	2410	G	A	4	4	--	--
CakTC02595	Ca(ICCV2/PI489777)SNP_25412	560	A	G	11	4	--	--
	Ca(ICCV2/PI489777)SNP_25413	752	C	T	8	4	--	--
	Ca(ICCV2/PI489777)SNP_25414	827	A	T	8	3	--	--
	Ca(ICCV2/PI489777)SNP_25415	893	A	G	10	4	--	--
	Ca(ICCV2/PI489777)SNP_25416	941	T	C	8	4	--	--
	Ca(ICCV2/PI489777)SNP_25417	1757	A	G	5	6	--	--
	Ca(ICCV2/PI489777)SNP_25418	1940	C	T	4	3	--	--
CakTC29192	Ca(ICCV2/PI489777)SNP_25419	688	T	C	21	27	--	--
	Ca(ICCV2/PI489777)SNP_25420	1161	C	T	7	5	--	--
CakTC33180	Ca(ICCV2/PI489777)SNP_25421	463	C	A	16	8	--	--
CakTC22483	Ca(ICCV2/PI489777)SNP_25422	346	A	G	7	3	--	--
	Ca(ICCV2/PI489777)SNP_25423	371	G	A	6	3	--	--
CakTC24595	Ca(ICCV2/PI489777)SNP_25424	470	C	T	11	7	--	--
CakTC36220	Ca(ICCV2/PI489777)SNP_25425	353	A	G	13	3	--	--
	Ca(ICCV2/PI489777)SNP_25426	360	T	C	9	3	--	--
	Ca(ICCV2/PI489777)SNP_25427	365	T	C	7	4	--	--
	Ca(ICCV2/PI489777)SNP_25428	860	T	C	15	6	--	--
	Ca(ICCV2/PI489777)SNP_25429	896	G	A	17	5	--	--
	Ca(ICCV2/PI489777)SNP_25430	941	C	T	22	5	--	--
	Ca(ICCV2/PI489777)SNP_25431	1217	G	A	21	4	--	--
	Ca(ICCV2/PI489777)SNP_25432	1220	T	C	21	4	--	--
	Ca(ICCV2/PI489777)SNP_25433	1352	C	T	15	7	--	--
	Ca(ICCV2/PI489777)SNP_25434	1431	A	G	10	6	--	--
	Ca(ICCV2/PI489777)SNP_25435	1437	G	C	10	6	--	--
	Ca(ICCV2/PI489777)SNP_25436	1820	A	G	16	4	--	--
	Ca(ICCV2/PI489777)SNP_25437	1930	T	C	19	4	--	--
	Ca(ICCV2/PI489777)SNP_25438	2092	A	C	23	5	--	--
	Ca(ICCV2/PI489777)SNP_25439	2174	C	G	20	5	--	--
	Ca(ICCV2/PI489777)SNP_25440	2433	C	T	14	3	--	--
	Ca(ICCV2/PI489777)SNP_25441	2606	G	A	12	7	--	--
	Ca(ICCV2/PI489777)SNP_25442	2685	A	G	8	8	--	--
	Ca(ICCV2/PI489777)SNP_25443	2830	G	A	9	6	--	--
	Ca(ICCV2/PI489777)SNP_25444	2843	A	G	12	6	--	--
CakTC26175	Ca(ICCV2/PI489777)SNP_25445	188	A	G	9	16	--	--
	Ca(ICCV2/PI489777)SNP_25446	734	A	G	6	3	--	--
CakTC33187	Ca(ICCV2/PI489777)SNP_25447	471	A	T	18	13	--	--
	Ca(ICCV2/PI489777)SNP_25448	1357	A	G	15	31	--	--
	Ca(ICCV2/PI489777)SNP_25449	1428	T	G	17	28	--	--
	Ca(ICCV2/PI489777)SNP_25450	1665	T	A	18	19	--	--
	Ca(ICCV2/PI489777)SNP_25451	1929	A	G	11	9	--	--

	Ca(ICCV2/PI489777)SNP_25452	1941	C	T	12	11	--	--
	Ca(ICCV2/PI489777)SNP_25453	2098	G	A	5	7	--	--
CakTC38271	Ca(ICCV2/PI489777)SNP_25454	495	C	G	6	6	--	--
	Ca(ICCV2/PI489777)SNP_25455	626	C	T	6	11	--	--
	Ca(ICCV2/PI489777)SNP_25456	733	G	A	7	8	--	--
	Ca(ICCV2/PI489777)SNP_25457	833	A	G	5	9	--	--
	Ca(ICCV2/PI489777)SNP_25458	855	T	C	5	9	--	--
	Ca(ICCV2/PI489777)SNP_25459	929	T	G	4	7	--	--
CakTC33160	Ca(ICCV2/PI489777)SNP_25460	51	A	G	33	18	--	--
	Ca(ICCV2/PI489777)SNP_25461	536	C	A	3	9	--	--
CakTC02827	Ca(ICCV2/PI489777)SNP_25462	1127	A	G	6	4	--	--
CakTC29368	Ca(ICCV2/PI489777)SNP_25463	154	T	C	24	16	--	--
	Ca(ICCV2/PI489777)SNP_25464	249	C	G	29	17	--	--
	Ca(ICCV2/PI489777)SNP_25465	264	C	T	28	18	--	--
CakTC28018	Ca(ICCV2/PI489777)SNP_25466	1035	G	A	22	10	--	GNAT
CakTC37831	Ca(ICCV2/PI489777)SNP_25467	203	G	T	4	5	--	--
	Ca(ICCV2/PI489777)SNP_25468	405	C	G	9	5	--	--
	Ca(ICCV2/PI489777)SNP_25469	429	G	T	9	4	--	--
	Ca(ICCV2/PI489777)SNP_25470	449	C	T	9	7	--	--
	Ca(ICCV2/PI489777)SNP_25471	617	T	C	17	3	--	--
	Ca(ICCV2/PI489777)SNP_25472	744	T	A	17	4	--	--
CakTC40915	Ca(ICCV2/PI489777)SNP_25473	1503	A	C	45	18	--	--
CakTC36386	Ca(ICCV2/PI489777)SNP_25474	642	A	G	3	3	--	--
CakTC08616	Ca(ICCV2/PI489777)SNP_25475	829	A	G	5	5	--	--
CakTC32375	Ca(ICCV2/PI489777)SNP_25476	1265	A	C	12	10	--	--
CakTC25026	Ca(ICCV2/PI489777)SNP_25477	1854	C	A	18	9	--	--
	Ca(ICCV2/PI489777)SNP_25478	2298	T	C	4	3	--	--
CakTC30532	Ca(ICCV2/PI489777)SNP_25479	912	T	C	16	5	--	SNF2
	Ca(ICCV2/PI489777)SNP_25480	1821	G	A	9	9	--	SNF2
	Ca(ICCV2/PI489777)SNP_25481	2232	C	G	11	6	--	SNF2
	Ca(ICCV2/PI489777)SNP_25482	2249	G	A	11	7	--	SNF2
CakTC24688	Ca(ICCV2/PI489777)SNP_25483	1997	C	T	15	10	--	--
	Ca(ICCV2/PI489777)SNP_25484	2181	G	C	24	22	--	--
	Ca(ICCV2/PI489777)SNP_25485	2388	C	T	22	18	--	--
	Ca(ICCV2/PI489777)SNP_25486	2559	A	G	23	21	--	--
	Ca(ICCV2/PI489777)SNP_25487	2655	A	T	17	13	--	--
	Ca(ICCV2/PI489777)SNP_25488	2761	C	T	15	7	--	--
	Ca(ICCV2/PI489777)SNP_25489	3384	G	A	13	20	--	--
	Ca(ICCV2/PI489777)SNP_25490	3953	T	A	6	3	--	--
CakTC31356	Ca(ICCV2/PI489777)SNP_25491	1244	T	G	11	4	Flower	bud
	Ca(ICCV2/PI489777)SNP_25492	1490	T	C	17	12	Flower	bud
	Ca(ICCV2/PI489777)SNP_25493	2366	T	C	14	5	Flower	bud
CakTC08536	Ca(ICCV2/PI489777)SNP_25494	222	T	C	8	4	Root	--
	Ca(ICCV2/PI489777)SNP_25495	362	A	G	9	4	Root	--
CakTC35370	Ca(ICCV2/PI489777)SNP_25496	87	C	A	10	3	--	--
	Ca(ICCV2/PI489777)SNP_25497	89	T	C	10	3	--	--
	Ca(ICCV2/PI489777)SNP_25498	106	G	A	11	4	--	--
	Ca(ICCV2/PI489777)SNP_25499	235	G	A	12	5	--	--
	Ca(ICCV2/PI489777)SNP_25500	1490	A	C	5	4	--	--
	Ca(ICCV2/PI489777)SNP_25501	1884	C	T	10	13	--	--
	Ca(ICCV2/PI489777)SNP_25502	2042	G	A	19	17	--	--
	Ca(ICCV2/PI489777)SNP_25503	2127	T	C	22	20	--	--
	Ca(ICCV2/PI489777)SNP_25504	2814	A	T	6	11	--	--
CakTC33905	Ca(ICCV2/PI489777)SNP_25505	109	G	A	4	4	--	--
	Ca(ICCV2/PI489777)SNP_25506	237	A	G	4	8	--	--
CakTC37883	Ca(ICCV2/PI489777)SNP_25507	245	A	C	6	4	--	--
CakTC38822	Ca(ICCV2/PI489777)SNP_25508	661	T	C	3	7	--	--
	Ca(ICCV2/PI489777)SNP_25509	781	C	T	3	7	--	--
	Ca(ICCV2/PI489777)SNP_25510	811	T	C	3	7	--	--
CakTC27192	Ca(ICCV2/PI489777)SNP_25511	1305	G	A	6	9	--	--
	Ca(ICCV2/PI489777)SNP_25512	1389	G	A	8	13	--	--
	Ca(ICCV2/PI489777)SNP_25513	1485	G	T	9	6	--	--
	Ca(ICCV2/PI489777)SNP_25514	1559	T	C	9	6	--	--
	Ca(ICCV2/PI489777)SNP_25515	1652	G	C	7	4	--	--
	Ca(ICCV2/PI489777)SNP_25516	1817	C	A	4	3	--	--
	Ca(ICCV2/PI489777)SNP_25517	1842	G	A	4	3	--	--
CakTC38432	Ca(ICCV2/PI489777)SNP_25518	688	G	T	11	8	--	GNAT
CakTC41434	Ca(ICCV2/PI489777)SNP_25519	222	G	A	19	7	--	--
	Ca(ICCV2/PI489777)SNP_25520	918	G	A	34	9	--	--
	Ca(ICCV2/PI489777)SNP_25521	1338	G	A	24	9	--	--
	Ca(ICCV2/PI489777)SNP_25522	1401	A	G	24	9	--	--
	Ca(ICCV2/PI489777)SNP_25523	1407	G	C	24	9	--	--
	Ca(ICCV2/PI489777)SNP_25524	2253	T	G	25	6	--	--
	Ca(ICCV2/PI489777)SNP_25525	2379	A	C	16	3	--	--

CakTC11319	Ca(ICCV2/PI489777)SNP_25526	676	G	A	20	4	--	--
	Ca(ICCV2/PI489777)SNP_25527	1009	G	A	28	6	--	--
CakTC25211	Ca(ICCV2/PI489777)SNP_25528	19	T	A	8	4	--	--
	Ca(ICCV2/PI489777)SNP_25529	44	T	C	12	5	--	--
	Ca(ICCV2/PI489777)SNP_25530	45	C	T	12	5	--	--
	Ca(ICCV2/PI489777)SNP_25531	687	T	C	5	5	--	--
	Ca(ICCV2/PI489777)SNP_25532	696	C	T	5	5	--	--
	Ca(ICCV2/PI489777)SNP_25533	849	G	A	7	4	--	--
CakTC41242	Ca(ICCV2/PI489777)SNP_25534	230	A	G	52	40	--	--
CakTC40172	Ca(ICCV2/PI489777)SNP_25535	23	A	G	4	3	--	MYB
	Ca(ICCV2/PI489777)SNP_25536	869	A	G	15	8	--	MYB
CakTC29871	Ca(ICCV2/PI489777)SNP_25537	734	G	T	21	21	--	--
	Ca(ICCV2/PI489777)SNP_25538	757	G	A	20	25	--	--
	Ca(ICCV2/PI489777)SNP_25539	1470	A	G	11	8	--	--
	Ca(ICCV2/PI489777)SNP_25540	1689	G	A	4	9	--	--
CakTC41941	Ca(ICCV2/PI489777)SNP_25541	269	G	A	21	6	--	--
	Ca(ICCV2/PI489777)SNP_25542	490	T	C	54	23	--	--
	Ca(ICCV2/PI489777)SNP_25543	1022	A	G	13	8	--	--
CakTC41960	Ca(ICCV2/PI489777)SNP_25544	118	C	G	15	11	--	--
	Ca(ICCV2/PI489777)SNP_25545	844	G	A	34	10	--	--
	Ca(ICCV2/PI489777)SNP_25546	904	C	G	16	4	--	--
	Ca(ICCV2/PI489777)SNP_25547	946	C	T	13	3	--	--
CakTC42832	Ca(ICCV2/PI489777)SNP_25548	235	T	C	24	6	--	--
	Ca(ICCV2/PI489777)SNP_25549	309	A	G	25	6	--	--
	Ca(ICCV2/PI489777)SNP_25550	421	A	T	16	3	--	--
	Ca(ICCV2/PI489777)SNP_25551	699	A	G	10	5	--	--
	Ca(ICCV2/PI489777)SNP_25552	844	A	G	21	7	--	--
	Ca(ICCV2/PI489777)SNP_25553	904	A	G	20	10	--	--
	Ca(ICCV2/PI489777)SNP_25554	954	G	A	22	8	--	--
	Ca(ICCV2/PI489777)SNP_25555	985	C	T	21	4	--	--
	Ca(ICCV2/PI489777)SNP_25556	1033	T	C	29	8	--	--
	Ca(ICCV2/PI489777)SNP_25557	1306	C	T	46	14	--	--
CakTC24321	Ca(ICCV2/PI489777)SNP_25558	1148	T	G	37	47	--	--
	Ca(ICCV2/PI489777)SNP_25559	1415	G	A	32	33	--	--
	Ca(ICCV2/PI489777)SNP_25560	1961	A	G	7	19	--	--
CakTC42501	Ca(ICCV2/PI489777)SNP_25561	418	A	G	6	9	--	--
CakTC32410	Ca(ICCV2/PI489777)SNP_25562	168	C	T	3	3	--	--
	Ca(ICCV2/PI489777)SNP_25563	392	C	T	3	3	--	--
	Ca(ICCV2/PI489777)SNP_25564	563	A	G	3	5	--	--
	Ca(ICCV2/PI489777)SNP_25565	629	G	C	4	5	--	--
	Ca(ICCV2/PI489777)SNP_25566	1685	A	G	5	5	--	--
	Ca(ICCV2/PI489777)SNP_25567	1767	G	T	10	7	--	--
CakTC39882	Ca(ICCV2/PI489777)SNP_25568	480	C	T	11	6	Young_pod	--
	Ca(ICCV2/PI489777)SNP_25569	1346	A	G	12	5	Young_pod	--
CakTC37123	Ca(ICCV2/PI489777)SNP_25570	398	C	G	3	12	--	--
	Ca(ICCV2/PI489777)SNP_25571	1052	C	G	8	12	--	--
	Ca(ICCV2/PI489777)SNP_25572	1383	G	A	14	13	--	--
CakTC38154	Ca(ICCV2/PI489777)SNP_25573	548	C	T	12	11	--	MADS
	Ca(ICCV2/PI489777)SNP_25574	641	T	C	16	13	--	MADS
	Ca(ICCV2/PI489777)SNP_25575	998	C	T	4	4	--	MADS
CakTC26143	Ca(ICCV2/PI489777)SNP_25576	2645	A	G	33	6	--	--
	Ca(ICCV2/PI489777)SNP_25577	3072	C	T	39	14	--	--
CakTC38140	Ca(ICCV2/PI489777)SNP_25578	930	G	A	7	5	--	--
CakTC31667	Ca(ICCV2/PI489777)SNP_25579	413	C	T	7	12	--	--
CakTC42194	Ca(ICCV2/PI489777)SNP_25580	256	G	T	21	7	--	--
	Ca(ICCV2/PI489777)SNP_25581	698	C	T	12	15	--	--
	Ca(ICCV2/PI489777)SNP_25582	710	A	G	10	15	--	--
	Ca(ICCV2/PI489777)SNP_25583	755	T	G	8	15	--	--
	Ca(ICCV2/PI489777)SNP_25584	894	G	T	5	15	--	--
CakTC20162	Ca(ICCV2/PI489777)SNP_25585	372	A	C	14	11	--	--
CakTC25251	Ca(ICCV2/PI489777)SNP_25586	1154	G	A	6	3	--	--
CakTC39480	Ca(ICCV2/PI489777)SNP_25587	253	C	A	12	3	--	--
	Ca(ICCV2/PI489777)SNP_25588	297	C	T	13	3	--	--
	Ca(ICCV2/PI489777)SNP_25589	392	A	G	9	6	--	--
	Ca(ICCV2/PI489777)SNP_25590	1247	A	G	16	7	--	--
	Ca(ICCV2/PI489777)SNP_25591	1248	T	A	16	7	--	--
CakTC26908	Ca(ICCV2/PI489777)SNP_25592	268	C	T	5	3	--	--
	Ca(ICCV2/PI489777)SNP_25593	858	T	C	8	3	--	--
CakTC29405	Ca(ICCV2/PI489777)SNP_25594	68	C	G	6	5	--	--
	Ca(ICCV2/PI489777)SNP_25595	78	T	G	6	5	--	--
	Ca(ICCV2/PI489777)SNP_25596	89	C	A	7	5	--	--
CakTC28467	Ca(ICCV2/PI489777)SNP_25597	1486	T	G	58	20	--	--
	Ca(ICCV2/PI489777)SNP_25598	2240	A	T	19	6	--	--
CakTC31802	Ca(ICCV2/PI489777)SNP_25599	123	T	C	23	16	--	--

	Ca(ICCV2/PI489777)SNP_25600	687	G	T	14	7	--	--
CakTC33925	Ca(ICCV2/PI489777)SNP_25601	106	C	T	11	10	--	--
	Ca(ICCV2/PI489777)SNP_25602	197	C	T	11	11	--	--
	Ca(ICCV2/PI489777)SNP_25603	492	C	G	10	6	--	--
	Ca(ICCV2/PI489777)SNP_25604	622	T	A	6	5	--	--
CakTC24163	Ca(ICCV2/PI489777)SNP_25605	667	T	C	15	10	--	--
	Ca(ICCV2/PI489777)SNP_25606	748	T	G	17	11	--	--
CakTC12624	Ca(ICCV2/PI489777)SNP_25607	47	G	A	19	7	--	--
	Ca(ICCV2/PI489777)SNP_25608	130	C	G	19	10	--	--
	Ca(ICCV2/PI489777)SNP_25609	223	T	C	30	13	--	--
	Ca(ICCV2/PI489777)SNP_25610	232	C	T	34	14	--	--
	Ca(ICCV2/PI489777)SNP_25611	523	T	C	9	10	--	--
CakTC34138	Ca(ICCV2/PI489777)SNP_25612	544	C	T	8	4	--	--
	Ca(ICCV2/PI489777)SNP_25613	545	G	A	8	4	--	--
CakTC31201	Ca(ICCV2/PI489777)SNP_25614	589	C	G	5	4	--	--
	Ca(ICCV2/PI489777)SNP_25615	613	A	C	5	5	--	--
	Ca(ICCV2/PI489777)SNP_25616	671	T	C	7	4	--	--
	Ca(ICCV2/PI489777)SNP_25617	2446	A	G	7	4	--	--
	Ca(ICCV2/PI489777)SNP_25618	2467	T	G	7	3	--	--
	Ca(ICCV2/PI489777)SNP_25619	2511	G	A	7	3	--	--
CakTC29930	Ca(ICCV2/PI489777)SNP_25620	483	T	C	40	15	--	--
	Ca(ICCV2/PI489777)SNP_25621	726	A	G	39	25	--	--
	Ca(ICCV2/PI489777)SNP_25622	798	T	G	32	27	--	--
	Ca(ICCV2/PI489777)SNP_25623	1045	G	A	48	34	--	--
CakTC37706	Ca(ICCV2/PI489777)SNP_25624	349	C	T	8	3	--	C2C2-GATA
	Ca(ICCV2/PI489777)SNP_25625	360	C	G	8	3	--	C2C2-GATA
	Ca(ICCV2/PI489777)SNP_25626	930	C	T	7	6	--	C2C2-GATA
	Ca(ICCV2/PI489777)SNP_25627	932	G	C	7	6	--	C2C2-GATA
CakTC26646	Ca(ICCV2/PI489777)SNP_25628	1146	T	A	35	11	--	--
	Ca(ICCV2/PI489777)SNP_25629	2177	T	C	29	15	--	--
	Ca(ICCV2/PI489777)SNP_25630	2353	G	A	33	11	--	--
	Ca(ICCV2/PI489777)SNP_25631	2481	G	A	27	7	--	--
	Ca(ICCV2/PI489777)SNP_25632	2514	T	C	33	7	--	--
	Ca(ICCV2/PI489777)SNP_25633	3134	C	G	25	8	--	--
	Ca(ICCV2/PI489777)SNP_25634	3288	G	A	9	5	--	--
CakTC26211	Ca(ICCV2/PI489777)SNP_25635	808	C	T	14	14	--	--
	Ca(ICCV2/PI489777)SNP_25636	1753	C	T	20	9	--	--
	Ca(ICCV2/PI489777)SNP_25637	2119	C	A	7	11	--	--
CakTC34733	Ca(ICCV2/PI489777)SNP_25638	353	A	G	7	6	--	--
	Ca(ICCV2/PI489777)SNP_25639	809	G	A	13	3	--	--
	Ca(ICCV2/PI489777)SNP_25640	1595	C	T	16	7	--	--
	Ca(ICCV2/PI489777)SNP_25641	1634	A	G	18	6	--	--
CakTC38597	Ca(ICCV2/PI489777)SNP_25642	890	A	G	8	5	--	--
CakTC26612	Ca(ICCV2/PI489777)SNP_25643	364	C	T	24	22	--	--
	Ca(ICCV2/PI489777)SNP_25644	642	A	G	62	87	--	--
CakTC34130	Ca(ICCV2/PI489777)SNP_25645	411	T	C	11	9	--	--
	Ca(ICCV2/PI489777)SNP_25646	576	A	G	9	8	--	--
	Ca(ICCV2/PI489777)SNP_25647	651	G	A	8	8	--	--
	Ca(ICCV2/PI489777)SNP_25648	660	A	G	8	8	--	--
CakTC34221	Ca(ICCV2/PI489777)SNP_25649	1657	A	T	3	3	--	--
CakTC26962	Ca(ICCV2/PI489777)SNP_25650	190	G	A	10	8	--	--
	Ca(ICCV2/PI489777)SNP_25651	321	A	G	13	17	--	--
	Ca(ICCV2/PI489777)SNP_25652	612	C	G	24	18	--	--
	Ca(ICCV2/PI489777)SNP_25653	763	A	C	15	12	--	--
	Ca(ICCV2/PI489777)SNP_25654	1062	T	C	19	13	--	--
	Ca(ICCV2/PI489777)SNP_25655	2068	T	C	11	5	--	--
	Ca(ICCV2/PI489777)SNP_25656	2725	G	A	7	5	--	--
	Ca(ICCV2/PI489777)SNP_25657	3549	C	G	10	9	--	--
CakTC23007	Ca(ICCV2/PI489777)SNP_25658	559	T	C	6	4	--	--
CakTC11932	Ca(ICCV2/PI489777)SNP_25659	645	C	T	5	3	--	--
	Ca(ICCV2/PI489777)SNP_25660	1825	T	C	7	3	--	--
	Ca(ICCV2/PI489777)SNP_25661	2718	C	A	4	5	--	--
	Ca(ICCV2/PI489777)SNP_25662	3195	G	C	3	3	--	--
CakTC28272	Ca(ICCV2/PI489777)SNP_25663	91	T	C	3	6	--	--
CakTC27031	Ca(ICCV2/PI489777)SNP_25664	495	T	C	26	14	--	--
	Ca(ICCV2/PI489777)SNP_25665	894	C	T	19	15	--	--
	Ca(ICCV2/PI489777)SNP_25666	1267	T	G	28	11	--	--
	Ca(ICCV2/PI489777)SNP_25667	1411	C	A	21	8	--	--
CakTC30232	Ca(ICCV2/PI489777)SNP_25668	124	C	G	4	6	--	--
CakTC08976	Ca(ICCV2/PI489777)SNP_25669	483	G	A	8	6	--	--
CakTC41862	Ca(ICCV2/PI489777)SNP_25670	312	T	G	5	3	--	--
	Ca(ICCV2/PI489777)SNP_25671	593	A	G	25	10	--	--
CakTC40060	Ca(ICCV2/PI489777)SNP_25672	441	A	G	11	4	--	--
	Ca(ICCV2/PI489777)SNP_25673	537	C	T	10	4	--	--

	Ca(ICCV2/PI489777)SNP_25674	624	A	G	8	8	--	--
	Ca(ICCV2/PI489777)SNP_25675	794	T	G	18	14	--	--
	Ca(ICCV2/PI489777)SNP_25676	799	G	T	18	15	--	--
CakTC12120	Ca(ICCV2/PI489777)SNP_25677	1004	T	G	18	3	--	--
	Ca(ICCV2/PI489777)SNP_25678	1012	C	T	17	6	--	--
	Ca(ICCV2/PI489777)SNP_25679	1019	A	G	17	5	--	--
	Ca(ICCV2/PI489777)SNP_25680	1187	C	T	23	19	--	--
	Ca(ICCV2/PI489777)SNP_25681	1239	A	G	30	24	--	--
	Ca(ICCV2/PI489777)SNP_25682	1286	A	G	26	23	--	--
	Ca(ICCV2/PI489777)SNP_25683	1298	G	A	28	23	--	--
	Ca(ICCV2/PI489777)SNP_25684	2227	A	G	10	18	--	--
CakTC27132	Ca(ICCV2/PI489777)SNP_25685	302	C	T	97	57	--	--
	Ca(ICCV2/PI489777)SNP_25686	329	T	C	90	56	--	--
	Ca(ICCV2/PI489777)SNP_25687	395	C	T	104	61	--	--
	Ca(ICCV2/PI489777)SNP_25688	630	T	G	35	31	--	--
CakTC37838	Ca(ICCV2/PI489777)SNP_25689	644	G	A	6	3	--	--
CakTC25195	Ca(ICCV2/PI489777)SNP_25690	539	C	A	3	5	--	--
	Ca(ICCV2/PI489777)SNP_25691	946	G	A	7	3	--	--
	Ca(ICCV2/PI489777)SNP_25692	983	T	C	12	6	--	--
	Ca(ICCV2/PI489777)SNP_25693	1012	G	C	15	5	--	--
	Ca(ICCV2/PI489777)SNP_25694	1026	T	A	15	6	--	--
	Ca(ICCV2/PI489777)SNP_25695	1122	T	C	17	6	--	--
	Ca(ICCV2/PI489777)SNP_25696	1128	C	A	18	6	--	--
	Ca(ICCV2/PI489777)SNP_25697	1194	G	A	27	11	--	--
	Ca(ICCV2/PI489777)SNP_25698	1731	C	A	13	8	--	--
	Ca(ICCV2/PI489777)SNP_25699	1734	A	T	14	9	--	--
CakTC38260	Ca(ICCV2/PI489777)SNP_25700	172	G	A	3	4	--	--
	Ca(ICCV2/PI489777)SNP_25701	544	A	G	10	6	--	--
CakTC40782	Ca(ICCV2/PI489777)SNP_25702	496	G	A	4	5	--	--
CakTC31700	Ca(ICCV2/PI489777)SNP_25703	46	G	T	3	4	--	TPR
	Ca(ICCV2/PI489777)SNP_25704	66	A	G	3	5	--	TPR
	Ca(ICCV2/PI489777)SNP_25705	72	A	C	3	5	--	TPR
	Ca(ICCV2/PI489777)SNP_25706	163	T	A	4	3	--	TPR
	Ca(ICCV2/PI489777)SNP_25707	199	T	A	3	3	--	TPR
	Ca(ICCV2/PI489777)SNP_25708	251	A	G	5	3	--	TPR
	Ca(ICCV2/PI489777)SNP_25709	307	A	C	5	3	--	TPR
	Ca(ICCV2/PI489777)SNP_25710	328	C	T	6	3	--	TPR
CakTC43162	Ca(ICCV2/PI489777)SNP_25711	679	T	A	5	9	--	--
CakTC12090	Ca(ICCV2/PI489777)SNP_25712	204	T	G	4	4	--	--
CakTC30432	Ca(ICCV2/PI489777)SNP_25713	1018	A	G	7	3	--	--
	Ca(ICCV2/PI489777)SNP_25714	1021	A	G	8	4	--	--
	Ca(ICCV2/PI489777)SNP_25715	1041	G	A	8	4	--	--
	Ca(ICCV2/PI489777)SNP_25716	1338	C	T	6	3	--	--
CakTC09735	Ca(ICCV2/PI489777)SNP_25717	2577	G	A	4	3	Mature	Leaf
	Ca(ICCV2/PI489777)SNP_25718	3545	A	G	6	3	Mature	Leaf
CakTC38993	Ca(ICCV2/PI489777)SNP_25719	955	A	T	17	16	--	bZIP
CakTC29985	Ca(ICCV2/PI489777)SNP_25720	319	C	G	28	4	--	--
	Ca(ICCV2/PI489777)SNP_25721	1337	A	C	46	8	--	--
	Ca(ICCV2/PI489777)SNP_25722	2959	A	C	19	9	--	--
CakTC30814	Ca(ICCV2/PI489777)SNP_25723	260	T	C	7	4	--	--
	Ca(ICCV2/PI489777)SNP_25724	281	A	C	7	5	--	--
CakTC30443	Ca(ICCV2/PI489777)SNP_25725	283	T	G	13	25	--	--
	Ca(ICCV2/PI489777)SNP_25726	353	C	T	12	25	--	--
	Ca(ICCV2/PI489777)SNP_25727	387	T	C	15	25	--	--
	Ca(ICCV2/PI489777)SNP_25728	1106	C	T	9	13	--	--
CakTC36286	Ca(ICCV2/PI489777)SNP_25729	348	T	C	9	3	--	--
	Ca(ICCV2/PI489777)SNP_25730	415	T	C	9	3	--	--
	Ca(ICCV2/PI489777)SNP_25731	480	C	T	6	4	--	--
CakTC41416	Ca(ICCV2/PI489777)SNP_25732	462	A	G	20	34	--	--
CakTC39602	Ca(ICCV2/PI489777)SNP_25733	60	C	T	58	24	--	--
	Ca(ICCV2/PI489777)SNP_25734	222	C	G	71	43	--	--
	Ca(ICCV2/PI489777)SNP_25735	530	G	T	43	49	--	--
	Ca(ICCV2/PI489777)SNP_25736	886	T	C	19	30	--	--
CakTC26323	Ca(ICCV2/PI489777)SNP_25737	47	A	G	6	3	--	--
	Ca(ICCV2/PI489777)SNP_25738	304	A	G	31	12	--	--
	Ca(ICCV2/PI489777)SNP_25739	540	T	G	26	10	--	--
CakTC39591	Ca(ICCV2/PI489777)SNP_25740	59	C	G	9	5	--	--
	Ca(ICCV2/PI489777)SNP_25741	203	A	C	15	15	--	--
	Ca(ICCV2/PI489777)SNP_25742	217	A	G	15	16	--	--
	Ca(ICCV2/PI489777)SNP_25743	496	A	G	14	16	--	--
	Ca(ICCV2/PI489777)SNP_25744	514	T	C	17	14	--	--
	Ca(ICCV2/PI489777)SNP_25745	880	A	T	5	5	--	--
	Ca(ICCV2/PI489777)SNP_25746	952	T	G	6	5	--	--
CakTC26069	Ca(ICCV2/PI489777)SNP_25747	678	C	A	28	26	--	--

	Ca(ICCV2/PI489777)SNP_25748	1305	A	G	20	13	--	--
	Ca(ICCV2/PI489777)SNP_25749	2062	G	A	27	44	--	--
	Ca(ICCV2/PI489777)SNP_25750	2124	T	C	31	46	--	--
	Ca(ICCV2/PI489777)SNP_25751	2136	A	T	30	43	--	--
	Ca(ICCV2/PI489777)SNP_25752	2562	C	G	26	25	--	--
	Ca(ICCV2/PI489777)SNP_25753	2580	G	T	20	15	--	--
	Ca(ICCV2/PI489777)SNP_25754	2678	T	C	11	8	--	--
CakTC11976	Ca(ICCV2/PI489777)SNP_25755	1262	G	T	7	5	--	--
CakTC29492	Ca(ICCV2/PI489777)SNP_25756	1018	G	A	4	3	--	--
CakTC40114	Ca(ICCV2/PI489777)SNP_25757	173	G	C	14	4	--	--
	Ca(ICCV2/PI489777)SNP_25758	187	A	G	13	4	--	--
	Ca(ICCV2/PI489777)SNP_25759	247	C	G	17	16	--	--
	Ca(ICCV2/PI489777)SNP_25760	454	T	C	21	19	--	--
	Ca(ICCV2/PI489777)SNP_25761	1076	A	G	24	11	--	--
CakTC17546	Ca(ICCV2/PI489777)SNP_25762	371	A	G	9	5	--	--
	Ca(ICCV2/PI489777)SNP_25763	502	C	T	7	7	--	--
CakTC28636	Ca(ICCV2/PI489777)SNP_25764	672	G	A	12	3	Root	--
	Ca(ICCV2/PI489777)SNP_25765	1038	A	G	7	4	Root	--
	Ca(ICCV2/PI489777)SNP_25766	1101	A	G	9	5	Root	--
	Ca(ICCV2/PI489777)SNP_25767	1188	G	T	23	10	Root	--
	Ca(ICCV2/PI489777)SNP_25768	1338	C	T	22	8	Root	--
	Ca(ICCV2/PI489777)SNP_25769	1593	T	C	24	13	Root	--
CakTC27301	Ca(ICCV2/PI489777)SNP_25770	740	C	A	45	10	--	--
	Ca(ICCV2/PI489777)SNP_25771	782	C	T	38	15	--	--
CakTC11332	Ca(ICCV2/PI489777)SNP_25772	35	G	T	18	11	--	--
	Ca(ICCV2/PI489777)SNP_25773	84	A	C	22	11	--	--
	Ca(ICCV2/PI489777)SNP_25774	134	A	C	25	10	--	--
	Ca(ICCV2/PI489777)SNP_25775	598	T	A	22	11	--	--
	Ca(ICCV2/PI489777)SNP_25776	658	G	A	26	16	--	--
	Ca(ICCV2/PI489777)SNP_25777	694	C	T	21	15	--	--
	Ca(ICCV2/PI489777)SNP_25778	778	T	C	24	11	--	--
	Ca(ICCV2/PI489777)SNP_25779	784	G	C	23	12	--	--
	Ca(ICCV2/PI489777)SNP_25780	793	G	A	21	11	--	--
	Ca(ICCV2/PI489777)SNP_25781	875	T	C	8	7	--	--
	Ca(ICCV2/PI489777)SNP_25782	879	C	A	11	7	--	--
	Ca(ICCV2/PI489777)SNP_25783	915	C	G	9	8	--	--
CakTC36132	Ca(ICCV2/PI489777)SNP_25784	373	G	T	6	7	Young_pod	--
	Ca(ICCV2/PI489777)SNP_25785	1074	G	T	5	3	Young_pod	--
	Ca(ICCV2/PI489777)SNP_25786	1276	T	C	5	3	Young_pod	--
CakTC14826	Ca(ICCV2/PI489777)SNP_25787	755	A	T	3	4	--	--
CakTC29773	Ca(ICCV2/PI489777)SNP_25788	902	A	G	7	6	--	--
	Ca(ICCV2/PI489777)SNP_25789	1041	T	A	5	7	--	--
	Ca(ICCV2/PI489777)SNP_25790	1160	G	A	4	8	--	--
CakTC41126	Ca(ICCV2/PI489777)SNP_25791	224	T	C	8	6	--	--
	Ca(ICCV2/PI489777)SNP_25792	1008	C	T	35	8	--	--
	Ca(ICCV2/PI489777)SNP_25793	1459	C	T	20	15	--	--
CakTC42333	Ca(ICCV2/PI489777)SNP_25794	80	G	T	123	118	--	--
CakTC42097	Ca(ICCV2/PI489777)SNP_25795	1280	T	C	3	3	--	SNF2
CakTC41012	Ca(ICCV2/PI489777)SNP_25796	62	A	G	8	7	--	--
	Ca(ICCV2/PI489777)SNP_25797	429	T	G	24	16	--	--
CakTC25752	Ca(ICCV2/PI489777)SNP_25798	1267	G	A	3	3	Flower	bud
	Ca(ICCV2/PI489777)SNP_25799	1457	T	A	3	4	Flower	bud
	Ca(ICCV2/PI489777)SNP_25800	1562	G	A	9	4	Flower	bud
	Ca(ICCV2/PI489777)SNP_25801	1573	T	C	9	3	Flower	bud
CakTC37551	Ca(ICCV2/PI489777)SNP_25802	769	T	G	21	5	--	--
	Ca(ICCV2/PI489777)SNP_25803	778	A	G	23	5	--	--
	Ca(ICCV2/PI489777)SNP_25804	844	T	G	18	3	--	--
	Ca(ICCV2/PI489777)SNP_25805	952	T	C	13	4	--	--
	Ca(ICCV2/PI489777)SNP_25806	1267	A	G	12	7	--	--
CakTC40867	Ca(ICCV2/PI489777)SNP_25807	358	C	A	30	32	--	--
	Ca(ICCV2/PI489777)SNP_25808	370	A	T	36	29	--	--
CakTC41661	Ca(ICCV2/PI489777)SNP_25809	325	A	T	17	6	--	--
	Ca(ICCV2/PI489777)SNP_25810	345	T	C	19	8	--	--
	Ca(ICCV2/PI489777)SNP_25811	1356	A	T	28	12	--	--
	Ca(ICCV2/PI489777)SNP_25812	1411	G	T	33	20	--	--
	Ca(ICCV2/PI489777)SNP_25813	1908	G	A	13	8	--	--
	Ca(ICCV2/PI489777)SNP_25814	2274	C	T	18	12	--	--
	Ca(ICCV2/PI489777)SNP_25815	2289	G	A	17	11	--	--
	Ca(ICCV2/PI489777)SNP_25816	2398	G	A	12	11	--	--
CakTC41614	Ca(ICCV2/PI489777)SNP_25817	214	C	T	33	12	--	--
	Ca(ICCV2/PI489777)SNP_25818	297	T	C	32	11	--	--
	Ca(ICCV2/PI489777)SNP_25819	445	G	A	67	22	--	--
	Ca(ICCV2/PI489777)SNP_25820	451	C	T	57	22	--	--
CakTC23449	Ca(ICCV2/PI489777)SNP_25821	767	A	G	7	4	--	--

CakTC32182	Ca(ICCV2/PI489777)SNP_25822	738	C	T	6	5	--	--
CakTC38117	Ca(ICCV2/PI489777)SNP_25823	1225	T	C	8	3	--	--
	Ca(ICCV2/PI489777)SNP_25824	1493	G	A	6	3	--	--
CakTC39026	Ca(ICCV2/PI489777)SNP_25825	1151	A	T	15	24	--	--
CakTC32708	Ca(ICCV2/PI489777)SNP_25826	497	C	T	12	9	--	--
	Ca(ICCV2/PI489777)SNP_25827	856	G	A	16	4	--	--
	Ca(ICCV2/PI489777)SNP_25828	1269	A	T	11	8	--	--
CakTC23969	Ca(ICCV2/PI489777)SNP_25829	265	G	C	33	32	--	--
	Ca(ICCV2/PI489777)SNP_25830	476	C	A	38	39	--	--
	Ca(ICCV2/PI489777)SNP_25831	543	G	T	33	31	--	--
	Ca(ICCV2/PI489777)SNP_25832	717	T	A	26	10	--	--
	Ca(ICCV2/PI489777)SNP_25833	740	G	T	26	7	--	--
CakTC42767	Ca(ICCV2/PI489777)SNP_25834	1092	C	G	107	37	--	--
	Ca(ICCV2/PI489777)SNP_25835	1272	T	C	77	29	--	--
	Ca(ICCV2/PI489777)SNP_25836	1487	G	A	50	21	--	--
	Ca(ICCV2/PI489777)SNP_25837	1514	C	T	48	19	--	--
CakTC40623	Ca(ICCV2/PI489777)SNP_25838	317	G	C	42	19	--	--
	Ca(ICCV2/PI489777)SNP_25839	347	G	T	44	19	--	--
	Ca(ICCV2/PI489777)SNP_25840	440	T	C	36	22	--	--
	Ca(ICCV2/PI489777)SNP_25841	482	T	C	48	25	--	--
	Ca(ICCV2/PI489777)SNP_25842	560	T	A	50	19	--	--
	Ca(ICCV2/PI489777)SNP_25843	783	A	G	50	18	--	--
	Ca(ICCV2/PI489777)SNP_25844	887	C	T	59	16	--	--
	Ca(ICCV2/PI489777)SNP_25845	1109	C	A	45	9	--	--
	Ca(ICCV2/PI489777)SNP_25846	1172	T	C	44	12	--	--
	Ca(ICCV2/PI489777)SNP_25847	1196	C	A	45	12	--	--
CakTC27933	Ca(ICCV2/PI489777)SNP_25848	1210	A	G	43	17	--	--
CakTC29655	Ca(ICCV2/PI489777)SNP_25849	141	G	A	12	7	--	--
	Ca(ICCV2/PI489777)SNP_25850	818	A	T	24	10	--	--
CakTC12442	Ca(ICCV2/PI489777)SNP_25851	1360	G	T	3	7	--	--
	Ca(ICCV2/PI489777)SNP_25852	1393	T	A	3	7	--	--
CakTC29103	Ca(ICCV2/PI489777)SNP_25853	426	T	C	20	7	--	--
	Ca(ICCV2/PI489777)SNP_25854	505	G	A	23	10	--	--
	Ca(ICCV2/PI489777)SNP_25855	1107	C	T	14	3	--	--
	Ca(ICCV2/PI489777)SNP_25856	1595	T	A	9	3	--	--
CakTC38414	Ca(ICCV2/PI489777)SNP_25857	427	C	G	14	3	--	--
	Ca(ICCV2/PI489777)SNP_25858	573	A	T	14	3	--	--
	Ca(ICCV2/PI489777)SNP_25859	625	T	C	18	4	--	--
	Ca(ICCV2/PI489777)SNP_25860	871	T	C	10	5	--	--
	Ca(ICCV2/PI489777)SNP_25861	991	T	A	15	4	--	--
	Ca(ICCV2/PI489777)SNP_25862	1052	T	A	19	4	--	--
	Ca(ICCV2/PI489777)SNP_25863	1327	T	C	16	4	--	--
	Ca(ICCV2/PI489777)SNP_25864	1576	G	A	10	3	--	--
CakTC36217	Ca(ICCV2/PI489777)SNP_25865	282	T	G	5	7	--	--
	Ca(ICCV2/PI489777)SNP_25866	319	A	G	5	7	--	--
	Ca(ICCV2/PI489777)SNP_25867	336	G	C	5	5	--	--
	Ca(ICCV2/PI489777)SNP_25868	348	C	T	5	5	--	--
CakTC34871	Ca(ICCV2/PI489777)SNP_25869	226	C	G	7	8	--	--
	Ca(ICCV2/PI489777)SNP_25870	1030	T	C	12	8	--	--
	Ca(ICCV2/PI489777)SNP_25871	1047	C	T	11	8	--	--
	Ca(ICCV2/PI489777)SNP_25872	1196	T	C	16	13	--	--
	Ca(ICCV2/PI489777)SNP_25873	1354	C	A	16	9	--	--
	Ca(ICCV2/PI489777)SNP_25874	1525	A	T	17	7	--	--
	Ca(ICCV2/PI489777)SNP_25875	2026	A	G	19	5	--	--
	Ca(ICCV2/PI489777)SNP_25876	2331	T	C	16	5	--	--
	Ca(ICCV2/PI489777)SNP_25877	2690	T	G	5	4	--	--
	Ca(ICCV2/PI489777)SNP_25878	2695	A	G	5	4	--	--
	Ca(ICCV2/PI489777)SNP_25879	2953	C	T	11	10	--	--
CakTC37143	Ca(ICCV2/PI489777)SNP_25880	528	C	T	11	3	--	--
CakTC28235	Ca(ICCV2/PI489777)SNP_25881	1064	C	T	20	15	--	--
	Ca(ICCV2/PI489777)SNP_25882	1742	A	G	8	9	--	--
CakTC27880	Ca(ICCV2/PI489777)SNP_25883	573	A	G	3	3	--	--
	Ca(ICCV2/PI489777)SNP_25884	821	G	A	4	4	--	--
CakTC40991	Ca(ICCV2/PI489777)SNP_25885	212	G	A	8	5	--	--
	Ca(ICCV2/PI489777)SNP_25886	839	T	C	46	21	--	--
CakTC42006	Ca(ICCV2/PI489777)SNP_25887	85	T	C	18	11	--	--
	Ca(ICCV2/PI489777)SNP_25888	869	C	A	8	7	--	--
CakTC29937	Ca(ICCV2/PI489777)SNP_25889	752	G	A	13	8	--	--
CakTC33934	Ca(ICCV2/PI489777)SNP_25890	435	T	G	26	9	--	--
CakTC32781	Ca(ICCV2/PI489777)SNP_25891	945	T	C	5	4	--	--
	Ca(ICCV2/PI489777)SNP_25892	3202	A	C	3	5	--	--
	Ca(ICCV2/PI489777)SNP_25893	3363	T	G	3	3	--	--
	Ca(ICCV2/PI489777)SNP_25894	3508	A	C	3	3	--	--
CakTC42467	Ca(ICCV2/PI489777)SNP_25895	1081	T	A	6	6	Flower	bud

	Ca(ICCV2/PI489777)SNP_25896	1320	T	C	3	3	Flower	bud
CakTC32282	Ca(ICCV2/PI489777)SNP_25897	140	T	G	4	5	--	--
CakTC38269	Ca(ICCV2/PI489777)SNP_25898	384	G	A	7	4	--	--
CakTC26805	Ca(ICCV2/PI489777)SNP_25899	941	A	G	23	15	--	Alfin-like
CakTC42149	Ca(ICCV2/PI489777)SNP_25900	400	C	T	41	20	--	--
	Ca(ICCV2/PI489777)SNP_25901	592	C	T	24	7	--	--
CakTC29160	Ca(ICCV2/PI489777)SNP_25902	562	G	T	10	6	--	--
CakTC25876	Ca(ICCV2/PI489777)SNP_25903	948	T	C	8	3	--	--
	Ca(ICCV2/PI489777)SNP_25904	1279	G	T	7	7	--	--
	Ca(ICCV2/PI489777)SNP_25905	1375	T	C	10	3	--	--
	Ca(ICCV2/PI489777)SNP_25906	1397	T	C	9	3	--	--
	Ca(ICCV2/PI489777)SNP_25907	1431	T	C	10	3	--	--
	Ca(ICCV2/PI489777)SNP_25908	2012	T	C	7	3	--	--
	Ca(ICCV2/PI489777)SNP_25909	2032	T	C	5	3	--	--
	Ca(ICCV2/PI489777)SNP_25910	2033	G	T	8	3	--	--
	Ca(ICCV2/PI489777)SNP_25911	2113	C	T	13	4	--	--
	Ca(ICCV2/PI489777)SNP_25912	2370	C	T	16	4	--	--
	Ca(ICCV2/PI489777)SNP_25913	2391	T	A	16	5	--	--
	Ca(ICCV2/PI489777)SNP_25914	2768	C	G	6	6	--	--
	Ca(ICCV2/PI489777)SNP_25915	2831	G	A	9	8	--	--
	Ca(ICCV2/PI489777)SNP_25916	2839	T	C	9	7	--	--
CakTC26522	Ca(ICCV2/PI489777)SNP_25917	58	T	A	14	4	--	--
	Ca(ICCV2/PI489777)SNP_25918	251	T	C	30	15	--	--
CakTC28822	Ca(ICCV2/PI489777)SNP_25919	231	C	T	6	7	--	--
	Ca(ICCV2/PI489777)SNP_25920	378	T	C	7	6	--	--
	Ca(ICCV2/PI489777)SNP_25921	570	T	A	9	4	--	--
CakTC24964	Ca(ICCV2/PI489777)SNP_25922	394	C	T	66	24	--	HB
CakTC31117	Ca(ICCV2/PI489777)SNP_25923	101	T	C	3	7	Young_pod	--
CakTC42329	Ca(ICCV2/PI489777)SNP_25924	465	C	T	7	6	--	--
CakTC12602	Ca(ICCV2/PI489777)SNP_25925	185	A	G	10	7	--	--
	Ca(ICCV2/PI489777)SNP_25926	250	G	A	13	8	--	--
CakTC42926	Ca(ICCV2/PI489777)SNP_25927	852	A	G	8	6	--	MBF1
CakTC27628	Ca(ICCV2/PI489777)SNP_25928	904	G	A	6	15	--	--
	Ca(ICCV2/PI489777)SNP_25929	1440	G	A	4	5	--	--
	Ca(ICCV2/PI489777)SNP_25930	1455	C	T	4	5	--	--
	Ca(ICCV2/PI489777)SNP_25931	1896	G	A	3	4	--	--
	Ca(ICCV2/PI489777)SNP_25932	2440	T	A	6	17	--	--
CakTC38071	Ca(ICCV2/PI489777)SNP_25933	240	C	T	20	9	--	--
	Ca(ICCV2/PI489777)SNP_25934	852	C	T	12	14	--	--
CakTC33831	Ca(ICCV2/PI489777)SNP_25935	616	G	A	3	11	--	--
CakTC35241	Ca(ICCV2/PI489777)SNP_25936	193	T	C	23	13	--	--
	Ca(ICCV2/PI489777)SNP_25937	274	C	T	27	16	--	--
	Ca(ICCV2/PI489777)SNP_25938	448	C	T	29	15	--	--
	Ca(ICCV2/PI489777)SNP_25939	466	C	A	30	21	--	--
	Ca(ICCV2/PI489777)SNP_25940	493	C	T	28	23	--	--
	Ca(ICCV2/PI489777)SNP_25941	589	T	G	31	21	--	--
	Ca(ICCV2/PI489777)SNP_25942	768	G	A	41	27	--	--
	Ca(ICCV2/PI489777)SNP_25943	1178	C	G	25	25	--	--
	Ca(ICCV2/PI489777)SNP_25944	1384	G	A	23	14	--	--
	Ca(ICCV2/PI489777)SNP_25945	1857	G	T	28	17	--	--
	Ca(ICCV2/PI489777)SNP_25946	2083	G	C	40	19	--	--
	Ca(ICCV2/PI489777)SNP_25947	2575	A	G	16	15	--	--
	Ca(ICCV2/PI489777)SNP_25948	3032	T	C	35	19	--	--
	Ca(ICCV2/PI489777)SNP_25949	3067	T	G	34	15	--	--
CakTC32612	Ca(ICCV2/PI489777)SNP_25950	1241	A	G	3	3	--	--
CakTC27869	Ca(ICCV2/PI489777)SNP_25951	409	G	A	4	4	--	--
	Ca(ICCV2/PI489777)SNP_25952	791	C	T	6	7	--	--
	Ca(ICCV2/PI489777)SNP_25953	1349	G	A	9	8	--	--
	Ca(ICCV2/PI489777)SNP_25954	1483	A	G	6	3	--	--
CakTC37191	Ca(ICCV2/PI489777)SNP_25955	146	C	A	9	7	--	--
	Ca(ICCV2/PI489777)SNP_25956	235	T	C	13	12	--	--
	Ca(ICCV2/PI489777)SNP_25957	394	A	T	13	14	--	--
	Ca(ICCV2/PI489777)SNP_25958	688	C	T	17	5	--	--
CakTC42651	Ca(ICCV2/PI489777)SNP_25959	698	G	A	9	7	--	--
	Ca(ICCV2/PI489777)SNP_25960	711	A	G	9	3	--	--
	Ca(ICCV2/PI489777)SNP_25961	808	A	G	7	4	--	--
	Ca(ICCV2/PI489777)SNP_25962	823	G	A	6	4	--	--
	Ca(ICCV2/PI489777)SNP_25963	834	T	G	6	3	--	--
	Ca(ICCV2/PI489777)SNP_25964	847	C	A	6	4	--	--
CakTC15321	Ca(ICCV2/PI489777)SNP_25965	2641	A	C	4	3	--	--
	Ca(ICCV2/PI489777)SNP_25966	2772	T	C	6	4	--	--
CakTC38381	Ca(ICCV2/PI489777)SNP_25967	1116	C	T	3	10	--	--
CakTC40360	Ca(ICCV2/PI489777)SNP_25968	277	T	C	20	17	--	MADS
CakTC34870	Ca(ICCV2/PI489777)SNP_25969	1787	A	G	3	3	--	--

CakTC09946	Ca(ICCV2/PI489777)SNP_25970	1308	T	C	60	59	--	AUX/IAA
CakTC29683	Ca(ICCV2/PI489777)SNP_25971	545	C	T	14	6	Flower	bud
CakTC41790	Ca(ICCV2/PI489777)SNP_25972	169	T	C	5	11	--	--
	Ca(ICCV2/PI489777)SNP_25973	413	T	C	17	10	--	--
	Ca(ICCV2/PI489777)SNP_25974	1703	T	C	21	12	--	--
CakTC08186	Ca(ICCV2/PI489777)SNP_25975	801	T	C	7	10	--	--
CakTC24138	Ca(ICCV2/PI489777)SNP_25976	918	A	G	7	4	--	--
CakTC38012	Ca(ICCV2/PI489777)SNP_25977	317	C	T	8	3	--	HSF
CakTC22636	Ca(ICCV2/PI489777)SNP_25978	1547	A	G	36	19	--	--
	Ca(ICCV2/PI489777)SNP_25979	1581	C	T	44	19	--	--
	Ca(ICCV2/PI489777)SNP_25980	1774	G	C	46	14	--	--
	Ca(ICCV2/PI489777)SNP_25981	1927	C	T	47	27	--	--
CakTC40753	Ca(ICCV2/PI489777)SNP_25982	1086	A	T	3	9	Young_pod	--
	Ca(ICCV2/PI489777)SNP_25983	1163	G	A	4	6	Young_pod	--
CakTC06236	Ca(ICCV2/PI489777)SNP_25984	737	C	G	3	6	--	--
CakTC42785	Ca(ICCV2/PI489777)SNP_25985	21	A	T	10	5	--	--
	Ca(ICCV2/PI489777)SNP_25986	44	T	C	10	7	--	--
	Ca(ICCV2/PI489777)SNP_25987	150	C	A	10	7	--	--
	Ca(ICCV2/PI489777)SNP_25988	239	T	A	11	7	--	--
	Ca(ICCV2/PI489777)SNP_25989	418	T	C	3	3	--	--
CakTC28655	Ca(ICCV2/PI489777)SNP_25990	56	T	C	42	26	--	--
	Ca(ICCV2/PI489777)SNP_25991	233	A	G	118	64	--	--
	Ca(ICCV2/PI489777)SNP_25992	266	C	T	158	71	--	--
	Ca(ICCV2/PI489777)SNP_25993	285	G	A	138	75	--	--
	Ca(ICCV2/PI489777)SNP_25994	286	T	A	145	76	--	--
	Ca(ICCV2/PI489777)SNP_25995	306	A	G	153	78	--	--
	Ca(ICCV2/PI489777)SNP_25996	443	A	G	160	95	--	--
	Ca(ICCV2/PI489777)SNP_25997	851	T	A	41	13	--	--
CakTC31446	Ca(ICCV2/PI489777)SNP_25998	159	T	C	3	8	--	--
CakTC39035	Ca(ICCV2/PI489777)SNP_25999	548	C	G	8	4	--	C3H
	Ca(ICCV2/PI489777)SNP_26000	994	C	T	8	14	--	C3H
CakTC26117	Ca(ICCV2/PI489777)SNP_26001	117	T	C	11	3	--	TPR
	Ca(ICCV2/PI489777)SNP_26002	146	C	T	11	3	--	TPR
	Ca(ICCV2/PI489777)SNP_26003	294	A	G	11	5	--	TPR
CakTC39144	Ca(ICCV2/PI489777)SNP_26004	610	T	C	5	3	--	--
	Ca(ICCV2/PI489777)SNP_26005	649	T	C	6	3	--	--
	Ca(ICCV2/PI489777)SNP_26006	683	T	A	4	3	--	--
	Ca(ICCV2/PI489777)SNP_26007	1099	C	T	10	8	--	--
CakTC41800	Ca(ICCV2/PI489777)SNP_26008	1904	T	C	8	4	--	--
CakTC42023	Ca(ICCV2/PI489777)SNP_26009	761	A	G	11	6	--	--
	Ca(ICCV2/PI489777)SNP_26010	1214	C	A	16	5	--	--
	Ca(ICCV2/PI489777)SNP_26011	1451	T	A	9	5	--	--
	Ca(ICCV2/PI489777)SNP_26012	1564	C	T	8	5	--	--
	Ca(ICCV2/PI489777)SNP_26013	1665	A	T	10	3	--	--
CakTC29456	Ca(ICCV2/PI489777)SNP_26014	22	C	A	7	3	--	--
	Ca(ICCV2/PI489777)SNP_26015	145	T	C	8	5	--	--
	Ca(ICCV2/PI489777)SNP_26016	237	A	G	9	6	--	--
	Ca(ICCV2/PI489777)SNP_26017	239	G	T	9	6	--	--
	Ca(ICCV2/PI489777)SNP_26018	262	A	C	8	6	--	--
	Ca(ICCV2/PI489777)SNP_26019	358	C	T	7	6	--	--
	Ca(ICCV2/PI489777)SNP_26020	391	C	A	6	4	--	--
	Ca(ICCV2/PI489777)SNP_26021	613	A	T	4	5	--	--
CakTC32858	Ca(ICCV2/PI489777)SNP_26022	248	C	T	9	4	--	--
	Ca(ICCV2/PI489777)SNP_26023	656	C	T	14	9	--	--
	Ca(ICCV2/PI489777)SNP_26024	794	C	A	10	14	--	--
	Ca(ICCV2/PI489777)SNP_26025	1808	T	A	12	8	--	--
	Ca(ICCV2/PI489777)SNP_26026	1917	T	C	8	8	--	--
CakTC34277	Ca(ICCV2/PI489777)SNP_26027	59	A	G	5	9	--	--
	Ca(ICCV2/PI489777)SNP_26028	91	A	G	7	10	--	--
CakTC03814	Ca(ICCV2/PI489777)SNP_26029	516	A	C	3	7	--	--
	Ca(ICCV2/PI489777)SNP_26030	791	C	T	5	8	--	--
CakTC39362	Ca(ICCV2/PI489777)SNP_26031	348	C	T	50	12	--	--
	Ca(ICCV2/PI489777)SNP_26032	357	T	C	49	11	--	--
	Ca(ICCV2/PI489777)SNP_26033	501	C	T	42	7	--	--
	Ca(ICCV2/PI489777)SNP_26034	776	G	A	36	14	--	--
	Ca(ICCV2/PI489777)SNP_26035	807	C	T	36	16	--	--
CakTC29174	Ca(ICCV2/PI489777)SNP_26036	71	C	G	11	8	--	--
CakTC24587	Ca(ICCV2/PI489777)SNP_26037	593	G	A	28	18	--	--
	Ca(ICCV2/PI489777)SNP_26038	731	T	C	35	20	--	--
	Ca(ICCV2/PI489777)SNP_26039	991	C	T	9	5	--	--
CakTC40618	Ca(ICCV2/PI489777)SNP_26040	130	G	C	9	9	--	--
	Ca(ICCV2/PI489777)SNP_26041	332	T	C	17	13	--	--
	Ca(ICCV2/PI489777)SNP_26042	420	C	A	15	13	--	--
	Ca(ICCV2/PI489777)SNP_26043	1667	C	T	28	10	--	--

	Ca(ICCV2/PI489777)SNP_26044	1679	A	G	29	10	--	--
CakTC10331	Ca(ICCV2/PI489777)SNP_26045	149	T	C	4	6	--	--
CakTC29085	Ca(ICCV2/PI489777)SNP_26046	118	A	G	14	4	--	--
CakTC23627	Ca(ICCV2/PI489777)SNP_26047	244	C	A	3	5	--	--
CakTC32566	Ca(ICCV2/PI489777)SNP_26048	381	T	G	10	6	--	--
CakTC41864	Ca(ICCV2/PI489777)SNP_26049	905	G	T	23	3	--	--
CakTC34953	Ca(ICCV2/PI489777)SNP_26050	734	A	G	4	3	Young_pod	--
CakTC39461	Ca(ICCV2/PI489777)SNP_26051	847	C	T	13	16	--	--
	Ca(ICCV2/PI489777)SNP_26052	948	A	C	6	12	--	--
	Ca(ICCV2/PI489777)SNP_26053	1043	A	G	10	12	--	--
	Ca(ICCV2/PI489777)SNP_26054	1050	T	C	12	12	--	--
CakTC11479	Ca(ICCV2/PI489777)SNP_26055	382	T	C	15	3	--	--
	Ca(ICCV2/PI489777)SNP_26056	562	A	G	8	3	--	--
CakTC31037	Ca(ICCV2/PI489777)SNP_26057	349	T	C	6	10	--	--
	Ca(ICCV2/PI489777)SNP_26058	781	G	A	10	13	--	--
	Ca(ICCV2/PI489777)SNP_26059	2038	T	C	5	4	--	--
CakTC28096	Ca(ICCV2/PI489777)SNP_26060	1895	C	T	26	15	--	--
	Ca(ICCV2/PI489777)SNP_26061	1916	C	T	25	16	--	--
	Ca(ICCV2/PI489777)SNP_26062	1928	G	A	22	11	--	--
	Ca(ICCV2/PI489777)SNP_26063	2096	C	T	24	9	--	--
	Ca(ICCV2/PI489777)SNP_26064	2482	C	A	24	17	--	--
	Ca(ICCV2/PI489777)SNP_26065	2600	T	C	13	9	--	--
	Ca(ICCV2/PI489777)SNP_26066	2636	G	A	15	6	--	--
CakTC38490	Ca(ICCV2/PI489777)SNP_26067	649	T	C	7	5	--	--
	Ca(ICCV2/PI489777)SNP_26068	776	A	G	9	4	--	--
	Ca(ICCV2/PI489777)SNP_26069	880	T	A	12	3	--	--
	Ca(ICCV2/PI489777)SNP_26070	883	A	G	12	3	--	--
	Ca(ICCV2/PI489777)SNP_26071	928	G	A	10	4	--	--
CakTC41272	Ca(ICCV2/PI489777)SNP_26072	357	A	G	79	252	--	--
	Ca(ICCV2/PI489777)SNP_26073	461	C	A	94	253	--	--
CakTC35471	Ca(ICCV2/PI489777)SNP_26074	2250	T	C	9	6	--	--
	Ca(ICCV2/PI489777)SNP_26075	3260	A	G	22	17	--	--
	Ca(ICCV2/PI489777)SNP_26076	3473	C	A	8	12	--	--
CakTC11581	Ca(ICCV2/PI489777)SNP_26077	436	T	G	4	3	--	--
	Ca(ICCV2/PI489777)SNP_26078	437	C	A	4	3	--	--
CakTC27729	Ca(ICCV2/PI489777)SNP_26079	1070	T	A	6	5	--	--
CakTC43241	Ca(ICCV2/PI489777)SNP_26080	483	T	A	11	15	--	--
	Ca(ICCV2/PI489777)SNP_26081	663	T	C	4	11	--	--
CakTC23430	Ca(ICCV2/PI489777)SNP_26082	415	T	G	7	4	Root	--
	Ca(ICCV2/PI489777)SNP_26083	421	A	G	6	5	Root	--
	Ca(ICCV2/PI489777)SNP_26084	432	G	C	7	5	Root	--
	Ca(ICCV2/PI489777)SNP_26085	434	T	A	8	4	Root	--
	Ca(ICCV2/PI489777)SNP_26086	663	G	A	10	6	Root	--
	Ca(ICCV2/PI489777)SNP_26087	715	T	C	15	3	Root	--
CakTC40134	Ca(ICCV2/PI489777)SNP_26088	480	C	A	26	7	--	--
	Ca(ICCV2/PI489777)SNP_26089	503	G	A	26	8	--	--
	Ca(ICCV2/PI489777)SNP_26090	556	A	G	28	15	--	--
	Ca(ICCV2/PI489777)SNP_26091	835	G	T	55	26	--	--
CakTC11110	Ca(ICCV2/PI489777)SNP_26092	702	T	C	30	18	--	--
	Ca(ICCV2/PI489777)SNP_26093	738	T	C	28	21	--	--
	Ca(ICCV2/PI489777)SNP_26094	1315	C	T	49	12	--	--
CakTC36388	Ca(ICCV2/PI489777)SNP_26095	124	T	C	4	6	--	--
	Ca(ICCV2/PI489777)SNP_26096	301	A	G	11	18	--	--
	Ca(ICCV2/PI489777)SNP_26097	317	G	C	11	19	--	--
CakTC40427	Ca(ICCV2/PI489777)SNP_26098	353	A	C	17	5	--	--
	Ca(ICCV2/PI489777)SNP_26099	583	A	G	15	12	--	--
	Ca(ICCV2/PI489777)SNP_26100	656	T	C	15	9	--	--
	Ca(ICCV2/PI489777)SNP_26101	963	T	G	36	8	--	--
	Ca(ICCV2/PI489777)SNP_26102	1791	G	C	6	10	--	--
CakTC34684	Ca(ICCV2/PI489777)SNP_26103	1127	A	C	9	6	--	--
	Ca(ICCV2/PI489777)SNP_26104	2073	T	C	6	4	--	--
	Ca(ICCV2/PI489777)SNP_26105	2729	A	T	9	9	--	--
CakTC37663	Ca(ICCV2/PI489777)SNP_26106	873	C	A	17	21	--	--
	Ca(ICCV2/PI489777)SNP_26107	1030	A	T	12	11	--	--
	Ca(ICCV2/PI489777)SNP_26108	1419	A	T	10	6	--	--
	Ca(ICCV2/PI489777)SNP_26109	1518	A	T	11	3	--	--
	Ca(ICCV2/PI489777)SNP_26110	1520	C	T	11	3	--	--
CakTC42954	Ca(ICCV2/PI489777)SNP_26111	524	T	G	3	3	--	--
CakTC26638	Ca(ICCV2/PI489777)SNP_26112	555	G	A	59	13	--	--
	Ca(ICCV2/PI489777)SNP_26113	743	C	T	26	4	--	--
CakTC41527	Ca(ICCV2/PI489777)SNP_26114	209	T	C	13	40	--	CCAAT
CakTC11858	Ca(ICCV2/PI489777)SNP_26115	244	A	G	6	3	Flower	bud
CakTC37781	Ca(ICCV2/PI489777)SNP_26116	1225	C	T	32	12	--	--
CakTC40016	Ca(ICCV2/PI489777)SNP_26117	124	A	T	5	8	--	--

	Ca(ICCV2/PI489777)SNP_26118	360	A	G	7	15	--	--
	Ca(ICCV2/PI489777)SNP_26119	452	C	G	9	15	--	--
	Ca(ICCV2/PI489777)SNP_26120	573	G	A	6	7	--	--
	Ca(ICCV2/PI489777)SNP_26121	1227	T	G	6	8	--	--
	Ca(ICCV2/PI489777)SNP_26122	1511	C	G	8	4	--	--
	Ca(ICCV2/PI489777)SNP_26123	1522	G	A	7	4	--	--
	Ca(ICCV2/PI489777)SNP_26124	1570	C	G	4	4	--	--
CakTC24064	Ca(ICCV2/PI489777)SNP_26125	422	G	C	6	3	--	AP2-EREBP
	Ca(ICCV2/PI489777)SNP_26126	433	C	T	7	3	--	AP2-EREBP
	Ca(ICCV2/PI489777)SNP_26127	1381	C	A	8	7	--	AP2-EREBP
	Ca(ICCV2/PI489777)SNP_26128	1390	A	G	6	7	--	AP2-EREBP
	Ca(ICCV2/PI489777)SNP_26129	1507	A	G	9	10	--	AP2-EREBP
	Ca(ICCV2/PI489777)SNP_26130	1675	A	G	5	15	--	AP2-EREBP
CakTC07500	Ca(ICCV2/PI489777)SNP_26131	929	T	A	5	10	--	--
CakTC34897	Ca(ICCV2/PI489777)SNP_26132	2442	T	C	10	5	--	--
	Ca(ICCV2/PI489777)SNP_26133	2919	T	C	10	5	--	--
CakTC09573	Ca(ICCV2/PI489777)SNP_26134	1325	C	T	3	3	--	--
CakTC22810	Ca(ICCV2/PI489777)SNP_26135	65	G	A	38	5	--	--
	Ca(ICCV2/PI489777)SNP_26136	99	T	C	104	35	--	--
	Ca(ICCV2/PI489777)SNP_26137	105	G	A	120	67	--	--
	Ca(ICCV2/PI489777)SNP_26138	151	T	A	152	74	--	--
	Ca(ICCV2/PI489777)SNP_26139	157	T	C	184	71	--	--
CakTC40082	Ca(ICCV2/PI489777)SNP_26140	233	A	G	22	3	--	--
	Ca(ICCV2/PI489777)SNP_26141	786	C	T	11	5	--	--
	Ca(ICCV2/PI489777)SNP_26142	795	T	C	10	4	--	--
CakTC00795	Ca(ICCV2/PI489777)SNP_26143	352	A	G	30	22	--	--
	Ca(ICCV2/PI489777)SNP_26144	364	G	A	31	24	--	--
	Ca(ICCV2/PI489777)SNP_26145	400	T	A	34	24	--	--
	Ca(ICCV2/PI489777)SNP_26146	406	G	A	34	31	--	--
	Ca(ICCV2/PI489777)SNP_26147	559	G	A	37	40	--	--
CakTC23727	Ca(ICCV2/PI489777)SNP_26148	622	C	T	85	51	--	--
	Ca(ICCV2/PI489777)SNP_26149	661	C	G	105	60	--	--
	Ca(ICCV2/PI489777)SNP_26150	750	A	C	103	52	--	--
CakTC25967	Ca(ICCV2/PI489777)SNP_26151	626	C	T	28	9	--	--
	Ca(ICCV2/PI489777)SNP_26152	637	A	C	26	7	--	--
	Ca(ICCV2/PI489777)SNP_26153	1406	C	T	28	20	--	--
	Ca(ICCV2/PI489777)SNP_26154	1856	G	T	20	3	--	--
	Ca(ICCV2/PI489777)SNP_26155	1913	A	C	14	3	--	--
CakTC32714	Ca(ICCV2/PI489777)SNP_26156	467	A	C	18	9	--	--
	Ca(ICCV2/PI489777)SNP_26157	1157	A	C	14	4	--	--
CakTC34885	Ca(ICCV2/PI489777)SNP_26158	556	C	A	12	4	--	--
	Ca(ICCV2/PI489777)SNP_26159	808	T	C	7	6	--	--
	Ca(ICCV2/PI489777)SNP_26160	1669	T	C	17	11	--	--
	Ca(ICCV2/PI489777)SNP_26161	2884	T	C	6	12	--	--
CakTC34635	Ca(ICCV2/PI489777)SNP_26162	580	A	T	3	7	--	--
	Ca(ICCV2/PI489777)SNP_26163	601	C	G	3	6	--	--
CakTC38088	Ca(ICCV2/PI489777)SNP_26164	864	A	C	14	6	--	--
	Ca(ICCV2/PI489777)SNP_26165	971	T	A	9	4	--	--
	Ca(ICCV2/PI489777)SNP_26166	972	G	T	9	4	--	--
CakTC30252	Ca(ICCV2/PI489777)SNP_26167	1093	T	G	12	3	--	--
CakTC34442	Ca(ICCV2/PI489777)SNP_26168	212	G	A	17	4	--	--
	Ca(ICCV2/PI489777)SNP_26169	311	G	C	20	4	--	--
CakTC22734	Ca(ICCV2/PI489777)SNP_26170	34	C	G	3	5	--	--
	Ca(ICCV2/PI489777)SNP_26171	1109	A	C	15	8	--	--
	Ca(ICCV2/PI489777)SNP_26172	1717	T	C	27	31	--	--
	Ca(ICCV2/PI489777)SNP_26173	1747	C	T	21	26	--	--
	Ca(ICCV2/PI489777)SNP_26174	2168	G	A	17	9	--	--
	Ca(ICCV2/PI489777)SNP_26175	2198	T	C	10	7	--	--
CakTC29473	Ca(ICCV2/PI489777)SNP_26176	1279	T	C	25	11	--	--
CakTC24020	Ca(ICCV2/PI489777)SNP_26177	62	A	G	11	10	--	--
	Ca(ICCV2/PI489777)SNP_26178	418	G	A	41	8	--	--
	Ca(ICCV2/PI489777)SNP_26179	508	T	C	39	8	--	--
	Ca(ICCV2/PI489777)SNP_26180	719	G	A	37	14	--	--
	Ca(ICCV2/PI489777)SNP_26181	861	T	C	52	12	--	--
	Ca(ICCV2/PI489777)SNP_26182	889	C	T	61	19	--	--
	Ca(ICCV2/PI489777)SNP_26183	1047	T	C	47	21	--	--
	Ca(ICCV2/PI489777)SNP_26184	1050	C	T	50	22	--	--
CakTC26988	Ca(ICCV2/PI489777)SNP_26185	288	T	C	9	4	--	--
	Ca(ICCV2/PI489777)SNP_26186	306	G	A	8	3	--	--
CakTC27943	Ca(ICCV2/PI489777)SNP_26187	1978	C	T	36	21	--	C3H
	Ca(ICCV2/PI489777)SNP_26188	2239	G	A	23	20	--	C3H
	Ca(ICCV2/PI489777)SNP_26189	2659	A	C	10	6	--	C3H
	Ca(ICCV2/PI489777)SNP_26190	2711	T	C	10	8	--	C3H
CakTC27976	Ca(ICCV2/PI489777)SNP_26191	1513	T	C	27	7	--	--

	Ca(ICCV2/PI489777)SNP_26192	2511	A	C	5	8	--	--
CakTC38553	Ca(ICCV2/PI489777)SNP_26193	296	G	A	10	5	--	--
	Ca(ICCV2/PI489777)SNP_26194	407	G	A	11	10	--	--
	Ca(ICCV2/PI489777)SNP_26195	518	G	C	9	7	--	--
	Ca(ICCV2/PI489777)SNP_26196	569	G	A	6	7	--	--
	Ca(ICCV2/PI489777)SNP_26197	878	G	A	6	10	--	--
CakTC38649	Ca(ICCV2/PI489777)SNP_26198	197	A	G	12	4	--	NAC
	Ca(ICCV2/PI489777)SNP_26199	608	A	G	40	5	--	NAC
	Ca(ICCV2/PI489777)SNP_26200	818	C	T	36	3	--	NAC
CakTC37375	Ca(ICCV2/PI489777)SNP_26201	648	C	T	8	10	--	--
	Ca(ICCV2/PI489777)SNP_26202	684	A	T	7	7	--	--
	Ca(ICCV2/PI489777)SNP_26203	804	C	A	14	9	--	--
	Ca(ICCV2/PI489777)SNP_26204	824	C	G	14	9	--	--
	Ca(ICCV2/PI489777)SNP_26205	1023	G	C	10	8	--	--
CakTC43023	Ca(ICCV2/PI489777)SNP_26206	168	C	T	3	8	--	--
	Ca(ICCV2/PI489777)SNP_26207	523	C	G	6	6	--	--
	Ca(ICCV2/PI489777)SNP_26208	699	A	G	3	3	--	--
CakTC30318	Ca(ICCV2/PI489777)SNP_26209	326	T	G	8	4	--	--
	Ca(ICCV2/PI489777)SNP_26210	1370	T	C	7	3	--	--
CakTC37872	Ca(ICCV2/PI489777)SNP_26211	359	A	C	38	23	--	--
	Ca(ICCV2/PI489777)SNP_26212	493	A	T	33	26	--	--
	Ca(ICCV2/PI489777)SNP_26213	589	A	C	25	10	--	--
	Ca(ICCV2/PI489777)SNP_26214	805	G	A	17	10	--	--
	Ca(ICCV2/PI489777)SNP_26215	935	G	A	3	3	--	--
CakTC43164	Ca(ICCV2/PI489777)SNP_26216	641	T	C	3	11	--	--
CakTC24286	Ca(ICCV2/PI489777)SNP_26217	1687	G	A	19	14	--	--
	Ca(ICCV2/PI489777)SNP_26218	1714	C	T	21	14	--	--
	Ca(ICCV2/PI489777)SNP_26219	1919	A	T	11	8	--	--
CakTC36730	Ca(ICCV2/PI489777)SNP_26220	398	C	A	4	3	--	--
	Ca(ICCV2/PI489777)SNP_26221	412	T	C	4	3	--	--
	Ca(ICCV2/PI489777)SNP_26222	1477	C	T	3	3	--	--
	Ca(ICCV2/PI489777)SNP_26223	1659	T	C	4	6	--	--
	Ca(ICCV2/PI489777)SNP_26224	1932	C	T	5	3	--	--
	Ca(ICCV2/PI489777)SNP_26225	2257	C	T	8	3	--	--
CakTC24811	Ca(ICCV2/PI489777)SNP_26226	1060	A	G	3	4	--	--
	Ca(ICCV2/PI489777)SNP_26227	1124	C	T	3	4	--	--
	Ca(ICCV2/PI489777)SNP_26228	1249	G	A	3	4	--	--
CakTC32303	Ca(ICCV2/PI489777)SNP_26229	468	G	A	7	3	--	--
	Ca(ICCV2/PI489777)SNP_26230	524	G	A	8	3	--	--
CakTC33819	Ca(ICCV2/PI489777)SNP_26231	1924	A	G	3	4	Flower	bud
	Ca(ICCV2/PI489777)SNP_26232	2002	A	G	5	4	Flower	bud
CakTC42188	Ca(ICCV2/PI489777)SNP_26233	204	C	T	14	10	--	--
CakTC38894	Ca(ICCV2/PI489777)SNP_26234	622	T	C	49	10	--	--
	Ca(ICCV2/PI489777)SNP_26235	722	C	G	38	14	--	--
CakTC39470	Ca(ICCV2/PI489777)SNP_26236	734	T	A	104	32	--	--
	Ca(ICCV2/PI489777)SNP_26237	750	A	G	103	29	--	--
	Ca(ICCV2/PI489777)SNP_26238	969	T	C	94	20	--	--
	Ca(ICCV2/PI489777)SNP_26239	1371	C	T	11	3	--	--
CakTC39206	Ca(ICCV2/PI489777)SNP_26240	258	G	A	33	17	--	AUX/IAA
	Ca(ICCV2/PI489777)SNP_26241	391	A	C	31	16	--	AUX/IAA
	Ca(ICCV2/PI489777)SNP_26242	633	G	C	16	20	--	AUX/IAA
	Ca(ICCV2/PI489777)SNP_26243	1198	T	C	18	4	--	AUX/IAA
CakTC39548	Ca(ICCV2/PI489777)SNP_26244	814	C	A	31	16	--	--
	Ca(ICCV2/PI489777)SNP_26245	868	A	G	43	21	--	--
CakTC12887	Ca(ICCV2/PI489777)SNP_26246	445	G	A	9	25	--	--
CakTC03352	Ca(ICCV2/PI489777)SNP_26247	537	G	C	12	8	--	--
	Ca(ICCV2/PI489777)SNP_26248	771	C	T	12	12	--	--
	Ca(ICCV2/PI489777)SNP_26249	804	A	G	10	9	--	--
	Ca(ICCV2/PI489777)SNP_26250	1272	T	C	16	15	--	--
CakTC41806	Ca(ICCV2/PI489777)SNP_26251	297	T	C	64	41	--	--
CakTC14298	Ca(ICCV2/PI489777)SNP_26252	154	C	T	4	7	--	--
CakTC03335	Ca(ICCV2/PI489777)SNP_26253	289	C	T	17	9	--	--
	Ca(ICCV2/PI489777)SNP_26254	308	T	C	13	9	--	--
	Ca(ICCV2/PI489777)SNP_26255	334	C	A	16	7	--	--
	Ca(ICCV2/PI489777)SNP_26256	362	T	C	15	7	--	--
	Ca(ICCV2/PI489777)SNP_26257	465	C	T	10	6	--	--
CakTC41738	Ca(ICCV2/PI489777)SNP_26258	1006	G	C	16	13	--	--
	Ca(ICCV2/PI489777)SNP_26259	1022	G	A	17	12	--	--
	Ca(ICCV2/PI489777)SNP_26260	1203	C	A	21	11	--	--
CakTC10136	Ca(ICCV2/PI489777)SNP_26261	1331	A	G	4	3	--	--
	Ca(ICCV2/PI489777)SNP_26262	1373	A	G	4	3	--	--
	Ca(ICCV2/PI489777)SNP_26263	1390	G	A	4	3	--	--
CakTC38228	Ca(ICCV2/PI489777)SNP_26264	153	G	C	3	5	--	--
	Ca(ICCV2/PI489777)SNP_26265	342	G	T	4	3	--	--

	Ca(ICCV2/PI489777)SNP_26266	359	T	A	3	3	--	--
CakTC39096	Ca(ICCV2/PI489777)SNP_26267	485	A	C	8	6	--	--
	Ca(ICCV2/PI489777)SNP_26268	554	T	C	8	5	--	--
	Ca(ICCV2/PI489777)SNP_26269	869	G	T	9	6	--	--
	Ca(ICCV2/PI489777)SNP_26270	875	A	G	9	7	--	--
	Ca(ICCV2/PI489777)SNP_26271	917	G	A	8	8	--	--
	Ca(ICCV2/PI489777)SNP_26272	975	C	T	8	8	--	--
CakTC26105	Ca(ICCV2/PI489777)SNP_26273	1229	G	A	8	3	--	--
	Ca(ICCV2/PI489777)SNP_26274	2128	T	C	3	3	--	--
	Ca(ICCV2/PI489777)SNP_26275	2129	G	A	5	3	--	--
CakTC37905	Ca(ICCV2/PI489777)SNP_26276	463	A	G	4	11	--	--
	Ca(ICCV2/PI489777)SNP_26277	560	T	C	5	6	--	--
	Ca(ICCV2/PI489777)SNP_26278	597	G	A	4	6	--	--
	Ca(ICCV2/PI489777)SNP_26279	693	G	A	5	5	--	--
	Ca(ICCV2/PI489777)SNP_26280	789	G	A	5	5	--	--
	Ca(ICCV2/PI489777)SNP_26281	843	G	A	4	3	--	--
CakTC30003	Ca(ICCV2/PI489777)SNP_26282	97	T	C	11	4	Flower	bud
	Ca(ICCV2/PI489777)SNP_26283	142	C	T	10	5	Flower	bud
	Ca(ICCV2/PI489777)SNP_26284	229	C	T	10	11	Flower	bud
	Ca(ICCV2/PI489777)SNP_26285	234	A	G	13	11	Flower	bud
	Ca(ICCV2/PI489777)SNP_26286	299	G	C	11	6	Flower	bud
	Ca(ICCV2/PI489777)SNP_26287	693	T	C	19	8	Flower	bud
CakTC26403	Ca(ICCV2/PI489777)SNP_26288	1294	C	T	18	4	--	--
	Ca(ICCV2/PI489777)SNP_26289	1902	G	C	10	3	--	--
CakTC06961	Ca(ICCV2/PI489777)SNP_26290	444	A	G	60	52	--	--
CakTC10124	Ca(ICCV2/PI489777)SNP_26291	305	G	C	28	12	--	--
	Ca(ICCV2/PI489777)SNP_26292	368	A	G	28	18	--	--
	Ca(ICCV2/PI489777)SNP_26293	423	A	G	27	16	--	--
	Ca(ICCV2/PI489777)SNP_26294	1136	C	T	12	6	--	--
CakTC38063	Ca(ICCV2/PI489777)SNP_26295	136	G	T	4	5	--	--
	Ca(ICCV2/PI489777)SNP_26296	211	T	G	6	6	--	--
	Ca(ICCV2/PI489777)SNP_26297	1043	A	G	6	4	--	--
	Ca(ICCV2/PI489777)SNP_26298	1273	A	G	6	6	--	--
	Ca(ICCV2/PI489777)SNP_26299	1582	G	T	4	4	--	--
CakTC25549	Ca(ICCV2/PI489777)SNP_26300	764	T	G	48	22	--	--
CakTC33866	Ca(ICCV2/PI489777)SNP_26301	77	C	T	4	3	--	--
	Ca(ICCV2/PI489777)SNP_26302	261	T	C	9	5	--	--
	Ca(ICCV2/PI489777)SNP_26303	459	G	C	7	5	--	--
	Ca(ICCV2/PI489777)SNP_26304	1554	T	G	9	6	--	--
	Ca(ICCV2/PI489777)SNP_26305	2046	C	T	15	3	--	--
CakTC26336	Ca(ICCV2/PI489777)SNP_26306	804	G	T	82	8	--	--
	Ca(ICCV2/PI489777)SNP_26307	999	G	A	107	19	--	--
	Ca(ICCV2/PI489777)SNP_26308	1882	A	G	94	21	--	--
	Ca(ICCV2/PI489777)SNP_26309	1887	A	G	89	20	--	--
	Ca(ICCV2/PI489777)SNP_26310	1900	T	A	84	18	--	--
	Ca(ICCV2/PI489777)SNP_26311	1960	G	T	52	10	--	--
CakTC34175	Ca(ICCV2/PI489777)SNP_26312	282	C	T	13	9	--	--
	Ca(ICCV2/PI489777)SNP_26313	1755	T	C	10	9	--	--
CakTC32953	Ca(ICCV2/PI489777)SNP_26314	154	G	A	11	3	--	--
	Ca(ICCV2/PI489777)SNP_26315	299	T	C	13	3	--	--
CakTC42064	Ca(ICCV2/PI489777)SNP_26316	445	C	G	27	11	--	--
	Ca(ICCV2/PI489777)SNP_26317	1456	G	C	9	9	--	--
	Ca(ICCV2/PI489777)SNP_26318	1823	A	T	8	11	--	--
CakTC42116	Ca(ICCV2/PI489777)SNP_26319	429	C	T	4	4	Flower	bud
	Ca(ICCV2/PI489777)SNP_26320	733	A	G	4	6	Flower	bud
CakTC24175	Ca(ICCV2/PI489777)SNP_26321	602	A	G	27	25	--	--
	Ca(ICCV2/PI489777)SNP_26322	1109	T	C	5	8	--	--
	Ca(ICCV2/PI489777)SNP_26323	1417	T	A	8	4	--	--
CakTC26591	Ca(ICCV2/PI489777)SNP_26324	480	C	A	58	43	--	--
CakTC37553	Ca(ICCV2/PI489777)SNP_26325	30	T	C	5	7	Flower	bud
	Ca(ICCV2/PI489777)SNP_26326	57	C	T	5	7	Flower	bud
	Ca(ICCV2/PI489777)SNP_26327	83	G	C	5	11	Flower	bud
	Ca(ICCV2/PI489777)SNP_26328	111	G	A	18	13	Flower	bud
	Ca(ICCV2/PI489777)SNP_26329	120	T	C	17	13	Flower	bud
	Ca(ICCV2/PI489777)SNP_26330	187	A	G	27	26	Flower	bud
CakTC38751	Ca(ICCV2/PI489777)SNP_26331	1611	A	G	5	3	--	--
CakTC39927	Ca(ICCV2/PI489777)SNP_26332	945	C	T	42	10	--	--
	Ca(ICCV2/PI489777)SNP_26333	1033	C	G	31	13	--	--
	Ca(ICCV2/PI489777)SNP_26334	1063	G	A	33	14	--	--
	Ca(ICCV2/PI489777)SNP_26335	1121	G	A	12	10	--	--
CakTC32646	Ca(ICCV2/PI489777)SNP_26336	730	G	A	20	22	--	--
	Ca(ICCV2/PI489777)SNP_26337	2131	A	G	6	13	--	--
CakTC37361	Ca(ICCV2/PI489777)SNP_26338	99	G	C	6	4	--	--
	Ca(ICCV2/PI489777)SNP_26339	584	C	G	7	3	--	--

	Ca(ICCV2/PI489777)SNP_26340	688	G	A	5	3	--	--
	Ca(ICCV2/PI489777)SNP_26341	690	T	C	5	3	--	--
	Ca(ICCV2/PI489777)SNP_26342	730	C	A	6	3	--	--
CakTC29792	Ca(ICCV2/PI489777)SNP_26343	271	T	C	31	6	--	--
	Ca(ICCV2/PI489777)SNP_26344	326	G	A	33	8	--	--
	Ca(ICCV2/PI489777)SNP_26345	941	G	A	42	11	--	--
CakTC28545	Ca(ICCV2/PI489777)SNP_26346	128	G	A	44	30	--	--
	Ca(ICCV2/PI489777)SNP_26347	227	T	C	51	39	--	--
	Ca(ICCV2/PI489777)SNP_26348	230	G	T	47	38	--	--
	Ca(ICCV2/PI489777)SNP_26349	287	C	T	52	42	--	--
	Ca(ICCV2/PI489777)SNP_26350	398	C	T	47	44	--	--
	Ca(ICCV2/PI489777)SNP_26351	674	C	T	25	14	--	--
	Ca(ICCV2/PI489777)SNP_26352	877	T	C	14	3	--	--
	Ca(ICCV2/PI489777)SNP_26353	949	G	A	32	16	--	--
	Ca(ICCV2/PI489777)SNP_26354	955	T	C	37	16	--	--
	Ca(ICCV2/PI489777)SNP_26355	1057	G	C	64	47	--	--
	Ca(ICCV2/PI489777)SNP_26356	1470	T	C	36	17	--	--
	Ca(ICCV2/PI489777)SNP_26357	1588	C	T	20	11	--	--
	Ca(ICCV2/PI489777)SNP_26358	1600	T	G	16	8	--	--
	Ca(ICCV2/PI489777)SNP_26359	1616	C	G	15	9	--	--
CakTC10504	Ca(ICCV2/PI489777)SNP_26360	1032	A	G	4	3	--	--
	Ca(ICCV2/PI489777)SNP_26361	2358	C	A	5	3	--	--
CakTC11592	Ca(ICCV2/PI489777)SNP_26362	221	G	C	30	5	--	--
	Ca(ICCV2/PI489777)SNP_26363	358	G	A	33	5	--	--
	Ca(ICCV2/PI489777)SNP_26364	445	A	G	35	12	--	--
	Ca(ICCV2/PI489777)SNP_26365	551	G	C	34	13	--	--
	Ca(ICCV2/PI489777)SNP_26366	772	C	T	30	13	--	--
CakTC37255	Ca(ICCV2/PI489777)SNP_26367	578	T	A	21	11	--	--
	Ca(ICCV2/PI489777)SNP_26368	788	A	G	48	30	--	--
CakTC28708	Ca(ICCV2/PI489777)SNP_26369	740	T	A	4	3	--	--
CakTC32097	Ca(ICCV2/PI489777)SNP_26370	699	T	C	16	8	--	--
CakTC42757	Ca(ICCV2/PI489777)SNP_26371	832	A	G	6	4	--	--
CakTC32049	Ca(ICCV2/PI489777)SNP_26372	56	A	C	26	8	Flower	bud
CakTC11058	Ca(ICCV2/PI489777)SNP_26373	188	G	A	7	3	--	--
CakTC42317	Ca(ICCV2/PI489777)SNP_26374	600	T	C	5	10	--	--
CakTC36694	Ca(ICCV2/PI489777)SNP_26375	546	A	G	21	18	--	--
	Ca(ICCV2/PI489777)SNP_26376	2276	A	C	9	5	--	--
	Ca(ICCV2/PI489777)SNP_26377	2366	A	G	15	8	--	--
	Ca(ICCV2/PI489777)SNP_26378	2576	C	T	21	11	--	--
CakTC29259	Ca(ICCV2/PI489777)SNP_26379	806	G	A	5	7	--	--
	Ca(ICCV2/PI489777)SNP_26380	870	T	A	6	8	--	--
	Ca(ICCV2/PI489777)SNP_26381	892	G	A	6	8	--	--
	Ca(ICCV2/PI489777)SNP_26382	982	G	C	3	7	--	--
	Ca(ICCV2/PI489777)SNP_26383	985	A	T	3	7	--	--
CakTC36687	Ca(ICCV2/PI489777)SNP_26384	189	C	T	9	17	--	--
	Ca(ICCV2/PI489777)SNP_26385	298	G	A	9	18	--	--
CakTC38412	Ca(ICCV2/PI489777)SNP_26386	76	C	T	5	3	--	--
	Ca(ICCV2/PI489777)SNP_26387	114	T	C	8	4	--	--
	Ca(ICCV2/PI489777)SNP_26388	430	T	C	14	10	--	--
	Ca(ICCV2/PI489777)SNP_26389	1459	C	T	10	4	--	--
	Ca(ICCV2/PI489777)SNP_26390	1469	A	G	11	4	--	--
	Ca(ICCV2/PI489777)SNP_26391	1532	A	C	11	4	--	--
CakTC41919	Ca(ICCV2/PI489777)SNP_26392	63	C	A	9	3	Shoot	--
	Ca(ICCV2/PI489777)SNP_26393	418	A	G	10	7	Shoot	--
	Ca(ICCV2/PI489777)SNP_26394	500	G	T	10	8	Shoot	--
	Ca(ICCV2/PI489777)SNP_26395	520	T	C	8	8	Shoot	--
	Ca(ICCV2/PI489777)SNP_26396	528	A	G	10	7	Shoot	--
	Ca(ICCV2/PI489777)SNP_26397	643	T	C	13	9	Shoot	--
	Ca(ICCV2/PI489777)SNP_26398	673	G	T	16	8	Shoot	--
CakTC40993	Ca(ICCV2/PI489777)SNP_26399	335	A	G	36	13	--	--
	Ca(ICCV2/PI489777)SNP_26400	663	C	G	36	10	--	--
CakTC15659	Ca(ICCV2/PI489777)SNP_26401	133	A	T	4	5	--	--
	Ca(ICCV2/PI489777)SNP_26402	139	A	C	3	5	--	--
CakTC26387	Ca(ICCV2/PI489777)SNP_26403	40	T	G	12	4	Shoot	--
	Ca(ICCV2/PI489777)SNP_26404	146	G	C	12	7	Shoot	--
	Ca(ICCV2/PI489777)SNP_26405	212	A	T	12	8	Shoot	--
	Ca(ICCV2/PI489777)SNP_26406	331	A	T	6	8	Shoot	--
CakTC40751	Ca(ICCV2/PI489777)SNP_26407	124	C	G	13	22	--	--
	Ca(ICCV2/PI489777)SNP_26408	583	T	C	12	21	--	--
CakTC29417	Ca(ICCV2/PI489777)SNP_26409	1105	T	A	11	5	--	--
	Ca(ICCV2/PI489777)SNP_26410	1198	T	C	12	3	--	--
CakTC33220	Ca(ICCV2/PI489777)SNP_26411	130	A	G	20	12	--	--
	Ca(ICCV2/PI489777)SNP_26412	146	G	A	20	13	--	--
	Ca(ICCV2/PI489777)SNP_26413	247	G	C	25	15	--	--

	Ca(ICCV2/PI489777)SNP_26414	1490	T	A	53	31	--	--
	Ca(ICCV2/PI489777)SNP_26415	1639	C	T	63	28	--	--
	Ca(ICCV2/PI489777)SNP_26416	1801	T	C	54	19	--	--
CakTC42866	Ca(ICCV2/PI489777)SNP_26417	136	C	T	6	4	--	--
	Ca(ICCV2/PI489777)SNP_26418	553	G	A	45	27	--	--
CakTC39216	Ca(ICCV2/PI489777)SNP_26419	519	G	T	9	27	--	--
CakTC25714	Ca(ICCV2/PI489777)SNP_26420	49	C	G	7	5	Root	--
	Ca(ICCV2/PI489777)SNP_26421	494	T	C	15	11	Root	--
	Ca(ICCV2/PI489777)SNP_26422	497	T	C	15	9	Root	--
	Ca(ICCV2/PI489777)SNP_26423	650	T	C	9	3	Root	--
CakTC27834	Ca(ICCV2/PI489777)SNP_26424	704	T	C	11	3	--	FHA
	Ca(ICCV2/PI489777)SNP_26425	822	T	C	19	3	--	FHA
	Ca(ICCV2/PI489777)SNP_26426	842	C	G	18	3	--	FHA
	Ca(ICCV2/PI489777)SNP_26427	929	C	A	17	3	--	FHA
CakTC43062	Ca(ICCV2/PI489777)SNP_26428	347	G	A	9	8	--	--
	Ca(ICCV2/PI489777)SNP_26429	1036	G	T	12	4	--	--
	Ca(ICCV2/PI489777)SNP_26430	1751	C	T	8	5	--	--
CakTC42640	Ca(ICCV2/PI489777)SNP_26431	496	T	C	16	6	--	--
	Ca(ICCV2/PI489777)SNP_26432	592	A	C	20	4	--	--
	Ca(ICCV2/PI489777)SNP_26433	784	A	T	7	6	--	--
	Ca(ICCV2/PI489777)SNP_26434	930	A	C	11	8	--	--
	Ca(ICCV2/PI489777)SNP_26435	1045	T	A	10	8	--	--
	Ca(ICCV2/PI489777)SNP_26436	1183	A	G	14	10	--	--
	Ca(ICCV2/PI489777)SNP_26437	1317	A	C	8	5	--	--
	Ca(ICCV2/PI489777)SNP_26438	1328	C	G	11	4	--	--
	Ca(ICCV2/PI489777)SNP_26439	1399	A	G	8	7	--	--
	Ca(ICCV2/PI489777)SNP_26440	1479	G	C	7	6	--	--
	Ca(ICCV2/PI489777)SNP_26441	1513	C	T	6	7	--	--
	Ca(ICCV2/PI489777)SNP_26442	1544	C	G	5	7	--	--
	Ca(ICCV2/PI489777)SNP_26443	1553	C	G	5	7	--	--
	Ca(ICCV2/PI489777)SNP_26444	1555	G	T	5	7	--	--
CakTC41180	Ca(ICCV2/PI489777)SNP_26445	367	G	A	62	63	--	--
CakTC11666	Ca(ICCV2/PI489777)SNP_26446	506	T	C	3	5	--	--
	Ca(ICCV2/PI489777)SNP_26447	1043	T	G	4	9	--	--
	Ca(ICCV2/PI489777)SNP_26448	1085	A	T	6	7	--	--
	Ca(ICCV2/PI489777)SNP_26449	1463	T	C	4	4	--	--
CakTC37985	Ca(ICCV2/PI489777)SNP_26450	392	G	A	7	7	--	--
	Ca(ICCV2/PI489777)SNP_26451	1355	C	G	6	7	--	--
CakTC30937	Ca(ICCV2/PI489777)SNP_26452	1408	G	A	3	4	--	--
CakTC41602	Ca(ICCV2/PI489777)SNP_26453	73	T	A	6	8	--	--
	Ca(ICCV2/PI489777)SNP_26454	75	A	T	5	8	--	--
	Ca(ICCV2/PI489777)SNP_26455	89	A	G	5	11	--	--
	Ca(ICCV2/PI489777)SNP_26456	114	T	C	15	13	--	--
	Ca(ICCV2/PI489777)SNP_26457	671	G	A	114	110	--	--
	Ca(ICCV2/PI489777)SNP_26458	675	A	C	108	119	--	--
CakTC25812	Ca(ICCV2/PI489777)SNP_26459	203	T	C	15	3	Shoot	--
	Ca(ICCV2/PI489777)SNP_26460	1100	C	A	13	3	Shoot	--
	Ca(ICCV2/PI489777)SNP_26461	1270	C	T	21	6	Shoot	--
CakTC39211	Ca(ICCV2/PI489777)SNP_26462	525	T	A	19	4	Root	--
	Ca(ICCV2/PI489777)SNP_26463	700	A	G	21	3	Root	--
	Ca(ICCV2/PI489777)SNP_26464	924	C	G	16	3	Root	--
	Ca(ICCV2/PI489777)SNP_26465	1260	T	C	18	3	Root	--
CakTC27599	Ca(ICCV2/PI489777)SNP_26466	418	A	G	10	13	--	--
	Ca(ICCV2/PI489777)SNP_26467	1058	G	T	4	3	--	--
	Ca(ICCV2/PI489777)SNP_26468	1130	T	C	7	4	--	--
	Ca(ICCV2/PI489777)SNP_26469	2165	A	G	22	11	--	--
	Ca(ICCV2/PI489777)SNP_26470	2231	T	C	17	17	--	--
	Ca(ICCV2/PI489777)SNP_26471	2618	G	A	13	7	--	--
	Ca(ICCV2/PI489777)SNP_26472	2755	A	G	10	16	--	--
	Ca(ICCV2/PI489777)SNP_26473	3202	T	G	4	3	--	--
CakTC22742	Ca(ICCV2/PI489777)SNP_26474	864	T	G	5	3	--	--
	Ca(ICCV2/PI489777)SNP_26475	895	T	C	4	3	--	--
CakTC29557	Ca(ICCV2/PI489777)SNP_26476	1642	C	T	14	5	--	--
CakTC34567	Ca(ICCV2/PI489777)SNP_26477	427	T	C	8	11	--	--
	Ca(ICCV2/PI489777)SNP_26478	561	A	G	19	15	--	--
	Ca(ICCV2/PI489777)SNP_26479	700	C	T	17	9	--	--
	Ca(ICCV2/PI489777)SNP_26480	931	C	T	15	11	--	--
	Ca(ICCV2/PI489777)SNP_26481	1242	T	G	8	20	--	--
	Ca(ICCV2/PI489777)SNP_26482	1278	C	A	7	18	--	--
	Ca(ICCV2/PI489777)SNP_26483	1655	G	A	13	8	--	--
	Ca(ICCV2/PI489777)SNP_26484	1850	T	C	12	8	--	--
	Ca(ICCV2/PI489777)SNP_26485	2161	G	A	9	4	--	--
CakTC32588	Ca(ICCV2/PI489777)SNP_26486	204	T	C	4	5	--	--
CakTC37562	Ca(ICCV2/PI489777)SNP_26487	599	G	A	12	12	--	--

	Ca(ICCV2/PI489777)SNP_26488	825	T	C	17	19	--	--
	Ca(ICCV2/PI489777)SNP_26489	969	C	T	15	16	--	--
CakTC32556	Ca(ICCV2/PI489777)SNP_26490	644	G	A	16	4	--	--
	Ca(ICCV2/PI489777)SNP_26491	1793	G	A	4	3	--	--
CakTC39434	Ca(ICCV2/PI489777)SNP_26492	474	A	G	16	42	--	--
	Ca(ICCV2/PI489777)SNP_26493	800	T	C	15	20	--	--
CakTC30128	Ca(ICCV2/PI489777)SNP_26494	461	G	C	4	3	--	--
CakTC32961	Ca(ICCV2/PI489777)SNP_26495	159	A	G	6	21	--	--
	Ca(ICCV2/PI489777)SNP_26496	300	T	C	7	21	--	--
	Ca(ICCV2/PI489777)SNP_26497	323	T	C	7	21	--	--
	Ca(ICCV2/PI489777)SNP_26498	410	C	T	5	20	--	--
CakTC38654	Ca(ICCV2/PI489777)SNP_26499	882	T	C	6	3	--	--
	Ca(ICCV2/PI489777)SNP_26500	929	G	T	5	3	--	--
	Ca(ICCV2/PI489777)SNP_26501	1256	A	C	4	4	--	--
CakTC22759	Ca(ICCV2/PI489777)SNP_26502	342	A	C	3	4	--	--
CakTC35046	Ca(ICCV2/PI489777)SNP_26503	830	T	A	4	6	--	--
	Ca(ICCV2/PI489777)SNP_26504	1202	A	T	9	8	--	--
CakTC39042	Ca(ICCV2/PI489777)SNP_26505	589	T	G	16	3	--	--
	Ca(ICCV2/PI489777)SNP_26506	765	G	A	16	3	--	--
CakTC40034	Ca(ICCV2/PI489777)SNP_26507	140	G	C	20	19	--	--
	Ca(ICCV2/PI489777)SNP_26508	204	T	C	15	22	--	--
	Ca(ICCV2/PI489777)SNP_26509	246	A	G	17	23	--	--
	Ca(ICCV2/PI489777)SNP_26510	444	T	C	12	20	--	--
	Ca(ICCV2/PI489777)SNP_26511	636	T	G	10	5	--	--
	Ca(ICCV2/PI489777)SNP_26512	1213	A	T	7	4	--	--
CakTC25636	Ca(ICCV2/PI489777)SNP_26513	1668	A	G	9	4	--	--
	Ca(ICCV2/PI489777)SNP_26514	2104	G	A	6	4	--	--
	Ca(ICCV2/PI489777)SNP_26515	2683	A	G	6	12	--	--
	Ca(ICCV2/PI489777)SNP_26516	2748	G	A	9	10	--	--
	Ca(ICCV2/PI489777)SNP_26517	2905	T	G	4	7	--	--
	Ca(ICCV2/PI489777)SNP_26518	2936	G	C	5	4	--	--
	Ca(ICCV2/PI489777)SNP_26519	3176	A	G	8	3	--	--
	Ca(ICCV2/PI489777)SNP_26520	3196	G	T	8	3	--	--
	Ca(ICCV2/PI489777)SNP_26521	3206	T	C	5	3	--	--
	Ca(ICCV2/PI489777)SNP_26522	4212	G	A	63	34	--	--
	Ca(ICCV2/PI489777)SNP_26523	4751	G	T	33	23	--	--
CakTC16840	Ca(ICCV2/PI489777)SNP_26524	294	A	G	4	3	--	--
CakTC41190	Ca(ICCV2/PI489777)SNP_26525	184	G	A	15	4	--	--
	Ca(ICCV2/PI489777)SNP_26526	193	C	G	13	5	--	--
	Ca(ICCV2/PI489777)SNP_26527	280	G	C	13	9	--	--
CakTC30879	Ca(ICCV2/PI489777)SNP_26528	340	A	G	11	3	--	--
	Ca(ICCV2/PI489777)SNP_26529	527	C	G	11	3	--	--
	Ca(ICCV2/PI489777)SNP_26530	1175	G	A	13	4	--	--
	Ca(ICCV2/PI489777)SNP_26531	1488	C	T	11	3	--	--
	Ca(ICCV2/PI489777)SNP_26532	1722	G	A	7	4	--	--
CakTC11388	Ca(ICCV2/PI489777)SNP_26533	169	C	T	69	47	--	--
	Ca(ICCV2/PI489777)SNP_26534	277	A	C	66	57	--	--
	Ca(ICCV2/PI489777)SNP_26535	436	T	A	66	50	--	--
	Ca(ICCV2/PI489777)SNP_26536	1372	C	T	38	36	--	--
CakTC41641	Ca(ICCV2/PI489777)SNP_26537	2064	A	G	9	7	--	--
	Ca(ICCV2/PI489777)SNP_26538	2820	A	G	5	5	--	--
CakTC26021	Ca(ICCV2/PI489777)SNP_26539	211	C	G	15	12	--	--
	Ca(ICCV2/PI489777)SNP_26540	239	G	A	17	13	--	--
	Ca(ICCV2/PI489777)SNP_26541	398	G	T	16	18	--	--
	Ca(ICCV2/PI489777)SNP_26542	490	A	G	17	21	--	--
	Ca(ICCV2/PI489777)SNP_26543	638	A	G	12	14	--	--
	Ca(ICCV2/PI489777)SNP_26544	764	T	G	20	12	--	--
	Ca(ICCV2/PI489777)SNP_26545	937	A	T	24	16	--	--
	Ca(ICCV2/PI489777)SNP_26546	987	T	C	34	17	--	--
	Ca(ICCV2/PI489777)SNP_26547	995	T	C	34	19	--	--
	Ca(ICCV2/PI489777)SNP_26548	1063	C	T	33	22	--	--
	Ca(ICCV2/PI489777)SNP_26549	1160	G	A	32	22	--	--
	Ca(ICCV2/PI489777)SNP_26550	1238	A	G	22	17	--	--
	Ca(ICCV2/PI489777)SNP_26551	1966	G	A	16	22	--	--
	Ca(ICCV2/PI489777)SNP_26552	2125	G	T	13	10	--	--
CakTC31418	Ca(ICCV2/PI489777)SNP_26553	320	C	T	15	4	Young_pod	--
	Ca(ICCV2/PI489777)SNP_26554	557	T	G	21	5	Young_pod	--
	Ca(ICCV2/PI489777)SNP_26555	630	T	G	21	5	Young_pod	--
	Ca(ICCV2/PI489777)SNP_26556	655	T	C	17	5	Young_pod	--
	Ca(ICCV2/PI489777)SNP_26557	730	T	G	14	5	Young_pod	--
CakTC39165	Ca(ICCV2/PI489777)SNP_26558	292	G	A	9	5	--	--
	Ca(ICCV2/PI489777)SNP_26559	1282	T	A	7	8	--	--
	Ca(ICCV2/PI489777)SNP_26560	1432	C	T	5	4	--	--
CakTC27992	Ca(ICCV2/PI489777)SNP_26561	183	A	G	5	5	--	--

	Ca(ICCV2/PI489777)SNP_26562	217	C	G	5	5	--	--
	Ca(ICCV2/PI489777)SNP_26563	220	G	A	6	5	--	--
CakTC26728	Ca(ICCV2/PI489777)SNP_26564	854	C	T	18	7	--	--
	Ca(ICCV2/PI489777)SNP_26565	1478	T	C	8	3	--	--
CakTC35530	Ca(ICCV2/PI489777)SNP_26566	2872	T	C	8	5	--	--
CakTC17629	Ca(ICCV2/PI489777)SNP_26567	614	T	C	18	8	--	--
	Ca(ICCV2/PI489777)SNP_26568	828	A	G	25	7	--	--
	Ca(ICCV2/PI489777)SNP_26569	950	C	T	20	5	--	--
CakTC42059	Ca(ICCV2/PI489777)SNP_26570	208	A	G	13	6	--	--
	Ca(ICCV2/PI489777)SNP_26571	340	G	T	12	7	--	--
CakTC39366	Ca(ICCV2/PI489777)SNP_26572	276	T	C	17	5	--	--
CakTC31241	Ca(ICCV2/PI489777)SNP_26573	418	C	A	7	4	--	--
	Ca(ICCV2/PI489777)SNP_26574	436	A	C	4	4	--	--
CakTC29151	Ca(ICCV2/PI489777)SNP_26575	208	G	C	5	5	--	--
	Ca(ICCV2/PI489777)SNP_26576	286	G	A	5	5	--	--
CakTC39281	Ca(ICCV2/PI489777)SNP_26577	623	G	C	11	14	--	--
CakTC24417	Ca(ICCV2/PI489777)SNP_26578	681	C	G	7	5	--	CCHC
CakTC25519	Ca(ICCV2/PI489777)SNP_26579	478	A	T	3	5	--	--
	Ca(ICCV2/PI489777)SNP_26580	1007	G	A	3	3	--	--
CakTC28489	Ca(ICCV2/PI489777)SNP_26581	1029	G	A	27	7	--	--
	Ca(ICCV2/PI489777)SNP_26582	1248	G	A	27	6	--	--
CakTC42685	Ca(ICCV2/PI489777)SNP_26583	245	T	C	10	9	--	--
	Ca(ICCV2/PI489777)SNP_26584	818	T	C	6	8	--	--
	Ca(ICCV2/PI489777)SNP_26585	888	T	A	6	8	--	--
CakTC41626	Ca(ICCV2/PI489777)SNP_26586	93	G	A	10	9	--	--
	Ca(ICCV2/PI489777)SNP_26587	229	T	C	12	13	--	--
	Ca(ICCV2/PI489777)SNP_26588	315	A	G	13	13	--	--
	Ca(ICCV2/PI489777)SNP_26589	1134	A	C	12	23	--	--
	Ca(ICCV2/PI489777)SNP_26590	1185	T	G	12	25	--	--
	Ca(ICCV2/PI489777)SNP_26591	1200	C	T	13	25	--	--
	Ca(ICCV2/PI489777)SNP_26592	1257	C	T	17	22	--	--
	Ca(ICCV2/PI489777)SNP_26593	1269	T	C	18	19	--	--
	Ca(ICCV2/PI489777)SNP_26594	1362	G	A	19	15	--	--
	Ca(ICCV2/PI489777)SNP_26595	1401	T	C	21	15	--	--
	Ca(ICCV2/PI489777)SNP_26596	1815	A	T	5	16	--	--
	Ca(ICCV2/PI489777)SNP_26597	1834	A	G	6	18	--	--
	Ca(ICCV2/PI489777)SNP_26598	2071	T	C	5	14	--	--
	Ca(ICCV2/PI489777)SNP_26599	2174	G	T	4	10	--	--
CakTC35924	Ca(ICCV2/PI489777)SNP_26600	283	G	A	13	8	--	--
	Ca(ICCV2/PI489777)SNP_26601	420	T	C	7	6	--	--
	Ca(ICCV2/PI489777)SNP_26602	2237	T	C	5	5	--	--
CakTC42857	Ca(ICCV2/PI489777)SNP_26603	676	G	A	7	9	--	ARID
	Ca(ICCV2/PI489777)SNP_26604	1387	T	G	5	6	--	ARID
	Ca(ICCV2/PI489777)SNP_26605	1604	G	C	3	5	--	ARID
CakTC23795	Ca(ICCV2/PI489777)SNP_26606	714	T	A	11	4	--	--
CakTC28922	Ca(ICCV2/PI489777)SNP_26607	2224	G	T	15	7	--	FHA
CakTC34402	Ca(ICCV2/PI489777)SNP_26608	292	A	T	10	6	--	--
CakTC42522	Ca(ICCV2/PI489777)SNP_26609	276	C	G	8	6	--	--
	Ca(ICCV2/PI489777)SNP_26610	553	T	C	6	5	--	--
CakTC37744	Ca(ICCV2/PI489777)SNP_26611	731	G	A	13	11	--	--
	Ca(ICCV2/PI489777)SNP_26612	930	T	G	11	6	--	--
CakTC33923	Ca(ICCV2/PI489777)SNP_26613	174	C	G	17	7	--	--
	Ca(ICCV2/PI489777)SNP_26614	254	A	G	19	11	--	--
	Ca(ICCV2/PI489777)SNP_26615	470	T	A	21	13	--	--
	Ca(ICCV2/PI489777)SNP_26616	643	G	A	10	4	--	--
CakTC12546	Ca(ICCV2/PI489777)SNP_26617	787	A	G	9	7	--	--
	Ca(ICCV2/PI489777)SNP_26618	3309	A	C	8	3	--	--
	Ca(ICCV2/PI489777)SNP_26619	3512	A	G	3	3	--	--
	Ca(ICCV2/PI489777)SNP_26620	3551	C	T	4	3	--	--
	Ca(ICCV2/PI489777)SNP_26621	4356	G	A	4	3	--	--
	Ca(ICCV2/PI489777)SNP_26622	4431	A	G	4	4	--	--
	Ca(ICCV2/PI489777)SNP_26623	4551	G	A	4	6	--	--
CakTC42234	Ca(ICCV2/PI489777)SNP_26624	71	G	C	10	5	--	--
	Ca(ICCV2/PI489777)SNP_26625	694	T	A	88	53	--	--
	Ca(ICCV2/PI489777)SNP_26626	700	A	T	87	54	--	--
	Ca(ICCV2/PI489777)SNP_26627	729	C	G	85	49	--	--
CakTC38495	Ca(ICCV2/PI489777)SNP_26628	12	G	C	5	3	--	--
CakTC33451	Ca(ICCV2/PI489777)SNP_26629	377	C	T	12	11	--	--
	Ca(ICCV2/PI489777)SNP_26630	538	C	A	11	13	--	--
CakTC11587	Ca(ICCV2/PI489777)SNP_26631	675	G	T	12	8	--	--
	Ca(ICCV2/PI489777)SNP_26632	777	G	A	5	3	--	--
CakTC11411	Ca(ICCV2/PI489777)SNP_26633	204	C	G	8	8	--	--
	Ca(ICCV2/PI489777)SNP_26634	987	A	G	17	7	--	--
	Ca(ICCV2/PI489777)SNP_26635	2210	C	T	29	11	--	--

CakTC29956	Ca(ICCV2/PI489777)SNP_26636	104	C	T	3	5	--	--
	Ca(ICCV2/PI489777)SNP_26637	240	T	C	3	7	--	--
	Ca(ICCV2/PI489777)SNP_26638	2295	G	A	11	5	--	--
	Ca(ICCV2/PI489777)SNP_26639	2403	T	G	15	7	--	--
	Ca(ICCV2/PI489777)SNP_26640	2505	G	A	17	9	--	--
	Ca(ICCV2/PI489777)SNP_26641	2818	G	A	16	5	--	--
CakTC09646	Ca(ICCV2/PI489777)SNP_26642	106	C	G	14	3	--	--
	Ca(ICCV2/PI489777)SNP_26643	120	T	C	16	3	--	--
	Ca(ICCV2/PI489777)SNP_26644	927	A	C	16	12	--	--
CakTC29860	Ca(ICCV2/PI489777)SNP_26645	265	C	T	14	6	--	--
	Ca(ICCV2/PI489777)SNP_26646	331	C	A	17	9	--	--
	Ca(ICCV2/PI489777)SNP_26647	1828	C	T	12	10	--	--
CakTC23583	Ca(ICCV2/PI489777)SNP_26648	358	A	G	4	5	--	--
	Ca(ICCV2/PI489777)SNP_26649	436	A	G	4	5	--	--
	Ca(ICCV2/PI489777)SNP_26650	475	C	G	4	3	--	--
	Ca(ICCV2/PI489777)SNP_26651	479	G	T	4	3	--	--
CakTC28883	Ca(ICCV2/PI489777)SNP_26652	1227	C	T	4	4	--	--
	Ca(ICCV2/PI489777)SNP_26653	1656	A	T	3	3	--	--
CakTC39225	Ca(ICCV2/PI489777)SNP_26654	456	A	G	16	7	--	--
	Ca(ICCV2/PI489777)SNP_26655	1624	G	A	4	6	--	--
CakTC39774	Ca(ICCV2/PI489777)SNP_26656	267	A	C	19	3	--	--
	Ca(ICCV2/PI489777)SNP_26657	339	G	A	17	6	--	--
CakTC28474	Ca(ICCV2/PI489777)SNP_26658	453	A	C	9	10	--	--
	Ca(ICCV2/PI489777)SNP_26659	524	A	C	6	8	--	--
	Ca(ICCV2/PI489777)SNP_26660	661	C	T	8	6	--	--
CakTC41174	Ca(ICCV2/PI489777)SNP_26661	413	C	T	10	5	--	--
CakTC22248	Ca(ICCV2/PI489777)SNP_26662	731	A	G	109	60	--	--
	Ca(ICCV2/PI489777)SNP_26663	1514	G	T	55	53	--	--
CakTC24410	Ca(ICCV2/PI489777)SNP_26664	988	A	C	34	21	--	--
	Ca(ICCV2/PI489777)SNP_26665	1060	G	A	29	14	--	--
	Ca(ICCV2/PI489777)SNP_26666	1433	A	G	20	16	--	--
CakTC26628	Ca(ICCV2/PI489777)SNP_26667	48	T	C	43	16	--	--
	Ca(ICCV2/PI489777)SNP_26668	101	T	A	49	20	--	--
	Ca(ICCV2/PI489777)SNP_26669	437	C	A	130	61	--	--
	Ca(ICCV2/PI489777)SNP_26670	470	C	A	127	59	--	--
CakTC37803	Ca(ICCV2/PI489777)SNP_26671	1167	G	A	87	71	--	--
CakTC43366	Ca(ICCV2/PI489777)SNP_26672	138	T	C	3	3	--	--
CakTC32513	Ca(ICCV2/PI489777)SNP_26673	700	C	T	9	9	--	--
	Ca(ICCV2/PI489777)SNP_26674	1048	A	G	14	9	--	--
	Ca(ICCV2/PI489777)SNP_26675	1438	G	A	25	20	--	--
	Ca(ICCV2/PI489777)SNP_26676	1945	A	G	9	4	--	--
CakTC43152	Ca(ICCV2/PI489777)SNP_26677	77	T	C	8	6	--	--
CakTC42828	Ca(ICCV2/PI489777)SNP_26678	164	A	T	11	10	--	--
	Ca(ICCV2/PI489777)SNP_26679	173	G	A	11	11	--	--
	Ca(ICCV2/PI489777)SNP_26680	311	A	G	14	13	--	--
	Ca(ICCV2/PI489777)SNP_26681	636	A	T	12	16	--	--
	Ca(ICCV2/PI489777)SNP_26682	661	C	T	10	16	--	--
	Ca(ICCV2/PI489777)SNP_26683	773	G	A	3	9	--	--
CakTC16343	Ca(ICCV2/PI489777)SNP_26684	1259	A	C	5	4	--	--
	Ca(ICCV2/PI489777)SNP_26685	1261	A	C	6	4	--	--
CakTC07843	Ca(ICCV2/PI489777)SNP_26686	281	C	T	5	3	--	--
CakTC37330	Ca(ICCV2/PI489777)SNP_26687	890	G	C	9	13	--	--
CakTC24038	Ca(ICCV2/PI489777)SNP_26688	597	A	T	34	6	--	SBP
	Ca(ICCV2/PI489777)SNP_26689	598	A	T	33	6	--	SBP
	Ca(ICCV2/PI489777)SNP_26690	1006	T	C	41	12	--	SBP
	Ca(ICCV2/PI489777)SNP_26691	1276	G	A	42	19	--	SBP
	Ca(ICCV2/PI489777)SNP_26692	1520	A	G	35	22	--	SBP
	Ca(ICCV2/PI489777)SNP_26693	2514	G	A	71	29	--	SBP
	Ca(ICCV2/PI489777)SNP_26694	2704	G	A	80	18	--	SBP
	Ca(ICCV2/PI489777)SNP_26695	3490	A	G	41	19	--	SBP
	Ca(ICCV2/PI489777)SNP_26696	3509	G	T	41	21	--	SBP
	Ca(ICCV2/PI489777)SNP_26697	3811	T	C	11	13	--	SBP
	Ca(ICCV2/PI489777)SNP_26698	3875	C	A	5	10	--	SBP
	Ca(ICCV2/PI489777)SNP_26699	3929	C	A	3	8	--	SBP
CakTC13870	Ca(ICCV2/PI489777)SNP_26700	475	C	T	6	41	--	--
	Ca(ICCV2/PI489777)SNP_26701	481	G	A	5	41	--	--
	Ca(ICCV2/PI489777)SNP_26702	489	G	T	5	41	--	--
	Ca(ICCV2/PI489777)SNP_26703	514	A	G	5	41	--	--
	Ca(ICCV2/PI489777)SNP_26704	524	C	T	5	38	--	--
	Ca(ICCV2/PI489777)SNP_26705	537	T	G	3	41	--	--
	Ca(ICCV2/PI489777)SNP_26706	544	G	A	4	39	--	--
CakTC24263	Ca(ICCV2/PI489777)SNP_26707	77	C	G	6	3	--	--
	Ca(ICCV2/PI489777)SNP_26708	309	A	C	57	25	--	--
	Ca(ICCV2/PI489777)SNP_26709	354	T	G	53	22	--	--

	Ca(ICCV2/PI489777)SNP_26710	549	C	A	70	61	--	--
CakTC25064	Ca(ICCV2/PI489777)SNP_26711	302	T	C	14	3	Shoot	--
	Ca(ICCV2/PI489777)SNP_26712	553	T	C	16	3	Shoot	--
	Ca(ICCV2/PI489777)SNP_26713	692	C	A	14	4	Shoot	--
CakTC28484	Ca(ICCV2/PI489777)SNP_26714	240	T	C	5	16	--	--
	Ca(ICCV2/PI489777)SNP_26715	250	T	C	6	19	--	--
	Ca(ICCV2/PI489777)SNP_26716	1965	C	T	17	11	--	--
CakTC10316	Ca(ICCV2/PI489777)SNP_26717	467	A	T	5	4	--	--
	Ca(ICCV2/PI489777)SNP_26718	711	C	A	6	4	--	--
	Ca(ICCV2/PI489777)SNP_26719	717	A	G	6	4	--	--
CakTC39971	Ca(ICCV2/PI489777)SNP_26720	212	T	G	6	7	--	--
	Ca(ICCV2/PI489777)SNP_26721	590	C	T	9	6	--	--
CakTC41187	Ca(ICCV2/PI489777)SNP_26722	1762	C	T	6	10	--	--
CakTC32094	Ca(ICCV2/PI489777)SNP_26723	231	G	A	15	6	--	--
	Ca(ICCV2/PI489777)SNP_26724	288	C	A	16	7	--	--
	Ca(ICCV2/PI489777)SNP_26725	967	C	T	13	3	--	--
	Ca(ICCV2/PI489777)SNP_26726	1437	T	C	10	10	--	--
CakTC42946	Ca(ICCV2/PI489777)SNP_26727	580	A	G	3	3	--	--
CakTC26589	Ca(ICCV2/PI489777)SNP_26728	244	A	T	5	5	--	--
	Ca(ICCV2/PI489777)SNP_26729	1090	T	C	45	29	--	--
CakTC33288	Ca(ICCV2/PI489777)SNP_26730	2537	T	C	12	8	--	TPR
	Ca(ICCV2/PI489777)SNP_26731	2724	A	C	4	4	--	TPR
CakTC40329	Ca(ICCV2/PI489777)SNP_26732	397	T	A	5	9	--	--
	Ca(ICCV2/PI489777)SNP_26733	558	A	T	5	8	--	--
	Ca(ICCV2/PI489777)SNP_26734	592	G	A	8	6	--	--
	Ca(ICCV2/PI489777)SNP_26735	1393	G	A	8	6	--	--
	Ca(ICCV2/PI489777)SNP_26736	1515	T	G	3	4	--	--
	Ca(ICCV2/PI489777)SNP_26737	1533	T	C	6	5	--	--
CakTC27239	Ca(ICCV2/PI489777)SNP_26738	790	C	T	31	16	--	--
	Ca(ICCV2/PI489777)SNP_26739	961	C	A	20	5	--	--
CakTC37461	Ca(ICCV2/PI489777)SNP_26740	28	C	A	3	5	--	--
	Ca(ICCV2/PI489777)SNP_26741	53	G	A	11	10	--	--
	Ca(ICCV2/PI489777)SNP_26742	107	T	C	29	32	--	--
	Ca(ICCV2/PI489777)SNP_26743	300	T	A	37	33	--	--
	Ca(ICCV2/PI489777)SNP_26744	307	T	C	36	34	--	--
	Ca(ICCV2/PI489777)SNP_26745	512	T	C	35	19	--	--
	Ca(ICCV2/PI489777)SNP_26746	915	C	T	10	8	--	--
CakTC43059	Ca(ICCV2/PI489777)SNP_26747	189	T	C	10	5	--	--
CakTC34619	Ca(ICCV2/PI489777)SNP_26748	183	C	T	6	14	--	NAC
	Ca(ICCV2/PI489777)SNP_26749	218	T	A	8	14	--	NAC
	Ca(ICCV2/PI489777)SNP_26750	1060	G	A	9	8	--	NAC
	Ca(ICCV2/PI489777)SNP_26751	1304	C	G	14	6	--	NAC
	Ca(ICCV2/PI489777)SNP_26752	1361	C	T	15	3	--	NAC
	Ca(ICCV2/PI489777)SNP_26753	1387	T	G	17	7	--	NAC
	Ca(ICCV2/PI489777)SNP_26754	1510	A	G	21	10	--	NAC
	Ca(ICCV2/PI489777)SNP_26755	1525	C	T	21	8	--	NAC
	Ca(ICCV2/PI489777)SNP_26756	1587	C	A	18	6	--	NAC
	Ca(ICCV2/PI489777)SNP_26757	1647	T	C	13	7	--	NAC
	Ca(ICCV2/PI489777)SNP_26758	1681	C	A	14	6	--	NAC
	Ca(ICCV2/PI489777)SNP_26759	1705	T	C	12	5	--	NAC
	Ca(ICCV2/PI489777)SNP_26760	1762	G	A	8	9	--	NAC
	Ca(ICCV2/PI489777)SNP_26761	1773	T	C	9	7	--	NAC
	Ca(ICCV2/PI489777)SNP_26762	1889	G	A	6	6	--	NAC
CakTC42423	Ca(ICCV2/PI489777)SNP_26763	245	T	C	13	48	--	--
	Ca(ICCV2/PI489777)SNP_26764	1279	G	A	78	66	--	--
	Ca(ICCV2/PI489777)SNP_26765	1379	T	C	76	57	--	--
	Ca(ICCV2/PI489777)SNP_26766	1757	T	C	30	32	--	--
CakTC30766	Ca(ICCV2/PI489777)SNP_26767	1022	C	A	17	3	--	--
	Ca(ICCV2/PI489777)SNP_26768	1026	C	T	15	3	--	--
	Ca(ICCV2/PI489777)SNP_26769	1497	A	G	6	3	--	--
	Ca(ICCV2/PI489777)SNP_26770	1946	C	G	14	7	--	--
	Ca(ICCV2/PI489777)SNP_26771	2790	A	G	3	4	--	--
	Ca(ICCV2/PI489777)SNP_26772	3018	G	A	4	6	--	--
	Ca(ICCV2/PI489777)SNP_26773	3264	C	A	11	4	--	--
	Ca(ICCV2/PI489777)SNP_26774	3901	T	C	3	7	--	--
CakTC22602	Ca(ICCV2/PI489777)SNP_26775	509	T	G	610	11	--	--
	Ca(ICCV2/PI489777)SNP_26776	526	G	A	584	10	--	--
CakTC40913	Ca(ICCV2/PI489777)SNP_26777	439	T	A	7	4	--	--
CakTC41098	Ca(ICCV2/PI489777)SNP_26778	107	T	A	4	5	--	--
	Ca(ICCV2/PI489777)SNP_26779	654	G	C	4	4	--	--
CakTC38207	Ca(ICCV2/PI489777)SNP_26780	302	G	A	21	19	--	--
	Ca(ICCV2/PI489777)SNP_26781	611	C	G	22	13	--	--
	Ca(ICCV2/PI489777)SNP_26782	875	G	A	22	12	--	--
	Ca(ICCV2/PI489777)SNP_26783	1031	T	A	10	8	--	--

CakTC40040	Ca(ICCV2/PI489777)SNP_26784	230	C	T	23	9	--	--
	Ca(ICCV2/PI489777)SNP_26785	336	C	T	37	12	--	--
	Ca(ICCV2/PI489777)SNP_26786	1133	A	G	23	19	--	--
	Ca(ICCV2/PI489777)SNP_26787	1313	T	A	18	18	--	--
CakTC29900	Ca(ICCV2/PI489777)SNP_26788	306	C	T	3	3	Flower	bud
	Ca(ICCV2/PI489777)SNP_26789	408	C	G	5	4	Flower	bud
	Ca(ICCV2/PI489777)SNP_26790	1200	A	G	10	8	Flower	bud
	Ca(ICCV2/PI489777)SNP_26791	2980	C	T	8	10	Flower	bud
	Ca(ICCV2/PI489777)SNP_26792	3346	A	G	12	4	Flower	bud
	Ca(ICCV2/PI489777)SNP_26793	3347	A	G	12	4	Flower	bud
	Ca(ICCV2/PI489777)SNP_26794	3378	C	T	10	4	Flower	bud
	Ca(ICCV2/PI489777)SNP_26795	3701	T	C	13	3	Flower	bud
CakTC25276	Ca(ICCV2/PI489777)SNP_26796	927	G	A	3	3	--	--
CakTC08345	Ca(ICCV2/PI489777)SNP_26797	655	C	A	10	5	--	--
	Ca(ICCV2/PI489777)SNP_26798	718	C	T	9	6	--	--
	Ca(ICCV2/PI489777)SNP_26799	1037	T	C	3	3	--	--
	Ca(ICCV2/PI489777)SNP_26800	1072	G	C	3	3	--	--
CakTC40546	Ca(ICCV2/PI489777)SNP_26801	40	A	G	19	18	--	--
	Ca(ICCV2/PI489777)SNP_26802	484	G	A	34	27	--	--
	Ca(ICCV2/PI489777)SNP_26803	508	C	T	33	25	--	--
	Ca(ICCV2/PI489777)SNP_26804	657	T	A	5	4	--	--
CakTC43015	Ca(ICCV2/PI489777)SNP_26805	138	T	C	10	10	--	--
	Ca(ICCV2/PI489777)SNP_26806	587	C	A	100	89	--	--
CakTC42983	Ca(ICCV2/PI489777)SNP_26807	950	C	T	20	5	--	--
	Ca(ICCV2/PI489777)SNP_26808	1031	T	C	21	6	--	--
	Ca(ICCV2/PI489777)SNP_26809	1151	C	T	14	5	--	--
	Ca(ICCV2/PI489777)SNP_26810	1158	C	T	13	4	--	--
	Ca(ICCV2/PI489777)SNP_26811	1274	C	T	8	5	--	--
	Ca(ICCV2/PI489777)SNP_26812	1334	C	A	18	7	--	--
	Ca(ICCV2/PI489777)SNP_26813	1466	A	G	30	8	--	--
	Ca(ICCV2/PI489777)SNP_26814	1505	C	T	33	8	--	--
	Ca(ICCV2/PI489777)SNP_26815	1634	C	T	21	6	--	--
	Ca(ICCV2/PI489777)SNP_26816	1798	T	C	5	3	--	--
CakTC34725	Ca(ICCV2/PI489777)SNP_26817	133	G	T	13	4	--	--
CakTC36364	Ca(ICCV2/PI489777)SNP_26818	399	G	A	3	4	--	--
CakTC26364	Ca(ICCV2/PI489777)SNP_26819	37	G	T	23	12	--	--
	Ca(ICCV2/PI489777)SNP_26820	829	G	A	24	12	--	--
CakTC29702	Ca(ICCV2/PI489777)SNP_26821	588	C	T	41	31	--	--
	Ca(ICCV2/PI489777)SNP_26822	630	C	T	41	25	--	--
CakTC43243	Ca(ICCV2/PI489777)SNP_26823	272	G	C	13	3	--	--
	Ca(ICCV2/PI489777)SNP_26824	479	C	A	9	4	--	--
CakTC04300	Ca(ICCV2/PI489777)SNP_26825	130	C	T	14	6	--	--
	Ca(ICCV2/PI489777)SNP_26826	133	T	A	14	6	--	--
	Ca(ICCV2/PI489777)SNP_26827	161	T	C	17	6	--	--
	Ca(ICCV2/PI489777)SNP_26828	184	C	T	26	7	--	--
	Ca(ICCV2/PI489777)SNP_26829	194	T	C	21	5	--	--
	Ca(ICCV2/PI489777)SNP_26830	207	A	G	27	8	--	--
	Ca(ICCV2/PI489777)SNP_26831	220	G	C	28	8	--	--
	Ca(ICCV2/PI489777)SNP_26832	263	C	T	31	8	--	--
CakTC22324	Ca(ICCV2/PI489777)SNP_26833	330	A	T	6	5	--	--
	Ca(ICCV2/PI489777)SNP_26834	342	C	T	6	5	--	--
	Ca(ICCV2/PI489777)SNP_26835	354	C	T	6	5	--	--
	Ca(ICCV2/PI489777)SNP_26836	378	C	G	6	5	--	--
	Ca(ICCV2/PI489777)SNP_26837	741	C	T	54	15	--	--
CakTC08995	Ca(ICCV2/PI489777)SNP_26838	345	C	T	35	42	--	--
	Ca(ICCV2/PI489777)SNP_26839	414	T	C	48	36	--	--
	Ca(ICCV2/PI489777)SNP_26840	532	C	A	55	38	--	--
	Ca(ICCV2/PI489777)SNP_26841	533	A	C	54	40	--	--
	Ca(ICCV2/PI489777)SNP_26842	914	G	C	17	6	--	--
CakTC28600	Ca(ICCV2/PI489777)SNP_26843	350	C	T	7	7	--	--
CakTC30779	Ca(ICCV2/PI489777)SNP_26844	1613	T	C	6	3	--	VARL
	Ca(ICCV2/PI489777)SNP_26845	3530	C	T	12	8	--	VARL
CakTC32165	Ca(ICCV2/PI489777)SNP_26846	144	T	A	3	3	--	--
	Ca(ICCV2/PI489777)SNP_26847	151	A	G	3	3	--	--
	Ca(ICCV2/PI489777)SNP_26848	173	T	C	3	3	--	--
	Ca(ICCV2/PI489777)SNP_26849	186	A	G	3	3	--	--
	Ca(ICCV2/PI489777)SNP_26850	187	G	A	3	3	--	--
	Ca(ICCV2/PI489777)SNP_26851	204	A	G	3	3	--	--
	Ca(ICCV2/PI489777)SNP_26852	208	A	G	4	3	--	--
	Ca(ICCV2/PI489777)SNP_26853	218	A	G	4	3	--	--
	Ca(ICCV2/PI489777)SNP_26854	282	G	A	4	3	--	--
	Ca(ICCV2/PI489777)SNP_26855	287	A	G	4	3	--	--
	Ca(ICCV2/PI489777)SNP_26856	307	C	T	4	3	--	--
	Ca(ICCV2/PI489777)SNP_26857	308	T	C	4	3	--	--

	Ca(ICCV2/PI489777)SNP_26858	320	C	T	3	3	--	--
	Ca(ICCV2/PI489777)SNP_26859	322	T	A	4	3	--	--
	Ca(ICCV2/PI489777)SNP_26860	379	C	T	4	5	--	--
	Ca(ICCV2/PI489777)SNP_26861	387	G	T	3	5	--	--
	Ca(ICCV2/PI489777)SNP_26862	445	C	T	4	3	--	--
CakTC37763	Ca(ICCV2/PI489777)SNP_26863	262	A	G	8	4	--	--
	Ca(ICCV2/PI489777)SNP_26864	263	G	T	8	5	--	--
	Ca(ICCV2/PI489777)SNP_26865	634	G	A	3	13	--	--
	Ca(ICCV2/PI489777)SNP_26866	1616	C	G	3	7	--	--
	Ca(ICCV2/PI489777)SNP_26867	1631	A	G	3	7	--	--
CakTC26547	Ca(ICCV2/PI489777)SNP_26868	403	T	C	9	3	--	--
	Ca(ICCV2/PI489777)SNP_26869	676	T	C	7	5	--	--
	Ca(ICCV2/PI489777)SNP_26870	678	A	G	7	5	--	--
	Ca(ICCV2/PI489777)SNP_26871	717	A	T	7	5	--	--
	Ca(ICCV2/PI489777)SNP_26872	720	T	C	7	5	--	--
	Ca(ICCV2/PI489777)SNP_26873	733	C	T	7	5	--	--
	Ca(ICCV2/PI489777)SNP_26874	792	A	G	5	5	--	--
	Ca(ICCV2/PI489777)SNP_26875	801	A	T	7	5	--	--
	Ca(ICCV2/PI489777)SNP_26876	809	T	C	8	5	--	--
	Ca(ICCV2/PI489777)SNP_26877	885	C	T	9	4	--	--
	Ca(ICCV2/PI489777)SNP_26878	894	G	C	9	4	--	--
CakTC41299	Ca(ICCV2/PI489777)SNP_26879	1426	G	A	14	12	--	--
	Ca(ICCV2/PI489777)SNP_26880	2103	A	G	10	14	--	--
CakTC30863	Ca(ICCV2/PI489777)SNP_26881	364	C	T	13	5	--	--
	Ca(ICCV2/PI489777)SNP_26882	613	C	G	5	3	--	--
	Ca(ICCV2/PI489777)SNP_26883	721	A	G	8	8	--	--
	Ca(ICCV2/PI489777)SNP_26884	787	T	C	8	8	--	--
CakTC32494	Ca(ICCV2/PI489777)SNP_26885	1538	T	C	6	3	--	C3H
CakTC08720	Ca(ICCV2/PI489777)SNP_26886	1949	C	G	4	5	--	--
	Ca(ICCV2/PI489777)SNP_26887	2002	T	A	3	4	--	--
	Ca(ICCV2/PI489777)SNP_26888	2040	T	G	3	4	--	--
CakTC27695	Ca(ICCV2/PI489777)SNP_26889	742	T	C	31	18	--	--
CakTC40197	Ca(ICCV2/PI489777)SNP_26890	476	C	T	23	20	--	--
	Ca(ICCV2/PI489777)SNP_26891	726	G	A	32	18	--	--
	Ca(ICCV2/PI489777)SNP_26892	1233	A	G	12	5	--	--
CakTC29665	Ca(ICCV2/PI489777)SNP_26893	106	A	G	4	4	--	--
	Ca(ICCV2/PI489777)SNP_26894	348	C	A	8	6	--	--
	Ca(ICCV2/PI489777)SNP_26895	791	A	T	10	4	--	--
	Ca(ICCV2/PI489777)SNP_26896	878	A	T	12	6	--	--
	Ca(ICCV2/PI489777)SNP_26897	1492	C	A	13	8	--	--
CakTC32727	Ca(ICCV2/PI489777)SNP_26898	115	C	A	6	7	--	--
CakTC31186	Ca(ICCV2/PI489777)SNP_26899	106	G	A	38	20	--	--
CakTC32601	Ca(ICCV2/PI489777)SNP_26900	68	A	T	54	20	--	--
	Ca(ICCV2/PI489777)SNP_26901	450	A	T	11	10	--	--
CakTC33787	Ca(ICCV2/PI489777)SNP_26902	377	A	C	4	8	--	--
	Ca(ICCV2/PI489777)SNP_26903	381	G	T	4	8	--	--
	Ca(ICCV2/PI489777)SNP_26904	479	T	C	5	7	--	--
CakTC41796	Ca(ICCV2/PI489777)SNP_26905	1251	T	C	20	5	--	--
	Ca(ICCV2/PI489777)SNP_26906	1725	A	G	9	4	--	--
	Ca(ICCV2/PI489777)SNP_26907	1827	G	A	15	3	--	--
	Ca(ICCV2/PI489777)SNP_26908	2069	G	A	4	3	--	--
	Ca(ICCV2/PI489777)SNP_26909	2072	C	G	6	3	--	--
	Ca(ICCV2/PI489777)SNP_26910	2086	C	T	6	3	--	--
CakTC28695	Ca(ICCV2/PI489777)SNP_26911	2720	A	G	6	3	--	--
CakTC39322	Ca(ICCV2/PI489777)SNP_26912	623	T	C	10	5	--	Tify
CakTC39108	Ca(ICCV2/PI489777)SNP_26913	326	C	T	27	20	--	--
	Ca(ICCV2/PI489777)SNP_26914	1118	C	T	19	9	--	--
	Ca(ICCV2/PI489777)SNP_26915	1239	A	G	23	10	--	--
CakTC39101	Ca(ICCV2/PI489777)SNP_26916	450	G	A	14	9	--	--
	Ca(ICCV2/PI489777)SNP_26917	867	T	C	29	24	--	--
	Ca(ICCV2/PI489777)SNP_26918	1005	G	A	24	27	--	--
	Ca(ICCV2/PI489777)SNP_26919	1008	G	A	27	24	--	--
	Ca(ICCV2/PI489777)SNP_26920	1083	T	C	26	22	--	--
	Ca(ICCV2/PI489777)SNP_26921	1167	G	A	24	22	--	--
	Ca(ICCV2/PI489777)SNP_26922	1201	A	G	21	21	--	--
CakTC26721	Ca(ICCV2/PI489777)SNP_26923	1408	C	T	12	5	--	--
	Ca(ICCV2/PI489777)SNP_26924	2856	C	T	13	3	--	--
CakTC39953	Ca(ICCV2/PI489777)SNP_26925	368	C	G	9	4	--	--
	Ca(ICCV2/PI489777)SNP_26926	579	G	A	11	4	--	--
CakTC28059	Ca(ICCV2/PI489777)SNP_26927	637	G	A	19	24	--	--
	Ca(ICCV2/PI489777)SNP_26928	1751	G	A	13	8	--	--
	Ca(ICCV2/PI489777)SNP_26929	1967	G	A	6	6	--	--
CakTC36426	Ca(ICCV2/PI489777)SNP_26930	197	A	G	15	4	--	--
	Ca(ICCV2/PI489777)SNP_26931	203	T	C	17	4	--	--

	Ca(ICCV2/PI489777)SNP_26932	209	G	T	18	4	--	--
CakTC39941	Ca(ICCV2/PI489777)SNP_26933	275	G	A	9	4	--	--
	Ca(ICCV2/PI489777)SNP_26934	536	C	T	6	4	--	--
	Ca(ICCV2/PI489777)SNP_26935	578	G	T	6	3	--	--
	Ca(ICCV2/PI489777)SNP_26936	584	G	A	6	4	--	--
CakTC17801	Ca(ICCV2/PI489777)SNP_26937	311	T	C	3	3	--	--
CakTC09296	Ca(ICCV2/PI489777)SNP_26938	797	T	C	3	3	--	--
CakTC28796	Ca(ICCV2/PI489777)SNP_26939	669	A	G	7	10	--	--
	Ca(ICCV2/PI489777)SNP_26940	758	A	G	8	7	--	--
	Ca(ICCV2/PI489777)SNP_26941	859	T	C	9	6	--	--
	Ca(ICCV2/PI489777)SNP_26942	1156	G	A	6	3	--	--
CakTC40208	Ca(ICCV2/PI489777)SNP_26943	190	T	A	8	3	--	--
	Ca(ICCV2/PI489777)SNP_26944	542	T	C	11	6	--	--
	Ca(ICCV2/PI489777)SNP_26945	545	G	C	11	8	--	--
	Ca(ICCV2/PI489777)SNP_26946	605	T	C	9	8	--	--
CakTC34305	Ca(ICCV2/PI489777)SNP_26947	545	A	C	9	7	--	--
CakTC38519	Ca(ICCV2/PI489777)SNP_26948	991	T	G	10	6	--	--
	Ca(ICCV2/PI489777)SNP_26949	1092	T	C	16	7	--	--
	Ca(ICCV2/PI489777)SNP_26950	1596	G	C	11	4	--	--
CakTC29971	Ca(ICCV2/PI489777)SNP_26951	573	G	T	9	7	Root	--
	Ca(ICCV2/PI489777)SNP_26952	793	G	A	12	4	Root	--
	Ca(ICCV2/PI489777)SNP_26953	922	C	T	8	3	Root	--
	Ca(ICCV2/PI489777)SNP_26954	972	C	T	7	3	Root	--
	Ca(ICCV2/PI489777)SNP_26955	1438	C	G	6	6	Root	--
	Ca(ICCV2/PI489777)SNP_26956	1718	C	T	7	6	Root	--
	Ca(ICCV2/PI489777)SNP_26957	1720	T	C	7	5	Root	--
	Ca(ICCV2/PI489777)SNP_26958	1842	G	A	9	3	Root	--
	Ca(ICCV2/PI489777)SNP_26959	3520	G	A	3	3	Root	--
	Ca(ICCV2/PI489777)SNP_26960	3625	G	A	9	4	Root	--
	Ca(ICCV2/PI489777)SNP_26961	4639	T	C	5	6	Root	--
CakTC22859	Ca(ICCV2/PI489777)SNP_26962	228	A	G	3	5	--	--
	Ca(ICCV2/PI489777)SNP_26963	342	C	A	4	5	--	--
	Ca(ICCV2/PI489777)SNP_26964	377	G	C	4	5	--	--
CakTC39704	Ca(ICCV2/PI489777)SNP_26965	211	G	C	36	19	--	--
	Ca(ICCV2/PI489777)SNP_26966	308	A	T	36	24	--	--
CakTC34702	Ca(ICCV2/PI489777)SNP_26967	1160	G	A	31	9	--	--
	Ca(ICCV2/PI489777)SNP_26968	1229	A	T	33	12	--	--
	Ca(ICCV2/PI489777)SNP_26969	2747	C	T	7	6	--	--
	Ca(ICCV2/PI489777)SNP_26970	2896	C	T	12	7	--	--
	Ca(ICCV2/PI489777)SNP_26971	3139	G	A	17	7	--	--
	Ca(ICCV2/PI489777)SNP_26972	3170	T	G	13	7	--	--
	Ca(ICCV2/PI489777)SNP_26973	3397	T	G	9	7	--	--
CakTC10006	Ca(ICCV2/PI489777)SNP_26974	274	G	A	18	10	--	--
	Ca(ICCV2/PI489777)SNP_26975	380	G	A	11	9	--	--
CakTC33380	Ca(ICCV2/PI489777)SNP_26976	220	C	T	7	4	--	--
	Ca(ICCV2/PI489777)SNP_26977	236	G	A	7	4	--	--
	Ca(ICCV2/PI489777)SNP_26978	1825	G	A	16	5	--	--
	Ca(ICCV2/PI489777)SNP_26979	1908	C	A	18	5	--	--
CakTC42590	Ca(ICCV2/PI489777)SNP_26980	388	C	T	39	14	--	--
	Ca(ICCV2/PI489777)SNP_26981	728	G	A	26	11	--	--
	Ca(ICCV2/PI489777)SNP_26982	889	G	A	25	4	--	--
	Ca(ICCV2/PI489777)SNP_26983	971	C	G	23	5	--	--
	Ca(ICCV2/PI489777)SNP_26984	1062	T	G	10	4	--	--
CakTC24448	Ca(ICCV2/PI489777)SNP_26985	103	C	T	27	18	--	--
	Ca(ICCV2/PI489777)SNP_26986	133	C	T	26	18	--	--
CakTC32681	Ca(ICCV2/PI489777)SNP_26987	473	T	C	11	9	Shoot	--
CakTC37193	Ca(ICCV2/PI489777)SNP_26988	408	G	A	78	12	--	--
	Ca(ICCV2/PI489777)SNP_26989	634	C	T	60	11	--	--
CakTC30195	Ca(ICCV2/PI489777)SNP_26990	608	A	T	6	3	Shoot	--
CakTC42838	Ca(ICCV2/PI489777)SNP_26991	517	T	C	14	10	--	--
CakTC38032	Ca(ICCV2/PI489777)SNP_26992	435	G	A	12	4	--	--
CakTC13051	Ca(ICCV2/PI489777)SNP_26993	308	T	C	14	14	--	--
	Ca(ICCV2/PI489777)SNP_26994	453	C	T	18	20	--	--
	Ca(ICCV2/PI489777)SNP_26995	474	A	G	20	21	--	--
	Ca(ICCV2/PI489777)SNP_26996	1245	T	C	16	6	--	--
	Ca(ICCV2/PI489777)SNP_26997	1787	A	G	13	12	--	--
	Ca(ICCV2/PI489777)SNP_26998	2315	C	T	40	26	--	--
	Ca(ICCV2/PI489777)SNP_26999	2558	T	C	33	16	--	--
	Ca(ICCV2/PI489777)SNP_27000	2579	G	A	34	14	--	--
	Ca(ICCV2/PI489777)SNP_27001	2724	A	G	22	11	--	--
CakTC41036	Ca(ICCV2/PI489777)SNP_27002	1319	C	A	8	3	--	--
	Ca(ICCV2/PI489777)SNP_27003	1328	G	A	8	4	--	--
CakTC10815	Ca(ICCV2/PI489777)SNP_27004	311	G	A	4	4	--	--
	Ca(ICCV2/PI489777)SNP_27005	805	A	G	24	4	--	--

	Ca(ICCV2/PI489777)SNP_27006	1279	C	T	10	4	--	--
CakTC38090	Ca(ICCV2/PI489777)SNP_27007	729	G	A	22	15	--	--
CakTC22961	Ca(ICCV2/PI489777)SNP_27008	72	A	G	13	9	--	--
CakTC39999	Ca(ICCV2/PI489777)SNP_27009	787	T	G	5	4	--	--
CakTC34132	Ca(ICCV2/PI489777)SNP_27010	222	T	A	19	3	--	--
	Ca(ICCV2/PI489777)SNP_27011	231	G	A	19	3	--	--
	Ca(ICCV2/PI489777)SNP_27012	437	G	A	21	4	--	--
	Ca(ICCV2/PI489777)SNP_27013	529	G	C	16	3	--	--
	Ca(ICCV2/PI489777)SNP_27014	697	G	A	18	3	--	--
	Ca(ICCV2/PI489777)SNP_27015	705	A	G	17	3	--	--
	Ca(ICCV2/PI489777)SNP_27016	1094	G	C	29	9	--	--
	Ca(ICCV2/PI489777)SNP_27017	1110	G	C	41	9	--	--
	Ca(ICCV2/PI489777)SNP_27018	1149	C	T	44	9	--	--
	Ca(ICCV2/PI489777)SNP_27019	1271	G	T	55	10	--	--
	Ca(ICCV2/PI489777)SNP_27020	1356	A	G	59	10	--	--
	Ca(ICCV2/PI489777)SNP_27021	1422	A	G	59	8	--	--
	Ca(ICCV2/PI489777)SNP_27022	1431	C	T	60	8	--	--
CakTC28572	Ca(ICCV2/PI489777)SNP_27023	650	G	A	7	6	Root	--
	Ca(ICCV2/PI489777)SNP_27024	752	C	G	9	5	Root	--
	Ca(ICCV2/PI489777)SNP_27025	1145	G	A	5	3	Root	--
CakTC32391	Ca(ICCV2/PI489777)SNP_27026	77	C	A	3	3	--	--
CakTC37696	Ca(ICCV2/PI489777)SNP_27027	286	C	T	8	6	--	--
	Ca(ICCV2/PI489777)SNP_27028	442	A	G	7	9	--	--
	Ca(ICCV2/PI489777)SNP_27029	704	G	T	7	6	--	--
	Ca(ICCV2/PI489777)SNP_27030	1121	A	G	10	3	--	--
CakTC24365	Ca(ICCV2/PI489777)SNP_27031	877	G	A	7	4	--	--
CakTC31229	Ca(ICCV2/PI489777)SNP_27032	183	A	G	12	10	--	--
	Ca(ICCV2/PI489777)SNP_27033	456	C	A	5	9	--	--
	Ca(ICCV2/PI489777)SNP_27034	477	G	C	5	8	--	--
	Ca(ICCV2/PI489777)SNP_27035	681	A	T	6	14	--	--
	Ca(ICCV2/PI489777)SNP_27036	978	G	A	14	19	--	--
	Ca(ICCV2/PI489777)SNP_27037	984	C	G	15	20	--	--
	Ca(ICCV2/PI489777)SNP_27038	1002	T	C	15	17	--	--
CakTC33145	Ca(ICCV2/PI489777)SNP_27039	242	A	G	21	18	--	--
	Ca(ICCV2/PI489777)SNP_27040	284	C	T	23	20	--	--
	Ca(ICCV2/PI489777)SNP_27041	422	T	C	24	17	--	--
	Ca(ICCV2/PI489777)SNP_27042	626	G	A	21	16	--	--
	Ca(ICCV2/PI489777)SNP_27043	724	A	T	21	11	--	--
	Ca(ICCV2/PI489777)SNP_27044	2322	A	G	10	5	--	--
CakTC40739	Ca(ICCV2/PI489777)SNP_27045	356	T	C	35	20	--	--
	Ca(ICCV2/PI489777)SNP_27046	397	A	G	34	17	--	--
	Ca(ICCV2/PI489777)SNP_27047	406	C	T	32	19	--	--
	Ca(ICCV2/PI489777)SNP_27048	499	T	G	25	15	--	--
	Ca(ICCV2/PI489777)SNP_27049	526	G	A	30	15	--	--
	Ca(ICCV2/PI489777)SNP_27050	1051	T	C	28	31	--	--
	Ca(ICCV2/PI489777)SNP_27051	1117	T	C	22	28	--	--
	Ca(ICCV2/PI489777)SNP_27052	1342	C	T	36	29	--	--
	Ca(ICCV2/PI489777)SNP_27053	1507	C	T	25	24	--	--
	Ca(ICCV2/PI489777)SNP_27054	1555	C	A	17	25	--	--
	Ca(ICCV2/PI489777)SNP_27055	1591	C	G	16	25	--	--
	Ca(ICCV2/PI489777)SNP_27056	1610	C	T	16	21	--	--
	Ca(ICCV2/PI489777)SNP_27057	1705	T	C	9	25	--	--
	Ca(ICCV2/PI489777)SNP_27058	1802	T	C	3	11	--	--
CakTC31605	Ca(ICCV2/PI489777)SNP_27059	368	C	T	15	5	--	TCP
	Ca(ICCV2/PI489777)SNP_27060	461	C	T	19	5	--	TCP
	Ca(ICCV2/PI489777)SNP_27061	660	T	C	12	4	--	TCP
CakTC43043	Ca(ICCV2/PI489777)SNP_27062	340	A	G	8	4	--	--
	Ca(ICCV2/PI489777)SNP_27063	344	G	C	8	5	--	--
	Ca(ICCV2/PI489777)SNP_27064	697	G	A	15	7	--	--
	Ca(ICCV2/PI489777)SNP_27065	1758	G	C	3	5	--	--
	Ca(ICCV2/PI489777)SNP_27066	1782	A	G	3	9	--	--
	Ca(ICCV2/PI489777)SNP_27067	1824	G	T	3	8	--	--
	Ca(ICCV2/PI489777)SNP_27068	1848	C	G	3	7	--	--
	Ca(ICCV2/PI489777)SNP_27069	1850	G	C	3	7	--	--
CakTC23967	Ca(ICCV2/PI489777)SNP_27070	231	T	C	25	6	--	--
CakTC22988	Ca(ICCV2/PI489777)SNP_27071	701	A	T	134	154	--	--
CakTC29269	Ca(ICCV2/PI489777)SNP_27072	1531	T	C	5	3	--	--
	Ca(ICCV2/PI489777)SNP_27073	1544	C	G	6	3	--	--
	Ca(ICCV2/PI489777)SNP_27074	1555	A	G	4	3	--	--
CakTC12147	Ca(ICCV2/PI489777)SNP_27075	424	A	G	3	3	--	--
CakTC06122	Ca(ICCV2/PI489777)SNP_27076	68	G	C	13	9	--	--
CakTC40535	Ca(ICCV2/PI489777)SNP_27077	1110	G	A	14	4	--	--
	Ca(ICCV2/PI489777)SNP_27078	1806	T	C	7	9	--	--
CakTC10032	Ca(ICCV2/PI489777)SNP_27079	1402	G	A	19	8	--	--

	Ca(ICCV2/PI489777)SNP_27080	1474	A	G	18	8	--	--
	Ca(ICCV2/PI489777)SNP_27081	1735	C	T	10	4	--	--
CakTC43341	Ca(ICCV2/PI489777)SNP_27082	480	A	G	13	6	--	--
	Ca(ICCV2/PI489777)SNP_27083	578	T	C	16	5	--	--
CakTC33750	Ca(ICCV2/PI489777)SNP_27084	138	C	A	10	4	--	--
	Ca(ICCV2/PI489777)SNP_27085	462	T	C	39	27	--	--
CakTC32088	Ca(ICCV2/PI489777)SNP_27086	1455	C	T	4	5	--	--
CakTC25669	Ca(ICCV2/PI489777)SNP_27087	265	A	C	36	8	--	--
	Ca(ICCV2/PI489777)SNP_27088	267	A	C	36	9	--	--
CakTC30133	Ca(ICCV2/PI489777)SNP_27089	266	C	A	3	9	--	--
	Ca(ICCV2/PI489777)SNP_27090	320	A	G	4	8	--	--
CakTC39764	Ca(ICCV2/PI489777)SNP_27091	935	T	C	4	5	--	--
	Ca(ICCV2/PI489777)SNP_27092	1048	C	G	3	4	--	--
CakTC39194	Ca(ICCV2/PI489777)SNP_27093	280	G	A	6	5	--	--
	Ca(ICCV2/PI489777)SNP_27094	586	T	C	6	3	--	--
CakTC27148	Ca(ICCV2/PI489777)SNP_27095	265	G	C	138	31	--	MYB-related
	Ca(ICCV2/PI489777)SNP_27096	1261	A	G	151	54	--	MYB-related
CakTC40246	Ca(ICCV2/PI489777)SNP_27097	103	G	A	5	6	--	--
	Ca(ICCV2/PI489777)SNP_27098	314	C	T	12	21	--	--
	Ca(ICCV2/PI489777)SNP_27099	464	T	A	16	17	--	--
	Ca(ICCV2/PI489777)SNP_27100	728	A	G	13	13	--	--
	Ca(ICCV2/PI489777)SNP_27101	1066	G	A	18	14	--	--
	Ca(ICCV2/PI489777)SNP_27102	1125	T	G	21	9	--	--
	Ca(ICCV2/PI489777)SNP_27103	1200	G	T	18	8	--	--
CakTC39015	Ca(ICCV2/PI489777)SNP_27104	437	A	G	20	8	--	--
	Ca(ICCV2/PI489777)SNP_27105	791	T	A	6	3	--	--
CakTC40093	Ca(ICCV2/PI489777)SNP_27106	878	C	T	22	4	--	BBR/BPC
CakTC24835	Ca(ICCV2/PI489777)SNP_27107	407	G	A	3	4	Flower	bud
	Ca(ICCV2/PI489777)SNP_27108	490	C	A	7	6	Flower	bud
	Ca(ICCV2/PI489777)SNP_27109	1880	G	A	14	5	Flower	bud
	Ca(ICCV2/PI489777)SNP_27110	1882	G	A	14	5	Flower	bud
CakTC42070	Ca(ICCV2/PI489777)SNP_27111	1530	G	T	7	3	--	--
CakTC39556	Ca(ICCV2/PI489777)SNP_27112	745	T	C	8	11	--	--
	Ca(ICCV2/PI489777)SNP_27113	943	G	A	7	13	--	--
	Ca(ICCV2/PI489777)SNP_27114	1069	A	G	8	13	--	--
	Ca(ICCV2/PI489777)SNP_27115	1109	A	G	7	11	--	--
CakTC23773	Ca(ICCV2/PI489777)SNP_27116	1507	A	G	62	33	--	C3H
CakTC26621	Ca(ICCV2/PI489777)SNP_27117	320	A	C	8	5	--	--
	Ca(ICCV2/PI489777)SNP_27118	337	C	A	9	5	--	--
	Ca(ICCV2/PI489777)SNP_27119	721	A	G	8	4	--	--
	Ca(ICCV2/PI489777)SNP_27120	771	G	A	10	5	--	--
	Ca(ICCV2/PI489777)SNP_27121	925	C	T	8	4	--	--
	Ca(ICCV2/PI489777)SNP_27122	979	G	A	9	5	--	--
	Ca(ICCV2/PI489777)SNP_27123	1015	G	C	8	5	--	--
CakTC28028	Ca(ICCV2/PI489777)SNP_27124	116	G	C	26	17	--	--
	Ca(ICCV2/PI489777)SNP_27125	950	G	A	49	16	--	--
	Ca(ICCV2/PI489777)SNP_27126	2465	A	G	26	9	--	--
CakTC35722	Ca(ICCV2/PI489777)SNP_27127	87	A	G	5	5	Mature	Leaf
	Ca(ICCV2/PI489777)SNP_27128	181	A	T	7	5	Mature	Leaf
	Ca(ICCV2/PI489777)SNP_27129	247	A	C	8	7	Mature	Leaf
	Ca(ICCV2/PI489777)SNP_27130	1932	T	C	9	3	Mature	Leaf
	Ca(ICCV2/PI489777)SNP_27131	2398	A	G	9	5	Mature	Leaf
	Ca(ICCV2/PI489777)SNP_27132	3037	G	A	3	7	Mature	Leaf
	Ca(ICCV2/PI489777)SNP_27133	3040	A	G	3	8	Mature	Leaf
	Ca(ICCV2/PI489777)SNP_27134	3622	C	G	4	3	Mature	Leaf
	Ca(ICCV2/PI489777)SNP_27135	3834	C	T	3	3	Mature	Leaf
	Ca(ICCV2/PI489777)SNP_27136	3909	C	T	5	3	Mature	Leaf
CakTC38891	Ca(ICCV2/PI489777)SNP_27137	495	T	C	22	18	--	--
	Ca(ICCV2/PI489777)SNP_27138	843	C	T	15	18	--	--
CakTC30523	Ca(ICCV2/PI489777)SNP_27139	753	A	G	28	3	--	--
	Ca(ICCV2/PI489777)SNP_27140	1350	T	A	6	3	--	--
	Ca(ICCV2/PI489777)SNP_27141	1896	C	T	6	4	--	--
	Ca(ICCV2/PI489777)SNP_27142	2107	C	A	4	6	--	--
	Ca(ICCV2/PI489777)SNP_27143	2794	C	A	7	3	--	--
CakTC35858	Ca(ICCV2/PI489777)SNP_27144	725	C	A	11	3	--	--
	Ca(ICCV2/PI489777)SNP_27145	1721	G	A	8	4	--	--
	Ca(ICCV2/PI489777)SNP_27146	1993	G	A	6	4	--	--
	Ca(ICCV2/PI489777)SNP_27147	2414	T	A	12	4	--	--
CakTC38641	Ca(ICCV2/PI489777)SNP_27148	232	A	G	8	3	--	--
	Ca(ICCV2/PI489777)SNP_27149	827	A	G	3	3	--	--
	Ca(ICCV2/PI489777)SNP_27150	828	A	G	3	3	--	--
CakTC09653	Ca(ICCV2/PI489777)SNP_27151	2380	G	A	16	9	--	--
CakTC40725	Ca(ICCV2/PI489777)SNP_27152	1641	G	A	8	11	--	--
CakTC40004	Ca(ICCV2/PI489777)SNP_27153	627	T	C	5	10	--	HB

	Ca(ICCV2/PI489777)SNP_27154	742	A	C	4	34	--	HB
	Ca(ICCV2/PI489777)SNP_27155	825	G	A	3	14	--	HB
	Ca(ICCV2/PI489777)SNP_27156	856	T	C	4	10	--	HB
CakTC38636	Ca(ICCV2/PI489777)SNP_27157	497	C	G	9	4	--	--
CakTC33897	Ca(ICCV2/PI489777)SNP_27158	157	C	G	26	12	Root	--
	Ca(ICCV2/PI489777)SNP_27159	159	T	A	26	13	Root	--
	Ca(ICCV2/PI489777)SNP_27160	685	A	T	3	10	Root	--
	Ca(ICCV2/PI489777)SNP_27161	725	A	C	3	10	Root	--
	Ca(ICCV2/PI489777)SNP_27162	770	G	A	3	6	Root	--
	Ca(ICCV2/PI489777)SNP_27163	802	A	T	3	5	Root	--
	Ca(ICCV2/PI489777)SNP_27164	804	G	C	3	7	Root	--
CakTC39126	Ca(ICCV2/PI489777)SNP_27165	515	T	C	6	5	--	--
CakTC42199	Ca(ICCV2/PI489777)SNP_27166	510	A	G	3	7	--	--
	Ca(ICCV2/PI489777)SNP_27167	1152	A	G	15	16	--	--
	Ca(ICCV2/PI489777)SNP_27168	1245	G	C	17	15	--	--
	Ca(ICCV2/PI489777)SNP_27169	1402	A	G	10	14	--	--
	Ca(ICCV2/PI489777)SNP_27170	1404	C	T	11	14	--	--
	Ca(ICCV2/PI489777)SNP_27171	1473	G	A	7	14	--	--
CakTC38903	Ca(ICCV2/PI489777)SNP_27172	441	C	T	10	10	--	--
	Ca(ICCV2/PI489777)SNP_27173	649	A	G	8	7	--	--
	Ca(ICCV2/PI489777)SNP_27174	841	A	C	5	4	--	--
	Ca(ICCV2/PI489777)SNP_27175	947	A	G	5	6	--	--
	Ca(ICCV2/PI489777)SNP_27176	1041	A	G	6	4	--	--
	Ca(ICCV2/PI489777)SNP_27177	1197	G	A	7	4	--	--
	Ca(ICCV2/PI489777)SNP_27178	1200	T	G	7	4	--	--
CakTC25591	Ca(ICCV2/PI489777)SNP_27179	930	T	G	36	19	--	--
CakTC36929	Ca(ICCV2/PI489777)SNP_27180	940	G	T	6	8	--	--
	Ca(ICCV2/PI489777)SNP_27181	981	A	G	6	9	--	--
	Ca(ICCV2/PI489777)SNP_27182	1418	C	T	9	9	--	--
	Ca(ICCV2/PI489777)SNP_27183	1653	C	G	6	7	--	--
	Ca(ICCV2/PI489777)SNP_27184	2006	C	T	8	6	--	--
	Ca(ICCV2/PI489777)SNP_27185	2157	T	C	12	4	--	--
	Ca(ICCV2/PI489777)SNP_27186	2240	T	C	8	4	--	--
	Ca(ICCV2/PI489777)SNP_27187	3431	T	C	17	9	--	--
	Ca(ICCV2/PI489777)SNP_27188	4106	T	G	11	5	--	--
CakTC04399	Ca(ICCV2/PI489777)SNP_27189	135	G	C	22	22	--	--
	Ca(ICCV2/PI489777)SNP_27190	161	C	G	28	23	--	--
	Ca(ICCV2/PI489777)SNP_27191	162	A	T	26	24	--	--
	Ca(ICCV2/PI489777)SNP_27192	837	A	G	26	6	--	--
	Ca(ICCV2/PI489777)SNP_27193	867	C	T	23	6	--	--
CakTC29463	Ca(ICCV2/PI489777)SNP_27194	53	A	G	5	5	--	--
	Ca(ICCV2/PI489777)SNP_27195	95	C	T	10	5	--	--
	Ca(ICCV2/PI489777)SNP_27196	601	T	C	19	16	--	--
	Ca(ICCV2/PI489777)SNP_27197	640	T	G	25	14	--	--
	Ca(ICCV2/PI489777)SNP_27198	678	A	C	20	14	--	--
CakTC26967	Ca(ICCV2/PI489777)SNP_27199	1357	C	T	33	26	--	--
CakTC42635	Ca(ICCV2/PI489777)SNP_27200	231	T	C	3	5	--	--
	Ca(ICCV2/PI489777)SNP_27201	887	A	G	5	3	--	--
	Ca(ICCV2/PI489777)SNP_27202	1184	A	G	17	13	--	--
	Ca(ICCV2/PI489777)SNP_27203	1679	A	G	6	8	--	--
	Ca(ICCV2/PI489777)SNP_27204	1694	C	T	6	6	--	--
	Ca(ICCV2/PI489777)SNP_27205	1701	A	G	6	5	--	--
CakTC17073	Ca(ICCV2/PI489777)SNP_27206	278	G	C	7	3	--	--
CakTC07851	Ca(ICCV2/PI489777)SNP_27207	897	C	T	3	4	--	--
	Ca(ICCV2/PI489777)SNP_27208	912	T	C	5	4	--	--
	Ca(ICCV2/PI489777)SNP_27209	1113	A	G	3	4	--	--
	Ca(ICCV2/PI489777)SNP_27210	1260	A	G	5	4	--	--
	Ca(ICCV2/PI489777)SNP_27211	1386	T	C	3	3	--	--
	Ca(ICCV2/PI489777)SNP_27212	1458	A	G	3	3	--	--
CakTC28367	Ca(ICCV2/PI489777)SNP_27213	424	T	C	5	5	--	--
CakTC37833	Ca(ICCV2/PI489777)SNP_27214	1026	C	T	16	23	--	--
	Ca(ICCV2/PI489777)SNP_27215	1386	T	C	8	3	--	--
CakTC41119	Ca(ICCV2/PI489777)SNP_27216	54	C	T	7	8	--	--
	Ca(ICCV2/PI489777)SNP_27217	87	T	C	19	10	--	--
	Ca(ICCV2/PI489777)SNP_27218	198	A	G	24	13	--	--
	Ca(ICCV2/PI489777)SNP_27219	434	C	T	22	16	--	--
	Ca(ICCV2/PI489777)SNP_27220	710	C	T	13	5	--	--
	Ca(ICCV2/PI489777)SNP_27221	974	C	T	29	14	--	--
	Ca(ICCV2/PI489777)SNP_27222	1013	C	T	32	16	--	--
	Ca(ICCV2/PI489777)SNP_27223	1052	A	T	38	15	--	--
	Ca(ICCV2/PI489777)SNP_27224	1336	C	T	32	19	--	--
	Ca(ICCV2/PI489777)SNP_27225	1643	T	C	39	21	--	--
CakTC28074	Ca(ICCV2/PI489777)SNP_27226	228	T	G	12	4	--	--
	Ca(ICCV2/PI489777)SNP_27227	263	A	T	18	8	--	--

	Ca(ICCV2/PI489777)SNP_27228	392	T	A	22	9	--	--
	Ca(ICCV2/PI489777)SNP_27229	512	T	C	17	10	--	--
	Ca(ICCV2/PI489777)SNP_27230	632	A	G	14	8	--	--
	Ca(ICCV2/PI489777)SNP_27231	890	T	C	16	9	--	--
	Ca(ICCV2/PI489777)SNP_27232	1097	A	T	21	11	--	--
	Ca(ICCV2/PI489777)SNP_27233	1254	C	A	15	6	--	--
	Ca(ICCV2/PI489777)SNP_27234	1362	T	A	11	5	--	--
CakTC32813	Ca(ICCV2/PI489777)SNP_27235	543	A	T	3	3	--	--
	Ca(ICCV2/PI489777)SNP_27236	1168	A	C	9	3	--	--
	Ca(ICCV2/PI489777)SNP_27237	1181	T	C	8	3	--	--
	Ca(ICCV2/PI489777)SNP_27238	1339	C	T	7	3	--	--
CakTC37635	Ca(ICCV2/PI489777)SNP_27239	322	T	A	19	3	--	--
CakTC37211	Ca(ICCV2/PI489777)SNP_27240	357	T	G	19	11	--	--
	Ca(ICCV2/PI489777)SNP_27241	366	C	T	19	10	--	--
	Ca(ICCV2/PI489777)SNP_27242	654	A	G	37	19	--	--
CakTC37804	Ca(ICCV2/PI489777)SNP_27243	332	C	T	5	4	--	--
CakTC23843	Ca(ICCV2/PI489777)SNP_27244	315	C	T	4	4	--	DBP
	Ca(ICCV2/PI489777)SNP_27245	347	T	G	5	3	--	DBP
	Ca(ICCV2/PI489777)SNP_27246	558	T	C	24	17	--	DBP
CakTC28270	Ca(ICCV2/PI489777)SNP_27247	378	G	A	7	10	--	WRKY
	Ca(ICCV2/PI489777)SNP_27248	576	T	C	7	7	--	WRKY
	Ca(ICCV2/PI489777)SNP_27249	1667	G	C	15	3	--	WRKY
	Ca(ICCV2/PI489777)SNP_27250	1891	C	T	7	3	--	WRKY
CakTC38428	Ca(ICCV2/PI489777)SNP_27251	282	G	T	3	3	--	--
CakTC37687	Ca(ICCV2/PI489777)SNP_27252	691	G	A	49	44	--	--
	Ca(ICCV2/PI489777)SNP_27253	858	A	G	53	42	--	--
	Ca(ICCV2/PI489777)SNP_27254	1069	G	T	25	21	--	--
	Ca(ICCV2/PI489777)SNP_27255	1171	C	T	15	13	--	--
CakTC22511	Ca(ICCV2/PI489777)SNP_27256	1541	T	C	92	36	Root	--
	Ca(ICCV2/PI489777)SNP_27257	1747	T	C	85	54	Root	--
	Ca(ICCV2/PI489777)SNP_27258	1790	A	G	64	49	Root	--
	Ca(ICCV2/PI489777)SNP_27259	1928	A	T	62	64	Root	--
CakTC09985	Ca(ICCV2/PI489777)SNP_27260	1373	G	A	48	17	--	--
	Ca(ICCV2/PI489777)SNP_27261	1663	G	A	23	15	--	--
CakTC21048	Ca(ICCV2/PI489777)SNP_27262	768	T	C	5	3	--	--
	Ca(ICCV2/PI489777)SNP_27263	849	A	T	7	3	--	--
	Ca(ICCV2/PI489777)SNP_27264	1378	C	T	3	3	--	--
CakTC26784	Ca(ICCV2/PI489777)SNP_27265	748	T	G	79	83	--	--
CakTC11079	Ca(ICCV2/PI489777)SNP_27266	247	C	T	4	9	--	--
	Ca(ICCV2/PI489777)SNP_27267	301	T	C	4	9	--	--
CakTC25871	Ca(ICCV2/PI489777)SNP_27268	222	C	T	5	5	--	--
CakTC30091	Ca(ICCV2/PI489777)SNP_27269	566	A	G	5	4	--	zf-HD
	Ca(ICCV2/PI489777)SNP_27270	703	C	T	4	4	--	zf-HD
	Ca(ICCV2/PI489777)SNP_27271	800	C	G	4	3	--	zf-HD
CakTC42077	Ca(ICCV2/PI489777)SNP_27272	360	T	C	5	5	--	--
	Ca(ICCV2/PI489777)SNP_27273	533	A	G	11	12	--	--
	Ca(ICCV2/PI489777)SNP_27274	971	G	C	16	11	--	--
CakTC28315	Ca(ICCV2/PI489777)SNP_27275	255	A	G	5	5	--	--
	Ca(ICCV2/PI489777)SNP_27276	651	T	C	23	9	--	--
	Ca(ICCV2/PI489777)SNP_27277	1069	A	G	16	11	--	--
	Ca(ICCV2/PI489777)SNP_27278	1125	T	A	9	9	--	--
	Ca(ICCV2/PI489777)SNP_27279	1148	A	G	14	11	--	--
	Ca(ICCV2/PI489777)SNP_27280	1255	T	C	16	8	--	--
	Ca(ICCV2/PI489777)SNP_27281	1470	C	A	11	6	--	--
	Ca(ICCV2/PI489777)SNP_27282	1489	A	G	12	6	--	--
	Ca(ICCV2/PI489777)SNP_27283	1772	C	T	11	4	--	--
CakTC40933	Ca(ICCV2/PI489777)SNP_27284	67	T	G	6	8	--	--
	Ca(ICCV2/PI489777)SNP_27285	91	T	C	8	9	--	--
	Ca(ICCV2/PI489777)SNP_27286	702	A	C	77	91	--	--
CakTC24646	Ca(ICCV2/PI489777)SNP_27287	573	T	C	61	32	--	HB
	Ca(ICCV2/PI489777)SNP_27288	1356	G	T	42	36	--	HB
	Ca(ICCV2/PI489777)SNP_27289	1513	G	T	23	7	--	HB
	Ca(ICCV2/PI489777)SNP_27290	1531	G	A	19	9	--	HB
CakTC32348	Ca(ICCV2/PI489777)SNP_27291	1049	A	C	9	6	--	--
	Ca(ICCV2/PI489777)SNP_27292	2050	T	C	8	5	--	--
CakTC41583	Ca(ICCV2/PI489777)SNP_27293	251	C	G	7	4	--	--
	Ca(ICCV2/PI489777)SNP_27294	471	A	G	4	5	--	--
	Ca(ICCV2/PI489777)SNP_27295	483	A	G	3	6	--	--
	Ca(ICCV2/PI489777)SNP_27296	747	C	T	12	4	--	--
	Ca(ICCV2/PI489777)SNP_27297	777	C	T	12	4	--	--
	Ca(ICCV2/PI489777)SNP_27298	1155	C	T	6	3	--	--
	Ca(ICCV2/PI489777)SNP_27299	1288	A	G	8	7	--	--
	Ca(ICCV2/PI489777)SNP_27300	1519	A	T	5	5	--	--
	Ca(ICCV2/PI489777)SNP_27301	1528	G	T	6	6	--	--

CakTC40335	Ca(ICCV2/PI489777)SNP_27302	199	T	G	44	13	--	TPR
	Ca(ICCV2/PI489777)SNP_27303	201	T	C	40	13	--	TPR
	Ca(ICCV2/PI489777)SNP_27304	641	G	C	12	9	--	TPR
CakTC38523	Ca(ICCV2/PI489777)SNP_27305	459	G	A	27	14	--	--
CakTC24764	Ca(ICCV2/PI489777)SNP_27306	407	G	A	24	4	--	--
	Ca(ICCV2/PI489777)SNP_27307	517	C	T	29	10	--	--
	Ca(ICCV2/PI489777)SNP_27308	654	C	T	34	12	--	--
CakTC24592	Ca(ICCV2/PI489777)SNP_27309	730	T	G	20	7	--	--
	Ca(ICCV2/PI489777)SNP_27310	831	A	T	16	6	--	--
	Ca(ICCV2/PI489777)SNP_27311	879	C	A	13	7	--	--
	Ca(ICCV2/PI489777)SNP_27312	953	G	A	11	5	--	--
CakTC08814	Ca(ICCV2/PI489777)SNP_27313	675	C	T	3	3	--	--
CakTC24219	Ca(ICCV2/PI489777)SNP_27314	593	T	C	16	3	--	--
CakTC37412	Ca(ICCV2/PI489777)SNP_27315	190	A	T	8	5	--	--
	Ca(ICCV2/PI489777)SNP_27316	305	A	G	10	8	--	--
	Ca(ICCV2/PI489777)SNP_27317	470	G	A	6	6	--	--
CakTC41742	Ca(ICCV2/PI489777)SNP_27318	1397	A	G	16	14	--	--
	Ca(ICCV2/PI489777)SNP_27319	1617	A	C	3	9	--	--
	Ca(ICCV2/PI489777)SNP_27320	1895	T	A	4	12	--	--
	Ca(ICCV2/PI489777)SNP_27321	1991	A	G	4	10	--	--
	Ca(ICCV2/PI489777)SNP_27322	2063	T	C	6	14	--	--
CakTC29038	Ca(ICCV2/PI489777)SNP_27323	413	G	A	3	6	Flower	bud
CakTC41232	Ca(ICCV2/PI489777)SNP_27324	449	C	G	3	3	--	HB
	Ca(ICCV2/PI489777)SNP_27325	858	A	G	8	6	--	HB
	Ca(ICCV2/PI489777)SNP_27326	887	G	A	9	7	--	HB
	Ca(ICCV2/PI489777)SNP_27327	896	A	G	9	7	--	HB
	Ca(ICCV2/PI489777)SNP_27328	899	G	A	9	7	--	HB
CakTC28108	Ca(ICCV2/PI489777)SNP_27329	212	A	C	31	13	--	--
	Ca(ICCV2/PI489777)SNP_27330	226	A	G	33	15	--	--
	Ca(ICCV2/PI489777)SNP_27331	518	T	C	22	16	--	--
	Ca(ICCV2/PI489777)SNP_27332	1539	T	C	38	8	--	--
	Ca(ICCV2/PI489777)SNP_27333	1586	G	A	50	6	--	--
CakTC41110	Ca(ICCV2/PI489777)SNP_27334	375	A	G	5	13	--	AP2-EREBP
	Ca(ICCV2/PI489777)SNP_27335	392	A	G	5	13	--	AP2-EREBP
	Ca(ICCV2/PI489777)SNP_27336	429	T	C	4	12	--	AP2-EREBP
	Ca(ICCV2/PI489777)SNP_27337	539	T	C	3	12	--	AP2-EREBP
CakTC42386	Ca(ICCV2/PI489777)SNP_27338	63	C	T	45	55	--	--
	Ca(ICCV2/PI489777)SNP_27339	85	C	G	76	68	--	--
	Ca(ICCV2/PI489777)SNP_27340	136	G	A	78	70	--	--
	Ca(ICCV2/PI489777)SNP_27341	728	G	A	10	14	--	--
CakTC27108	Ca(ICCV2/PI489777)SNP_27342	465	C	A	6	4	--	--
	Ca(ICCV2/PI489777)SNP_27343	633	A	G	7	6	--	--
	Ca(ICCV2/PI489777)SNP_27344	713	T	C	8	5	--	--
	Ca(ICCV2/PI489777)SNP_27345	1011	A	G	8	3	--	--
CakTC42748	Ca(ICCV2/PI489777)SNP_27346	530	A	G	4	4	--	--
	Ca(ICCV2/PI489777)SNP_27347	535	T	A	3	5	--	--
	Ca(ICCV2/PI489777)SNP_27348	635	A	T	4	3	--	--
CakTC02338	Ca(ICCV2/PI489777)SNP_27349	1008	G	A	7	11	--	--
CakTC24597	Ca(ICCV2/PI489777)SNP_27350	260	T	G	11	6	Root	--
CakTC25649	Ca(ICCV2/PI489777)SNP_27351	602	T	G	4	5	Flower	bud
	Ca(ICCV2/PI489777)SNP_27352	610	C	T	4	5	Flower	bud
	Ca(ICCV2/PI489777)SNP_27353	652	G	A	4	6	Flower	bud
CakTC37339	Ca(ICCV2/PI489777)SNP_27354	45	C	G	36	19	--	--
	Ca(ICCV2/PI489777)SNP_27355	127	C	G	47	28	--	--
	Ca(ICCV2/PI489777)SNP_27356	143	C	T	46	28	--	--
	Ca(ICCV2/PI489777)SNP_27357	302	C	T	56	38	--	--
	Ca(ICCV2/PI489777)SNP_27358	407	G	C	43	40	--	--
	Ca(ICCV2/PI489777)SNP_27359	416	T	C	54	37	--	--
	Ca(ICCV2/PI489777)SNP_27360	524	C	T	48	29	--	--
	Ca(ICCV2/PI489777)SNP_27361	788	T	C	45	17	--	--
	Ca(ICCV2/PI489777)SNP_27362	983	C	T	22	8	--	--
	Ca(ICCV2/PI489777)SNP_27363	1040	G	A	17	4	--	--
CakTC31659	Ca(ICCV2/PI489777)SNP_27364	1512	G	T	4	6	--	--
	Ca(ICCV2/PI489777)SNP_27365	2308	A	G	3	4	--	--
CakTC36577	Ca(ICCV2/PI489777)SNP_27366	273	A	G	8	6	--	--
	Ca(ICCV2/PI489777)SNP_27367	295	G	C	8	6	--	--
	Ca(ICCV2/PI489777)SNP_27368	316	C	G	6	6	--	--
CakTC23505	Ca(ICCV2/PI489777)SNP_27369	597	A	G	18	7	--	--
	Ca(ICCV2/PI489777)SNP_27370	618	G	T	16	5	--	--
	Ca(ICCV2/PI489777)SNP_27371	695	G	A	10	10	--	--
	Ca(ICCV2/PI489777)SNP_27372	854	A	G	16	8	--	--
CakTC11732	Ca(ICCV2/PI489777)SNP_27373	280	G	C	6	6	--	--
	Ca(ICCV2/PI489777)SNP_27374	354	T	C	4	4	--	--
	Ca(ICCV2/PI489777)SNP_27375	513	T	A	4	6	--	--

	Ca(ICCV2/PI489777)SNP_27376	948	C	G	20	9	--	--
	Ca(ICCV2/PI489777)SNP_27377	1413	A	G	6	3	--	--
	Ca(ICCV2/PI489777)SNP_27378	1725	C	A	4	4	--	--
CakTC36878	Ca(ICCV2/PI489777)SNP_27379	253	C	T	14	10	--	--
CakTC31284	Ca(ICCV2/PI489777)SNP_27380	820	T	C	21	19	--	--
	Ca(ICCV2/PI489777)SNP_27381	883	G	A	20	18	--	--
	Ca(ICCV2/PI489777)SNP_27382	966	T	C	14	11	--	--
CakTC39043	Ca(ICCV2/PI489777)SNP_27383	621	A	G	6	8	--	--
	Ca(ICCV2/PI489777)SNP_27384	720	T	C	6	8	--	--
	Ca(ICCV2/PI489777)SNP_27385	922	G	T	4	7	--	--
CakTC24999	Ca(ICCV2/PI489777)SNP_27386	570	T	C	214	26	--	--
	Ca(ICCV2/PI489777)SNP_27387	921	C	T	207	81	--	--
	Ca(ICCV2/PI489777)SNP_27388	996	G	A	106	91	--	--
CakTC27364	Ca(ICCV2/PI489777)SNP_27389	859	T	C	10	4	--	--
CakTC37064	Ca(ICCV2/PI489777)SNP_27390	2035	C	A	3	3	--	--
CakTC42960	Ca(ICCV2/PI489777)SNP_27391	198	T	C	18	30	--	--
	Ca(ICCV2/PI489777)SNP_27392	215	C	G	22	30	--	--
	Ca(ICCV2/PI489777)SNP_27393	381	C	G	26	41	--	--
	Ca(ICCV2/PI489777)SNP_27394	433	T	G	26	42	--	--
	Ca(ICCV2/PI489777)SNP_27395	486	T	C	22	46	--	--
	Ca(ICCV2/PI489777)SNP_27396	529	C	G	24	41	--	--
	Ca(ICCV2/PI489777)SNP_27397	567	C	T	17	39	--	--
	Ca(ICCV2/PI489777)SNP_27398	646	C	G	14	21	--	--
	Ca(ICCV2/PI489777)SNP_27399	696	C	G	10	17	--	--
	Ca(ICCV2/PI489777)SNP_27400	807	G	A	7	5	--	--
CakTC42485	Ca(ICCV2/PI489777)SNP_27401	54	T	C	9	5	--	--
	Ca(ICCV2/PI489777)SNP_27402	174	T	C	9	7	--	--
	Ca(ICCV2/PI489777)SNP_27403	192	T	C	9	12	--	--
	Ca(ICCV2/PI489777)SNP_27404	252	C	T	9	14	--	--
	Ca(ICCV2/PI489777)SNP_27405	302	C	G	10	16	--	--
	Ca(ICCV2/PI489777)SNP_27406	324	T	C	10	16	--	--
	Ca(ICCV2/PI489777)SNP_27407	363	C	G	10	15	--	--
	Ca(ICCV2/PI489777)SNP_27408	735	C	T	6	3	--	--
	Ca(ICCV2/PI489777)SNP_27409	738	G	A	6	3	--	--
CakTC38991	Ca(ICCV2/PI489777)SNP_27410	526	C	T	36	12	--	MYB
	Ca(ICCV2/PI489777)SNP_27411	720	G	C	41	21	--	MYB
	Ca(ICCV2/PI489777)SNP_27412	774	A	C	39	21	--	MYB
CakTC10052	Ca(ICCV2/PI489777)SNP_27413	2920	T	C	12	7	--	Jumonji
CakTC33173	Ca(ICCV2/PI489777)SNP_27414	158	G	A	6	4	--	--
	Ca(ICCV2/PI489777)SNP_27415	172	A	C	6	4	--	--
	Ca(ICCV2/PI489777)SNP_27416	181	G	T	6	4	--	--
	Ca(ICCV2/PI489777)SNP_27417	183	A	T	6	4	--	--
	Ca(ICCV2/PI489777)SNP_27418	217	A	G	7	4	--	--
	Ca(ICCV2/PI489777)SNP_27419	700	C	T	31	9	--	--
CakTC03257	Ca(ICCV2/PI489777)SNP_27420	1627	G	A	3	3	--	--
CakTC42490	Ca(ICCV2/PI489777)SNP_27421	845	C	G	58	22	--	--
	Ca(ICCV2/PI489777)SNP_27422	915	G	A	55	19	--	--
	Ca(ICCV2/PI489777)SNP_27423	976	C	A	10	7	--	--
CakTC31391	Ca(ICCV2/PI489777)SNP_27424	1075	G	A	4	4	--	--
	Ca(ICCV2/PI489777)SNP_27425	1184	T	G	10	3	--	--
	Ca(ICCV2/PI489777)SNP_27426	1191	C	T	11	5	--	--
	Ca(ICCV2/PI489777)SNP_27427	1233	A	G	11	3	--	--
	Ca(ICCV2/PI489777)SNP_27428	1263	A	G	6	3	--	--
	Ca(ICCV2/PI489777)SNP_27429	2053	T	G	3	6	--	--
CakTC09901	Ca(ICCV2/PI489777)SNP_27430	475	C	T	3	3	--	--
CakTC25744	Ca(ICCV2/PI489777)SNP_27431	923	A	G	41	24	--	--
CakTC23642	Ca(ICCV2/PI489777)SNP_27432	155	C	G	13	7	--	--
	Ca(ICCV2/PI489777)SNP_27433	200	A	T	14	9	--	--
	Ca(ICCV2/PI489777)SNP_27434	202	A	G	13	9	--	--
CakTC25586	Ca(ICCV2/PI489777)SNP_27435	489	T	A	8	5	--	--
CakTC30746	Ca(ICCV2/PI489777)SNP_27436	815	A	T	5	7	--	--
	Ca(ICCV2/PI489777)SNP_27437	818	A	G	5	7	--	--
CakTC33058	Ca(ICCV2/PI489777)SNP_27438	1354	A	G	9	5	--	MYB-related
	Ca(ICCV2/PI489777)SNP_27439	2341	G	A	5	8	--	MYB-related
	Ca(ICCV2/PI489777)SNP_27440	2599	G	A	19	7	--	MYB-related
CakTC27353	Ca(ICCV2/PI489777)SNP_27441	225	C	G	12	7	--	--
	Ca(ICCV2/PI489777)SNP_27442	394	A	G	16	10	--	--
	Ca(ICCV2/PI489777)SNP_27443	439	G	A	18	10	--	--
	Ca(ICCV2/PI489777)SNP_27444	838	A	G	20	14	--	--
	Ca(ICCV2/PI489777)SNP_27445	1159	A	G	6	12	--	--
	Ca(ICCV2/PI489777)SNP_27446	1252	T	C	6	16	--	--
	Ca(ICCV2/PI489777)SNP_27447	1270	C	A	3	14	--	--
CakTC25289	Ca(ICCV2/PI489777)SNP_27448	598	G	A	49	16	--	C2C2-GATA
	Ca(ICCV2/PI489777)SNP_27449	730	T	C	53	15	--	C2C2-GATA

	Ca(ICCV2/PI489777)SNP_27450	786	T	C	45	15	--	C2C2-GATA
	Ca(ICCV2/PI489777)SNP_27451	812	T	C	49	17	--	C2C2-GATA
	Ca(ICCV2/PI489777)SNP_27452	875	A	C	51	21	--	C2C2-GATA
	Ca(ICCV2/PI489777)SNP_27453	1288	A	C	38	17	--	C2C2-GATA
CakTC38402	Ca(ICCV2/PI489777)SNP_27454	573	T	C	10	4	--	--
	Ca(ICCV2/PI489777)SNP_27455	729	T	C	9	8	--	--
CakTC41636	Ca(ICCV2/PI489777)SNP_27456	227	G	C	5	8	--	--
CakTC28727	Ca(ICCV2/PI489777)SNP_27457	1687	C	A	19	6	--	--
CakTC10966	Ca(ICCV2/PI489777)SNP_27458	337	T	C	5	3	--	--
CakTC26716	Ca(ICCV2/PI489777)SNP_27459	75	A	G	22	21	--	--
	Ca(ICCV2/PI489777)SNP_27460	113	G	C	21	23	--	--
CakTC30852	Ca(ICCV2/PI489777)SNP_27461	878	T	C	5	3	--	--
CakTC25100	Ca(ICCV2/PI489777)SNP_27462	1798	T	A	98	20	--	--
CakTC28595	Ca(ICCV2/PI489777)SNP_27463	311	G	C	17	7	--	--
	Ca(ICCV2/PI489777)SNP_27464	1503	A	T	14	8	--	--
	Ca(ICCV2/PI489777)SNP_27465	1600	A	C	11	8	--	--
	Ca(ICCV2/PI489777)SNP_27466	2695	A	G	5	6	--	--
CakTC26576	Ca(ICCV2/PI489777)SNP_27467	412	T	C	46	6	--	--
	Ca(ICCV2/PI489777)SNP_27468	436	A	G	43	6	--	--
	Ca(ICCV2/PI489777)SNP_27469	502	A	T	72	8	--	--
	Ca(ICCV2/PI489777)SNP_27470	629	A	G	60	7	--	--
CakTC39176	Ca(ICCV2/PI489777)SNP_27471	546	A	T	6	4	--	--
CakTC25070	Ca(ICCV2/PI489777)SNP_27472	344	C	G	6	6	--	--
	Ca(ICCV2/PI489777)SNP_27473	454	C	T	11	10	--	--
	Ca(ICCV2/PI489777)SNP_27474	1055	T	C	23	10	--	--
	Ca(ICCV2/PI489777)SNP_27475	1192	T	A	16	7	--	--
	Ca(ICCV2/PI489777)SNP_27476	1496	C	G	24	6	--	--
CakTC32449	Ca(ICCV2/PI489777)SNP_27477	293	G	T	3	12	--	BSD
	Ca(ICCV2/PI489777)SNP_27478	2043	G	A	6	3	--	BSD
	Ca(ICCV2/PI489777)SNP_27479	2064	A	C	6	3	--	BSD
CakTC41939	Ca(ICCV2/PI489777)SNP_27480	393	G	A	15	6	--	--
	Ca(ICCV2/PI489777)SNP_27481	1084	A	C	32	6	--	--
CakTC39810	Ca(ICCV2/PI489777)SNP_27482	250	C	T	8	12	--	--
CakTC41018	Ca(ICCV2/PI489777)SNP_27483	507	C	A	61	54	--	--
	Ca(ICCV2/PI489777)SNP_27484	1164	T	C	91	56	--	--
CakTC35735	Ca(ICCV2/PI489777)SNP_27485	852	C	T	9	4	--	--
CakTC37383	Ca(ICCV2/PI489777)SNP_27486	994	G	A	10	11	--	--
CakTC22561	Ca(ICCV2/PI489777)SNP_27487	1575	A	C	4	5	--	--
CakTC09680	Ca(ICCV2/PI489777)SNP_27488	324	A	G	10	4	--	--
	Ca(ICCV2/PI489777)SNP_27489	562	A	C	11	4	--	--
	Ca(ICCV2/PI489777)SNP_27490	563	G	T	11	4	--	--
	Ca(ICCV2/PI489777)SNP_27491	612	A	C	8	3	--	--
CakTC29340	Ca(ICCV2/PI489777)SNP_27492	278	G	A	38	42	--	--
	Ca(ICCV2/PI489777)SNP_27493	416	C	T	76	39	--	--
CakTC02565	Ca(ICCV2/PI489777)SNP_27494	438	C	G	3	13	--	--
CakTC24713	Ca(ICCV2/PI489777)SNP_27495	1820	G	A	9	3	Shoot	--
CakTC42497	Ca(ICCV2/PI489777)SNP_27496	225	A	G	12	3	--	--
	Ca(ICCV2/PI489777)SNP_27497	258	G	C	20	3	--	--
	Ca(ICCV2/PI489777)SNP_27498	336	T	C	20	3	--	--
	Ca(ICCV2/PI489777)SNP_27499	1715	T	C	3	4	--	--
CakTC36647	Ca(ICCV2/PI489777)SNP_27500	1201	A	T	6	3	--	--
	Ca(ICCV2/PI489777)SNP_27501	2573	G	C	3	3	--	--
CakTC39422	Ca(ICCV2/PI489777)SNP_27502	964	A	G	13	7	--	--
	Ca(ICCV2/PI489777)SNP_27503	1197	A	G	14	5	--	--
	Ca(ICCV2/PI489777)SNP_27504	1213	G	A	13	6	--	--
CakTC23677	Ca(ICCV2/PI489777)SNP_27505	698	C	T	11	4	--	--
	Ca(ICCV2/PI489777)SNP_27506	699	A	G	11	5	--	--
CakTC25143	Ca(ICCV2/PI489777)SNP_27507	180	C	G	27	3	--	--
	Ca(ICCV2/PI489777)SNP_27508	189	G	C	31	3	--	--
	Ca(ICCV2/PI489777)SNP_27509	281	C	G	33	4	--	--
	Ca(ICCV2/PI489777)SNP_27510	467	G	A	19	3	--	--
	Ca(ICCV2/PI489777)SNP_27511	604	T	C	11	3	--	--
CakTC34271	Ca(ICCV2/PI489777)SNP_27512	594	G	C	6	7	--	--
	Ca(ICCV2/PI489777)SNP_27513	1074	A	G	10	8	--	--
	Ca(ICCV2/PI489777)SNP_27514	1484	T	A	6	11	--	--
	Ca(ICCV2/PI489777)SNP_27515	2131	T	G	3	3	--	--
	Ca(ICCV2/PI489777)SNP_27516	2175	G	A	3	4	--	--
CakTC27055	Ca(ICCV2/PI489777)SNP_27517	920	T	C	102	35	--	NAC
CakTC33278	Ca(ICCV2/PI489777)SNP_27518	183	T	C	5	4	Root	--
	Ca(ICCV2/PI489777)SNP_27519	360	G	A	6	6	Root	--
	Ca(ICCV2/PI489777)SNP_27520	417	T	C	8	6	Root	--
	Ca(ICCV2/PI489777)SNP_27521	498	G	T	8	6	Root	--
CakTC28115	Ca(ICCV2/PI489777)SNP_27522	130	T	C	5	7	--	--
	Ca(ICCV2/PI489777)SNP_27523	375	T	C	12	12	--	--

	Ca(ICCV2/PI489777)SNP_27524	400	T	C	12	13	--	--
CakTC37927	Ca(ICCV2/PI489777)SNP_27525	262	C	T	17	9	--	--
	Ca(ICCV2/PI489777)SNP_27526	445	C	A	14	9	--	--
CakTC39300	Ca(ICCV2/PI489777)SNP_27527	258	T	C	19	7	--	Tify
	Ca(ICCV2/PI489777)SNP_27528	374	T	A	25	9	--	Tify
	Ca(ICCV2/PI489777)SNP_27529	444	T	C	27	11	--	Tify
	Ca(ICCV2/PI489777)SNP_27530	501	G	T	18	14	--	Tify
	Ca(ICCV2/PI489777)SNP_27531	675	A	G	16	12	--	Tify
CakTC35910	Ca(ICCV2/PI489777)SNP_27532	160	C	T	12	6	--	--
	Ca(ICCV2/PI489777)SNP_27533	748	G	C	22	12	--	--
	Ca(ICCV2/PI489777)SNP_27534	858	G	C	25	16	--	--
	Ca(ICCV2/PI489777)SNP_27535	947	A	G	20	15	--	--
	Ca(ICCV2/PI489777)SNP_27536	1146	G	A	18	18	--	--
	Ca(ICCV2/PI489777)SNP_27537	1173	C	T	19	15	--	--
	Ca(ICCV2/PI489777)SNP_27538	1254	A	G	16	14	--	--
	Ca(ICCV2/PI489777)SNP_27539	1676	C	A	18	10	--	--
	Ca(ICCV2/PI489777)SNP_27540	1677	A	G	21	10	--	--
	Ca(ICCV2/PI489777)SNP_27541	1826	A	G	19	16	--	--
	Ca(ICCV2/PI489777)SNP_27542	1828	A	T	17	16	--	--
	Ca(ICCV2/PI489777)SNP_27543	1851	A	T	16	13	--	--
	Ca(ICCV2/PI489777)SNP_27544	1928	T	C	17	13	--	--
	Ca(ICCV2/PI489777)SNP_27545	1993	A	G	18	19	--	--
	Ca(ICCV2/PI489777)SNP_27546	2020	T	C	21	17	--	--
	Ca(ICCV2/PI489777)SNP_27547	2077	T	C	16	17	--	--
	Ca(ICCV2/PI489777)SNP_27548	2117	T	A	18	17	--	--
	Ca(ICCV2/PI489777)SNP_27549	2299	A	T	21	19	--	--
	Ca(ICCV2/PI489777)SNP_27550	2349	G	A	20	10	--	--
	Ca(ICCV2/PI489777)SNP_27551	2587	A	T	47	17	--	--
	Ca(ICCV2/PI489777)SNP_27552	2764	C	T	27	11	--	--
	Ca(ICCV2/PI489777)SNP_27553	2808	G	A	21	9	--	--
CakTC17861	Ca(ICCV2/PI489777)SNP_27554	165	G	C	18	12	--	--
	Ca(ICCV2/PI489777)SNP_27555	174	G	T	18	10	--	--
	Ca(ICCV2/PI489777)SNP_27556	194	C	G	23	16	--	--
	Ca(ICCV2/PI489777)SNP_27557	205	T	C	24	16	--	--
	Ca(ICCV2/PI489777)SNP_27558	231	G	C	43	16	--	--
	Ca(ICCV2/PI489777)SNP_27559	260	T	A	37	14	--	--
	Ca(ICCV2/PI489777)SNP_27560	295	A	G	51	17	--	--
	Ca(ICCV2/PI489777)SNP_27561	313	G	A	49	17	--	--
CakTC26353	Ca(ICCV2/PI489777)SNP_27562	360	G	C	21	3	--	--
	Ca(ICCV2/PI489777)SNP_27563	690	C	G	34	5	--	--
	Ca(ICCV2/PI489777)SNP_27564	828	A	T	39	10	--	--
	Ca(ICCV2/PI489777)SNP_27565	836	G	A	39	10	--	--
	Ca(ICCV2/PI489777)SNP_27566	1113	A	G	20	4	--	--
	Ca(ICCV2/PI489777)SNP_27567	1399	T	C	37	4	--	--
	Ca(ICCV2/PI489777)SNP_27568	1443	A	G	37	4	--	--
	Ca(ICCV2/PI489777)SNP_27569	1485	A	G	36	3	--	--
	Ca(ICCV2/PI489777)SNP_27570	1978	C	T	13	6	--	--
	Ca(ICCV2/PI489777)SNP_27571	2030	C	T	16	7	--	--
	Ca(ICCV2/PI489777)SNP_27572	2057	T	C	17	7	--	--
	Ca(ICCV2/PI489777)SNP_27573	2170	T	C	16	6	--	--
	Ca(ICCV2/PI489777)SNP_27574	2178	G	A	17	7	--	--
	Ca(ICCV2/PI489777)SNP_27575	2214	T	A	19	10	--	--
CakTC39893	Ca(ICCV2/PI489777)SNP_27576	2028	T	C	4	4	--	--
	Ca(ICCV2/PI489777)SNP_27577	2277	C	T	5	3	--	--
	Ca(ICCV2/PI489777)SNP_27578	2384	A	T	7	4	--	--
	Ca(ICCV2/PI489777)SNP_27579	2487	A	G	5	4	--	--
	Ca(ICCV2/PI489777)SNP_27580	3012	A	C	3	4	--	--
	Ca(ICCV2/PI489777)SNP_27581	3066	G	C	4	3	--	--
CakTC31258	Ca(ICCV2/PI489777)SNP_27582	819	G	A	9	11	--	--
	Ca(ICCV2/PI489777)SNP_27583	852	C	T	12	9	--	--
	Ca(ICCV2/PI489777)SNP_27584	1281	T	C	17	17	--	--
CakTC29532	Ca(ICCV2/PI489777)SNP_27585	437	T	A	5	5	--	Trihelix
	Ca(ICCV2/PI489777)SNP_27586	566	A	C	3	4	--	Trihelix
	Ca(ICCV2/PI489777)SNP_27587	608	T	C	3	3	--	Trihelix
CakTC07052	Ca(ICCV2/PI489777)SNP_27588	480	C	T	3	4	--	--
	Ca(ICCV2/PI489777)SNP_27589	617	T	C	4	3	--	--
CakTC28965	Ca(ICCV2/PI489777)SNP_27590	279	A	G	10	9	--	--
CakTC27503	Ca(ICCV2/PI489777)SNP_27591	1439	T	A	11	4	--	C2C2-CO-like
CakTC26654	Ca(ICCV2/PI489777)SNP_27592	552	C	T	5	3	--	--
CakTC37422	Ca(ICCV2/PI489777)SNP_27593	353	C	T	5	4	--	--
	Ca(ICCV2/PI489777)SNP_27594	949	C	G	9	6	--	--
	Ca(ICCV2/PI489777)SNP_27595	952	C	T	9	6	--	--
CakTC35164	Ca(ICCV2/PI489777)SNP_27596	341	C	A	9	3	--	--
	Ca(ICCV2/PI489777)SNP_27597	357	C	T	9	3	--	--

	Ca(ICCV2/PI489777)SNP_27598	392	A	T	8	4	--	--
	Ca(ICCV2/PI489777)SNP_27599	394	T	C	8	4	--	--
	Ca(ICCV2/PI489777)SNP_27600	432	A	G	7	4	--	--
	Ca(ICCV2/PI489777)SNP_27601	567	A	G	11	7	--	--
	Ca(ICCV2/PI489777)SNP_27602	582	G	A	12	7	--	--
	Ca(ICCV2/PI489777)SNP_27603	627	A	C	10	7	--	--
	Ca(ICCV2/PI489777)SNP_27604	642	G	A	15	6	--	--
CakTC35913	Ca(ICCV2/PI489777)SNP_27605	1114	A	G	42	4	--	--
	Ca(ICCV2/PI489777)SNP_27606	2353	A	G	17	4	--	--
	Ca(ICCV2/PI489777)SNP_27607	2481	G	A	9	5	--	--
	Ca(ICCV2/PI489777)SNP_27608	2512	G	A	11	6	--	--
CakTC12578	Ca(ICCV2/PI489777)SNP_27609	1246	A	C	10	6	--	--
CakTC32275	Ca(ICCV2/PI489777)SNP_27610	1488	A	G	8	5	Flower	bud
CakTC34985	Ca(ICCV2/PI489777)SNP_27611	282	G	A	5	13	--	--
	Ca(ICCV2/PI489777)SNP_27612	304	C	G	4	15	--	--
CakTC42900	Ca(ICCV2/PI489777)SNP_27613	202	T	C	14	6	--	--
	Ca(ICCV2/PI489777)SNP_27614	303	C	G	21	13	--	--
	Ca(ICCV2/PI489777)SNP_27615	317	G	T	21	13	--	--
	Ca(ICCV2/PI489777)SNP_27616	1742	G	A	4	13	--	--
CakTC37458	Ca(ICCV2/PI489777)SNP_27617	922	C	T	9	3	--	--
CakTC26474	Ca(ICCV2/PI489777)SNP_27618	517	G	T	27	20	--	--
	Ca(ICCV2/PI489777)SNP_27619	586	T	C	15	20	--	--
	Ca(ICCV2/PI489777)SNP_27620	823	T	C	51	22	--	--
	Ca(ICCV2/PI489777)SNP_27621	975	T	C	51	21	--	--
	Ca(ICCV2/PI489777)SNP_27622	1000	C	T	48	20	--	--
	Ca(ICCV2/PI489777)SNP_27623	1111	A	T	38	16	--	--
	Ca(ICCV2/PI489777)SNP_27624	1112	A	G	38	16	--	--
CakTC29809	Ca(ICCV2/PI489777)SNP_27625	225	T	C	17	3	--	--
	Ca(ICCV2/PI489777)SNP_27626	1210	C	T	18	5	--	--
	Ca(ICCV2/PI489777)SNP_27627	1232	T	C	18	4	--	--
CakTC40311	Ca(ICCV2/PI489777)SNP_27628	342	T	G	41	17	--	--
	Ca(ICCV2/PI489777)SNP_27629	442	G	A	44	20	--	--
	Ca(ICCV2/PI489777)SNP_27630	656	G	C	38	18	--	--
CakTC37771	Ca(ICCV2/PI489777)SNP_27631	685	A	T	8	3	Shoot	--
	Ca(ICCV2/PI489777)SNP_27632	689	C	G	8	3	Shoot	--
	Ca(ICCV2/PI489777)SNP_27633	727	T	C	5	3	Shoot	--
	Ca(ICCV2/PI489777)SNP_27634	912	G	C	5	6	Shoot	--
CakTC17796	Ca(ICCV2/PI489777)SNP_27635	1699	G	A	26	15	--	--
CakTC00959	Ca(ICCV2/PI489777)SNP_27636	1207	C	T	3	4	--	mTERF
	Ca(ICCV2/PI489777)SNP_27637	1252	C	G	3	4	--	mTERF
	Ca(ICCV2/PI489777)SNP_27638	1261	T	C	3	3	--	mTERF
CakTC33105	Ca(ICCV2/PI489777)SNP_27639	1840	A	T	12	8	--	--
	Ca(ICCV2/PI489777)SNP_27640	2161	A	G	4	6	--	--
CakTC31787	Ca(ICCV2/PI489777)SNP_27641	577	G	C	3	3	--	GeBP
CakTC01704	Ca(ICCV2/PI489777)SNP_27642	972	C	T	7	5	--	--
CakTC40646	Ca(ICCV2/PI489777)SNP_27643	326	A	G	72	60	--	--
CakTC32058	Ca(ICCV2/PI489777)SNP_27644	204	T	C	5	28	--	--
	Ca(ICCV2/PI489777)SNP_27645	252	A	C	6	27	--	--
	Ca(ICCV2/PI489777)SNP_27646	275	C	A	7	28	--	--
CakTC30158	Ca(ICCV2/PI489777)SNP_27647	716	G	A	3	4	--	--
	Ca(ICCV2/PI489777)SNP_27648	1977	G	A	5	3	--	--
CakTC42523	Ca(ICCV2/PI489777)SNP_27649	197	T	C	16	24	--	--
	Ca(ICCV2/PI489777)SNP_27650	208	A	T	16	24	--	--
	Ca(ICCV2/PI489777)SNP_27651	220	T	C	16	21	--	--
	Ca(ICCV2/PI489777)SNP_27652	300	A	G	18	24	--	--
	Ca(ICCV2/PI489777)SNP_27653	342	T	G	20	23	--	--
	Ca(ICCV2/PI489777)SNP_27654	1319	T	C	17	32	--	--
	Ca(ICCV2/PI489777)SNP_27655	1459	G	C	16	19	--	--
	Ca(ICCV2/PI489777)SNP_27656	1525	T	C	15	19	--	--
CakTC31789	Ca(ICCV2/PI489777)SNP_27657	409	C	T	165	107	--	--
	Ca(ICCV2/PI489777)SNP_27658	493	G	C	183	102	--	--
	Ca(ICCV2/PI489777)SNP_27659	1017	G	C	18	8	--	--
	Ca(ICCV2/PI489777)SNP_27660	1058	A	G	13	7	--	--
CakTC24539	Ca(ICCV2/PI489777)SNP_27661	169	C	T	5	3	--	--
	Ca(ICCV2/PI489777)SNP_27662	217	C	T	7	3	--	--
	Ca(ICCV2/PI489777)SNP_27663	748	T	G	10	4	--	--
	Ca(ICCV2/PI489777)SNP_27664	2929	C	G	10	3	--	--
CakTC40460	Ca(ICCV2/PI489777)SNP_27665	1051	G	A	5	3	--	PHD
	Ca(ICCV2/PI489777)SNP_27666	2805	T	A	17	13	--	PHD
	Ca(ICCV2/PI489777)SNP_27667	2807	A	G	16	13	--	PHD
	Ca(ICCV2/PI489777)SNP_27668	3060	A	C	22	25	--	PHD
CakTC34907	Ca(ICCV2/PI489777)SNP_27669	945	C	T	16	9	--	SNF2
	Ca(ICCV2/PI489777)SNP_27670	3434	A	G	5	12	--	SNF2
CakTC34102	Ca(ICCV2/PI489777)SNP_27671	314	A	G	5	12	--	--

CakTC42666	Ca(ICCV2/PI489777)SNP_27672	372	C	T	7	8	--	--
CakTC14366	Ca(ICCV2/PI489777)SNP_27673	209	G	A	6	3	--	Jumonji
CakTC24210	Ca(ICCV2/PI489777)SNP_27674	77	C	T	5	3	--	--
	Ca(ICCV2/PI489777)SNP_27675	232	T	A	9	4	--	--
	Ca(ICCV2/PI489777)SNP_27676	261	G	A	7	5	--	--
	Ca(ICCV2/PI489777)SNP_27677	402	C	T	5	5	--	--
CakTC42104	Ca(ICCV2/PI489777)SNP_27678	552	T	C	5	3	--	MYB
CakTC24276	Ca(ICCV2/PI489777)SNP_27679	141	T	A	5	3	--	--
	Ca(ICCV2/PI489777)SNP_27680	174	C	A	4	3	--	--
	Ca(ICCV2/PI489777)SNP_27681	533	A	C	10	6	--	--
	Ca(ICCV2/PI489777)SNP_27682	565	G	C	10	6	--	--
	Ca(ICCV2/PI489777)SNP_27683	713	A	G	23	8	--	--
	Ca(ICCV2/PI489777)SNP_27684	801	C	T	26	11	--	--
	Ca(ICCV2/PI489777)SNP_27685	817	T	C	21	11	--	--
	Ca(ICCV2/PI489777)SNP_27686	928	A	G	49	14	--	--
	Ca(ICCV2/PI489777)SNP_27687	1114	G	A	37	28	--	--
	Ca(ICCV2/PI489777)SNP_27688	1184	T	C	34	26	--	--
	Ca(ICCV2/PI489777)SNP_27689	1701	C	A	9	13	--	--
CakTC10754	Ca(ICCV2/PI489777)SNP_27690	426	C	T	23	13	--	--
	Ca(ICCV2/PI489777)SNP_27691	846	A	G	9	9	--	--
	Ca(ICCV2/PI489777)SNP_27692	1047	A	T	7	5	--	--
	Ca(ICCV2/PI489777)SNP_27693	1107	A	C	8	9	--	--
	Ca(ICCV2/PI489777)SNP_27694	1710	T	C	3	3	--	--
CakTC37451	Ca(ICCV2/PI489777)SNP_27695	205	C	T	13	19	--	--
	Ca(ICCV2/PI489777)SNP_27696	280	C	T	19	20	--	--
	Ca(ICCV2/PI489777)SNP_27697	424	A	G	24	16	--	--
	Ca(ICCV2/PI489777)SNP_27698	595	T	C	22	24	--	--
	Ca(ICCV2/PI489777)SNP_27699	749	T	C	24	26	--	--
	Ca(ICCV2/PI489777)SNP_27700	1054	T	C	12	24	--	--
	Ca(ICCV2/PI489777)SNP_27701	1144	G	A	17	22	--	--
	Ca(ICCV2/PI489777)SNP_27702	1184	C	T	22	18	--	--
	Ca(ICCV2/PI489777)SNP_27703	1396	C	A	19	15	--	--
	Ca(ICCV2/PI489777)SNP_27704	1474	C	T	18	14	--	--
	Ca(ICCV2/PI489777)SNP_27705	1531	A	G	19	15	--	--
	Ca(ICCV2/PI489777)SNP_27706	1582	T	A	11	9	--	--
	Ca(ICCV2/PI489777)SNP_27707	1598	A	G	10	8	--	--
CakTC10677	Ca(ICCV2/PI489777)SNP_27708	71	A	C	3	7	--	--
CakTC30697	Ca(ICCV2/PI489777)SNP_27709	519	G	A	5	3	--	TPR
	Ca(ICCV2/PI489777)SNP_27710	643	T	A	7	4	--	TPR
	Ca(ICCV2/PI489777)SNP_27711	762	T	G	5	5	--	TPR
	Ca(ICCV2/PI489777)SNP_27712	763	C	T	6	5	--	TPR
	Ca(ICCV2/PI489777)SNP_27713	785	G	T	5	4	--	TPR
	Ca(ICCV2/PI489777)SNP_27714	787	T	C	5	4	--	TPR
	Ca(ICCV2/PI489777)SNP_27715	879	C	T	3	3	--	TPR
	Ca(ICCV2/PI489777)SNP_27716	901	T	C	3	3	--	TPR
CakTC29378	Ca(ICCV2/PI489777)SNP_27717	1023	A	T	3	3	--	--
CakTC28228	Ca(ICCV2/PI489777)SNP_27718	561	A	G	23	13	--	--
	Ca(ICCV2/PI489777)SNP_27719	615	G	T	14	13	--	--
CakTC26956	Ca(ICCV2/PI489777)SNP_27720	550	C	T	105	103	--	--
	Ca(ICCV2/PI489777)SNP_27721	733	C	T	13	5	--	--
CakTC25695	Ca(ICCV2/PI489777)SNP_27722	1644	T	C	9	7	--	--
	Ca(ICCV2/PI489777)SNP_27723	2055	T	G	8	7	--	--
CakTC30184	Ca(ICCV2/PI489777)SNP_27724	301	T	C	9	3	--	--
	Ca(ICCV2/PI489777)SNP_27725	732	T	A	6	6	--	--
	Ca(ICCV2/PI489777)SNP_27726	901	C	A	4	3	--	--
CakTC41209	Ca(ICCV2/PI489777)SNP_27727	619	A	C	29	11	--	--
	Ca(ICCV2/PI489777)SNP_27728	1570	A	G	19	11	--	--
CakTC23661	Ca(ICCV2/PI489777)SNP_27729	138	G	A	19	15	--	--
	Ca(ICCV2/PI489777)SNP_27730	1040	G	T	41	23	--	--
	Ca(ICCV2/PI489777)SNP_27731	2006	C	T	59	30	--	--
CakTC24248	Ca(ICCV2/PI489777)SNP_27732	232	C	T	15	9	--	--
	Ca(ICCV2/PI489777)SNP_27733	450	G	A	9	3	--	--
	Ca(ICCV2/PI489777)SNP_27734	1373	C	T	32	27	--	--
	Ca(ICCV2/PI489777)SNP_27735	1458	A	C	9	16	--	--
	Ca(ICCV2/PI489777)SNP_27736	1473	T	A	6	13	--	--
CakTC36588	Ca(ICCV2/PI489777)SNP_27737	74	C	A	5	9	--	--
	Ca(ICCV2/PI489777)SNP_27738	318	C	T	15	17	--	--
CakTC29968	Ca(ICCV2/PI489777)SNP_27739	182	C	T	5	6	--	--
	Ca(ICCV2/PI489777)SNP_27740	256	G	A	5	7	--	--
	Ca(ICCV2/PI489777)SNP_27741	617	C	A	6	7	--	--
	Ca(ICCV2/PI489777)SNP_27742	3989	G	A	13	3	--	--
	Ca(ICCV2/PI489777)SNP_27743	4414	G	C	15	4	--	--
	Ca(ICCV2/PI489777)SNP_27744	5656	G	A	4	5	--	--
	Ca(ICCV2/PI489777)SNP_27745	5665	G	A	5	4	--	--

	Ca(ICCV2/PI489777)SNP_27746	5756	G	A	6	4	--	--
	Ca(ICCV2/PI489777)SNP_27747	6385	G	A	3	3	--	--
	Ca(ICCV2/PI489777)SNP_27748	6930	G	A	6	3	--	--
CakTC42050	Ca(ICCV2/PI489777)SNP_27749	1082	A	G	5	5	Shoot	--
	Ca(ICCV2/PI489777)SNP_27750	1660	G	A	4	7	Shoot	--
CakTC34807	Ca(ICCV2/PI489777)SNP_27751	1319	T	C	6	3	--	--
	Ca(ICCV2/PI489777)SNP_27752	1558	A	G	4	3	--	--
CakTC37730	Ca(ICCV2/PI489777)SNP_27753	322	G	T	33	12	--	--
	Ca(ICCV2/PI489777)SNP_27754	444	G	T	29	12	--	--
	Ca(ICCV2/PI489777)SNP_27755	470	G	A	39	11	--	--
	Ca(ICCV2/PI489777)SNP_27756	584	T	A	28	17	--	--
CakTC41029	Ca(ICCV2/PI489777)SNP_27757	1554	C	G	74	64	--	--
CakTC31811	Ca(ICCV2/PI489777)SNP_27758	585	G	A	3	7	--	--
	Ca(ICCV2/PI489777)SNP_27759	977	A	G	7	10	--	--
CakTC33842	Ca(ICCV2/PI489777)SNP_27760	440	A	G	14	9	--	--
	Ca(ICCV2/PI489777)SNP_27761	484	G	T	8	8	--	--
CakTC42544	Ca(ICCV2/PI489777)SNP_27762	256	A	C	5	8	--	--
CakTC25228	Ca(ICCV2/PI489777)SNP_27763	120	C	T	8	12	--	--
	Ca(ICCV2/PI489777)SNP_27764	212	C	T	58	19	--	--
	Ca(ICCV2/PI489777)SNP_27765	241	A	T	82	31	--	--
	Ca(ICCV2/PI489777)SNP_27766	357	T	C	124	43	--	--
	Ca(ICCV2/PI489777)SNP_27767	520	C	T	151	37	--	--
	Ca(ICCV2/PI489777)SNP_27768	730	A	C	98	15	--	--
	Ca(ICCV2/PI489777)SNP_27769	763	C	T	96	13	--	--
	Ca(ICCV2/PI489777)SNP_27770	781	C	T	95	12	--	--
	Ca(ICCV2/PI489777)SNP_27771	793	C	A	61	10	--	--
	Ca(ICCV2/PI489777)SNP_27772	1222	G	C	28	3	--	--
CakTC35960	Ca(ICCV2/PI489777)SNP_27773	1349	C	T	24	6	--	bHLH
	Ca(ICCV2/PI489777)SNP_27774	2111	G	C	14	11	--	bHLH
CakTC28112	Ca(ICCV2/PI489777)SNP_27775	869	C	A	7	4	--	MYB
	Ca(ICCV2/PI489777)SNP_27776	1532	G	C	7	3	--	MYB
	Ca(ICCV2/PI489777)SNP_27777	1899	G	A	13	8	--	MYB
	Ca(ICCV2/PI489777)SNP_27778	1982	C	T	14	9	--	MYB
	Ca(ICCV2/PI489777)SNP_27779	2624	C	T	13	5	--	MYB
	Ca(ICCV2/PI489777)SNP_27780	2961	A	G	9	3	--	MYB
	Ca(ICCV2/PI489777)SNP_27781	3371	A	G	6	9	--	MYB
	Ca(ICCV2/PI489777)SNP_27782	3423	G	T	8	11	--	MYB
	Ca(ICCV2/PI489777)SNP_27783	3700	C	T	22	14	--	MYB
	Ca(ICCV2/PI489777)SNP_27784	4046	C	A	14	15	--	MYB
	Ca(ICCV2/PI489777)SNP_27785	4706	C	A	13	7	--	MYB
	Ca(ICCV2/PI489777)SNP_27786	5078	C	T	10	8	--	MYB
CakTC28252	Ca(ICCV2/PI489777)SNP_27787	1366	T	C	33	21	--	--
CakTC30626	Ca(ICCV2/PI489777)SNP_27788	281	C	G	9	12	--	--
	Ca(ICCV2/PI489777)SNP_27789	1035	A	G	3	5	--	--
	Ca(ICCV2/PI489777)SNP_27790	1052	C	G	3	5	--	--
CakTC30300	Ca(ICCV2/PI489777)SNP_27791	43	A	T	4	5	--	--
	Ca(ICCV2/PI489777)SNP_27792	374	T	C	12	7	--	--
	Ca(ICCV2/PI489777)SNP_27793	1445	T	C	3	3	--	--
	Ca(ICCV2/PI489777)SNP_27794	1793	G	T	6	3	--	--
CakTC39735	Ca(ICCV2/PI489777)SNP_27795	359	A	G	5	3	--	--
CakTC24624	Ca(ICCV2/PI489777)SNP_27796	4220	T	C	4	4	--	--
	Ca(ICCV2/PI489777)SNP_27797	4263	G	T	4	4	--	--
CakTC41899	Ca(ICCV2/PI489777)SNP_27798	246	G	A	9	7	--	--
	Ca(ICCV2/PI489777)SNP_27799	959	A	G	27	7	--	--
	Ca(ICCV2/PI489777)SNP_27800	995	G	A	22	8	--	--
	Ca(ICCV2/PI489777)SNP_27801	1256	C	T	34	14	--	--
CakTC41179	Ca(ICCV2/PI489777)SNP_27802	612	A	T	99	10	--	--
	Ca(ICCV2/PI489777)SNP_27803	636	A	C	72	8	--	--
CakTC11798	Ca(ICCV2/PI489777)SNP_27804	400	G	T	6	6	--	--
	Ca(ICCV2/PI489777)SNP_27805	892	C	G	3	4	--	--
	Ca(ICCV2/PI489777)SNP_27806	1054	T	G	6	3	--	--
CakTC29287	Ca(ICCV2/PI489777)SNP_27807	342	T	C	6	3	--	--
CakTC37998	Ca(ICCV2/PI489777)SNP_27808	1161	A	G	5	6	--	--
CakTC41066	Ca(ICCV2/PI489777)SNP_27809	228	G	C	19	5	--	--
CakTC38997	Ca(ICCV2/PI489777)SNP_27810	1196	T	C	10	7	--	WI/SNF-BAF60
CakTC31171	Ca(ICCV2/PI489777)SNP_27811	1363	T	G	12	6	--	--
	Ca(ICCV2/PI489777)SNP_27812	2301	C	T	3	8	--	--
CakTC33768	Ca(ICCV2/PI489777)SNP_27813	570	C	T	76	52	--	--
CakTC33861	Ca(ICCV2/PI489777)SNP_27814	325	A	G	11	11	--	--
	Ca(ICCV2/PI489777)SNP_27815	333	C	A	12	10	--	--
	Ca(ICCV2/PI489777)SNP_27816	1716	T	C	17	5	--	--
	Ca(ICCV2/PI489777)SNP_27817	2312	T	A	14	15	--	--
CakTC42267	Ca(ICCV2/PI489777)SNP_27818	594	C	G	8	15	--	--
CakTC29044	Ca(ICCV2/PI489777)SNP_27819	762	A	T	91	65	--	--

	Ca(ICCV2/PI489777)SNP_27820	885	G	A	104	94	--	--
CakTC07771	Ca(ICCV2/PI489777)SNP_27821	1213	T	C	7	5	--	--
CakTC26677	Ca(ICCV2/PI489777)SNP_27822	933	G	A	16	12	--	bZIP
CakTC39675	Ca(ICCV2/PI489777)SNP_27823	167	T	C	8	34	--	--
	Ca(ICCV2/PI489777)SNP_27824	278	C	T	19	43	--	--
[truncated: 727,674 more chars]
