# Supplementary material for: Comparative Analysis of Kabuli Chickpea Transcriptome with Desi and Wild Chickpea Provides a Rich Resource for Development of Functional Markers
Source: PLoS One. 2012 Dec 27;7(12):e52443. doi: 10.1371/journal.pone.0052443 (PMC3531472; doi:10.1371/journal.pone.0052443)
Supplement: Table S12 — List of selected SSRs validated for polymorphism in chickpea genotypes. (PDF) [file pone.0052443.s022.pdf]

Table S12. List of selected SSRs validated for polymorphism in chickpea genotypes.

SSR ID	Transcript ID	SSR repeat motif	Kabuli repeat unit size	Desi repeat unit size	Wild repeat unit size	Primer sequence (5'-3')	Annealing temperature (°C)	Product size (bp)	A/NA[1] in at least two genotypes	M/P[2] SSR in Kabuli/desi	M/P[2] SSR in Kabuli/ wild
CakTpSSR00428	CakTC09678	TCAC	9	7	6	F- AGGGTTTGTGAAATTGCGAA<br>R- GACTGAGAAGGAGCGGTGAC	60.0	168	A	M	P
CakTpSSR03117*#	CakTC34751	AT	10	12	7	F- CCAACCAATGAAAGCTAGGC<br>R- TCCTATACCAATCCCCCACA	60.0	134	A	P	P
CakTpSSR01214	CakTC22634	TA	11	12	8	F- CGAAGCATACCAGGTTGATG<br>R- GGGATCTTGTTTCACGCTGT	60.1	121	A	M	P
CakTpSSR01352	CakTC23616	CT	9	8	6	F- ATTCGGTCGTATCGTCTTGG<br>R- GCGTGTGAGCGTGTAAAAGA	60.1	279	A	M	M
CakTpSSR01506	CakTC24680	AT	7	8	-	F- TTTCGTCCCCAAATCCAATA<br>R- ATTGGTCCCCAATGTCTTGA	60.2	226	A	P	M
CakTpSSR03923	CakTC39799	GAA	19	18	15	F- ACAGAGAGGGAGGTTCGTCA<br>R- CCCTTCTTCCATGTACCACC	59.3	279	A	M	P
CakTpSSR01486	CakTC24535	TGGA	8	7	6	F- ATCCACCTCCTTTTGCCTTT<br>R- CCTCAGTCTAGGTGGGCAGA	60.4	275	A	M	M
CakTpSSR04389	CakTC42648	AG	6	7	8	F- AGAGTCAAATTAACAAAGAGCATAGA<br>R- AAAATGCCCACAAGAGCAAG	60.2	122	A	P	P
CakTpSSR02813	CakTC32488	CTT	8	9	7	F- ATCACAACGTGGTGGTAGCC<br>R- TCTTCTTCTCCTGGGGATCA	59.7	265	A	M	P
CakTpSSR01394	CakTC23803	TGA	15	14	6	F- CGAGGATCTCTGGGAAATGA<br>R- AAGCGTGCTGCTTCAGAAAT	60.2	236	A	M	P
CakTpSSR02497	CakTC30675	AT	7	10	6	F- TCCATTCCAACCTTAACCACA<br>R- GGAATGGAAGAAGAGAAGGGA	59.6	152	A	P	M
CakTpSSR02142	CakTC28476	TA	6	7	-	F- GGAATGGAAGAAGAGAAGGGA<br>R- TGACCCTCTTGTCATACTCAT	59.0	153	A	M	P
CakTpSSR03532	CakTC37598	TTG	6	5	7	F- TCACCATCGTGTGATGGACT<br>R- TTGTTGGGTCCTCTTTTGTTTT	59.9	220	A	P	P
CakTpSSR03684	CakTC38539	TC	7	8	-	F- CCCAGAAAAGAGAAAACGCA<br>R- ATTTCTCAACTGTCTCGCCG	60.4	269	A	M	P
CakTpSSR00219	CakTC05322	CAT	5	7	7	F- CATTATCAGCAGATACGGACT<br>R- GATTCTGCTGGAAGTGAAGAT	55.2	148	A	M	M
CakTpSSR00867*#ǂ	CakTC15258	GA	14	16	16	F- AGTCAATCCGTAAGAAGAAGC<br>R- ATAGCGAAAACAACTGAAACA	55.3	160	A	P	P
CakTpSSR01928	CakTC27222	CT	13	12	12	F- GGTTCATGGCTAAAAATCTTC<br>R- TTTGCATTACAAAAGCTCCTA	55.5	149	A	M	M
CakTpSSR02222	CakTC29000	TTC	10	5	5	F- CACCAATTGTAACGAATCAAC<br>R- AGAGGTTGAAAAATGGAAGAG	55.1	149	A	M	P
CakTpSSR02719	CakTC32010	CAG	5	6	6	F- ATCAACAGCATTCTCAACAAC<br>R- CACATGTCCAATACCACCTAT	55.0	155	A	P	P
CakTpSSR02637*#	CakTC31492	GATA	7	8	8	F- AGTTAGTGGTGTGCTGAAGAA<br>R- ACATTGTAGGGGTAGGTTAGC	55.0	142	A	P	P
CakTpSSR03815	CakTC39206	CT	7	6	6	F- GCCCTTGAGTTGAGTTAAGAC<br>R- AAACTATGGAAATGGAAGGTT	55.0	147	A	M	P
CakTpSSR04076	CakTC40711	CCA	5	6	6	F- AATATGCCTATGAGCATCTGA<br>R- TTGAAGGTGTTAAATTGGTTG	55.1	148	A	P	M
CakTpSSR01542*#	CakTC24891	AAT	11	13	-	F- CCTTGCTCTTCTTTCACTTCT<br>R- AGCTAAAAAGGTGAATTCGTT	53.8	165	A	P	P
CakTpSSR01660	CakTC25584	AG	6	13	-	F- TTGTTAATCAAGTTGGTTGCT<br>R- CCATCCCATACCAATAACATA	53.6	132	A	M	M

CakTpSSR03317	CakTC36179	GA	19	24	-	F- GGTGATTTGAGATTTGGAGAT<br>R- ATATGATCTATCCTCGCCAGT	54.2	155	A	P	P
CakTpSSR03090*#	CakTC34556	ATTCAT	7	6	-	F- AACTCACCCTTTTTCTTCAAA<br>R- AGGATAAAGGTGAAGCAAATC	53.8	148	A	P	P
CakTpSSR03637*#ǂ	CakTC38143	TCC	12	8	9	F- AAAGTTGTTGTTGAGCGTTT<br>R- CAAAGTTCACTCTCGTTGATT	53.7	167	A	P	P
CakTpSSR02074	CakTC28116	GTG	8	6	-	F- GTTGAGAAATTGAGGAGTGTG<br>R- TAACCACCACAATTTTCTTTC	53.4	156	A	P	P
CakTpSSR03639*#ǂ	CakTC38187	GA	14	11	-	F- CCAAAGTAAAACCATGATGAG<br>R- CACCACACTTCTTTTCTTTCA	53.9	169	A	P	P
CakTpSSR00752*#ǂ	CakTC13120	AGA	6	8	-	F- AATAAAGGAGGATTTTGATGG<br>R- CTTGTTCGTCTACTTCCATTG	53.6	159	A	P	P
CakTpSSR01488*#ǂ	CakTC24545	AG	9	6	-	F- CGAAAGAGAAAGAGAGTGTGA<br>R- GTAAATGAAATTCGGGTTACG	54.3	152	A	P	P
CakTpSSR03485#	CakTC37408	AAG	13	5	5	F- CATTTTTAATCTCGATTTCACC<br>R- ATTCTCCGATTAAATCTTCCA	54	129	A	P	P
CakTpSSR04265#	CakTC41944	TAA	17	14	-	F- CAAGATCTTAATCTTTCTTTTCC<br>R- TAGGTCAAGAGCTGAAAGTGA	53.8	147	A	P	P
CakTpSSR01720	CakTC25855	TAA	11	9	8	F- AAGCATACATGAAAGAAAACC<br>R- AGTTTATAAGTTGACGCGATG	52.9	120	A	P	P
CakTpSSR02499*#	CakTC30698	ATG	7	5	-	F- AAAAAGCAACAAGAAACACAG<br>R- CAACCATAGCAAAATTGAGAG	53.7	157	A	P	M
CakTpSSR03521*#	CakTC37545	GA	17	22	-	F- GAATGGTAATGTGAATTGGAA<br>R- GGTATATTTATGCAGCGTTTG	53.7	141	A	P	P
CakTpSSR03891	CakTC39656	TTC	17	21	7	F- TTAATGTTTGTGTGAGGTG<br>R- GTTGAATCCCTCTTTCCTT	53.5	119	A	P	P
CakTpSSR03970	CakTC40146	TGA	9	12	-	F- GAGTTGTGGTTGGTAGTTTGA<br>R- CTCACTTCTCATCACCATCAT	55	157	A	P	M
CakTpSSR04045	CakTC40557	TC	12	11	7	F- TTCTACTTCTCGATGGAAACA<br>R- AAAGGTAGGTAGCTAGCAAGG	53.5	147	A	M	M
Cak TpSSR2543#	CakTC30946	TAT	8	16	-	F- GGGAAGTTACGGTTAGAT<br>R- CCACCATTTTCATTTTCAT	53.6	164	A	P	P

*12 SSRs shown in Figure 6A.
#14 SSRs validated for polymorphic potential in 21 kabuli and desi genotypes
ǂ5 SSRs validated for polymorphic potential in 21 kabuli and desi genotypes shown in Figure 6B.
