# Supplementary material for: Comparative Analysis of Kabuli Chickpea Transcriptome with Desi and Wild Chickpea Provides a Rich Resource for Development of Functional Markers
Source: PLoS One. 2012 Dec 27;7(12):e52443. doi: 10.1371/journal.pone.0052443 (PMC3531472; doi:10.1371/journal.pone.0052443)
Supplement: Table S13 — List of 22 selected SNPs between kabuli and desi validated using the allele-specific PCR. (PDF) [file pone.0052443.s023.pdf]

Table S13. List of 22 selected SNPs between kabuli and desi validated using the allele-specific PCR.

***Kabuli* transcript sequence IDS**	**SNP IDs**	**Position (bp) of SNP loci on *Kabuli* transcripts**	**SNPs between *Kabuli* and *desi***	**SNP-flanking primers (5'-3')**	**SNP allele specific primers (5'-3')**	**Traget SNP loci**	**Annealing temperature (°C)**	**Product size (bp)**	**SNPs Validated (V)/Not Validated (NV)**
CakTC11127	Ca(ICCV2/ICC4958)SNP_00049	318	(C/T)	F- CCGTTGTAATCGGAGGTGTT<br>R- TCAAAAGACGTCGGTGAGG	CCAGTAAAGAAAACCCTAACAACATT	T	59.8	148	NV
CakTC30832*	Ca(ICCV2/ICC4958)SNP_00060	1610	(T/G)	F- TCAACTCAACCTCCTTGTCAAA<br>R-TGAAGGCACCGACTCTTACA	CACAAATCTCTCTCAAATATCCTTCC	G	58.4	166	V
CakTC34113*	Ca(ICCV2/ICC4958)SNP_00062	867	(G/A)	F- TAGACGATTGGCCAAAATCC<br>R- TCTCGCCTAGCAATTCAATG	ATTATTTTCTTCATCTCCTCCGAA	A	59.0	170	V
CakTC38380	Ca(ICCV2/ICC4958)SNP_00107	1009	(T/C)	F- TCTGAGTCTGAACCTGAGCTTG<br>R- CTCTCAATTGGCACCAACAA	TGAGTCTGAACCTGAGCTTGATT	T	58.7	147	NV
CakTC29297	Ca(ICCV2/ICC4958)SNP_00203	200	(C/G)	F- GGCCCCTCATTAAGGACTTT<br>R- GACCAGCTCACGTTTTCTGA	GGGATTCTCTCTCCCAACG	G	59.0	154	V
CakTC30888*	Ca(ICCV2/ICC4958)SNP_00316	1470	(C/T)	F- TGCCTCACCACTTGACTAGC<br>R- AAAGATTTTCGGGCAGGAAT	CAGCAAGTTTTTCCCTAGAAGGT	T	57.9	171	V
CakTC23861	Ca(ICCV2/ICC4958)SNP_00371	376	(A/C)	F- CACCAGCCATGCTCACTTT<br>R- TGACCAAGACAATGCTCCAC	TGGTGGCCATCCCACTAA	A	59.7	134	NV
CakTC29356*	Ca(ICCV2/ICC4958)SNP_00374	287	(T/C)	F- CCATCACCTTGCACCTCTAAG<br>R- TGGAATTGGTCATAGTAAAAATGC	CATTGTAGCATTCCACATGAGT	T	59.3	159	V
CakTC33643*	Ca(ICCV2/ICC4958)SNP_00428	2996	(T/G)	F- AGGTGGTGACTGGAAGAGGA<br>R- TCAAGCTTCCTGCTGTGAGA	GTCAAATGCCTAAGTCTGGATG	G	56.9	168	V
CakTC43107*	Ca(ICCV2/ICC4958)SNP_00637	1386	(A/G)	F- CACAATAGGGTGTTGTTGTGG<br>R- TTCTTCAAGTGGAAACAACCAA	TTCAAGTGGAAACAACCAACAC	G	58.6	154	V
CakTC10926*	Ca(ICCV2/ICC4958)SNP_00649	883	(G/A)	F- CAGCAACAACCTCATTCTGC<br>R- TCGACCAGCTGAGAGAATTG	CAACAACCTCATTCTGCCTAATA	A	59.1	155	V
CakTC36652*	Ca(ICCV2/ICC4958)SNP_00830	2548	(A/G)	F- GCATCCATCCCAATCTCAAT<br>R- TGTTGTTGTTGTTGCTGCTG	CATCATCTACAGCAGCAGCAA	A	58.5	150	V
CakTC10919	Ca(ICCV2/ICC4958)SNP_00936	149	(C/G)	F- AAGACATGCCGTGAACGAT<br>R- GGATGATTTTTGGGGTGAGA	CGGAGCTTATGAATGGAACC	G	59.7	130	NV
CakTC30819	Ca(ICCV2/ICC4958)SNP_00948	184	(T/C)	F- GAAATGGAAGCAGAGCATGA<br>R- TGTGCATCTGAACAAGGTGA	GATTCCTTTATGTGAAAGTTATGACC	C	58.8	151	V
CakTC40460*	Ca(ICCV2/ICC4958)SNP_01291	2458	(G/T)	F- TGTTCTTCATCGTCGTCGTC<br>R- CAAGTAAAACCTCGCGGAAG	TTCTTGTTCTTGTTCTTCTAGCTGTG	G	59.9	142	V
CakTC40874	Ca(ICCV2/ICC4958)SNP_01379	447	(C/T)	F- ACCGCCGTCGATAGTAAAGA<br>R- CCAAAGGGACTCTGAGGAGA	GATAGTAAAGAAGATGATGGGACCT	T	57.4	164	V
CakTC31552	Ca(ICCV2/ICC4958)SNP_01427	754	(C/T)	F- GGTGGTGGTGAGGTTGAGTT<br>R- AACCACCGAGTCATTGAACC	CGGCCGCCTTCAGAT	T	59.8	153	V
CakTC39741	Ca(ICCV2/ICC4958)SNP_01729	457	(A/G)	F- TTCATGGGATTTAGGGGGTA<br>R- GGAAGTGGTGATTCATTGGAA	TGATTCATTGGAAGAATTAACCAC	G	55.0	162	V
CakTC39748*	Ca(ICCV2/ICC4958)SNP_01832	1221	(A/G)	F- CGATGGGTTGAATGACACAG<br>R- TCTTCGCCAAACTCATTTCA	GTGGGCCAGACACCAGAG	G	58.4	149	V
CakTC34197	Ca(ICCV2/ICC4958)SNP_01876	863	(A/G)	F- AGTTCGCAGTCAAGGATGGT<br>R- TGGAACTGCACCTTCATCAC	CACAGTATGGATTTGGTGAGAAG	G	59.7	152	NV
CakTC38606*	Ca(ICCV2/ICC4958)SNP_01935	490	(C/G)	F- TGTGGAGTTTTCTGCACCTG<br>R- GACCAGAAAGCCCAACTGTC	CACCTGGCAATGGCTACAG	G	57.7	144	V
CakTC42135*	Ca(ICCV2/ICC4958)SNP_01956	325	(C/T)	F- CCTCACCTAGCTTTGGCAAT<br>R- GCGTTGTTGTTGTTGGTTCT	TTGTTGGTTCTGTTTCGTATGATTA	T	58.7	152	V

*12 SNPs shown in Figure 6C.
